# Biodiversity dataset and atlas of the special area of conservation Montesinho/Nogueira, Portugal

**DOI:** 10.3897/BDJ.12.e118854

**Published:** 2024-04-08

**Authors:** Nuno Garcia, João C. Campos, Daniel Silva, João Alírio, Lia B. Duarte, Salvador Arenas-Castro, Isabel Pôças, Armando Loureiro, Ana C. Teodoro, Neftalí Sillero

**Affiliations:** 1 CICGE - Centro de Investigação em Ciências GeoEespaciais, Faculdade de Ciências da Universidade do Porto, Porto, Portugal CICGE - Centro de Investigação em Ciências GeoEespaciais, Faculdade de Ciências da Universidade do Porto Porto Portugal; 2 Department of Geosciences, Environment and Land Planning, Faculty of Sciences, University of Porto, Porto, Portugal Department of Geosciences, Environment and Land Planning, Faculty of Sciences, University of Porto Porto Portugal; 3 Área de Ecología – Departamento de Botánica, Ecología y Fisiología Vegetal, Facultad de Ciencias (Universidad de Córdoba)., Córdoba, Spain Área de Ecología – Departamento de Botánica, Ecología y Fisiología Vegetal, Facultad de Ciencias (Universidad de Córdoba). Córdoba Spain; 4 ForestWISE – Collaborative Laboratory for Integrated Forest and Fire Management, Vila Real, Portugal ForestWISE – Collaborative Laboratory for Integrated Forest and Fire Management Vila Real Portugal; 5 Institute for the Conservation of Nature and Forests (ICNF), Lisbon, Portugal Institute for the Conservation of Nature and Forests (ICNF) Lisbon Portugal

**Keywords:** biodiversity conservation, biodiversity dataset, conservation status, open science, occurrence records, species distributions, Web GIS

## Abstract

**Background:**

The special area of conservation Montesinho/Nogueira (SAC-MN) is a key area for biodiversity conservation in the Iberian Peninsula. Covering an area of approximately 1081 km² in the northeast of Portugal mainland, the SAC-MN is home to a wide range of species, including several endemic and endangered species and priority habitats. Despite its ecological significance and importance for conservation, there is a lack of publicly available biodiversity data, which urges the need to create a comprehensive and up-to-date biodiversity dataset for the SAC-MN.

**New information:**

To bridge the knowledge gap on biodiversity in SAC-MN, we undertook a thorough data collection process, including species occurrence records and conservation status information at regional (Portugal) and European levels, from multiple sources. We collected and compiled this information for five major taxonomic groups (amphibians, birds, flora - vascular plants, mammals and reptiles) in SAC-MN, resulting in a total of 31,871 records with 1,312 documented species. In addition, we developed an easy-to-navigate web-based geographic information system (Web GIS). In this article, we present an in-depth report on the process of compiling and preparing data, as well as the development and design of our Web GIS to increase awareness and enhance understanding of the importance of preserving biodiversity in SAC-MN.

## Introduction

Guaranteeing biodiversity conservation from local to global scale is a critical challenge that requires urgent attention. Nonetheless, biodiversity has been declining at an unprecedented rate in recent decades due to negative drivers working at various scales, such as human-related activities (e.g. deforestation, land-use change, habitat destruction, wildfires and climate change) (Waldron et al. 2017). Biodiversity monitoring is essential to measure species conservation status and to plan evidence-based and impactful conservation actions (Jetz et al. 2019). According to recent studies, inadequate biodiversity monitoring has led to significant biases in our understanding of species distribution and abundance, particularly in under-studied taxa and ecosystems (Speed et al. 2018, Guerra et al. 2019, Johnston et al. 2023).

Accurate and up-to-date species occurrence records data are crucial for assessing and monitoring the condition of biodiversity (Jetz et al. 2019). There are multiple sources from which species occurrence data can be retrieved (Bloom et al. 2018). Platforms such as the Global Biodiversity Information Facility (GBIF; https://www.gbif.org/) and iNaturalist (https://www.inaturalist.org/) and historical collection data (Sillero et al. 2014) are commonly used. Nevertheless, the availability and accessibility of these records are often limited (e.g. no processing and integration in some citizens' reports), hampering the efforts to maintain up-to-date information on species diversity (Sillero et al. 2018, Petersen et al. 2021).

Web GIS provides an effective way to map and visualise species occurrence data (Asaad et al. 2019, Hancock et al. 2022) and make them accessible to a wider audience (Steiniger and Hay 2009). By utilising these systems, conservationists and policy-makers can easily identify areas of high biodiversity value, assess the effectiveness of conservation measures and monitor changes in biodiversity over time (Arkhipova 2020, Frans et al. 2022, Hancock et al. 2022).

This is particularly important for protected areas and conservation sites, such as the SAC-MN which is notable for its diverse characteristics. Located in north-eastern mainland Portugal, SAC-MN is an EU-designated Natura 2000 site featuring elements typical of Atlantic and/or Mediterranean ecosystems—forests, pastures and mountainous terrain. Comprising Montesinho Natural Park (MNP) and the Nogueira Mountains (NM), MNP's landscape is primarily defined by a mosaic of habitats shaped by mountain agriculture, predominantly centred on extensive livestock farming and chestnut crops (*Castaneasativa* Mill. (1768)) (Castro 2010), while NM is characterised by dense and homogenous oak forests (e.g. the black oak, *Quercuspyrenaica* Willd (1805)). Nonetheless, SAC-MN’s region encapsulates unique supra- and oro-mediterranean bioclimatic conditions, making it a biodiversity hotspot, particularly for Iberian and Mediterranean species, such as the Iberian wolf (*Canislupussignatus* Cabrera (1907)), roe deer (*Capreoluscapreolus* Linnaeus (1758)) and Iberian’s wall and emerald lizards (*Podarcishispanicus* Steindachner (1870) and *Lacertaschreiberi* Bedriaga (1878)).

Herein, we aim to provide a comprehensive dataset of biodiversity data with high spatial resolution and a Web GIS for monitoring biodiversity in the SAC-MN in Portugal. The dataset contains a total of 31,871 species occurrence records from the mentioned area, documented between 2000 and 2022, covering five taxonomic groups: flora (vascular plants), amphibians, reptiles, birds and mammals. Additionally, we developed a Web GIS to visualise and monitor the local biodiversity distribution of 1,312 individual species documented in the mentioned records, as Web GIS’s can be a valuable tool for ecologists, conservationists and other stakeholders to gain enhanced insights into species distribution.

## General description

### Purpose

The creation of this dataset stems from the lack of standardised records of species occurrence in the region. Recognising this gap, the dataset was developed to fulfil the need for detailed records of various species, including vascular plants, amphibians, reptiles, birds and mammals. The goal is to present an all-encompassing and previously unavailable resource, providing high spatial resolution records tailored to a grid of 1 km, thereby facilitating modelling (e.g. ecological niche models). Furthermore, a Web GIS was developed to map the records within the dataset.

### Additional information

**Figure 1.**
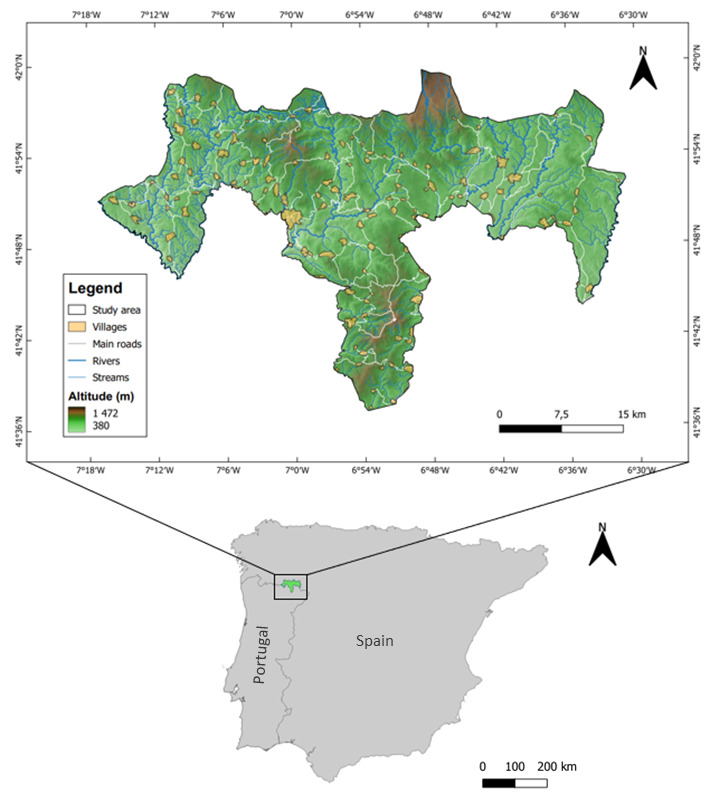
Overview of the study area: Special area of conservation Montesinho/Nogueira (SAC-MN). Elevation for SAC-MN from the digital model terrain of Portuguese Directorate General of the Territory (DGT; https://www.dgterritorio.gov.pt/). Figure was created using QGIS software Version 3.28.1 (https://www.qgis.org/).

The records were collected and assembled specifically for the SAC-MN (Fig. 1), which is a European Union’s Natura 2000 site (https://natura2000.eea.europa.eu/Natura2000/SDF.aspx?site=SAC-MN). The data collection period spans from 2000 to 2022. All the species distribution maps are available through our Web GIS (https://montobeo.shinyapps.io/MN-SPA_WebGIS/).

## Sampling methods

### Sampling description

We compiled biodiversity data from various sources spanning from 2000 to 2022. We focused on five major taxonomic groups with the highest proportions of endangered species in SAC-MN: flora - vascular plants (42%), amphibians (41%), reptiles (21%), birds (13%) and mammals (27%) (IUCN 2022a). Each dataset was obtained from different sources, such as biological surveys, museum vouchers, historical records and visual registrations. Most of the data were acquired by directly exporting them from online databases (e.g. GBIF and iNaturalist) (Table 1), which are vulnerable to nomenclature errors, coordinate absence, accuracy issues and data entry errors (Zizka et al. 2019). Thus, we filtered the datasets by manually removing nomenclature errors and records with absences and/or errors. The occurrence records were sorted according to their spatial resolution (e.g. <1 km, 1 km, 2 km, 10 km limit resolutions) and only georeferenced and aggregated (1 km) records were included in the dataset. Due to the coordinated uncertainty field (e.g. Darwin Core), certain adjustments were required and executed using QGIS software Version 3.28.1 (https://www.qgis.org/). Furthermore, we verified the conservation status of each species at both regional (Portugal) and European levels, in accordance with the International Union for Conservation of Nature (IUCN; https://www.iucnredlist.org/) and followed the most up-to-date taxonomy for each species (Cabral et al. 2005, Bencatel et al. 2019, Carapeto et al. 2020, Speybroeck et al. 2020, SPB 2021, Almeida et al. 2022, IUCN 2022b, Mathias et al. 2023). We built species distribution maps through the R programme version 4.2.2 (https://www.r-project.org/) (consult Suppl. materials 1, 2). Then, we assessed each map to exclude any doubtful observations by cross-referencing species occurrence records with other databases/datasets and excluding records that do not occur in the region.

Finally, we developed a Web GIS with R programme to display individual species’ distributions (see Suppl. material 2). We used several R packages, including "shiny" and "shinydashboard" packages (Chang et al. 2021, Chang and Ribeiro 2021), to connect the web user interface (UI) and the server through web widgets and to enhance the UI performance, respectively. Additionally, we used the "leaflet" and "leaflet.extras" packages (Cheng et al. 2022, Karambelkar and Schloerke 2022) to create mobile-friendly interactive maps and the "raster" and "terra" packages (Hijmans 2022a, Hijmans 2022b) to visualise the compiled biodiversity data within the boundaries of the study area. The code and materials used in the Shiny app development can be found in the following GitHub repositories: **Link 1**: https://github.com/BravoAlpha2/WebGIS; or **Link 2**: https://github.com/SpatialBioLab/MontObEO-WebGIS.

## Geographic coverage

### Description

The geographic range of the data covers the entire SAC-MN region.

**Coordinates**: 41.618968° and 41.992493° Latitude; -7.285387° and -6.515783° Longitude. (EPSG: 4326; WGS84 - World Geodetic System 1984)

## Taxonomic coverage

### Description

**Figure 2.**
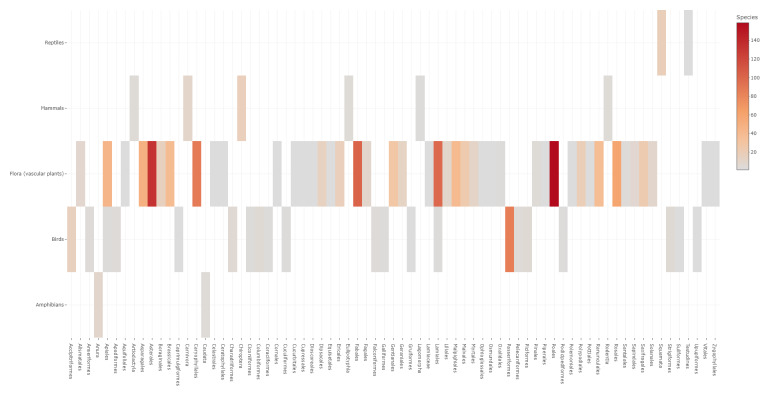
Number of species per order, according to taxonomic groups (flora - vascular plants, amphibians, reptiles, birds and mammals), in the dataset of SAC-MN.

**Figure 3.**
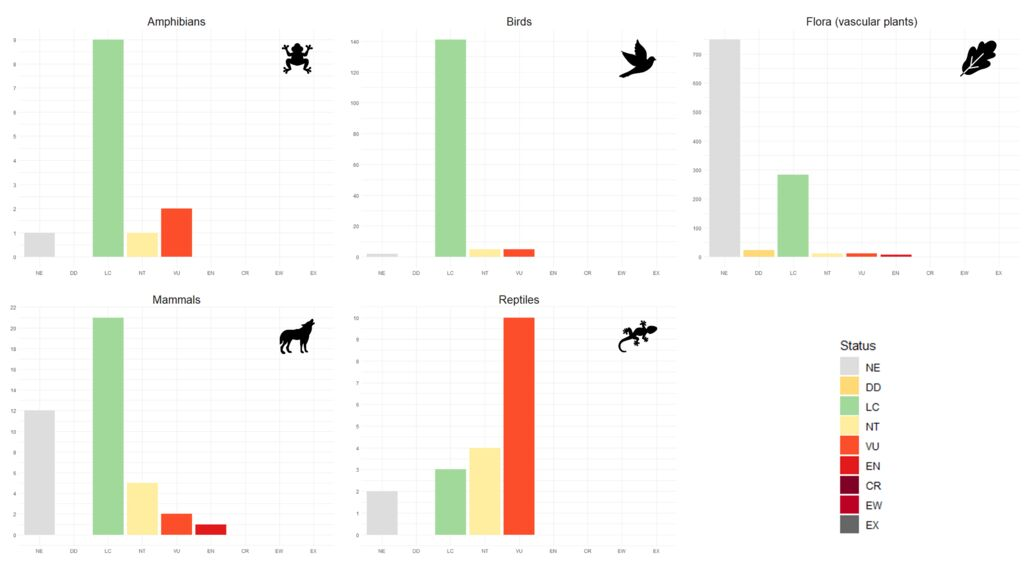
Number of species per European conservation status in the dataset of SAC-MN. Conservation statuses are represented as follows: Critically Endangered (CR), Data Deficient (DD), Endangered (EN), Least Concern (LC), Near Threatened (NT), Not Evaluated (NE) and Vulnerable (VU).

**Figure 4.**
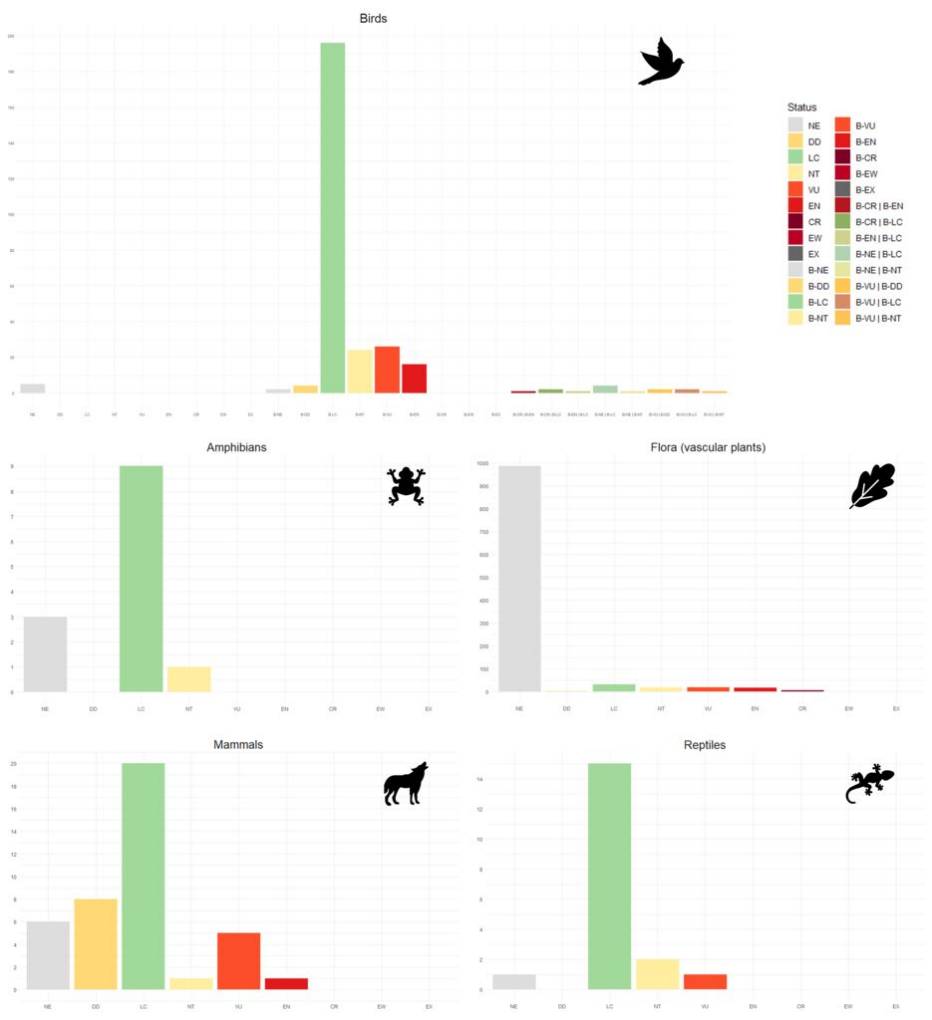
Number of species per regional (Portugal) conservation status in the dataset of SAC-MN. Conservation statuses are represented as follows: Critically Endangered (CR), Data Deficient (DD), Endangered (EN), Least Concern (LC), Near Threatened (NT), Not Evaluated (NE) and Vulnerable (VU). For birds, the regional status is indicated by their breeding (B) or wintering (W) seasons.

Overall, the present dataset consists of 31,871 species occurrence records, documenting 1,312 species in the region, compiled from different sources (Tables 1, 2, and Suppl. material 3). The SAC-MN biodiversity dataset encompasses species occurrence records from five major taxonomic group (flora – vascular plants, amphibians, reptiles, birds and mammals) with a total of 1,312 species: flora - vascular plants (n = 1,086), amphibians (n = 13), reptiles (n = 19), birds (n = 153) and mammals (n = 41). These species are part of two primary kingdoms, *Metazoa* and *Viridiplantae*, as well as two major phyla, *Chordata* and *Streptophyta*, highlighting a richness that spans over 72 orders (Fig. 2) and 183 families. In addition to the occurrence records, our dataset contains information on the current conservation status of each species at both the European (Fig. 3) and regional (Portugal) (Fig. 4) levels, in accordance with the IUCN (Cabral et al. 2005, Carapeto et al. 2020, Almeida et al. 2022, IUCN 2022b, Mathias et al. 2023) and the source and spatial resolution of the records.

**Figure 5.**
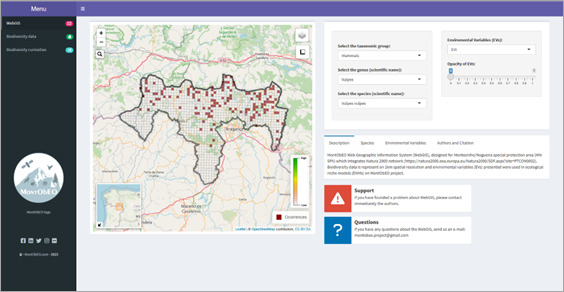
View of the Shiny app (English version) incorporating the Web GIS of SAC-MN. The Shiny app contains other elements (“Biodiversity data”; “Biodiversity curiosities”) for users to explore more about the biodiversity in SAC-MN. The app was created for the MontObEO project - Montesinho biodiversity observatory: an Earth Observation tool for biodiversity conservation, founded by the Portuguese Foundation for Science and Technology (FCT: MTS/BRB/0091/2020).

The Shiny app, which provides access to 1,312 individual species' distributions, is available in both Portuguese and English languages. It offers a user-friendly experience with quick 2-second access to individual species' distributions. While the app can support up to 50 users simultaneously, it may take up to 10 seconds to load. The app allows users to explore biodiversity data and environmental factors that influence species distributions within the SAC-MN in a comprehensive and accessible way. Fig. 5 presents a glimpse of the individual species' distributions available for exploration on the Web GIS. The app was originally designed for the MontObEO project - Montesinho biodiversity observatory: an Earth Observation tool for biodiversity conservation, founded by the Portuguese Foundation for Science and Technology (FCT: MTS/BRB/0091/2020) and it can be accessed through the MontObEO project website (https://montobeo.wordpress.com/). In addition, direct access to the app is available through the following URLs: **English (EN) version**: https://montobeo.shinyapps.io/MN-SPA_WebGIS/; or **Portuguese (PT) version**: https://montobeo.shinyapps.io/MN-SPA_WebSIG/

## Temporal coverage

### Notes

From 2000 to 2022.

## Usage licence

### Usage licence

Creative Commons Public Domain Waiver (CC-Zero)

## Data resources

### Data package title

Species occurrence records of special areas of conservation Montesinho/Nogueira.

### Number of data sets

1

### Data set 1.

#### Data set name

Species occurrence records of special areas of conservation Montesinho/Nogueira.

#### Data format

Comma-separated values (.csv)

#### Download URL

https://zenodo.org/doi/10.5281/zenodo.7657330

#### Description

The dataset contains biodiversity data for significant taxonomic groups (flora - vascular plants, amphibians, reptiles, birds and mammals) in special areas of conservation Montesinho/Nogueira (Portugal). It covers the period from 2000 to 2022 and has a high spatial resolution (e.g. georeferenced and aggregated (1 km) records. Additionally, the dataset offers details on the conservation status of each species at both regional (Portugal) and European levels, as well as the sources of the records and their corresponding spatial resolution. The dataset was developed in response to the absence of standardised species occurrence records in the region and to facilitate modelling (e.g. development of ecological niche models).

**Data set 1.** 

Column label	Column description
Taxonomic groups	Taxonomic groups in the dataset.
Kingdom	Kingdom of species in the dataset.
Phylum	Phylum of species in the dataset.
Order	Order of species in the dataset.
Family	Family of species in the dataset.
Genus	Genus of species in the dataset.
Species	Scientific name of the species in the dataset.
European (EU) status	European status is in accordance with the International Union for Conservation of Nature (IUCN; https://www.iucnredlist.org/) 2021-3 version of the Red List.
Regional (Portugal) status	Regional (Portugal) status of amphibians and reptiles in accordance with the 2005 version of the national Red List. For vascular plants, birds and mammals, the status is in accordance with the 2020, 2022 and 2023 versions of the national Red List, respectively.
Latitude	Latitude coordinates in EPSG: 4326 (WGS84 - World Geodetic System 1984).
Longitude	Longitude coordinates in EPSG: 4326 (WGS84 - World Geodetic System 1984).
Sources	Name of the source where the record was obtained.
Resolution	Code description – GPS indicates georeferenced records; 1 km refers to aggregated records with precisely one-kilometre spatial resolution.

## Supplementary Material

770B8AD7-2240-532D-8960-DEDCCD013D6D 10.3897/BDJ.12.e118854.suppl1 Supplementary material 1 R script used to plot distribution maps for each species. Data type Script (.pdf) File: oo_983198.pdf https://binary.pensoft.net/file/983198 Nuno Garcia, João C. Campos, Daniel Silva, João Alírio, Lia Duarte, Salvador Arenas-Castro, Isabel Pôças, Armando Loureiro, Ana C. Teodoro, Neftalí Sillero

B4789587-FAC3-5B9C-BFEA-E7A481569E38 10.3897/BDJ.12.e118854.suppl2 Supplementary material 2 Species distribution maps. Data type Maps (.pdf) File: oo_984417.pdf https://binary.pensoft.net/file/984417 Nuno Garcia, João C. Campos, Daniel Silva, João Alírio, Lia Duarte, Salvador Arenas-Castro, Isabel Pôças, Armando Loureiro, Ana C. Teodoro, Neftalí Sillero

808A800D-222A-570E-8BDB-A60C287CF345 10.3897/BDJ.12.e118854.suppl3 Supplementary material 3 Summary of compiled biodiversity data. Data type Table (.pdf) File: oo_984416.pdf https://binary.pensoft.net/file/984416 Nuno Garcia, João C. Campos, Daniel Silva, João Alírio, Lia Duarte, Salvador Arenas-Castro, Isabel Pôças, Armando Loureiro, Ana C. Teodoro, Neftalí Sillero

## Figures and Tables

**Table 1.** Summary of the data sources used to compile the biodiversity data in the SAC-MN indicating source link, number of occurrence records compiled, spatial resolution and original timespan.

**Sources**	**Records**	**Data type**	**Original timespan**	**Citations**
Atlas of amphibians and reptiles of Portugal	1,905	Georeferenced and aggregated (1 km)	2000 – 2020	GBIF.org (17 January 2022) GBIF Occurrence Download https://doi.org/10.15468/dl.eza8dq
Field collected data	839	Georeferenced	2020 and 2021	Not available
Floradata	3,508	Aggregated (1 km)	2021	Flora-On: Flora de Portugal Interactiva (2021). Portuguese Botanic Society, Lisbon, Portugal (www.flora-on.pt)
Biodiversity.eu	86	Georeferenced and aggregated (1 km)	2000 – 2020	Biodiversidade.eu project (https://biodiversidade.eu/)
Global Biodiversity Information Facility (GBIF)	8,459	Georeferenced and aggregated (1 km)		GBIF.org (17 January 2022) GBIF Occurrence Download https://doi.org/10.15468/dl.jubg6p GBIF.org (17 January 2022) GBIF Occurrence Download https://doi.org/10.15468/dl.hvzbgh GBIF.org (17 January 2022) GBIF Occurrence Download https://doi.org/10.15468/dl.s6yzny GBIF.org (17 January 2022) GBIF Occurrence Download https://doi.org/10.15468/dl.9amkc6 GBIF.org (17 January 2022) GBIF Occurrence Download https://doi.org/10.15468/dl.9kqs2x
EOD – Dataset eBird (GBIF dataset)	7,958	Georeferenced and aggregated (1 km)		GBIF.org (17 January 2022) GBIF Occurrence Download https://doi.org/10.15468/dl.jfq4jn
INaturalist	906	Georeferenced		iNaturalist community. Observations of amphibians, birds, mammals, plants and reptiles from Montesinho, Bragança, Portugal, observed on/between 2000 and 2020. Exported from https://www.inaturalist.org on 2022.
Portuguese Botanic Society	6,040	Georeferenced		Portuguese Botanic Society, Lisbon, Portugal (https://spbotanica.pt/)
ICNF’s monitoring programme	2,170	Georeferenced	2000 – 2022	Institute for the Conservation of Nature and Forests (ICNF), Lisbon, Portugal (https://www.icnf.pt/)

**Table 2.** Number of occurrence records and species documented in the SAC-MN by taxonomic group.

**Taxonomic group**	**Occurrence records**	**Species**
Amphibians	1,841	13
Birds	16,224	153
Flora (vascular plants)	11,405	1,086
Mammals	1,439	41
Reptiles	962	19
