# Supplementary material for: Biodiversity dataset and atlas of the special area of conservation Montesinho/Nogueira, Portugal
Source: Biodivers Data J. 2024 Apr 8;12:e118854. doi: 10.3897/BDJ.12.e118854 (PMC11019261; doi:10.3897/BDJ.12.e118854)
Supplement: Supplementary material 1 — R script used to plot distribution maps for each species. [file bdj-12-e118854-s001.pdf]

```
################## Script: Species standard maps ##################


## Packages required

require(rgdal)
require(raster)
require(sp)
require(latticeExtra)
require(maps)
require(GISTools)


## Set working directory
setwd("Path_of_working_directory")

## load digital model terrain (DEM)(.tif format)
DEM <- raster("Path_of_DEM_.tif")

## load 1x1km grid of the study area(.shp format)
grid <- shapefile("Path_of_grid.shp")

## Import species list (.csv format)
names<- read.csv2("Path_of_ species_list.csv")
row.names(names) <- names$species
species <- row.names(names)

## Generate pdf
pdf("Species_maps.pdf", width = 8,height = 8, pointsize = 12,
compress = T)

## Generate loop for species maps
for (i.spp in species){
  ## Plot study area DEM
  plot(DEM,  col = hsv(0.532, .75, seq(.95,.10,length.out = 20)),
       font.main = 3, main = paste(names$taxon[names$species ==
i.spp], ": ", i.spp, sep=""),
       legend.args = list(text = "Altitude (m)",
                          side = 2,
                          font = 2,
                          line = 0.2,
                          cex = 1),legend.shrink = 0.4,
       xlab = "Longitude (UTM)", ylab = "Latitude (UTM)",
axes=TRUE)

  ## Include scale bar
  maps::map.scale(x=-6.73, y=41.585, ratio=FALSE, relwidth=0.15,
metric = TRUE)
  ## North arrow
  GISTools::north.arrow(xb=-6.6, yb=42.025, len=0.010, lab="N", col
= "black")
  ## Load species shp
  species <- shapefile(paste("Species_", i.spp, ".shp", sep=""))
  ## Plot grid with absences
  grid_absences <- grid[is.na(sp::over(grid,
sp::geometry(species))), ]
  plot(grid_absences, add=T)
  ## Plot presences with different color (add to previous map)
```

```
  grid_presences <- grid[!is.na(sp::over(grid,
sp::geometry(species))), ]
  plot(grid_presences, add=T, col = "burlywood1")
}

dev.off()


########################## End of script ##########################
```
