# Supplementary material for: Biodiversity dataset and atlas of the special area of conservation Montesinho/Nogueira, Portugal
Source: Biodivers Data J. 2024 Apr 8;12:e118854. doi: 10.3897/BDJ.12.e118854 (PMC11019261; doi:10.3897/BDJ.12.e118854)
Supplement: Supplementary material 3 — Summary of compiled biodiversity data. [file bdj-12-e118854-s003.pdf]

Table SM1. Summary of compiled biodiversity data, indicating the taxonomic group (only vascular plants were considered in the flora group), the scientific name of the species, the number of occurrences (N), and the conservation status of the species at the European (EU status) and regional (Portugal; Reg. status) level in the special area of conservation Montesinho/Nogueira. The European status is in accordance with the International Union for Conservation of Nature (IUCN; https://www.iucnredlist.org/) 2022-2 version of the red list. The regional (Portugal) status is in accordance with the national Red Lists, with amphibians and reptiles following the 2005 edition, vascular plants to the 2020 edition, birds to the 2022 edition, and mammals to the 2023 edition. Conservation statuses are represented as follows: Critically Endangered (CR), Data Deficient (DD), Endangered (EN), Least Concern (LC), Near Threatened (NT), Not Evaluated (NE), and Vulnerable (VU). For birds, the regional status is indicated by their breeding (B) or wintering (W) seasons.

**Taxa**	***Species (scientific name)***	**N**	**EU status[1]**	**Reg. status[2,3,4,5]**
Amphibians	*Alytes cisternasii*	11	LC	LC
Amphibians	*Alytes obstetricans*	179	LC	LC
Amphibians	*Bufo spinosus*	187	NE	NE
Amphibians	*Discoglossus galganoi*	26	LC	NT
Amphibians	*Epidalea calamita*	63	LC	LC
Amphibians	*Hyla molleri*	62	LC	NE
Amphibians	*Lissotriton boscai*	319	LC	LC
Amphibians	*Pelobates cultripes*	2	VU	LC
Amphibians	*Pelophylax perezi*	430	LC	NE
Amphibians	*Pleurodeles waltl*	4	NT	LC
Amphibians	*Rana iberica*	190	VU	LC
Amphibians	*Salamandra salamandra*	311	LC	LC
Amphibians	*Triturus marmoratus*	283	LC	LC
Birds	*Accipiter gentilis*	19	LC	B-VU
Birds	*Accipiter nisus*	38	LC	B-LC
Birds	*Actitis hypoleucos*	14	LC	B-VU | W-NT
Birds	*Aegithalos caudatus*	238	LC	B-LC
Birds	*Aegypius monachus*	20	LC	B-EN
Birds	*Alauda arvensis*	113	LC	B-LC
Birds	*Alcedo atthis*	21	LC	B-LC
Birds	*Alectoris rufa*	140	NT	B-LC
Birds	*Anas platyrhynchos*	27	LC	B-LC
Birds	*Anthus campestris*	63	LC	B-LC
Birds	*Anthus pratensis*	48	LC	W-LC
Birds	*Anthus spinoletta*	13	LC	B-EN | W-LC
Birds	*Anthus trivialis*	41	LC	B-NT
Birds	*Apus apus*	166	NT	B-LC
Birds	*Apus pallidus*	8	LC	B-LC
Birds	*Aquila chrysaetos*	36	LC	B-EN
Birds	*Aquila fasciata*	1	LC	B-VU
Birds	*Ardea alba*	2	LC	B-NE | W-NT
Birds	*Ardea cinerea*	21	LC	B-LC
Birds	*Asio otus*	1	LC	B-VU
Birds	*Athene noctua*	3	LC	B-LC

Birds	*Buteo buteo*	236	LC	B-LC
Birds	*Calandrella brachydactyla*	2	LC	B-LC
Birds	*Caprimulgus europaeus*	27	LC	B-LC
Birds	*Carduelis carduelis*	221	LC	B-LC
Birds	*Cecropis daurica*	114	LC	B-LC
Birds	*Certhia brachydactyla*	312	LC	B-LC
Birds	*Cettia cetti*	108	LC	B-LC
Birds	*Chloris chloris*	204	LC	B-LC
Birds	*Ciconia ciconia*	70	LC	B-LC
Birds	*Ciconia nigra*	6	LC	B-VU
Birds	*Cinclus cinclus*	73	LC	B-NT
Birds	*Circaetus gallicus*	68	LC	B-NT
Birds	*Circus cyaneus*	30	LC	B-CR | W-EN
Birds	*Circus pygargus*	80	LC	B-EN
Birds	*Cisticola juncidis*	14	LC	B-LC
Birds	*Clamator glandarius*	6	VU	B-NT
Birds	*Coccothraustes coccothraustes*	13	LC	B-LC
Birds	*Columba livia*	57	LC	B-DD
Birds	*Columba oenas*	21	LC	B-VU | W-DD
Birds	*Columba palumbus*	283	LC	B-LC
Birds	*Corvus corax*	95	LC	B-LC
Birds	*Corvus corone*	275	LC	B-LC
Birds	*Coturnix coturnix*	75	NT	B-LC
Birds	*Cuculus canorus*	253	LC	B-LC
Birds	*Curruca cantillans*	188	LC	B-LC
Birds	*Curruca communis*	181	LC	B-LC
Birds	*Curruca conspicillata*	6	LC	B-NT
Birds	*Curruca melanocephala*	72	LC	B-LC
Birds	*Curruca undata*	240	NT	B-LC
Birds	*Cyanistes caeruleus*	369	LC	B-LC
Birds	*Delichon urbicum*	179	LC	B-LC
Birds	*Dendrocopos major*	219	LC	B-LC
Birds	*Dryobates minor*	29	LC	B-LC
Birds	*Egretta garzetta*	2	LC	B-LC
Birds	*Elanus caeruleus*	8	LC	B-LC
Birds	*Emberiza calandra*	186	LC	B-LC
Birds	*Emberiza cia*	319	LC	B-LC
Birds	*Emberiza cirlus*	183	LC	B-LC
Birds	*Emberiza citrinella*	9	LC	B-EN
Birds	*Emberiza hortulana*	20	LC	B-VU
Birds	*Erithacus rubecula*	456	LC	B-LC
Birds	*Estrilda astrild*	2	NE	B-NE
Birds	*Falco peregrinus*	36	LC	B-VU
Birds	*Falco subbuteo*	27	LC	B-VU
Birds	*Falco tinnunculus*	43	LC	B-VU
Birds	*Ficedula hypoleuca*	104	LC	NE

Birds	*Fringilla coelebs*	458	LC	B-LC
Birds	*Fringilla montifringilla*	3	LC	B-LC
Birds	*Galerida cristata*	14	LC	B-LC
Birds	*Galerida theklae*	27	LC	B-LC
Birds	*Gallinago gallinago*	3	VU	B-CR | W-LC
Birds	*Gallinula chloropus*	8	LC	B-LC
Birds	*Garrulus glandarius*	326	LC	B-LC
Birds	*Gyps fulvus*	66	LC	B-LC
Birds	*Hieraaetus pennatus*	117	LC	B-LC
Birds	*Hippolais polyglotta*	145	LC	B-LC
Birds	*Hirundo rustica*	262	LC	B-LC
Birds	*Jynx torquilla*	40	LC	B-LC
Birds	*Lanius collurio*	62	LC	B-VU
Birds	*Lanius meridionalis*	100	VU	B-VU
Birds	*Lanius senator*	47	NT	B-VU
Birds	*Larus fuscus*	1	LC	B-VU | W-LC
Birds	*Linaria cannabina*	292	LC	B-LC
Birds	*Locustella naevia*	2	LC	NE
Birds	*Lophophanes cristatus*	164	LC	B-LC
Birds	*Loxia curvirostra*	25	LC	B-VU | W-DD
Birds	*Lullula arborea*	296	LC	B-LC
Birds	*Luscinia megarhynchos*	276	LC	B-LC
Birds	*Luscinia svecica*	7	LC	B-NE | W-LC
Birds	*Merops apiaster*	94	LC	B-LC
Birds	*Milvus migrans*	67	LC	B-LC
Birds	*Milvus milvus*	54	LC	B-CR | W-LC
Birds	*Monticola saxatilis*	12	LC	B-EN
Birds	*Monticola solitarius*	13	LC	B-LC
Birds	*Motacilla alba*	263	LC	B-LC
Birds	*Motacilla cinerea*	123	LC	B-LC
Birds	*Motacilla flava*	7	LC	B-LC
Birds	*Muscicapa striata*	19	LC	B-NT
Birds	*Neophron percnopterus*	4	VU	B-EN
Birds	*Oenanthe hispanica*	10	LC	B -VU
Birds	*Oenanthe oenanthe*	69	LC	B -LC
Birds	*Oriolus oriolus*	205	LC	B-LC
Birds	*Otus scops*	21	LC	B- VU
Birds	*Parus major*	365	LC	B-LC
Birds	*Passer domesticus*	320	LC	B-LC
Birds	*Passer montanus*	46	LC	B-NT
Birds	*Periparus ater*	174	LC	B-LC
Birds	*Pernis apivorus*	56	LC	B-NT
Birds	*Petronia petronia*	87	LC	B-LC
Birds	*Phalacrocorax carbo*	17	LC	B-NE | W-LC
Birds	*Phoenicurus ochruros*	268	LC	B-LC
Birds	*Phoenicurus phoenicurus*	67	LC	B-LC

Birds	*Phylloscopus bonelli*	278	LC	B-LC
Birds	*Phylloscopus collybita*	74	LC	B-NE | W-LC
Birds	*Phylloscopus ibericus*	207	LC	B-LC
Birds	*Phylloscopus trochilus*	14	LC	NE
Birds	*Pica pica*	121	LC	B-LC
Birds	*Picus sharpei*	88	LC	B-LC
Birds	*Picus viridis*	79	LC	NE
Birds	*Podiceps cristatus*	29	LC	B-LC
Birds	*Prunella modularis*	170	LC	B-LC
Birds	*Ptyonoprogne rupestris*	98	LC	B-LC
Birds	*Pyrrhula pyrrhula*	48	LC	B-LC
Birds	*Regulus ignicapilla*	183	LC	B-LC
Birds	*Regulus regulus*	6	LC	B-LC
Birds	*Riparia riparia*	6	LC	B-LC
Birds	*Saxicola rubetra*	20	LC	B-EN
Birds	*Saxicola rubicola*	258	NE	NE
Birds	*Scolopax rusticola*	2	LC	B-DD
Birds	*Serinus serinus*	385	LC	B-LC
Birds	*Sitta europaea*	228	LC	B-LC
Birds	*Spatula clypeata*	2	LC	B-VU | W-LC
Birds	*Spatula querquedula*	2	LC	B-NE | W-LC
Birds	*Spinus spinus*	19	LC	B-LC
Birds	*Sterna hirundo*	4	LC	B-EN
Birds	*Streptopelia decaocto*	46	LC	B-LC
Birds	*Streptopelia turtur*	216	VU	B-NT
Birds	*Strix aluco*	42	LC	B-LC
Birds	*Sturnus unicolor*	261	LC	B-LC
Birds	*Sturnus vulgaris*	12	LC	B-LC
Birds	*Sylvia atricapilla*	381	LC	B-LC
Birds	*Tachybaptus ruficollis*	17	LC	B-LC
Birds	*Tachymarptis melba*	4	LC	B-NT
Birds	*Tringa ochropus*	6	LC	B-NT
Birds	*Troglodytes troglodytes*	388	LC	B-LC
Birds	*Turdus iliacus*	8	LC	B-LC
Birds	*Turdus merula*	467	LC	B-LC
Birds	*Turdus philomelos*	182	LC	B-LC
Birds	*Turdus pilaris*	2	LC	W-LC
Birds	*Turdus viscivorus*	154	LC	B-LC
Birds	*Tyto alba*	8	LC	B-NT
Birds	*Upupa epops*	53	LC	B-LC
Flora (vascular plants)	*Abutilon theophrasti*	1	NE	NE
Flora (vascular plants)	*Acacia dealbata*	6	NE	NE
Flora (vascular plants)	*Acanthus mollis*	1	NE	NE
Flora (vascular plants)	*Acer monspessulanum*	10	LC	NE
Flora (vascular plants)	*Acer pseudoplatanus*	1	LC	NE
Flora (vascular plants)	*Achillea millefolium*	75	LC	NE

Flora (vascular plants)	*Acinos alpinus*	5	LC	NE
Flora (vascular plants)	*Adenocarpus complicatus*	19	LC	NE
Flora (vascular plants)	*Aegilops geniculata*	10	LC	NE
Flora (vascular plants)	*Aegilops neglecta*	2	NE	NE
Flora (vascular plants)	*Aegilops triuncialis*	5	LC	NE
Flora (vascular plants)	*Agave americana*	1	NE	NE
Flora (vascular plants)	*Agrimonia eupatoria*	38	LC	NE
Flora (vascular plants)	*Agrimonia procera*	3	NE	NE
Flora (vascular plants)	*Agrostemma githago*	7	NE	NE
Flora (vascular plants)	*Agrostis castellana*	41	NE	NE
Flora (vascular plants)	*Agrostis curtisii*	17	NE	NE
Flora (vascular plants)	*Agrostis hesperica*	4	NE	NE
Flora (vascular plants)	*Agrostis stolonifera*	7	LC	NE
Flora (vascular plants)	*Agrostis truncatula*	26	NE	NE
Flora (vascular plants)	*Ailanthus altissima*	11	NE	NE
Flora (vascular plants)	*Aira caryophyllea*	31	NE	NE
Flora (vascular plants)	*Aira praecox*	5	NE	NE
Flora (vascular plants)	*Airopsis tenella*	1	NE	NE
Flora (vascular plants)	*Ajuga pyramidalis*	6	NE	NE
Flora (vascular plants)	*Ajuga reptans*	7	NE	NE
Flora (vascular plants)	*Alisma lanceolatum*	1	NE	NE
Flora (vascular plants)	*Alliaria petiolata*	41	NE	NE
Flora (vascular plants)	*Allium guttatum*	4	LC	NE
Flora (vascular plants)	*Allium oleraceum*	4	LC	NE
Flora (vascular plants)	*Allium pallens*	2	DD	NE
Flora (vascular plants)	*Allium paniculatum*	2	LC	NE
Flora (vascular plants)	*Allium scorzonerifolium*	5	LC	NE
Flora (vascular plants)	*Allium sphaerocephalon*	7	LC	NE
Flora (vascular plants)	*Allium ursinum*	1	NE	NE
Flora (vascular plants)	*Allium vineale*	3	LC	NE
Flora (vascular plants)	*Alnus glutinosa*	53	LC	NE
Flora (vascular plants)	*Alopecurus aequalis*	1	LC	NE
Flora (vascular plants)	*Alopecurus arundinaceus*	8	NE	NE
Flora (vascular plants)	*Alopecurus geniculatus*	2	LC	NE
Flora (vascular plants)	*Alyssum granatense*	2	NE	NE
Flora (vascular plants)	*Alyssum minutum*	2	NE	NE
Flora (vascular plants)	*Alyssum serpyllifolium*	57	DD	LC
Flora (vascular plants)	*Amaranthus albus*	1	NE	NE
Flora (vascular plants)	*Amaranthus deflexus*	9	NE	NE
Flora (vascular plants)	*Amaranthus hybridus*	6	NE	NE
Flora (vascular plants)	*Amaranthus retroflexus*	1	NE	NE
Flora (vascular plants)	*Amelanchier ovalis*	9	NE	NE
Flora (vascular plants)	*Ammi majus*	1	LC	NE
Flora (vascular plants)	*Anagallis arvensis*	29	LC	NE
Flora (vascular plants)	*Anagallis tenella*	1	NE	NE
Flora (vascular plants)	*Anarrhinum bellidifolium*	55	NE	NE

Flora (vascular plants)	*Anarrhinum duriminium*	1	NE	NE
Flora (vascular plants)	*Anchusa arvensis*	6	NE	NE
Flora (vascular plants)	*Andryala integrifolia*	44	NE	NE
Flora (vascular plants)	*Andryala rothia*	1	NE	NE
Flora (vascular plants)	*Anemone palmata*	1	NE	NE
Flora (vascular plants)	*Angelica major*	6	NE	NE
Flora (vascular plants)	*Angelica sylvestris*	6	NE	NE
Flora (vascular plants)	*Anogramma leptophylla*	8	LC	NE
Flora (vascular plants)	*Anthemis alpestris*	20	NE	NT
Flora (vascular plants)	*Anthemis arvensis*	24	NE	NE
Flora (vascular plants)	*Anthemis canescens*	3	NE	EN
Flora (vascular plants)	*Anthemis cotula*	4	NE	NE
Flora (vascular plants)	*Anthericum liliago*	1	NE	VU
Flora (vascular plants)	*Anthoxanthum amarum*	15	NE	NE
Flora (vascular plants)	*Anthoxanthum aristatum*	9	NE	NE
Flora (vascular plants)	*Anthoxanthum odoratum*	27	NE	NE
Flora (vascular plants)	*Anthoxanthum ovatum*	20	NE	NE
Flora (vascular plants)	*Anthriscus caucalis*	2	NE	NE
Flora (vascular plants)	*Anthriscus sylvestris*	4	NE	NE
Flora (vascular plants)	*Anthyllis vulneraria*	64	DD	LC
Flora (vascular plants)	*Antinoria agrostidea*	4	NE	NE
Flora (vascular plants)	*Antirrhinum graniticum*	3	NE	NE
Flora (vascular plants)	*Antirrhinum rothmaleri*	9	NE	EN
Flora (vascular plants)	*Aphanes australis*	11	NE	NE
Flora (vascular plants)	*Aphanes cornucopioides*	2	NE	NE
Flora (vascular plants)	*Apium nodiflorum*	11	LC	NE
Flora (vascular plants)	*Aquilegia vulgaris*	24	NE	NE
Flora (vascular plants)	*Arabidopsis thaliana*	2	NE	NE
Flora (vascular plants)	*Arabis glabra*	9	NE	NE
Flora (vascular plants)	*Arabis juressi*	3	NE	NE
Flora (vascular plants)	*Arabis stenocarpa*	3	NE	NE
Flora (vascular plants)	*Arbutus unedo*	18	LC	NE
Flora (vascular plants)	*Arctium minus*	29	NE	NE
Flora (vascular plants)	*Arenaria leptoclados*	3	NE	NE
Flora (vascular plants)	*Arenaria montana*	58	NE	NE
Flora (vascular plants)	*Arenaria querioides*	32	NE	LC
Flora (vascular plants)	*Arisarum simorrhinum*	1	LC	NE
Flora (vascular plants)	*Aristolochia paucinervis*	26	NE	NE
Flora (vascular plants)	*Armeria eriophylla*	29	NE	NT
Flora (vascular plants)	*Armeria langei*	14	NE	NT
Flora (vascular plants)	*Armeria transmontana*	5	NE	NE
Flora (vascular plants)	*Arnica montana*	6	LC	NT
Flora (vascular plants)	*Arnoseris minima*	18	NE	NE
Flora (vascular plants)	*Arrhenatherum album*	19	NE	NE
Flora (vascular plants)	*Arrhenatherum elatius*	32	LC	NE
Flora (vascular plants)	*Artemisia absinthium*	1	LC	NE

Flora (vascular plants)	*Arum cylindraceum*	4	DD	NE
Flora (vascular plants)	*Arum italicum*	7	NE	LC
Flora (vascular plants)	*Arundo donax*	1	LC	NE
Flora (vascular plants)	*Asperula aristata*	2	NE	NE
Flora (vascular plants)	*Asphodelus albus*	2	NE	NE
Flora (vascular plants)	*Asphodelus macrocarpus*	8	NE	NE
Flora (vascular plants)	*Asphodelus serotinus*	3	NE	NE
Flora (vascular plants)	*Asplenium adiantum-nigrum*	13	LC	NE
Flora (vascular plants)	*Asplenium billotii*	10	NE	NE
Flora (vascular plants)	*Asplenium onopteris*	8	NE	NE
Flora (vascular plants)	*Asplenium septentrionale*	8	LC	EN
Flora (vascular plants)	*Asplenium trichomanes*	24	LC	NE
Flora (vascular plants)	*Aster aragonensis*	11	NE	EN
Flora (vascular plants)	*Aster lanceolatus*	1	NE	NE
Flora (vascular plants)	*Asterolinon linum-stellatum*	10	NE	NE
Flora (vascular plants)	*Astragalus glycyphyllos*	2	LC	EN
Flora (vascular plants)	*Astragalus incanus*	10	NE	EN
Flora (vascular plants)	*Astragalus pelecinus*	1	NE	NE
Flora (vascular plants)	*Athyrium filix-femina*	29	LC	NE
Flora (vascular plants)	*Avena barbata*	30	LC	NE
Flora (vascular plants)	*Avena fatua*	1	LC	NE
Flora (vascular plants)	*Avena sterilis*	3	LC	NE
Flora (vascular plants)	*Avenula pratensis*	11	NE	VU
Flora (vascular plants)	*Avenula pubescens*	3	NE	CR
Flora (vascular plants)	*Avenula sulcata*	11	NE	NE
Flora (vascular plants)	*Baldellia alpestris*	1	NT	NE
Flora (vascular plants)	*Ballota nigra*	11	LC	NE
Flora (vascular plants)	*Barbarea intermedia*	6	DD	NE
Flora (vascular plants)	*Bellardia trixago*	2	NE	NE
Flora (vascular plants)	*Bellis perennis*	6	NE	NE
Flora (vascular plants)	*Bellis sylvestris*	4	NE	NE
Flora (vascular plants)	*Betula pubescens*	12	LC	NE
Flora (vascular plants)	*Bidens frondosa*	1	NE	NE
Flora (vascular plants)	*Bidens tripartita*	2	LC	NE
Flora (vascular plants)	*Blechnum spicant*	7	LC	NE
Flora (vascular plants)	*Brachypodium rupestre*	31	NE	NE
Flora (vascular plants)	*Brachypodium sylvaticum*	30	NE	NE
Flora (vascular plants)	*Brassica barrelieri*	6	LC	NE
Flora (vascular plants)	*Briza maxima*	37	NE	NE
Flora (vascular plants)	*Briza media*	10	NE	NE
Flora (vascular plants)	*Briza minor*	13	NE	NE
Flora (vascular plants)	*Bromus diandrus*	20	NE	NE
Flora (vascular plants)	*Bromus hordeaceus*	34	NE	NE
Flora (vascular plants)	*Bromus madritensis*	16	NE	NE
Flora (vascular plants)	*Bromus racemosus*	4	NE	NE
Flora (vascular plants)	*Bromus ramosus*	6	NE	NT

Flora (vascular plants)	*Bromus rubens*	1	NE	NE
Flora (vascular plants)	*Bromus scoparius*	3	NE	NE
Flora (vascular plants)	*Bromus squarrosus*	2	VU	NE
Flora (vascular plants)	*Bromus sterilis*	20	NE	NE
Flora (vascular plants)	*Bromus tectorum*	17	NE	NE
Flora (vascular plants)	*Bryonia dioica*	43	NE	NE
Flora (vascular plants)	*Bunias erucago*	14	NE	NE
Flora (vascular plants)	*Bupleurum gerardi*	3	NE	NE
Flora (vascular plants)	*Calamintha baetica*	1	NE	NE
Flora (vascular plants)	*Calamintha nepeta*	3	NE	NE
Flora (vascular plants)	*Calendula arvensis*	4	NE	NE
Flora (vascular plants)	*Calepina irregularis*	8	NE	NE
Flora (vascular plants)	*Callitriche stagnalis*	1	LC	NE
Flora (vascular plants)	*Calluna vulgaris*	22	LC	NE
Flora (vascular plants)	*Caltha palustris*	2	LC	NE
Flora (vascular plants)	*Calystegia silvatica*	3	NE	NE
Flora (vascular plants)	*Campanula erinus*	1	NE	NE
Flora (vascular plants)	*Campanula lusitanica*	18	NE	NE
Flora (vascular plants)	*Campanula rapunculus*	36	NE	NE
Flora (vascular plants)	*Capsella bursa-pastoris*	9	LC	NE
Flora (vascular plants)	*Cardamine flexuosa*	3	NE	NE
Flora (vascular plants)	*Cardamine hirsuta*	5	NE	NE
Flora (vascular plants)	*Cardaria draba*	1	NE	NE
Flora (vascular plants)	*Carduus carpetanus*	35	NE	NE
Flora (vascular plants)	*Carduus pycnocephalus*	7	NE	NE
Flora (vascular plants)	*Carduus tenuiflorus*	27	NE	NE
Flora (vascular plants)	*Carex binervis*	9	NE	NE
Flora (vascular plants)	*Carex caryophyllea*	7	NE	NE
Flora (vascular plants)	*Carex cuprina*	1	NE	NE
Flora (vascular plants)	*Carex demissa*	1	NE	NE
Flora (vascular plants)	*Carex distachya*	3	NE	NE
Flora (vascular plants)	*Carex divulsa*	4	NE	NE
Flora (vascular plants)	*Carex echinata*	4	NE	NE
Flora (vascular plants)	*Carex elata*	31	LC	NE
Flora (vascular plants)	*Carex flacca*	4	NE	NE
Flora (vascular plants)	*Carex hirta*	9	NE	NE
Flora (vascular plants)	*Carex laevigata*	10	NE	NE
Flora (vascular plants)	*Carex leporina*	17	NE	NE
Flora (vascular plants)	*Carex muricata*	23	NE	NE
Flora (vascular plants)	*Carex nigra*	4	NE	NE
Flora (vascular plants)	*Carex paniculata*	2	LC	NE
Flora (vascular plants)	*Carex remota*	3	NE	NE
Flora (vascular plants)	*Carex sylvatica*	3	EN	NE
Flora (vascular plants)	*Carlina corymbosa*	6	NE	NE
Flora (vascular plants)	*Carlina hispanica*	5	NE	NE
Flora (vascular plants)	*Carlina vulgaris*	3	NE	NE

Flora (vascular plants)	*Carthamus lanatus*	6	NE	NE
Flora (vascular plants)	*Carum verticillatum*	26	LC	NE
Flora (vascular plants)	*Castanea sativa*	61	LC	NE
Flora (vascular plants)	*Catapodium rigidum*	1	NE	NE
Flora (vascular plants)	*Cedrus atlantica*	2	NE	NE
Flora (vascular plants)	*Centaurea aristata*	9	NE	LC
Flora (vascular plants)	*Centaurea calcitrapa*	1	NE	NE
Flora (vascular plants)	*Centaurea cyanus*	7	NE	NE
Flora (vascular plants)	*Centaurea graminifolia*	10	NE	NE
Flora (vascular plants)	*Centaurea langei*	14	NE	NE
Flora (vascular plants)	*Centaurea melitensis*	1	NE	NE
Flora (vascular plants)	*Centaurea micrantha*	1	LC	NE
Flora (vascular plants)	*Centaurea nigra*	30	NE	NE
Flora (vascular plants)	*Centaurea ornata*	4	NE	NE
Flora (vascular plants)	*Centaurium erythraea*	13	LC	NE
Flora (vascular plants)	*Centaurium grandiflorum*	1	NE	NE
Flora (vascular plants)	*Centaurium maritimum*	1	NE	NE
Flora (vascular plants)	*Centranthus calcitrapae*	12	NE	NE
Flora (vascular plants)	*Centranthus ruber*	2	NE	NE
Flora (vascular plants)	*Cephalanthera longifolia*	8	LC	NE
Flora (vascular plants)	*Cephalanthera rubra*	17	LC	EN
Flora (vascular plants)	*Cerastium brachypetalum*	5	NE	NE
Flora (vascular plants)	*Cerastium diffusum*	4	NE	NE
Flora (vascular plants)	*Cerastium fontanum*	24	NE	NE
Flora (vascular plants)	*Cerastium glomeratum*	12	NE	NE
Flora (vascular plants)	*Cerastium ramosissimum*	2	NE	NE
Flora (vascular plants)	*Ceratocapnos claviculata*	15	NE	NE
Flora (vascular plants)	*Ceratophyllum demersum*	1	LC	NE
Flora (vascular plants)	*Ceterach officinarum*	3	LC	NE
Flora (vascular plants)	*Chaerophyllum temulum*	32	NE	NE
Flora (vascular plants)	*Chaetonychia cymosa*	1	NE	NE
Flora (vascular plants)	*Chamaemelum fuscatum*	1	NE	NE
Flora (vascular plants)	*Chamaemelum mixtum*	2	NE	NE
Flora (vascular plants)	*Chamaemelum nobile*	36	LC	NE
Flora (vascular plants)	*Chamomilla suaveolens*	8	NE	NE
Flora (vascular plants)	*Cheilanthes hispanica*	1	LC	NE
Flora (vascular plants)	*Chelidonium majus*	45	LC	NE
Flora (vascular plants)	*Chenopodium album*	13	NE	NE
Flora (vascular plants)	*Chondrilla juncea*	39	NE	NE
Flora (vascular plants)	*Chrysosplenium oppositifolium*	4	NE	NE
Flora (vascular plants)	*Cicendia filiformis*	2	NE	NE
Flora (vascular plants)	*Circaea lutetiana*	4	NE	NE
Flora (vascular plants)	*Cirsium arvense*	17	NE	NE
Flora (vascular plants)	*Cirsium palustre*	18	NE	NE
Flora (vascular plants)	*Cirsium vulgare*	24	NE	NE
Flora (vascular plants)	*Cistus ladanifer*	85	NE	NE

Flora (vascular plants)	*Cistus laurifolius*	13	NE	LC
Flora (vascular plants)	*Cistus populifolius*	6	NE	NE
Flora (vascular plants)	*Cistus psilosepalus*	61	LC	LC
Flora (vascular plants)	*Cistus salviifolius*	12	NE	NE
Flora (vascular plants)	*Clematis campaniflora*	7	NE	NE
Flora (vascular plants)	*Clematis vitalba*	6	NE	NE
Flora (vascular plants)	*Cleome violacea*	2	NE	NE
Flora (vascular plants)	*Clinopodium vulgare*	58	NE	NE
Flora (vascular plants)	*Coincya monensis*	6	DD	NE
Flora (vascular plants)	*Colchicum multiflorum*	6	NE	NE
Flora (vascular plants)	*Coleostephus myconis*	1	NE	NE
Flora (vascular plants)	*Conium maculatum*	14	NE	NE
Flora (vascular plants)	*Conopodium majus*	14	NE	NE
Flora (vascular plants)	*Conopodium pyrenaeum*	2	NE	NE
Flora (vascular plants)	*Convolvulus arvensis*	24	NE	NE
Flora (vascular plants)	*Conyza canadensis*	2	NE	NE
Flora (vascular plants)	*Conyza sumatrensis*	8	NE	NE
Flora (vascular plants)	*Cornus sanguinea*	5	NE	NE
Flora (vascular plants)	*Coronilla repanda*	5	NE	NE
Flora (vascular plants)	*Corrigiola litoralis*	2	NE	NE
Flora (vascular plants)	*Corrigiola telephiifolia*	9	NE	NE
Flora (vascular plants)	*Corydalis cava*	7	NE	VU
Flora (vascular plants)	*Corylus avellana*	19	LC	NE
Flora (vascular plants)	*Corynephorus canescens*	2	NE	NE
Flora (vascular plants)	*Crassula tillaea*	5	LC	NE
Flora (vascular plants)	*Crataegus monogyna*	58	LC	NE
Flora (vascular plants)	*Crepis capillaris*	32	NE	NE
Flora (vascular plants)	*Crepis lampsanoides*	18	NE	NE
Flora (vascular plants)	*Crepis pulchra*	1	NE	NE
Flora (vascular plants)	*Crepis vesicaria*	15	NE	NE
Flora (vascular plants)	*Crocus serotinus*	5	NE	NE
Flora (vascular plants)	*Crucianella angustifolia*	15	NE	NE
Flora (vascular plants)	*Cruciata glabra*	17	NE	NE
Flora (vascular plants)	*Cruciata laevipes*	21	NE	NE
Flora (vascular plants)	*Cruciata pedemontana*	4	NE	NE
Flora (vascular plants)	*Crupina vulgaris*	1	NE	NE
Flora (vascular plants)	*Crypsis schoenoides*	2	NE	NE
Flora (vascular plants)	*Cucubalus baccifer*	16	NE	NE
Flora (vascular plants)	*Cupressus lusitanica*	1	NE	NE
Flora (vascular plants)	*Cuscuta approximata*	1	NE	NE
Flora (vascular plants)	*Cuscuta epithymum*	1	NE	NE
Flora (vascular plants)	*Cymbalaria muralis*	6	NE	NE
Flora (vascular plants)	*Cynodon dactylon*	8	NE	LC
Flora (vascular plants)	*Cynosurus cristatus*	19	NE	NE
Flora (vascular plants)	*Cynosurus echinatus*	25	NE	NE
Flora (vascular plants)	*Cyperus fuscus*	2	LC	NE

Flora (vascular plants)	*Cyperus longus*	7	LC	NE
Flora (vascular plants)	*Cystopteris dickieana*	1	LC	NE
Flora (vascular plants)	*Cystopteris viridula*	4	LC	NE
Flora (vascular plants)	*Cytinus hypocistis*	6	NE	NE
Flora (vascular plants)	*Cytisus multiflorus*	75	LC	NE
Flora (vascular plants)	*Cytisus scoparius*	35	NE	NE
Flora (vascular plants)	*Cytisus striatus*	10	NE	NE
Flora (vascular plants)	*Dactylis glomerata*	43	NE	NE
Flora (vascular plants)	*Dactylorhiza elata*	23	LC	NT
Flora (vascular plants)	*Dactylorhiza insularis*	2	EN	NE
Flora (vascular plants)	*Dactylorhiza maculata*	12	LC	NE
Flora (vascular plants)	*Dactylorhiza sulphurea*	1	NT	NE
Flora (vascular plants)	*Danthonia decumbens*	8	NE	NE
Flora (vascular plants)	*Daphne gnidium*	26	NE	NE
Flora (vascular plants)	*Datura stramonium*	7	NE	NE
Flora (vascular plants)	*Daucus carota*	36	LC	NT
Flora (vascular plants)	*Delphinium halteratum*	2	NE	NE
Flora (vascular plants)	*Deschampsia cespitosa*	2	NE	NE
Flora (vascular plants)	*Dianthus armeria*	15	NE	LC
Flora (vascular plants)	*Dianthus hyssopifolius*	16	NE	NT
Flora (vascular plants)	*Dianthus langeanus*	12	NE	NE
Flora (vascular plants)	*Dianthus laricifolius*	37	LC	NE
Flora (vascular plants)	*Dianthus lusitanus*	1	NE	NE
Flora (vascular plants)	*Digitalis purpurea*	63	LC	LC
Flora (vascular plants)	*Digitaria sanguinalis*	5	NE	NE
Flora (vascular plants)	*Diplotaxis virgata*	1	LC	NE
Flora (vascular plants)	*Dipsacus fullonum*	10	NE	NE
Flora (vascular plants)	*Dittrichia graveolens*	2	NE	NE
Flora (vascular plants)	*Dittrichia viscosa*	1	NE	NE
Flora (vascular plants)	*Doronicum carpetanum*	28	NE	VU
Flora (vascular plants)	*Doronicum plantagineum*	2	DD	NE
Flora (vascular plants)	*Dorycnium pentaphyllum*	35	LC	NE
Flora (vascular plants)	*Dorycnopsis gerardi*	1	NE	NE
Flora (vascular plants)	*Draba muralis*	19	NE	NE
Flora (vascular plants)	*Drosera intermedia*	1	NT	NE
Flora (vascular plants)	*Drosera rotundifolia*	3	LC	NE
Flora (vascular plants)	*Dryopteris affinis*	8	LC	NE
Flora (vascular plants)	*Dryopteris filix-mas*	9	LC	NE
Flora (vascular plants)	*Echinochloa crus-galli*	7	NE	NE
Flora (vascular plants)	*Echinospartum ibericum*	25	LC	NE
Flora (vascular plants)	*Echium plantagineum*	6	NE	NE
Flora (vascular plants)	*Echium rosulatum*	26	NE	NT
Flora (vascular plants)	*Echium vulgare*	14	NE	NE
Flora (vascular plants)	*Eleocharis palustris*	7	LC	NE
Flora (vascular plants)	*Elymus caninus*	7	LC	NE
Flora (vascular plants)	*Epilobium angustifolium*	1	NE	NE

Flora (vascular plants)	*Epilobium brachycarpum*	2	NE	NE
Flora (vascular plants)	*Epilobium hirsutum*	6	NE	NE
Flora (vascular plants)	*Epilobium lanceolatum*	5	NE	NE
Flora (vascular plants)	*Epilobium montanum*	1	NE	NE
Flora (vascular plants)	*Epilobium obscurum*	9	NE	NE
Flora (vascular plants)	*Epilobium parviflorum*	1	NE	NE
Flora (vascular plants)	*Epipactis fageticola*	5	NE	EN
Flora (vascular plants)	*Epipactis tremolsii*	6	NE	NE
Flora (vascular plants)	*Equisetum arvense*	13	LC	NE
Flora (vascular plants)	*Eragrostis minor*	1	NE	NE
Flora (vascular plants)	*Erica arborea*	39	LC	NE
Flora (vascular plants)	*Erica australis*	42	NE	NE
Flora (vascular plants)	*Erica cinerea*	15	LC	NE
Flora (vascular plants)	*Erica scoparia*	7	NE	NE
Flora (vascular plants)	*Erica tetralix*	24	NE	NE
Flora (vascular plants)	*Erica umbellata*	18	NE	NE
Flora (vascular plants)	*Erigeron acris*	2	NE	NE
Flora (vascular plants)	*Eriophorum angustifolium*	4	LC	VU
Flora (vascular plants)	*Erodium botrys*	2	NE	NE
Flora (vascular plants)	*Erodium cicutarium*	24	NE	NE
Flora (vascular plants)	*Erodium malacoides*	1	NE	NE
Flora (vascular plants)	*Erodium moschatum*	3	NE	NE
Flora (vascular plants)	*Erophila verna*	1	NE	NE
Flora (vascular plants)	*Eryngium campestre*	20	NE	NE
Flora (vascular plants)	*Eryngium tenue*	14	NE	NE
Flora (vascular plants)	*Eryngium viviparum*	6	EN	CR
Flora (vascular plants)	*Erysimum linifolium*	25	NE	NE
Flora (vascular plants)	*Erysimum portugalense*	1	NE	NE
Flora (vascular plants)	*Erythronium dens-canis*	6	NE	NE
Flora (vascular plants)	*Eucalyptus globulus*	1	NE	NE
Flora (vascular plants)	*Euonymus europaeus*	14	LC	VU
Flora (vascular plants)	*Eupatorium cannabinum*	13	NE	NE
Flora (vascular plants)	*Euphorbia amygdaloides*	30	NE	NE
Flora (vascular plants)	*Euphorbia angulata*	1	NE	NE
Flora (vascular plants)	*Euphorbia dulcis*	2	NE	NE
Flora (vascular plants)	*Euphorbia exigua*	5	NE	NE
Flora (vascular plants)	*Euphorbia falcata*	1	NE	NE
Flora (vascular plants)	*Euphorbia hyberna*	1	NE	NE
Flora (vascular plants)	*Euphorbia lathyris*	3	NE	NE
Flora (vascular plants)	*Euphorbia oxyphylla*	2	NE	NE
Flora (vascular plants)	*Euphorbia pterococca*	1	NE	NE
Flora (vascular plants)	*Euphorbia segetalis*	6	NE	NE
Flora (vascular plants)	*Euphrasia hirtella*	5	NE	EN
Flora (vascular plants)	*Evax carpetana*	1	NE	NE
Flora (vascular plants)	*Fallopia convolvulus*	12	NE	NE
Flora (vascular plants)	*Ferula communis*	1	NE	NE

Flora (vascular plants)	*Festuca ampla*	8	NE	NE
Flora (vascular plants)	*Festuca arundinacea*	5	NE	NE
Flora (vascular plants)	*Festuca brigantina*	22	VU	VU
Flora (vascular plants)	*Festuca durandoi*	2	NE	NE
Flora (vascular plants)	*Festuca elegans*	57	LC	LC
Flora (vascular plants)	*Festuca nigrescens*	1	NE	NE
Flora (vascular plants)	*Festuca paniculata*	1	NE	NE
Flora (vascular plants)	*Festuca pseudotrichophylla*	1	NE	NE
Flora (vascular plants)	*Festuca rivularis*	1	NE	NE
Flora (vascular plants)	*Festuca rothmaleri*	13	NE	NE
Flora (vascular plants)	*Festuca summilusitana*	8	NE	LC
Flora (vascular plants)	*Ficus carica*	2	LC	NE
Flora (vascular plants)	*Filago albicans*	10	NE	NE
Flora (vascular plants)	*Filago pyramidata*	8	NE	NE
Flora (vascular plants)	*Filipendula ulmaria*	26	LC	NE
Flora (vascular plants)	*Filipendula vulgaris*	11	LC	NE
Flora (vascular plants)	*Foeniculum vulgare*	6	LC	NE
Flora (vascular plants)	*Fragaria vesca*	22	LC	NE
Flora (vascular plants)	*Frangula alnus*	41	LC	NE
Flora (vascular plants)	*Fraxinus angustifolia*	65	LC	NE
Flora (vascular plants)	*Fritillaria nervosa*	2	NT	NE
Flora (vascular plants)	*Fumaria bastardii*	1	NE	NE
Flora (vascular plants)	*Fumaria muralis*	5	NE	NE
Flora (vascular plants)	*Fumaria officinalis*	2	LC	NE
Flora (vascular plants)	*Fumaria parviflora*	2	NE	NE
Flora (vascular plants)	*Fumaria reuteri*	4	NE	NE
Flora (vascular plants)	*Gagea bohemica*	1	NE	NE
Flora (vascular plants)	*Gagea pratensis*	2	VU	NE
Flora (vascular plants)	*Gagea soleirolii*	2	LC	NE
Flora (vascular plants)	*Galactites tomentosus*	2	NE	NE
Flora (vascular plants)	*Galeopsis tetrahit*	8	NE	NE
Flora (vascular plants)	*Galinsoga parviflora*	8	NE	NE
Flora (vascular plants)	*Galium aparine*	33	LC	NE
Flora (vascular plants)	*Galium broterianum*	37	NE	NE
Flora (vascular plants)	*Galium debile*	10	NE	NE
Flora (vascular plants)	*Galium glaucum*	12	NE	LC
Flora (vascular plants)	*Galium lucidum*	28	NE	NE
Flora (vascular plants)	*Galium palustre*	8	NE	NE
Flora (vascular plants)	*Galium papillosum*	20	NE	NE
Flora (vascular plants)	*Galium parisiense*	10	NE	NE
Flora (vascular plants)	*Galium rotundifolium*	5	NE	NE
Flora (vascular plants)	*Galium saxatile*	2	NE	NE
Flora (vascular plants)	*Galium verrucosum*	3	NE	NE
Flora (vascular plants)	*Galium verum*	27	LC	NE
Flora (vascular plants)	*Gastridium ventricosum*	1	NE	NE
Flora (vascular plants)	*Gaudinia fragilis*	3	NE	NE

Flora (vascular plants)	*Genista anglica*	11	NE	NE
Flora (vascular plants)	*Genista falcata*	36	LC	NE
Flora (vascular plants)	*Genista florida*	31	NE	NE
Flora (vascular plants)	*Genista hystrix*	6	LC	NE
Flora (vascular plants)	*Genista micrantha*	9	NE	NE
Flora (vascular plants)	*Genista tridentata*	5	NE	NE
Flora (vascular plants)	*Gentiana pneumonanthe*	1	LC	NE
Flora (vascular plants)	*Geranium columbinum*	11	NE	NE
Flora (vascular plants)	*Geranium dissectum*	6	NE	NE
Flora (vascular plants)	*Geranium lucidum*	28	NE	NE
Flora (vascular plants)	*Geranium molle*	11	NE	NE
Flora (vascular plants)	*Geranium purpureum*	13	NE	NE
Flora (vascular plants)	*Geranium pyrenaicum*	16	NE	NE
Flora (vascular plants)	*Geranium robertianum*	15	NE	NE
Flora (vascular plants)	*Geranium rotundifolium*	5	NE	NE
Flora (vascular plants)	*Geranium sanguineum*	6	LC	NE
Flora (vascular plants)	*Geum hispidum*	3	NE	NE
Flora (vascular plants)	*Geum sylvaticum*	15	NE	NE
Flora (vascular plants)	*Geum urbanum*	34	LC	NE
Flora (vascular plants)	*Gladiolus illyricus*	6	NE	NE
Flora (vascular plants)	*Glechoma hederacea*	12	LC	NE
Flora (vascular plants)	*Glyceria declinata*	7	LC	NE
Flora (vascular plants)	*Halimium lasianthum*	36	NE	NE
Flora (vascular plants)	*Halimium umbellatum*	18	DD	LC
Flora (vascular plants)	*Hedera hibernica*	7	NE	NE
Flora (vascular plants)	*Hedypnois cretica*	1	NE	NE
Flora (vascular plants)	*Helianthemum aegyptiacum*	4	NE	NE
Flora (vascular plants)	*Helianthemum apenninum*	13	NE	NT
Flora (vascular plants)	*Helianthemum ledifolium*	1	NE	NE
Flora (vascular plants)	*Helianthemum nummularium*	6	NE	NE
Flora (vascular plants)	*Helichrysum petiolare*	1	NE	NE
Flora (vascular plants)	*Helichrysum stoechas*	19	LC	NE
Flora (vascular plants)	*Helictochloa marginata*	4	NE	NE
Flora (vascular plants)	*Heliotropium europaeum*	1	NE	NE
Flora (vascular plants)	*Helleborus foetidus*	22	LC	NE
Flora (vascular plants)	*Heracleum sphondylium*	25	NE	NE
Flora (vascular plants)	*Herniaria lusitanica*	4	EN	EN
Flora (vascular plants)	*Herniaria scabrida*	9	NE	NE
Flora (vascular plants)	*Hieracium murorum*	3	NE	NE
Flora (vascular plants)	*Hieracium sabaudum*	4	NE	NE
Flora (vascular plants)	*Hieracium umbellatum*	1	NE	NE
Flora (vascular plants)	*Hirschfeldia incana*	1	NE	NE
Flora (vascular plants)	*Hispidella hispanica*	20	NE	NE
Flora (vascular plants)	*Holcus annuus*	1	NT	LC
Flora (vascular plants)	*Holcus lanatus*	44	NE	NE
Flora (vascular plants)	*Holcus mollis*	33	NE	NE

Flora (vascular plants)	*Hordeum geniculatum*	4	LC	NE
Flora (vascular plants)	*Hordeum marinum*	1	LC	NE
Flora (vascular plants)	*Hordeum murinum*	20	LC	NE
Flora (vascular plants)	*Humulus lupulus*	16	LC	NE
Flora (vascular plants)	*Hyacinthoides hispanica*	9	NE	NE
Flora (vascular plants)	*Hyacinthoides paivae*	1	NE	NE
Flora (vascular plants)	*Hymenocarpos cornicina*	4	NE	NE
Flora (vascular plants)	*Hymenocarpos lotoides*	18	NE	NE
Flora (vascular plants)	*Hypericum androsaemum*	6	NE	NE
Flora (vascular plants)	*Hypericum humifusum*	9	NE	NE
Flora (vascular plants)	*Hypericum linariifolium*	26	NE	NE
Flora (vascular plants)	*Hypericum montanum*	10	NE	NT
Flora (vascular plants)	*Hypericum perfoliatum*	2	NE	NE
Flora (vascular plants)	*Hypericum perforatum*	47	NE	NE
Flora (vascular plants)	*Hypericum pulchrum*	2	NE	NE
Flora (vascular plants)	*Hypericum undulatum*	19	NE	NE
Flora (vascular plants)	*Hypochaeris glabra*	7	NE	NE
Flora (vascular plants)	*Hypochaeris radicata*	44	NE	NE
Flora (vascular plants)	*Ilex aquifolium*	2	LC	NE
Flora (vascular plants)	*Illecebrum verticillatum*	9	NE	NE
Flora (vascular plants)	*Inula conyza*	4	NE	NE
Flora (vascular plants)	*Inula salicina*	2	NE	NE
Flora (vascular plants)	*Iris pseudacorus*	4	LC	NE
Flora (vascular plants)	*Isolepis setacea*	2	NE	NE
Flora (vascular plants)	*Jasione montana*	35	NE	NE
Flora (vascular plants)	*Jasione sessiliflora*	22	NE	LC
Flora (vascular plants)	*Jasminum fruticans*	4	NE	NE
Flora (vascular plants)	*Jasonia tuberosa*	5	NT	NE
Flora (vascular plants)	*Jonopsidium abulense*	3	NE	NE
Flora (vascular plants)	*Juncus acutiflorus*	12	LC	NE
Flora (vascular plants)	*Juncus articulatus*	4	LC	NE
Flora (vascular plants)	*Juncus bufonius*	11	LC	NE
Flora (vascular plants)	*Juncus bulbosus*	8	LC	NE
Flora (vascular plants)	*Juncus capitatus*	6	NE	NE
Flora (vascular plants)	*Juncus conglomeratus*	1	NE	NE
Flora (vascular plants)	*Juncus effusus*	48	LC	NE
Flora (vascular plants)	*Juncus inflexus*	3	NE	NE
Flora (vascular plants)	*Juncus pygmaeus*	3	NE	NE
Flora (vascular plants)	*Juncus squarrosus*	14	NE	NE
Flora (vascular plants)	*Juncus tenageia*	7	LC	NE
Flora (vascular plants)	*Knautia nevadensis*	2	NE	VU
Flora (vascular plants)	*Koeleria crassipes*	5	NE	NE
Flora (vascular plants)	*Lactuca serriola*	20	LC	NE
Flora (vascular plants)	*Lactuca viminea*	25	LC	NE
Flora (vascular plants)	*Lactuca virosa*	11	DD	NE
Flora (vascular plants)	*Lamium amplexicaule*	8	NE	NE

Flora (vascular plants)	*Lamium coutinhoi*	1	NE	NT
Flora (vascular plants)	*Lamium hybridum*	2	NE	NE
Flora (vascular plants)	*Lamium maculatum*	19	NE	NE
Flora (vascular plants)	*Lamium purpureum*	7	NE	NE
Flora (vascular plants)	*Lapsana communis*	32	NE	NE
Flora (vascular plants)	*Laserpitium eliasii*	2	NE	NE
Flora (vascular plants)	*Lathyrus angulatus*	3	NE	NE
Flora (vascular plants)	*Lathyrus cicera*	2	LC	NE
Flora (vascular plants)	*Lathyrus hirsutus*	1	LC	NE
Flora (vascular plants)	*Lathyrus latifolius*	5	LC	NE
Flora (vascular plants)	*Lathyrus linifolius*	6	NE	NE
Flora (vascular plants)	*Lathyrus niger*	24	NE	NE
Flora (vascular plants)	*Lathyrus nissolia*	2	NE	NT
Flora (vascular plants)	*Lathyrus pratensis*	20	NE	NT
Flora (vascular plants)	*Lathyrus sphaericus*	7	NE	NE
Flora (vascular plants)	*Lavandula pedunculata*	58	NE	NE
Flora (vascular plants)	*Lavandula stoechas*	6	NE	NE
Flora (vascular plants)	*Lavatera cretica*	1	NE	NE
Flora (vascular plants)	*Legousia scabra*	1	NE	NE
Flora (vascular plants)	*Lemna minor*	2	LC	NE
Flora (vascular plants)	*Lens nigricans*	1	LC	NE
Flora (vascular plants)	*Leontodon carpetanus*	2	NE	NE
Flora (vascular plants)	*Leontodon hispidus*	8	NE	NT
Flora (vascular plants)	*Leontodon saxatilis*	22	NE	NE
Flora (vascular plants)	*Leontodon tuberosa*	1	NE	NE
Flora (vascular plants)	*Lepidium heterophyllum*	24	LC	NE
Flora (vascular plants)	*Leucanthemopsis flaveola*	2	NE	NE
Flora (vascular plants)	*Leucanthemum ircutianum*	2	NE	NE
Flora (vascular plants)	*Leuzea rhaponticoides*	30	NT	CR
Flora (vascular plants)	*Ligustrum vulgare*	4	NE	NE
Flora (vascular plants)	*Lilium martagon*	33	LC	VU
Flora (vascular plants)	*Linaria aeruginea*	3	NE	NE
Flora (vascular plants)	*Linaria amethystea*	5	NE	DD
Flora (vascular plants)	*Linaria elegans*	14	NE	NE
Flora (vascular plants)	*Linaria intricata*	57	NE	DD
Flora (vascular plants)	*Linaria saxatilis*	10	NE	NE
Flora (vascular plants)	*Linaria spartea*	9	NE	NE
Flora (vascular plants)	*Linaria triornithophora*	18	NE	NE
Flora (vascular plants)	*Linum bienne*	17	NE	NE
Flora (vascular plants)	*Linum catharticum*	12	NE	LC
Flora (vascular plants)	*Lobelia urens*	4	NE	NE
Flora (vascular plants)	*Logfia gallica*	5	NE	NE
Flora (vascular plants)	*Logfia minima*	40	NE	NE
Flora (vascular plants)	*Lolium multiflorum*	1	LC	NE
Flora (vascular plants)	*Lolium perenne*	15	LC	NE
Flora (vascular plants)	*Lolium rigidum*	7	LC	NE

Flora (vascular plants)	*Lonicera etrusca*	1	NE	NE
Flora (vascular plants)	*Lonicera periclymenum*	47	NE	NE
Flora (vascular plants)	*Lotus angustissimus*	1	NE	NE
Flora (vascular plants)	*Lotus corniculatus*	15	LC	NE
Flora (vascular plants)	*Lotus dorycnium*	10	NE	LC
Flora (vascular plants)	*Lotus glaber*	3	NE	NE
Flora (vascular plants)	*Lotus hispidus*	2	NE	NE
Flora (vascular plants)	*Lotus pedunculatus*	39	LC	NE
Flora (vascular plants)	*Lupinus angustifolius*	3	LC	NE
Flora (vascular plants)	*Lupinus gredensis*	3	NE	NE
Flora (vascular plants)	*Lupinus hispanicus*	1	LC	NE
Flora (vascular plants)	*Luzula campestris*	3	NE	NE
Flora (vascular plants)	*Luzula forsteri*	13	NE	NE
Flora (vascular plants)	*Luzula lactea*	15	LC	NE
Flora (vascular plants)	*Luzula multiflora*	10	NE	NE
Flora (vascular plants)	*Luzula sylvatica*	11	NE	NE
Flora (vascular plants)	*Lycopus europaeus*	9	LC	NE
Flora (vascular plants)	*Lysimachia ephemerum*	5	VU	NE
Flora (vascular plants)	*Lysimachia monelli*	1	NE	NE
Flora (vascular plants)	*Lysimachia vulgaris*	11	LC	NE
Flora (vascular plants)	*Lythrum portula*	8	LC	NE
Flora (vascular plants)	*Lythrum salicaria*	37	LC	NE
Flora (vascular plants)	*Magydaris panacifolia*	2	NE	NE
Flora (vascular plants)	*Malus sylvestris*	1	NE	NE
Flora (vascular plants)	*Malva neglecta*	5	LC	NE
Flora (vascular plants)	*Malva sylvestris*	23	LC	NE
Flora (vascular plants)	*Malva tournefortiana*	29	NE	NE
Flora (vascular plants)	*Margotia gummifera*	2	NE	NE
Flora (vascular plants)	*Marrubium vulgare*	3	NT	NE
Flora (vascular plants)	*Matricaria discoidea*	1	NE	NE
Flora (vascular plants)	*Medicago arabica*	9	LC	NE
Flora (vascular plants)	*Medicago lupulina*	8	LC	NE
Flora (vascular plants)	*Medicago minima*	3	LC	NE
Flora (vascular plants)	*Medicago polymorpha*	5	LC	NE
Flora (vascular plants)	*Medicago rigidula*	5	LC	NE
Flora (vascular plants)	*Medicago sativa*	1	LC	NE
Flora (vascular plants)	*Melampyrum pratense*	9	NE	NE
Flora (vascular plants)	*Melica ciliata*	8	NE	NE
Flora (vascular plants)	*Melica uniflora*	12	NE	NE
Flora (vascular plants)	*Melilotus albus*	1	LC	NE
Flora (vascular plants)	*Melissa officinalis*	2	LC	NE
Flora (vascular plants)	*Melittis melissophyllum*	7	NE	NE
Flora (vascular plants)	*Mentha aquatica*	1	LC	NE
Flora (vascular plants)	*Mentha longifolia*	2	EN	NE
Flora (vascular plants)	*Mentha pulegium*	11	LC	NE
Flora (vascular plants)	*Mentha suaveolens*	55	LC	NE

Flora (vascular plants)	*Merendera montana*	6	NE	NE
Flora (vascular plants)	*Mibora minima*	4	NE	NE
Flora (vascular plants)	*Micropyrum patens*	4	NE	NE
Flora (vascular plants)	*Micropyrum tenellum*	23	NE	NE
Flora (vascular plants)	*Milium vernale*	2	NE	NE
Flora (vascular plants)	*Misopates orontium*	2	NE	NE
Flora (vascular plants)	*Moehringia pentandra*	1	NE	NE
Flora (vascular plants)	*Moehringia trinervia*	1	NE	NE
Flora (vascular plants)	*Moenchia erecta*	5	NE	NE
Flora (vascular plants)	*Molineriella laevis*	5	NE	NE
Flora (vascular plants)	*Molinia caerulea*	8	NE	NE
Flora (vascular plants)	*Monotropa hypopitys*	3	NE	VU
Flora (vascular plants)	*Montia fontana*	4	LC	NE
Flora (vascular plants)	*Muscari comosum*	4	NE	NE
Flora (vascular plants)	*Myosotis arvensis*	5	NE	NE
Flora (vascular plants)	*Myosotis debilis*	1	NE	NE
Flora (vascular plants)	*Myosotis discolor*	18	NE	NE
Flora (vascular plants)	*Myosotis ramosissima*	28	NE	NE
Flora (vascular plants)	*Myosotis secunda*	7	LC	NE
Flora (vascular plants)	*Myosotis stolonifera*	1	NE	NE
Flora (vascular plants)	*Myosotis welwitschii*	3	NE	NE
Flora (vascular plants)	*Myriophyllum alterniflorum*	2	NE	LC
Flora (vascular plants)	*Narcissus asturiensis*	8	LC	NT
Flora (vascular plants)	*Narcissus bulbocodium*	22	LC	LC
Flora (vascular plants)	*Narcissus triandrus*	34	LC	LC
Flora (vascular plants)	*Nardus stricta*	17	NE	NE
Flora (vascular plants)	*Neottia nidus-avis*	14	VU	NE
Flora (vascular plants)	*Nepeta caerulea*	7	NE	EN
Flora (vascular plants)	*Nerium oleander*	4	LC	NE
Flora (vascular plants)	*Nigella damascena*	1	NE	NE
Flora (vascular plants)	*Notholaena marantae*	9	NT	NT
Flora (vascular plants)	*Odontitella virgata*	3	NE	NE
Flora (vascular plants)	*Odontites vernus*	4	NE	NE
Flora (vascular plants)	*Oenanthe crocata*	39	LC	NE
Flora (vascular plants)	*Omphalodes nitida*	56	NE	NE
Flora (vascular plants)	*Ononis spinosa*	9	LC	NE
Flora (vascular plants)	*Onopordum acanthium*	13	NE	NE
Flora (vascular plants)	*Ophioglossum vulgatum*	7	LC	VU
Flora (vascular plants)	*Orchis anthropophora*	1	LC	NE
Flora (vascular plants)	*Orchis coriophora*	8	LC	NE
Flora (vascular plants)	*Orchis langei*	1	NE	NE
Flora (vascular plants)	*Orchis mascula*	2	LC	NE
Flora (vascular plants)	*Orchis morio*	5	NT	NE
Flora (vascular plants)	*Origanum vulgare*	13	LC	NE
Flora (vascular plants)	*Ornithogalum concinnum*	8	NE	NE
Flora (vascular plants)	*Ornithogalum orthophyllum*	2	NE	NE

Flora (vascular plants)	*Ornithogalum pyrenaicum*	14	NE	NE
Flora (vascular plants)	*Ornithopus compressus*	9	NE	NE
Flora (vascular plants)	*Ornithopus perpusillus*	11	NE	NE
Flora (vascular plants)	*Ornithopus pinnatus*	1	NE	NE
Flora (vascular plants)	*Ornithopus sativus*	3	LC	NE
Flora (vascular plants)	*Orobanche gracilis*	3	NE	NE
Flora (vascular plants)	*Orobanche hederae*	1	NE	NE
Flora (vascular plants)	*Orobanche minor*	4	NE	NE
Flora (vascular plants)	*Orobanche rapum-genistae*	3	NE	NE
Flora (vascular plants)	*Ortegia hispanica*	3	NE	NE
Flora (vascular plants)	*Osmunda regalis*	32	LC	NE
Flora (vascular plants)	*Osyris alba*	6	NE	NE
Flora (vascular plants)	*Oxalis corniculata*	1	NE	NE
Flora (vascular plants)	*Oxalis latifolia*	2	NE	NE
Flora (vascular plants)	*Paeonia broteri*	26	NE	LC
Flora (vascular plants)	*Paeonia officinalis*	5	LC	NE
Flora (vascular plants)	*Panicum capillare*	2	NE	NE
Flora (vascular plants)	*Papaver dubium*	5	NE	NE
Flora (vascular plants)	*Papaver rhoeas*	12	LC	NE
Flora (vascular plants)	*Papaver somniferum*	2	LC	NE
Flora (vascular plants)	*Paradisea lusitanica*	1	NE	NE
Flora (vascular plants)	*Parentucellia latifolia*	5	NE	NE
Flora (vascular plants)	*Parentucellia viscosa*	2	NE	NE
Flora (vascular plants)	*Parietaria judaica*	2	NE	NE
Flora (vascular plants)	*Paronychia argentea*	3	NE	NE
Flora (vascular plants)	*Paspalum dilatatum*	1	NE	NE
Flora (vascular plants)	*Paspalum distichum*	1	NE	NE
Flora (vascular plants)	*Pedicularis sylvatica*	9	NE	NE
Flora (vascular plants)	*Pentaglottis sempervirens*	46	NE	NE
Flora (vascular plants)	*Pentanema salicinum*	2	NE	NE
Flora (vascular plants)	*Periballia involucrata*	8	NE	NE
Flora (vascular plants)	*Petrorhagia nanteuilii*	33	NE	NE
Flora (vascular plants)	*Petroselinum crispum*	1	NE	NE
Flora (vascular plants)	*Peucedanum carvifolia*	4	EN	NE
Flora (vascular plants)	*Peucedanum gallicum*	11	NE	NE
Flora (vascular plants)	*Peucedanum lancifolium*	5	NE	NE
Flora (vascular plants)	*Peucedanum oreoselinum*	6	NE	VU
Flora (vascular plants)	*Phagnalon saxatile*	8	NE	NE
Flora (vascular plants)	*Phalacrocarpum hoffmannseggii*	9	NE	NE
Flora (vascular plants)	*Phalacrocarpum oppositifolium*	7	NE	NE
Flora (vascular plants)	*Phalaris aquatica*	1	LC	NE
Flora (vascular plants)	*Phaseolus vulgaris*	1	NE	NE
Flora (vascular plants)	*Phillyrea angustifolia*	11	NE	NE
Flora (vascular plants)	*Phleum phleoides*	3	NE	EN
Flora (vascular plants)	*Phleum pratense*	5	LC	NE
Flora (vascular plants)	*Physospermum cornubiense*	16	NE	NE

Flora (vascular plants)	*Phytolacca americana*	1	NE	NE
Flora (vascular plants)	*Picris echioides*	1	NE	NE
Flora (vascular plants)	*Picris hieracioides*	16	NE	NE
Flora (vascular plants)	*Pilosella castellana*	3	NE	NE
Flora (vascular plants)	*Pilosella officinarum*	2	NE	NE
Flora (vascular plants)	*Pilosella pseudopilosella*	32	NE	NE
Flora (vascular plants)	*Pimpinella major*	12	NE	NE
Flora (vascular plants)	*Pimpinella villosa*	19	NE	NE
Flora (vascular plants)	*Pinus pinaster*	11	LC	NE
Flora (vascular plants)	*Pinus sylvestris*	12	LC	EN
Flora (vascular plants)	*Piptatherum paradoxum*	1	NE	NE
Flora (vascular plants)	*Pistacia terebinthus*	3	LC	NE
Flora (vascular plants)	*Plantago coronopus*	35	NE	NE
Flora (vascular plants)	*Plantago holosteum*	19	NE	NE
Flora (vascular plants)	*Plantago lanceolata*	26	LC	NE
Flora (vascular plants)	*Plantago major*	36	LC	NE
Flora (vascular plants)	*Poa annua*	12	NE	NE
Flora (vascular plants)	*Poa bulbosa*	18	NE	NE
Flora (vascular plants)	*Poa nemoralis*	11	NE	NE
Flora (vascular plants)	*Poa pratensis*	4	LC	NE
Flora (vascular plants)	*Poa trivialis*	27	NE	NE
Flora (vascular plants)	*Polycarpon tetraphyllum*	2	NE	NE
Flora (vascular plants)	*Polygala microphylla*	25	NE	LC
Flora (vascular plants)	*Polygala serpyllifolia*	4	NE	NE
Flora (vascular plants)	*Polygala vulgaris*	24	NE	NE
Flora (vascular plants)	*Polygonatum odoratum*	8	LC	NE
Flora (vascular plants)	*Polygonum amphibium*	4	LC	NT
Flora (vascular plants)	*Polygonum aviculare*	14	LC	NE
Flora (vascular plants)	*Polygonum hydropiper*	4	LC	NE
Flora (vascular plants)	*Polygonum lapathifolium*	5	LC	NE
Flora (vascular plants)	*Polygonum rurivagum*	1	NE	NE
Flora (vascular plants)	*Polypodium interjectum*	1	LC	NE
Flora (vascular plants)	*Polypodium vulgare*	2	LC	NE
Flora (vascular plants)	*Polypogon viridis*	1	LC	NE
Flora (vascular plants)	*Polystichum setiferum*	13	LC	NE
Flora (vascular plants)	*Populus nigra*	5	DD	NE
Flora (vascular plants)	*Populus tremula*	1	LC	NE
Flora (vascular plants)	*Populus x canadensis*	2	NE	NE
Flora (vascular plants)	*Portulaca oleracea*	4	LC	NE
Flora (vascular plants)	*Potamogeton natans*	3	LC	NE
Flora (vascular plants)	*Potamogeton polygonifolius*	1	LC	NE
Flora (vascular plants)	*Potentilla erecta*	18	LC	NE
Flora (vascular plants)	*Potentilla neumanniana*	4	NT	NE
Flora (vascular plants)	*Potentilla reptans*	14	NE	NE
Flora (vascular plants)	*Potentilla rupestris*	1	NE	NE
Flora (vascular plants)	*Potentilla sterilis*	18	NE	NE

Flora (vascular plants)	*Primula acaulis*	33	NE	NE
Flora (vascular plants)	*Primula vulgaris*	5	NE	NE
Flora (vascular plants)	*Pritzelago alpina*	3	EN	NE
Flora (vascular plants)	*Prunella grandiflora*	18	NE	NE
Flora (vascular plants)	*Prunella laciniata*	11	NE	NE
Flora (vascular plants)	*Prunella vulgaris*	23	LC	NE
Flora (vascular plants)	*Prunus avium*	23	LC	NE
Flora (vascular plants)	*Prunus cerasifera*	1	DD	NE
Flora (vascular plants)	*Prunus domestica*	6	DD	NE
Flora (vascular plants)	*Prunus laurocerasus*	2	LC	NE
Flora (vascular plants)	*Prunus lusitanica*	10	NE	NT
Flora (vascular plants)	*Prunus mahaleb*	1	LC	NE
Flora (vascular plants)	*Prunus spinosa*	18	LC	NE
Flora (vascular plants)	*Pseudotsuga menziesii*	2	NE	NE
Flora (vascular plants)	*Pteridium aquilinum*	36	LC	NE
Flora (vascular plants)	*Pterocephalidium diandrum*	6	NE	NE
Flora (vascular plants)	*Pterospartum tridentatum*	27	NE	NE
Flora (vascular plants)	*Pulmonaria longifolia*	5	NE	NE
Flora (vascular plants)	*Pycreus flavescens*	1	LC	NE
Flora (vascular plants)	*Pyrus cordata*	2	DD	NE
Flora (vascular plants)	*Quercus faginea*	7	LC	NE
Flora (vascular plants)	*Quercus ilex*	3	LC	NE
Flora (vascular plants)	*Quercus pyrenaica*	90	LC	NE
Flora (vascular plants)	*Quercus robur*	5	LC	NE
Flora (vascular plants)	*Quercus rotundifolia*	59	LC	NE
Flora (vascular plants)	*Radiola linoides*	3	NE	NE
Flora (vascular plants)	*Ranunculus abnormis*	8	NE	VU
Flora (vascular plants)	*Ranunculus arvensis*	1	NE	NE
Flora (vascular plants)	*Ranunculus bulbosus*	10	NE	NE
Flora (vascular plants)	*Ranunculus ficaria*	8	LC	NE
Flora (vascular plants)	*Ranunculus flammula*	8	LC	NE
Flora (vascular plants)	*Ranunculus hederaceus*	2	LC	NE
Flora (vascular plants)	*Ranunculus longipes*	2	NE	NE
Flora (vascular plants)	*Ranunculus muricatus*	1	NE	NE
Flora (vascular plants)	*Ranunculus nigrescens*	2	NE	NE
Flora (vascular plants)	*Ranunculus nodiflorus*	2	LC	NE
Flora (vascular plants)	*Ranunculus ollissiponensis*	9	NE	NE
Flora (vascular plants)	*Ranunculus ololeucos*	1	DD	NE
Flora (vascular plants)	*Ranunculus omiophyllus*	4	LC	NE
Flora (vascular plants)	*Ranunculus ophioglossifolius*	2	LC	NE
Flora (vascular plants)	*Ranunculus paludosus*	7	NE	NE
Flora (vascular plants)	*Ranunculus parviflorus*	3	NE	NE
Flora (vascular plants)	*Ranunculus peltatus*	3	LC	NE
Flora (vascular plants)	*Ranunculus repens*	22	LC	NE
Flora (vascular plants)	*Raphanus raphanistrum*	3	LC	NE
Flora (vascular plants)	*Reseda barrelieri*	1	NE	EN

Flora (vascular plants)	*Reseda luteola*	14	NE	NE
Flora (vascular plants)	*Reseda virgata*	3	NE	NE
Flora (vascular plants)	*Rhamnus catharticus*	4	LC	LC
Flora (vascular plants)	*Rhinanthus minor*	14	NE	NE
Flora (vascular plants)	*Rhus coriaria*	1	LC	NE
Flora (vascular plants)	*Robinia pseudoacacia*	5	NE	NE
Flora (vascular plants)	*Rorippa nasturtium-aquaticum*	4	LC	NE
Flora (vascular plants)	*Rorippa palustris*	4	LC	NE
Flora (vascular plants)	*Rorippa pyrenaica*	9	LC	NE
Flora (vascular plants)	*Rosa andegavensis*	1	NE	NE
Flora (vascular plants)	*Rosa blondaeana*	1	NE	NE
Flora (vascular plants)	*Rosa canina*	8	LC	NE
Flora (vascular plants)	*Rosa corymbifera*	14	NE	NE
Flora (vascular plants)	*Rosa deseglisei*	1	NE	NE
Flora (vascular plants)	*Rosa micrantha*	22	NE	NE
Flora (vascular plants)	*Rosa pouzinii*	14	NE	NE
Flora (vascular plants)	*Rosa squarrosa*	4	NE	NE
Flora (vascular plants)	*Rubia peregrina*	12	NE	NE
Flora (vascular plants)	*Rubus brigantinus*	16	NE	NE
Flora (vascular plants)	*Rubus fruticosus*	1	LC	NE
Flora (vascular plants)	*Rubus galloecicus*	2	NE	NE
Flora (vascular plants)	*Rubus genevieri*	8	DD	VU
Flora (vascular plants)	*Rubus henriquesii*	8	NE	NE
Flora (vascular plants)	*Rubus lainzii*	11	NE	NE
Flora (vascular plants)	*Rubus praecox*	2	NE	NE
Flora (vascular plants)	*Rubus radula*	3	NE	NE
Flora (vascular plants)	*Rubus sampaioanus*	1	NE	NE
Flora (vascular plants)	*Rubus ulmifolius*	38	NE	NE
Flora (vascular plants)	*Rubus vagabundus*	16	NE	NE
Flora (vascular plants)	*Rubus vestitus*	2	DD	NE
Flora (vascular plants)	*Rubus vigoi*	7	NE	NE
Flora (vascular plants)	*Rumex acetosa*	15	NE	NE
Flora (vascular plants)	*Rumex acetosella*	24	LC	NE
Flora (vascular plants)	*Rumex bucephalophorus*	11	NE	NE
Flora (vascular plants)	*Rumex conglomeratus*	9	NE	NE
Flora (vascular plants)	*Rumex crispus*	17	LC	NE
Flora (vascular plants)	*Rumex induratus*	6	NE	NE
Flora (vascular plants)	*Rumex longifolius*	6	NE	CR
Flora (vascular plants)	*Rumex obtusifolius*	6	NE	NE
Flora (vascular plants)	*Rumex papillaris*	1	NE	NE
Flora (vascular plants)	*Rumex pulcher*	8	NE	NE
Flora (vascular plants)	*Ruscus aculeatus*	50	LC	LC
Flora (vascular plants)	*Ruta angustifolia*	2	NE	NE
Flora (vascular plants)	*Ruta montana*	12	NE	NE
Flora (vascular plants)	*Sagina apetala*	4	NE	NE
Flora (vascular plants)	*Sagina procumbens*	2	NE	NE

Flora (vascular plants)	*Salix alba*	3	LC	NE
Flora (vascular plants)	*Salix atrocinerea*	40	LC	NE
Flora (vascular plants)	*Salix caprea*	1	LC	NE
Flora (vascular plants)	*Salix repens*	1	EN	NE
Flora (vascular plants)	*Salix rubens*	2	NE	NE
Flora (vascular plants)	*Salix salviifolia*	23	LC	LC
Flora (vascular plants)	*Salix x alopecuroides*	3	NE	NE
Flora (vascular plants)	*Salvia officinalis*	1	LC	NE
Flora (vascular plants)	*Salvia sclarea*	2	LC	NE
Flora (vascular plants)	*Salvia verbenaca*	10	NE	NE
Flora (vascular plants)	*Sambucus ebulus*	5	LC	NE
Flora (vascular plants)	*Sambucus nigra*	29	LC	NE
Flora (vascular plants)	*Sanguisorba minor*	47	NE	NE
Flora (vascular plants)	*Sanicula europaea*	1	LC	NE
Flora (vascular plants)	*Santolina semidentata*	53	LC	LC
Flora (vascular plants)	*Saponaria officinalis*	41	LC	NE
Flora (vascular plants)	*Saxifraga dichotoma*	6	VU	NE
Flora (vascular plants)	*Saxifraga fragosoi*	18	NE	NE
Flora (vascular plants)	*Saxifraga granulata*	8	NE	NE
Flora (vascular plants)	*Saxifraga lepismigena*	5	NE	NE
Flora (vascular plants)	*Saxifraga spathularis*	5	NE	NE
Flora (vascular plants)	*Scabiosa columbaria*	2	NE	NE
Flora (vascular plants)	*Scilla autumnalis*	4	NE	NE
Flora (vascular plants)	*Scilla monophyllos*	1	NE	NE
Flora (vascular plants)	*Scilla ramburei*	2	DD	LC
Flora (vascular plants)	*Scilla verna*	1	DD	NE
Flora (vascular plants)	*Scirpoides holoschoenus*	3	LC	NE
Flora (vascular plants)	*Scleranthus annuus*	7	NE	NE
Flora (vascular plants)	*Scleranthus polycarpos*	1	NE	NE
Flora (vascular plants)	*Scorzonera graminifolia*	3	NE	NE
Flora (vascular plants)	*Scorzonera laciniata*	1	NE	NE
Flora (vascular plants)	*Scrophularia auriculata*	7	NE	NE
Flora (vascular plants)	*Scrophularia canina*	10	NE	NE
Flora (vascular plants)	*Scrophularia herminii*	7	DD	LC
Flora (vascular plants)	*Scrophularia scorodonia*	16	NE	NE
Flora (vascular plants)	*Scutellaria minor*	4	NE	NE
Flora (vascular plants)	*Secale cereale*	1	NE	NE
Flora (vascular plants)	*Sedum acre*	1	LC	NE
Flora (vascular plants)	*Sedum album*	2	NE	NE
Flora (vascular plants)	*Sedum amplexicaule*	13	NE	NE
Flora (vascular plants)	*Sedum andegavense*	1	NE	NE
Flora (vascular plants)	*Sedum arenarium*	20	NE	NE
Flora (vascular plants)	*Sedum brevifolium*	28	NE	NE
Flora (vascular plants)	*Sedum caespitosum*	1	NE	NE
Flora (vascular plants)	*Sedum forsterianum*	57	NE	NE
Flora (vascular plants)	*Sedum hirsutum*	24	NE	NE

Flora (vascular plants)	*Sedum maireanum*	5	NE	NE
Flora (vascular plants)	*Sedum pedicellatum*	11	NE	LC
Flora (vascular plants)	*Selinum broteri*	10	NE	VU
Flora (vascular plants)	*Senecio jacobaea*	20	DD	NE
Flora (vascular plants)	*Senecio lividus*	4	NE	NE
Flora (vascular plants)	*Senecio sylvaticus*	19	NE	NE
Flora (vascular plants)	*Senecio vulgaris*	8	NE	NE
Flora (vascular plants)	*Senencio jacobaea*	4	DD	NE
Flora (vascular plants)	*Serapias lingua*	5	LC	NE
Flora (vascular plants)	*Serapias parviflora*	1	LC	NE
Flora (vascular plants)	*Serratula tinctoria*	1	NE	NE
Flora (vascular plants)	*Sesamoides purpurascens*	14	NE	NE
Flora (vascular plants)	*Seseli montanum*	35	NE	LC
Flora (vascular plants)	*Setaria verticillata*	3	NE	NE
Flora (vascular plants)	*Setaria viridis*	2	NE	NE
Flora (vascular plants)	*Sherardia arvensis*	13	NE	NE
Flora (vascular plants)	*Sibthorpia europaea*	5	NE	NE
Flora (vascular plants)	*Silene alba*	9	NE	NE
Flora (vascular plants)	*Silene baccifera*	1	NE	NE
Flora (vascular plants)	*Silene colorata*	2	NE	NE
Flora (vascular plants)	*Silene coutinhoi*	4	NE	NE
Flora (vascular plants)	*Silene dioica*	5	NE	NE
Flora (vascular plants)	*Silene gallica*	6	NE	NE
Flora (vascular plants)	*Silene inaperta*	3	NE	NE
Flora (vascular plants)	*Silene latifolia*	12	NE	NE
Flora (vascular plants)	*Silene legionensis*	31	VU	NE
Flora (vascular plants)	*Silene nutans*	11	NE	NE
Flora (vascular plants)	*Silene portensis*	3	NE	NE
Flora (vascular plants)	*Silene scabriflora*	2	NE	NE
Flora (vascular plants)	*Silene vulgaris*	9	NE	NE
Flora (vascular plants)	*Silybum marianum*	2	LC	NE
Flora (vascular plants)	*Simethis mattiazzi*	7	NE	NE
Flora (vascular plants)	*Sisymbrella aspera*	3	NE	NE
Flora (vascular plants)	*Sisymbrium austriacum*	2	NE	NE
Flora (vascular plants)	*Sisymbrium officinale*	13	LC	NE
Flora (vascular plants)	*Smyrnium olusatrum*	1	NE	NE
Flora (vascular plants)	*Solanum chenopodioides*	2	NE	NE
Flora (vascular plants)	*Solanum dulcamara*	11	LC	NE
Flora (vascular plants)	*Solanum nigrum*	4	NE	NE
Flora (vascular plants)	*Solanum pseudocapsicum*	2	NE	NE
Flora (vascular plants)	*Soleirolia soleirolii*	1	NE	NE
Flora (vascular plants)	*Solidago virgaurea*	5	LC	NE
Flora (vascular plants)	*Sonchus asper*	17	NE	NE
Flora (vascular plants)	*Sonchus oleraceus*	7	NE	NE
Flora (vascular plants)	*Sonchus tenerrimus*	2	NE	NE
Flora (vascular plants)	*Sorbus aucuparia*	9	LC	NE

Flora (vascular plants)	*Sorbus torminalis*	4	VU	NE
Flora (vascular plants)	*Sparganium erectum*	1	LC	NE
Flora (vascular plants)	*Spergula arvensis*	7	NE	NE
Flora (vascular plants)	*Spergula morisonii*	4	NE	NE
Flora (vascular plants)	*Spergula pentandra*	1	NE	NE
Flora (vascular plants)	*Spergularia capillacea*	2	NE	NE
Flora (vascular plants)	*Spergularia purpurea*	7	NE	NE
Flora (vascular plants)	*Spergularia rubra*	2	NE	NE
Flora (vascular plants)	*Spergularia segetalis*	2	NE	NE
Flora (vascular plants)	*Spiraea hypericifolia*	2	NE	VU
Flora (vascular plants)	*Stachys arvensis*	7	NE	NE
Flora (vascular plants)	*Stachys officinalis*	7	LC	NE
Flora (vascular plants)	*Stachys sylvatica*	28	NE	LC
Flora (vascular plants)	*Staehelina dubia*	1	NE	NE
Flora (vascular plants)	*Stellaria alsine*	7	NE	NE
Flora (vascular plants)	*Stellaria graminea*	37	NE	NE
Flora (vascular plants)	*Stellaria holostea*	24	NE	NE
Flora (vascular plants)	*Stellaria media*	12	LC	NE
Flora (vascular plants)	*Symphyotrichum lanceolatum*	1	NE	NE
Flora (vascular plants)	*Symphytum tuberosum*	1	NE	NE
Flora (vascular plants)	*Syntrichia ruralis*	2	LC	NE
Flora (vascular plants)	*Taeniatherum caput-medusae*	8	NE	NE
Flora (vascular plants)	*Tagetes erecta*	1	NE	NE
Flora (vascular plants)	*Tamus communis*	42	LC	NE
Flora (vascular plants)	*Tanacetum corymbosum*	15	NE	NE
Flora (vascular plants)	*Tanacetum mucronulatum*	3	NE	NE
Flora (vascular plants)	*Tanacetum parthenium*	3	LC	NE
Flora (vascular plants)	*Teesdalia coronopifolia*	2	NE	NE
Flora (vascular plants)	*Teesdalia nudicaulis*	7	NE	NE
Flora (vascular plants)	*Teucrium scordium*	2	LC	NE
Flora (vascular plants)	*Teucrium scorodonia*	65	LC	NE
Flora (vascular plants)	*Thalictrum speciosissimum*	17	NE	NE
Flora (vascular plants)	*Thapsia minor*	8	NE	NE
Flora (vascular plants)	*Thapsia nitida*	1	NE	NE
Flora (vascular plants)	*Thapsia villosa*	3	NE	NE
Flora (vascular plants)	*Thesium humifusum*	6	NE	NE
Flora (vascular plants)	*Thesium pyrenaicum*	9	NE	NE
Flora (vascular plants)	*Thymelaea ruizii*	4	NE	CR
Flora (vascular plants)	*Thymus mastichina*	31	LC	NE
Flora (vascular plants)	*Thymus pulegioides*	31	NE	NE
Flora (vascular plants)	*Thymus zygis*	3	LC	NE
Flora (vascular plants)	*Tilia cordata*	1	LC	NE
Flora (vascular plants)	*Tolpis barbata*	7	NE	NE
Flora (vascular plants)	*Tolpis umbellata*	8	NE	NE
Flora (vascular plants)	*Tordylium maximum*	36	NE	NE
Flora (vascular plants)	*Torilis arvensis*	15	NE	NE

Flora (vascular plants)	*Torilis elongata*	6	NE	NE
Flora (vascular plants)	*Torilis japonica*	2	NE	NE
Flora (vascular plants)	*Torilis leptophylla*	3	NE	NE
Flora (vascular plants)	*Torilis nodosa*	2	NE	NE
Flora (vascular plants)	*Tragopogon crocifolius*	1	NE	NE
Flora (vascular plants)	*Tragopogon dubius*	9	NE	NE
Flora (vascular plants)	*Tribulus terrestris*	1	LC	NE
Flora (vascular plants)	*Trichophorum cespitosum*	2	NE	EN
Flora (vascular plants)	*Trifolium angustifolium*	18	LC	NE
Flora (vascular plants)	*Trifolium arvense*	25	LC	NE
Flora (vascular plants)	*Trifolium bocconei*	1	NE	NE
Flora (vascular plants)	*Trifolium campestre*	39	NE	LC
Flora (vascular plants)	*Trifolium cernuum*	3	NE	NE
Flora (vascular plants)	*Trifolium cherleri*	2	NE	NE
Flora (vascular plants)	*Trifolium dubium*	22	NE	NE
Flora (vascular plants)	*Trifolium fragiferum*	1	NE	NE
Flora (vascular plants)	*Trifolium glomeratum*	10	NE	NE
Flora (vascular plants)	*Trifolium hirtum*	8	NE	NE
Flora (vascular plants)	*Trifolium incarnatum*	1	LC	NE
Flora (vascular plants)	*Trifolium leucanthum*	2	NE	NE
Flora (vascular plants)	*Trifolium ligusticum*	1	NE	NE
Flora (vascular plants)	*Trifolium medium*	4	NE	NE
Flora (vascular plants)	*Trifolium michelianum*	2	NE	NE
Flora (vascular plants)	*Trifolium micranthum*	1	NE	NE
Flora (vascular plants)	*Trifolium ochroleucon*	2	NE	NE
Flora (vascular plants)	*Trifolium ornithopodioides*	1	NE	NE
Flora (vascular plants)	*Trifolium phleoides*	3	NE	NE
Flora (vascular plants)	*Trifolium pratense*	40	LC	NE
Flora (vascular plants)	*Trifolium repens*	56	LC	NE
Flora (vascular plants)	*Trifolium scabrum*	7	NE	NE
Flora (vascular plants)	*Trifolium striatum*	16	NE	NE
Flora (vascular plants)	*Trifolium strictum*	7	NE	NE
Flora (vascular plants)	*Trifolium subterraneum*	9	LC	NE
Flora (vascular plants)	*Trifolium suffocatum*	1	NE	NE
Flora (vascular plants)	*Trifolium sylvaticum*	6	NE	NE
Flora (vascular plants)	*Trifolium vesiculosum*	1	LC	NE
Flora (vascular plants)	*Trisetaria ovata*	4	NE	NE
Flora (vascular plants)	*Trisetum flavescens*	5	NE	NE
Flora (vascular plants)	*Tuberaria guttata*	38	NE	NE
Flora (vascular plants)	*Tuberaria lignosa*	4	NE	NE
Flora (vascular plants)	*Tuberaria plantaginea*	2	NE	NE
Flora (vascular plants)	*Tulipa sylvestris*	1	NE	NE
Flora (vascular plants)	*Typha domingensis*	1	LC	NE
Flora (vascular plants)	*Typha latifolia*	4	LC	NE
Flora (vascular plants)	*Ulex europaeus*	4	LC	NE
Flora (vascular plants)	*Ulex minor*	9	LC	NE

Flora (vascular plants)	*Ulmus minor*	24	DD	NE
Flora (vascular plants)	*Umbilicus heylandianus*	1	DD	NE
Flora (vascular plants)	*Umbilicus rupestris*	16	NE	NE
Flora (vascular plants)	*Urospermum picroides*	1	NE	NE
Flora (vascular plants)	*Urtica dioica*	47	LC	NE
Flora (vascular plants)	*Urtica urens*	5	LC	NE
Flora (vascular plants)	*Vaccaria hispanica*	1	VU	NE
Flora (vascular plants)	*Valeriana officinalis*	5	LC	EN
Flora (vascular plants)	*Valerianella dentata*	1	NE	NE
Flora (vascular plants)	*Valerianella locusta*	3	NE	NE
Flora (vascular plants)	*Velezia rigida*	1	NE	NE
Flora (vascular plants)	*Verbascum pulverulentum*	4	NE	NE
Flora (vascular plants)	*Verbascum simplex*	3	NE	NE
Flora (vascular plants)	*Verbascum thapsus*	9	LC	NE
Flora (vascular plants)	*Verbascum virgatum*	8	NE	NE
Flora (vascular plants)	*Verbena officinalis*	11	LC	NE
Flora (vascular plants)	*Veronica anagallis-aquatica*	7	LC	NE
Flora (vascular plants)	*Veronica anagalloides*	3	LC	NE
Flora (vascular plants)	*Veronica arvensis*	7	NE	NE
Flora (vascular plants)	*Veronica beccabunga*	1	LC	NE
Flora (vascular plants)	*Veronica chamaedrys*	3	NE	NE
Flora (vascular plants)	*Veronica hederifolia*	3	NE	NE
Flora (vascular plants)	*Veronica micrantha*	19	VU	NT
Flora (vascular plants)	*Veronica montana*	1	NE	NE
Flora (vascular plants)	*Veronica officinalis*	2	LC	NE
Flora (vascular plants)	*Veronica peregrina*	1	LC	NE
Flora (vascular plants)	*Veronica persica*	5	NE	NE
Flora (vascular plants)	*Veronica serpyllifolia*	5	NE	NE
Flora (vascular plants)	*Viburnum lantana*	4	NE	CR
Flora (vascular plants)	*Viburnum opulus*	21	LC	VU
Flora (vascular plants)	*Vicia angustifolia*	11	NE	NE
Flora (vascular plants)	*Vicia dasycarpa*	1	NE	NE
Flora (vascular plants)	*Vicia disperma*	4	NE	NE
Flora (vascular plants)	*Vicia hirsuta*	2	NE	NE
Flora (vascular plants)	*Vicia lathyroides*	2	LC	NE
Flora (vascular plants)	*Vicia lutea*	9	LC	NE
Flora (vascular plants)	*Vicia narbonensis*	2	NT	NE
Flora (vascular plants)	*Vicia onobrychioides*	3	NE	VU
Flora (vascular plants)	*Vicia orobus*	2	NE	VU
Flora (vascular plants)	*Vicia parviflora*	2	NE	NE
Flora (vascular plants)	*Vicia sativa*	1	LC	NE
Flora (vascular plants)	*Vicia sepium*	8	LC	NE
Flora (vascular plants)	*Vicia tenuifolia*	4	NE	NE
Flora (vascular plants)	*Vicia villosa*	1	NE	NE
Flora (vascular plants)	*Vinca major*	5	NE	NE
Flora (vascular plants)	*Vinca minor*	2	LC	NE

Flora (vascular plants)	*Vincetoxicum nigrum*	16	NE	NE
Flora (vascular plants)	*Viola bubanii*	3	NE	CR
Flora (vascular plants)	*Viola canina*	1	LC	NE
Flora (vascular plants)	*Viola cornuta*	1	NE	NE
Flora (vascular plants)	*Viola hirta*	9	NE	EN
Flora (vascular plants)	*Viola kitaibeliana*	11	NE	NE
Flora (vascular plants)	*Viola lactea*	1	NE	NE
Flora (vascular plants)	*Viola palustris*	3	NE	NE
Flora (vascular plants)	*Viola parvula*	3	NE	VU
Flora (vascular plants)	*Viola riviniana*	41	NE	NE
Flora (vascular plants)	*Viola suavis*	1	NE	NE
Flora (vascular plants)	*Vitis x instabilis*	1	NE	NE
Flora (vascular plants)	*Vulpia bromoides*	10	NE	NE
Flora (vascular plants)	*Vulpia ciliata*	11	NE	NE
Flora (vascular plants)	*Vulpia muralis*	16	NE	NE
Flora (vascular plants)	*Vulpia myuros*	15	NE	NE
Flora (vascular plants)	*Wahlenbergia hederacea*	12	NE	NE
Flora (vascular plants)	*Xanthium spinosum*	1	NE	NE
Flora (vascular plants)	*Xeranthemum cylindraceum*	1	VU	NE
Flora (vascular plants)	*Xolantha guttata*	14	NE	NE
Flora (vascular plants)	*Zea mays*	2	NE	NE
Flora (vascular plants)	*Zinnia elegans*	1	NE	NE
Mammals	*Apodemus sylvaticus*	2	LC	LC
Mammals	*Arvicola sapidus*	2	VU	NE
Mammals	*Barbastella barbastellus*	11	VU	DD
Mammals	*Canis lupus*	235	LC	EN
Mammals	*Capreolus capreolus*	210	LC	LC
Mammals	*Cervus elaphus*	195	NE	LC
Mammals	*Eptesicus serotinus*	3	NE	LC
Mammals	*Erinaceus europaeus*	8	LC	LC
Mammals	*Felis silvestris*	29	NE	VU
Mammals	*Galemys pyrenaicus*	6	EN	VU
Mammals	*Genetta genetta*	64	LC	LC
Mammals	*Hypsugo savii*	8	LC	DD
Mammals	*Lepus granatensis*	38	LC	LC
Mammals	*Lutra lutra*	8	NT	LC
Mammals	*Martes foina*	50	LC	LC
Mammals	*Martes martes*	42	LC	DD
Mammals	*Meles meles*	64	NE	LC
Mammals	*Microtus agrestis*	2	LC	NE
Mammals	*Miniopterus schreibersii*	2	NE	VU
Mammals	*Mustela nivalis*	3	LC	LC
Mammals	*Myotis daubentonii*	10	NE	LC
Mammals	*Myotis emarginatus*	1	LC	NE
Mammals	*Myotis escalerai*	1	NE	NE
Mammals	*Myotis mystacinus*	1	LC	DD

Mammals	*Neomys anomalus*	1	LC	DD
Mammals	*Neovison vison*	3	NE	NE
Mammals	*Nyctalus leisleri*	10	NE	DD
Mammals	*Oryctolagus cuniculus*	20	NT	NT
Mammals	*Ovis aries*	1	NE	NE
Mammals	*Pipistrellus kuhlii*	6	LC	LC
Mammals	*Pipistrellus pipistrellus*	18	NE	LC
Mammals	*Pipistrellus pygmaeus*	5	LC	LC
Mammals	*Plecotus auritus*	4	NE	DD
Mammals	*Plecotus austriacus*	5	NT	LC
Mammals	*Rhinolophus ferrumequinum*	6	NT	VU
Mammals	*Rhinolophus hipposideros*	3	NT	VU
Mammals	*Sciurus vulgaris*	34	LC	LC
Mammals	*Sus scrofa*	134	LC	LC
Mammals	*Tadarida teniotis*	1	LC	DD
Mammals	*Talpa occidentalis*	9	LC	LC
Mammals	*Vulpes vulpes*	191	LC	LC
Reptiles	*Acanthodactylus erythrurus*	1	LC	NT
Reptiles	*Anguis fragilis*	17	NE	LC
Reptiles	*Blanus cinereus*	20	LC	LC
Reptiles	*Chalcides bedriagai*	1	NT	LC
Reptiles	*Chalcides striatus*	3	LC	LC
Reptiles	*Coronella girondica*	12	LC	LC
Reptiles	*Lacerta schreiberi*	54	NT	LC
Reptiles	*Malpolon monspessulanus*	62	LC	LC
Reptiles	*Mauremys leprosa*	2	VU	LC
Reptiles	*Natrix astreptophora*	26	NE	LC
Reptiles	*Natrix maura*	79	LC	LC
Reptiles	*Podarcis bocagei*	24	LC	LC
Reptiles	*Podarcis guadarramae*	97	NE	NE
Reptiles	*Psammodromus algirus*	283	NE	LC
Reptiles	*Psammodromus occidentalis*	13	LC	NT
Reptiles	*Tarentola mauritanica*	5	LC	LC
Reptiles	*Timon lepidus*	185	NT	LC
Reptiles	*Vipera latastei*	14	VU	VU
Reptiles	*Zamenis scalaris*	71	LC	LC

[1] IUCN (International Union for Conservation of Nature), 2022b. The IUCN Red List of Threatened Species. Version 2022-2. https://www.iucnredlist.org

[2] Cabral MJ (coord.), Almeida J, Almeida PR, Dellinger T, Ferrand de Almeida N, Oliveira ME, Palmeirim JM, Queiroz AI, Rogado L & Santos-Reis M (eds.) (2005). Livro Vermelho dos Vertebrados de Portugal. Instituto da Conservação da Natureza. Lisboa. ???? p

[3] Carapeto A., Francisco A., Pereira P., Porto M. (eds.). (2020). Lista Vermelha da Flora Vascular de Portugal Continental. Sociedade Portuguesa de Botânica, Associação Portuguesa de Ciência da Vegetação – PHYTOS e Instituto da Conservação da Natureza e das Florestas (coord.). Coleção «Botânica em Português», Volume 7. Lisboa: Imprensa Nacional, 374 pp

[4] Almeida J, Godinho C, Leitão D, Lopes RJ (2022) Lista Vermelha das Aves de Portugal Continental. SPEA, ICNF, LabOR/UÉ, CIBIO/BIOPOLIS, Portuga

[5] Mathias ML (coord.), Fonseca C, Rodrigues L, Grilo C, Lopes-Fernandes M, Palmeirim JM, Santos-Reis M, Alves PC, Cabral JA, Ferreira M, Mira A, Eira C, Negrões N, Pauperio J, Pita R, Rainho A, Rosalino LM, Tapisso JT & Vingada J (eds.) (2023). Livro Vermelho dos Mamíferos de Portugal Continental, FCiências. ID, ICNF, Lisboa.
