# Supplementary material for: Biodiversity dataset and atlas of the special area of conservation Montesinho/Nogueira, Portugal
Source: Biodivers Data J. 2024 Apr 8;12:e118854. doi: 10.3897/BDJ.12.e118854 (PMC11019261; doi:10.3897/BDJ.12.e118854)
Supplement: Supplementary material 2 — Species distribution maps. [file bdj-12-e118854-s002.pdf]

Flora (vascular plants): Abutilon theophrasti

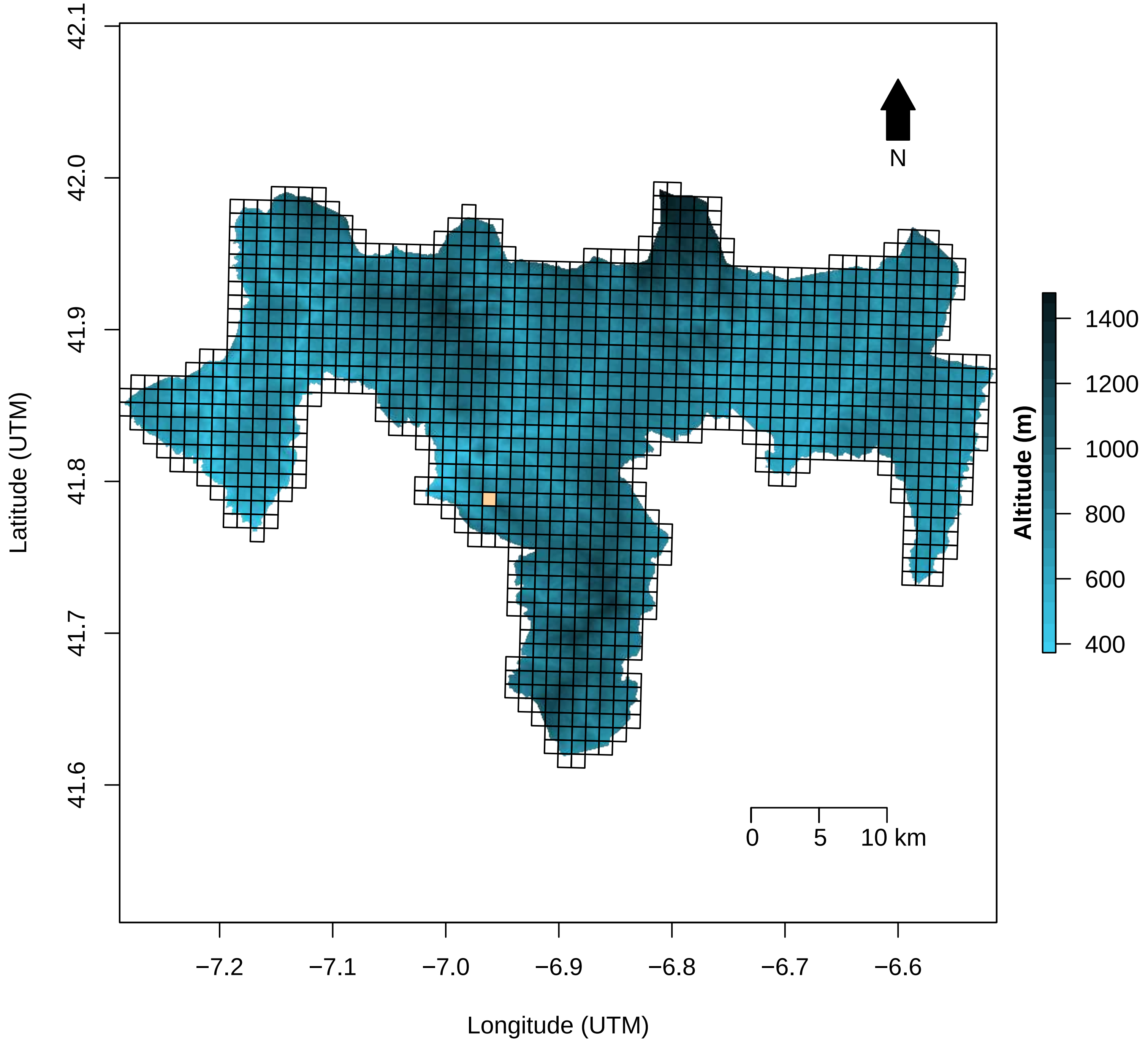

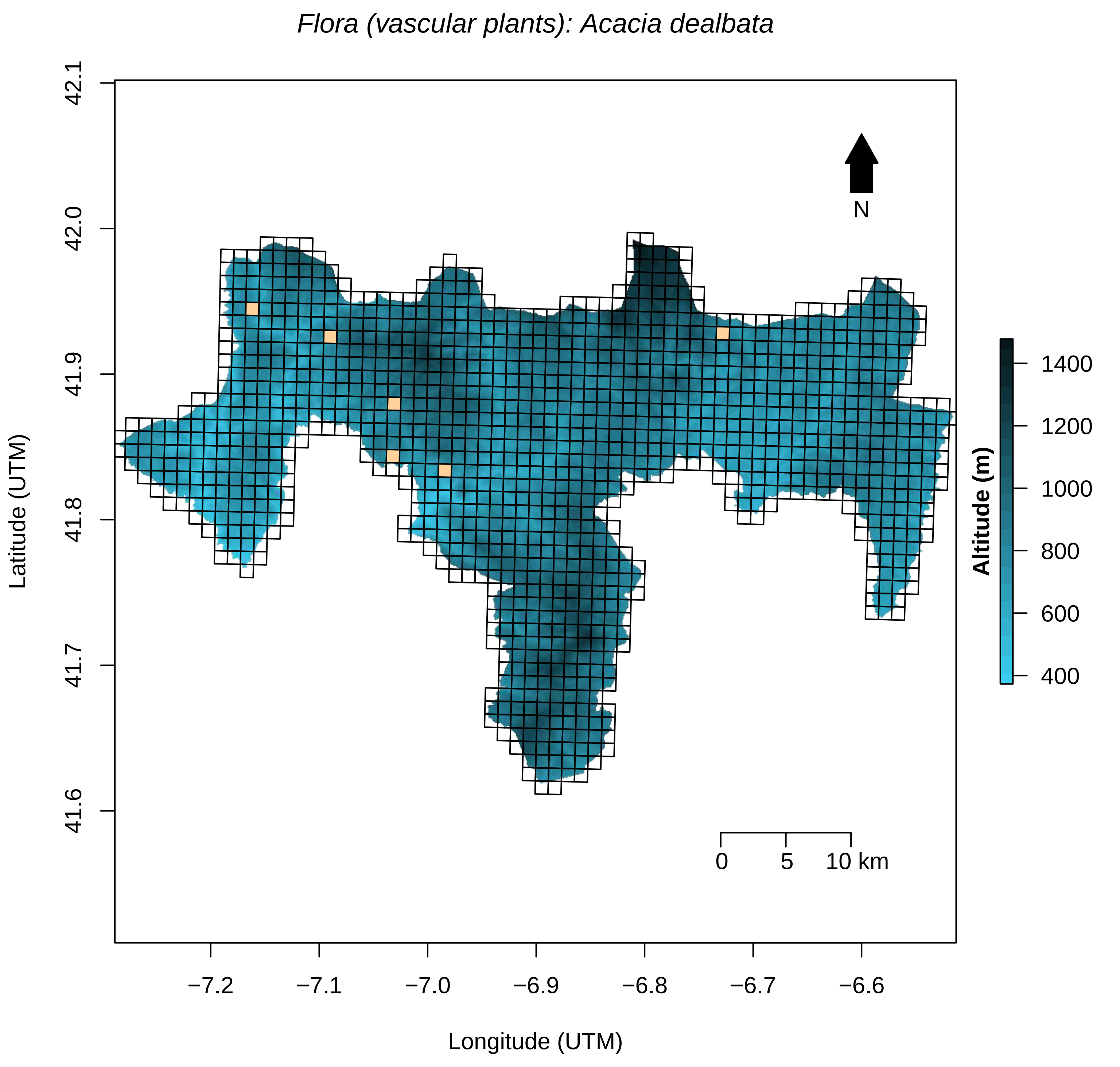

Flora (vascular plants): Acacia dealbata
N
0
5
10 km
Altitude (m)
1400
1200
1000
800
600
400
Latitude (UTM)
42.1
42.0
41.9
41.8
41.7
41.6
Longitude (UTM)
−7.2
−7.1
−7.0
−6.9
−6.8
−6.7
−6.6

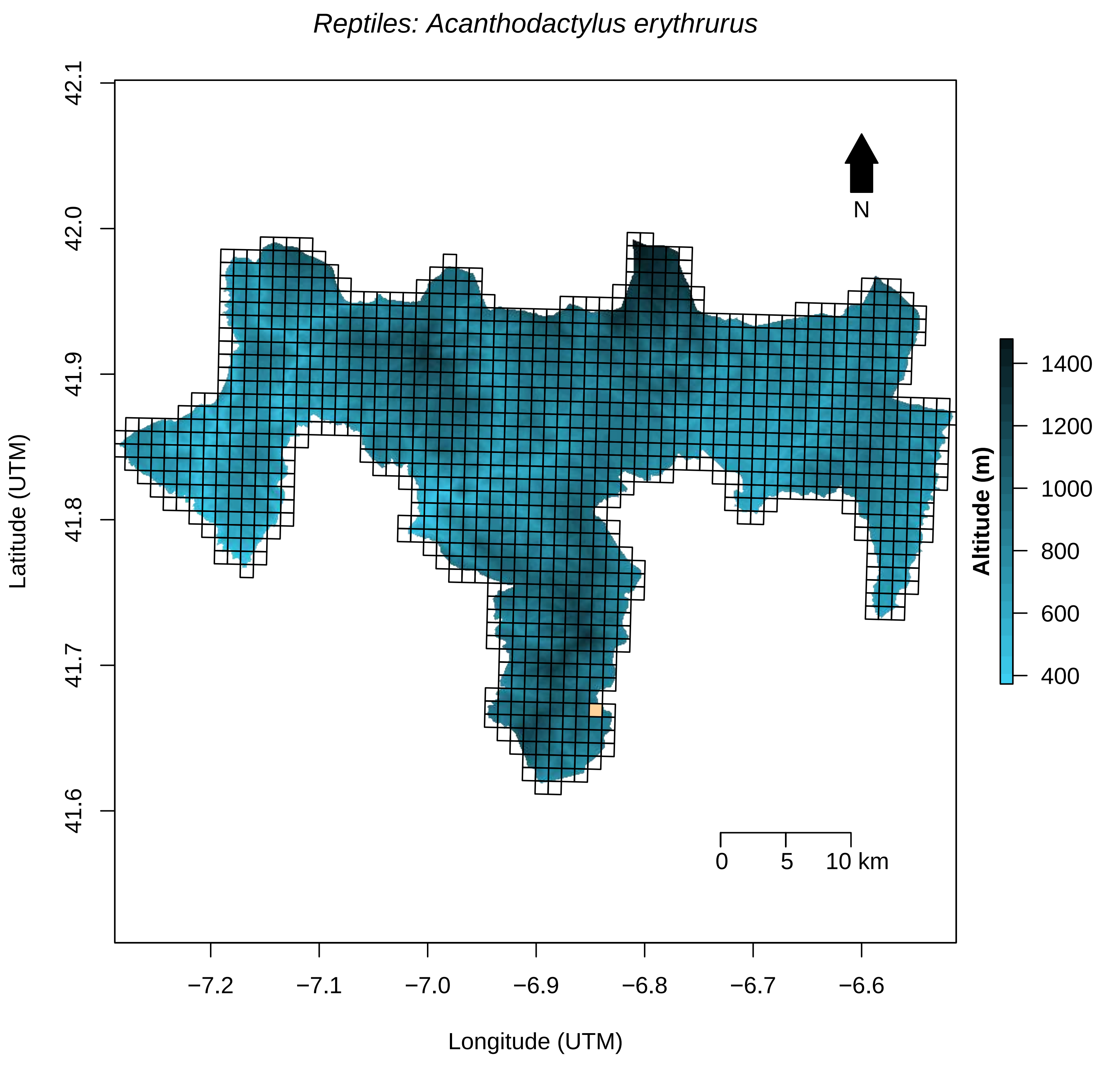

Reptiles: Acanthodactylus erythrurus
Latitude (UTM)
42.1
42.0
41.9
41.8
41.7
41.6
N
0
5
10 km
−7.2
−7.1
−7.0
−6.9
−6.8
−6.7
−6.6
Longitude (UTM)
Altitude (m)
1400
1200
1000
800
600
400

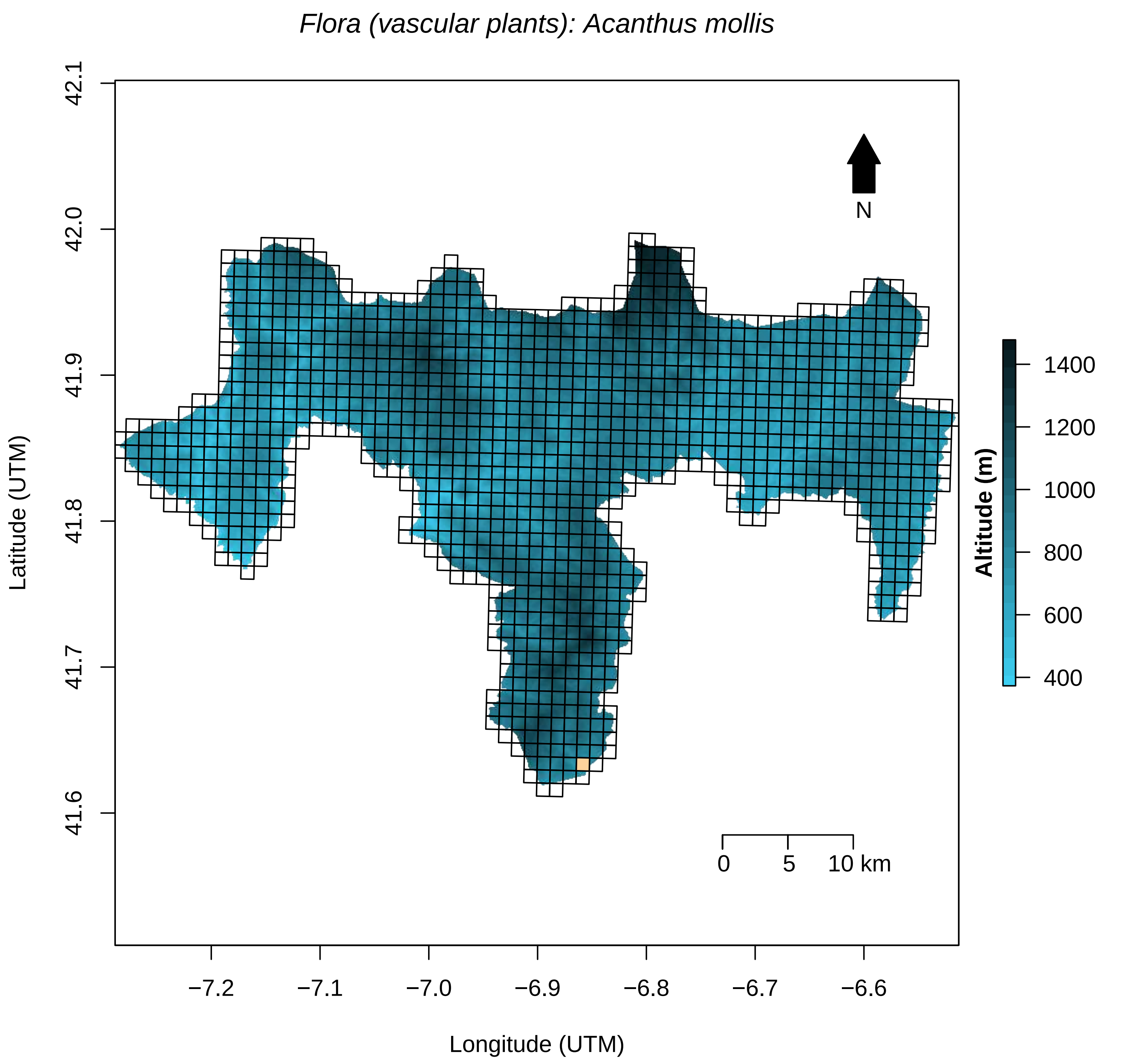

Flora (vascular plants): Acanthus mollis
42.1
42.0
41.9
41.8
41.7
41.6
Latitude (UTM)
−7.2
−7.1
−7.0
−6.9
−6.8
−6.7
−6.6
Longitude (UTM)
N
0
5
10 km
1400
1200
1000
800
600
400
Altitude (m)

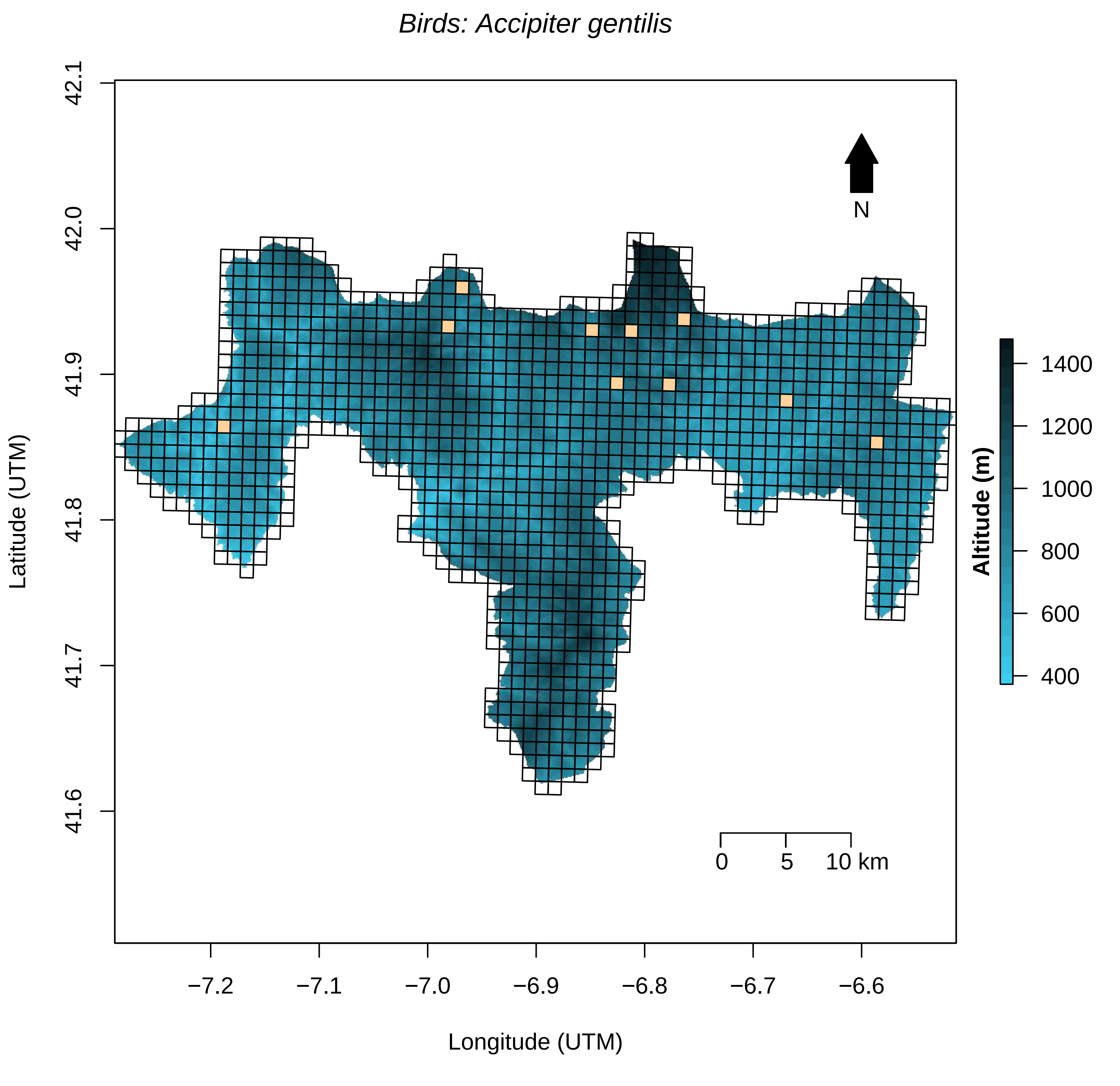
Birds: Accipiter gentilis
N
0
5
10 km
Latitude (UTM)
Longitude (UTM)
42.1
42.0
41.9
41.8
41.7
41.6
−7.2
−7.1
−7.0
−6.9
−6.8
−6.7
−6.6
Altitude (m)
1400
1200
1000
800
600
400

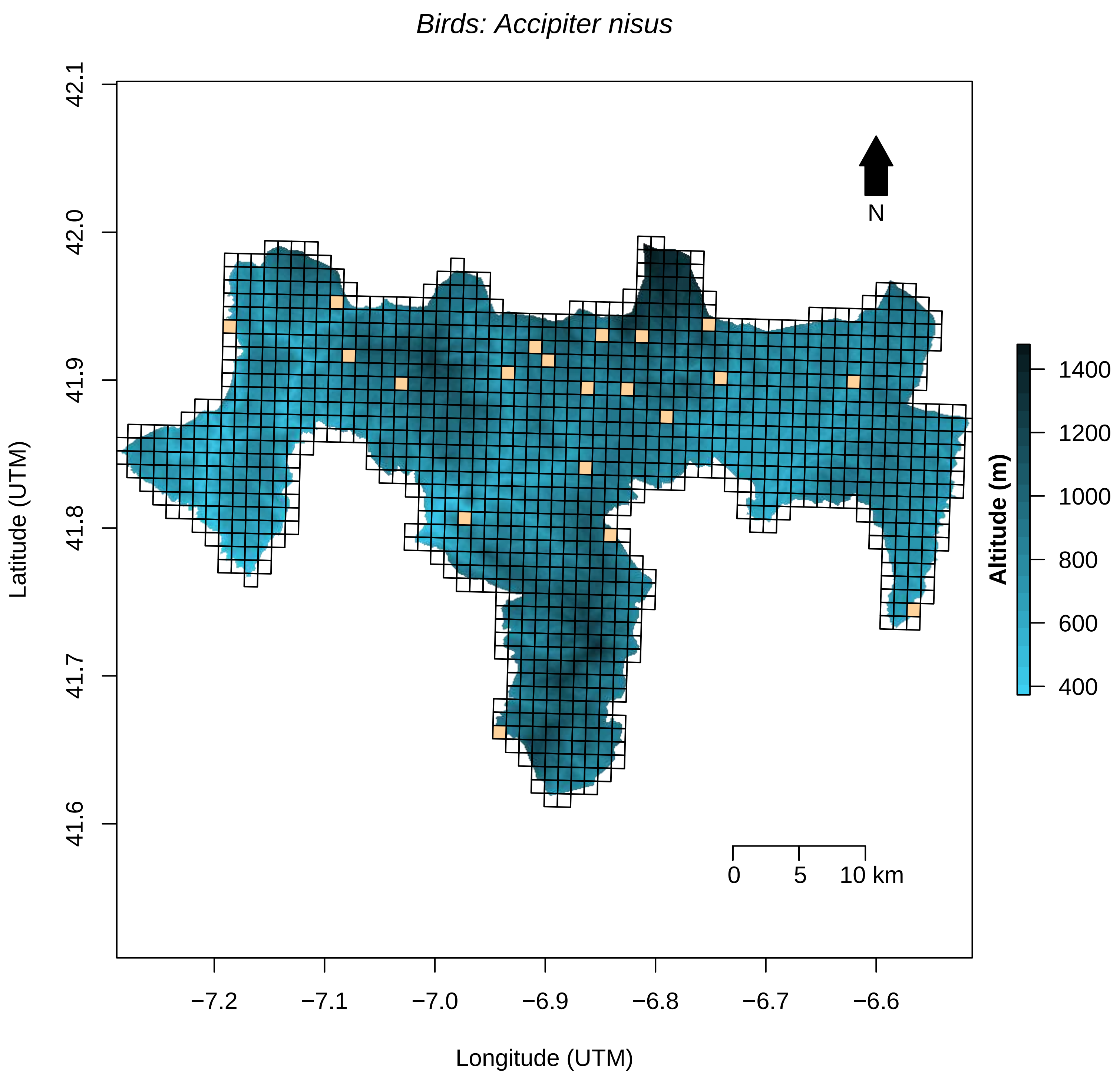

Birds: Accipiter nisus
Latitude (UTM)
42.1
42.0
41.9
41.8
41.7
41.6
Longitude (UTM)
−7.2
−7.1
−7.0
−6.9
−6.8
−6.7
−6.6
N
0
5
10 km
Altitude (m)
1400
1200
1000
800
600
400

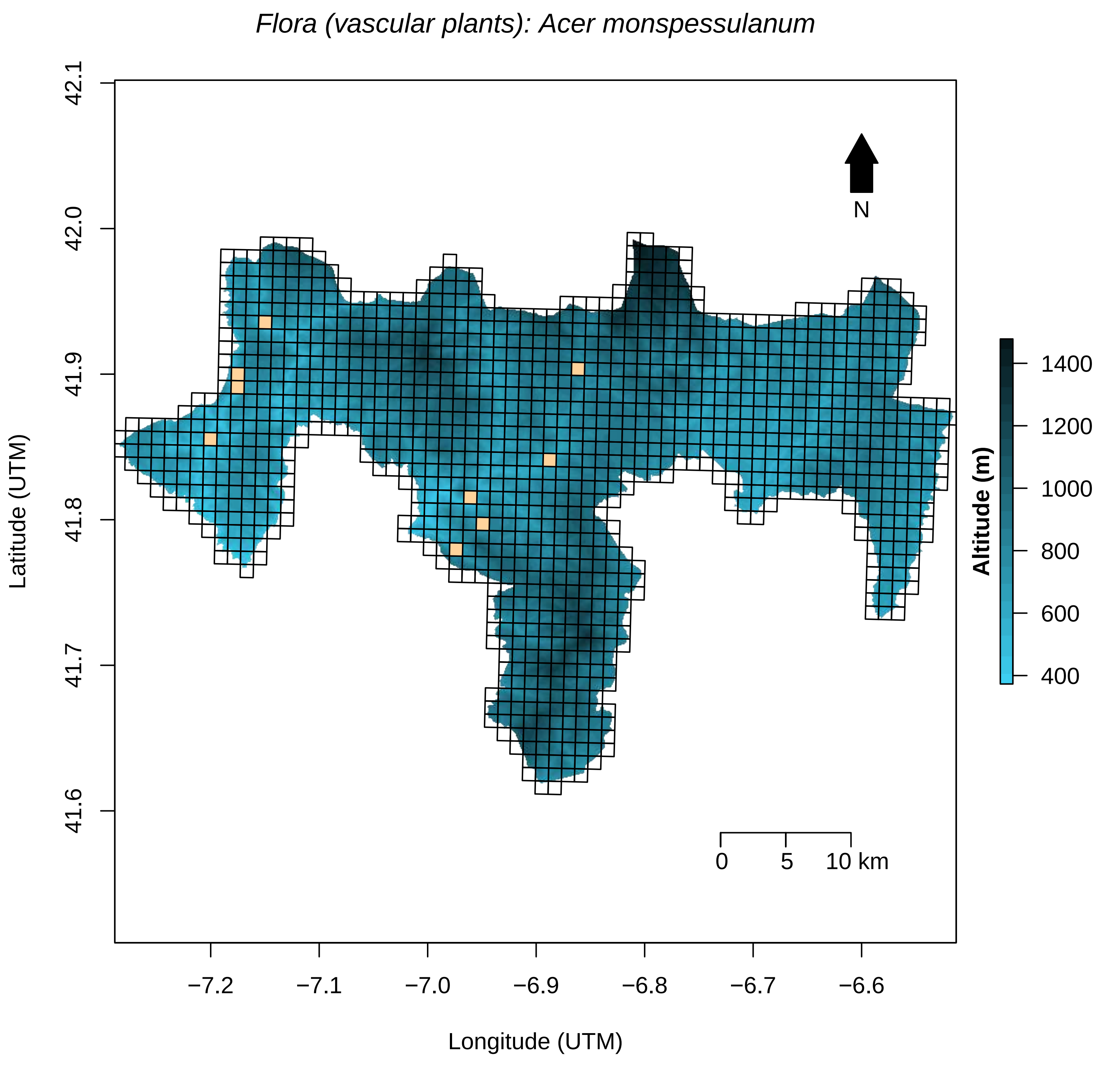

Flora (vascular plants): Acer monspessulanum
42.1
42.0
41.9
41.8
41.7
41.6
Latitude (UTM)
−7.2
−7.1
−7.0
−6.9
−6.8
−6.7
−6.6
Longitude (UTM)
N
0
5
10 km
1400
1200
1000
800
600
400
Altitude (m)

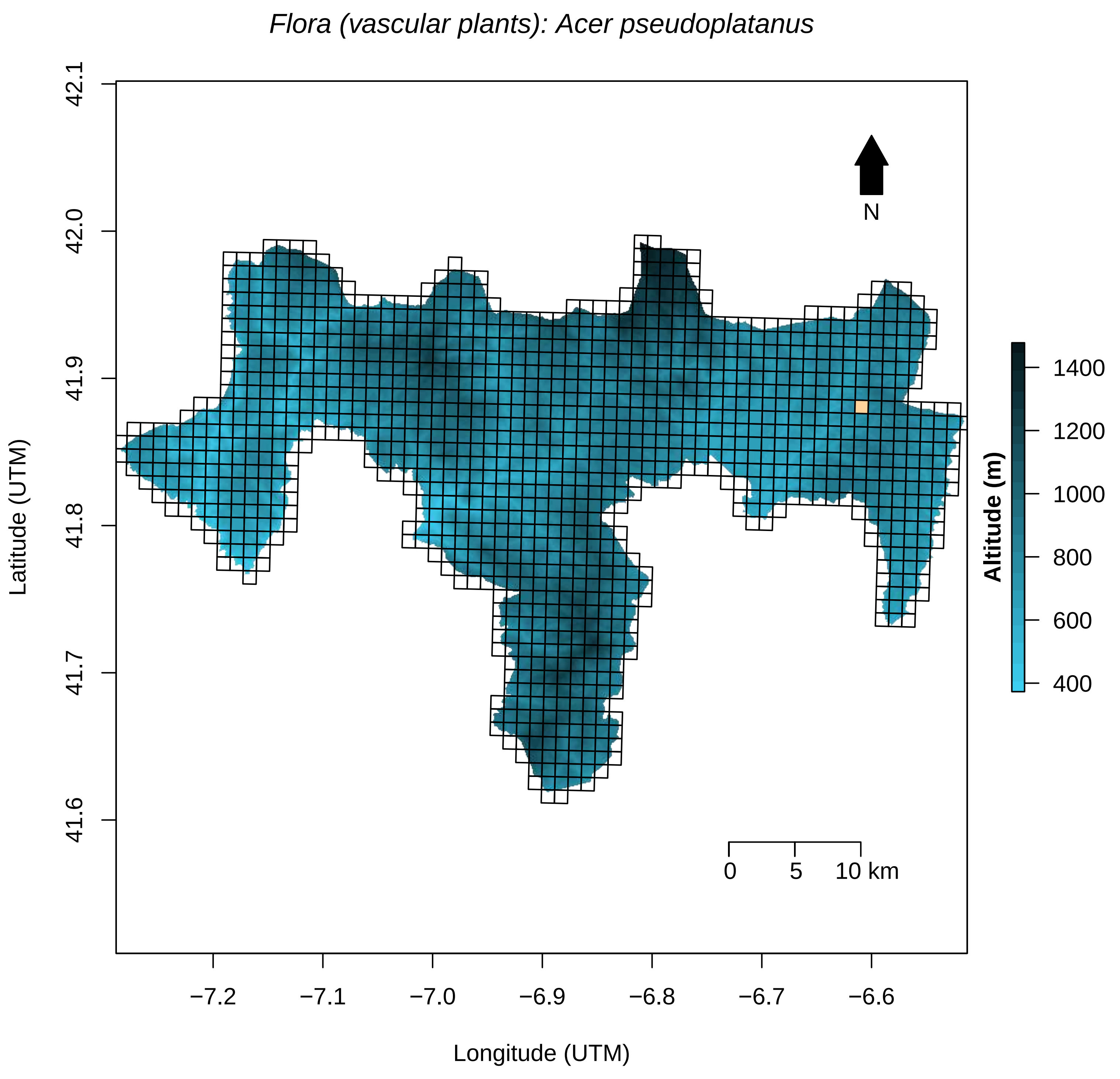
Flora (vascular plants): Acer pseudoplatanus
42.1
42.0
41.9
41.8
41.7
41.6
Latitude (UTM)
−7.2
−7.1
−7.0
−6.9
−6.8
−6.7
−6.6
Longitude (UTM)
N
0
5
10 km
Altitude (m)
1400
1200
1000
800
600
400

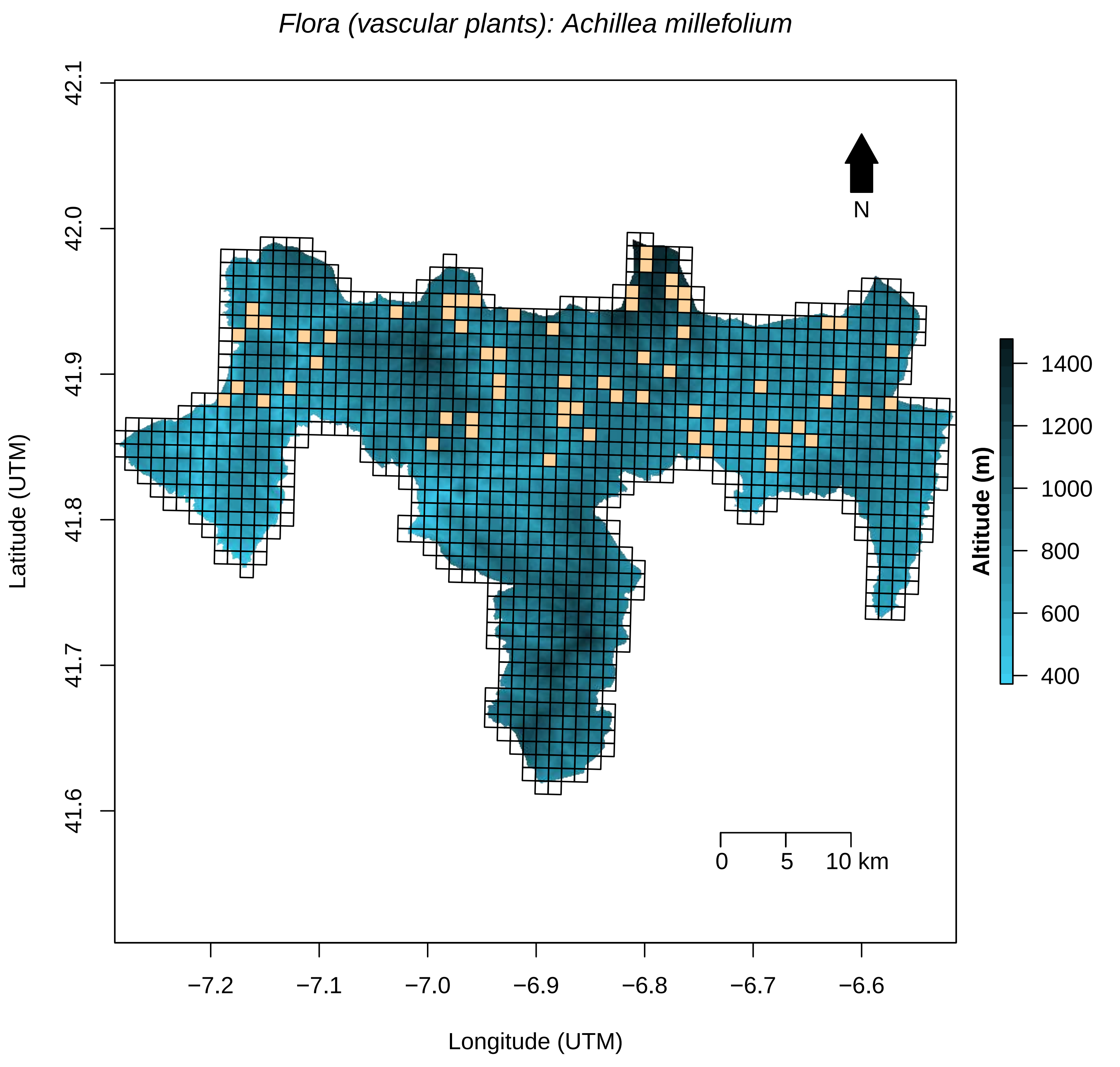
Flora (vascular plants): Achillea millefolium
Latitude (UTM)
Longitude (UTM)
42.1
42.0
41.9
41.8
41.7
41.6
−7.2
−7.1
−7.0
−6.9
−6.8
−6.7
−6.6
N
0
5
10 km
Altitude (m)
1400
1200
1000
800
600
400

Flora (vascular plants): Acinos alpinus

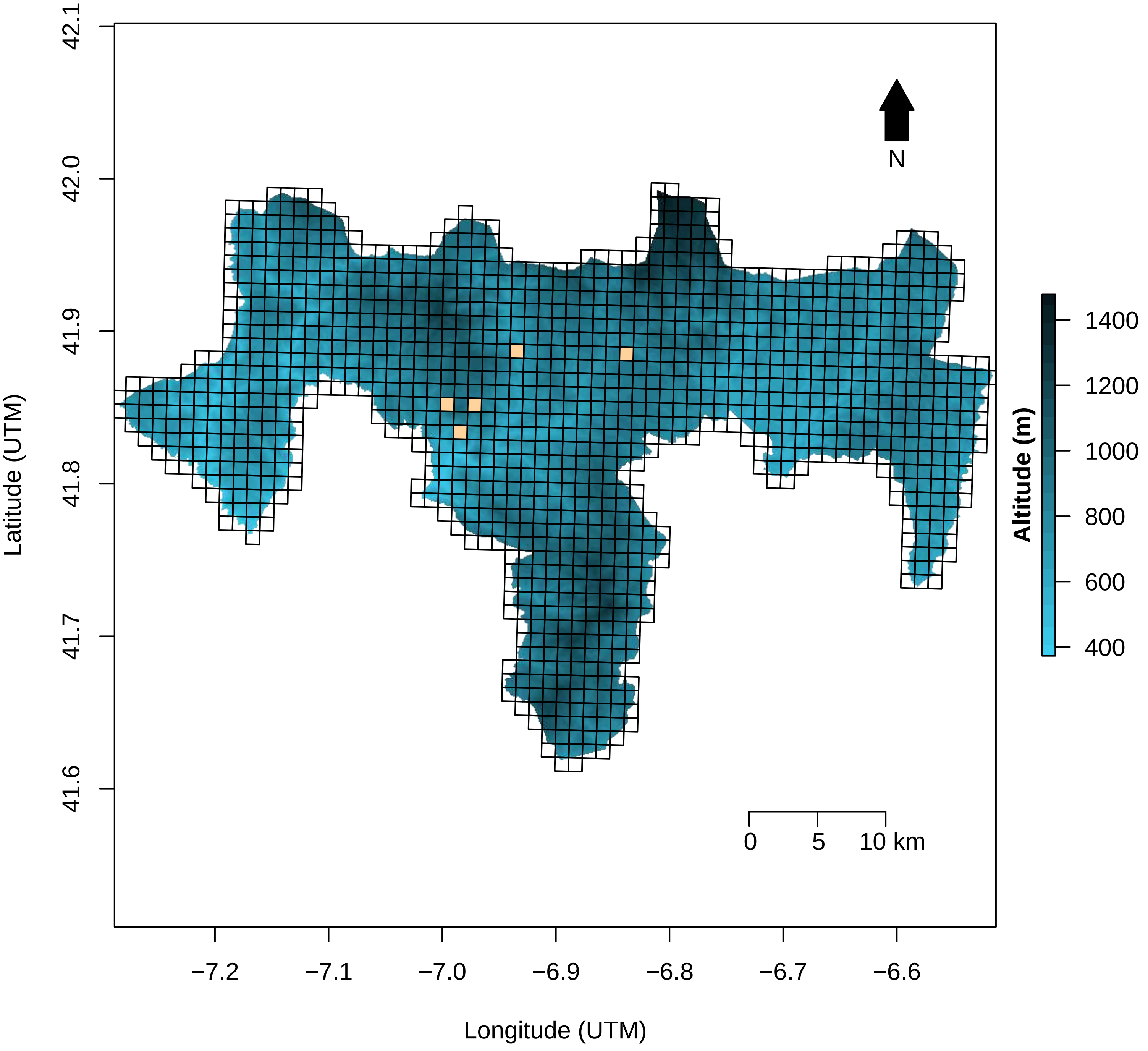

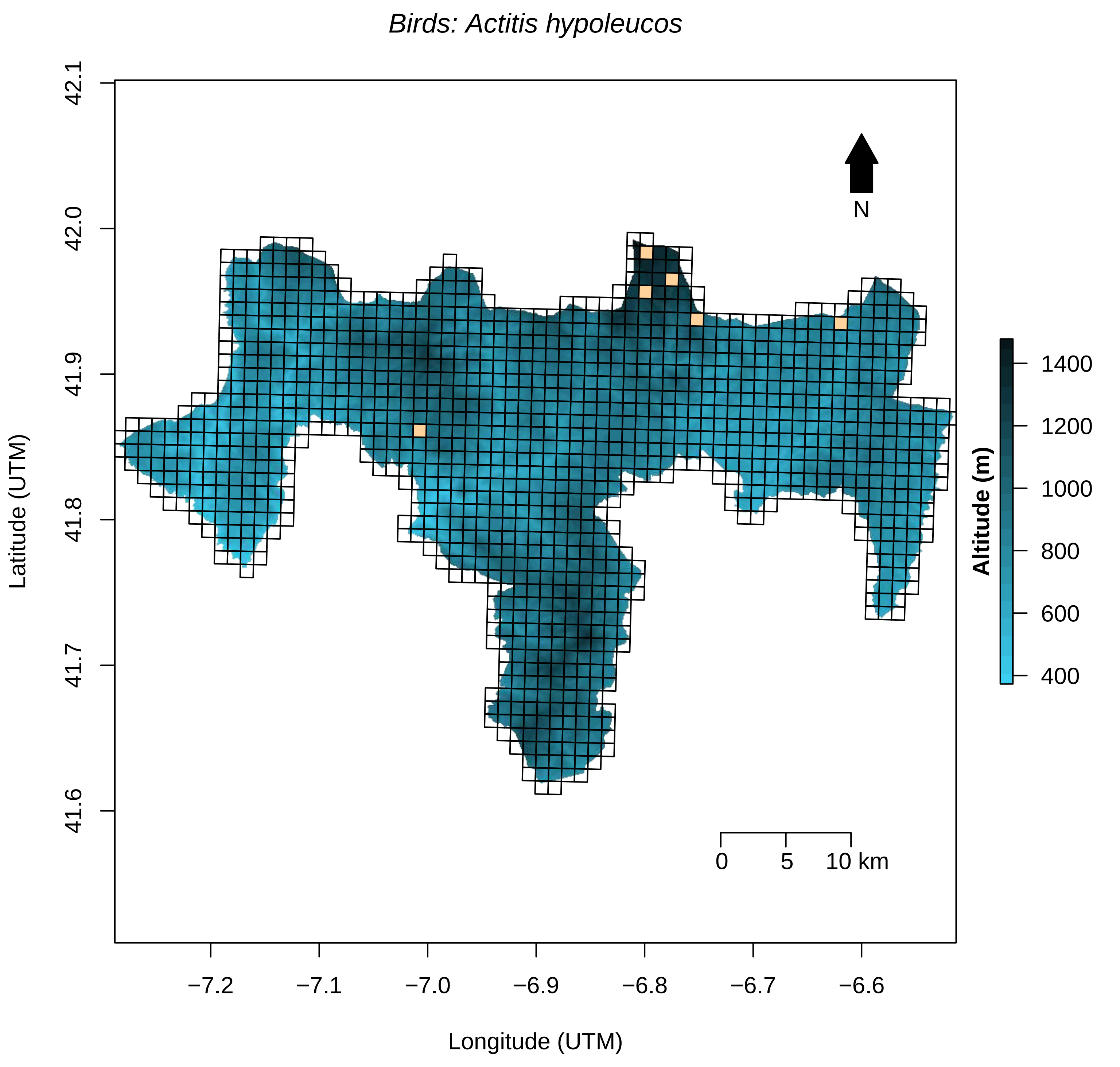

Birds: Actitis hypoleucos
N
0
5
10 km
Altitude (m)
1400
1200
1000
800
600
400
42.1
42.0
41.9
41.8
41.7
41.6
Latitude (UTM)
−7.2
−7.1
−7.0
−6.9
−6.8
−6.7
−6.6
Longitude (UTM)

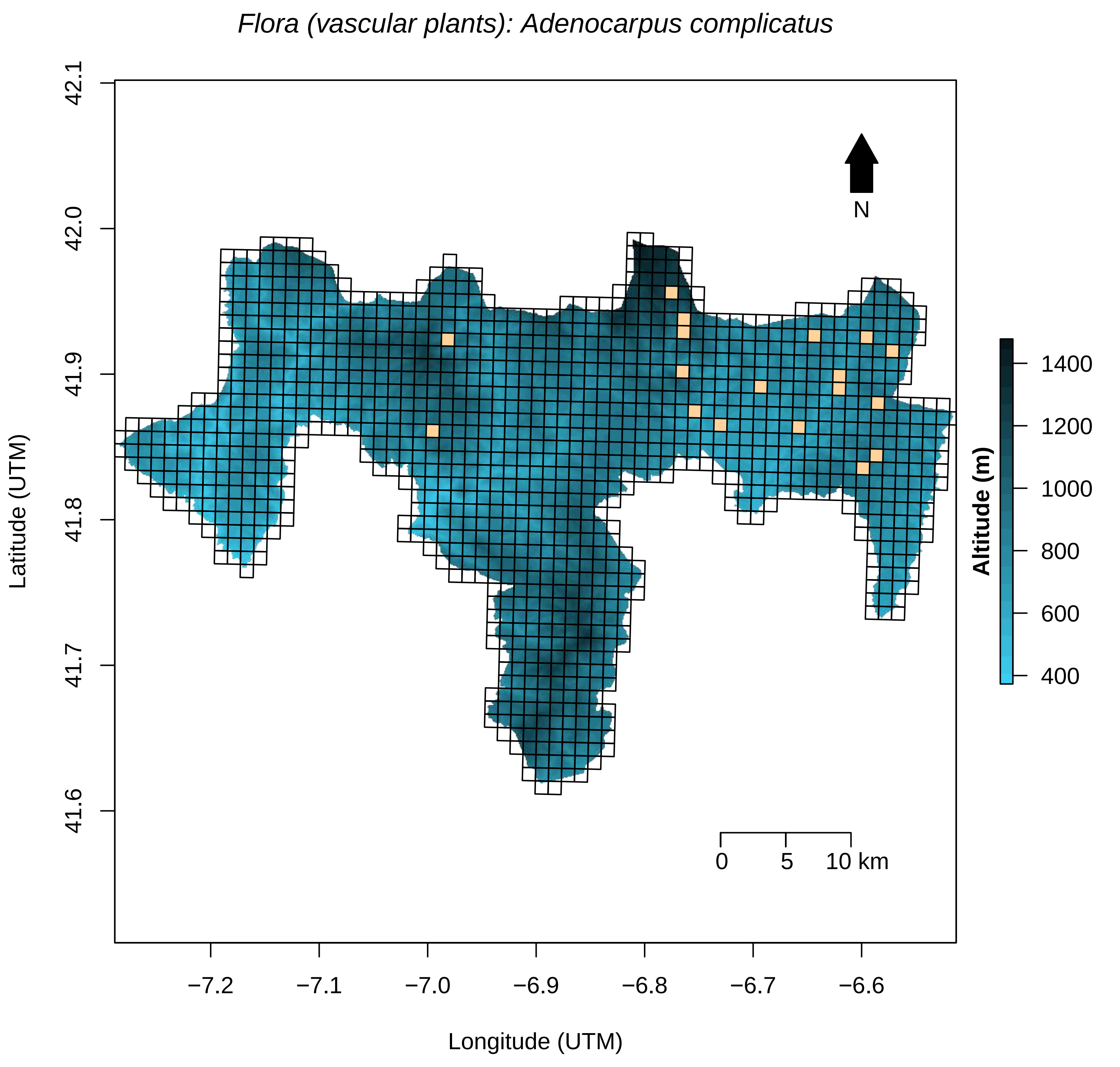
Flora (vascular plants): Adenocarpus complicatus
42.1
42.0
41.9
41.8
41.7
41.6
Latitude (UTM)
−7.2
−7.1
−7.0
−6.9
−6.8
−6.7
−6.6
Longitude (UTM)
N
0
5
10 km
Altitude (m)
1400
1200
1000
800
600
400

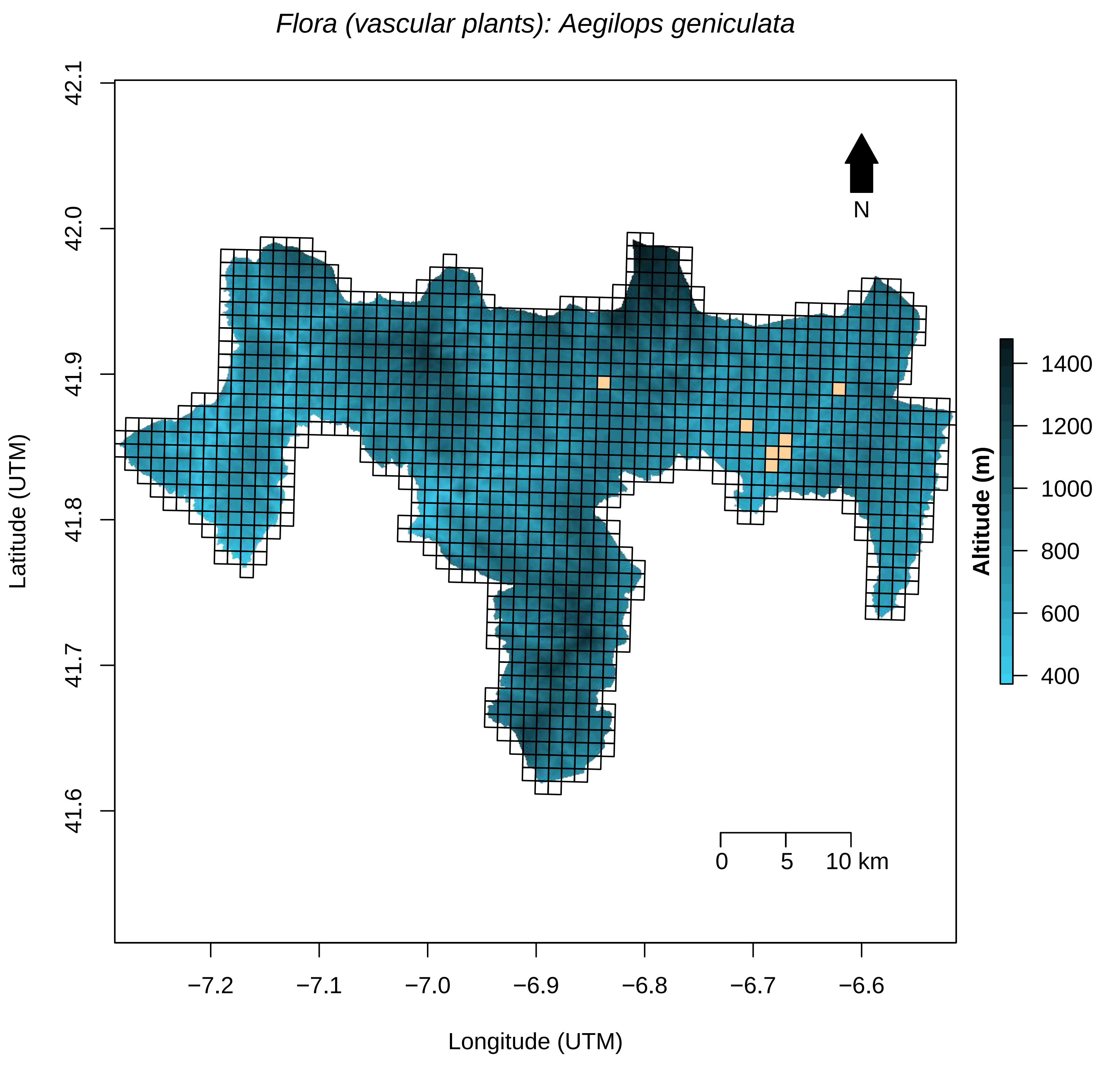

Flora (vascular plants): Aegilops geniculata
42.1
42.0
41.9
41.8
41.7
41.6
Latitude (UTM)
N
0
5
10 km
1400
1200
1000
800
600
400
Altitude (m)
−7.2
−7.1
−7.0
−6.9
−6.8
−6.7
−6.6
Longitude (UTM)

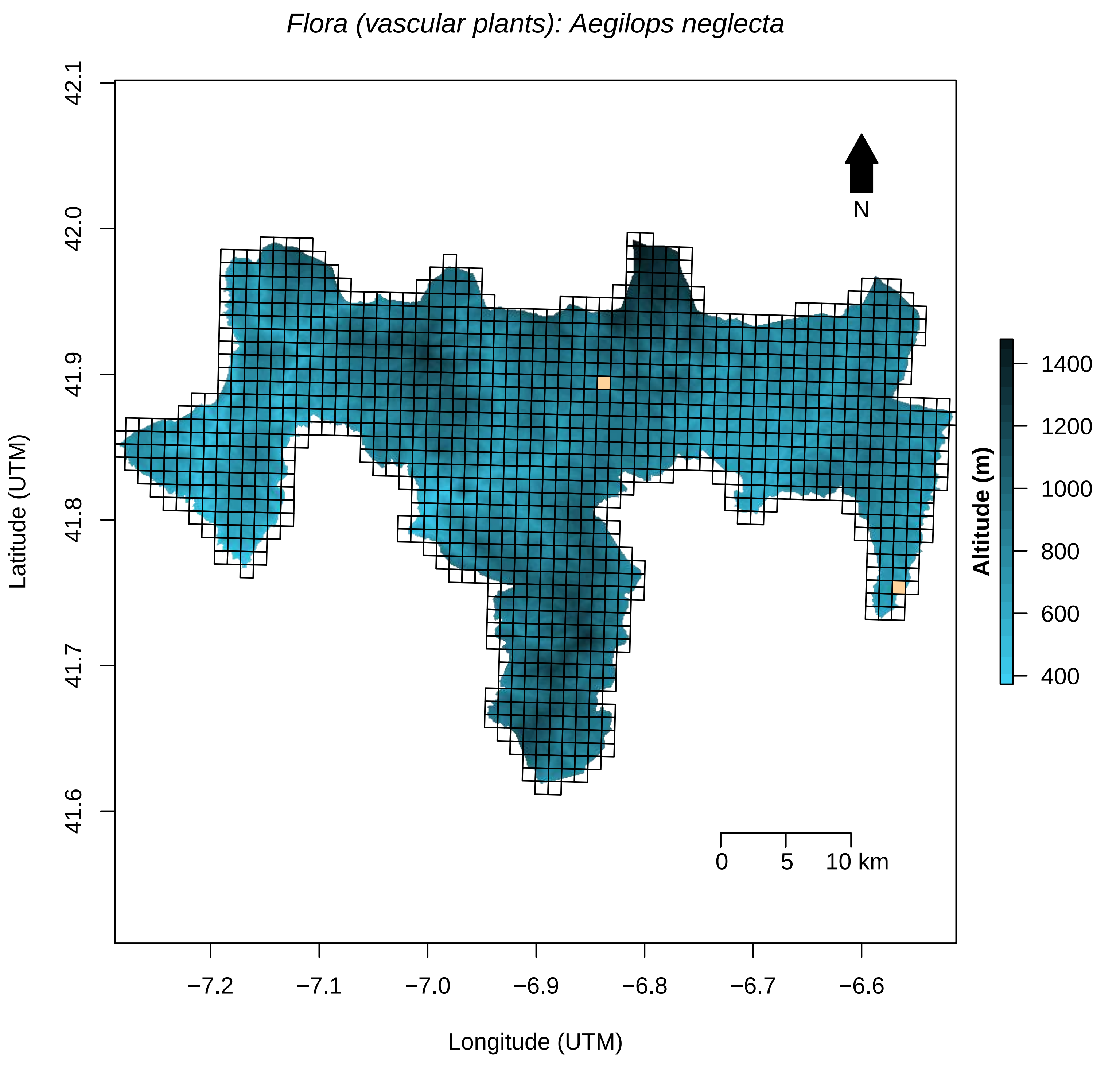

Flora (vascular plants): Aegilops neglecta
42.1
42.0
41.9
41.8
41.7
41.6
Latitude (UTM)
−7.2
−7.1
−7.0
−6.9
−6.8
−6.7
−6.6
Longitude (UTM)
N
0
5
10 km
Altitude (m)
1400
1200
1000
800
600
400

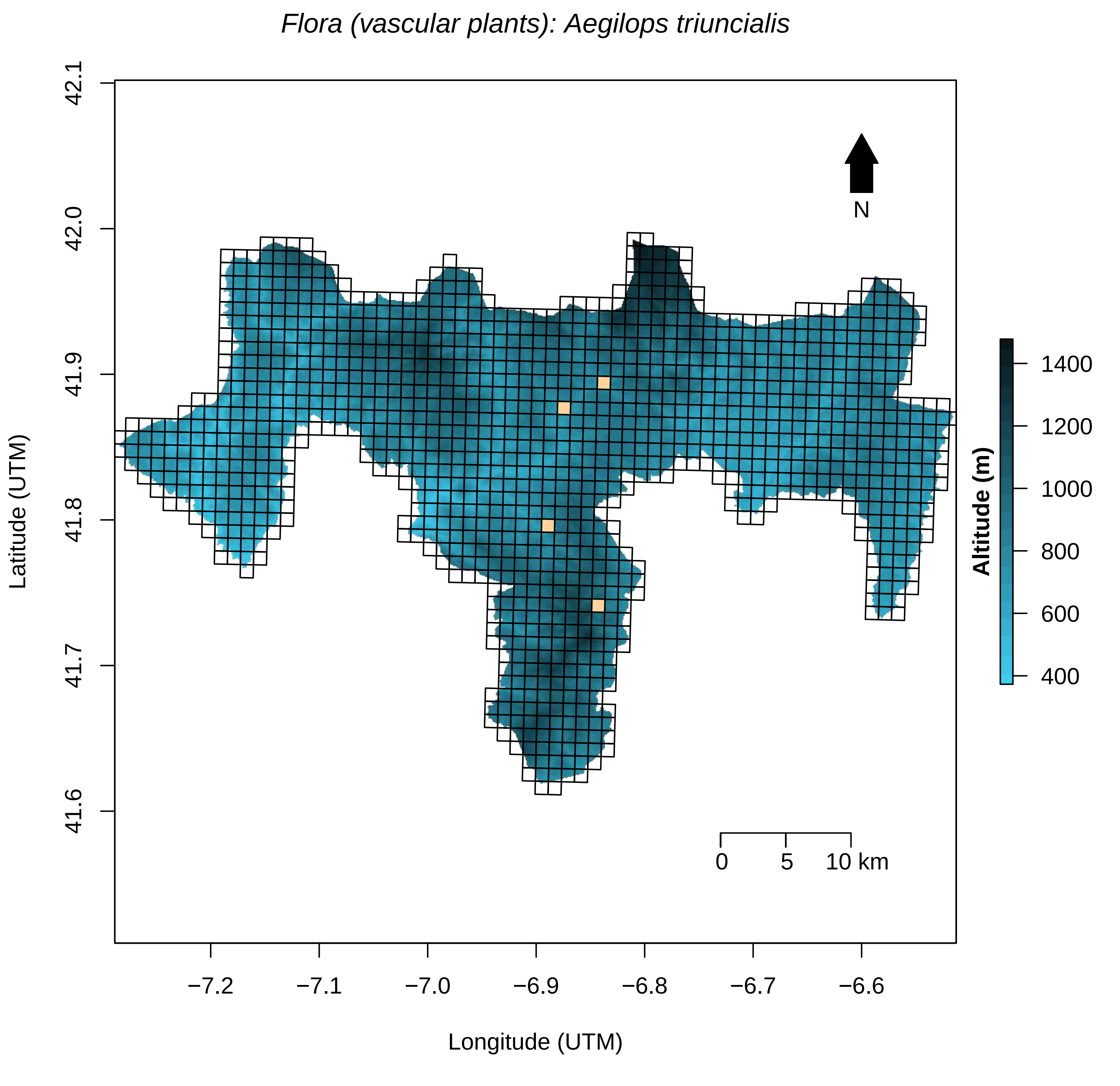

Flora (vascular plants): Aegilops triuncialis
42.1
42.0
41.9
41.8
41.7
41.6
Latitude (UTM)
−7.2
−7.1
−7.0
−6.9
−6.8
−6.7
−6.6
Longitude (UTM)
N
0
5
10 km
Altitude (m)
1400
1200
1000
800
600
400

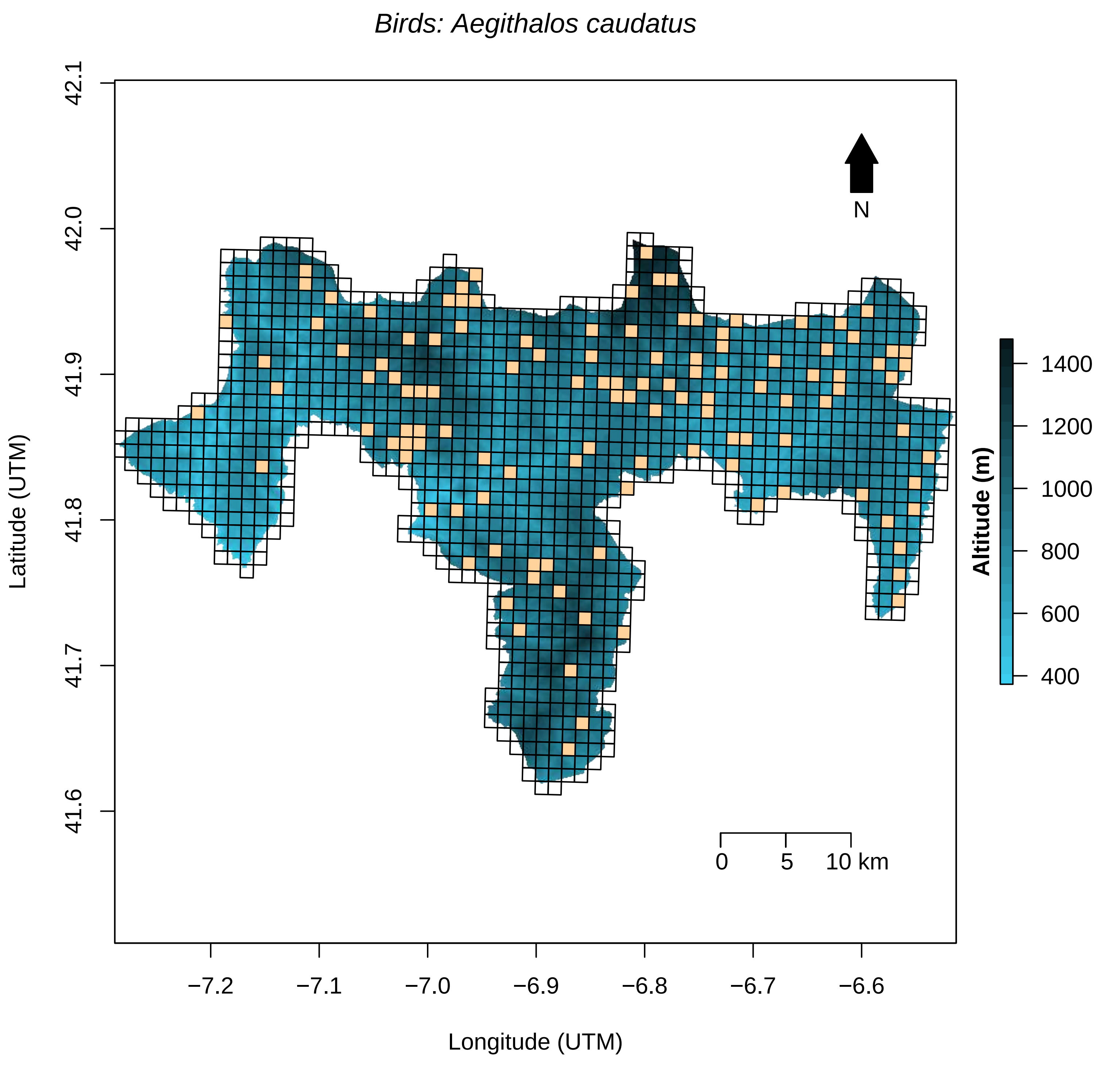

Birds: Aegithalos caudatus
42.1
42.0
41.9
41.8
41.7
41.6
Latitude (UTM)
−7.2
−7.1
−7.0
−6.9
−6.8
−6.7
−6.6
Longitude (UTM)
N
0
5
10 km
Altitude (m)
1400
1200
1000
800
600
400

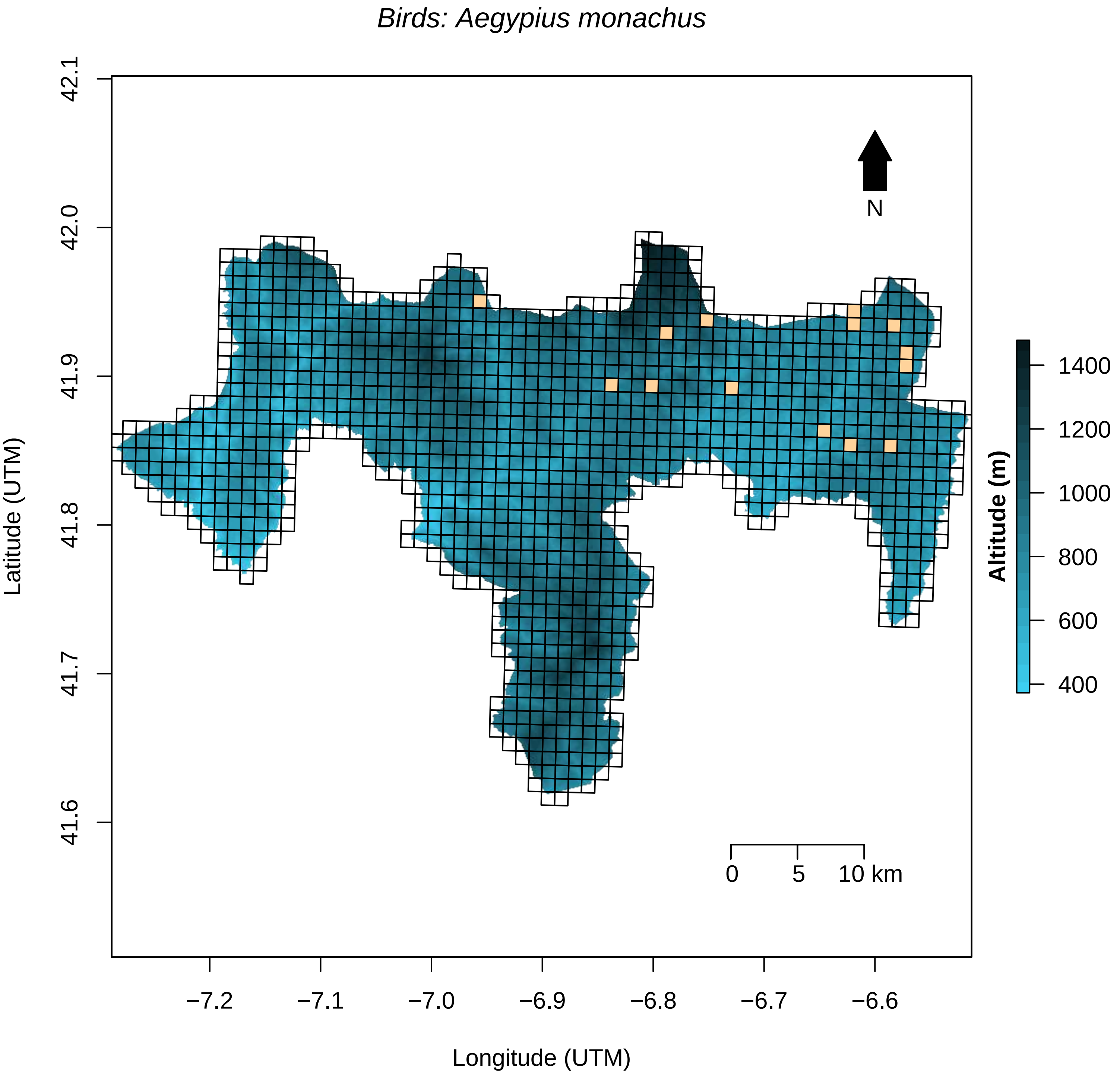
Birds: Aegypius monachus
42.1
42.0
41.9
41.8
41.7
41.6
Latitude (UTM)
−7.2
−7.1
−7.0
−6.9
−6.8
−6.7
−6.6
Longitude (UTM)
N
0
5
10 km
Altitude (m)
1400
1200
1000
800
600
400

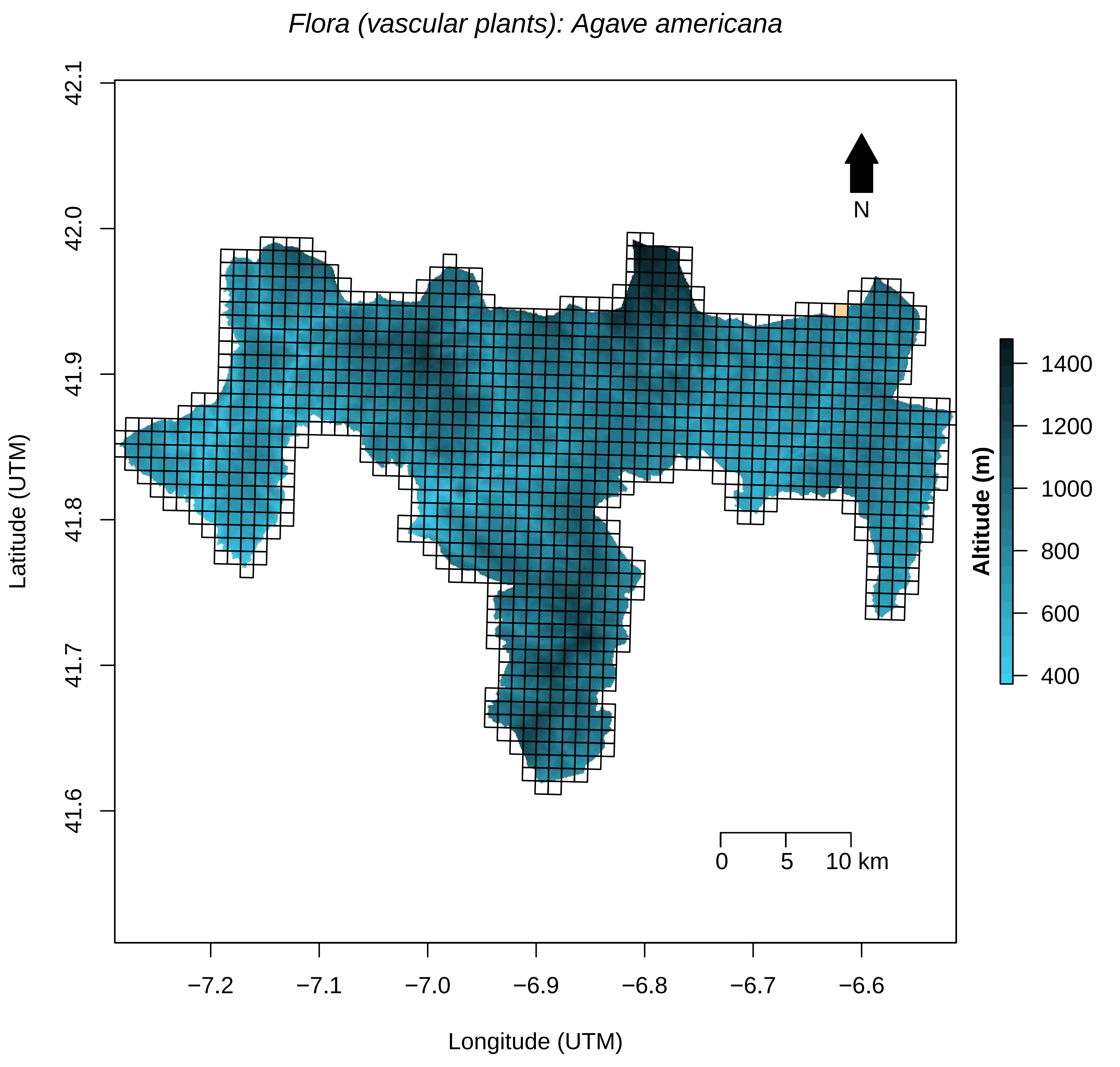

Flora (vascular plants): Agave americana
N
0
5
10 km
Altitude (m)
1400
1200
1000
800
600
400
Latitude (UTM)
42.1
42.0
41.9
41.8
41.7
41.6
Longitude (UTM)
−7.2
−7.1
−7.0
−6.9
−6.8
−6.7
−6.6

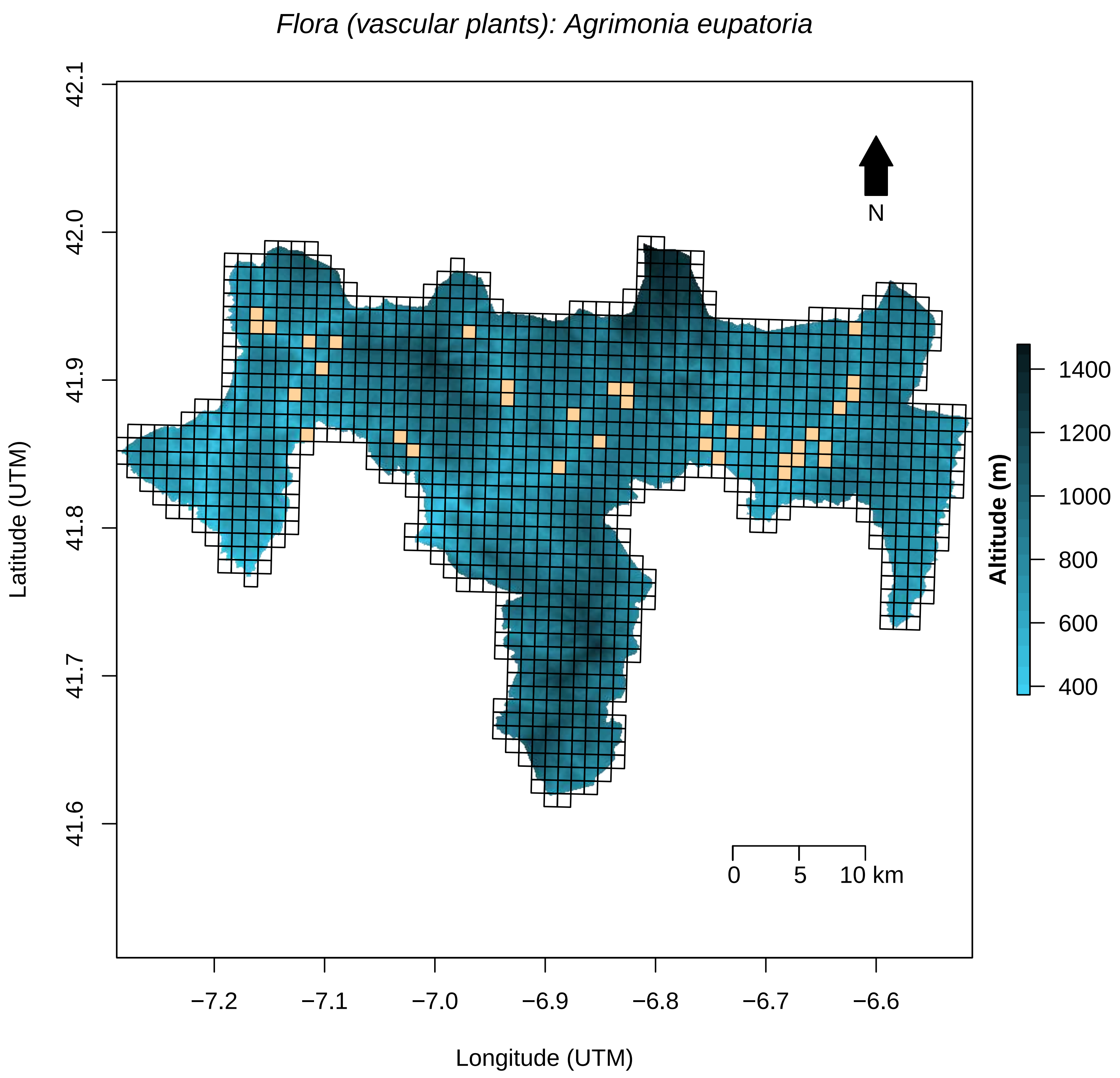

Flora (vascular plants): Agrimonia eupatoria
Latitude (UTM)
Longitude (UTM)
42.1
42.0
41.9
41.8
41.7
41.6
−7.2
−7.1
−7.0
−6.9
−6.8
−6.7
−6.6
N
0
5
10 km
Altitude (m)
1400
1200
1000
800
600
400

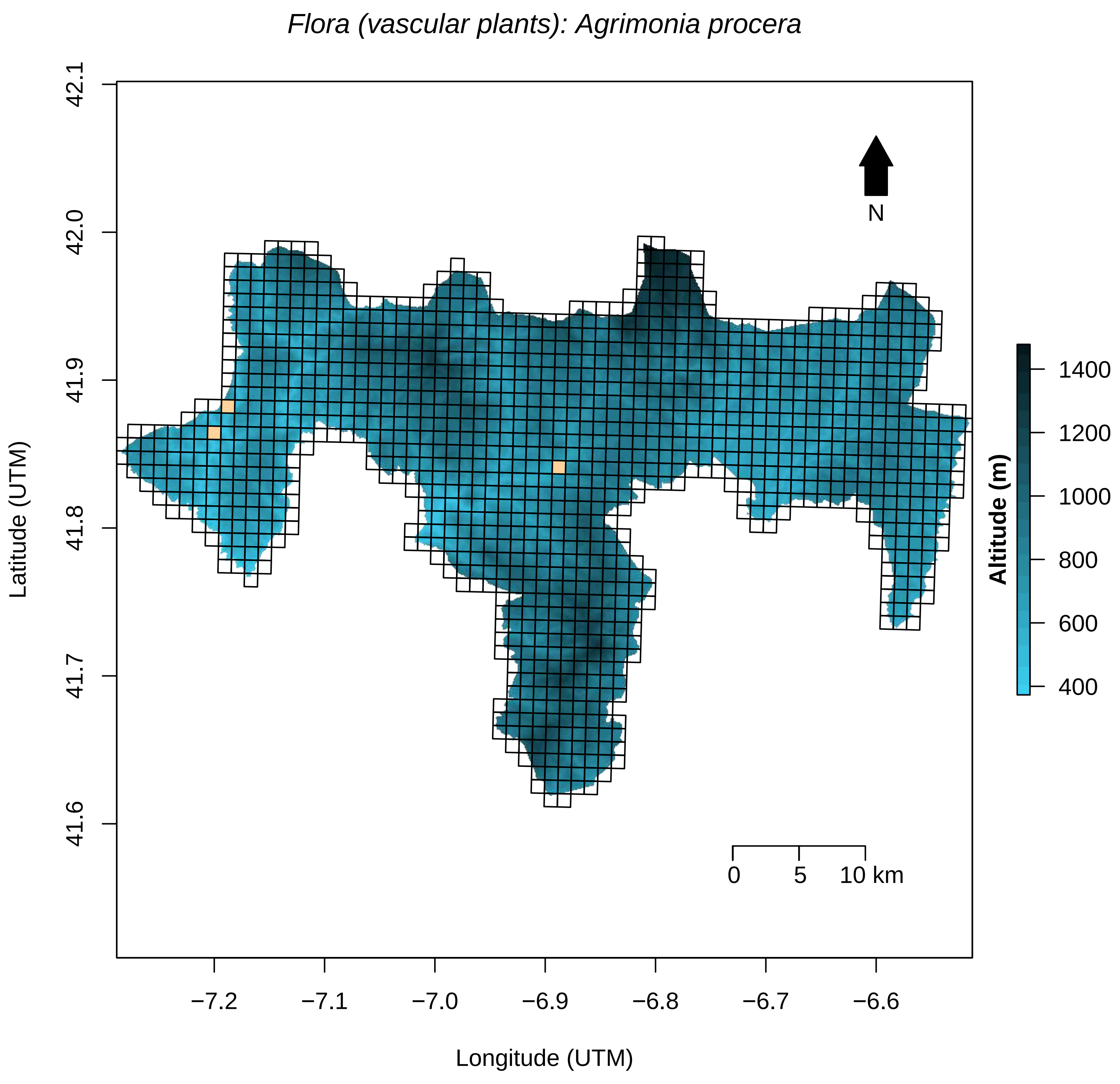
Flora (vascular plants): Agrimonia procera
42.1
42.0
41.9
41.8
41.7
41.6
Latitude (UTM)
N
0 5 10 km
−7.2
−7.1
−7.0
−6.9
−6.8
−6.7
−6.6
Longitude (UTM)
1400
1200
1000
800
600
400
Altitude (m)

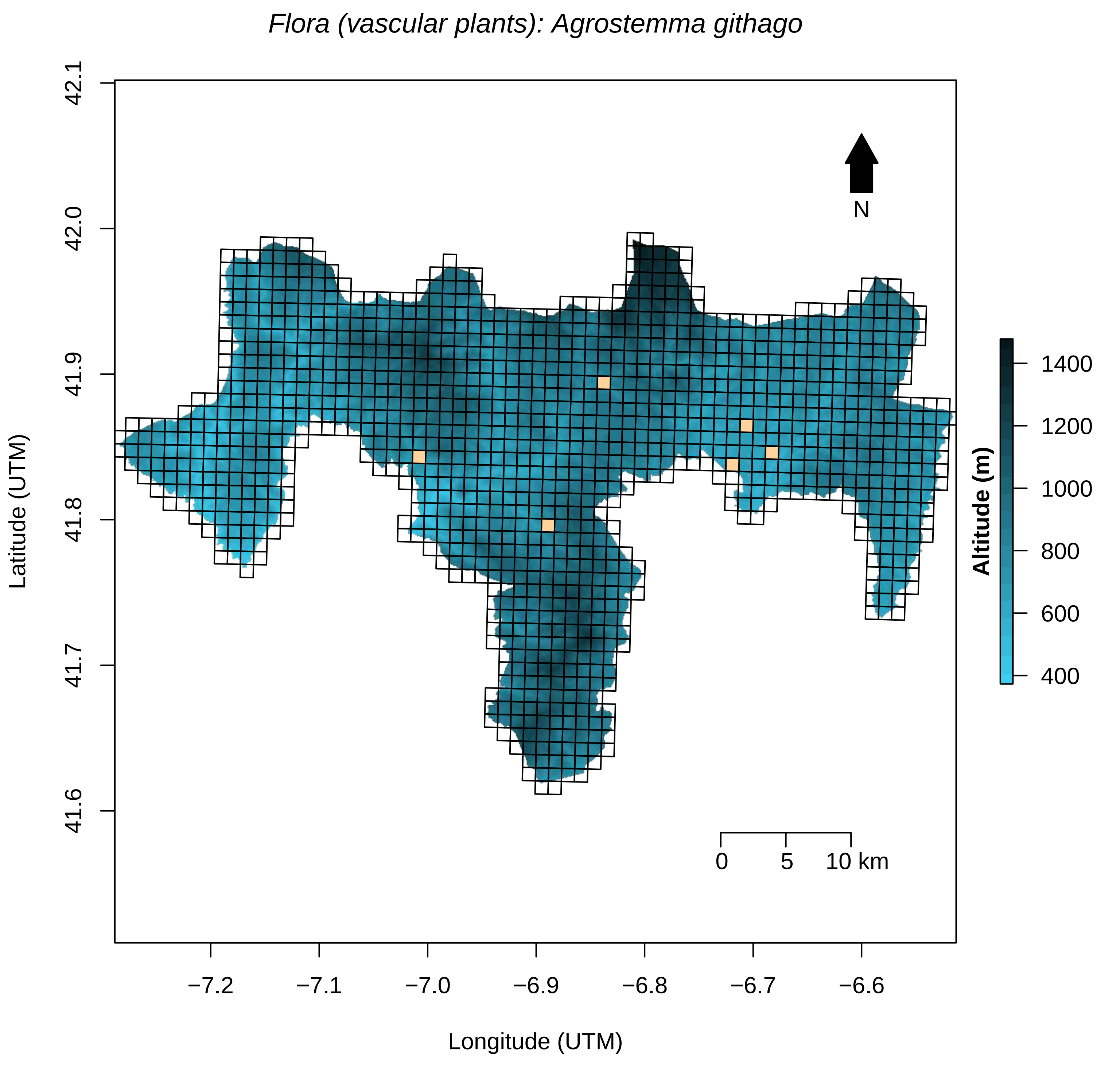
Flora (vascular plants): Agrostemma githago
N
0
5
10 km
Altitude (m)
1400
1200
1000
800
600
400
Latitude (UTM)
42.1
42.0
41.9
41.8
41.7
41.6
Longitude (UTM)
−7.2
−7.1
−7.0
−6.9
−6.8
−6.7
−6.6

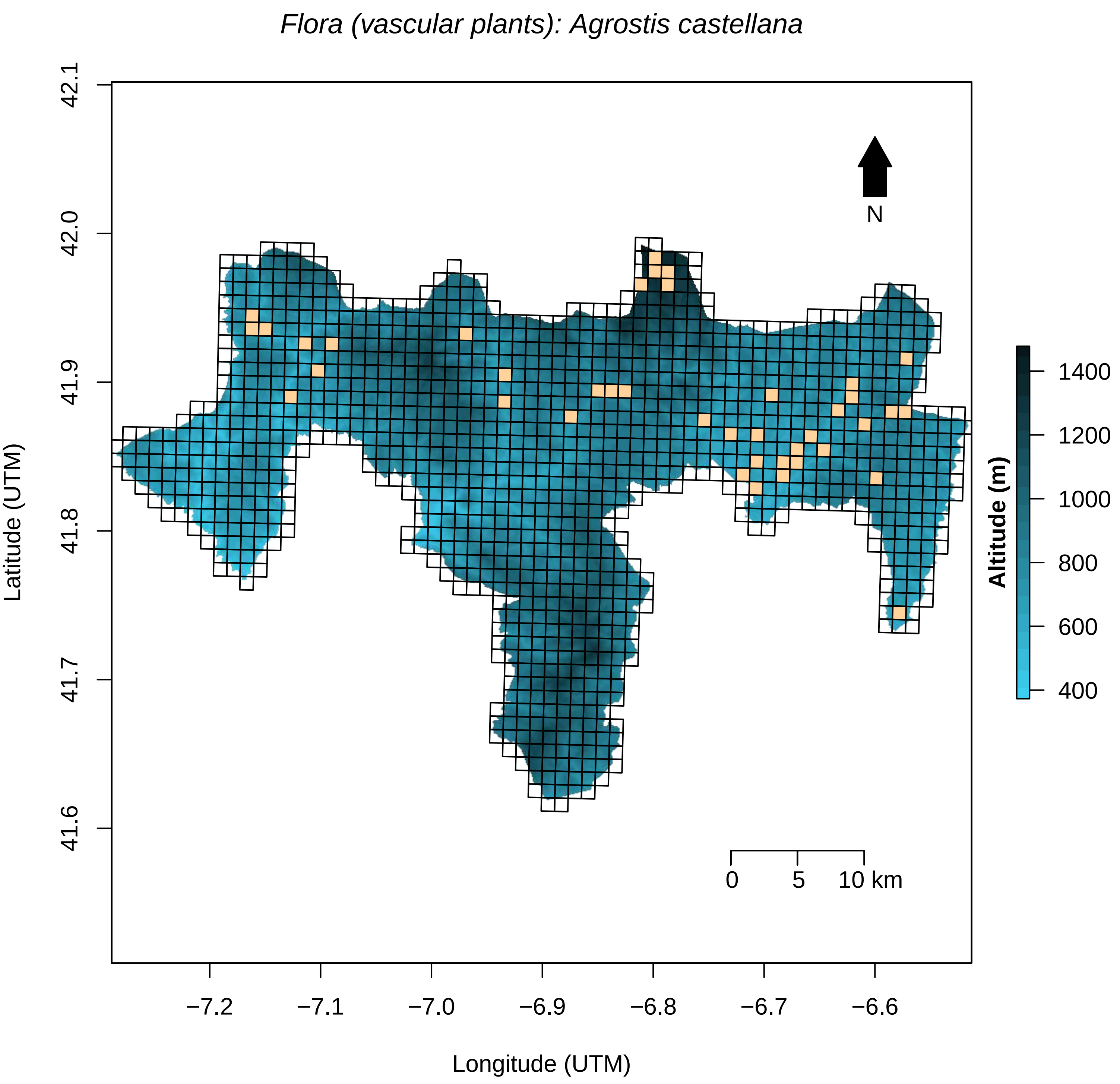

Flora (vascular plants): Agrostis castellana
Latitude (UTM)
Longitude (UTM)
42.1
42.0
41.9
41.8
41.7
41.6
−7.2
−7.1
−7.0
−6.9
−6.8
−6.7
−6.6
N
0
5
10 km
Altitude (m)
1400
1200
1000
800
600
400

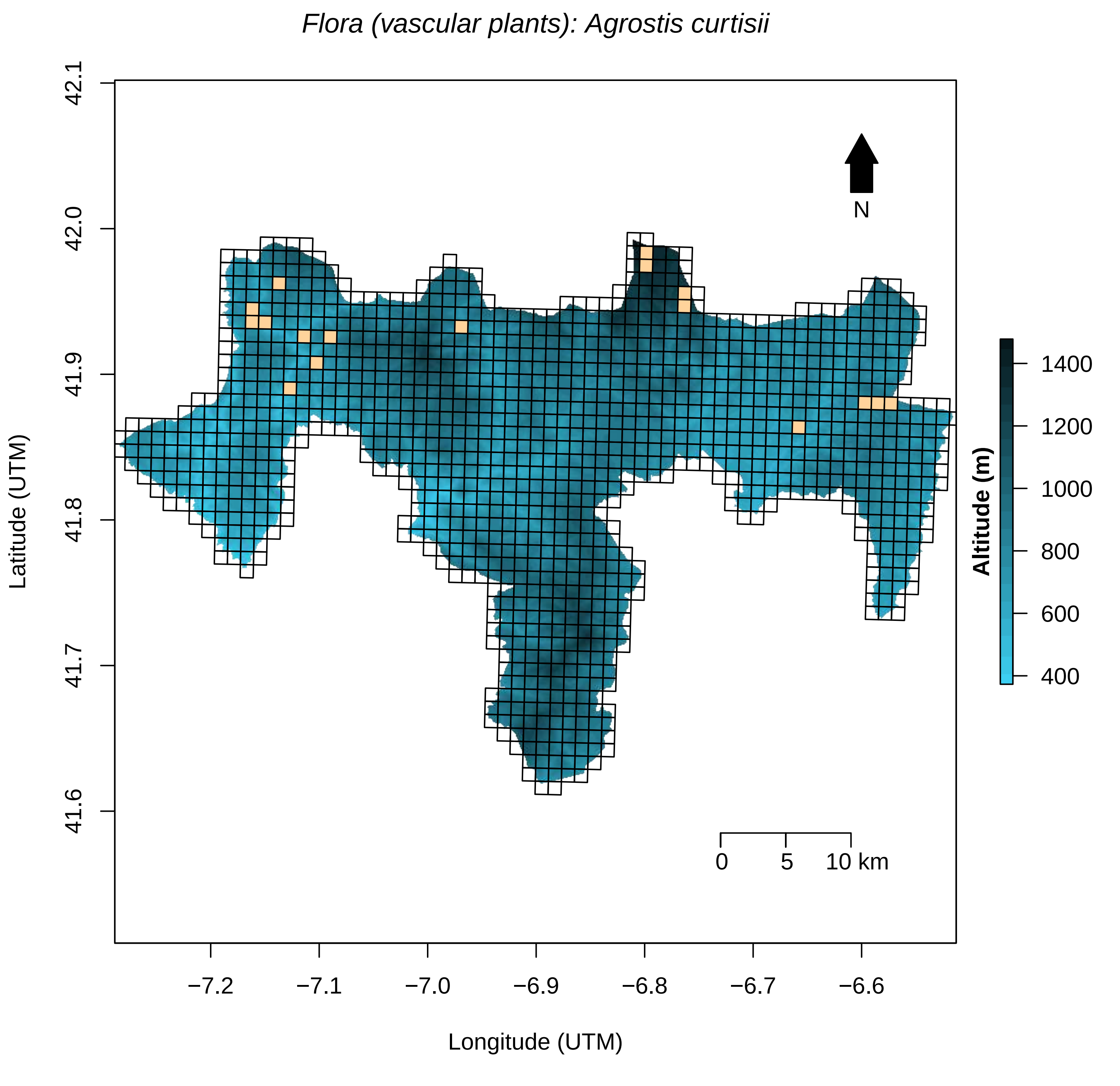

Flora (vascular plants): Agrostis curtisii
N
0
5
10 km
Altitude (m)
1400
1200
1000
800
600
400
42.1
42.0
41.9
41.8
41.7
41.6
Latitude (UTM)
−7.2
−7.1
−7.0
−6.9
−6.8
−6.7
−6.6
Longitude (UTM)

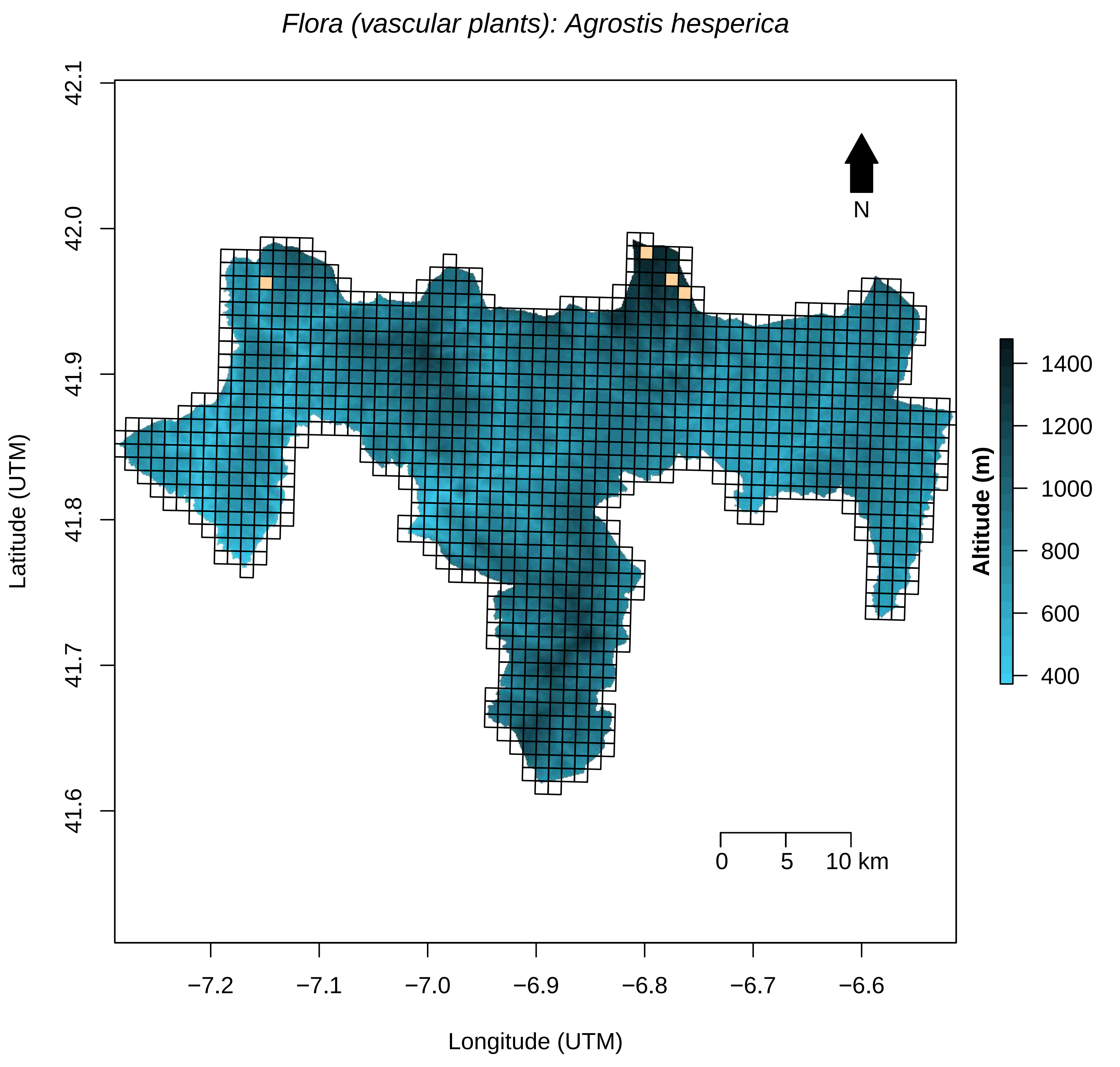

Flora (vascular plants): Agrostis hesperica
42.1
42.0
41.9
41.8
41.7
41.6
Latitude (UTM)
−7.2
−7.1
−7.0
−6.9
−6.8
−6.7
−6.6
Longitude (UTM)
N
0
5
10 km
Altitude (m)
1400
1200
1000
800
600
400

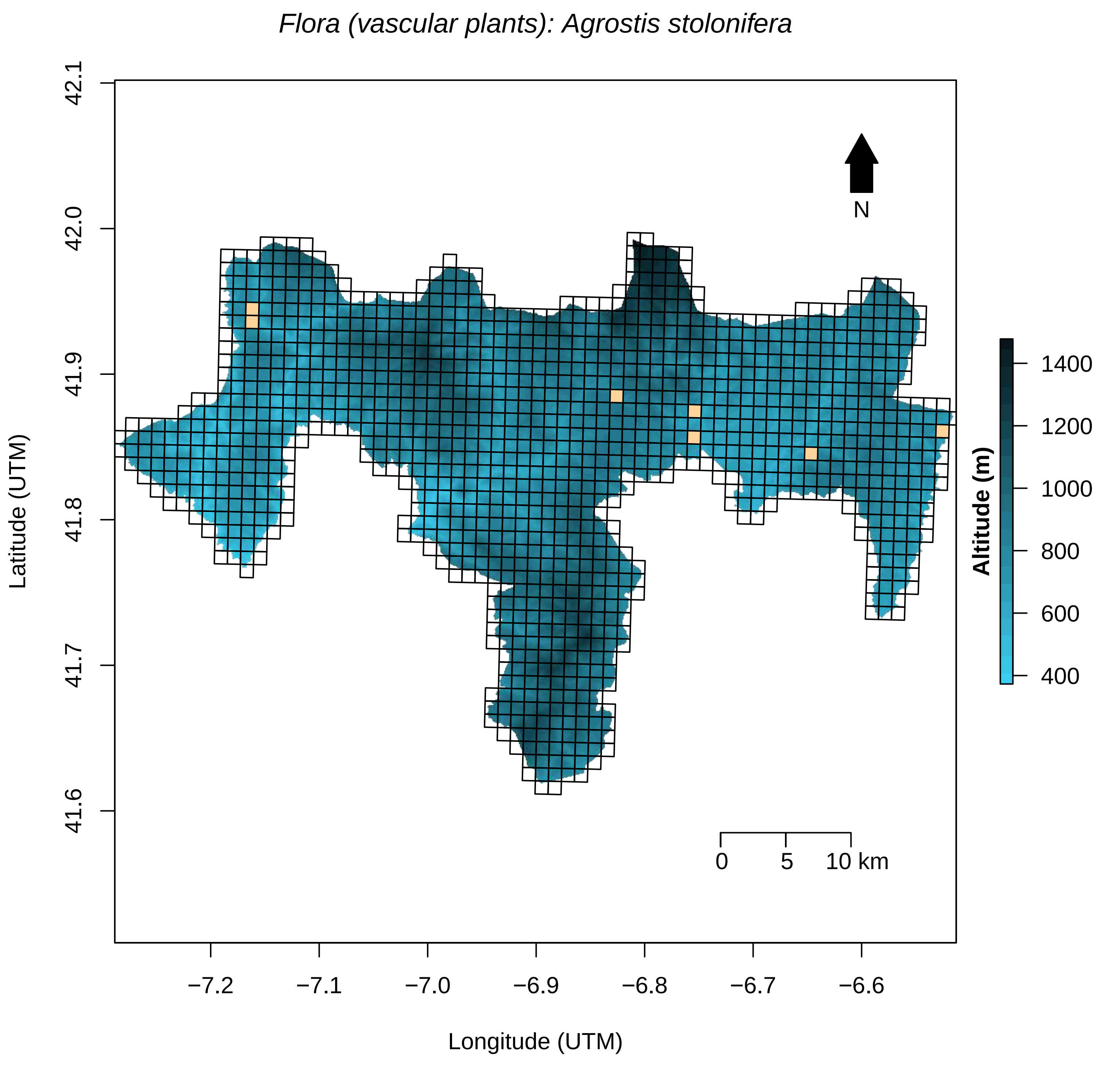

Flora (vascular plants): Agrostis stolonifera
N
0
5
10 km
Latitude (UTM)
Longitude (UTM)
Altitude (m)
42.1
42.0
41.9
41.8
41.7
41.6
−7.2
−7.1
−7.0
−6.9
−6.8
−6.7
−6.6
1400
1200
1000
800
600
400

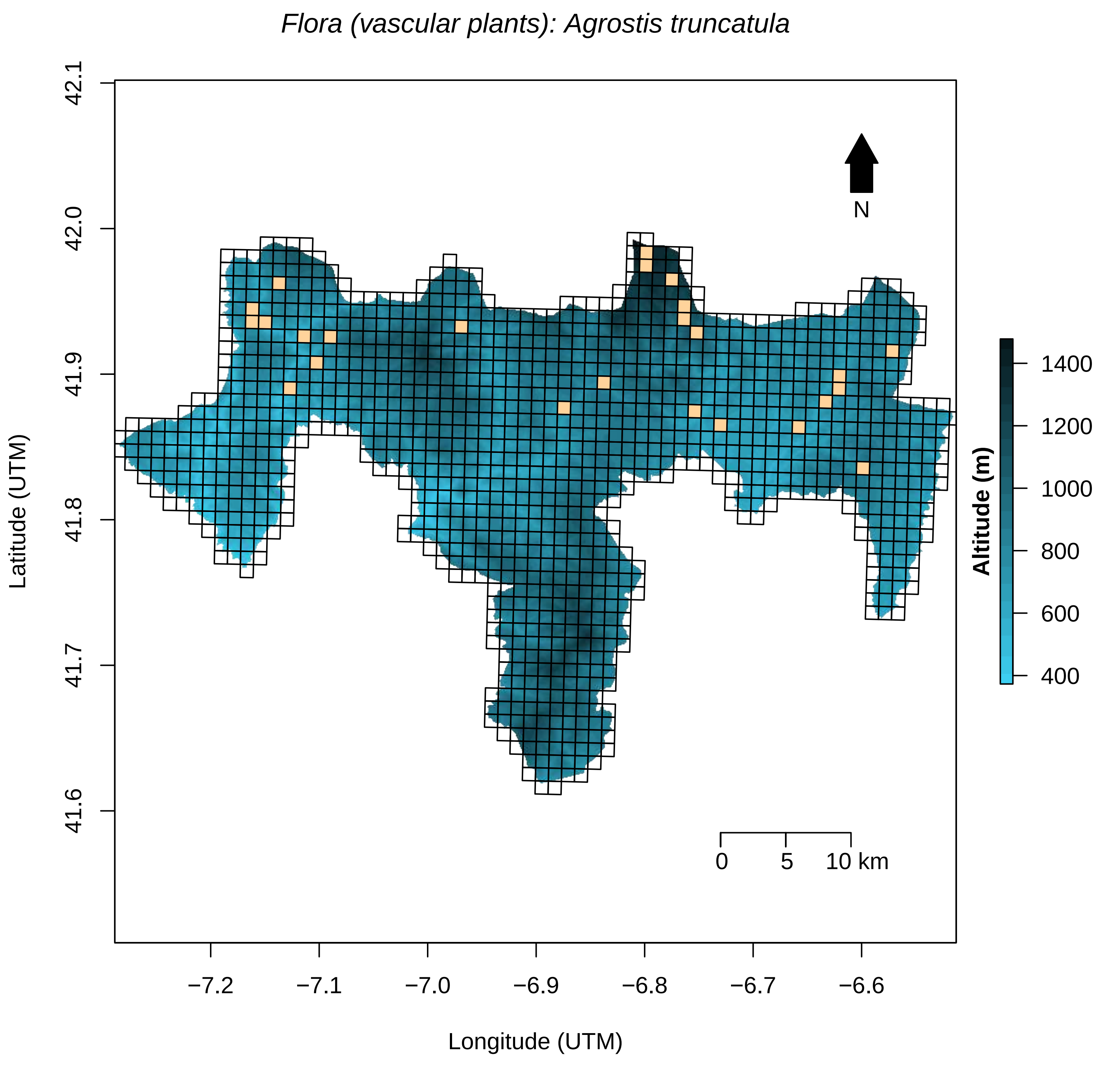

Flora (vascular plants): Agrostis truncatula
42.1
42.0
41.9
41.8
41.7
41.6
Latitude (UTM)
−7.2
−7.1
−7.0
−6.9
−6.8
−6.7
−6.6
Longitude (UTM)
N
0
5
10 km
Altitude (m)
1400
1200
1000
800
600
400

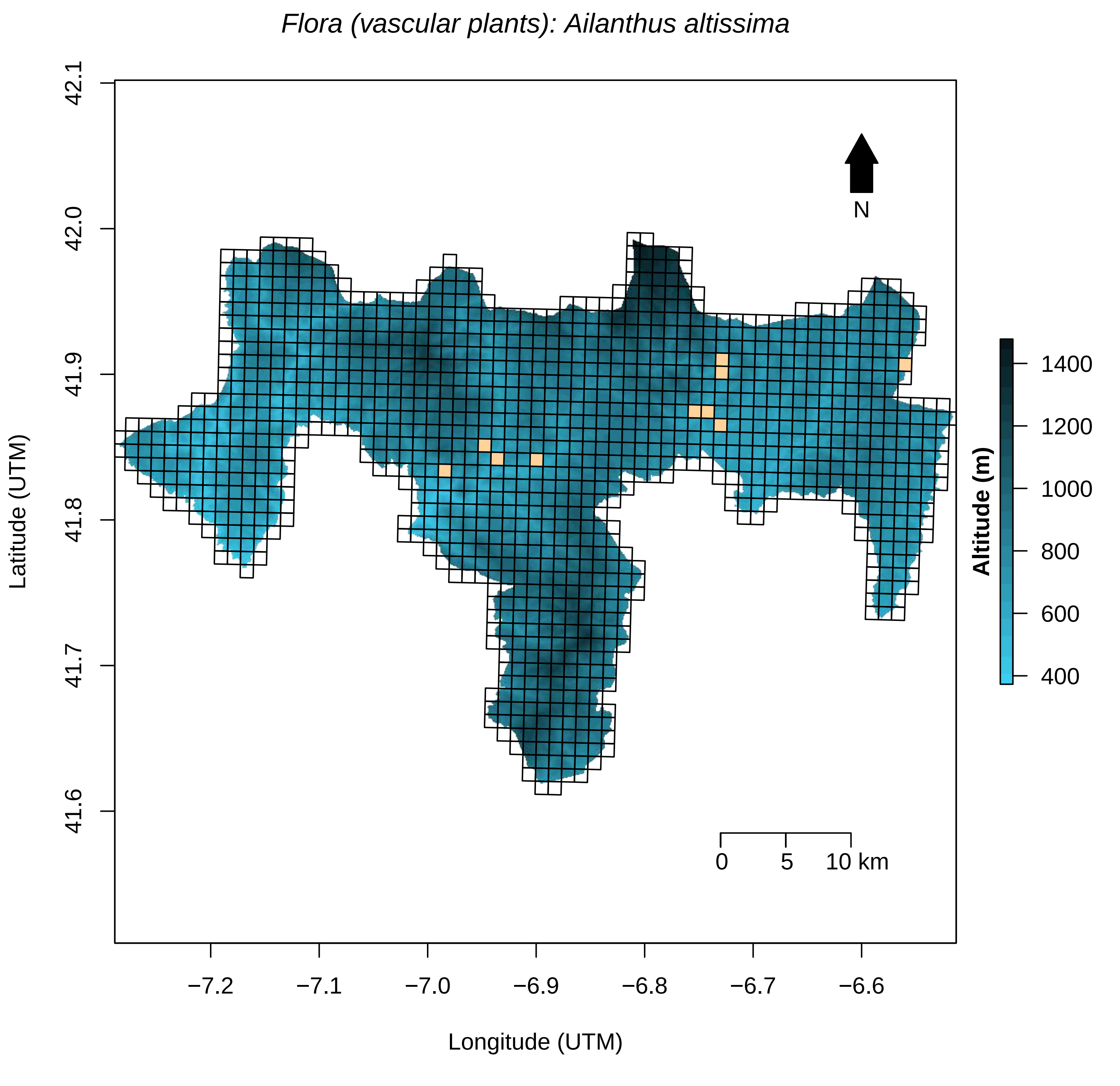

Flora (vascular plants): Ailanthus altissima
42.1
42.0
41.9
41.8
41.7
41.6
Latitude (UTM)
N
0
5
10 km
−7.2
−7.1
−7.0
−6.9
−6.8
−6.7
−6.6
Longitude (UTM)
Altitude (m)
1400
1200
1000
800
600
400

Flora (vascular plants): Aira caryophyllea

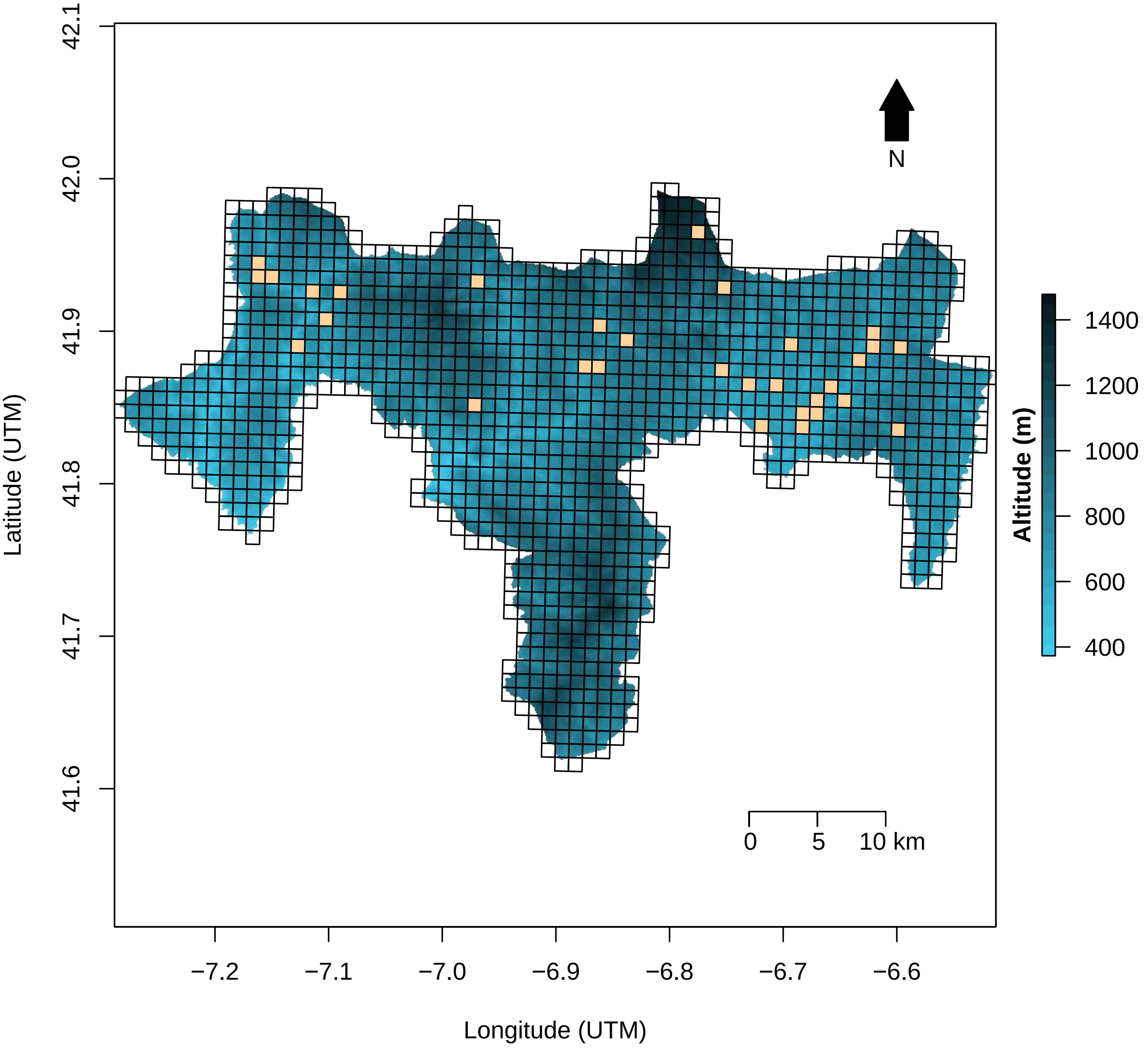

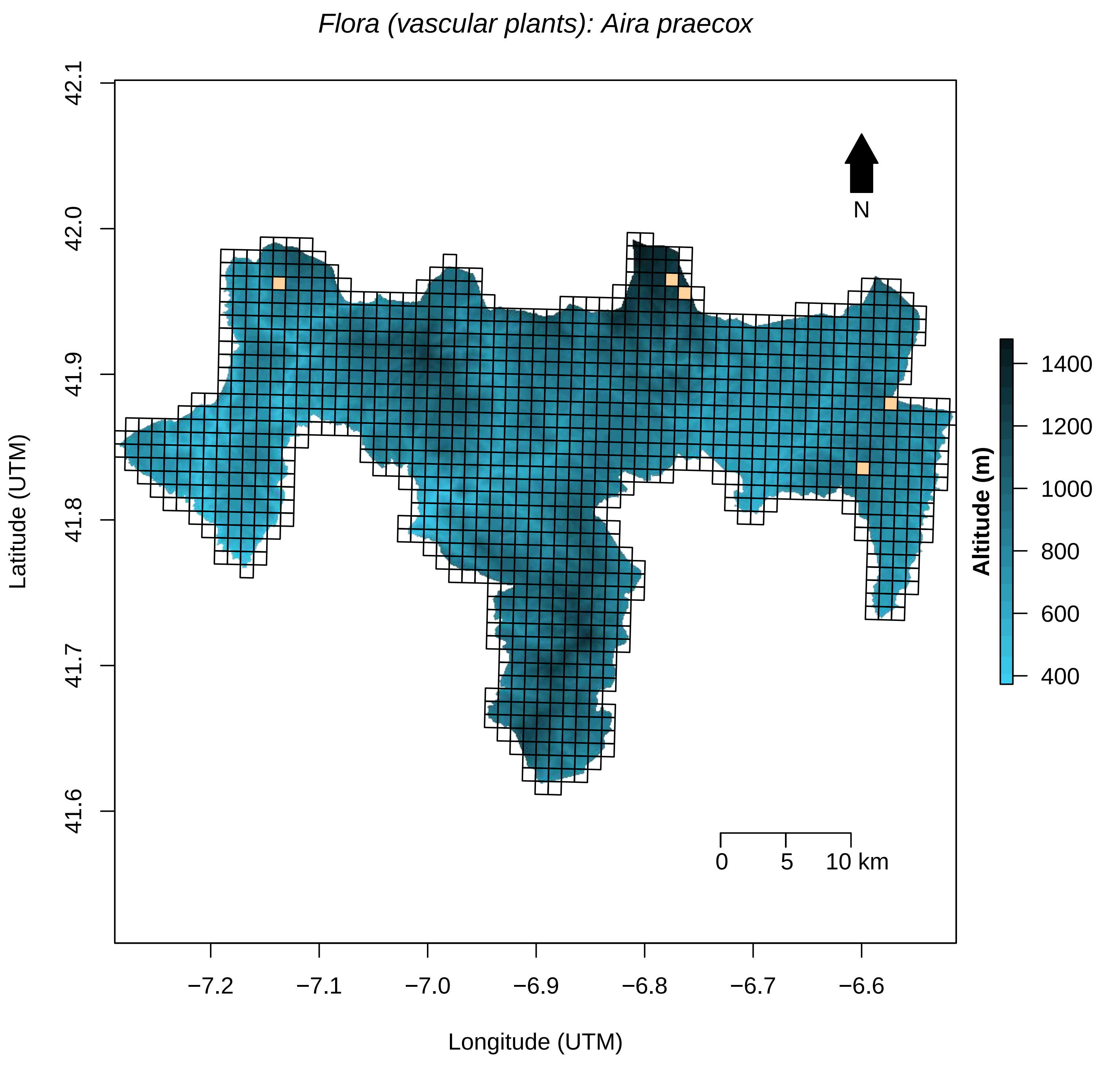

Flora (vascular plants): Aira praecox
42.1
42.0
41.9
41.8
41.7
41.6
Latitude (UTM)
−7.2
−7.1
−7.0
−6.9
−6.8
−6.7
−6.6
Longitude (UTM)
N
0
5
10 km
Altitude (m)
1400
1200
1000
800
600
400

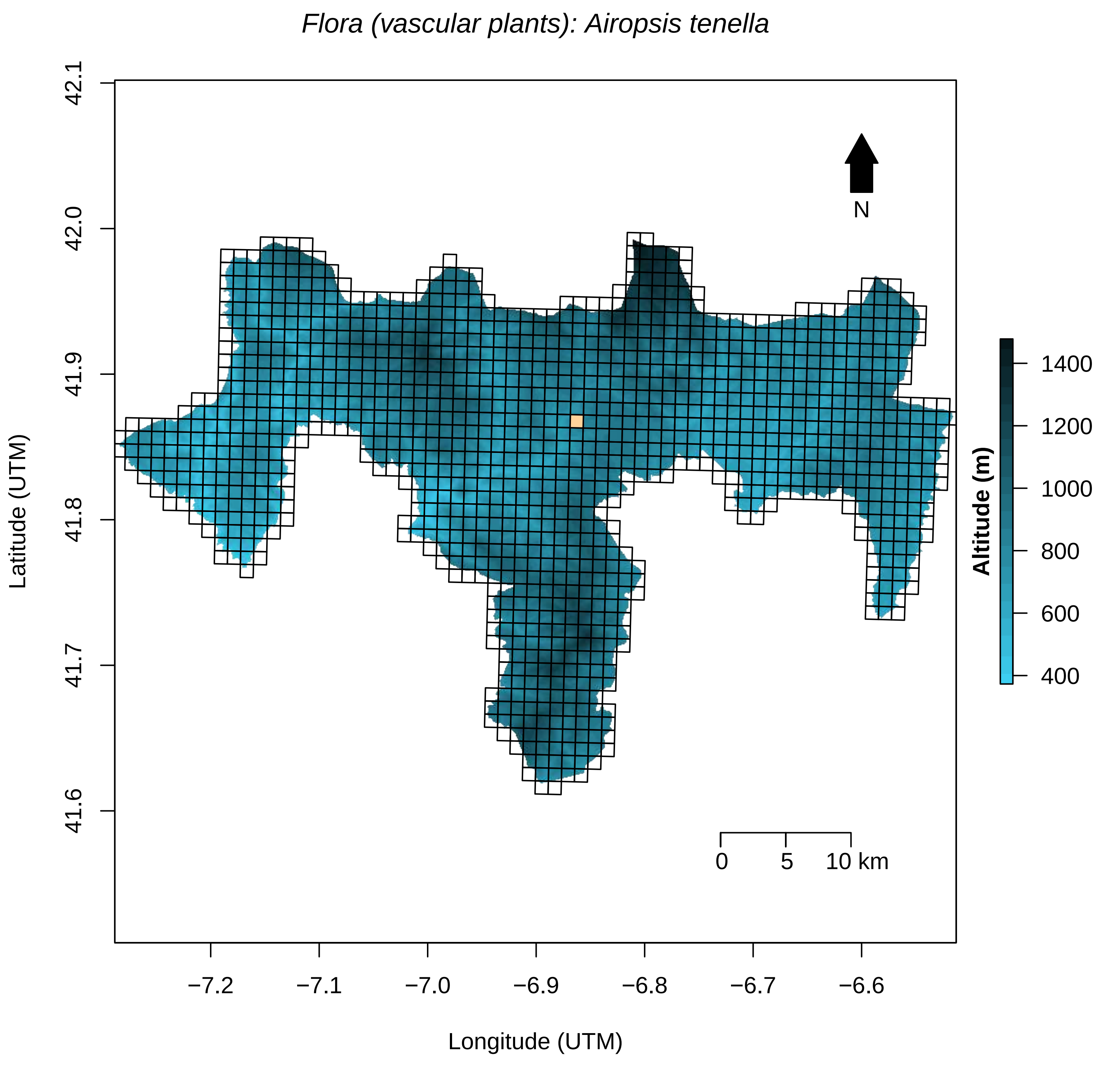

Flora (vascular plants): Airopsis tenella
42.1
42.0
41.9
41.8
41.7
41.6
Latitude (UTM)
N
0
5
10 km
−7.2
−7.1
−7.0
−6.9
−6.8
−6.7
−6.6
Longitude (UTM)
1400
1200
1000
800
600
400
Altitude (m)

Flora (vascular plants): Ajuga pyramidalis

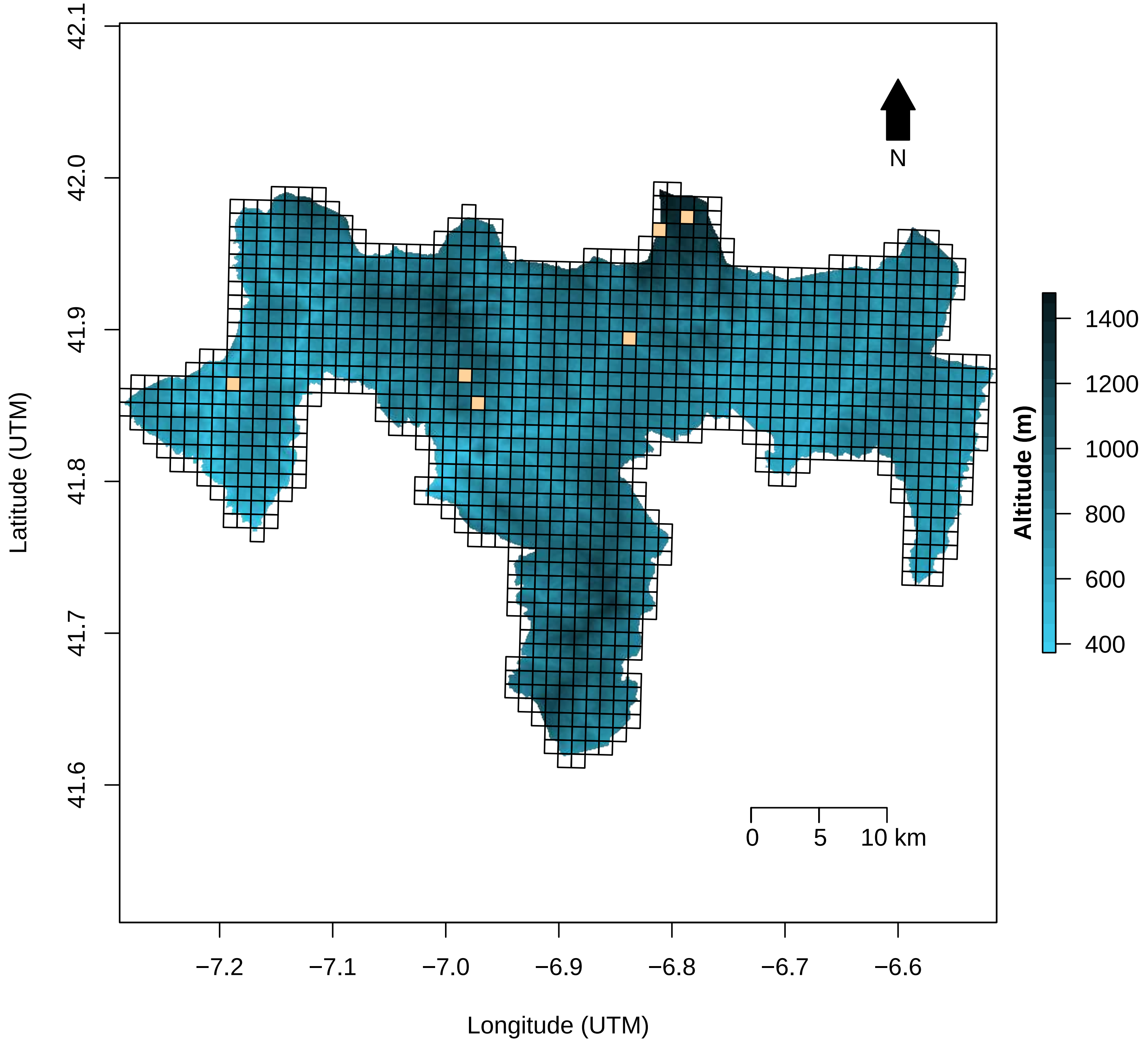

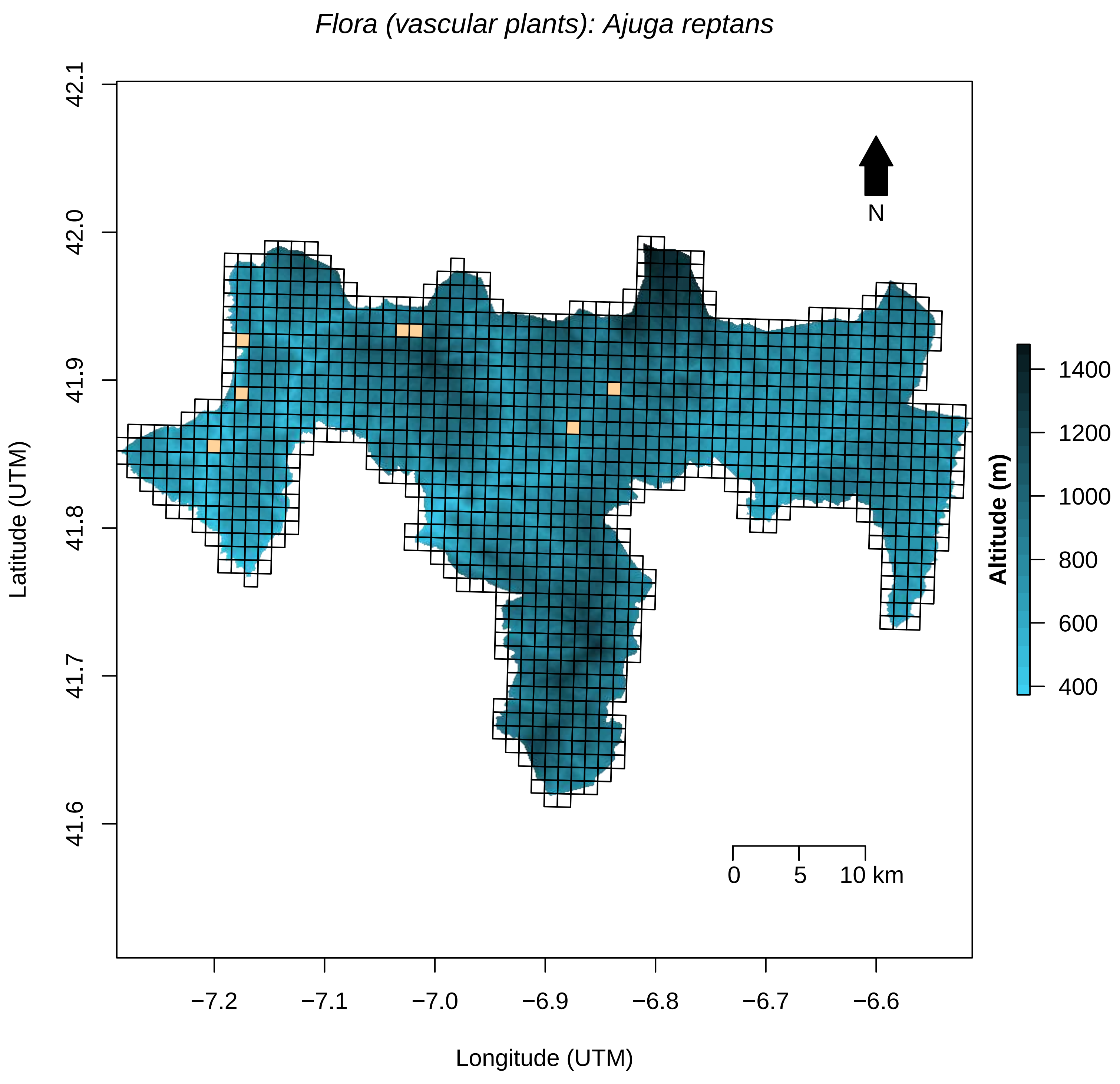

Flora (vascular plants): Ajuga reptans
42.1
42.0
41.9
41.8
41.7
41.6
Latitude (UTM)
−7.2
−7.1
−7.0
−6.9
−6.8
−6.7
−6.6
Longitude (UTM)
N
0
5
10 km
Altitude (m)
1400
1200
1000
800
600
400

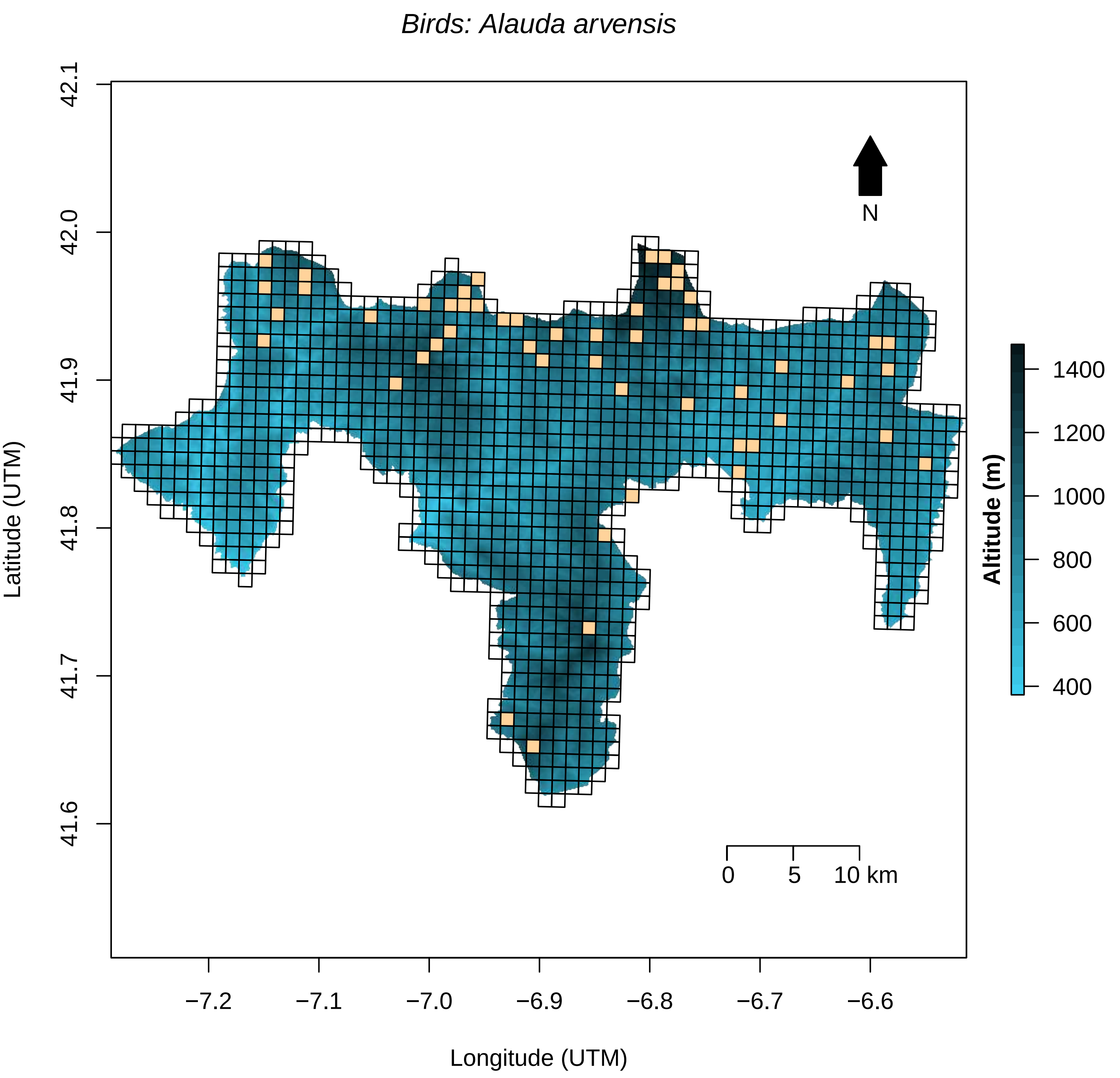

Birds: Alauda arvensis
42.1
42.0
41.9
41.8
41.7
41.6
Latitude (UTM)
−7.2
−7.1
−7.0
−6.9
−6.8
−6.7
−6.6
Longitude (UTM)
N
0
5
10 km
1400
1200
1000
800
600
400
Altitude (m)

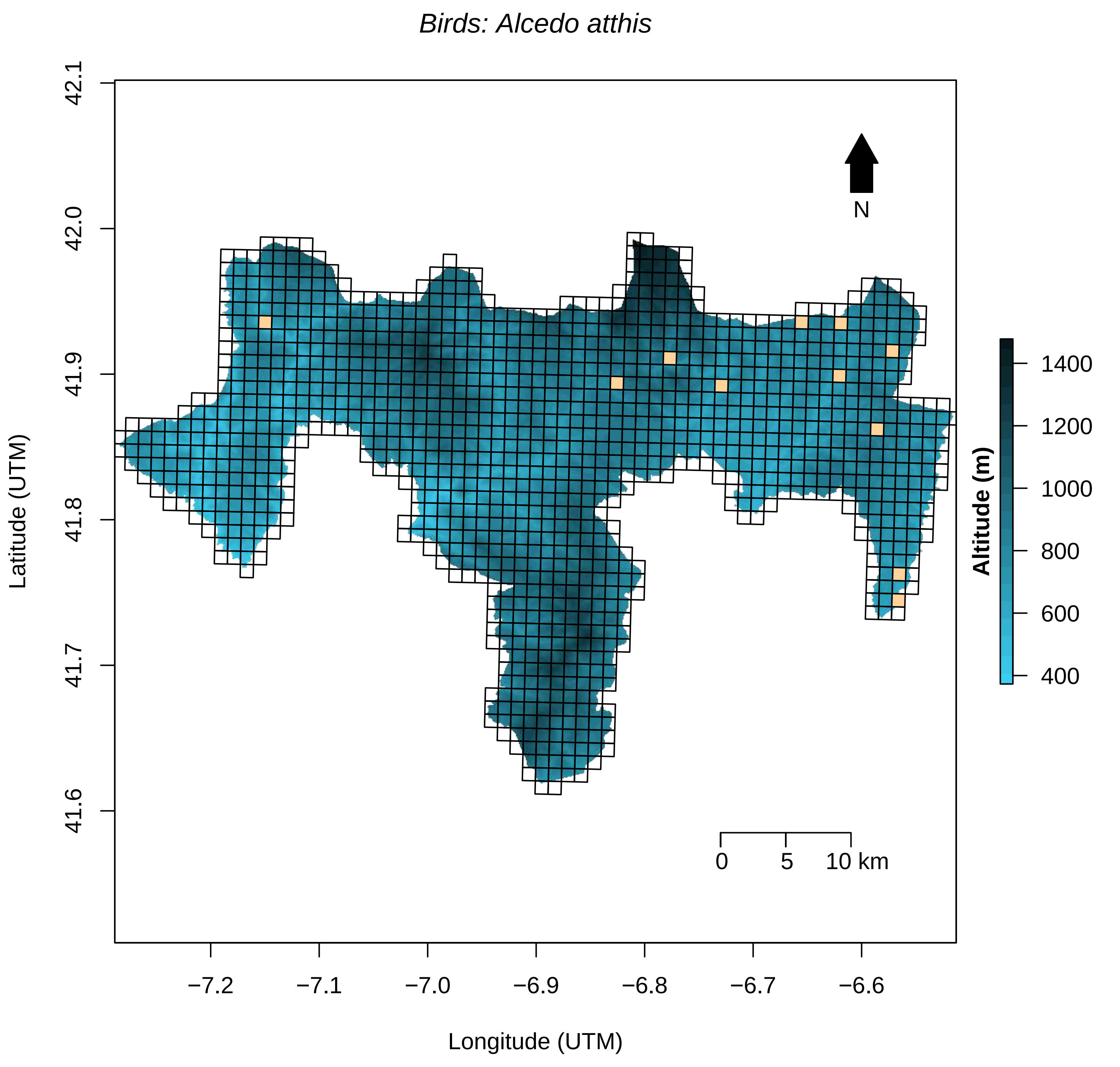

Birds: Alcedo atthis
42.1
42.0
41.9
41.8
41.7
41.6
Latitude (UTM)
N
0
5
10 km
−7.2
−7.1
−7.0
−6.9
−6.8
−6.7
−6.6
Longitude (UTM)
1400
1200
1000
800
600
400
Altitude (m)

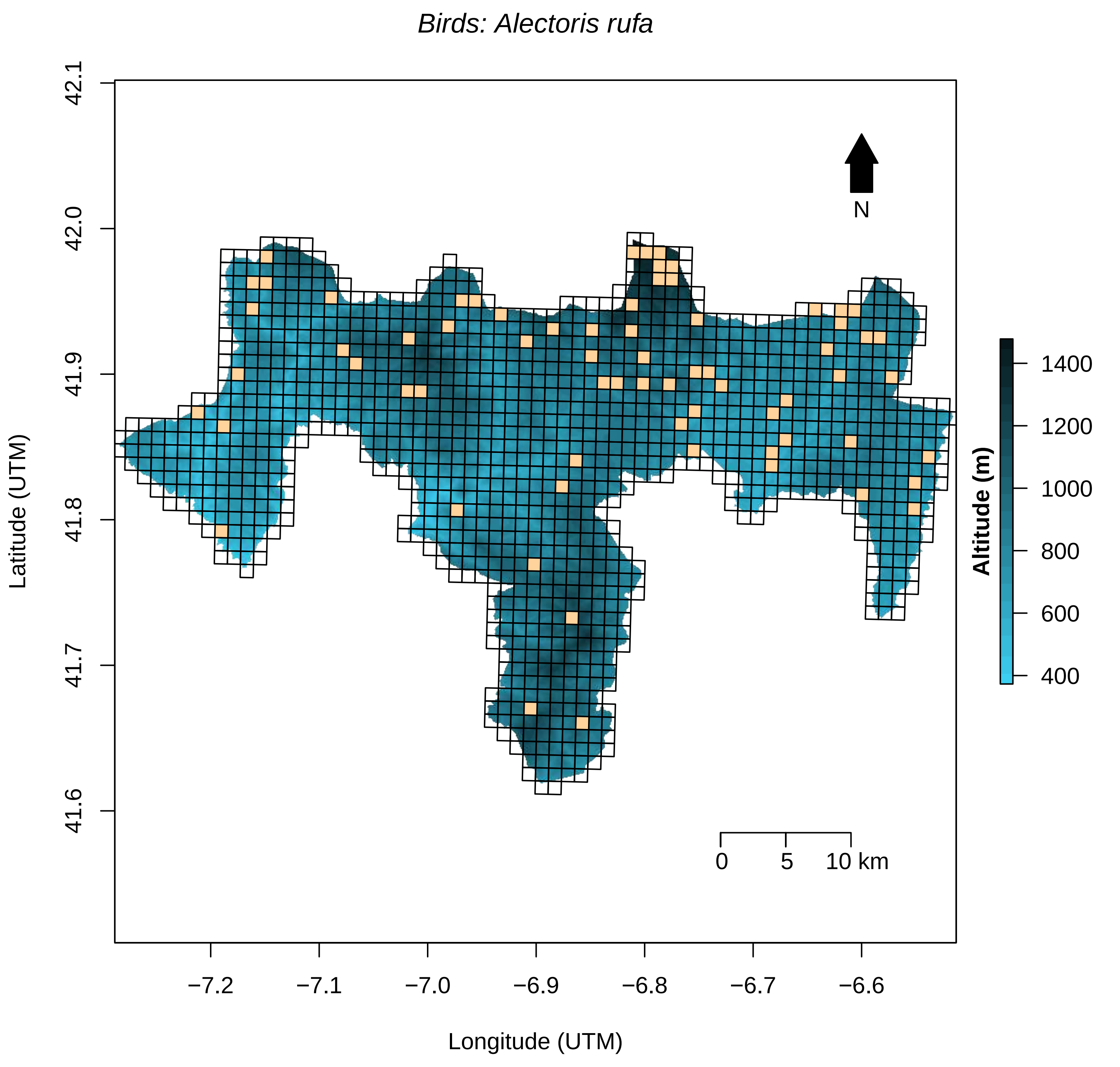

Birds: Alectoris rufa
42.1
42.0
41.9
41.8
41.7
41.6
Latitude (UTM)
−7.2
−7.1
−7.0
−6.9
−6.8
−6.7
−6.6
Longitude (UTM)
N
0
5
10 km
1400
1200
1000
800
600
400
Altitude (m)

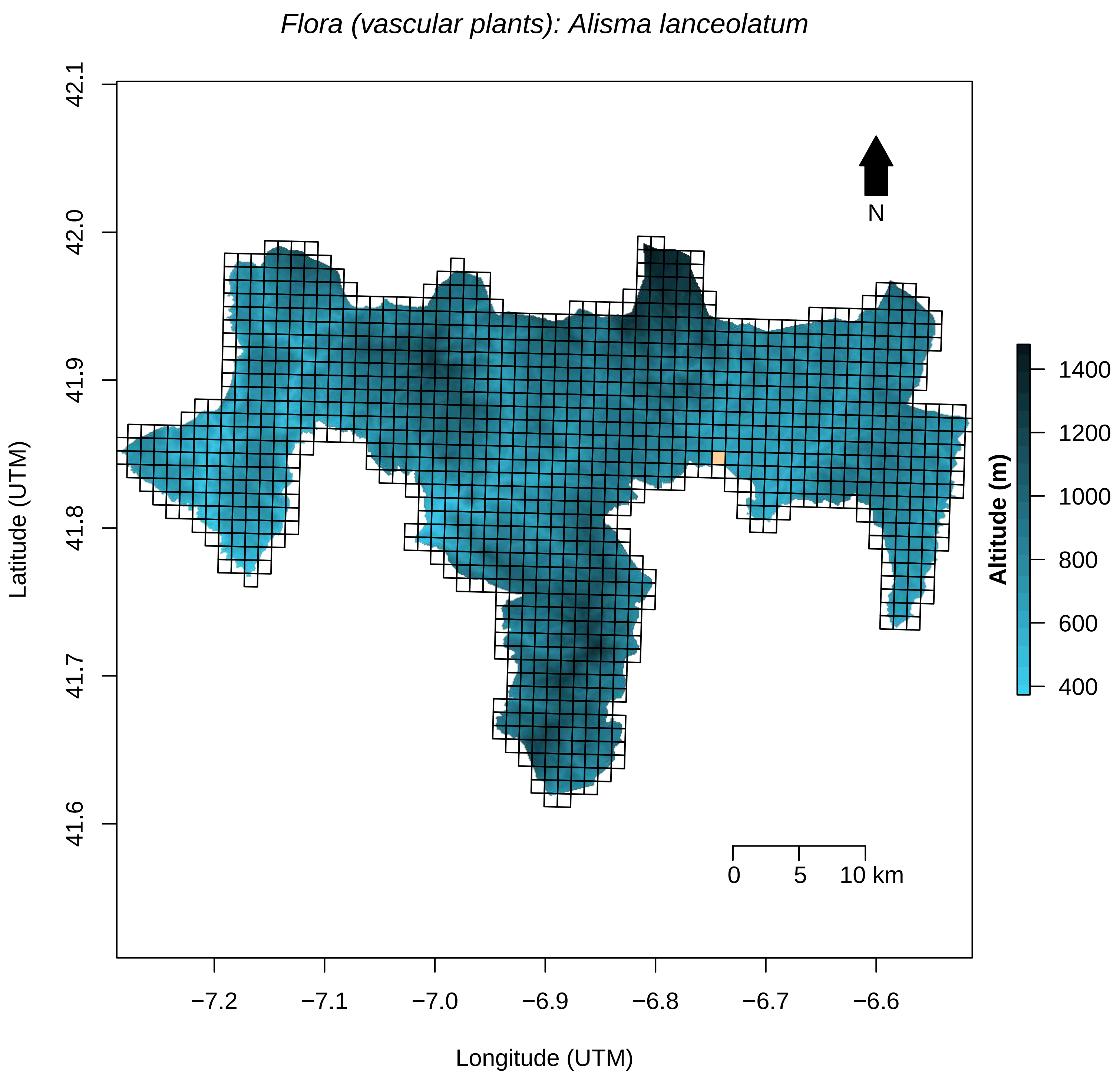

Flora (vascular plants): Alisma lanceolatum
Latitude (UTM)
42.1
42.0
41.9
41.8
41.7
41.6
−7.2
−7.1
−7.0
−6.9
−6.8
−6.7
−6.6
Longitude (UTM)
N
0
5
10 km
Altitude (m)
1400
1200
1000
800
600
400

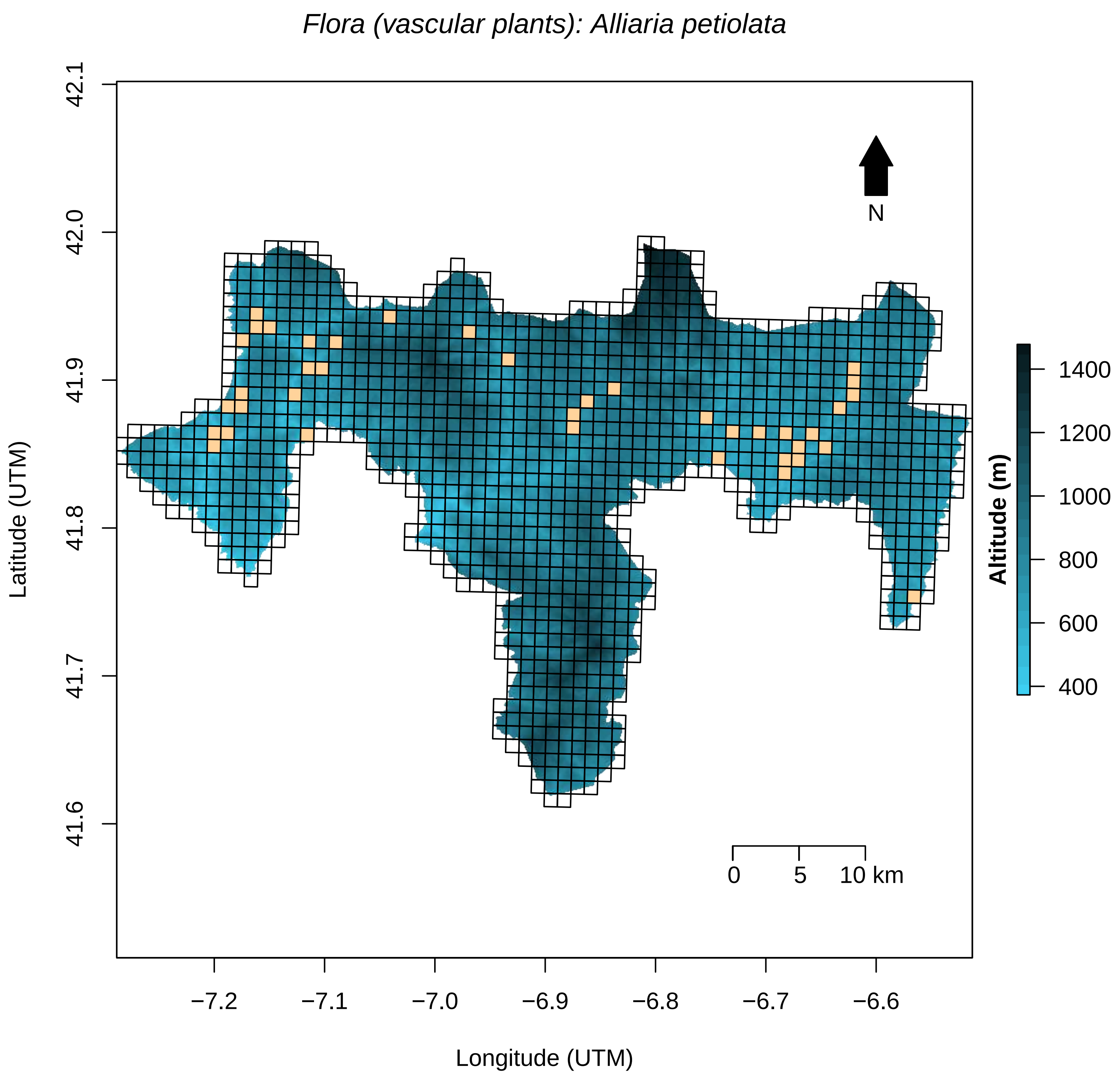
Flora (vascular plants): Alliaria petiolata
42.1
42.0
41.9
41.8
41.7
41.6
Latitude (UTM)
−7.2
−7.1
−7.0
−6.9
−6.8
−6.7
−6.6
Longitude (UTM)
N
0
5
10 km
1400
1200
1000
800
600
400
Altitude (m)

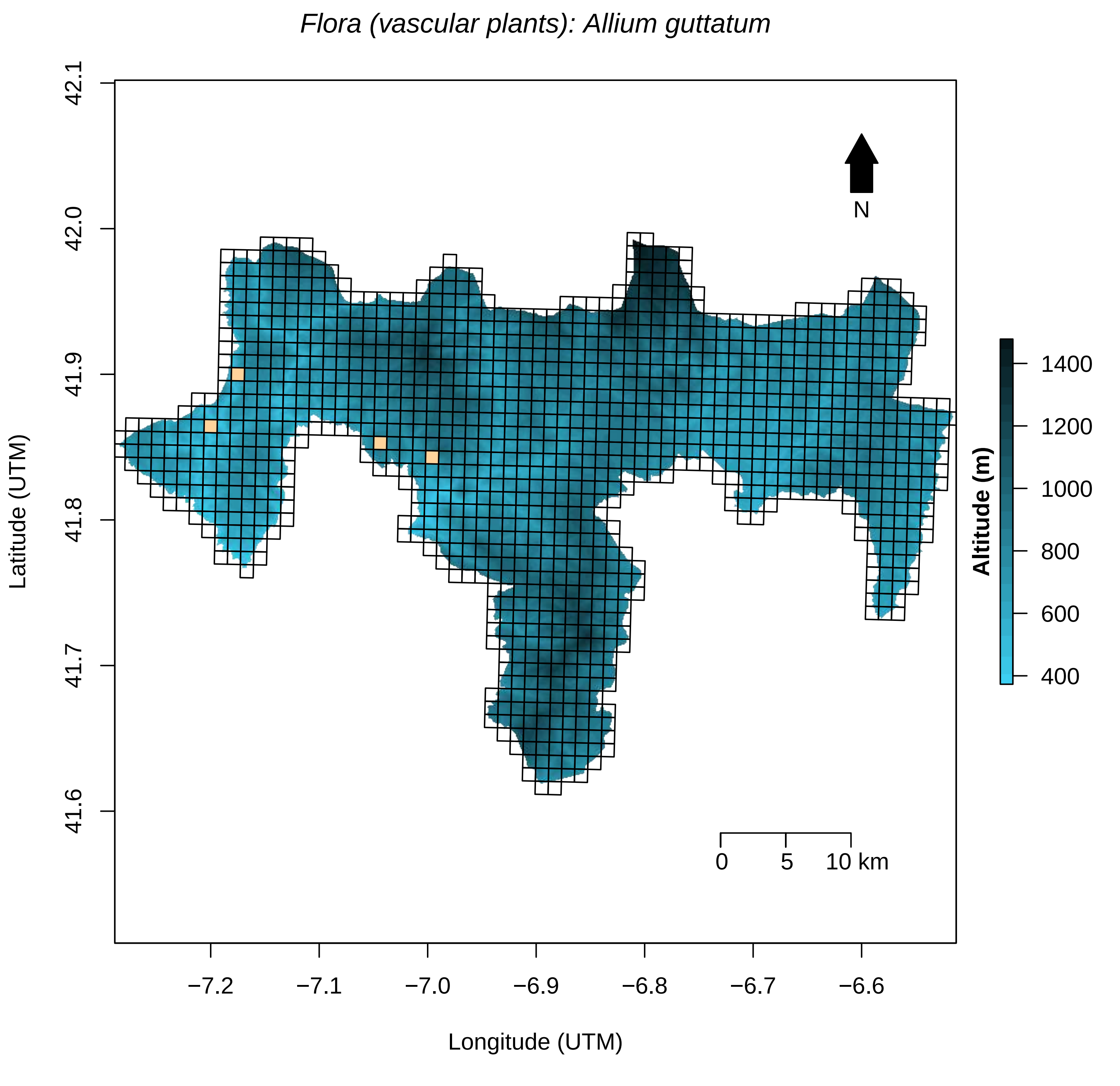

Flora (vascular plants): Allium guttatum
42.1
42.0
41.9
41.8
41.7
41.6
Latitude (UTM)
−7.2
−7.1
−7.0
−6.9
−6.8
−6.7
−6.6
Longitude (UTM)
N
0
5
10 km
Altitude (m)
1400
1200
1000
800
600
400

Flora (vascular plants): Allium oleraceum

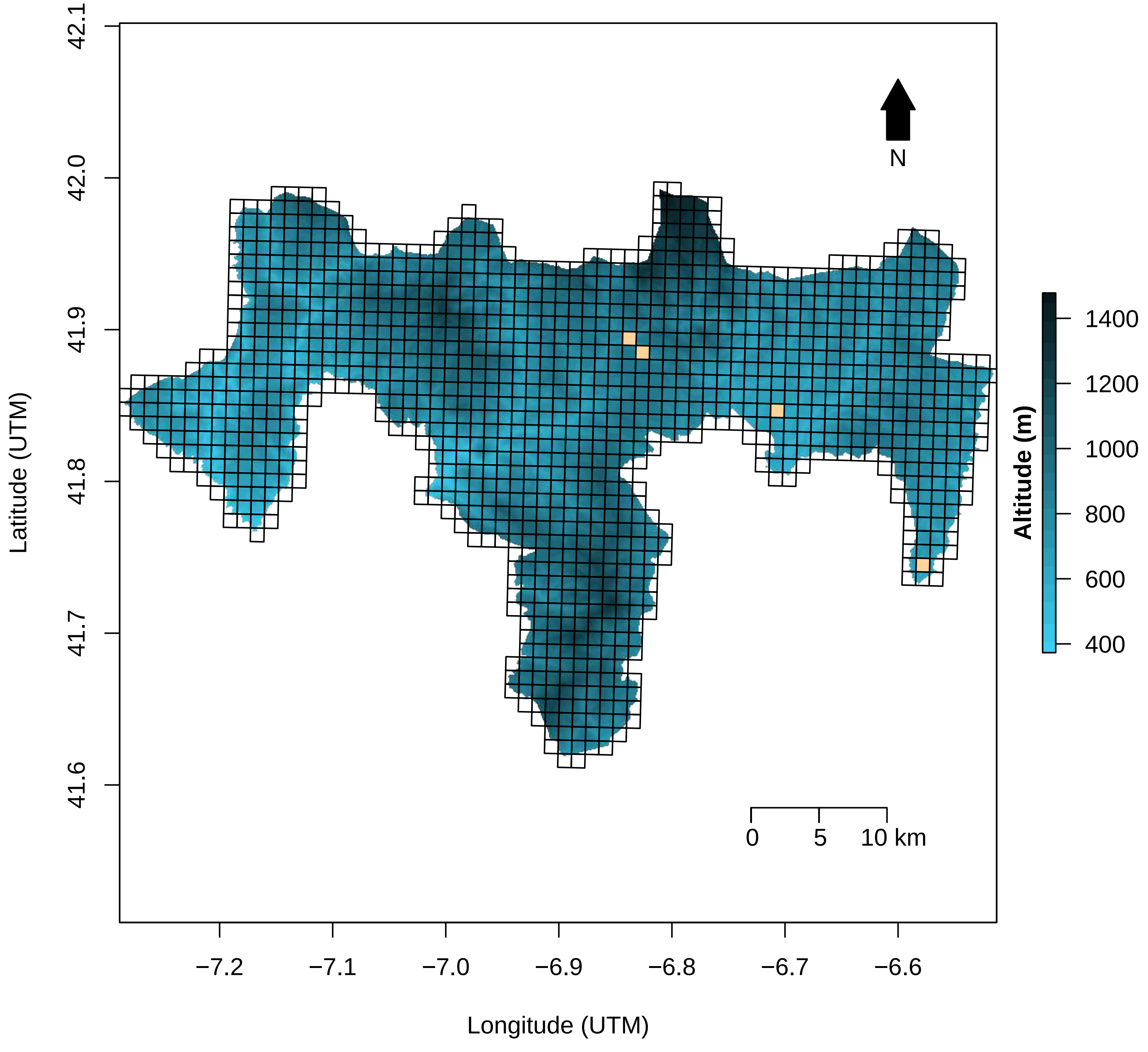

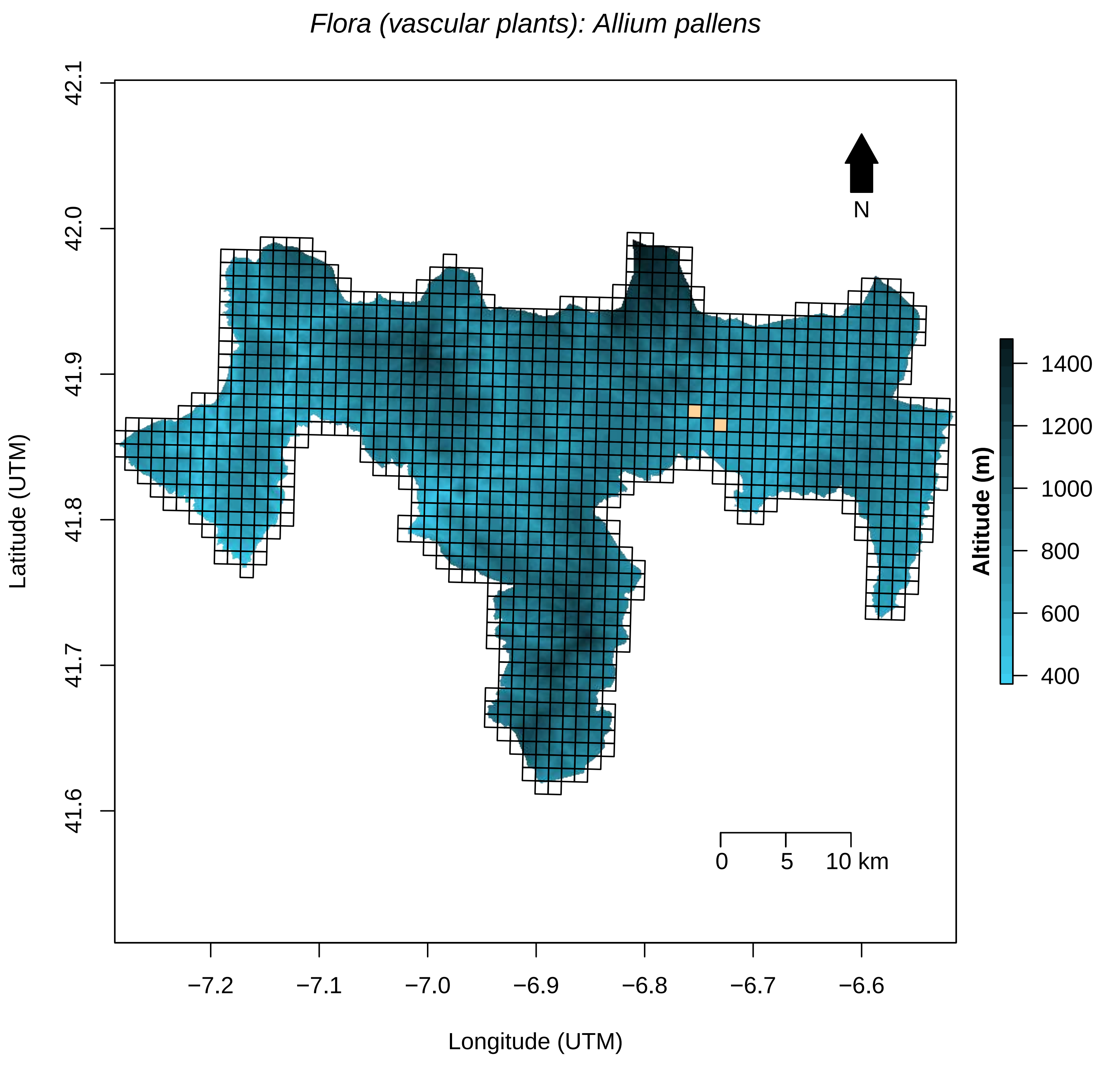
Flora (vascular plants): Allium pallens
42.1
42.0
41.9
41.8
41.7
41.6
Latitude (UTM)
N
0
5
10 km
−7.2
−7.1
−7.0
−6.9
−6.8
−6.7
−6.6
Longitude (UTM)
Altitude (m)
1400
1200
1000
800
600
400

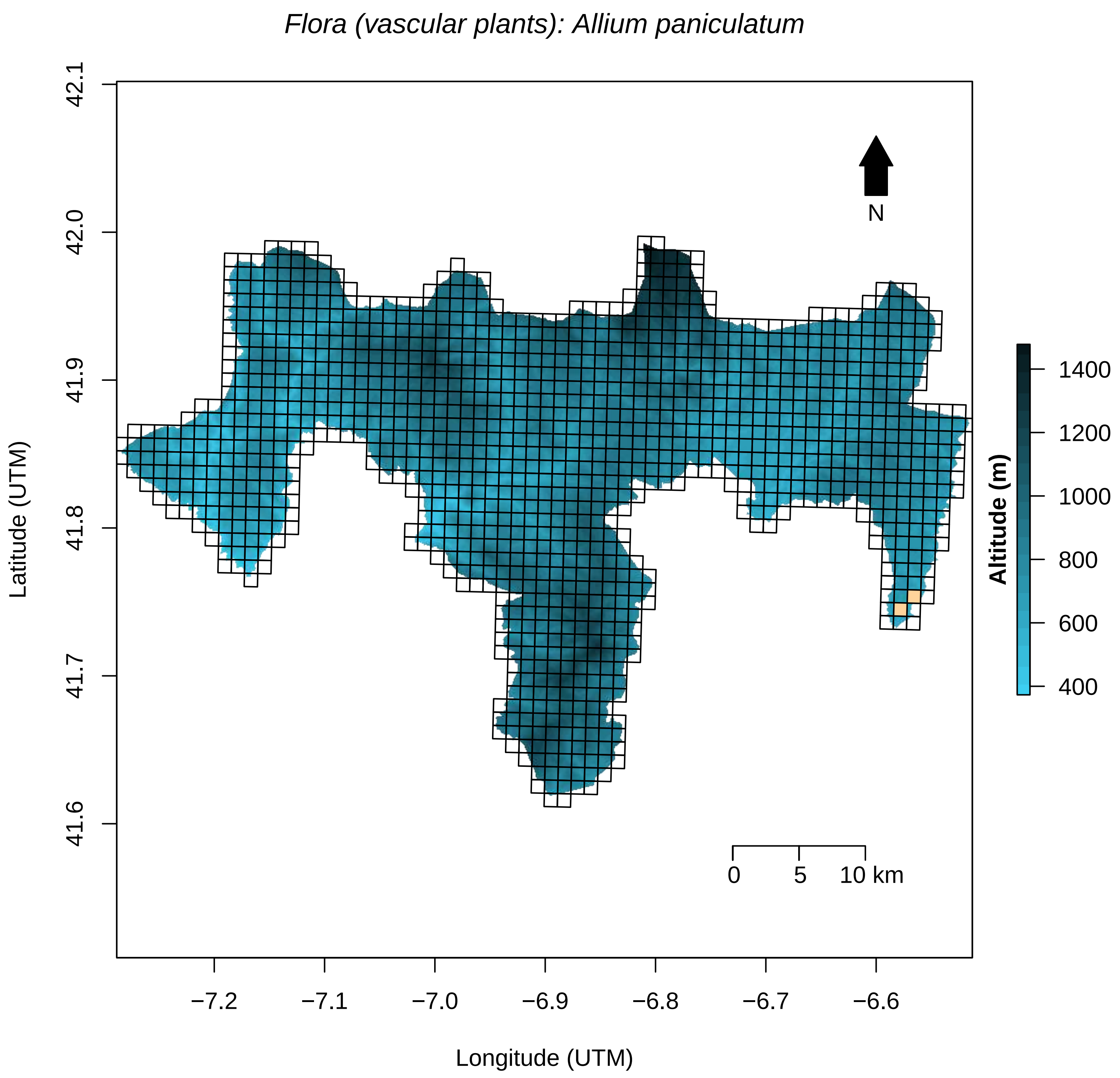

Flora (vascular plants): Allium paniculatum
42.1
42.0
41.9
41.8
41.7
41.6
Latitude (UTM)
N
0
5
10 km
1400
1200
1000
800
600
400
Altitude (m)
−7.2
−7.1
−7.0
−6.9
−6.8
−6.7
−6.6
Longitude (UTM)

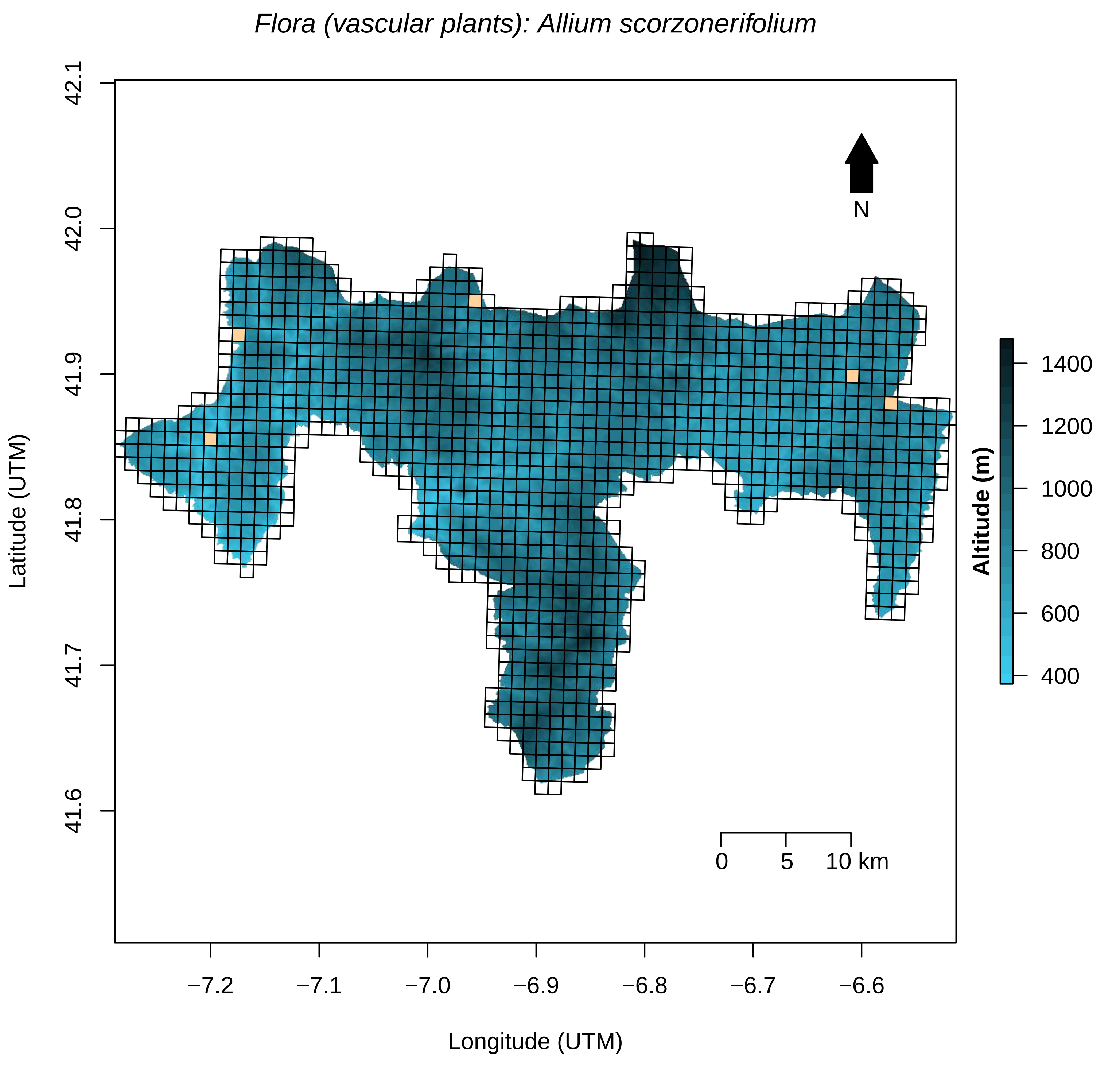
Flora (vascular plants): Allium scorzonerifolium
42.1
42.0
41.9
41.8
41.7
41.6
Latitude (UTM)
−7.2
−7.1
−7.0
−6.9
−6.8
−6.7
−6.6
Longitude (UTM)
N
0
5
10 km
1400
1200
1000
800
600
400
Altitude (m)

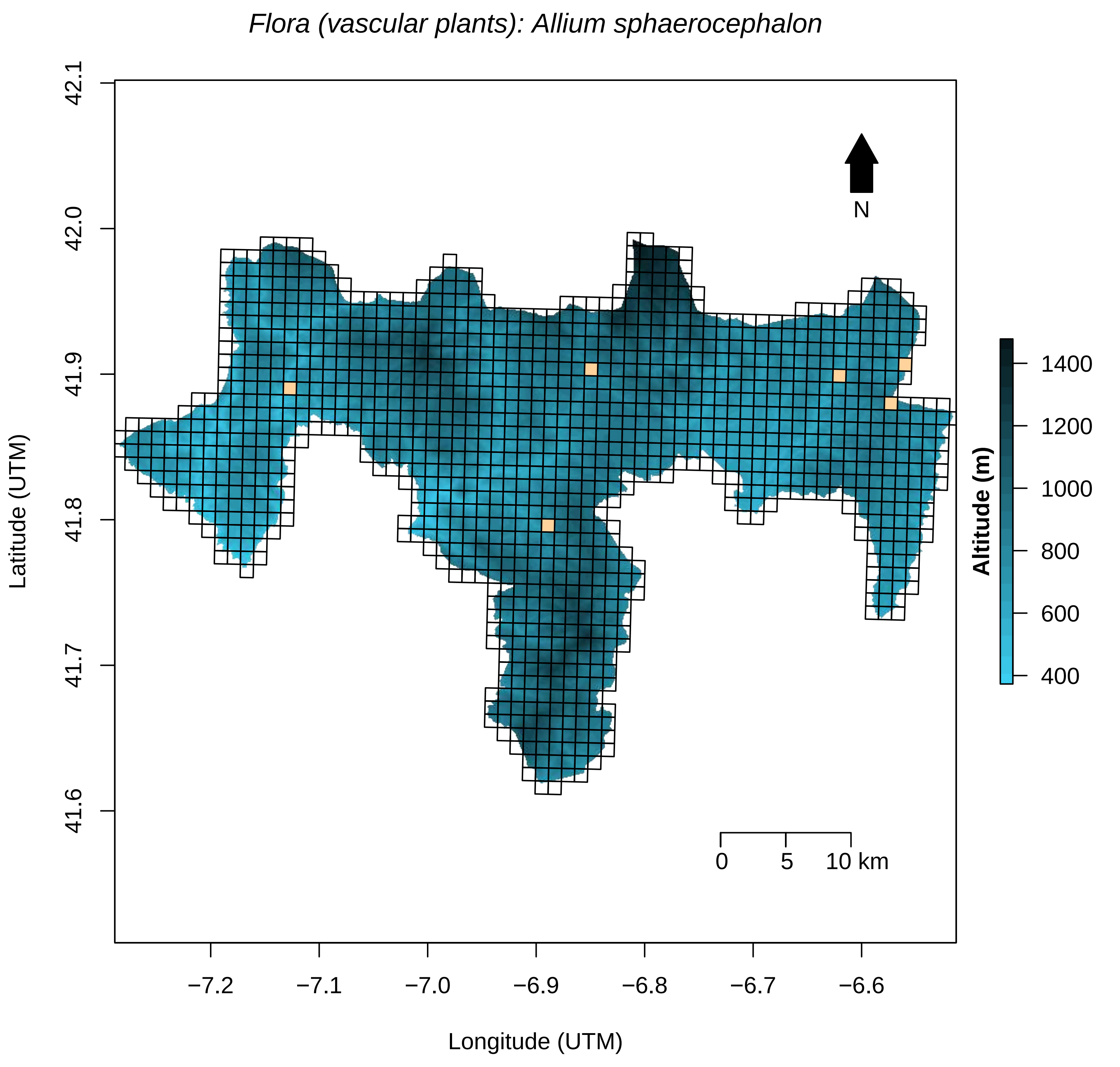

Flora (vascular plants): Allium sphaerocephalon
42.1
42.0
41.9
41.8
41.7
41.6
Latitude (UTM)
−7.2
−7.1
−7.0
−6.9
−6.8
−6.7
−6.6
Longitude (UTM)
N
0
5
10 km
Altitude (m)
1400
1200
1000
800
600
400

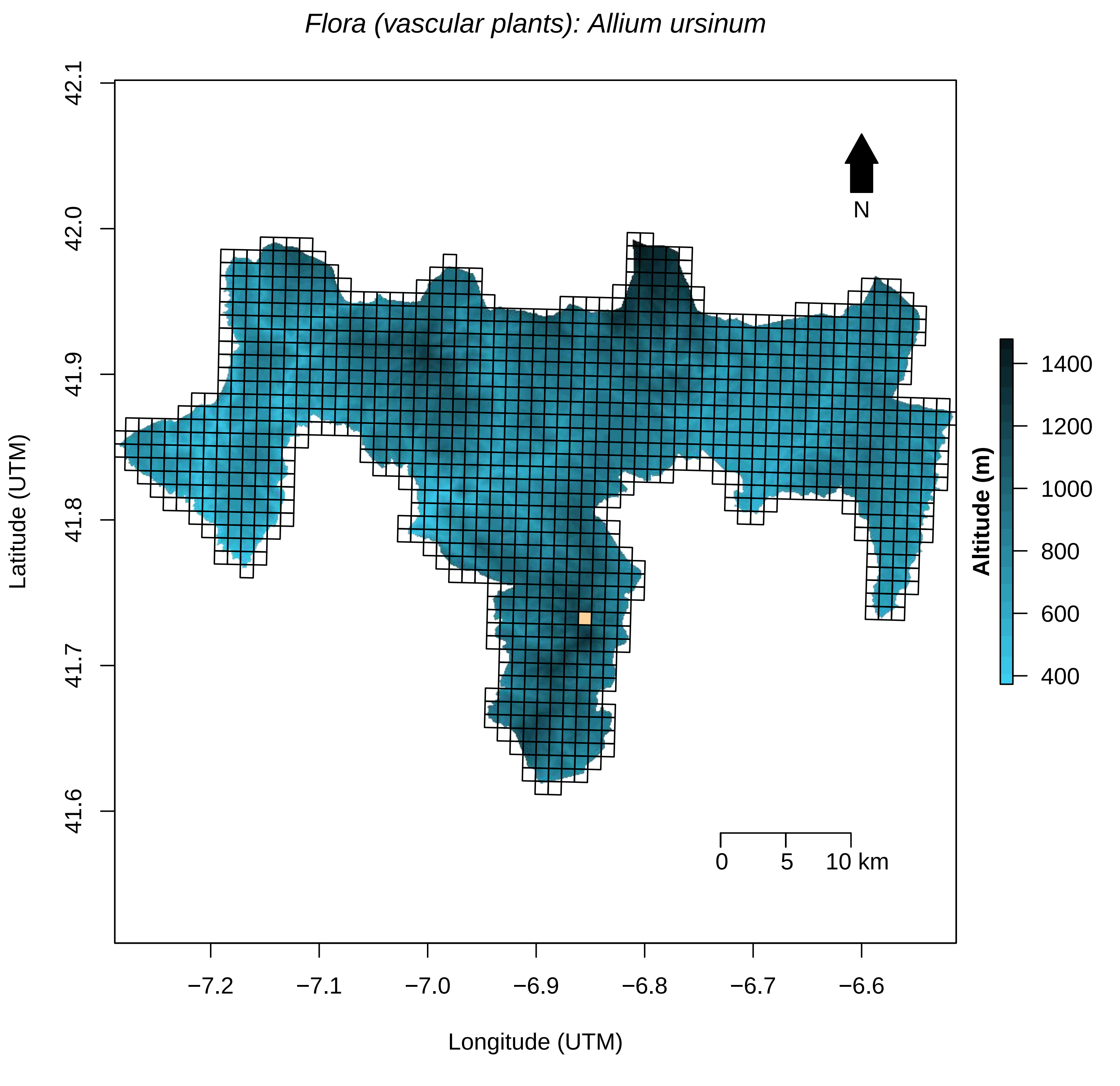
Flora (vascular plants): Allium ursinum
42.1
42.0
41.9
41.8
41.7
41.6
Latitude (UTM)
−7.2
−7.1
−7.0
−6.9
−6.8
−6.7
−6.6
Longitude (UTM)
N
0
5
10 km
1400
1200
1000
800
600
400
Altitude (m)

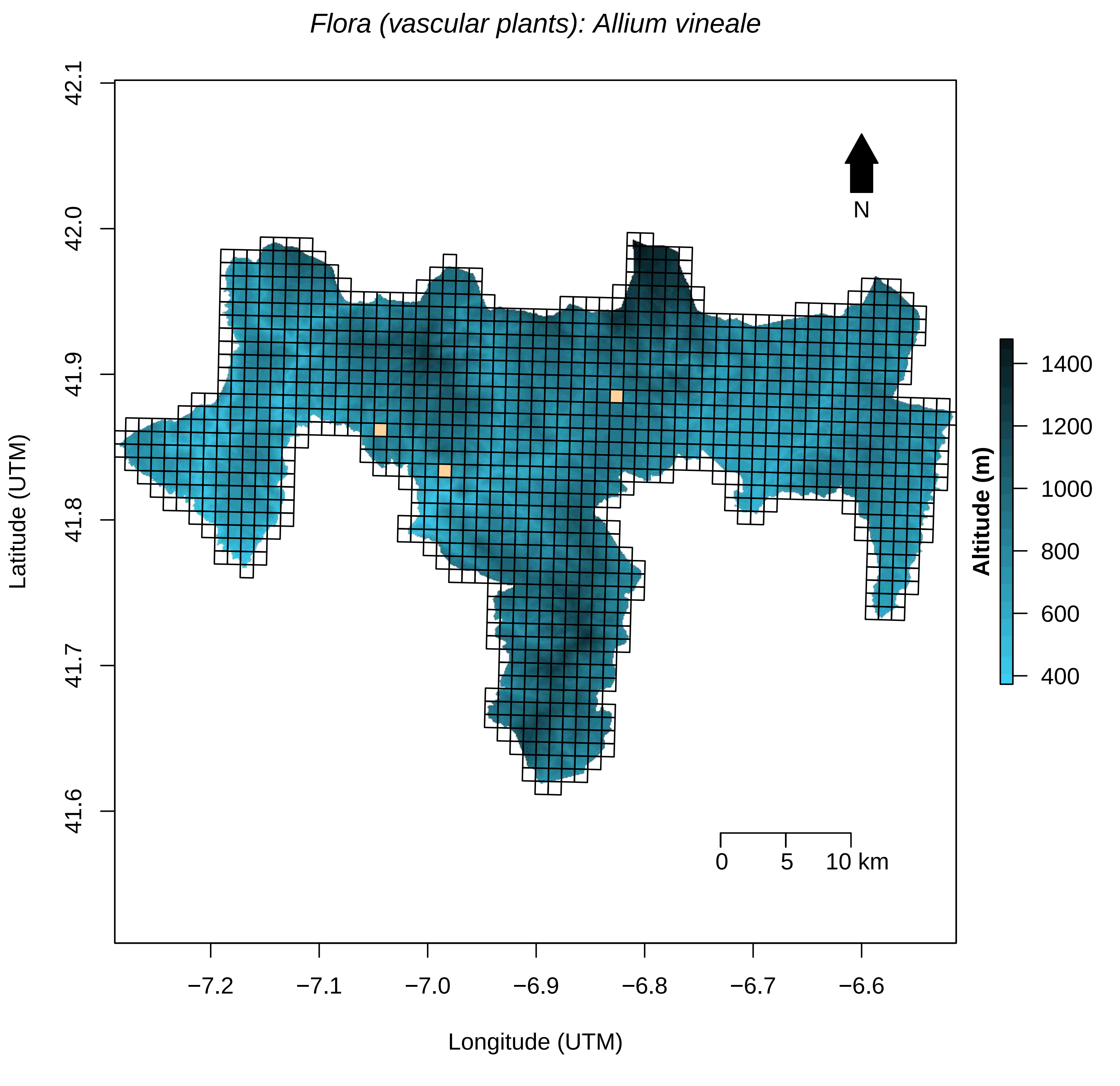
Flora (vascular plants): Allium vineale
42.1
42.0
41.9
41.8
41.7
41.6
Latitude (UTM)
−7.2
−7.1
−7.0
−6.9
−6.8
−6.7
−6.6
Longitude (UTM)
N
0
5
10 km
Altitude (m)
1400
1200
1000
800
600
400

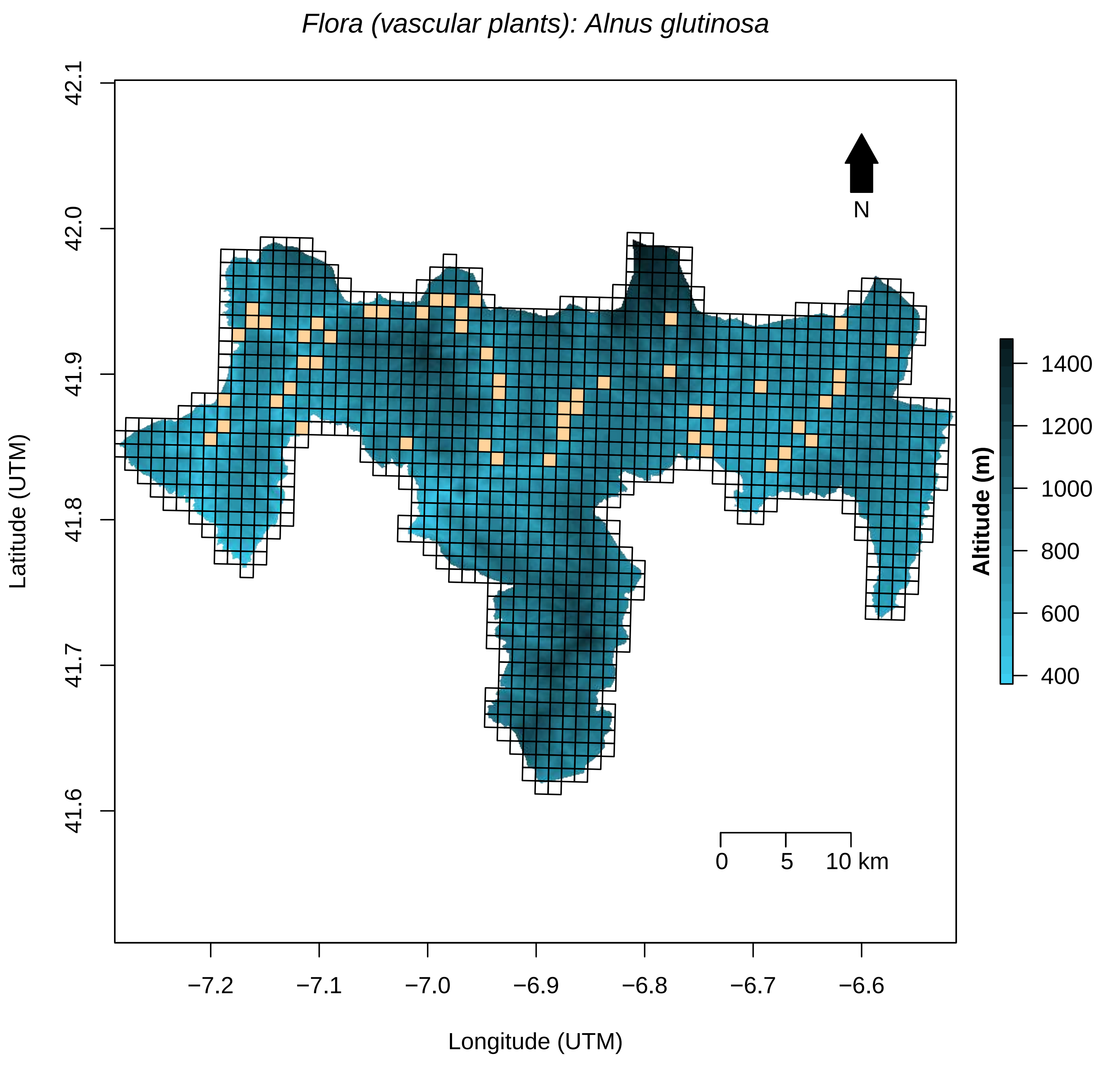

Flora (vascular plants): Alnus glutinosa
42.1
42.0
41.9
41.8
41.7
41.6
Latitude (UTM)
−7.2
−7.1
−7.0
−6.9
−6.8
−6.7
−6.6
Longitude (UTM)
N
0
5
10 km
Altitude (m)
1400
1200
1000
800
600
400

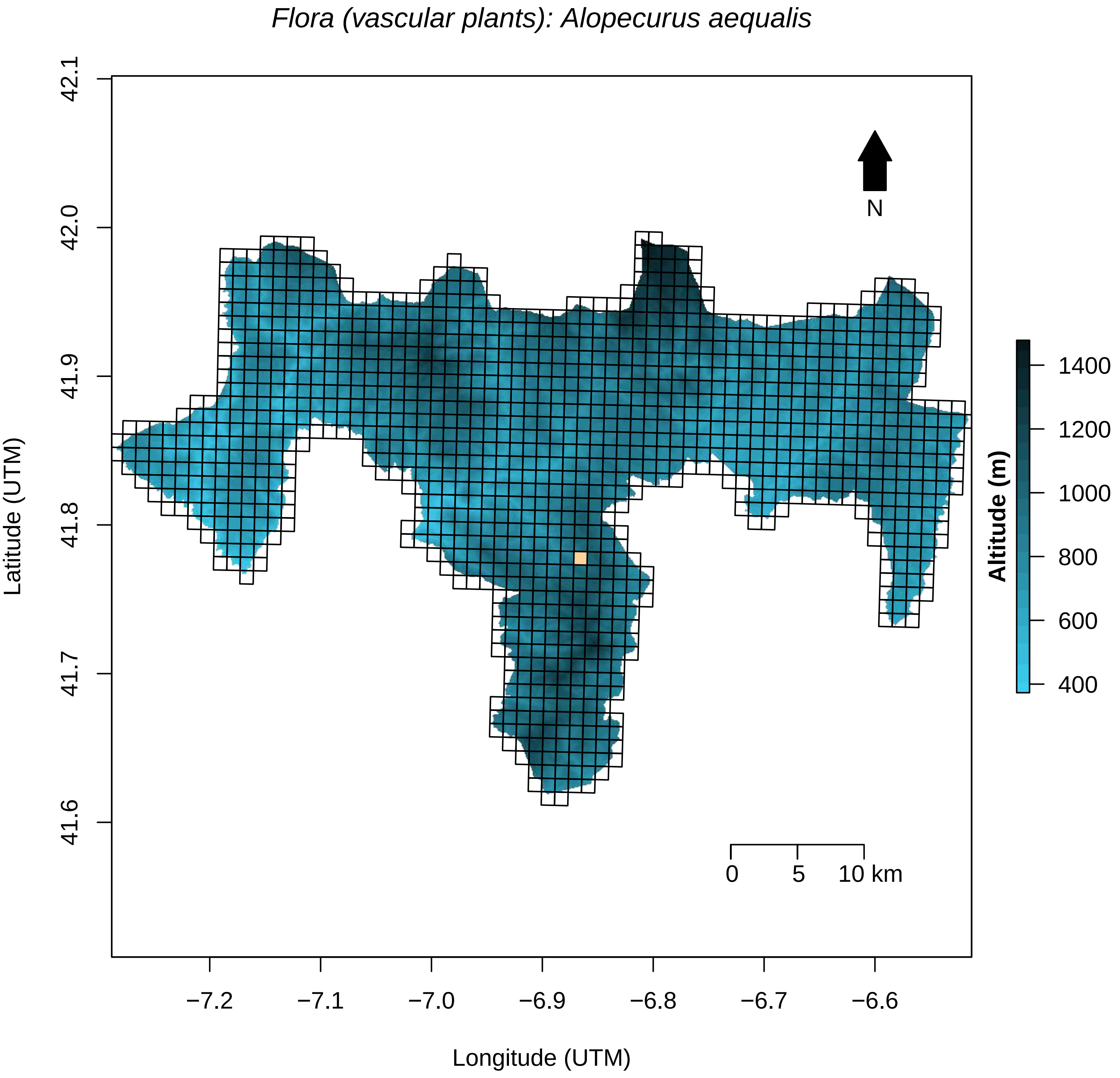

Flora (vascular plants): Alopecurus aequalis
N
0 5 10 km
Altitude (m)
1400
1200
1000
800
600
400
Latitude (UTM)
42.1
42.0
41.9
41.8
41.7
41.6
Longitude (UTM)
−7.2
−7.1
−7.0
−6.9
−6.8
−6.7
−6.6

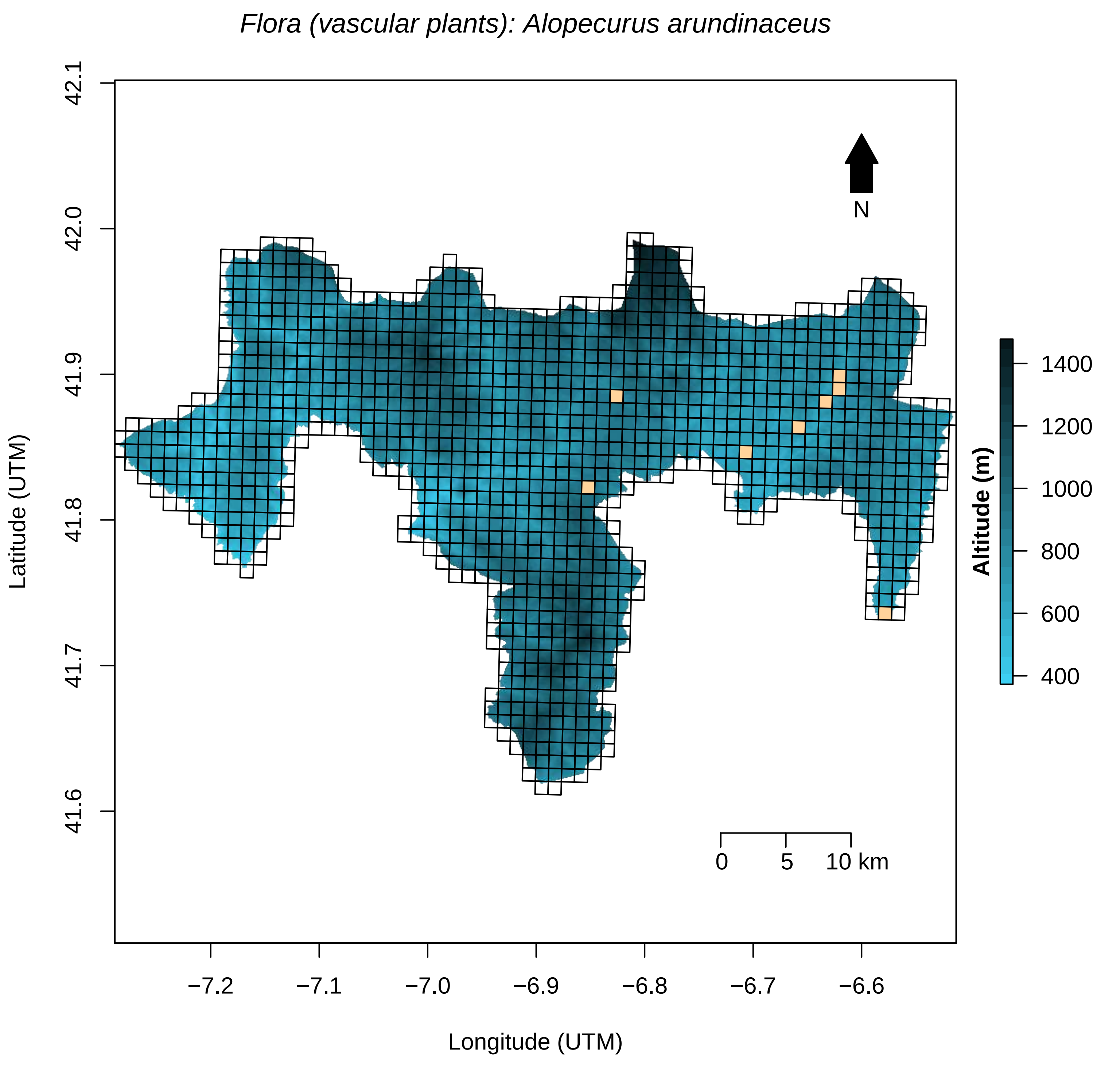

Flora (vascular plants): Alopecurus arundinaceus
42.1
42.0
41.9
41.8
41.7
41.6
Latitude (UTM)
N
0
5
10 km
1400
1200
1000
800
600
400
Altitude (m)
−7.2
−7.1
−7.0
−6.9
−6.8
−6.7
−6.6
Longitude (UTM)

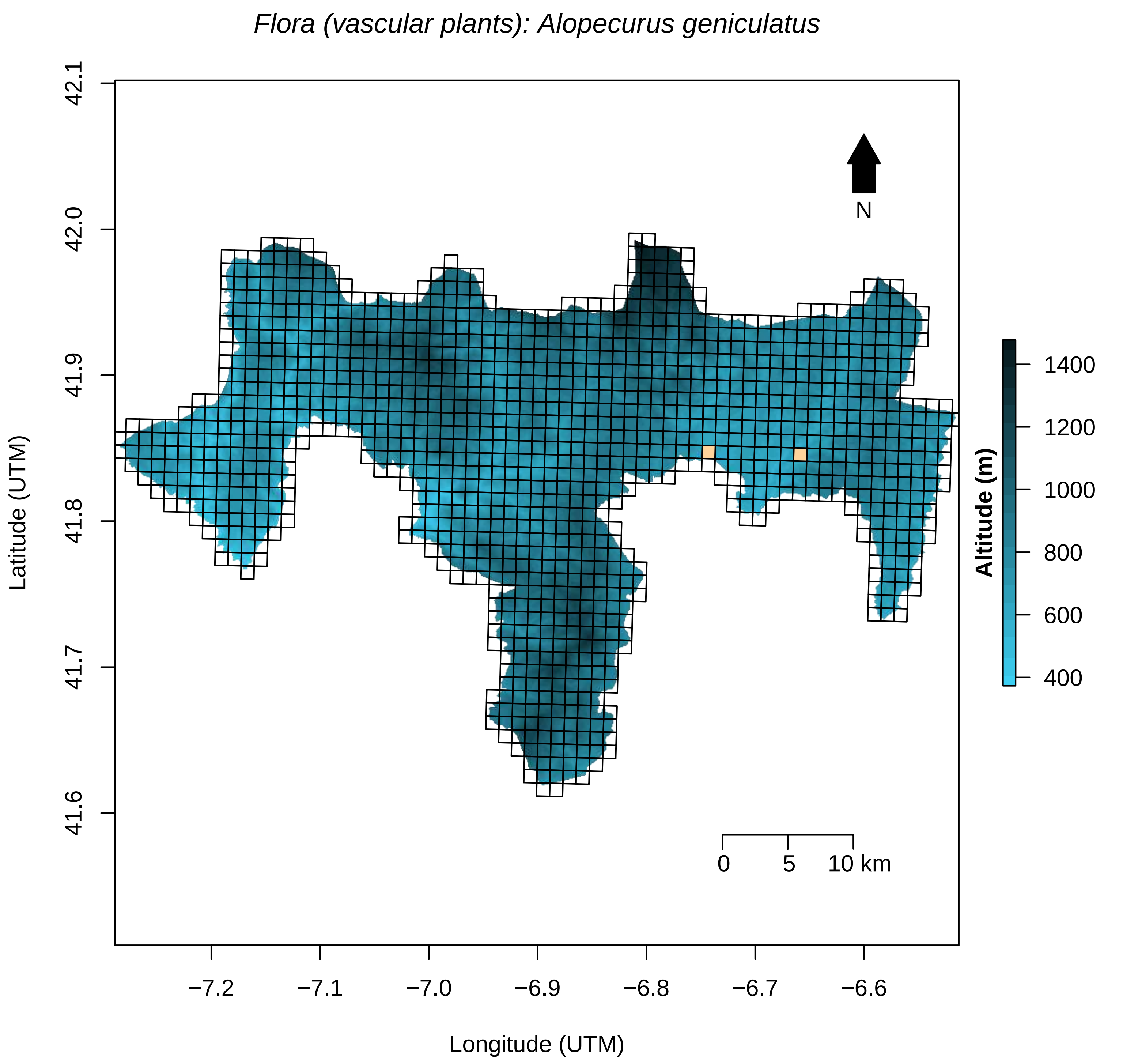
Flora (vascular plants): Alopecurus geniculatus
42.1
42.0
41.9
41.8
41.7
41.6
Latitude (UTM)
−7.2
−7.1
−7.0
−6.9
−6.8
−6.7
−6.6
Longitude (UTM)
N
0
5
10 km
Altitude (m)
1400
1200
1000
800
600
400

Flora (vascular plants): Alyssum granatense

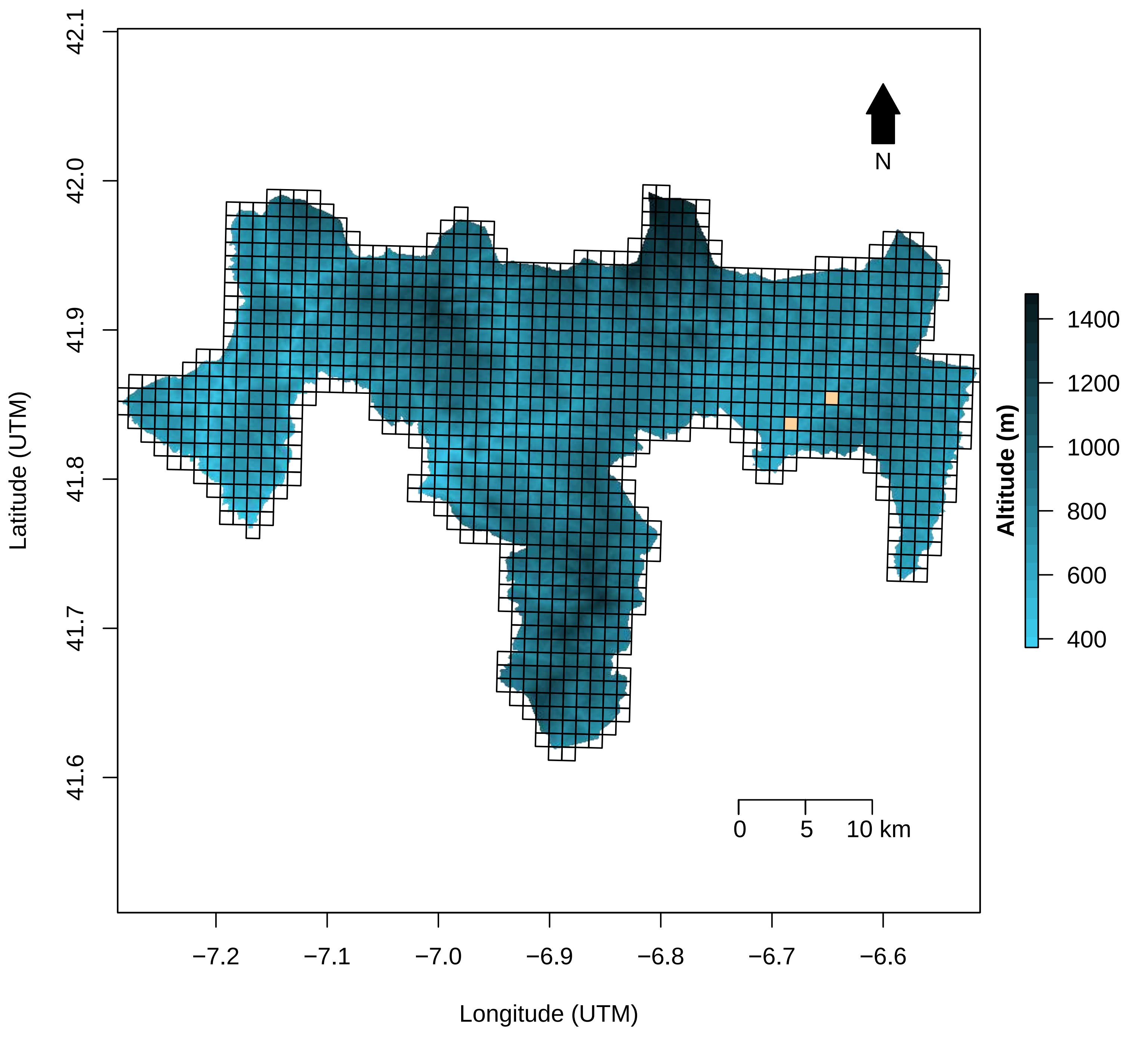

Flora (vascular plants): Alyssum minutum

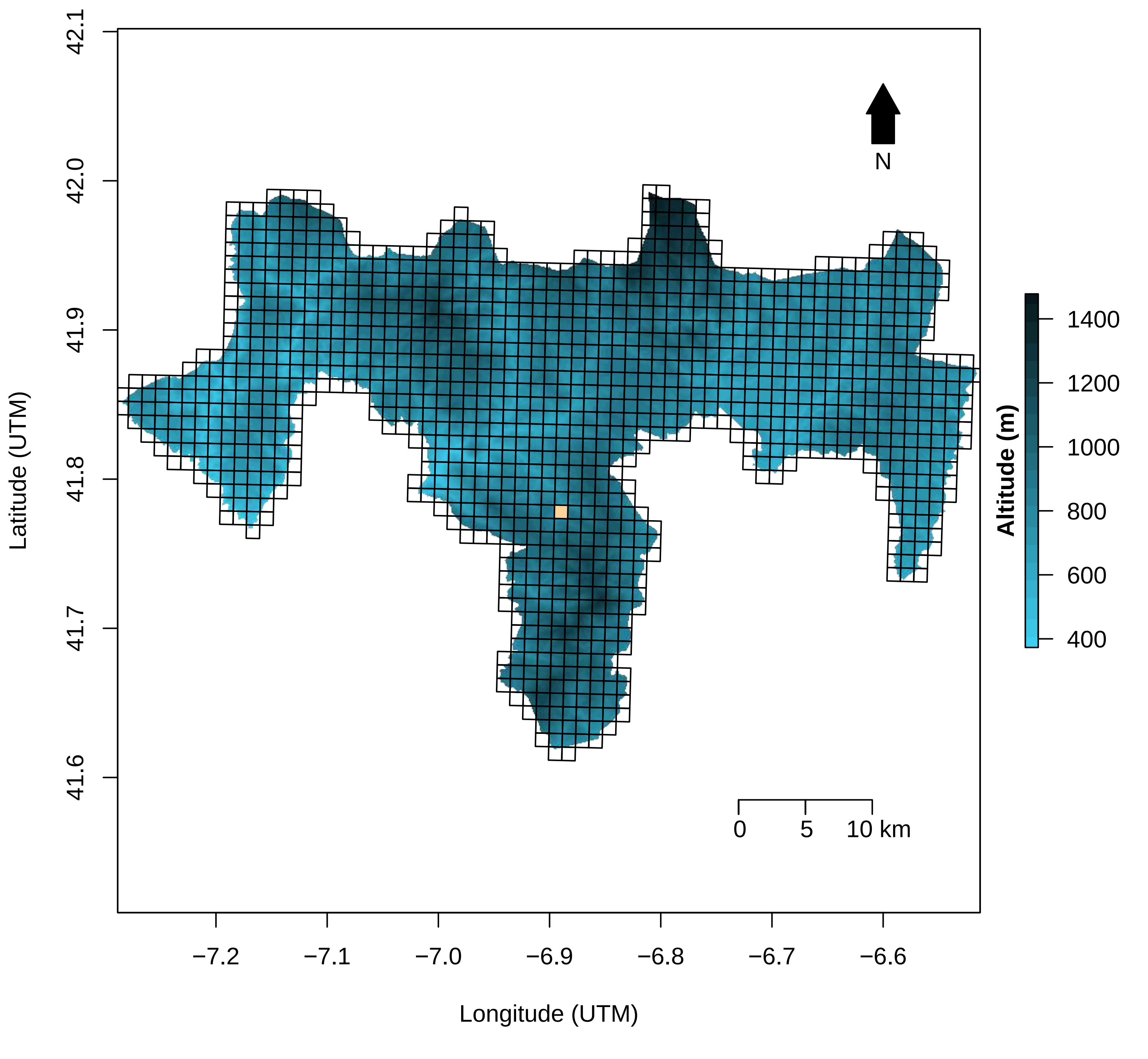

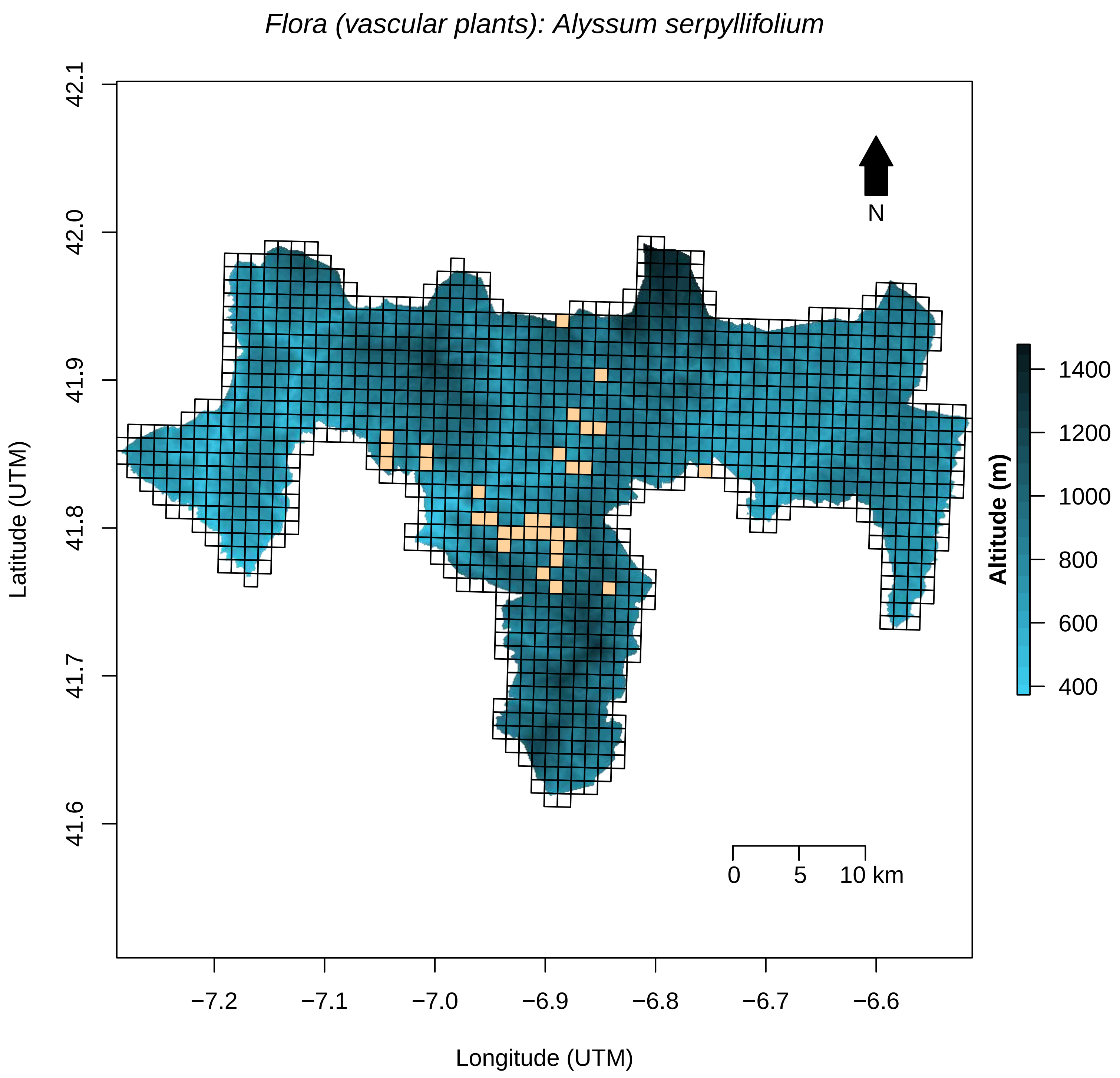

Flora (vascular plants): Alyssum serpyllifolium
Latitude (UTM)
42.1
42.0
41.9
41.8
41.7
41.6
N
0
5
10 km
Altitude (m)
1400
1200
1000
800
600
400
−7.2
−7.1
−7.0
−6.9
−6.8
−6.7
−6.6
Longitude (UTM)

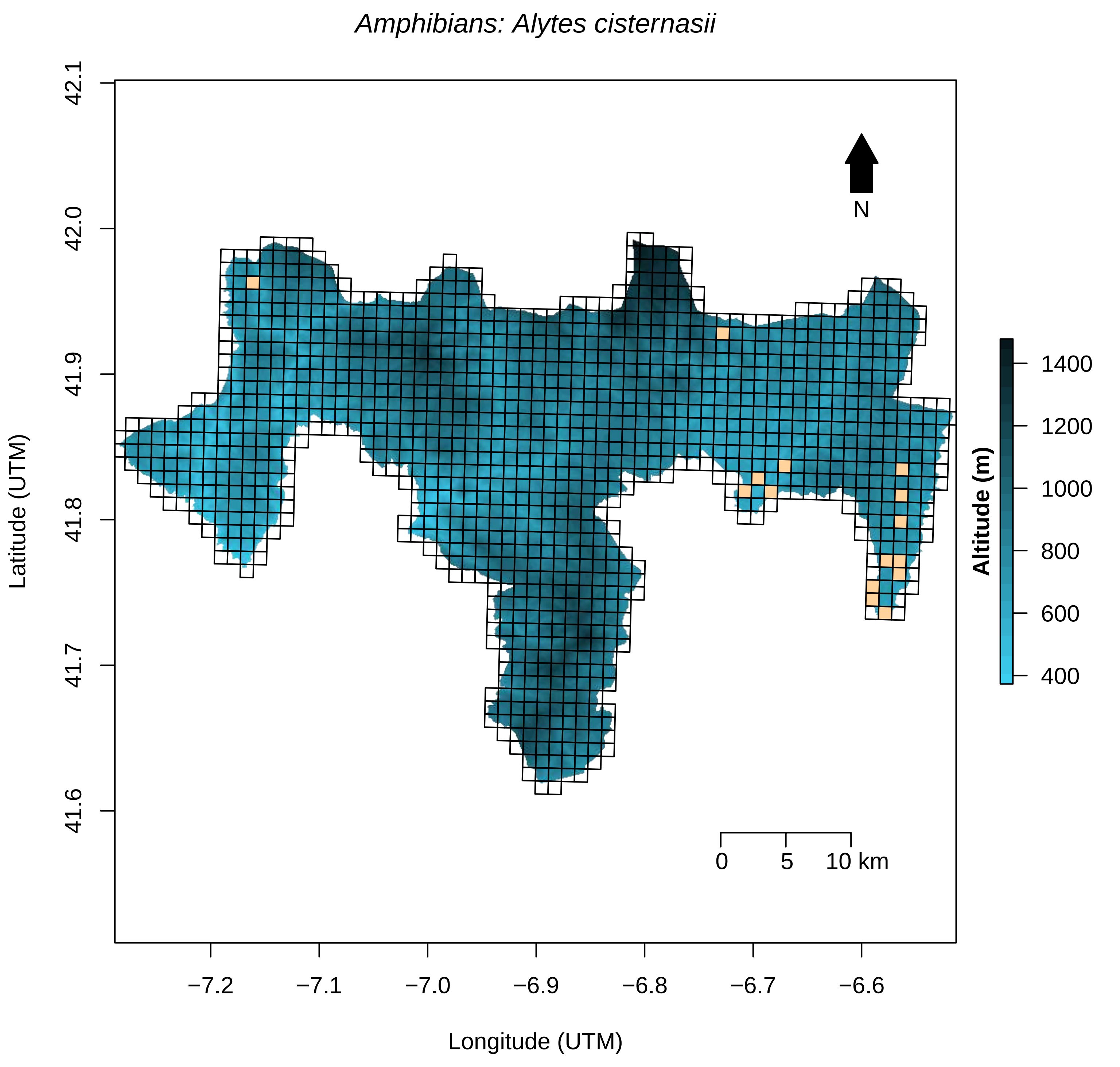

Amphibians: Alytes cisternasii
42.1
42.0
41.9
41.8
41.7
41.6
Latitude (UTM)
−7.2
−7.1
−7.0
−6.9
−6.8
−6.7
−6.6
Longitude (UTM)
N
0
5
10 km
Altitude (m)
1400
1200
1000
800
600
400

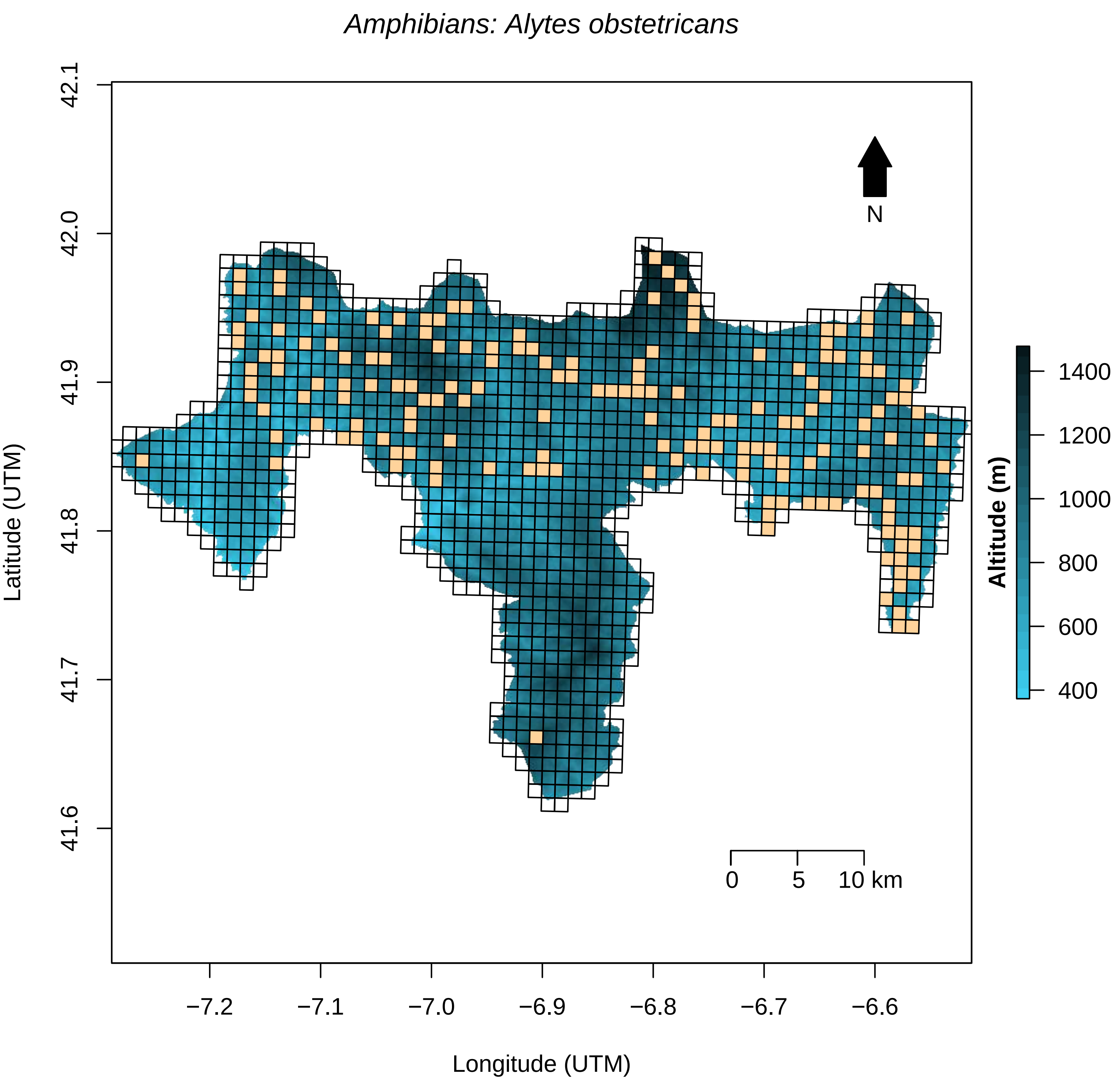
Amphibians: Alytes obstetricans
N
0 5 10 km
Latitude (UTM)
42.1
42.0
41.9
41.8
41.7
41.6
Longitude (UTM)
−7.2
−7.1
−7.0
−6.9
−6.8
−6.7
−6.6
Altitude (m)
1400
1200
1000
800
600
400

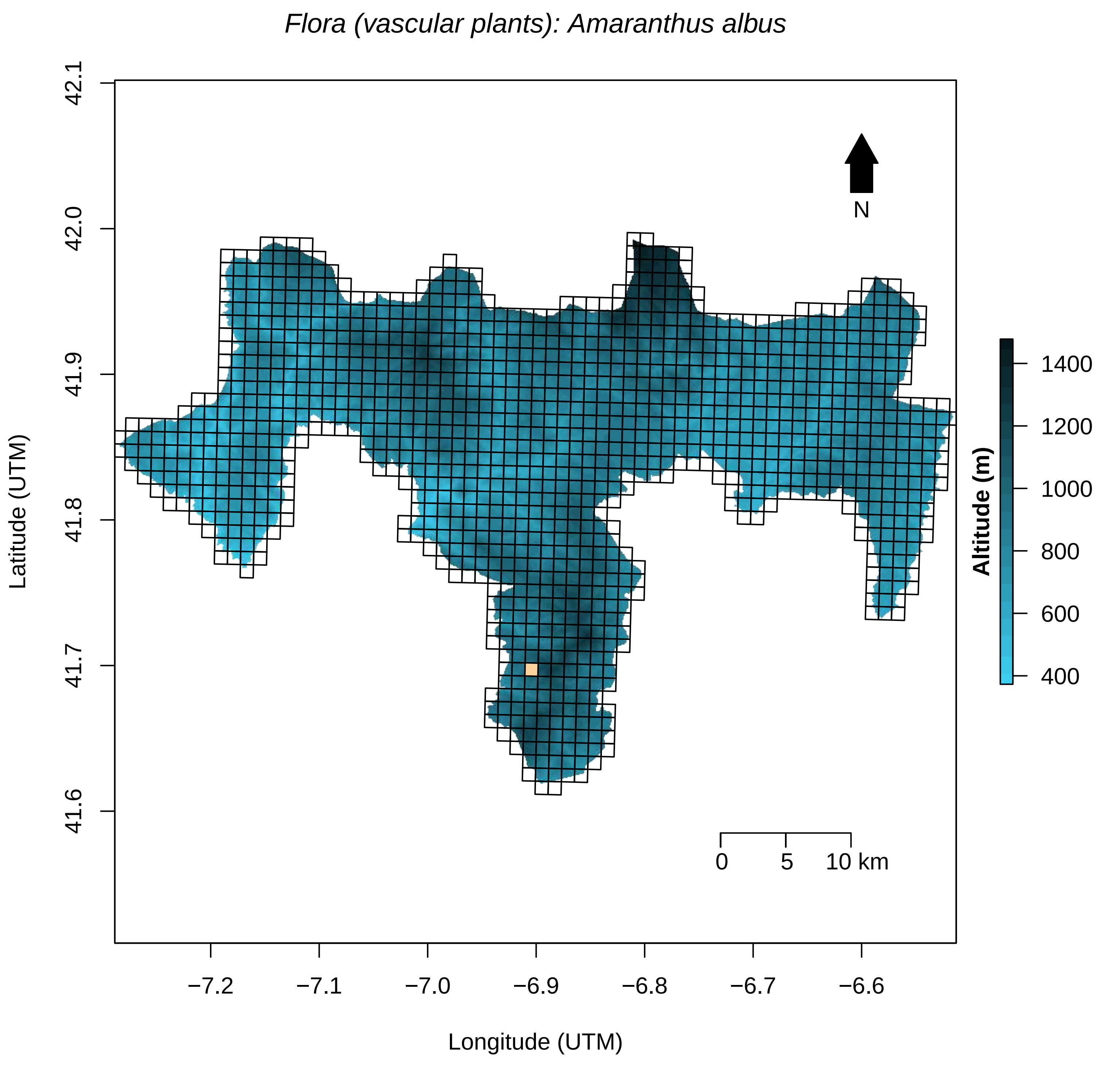
Flora (vascular plants): Amaranthus albus
42.1
42.0
41.9
41.8
41.7
41.6
Latitude (UTM)
−7.2
−7.1
−7.0
−6.9
−6.8
−6.7
−6.6
Longitude (UTM)
N
0
5
10 km
Altitude (m)
1400
1200
1000
800
600
400

Flora (vascular plants): Amaranthus deflexus

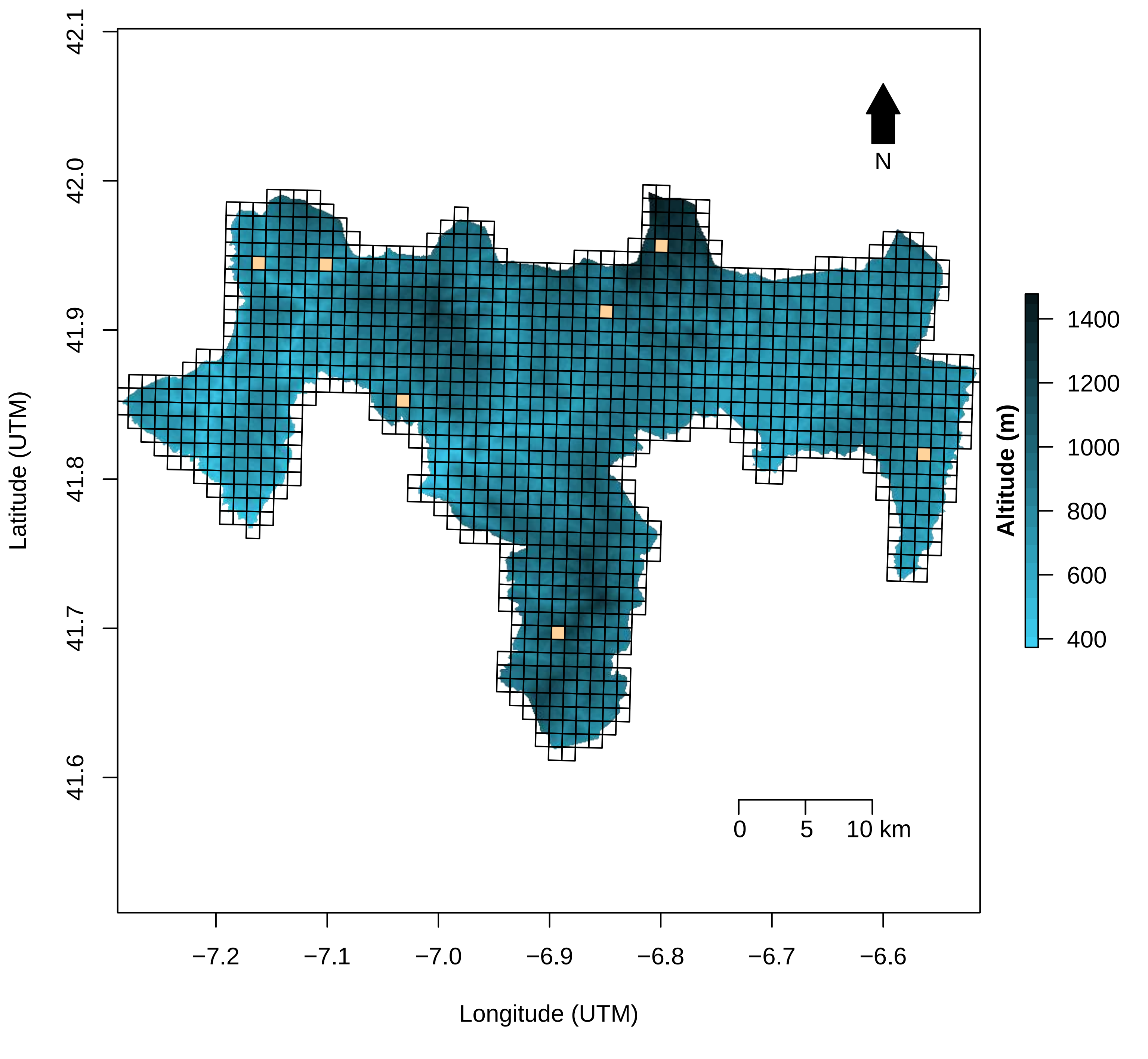

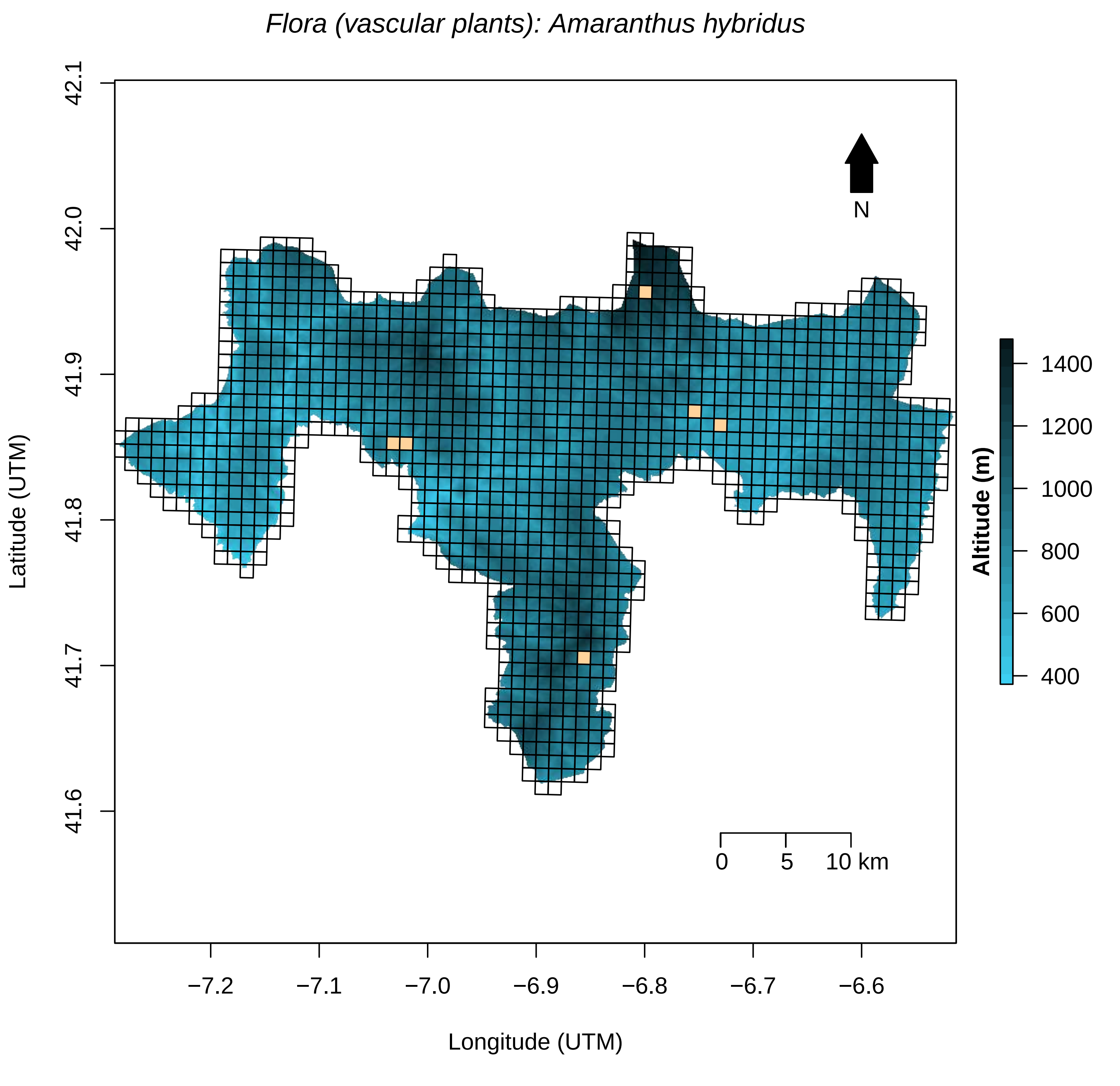

Flora (vascular plants): Amaranthus hybridus
42.1
42.0
41.9
41.8
41.7
41.6
Latitude (UTM)
−7.2
−7.1
−7.0
−6.9
−6.8
−6.7
−6.6
Longitude (UTM)
N
0
5
10 km
1400
1200
1000
800
600
400
Altitude (m)

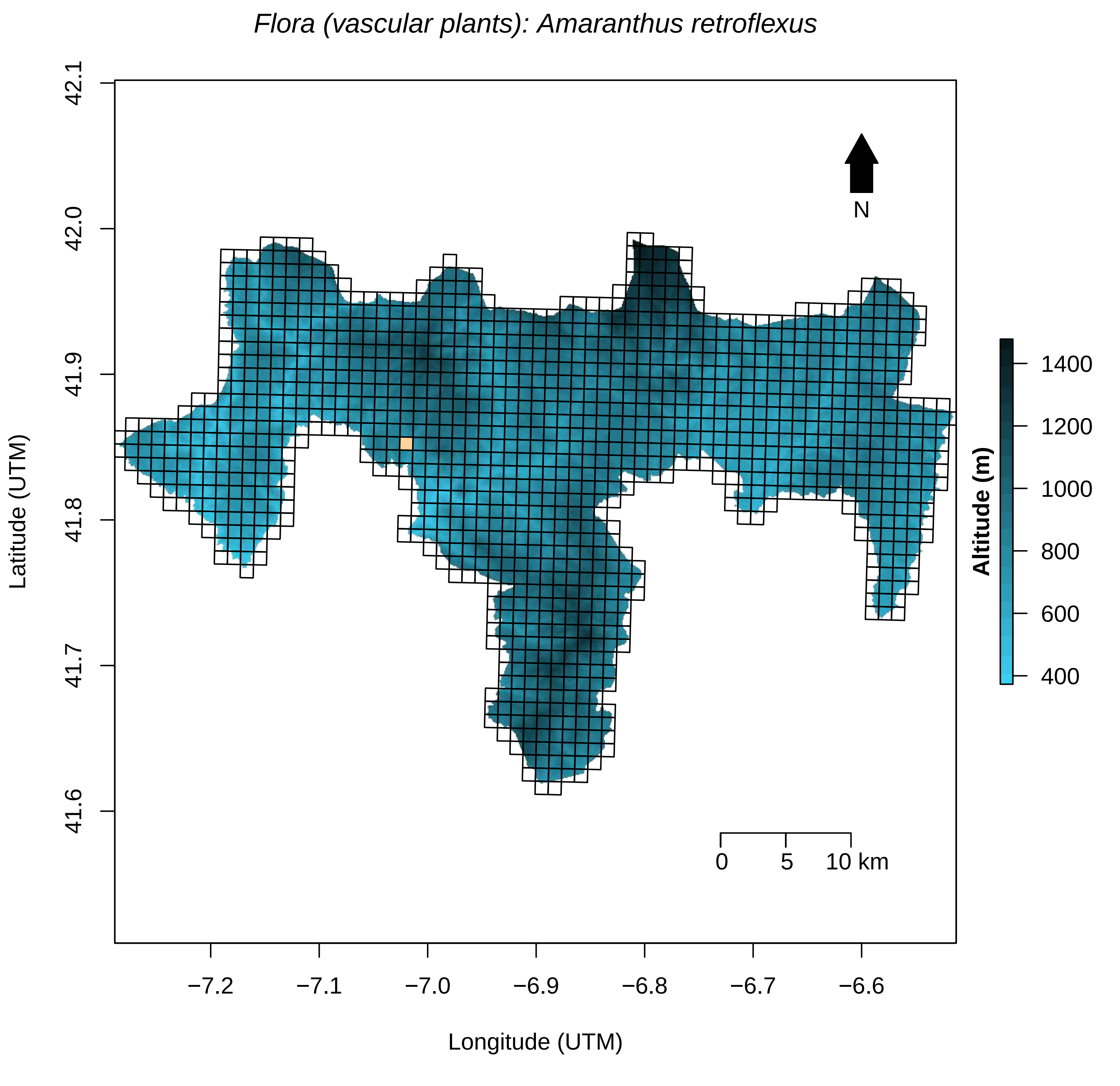

Flora (vascular plants): Amaranthus retroflexus
N
0
5
10 km
Altitude (m)
1400
1200
1000
800
600
400
Latitude (UTM)
42.1
42.0
41.9
41.8
41.7
41.6
Longitude (UTM)
−7.2
−7.1
−7.0
−6.9
−6.8
−6.7
−6.6

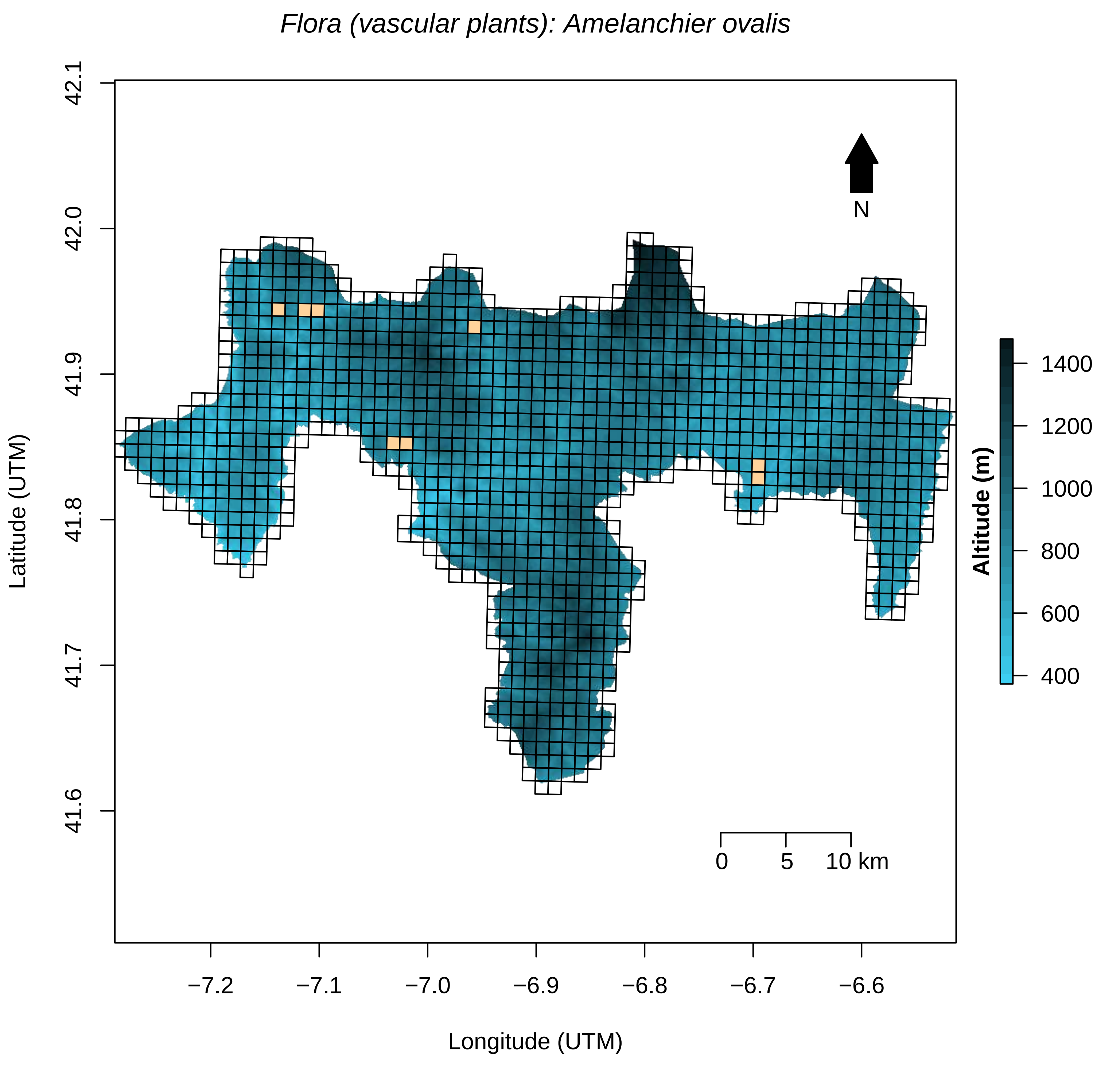

Flora (vascular plants): Amelanchier ovalis
42.1
42.0
41.9
41.8
41.7
41.6
Latitude (UTM)
−7.2
−7.1
−7.0
−6.9
−6.8
−6.7
−6.6
Longitude (UTM)
N
0
5
10 km
Altitude (m)
1400
1200
1000
800
600
400

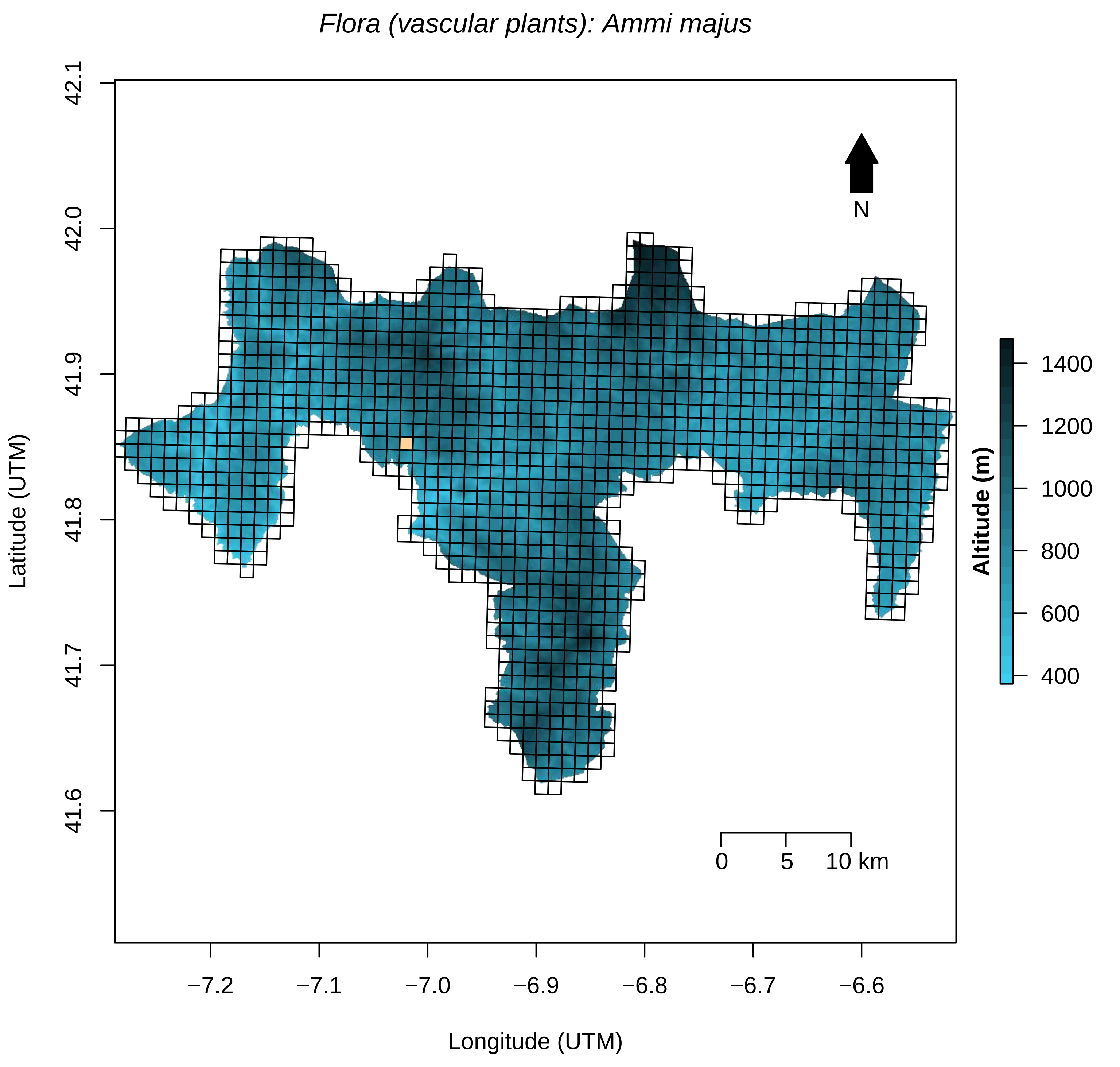
Flora (vascular plants): Ammi majus
42.1
42.0
41.9
41.8
41.7
41.6
Latitude (UTM)
−7.2
−7.1
−7.0
−6.9
−6.8
−6.7
−6.6
Longitude (UTM)
N
0
5
10 km
Altitude (m)
1400
1200
1000
800
600
400

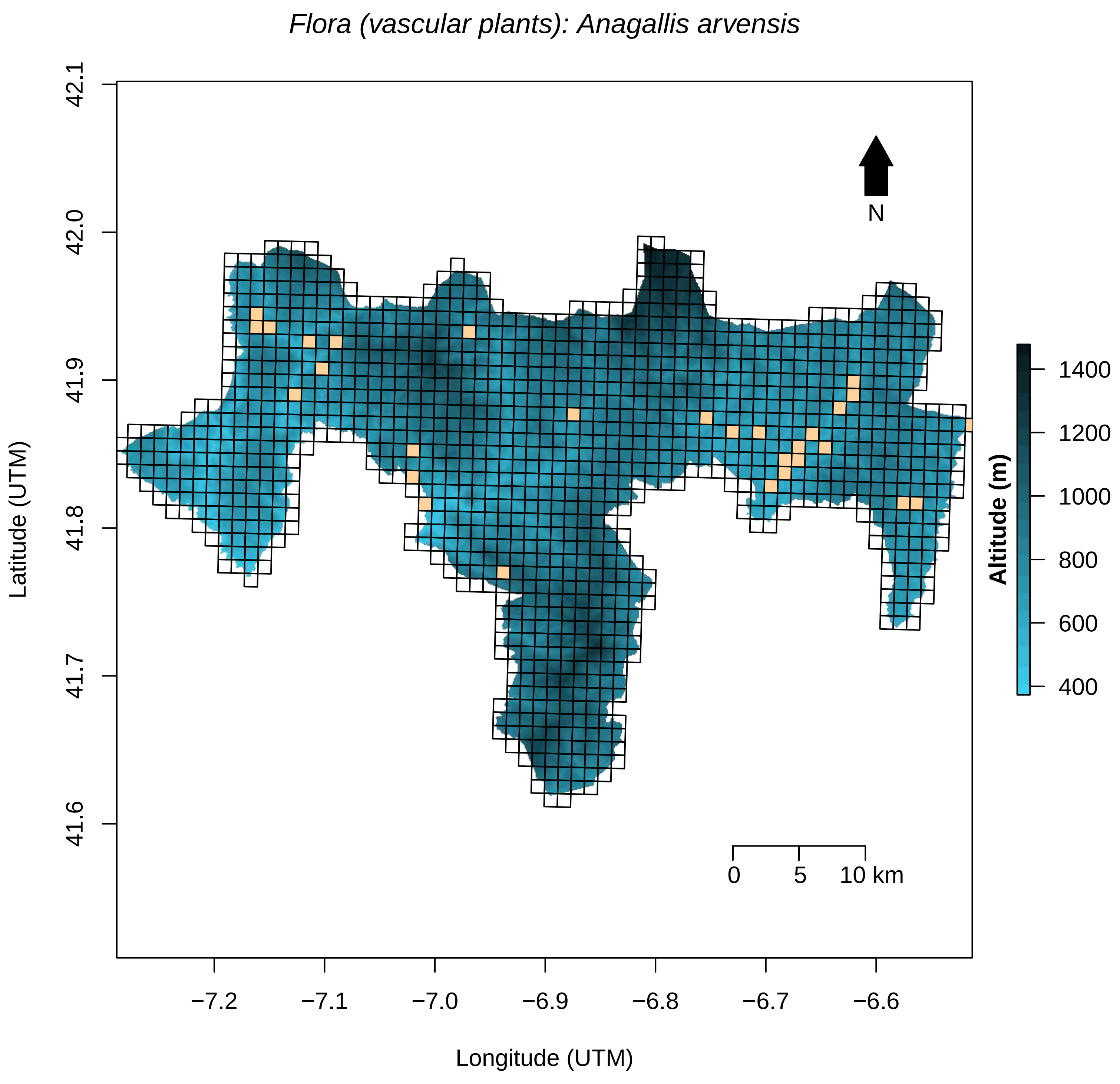

Flora (vascular plants): *Anagallis arvensis*
42.1
42.0
41.9
41.8
41.7
41.6
Latitude (UTM)
N
0
5
10 km
−7.2
−7.1
−7.0
−6.9
−6.8
−6.7
−6.6
Longitude (UTM)
1400
1200
1000
800
600
400
Altitude (m)

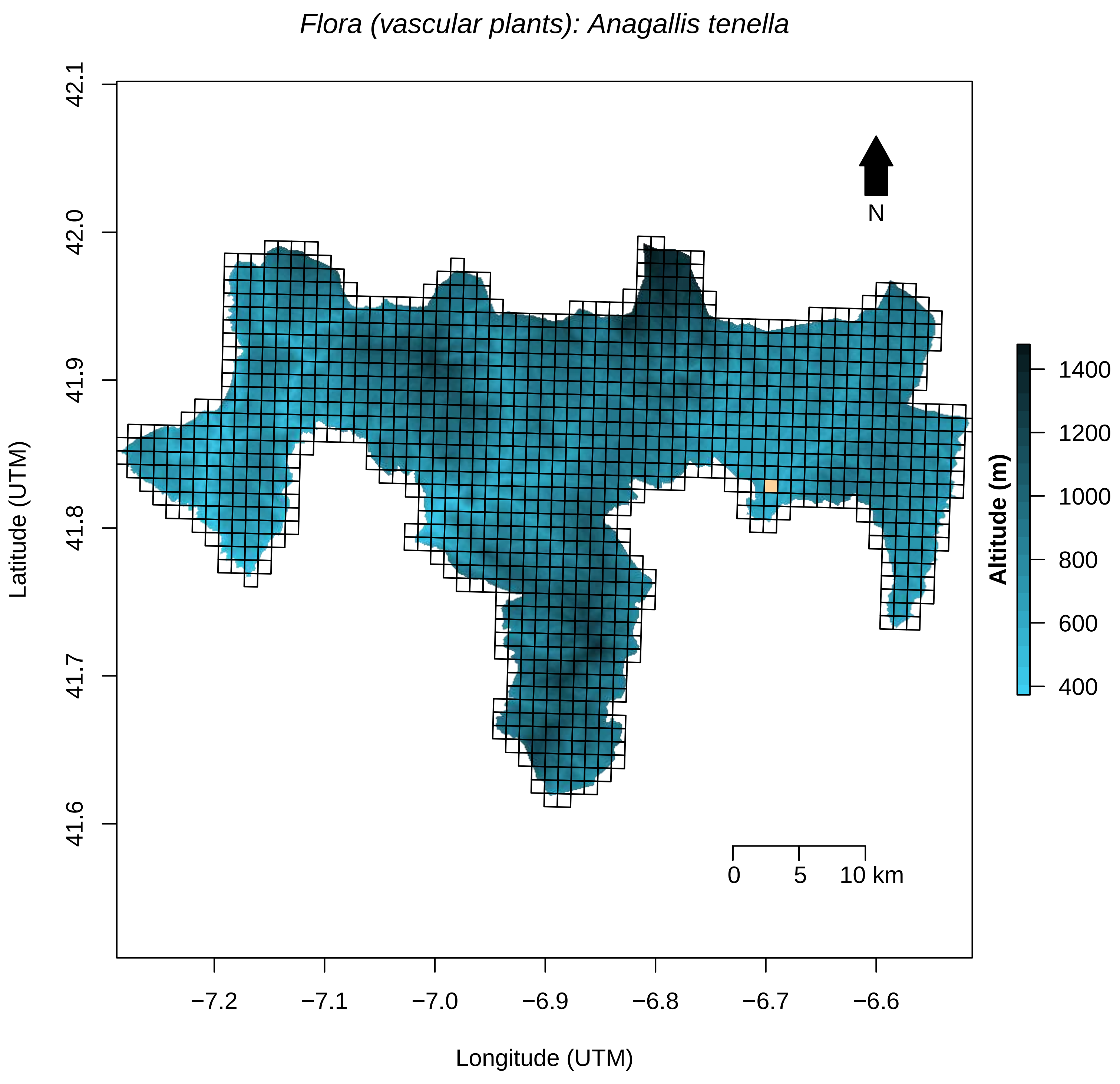

Flora (vascular plants): Anagallis tenella
42.1
42.0
41.9
41.8
41.7
41.6
Latitude (UTM)
−7.2
−7.1
−7.0
−6.9
−6.8
−6.7
−6.6
Longitude (UTM)
N
0
5
10 km
Altitude (m)
1400
1200
1000
800
600
400

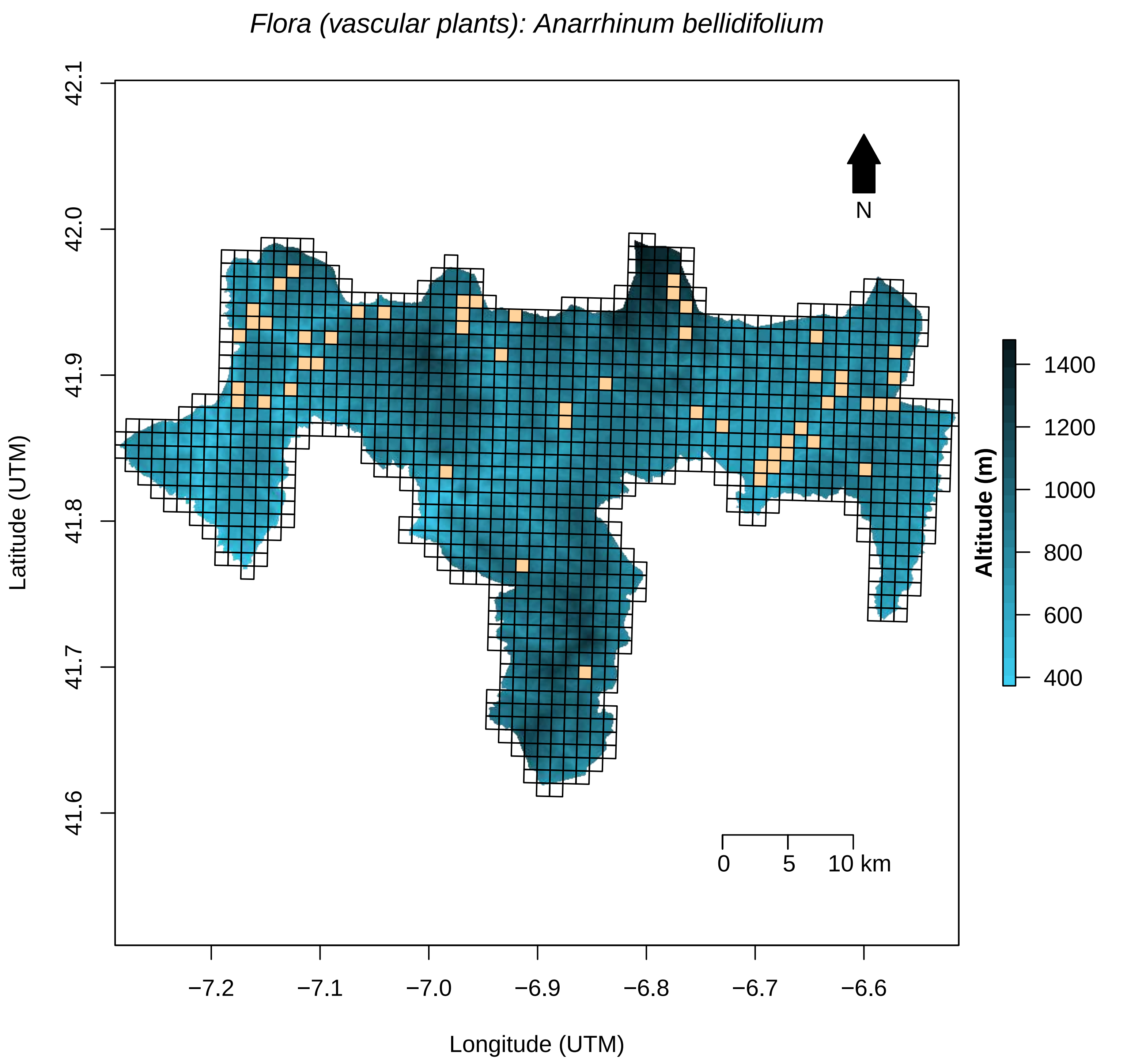

Flora (vascular plants): Anarrhinum bellidifolium
N
0 5 10 km
Altitude (m)
1400
1200
1000
800
600
400
Latitude (UTM)
42.1
42.0
41.9
41.8
41.7
41.6
Longitude (UTM)
−7.2
−7.1
−7.0
−6.9
−6.8
−6.7
−6.6

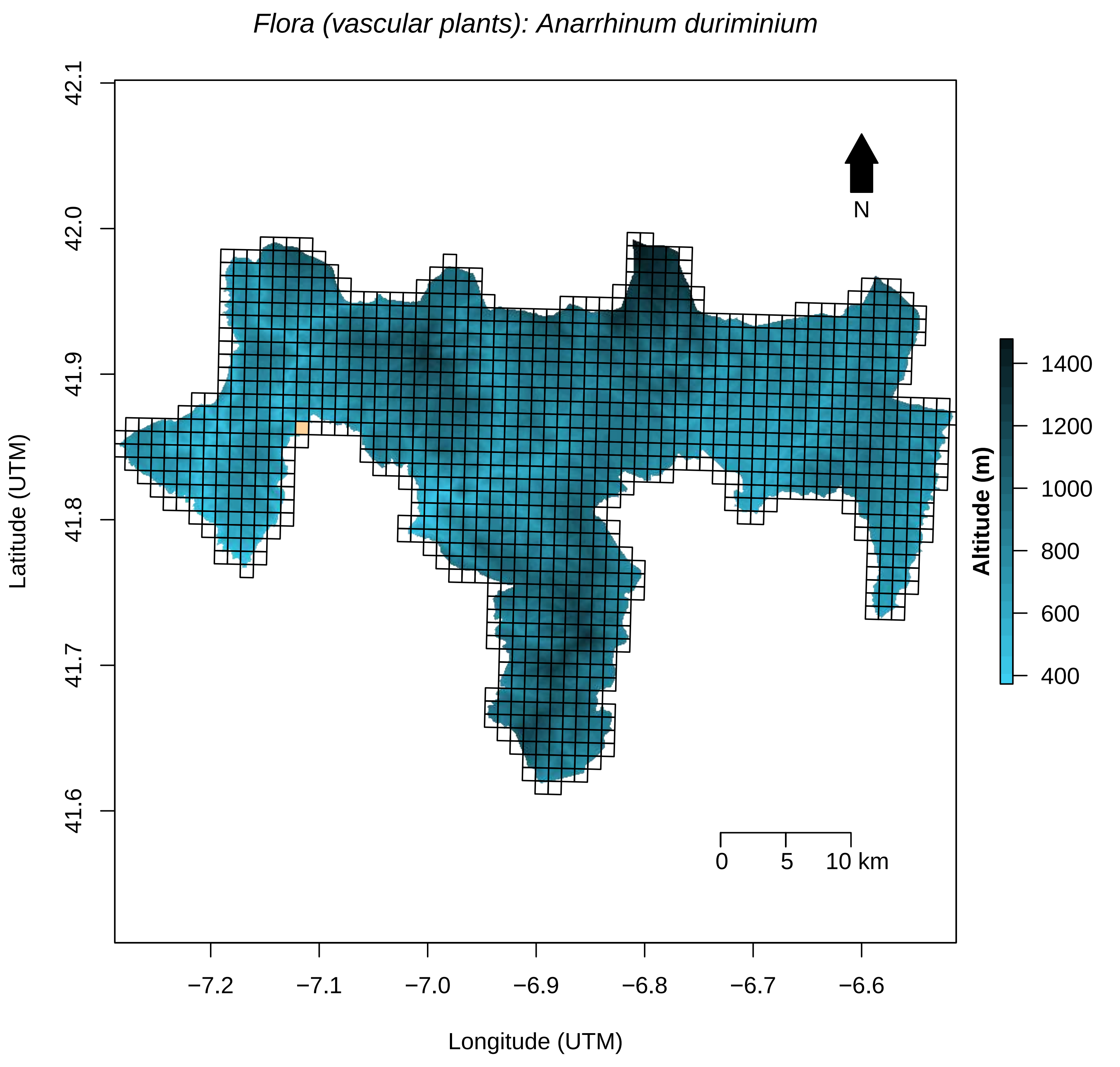

Flora (vascular plants): Anarrhinum duriminium
42.1
42.0
41.9
41.8
41.7
41.6
Latitude (UTM)
−7.2
−7.1
−7.0
−6.9
−6.8
−6.7
−6.6
Longitude (UTM)
N
0
5
10 km
Altitude (m)
1400
1200
1000
800
600
400

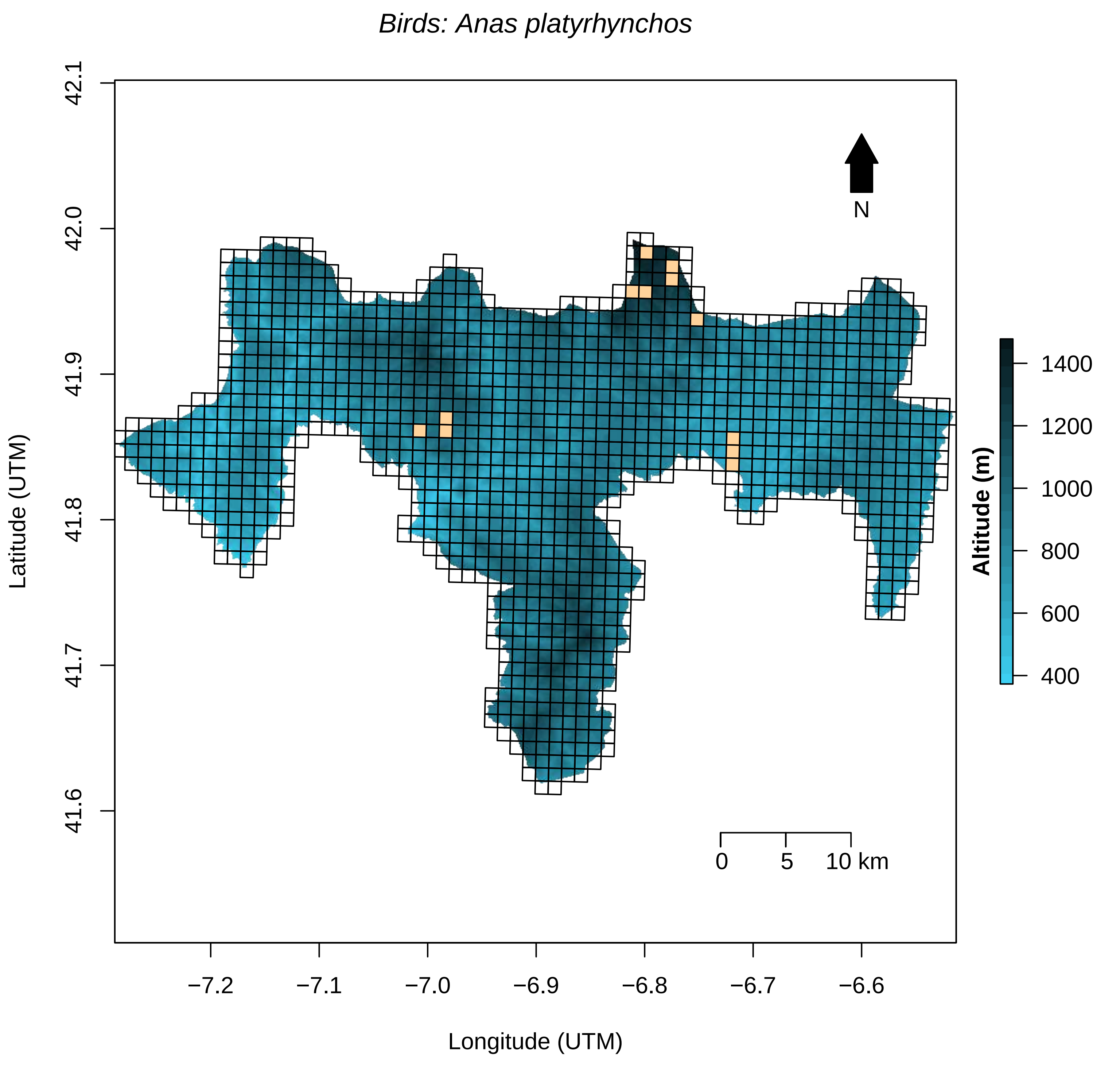

Birds: Anas platyrhynchos
Latitude (UTM)
42.1
42.0
41.9
41.8
41.7
41.6
N
0
5
10 km
−7.2
−7.1
−7.0
−6.9
−6.8
−6.7
−6.6
Longitude (UTM)
Altitude (m)
1400
1200
1000
800
600
400

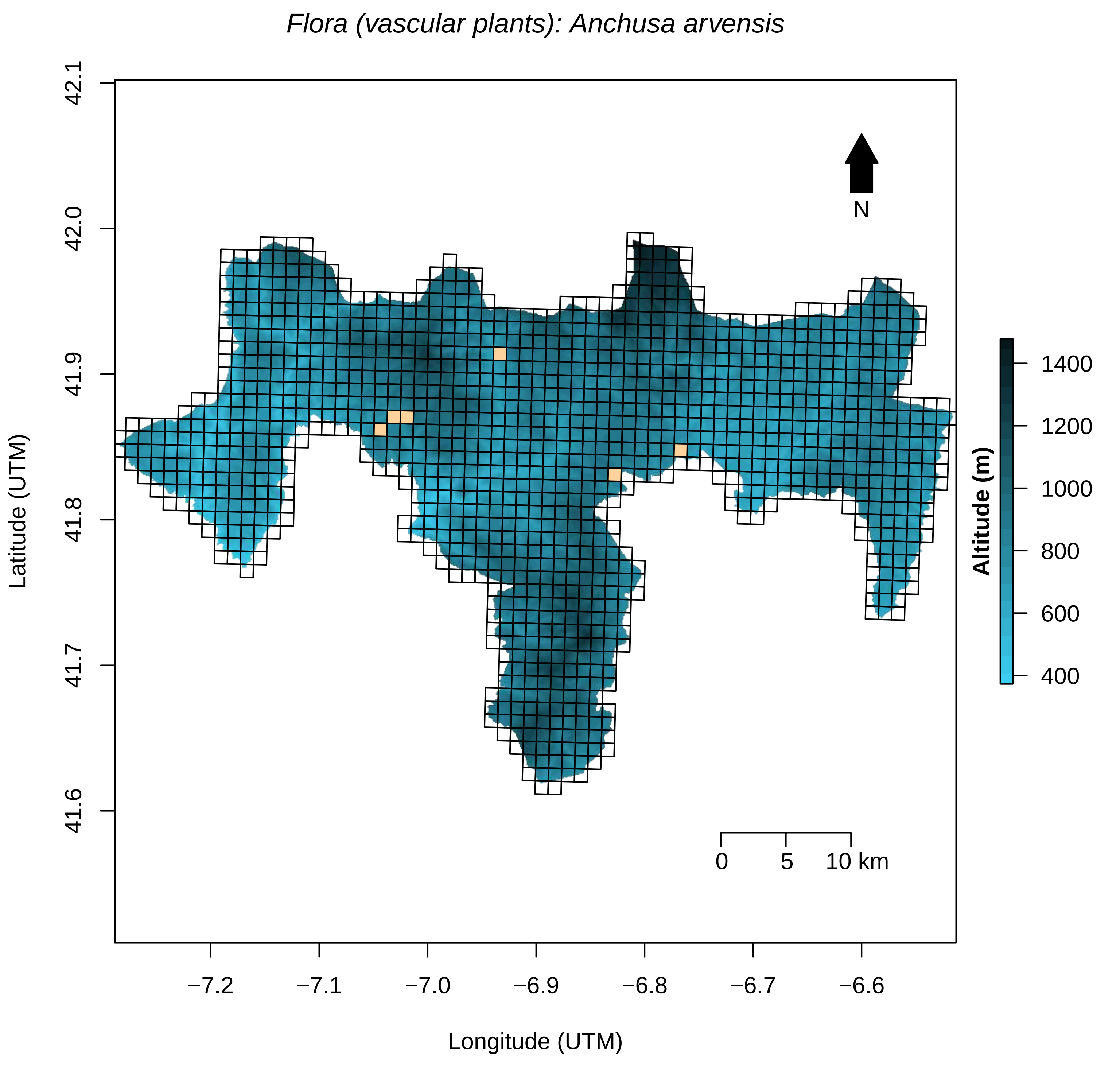

Flora (vascular plants): Anchusa arvensis
N
0
5
10 km
Altitude (m)
1400
1200
1000
800
600
400
Latitude (UTM)
42.1
42.0
41.9
41.8
41.7
41.6
Longitude (UTM)
−7.2
−7.1
−7.0
−6.9
−6.8
−6.7
−6.6

Flora (vascular plants): Andryala integrifolia

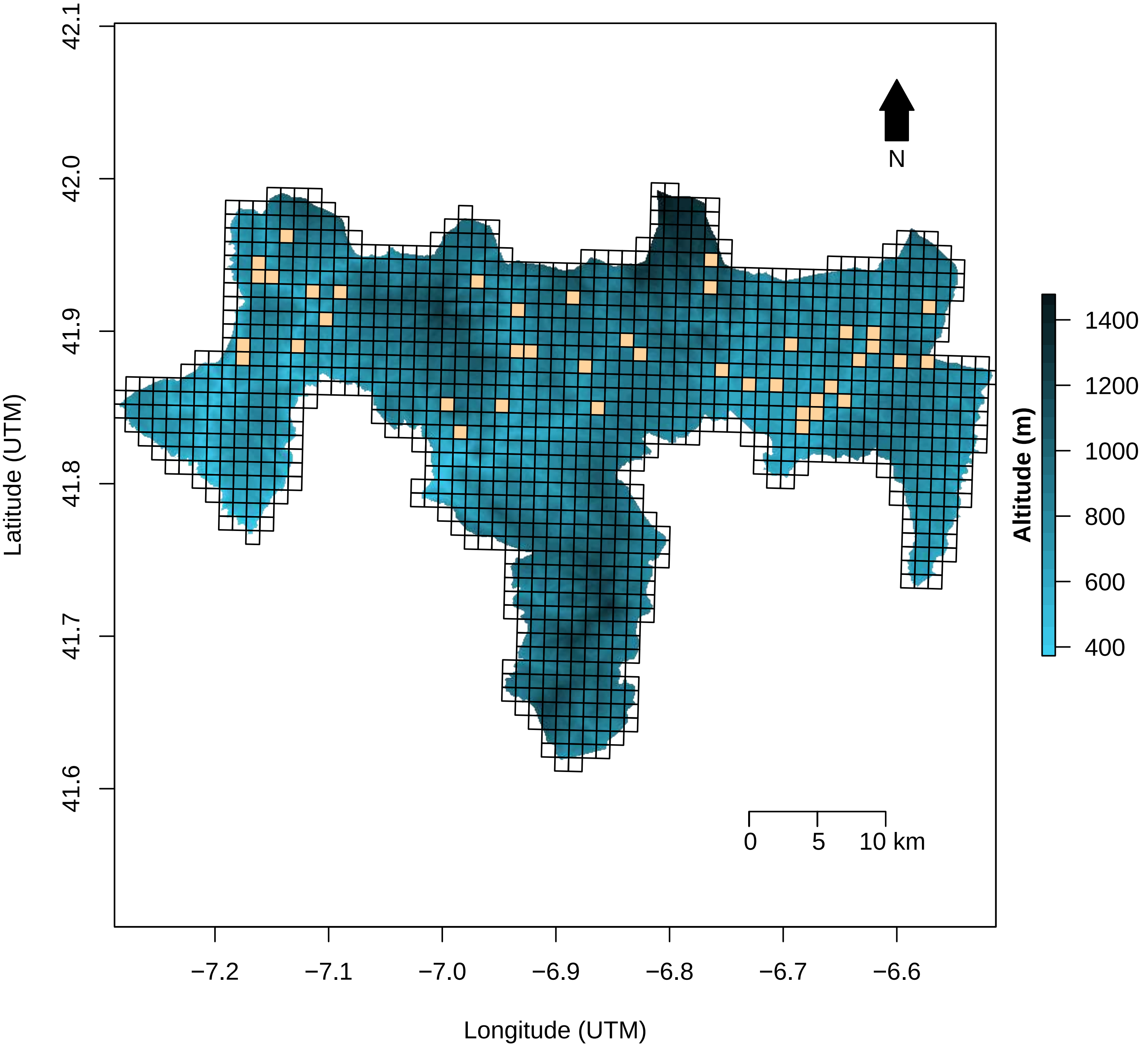

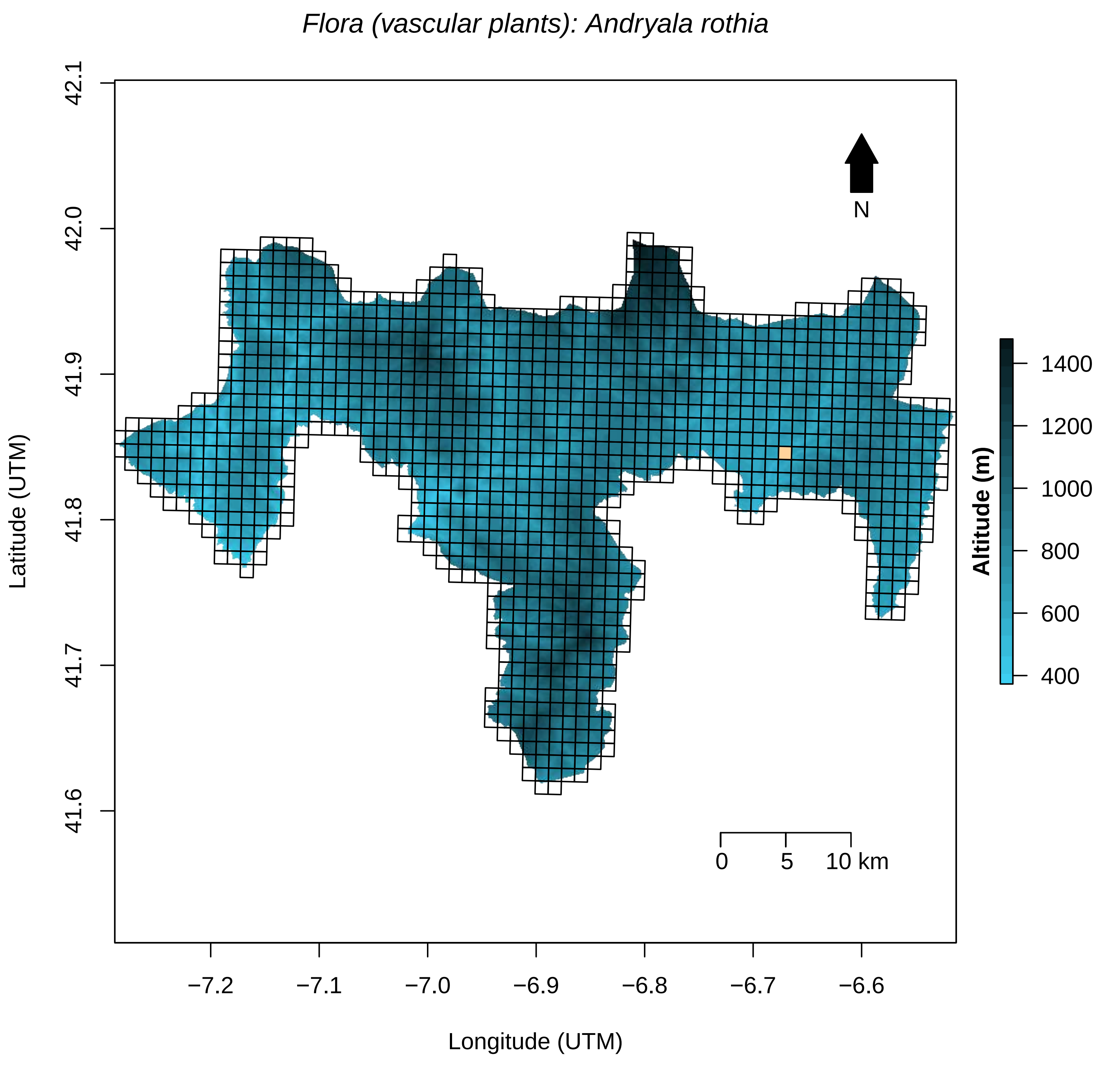
Flora (vascular plants): Andryala rothia
42.1
42.0
41.9
41.8
41.7
41.6
Latitude (UTM)
−7.2
−7.1
−7.0
−6.9
−6.8
−6.7
−6.6
Longitude (UTM)
N
0
5
10 km
Altitude (m)
1400
1200
1000
800
600
400

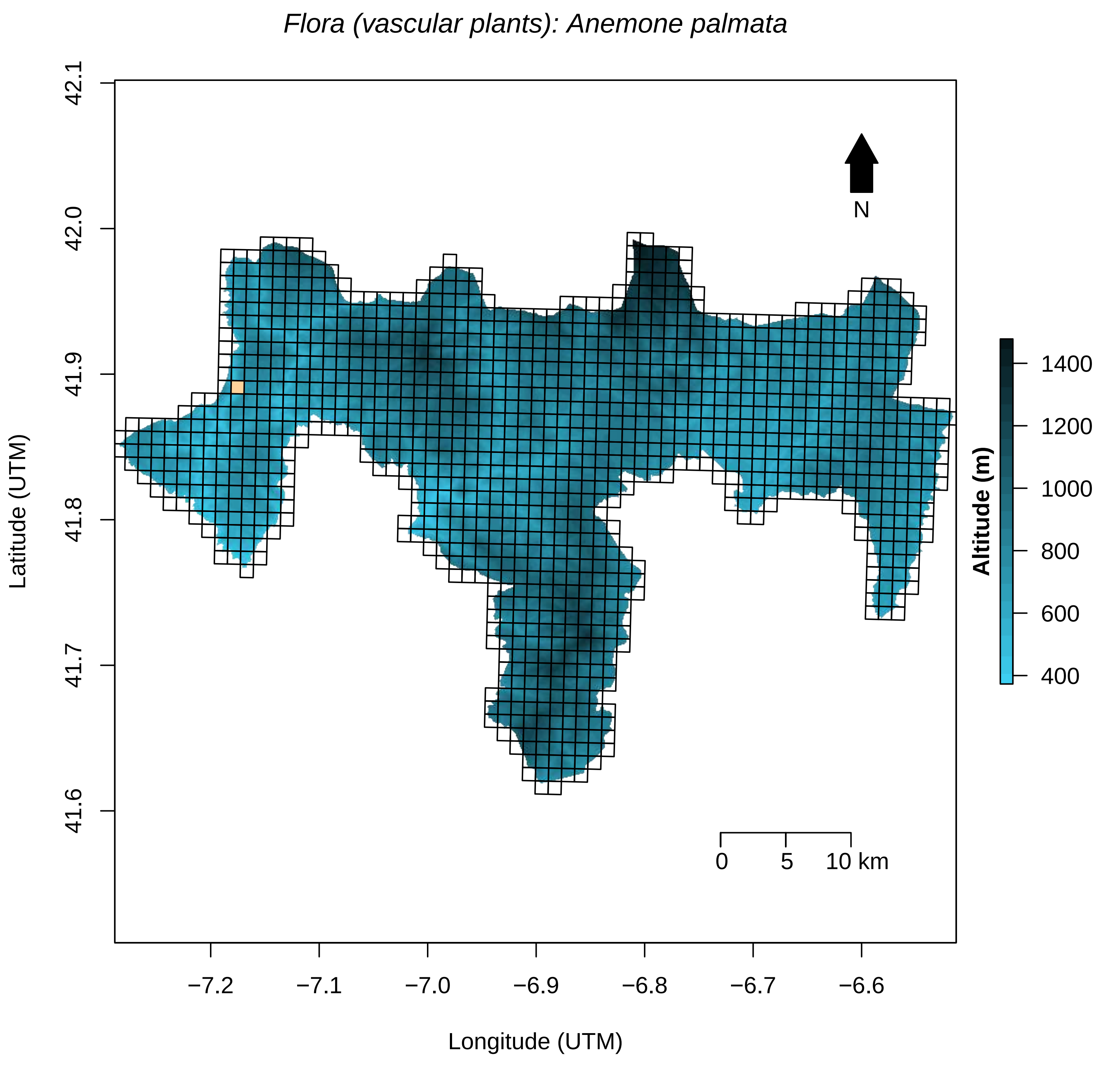

Flora (vascular plants): Anemone palmata
N
0
5
10 km
42.1
42.0
41.9
41.8
41.7
41.6
Latitude (UTM)
−7.2
−7.1
−7.0
−6.9
−6.8
−6.7
−6.6
Longitude (UTM)
Altitude (m)
1400
1200
1000
800
600
400

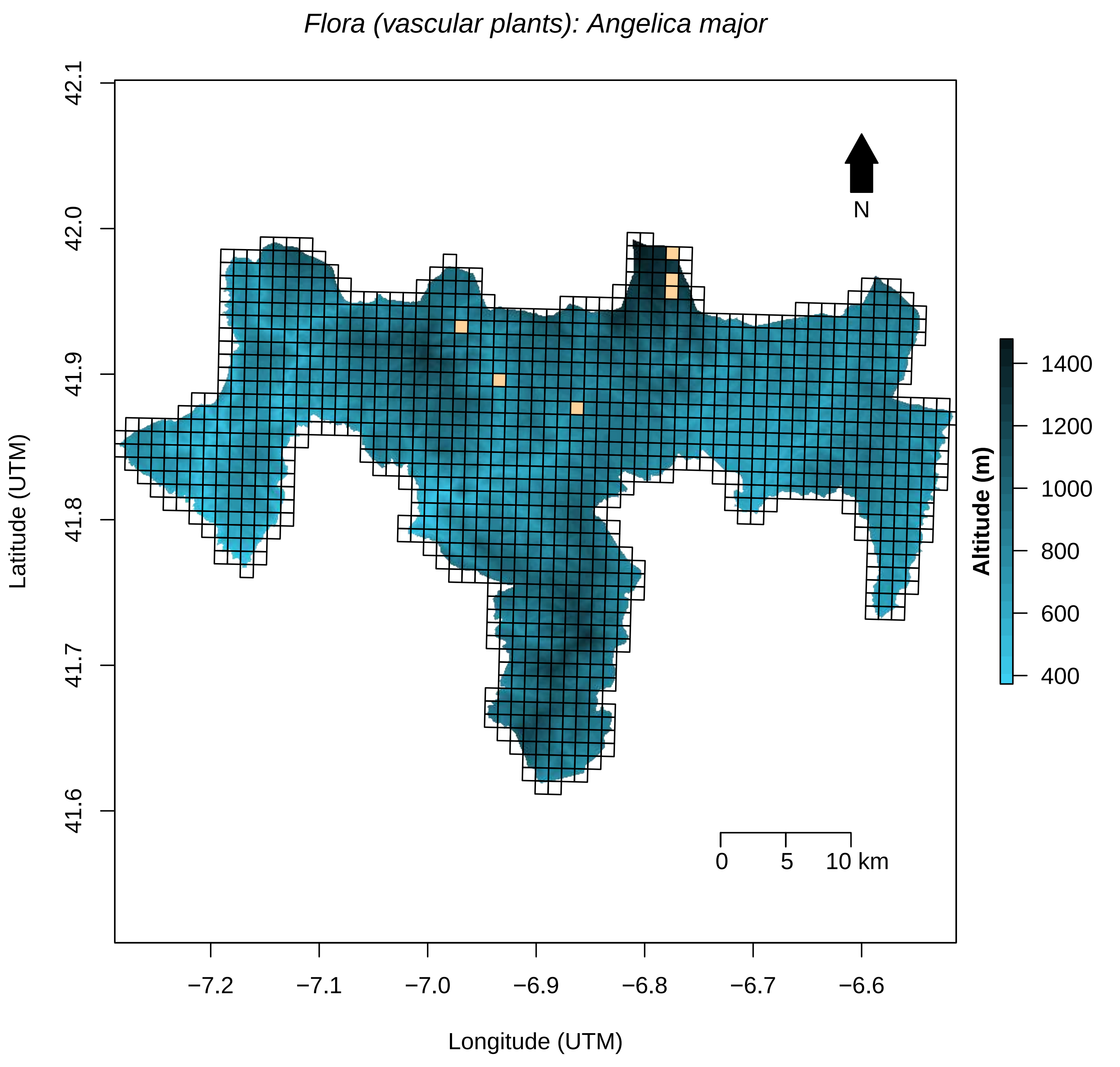
Flora (vascular plants): Angelica major
Latitude (UTM)
42.1
42.0
41.9
41.8
41.7
41.6
Longitude (UTM)
−7.2
−7.1
−7.0
−6.9
−6.8
−6.7
−6.6
N
0
5
10 km
Altitude (m)
1400
1200
1000
800
600
400

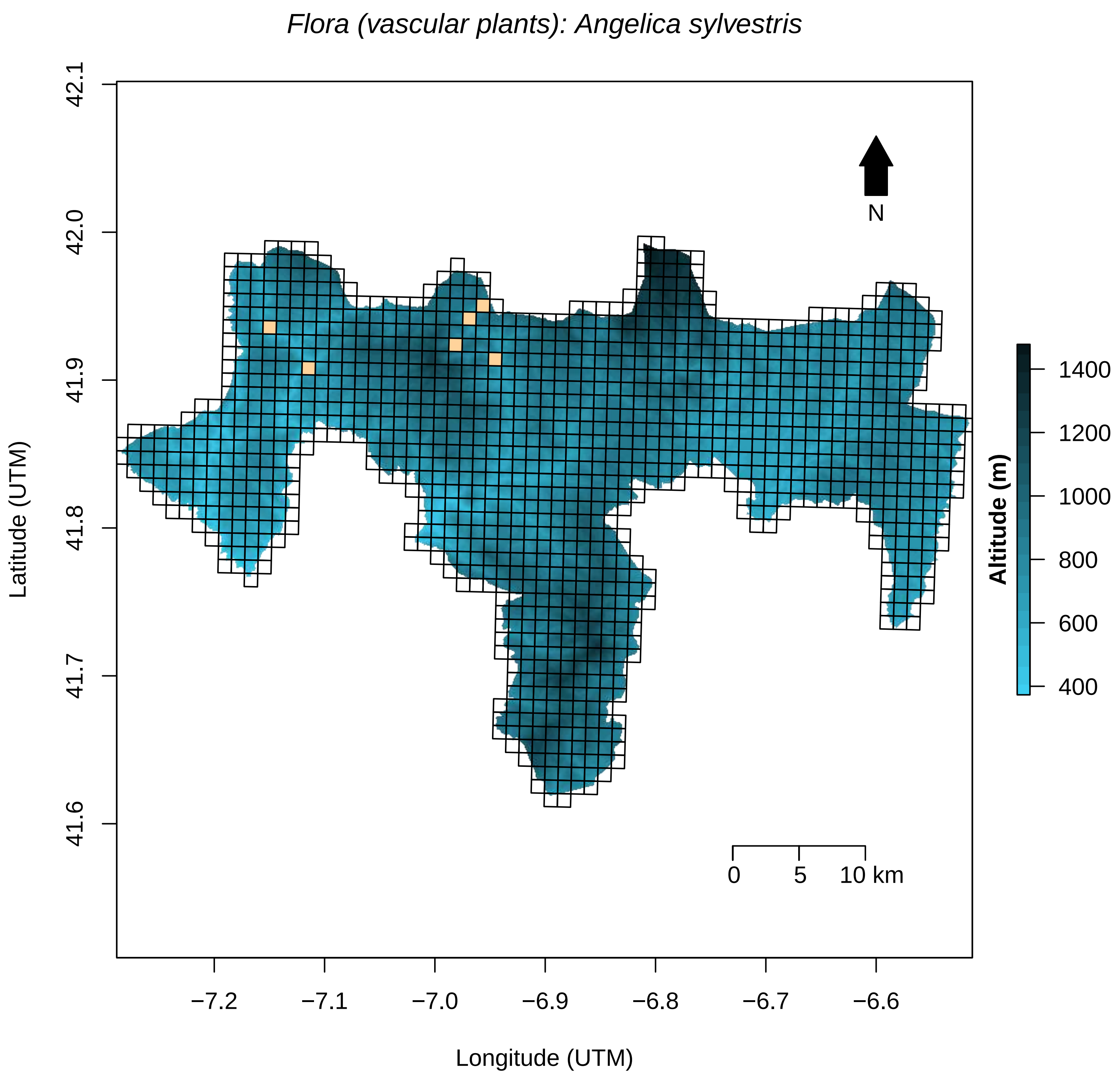

Flora (vascular plants): Angelica sylvestris
42.1
42.0
41.9
41.8
41.7
41.6
Latitude (UTM)
N
0 5 10 km
−7.2 −7.1 −7.0 −6.9 −6.8 −6.7 −6.6
Longitude (UTM)
1400
1200
1000
800
600
400
Altitude (m)

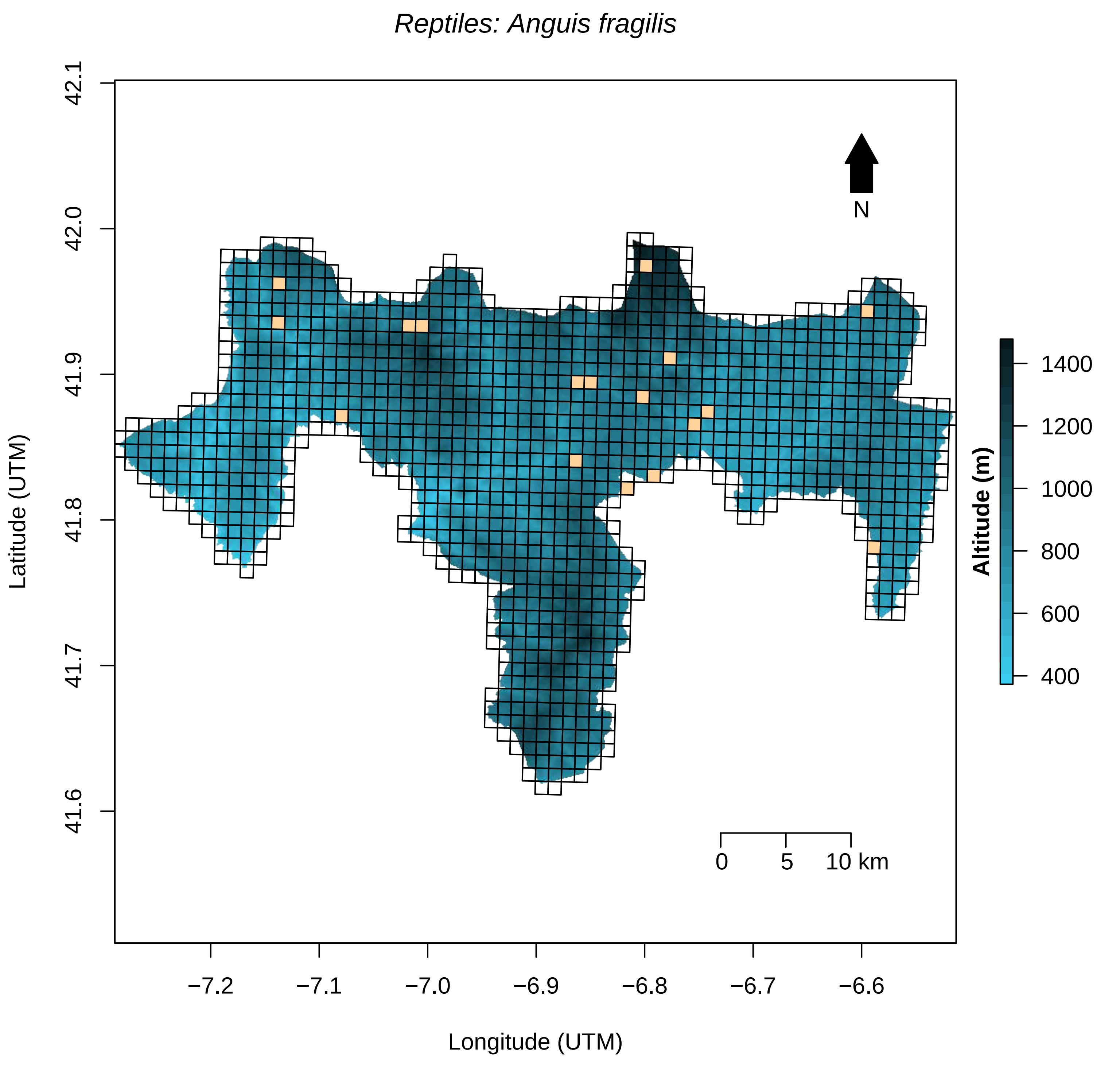

Reptiles: Anguis fragilis
Latitude (UTM)
42.1
42.0
41.9
41.8
41.7
41.6
N
0
5
10 km
−7.2
−7.1
−7.0
−6.9
−6.8
−6.7
−6.6
Longitude (UTM)
Altitude (m)
1400
1200
1000
800
600
400

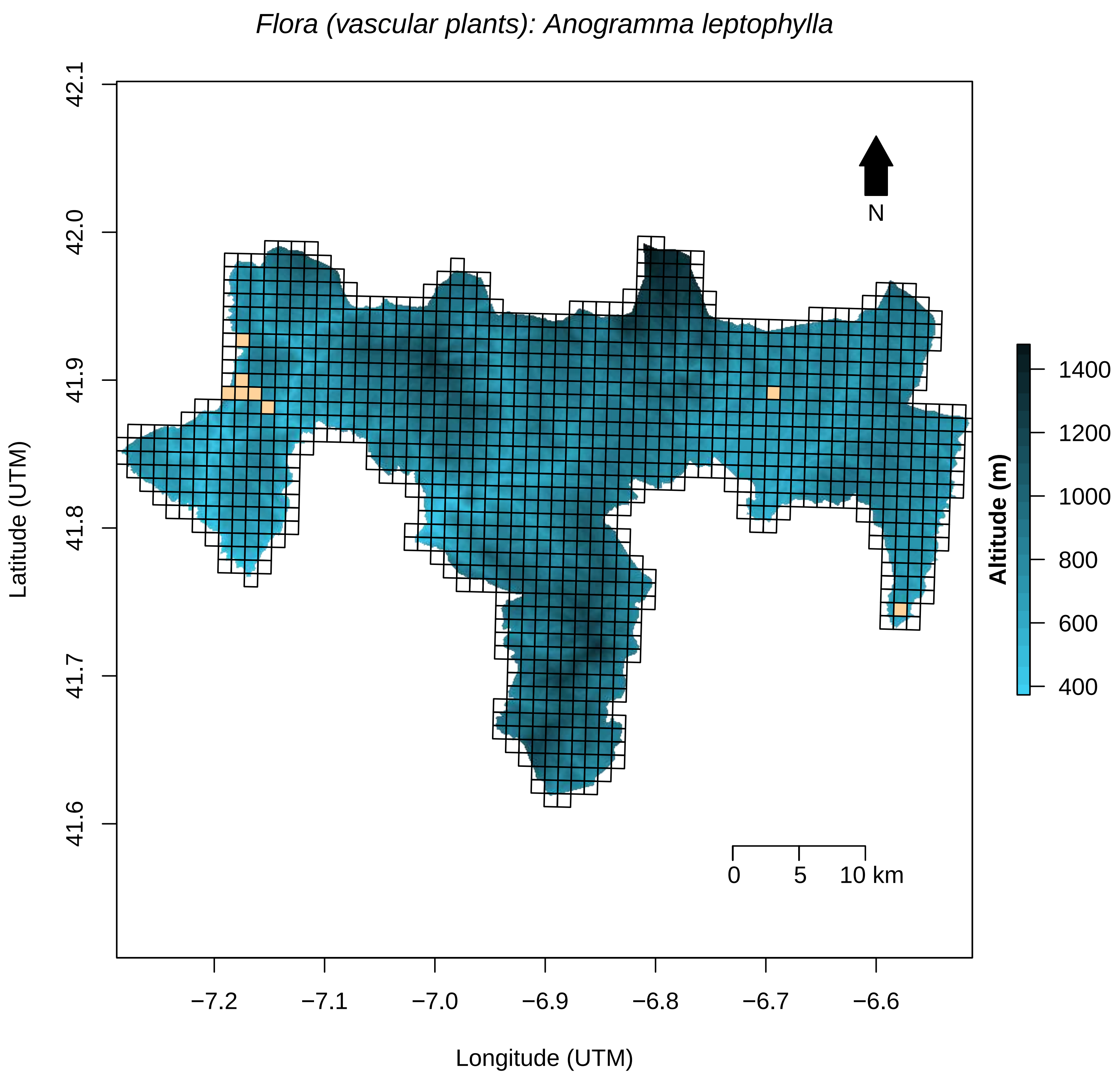
Flora (vascular plants): Anogramma leptophylla
42.1
42.0
41.9
41.8
41.7
41.6
Latitude (UTM)
−7.2
−7.1
−7.0
−6.9
−6.8
−6.7
−6.6
Longitude (UTM)
N
0
5
10 km
Altitude (m)
1400
1200
1000
800
600
400

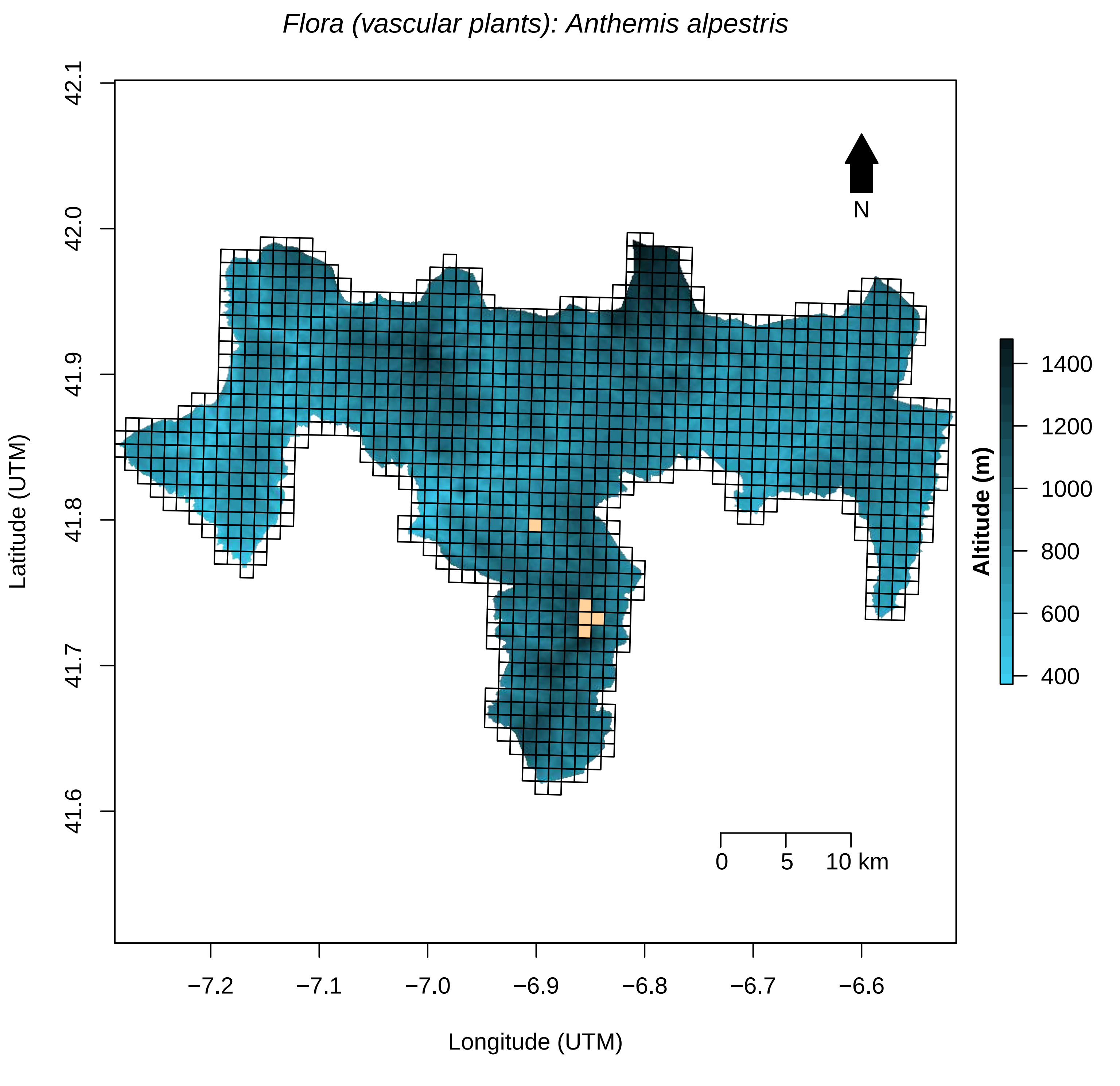

Flora (vascular plants): Anthemis alpestris
42.1
42.0
41.9
41.8
41.7
41.6
Latitude (UTM)
N
0
5
10 km
−7.2
−7.1
−7.0
−6.9
−6.8
−6.7
−6.6
Longitude (UTM)
1400
1200
1000
800
600
400
Altitude (m)

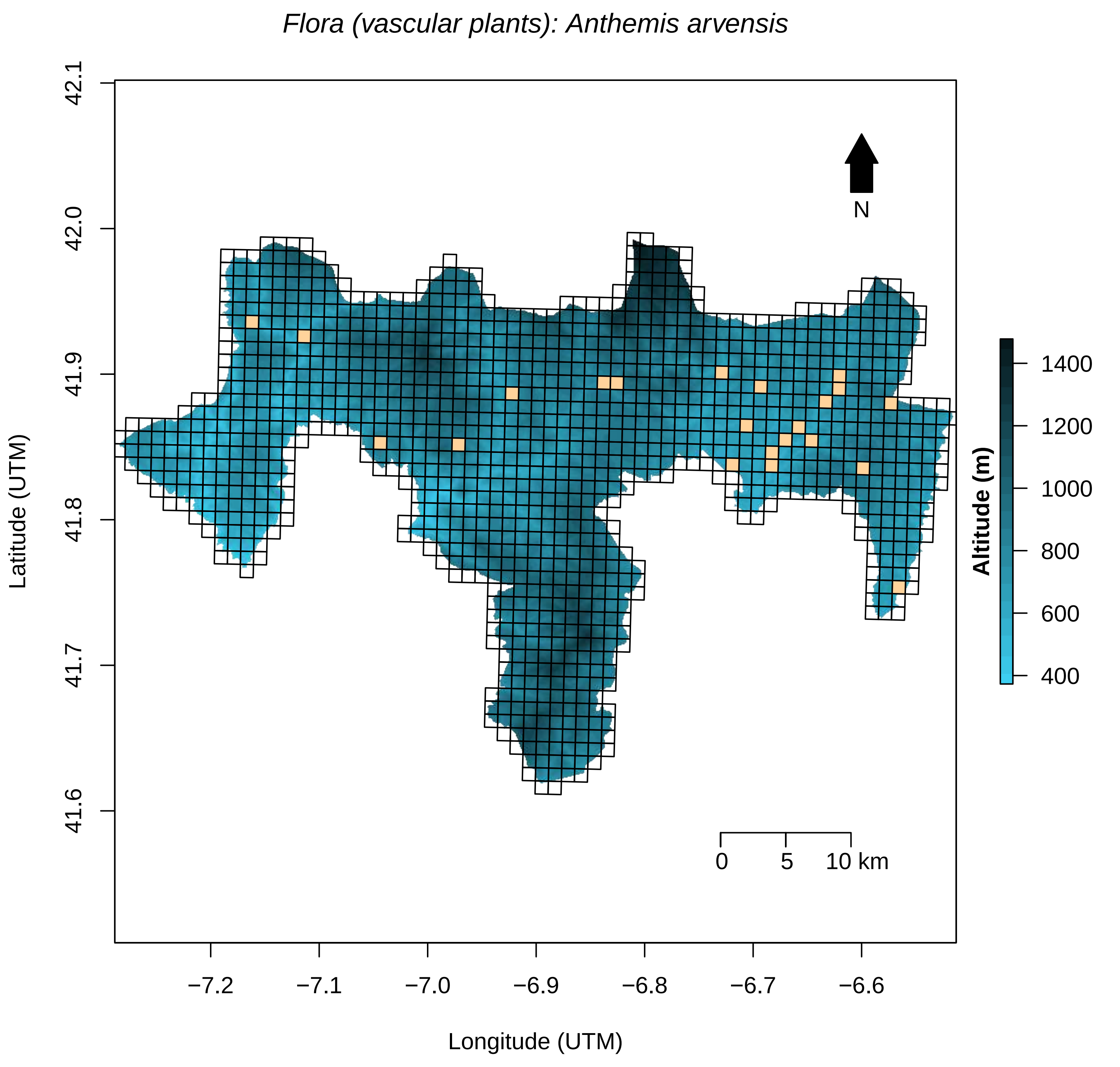
Flora (vascular plants): Anthemis arvensis
42.1
42.0
41.9
41.8
41.7
41.6
Latitude (UTM)
−7.2
−7.1
−7.0
−6.9
−6.8
−6.7
−6.6
Longitude (UTM)
N
0
5
10 km
Altitude (m)
1400
1200
1000
800
600
400

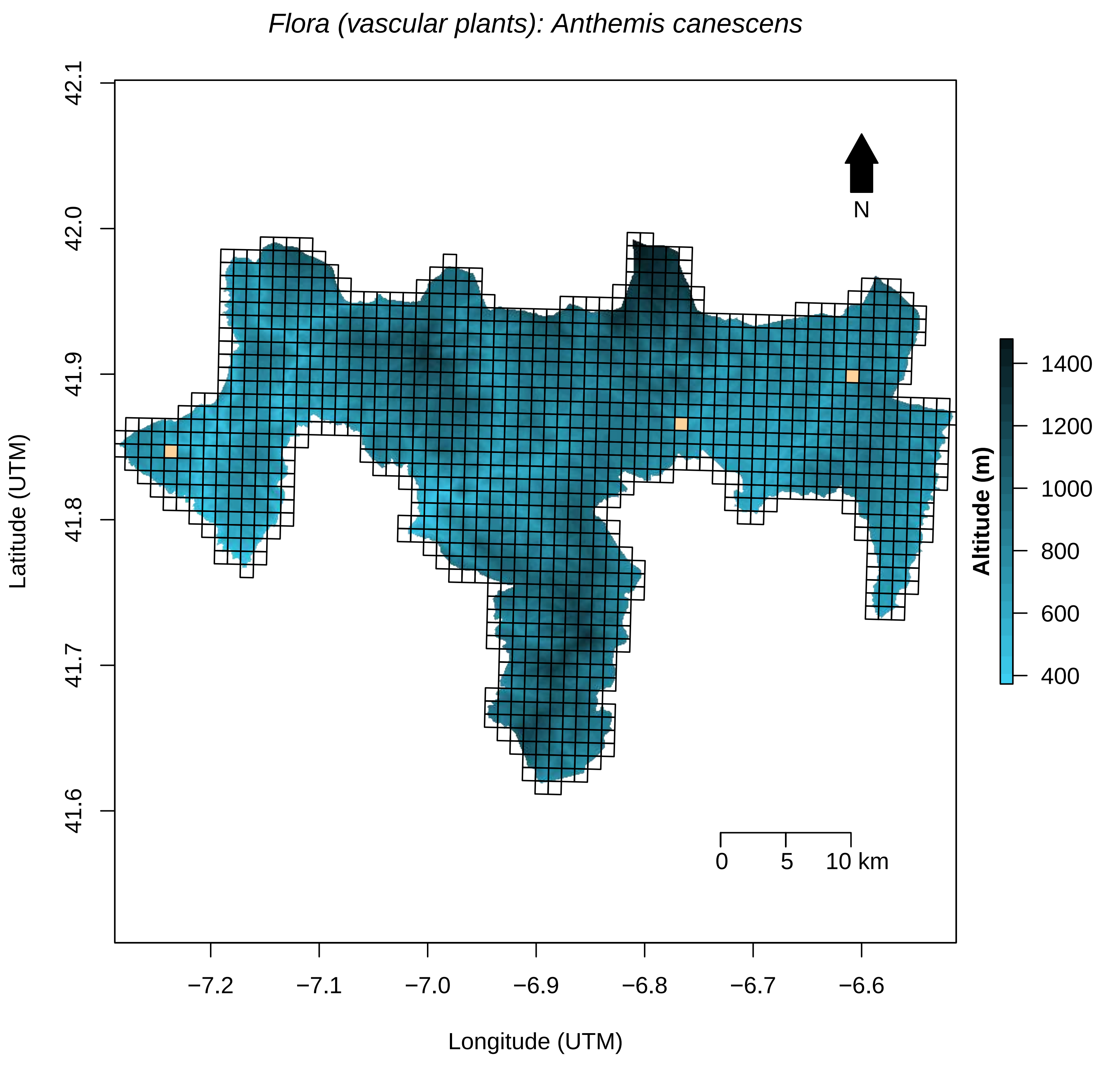
Flora (vascular plants): Anthemis canescens
Latitude (UTM)
Longitude (UTM)
42.1
42.0
41.9
41.8
41.7
41.6
−7.2
−7.1
−7.0
−6.9
−6.8
−6.7
−6.6
N
0
5
10 km
Altitude (m)
1400
1200
1000
800
600
400

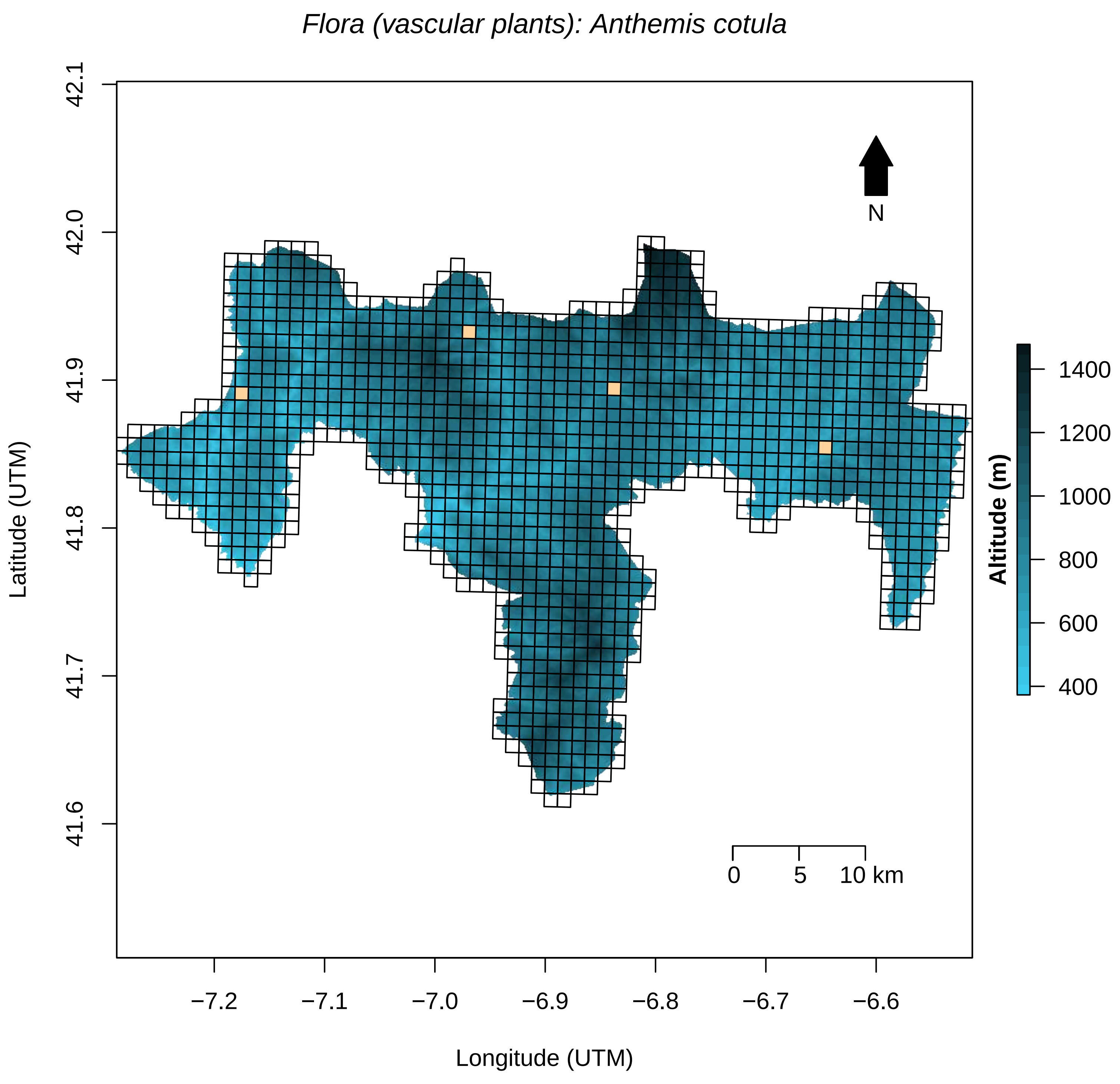

Flora (vascular plants): Anthemis cotula
42.1
42.0
41.9
41.8
41.7
41.6
Latitude (UTM)
−7.2
−7.1
−7.0
−6.9
−6.8
−6.7
−6.6
Longitude (UTM)
N
0
5
10 km
Altitude (m)
1400
1200
1000
800
600
400

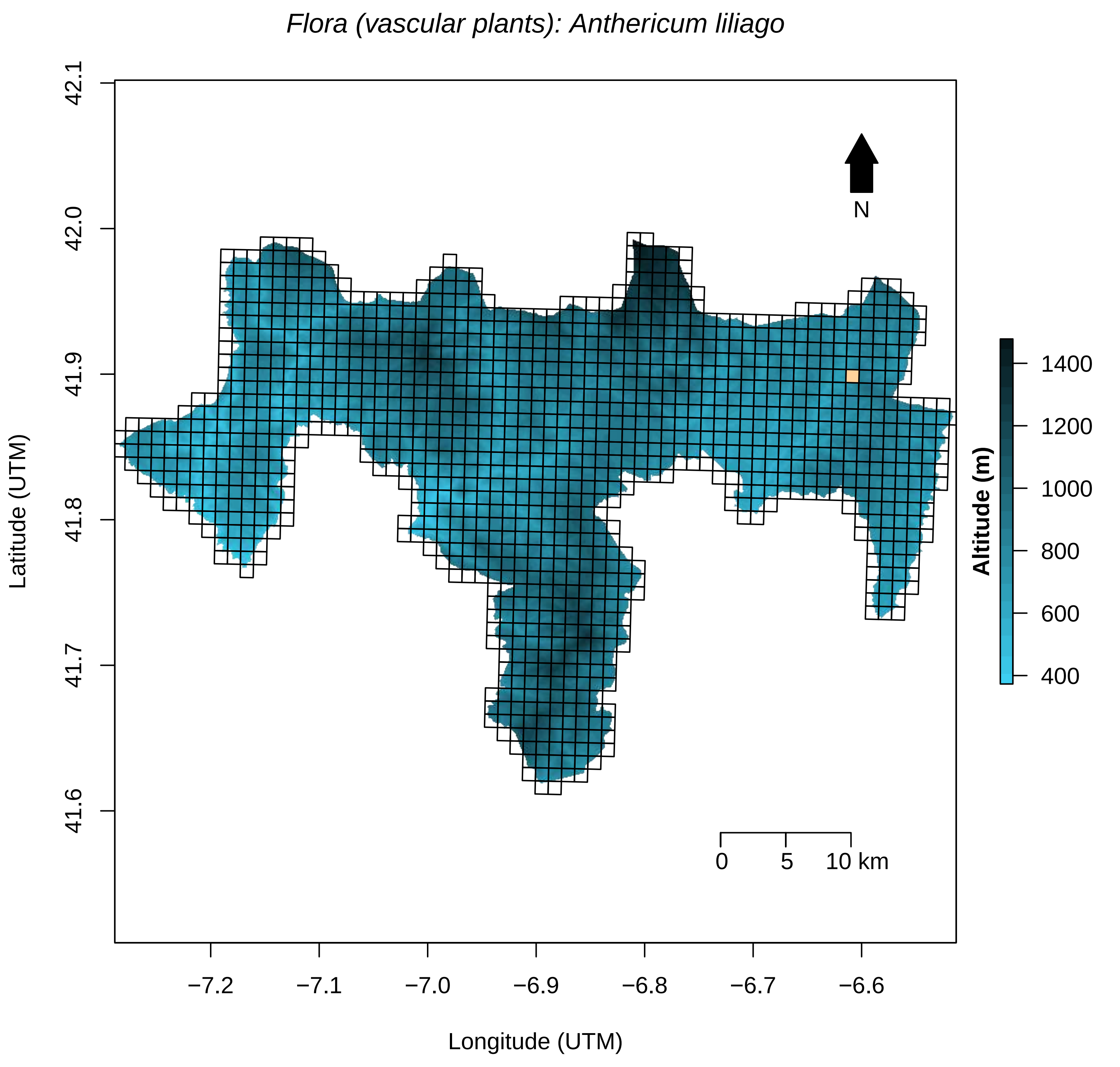

Flora (vascular plants): Anthericum liliago
42.1
42.0
41.9
41.8
41.7
41.6
Latitude (UTM)
−7.2
−7.1
−7.0
−6.9
−6.8
−6.7
−6.6
Longitude (UTM)
N
0
5
10 km
1400
1200
1000
800
600
400
Altitude (m)

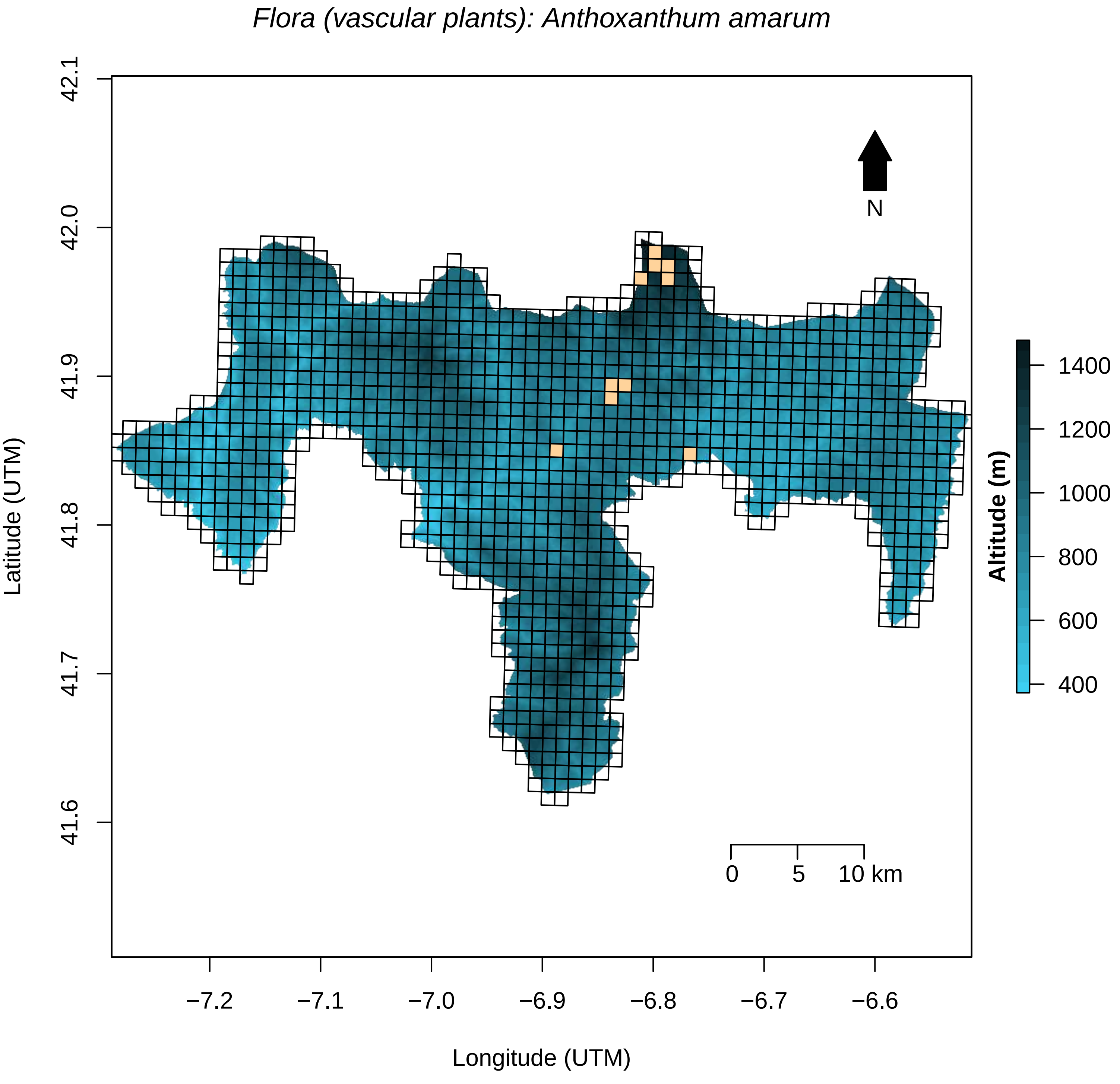
Flora (vascular plants): Anthoxanthum amarum
N
0 5 10 km
Altitude (m)
1400
1200
1000
800
600
400
Latitude (UTM)
42.1
42.0
41.9
41.8
41.7
41.6
Longitude (UTM)
−7.2
−7.1
−7.0
−6.9
−6.8
−6.7
−6.6

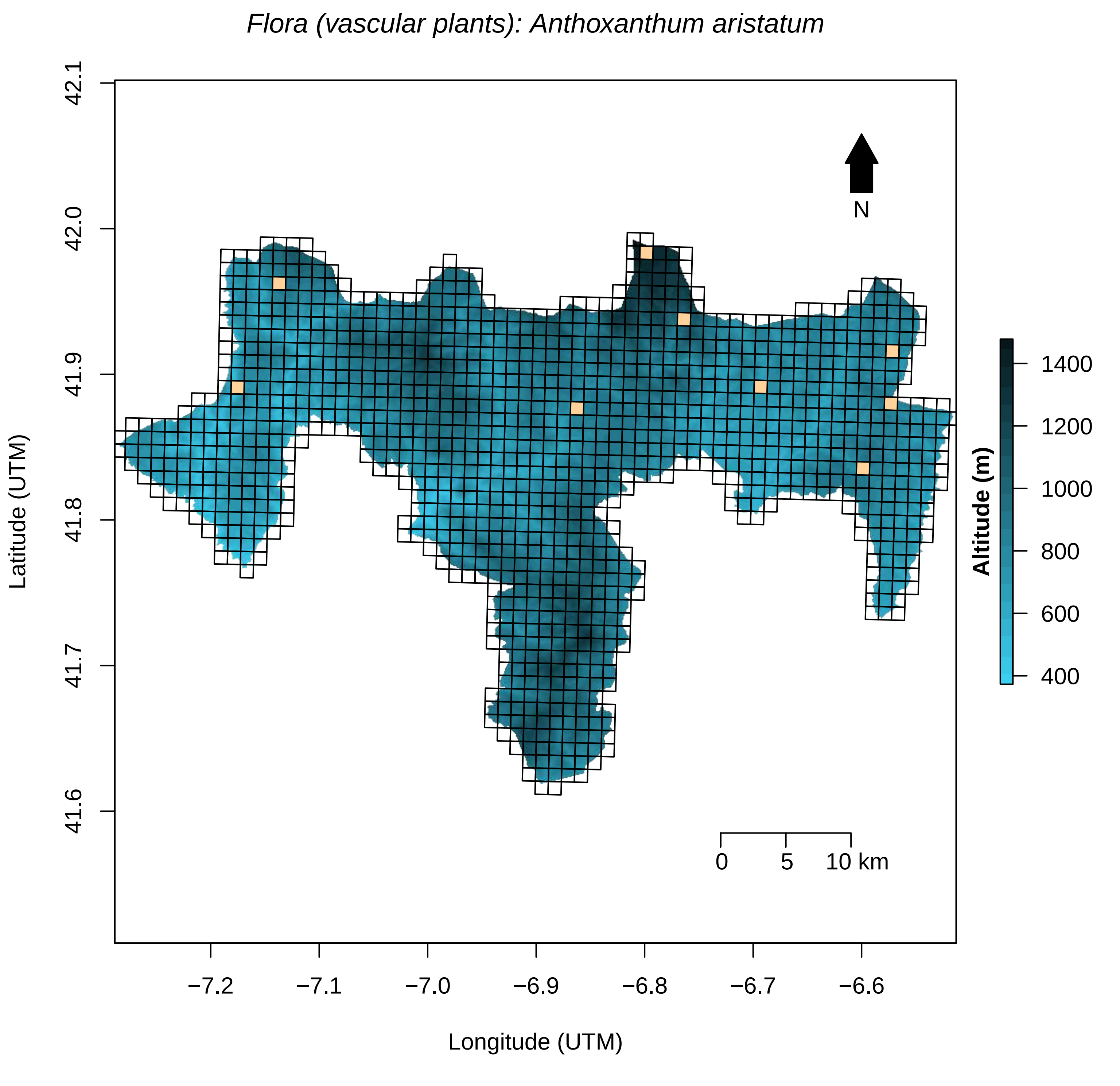

Flora (vascular plants): Anthoxanthum aristatum
Latitude (UTM)
42.1
42.0
41.9
41.8
41.7
41.6
N
0 5 10 km
−7.2
−7.1
−7.0
−6.9
−6.8
−6.7
−6.6
Longitude (UTM)
Altitude (m)
1400
1200
1000
800
600
400

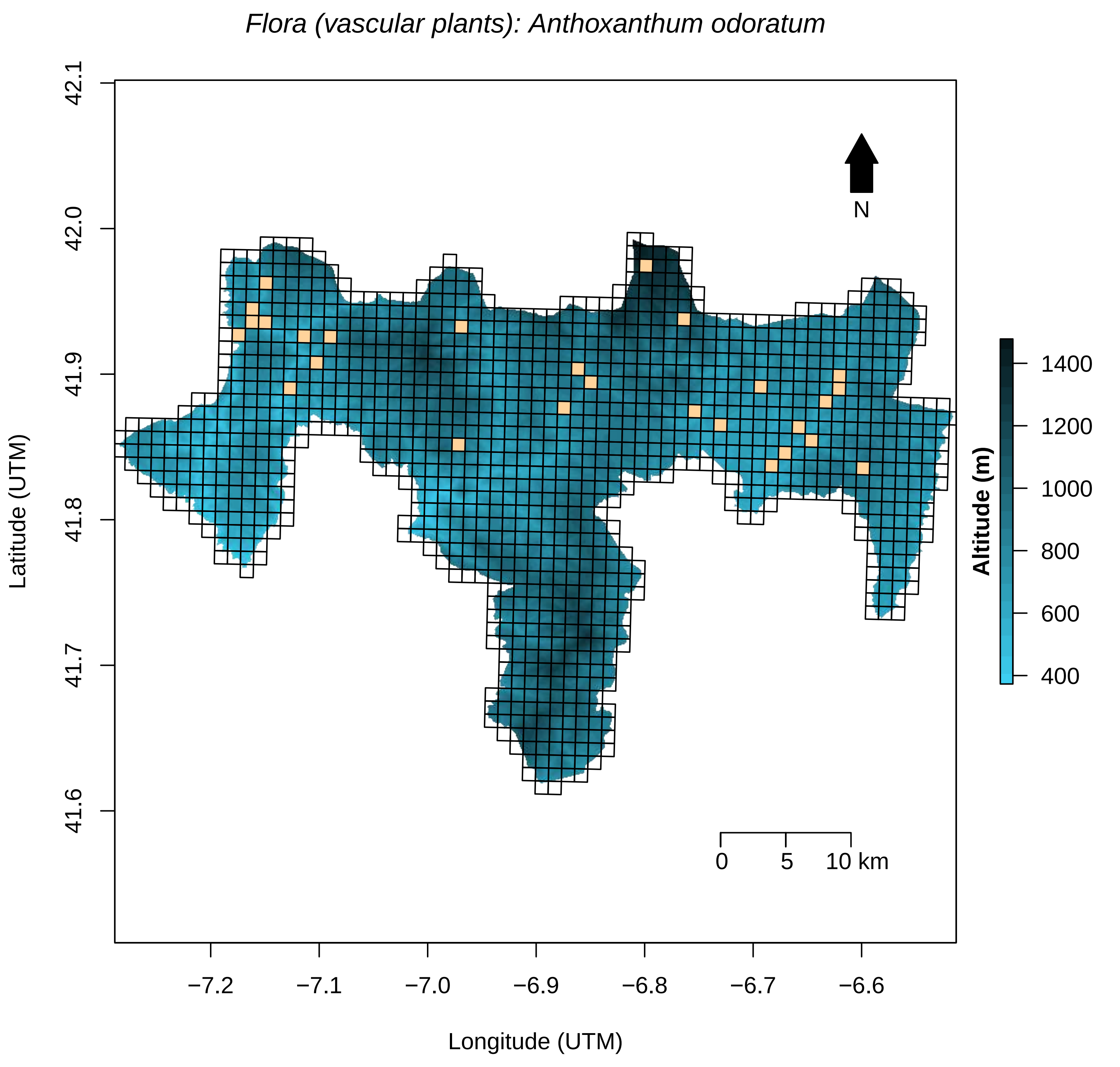

Flora (vascular plants): Anthoxanthum odoratum
42.1
42.0
41.9
41.8
41.7
41.6
Latitude (UTM)
−7.2
−7.1
−7.0
−6.9
−6.8
−6.7
−6.6
Longitude (UTM)
N
0
5
10 km
Altitude (m)
1400
1200
1000
800
600
400

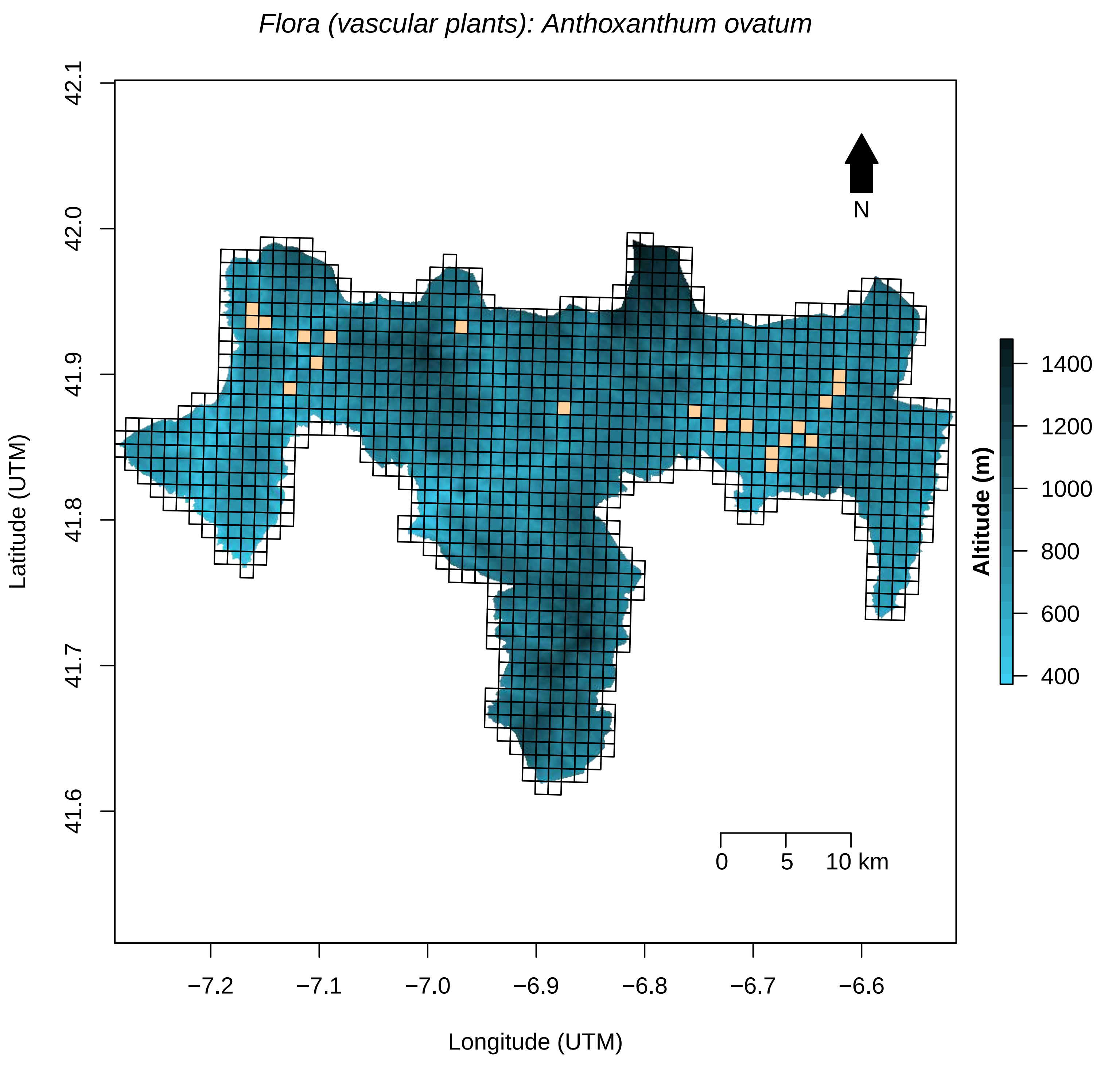

Flora (vascular plants): Anthoxanthum ovatum
Latitude (UTM)
42.1
42.0
41.9
41.8
41.7
41.6
Longitude (UTM)
−7.2
−7.1
−7.0
−6.9
−6.8
−6.7
−6.6
N
0
5
10 km
Altitude (m)
1400
1200
1000
800
600
400

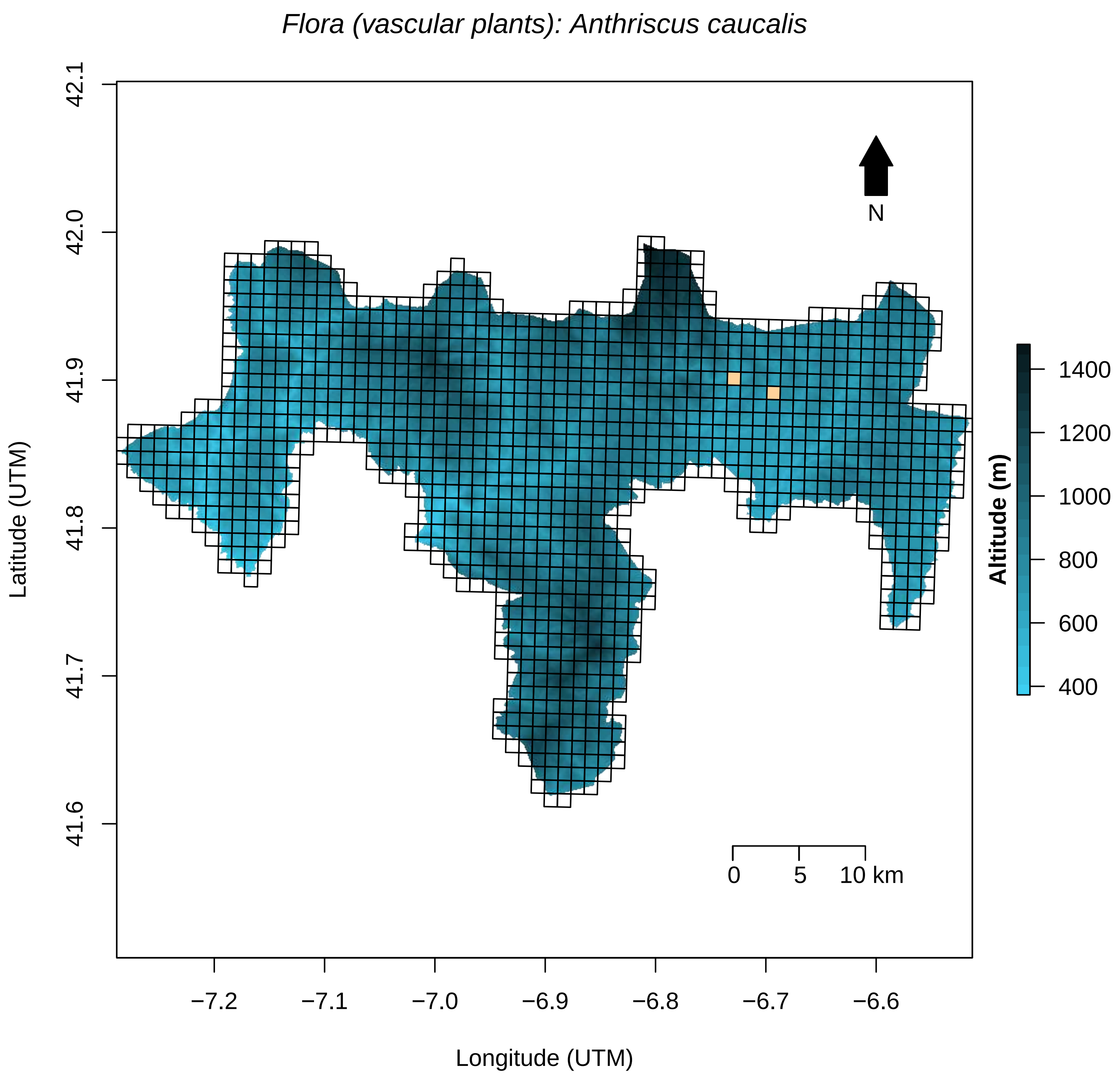

Flora (vascular plants): Anthriscus caucalis
Latitude (UTM)
Longitude (UTM)
42.1
42.0
41.9
41.8
41.7
41.6
−7.2
−7.1
−7.0
−6.9
−6.8
−6.7
−6.6
N
0
5
10 km
Altitude (m)
1400
1200
1000
800
600
400

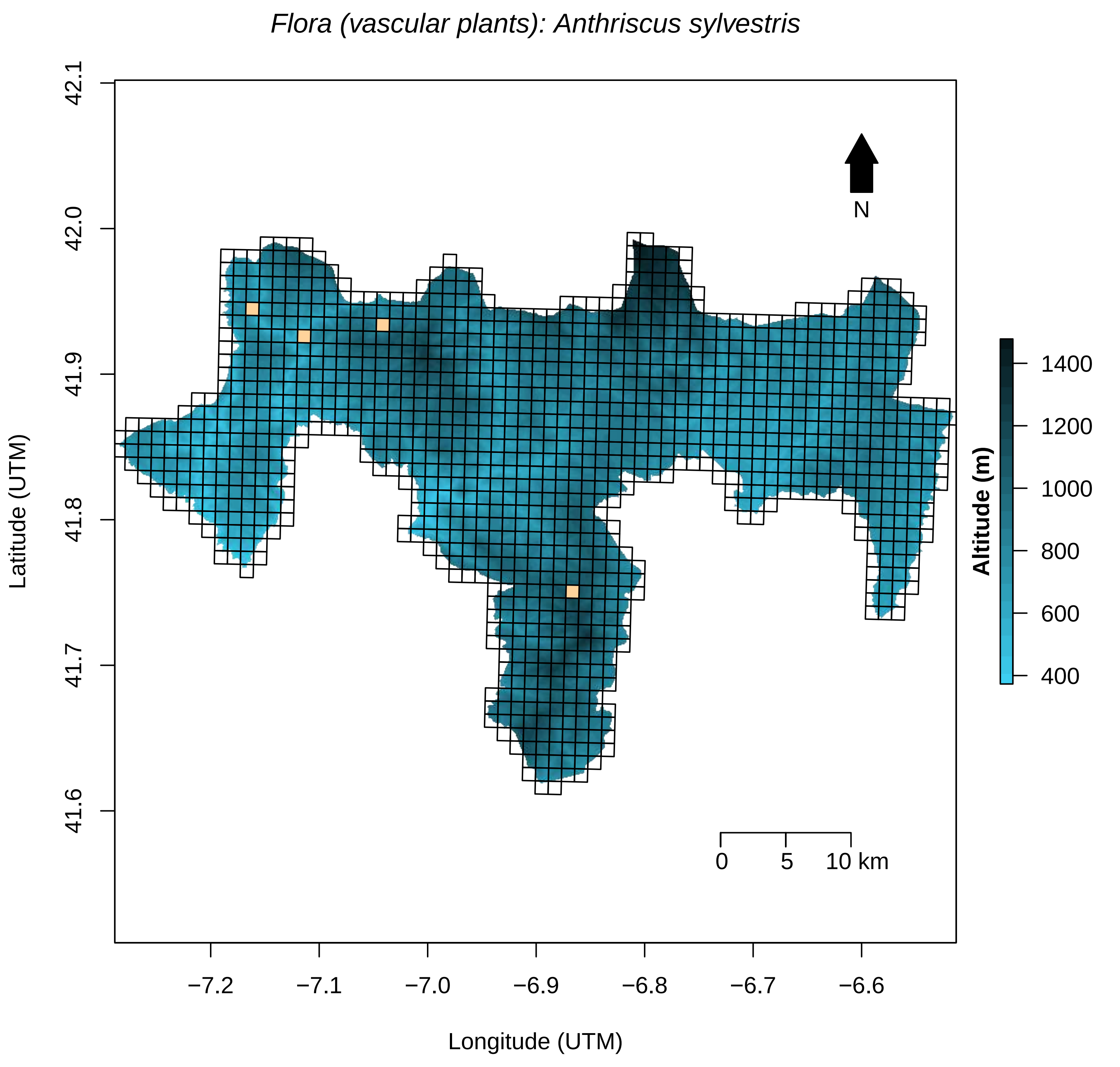

Flora (vascular plants): Anthriscus sylvestris
Latitude (UTM)
Longitude (UTM)
42.1
42.0
41.9
41.8
41.7
41.6
−7.2
−7.1
−7.0
−6.9
−6.8
−6.7
−6.6
N
0
5
10 km
Altitude (m)
1400
1200
1000
800
600
400

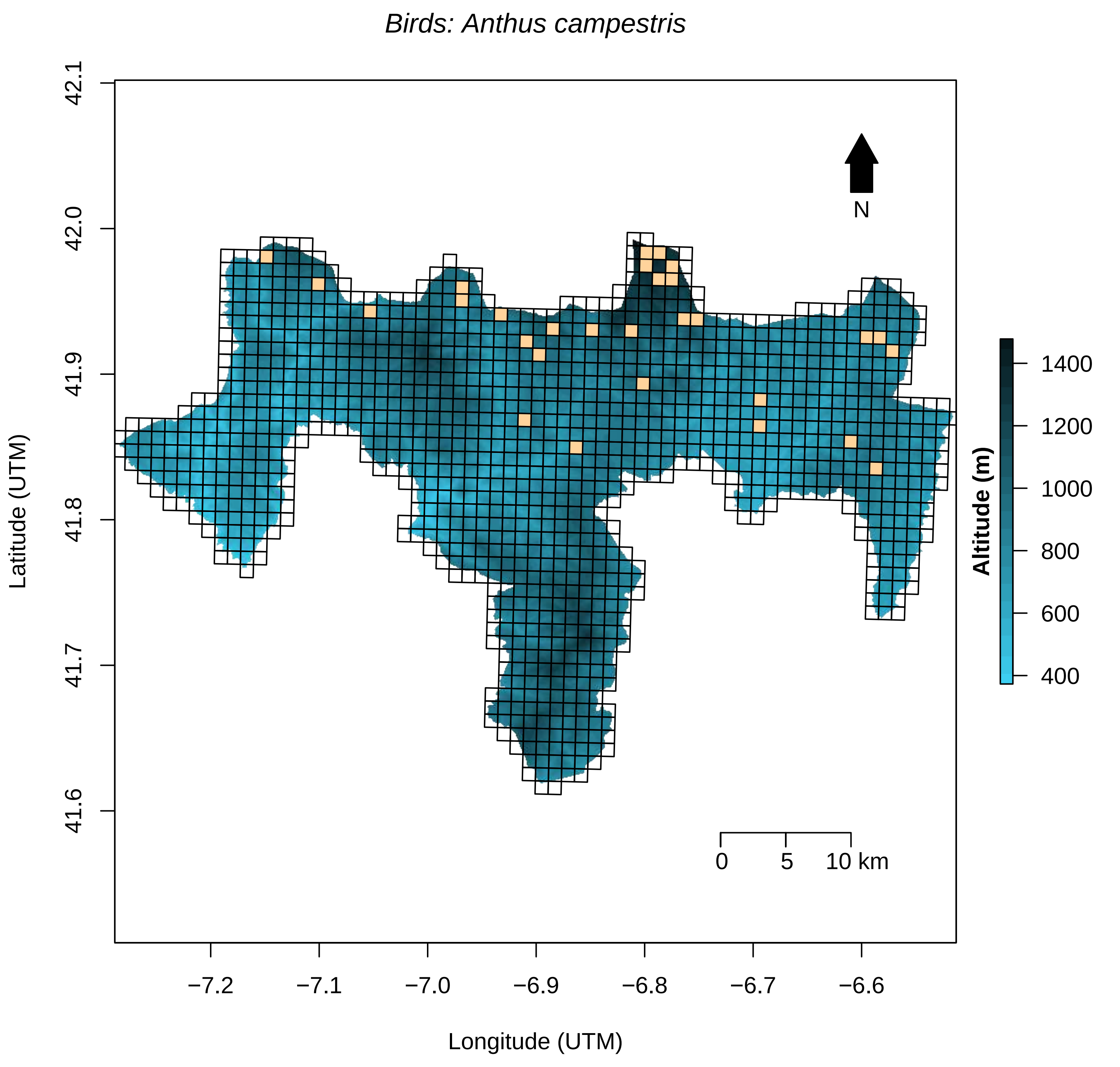
Birds: Anthus campestris
42.1
42.0
41.9
41.8
41.7
41.6
Latitude (UTM)
−7.2
−7.1
−7.0
−6.9
−6.8
−6.7
−6.6
Longitude (UTM)
N
0
5
10 km
Altitude (m)
1400
1200
1000
800
600
400

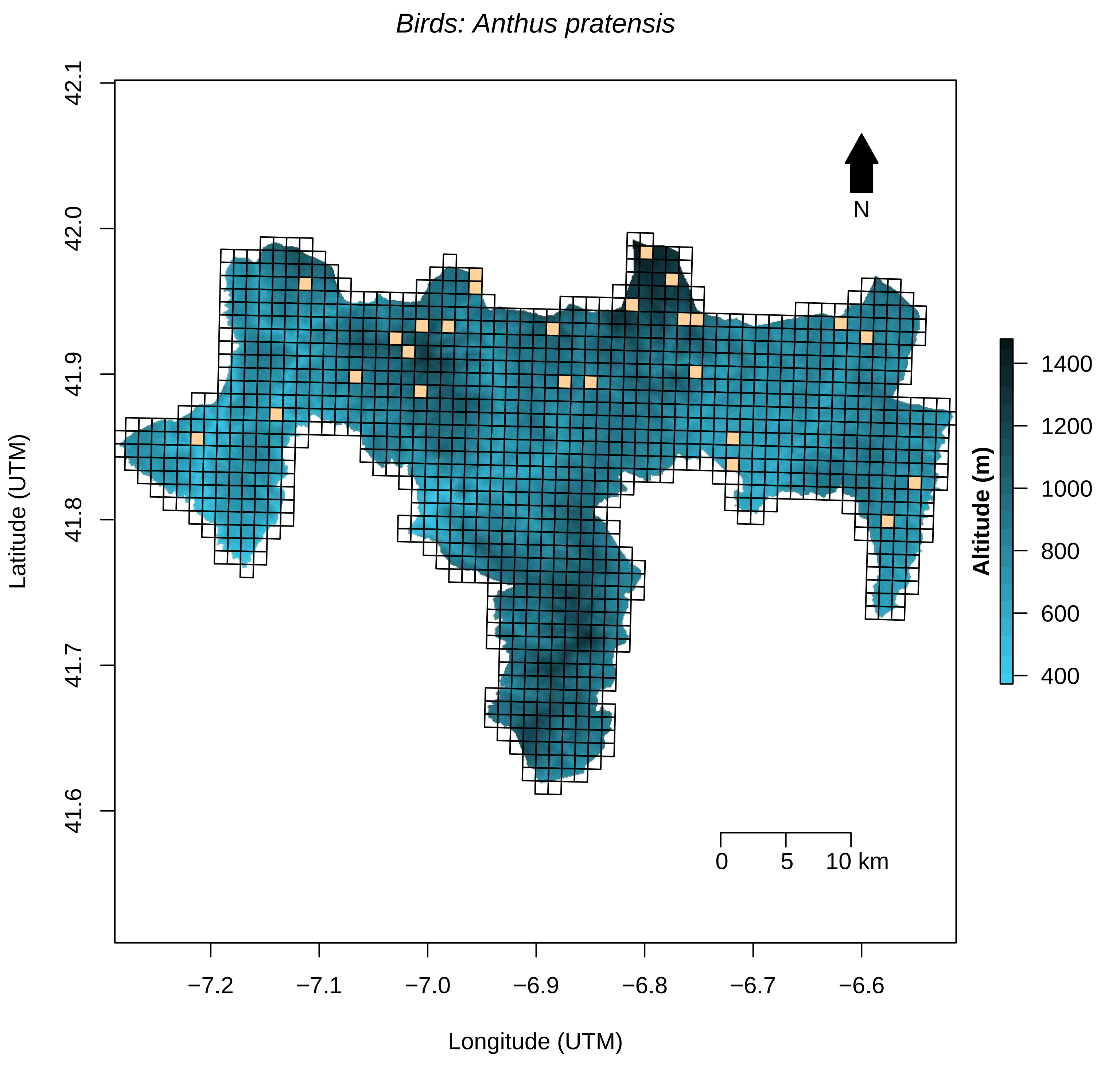

Birds: Anthus pratensis
42.1
42.0
41.9
41.8
41.7
41.6
Latitude (UTM)
−7.2
−7.1
−7.0
−6.9
−6.8
−6.7
−6.6
Longitude (UTM)
N
0
5
10 km
1400
1200
1000
800
600
400
Altitude (m)

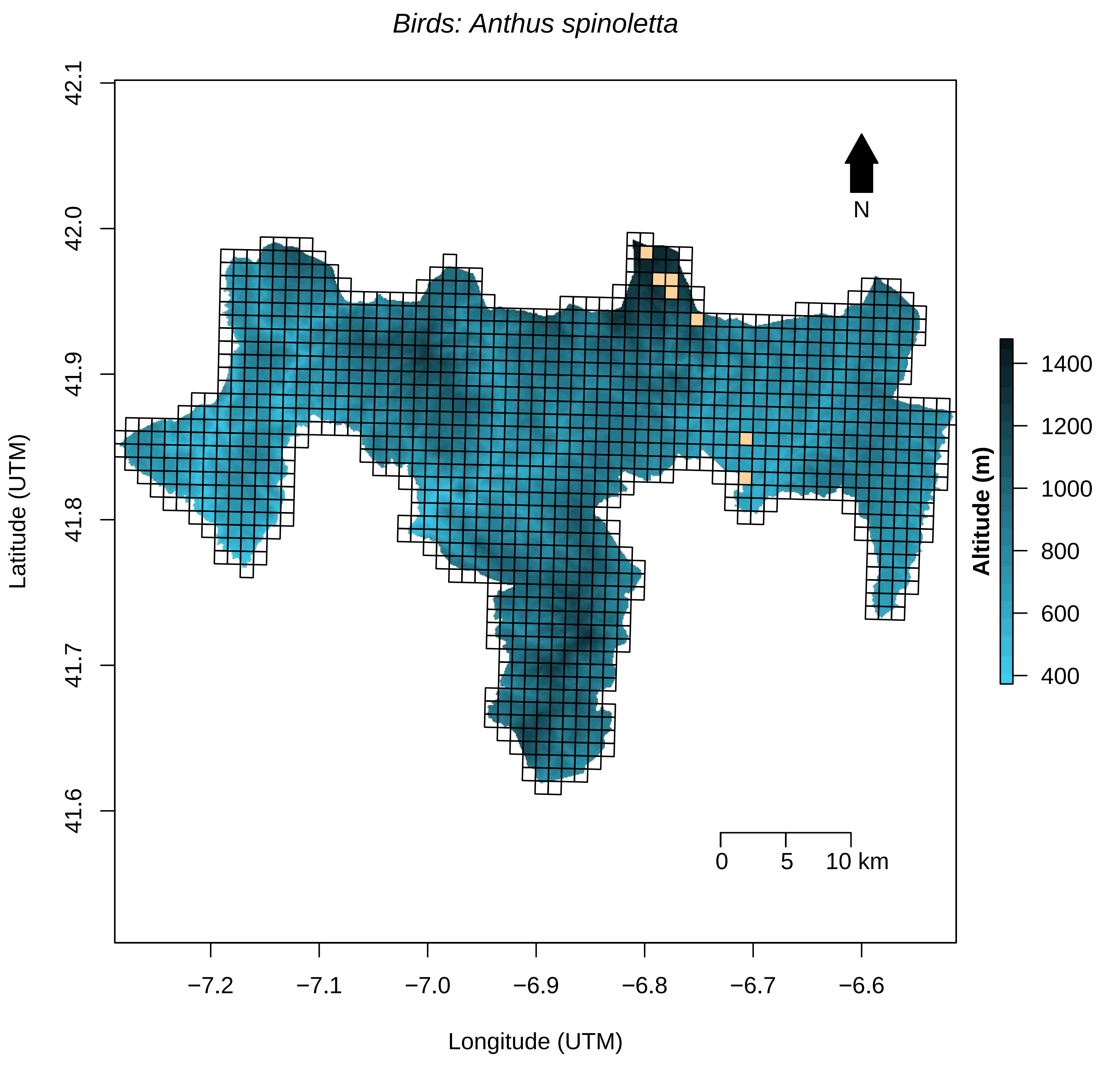

Birds: Anthus spinoletta
N
0
5
10 km
Altitude (m)
1400
1200
1000
800
600
400
Latitude (UTM)
42.1
42.0
41.9
41.8
41.7
41.6
Longitude (UTM)
−7.2
−7.1
−7.0
−6.9
−6.8
−6.7
−6.6

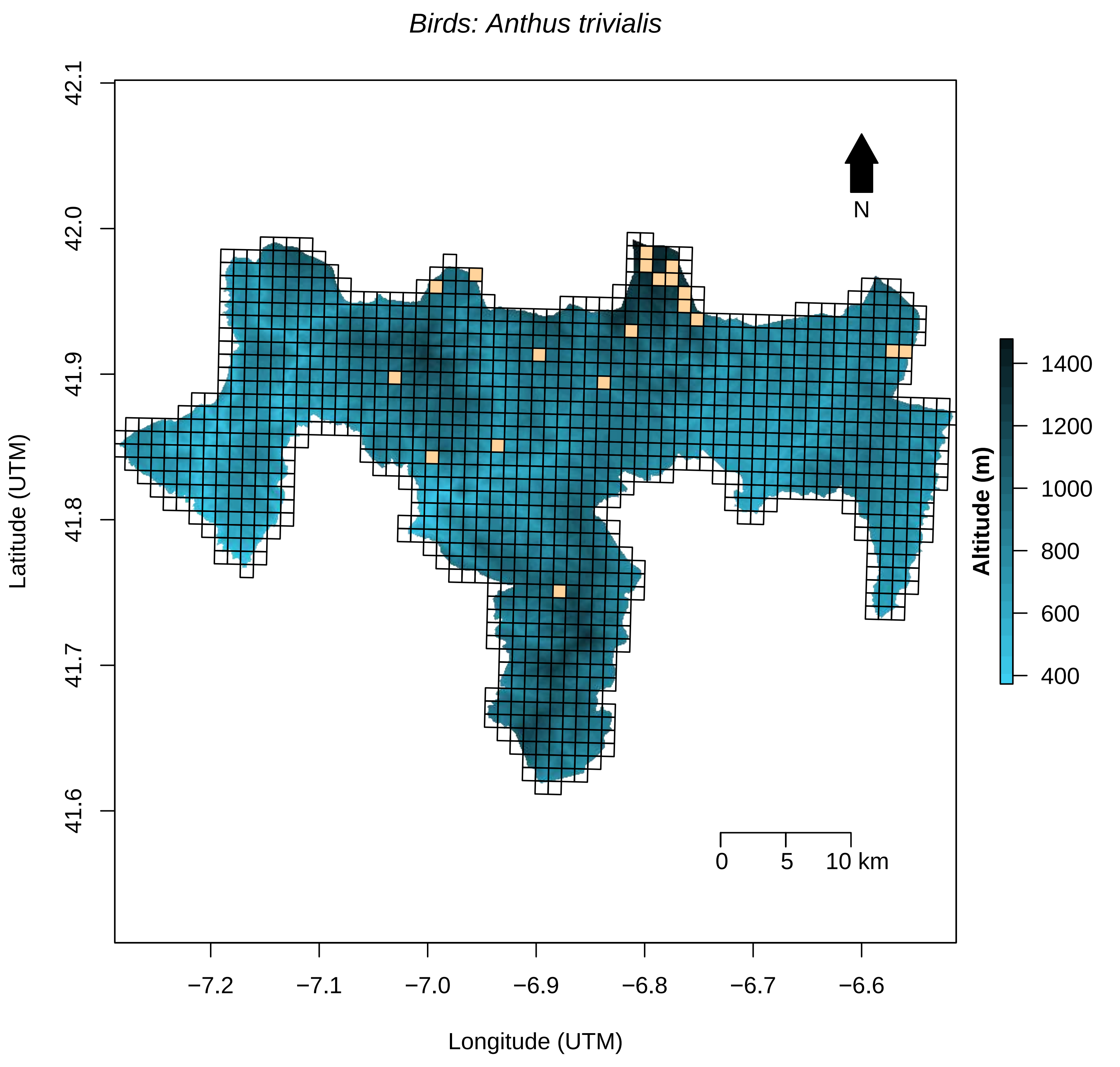
Birds: Anthus trivialis
Latitude (UTM)
Longitude (UTM)
42.1
42.0
41.9
41.8
41.7
41.6
−7.2
−7.1
−7.0
−6.9
−6.8
−6.7
−6.6
N
0
5
10 km
Altitude (m)
1400
1200
1000
800
600
400

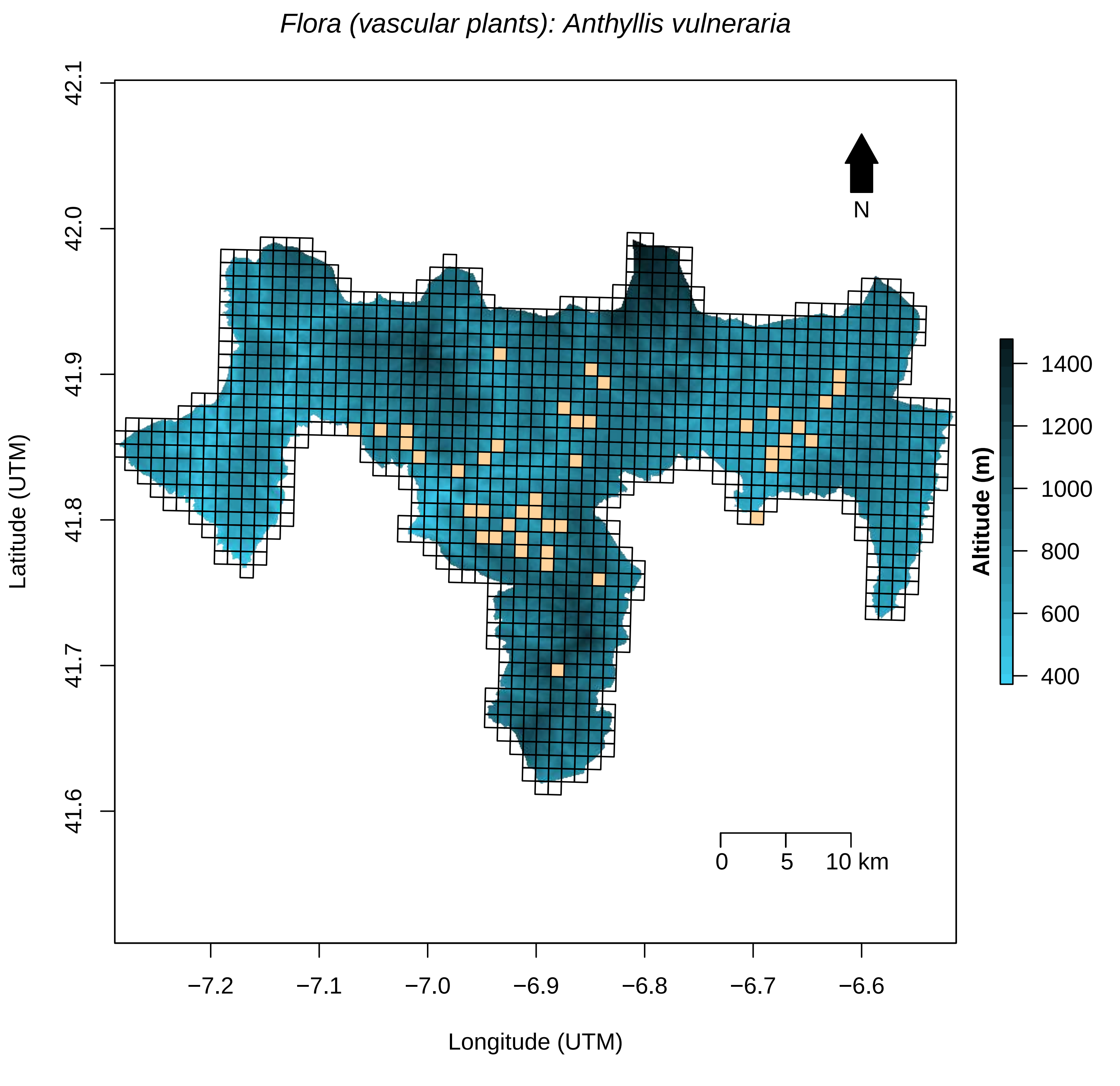

Flora (vascular plants): Anthyllis vulneraria
Latitude (UTM)
42.1
42.0
41.9
41.8
41.7
41.6
N
0
5
10 km
Altitude (m)
1400
1200
1000
800
600
400
−7.2
−7.1
−7.0
−6.9
−6.8
−6.7
−6.6
Longitude (UTM)

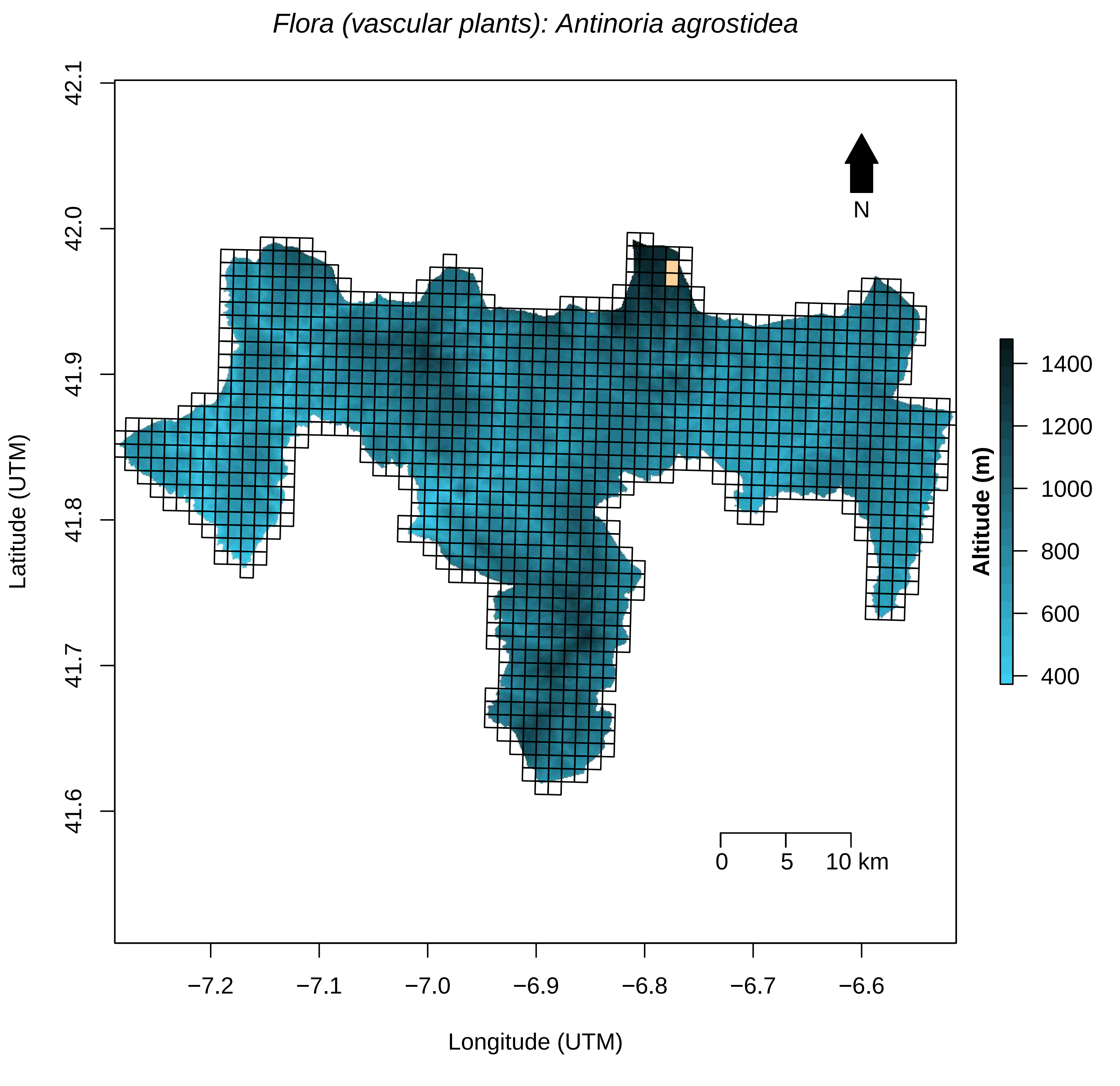

Flora (vascular plants): Antinoria agrostidea
42.1
42.0
41.9
41.8
41.7
41.6
Latitude (UTM)
−7.2
−7.1
−7.0
−6.9
−6.8
−6.7
−6.6
Longitude (UTM)
N
0
5
10 km
Altitude (m)
1400
1200
1000
800
600
400

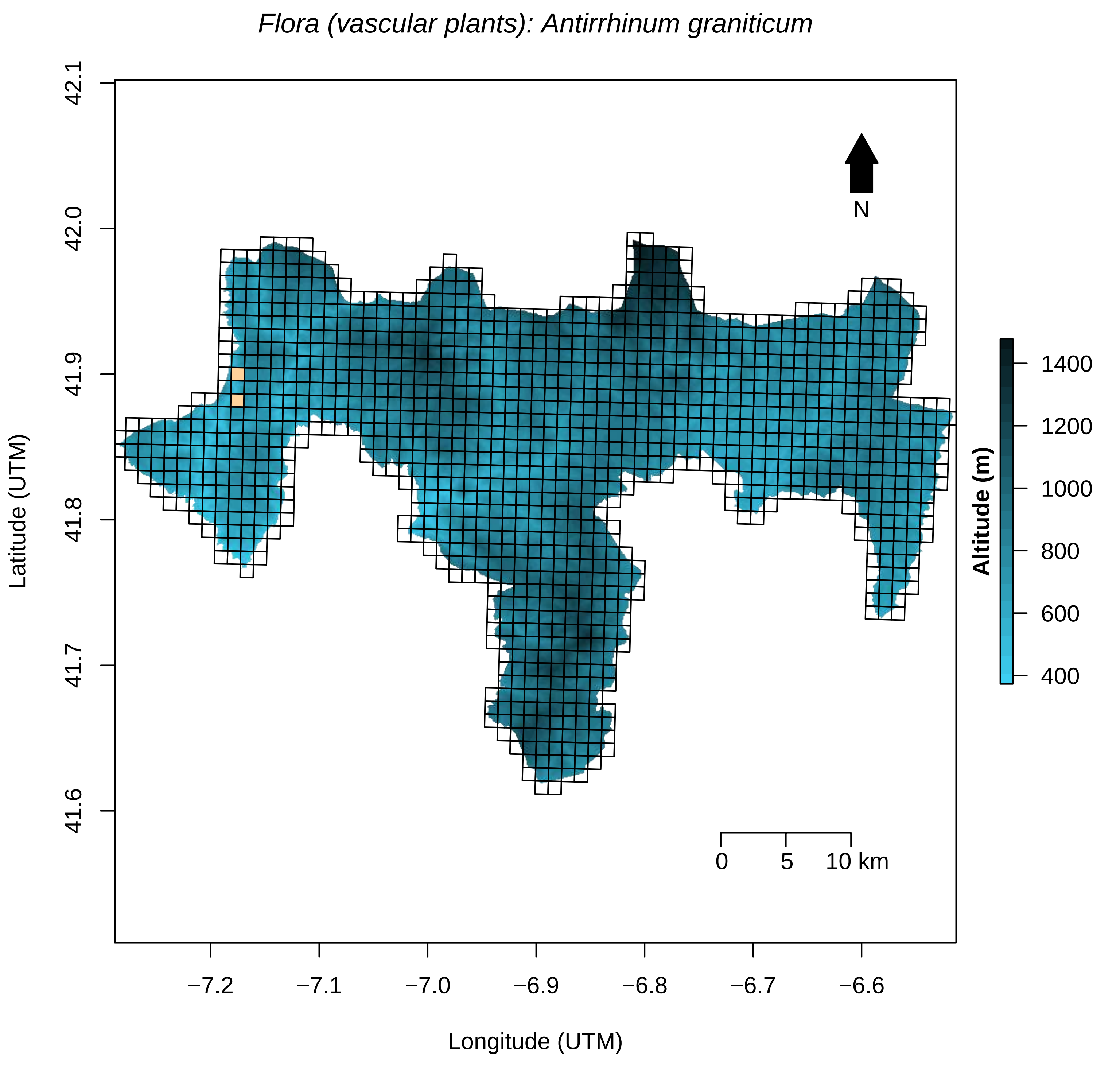
Flora (vascular plants): Antirrhinum graniticum
N
0
5
10 km
Altitude (m)
1400
1200
1000
800
600
400
Latitude (UTM)
42.1
42.0
41.9
41.8
41.7
41.6
Longitude (UTM)
−7.2
−7.1
−7.0
−6.9
−6.8
−6.7
−6.6

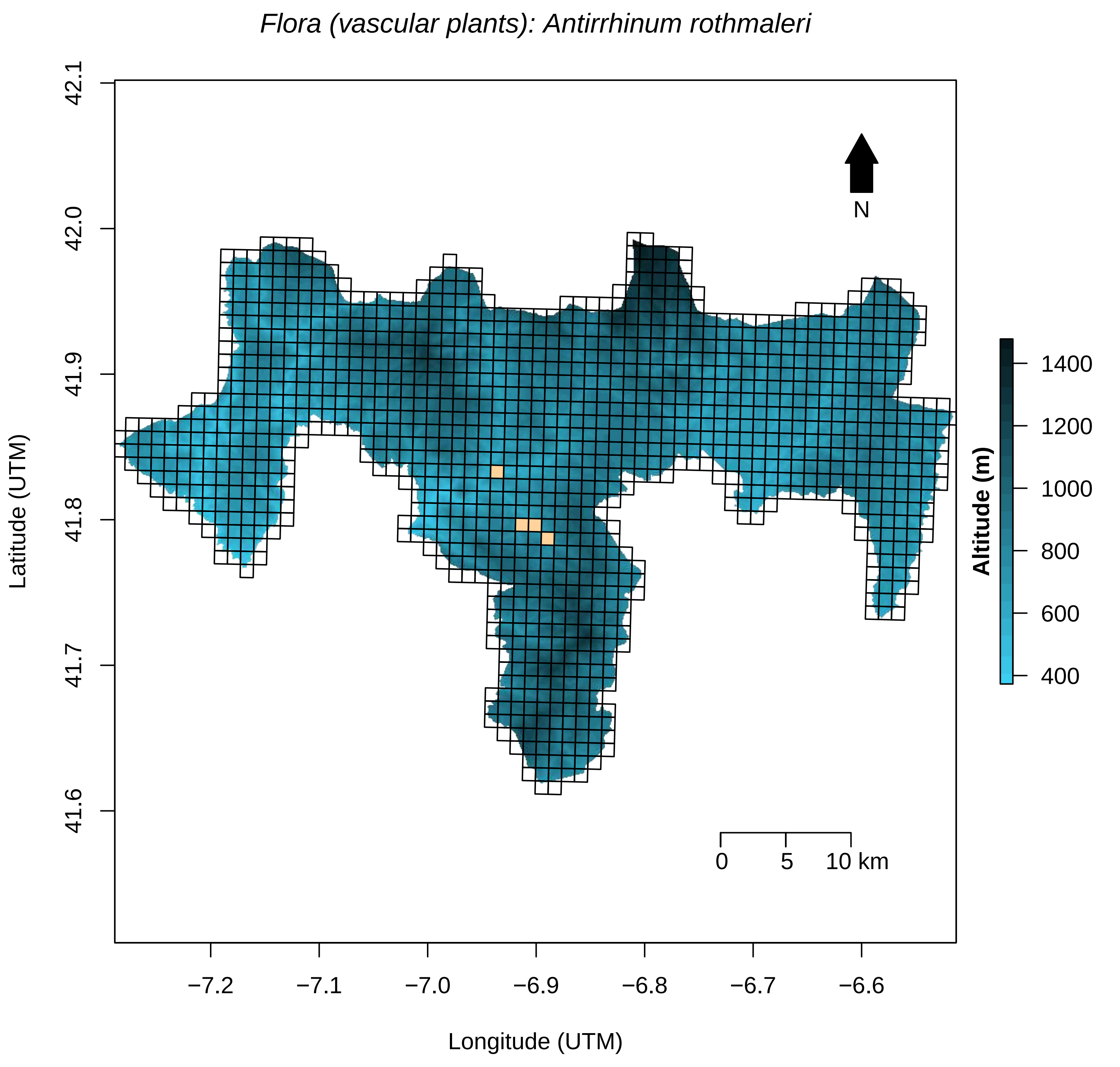
Flora (vascular plants): Antirrhinum rothmaleri
42.1
42.0
41.9
41.8
41.7
41.6
Latitude (UTM)
−7.2
−7.1
−7.0
−6.9
−6.8
−6.7
−6.6
Longitude (UTM)
N
0
5
10 km
Altitude (m)
1400
1200
1000
800
600
400

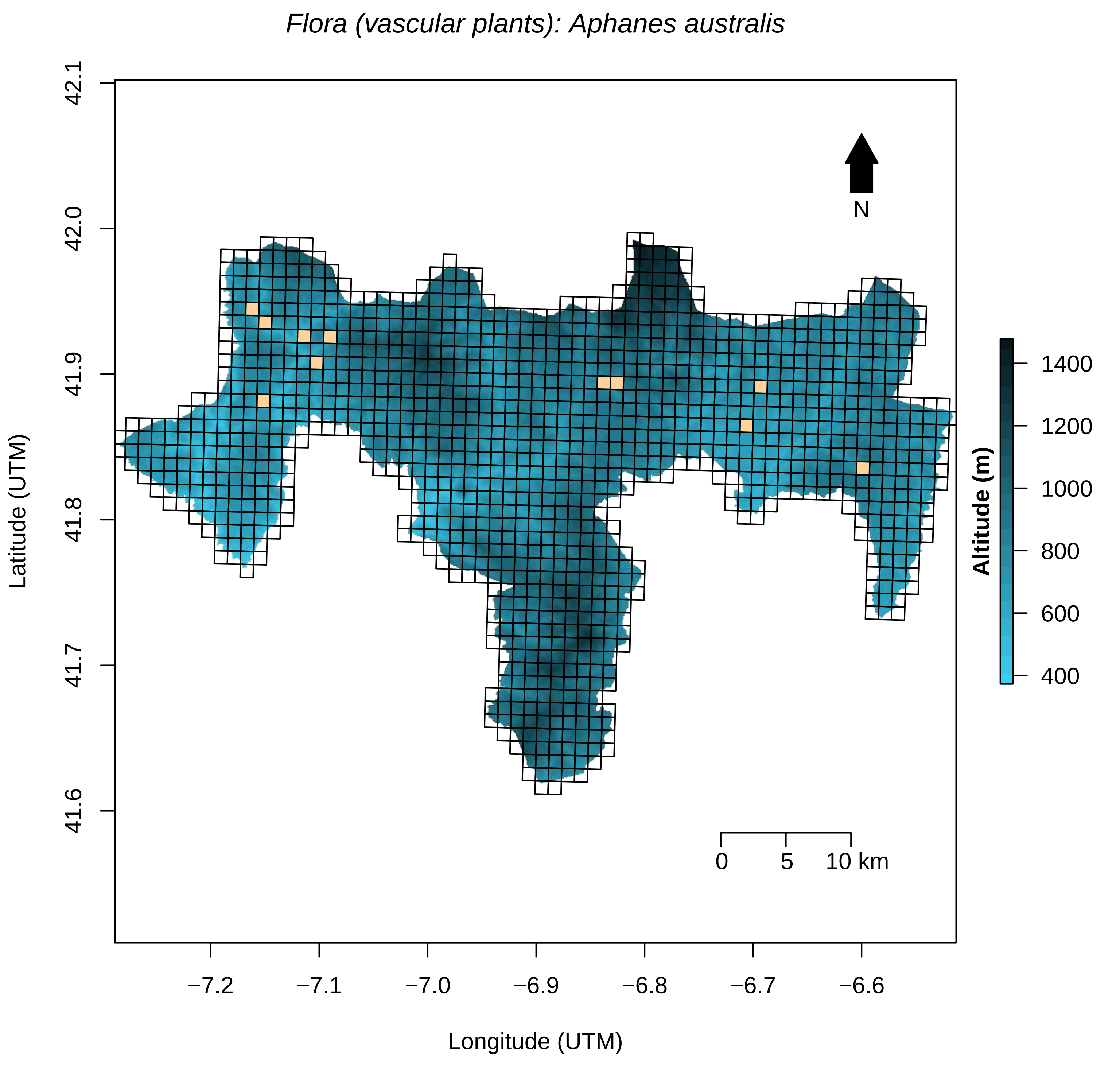

Flora (vascular plants): Aphanes australis
42.1
42.0
41.9
41.8
41.7
41.6
Latitude (UTM)
−7.2
−7.1
−7.0
−6.9
−6.8
−6.7
−6.6
Longitude (UTM)
N
0
5
10 km
Altitude (m)
1400
1200
1000
800
600
400

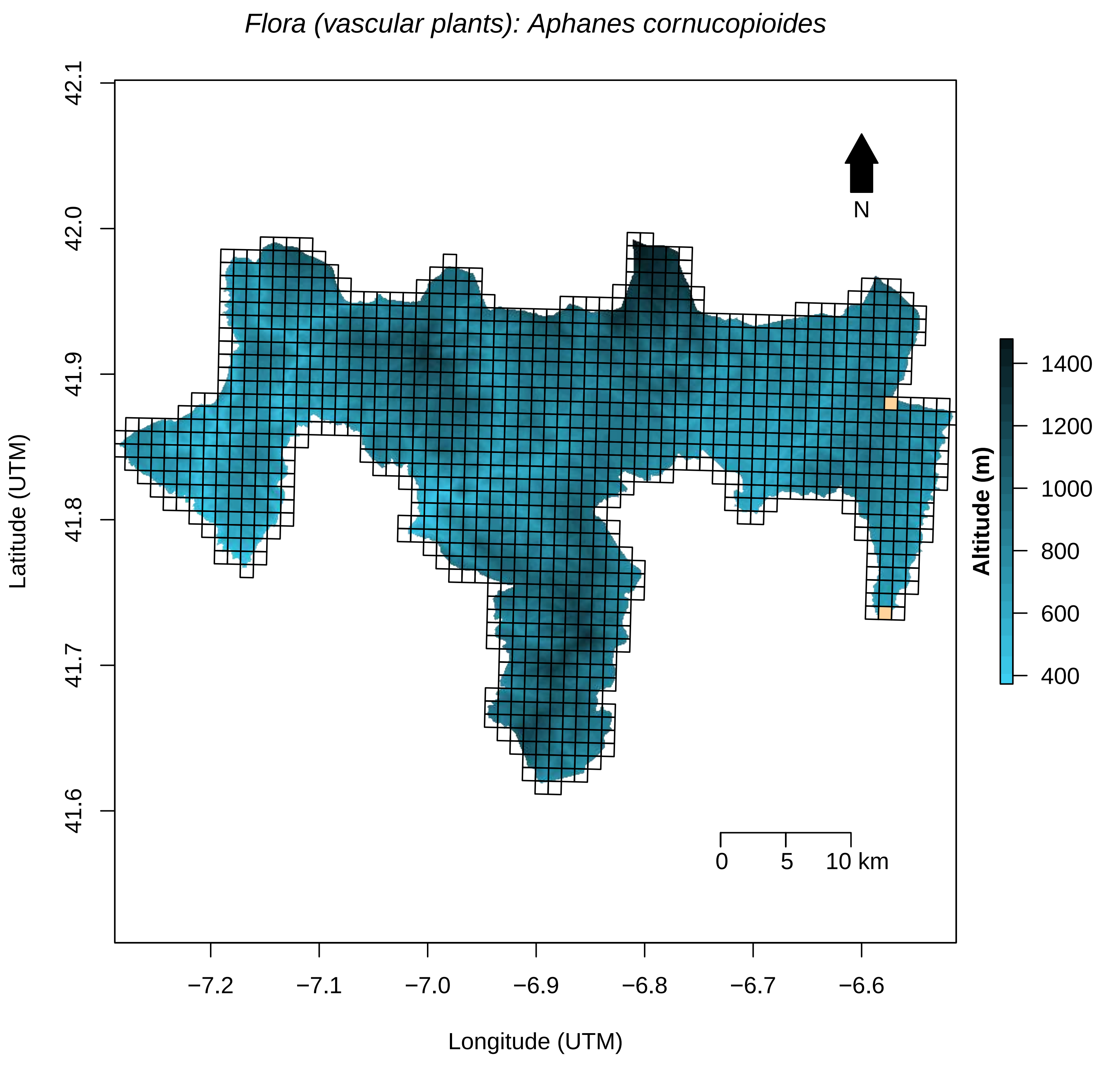

Flora (vascular plants): Aphanes cornucopioides
42.1
42.0
41.9
41.8
41.7
41.6
Latitude (UTM)
−7.2
−7.1
−7.0
−6.9
−6.8
−6.7
−6.6
Longitude (UTM)
N
0
5
10 km
Altitude (m)
1400
1200
1000
800
600
400

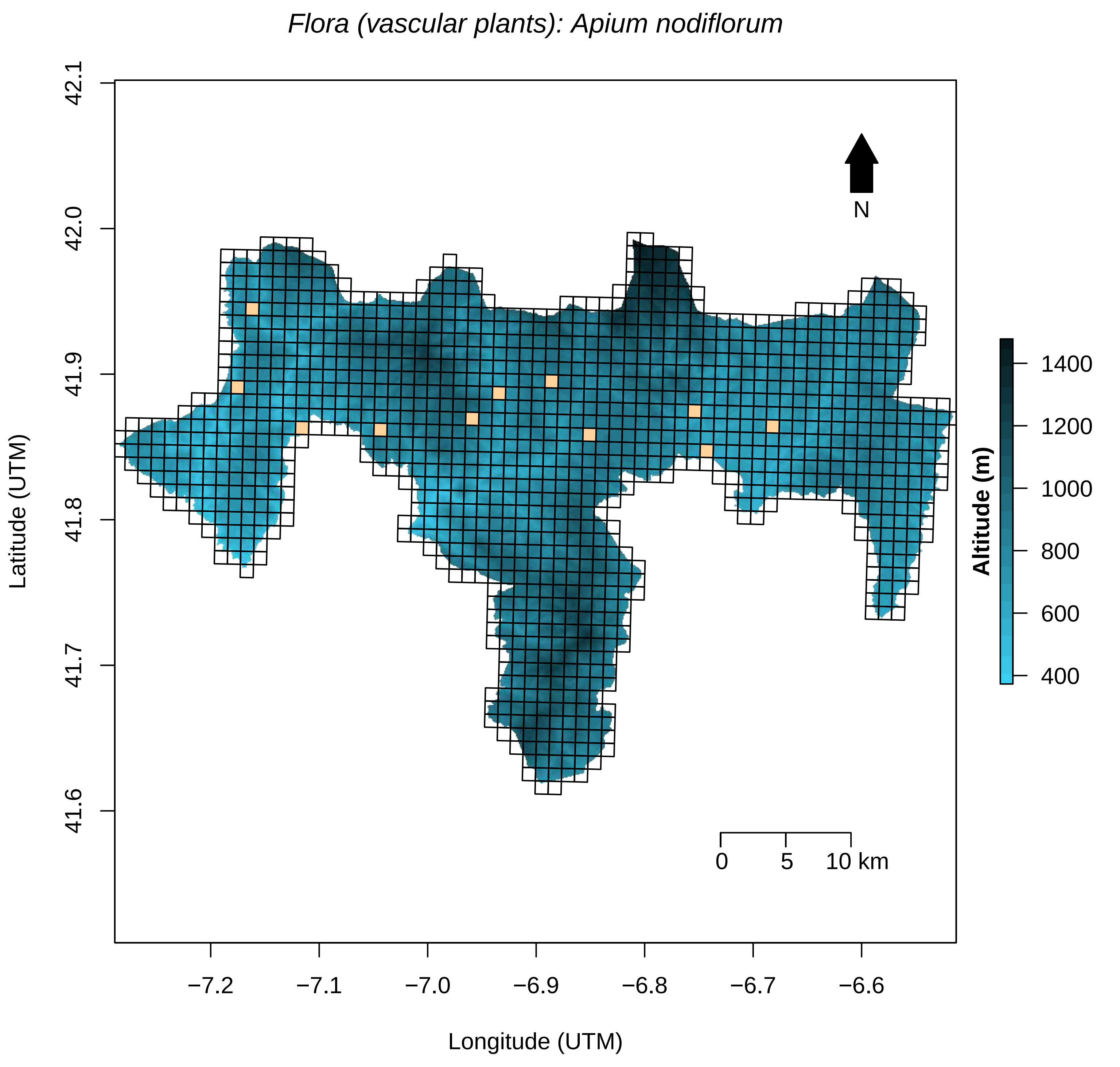

Flora (vascular plants): Apium nodiflorum
N
0
5
10 km
Altitude (m)
1400
1200
1000
800
600
400
Latitude (UTM)
42.1
42.0
41.9
41.8
41.7
41.6
Longitude (UTM)
−7.2
−7.1
−7.0
−6.9
−6.8
−6.7
−6.6

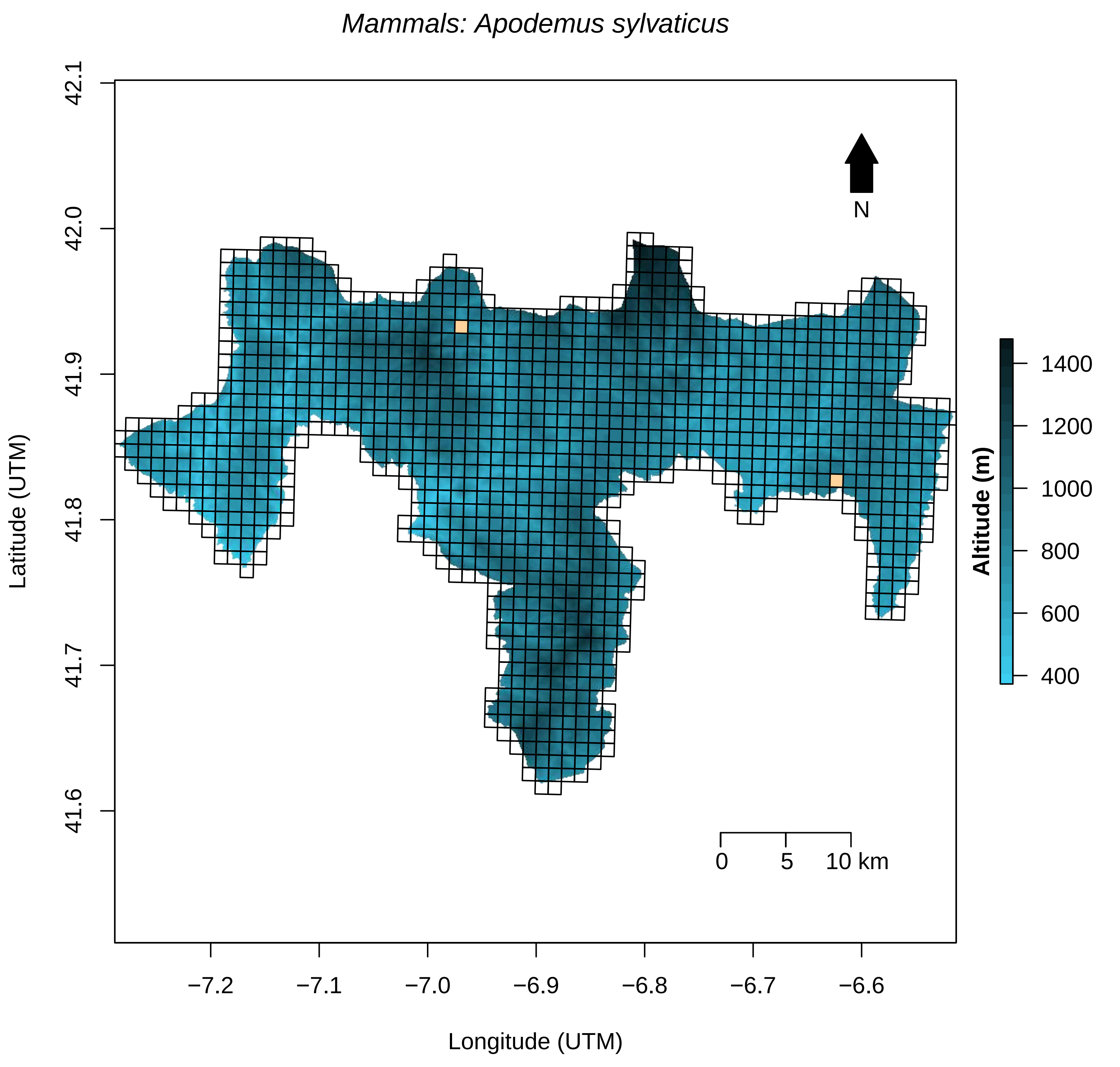
Mammals: Apodemus sylvaticus
N
0
5
10 km
Altitude (m)
1400
1200
1000
800
600
400
Latitude (UTM)
42.1
42.0
41.9
41.8
41.7
41.6
Longitude (UTM)
−7.2
−7.1
−7.0
−6.9
−6.8
−6.7
−6.6

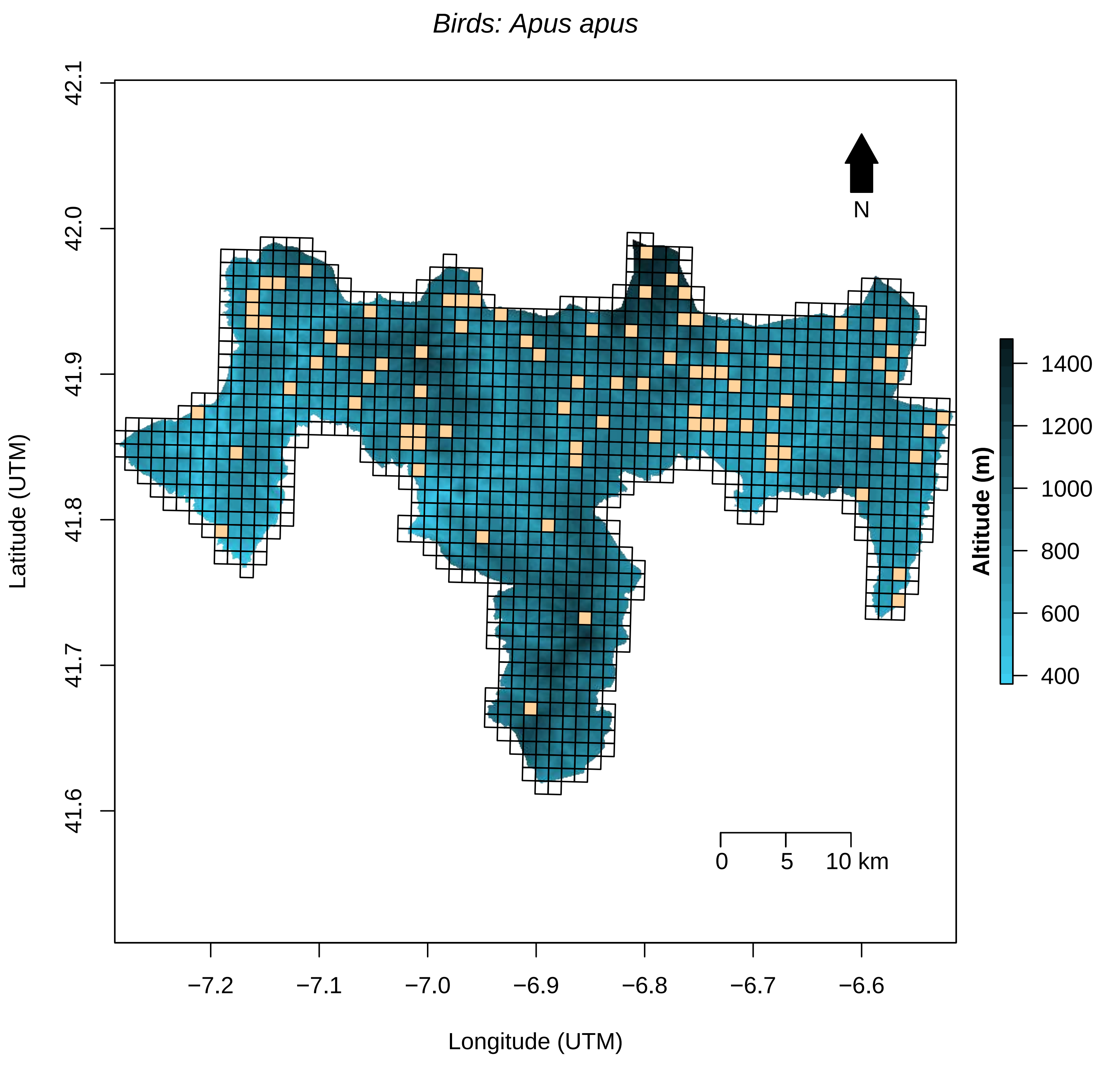

Birds: Apus apus
42.1
42.0
41.9
41.8
41.7
41.6
Latitude (UTM)
−7.2
−7.1
−7.0
−6.9
−6.8
−6.7
−6.6
Longitude (UTM)
N
0
5
10 km
Altitude (m)
1400
1200
1000
800
600
400

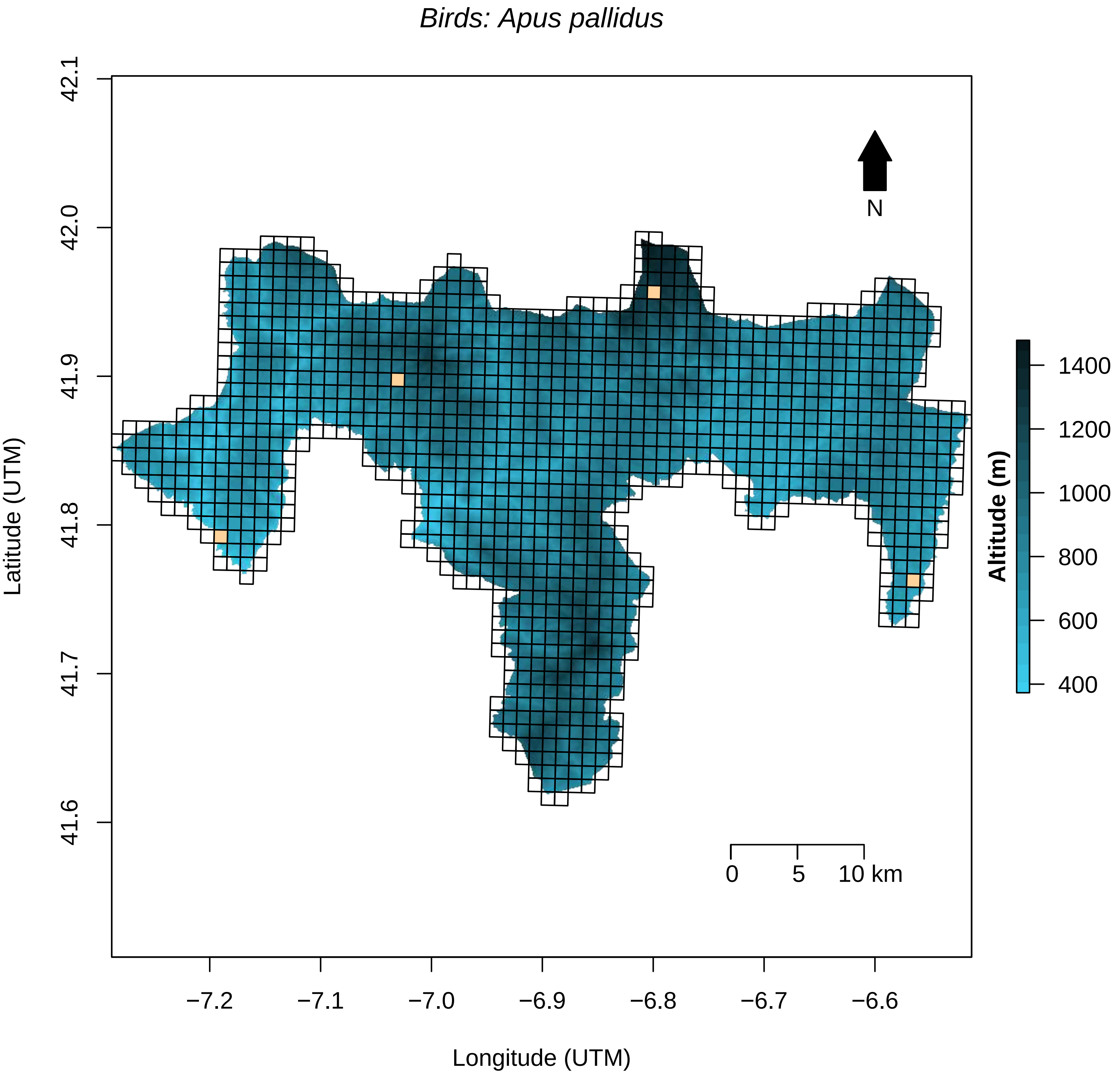

Birds: Apus pallidus
N
0
5
10 km
Altitude (m)
1400
1200
1000
800
600
400
Latitude (UTM)
42.1
42.0
41.9
41.8
41.7
41.6
Longitude (UTM)
−7.2
−7.1
−7.0
−6.9
−6.8
−6.7
−6.6

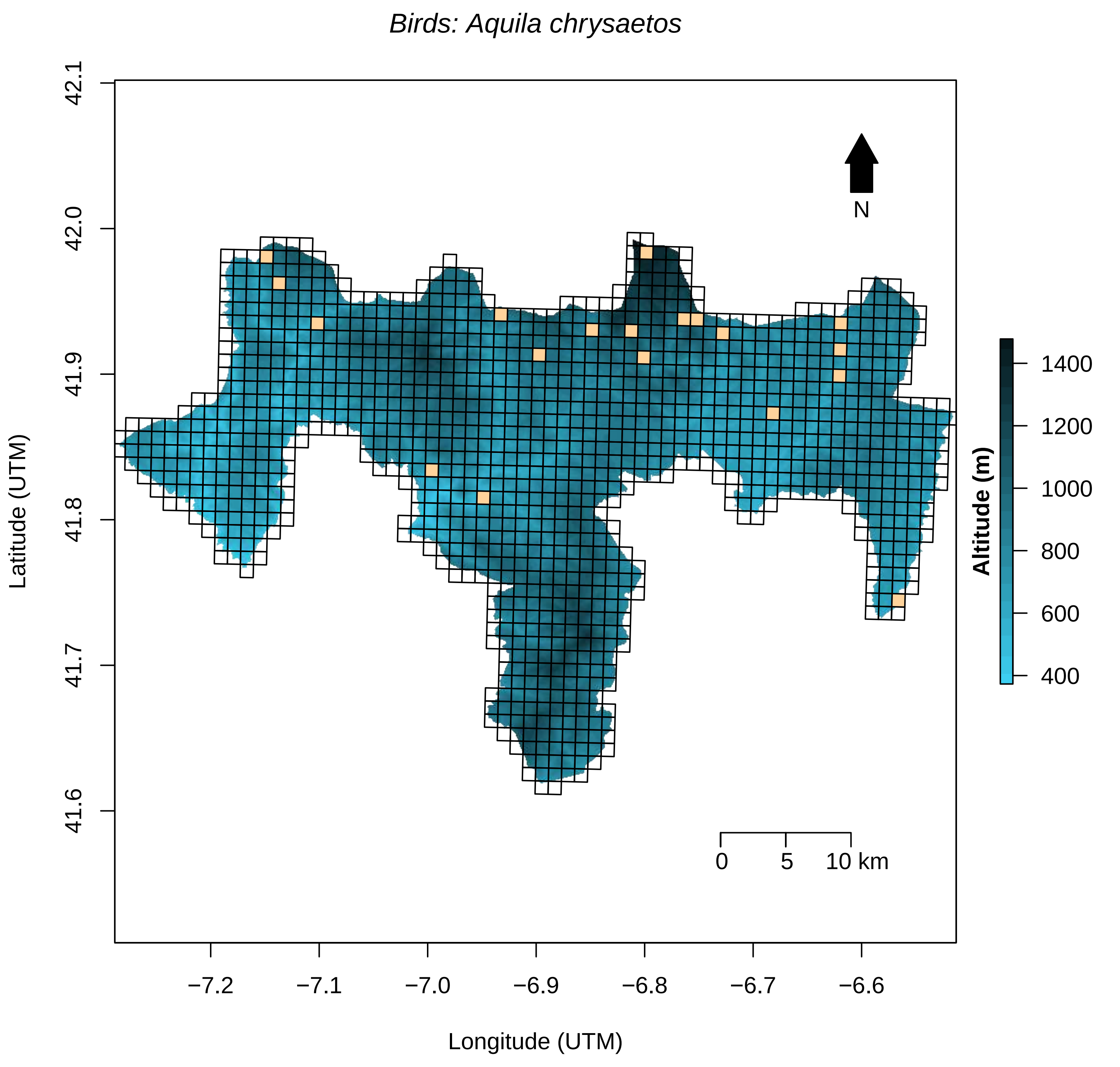

Birds: Aquila chrysaetos
N
0 5 10 km
Altitude (m)
1400
1200
1000
800
600
400
Latitude (UTM)
42.1
42.0
41.9
41.8
41.7
41.6
Longitude (UTM)
−7.2
−7.1
−7.0
−6.9
−6.8
−6.7
−6.6

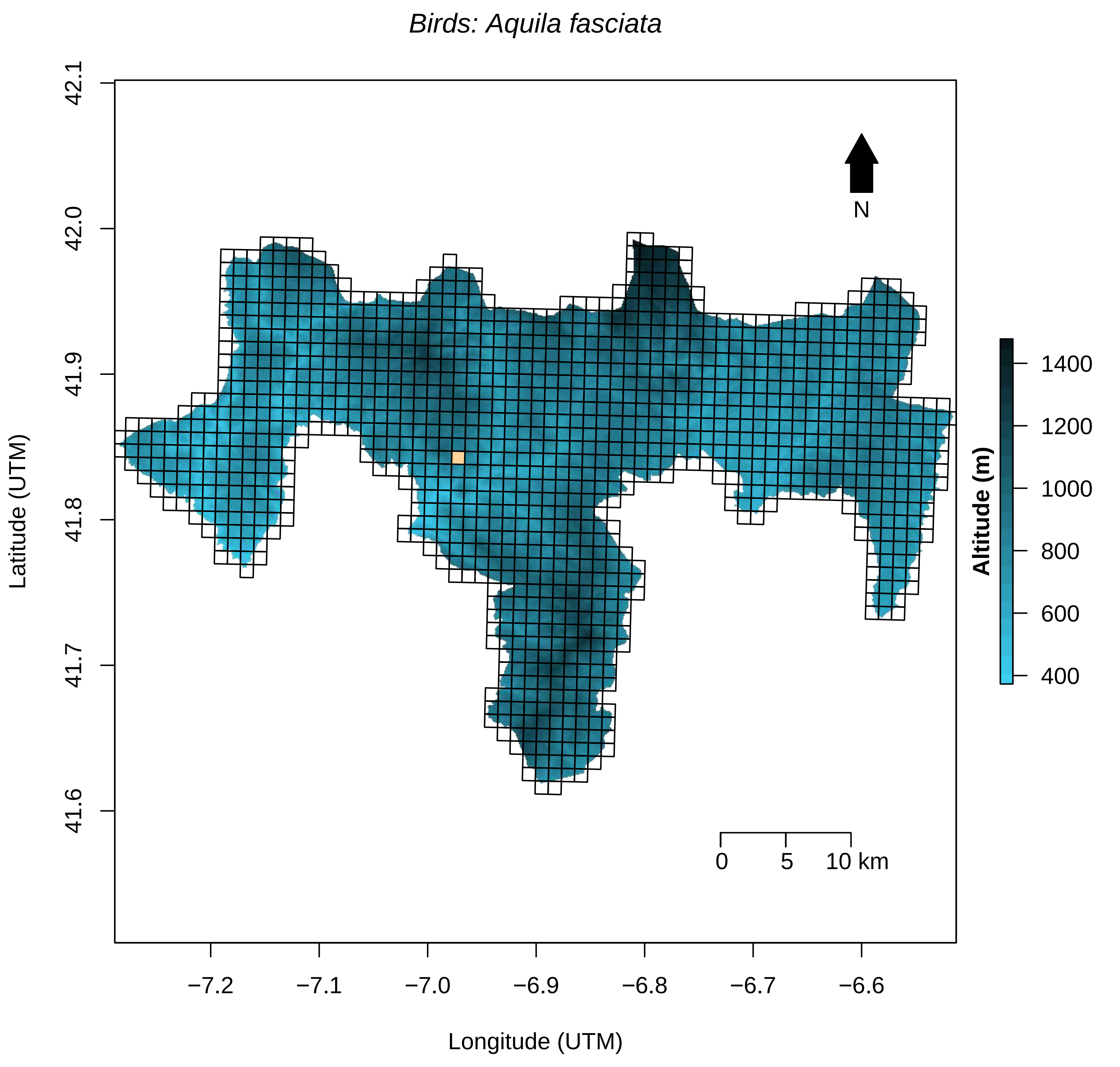

Birds: Aquila fasciata
42.1
42.0
41.9
41.8
41.7
41.6
Latitude (UTM)
−7.2
−7.1
−7.0
−6.9
−6.8
−6.7
−6.6
Longitude (UTM)
N
0
5
10 km
Altitude (m)
1400
1200
1000
800
600
400

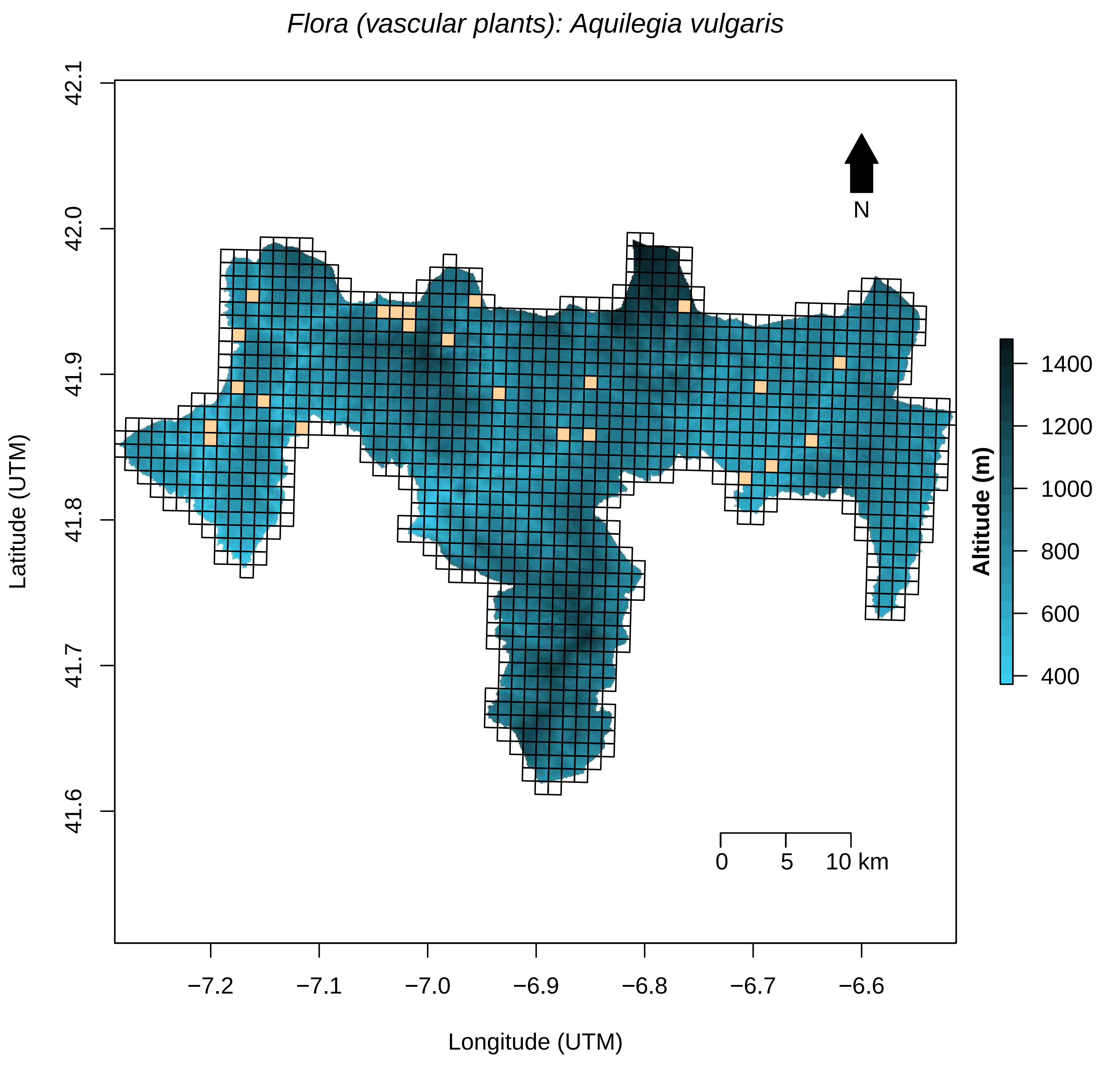
Flora (vascular plants): Aquilegia vulgaris
Latitude (UTM)
42.1
42.0
41.9
41.8
41.7
41.6
−7.2
−7.1
−7.0
−6.9
−6.8
−6.7
−6.6
Longitude (UTM)
N
0
5
10 km
Altitude (m)
1400
1200
1000
800
600
400

Flora (vascular plants): Arabidopsis thaliana

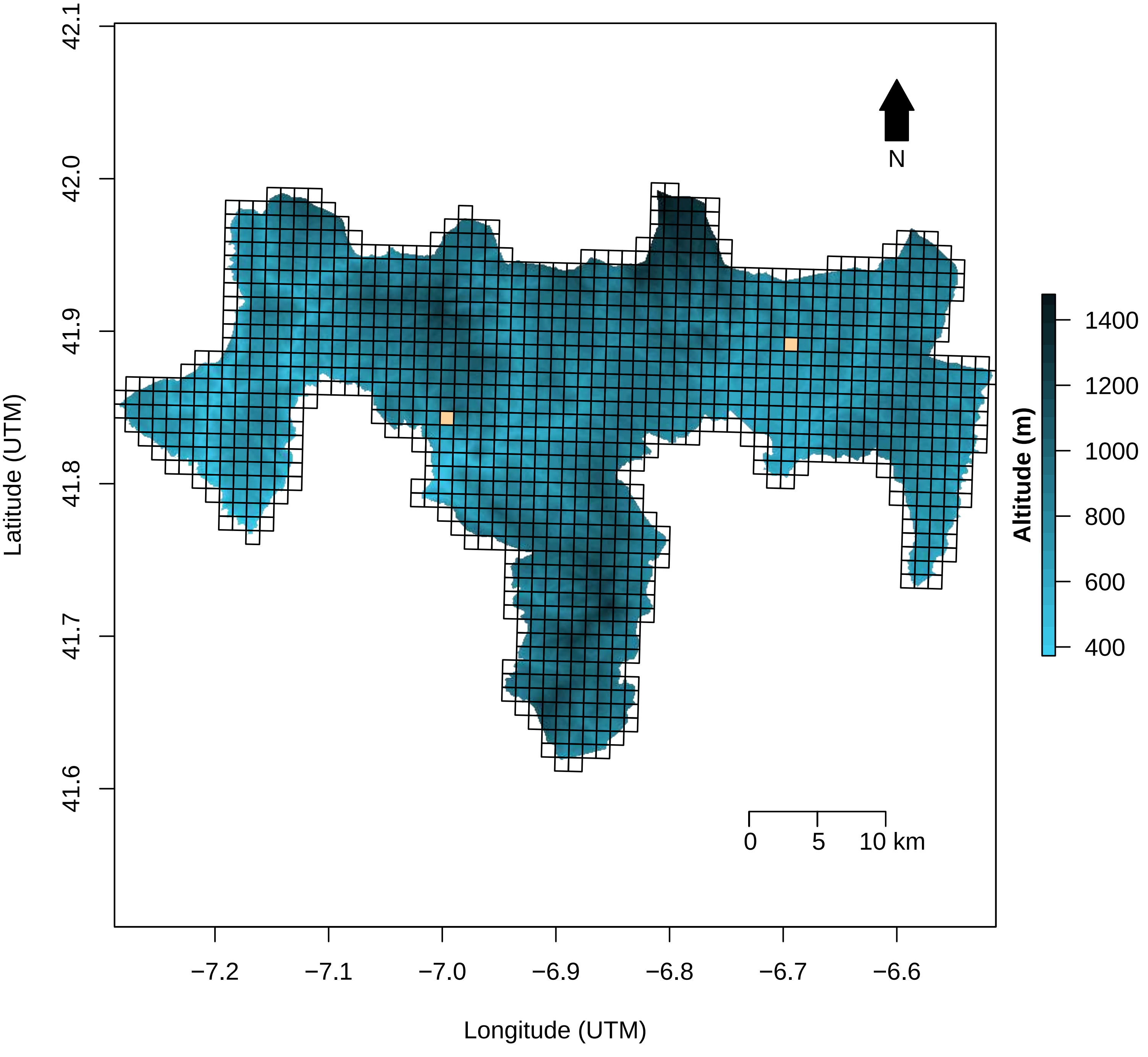

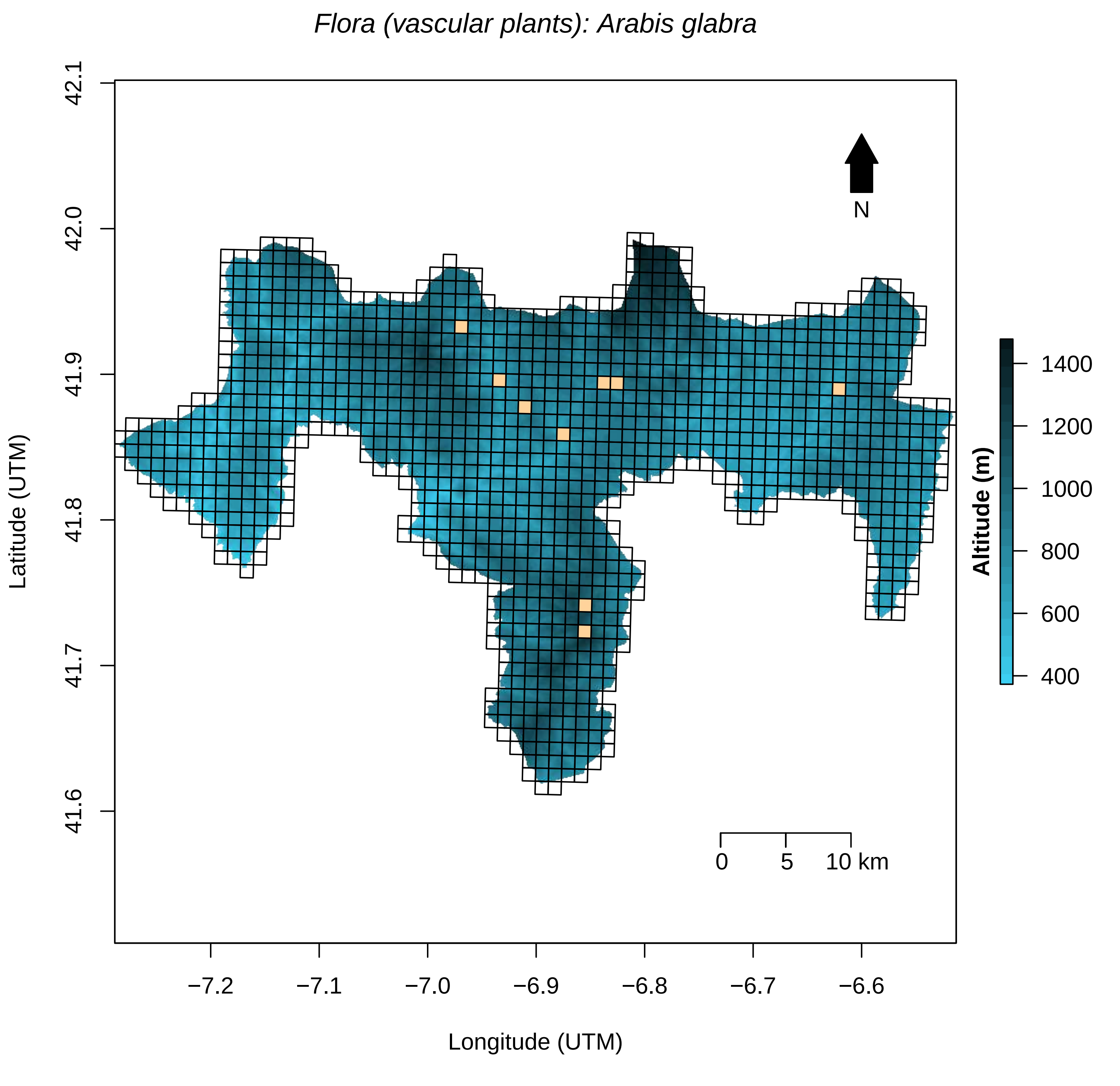

Flora (vascular plants): Arabis glabra
42.1
42.0
41.9
41.8
41.7
41.6
Latitude (UTM)
−7.2
−7.1
−7.0
−6.9
−6.8
−6.7
−6.6
Longitude (UTM)
N
0
5
10 km
Altitude (m)
1400
1200
1000
800
600
400

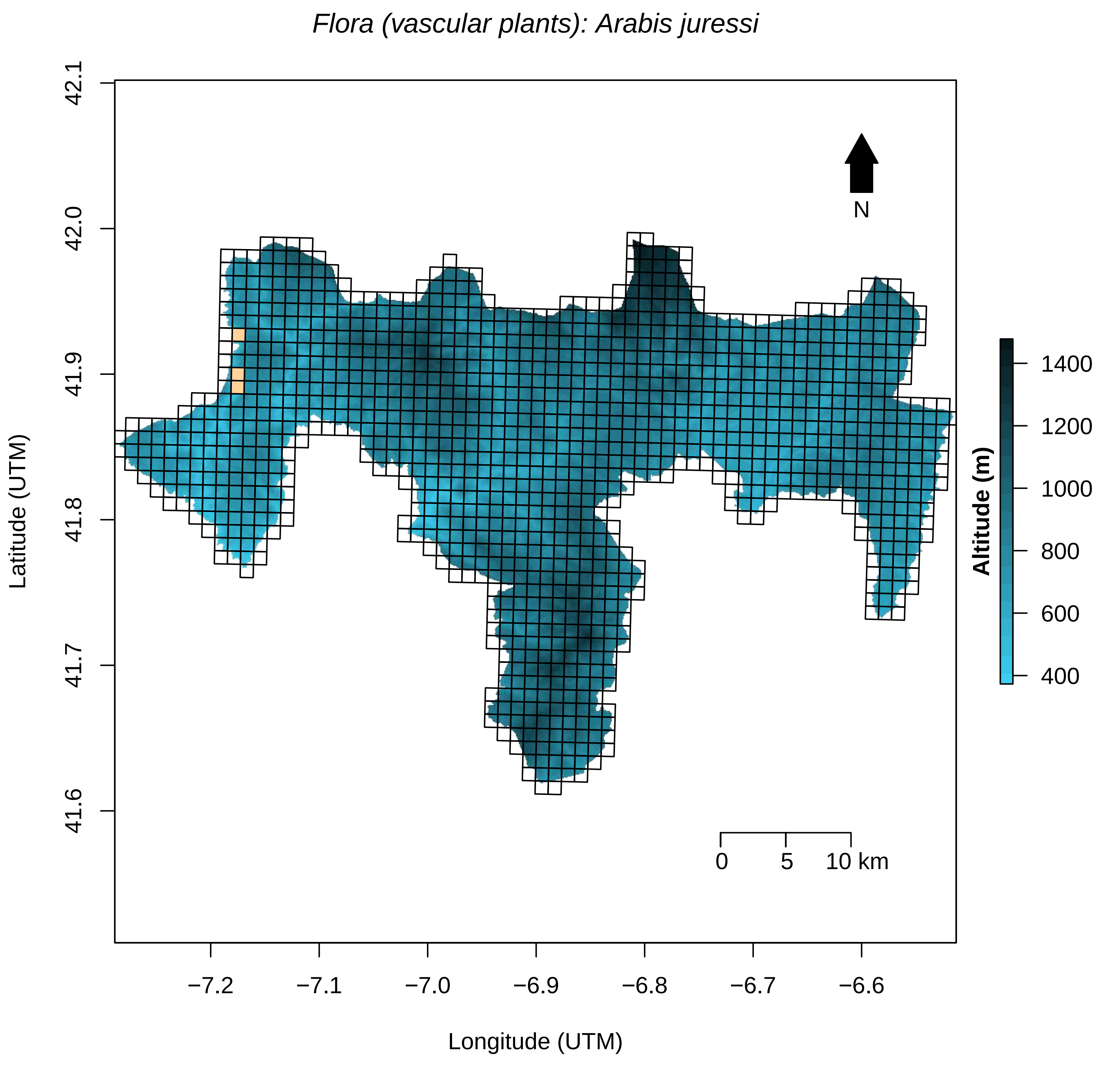

Flora (vascular plants): Arabis juressi
N
0
5
10 km
Altitude (m)
1400
1200
1000
800
600
400
Latitude (UTM)
42.1
42.0
41.9
41.8
41.7
41.6
Longitude (UTM)
−7.2
−7.1
−7.0
−6.9
−6.8
−6.7
−6.6

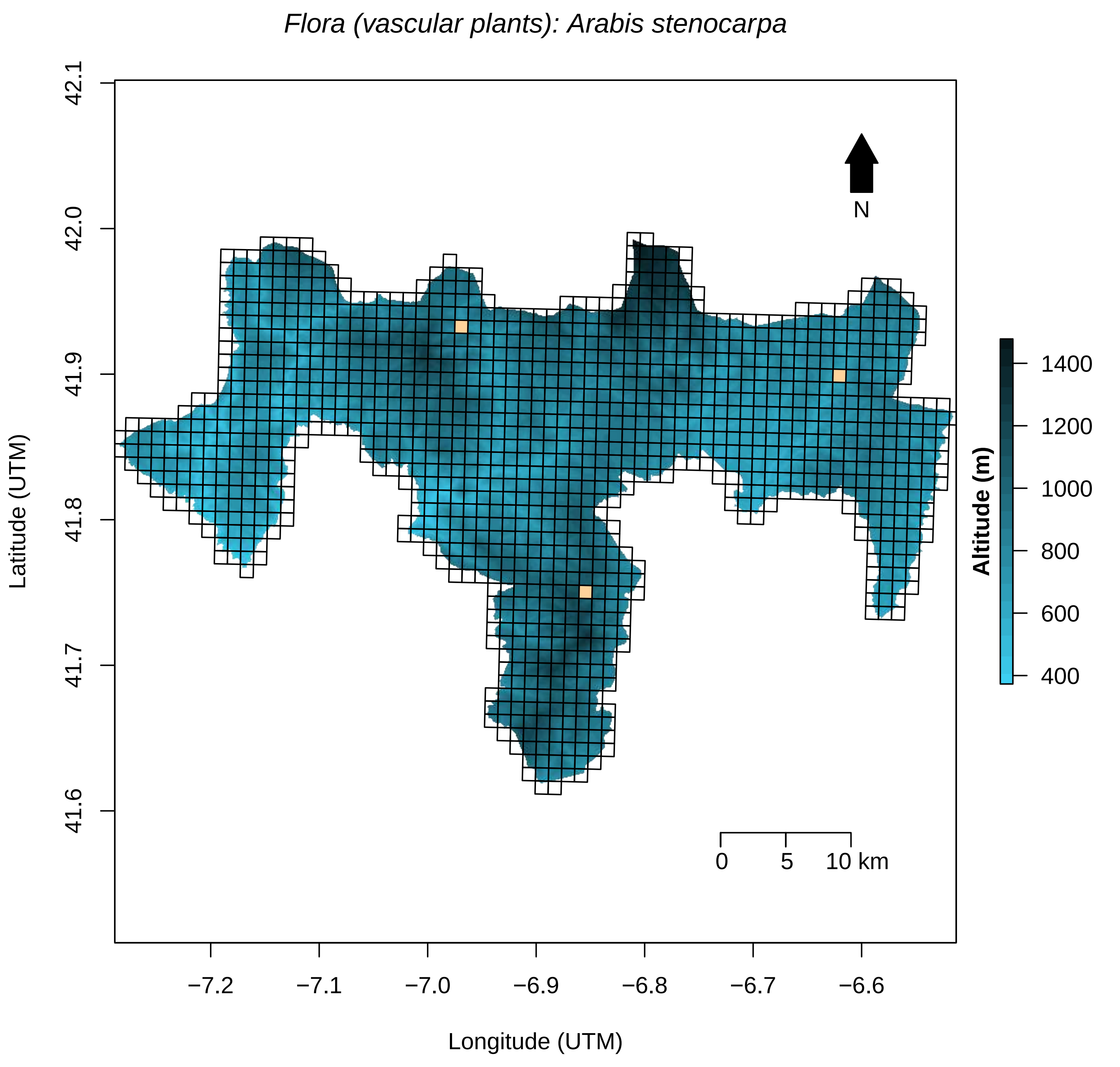

Flora (vascular plants): Arabis stenocarpa
42.1
42.0
41.9
41.8
41.7
41.6
Latitude (UTM)
−7.2
−7.1
−7.0
−6.9
−6.8
−6.7
−6.6
Longitude (UTM)
N
0
5
10 km
1400
1200
1000
800
600
400
Altitude (m)

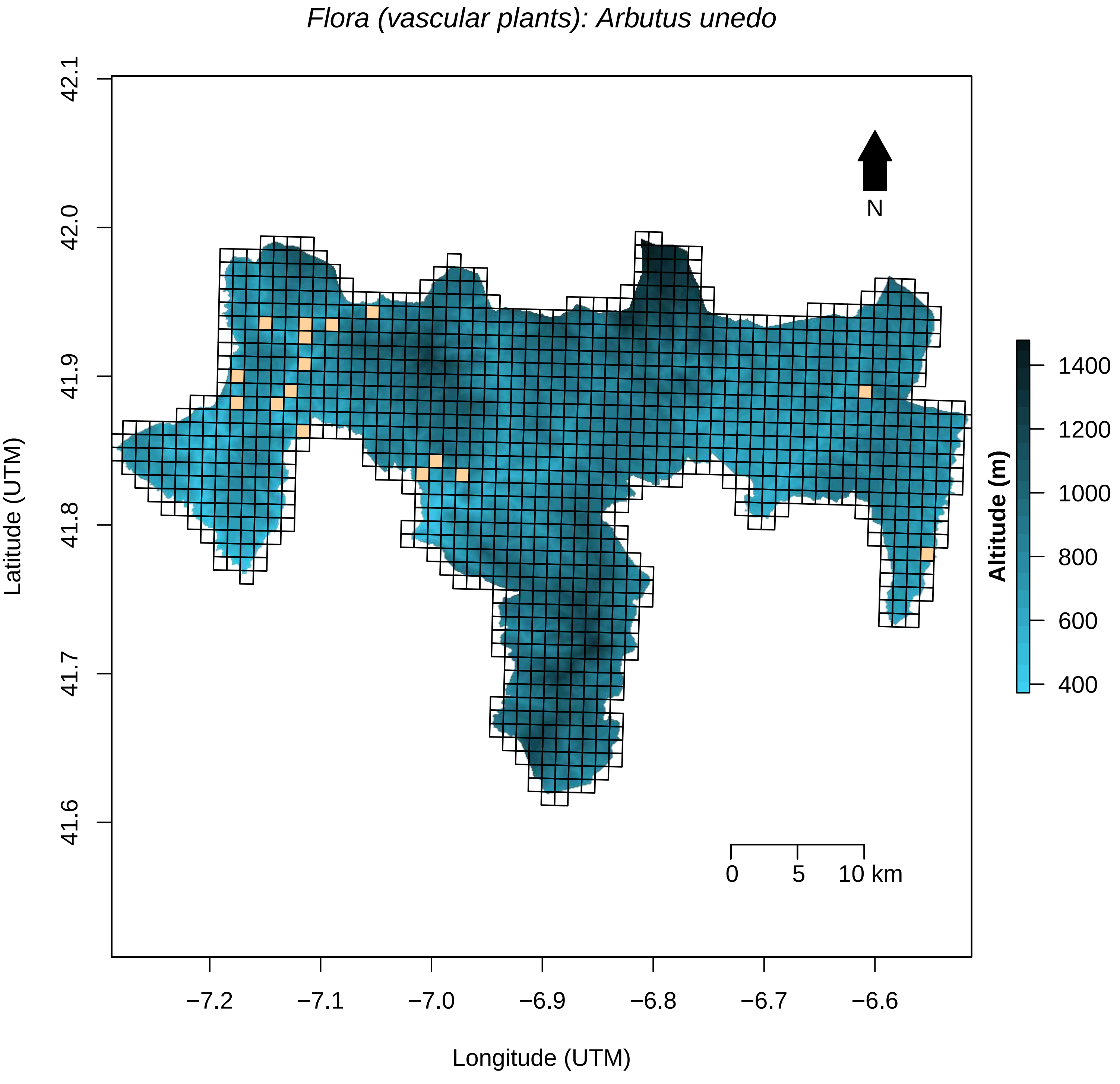

Flora (vascular plants): Arbutus unedo
N
0
5
10 km
Altitude (m)
1400
1200
1000
800
600
400
Latitude (UTM)
42.1
42.0
41.9
41.8
41.7
41.6
−7.2
−7.1
−7.0
−6.9
−6.8
−6.7
−6.6
Longitude (UTM)

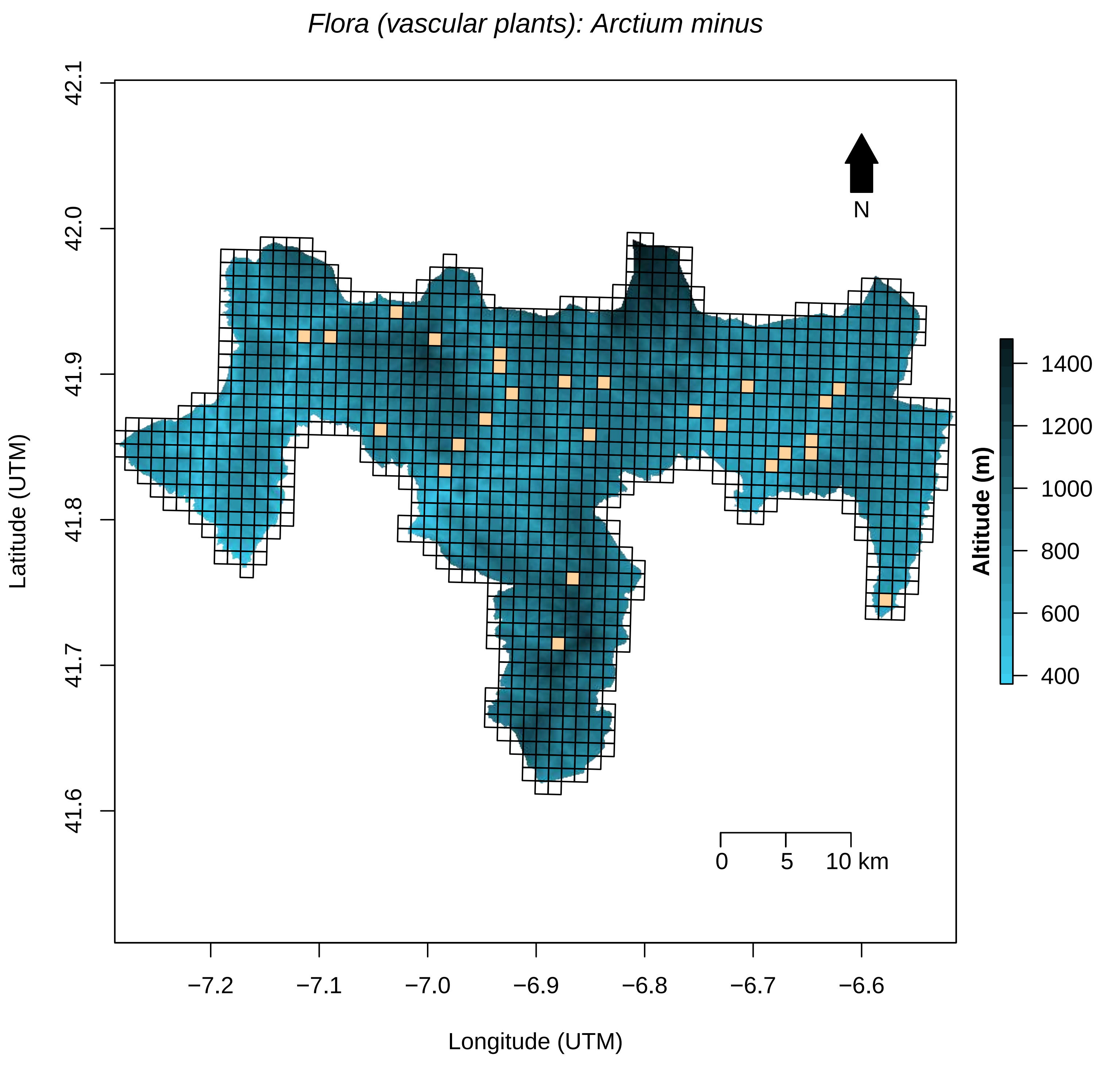
Flora (vascular plants): Arctium minus
42.1
42.0
41.9
41.8
41.7
41.6
Latitude (UTM)
−7.2
−7.1
−7.0
−6.9
−6.8
−6.7
−6.6
Longitude (UTM)
N
0
5
10 km
Altitude (m)
1400
1200
1000
800
600
400

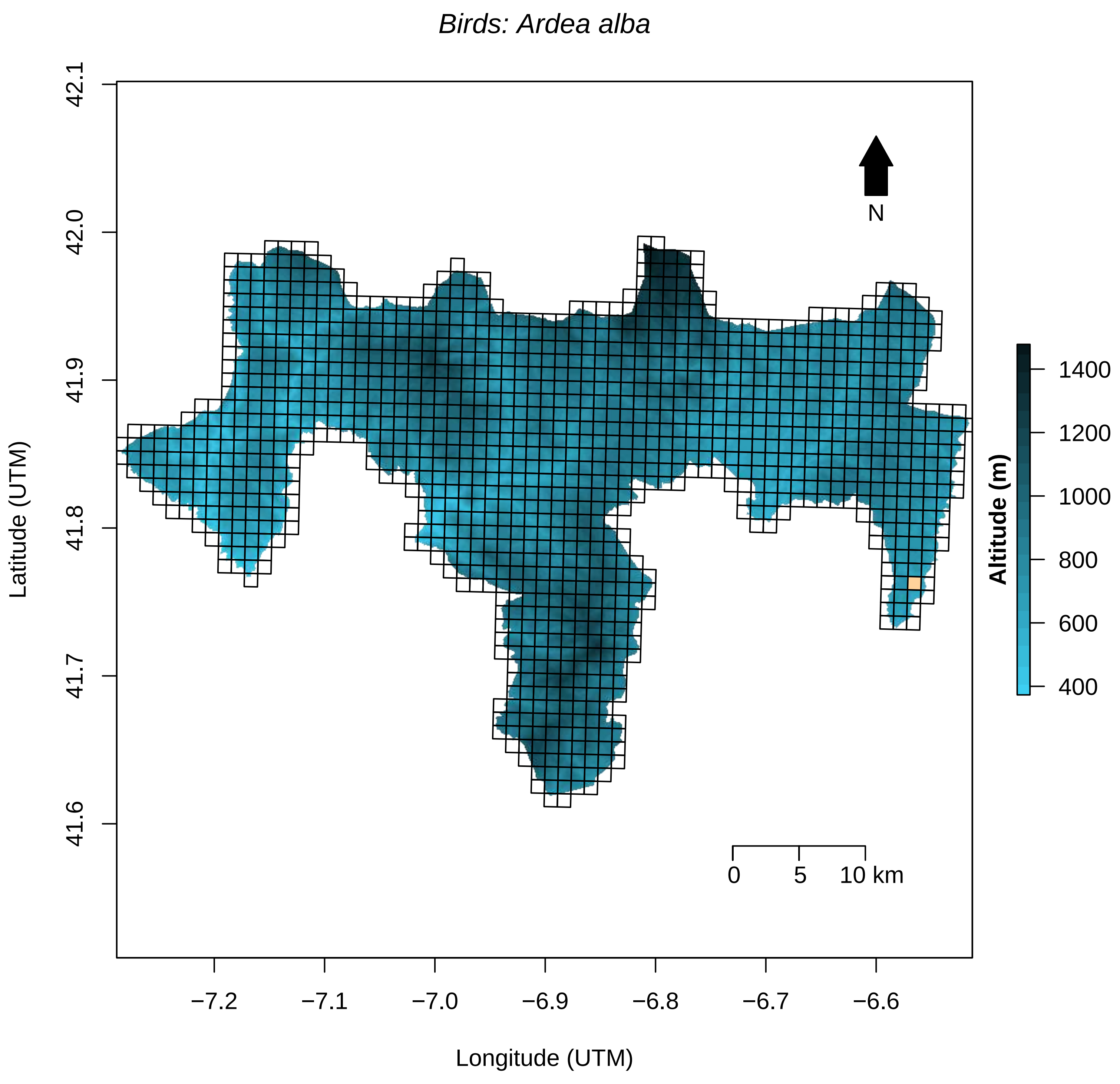

Birds: Ardea alba
N
0 5 10 km
Altitude (m)
1400
1200
1000
800
600
400
Latitude (UTM)
42.1
42.0
41.9
41.8
41.7
41.6
Longitude (UTM)
−7.2
−7.1
−7.0
−6.9
−6.8
−6.7
−6.6

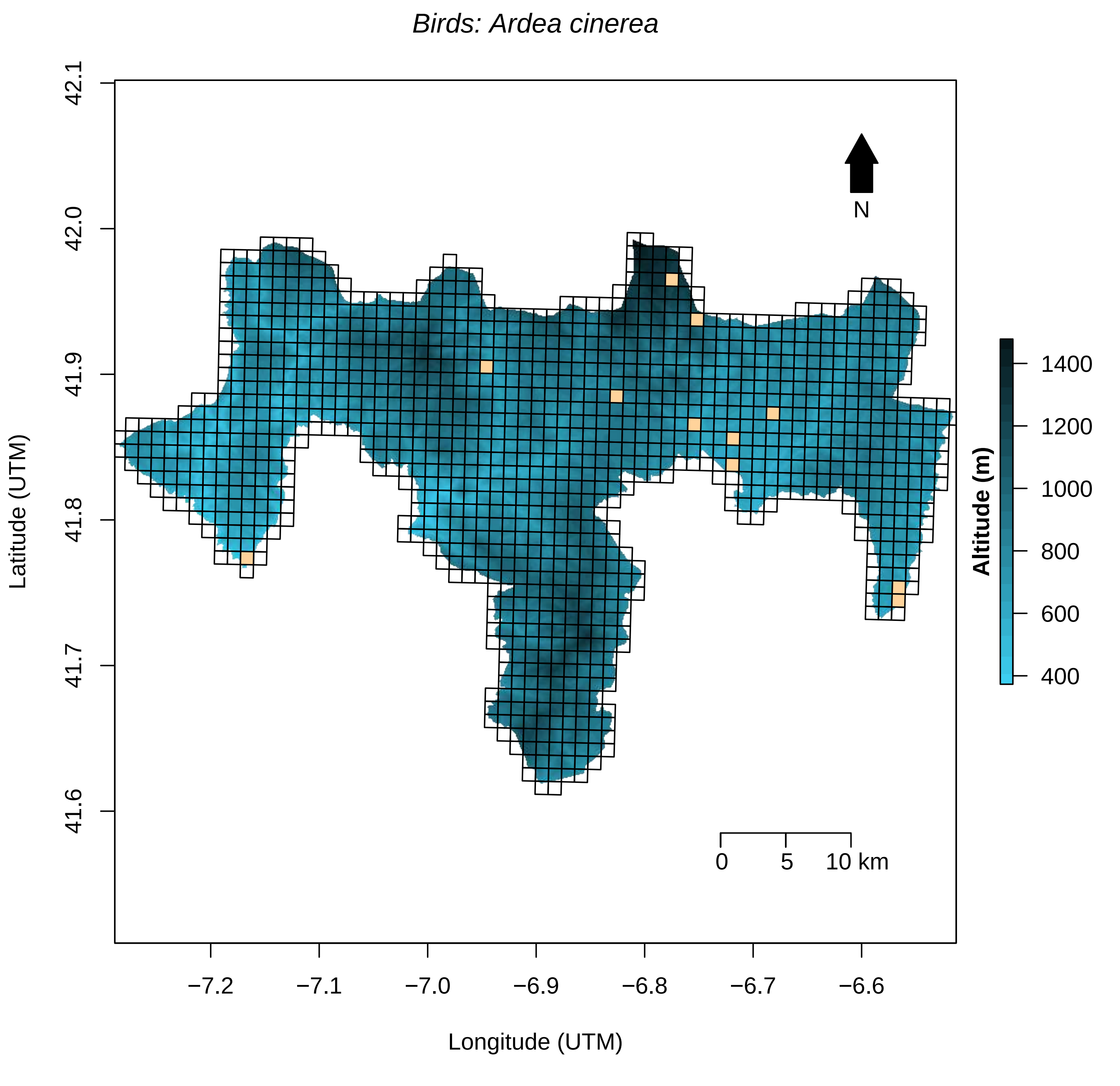

Birds: Ardea cinerea
N
0
5
10 km
Altitude (m)
1400
1200
1000
800
600
400
42.1
42.0
41.9
41.8
41.7
41.6
Latitude (UTM)
−7.2
−7.1
−7.0
−6.9
−6.8
−6.7
−6.6
Longitude (UTM)

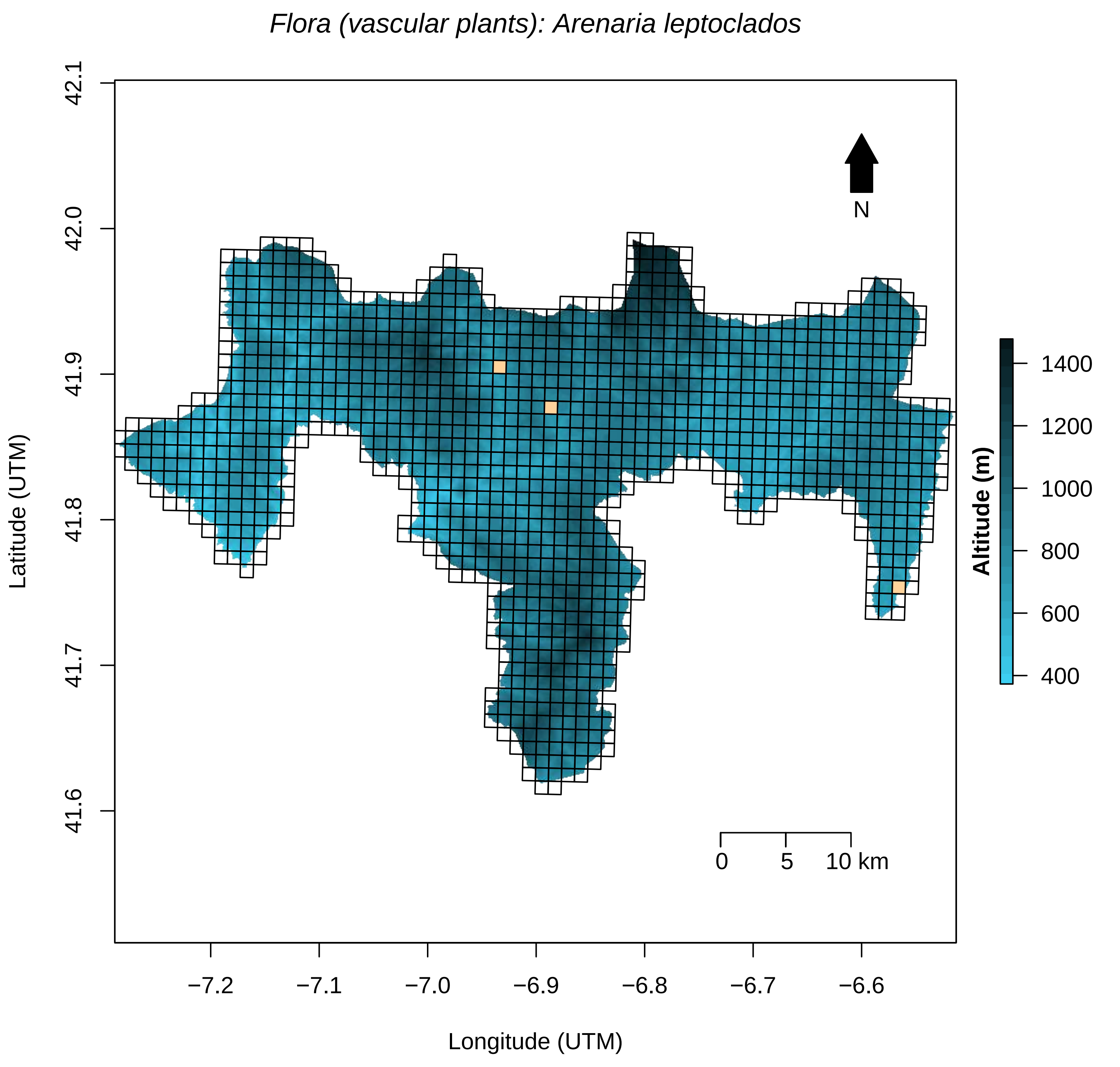

Flora (vascular plants): Arenaria leptoclados
42.1
42.0
41.9
41.8
41.7
41.6
Latitude (UTM)
−7.2
−7.1
−7.0
−6.9
−6.8
−6.7
−6.6
Longitude (UTM)
N
0
5
10 km
1400
1200
1000
800
600
400
Altitude (m)

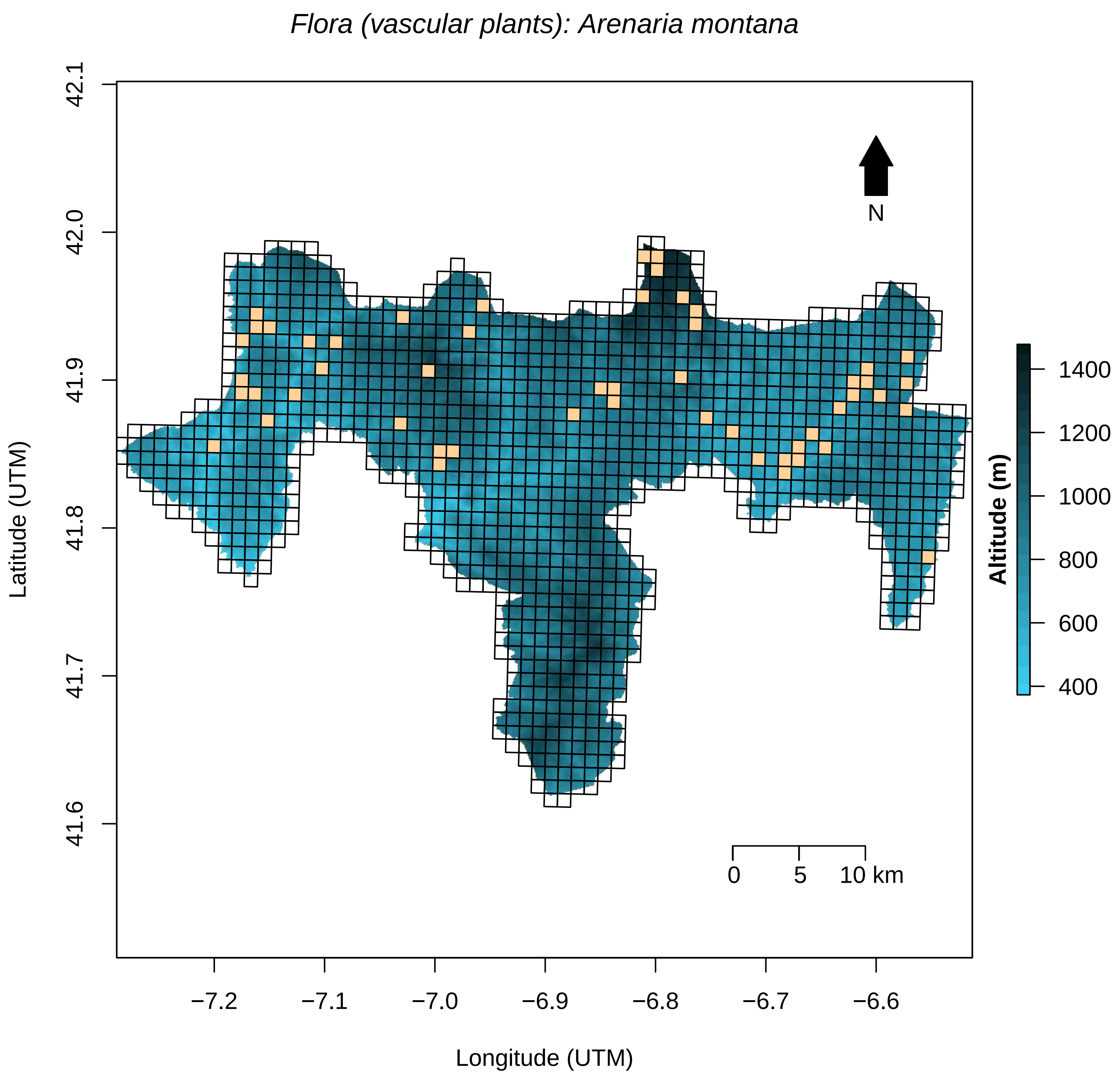

Flora (vascular plants): Arenaria montana
N
0
5
10 km
Latitude (UTM)
42.1
42.0
41.9
41.8
41.7
41.6
Longitude (UTM)
−7.2
−7.1
−7.0
−6.9
−6.8
−6.7
−6.6
Altitude (m)
1400
1200
1000
800
600
400

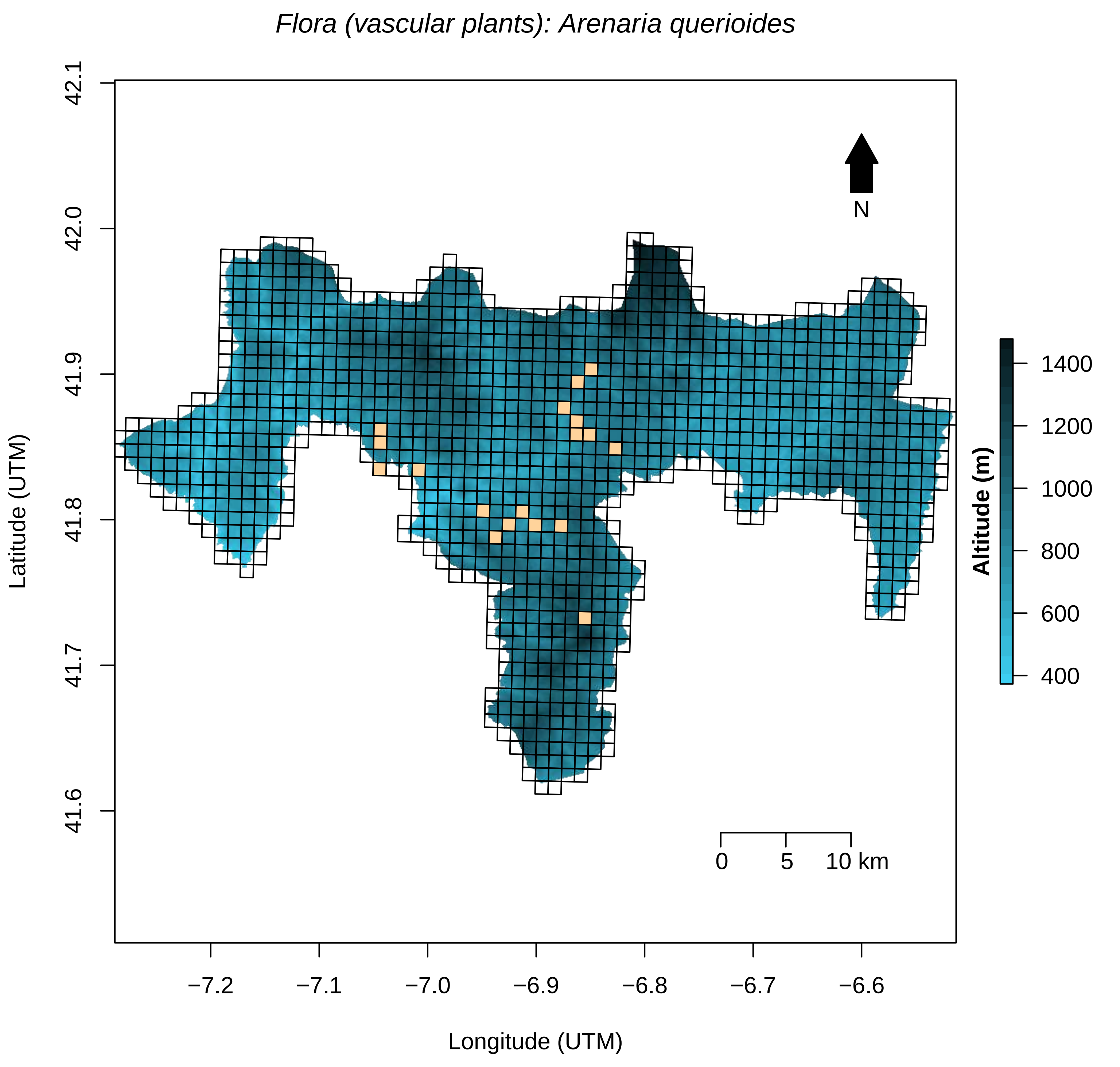

Flora (vascular plants): Arenaria querioides
42.1
42.0
41.9
41.8
41.7
41.6
Latitude (UTM)
−7.2
−7.1
−7.0
−6.9
−6.8
−6.7
−6.6
Longitude (UTM)
N
0
5
10 km
Altitude (m)
1400
1200
1000
800
600
400

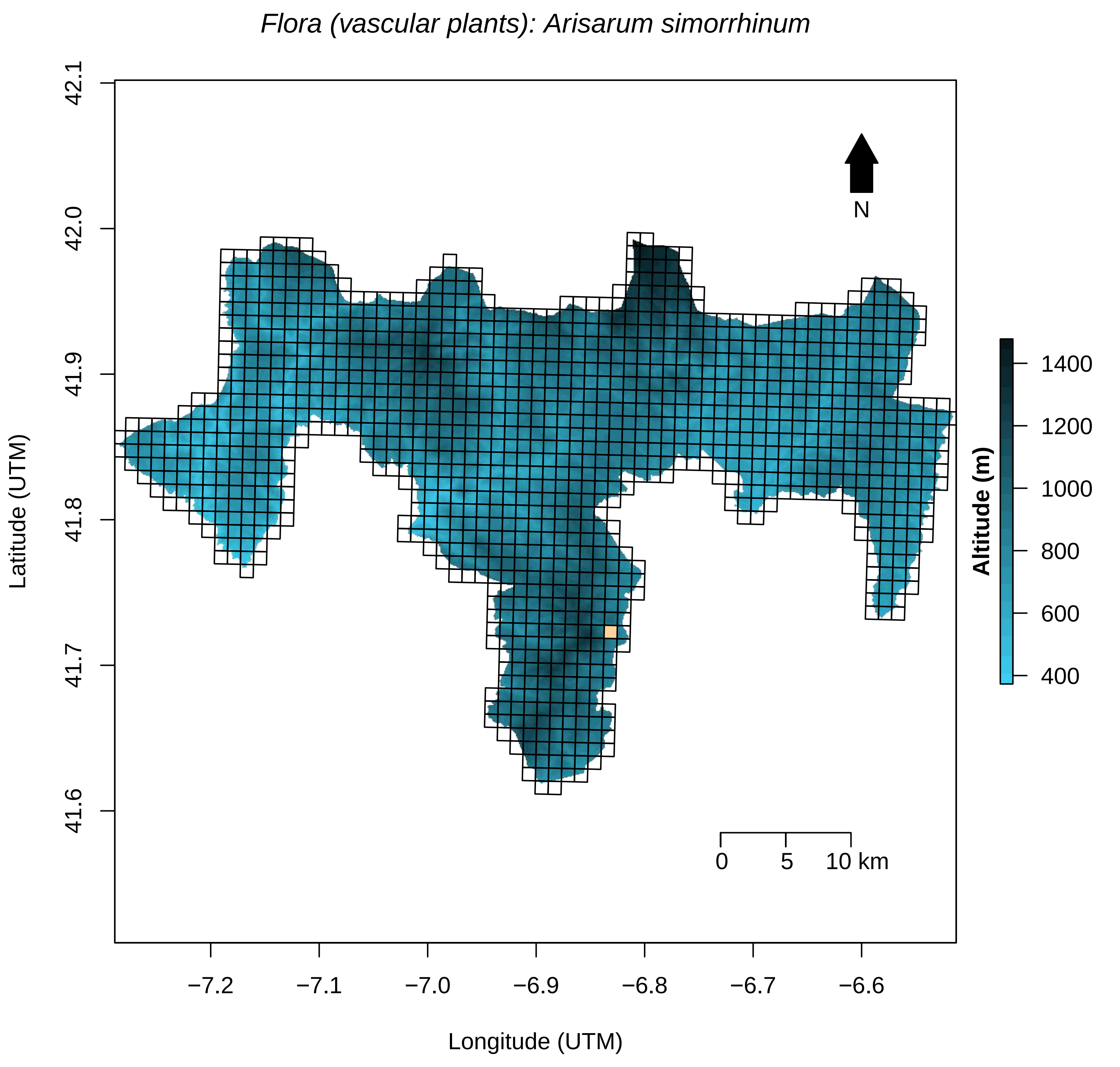

Flora (vascular plants): Arisarum simorrhinum
42.1
42.0
41.9
41.8
41.7
41.6
Latitude (UTM)
−7.2
−7.1
−7.0
−6.9
−6.8
−6.7
−6.6
Longitude (UTM)
N
0
5
10 km
Altitude (m)
1400
1200
1000
800
600
400

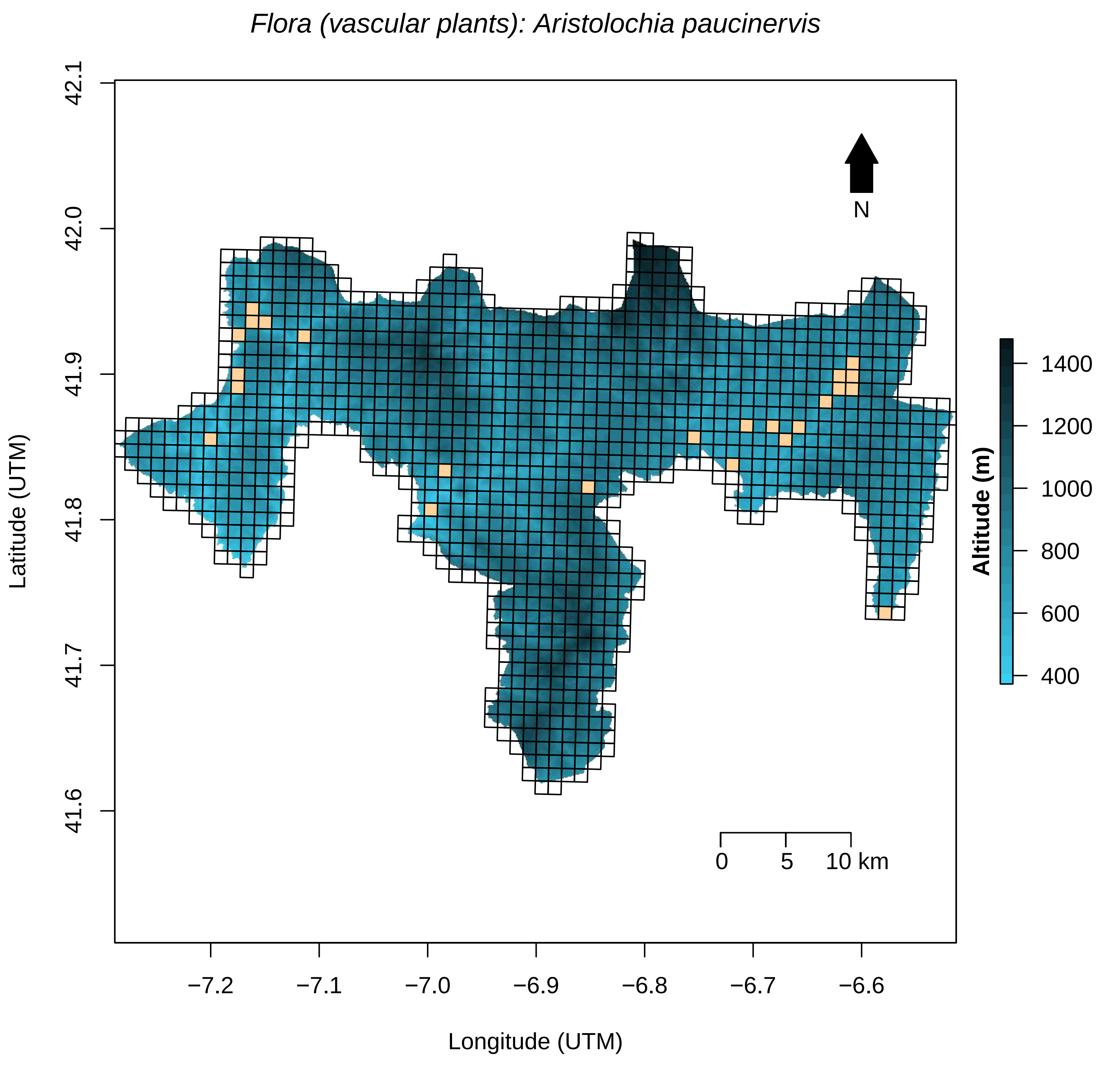
Flora (vascular plants): Aristolochia paucinervis
Latitude (UTM)
Longitude (UTM)
42.1
42.0
41.9
41.8
41.7
41.6
−7.2
−7.1
−7.0
−6.9
−6.8
−6.7
−6.6
N
0
5
10 km
Altitude (m)
1400
1200
1000
800
600
400

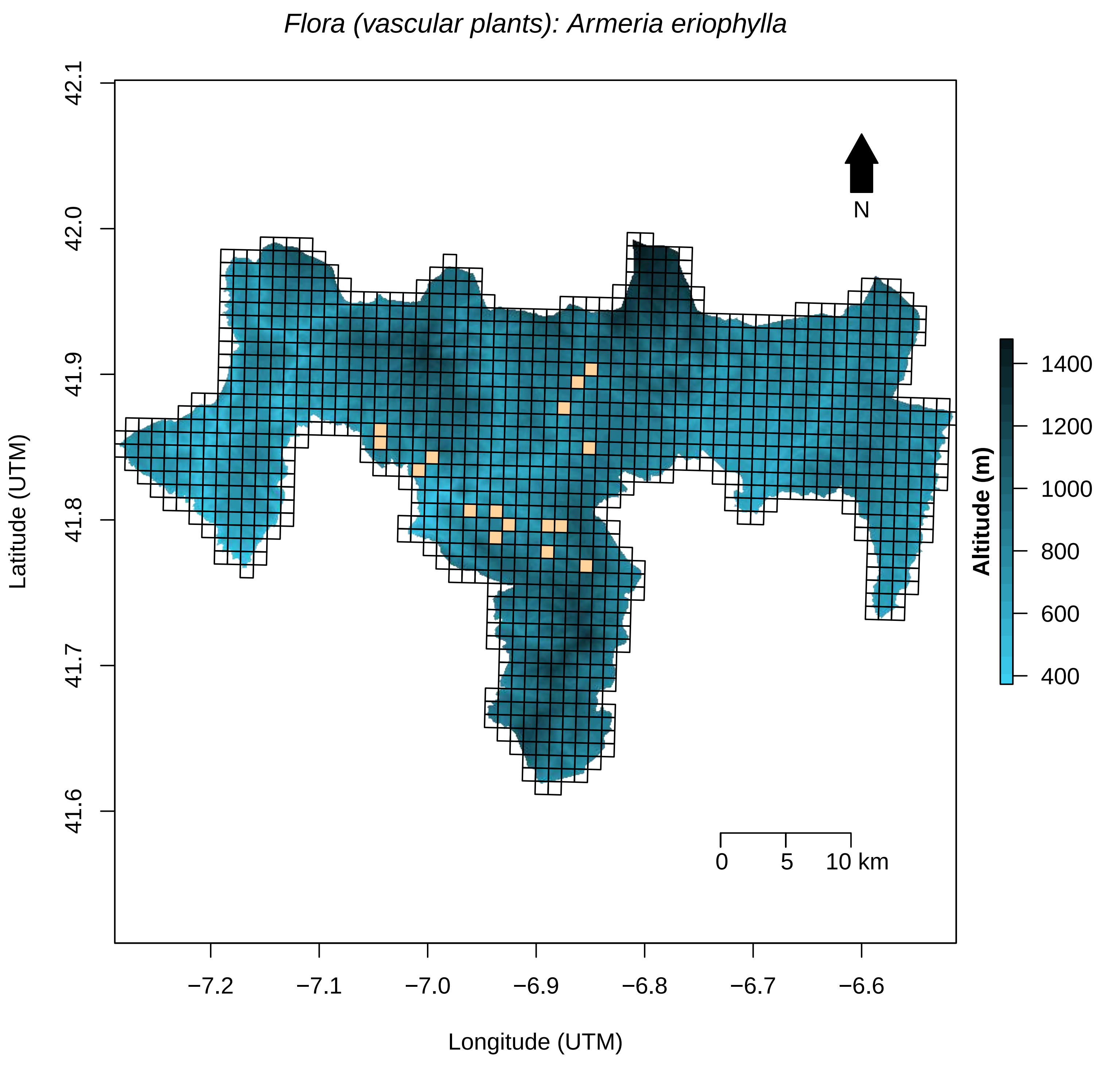

Flora (vascular plants): Armeria eriophylla
N
0
5
10 km
Altitude (m)
1400
1200
1000
800
600
400
42.1
42.0
41.9
41.8
41.7
41.6
Latitude (UTM)
−7.2
−7.1
−7.0
−6.9
−6.8
−6.7
−6.6
Longitude (UTM)

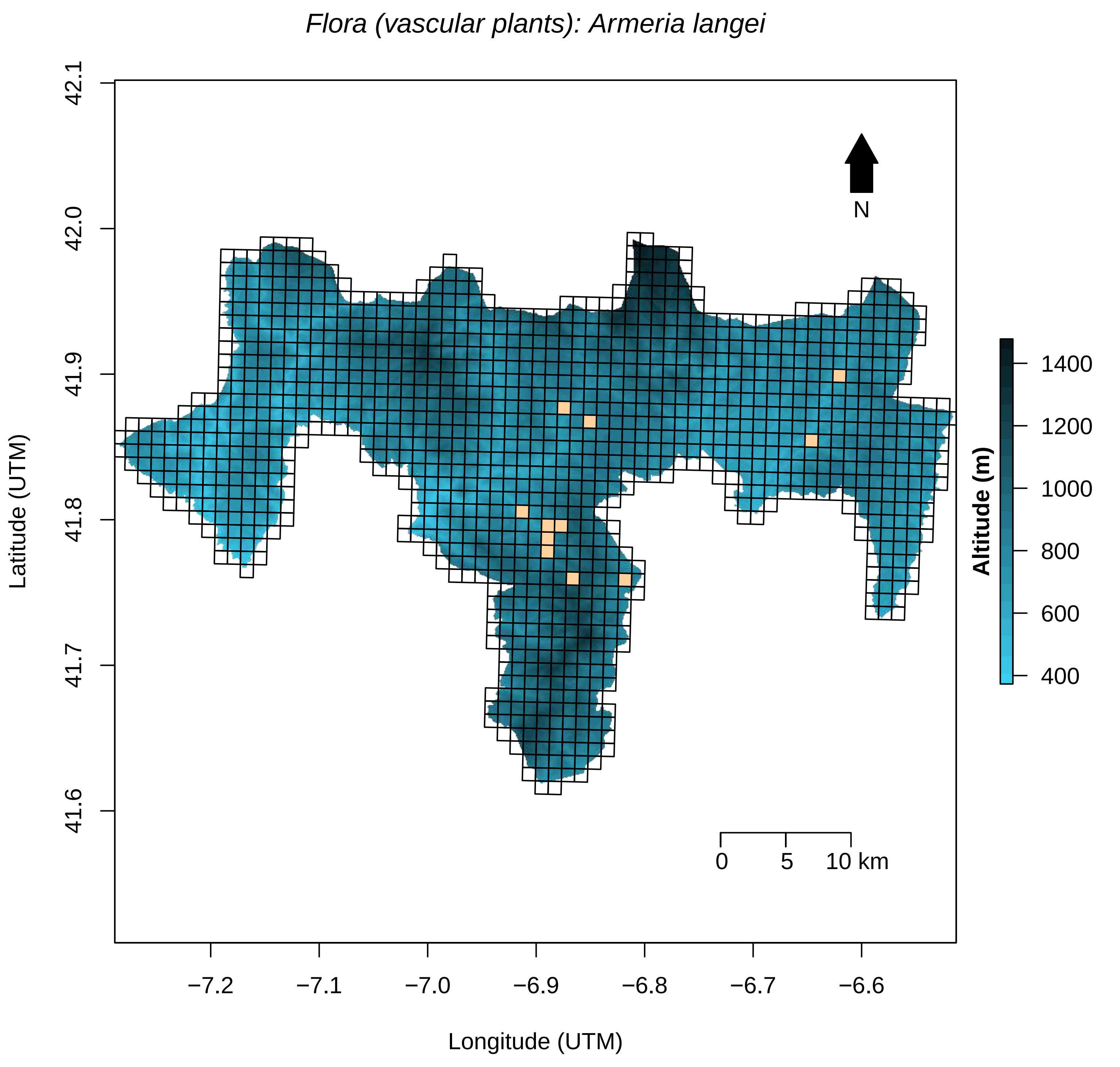

Flora (vascular plants): Armeria langei
42.1
42.0
41.9
41.8
41.7
41.6
Latitude (UTM)
−7.2
−7.1
−7.0
−6.9
−6.8
−6.7
−6.6
Longitude (UTM)
N
0
5
10 km
Altitude (m)
1400
1200
1000
800
600
400

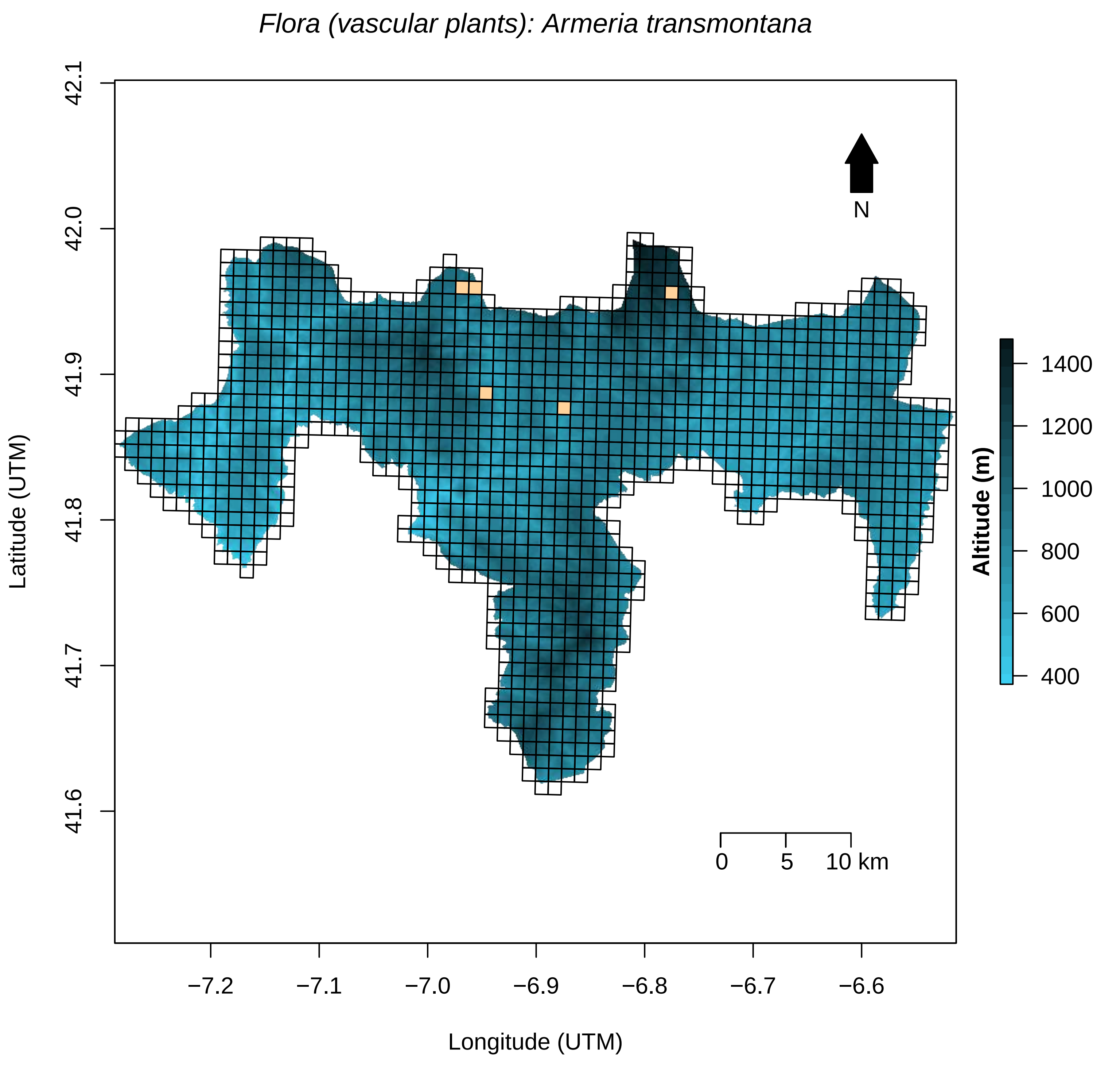

Flora (vascular plants): Armeria transmontana
N
0
5
10 km
Latitude (UTM)
Longitude (UTM)
Altitude (m)
42.1
42.0
41.9
41.8
41.7
41.6
−7.2
−7.1
−7.0
−6.9
−6.8
−6.7
−6.6
1400
1200
1000
800
600
400

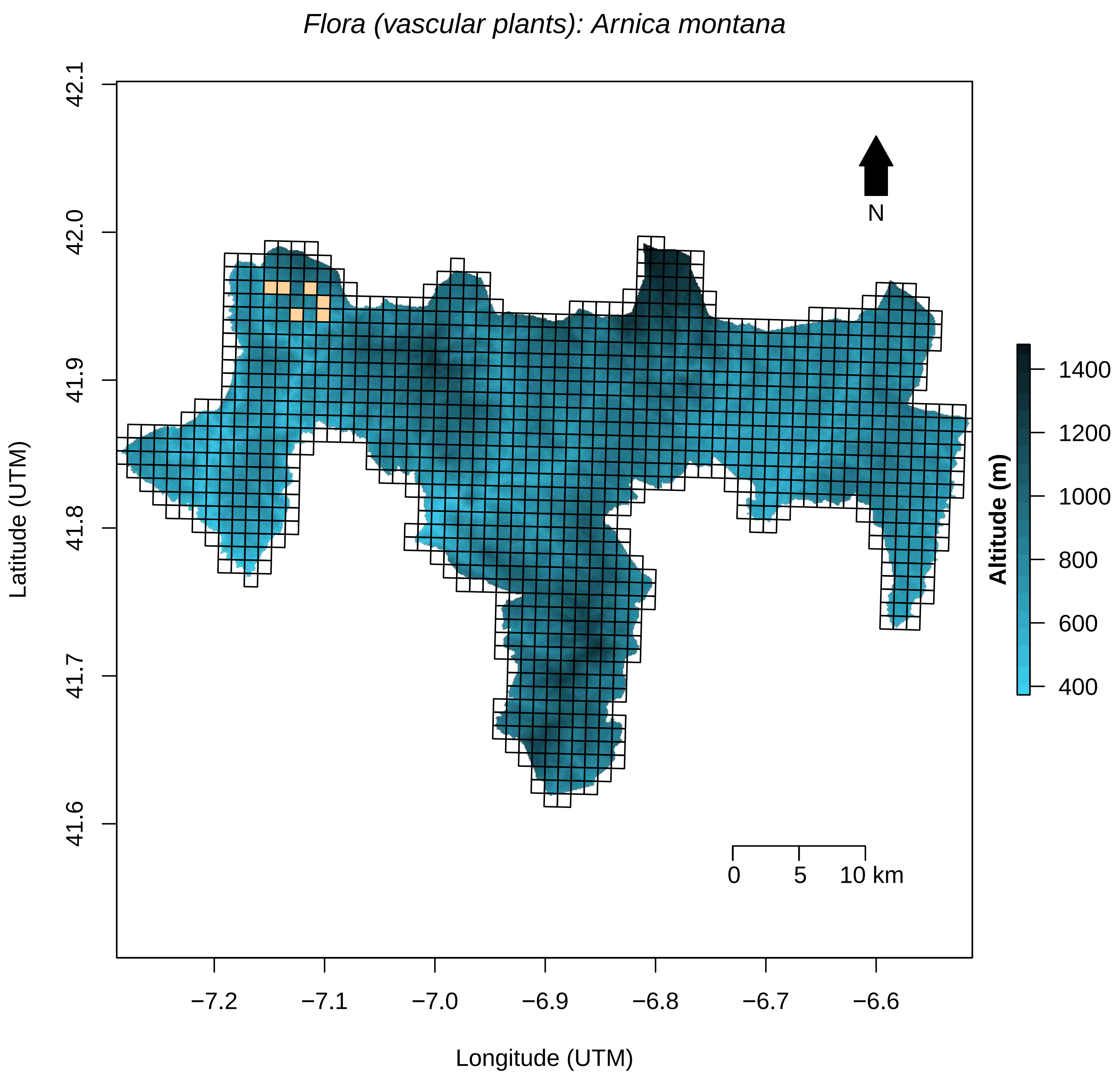

Flora (vascular plants): Arnica montana
N
0
5
10 km
Altitude (m)
1400
1200
1000
800
600
400
Latitude (UTM)
42.1
42.0
41.9
41.8
41.7
41.6
Longitude (UTM)
−7.2
−7.1
−7.0
−6.9
−6.8
−6.7
−6.6

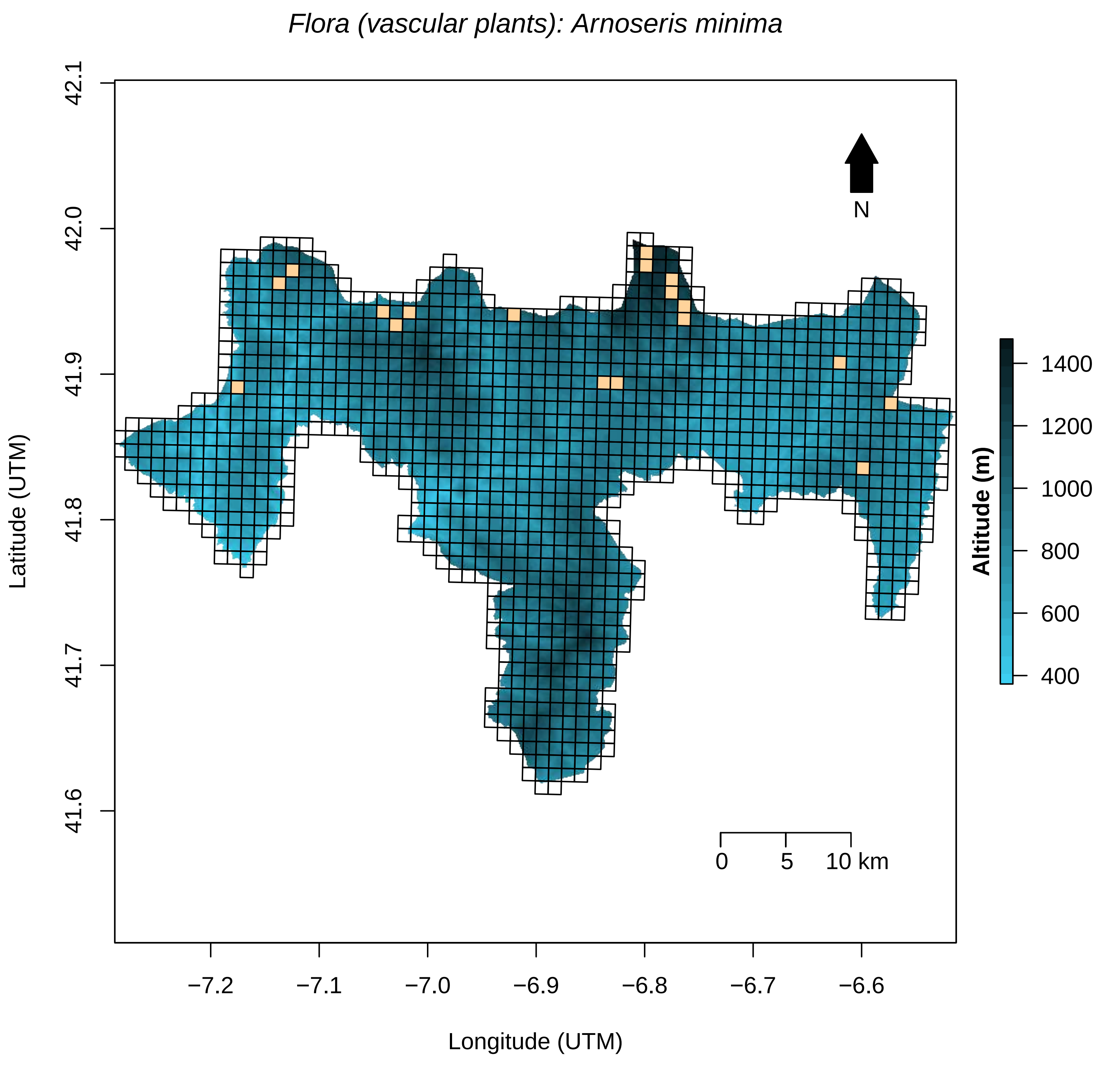

Flora (vascular plants): Arnoseris minima
42.1
42.0
41.9
41.8
41.7
41.6
Latitude (UTM)
N
0 5 10 km
1400
1200
1000
800
600
400
Altitude (m)
−7.2 −7.1 −7.0 −6.9 −6.8 −6.7 −6.6
Longitude (UTM)

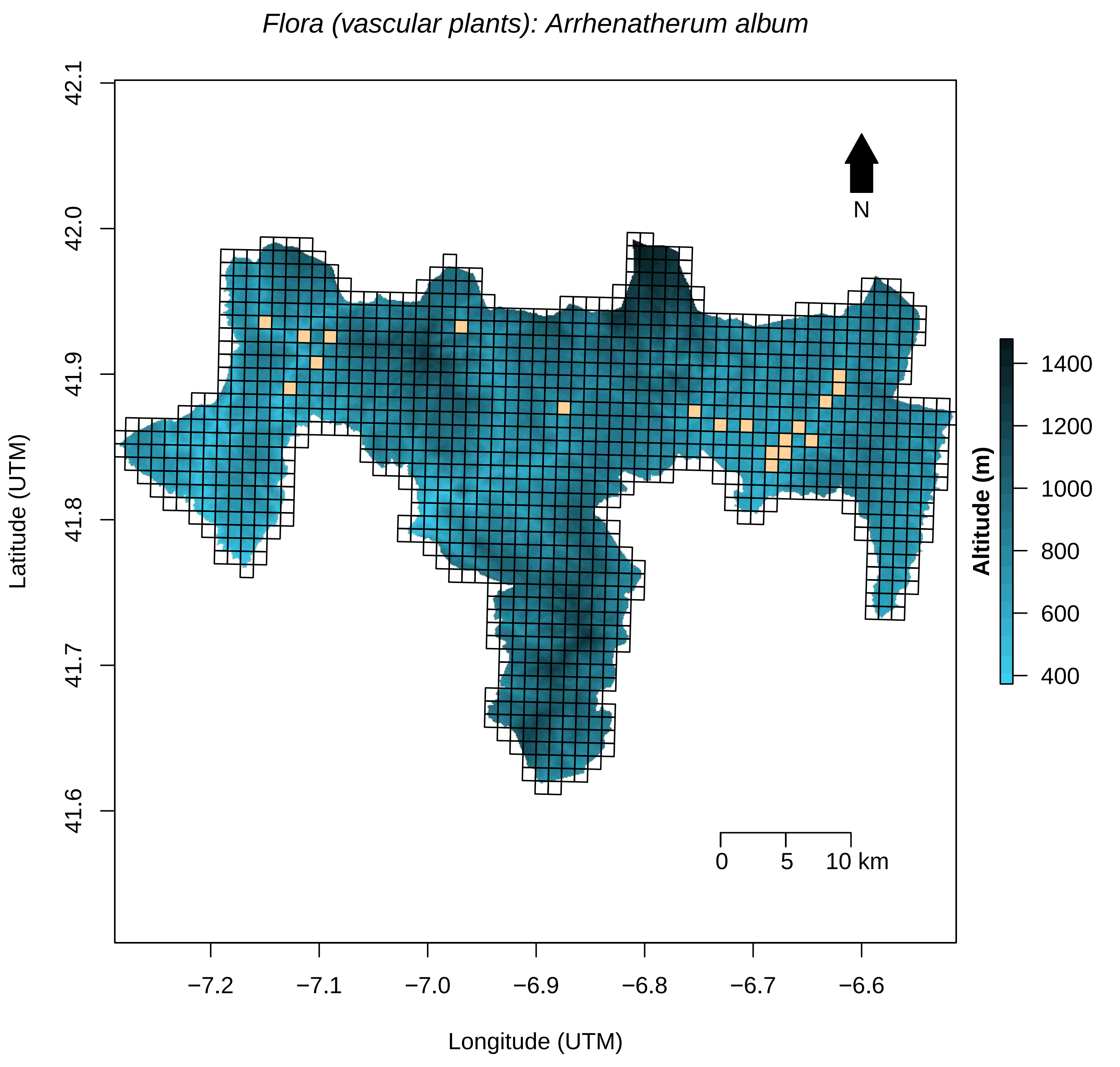

Flora (vascular plants): Arrhenatherum album
42.1
42.0
41.9
41.8
41.7
41.6
Latitude (UTM)
−7.2
−7.1
−7.0
−6.9
−6.8
−6.7
−6.6
Longitude (UTM)
N
0
5
10 km
Altitude (m)
1400
1200
1000
800
600
400

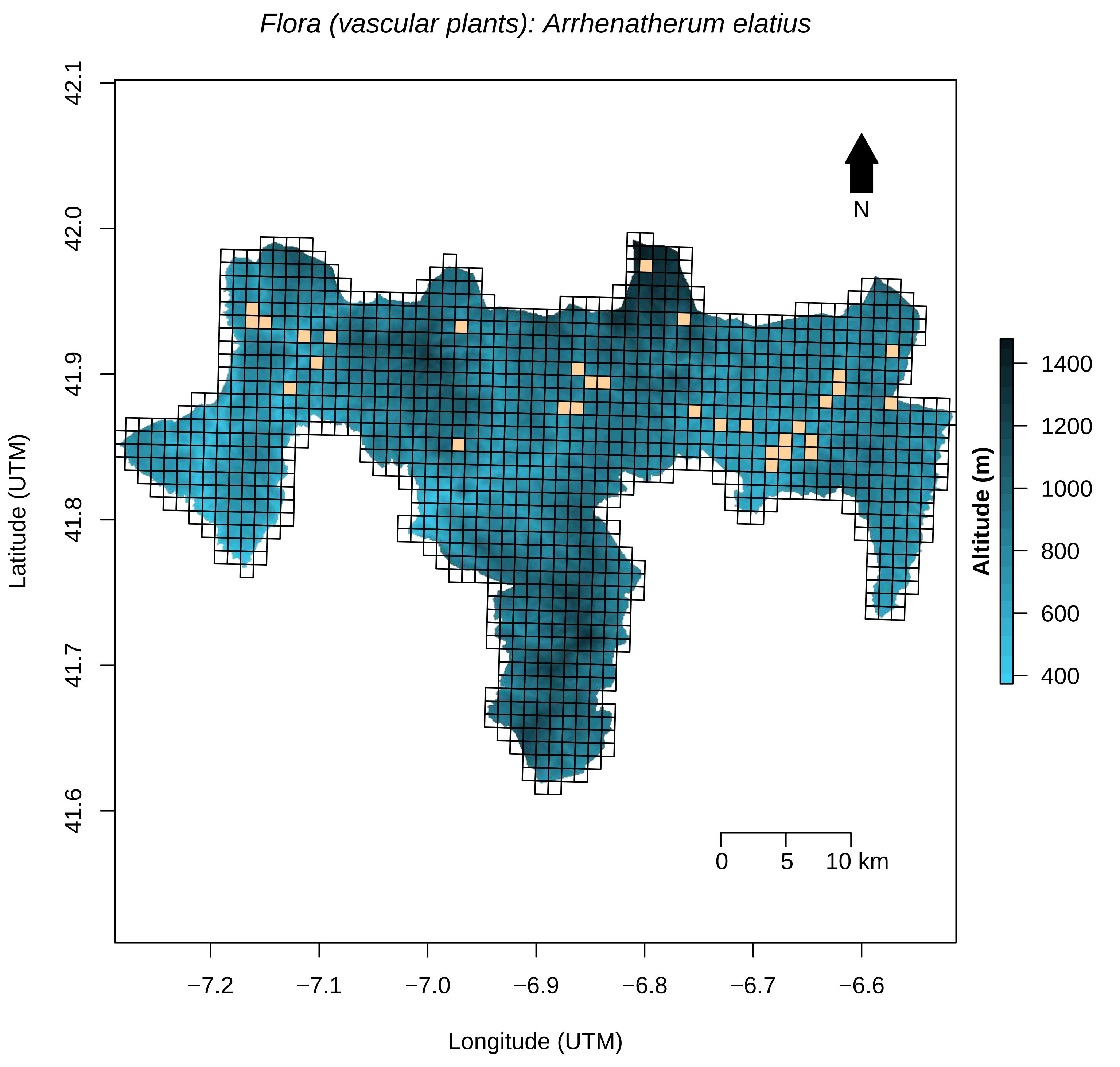

Flora (vascular plants): Arrhenatherum elatius
42.1
42.0
41.9
41.8
41.7
41.6
Latitude (UTM)
−7.2
−7.1
−7.0
−6.9
−6.8
−6.7
−6.6
Longitude (UTM)
N
0
5
10 km
Altitude (m)
1400
1200
1000
800
600
400

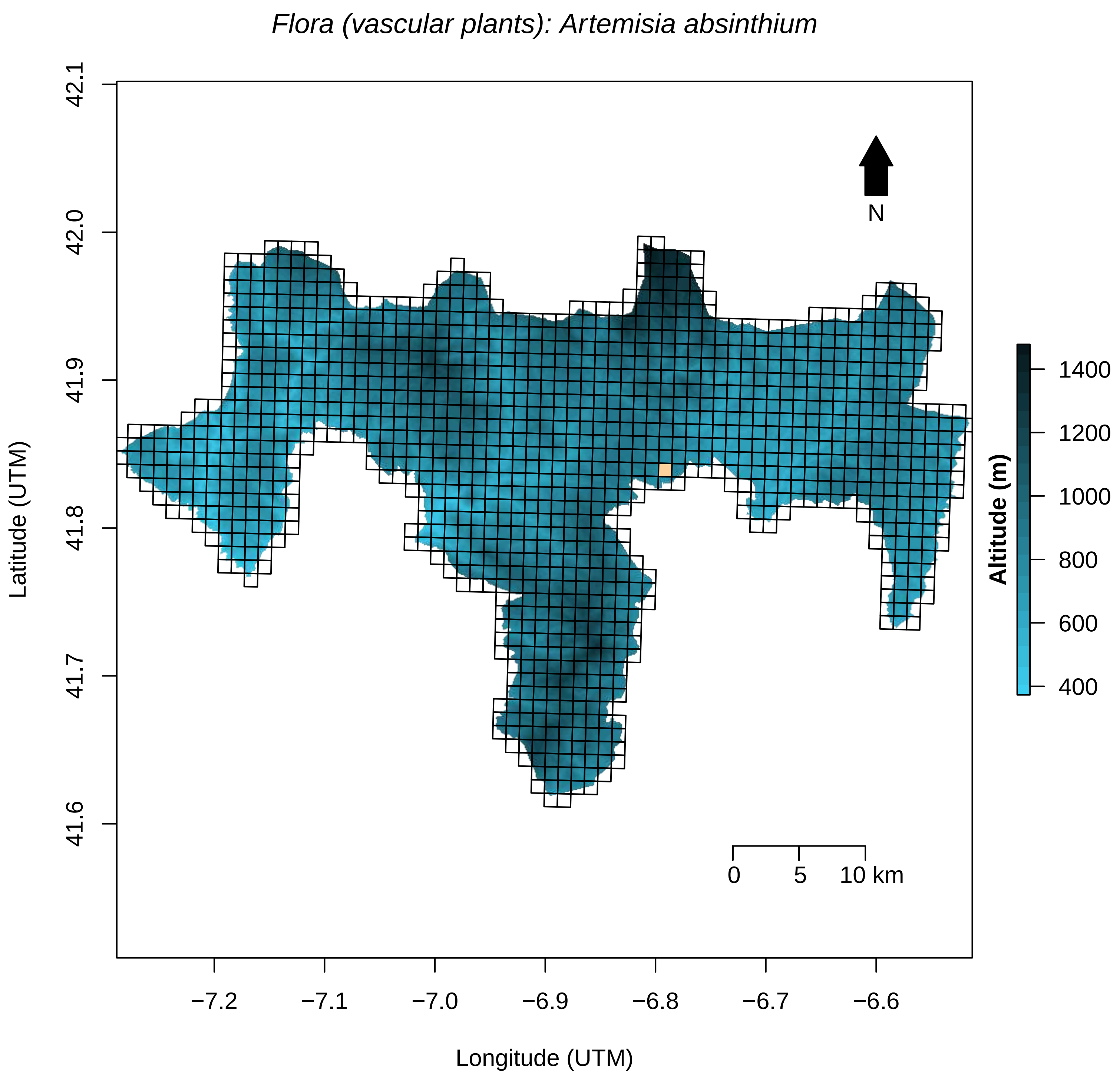

Flora (vascular plants): Artemisia absinthium
42.1
42.0
41.9
41.8
41.7
41.6
Latitude (UTM)
−7.2
−7.1
−7.0
−6.9
−6.8
−6.7
−6.6
Longitude (UTM)
N
0
5
10 km
Altitude (m)
1400
1200
1000
800
600
400

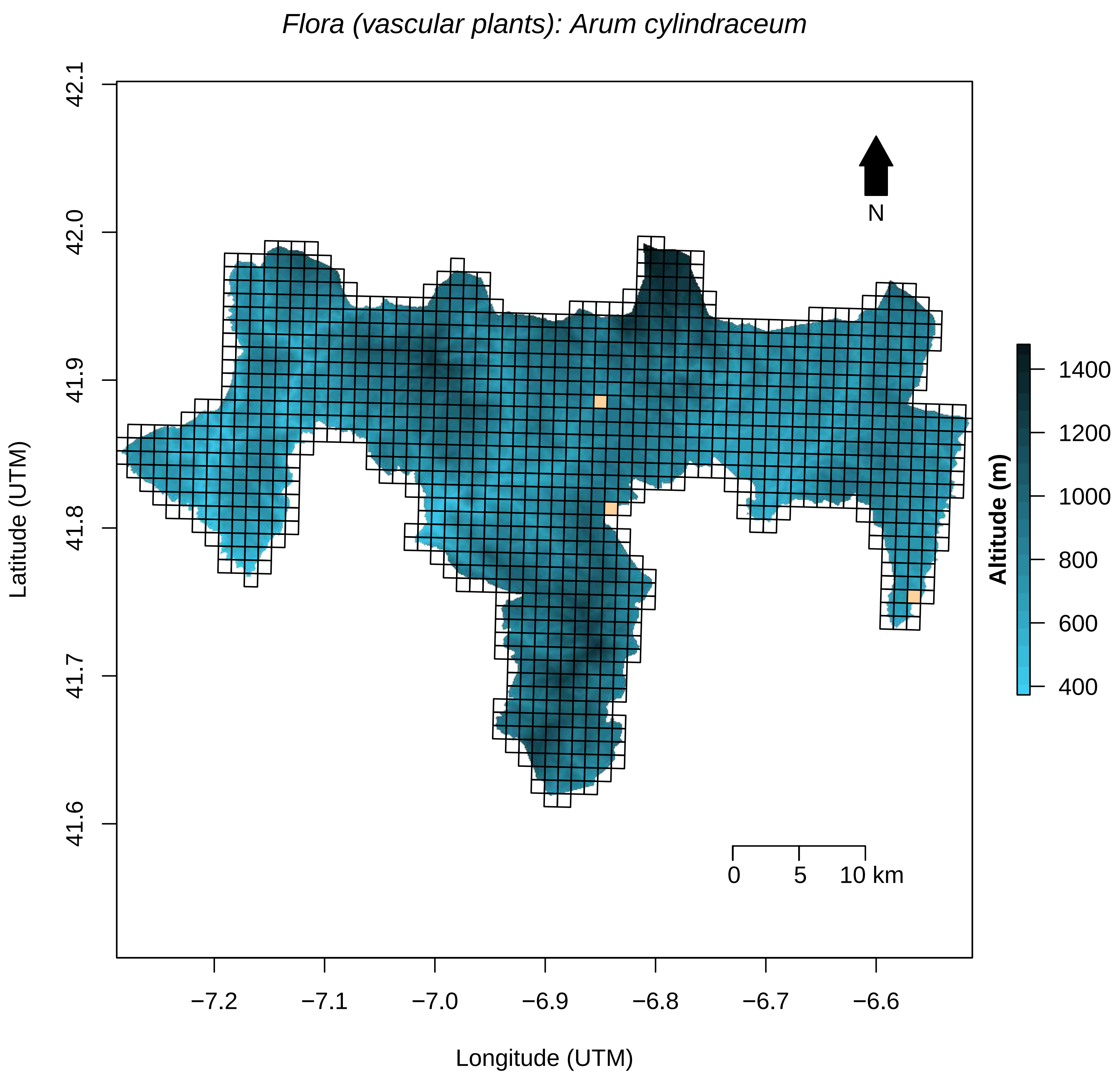

Flora (vascular plants): Arum cylindraceum
42.1
42.0
41.9
41.8
41.7
41.6
Latitude (UTM)
−7.2
−7.1
−7.0
−6.9
−6.8
−6.7
−6.6
Longitude (UTM)
N
0
5
10 km
Altitude (m)
1400
1200
1000
800
600
400

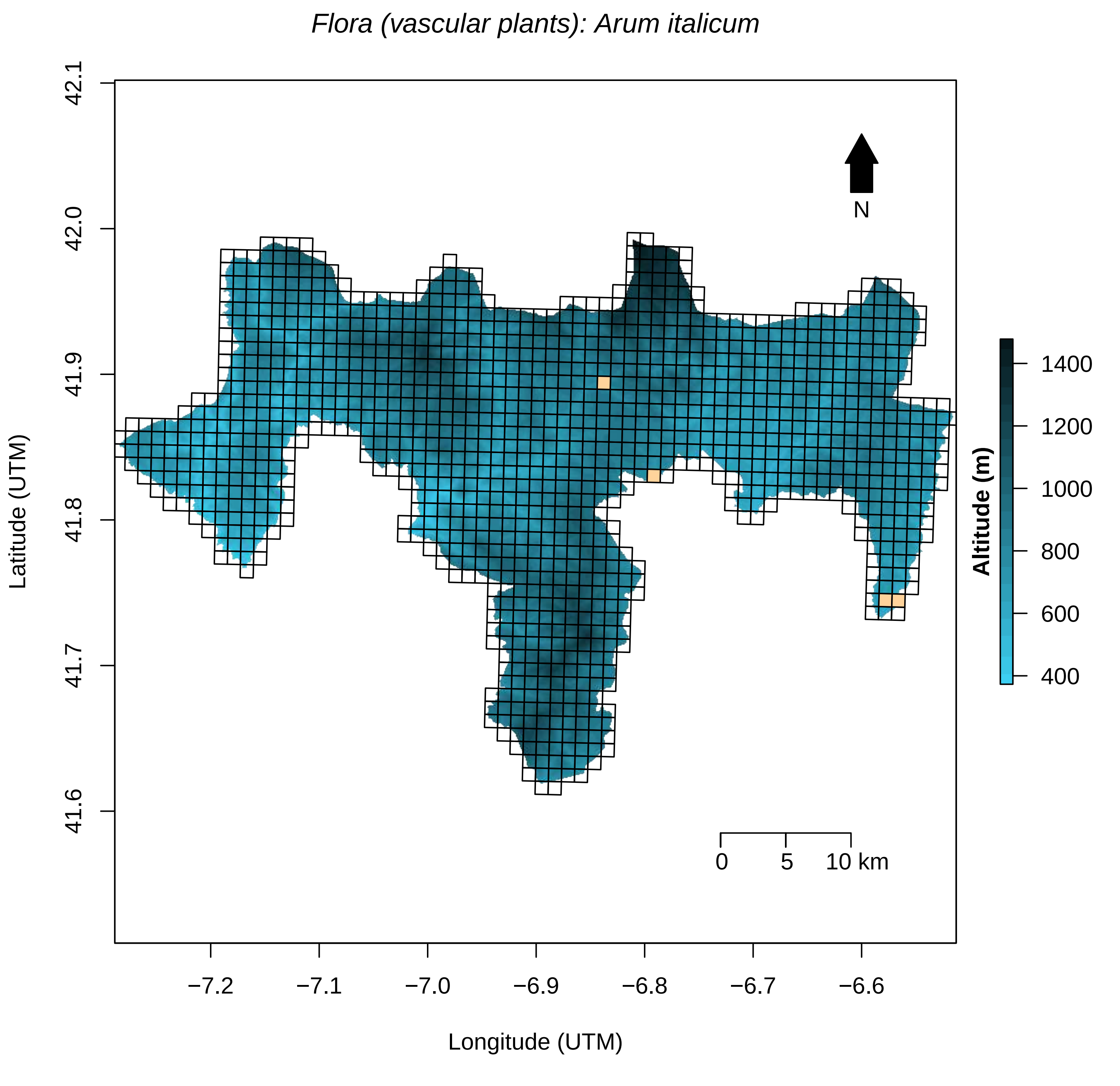

Flora (vascular plants): Arum italicum
N
0
5
10 km
Altitude (m)
1400
1200
1000
800
600
400
Latitude (UTM)
42.1
42.0
41.9
41.8
41.7
41.6
Longitude (UTM)
−7.2
−7.1
−7.0
−6.9
−6.8
−6.7
−6.6

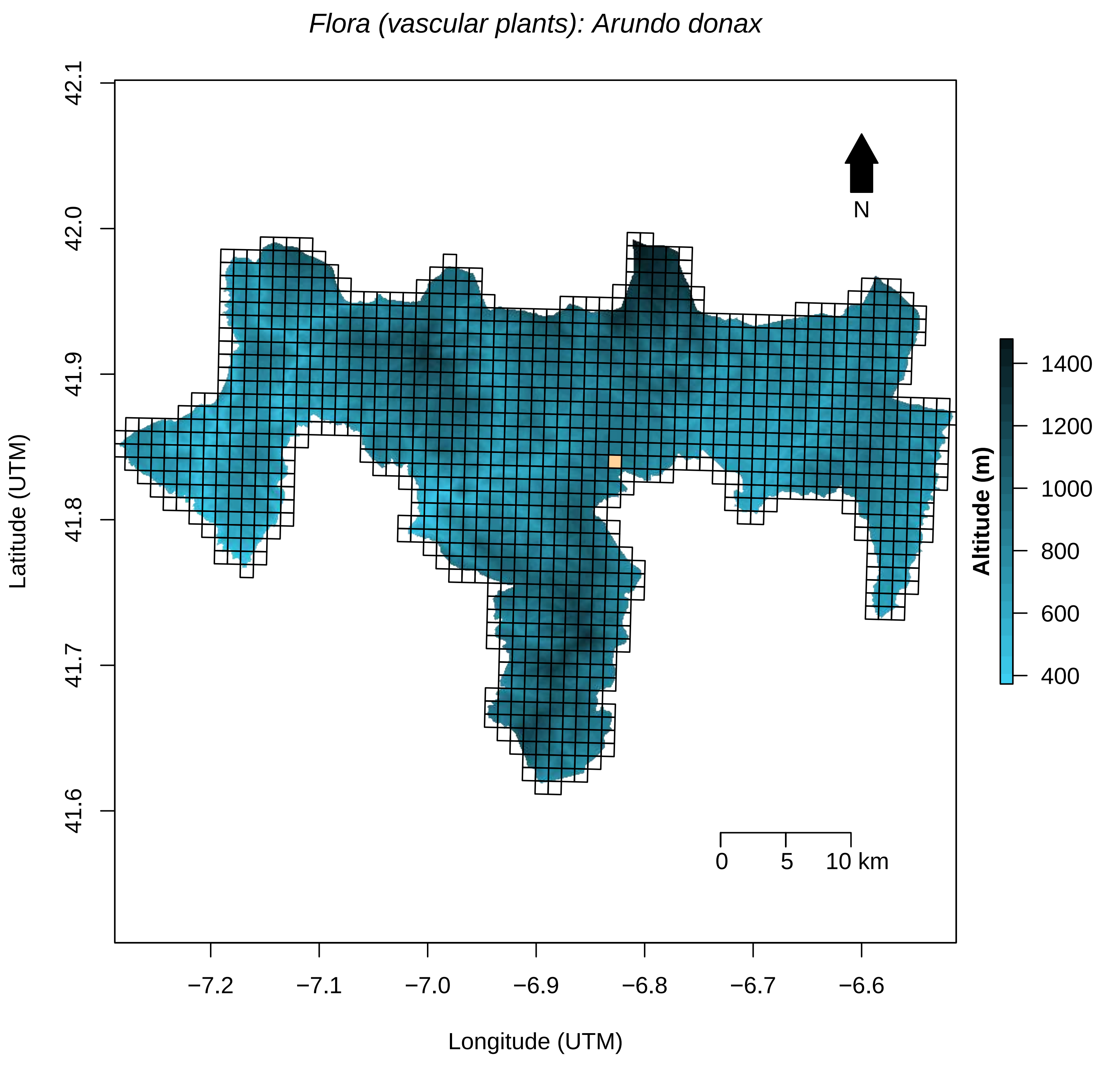

Flora (vascular plants): Arundo donax
42.1
42.0
41.9
41.8
41.7
41.6
Latitude (UTM)
−7.2
−7.1
−7.0
−6.9
−6.8
−6.7
−6.6
Longitude (UTM)
N
0
5
10 km
Altitude (m)
1400
1200
1000
800
600
400

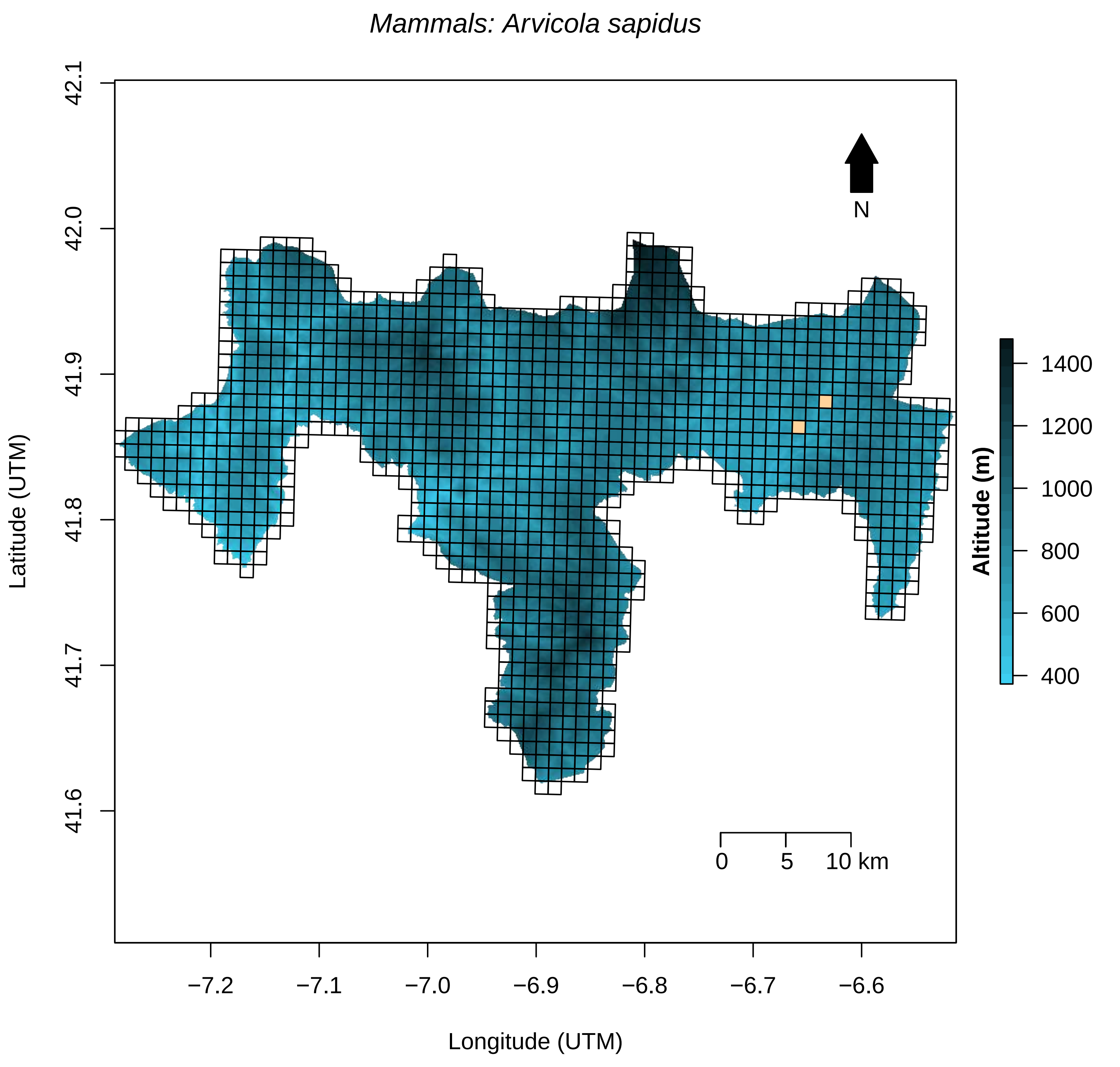

Mammals: Arvicola sapidus
42.1
42.0
41.9
41.8
41.7
41.6
Latitude (UTM)
−7.2
−7.1
−7.0
−6.9
−6.8
−6.7
−6.6
Longitude (UTM)
N
0
5
10 km
Altitude (m)
1400
1200
1000
800
600
400

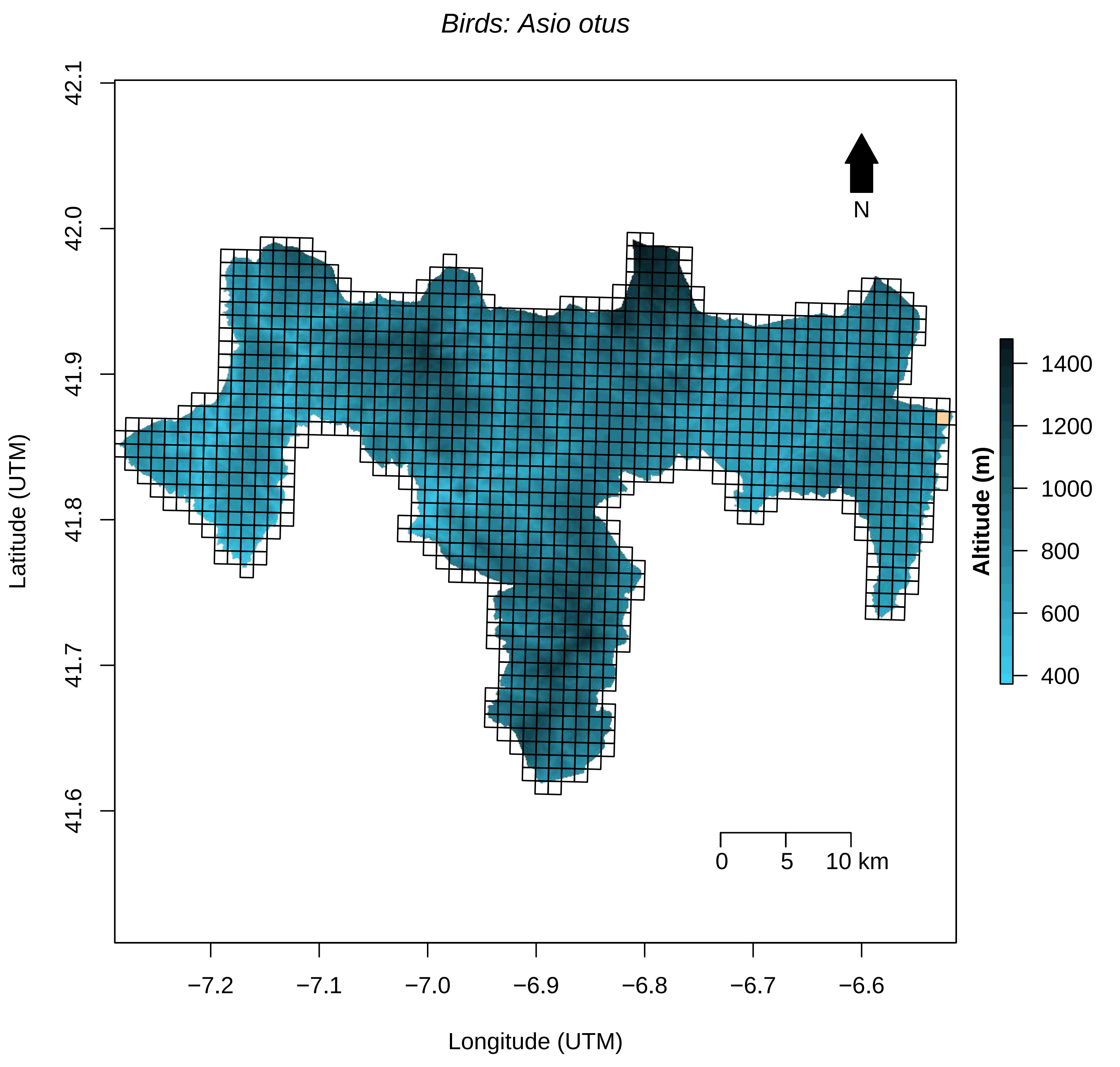

Birds: Asio otus
42.1
42.0
41.9
41.8
41.7
41.6
Latitude (UTM)
−7.2
−7.1
−7.0
−6.9
−6.8
−6.7
−6.6
Longitude (UTM)
N
0
5
10 km
1400
1200
1000
800
600
400
Altitude (m)

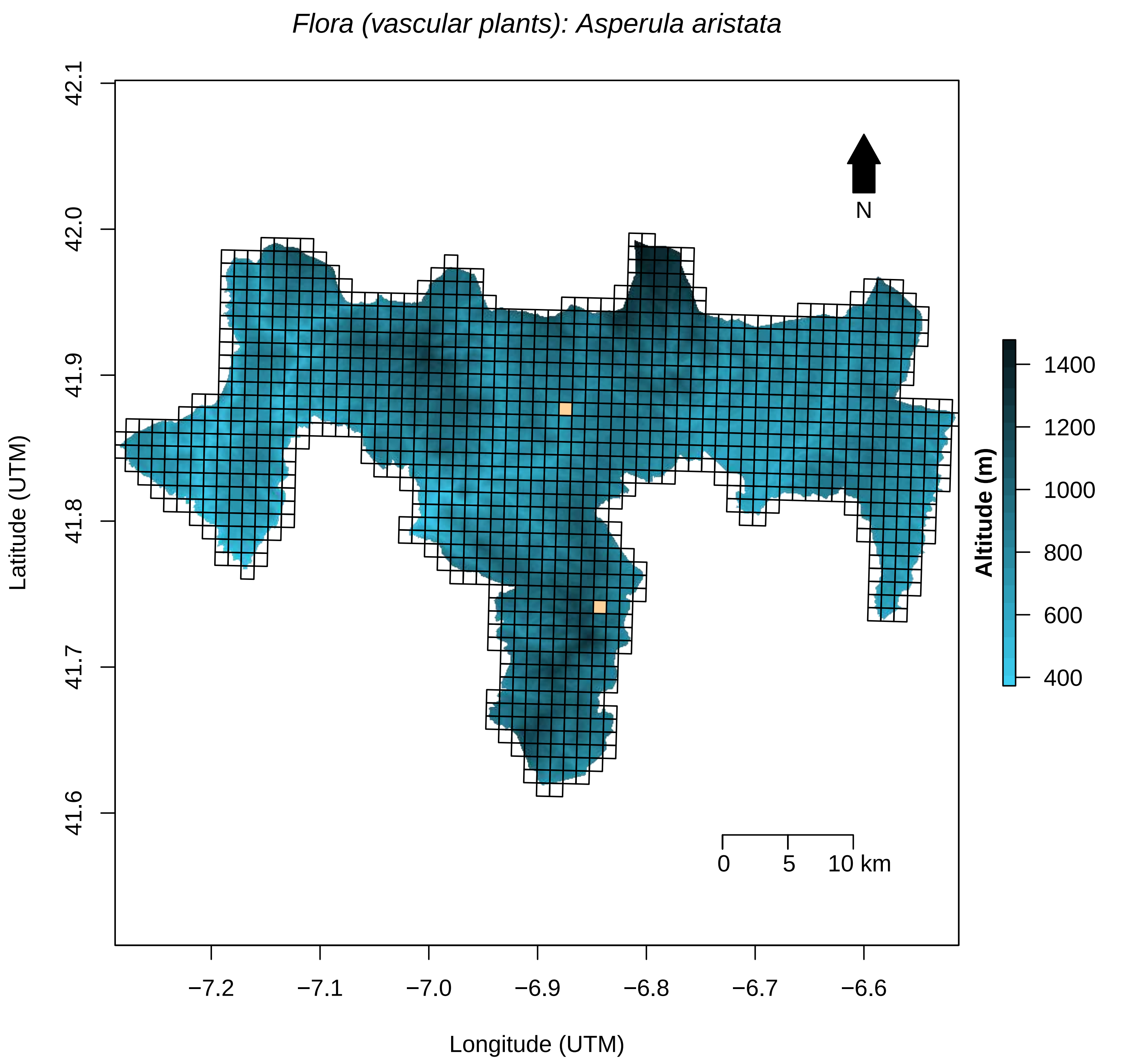

Flora (vascular plants): Asperula aristata
42.1
42.0
41.9
41.8
41.7
41.6
Latitude (UTM)
−7.2
−7.1
−7.0
−6.9
−6.8
−6.7
−6.6
Longitude (UTM)
N
0
5
10 km
Altitude (m)
1400
1200
1000
800
600
400

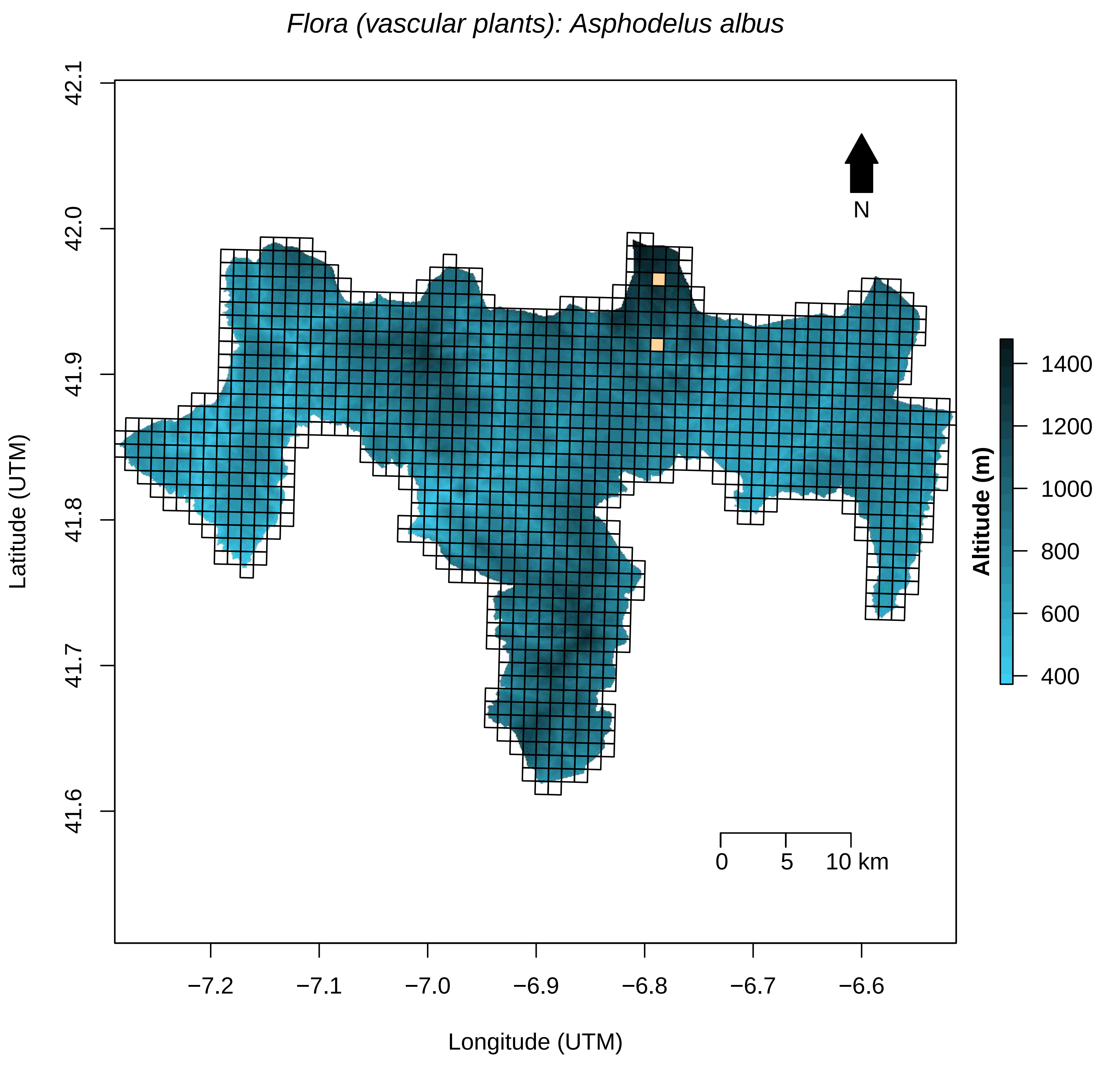

Flora (vascular plants): Asphodelus albus
42.1
42.0
41.9
41.8
41.7
41.6
Latitude (UTM)
−7.2
−7.1
−7.0
−6.9
−6.8
−6.7
−6.6
Longitude (UTM)
N
0
5
10 km
Altitude (m)
1400
1200
1000
800
600
400

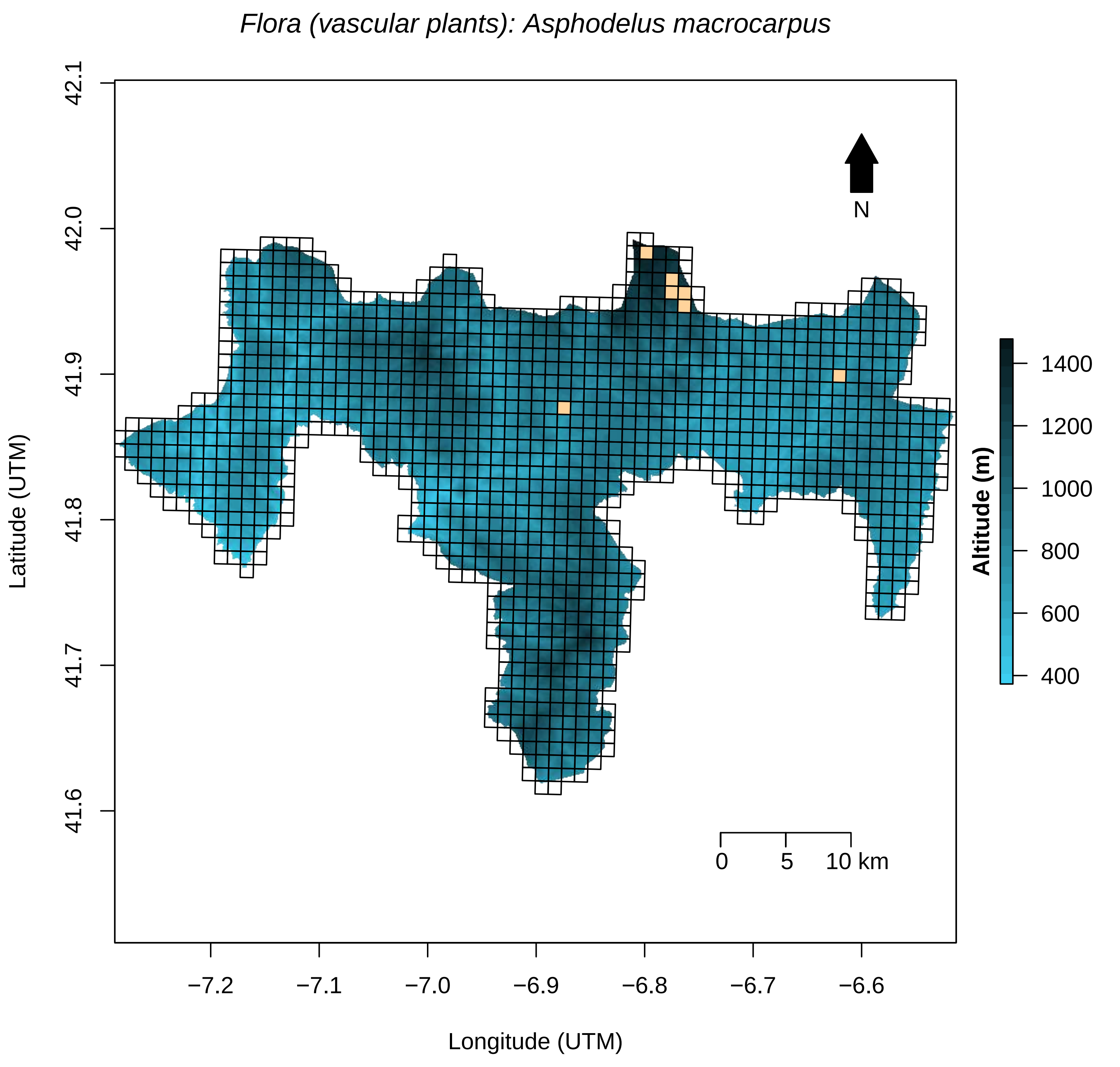

Flora (vascular plants): Asphodelus macrocarpus
Latitude (UTM)
42.1
42.0
41.9
41.8
41.7
41.6
Longitude (UTM)
−7.2
−7.1
−7.0
−6.9
−6.8
−6.7
−6.6
N
0
5
10 km
Altitude (m)
1400
1200
1000
800
600
400

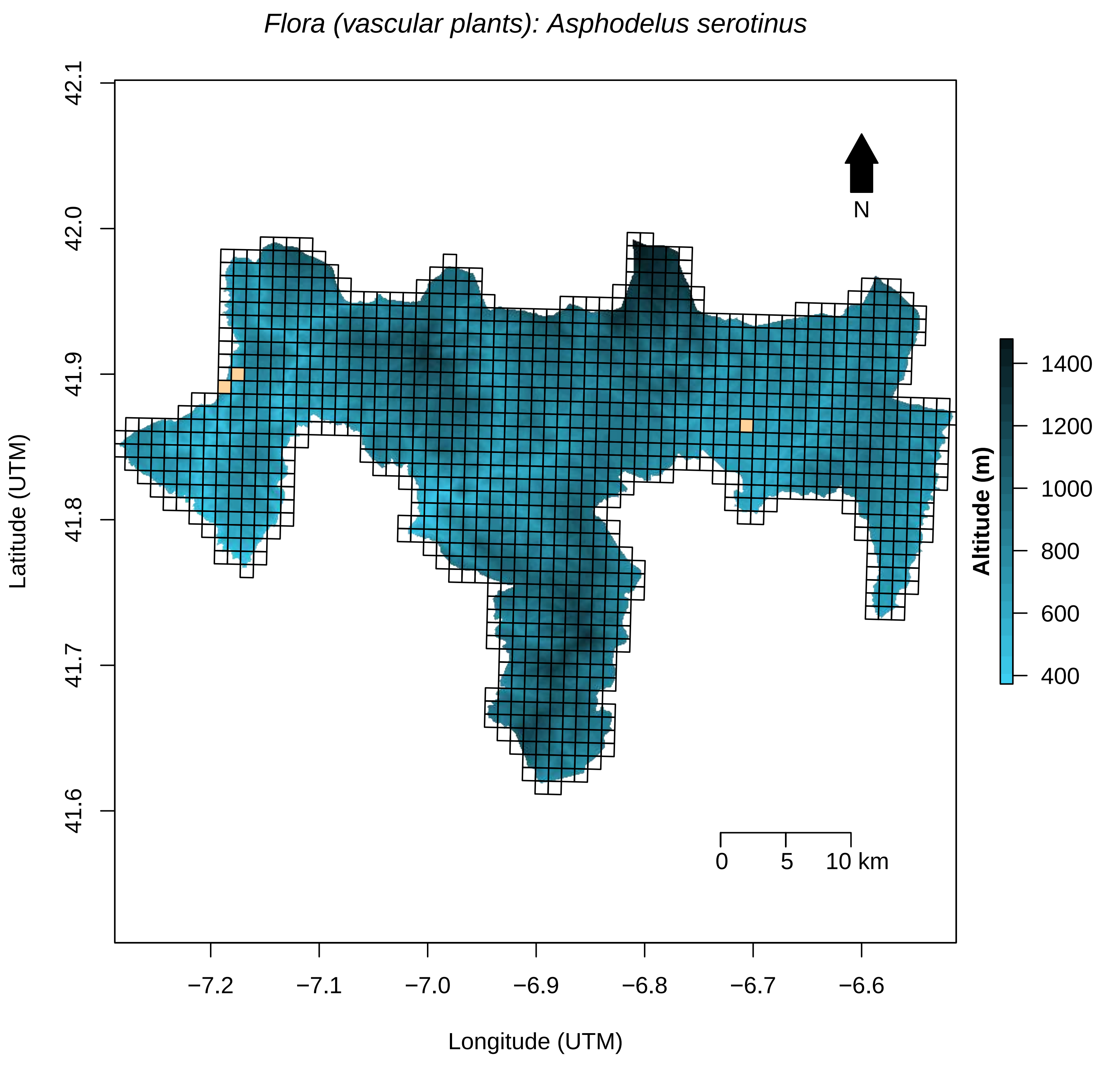

Flora (vascular plants): Asphodelus serotinus
N
0
5
10 km
Altitude (m)
1400
1200
1000
800
600
400
Latitude (UTM)
42.1
42.0
41.9
41.8
41.7
41.6
Longitude (UTM)
−7.2
−7.1
−7.0
−6.9
−6.8
−6.7
−6.6

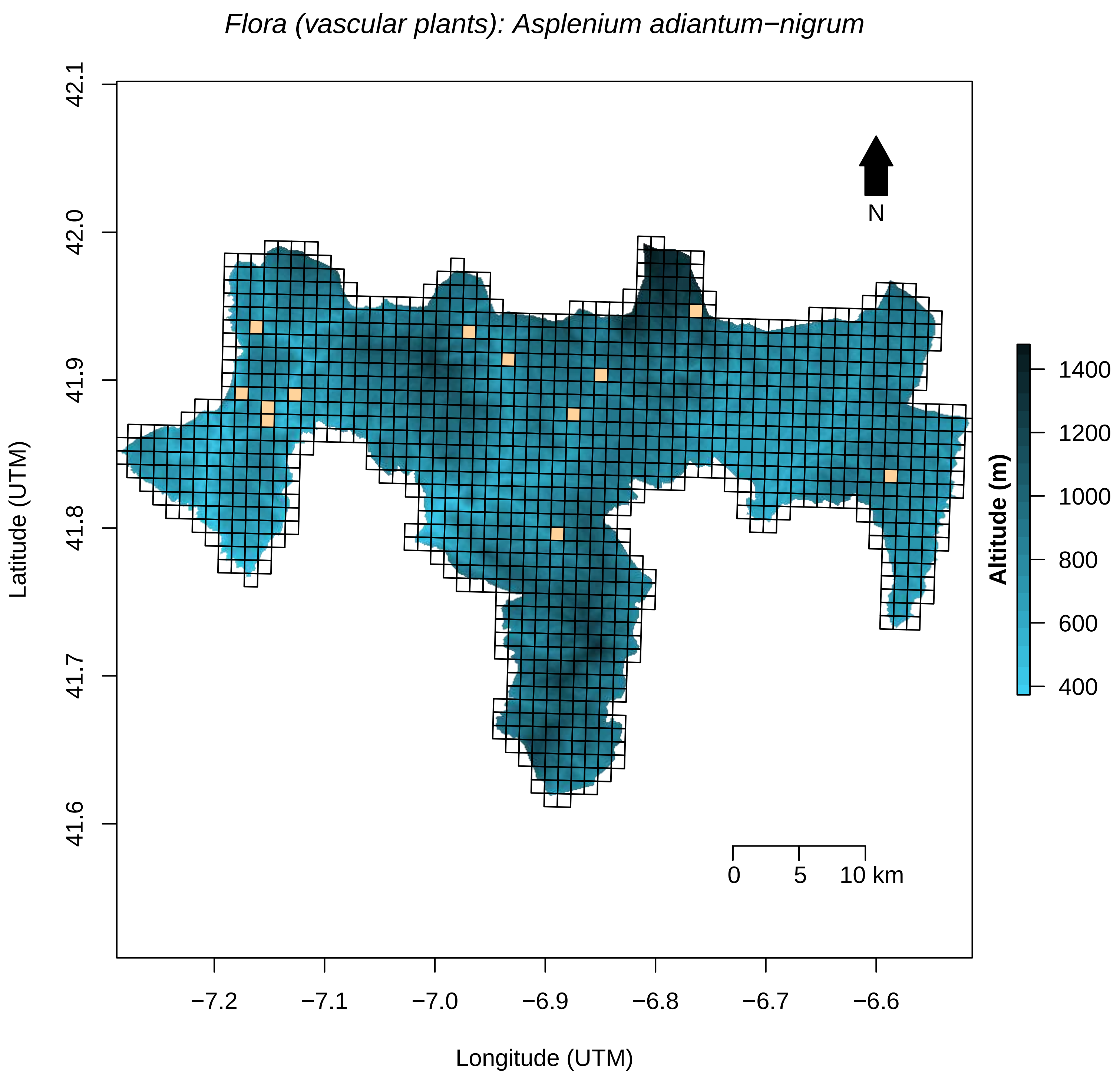
Flora (vascular plants): Asplenium adiantum−nigrum
42.1
42.0
41.9
41.8
41.7
41.6
Latitude (UTM)
−7.2
−7.1
−7.0
−6.9
−6.8
−6.7
−6.6
Longitude (UTM)
N
0
5
10 km
Altitude (m)
1400
1200
1000
800
600
400

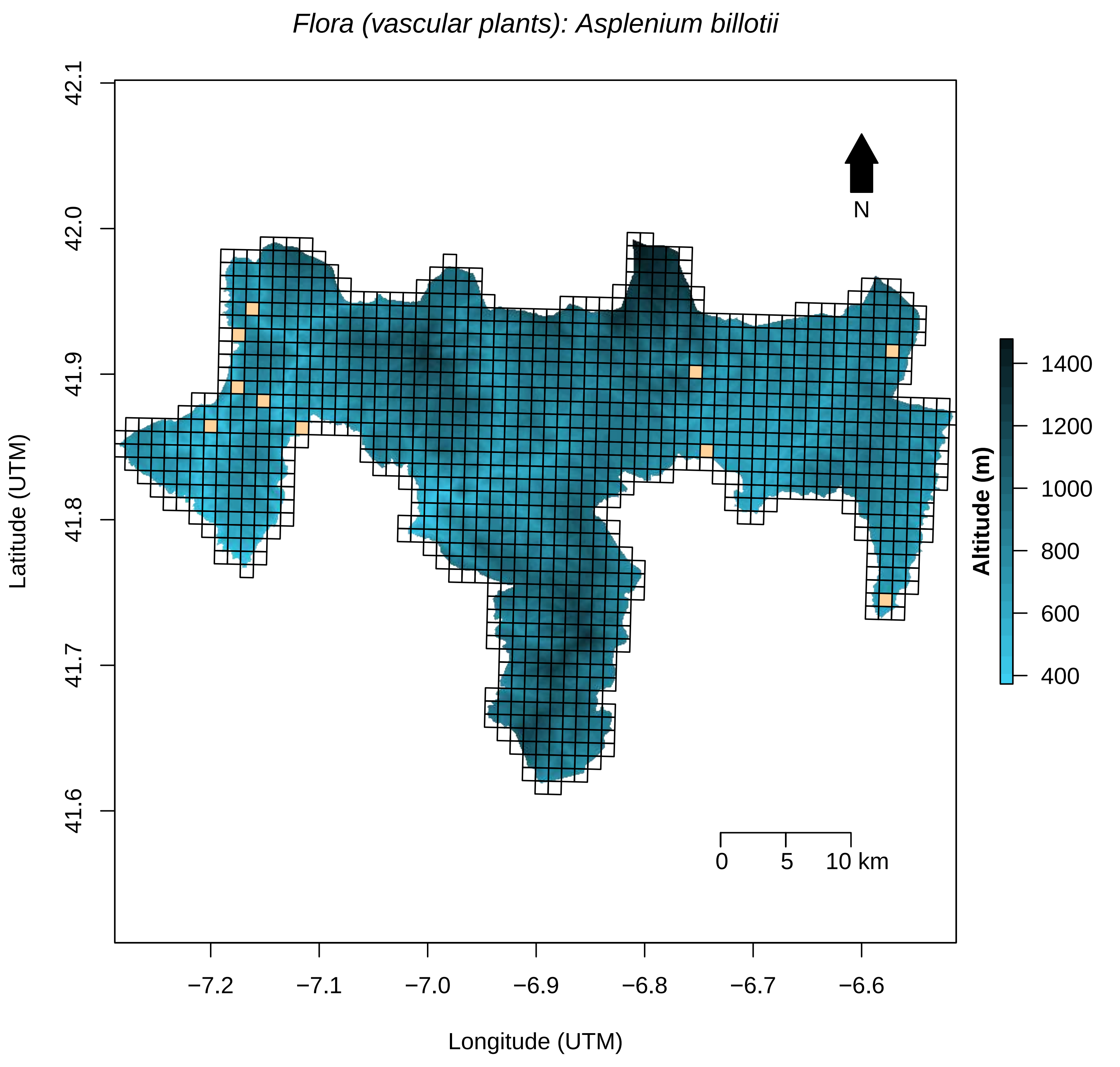

Flora (vascular plants): Asplenium billotii
42.1
42.0
41.9
41.8
41.7
41.6
Latitude (UTM)
−7.2
−7.1
−7.0
−6.9
−6.8
−6.7
−6.6
Longitude (UTM)
N
0
5
10 km
Altitude (m)
1400
1200
1000
800
600
400

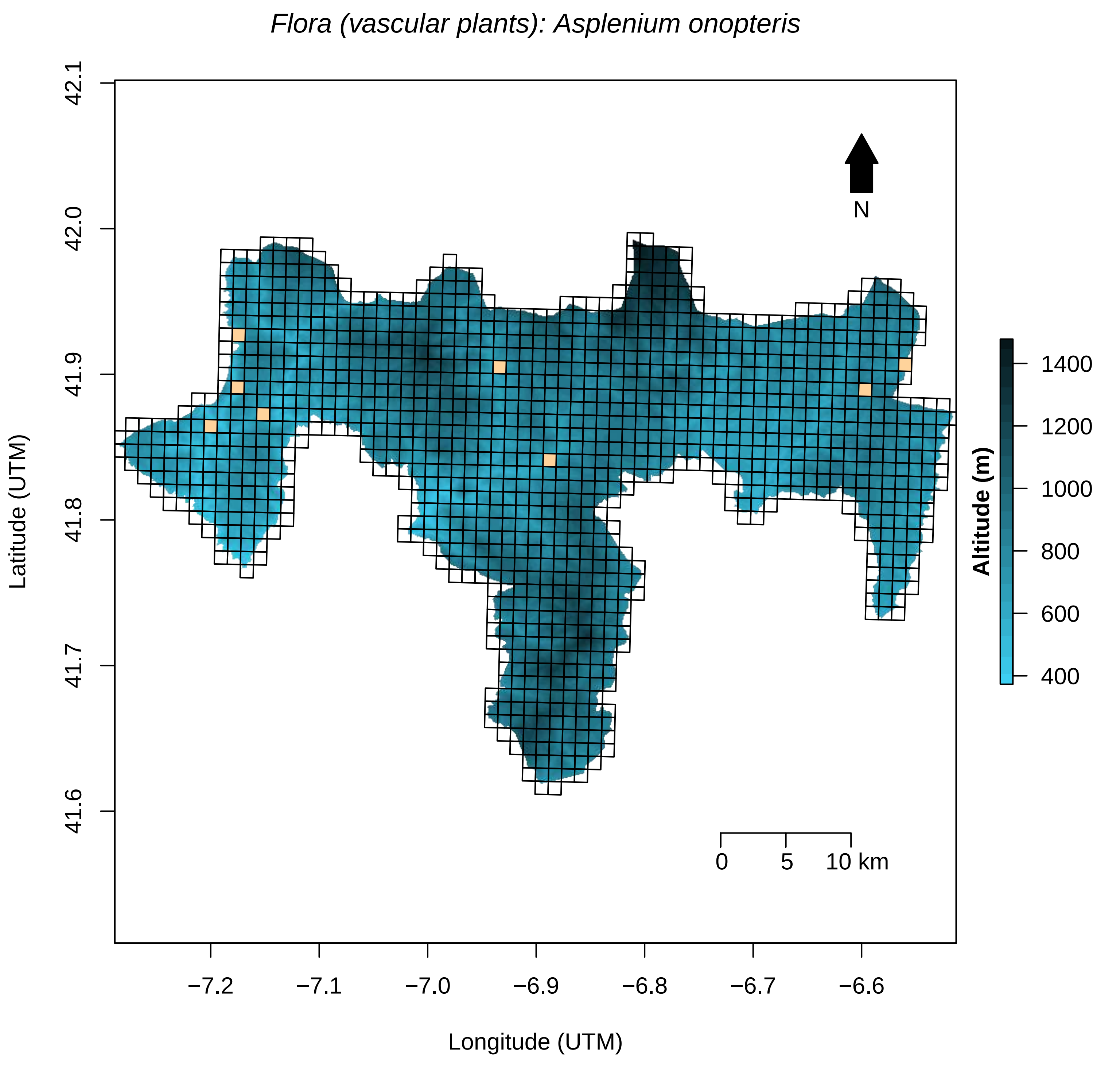

Flora (vascular plants): Asplenium onopteris
42.1
42.0
41.9
41.8
41.7
41.6
Latitude (UTM)
−7.2
−7.1
−7.0
−6.9
−6.8
−6.7
−6.6
Longitude (UTM)
N
0
5
10 km
Altitude (m)
1400
1200
1000
800
600
400

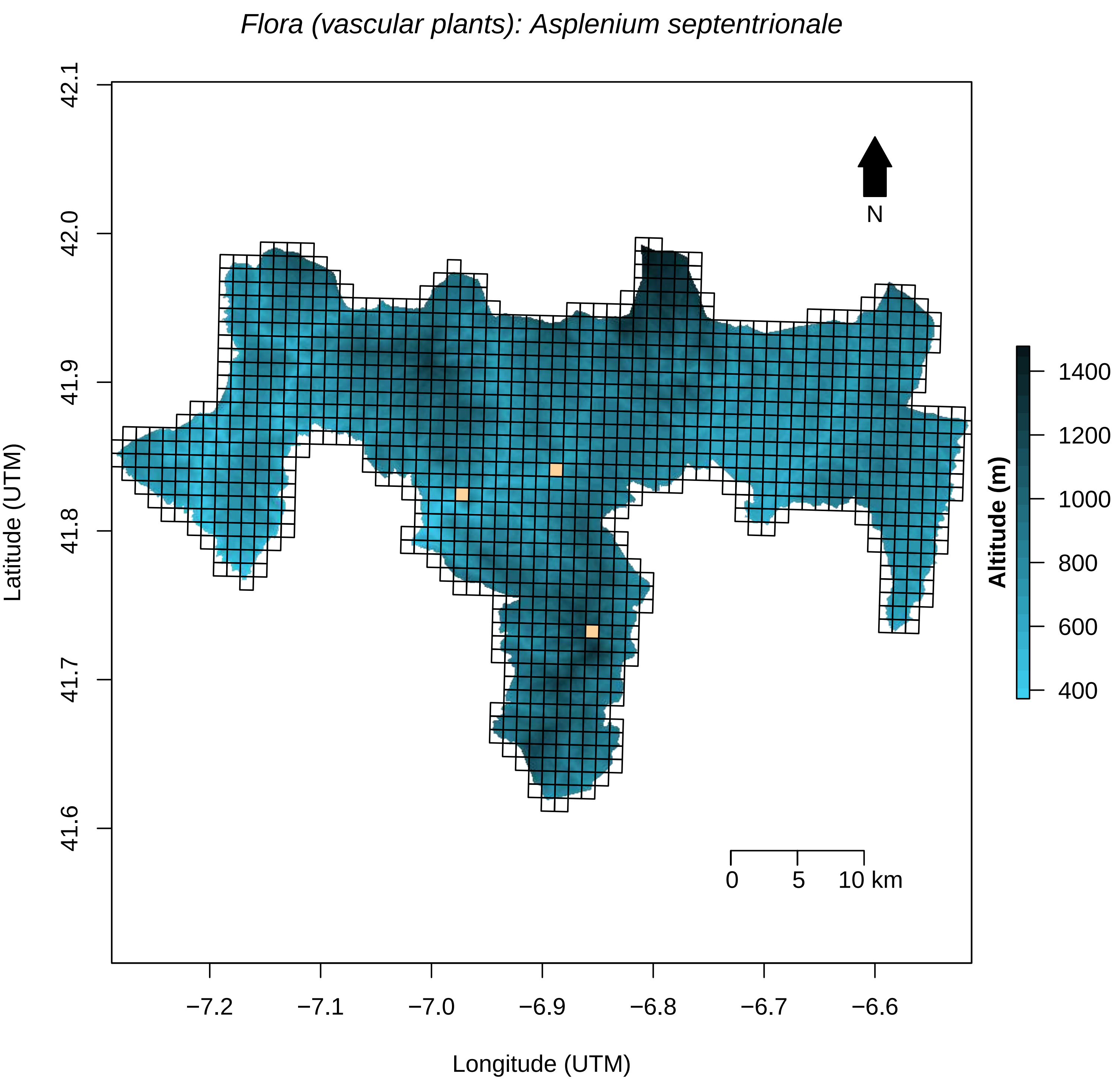

Flora (vascular plants): Asplenium septentrionale
42.1
42.0
41.9
41.8
41.7
41.6
Latitude (UTM)
−7.2
−7.1
−7.0
−6.9
−6.8
−6.7
−6.6
Longitude (UTM)
N
0
5
10 km
1400
1200
1000
800
600
400
Altitude (m)

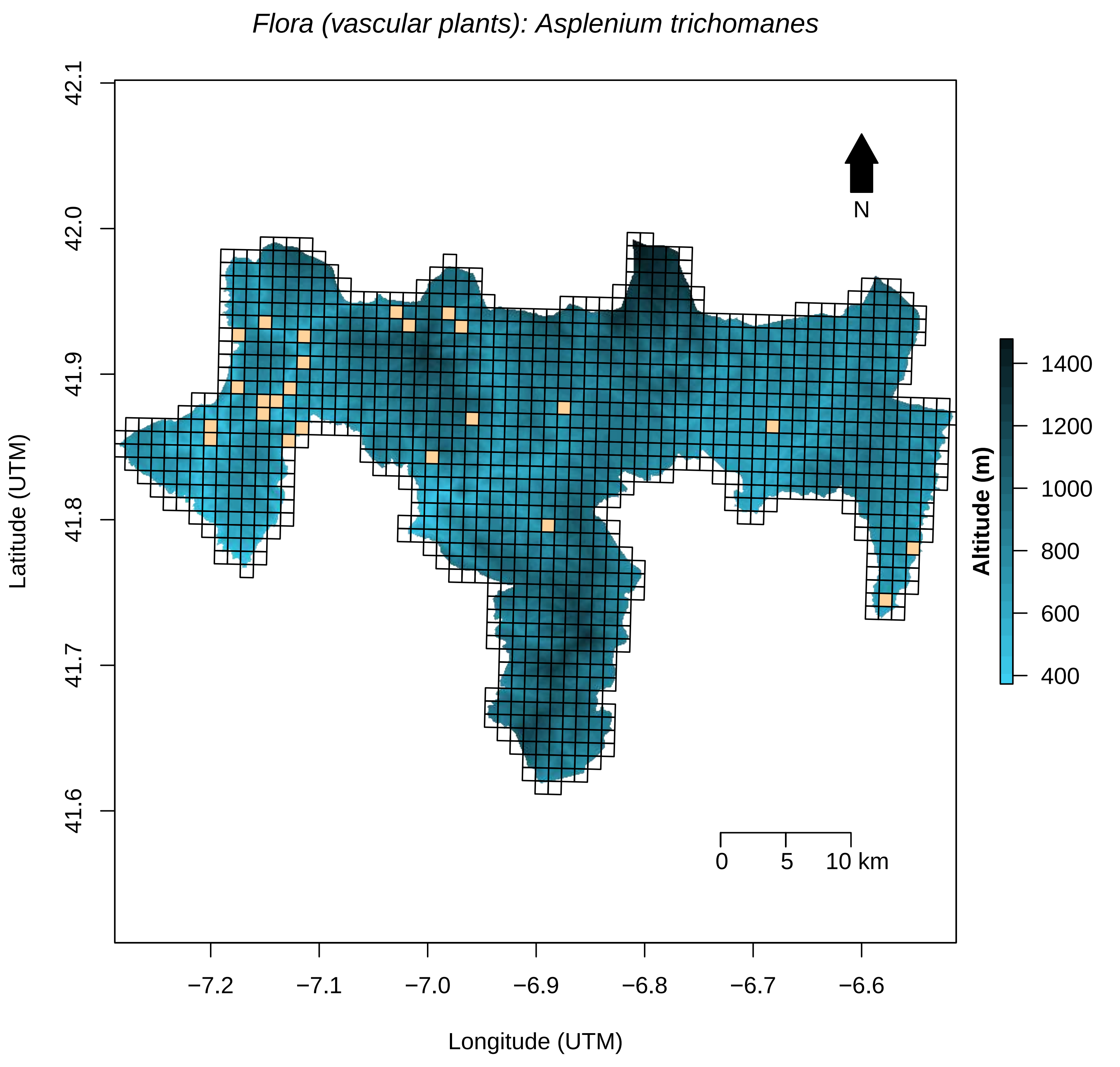

Flora (vascular plants): Asplenium trichomanes
42.1
42.0
41.9
41.8
41.7
41.6
Latitude (UTM)
−7.2
−7.1
−7.0
−6.9
−6.8
−6.7
−6.6
Longitude (UTM)
N
0
5
10 km
1400
1200
1000
800
600
400
Altitude (m)

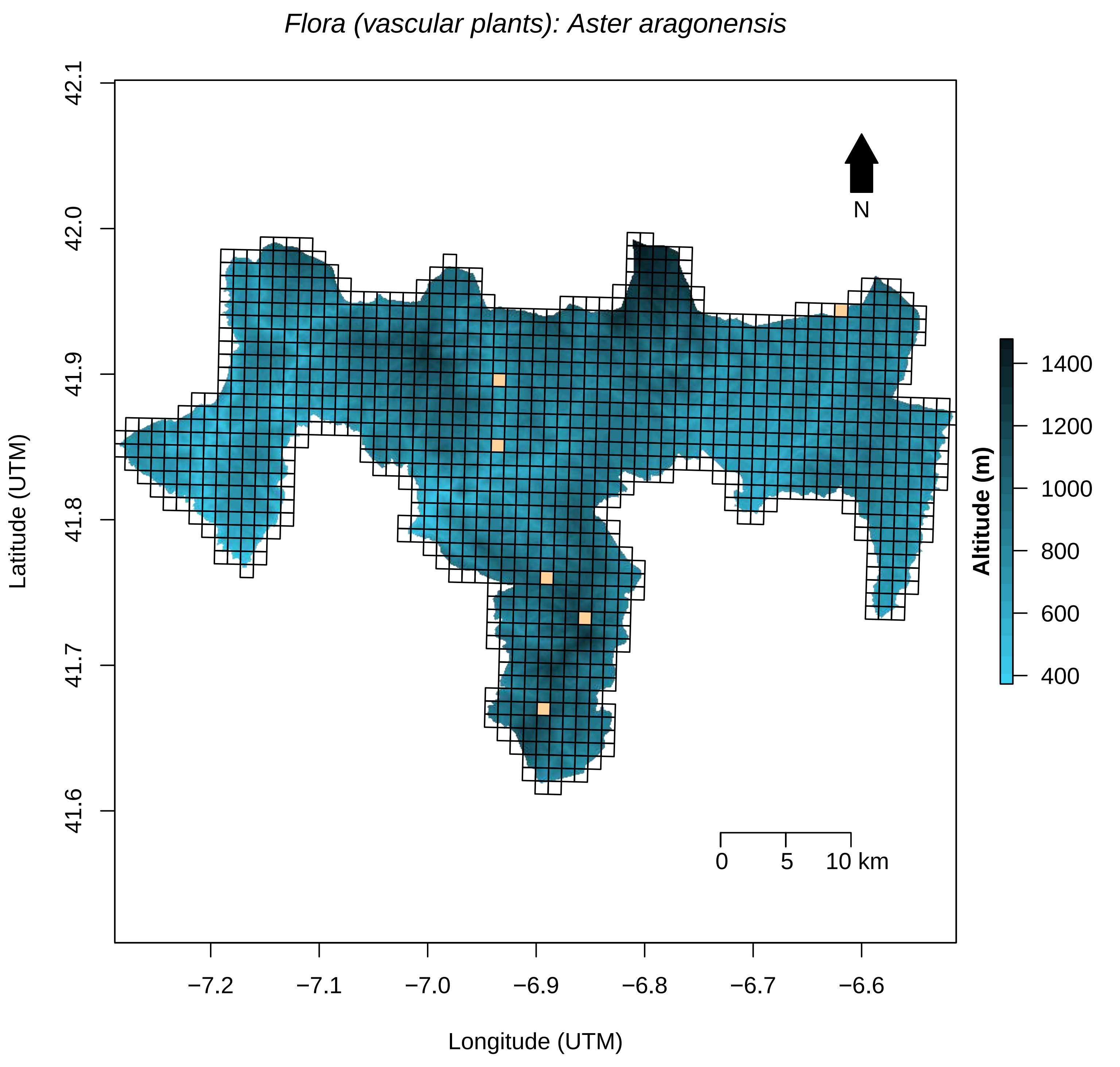
Flora (vascular plants): Aster aragonensis
N
0
5
10 km
Altitude (m)
1400
1200
1000
800
600
400
Latitude (UTM)
42.1
42.0
41.9
41.8
41.7
41.6
Longitude (UTM)
−7.2
−7.1
−7.0
−6.9
−6.8
−6.7
−6.6

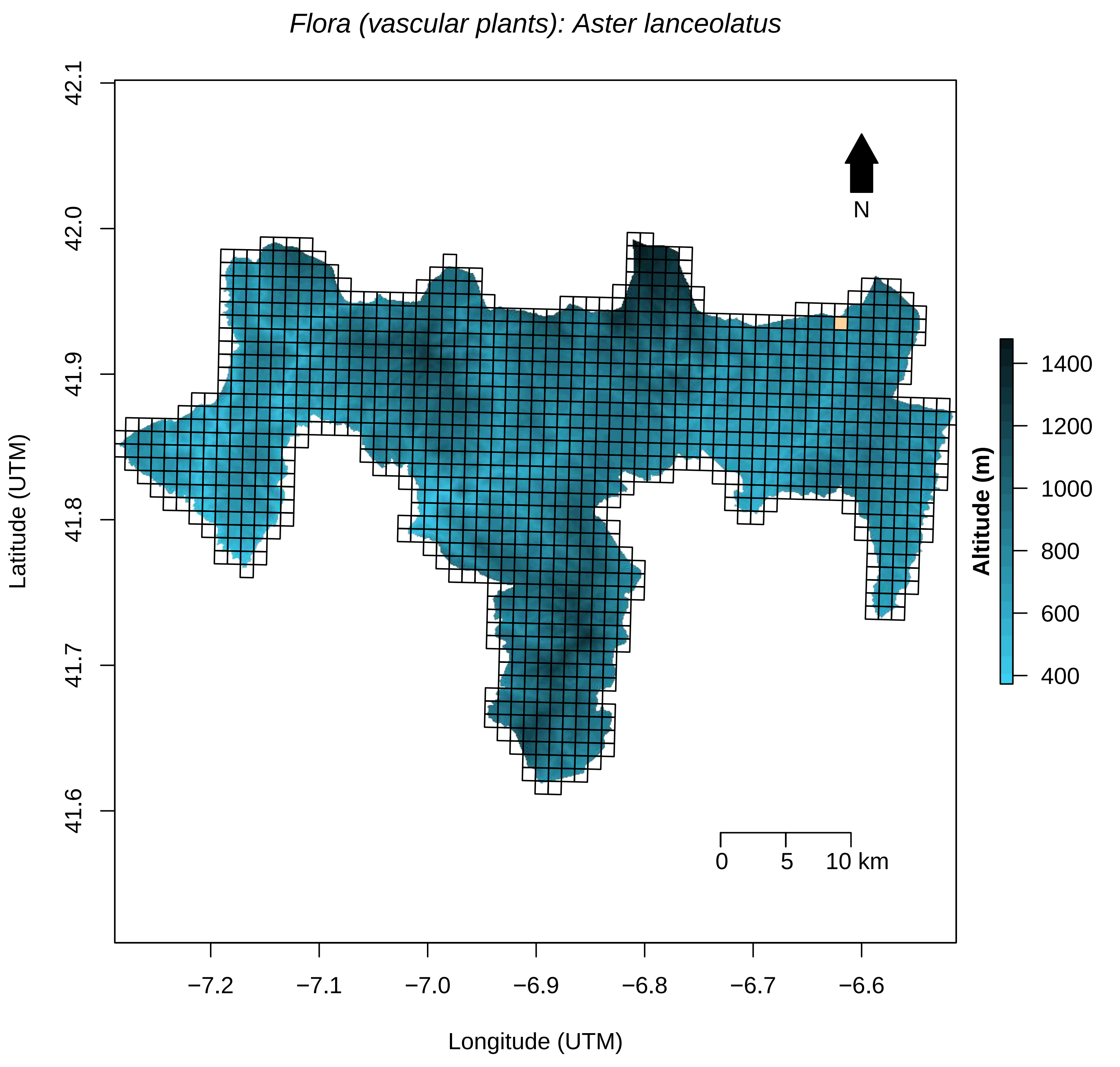

Flora (vascular plants): Aster lanceolatus
Latitude (UTM)
42.1
42.0
41.9
41.8
41.7
41.6
Longitude (UTM)
−7.2
−7.1
−7.0
−6.9
−6.8
−6.7
−6.6
N
0
5
10 km
Altitude (m)
1400
1200
1000
800
600
400

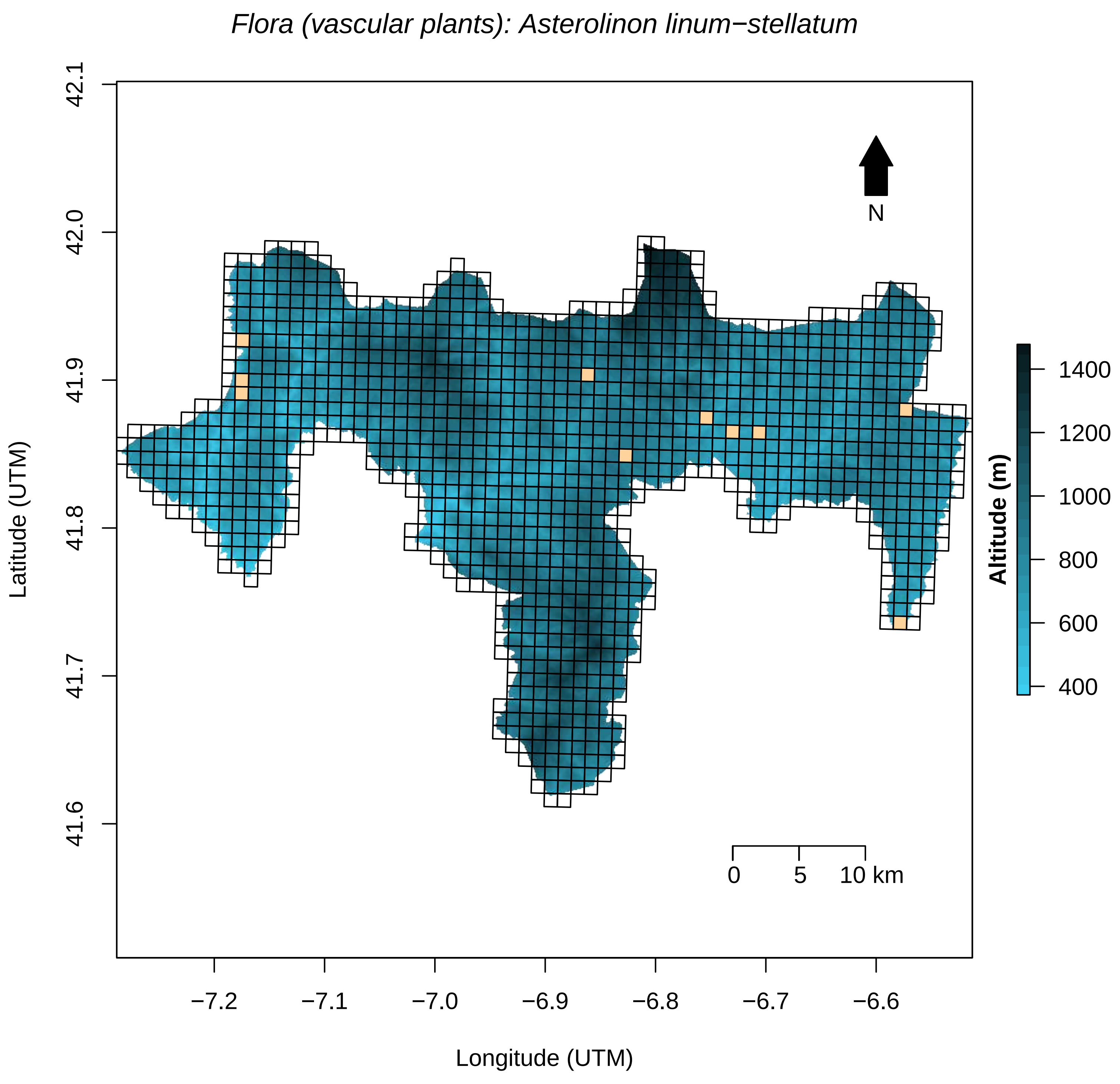
Flora (vascular plants): Asterolinon linum−stellatum
Latitude (UTM)
Longitude (UTM)
42.1
42.0
41.9
41.8
41.7
41.6
−7.2
−7.1
−7.0
−6.9
−6.8
−6.7
−6.6
N
0
5
10 km
Altitude (m)
1400
1200
1000
800
600
400

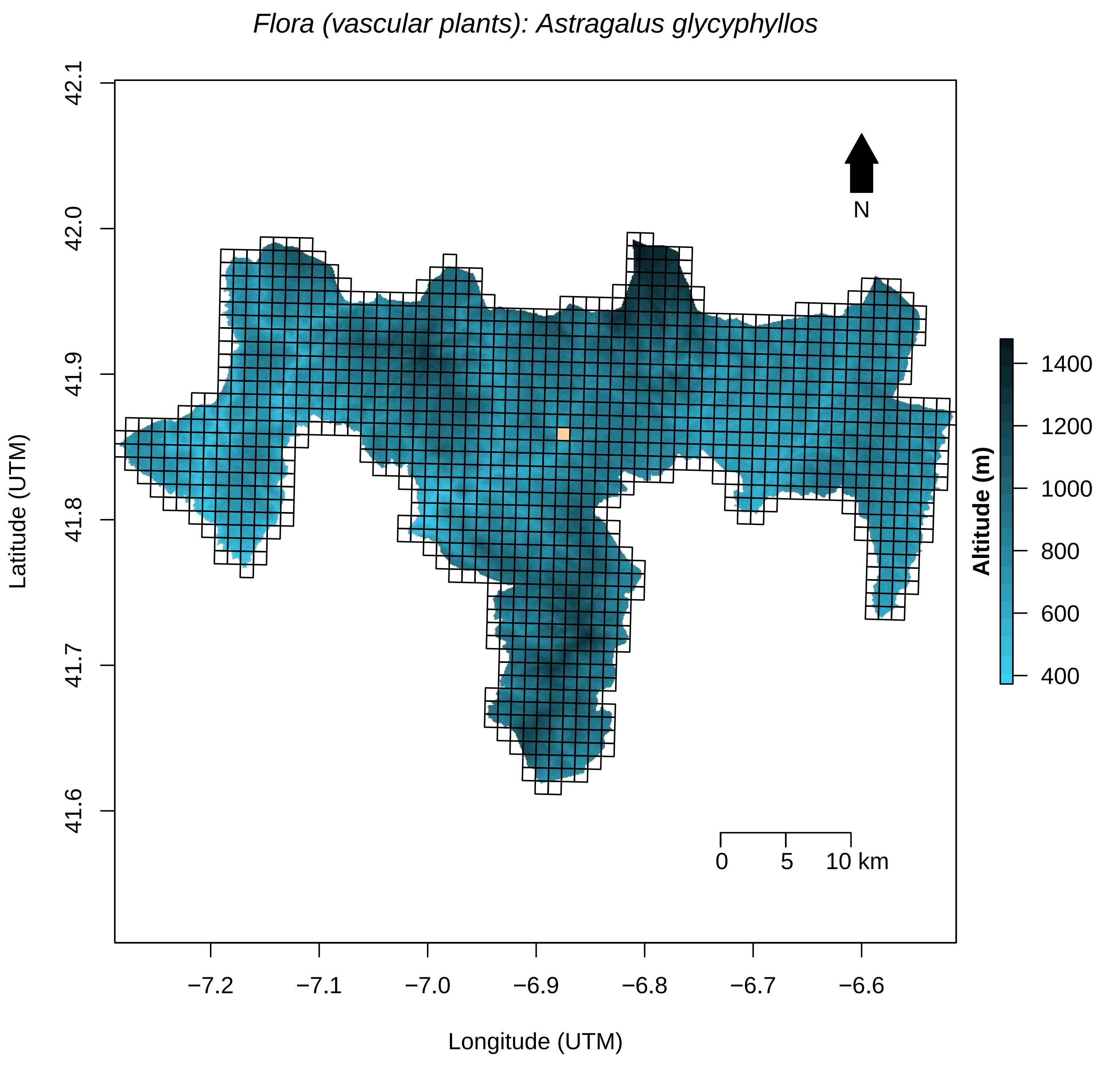

Flora (vascular plants): Astragalus glycyphyllos
Latitude (UTM)
Longitude (UTM)
42.1
42.0
41.9
41.8
41.7
41.6
−7.2
−7.1
−7.0
−6.9
−6.8
−6.7
−6.6
N
0
5
10 km
Altitude (m)
1400
1200
1000
800
600
400

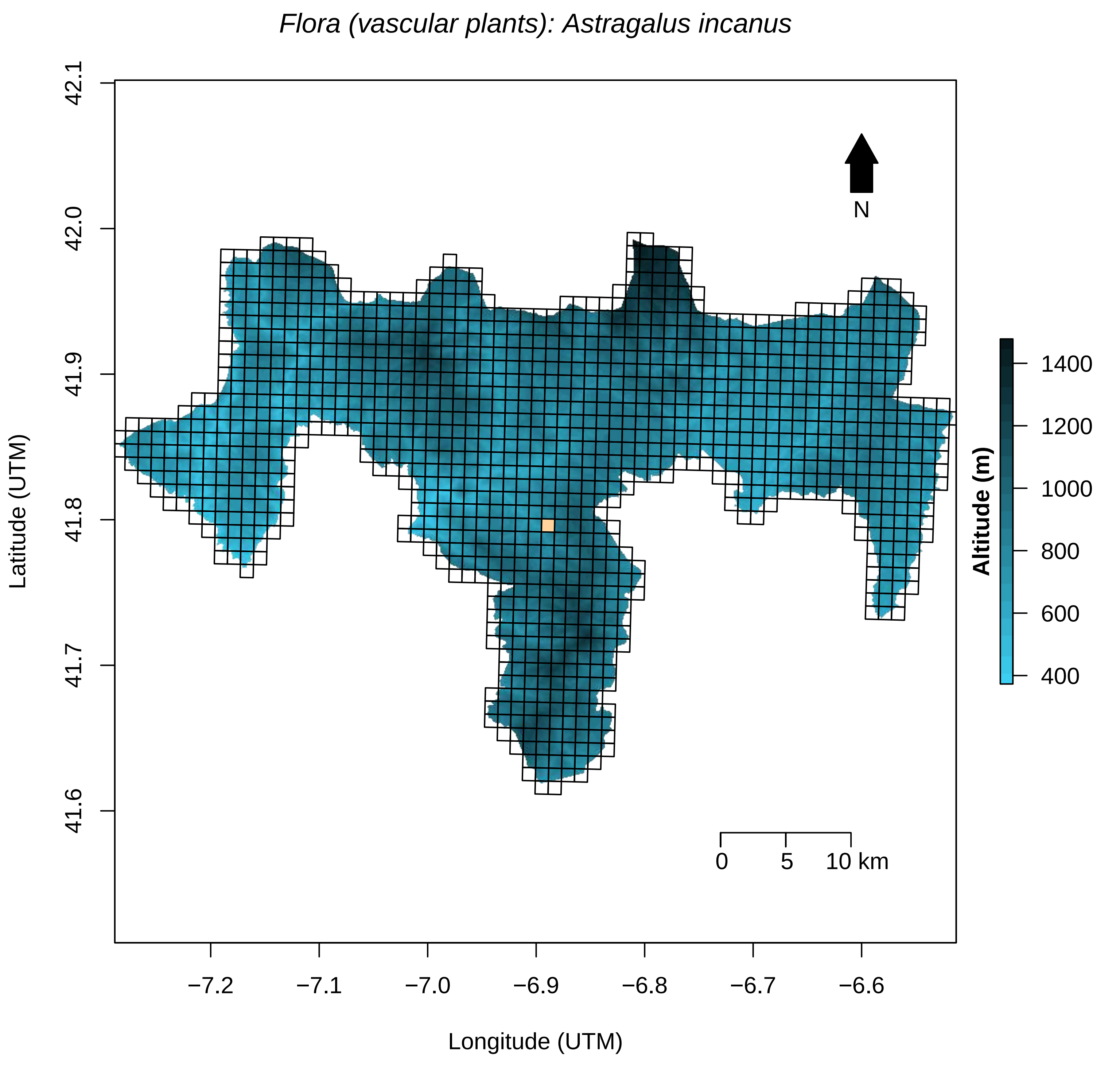

Flora (vascular plants): Astragalus incanus
Latitude (UTM)
42.1
42.0
41.9
41.8
41.7
41.6
Longitude (UTM)
−7.2
−7.1
−7.0
−6.9
−6.8
−6.7
−6.6
N
0 5 10 km
Altitude (m)
1400
1200
1000
800
600
400

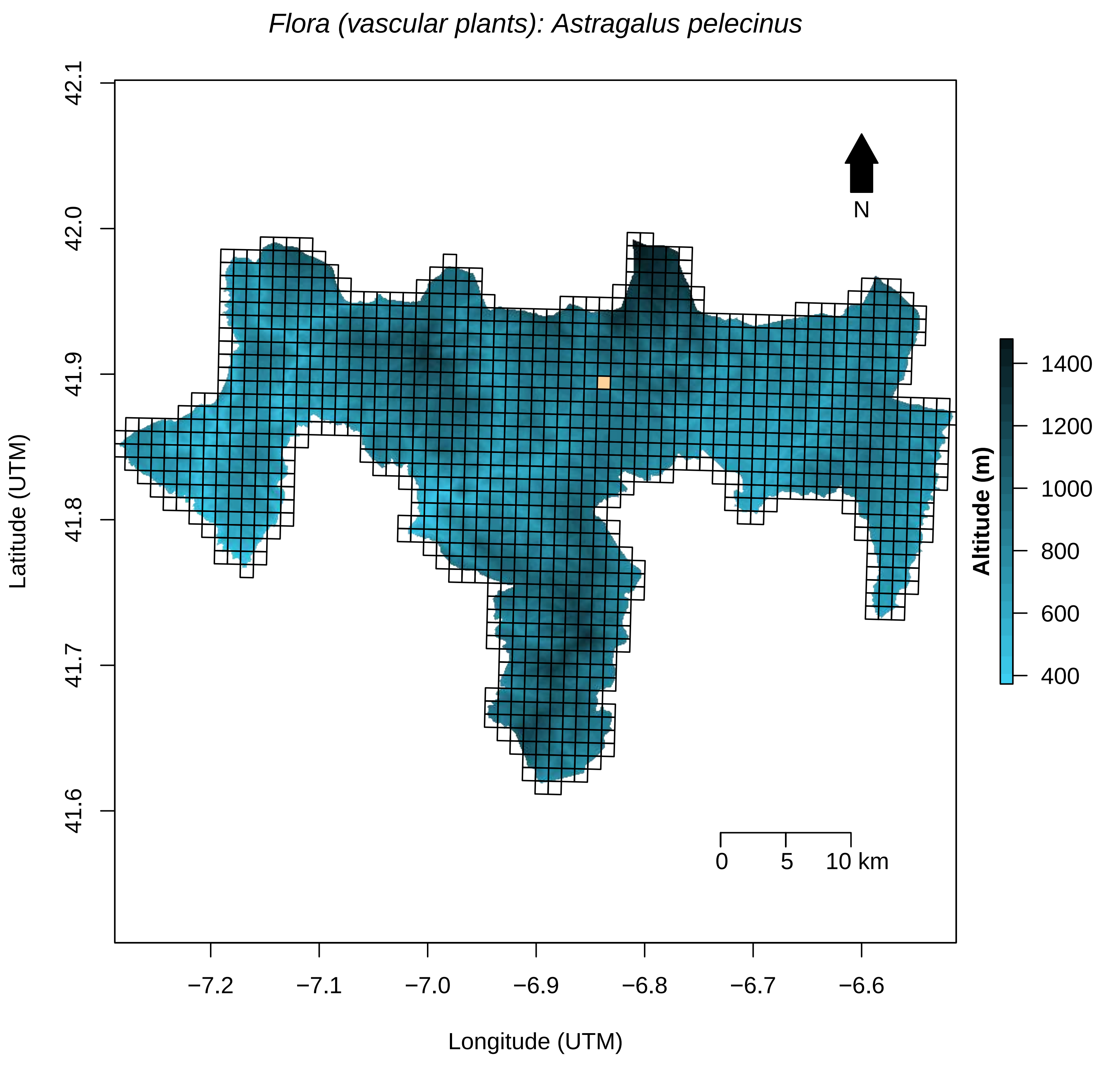

Flora (vascular plants): Astragalus pelecinus
42.1
42.0
41.9
41.8
41.7
41.6
Latitude (UTM)
−7.2
−7.1
−7.0
−6.9
−6.8
−6.7
−6.6
Longitude (UTM)
N
0
5
10 km
Altitude (m)
1400
1200
1000
800
600
400

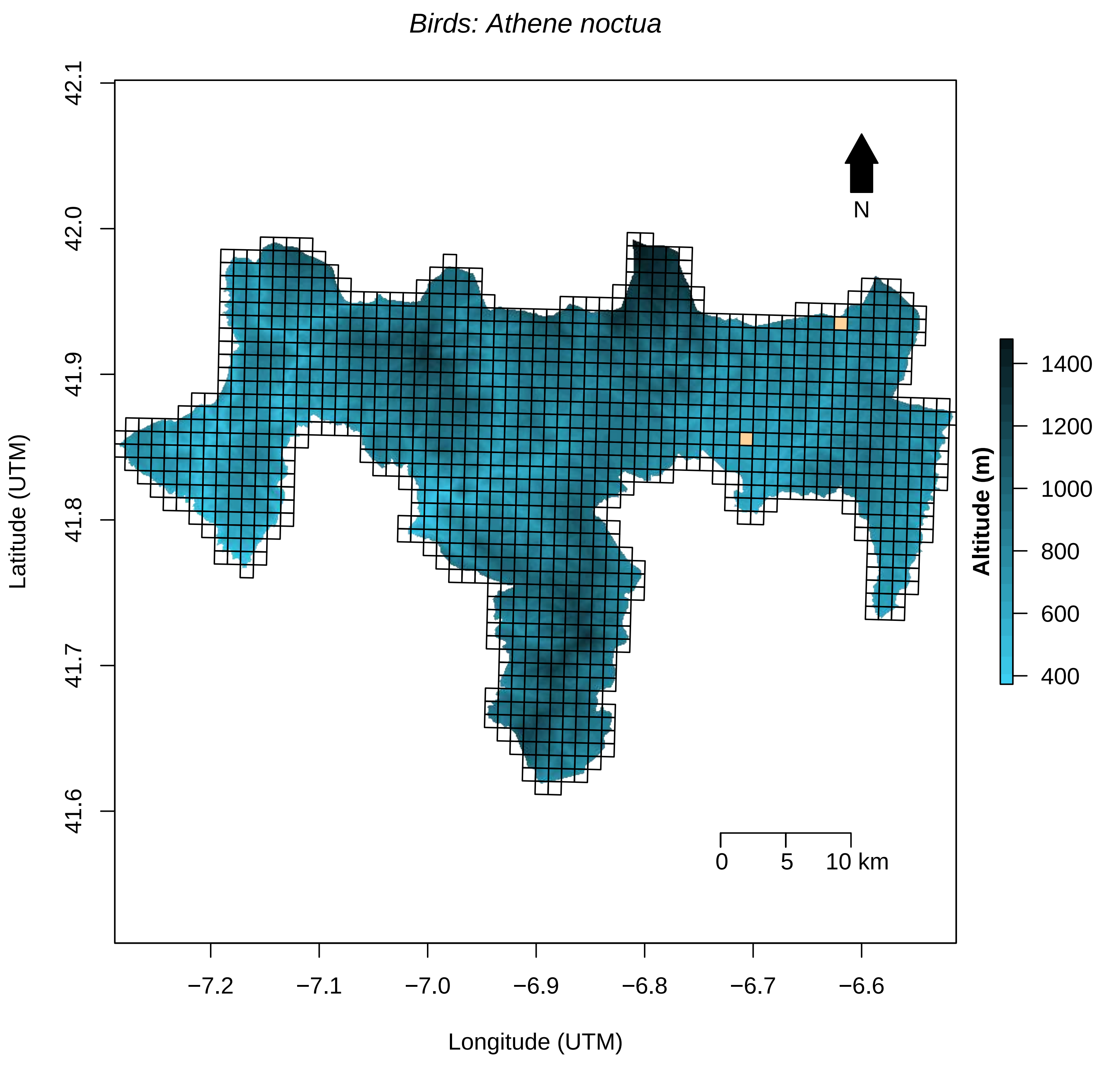

Birds: Athene noctua
N
0
5
10 km
Altitude (m)
1400
1200
1000
800
600
400
Latitude (UTM)
42.1
42.0
41.9
41.8
41.7
41.6
Longitude (UTM)
−7.2
−7.1
−7.0
−6.9
−6.8
−6.7
−6.6

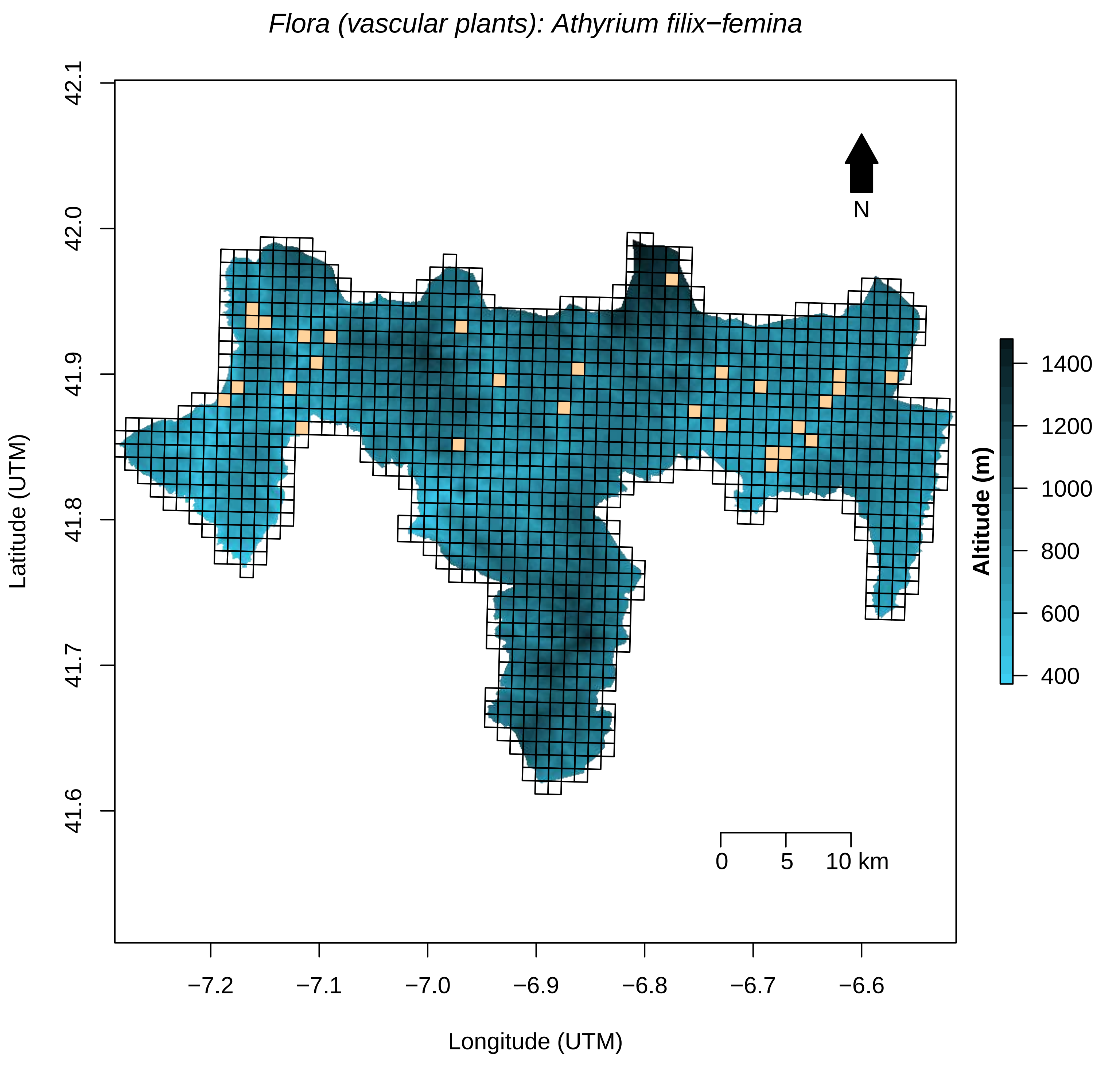
Flora (vascular plants): Athyrium filix−femina
N
0 5 10 km
Altitude (m)
1400
1200
1000
800
600
400
Latitude (UTM)
42.1
42.0
41.9
41.8
41.7
41.6
Longitude (UTM)
−7.2
−7.1
−7.0
−6.9
−6.8
−6.7
−6.6

Flora (vascular plants): *Avena barbata*

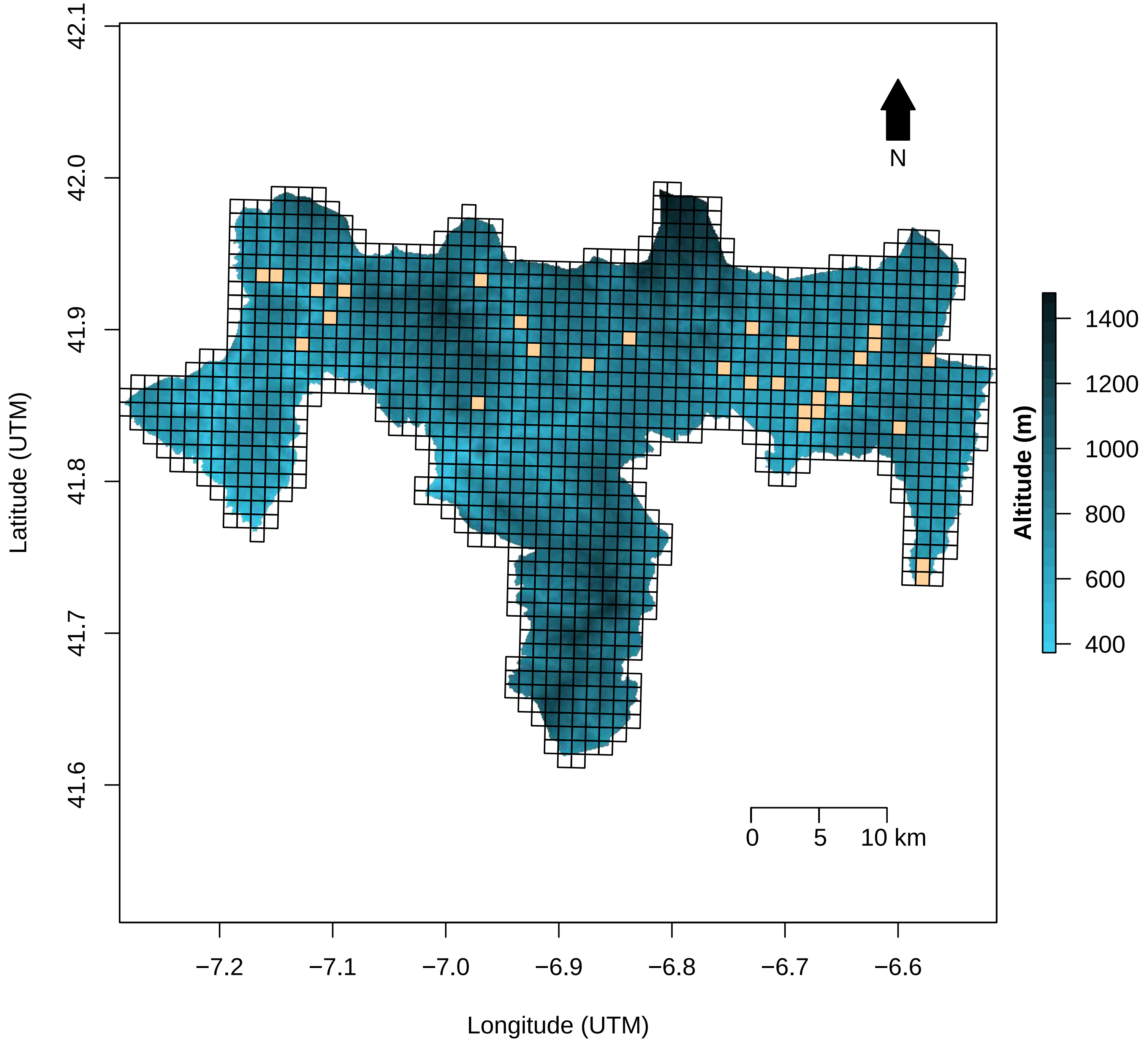

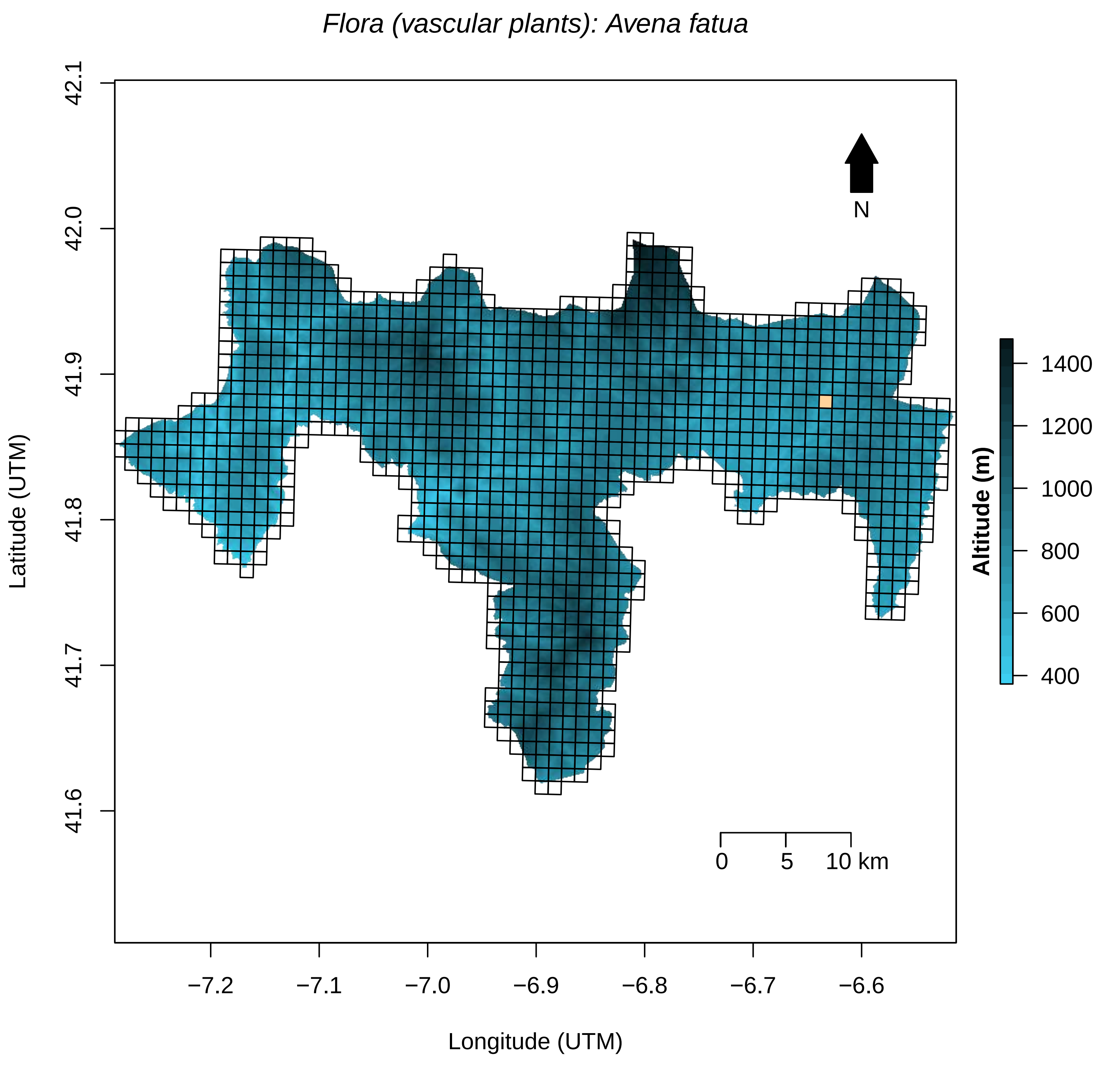

Flora (vascular plants): Avena fatua
42.1
42.0
41.9
41.8
41.7
41.6
Latitude (UTM)
−7.2
−7.1
−7.0
−6.9
−6.8
−6.7
−6.6
Longitude (UTM)
N
0
5
10 km
1400
1200
1000
800
600
400
Altitude (m)

Flora (vascular plants): Avena sterilis

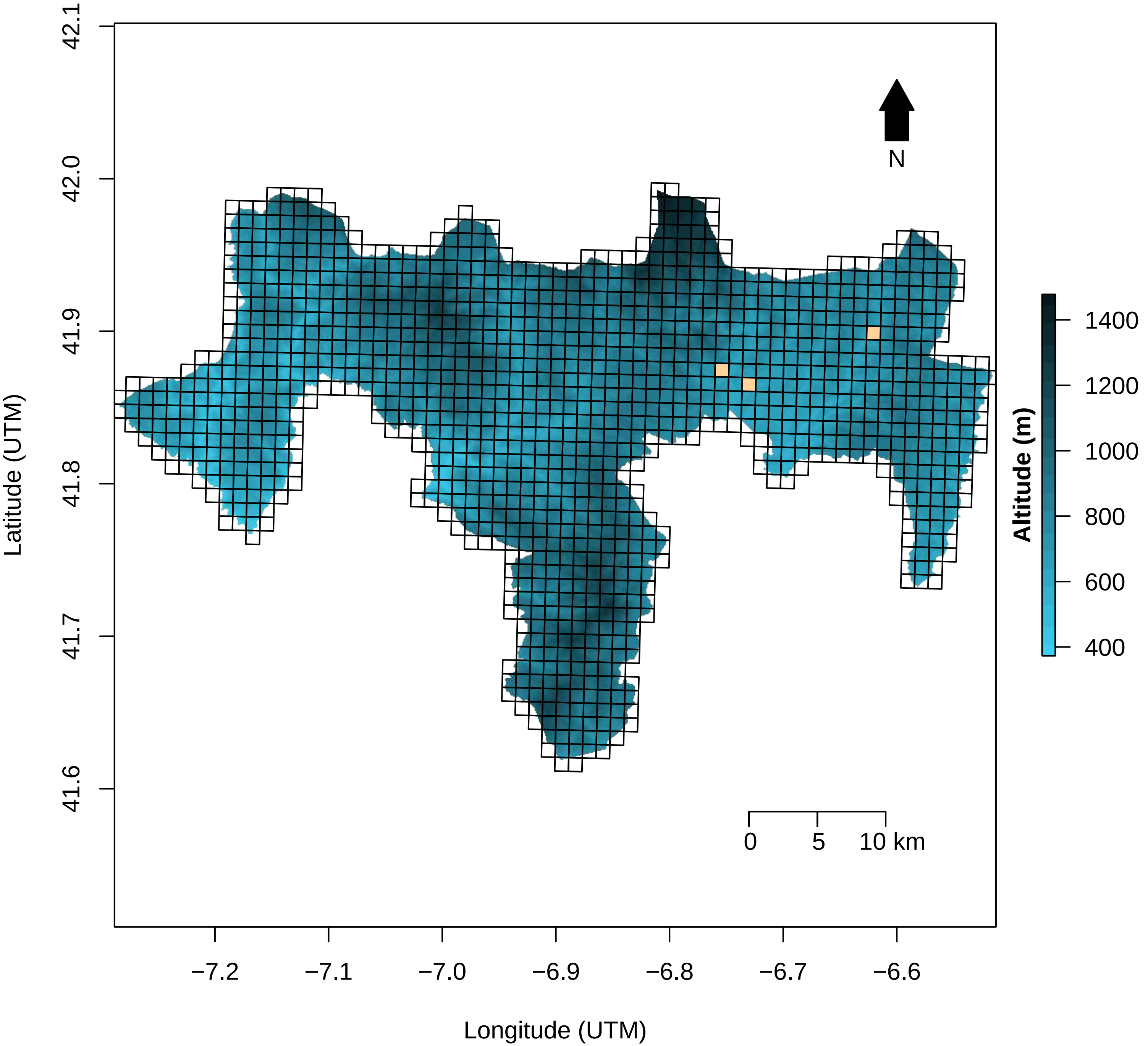

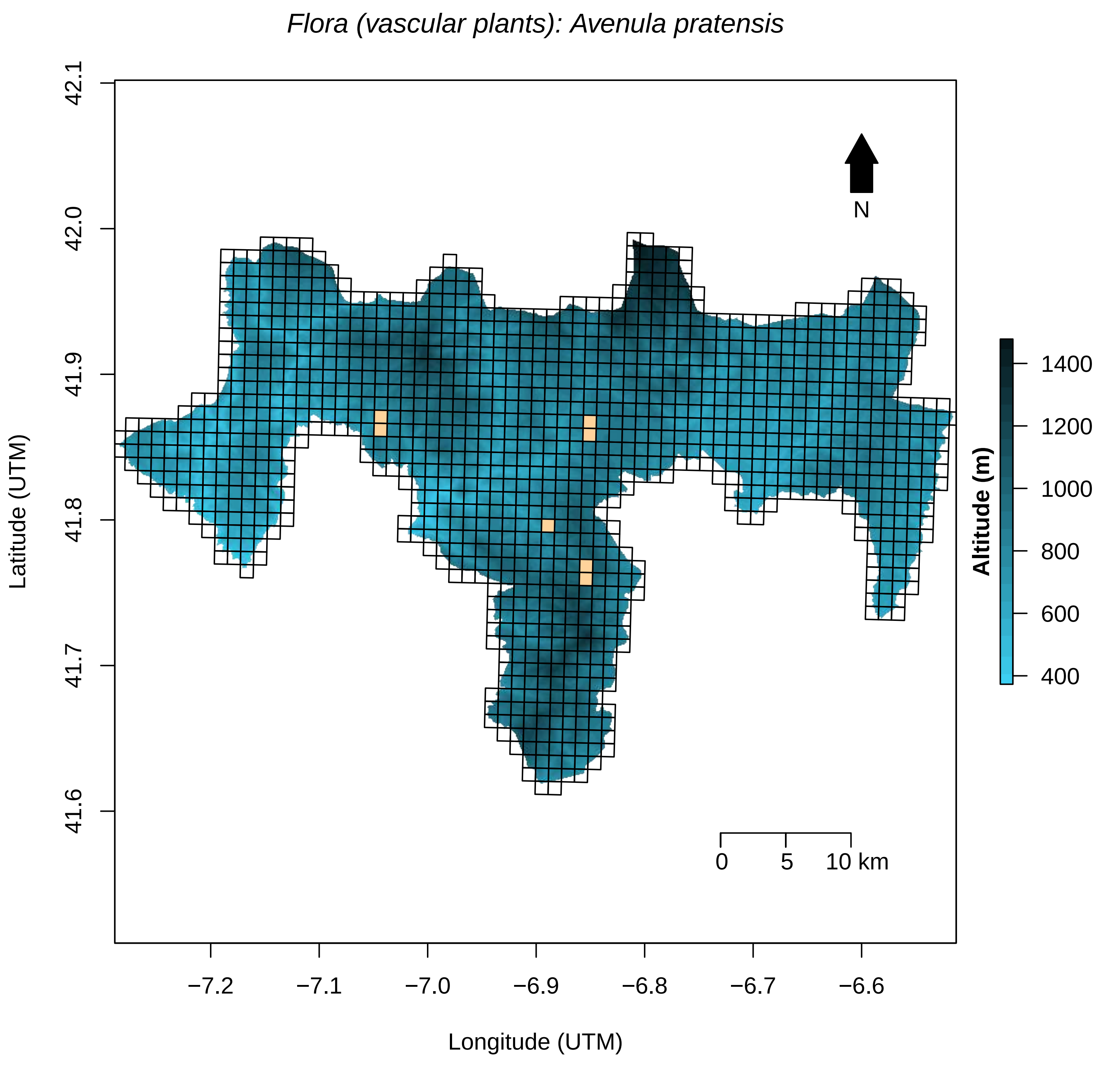

Flora (vascular plants): Avenula pratensis
42.1
42.0
41.9
41.8
41.7
41.6
Latitude (UTM)
N
0
5
10 km
1400
1200
1000
800
600
400
Altitude (m)
−7.2
−7.1
−7.0
−6.9
−6.8
−6.7
−6.6
Longitude (UTM)

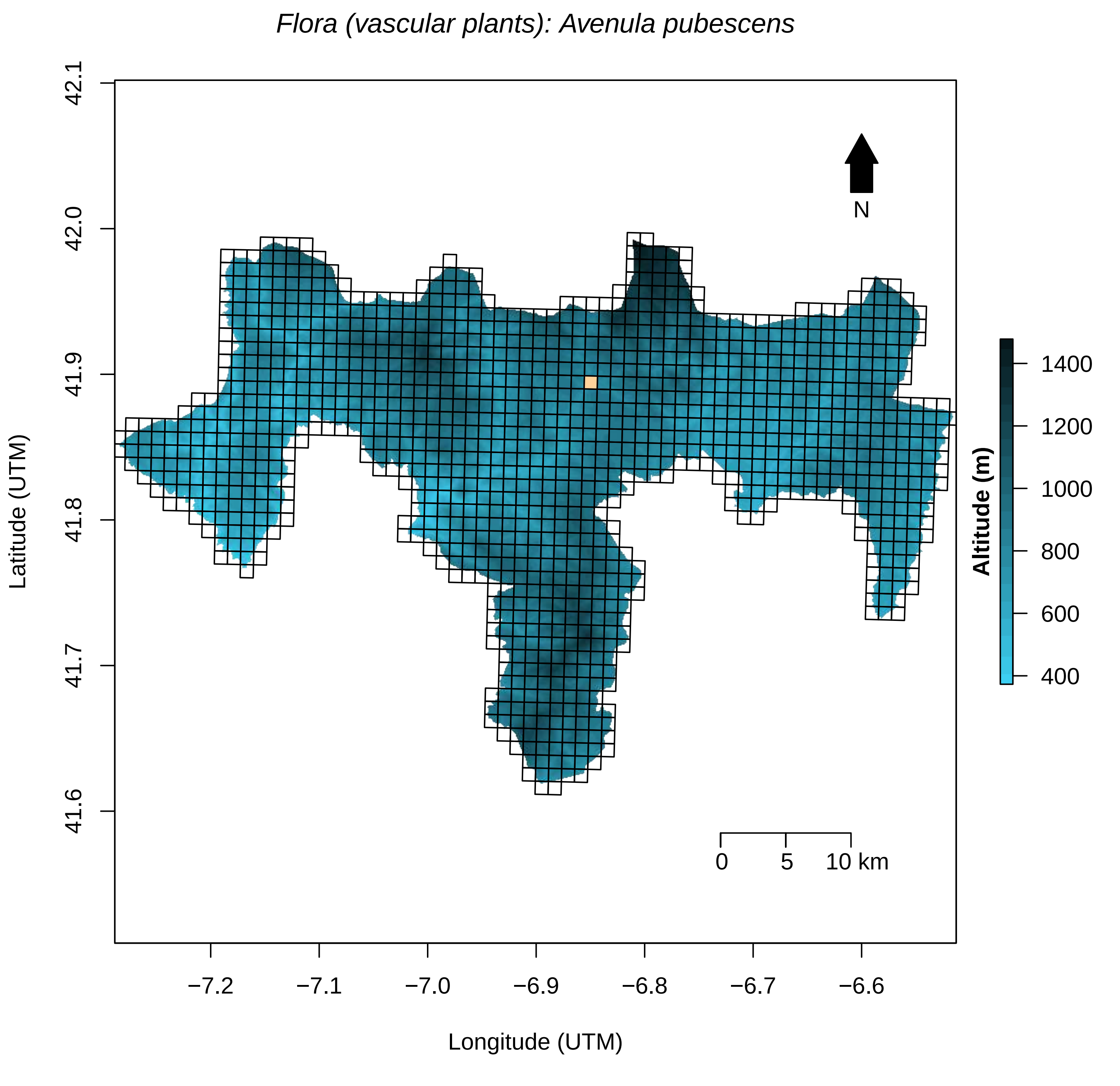

Flora (vascular plants): Avenula pubescens
42.1
42.0
41.9
41.8
41.7
41.6
Latitude (UTM)
−7.2
−7.1
−7.0
−6.9
−6.8
−6.7
−6.6
Longitude (UTM)
N
0
5
10 km
Altitude (m)
1400
1200
1000
800
600
400

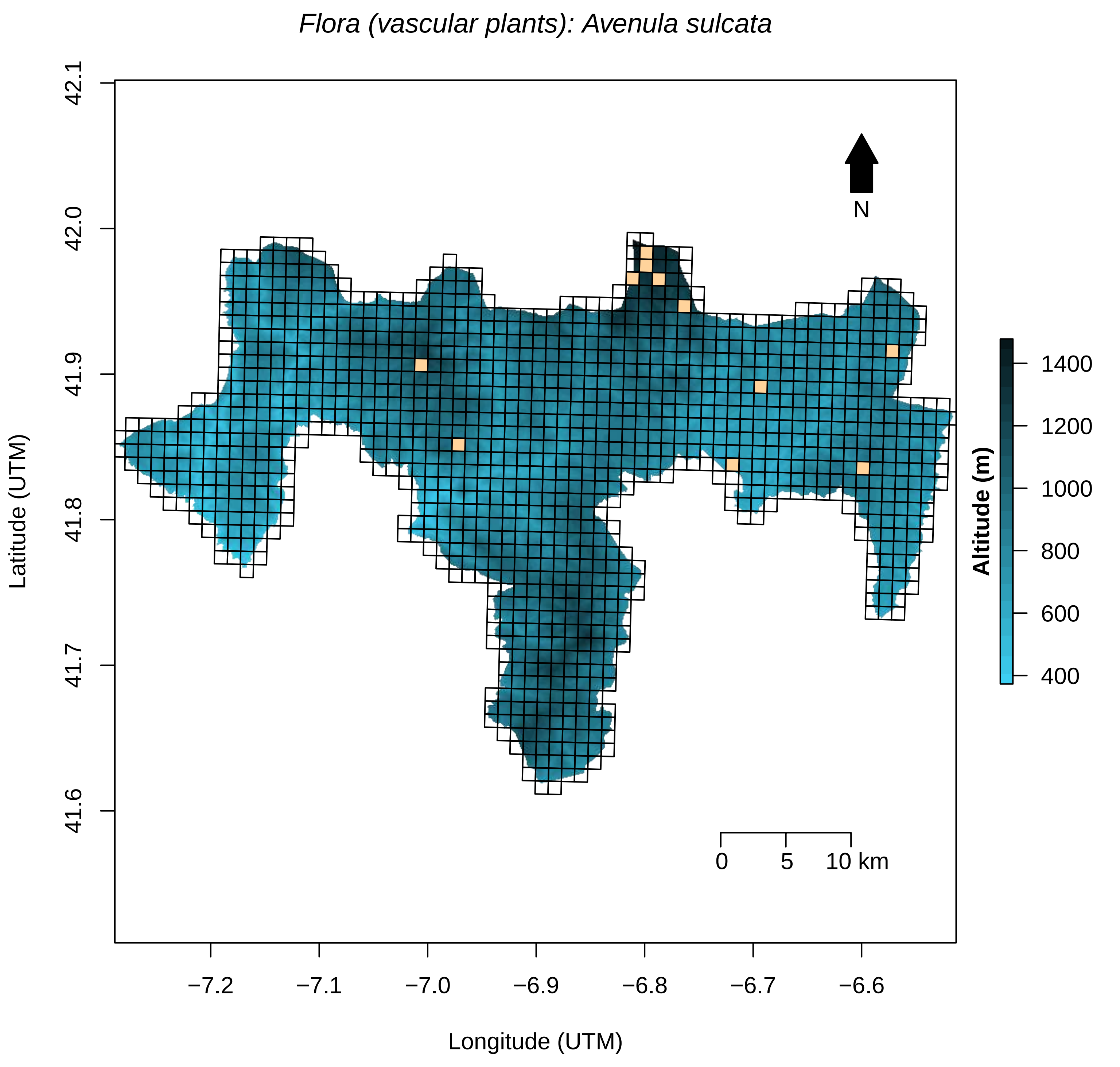
Flora (vascular plants): Avenula sulcata
42.1
42.0
41.9
41.8
41.7
41.6
Latitude (UTM)
−7.2
−7.1
−7.0
−6.9
−6.8
−6.7
−6.6
Longitude (UTM)
N
0
5
10 km
1400
1200
1000
800
600
400
Altitude (m)

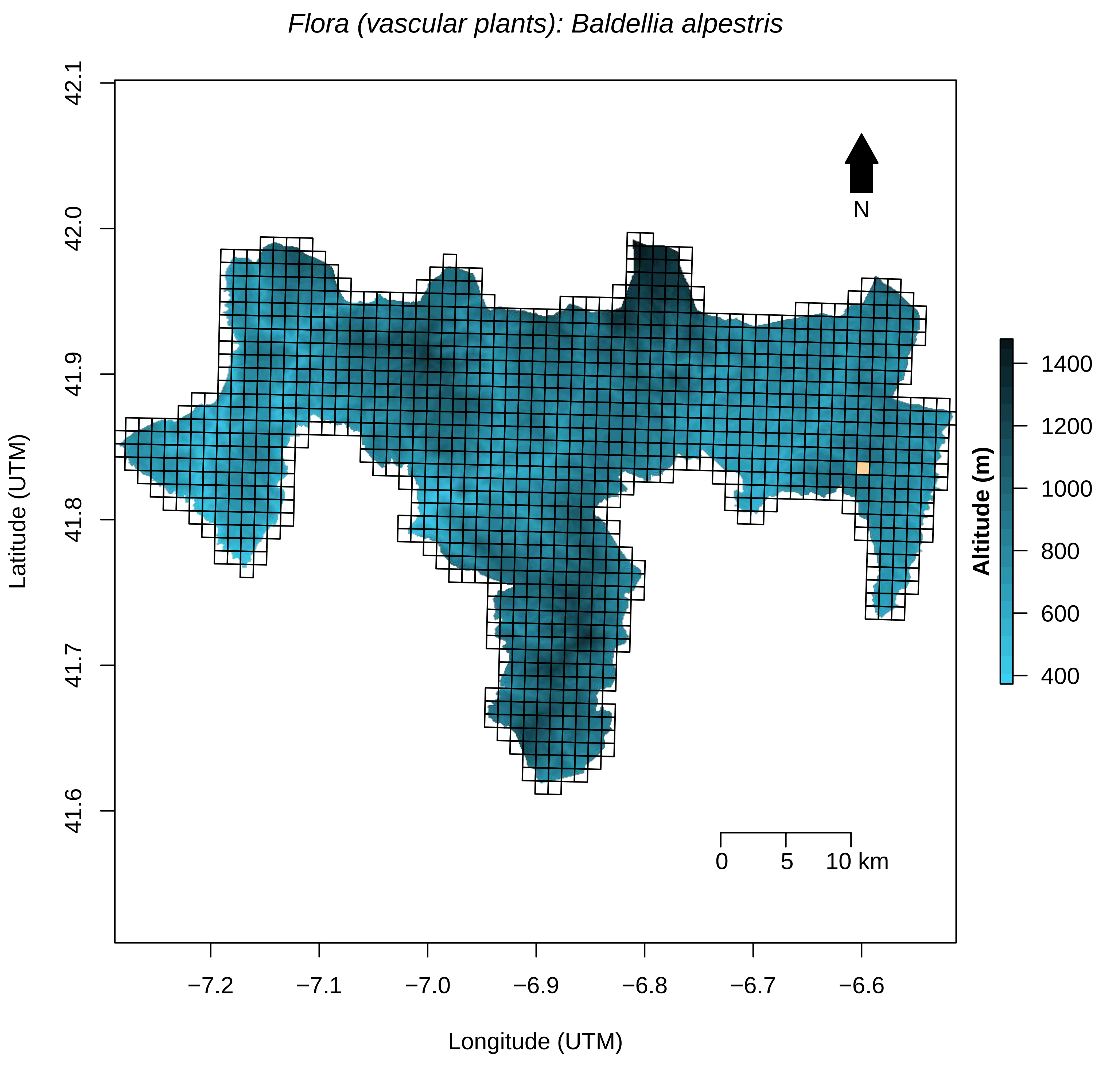

Flora (vascular plants): Baldellia alpestris
N
0 5 10 km
Altitude (m)
1400
1200
1000
800
600
400
Latitude (UTM)
42.1
42.0
41.9
41.8
41.7
41.6
Longitude (UTM)
−7.2
−7.1
−7.0
−6.9
−6.8
−6.7
−6.6

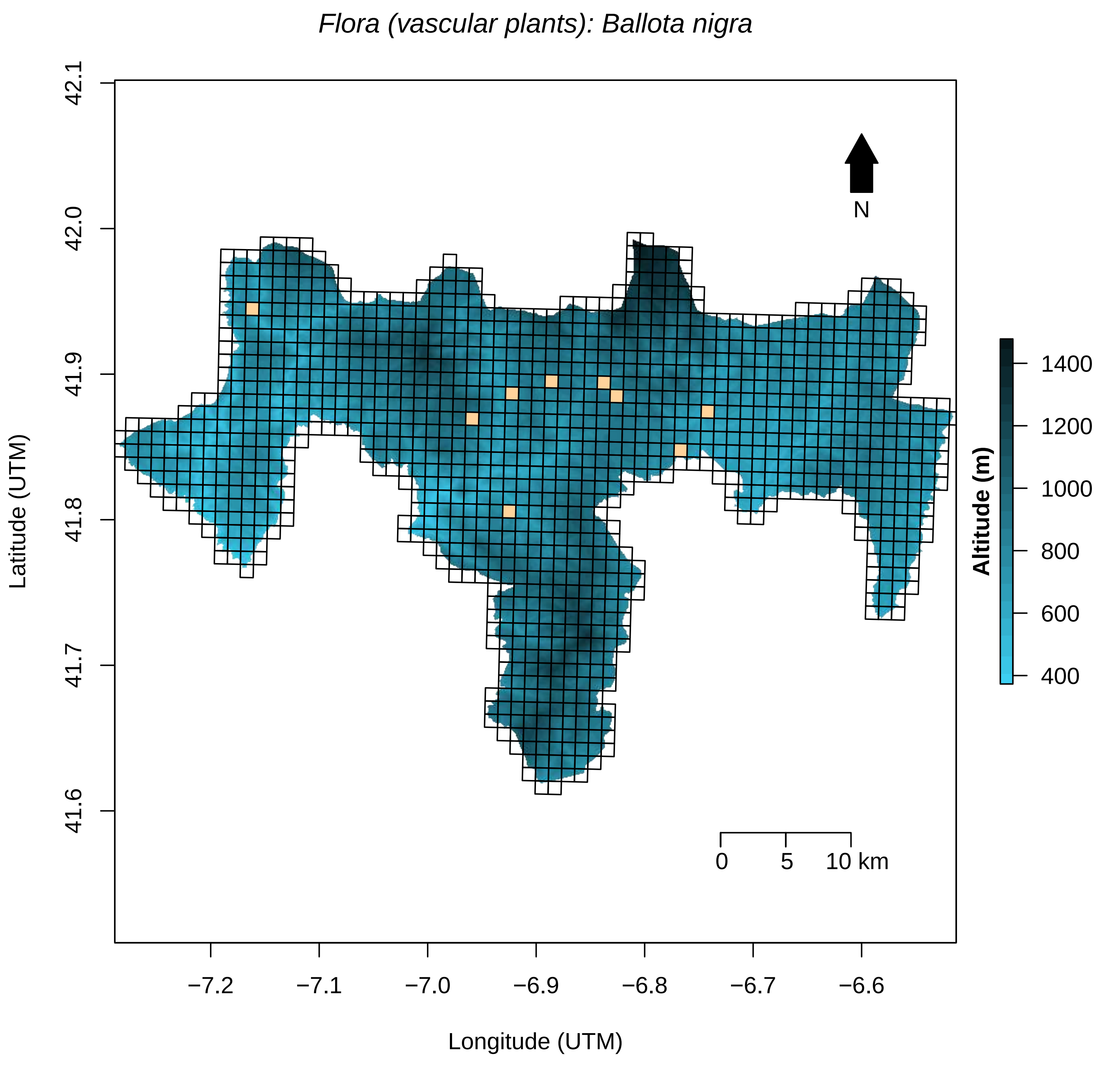
Flora (vascular plants): Ballota nigra
N
0 5 10 km
Altitude (m)
1400
1200
1000
800
600
400
Latitude (UTM)
42.1
42.0
41.9
41.8
41.7
41.6
Longitude (UTM)
−7.2
−7.1
−7.0
−6.9
−6.8
−6.7
−6.6

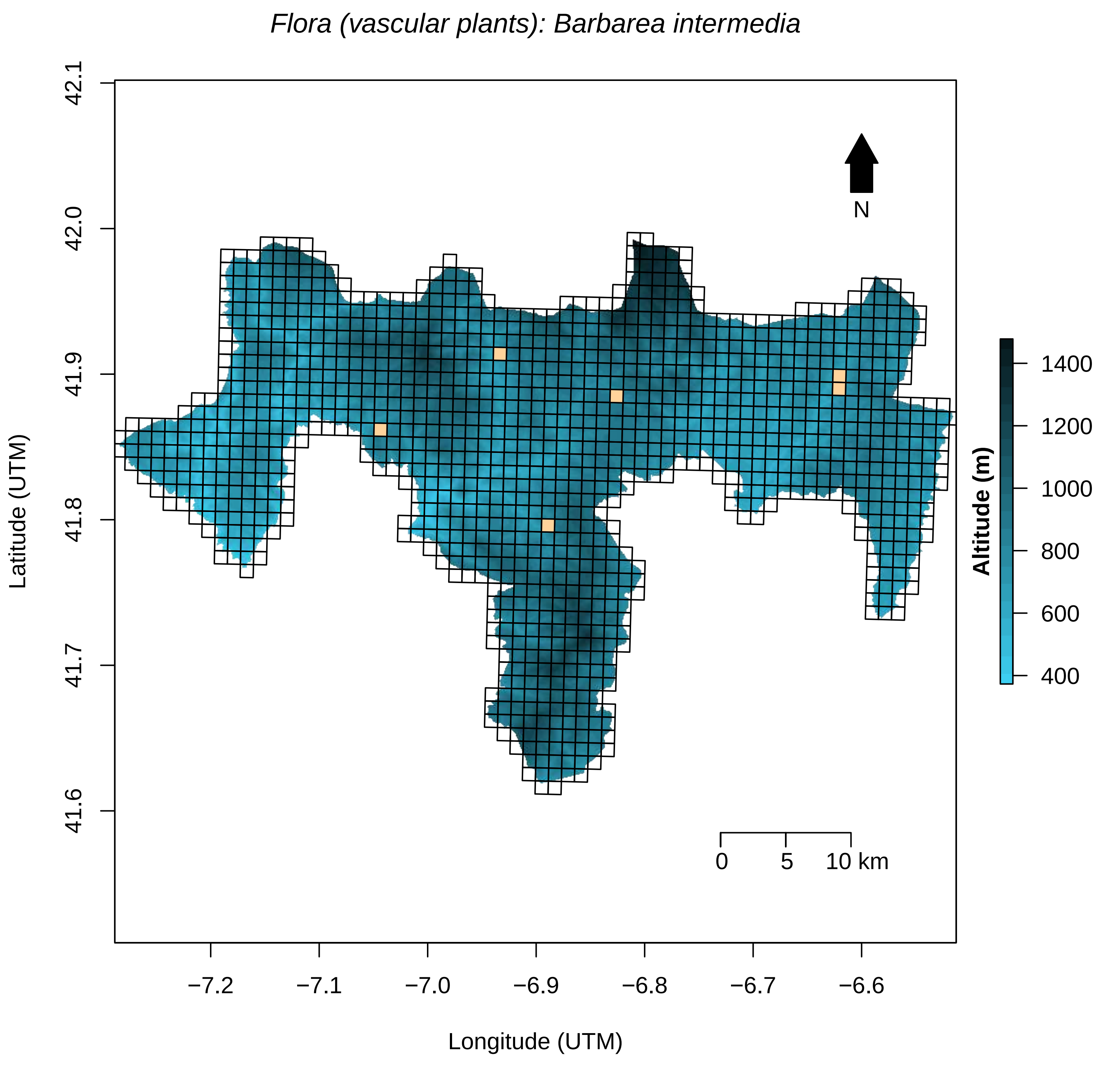

Flora (vascular plants): Barbarea intermedia
Latitude (UTM)
Longitude (UTM)
42.1
42.0
41.9
41.8
41.7
41.6
−7.2
−7.1
−7.0
−6.9
−6.8
−6.7
−6.6
N
0
5
10 km
Altitude (m)
1400
1200
1000
800
600
400

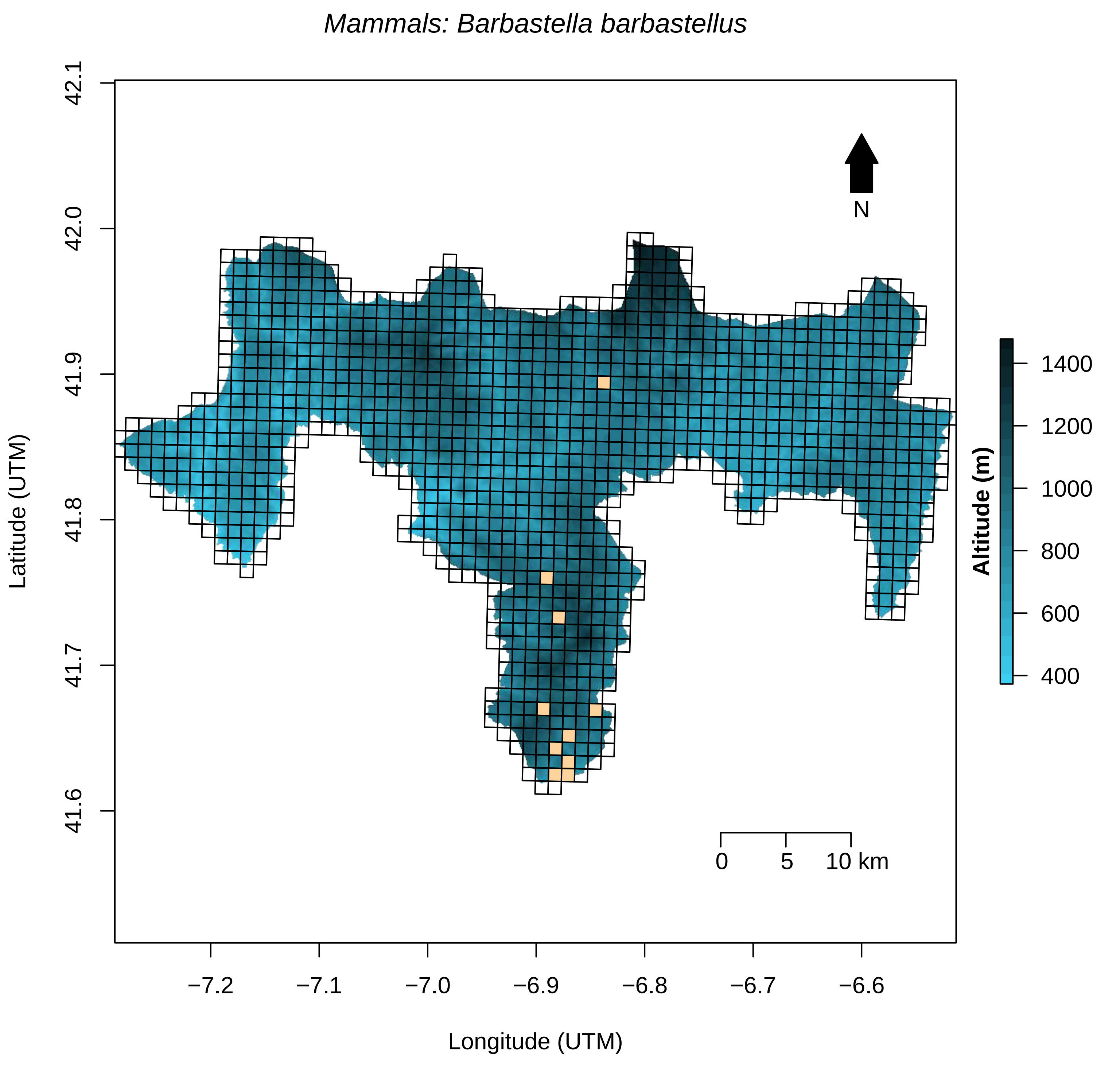
Mammals: Barbastella barbastellus
42.1
42.0
41.9
41.8
41.7
41.6
Latitude (UTM)
−7.2
−7.1
−7.0
−6.9
−6.8
−6.7
−6.6
Longitude (UTM)
N
0
5
10 km
Altitude (m)
1400
1200
1000
800
600
400

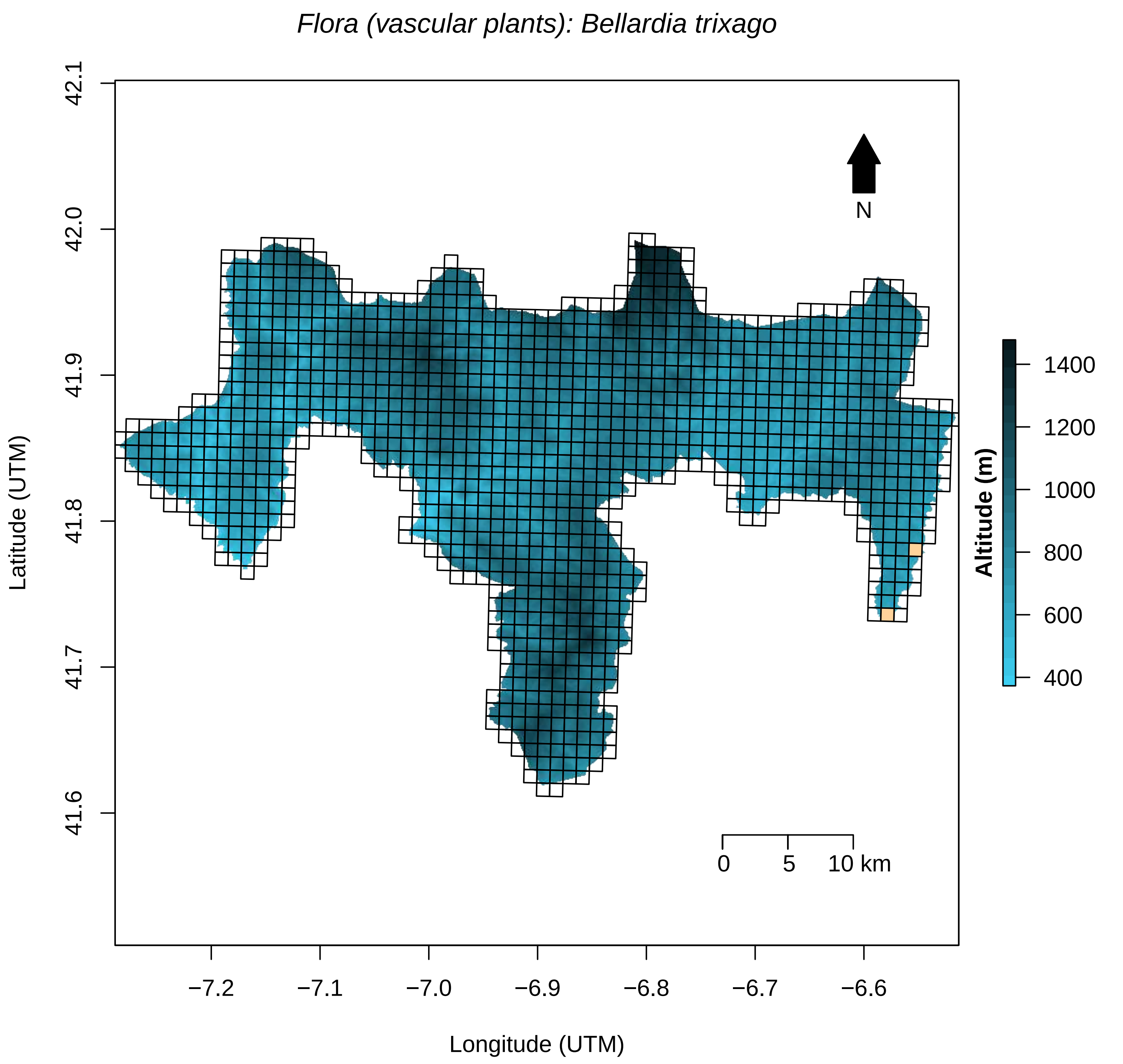

Flora (vascular plants): Bellardia trixago
N
0
5
10 km
Altitude (m)
1400
1200
1000
800
600
400
42.1
42.0
41.9
41.8
41.7
41.6
Latitude (UTM)
−7.2
−7.1
−7.0
−6.9
−6.8
−6.7
−6.6
Longitude (UTM)

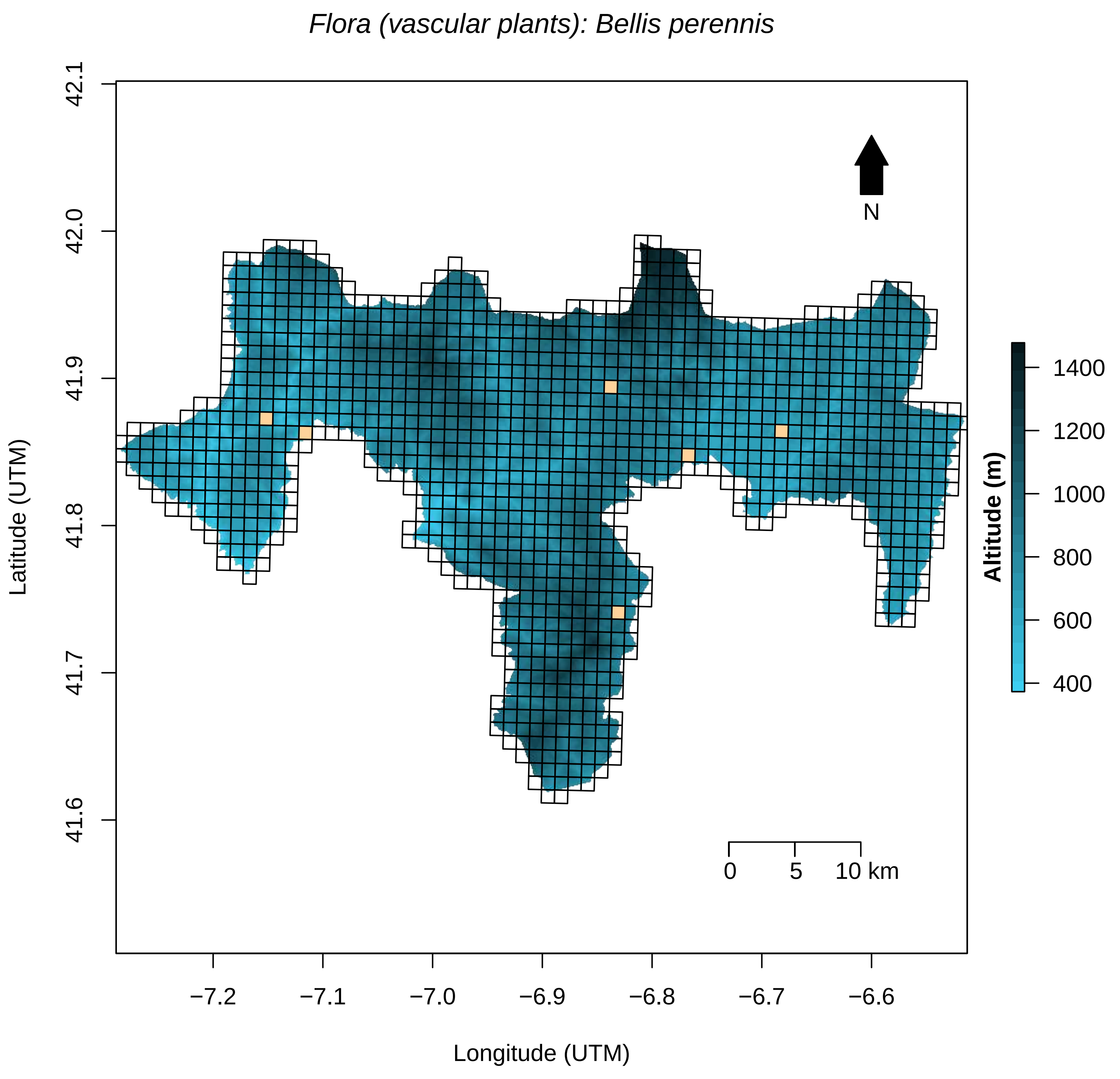

Flora (vascular plants): Bellis perennis
N
0
5
10 km
Latitude (UTM)
Longitude (UTM)
42.1
42.0
41.9
41.8
41.7
41.6
−7.2
−7.1
−7.0
−6.9
−6.8
−6.7
−6.6
Altitude (m)
1400
1200
1000
800
600
400

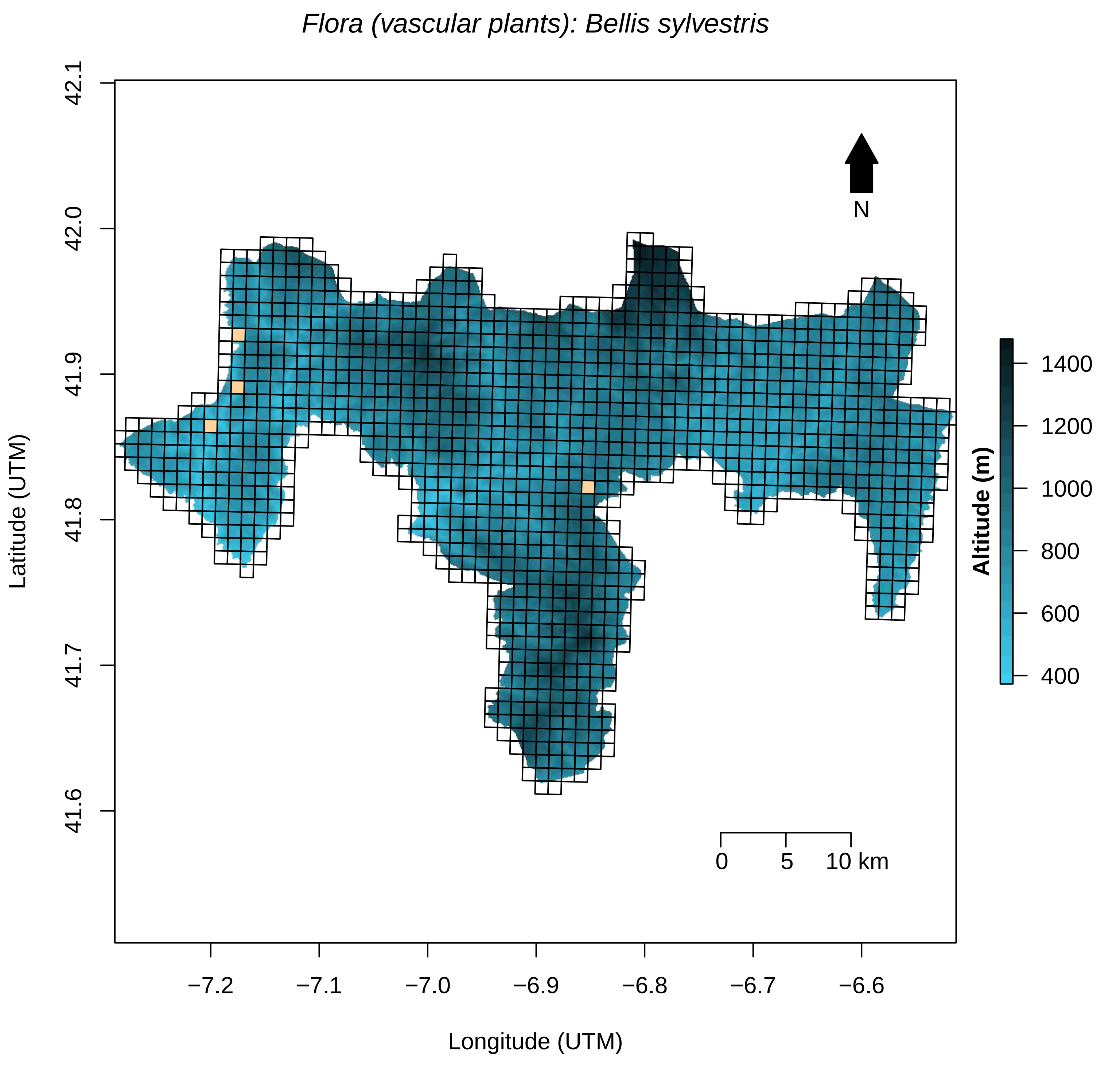

Flora (vascular plants): Bellis sylvestris
Latitude (UTM)
42.1
42.0
41.9
41.8
41.7
41.6
N
0
5
10 km
Altitude (m)
1400
1200
1000
800
600
400
−7.2
−7.1
−7.0
−6.9
−6.8
−6.7
−6.6
Longitude (UTM)

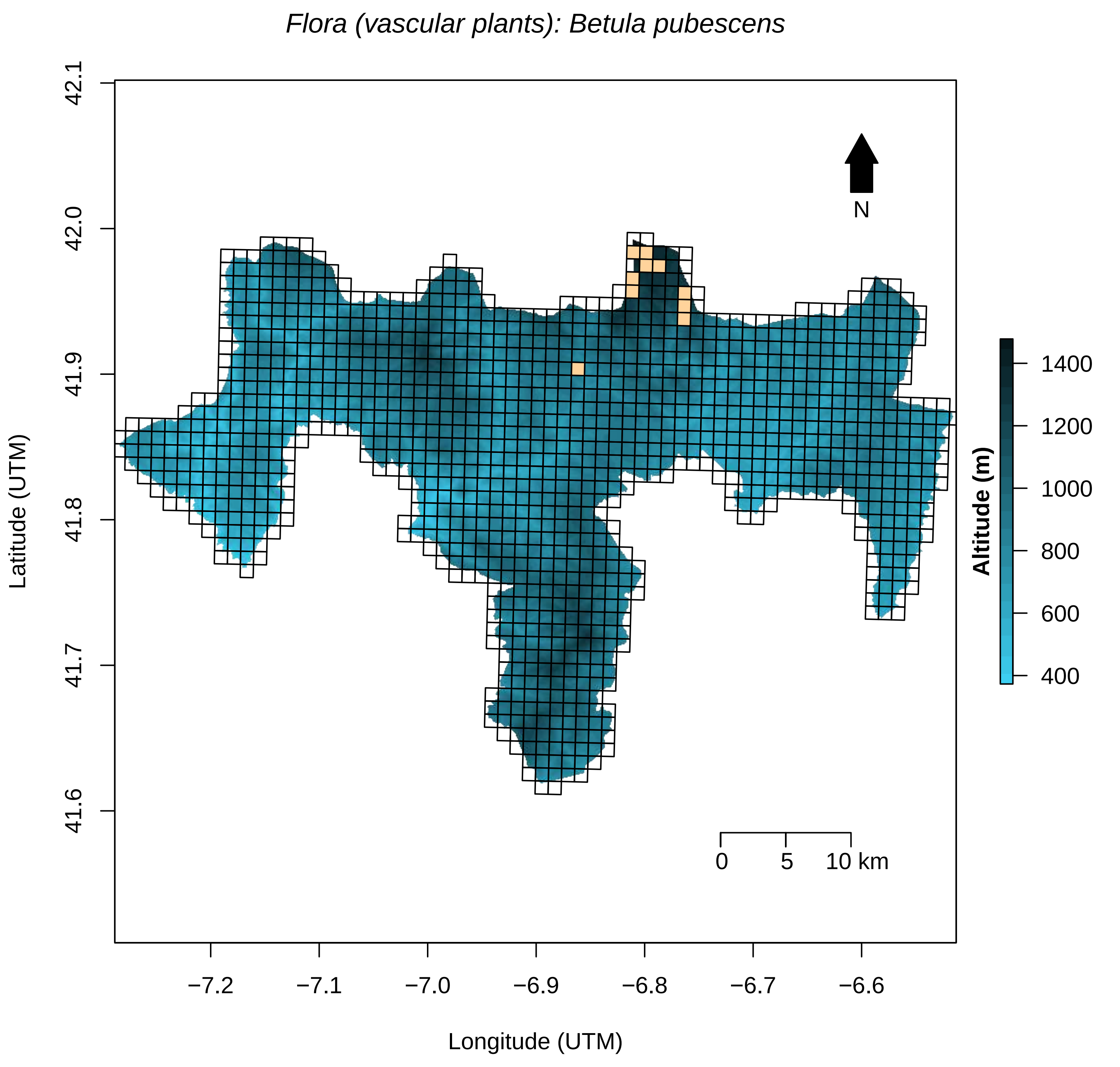
Flora (vascular plants): Betula pubescens
42.1
42.0
41.9
41.8
41.7
41.6
Latitude (UTM)
−7.2
−7.1
−7.0
−6.9
−6.8
−6.7
−6.6
Longitude (UTM)
N
0
5
10 km
Altitude (m)
1400
1200
1000
800
600
400

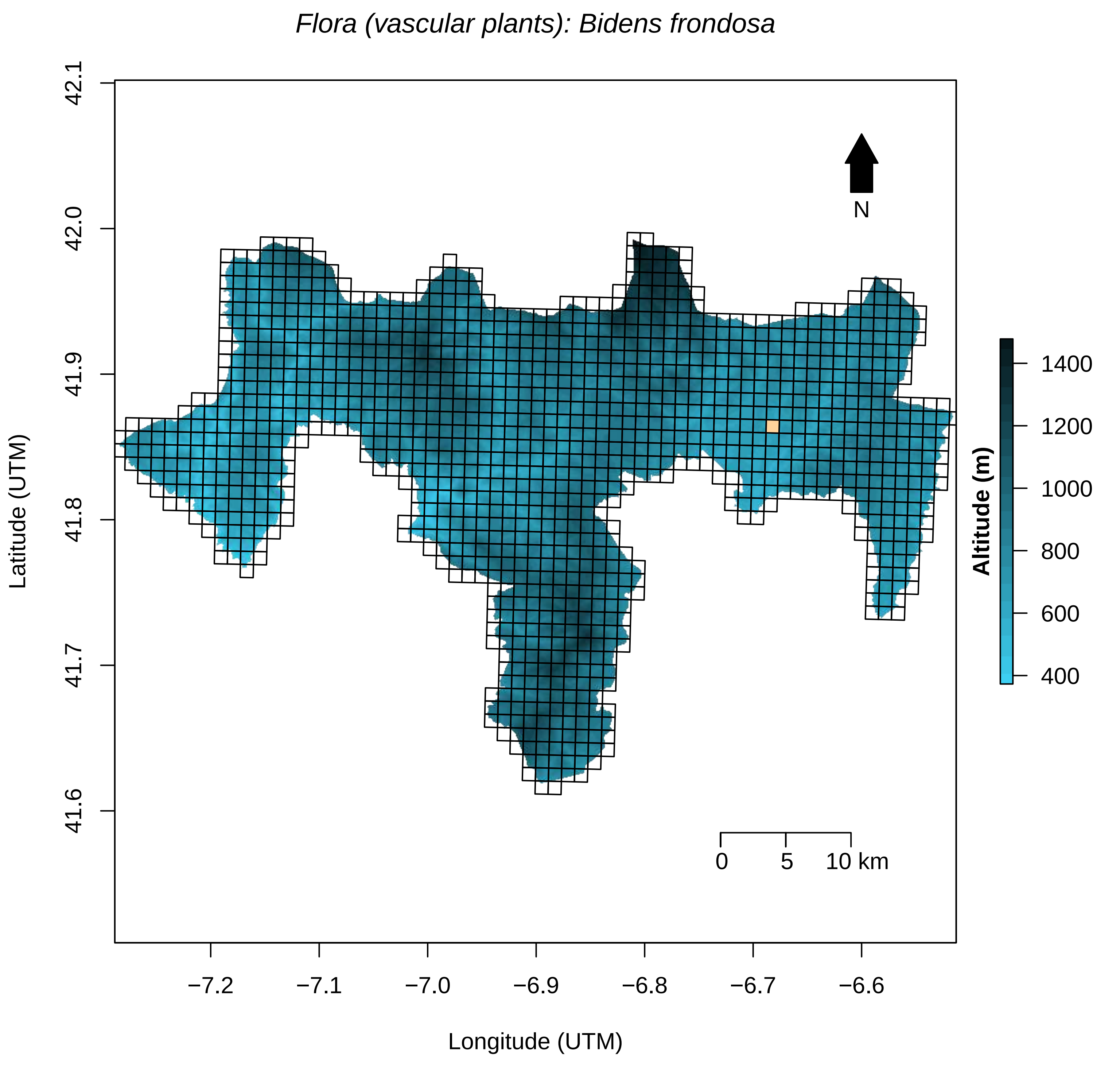
Flora (vascular plants): Bidens frondosa
42.1
42.0
41.9
41.8
41.7
41.6
Latitude (UTM)
−7.2
−7.1
−7.0
−6.9
−6.8
−6.7
−6.6
Longitude (UTM)
N
0
5
10 km
Altitude (m)
1400
1200
1000
800
600
400

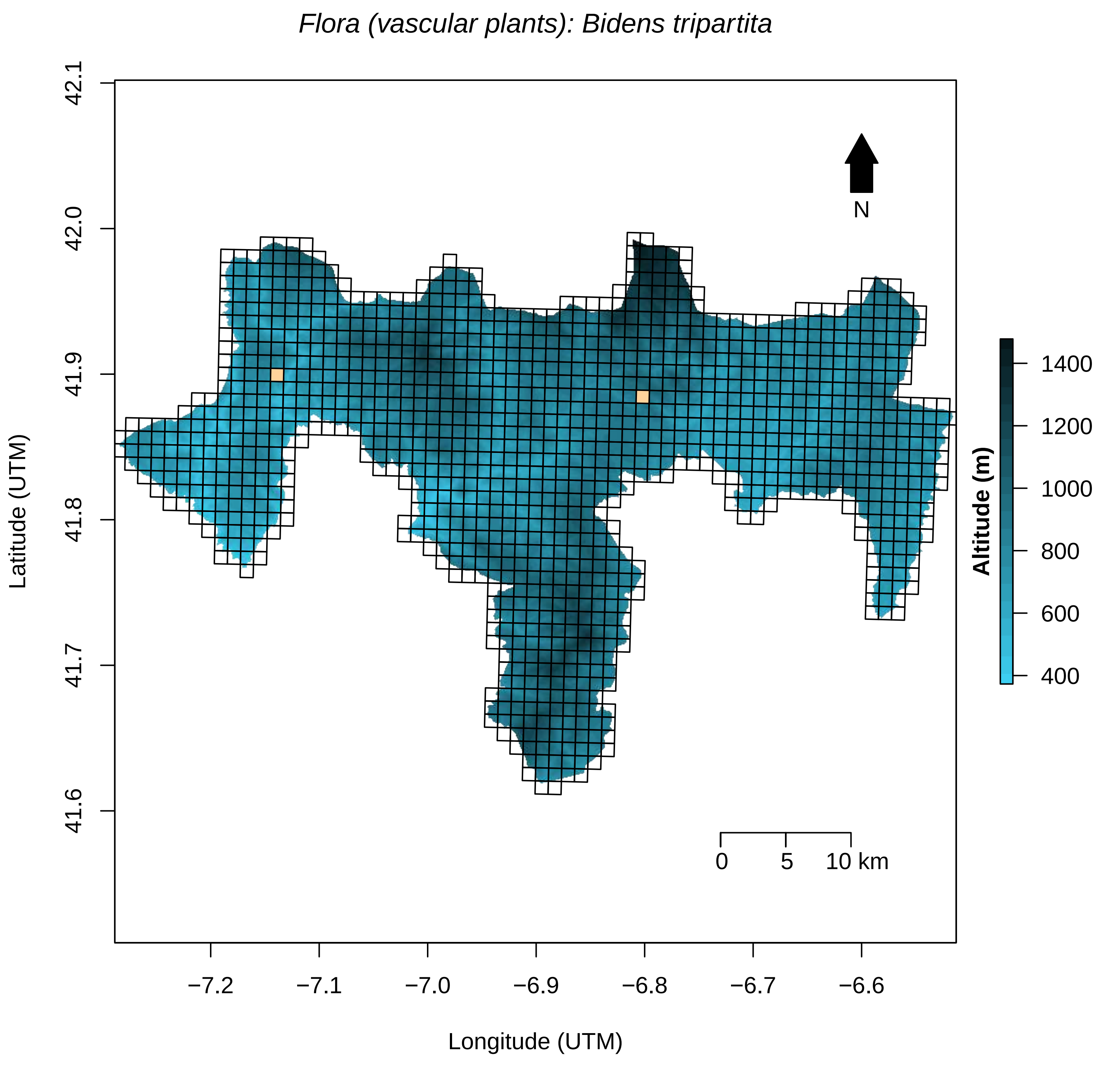

Flora (vascular plants): Bidens tripartita
Latitude (UTM)
Longitude (UTM)
42.1
42.0
41.9
41.8
41.7
41.6
−7.2
−7.1
−7.0
−6.9
−6.8
−6.7
−6.6
N
0
5
10 km
Altitude (m)
1400
1200
1000
800
600
400

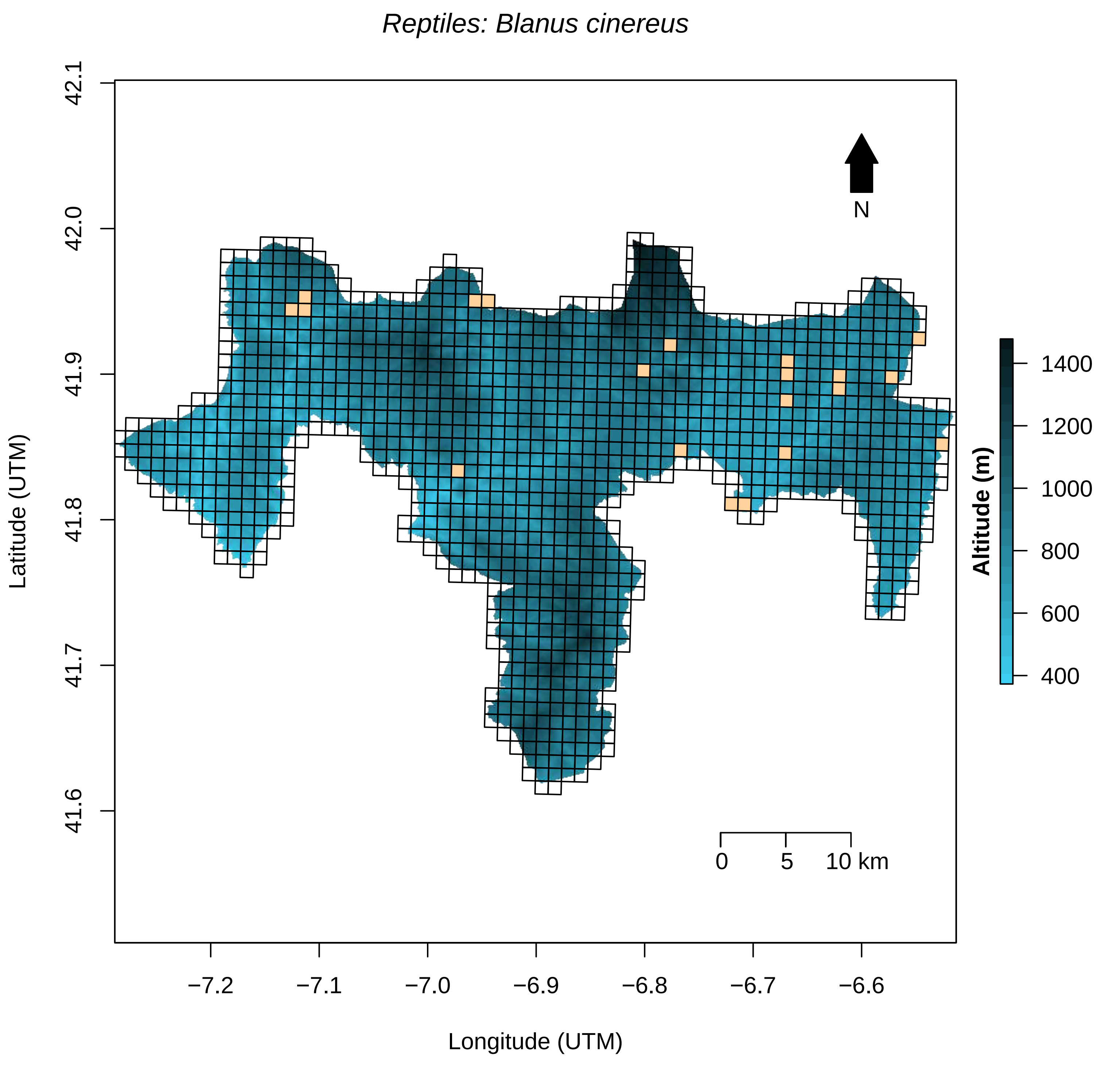

Reptiles: Blanus cinereus
Latitude (UTM)
Longitude (UTM)
42.1
42.0
41.9
41.8
41.7
41.6
−7.2
−7.1
−7.0
−6.9
−6.8
−6.7
−6.6
N
0
5
10 km
Altitude (m)
1400
1200
1000
800
600
400

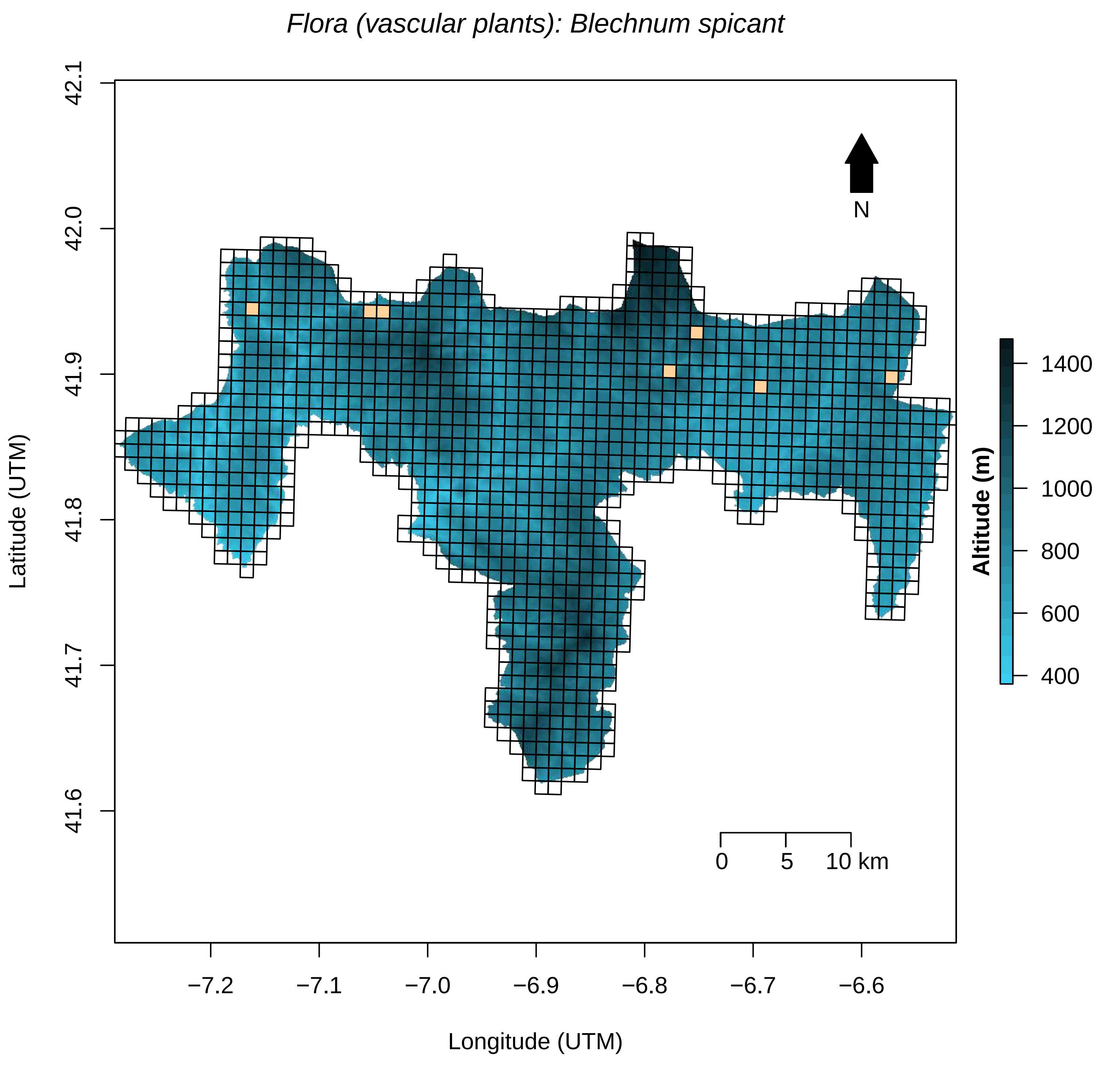
Flora (vascular plants): Blechnum spicant
42.1
42.0
41.9
41.8
41.7
41.6
Latitude (UTM)
−7.2
−7.1
−7.0
−6.9
−6.8
−6.7
−6.6
Longitude (UTM)
N
0
5
10 km
Altitude (m)
1400
1200
1000
800
600
400

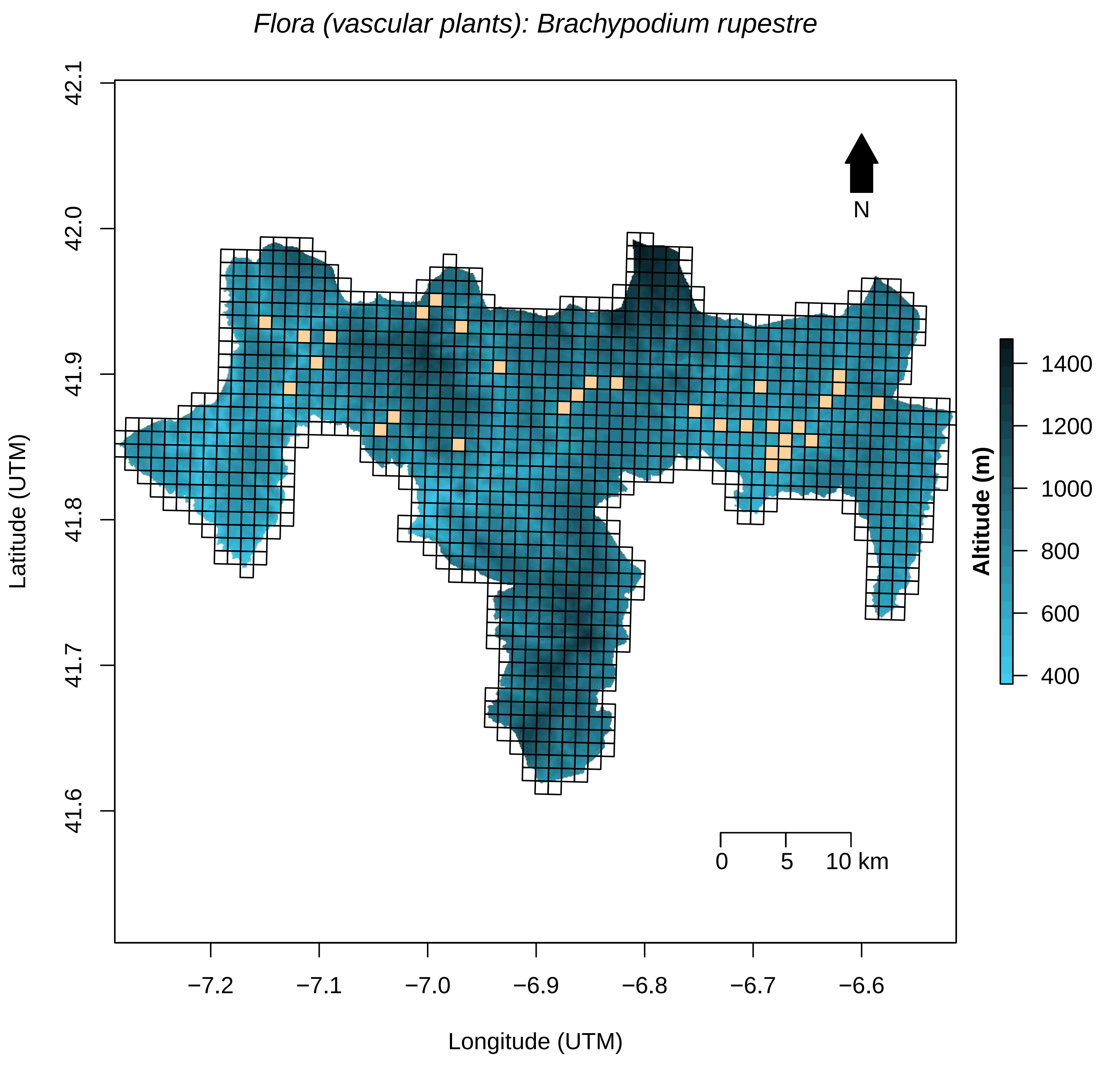

Flora (vascular plants): Brachypodium rupestre
42.1
42.0
41.9
41.8
41.7
41.6
Latitude (UTM)
−7.2
−7.1
−7.0
−6.9
−6.8
−6.7
−6.6
Longitude (UTM)
N
0
5
10 km
Altitude (m)
1400
1200
1000
800
600
400

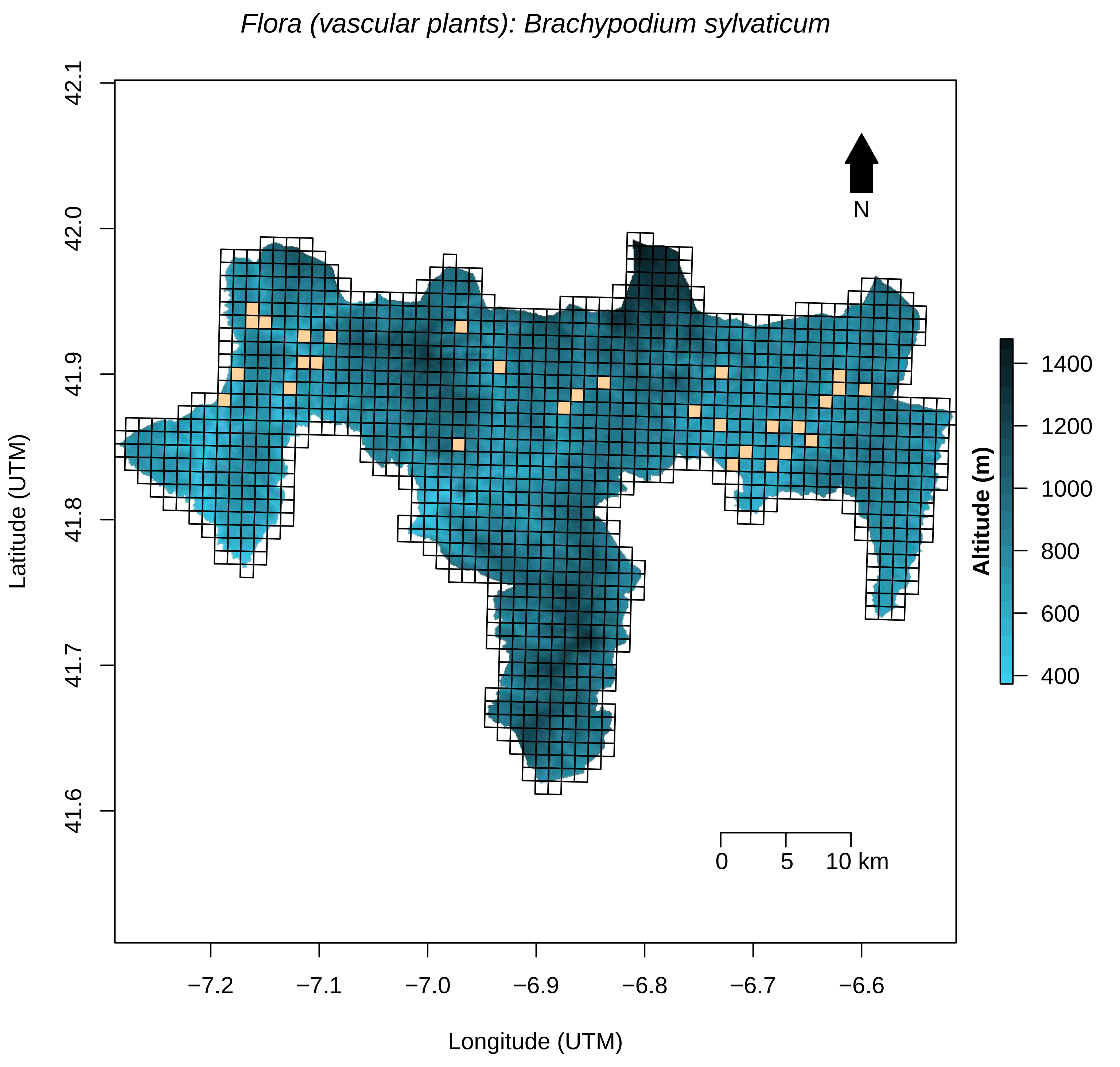

Flora (vascular plants): Brachypodium sylvaticum
42.1
42.0
41.9
41.8
41.7
41.6
Latitude (UTM)
−7.2
−7.1
−7.0
−6.9
−6.8
−6.7
−6.6
Longitude (UTM)
N
0
5
10 km
Altitude (m)
1400
1200
1000
800
600
400

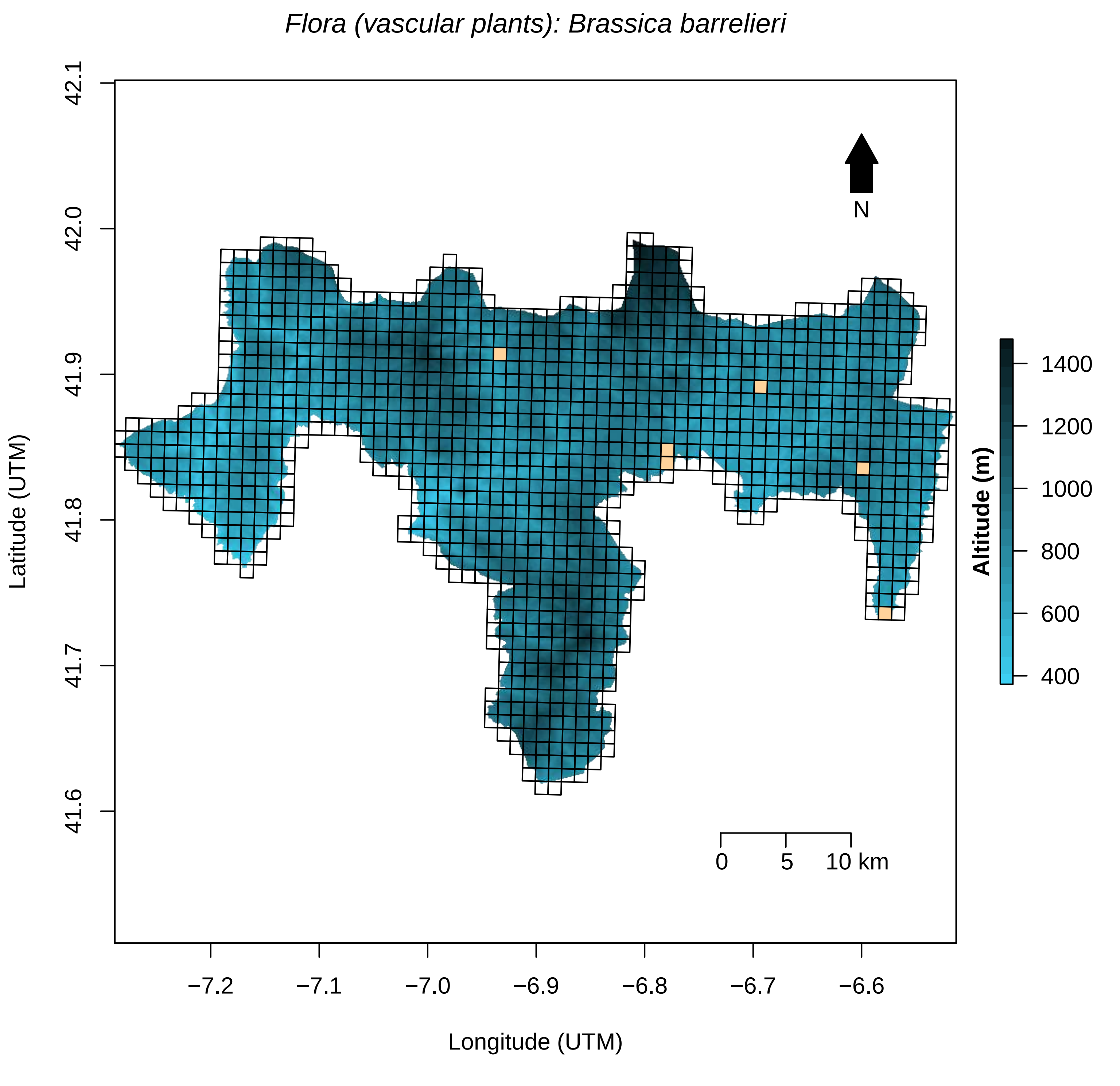

Flora (vascular plants): Brassica barrelieri
Latitude (UTM)
42.1
42.0
41.9
41.8
41.7
41.6
N
Altitude (m)
1400
1200
1000
800
600
400
0
5
10 km
−7.2
−7.1
−7.0
−6.9
−6.8
−6.7
−6.6
Longitude (UTM)

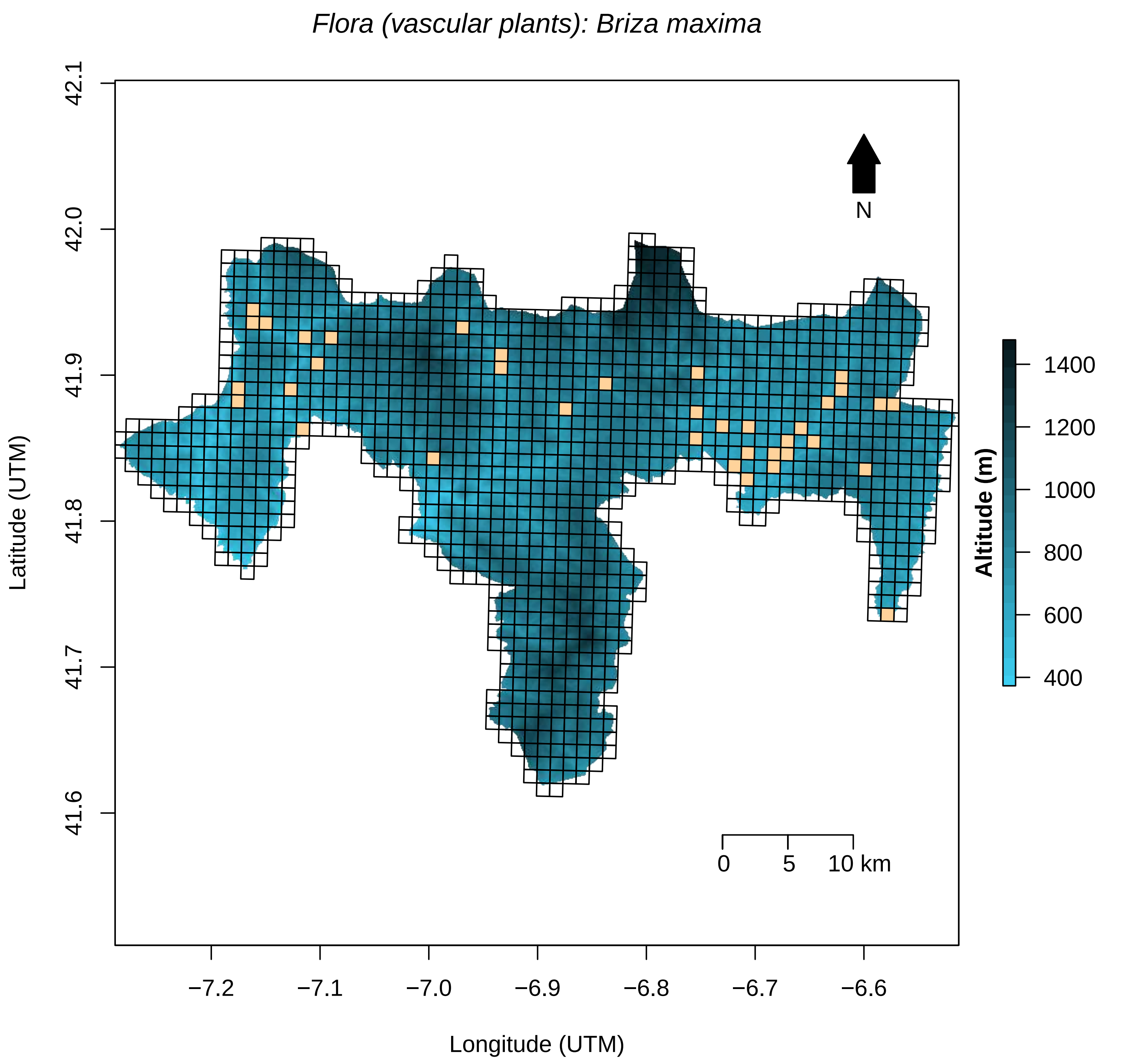

Flora (vascular plants): Briza maxima
Latitude (UTM)
42.1
42.0
41.9
41.8
41.7
41.6
Longitude (UTM)
−7.2
−7.1
−7.0
−6.9
−6.8
−6.7
−6.6
N
0
5
10 km
Altitude (m)
1400
1200
1000
800
600
400

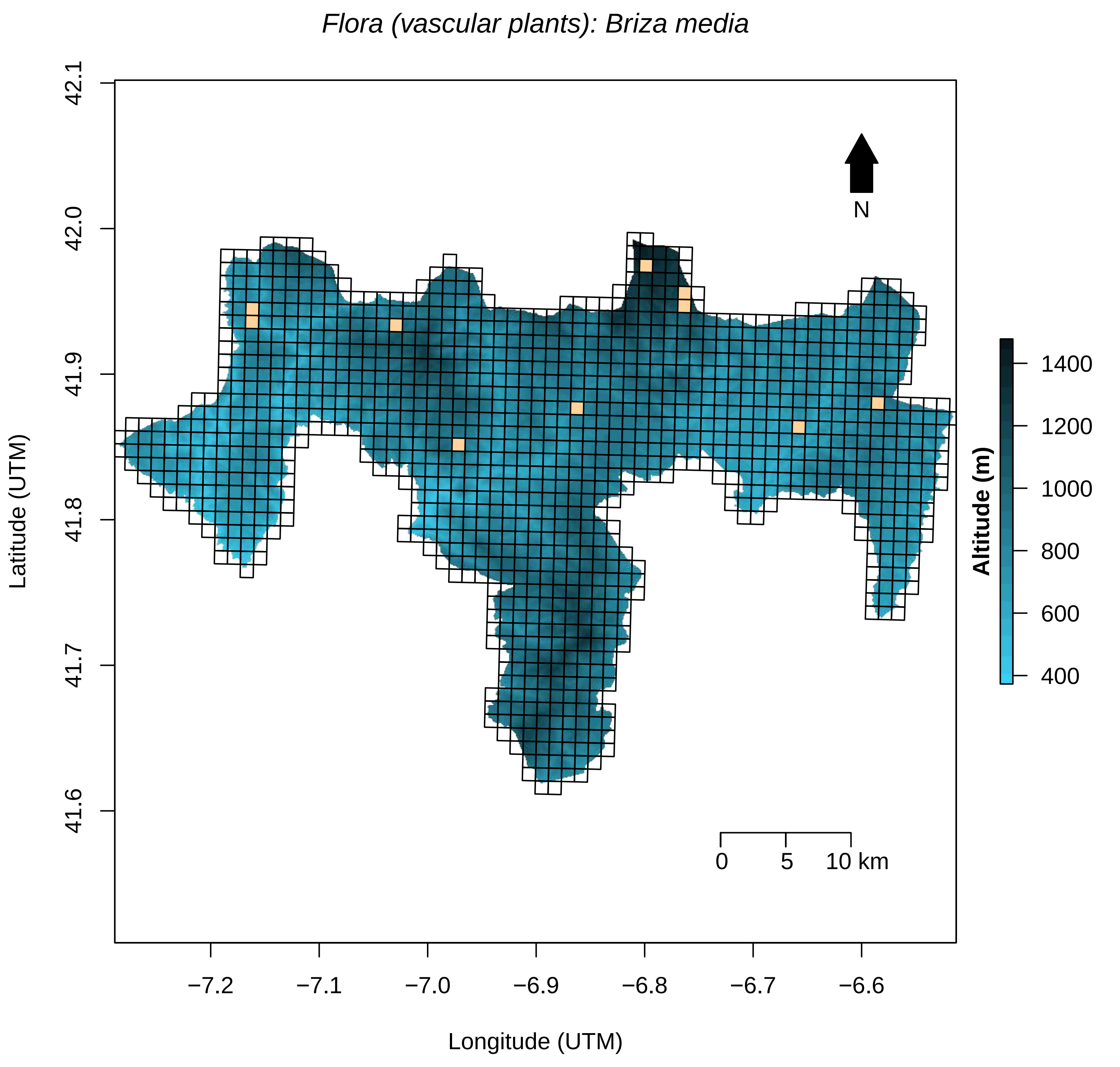

Flora (vascular plants): Briza media
42.1
42.0
41.9
41.8
41.7
41.6
Latitude (UTM)
−7.2
−7.1
−7.0
−6.9
−6.8
−6.7
−6.6
Longitude (UTM)
N
0
5
10 km
Altitude (m)
1400
1200
1000
800
600
400

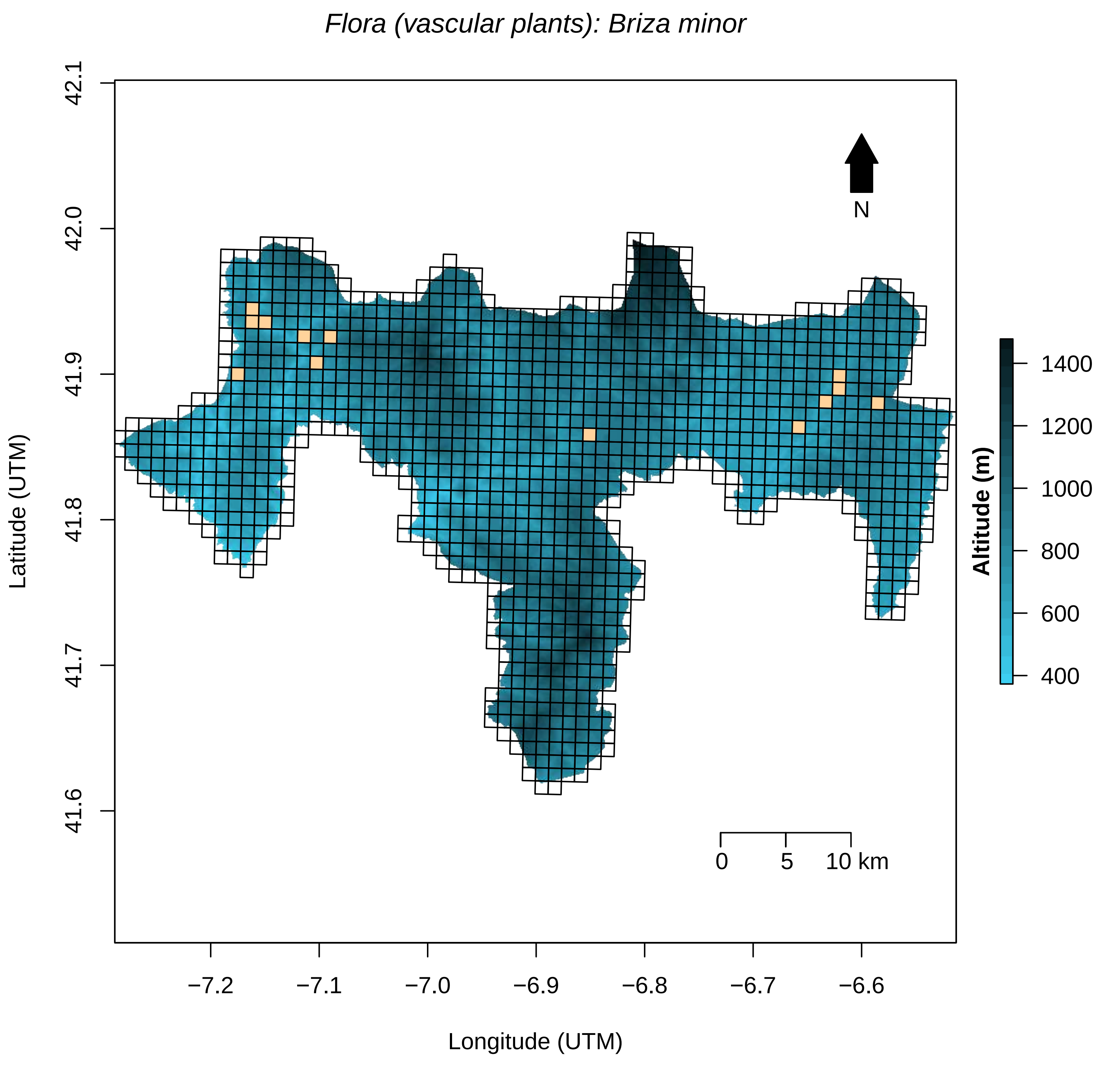

Flora (vascular plants): Briza minor
42.1
42.0
41.9
41.8
41.7
41.6
Latitude (UTM)
−7.2
−7.1
−7.0
−6.9
−6.8
−6.7
−6.6
Longitude (UTM)
N
0
5
10 km
Altitude (m)
1400
1200
1000
800
600
400

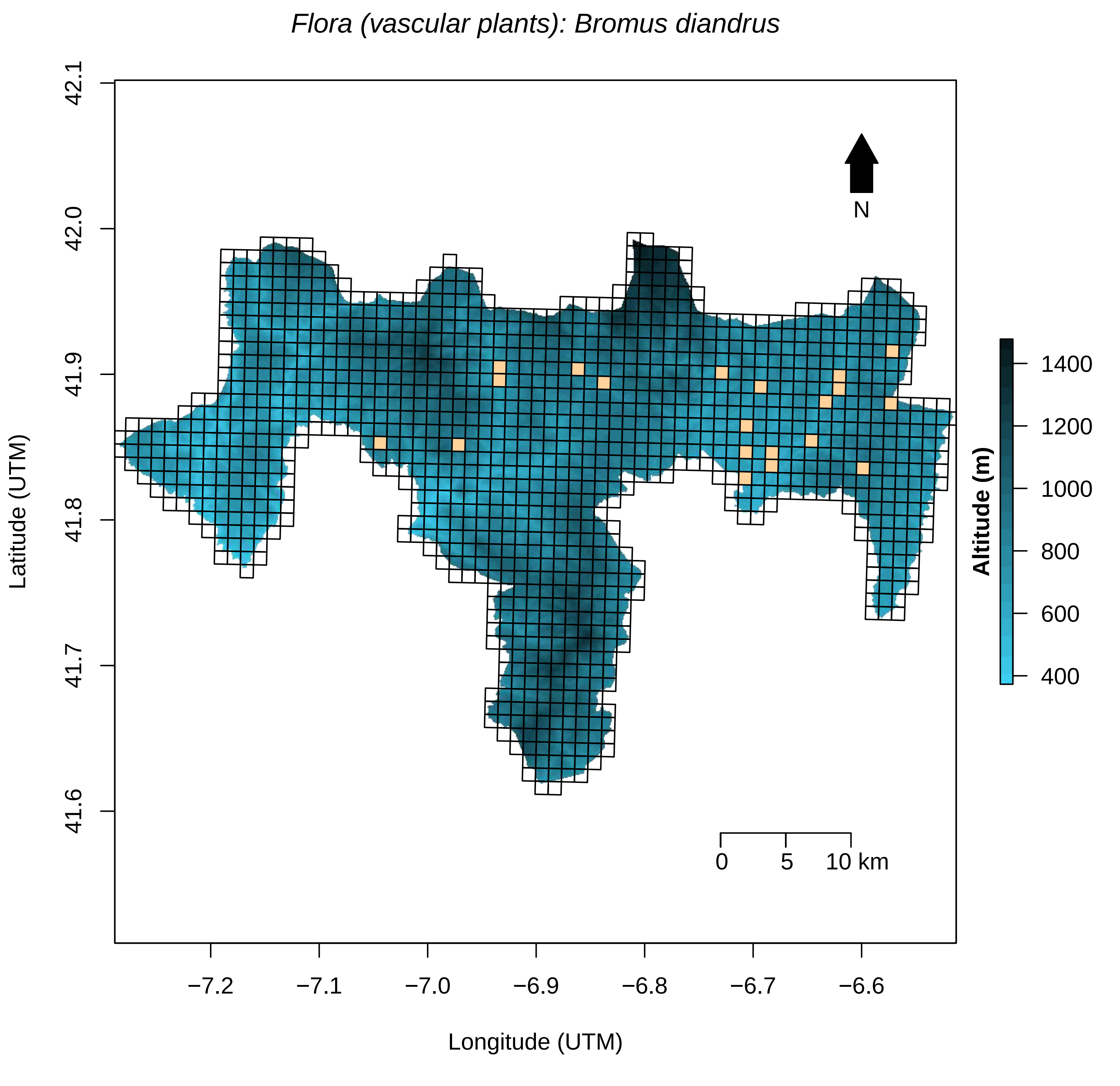

Flora (vascular plants): Bromus diandrus
42.1
42.0
41.9
41.8
41.7
41.6
Latitude (UTM)
−7.2
−7.1
−7.0
−6.9
−6.8
−6.7
−6.6
Longitude (UTM)
N
0
5
10 km
1400
1200
1000
800
600
400
Altitude (m)

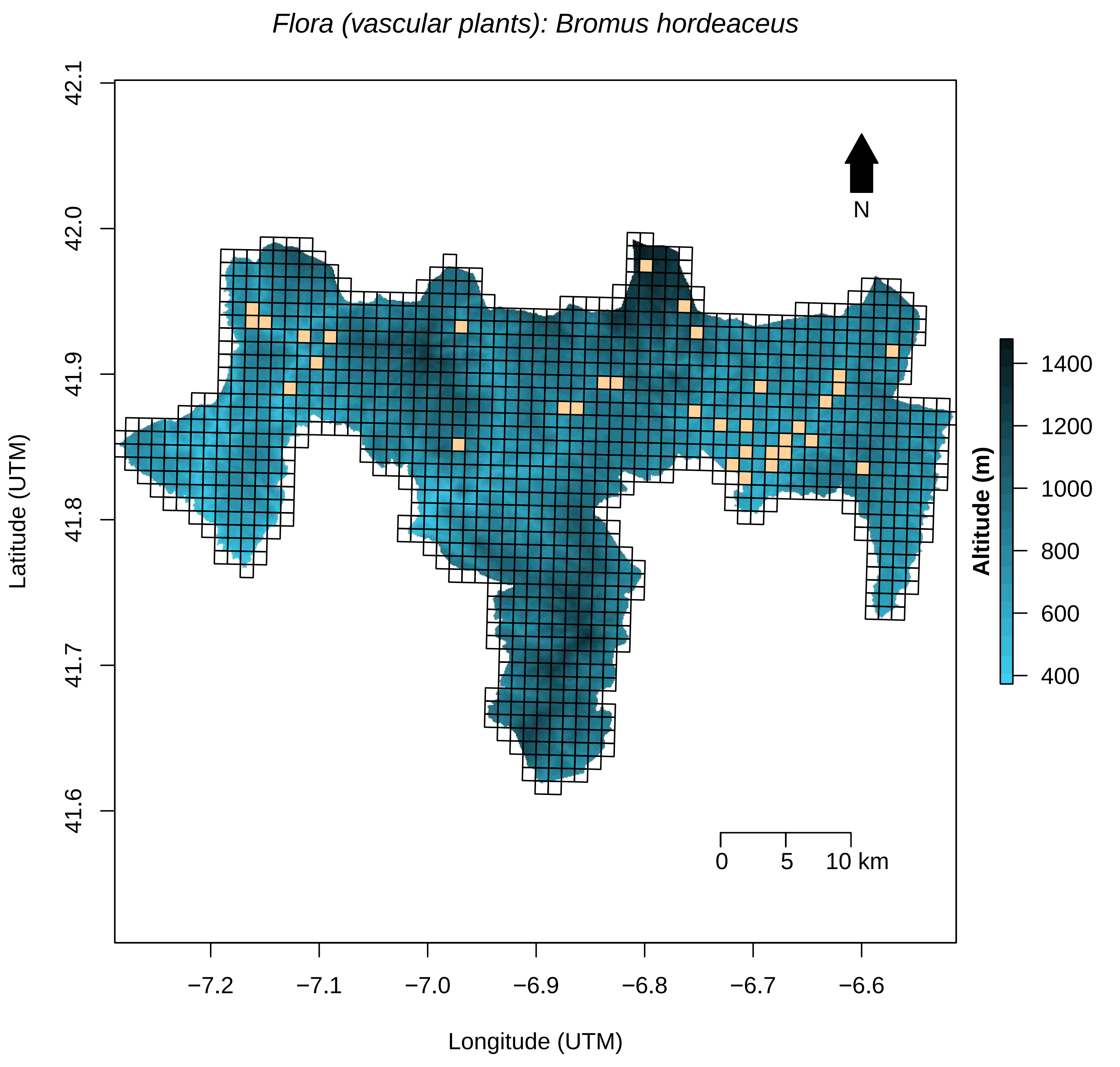
Flora (vascular plants): Bromus hordeaceus
42.1
42.0
41.9
41.8
41.7
41.6
Latitude (UTM)
−7.2
−7.1
−7.0
−6.9
−6.8
−6.7
−6.6
Longitude (UTM)
N
0
5
10 km
Altitude (m)
1400
1200
1000
800
600
400

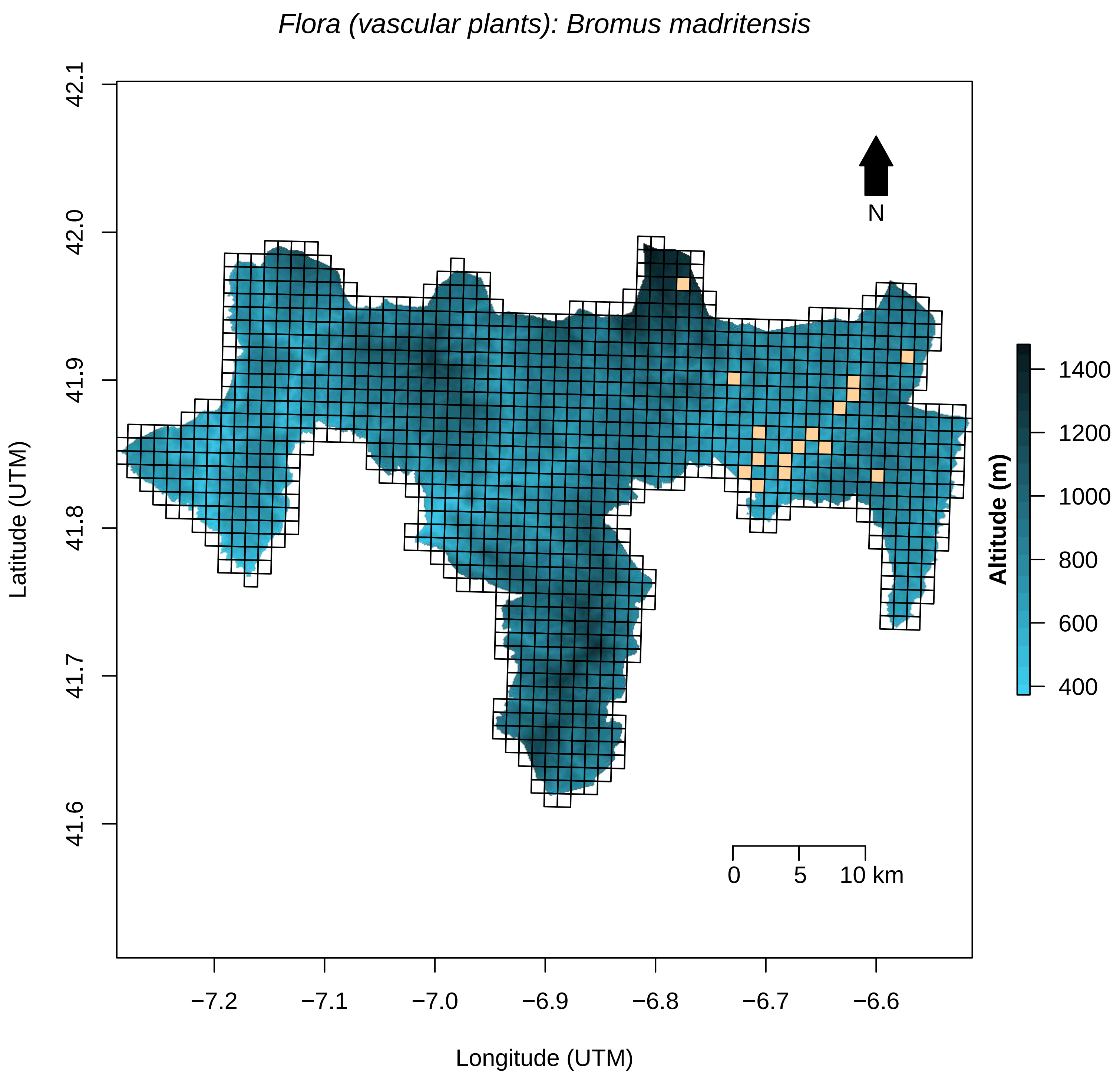
Flora (vascular plants): Bromus madritensis
Latitude (UTM)
42.1
42.0
41.9
41.8
41.7
41.6
Longitude (UTM)
−7.2
−7.1
−7.0
−6.9
−6.8
−6.7
−6.6
N
0
5
10 km
Altitude (m)
1400
1200
1000
800
600
400

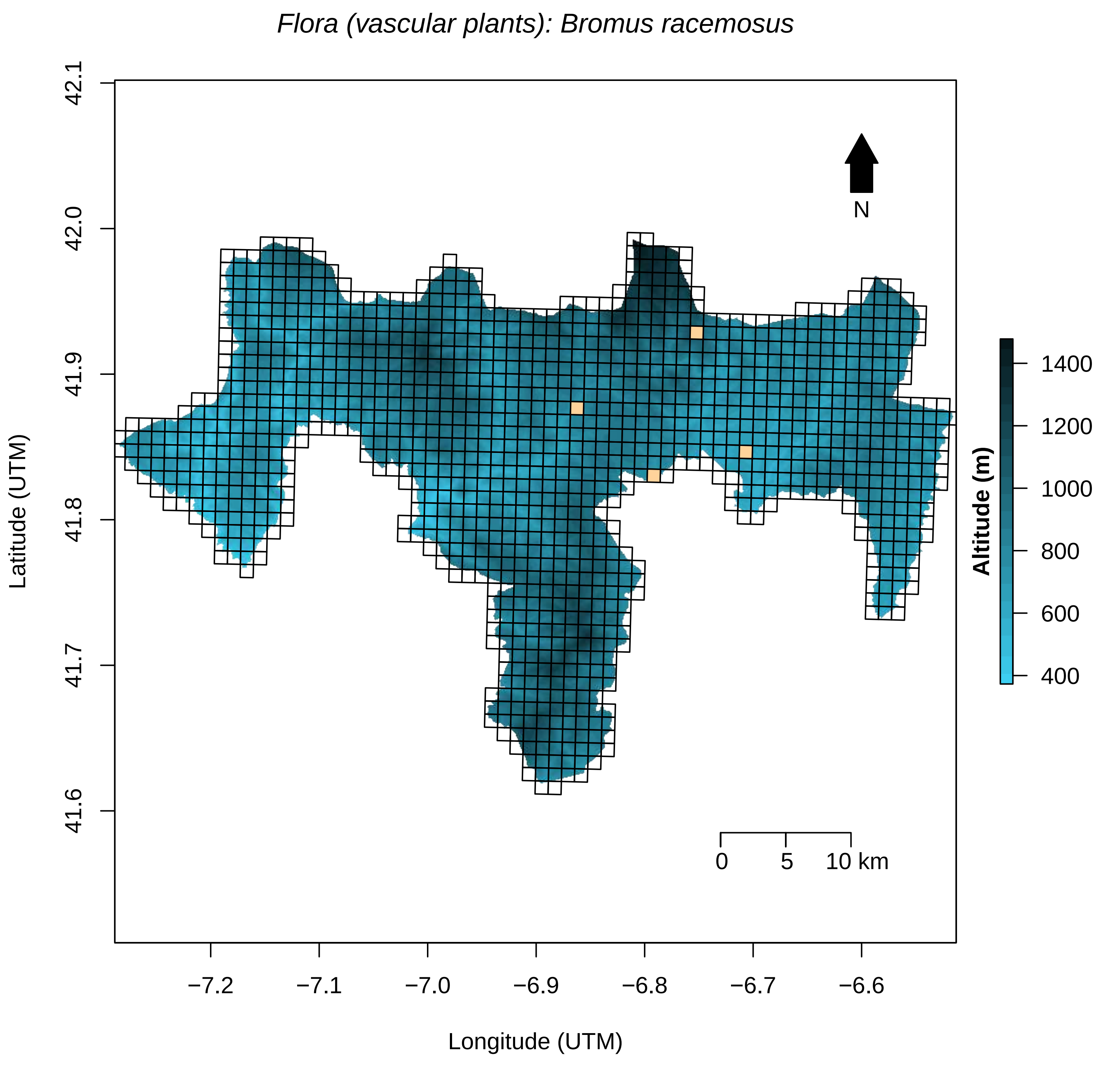
Flora (vascular plants): Bromus racemosus
N
0
5
10 km
Altitude (m)
1400
1200
1000
800
600
400
42.1
42.0
41.9
41.8
41.7
41.6
−7.2
−7.1
−7.0
−6.9
−6.8
−6.7
−6.6
Latitude (UTM)
Longitude (UTM)

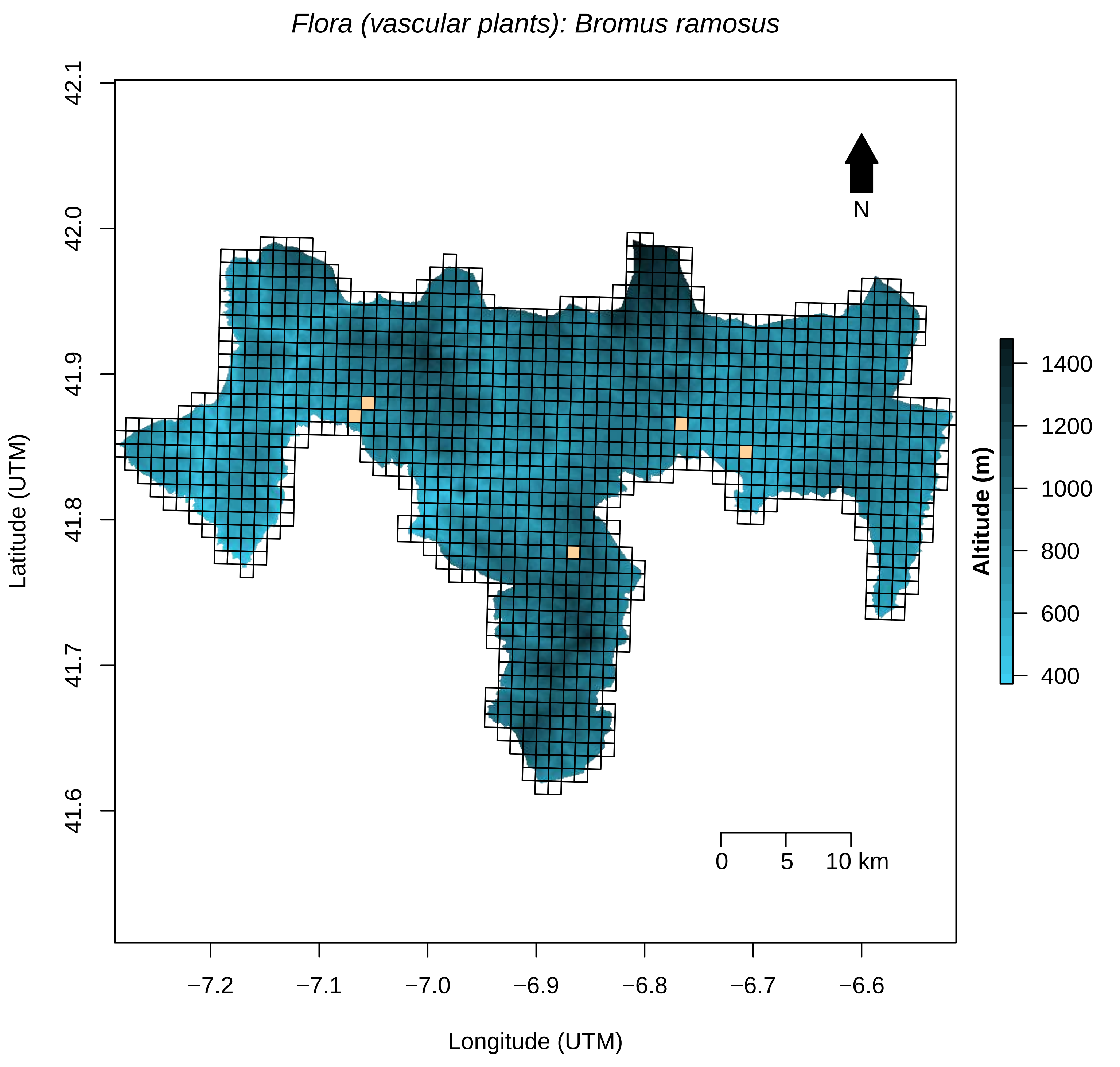

Flora (vascular plants): Bromus ramosus
N
0
5
10 km
Altitude (m)
1400
1200
1000
800
600
400
42.1
42.0
41.9
41.8
41.7
41.6
Latitude (UTM)
−7.2
−7.1
−7.0
−6.9
−6.8
−6.7
−6.6
Longitude (UTM)

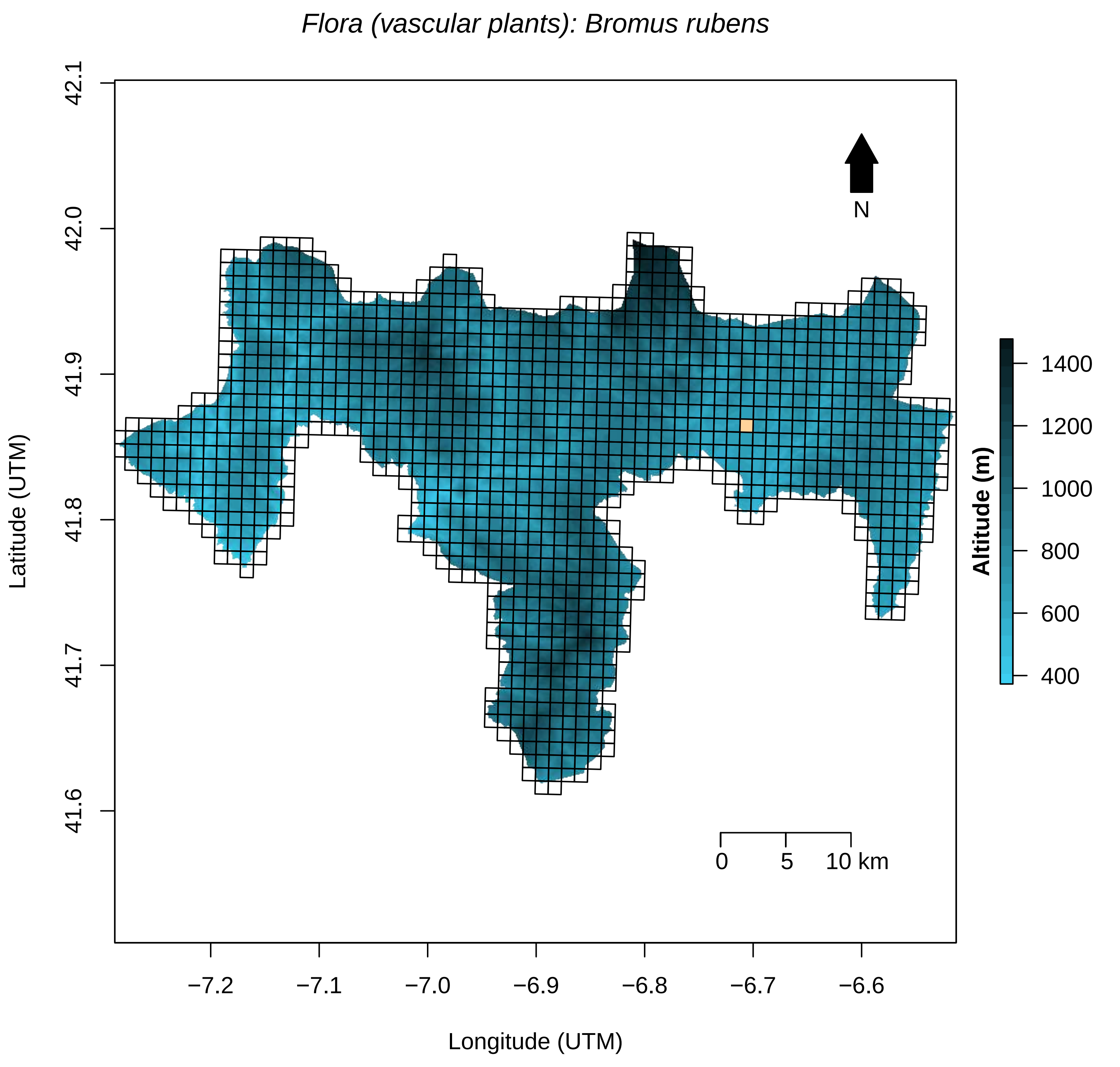
Flora (vascular plants): Bromus rubens
Latitude (UTM)
Longitude (UTM)
42.1
42.0
41.9
41.8
41.7
41.6
−7.2
−7.1
−7.0
−6.9
−6.8
−6.7
−6.6
N
0
5
10 km
Altitude (m)
1400
1200
1000
800
600
400

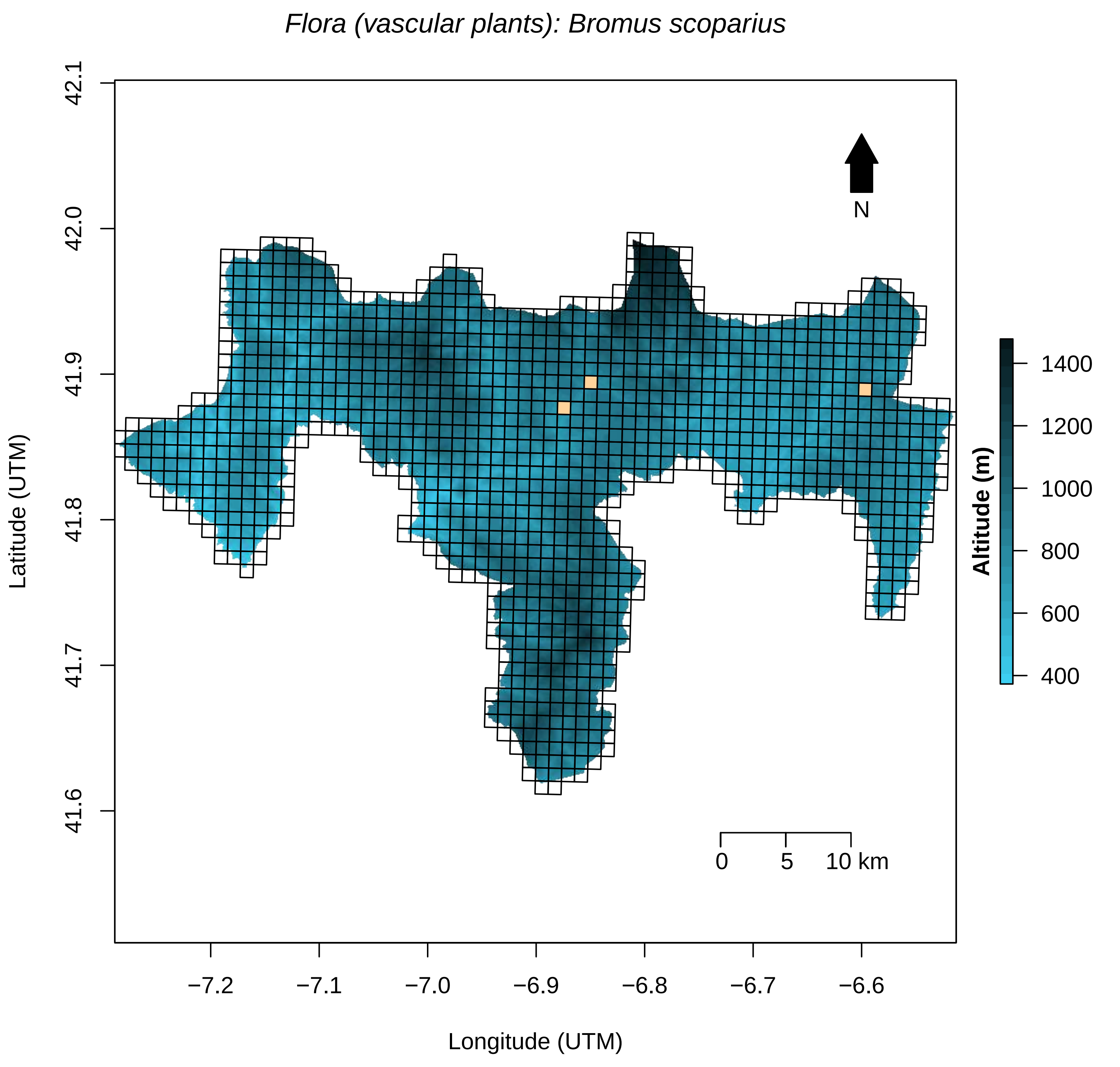

Flora (vascular plants): Bromus scoparius
Latitude (UTM)
42.1
42.0
41.9
41.8
41.7
41.6
Longitude (UTM)
−7.2
−7.1
−7.0
−6.9
−6.8
−6.7
−6.6
N
0
5
10 km
Altitude (m)
1400
1200
1000
800
600
400

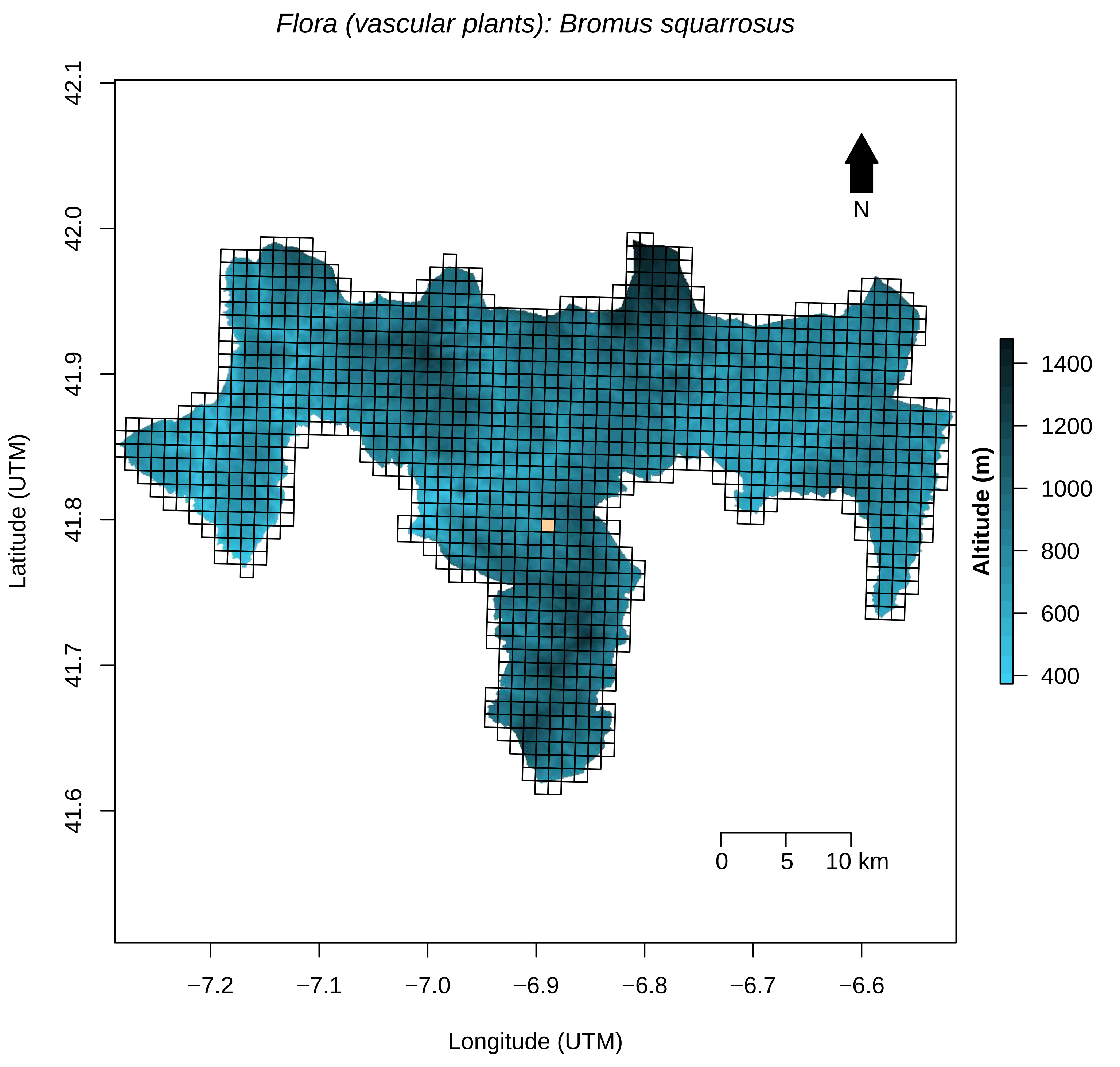

Flora (vascular plants): Bromus squarrosus
42.1
42.0
41.9
41.8
41.7
41.6
Latitude (UTM)
−7.2
−7.1
−7.0
−6.9
−6.8
−6.7
−6.6
Longitude (UTM)
N
0
5
10 km
Altitude (m)
1400
1200
1000
800
600
400

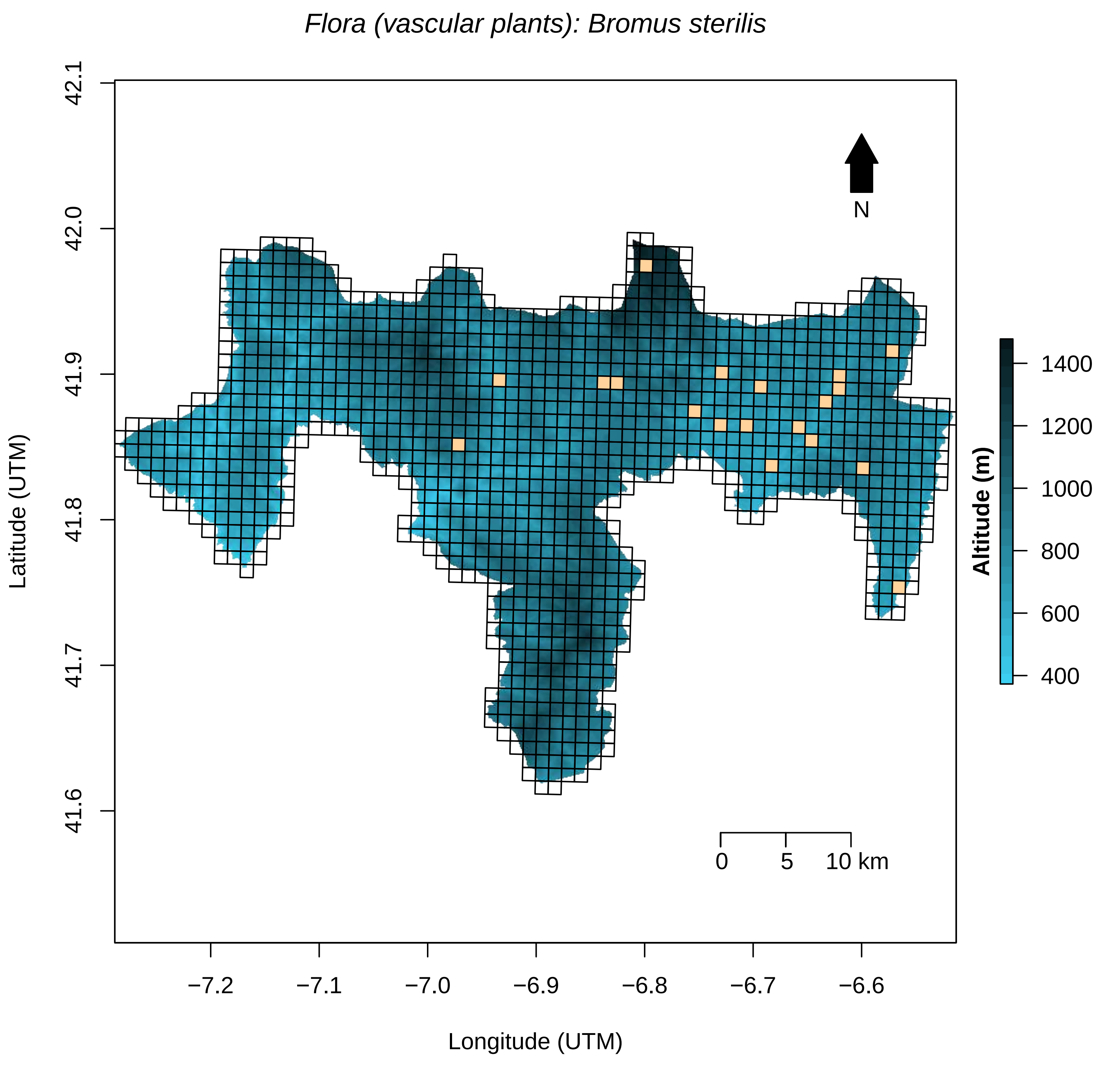

Flora (vascular plants): Bromus sterilis
N
0
5
10 km
Altitude (m)
1400
1200
1000
800
600
400
Latitude (UTM)
42.1
42.0
41.9
41.8
41.7
41.6
Longitude (UTM)
−7.2
−7.1
−7.0
−6.9
−6.8
−6.7
−6.6

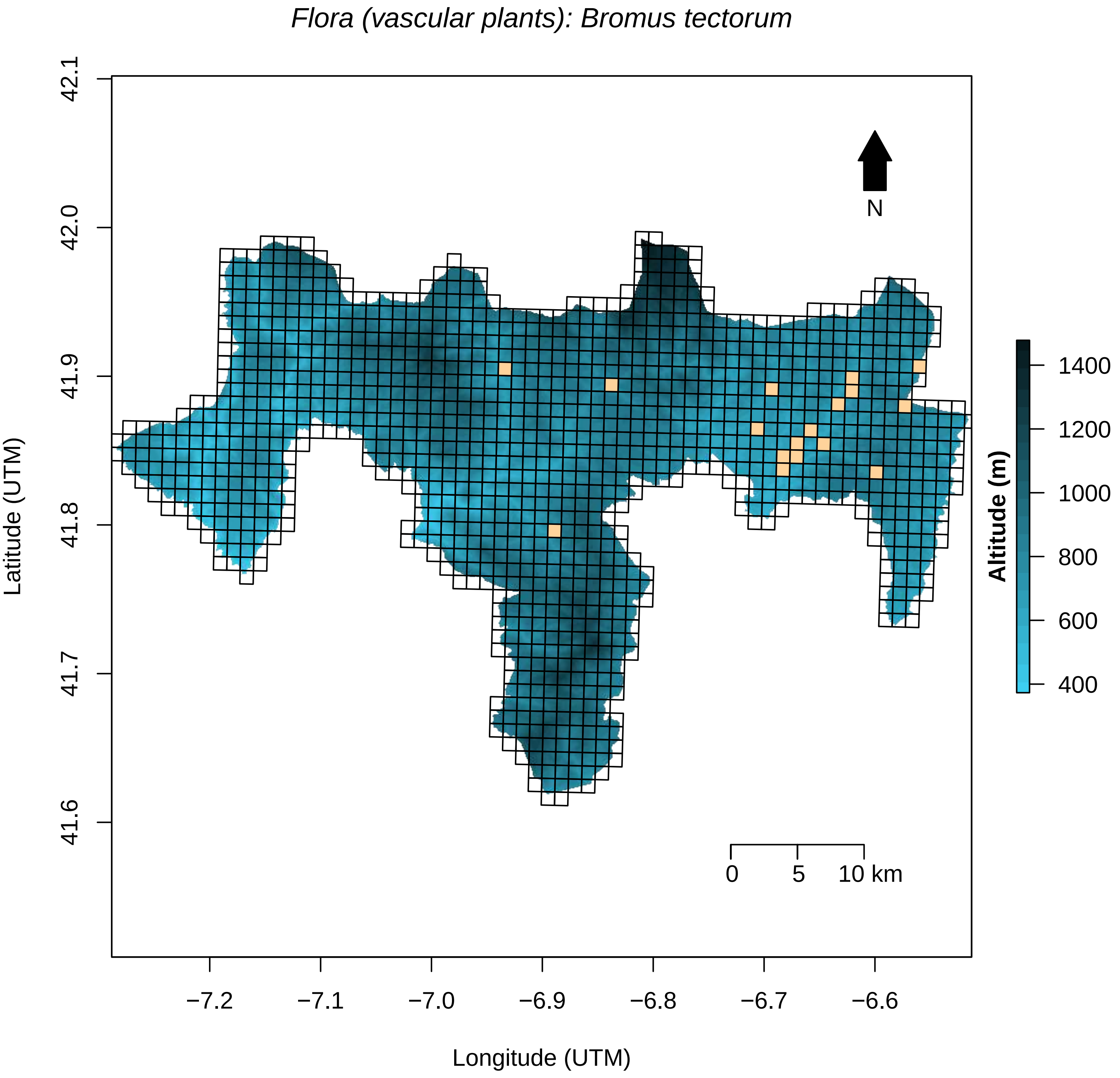

Flora (vascular plants): Bromus tectorum
N
0 5 10 km
Altitude (m)
1400
1200
1000
800
600
400
42.1
42.0
41.9
41.8
41.7
41.6
Latitude (UTM)
−7.2
−7.1
−7.0
−6.9
−6.8
−6.7
−6.6
Longitude (UTM)

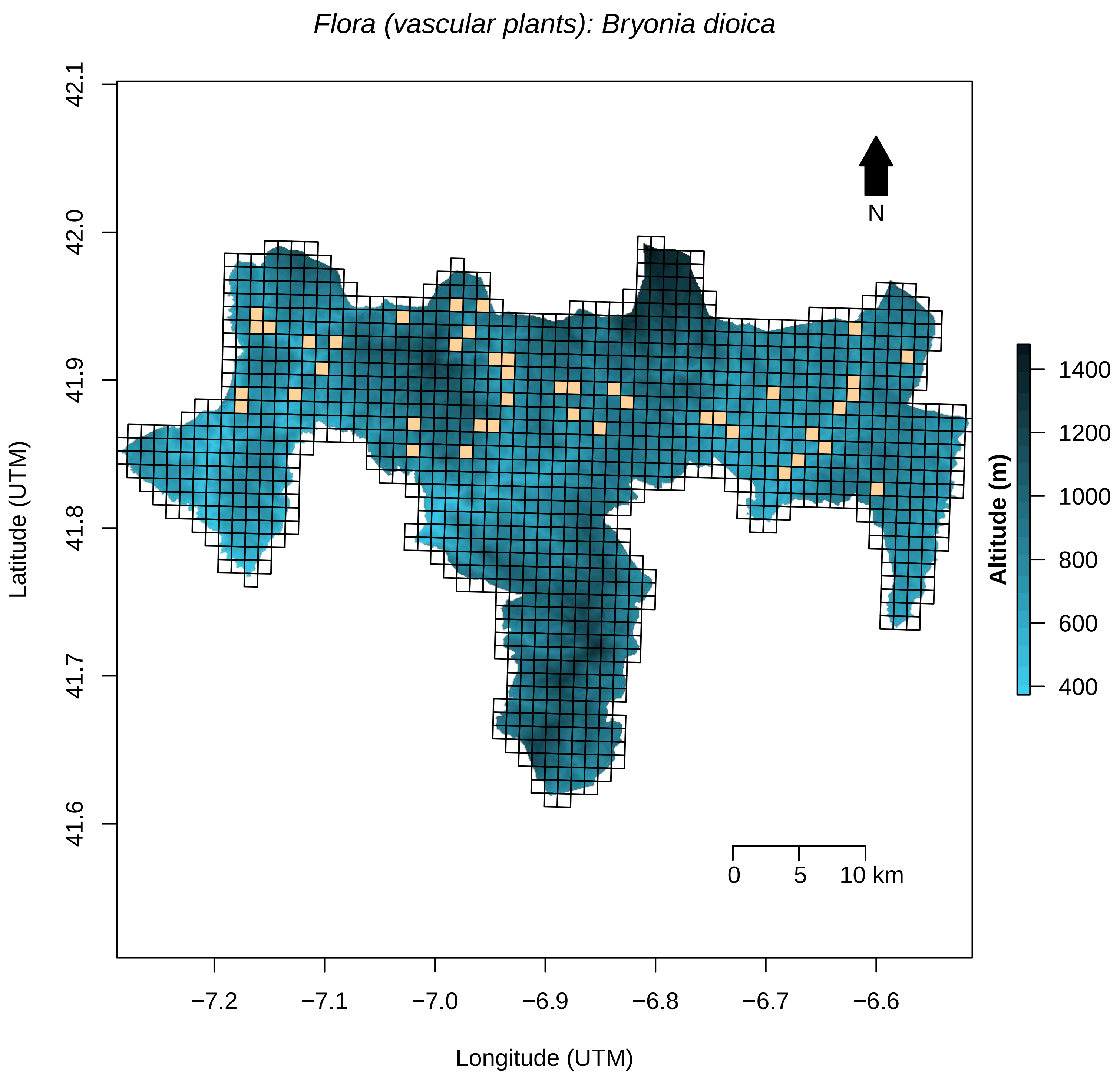

Flora (vascular plants): Bryonia dioica
Latitude (UTM)
42.1
42.0
41.9
41.8
41.7
41.6
Longitude (UTM)
−7.2
−7.1
−7.0
−6.9
−6.8
−6.7
−6.6
N
0
5
10 km
Altitude (m)
1400
1200
1000
800
600
400

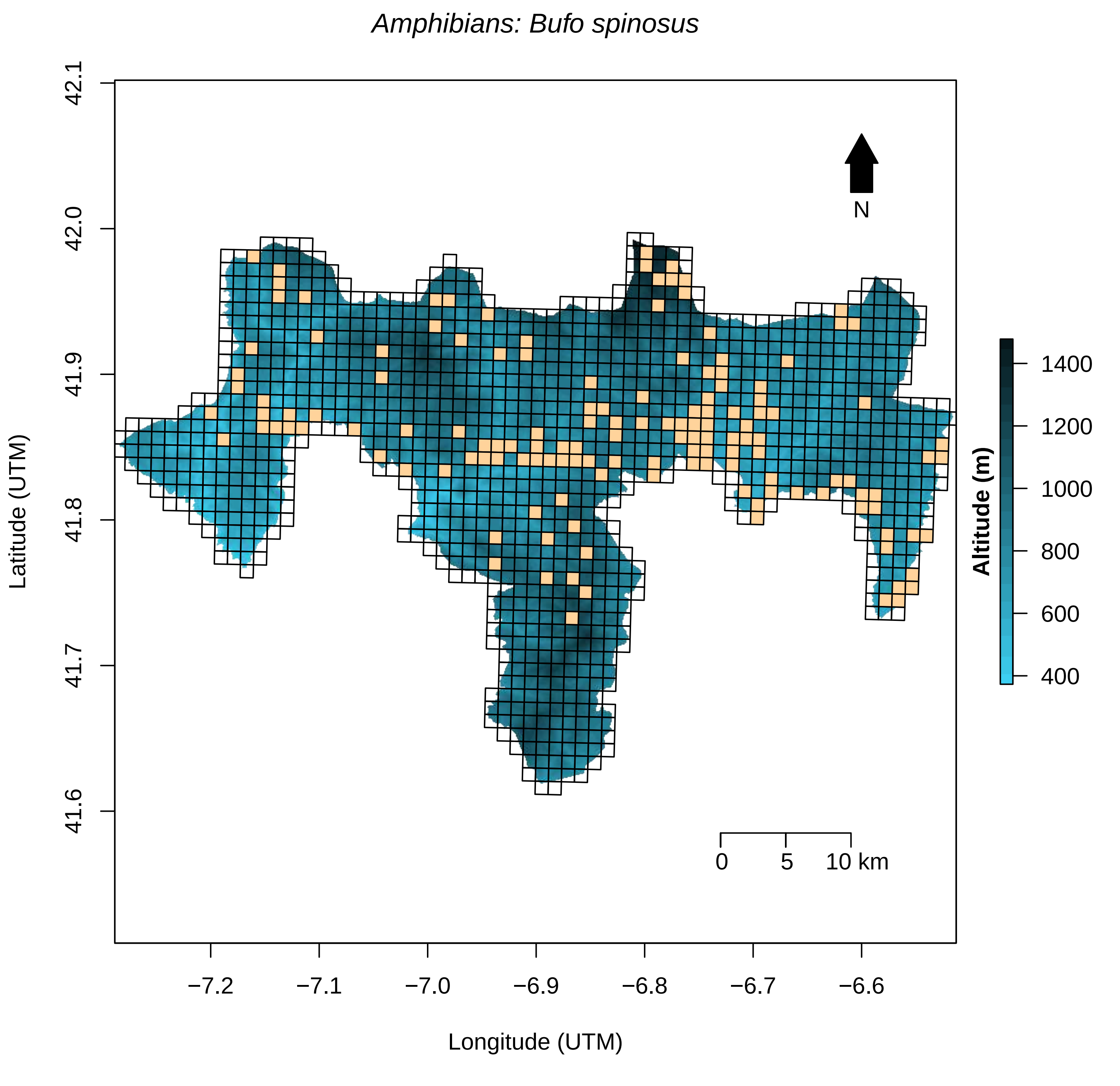

Amphibians: Bufo spinosus
42.1
42.0
41.9
41.8
41.7
41.6
Latitude (UTM)
−7.2
−7.1
−7.0
−6.9
−6.8
−6.7
−6.6
Longitude (UTM)
N
0
5
10 km
Altitude (m)
1400
1200
1000
800
600
400

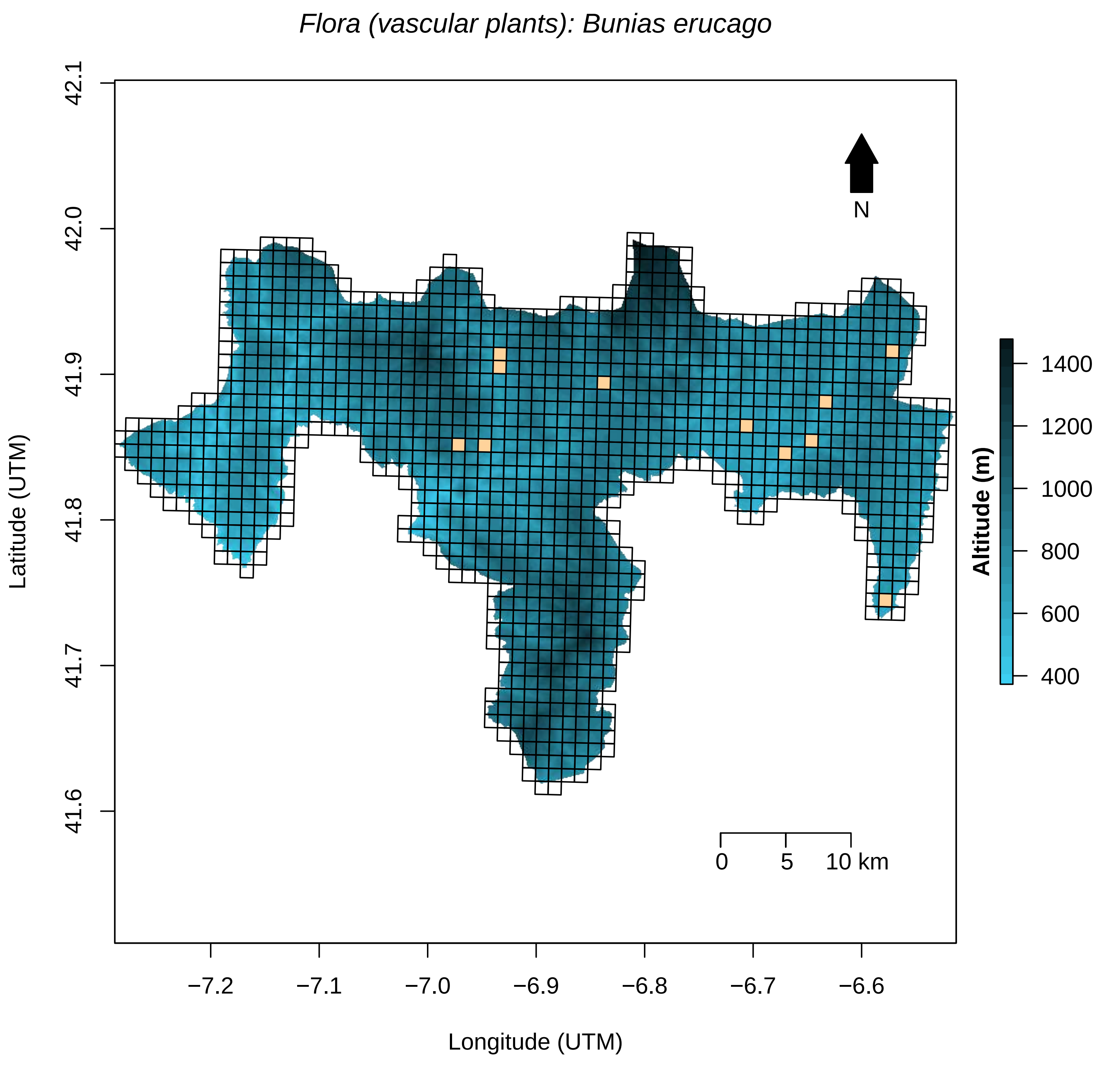
Flora (vascular plants): Bunias erucago
N
0 5 10 km
Altitude (m)
1400
1200
1000
800
600
400
Latitude (UTM)
42.1
42.0
41.9
41.8
41.7
41.6
Longitude (UTM)
−7.2
−7.1
−7.0
−6.9
−6.8
−6.7
−6.6

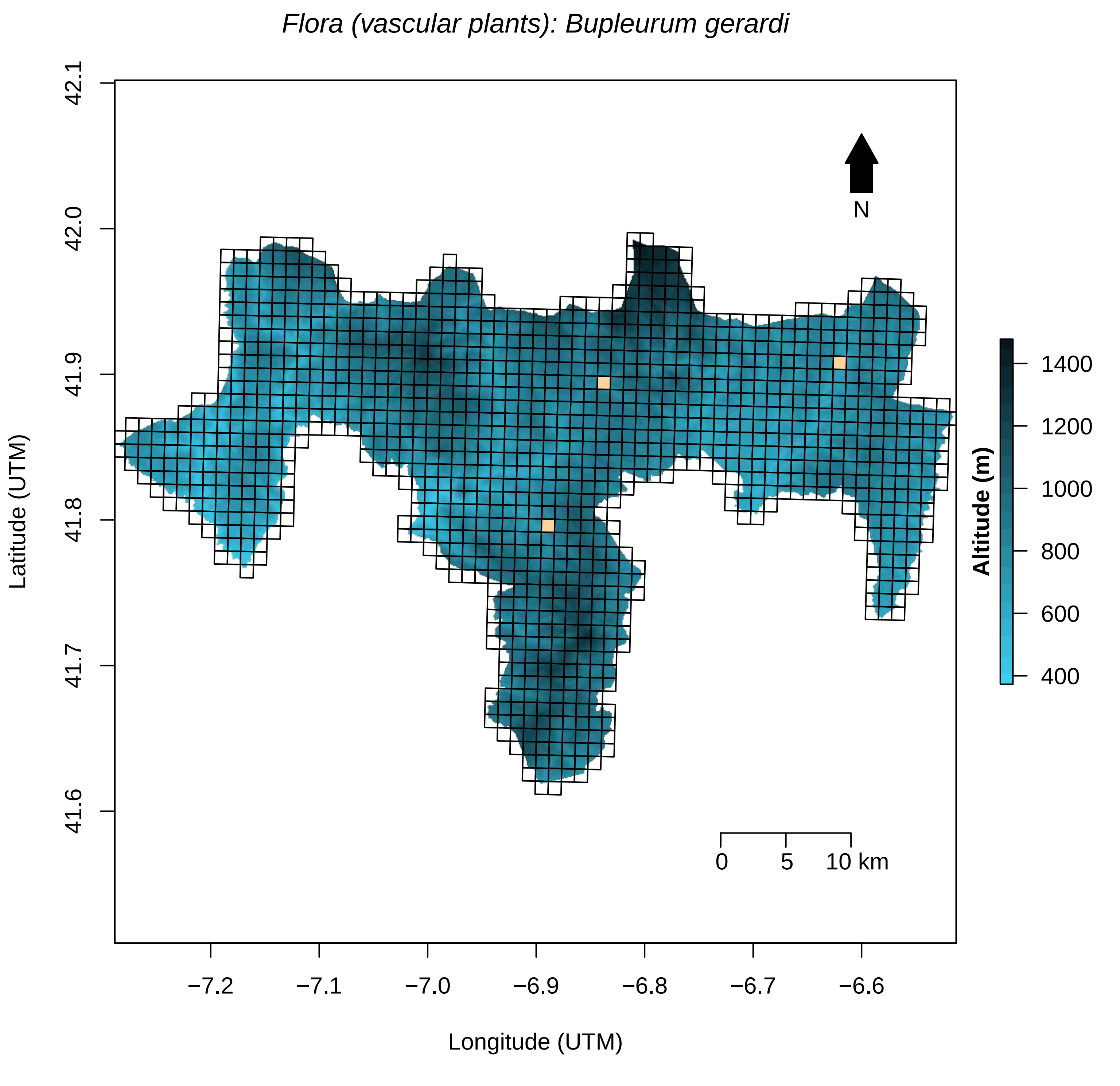

Flora (vascular plants): Bupleurum gerardi
42.1
42.0
41.9
41.8
41.7
41.6
Latitude (UTM)
−7.2
−7.1
−7.0
−6.9
−6.8
−6.7
−6.6
Longitude (UTM)
N
0
5
10 km
Altitude (m)
1400
1200
1000
800
600
400

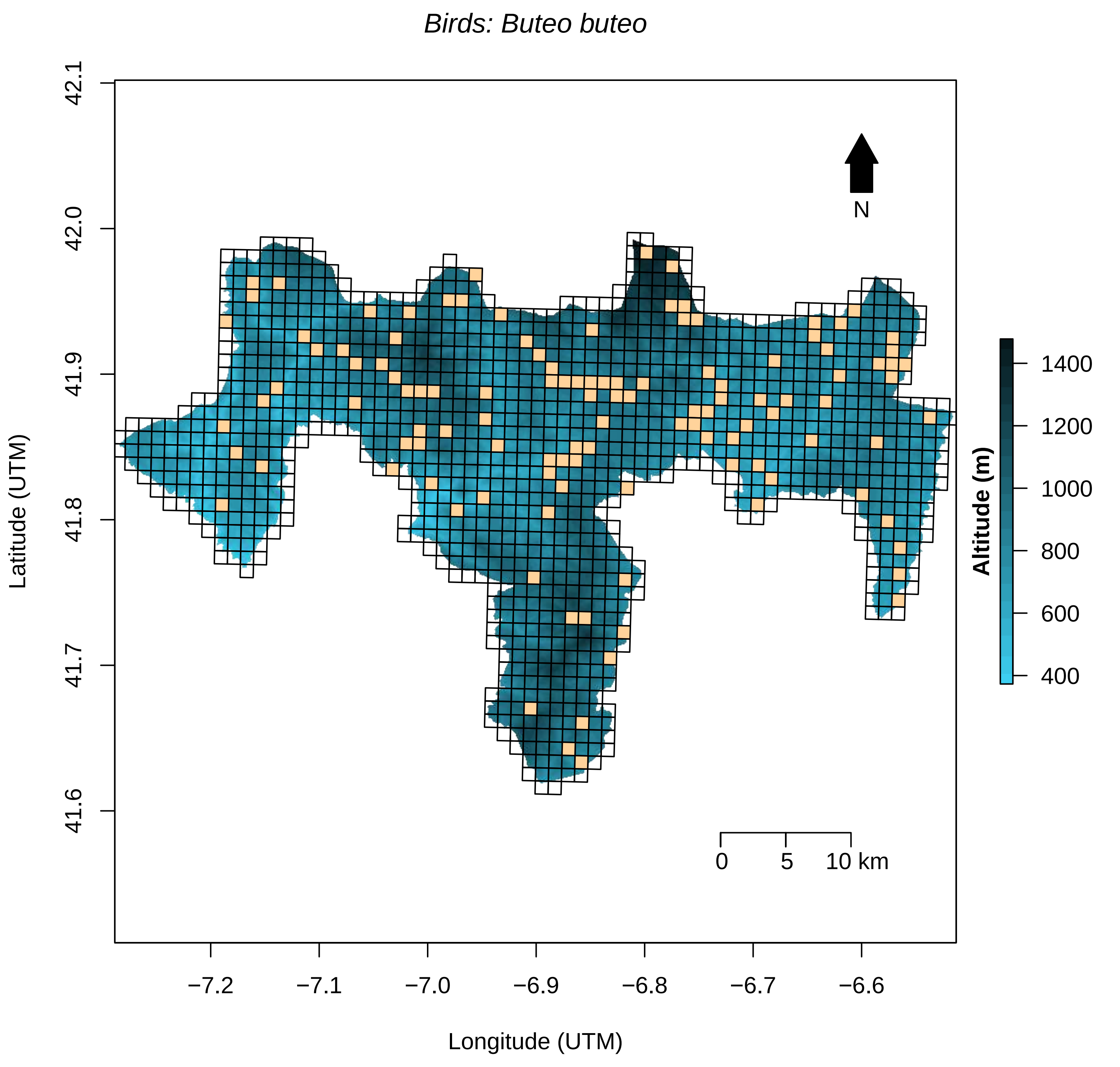

Birds: Buteo buteo
N
0 5 10 km
Altitude (m)
1400
1200
1000
800
600
400
Latitude (UTM)
42.1
42.0
41.9
41.8
41.7
41.6
Longitude (UTM)
−7.2
−7.1
−7.0
−6.9
−6.8
−6.7
−6.6

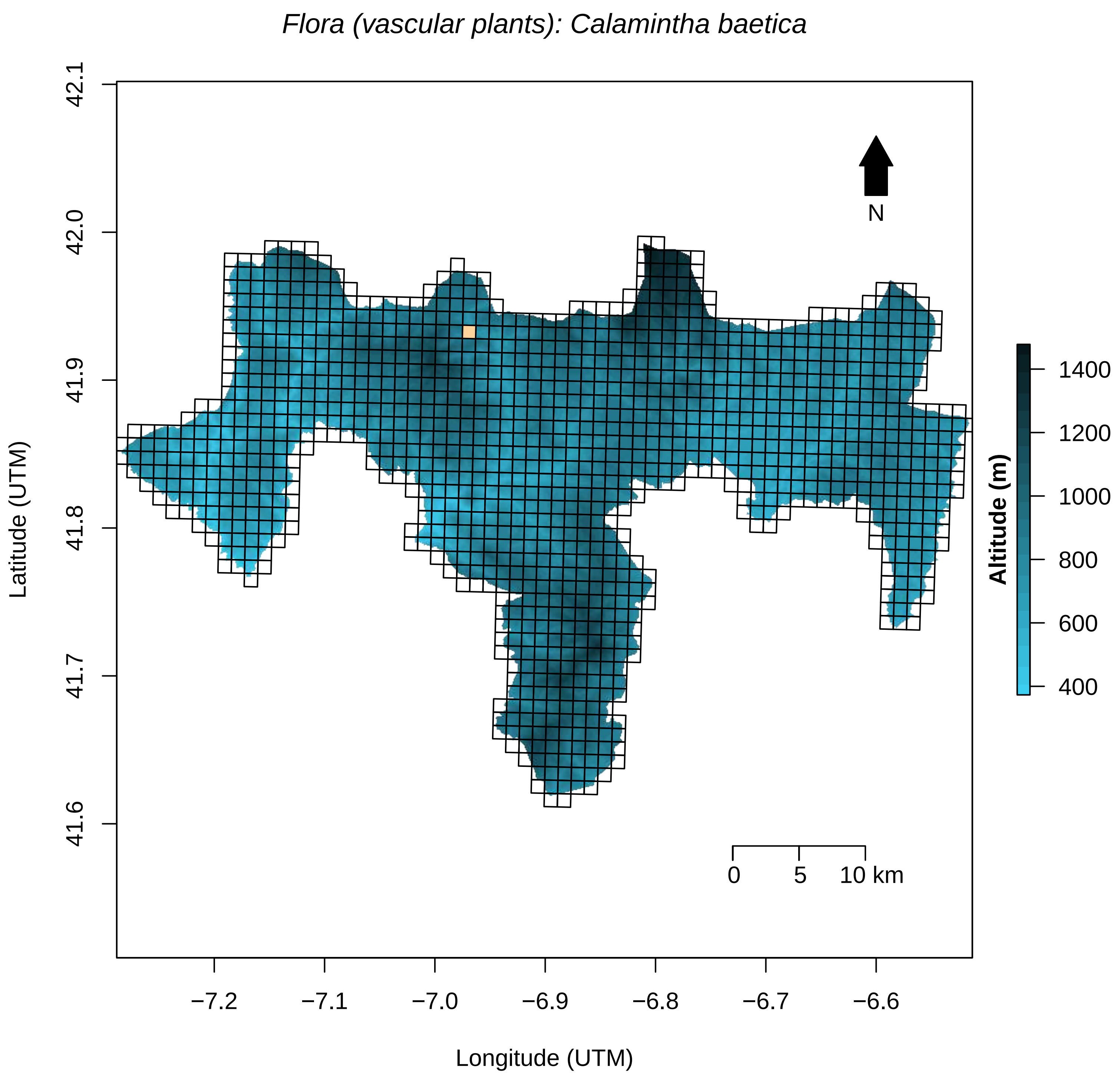
Flora (vascular plants): Calamintha baetica
N
0 5 10 km
Altitude (m)
400
600
800
1000
1200
1400
Latitude (UTM)
41.6
41.7
41.8
41.9
42.0
42.1
Longitude (UTM)
−7.2
−7.1
−7.0
−6.9
−6.8
−6.7
−6.6

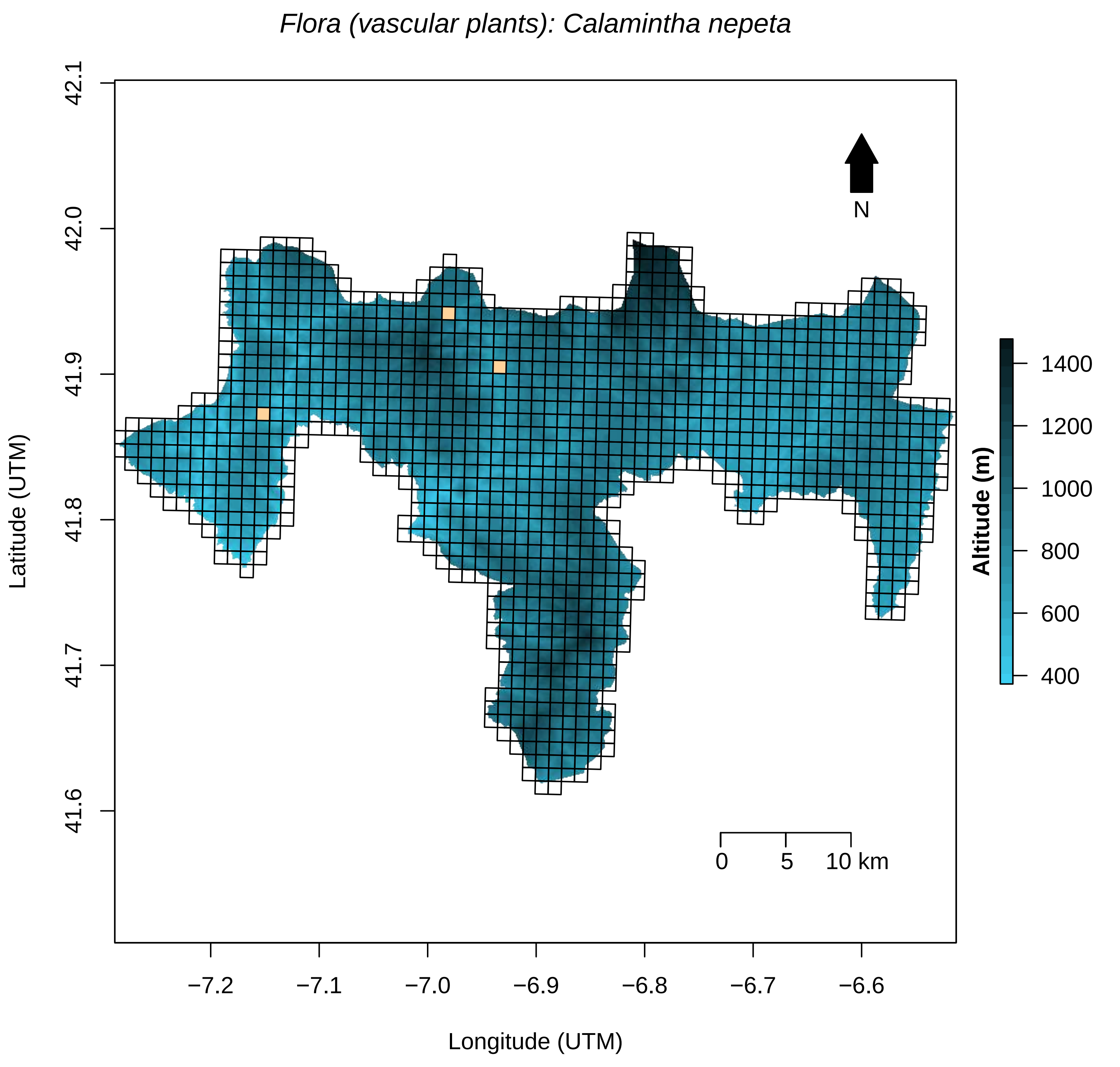

Flora (vascular plants): Calamintha nepeta
42.1
42.0
41.9
41.8
41.7
41.6
Latitude (UTM)
−7.2
−7.1
−7.0
−6.9
−6.8
−6.7
−6.6
Longitude (UTM)
N
0
5
10 km
1400
1200
1000
800
600
400
Altitude (m)

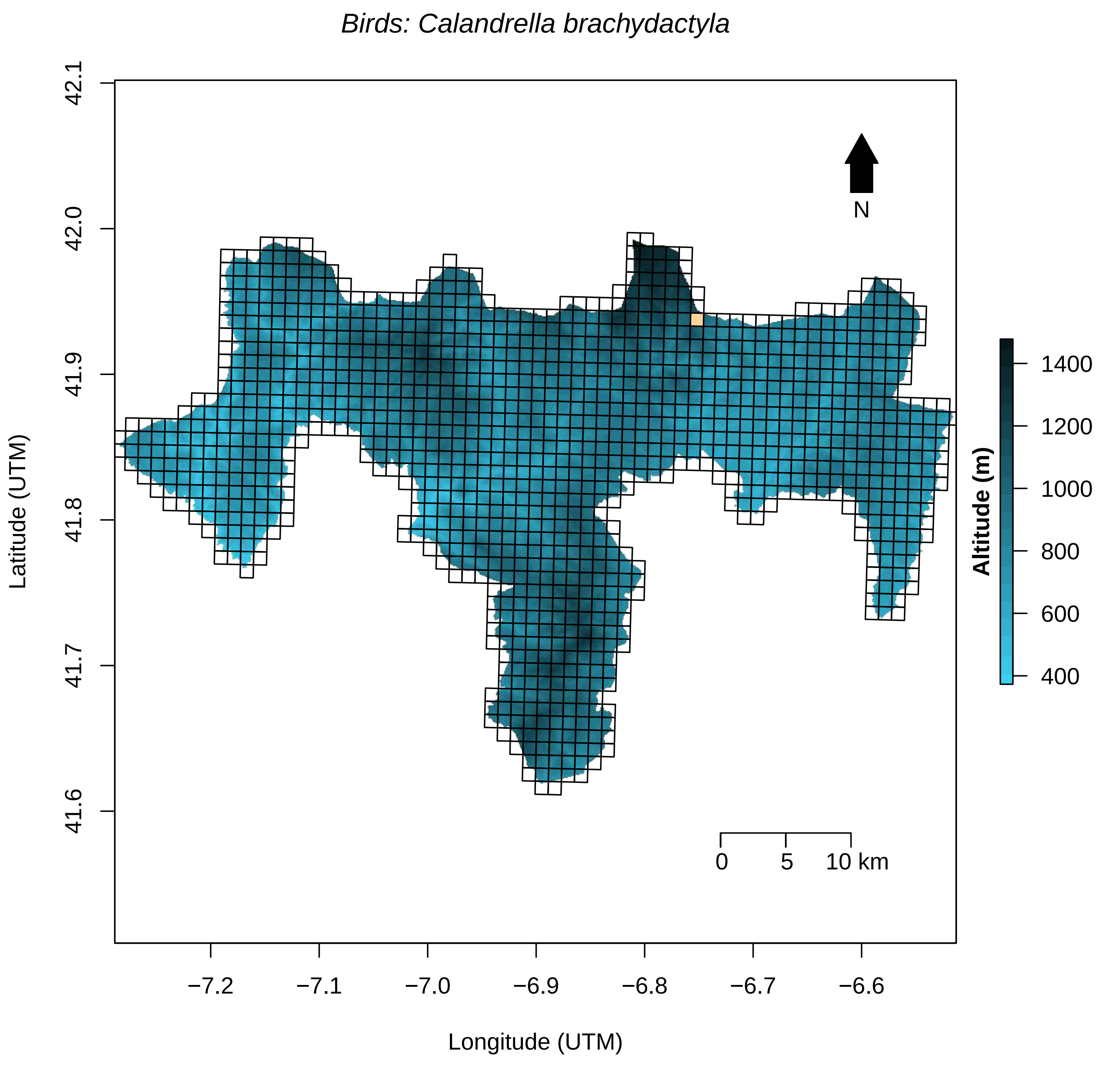

Birds: Calandrella brachydactyla
N
0 5 10 km
Altitude (m)
1400
1200
1000
800
600
400
42.1
42.0
41.9
41.8
41.7
41.6
Latitude (UTM)
−7.2
−7.1
−7.0
−6.9
−6.8
−6.7
−6.6
Longitude (UTM)

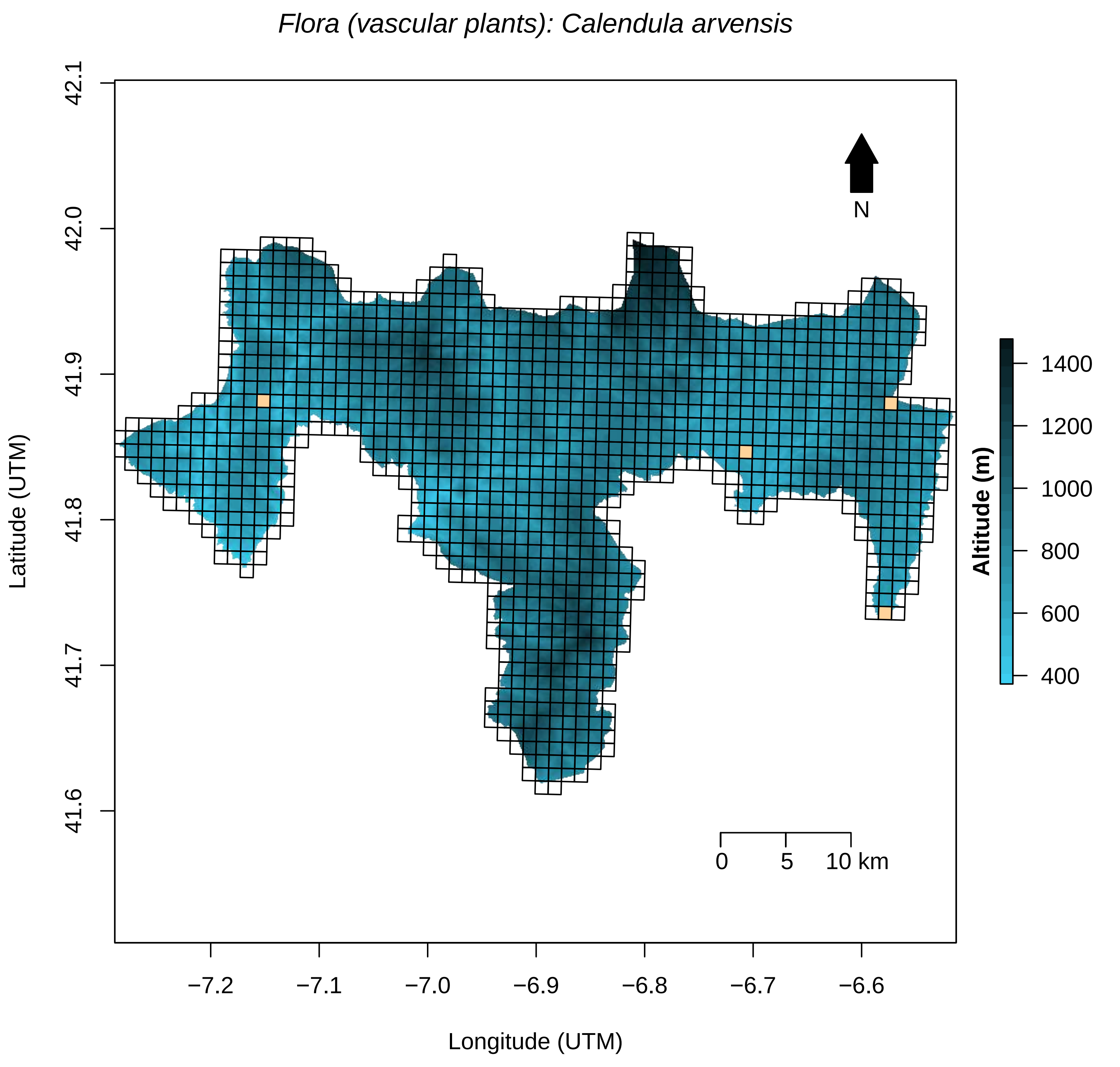
Flora (vascular plants): Calendula arvensis
42.1
42.0
41.9
41.8
41.7
41.6
Latitude (UTM)
N
0
5
10 km
−7.2
−7.1
−7.0
−6.9
−6.8
−6.7
−6.6
Longitude (UTM)
Altitude (m)
1400
1200
1000
800
600
400

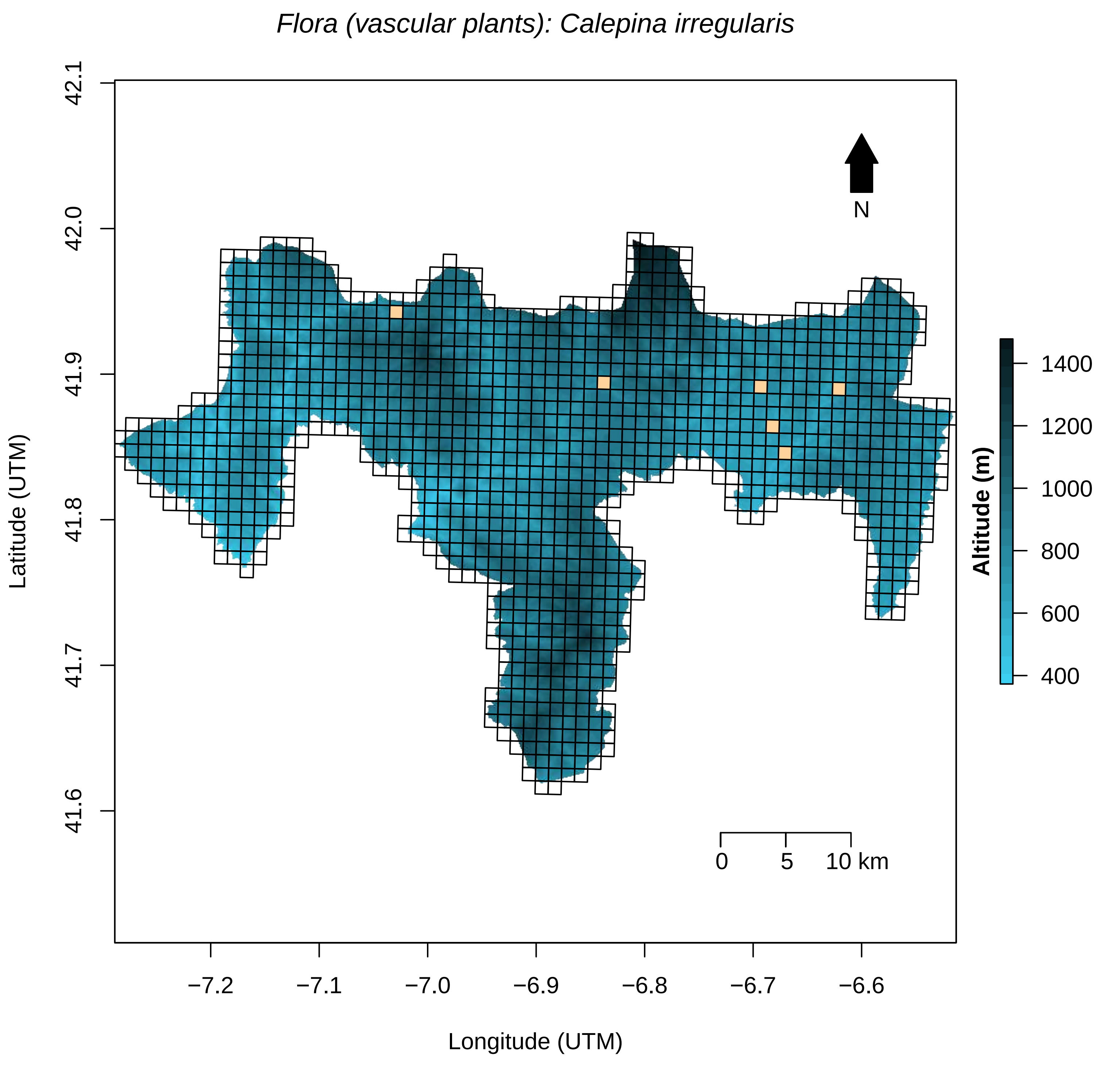

Flora (vascular plants): Calepina irregularis
N
0
5
10 km
Latitude (UTM)
Longitude (UTM)
42.1
42.0
41.9
41.8
41.7
41.6
−7.2
−7.1
−7.0
−6.9
−6.8
−6.7
−6.6
Altitude (m)
1400
1200
1000
800
600
400

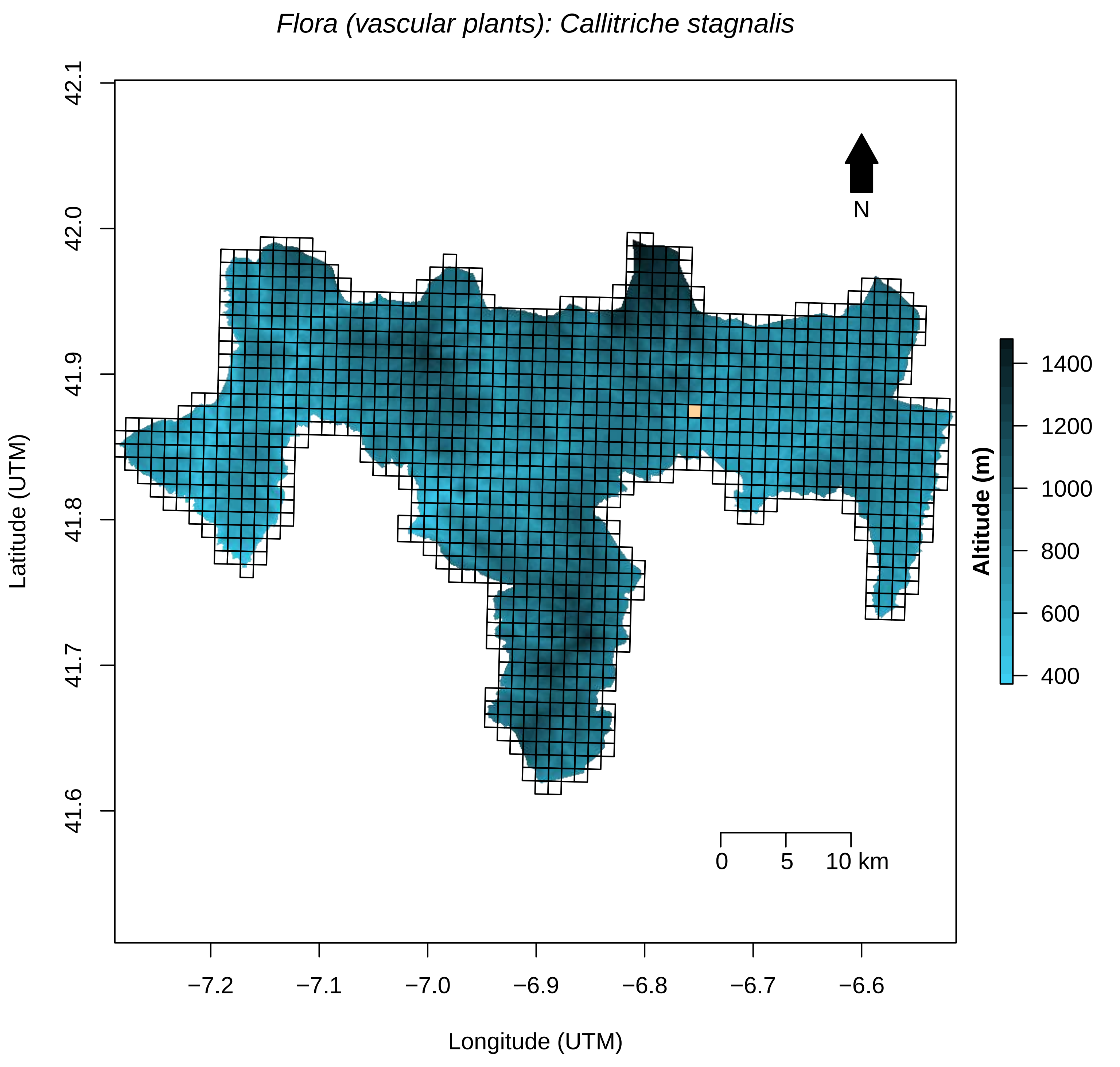
Flora (vascular plants): Callitriche stagnalis
42.1
42.0
41.9
41.8
41.7
41.6
Latitude (UTM)
−7.2
−7.1
−7.0
−6.9
−6.8
−6.7
−6.6
Longitude (UTM)
N
0
5
10 km
Altitude (m)
1400
1200
1000
800
600
400

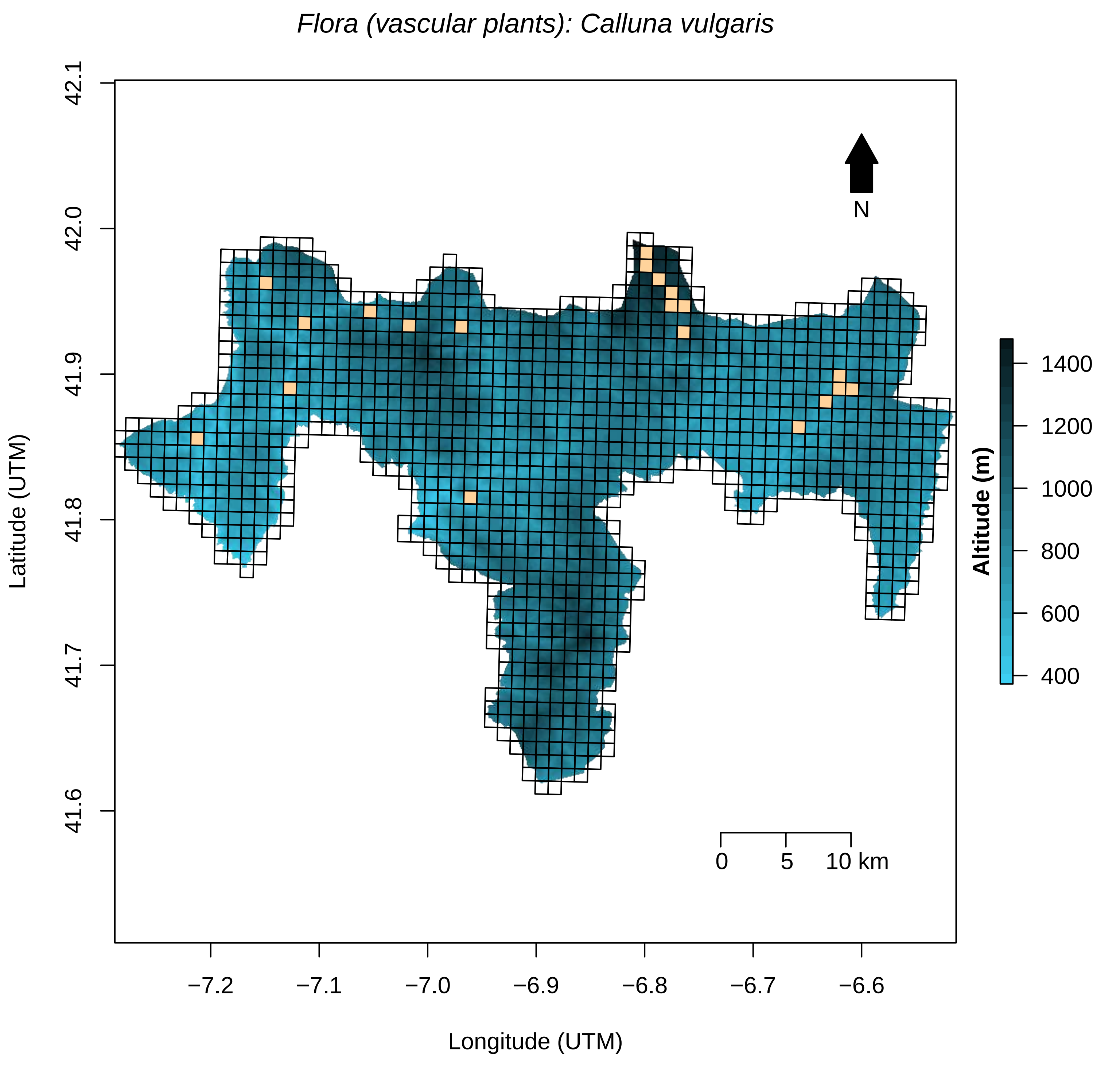

Flora (vascular plants): Calluna vulgaris
42.1
42.0
41.9
41.8
41.7
41.6
Latitude (UTM)
−7.2
−7.1
−7.0
−6.9
−6.8
−6.7
−6.6
Longitude (UTM)
N
0
5
10 km
Altitude (m)
1400
1200
1000
800
600
400

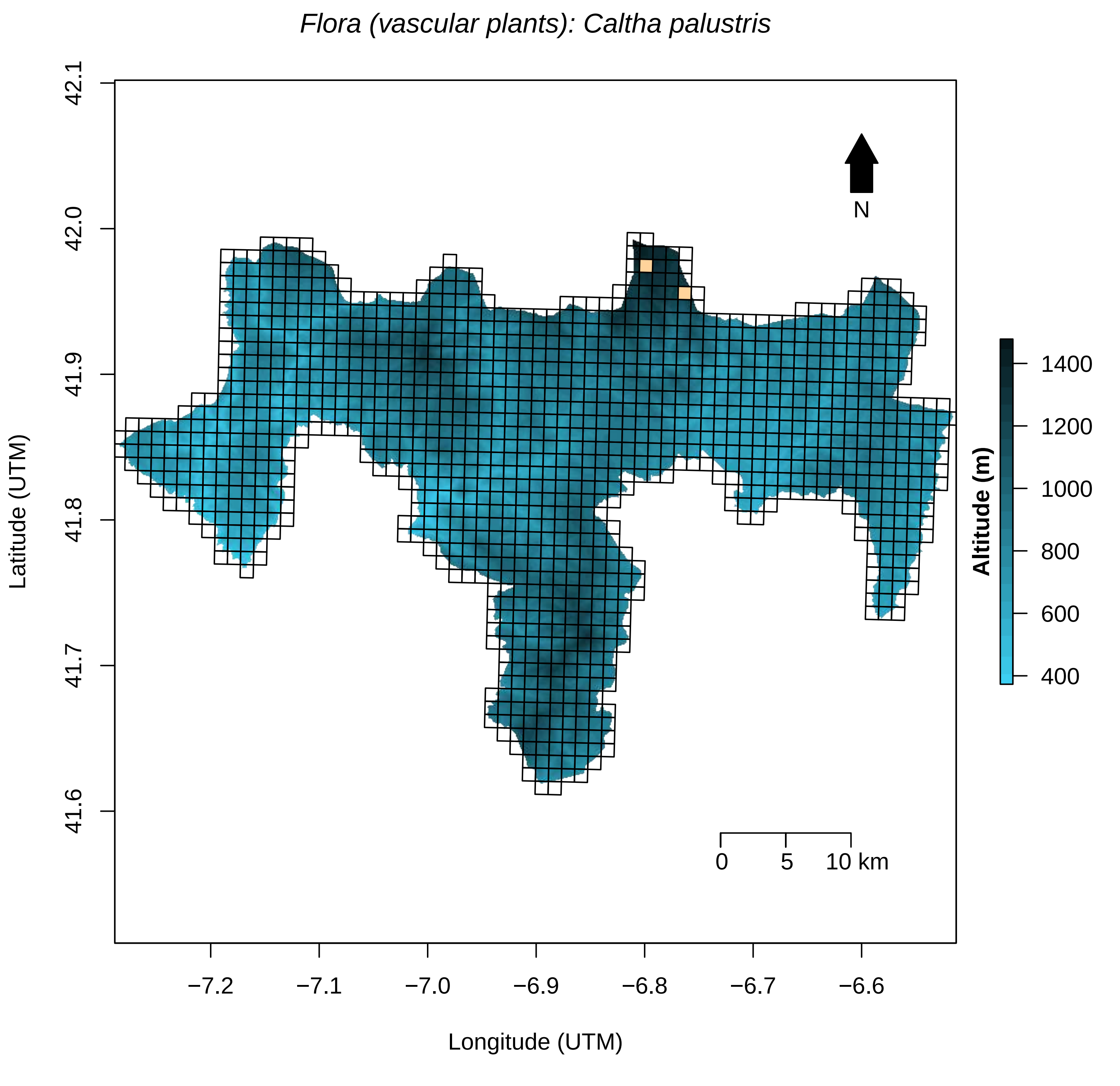

Flora (vascular plants): Caltha palustris
42.1
42.0
41.9
41.8
41.7
41.6
Latitude (UTM)
−7.2
−7.1
−7.0
−6.9
−6.8
−6.7
−6.6
Longitude (UTM)
N
0
5
10 km
1400
1200
1000
800
600
400
Altitude (m)

Flora (vascular plants): Calystegia silvatica

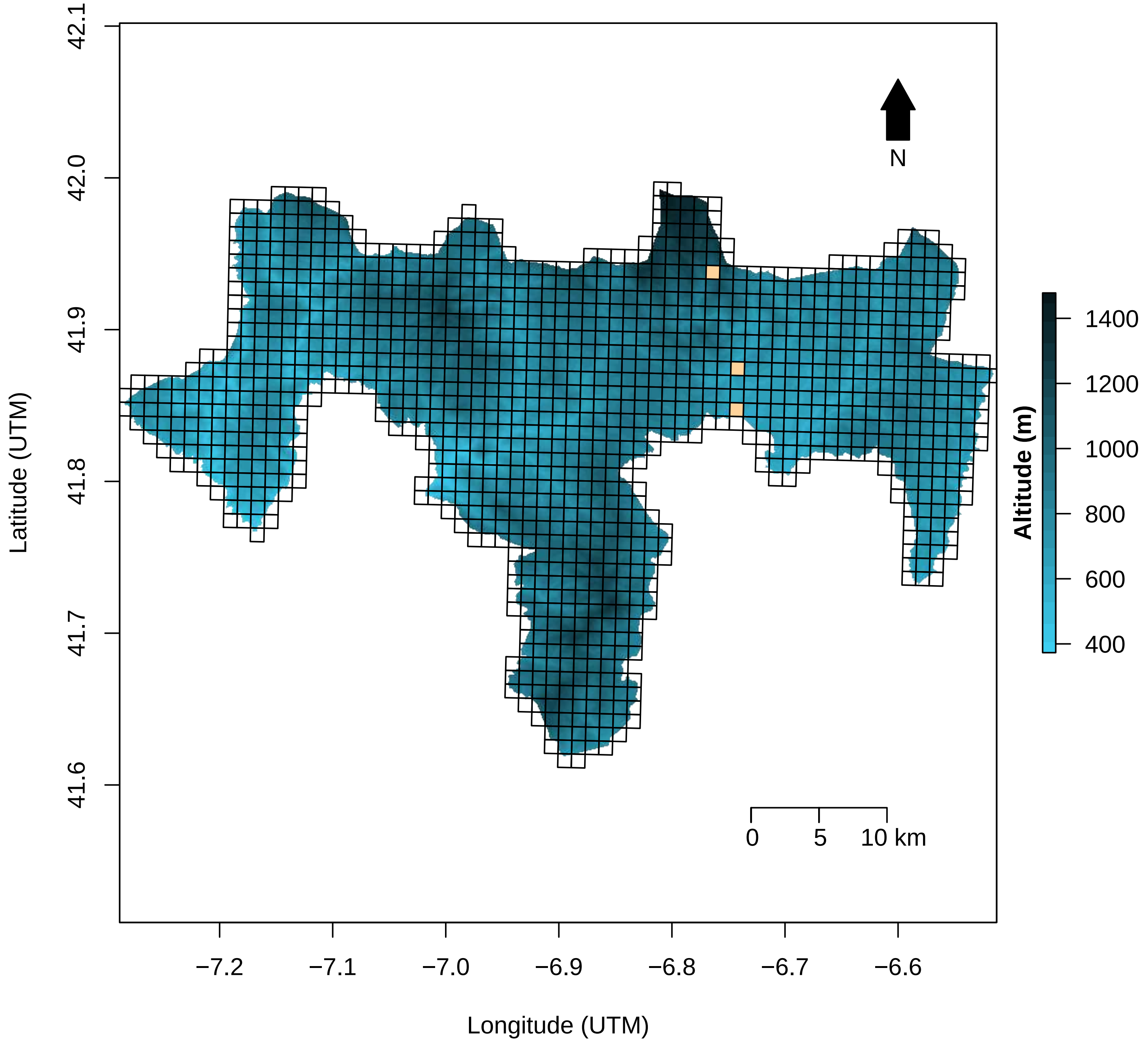

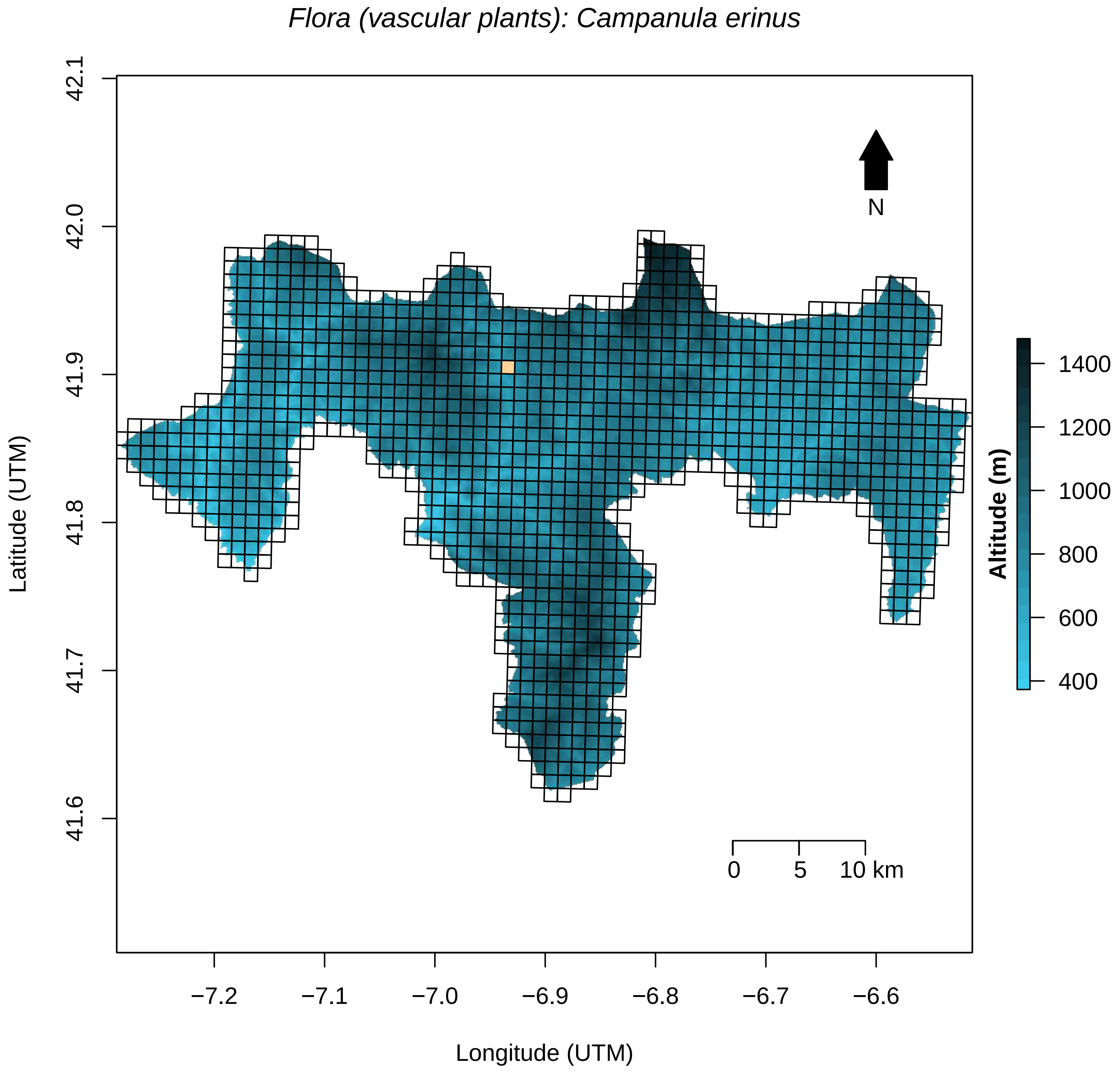
Flora (vascular plants): Campanula erinus
Latitude (UTM)
42.1
42.0
41.9
41.8
41.7
41.6
Longitude (UTM)
−7.2
−7.1
−7.0
−6.9
−6.8
−6.7
−6.6
N
0
5
10 km
Altitude (m)
1400
1200
1000
800
600
400

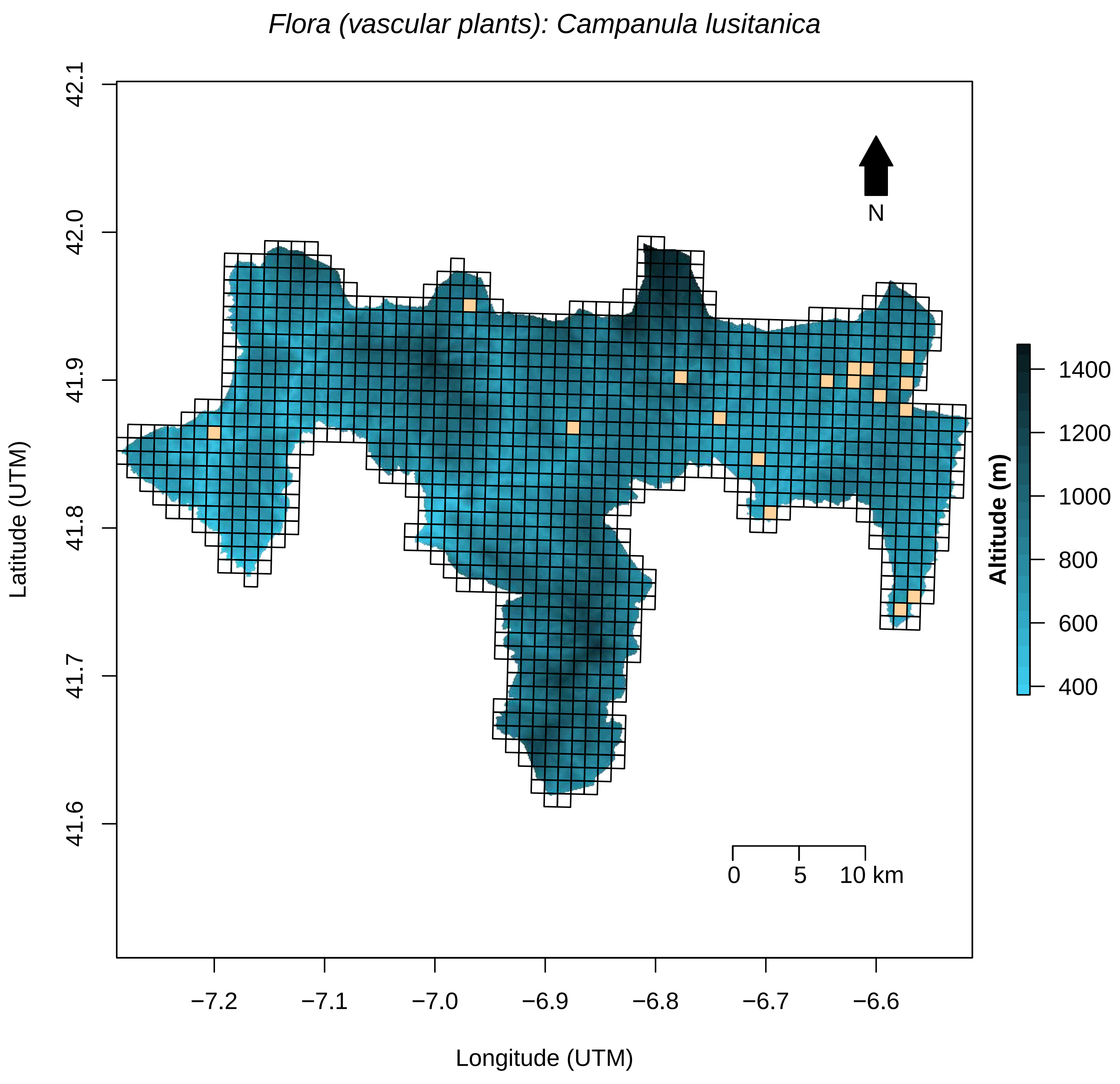

Flora (vascular plants): Campanula lusitanica
N
0
5
10 km
Latitude (UTM)
Longitude (UTM)
Altitude (m)
42.1
42.0
41.9
41.8
41.7
41.6
−7.2
−7.1
−7.0
−6.9
−6.8
−6.7
−6.6
1400
1200
1000
800
600
400

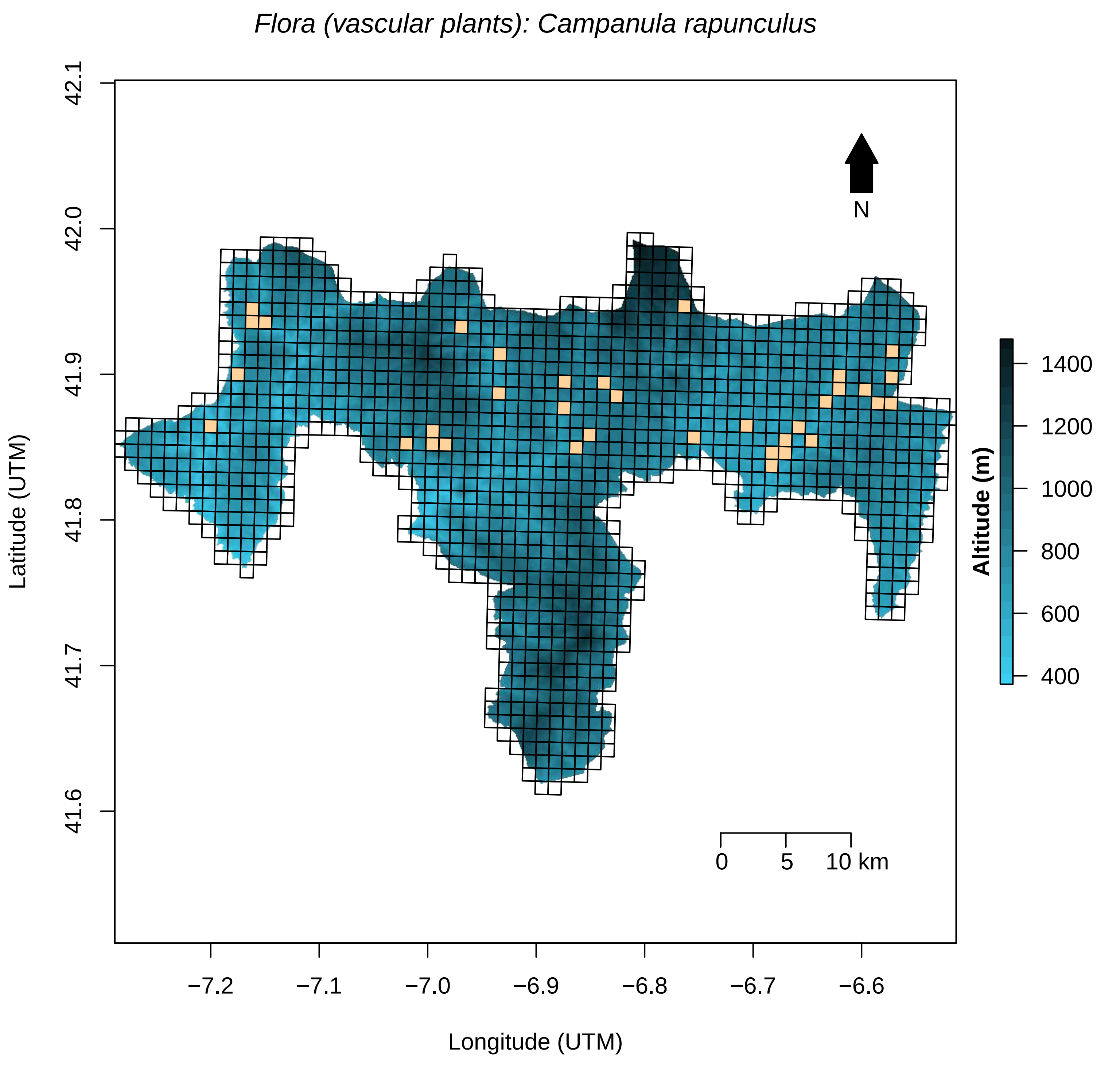
Flora (vascular plants): Campanula rapunculus
42.1
42.0
41.9
41.8
41.7
41.6
Latitude (UTM)
−7.2
−7.1
−7.0
−6.9
−6.8
−6.7
−6.6
Longitude (UTM)
N
0
5
10 km
1400
1200
1000
800
600
400
Altitude (m)

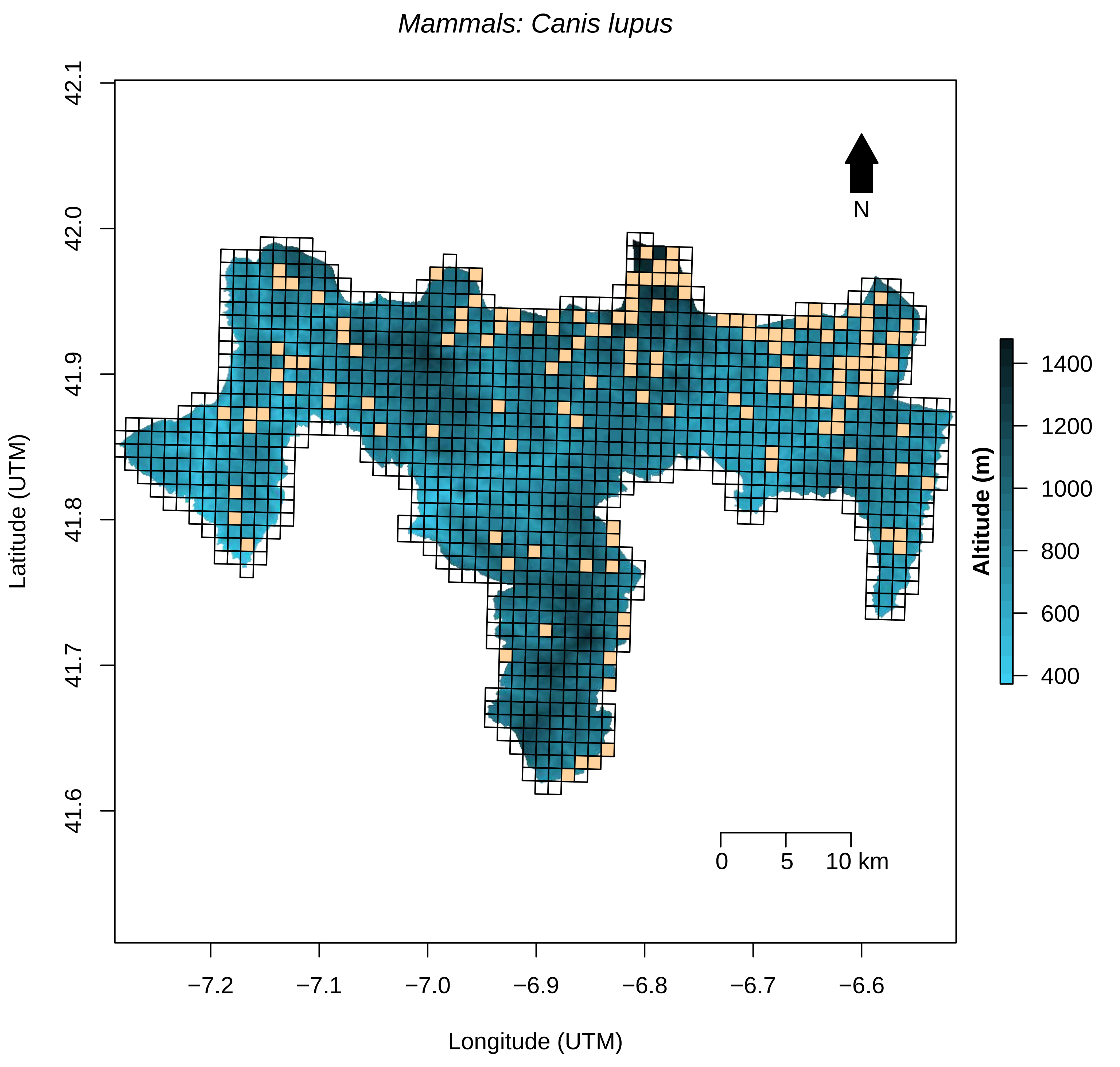

Mammals: Canis lupus
N
0 5 10 km
Altitude (m)
1400
1200
1000
800
600
400
Latitude (UTM)
42.1
42.0
41.9
41.8
41.7
41.6
Longitude (UTM)
−7.2
−7.1
−7.0
−6.9
−6.8
−6.7
−6.6

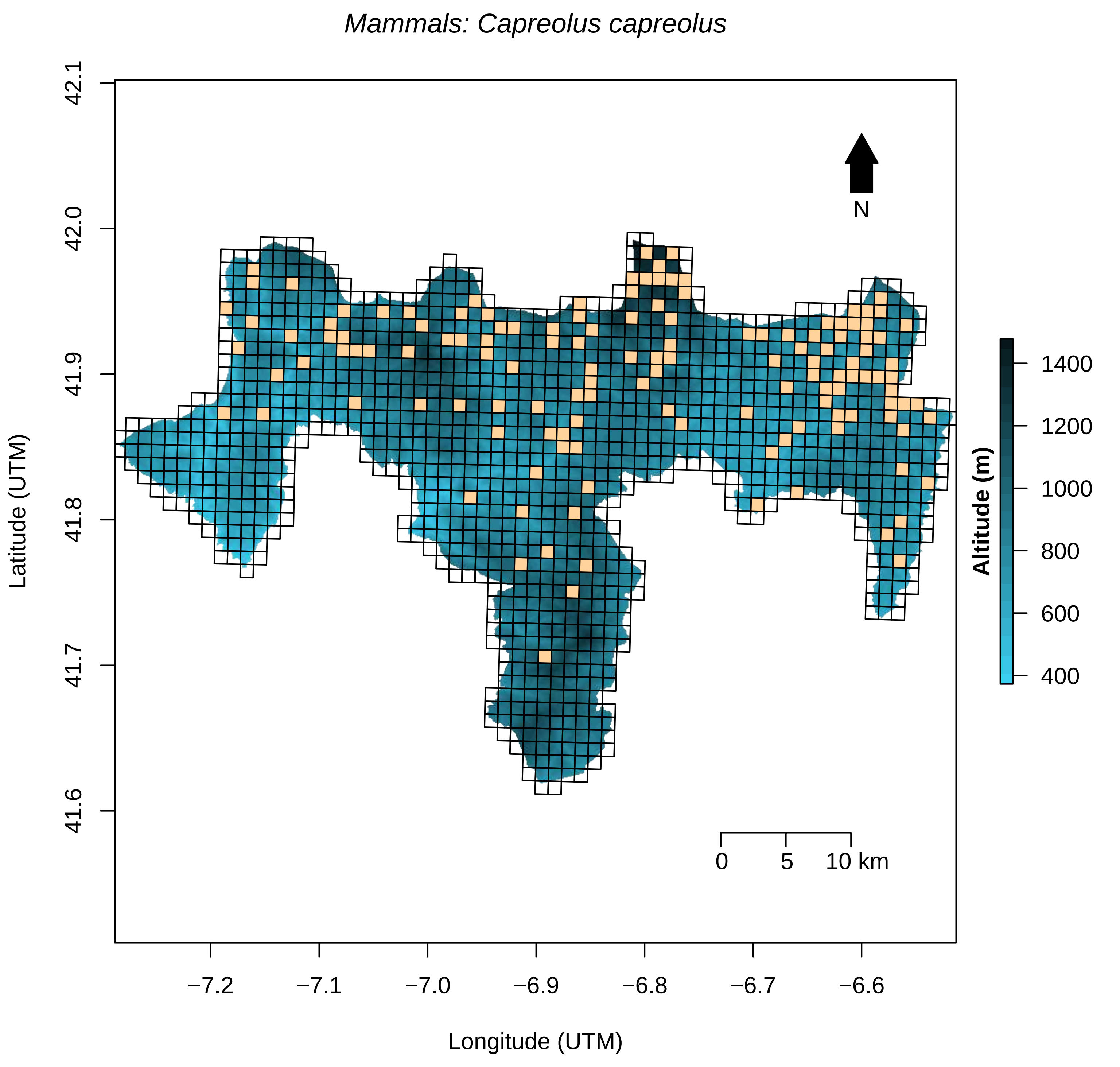

Mammals: Capreolus capreolus
N
0 5 10 km
Altitude (m)
400
600
800
1000
1200
1400
Latitude (UTM)
42.1
42.0
41.9
41.8
41.7
41.6
Longitude (UTM)
−7.2
−7.1
−7.0
−6.9
−6.8
−6.7
−6.6

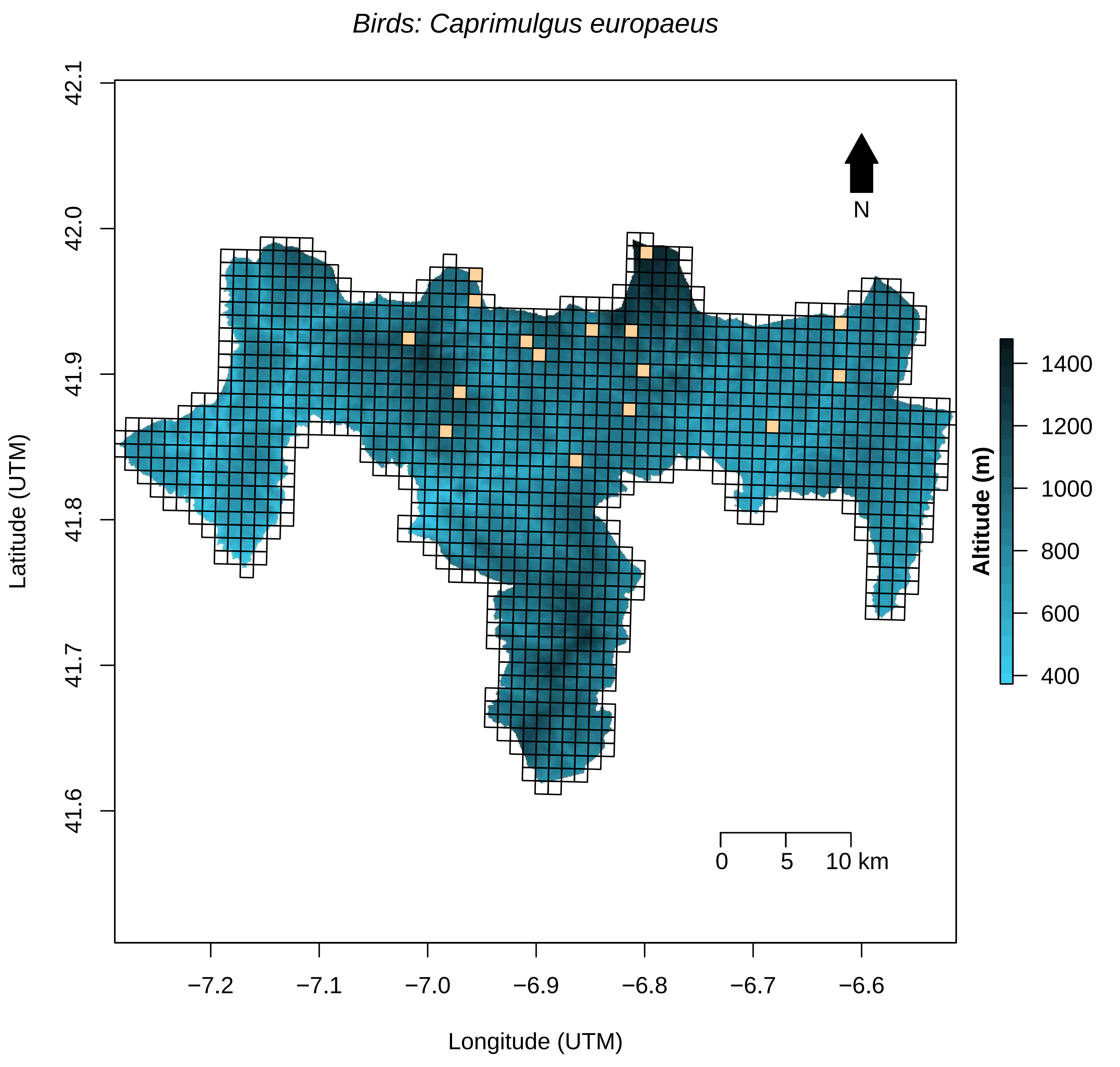

Birds: Caprimulgus europaeus
N
0
5
10 km
Altitude (m)
1400
1200
1000
800
600
400
Latitude (UTM)
42.1
42.0
41.9
41.8
41.7
41.6
Longitude (UTM)
−7.2
−7.1
−7.0
−6.9
−6.8
−6.7
−6.6

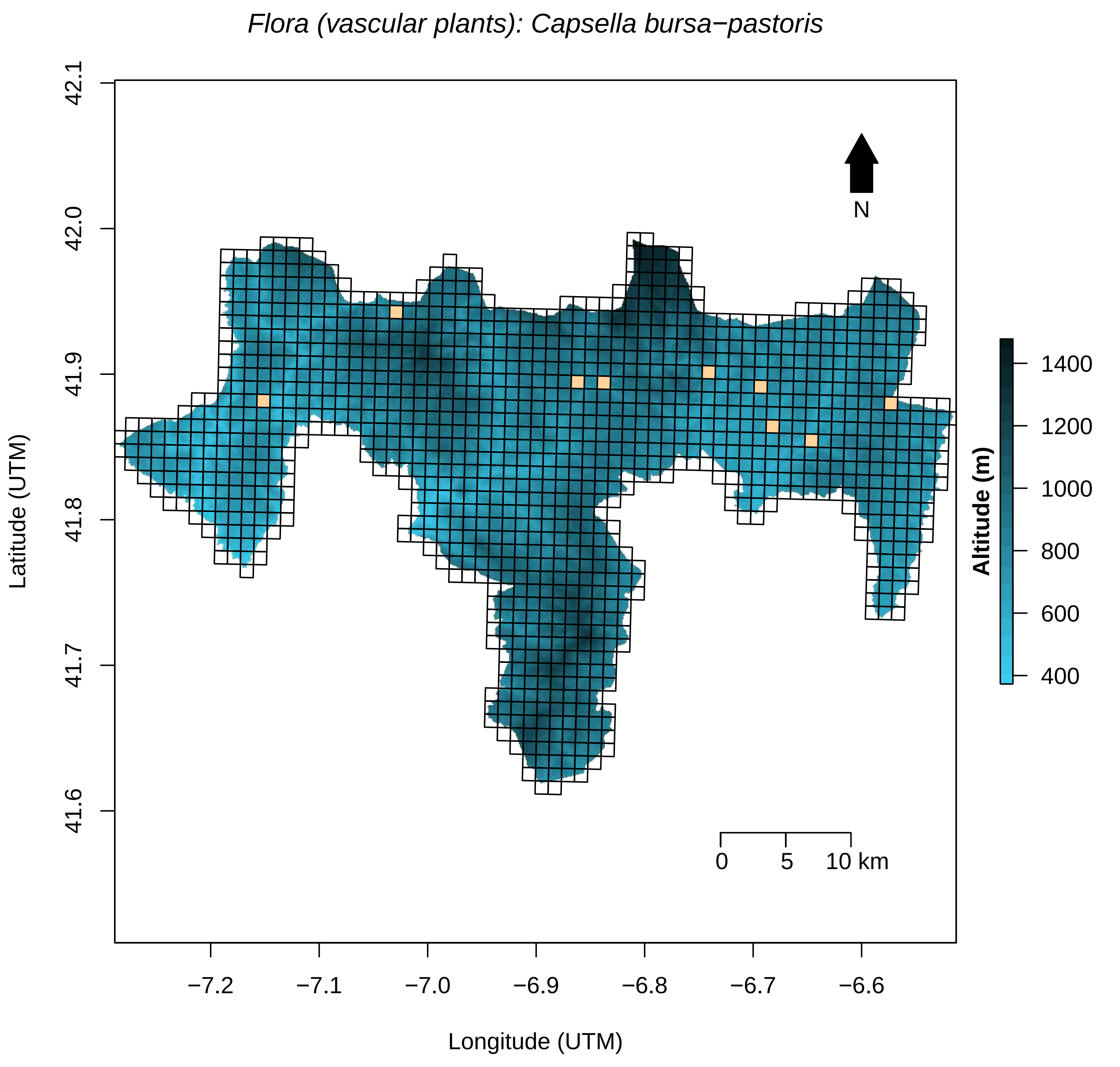

Flora (vascular plants): Capsella bursa−pastoris
42.1
42.0
41.9
41.8
41.7
41.6
Latitude (UTM)
−7.2
−7.1
−7.0
−6.9
−6.8
−6.7
−6.6
Longitude (UTM)
N
0
5
10 km
Altitude (m)
1400
1200
1000
800
600
400

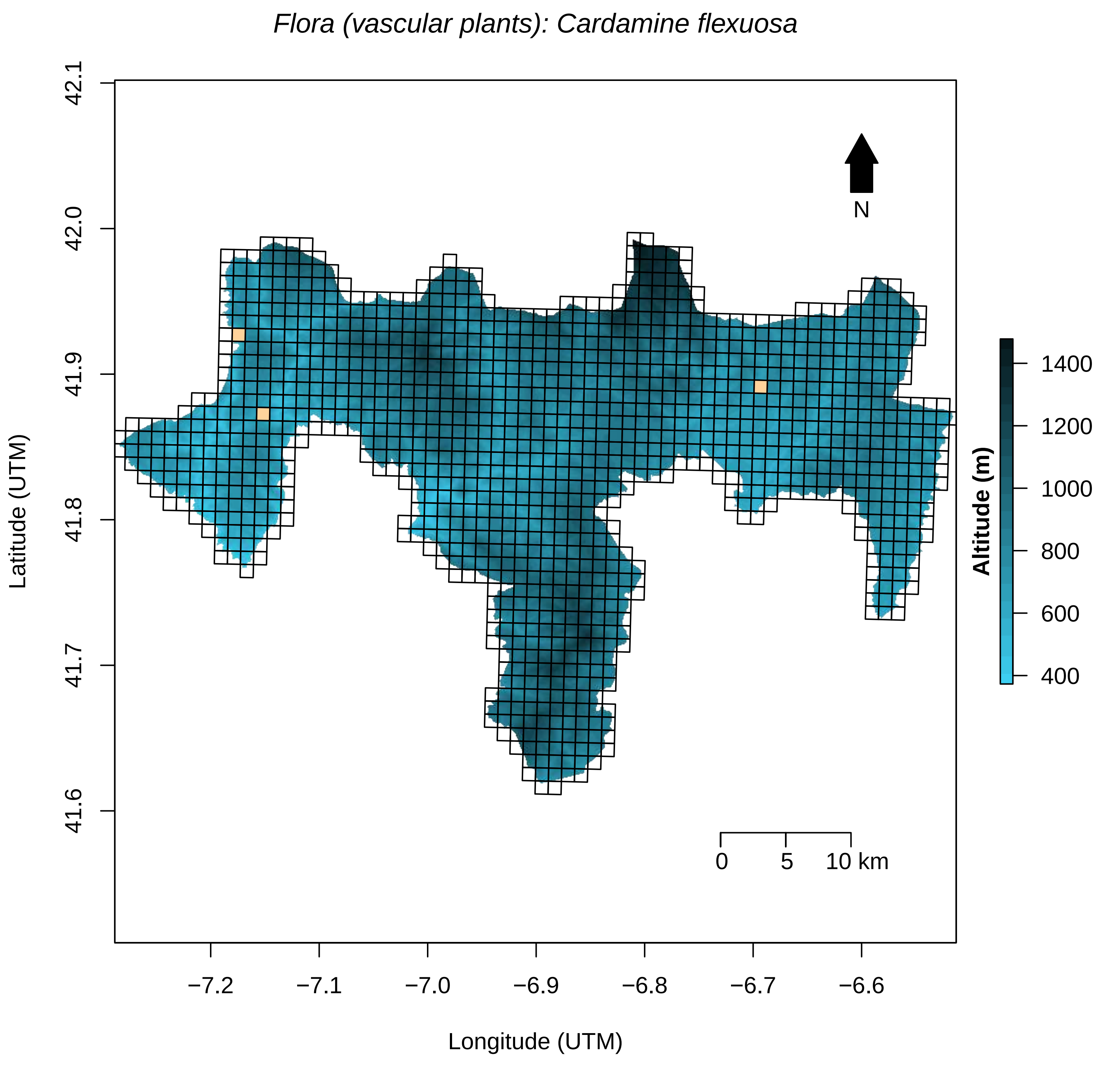

Flora (vascular plants): Cardamine flexuosa
N
0
5
10 km
Altitude (m)
1400
1200
1000
800
600
400
Latitude (UTM)
42.1
42.0
41.9
41.8
41.7
41.6
Longitude (UTM)
−7.2
−7.1
−7.0
−6.9
−6.8
−6.7
−6.6

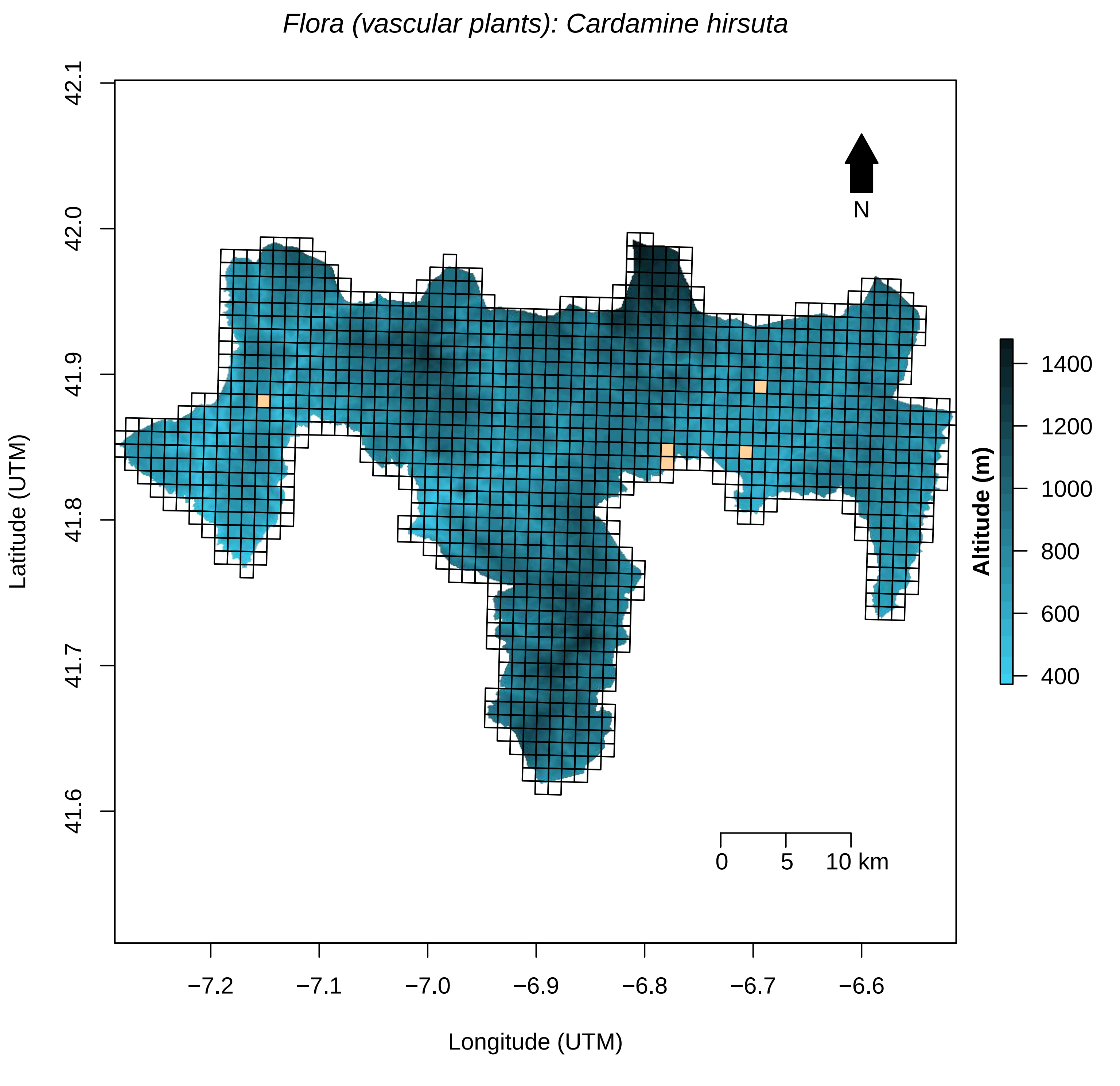

Flora (vascular plants): Cardamine hirsuta
42.1
42.0
41.9
41.8
41.7
41.6
Latitude (UTM)
−7.2
−7.1
−7.0
−6.9
−6.8
−6.7
−6.6
Longitude (UTM)
N
0
5
10 km
1400
1200
1000
800
600
400
Altitude (m)

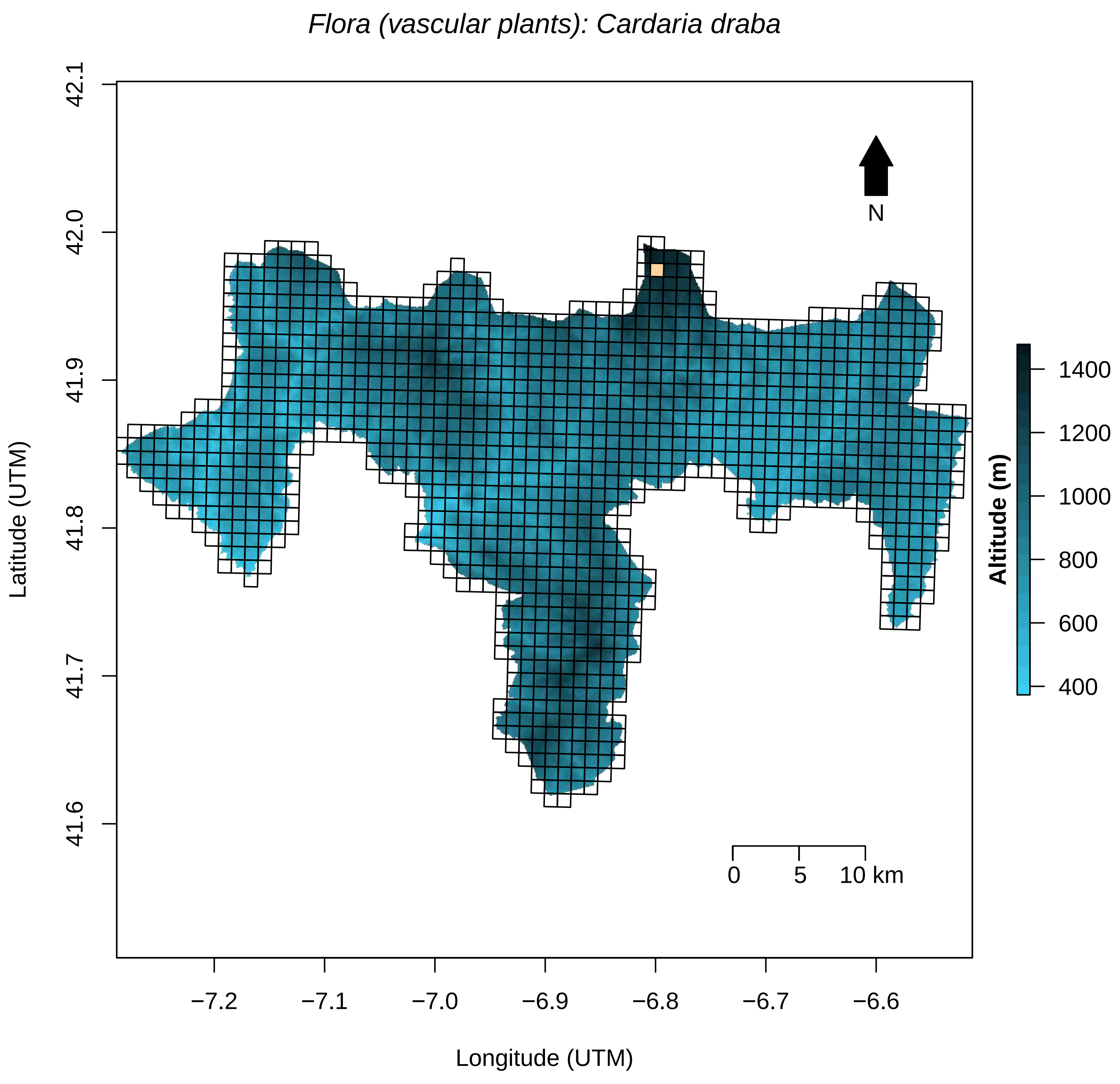

Flora (vascular plants): Cardaria draba
42.1
42.0
41.9
41.8
41.7
41.6
Latitude (UTM)
−7.2
−7.1
−7.0
−6.9
−6.8
−6.7
−6.6
Longitude (UTM)
N
0
5
10 km
Altitude (m)
1400
1200
1000
800
600
400

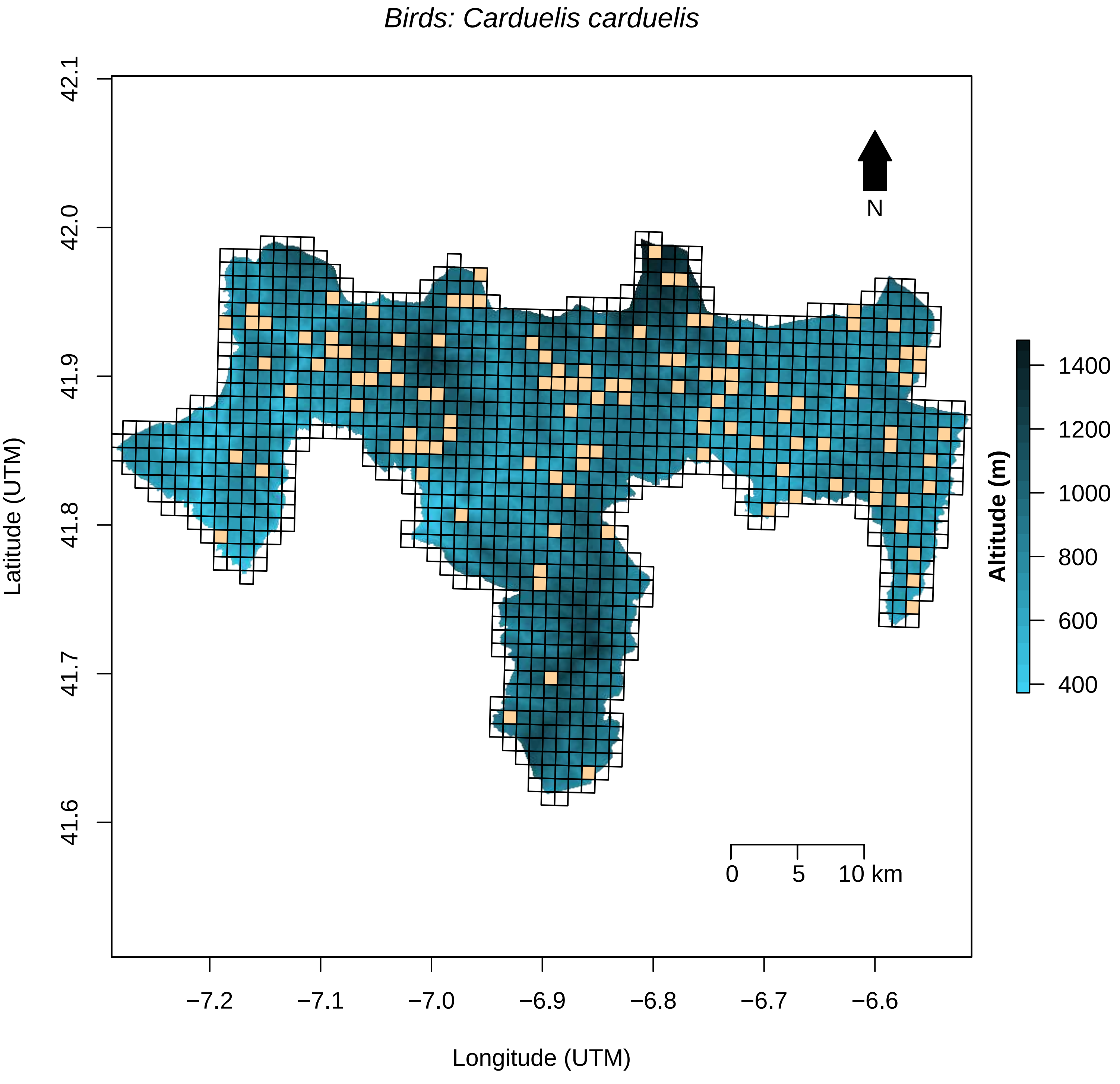

Birds: Carduelis carduelis
Latitude (UTM)
42.1
42.0
41.9
41.8
41.7
41.6
Longitude (UTM)
−7.2
−7.1
−7.0
−6.9
−6.8
−6.7
−6.6
N
0
5
10 km
Altitude (m)
1400
1200
1000
800
600
400

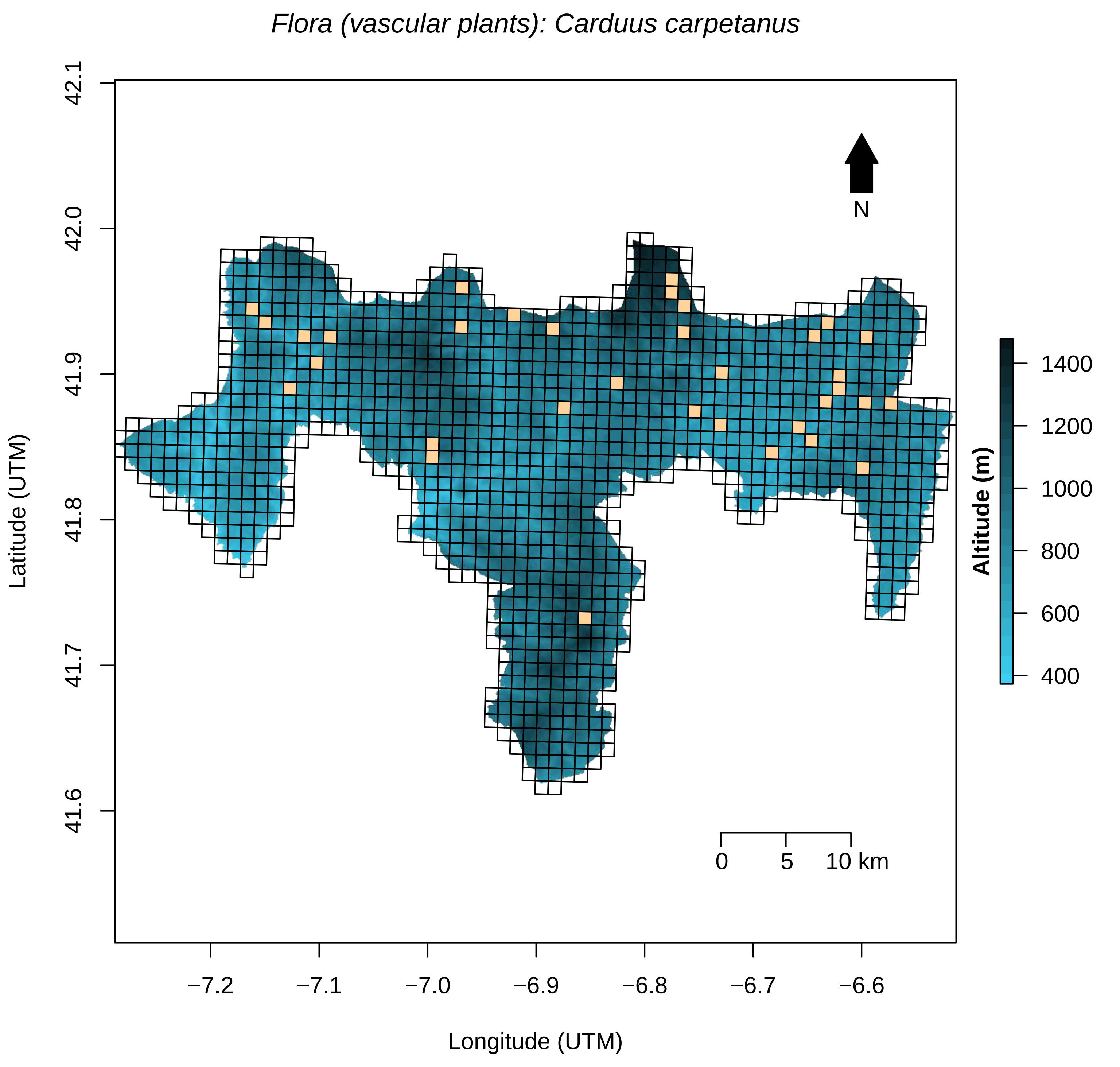

Flora (vascular plants): Carduus carpetanus
N
0 5 10 km
Altitude (m)
1400
1200
1000
800
600
400
Latitude (UTM)
42.1
42.0
41.9
41.8
41.7
41.6
Longitude (UTM)
−7.2
−7.1
−7.0
−6.9
−6.8
−6.7
−6.6

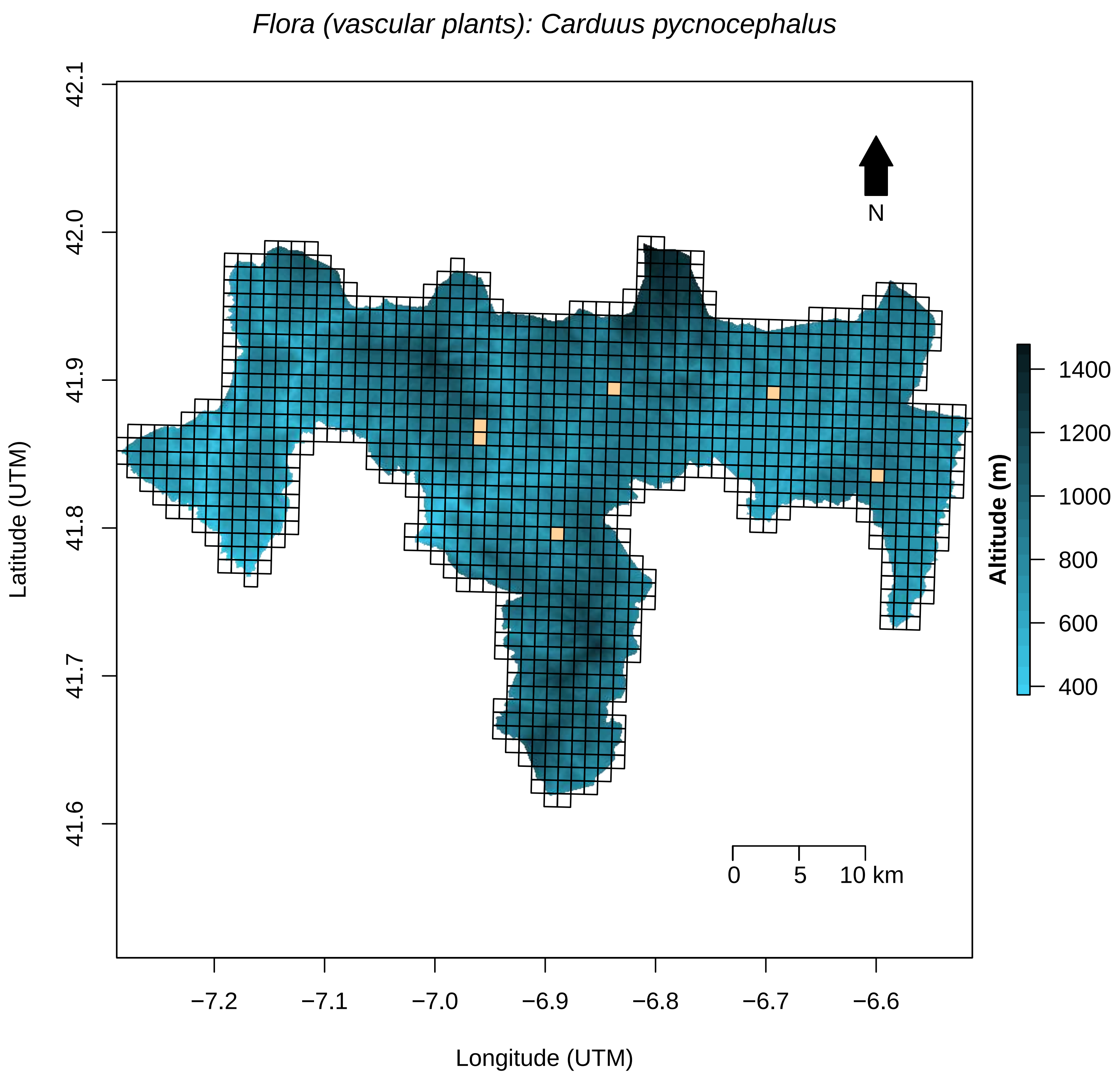

Flora (vascular plants): Carduus pycnocephalus
42.1
42.0
41.9
41.8
41.7
41.6
Latitude (UTM)
−7.2
−7.1
−7.0
−6.9
−6.8
−6.7
−6.6
Longitude (UTM)
N
0
5
10 km
Altitude (m)
1400
1200
1000
800
600
400

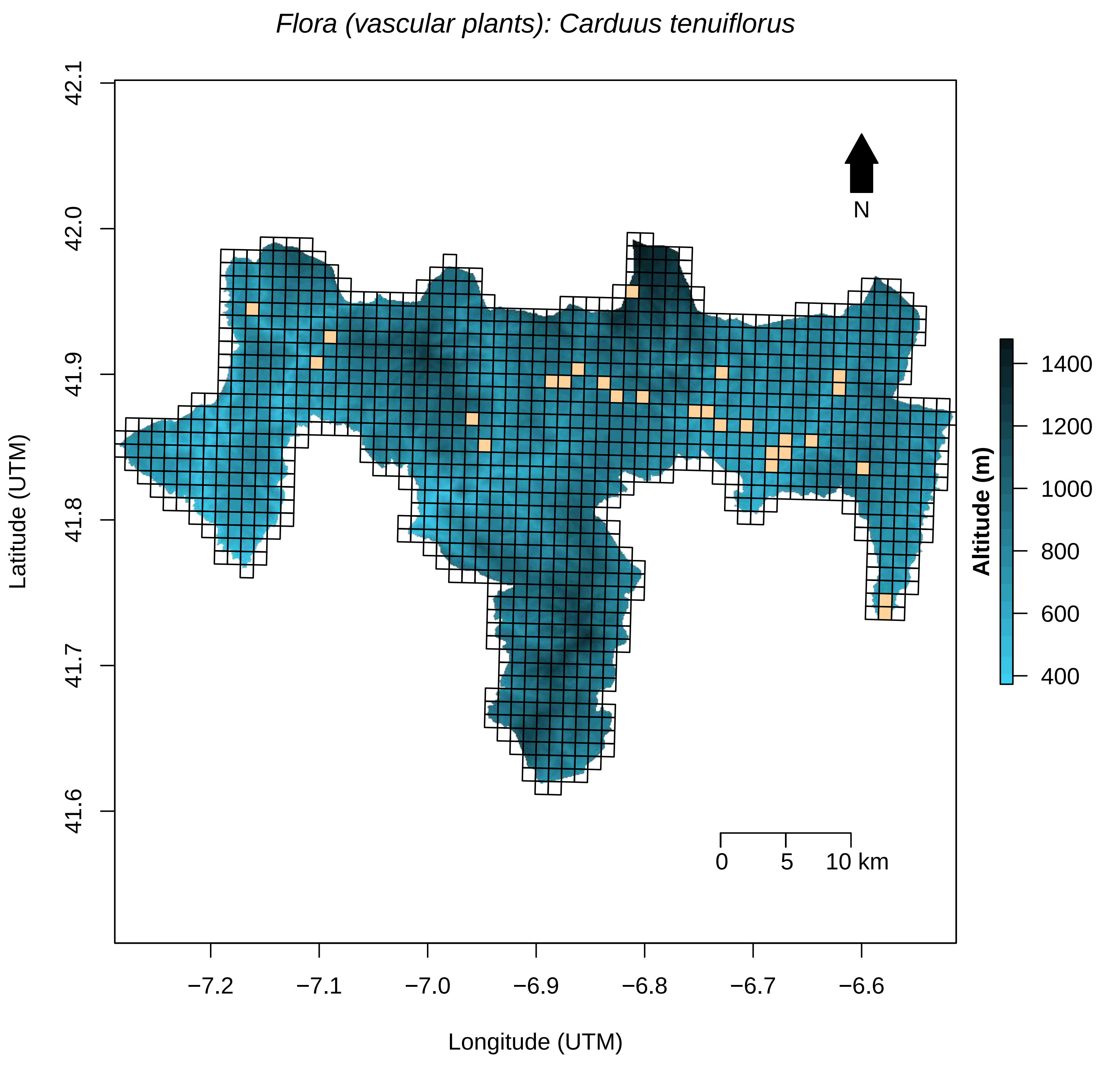

Flora (vascular plants): Carduus tenuiflorus
42.1
42.0
41.9
41.8
41.7
41.6
Latitude (UTM)
−7.2
−7.1
−7.0
−6.9
−6.8
−6.7
−6.6
Longitude (UTM)
N
0
5
10 km
Altitude (m)
1400
1200
1000
800
600
400

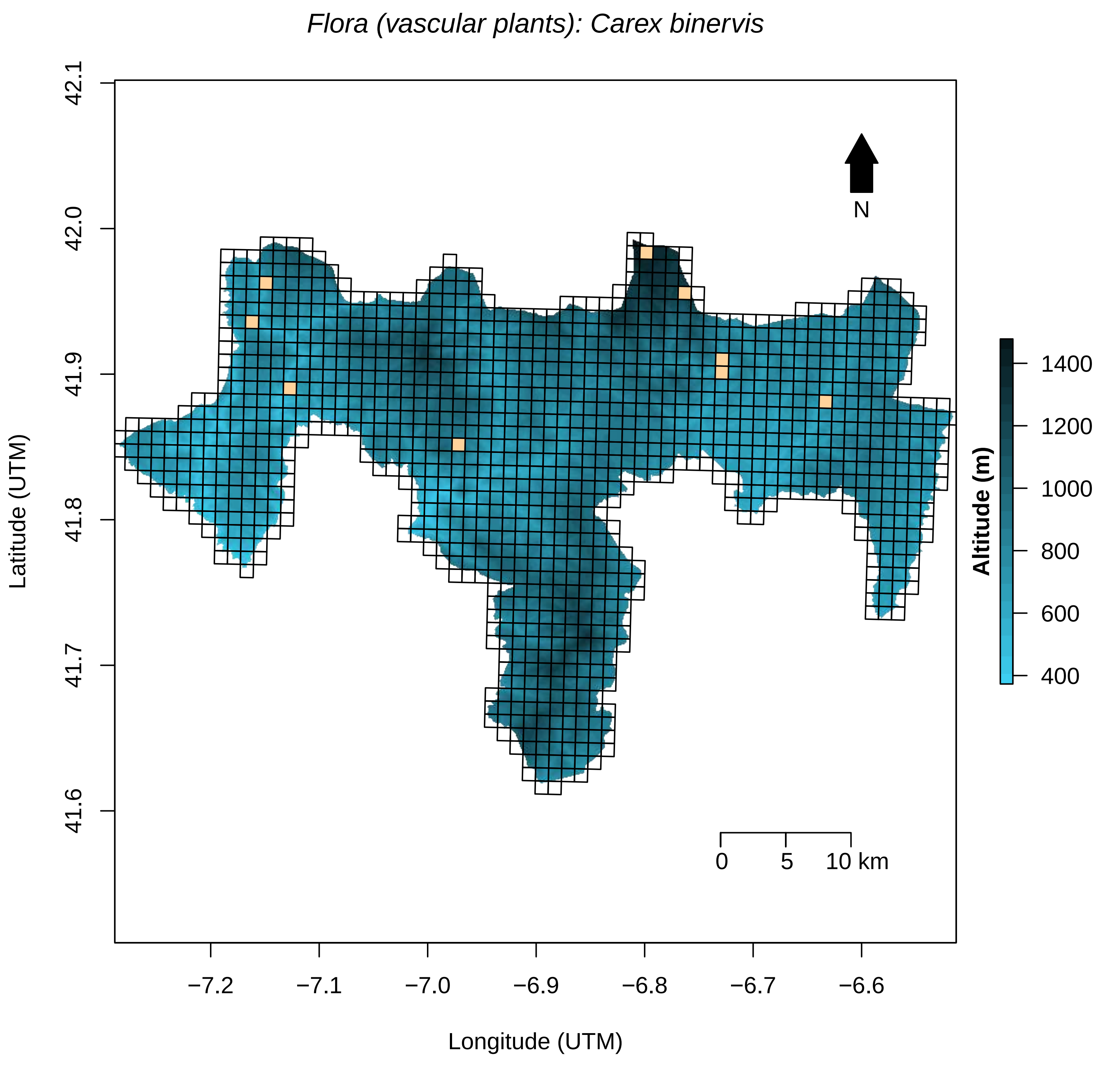

Flora (vascular plants): Carex binervis
42.1
42.0
41.9
41.8
41.7
41.6
Latitude (UTM)
−7.2
−7.1
−7.0
−6.9
−6.8
−6.7
−6.6
Longitude (UTM)
N
0
5
10 km
Altitude (m)
1400
1200
1000
800
600
400

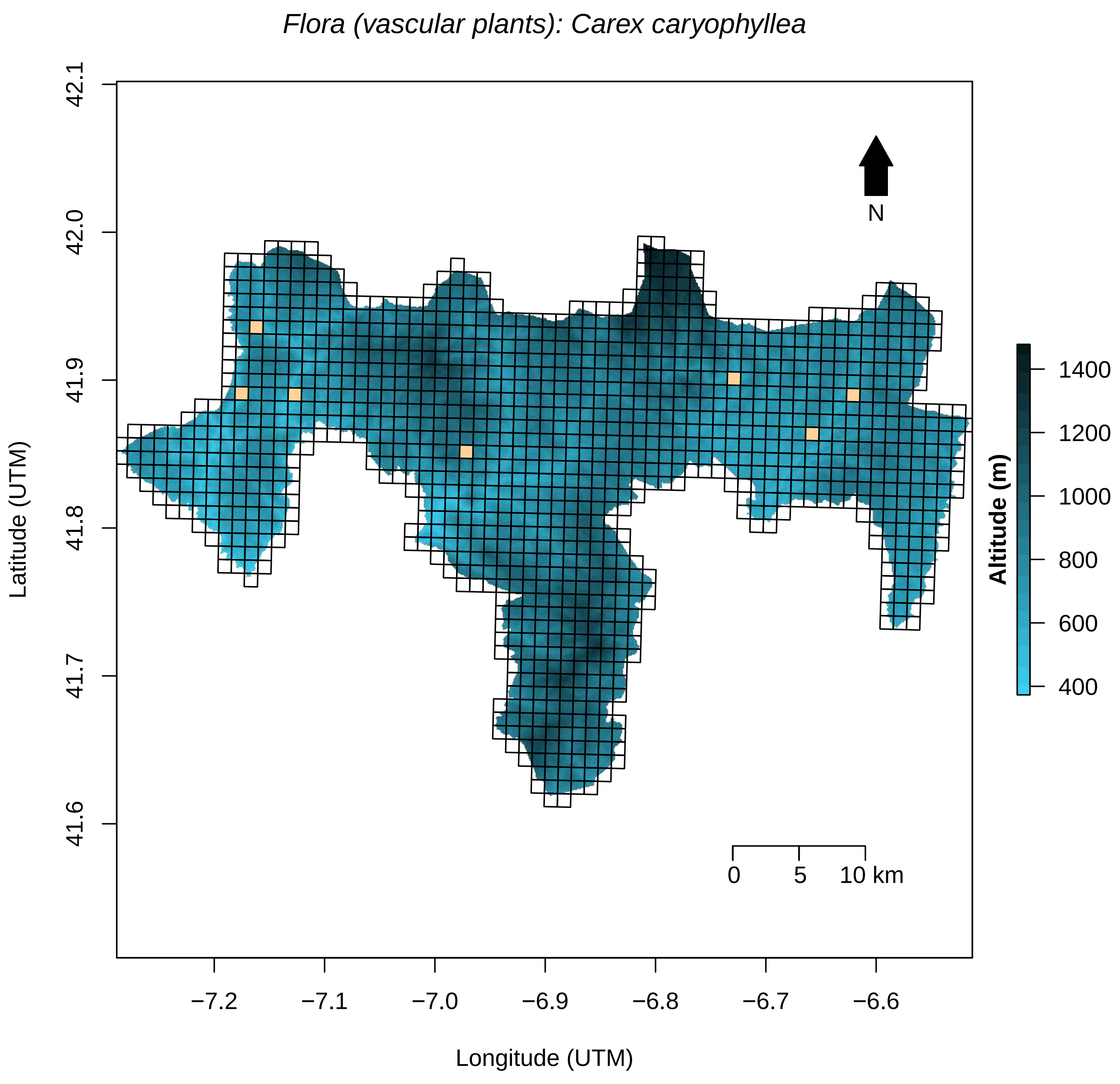

Flora (vascular plants): Carex caryophyllea
42.1
42.0
41.9
41.8
41.7
41.6
Latitude (UTM)
−7.2
−7.1
−7.0
−6.9
−6.8
−6.7
−6.6
Longitude (UTM)
N
0
5
10 km
Altitude (m)
1400
1200
1000
800
600
400

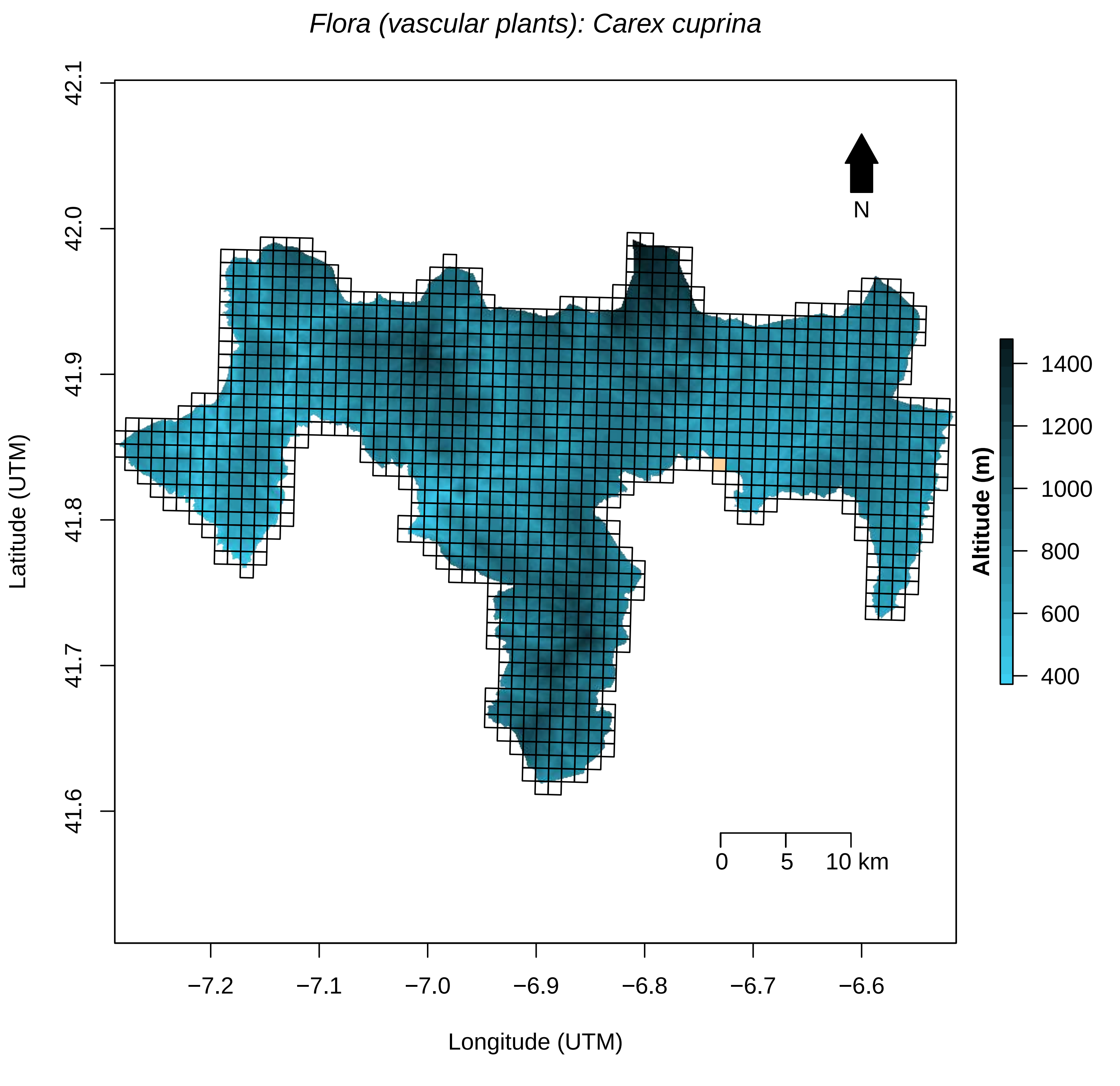

Flora (vascular plants): Carex cuprina
42.1
42.0
41.9
41.8
41.7
41.6
Latitude (UTM)
−7.2
−7.1
−7.0
−6.9
−6.8
−6.7
−6.6
Longitude (UTM)
N
0
5
10 km
Altitude (m)
1400
1200
1000
800
600
400

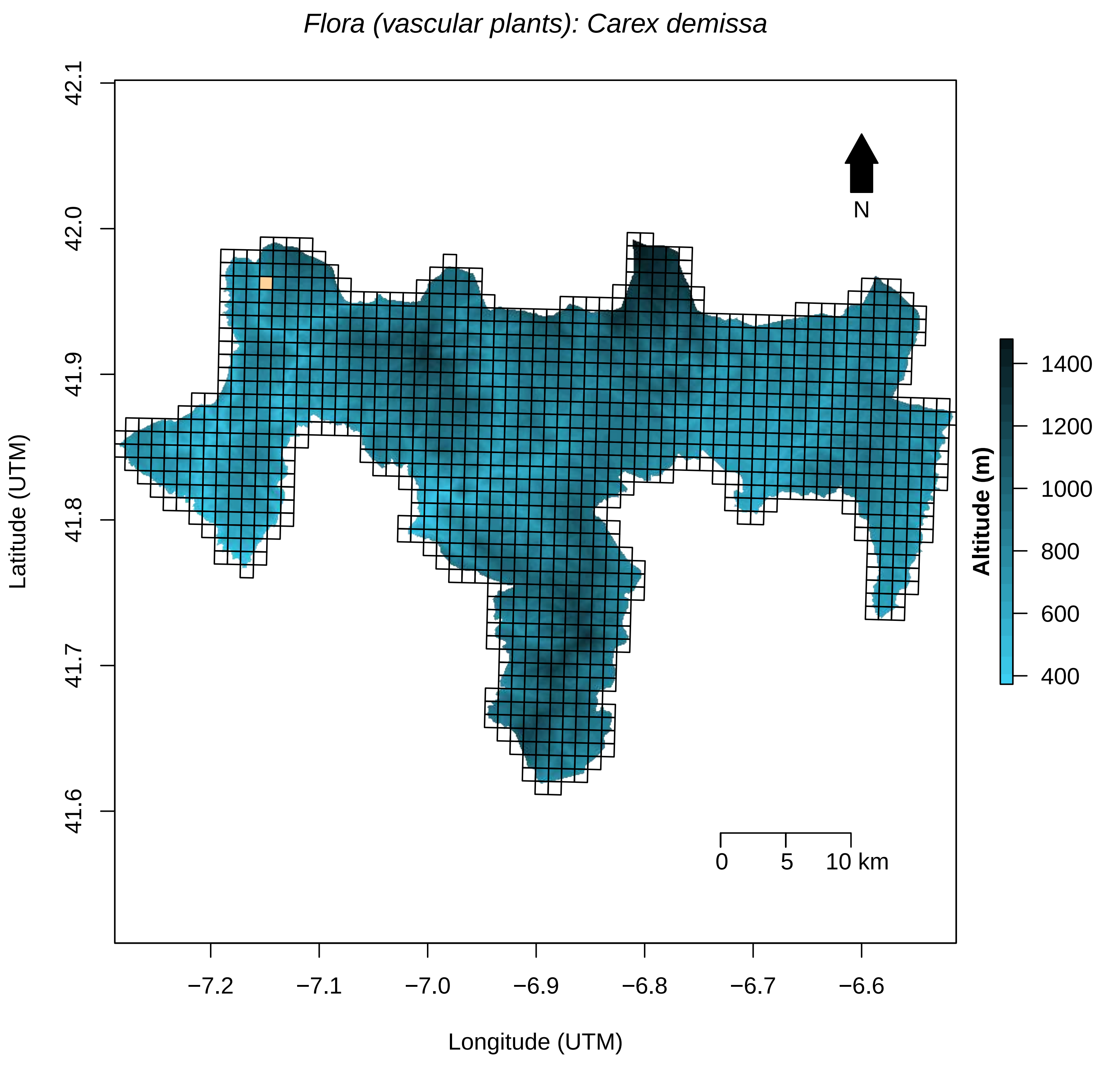

Flora (vascular plants): Carex demissa
42.1
42.0
41.9
41.8
41.7
41.6
Latitude (UTM)
−7.2
−7.1
−7.0
−6.9
−6.8
−6.7
−6.6
Longitude (UTM)
N
0
5
10 km
1400
1200
1000
800
600
400
Altitude (m)

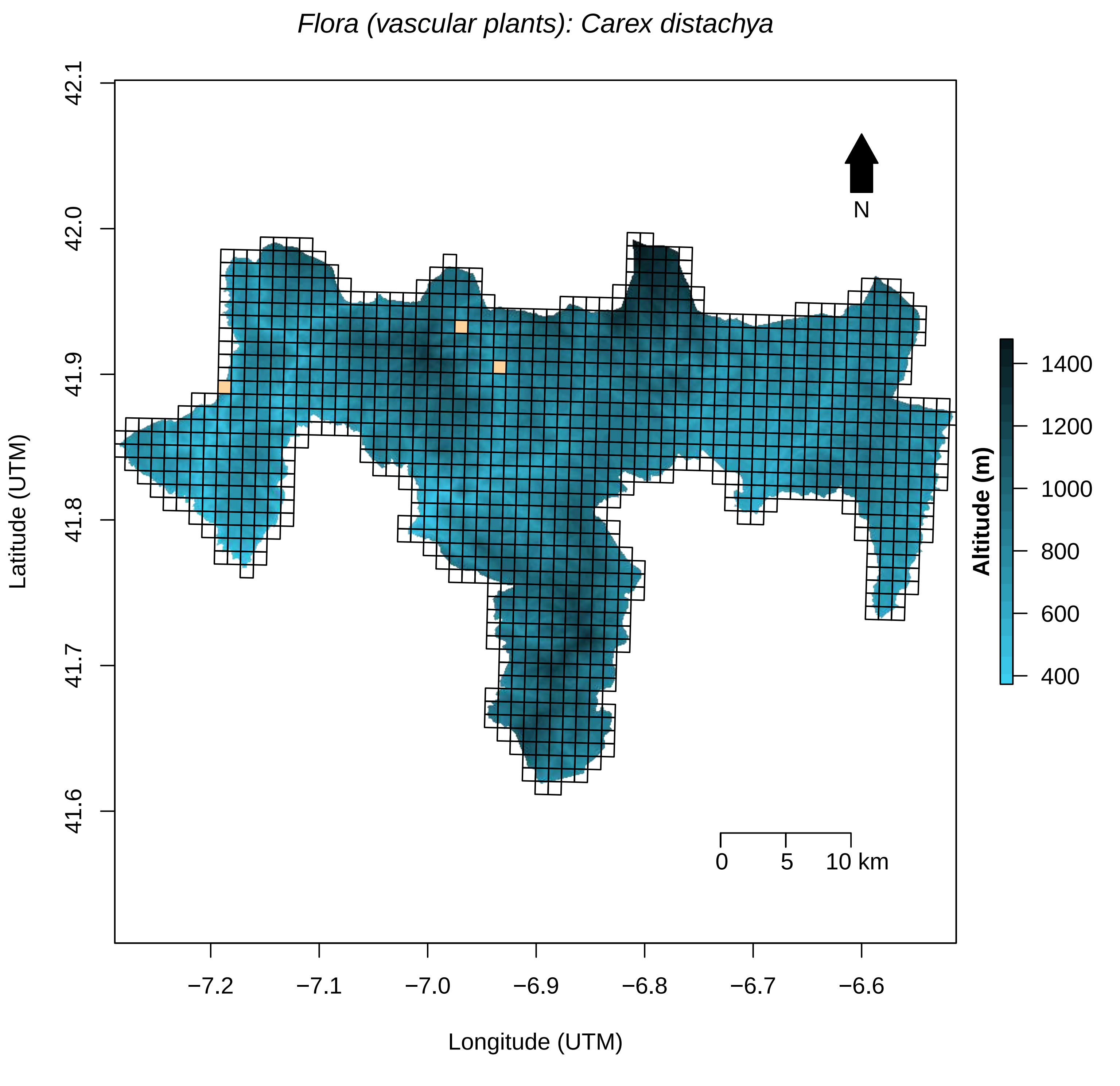

Flora (vascular plants): Carex distachya
42.1
42.0
41.9
41.8
41.7
41.6
Latitude (UTM)
−7.2
−7.1
−7.0
−6.9
−6.8
−6.7
−6.6
Longitude (UTM)
N
0
5
10 km
Altitude (m)
1400
1200
1000
800
600
400

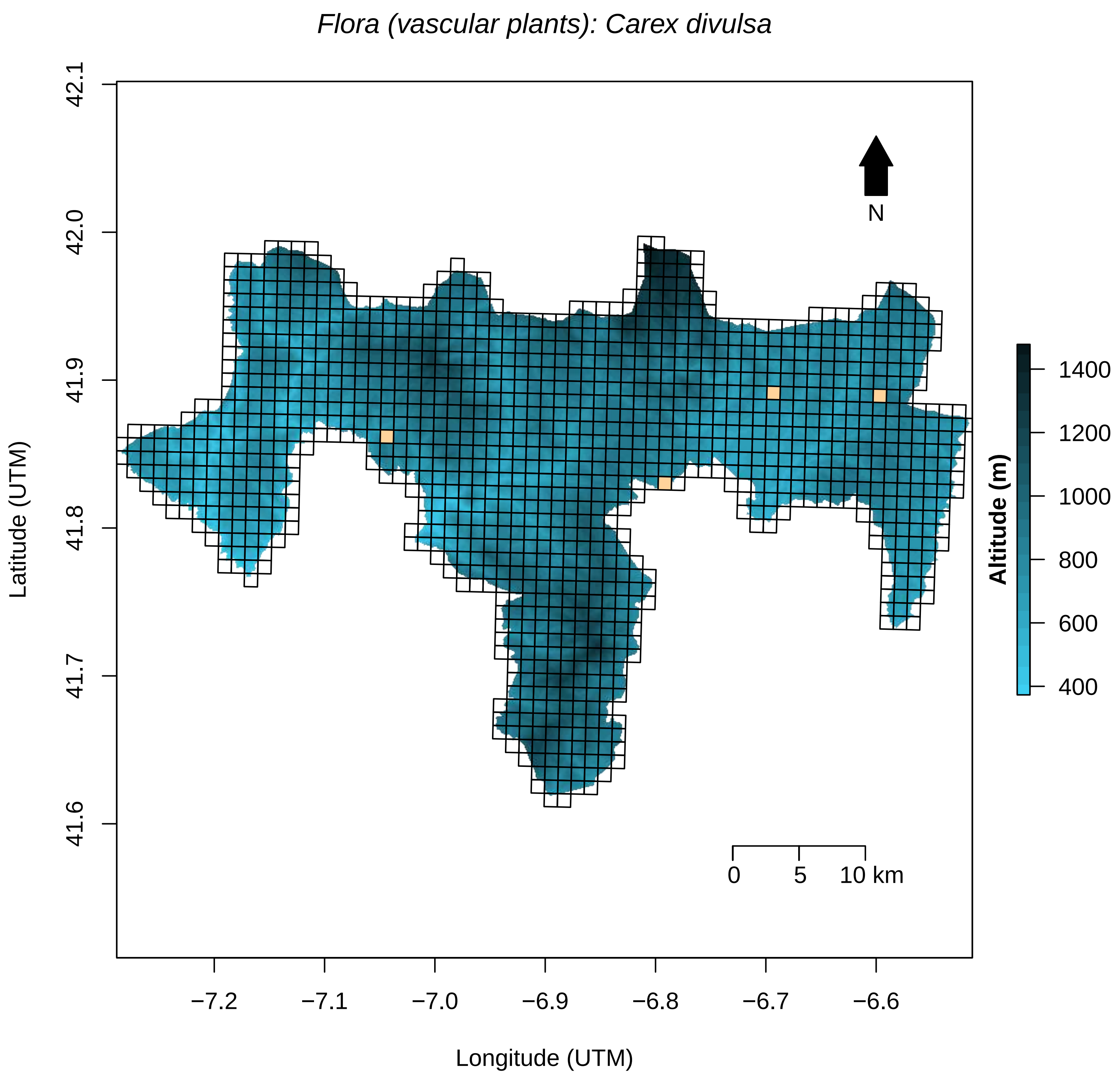

Flora (vascular plants): Carex divulsa
42.1
42.0
41.9
41.8
41.7
41.6
Latitude (UTM)
−7.2
−7.1
−7.0
−6.9
−6.8
−6.7
−6.6
Longitude (UTM)
N
0
5
10 km
Altitude (m)
1400
1200
1000
800
600
400

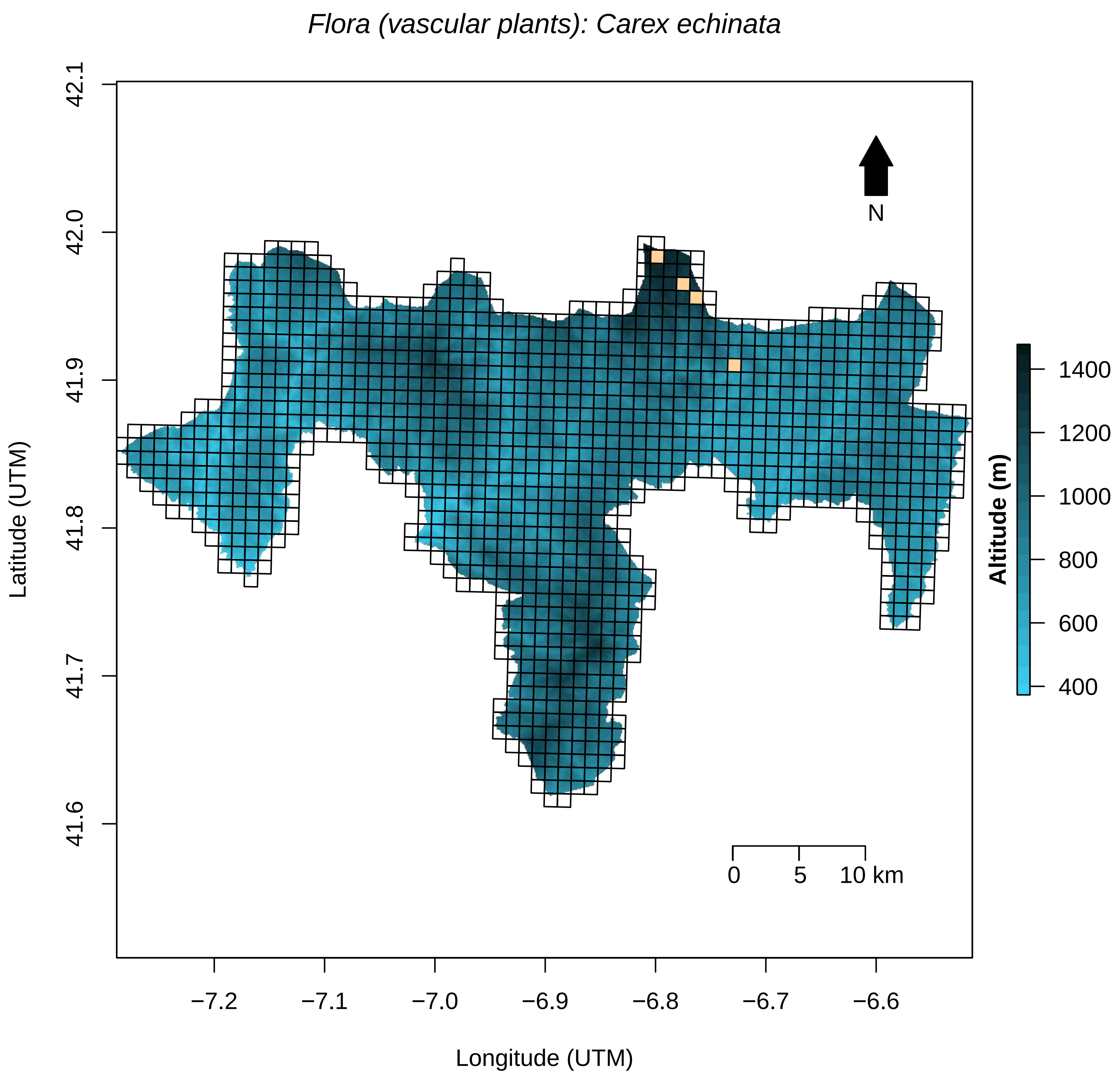

Flora (vascular plants): Carex echinata
42.1
42.0
41.9
41.8
41.7
41.6
Latitude (UTM)
−7.2
−7.1
−7.0
−6.9
−6.8
−6.7
−6.6
Longitude (UTM)
N
0
5
10 km
1400
1200
1000
800
600
400
Altitude (m)

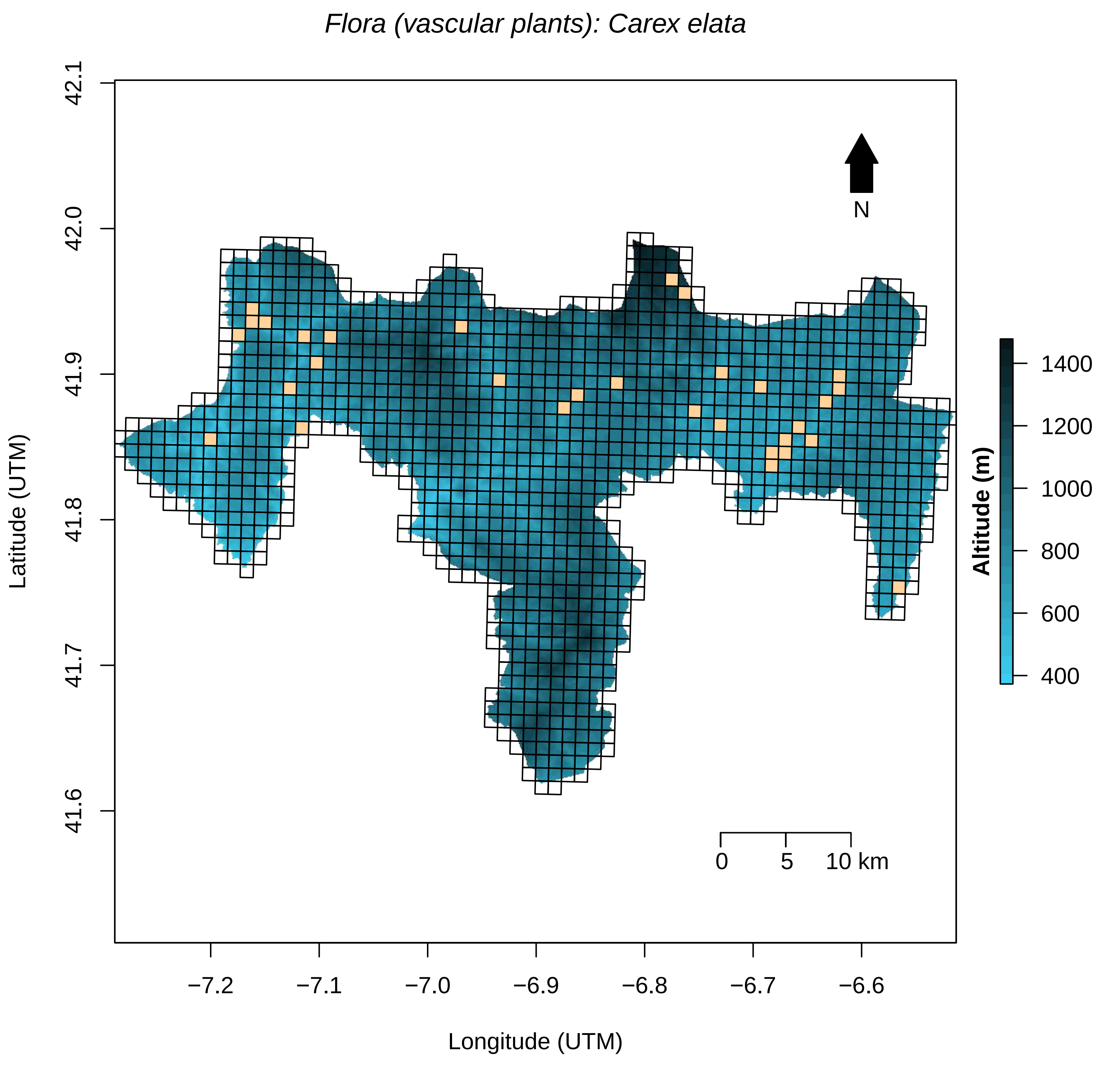

Flora (vascular plants): Carex elata
Latitude (UTM)
42.1
42.0
41.9
41.8
41.7
41.6
−7.2
−7.1
−7.0
−6.9
−6.8
−6.7
−6.6
Longitude (UTM)
N
0
5
10 km
Altitude (m)
1400
1200
1000
800
600
400

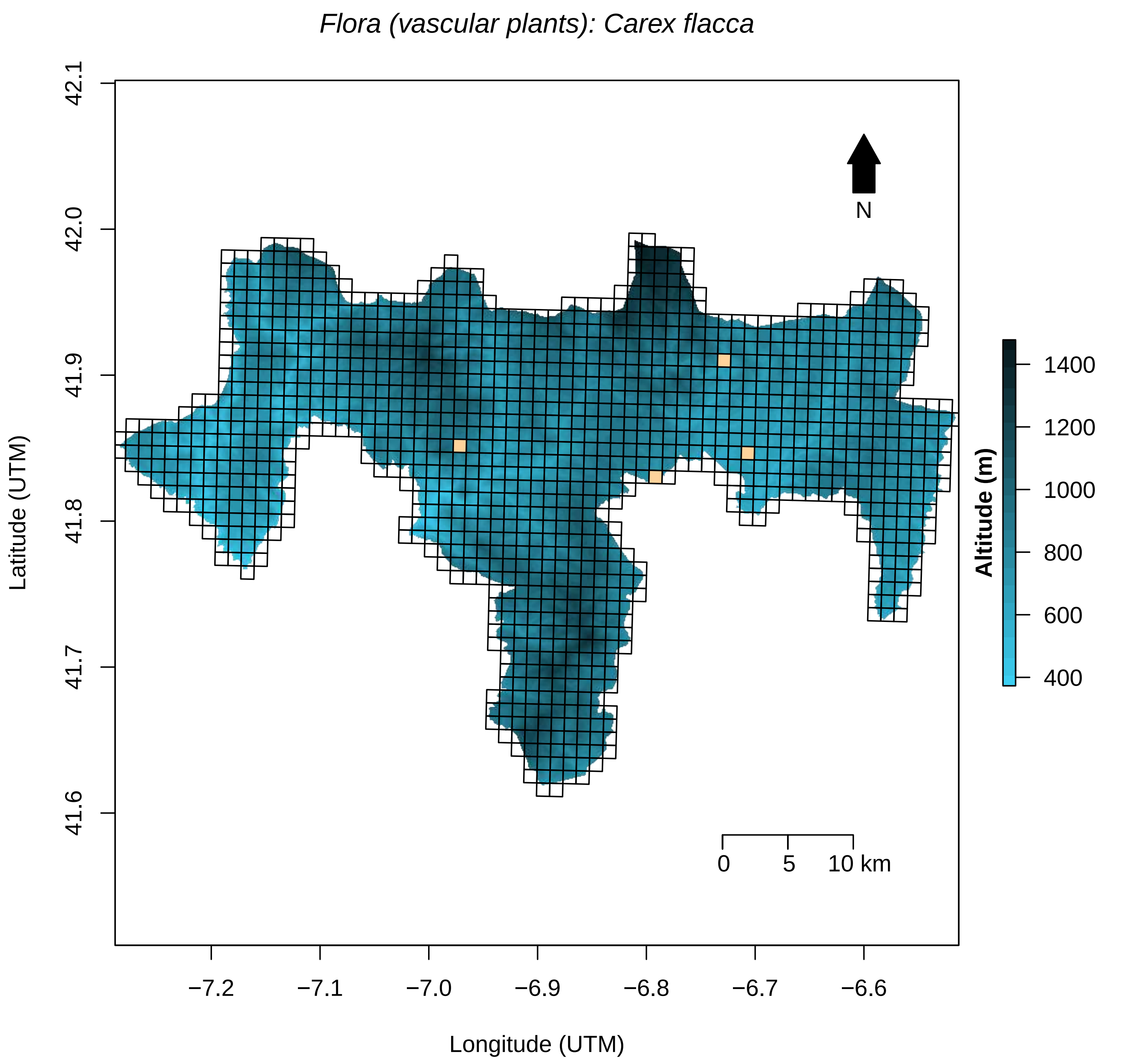
Flora (vascular plants): Carex flacca
42.1
42.0
41.9
41.8
41.7
41.6
Latitude (UTM)
−7.2
−7.1
−7.0
−6.9
−6.8
−6.7
−6.6
Longitude (UTM)
N
0
5
10 km
1400
1200
1000
800
600
400
Altitude (m)

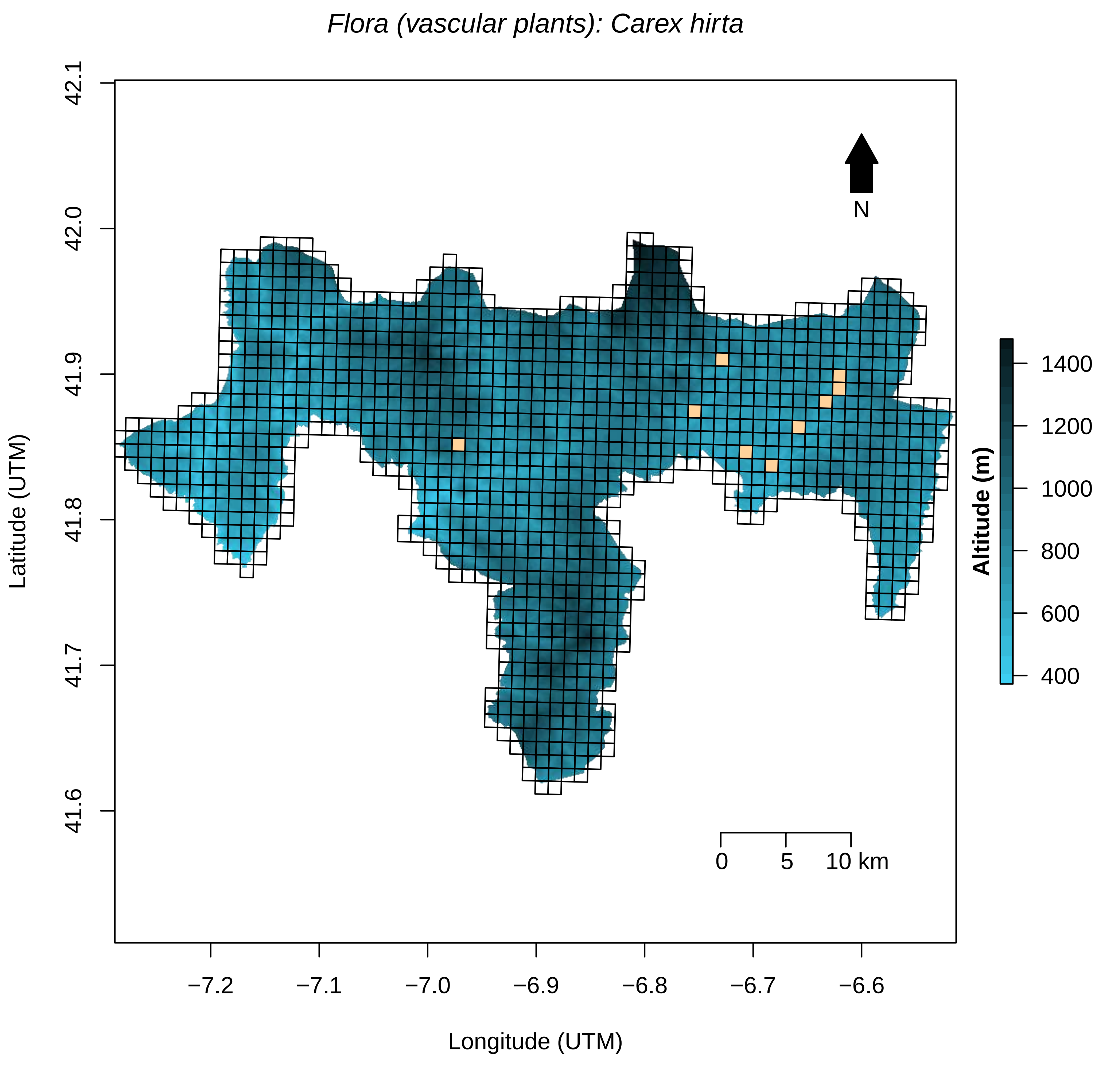

Flora (vascular plants): Carex hirta
42.1
42.0
41.9
41.8
41.7
41.6
Latitude (UTM)
−7.2
−7.1
−7.0
−6.9
−6.8
−6.7
−6.6
Longitude (UTM)
N
0
5
10 km
Altitude (m)
1400
1200
1000
800
600
400

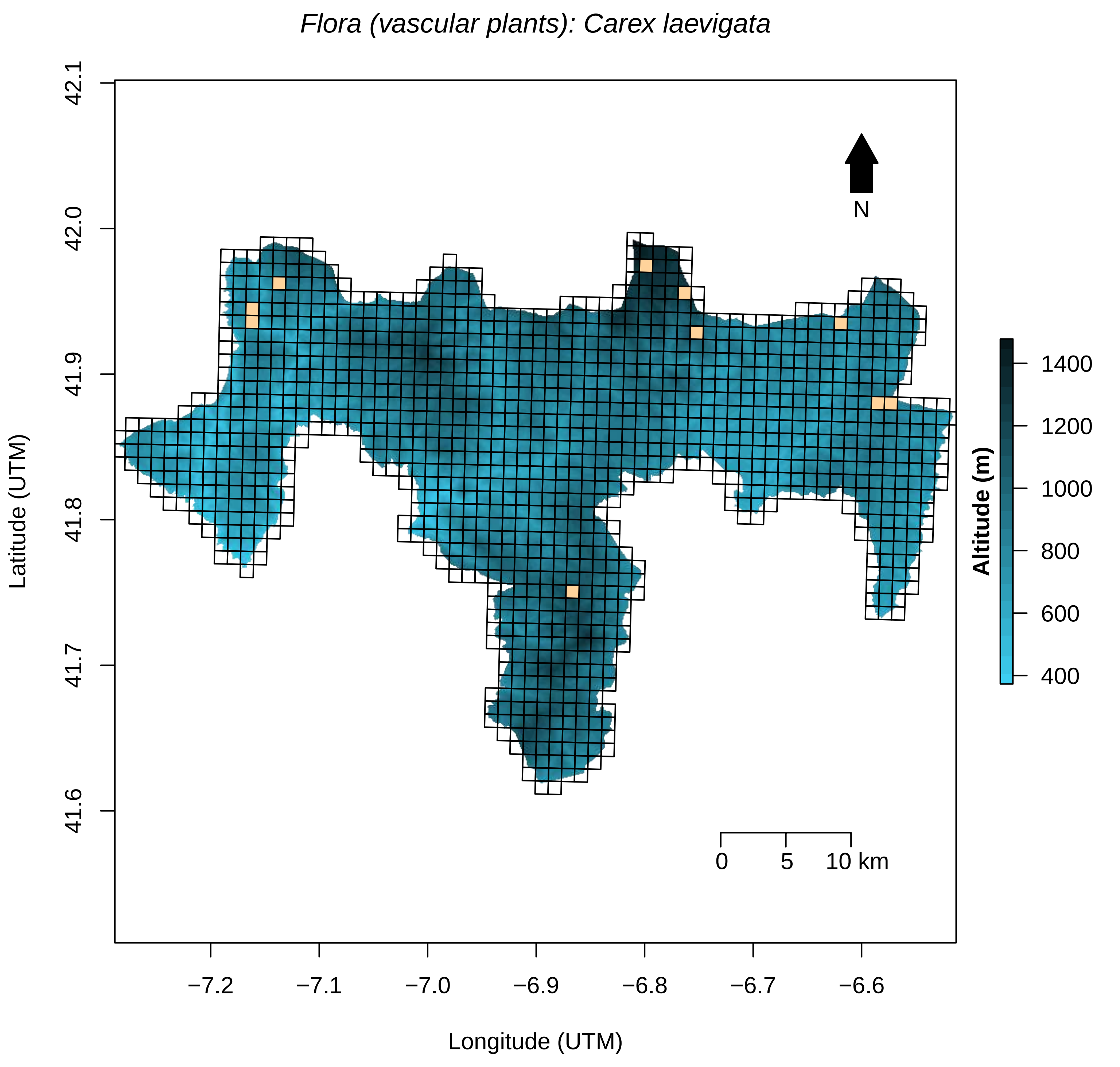

Flora (vascular plants): Carex laevigata
42.1
42.0
41.9
41.8
41.7
41.6
Latitude (UTM)
−7.2
−7.1
−7.0
−6.9
−6.8
−6.7
−6.6
Longitude (UTM)
N
0
5
10 km
1400
1200
1000
800
600
400
Altitude (m)

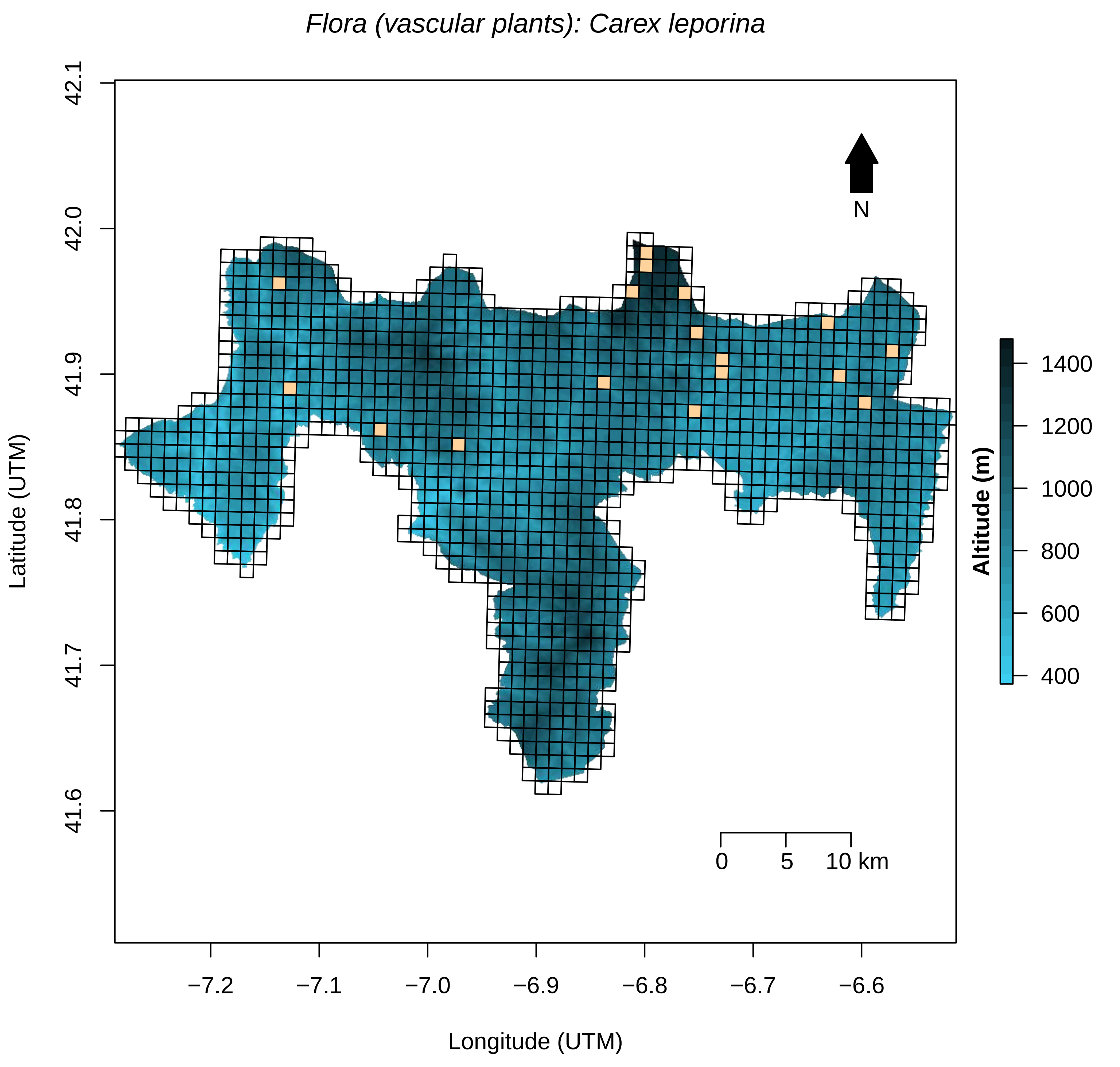

Flora (vascular plants): Carex leporina
42.1
42.0
41.9
41.8
41.7
41.6
Latitude (UTM)
−7.2
−7.1
−7.0
−6.9
−6.8
−6.7
−6.6
Longitude (UTM)
N
0
5
10 km
Altitude (m)
1400
1200
1000
800
600
400

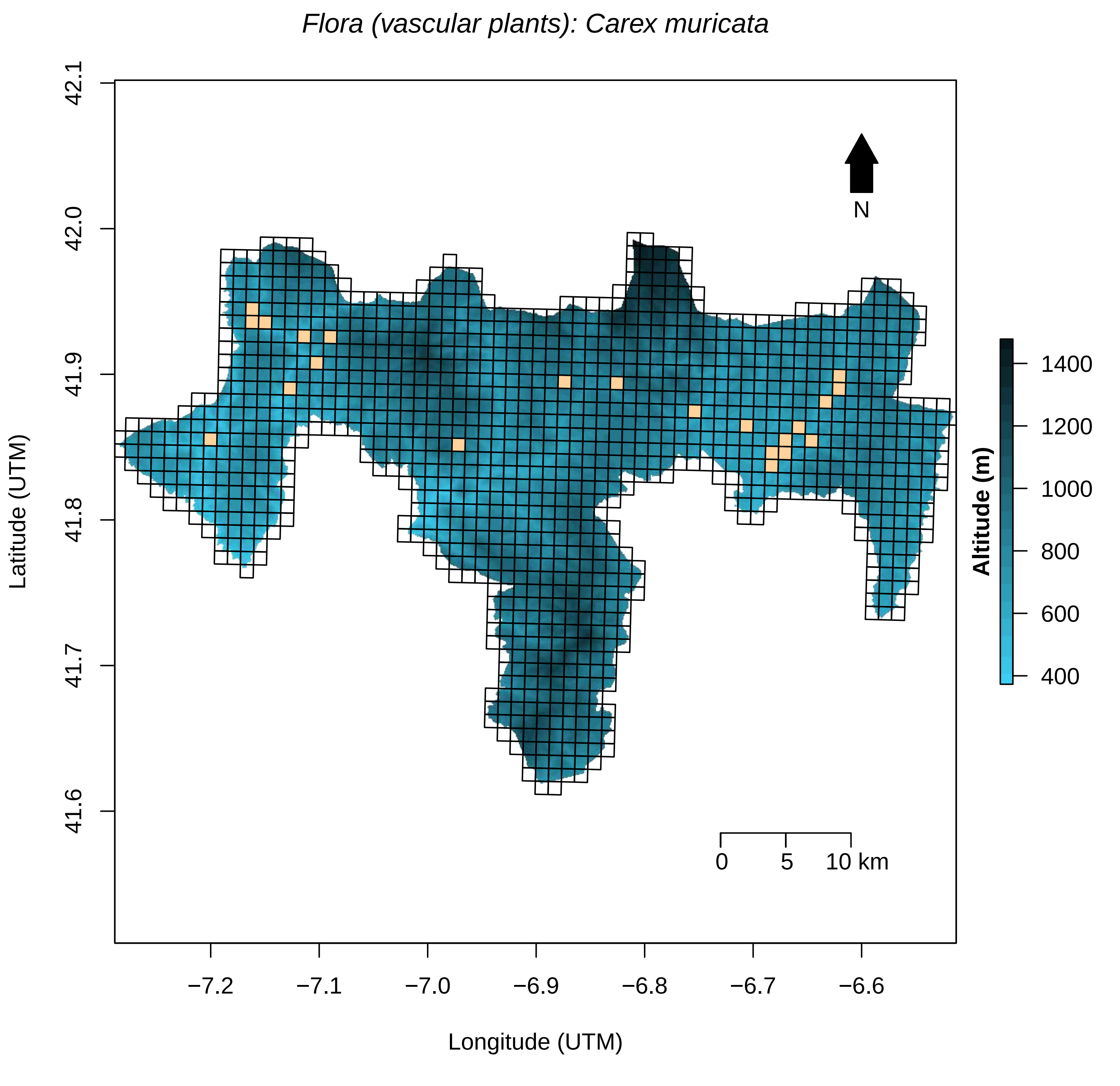

Flora (vascular plants): Carex muricata
N
0
5
10 km
Altitude (m)
1400
1200
1000
800
600
400
Latitude (UTM)
42.1
42.0
41.9
41.8
41.7
41.6
Longitude (UTM)
−7.2
−7.1
−7.0
−6.9
−6.8
−6.7
−6.6

Flora (vascular plants): Carex nigra

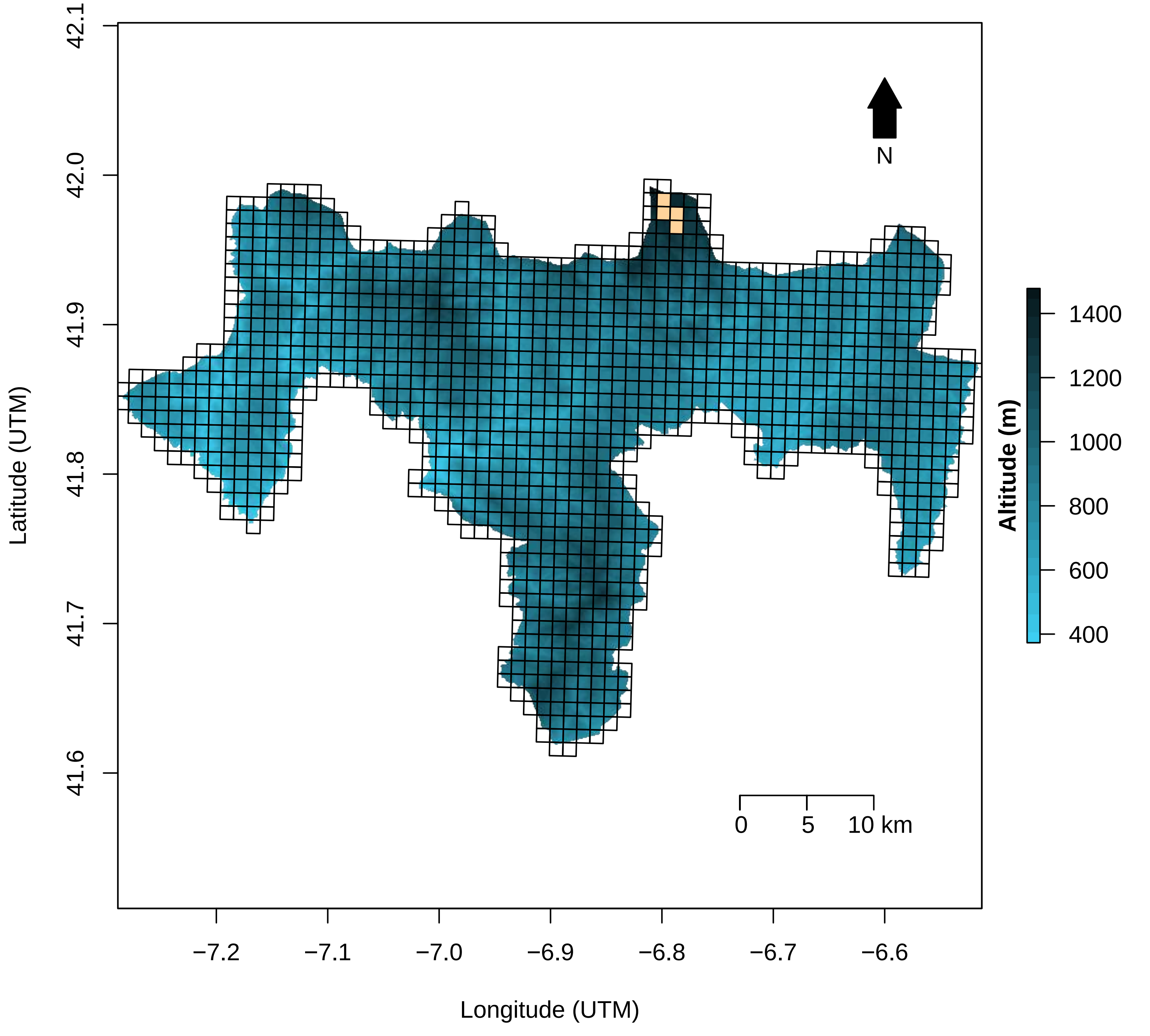

Flora (vascular plants): Carex paniculata

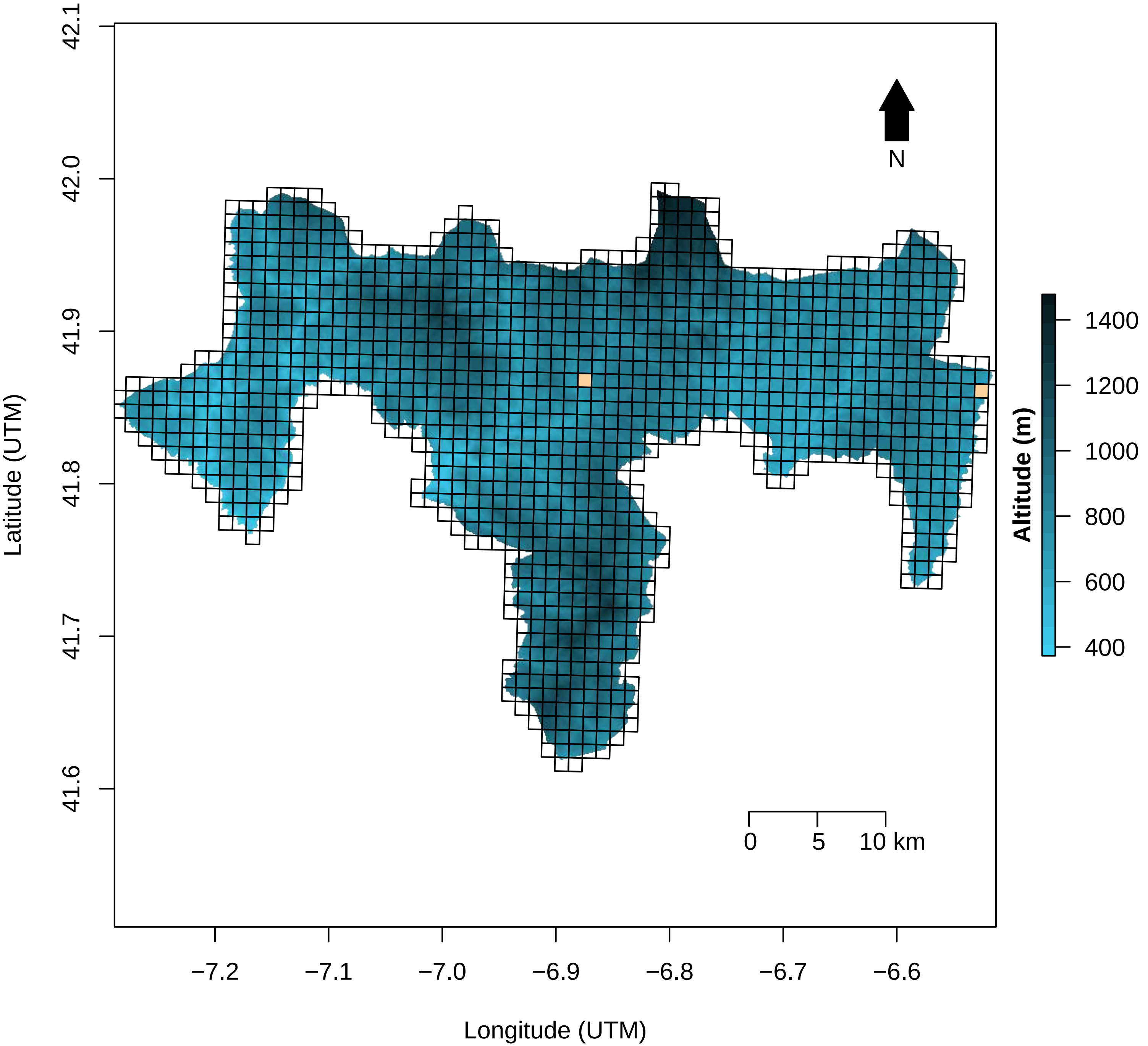

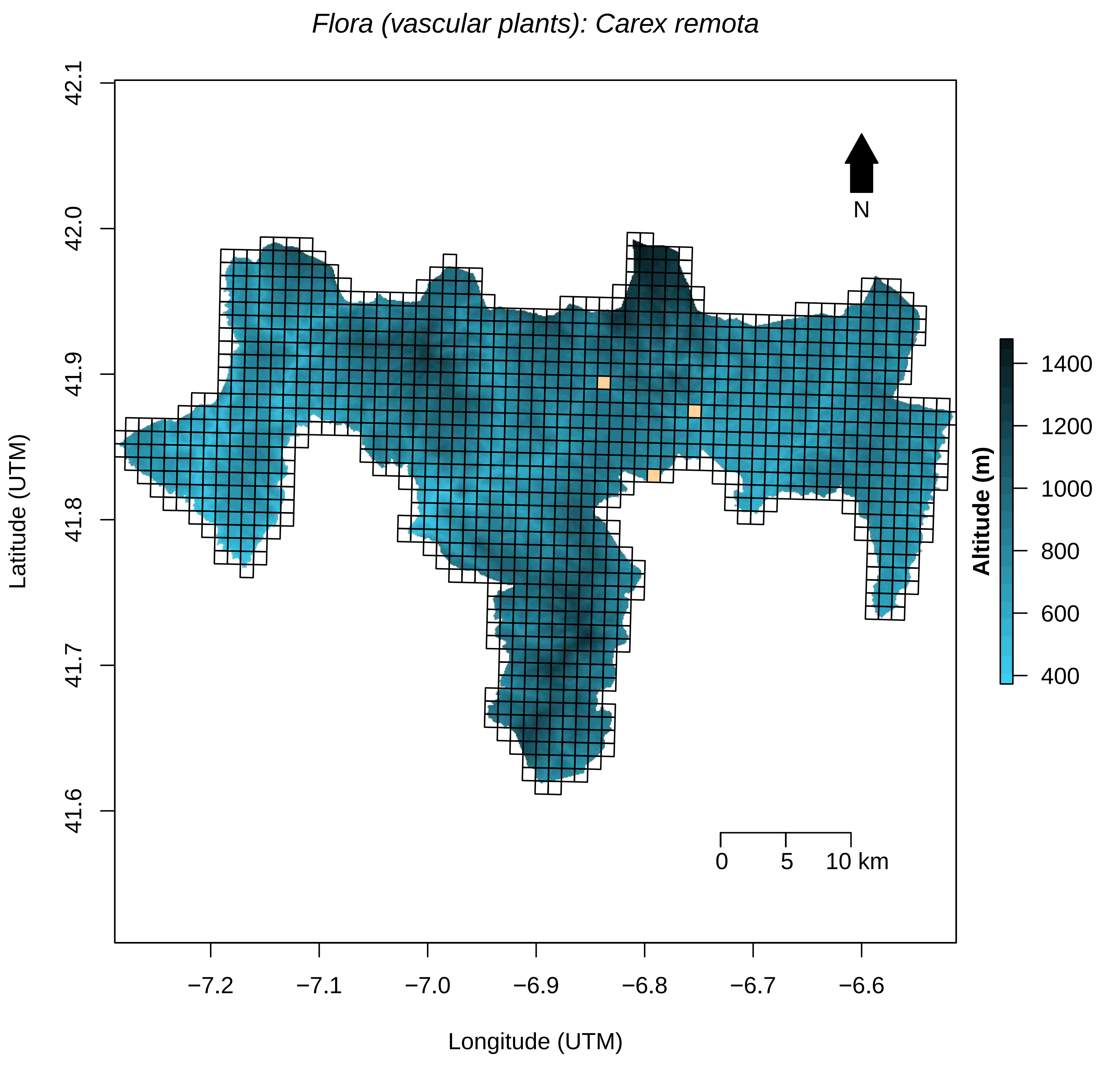

Flora (vascular plants): Carex remota
N
0
5
10 km
Altitude (m)
1400
1200
1000
800
600
400
Latitude (UTM)
42.1
42.0
41.9
41.8
41.7
41.6
Longitude (UTM)
−7.2
−7.1
−7.0
−6.9
−6.8
−6.7
−6.6

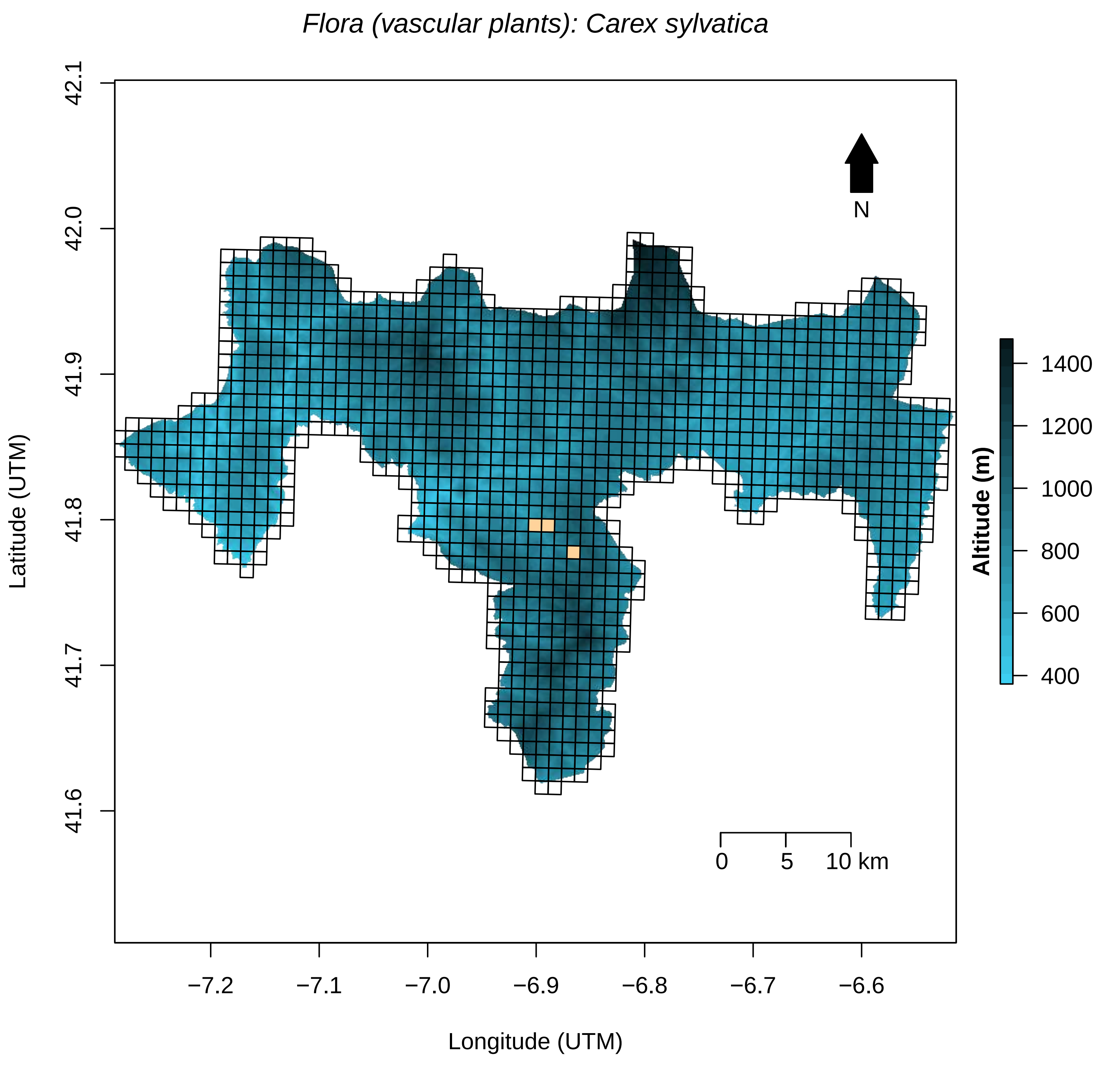

Flora (vascular plants): Carex sylvatica
N
0
5
10 km
Altitude (m)
1400
1200
1000
800
600
400
Latitude (UTM)
42.1
42.0
41.9
41.8
41.7
41.6
Longitude (UTM)
−7.2
−7.1
−7.0
−6.9
−6.8
−6.7
−6.6

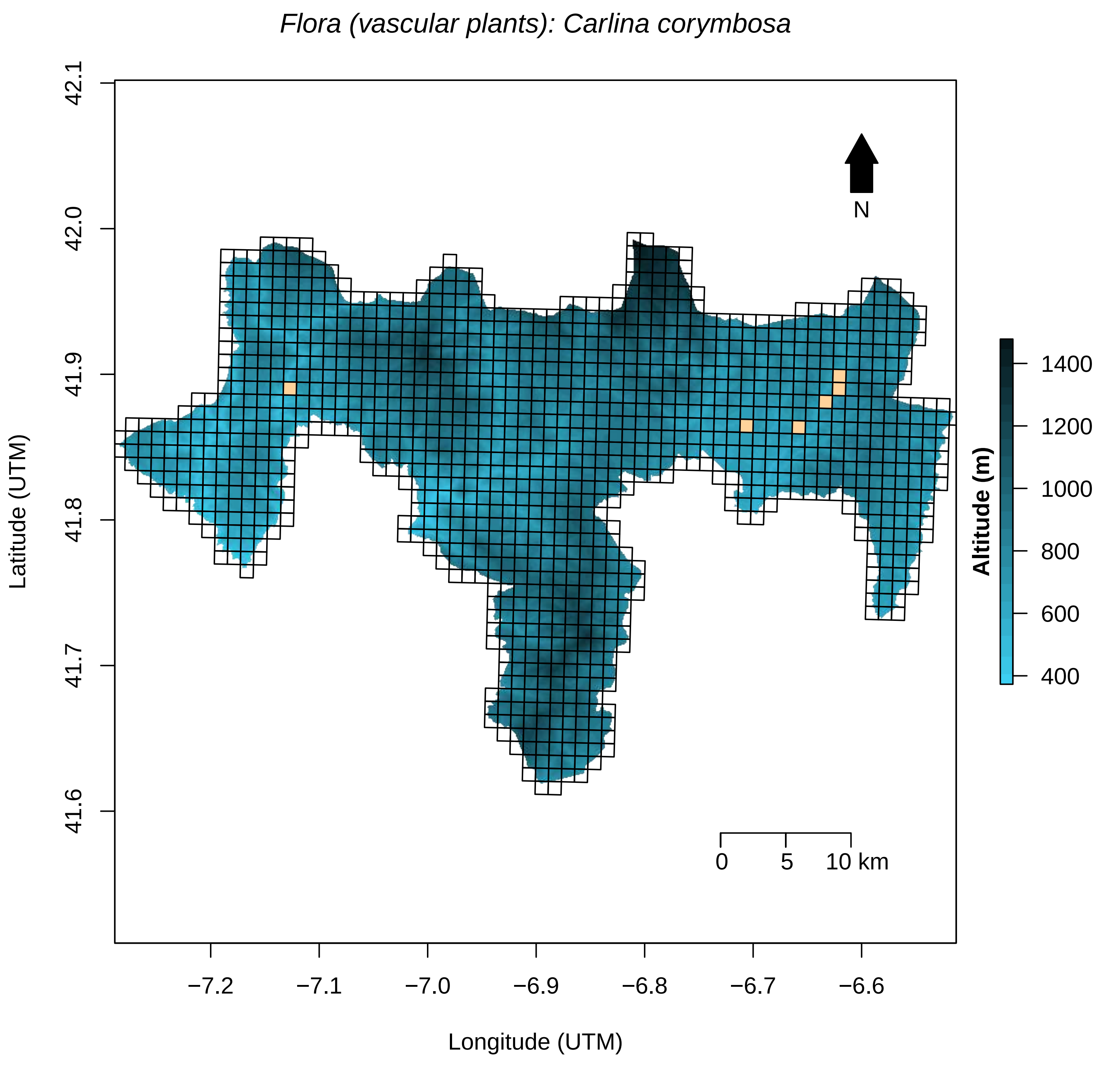
Flora (vascular plants): Carlina corymbosa
42.1
42.0
41.9
41.8
41.7
41.6
Latitude (UTM)
−7.2
−7.1
−7.0
−6.9
−6.8
−6.7
−6.6
Longitude (UTM)
N
0
5
10 km
1400
1200
1000
800
600
400
Altitude (m)

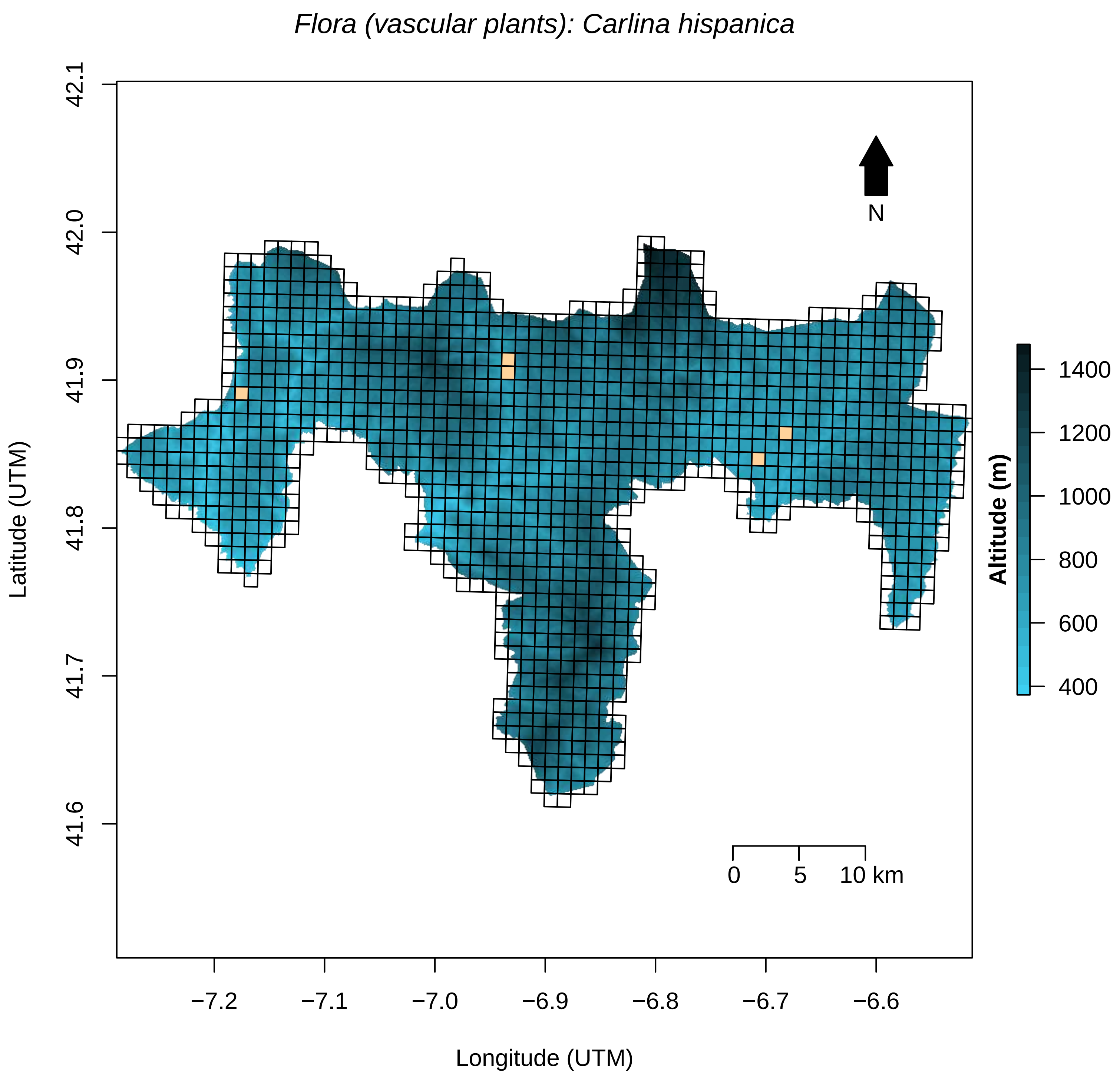

Flora (vascular plants): Carlina hispanica
N
0
5
10 km
Latitude (UTM)
42.1
42.0
41.9
41.8
41.7
41.6
Longitude (UTM)
−7.2
−7.1
−7.0
−6.9
−6.8
−6.7
−6.6
Altitude (m)
1400
1200
1000
800
600
400

Flora (vascular plants): Carlina vulgaris

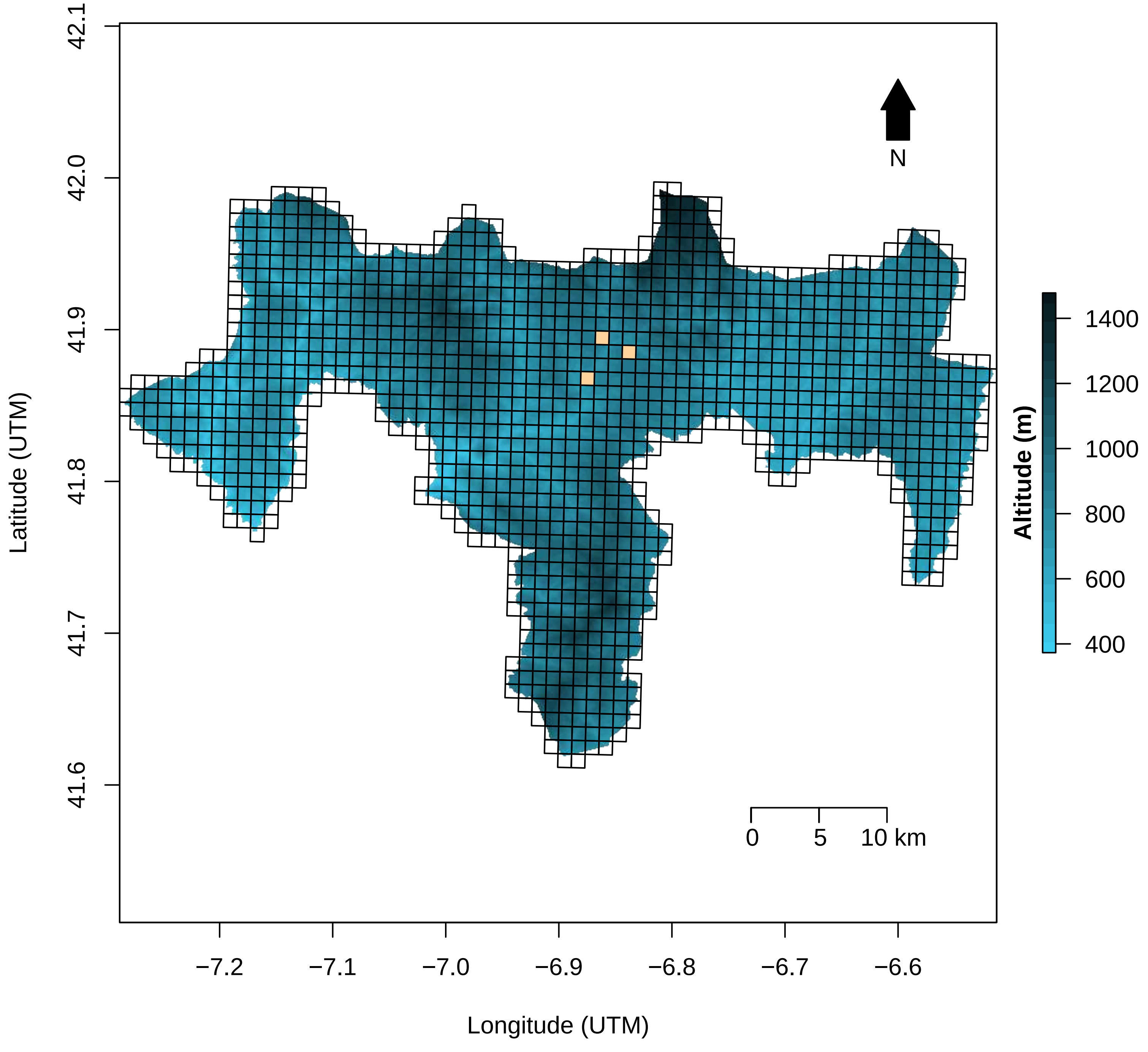

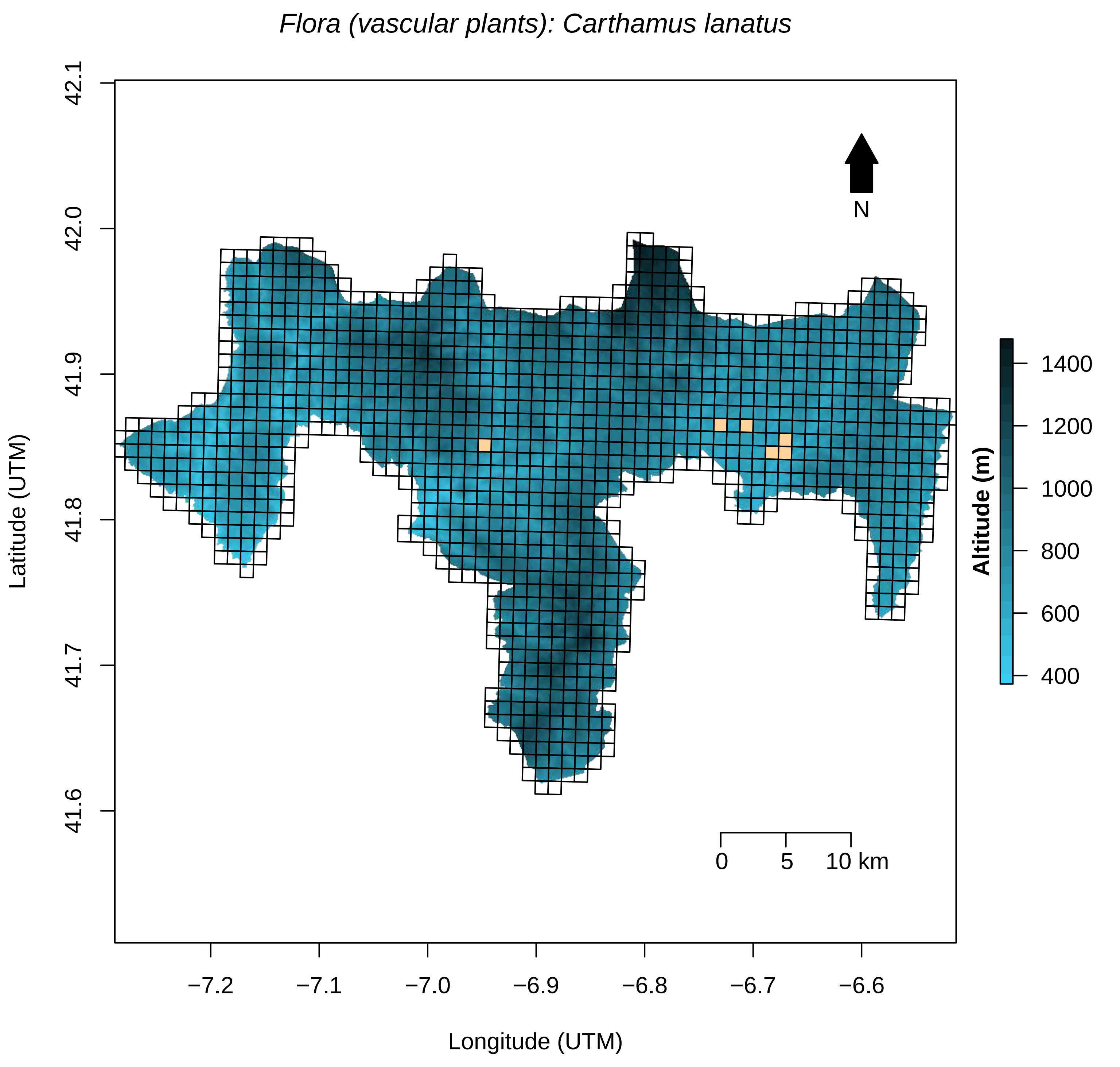

Flora (vascular plants): Carthamus lanatus
N
0 5 10 km
Latitude (UTM)
42.1
42.0
41.9
41.8
41.7
41.6
Longitude (UTM)
−7.2
−7.1
−7.0
−6.9
−6.8
−6.7
−6.6
Altitude (m)
1400
1200
1000
800
600
400

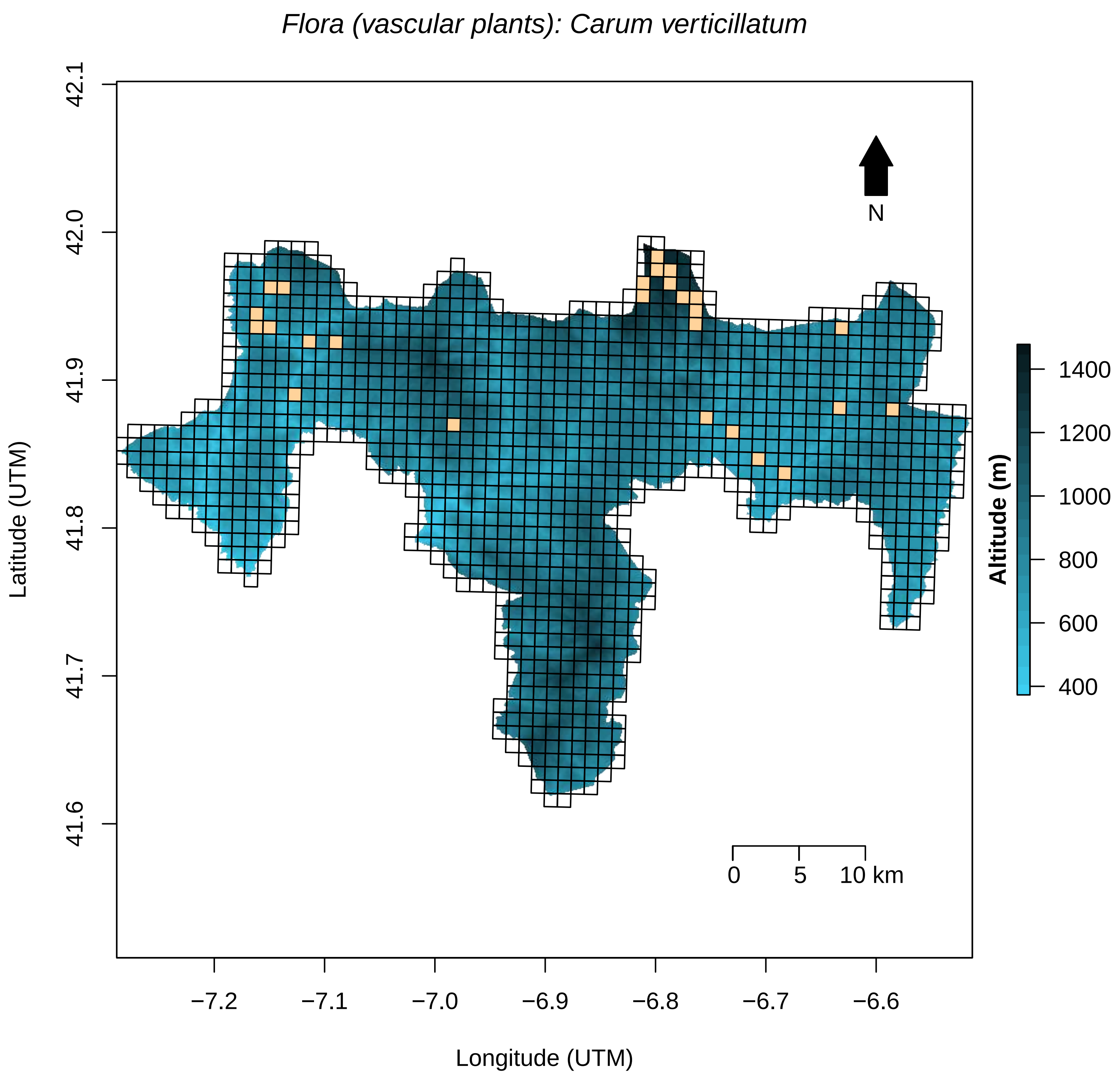

Flora (vascular plants): Carum verticillatum
42.1
42.0
41.9
41.8
41.7
41.6
Latitude (UTM)
−7.2
−7.1
−7.0
−6.9
−6.8
−6.7
−6.6
Longitude (UTM)
N
0
5
10 km
Altitude (m)
1400
1200
1000
800
600
400

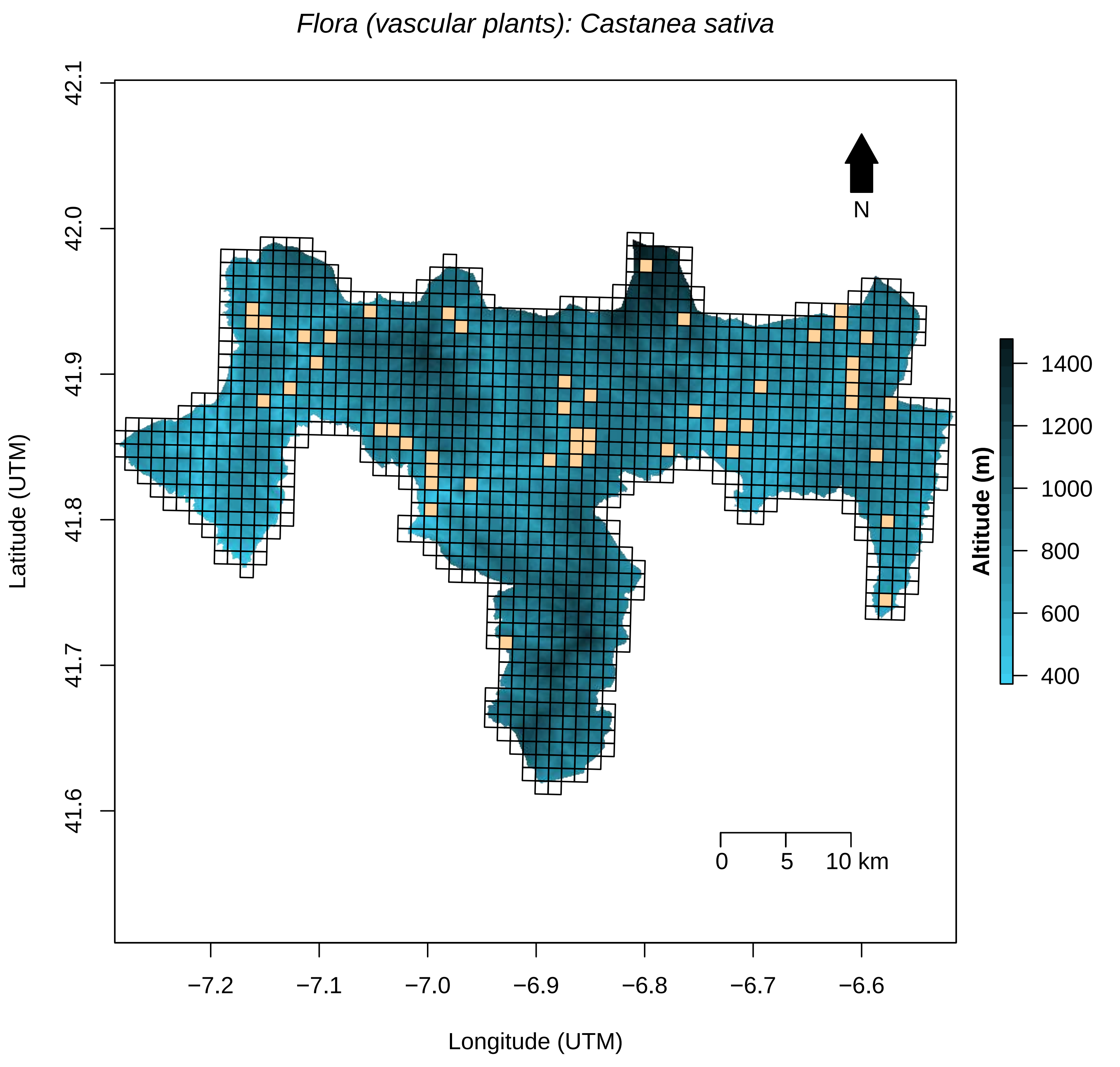

Flora (vascular plants): Castanea sativa
N
0
5
10 km
Altitude (m)
1400
1200
1000
800
600
400
Latitude (UTM)
42.1
42.0
41.9
41.8
41.7
41.6
Longitude (UTM)
−7.2
−7.1
−7.0
−6.9
−6.8
−6.7
−6.6

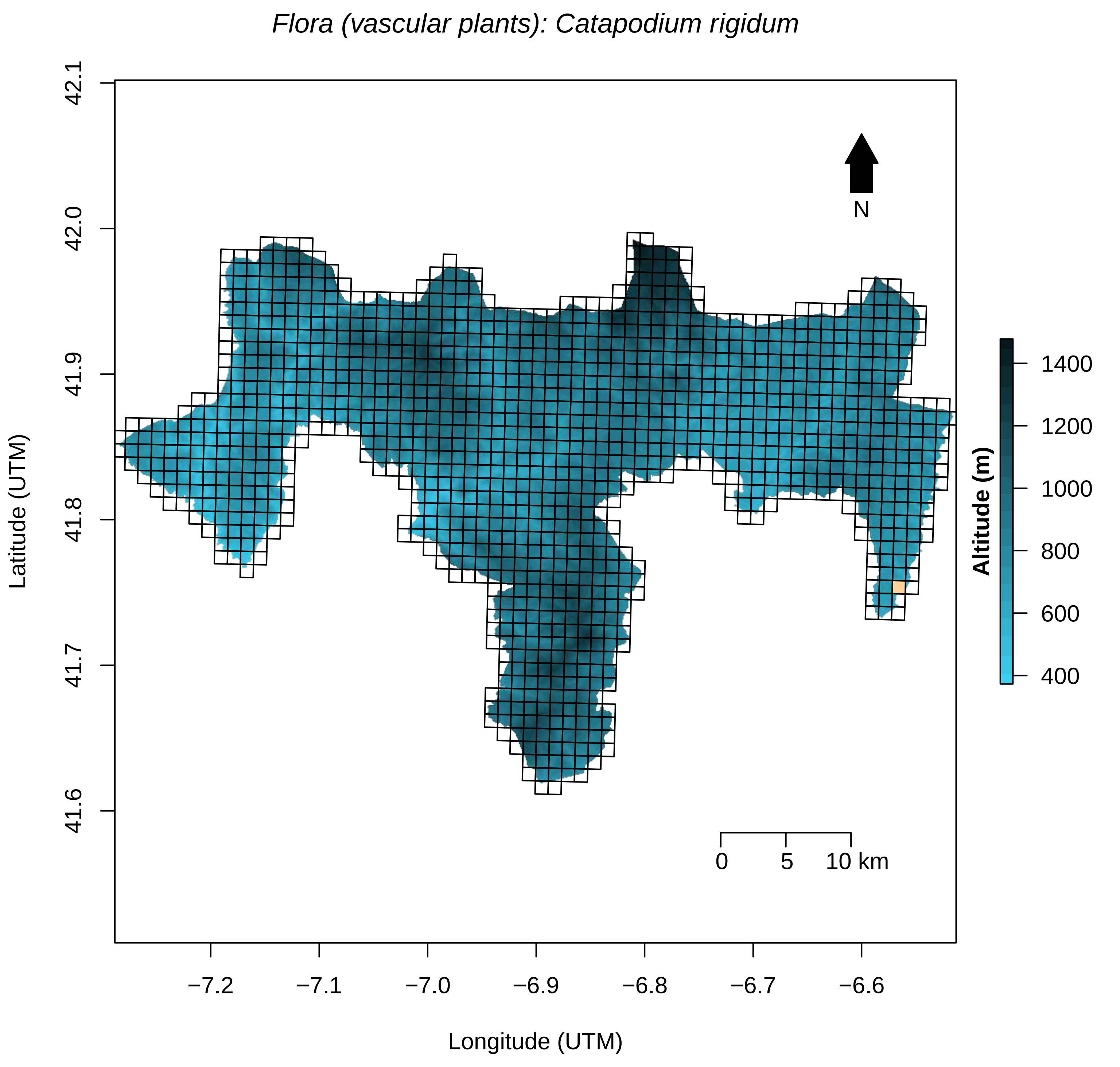

Flora (vascular plants): Catapodium rigidum
N
0
5
10 km
Altitude (m)
1400
1200
1000
800
600
400
42.1
42.0
41.9
41.8
41.7
41.6
Latitude (UTM)
−7.2
−7.1
−7.0
−6.9
−6.8
−6.7
−6.6
Longitude (UTM)

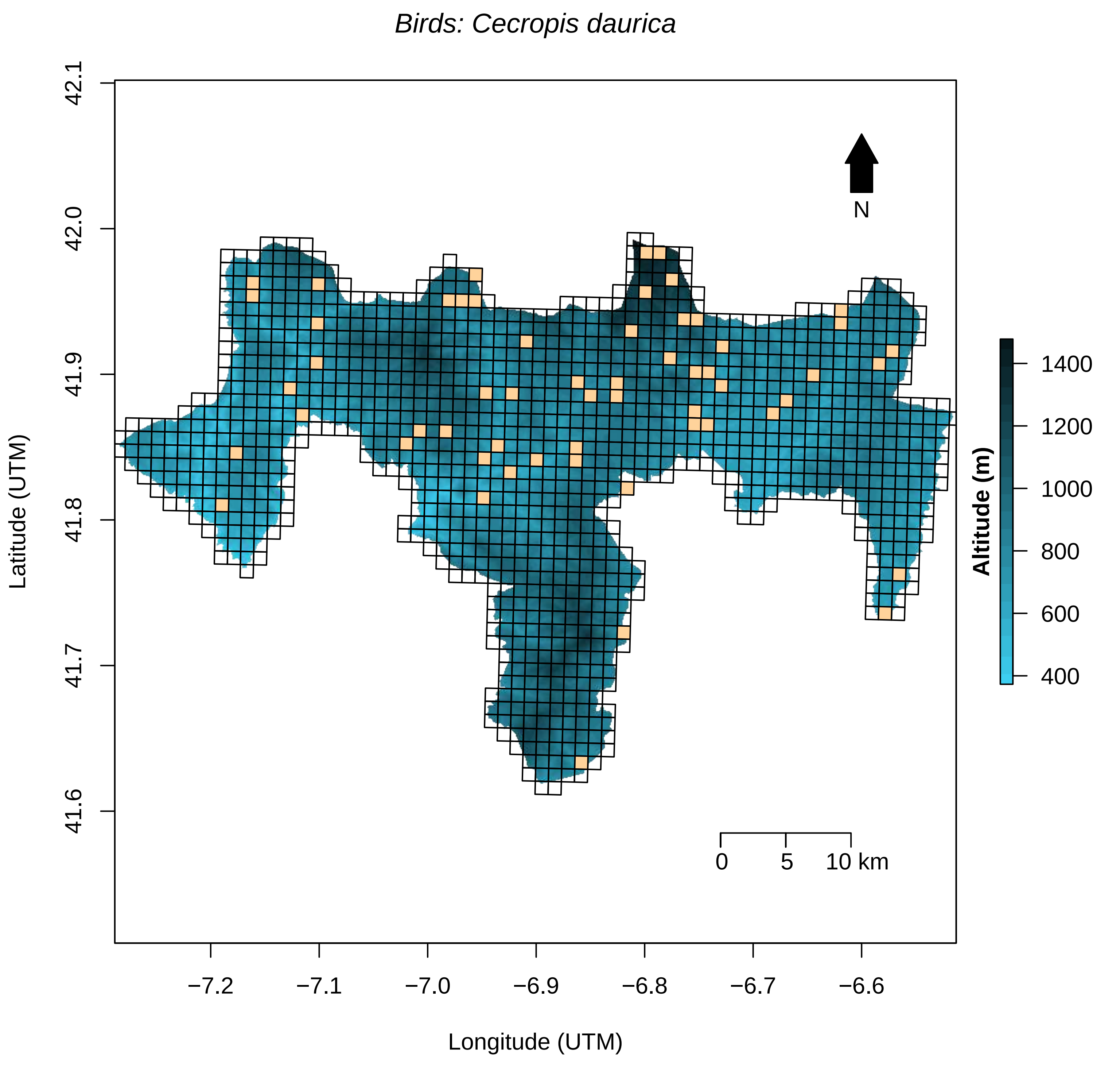

Birds: Cecropis daurica
N
0
5
10 km
Altitude (m)
1400
1200
1000
800
600
400
Latitude (UTM)
42.1
42.0
41.9
41.8
41.7
41.6
Longitude (UTM)
−7.2
−7.1
−7.0
−6.9
−6.8
−6.7
−6.6

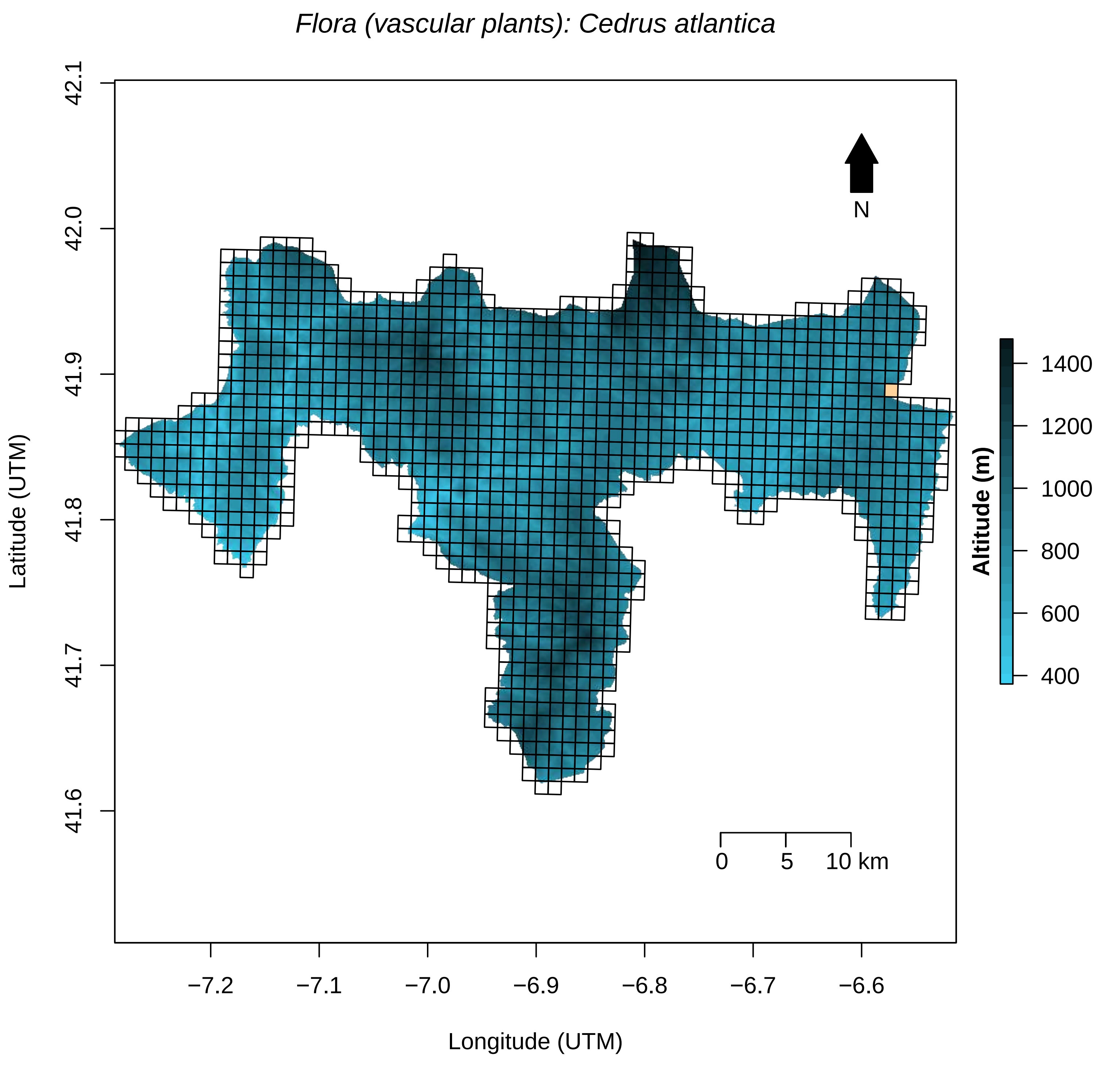

Flora (vascular plants): Cedrus atlantica
N
0
5
10 km
Altitude (m)
1400
1200
1000
800
600
400
Latitude (UTM)
42.1
42.0
41.9
41.8
41.7
41.6
Longitude (UTM)
−7.2
−7.1
−7.0
−6.9
−6.8
−6.7
−6.6

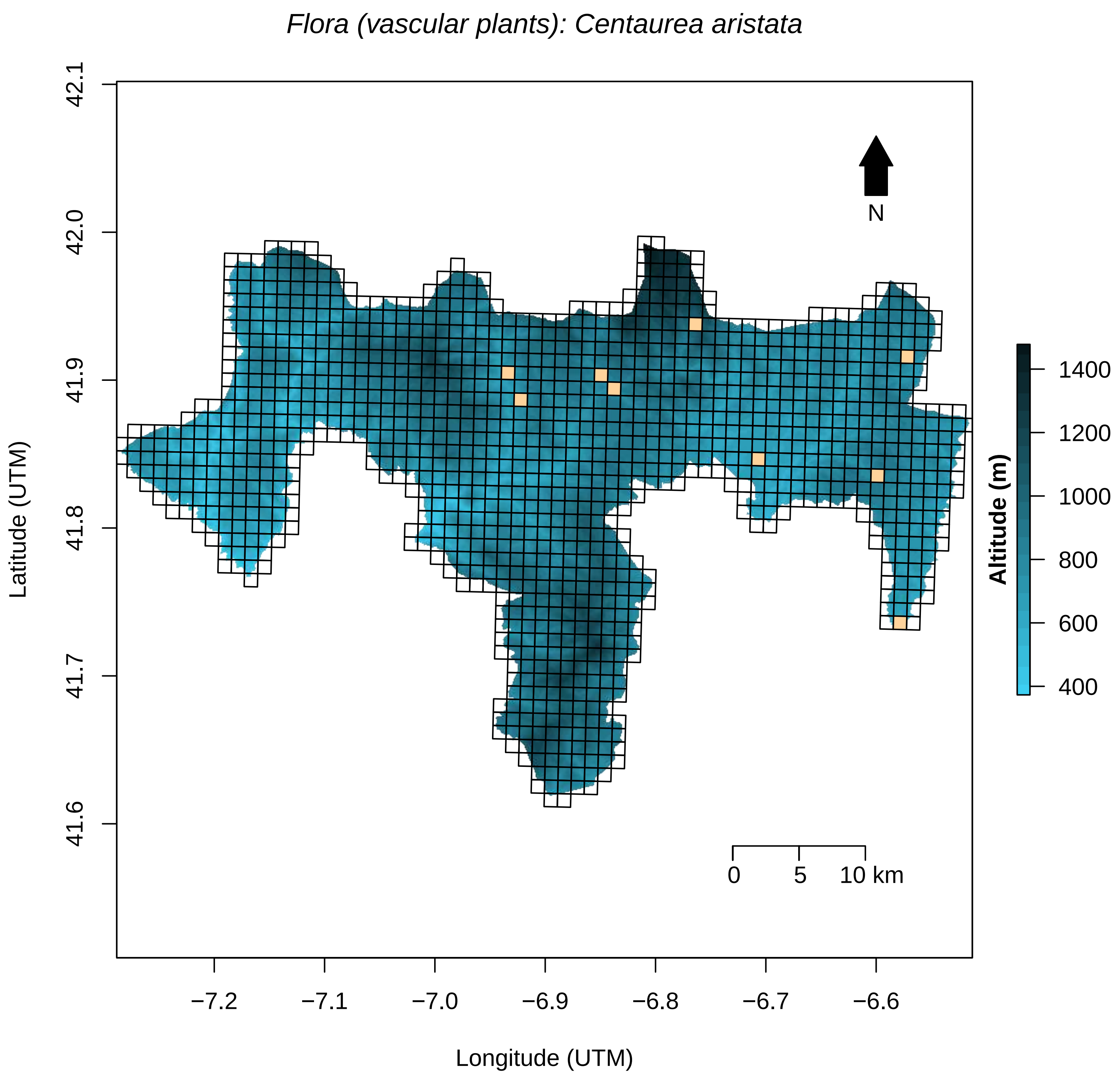

Flora (vascular plants): Centaurea aristata
42.1
42.0
41.9
41.8
41.7
41.6
Latitude (UTM)
−7.2
−7.1
−7.0
−6.9
−6.8
−6.7
−6.6
Longitude (UTM)
N
0
5
10 km
1400
1200
1000
800
600
400
Altitude (m)

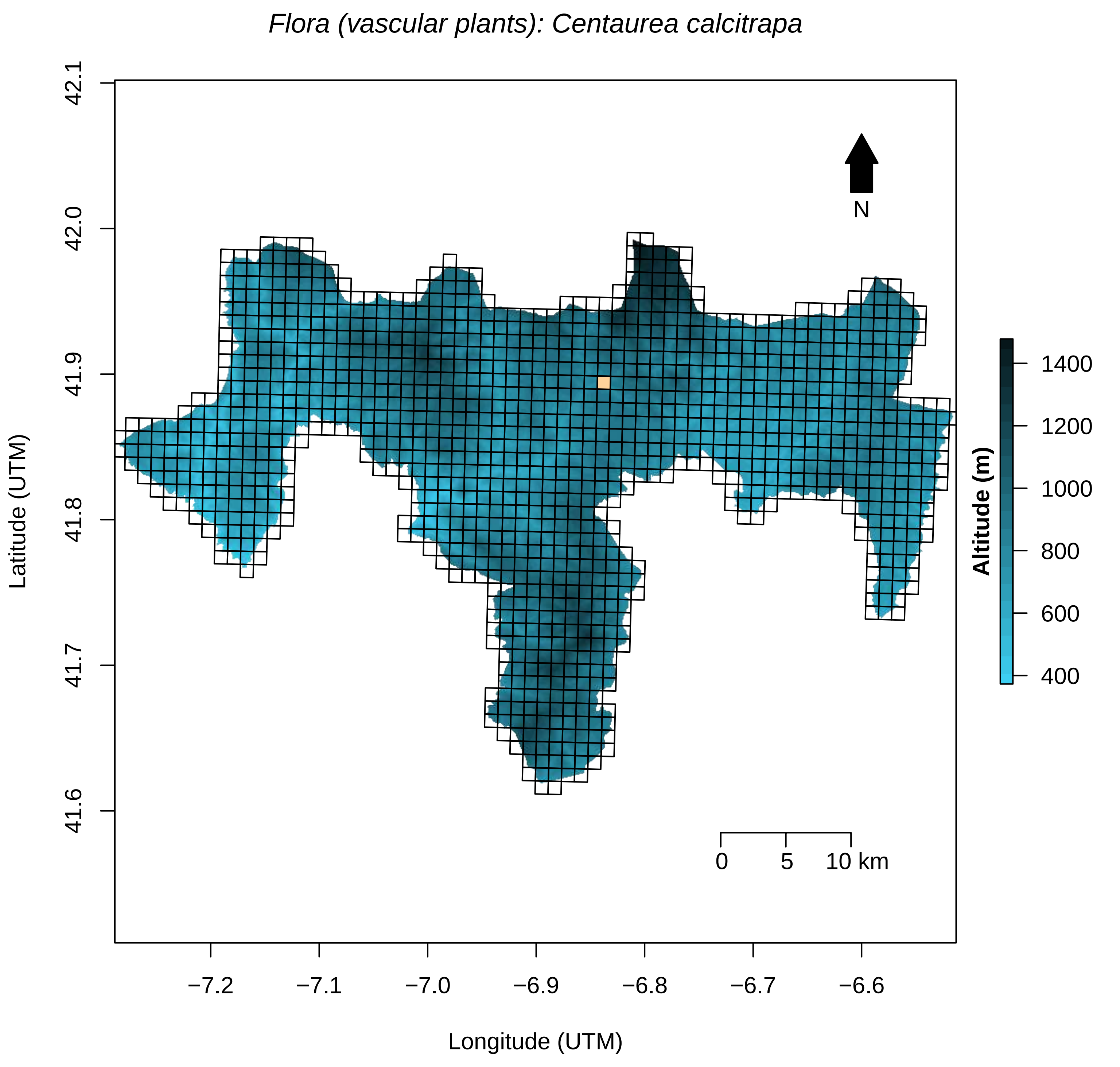

Flora (vascular plants): Centaurea calcitrapa
42.1
42.0
41.9
41.8
41.7
41.6
Latitude (UTM)
−7.2
−7.1
−7.0
−6.9
−6.8
−6.7
−6.6
Longitude (UTM)
N
0
5
10 km
Altitude (m)
1400
1200
1000
800
600
400

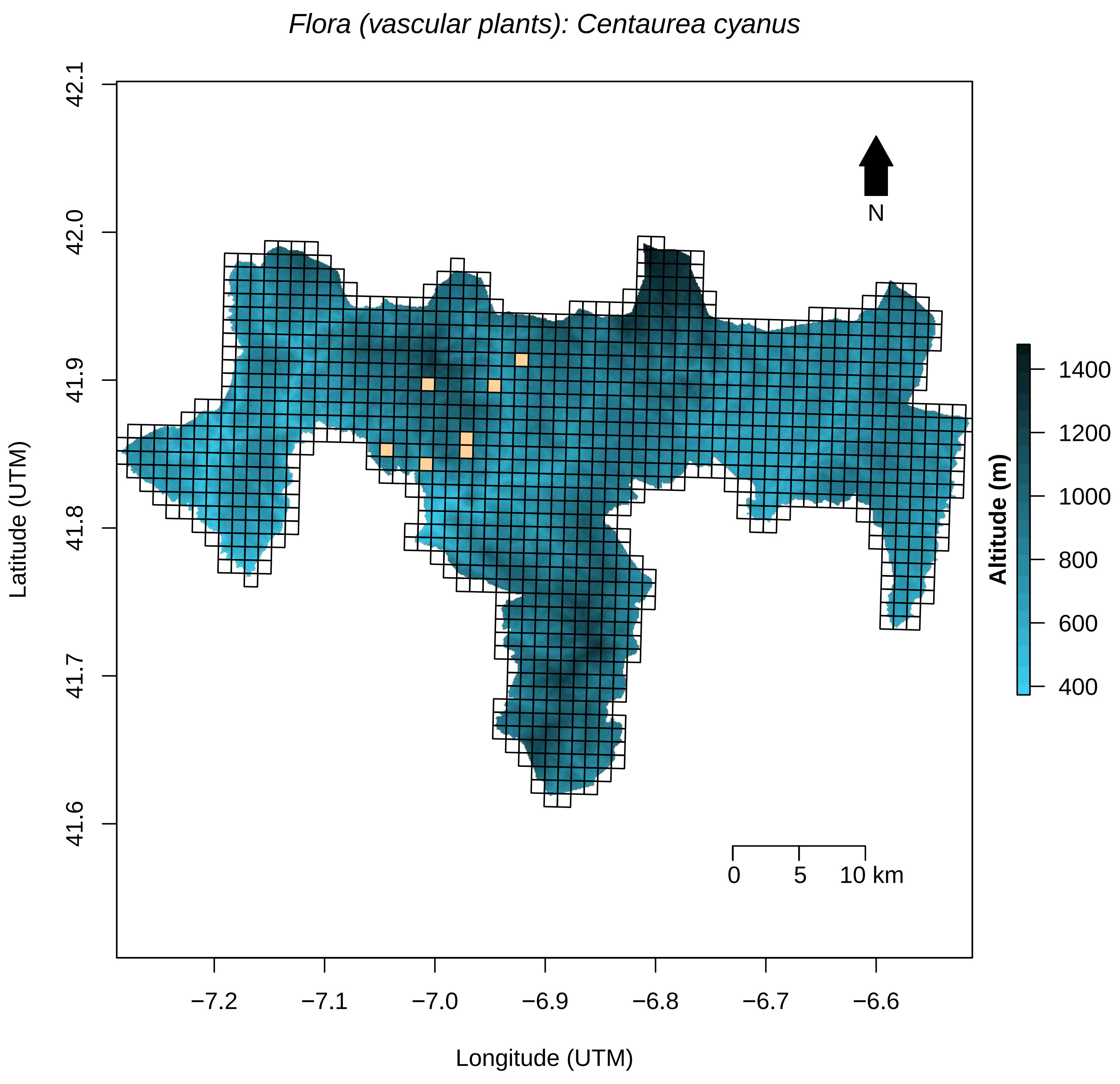

Flora (vascular plants): Centaurea cyanus
42.1
42.0
41.9
41.8
41.7
41.6
Latitude (UTM)
−7.2
−7.1
−7.0
−6.9
−6.8
−6.7
−6.6
Longitude (UTM)
N
0
5
10 km
Altitude (m)
1400
1200
1000
800
600
400

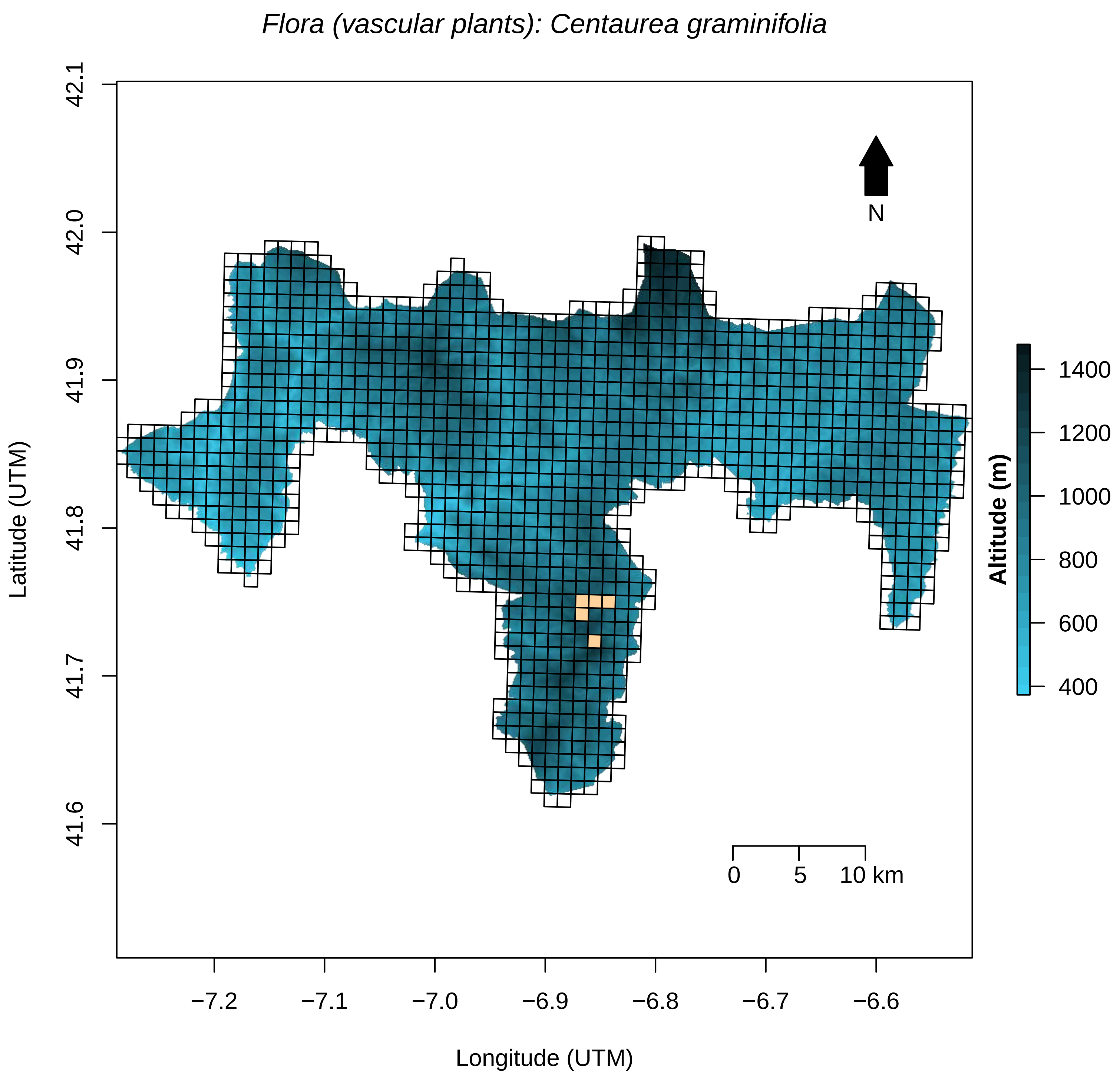

Flora (vascular plants): Centaurea graminifolia
42.1
42.0
41.9
41.8
41.7
41.6
Latitude (UTM)
−7.2
−7.1
−7.0
−6.9
−6.8
−6.7
−6.6
Longitude (UTM)
N
0
5
10 km
Altitude (m)
1400
1200
1000
800
600
400

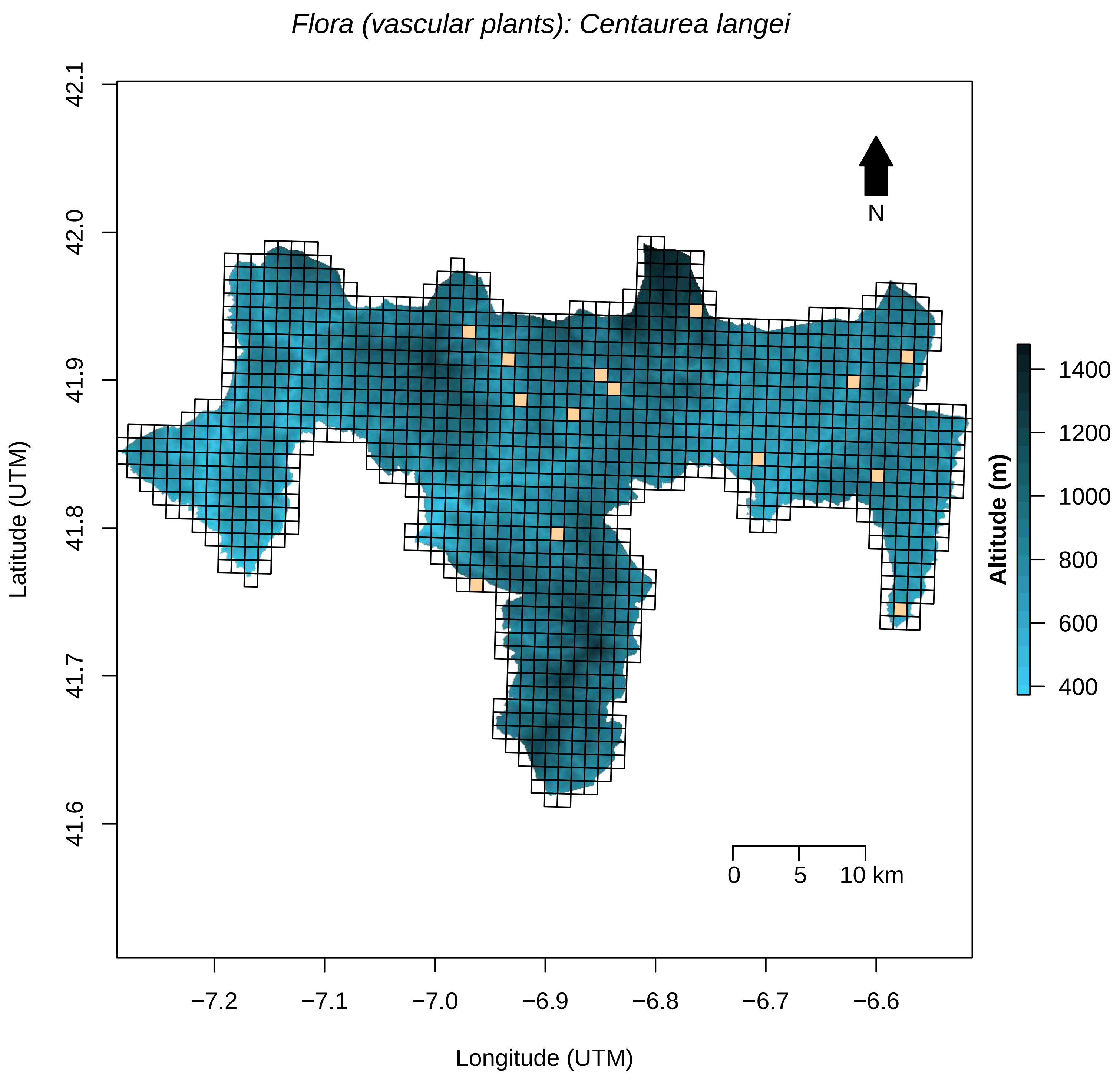

Flora (vascular plants): Centaurea langei
42.1
42.0
41.9
41.8
41.7
41.6
N
0
5
10 km
1400
1200
1000
800
600
400
Altitude (m)
−7.2
−7.1
−7.0
−6.9
−6.8
−6.7
−6.6
Latitude (UTM)
Longitude (UTM)

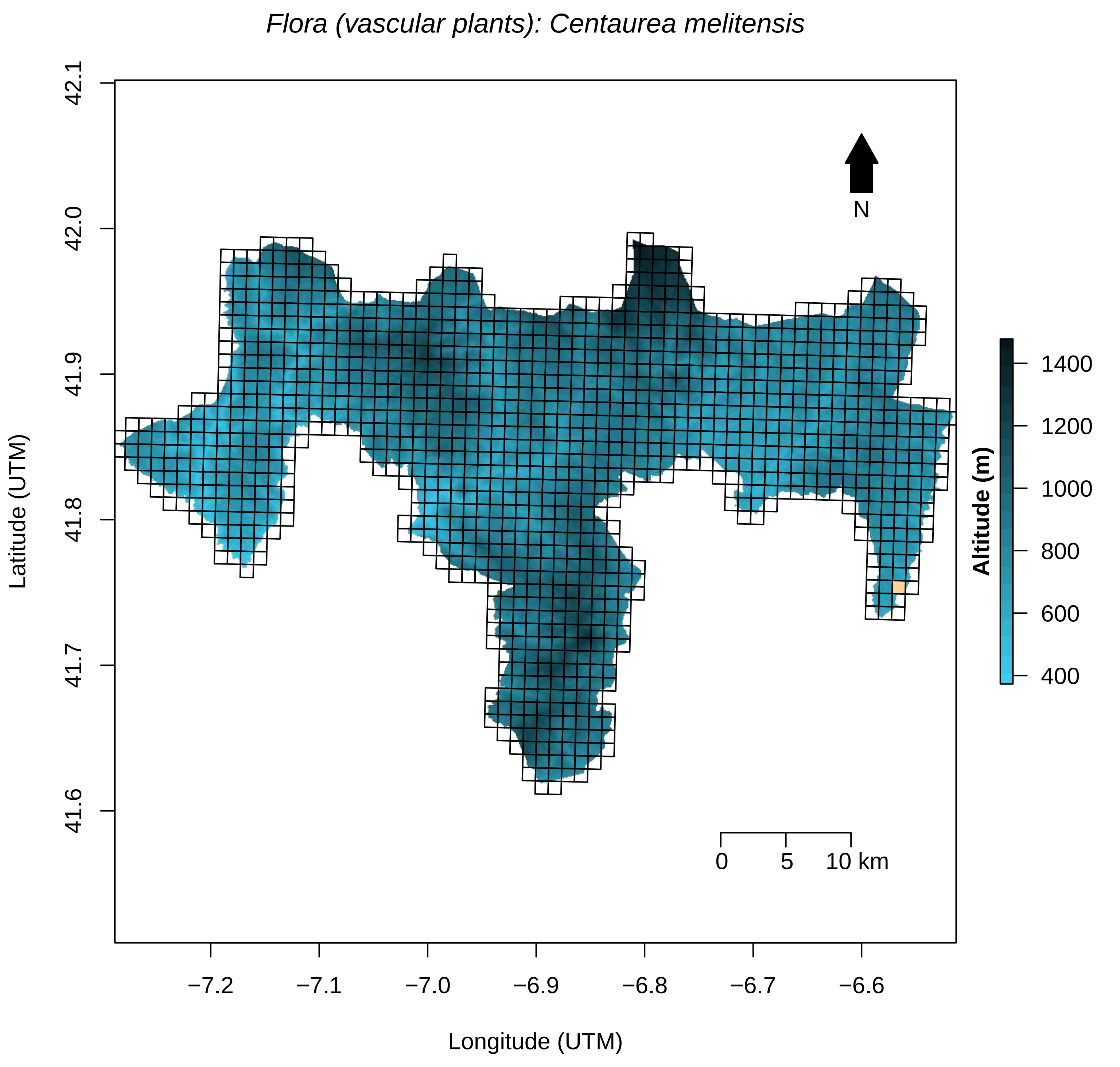
Flora (vascular plants): Centaurea melitensis
N
0 5 10 km
Latitude (UTM)
Longitude (UTM)
Altitude (m)
42.1
42.0
41.9
41.8
41.7
41.6
−7.2
−7.1
−7.0
−6.9
−6.8
−6.7
−6.6
1400
1200
1000
800
600
400

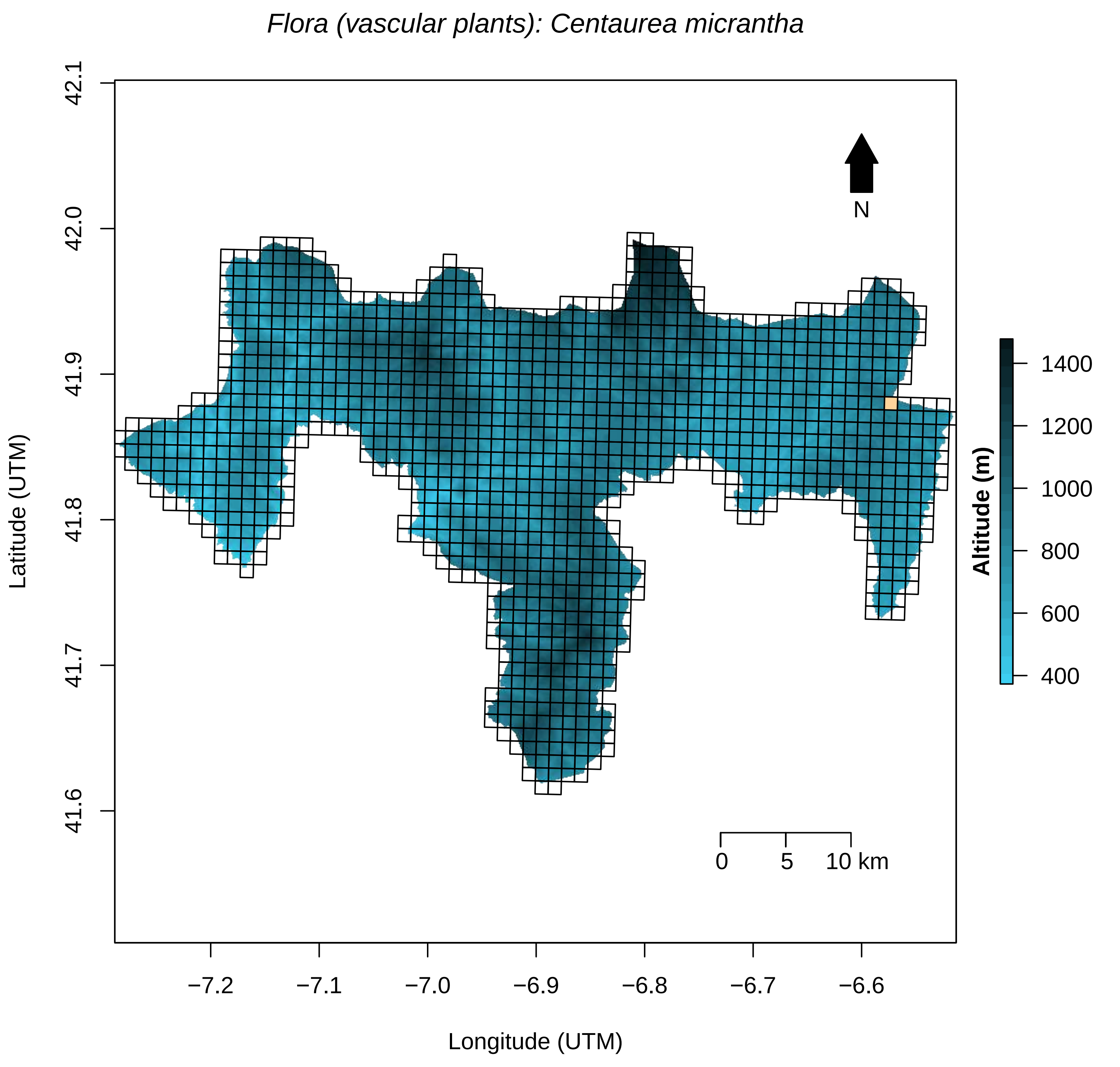

Flora (vascular plants): Centaurea micrantha
N
0
5
10 km
Altitude (m)
1400
1200
1000
800
600
400
Latitude (UTM)
42.1
42.0
41.9
41.8
41.7
41.6
Longitude (UTM)
−7.2
−7.1
−7.0
−6.9
−6.8
−6.7
−6.6

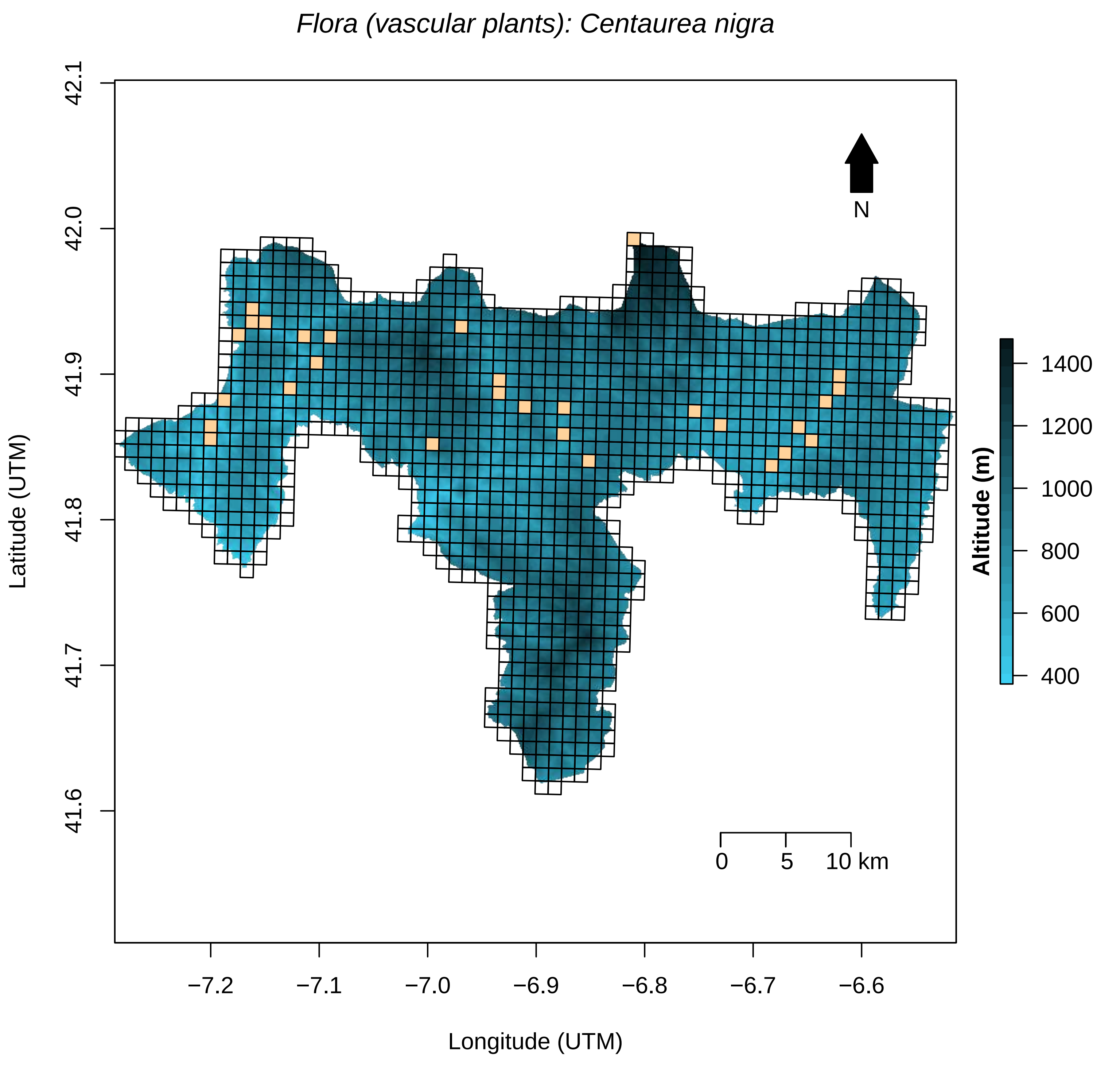
Flora (vascular plants): Centaurea nigra
N
0 5 10 km
Altitude (m)
1400
1200
1000
800
600
400
Latitude (UTM)
42.1
42.0
41.9
41.8
41.7
41.6
Longitude (UTM)
−7.2
−7.1
−7.0
−6.9
−6.8
−6.7
−6.6

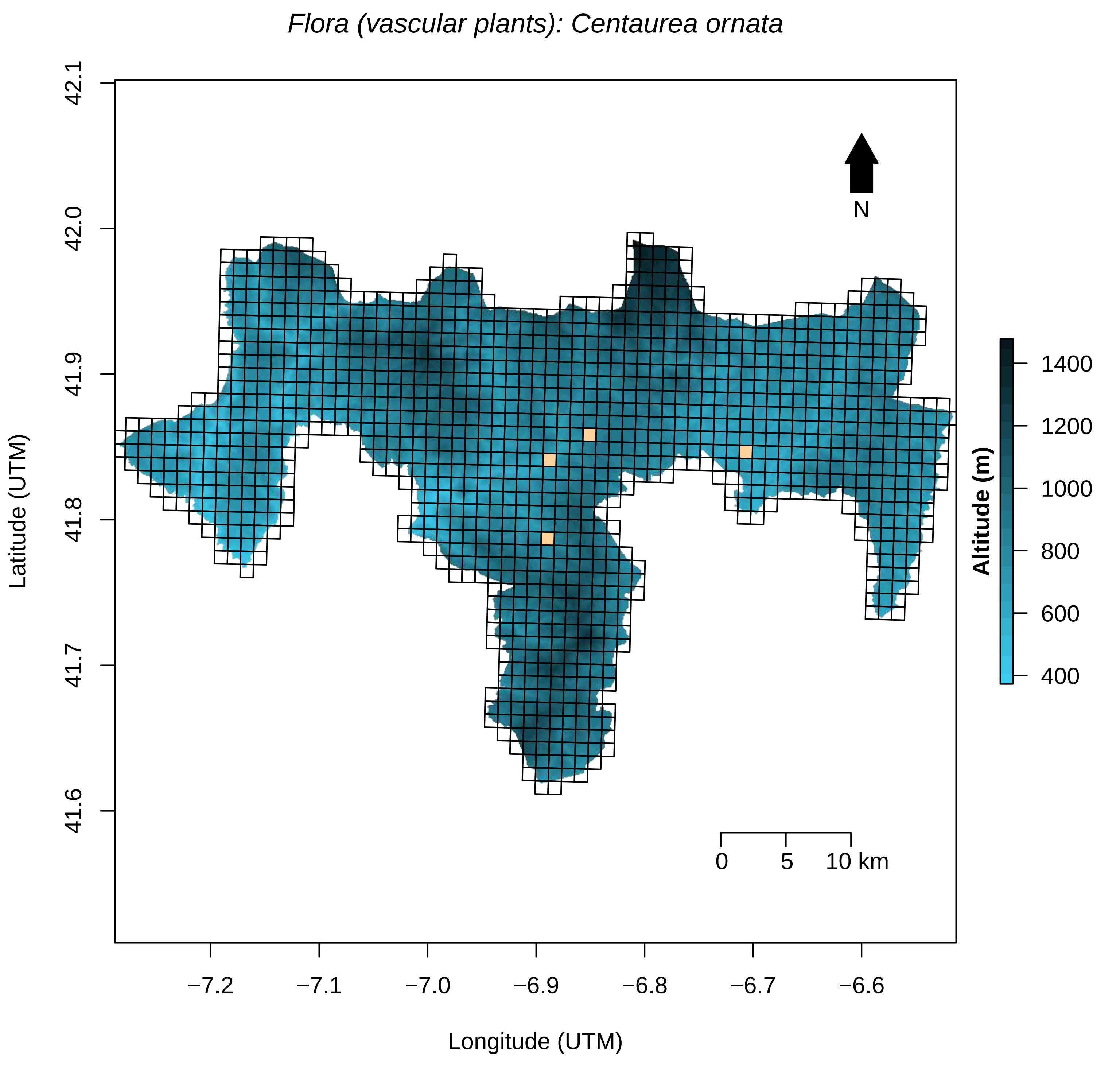
Flora (vascular plants): Centaurea ornata
42.1
42.0
41.9
41.8
41.7
41.6
Latitude (UTM)
−7.2
−7.1
−7.0
−6.9
−6.8
−6.7
−6.6
Longitude (UTM)
N
0
5
10 km
Altitude (m)
1400
1200
1000
800
600
400

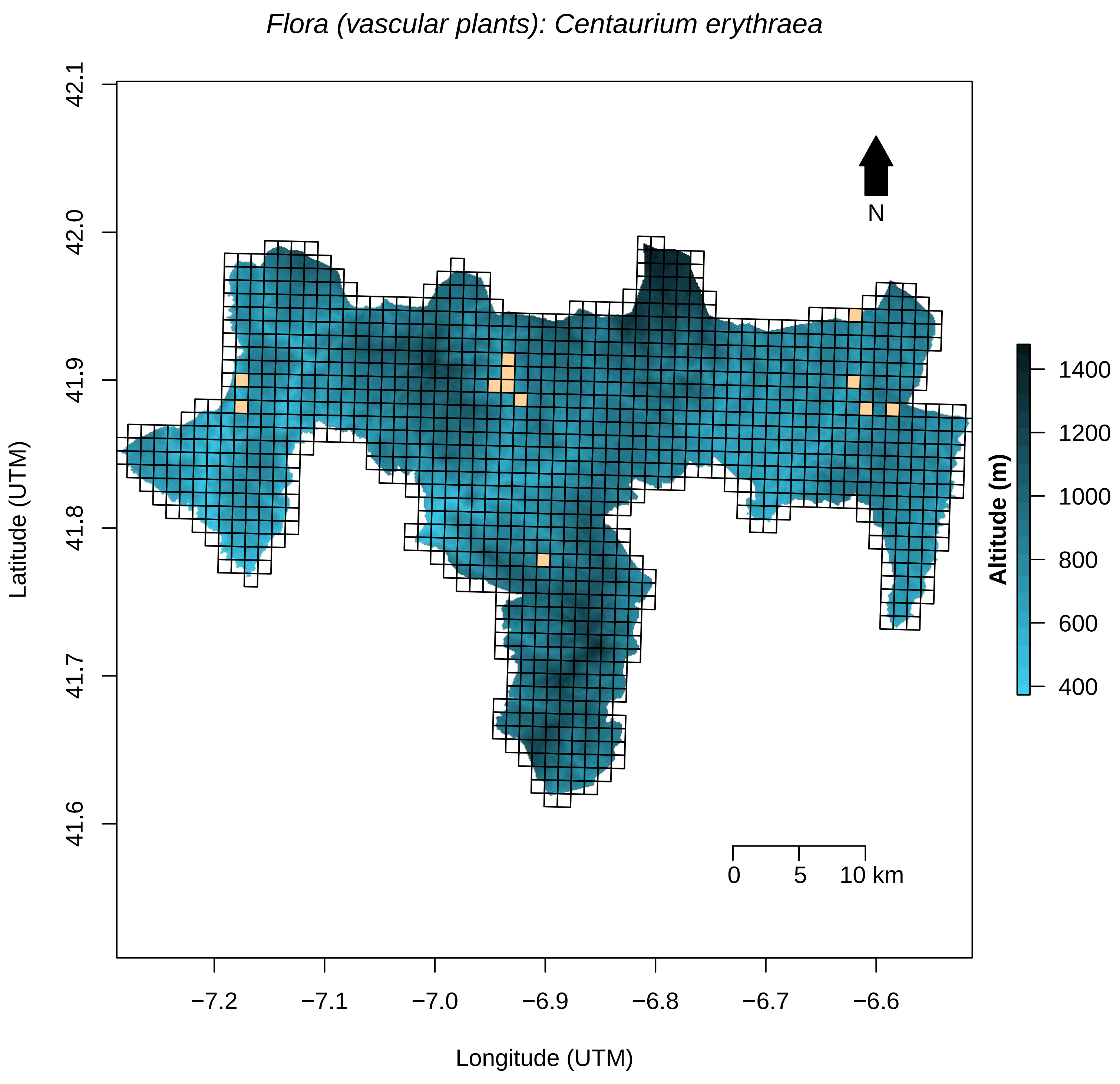

Flora (vascular plants): Centaurium erythraea
42.1
42.0
41.9
41.8
41.7
41.6
Latitude (UTM)
−7.2
−7.1
−7.0
−6.9
−6.8
−6.7
−6.6
Longitude (UTM)
N
0
5
10 km
1400
1200
1000
800
600
400
Altitude (m)

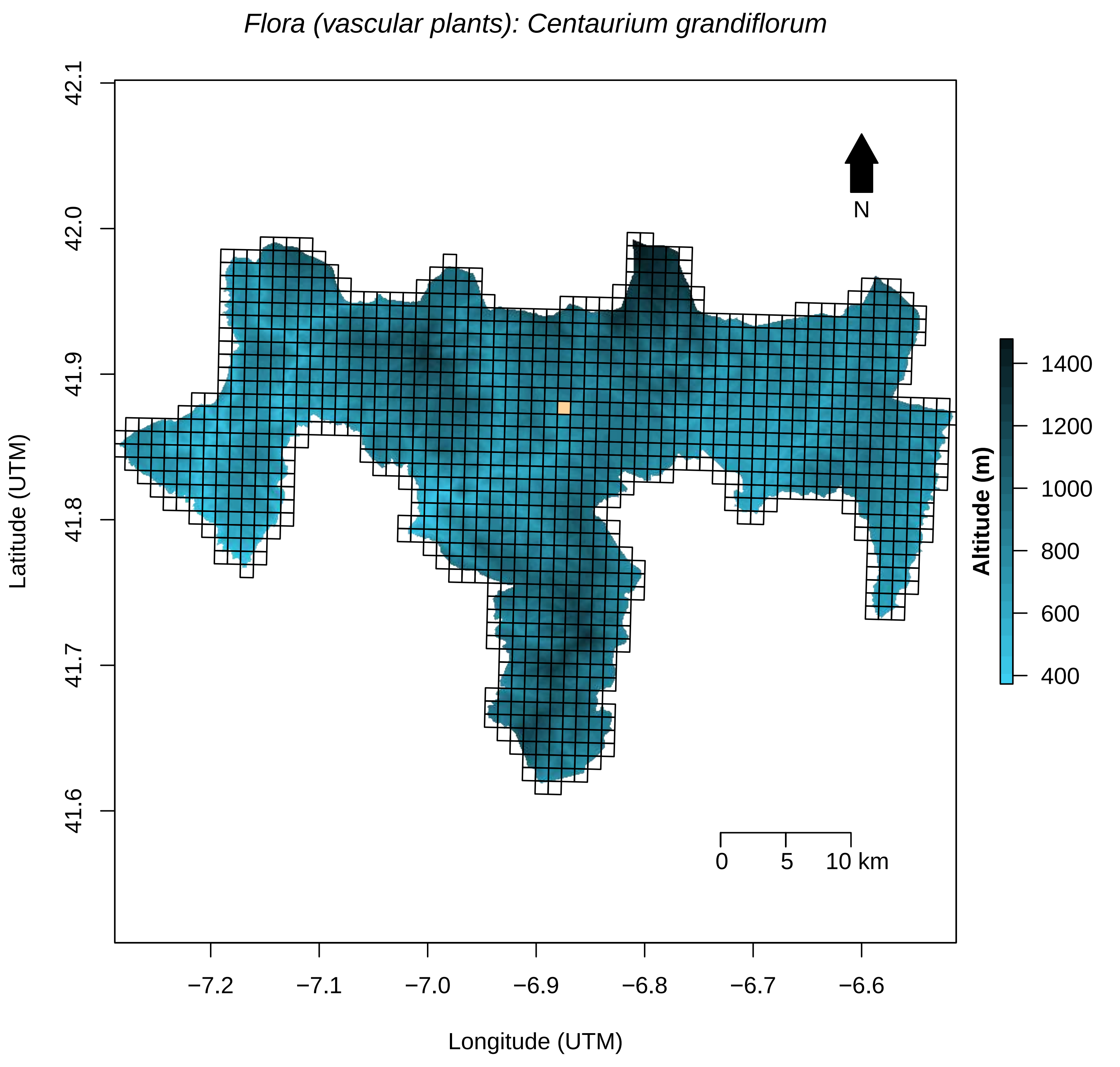

Flora (vascular plants): Centaurium grandiflorum
42.1
42.0
41.9
41.8
41.7
41.6
Latitude (UTM)
−7.2
−7.1
−7.0
−6.9
−6.8
−6.7
−6.6
Longitude (UTM)
N
0
5
10 km
1400
1200
1000
800
600
400
Altitude (m)

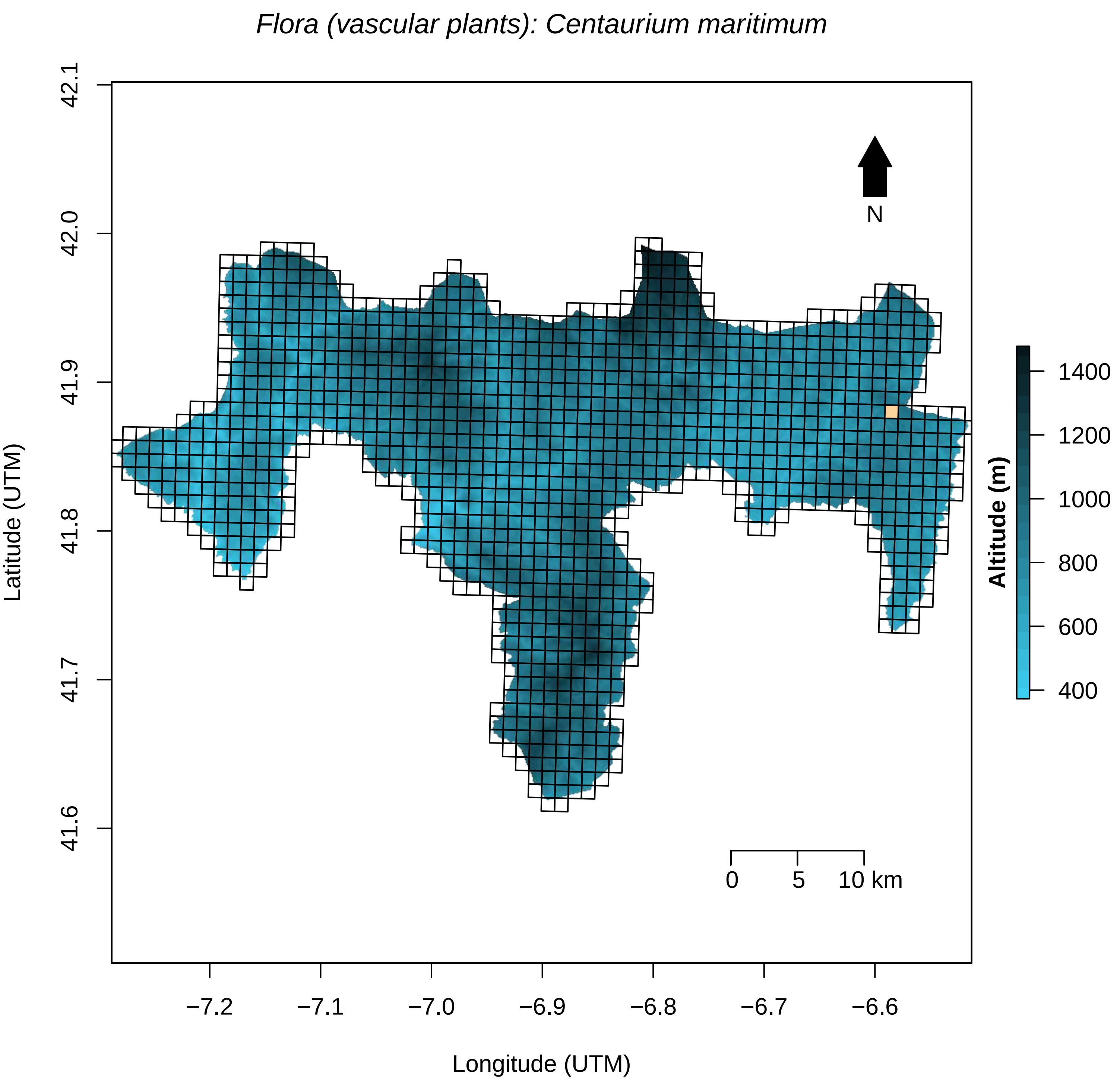

Flora (vascular plants): Centaurium maritimum
42.1
42.0
41.9
41.8
41.7
41.6
Latitude (UTM)
N
1400
1200
1000
800
600
400
Altitude (m)
0
5
10 km
−7.2
−7.1
−7.0
−6.9
−6.8
−6.7
−6.6
Longitude (UTM)

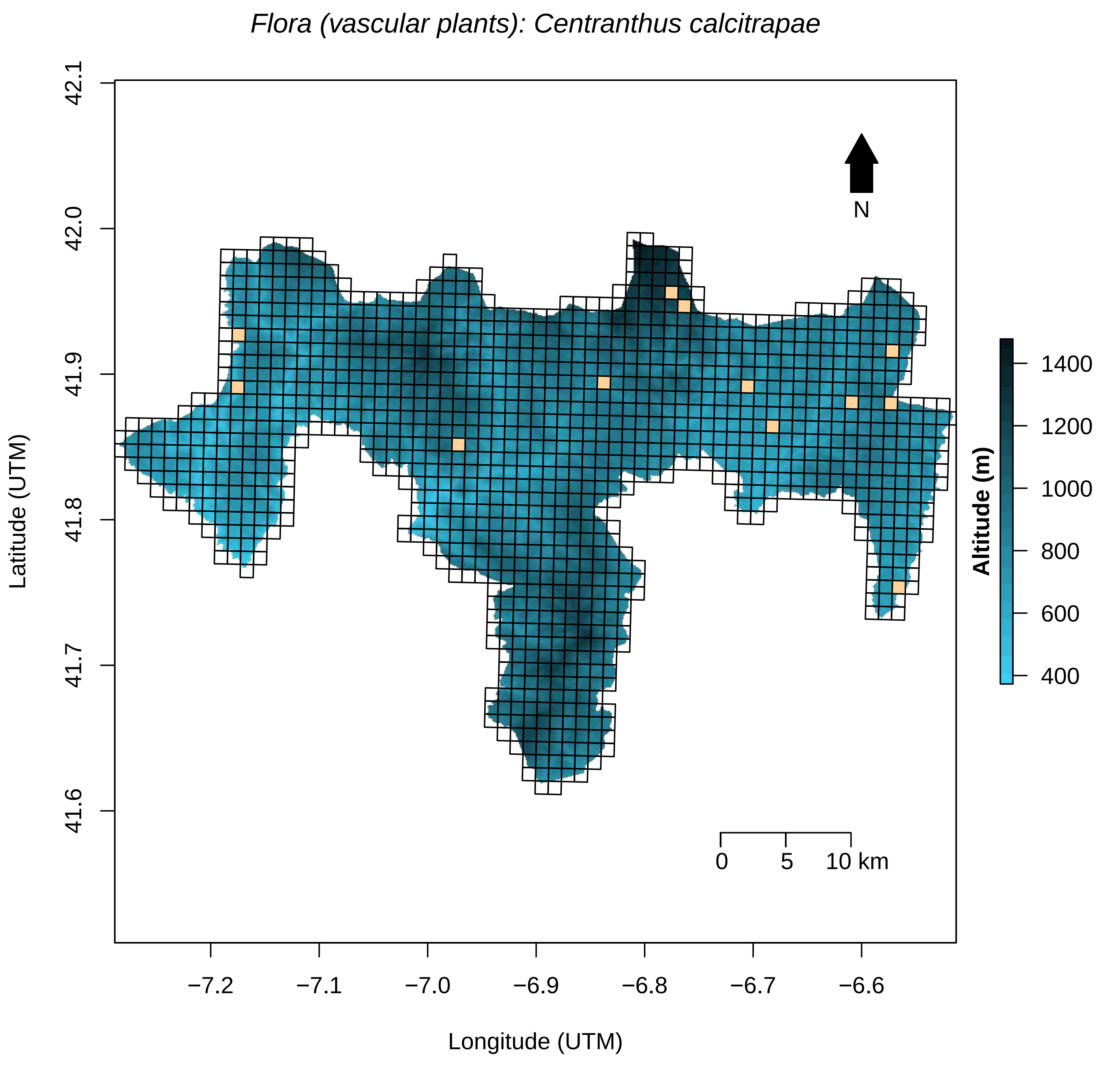

Flora (vascular plants): Centranthus calcitrapae
N
0
5
10 km
Latitude (UTM)
Longitude (UTM)
Altitude (m)
42.1
42.0
41.9
41.8
41.7
41.6
−7.2
−7.1
−7.0
−6.9
−6.8
−6.7
−6.6
1400
1200
1000
800
600
400

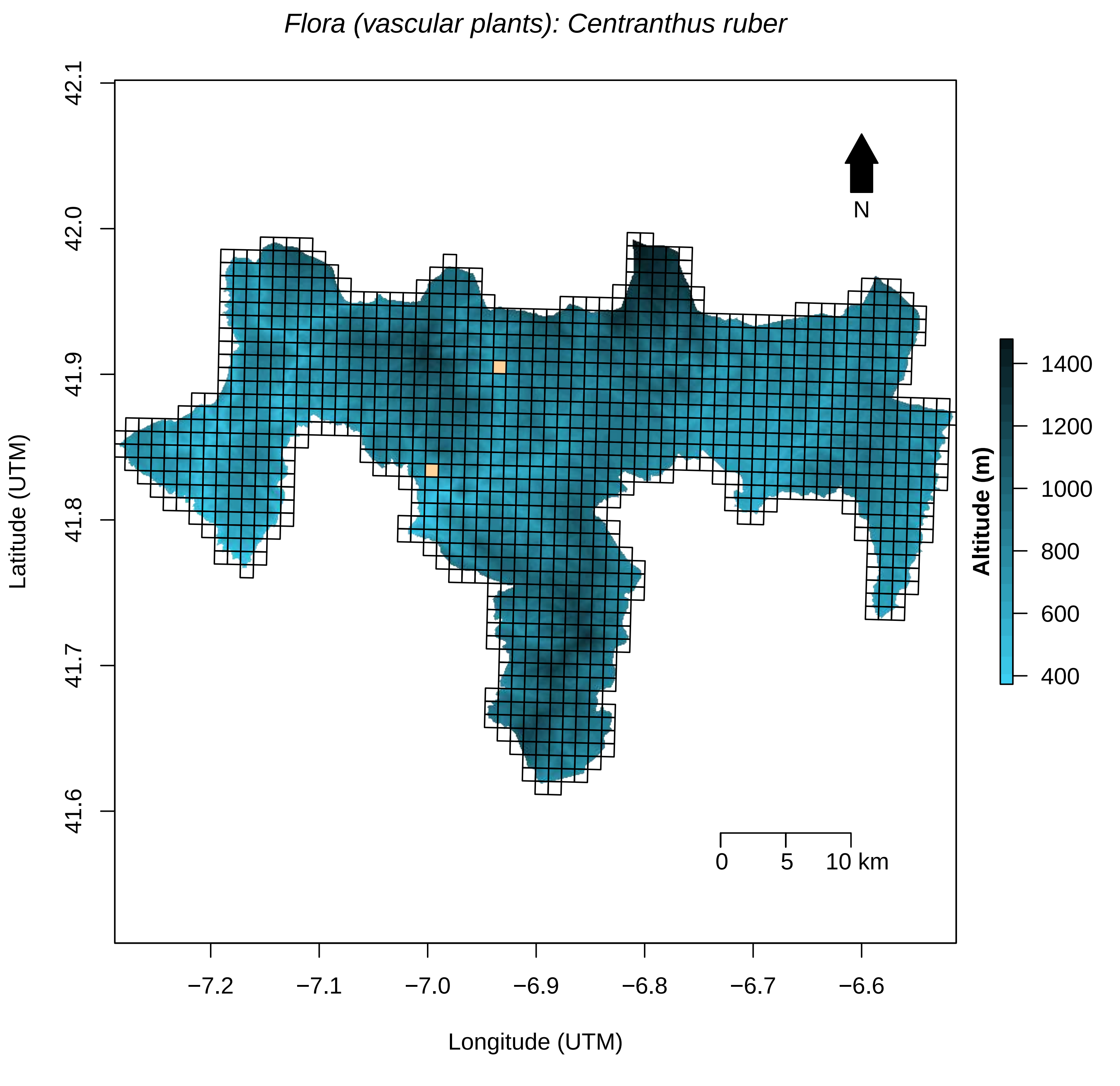

Flora (vascular plants): Centranthus ruber
42.1
42.0
41.9
41.8
41.7
41.6
Latitude (UTM)
−7.2
−7.1
−7.0
−6.9
−6.8
−6.7
−6.6
Longitude (UTM)
N
0
5
10 km
Altitude (m)
1400
1200
1000
800
600
400

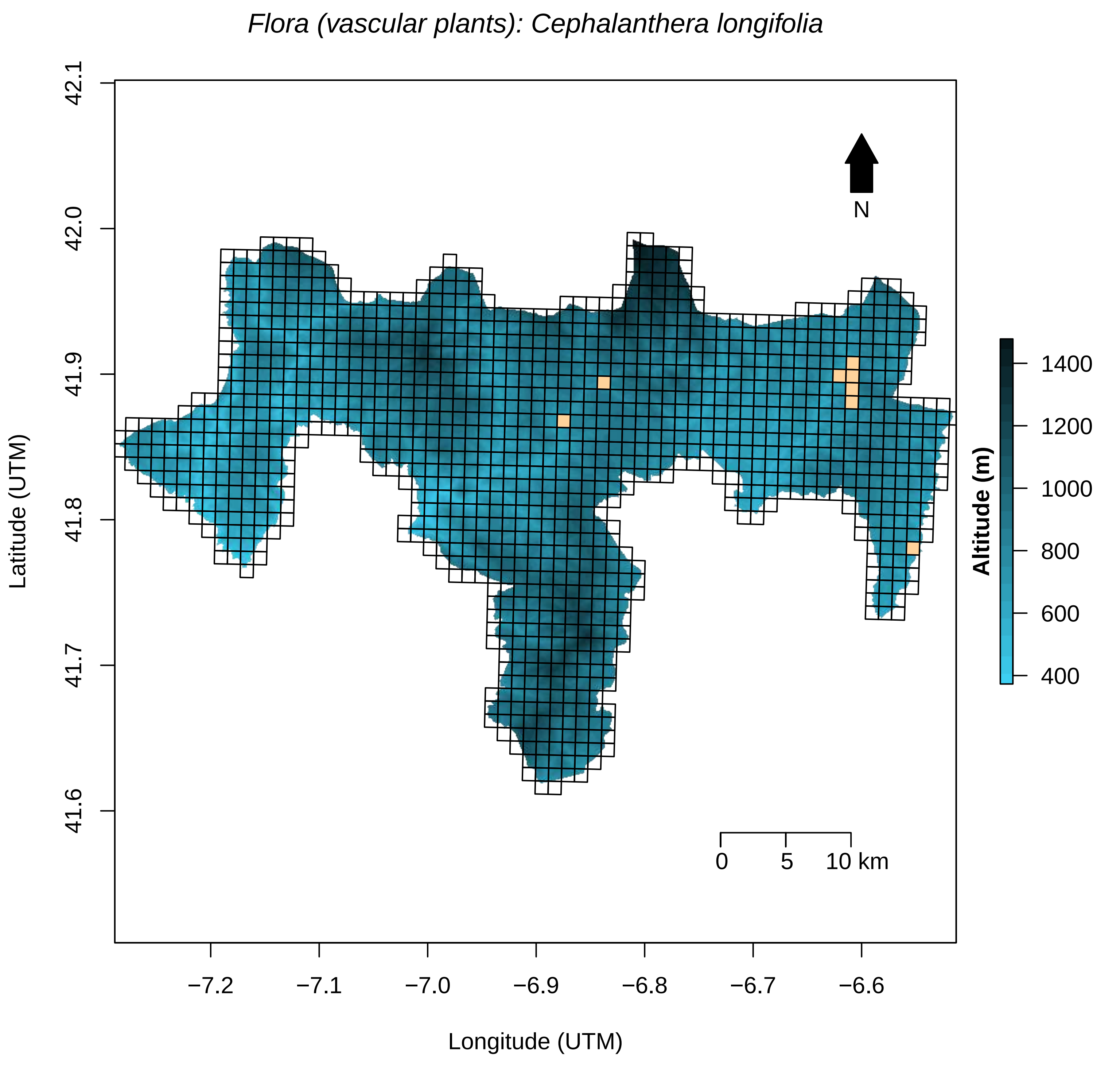

Flora (vascular plants): Cephalanthera longifolia
N
0 5 10 km
Altitude (m)
1400
1200
1000
800
600
400
42.1
42.0
41.9
41.8
41.7
41.6
Latitude (UTM)
−7.2
−7.1
−7.0
−6.9
−6.8
−6.7
−6.6
Longitude (UTM)

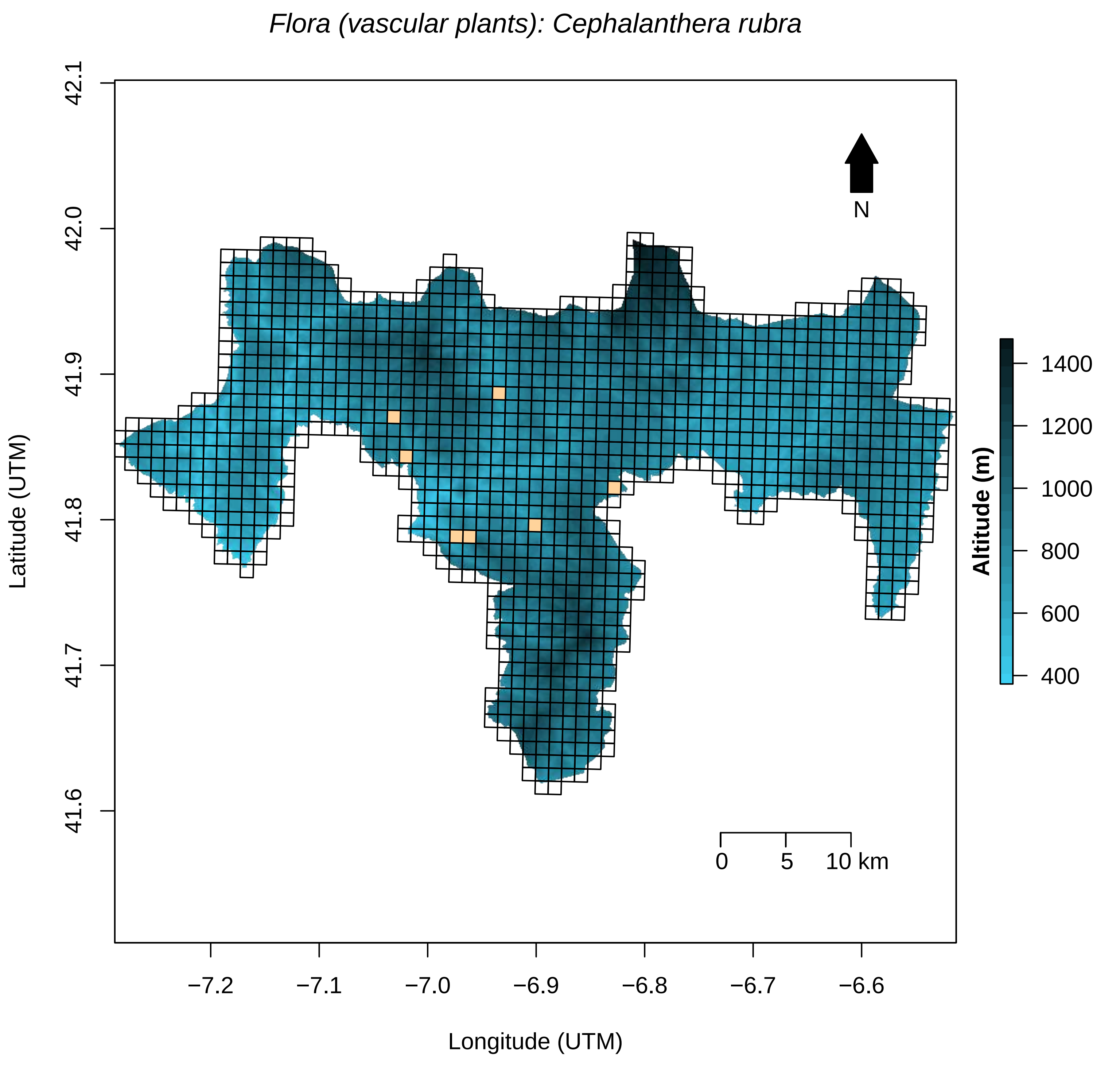

Flora (vascular plants): Cephalanthera rubra
N
0
5
10 km
Altitude (m)
1400
1200
1000
800
600
400
Latitude (UTM)
42.1
42.0
41.9
41.8
41.7
41.6
Longitude (UTM)
−7.2
−7.1
−7.0
−6.9
−6.8
−6.7
−6.6

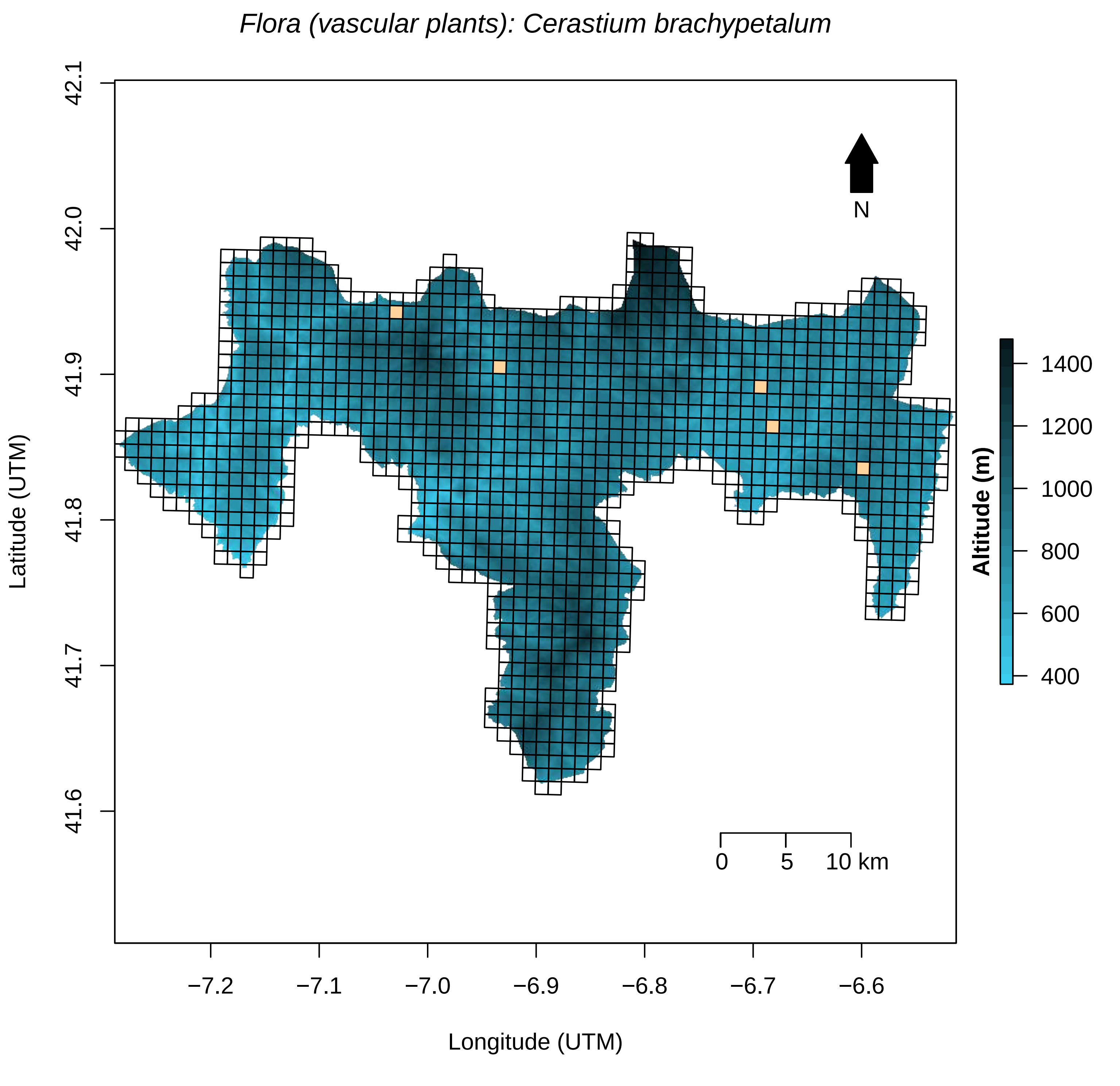

Flora (vascular plants): Cerastium brachypetalum
N
0
5
10 km
Altitude (m)
1400
1200
1000
800
600
400
42.1
42.0
41.9
41.8
41.7
41.6
Latitude (UTM)
−7.2
−7.1
−7.0
−6.9
−6.8
−6.7
−6.6
Longitude (UTM)

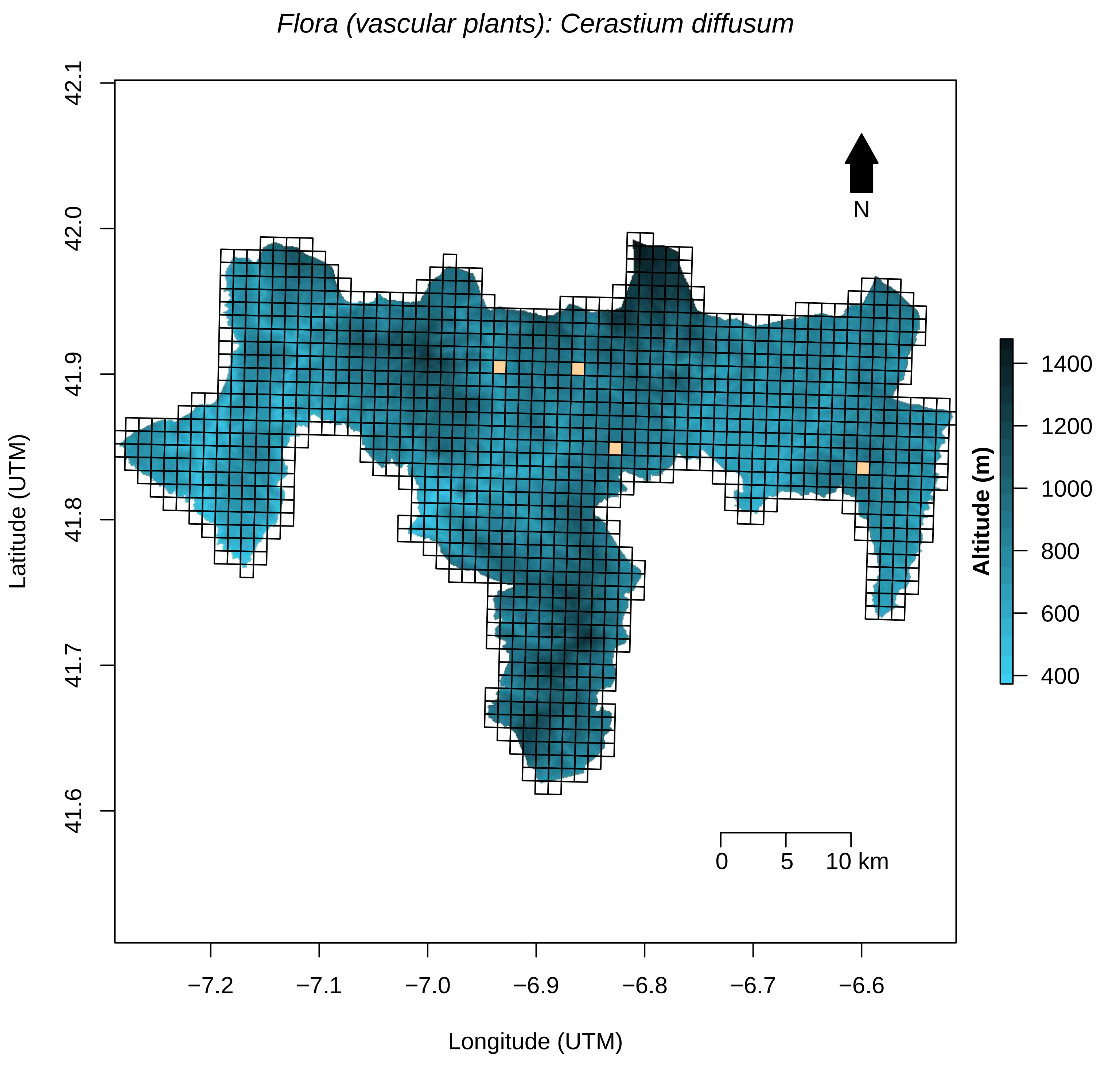

Flora (vascular plants): Cerastium diffusum
N
0
5
10 km
Altitude (m)
1400
1200
1000
800
600
400
Latitude (UTM)
42.1
42.0
41.9
41.8
41.7
41.6
Longitude (UTM)
−7.2
−7.1
−7.0
−6.9
−6.8
−6.7
−6.6

Flora (vascular plants): Cerastium fontanum

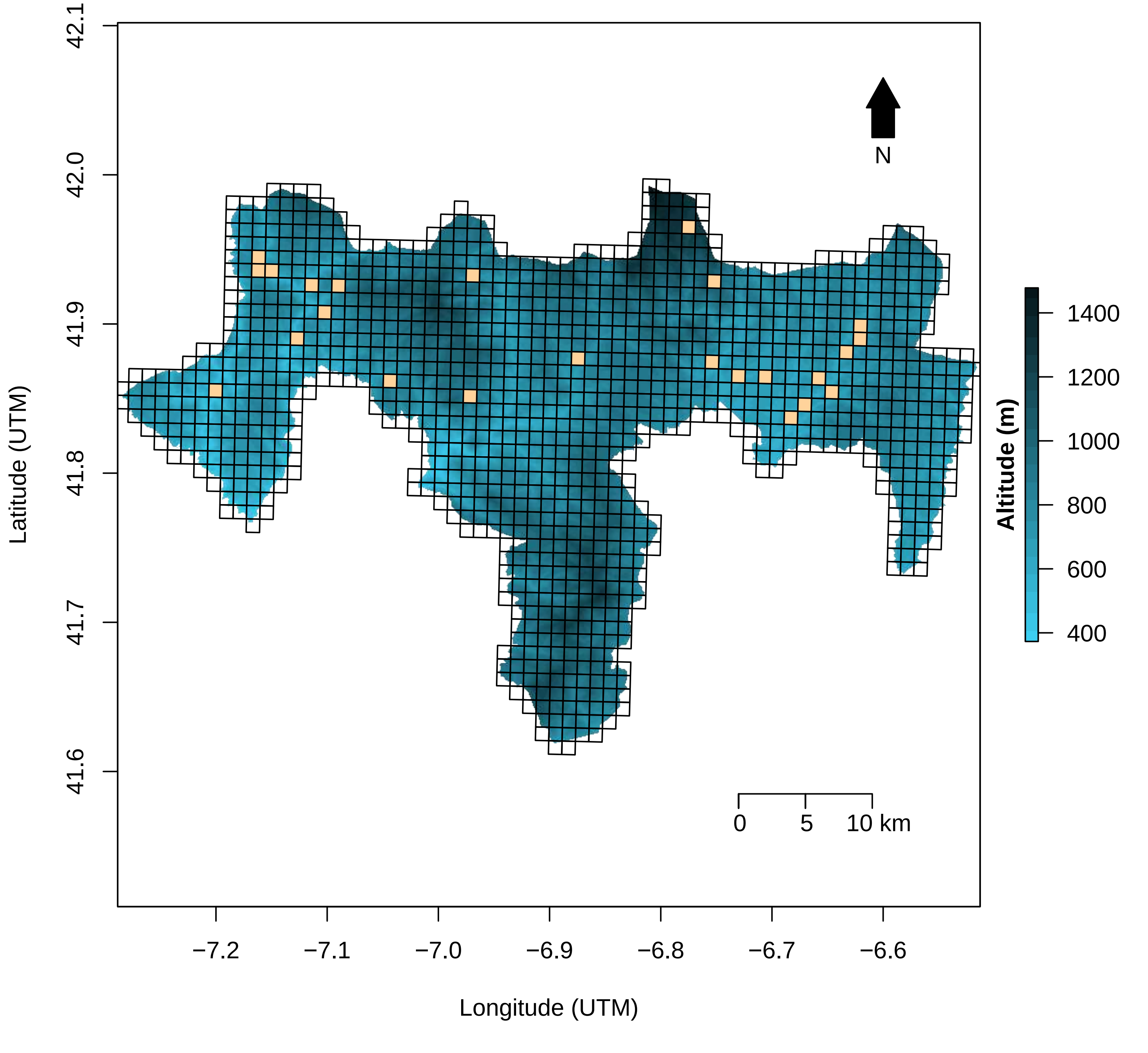

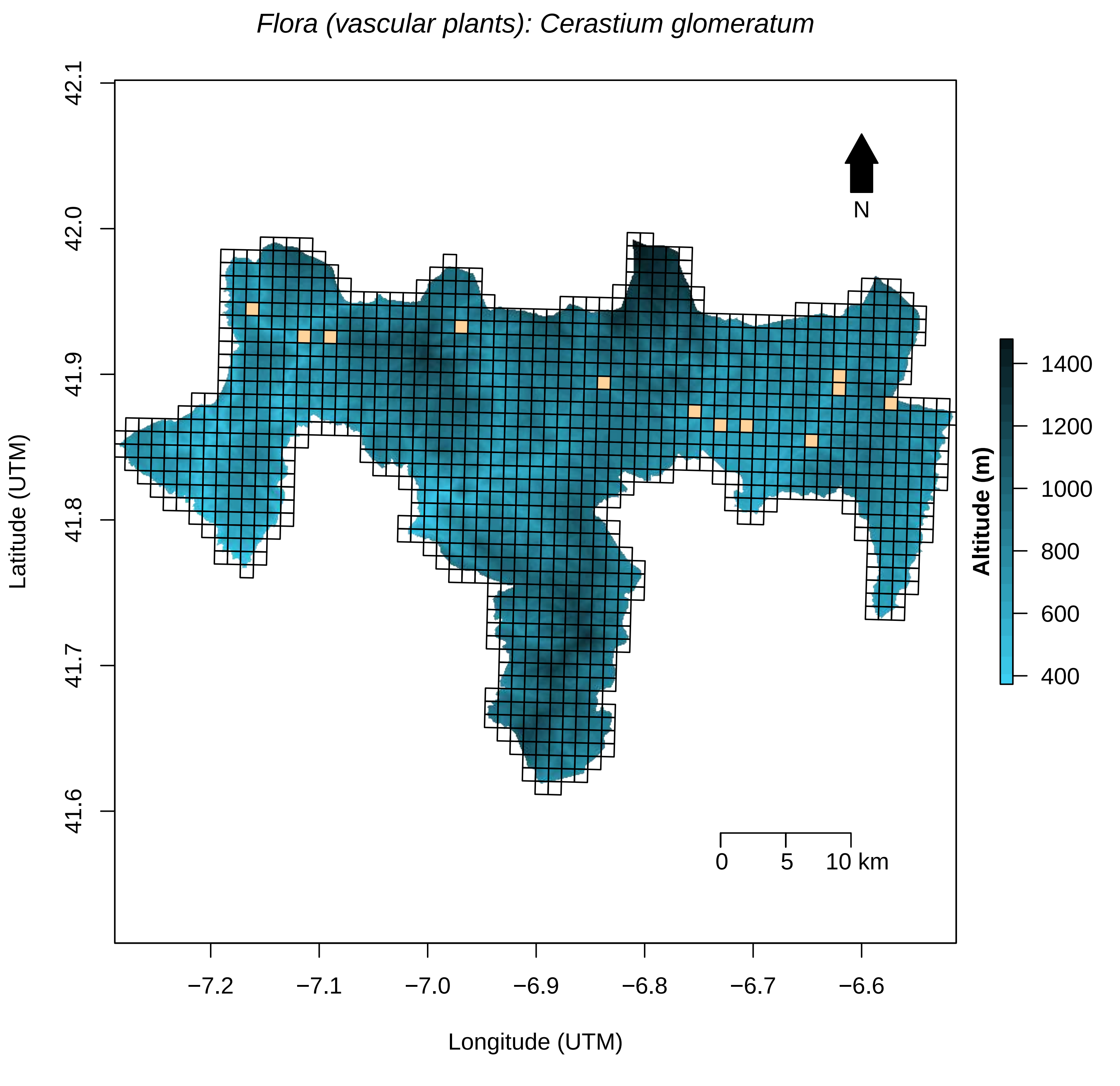

Flora (vascular plants): Cerastium glomeratum
42.1
42.0
41.9
41.8
41.7
41.6
Latitude (UTM)
−7.2
−7.1
−7.0
−6.9
−6.8
−6.7
−6.6
Longitude (UTM)
N
0
5
10 km
Altitude (m)
1400
1200
1000
800
600
400

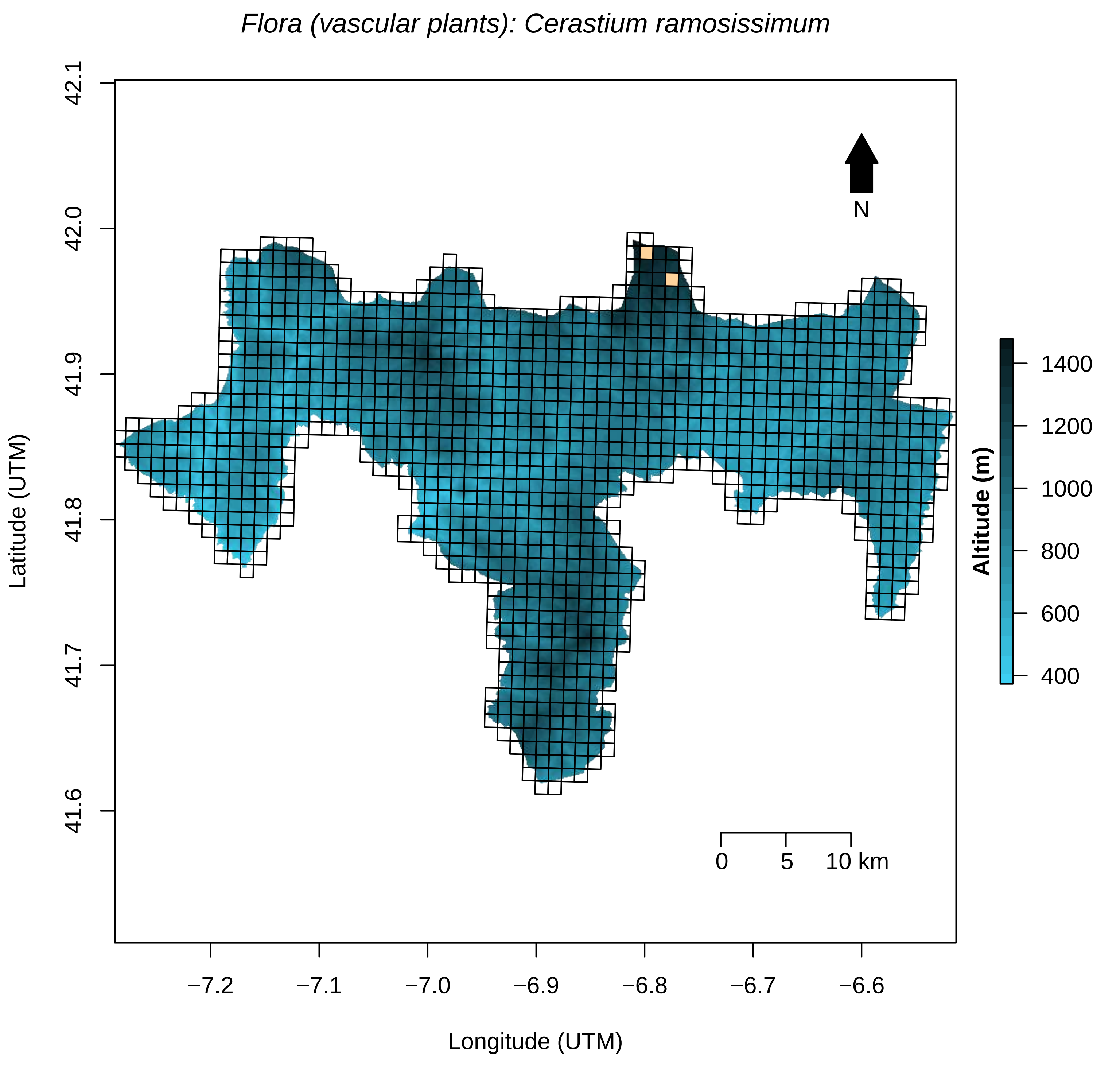

Flora (vascular plants): Cerastium ramosissimum
42.1
42.0
41.9
41.8
41.7
41.6
Latitude (UTM)
−7.2
−7.1
−7.0
−6.9
−6.8
−6.7
−6.6
Longitude (UTM)
N
0
5
10 km
1400
1200
1000
800
600
400
Altitude (m)

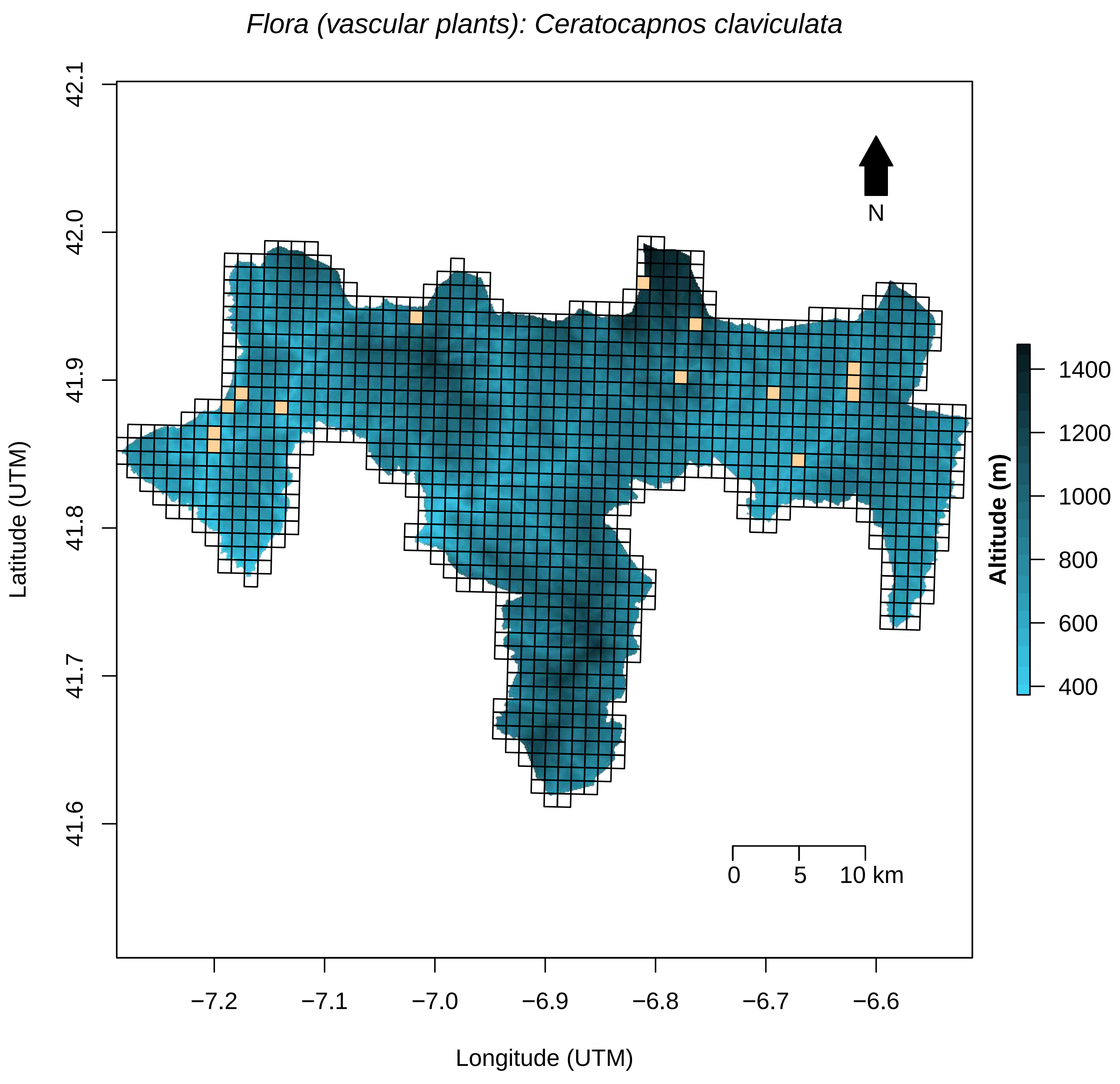

Flora (vascular plants): Ceratocapnos claviculata
Latitude (UTM)
42.1
42.0
41.9
41.8
41.7
41.6
Longitude (UTM)
−7.2
−7.1
−7.0
−6.9
−6.8
−6.7
−6.6
N
0
5
10 km
Altitude (m)
1400
1200
1000
800
600
400

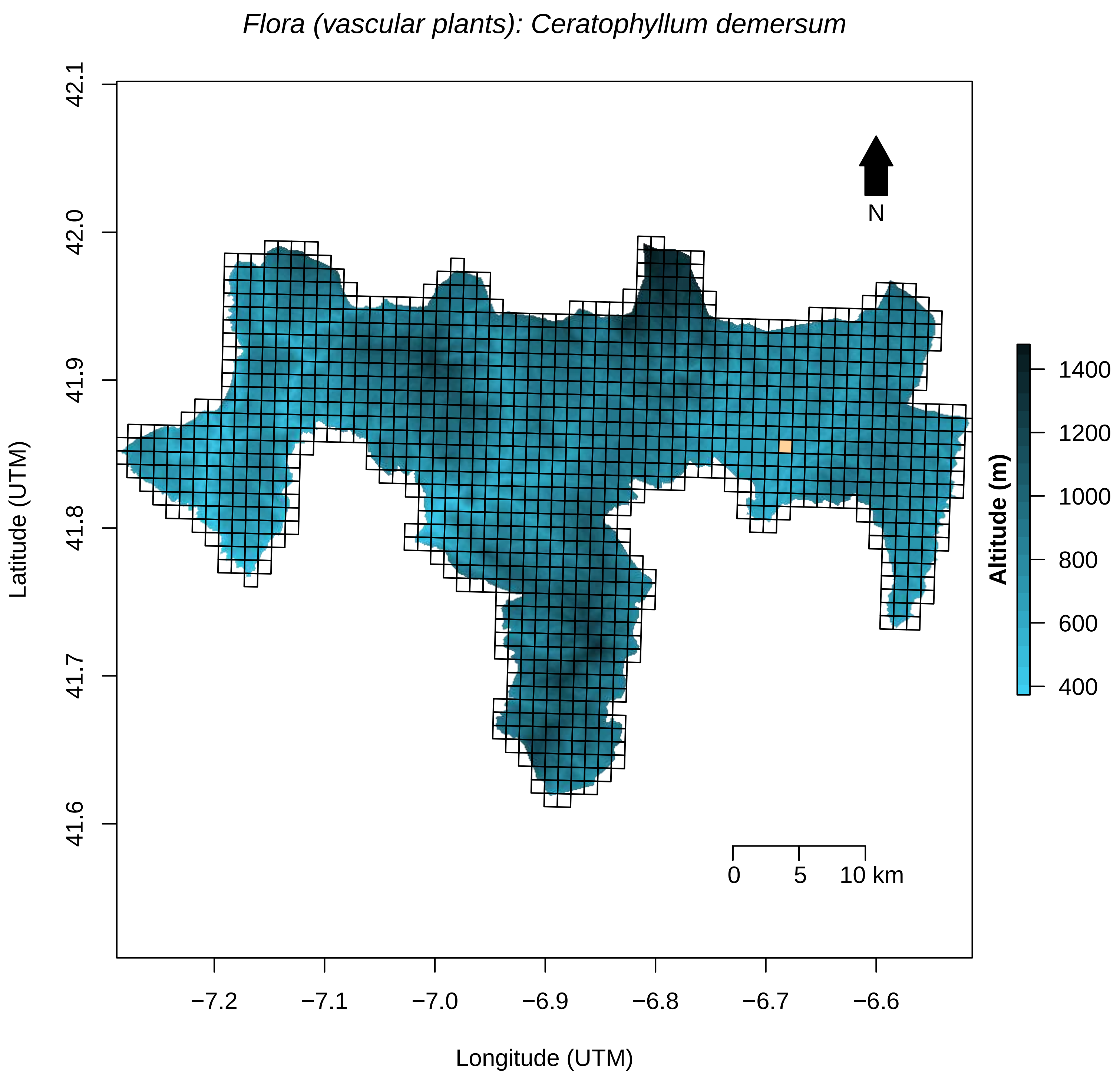
Flora (vascular plants): Ceratophyllum demersum
N
0 5 10 km
Altitude (m)
1400
1200
1000
800
600
400
Latitude (UTM)
42.1
42.0
41.9
41.8
41.7
41.6
Longitude (UTM)
−7.2
−7.1
−7.0
−6.9
−6.8
−6.7
−6.6

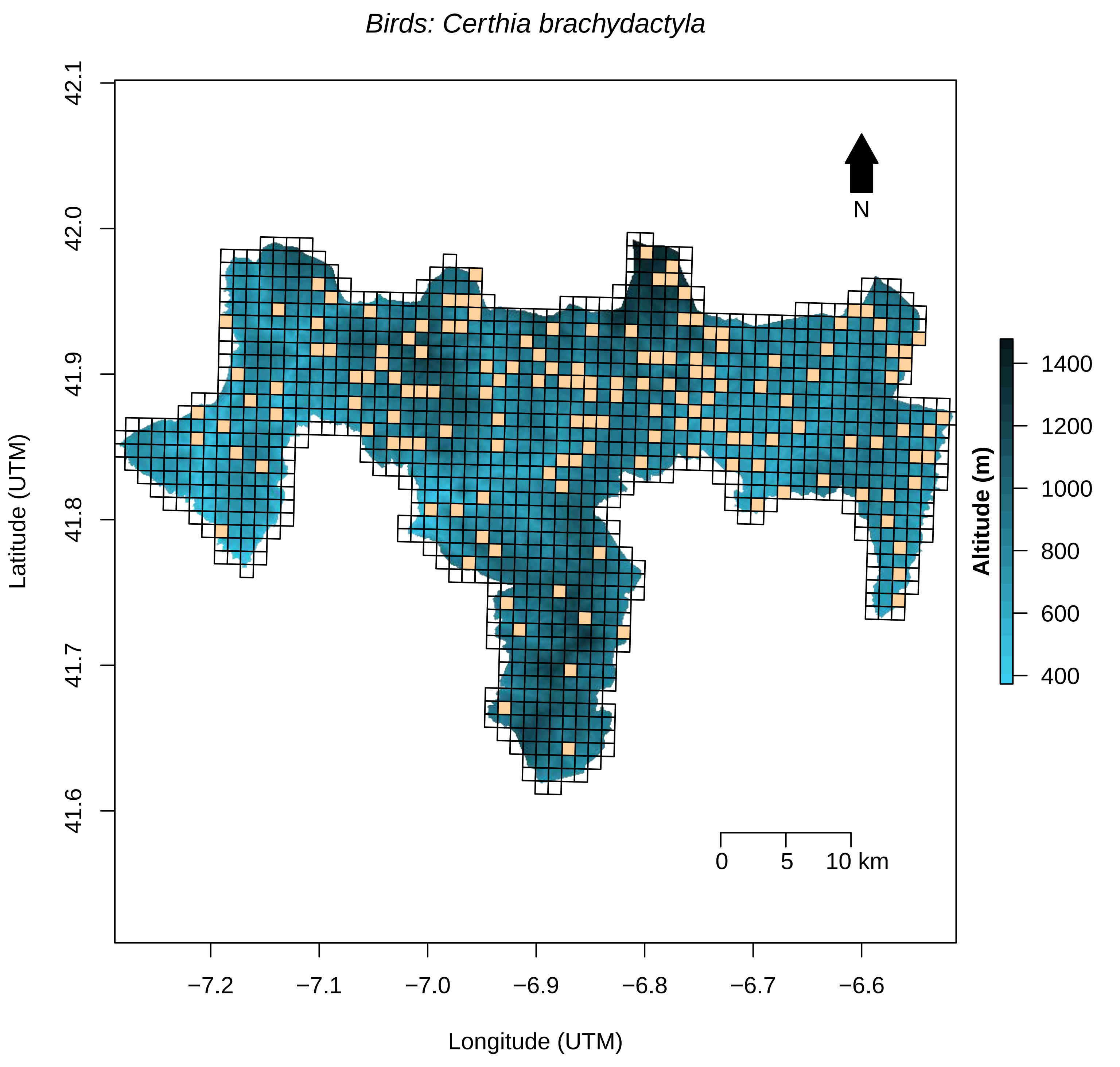

Birds: Certhia brachydactyla
Latitude (UTM)
Longitude (UTM)
42.1
42.0
41.9
41.8
41.7
41.6
−7.2
−7.1
−7.0
−6.9
−6.8
−6.7
−6.6
N
0
5
10 km
Altitude (m)
1400
1200
1000
800
600
400

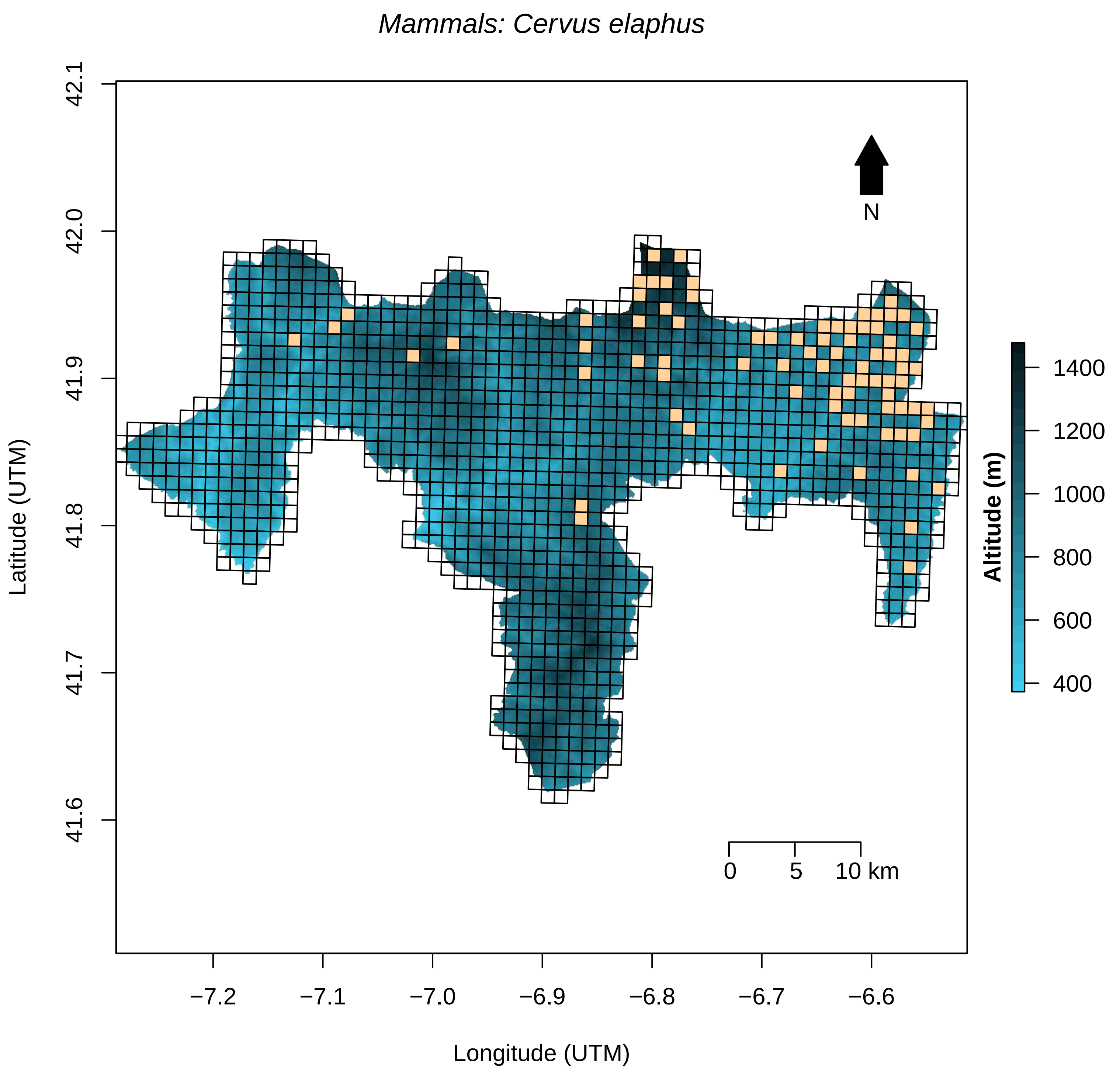
Mammals: Cervus elaphus
42.1
42.0
41.9
41.8
41.7
41.6
Latitude (UTM)
−7.2
−7.1
−7.0
−6.9
−6.8
−6.7
−6.6
Longitude (UTM)
N
0
5
10 km
1400
1200
1000
800
600
400
Altitude (m)

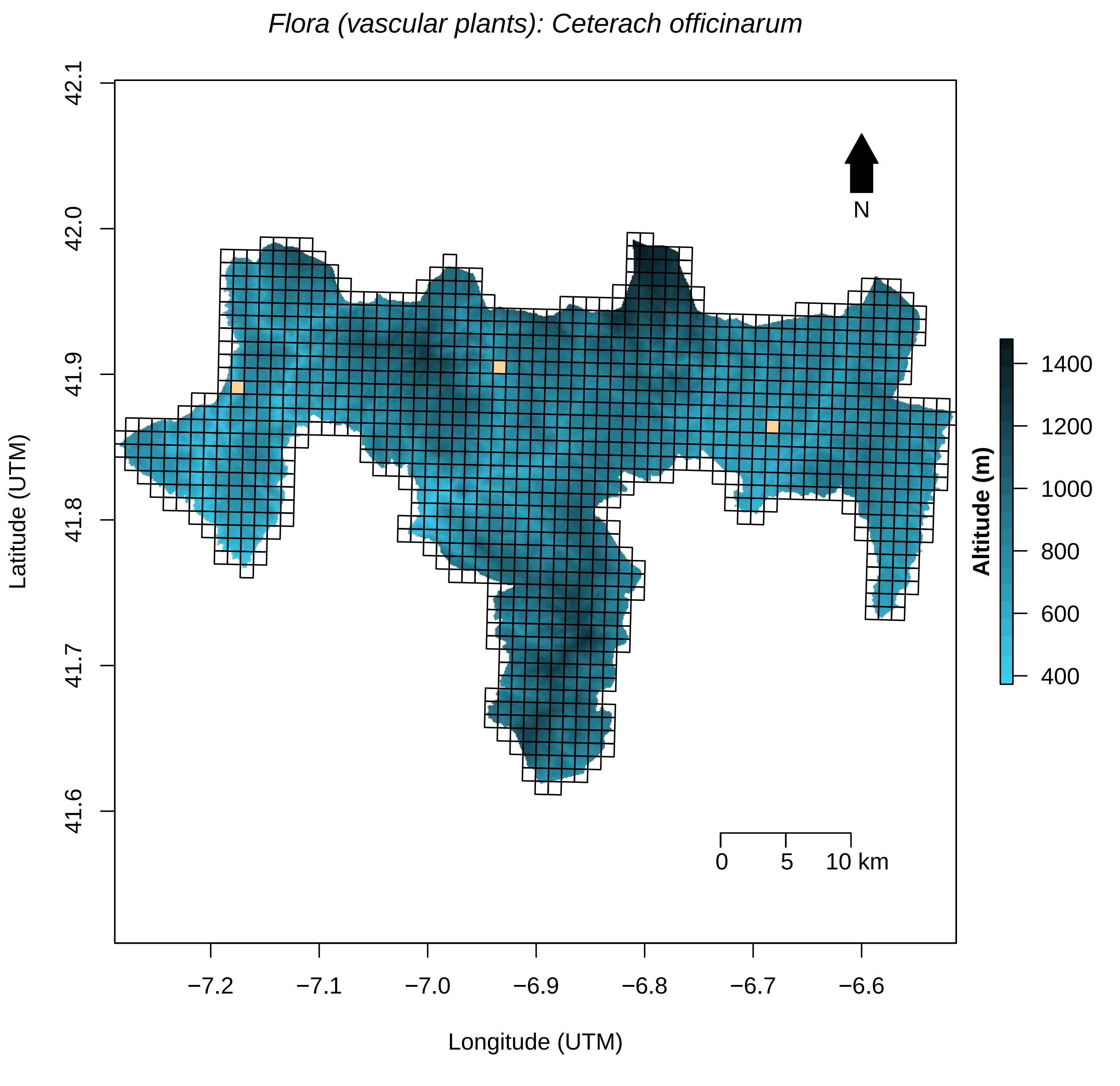
Flora (vascular plants): Ceterach officinarum
42.1
42.0
41.9
41.8
41.7
41.6
Latitude (UTM)
−7.2
−7.1
−7.0
−6.9
−6.8
−6.7
−6.6
Longitude (UTM)
N
0
5
10 km
1400
1200
1000
800
600
400
Altitude (m)

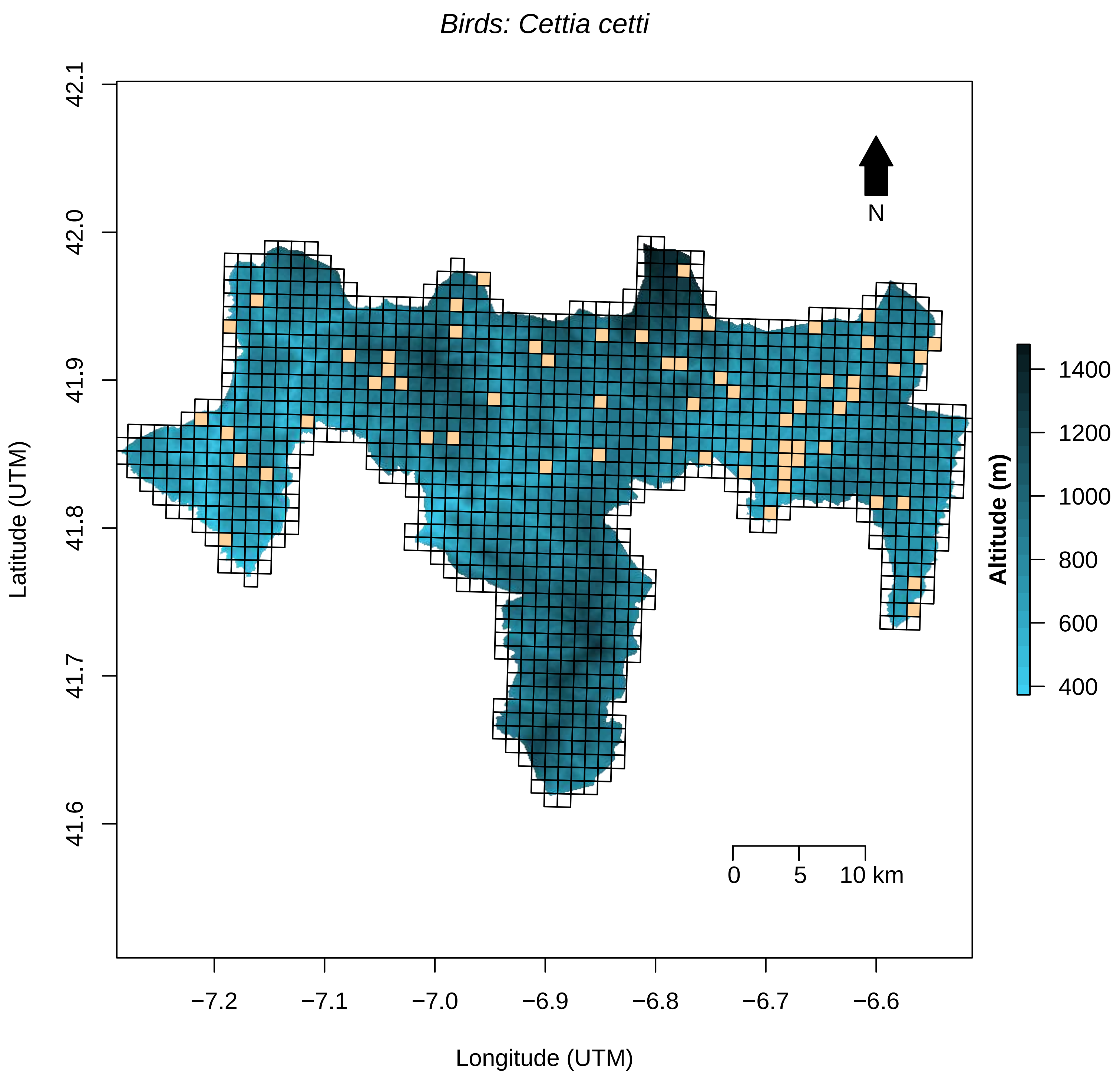
Birds: Cettia cetti
Latitude (UTM)
42.1
42.0
41.9
41.8
41.7
41.6
Longitude (UTM)
−7.2
−7.1
−7.0
−6.9
−6.8
−6.7
−6.6
N
0
5
10 km
Altitude (m)
1400
1200
1000
800
600
400

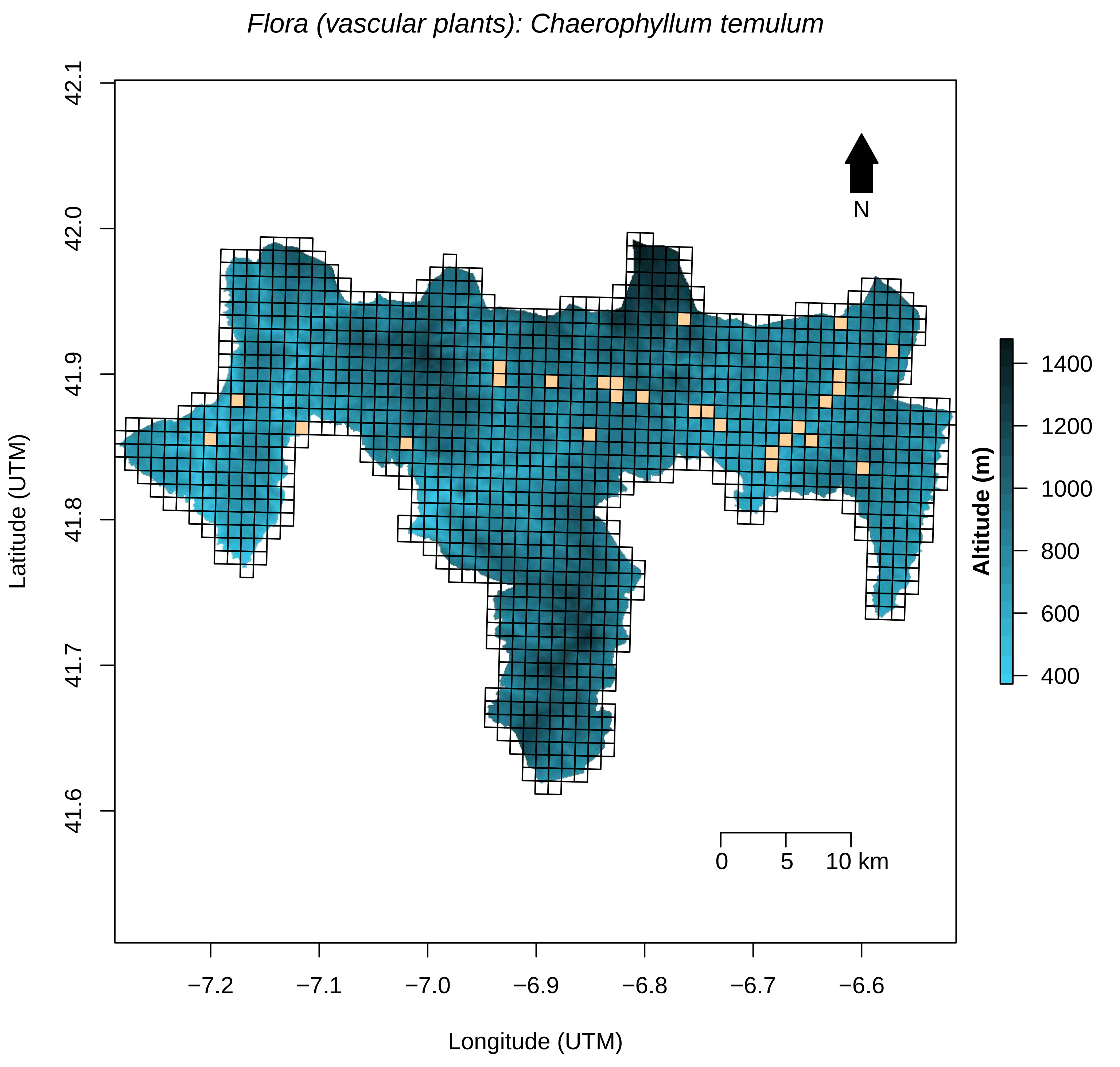

Flora (vascular plants): Chaerophyllum temulum
Latitude (UTM)
Longitude (UTM)
42.1
42.0
41.9
41.8
41.7
41.6
−7.2
−7.1
−7.0
−6.9
−6.8
−6.7
−6.6
N
0
5
10 km
Altitude (m)
1400
1200
1000
800
600
400

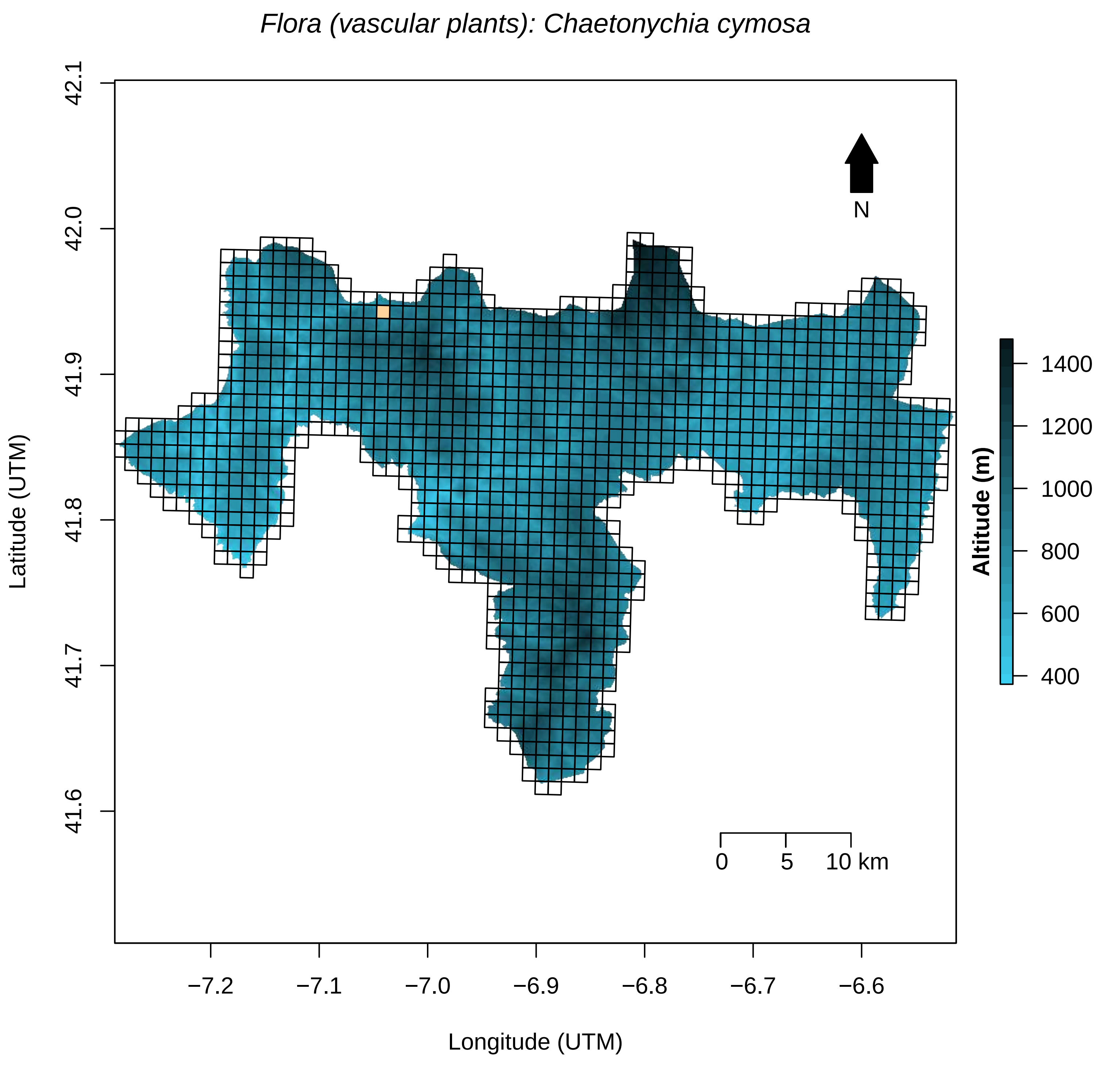

Flora (vascular plants): Chaetonychia cymosa
42.1
42.0
41.9
41.8
41.7
41.6
Latitude (UTM)
−7.2
−7.1
−7.0
−6.9
−6.8
−6.7
−6.6
Longitude (UTM)
N
0
5
10 km
Altitude (m)
1400
1200
1000
800
600
400

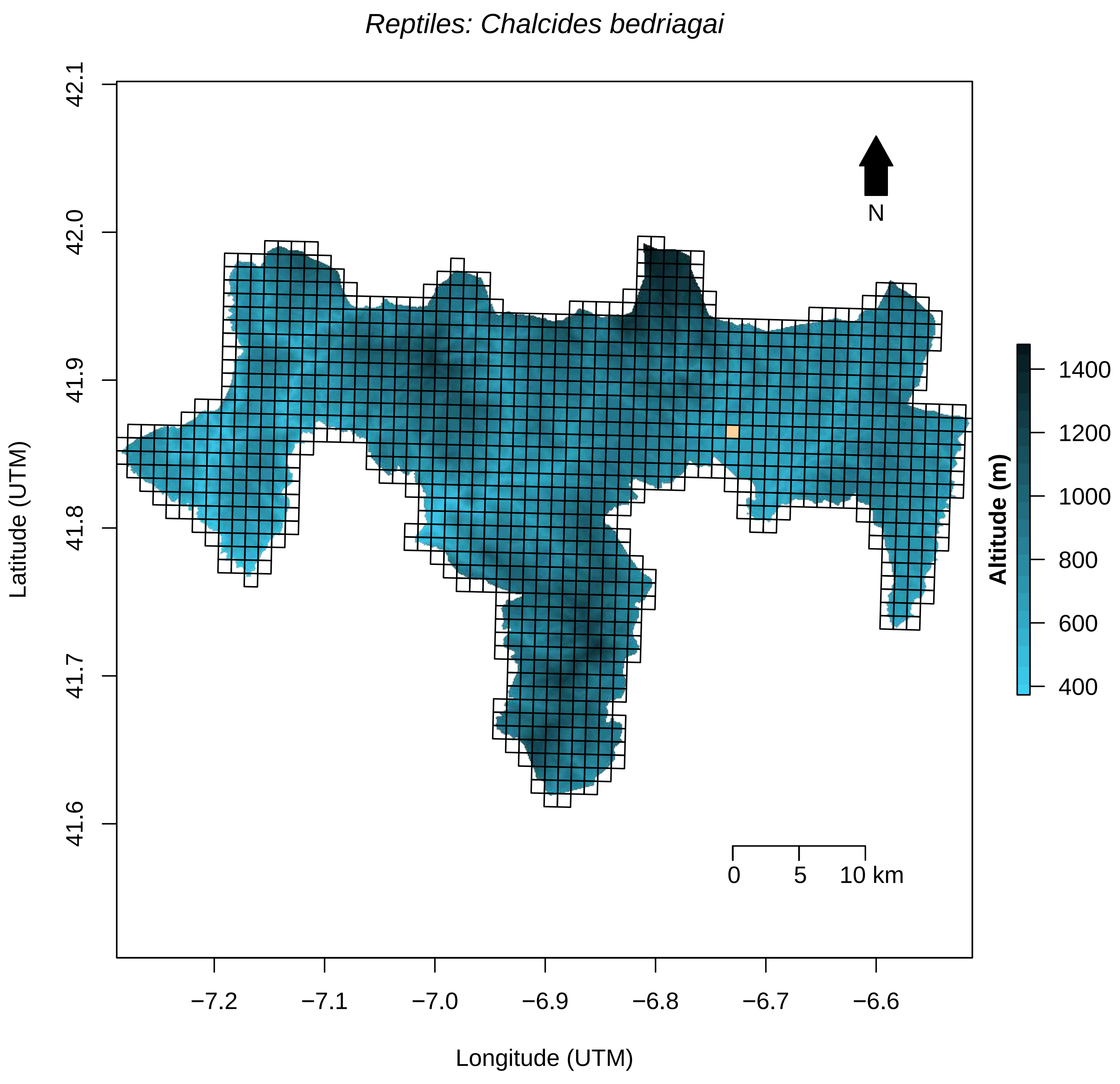

Reptiles: Chalcides bedriagai
Latitude (UTM)
42.1
42.0
41.9
41.8
41.7
41.6
−7.2
−7.1
−7.0
−6.9
−6.8
−6.7
−6.6
Longitude (UTM)
N
0
5
10 km
Altitude (m)
1400
1200
1000
800
600
400

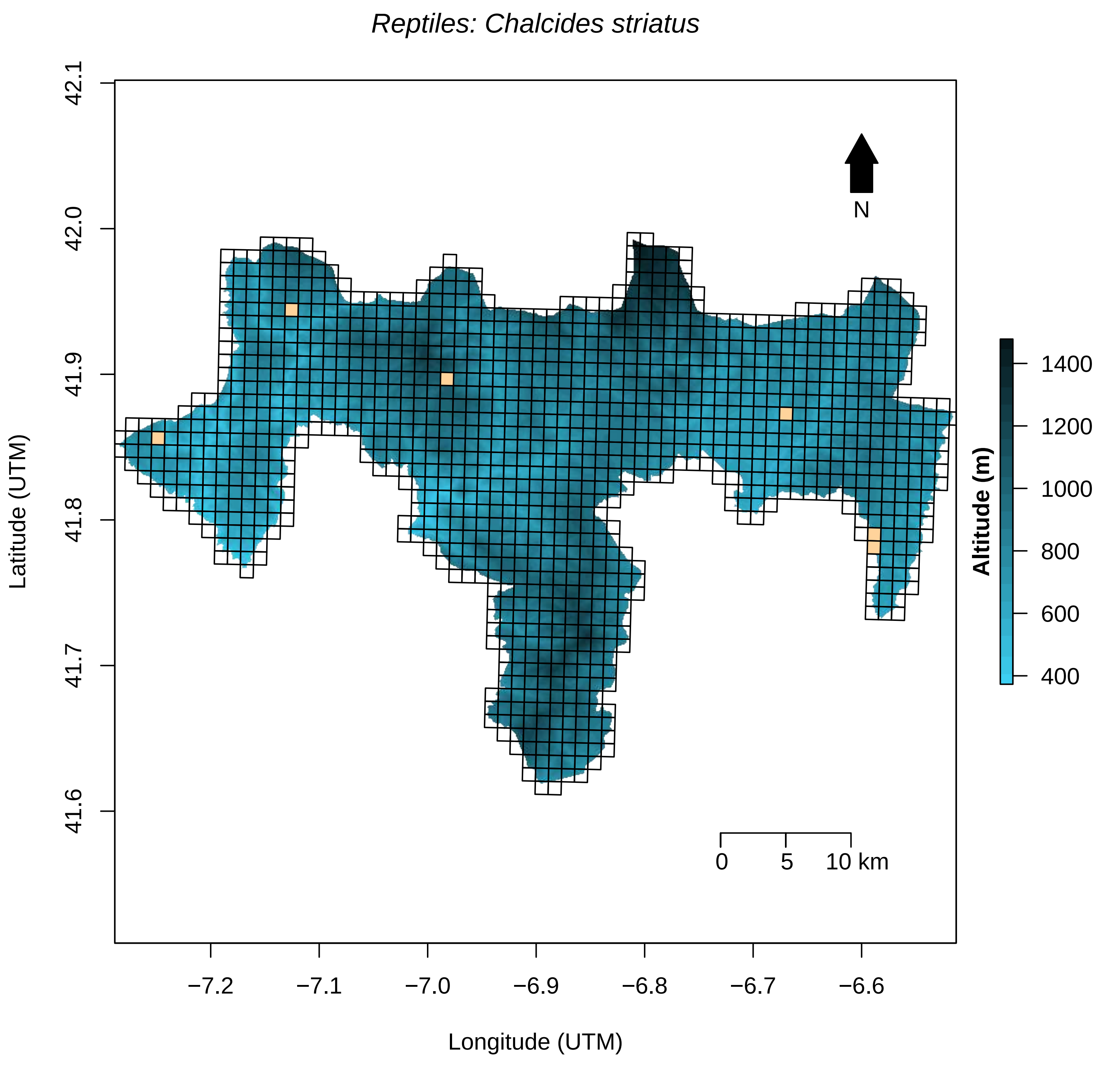

Reptiles: Chalcides striatus
N
0
5
10 km
Altitude (m)
1400
1200
1000
800
600
400
42.1
42.0
41.9
41.8
41.7
41.6
Latitude (UTM)
−7.2
−7.1
−7.0
−6.9
−6.8
−6.7
−6.6
Longitude (UTM)

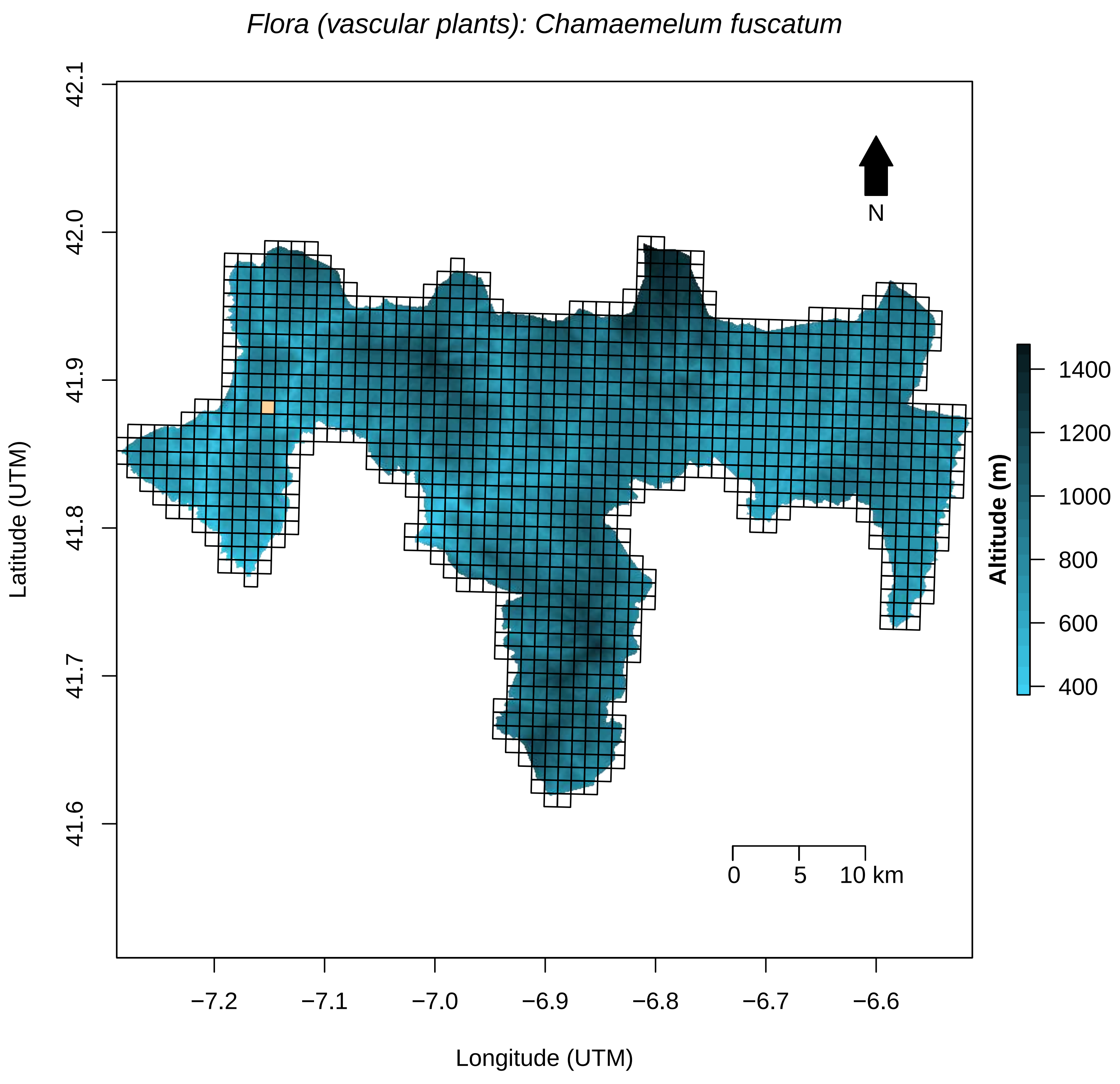

Flora (vascular plants): Chamaemelum fuscatum
42.1
42.0
41.9
41.8
41.7
41.6
Latitude (UTM)
−7.2
−7.1
−7.0
−6.9
−6.8
−6.7
−6.6
Longitude (UTM)
N
0
5
10 km
Altitude (m)
1400
1200
1000
800
600
400

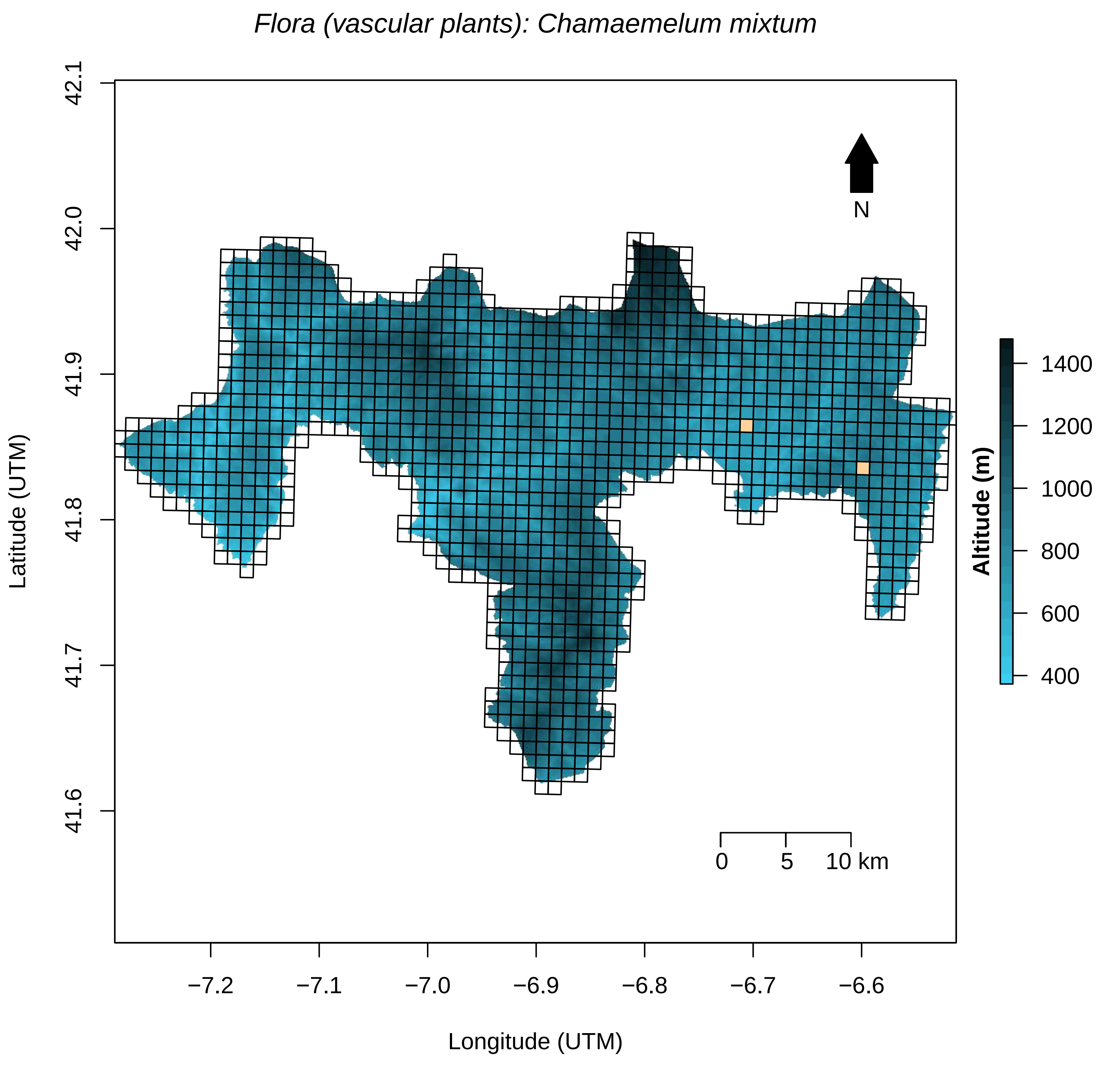

Flora (vascular plants): Chamaemelum mixtum
42.1
42.0
41.9
41.8
41.7
41.6
Latitude (UTM)
N
0 5 10 km
−7.2 −7.1 −7.0 −6.9 −6.8 −6.7 −6.6
Longitude (UTM)
Altitude (m)
1400
1200
1000
800
600
400

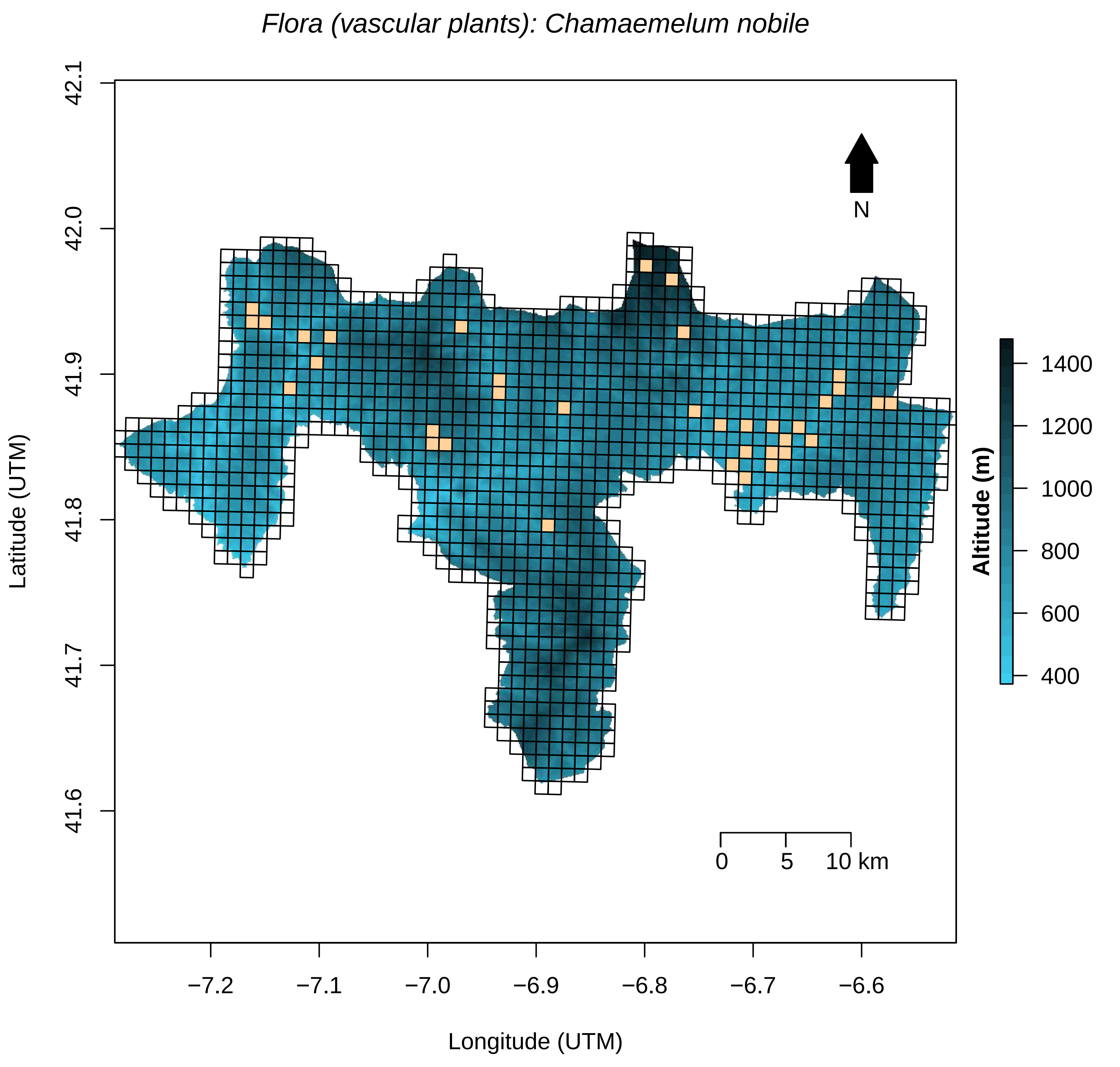

Flora (vascular plants): Chamaemelum nobile
N
0 5 10 km
Altitude (m)
1400
1200
1000
800
600
400
Latitude (UTM)
42.1
42.0
41.9
41.8
41.7
41.6
Longitude (UTM)
−7.2
−7.1
−7.0
−6.9
−6.8
−6.7
−6.6

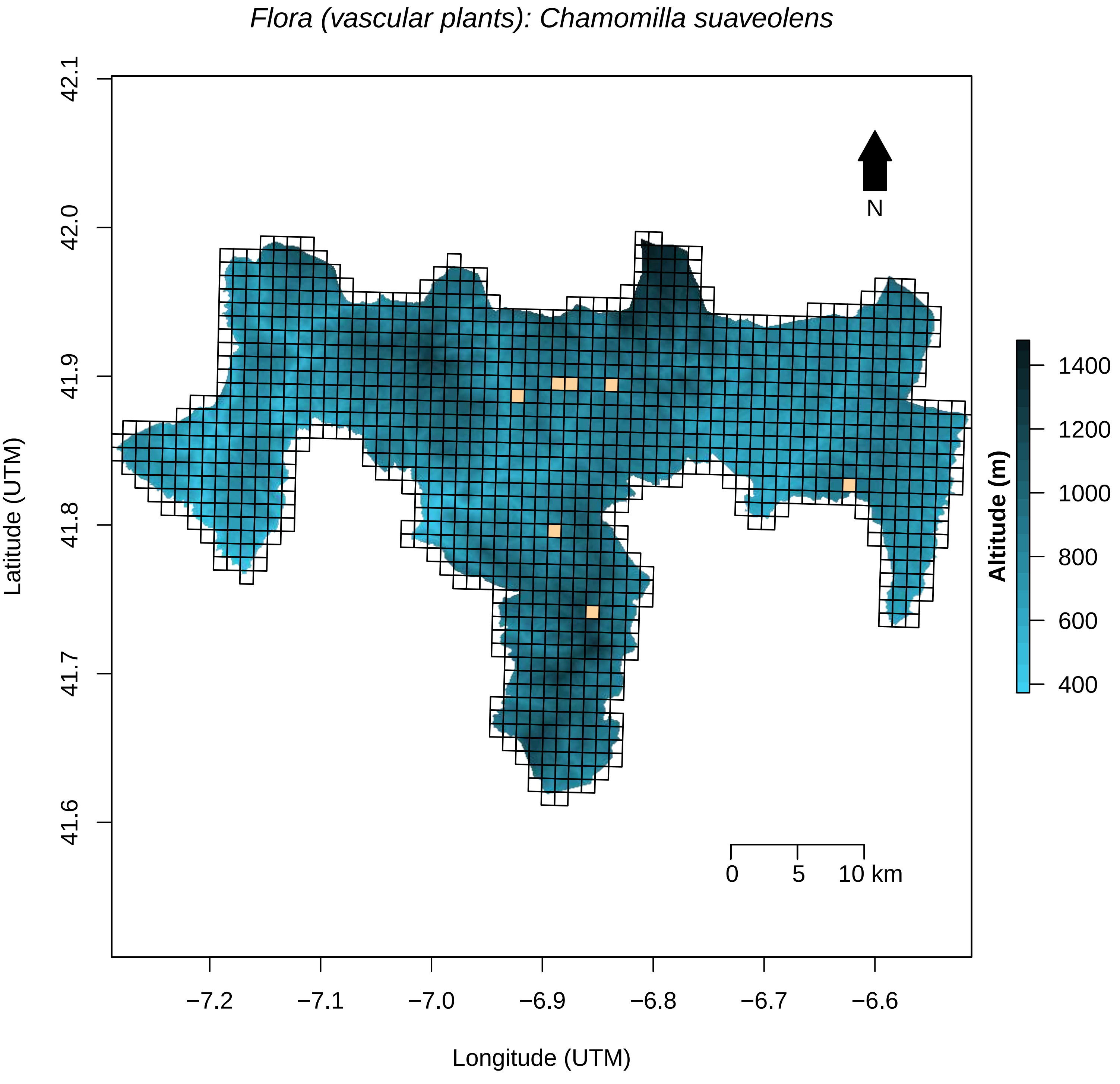

Flora (vascular plants): Chamomilla suaveolens
42.1
42.0
41.9
41.8
41.7
41.6
Latitude (UTM)
N
0
5
10 km
−7.2
−7.1
−7.0
−6.9
−6.8
−6.7
−6.6
Longitude (UTM)
Altitude (m)
1400
1200
1000
800
600
400

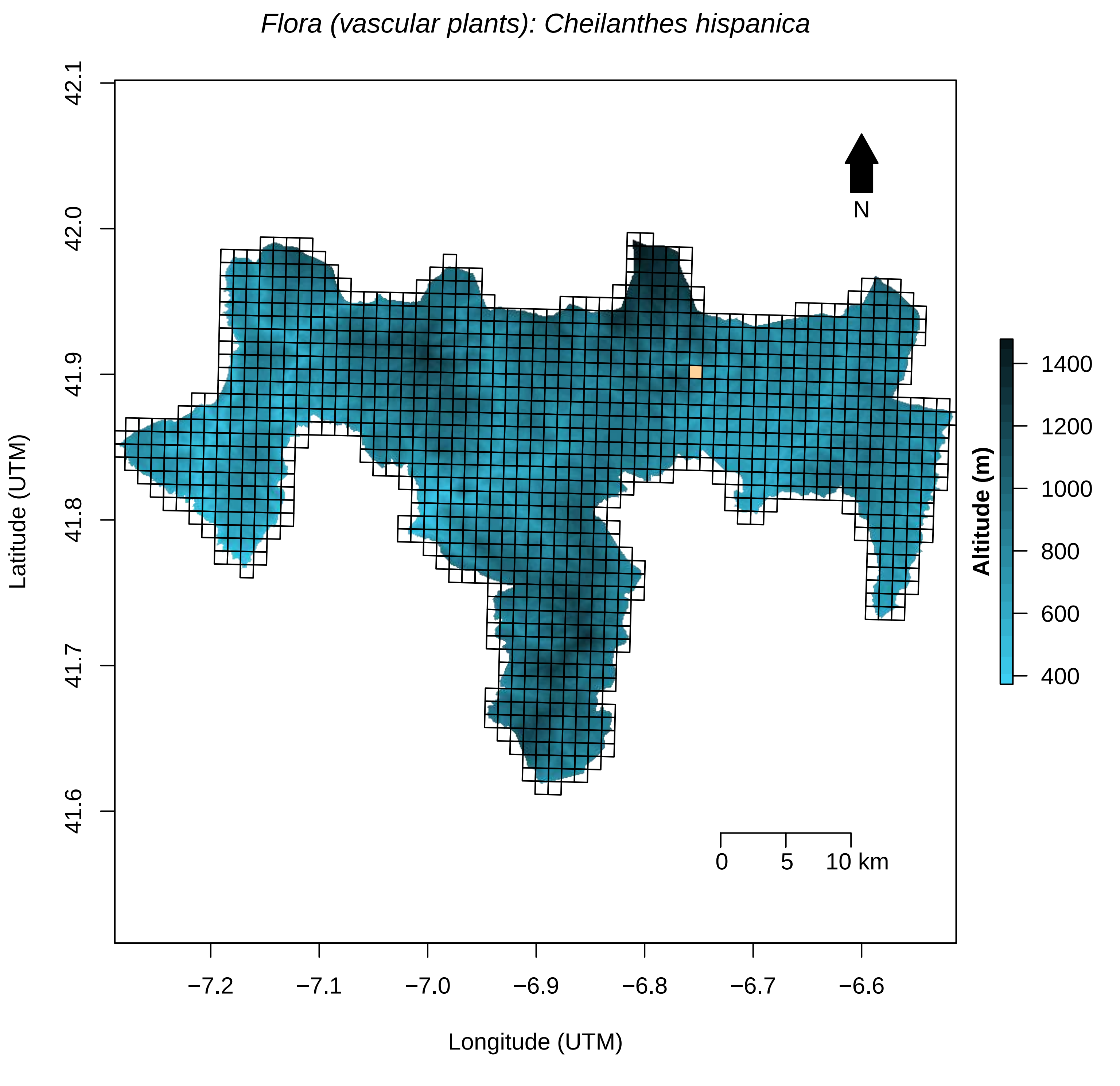

Flora (vascular plants): Cheilanthes hispanica
42.1
42.0
41.9
41.8
41.7
41.6
Latitude (UTM)
−7.2
−7.1
−7.0
−6.9
−6.8
−6.7
−6.6
Longitude (UTM)
N
0
5
10 km
Altitude (m)
1400
1200
1000
800
600
400

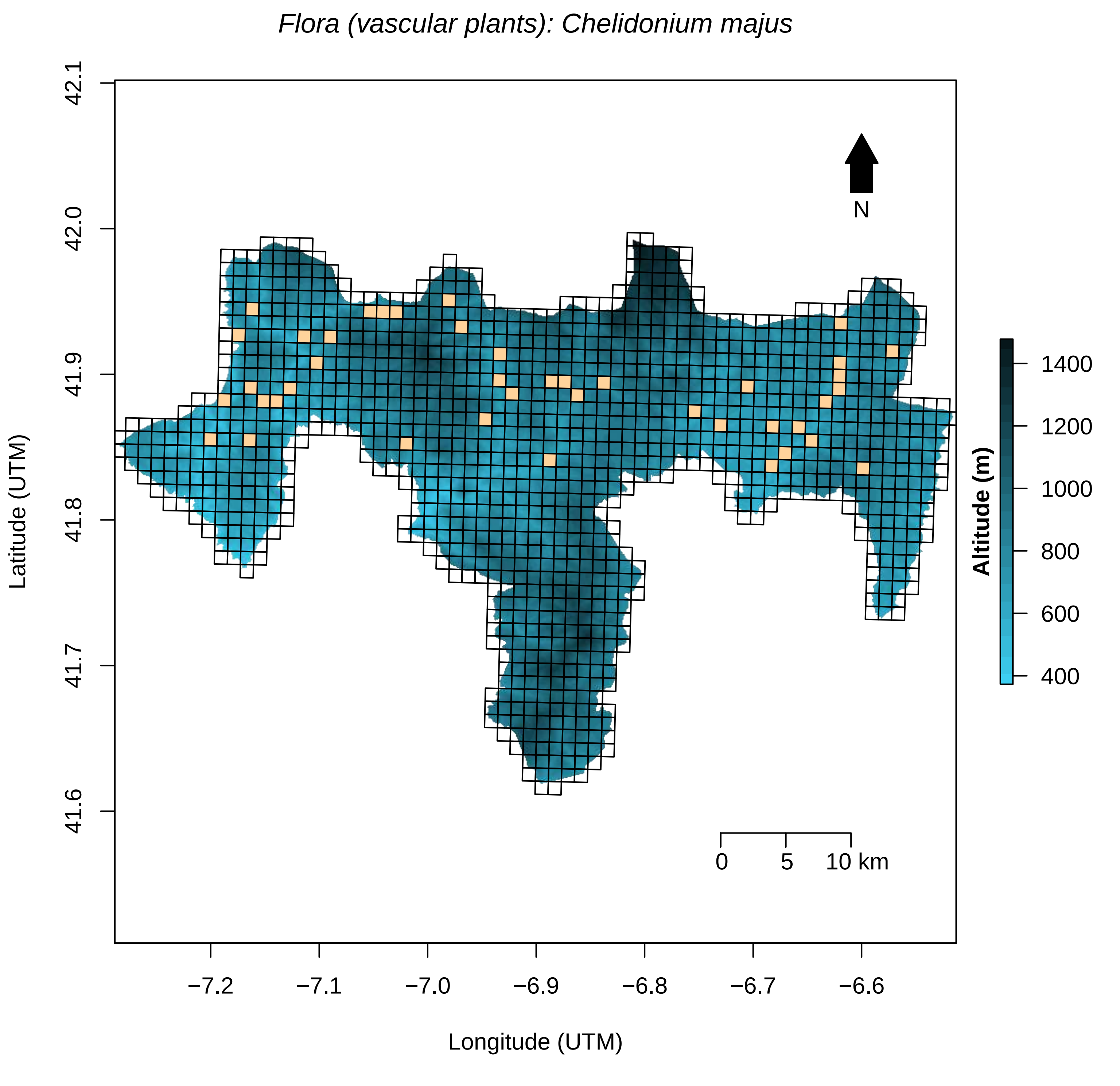
Flora (vascular plants): Chelidonium majus
42.1
42.0
41.9
41.8
41.7
41.6
Latitude (UTM)
−7.2
−7.1
−7.0
−6.9
−6.8
−6.7
−6.6
Longitude (UTM)
N
0
5
10 km
Altitude (m)
1400
1200
1000
800
600
400

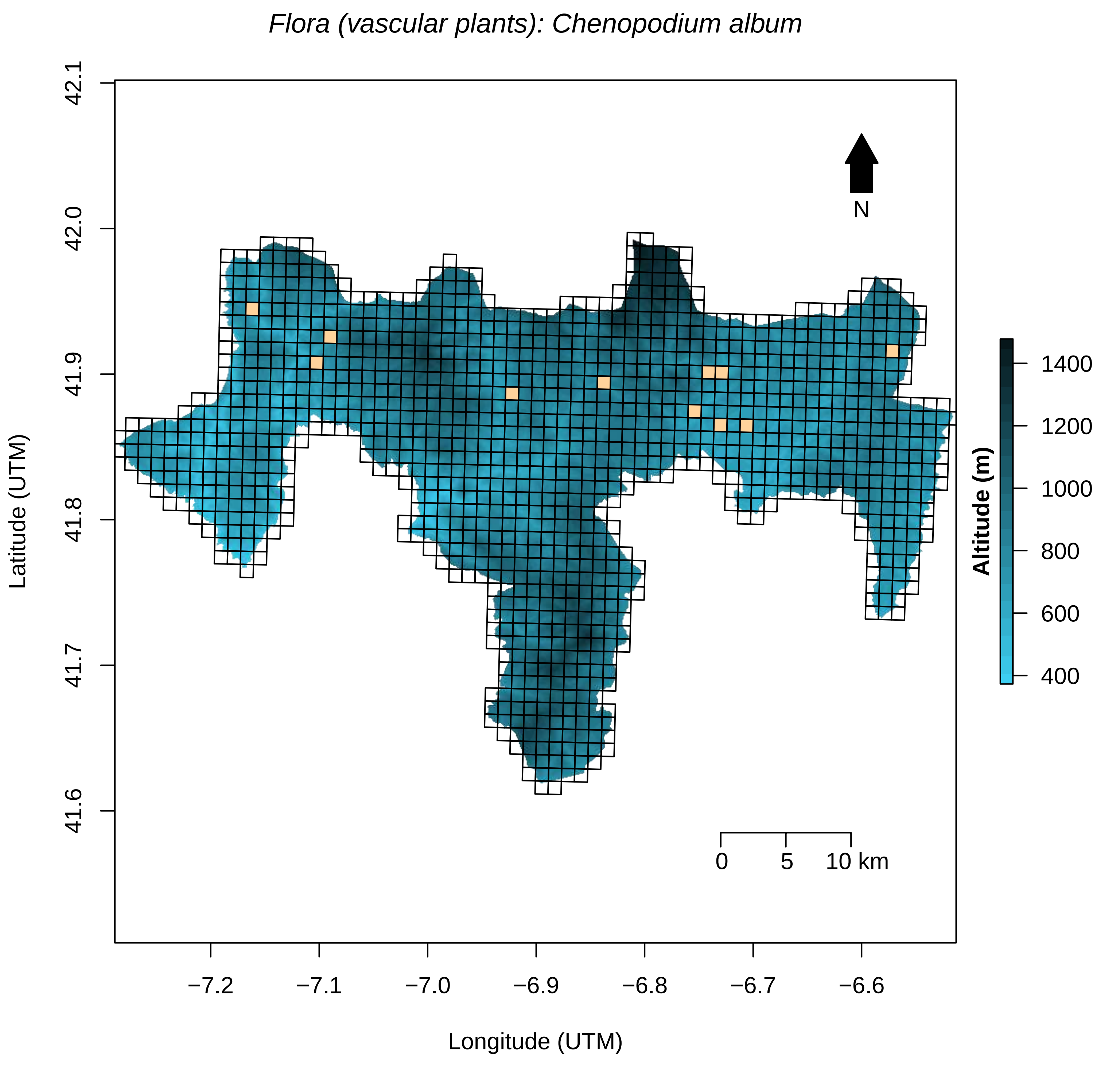

Flora (vascular plants): Chenopodium album
42.1
42.0
41.9
41.8
41.7
41.6
Latitude (UTM)
−7.2
−7.1
−7.0
−6.9
−6.8
−6.7
−6.6
Longitude (UTM)
N
0
5
10 km
1400
1200
1000
800
600
400
Altitude (m)

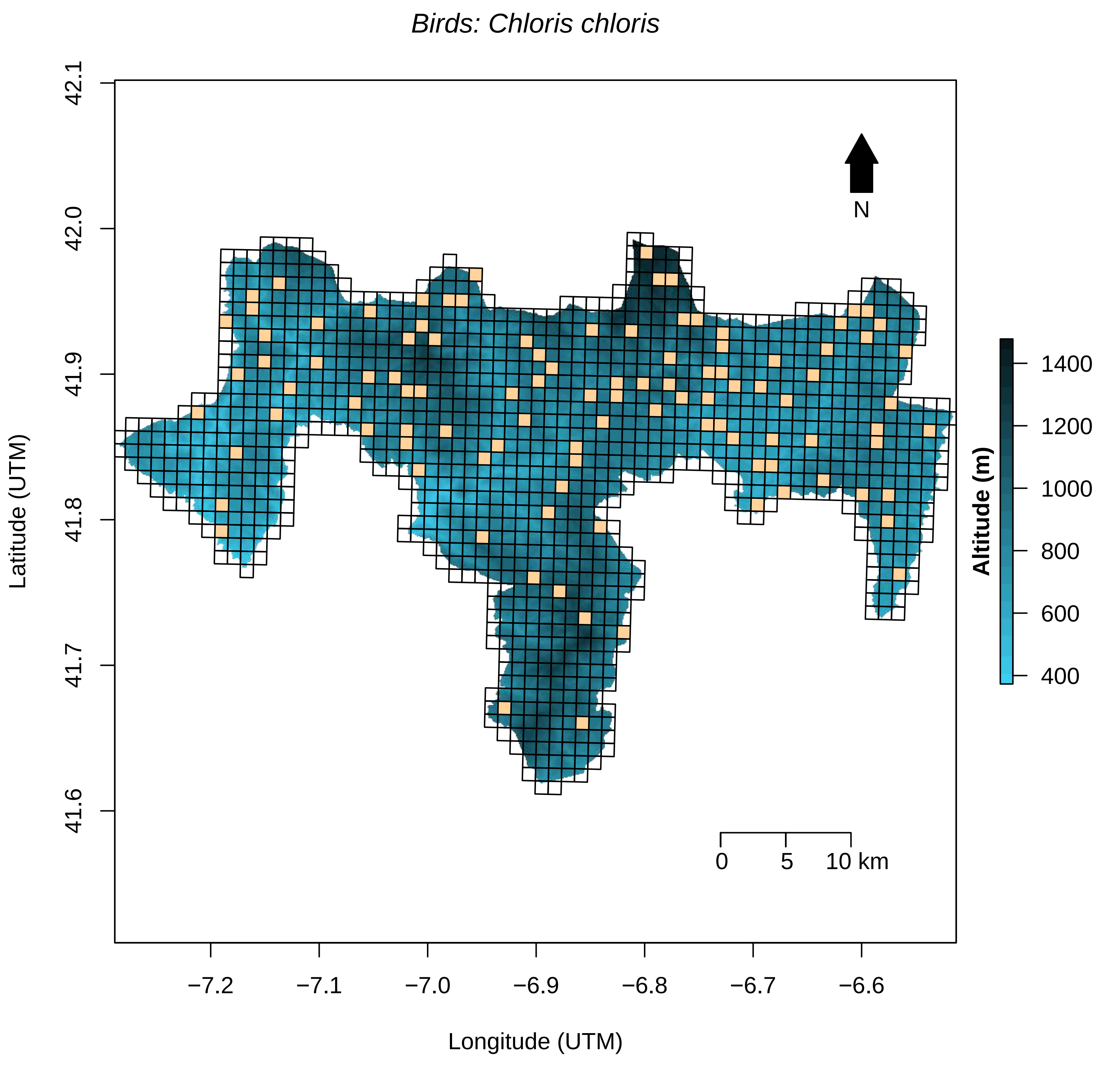

Birds: Chloris chloris
42.1
42.0
41.9
41.8
41.7
41.6
Latitude (UTM)
−7.2
−7.1
−7.0
−6.9
−6.8
−6.7
−6.6
Longitude (UTM)
N
0
5
10 km
1400
1200
1000
800
600
400
Altitude (m)

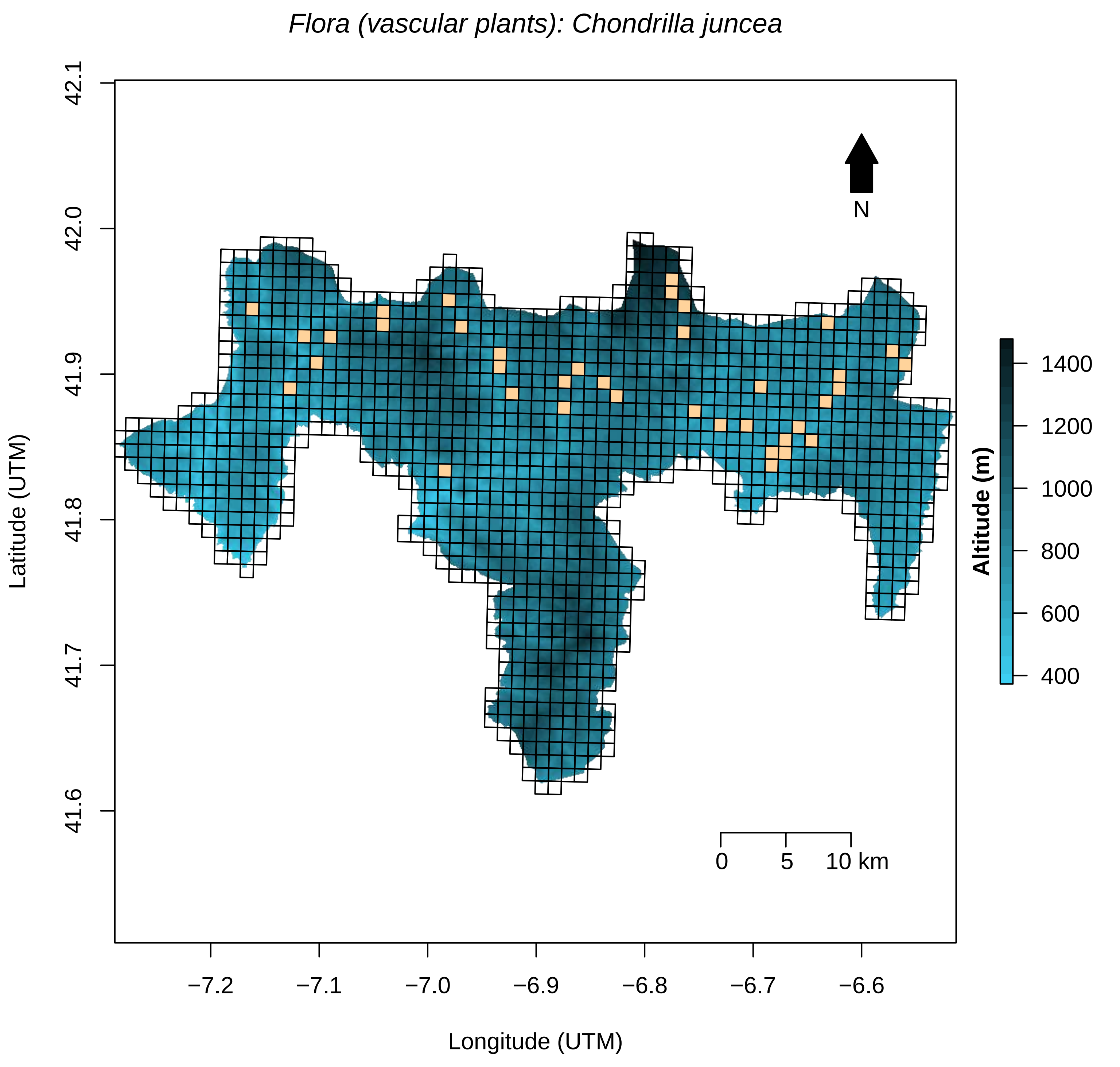
Flora (vascular plants): Chondrilla juncea
42.1
42.0
41.9
41.8
41.7
41.6
Latitude (UTM)
N
0
5
10 km
−7.2
−7.1
−7.0
−6.9
−6.8
−6.7
−6.6
Longitude (UTM)
Altitude (m)
1400
1200
1000
800
600
400

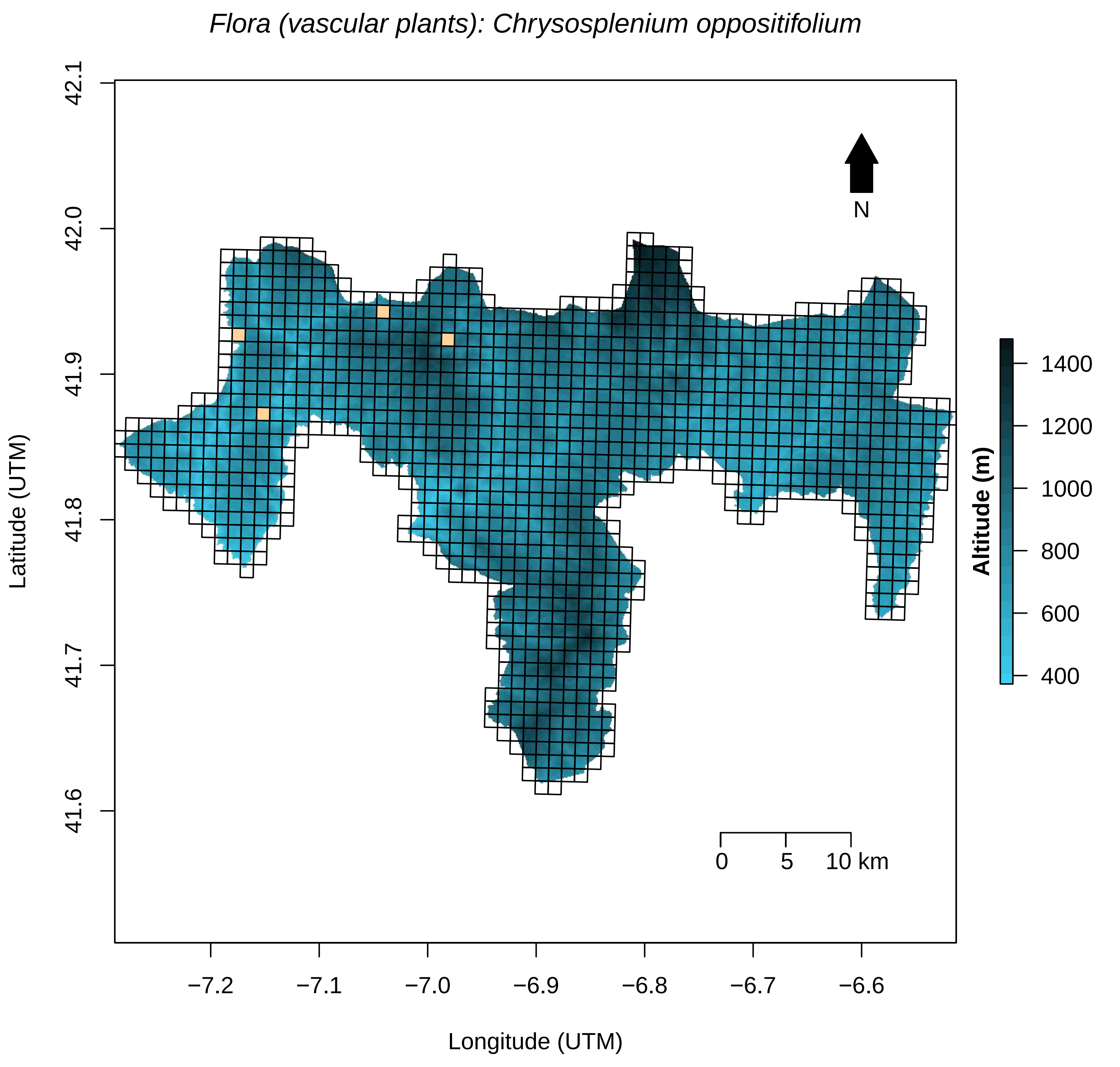

Flora (vascular plants): Chrysosplenium oppositifolium
N
0 5 10 km
Altitude (m)
1400
1200
1000
800
600
400
Latitude (UTM)
42.1
42.0
41.9
41.8
41.7
41.6
−7.2
−7.1
−7.0
−6.9
−6.8
−6.7
−6.6
Longitude (UTM)

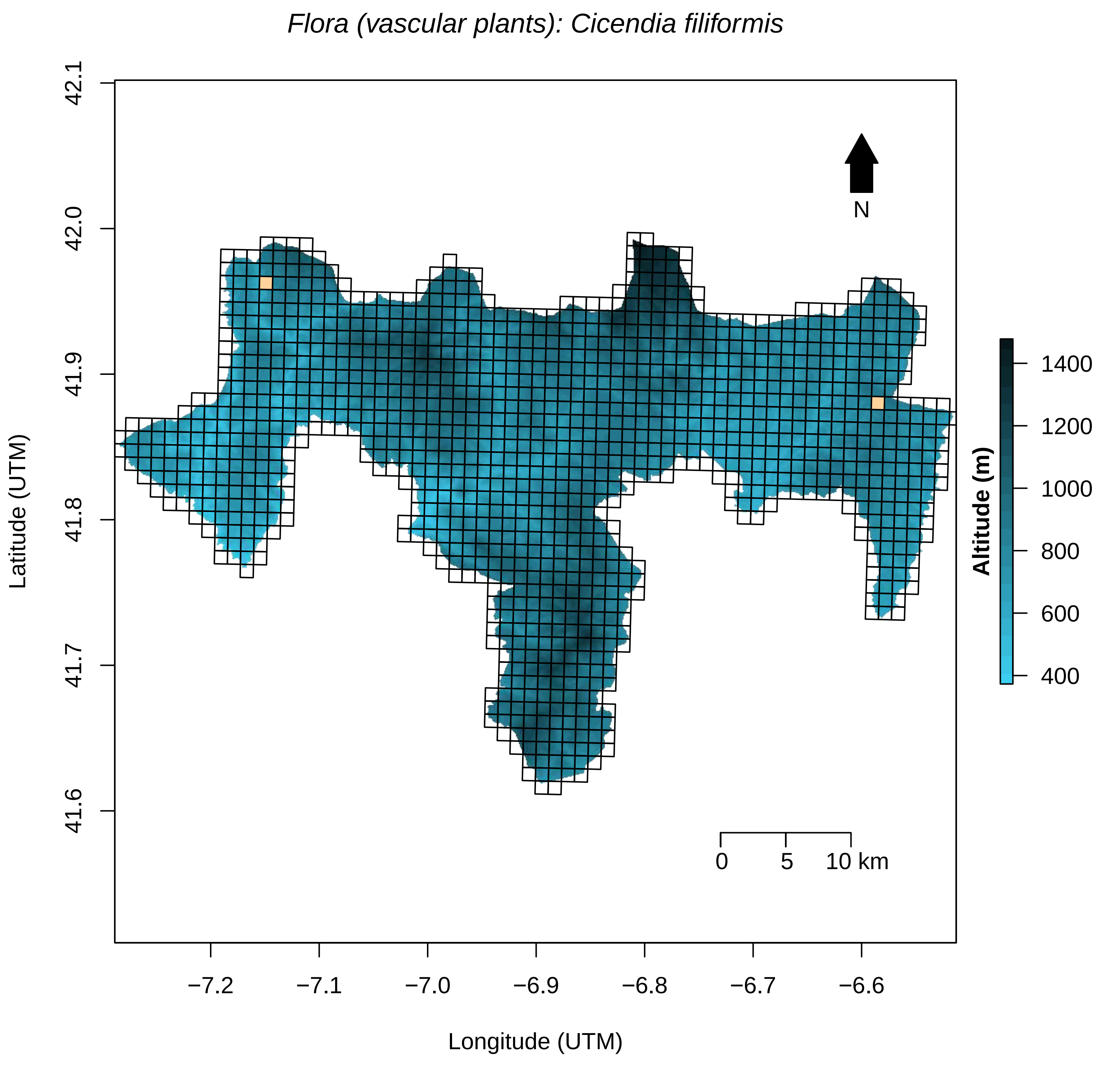

Flora (vascular plants): Cicendia filiformis
42.1
42.0
41.9
41.8
41.7
41.6
Latitude (UTM)
−7.2
−7.1
−7.0
−6.9
−6.8
−6.7
−6.6
Longitude (UTM)
N
0
5
10 km
1400
1200
1000
800
600
400
Altitude (m)

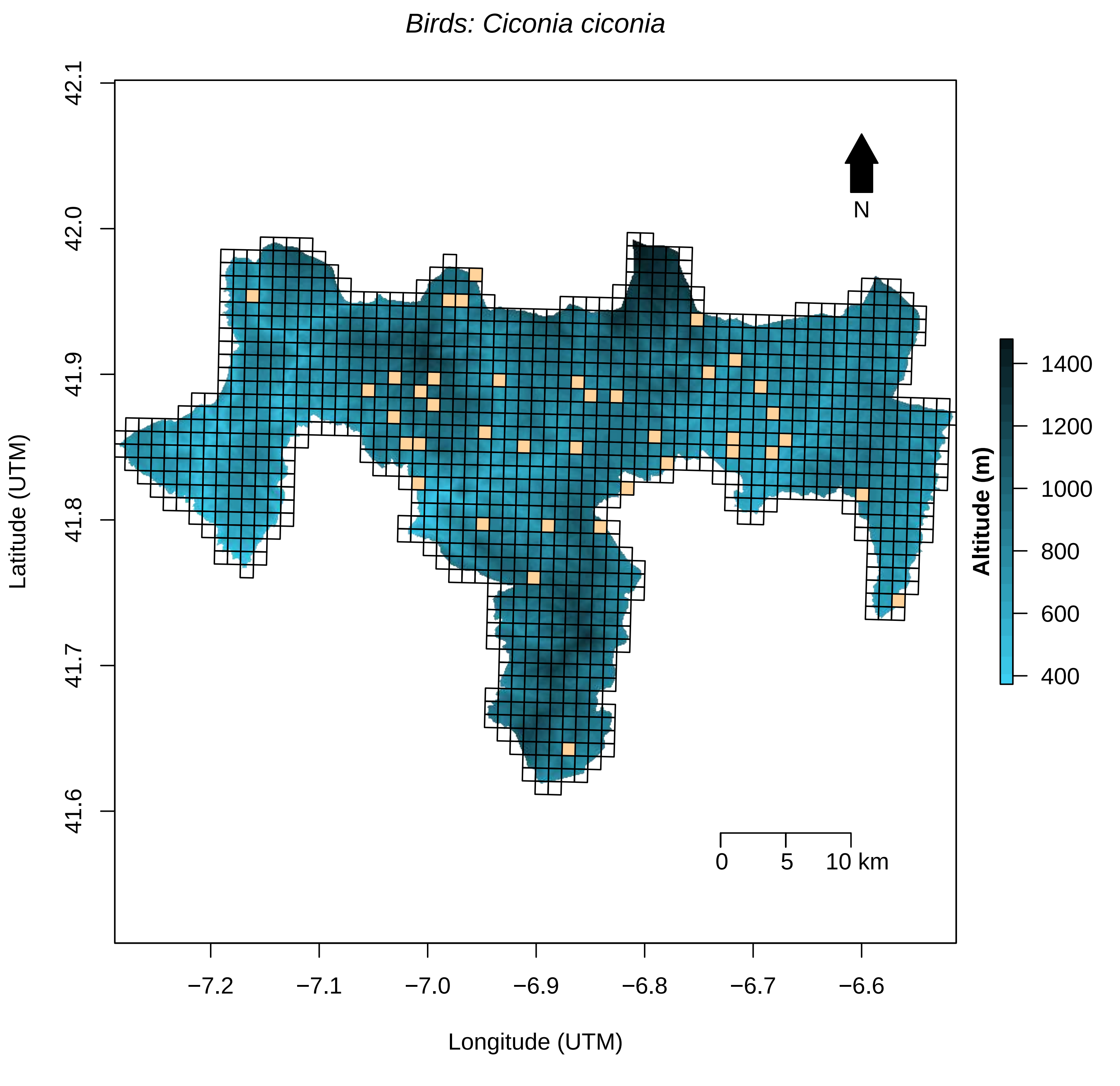
Birds: Ciconia ciconia
N
0
5
10 km
Altitude (m)
1400
1200
1000
800
600
400
Latitude (UTM)
42.1
42.0
41.9
41.8
41.7
41.6
Longitude (UTM)
−7.2
−7.1
−7.0
−6.9
−6.8
−6.7
−6.6

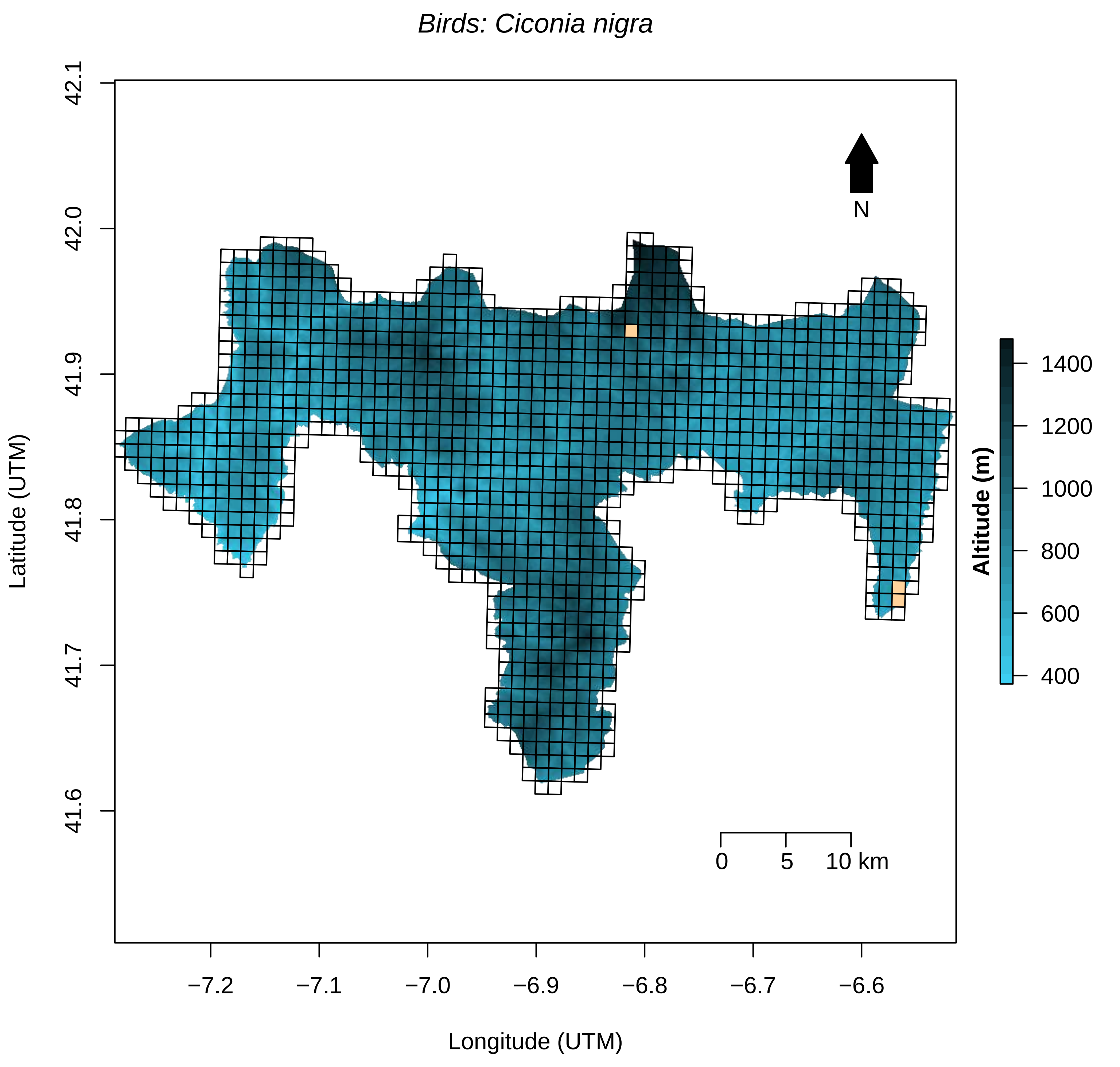

Birds: Ciconia nigra
42.1
42.0
41.9
41.8
41.7
41.6
Latitude (UTM)
−7.2
−7.1
−7.0
−6.9
−6.8
−6.7
−6.6
Longitude (UTM)
N
0
5
10 km
Altitude (m)
1400
1200
1000
800
600
400

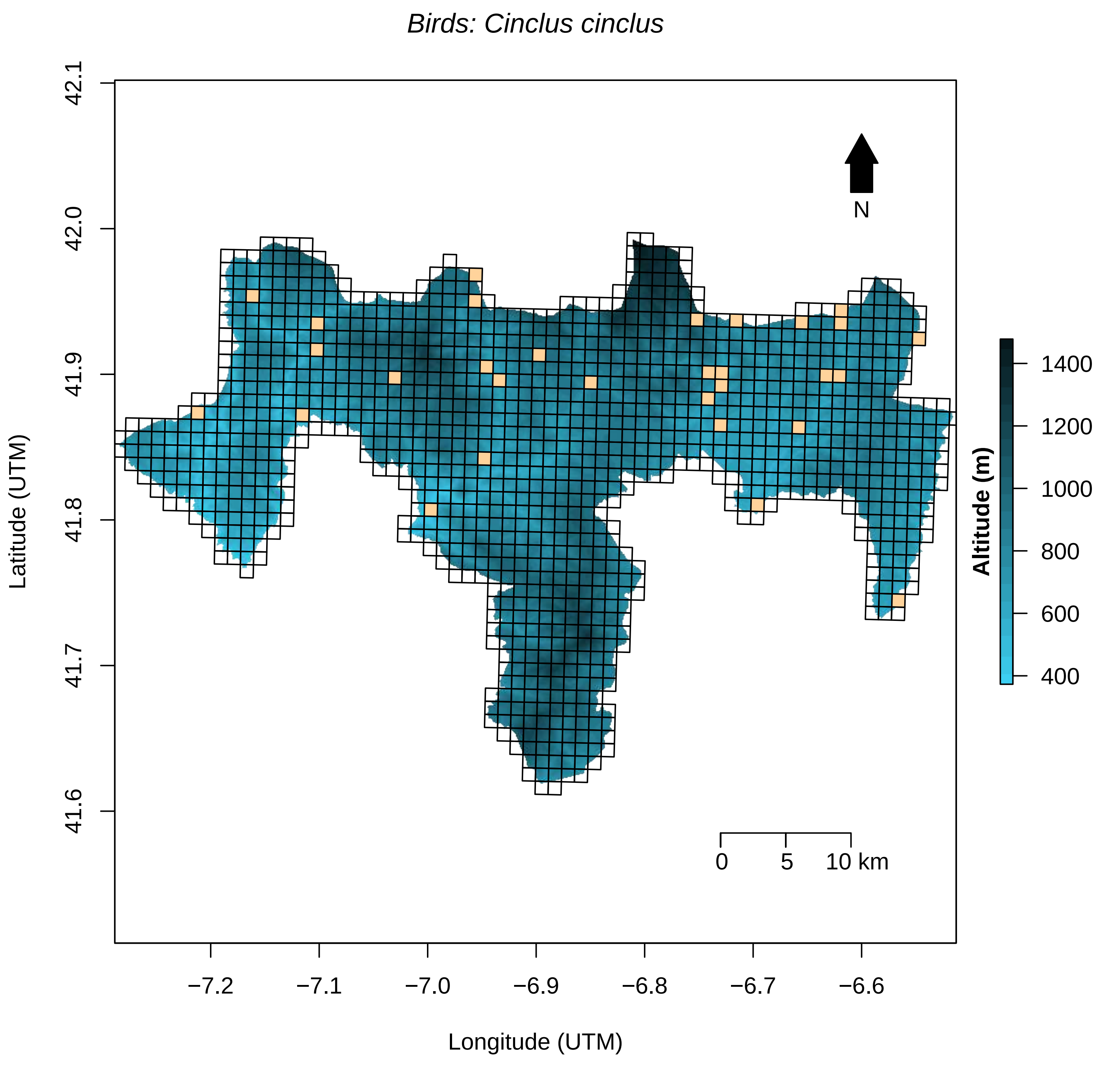

Birds: Cinclus cinclus
42.1
42.0
41.9
41.8
41.7
41.6
Latitude (UTM)
−7.2
−7.1
−7.0
−6.9
−6.8
−6.7
−6.6
Longitude (UTM)
N
0
5
10 km
1400
1200
1000
800
600
400
Altitude (m)

Flora (vascular plants): Circaea lutetiana

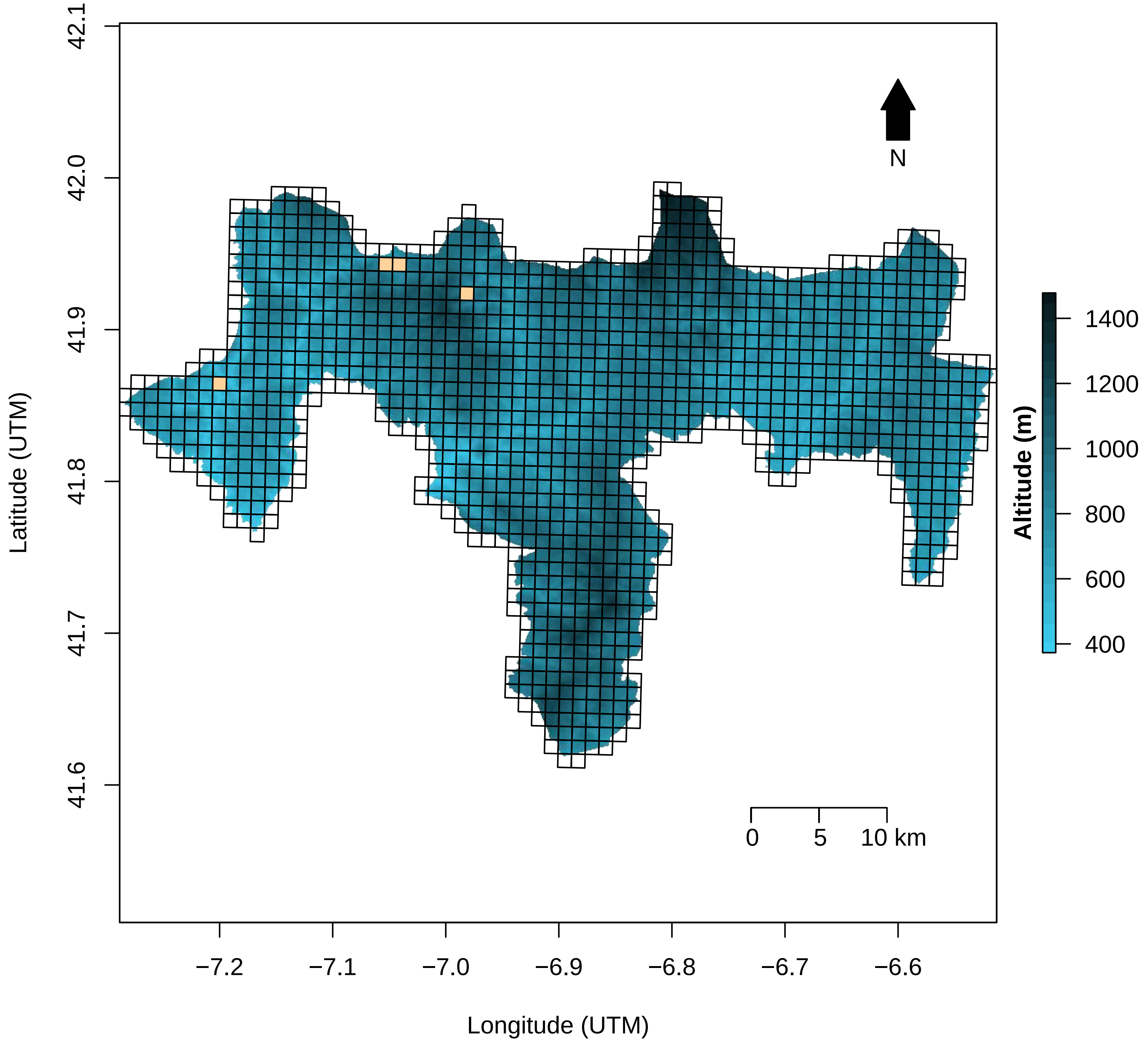

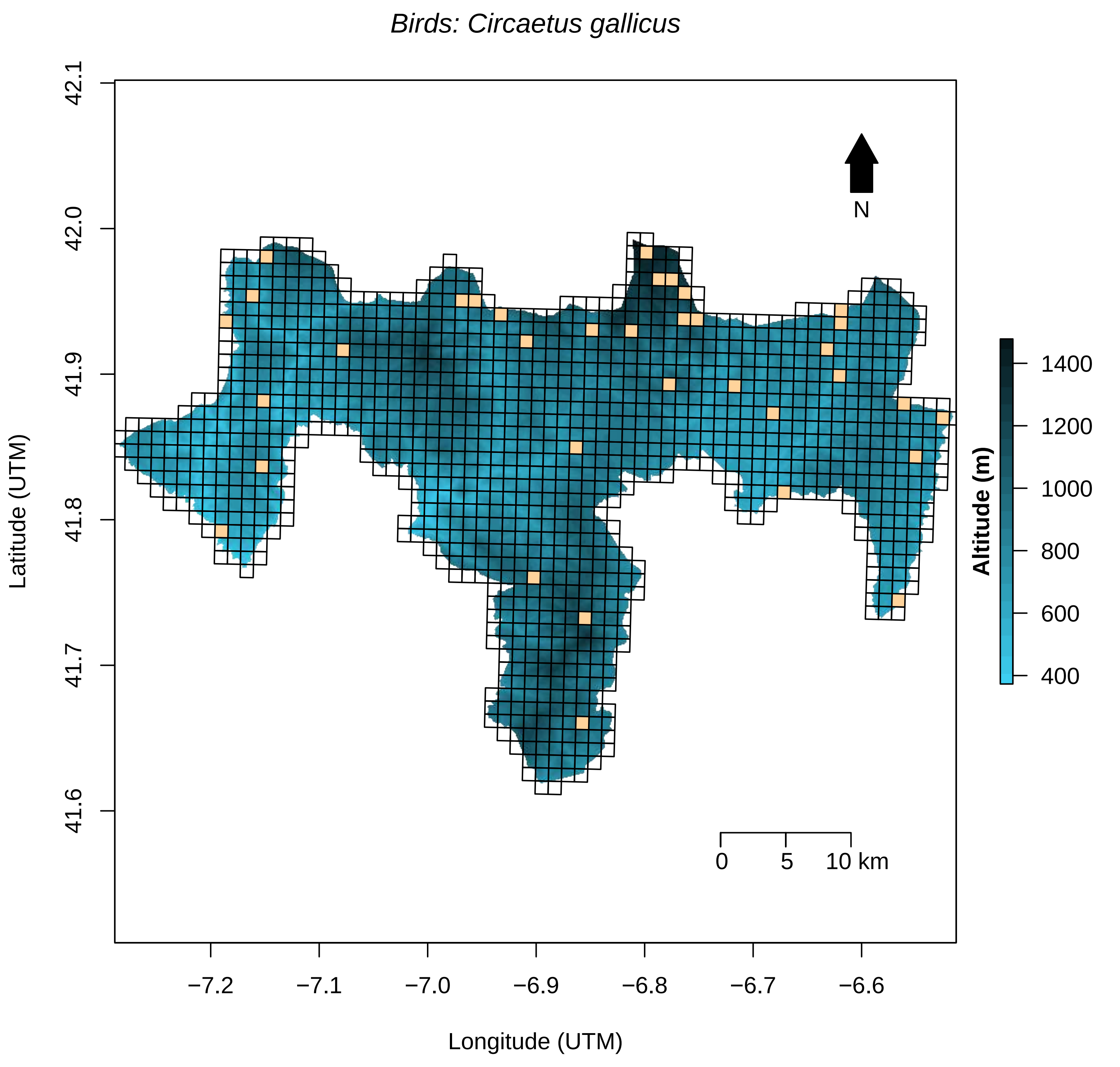

Birds: Circaetus gallicus
42.1
42.0
41.9
41.8
41.7
41.6
Latitude (UTM)
−7.2
−7.1
−7.0
−6.9
−6.8
−6.7
−6.6
Longitude (UTM)
N
0
5
10 km
1400
1200
1000
800
600
400
Altitude (m)

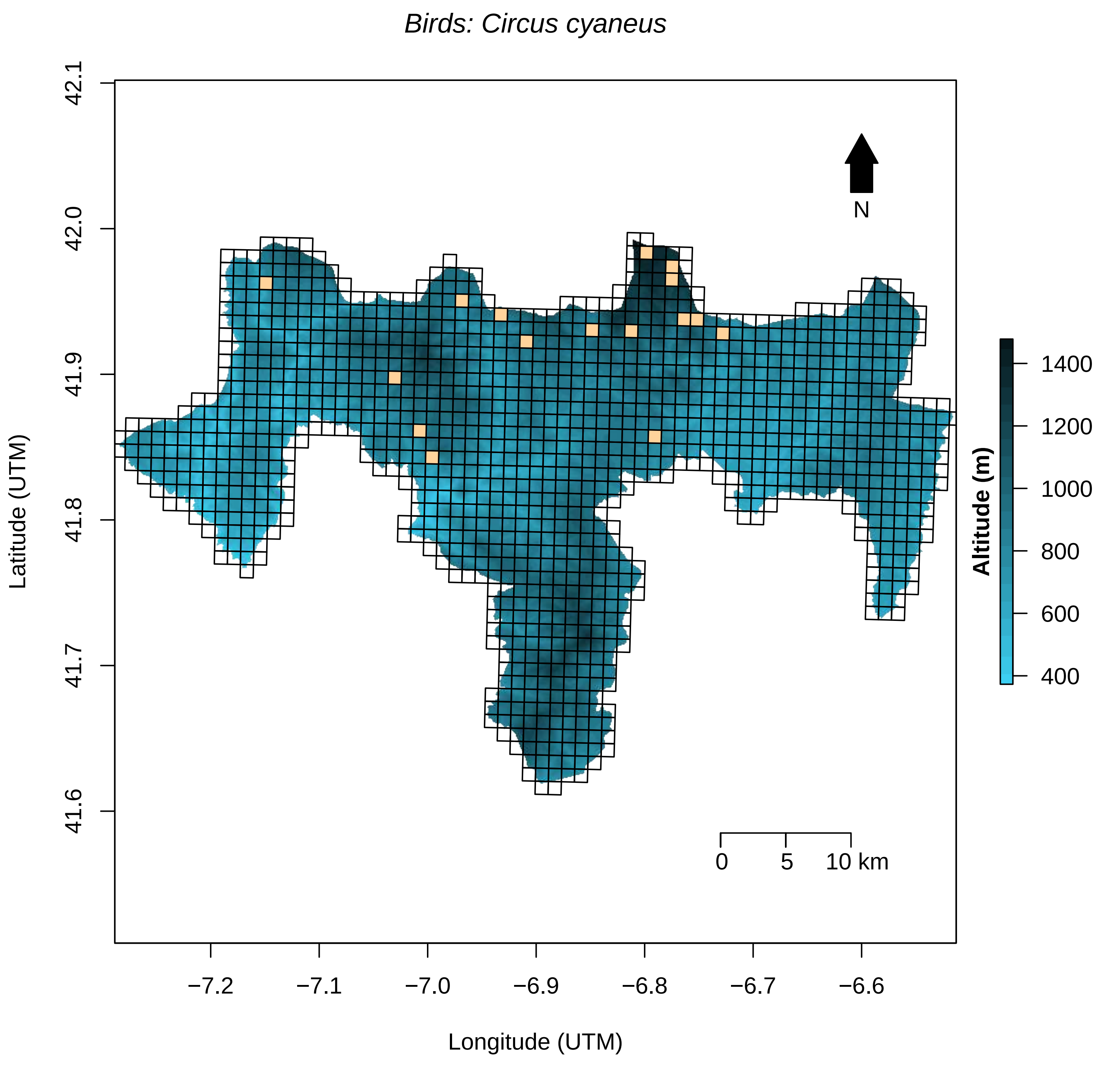
Birds: Circus cyaneus
42.1
42.0
41.9
41.8
41.7
41.6
Latitude (UTM)
−7.2
−7.1
−7.0
−6.9
−6.8
−6.7
−6.6
Longitude (UTM)
N
0
5
10 km
Altitude (m)
1400
1200
1000
800
600
400

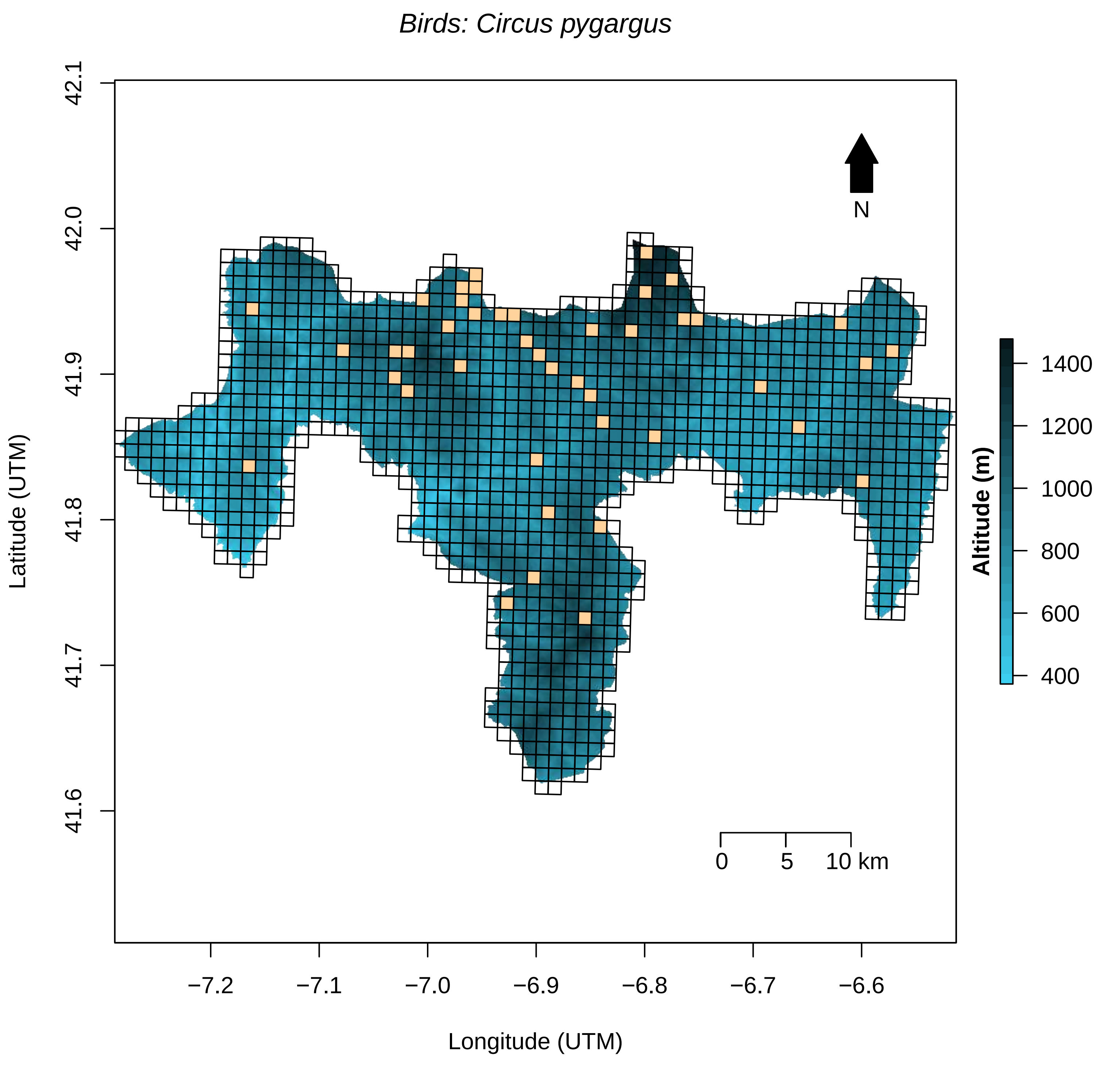

Birds: Circus pygargus
42.1
42.0
41.9
41.8
41.7
41.6
Latitude (UTM)
−7.2
−7.1
−7.0
−6.9
−6.8
−6.7
−6.6
Longitude (UTM)
N
0
5
10 km
1400
1200
1000
800
600
400
Altitude (m)

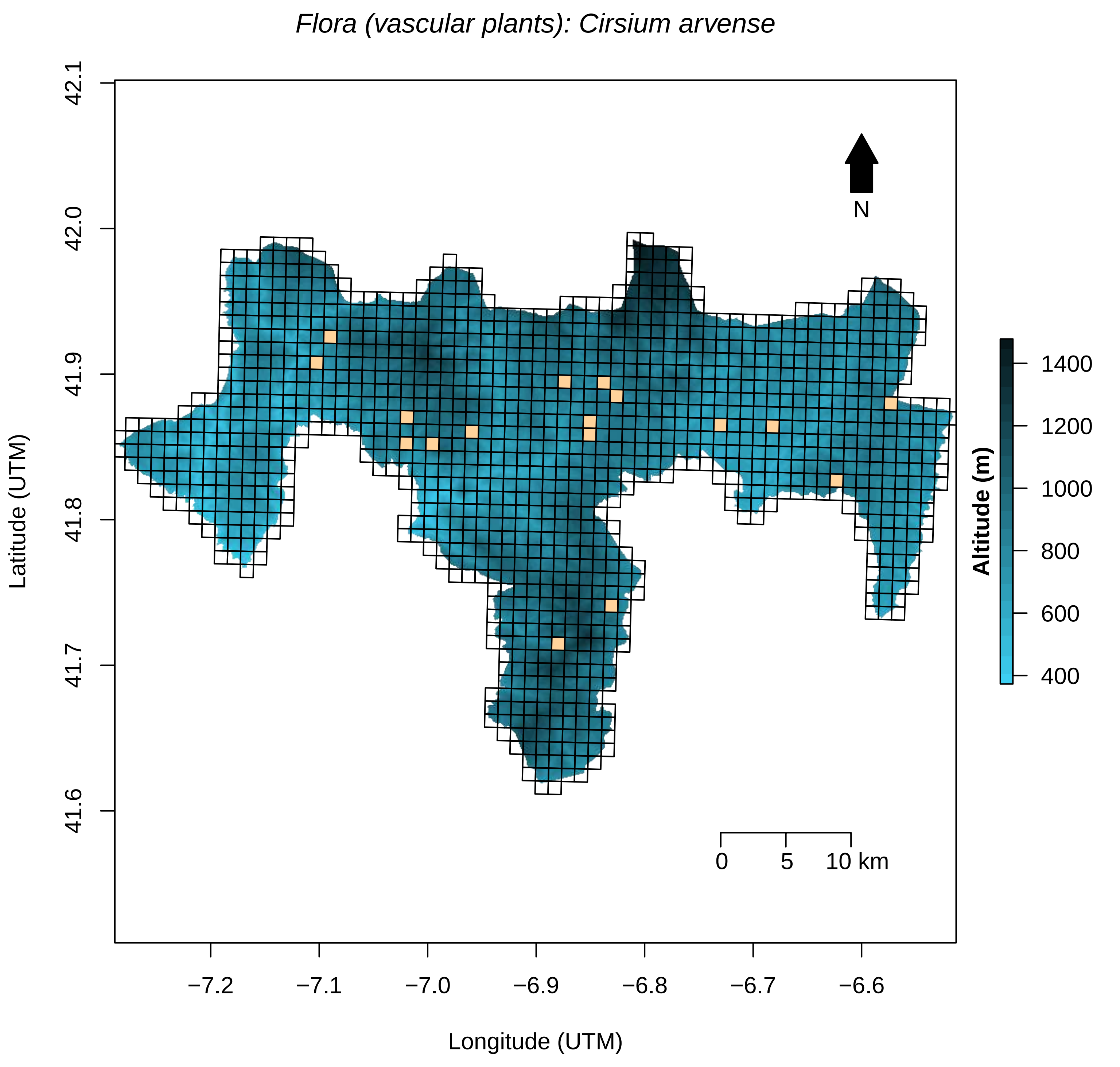

Flora (vascular plants): Cirsium arvense
N
0
5
10 km
Altitude (m)
1400
1200
1000
800
600
400
42.1
42.0
41.9
41.8
41.7
41.6
Latitude (UTM)
−7.2
−7.1
−7.0
−6.9
−6.8
−6.7
−6.6
Longitude (UTM)

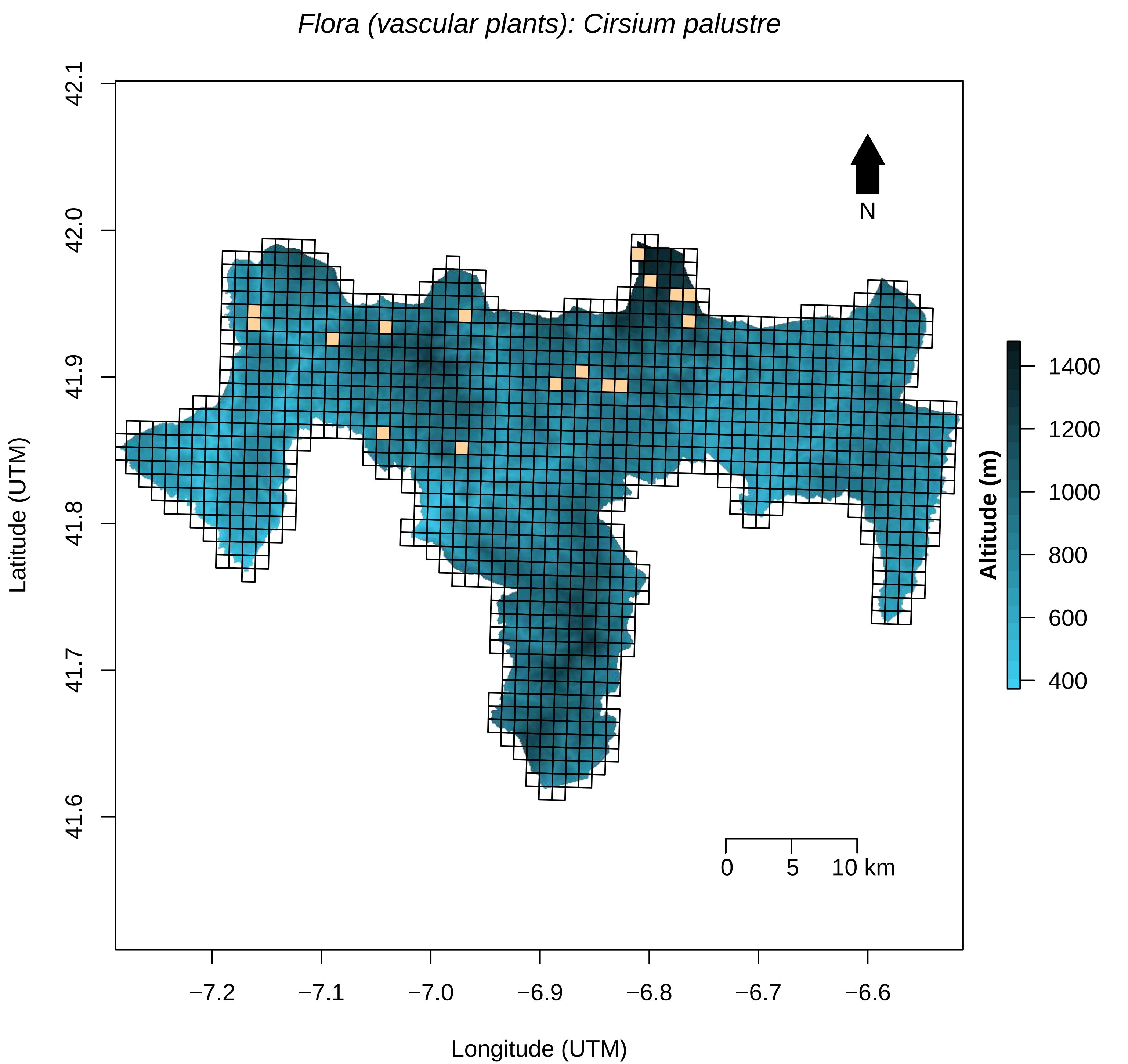

Flora (vascular plants): Cirsium palustre
N
0
5
10 km
42.1
42.0
41.9
41.8
41.7
41.6
Latitude (UTM)
−7.2
−7.1
−7.0
−6.9
−6.8
−6.7
−6.6
Longitude (UTM)
Altitude (m)
1400
1200
1000
800
600
400

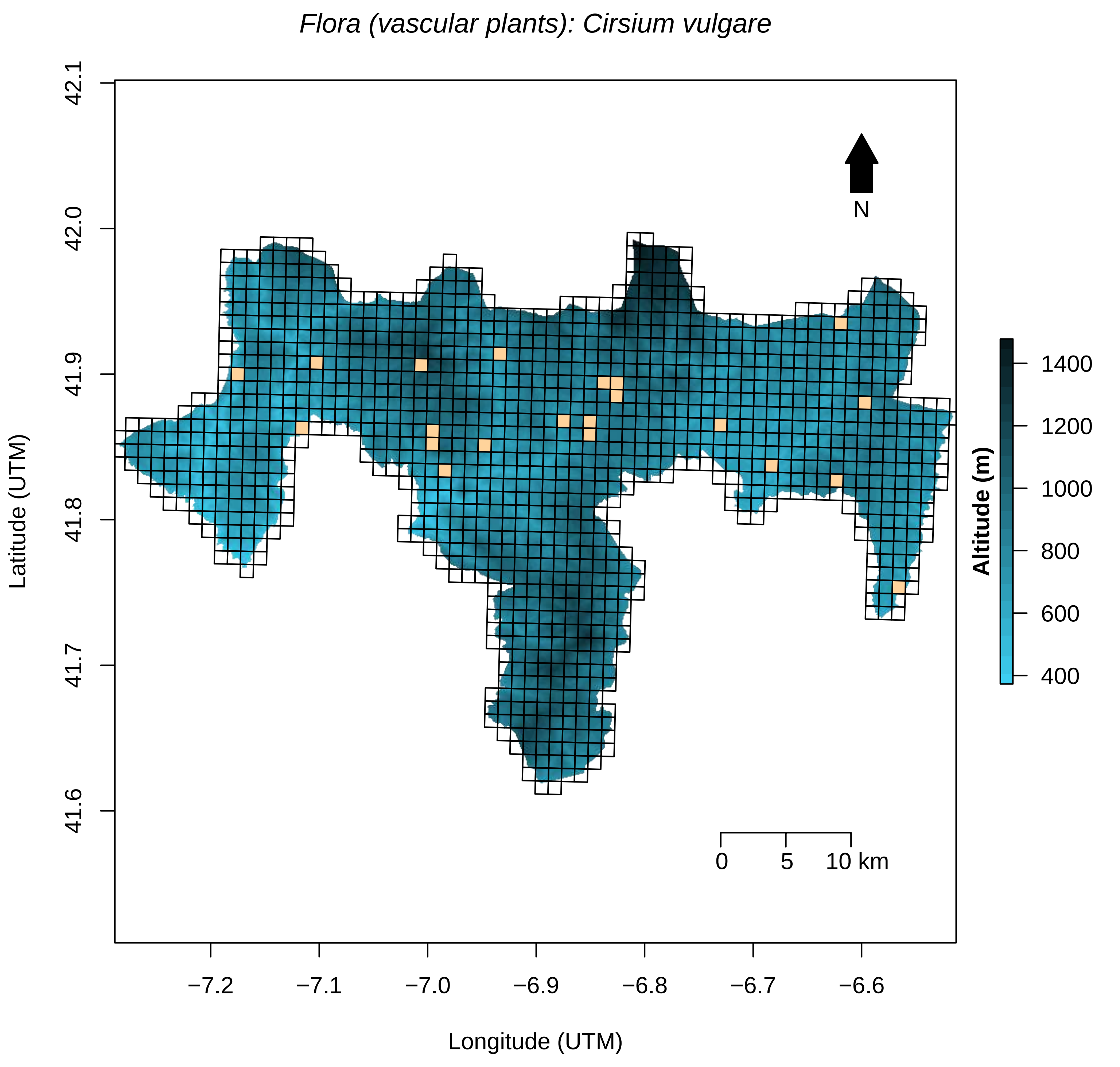
Flora (vascular plants): Cirsium vulgare
N
0
5
10 km
Altitude (m)
1400
1200
1000
800
600
400
Latitude (UTM)
42.1
42.0
41.9
41.8
41.7
41.6
Longitude (UTM)
−7.2
−7.1
−7.0
−6.9
−6.8
−6.7
−6.6

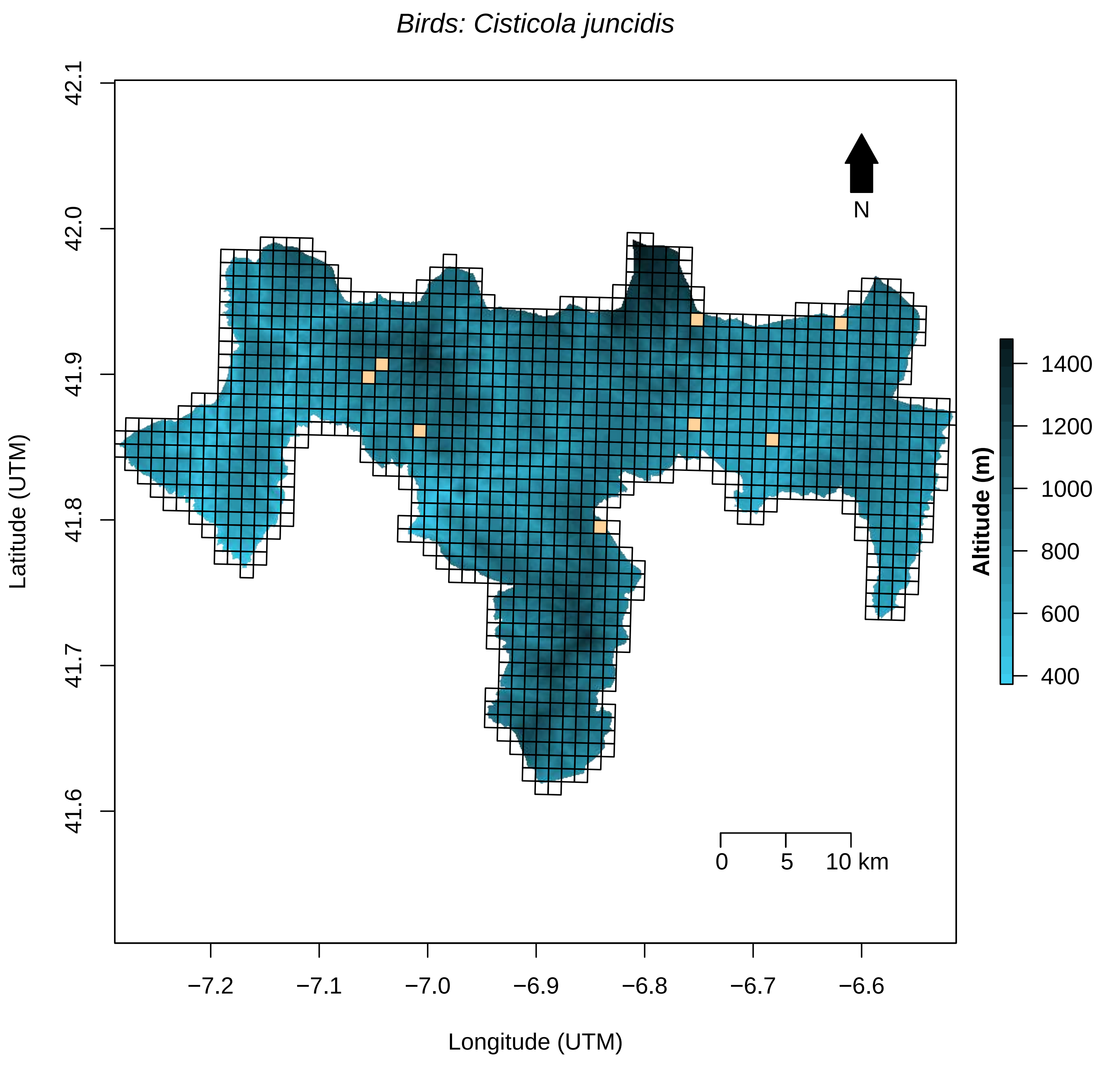

Birds: Cisticola juncidis
N
0 5 10 km
Altitude (m)
1400
1200
1000
800
600
400
Latitude (UTM)
42.1
42.0
41.9
41.8
41.7
41.6
Longitude (UTM)
−7.2
−7.1
−7.0
−6.9
−6.8
−6.7
−6.6

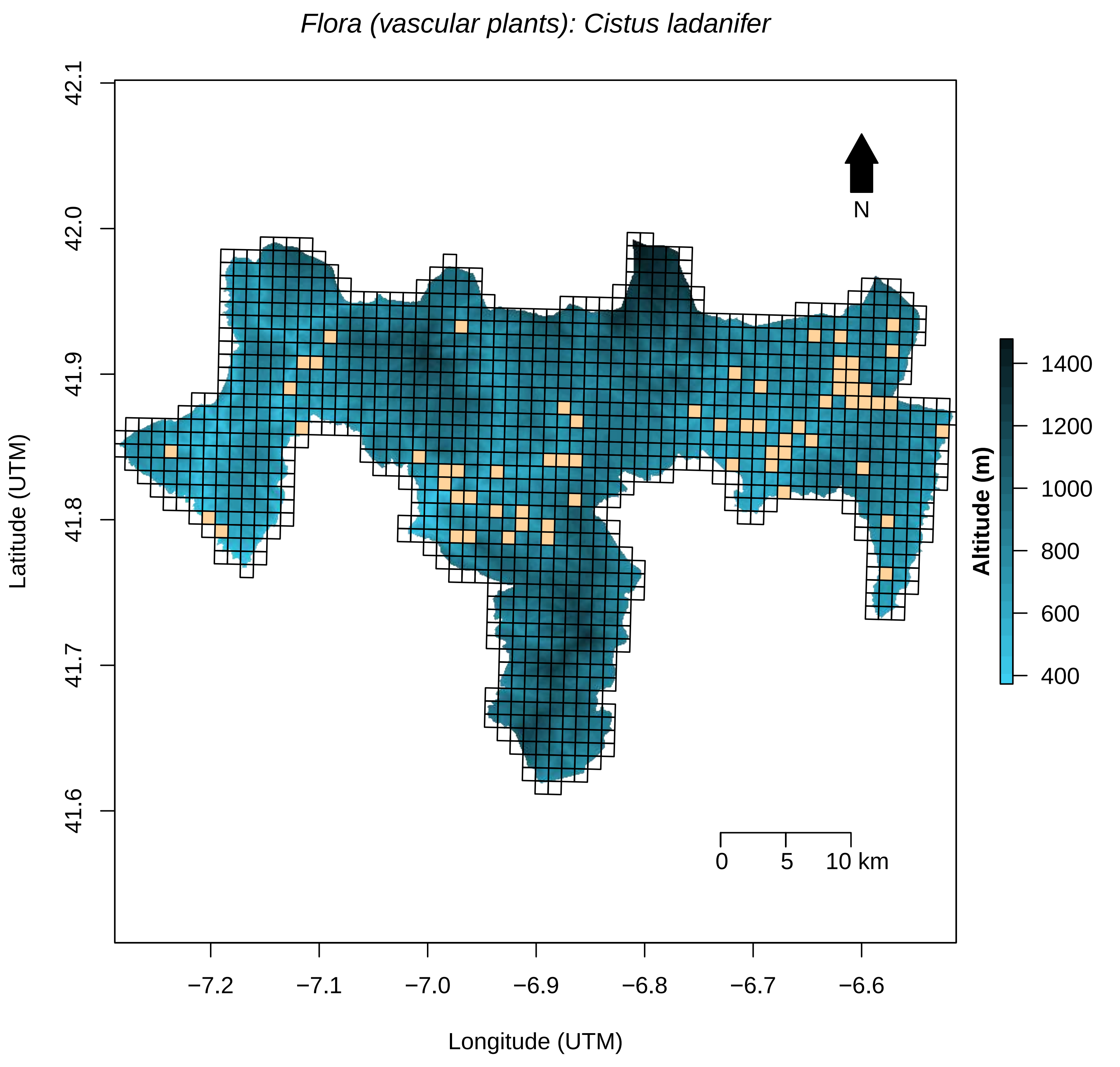
Flora (vascular plants): Cistus ladanifer
42.1
42.0
41.9
41.8
41.7
41.6
Latitude (UTM)
−7.2
−7.1
−7.0
−6.9
−6.8
−6.7
−6.6
Longitude (UTM)
N
0
5
10 km
Altitude (m)
1400
1200
1000
800
600
400

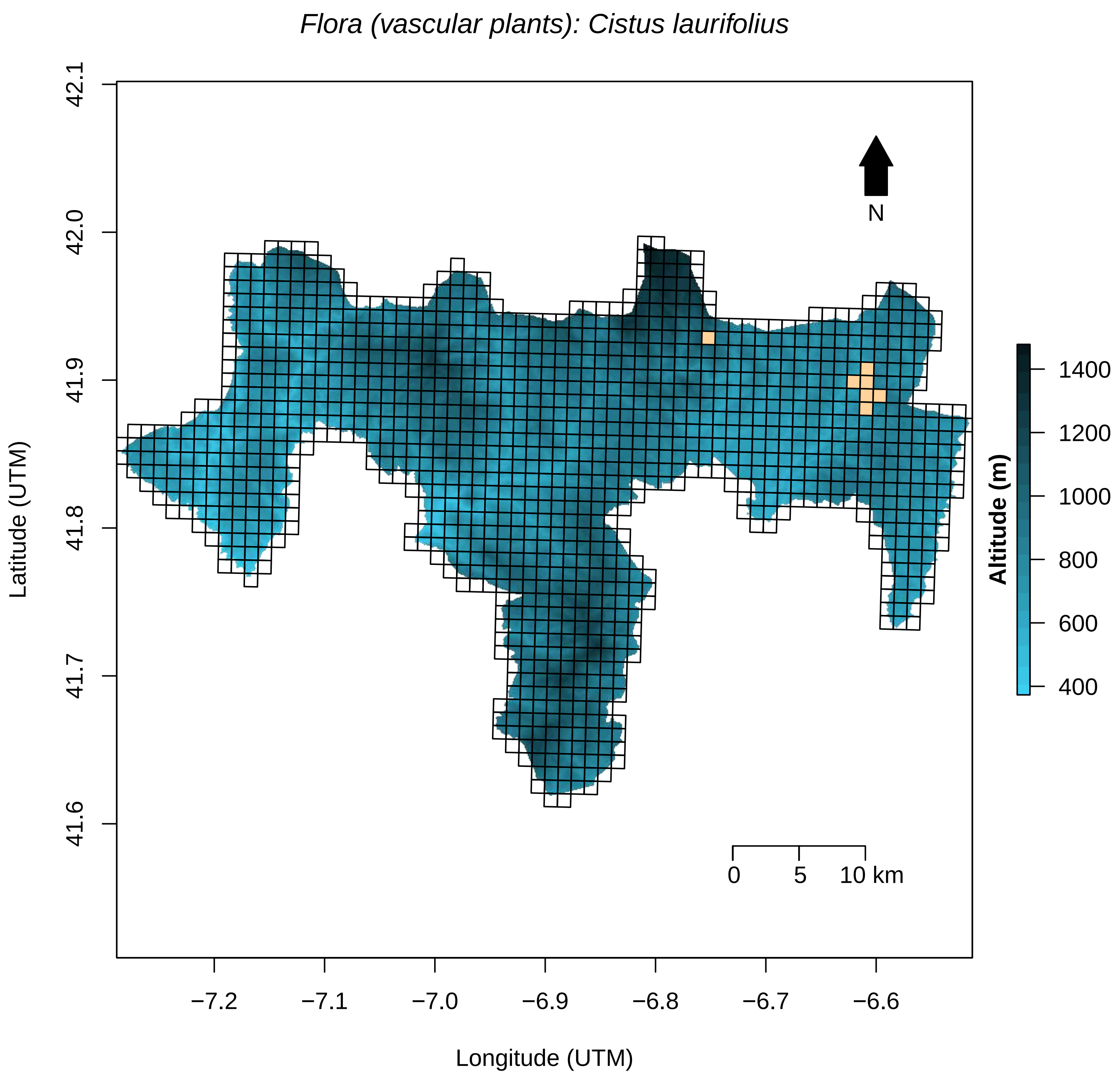

Flora (vascular plants): Cistus laurifolius
42.1
42.0
41.9
41.8
41.7
41.6
Latitude (UTM)
−7.2
−7.1
−7.0
−6.9
−6.8
−6.7
−6.6
Longitude (UTM)
N
0
5
10 km
Altitude (m)
1400
1200
1000
800
600
400

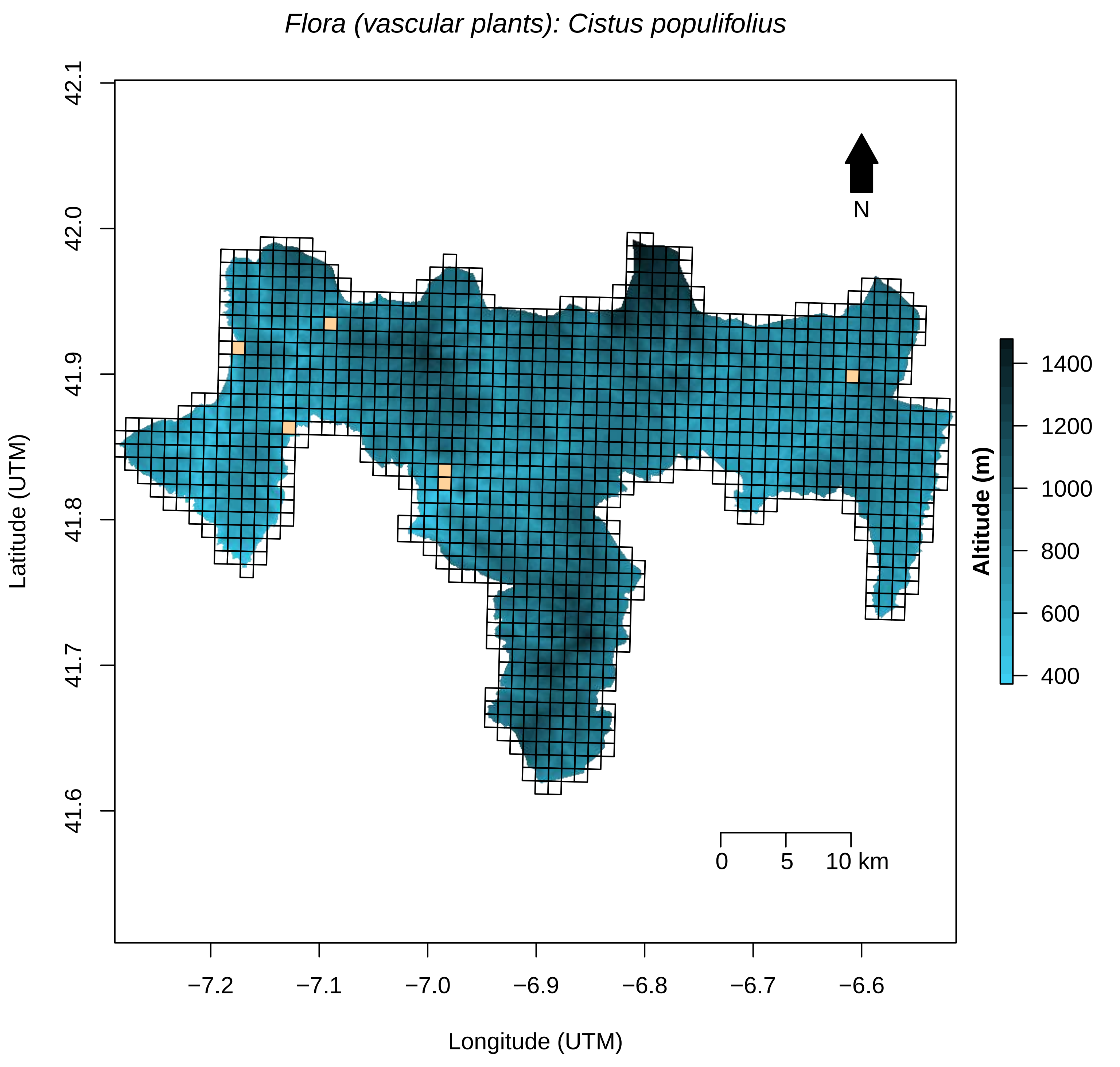

Flora (vascular plants): Cistus populifolius
42.1
42.0
41.9
41.8
41.7
41.6
Latitude (UTM)
−7.2
−7.1
−7.0
−6.9
−6.8
−6.7
−6.6
Longitude (UTM)
N
0
5
10 km
Altitude (m)
1400
1200
1000
800
600
400

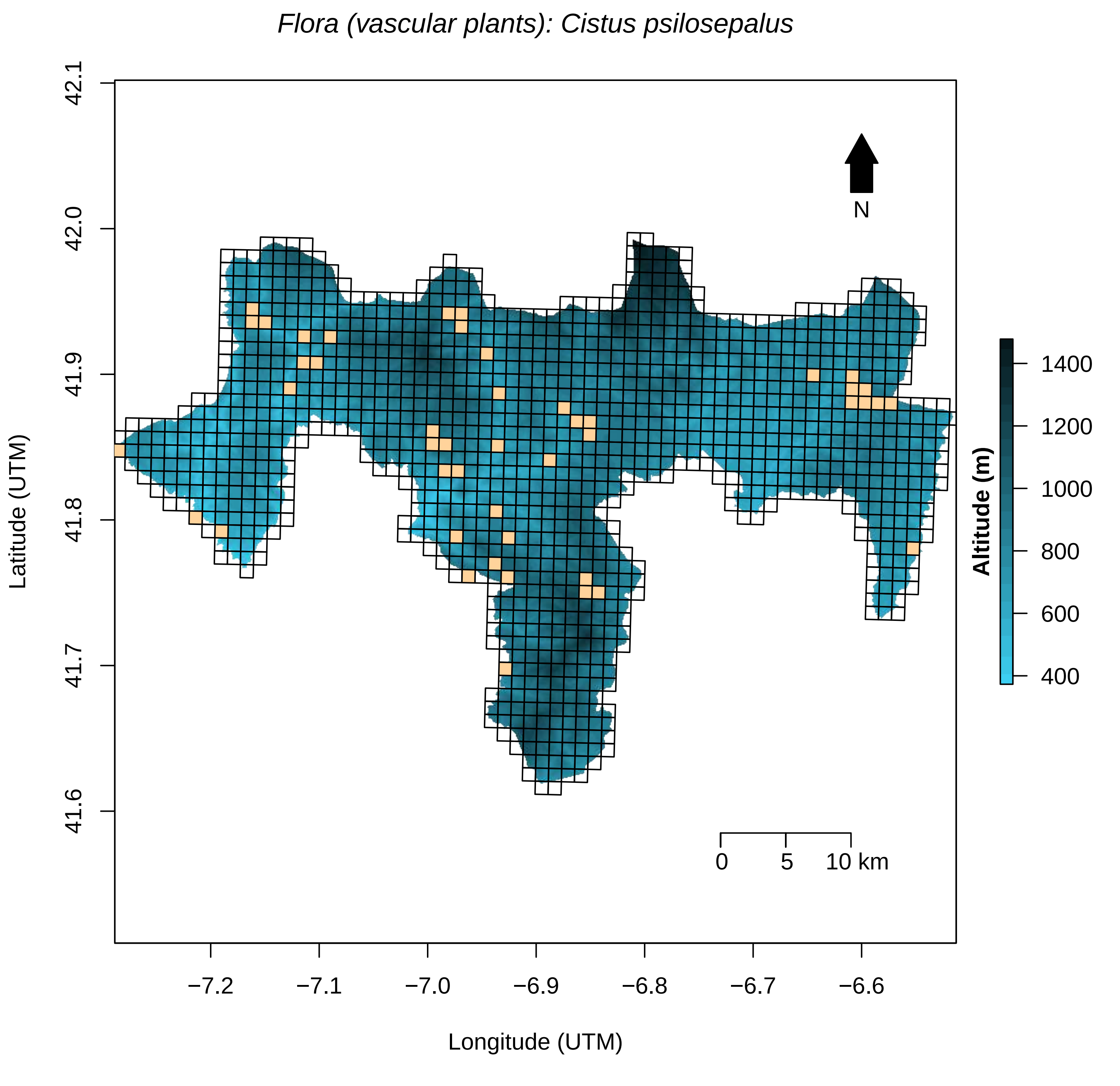
Flora (vascular plants): Cistus psilosepalus
N
0 5 10 km
Altitude (m)
1400
1200
1000
800
600
400
42.1
42.0
41.9
41.8
41.7
41.6
Latitude (UTM)
−7.2 −7.1 −7.0 −6.9 −6.8 −6.7 −6.6
Longitude (UTM)

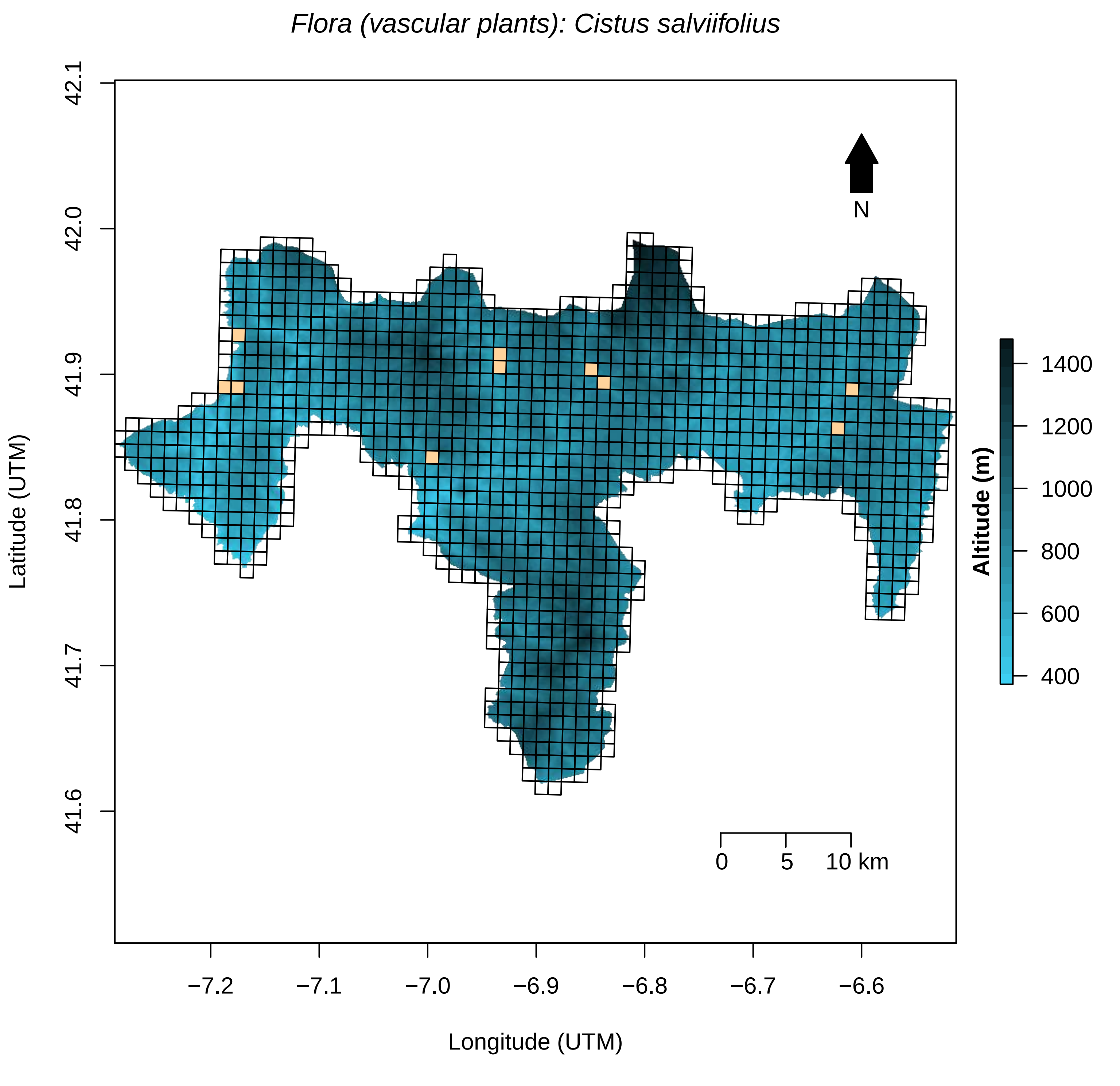
Flora (vascular plants): Cistus salviifolius
42.1
42.0
41.9
41.8
41.7
41.6
Latitude (UTM)
N
0
5
10 km
−7.2
−7.1
−7.0
−6.9
−6.8
−6.7
−6.6
Longitude (UTM)
1400
1200
1000
800
600
400
Altitude (m)

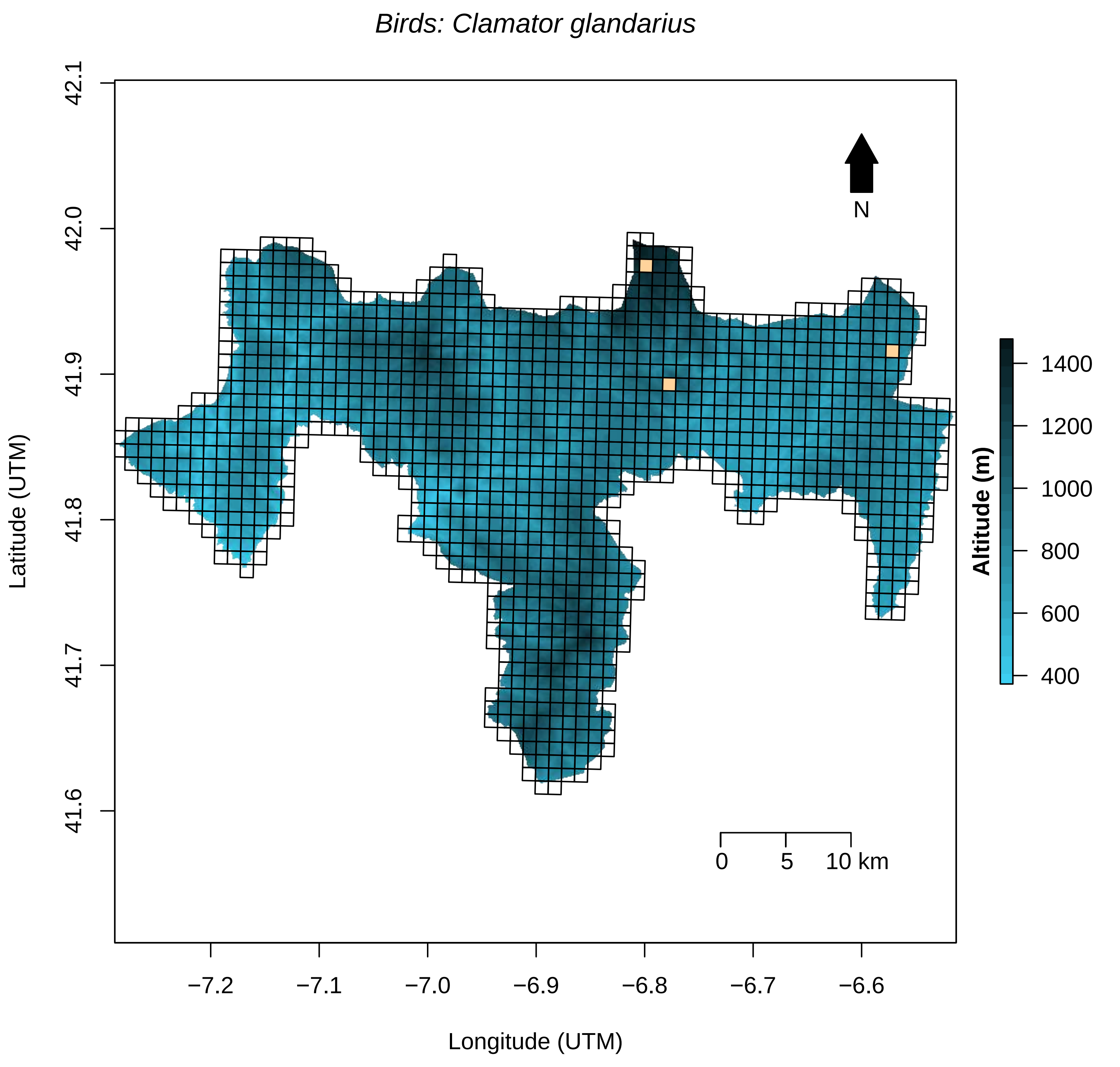

Birds: Clamator glandarius
42.1
42.0
41.9
41.8
41.7
41.6
Latitude (UTM)
N
0
5
10 km
1400
1200
1000
800
600
400
Altitude (m)
−7.2
−7.1
−7.0
−6.9
−6.8
−6.7
−6.6
Longitude (UTM)

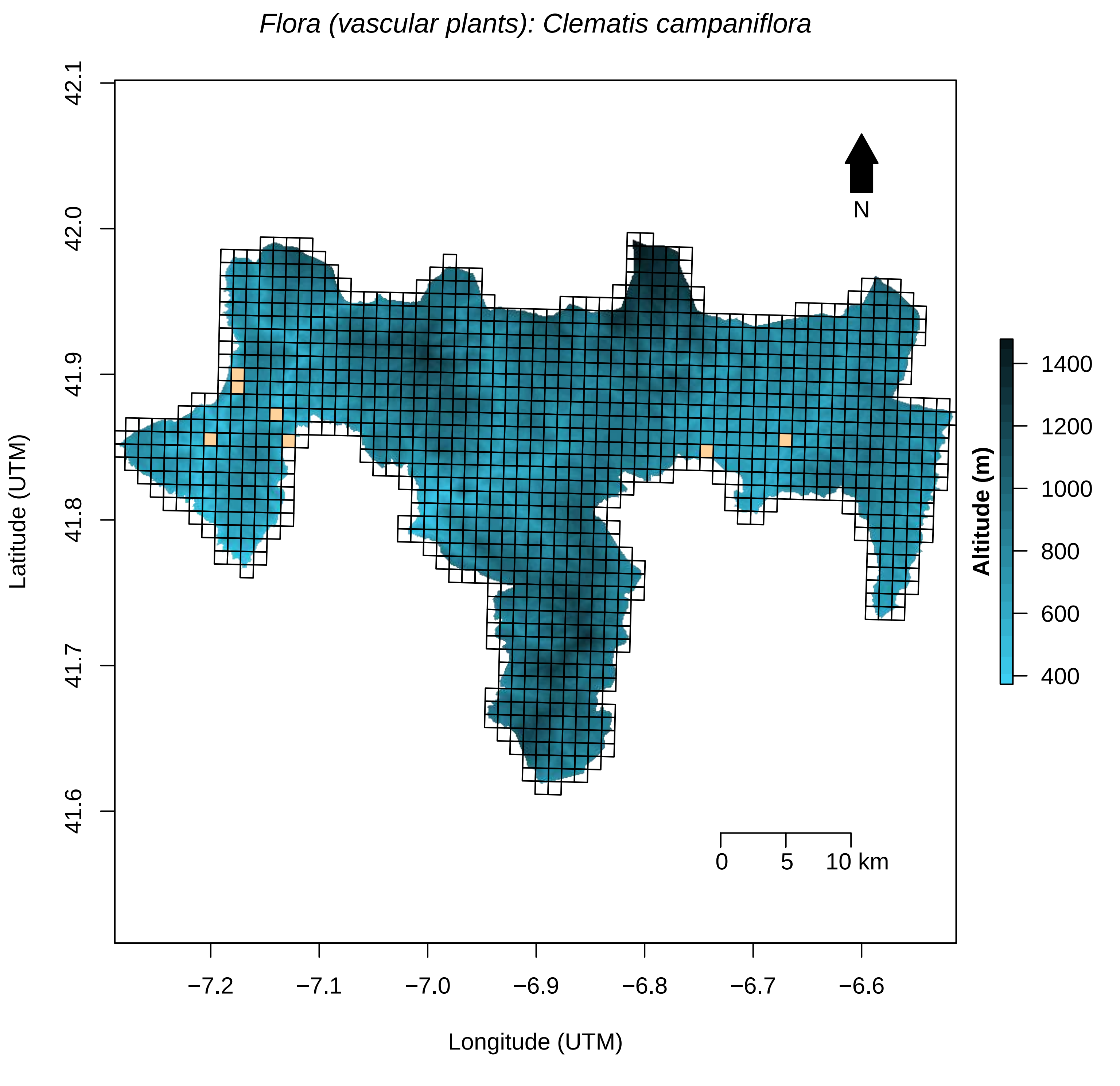

Flora (vascular plants): Clematis campaniflora
42.1
42.0
41.9
41.8
41.7
41.6
Latitude (UTM)
−7.2
−7.1
−7.0
−6.9
−6.8
−6.7
−6.6
Longitude (UTM)
N
0
5
10 km
1400
1200
1000
800
600
400
Altitude (m)

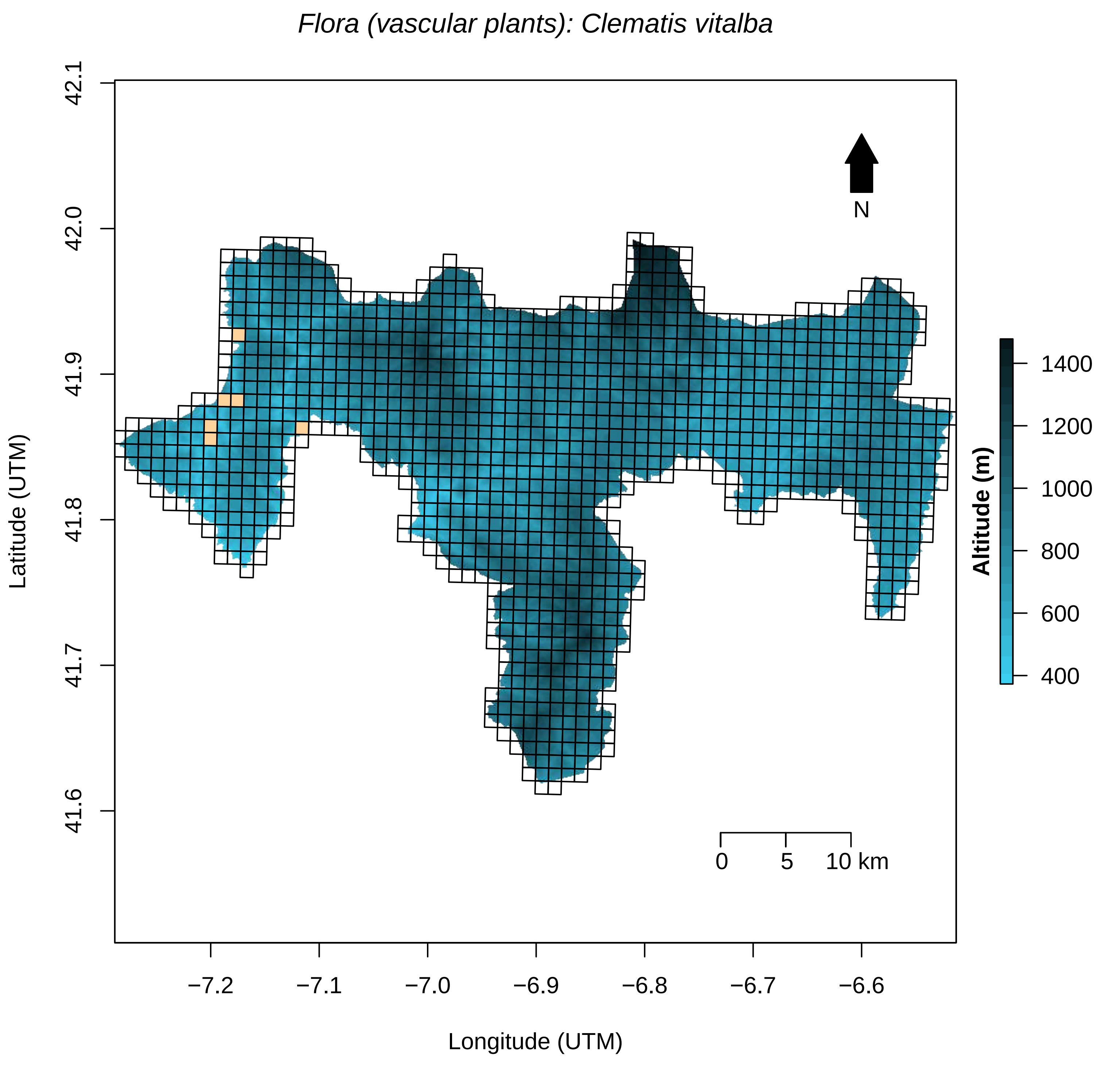

Flora (vascular plants): Clematis vitalba
42.1
42.0
41.9
41.8
41.7
41.6
Latitude (UTM)
−7.2
−7.1
−7.0
−6.9
−6.8
−6.7
−6.6
Longitude (UTM)
N
0
5
10 km
Altitude (m)
1400
1200
1000
800
600
400

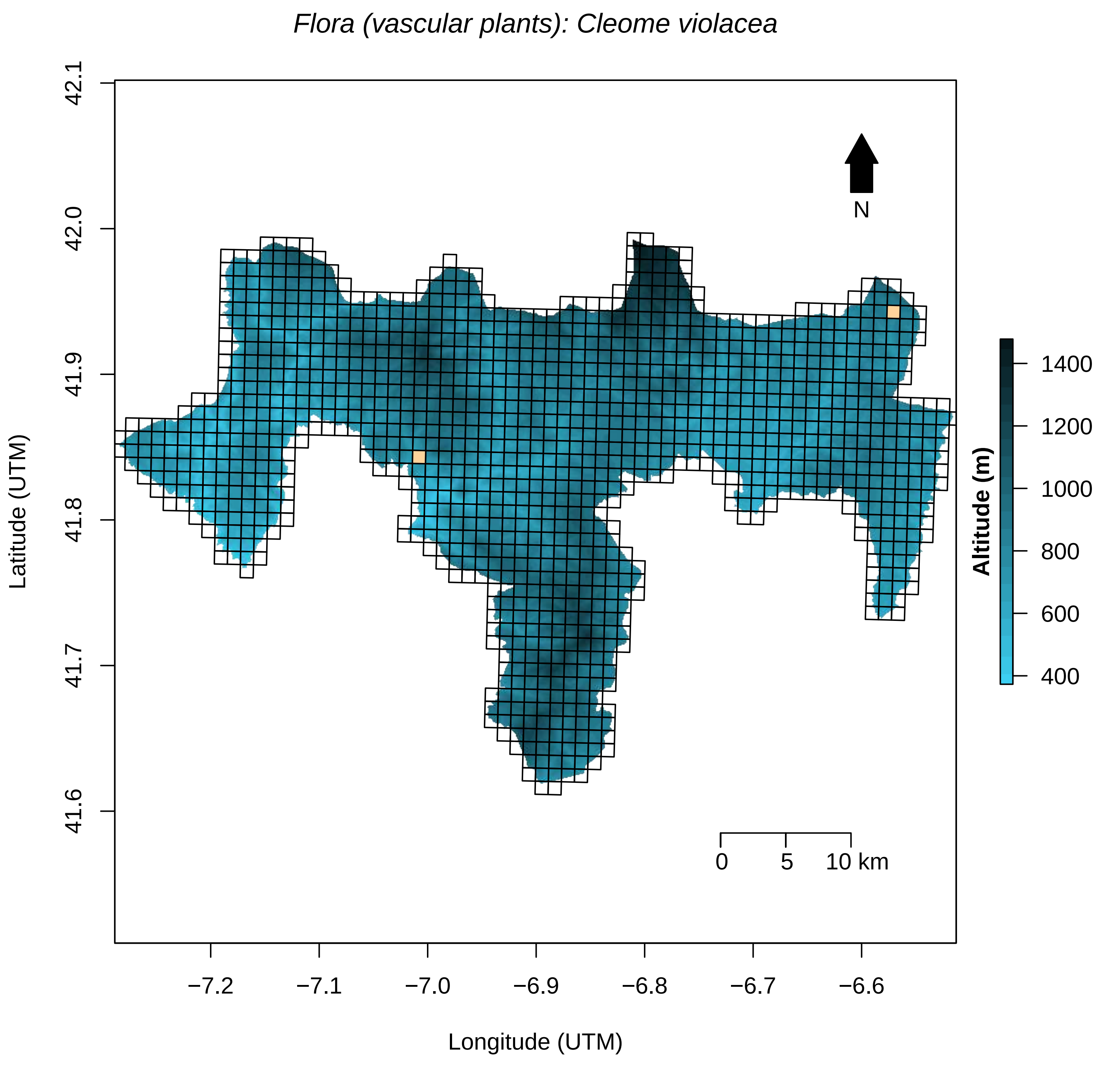

Flora (vascular plants): Cleome violacea
42.1
42.0
41.9
41.8
41.7
41.6
Latitude (UTM)
−7.2
−7.1
−7.0
−6.9
−6.8
−6.7
−6.6
Longitude (UTM)
N
0
5
10 km
Altitude (m)
1400
1200
1000
800
600
400

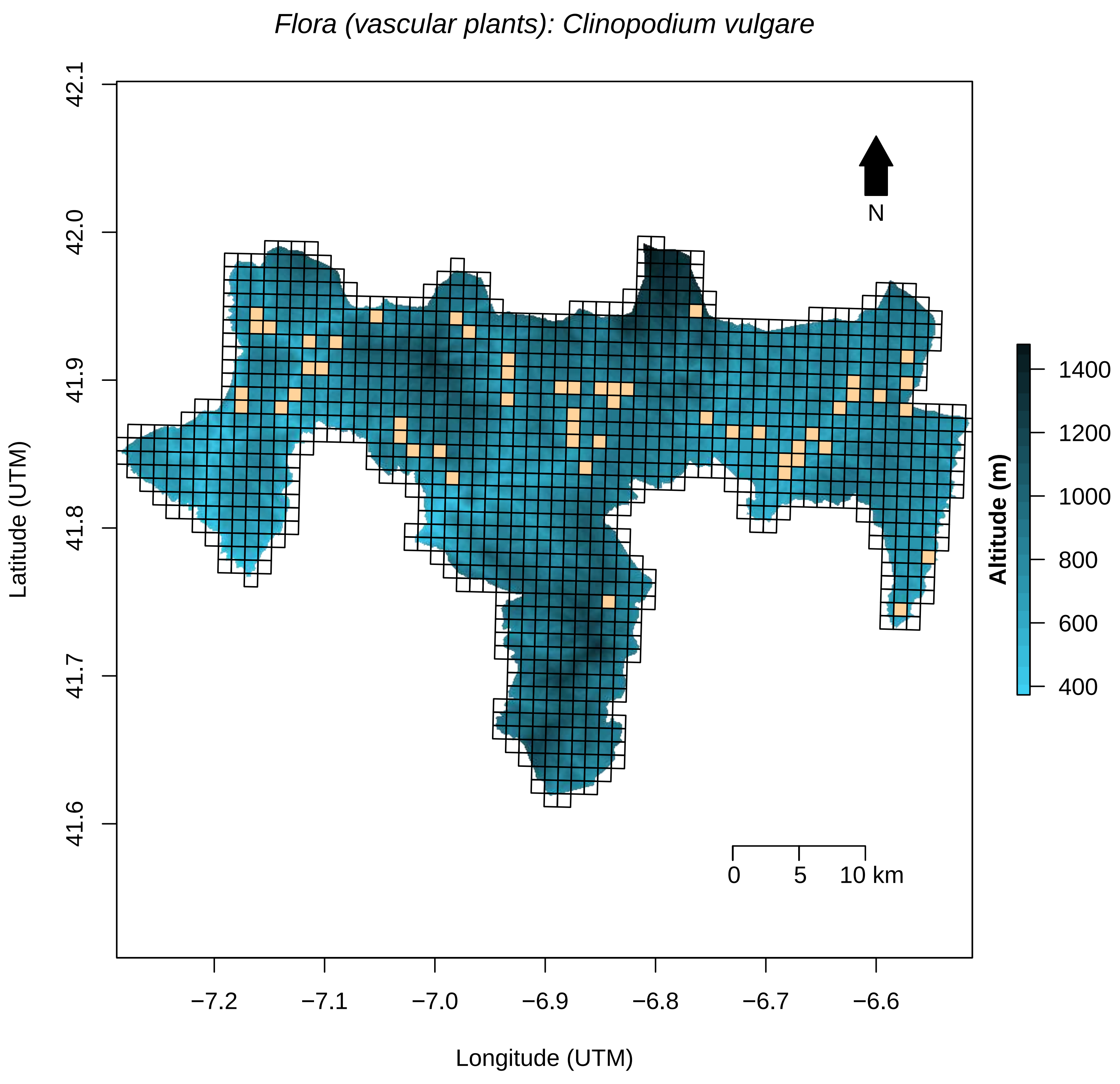
Flora (vascular plants): Clinopodium vulgare
Latitude (UTM)
42.1
42.0
41.9
41.8
41.7
41.6
Longitude (UTM)
−7.2
−7.1
−7.0
−6.9
−6.8
−6.7
−6.6
N
0
5
10 km
Altitude (m)
1400
1200
1000
800
600
400

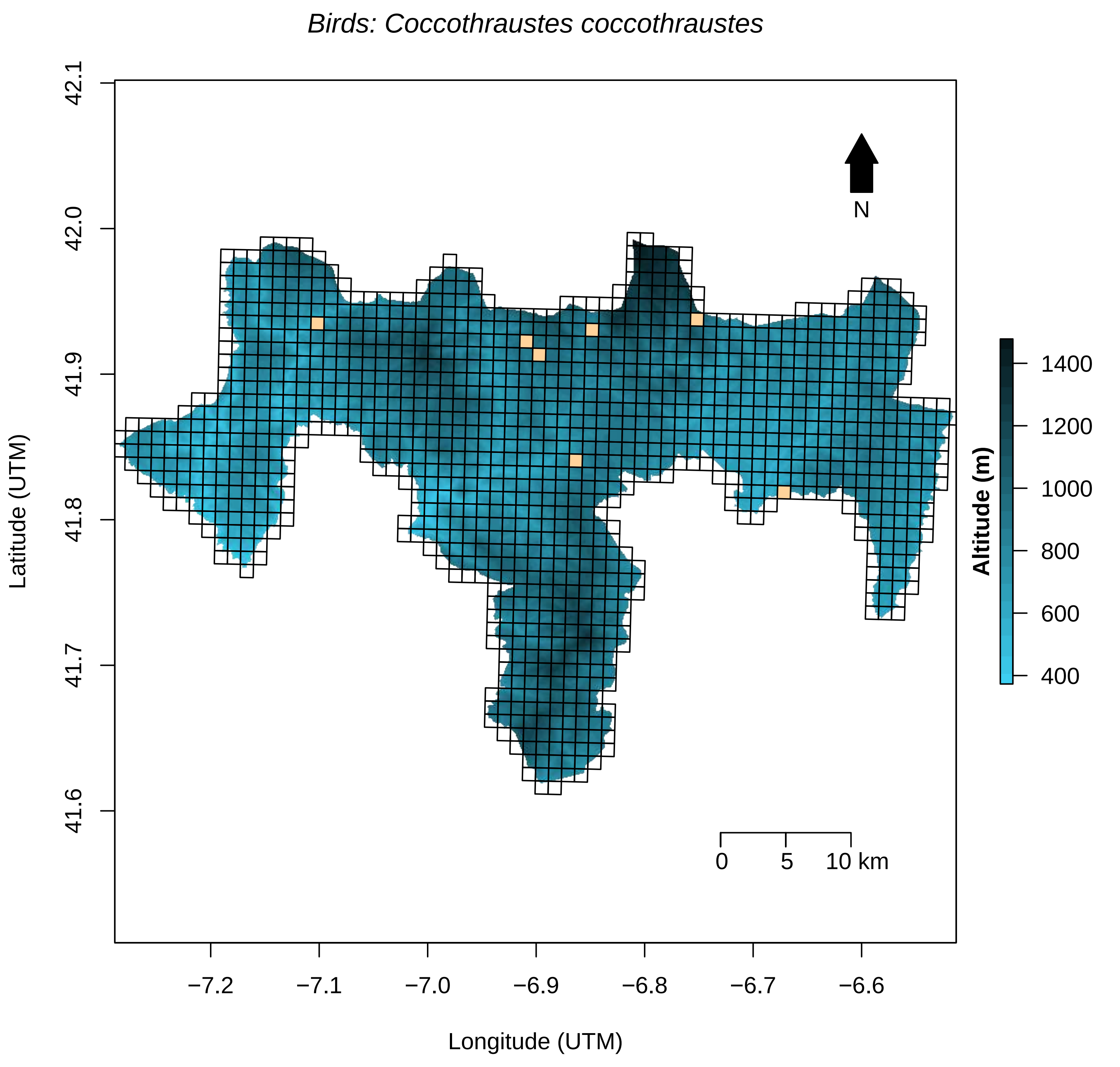

Birds: Coccothraustes coccothraustes
42.1
42.0
41.9
41.8
41.7
41.6
Latitude (UTM)
−7.2
−7.1
−7.0
−6.9
−6.8
−6.7
−6.6
Longitude (UTM)
N
0
5
10 km
1400
1200
1000
800
600
400
Altitude (m)

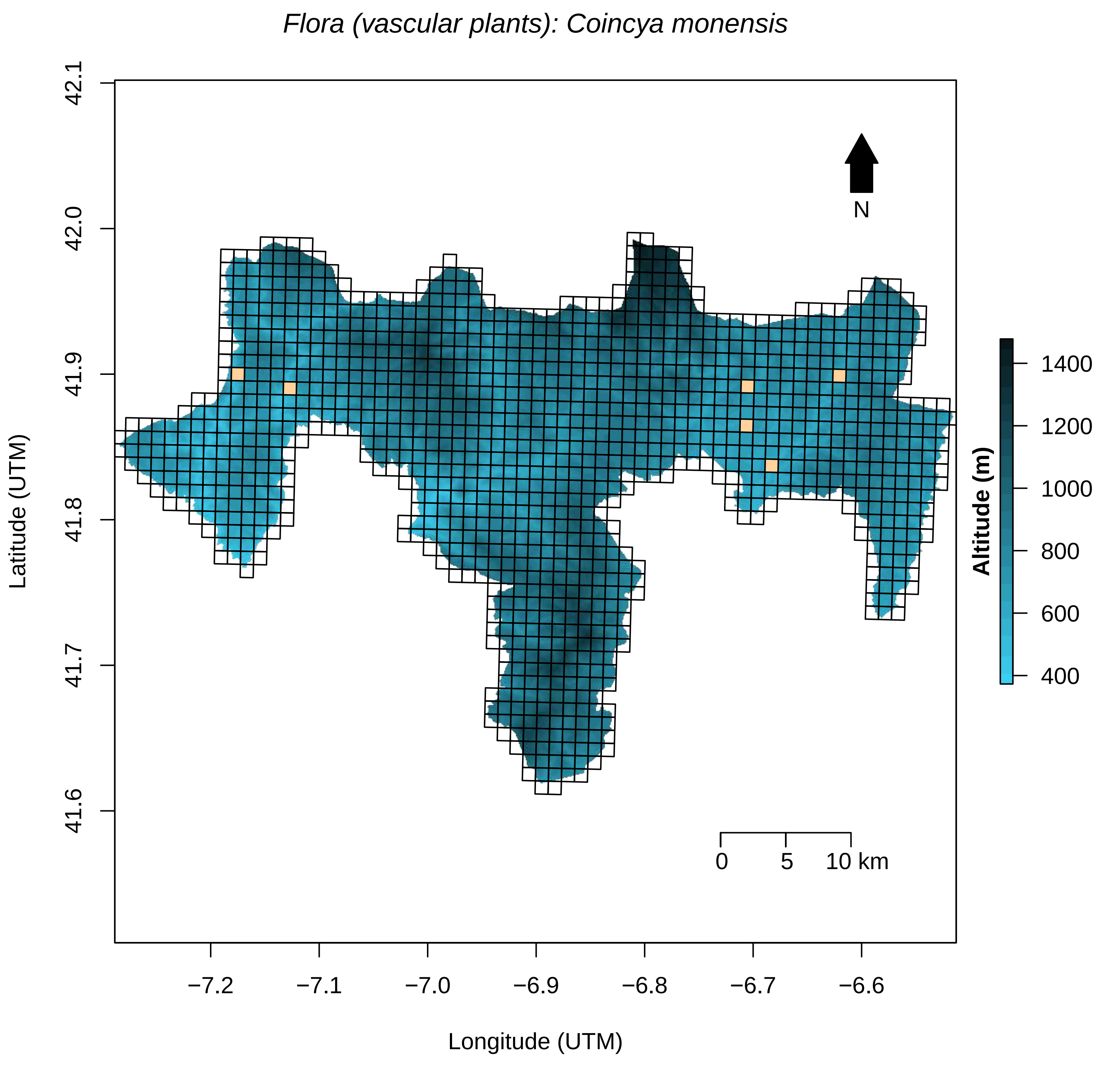

Flora (vascular plants): Coincya monensis
Latitude (UTM)
42.1
42.0
41.9
41.8
41.7
41.6
Longitude (UTM)
−7.2
−7.1
−7.0
−6.9
−6.8
−6.7
−6.6
N
0
5
10 km
Altitude (m)
1400
1200
1000
800
600
400

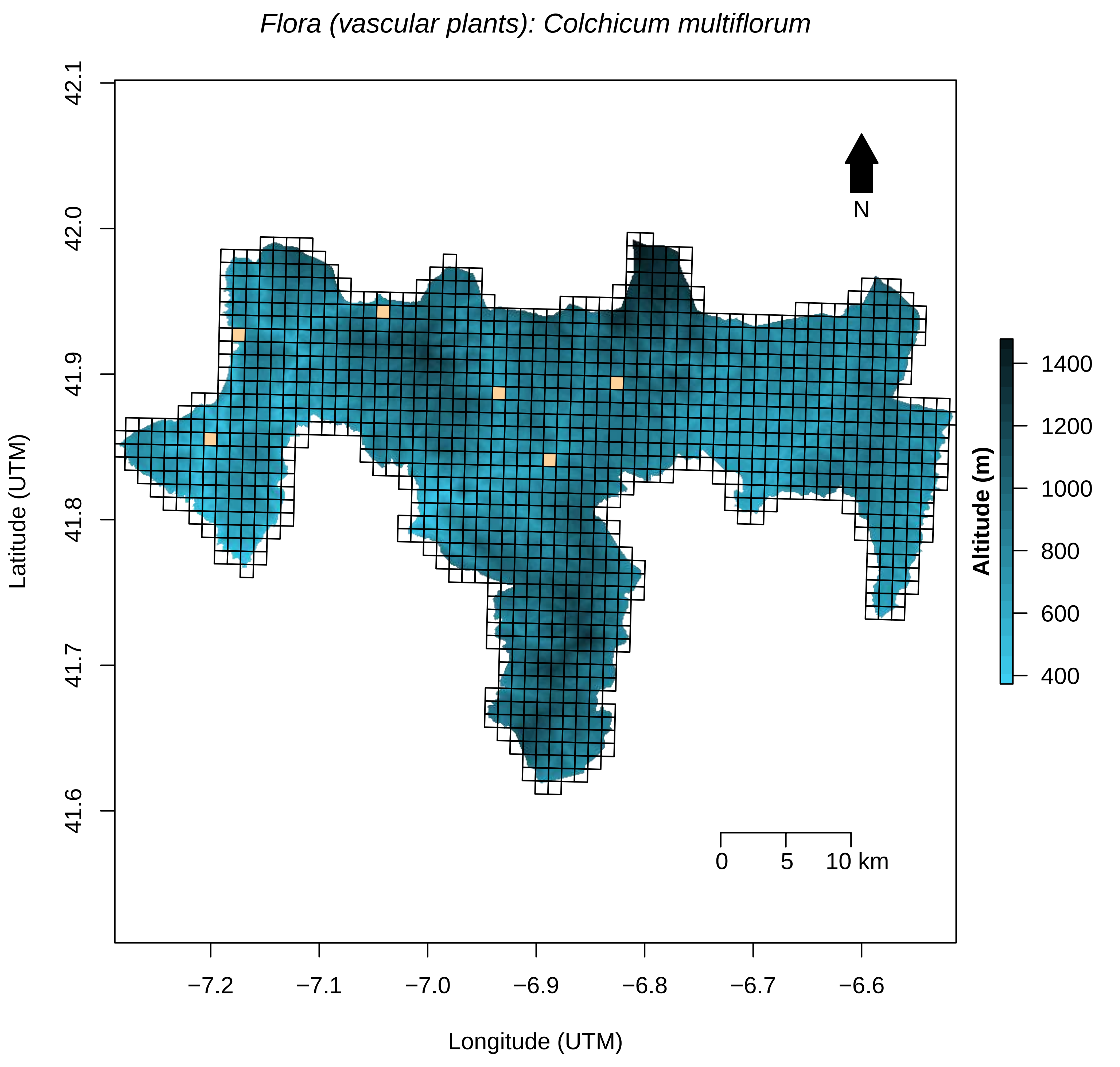

Flora (vascular plants): Colchicum multiflorum
42.1
42.0
41.9
41.8
41.7
41.6
Latitude (UTM)
N
0 5 10 km
−7.2 −7.1 −7.0 −6.9 −6.8 −6.7 −6.6
Longitude (UTM)
Altitude (m)
1400
1200
1000
800
600
400

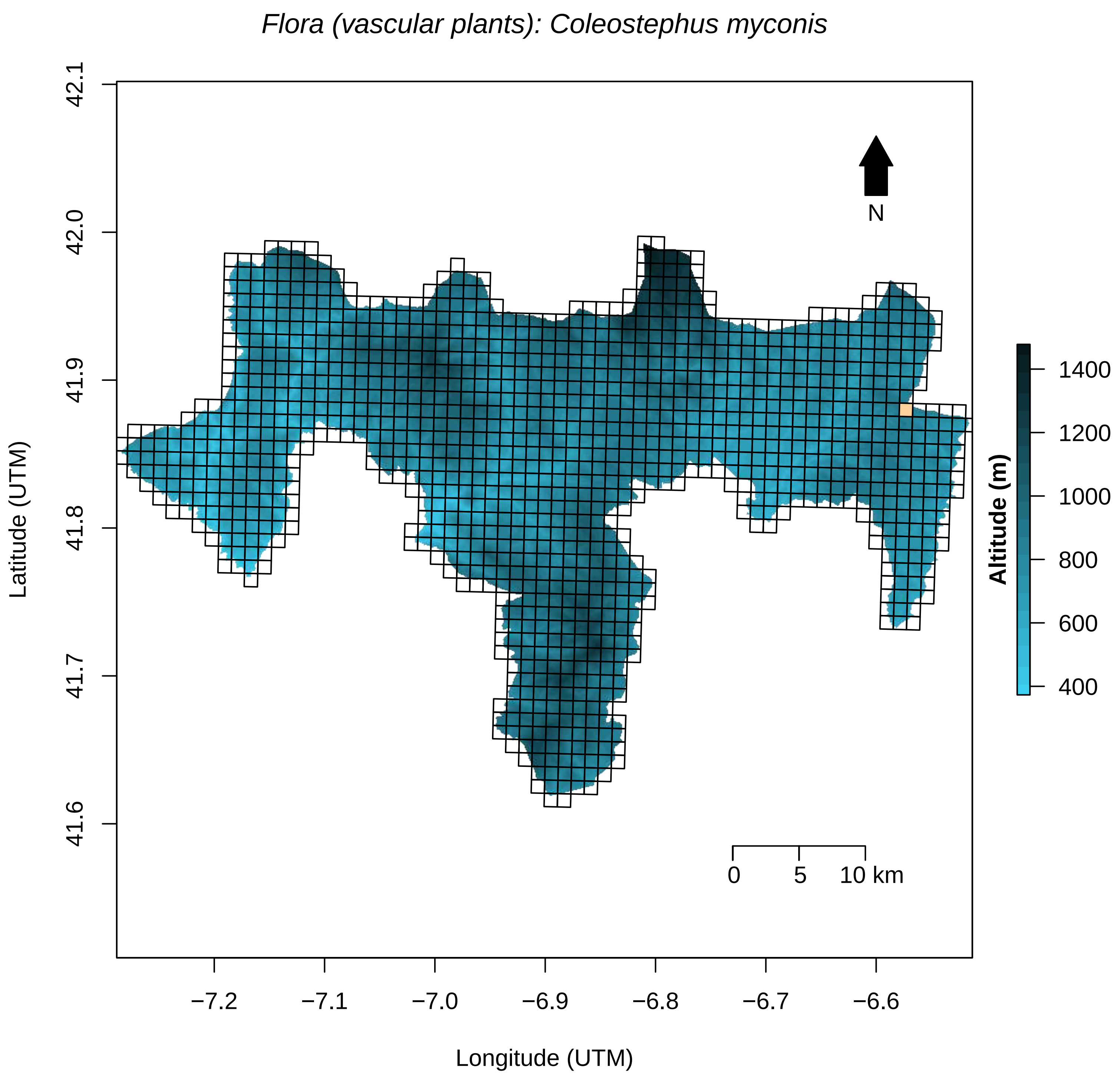

Flora (vascular plants): Coleostephus myconis
42.1
42.0
41.9
41.8
41.7
41.6
Latitude (UTM)
−7.2
−7.1
−7.0
−6.9
−6.8
−6.7
−6.6
Longitude (UTM)
N
0
5
10 km
Altitude (m)
1400
1200
1000
800
600
400

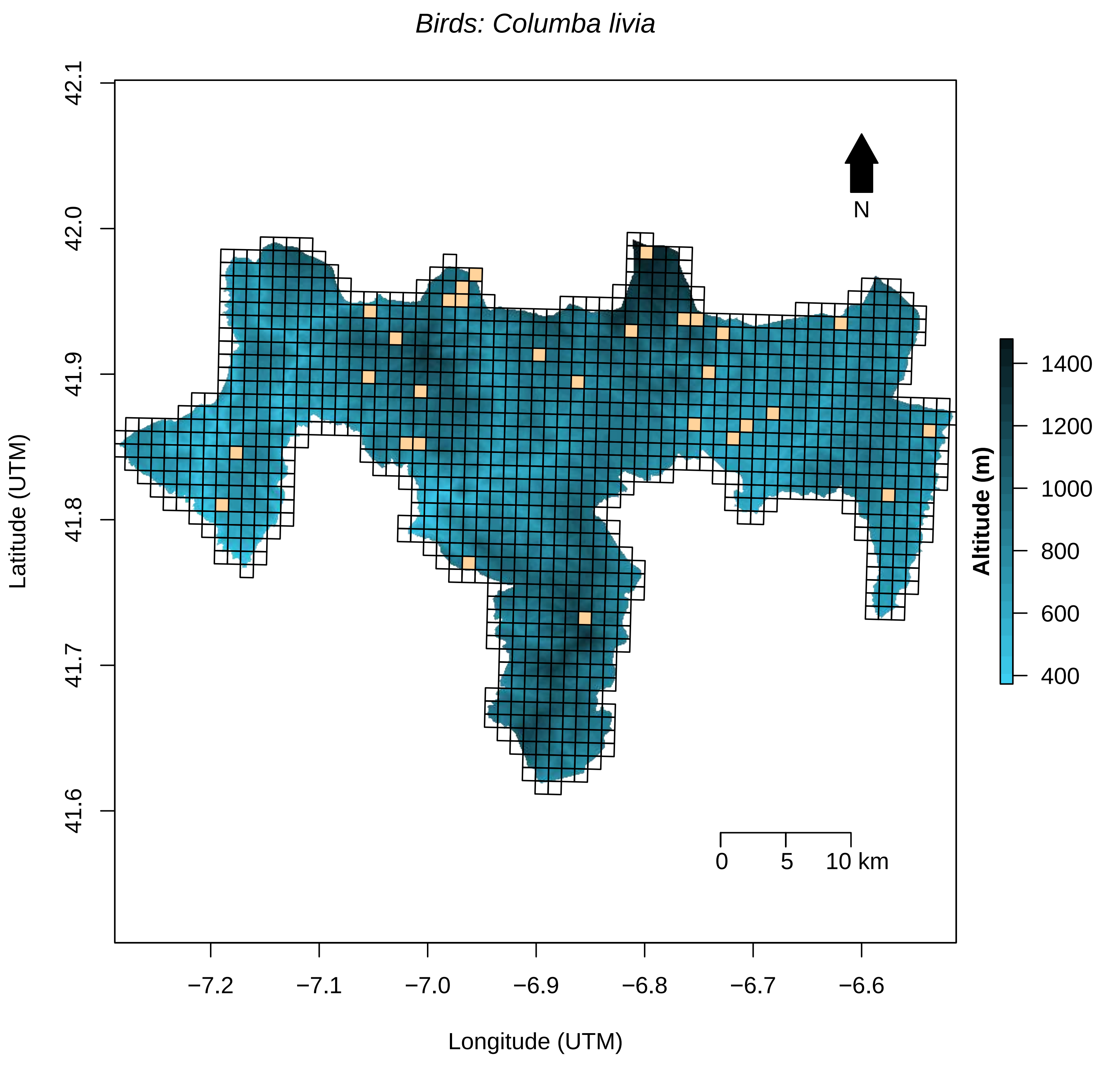

Birds: Columba livia
42.1
42.0
41.9
41.8
41.7
41.6
Latitude (UTM)
−7.2
−7.1
−7.0
−6.9
−6.8
−6.7
−6.6
Longitude (UTM)
N
0
5
10 km
1400
1200
1000
800
600
400
Altitude (m)

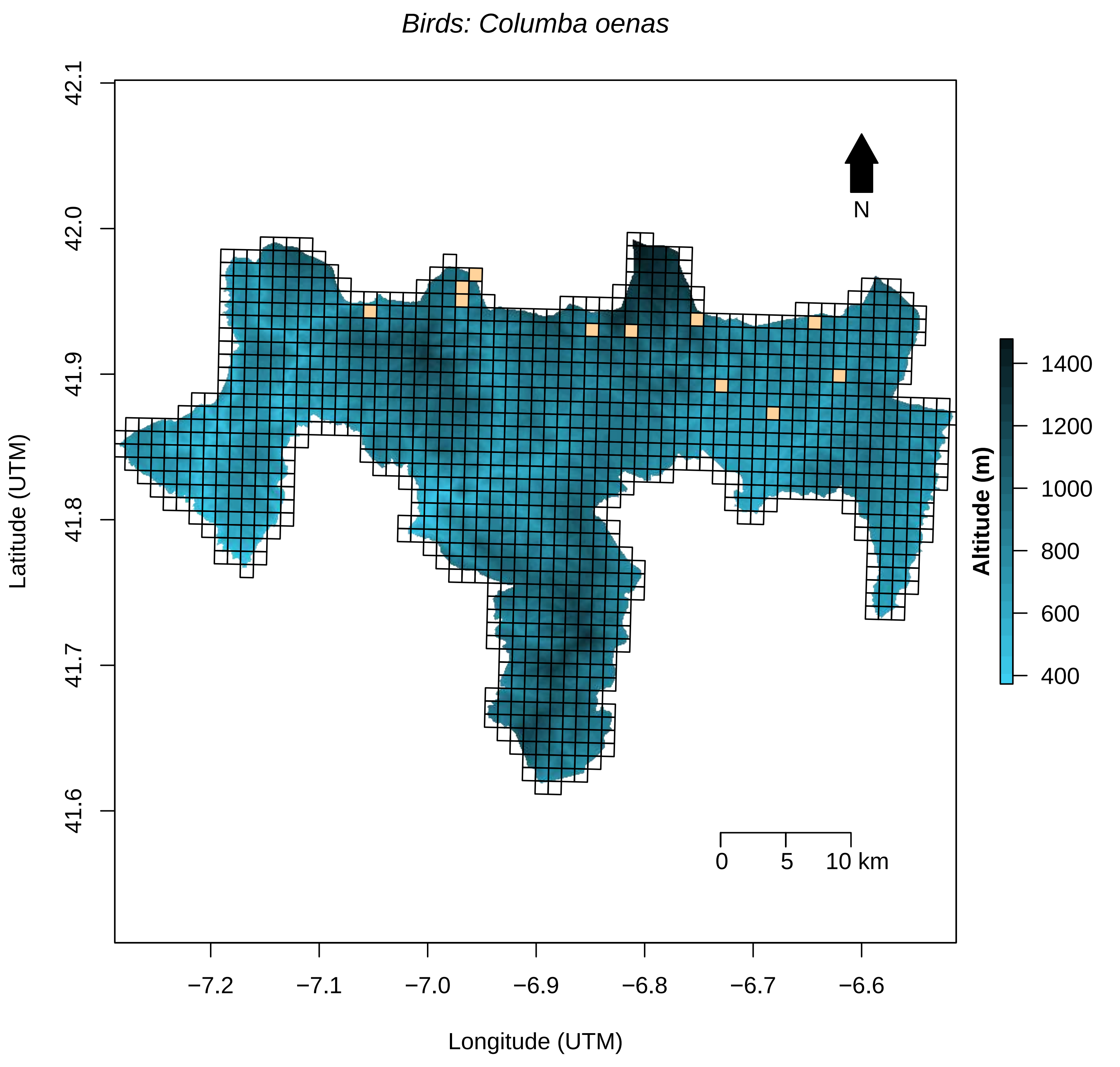

Birds: Columba oenas
42.1
42.0
41.9
41.8
41.7
41.6
Latitude (UTM)
−7.2
−7.1
−7.0
−6.9
−6.8
−6.7
−6.6
Longitude (UTM)
N
0
5
10 km
Altitude (m)
1400
1200
1000
800
600
400

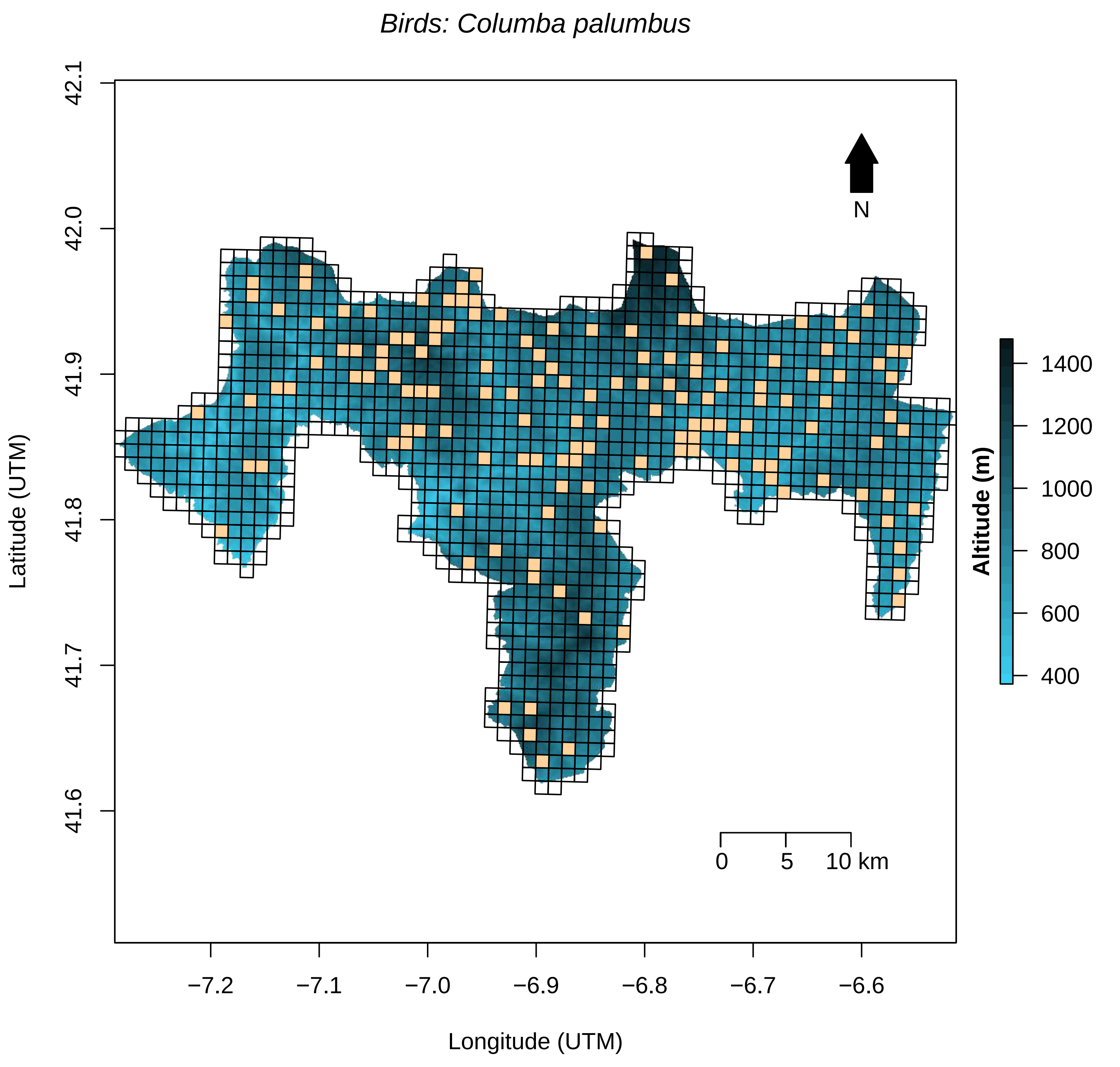
Birds: Columba palumbus
N
0 5 10 km
Altitude (m)
1400
1200
1000
800
600
400
42.1
42.0
41.9
41.8
41.7
41.6
Latitude (UTM)
−7.2
−7.1
−7.0
−6.9
−6.8
−6.7
−6.6
Longitude (UTM)

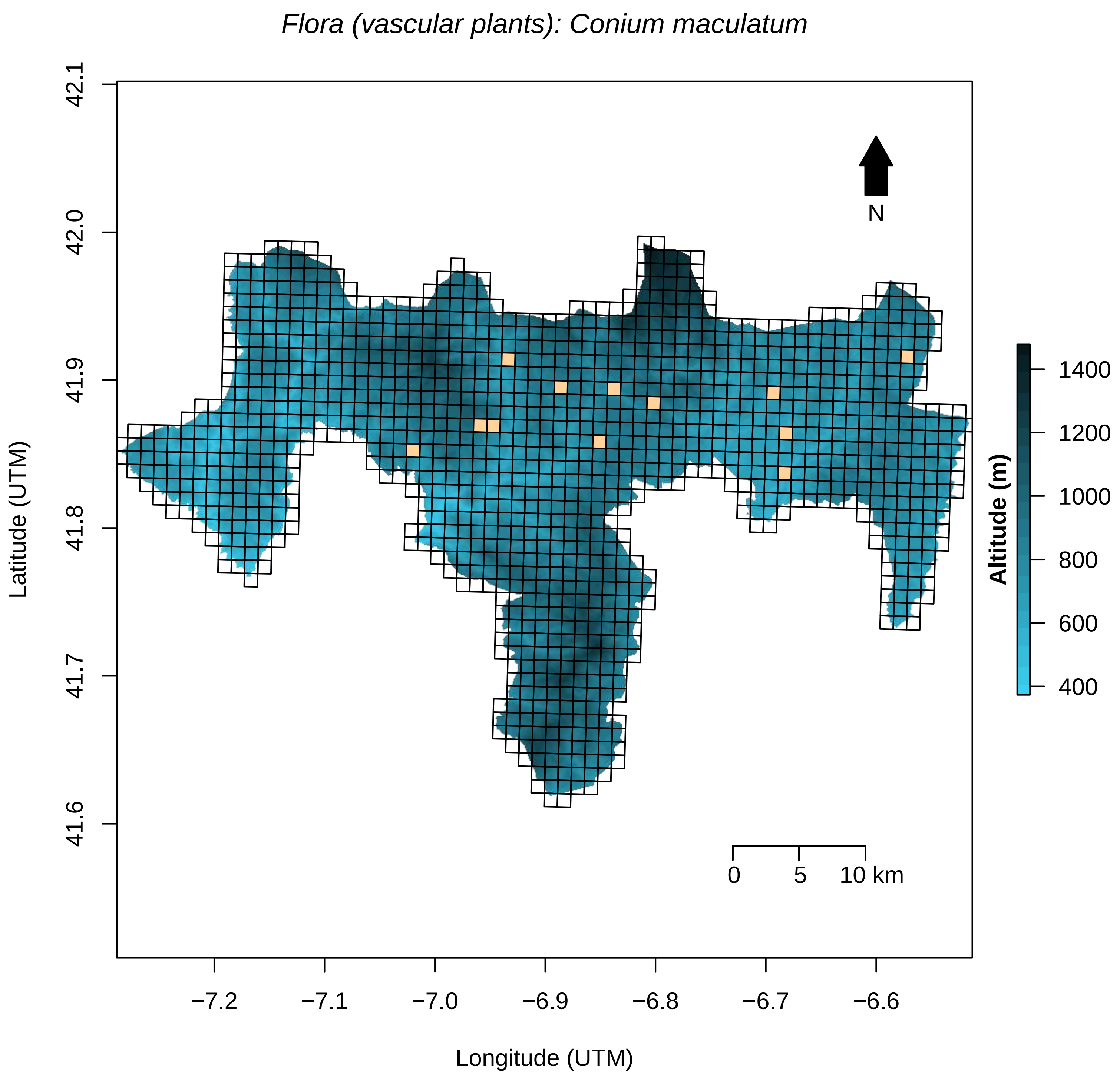

Flora (vascular plants): Conium maculatum
42.1
42.0
41.9
41.8
41.7
41.6
Latitude (UTM)
−7.2
−7.1
−7.0
−6.9
−6.8
−6.7
−6.6
Longitude (UTM)
N
0
5
10 km
Altitude (m)
1400
1200
1000
800
600
400

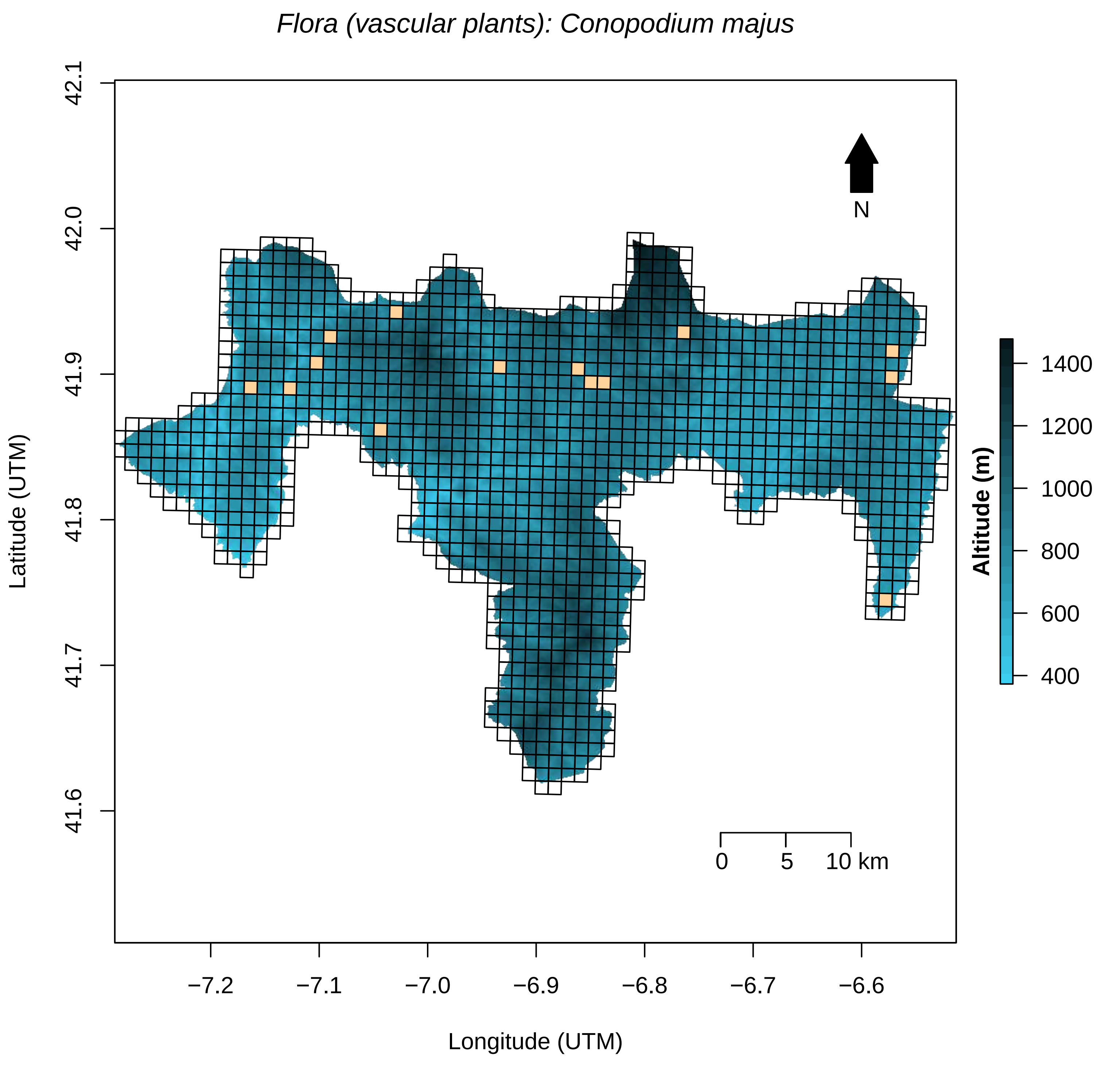

Flora (vascular plants): Conopodium majus
42.1
42.0
41.9
41.8
41.7
41.6
Latitude (UTM)
−7.2
−7.1
−7.0
−6.9
−6.8
−6.7
−6.6
Longitude (UTM)
N
0
5
10 km
Altitude (m)
1400
1200
1000
800
600
400

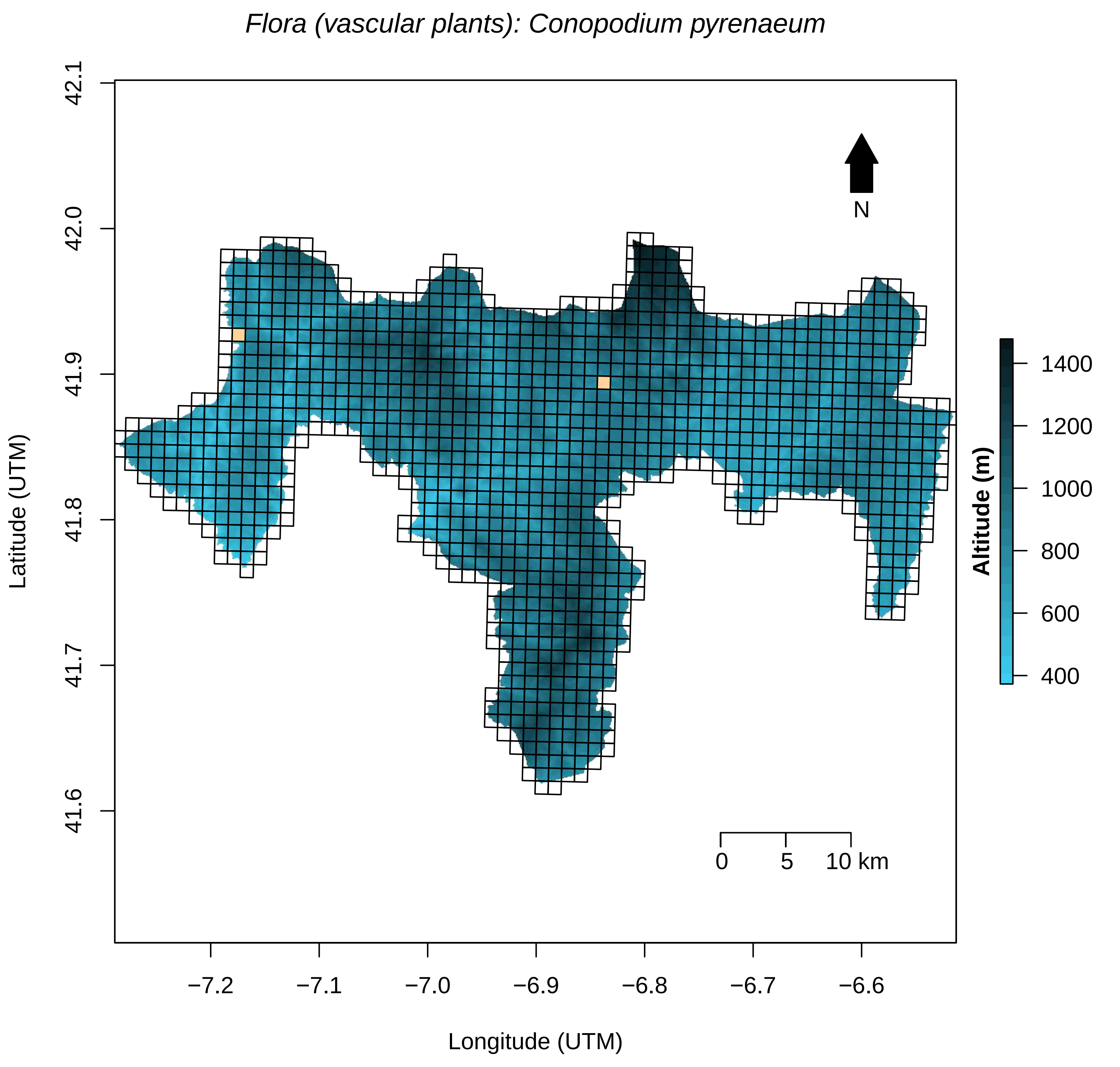

Flora (vascular plants): Conopodium pyrenaeum
N
0 5 10 km
Altitude (m)
1400
1200
1000
800
600
400
Latitude (UTM)
42.1
42.0
41.9
41.8
41.7
41.6
Longitude (UTM)
−7.2
−7.1
−7.0
−6.9
−6.8
−6.7
−6.6

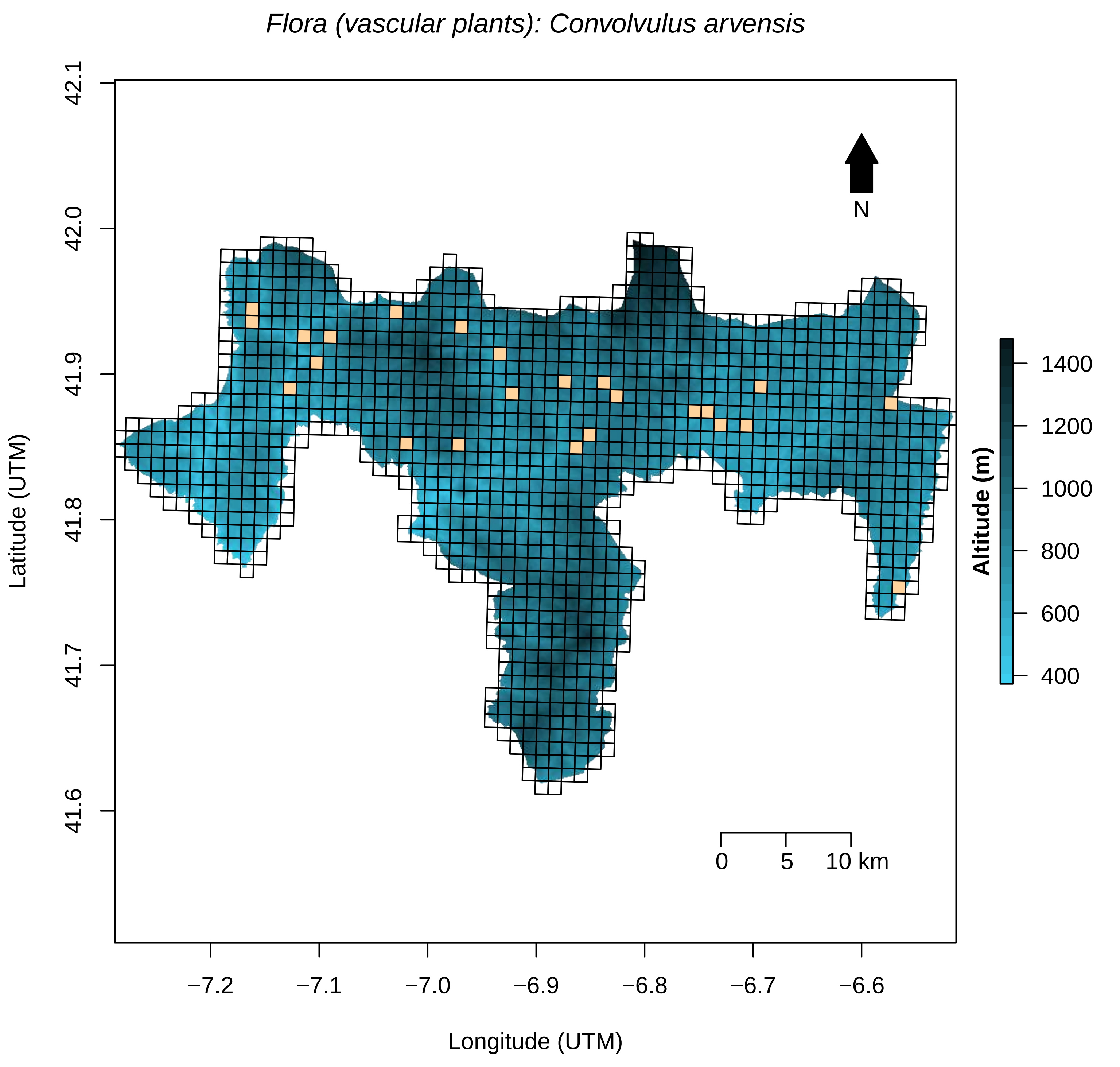

Flora (vascular plants): Convolvulus arvensis
42.1
42.0
41.9
41.8
41.7
41.6
Latitude (UTM)
N
0 5 10 km
−7.2 −7.1 −7.0 −6.9 −6.8 −6.7 −6.6
Longitude (UTM)
1400
1200
1000
800
600
400
Altitude (m)

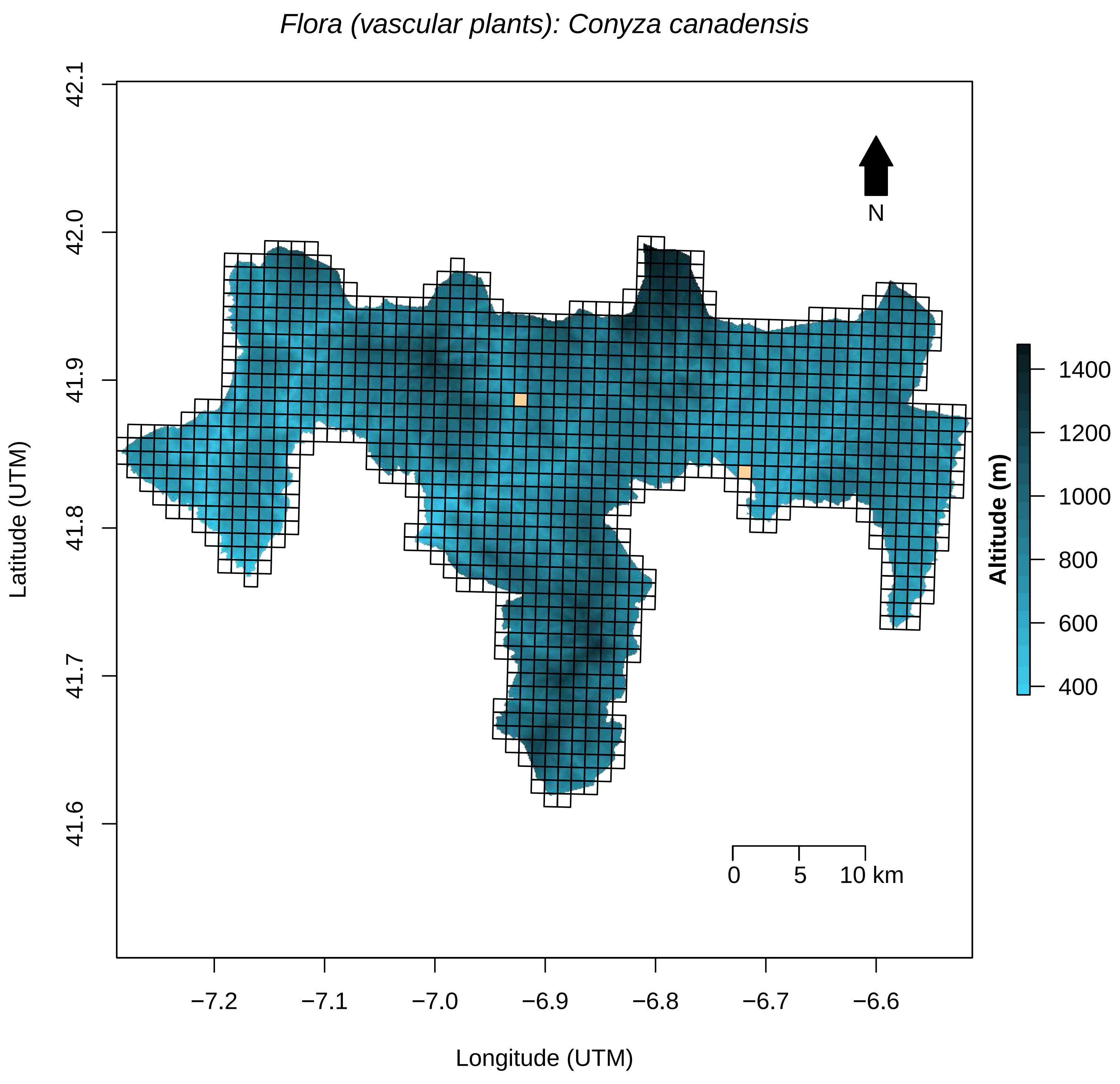

Flora (vascular plants): Conyza canadensis
42.1
42.0
41.9
41.8
41.7
41.6
Latitude (UTM)
N
0
5
10 km
−7.2
−7.1
−7.0
−6.9
−6.8
−6.7
−6.6
Longitude (UTM)
1400
1200
1000
800
600
400
Altitude (m)

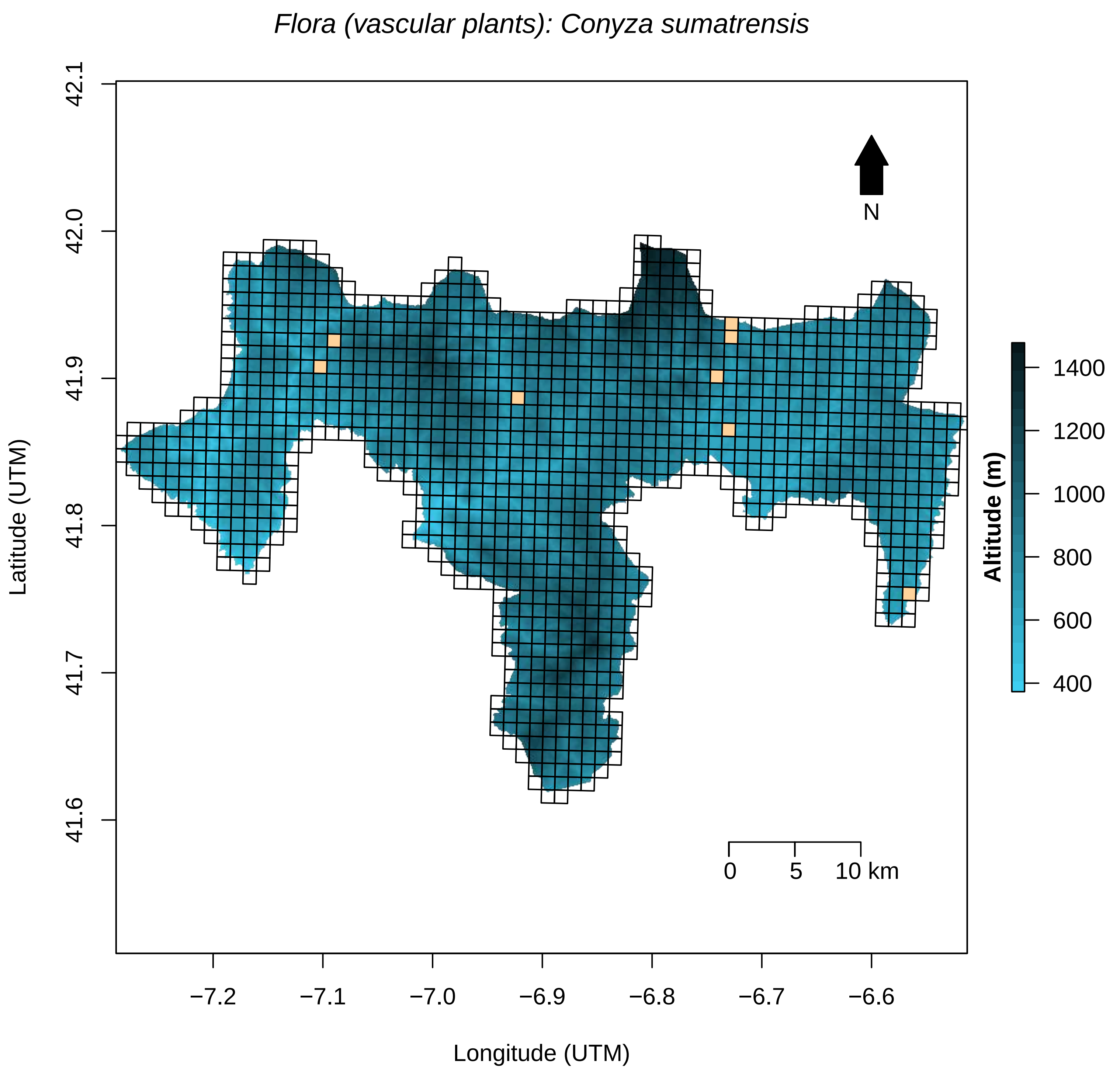
Flora (vascular plants): Conyza sumatrensis
Latitude (UTM)
42.1
42.0
41.9
41.8
41.7
41.6
Longitude (UTM)
−7.2
−7.1
−7.0
−6.9
−6.8
−6.7
−6.6
N
0
5
10 km
Altitude (m)
1400
1200
1000
800
600
400

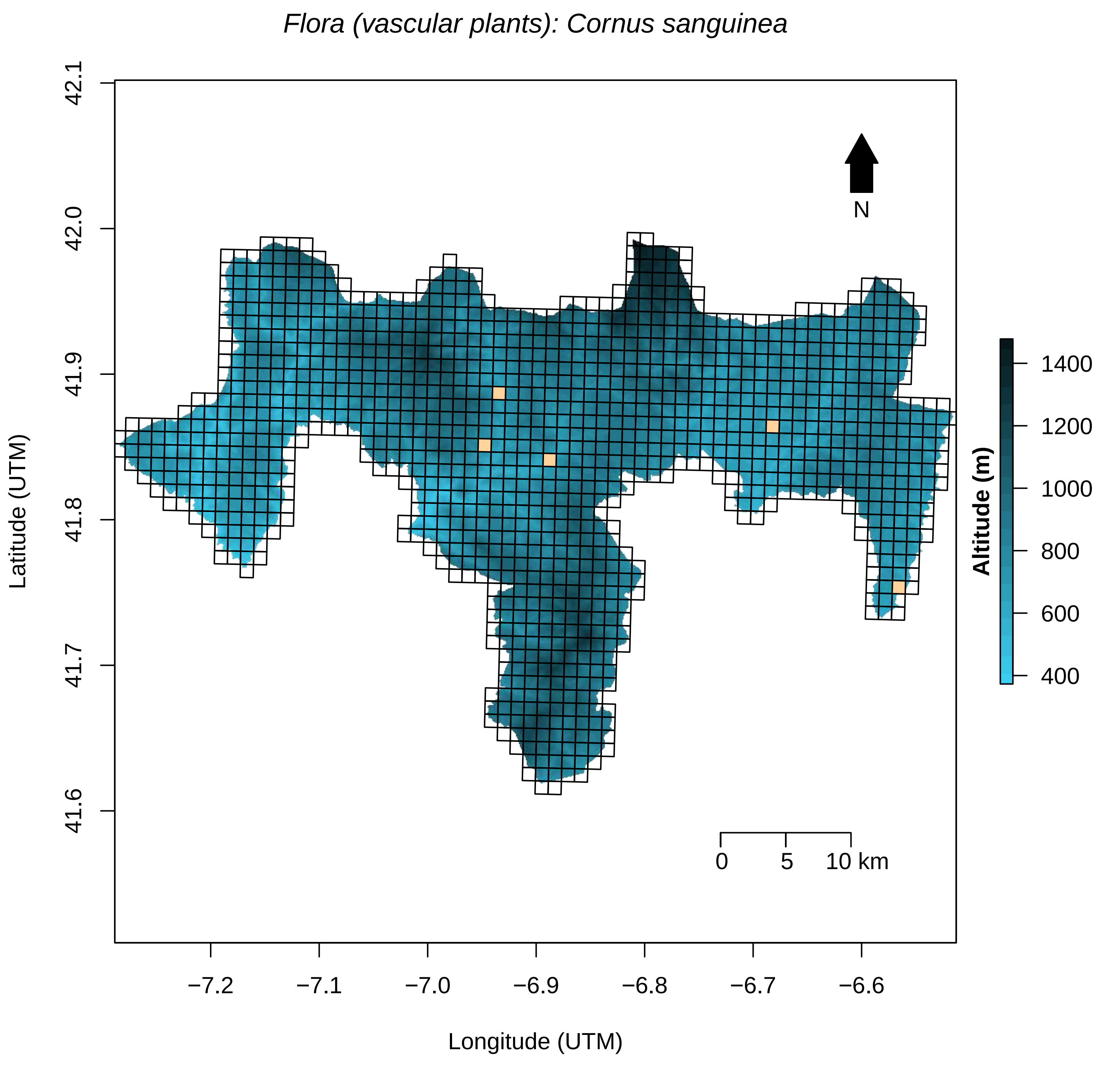

Flora (vascular plants): Cornus sanguinea
42.1
42.0
41.9
41.8
41.7
41.6
Latitude (UTM)
−7.2
−7.1
−7.0
−6.9
−6.8
−6.7
−6.6
Longitude (UTM)
N
0
5
10 km
Altitude (m)
1400
1200
1000
800
600
400

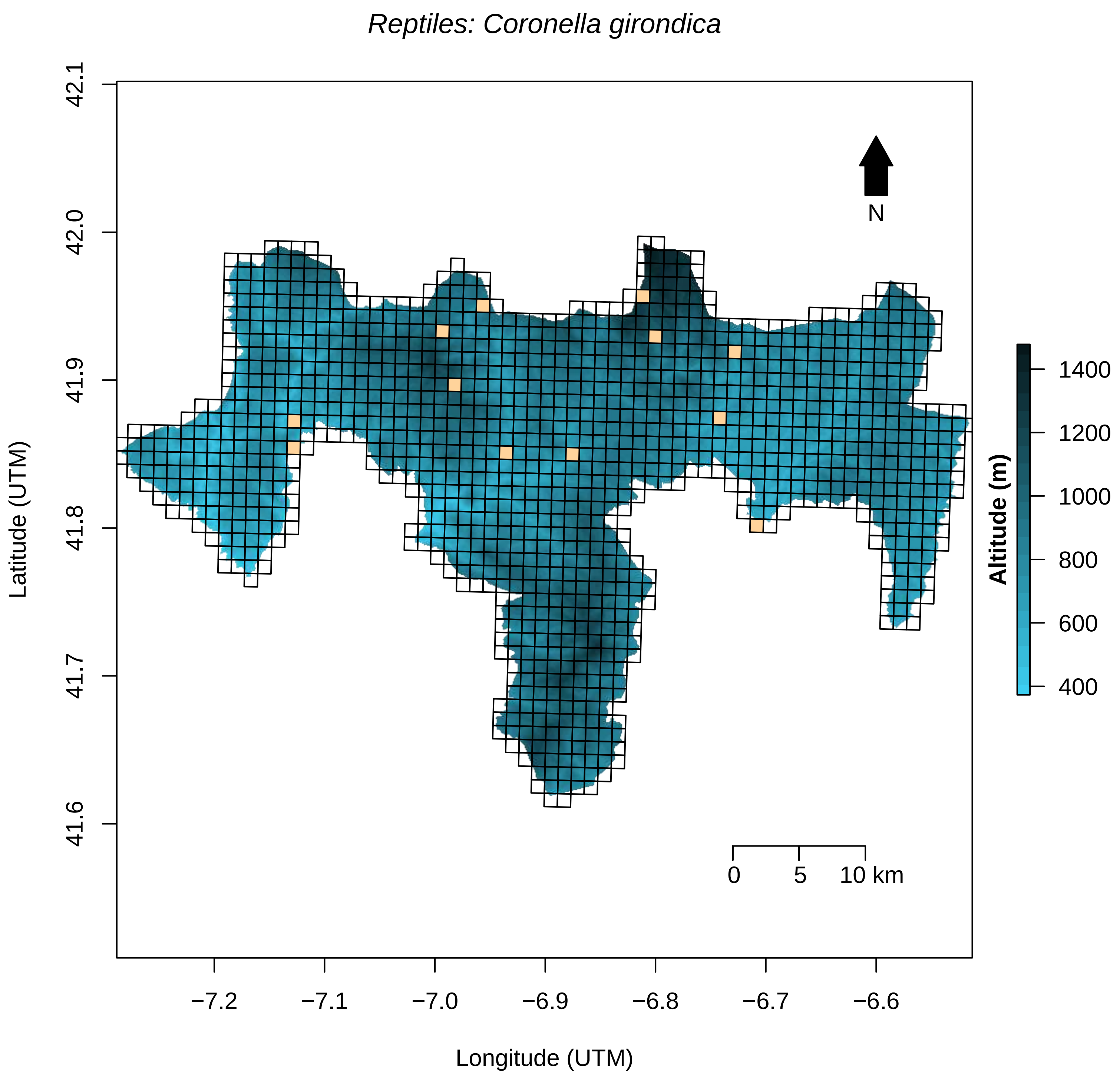

Reptiles: Coronella girondica
N
0 5 10 km
Altitude (m)
1400
1200
1000
800
600
400
Latitude (UTM)
42.1
42.0
41.9
41.8
41.7
41.6
Longitude (UTM)
−7.2
−7.1
−7.0
−6.9
−6.8
−6.7
−6.6

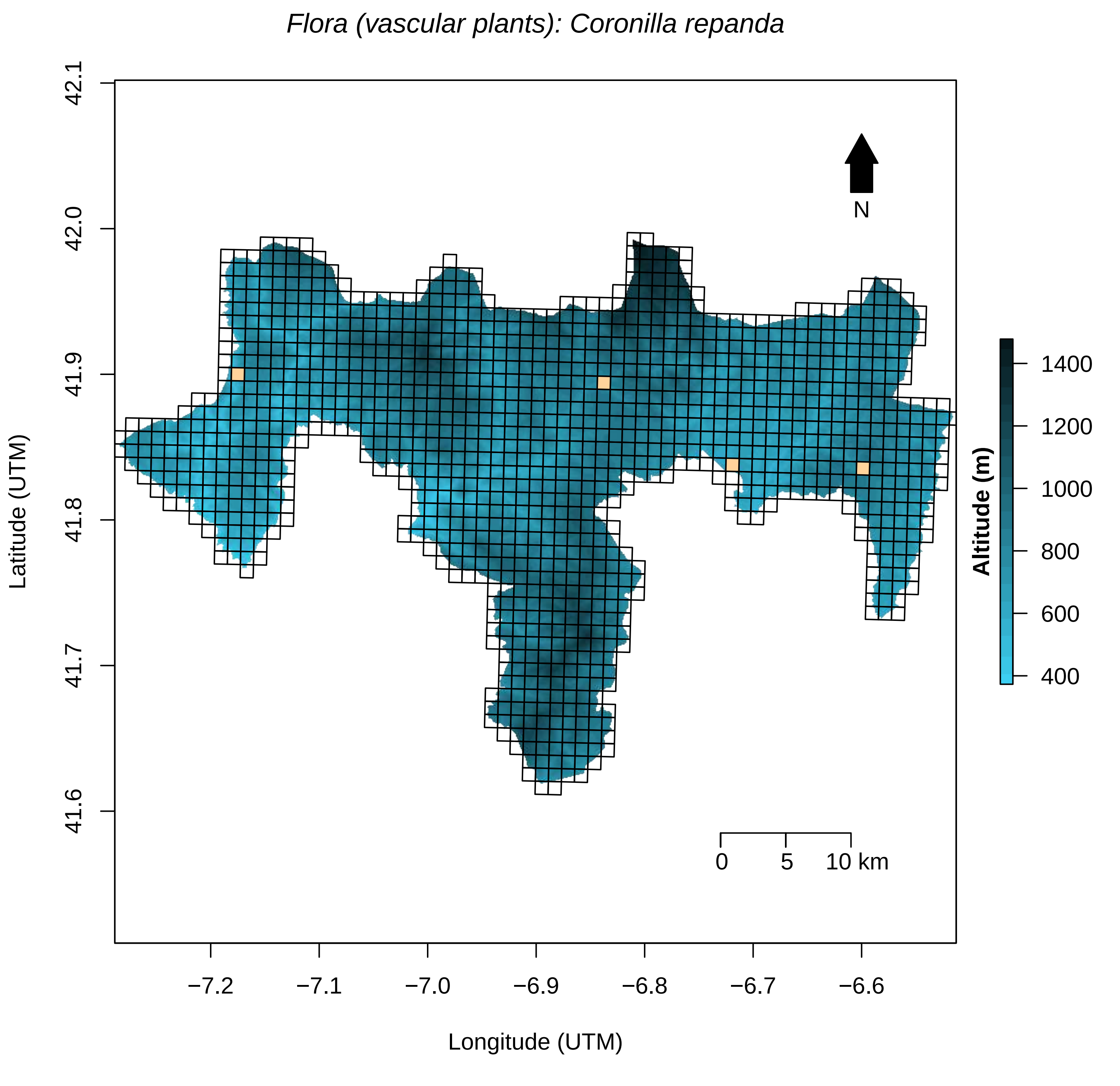

Flora (vascular plants): Coronilla repanda
42.1
42.0
41.9
41.8
41.7
41.6
Latitude (UTM)
−7.2
−7.1
−7.0
−6.9
−6.8
−6.7
−6.6
Longitude (UTM)
N
0
5
10 km
1400
1200
1000
800
600
400
Altitude (m)

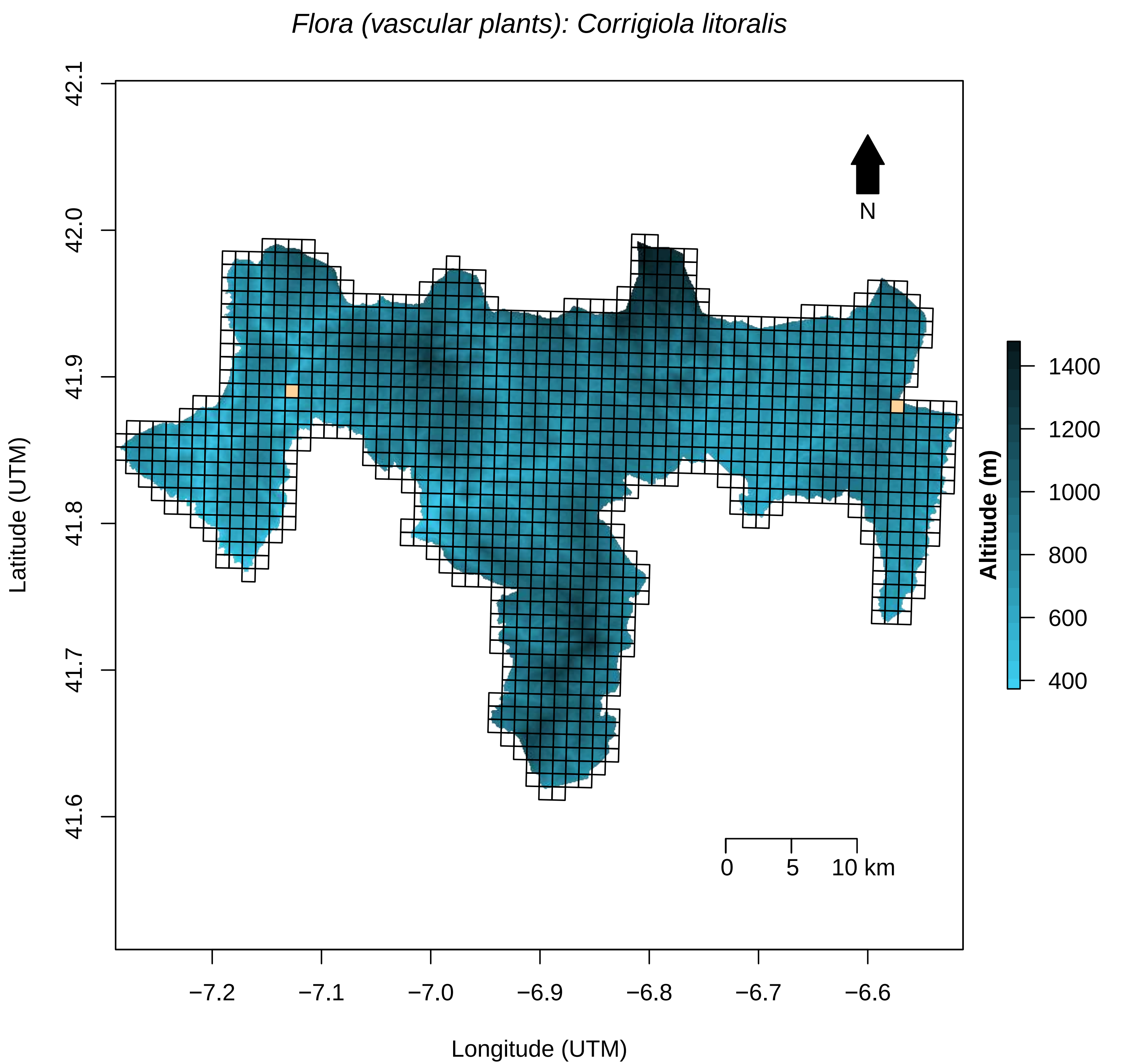

Flora (vascular plants): Corrigiola litoralis
42.1
42.0
41.9
41.8
41.7
41.6
Latitude (UTM)
−7.2
−7.1
−7.0
−6.9
−6.8
−6.7
−6.6
Longitude (UTM)
N
0
5
10 km
Altitude (m)
1400
1200
1000
800
600
400

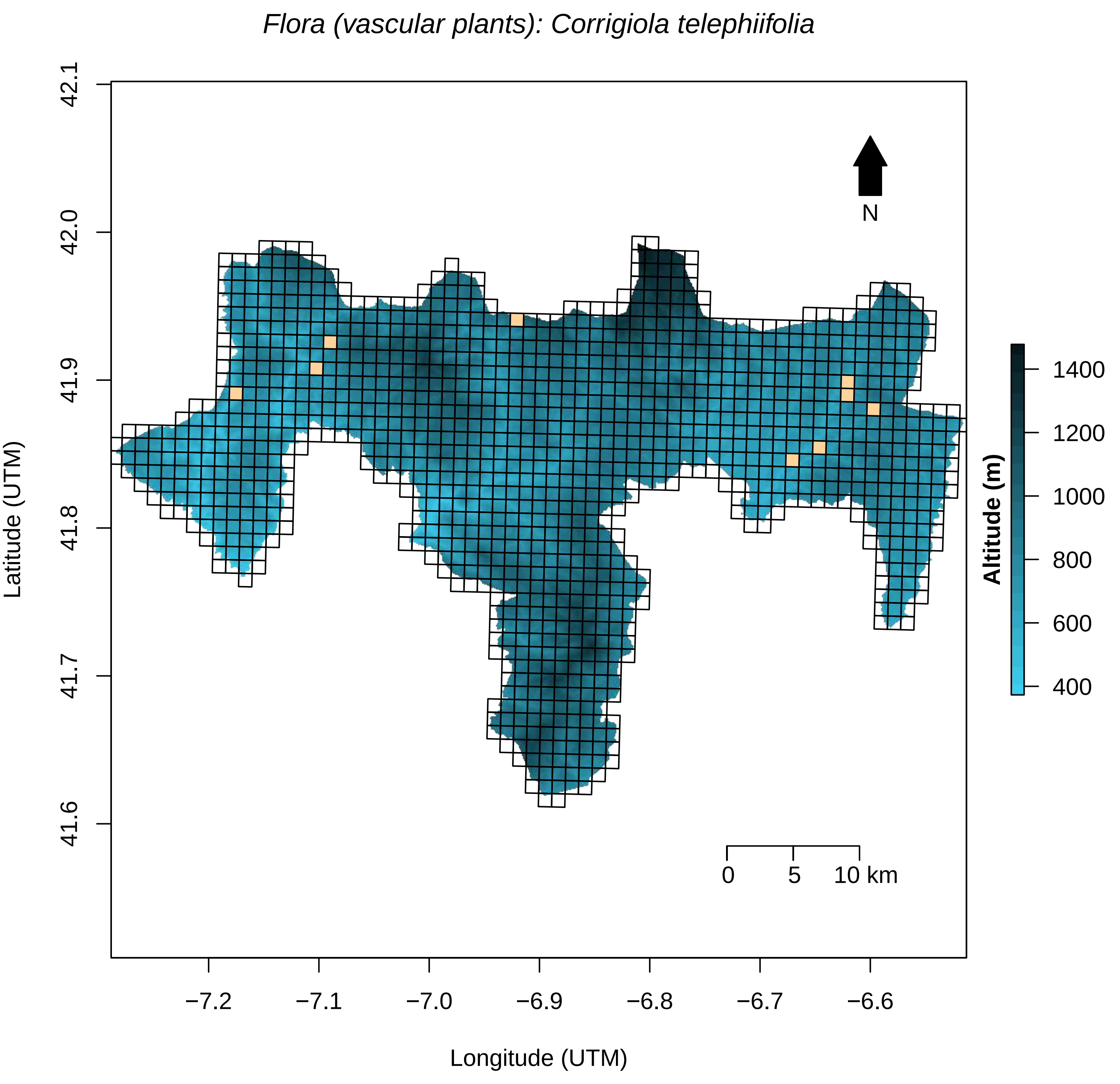

Flora (vascular plants): Corrigiola telephiifolia
42.1
42.0
41.9
41.8
41.7
41.6
Latitude (UTM)
−7.2
−7.1
−7.0
−6.9
−6.8
−6.7
−6.6
Longitude (UTM)
N
0
5
10 km
1400
1200
1000
800
600
400
Altitude (m)

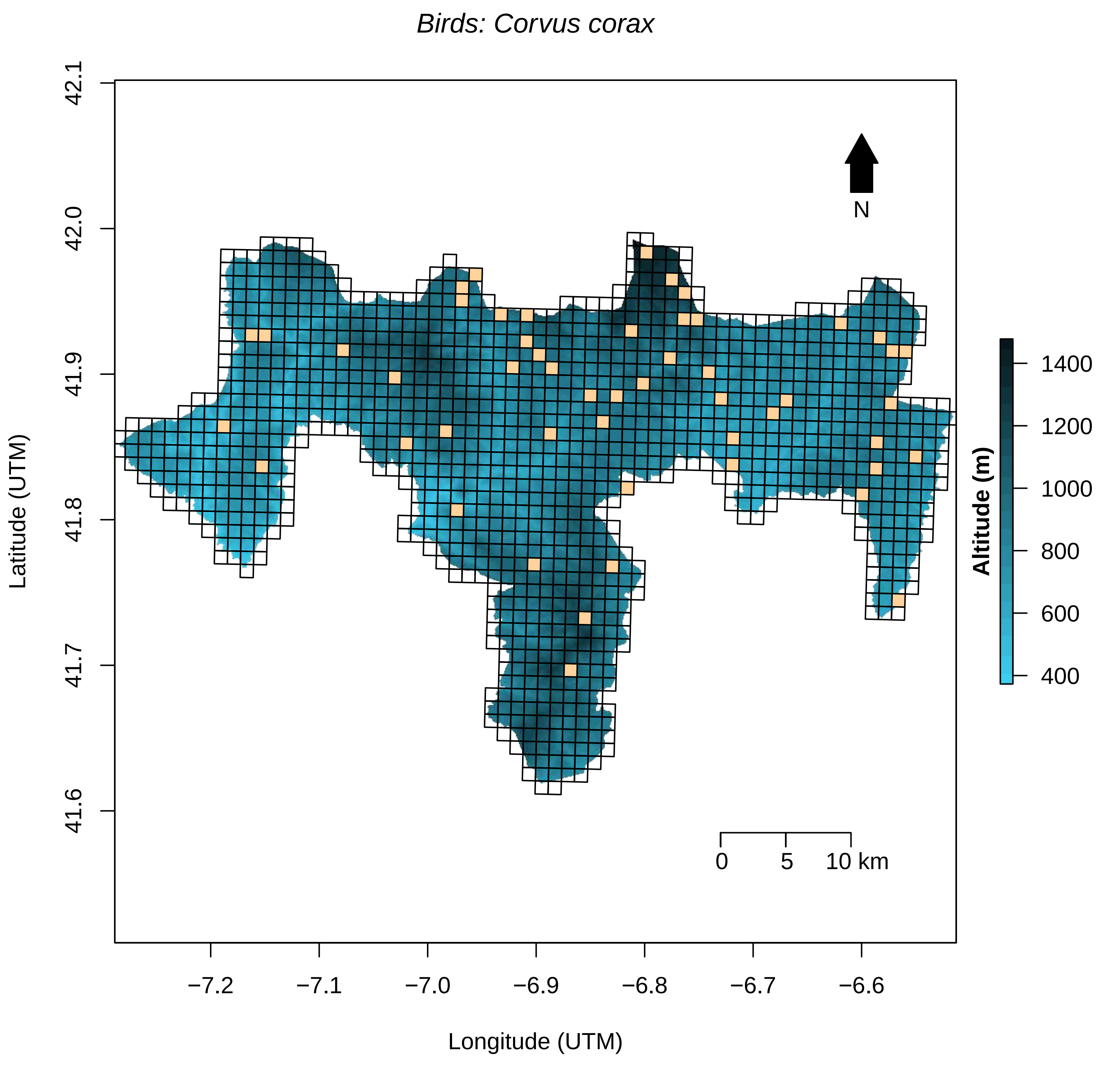
Birds: Corvus corax
42.1
42.0
41.9
41.8
41.7
41.6
Latitude (UTM)
−7.2
−7.1
−7.0
−6.9
−6.8
−6.7
−6.6
Longitude (UTM)
N
0
5
10 km
Altitude (m)
1400
1200
1000
800
600
400

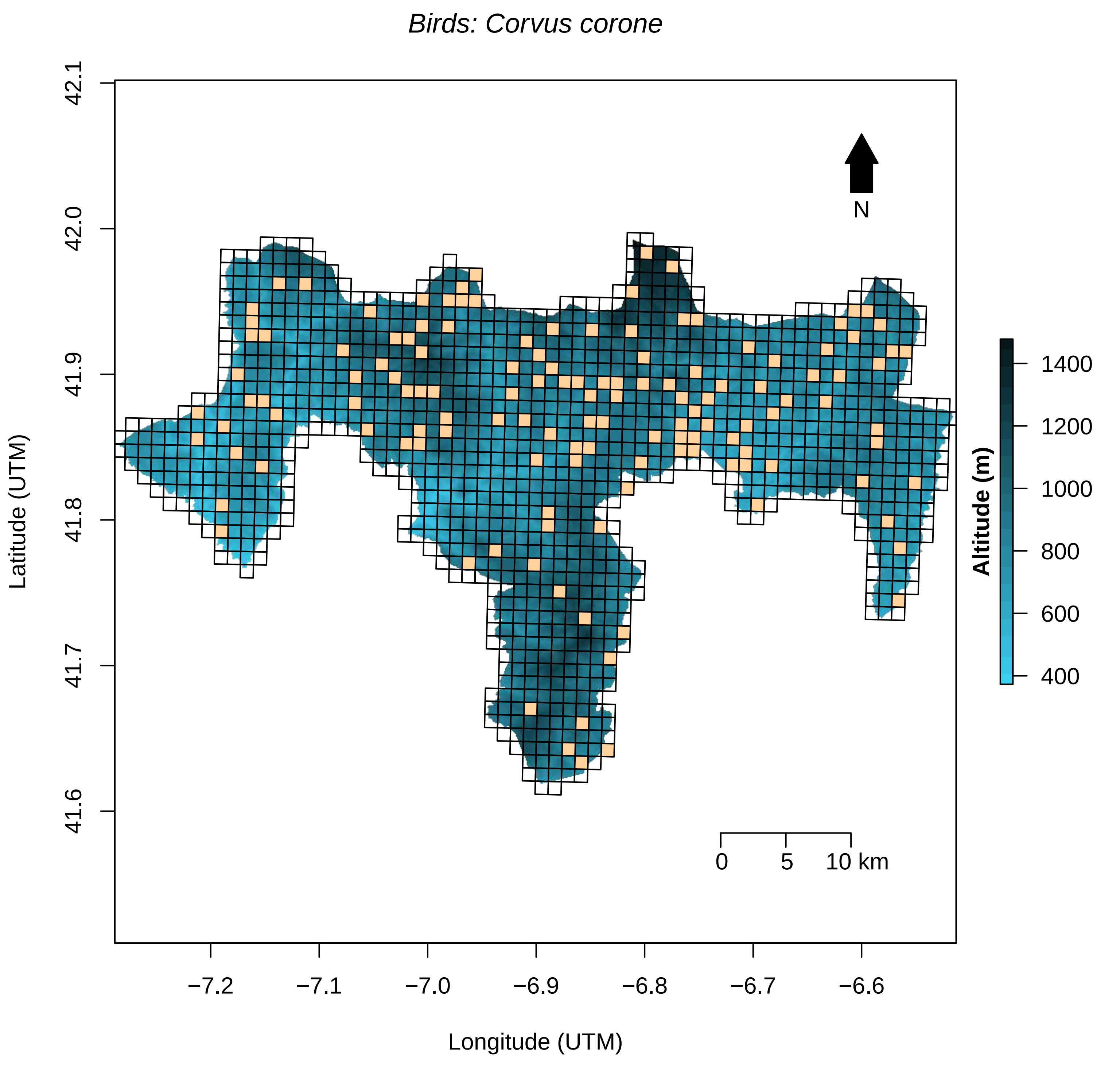
Birds: Corvus corone
42.1
42.0
41.9
41.8
41.7
41.6
Latitude (UTM)
−7.2
−7.1
−7.0
−6.9
−6.8
−6.7
−6.6
Longitude (UTM)
N
0
5
10 km
Altitude (m)
1400
1200
1000
800
600
400

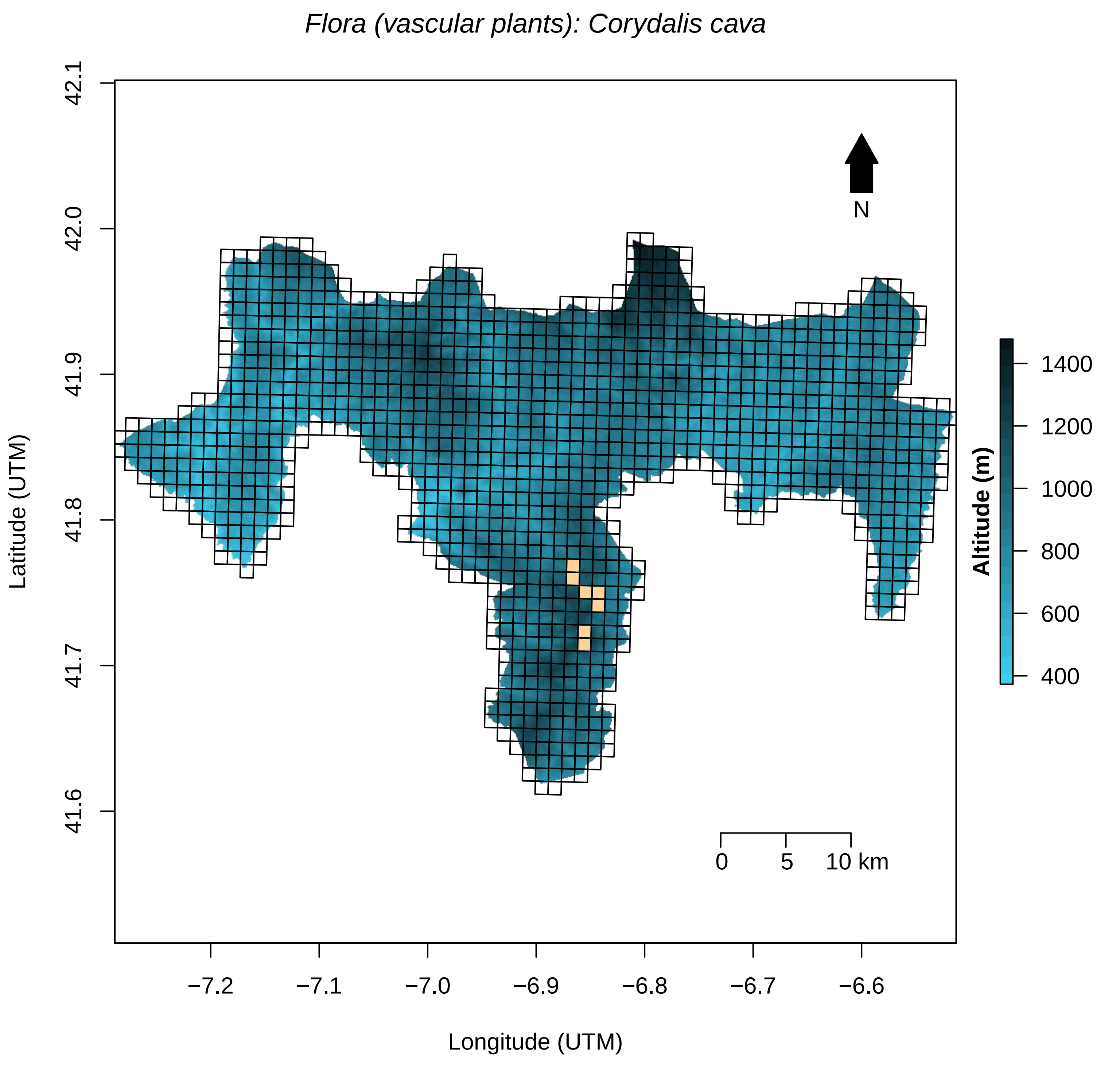

Flora (vascular plants): Corydalis cava
N
0
5
10 km
Latitude (UTM)
42.1
42.0
41.9
41.8
41.7
41.6
Longitude (UTM)
−7.2
−7.1
−7.0
−6.9
−6.8
−6.7
−6.6
Altitude (m)
1400
1200
1000
800
600
400

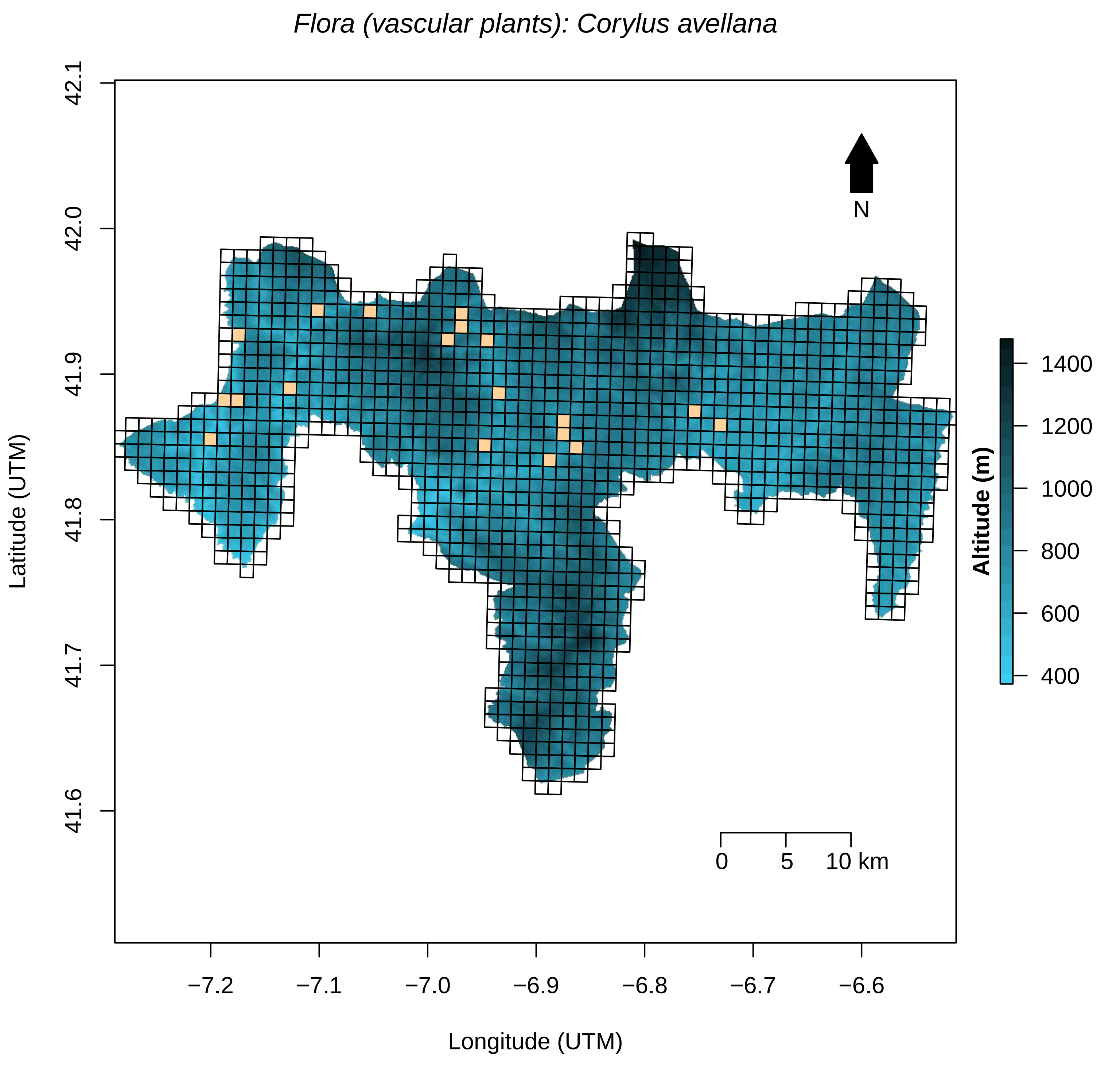

Flora (vascular plants): Corylus avellana
N
0
5
10 km
Altitude (m)
1400
1200
1000
800
600
400
Latitude (UTM)
42.1
42.0
41.9
41.8
41.7
41.6
Longitude (UTM)
−7.2
−7.1
−7.0
−6.9
−6.8
−6.7
−6.6

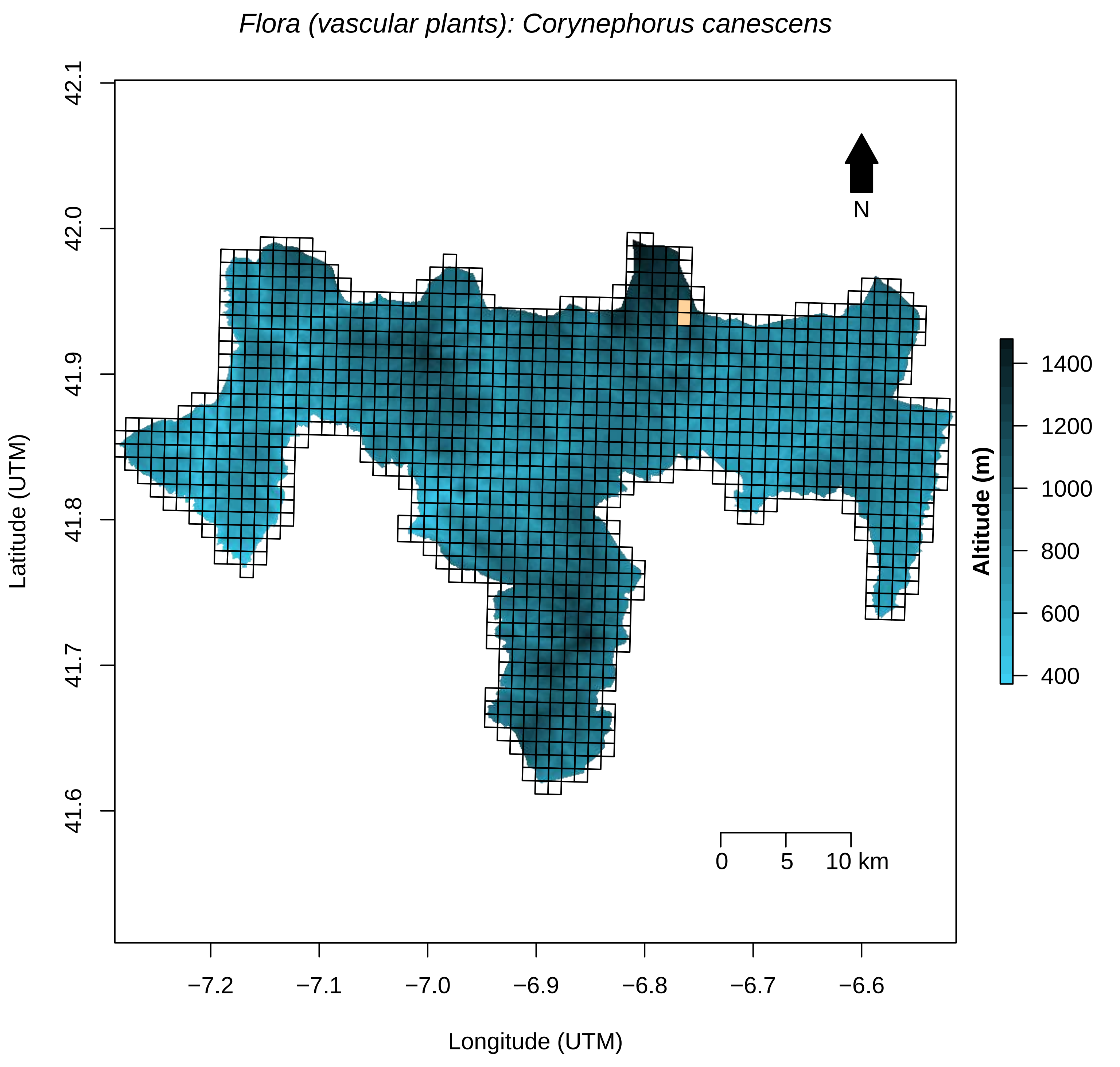

Flora (vascular plants): Corynephorus canescens
42.1
42.0
41.9
41.8
41.7
41.6
Latitude (UTM)
−7.2
−7.1
−7.0
−6.9
−6.8
−6.7
−6.6
Longitude (UTM)
N
0
5
10 km
1400
1200
1000
800
600
400
Altitude (m)

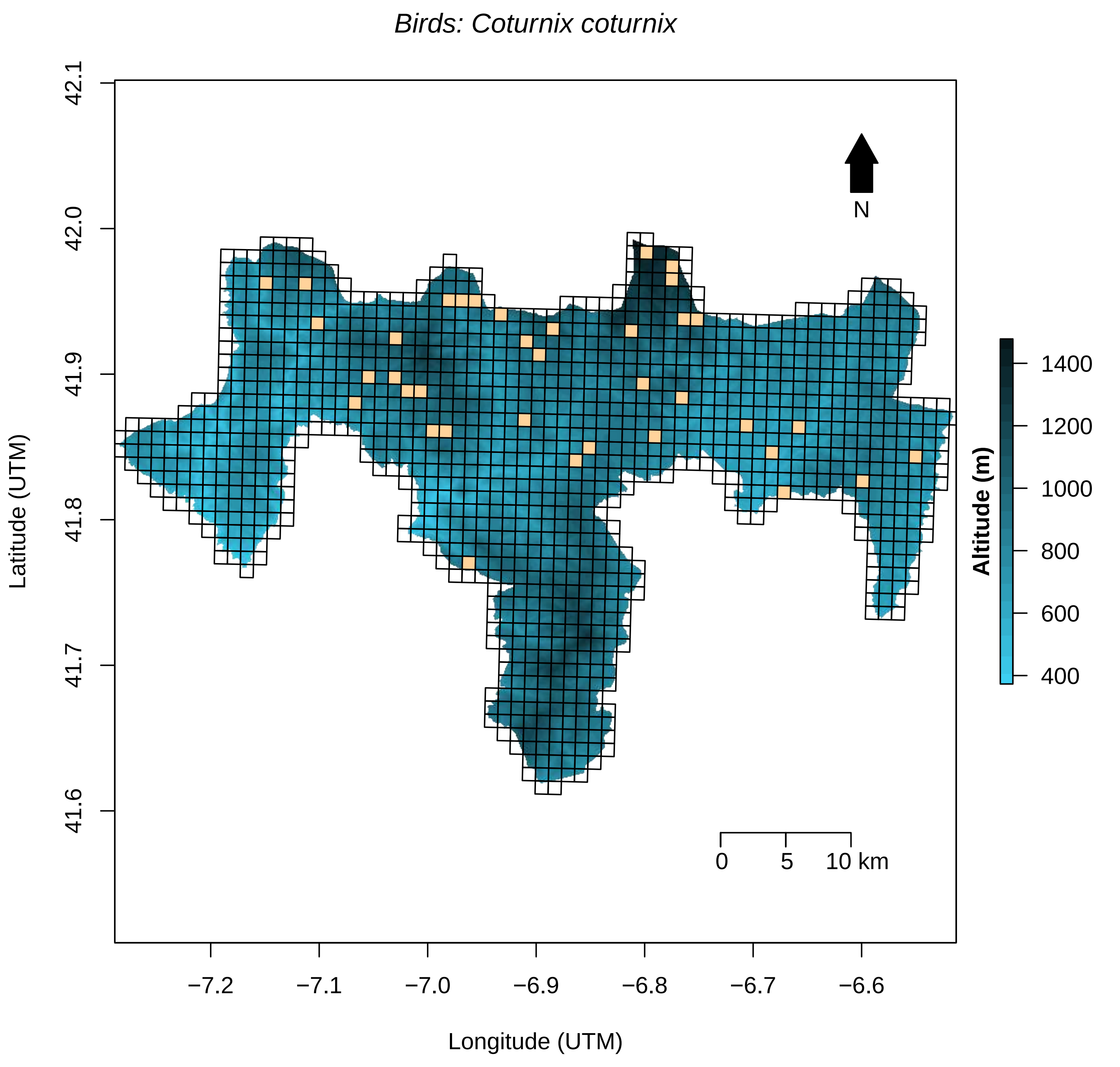

Birds: Coturnix coturnix
42.1
42.0
41.9
41.8
41.7
41.6
Latitude (UTM)
−7.2
−7.1
−7.0
−6.9
−6.8
−6.7
−6.6
Longitude (UTM)
N
0
5
10 km
1400
1200
1000
800
600
400
Altitude (m)

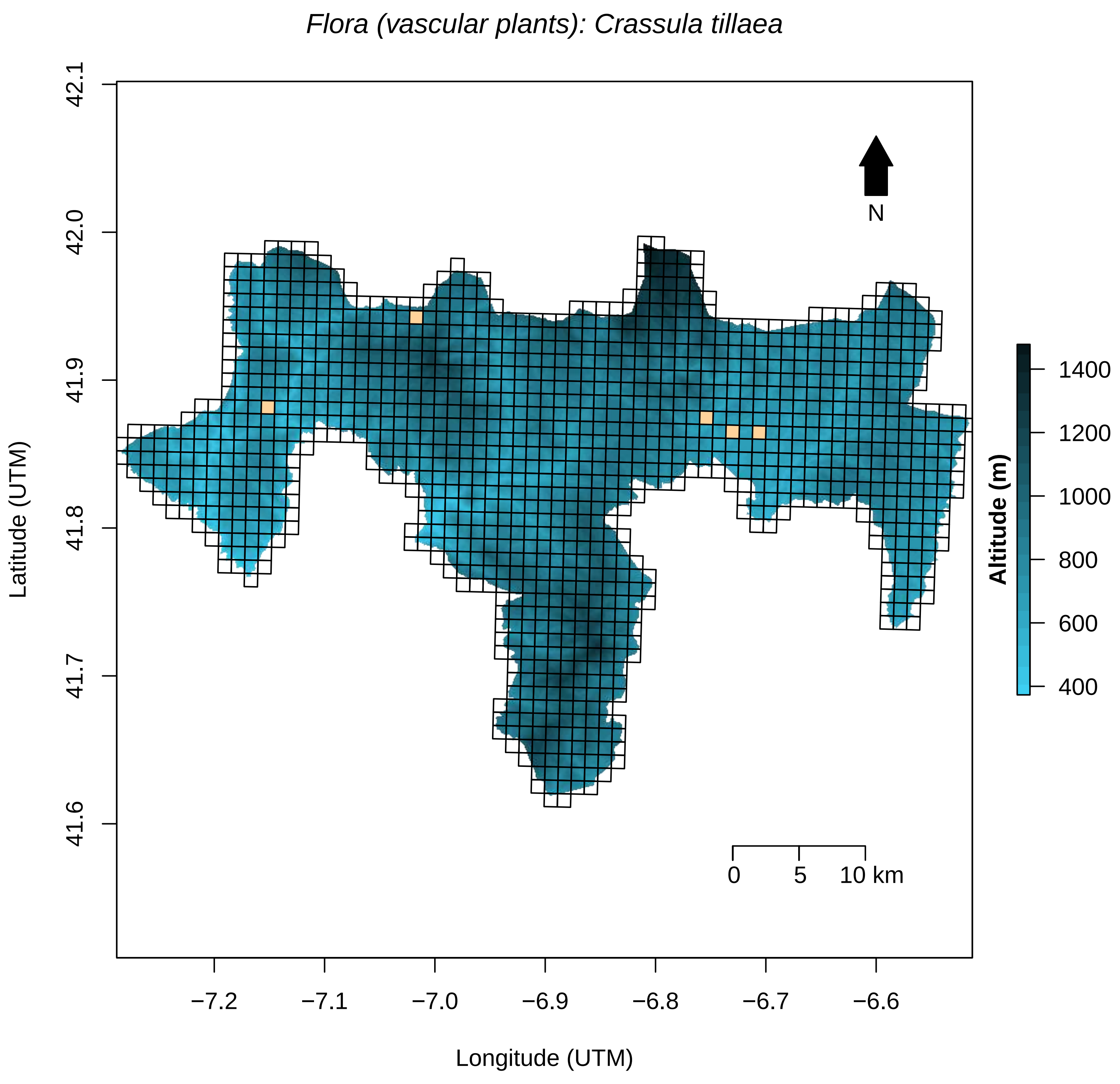

Flora (vascular plants): Crassula tillaea
42.1
42.0
41.9
41.8
41.7
41.6
Latitude (UTM)
−7.2
−7.1
−7.0
−6.9
−6.8
−6.7
−6.6
Longitude (UTM)
N
0
5
10 km
Altitude (m)
1400
1200
1000
800
600
400

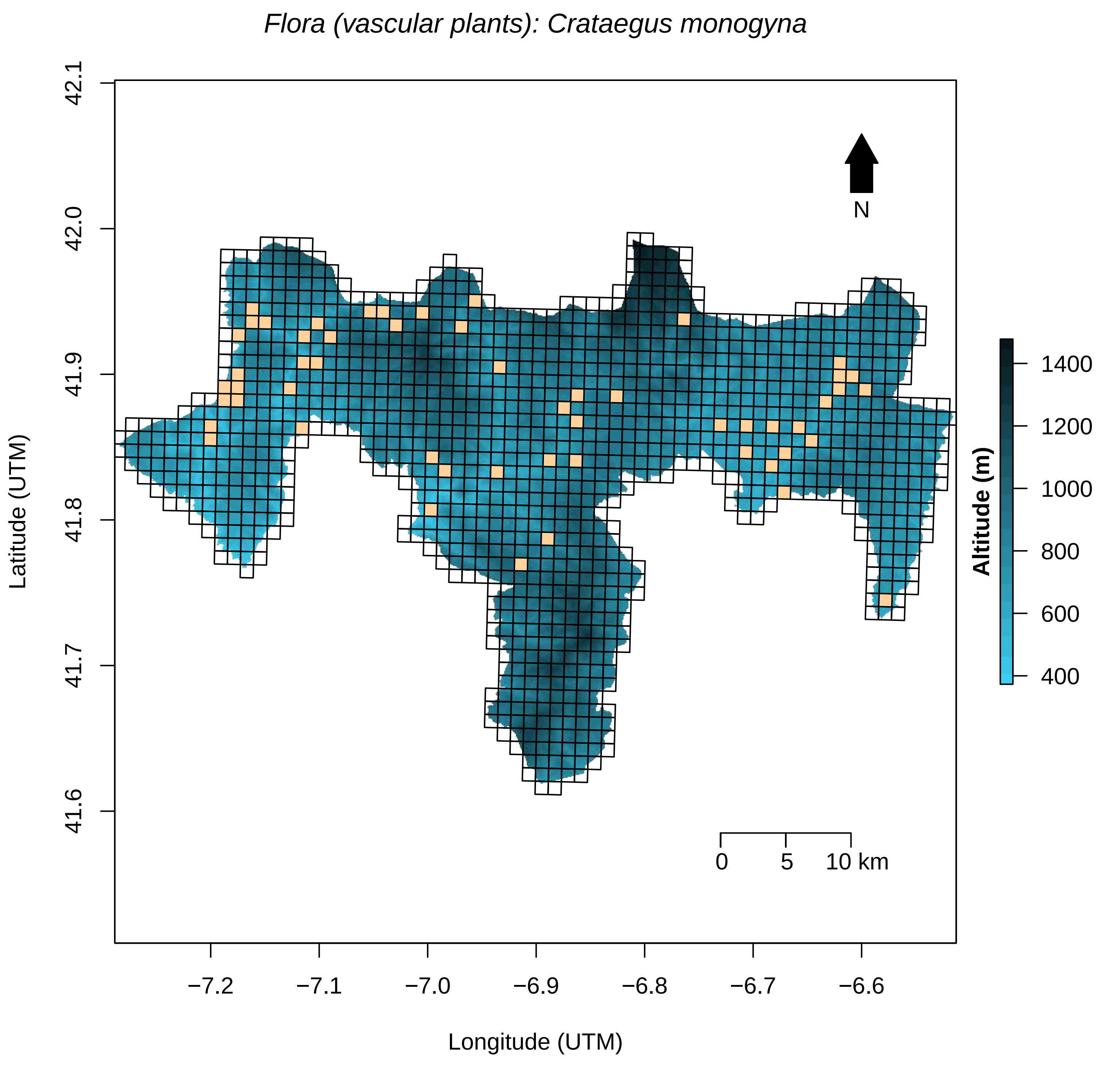

Flora (vascular plants): Crataegus monogyna
Latitude (UTM)
42.1
42.0
41.9
41.8
41.7
41.6
N
0
5
10 km
Altitude (m)
1400
1200
1000
800
600
400
−7.2
−7.1
−7.0
−6.9
−6.8
−6.7
−6.6
Longitude (UTM)

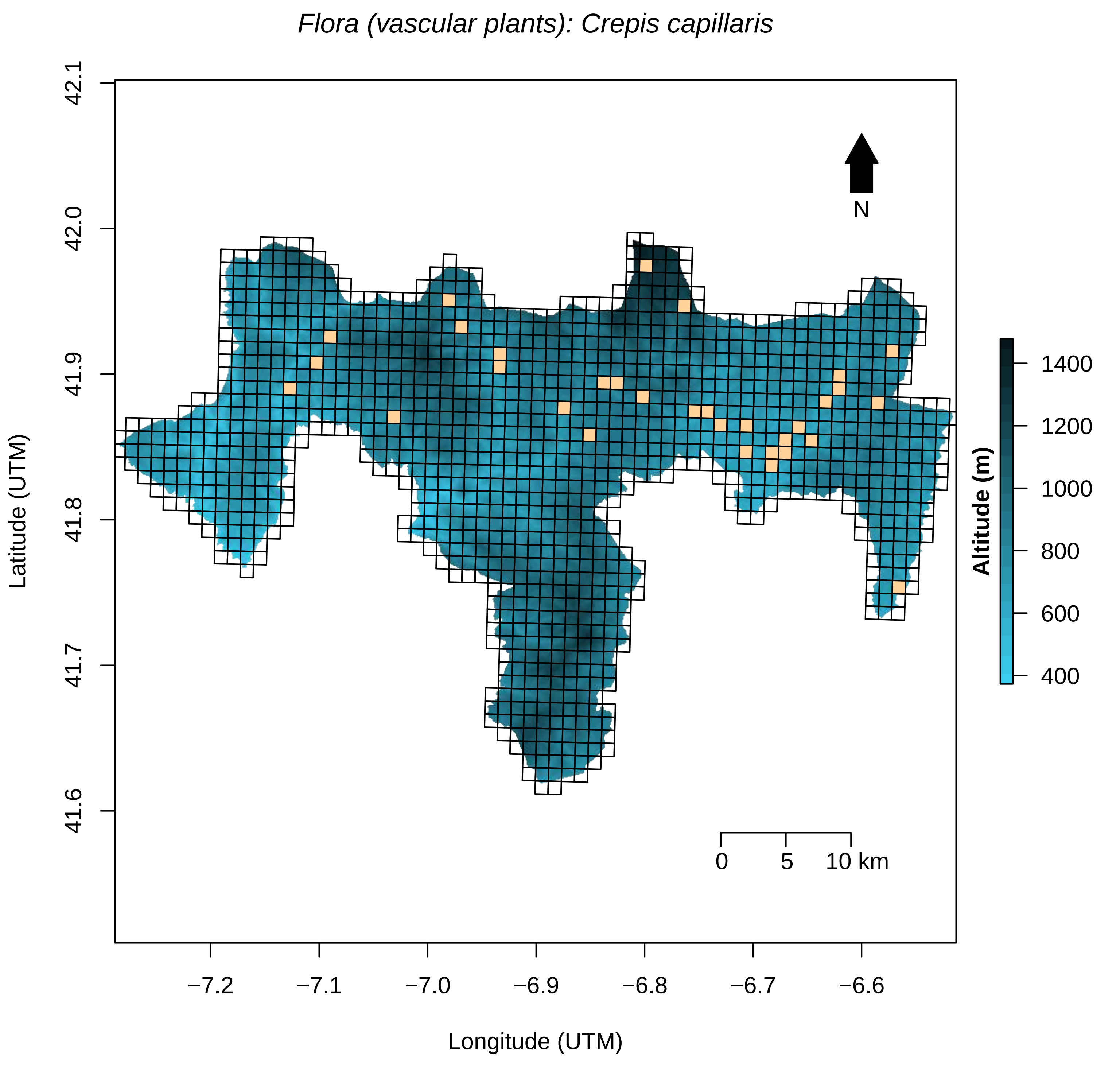

Flora (vascular plants): Crepis capillaris
42.1
42.0
41.9
41.8
41.7
41.6
Latitude (UTM)
−7.2
−7.1
−7.0
−6.9
−6.8
−6.7
−6.6
Longitude (UTM)
N
0
5
10 km
Altitude (m)
1400
1200
1000
800
600
400

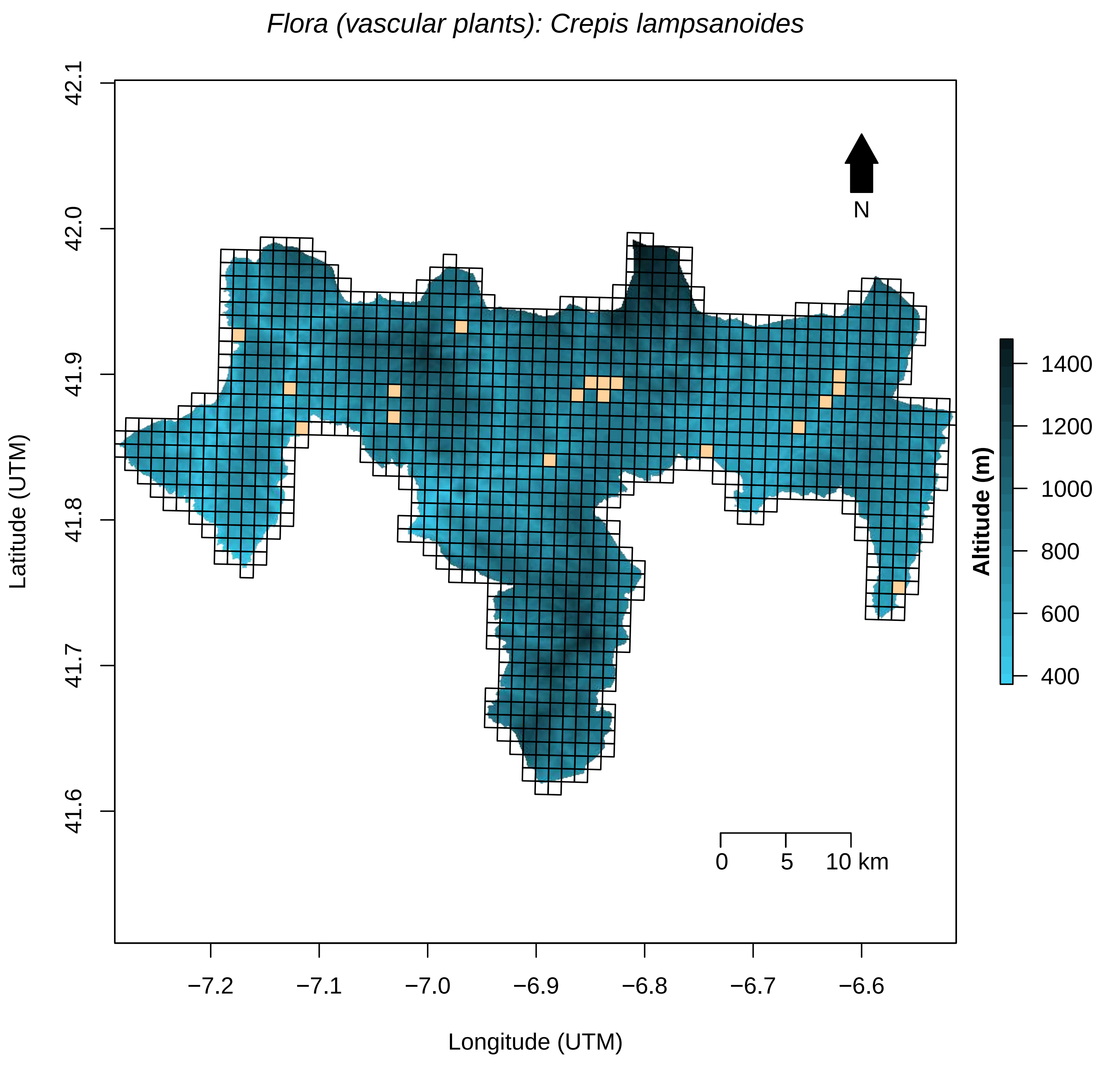
Flora (vascular plants): Crepis lampsanoides
Latitude (UTM)
Longitude (UTM)
42.1
42.0
41.9
41.8
41.7
41.6
−7.2
−7.1
−7.0
−6.9
−6.8
−6.7
−6.6
N
0
5
10 km
Altitude (m)
1400
1200
1000
800
600
400

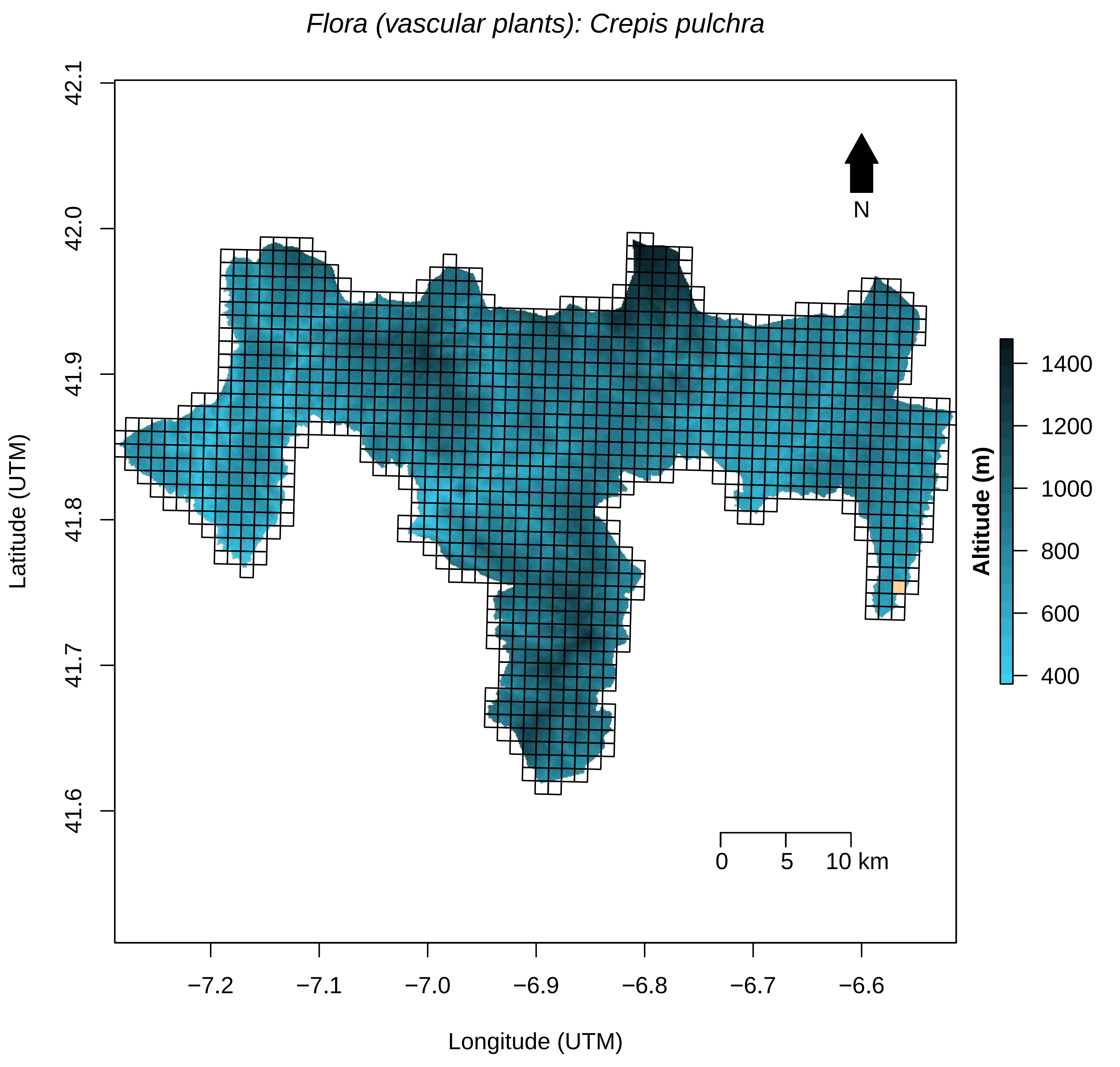
Flora (vascular plants): Crepis pulchra
N
0
5
10 km
Latitude (UTM)
42.1
42.0
41.9
41.8
41.7
41.6
Longitude (UTM)
−7.2
−7.1
−7.0
−6.9
−6.8
−6.7
−6.6
Altitude (m)
1400
1200
1000
800
600
400

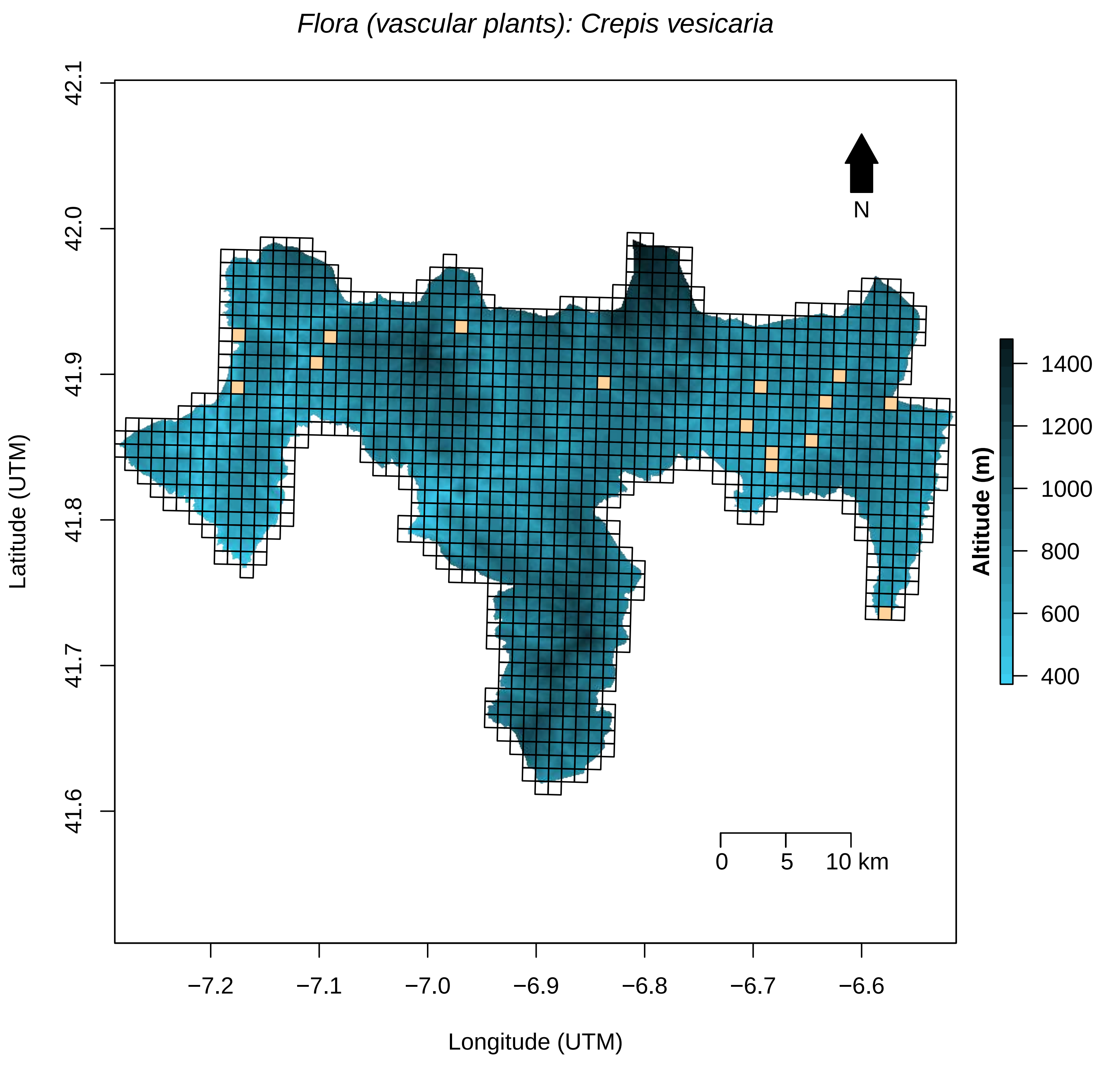

Flora (vascular plants): Crepis vesicaria
Latitude (UTM)
42.1
42.0
41.9
41.8
41.7
41.6
Longitude (UTM)
−7.2
−7.1
−7.0
−6.9
−6.8
−6.7
−6.6
N
0
5
10 km
Altitude (m)
1400
1200
1000
800
600
400

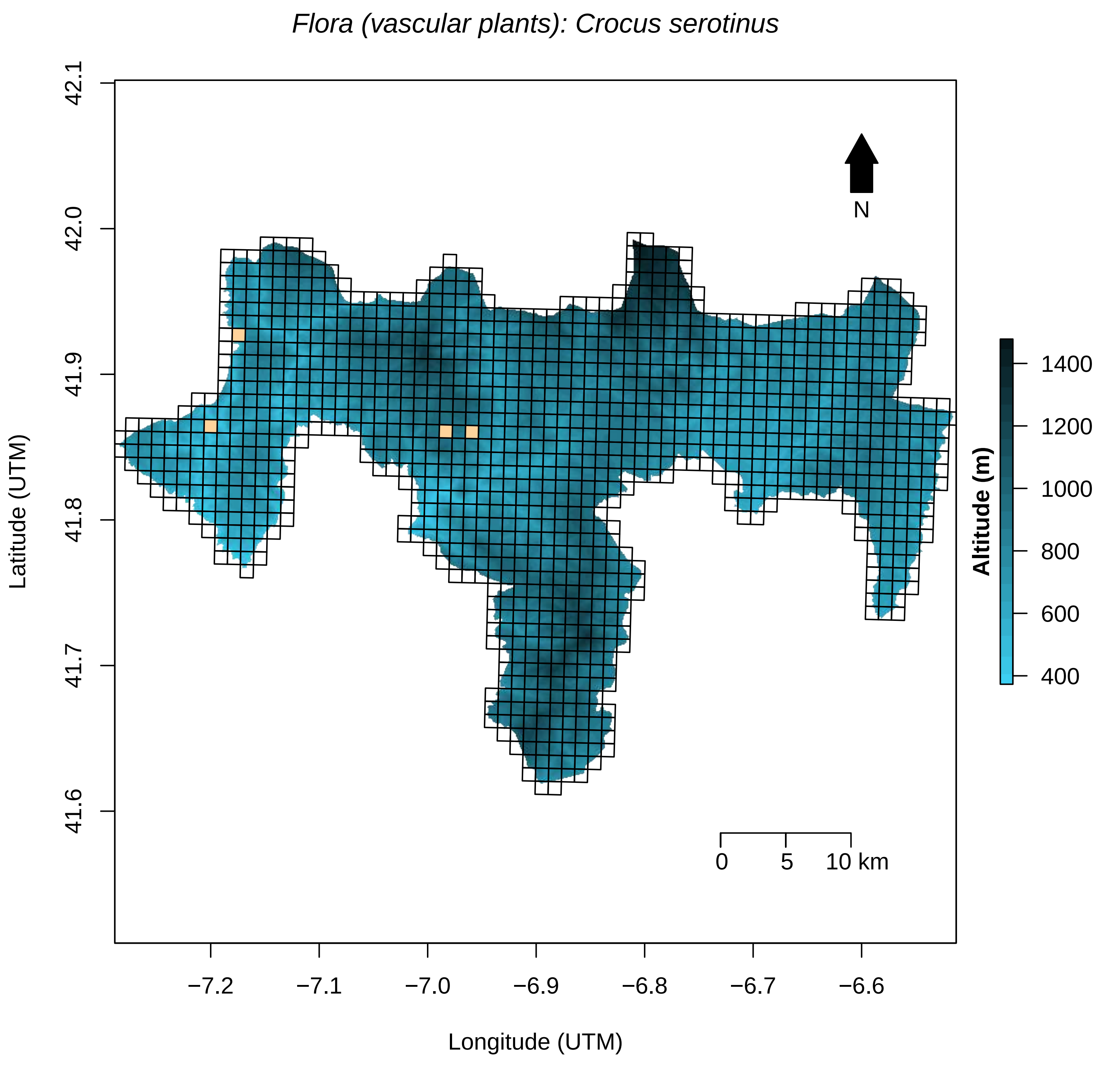
Flora (vascular plants): Crocus serotinus
N
0
5
10 km
Altitude (m)
1400
1200
1000
800
600
400
Latitude (UTM)
42.1
42.0
41.9
41.8
41.7
41.6
Longitude (UTM)
−7.2
−7.1
−7.0
−6.9
−6.8
−6.7
−6.6

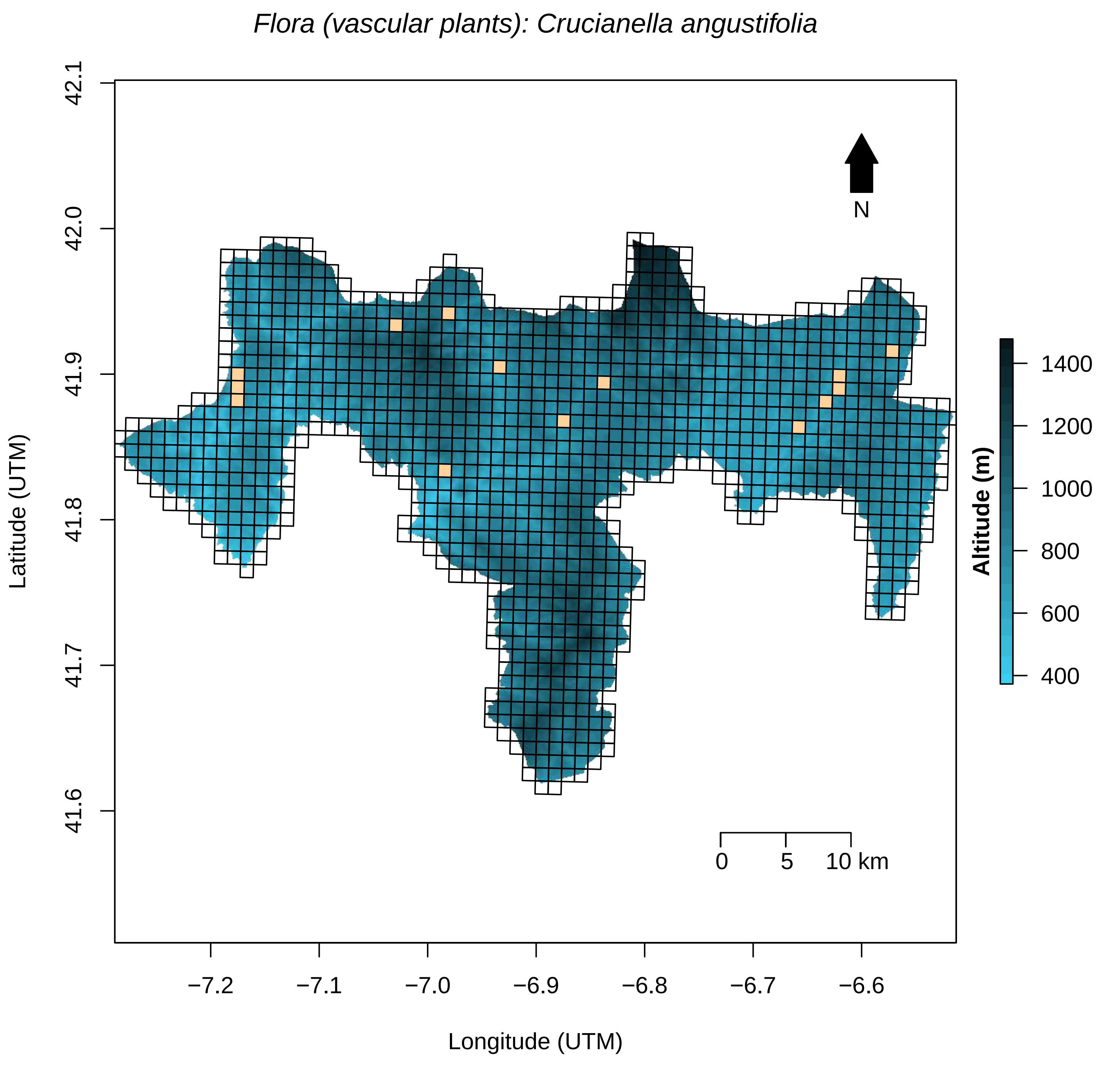
Flora (vascular plants): Crucianella angustifolia
42.1
42.0
41.9
41.8
41.7
41.6
Latitude (UTM)
N
0
5
10 km
−7.2
−7.1
−7.0
−6.9
−6.8
−6.7
−6.6
Longitude (UTM)
Altitude (m)
1400
1200
1000
800
600
400

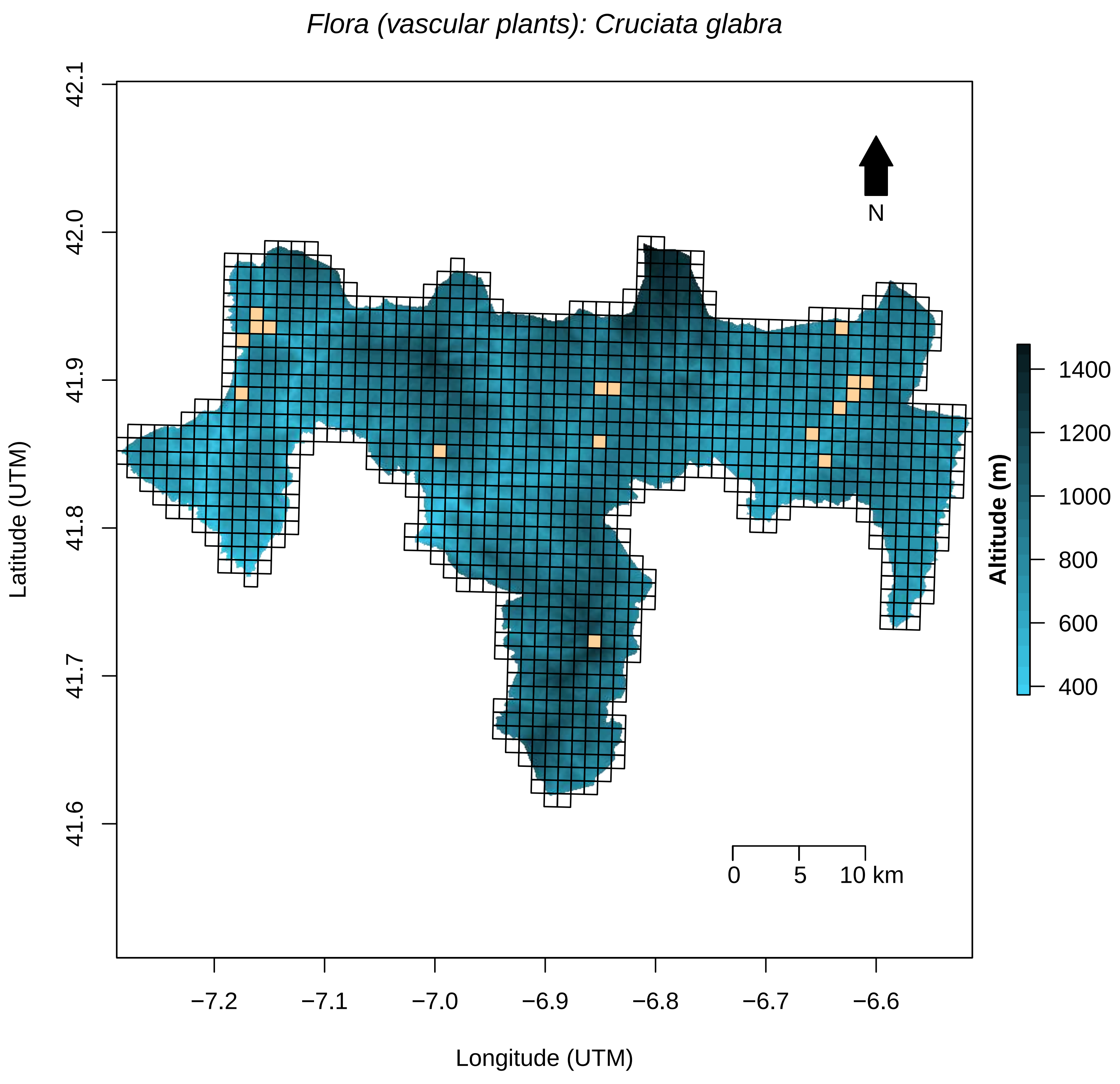
Flora (vascular plants): Cruciata glabra
Latitude (UTM)
42.1
42.0
41.9
41.8
41.7
41.6
Longitude (UTM)
−7.2
−7.1
−7.0
−6.9
−6.8
−6.7
−6.6
N
0
5
10 km
Altitude (m)
1400
1200
1000
800
600
400

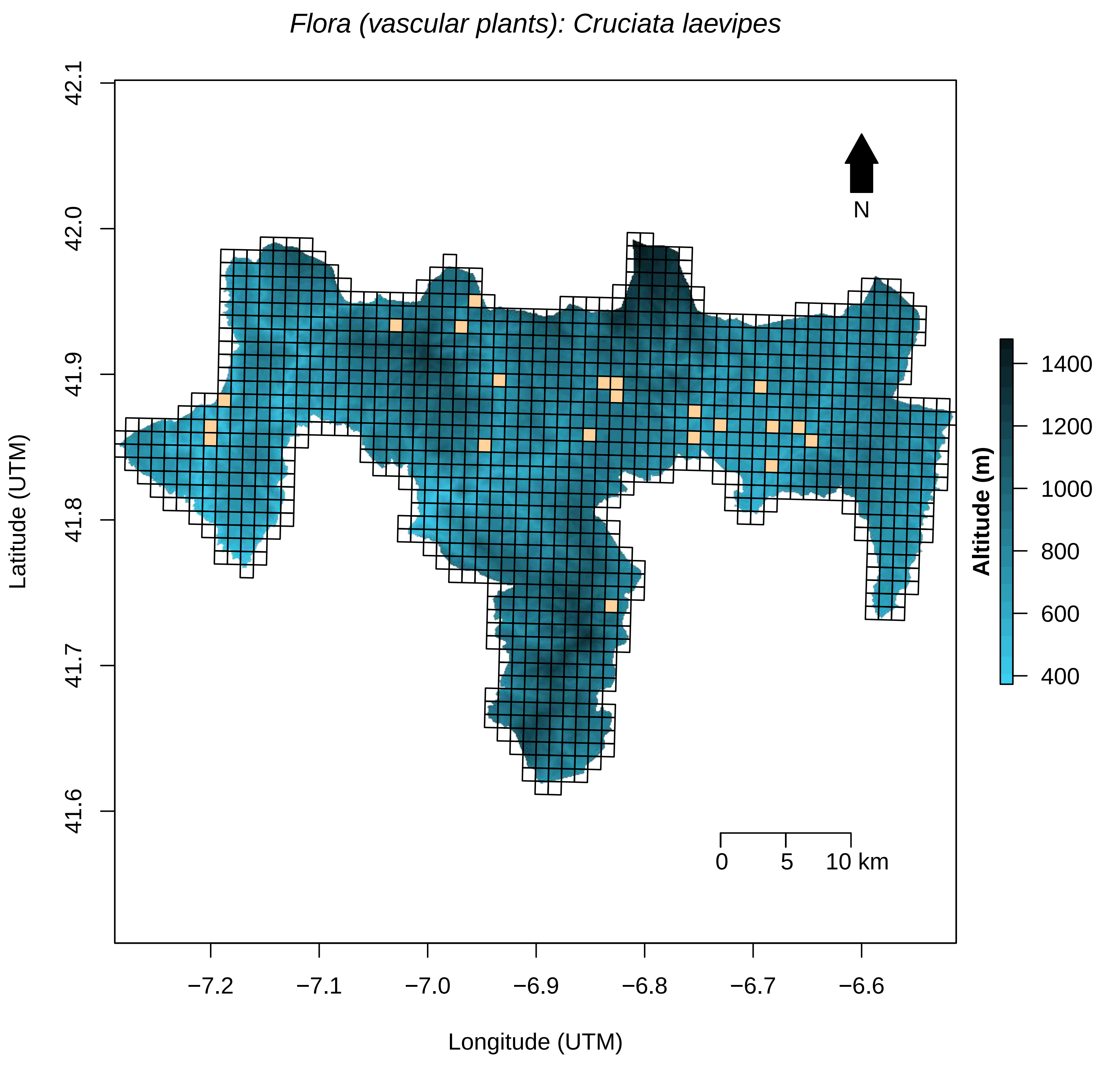

Flora (vascular plants): Cruciata laevipes
42.1
42.0
41.9
41.8
41.7
41.6
Latitude (UTM)
−7.2
−7.1
−7.0
−6.9
−6.8
−6.7
−6.6
Longitude (UTM)
N
0
5
10 km
1400
1200
1000
800
600
400
Altitude (m)

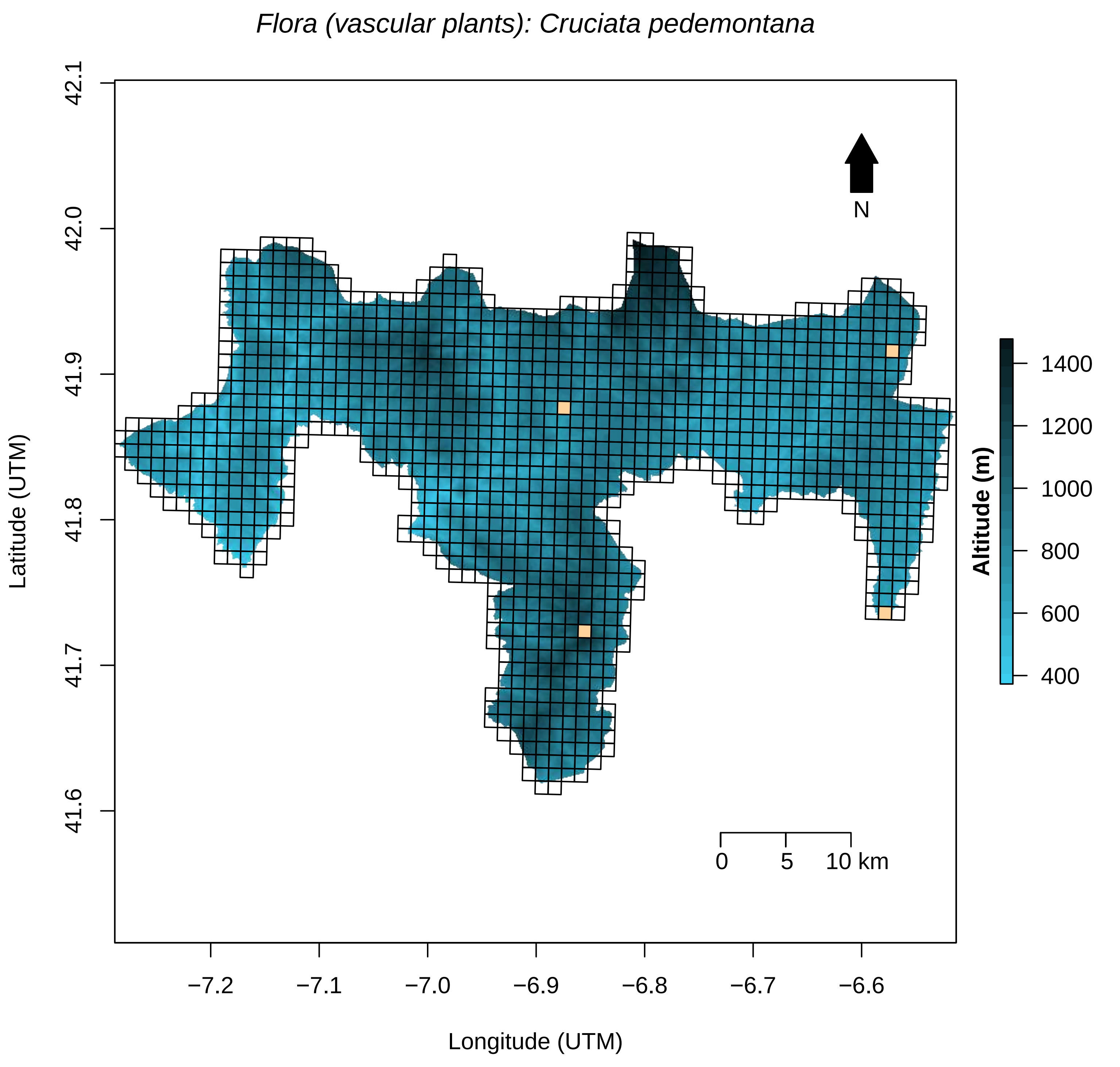
Flora (vascular plants): Cruciata pedemontana
Latitude (UTM)
Longitude (UTM)
42.1
42.0
41.9
41.8
41.7
41.6
−7.2
−7.1
−7.0
−6.9
−6.8
−6.7
−6.6
N
0
5
10 km
Altitude (m)
1400
1200
1000
800
600
400

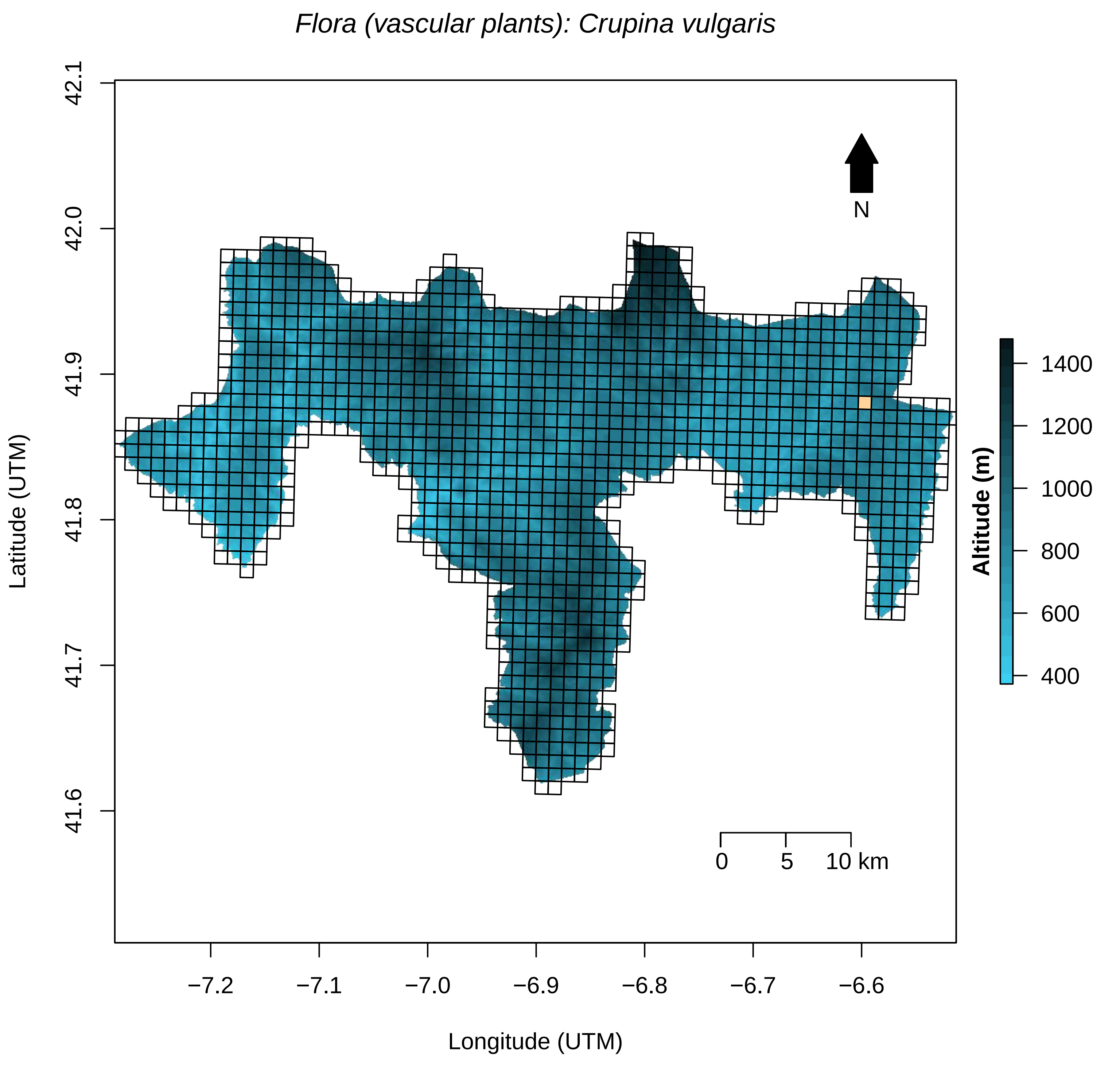
Flora (vascular plants): Crupina vulgaris
N
0
5
10 km
Latitude (UTM)
42.1
42.0
41.9
41.8
41.7
41.6
Longitude (UTM)
−7.2
−7.1
−7.0
−6.9
−6.8
−6.7
−6.6
Altitude (m)
1400
1200
1000
800
600
400

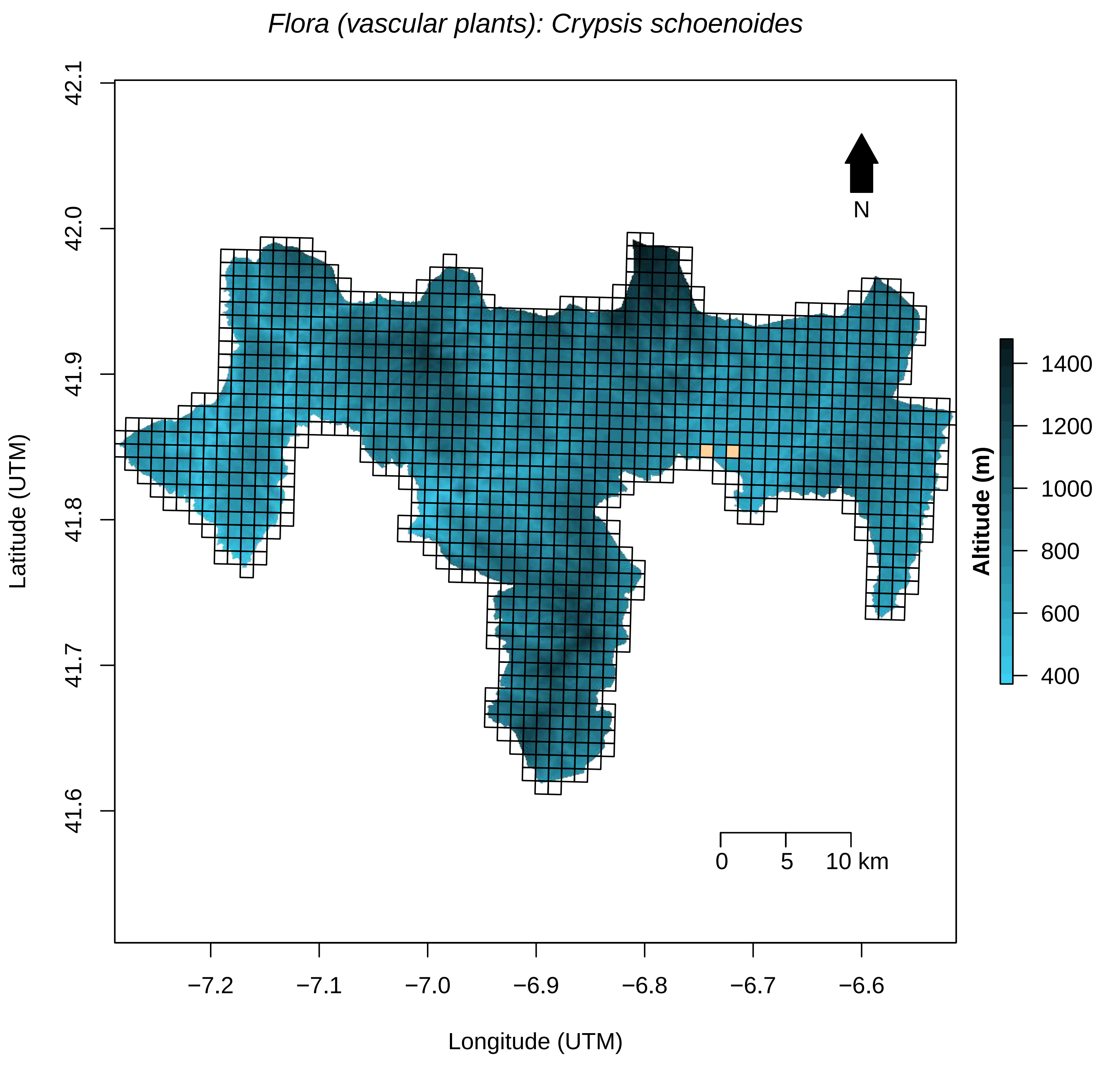
Flora (vascular plants): Crypsis schoenoides
42.1
42.0
41.9
41.8
41.7
41.6
Latitude (UTM)
−7.2
−7.1
−7.0
−6.9
−6.8
−6.7
−6.6
Longitude (UTM)
N
0
5
10 km
Altitude (m)
1400
1200
1000
800
600
400

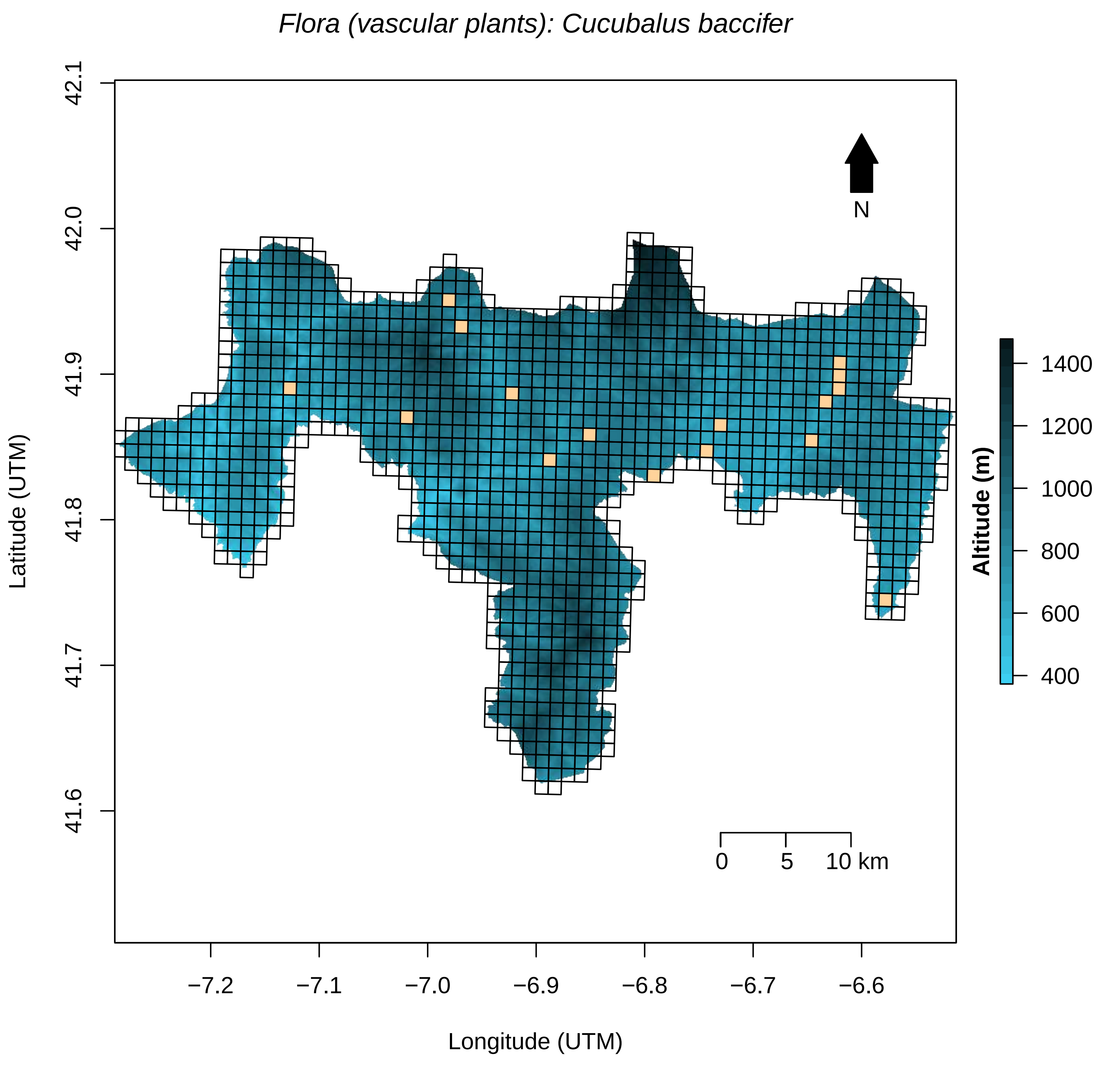
Flora (vascular plants): Cucubalus baccifer
N
0
5
10 km
Altitude (m)
1400
1200
1000
800
600
400
42.1
42.0
41.9
41.8
41.7
41.6
Latitude (UTM)
−7.2
−7.1
−7.0
−6.9
−6.8
−6.7
−6.6
Longitude (UTM)

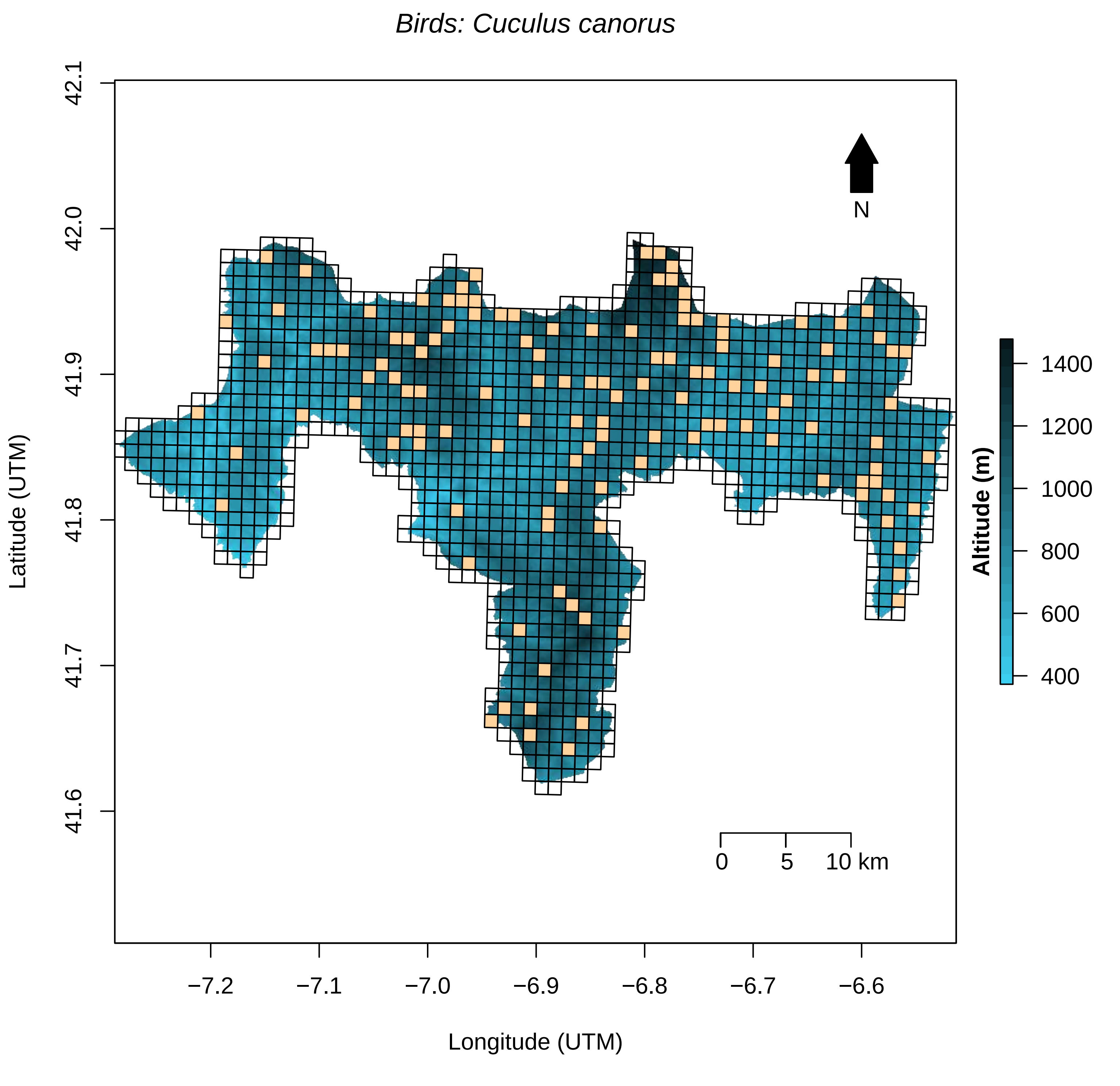

Birds: Cuculus canorus
N
0 5 10 km
Latitude (UTM)
42.1
42.0
41.9
41.8
41.7
41.6
Longitude (UTM)
−7.2
−7.1
−7.0
−6.9
−6.8
−6.7
−6.6
Altitude (m)
1400
1200
1000
800
600
400

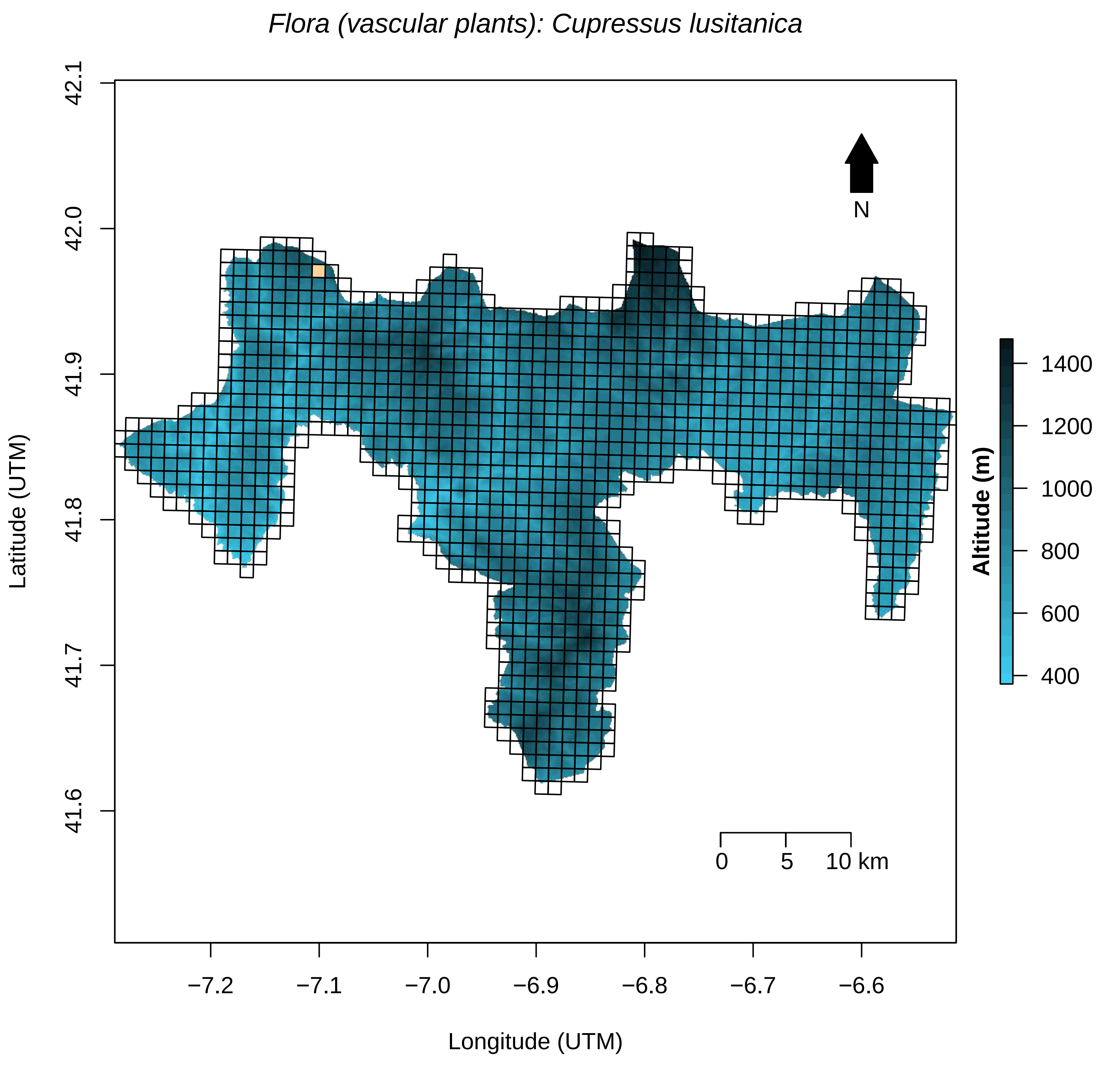

Flora (vascular plants): Cupressus lusitanica
N
0
5
10 km
Altitude (m)
1400
1200
1000
800
600
400
Latitude (UTM)
42.1
42.0
41.9
41.8
41.7
41.6
Longitude (UTM)
−7.2
−7.1
−7.0
−6.9
−6.8
−6.7
−6.6

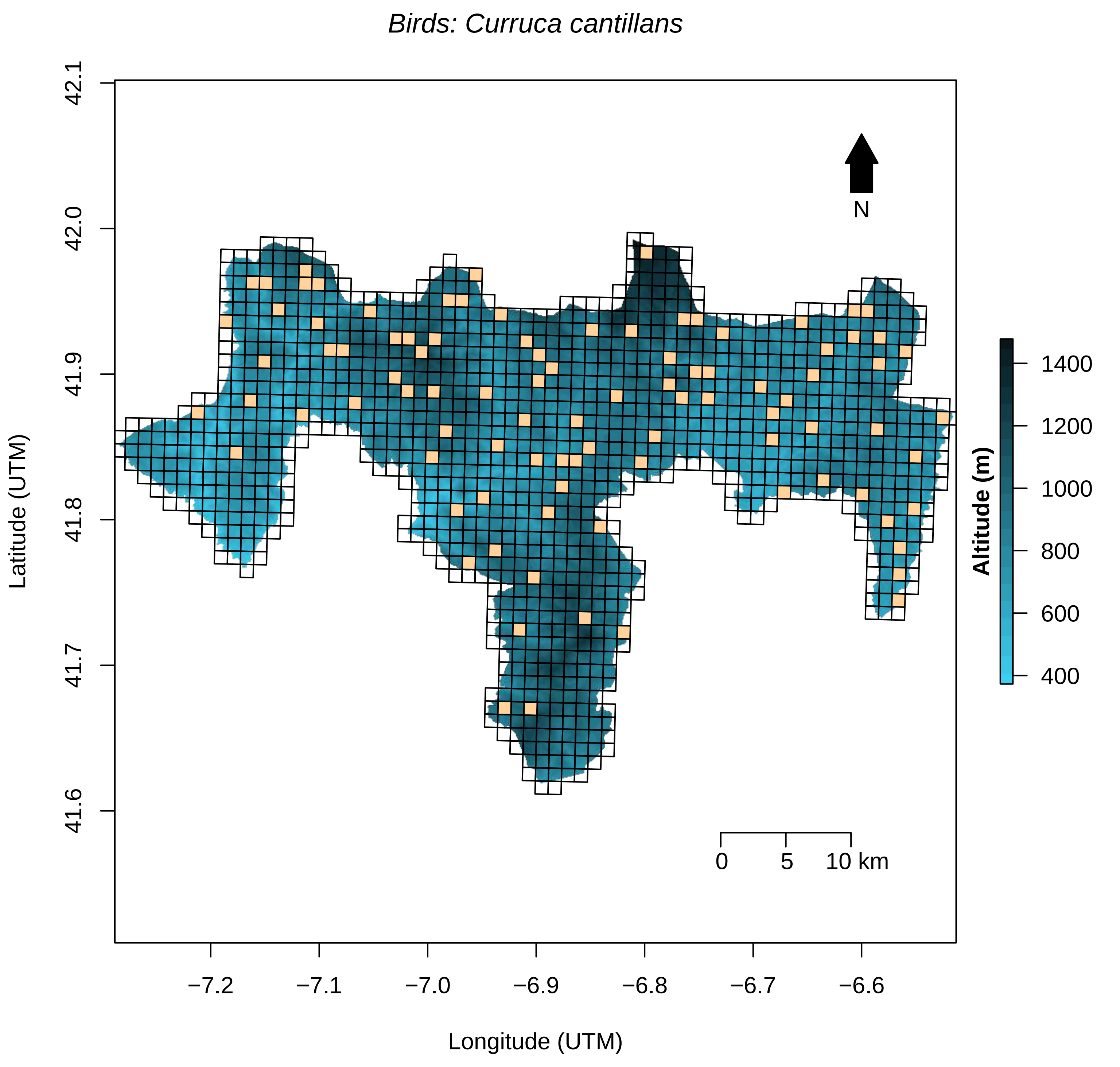

Birds: Curruca cantillans
N
0
5
10 km
Altitude (m)
1400
1200
1000
800
600
400
Latitude (UTM)
42.1
42.0
41.9
41.8
41.7
41.6
Longitude (UTM)
−7.2
−7.1
−7.0
−6.9
−6.8
−6.7
−6.6

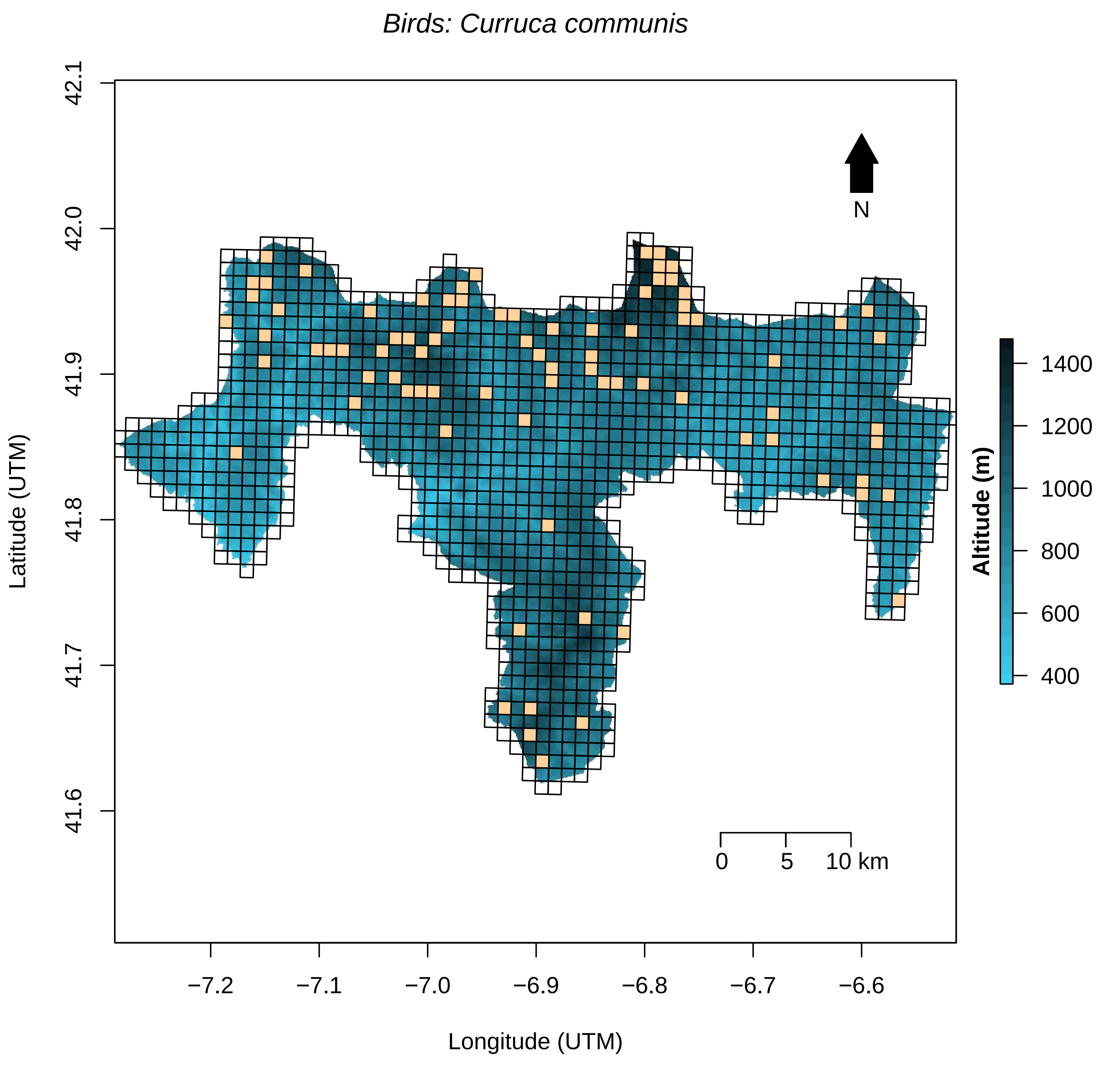

Birds: Curruca communis
N
0
5
10 km
Altitude (m)
1400
1200
1000
800
600
400
42.1
42.0
41.9
41.8
41.7
41.6
Latitude (UTM)
−7.2
−7.1
−7.0
−6.9
−6.8
−6.7
−6.6
Longitude (UTM)

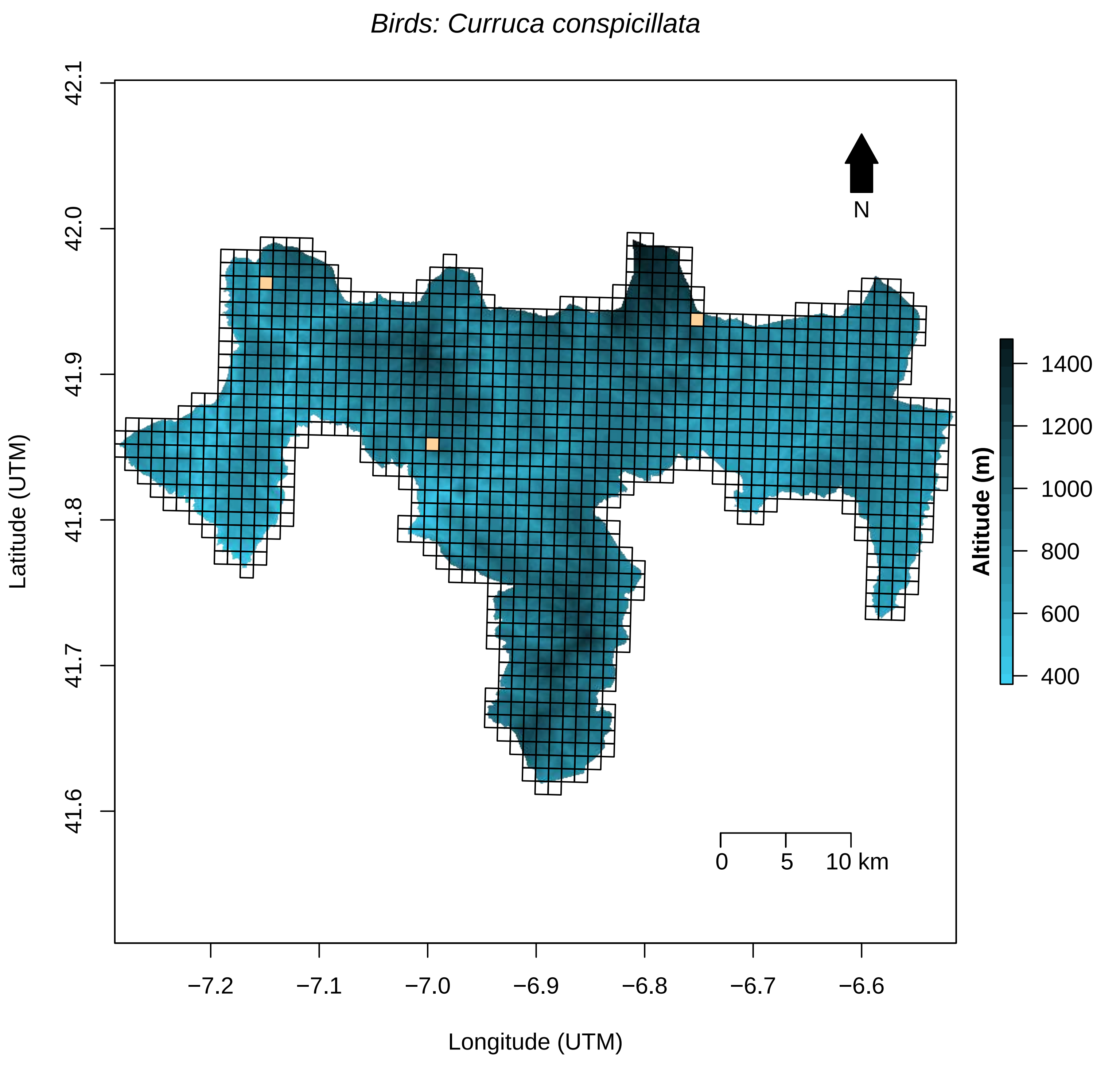

Birds: Curruca conspicillata
42.1
42.0
41.9
41.8
41.7
41.6
Latitude (UTM)
−7.2
−7.1
−7.0
−6.9
−6.8
−6.7
−6.6
Longitude (UTM)
N
0
5
10 km
1400
1200
1000
800
600
400
Altitude (m)

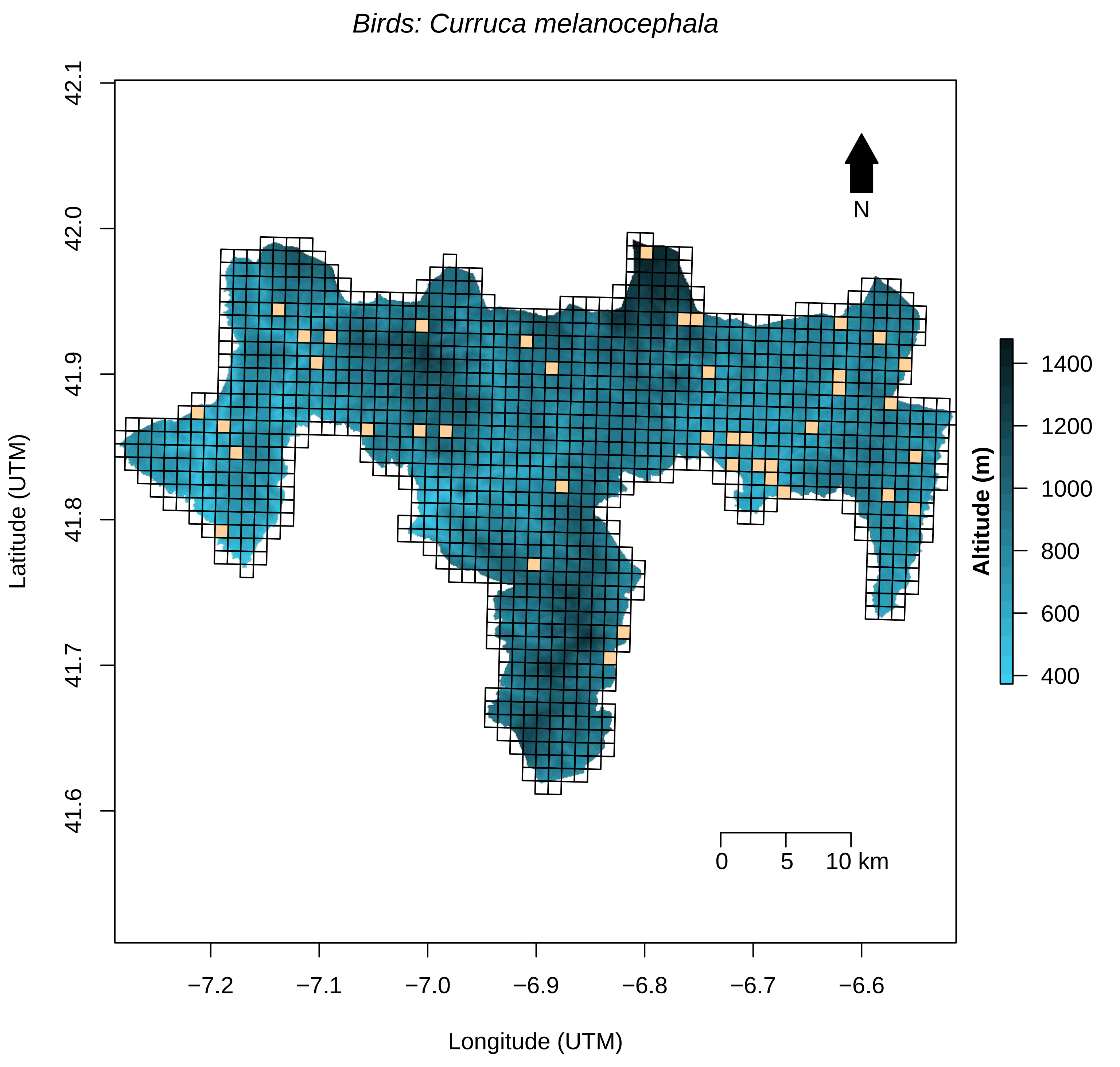

Birds: Curruca melanocephala
N
0 5 10 km
Altitude (m)
1400
1200
1000
800
600
400
42.1
42.0
41.9
41.8
41.7
41.6
Latitude (UTM)
−7.2
−7.1
−7.0
−6.9
−6.8
−6.7
−6.6
Longitude (UTM)

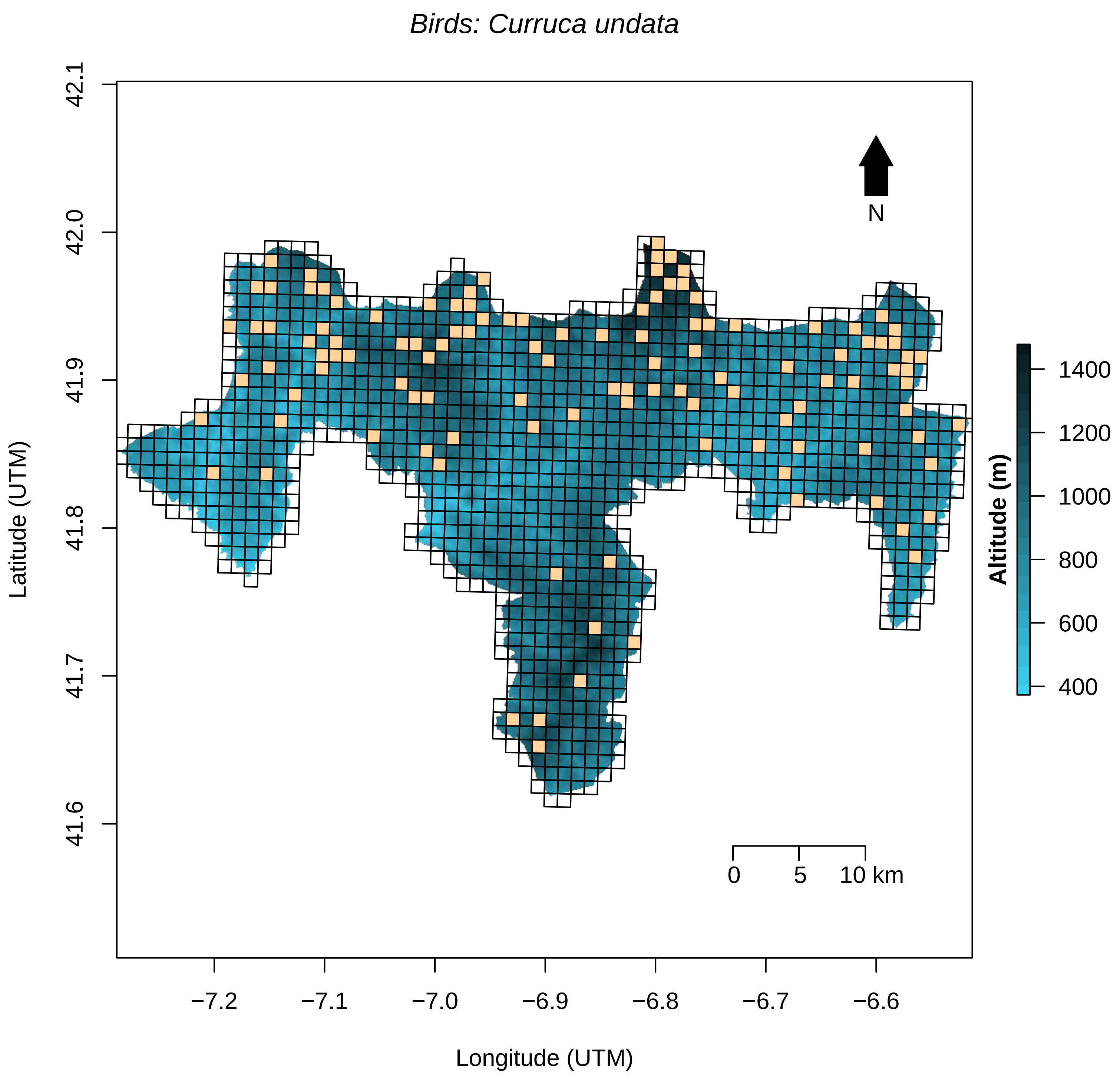

Birds: Curruca undata
42.1
42.0
41.9
41.8
41.7
41.6
Latitude (UTM)
−7.2
−7.1
−7.0
−6.9
−6.8
−6.7
−6.6
Longitude (UTM)
N
0
5
10 km
1400
1200
1000
800
600
400
Altitude (m)

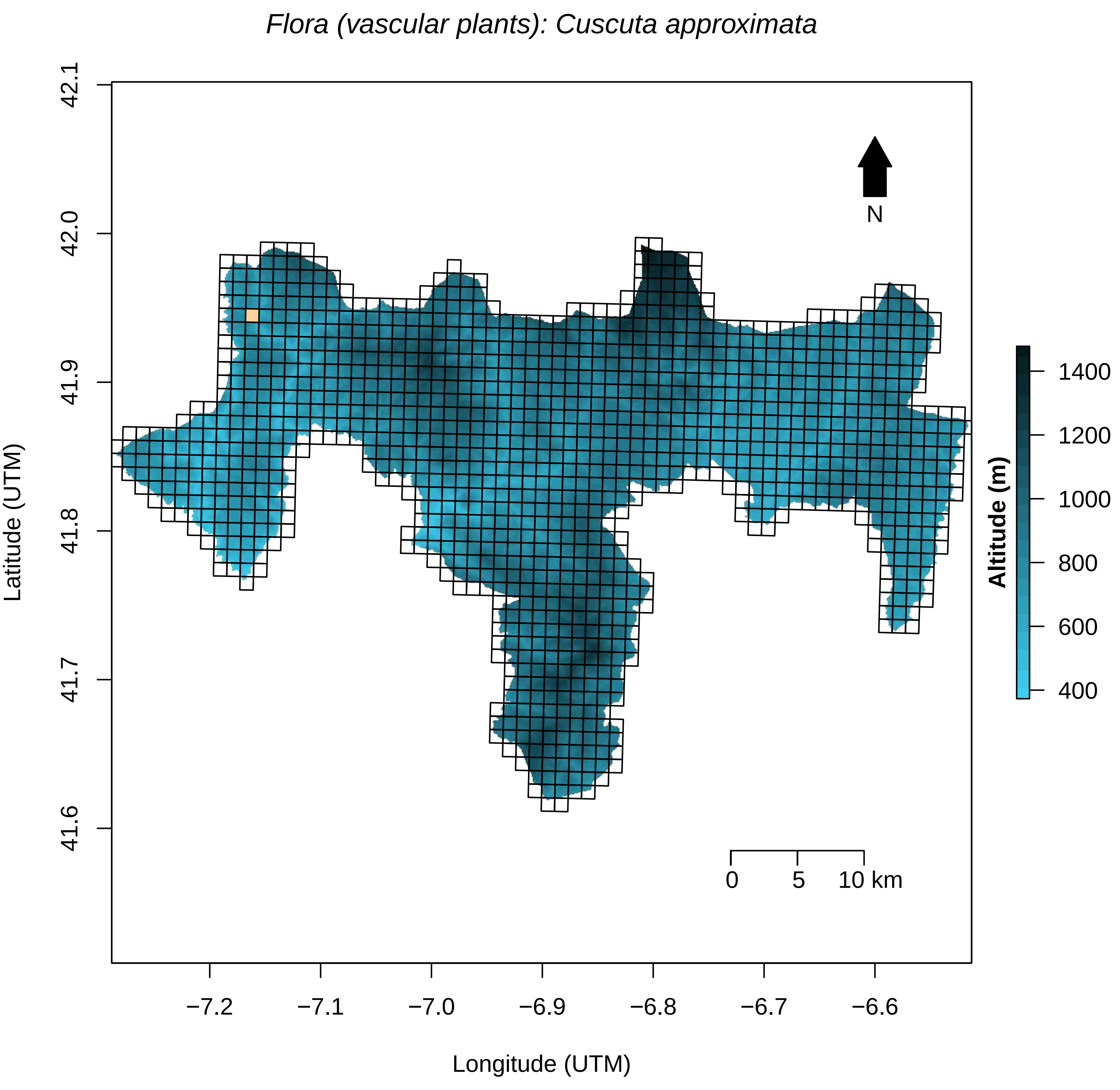

Flora (vascular plants): Cuscuta approximata
N
0 5 10 km
Latitude (UTM)
Longitude (UTM)
42.1
42.0
41.9
41.8
41.7
41.6
−7.2 −7.1 −7.0 −6.9 −6.8 −6.7 −6.6
Altitude (m)
1400
1200
1000
800
600
400

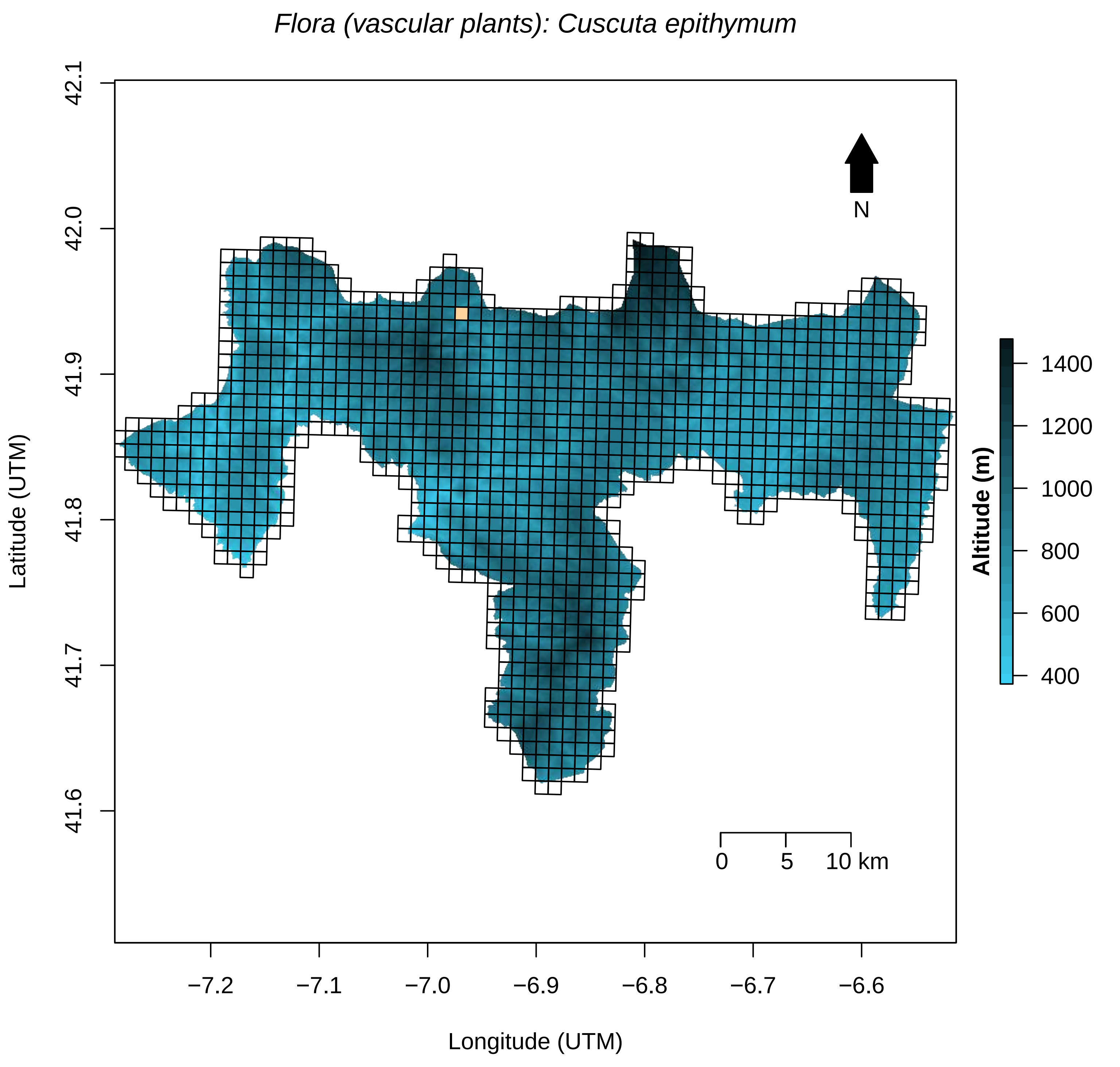

Flora (vascular plants): Cuscuta epithymum
Latitude (UTM)
42.1
42.0
41.9
41.8
41.7
41.6
Longitude (UTM)
−7.2
−7.1
−7.0
−6.9
−6.8
−6.7
−6.6
N
0
5
10 km
Altitude (m)
1400
1200
1000
800
600
400

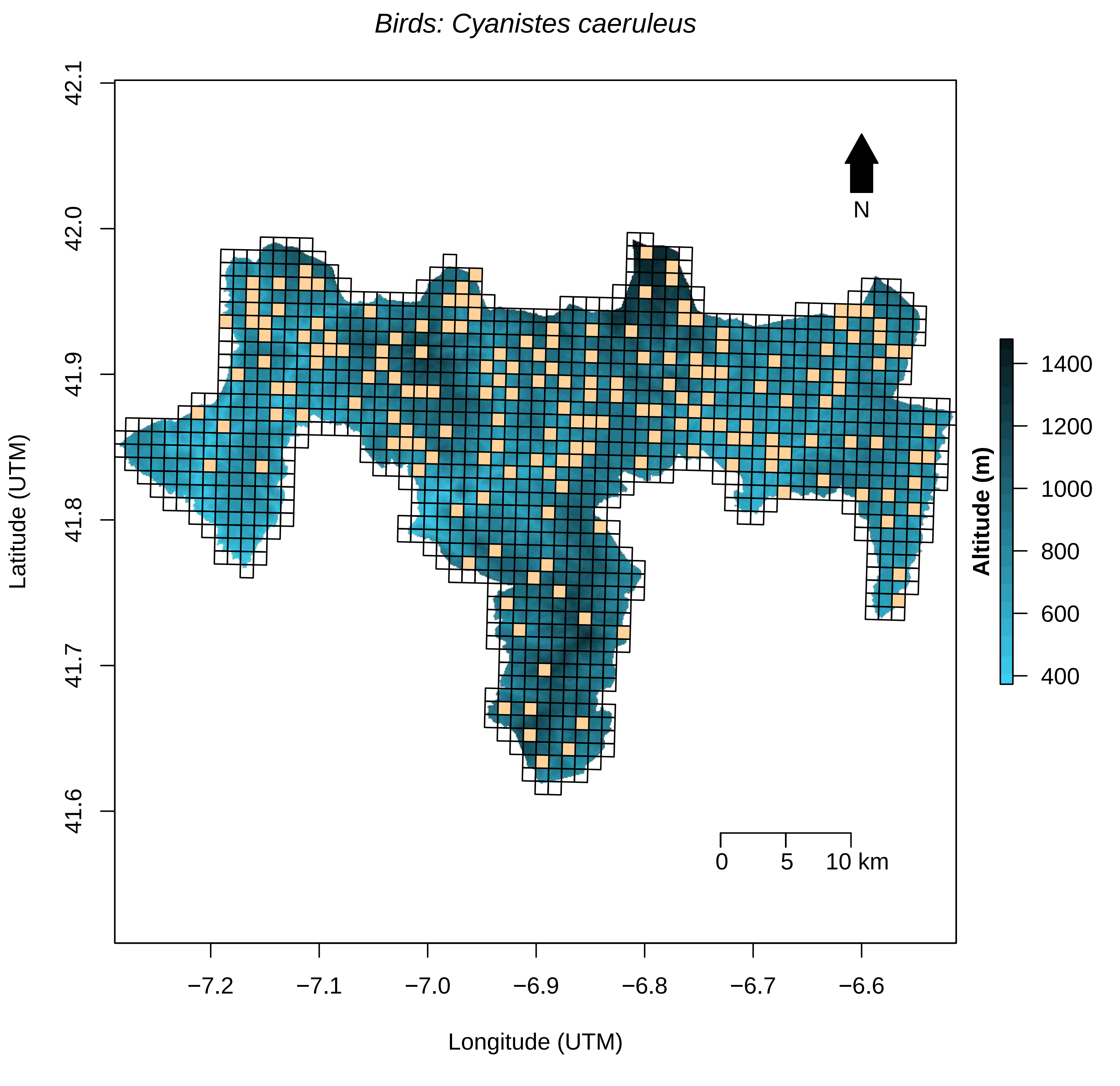

Birds: Cyanistes caeruleus
42.1
42.0
41.9
41.8
41.7
41.6
Latitude (UTM)
−7.2
−7.1
−7.0
−6.9
−6.8
−6.7
−6.6
Longitude (UTM)
N
0
5
10 km
Altitude (m)
1400
1200
1000
800
600
400

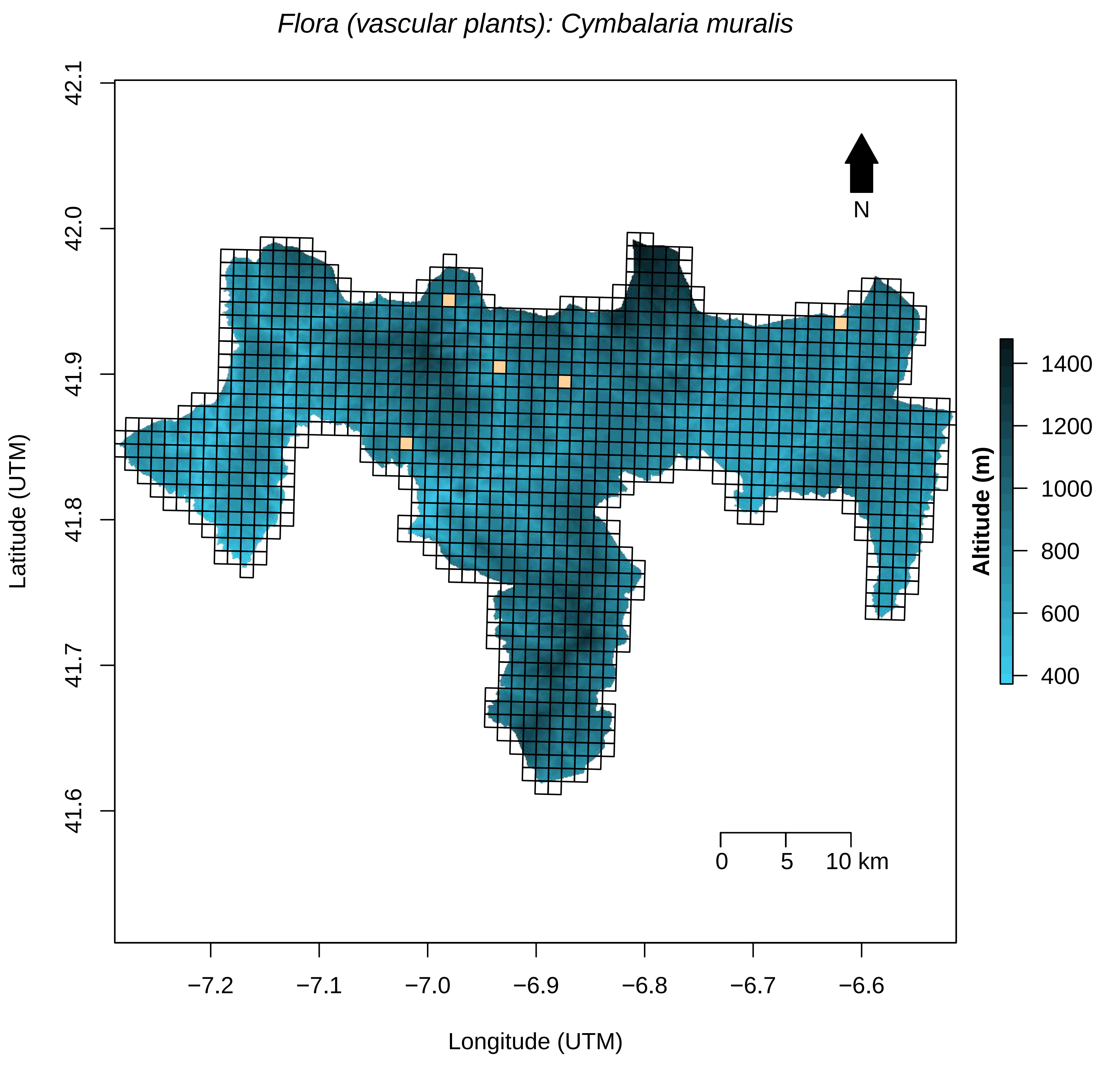

Flora (vascular plants): Cymbalaria muralis
42.1
42.0
41.9
41.8
41.7
41.6
Latitude (UTM)
N
0
5
10 km
1400
1200
1000
800
600
400
Altitude (m)
−7.2
−7.1
−7.0
−6.9
−6.8
−6.7
−6.6
Longitude (UTM)

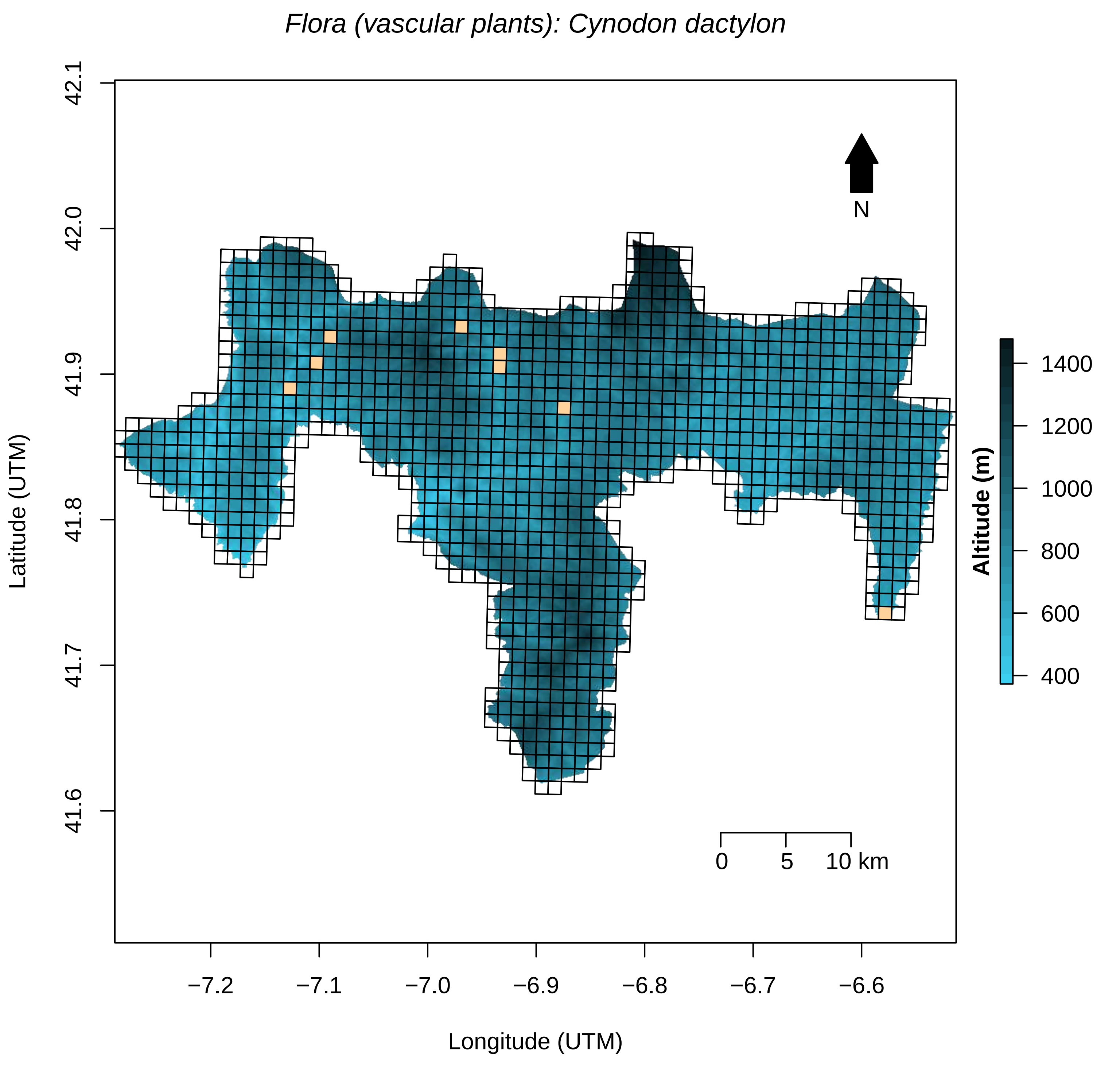

Flora (vascular plants): Cynodon dactylon
42.1
42.0
41.9
41.8
41.7
41.6
Latitude (UTM)
−7.2
−7.1
−7.0
−6.9
−6.8
−6.7
−6.6
Longitude (UTM)
N
0
5
10 km
Altitude (m)
1400
1200
1000
800
600
400

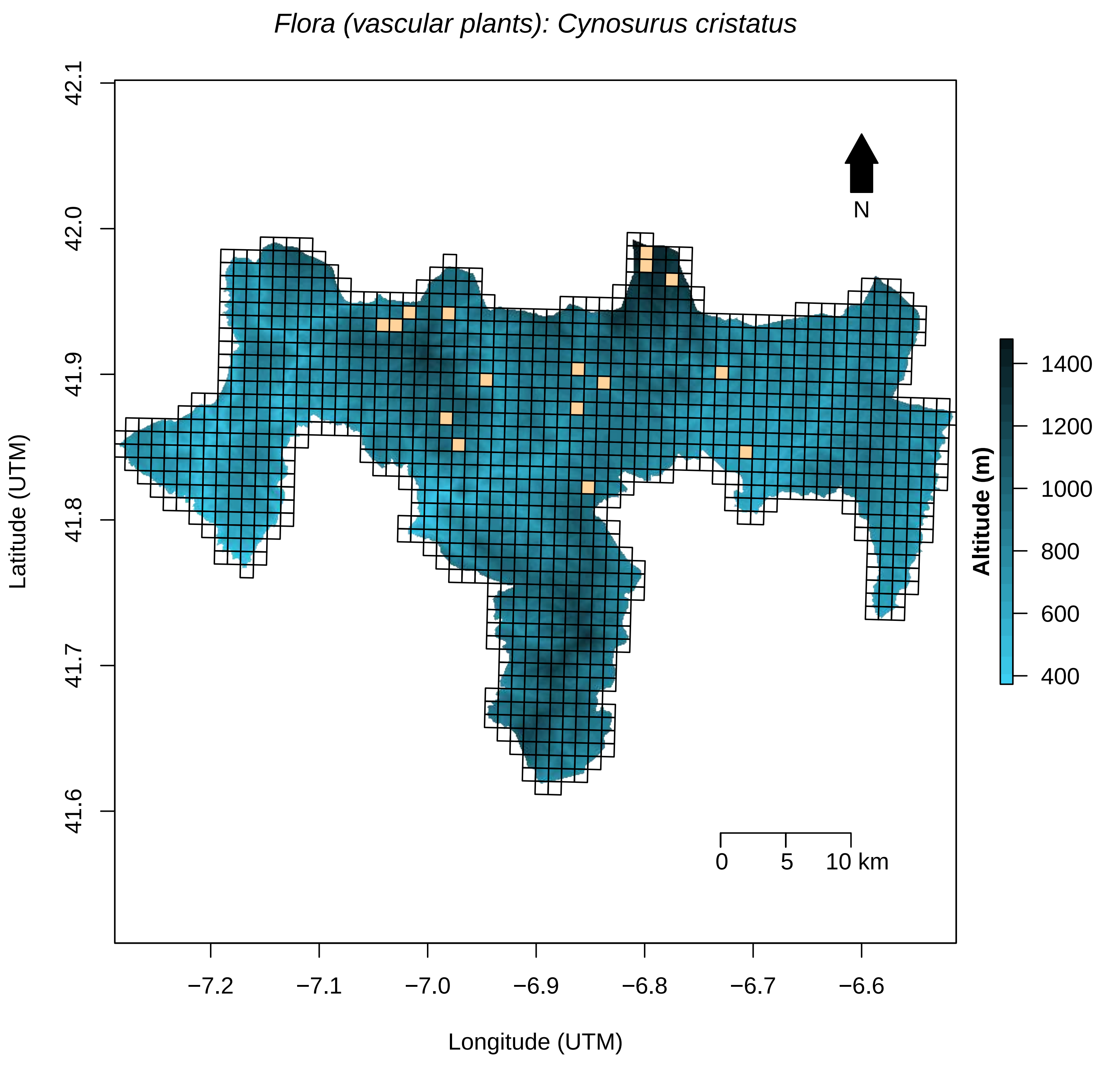

Flora (vascular plants): Cynosurus cristatus
N
0
5
10 km
Altitude (m)
1400
1200
1000
800
600
400
Latitude (UTM)
42.1
42.0
41.9
41.8
41.7
41.6
Longitude (UTM)
−7.2
−7.1
−7.0
−6.9
−6.8
−6.7
−6.6

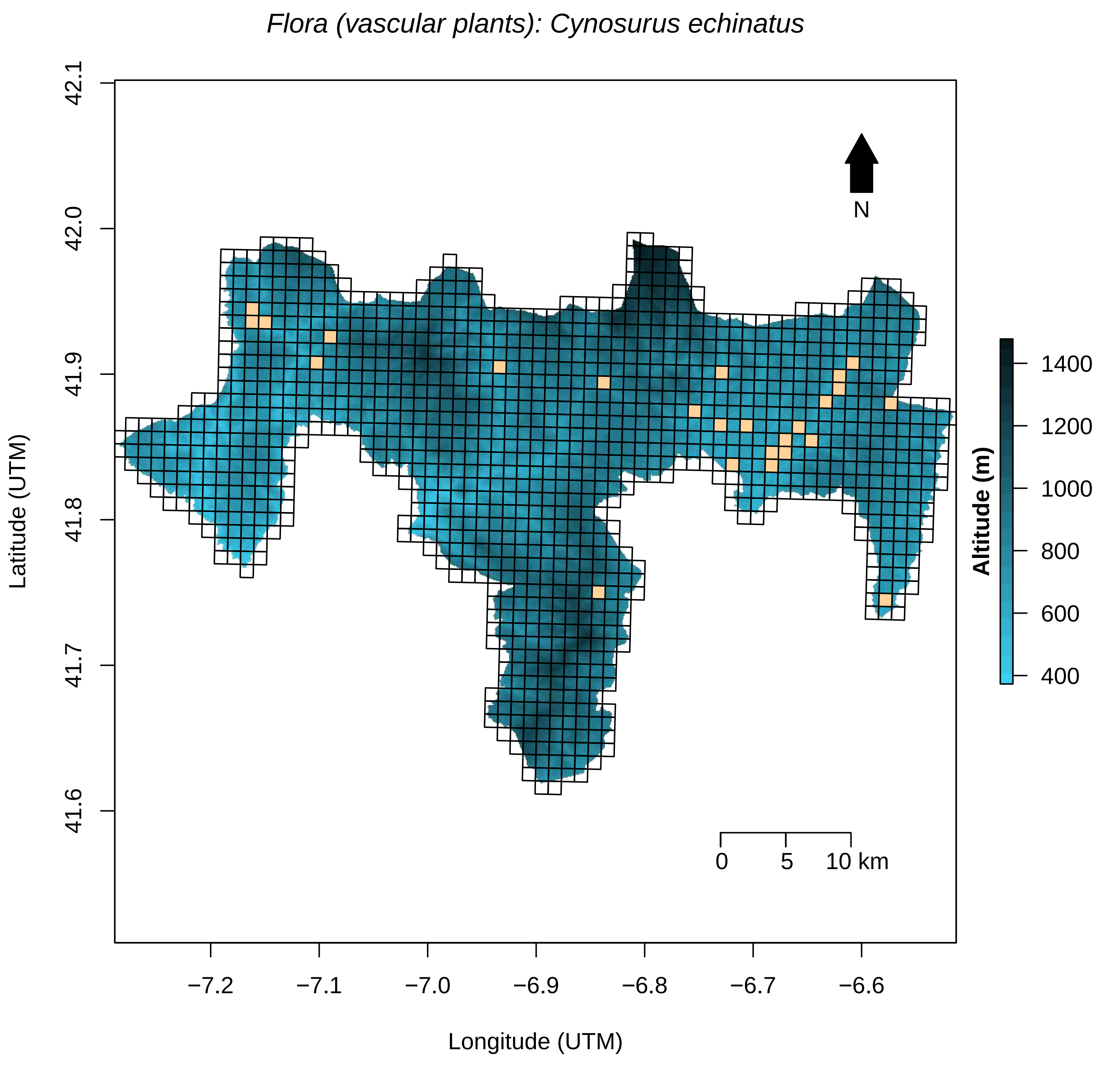

Flora (vascular plants): Cynosurus echinatus
Latitude (UTM)
42.1
42.0
41.9
41.8
41.7
41.6
Longitude (UTM)
−7.2
−7.1
−7.0
−6.9
−6.8
−6.7
−6.6
N
0
5
10 km
Altitude (m)
1400
1200
1000
800
600
400

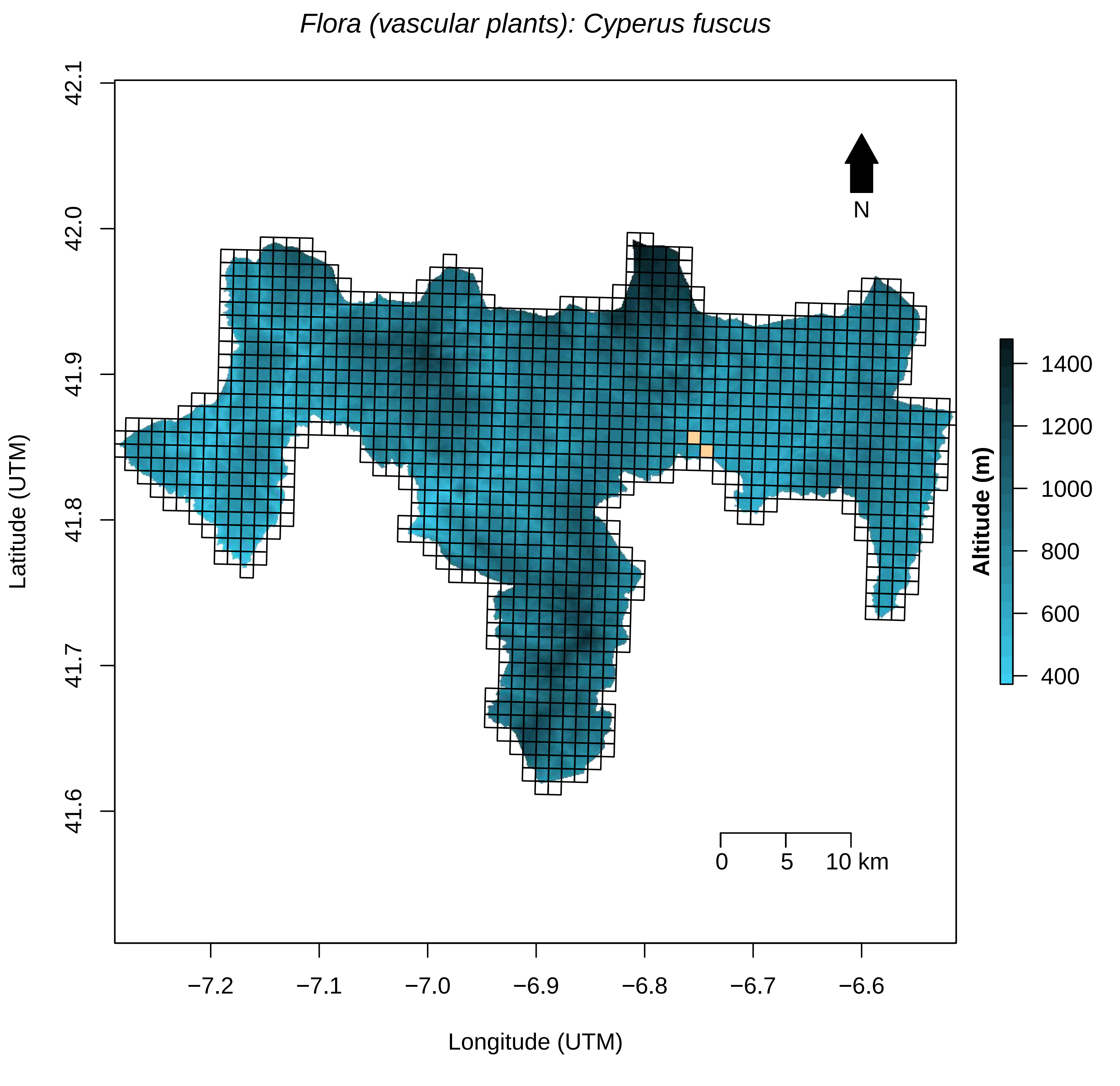

Flora (vascular plants): Cyperus fuscus
42.1
42.0
41.9
41.8
41.7
41.6
Latitude (UTM)
N
0
5
10 km
−7.2
−7.1
−7.0
−6.9
−6.8
−6.7
−6.6
Longitude (UTM)
1400
1200
1000
800
600
400
Altitude (m)

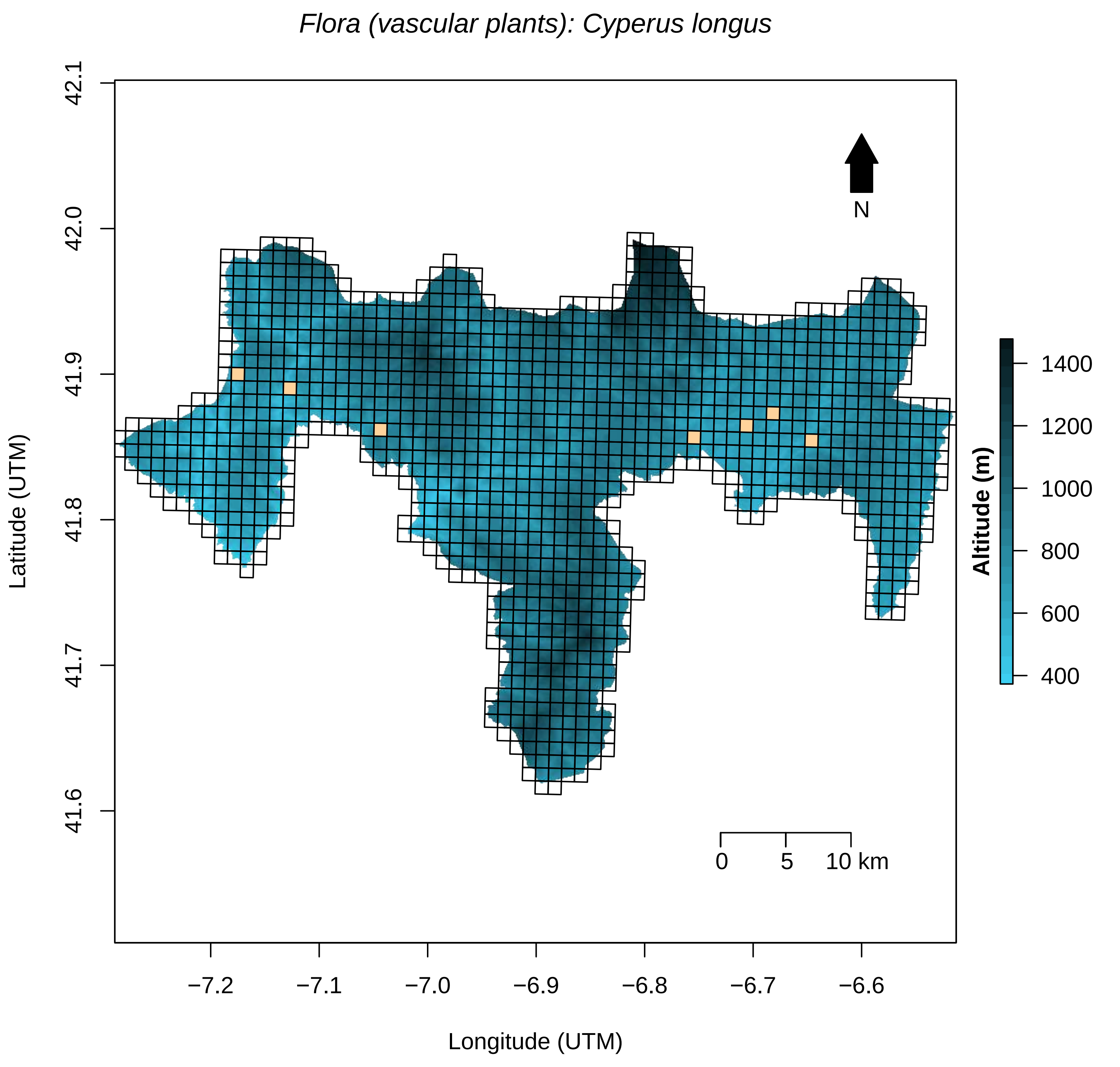
Flora (vascular plants): Cyperus longus
N
0
5
10 km
Altitude (m)
1400
1200
1000
800
600
400
Latitude (UTM)
42.1
42.0
41.9
41.8
41.7
41.6
Longitude (UTM)
−7.2
−7.1
−7.0
−6.9
−6.8
−6.7
−6.6

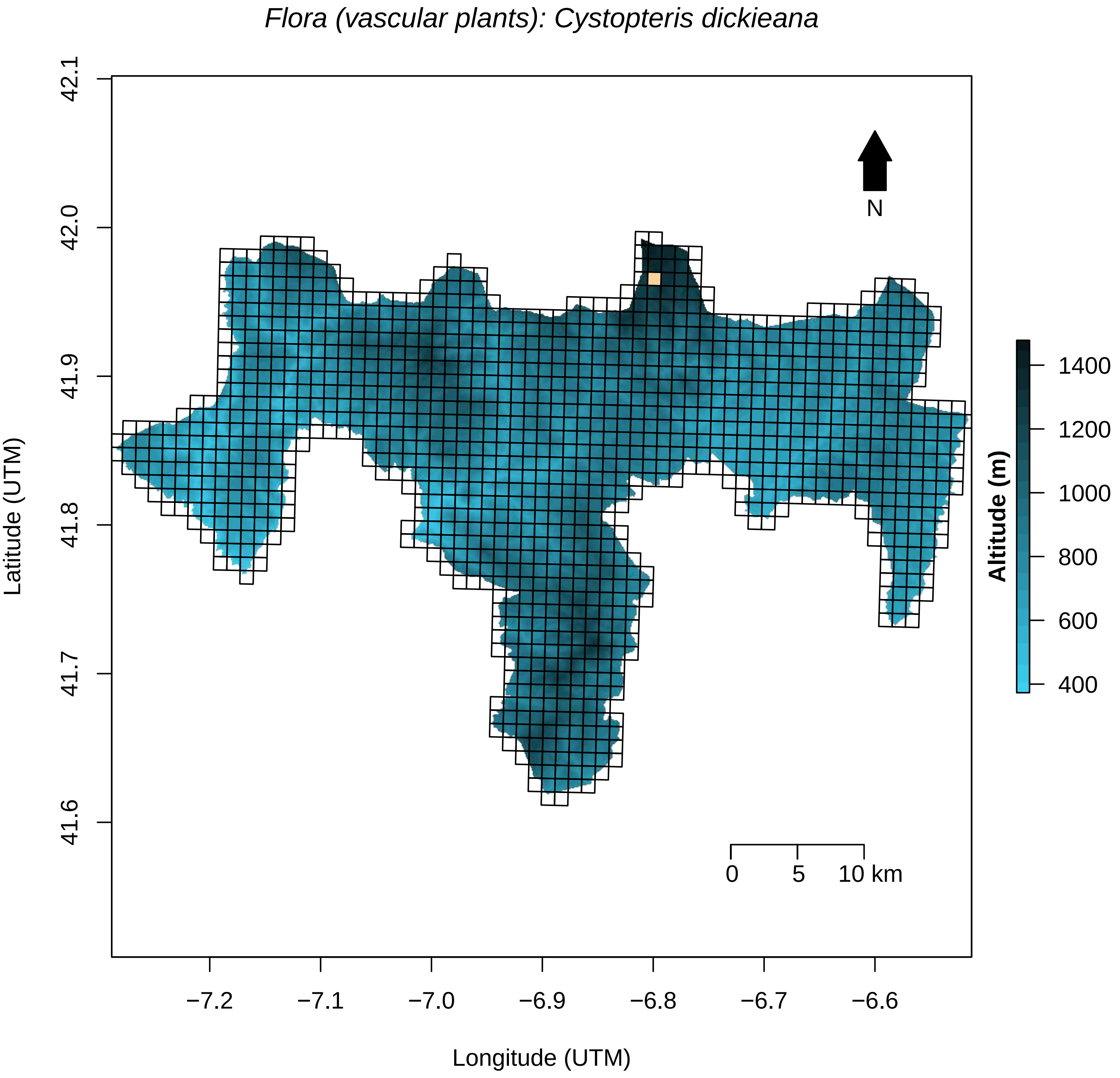

Flora (vascular plants): Cystopteris dickieana
N
0 5 10 km
Altitude (m)
1400
1200
1000
800
600
400
Latitude (UTM)
42.1
42.0
41.9
41.8
41.7
41.6
Longitude (UTM)
−7.2
−7.1
−7.0
−6.9
−6.8
−6.7
−6.6

Flora (vascular plants): Cystopteris viridula

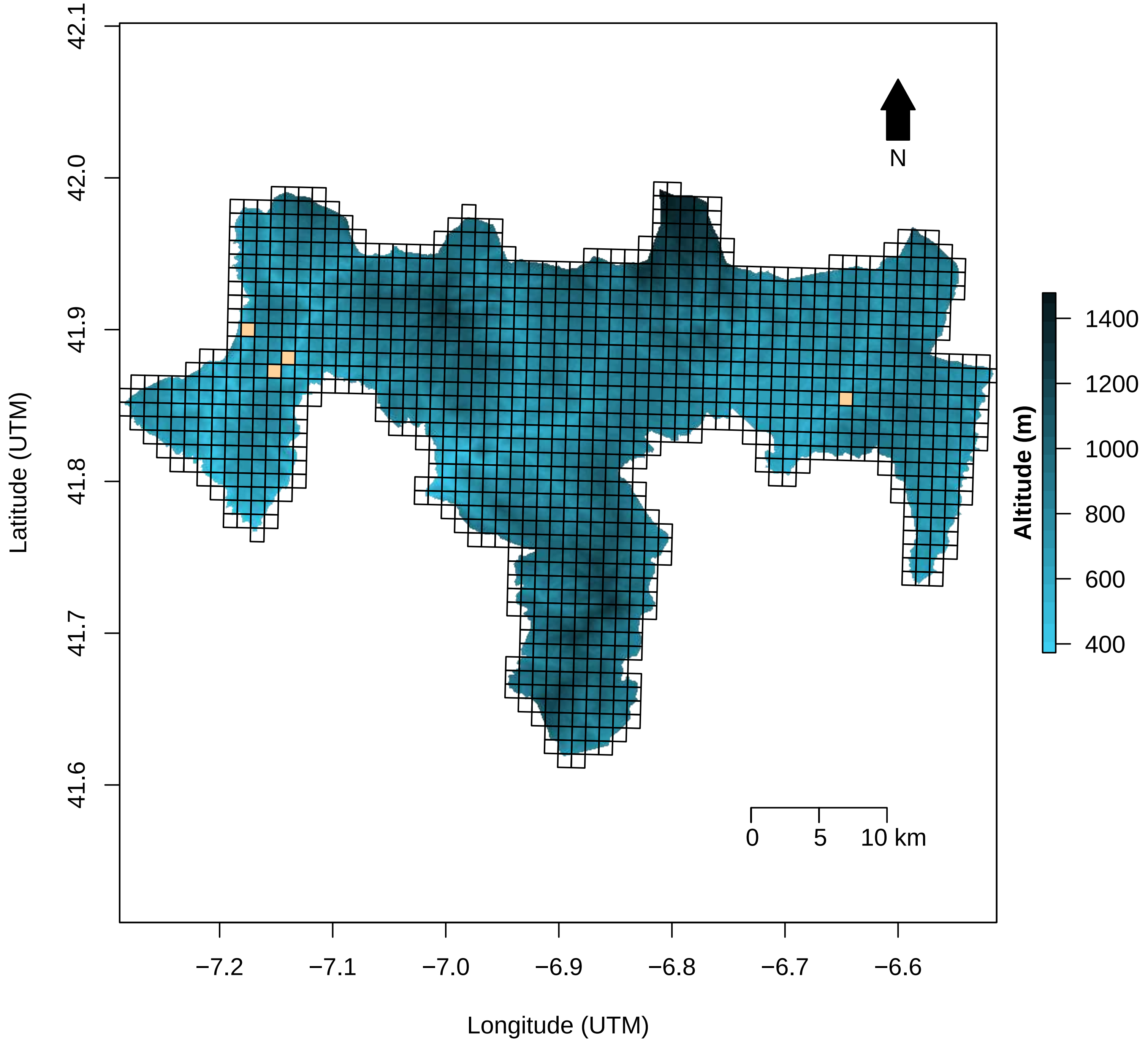

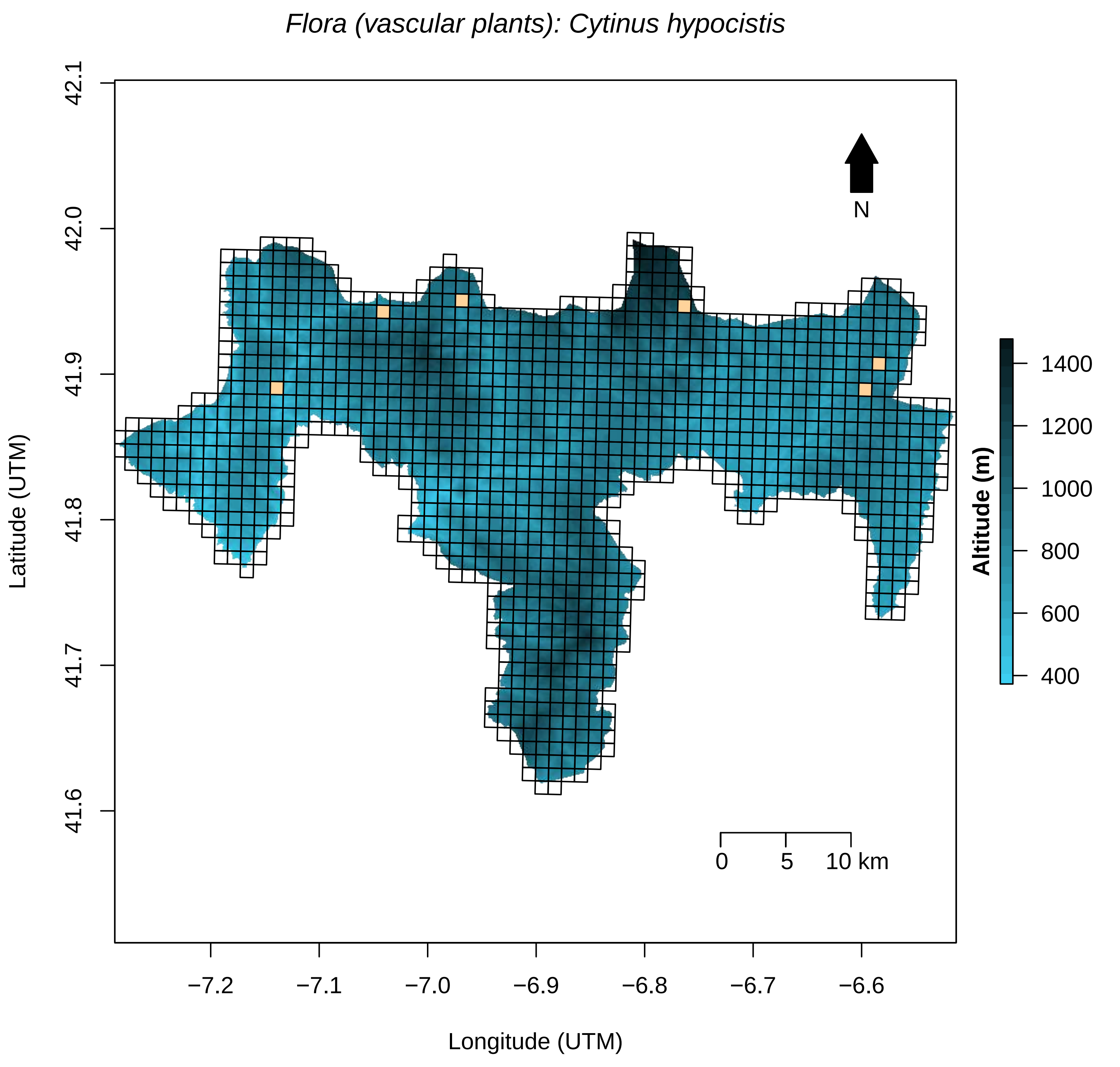

Flora (vascular plants): Cytinus hypocistis
N
0
5
10 km
Altitude (m)
1400
1200
1000
800
600
400
42.1
42.0
41.9
41.8
41.7
41.6
Latitude (UTM)
−7.2
−7.1
−7.0
−6.9
−6.8
−6.7
−6.6
Longitude (UTM)

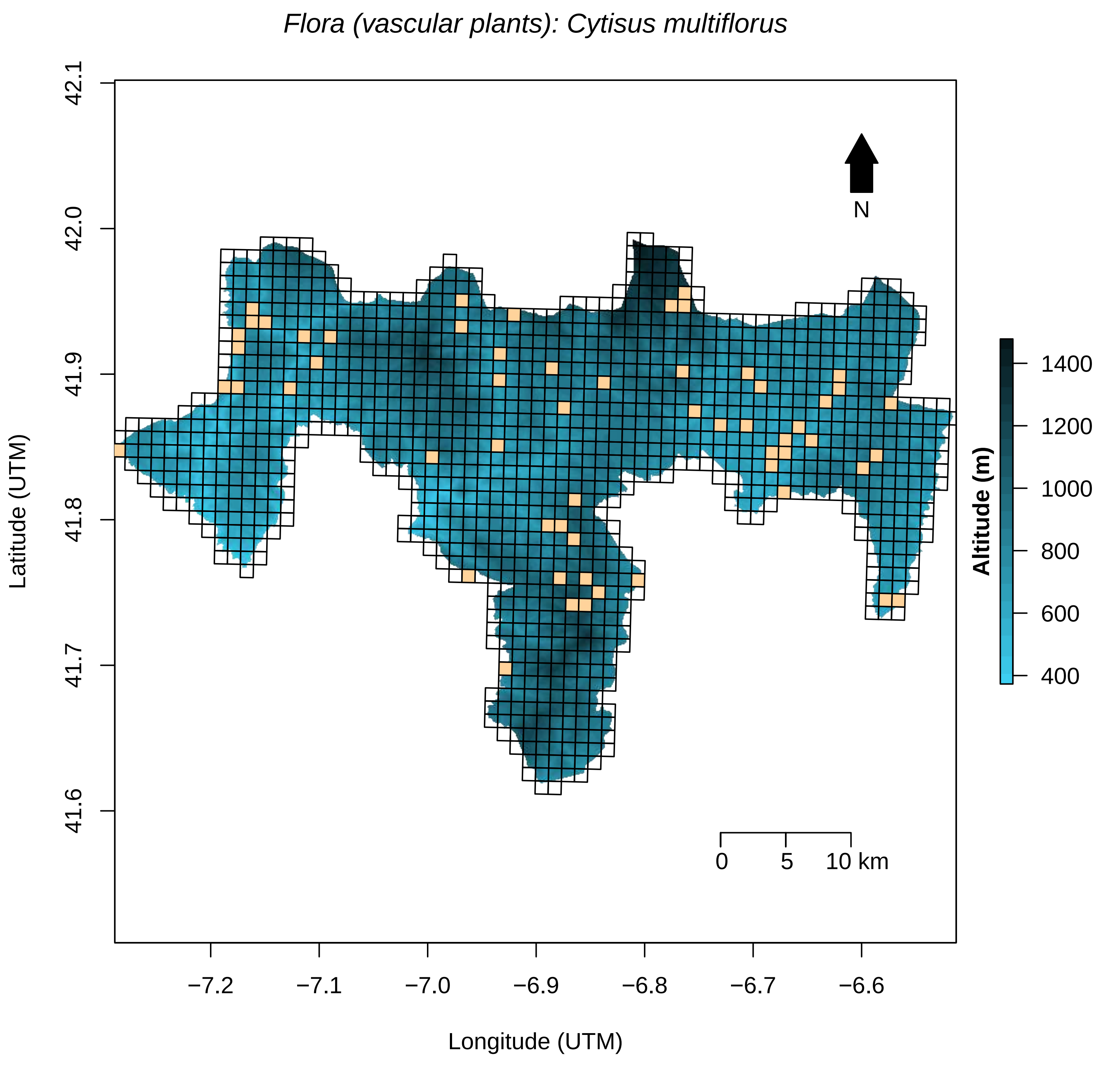

Flora (vascular plants): Cytisus multiflorus
N
0
5
10 km
Altitude (m)
1400
1200
1000
800
600
400
42.1
42.0
41.9
41.8
41.7
41.6
Latitude (UTM)
−7.2
−7.1
−7.0
−6.9
−6.8
−6.7
−6.6
Longitude (UTM)

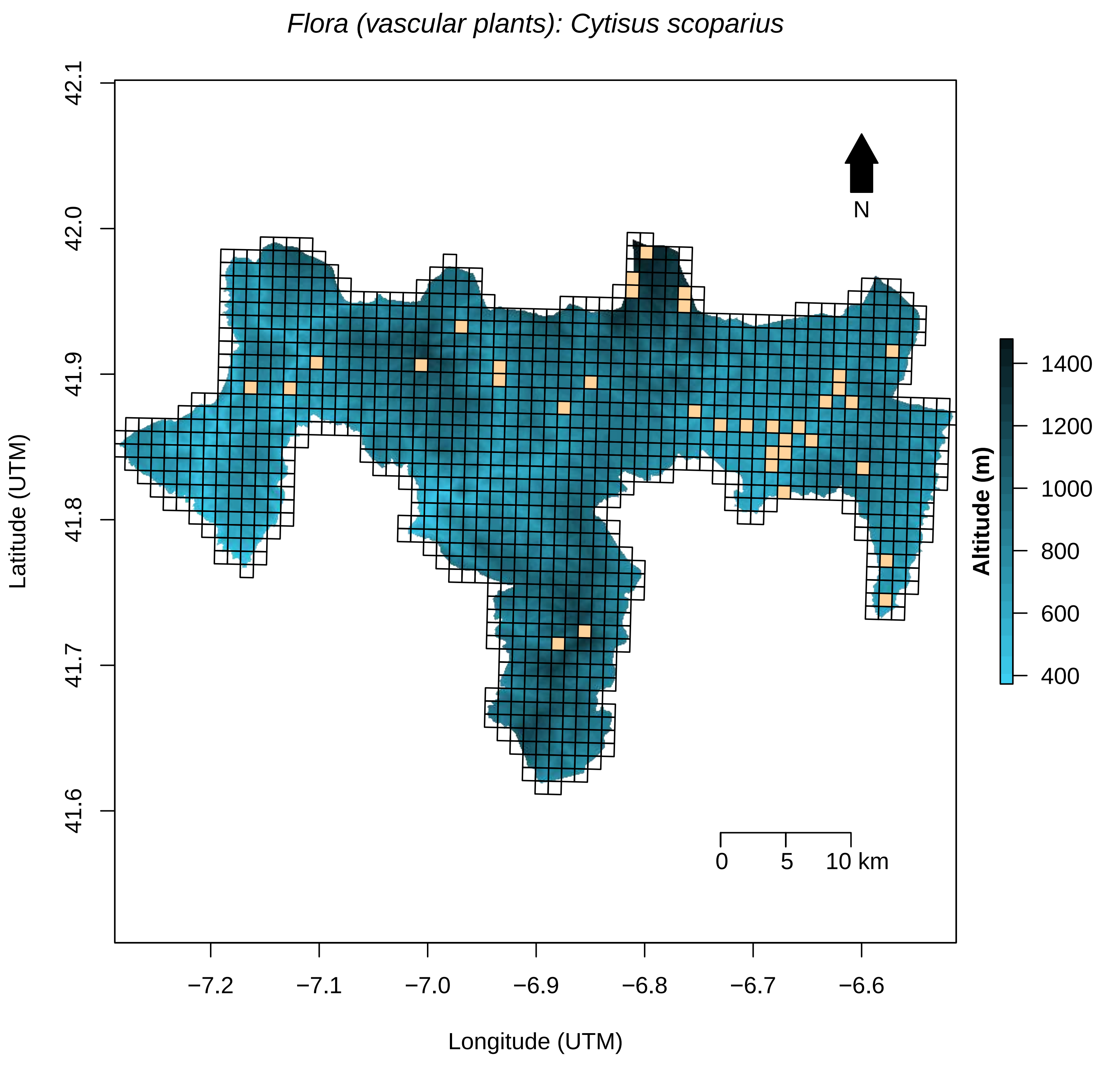

Flora (vascular plants): Cytisus scoparius
42.1
42.0
41.9
41.8
41.7
41.6
Latitude (UTM)
−7.2
−7.1
−7.0
−6.9
−6.8
−6.7
−6.6
Longitude (UTM)
N
0
5
10 km
Altitude (m)
1400
1200
1000
800
600
400

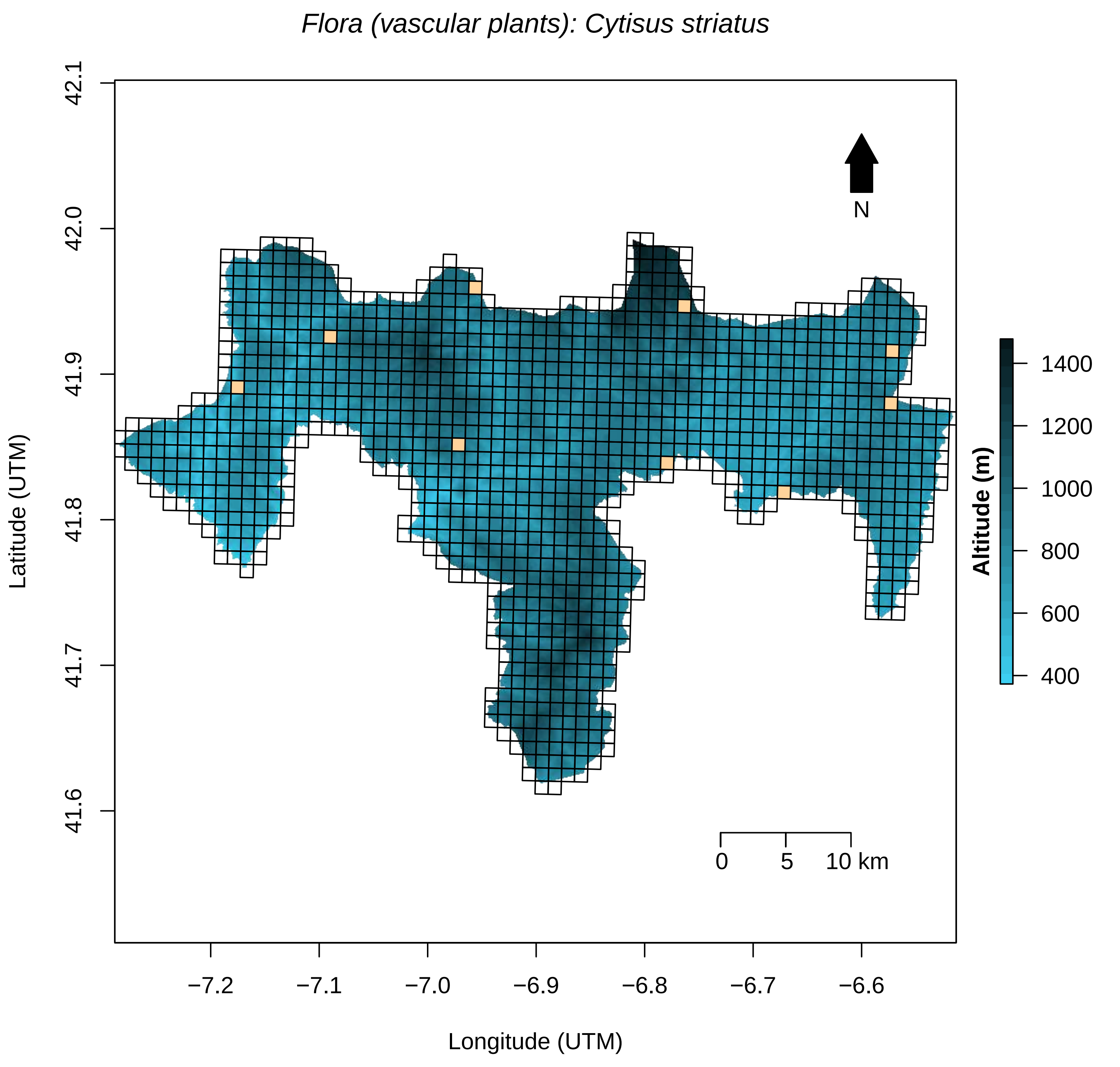

Flora (vascular plants): Cytisus striatus
42.1
42.0
41.9
41.8
41.7
41.6
Latitude (UTM)
−7.2
−7.1
−7.0
−6.9
−6.8
−6.7
−6.6
Longitude (UTM)
N
0
5
10 km
Altitude (m)
1400
1200
1000
800
600
400

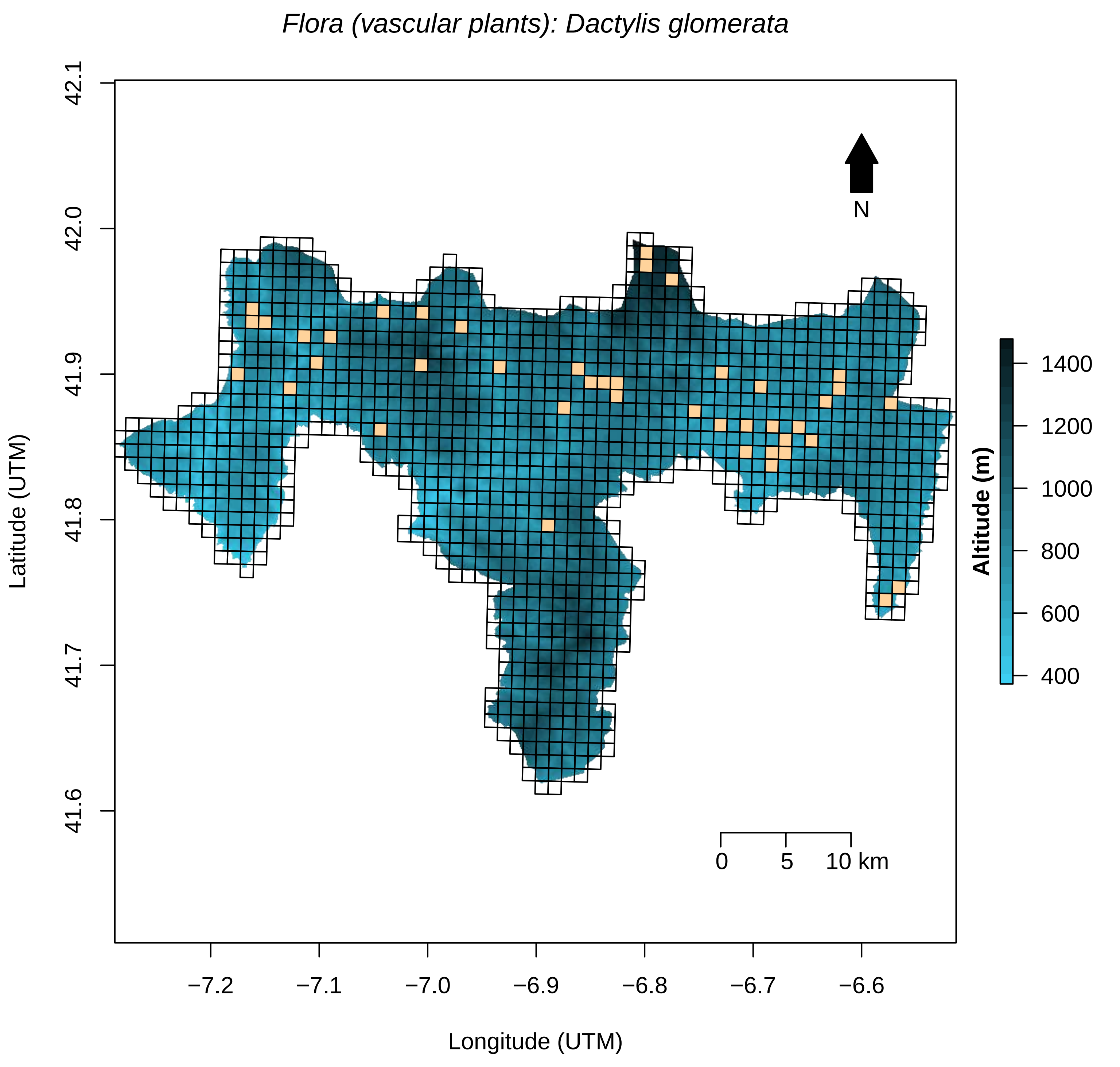

Flora (vascular plants): Dactylis glomerata
Latitude (UTM)
Longitude (UTM)
42.1
42.0
41.9
41.8
41.7
41.6
−7.2
−7.1
−7.0
−6.9
−6.8
−6.7
−6.6
N
0
5
10 km
Altitude (m)
1400
1200
1000
800
600
400

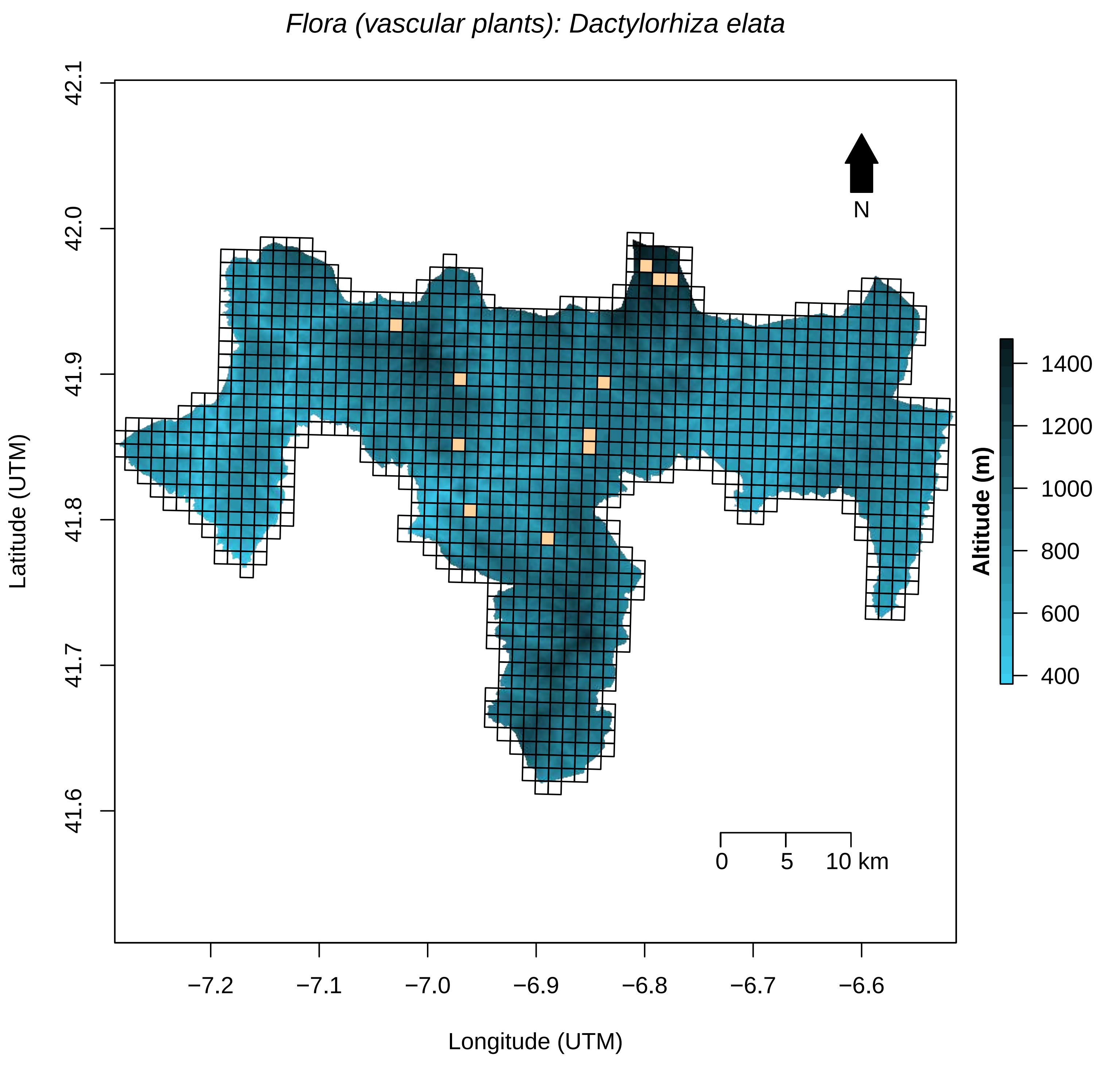

Flora (vascular plants): Dactylorhiza elata
42.1
42.0
41.9
41.8
41.7
41.6
Latitude (UTM)
−7.2
−7.1
−7.0
−6.9
−6.8
−6.7
−6.6
Longitude (UTM)
N
0
5
10 km
1400
1200
1000
800
600
400
Altitude (m)

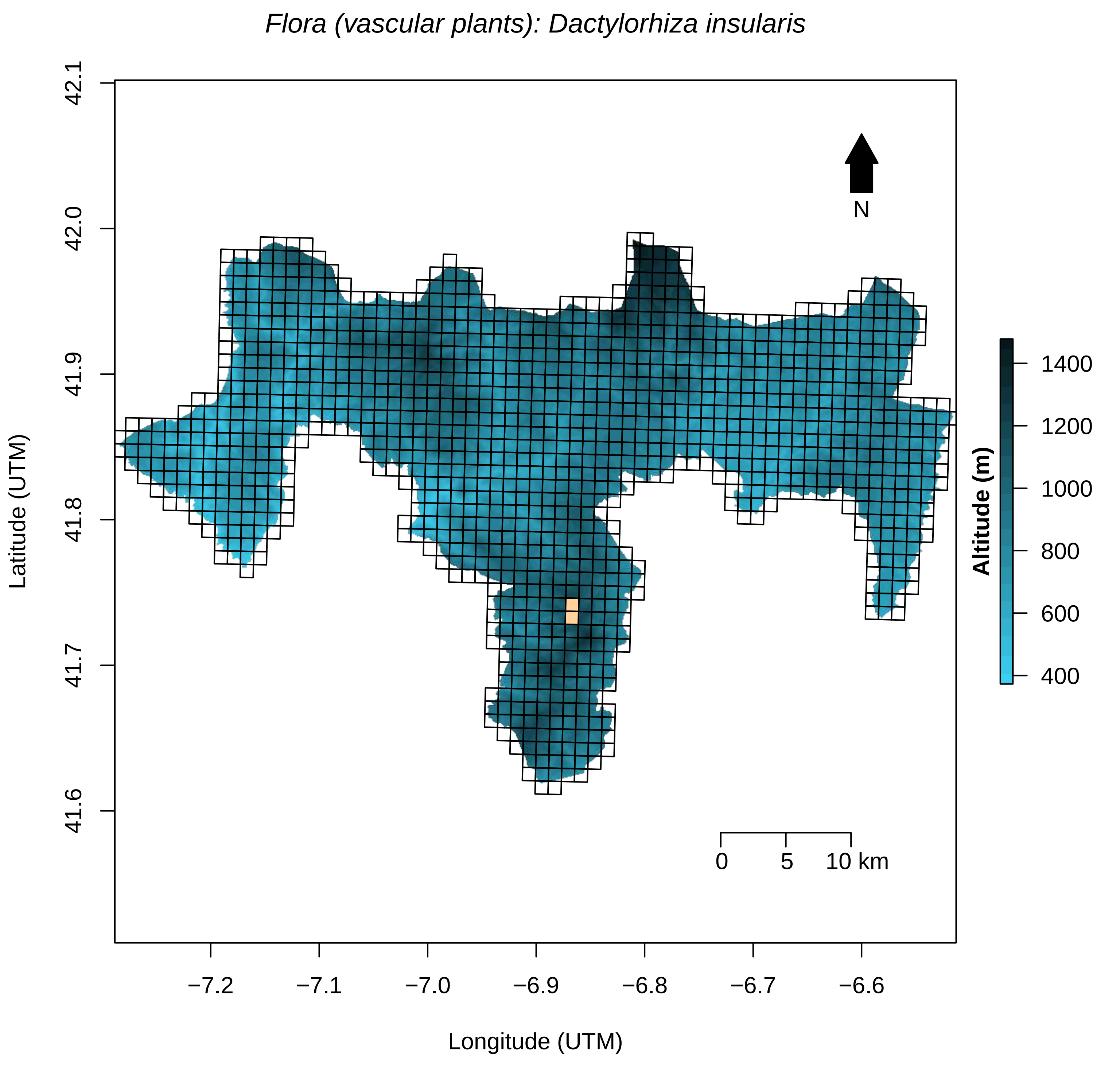

Flora (vascular plants): Dactylorhiza insularis
42.1
42.0
41.9
41.8
41.7
41.6
Latitude (UTM)
−7.2
−7.1
−7.0
−6.9
−6.8
−6.7
−6.6
Longitude (UTM)
N
0
5
10 km
1400
1200
1000
800
600
400
Altitude (m)

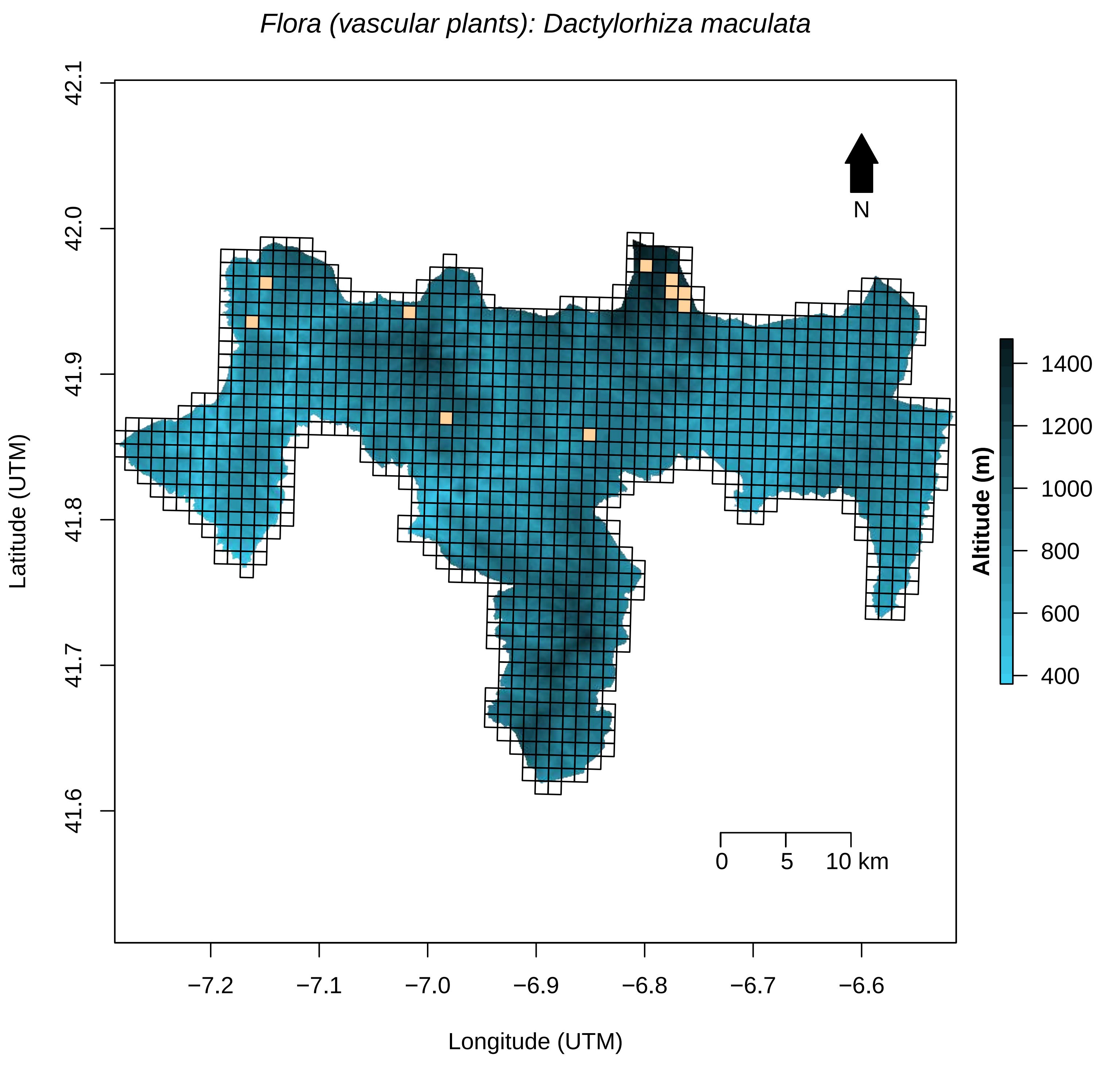

Flora (vascular plants): Dactylorhiza maculata
42.1
42.0
41.9
41.8
41.7
41.6
Latitude (UTM)
N
0
5
10 km
−7.2
−7.1
−7.0
−6.9
−6.8
−6.7
−6.6
Longitude (UTM)
Altitude (m)
1400
1200
1000
800
600
400

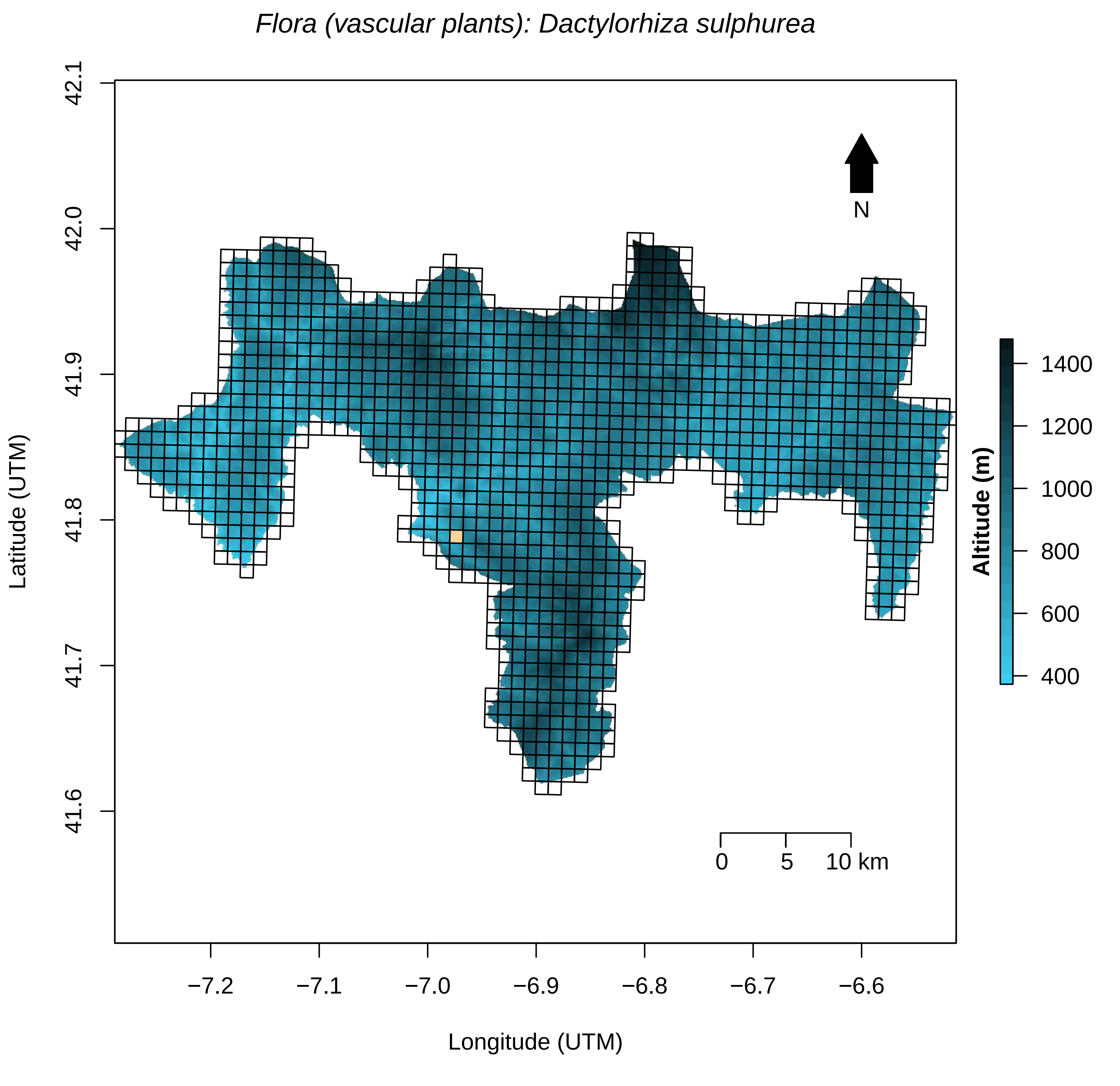

Flora (vascular plants): Dactylorhiza sulphurea
42.1
42.0
41.9
41.8
41.7
41.6
Latitude (UTM)
−7.2
−7.1
−7.0
−6.9
−6.8
−6.7
−6.6
Longitude (UTM)
N
0
5
10 km
1400
1200
1000
800
600
400
Altitude (m)

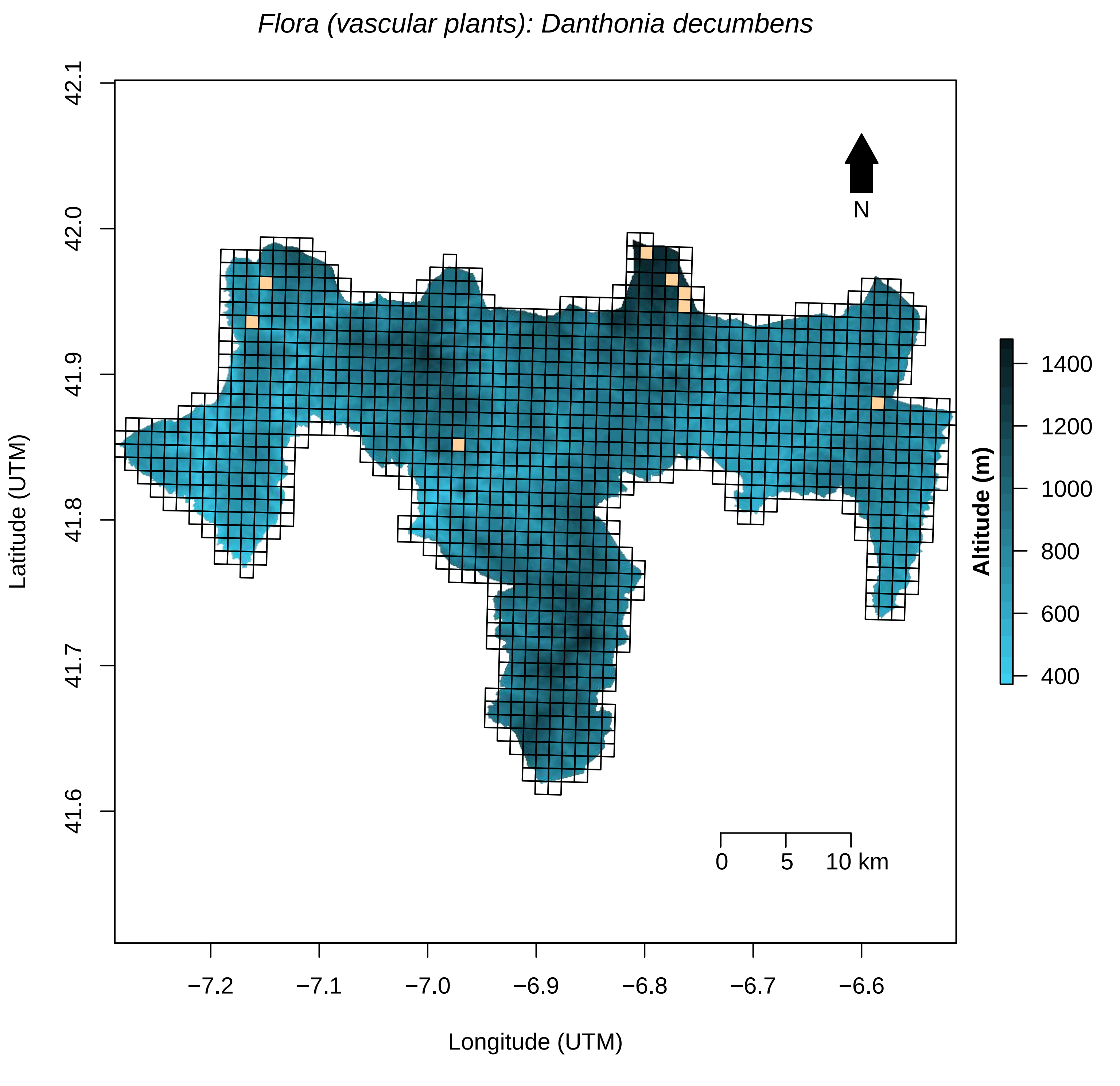

Flora (vascular plants): Danthonia decumbens
42.1
42.0
41.9
41.8
41.7
41.6
Latitude (UTM)
−7.2
−7.1
−7.0
−6.9
−6.8
−6.7
−6.6
Longitude (UTM)
N
0
5
10 km
Altitude (m)
1400
1200
1000
800
600
400

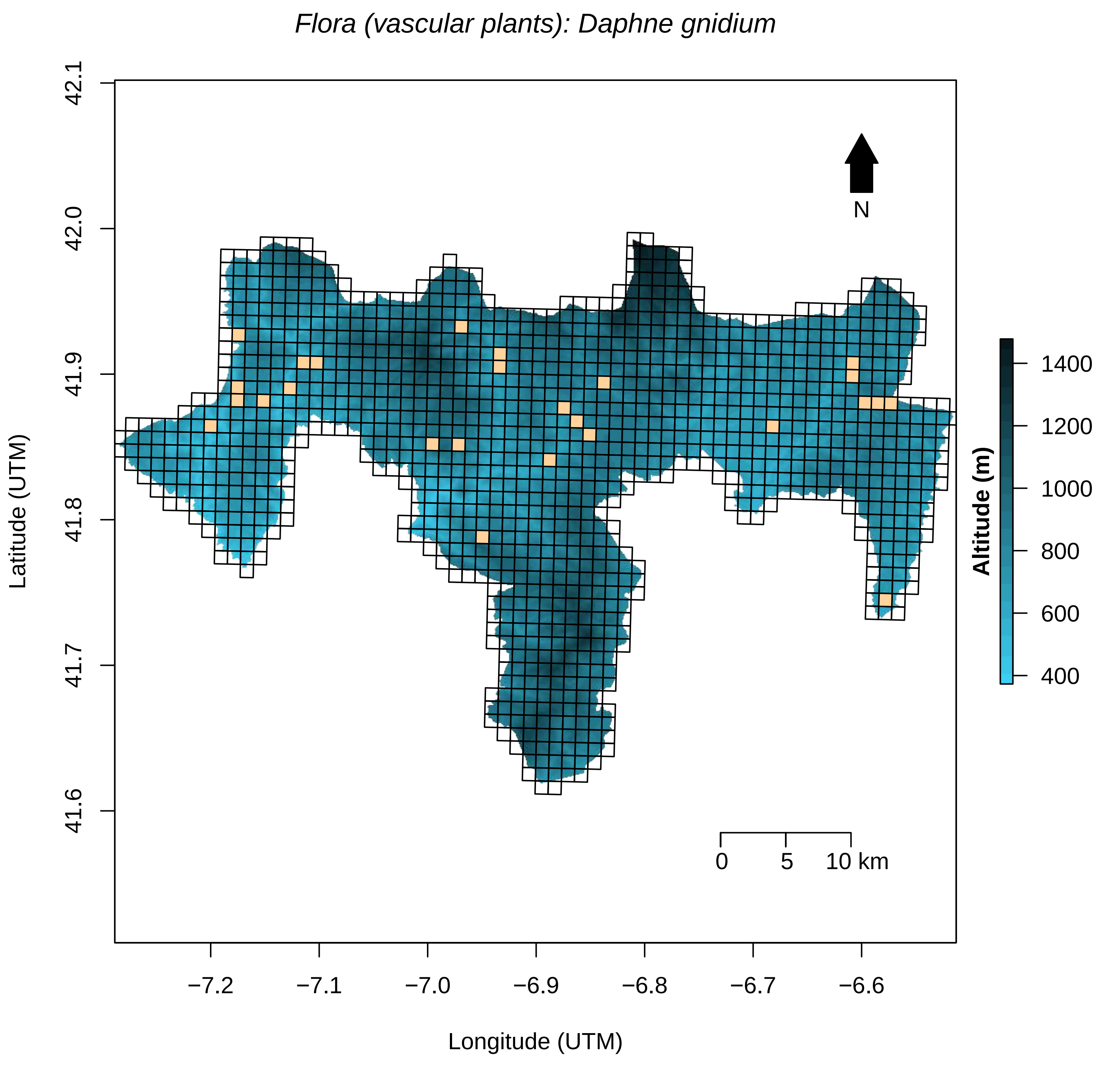

Flora (vascular plants): Daphne gnidium
N
0
5
10 km
Altitude (m)
1400
1200
1000
800
600
400
Latitude (UTM)
42.1
42.0
41.9
41.8
41.7
41.6
Longitude (UTM)
−7.2
−7.1
−7.0
−6.9
−6.8
−6.7
−6.6

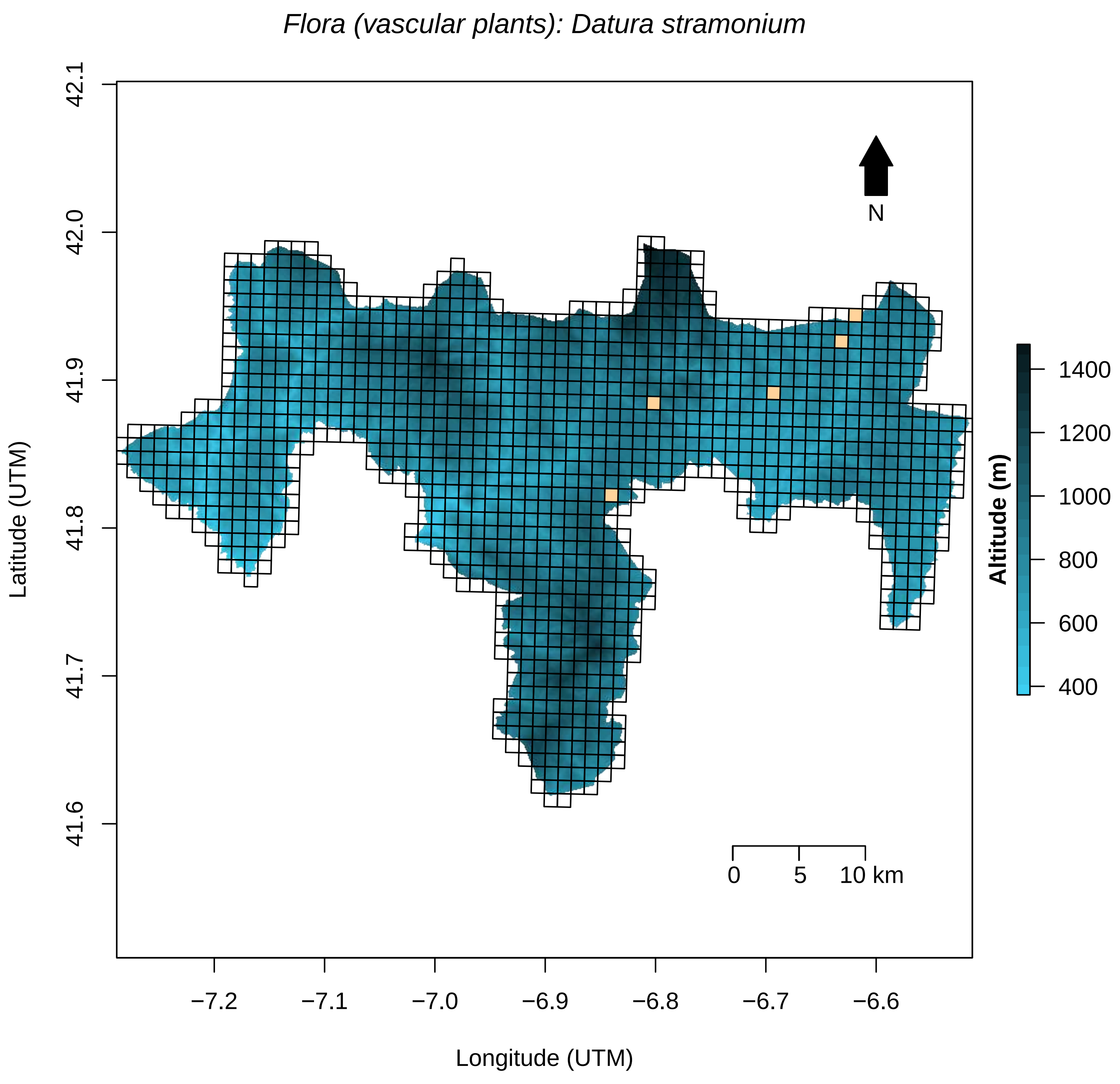

Flora (vascular plants): Datura stramonium
42.1
42.0
41.9
41.8
41.7
41.6
Latitude (UTM)
N
0
5
10 km
−7.2
−7.1
−7.0
−6.9
−6.8
−6.7
−6.6
Longitude (UTM)
Altitude (m)
1400
1200
1000
800
600
400

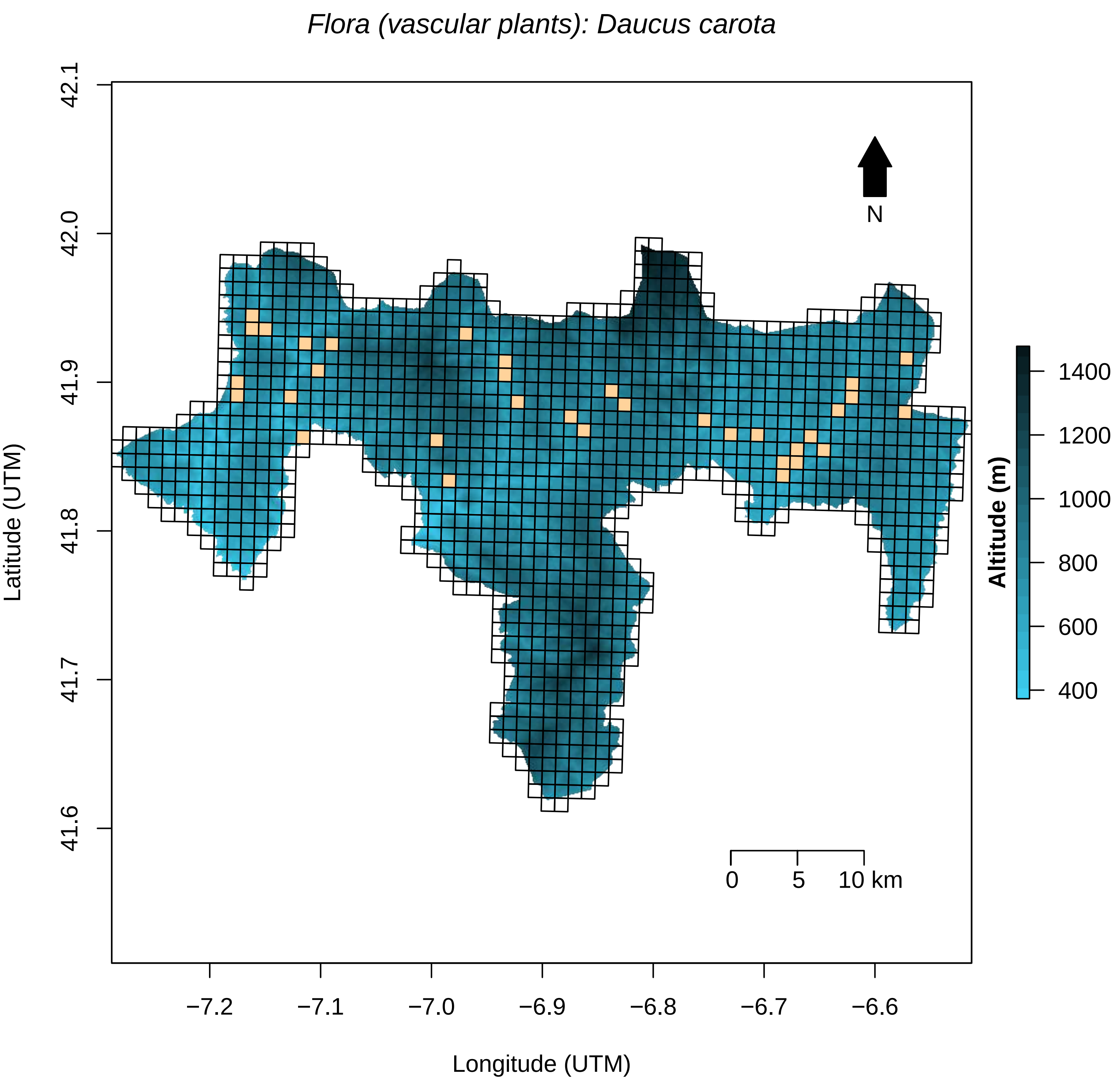
Flora (vascular plants): Daucus carota
42.1
42.0
41.9
41.8
41.7
41.6
Latitude (UTM)
−7.2
−7.1
−7.0
−6.9
−6.8
−6.7
−6.6
Longitude (UTM)
N
0
5
10 km
Altitude (m)
1400
1200
1000
800
600
400

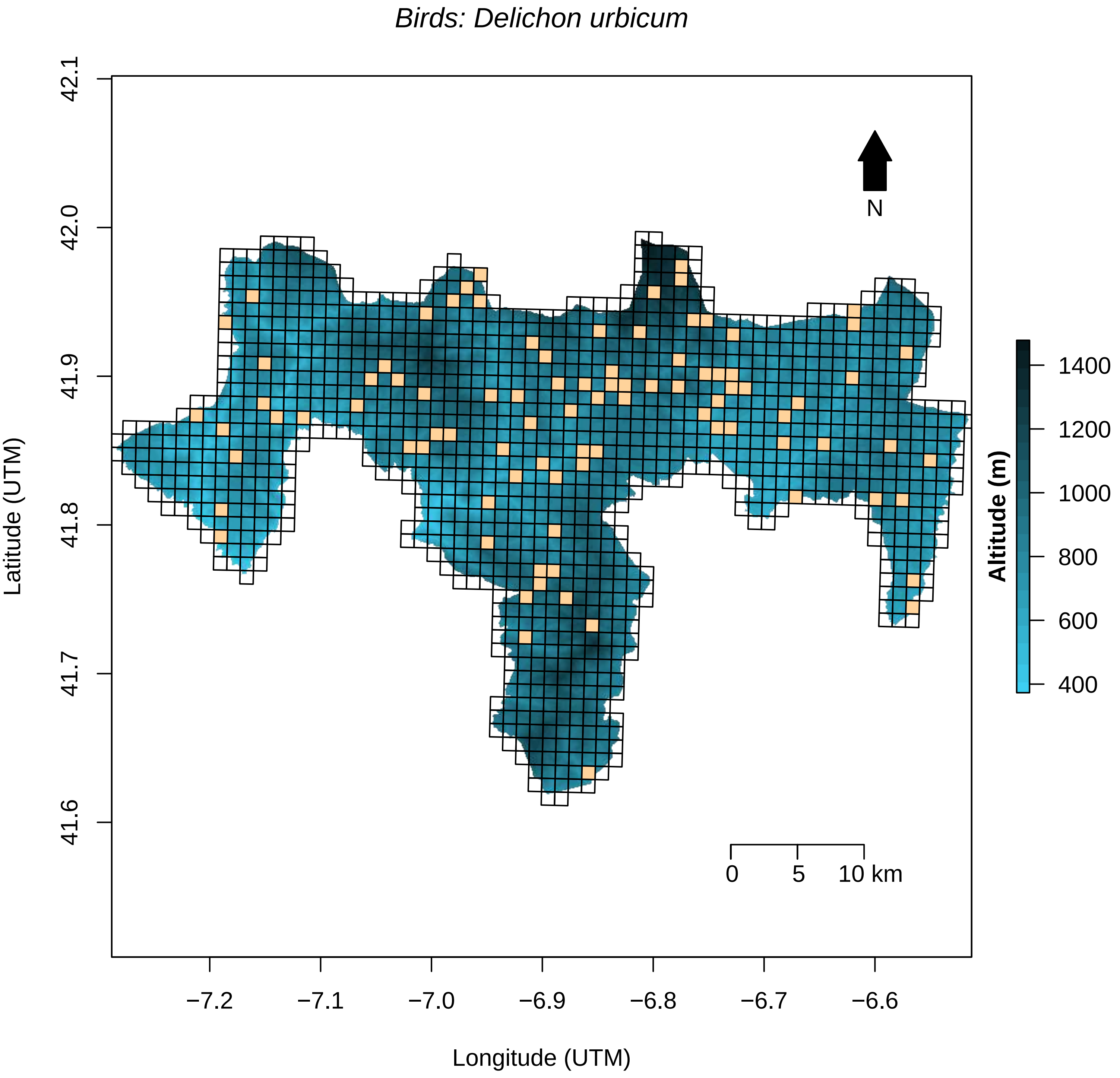
Birds: Delichon urbicum
42.1
42.0
41.9
41.8
41.7
41.6
Latitude (UTM)
−7.2
−7.1
−7.0
−6.9
−6.8
−6.7
−6.6
Longitude (UTM)
N
0
5
10 km
1400
1200
1000
800
600
400
Altitude (m)

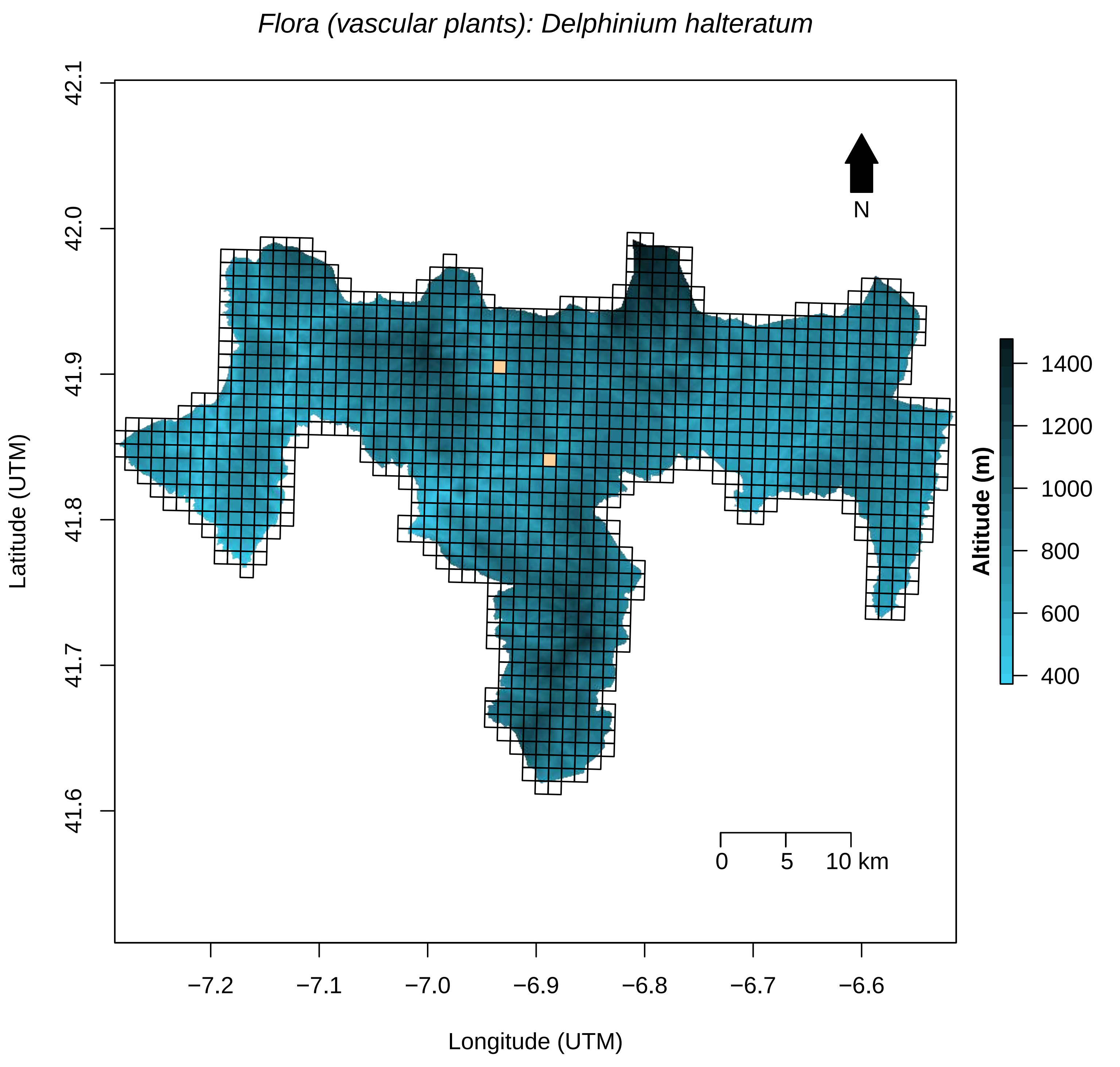
Flora (vascular plants): Delphinium halterratum
N
0
5
10 km
42.1
42.0
41.9
41.8
41.7
41.6
Latitude (UTM)
−7.2
−7.1
−7.0
−6.9
−6.8
−6.7
−6.6
Longitude (UTM)
Altitude (m)
1400
1200
1000
800
600
400

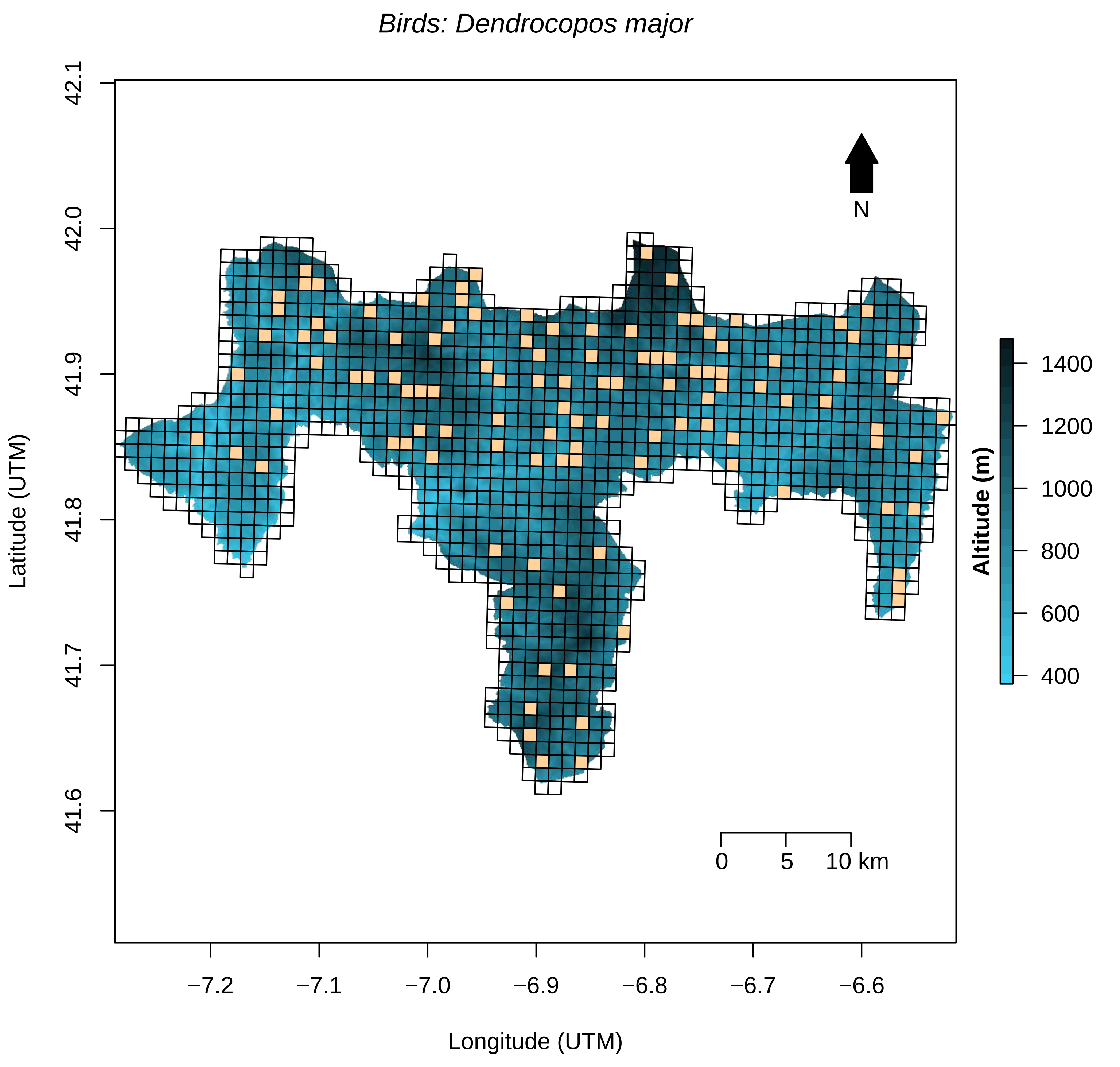
Birds: Dendrocopos major
Latitude (UTM)
42.1
42.0
41.9
41.8
41.7
41.6
Longitude (UTM)
−7.2
−7.1
−7.0
−6.9
−6.8
−6.7
−6.6
N
0
5
10 km
Altitude (m)
1400
1200
1000
800
600
400

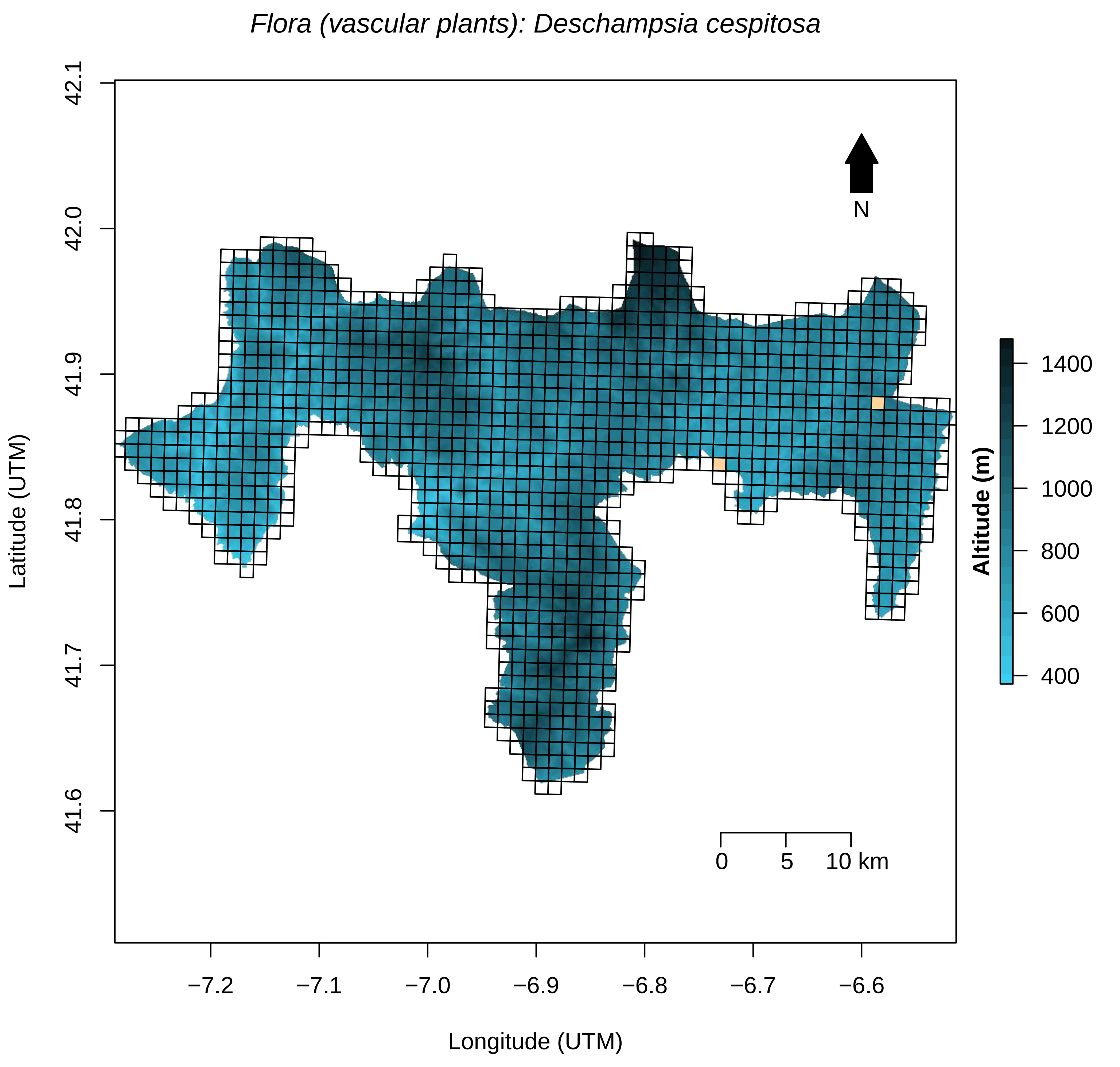

Flora (vascular plants): Deschampsia cespitosa
42.1
42.0
41.9
41.8
41.7
41.6
Latitude (UTM)
−7.2
−7.1
−7.0
−6.9
−6.8
−6.7
−6.6
Longitude (UTM)
N
0
5
10 km
Altitude (m)
1400
1200
1000
800
600
400

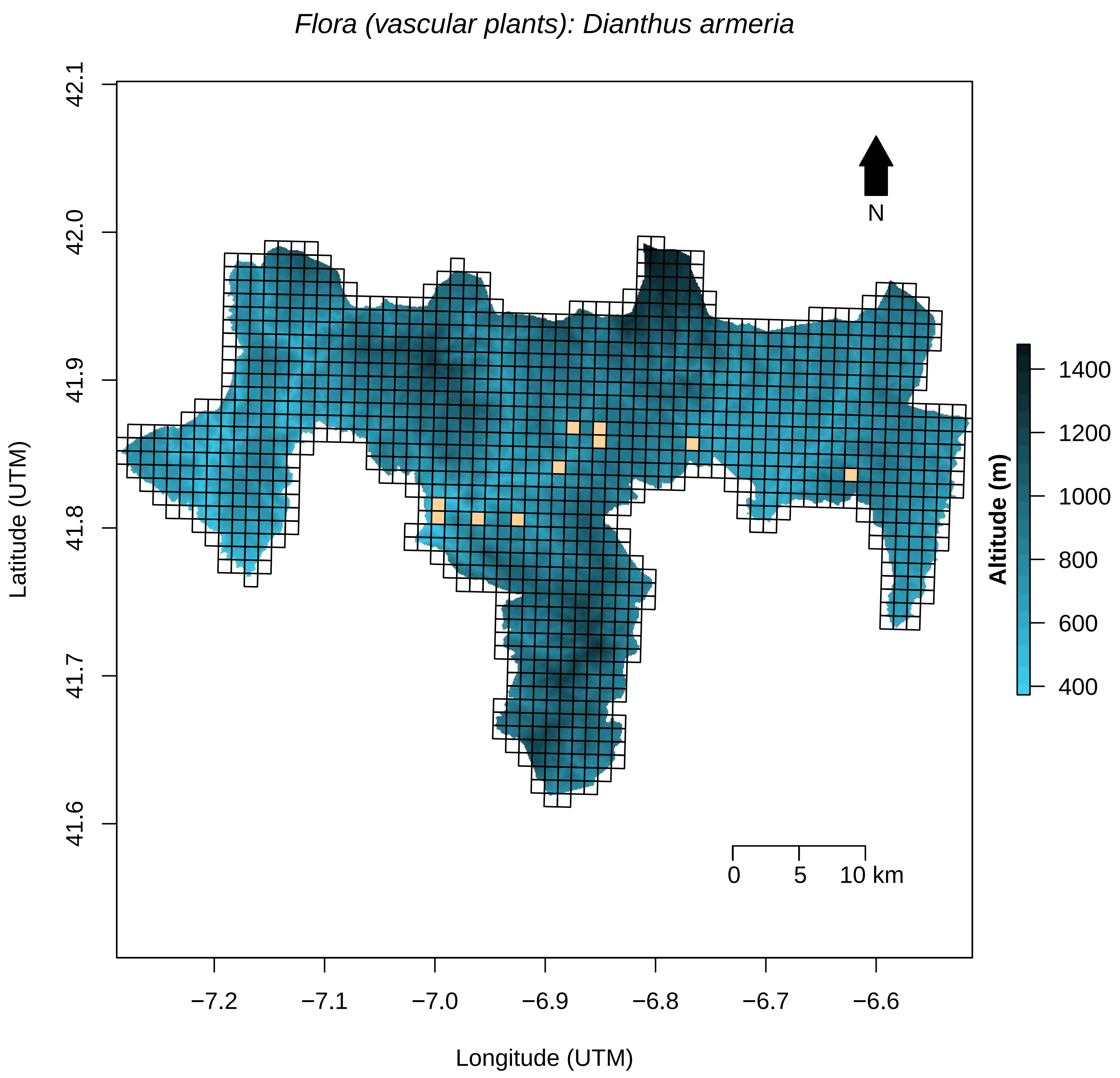

Flora (vascular plants): Dianthus armeria
42.1
42.0
41.9
41.8
41.7
41.6
Latitude (UTM)
−7.2
−7.1
−7.0
−6.9
−6.8
−6.7
−6.6
Longitude (UTM)
N
0
5
10 km
Altitude (m)
1400
1200
1000
800
600
400

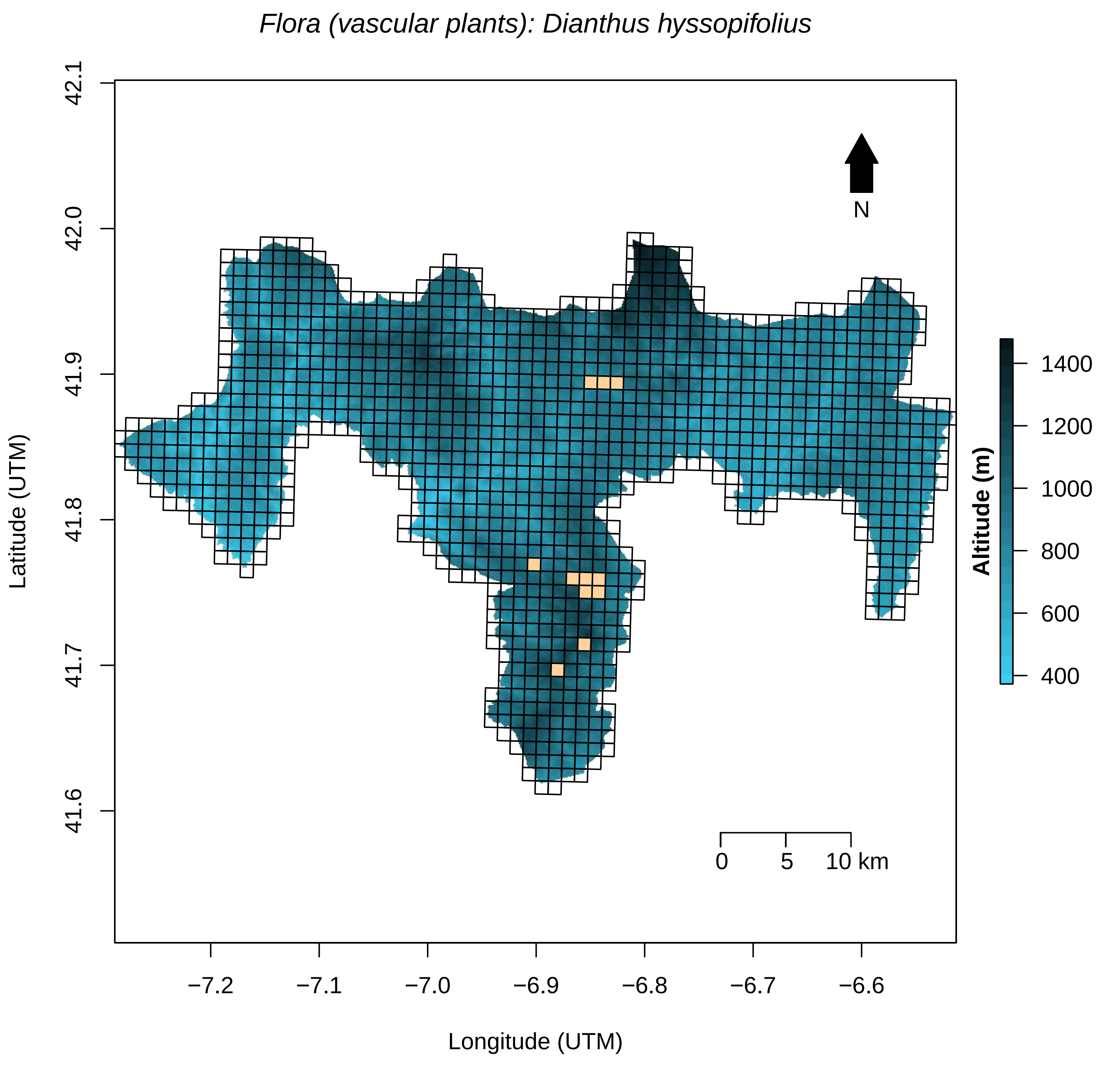

Flora (vascular plants): Dianthus hyssopifolius
Latitude (UTM)
42.1
42.0
41.9
41.8
41.7
41.6
Longitude (UTM)
−7.2
−7.1
−7.0
−6.9
−6.8
−6.7
−6.6
N
0
5
10 km
Altitude (m)
1400
1200
1000
800
600
400

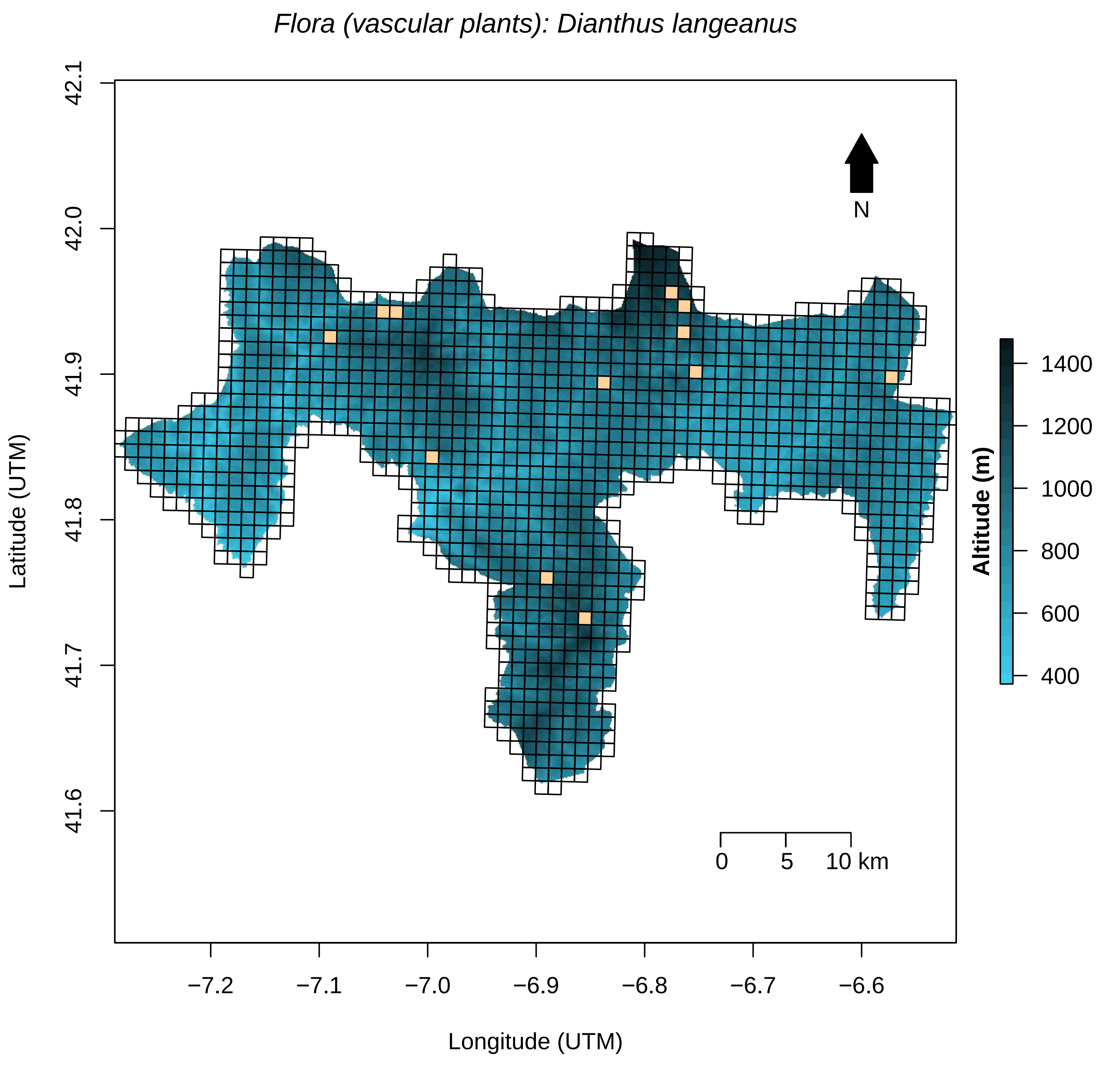

Flora (vascular plants): Dianthus langeanus
Latitude (UTM)
Longitude (UTM)
42.1
42.0
41.9
41.8
41.7
41.6
−7.2
−7.1
−7.0
−6.9
−6.8
−6.7
−6.6
N
0
5
10 km
Altitude (m)
1400
1200
1000
800
600
400

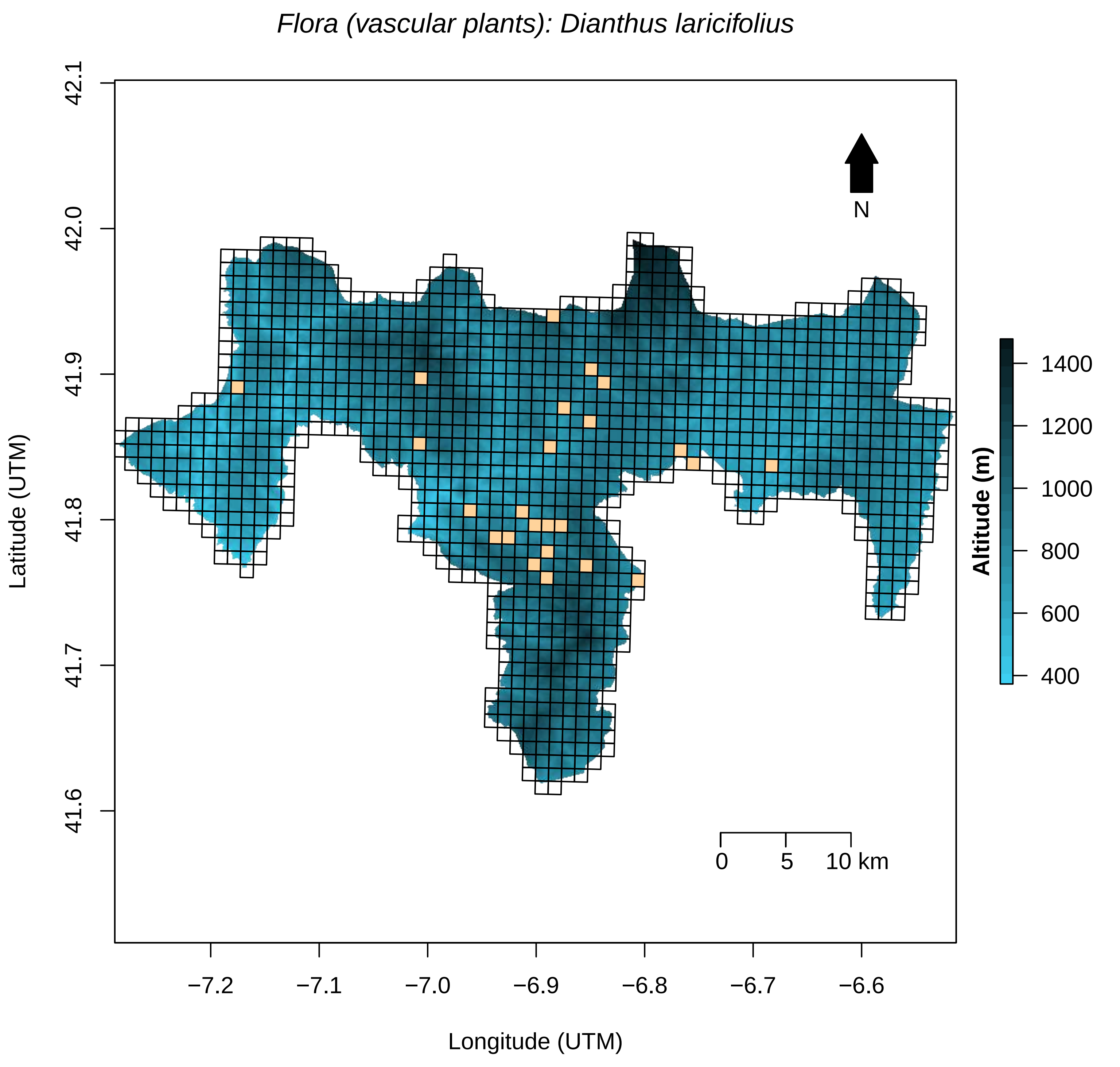

Flora (vascular plants): Dianthus laricifolius
Latitude (UTM)
42.1
42.0
41.9
41.8
41.7
41.6
Longitude (UTM)
−7.2
−7.1
−7.0
−6.9
−6.8
−6.7
−6.6
N
0
5
10 km
Altitude (m)
1400
1200
1000
800
600
400

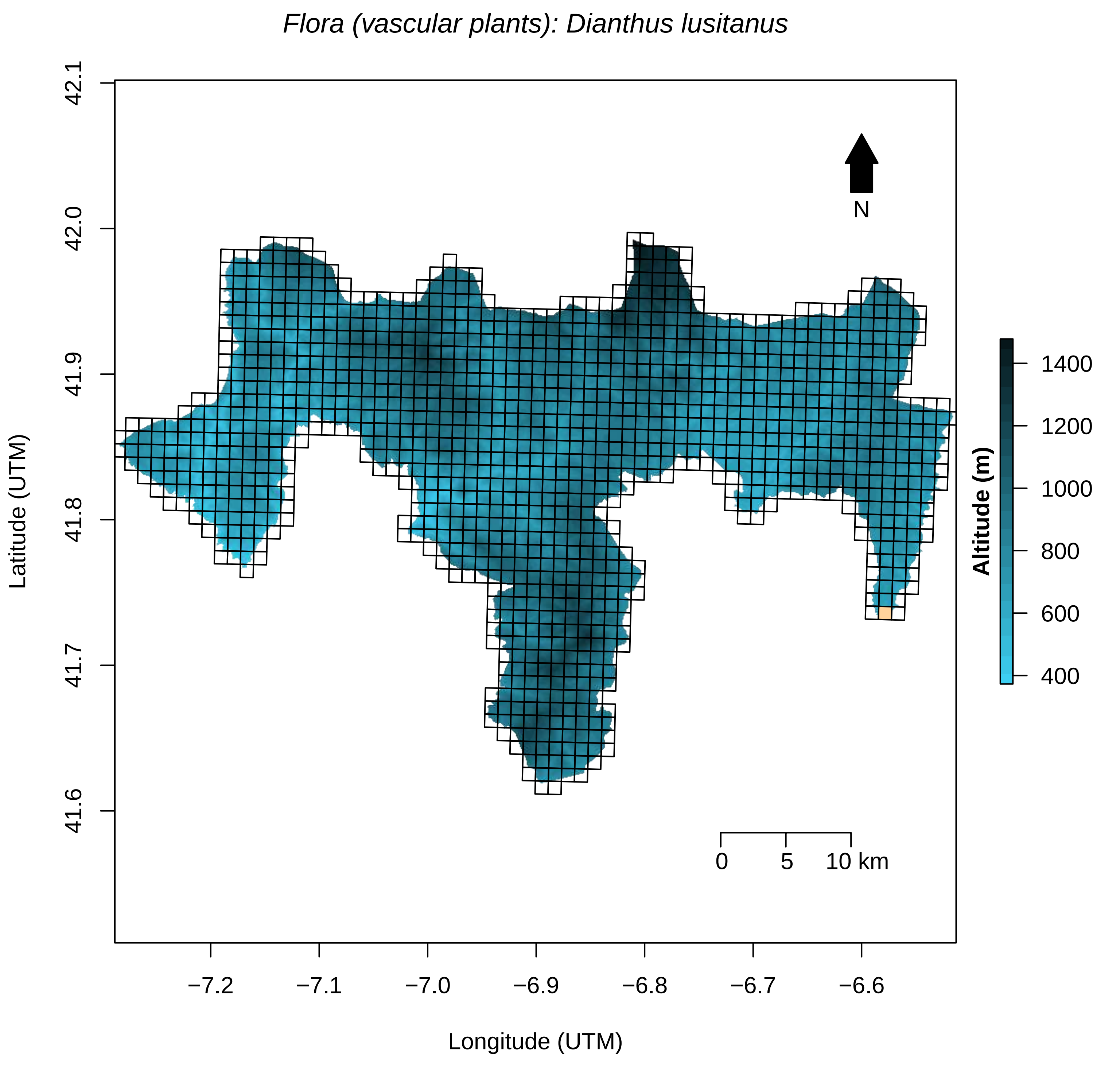

Flora (vascular plants): Dianthus lusitanus
N
0
5
10 km
Altitude (m)
1400
1200
1000
800
600
400
Latitude (UTM)
42.1
42.0
41.9
41.8
41.7
41.6
Longitude (UTM)
−7.2
−7.1
−7.0
−6.9
−6.8
−6.7
−6.6

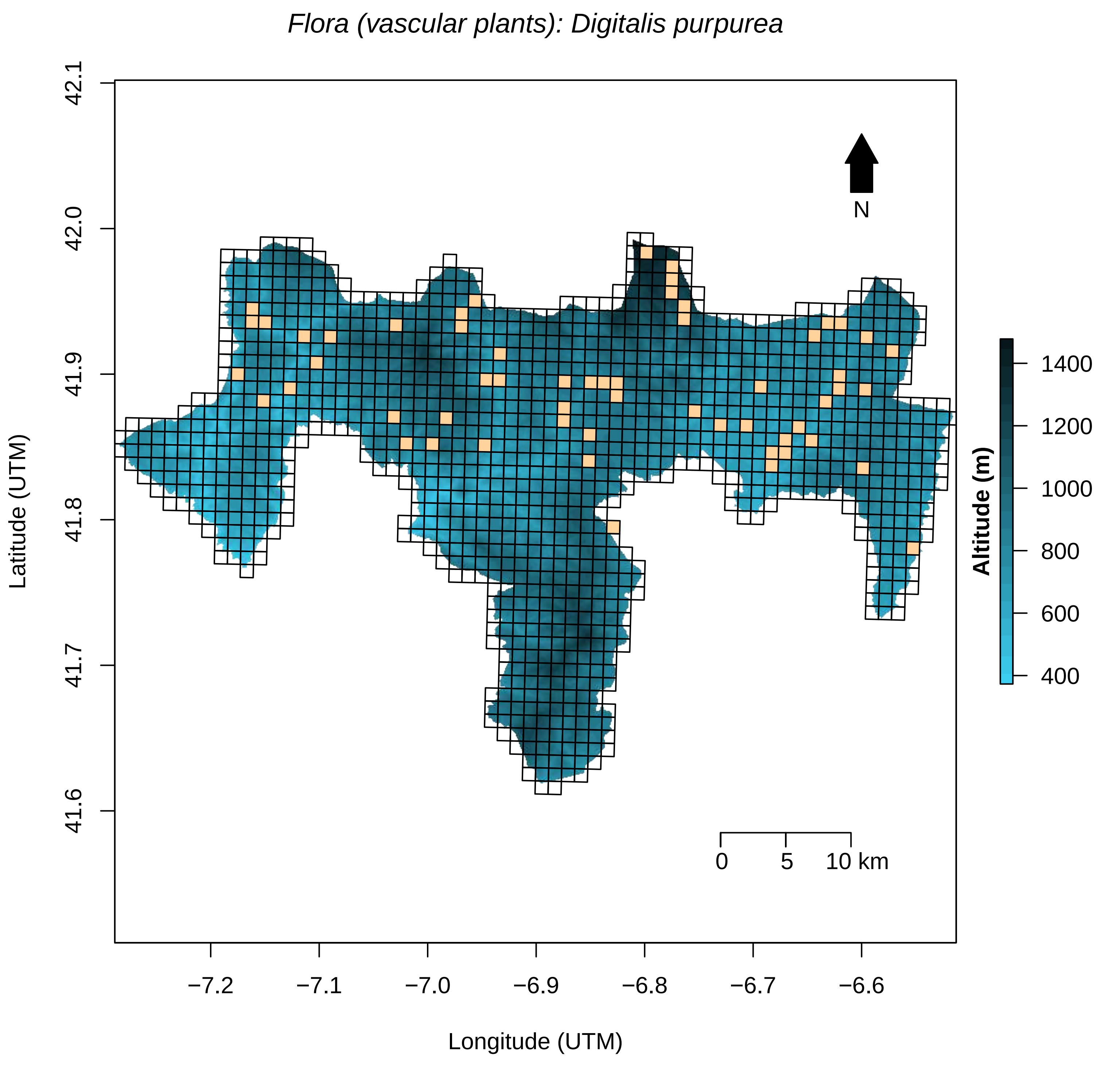

Flora (vascular plants): Digitalis purpurea
42.1
42.0
41.9
41.8
41.7
41.6
Latitude (UTM)
−7.2
−7.1
−7.0
−6.9
−6.8
−6.7
−6.6
Longitude (UTM)
N
0
5
10 km
Altitude (m)
1400
1200
1000
800
600
400

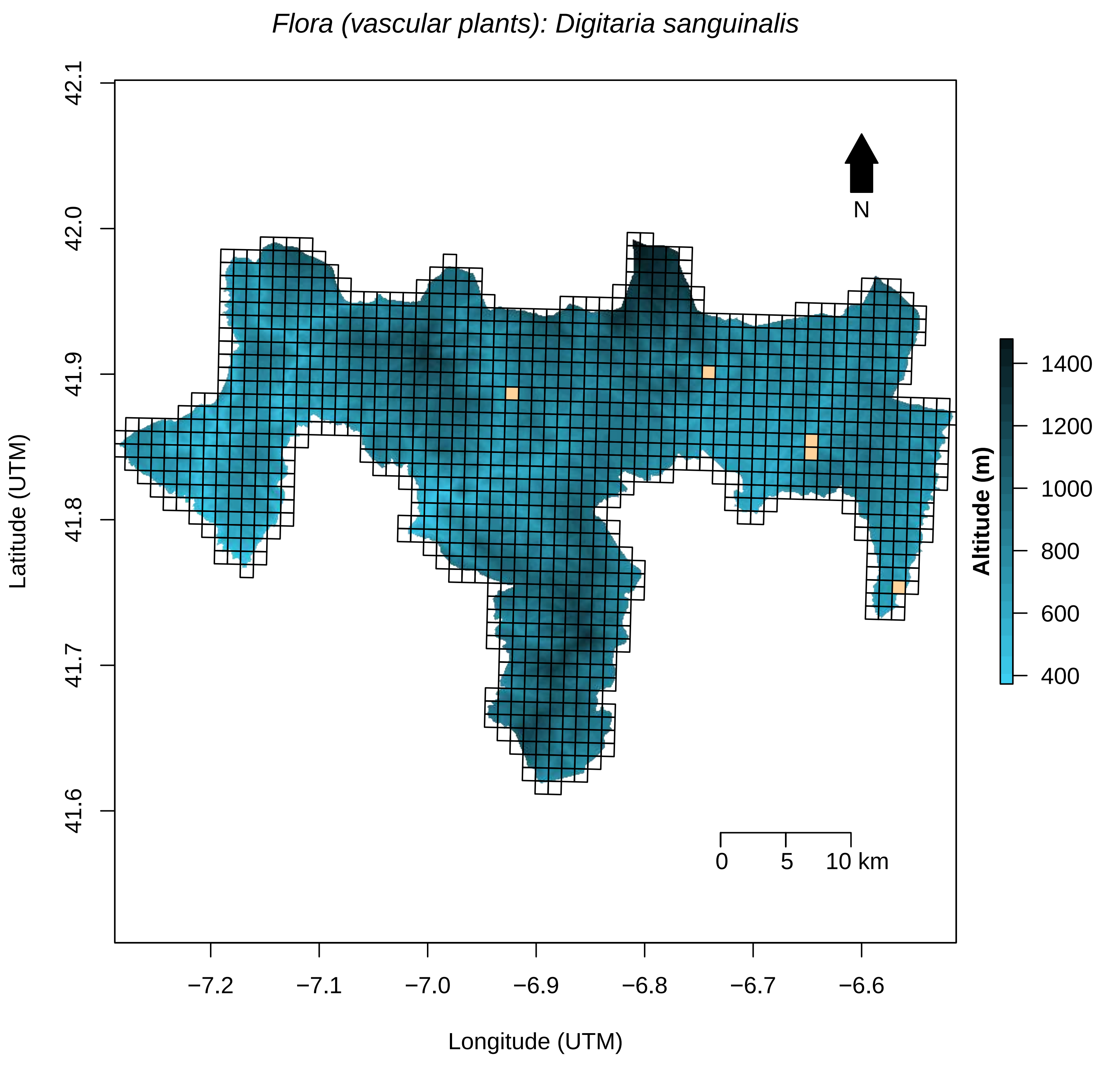

Flora (vascular plants): Digitaria sanguinalis
N
0 5 10 km
Altitude (m)
1400
1200
1000
800
600
400
Latitude (UTM)
42.1
42.0
41.9
41.8
41.7
41.6
Longitude (UTM)
−7.2
−7.1
−7.0
−6.9
−6.8
−6.7
−6.6

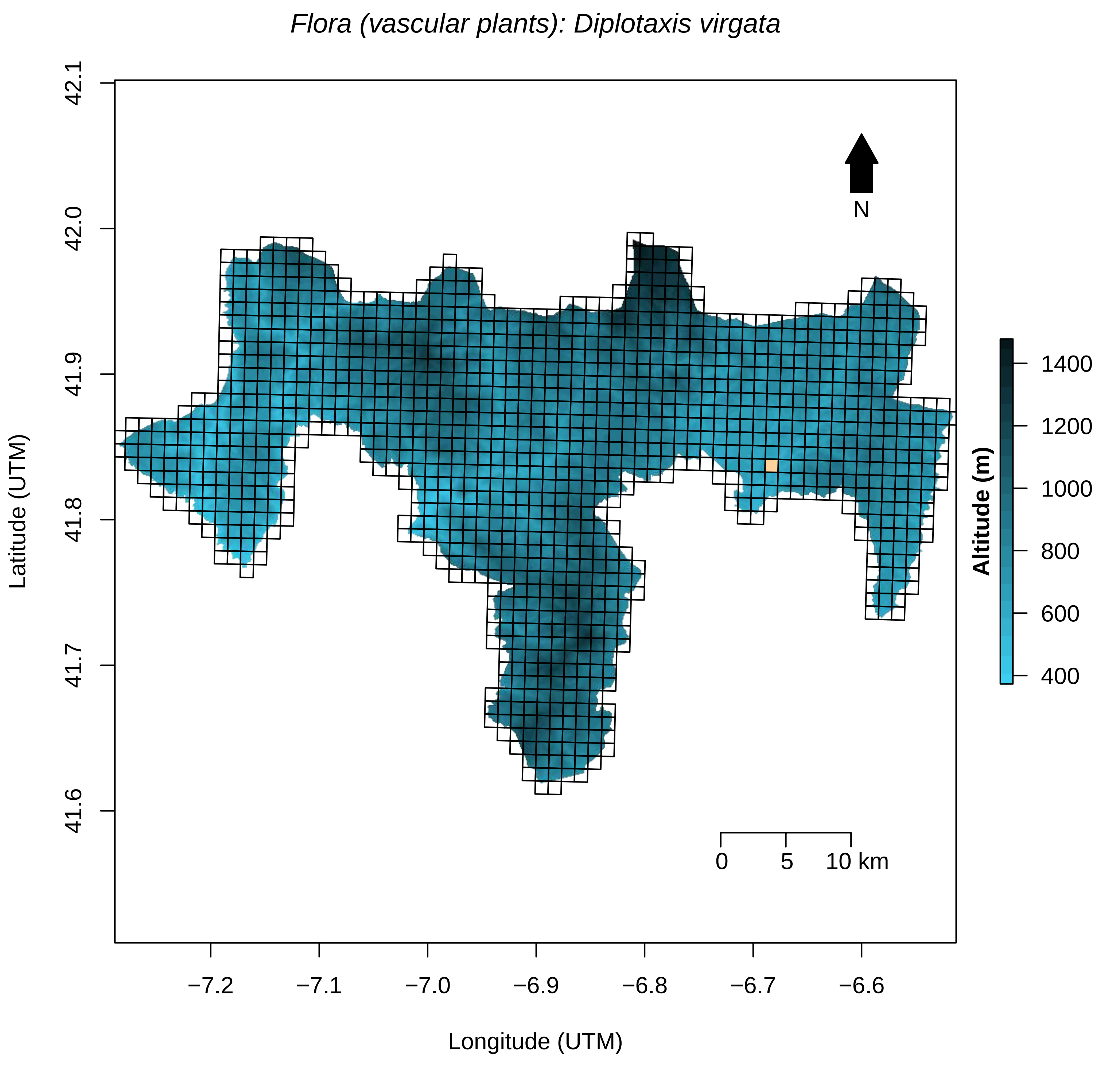
Flora (vascular plants): Diplotaxis virgata
Latitude (UTM)
42.1
42.0
41.9
41.8
41.7
41.6
Longitude (UTM)
−7.2
−7.1
−7.0
−6.9
−6.8
−6.7
−6.6
N
0
5
10 km
Altitude (m)
1400
1200
1000
800
600
400

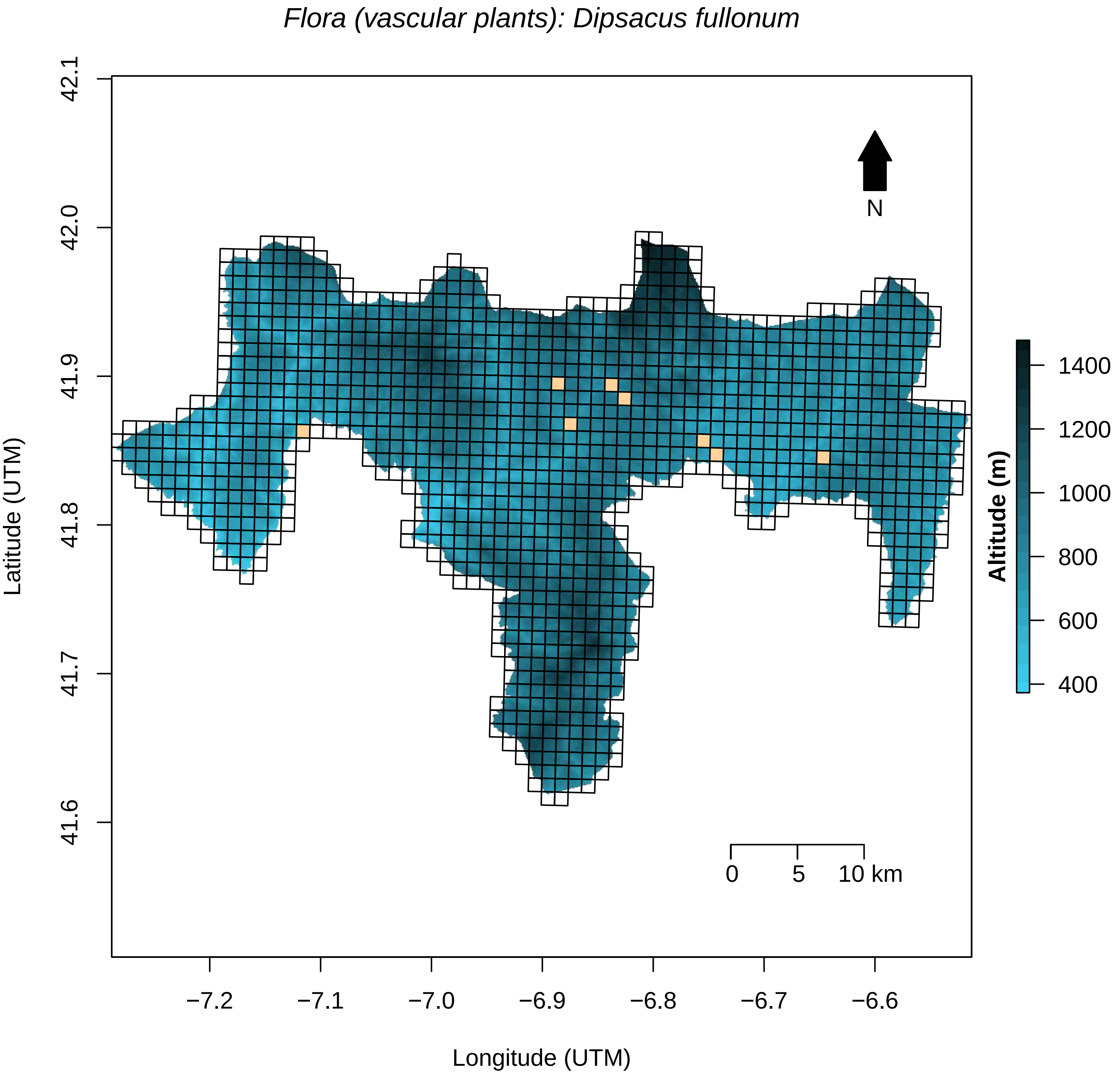
Flora (vascular plants): Dipsacus fullonum
42.1
42.0
41.9
41.8
41.7
41.6
Latitude (UTM)
−7.2
−7.1
−7.0
−6.9
−6.8
−6.7
−6.6
Longitude (UTM)
N
0
5
10 km
Altitude (m)
1400
1200
1000
800
600
400

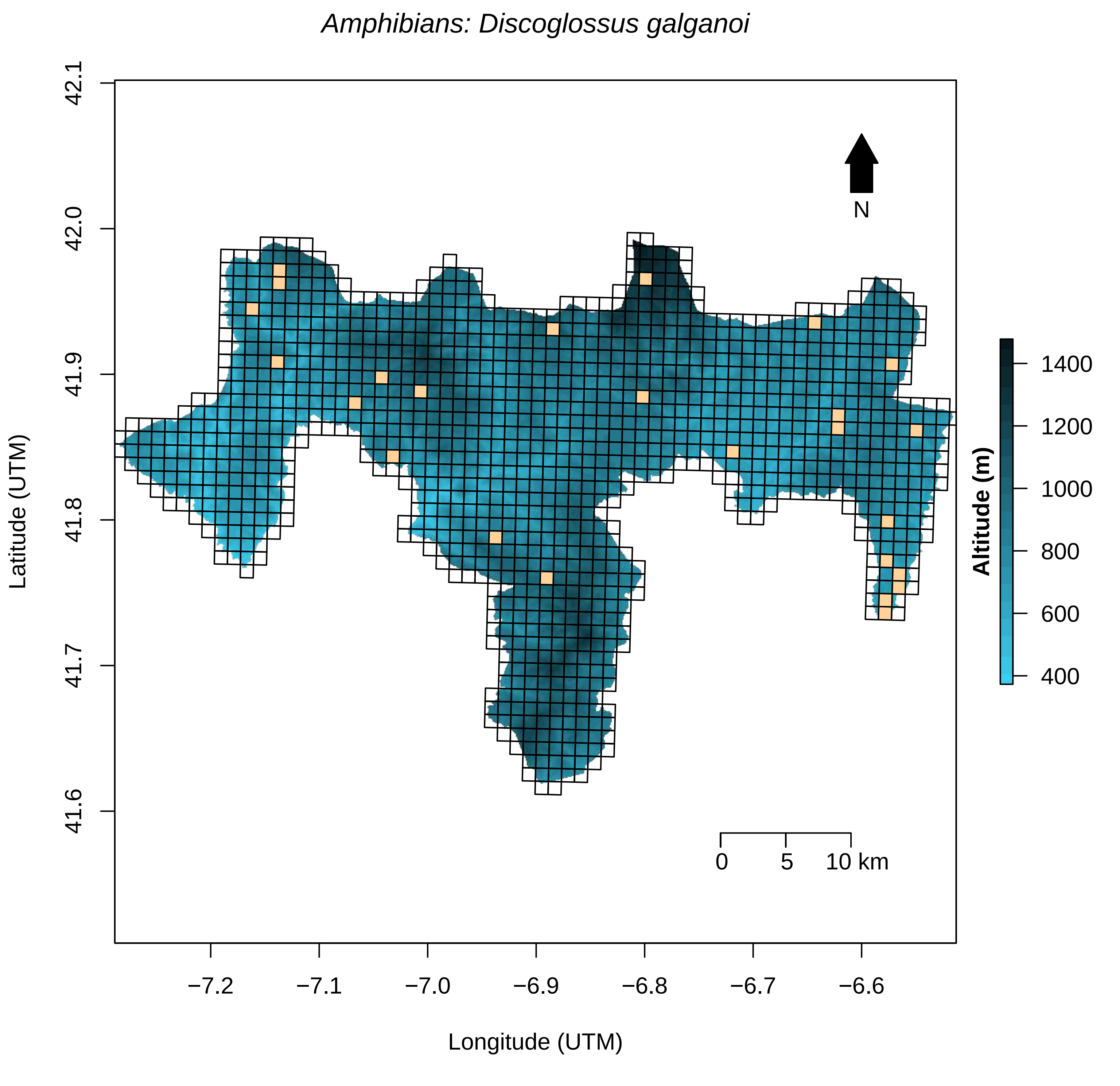

Amphibians: Discoglossus galganoi
42.1
42.0
41.9
41.8
41.7
41.6
Latitude (UTM)
N
0
5
10 km
−7.2
−7.1
−7.0
−6.9
−6.8
−6.7
−6.6
Longitude (UTM)
1400
1200
1000
800
600
400
Altitude (m)

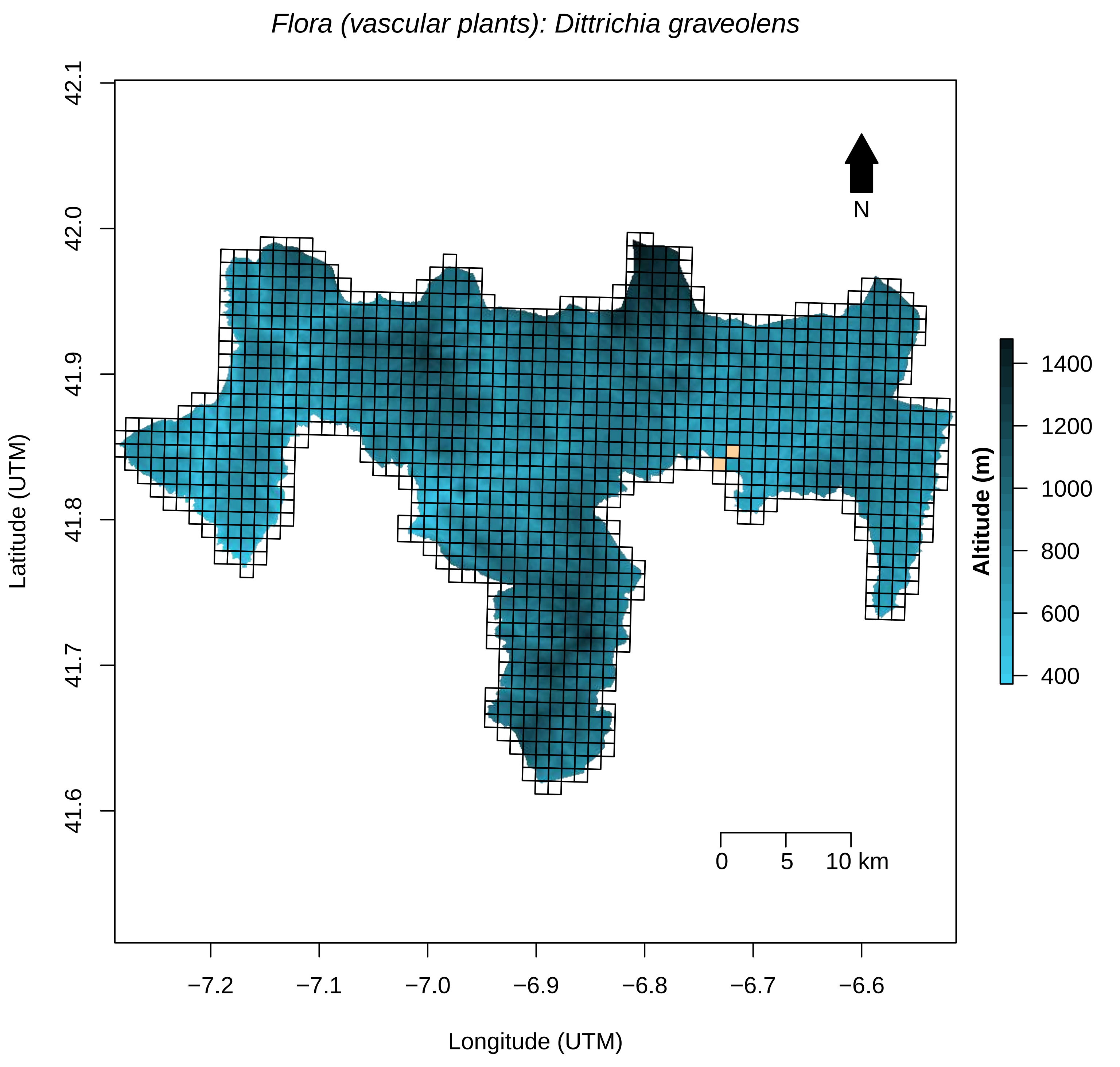

Flora (vascular plants): Dittrichia graveolens
N
0
5
10 km
Altitude (m)
1400
1200
1000
800
600
400
42.1
42.0
41.9
41.8
41.7
41.6
Latitude (UTM)
−7.2
−7.1
−7.0
−6.9
−6.8
−6.7
−6.6
Longitude (UTM)

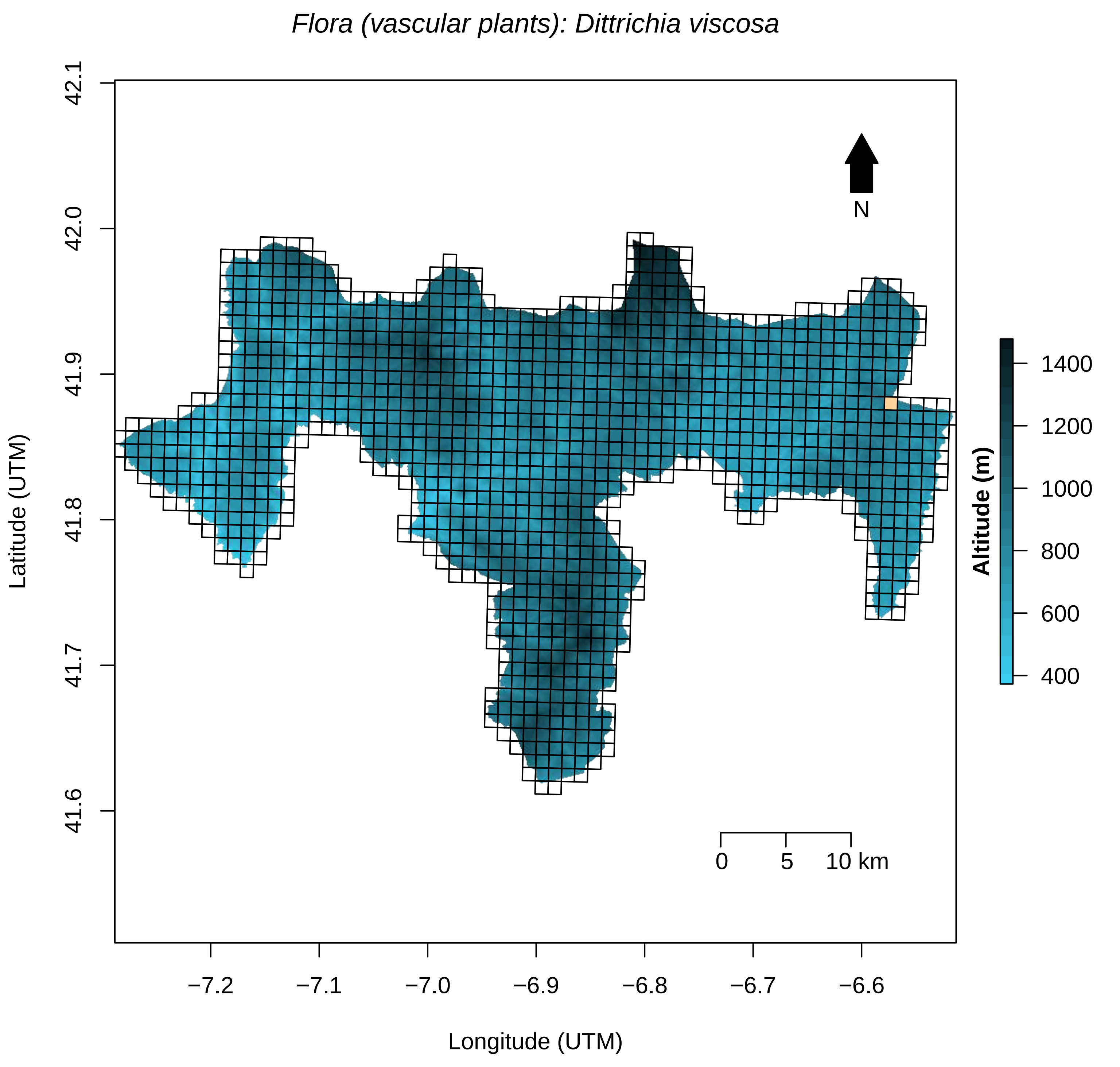
Flora (vascular plants): Dittrichia viscosa
42.1
42.0
41.9
41.8
41.7
41.6
Latitude (UTM)
−7.2
−7.1
−7.0
−6.9
−6.8
−6.7
−6.6
Longitude (UTM)
N
0
5
10 km
1400
1200
1000
800
600
400
Altitude (m)

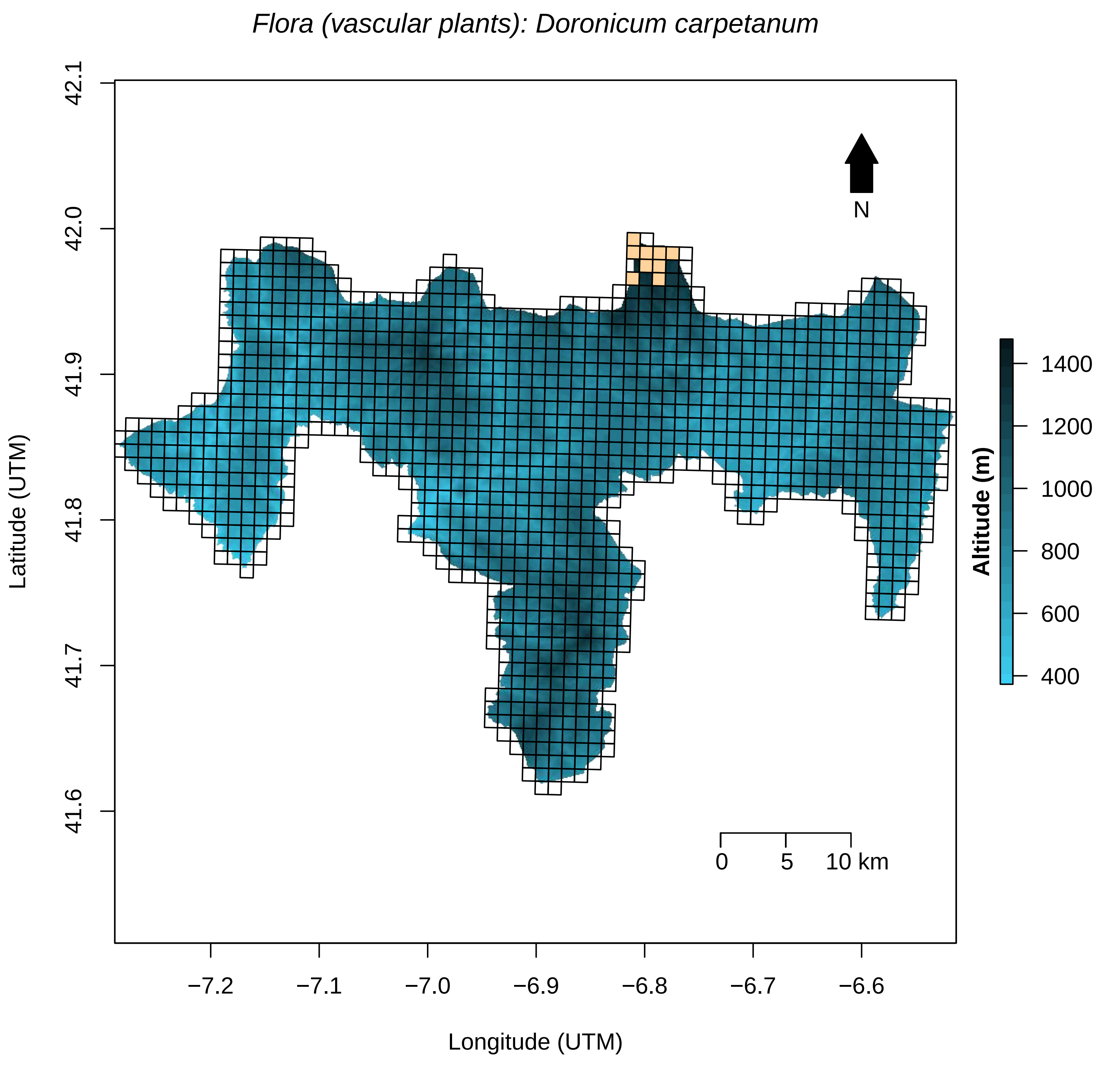

Flora (vascular plants): Doronicum carpetanum
42.1
42.0
41.9
41.8
41.7
41.6
Latitude (UTM)
−7.2
−7.1
−7.0
−6.9
−6.8
−6.7
−6.6
Longitude (UTM)
N
0
5
10 km
1400
1200
1000
800
600
400
Altitude (m)

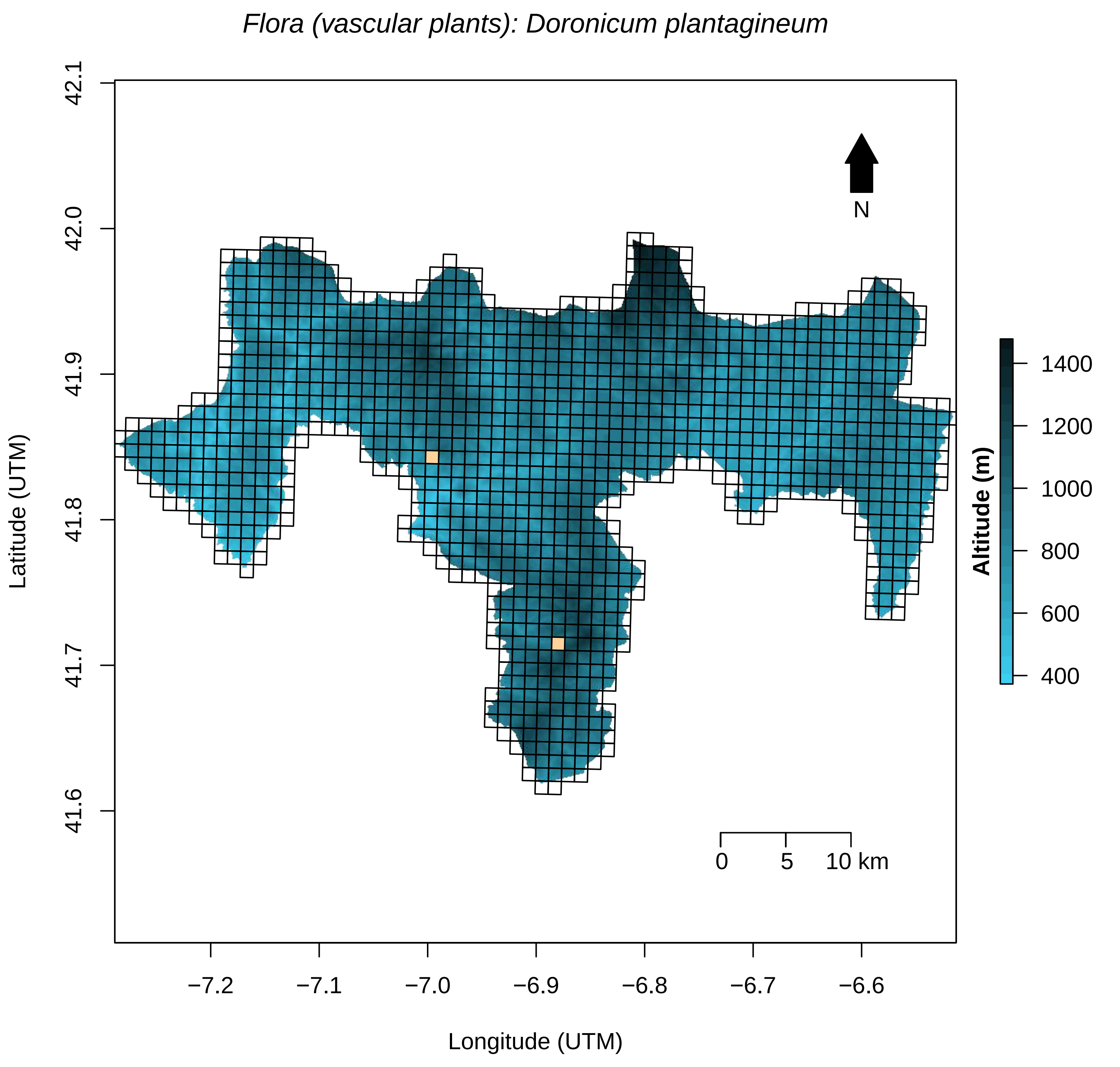
Flora (vascular plants): Doronicum plantagineum
42.1
42.0
41.9
41.8
41.7
41.6
Latitude (UTM)
−7.2
−7.1
−7.0
−6.9
−6.8
−6.7
−6.6
Longitude (UTM)
N
0
5
10 km
Altitude (m)
1400
1200
1000
800
600
400

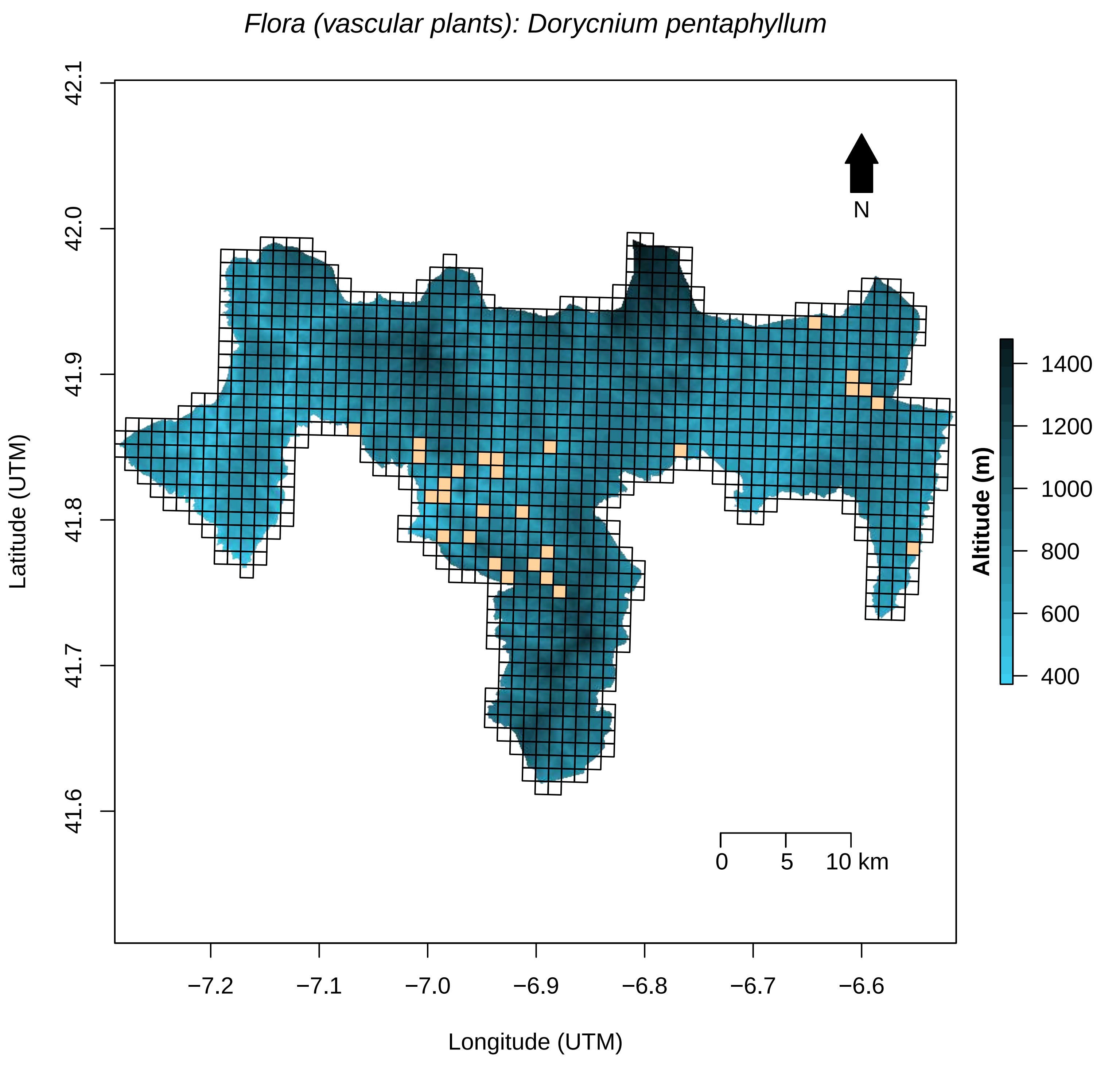

Flora (vascular plants): Dorycnium pentaphyllum
42.1
42.0
41.9
41.8
41.7
41.6
Latitude (UTM)
−7.2
−7.1
−7.0
−6.9
−6.8
−6.7
−6.6
Longitude (UTM)
N
0
5
10 km
1400
1200
1000
800
600
400
Altitude (m)

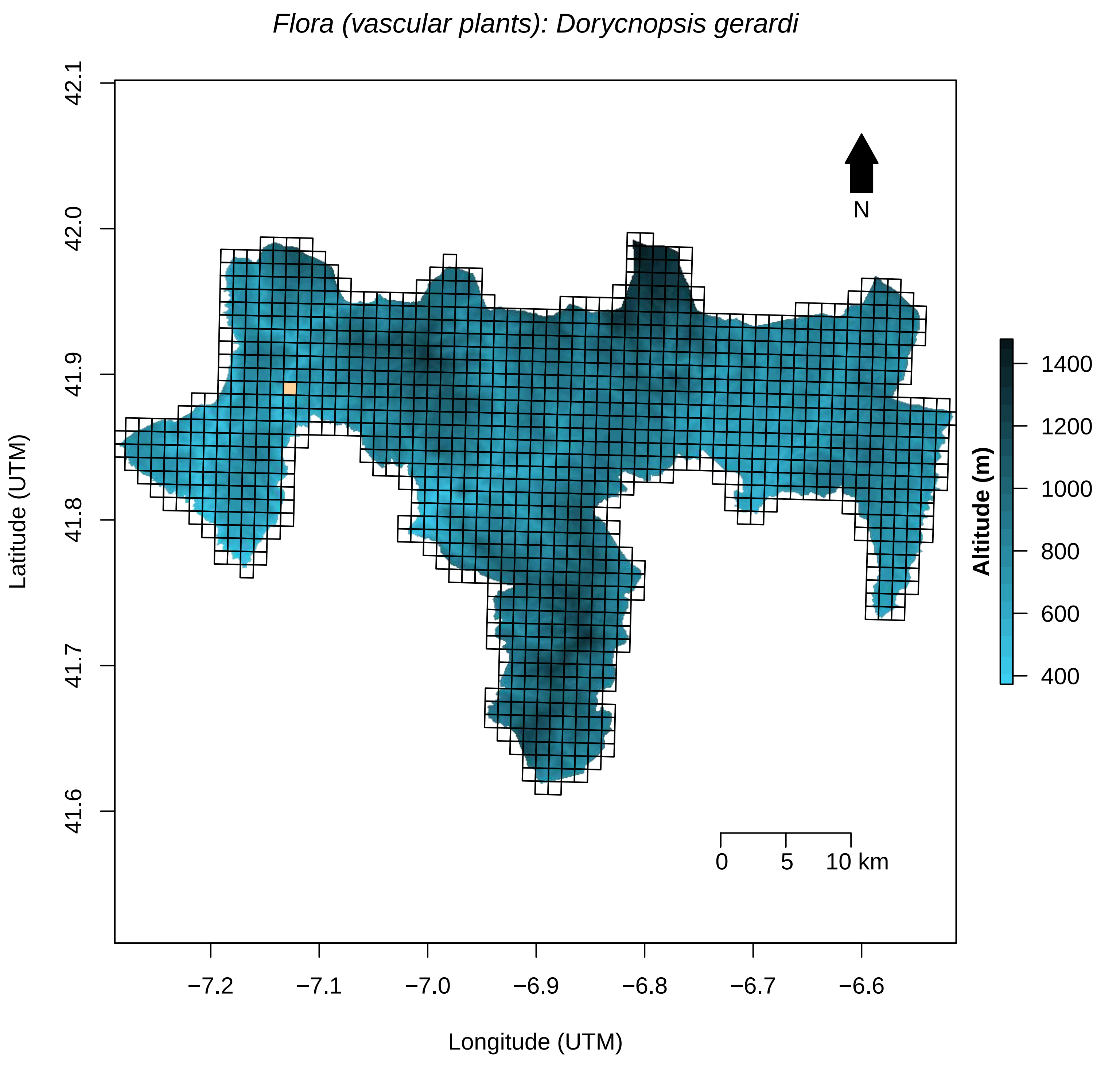

Flora (vascular plants): Dorycnopsis gerardi
42.1
42.0
41.9
41.8
41.7
41.6
Latitude (UTM)
−7.2
−7.1
−7.0
−6.9
−6.8
−6.7
−6.6
Longitude (UTM)
N
0
5
10 km
1400
1200
1000
800
600
400
Altitude (m)

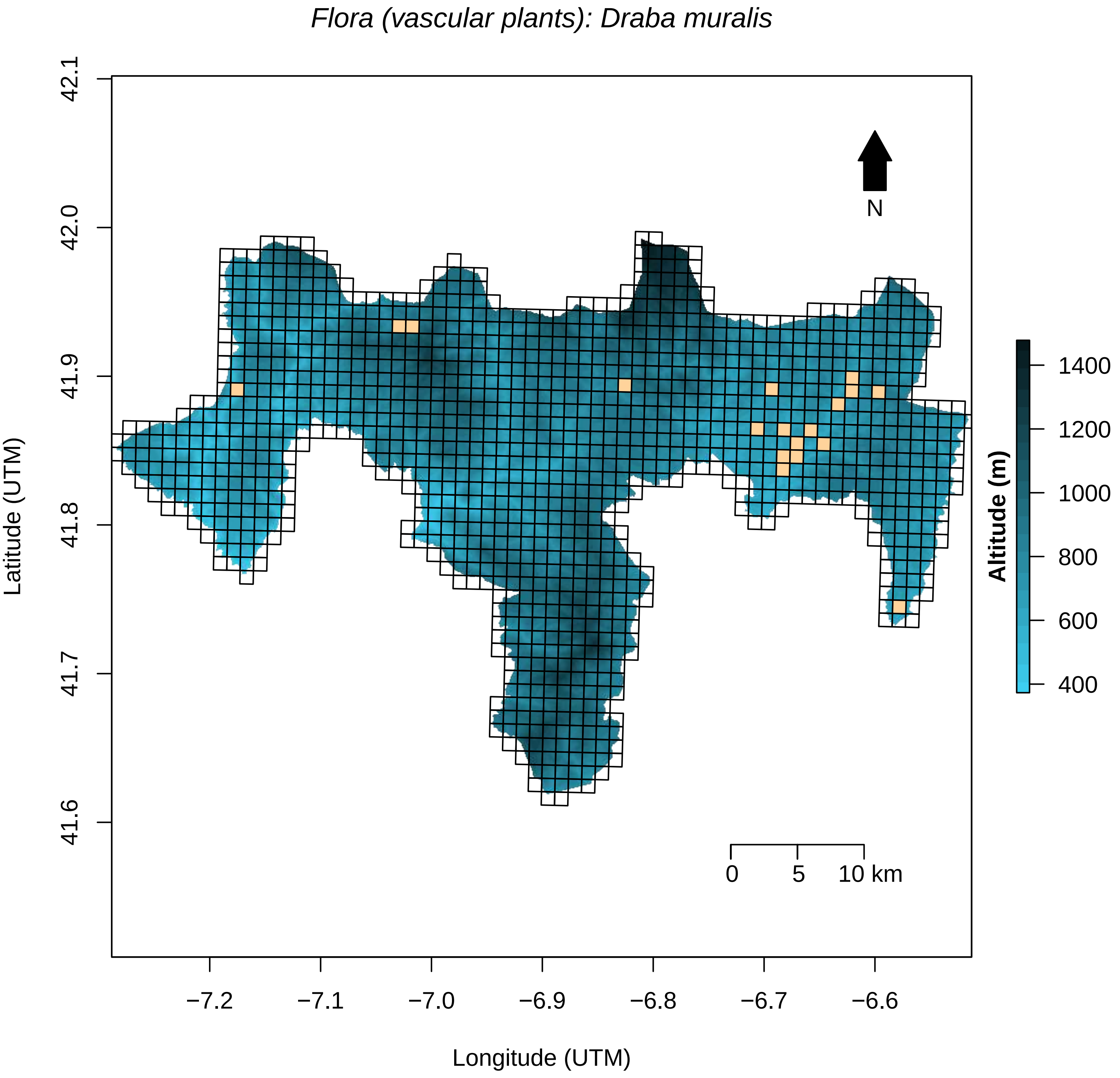

Flora (vascular plants): Draba muralis
42.1
42.0
41.9
41.8
41.7
41.6
Latitude (UTM)
−7.2
−7.1
−7.0
−6.9
−6.8
−6.7
−6.6
Longitude (UTM)
N
0
5
10 km
Altitude (m)
1400
1200
1000
800
600
400

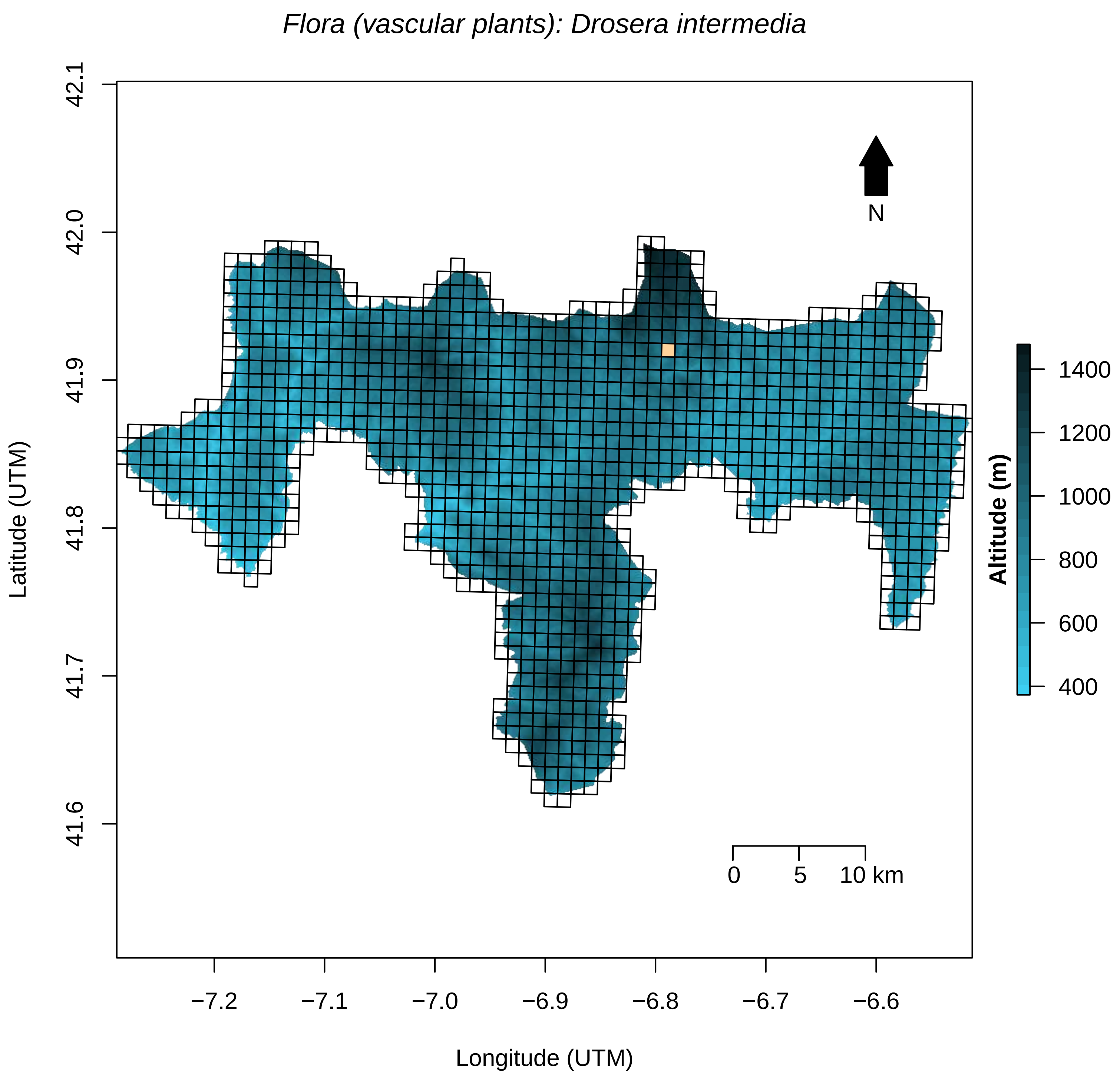
Flora (vascular plants): Drosera intermedia
42.1
42.0
41.9
41.8
41.7
41.6
Latitude (UTM)
−7.2
−7.1
−7.0
−6.9
−6.8
−6.7
−6.6
Longitude (UTM)
N
0
5
10 km
Altitude (m)
1400
1200
1000
800
600
400

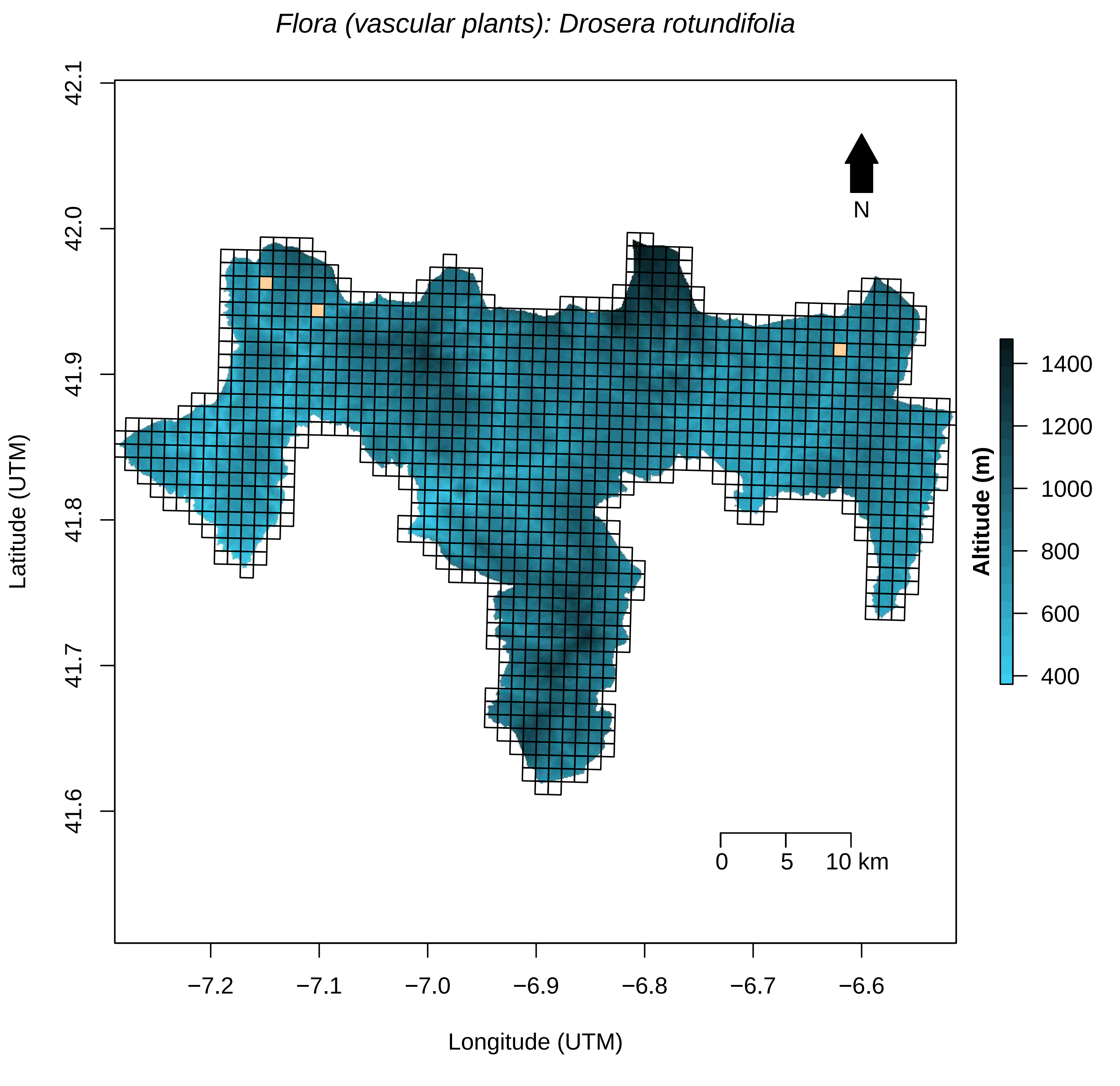

Flora (vascular plants): Drosera rotundifolia
42.1
42.0
41.9
41.8
41.7
41.6
Latitude (UTM)
−7.2
−7.1
−7.0
−6.9
−6.8
−6.7
−6.6
Longitude (UTM)
N
0
5
10 km
Altitude (m)
1400
1200
1000
800
600
400

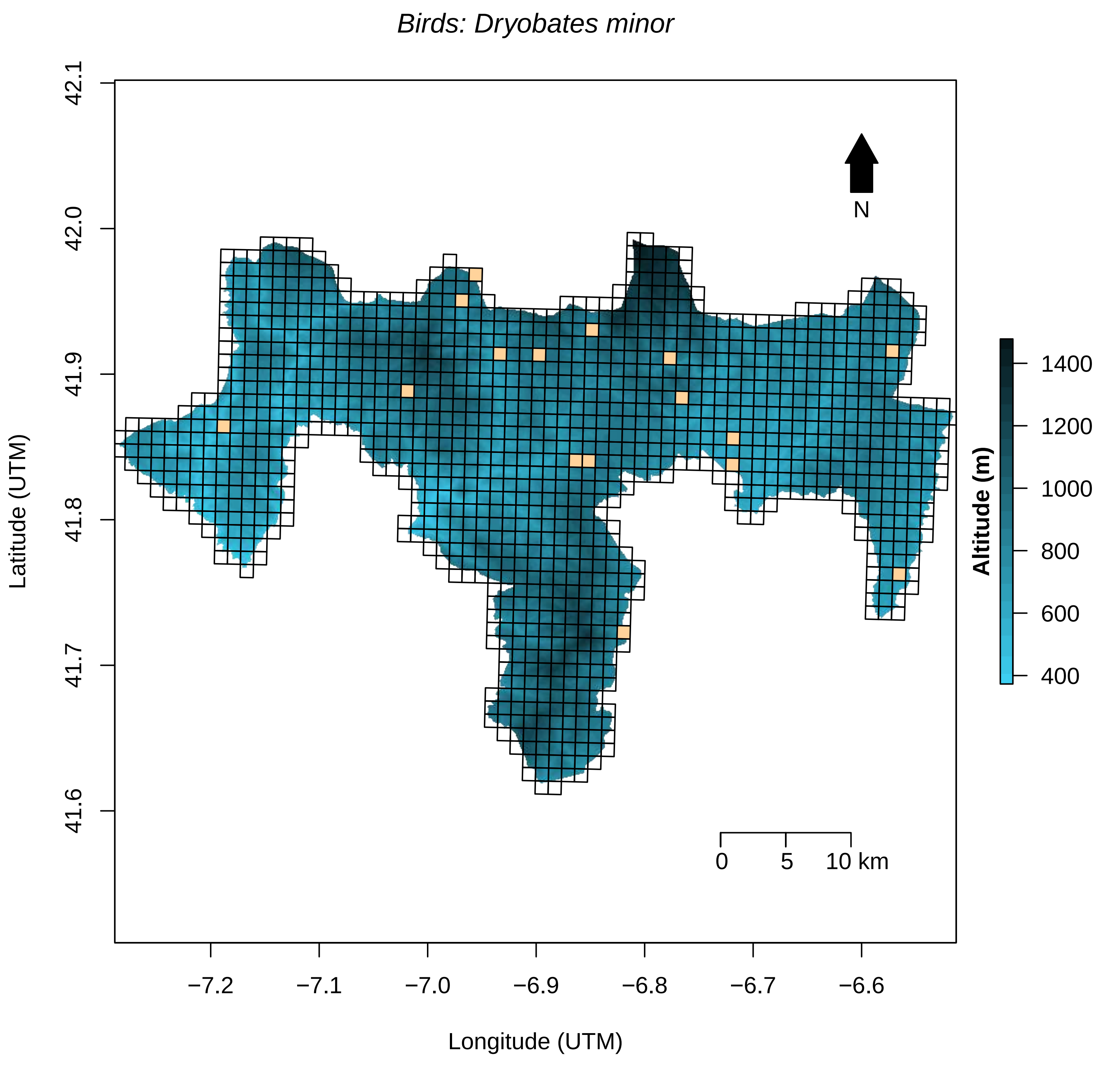
Birds: Dryobates minor
Latitude (UTM)
42.1
42.0
41.9
41.8
41.7
41.6
Longitude (UTM)
−7.2
−7.1
−7.0
−6.9
−6.8
−6.7
−6.6
N
0
5
10 km
Altitude (m)
1400
1200
1000
800
600
400

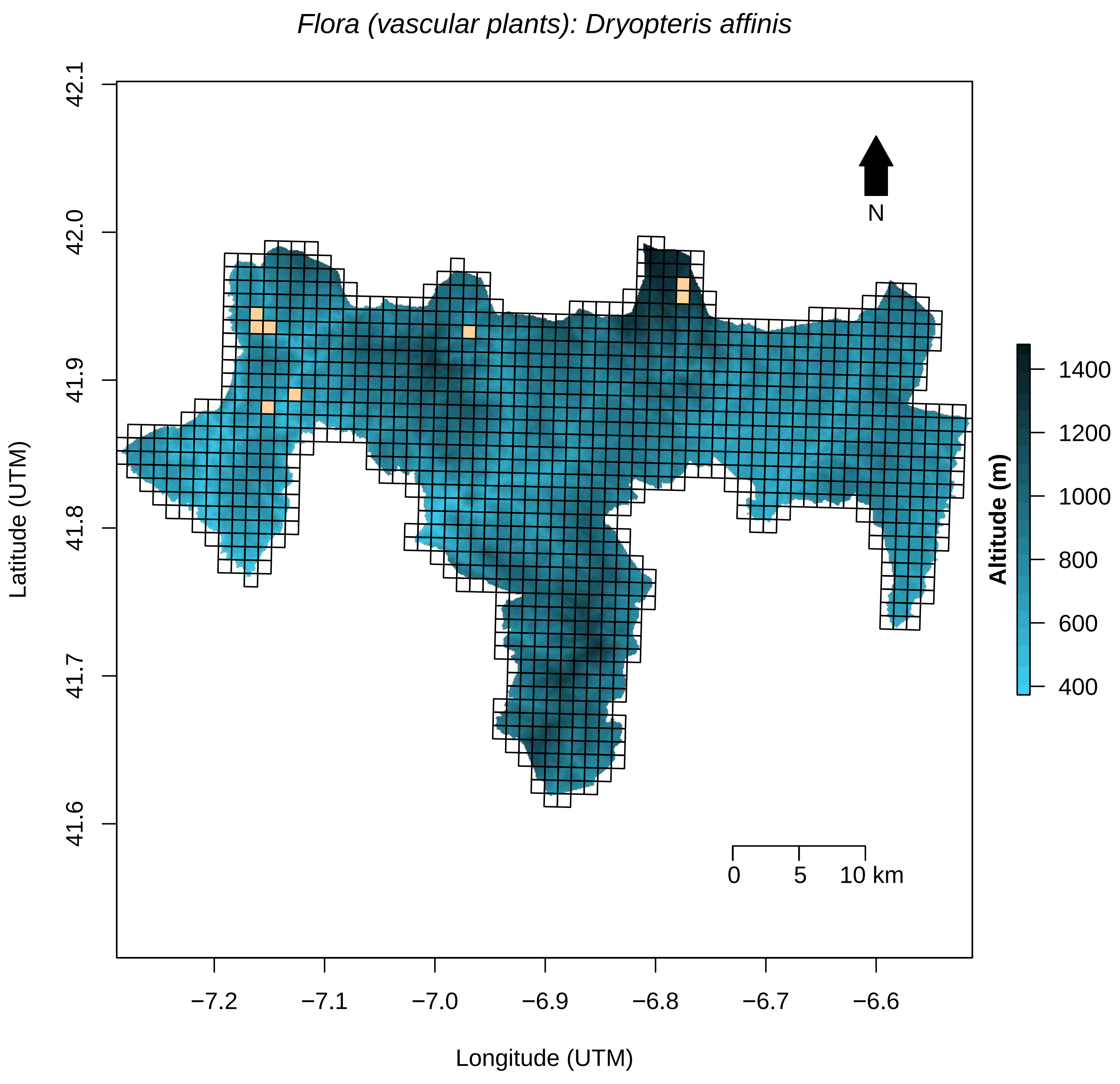

Flora (vascular plants): Dryopteris affinis
42.1
42.0
41.9
41.8
41.7
41.6
Latitude (UTM)
−7.2
−7.1
−7.0
−6.9
−6.8
−6.7
−6.6
Longitude (UTM)
N
0
5
10 km
Altitude (m)
1400
1200
1000
800
600
400

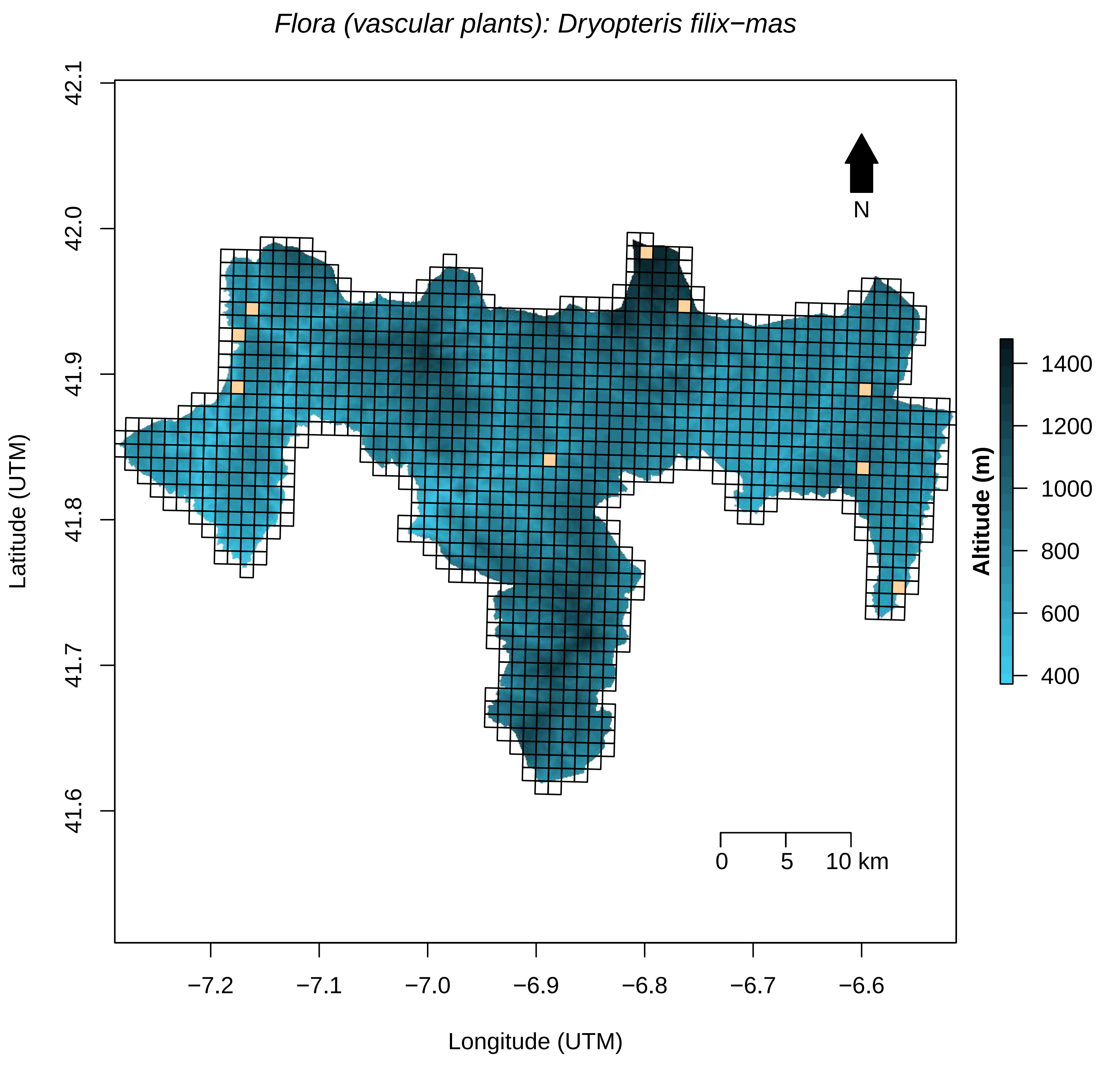

Flora (vascular plants): Dryopteris filix−mas
42.1
42.0
41.9
41.8
41.7
41.6
Latitude (UTM)
−7.2
−7.1
−7.0
−6.9
−6.8
−6.7
−6.6
Longitude (UTM)
N
0
5
10 km
Altitude (m)
1400
1200
1000
800
600
400

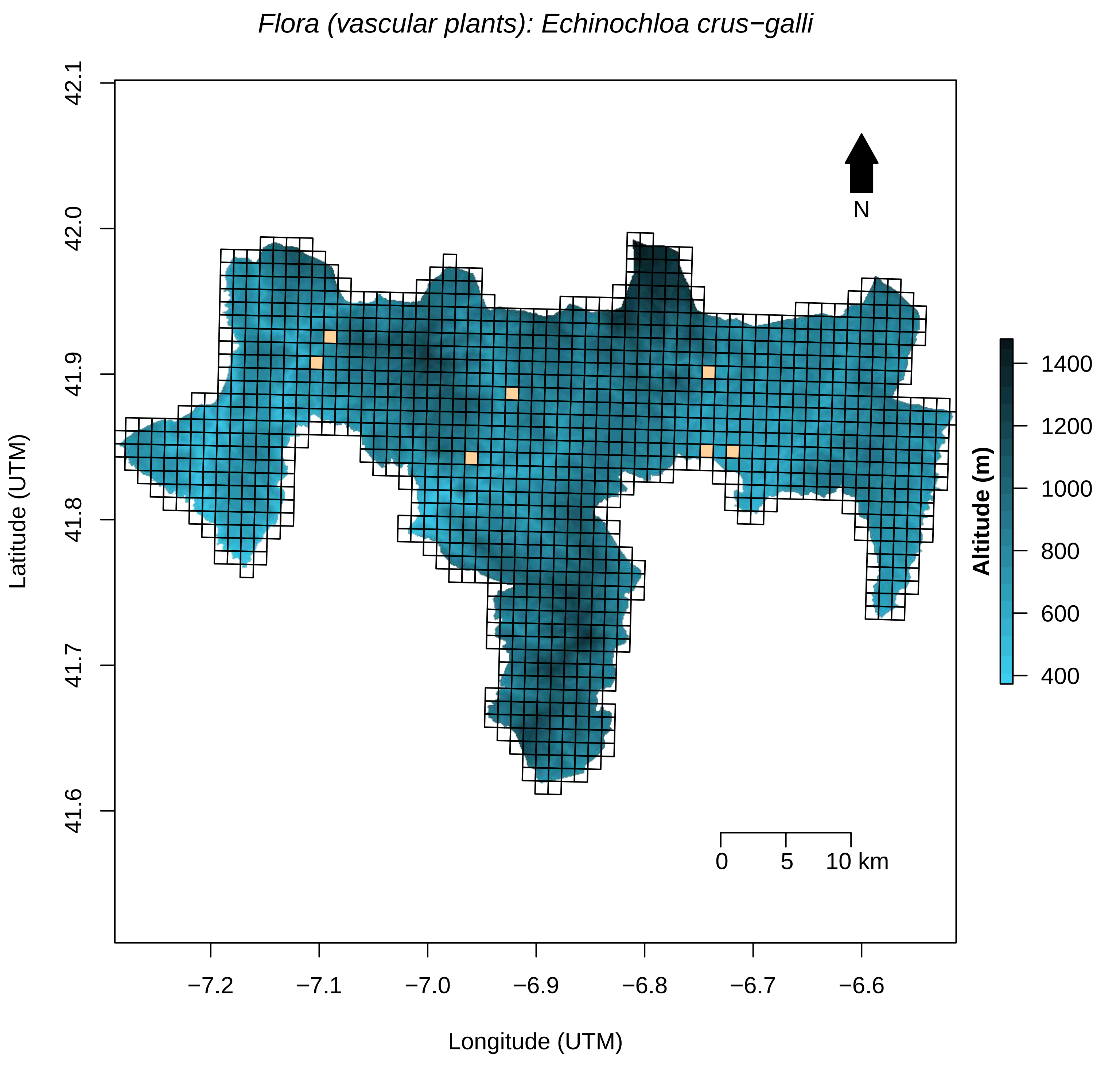
Flora (vascular plants): Echinochloa crus−galli
42.1
42.0
41.9
41.8
41.7
41.6
Latitude (UTM)
−7.2
−7.1
−7.0
−6.9
−6.8
−6.7
−6.6
Longitude (UTM)
N
0
5
10 km
Altitude (m)
1400
1200
1000
800
600
400

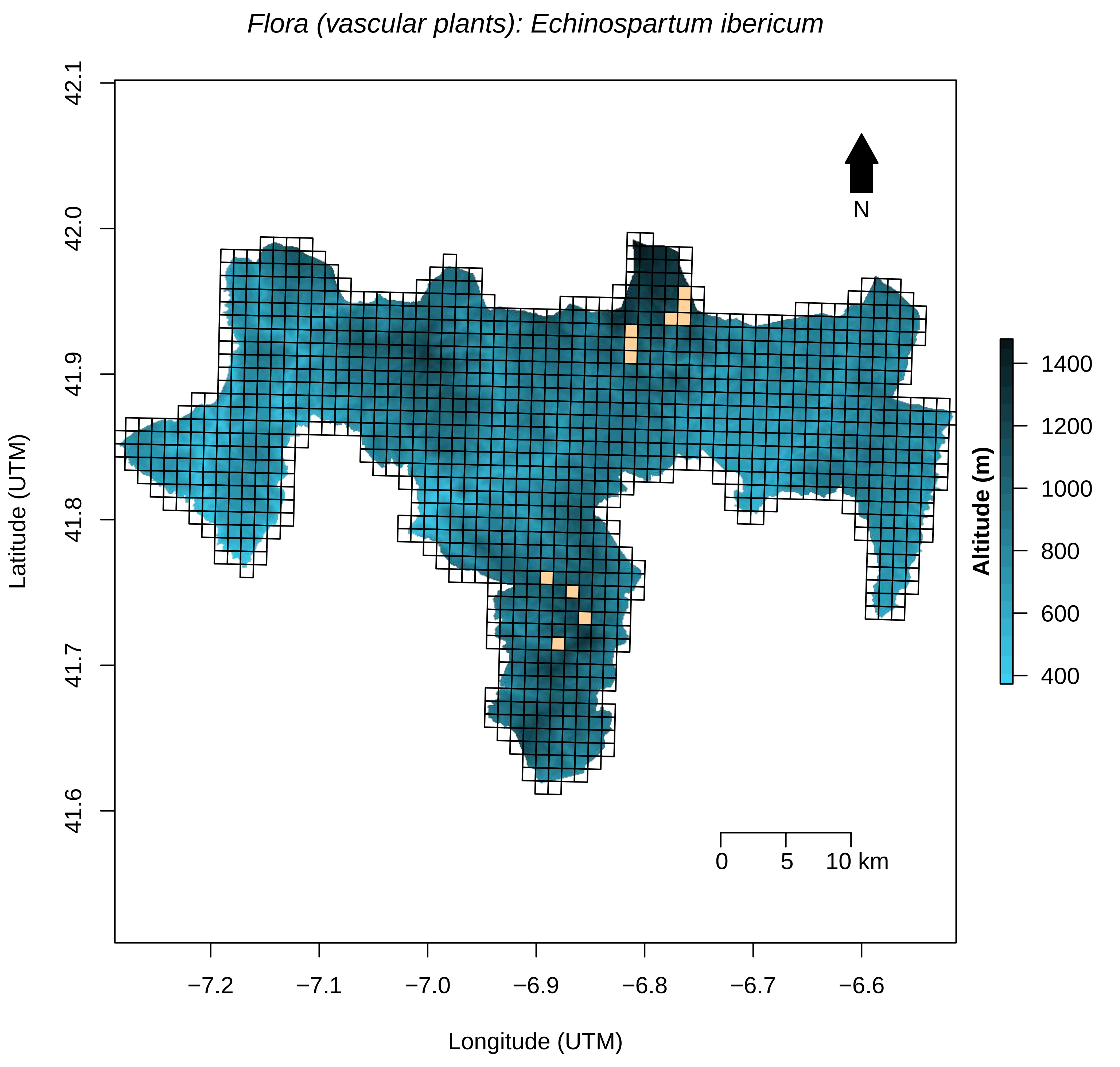

Flora (vascular plants): Echinospartum ibericum
42.1
42.0
41.9
41.8
41.7
41.6
Latitude (UTM)
−7.2
−7.1
−7.0
−6.9
−6.8
−6.7
−6.6
Longitude (UTM)
N
0
5
10 km
Altitude (m)
1400
1200
1000
800
600
400

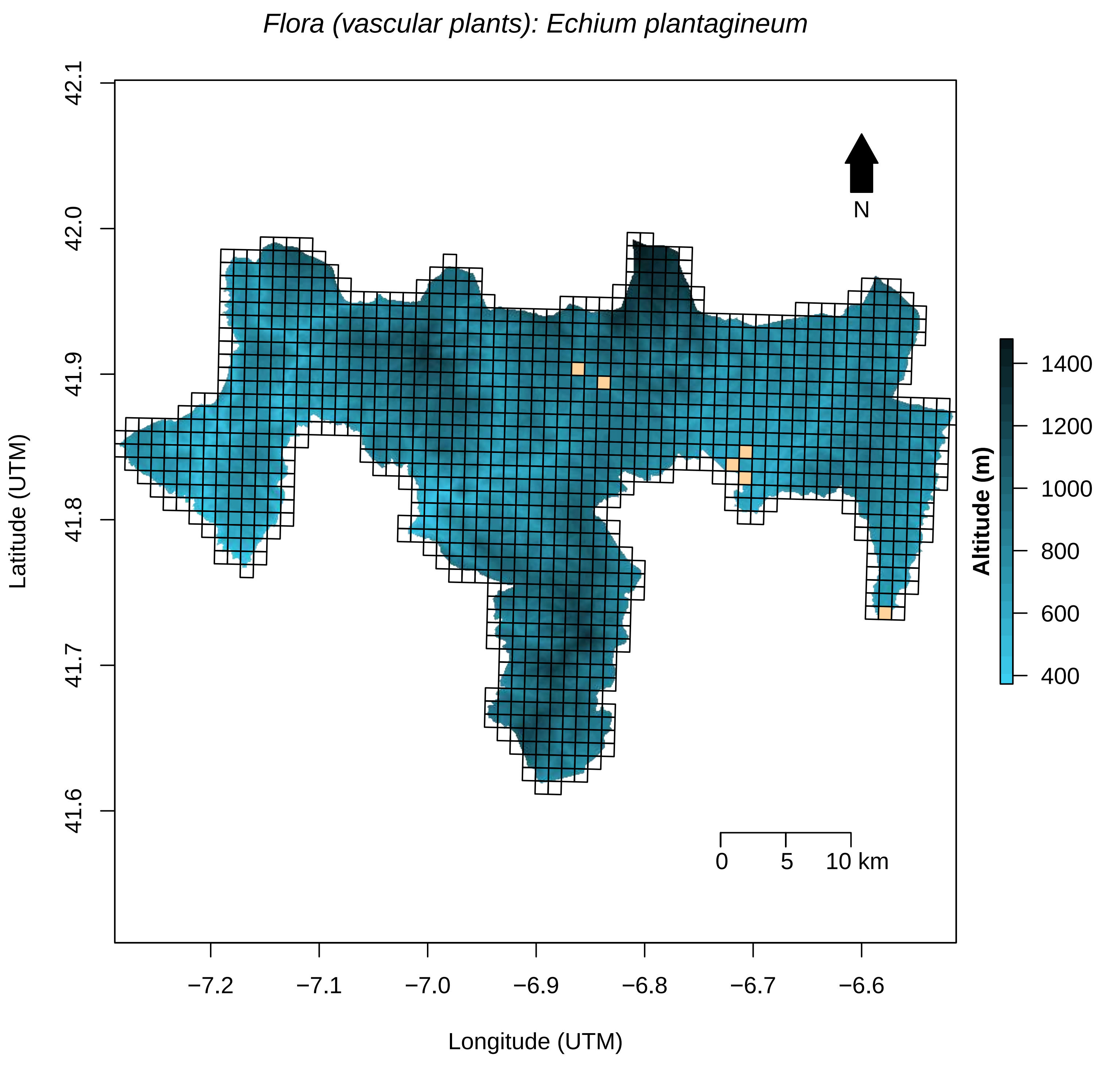

Flora (vascular plants): Echium plantagineum
42.1
42.0
41.9
41.8
41.7
41.6
Latitude (UTM)
−7.2
−7.1
−7.0
−6.9
−6.8
−6.7
−6.6
Longitude (UTM)
N
0
5
10 km
Altitude (m)
1400
1200
1000
800
600
400

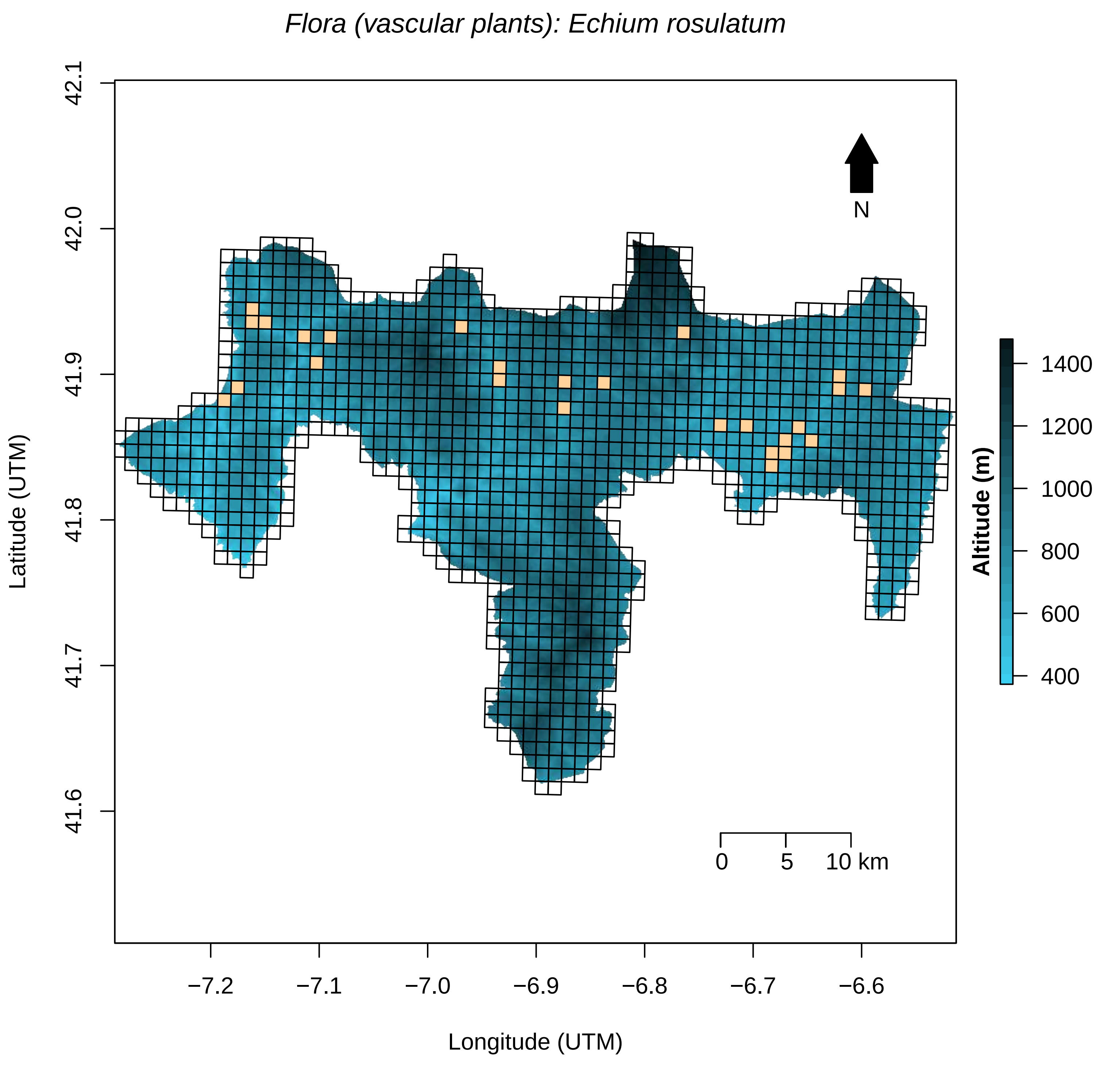

Flora (vascular plants): Echium rosulatum
Latitude (UTM)
Longitude (UTM)
42.1
42.0
41.9
41.8
41.7
41.6
−7.2
−7.1
−7.0
−6.9
−6.8
−6.7
−6.6
N
0
5
10 km
Altitude (m)
1400
1200
1000
800
600
400

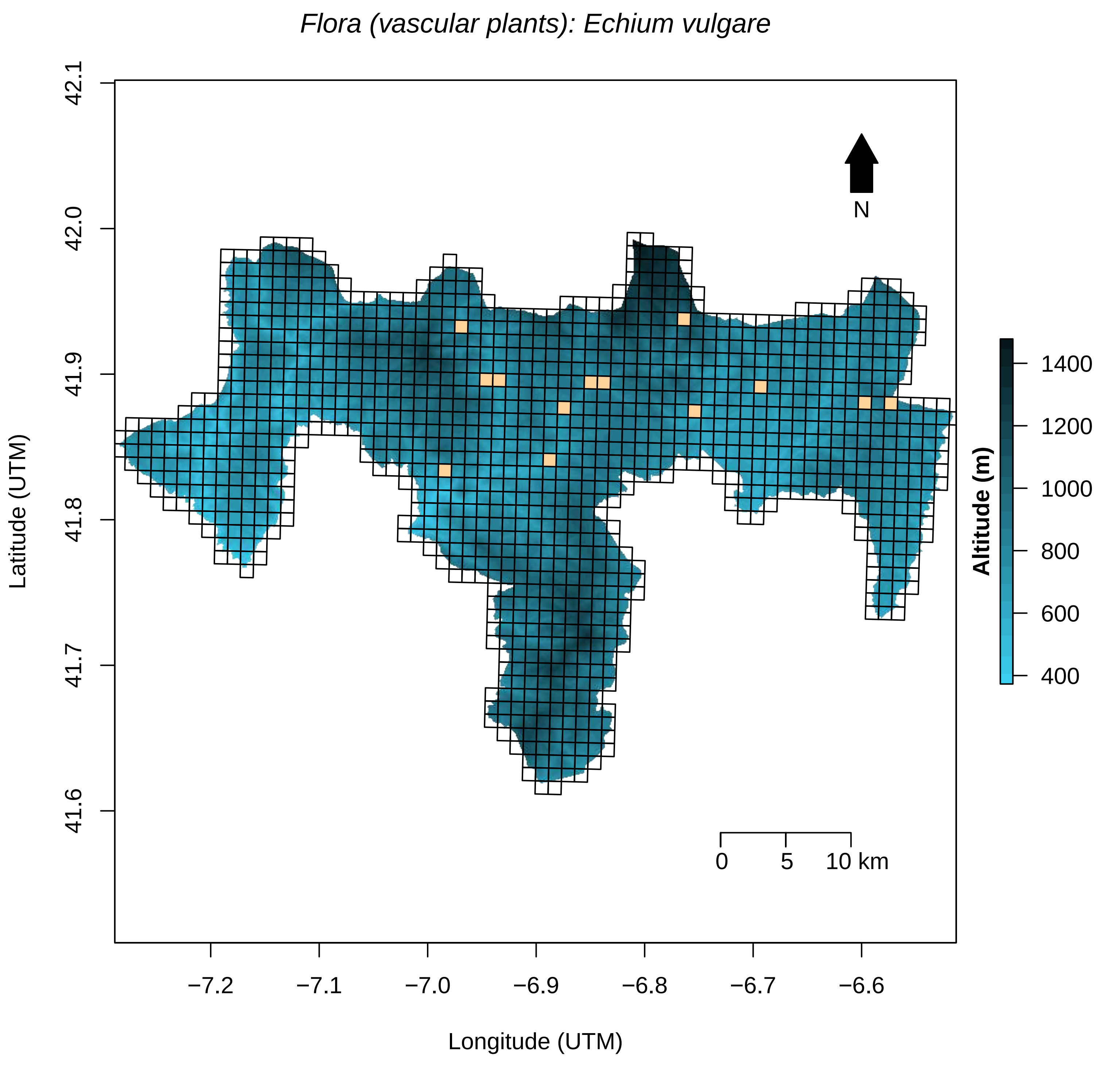

Flora (vascular plants): Echium vulgare
N
0 5 10 km
Altitude (m)
1400
1200
1000
800
600
400
42.1
42.0
41.9
41.8
41.7
41.6
Latitude (UTM)
−7.2
−7.1
−7.0
−6.9
−6.8
−6.7
−6.6
Longitude (UTM)

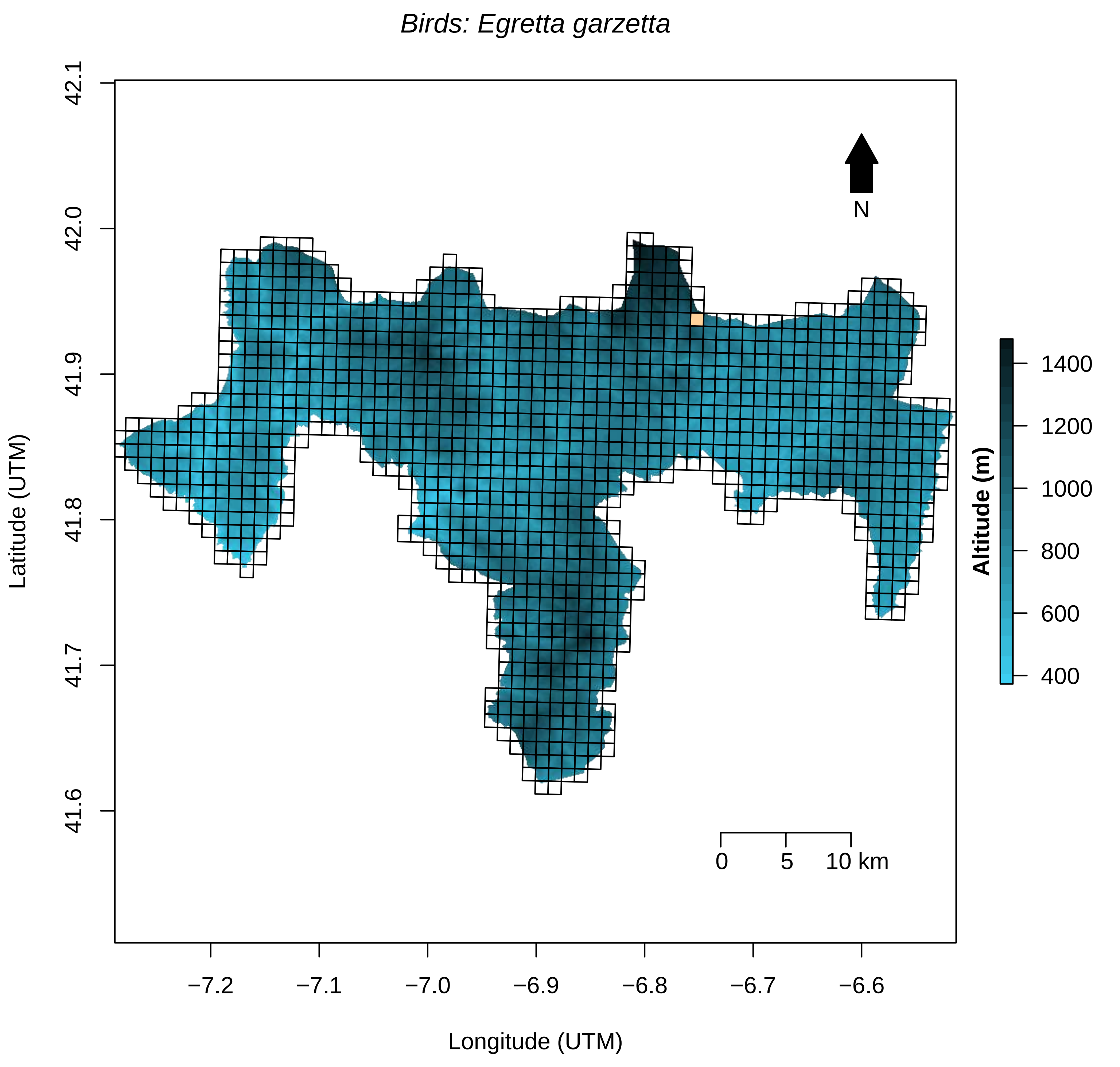

Birds: Egretta garzetta
Latitude (UTM)
42.1
42.0
41.9
41.8
41.7
41.6
Longitude (UTM)
−7.2
−7.1
−7.0
−6.9
−6.8
−6.7
−6.6
N
0
5
10 km
Altitude (m)
1400
1200
1000
800
600
400

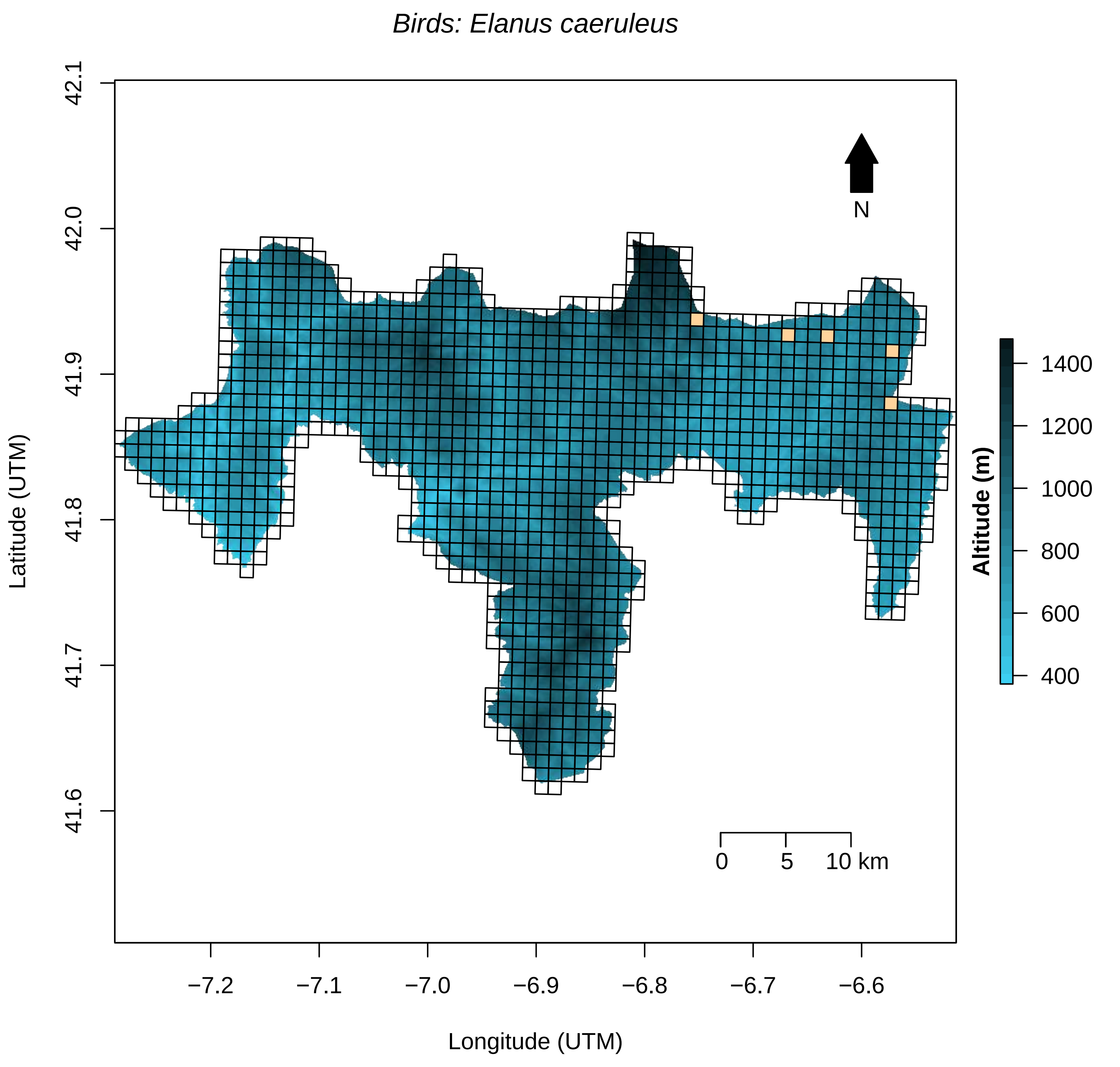

Birds: Elanus caeruleus
42.1
42.0
41.9
41.8
41.7
41.6
Latitude (UTM)
−7.2
−7.1
−7.0
−6.9
−6.8
−6.7
−6.6
Longitude (UTM)
N
0
5
10 km
Altitude (m)
1400
1200
1000
800
600
400

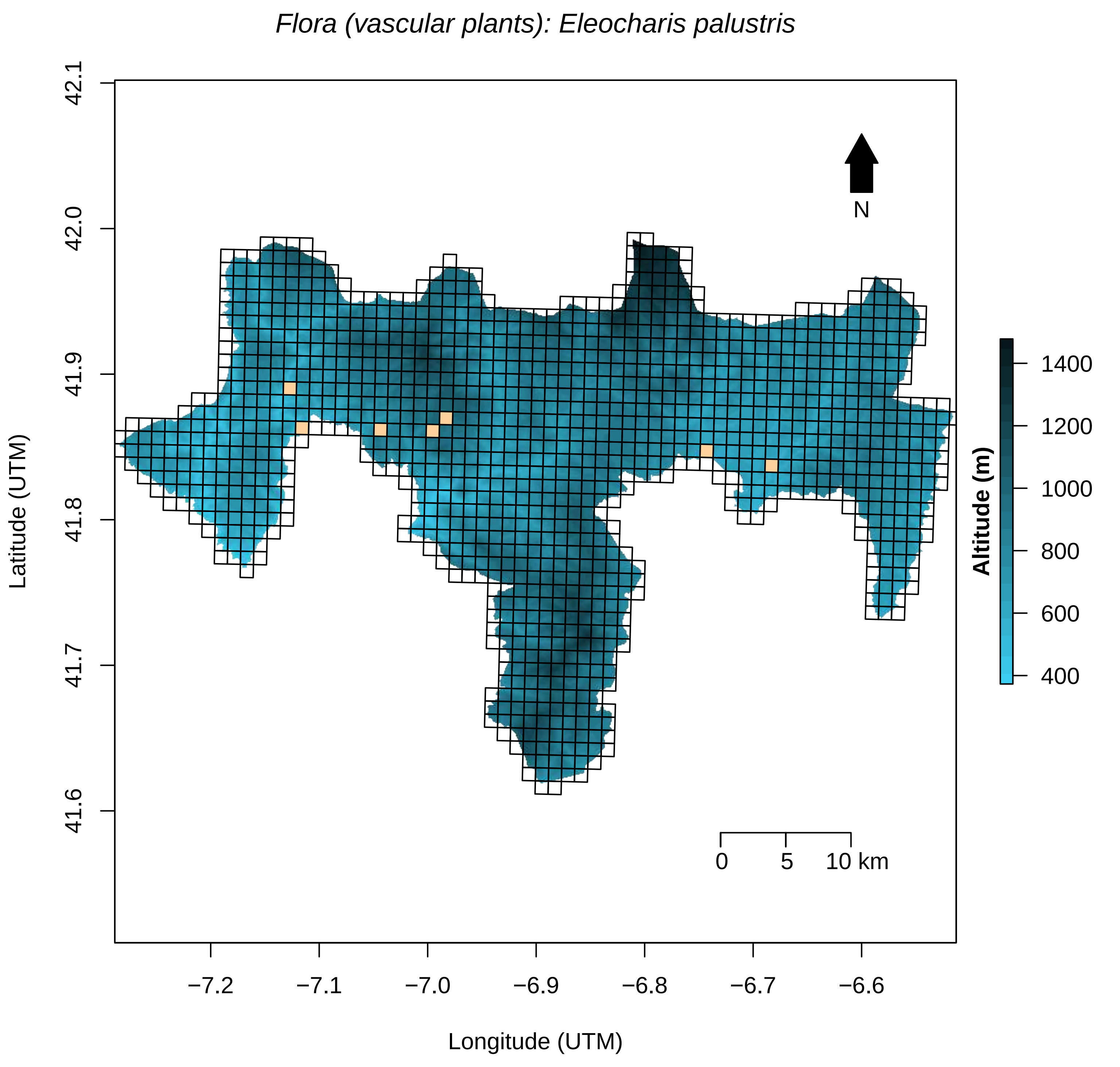

Flora (vascular plants): Eleocharis palustris
42.1
42.0
41.9
41.8
41.7
41.6
Latitude (UTM)
−7.2
−7.1
−7.0
−6.9
−6.8
−6.7
−6.6
Longitude (UTM)
N
0
5
10 km
Altitude (m)
1400
1200
1000
800
600
400

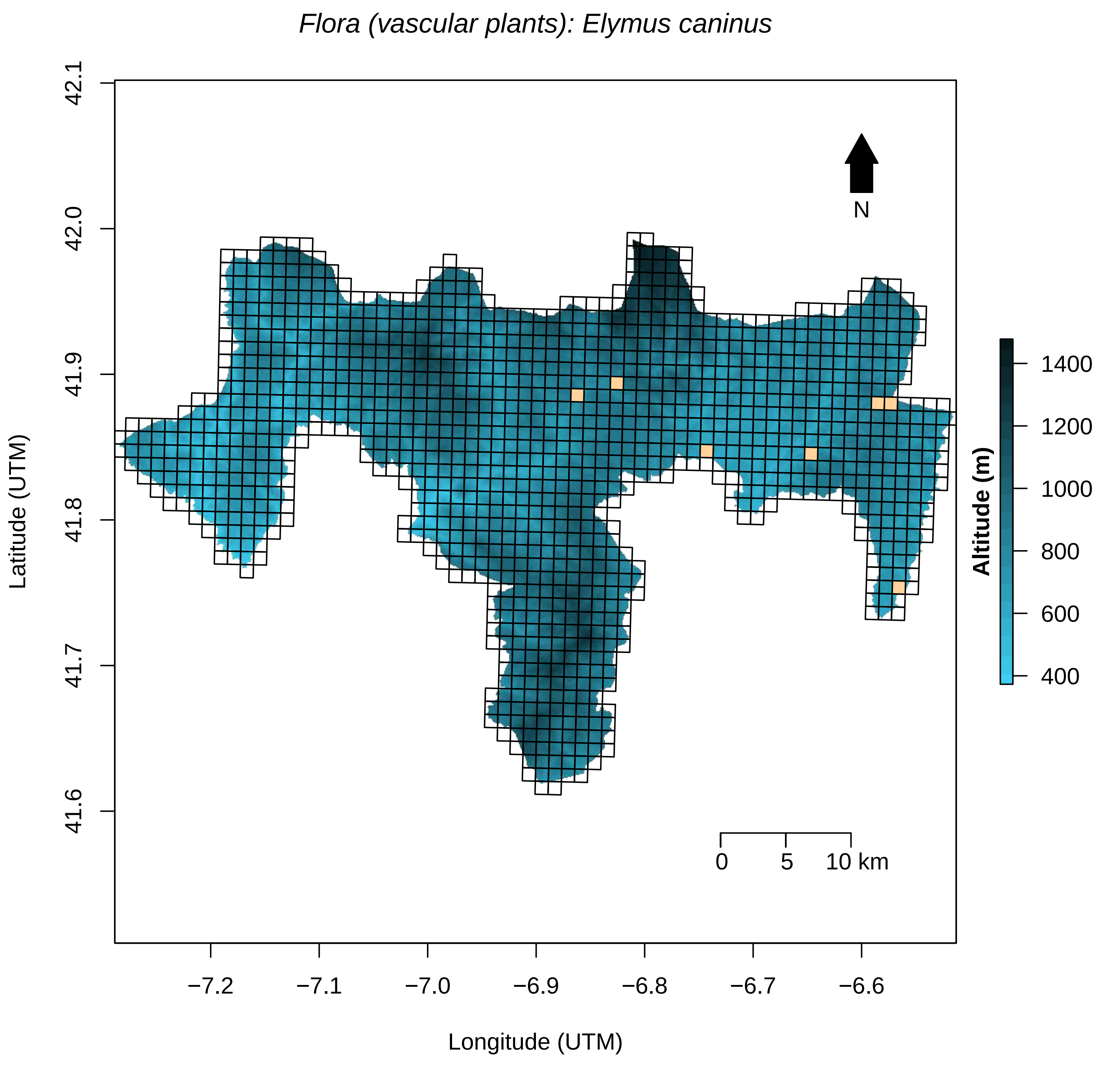

Flora (vascular plants): Elymus caninus
42.1
42.0
41.9
41.8
41.7
41.6
Latitude (UTM)
−7.2
−7.1
−7.0
−6.9
−6.8
−6.7
−6.6
Longitude (UTM)
N
0
5
10 km
1400
1200
1000
800
600
400
Altitude (m)

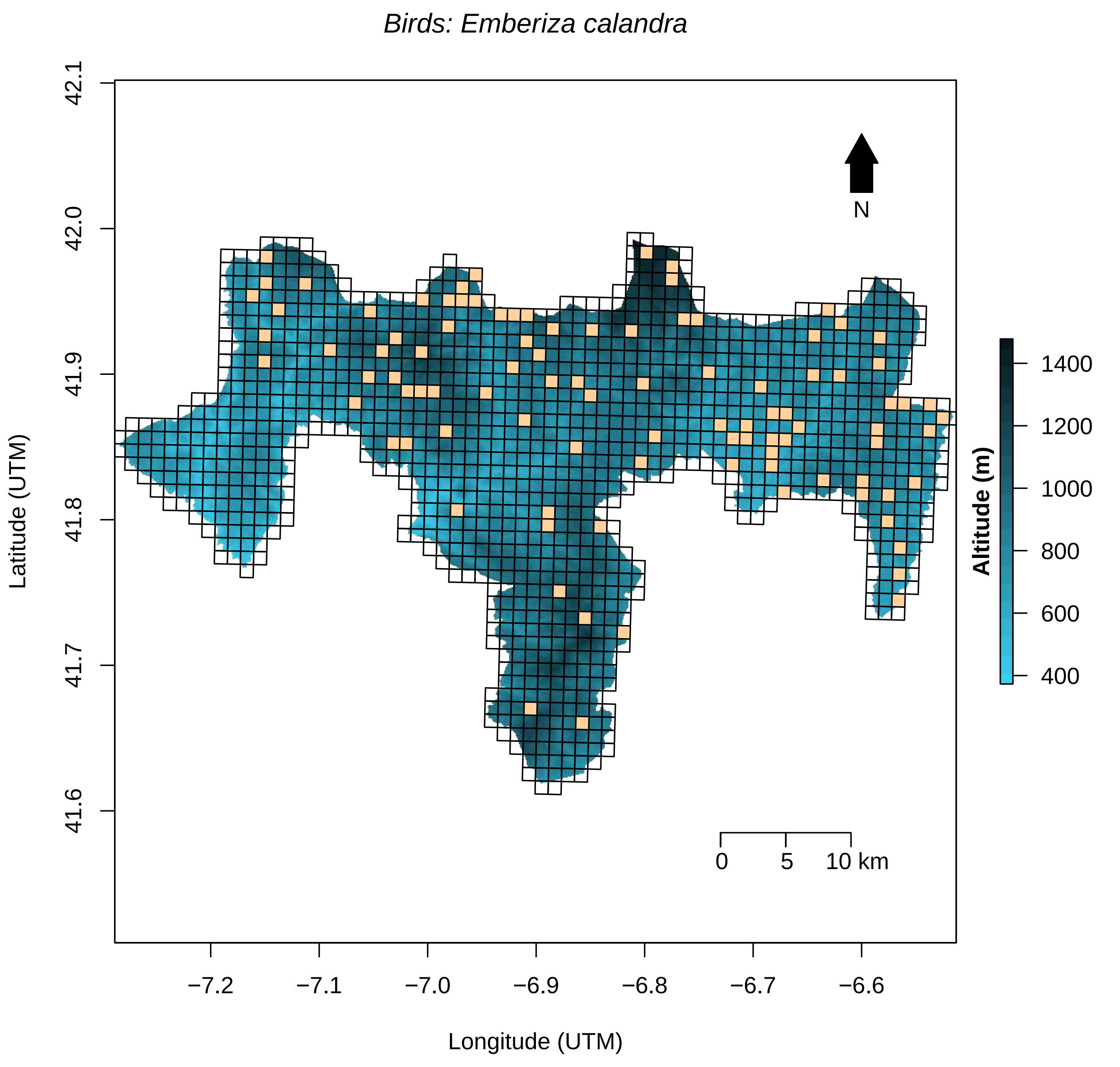

Birds: Emberiza calandra
42.1
42.0
41.9
41.8
41.7
41.6
Latitude (UTM)
−7.2
−7.1
−7.0
−6.9
−6.8
−6.7
−6.6
Longitude (UTM)
N
0
5
10 km
Altitude (m)
1400
1200
1000
800
600
400

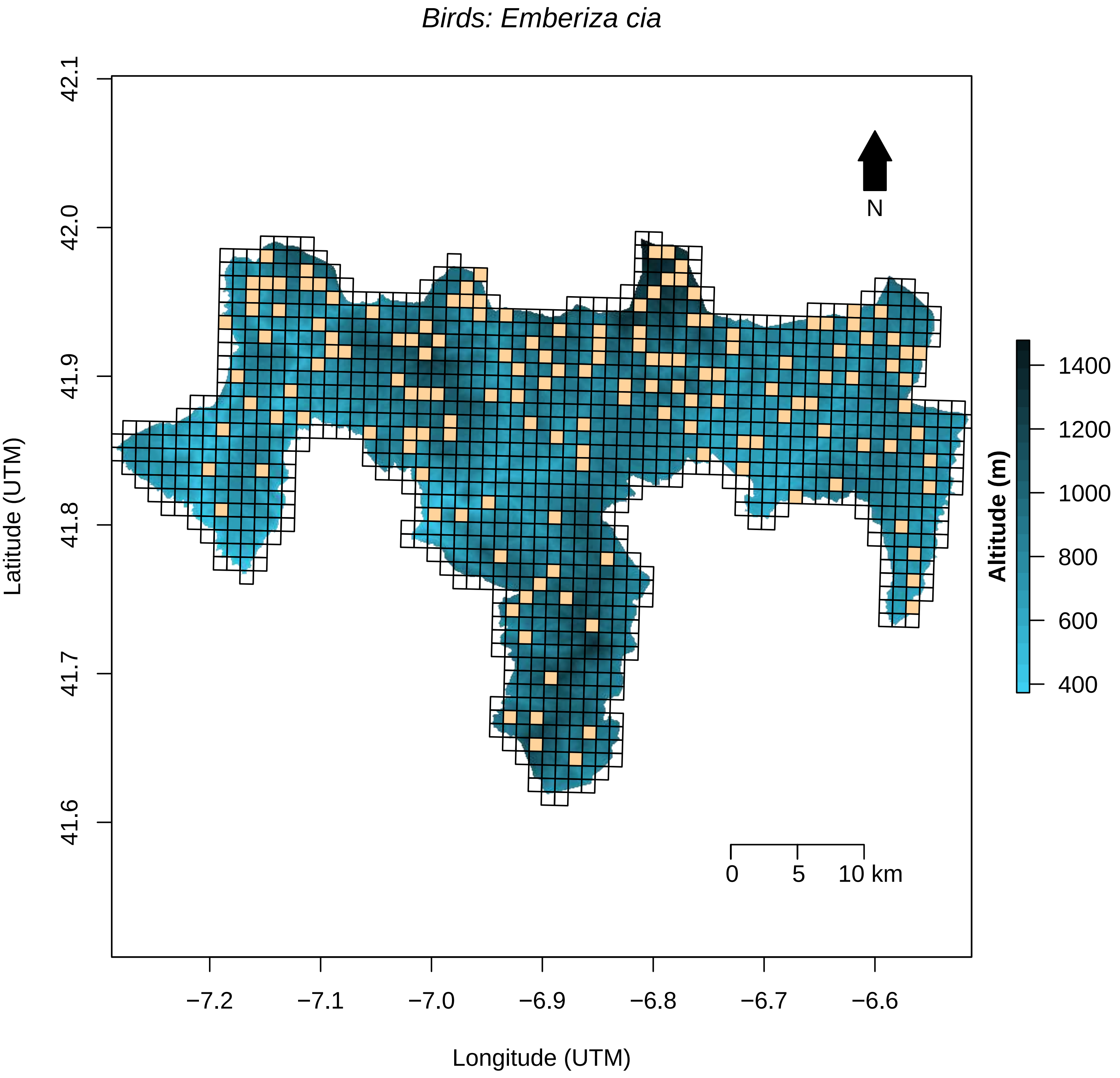

Birds: Emberiza cia
42.1
42.0
41.9
41.8
41.7
41.6
Latitude (UTM)
−7.2
−7.1
−7.0
−6.9
−6.8
−6.7
−6.6
Longitude (UTM)
N
0
5
10 km
1400
1200
1000
800
600
400
Altitude (m)

Birds: Emberiza cirlus

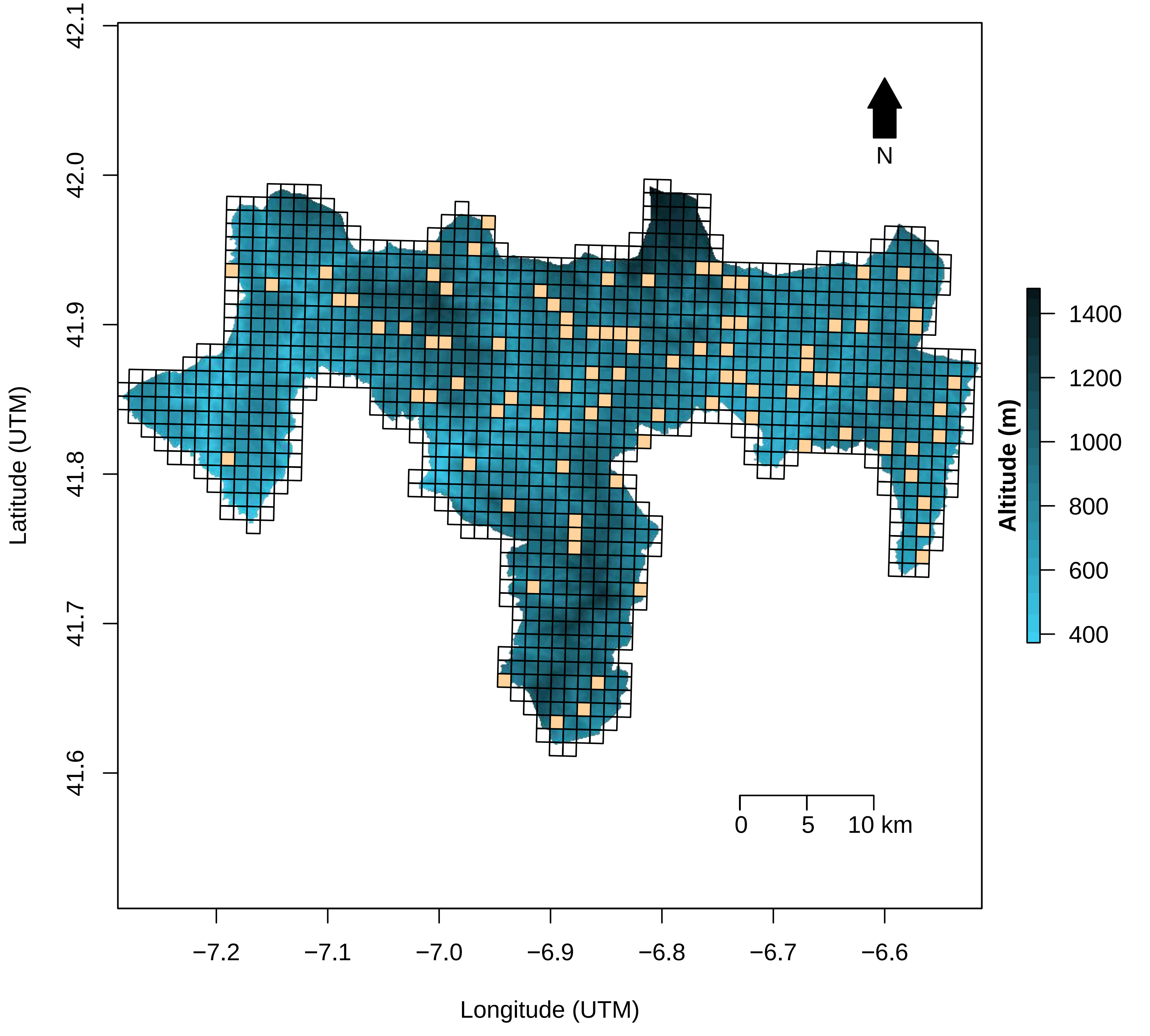

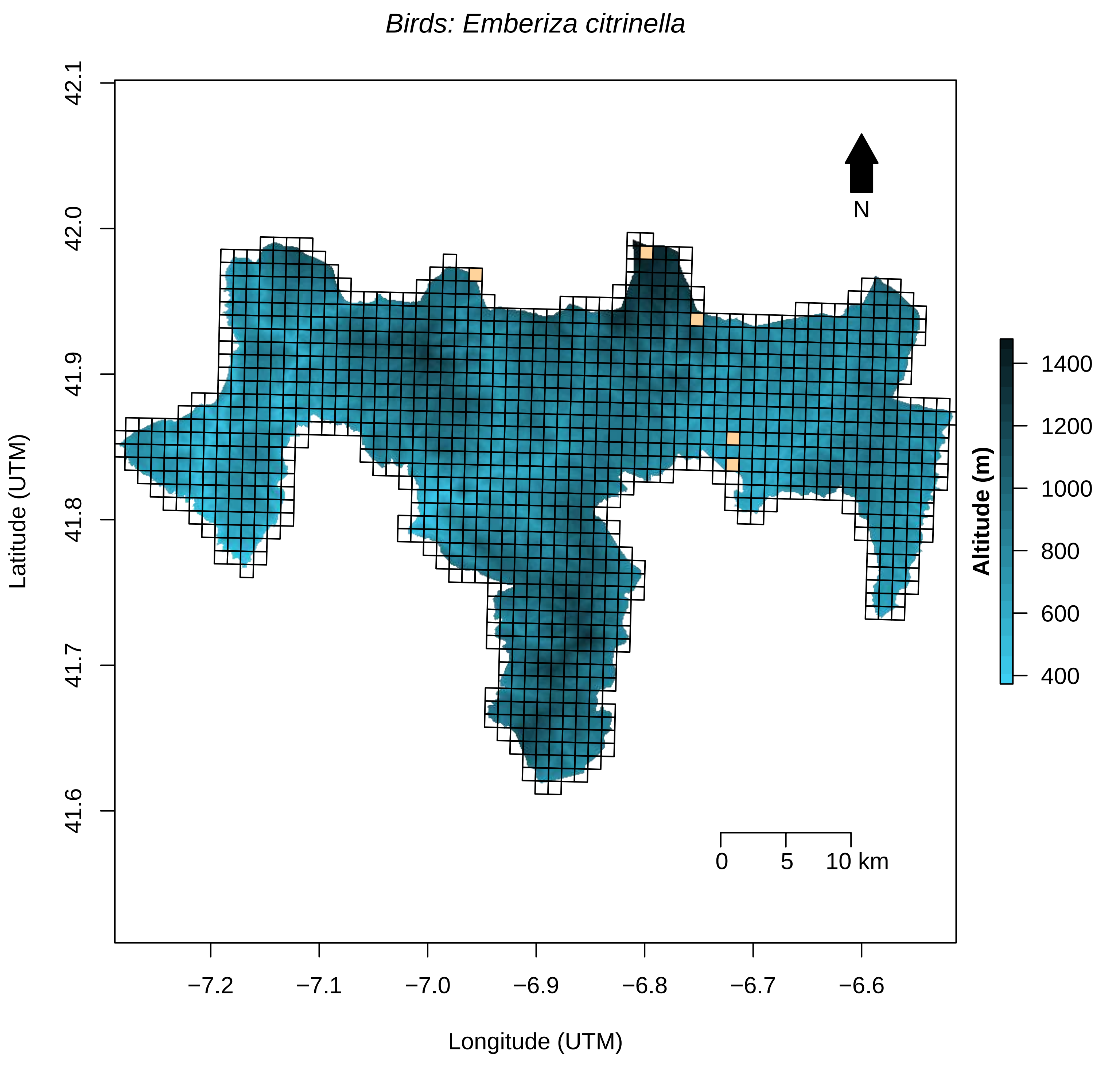

Birds: Emberiza citrinella
42.1
42.0
41.9
41.8
41.7
41.6
Latitude (UTM)
−7.2
−7.1
−7.0
−6.9
−6.8
−6.7
−6.6
Longitude (UTM)
N
0
5
10 km
1400
1200
1000
800
600
400
Altitude (m)

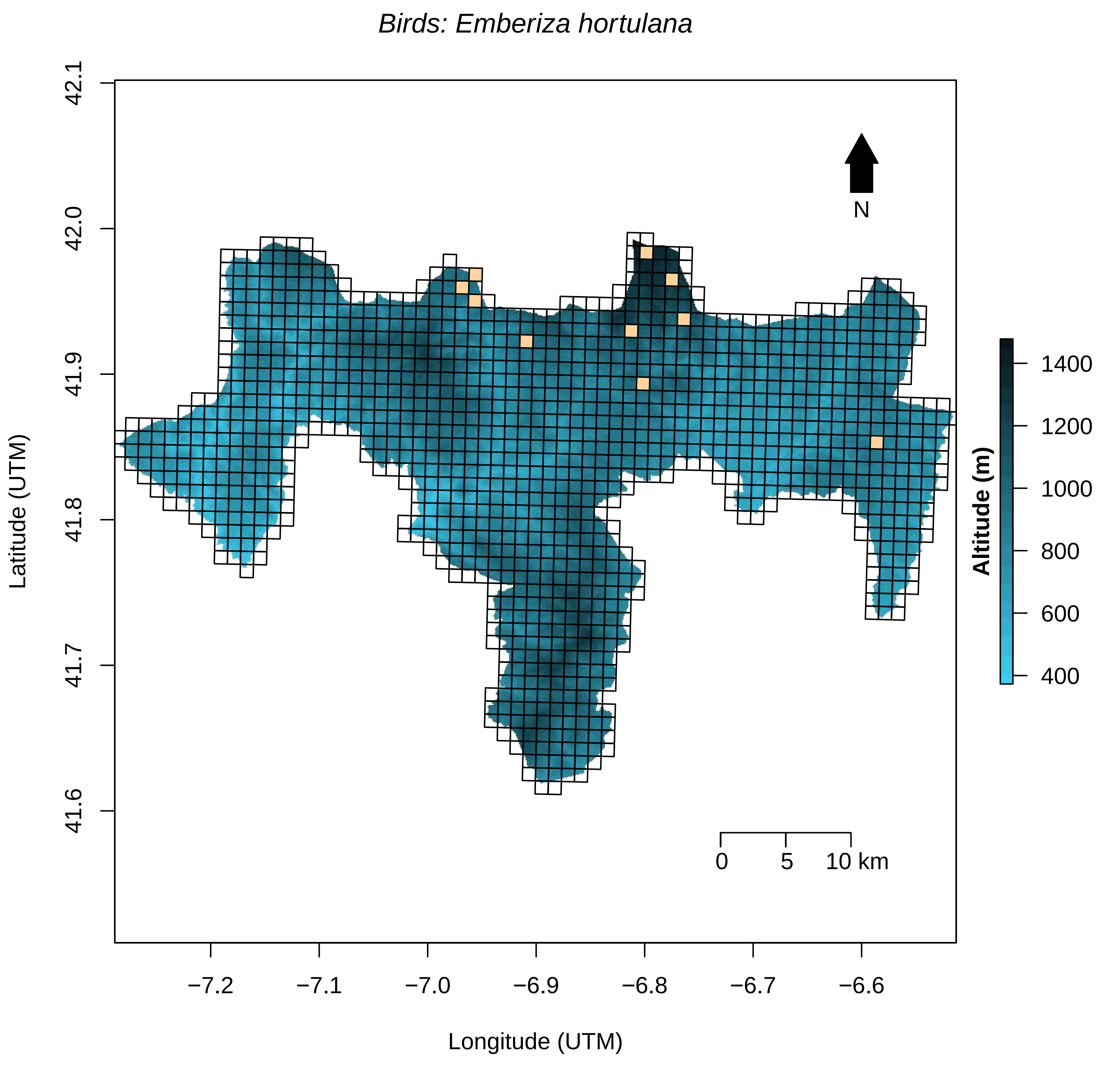
Birds: Emberiza hortulana
Latitude (UTM)
42.1
42.0
41.9
41.8
41.7
41.6
Longitude (UTM)
−7.2
−7.1
−7.0
−6.9
−6.8
−6.7
−6.6
N
0
5
10 km
Altitude (m)
1400
1200
1000
800
600
400

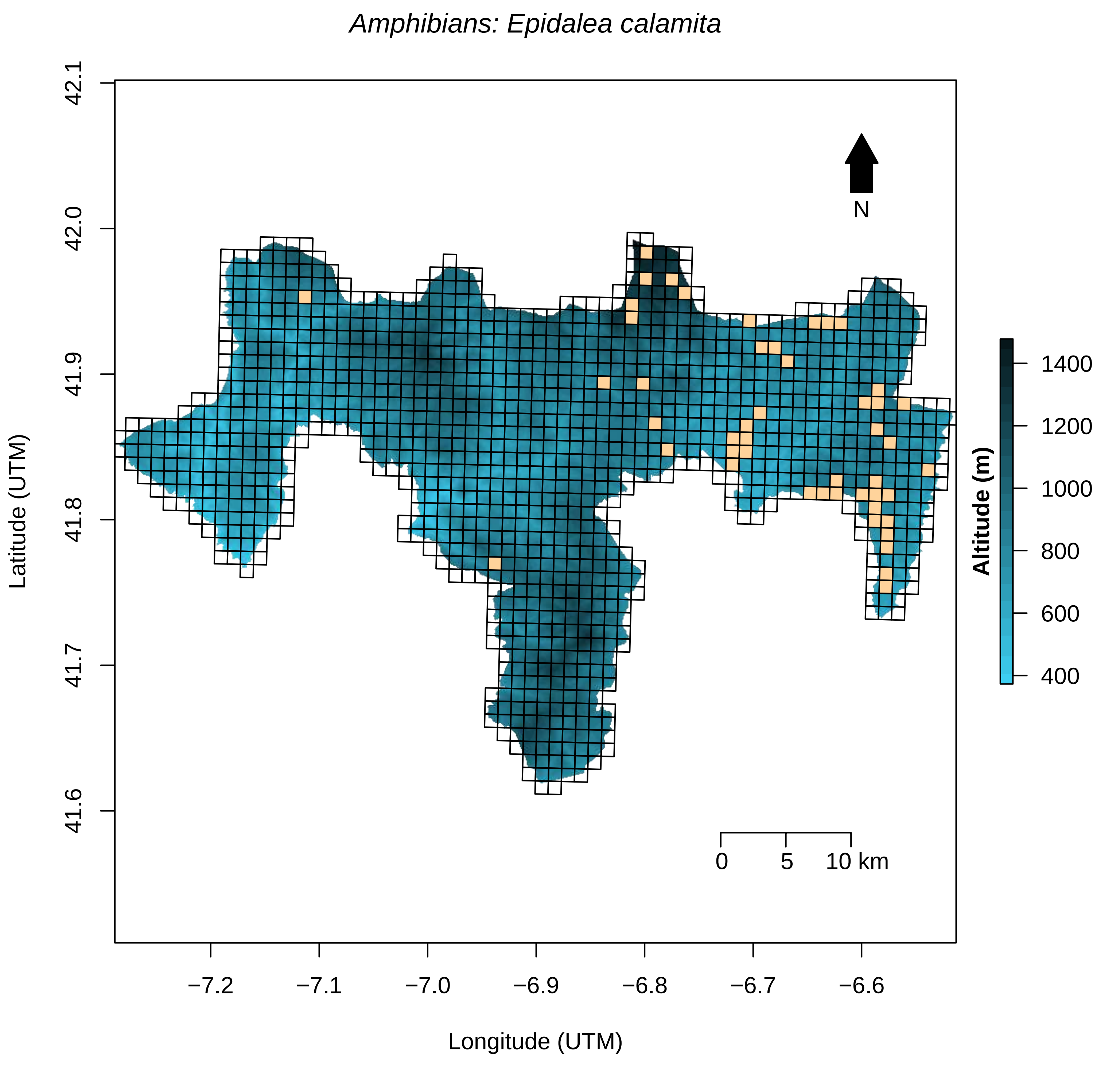
Amphibians: Epidalea calamita
N
0 5 10 km
Altitude (m)
400
600
800
1000
1200
1400
Latitude (UTM)
41.6
41.7
41.8
41.9
42.0
42.1
Longitude (UTM)
−7.2
−7.1
−7.0
−6.9
−6.8
−6.7
−6.6

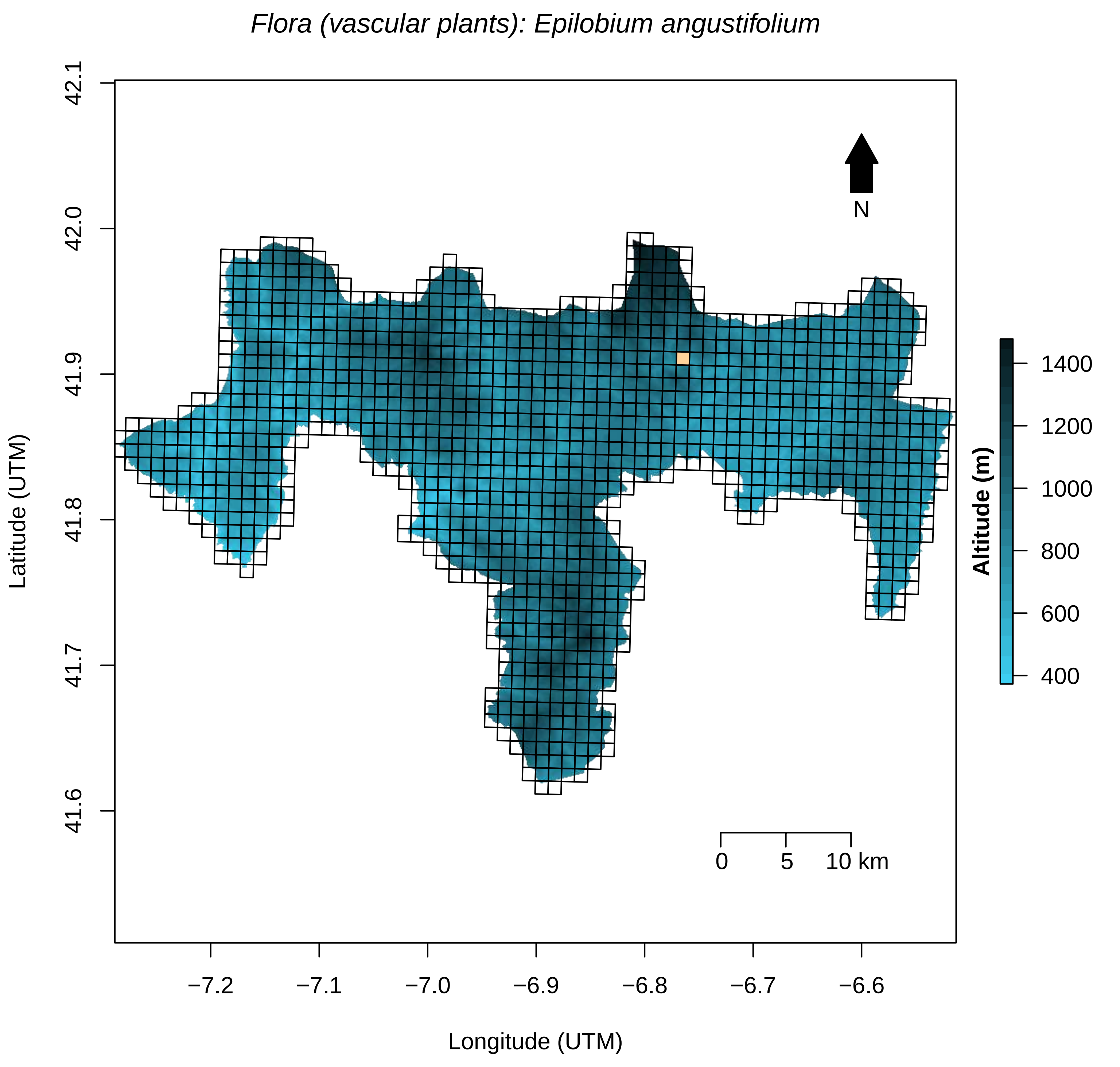

Flora (vascular plants): Epilobium angustifolium
Latitude (UTM)
Longitude (UTM)
42.1
42.0
41.9
41.8
41.7
41.6
−7.2
−7.1
−7.0
−6.9
−6.8
−6.7
−6.6
N
0
5
10 km
Altitude (m)
1400
1200
1000
800
600
400

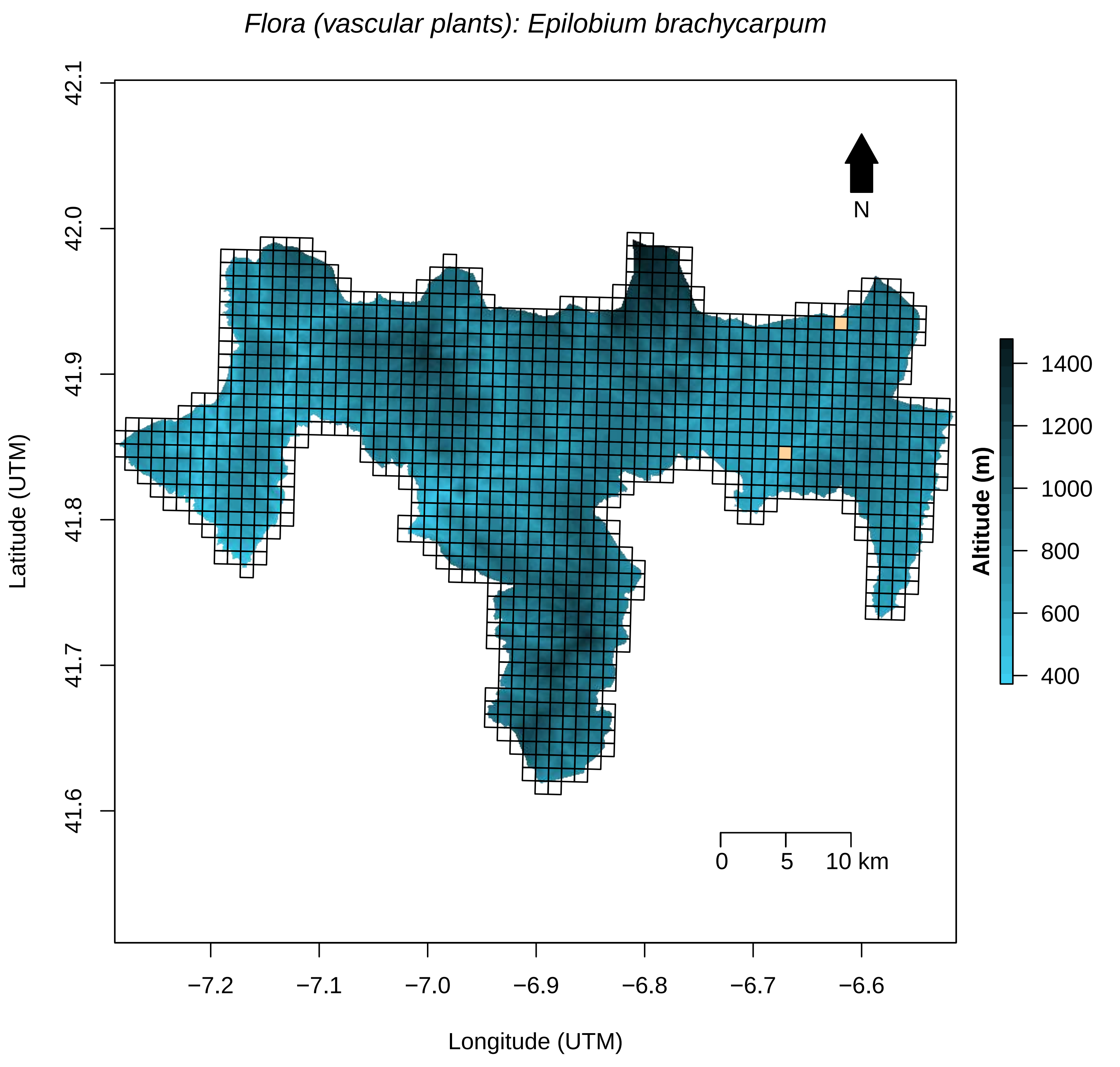

Flora (vascular plants): Epilobium brachycarpum
42.1
42.0
41.9
41.8
41.7
41.6
Latitude (UTM)
−7.2
−7.1
−7.0
−6.9
−6.8
−6.7
−6.6
Longitude (UTM)
N
0
5
10 km
1400
1200
1000
800
600
400
Altitude (m)

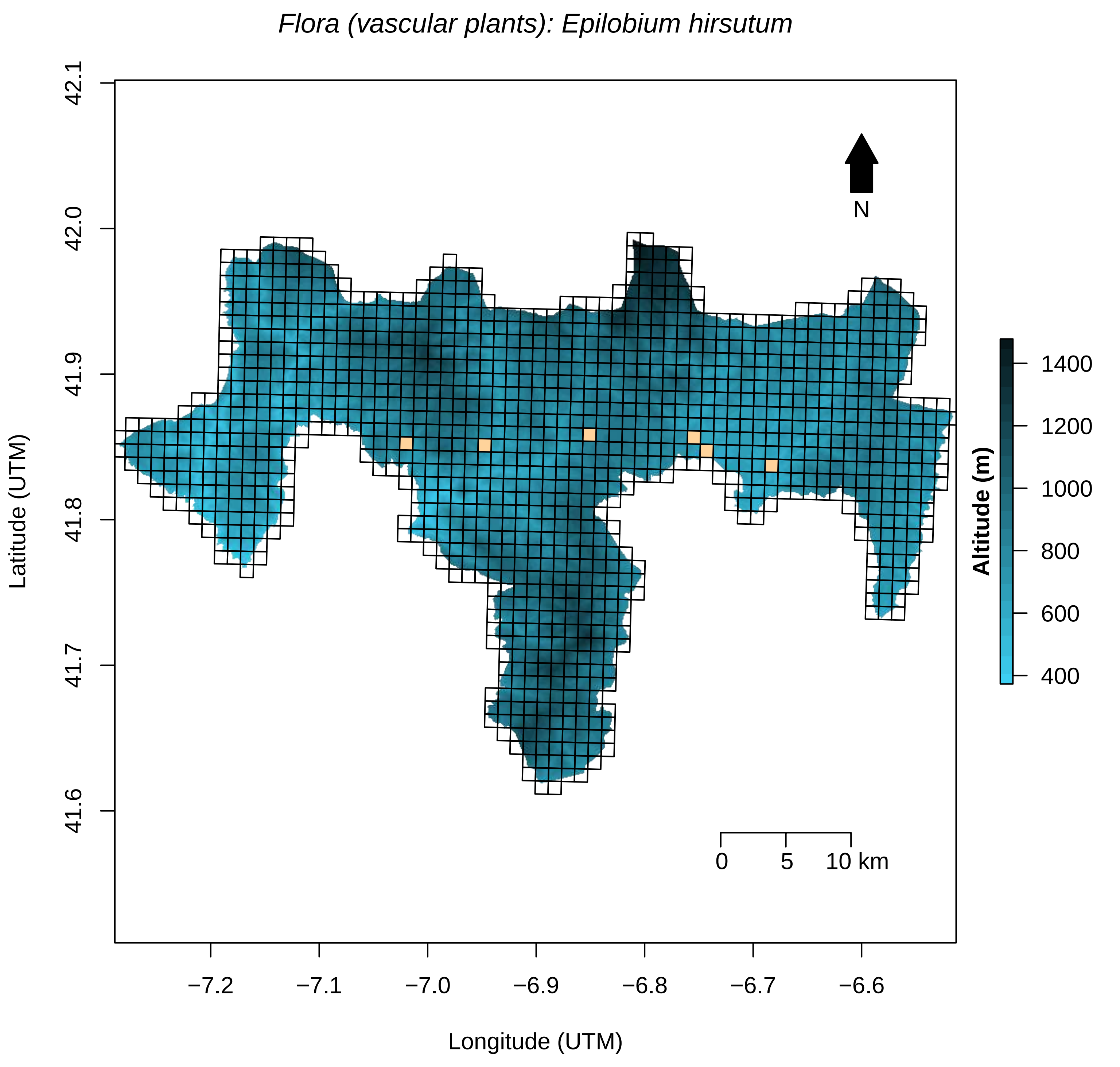

Flora (vascular plants): Epilobium hirsutum
Latitude (UTM)
Longitude (UTM)
42.1
42.0
41.9
41.8
41.7
41.6
−7.2
−7.1
−7.0
−6.9
−6.8
−6.7
−6.6
N
0
5
10 km
Altitude (m)
1400
1200
1000
800
600
400

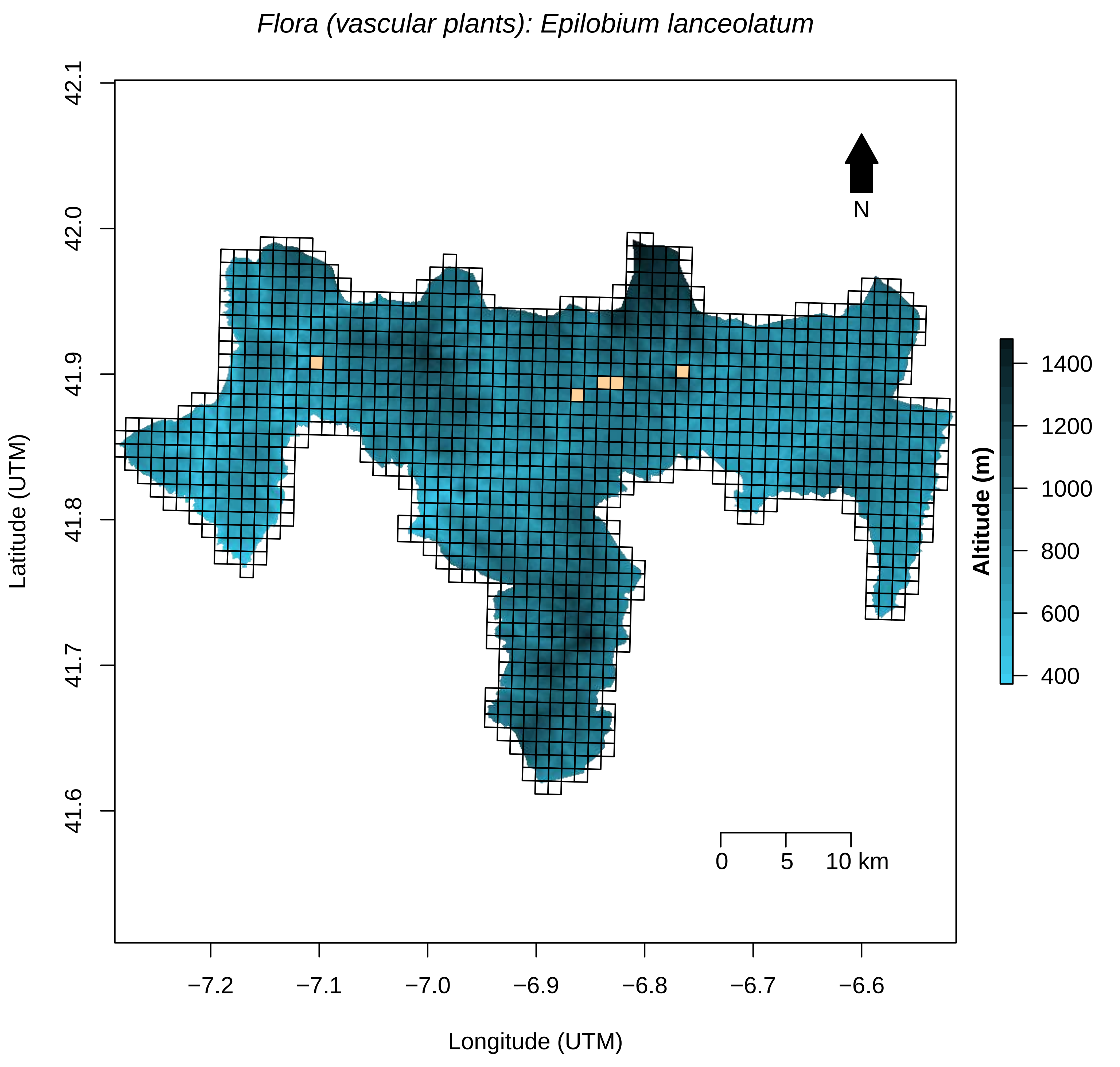

Flora (vascular plants): Epilobium lanceolatum
42.1
42.0
41.9
41.8
41.7
41.6
Latitude (UTM)
−7.2
−7.1
−7.0
−6.9
−6.8
−6.7
−6.6
Longitude (UTM)
N
0
5
10 km
1400
1200
1000
800
600
400
Altitude (m)

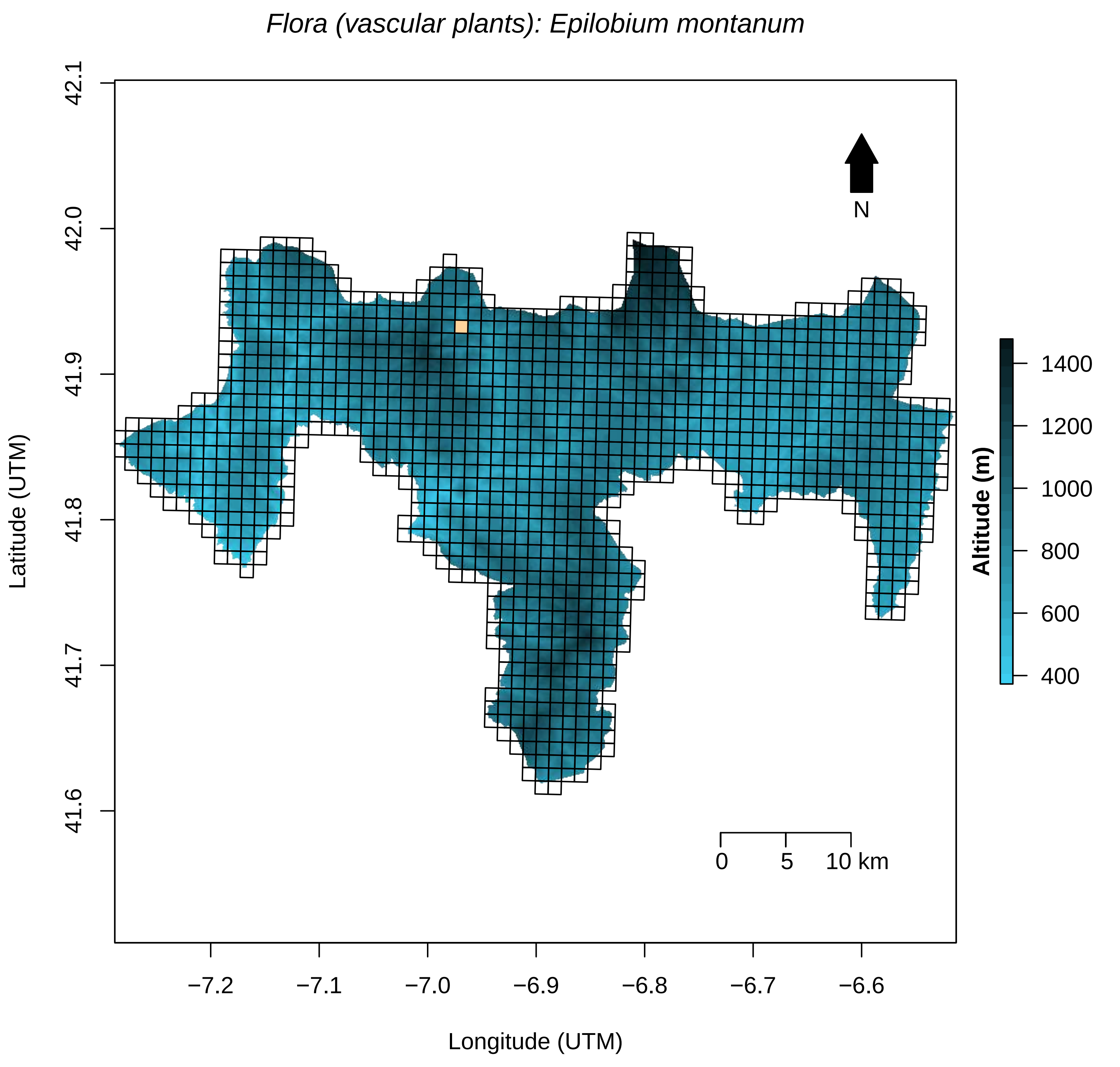

Flora (vascular plants): Epilobium montanum
42.1
42.0
41.9
41.8
41.7
41.6
Latitude (UTM)
−7.2
−7.1
−7.0
−6.9
−6.8
−6.7
−6.6
Longitude (UTM)
N
0
5
10 km
1400
1200
1000
800
600
400
Altitude (m)

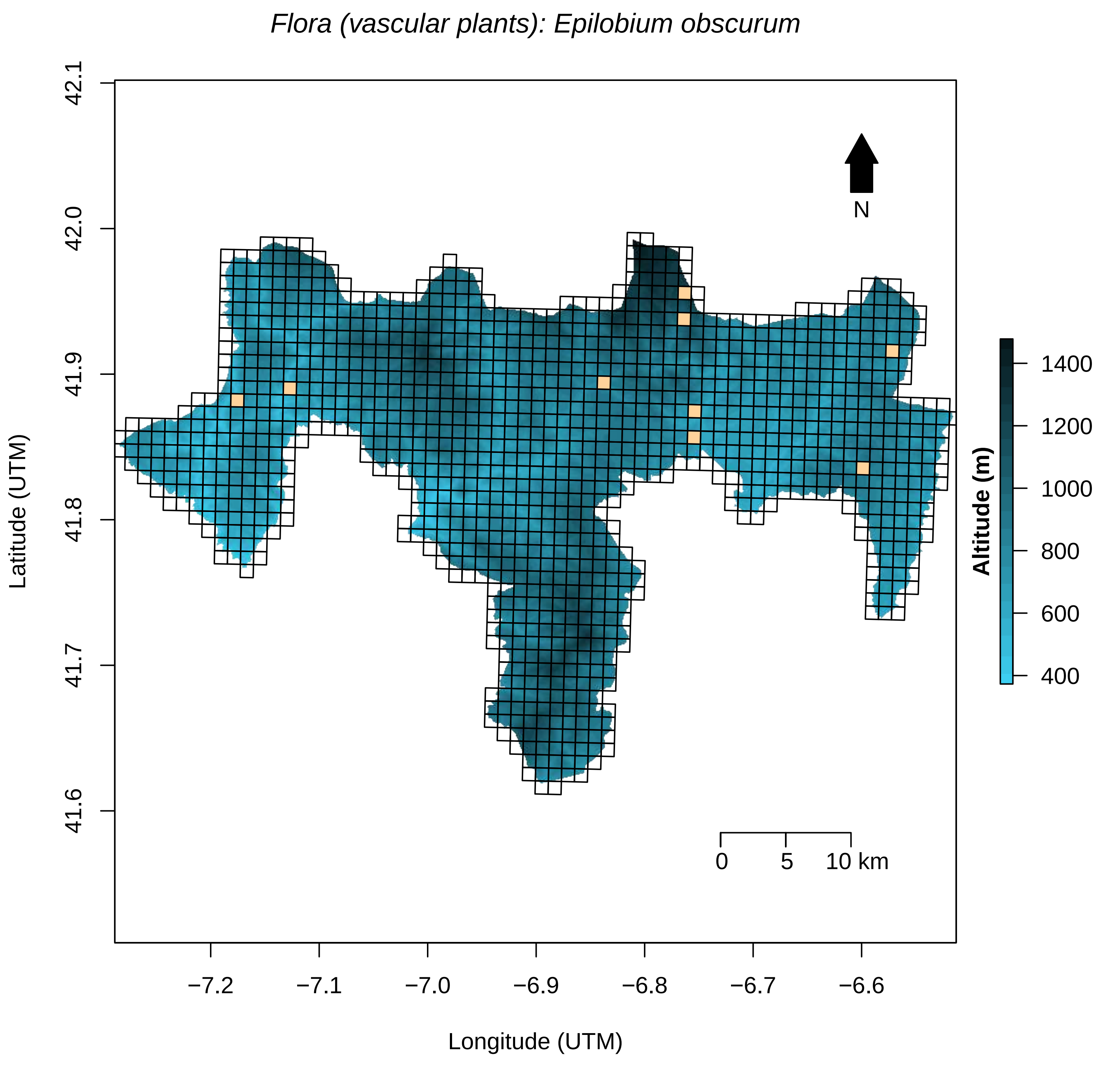

Flora (vascular plants): Epilobium obscurum
Latitude (UTM)
42.1
42.0
41.9
41.8
41.7
41.6
Longitude (UTM)
−7.2
−7.1
−7.0
−6.9
−6.8
−6.7
−6.6
N
0
5
10 km
Altitude (m)
1400
1200
1000
800
600
400

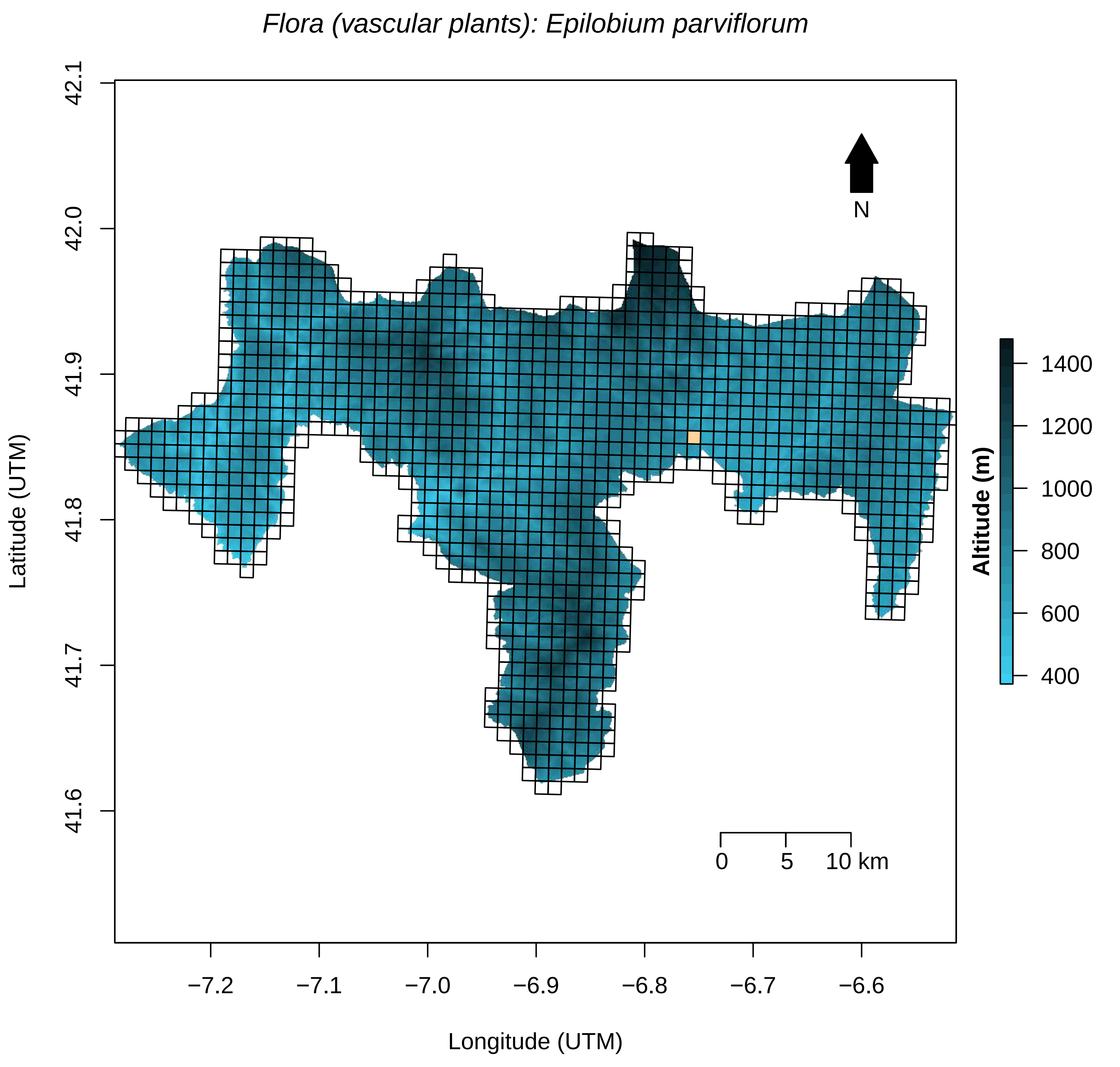

Flora (vascular plants): Epilobium parviflorum
Latitude (UTM)
42.1
42.0
41.9
41.8
41.7
41.6
N
0
5
10 km
Altitude (m)
1400
1200
1000
800
600
400
−7.2
−7.1
−7.0
−6.9
−6.8
−6.7
−6.6
Longitude (UTM)

Flora (vascular plants): *Epipactis fageticola*

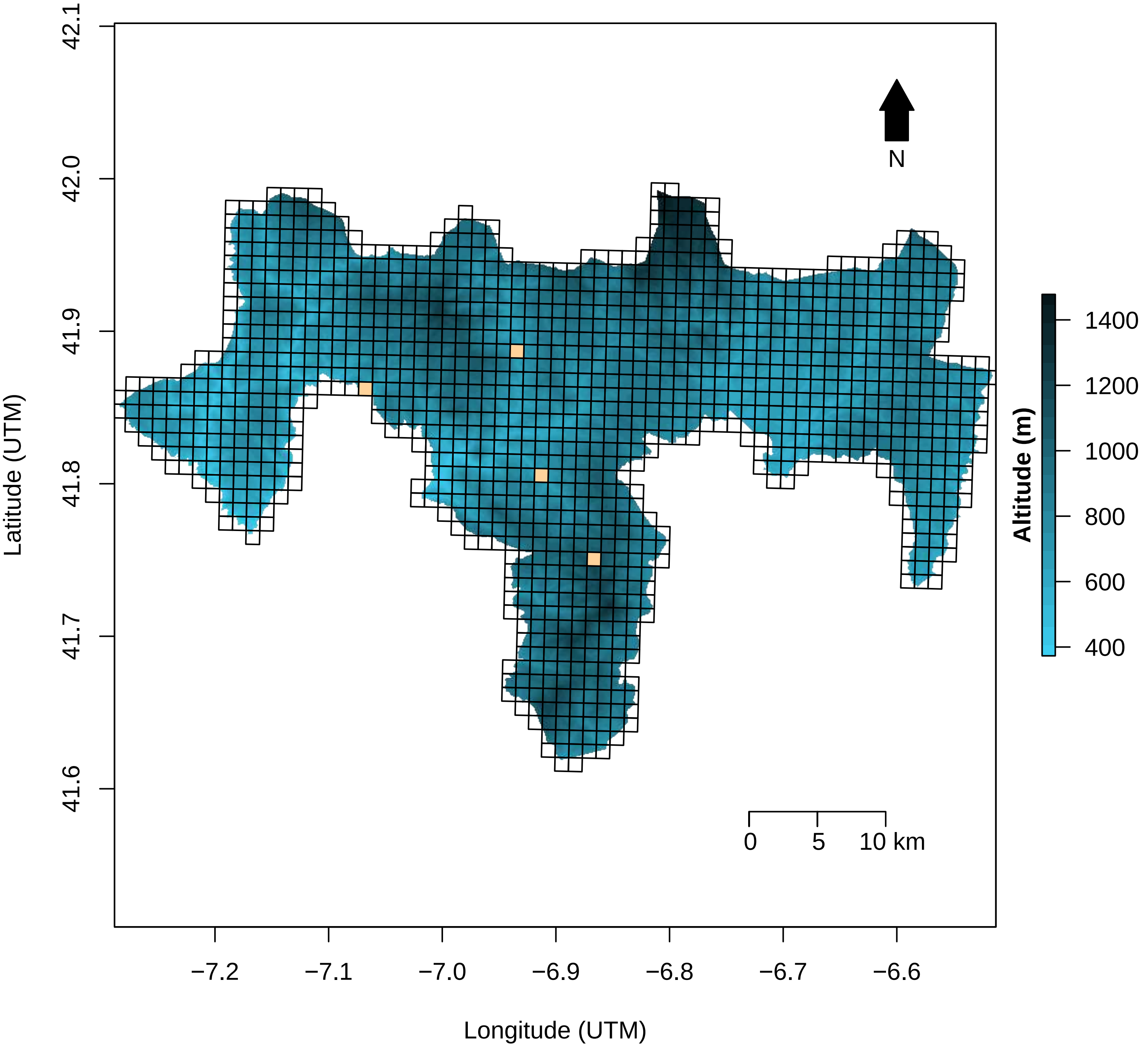

Flora (vascular plants): Epipactis tremolsii

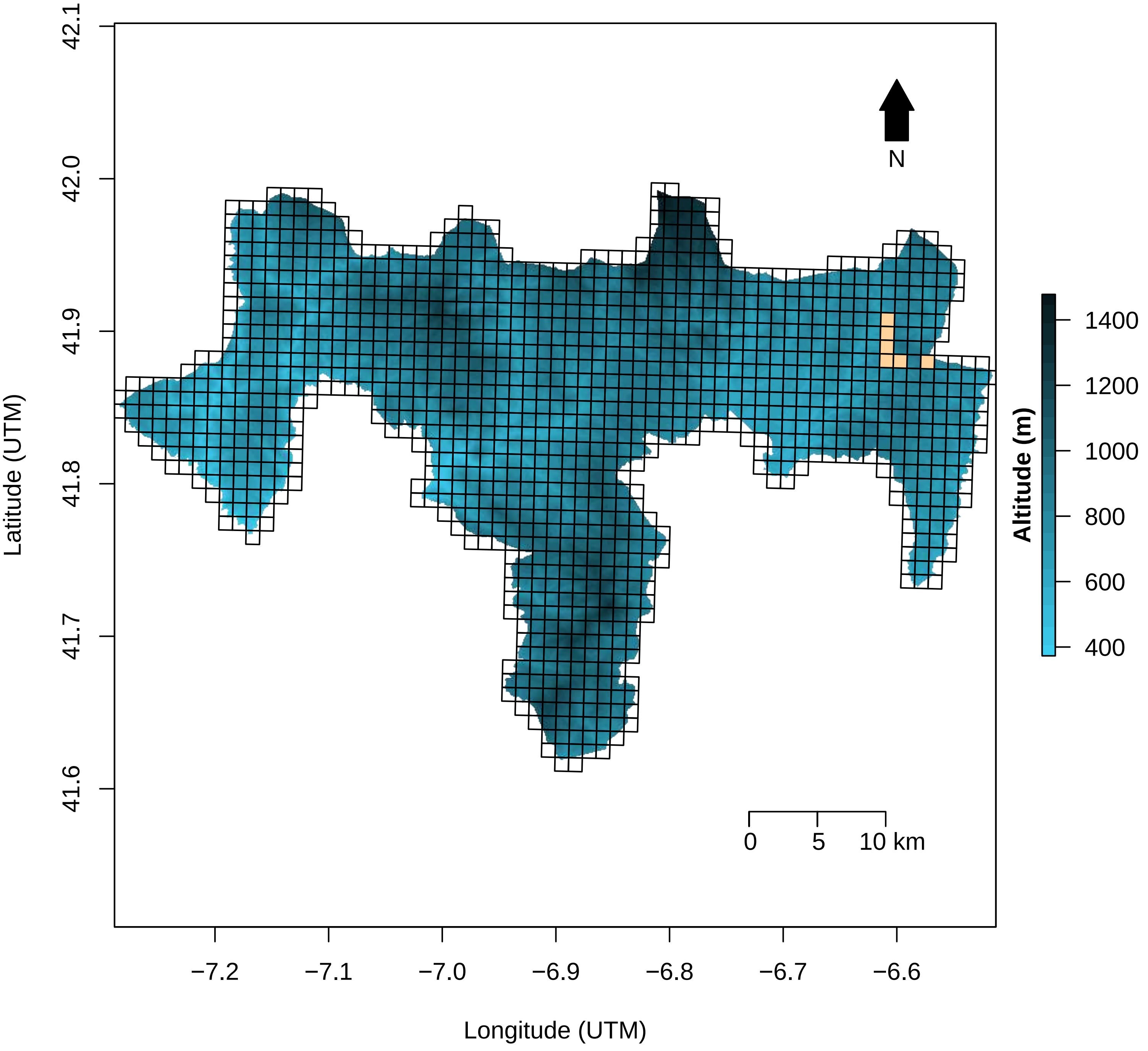

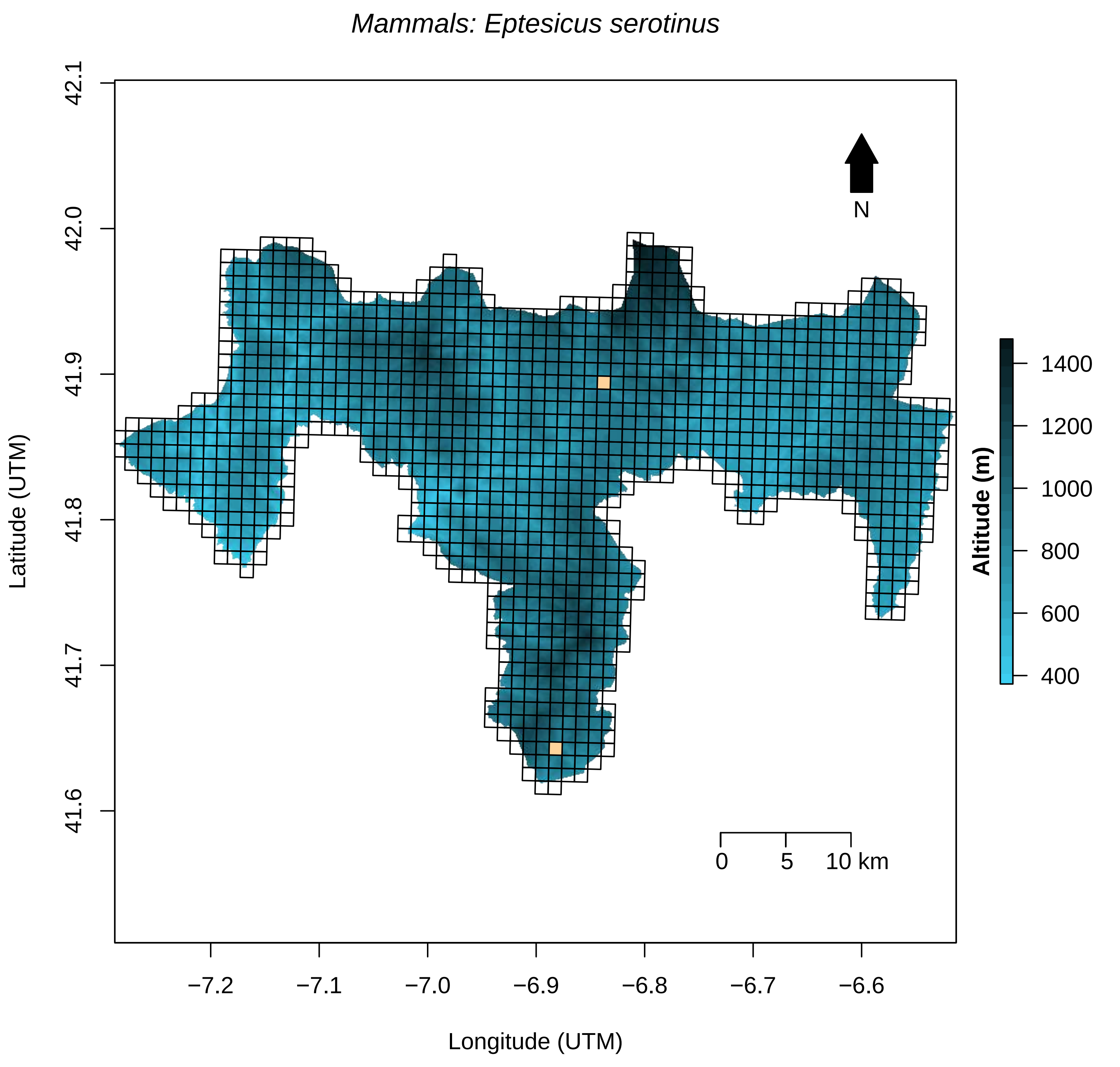
Mammals: Eptesicus serotinus
Latitude (UTM)
Longitude (UTM)
42.1
42.0
41.9
41.8
41.7
41.6
−7.2
−7.1
−7.0
−6.9
−6.8
−6.7
−6.6
N
0
5
10 km
Altitude (m)
1400
1200
1000
800
600
400

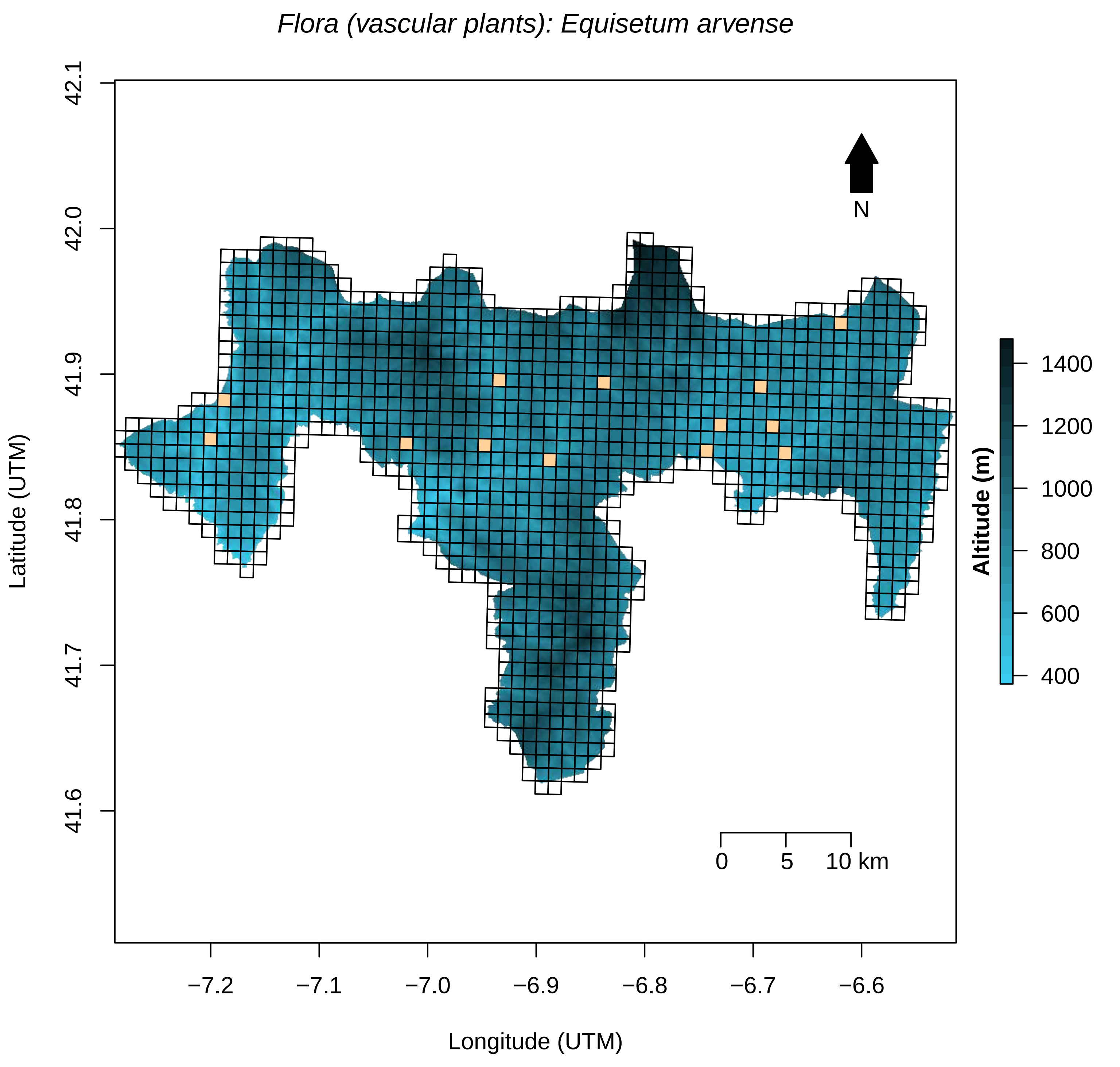

Flora (vascular plants): Equisetum arvense
42.1
42.0
41.9
41.8
41.7
41.6
Latitude (UTM)
−7.2
−7.1
−7.0
−6.9
−6.8
−6.7
−6.6
Longitude (UTM)
N
0
5
10 km
1400
1200
1000
800
600
400
Altitude (m)

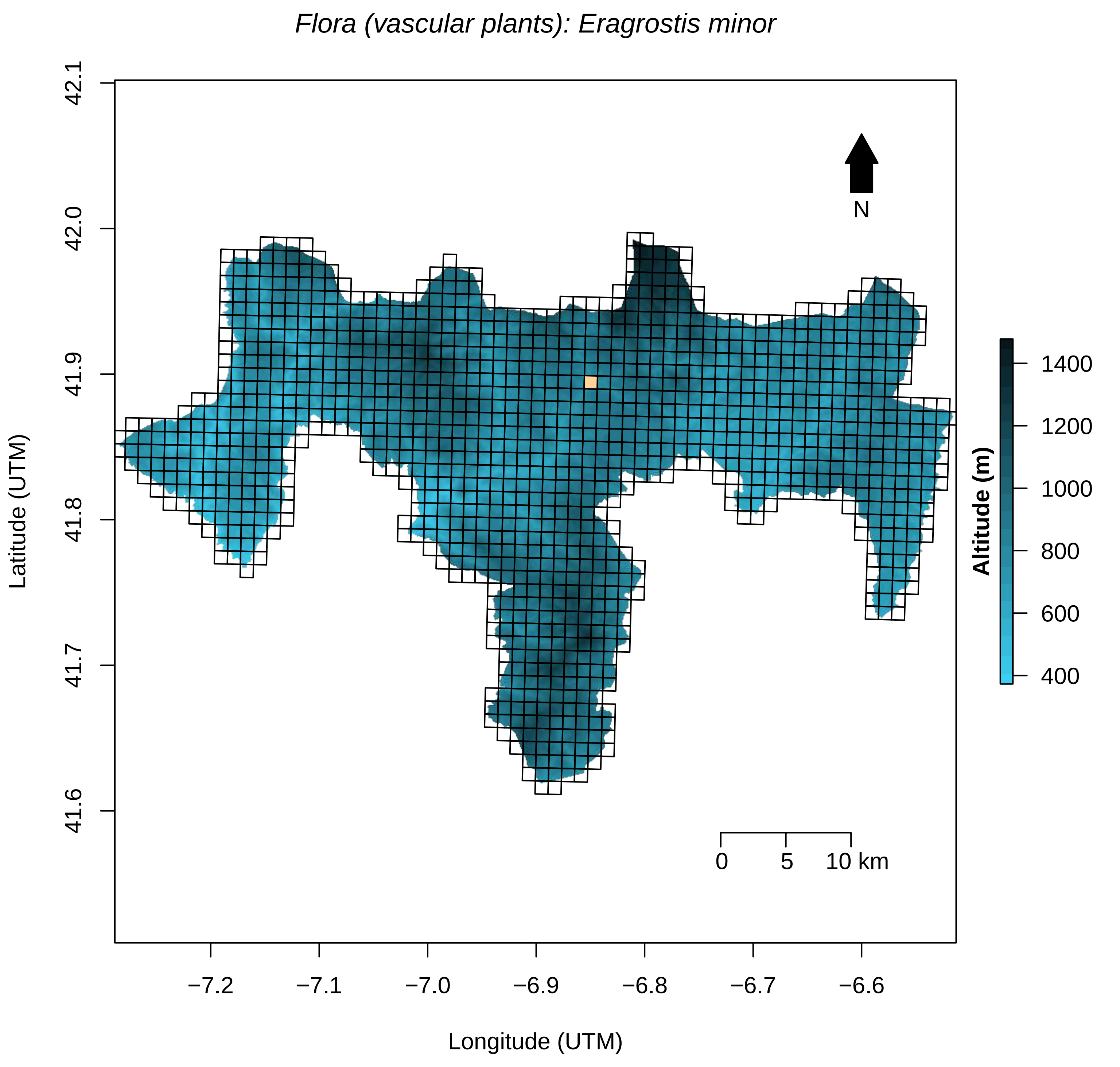

Flora (vascular plants): Eragrostis minor
42.1
42.0
41.9
41.8
41.7
41.6
Latitude (UTM)
−7.2
−7.1
−7.0
−6.9
−6.8
−6.7
−6.6
Longitude (UTM)
N
0
5
10 km
Altitude (m)
1400
1200
1000
800
600
400

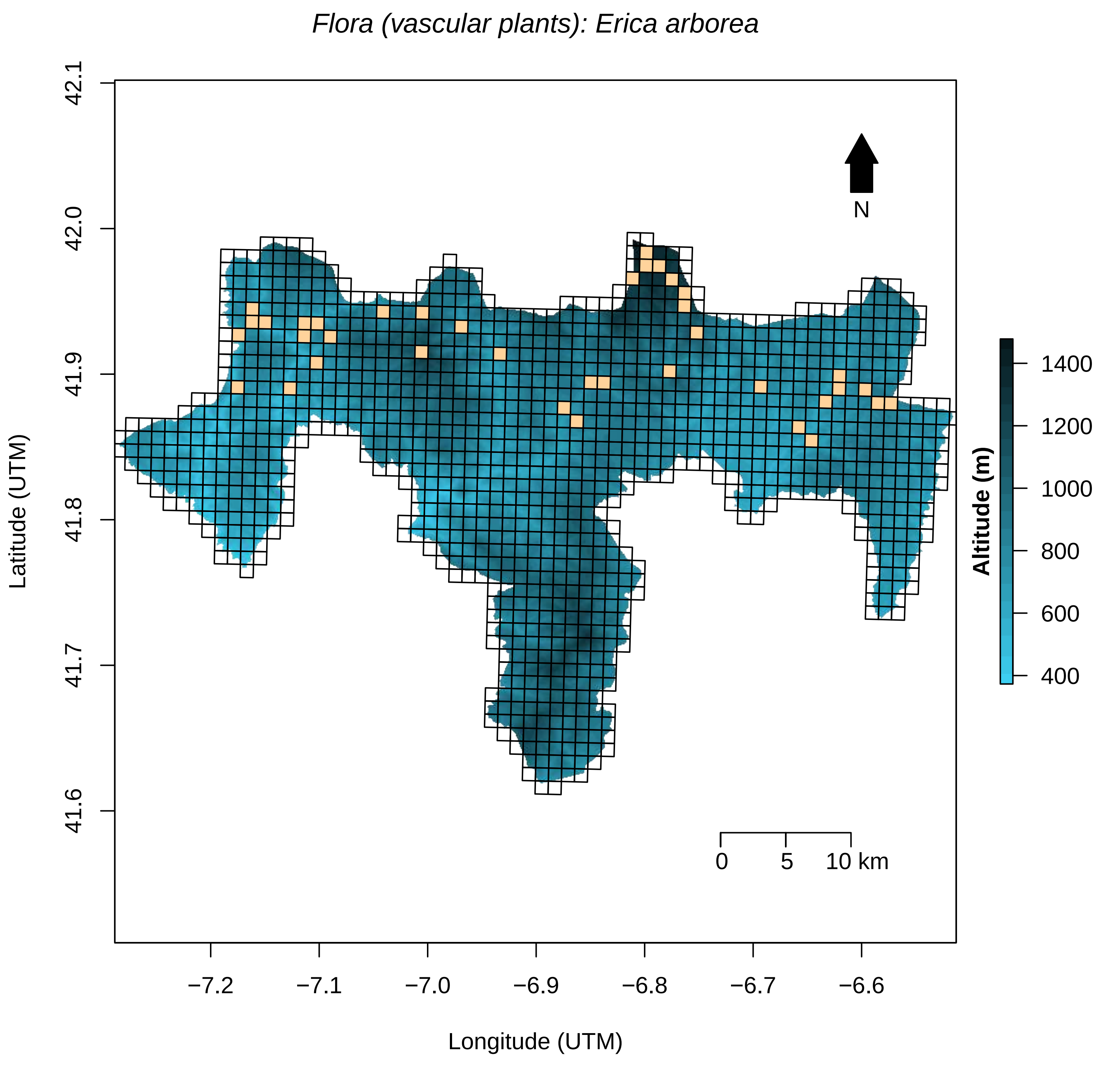
Flora (vascular plants): Erica arborea
Latitude (UTM)
42.1
42.0
41.9
41.8
41.7
41.6
Longitude (UTM)
−7.2
−7.1
−7.0
−6.9
−6.8
−6.7
−6.6
N
0
5
10 km
Altitude (m)
1400
1200
1000
800
600
400

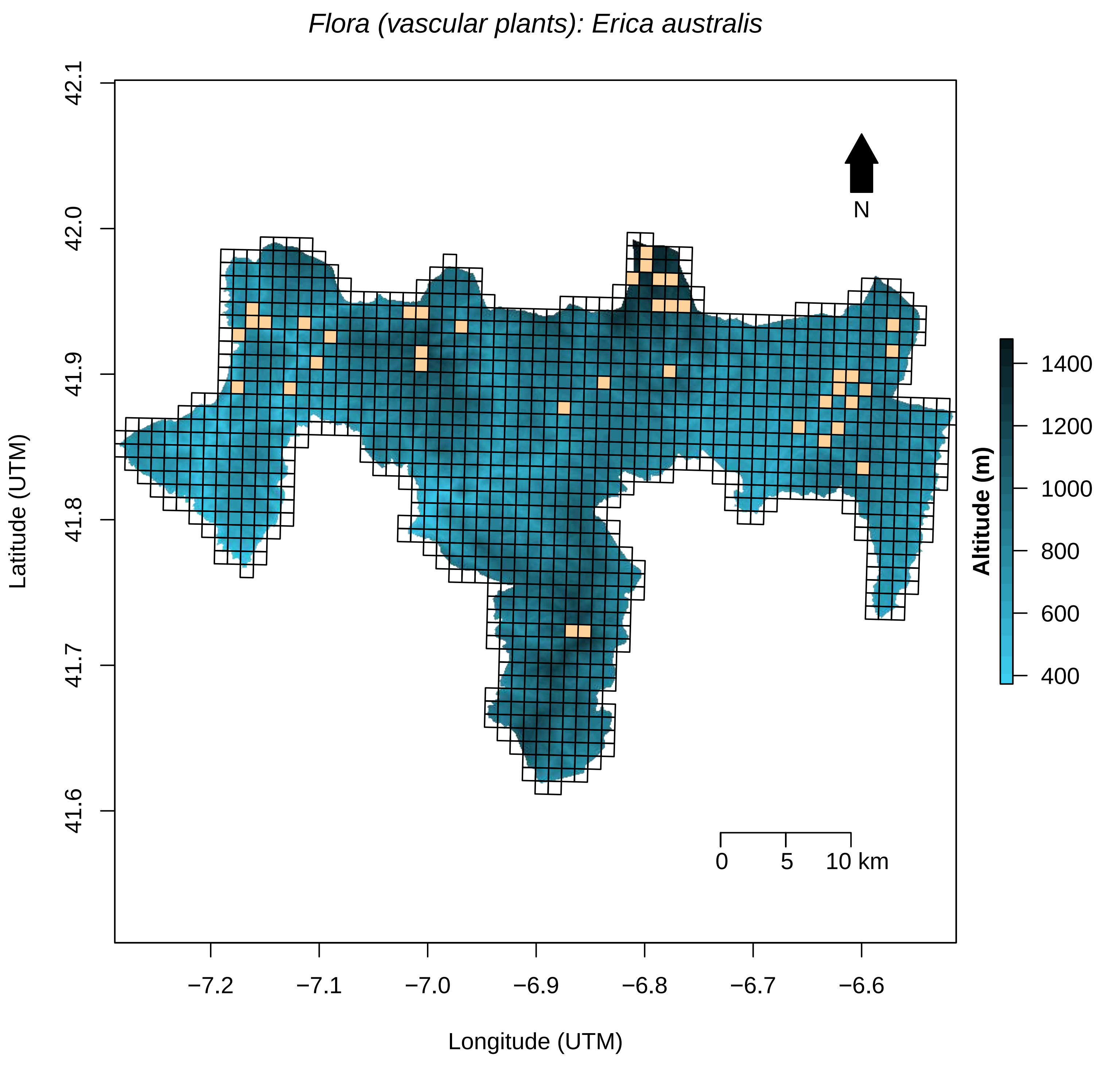

Flora (vascular plants): Erica australis
N
0
5
10 km
Altitude (m)
1400
1200
1000
800
600
400
Latitude (UTM)
42.1
42.0
41.9
41.8
41.7
41.6
Longitude (UTM)
−7.2
−7.1
−7.0
−6.9
−6.8
−6.7
−6.6

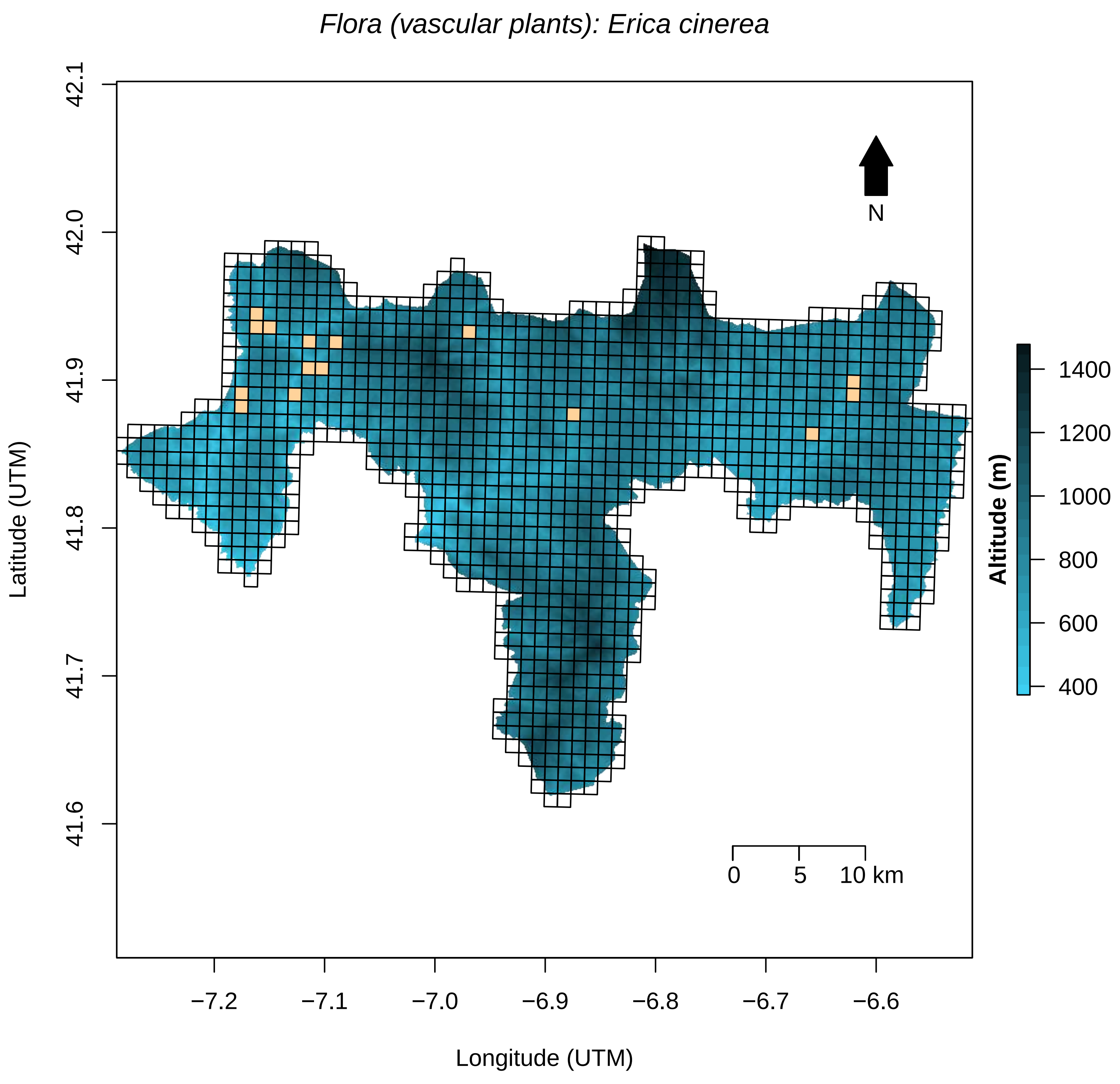

Flora (vascular plants): Erica cinerea
N
0
5
10 km
Altitude (m)
1400
1200
1000
800
600
400
Latitude (UTM)
42.1
42.0
41.9
41.8
41.7
41.6
Longitude (UTM)
−7.2
−7.1
−7.0
−6.9
−6.8
−6.7
−6.6

Flora (vascular plants): Erica scoparia

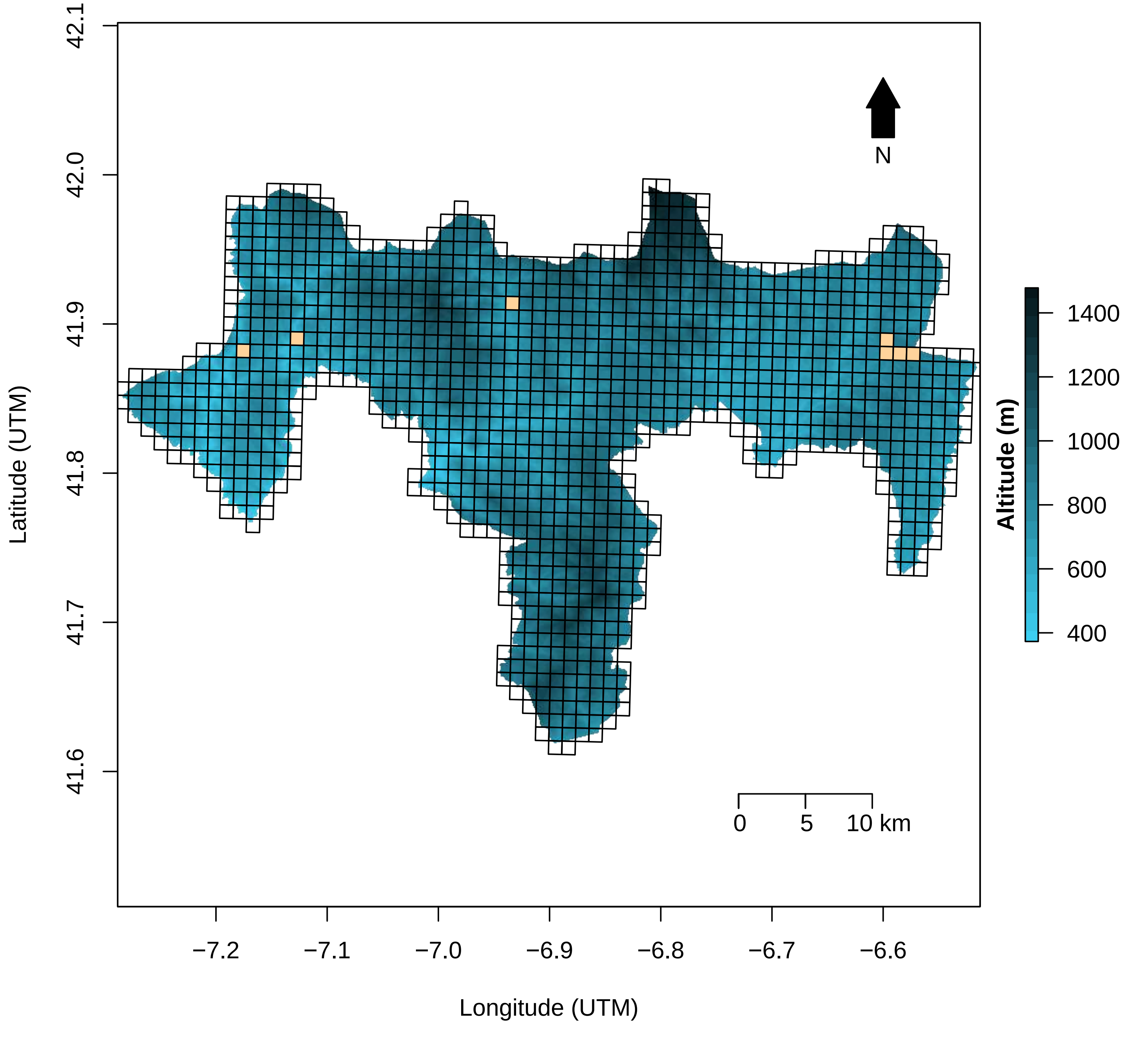

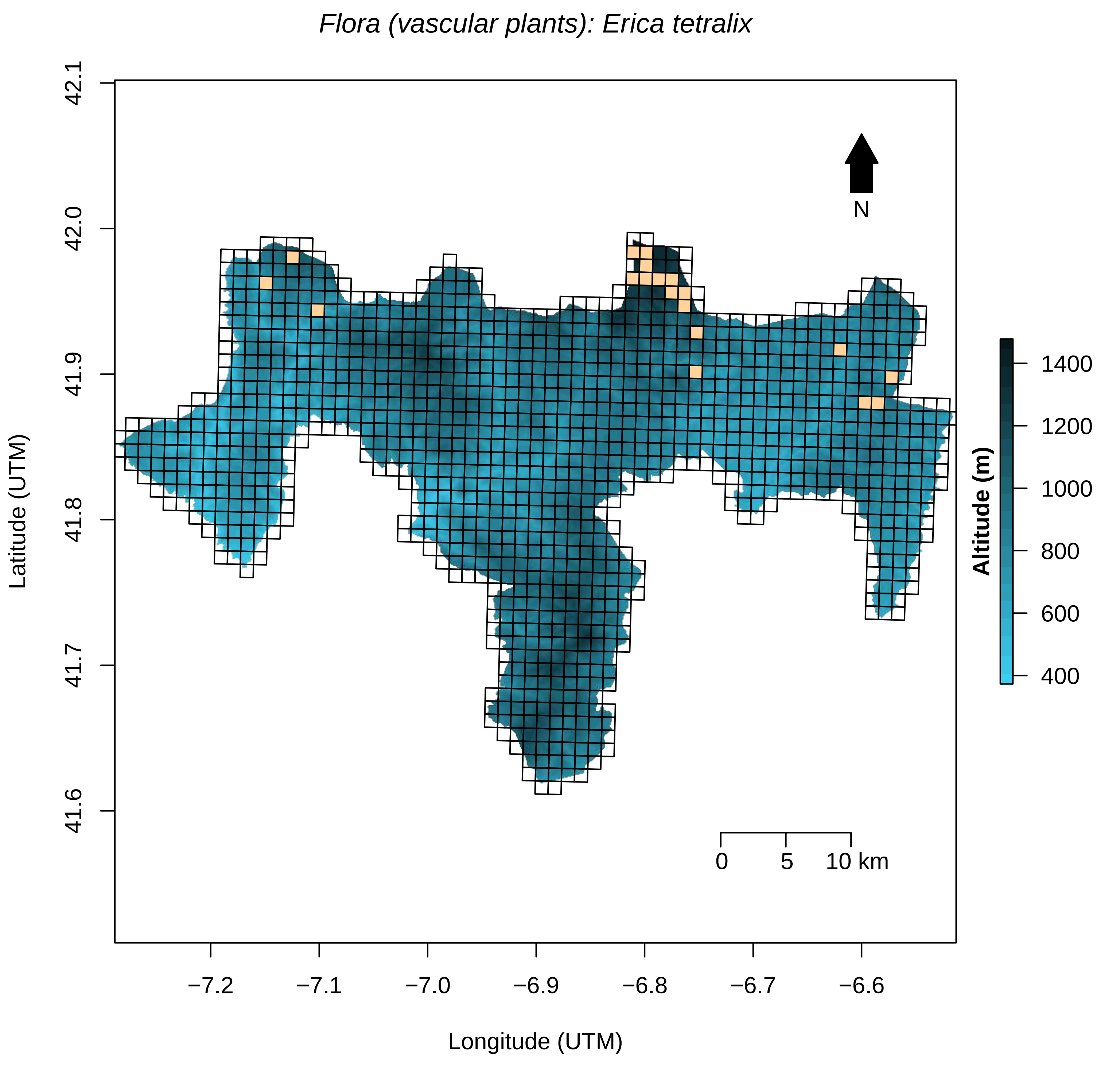
Flora (vascular plants): Erica tetralix
Latitude (UTM)
Longitude (UTM)
42.1
42.0
41.9
41.8
41.7
41.6
−7.2
−7.1
−7.0
−6.9
−6.8
−6.7
−6.6
N
0
5
10 km
Altitude (m)
1400
1200
1000
800
600
400

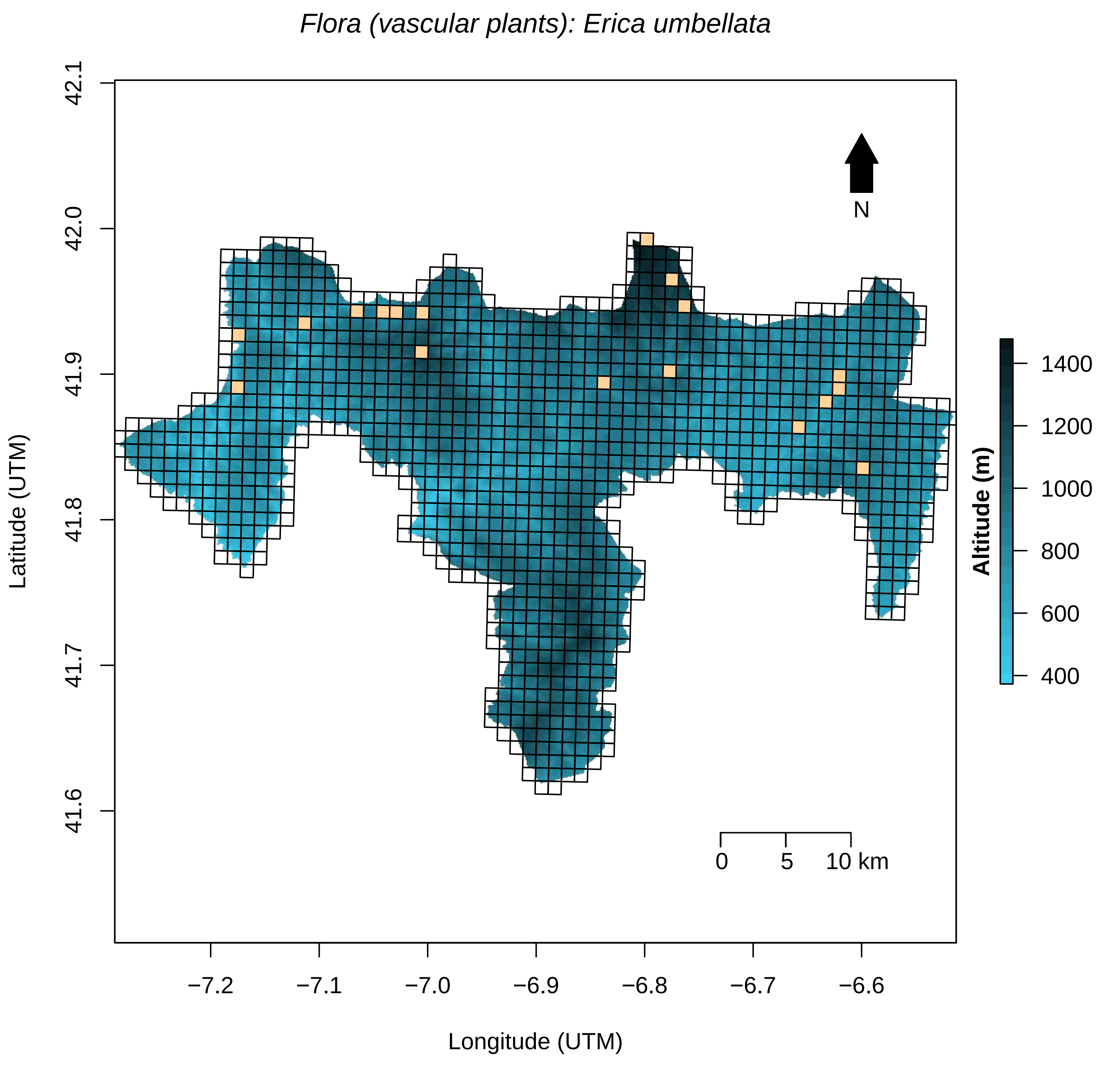

Flora (vascular plants): Erica umbellata
Latitude (UTM)
42.1
42.0
41.9
41.8
41.7
41.6
N
0
5
10 km
Altitude (m)
1400
1200
1000
800
600
400
−7.2
−7.1
−7.0
−6.9
−6.8
−6.7
−6.6
Longitude (UTM)

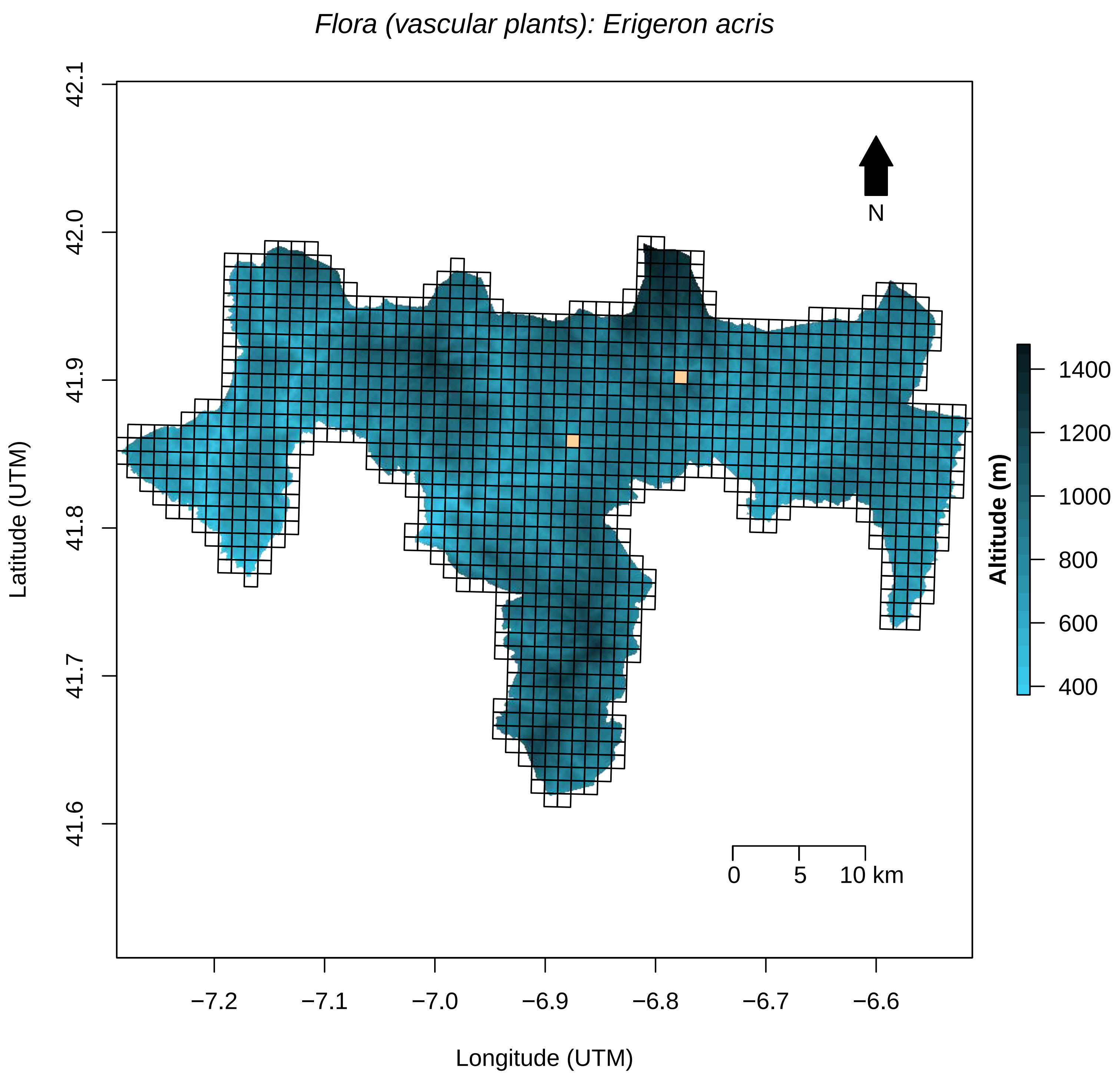

Flora (vascular plants): Erigeron acris
42.1
42.0
41.9
41.8
41.7
41.6
Latitude (UTM)
−7.2
−7.1
−7.0
−6.9
−6.8
−6.7
−6.6
Longitude (UTM)
N
0
5
10 km
Altitude (m)
1400
1200
1000
800
600
400

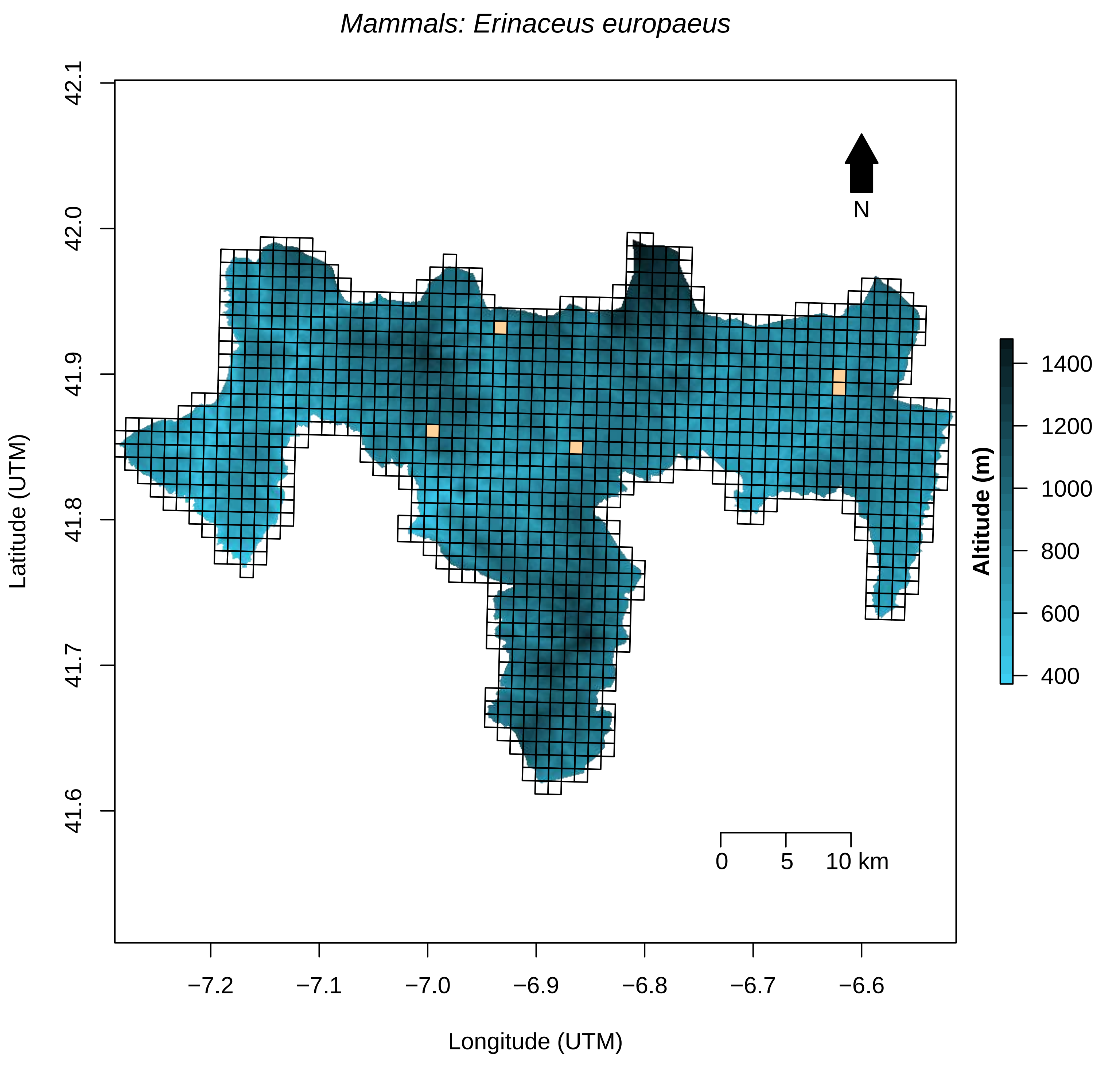

Mammals: Erinaceus europaeus
42.1
42.0
41.9
41.8
41.7
41.6
Latitude (UTM)
−7.2
−7.1
−7.0
−6.9
−6.8
−6.7
−6.6
Longitude (UTM)
N
0
5
10 km
1400
1200
1000
800
600
400
Altitude (m)

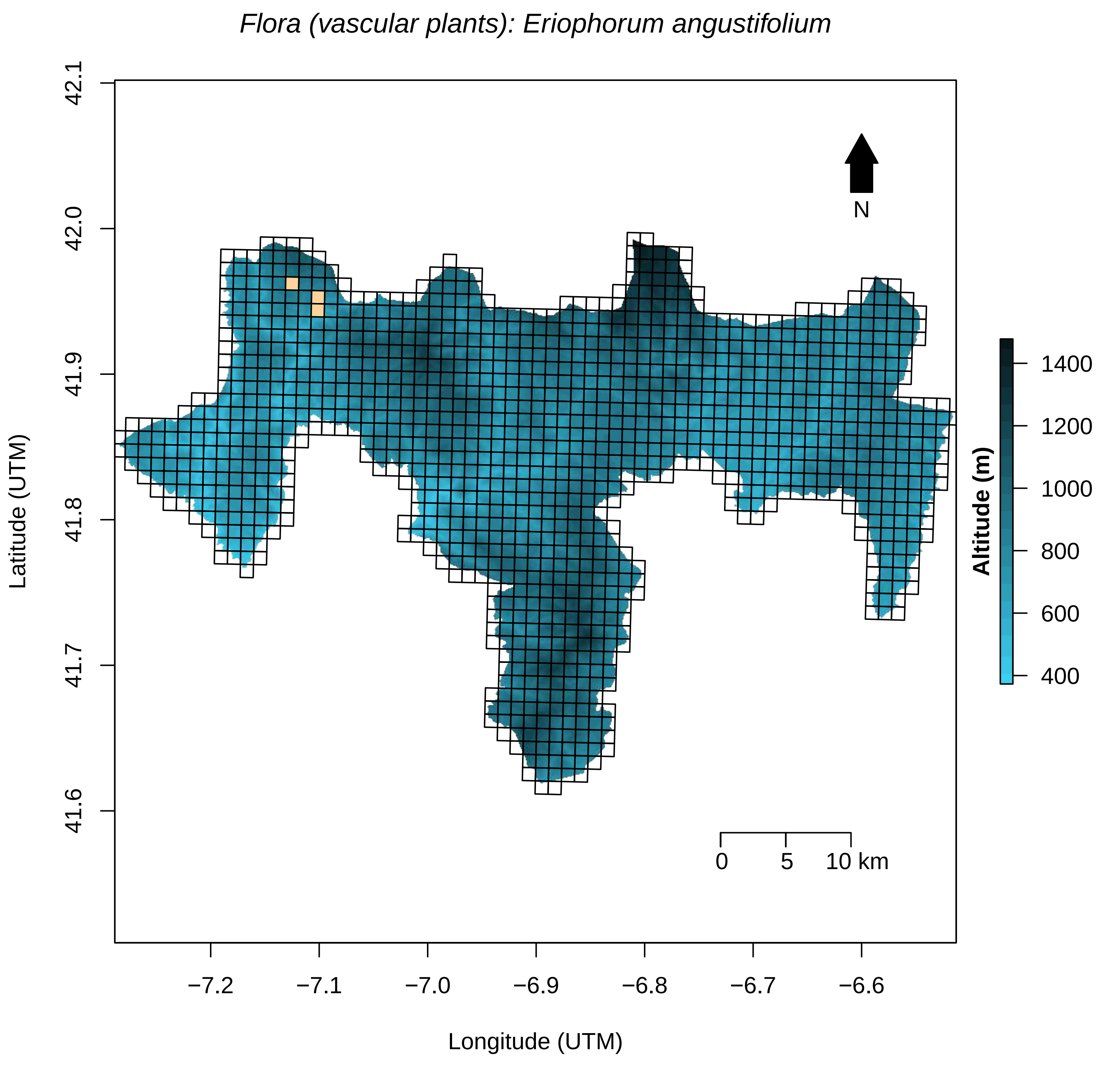
Flora (vascular plants): Eriophorum angustifolium
N
0
5
10 km
Altitude (m)
1400
1200
1000
800
600
400
42.1
42.0
41.9
41.8
41.7
41.6
Latitude (UTM)
−7.2
−7.1
−7.0
−6.9
−6.8
−6.7
−6.6
Longitude (UTM)

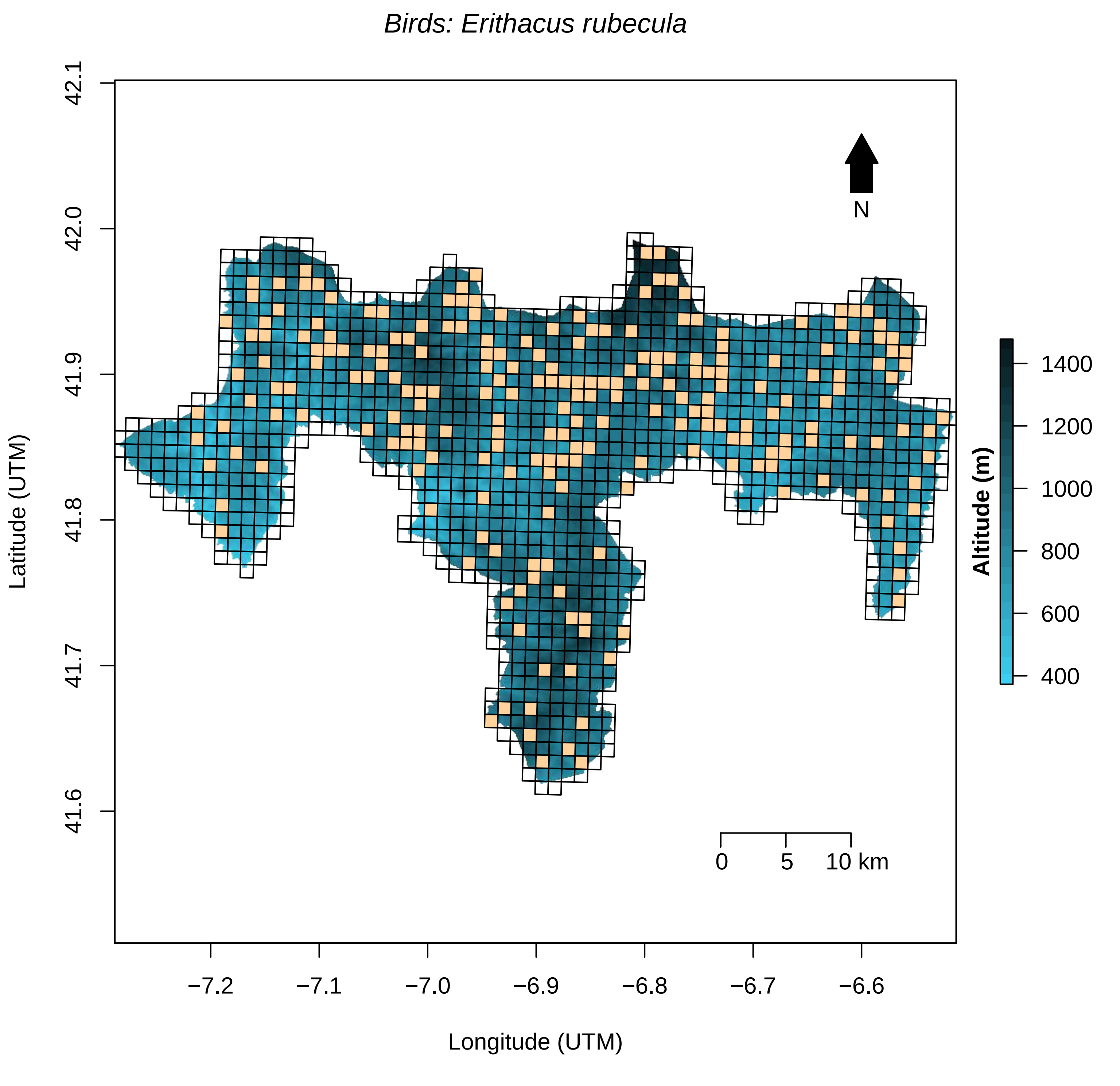

Birds: Erithacus rubecula
42.1
42.0
41.9
41.8
41.7
41.6
Latitude (UTM)
−7.2
−7.1
−7.0
−6.9
−6.8
−6.7
−6.6
Longitude (UTM)
N
0
5
10 km
Altitude (m)
1400
1200
1000
800
600
400

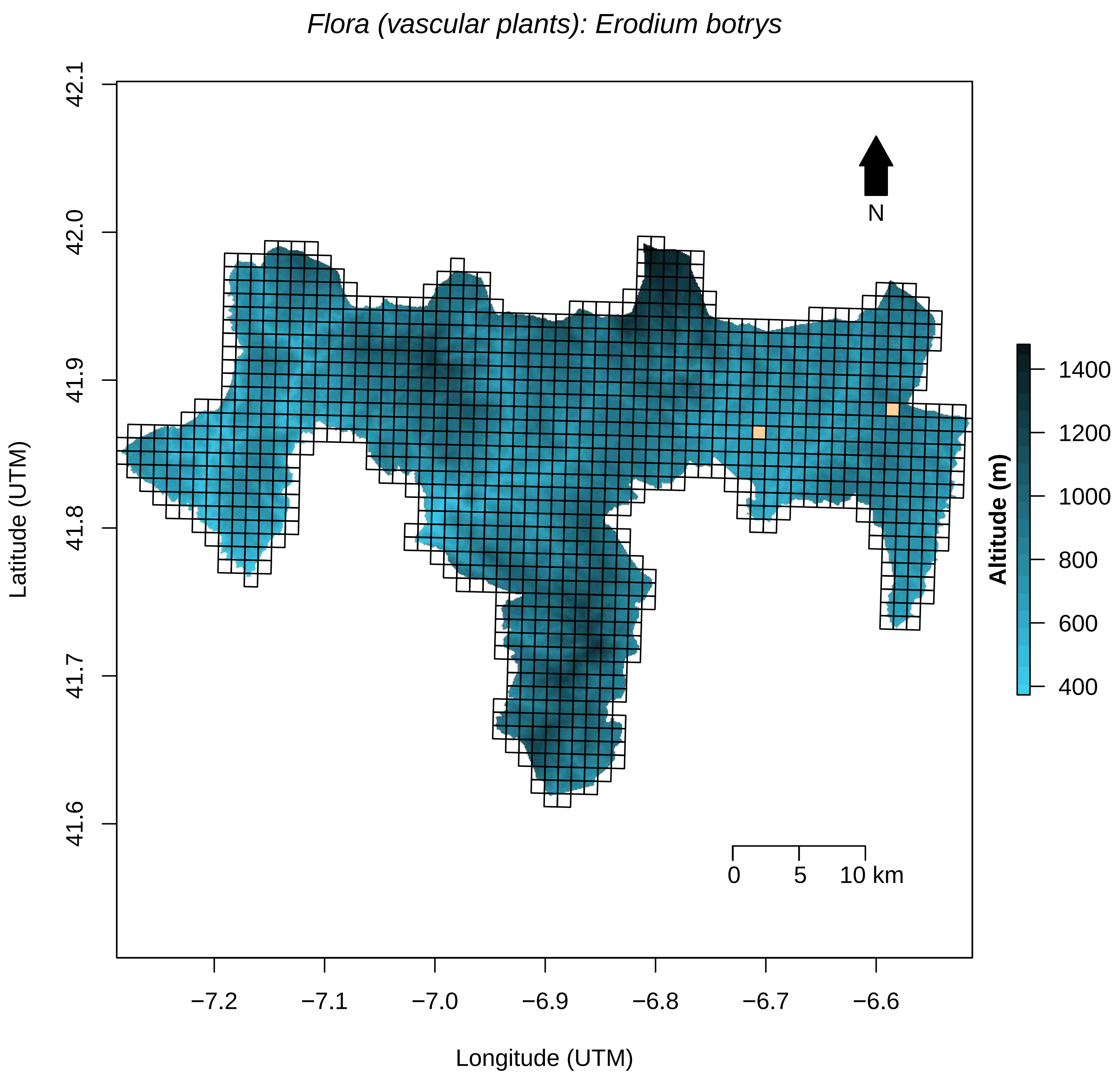

Flora (vascular plants): Erodium botrys
42.1
42.0
41.9
41.8
41.7
41.6
Latitude (UTM)
−7.2
−7.1
−7.0
−6.9
−6.8
−6.7
−6.6
Longitude (UTM)
N
0
5
10 km
Altitude (m)
1400
1200
1000
800
600
400

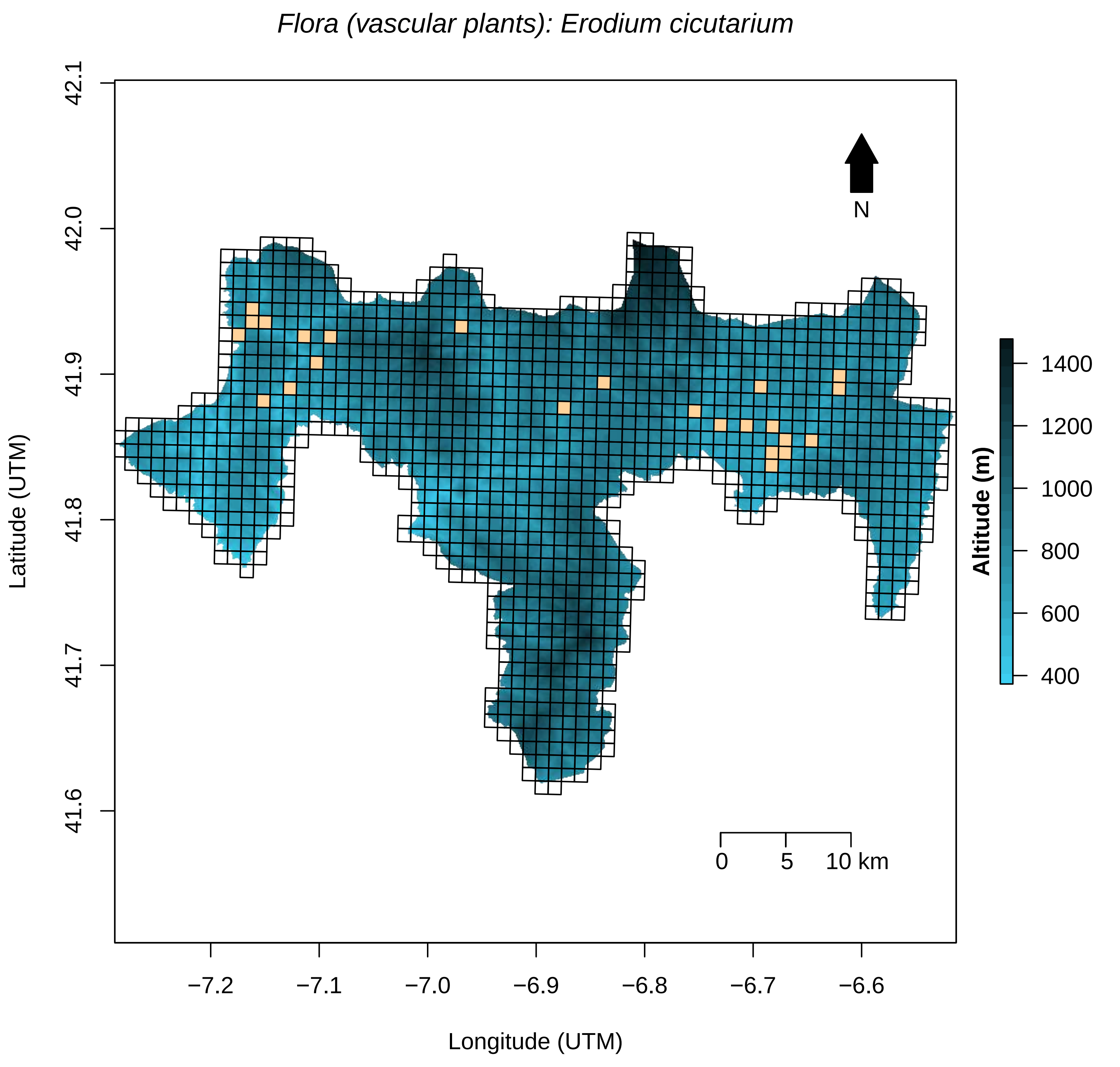

Flora (vascular plants): Erodium cicutarium
Latitude (UTM)
42.1
42.0
41.9
41.8
41.7
41.6
Longitude (UTM)
−7.2
−7.1
−7.0
−6.9
−6.8
−6.7
−6.6
N
0
5
10 km
Altitude (m)
1400
1200
1000
800
600
400

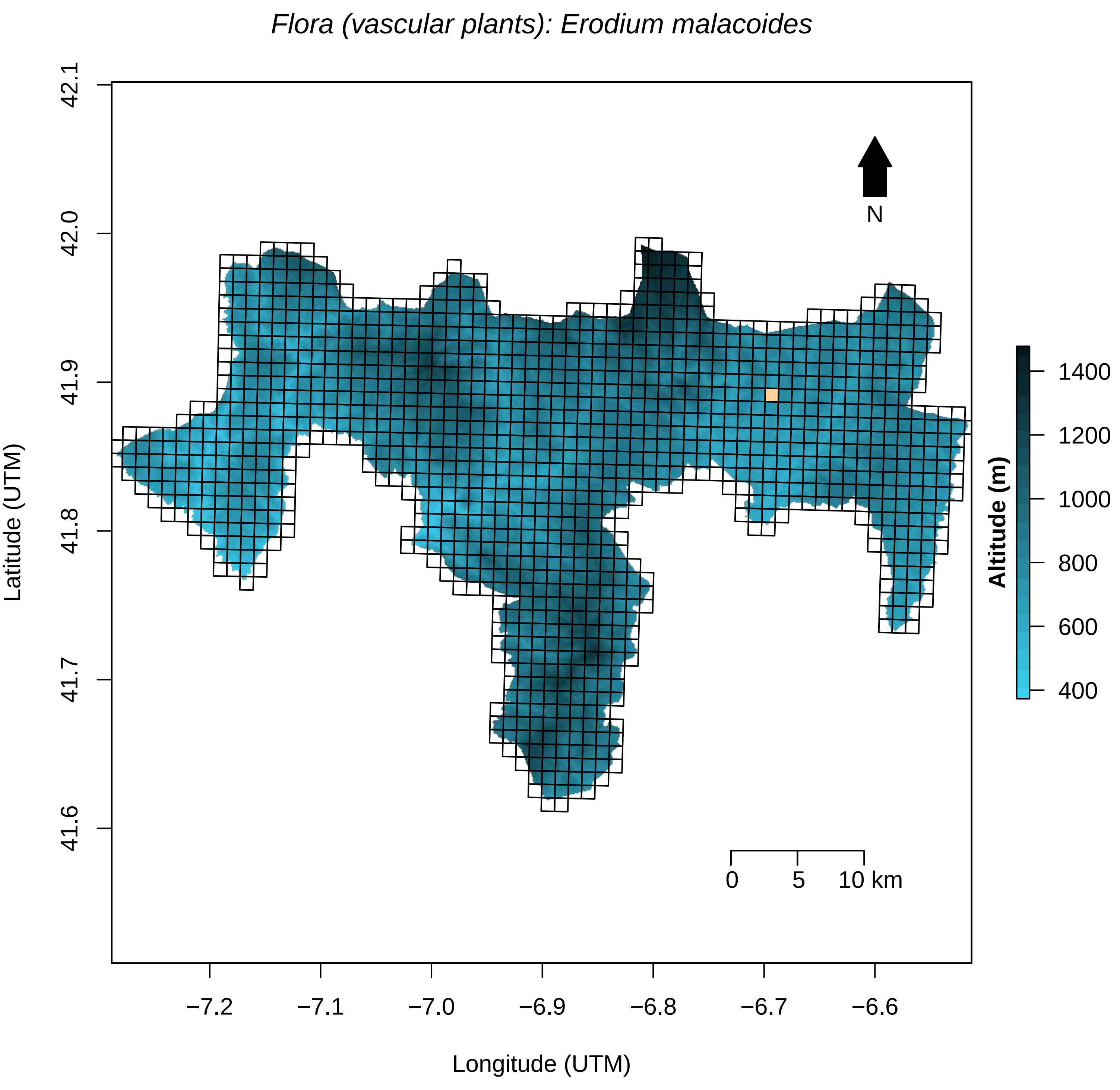

Flora (vascular plants): Erodium malacoides
42.1
42.0
41.9
41.8
41.7
41.6
Latitude (UTM)
N
0
5
10 km
−7.2
−7.1
−7.0
−6.9
−6.8
−6.7
−6.6
Longitude (UTM)
Altitude (m)
1400
1200
1000
800
600
400

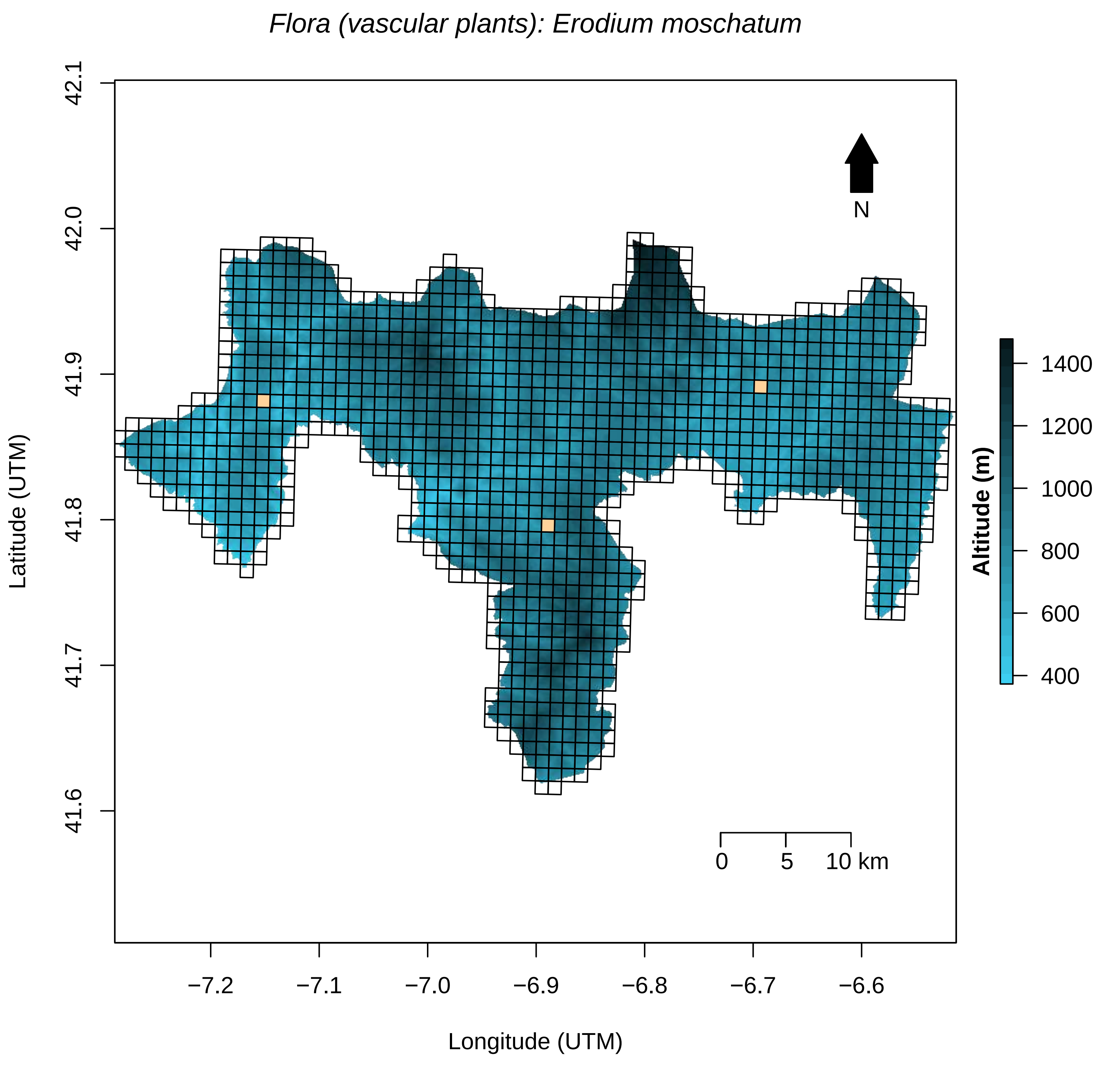
Flora (vascular plants): Erodium moschatum
42.1
42.0
41.9
41.8
41.7
41.6
Latitude (UTM)
N
0
5
10 km
−7.2
−7.1
−7.0
−6.9
−6.8
−6.7
−6.6
Longitude (UTM)
Altitude (m)
1400
1200
1000
800
600
400

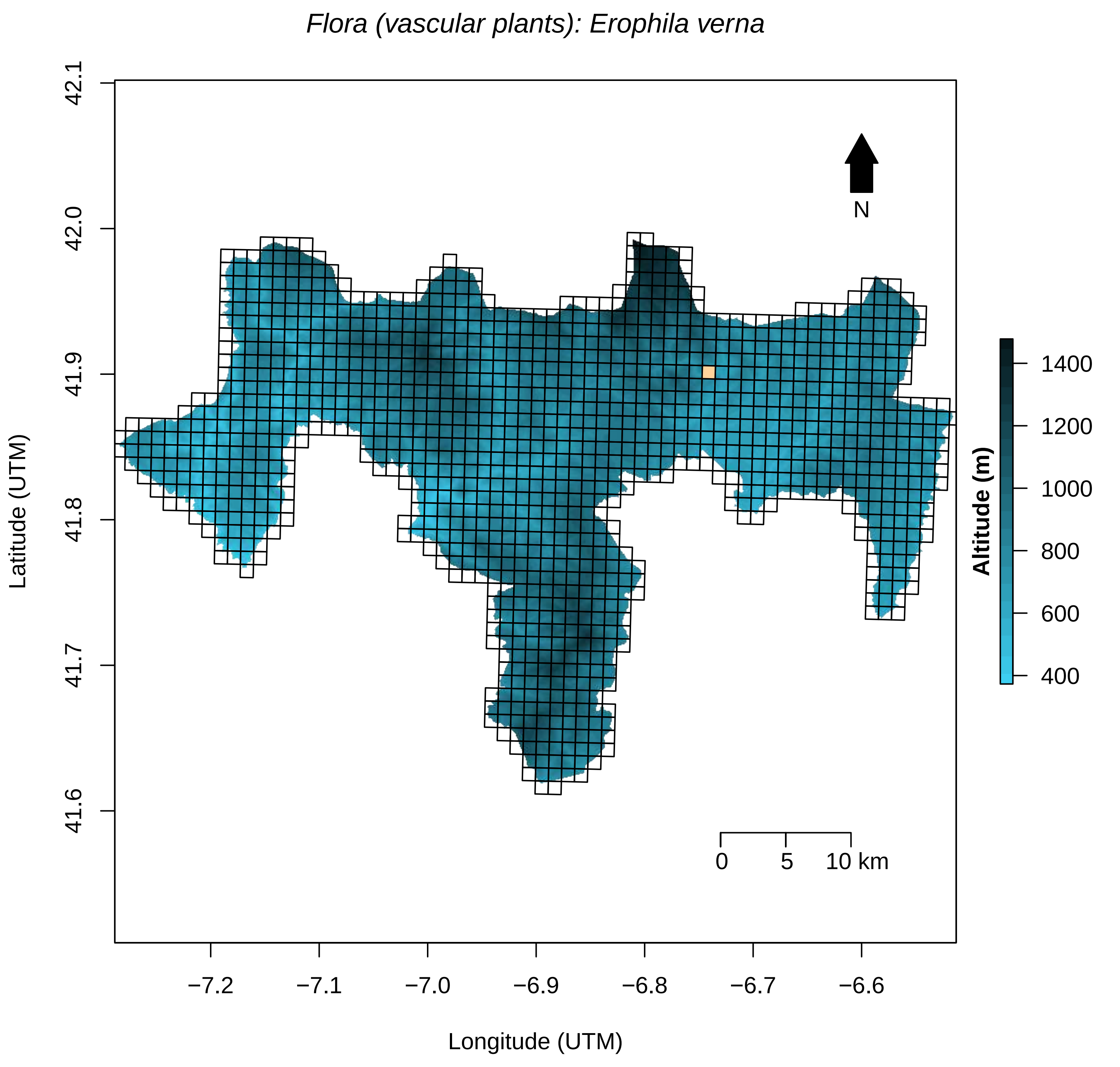

Flora (vascular plants): Erophila verna
42.1
42.0
41.9
41.8
41.7
41.6
Latitude (UTM)
−7.2
−7.1
−7.0
−6.9
−6.8
−6.7
−6.6
Longitude (UTM)
N
0
5
10 km
Altitude (m)
1400
1200
1000
800
600
400

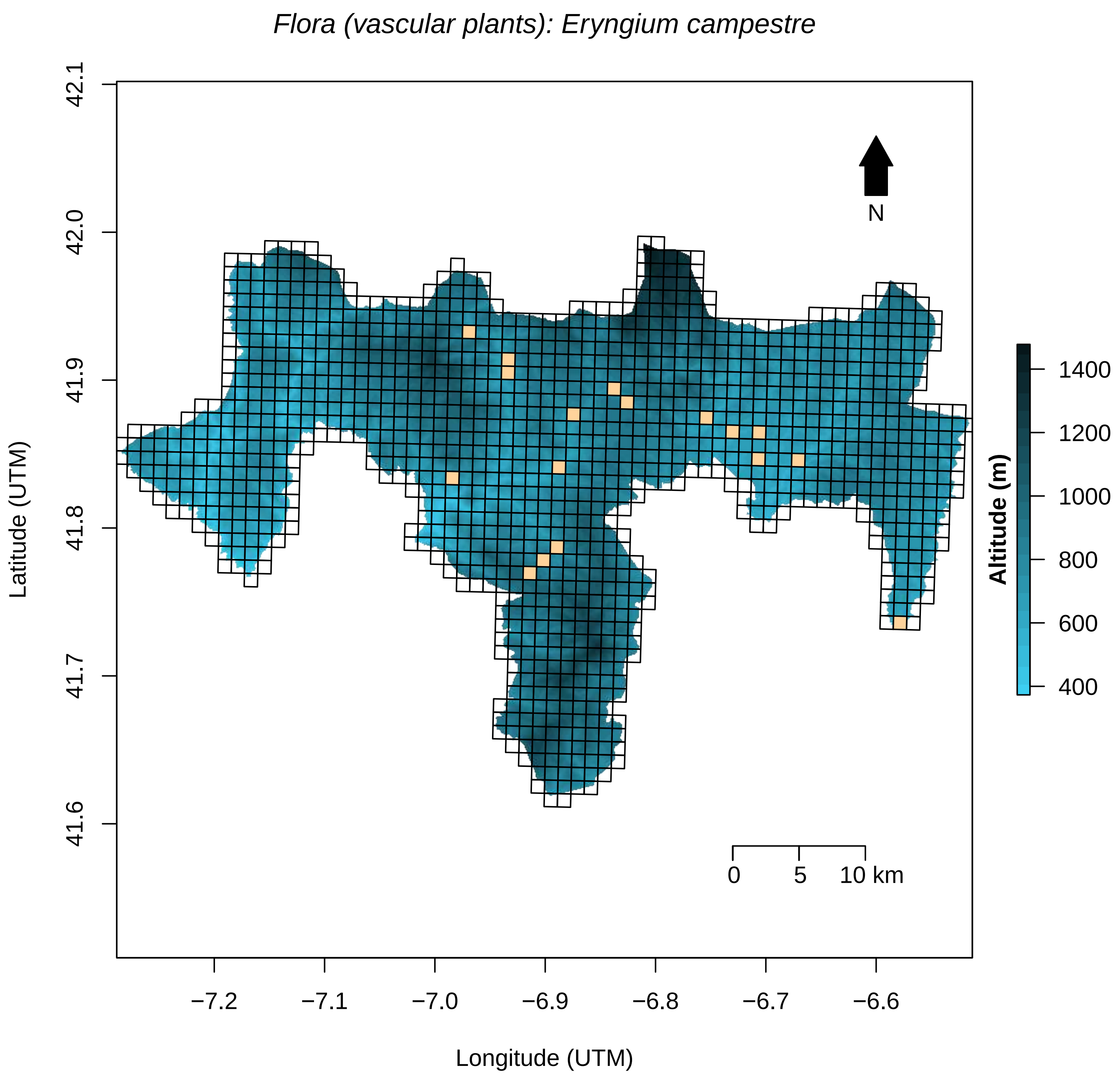

Flora (vascular plants): Eryngium campestre
42.1
42.0
41.9
41.8
41.7
41.6
Latitude (UTM)
−7.2
−7.1
−7.0
−6.9
−6.8
−6.7
−6.6
Longitude (UTM)
N
0
5
10 km
Altitude (m)
1400
1200
1000
800
600
400

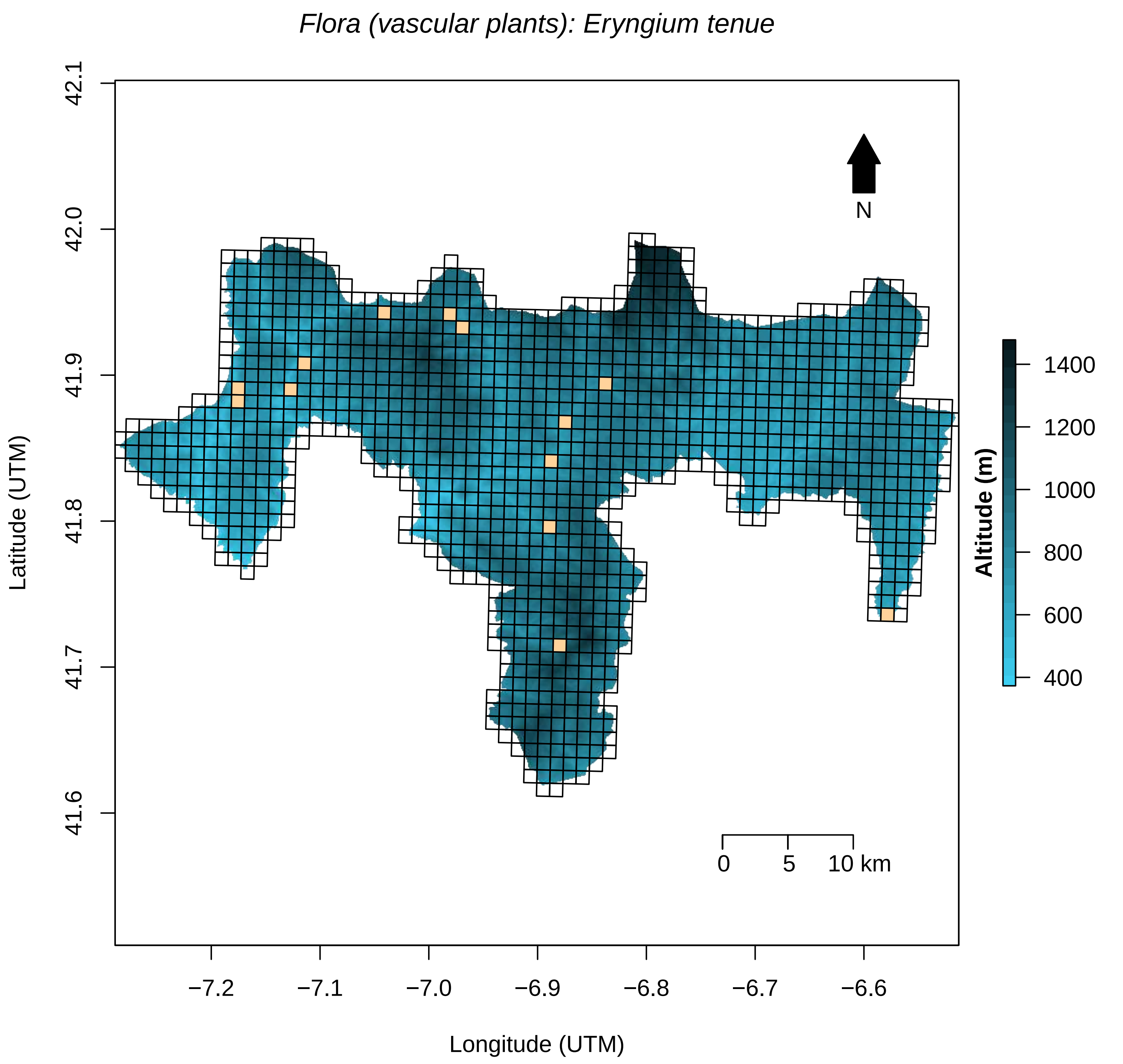

Flora (vascular plants): Eryngium tenue
42.1
42.0
41.9
41.8
41.7
41.6
Latitude (UTM)
−7.2
−7.1
−7.0
−6.9
−6.8
−6.7
−6.6
Longitude (UTM)
N
0
5
10 km
1400
1200
1000
800
600
400
Altitude (m)

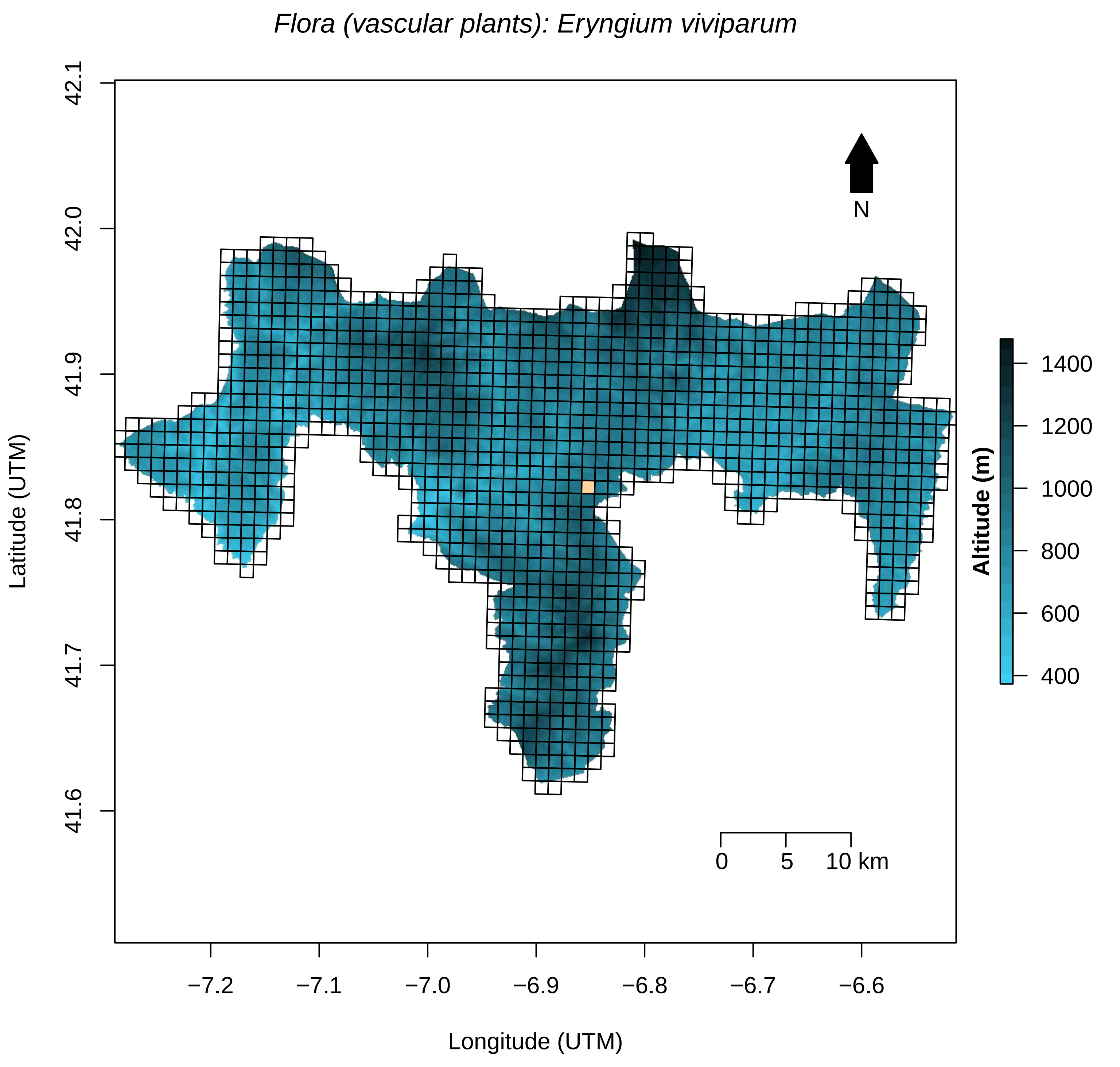

Flora (vascular plants): Eryngium viviparum
N
0
5
10 km
Altitude (m)
1400
1200
1000
800
600
400
Latitude (UTM)
42.1
42.0
41.9
41.8
41.7
41.6
Longitude (UTM)
−7.2
−7.1
−7.0
−6.9
−6.8
−6.7
−6.6

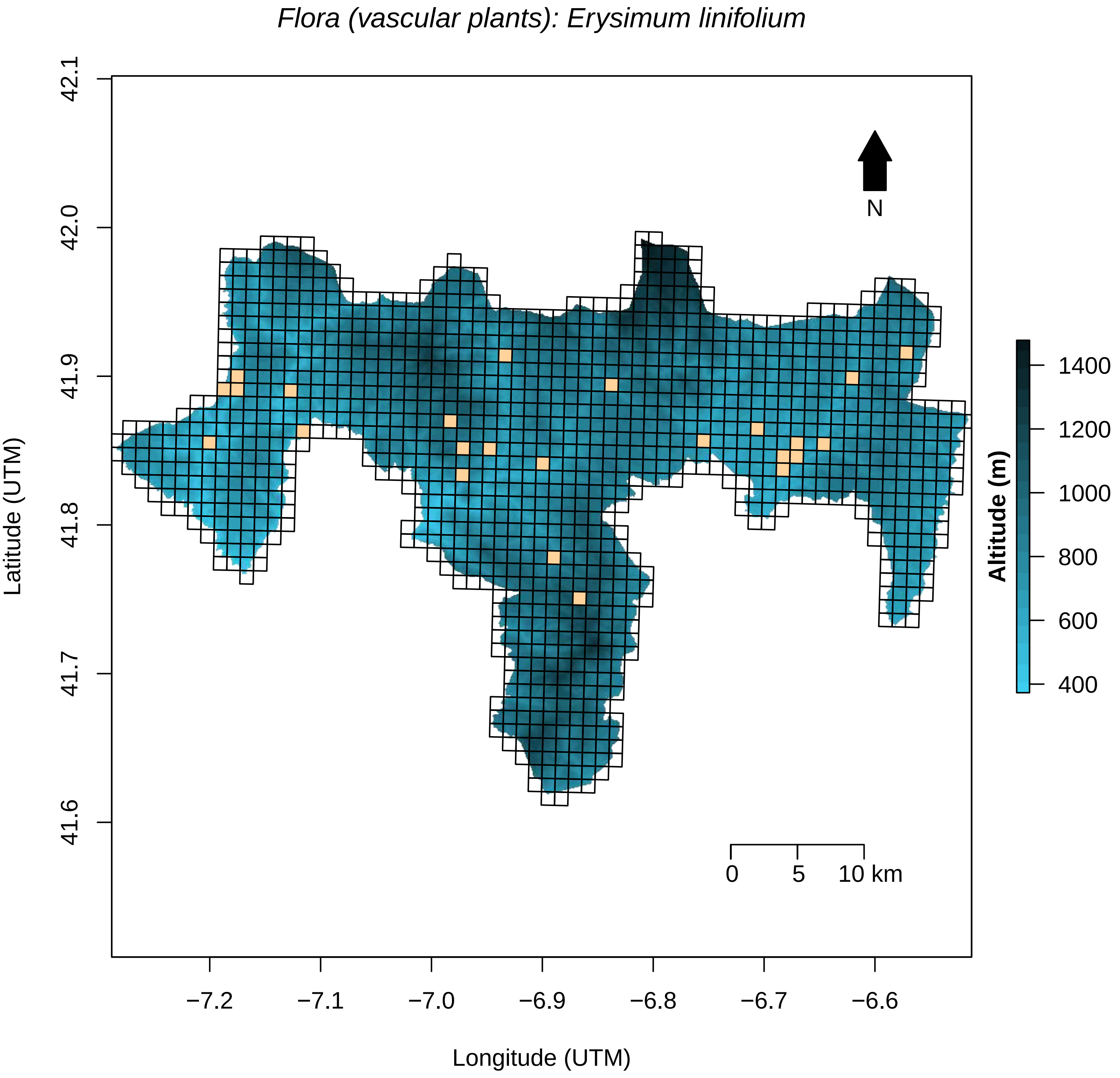

Flora (vascular plants): Erysimum linifolium
42.1
42.0
41.9
41.8
41.7
41.6
Latitude (UTM)
−7.2
−7.1
−7.0
−6.9
−6.8
−6.7
−6.6
Longitude (UTM)
N
0
5
10 km
Altitude (m)
1400
1200
1000
800
600
400

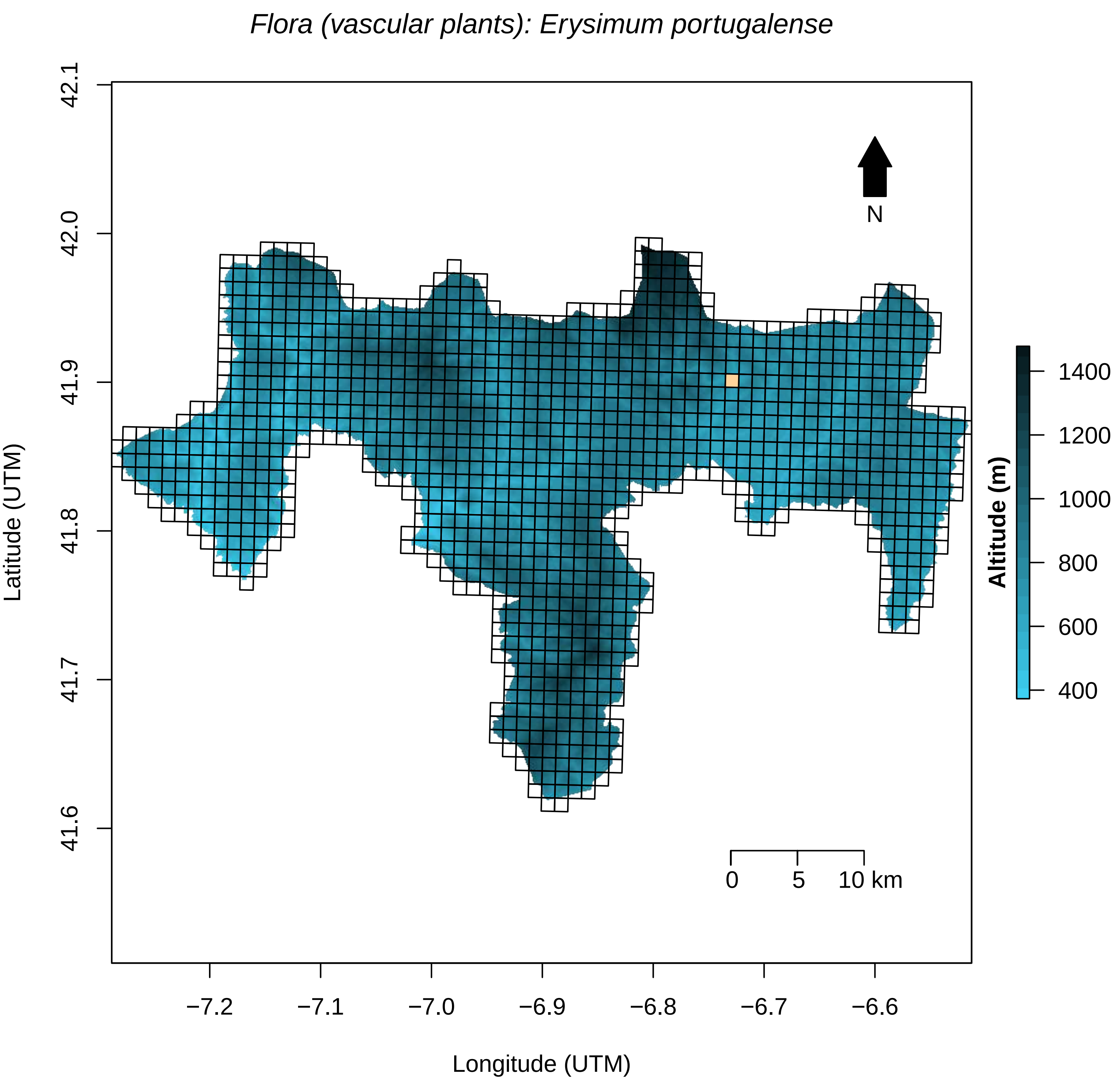

Flora (vascular plants): Erysimum portugalense
N
0
5
10 km
Latitude (UTM)
42.1
42.0
41.9
41.8
41.7
41.6
Longitude (UTM)
−7.2
−7.1
−7.0
−6.9
−6.8
−6.7
−6.6
Altitude (m)
1400
1200
1000
800
600
400

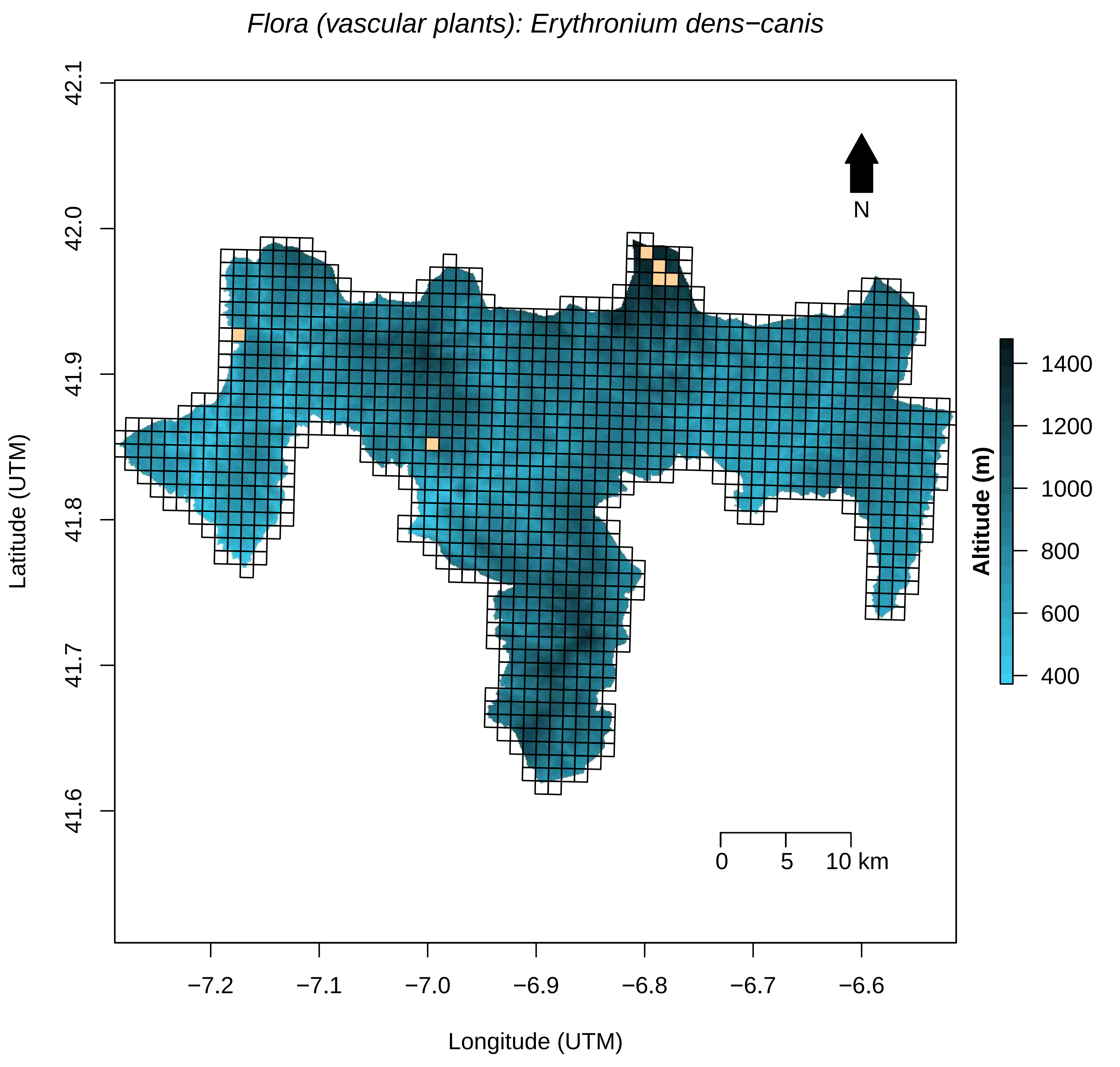
Flora (vascular plants): Erythronium dens−canis
42.1
42.0
41.9
41.8
41.7
41.6
Latitude (UTM)
−7.2
−7.1
−7.0
−6.9
−6.8
−6.7
−6.6
Longitude (UTM)
N
0
5
10 km
Altitude (m)
1400
1200
1000
800
600
400

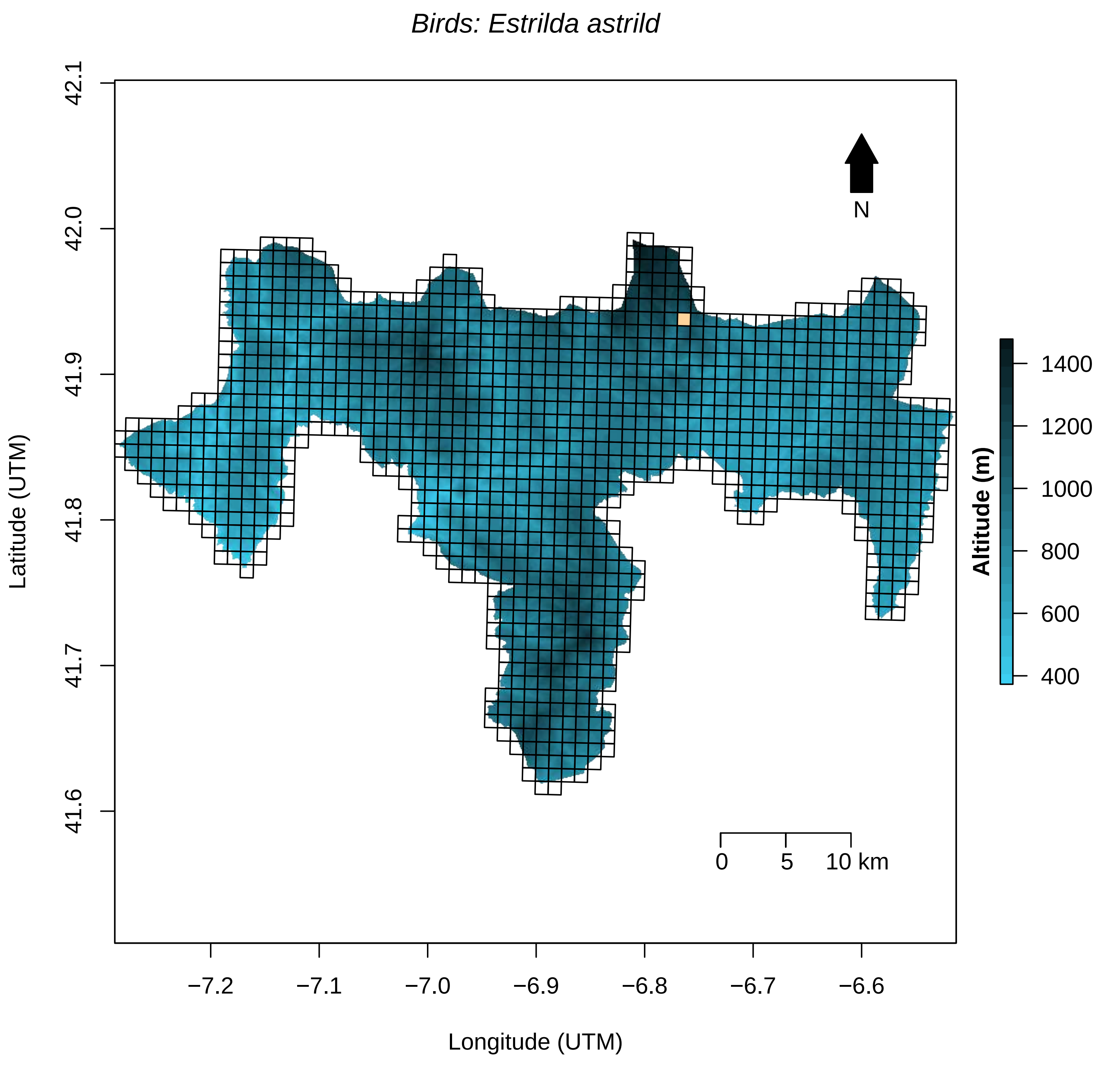
Birds: Estrilda astrild
42.1
42.0
41.9
41.8
41.7
41.6
Latitude (UTM)
−7.2
−7.1
−7.0
−6.9
−6.8
−6.7
−6.6
Longitude (UTM)
N
0
5
10 km
Altitude (m)
1400
1200
1000
800
600
400

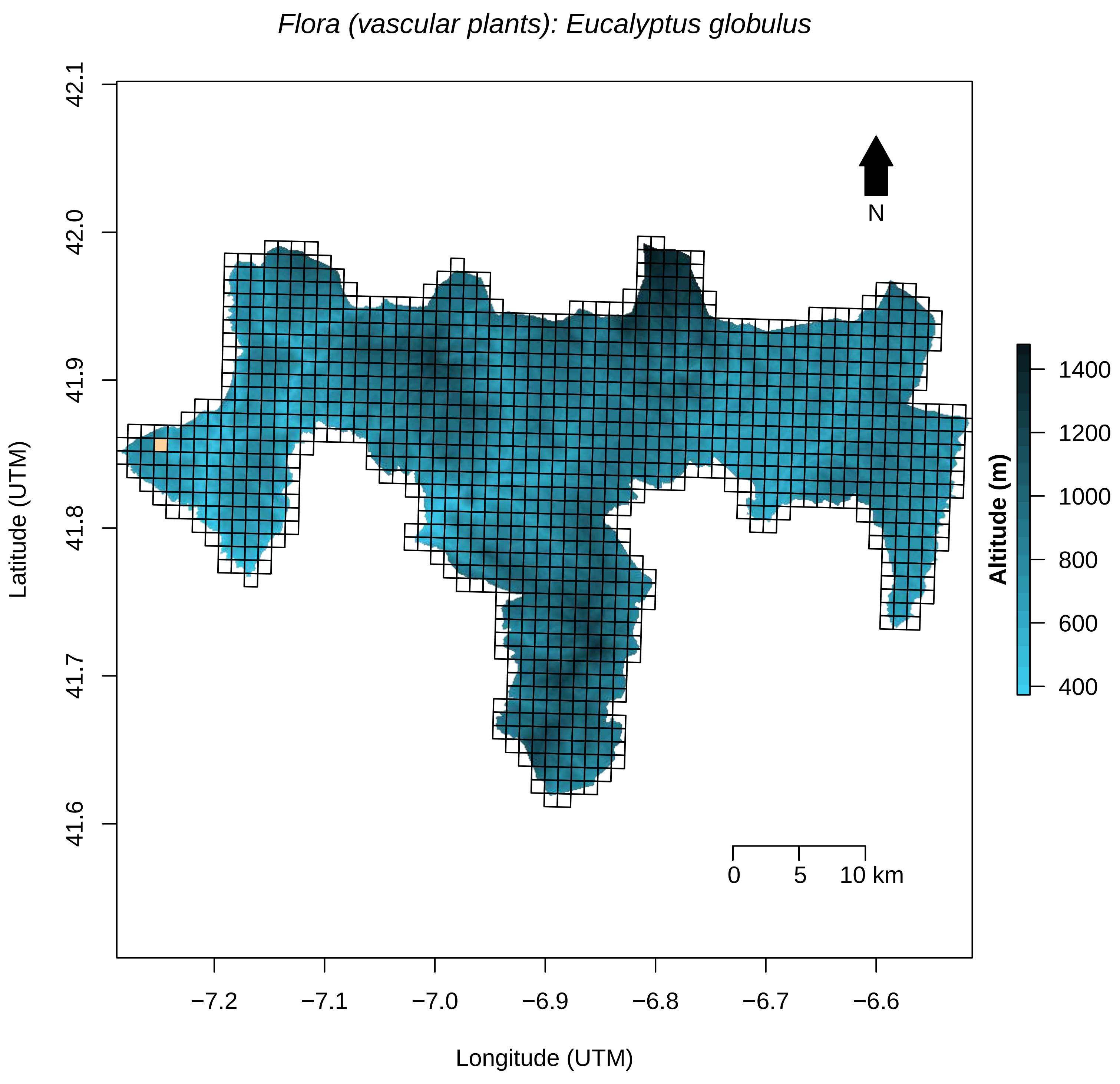

Flora (vascular plants): Eucalyptus globulus
42.1
42.0
41.9
41.8
41.7
41.6
Latitude (UTM)
−7.2
−7.1
−7.0
−6.9
−6.8
−6.7
−6.6
Longitude (UTM)
N
0
5
10 km
Altitude (m)
1400
1200
1000
800
600
400

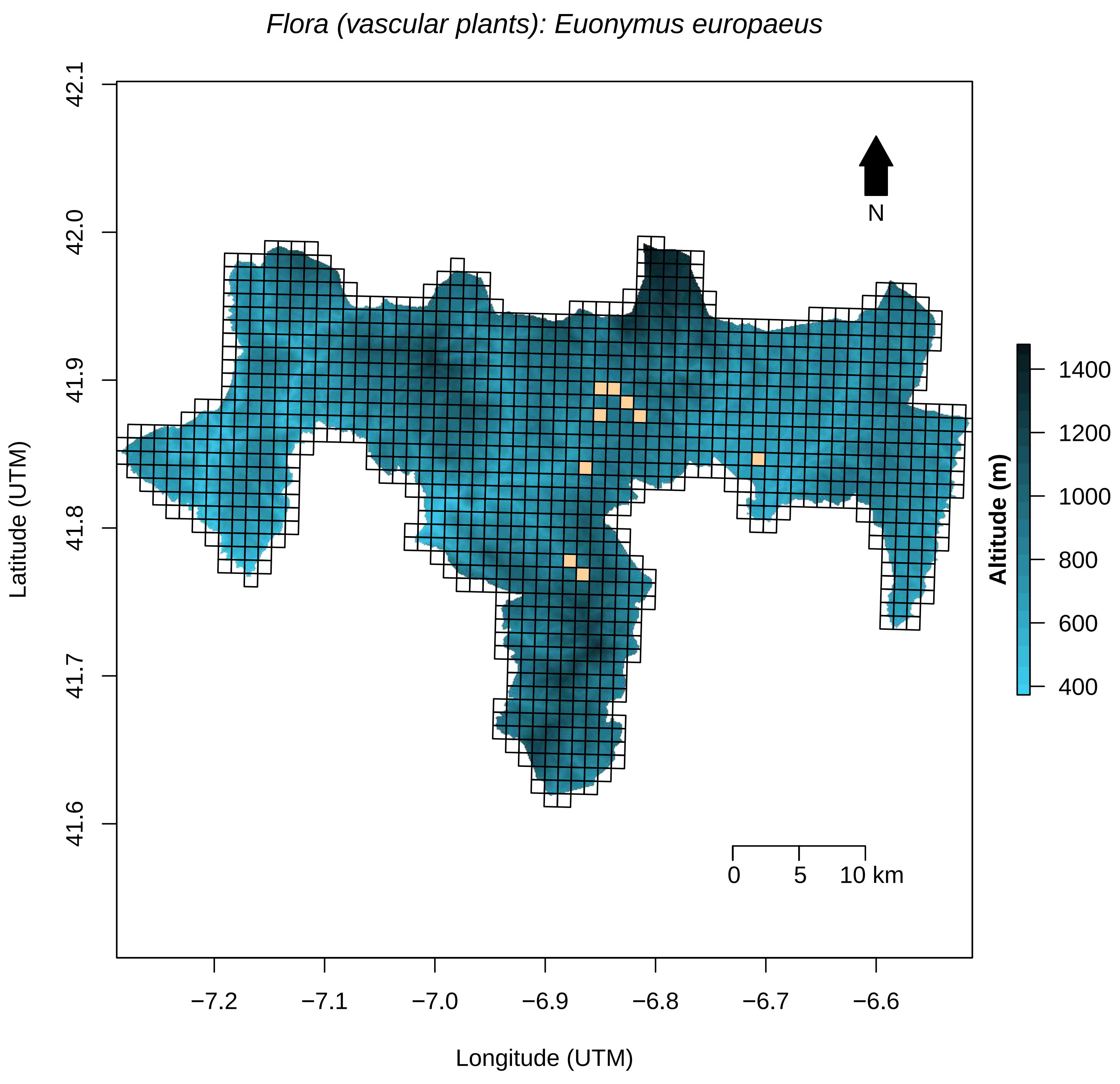
Flora (vascular plants): Euonymus europaeus
Latitude (UTM)
Longitude (UTM)
42.1
42.0
41.9
41.8
41.7
41.6
−7.2
−7.1
−7.0
−6.9
−6.8
−6.7
−6.6
N
0
5
10 km
Altitude (m)
1400
1200
1000
800
600
400

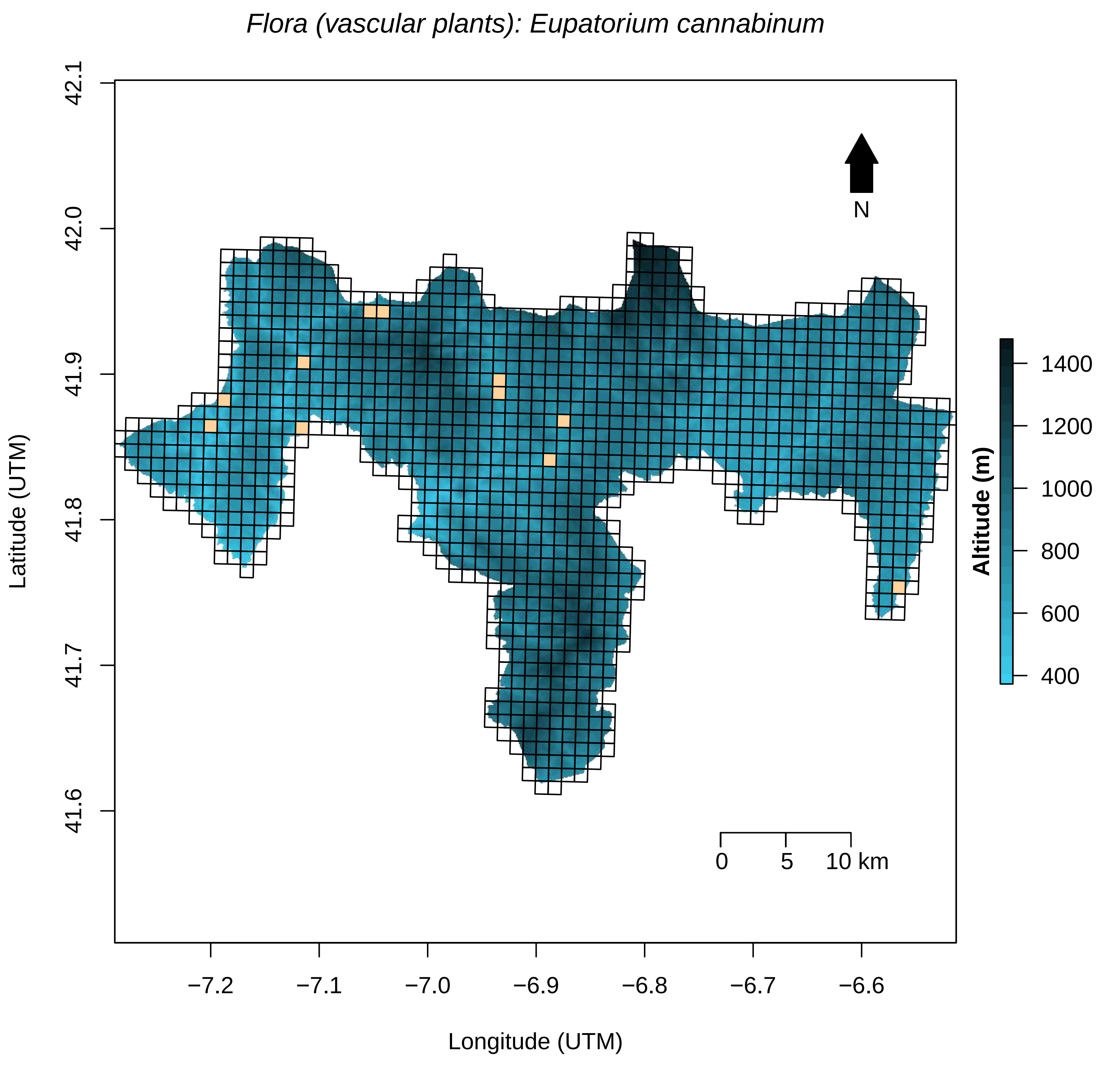

Flora (vascular plants): Eupatorium cannabinum
N
0
5
10 km
Altitude (m)
1400
1200
1000
800
600
400
Latitude (UTM)
42.1
42.0
41.9
41.8
41.7
41.6
Longitude (UTM)
−7.2
−7.1
−7.0
−6.9
−6.8
−6.7
−6.6

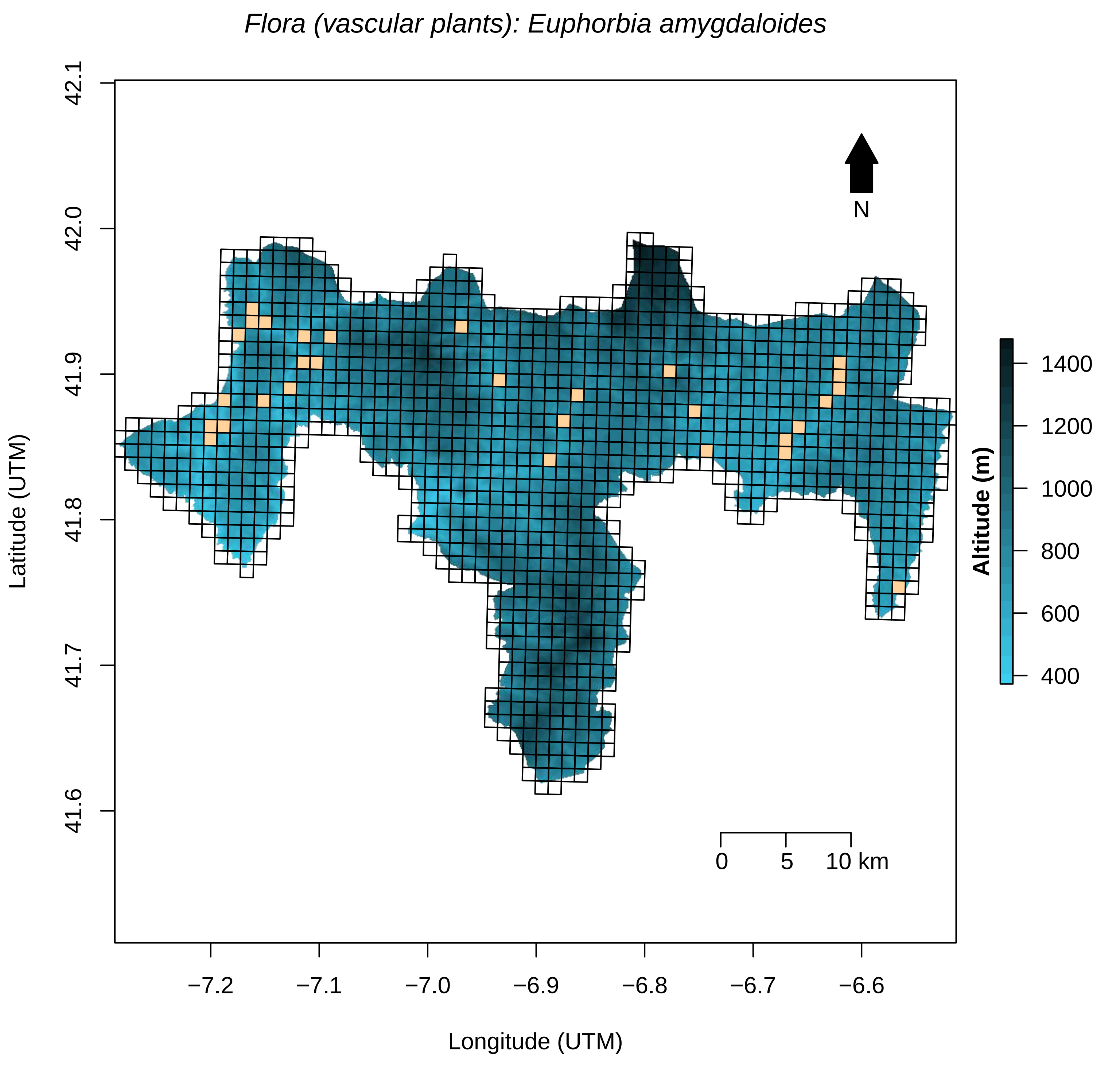

Flora (vascular plants): Euphorbia amygdaloides
Latitude (UTM)
42.1
42.0
41.9
41.8
41.7
41.6
Longitude (UTM)
−7.2
−7.1
−7.0
−6.9
−6.8
−6.7
−6.6
N
0
5
10 km
Altitude (m)
1400
1200
1000
800
600
400

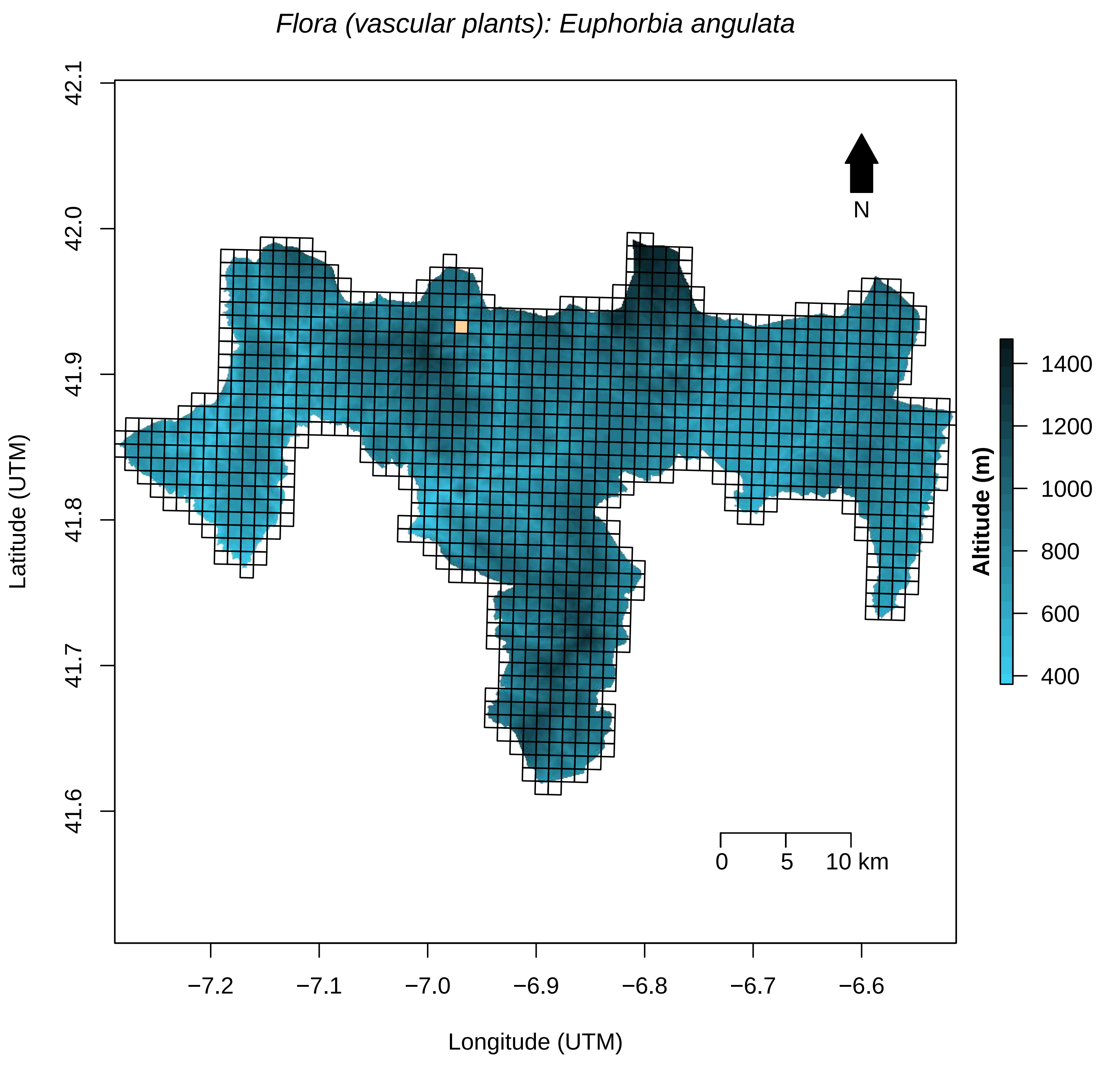

Flora (vascular plants): Euphorbia angulata
42.1
42.0
41.9
41.8
41.7
41.6
Latitude (UTM)
−7.2
−7.1
−7.0
−6.9
−6.8
−6.7
−6.6
Longitude (UTM)
N
0
5
10 km
Altitude (m)
1400
1200
1000
800
600
400

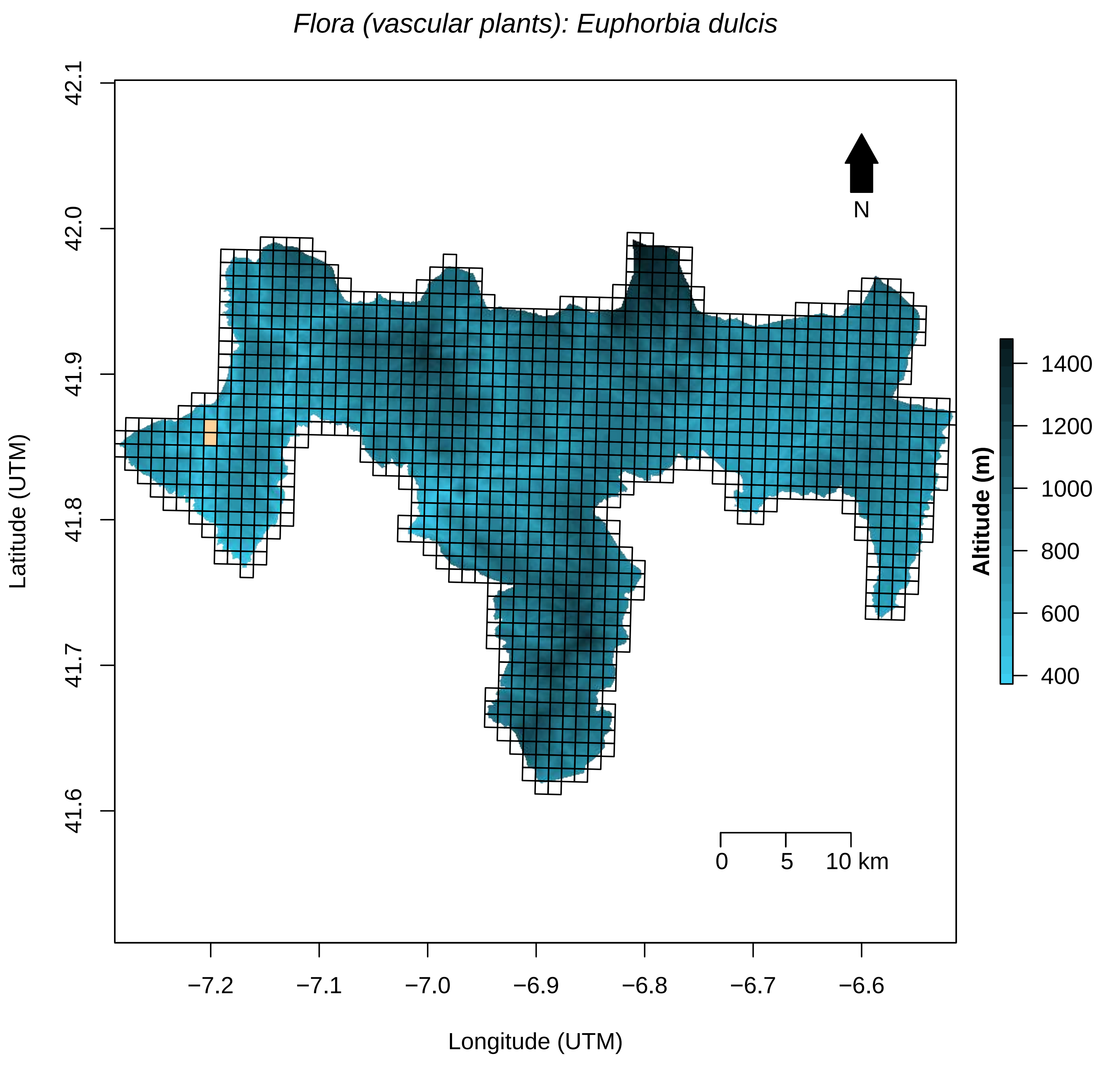

Flora (vascular plants): Euphorbia dulcis
42.1
42.0
41.9
41.8
41.7
41.6
Latitude (UTM)
−7.2
−7.1
−7.0
−6.9
−6.8
−6.7
−6.6
Longitude (UTM)
N
0
5
10 km
Altitude (m)
1400
1200
1000
800
600
400

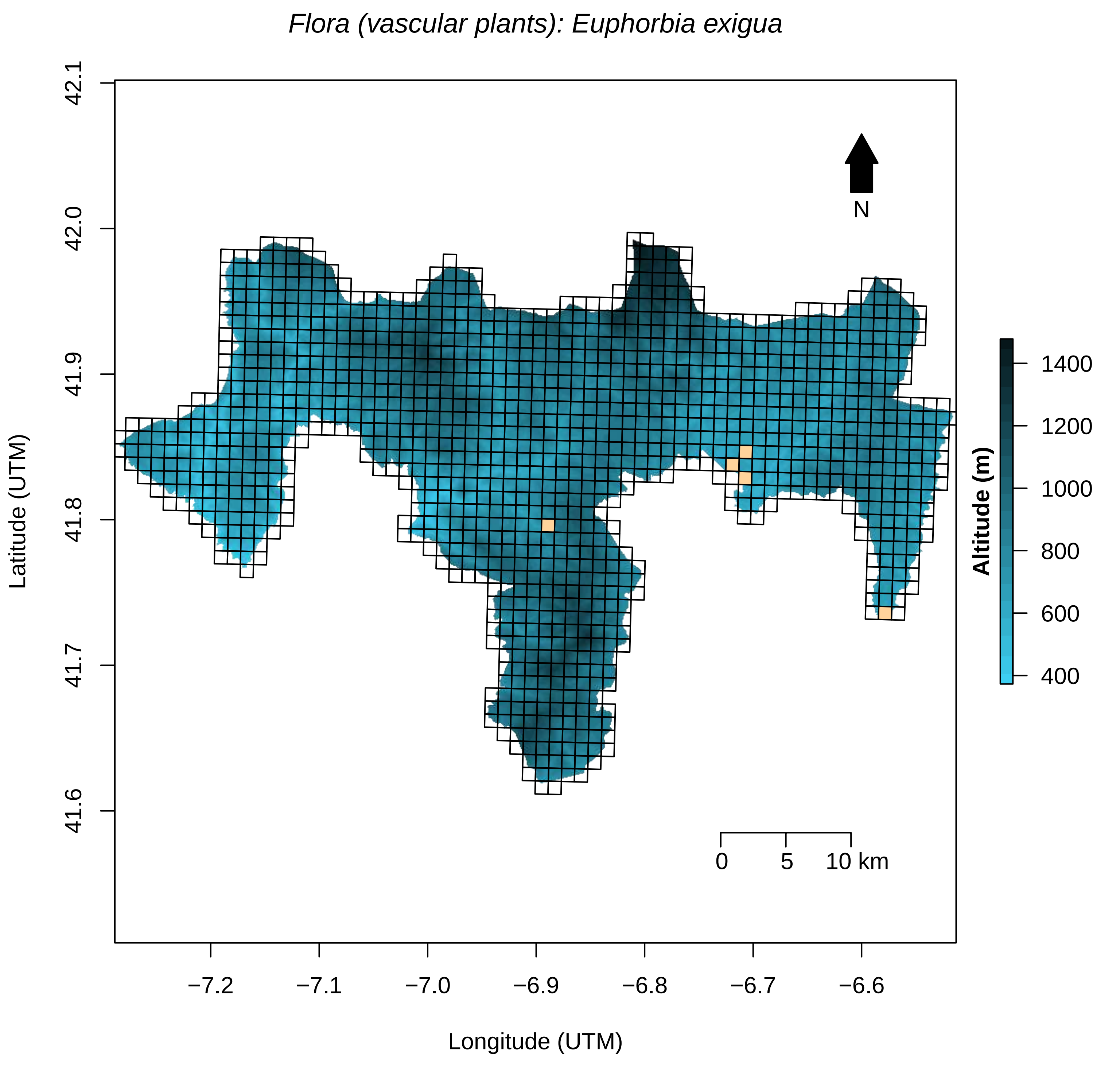

Flora (vascular plants): Euphorbia exigua
42.1
42.0
41.9
41.8
41.7
41.6
Latitude (UTM)
−7.2
−7.1
−7.0
−6.9
−6.8
−6.7
−6.6
Longitude (UTM)
N
0
5
10 km
Altitude (m)
1400
1200
1000
800
600
400

Flora (vascular plants): Euphorbia falcata

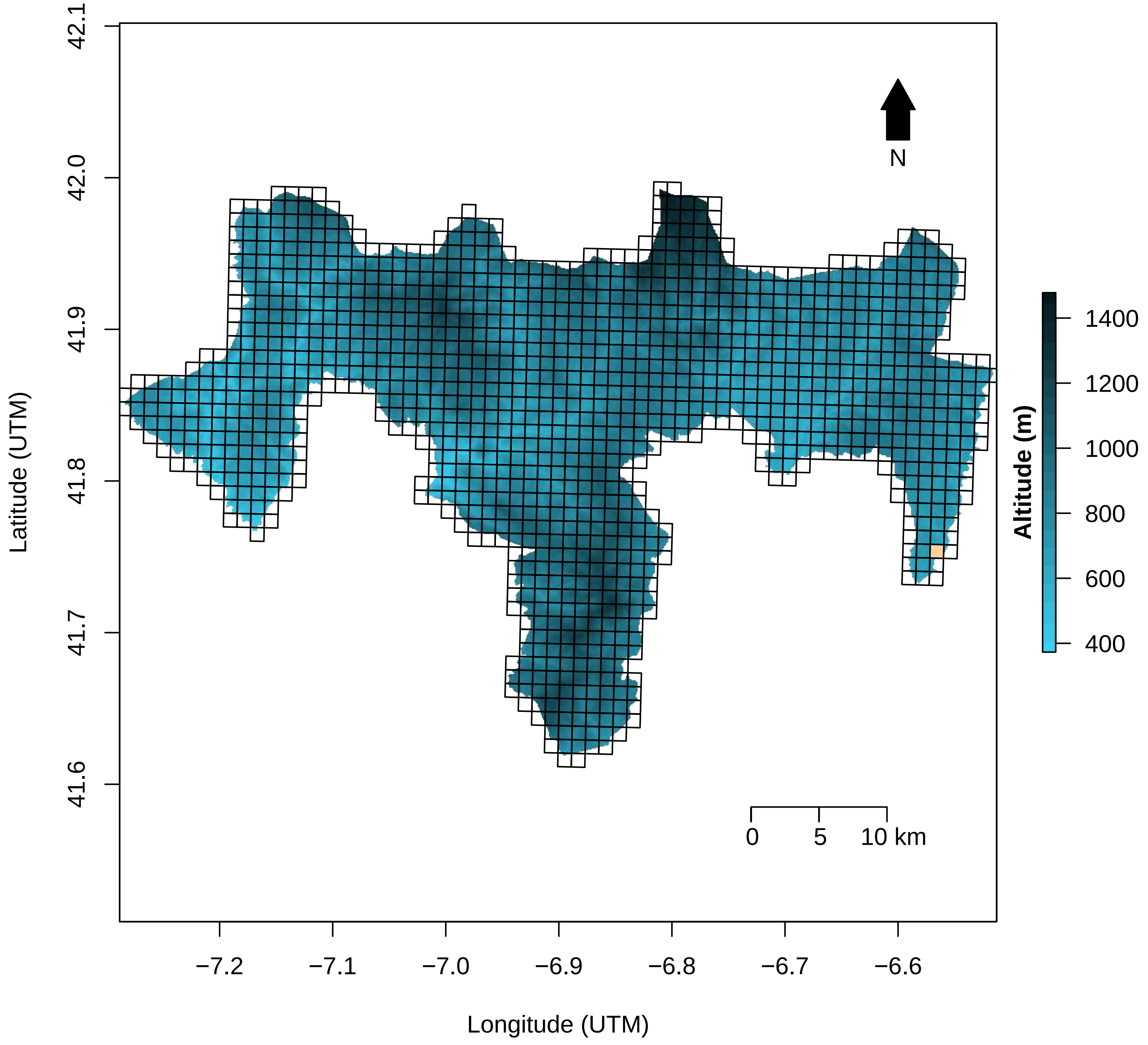

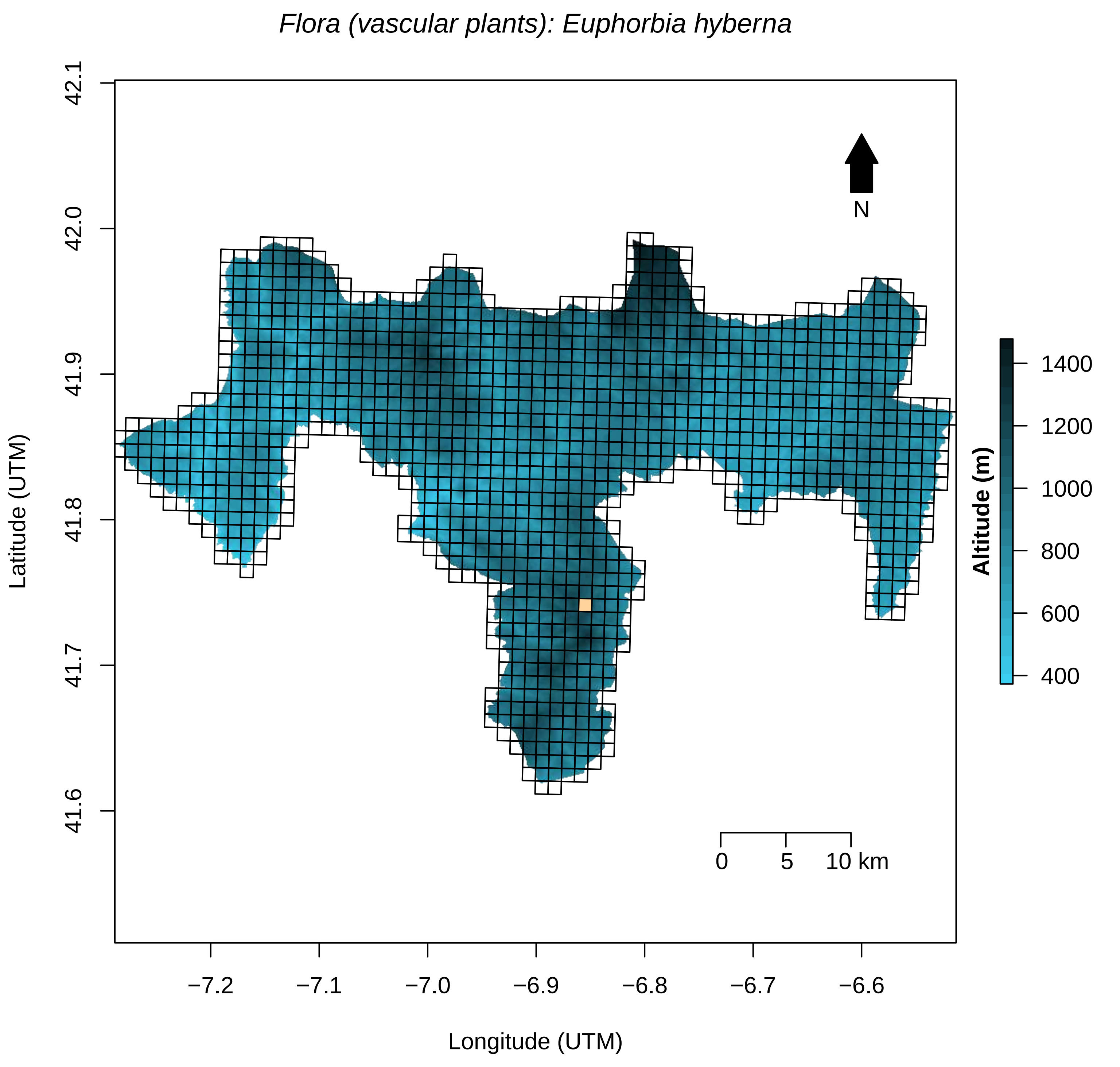

Flora (vascular plants): Euphorbia hyberna
42.1
42.0
41.9
41.8
41.7
41.6
Latitude (UTM)
−7.2
−7.1
−7.0
−6.9
−6.8
−6.7
−6.6
Longitude (UTM)
N
0
5
10 km
Altitude (m)
1400
1200
1000
800
600
400

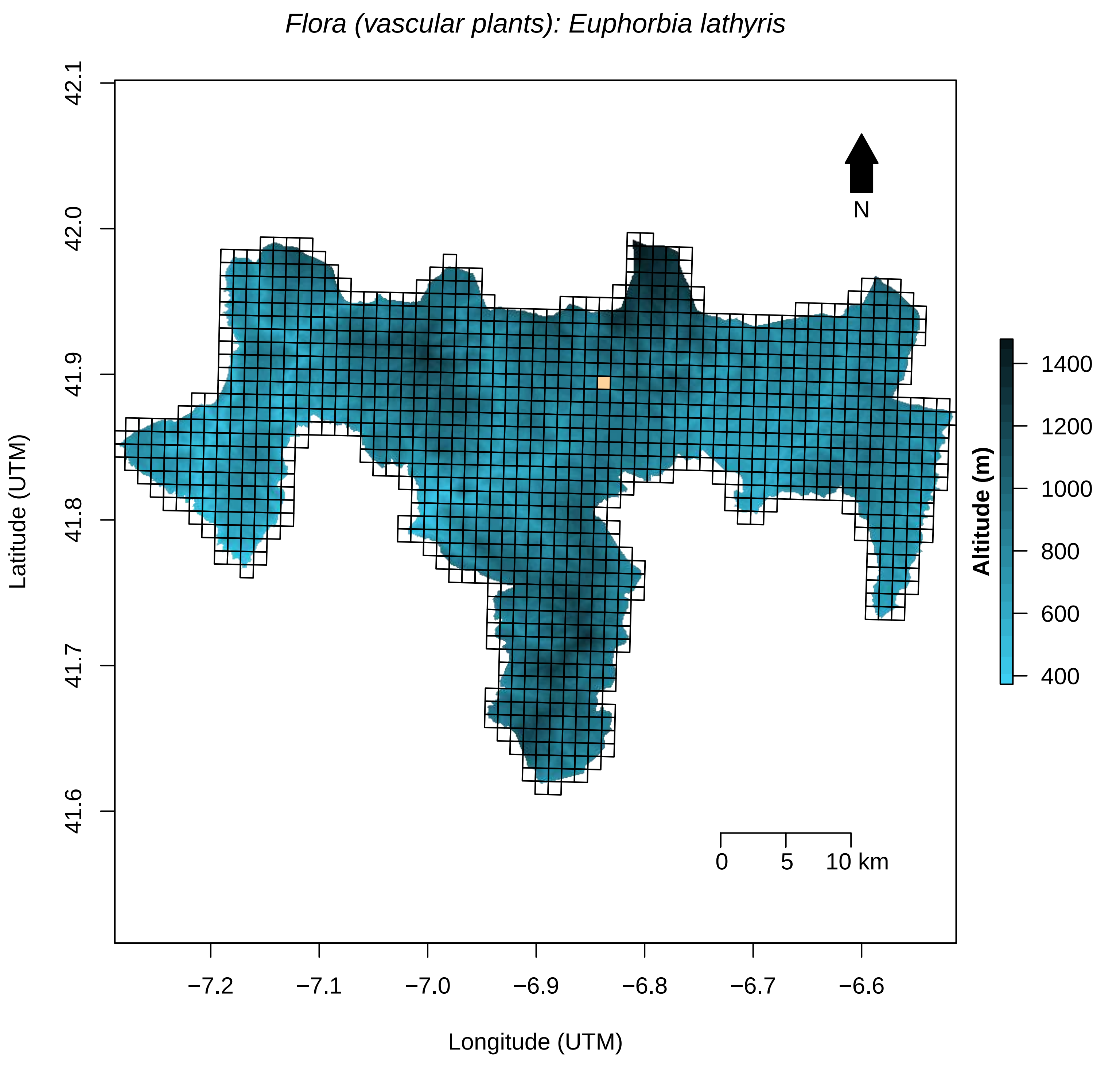

Flora (vascular plants): Euphorbia lathyris
Latitude (UTM)
42.1
42.0
41.9
41.8
41.7
41.6
Longitude (UTM)
−7.2
−7.1
−7.0
−6.9
−6.8
−6.7
−6.6
N
0
5
10 km
Altitude (m)
1400
1200
1000
800
600
400

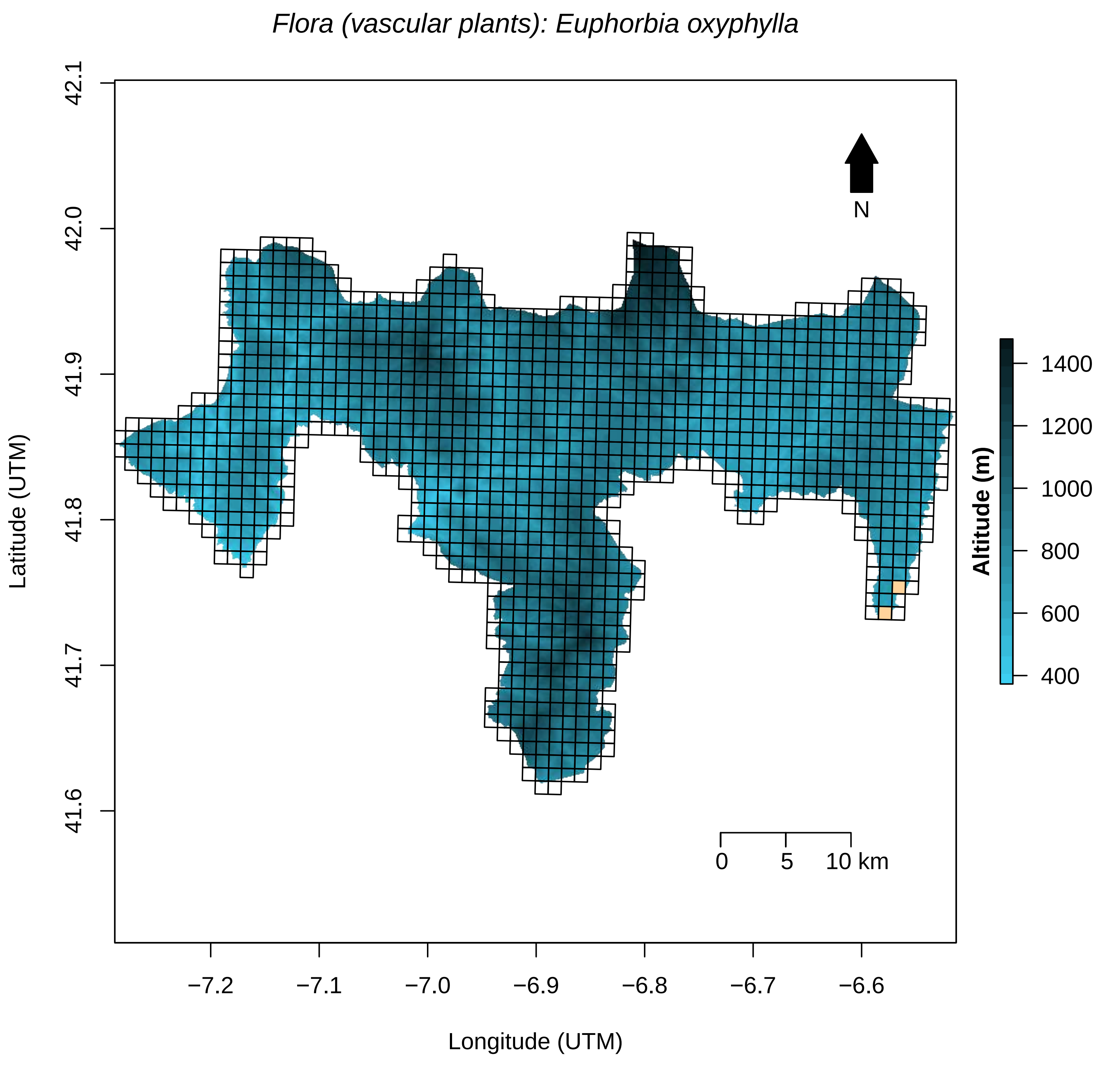
Flora (vascular plants): Euphorbia oxyphylla
N
0
5
10 km
Altitude (m)
1400
1200
1000
800
600
400
Latitude (UTM)
42.1
42.0
41.9
41.8
41.7
41.6
Longitude (UTM)
−7.2
−7.1
−7.0
−6.9
−6.8
−6.7
−6.6

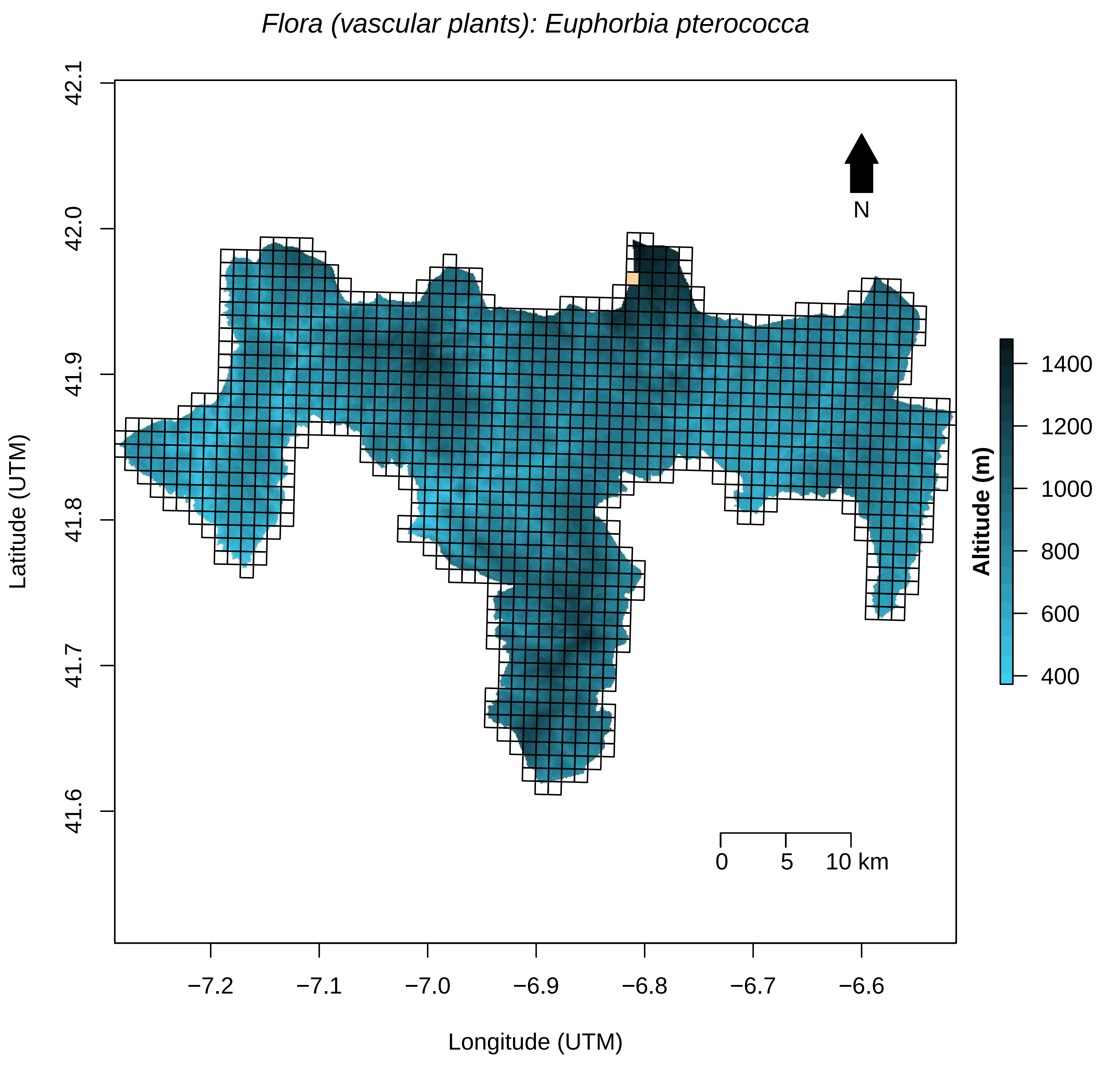

Flora (vascular plants): Euphorbia pterococca
42.1
42.0
41.9
41.8
41.7
41.6
Latitude (UTM)
−7.2
−7.1
−7.0
−6.9
−6.8
−6.7
−6.6
Longitude (UTM)
N
0
5
10 km
Altitude (m)
1400
1200
1000
800
600
400

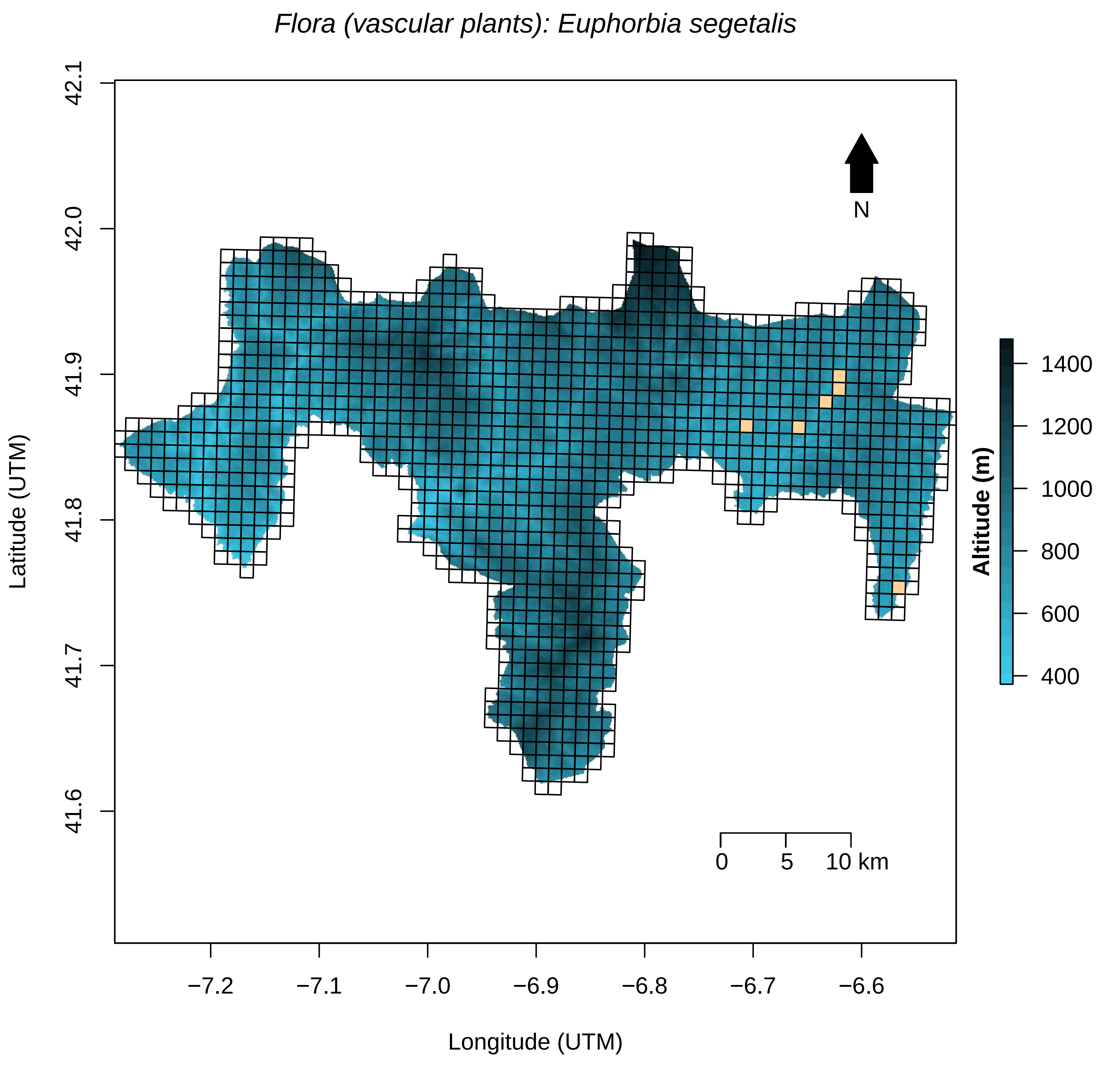

Flora (vascular plants): Euphorbia segetalis
N
0 5 10 km
Altitude (m)
1400
1200
1000
800
600
400
Latitude (UTM)
42.1
42.0
41.9
41.8
41.7
41.6
Longitude (UTM)
−7.2
−7.1
−7.0
−6.9
−6.8
−6.7
−6.6

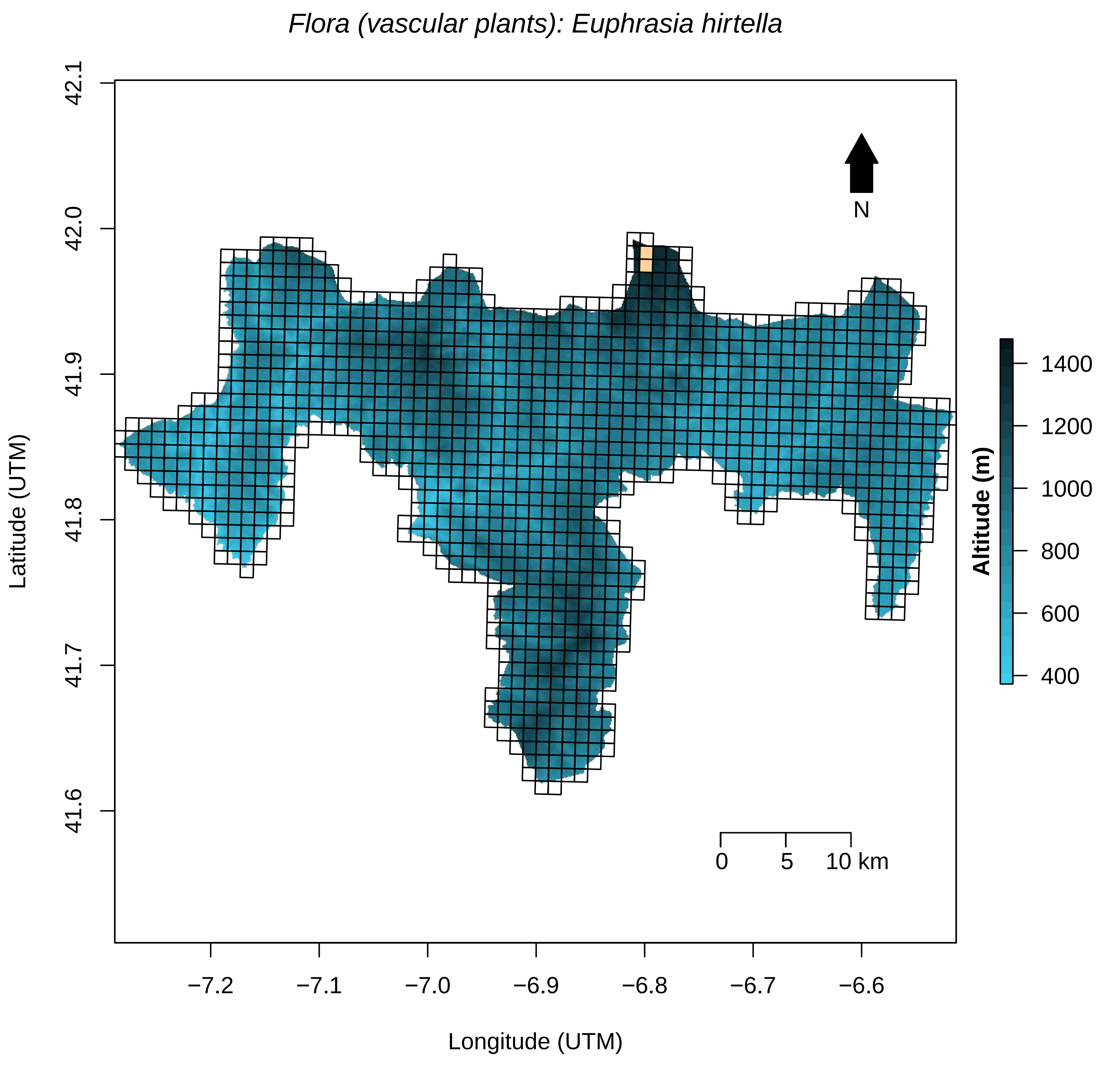

Flora (vascular plants): Euphrasia hirtella
42.1
42.0
41.9
41.8
41.7
41.6
Latitude (UTM)
−7.2
−7.1
−7.0
−6.9
−6.8
−6.7
−6.6
Longitude (UTM)
N
0
5
10 km
Altitude (m)
1400
1200
1000
800
600
400

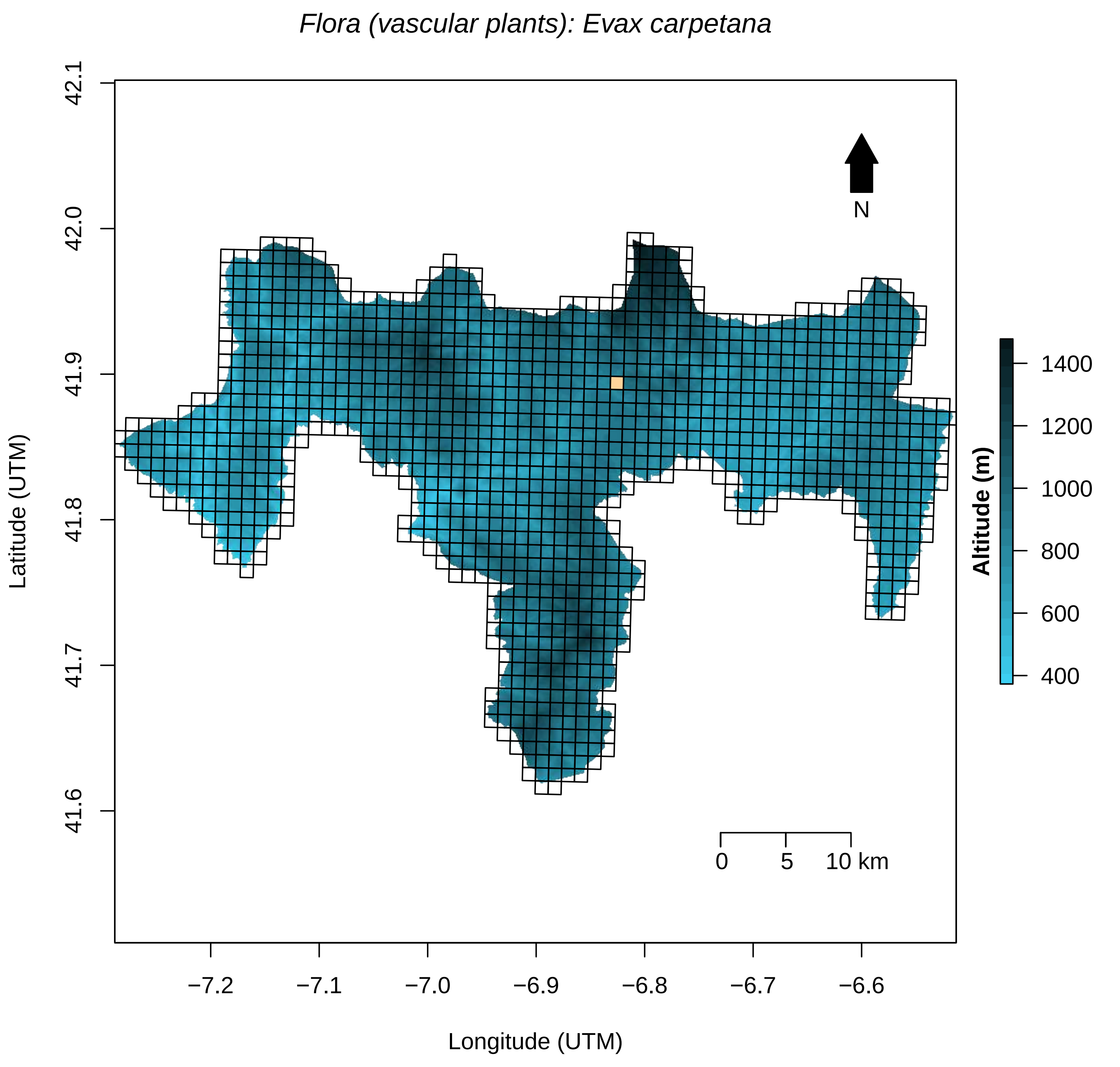

Flora (vascular plants): Evax carpetana
N
0 5 10 km
Altitude (m)
1400
1200
1000
800
600
400
42.1
42.0
41.9
41.8
41.7
41.6
Latitude (UTM)
−7.2
−7.1
−7.0
−6.9
−6.8
−6.7
−6.6
Longitude (UTM)

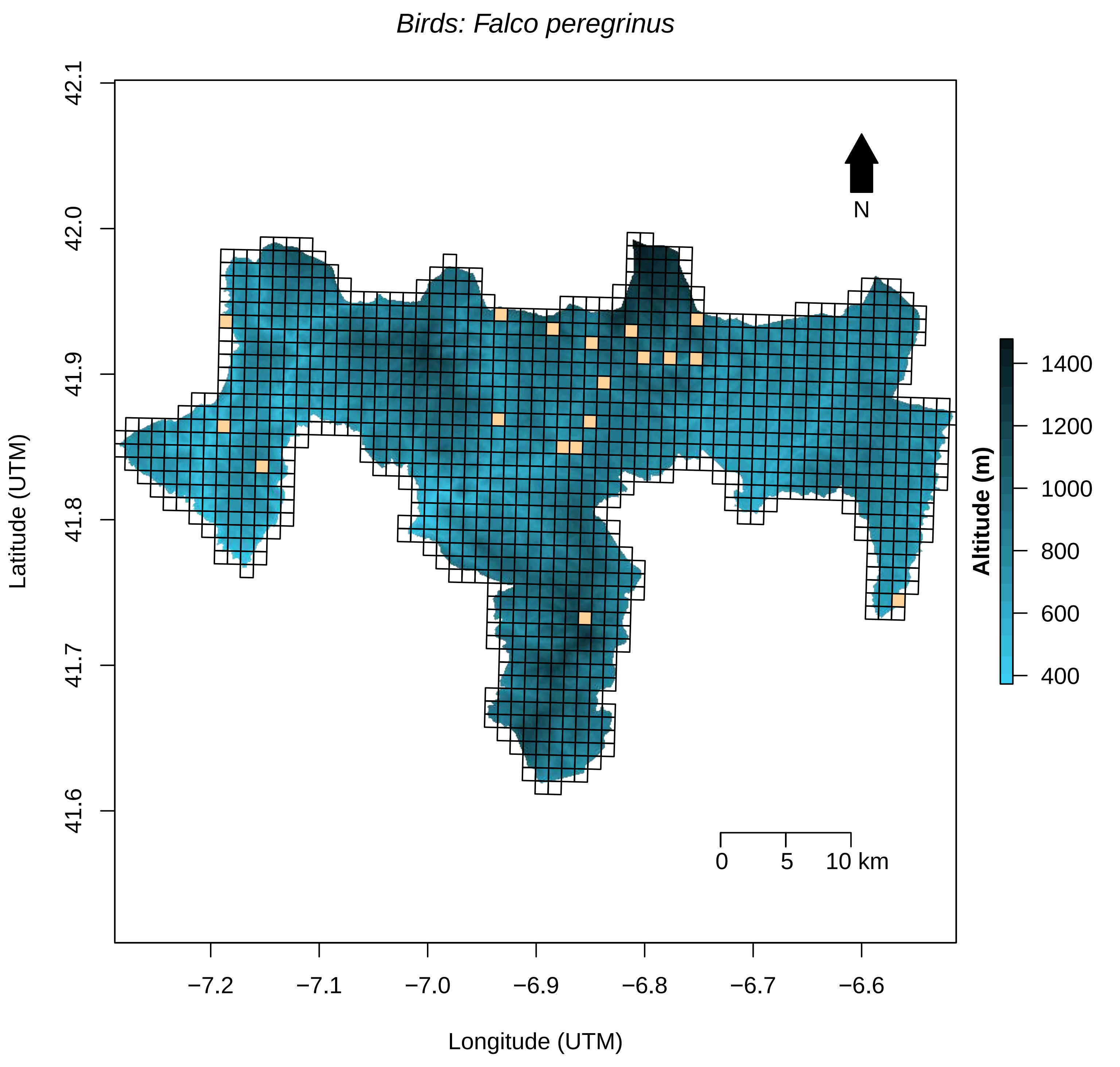

Birds: Falco peregrinus
N
0 5 10 km
Altitude (m)
1400
1200
1000
800
600
400
Latitude (UTM)
42.1
42.0
41.9
41.8
41.7
41.6
Longitude (UTM)
−7.2
−7.1
−7.0
−6.9
−6.8
−6.7
−6.6

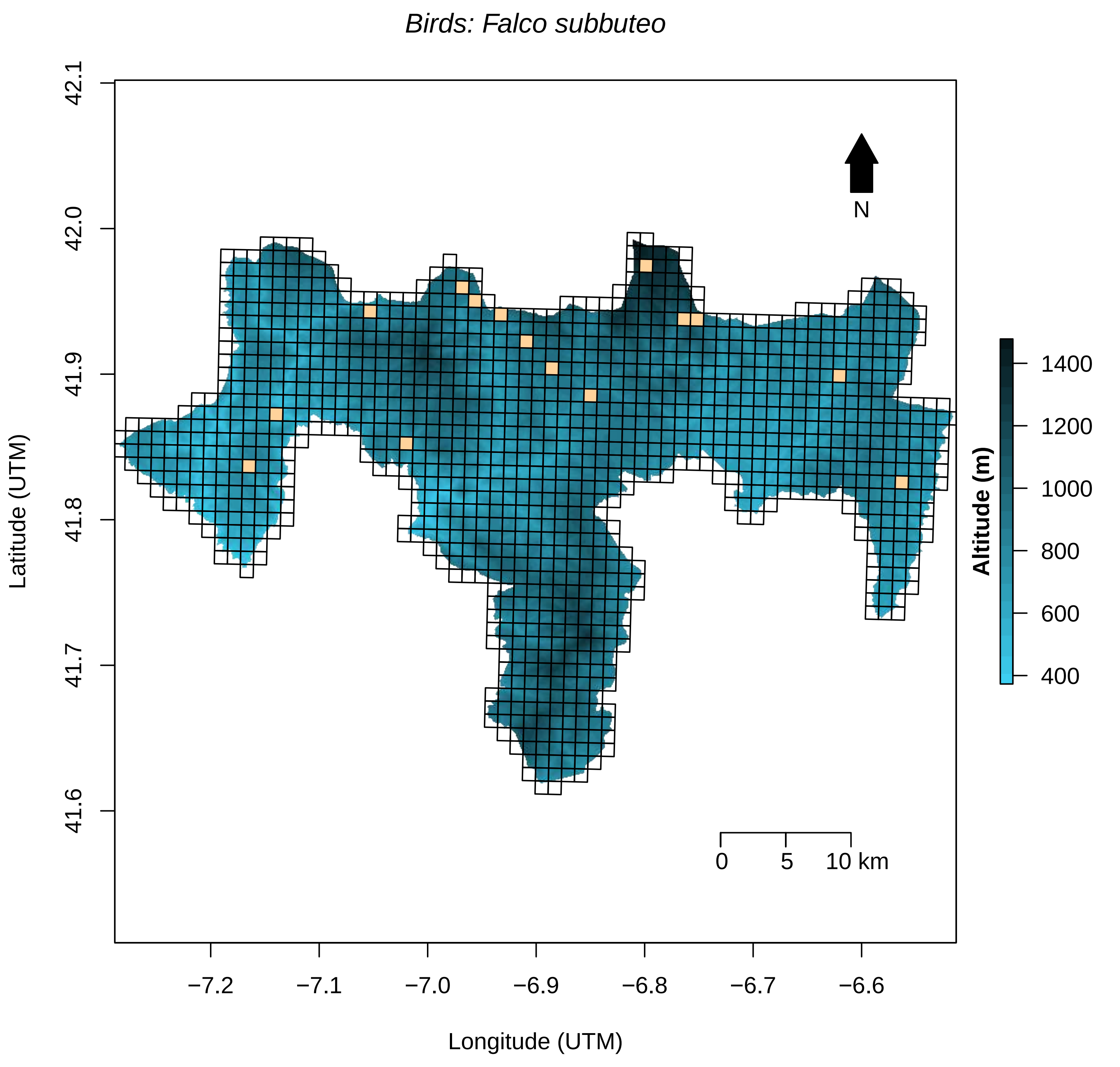

Birds: Falco subbuteo
42.1
42.0
41.9
41.8
41.7
41.6
Latitude (UTM)
−7.2
−7.1
−7.0
−6.9
−6.8
−6.7
−6.6
Longitude (UTM)
N
0
5
10 km
1400
1200
1000
800
600
400
Altitude (m)

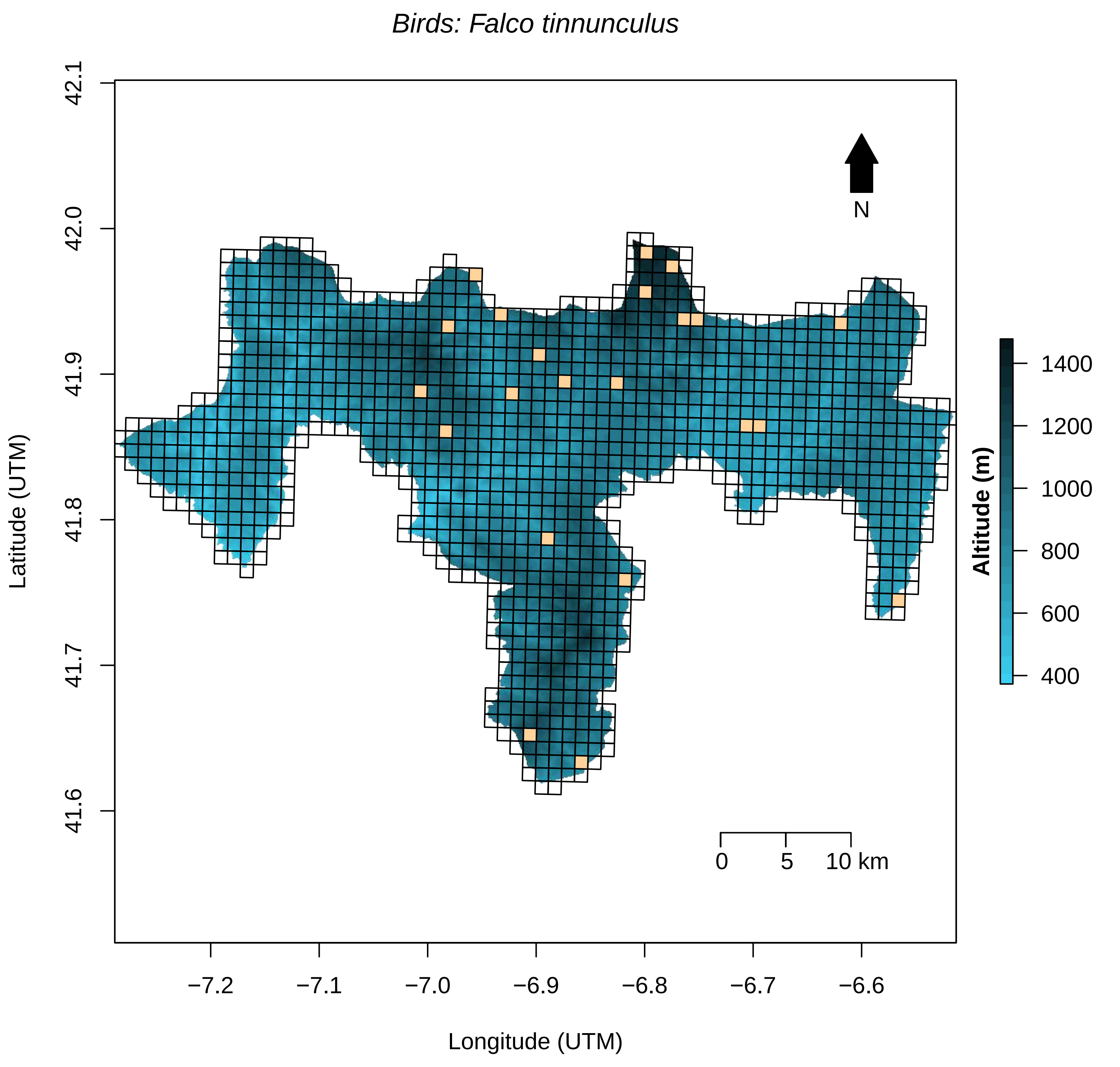

Birds: Falco tinnunculus
42.1
42.0
41.9
41.8
41.7
41.6
Latitude (UTM)
−7.2
−7.1
−7.0
−6.9
−6.8
−6.7
−6.6
Longitude (UTM)
N
0
5
10 km
Altitude (m)
1400
1200
1000
800
600
400

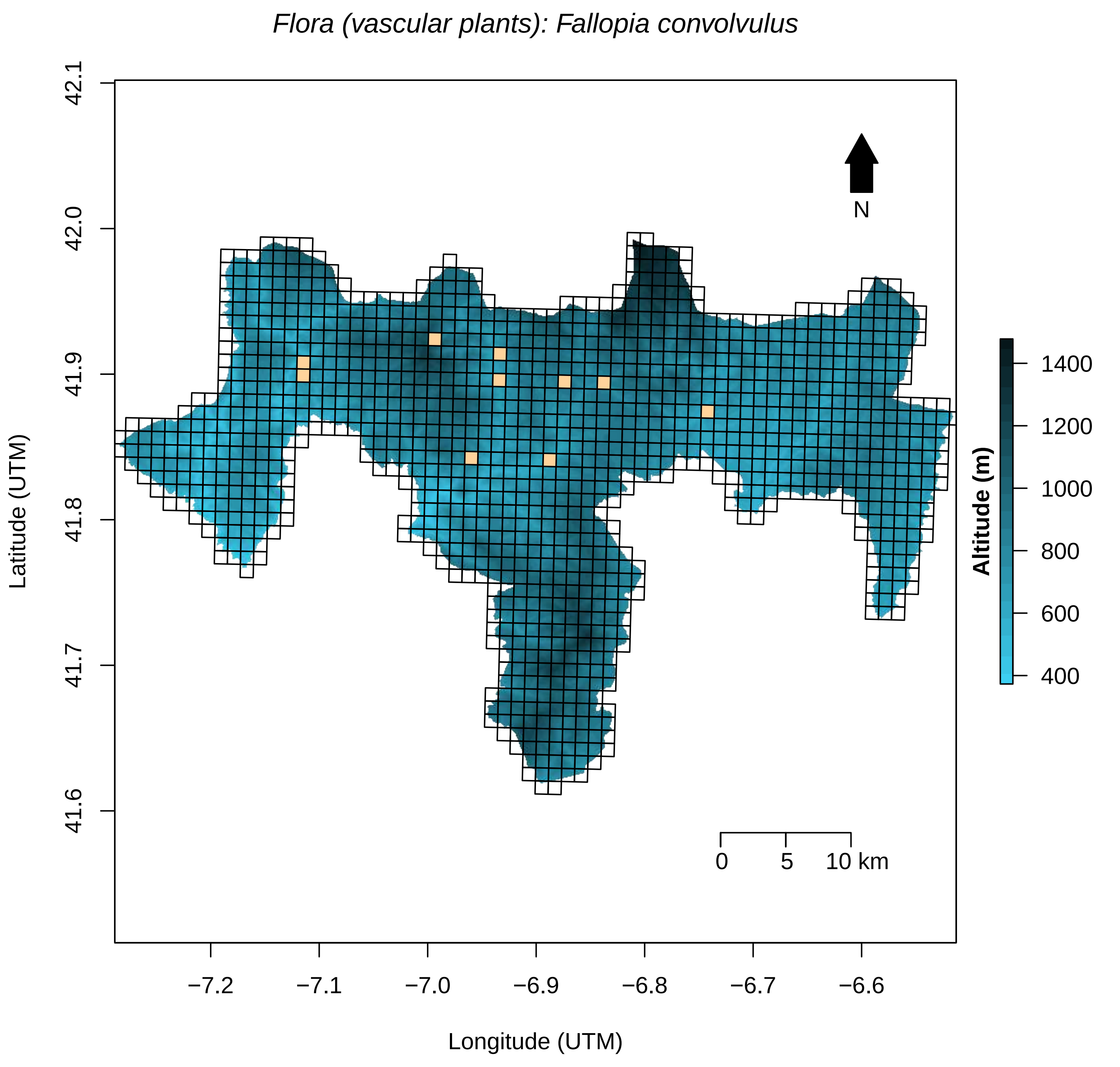

Flora (vascular plants): Fallopia convolvulus
N
0
5
10 km
Altitude (m)
1400
1200
1000
800
600
400
Latitude (UTM)
42.1
42.0
41.9
41.8
41.7
41.6
Longitude (UTM)
−7.2
−7.1
−7.0
−6.9
−6.8
−6.7
−6.6

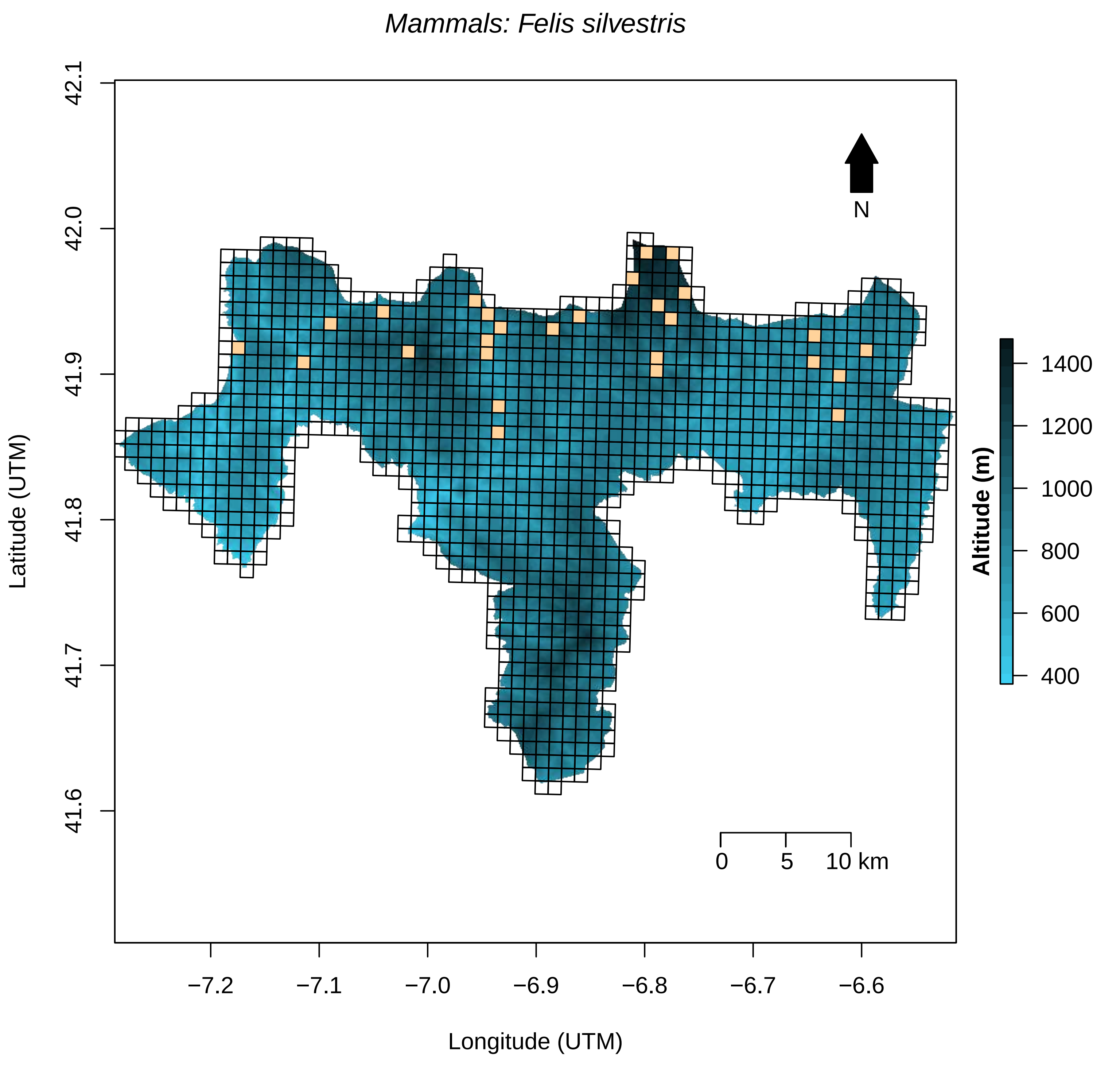

Mammals: Felis silvestris
N
0
5
10 km
Altitude (m)
1400
1200
1000
800
600
400
Latitude (UTM)
42.1
42.0
41.9
41.8
41.7
41.6
Longitude (UTM)
−7.2
−7.1
−7.0
−6.9
−6.8
−6.7
−6.6

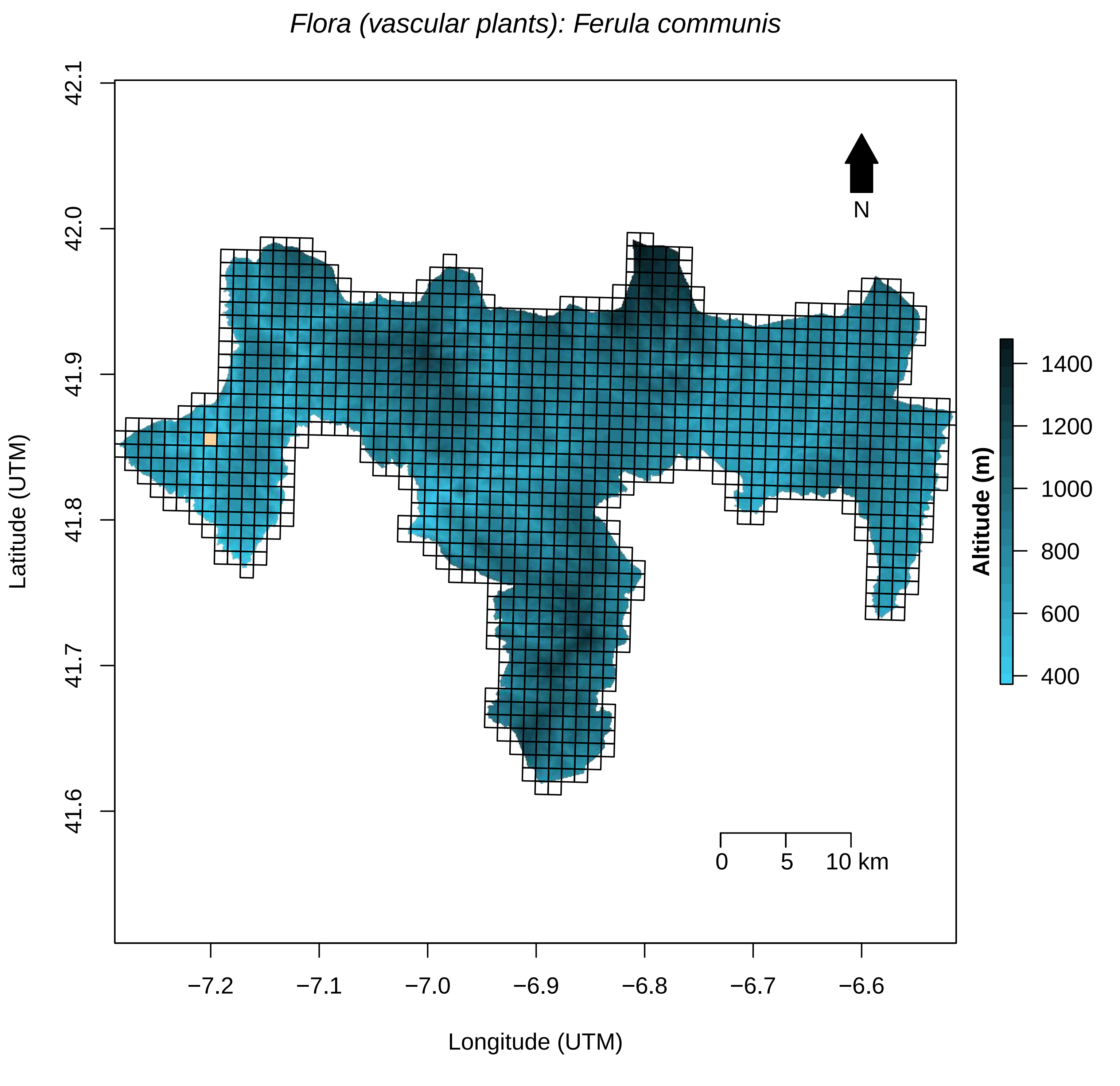

Flora (vascular plants): Ferula communis
42.1
42.0
41.9
41.8
41.7
41.6
Latitude (UTM)
−7.2
−7.1
−7.0
−6.9
−6.8
−6.7
−6.6
Longitude (UTM)
N
0
5
10 km
1400
1200
1000
800
600
400
Altitude (m)

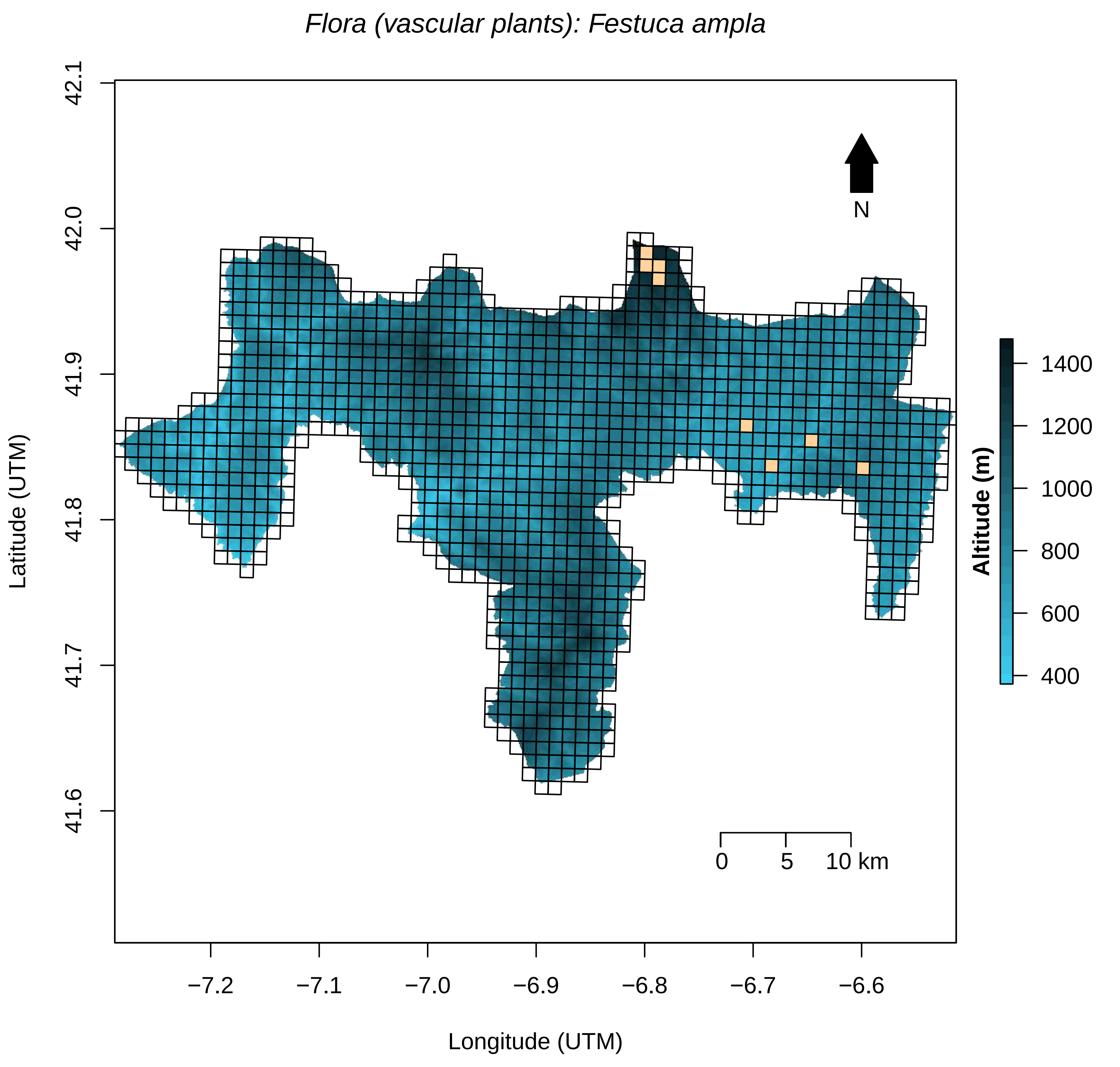
Flora (vascular plants): Festuca ampla
Latitude (UTM)
42.1
42.0
41.9
41.8
41.7
41.6
Longitude (UTM)
−7.2
−7.1
−7.0
−6.9
−6.8
−6.7
−6.6
N
0
5
10 km
Altitude (m)
1400
1200
1000
800
600
400

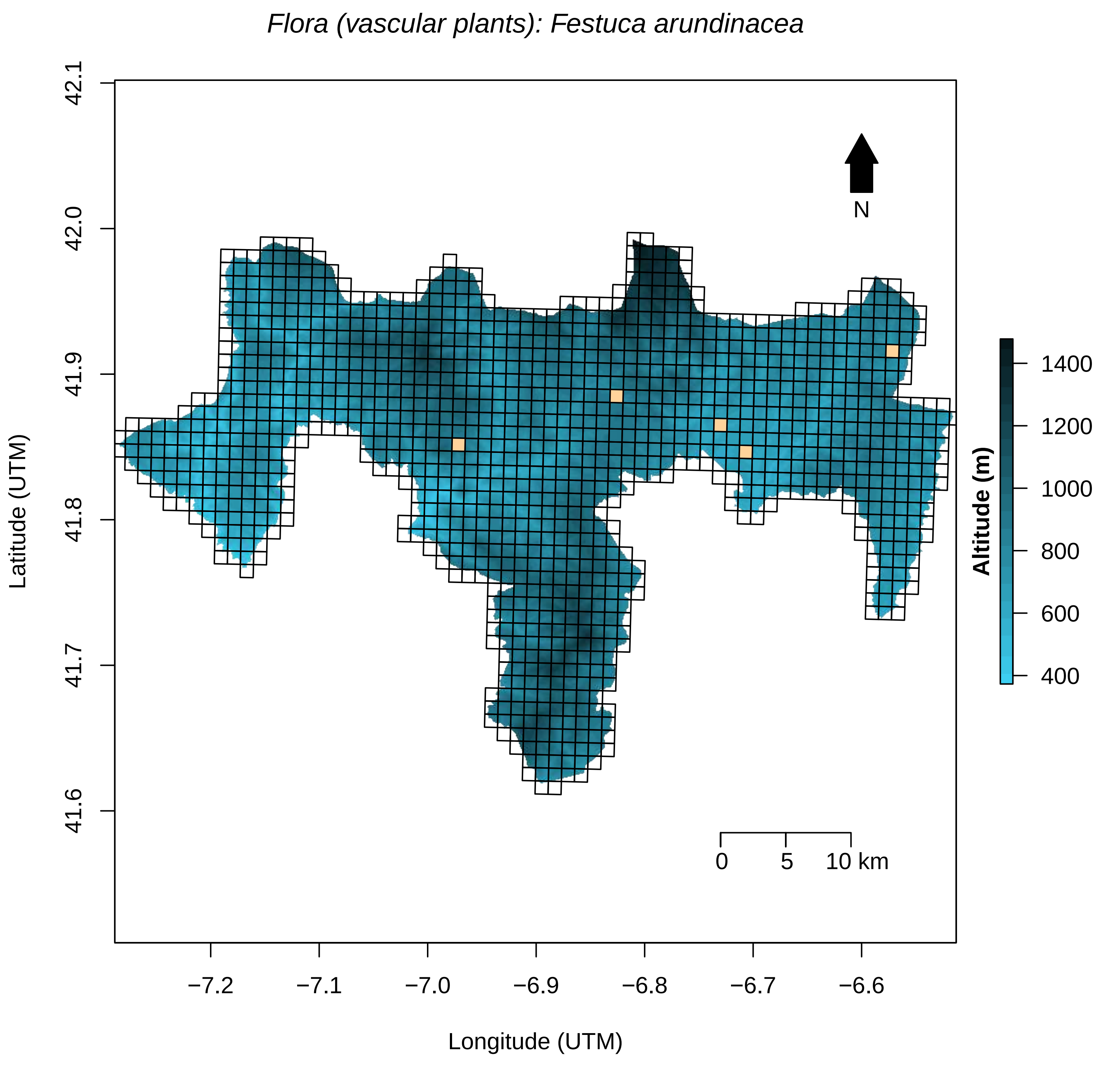

Flora (vascular plants): Festuca arundinacea
42.1
42.0
41.9
41.8
41.7
41.6
Latitude (UTM)
−7.2
−7.1
−7.0
−6.9
−6.8
−6.7
−6.6
Longitude (UTM)
N
0
5
10 km
Altitude (m)
1400
1200
1000
800
600
400

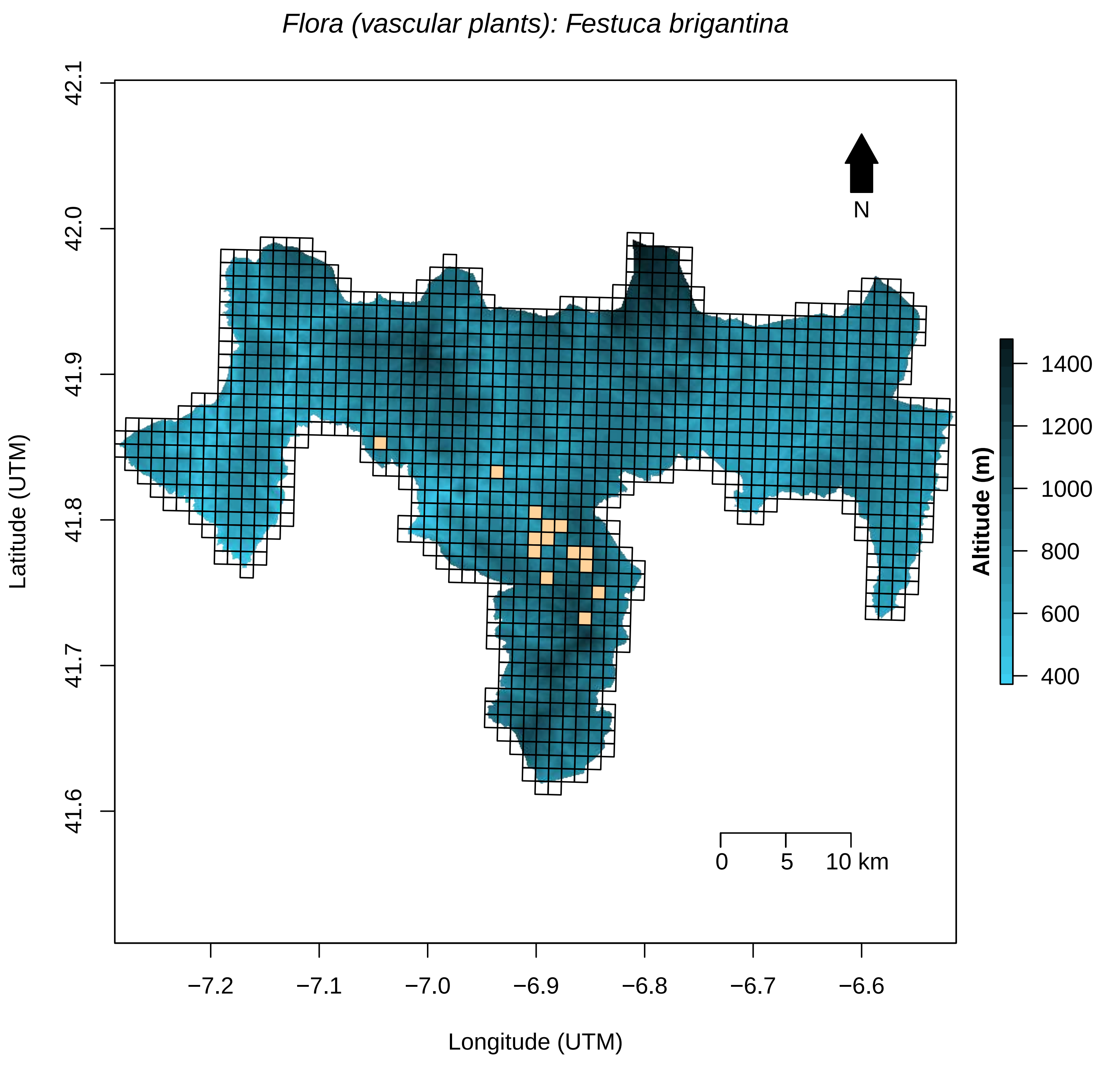

Flora (vascular plants): Festuca brigantina
42.1
42.0
41.9
41.8
41.7
41.6
Latitude (UTM)
−7.2
−7.1
−7.0
−6.9
−6.8
−6.7
−6.6
Longitude (UTM)
N
0
5
10 km
1400
1200
1000
800
600
400
Altitude (m)

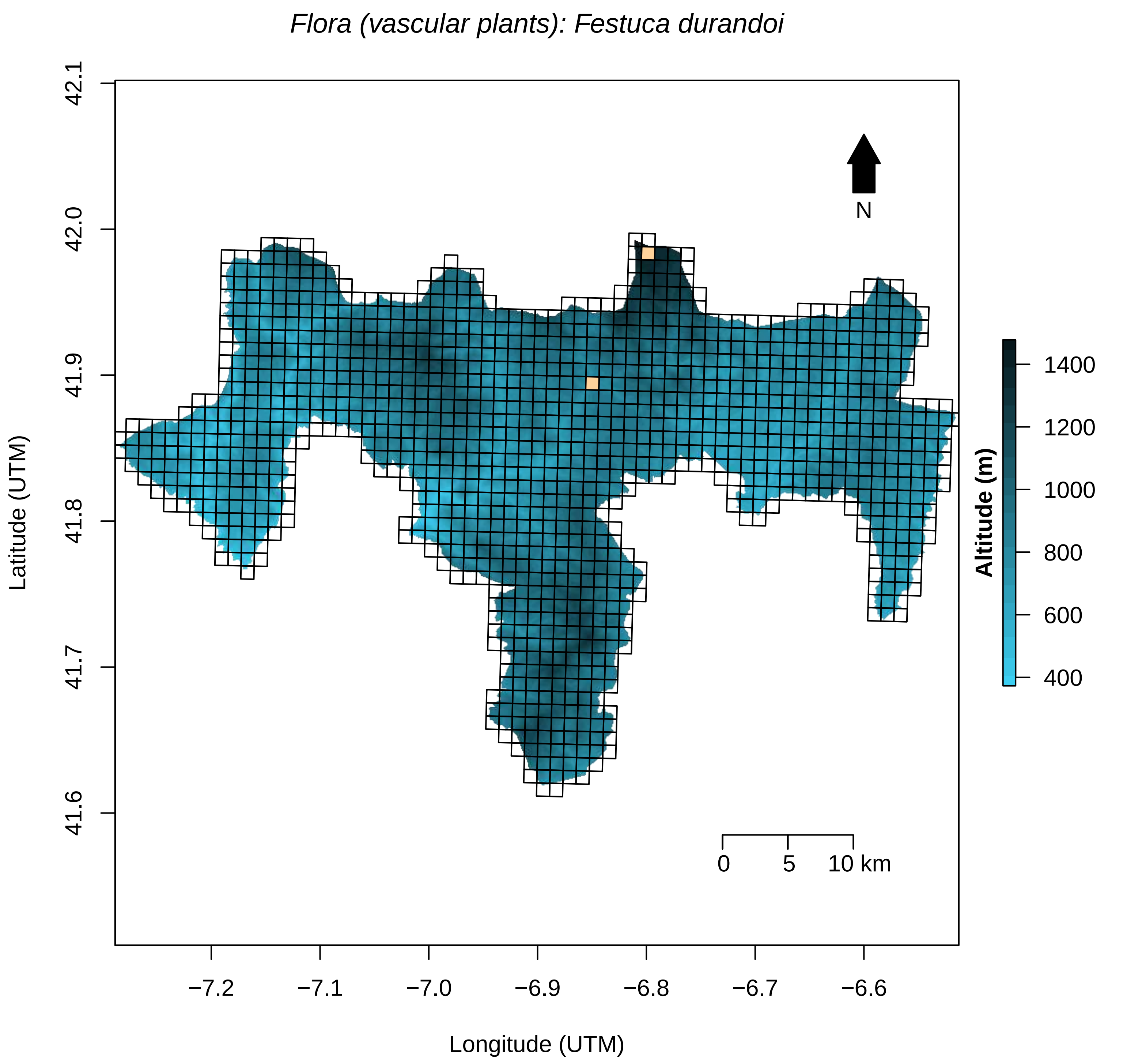
Flora (vascular plants): Festuca durandoi
N
0
5
10 km
Altitude (m)
1400
1200
1000
800
600
400
Latitude (UTM)
42.1
42.0
41.9
41.8
41.7
41.6
Longitude (UTM)
−7.2
−7.1
−7.0
−6.9
−6.8
−6.7
−6.6

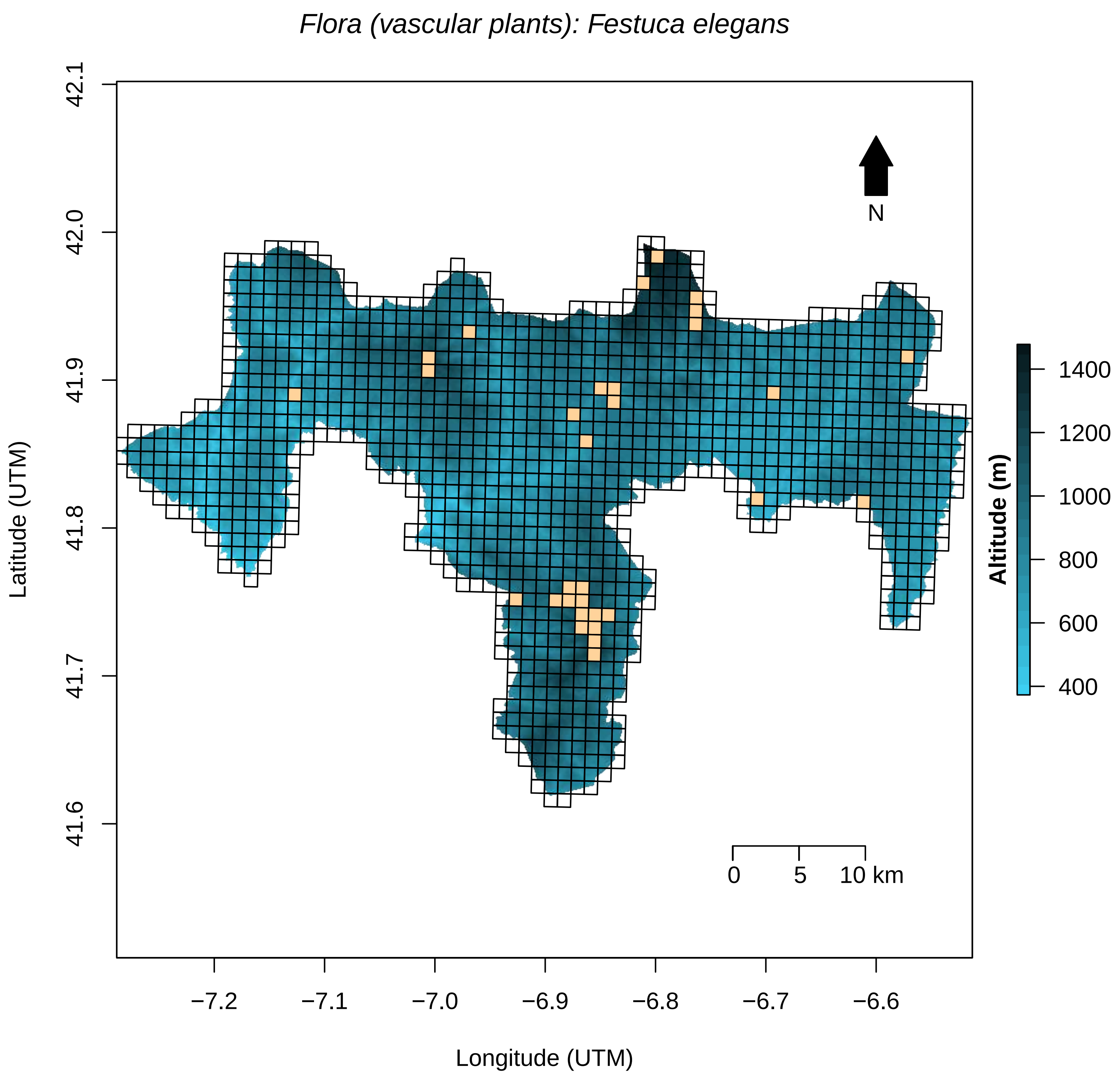
Flora (vascular plants): Festuca elegans
Latitude (UTM)
Longitude (UTM)
42.1
42.0
41.9
41.8
41.7
41.6
−7.2
−7.1
−7.0
−6.9
−6.8
−6.7
−6.6
N
0
5
10 km
Altitude (m)
1400
1200
1000
800
600
400

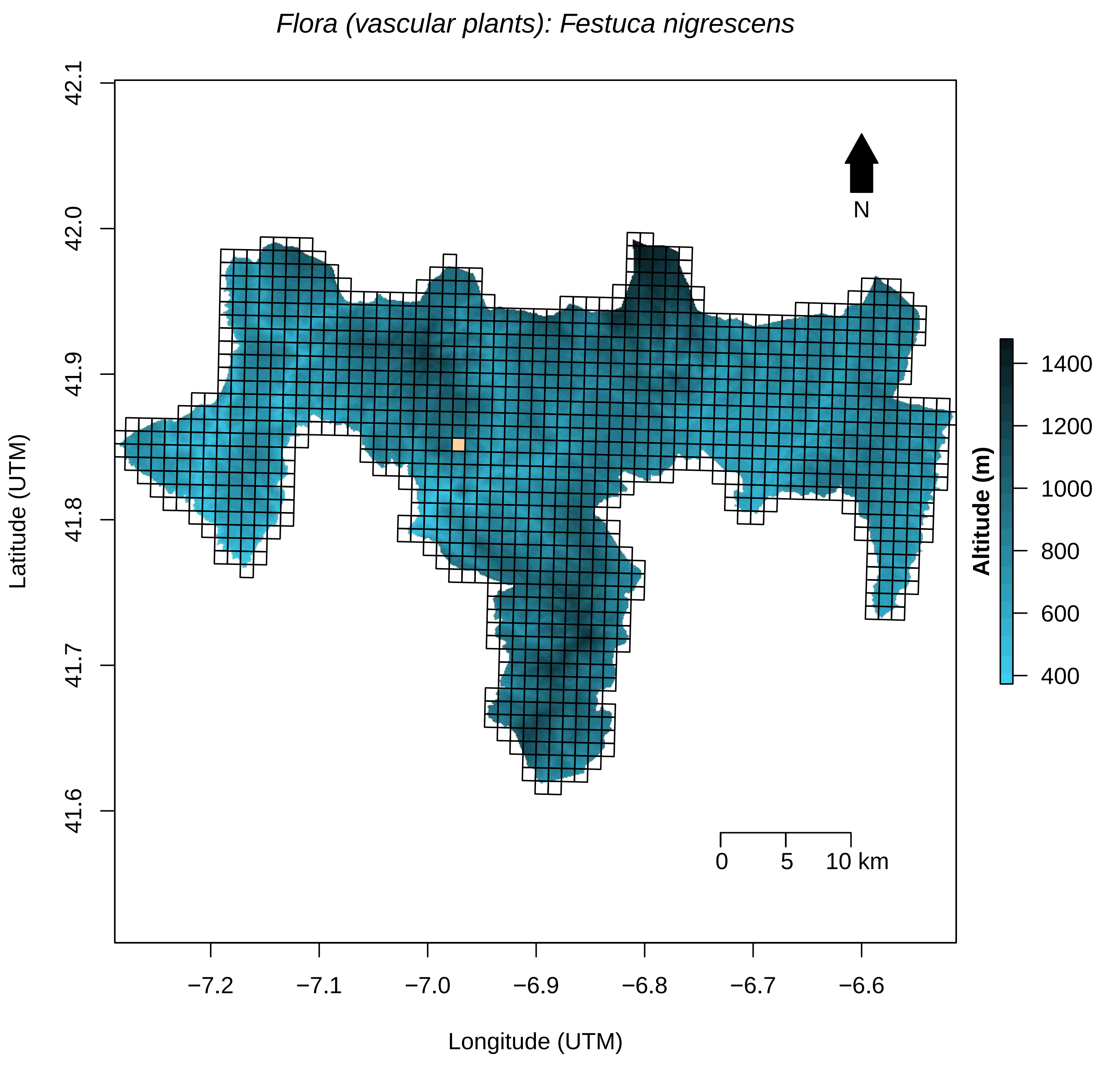
Flora (vascular plants): Festuca nigrescens
N
0
5
10 km
Latitude (UTM)
Longitude (UTM)
Altitude (m)
42.1
42.0
41.9
41.8
41.7
41.6
−7.2
−7.1
−7.0
−6.9
−6.8
−6.7
−6.6
1400
1200
1000
800
600
400

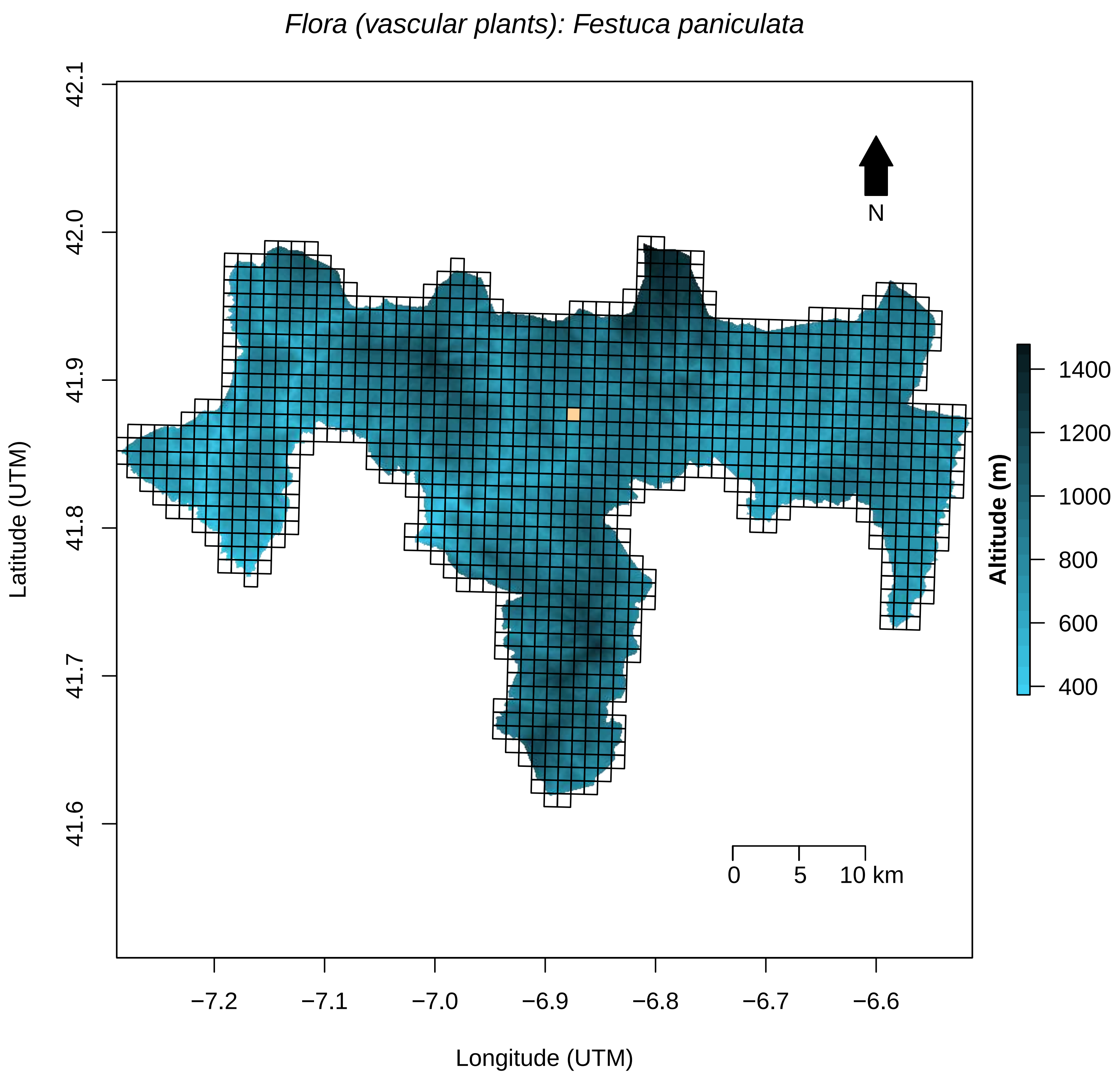
Flora (vascular plants): Festuca paniculata
42.1
42.0
41.9
41.8
41.7
41.6
Latitude (UTM)
−7.2
−7.1
−7.0
−6.9
−6.8
−6.7
−6.6
Longitude (UTM)
N
0
5
10 km
Altitude (m)
1400
1200
1000
800
600
400

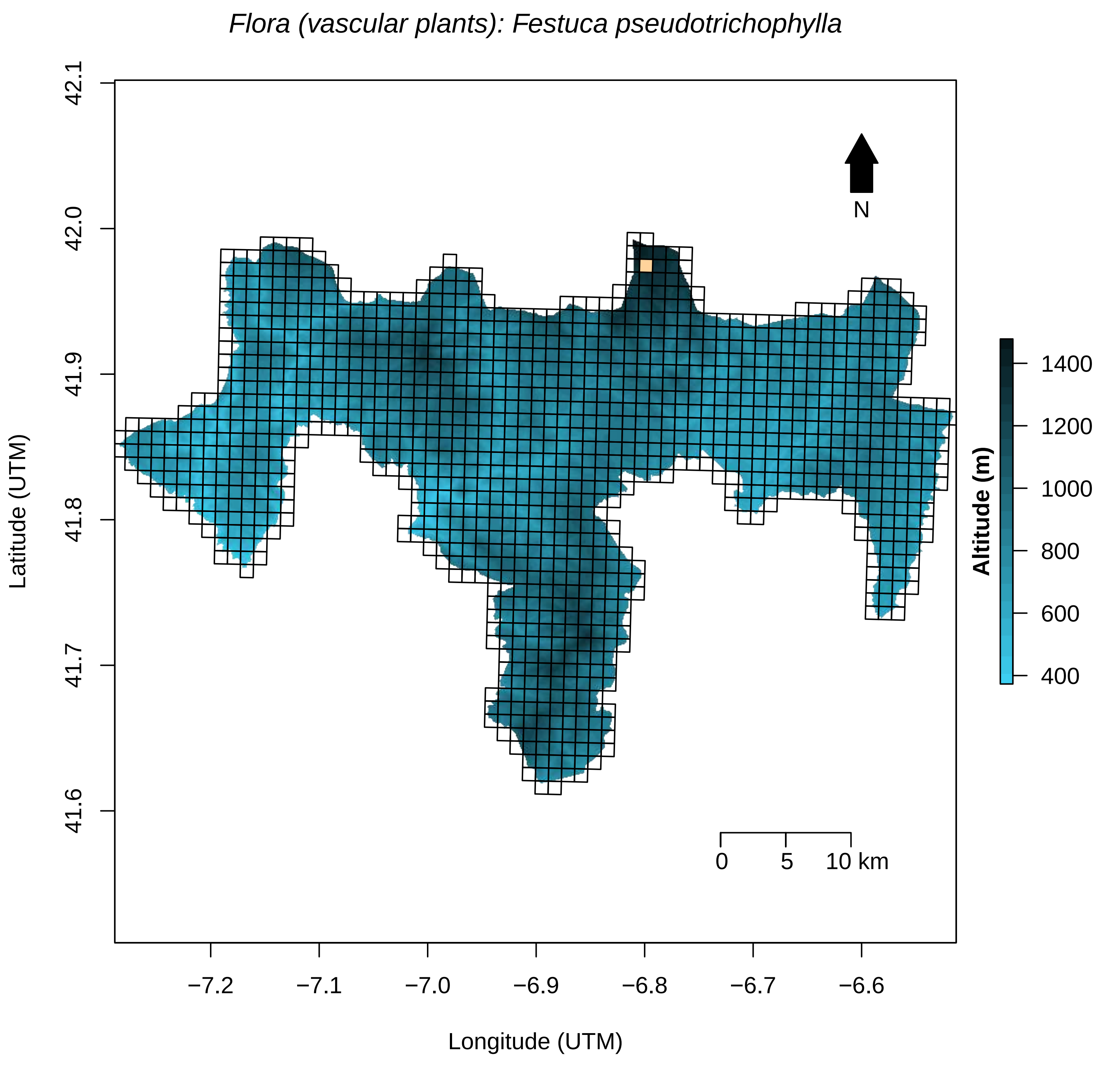

Flora (vascular plants): Festuca pseudotrichophylla
N
0
5
10 km
Altitude (m)
1400
1200
1000
800
600
400
42.1
42.0
41.9
41.8
41.7
41.6
Latitude (UTM)
−7.2
−7.1
−7.0
−6.9
−6.8
−6.7
−6.6
Longitude (UTM)

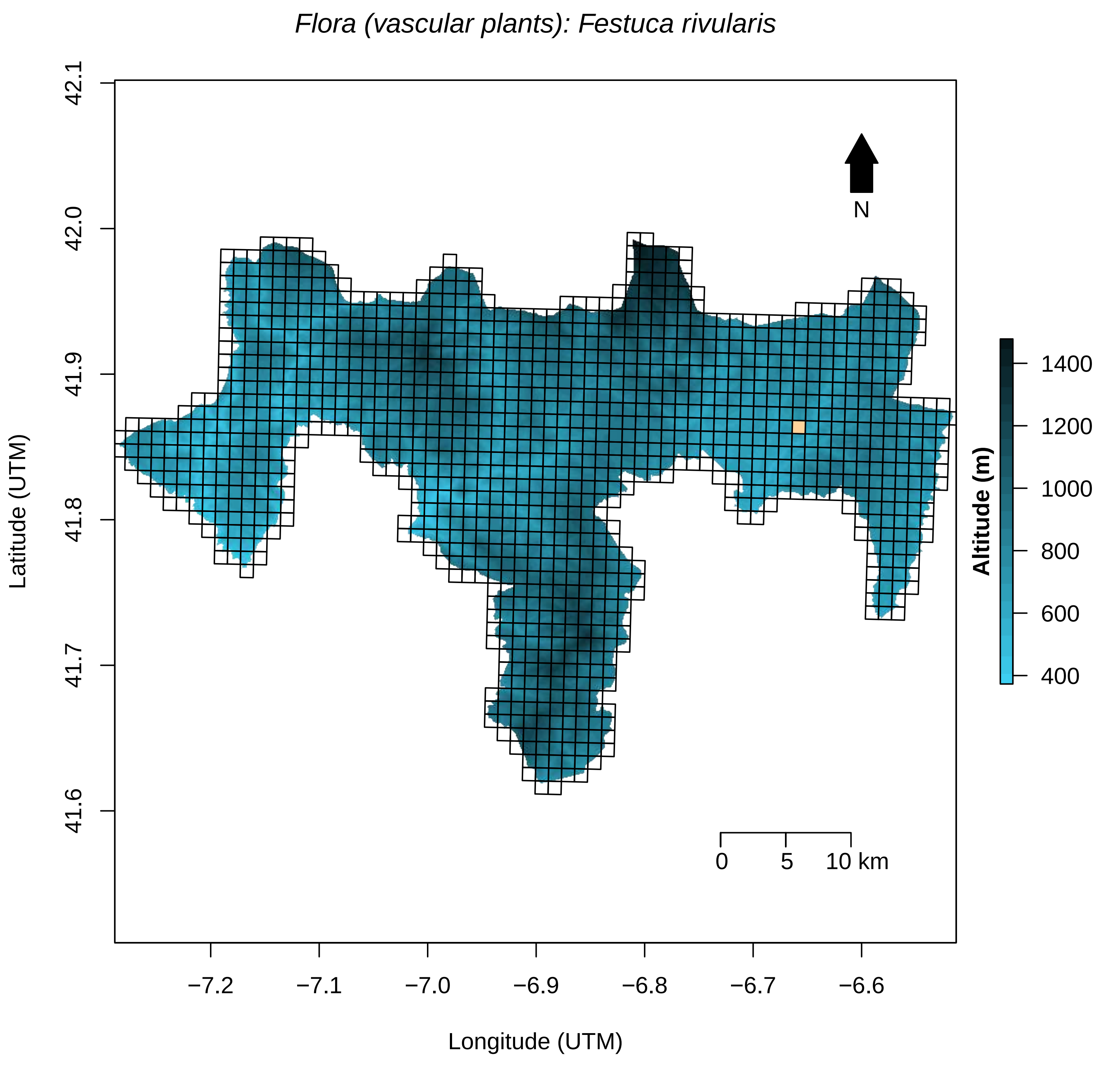
Flora (vascular plants): Festuca rivularis
42.1
42.0
41.9
41.8
41.7
41.6
Latitude (UTM)
N
0 5 10 km
−7.2 −7.1 −7.0 −6.9 −6.8 −6.7 −6.6
Longitude (UTM)
Altitude (m)
1400
1200
1000
800
600
400

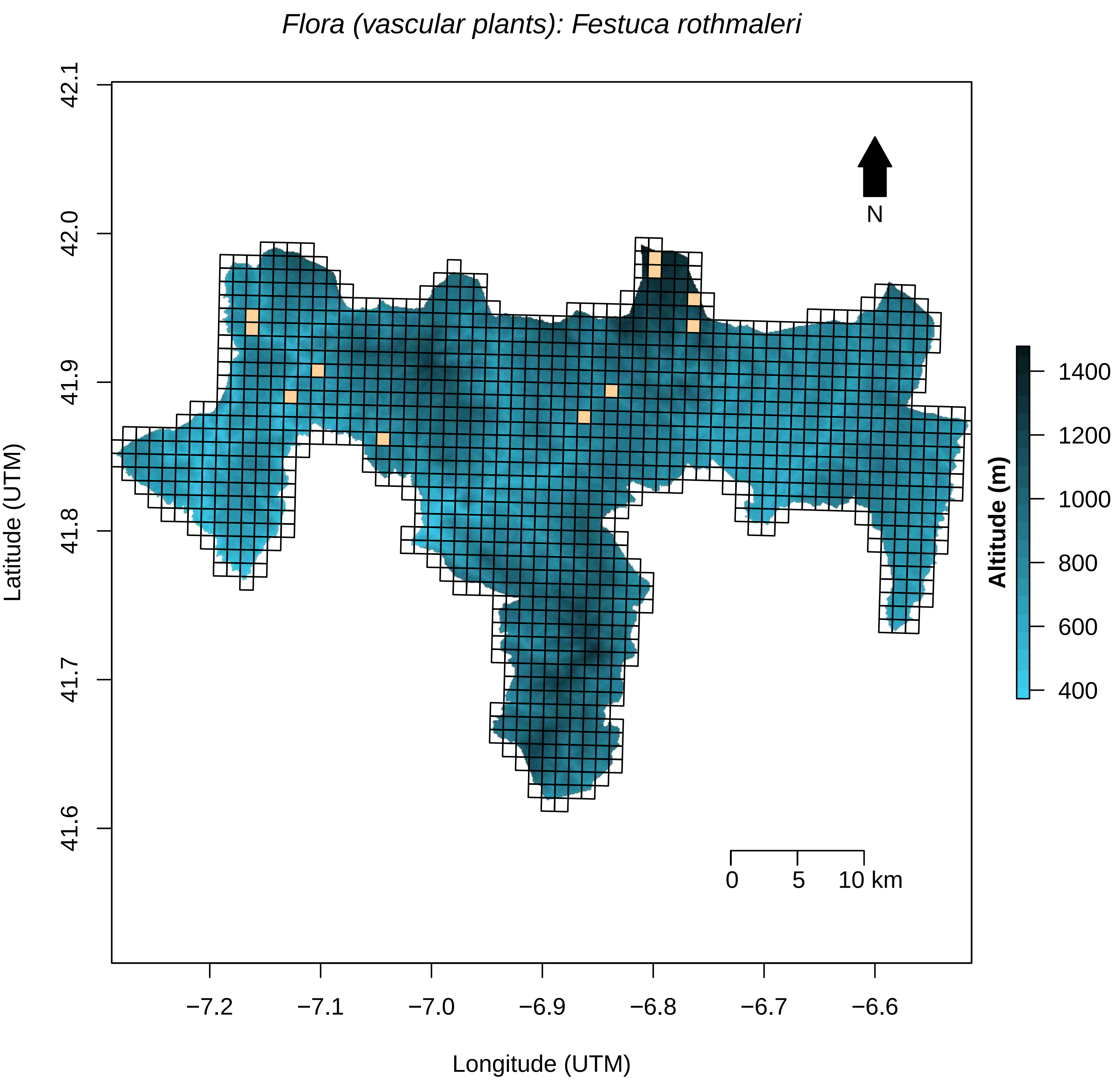

Flora (vascular plants): Festuca rothmaleri
Latitude (UTM)
42.1
42.0
41.9
41.8
41.7
41.6
Longitude (UTM)
−7.2
−7.1
−7.0
−6.9
−6.8
−6.7
−6.6
N
0
5
10 km
Altitude (m)
1400
1200
1000
800
600
400

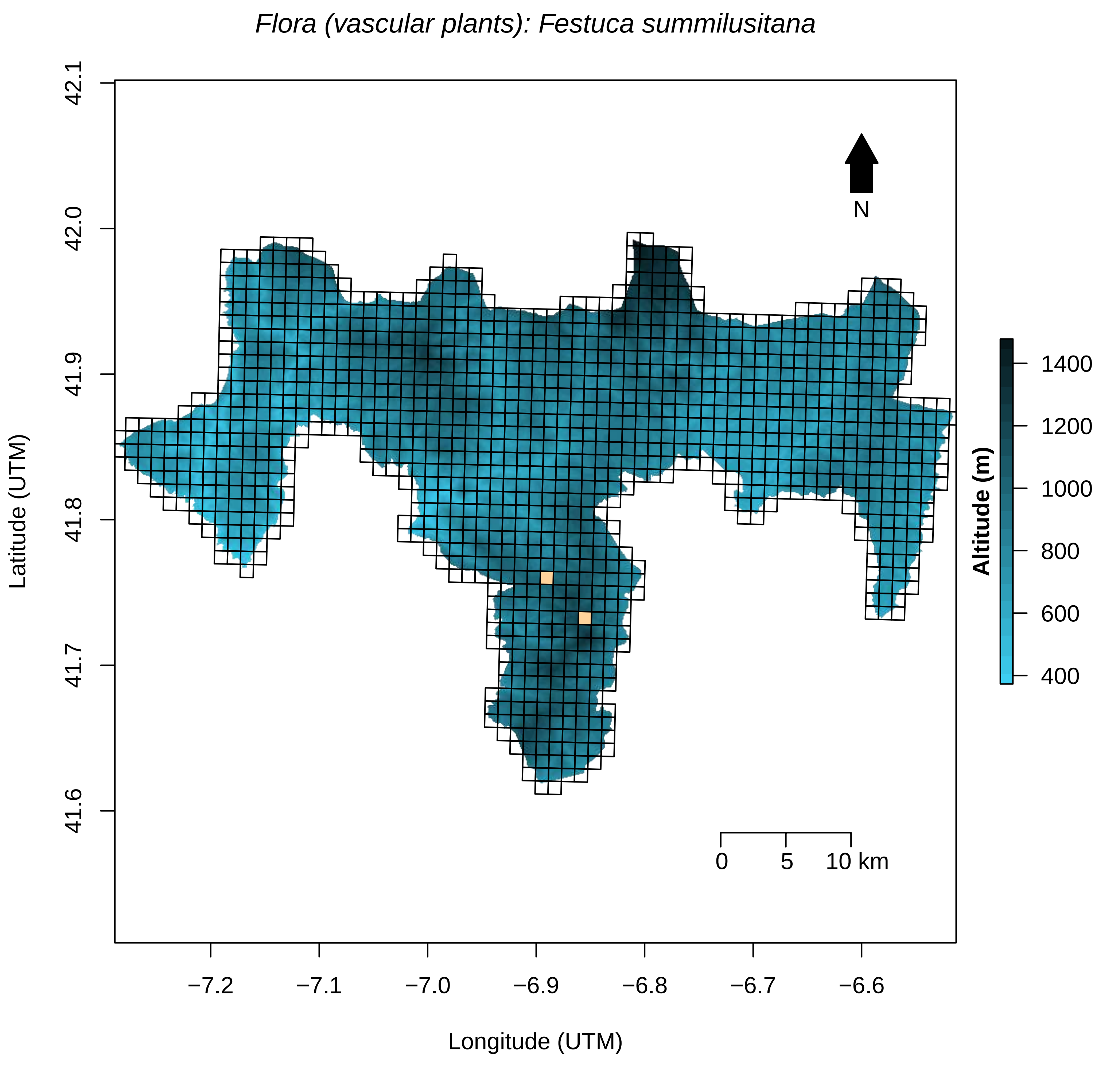

Flora (vascular plants): Festuca summilusitana
N
0
5
10 km
Latitude (UTM)
42.1
42.0
41.9
41.8
41.7
41.6
Longitude (UTM)
−7.2
−7.1
−7.0
−6.9
−6.8
−6.7
−6.6
Altitude (m)
1400
1200
1000
800
600
400

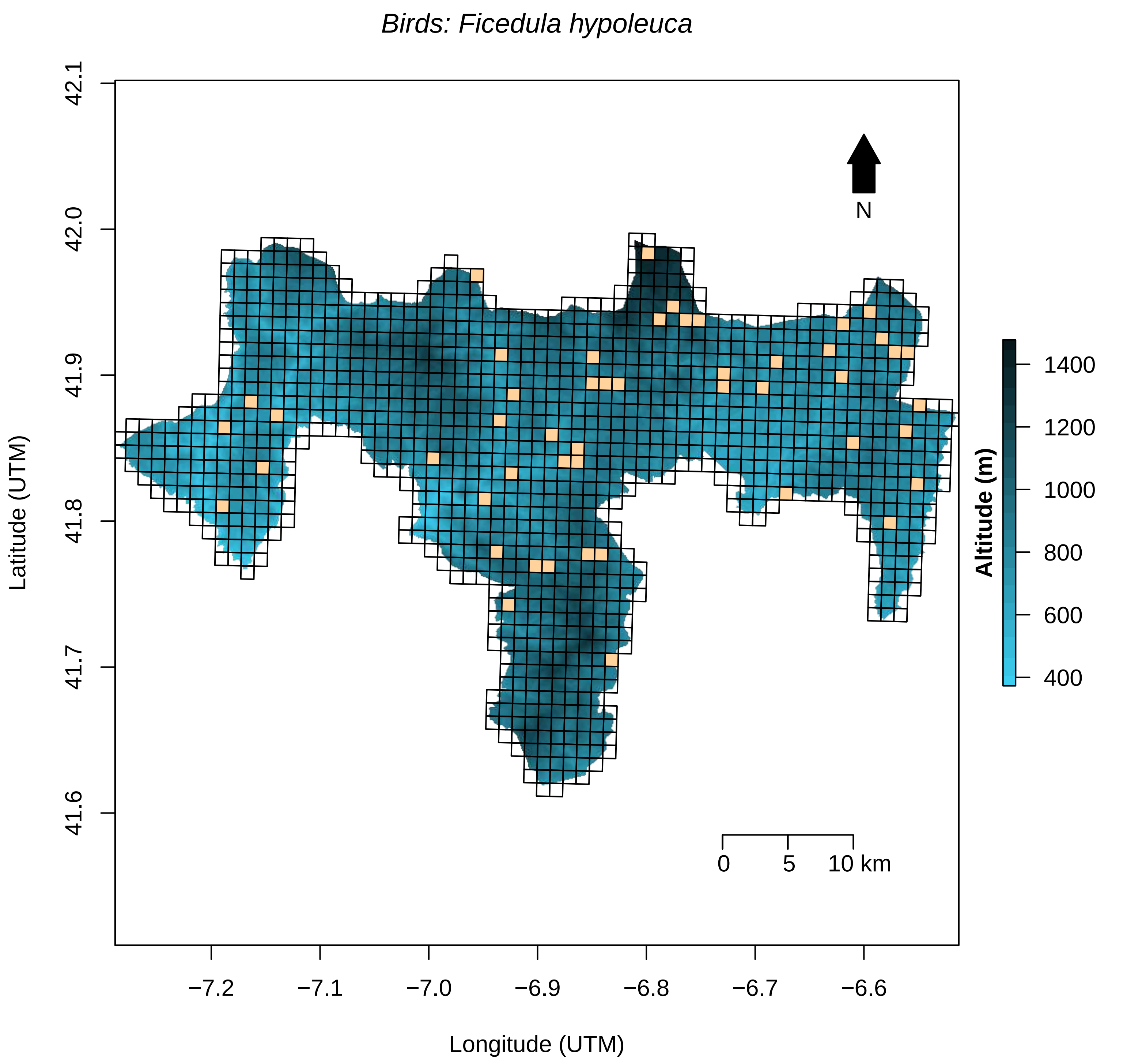

Birds: Ficedula hypoleuca
42.1
42.0
41.9
41.8
41.7
41.6
Latitude (UTM)
−7.2
−7.1
−7.0
−6.9
−6.8
−6.7
−6.6
Longitude (UTM)
N
0
5
10 km
Altitude (m)
1400
1200
1000
800
600
400

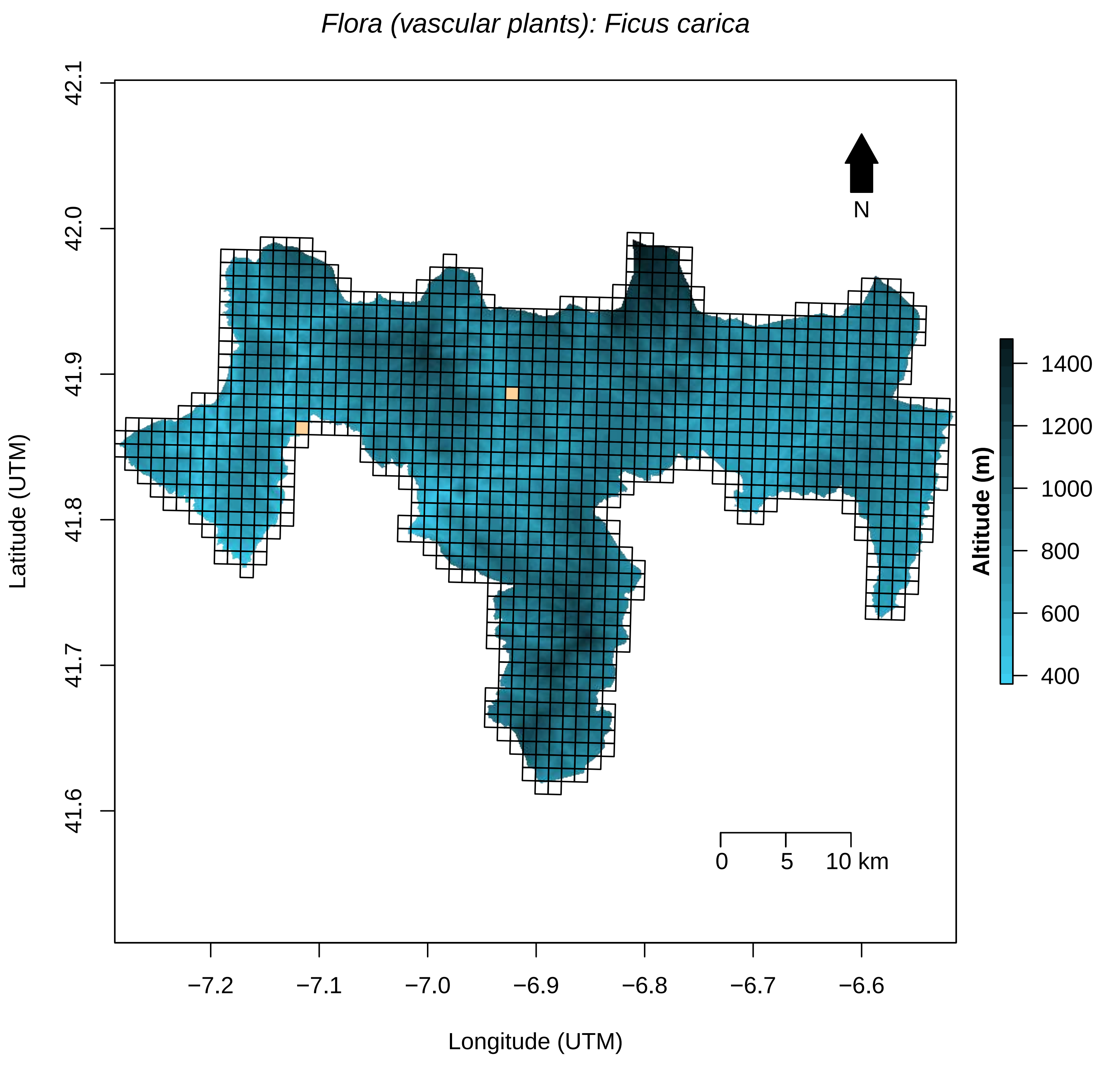

Flora (vascular plants): Ficus carica
42.1
42.0
41.9
41.8
41.7
41.6
Latitude (UTM)
N
0
5
10 km
−7.2
−7.1
−7.0
−6.9
−6.8
−6.7
−6.6
Longitude (UTM)
1400
1200
1000
800
600
400
Altitude (m)

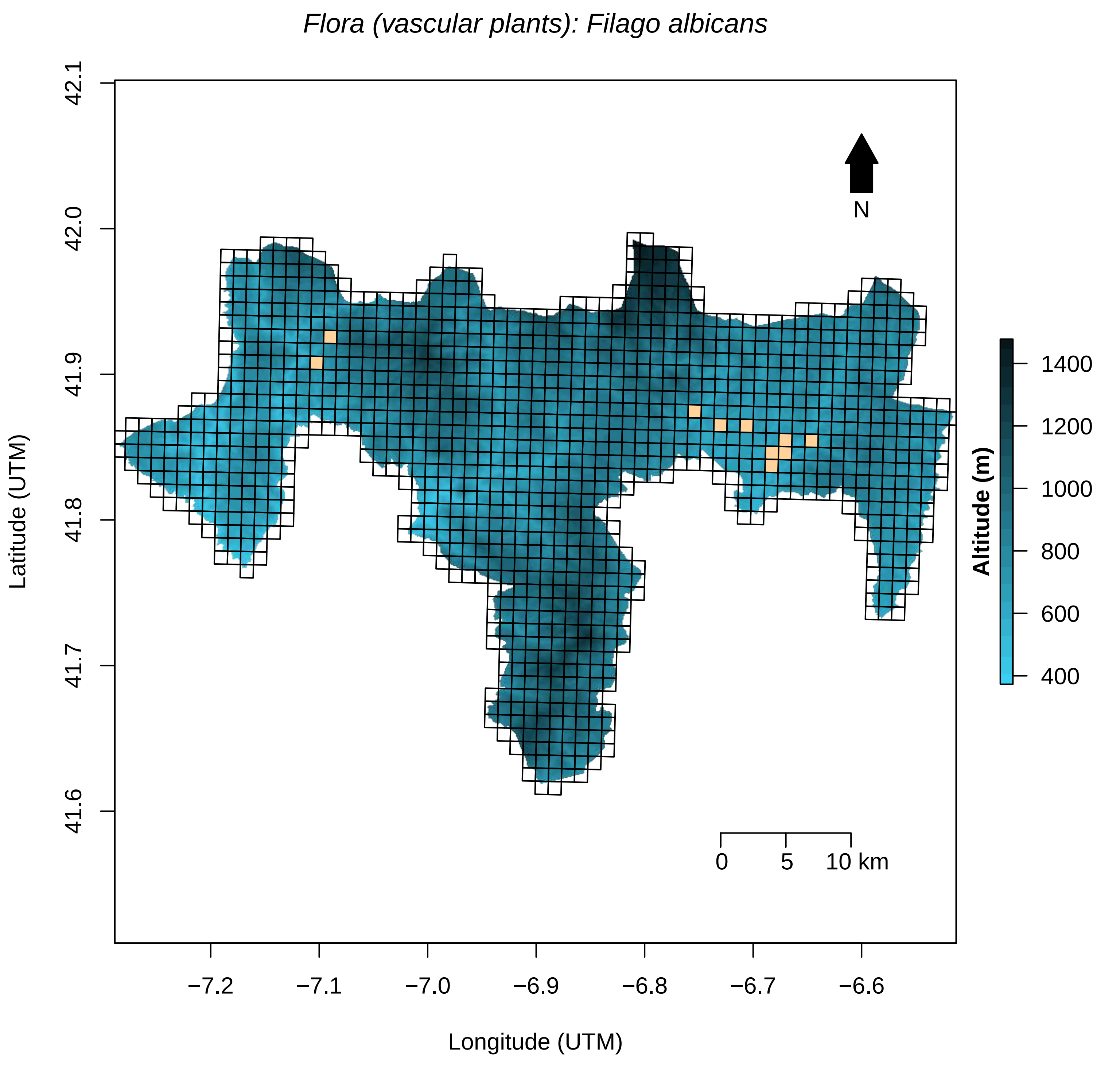

Flora (vascular plants): Filago albicans
42.1
42.0
41.9
41.8
41.7
41.6
Latitude (UTM)
−7.2
−7.1
−7.0
−6.9
−6.8
−6.7
−6.6
Longitude (UTM)
N
0
5
10 km
1400
1200
1000
800
600
400
Altitude (m)

Flora (vascular plants): Filago pyramidata

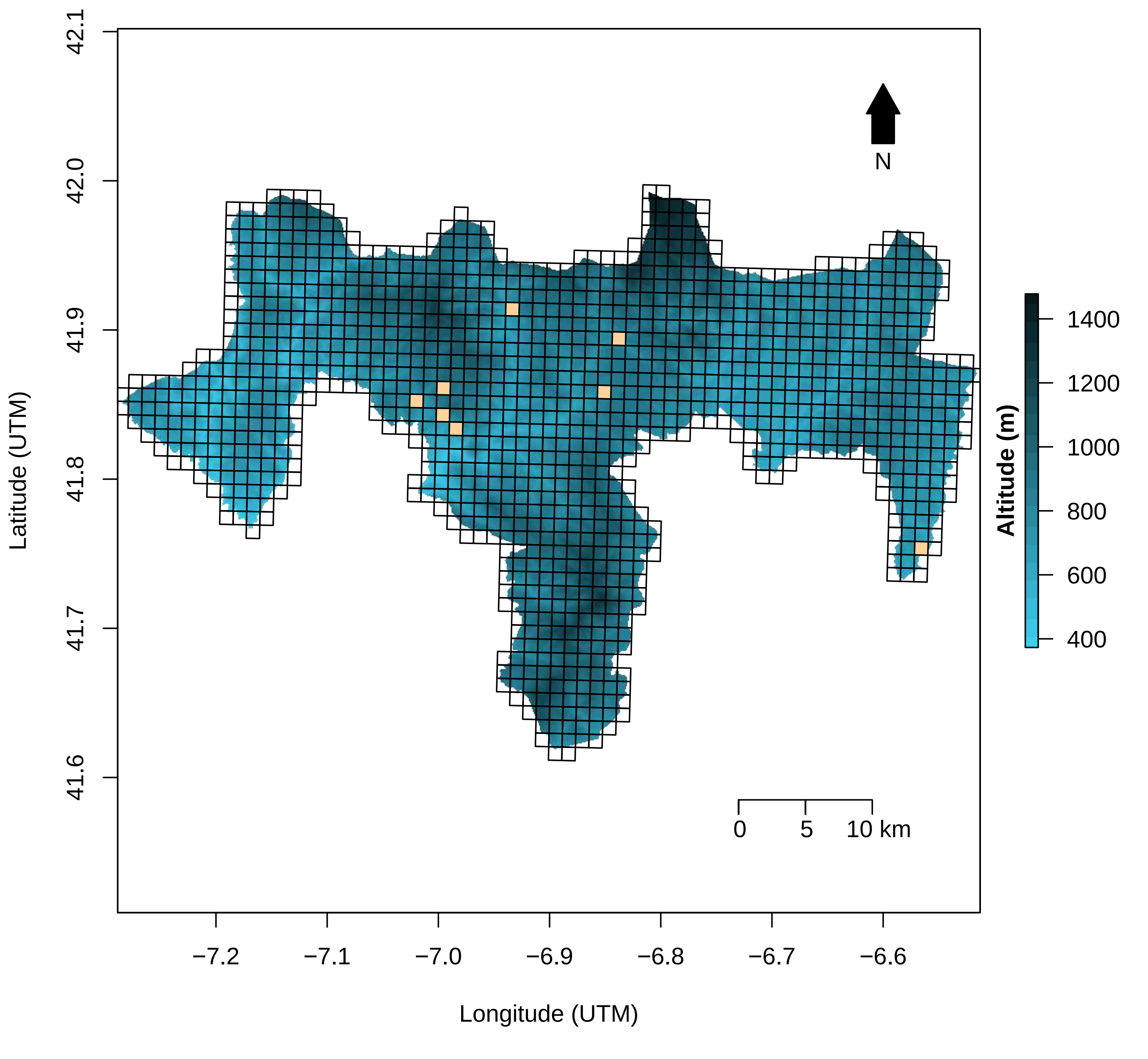

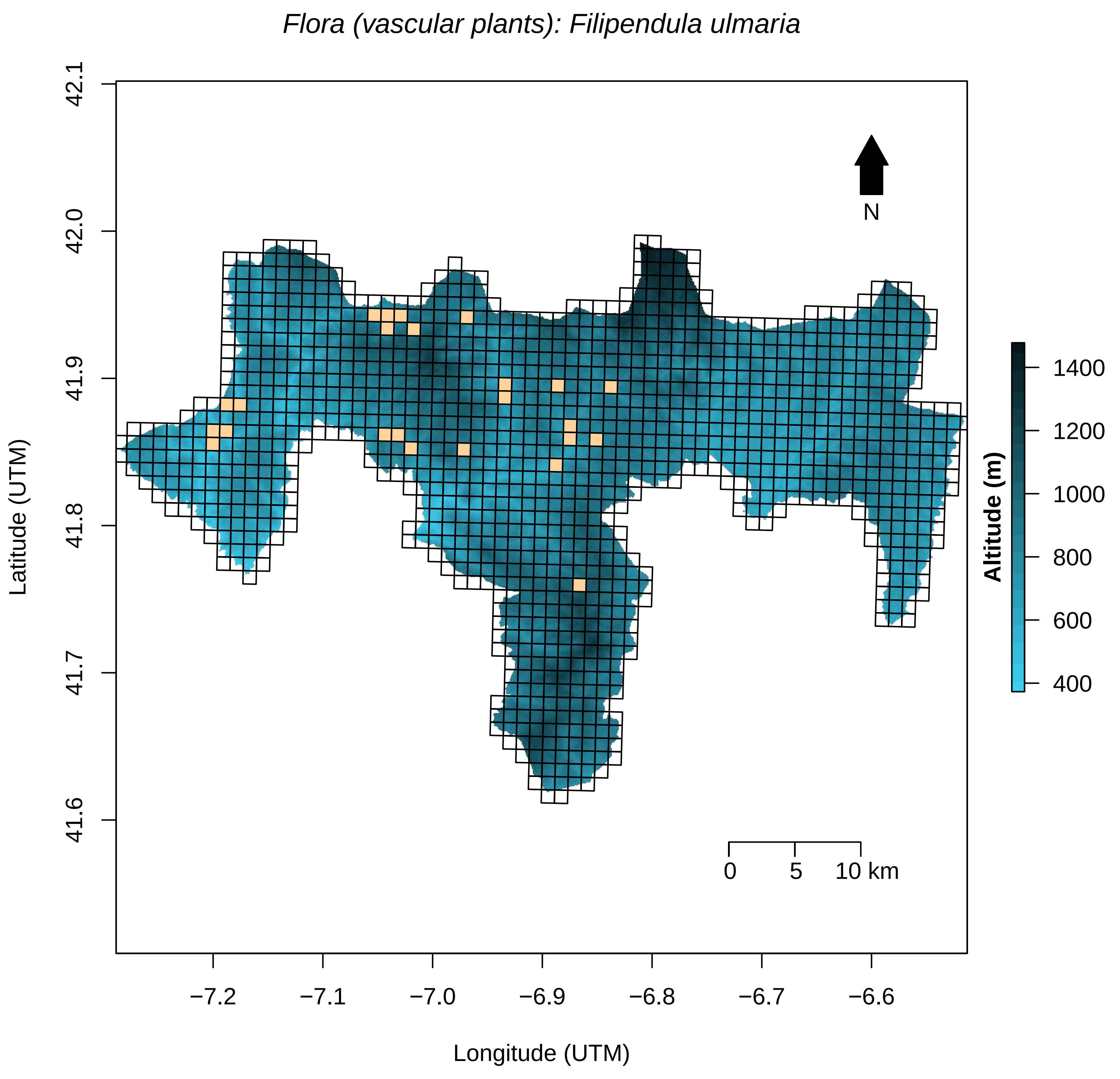

Flora (vascular plants): Filipendula ulmaria
N
0
5
10 km
Altitude (m)
1400
1200
1000
800
600
400
Latitude (UTM)
42.1
42.0
41.9
41.8
41.7
41.6
Longitude (UTM)
−7.2
−7.1
−7.0
−6.9
−6.8
−6.7
−6.6

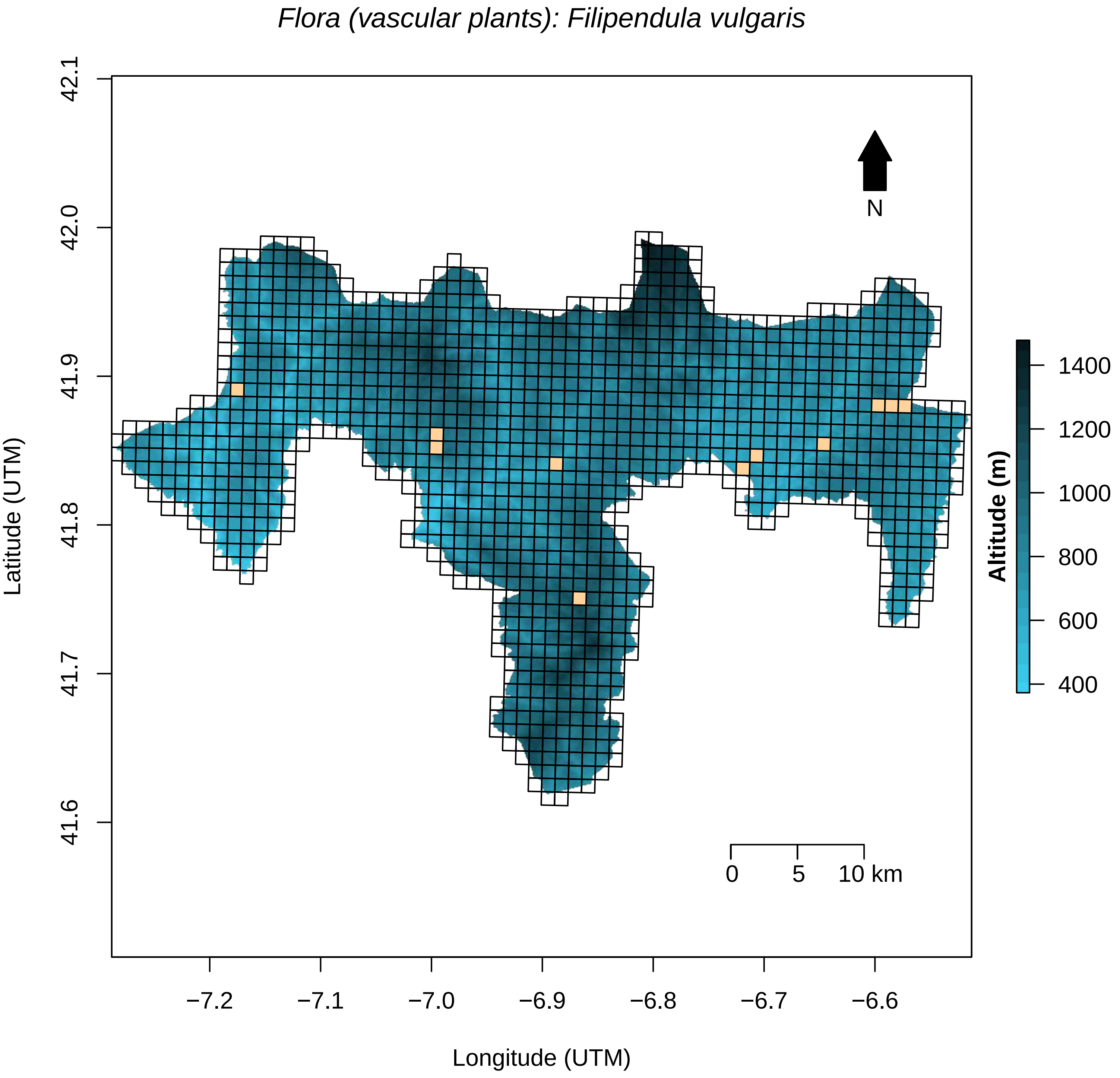

Flora (vascular plants): Filipendula vulgaris
Latitude (UTM)
42.1
42.0
41.9
41.8
41.7
41.6
−7.2
−7.1
−7.0
−6.9
−6.8
−6.7
−6.6
Longitude (UTM)
N
0
5
10 km
Altitude (m)
1400
1200
1000
800
600
400

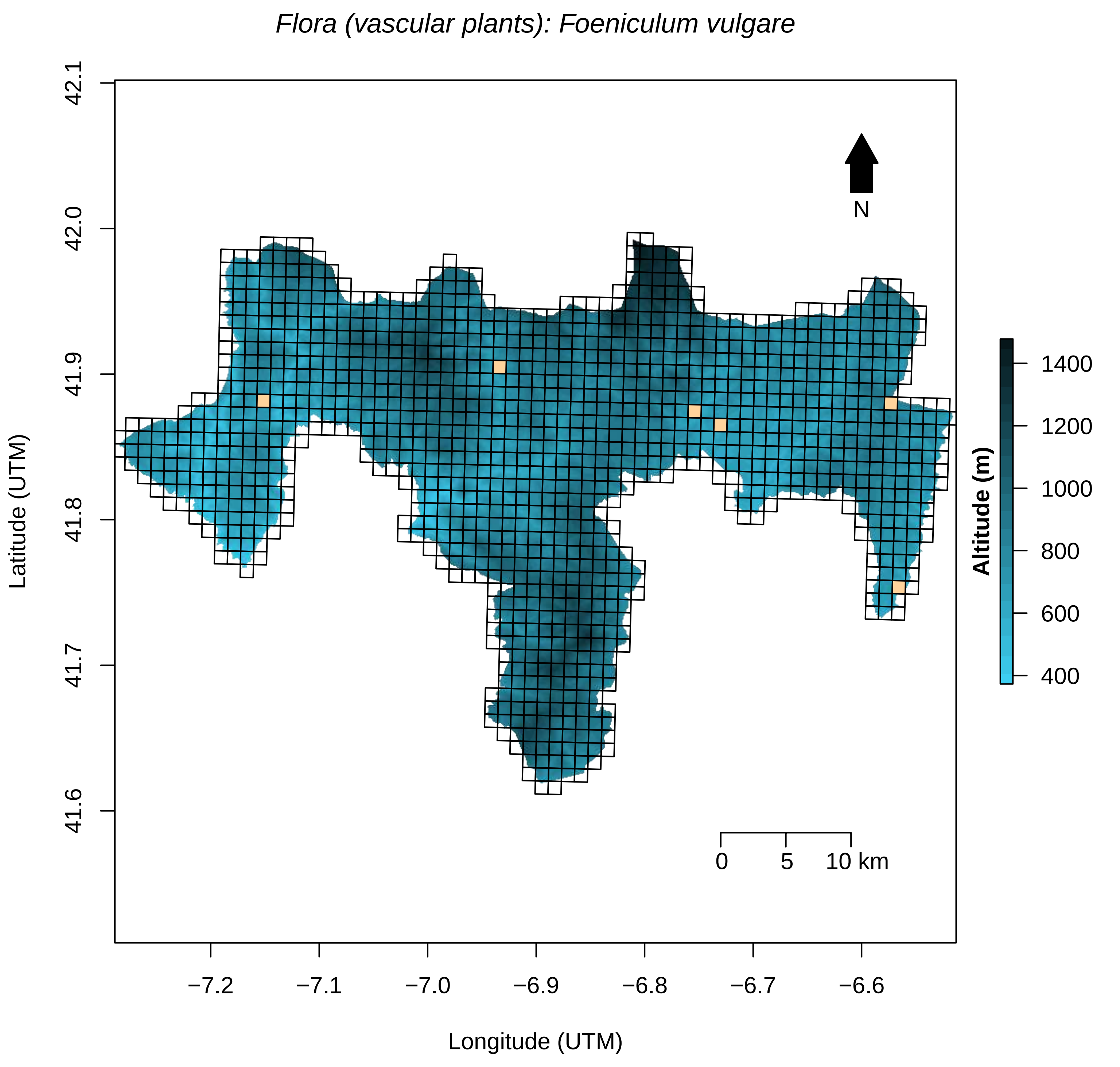

Flora (vascular plants): Foeniculum vulgare
42.1
42.0
41.9
41.8
41.7
41.6
Latitude (UTM)
−7.2
−7.1
−7.0
−6.9
−6.8
−6.7
−6.6
Longitude (UTM)
N
0
5
10 km
Altitude (m)
1400
1200
1000
800
600
400

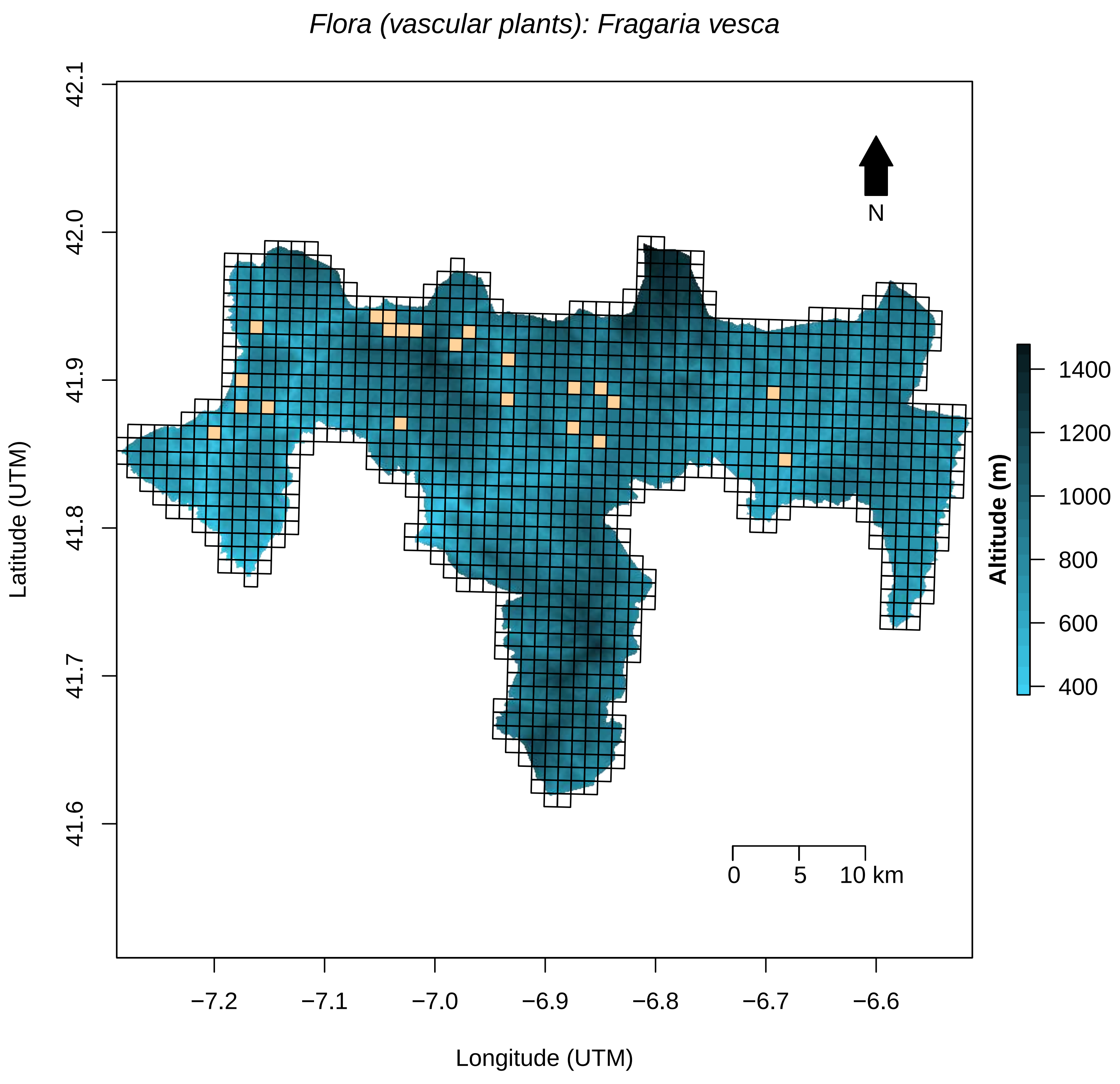

Flora (vascular plants): Fragaria vesca
N
0
5
10 km
Altitude (m)
1400
1200
1000
800
600
400
42.1
42.0
41.9
41.8
41.7
41.6
Latitude (UTM)
−7.2
−7.1
−7.0
−6.9
−6.8
−6.7
−6.6
Longitude (UTM)

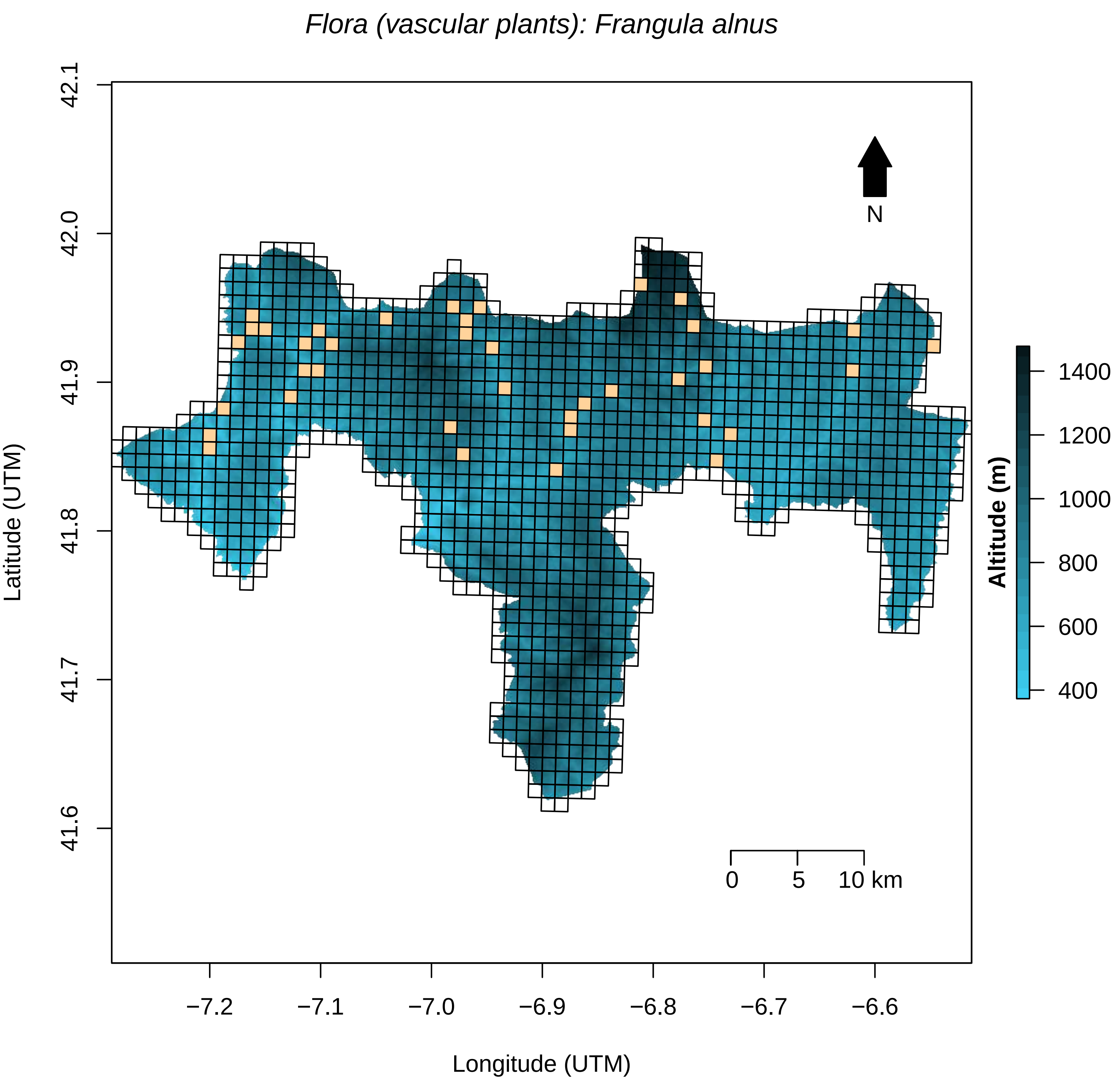

Flora (vascular plants): Frangula alnus
42.1
42.0
41.9
41.8
41.7
41.6
Latitude (UTM)
−7.2
−7.1
−7.0
−6.9
−6.8
−6.7
−6.6
Longitude (UTM)
N
0
5
10 km
1400
1200
1000
800
600
400
Altitude (m)

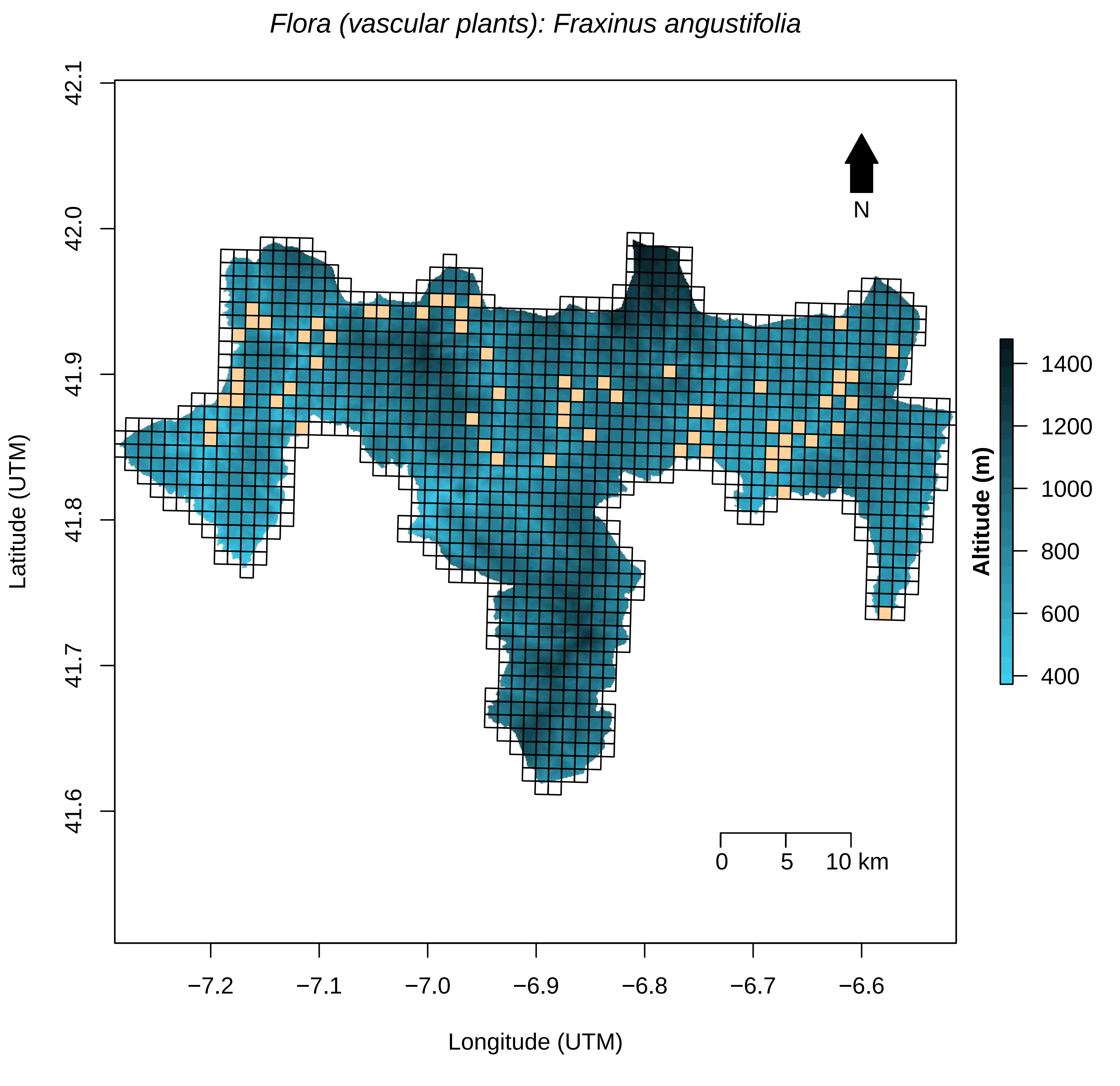

Flora (vascular plants): Fraxinus angustifolia
N
0
5
10 km
Altitude (m)
1400
1200
1000
800
600
400
Latitude (UTM)
41.6
41.7
41.8
41.9
42.0
42.1
Longitude (UTM)
−7.2
−7.1
−7.0
−6.9
−6.8
−6.7
−6.6

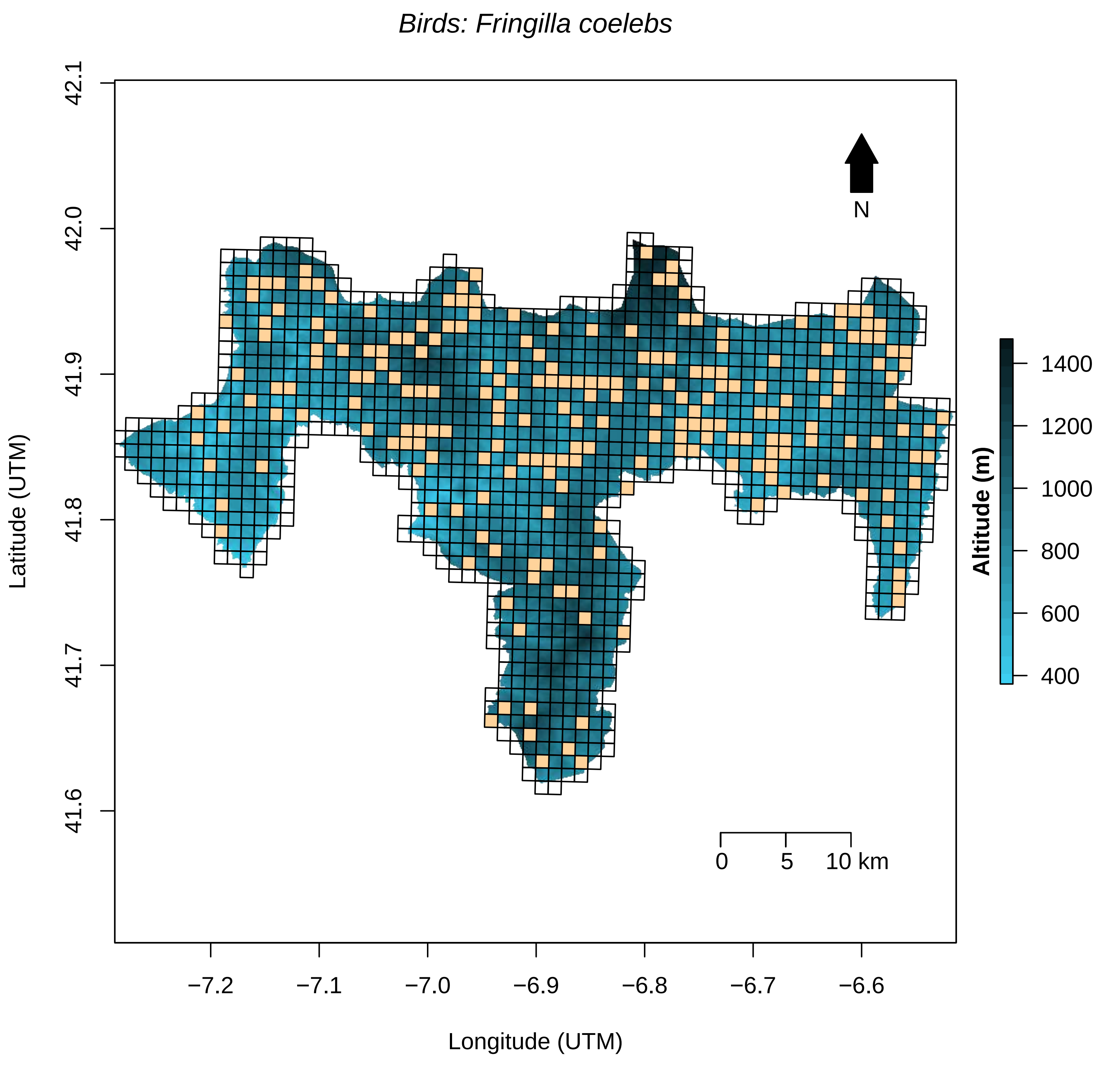

Birds: Fringilla coelebs
42.1
42.0
41.9
41.8
41.7
41.6
Latitude (UTM)
−7.2
−7.1
−7.0
−6.9
−6.8
−6.7
−6.6
Longitude (UTM)
N
0
5
10 km
1400
1200
1000
800
600
400
Altitude (m)

Birds: Fringilla montifringilla

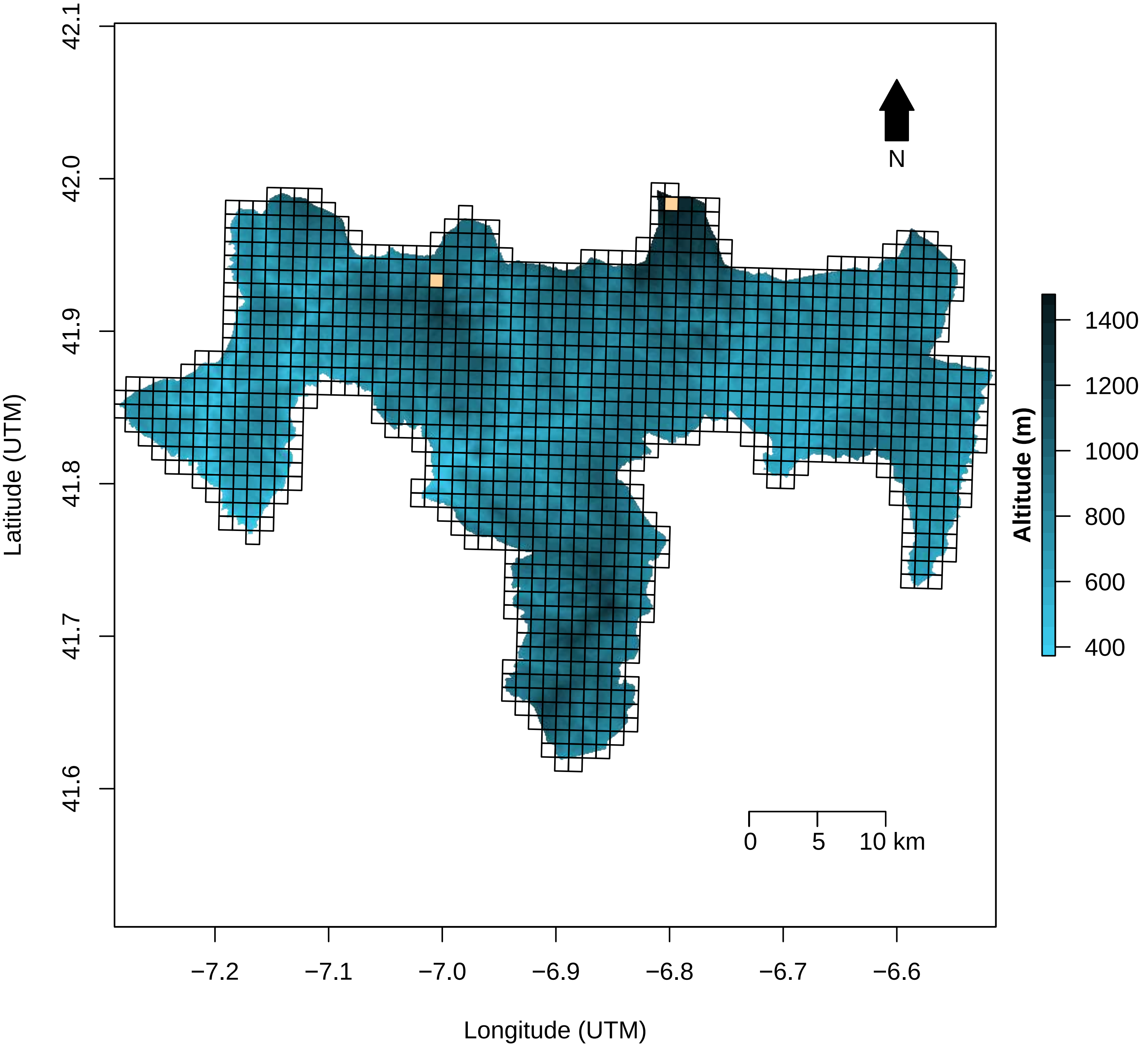

Flora (vascular plants): Fritillaria nervosa

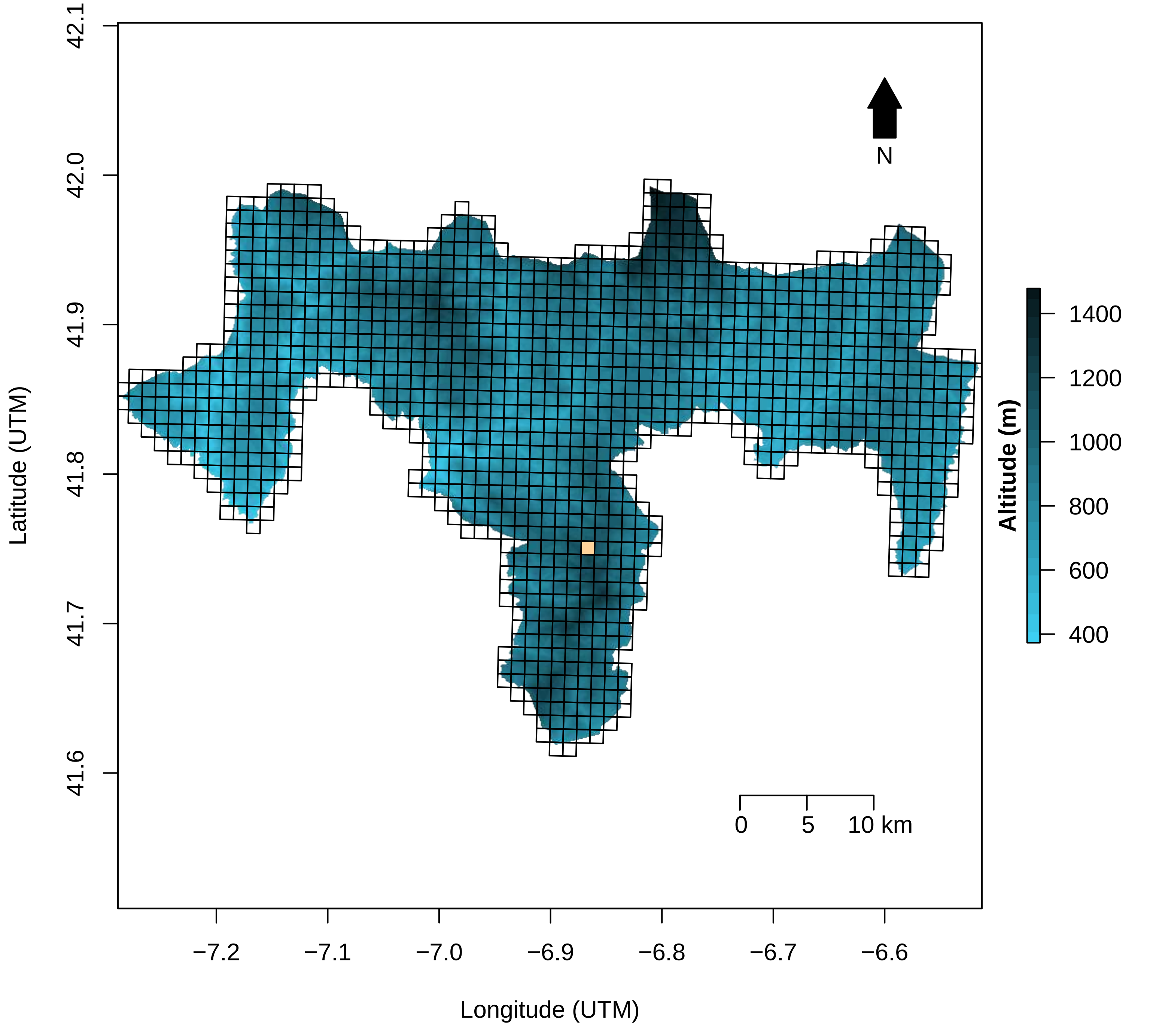

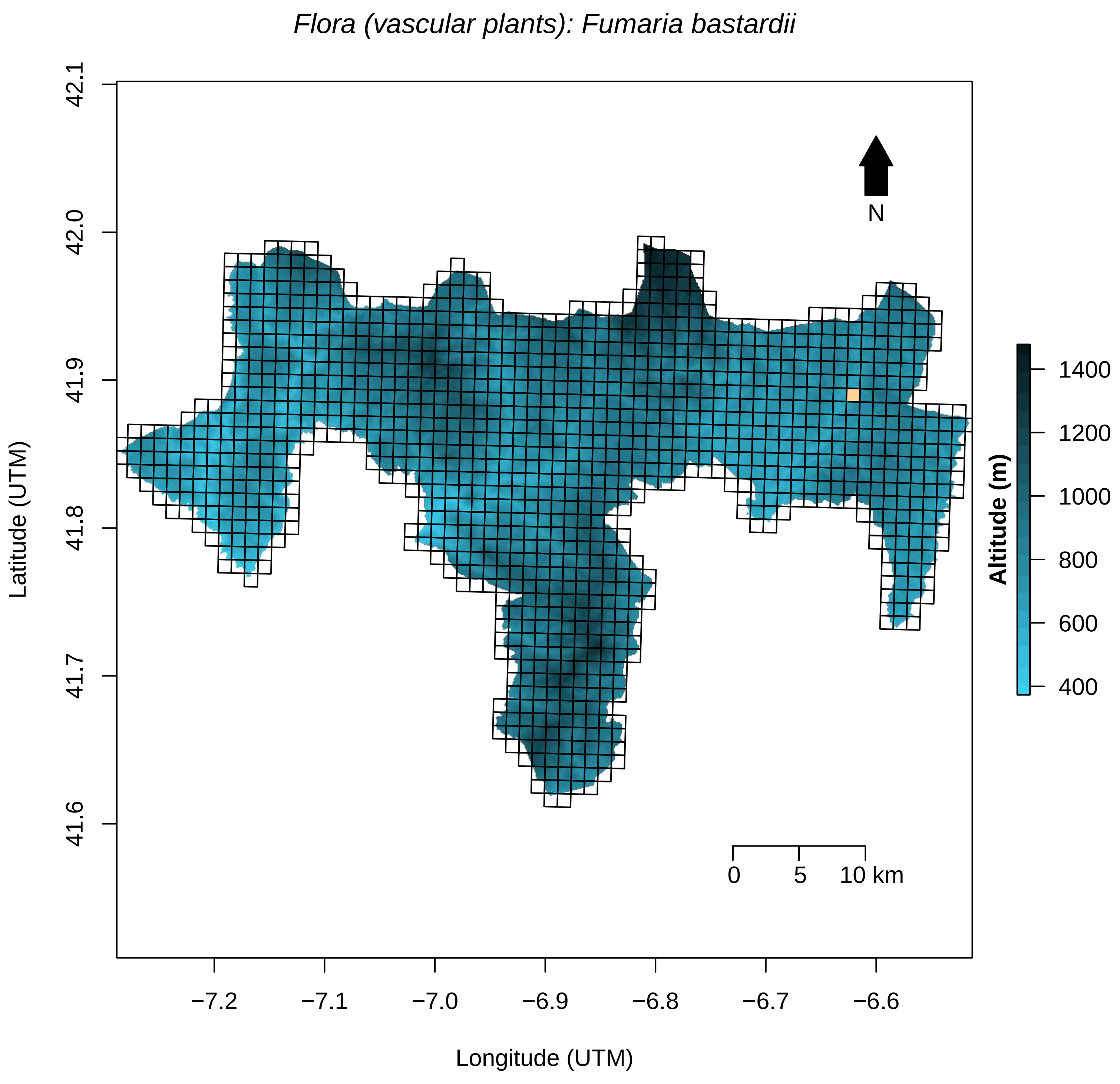

Flora (vascular plants): Fumaria bastardii
42.1
42.0
41.9
41.8
41.7
41.6
Latitude (UTM)
−7.2
−7.1
−7.0
−6.9
−6.8
−6.7
−6.6
Longitude (UTM)
N
0
5
10 km
Altitude (m)
1400
1200
1000
800
600
400

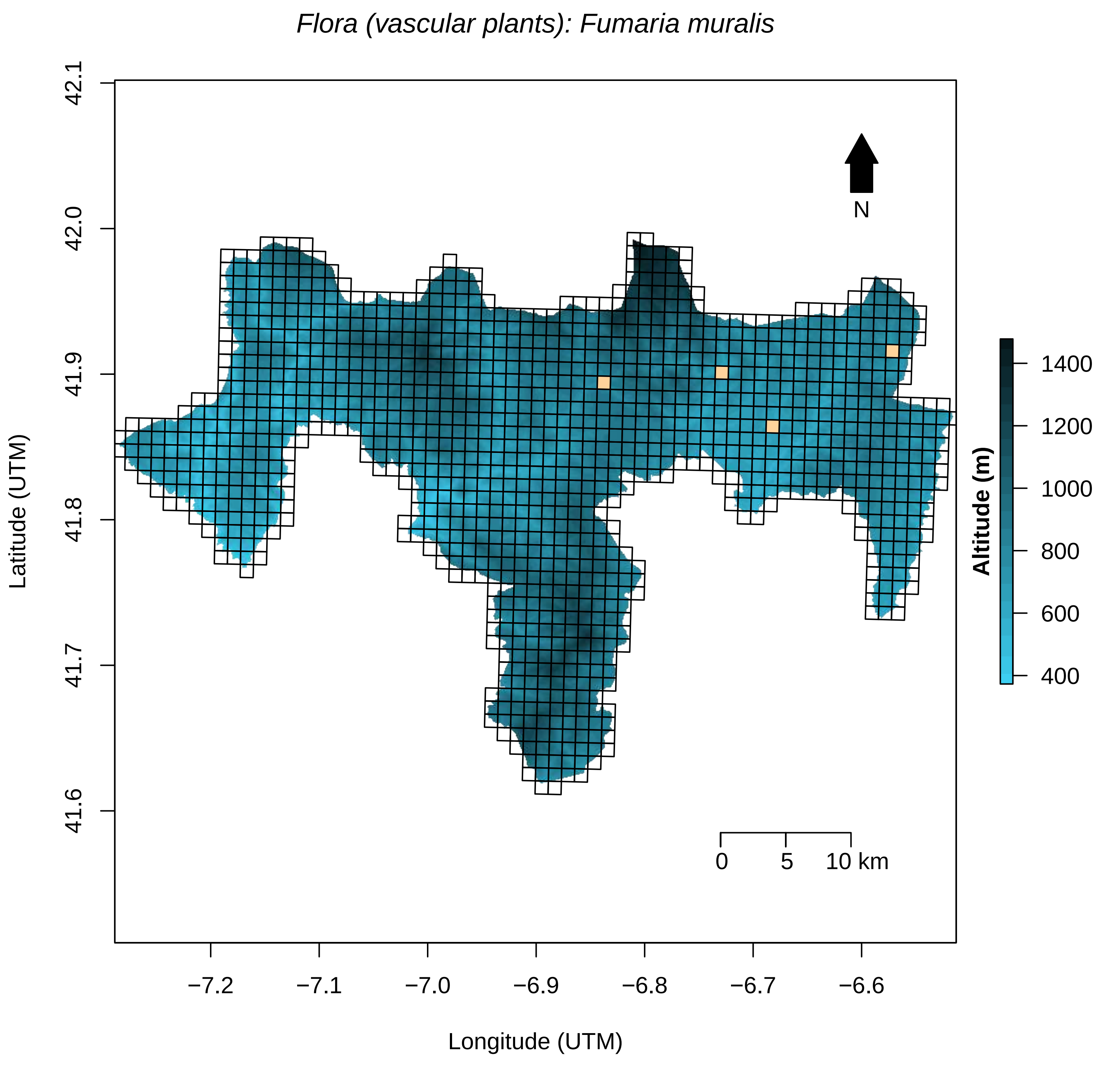
Flora (vascular plants): Fumaria muralis
42.1
42.0
41.9
41.8
41.7
41.6
Latitude (UTM)
−7.2
−7.1
−7.0
−6.9
−6.8
−6.7
−6.6
Longitude (UTM)
N
0
5
10 km
Altitude (m)
1400
1200
1000
800
600
400

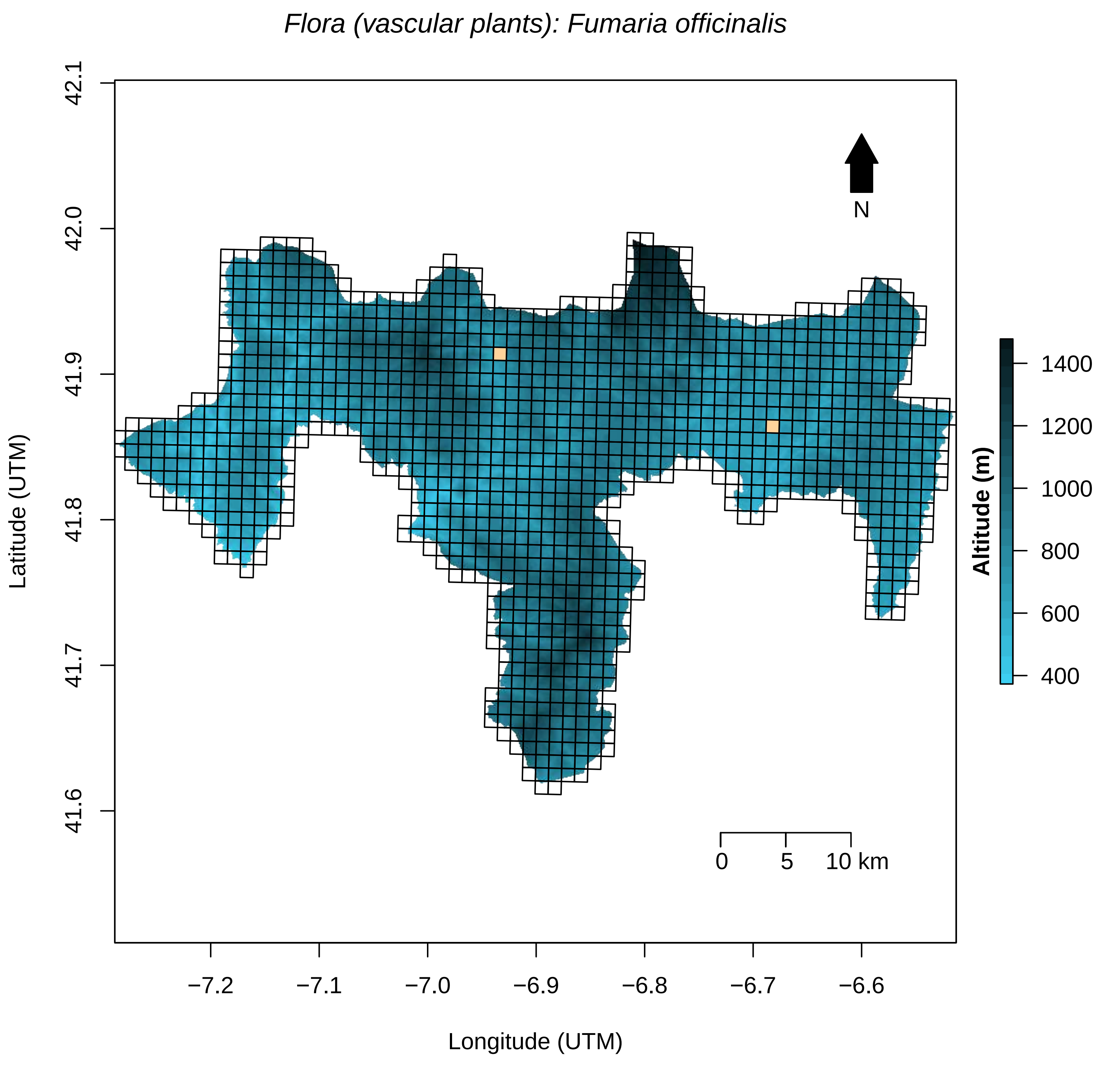

Flora (vascular plants): Fumaria officinalis
42.1
42.0
41.9
41.8
41.7
41.6
Latitude (UTM)
N
0
5
10 km
−7.2
−7.1
−7.0
−6.9
−6.8
−6.7
−6.6
Longitude (UTM)
Altitude (m)
1400
1200
1000
800
600
400

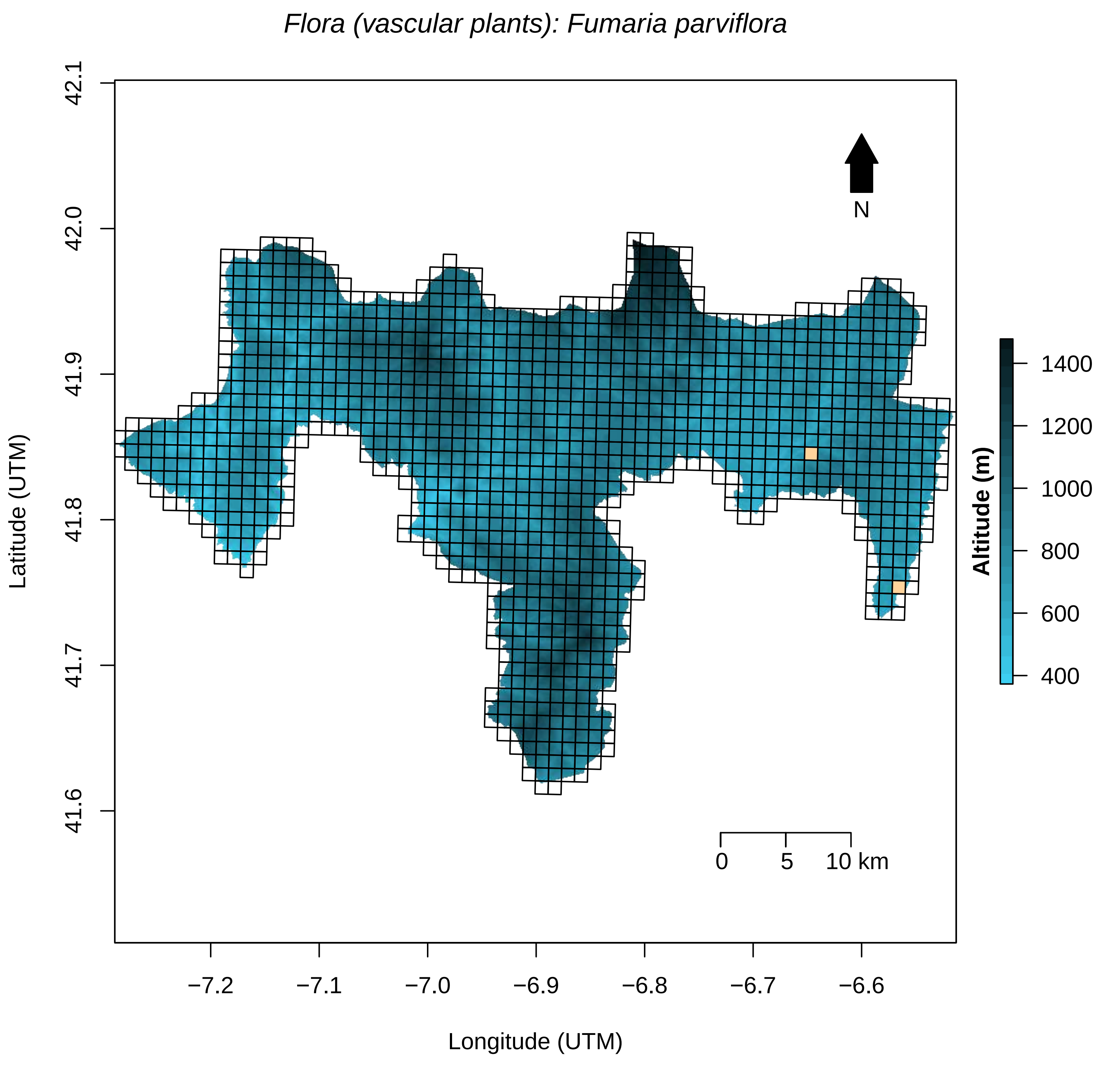
Flora (vascular plants): Fumaria parviflora
42.1
42.0
41.9
41.8
41.7
41.6
Latitude (UTM)
−7.2
−7.1
−7.0
−6.9
−6.8
−6.7
−6.6
Longitude (UTM)
N
0
5
10 km
Altitude (m)
1400
1200
1000
800
600
400

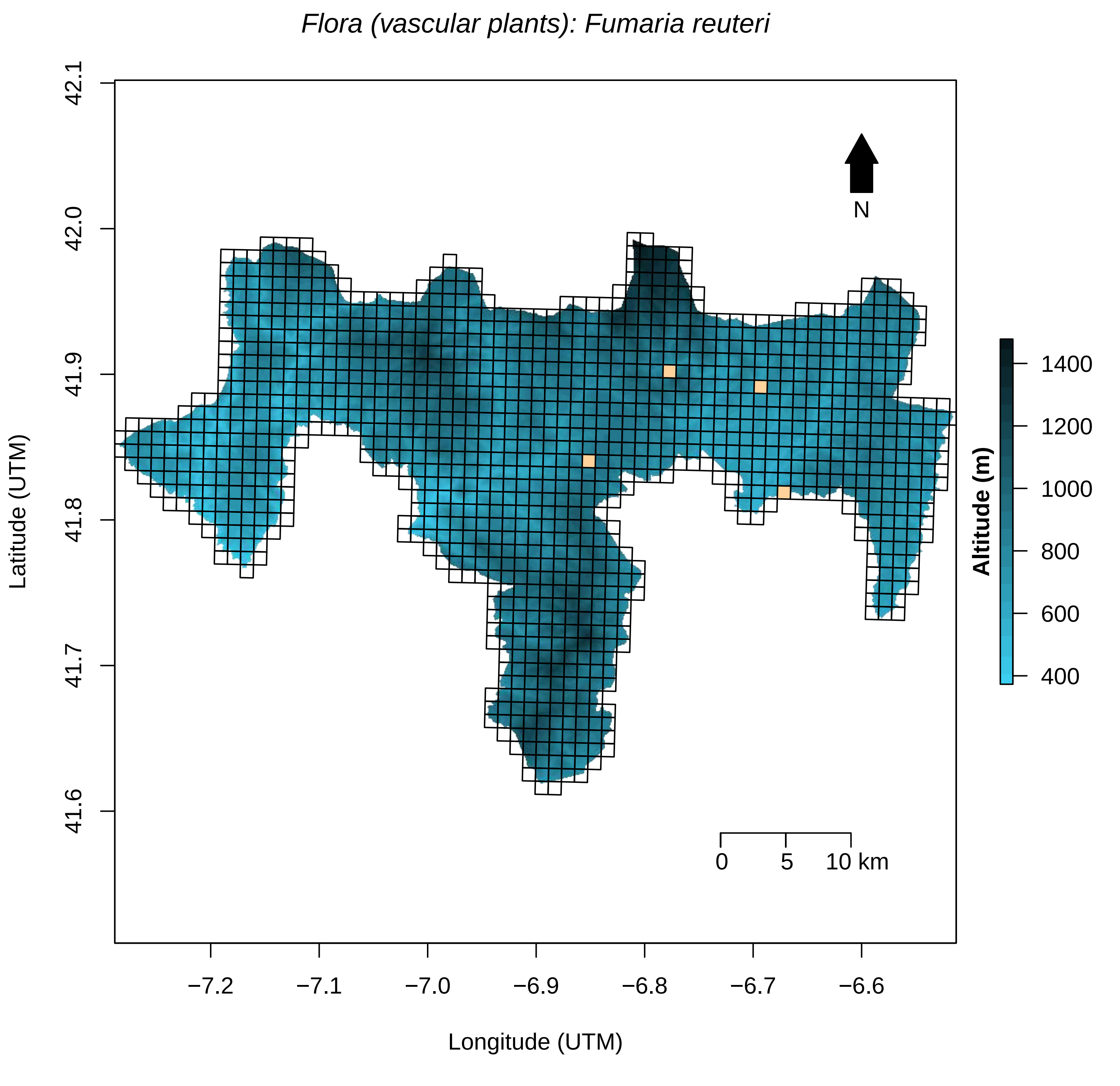

Flora (vascular plants): Fumaria reuteri
42.1
42.0
41.9
41.8
41.7
41.6
Latitude (UTM)
−7.2
−7.1
−7.0
−6.9
−6.8
−6.7
−6.6
Longitude (UTM)
N
0
5
10 km
Altitude (m)
1400
1200
1000
800
600
400

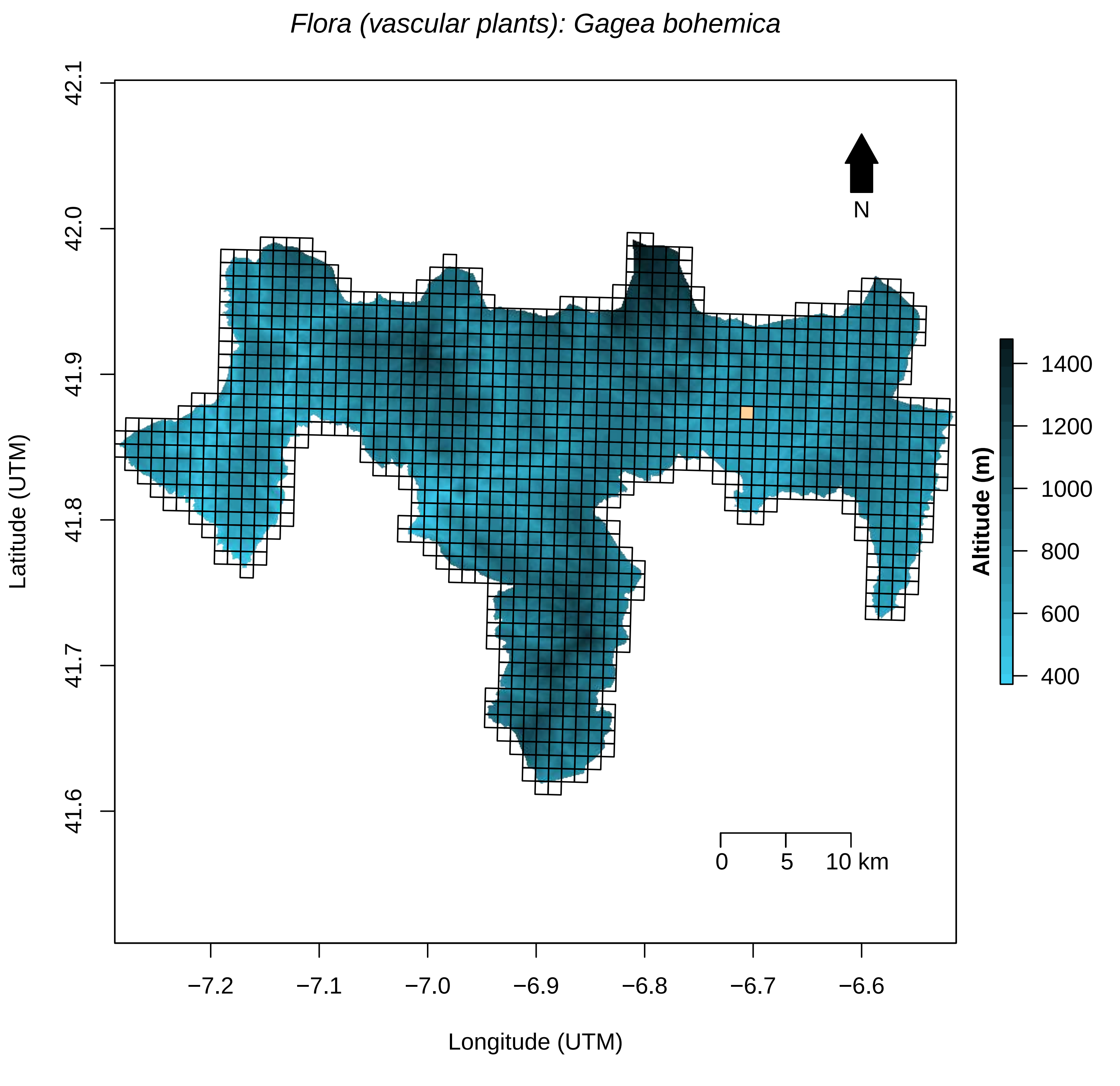

Flora (vascular plants): Gagea bohemica
42.1
42.0
41.9
41.8
41.7
41.6
Latitude (UTM)
N
0
5
10 km
1400
1200
1000
800
600
400
Altitude (m)
−7.2
−7.1
−7.0
−6.9
−6.8
−6.7
−6.6
Longitude (UTM)

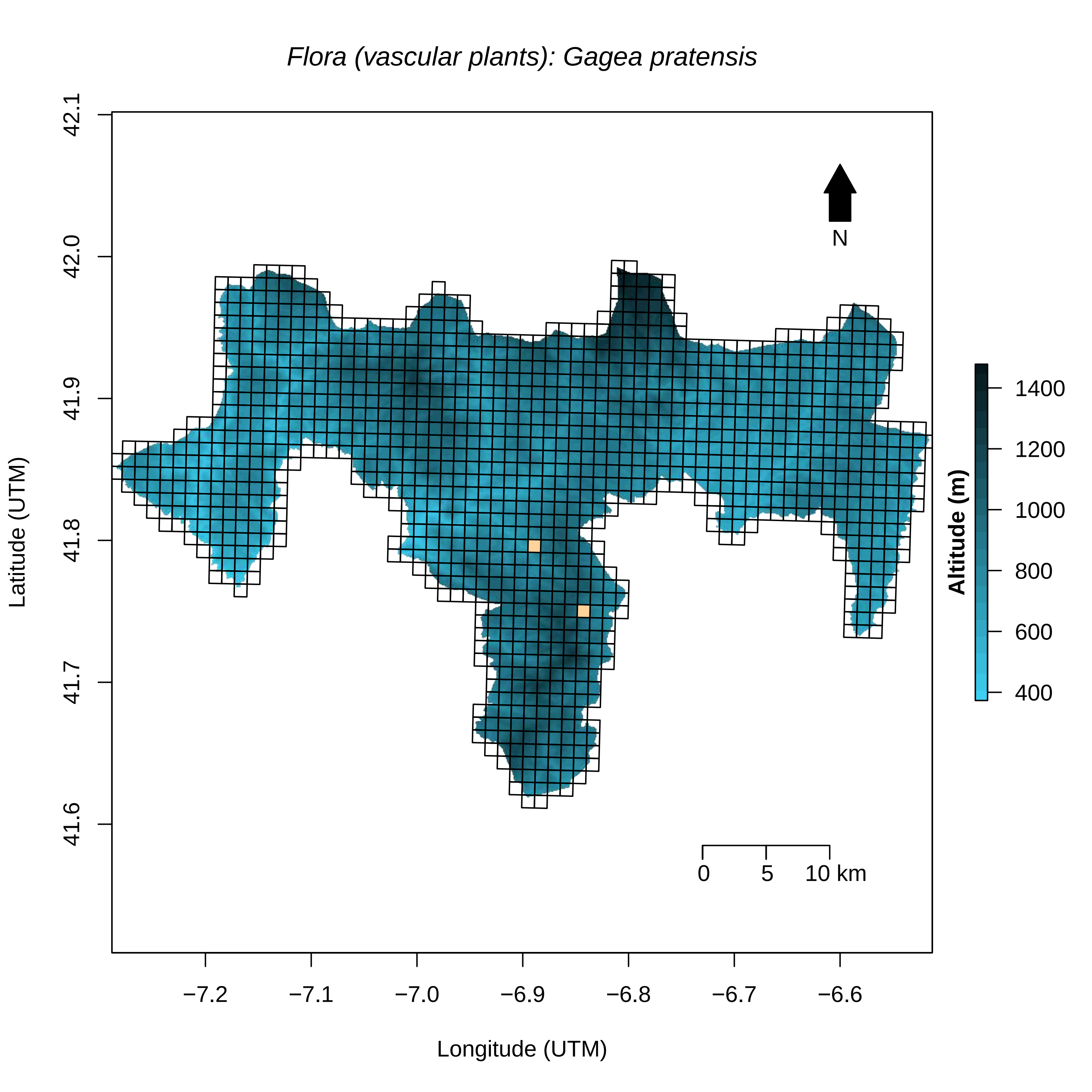
Flora (vascular plants): Gagea pratensis
Latitude (UTM)
42.1
42.0
41.9
41.8
41.7
41.6
Longitude (UTM)
−7.2
−7.1
−7.0
−6.9
−6.8
−6.7
−6.6
N
0
5
10 km
Altitude (m)
1400
1200
1000
800
600
400

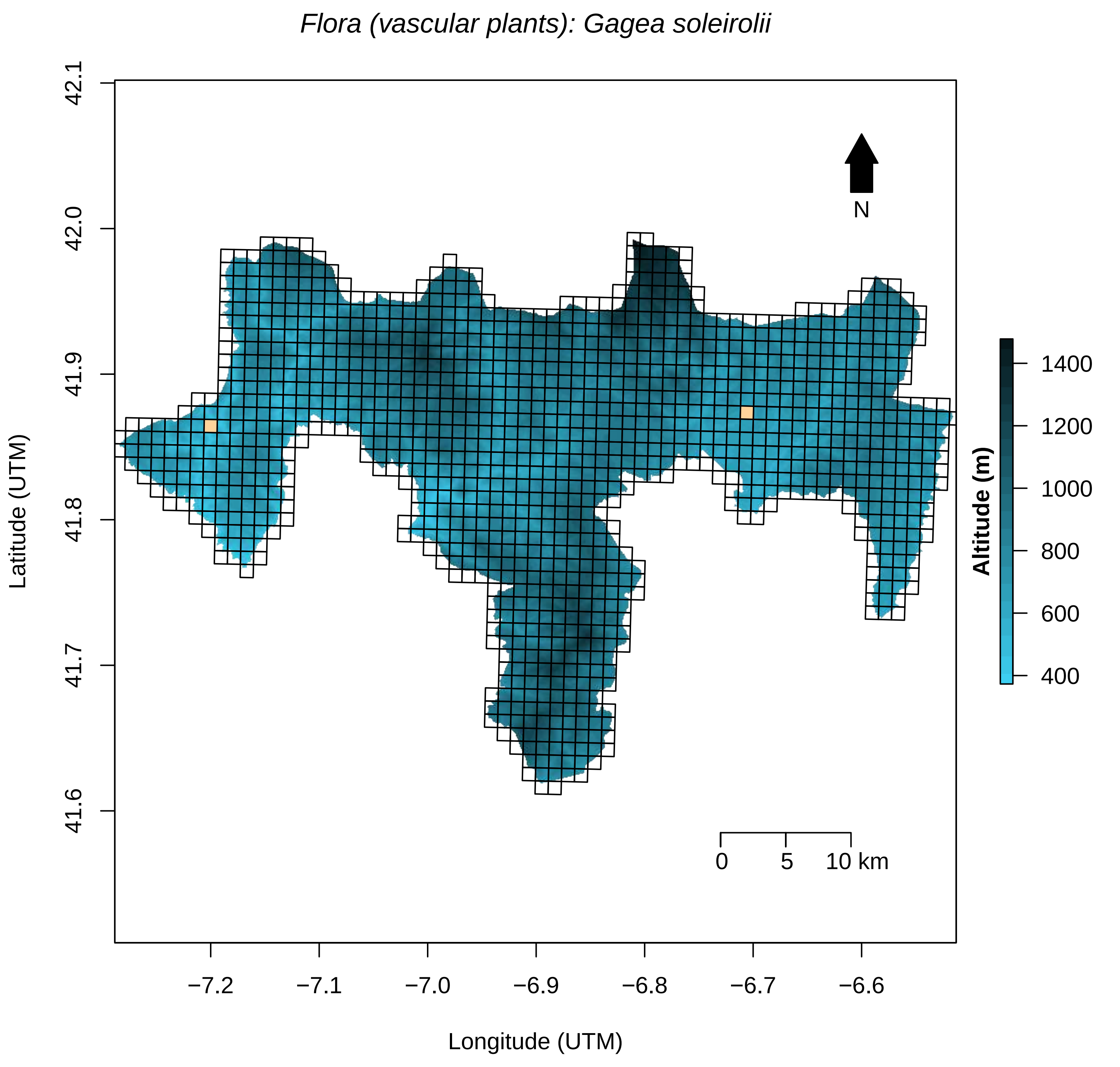
Flora (vascular plants): Gagea soleirolii
N
0
5
10 km
Latitude (UTM)
Longitude (UTM)
42.1
42.0
41.9
41.8
41.7
41.6
−7.2
−7.1
−7.0
−6.9
−6.8
−6.7
−6.6
Altitude (m)
1400
1200
1000
800
600
400

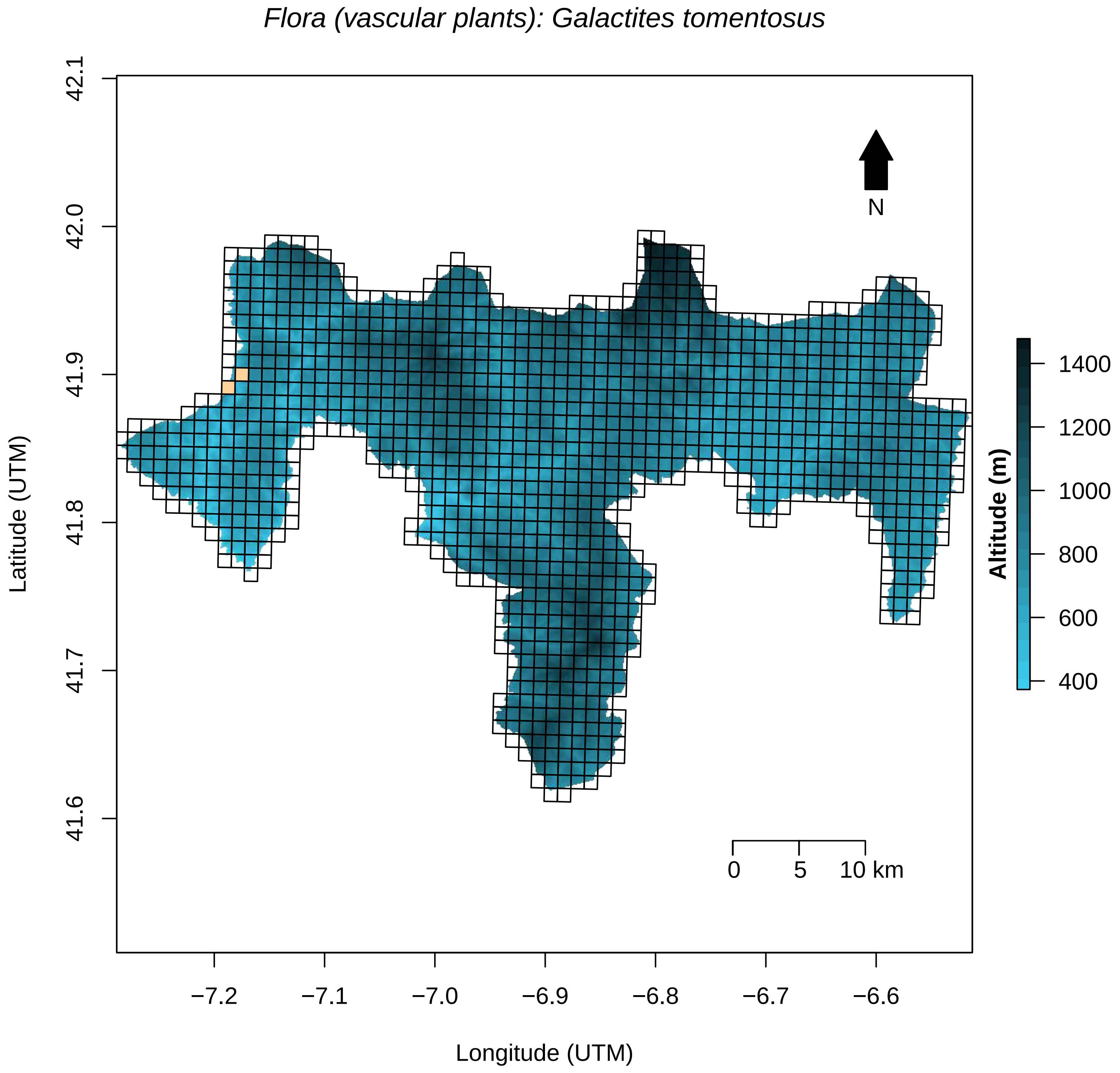

Flora (vascular plants): Galactites tomentosus
42.1
42.0
41.9
41.8
41.7
41.6
Latitude (UTM)
N
0
5
10 km
−7.2
−7.1
−7.0
−6.9
−6.8
−6.7
−6.6
Longitude (UTM)
1400
1200
1000
800
600
400
Altitude (m)

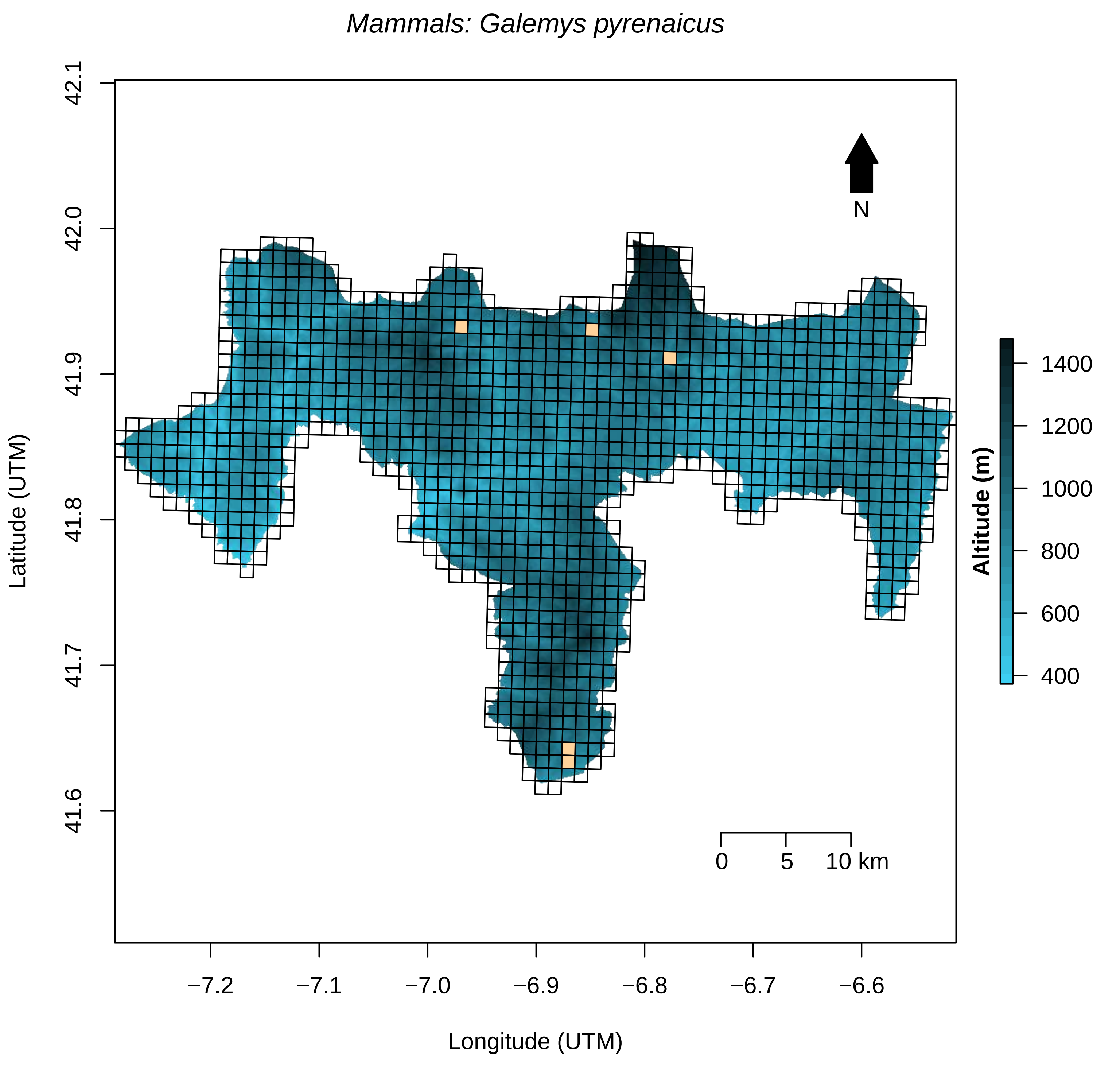

Mammals: Galemys pyrenaicus
N
0
5
10 km
Altitude (m)
1400
1200
1000
800
600
400
Latitude (UTM)
42.1
42.0
41.9
41.8
41.7
41.6
Longitude (UTM)
−7.2
−7.1
−7.0
−6.9
−6.8
−6.7
−6.6

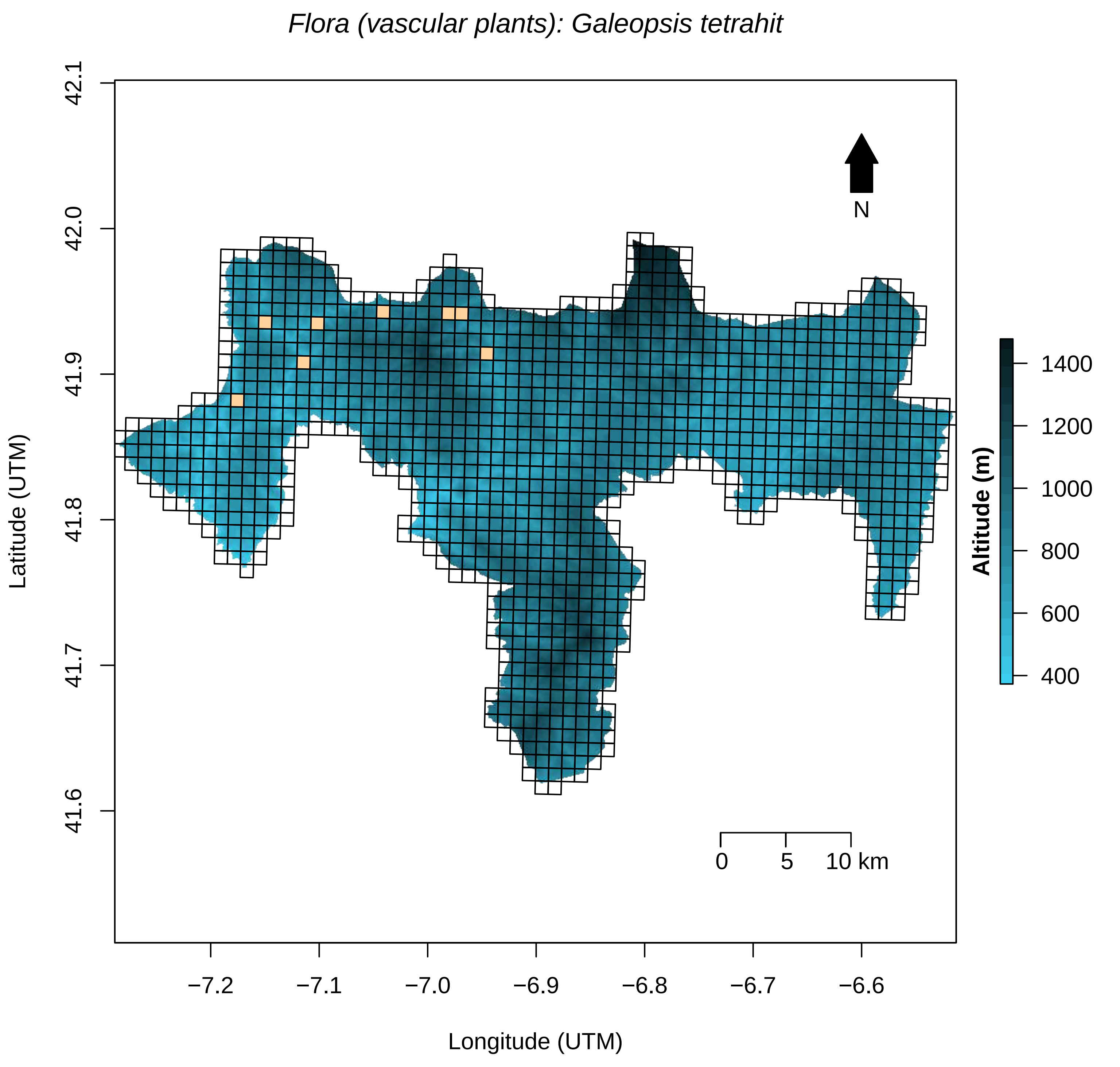

Flora (vascular plants): Galeopsis tetrahit
42.1
42.0
41.9
41.8
41.7
41.6
Latitude (UTM)
N
0
5
10 km
1400
1200
1000
800
600
400
Altitude (m)
−7.2
−7.1
−7.0
−6.9
−6.8
−6.7
−6.6
Longitude (UTM)

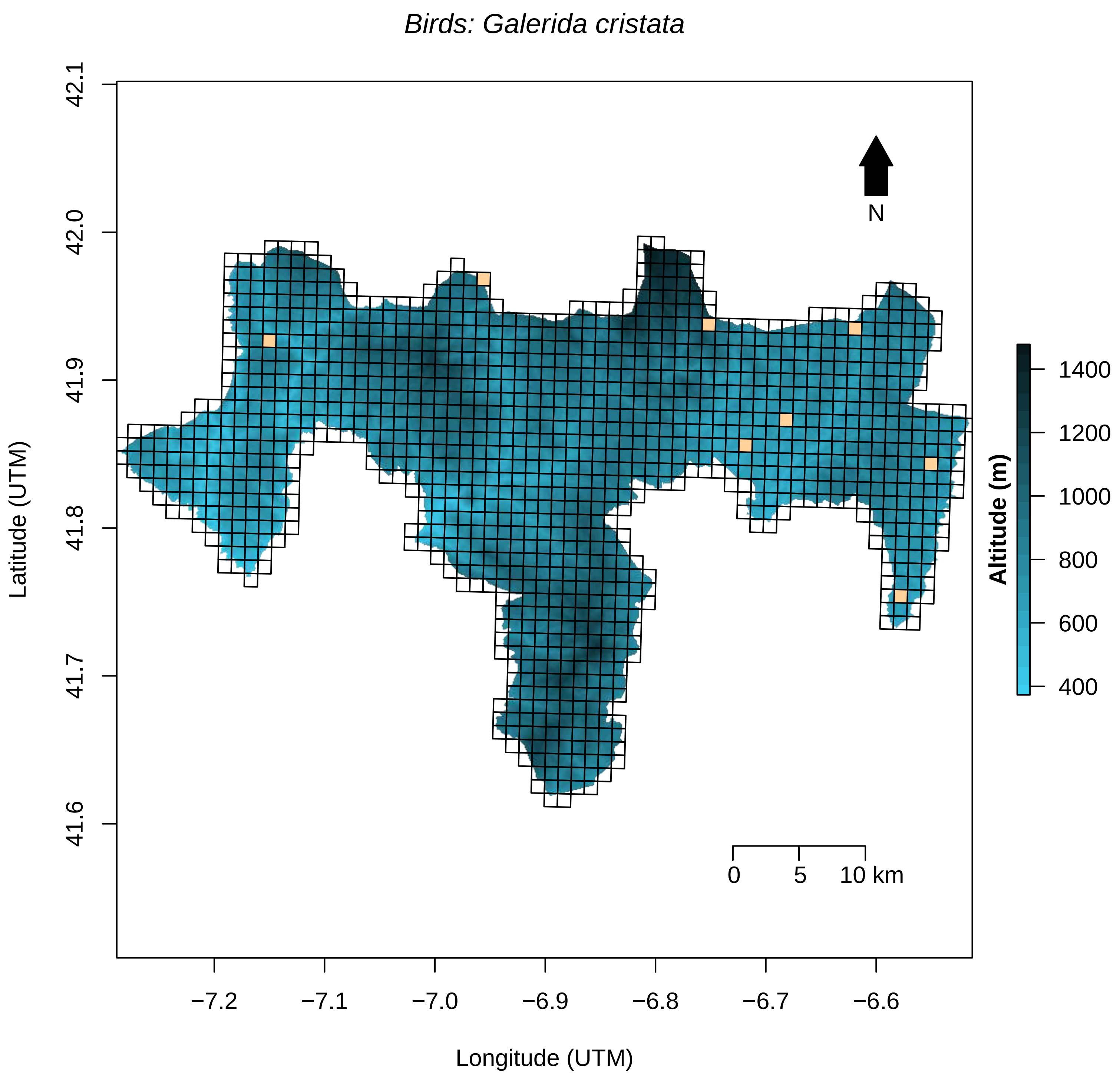

Birds: Galerida cristata
42.1
42.0
41.9
41.8
41.7
41.6
Latitude (UTM)
−7.2
−7.1
−7.0
−6.9
−6.8
−6.7
−6.6
Longitude (UTM)
N
0
5
10 km
1400
1200
1000
800
600
400
Altitude (m)

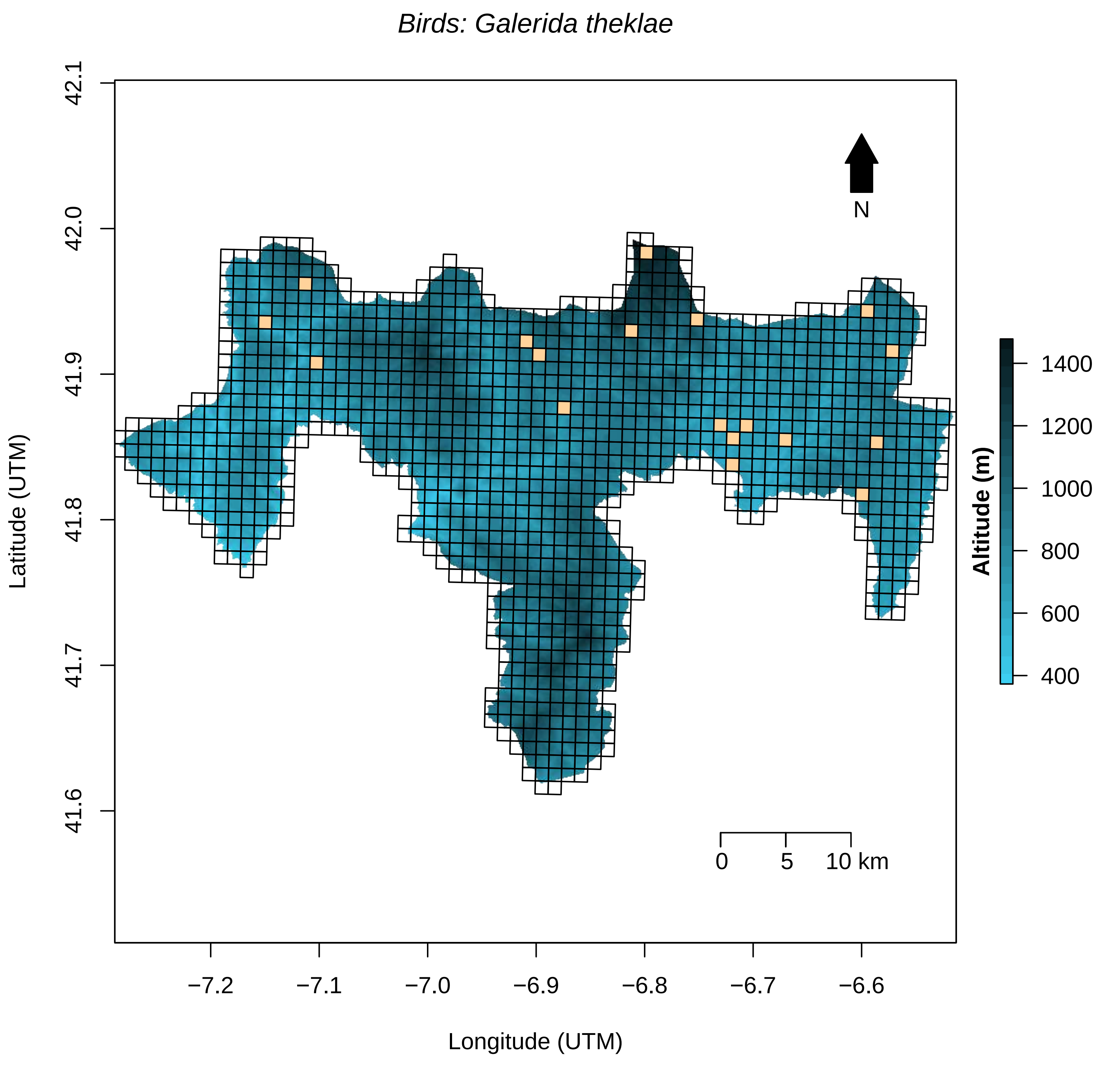

Birds: Galerida theklae
42.1
42.0
41.9
41.8
41.7
41.6
Latitude (UTM)
−7.2
−7.1
−7.0
−6.9
−6.8
−6.7
−6.6
Longitude (UTM)
N
0
5
10 km
Altitude (m)
1400
1200
1000
800
600
400

Flora (vascular plants): Galinsoga parviflora

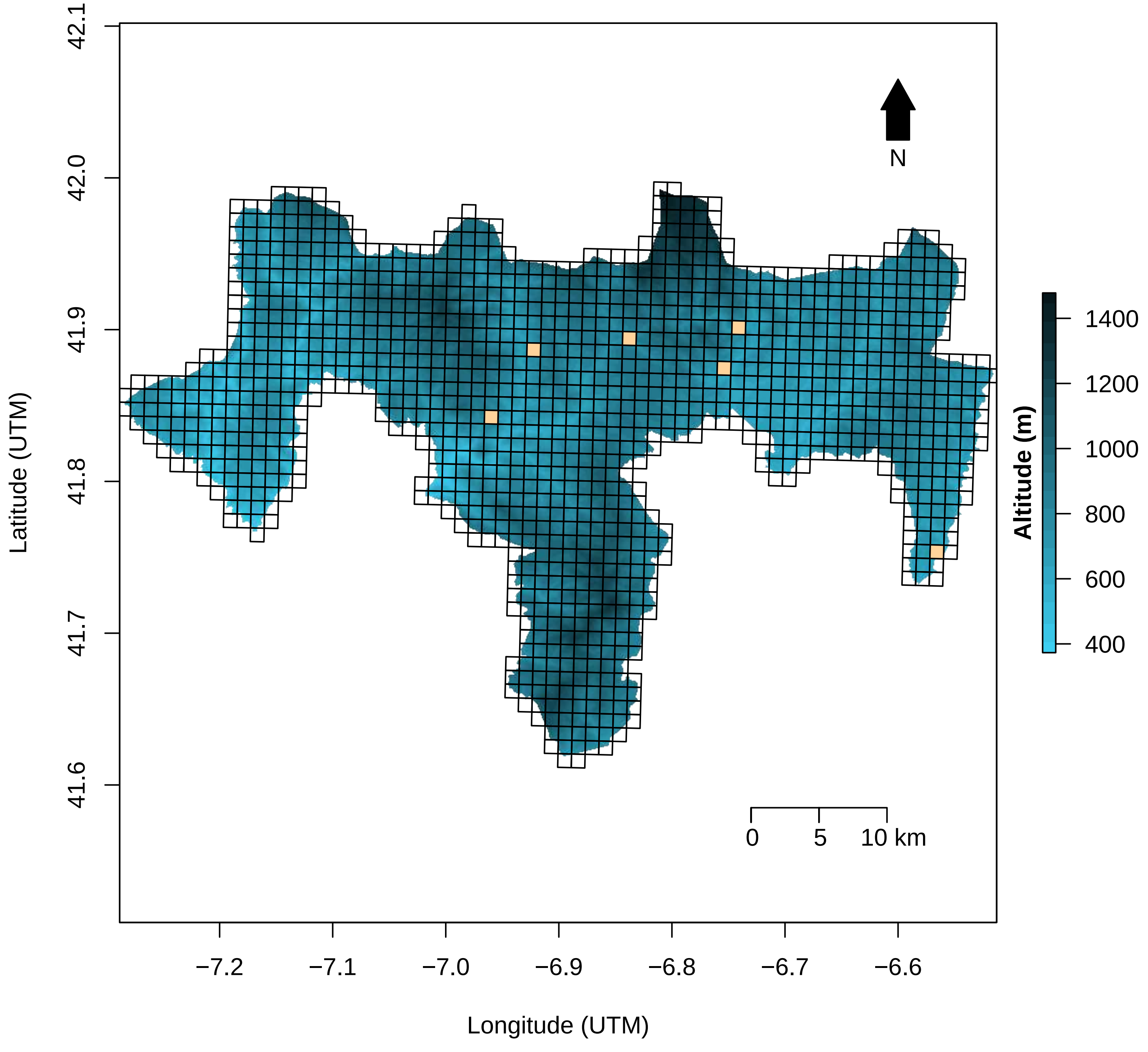

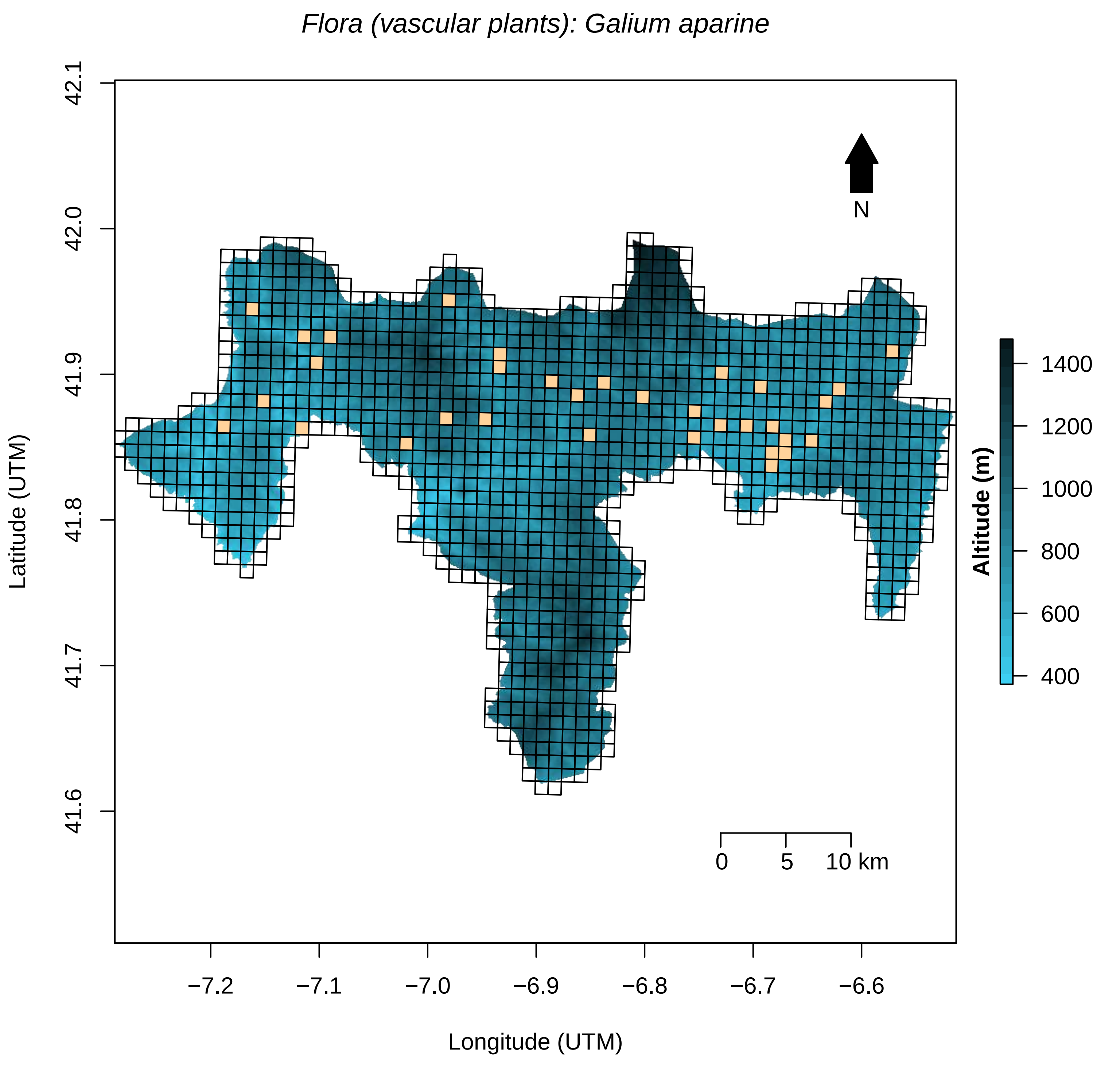

Flora (vascular plants): Galium aparine
42.1
42.0
41.9
41.8
41.7
41.6
Latitude (UTM)
−7.2
−7.1
−7.0
−6.9
−6.8
−6.7
−6.6
Longitude (UTM)
N
0
5
10 km
Altitude (m)
1400
1200
1000
800
600
400

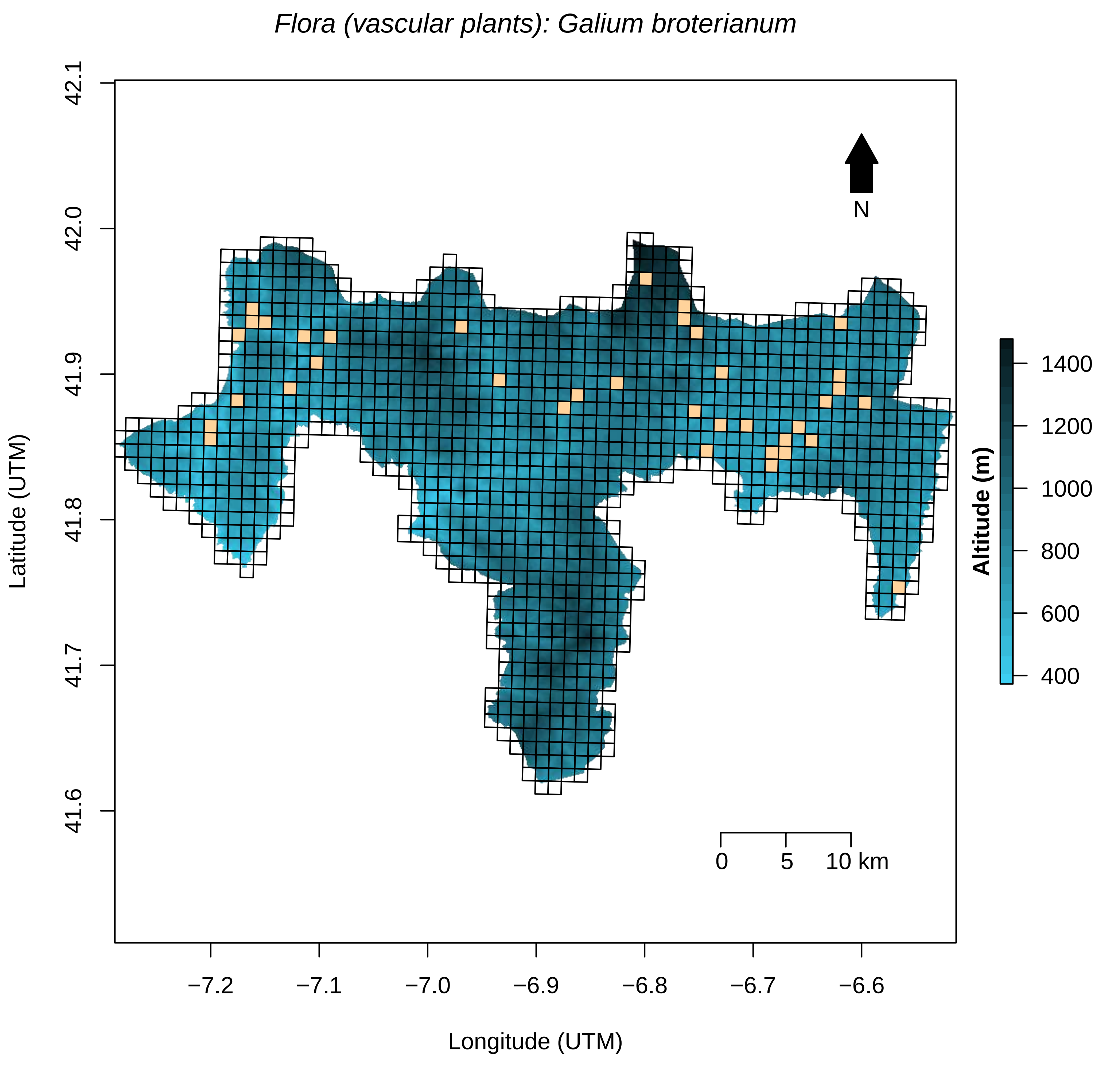

Flora (vascular plants): Galium broterianum
Latitude (UTM)
42.1
42.0
41.9
41.8
41.7
41.6
Longitude (UTM)
−7.2
−7.1
−7.0
−6.9
−6.8
−6.7
−6.6
N
0
5
10 km
Altitude (m)
1400
1200
1000
800
600
400

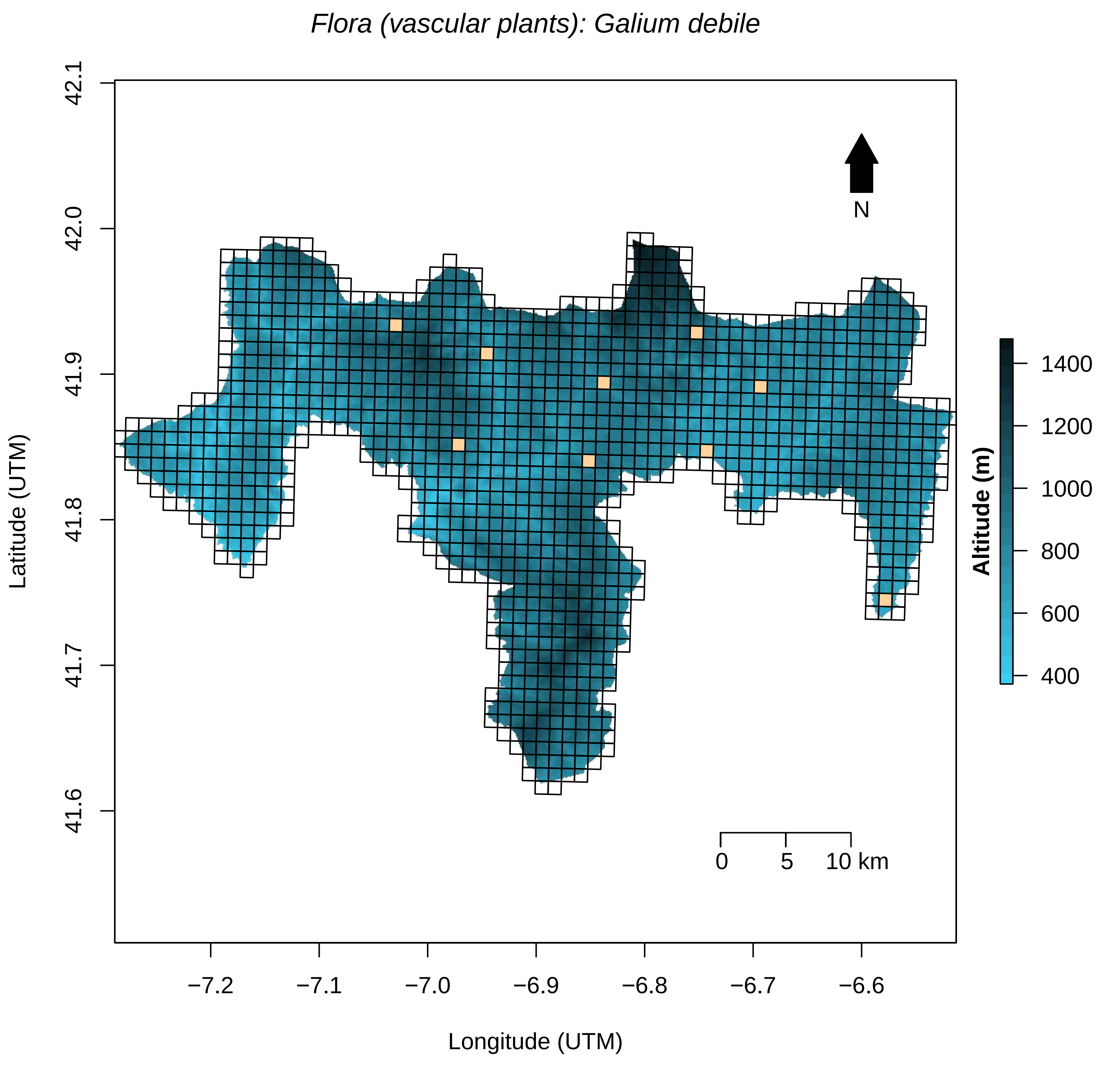

Flora (vascular plants): Galium debile
N
0
5
10 km
Altitude (m)
1400
1200
1000
800
600
400
42.1
42.0
41.9
41.8
41.7
41.6
Latitude (UTM)
−7.2
−7.1
−7.0
−6.9
−6.8
−6.7
−6.6
Longitude (UTM)

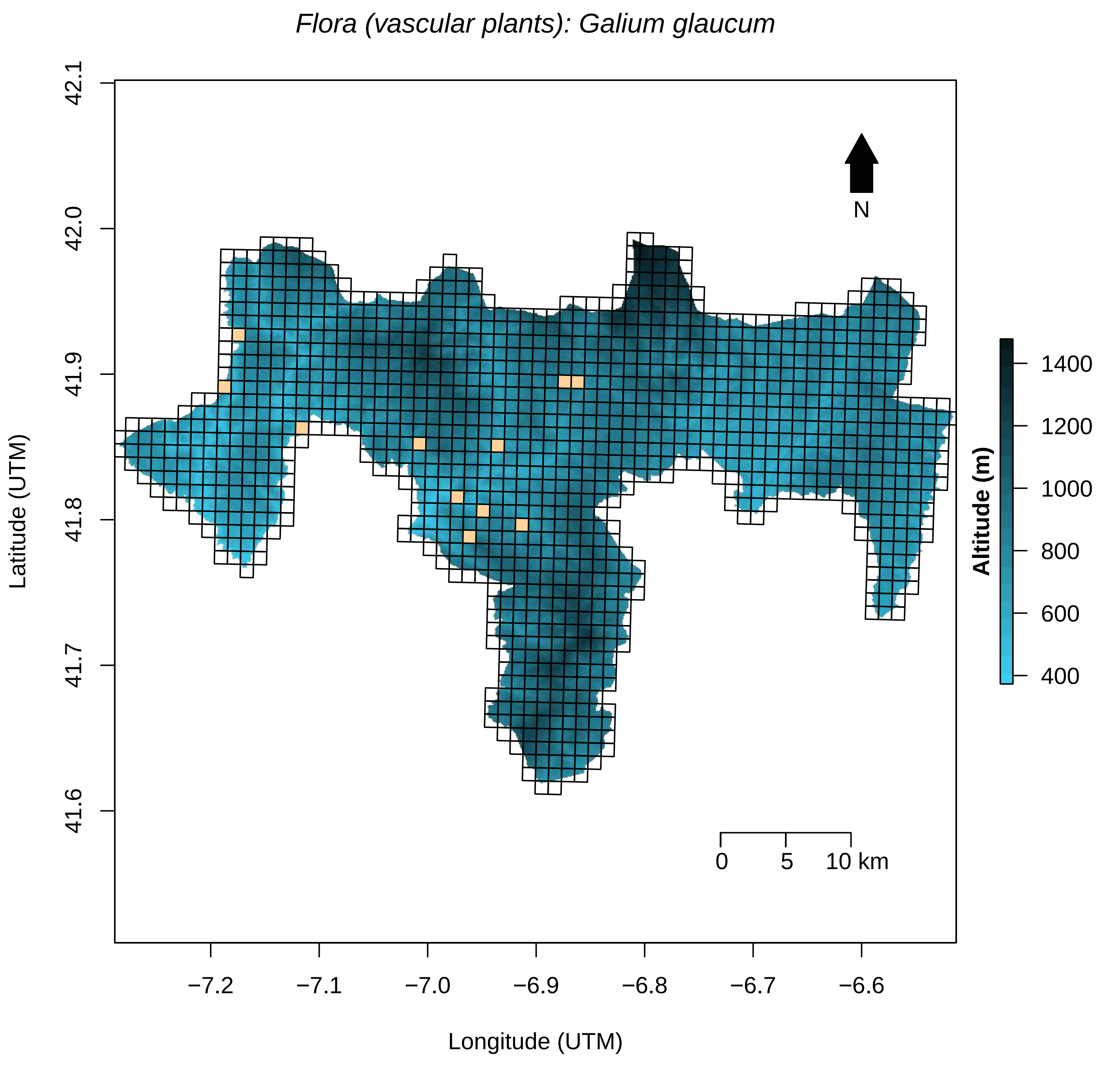

Flora (vascular plants): Galium glaucum
42.1
42.0
41.9
41.8
41.7
41.6
Latitude (UTM)
−7.2
−7.1
−7.0
−6.9
−6.8
−6.7
−6.6
Longitude (UTM)
N
0
5
10 km
Altitude (m)
1400
1200
1000
800
600
400

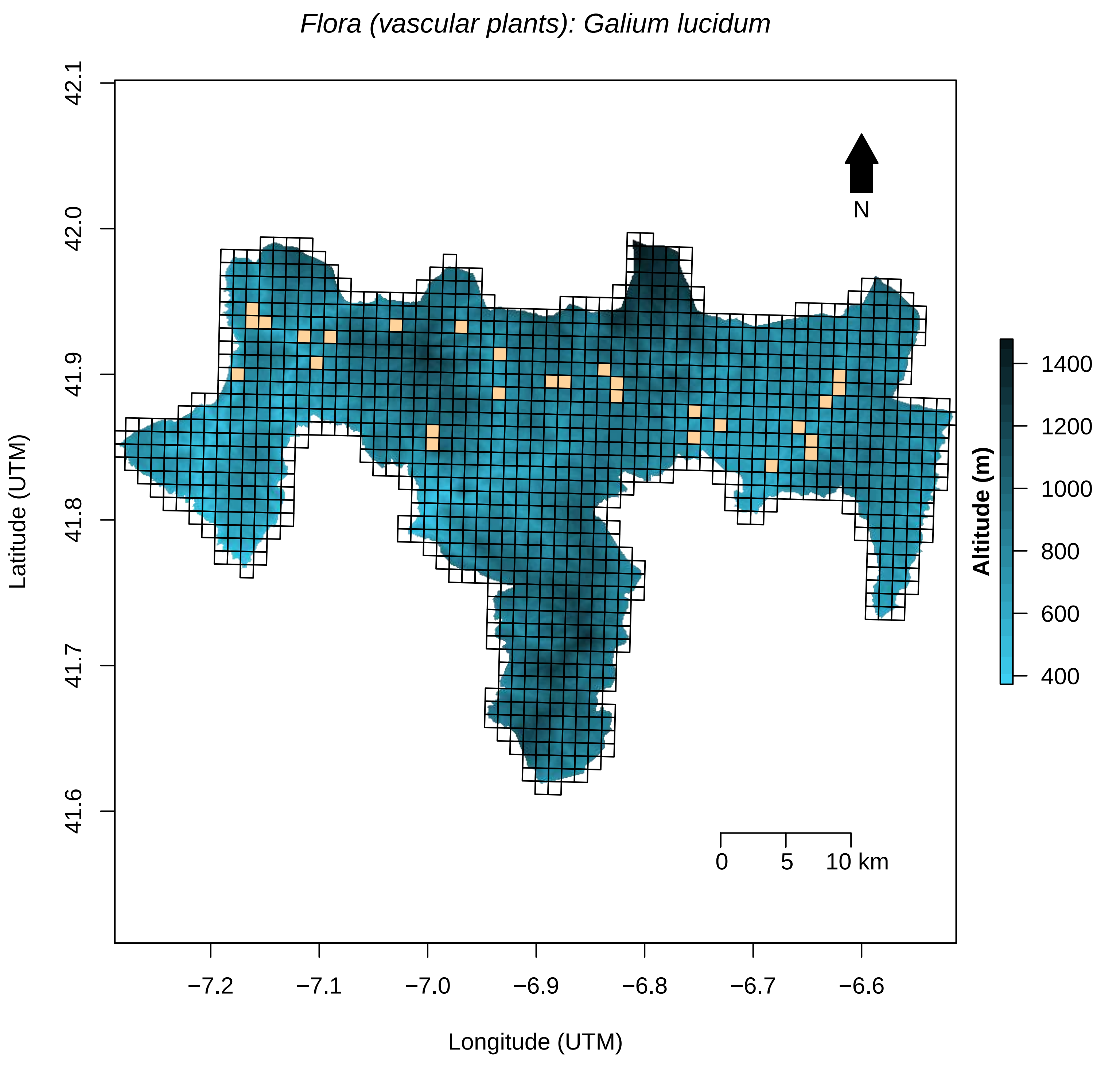

Flora (vascular plants): Galium lucidum
Latitude (UTM)
42.1
42.0
41.9
41.8
41.7
41.6
N
0
5
10 km
−7.2
−7.1
−7.0
−6.9
−6.8
−6.7
−6.6
Longitude (UTM)
Altitude (m)
1400
1200
1000
800
600
400

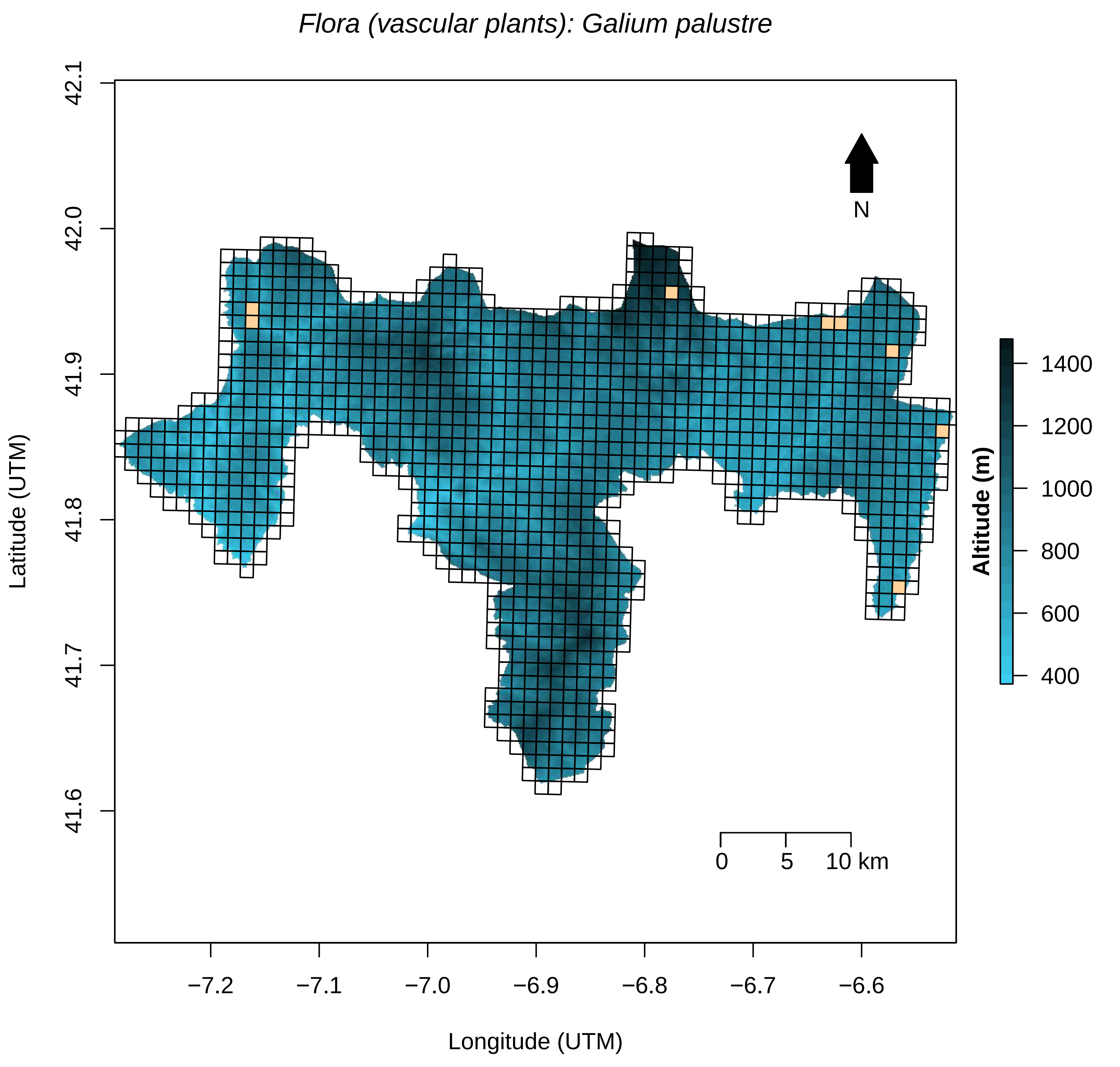
Flora (vascular plants): Galium palustre
N
0
5
10 km
Altitude (m)
1400
1200
1000
800
600
400
Latitude (UTM)
42.1
42.0
41.9
41.8
41.7
41.6
Longitude (UTM)
−7.2
−7.1
−7.0
−6.9
−6.8
−6.7
−6.6

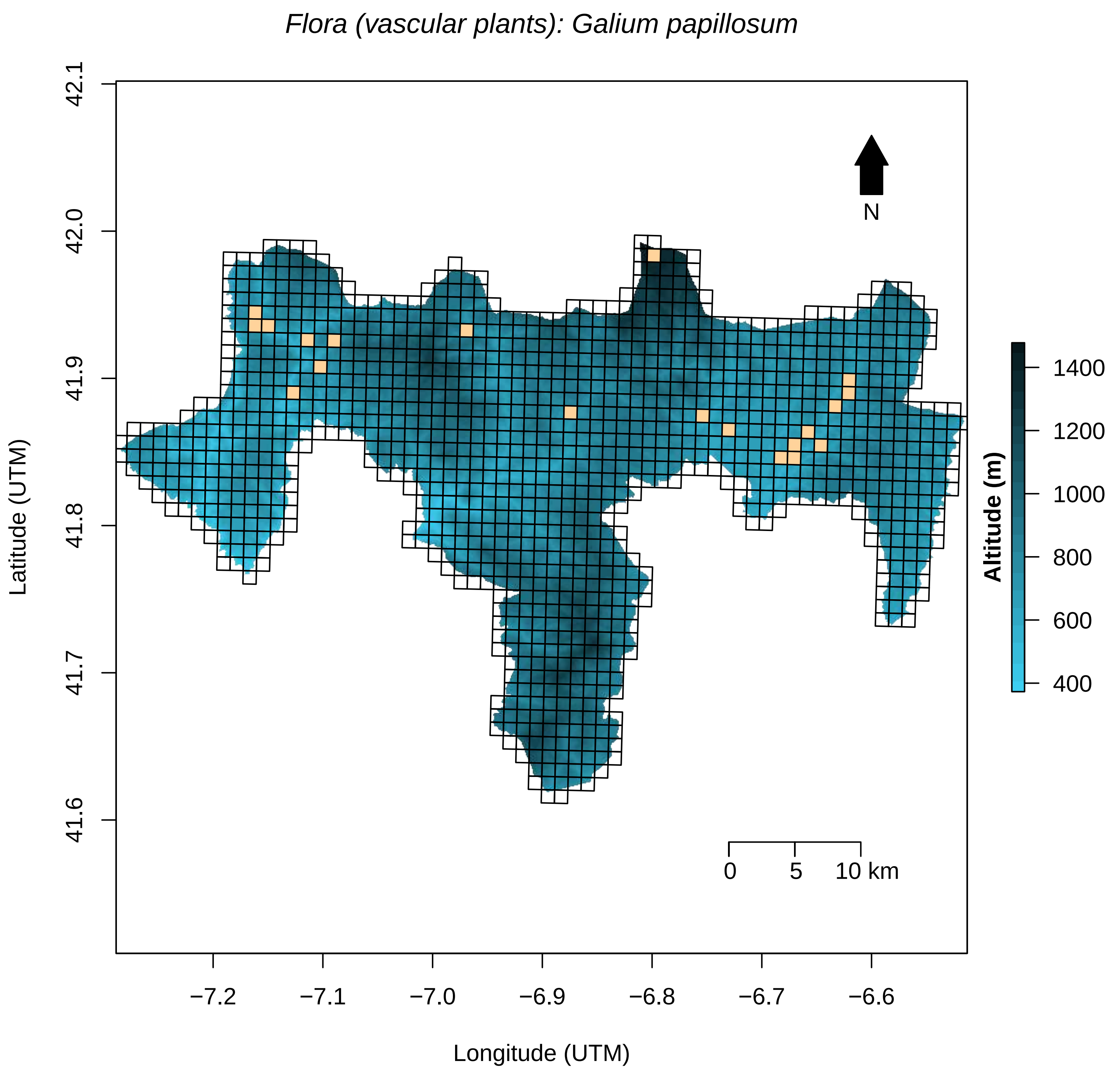
Flora (vascular plants): Galium papillosum
Latitude (UTM)
42.1
42.0
41.9
41.8
41.7
41.6
Longitude (UTM)
−7.2
−7.1
−7.0
−6.9
−6.8
−6.7
−6.6
N
0
5
10 km
Altitude (m)
1400
1200
1000
800
600
400

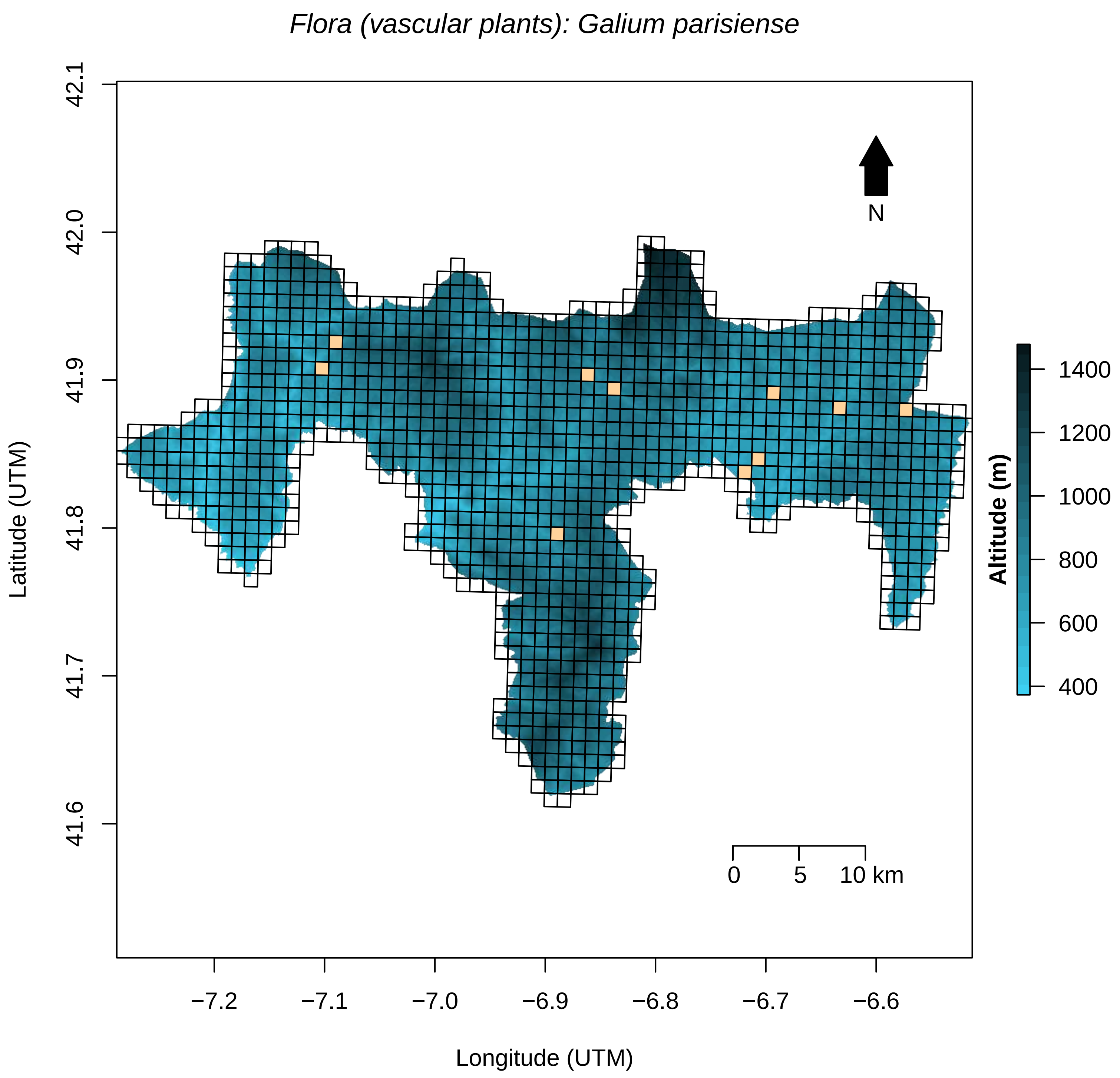

Flora (vascular plants): Galium parisiense
N
0 5 10 km
Altitude (m)
1400
1200
1000
800
600
400
42.1
42.0
41.9
41.8
41.7
41.6
Latitude (UTM)
−7.2
−7.1
−7.0
−6.9
−6.8
−6.7
−6.6
Longitude (UTM)

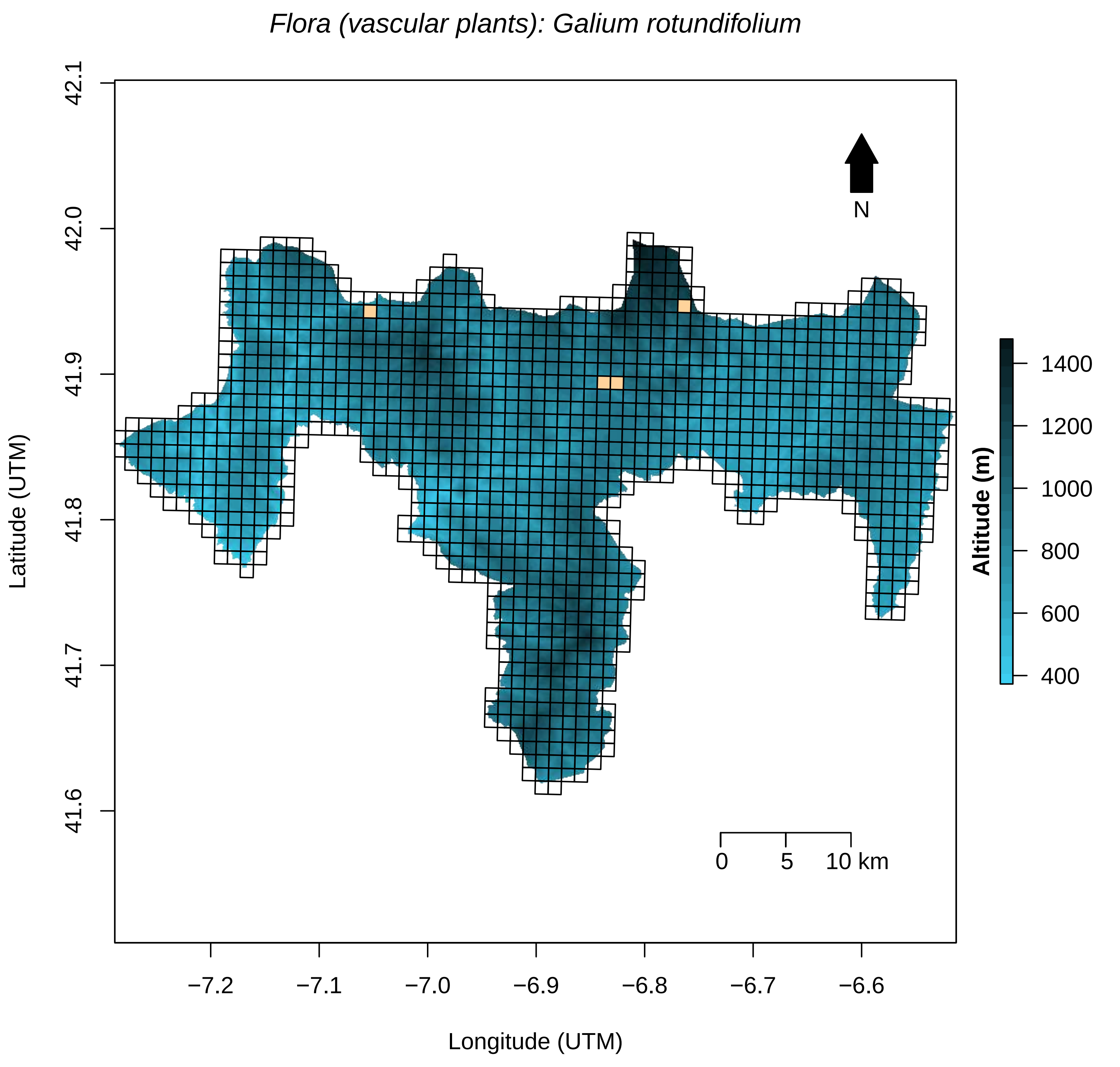
Flora (vascular plants): Galium rotundifolium
42.1
42.0
41.9
41.8
41.7
41.6
Latitude (UTM)
N
1400
1200
1000
800
600
400
Altitude (m)
0
5
10 km
−7.2
−7.1
−7.0
−6.9
−6.8
−6.7
−6.6
Longitude (UTM)

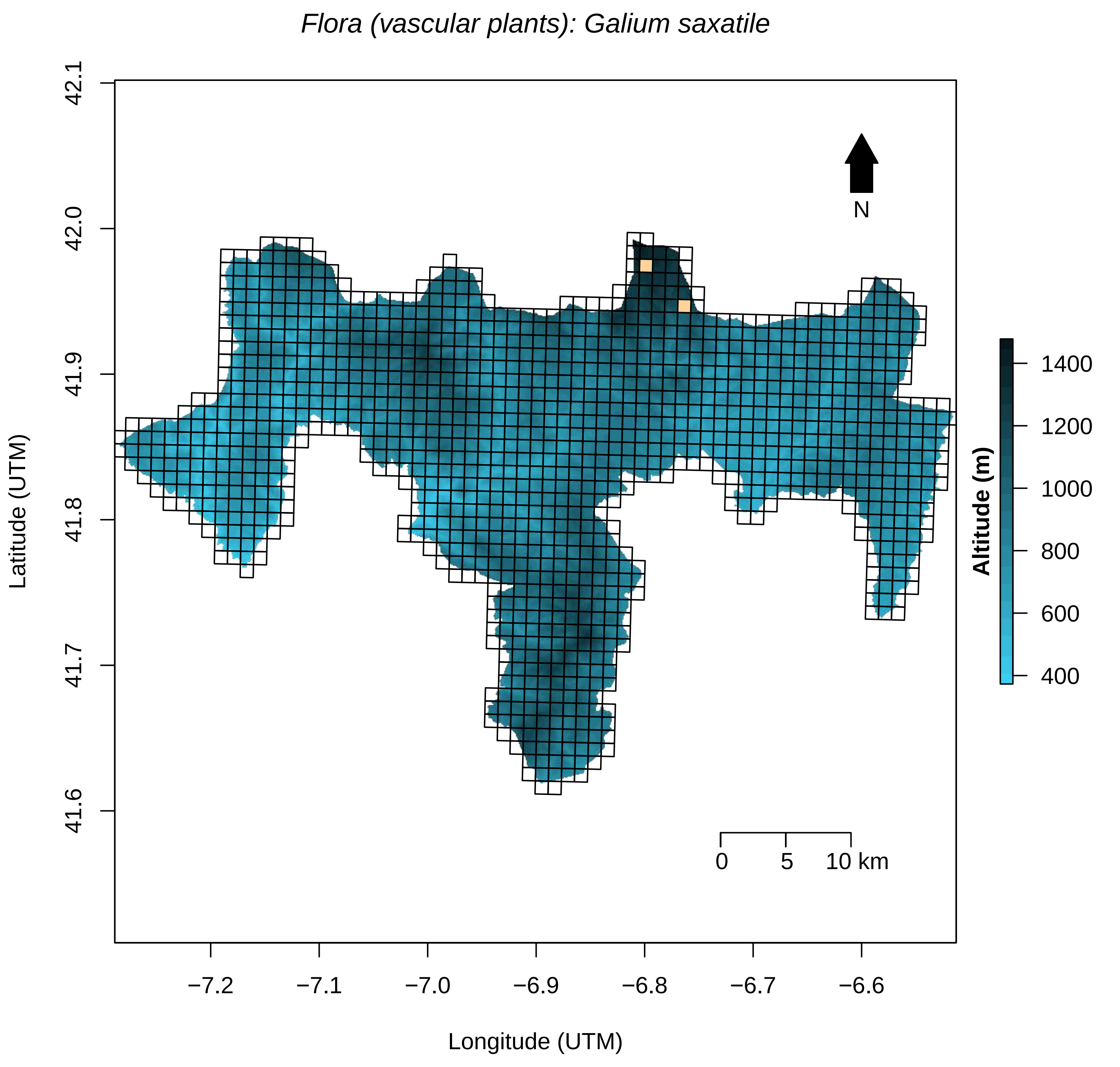

Flora (vascular plants): Galium saxatile
42.1
42.0
41.9
41.8
41.7
41.6
Latitude (UTM)
N
0 5 10 km
−7.2
−7.1
−7.0
−6.9
−6.8
−6.7
−6.6
Longitude (UTM)
1400
1200
1000
800
600
400
Altitude (m)

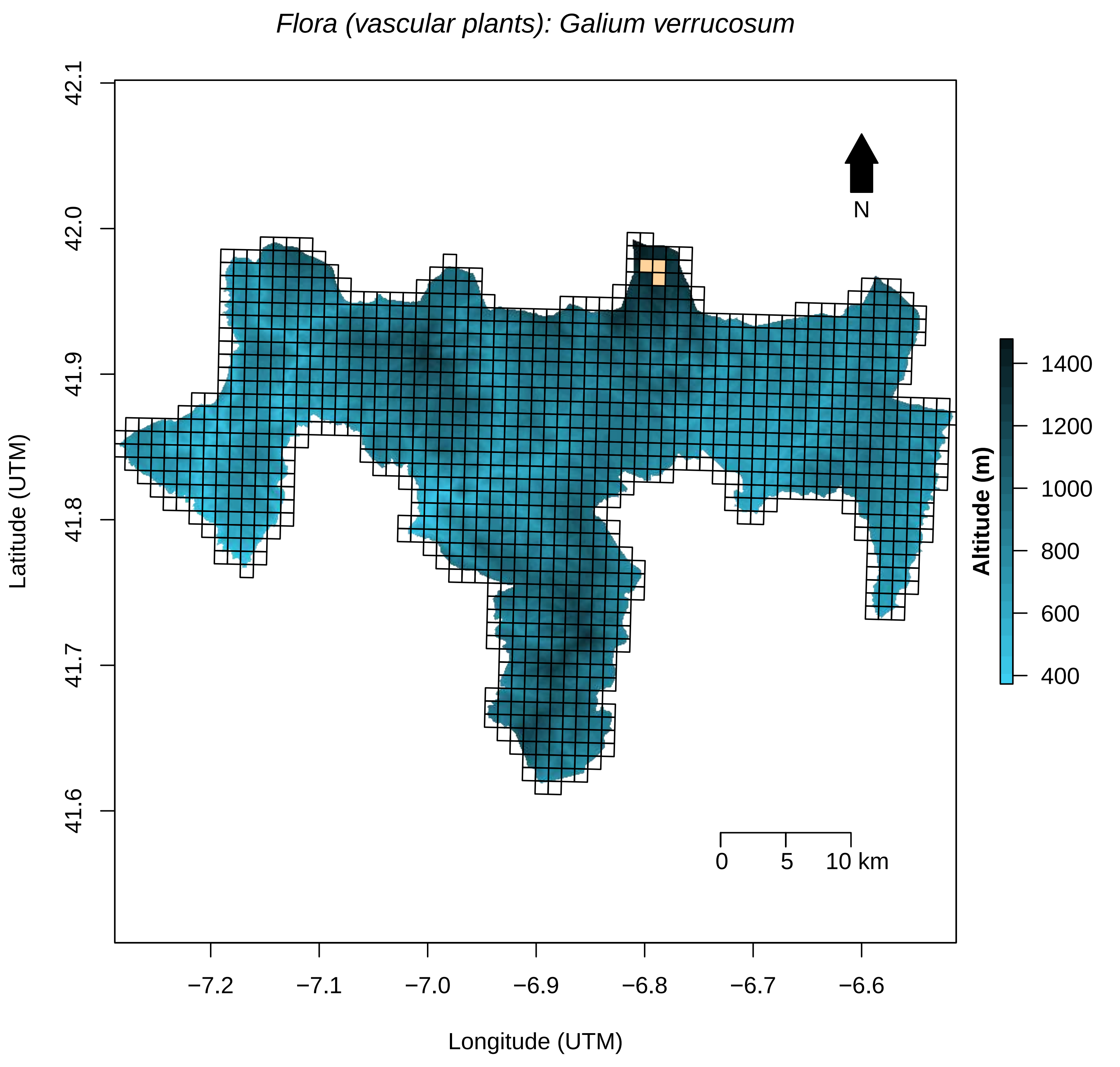

Flora (vascular plants): Galium verrucosum
42.1
42.0
41.9
41.8
41.7
41.6
Latitude (UTM)
−7.2
−7.1
−7.0
−6.9
−6.8
−6.7
−6.6
Longitude (UTM)
N
0
5
10 km
Altitude (m)
1400
1200
1000
800
600
400

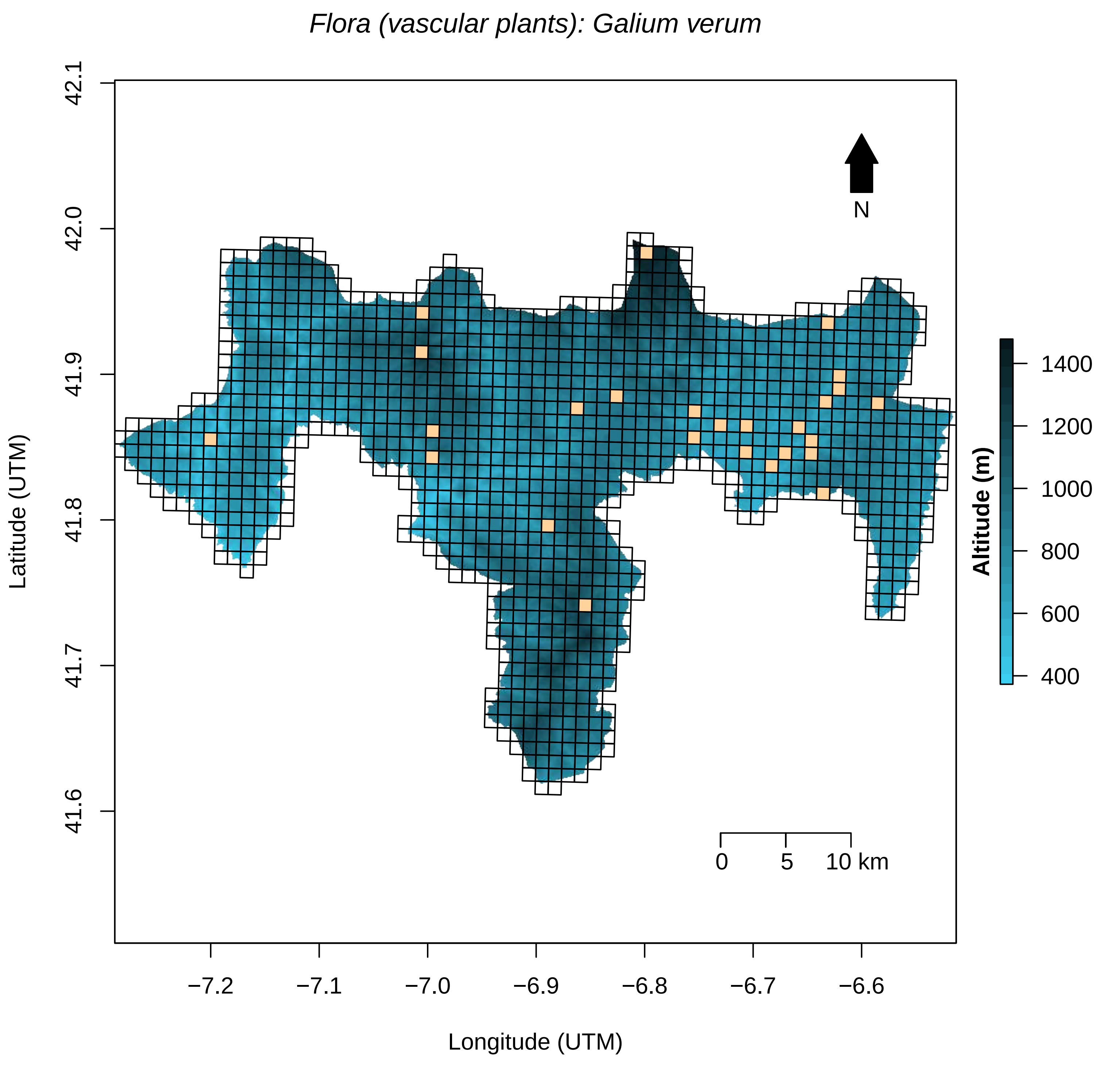
Flora (vascular plants): Galium verum
Latitude (UTM)
42.1
42.0
41.9
41.8
41.7
41.6
Longitude (UTM)
−7.2
−7.1
−7.0
−6.9
−6.8
−6.7
−6.6
N
0
5
10 km
Altitude (m)
1400
1200
1000
800
600
400

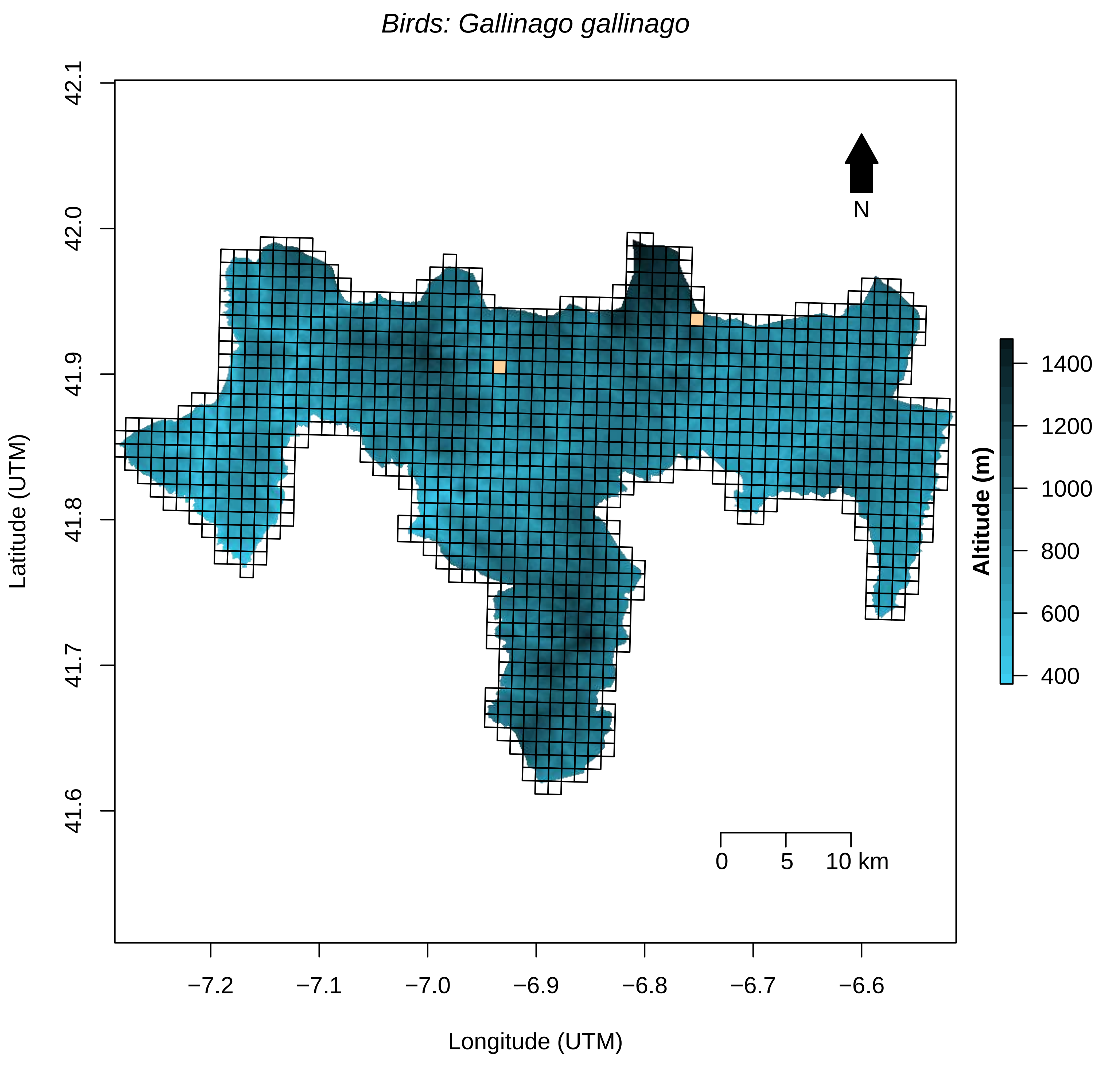
Birds: Gallinago gallinago
N
0
5
10 km
Altitude (m)
1400
1200
1000
800
600
400
Latitude (UTM)
42.1
42.0
41.9
41.8
41.7
41.6
Longitude (UTM)
−7.2
−7.1
−7.0
−6.9
−6.8
−6.7
−6.6

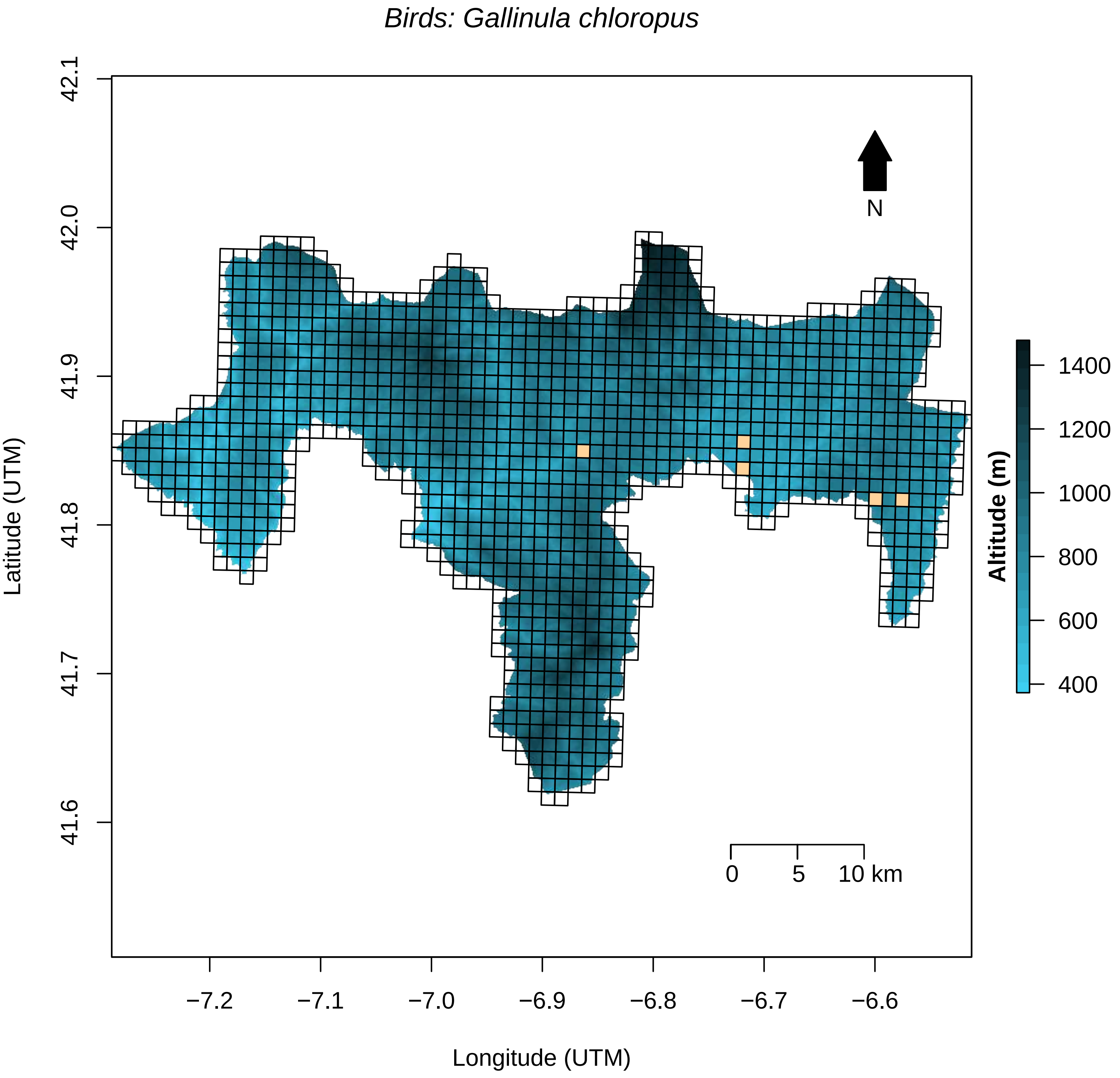

Birds: Gallinula chloropus
N
0 5 10 km
Altitude (m)
1400
1200
1000
800
600
400
Latitude (UTM)
42.1
42.0
41.9
41.8
41.7
41.6
Longitude (UTM)
−7.2
−7.1
−7.0
−6.9
−6.8
−6.7
−6.6

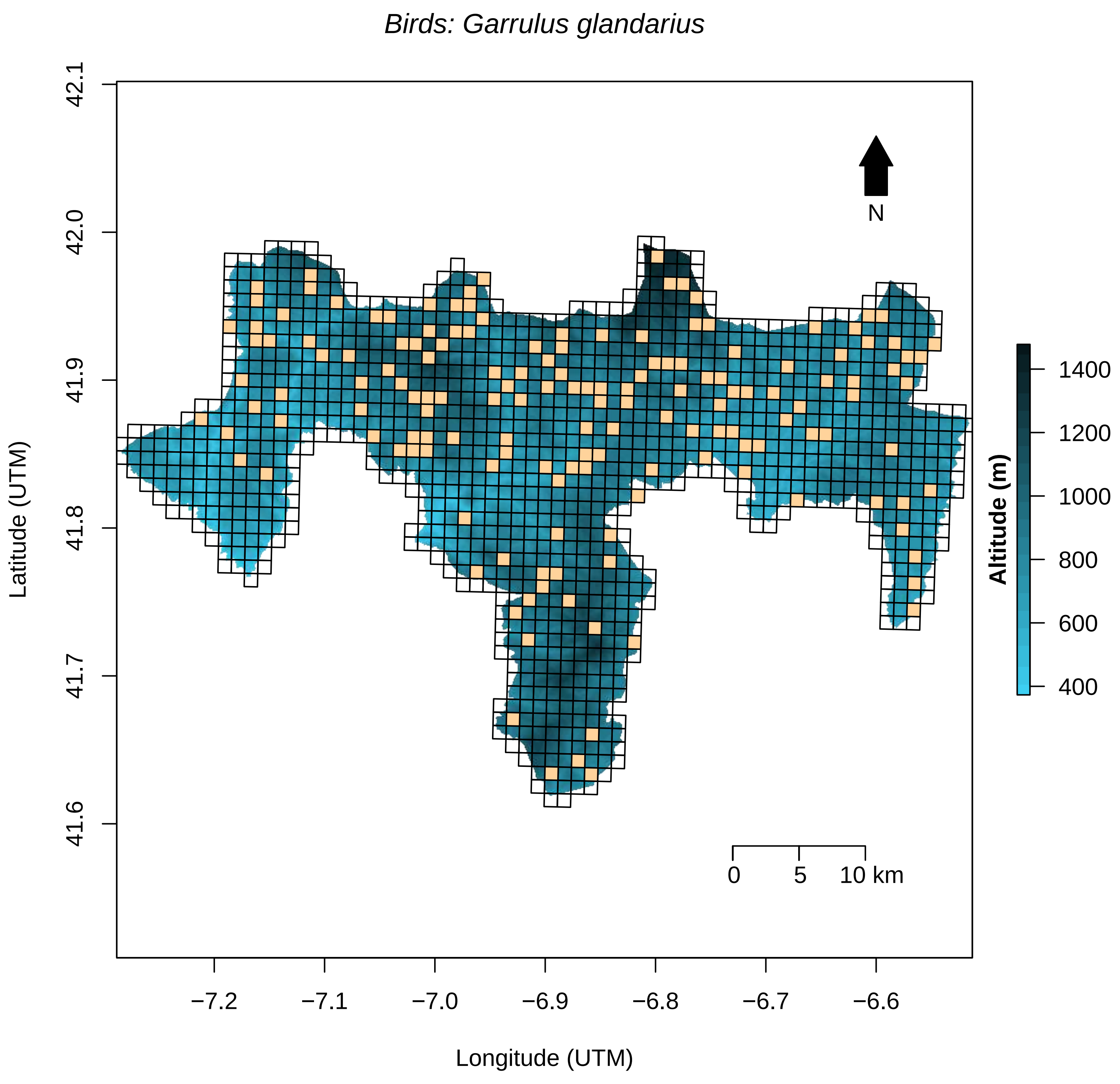

Birds: Garrulus glandarius
Latitude (UTM)
42.1
42.0
41.9
41.8
41.7
41.6
Longitude (UTM)
−7.2
−7.1
−7.0
−6.9
−6.8
−6.7
−6.6
N
0
5
10 km
Altitude (m)
1400
1200
1000
800
600
400

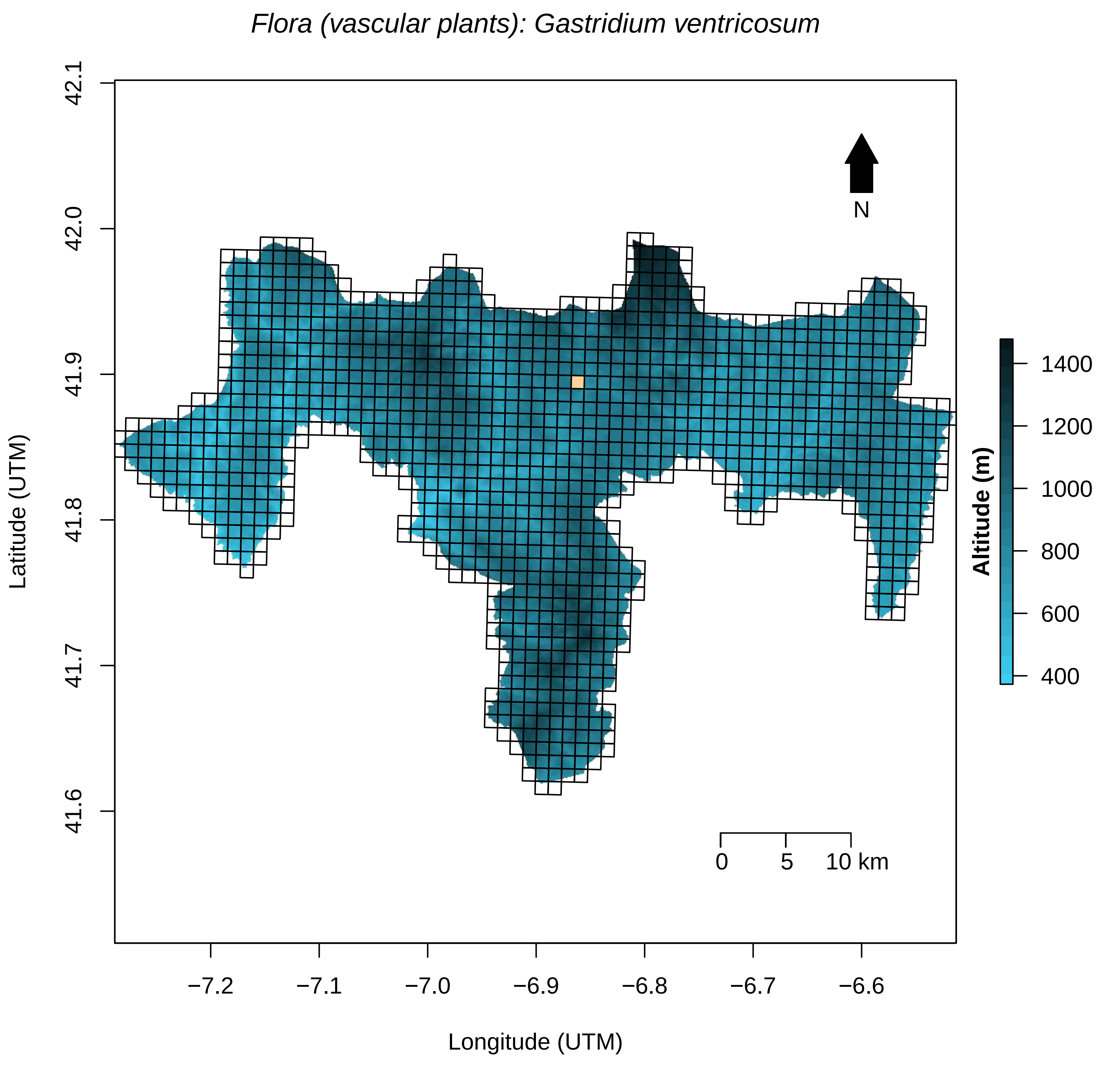

Flora (vascular plants): Gastridium ventricosum
Latitude (UTM)
Longitude (UTM)
42.1
42.0
41.9
41.8
41.7
41.6
−7.2
−7.1
−7.0
−6.9
−6.8
−6.7
−6.6
N
0
5
10 km
Altitude (m)
1400
1200
1000
800
600
400

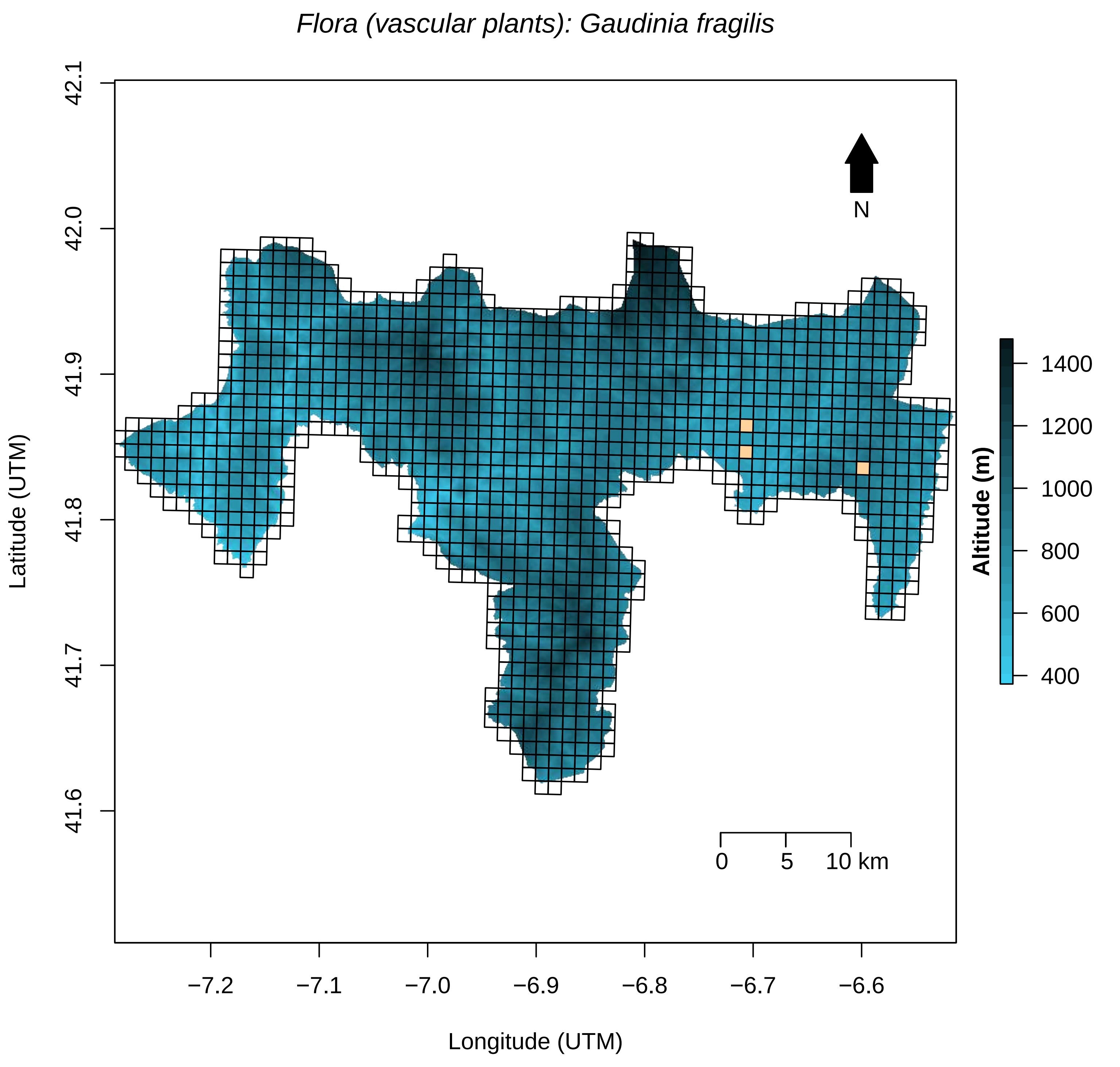

Flora (vascular plants): Gaudinia fragilis
Latitude (UTM)
42.1
42.0
41.9
41.8
41.7
41.6
Longitude (UTM)
−7.2
−7.1
−7.0
−6.9
−6.8
−6.7
−6.6
N
0
5
10 km
Altitude (m)
1400
1200
1000
800
600
400

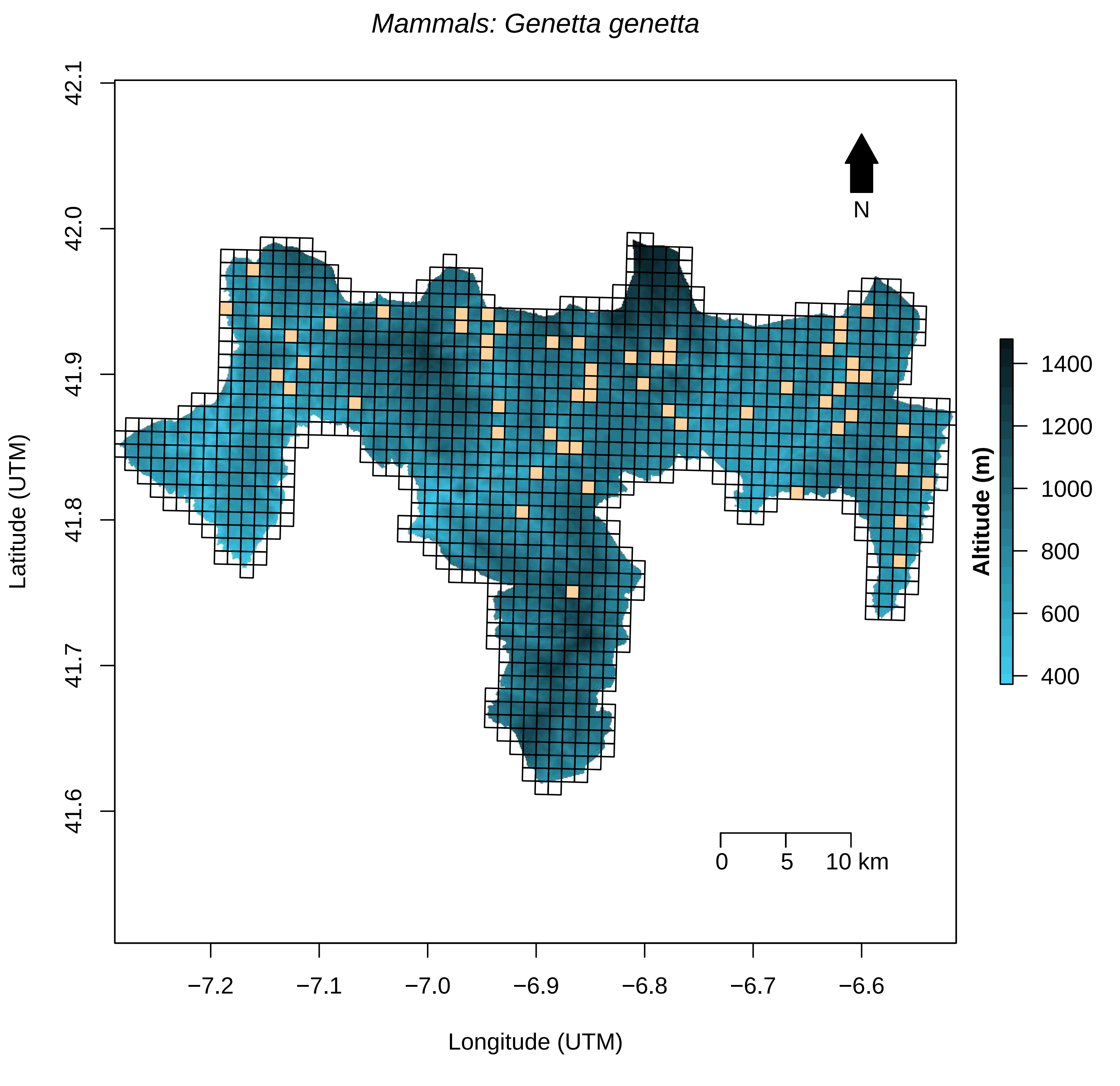
Mammals: Genetta genetta
Latitude (UTM)
Longitude (UTM)
42.1
42.0
41.9
41.8
41.7
41.6
−7.2
−7.1
−7.0
−6.9
−6.8
−6.7
−6.6
N
0
5
10 km
Altitude (m)
1400
1200
1000
800
600
400

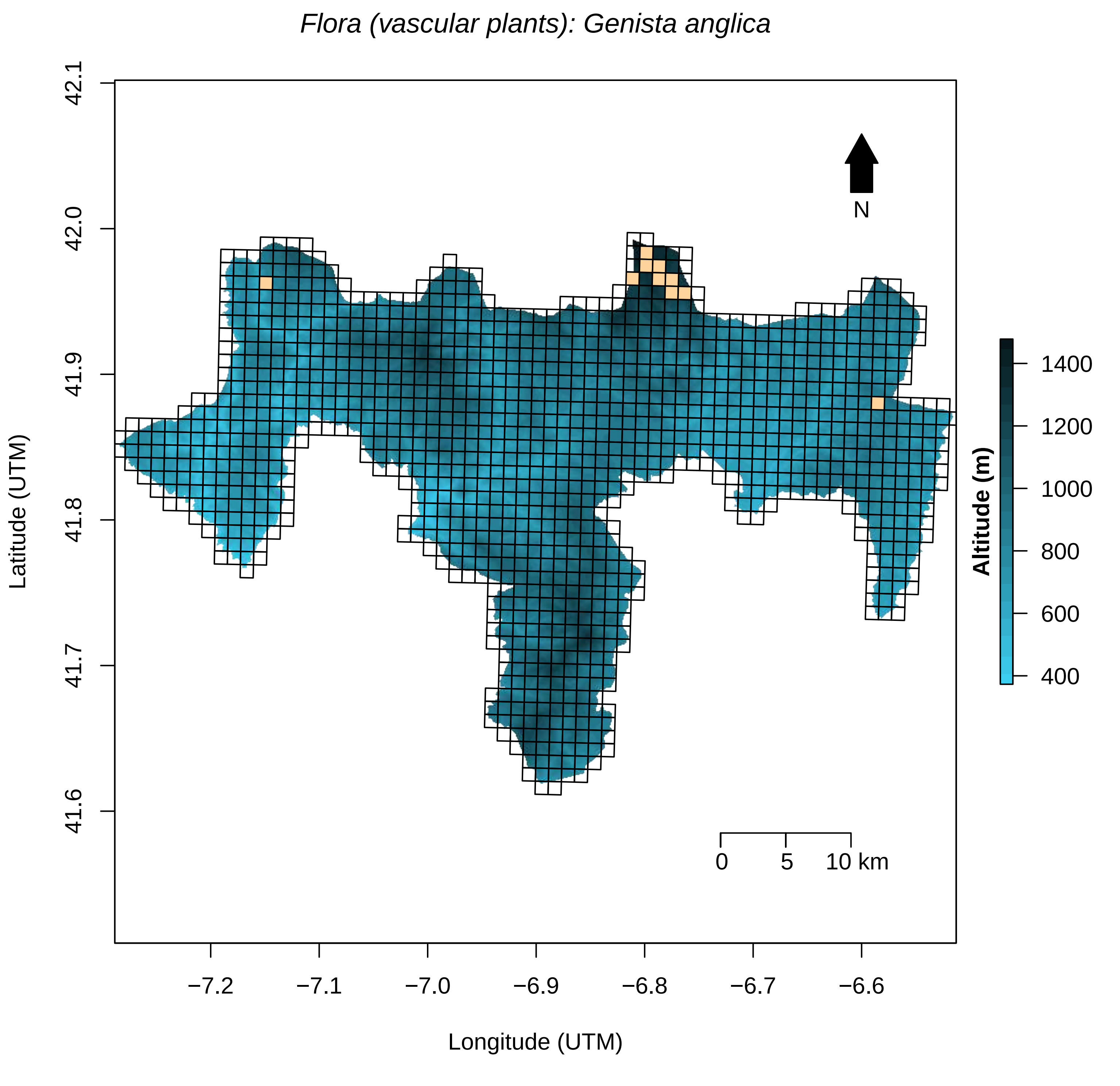

Flora (vascular plants): Genista anglica
Latitude (UTM)
42.1
42.0
41.9
41.8
41.7
41.6
Longitude (UTM)
−7.2
−7.1
−7.0
−6.9
−6.8
−6.7
−6.6
N
0
5
10 km
Altitude (m)
1400
1200
1000
800
600
400

Flora (vascular plants): Genista falcata

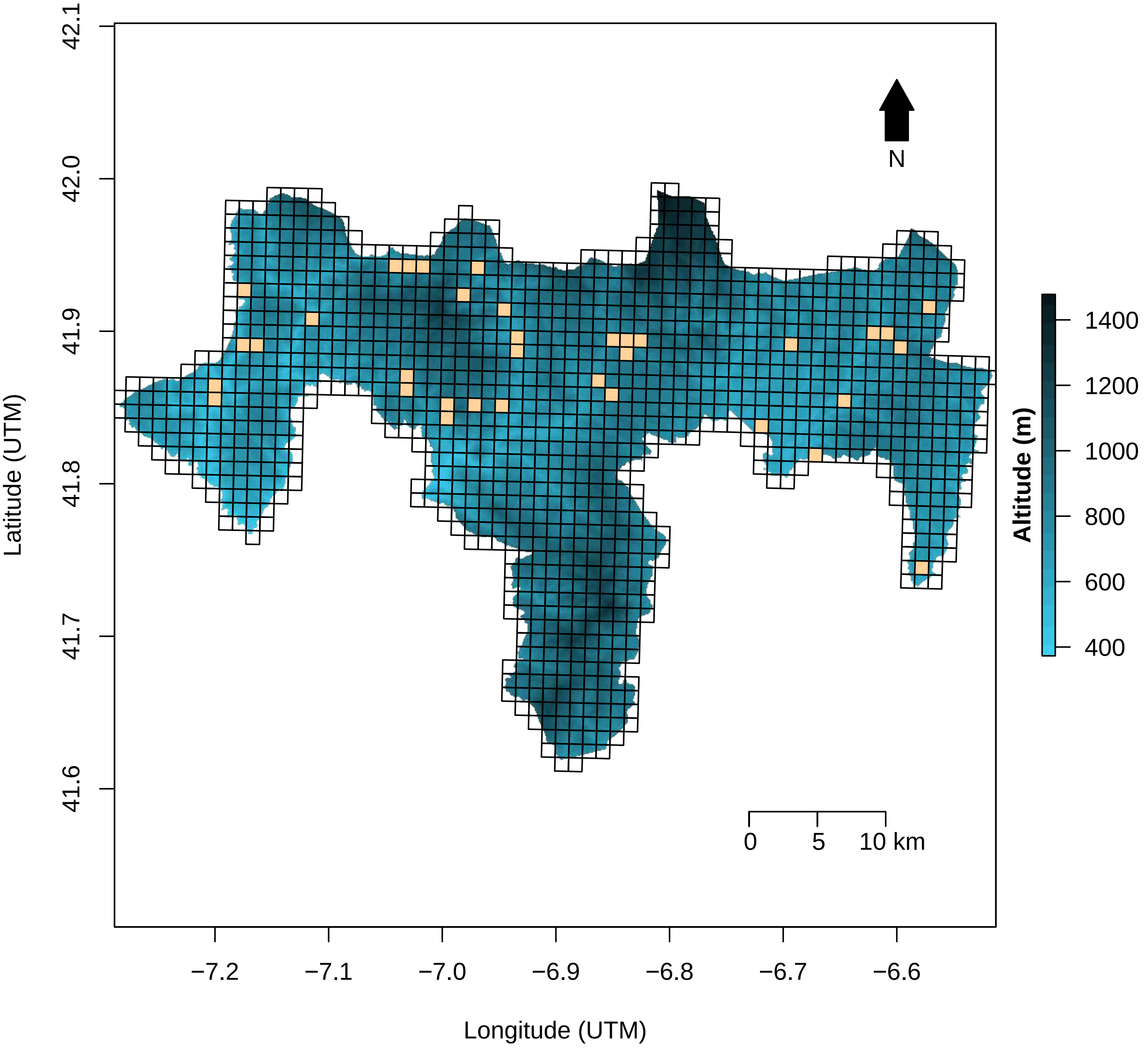

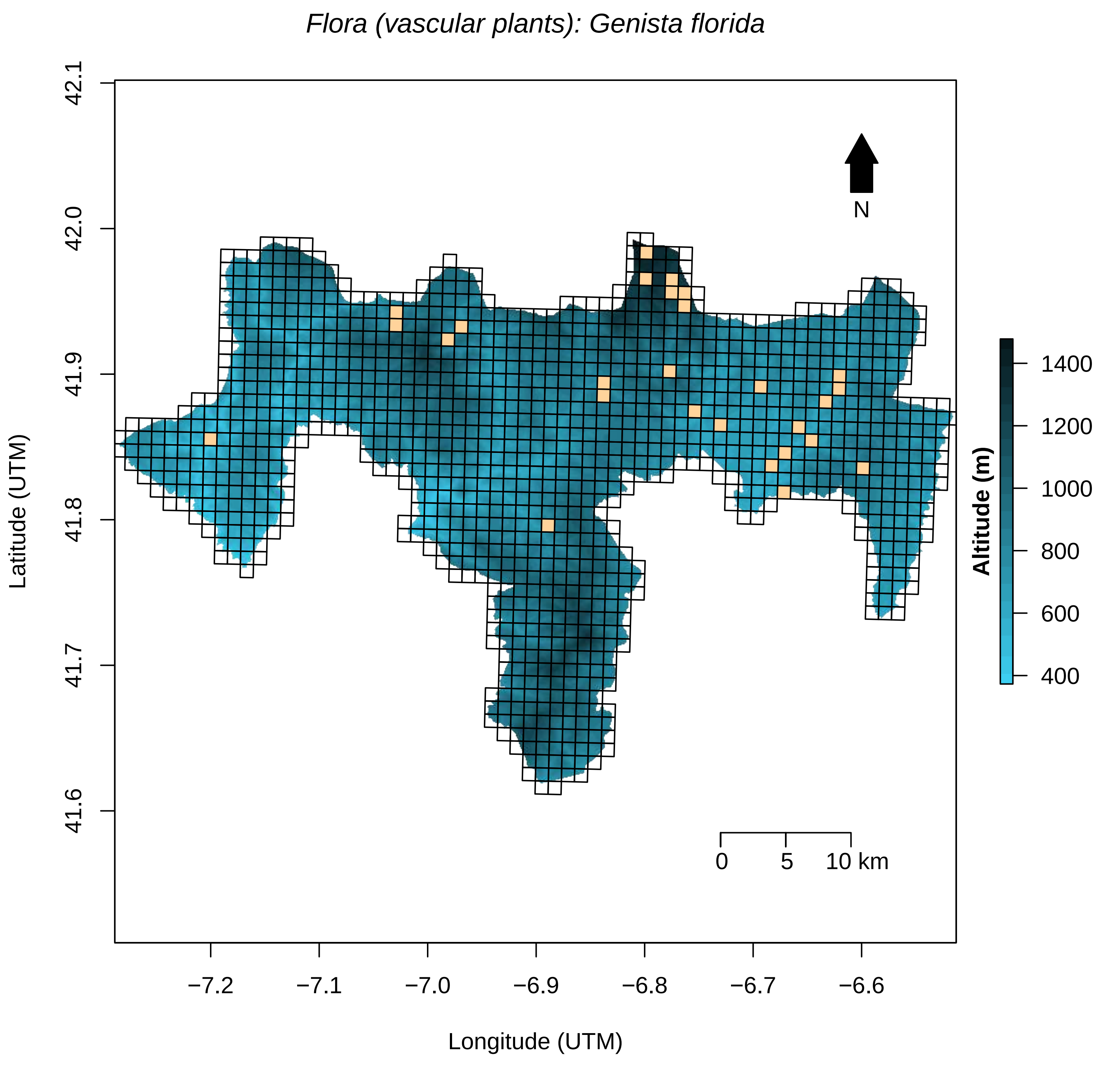

Flora (vascular plants): Genista florida
N
0
5
10 km
Altitude (m)
1400
1200
1000
800
600
400
Latitude (UTM)
42.1
42.0
41.9
41.8
41.7
41.6
Longitude (UTM)
−7.2
−7.1
−7.0
−6.9
−6.8
−6.7
−6.6

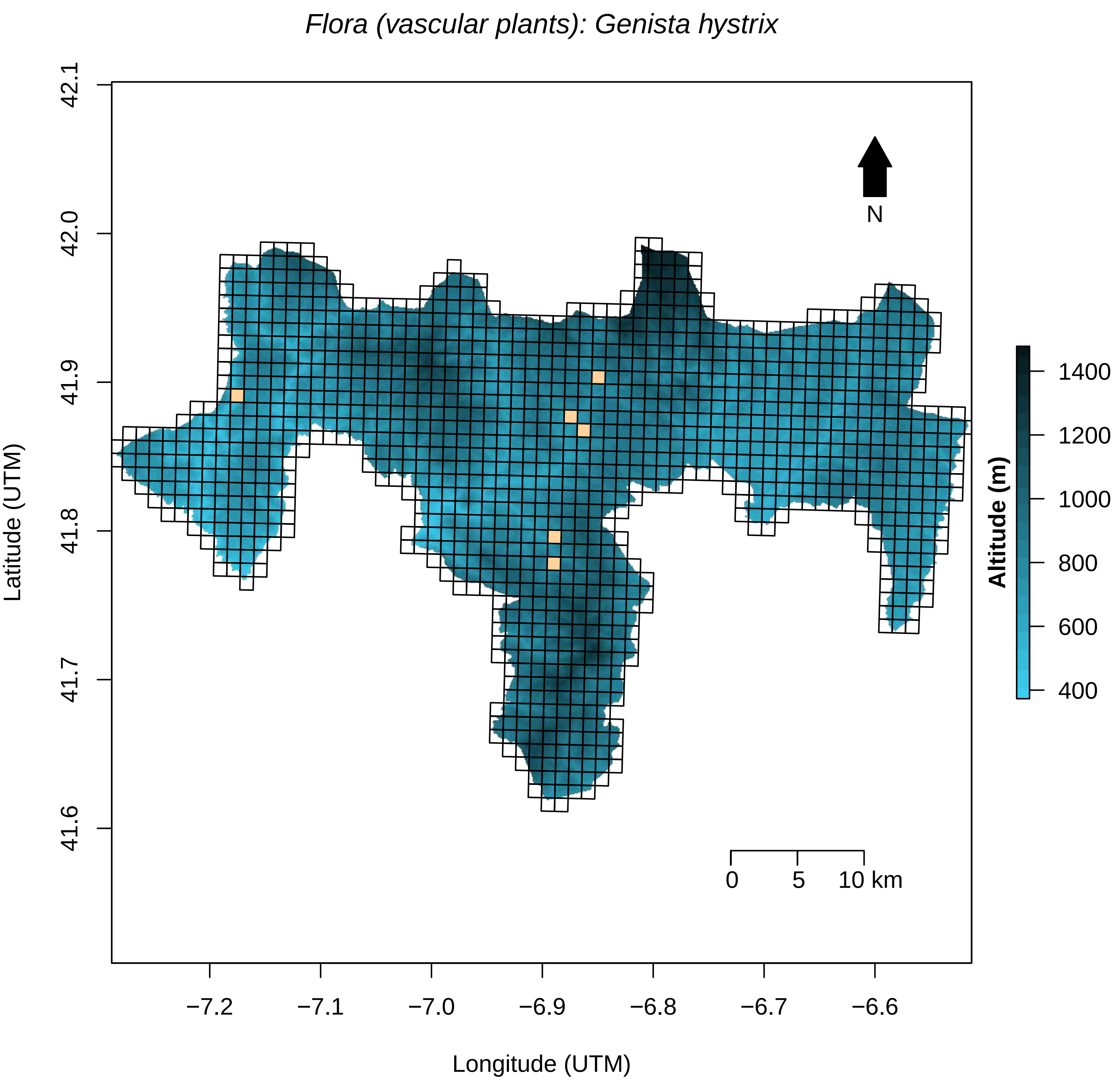

Flora (vascular plants): Genista hystrix
42.1
42.0
41.9
41.8
41.7
41.6
Latitude (UTM)
−7.2
−7.1
−7.0
−6.9
−6.8
−6.7
−6.6
Longitude (UTM)
N
0
5
10 km
Altitude (m)
1400
1200
1000
800
600
400

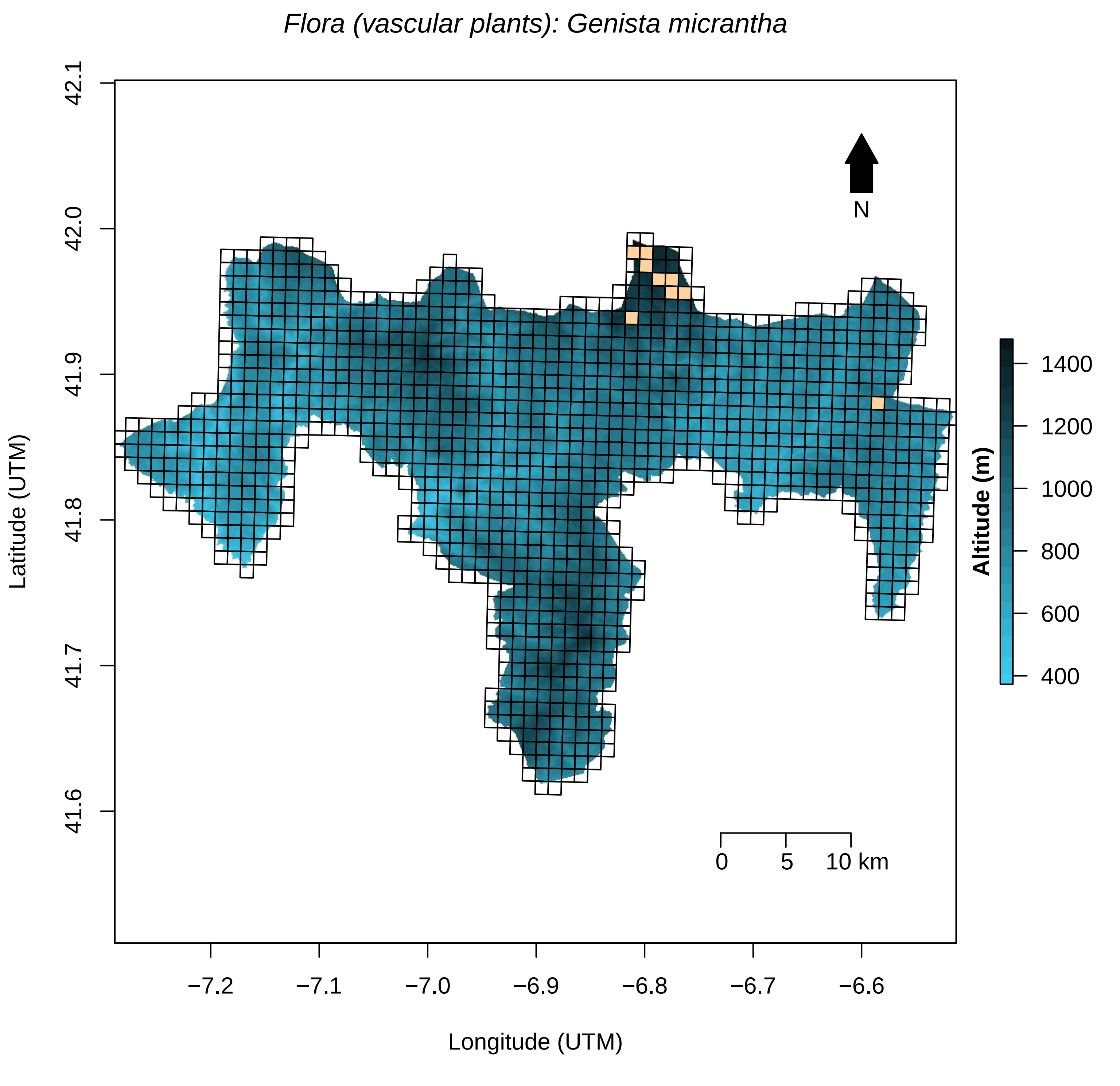

Flora (vascular plants): Genista micrantha
N
0
5
10 km
Altitude (m)
1400
1200
1000
800
600
400
Latitude (UTM)
42.1
42.0
41.9
41.8
41.7
41.6
Longitude (UTM)
−7.2
−7.1
−7.0
−6.9
−6.8
−6.7
−6.6

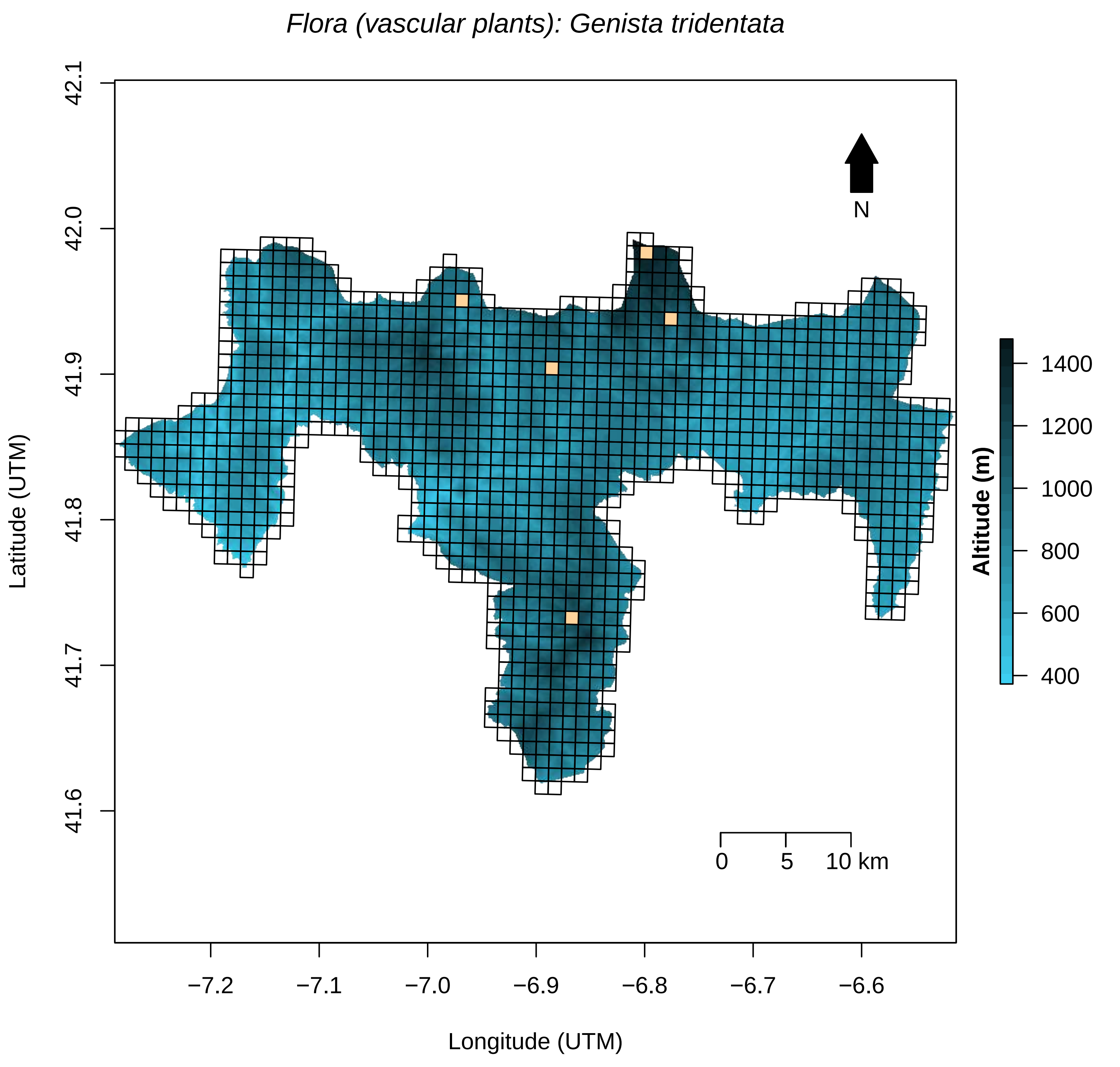

Flora (vascular plants): Genista tridentata
N
0
5
10 km
Latitude (UTM)
Longitude (UTM)
Altitude (m)
1400
1200
1000
800
600
400
42.1
42.0
41.9
41.8
41.7
41.6
−7.2
−7.1
−7.0
−6.9
−6.8
−6.7
−6.6

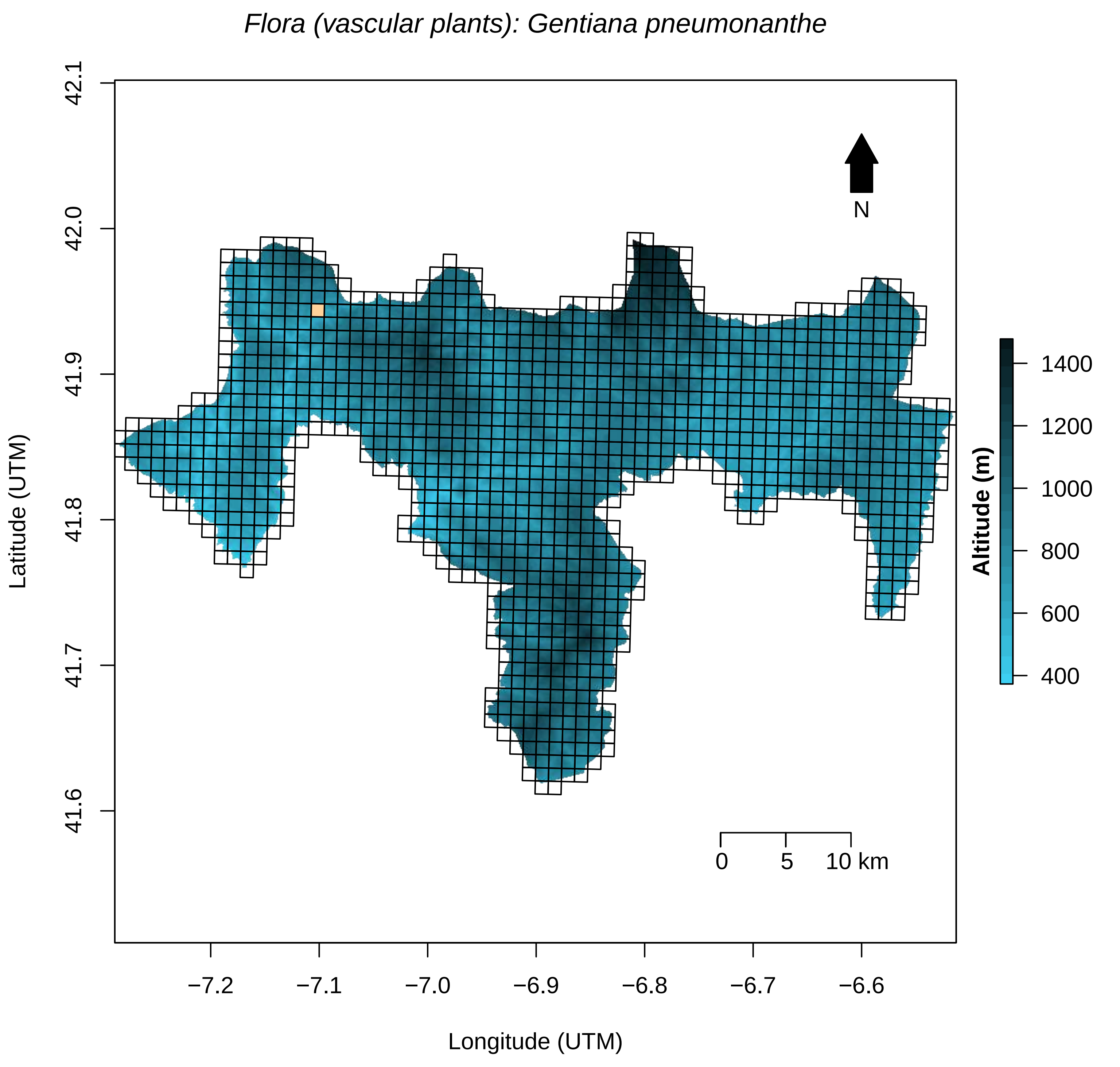

Flora (vascular plants): Gentiana pneumonanthe
42.1
42.0
41.9
41.8
41.7
41.6
Latitude (UTM)
−7.2
−7.1
−7.0
−6.9
−6.8
−6.7
−6.6
Longitude (UTM)
N
0
5
10 km
Altitude (m)
1400
1200
1000
800
600
400

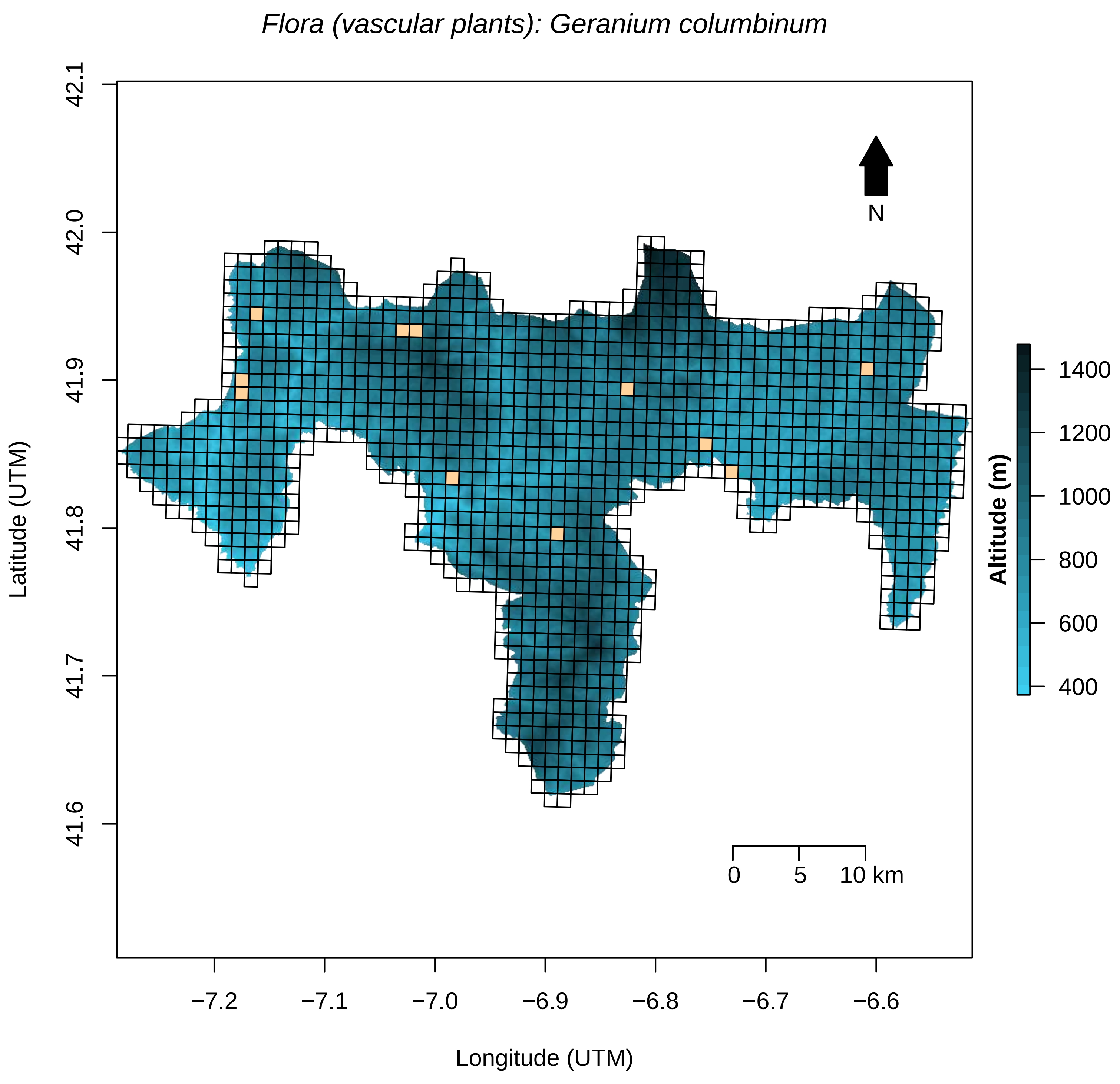
Flora (vascular plants): Geranium columbinum
Latitude (UTM)
42.1
42.0
41.9
41.8
41.7
41.6
Longitude (UTM)
−7.2
−7.1
−7.0
−6.9
−6.8
−6.7
−6.6
N
0
5
10 km
Altitude (m)
1400
1200
1000
800
600
400

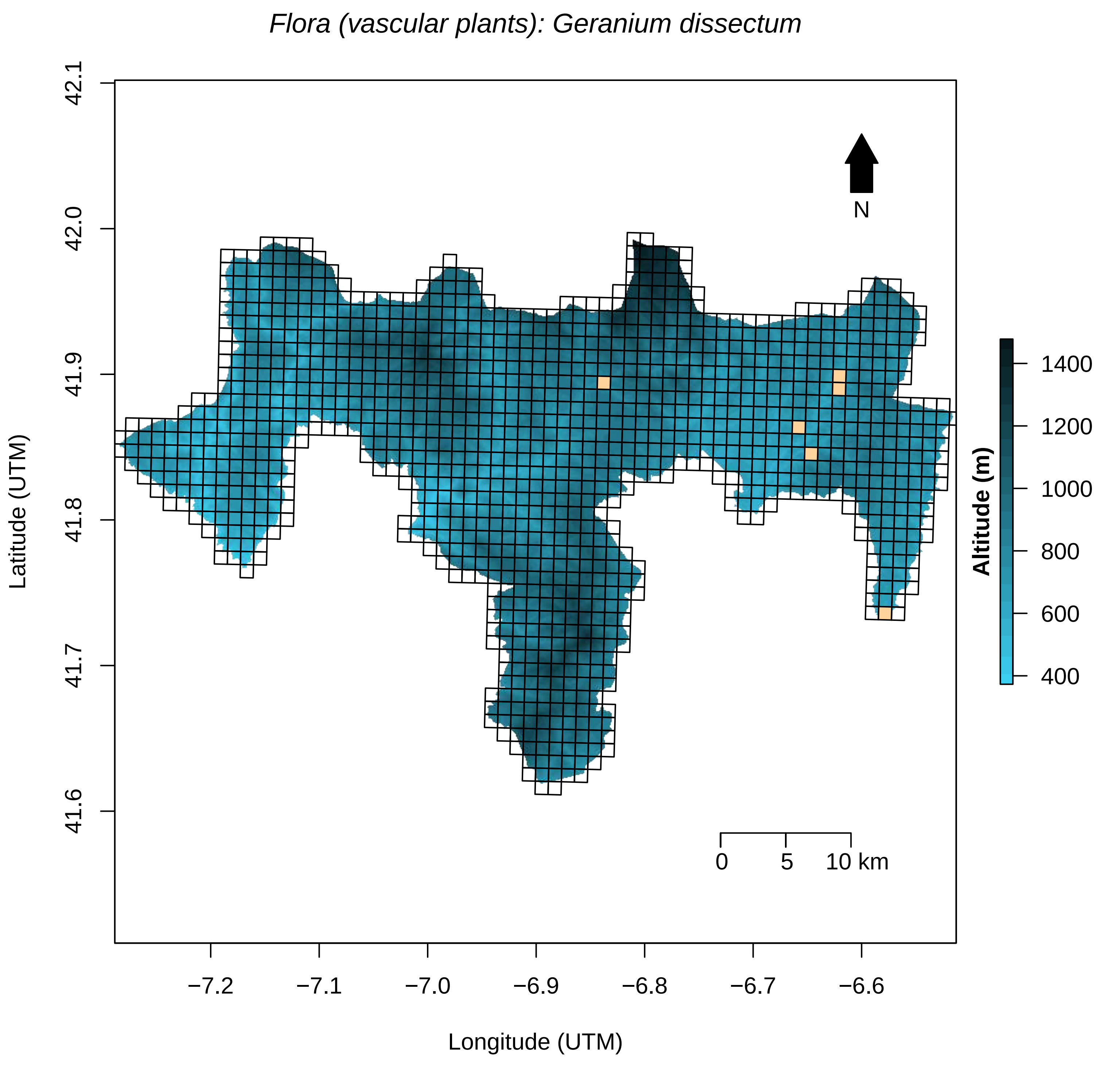
Flora (vascular plants): Geranium dissectum
42.1
42.0
41.9
41.8
41.7
41.6
Latitude (UTM)
−7.2
−7.1
−7.0
−6.9
−6.8
−6.7
−6.6
Longitude (UTM)
N
0
5
10 km
Altitude (m)
1400
1200
1000
800
600
400

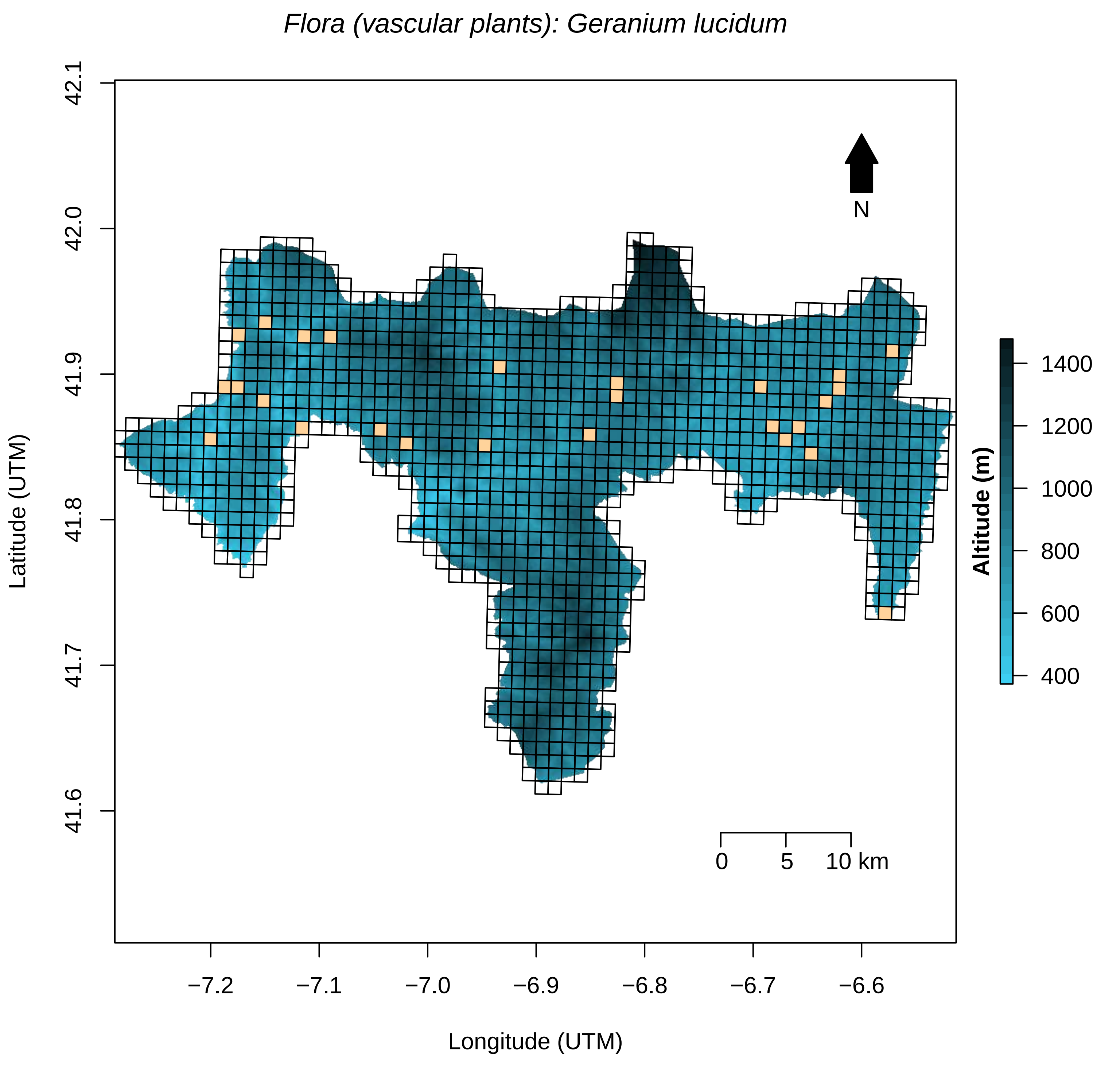

Flora (vascular plants): Geranium lucidum
N
0
5
10 km
Altitude (m)
1400
1200
1000
800
600
400
Latitude (UTM)
42.1
42.0
41.9
41.8
41.7
41.6
Longitude (UTM)
−7.2
−7.1
−7.0
−6.9
−6.8
−6.7
−6.6

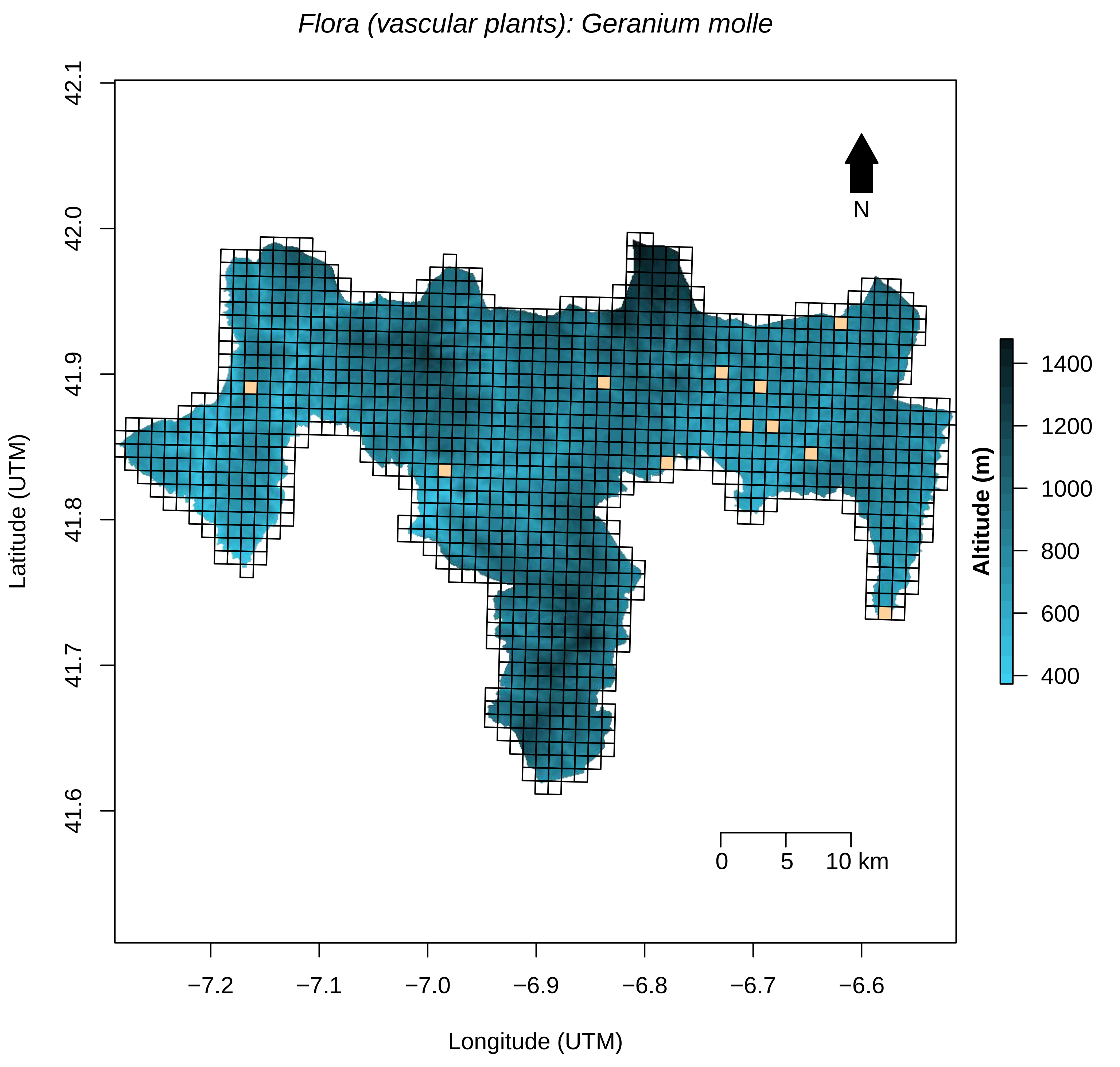

Flora (vascular plants): Geranium molle
42.1
42.0
41.9
41.8
41.7
41.6
Latitude (UTM)
−7.2
−7.1
−7.0
−6.9
−6.8
−6.7
−6.6
Longitude (UTM)
N
0
5
10 km
1400
1200
1000
800
600
400
Altitude (m)

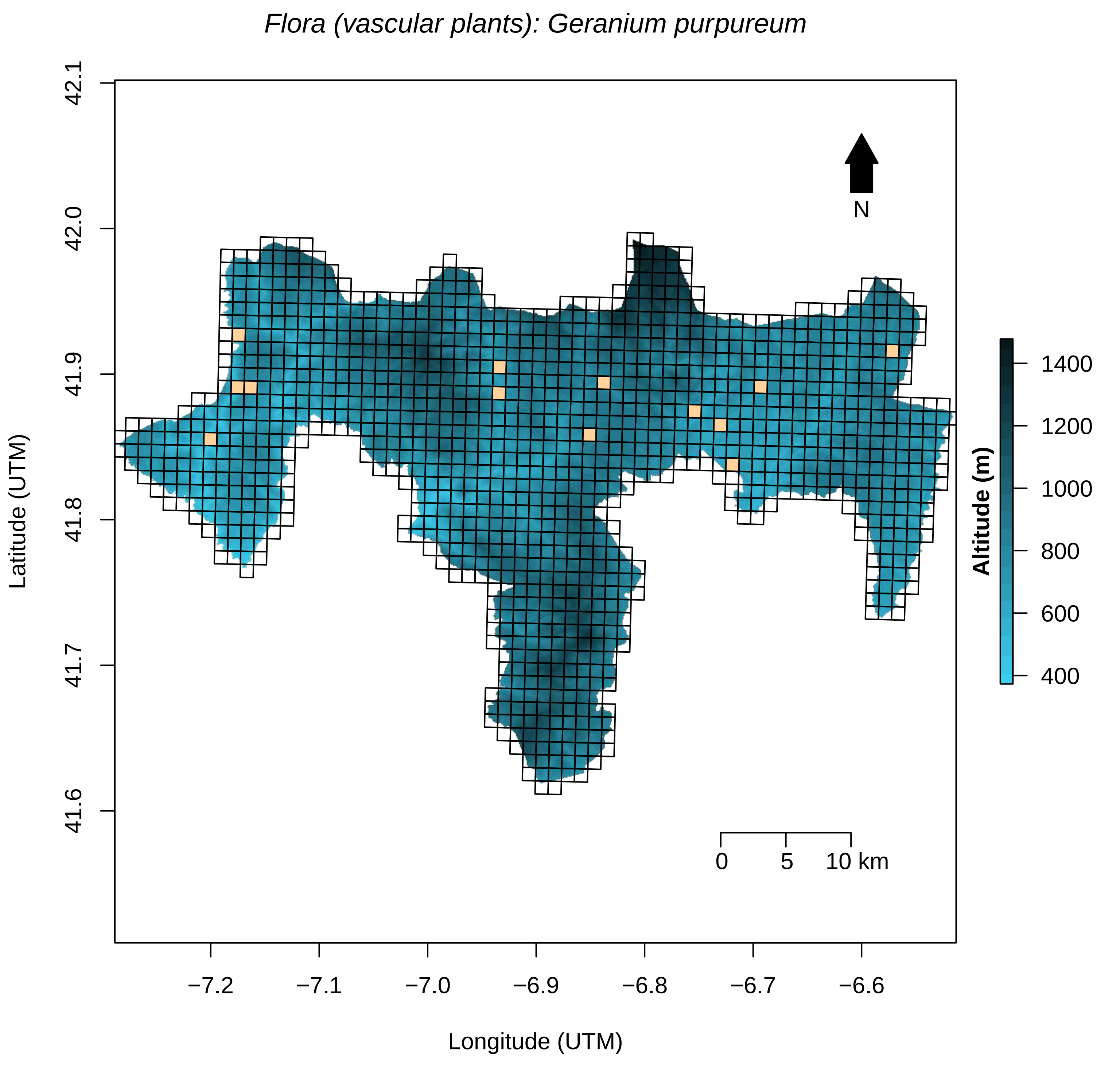

Flora (vascular plants): Geranium purpureum
42.1
42.0
41.9
41.8
41.7
41.6
Latitude (UTM)
−7.2
−7.1
−7.0
−6.9
−6.8
−6.7
−6.6
Longitude (UTM)
N
0
5
10 km
Altitude (m)
1400
1200
1000
800
600
400

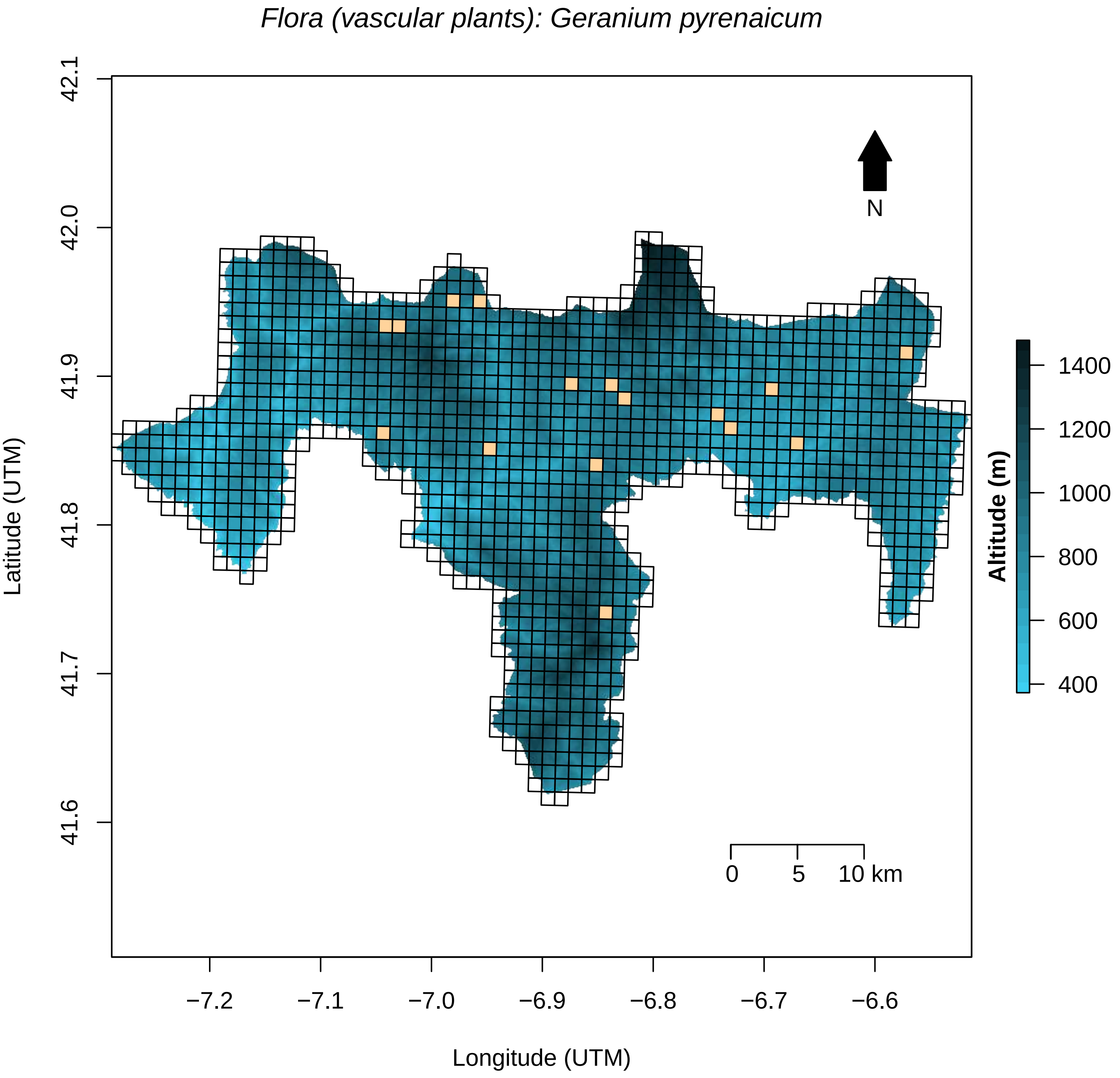
Flora (vascular plants): Geranium pyrenaicum
Latitude (UTM)
42.1
42.0
41.9
41.8
41.7
41.6
−7.2
−7.1
−7.0
−6.9
−6.8
−6.7
−6.6
Longitude (UTM)
N
0
5
10 km
Altitude (m)
1400
1200
1000
800
600
400

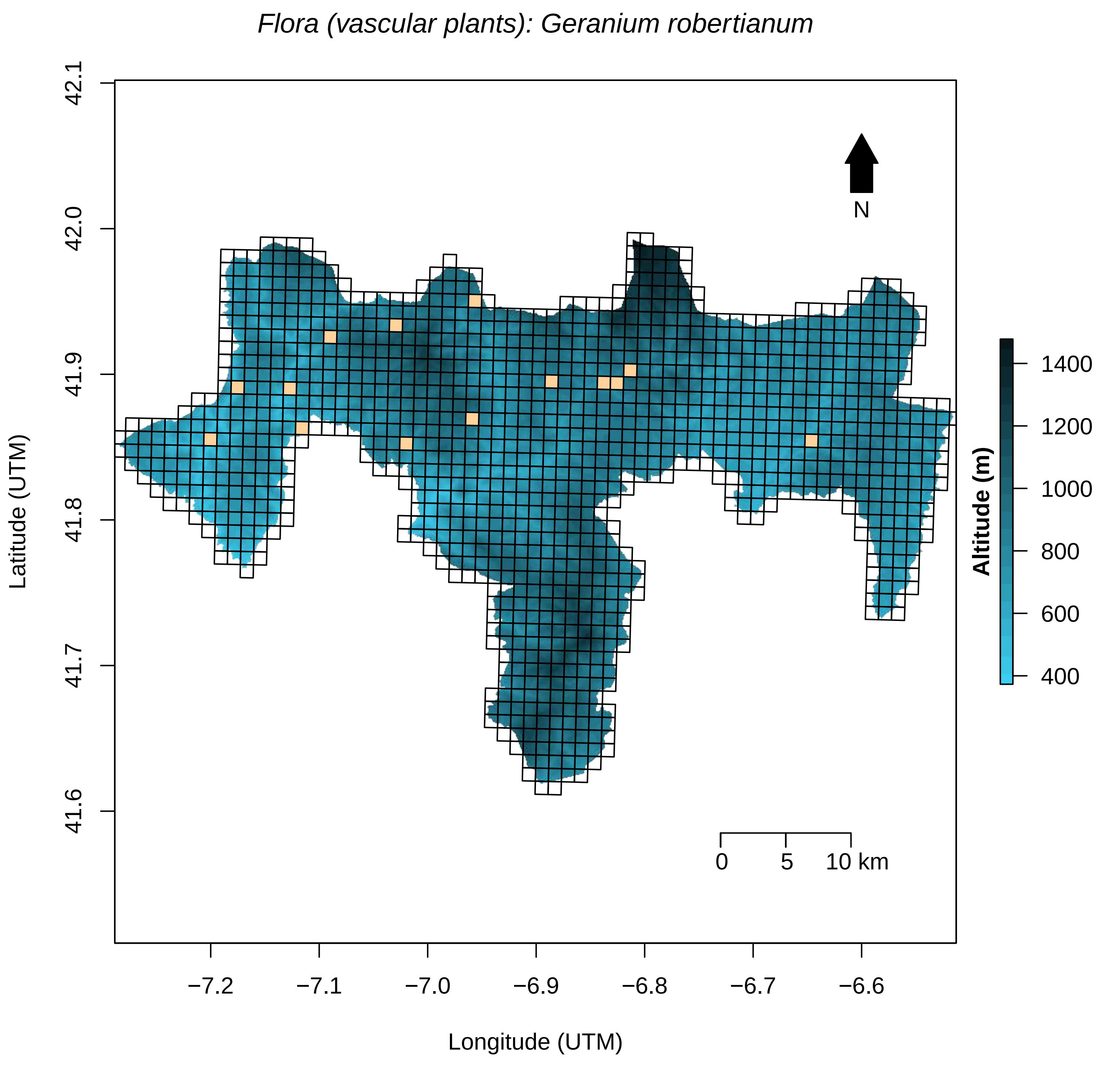

Flora (vascular plants): Geranium robertianum
N
0 5 10 km
Altitude (m)
1400
1200
1000
800
600
400
Latitude (UTM)
42.1
42.0
41.9
41.8
41.7
41.6
Longitude (UTM)
−7.2
−7.1
−7.0
−6.9
−6.8
−6.7
−6.6

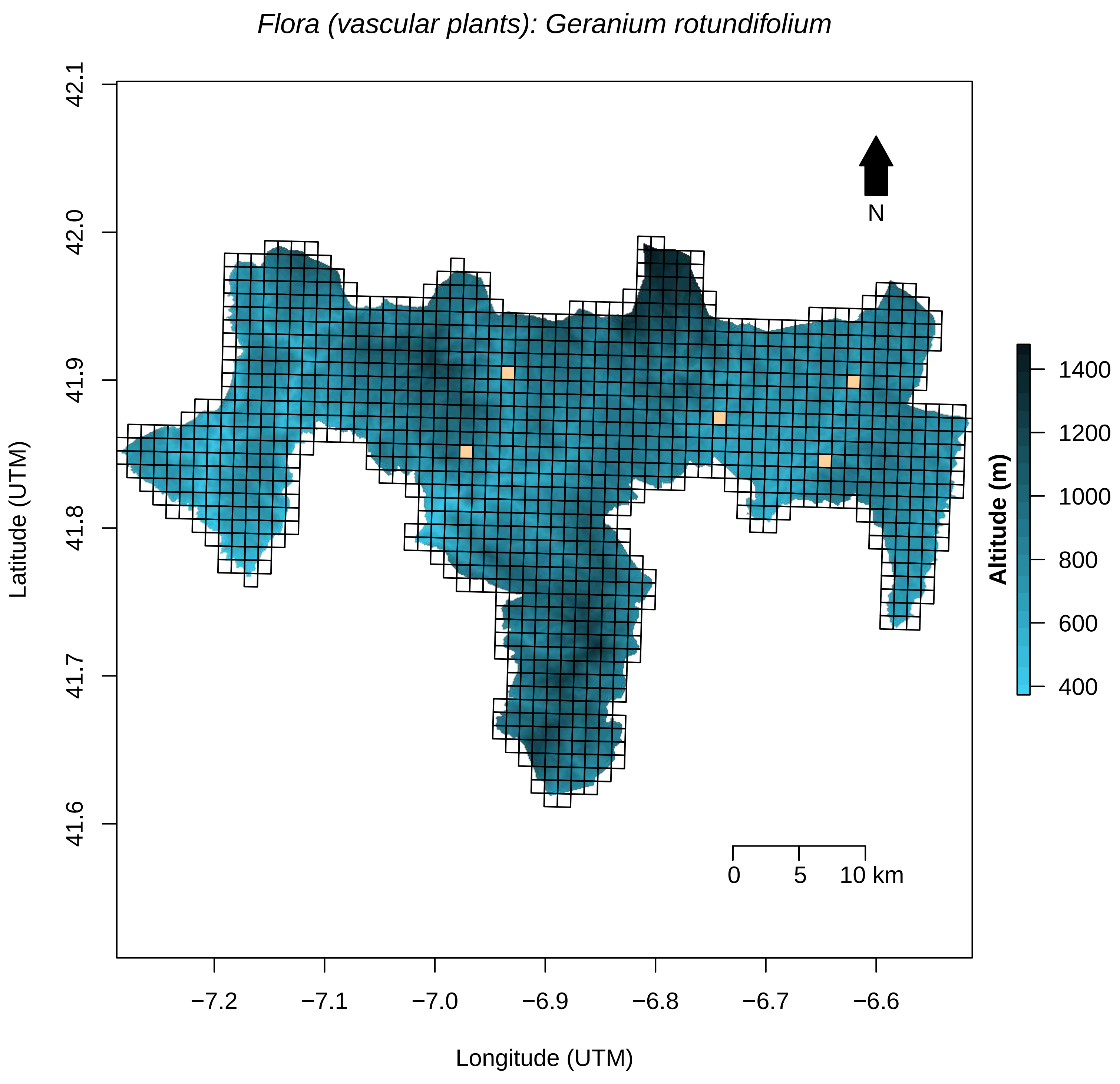

Flora (vascular plants): Geranium rotundifolium
Latitude (UTM)
42.1
42.0
41.9
41.8
41.7
41.6
Longitude (UTM)
−7.2
−7.1
−7.0
−6.9
−6.8
−6.7
−6.6
N
0
5
10 km
Altitude (m)
1400
1200
1000
800
600
400

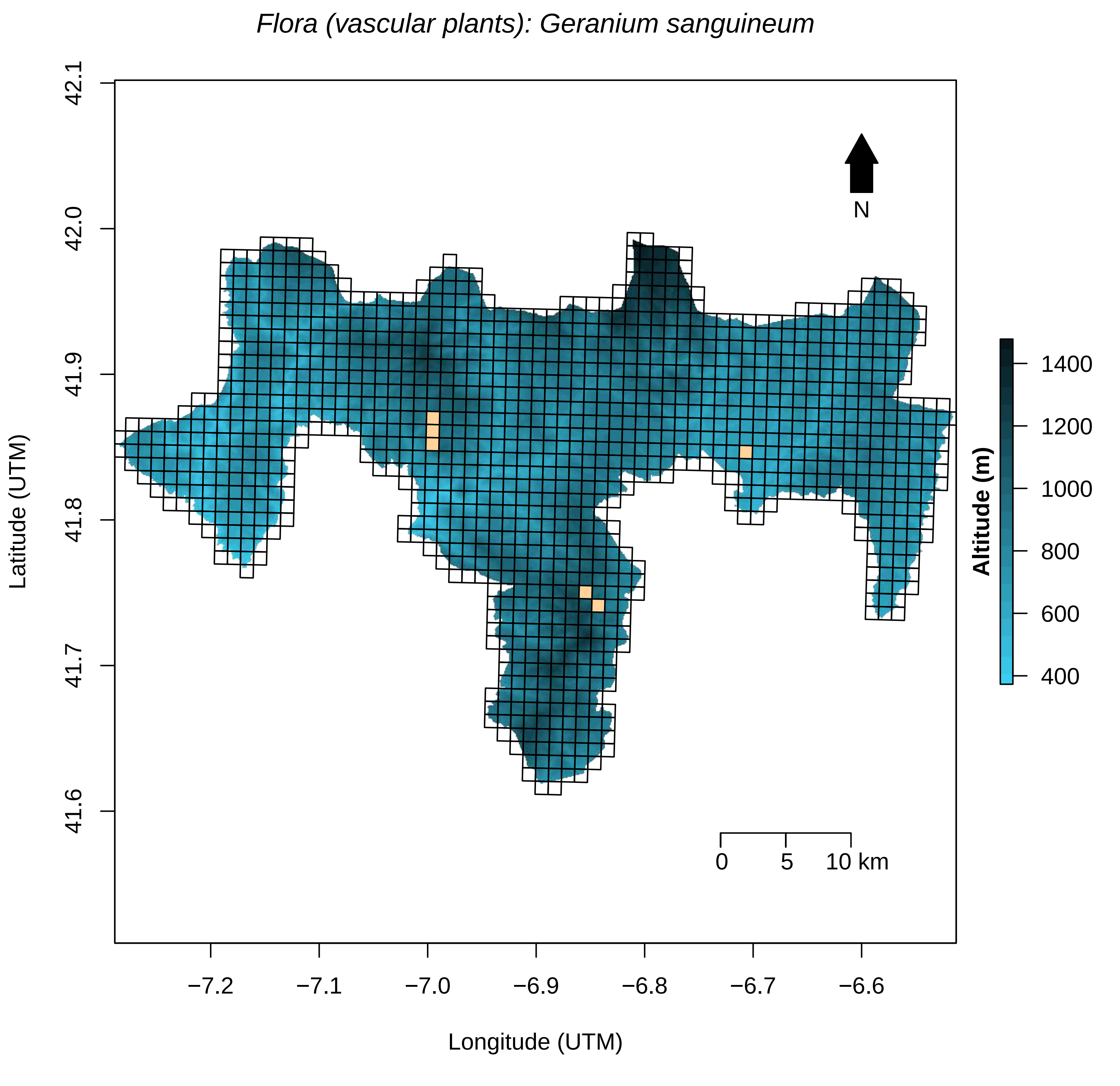

Flora (vascular plants): Geranium sanguineum
42.1
42.0
41.9
41.8
41.7
41.6
Latitude (UTM)
−7.2
−7.1
−7.0
−6.9
−6.8
−6.7
−6.6
Longitude (UTM)
N
0
5
10 km
Altitude (m)
1400
1200
1000
800
600
400

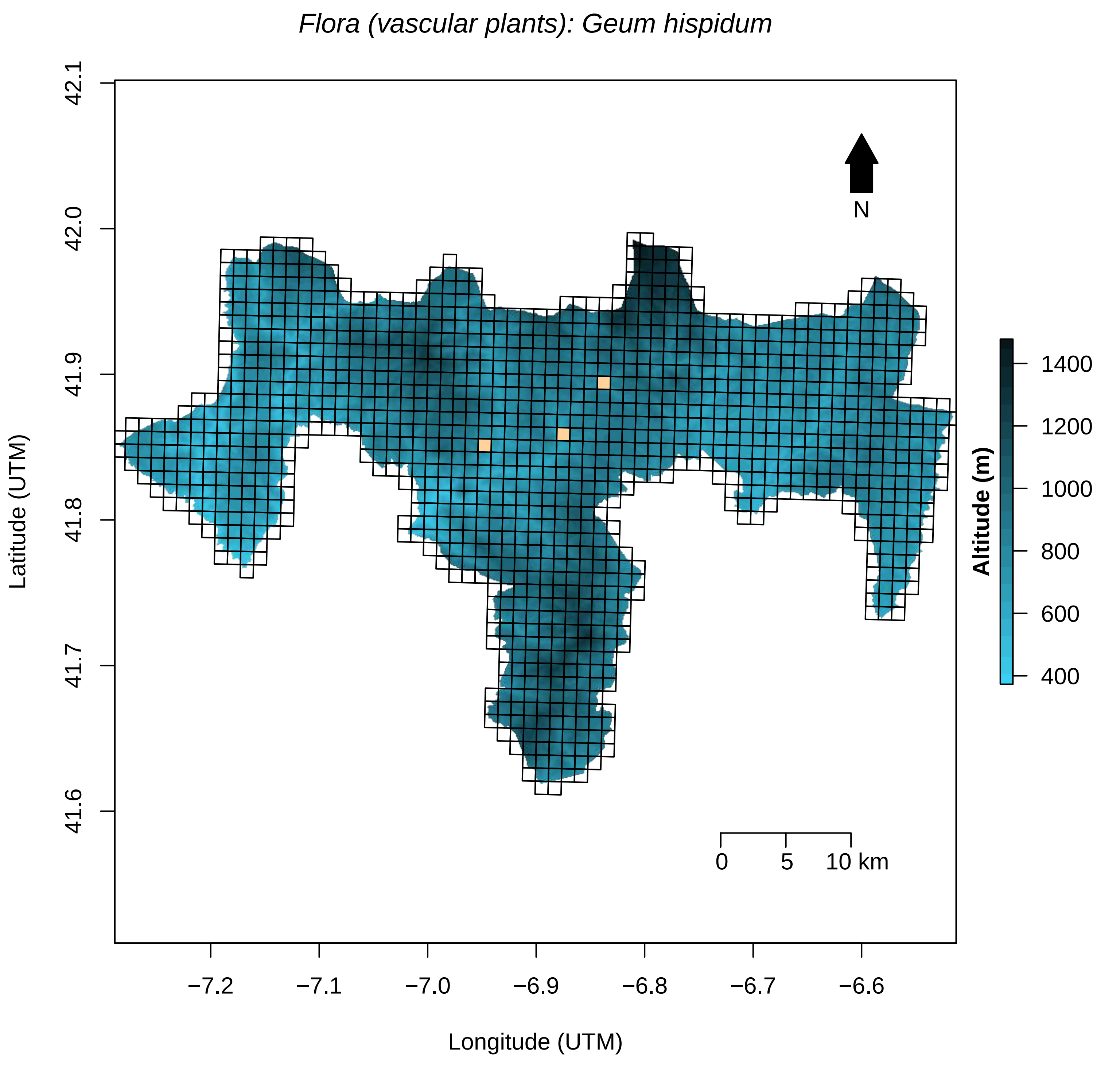

Flora (vascular plants): Geum hispidum
42.1
42.0
41.9
41.8
41.7
41.6
Latitude (UTM)
−7.2
−7.1
−7.0
−6.9
−6.8
−6.7
−6.6
Longitude (UTM)
N
0
5
10 km
Altitude (m)
1400
1200
1000
800
600
400

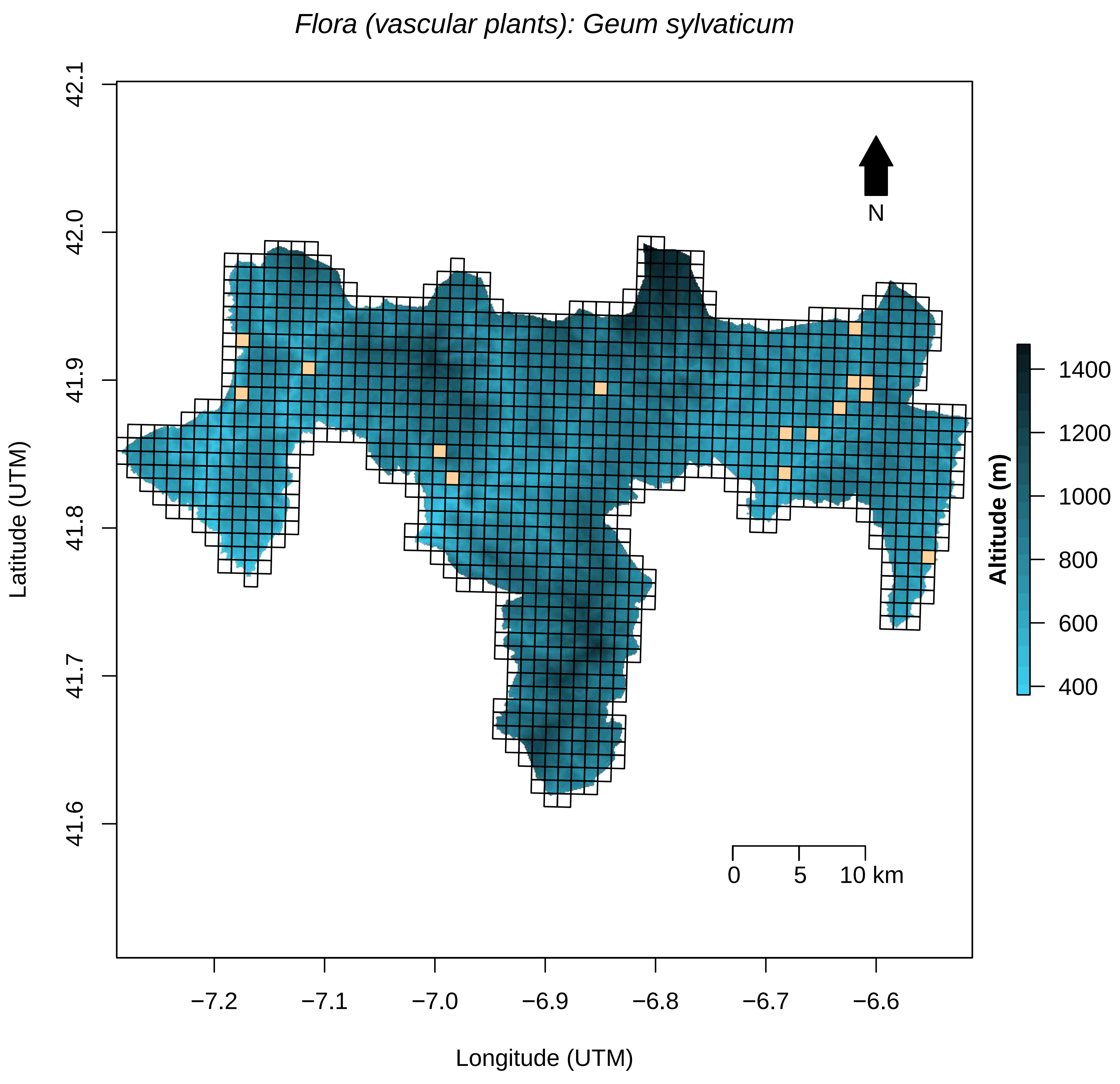

Flora (vascular plants): Geum sylvaticum
42.1
42.0
41.9
41.8
41.7
41.6
Latitude (UTM)
N
0
5
10 km
−7.2
−7.1
−7.0
−6.9
−6.8
−6.7
−6.6
Longitude (UTM)
1400
1200
1000
800
600
400
Altitude (m)

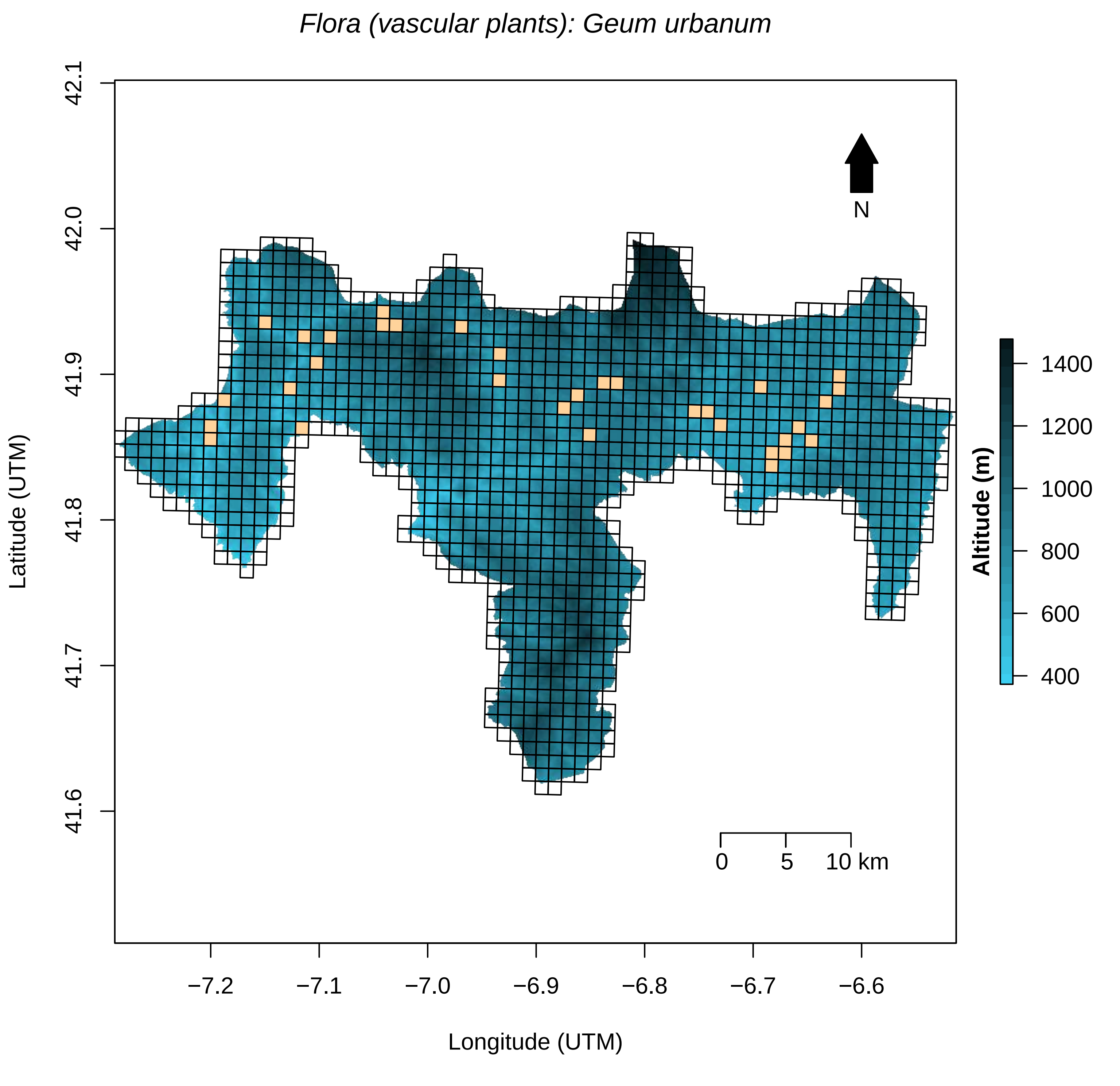

Flora (vascular plants): Geum urbanum
42.1
42.0
41.9
41.8
41.7
41.6
Latitude (UTM)
−7.2
−7.1
−7.0
−6.9
−6.8
−6.7
−6.6
Longitude (UTM)
N
0
5
10 km
1400
1200
1000
800
600
400
Altitude (m)

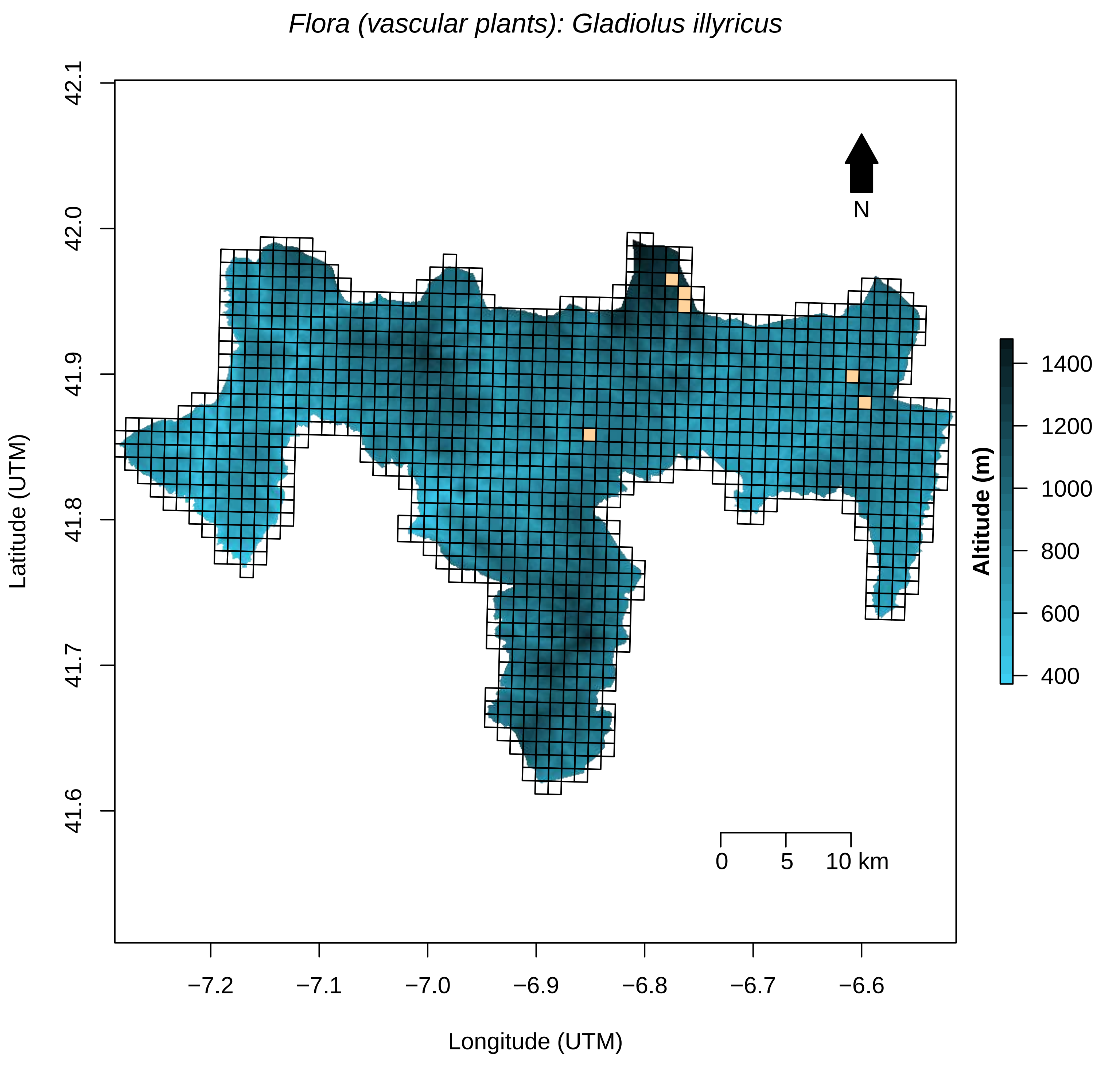
Flora (vascular plants): Gladiolus illyricus
Latitude (UTM)
Longitude (UTM)
42.1
42.0
41.9
41.8
41.7
41.6
−7.2
−7.1
−7.0
−6.9
−6.8
−6.7
−6.6
N
0
5
10 km
Altitude (m)
1400
1200
1000
800
600
400

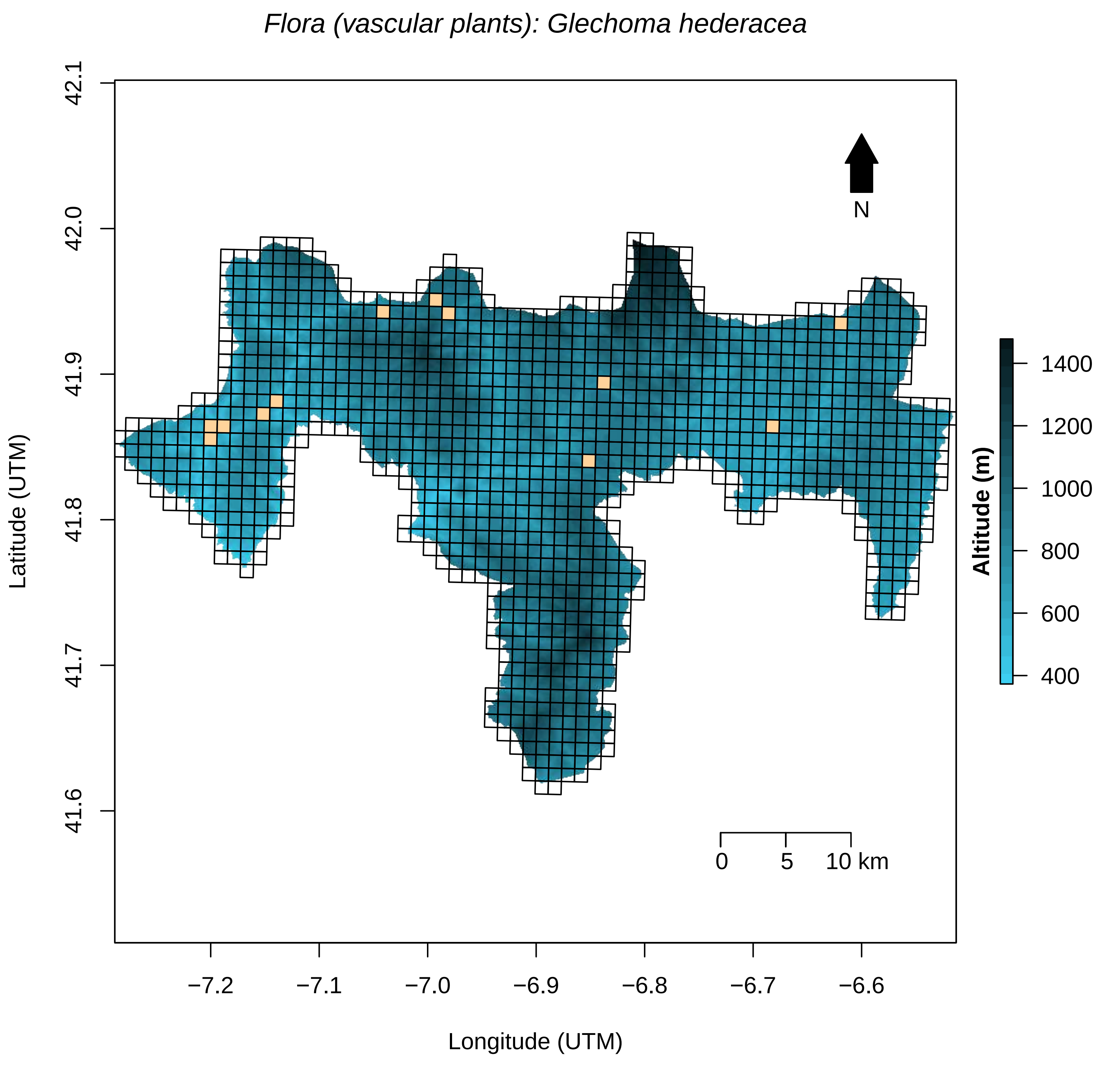

Flora (vascular plants): Glechoma hederacea
42.1
42.0
41.9
41.8
41.7
41.6
Latitude (UTM)
−7.2
−7.1
−7.0
−6.9
−6.8
−6.7
−6.6
Longitude (UTM)
N
0
5
10 km
Altitude (m)
1400
1200
1000
800
600
400

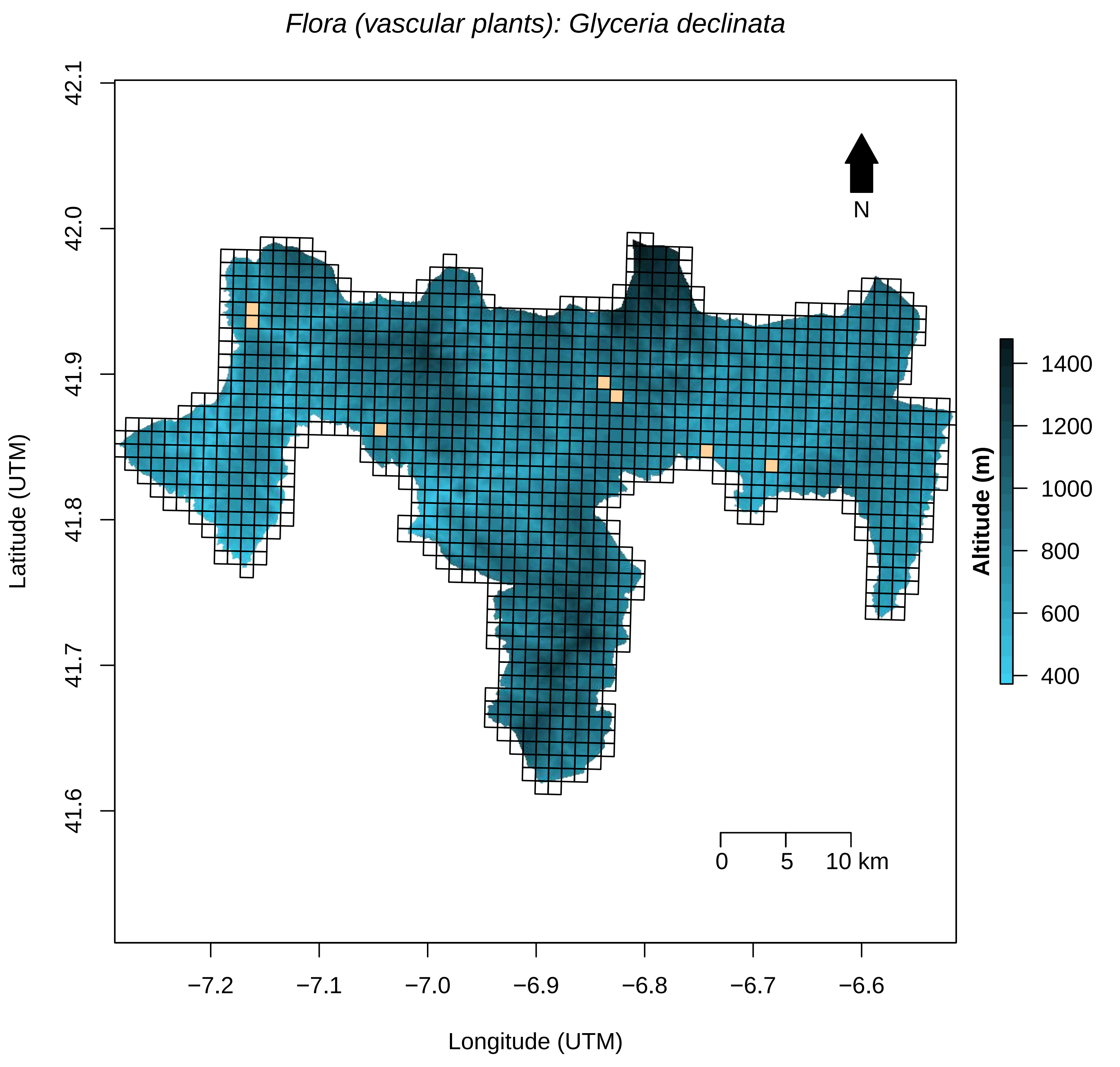
Flora (vascular plants): Glyceria declinata
N
0 5 10 km
Altitude (m)
1400
1200
1000
800
600
400
Latitude (UTM)
42.1
42.0
41.9
41.8
41.7
41.6
Longitude (UTM)
−7.2
−7.1
−7.0
−6.9
−6.8
−6.7
−6.6

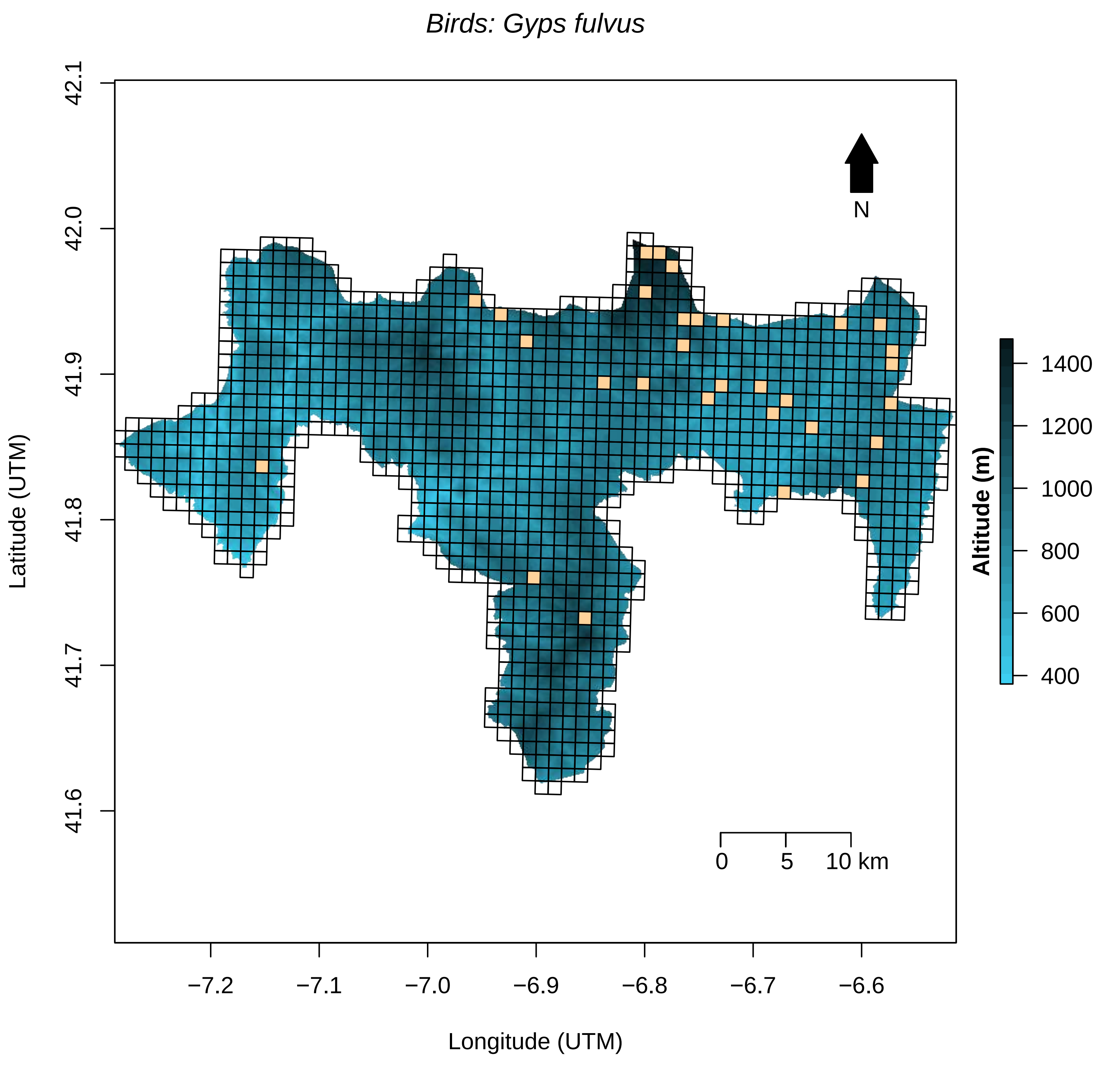
Birds: Gyps fulvus
N
0
5
10 km
Altitude (m)
1400
1200
1000
800
600
400
Latitude (UTM)
42.1
42.0
41.9
41.8
41.7
41.6
Longitude (UTM)
−7.2
−7.1
−7.0
−6.9
−6.8
−6.7
−6.6

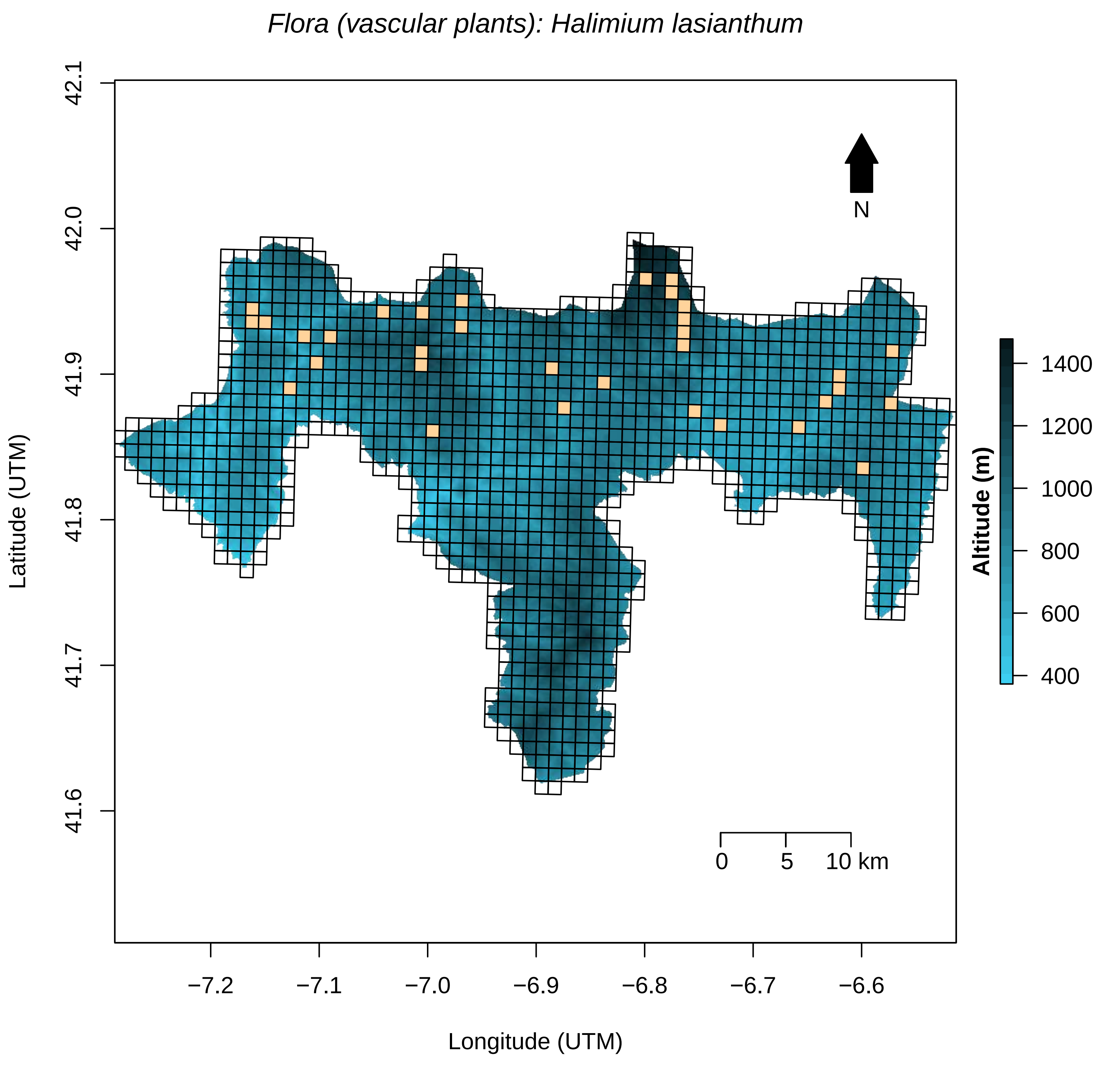

Flora (vascular plants): Halimium lasianthum
42.1
42.0
41.9
41.8
41.7
41.6
Latitude (UTM)
−7.2
−7.1
−7.0
−6.9
−6.8
−6.7
−6.6
Longitude (UTM)
N
0
5
10 km
Altitude (m)
1400
1200
1000
800
600
400

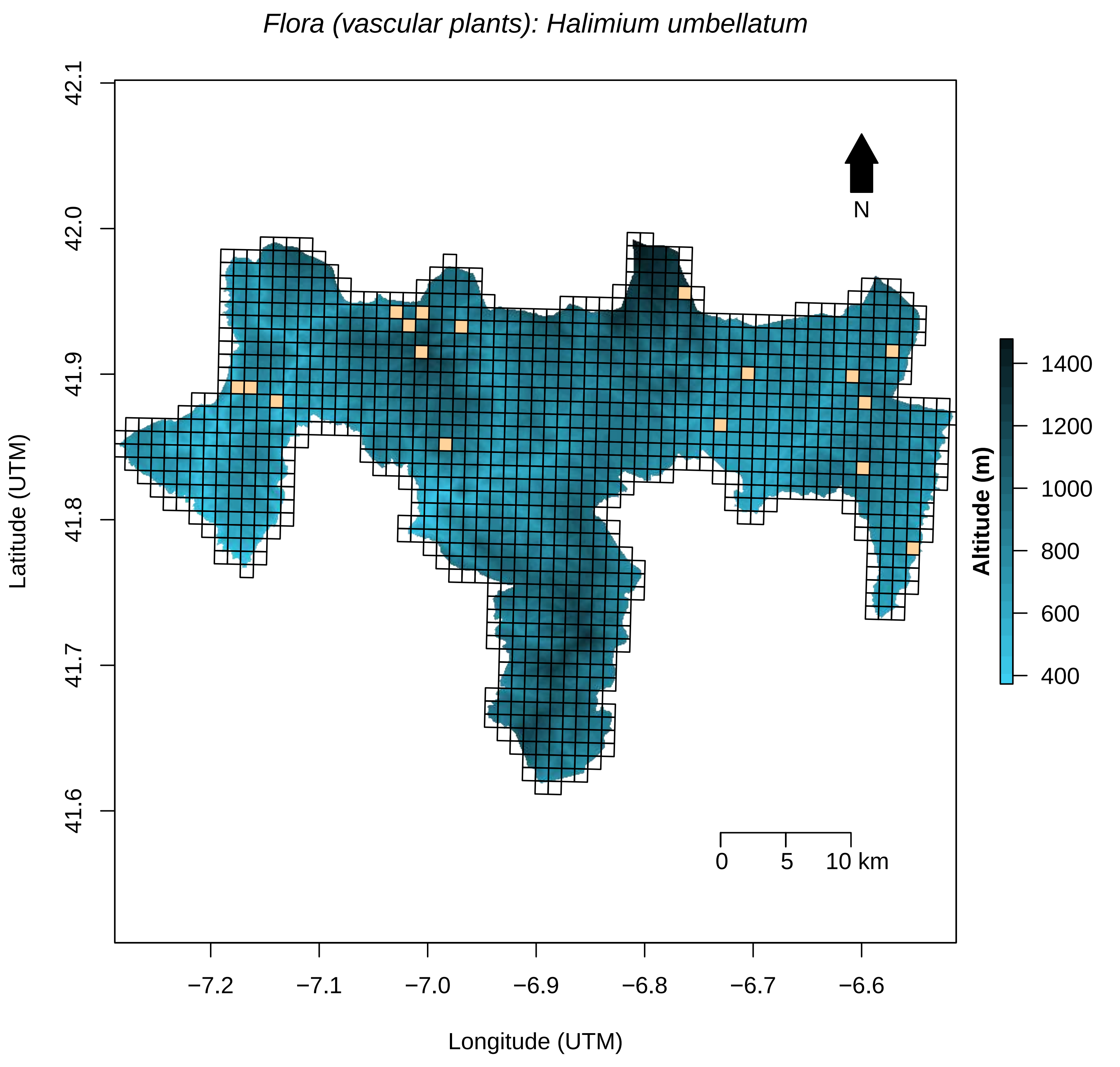

Flora (vascular plants): Halimium umbellatum
N
0
5
10 km
Altitude (m)
1400
1200
1000
800
600
400
Latitude (UTM)
42.1
42.0
41.9
41.8
41.7
41.6
Longitude (UTM)
−7.2
−7.1
−7.0
−6.9
−6.8
−6.7
−6.6

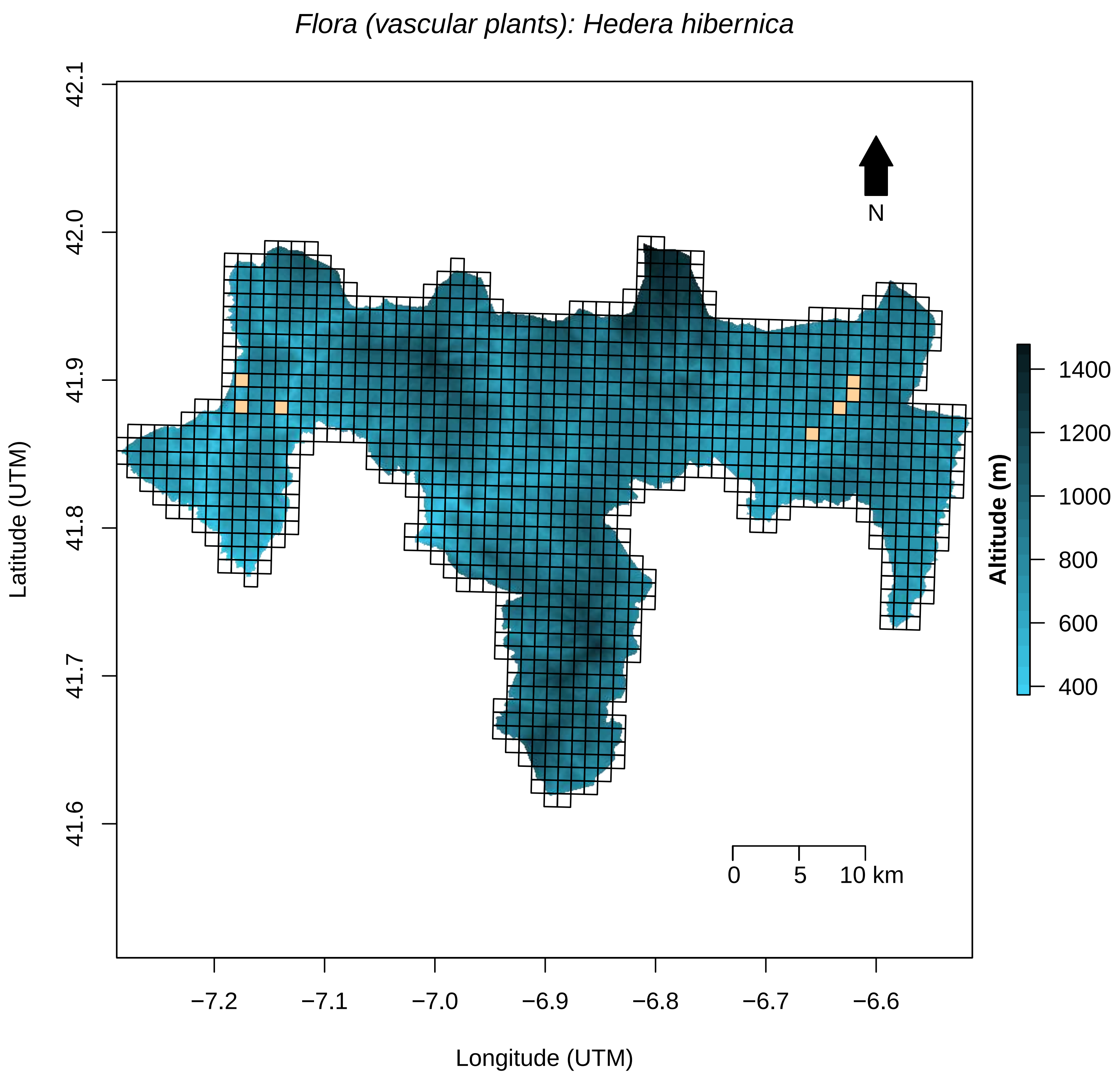

Flora (vascular plants): Hedera hibernica
42.1
42.0
41.9
41.8
41.7
41.6
Latitude (UTM)
−7.2
−7.1
−7.0
−6.9
−6.8
−6.7
−6.6
Longitude (UTM)
N
0
5
10 km
Altitude (m)
1400
1200
1000
800
600
400

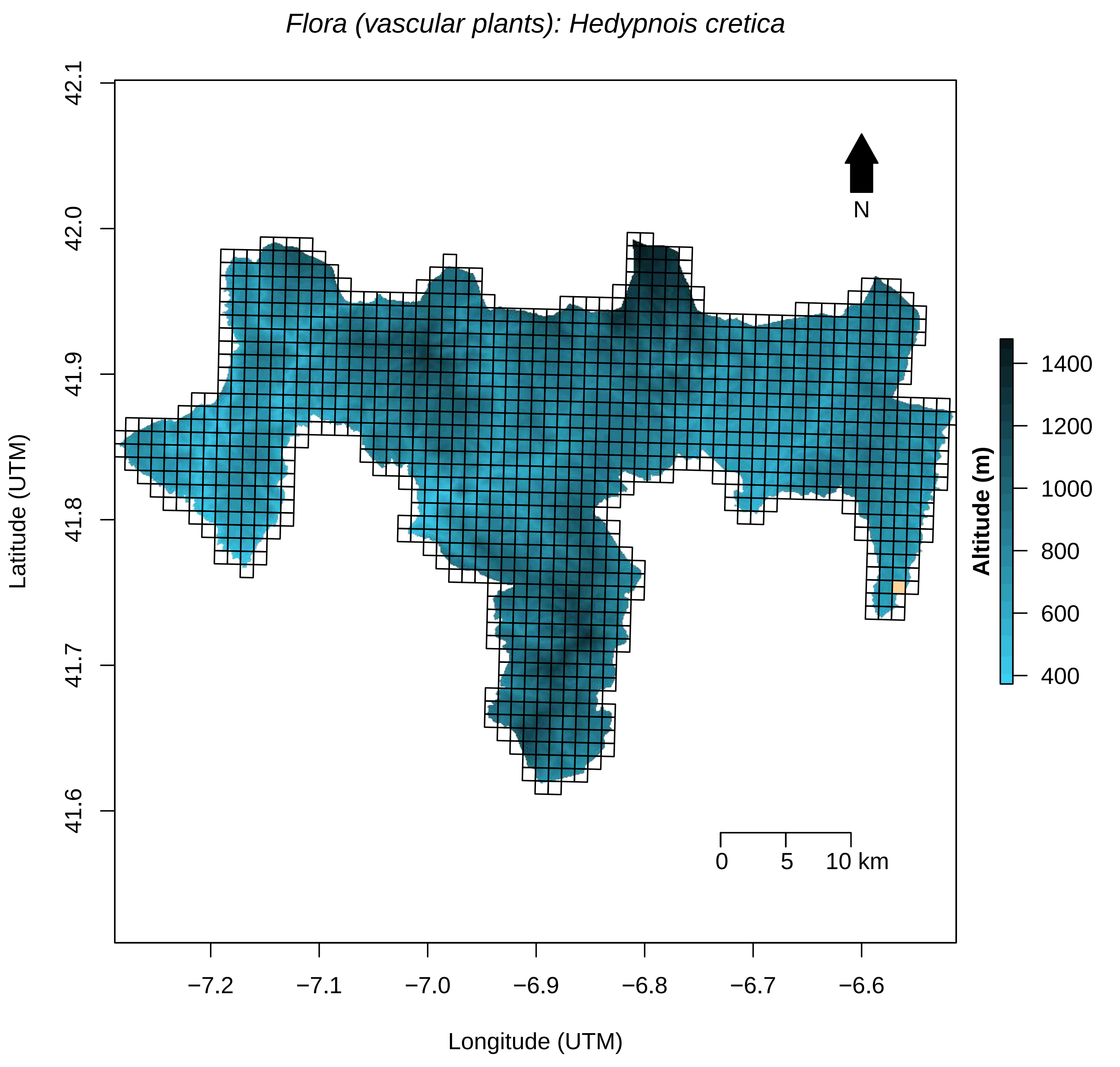

Flora (vascular plants): Hedypnois cretica
42.1
42.0
41.9
41.8
41.7
41.6
Latitude (UTM)
−7.2
−7.1
−7.0
−6.9
−6.8
−6.7
−6.6
Longitude (UTM)
N
0
5
10 km
Altitude (m)
1400
1200
1000
800
600
400

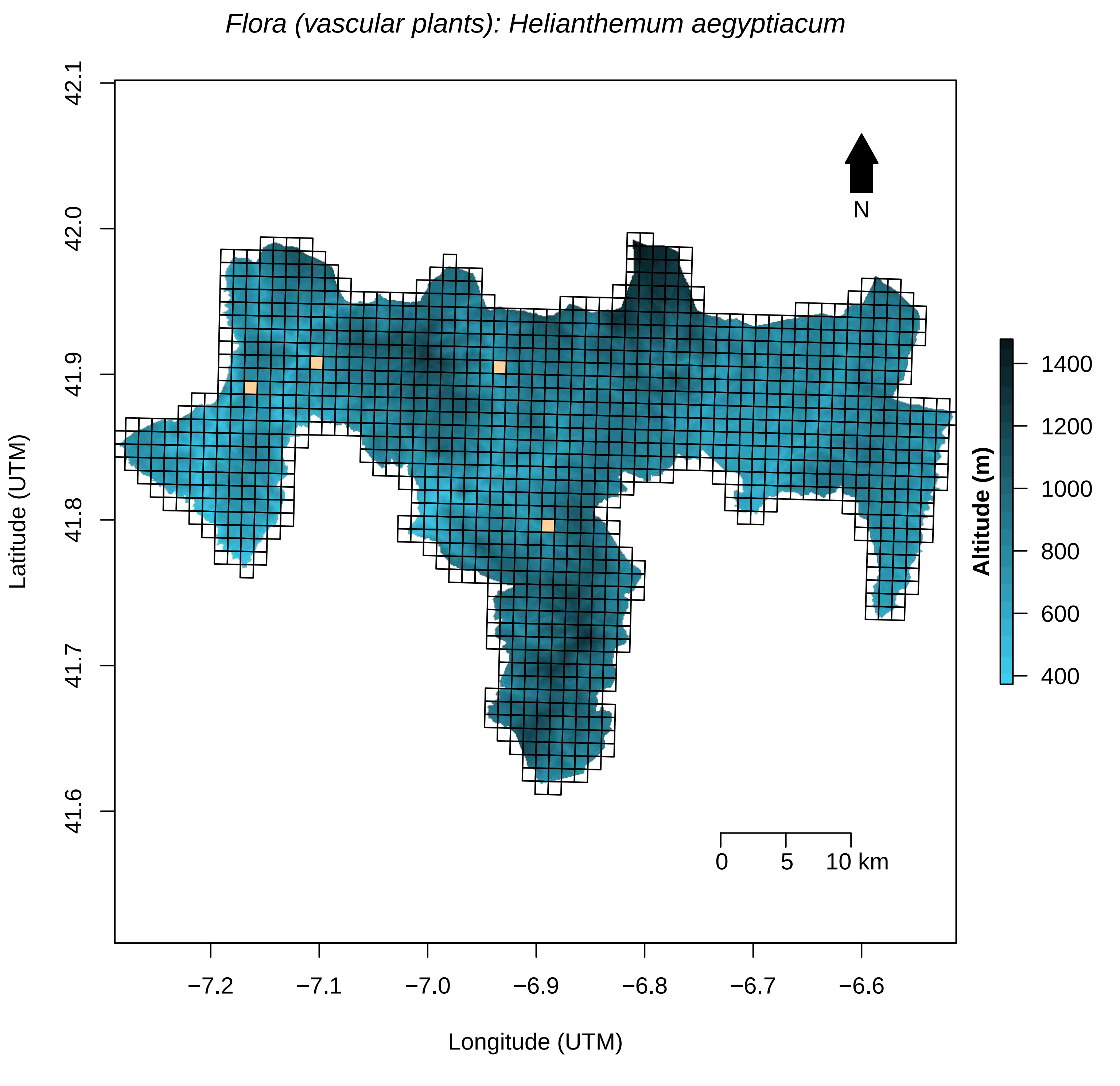

Flora (vascular plants): Helianthemum aegyptiacum
42.1
42.0
41.9
41.8
41.7
41.6
Latitude (UTM)
−7.2
−7.1
−7.0
−6.9
−6.8
−6.7
−6.6
Longitude (UTM)
N
0
5
10 km
Altitude (m)
1400
1200
1000
800
600
400

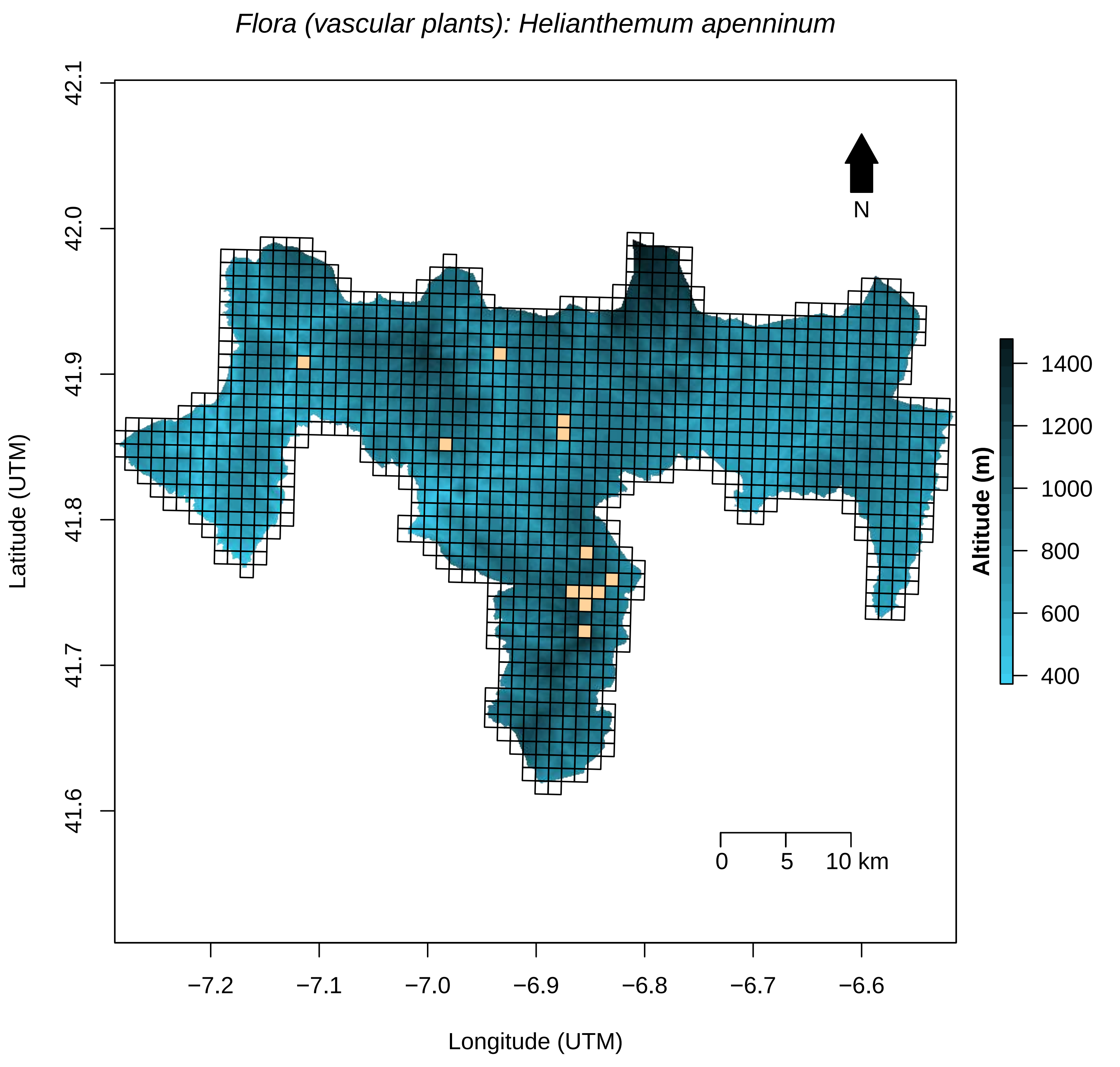

Flora (vascular plants): Helianthemum apenninum
N
0
5
10 km
Altitude (m)
1400
1200
1000
800
600
400
42.1
42.0
41.9
41.8
41.7
41.6
Latitude (UTM)
−7.2
−7.1
−7.0
−6.9
−6.8
−6.7
−6.6
Longitude (UTM)

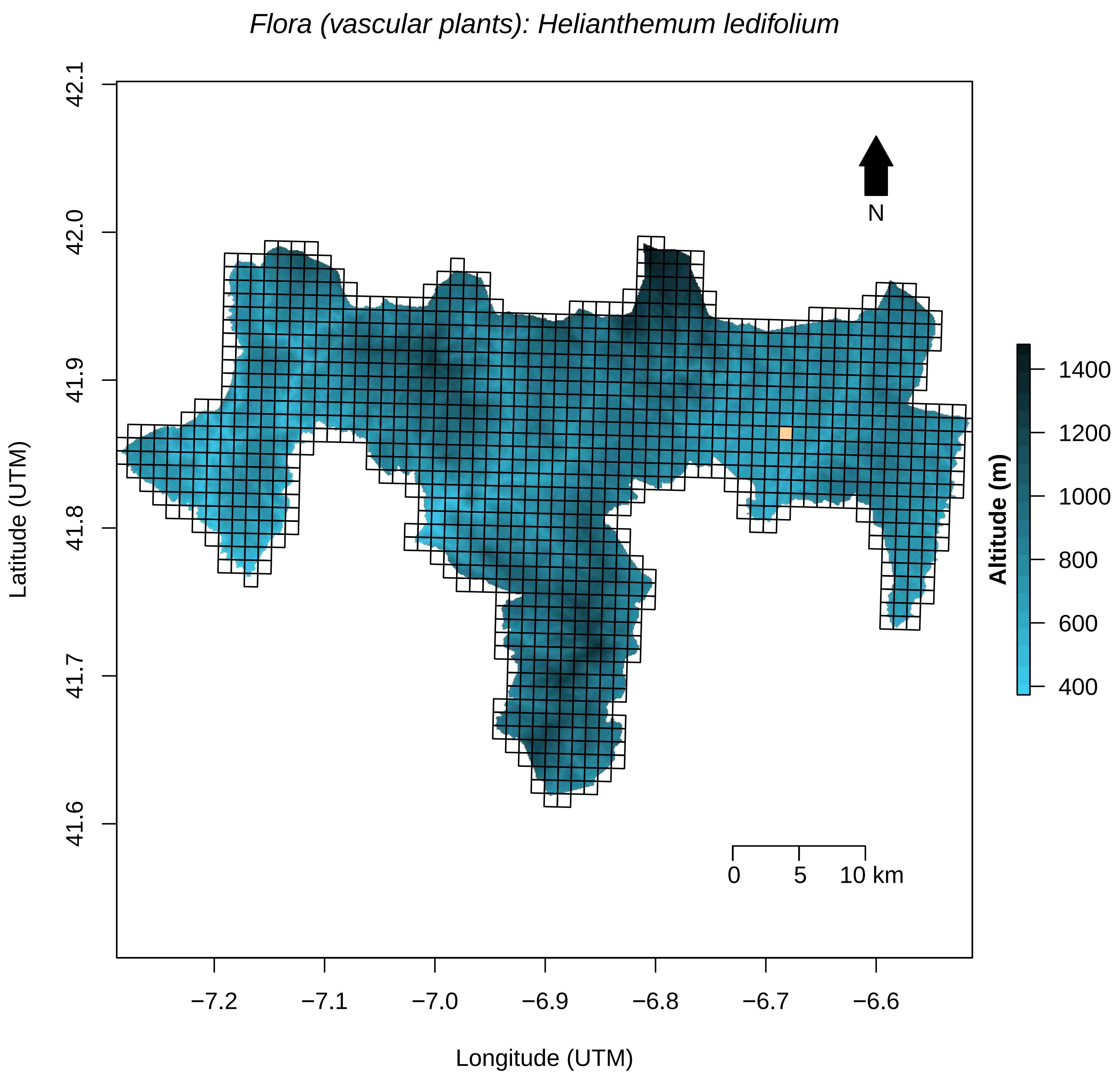

Flora (vascular plants): Helianthemum ledifolium
42.1
42.0
41.9
41.8
41.7
41.6
Latitude (UTM)
−7.2
−7.1
−7.0
−6.9
−6.8
−6.7
−6.6
Longitude (UTM)
N
0
5
10 km
Altitude (m)
1400
1200
1000
800
600
400

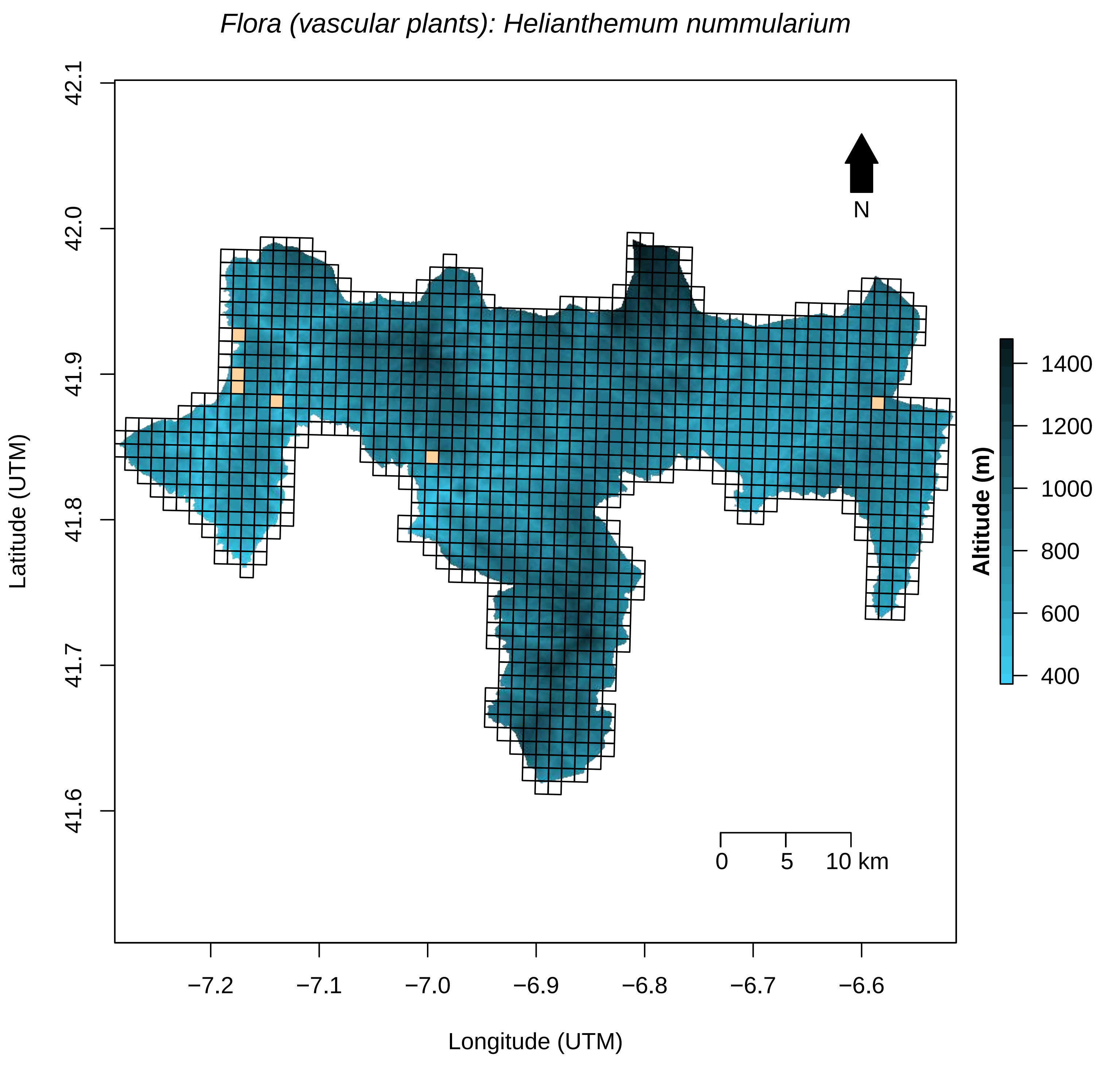

Flora (vascular plants): Helianthemum nummularium
Latitude (UTM)
Longitude (UTM)
42.1
42.0
41.9
41.8
41.7
41.6
−7.2
−7.1
−7.0
−6.9
−6.8
−6.7
−6.6
N
0
5
10 km
Altitude (m)
1400
1200
1000
800
600
400

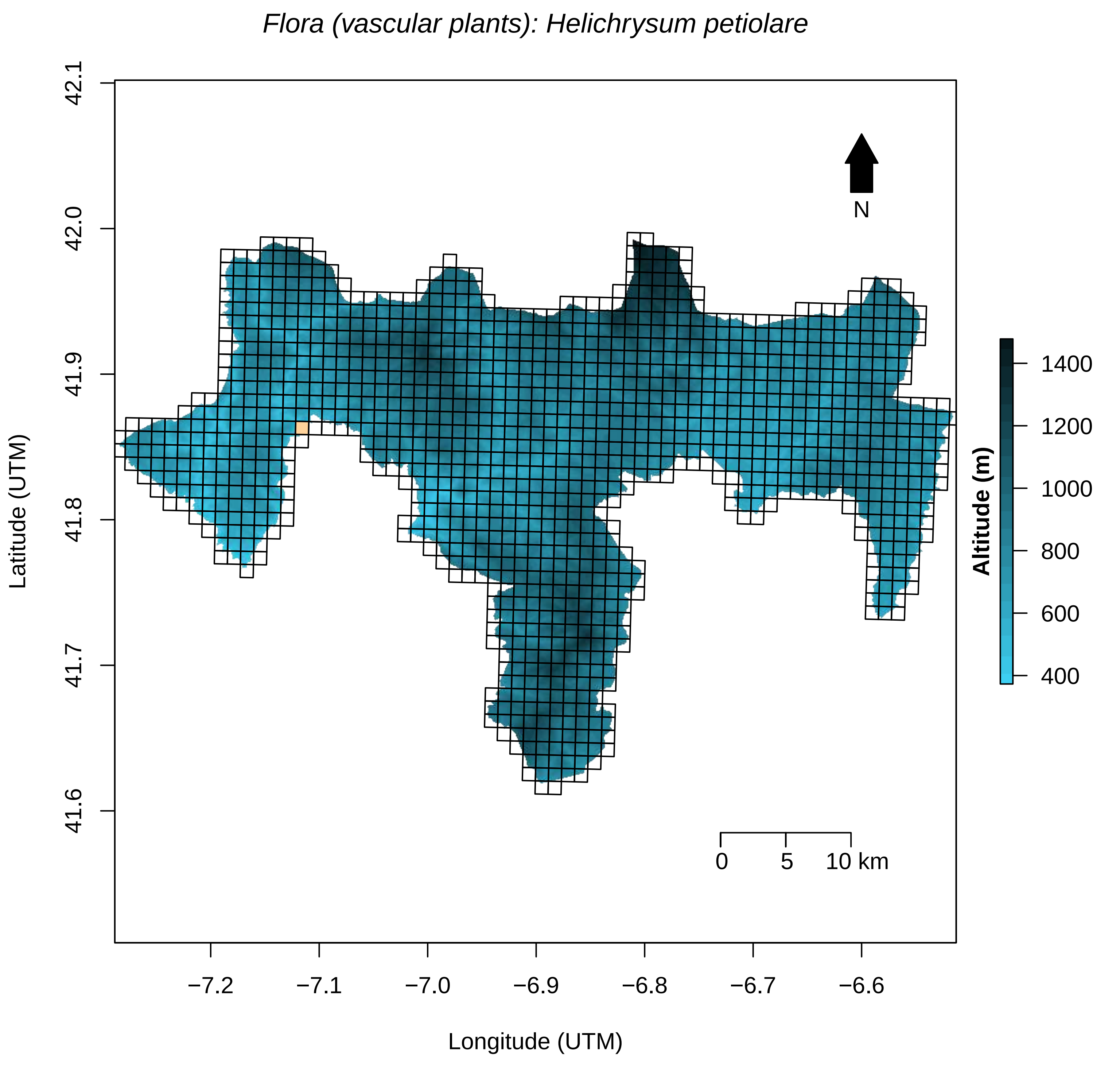

Flora (vascular plants): Helichrysum petiolare
42.1
42.0
41.9
41.8
41.7
41.6
Latitude (UTM)
−7.2
−7.1
−7.0
−6.9
−6.8
−6.7
−6.6
Longitude (UTM)
N
0
5
10 km
Altitude (m)
1400
1200
1000
800
600
400

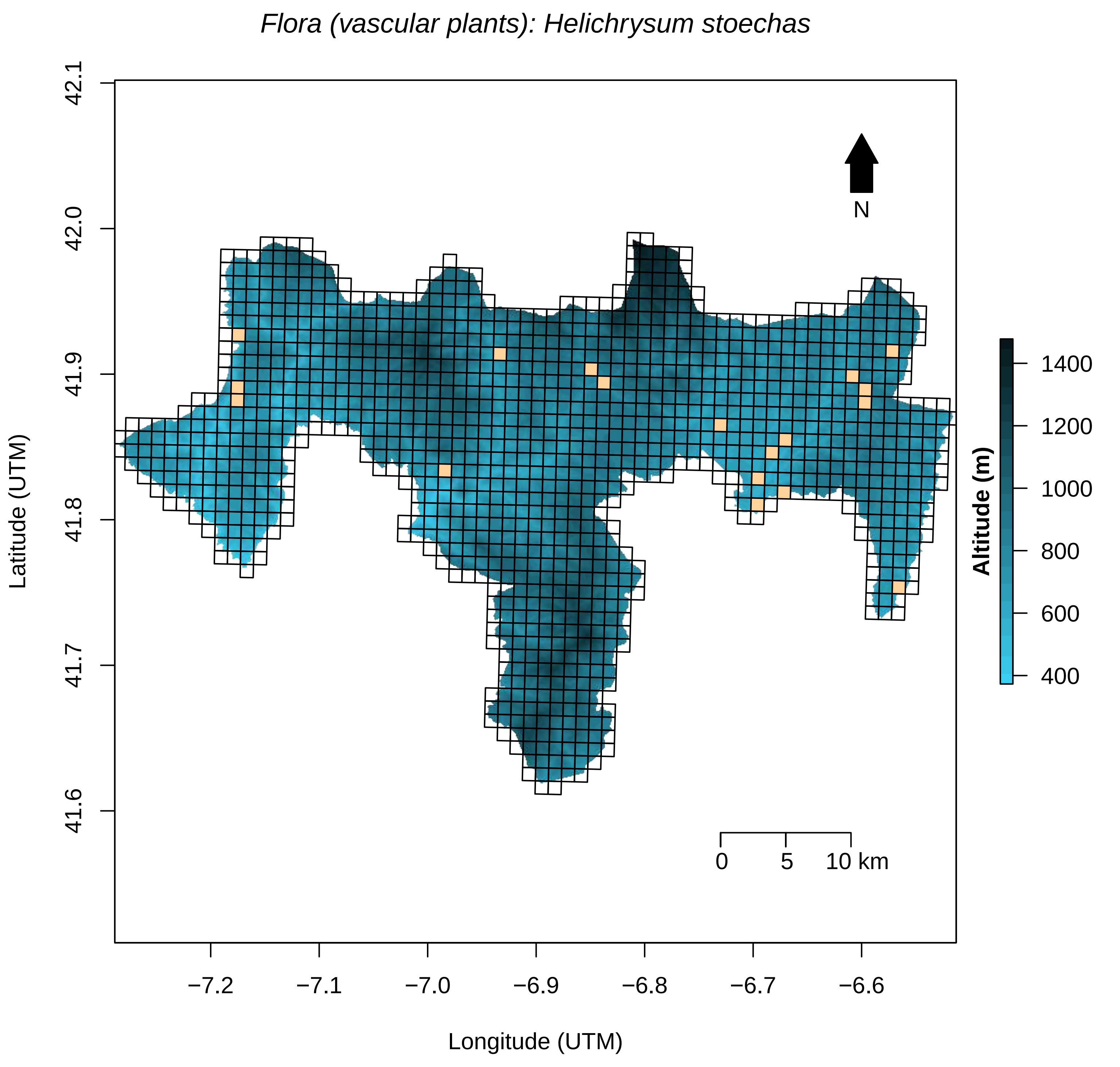
Flora (vascular plants): Helichrysum stoechas
Latitude (UTM)
42.1
42.0
41.9
41.8
41.7
41.6
Longitude (UTM)
−7.2
−7.1
−7.0
−6.9
−6.8
−6.7
−6.6
N
0
5
10 km
Altitude (m)
1400
1200
1000
800
600
400

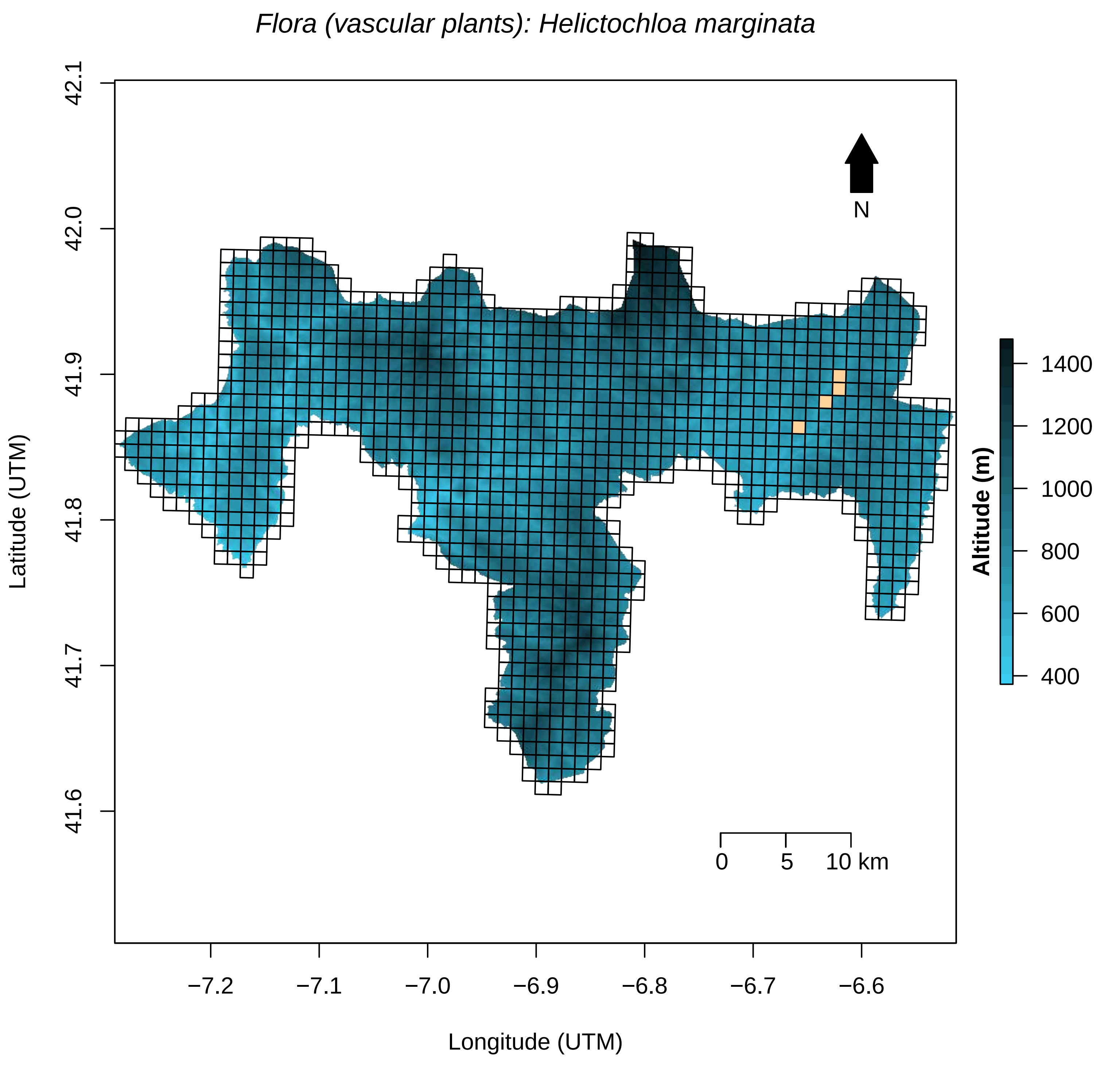

Flora (vascular plants): Helictochloa marginata
42.1
42.0
41.9
41.8
41.7
41.6
Latitude (UTM)
−7.2
−7.1
−7.0
−6.9
−6.8
−6.7
−6.6
Longitude (UTM)
N
0
5
10 km
Altitude (m)
1400
1200
1000
800
600
400

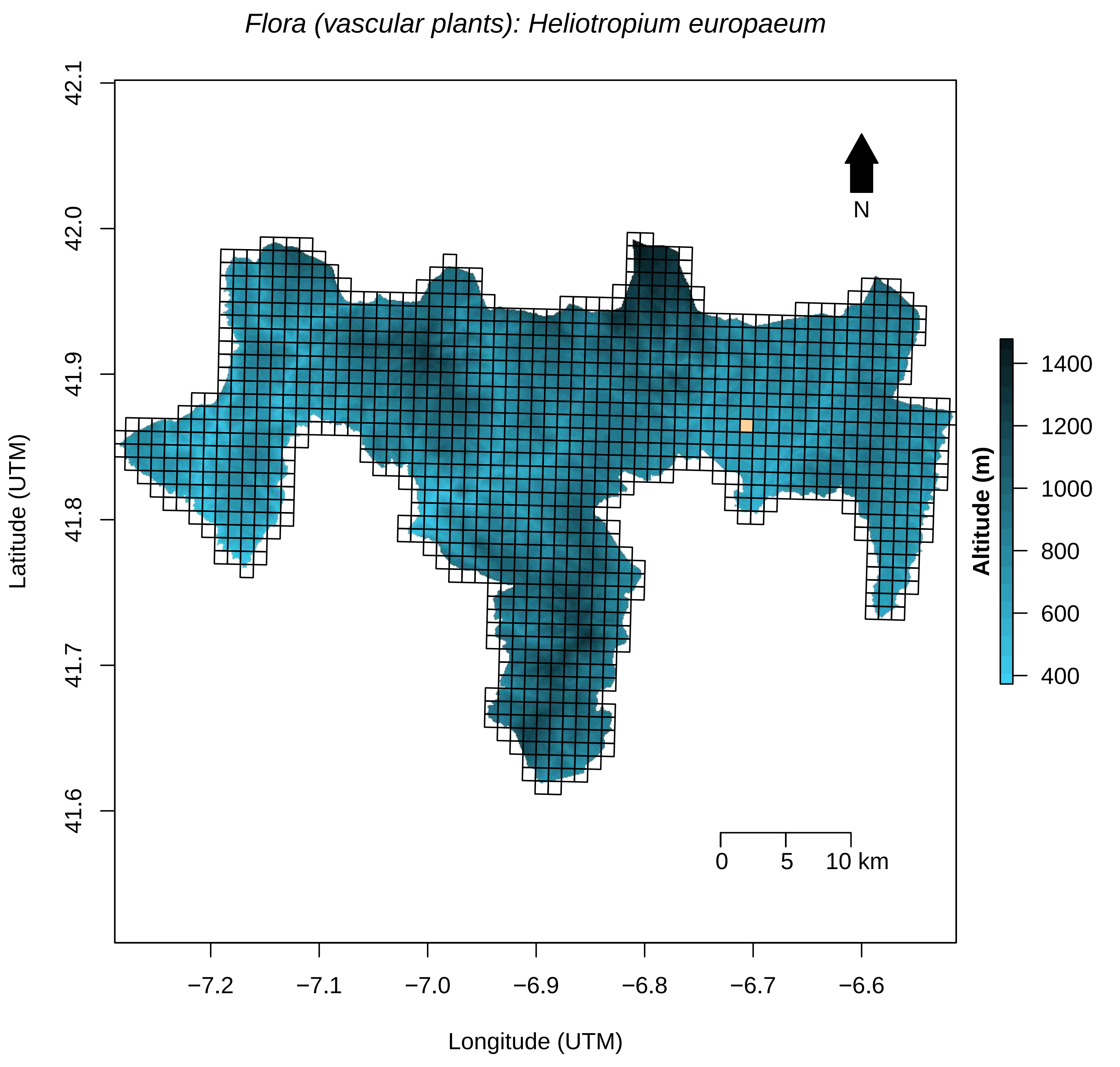

Flora (vascular plants): Heliotropium europaeum
Latitude (UTM)
42.1
42.0
41.9
41.8
41.7
41.6
Longitude (UTM)
−7.2
−7.1
−7.0
−6.9
−6.8
−6.7
−6.6
N
0
5
10 km
Altitude (m)
1400
1200
1000
800
600
400

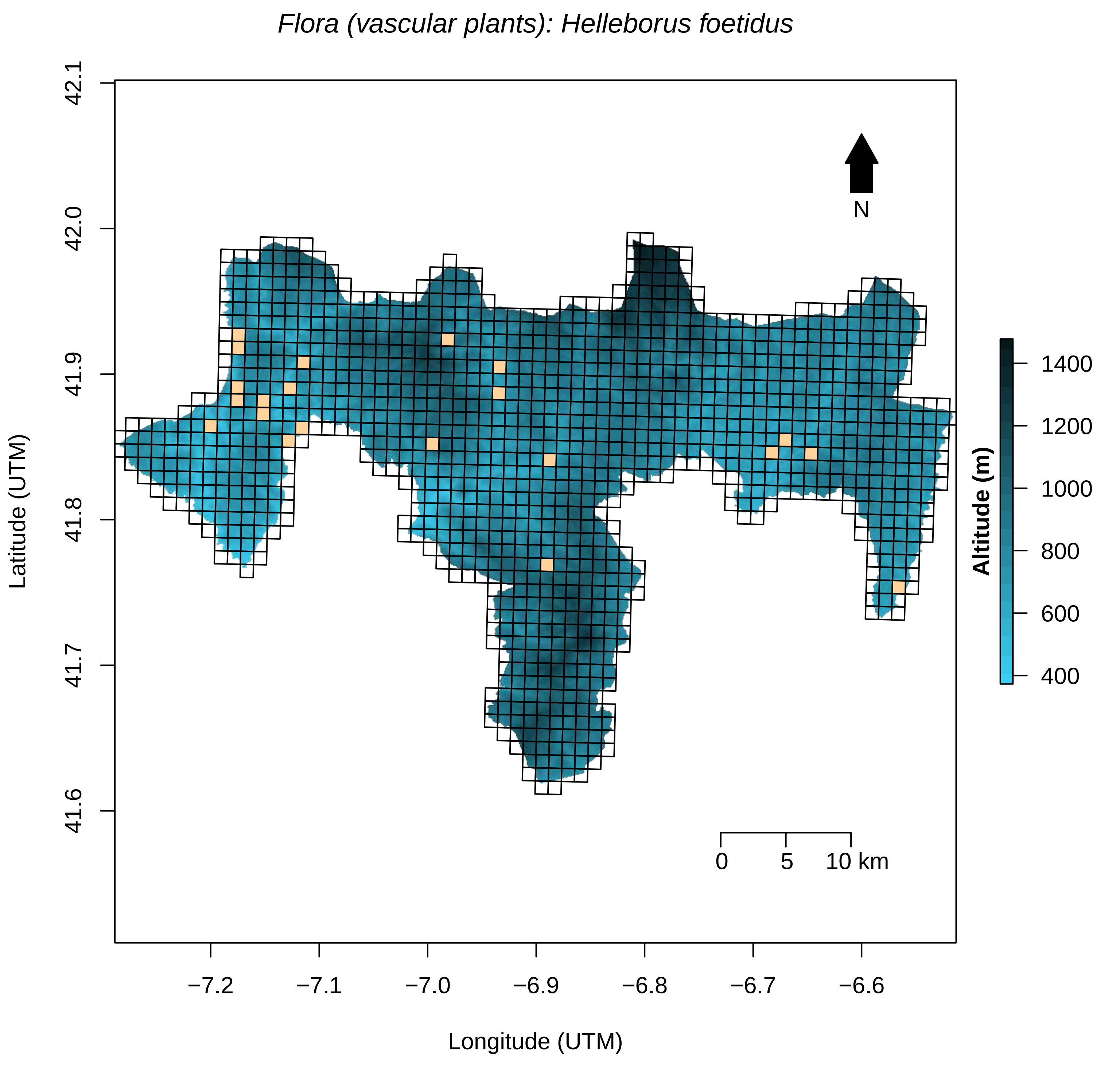

Flora (vascular plants): Helleborus foetidus
N
0
5
10 km
Altitude (m)
1400
1200
1000
800
600
400
42.1
42.0
41.9
41.8
41.7
41.6
Latitude (UTM)
−7.2
−7.1
−7.0
−6.9
−6.8
−6.7
−6.6
Longitude (UTM)

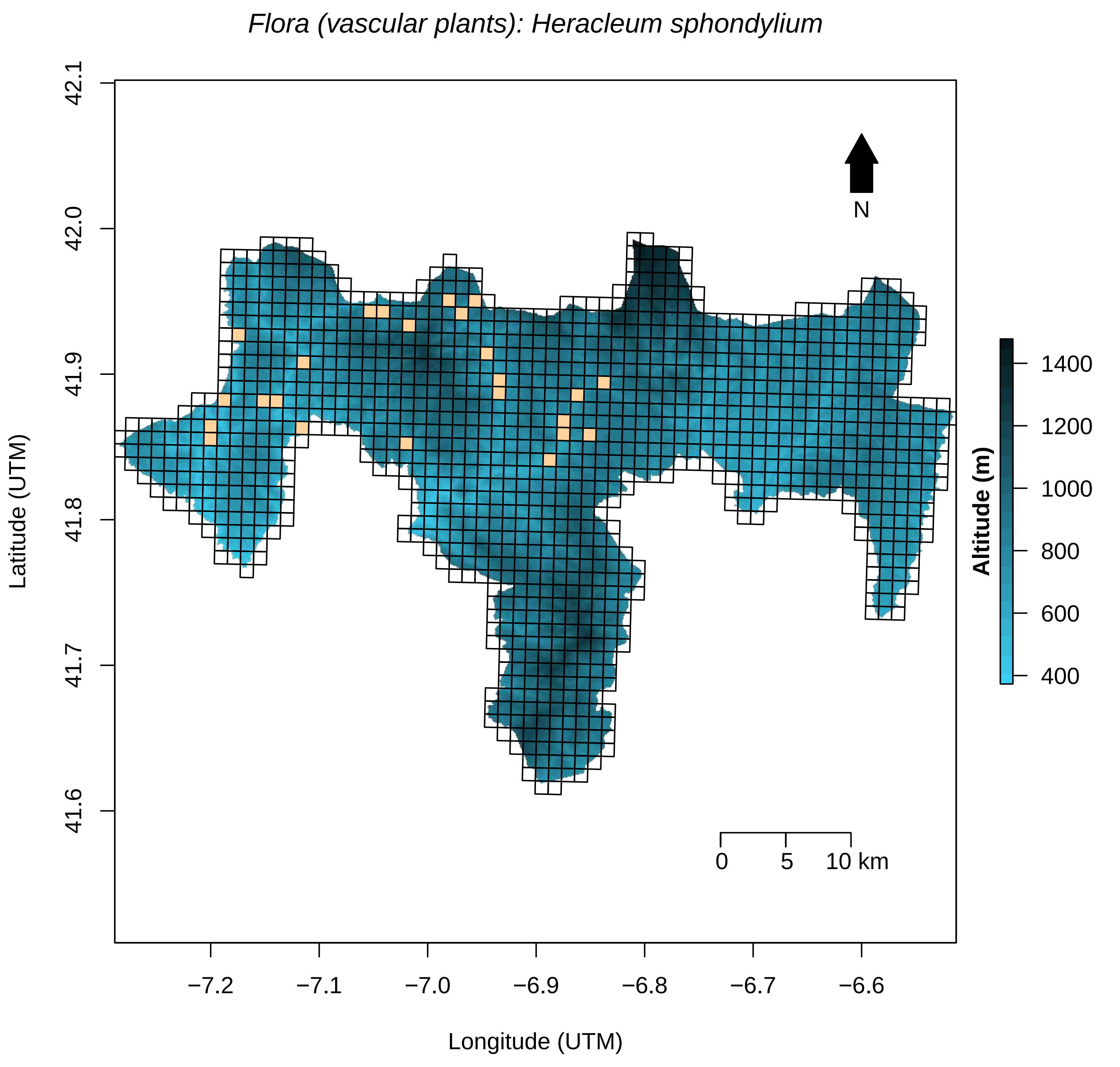

Flora (vascular plants): Heracleum sphondylium
N
0
5
10 km
Altitude (m)
1400
1200
1000
800
600
400
Latitude (UTM)
42.1
42.0
41.9
41.8
41.7
41.6
Longitude (UTM)
−7.2
−7.1
−7.0
−6.9
−6.8
−6.7
−6.6

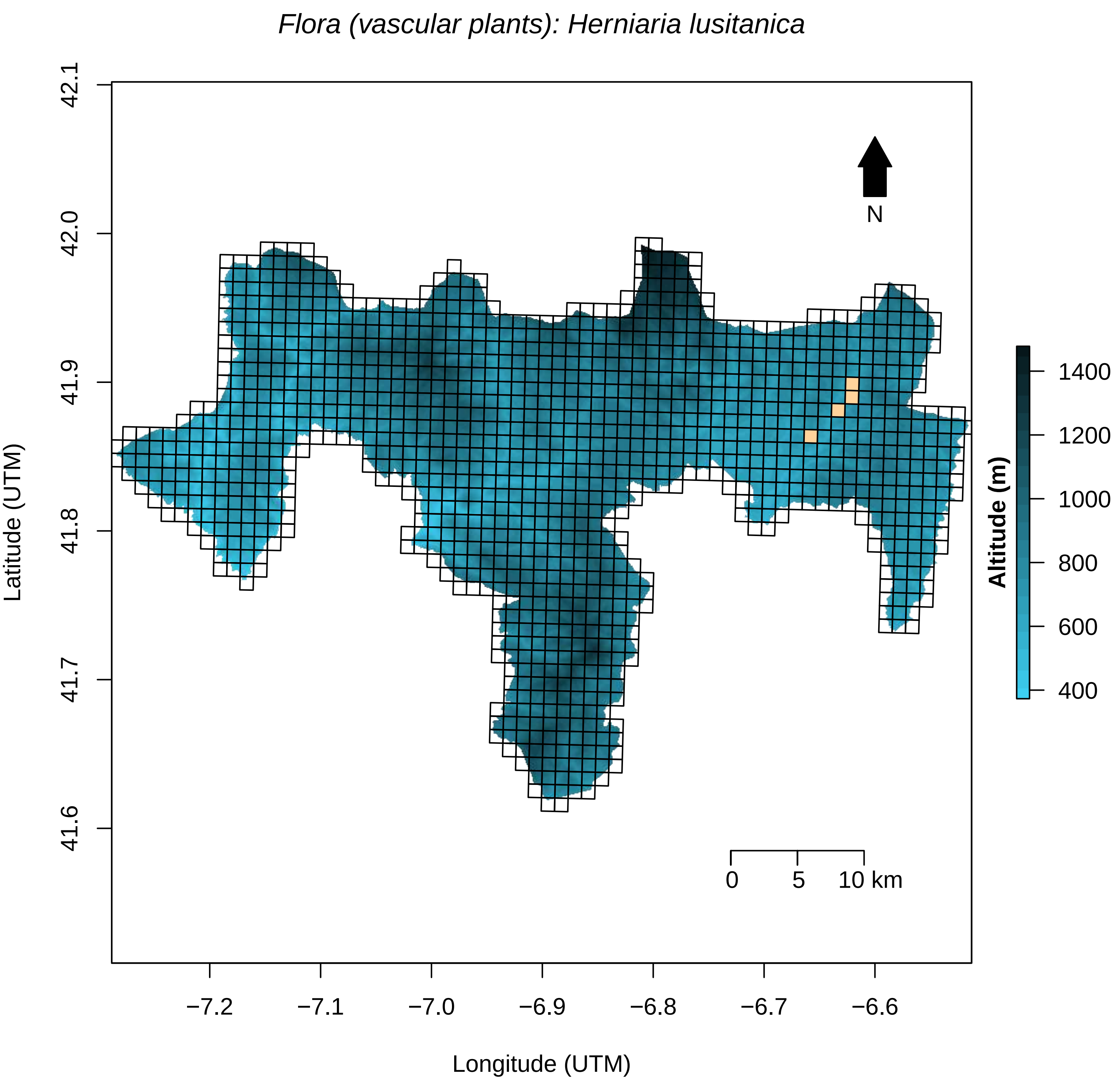

Flora (vascular plants): Herniaria lusitanica
N
0 5 10 km
Altitude (m)
1400
1200
1000
800
600
400
42.1
42.0
41.9
41.8
41.7
41.6
Latitude (UTM)
−7.2
−7.1
−7.0
−6.9
−6.8
−6.7
−6.6
Longitude (UTM)

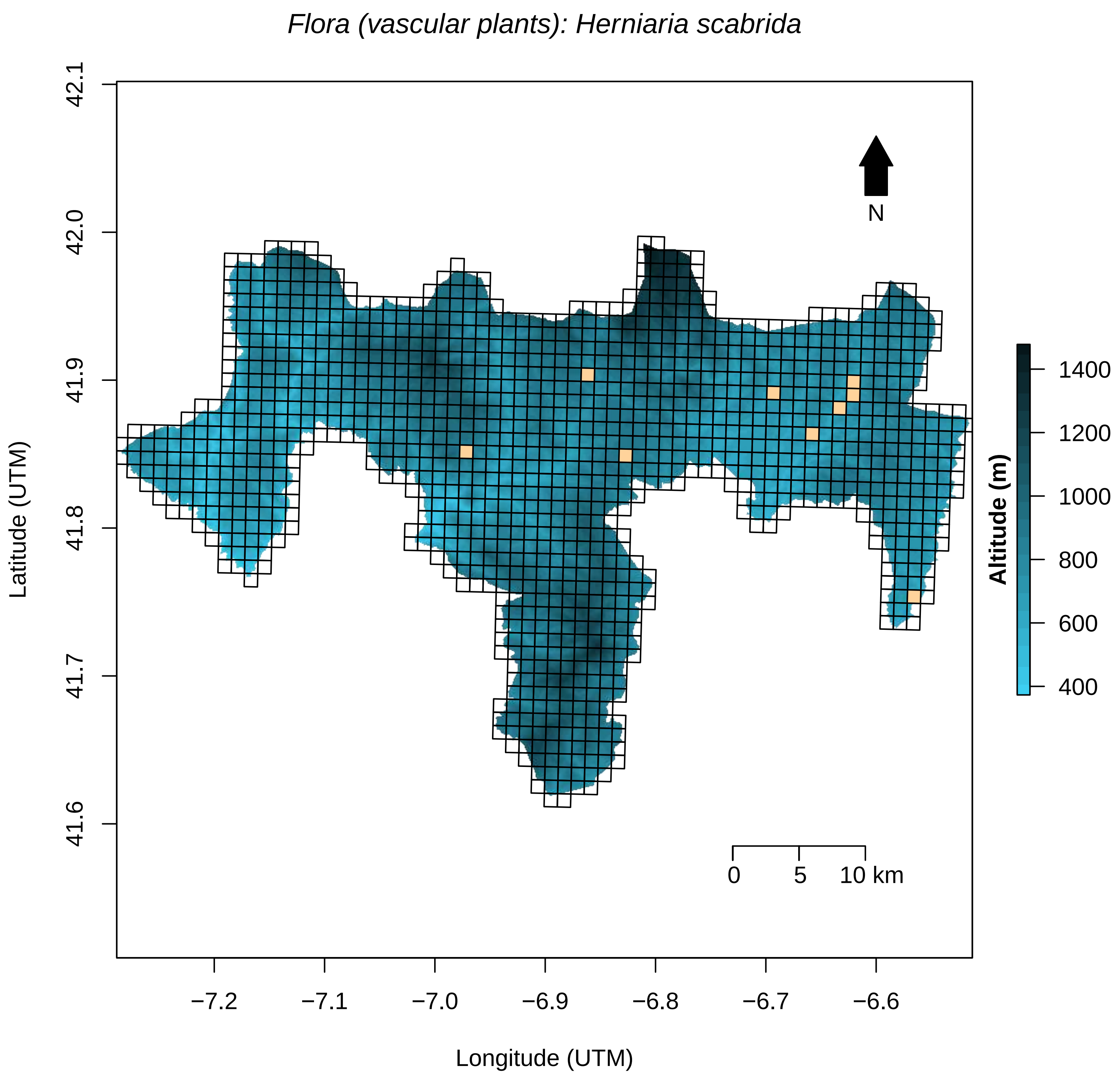

Flora (vascular plants): Herniaria scabrida
42.1
42.0
41.9
41.8
41.7
41.6
Latitude (UTM)
−7.2
−7.1
−7.0
−6.9
−6.8
−6.7
−6.6
Longitude (UTM)
N
0
5
10 km
Altitude (m)
1400
1200
1000
800
600
400

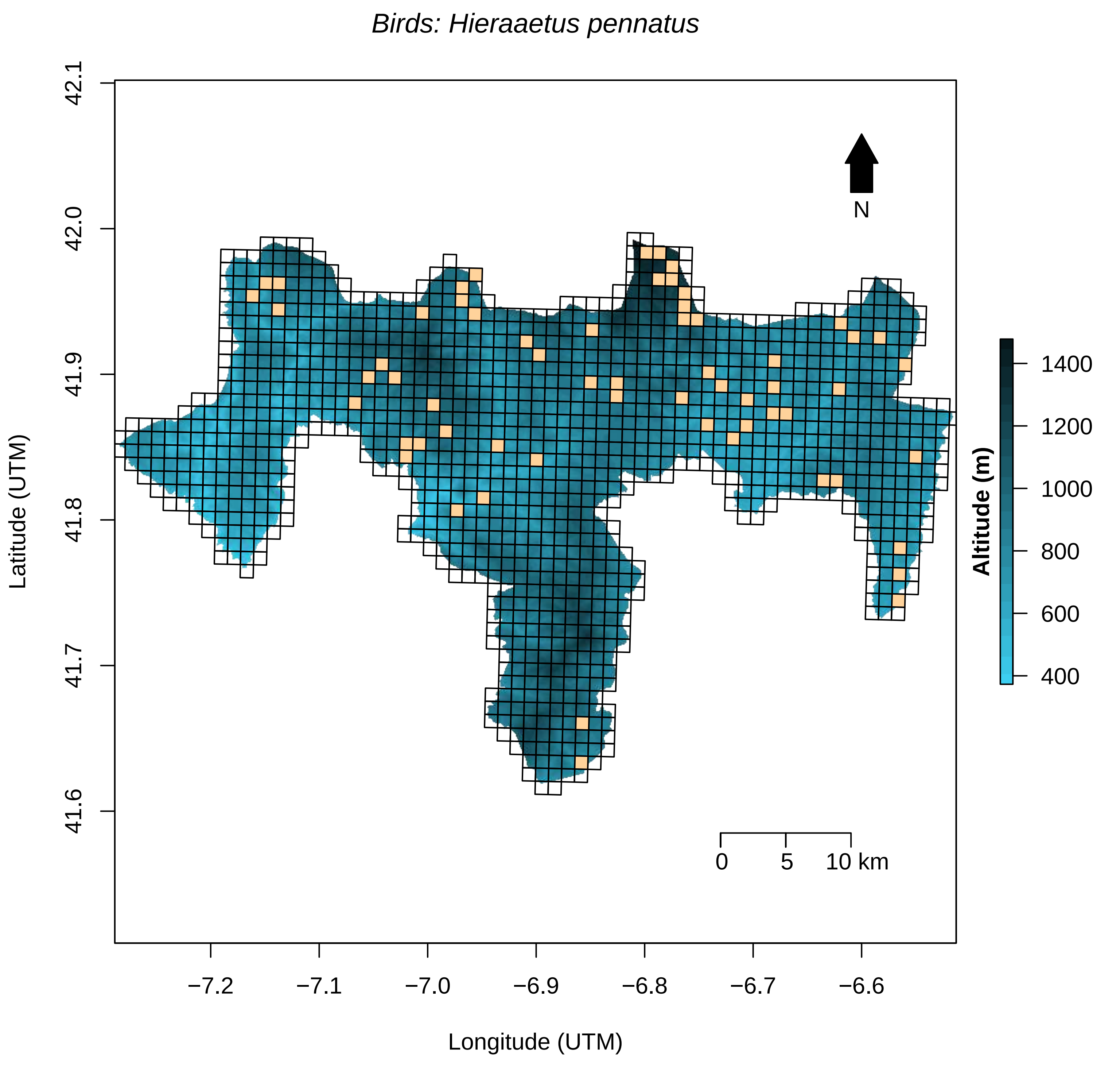
Birds: Hieraaetus pennatus
N
0 5 10 km
Altitude (m)
1400
1200
1000
800
600
400
Latitude (UTM)
42.1
42.0
41.9
41.8
41.7
41.6
Longitude (UTM)
−7.2
−7.1
−7.0
−6.9
−6.8
−6.7
−6.6

Flora (vascular plants): *Hieracium murorum*

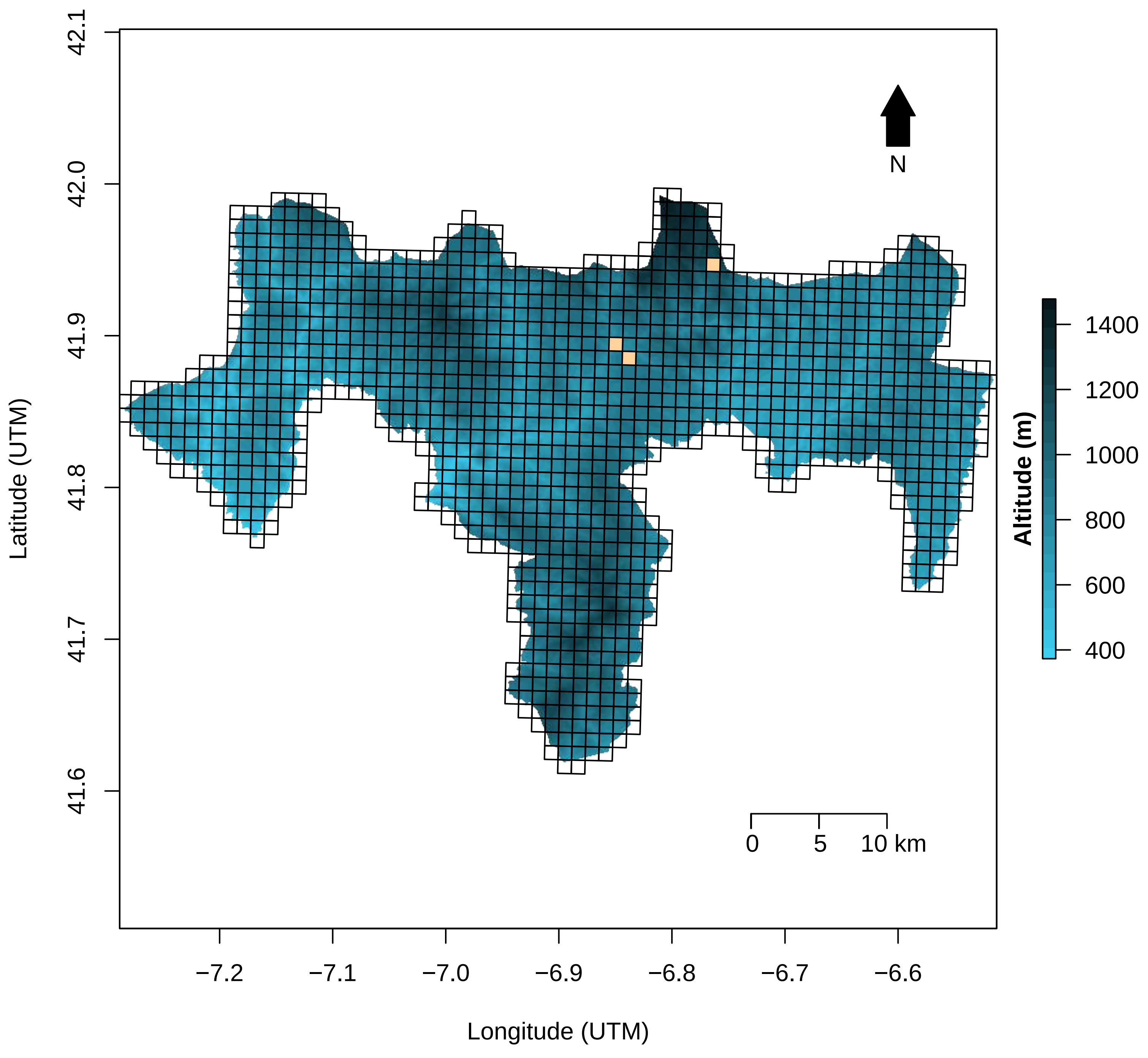

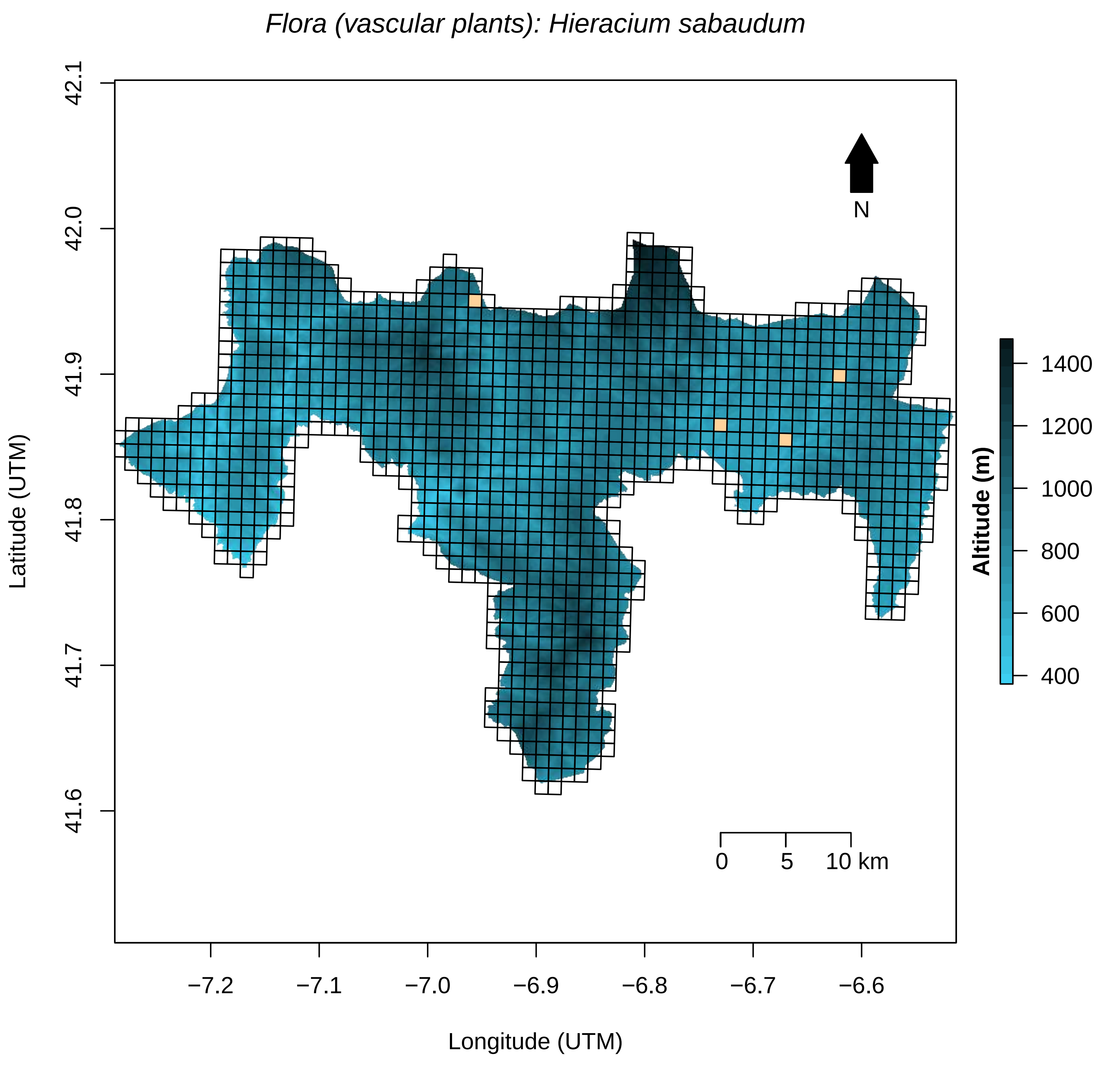

Flora (vascular plants): Hieracium sabaudum
Latitude (UTM)
Longitude (UTM)
42.1
42.0
41.9
41.8
41.7
41.6
−7.2
−7.1
−7.0
−6.9
−6.8
−6.7
−6.6
N
0
5
10 km
Altitude (m)
1400
1200
1000
800
600
400

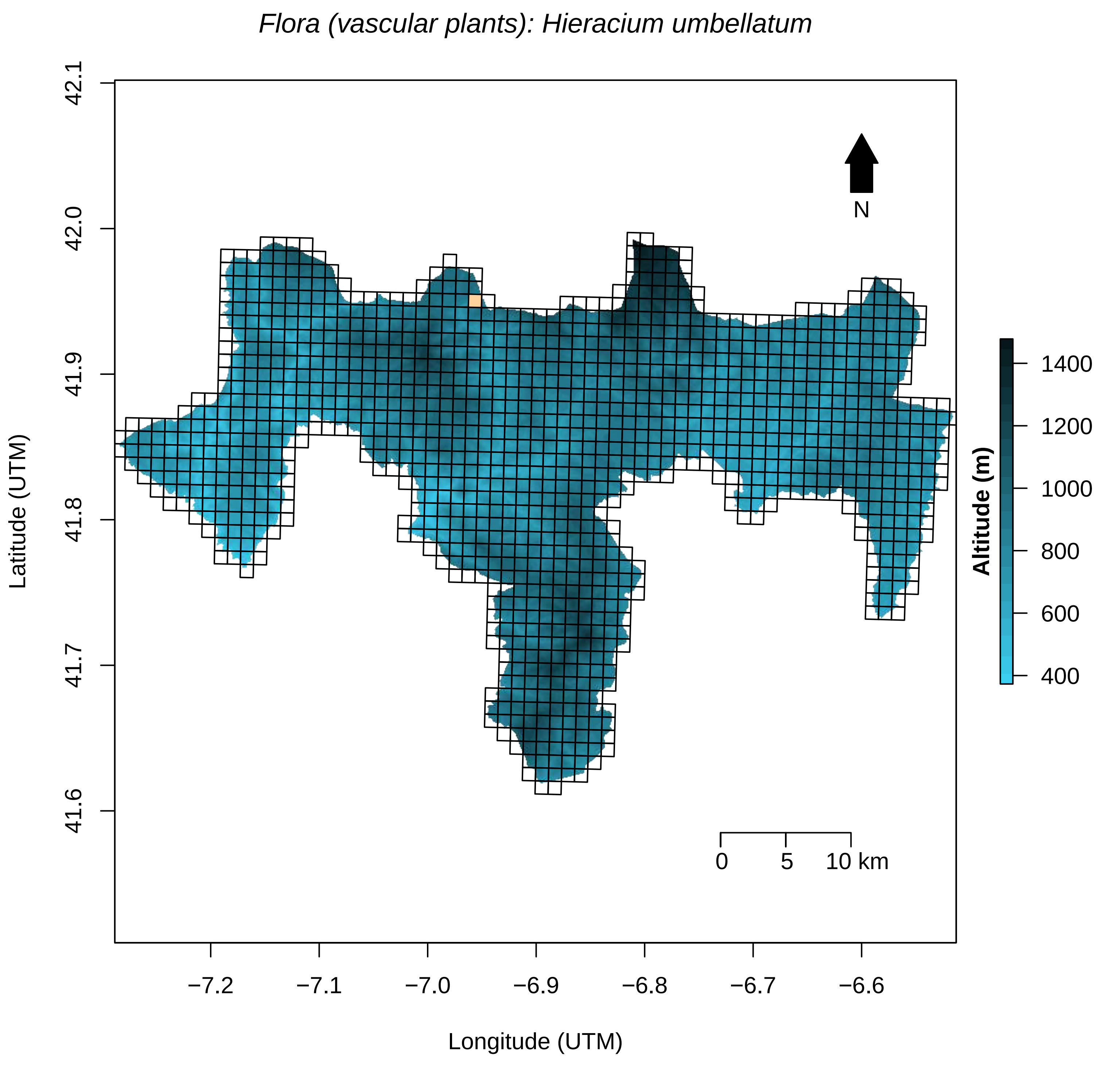
Flora (vascular plants): Hieracium umbellatum
Latitude (UTM)
Longitude (UTM)
42.1
42.0
41.9
41.8
41.7
41.6
−7.2
−7.1
−7.0
−6.9
−6.8
−6.7
−6.6
N
0
5
10 km
Altitude (m)
1400
1200
1000
800
600
400

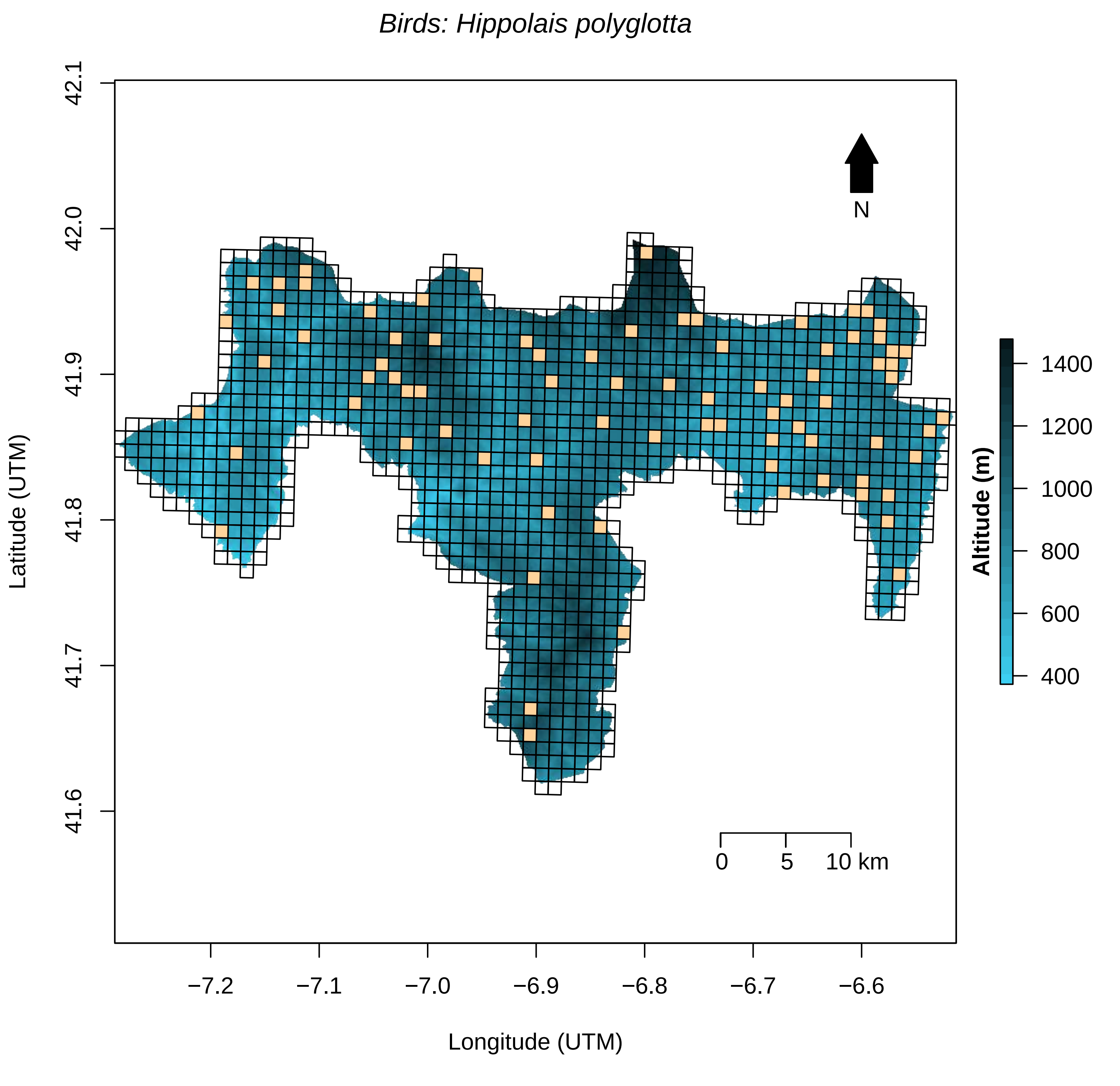

Birds: Hippolais polyglotta
Latitude (UTM)
Longitude (UTM)
42.1
42.0
41.9
41.8
41.7
41.6
−7.2
−7.1
−7.0
−6.9
−6.8
−6.7
−6.6
N
0
5
10 km
Altitude (m)
1400
1200
1000
800
600
400

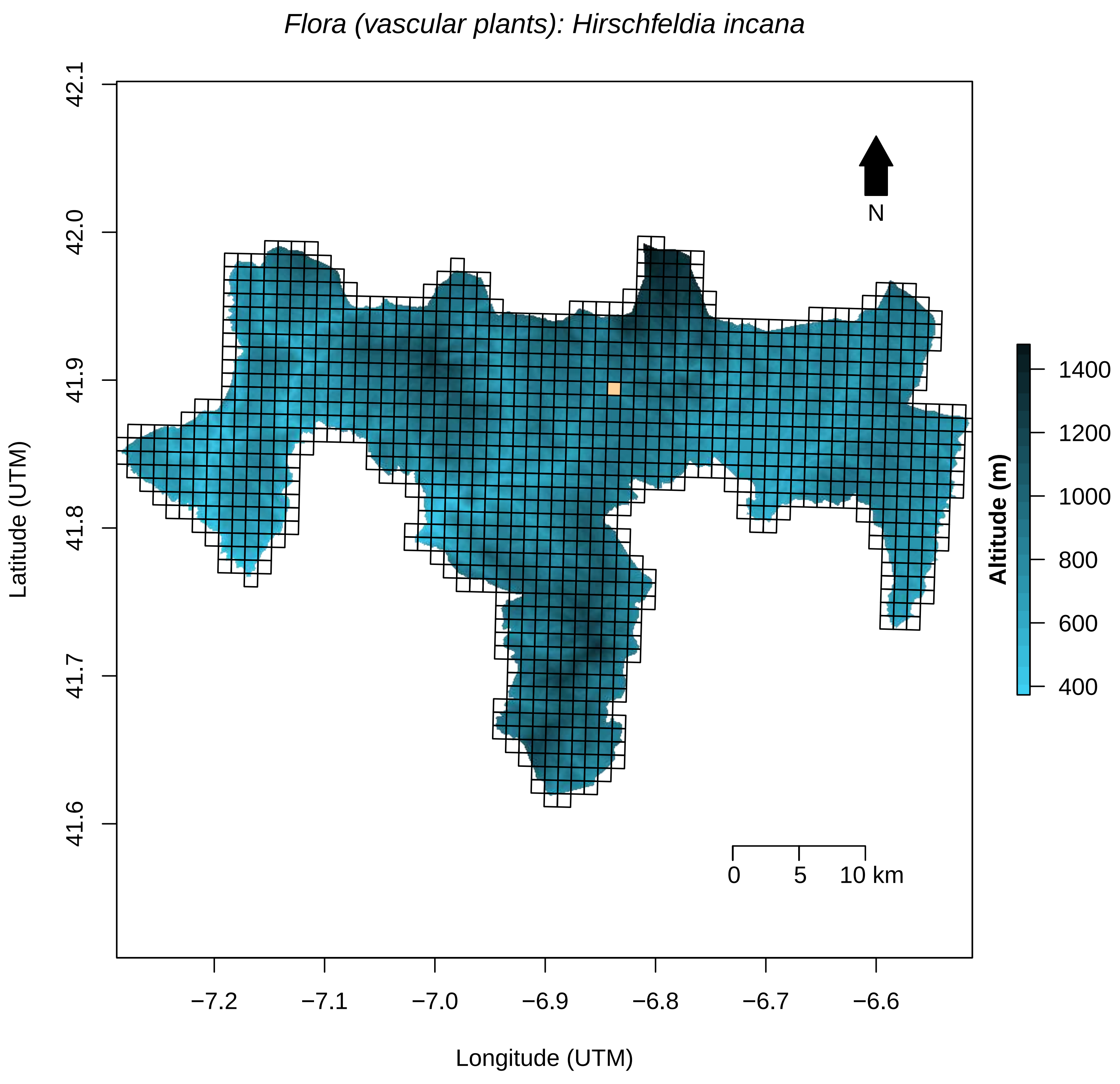

Flora (vascular plants): Hirschfeldia incana
42.1
42.0
41.9
41.8
41.7
41.6
Latitude (UTM)
−7.2
−7.1
−7.0
−6.9
−6.8
−6.7
−6.6
Longitude (UTM)
N
0
5
10 km
Altitude (m)
1400
1200
1000
800
600
400

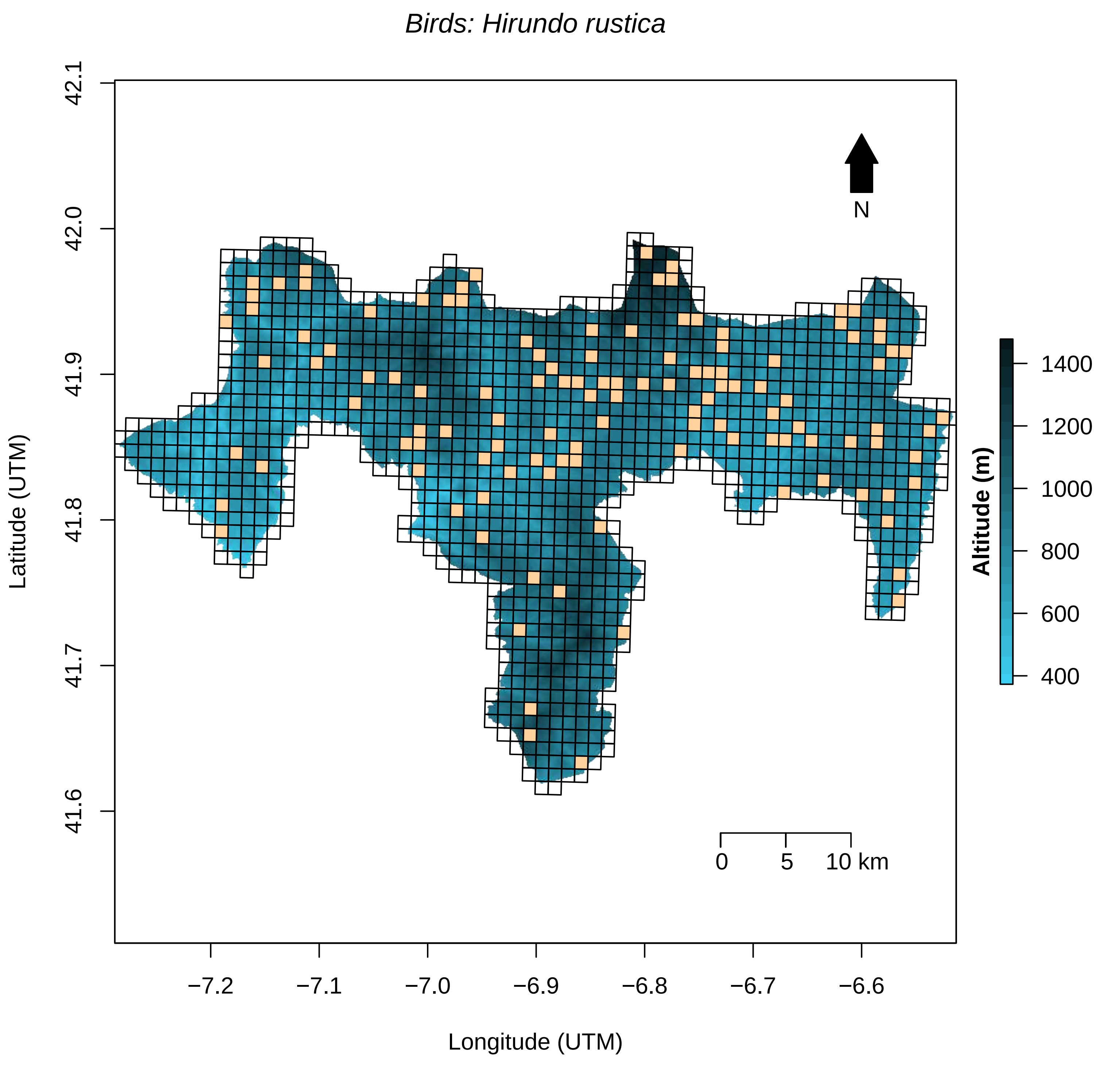
Birds: Hirundo rustica
N
0 5 10 km
Altitude (m)
1400
1200
1000
800
600
400
Latitude (UTM)
42.1
42.0
41.9
41.8
41.7
41.6
Longitude (UTM)
−7.2
−7.1
−7.0
−6.9
−6.8
−6.7
−6.6

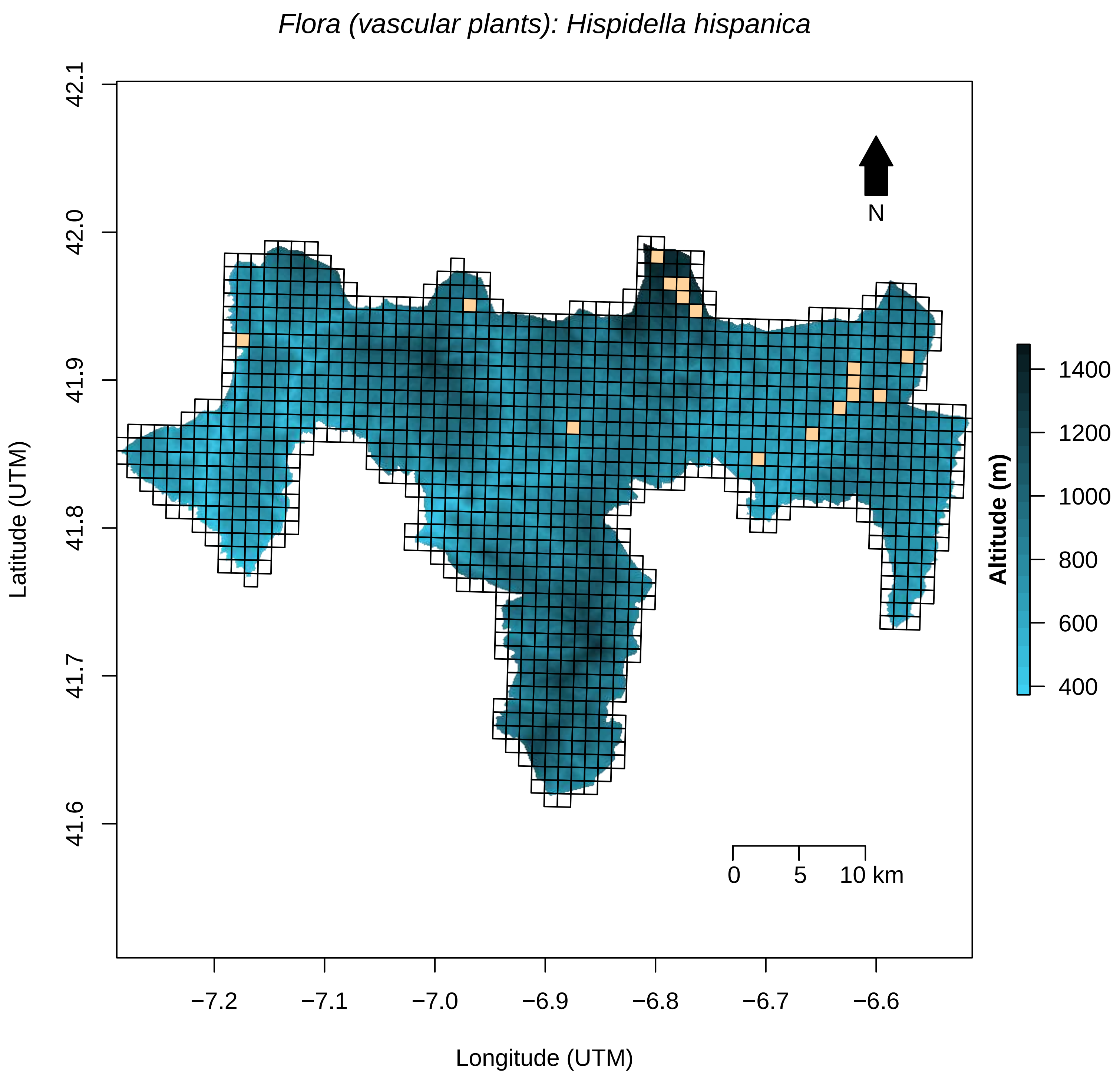

Flora (vascular plants): Hispidella hispanica
N
0 5 10 km
Altitude (m)
1400
1200
1000
800
600
400
Latitude (UTM)
42.1
42.0
41.9
41.8
41.7
41.6
Longitude (UTM)
−7.2
−7.1
−7.0
−6.9
−6.8
−6.7
−6.6

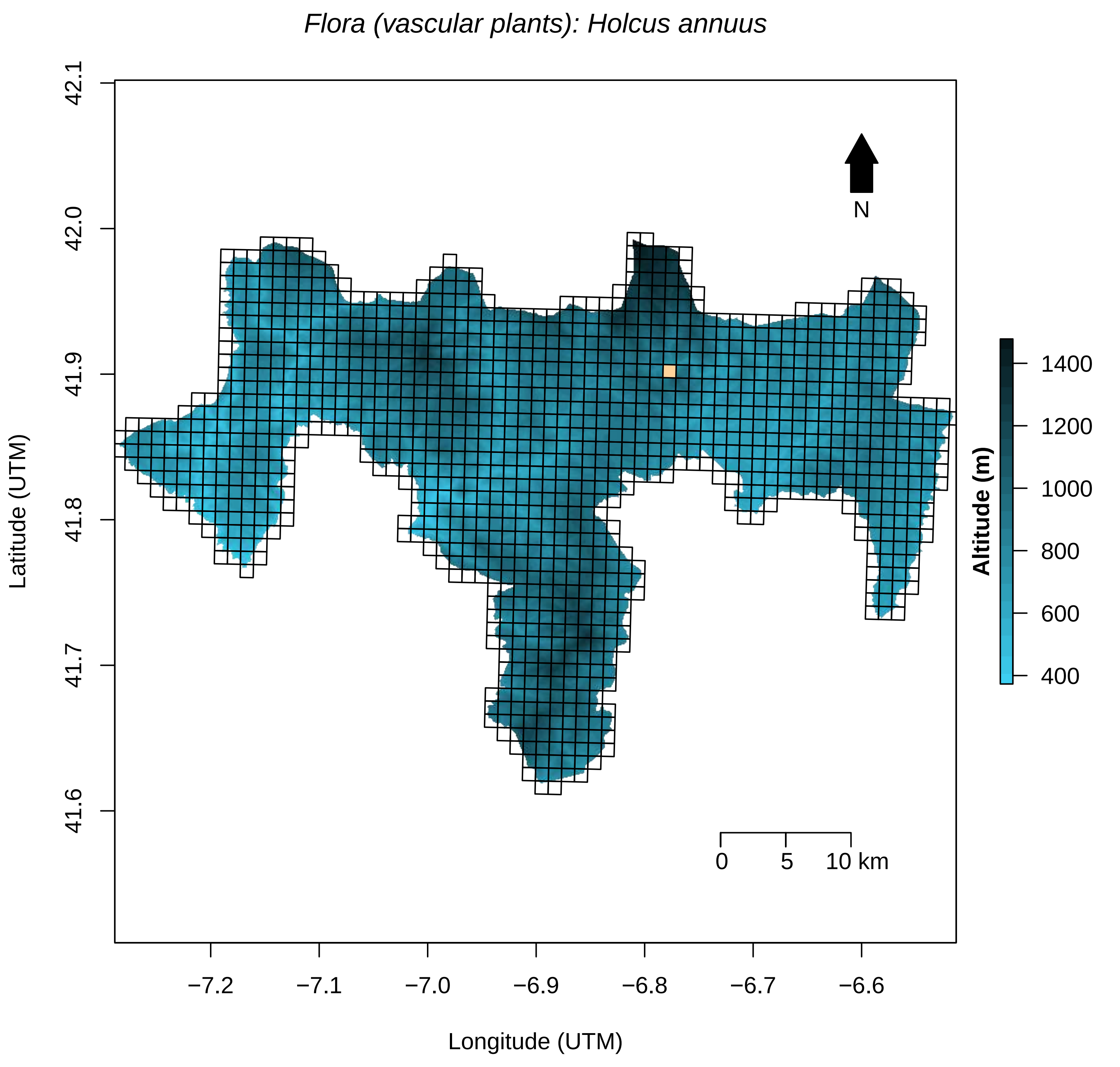

Flora (vascular plants): Holcus annuus
Latitude (UTM)
42.1
42.0
41.9
41.8
41.7
41.6
Longitude (UTM)
−7.2
−7.1
−7.0
−6.9
−6.8
−6.7
−6.6
N
0
5
10 km
Altitude (m)
1400
1200
1000
800
600
400

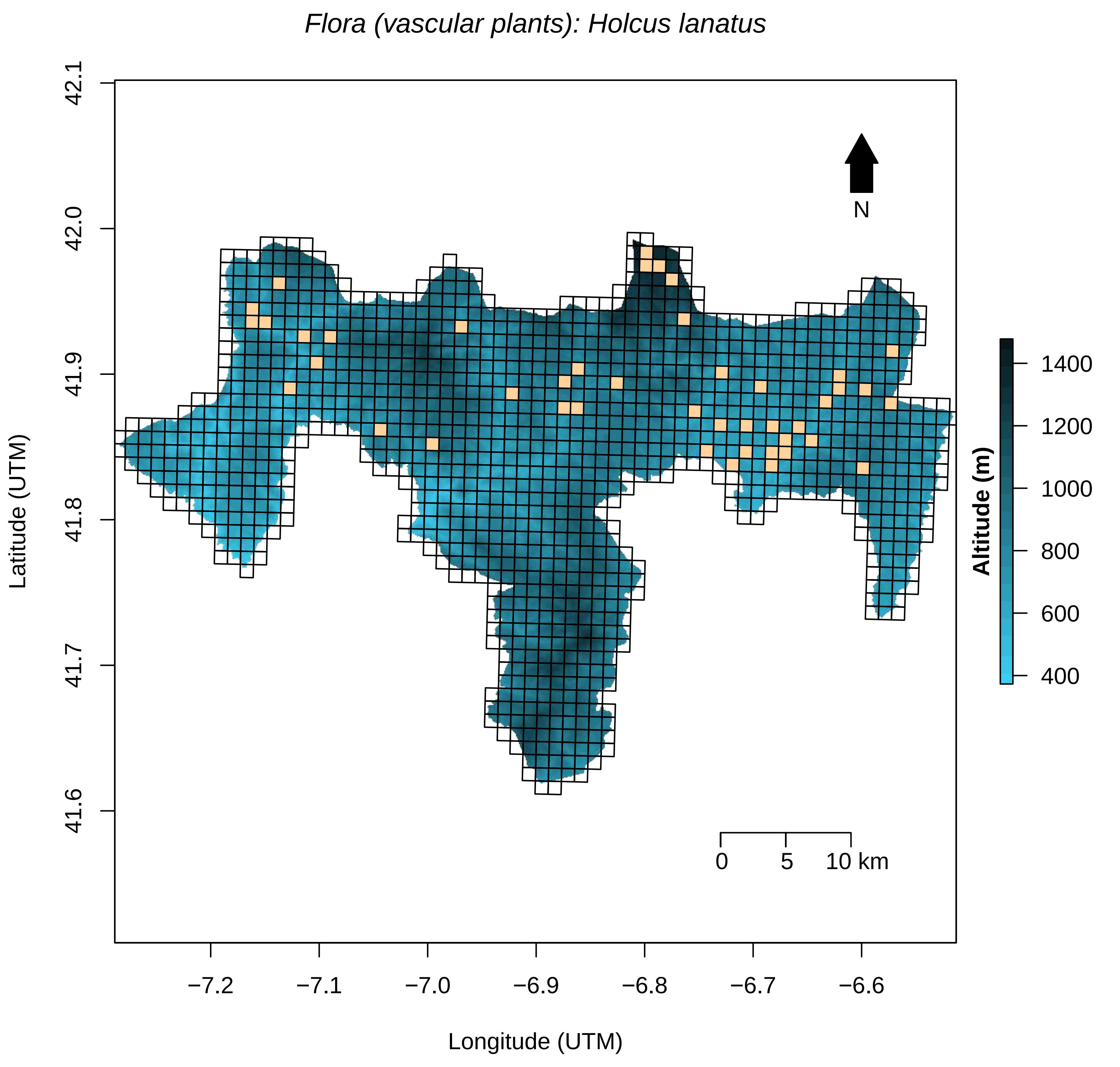

Flora (vascular plants): Holcus lanatus
Latitude (UTM)
Longitude (UTM)
42.1
42.0
41.9
41.8
41.7
41.6
−7.2
−7.1
−7.0
−6.9
−6.8
−6.7
−6.6
N
0
5
10 km
Altitude (m)
1400
1200
1000
800
600
400

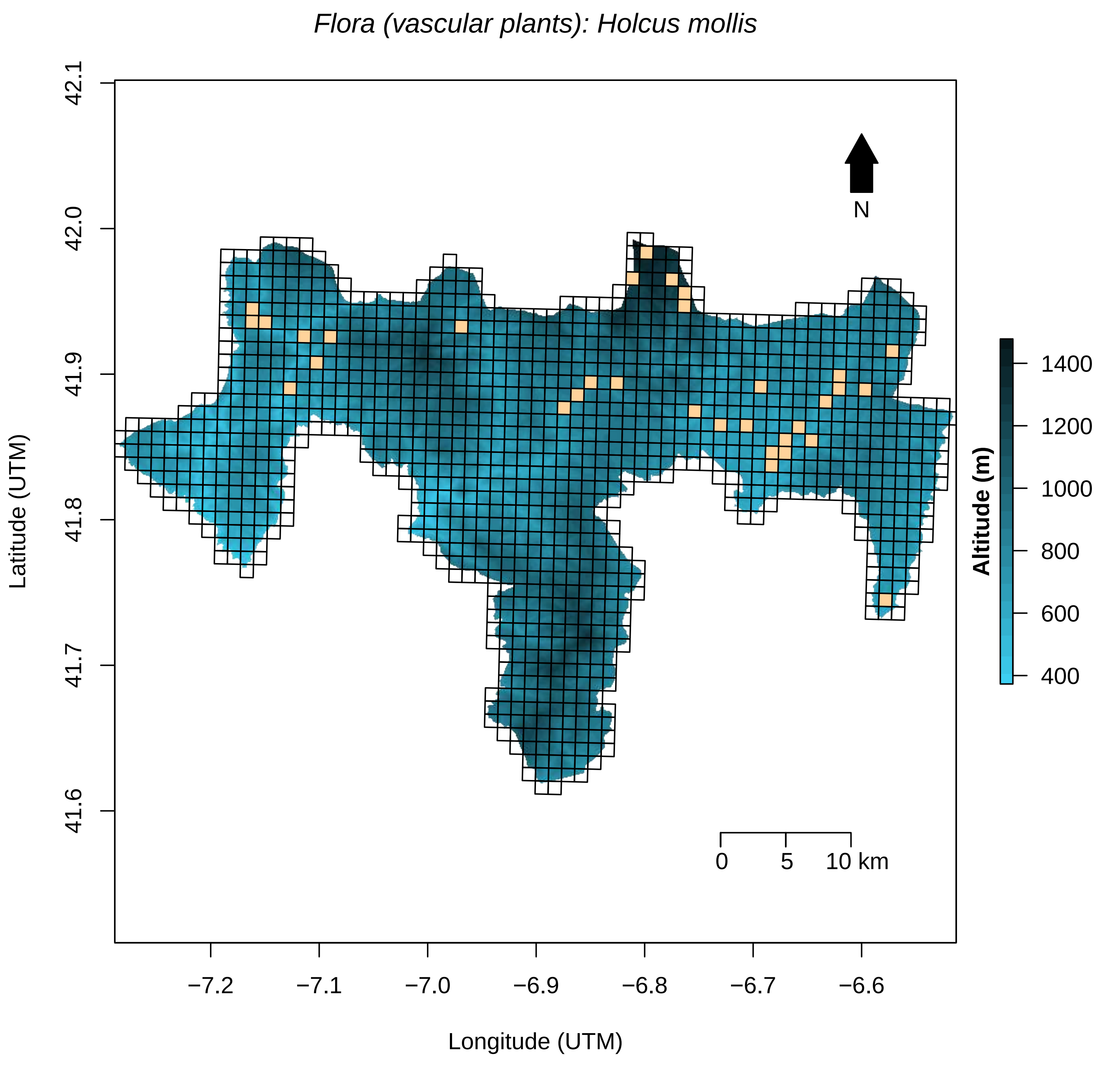
Flora (vascular plants): Holcus mollis
42.1
42.0
41.9
41.8
41.7
41.6
Latitude (UTM)
−7.2
−7.1
−7.0
−6.9
−6.8
−6.7
−6.6
Longitude (UTM)
N
0
5
10 km
Altitude (m)
1400
1200
1000
800
600
400

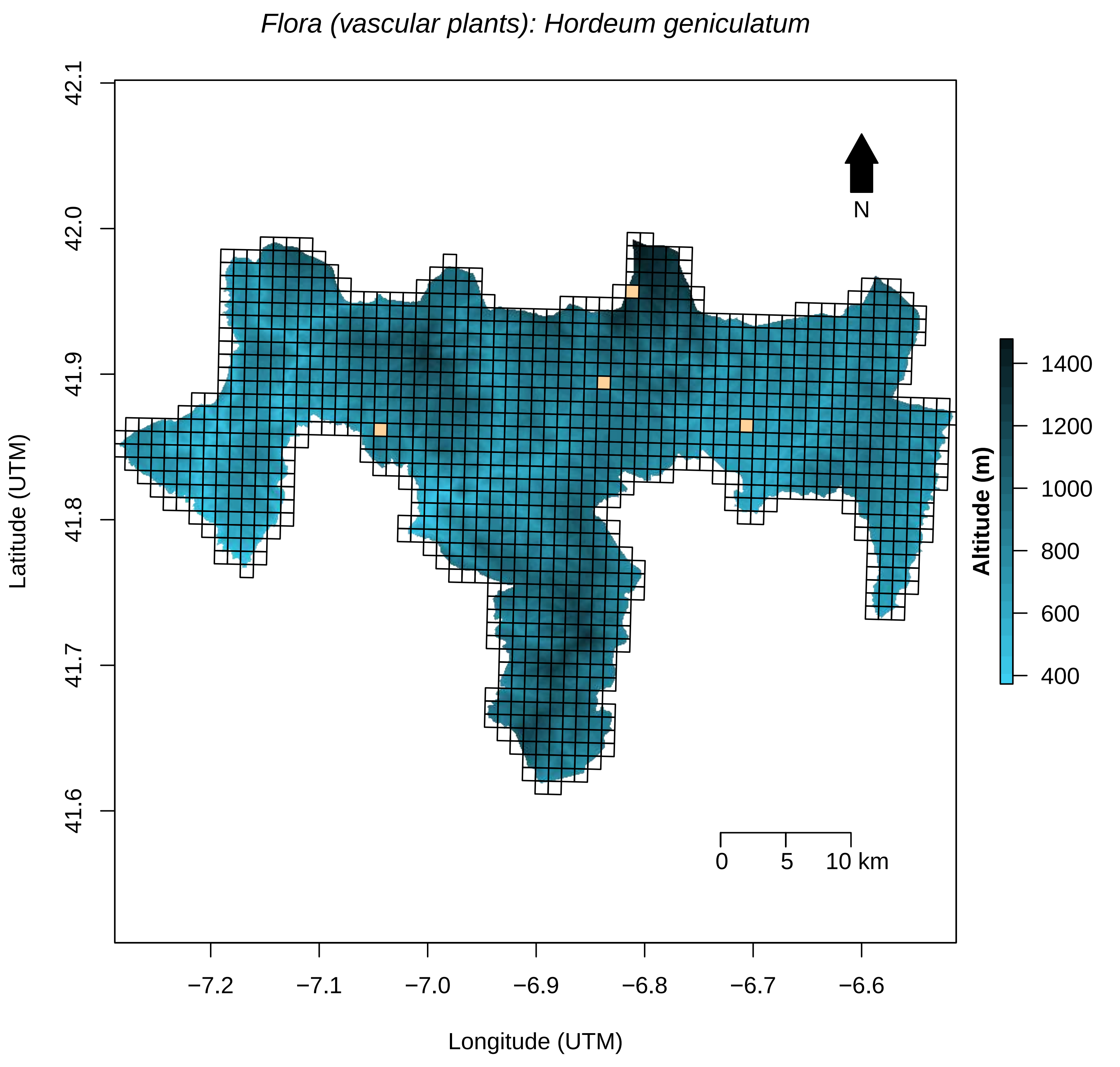

Flora (vascular plants): Hordeum geniculatum
42.1
42.0
41.9
41.8
41.7
41.6
Latitude (UTM)
−7.2
−7.1
−7.0
−6.9
−6.8
−6.7
−6.6
Longitude (UTM)
N
0
5
10 km
1400
1200
1000
800
600
400
Altitude (m)

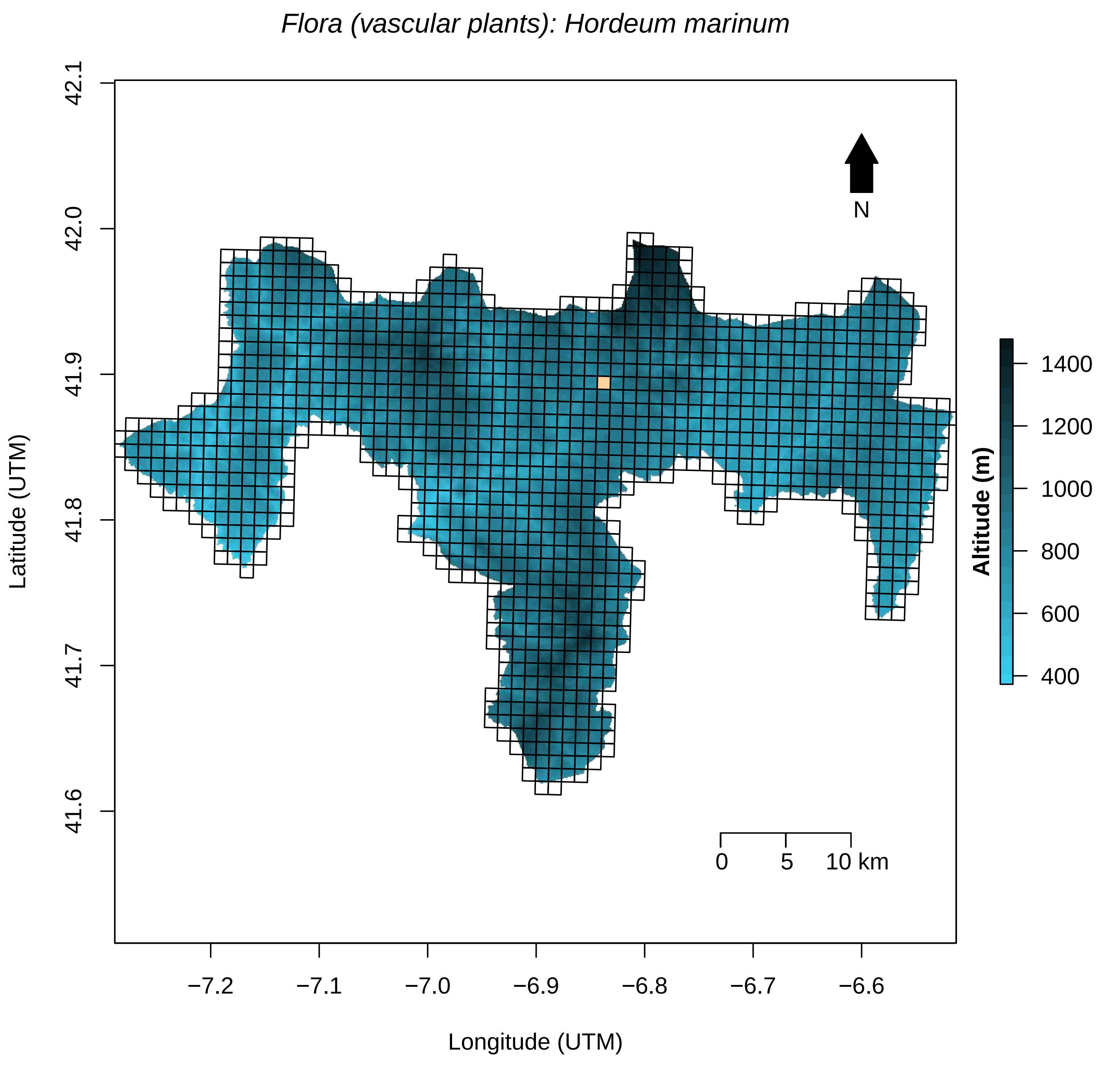

Flora (vascular plants): Hordeum marinum
42.1
42.0
41.9
41.8
41.7
41.6
Latitude (UTM)
−7.2
−7.1
−7.0
−6.9
−6.8
−6.7
−6.6
Longitude (UTM)
N
0
5
10 km
Altitude (m)
1400
1200
1000
800
600
400

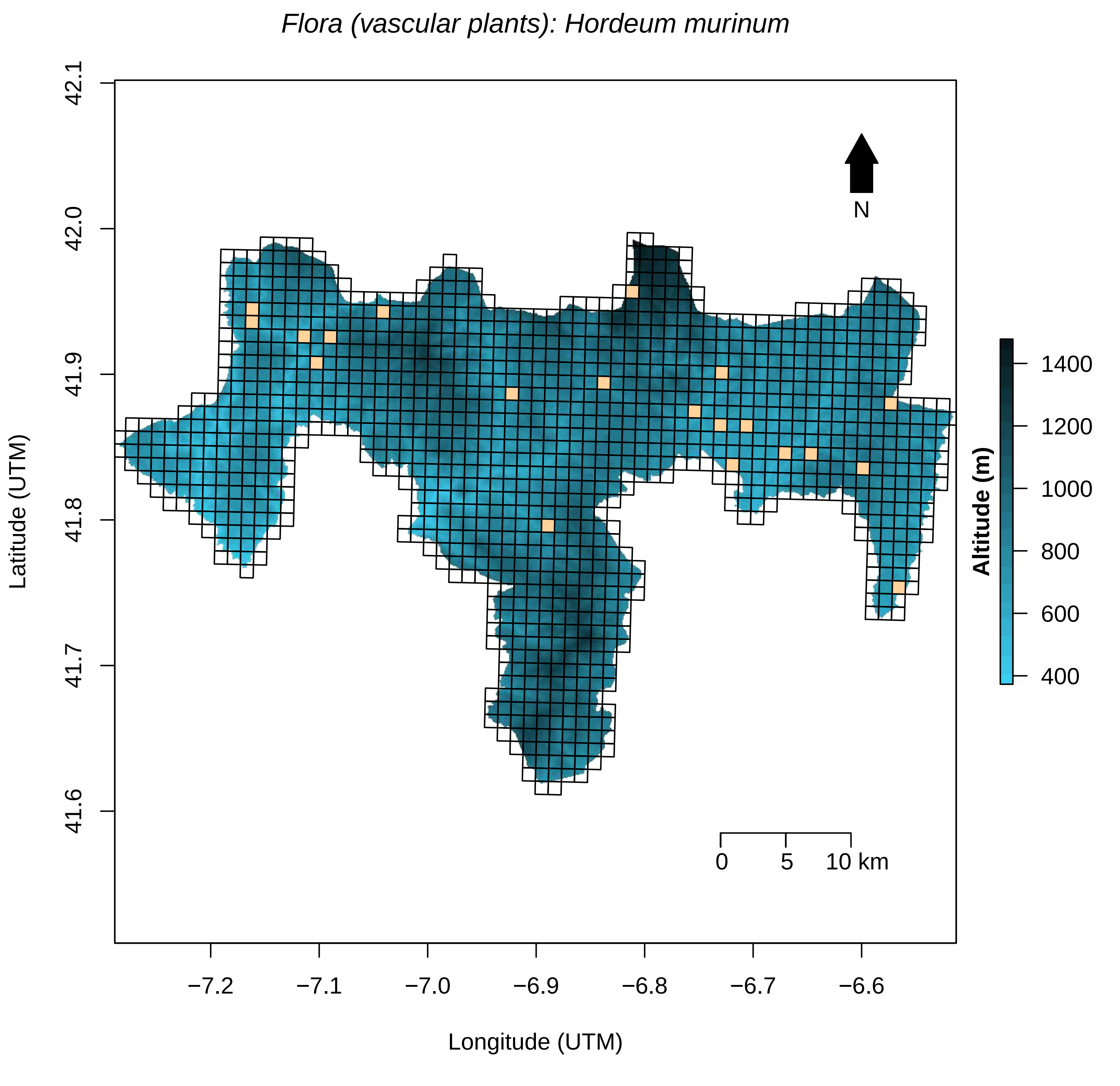

Flora (vascular plants): Hordeum murinum
42.1
42.0
41.9
41.8
41.7
41.6
Latitude (UTM)
−7.2
−7.1
−7.0
−6.9
−6.8
−6.7
−6.6
Longitude (UTM)
N
0
5
10 km
Altitude (m)
1400
1200
1000
800
600
400

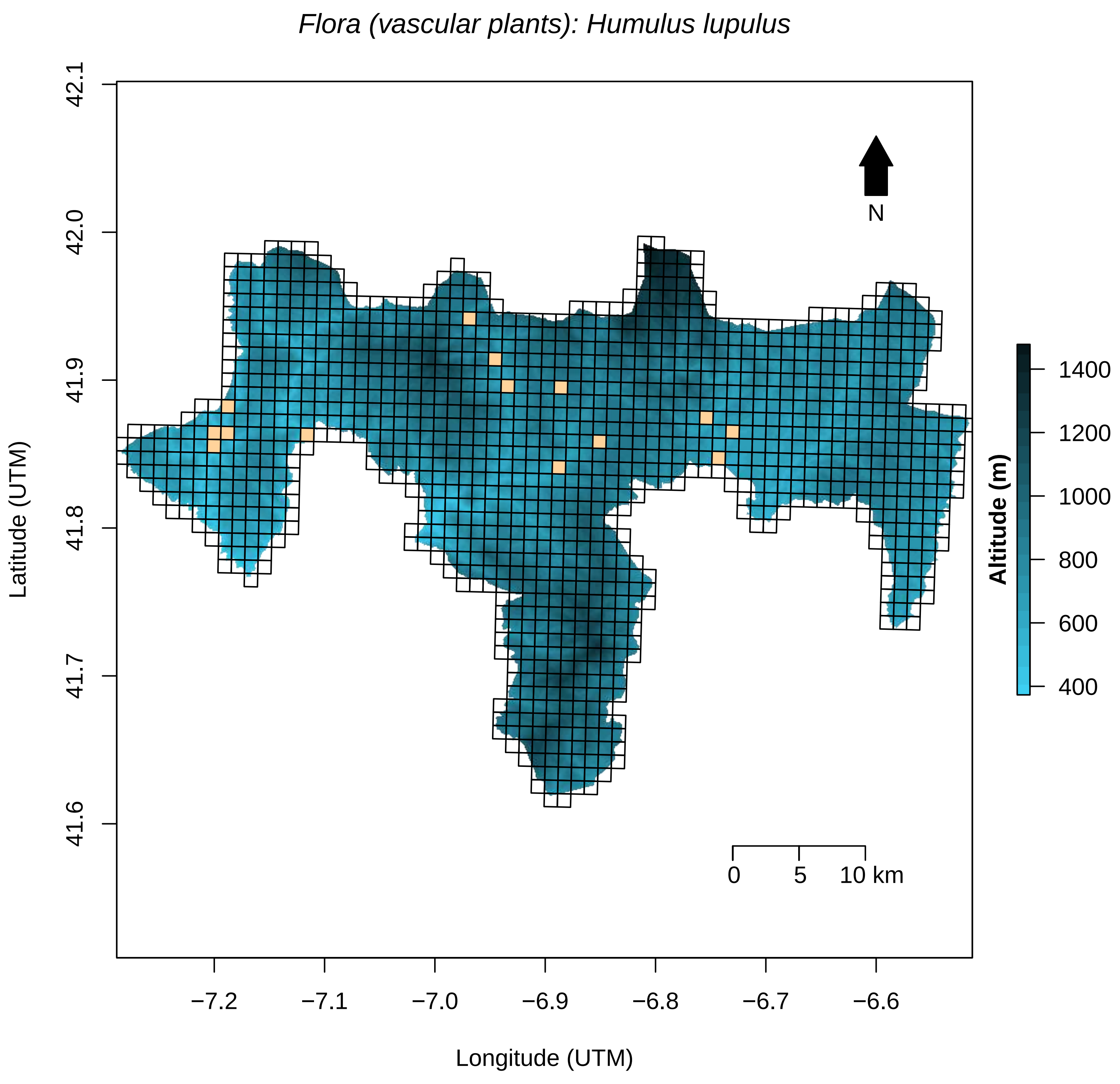
Flora (vascular plants): Humulus lupulus
42.1
42.0
41.9
41.8
41.7
41.6
Latitude (UTM)
−7.2
−7.1
−7.0
−6.9
−6.8
−6.7
−6.6
Longitude (UTM)
N
0
5
10 km
1400
1200
1000
800
600
400
Altitude (m)

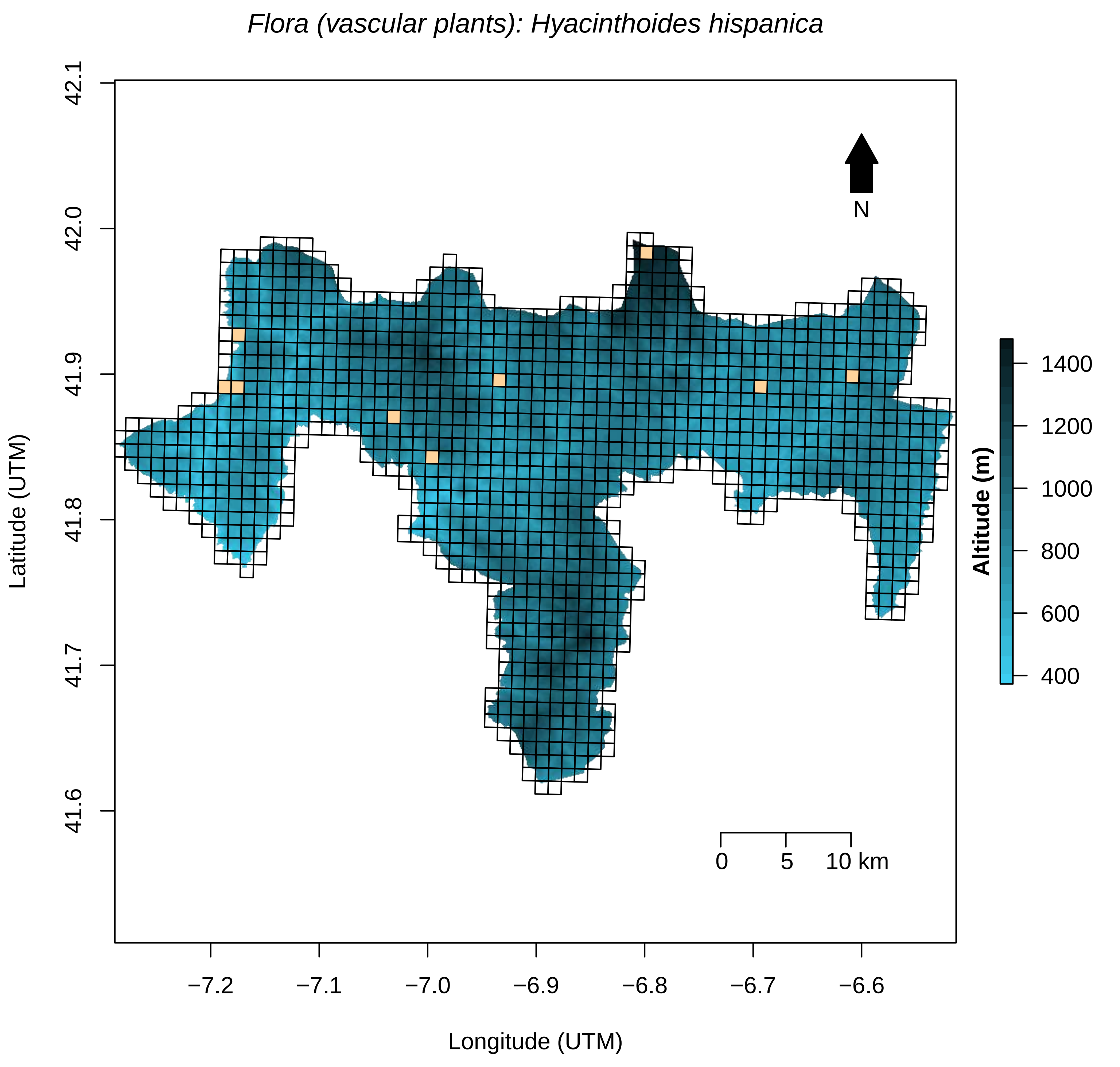
Flora (vascular plants): Hyacinthoides hispanica
42.1
42.0
41.9
41.8
41.7
41.6
Latitude (UTM)
−7.2
−7.1
−7.0
−6.9
−6.8
−6.7
−6.6
Longitude (UTM)
N
0
5
10 km
Altitude (m)
1400
1200
1000
800
600
400

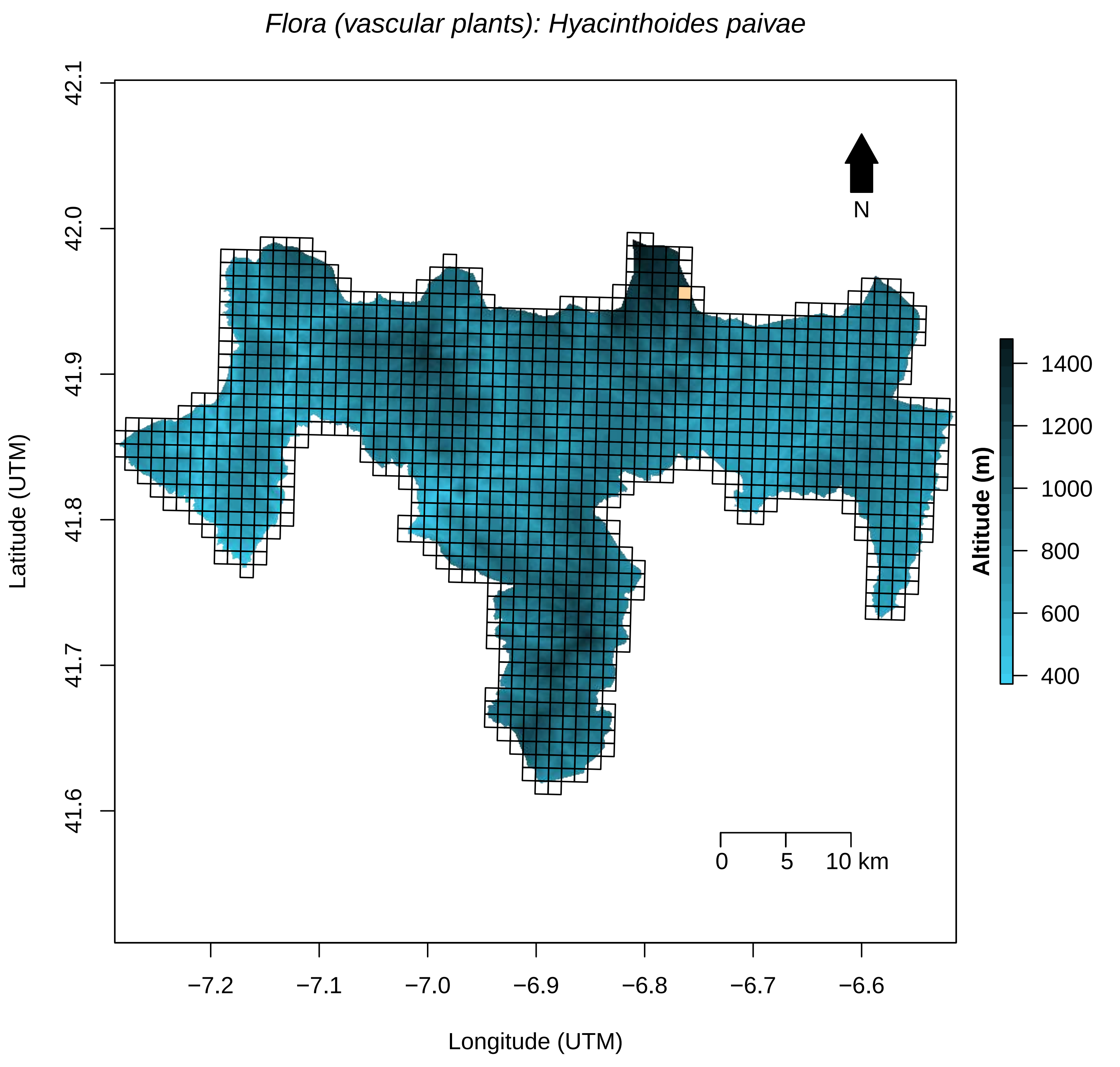

Flora (vascular plants): Hyacinthoides paivae
42.1
42.0
41.9
41.8
41.7
41.6
Latitude (UTM)
−7.2
−7.1
−7.0
−6.9
−6.8
−6.7
−6.6
Longitude (UTM)
N
0
5
10 km
Altitude (m)
1400
1200
1000
800
600
400

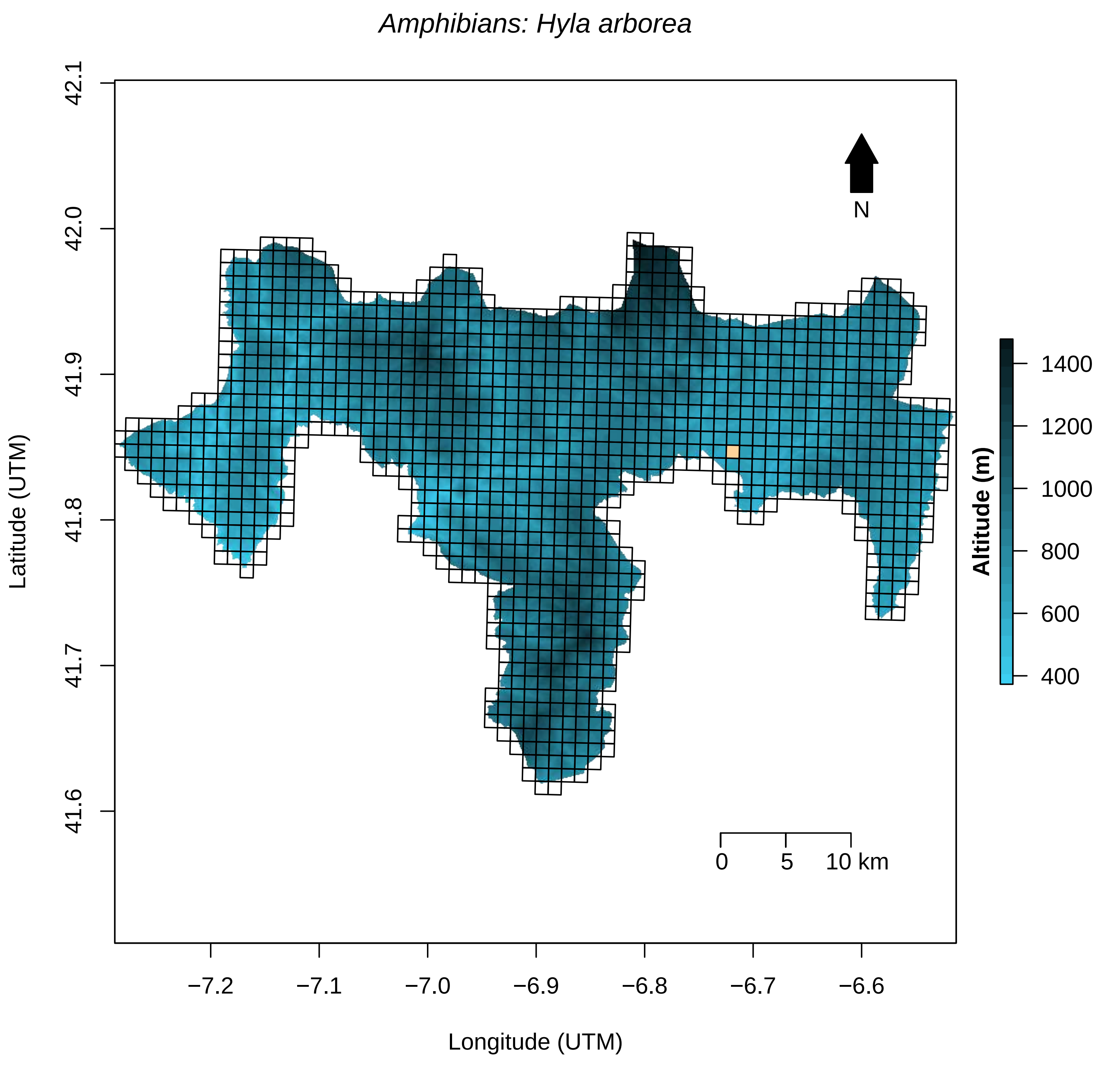

Amphibians: Hyla arborea
N
0 5 10 km
Altitude (m)
1400
1200
1000
800
600
400
42.1
42.0
41.9
41.8
41.7
41.6
Latitude (UTM)
−7.2
−7.1
−7.0
−6.9
−6.8
−6.7
−6.6
Longitude (UTM)

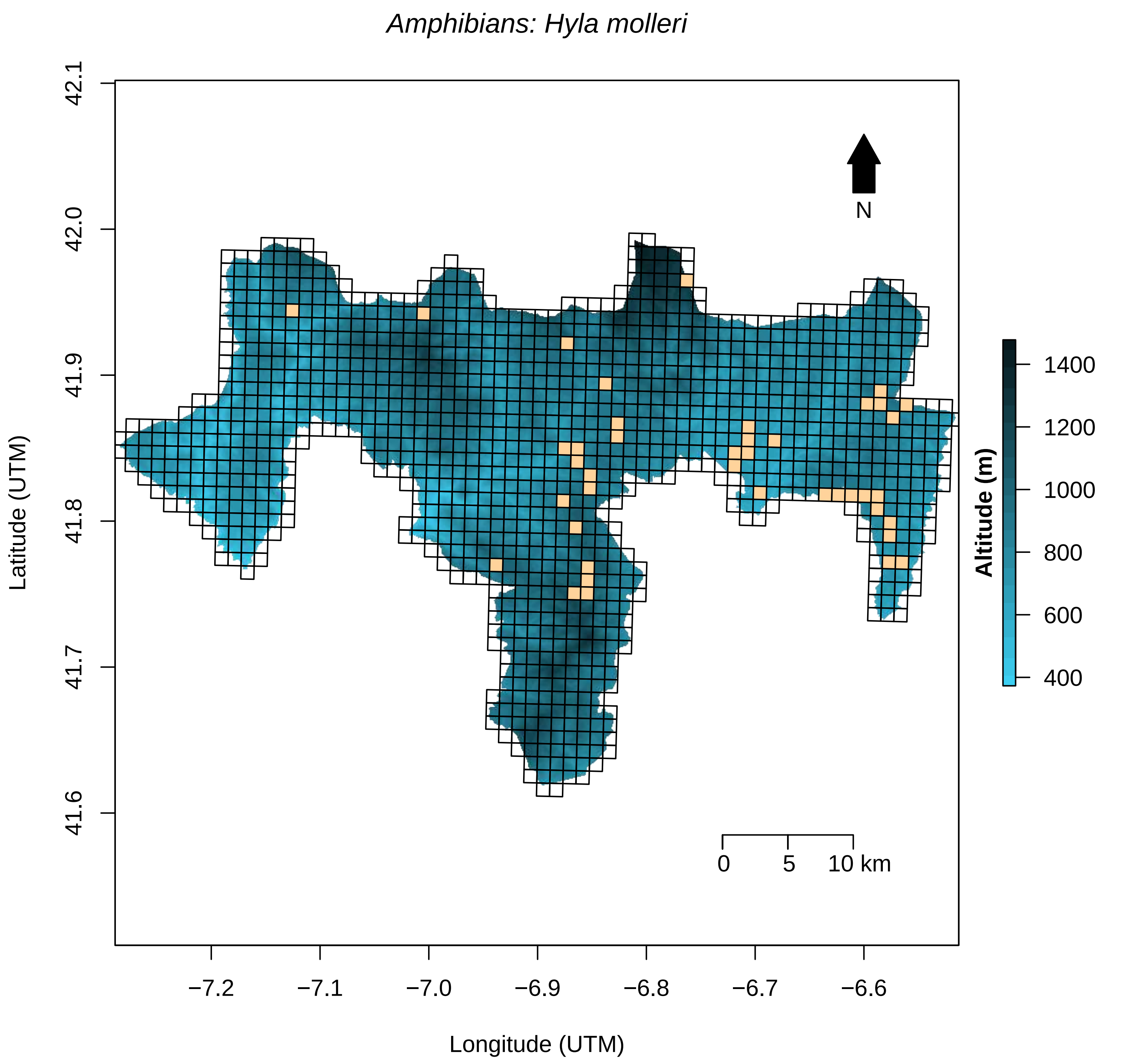
Amphibians: Hyla molleri
N
0 5 10 km
Latitude (UTM)
Longitude (UTM)
Altitude (m)
42.1
42.0
41.9
41.8
41.7
41.6
−7.2
−7.1
−7.0
−6.9
−6.8
−6.7
−6.6
1400
1200
1000
800
600
400

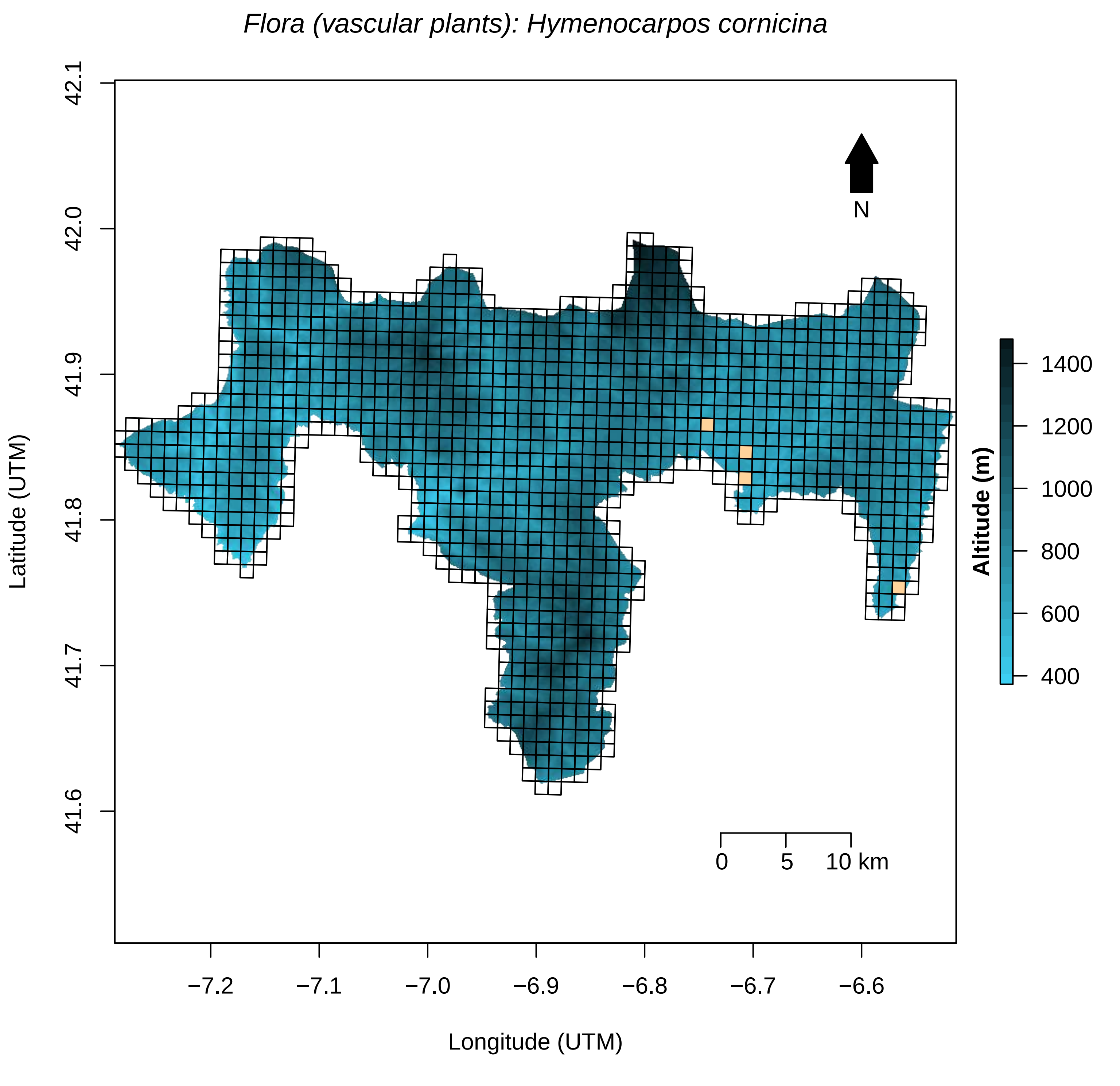

Flora (vascular plants): Hymenocarpos cornicina
42.1
42.0
41.9
41.8
41.7
41.6
Latitude (UTM)
−7.2
−7.1
−7.0
−6.9
−6.8
−6.7
−6.6
Longitude (UTM)
N
0
5
10 km
Altitude (m)
1400
1200
1000
800
600
400

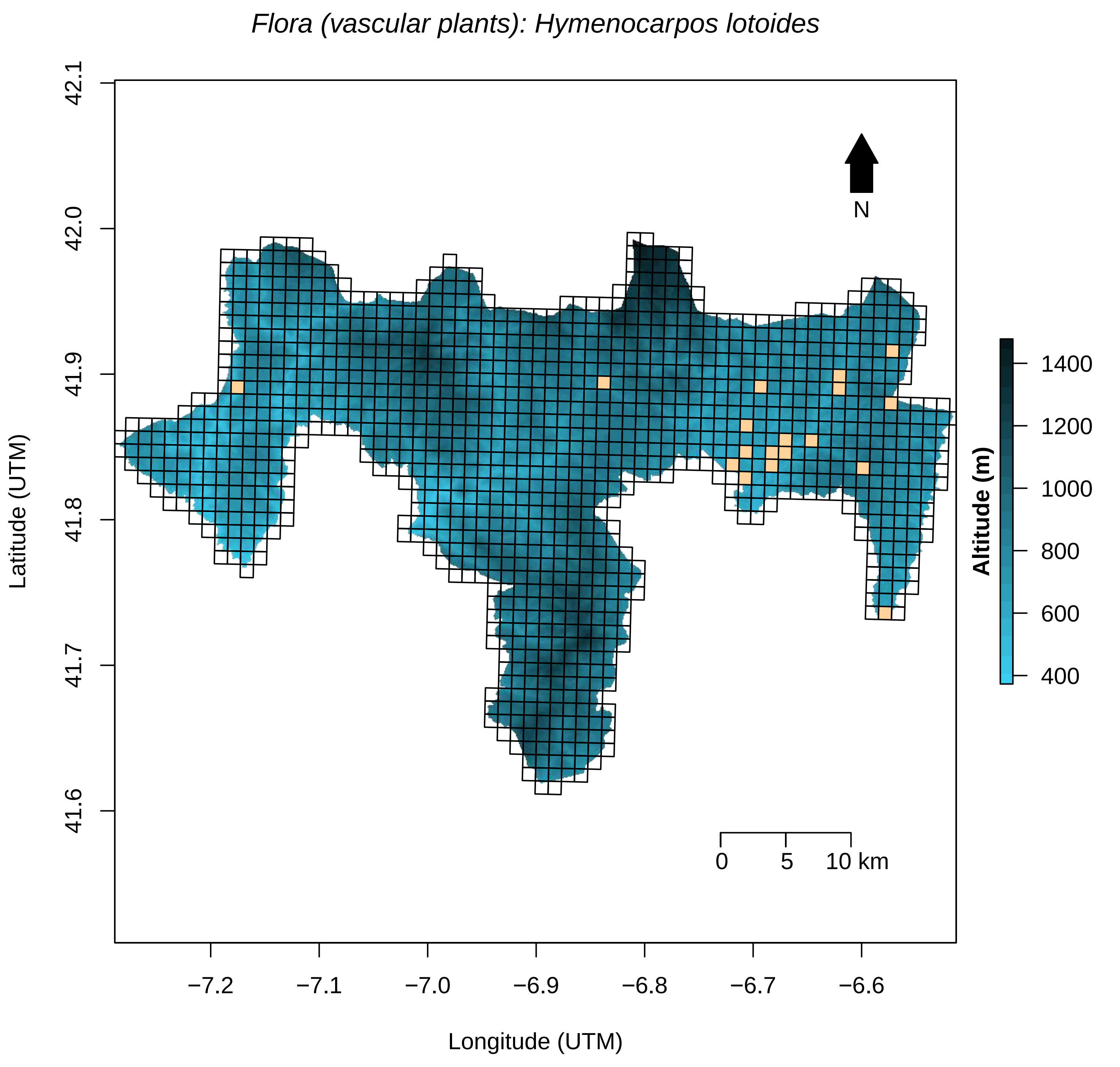

Flora (vascular plants): Hymenocarpos lotoides
Latitude (UTM)
Longitude (UTM)
42.1
42.0
41.9
41.8
41.7
41.6
−7.2
−7.1
−7.0
−6.9
−6.8
−6.7
−6.6
N
0
5
10 km
Altitude (m)
1400
1200
1000
800
600
400

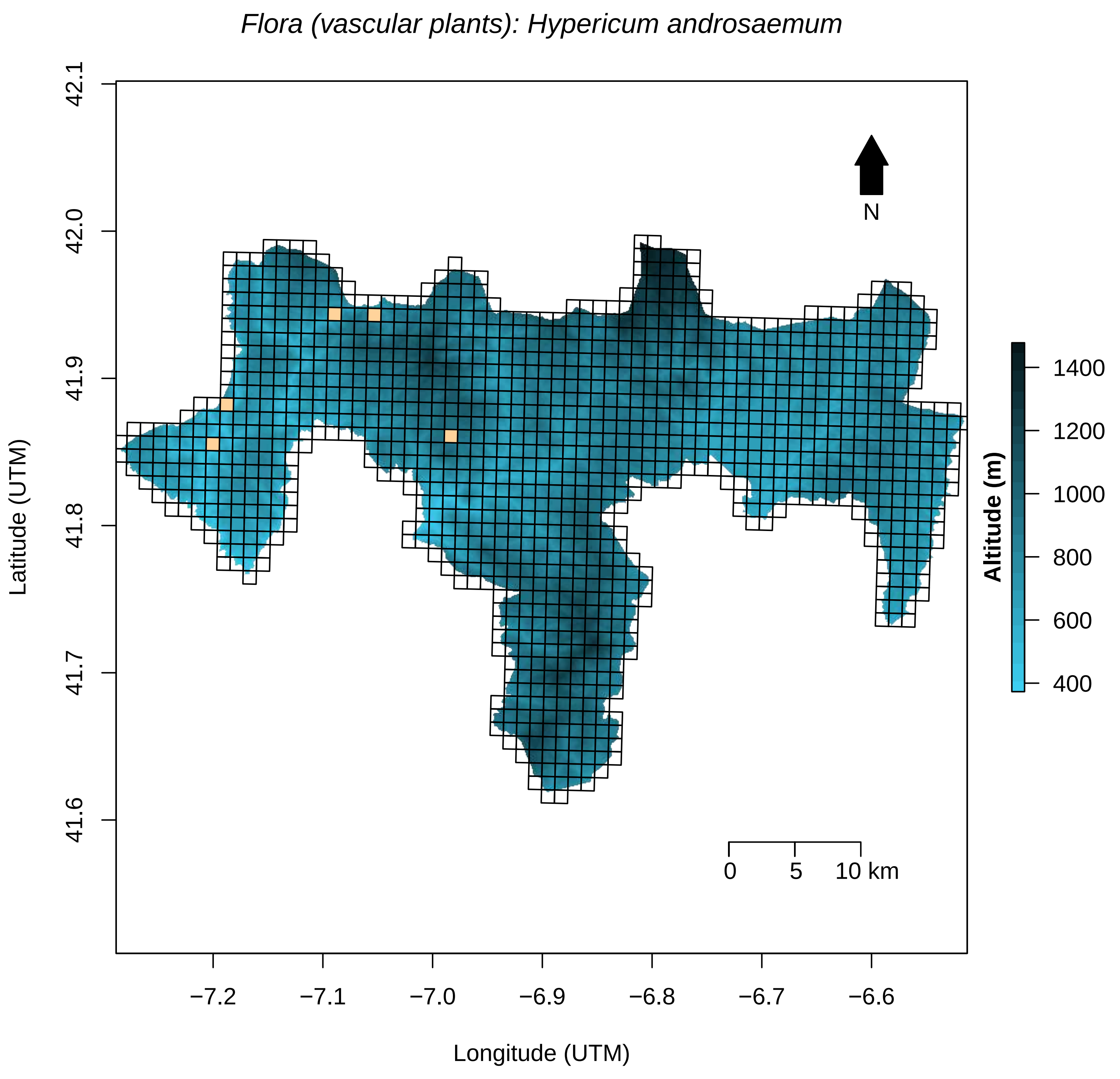

Flora (vascular plants): Hypericum androsaemum
42.1
42.0
41.9
41.8
41.7
41.6
Latitude (UTM)
−7.2
−7.1
−7.0
−6.9
−6.8
−6.7
−6.6
Longitude (UTM)
N
0
5
10 km
Altitude (m)
1400
1200
1000
800
600
400

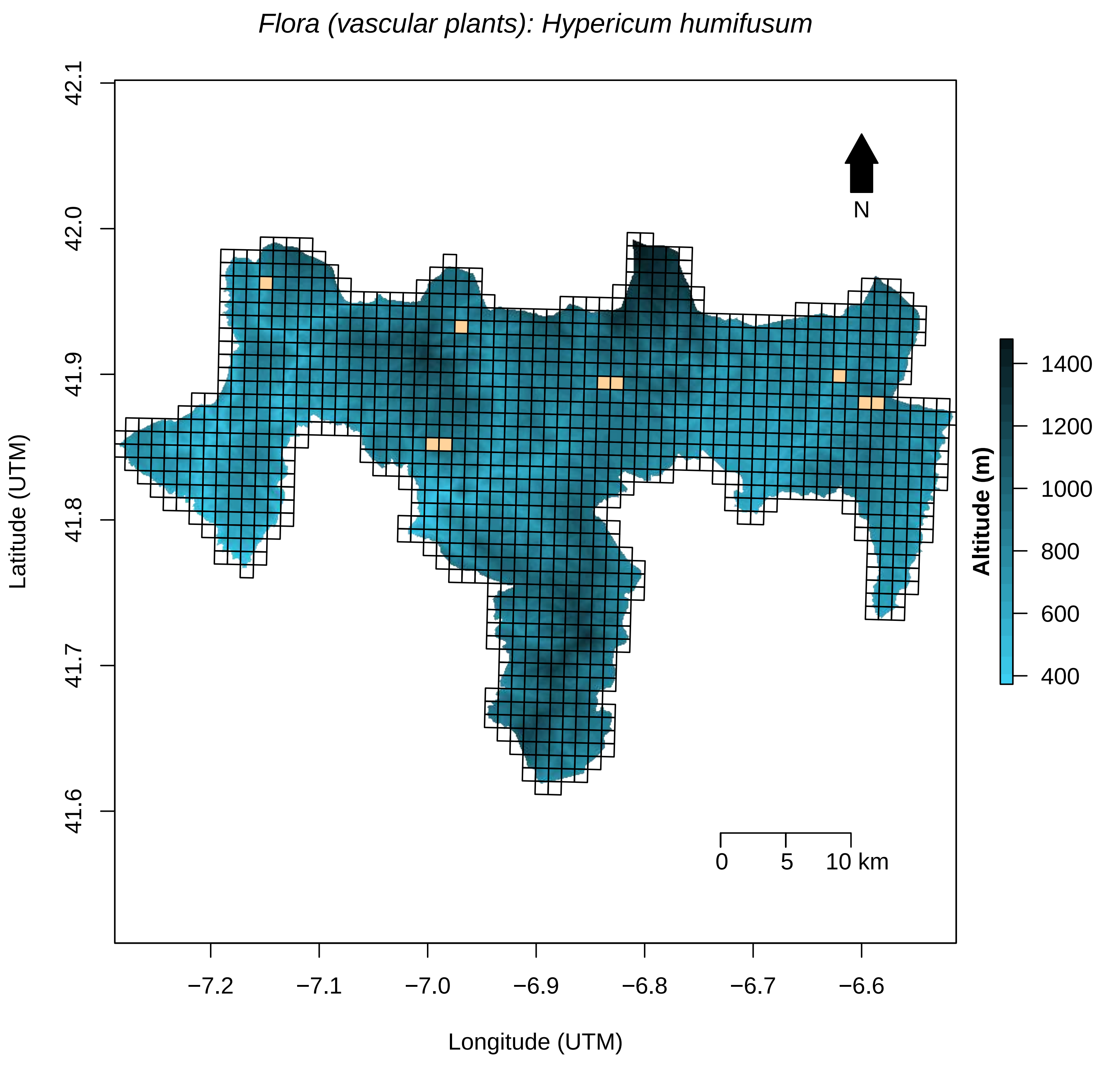

Flora (vascular plants): Hypericum humifusum
N
0
5
10 km
Altitude (m)
1400
1200
1000
800
600
400
Latitude (UTM)
42.1
42.0
41.9
41.8
41.7
41.6
Longitude (UTM)
−7.2
−7.1
−7.0
−6.9
−6.8
−6.7
−6.6

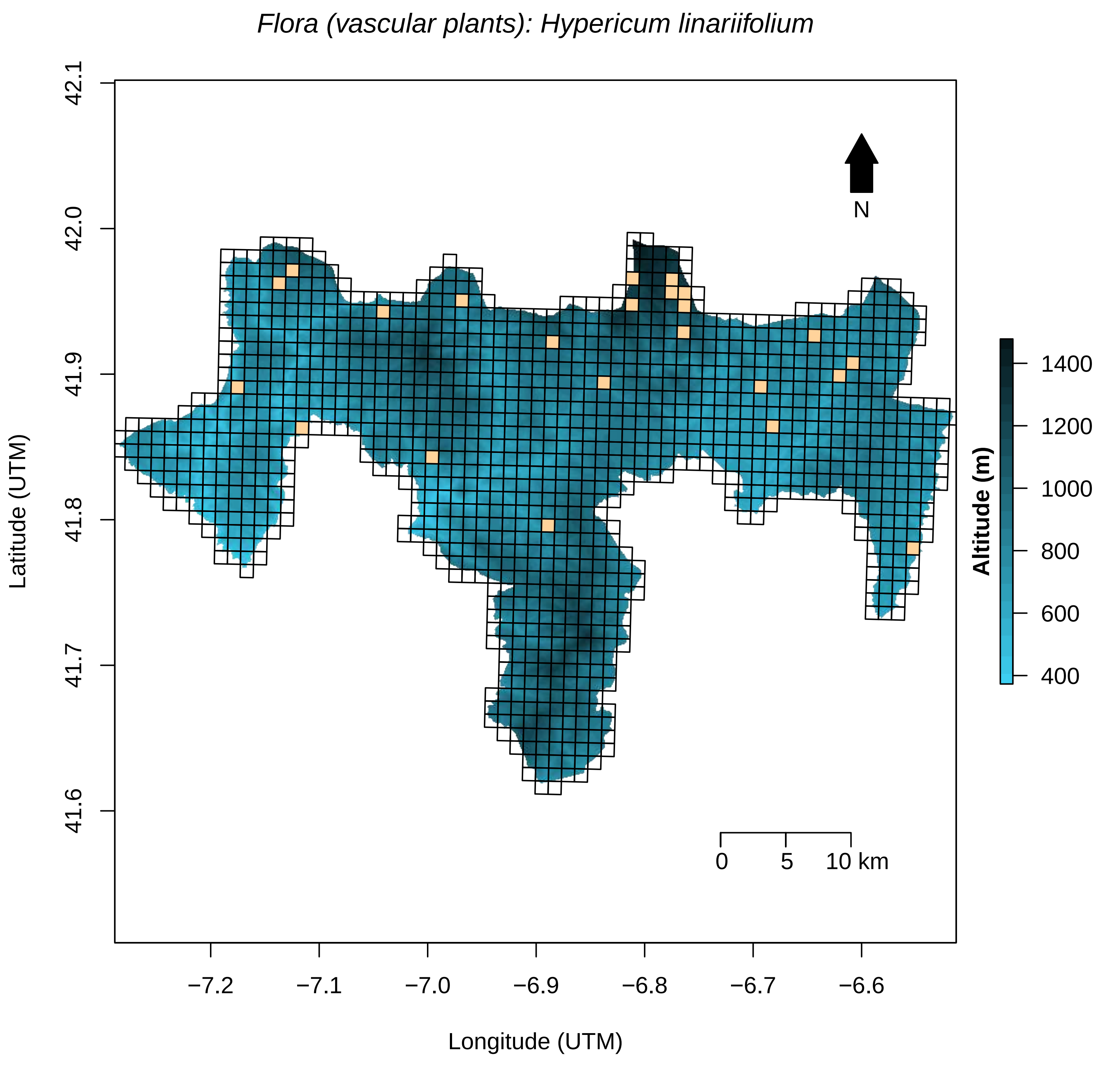

Flora (vascular plants): Hypericum linariifolium
N
0
5
10 km
Altitude (m)
1400
1200
1000
800
600
400
42.1
42.0
41.9
41.8
41.7
41.6
−7.2
−7.1
−7.0
−6.9
−6.8
−6.7
−6.6
Latitude (UTM)
Longitude (UTM)

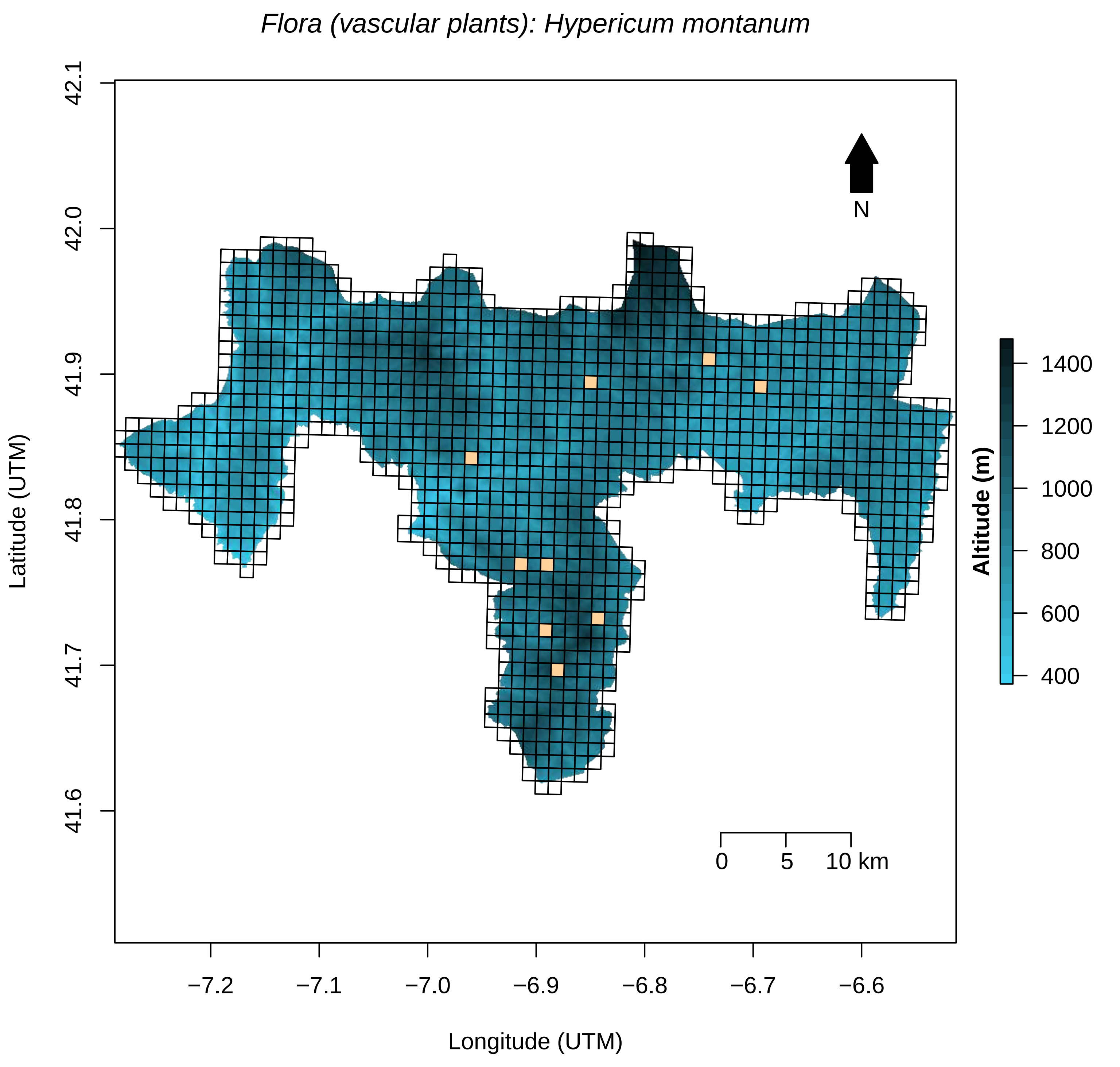

Flora (vascular plants): Hypericum montanum
42.1
42.0
41.9
41.8
41.7
41.6
Latitude (UTM)
−7.2
−7.1
−7.0
−6.9
−6.8
−6.7
−6.6
Longitude (UTM)
N
0
5
10 km
1400
1200
1000
800
600
400
Altitude (m)

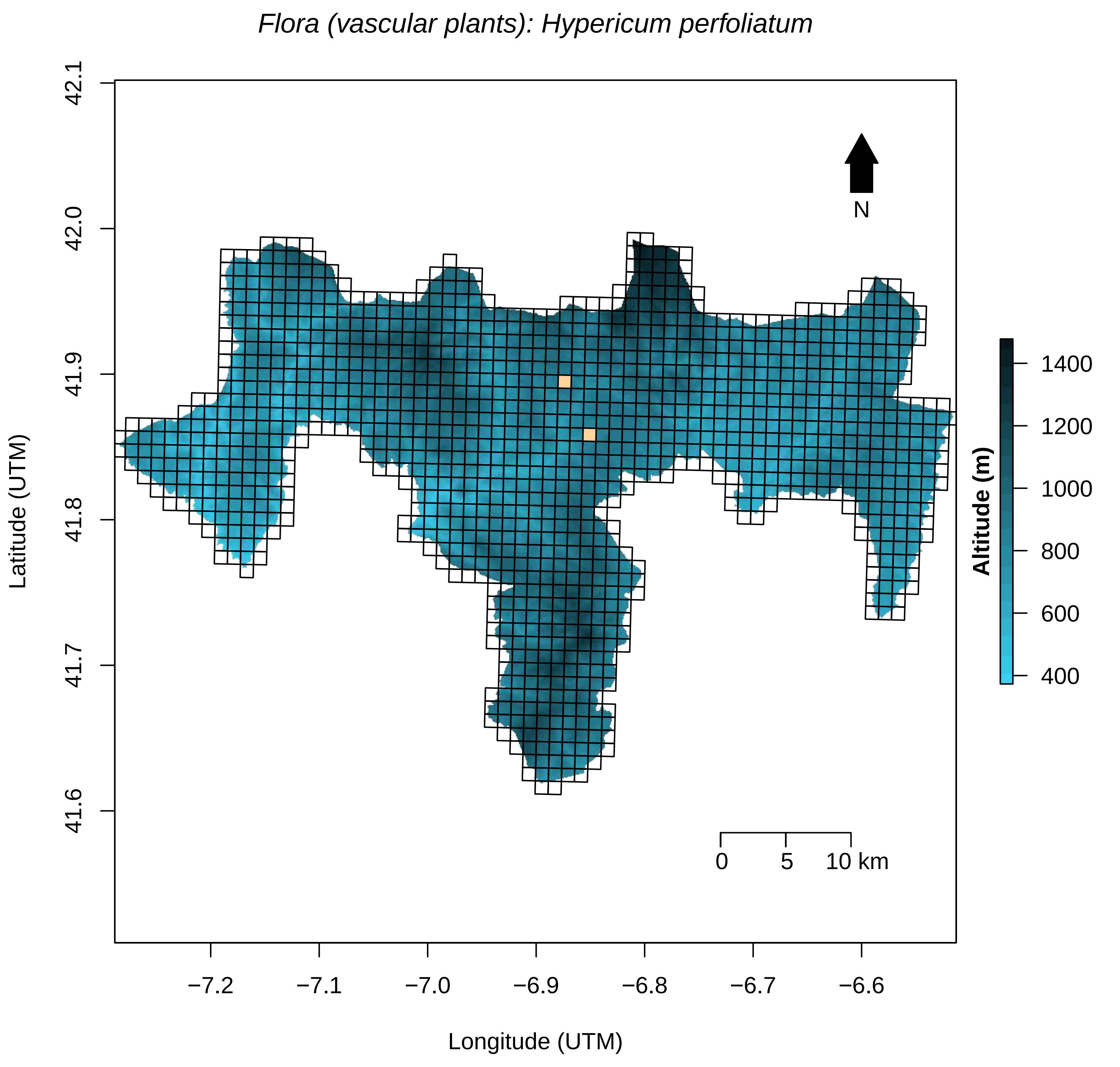

Flora (vascular plants): Hypericum perfoliatum
N
0
5
10 km
Altitude (m)
1400
1200
1000
800
600
400
42.1
42.0
41.9
41.8
41.7
41.6
Latitude (UTM)
−7.2
−7.1
−7.0
−6.9
−6.8
−6.7
−6.6
Longitude (UTM)

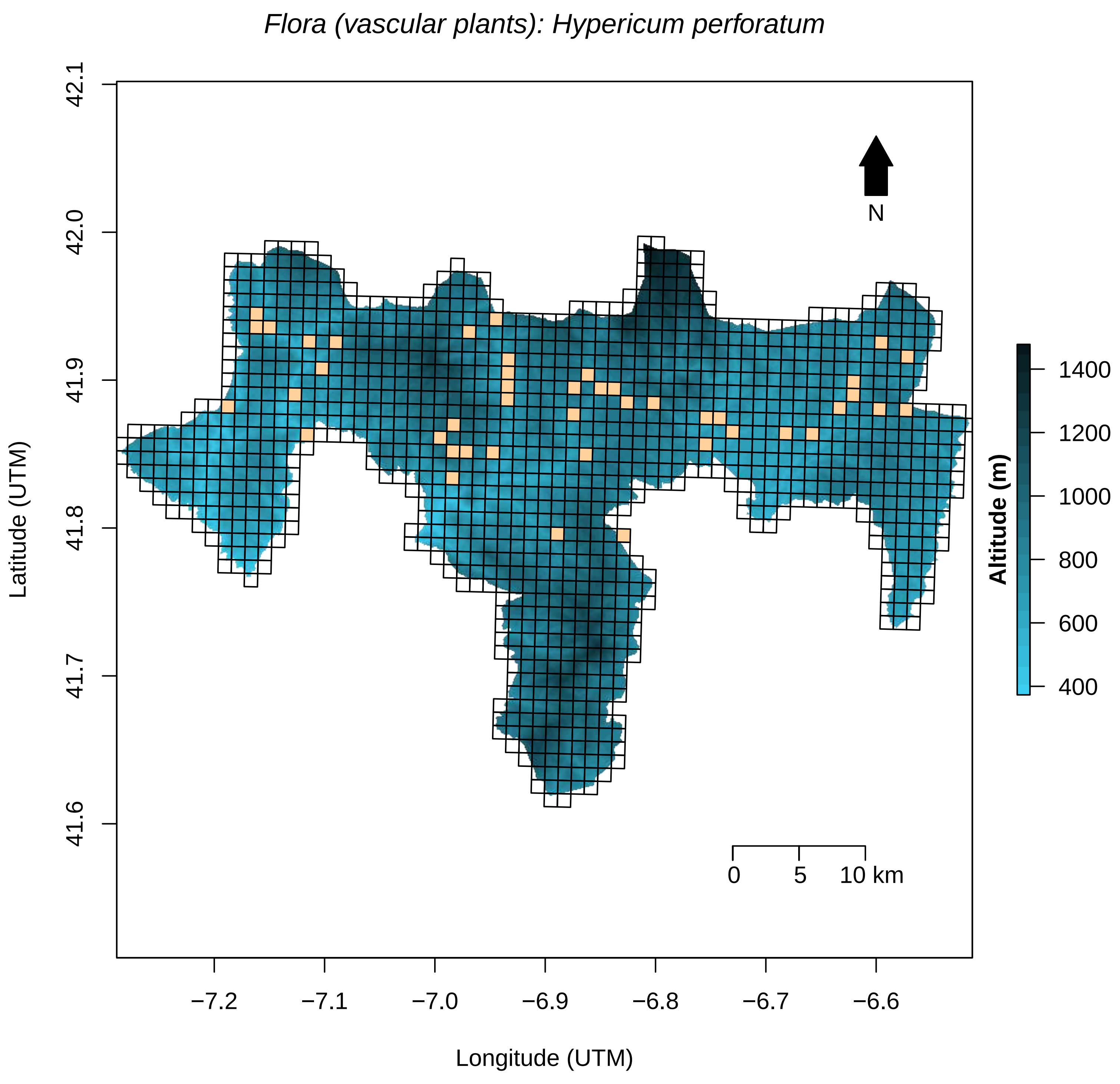

Flora (vascular plants): Hypericum perforatum
42.1
42.0
41.9
41.8
41.7
41.6
Latitude (UTM)
−7.2
−7.1
−7.0
−6.9
−6.8
−6.7
−6.6
Longitude (UTM)
N
0
5
10 km
1400
1200
1000
800
600
400
Altitude (m)

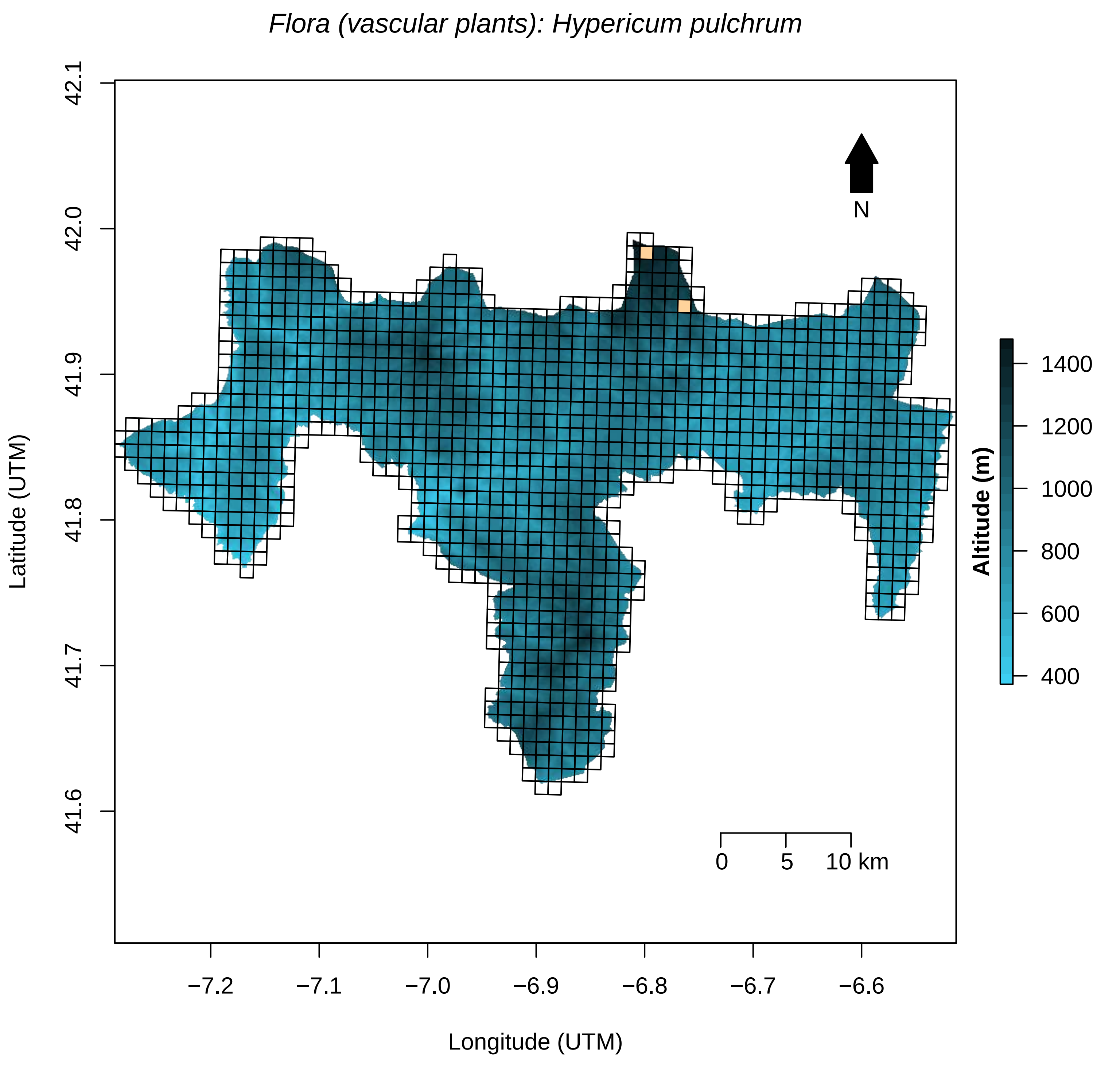

Flora (vascular plants): Hypericum pulchrum
42.1
42.0
41.9
41.8
41.7
41.6
Latitude (UTM)
N
0 5 10 km
−7.2 −7.1 −7.0 −6.9 −6.8 −6.7 −6.6
Longitude (UTM)
Altitude (m)
1400
1200
1000
800
600
400

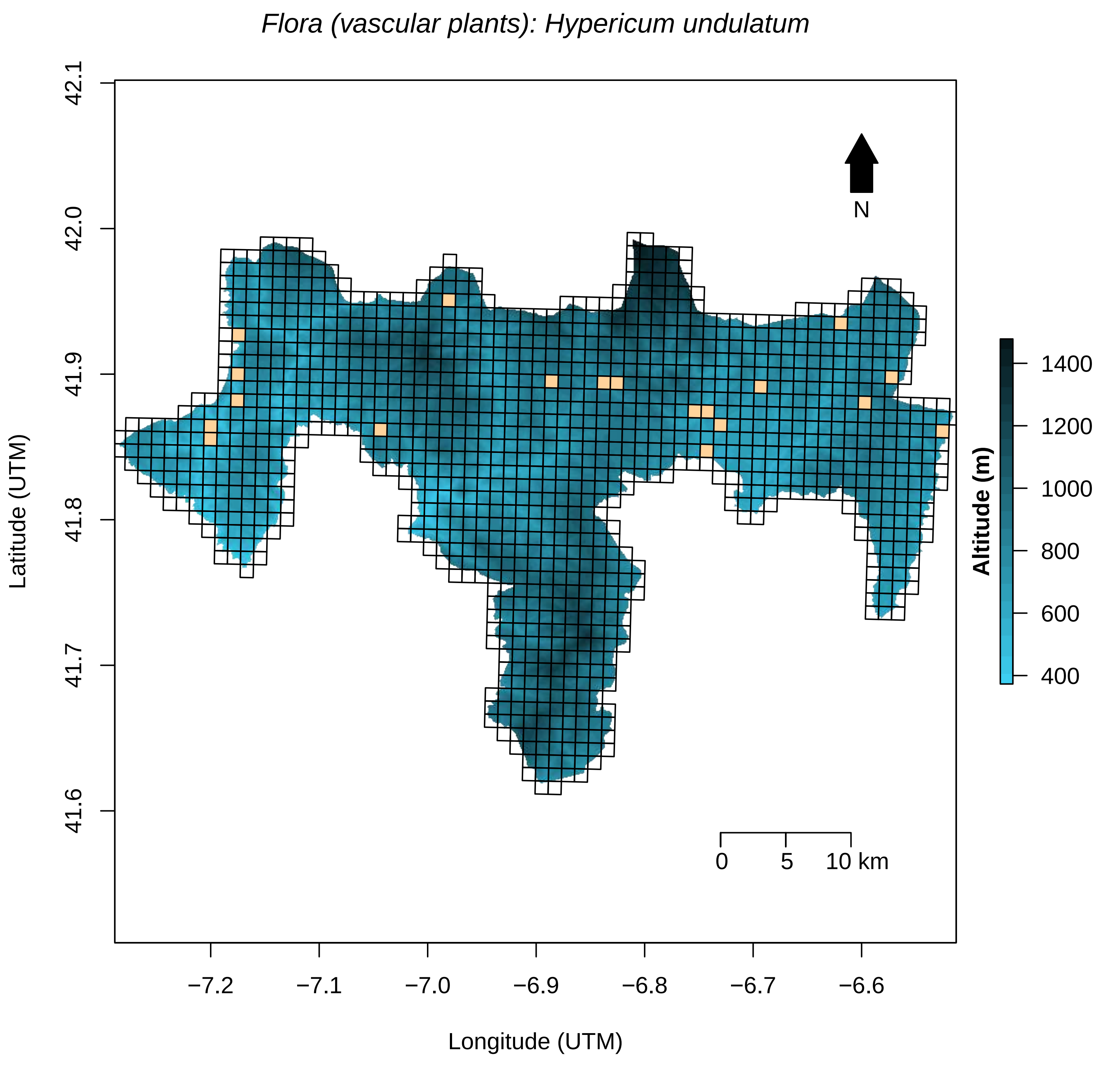
Flora (vascular plants): Hypericum undulatum
N
0
5
10 km
Altitude (m)
1400
1200
1000
800
600
400
Latitude (UTM)
42.1
42.0
41.9
41.8
41.7
41.6
Longitude (UTM)
−7.2
−7.1
−7.0
−6.9
−6.8
−6.7
−6.6

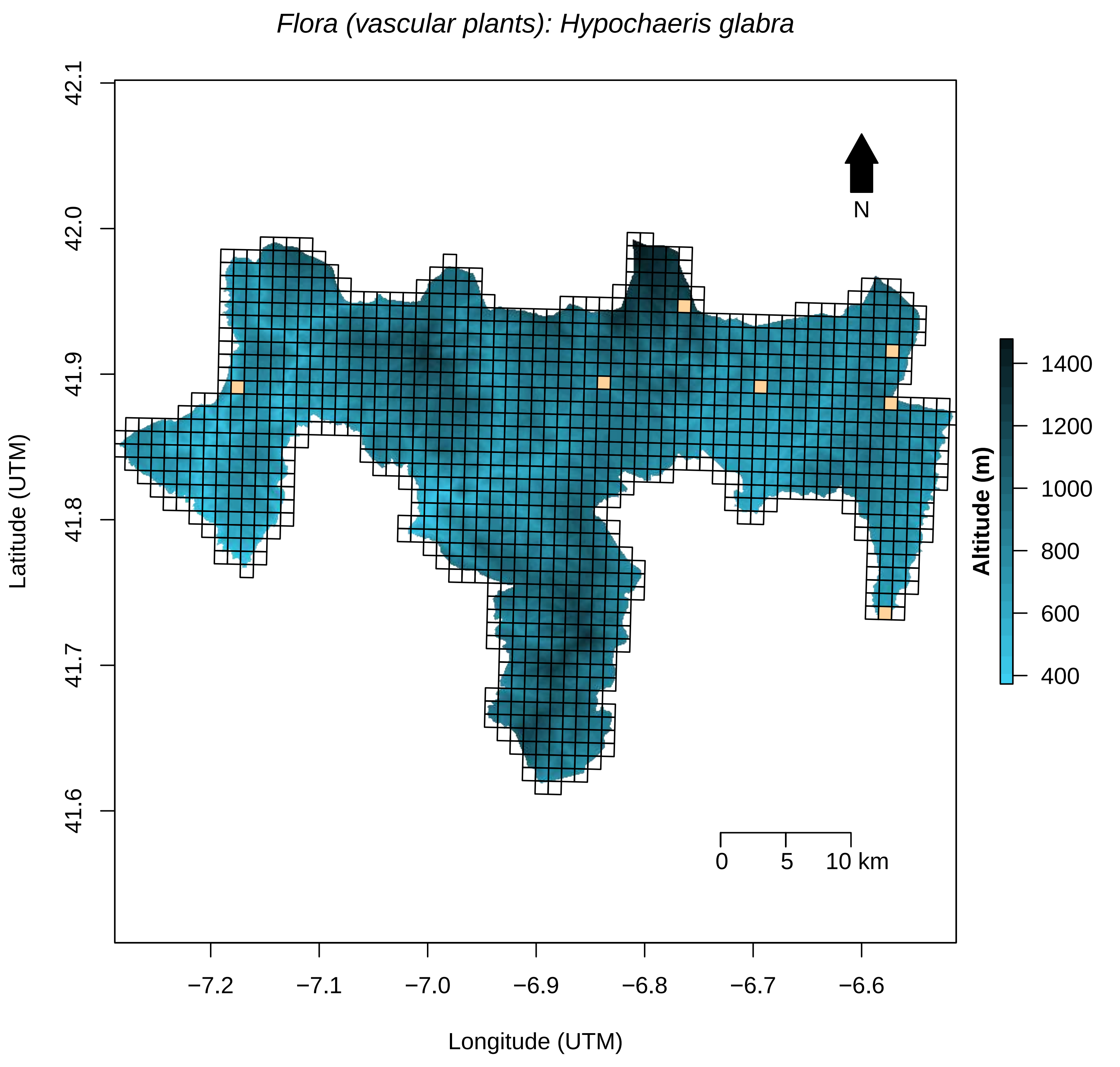

Flora (vascular plants): Hypochaeris glabra
42.1
42.0
41.9
41.8
41.7
41.6
Latitude (UTM)
N
0
5
10 km
1400
1200
1000
800
600
400
Altitude (m)
−7.2
−7.1
−7.0
−6.9
−6.8
−6.7
−6.6
Longitude (UTM)

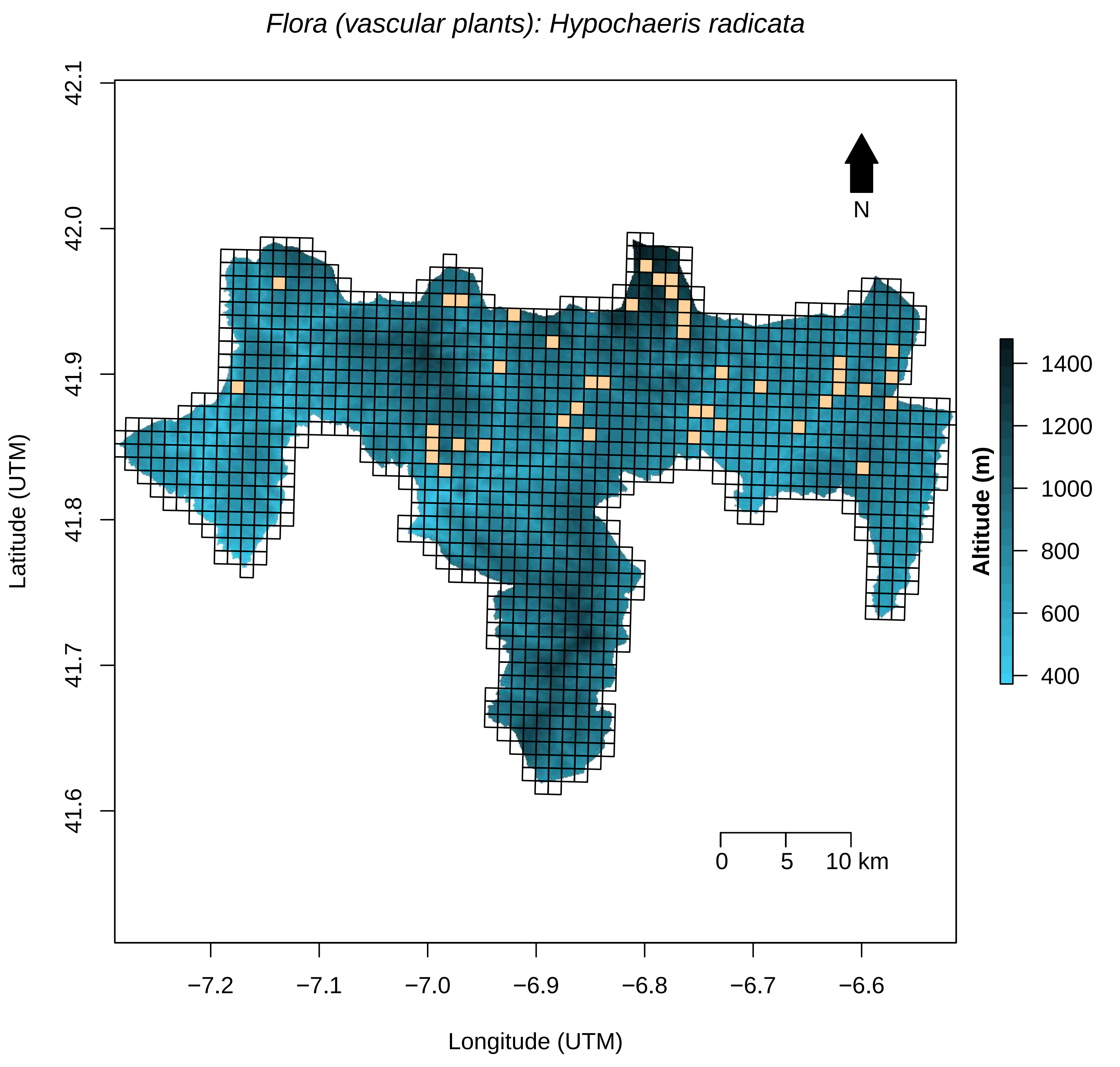
Flora (vascular plants): Hypochaeris radicata
N
0
5
10 km
Altitude (m)
1400
1200
1000
800
600
400
Latitude (UTM)
42.1
42.0
41.9
41.8
41.7
41.6
Longitude (UTM)
−7.2
−7.1
−7.0
−6.9
−6.8
−6.7
−6.6

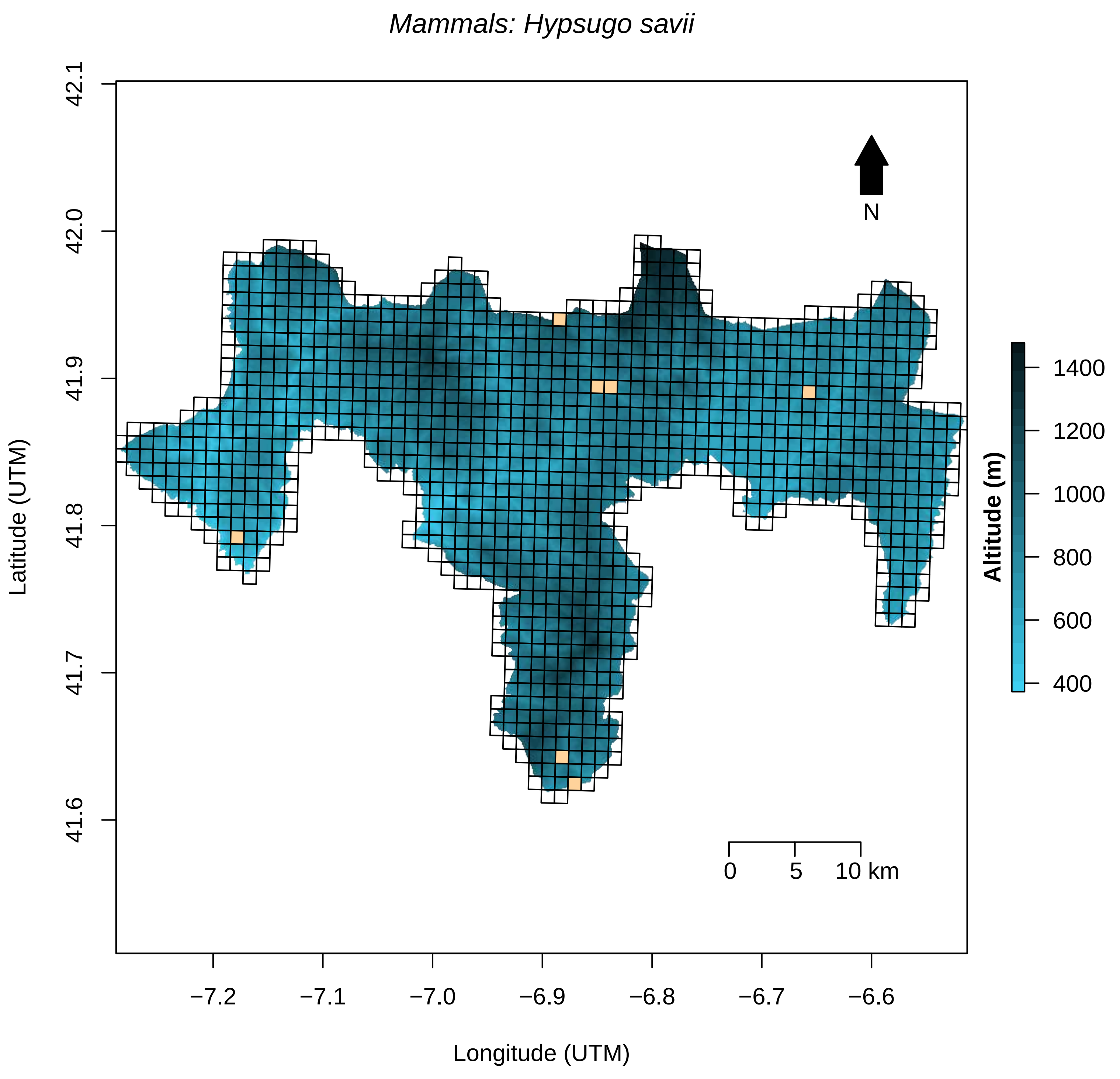

Mammals: Hypsugo savii
42.1
42.0
41.9
41.8
41.7
41.6
Latitude (UTM)
N
0
5
10 km
−7.2
−7.1
−7.0
−6.9
−6.8
−6.7
−6.6
Longitude (UTM)
Altitude (m)
1400
1200
1000
800
600
400

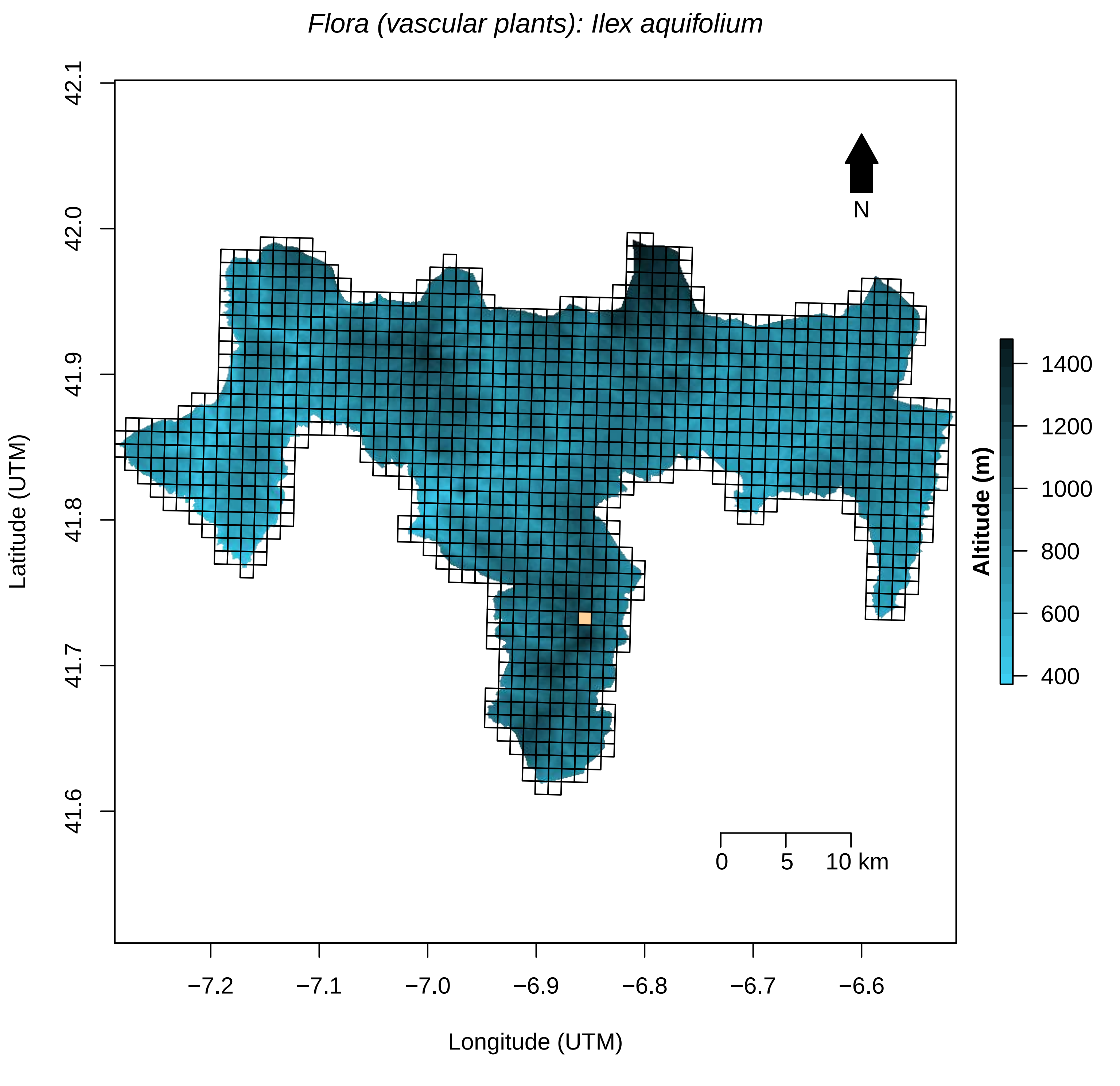

Flora (vascular plants): Ilex aquifolium
42.1
42.0
41.9
41.8
41.7
41.6
Latitude (UTM)
−7.2
−7.1
−7.0
−6.9
−6.8
−6.7
−6.6
Longitude (UTM)
N
0
5
10 km
Altitude (m)
1400
1200
1000
800
600
400

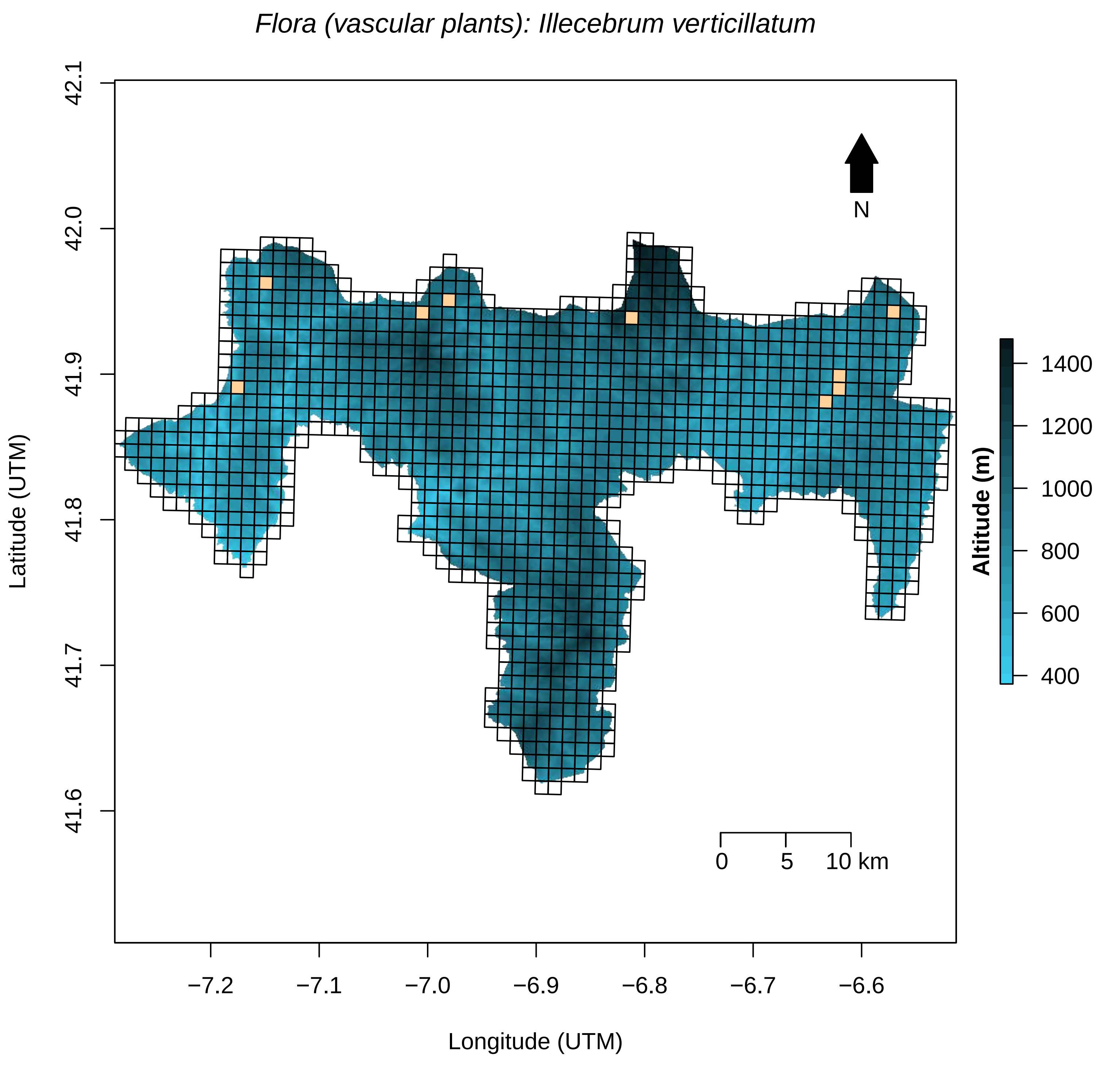

Flora (vascular plants): Illecebrum verticillatum
Latitude (UTM)
42.1
42.0
41.9
41.8
41.7
41.6
Longitude (UTM)
−7.2
−7.1
−7.0
−6.9
−6.8
−6.7
−6.6
N
0
5
10 km
Altitude (m)
1400
1200
1000
800
600
400

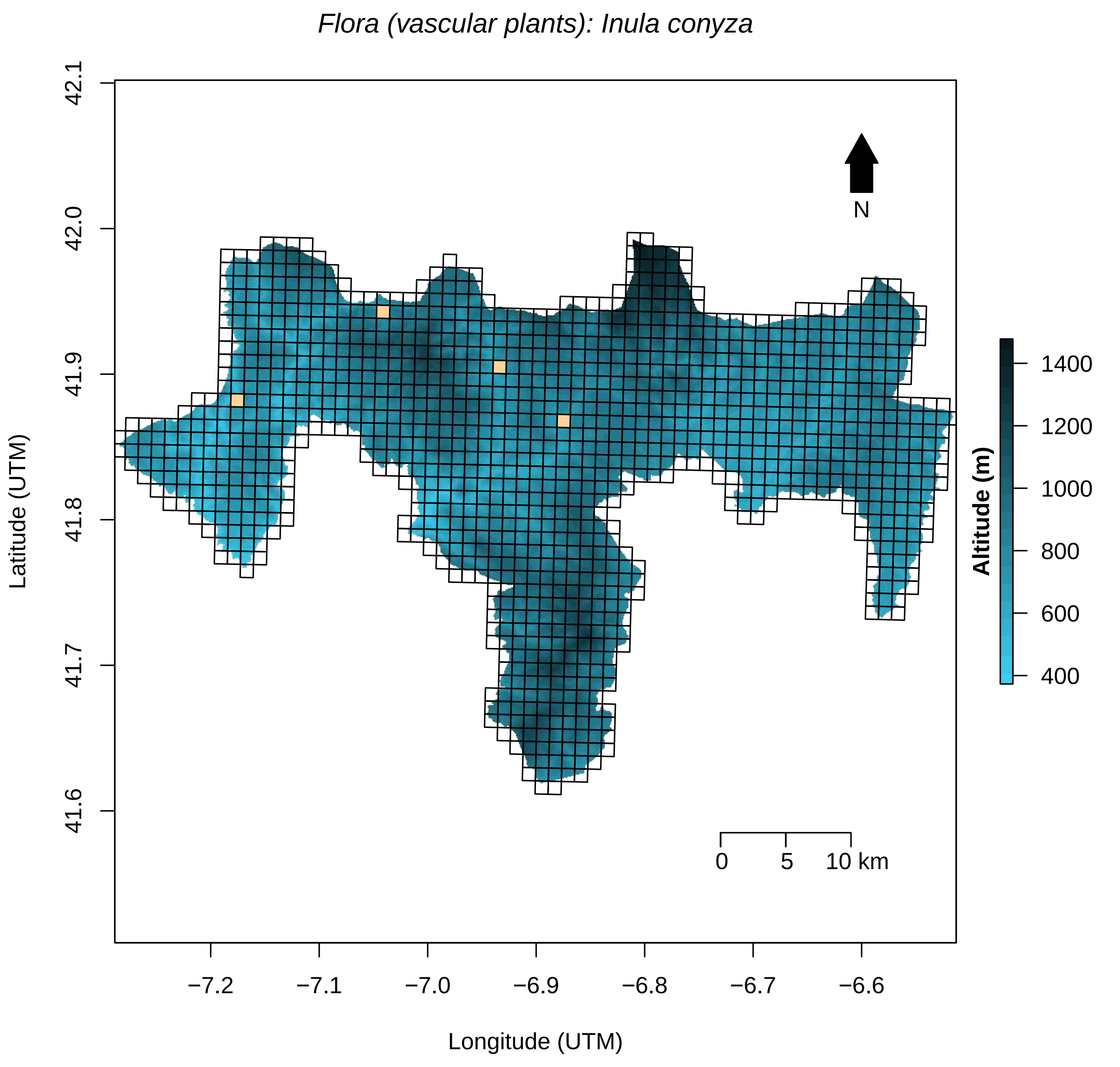
Flora (vascular plants): Inula conyza
42.1
42.0
41.9
41.8
41.7
41.6
Latitude (UTM)
−7.2
−7.1
−7.0
−6.9
−6.8
−6.7
−6.6
Longitude (UTM)
N
0
5
10 km
Altitude (m)
1400
1200
1000
800
600
400

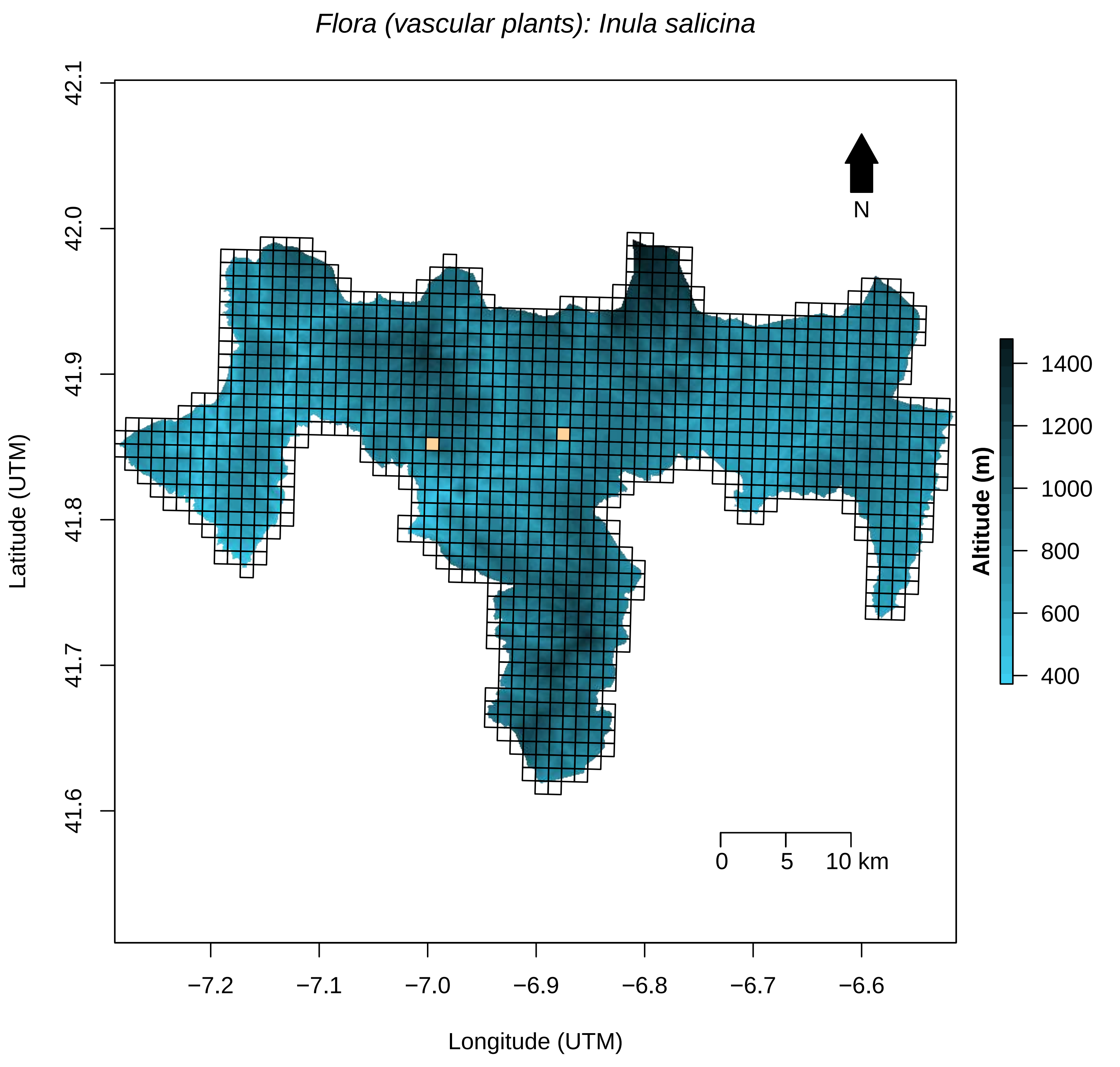

Flora (vascular plants): Inula salicina
N
0
5
10 km
Altitude (m)
1400
1200
1000
800
600
400
42.1
42.0
41.9
41.8
41.7
41.6
Latitude (UTM)
−7.2
−7.1
−7.0
−6.9
−6.8
−6.7
−6.6
Longitude (UTM)

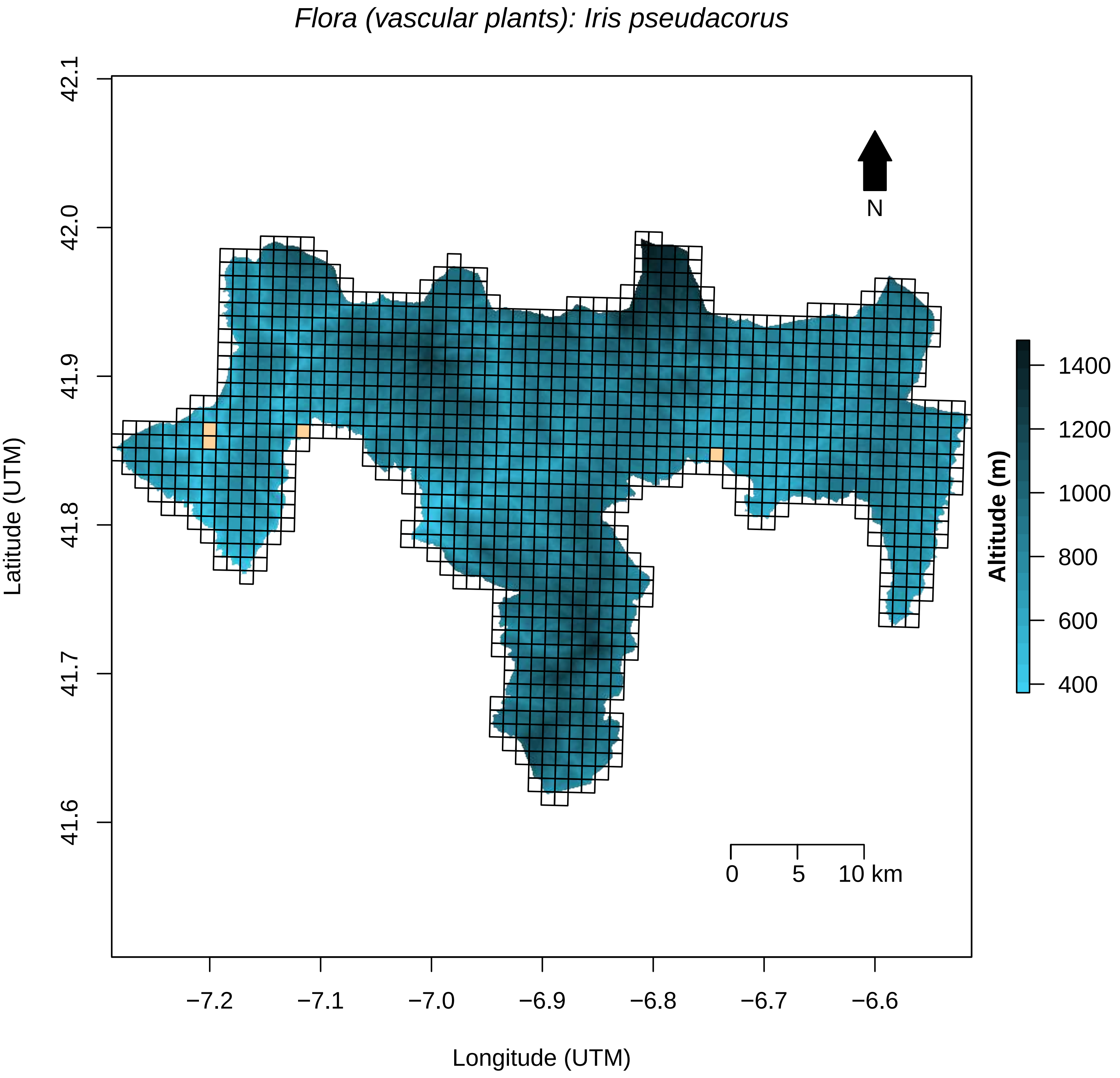

Flora (vascular plants): Iris pseudacorus
42.1
42.0
41.9
41.8
41.7
41.6
Latitude (UTM)
−7.2
−7.1
−7.0
−6.9
−6.8
−6.7
−6.6
Longitude (UTM)
N
0
5
10 km
Altitude (m)
1400
1200
1000
800
600
400

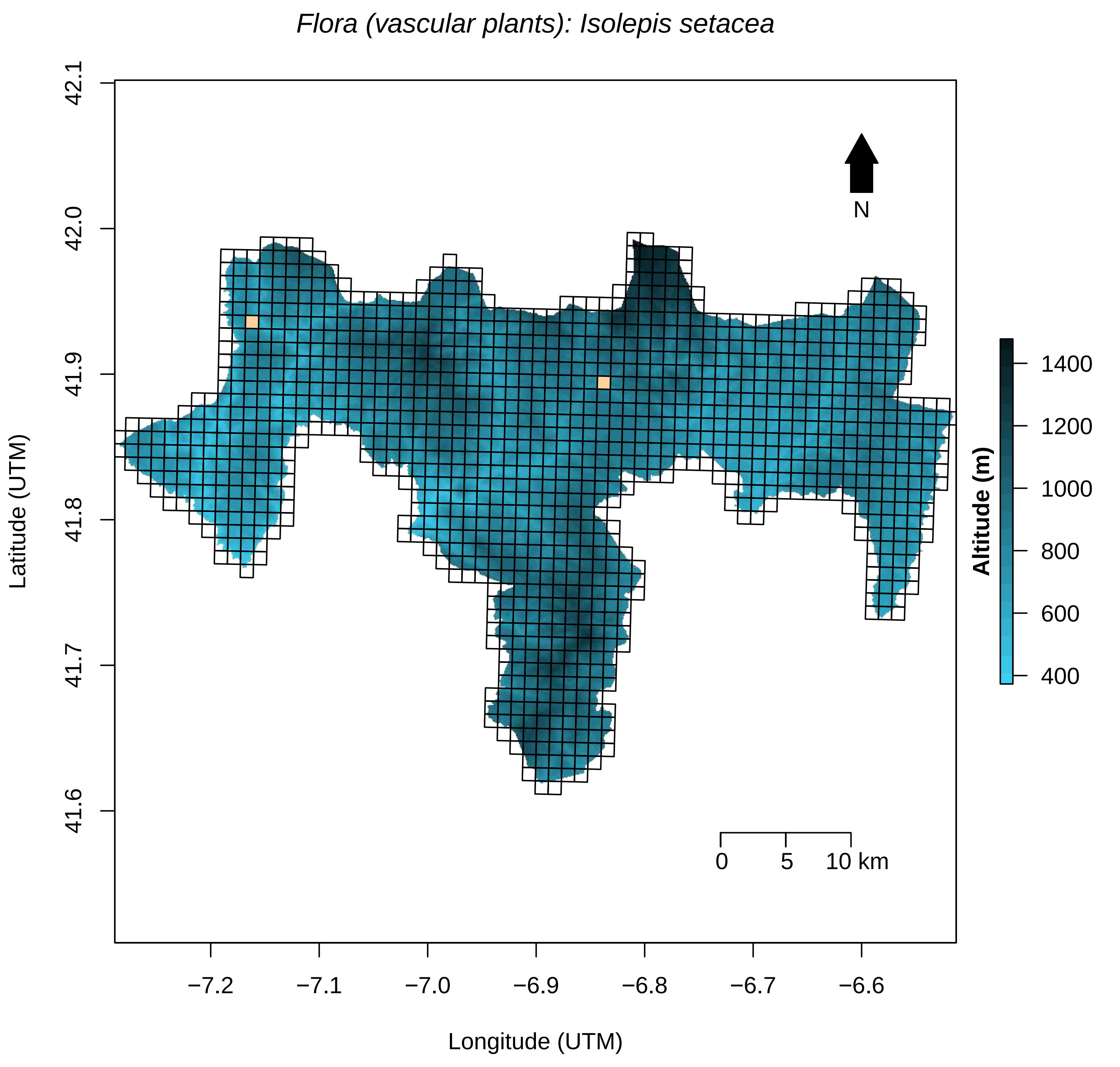
Flora (vascular plants): Isolepis setacea
Latitude (UTM)
Longitude (UTM)
42.1
42.0
41.9
41.8
41.7
41.6
−7.2
−7.1
−7.0
−6.9
−6.8
−6.7
−6.6
N
0
5
10 km
Altitude (m)
1400
1200
1000
800
600
400

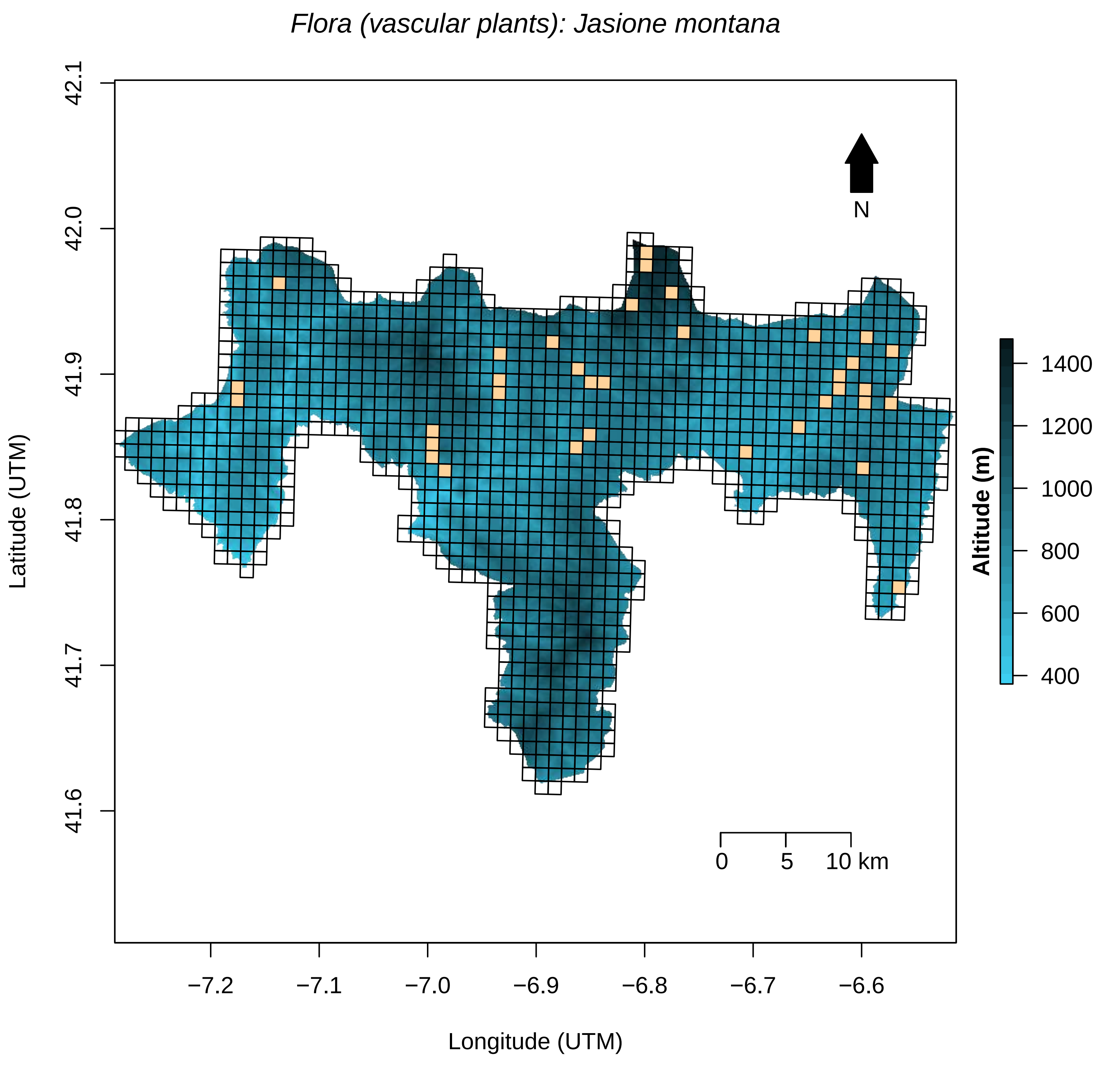

Flora (vascular plants): Jasione montana
Latitude (UTM)
42.1
42.0
41.9
41.8
41.7
41.6
Longitude (UTM)
−7.2
−7.1
−7.0
−6.9
−6.8
−6.7
−6.6
N
0
5
10 km
Altitude (m)
1400
1200
1000
800
600
400

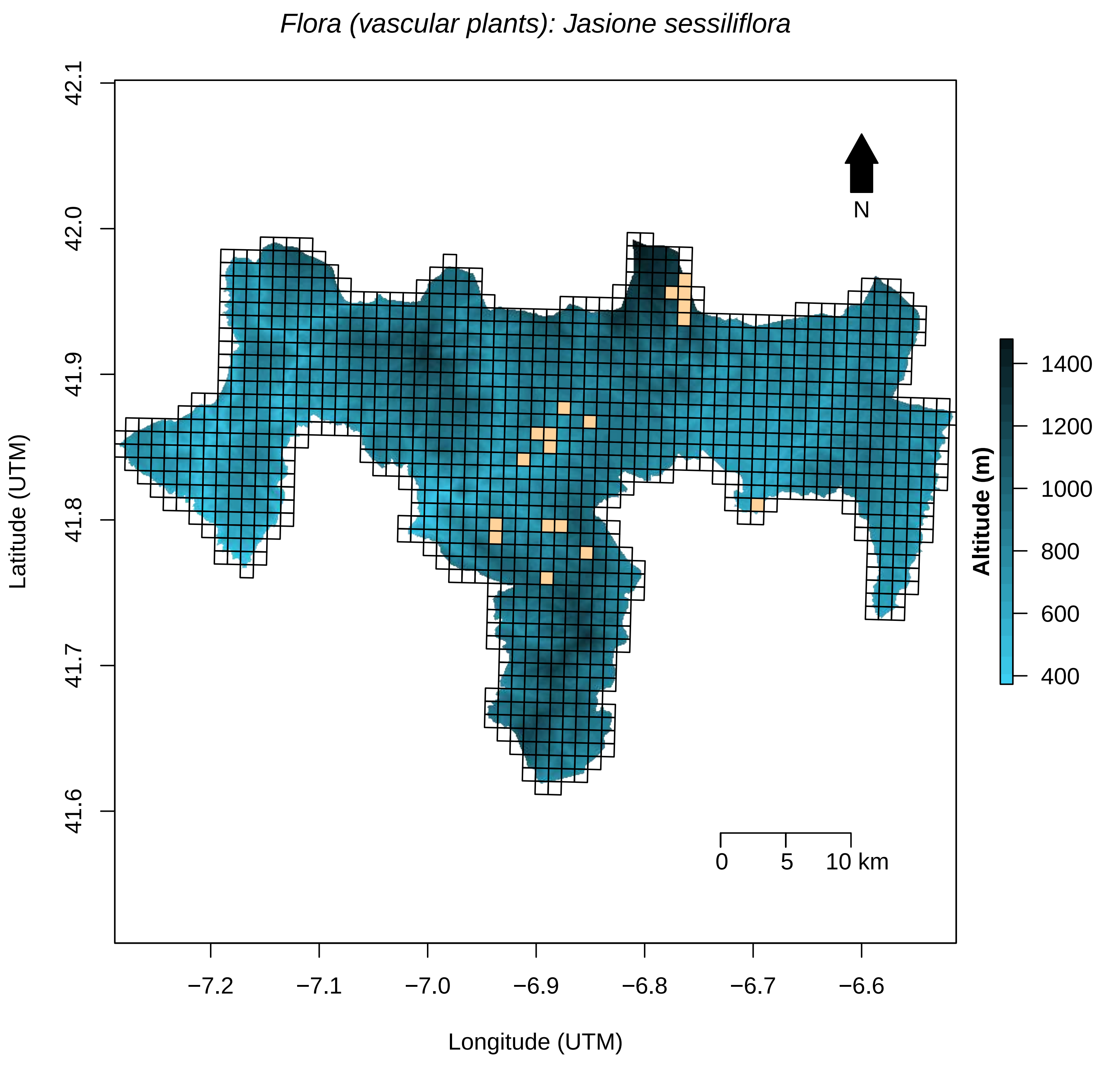

Flora (vascular plants): Jasione sessiliflora
N
0
5
10 km
Altitude (m)
1400
1200
1000
800
600
400
Latitude (UTM)
42.1
42.0
41.9
41.8
41.7
41.6
Longitude (UTM)
−7.2
−7.1
−7.0
−6.9
−6.8
−6.7
−6.6

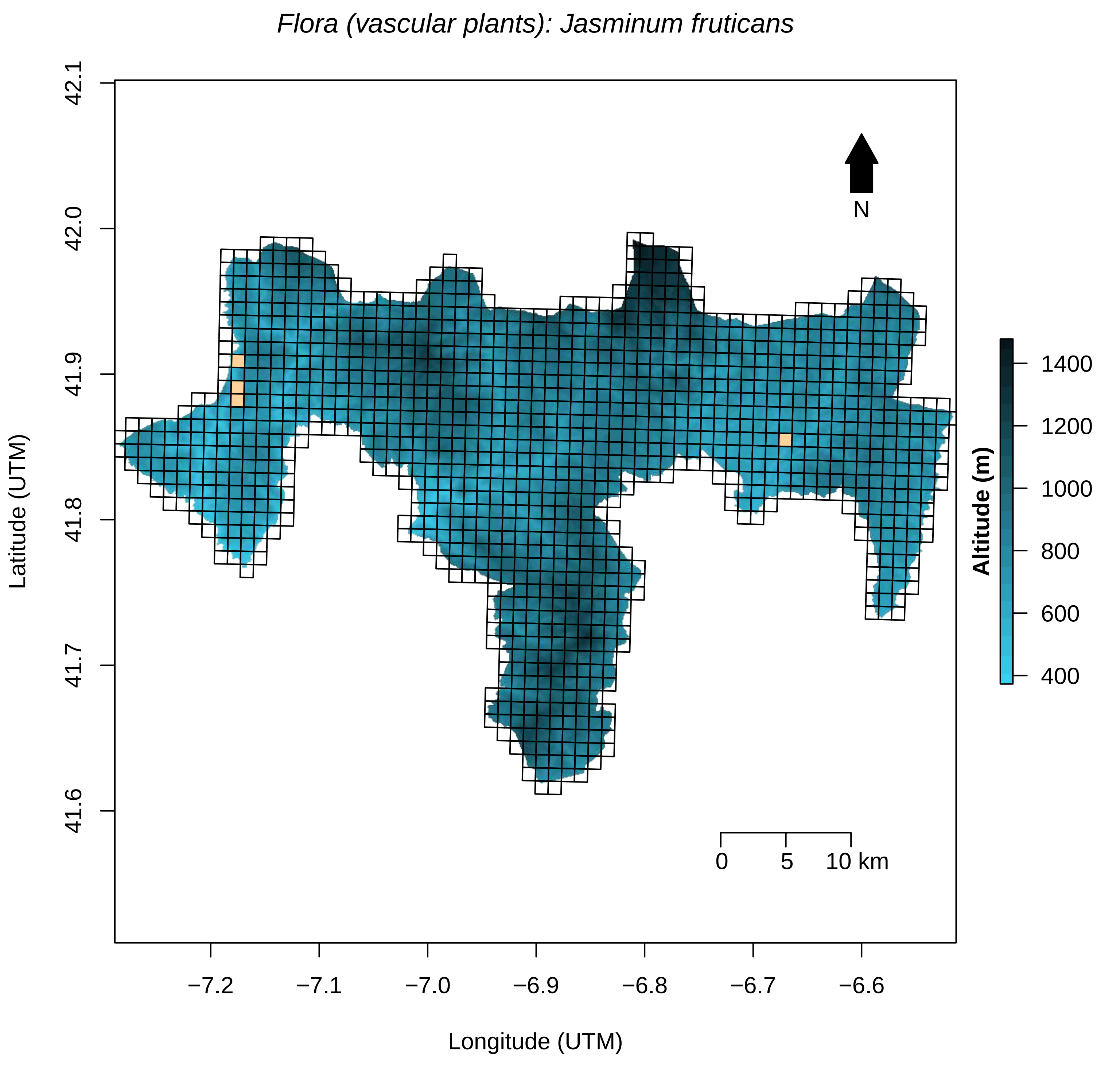

Flora (vascular plants): Jasminum fruticans
42.1
42.0
41.9
41.8
41.7
41.6
Latitude (UTM)
−7.2
−7.1
−7.0
−6.9
−6.8
−6.7
−6.6
Longitude (UTM)
N
0
5
10 km
Altitude (m)
1400
1200
1000
800
600
400

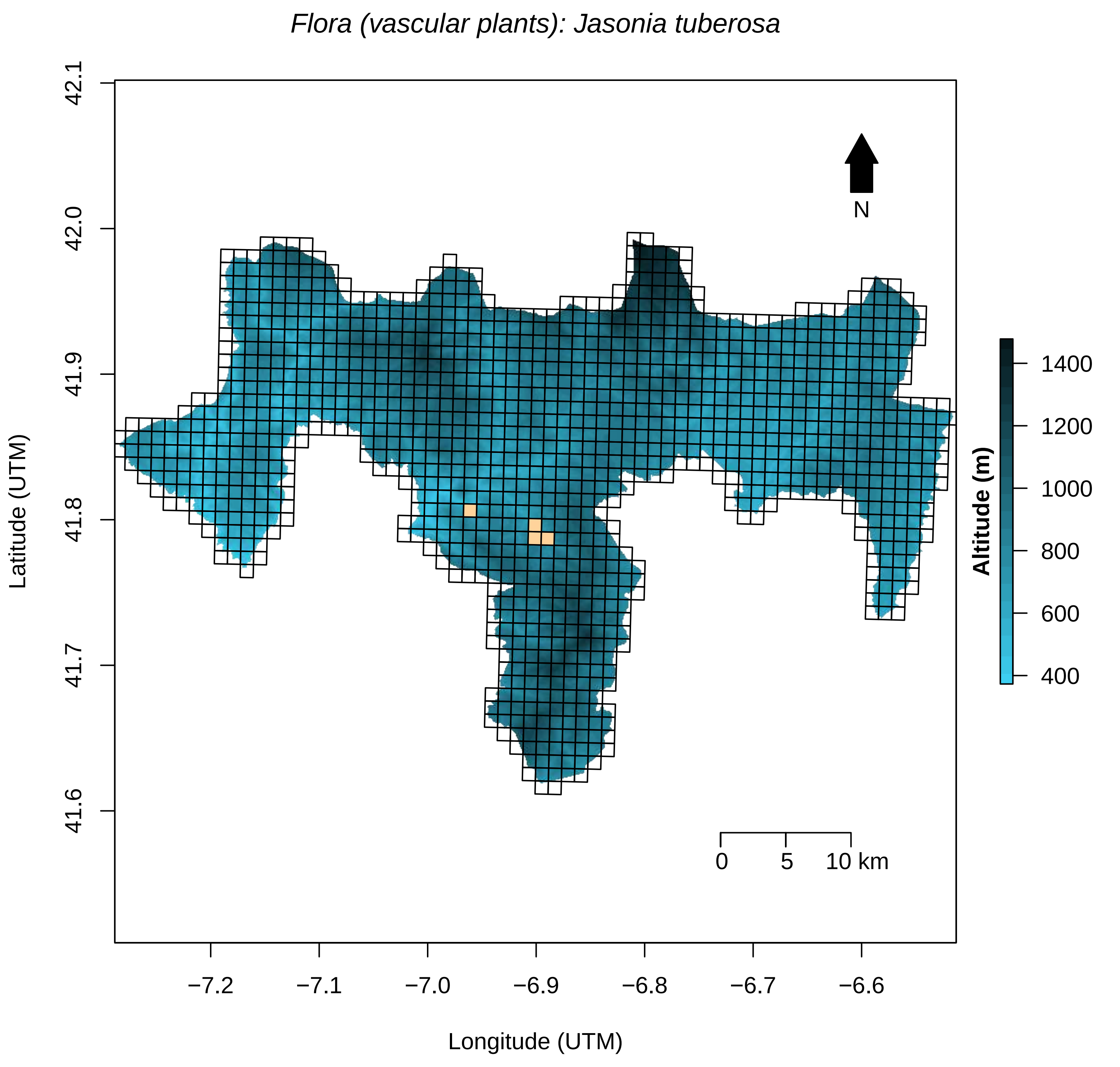
Flora (vascular plants): Jasonia tuberosa
N
0
5
10 km
Altitude (m)
1400
1200
1000
800
600
400
Latitude (UTM)
42.1
42.0
41.9
41.8
41.7
41.6
Longitude (UTM)
−7.2
−7.1
−7.0
−6.9
−6.8
−6.7
−6.6

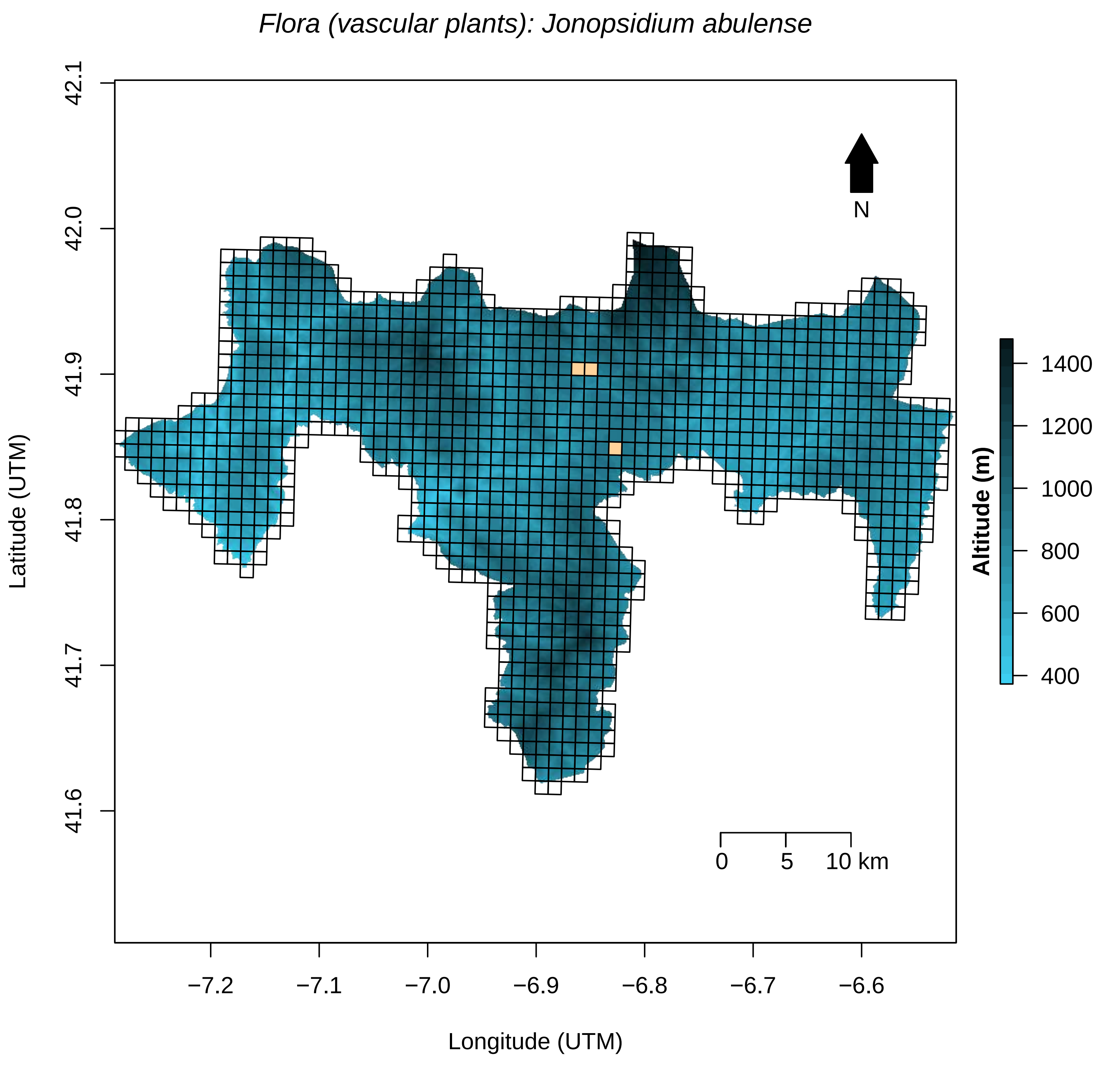

Flora (vascular plants): Jonopsidium abulense
42.1
42.0
41.9
41.8
41.7
41.6
Latitude (UTM)
−7.2
−7.1
−7.0
−6.9
−6.8
−6.7
−6.6
Longitude (UTM)
N
0
5
10 km
Altitude (m)
1400
1200
1000
800
600
400

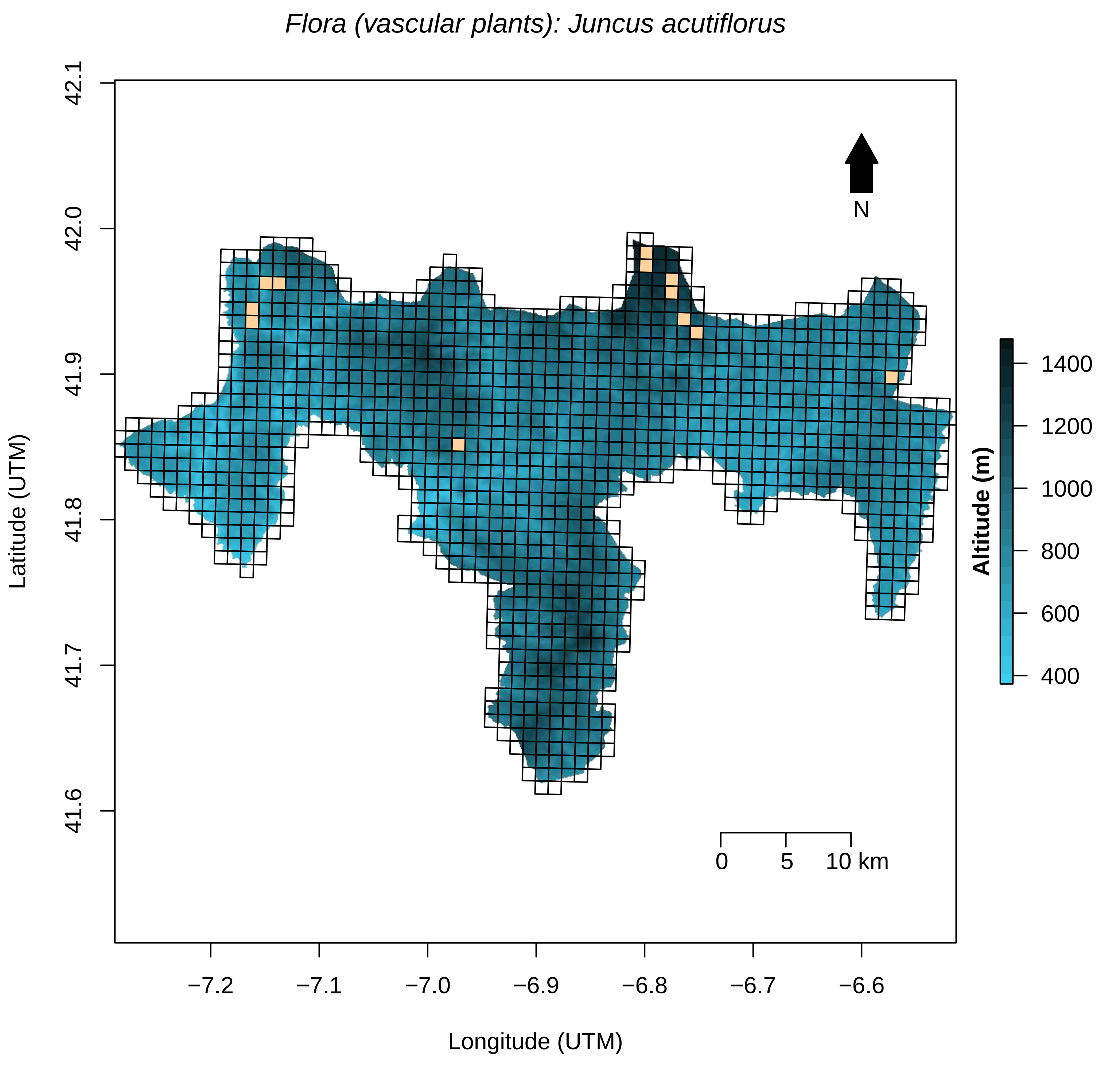

Flora (vascular plants): Juncus acutiflorus
Latitude (UTM)
42.1
42.0
41.9
41.8
41.7
41.6
Longitude (UTM)
−7.2
−7.1
−7.0
−6.9
−6.8
−6.7
−6.6
N
0
5
10 km
Altitude (m)
1400
1200
1000
800
600
400

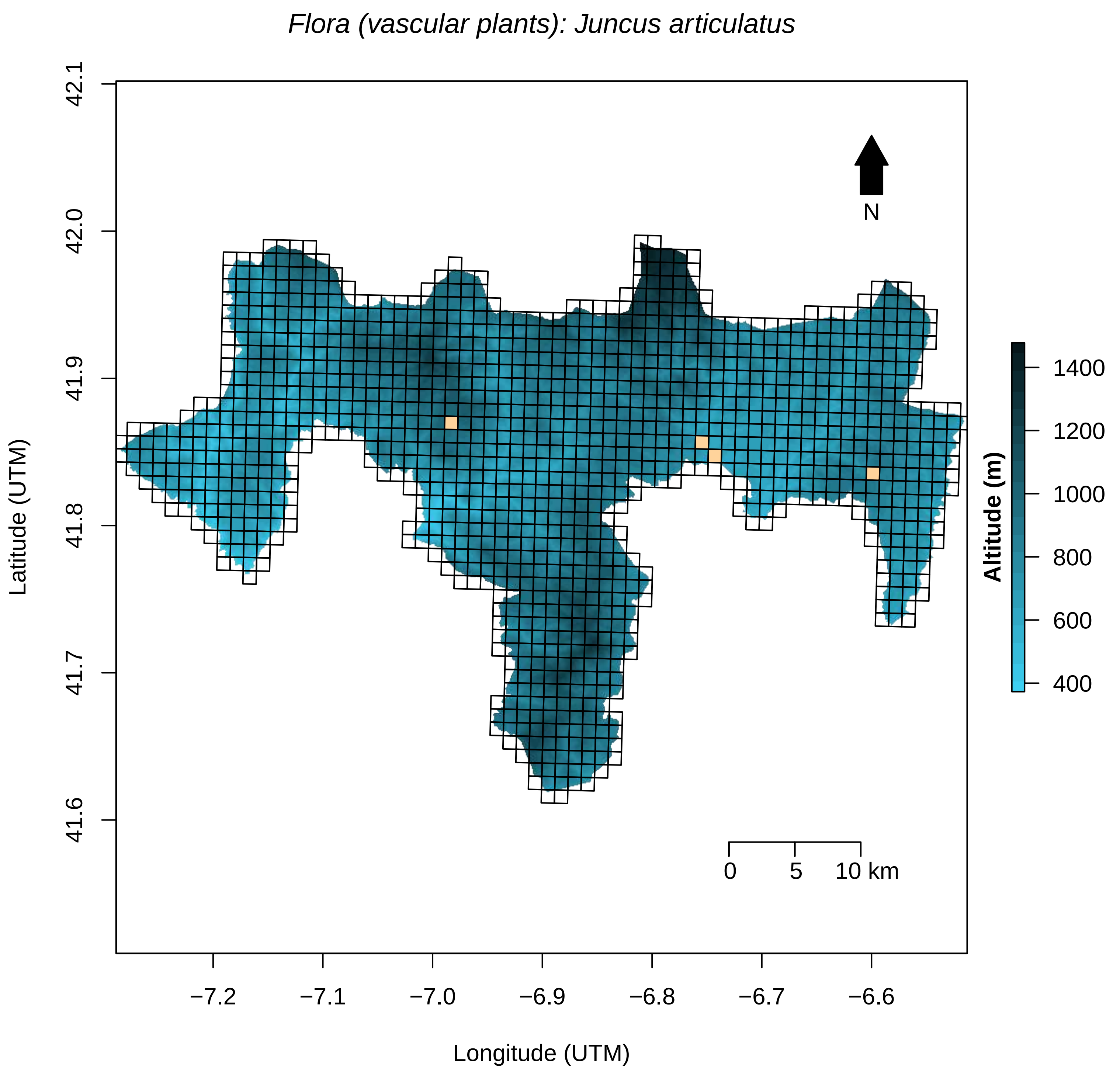
Flora (vascular plants): Juncus articulatus
42.1
42.0
41.9
41.8
41.7
41.6
Latitude (UTM)
−7.2
−7.1
−7.0
−6.9
−6.8
−6.7
−6.6
Longitude (UTM)
N
0
5
10 km
Altitude (m)
1400
1200
1000
800
600
400

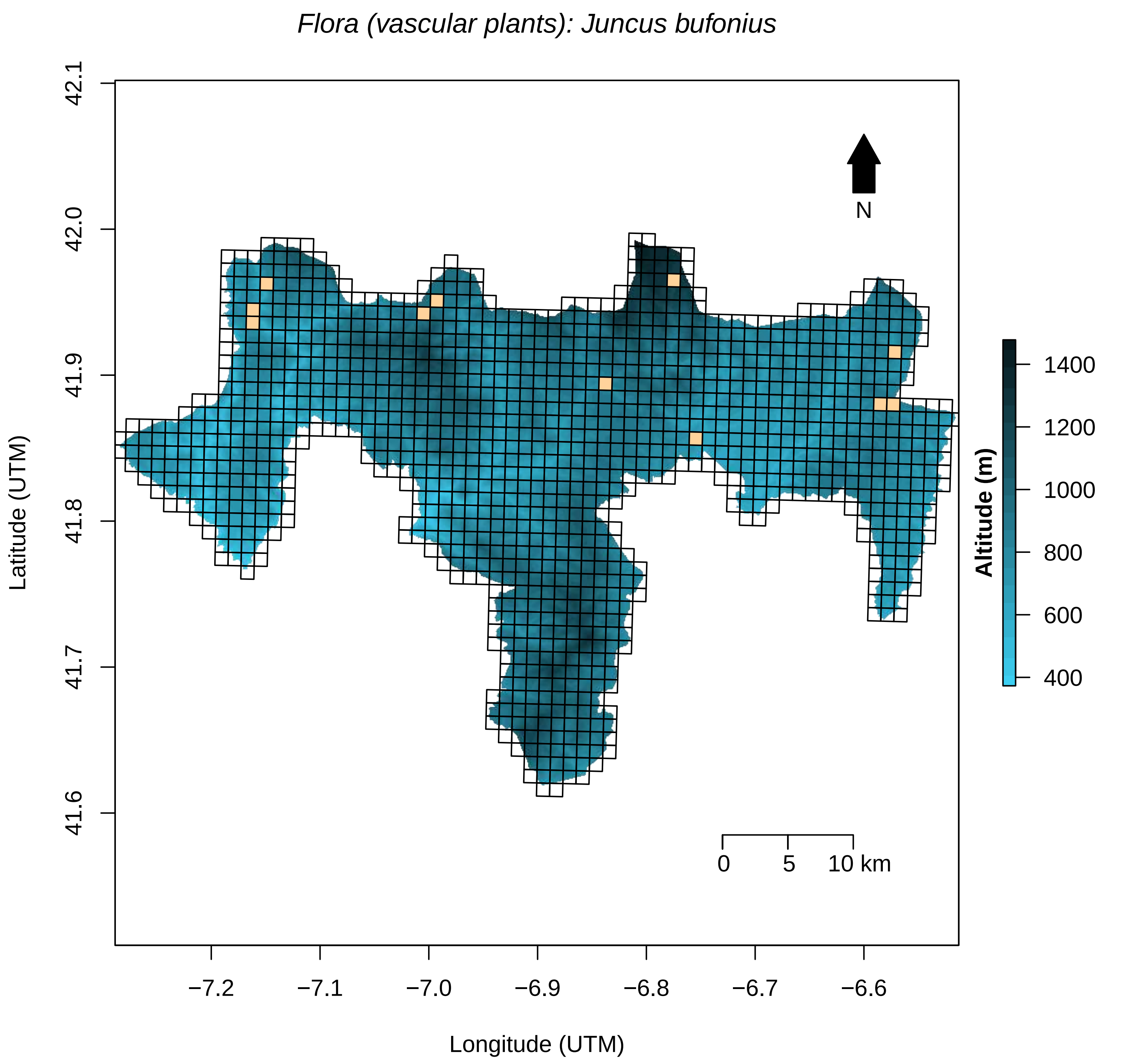

Flora (vascular plants): Juncus bufonius
42.1
42.0
41.9
41.8
41.7
41.6
Latitude (UTM)
−7.2
−7.1
−7.0
−6.9
−6.8
−6.7
−6.6
Longitude (UTM)
N
0
5
10 km
Altitude (m)
1400
1200
1000
800
600
400

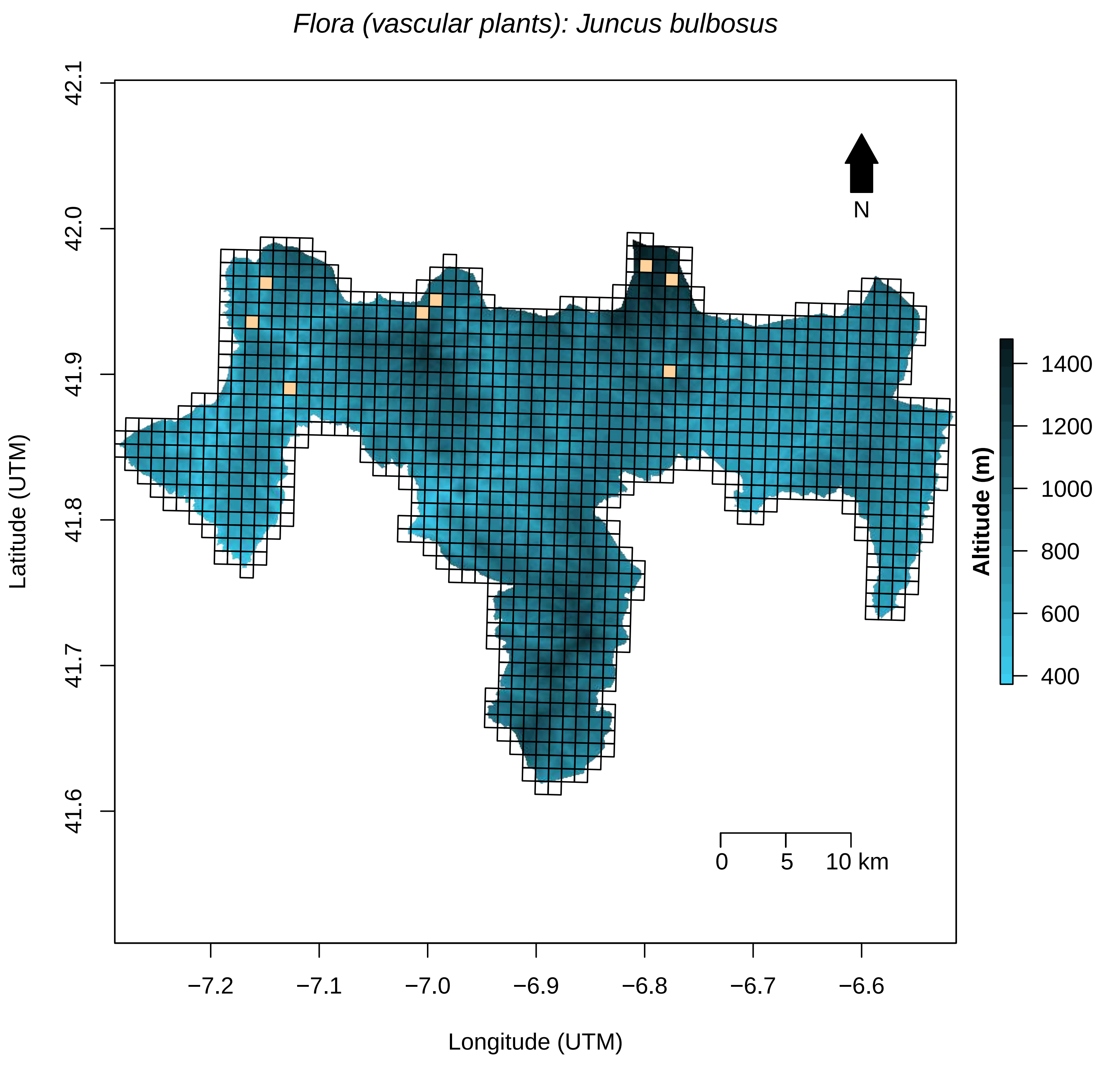

Flora (vascular plants): Juncus bulbosus
Latitude (UTM)
Longitude (UTM)
42.1
42.0
41.9
41.8
41.7
41.6
−7.2
−7.1
−7.0
−6.9
−6.8
−6.7
−6.6
N
0
5
10 km
Altitude (m)
1400
1200
1000
800
600
400

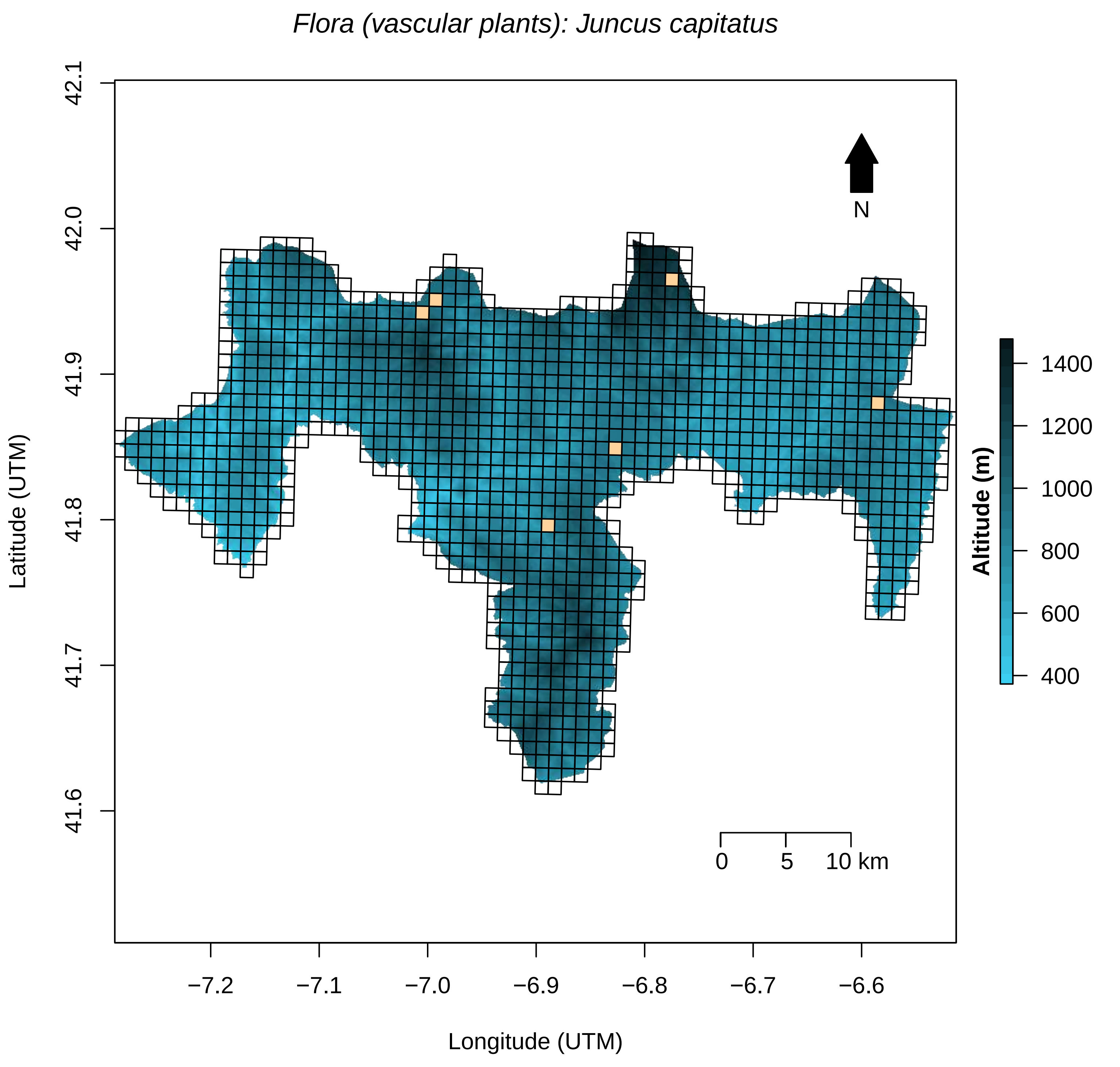

Flora (vascular plants): Juncus capitatus
42.1
42.0
41.9
41.8
41.7
41.6
Latitude (UTM)
−7.2
−7.1
−7.0
−6.9
−6.8
−6.7
−6.6
Longitude (UTM)
N
0
5
10 km
Altitude (m)
1400
1200
1000
800
600
400

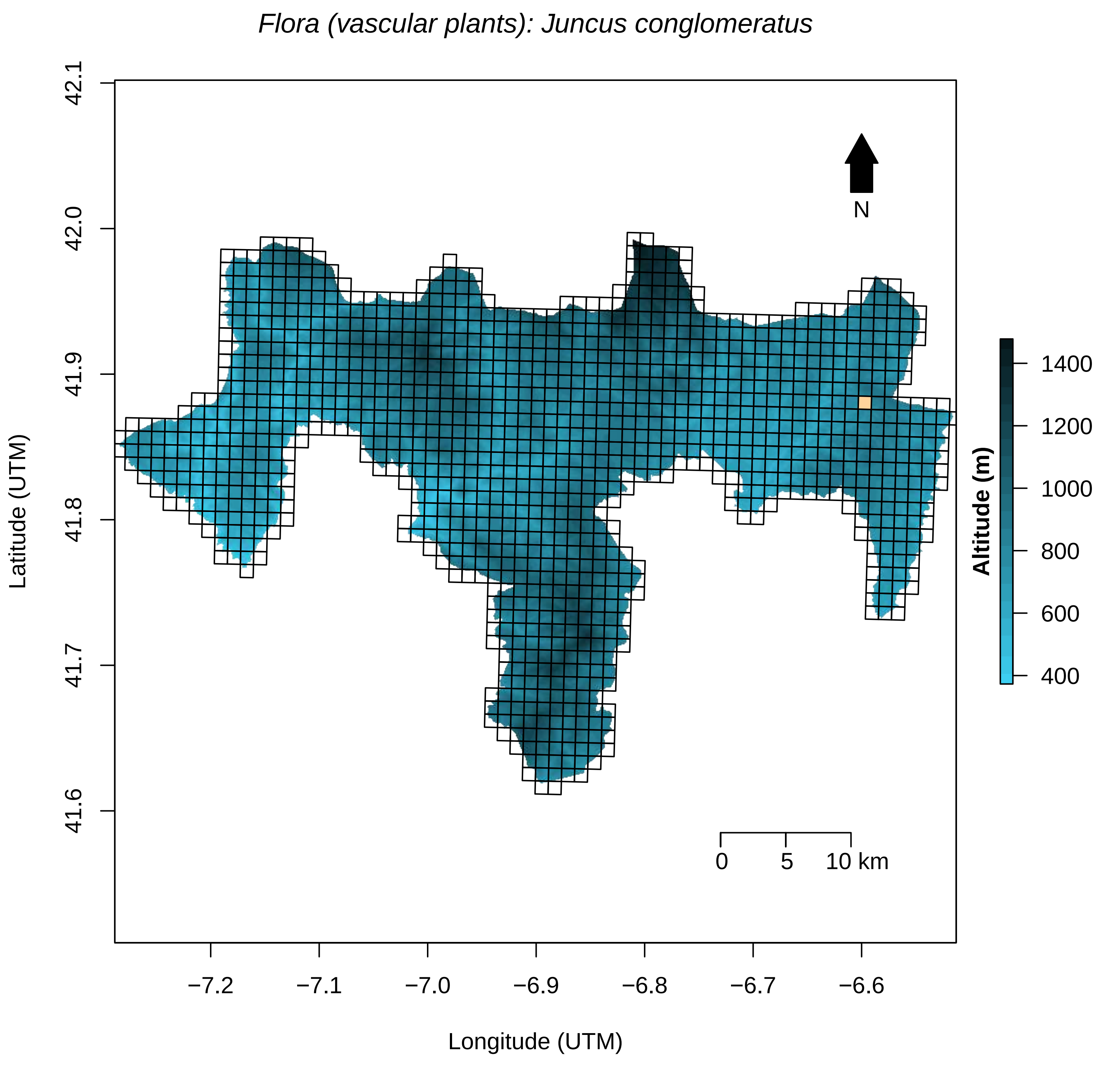

Flora (vascular plants): Juncus conglomeratus
N
0
5
10 km
Altitude (m)
1400
1200
1000
800
600
400
42.1
42.0
41.9
41.8
41.7
41.6
Latitude (UTM)
−7.2
−7.1
−7.0
−6.9
−6.8
−6.7
−6.6
Longitude (UTM)

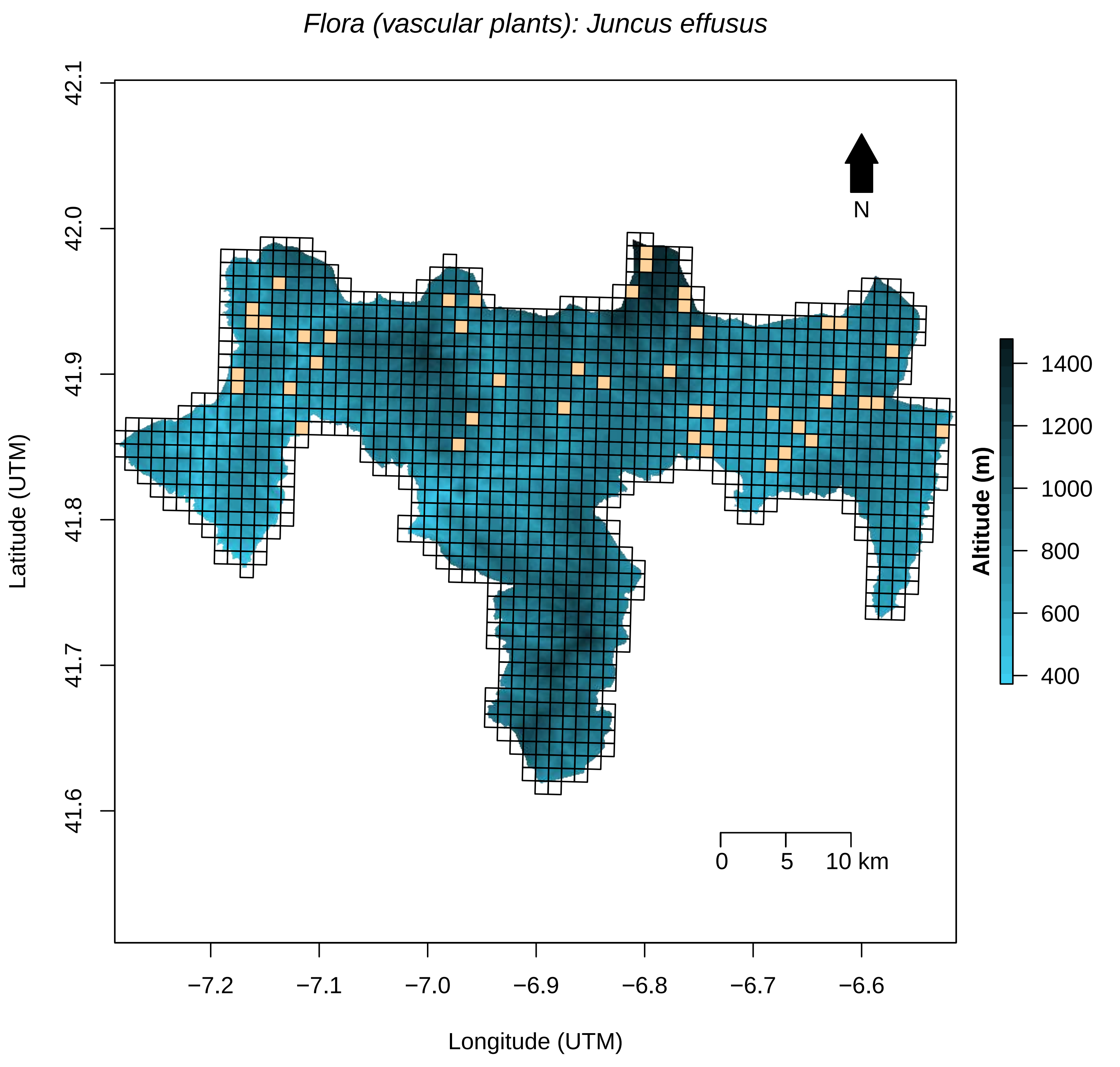

Flora (vascular plants): Juncus effusus
42.1
42.0
41.9
41.8
41.7
41.6
Latitude (UTM)
−7.2
−7.1
−7.0
−6.9
−6.8
−6.7
−6.6
Longitude (UTM)
N
0
5
10 km
Altitude (m)
1400
1200
1000
800
600
400

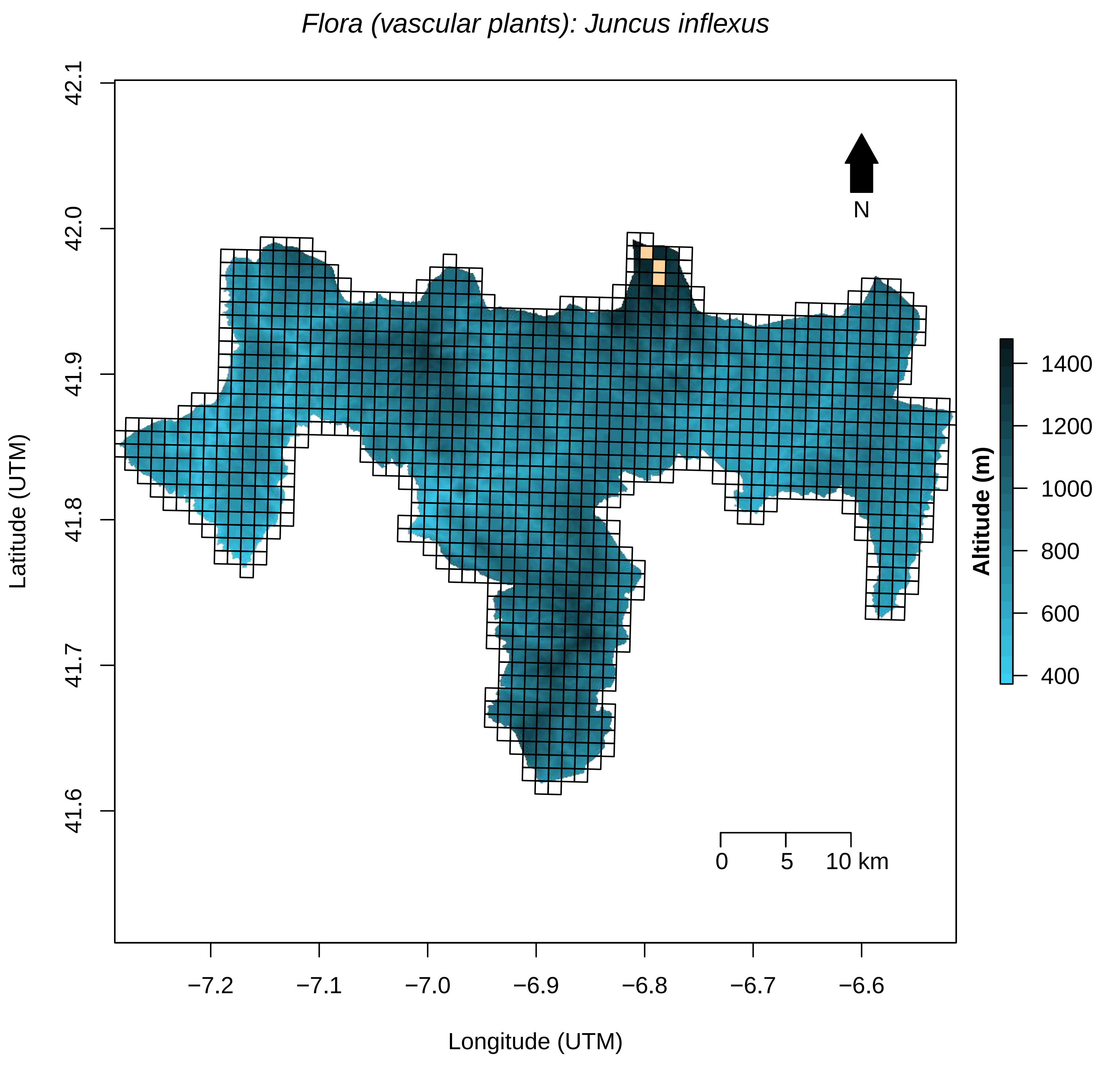

Flora (vascular plants): Juncus inflexus
Latitude (UTM)
Longitude (UTM)
42.1
42.0
41.9
41.8
41.7
41.6
−7.2
−7.1
−7.0
−6.9
−6.8
−6.7
−6.6
N
0
5
10 km
Altitude (m)
1400
1200
1000
800
600
400

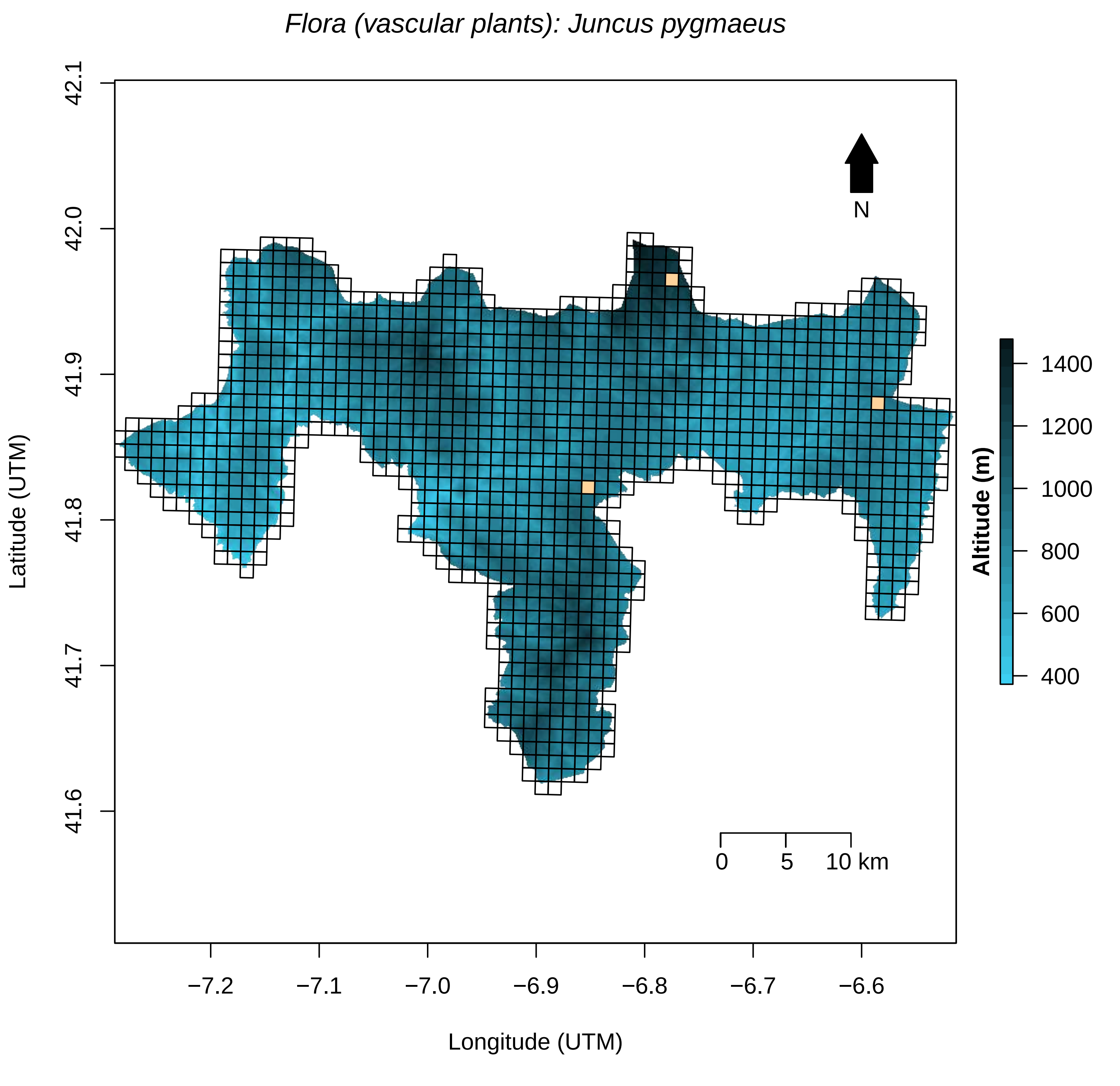

Flora (vascular plants): Juncus pygmaeus
42.1
42.0
41.9
41.8
41.7
41.6
Latitude (UTM)
−7.2
−7.1
−7.0
−6.9
−6.8
−6.7
−6.6
Longitude (UTM)
N
0
5
10 km
Altitude (m)
1400
1200
1000
800
600
400

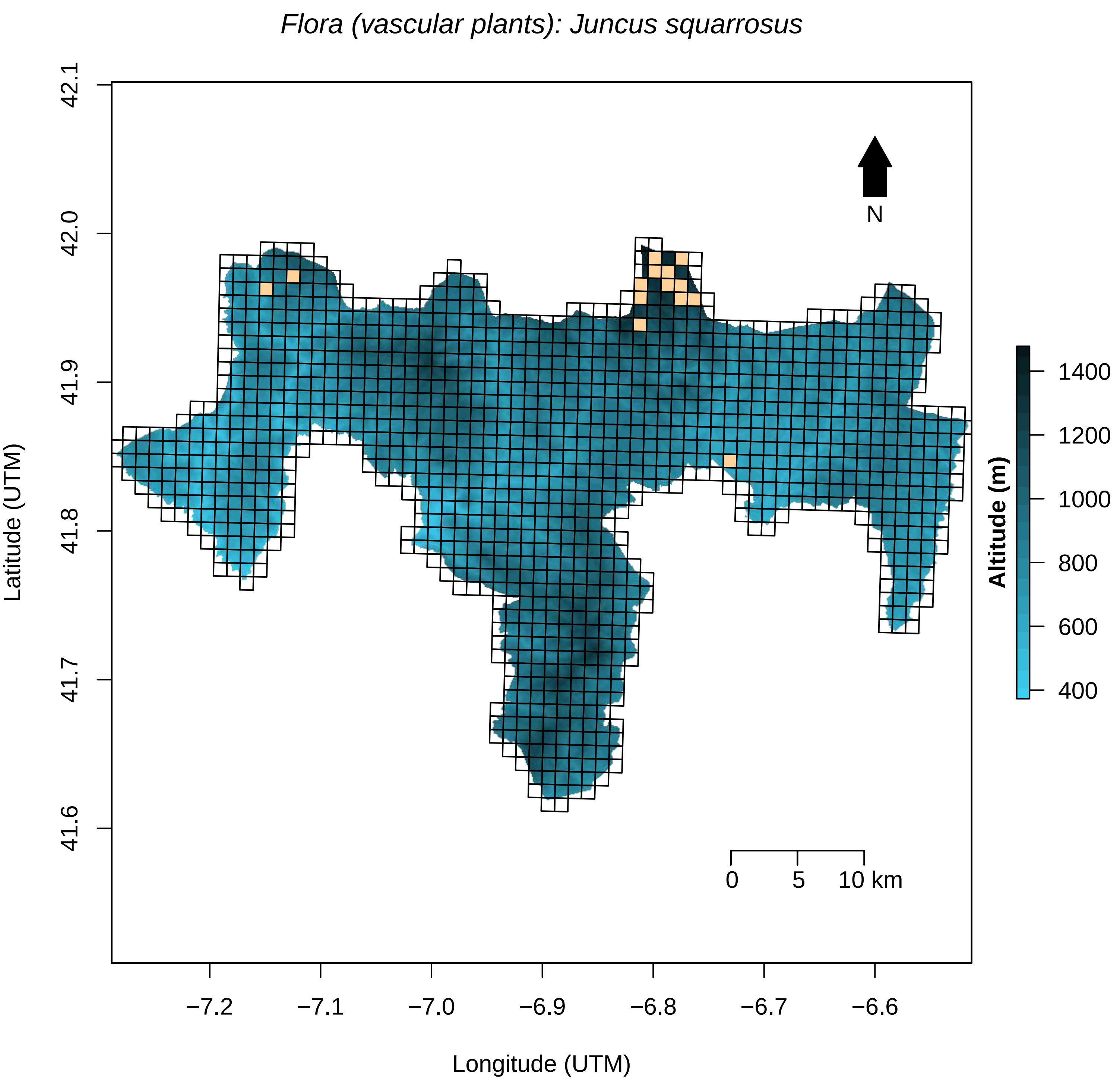

Flora (vascular plants): Juncus squarrosus
Latitude (UTM)
Longitude (UTM)
42.1
42.0
41.9
41.8
41.7
41.6
−7.2
−7.1
−7.0
−6.9
−6.8
−6.7
−6.6
N
0
5
10 km
Altitude (m)
1400
1200
1000
800
600
400

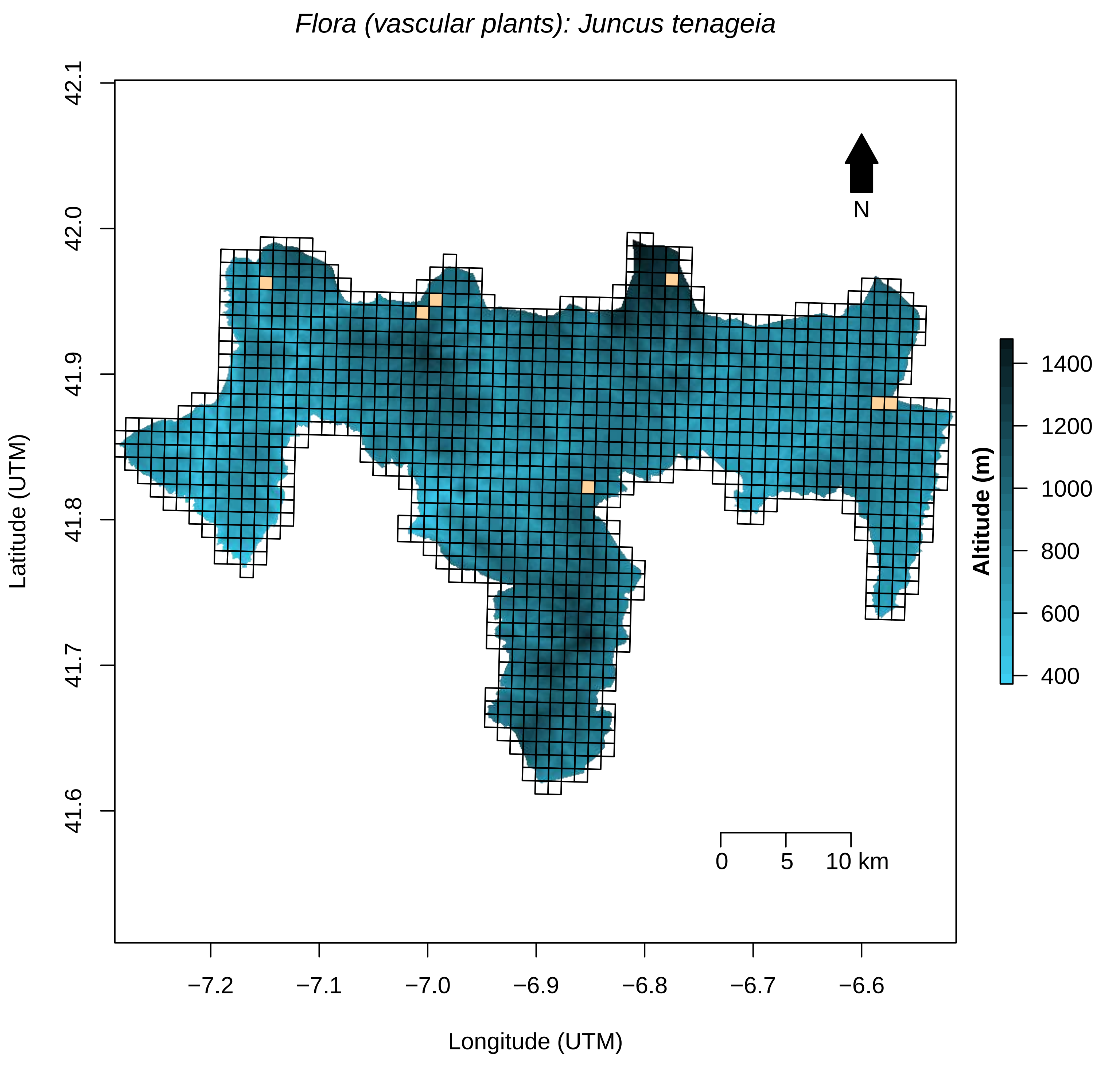

Flora (vascular plants): Juncus tenageia
Latitude (UTM)
Longitude (UTM)
42.1
42.0
41.9
41.8
41.7
41.6
−7.2
−7.1
−7.0
−6.9
−6.8
−6.7
−6.6
N
0
5
10 km
Altitude (m)
1400
1200
1000
800
600
400

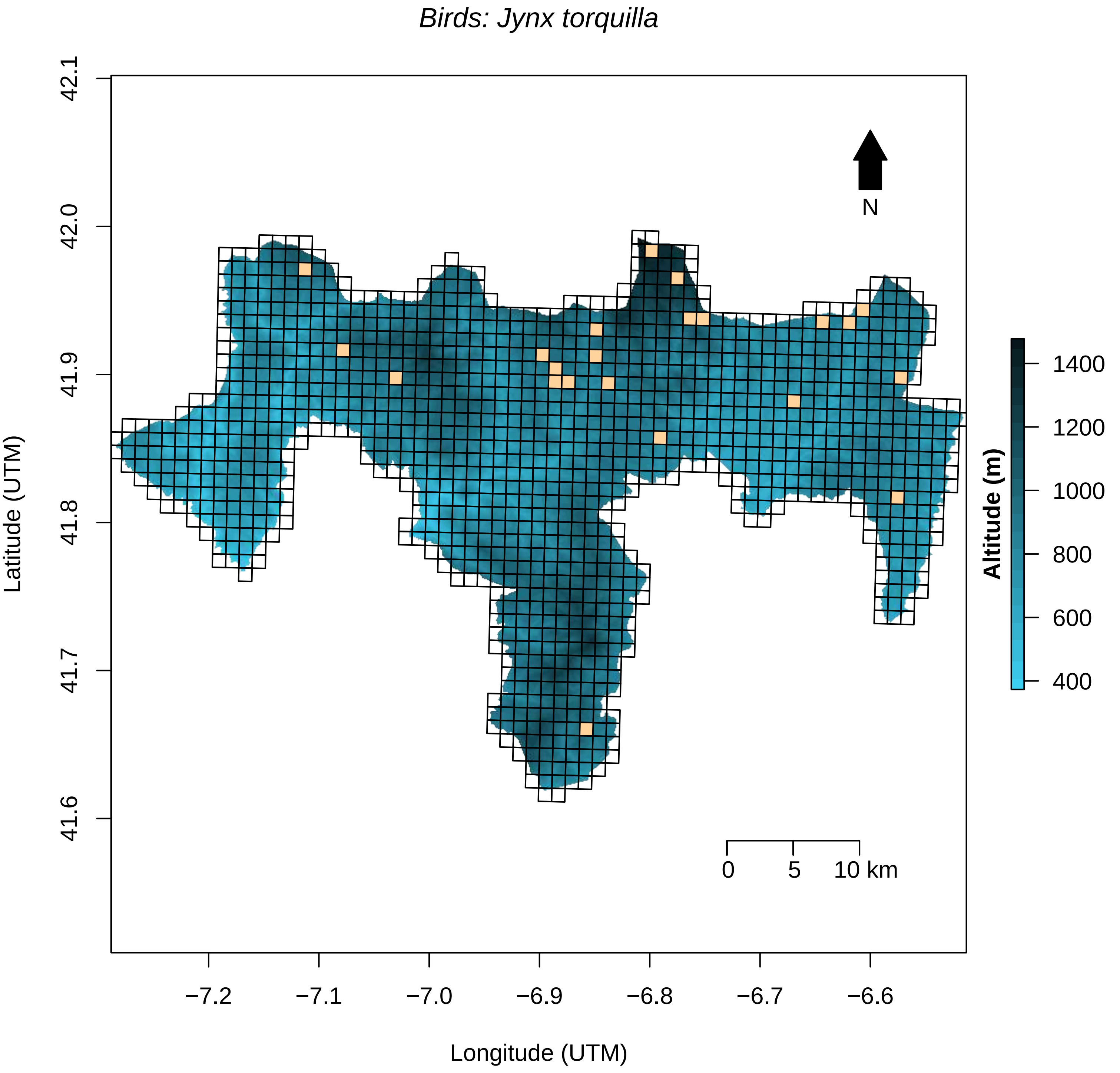
Birds: Jynx torquilla
42.1
42.0
41.9
41.8
41.7
41.6
Latitude (UTM)
−7.2
−7.1
−7.0
−6.9
−6.8
−6.7
−6.6
Longitude (UTM)
N
0
5
10 km
Altitude (m)
1400
1200
1000
800
600
400

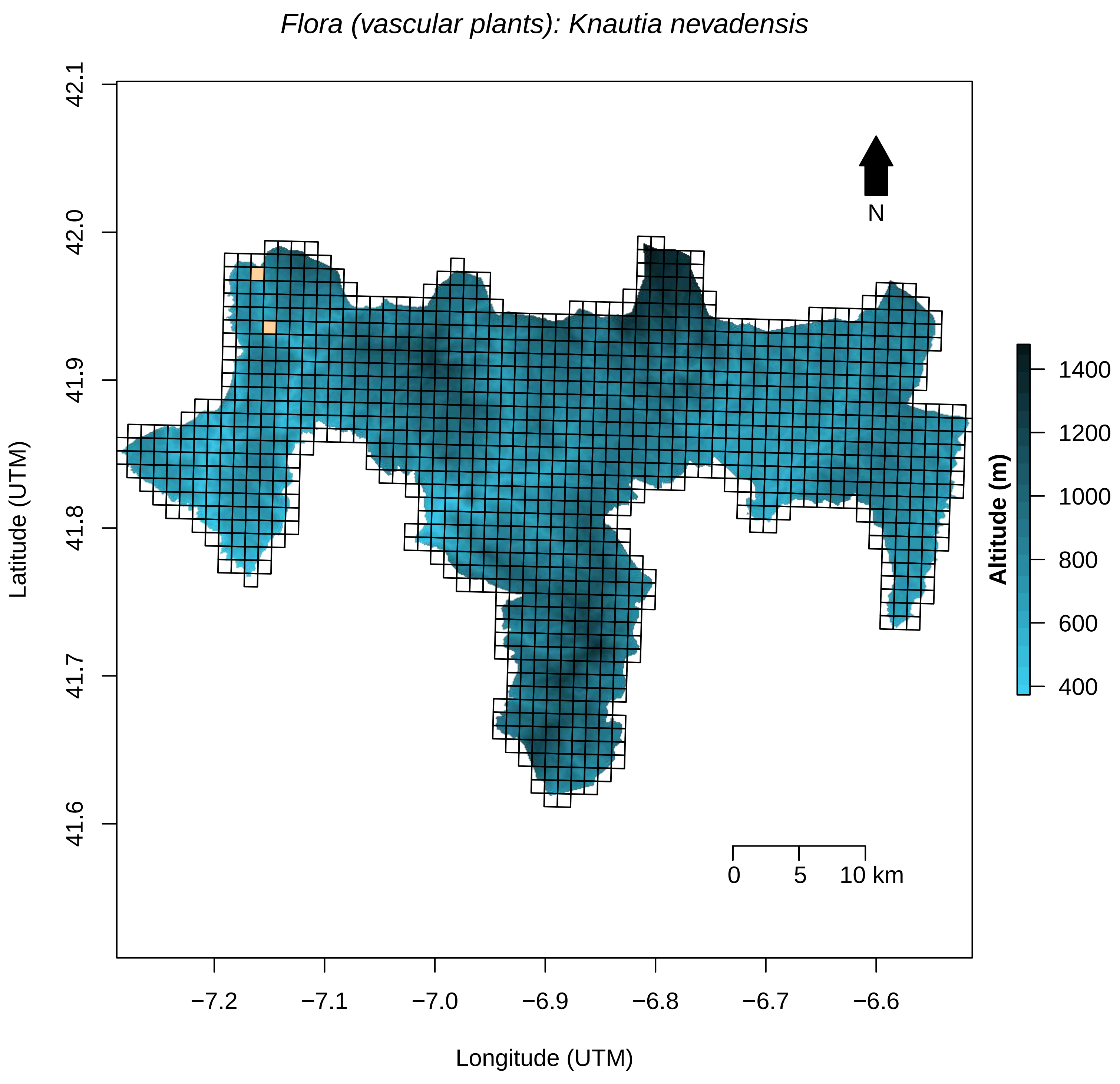

Flora (vascular plants): Knautia nevadensis
Latitude (UTM)
Longitude (UTM)
42.1
42.0
41.9
41.8
41.7
41.6
−7.2
−7.1
−7.0
−6.9
−6.8
−6.7
−6.6
N
0
5
10 km
Altitude (m)
1400
1200
1000
800
600
400

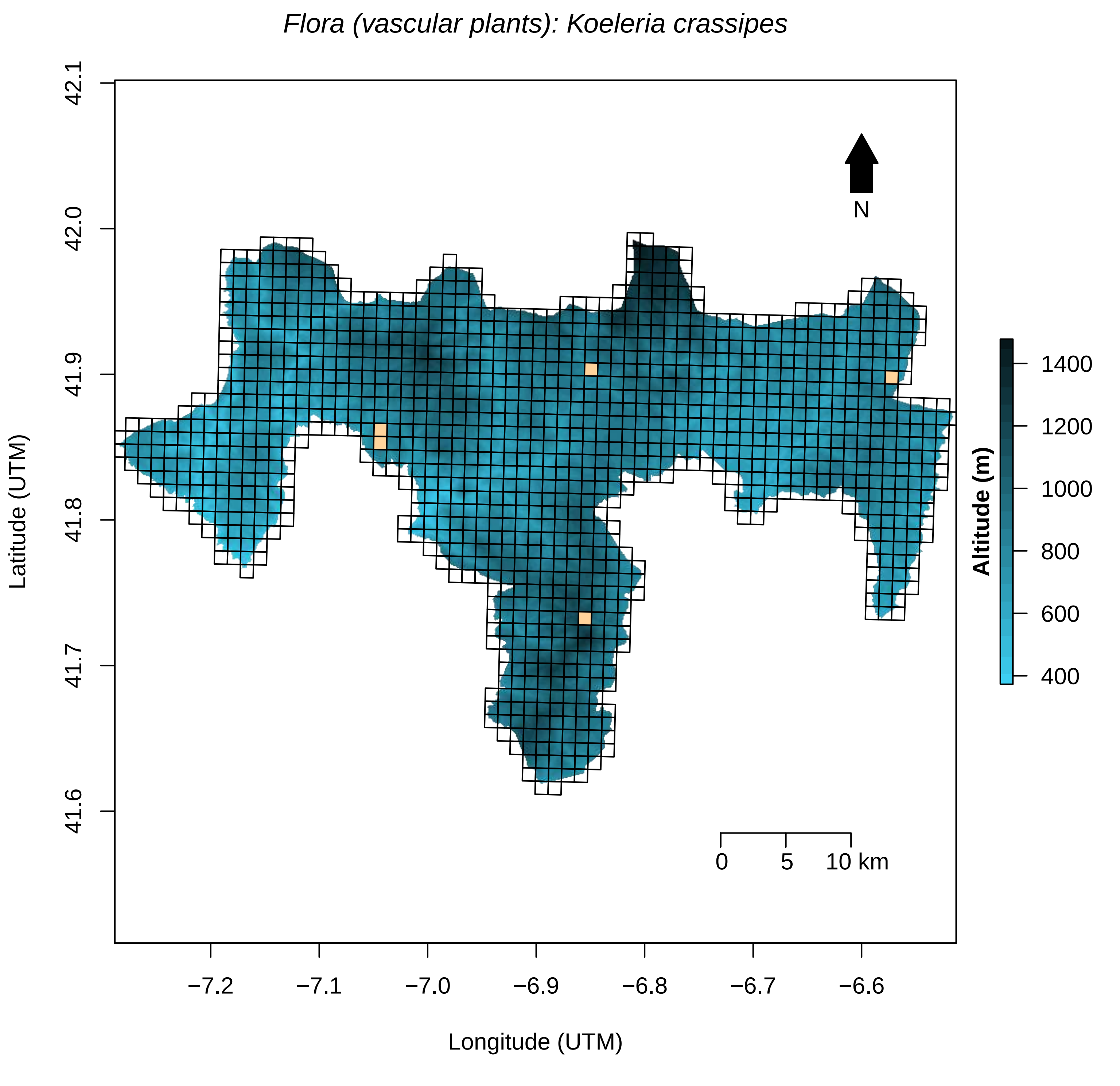
Flora (vascular plants): Koeleria crassipes
42.1
42.0
41.9
41.8
41.7
41.6
Latitude (UTM)
−7.2
−7.1
−7.0
−6.9
−6.8
−6.7
−6.6
Longitude (UTM)
N
0
5
10 km
1400
1200
1000
800
600
400
Altitude (m)

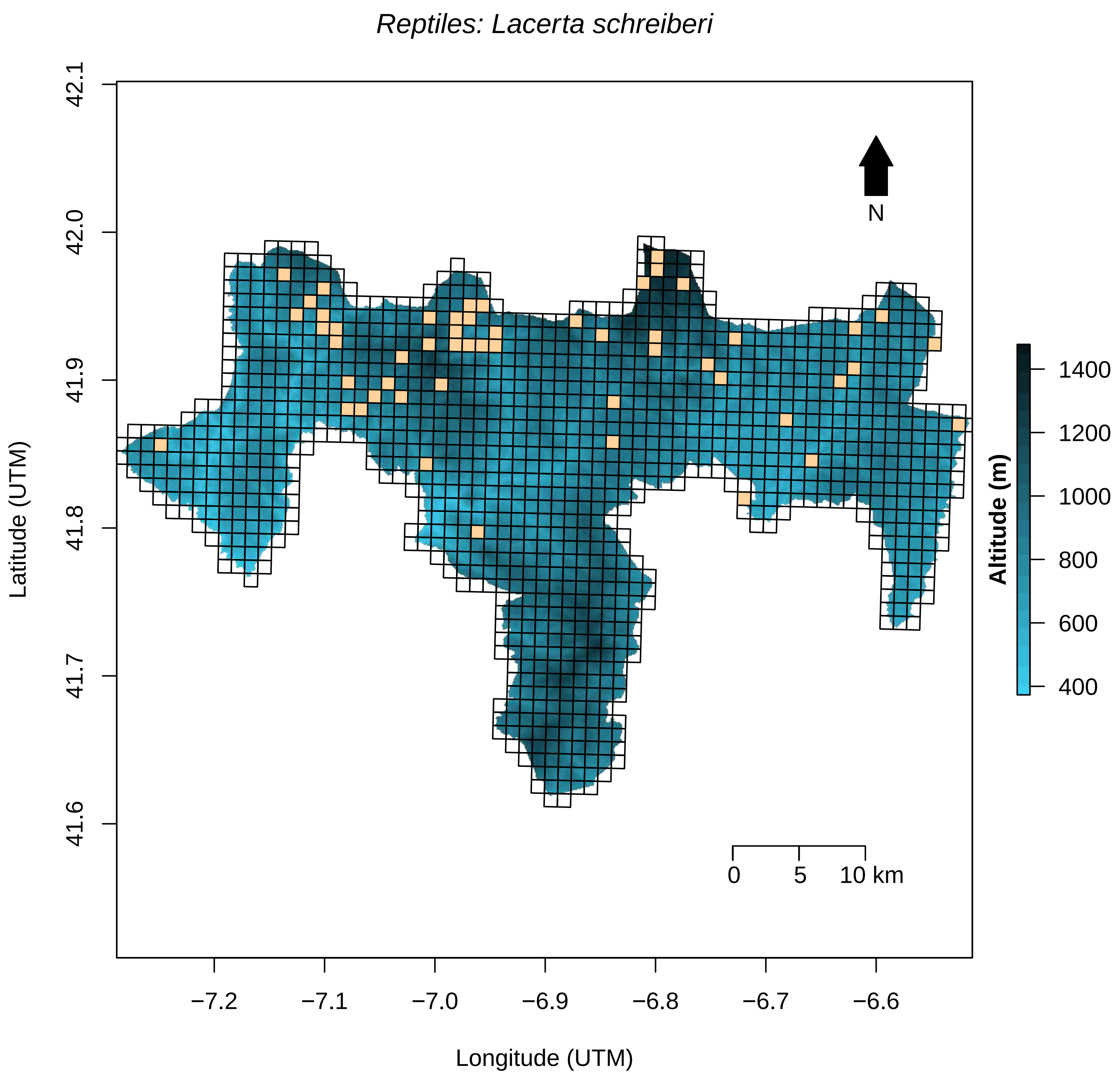

Reptiles: Lacerta schreiberi
42.1
42.0
41.9
41.8
41.7
41.6
Latitude (UTM)
−7.2
−7.1
−7.0
−6.9
−6.8
−6.7
−6.6
Longitude (UTM)
N
0
5
10 km
Altitude (m)
1400
1200
1000
800
600
400

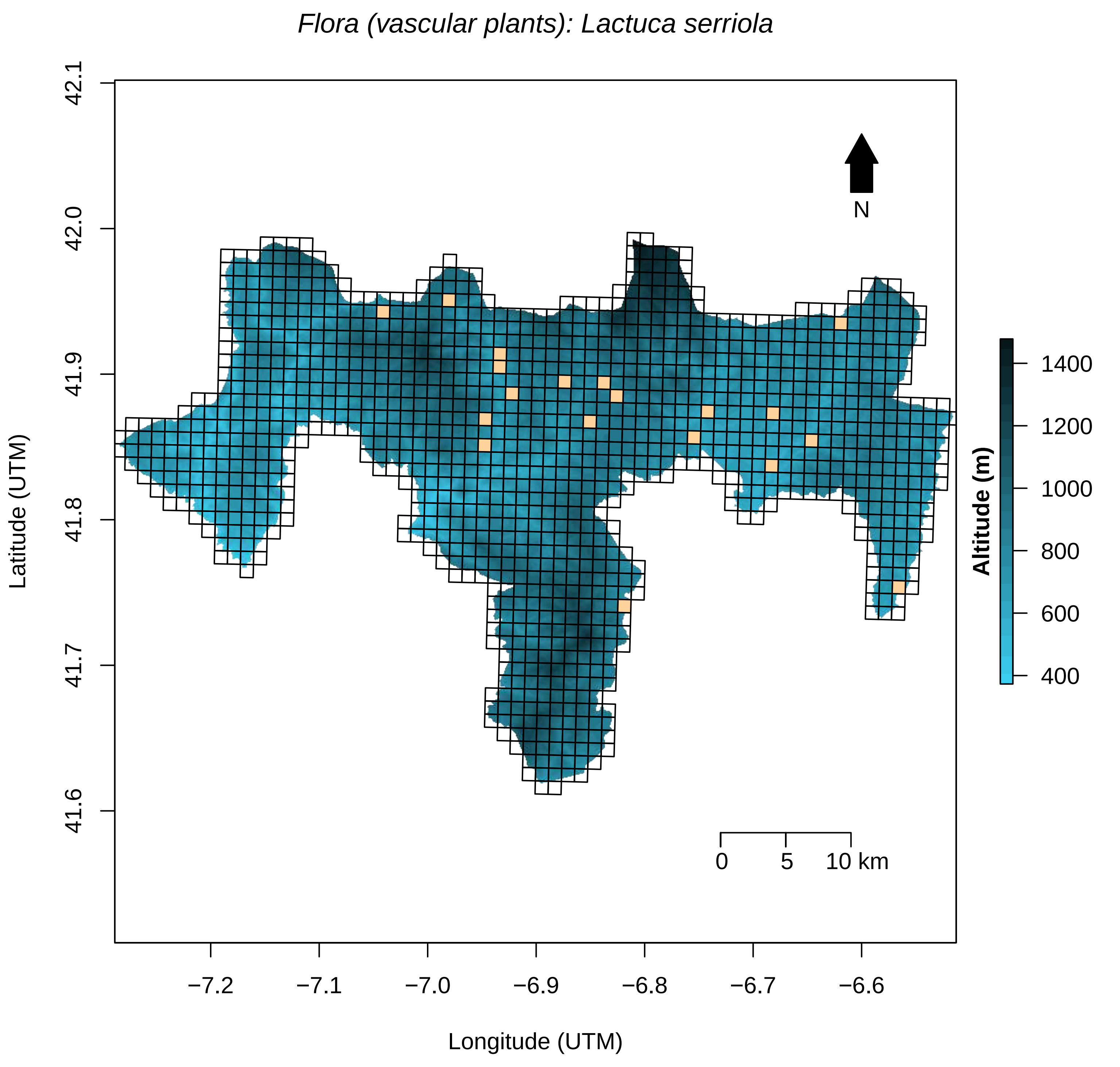

Flora (vascular plants): Lactuca serriola
Latitude (UTM)
42.1
42.0
41.9
41.8
41.7
41.6
−7.2
−7.1
−7.0
−6.9
−6.8
−6.7
−6.6
Longitude (UTM)
N
0
5
10 km
Altitude (m)
1400
1200
1000
800
600
400

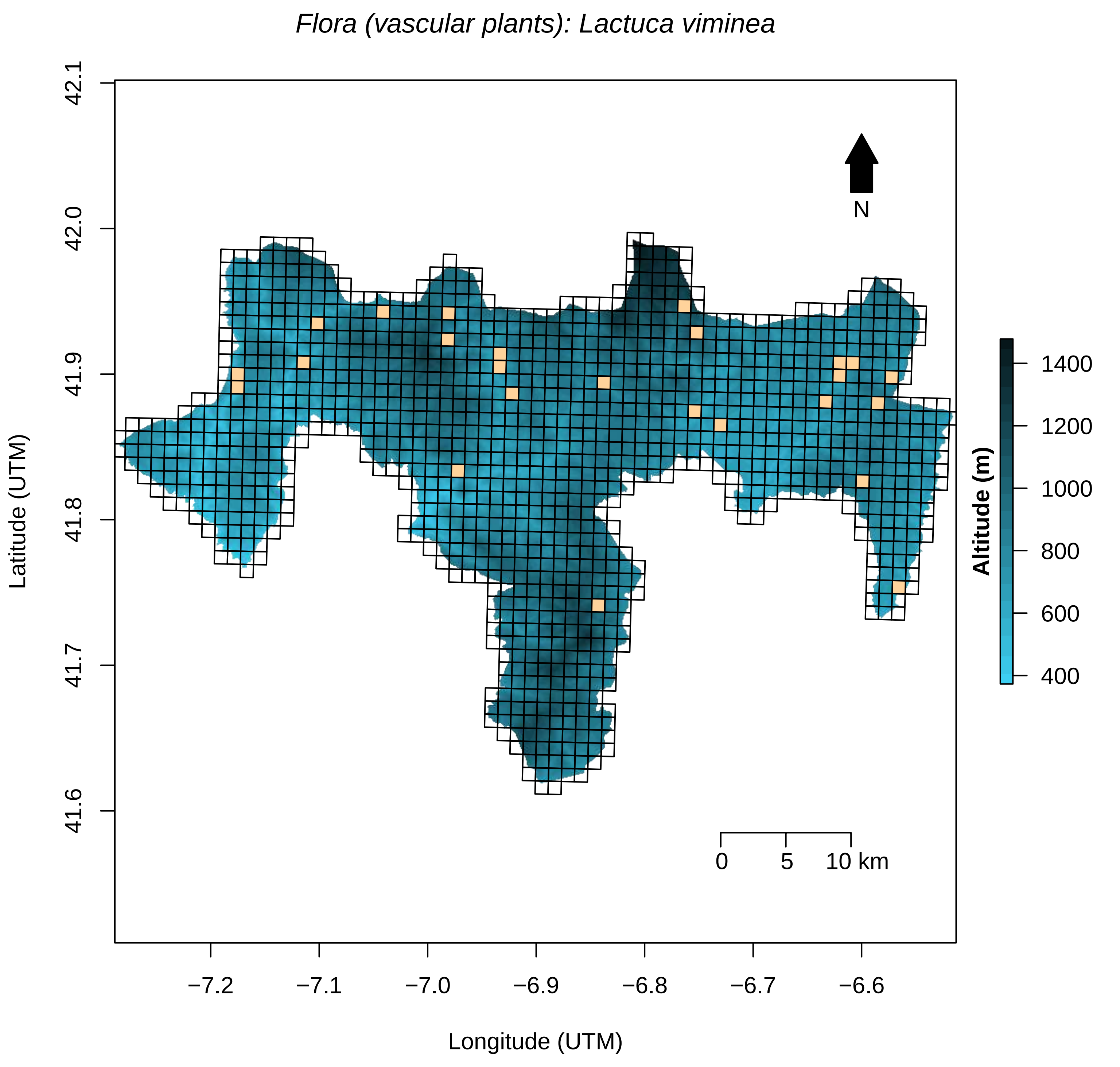

Flora (vascular plants): Lactuca viminea
N
0
5
10 km
Altitude (m)
1400
1200
1000
800
600
400
42.1
42.0
41.9
41.8
41.7
41.6
Latitude (UTM)
−7.2
−7.1
−7.0
−6.9
−6.8
−6.7
−6.6
Longitude (UTM)

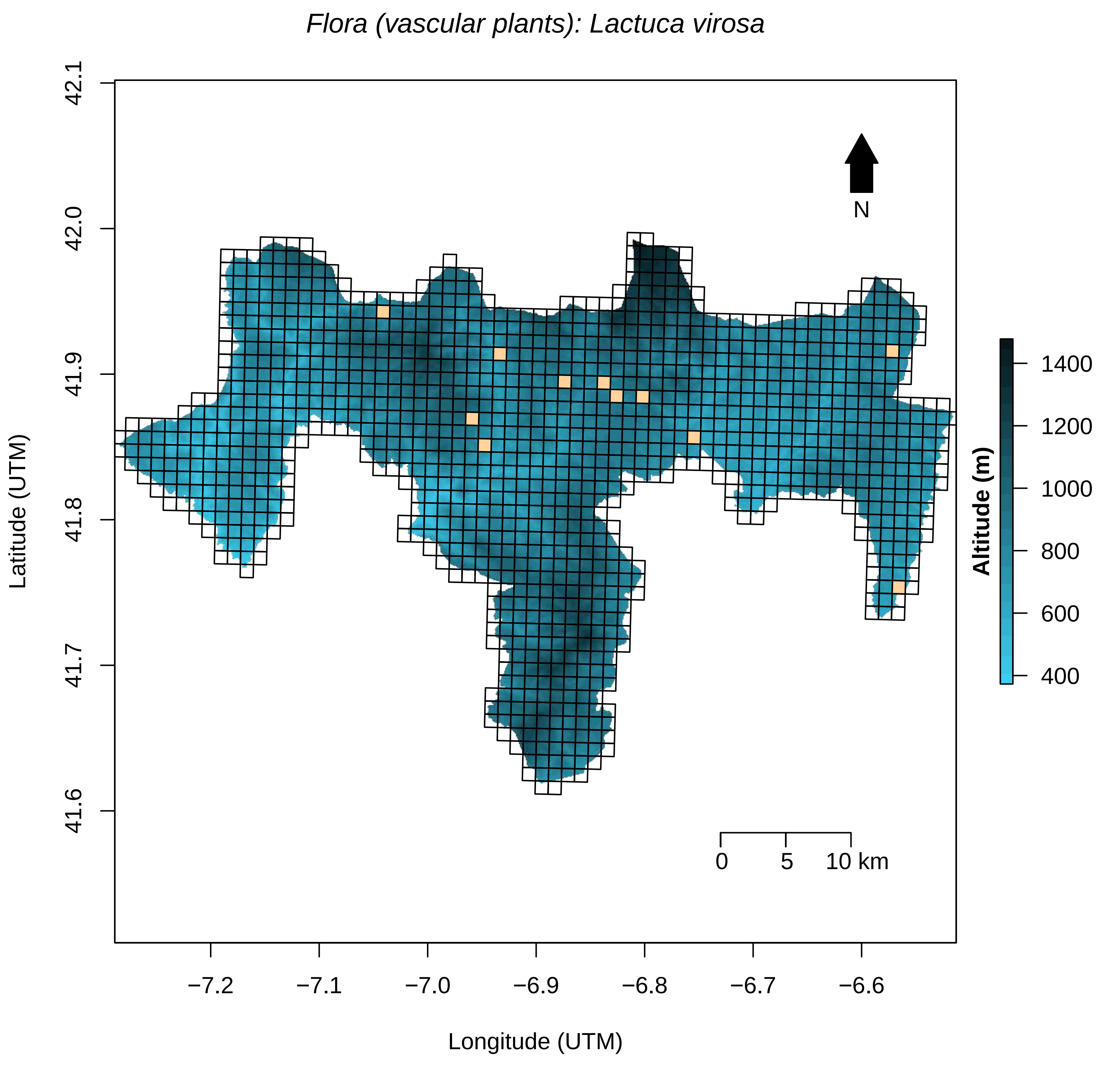

Flora (vascular plants): Lactuca virosa
42.1
42.0
41.9
41.8
41.7
41.6
Latitude (UTM)
N
0
5
10 km
−7.2
−7.1
−7.0
−6.9
−6.8
−6.7
−6.6
Longitude (UTM)
Altitude (m)
1400
1200
1000
800
600
400

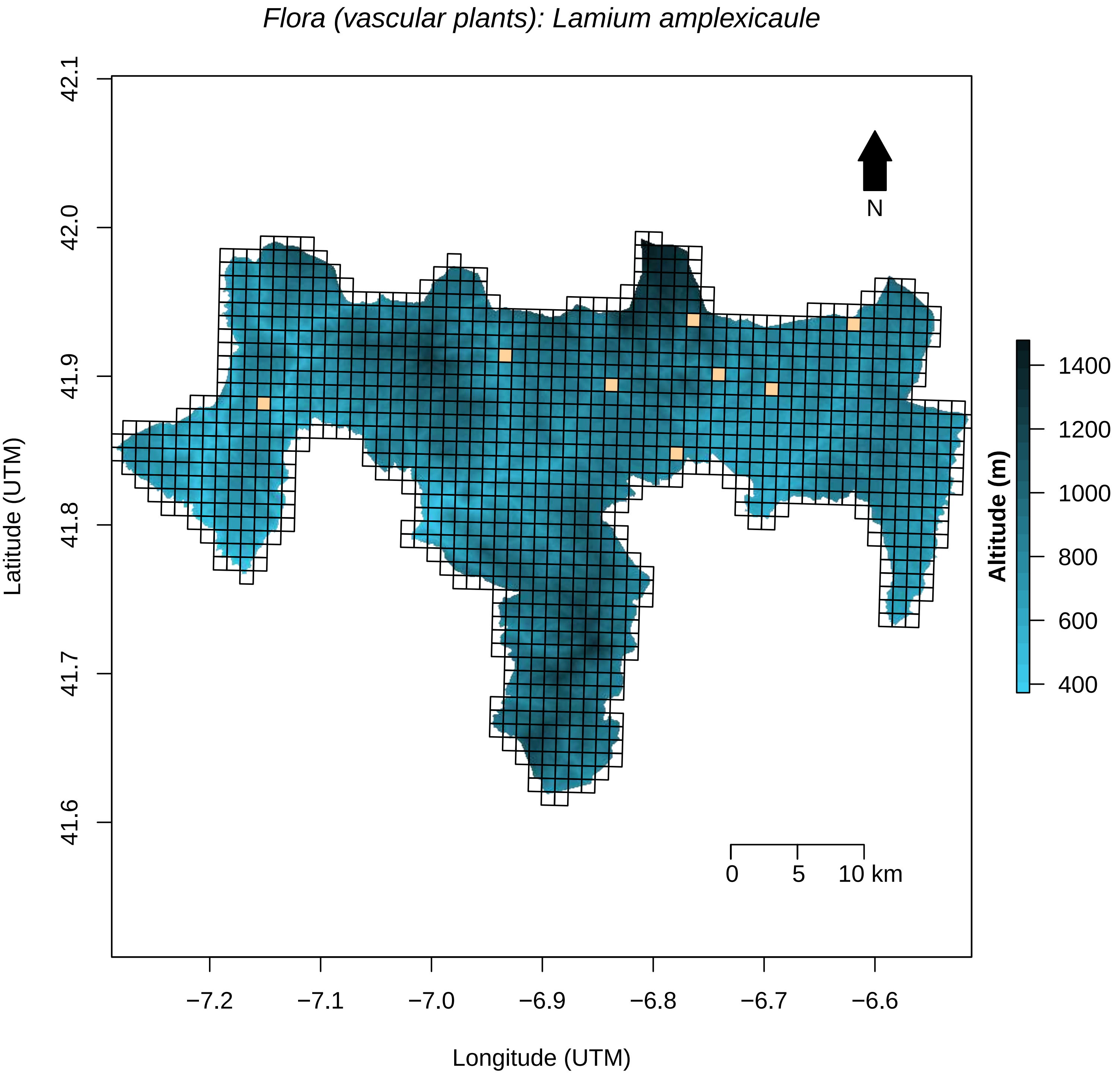
Flora (vascular plants): Lamium amplexicaule
Latitude (UTM)
Longitude (UTM)
42.1
42.0
41.9
41.8
41.7
41.6
−7.2
−7.1
−7.0
−6.9
−6.8
−6.7
−6.6
N
0
5
10 km
Altitude (m)
1400
1200
1000
800
600
400

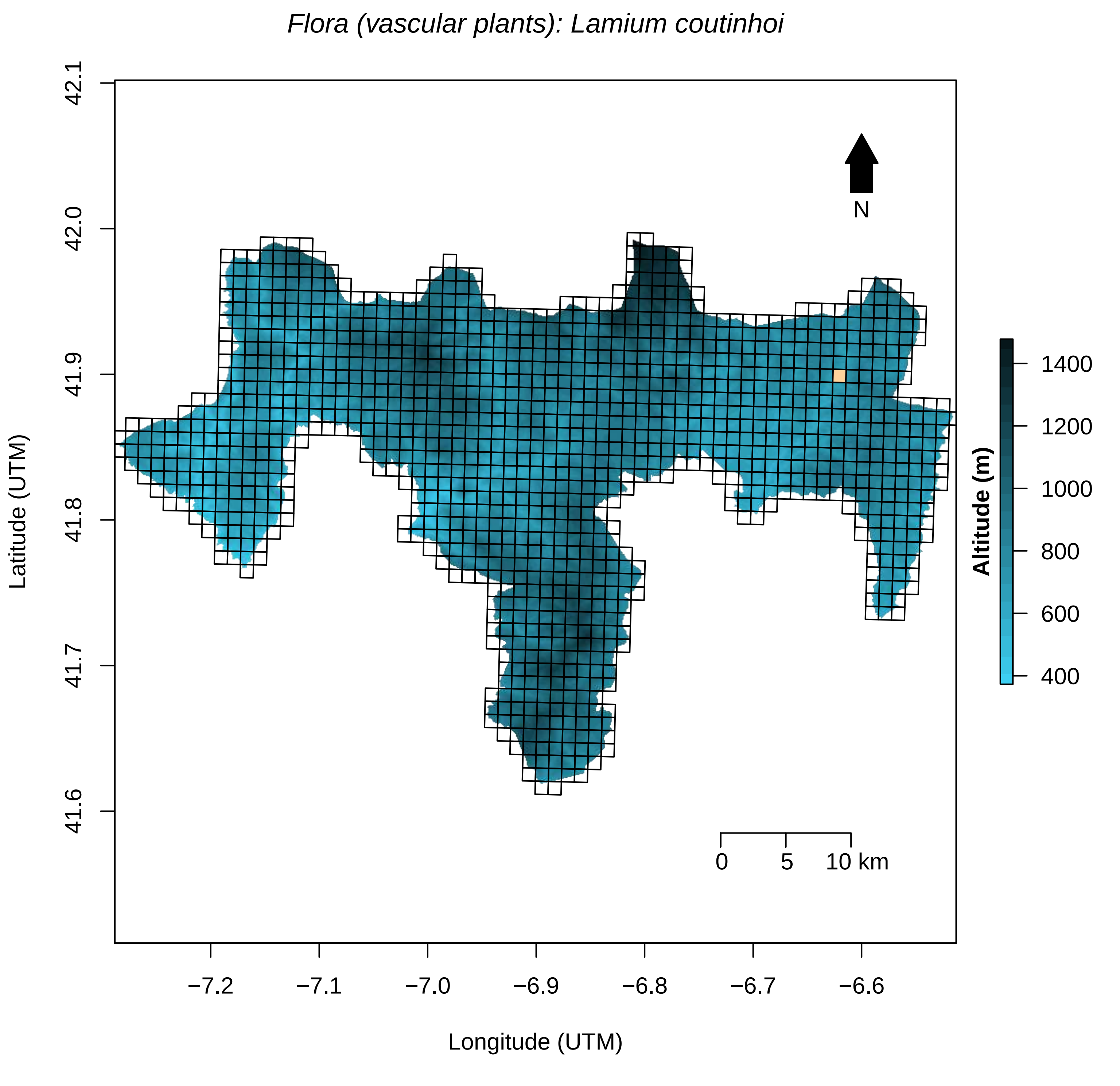

Flora (vascular plants): Lamium coutinhoi
N
0
5
10 km
Altitude (m)
1400
1200
1000
800
600
400
42.1
42.0
41.9
41.8
41.7
41.6
Latitude (UTM)
−7.2
−7.1
−7.0
−6.9
−6.8
−6.7
−6.6
Longitude (UTM)

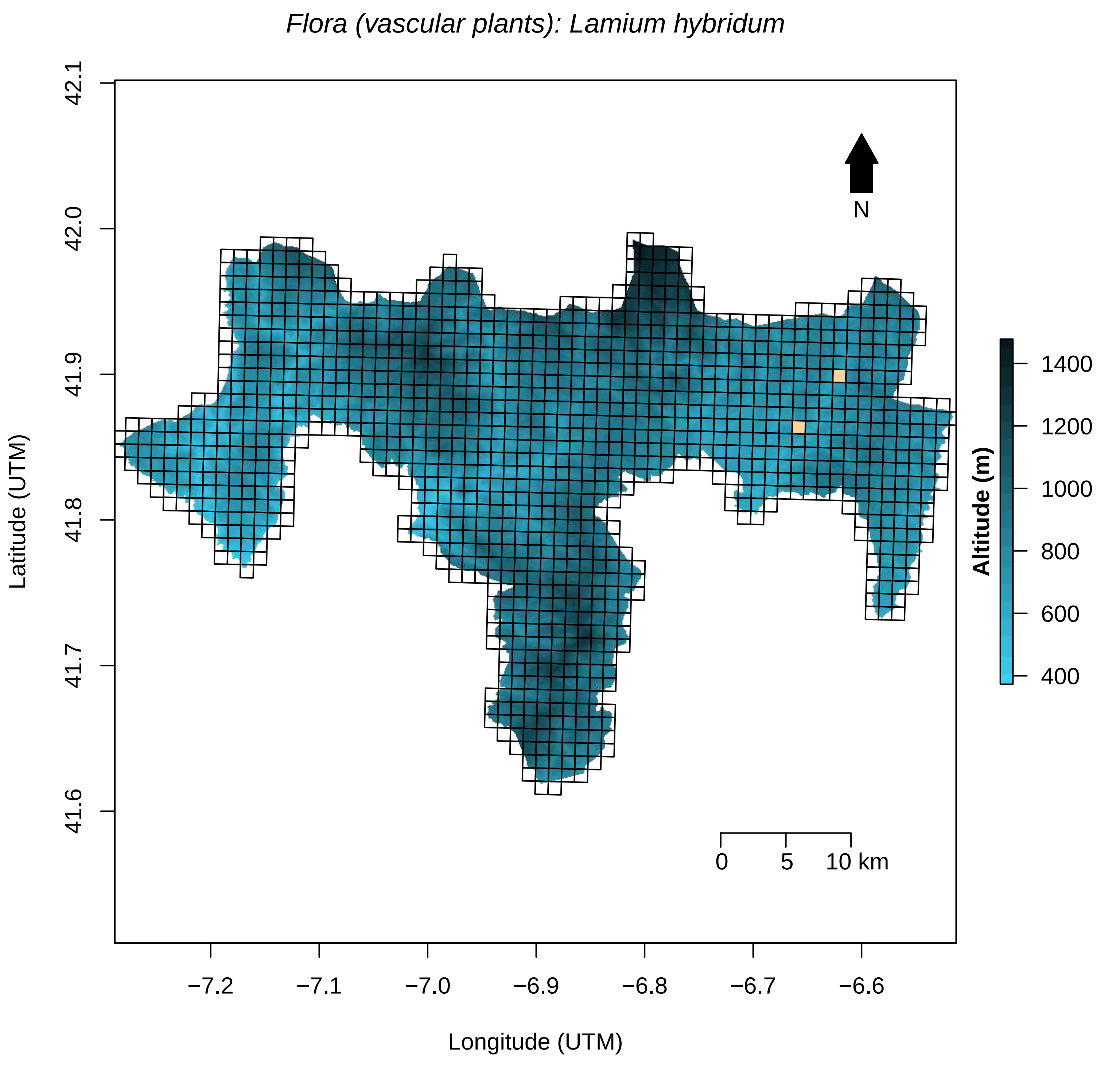
Flora (vascular plants): Lamium hybridum
42.1
42.0
41.9
41.8
41.7
41.6
Latitude (UTM)
−7.2
−7.1
−7.0
−6.9
−6.8
−6.7
−6.6
Longitude (UTM)
N
0
5
10 km
Altitude (m)
1400
1200
1000
800
600
400

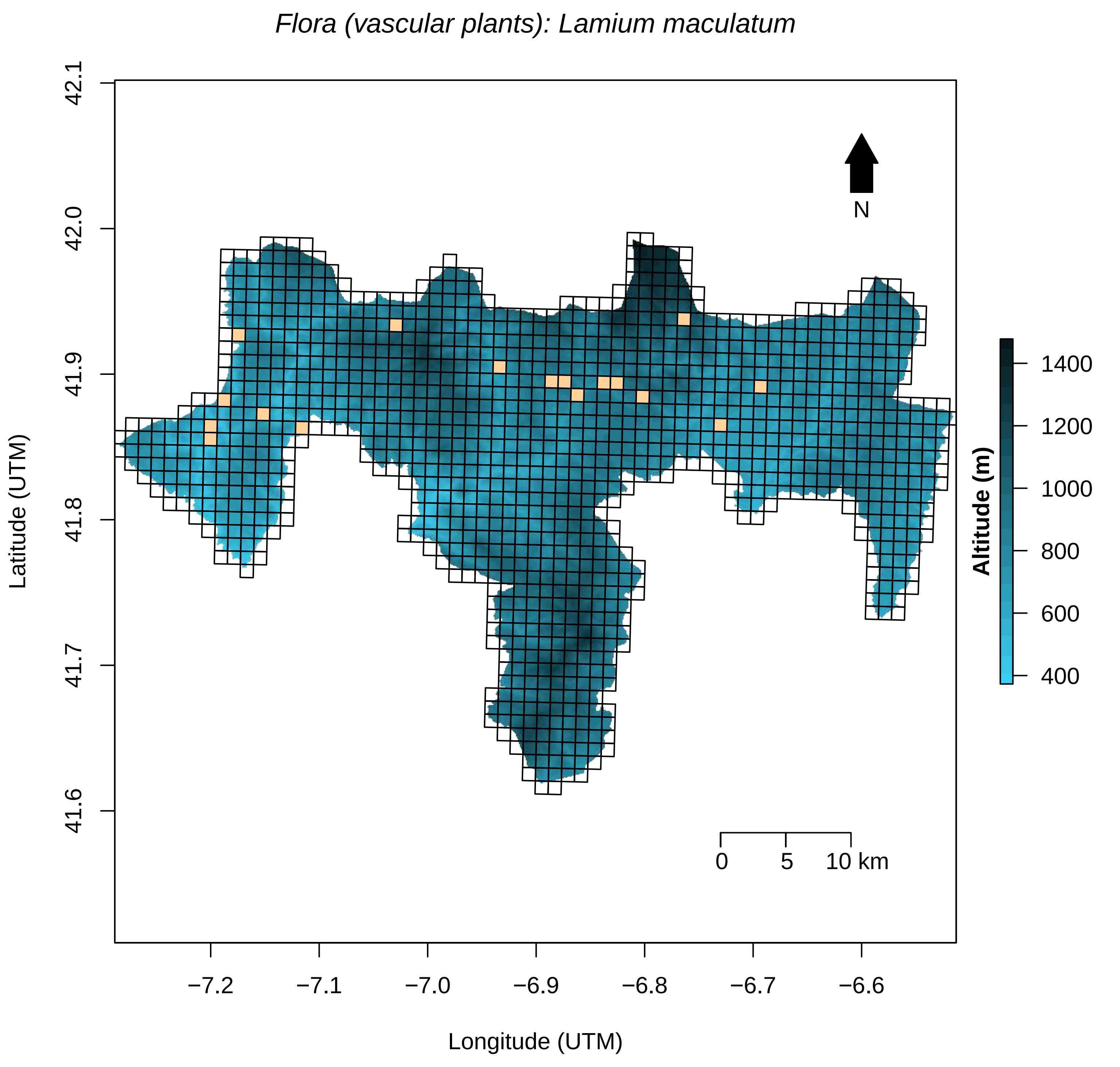

Flora (vascular plants): Lamium maculatum
42.1
42.0
41.9
41.8
41.7
41.6
Latitude (UTM)
−7.2
−7.1
−7.0
−6.9
−6.8
−6.7
−6.6
Longitude (UTM)
N
0
5
10 km
Altitude (m)
1400
1200
1000
800
600
400

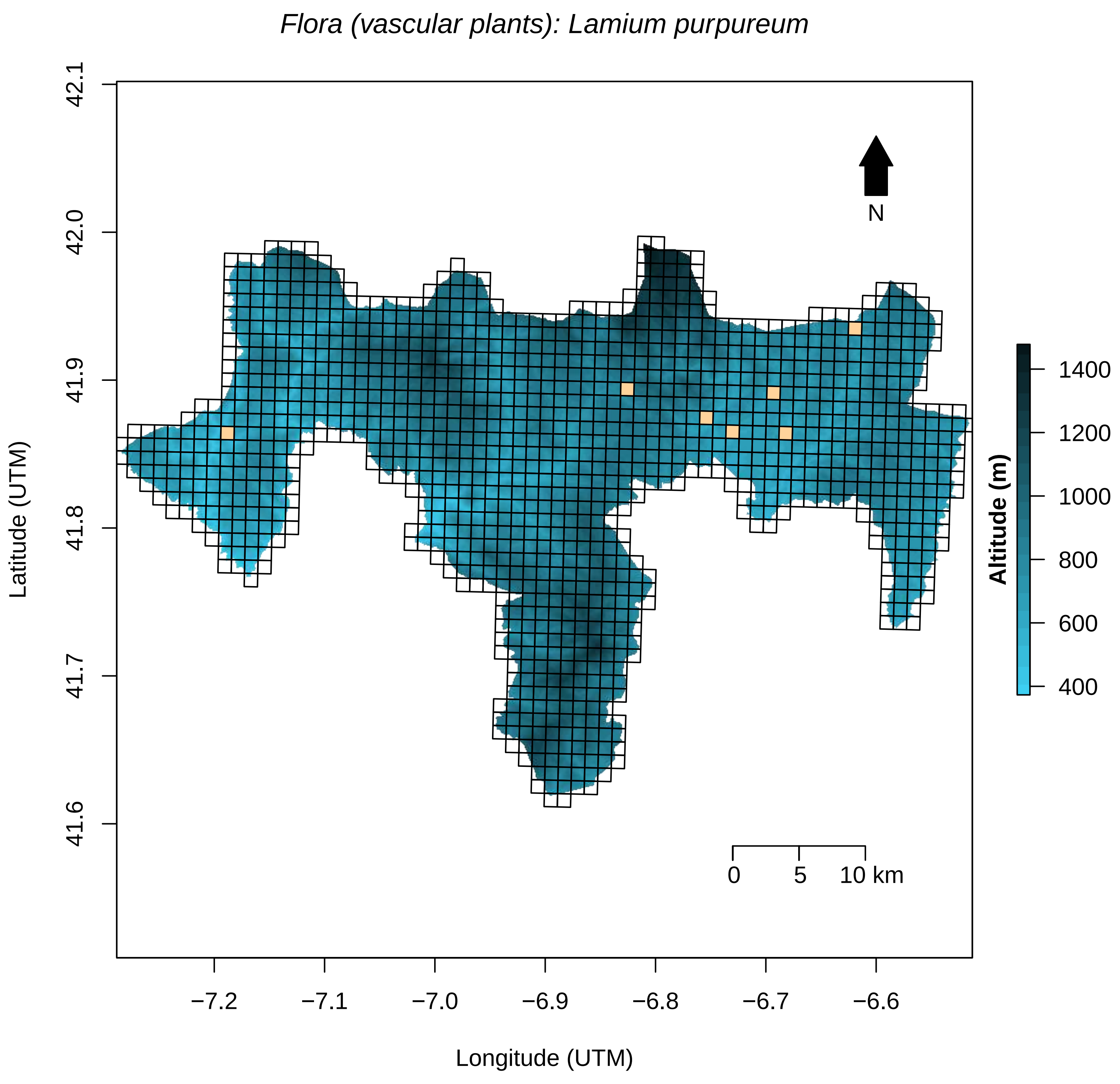

Flora (vascular plants): Lamium purpureum
Latitude (UTM)
42.1
42.0
41.9
41.8
41.7
41.6
Longitude (UTM)
−7.2
−7.1
−7.0
−6.9
−6.8
−6.7
−6.6
N
0
5
10 km
Altitude (m)
1400
1200
1000
800
600
400

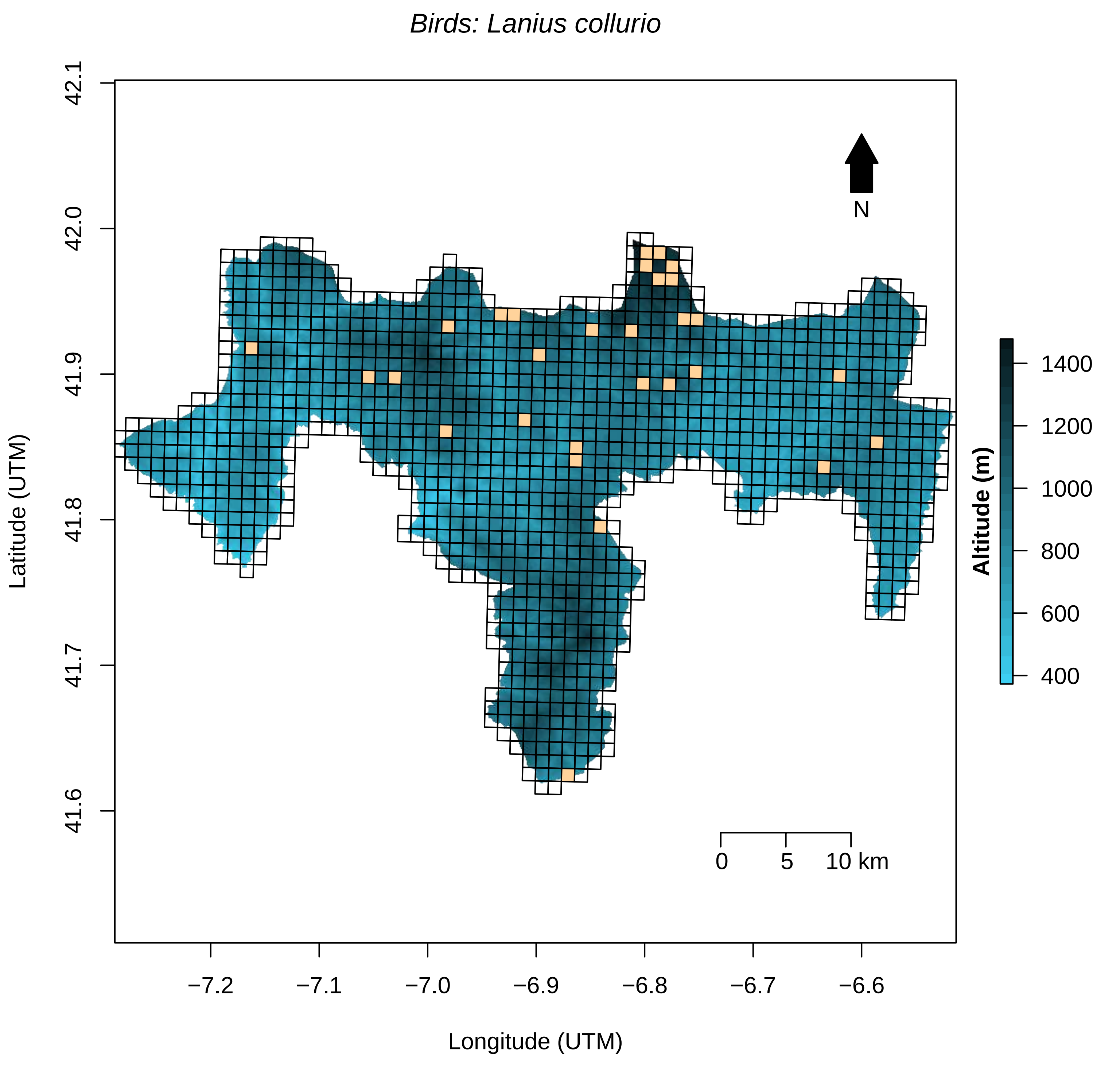

Birds: Lanius collurio
Latitude (UTM)
42.1
42.0
41.9
41.8
41.7
41.6
−7.2
−7.1
−7.0
−6.9
−6.8
−6.7
−6.6
Longitude (UTM)
N
0
5
10 km
Altitude (m)
1400
1200
1000
800
600
400

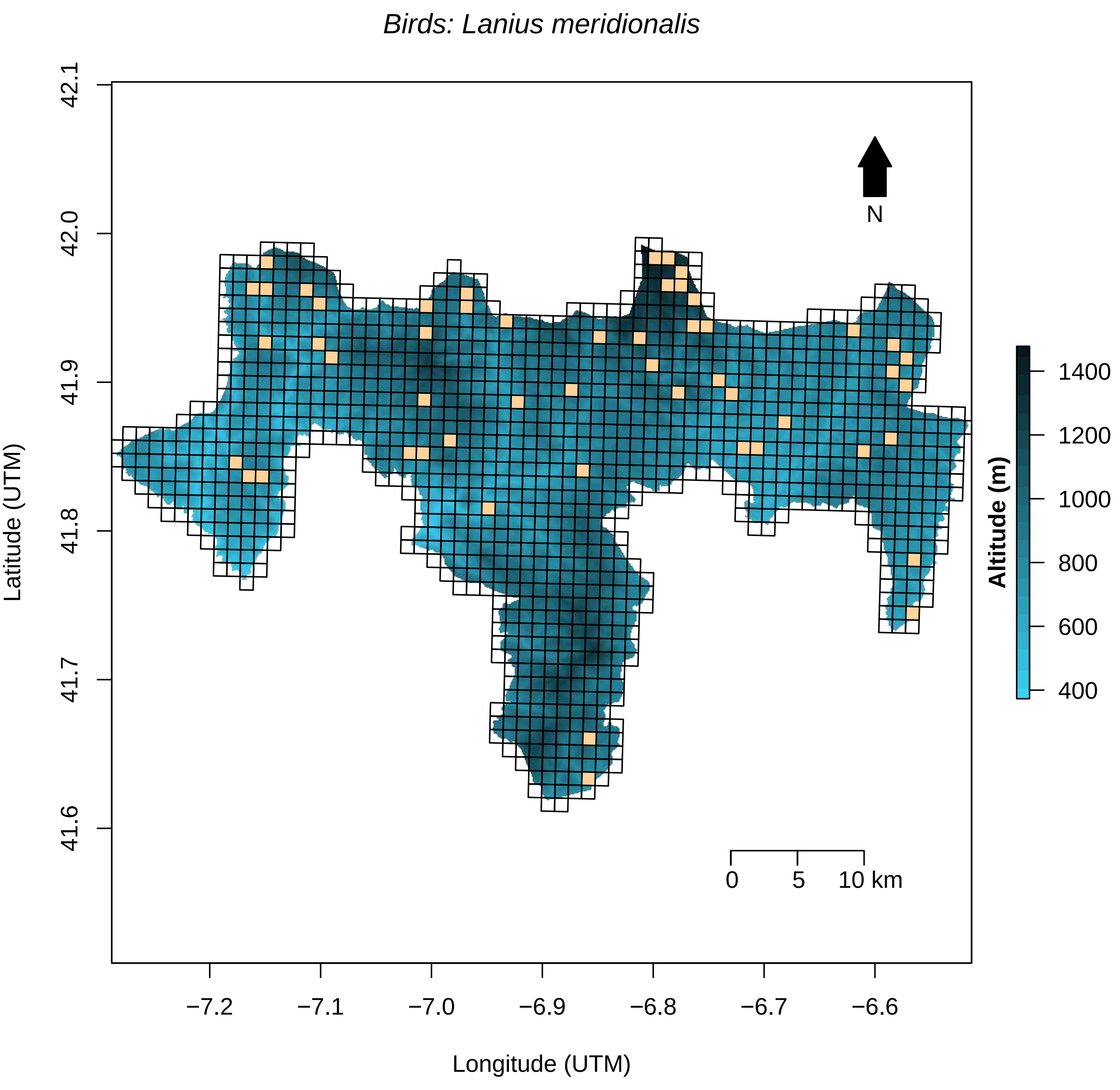

Birds: Lanius meridionalis
N
0 5 10 km
Altitude (m)
1400
1200
1000
800
600
400
Latitude (UTM)
42.1
42.0
41.9
41.8
41.7
41.6
Longitude (UTM)
−7.2
−7.1
−7.0
−6.9
−6.8
−6.7
−6.6

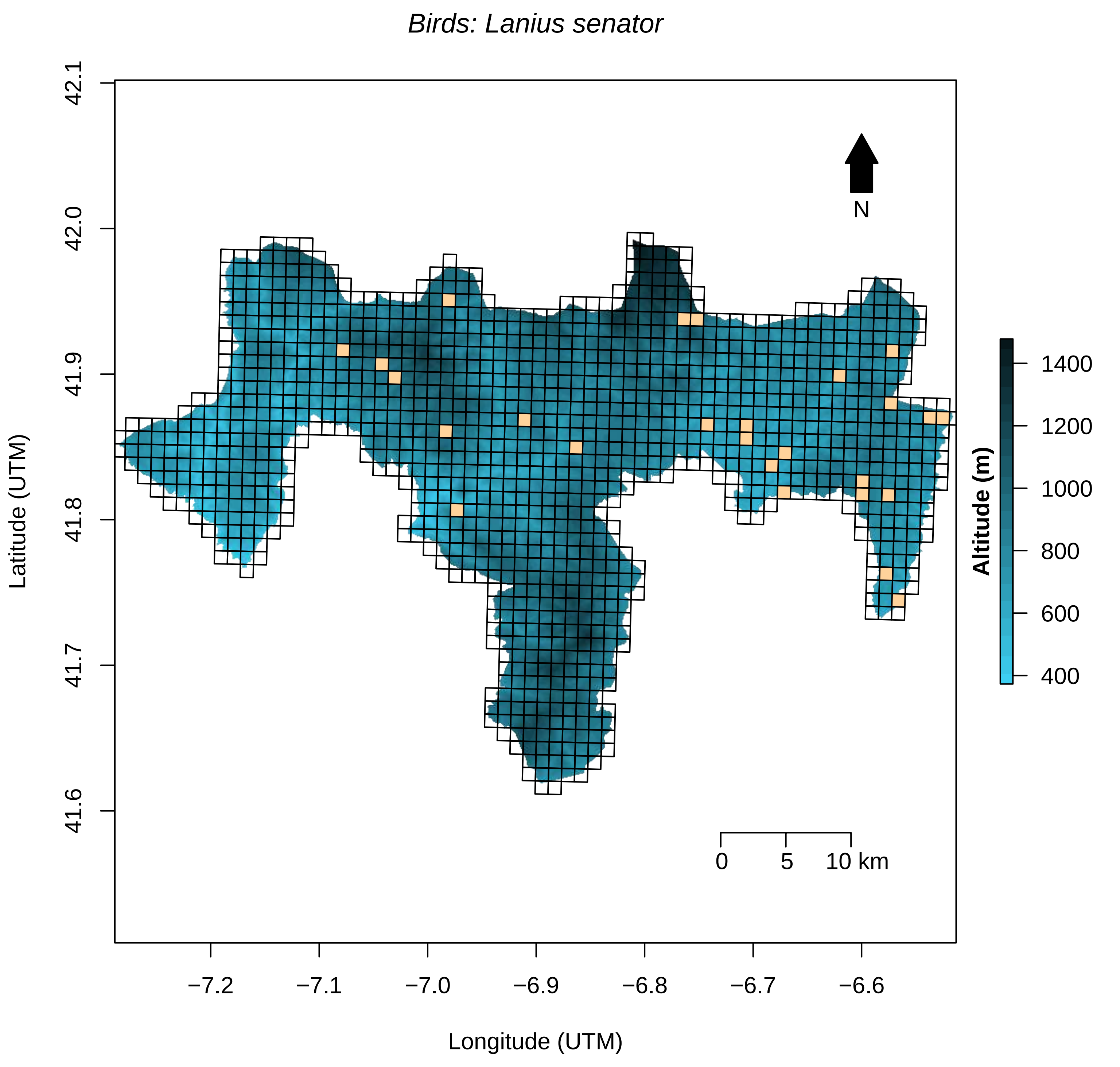

Birds: Lanius senator
N
0
5
10 km
Altitude (m)
1400
1200
1000
800
600
400
42.1
42.0
41.9
41.8
41.7
41.6
Latitude (UTM)
−7.2
−7.1
−7.0
−6.9
−6.8
−6.7
−6.6
Longitude (UTM)

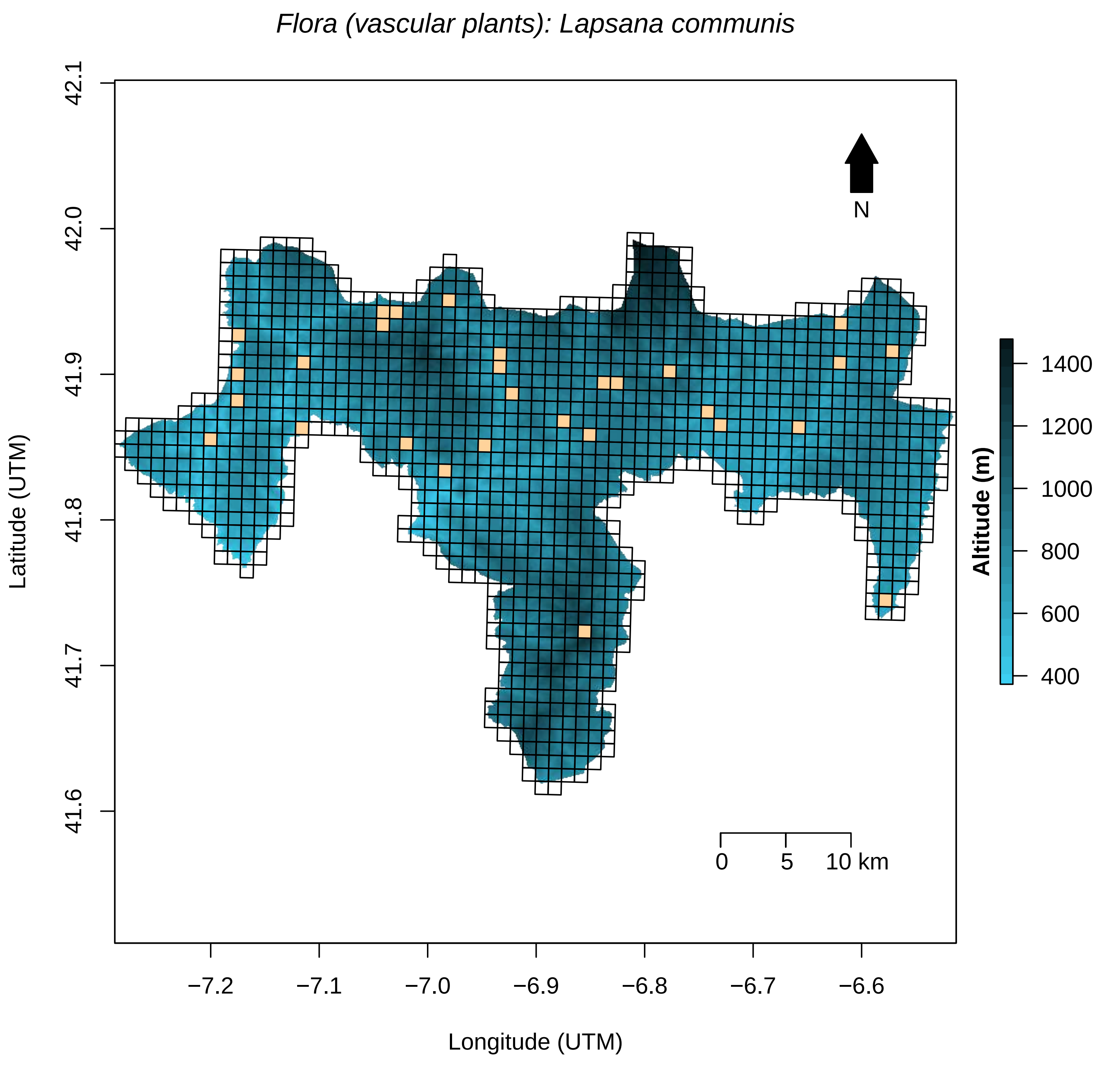

Flora (vascular plants): Lapsana communis
Latitude (UTM)
Longitude (UTM)
42.1
42.0
41.9
41.8
41.7
41.6
−7.2
−7.1
−7.0
−6.9
−6.8
−6.7
−6.6
N
0
5
10 km
Altitude (m)
1400
1200
1000
800
600
400

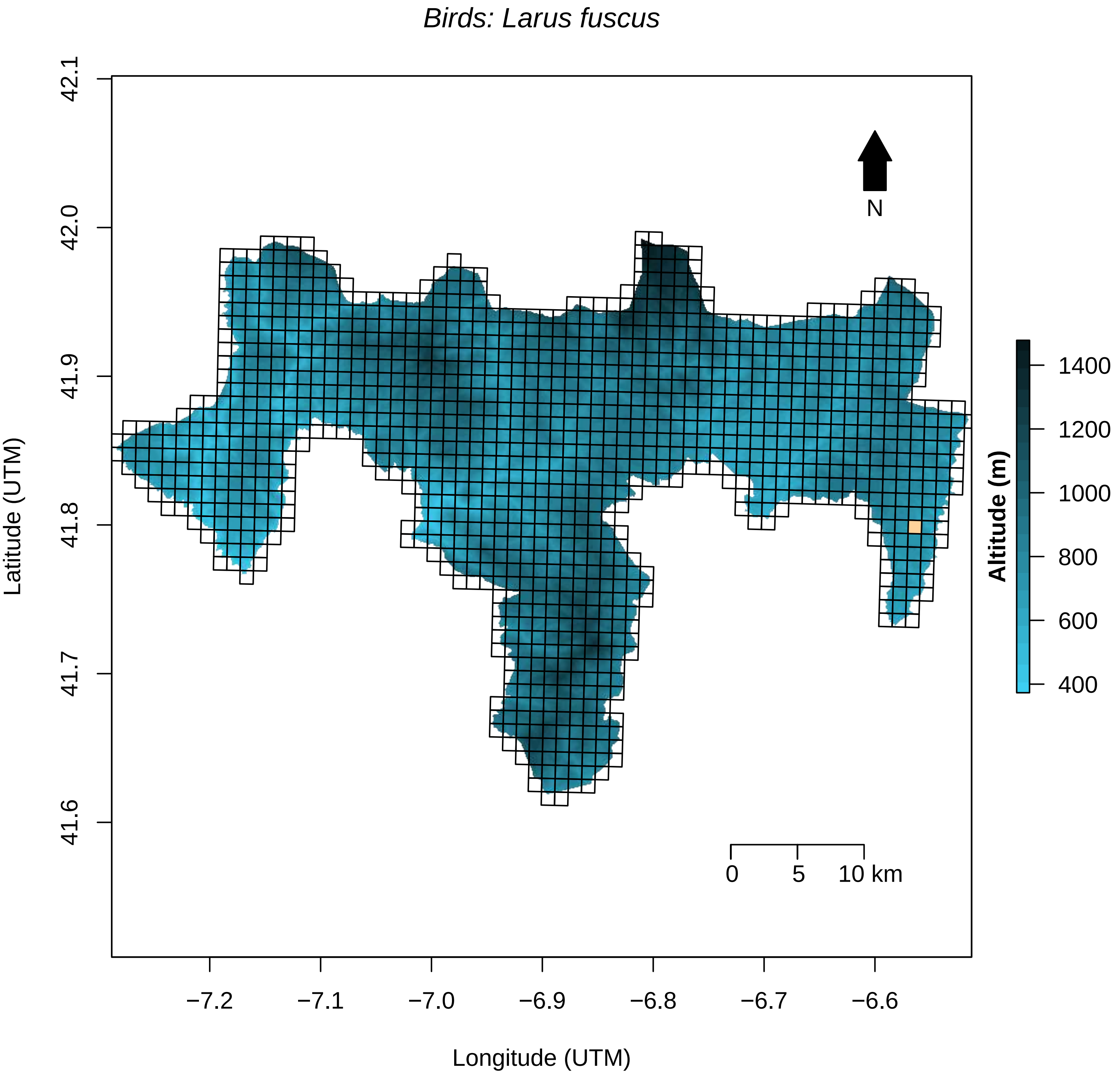

Birds: Larus fuscus
42.1
42.0
41.9
41.8
41.7
41.6
Latitude (UTM)
−7.2
−7.1
−7.0
−6.9
−6.8
−6.7
−6.6
Longitude (UTM)
N
0
5
10 km
1400
1200
1000
800
600
400
Altitude (m)

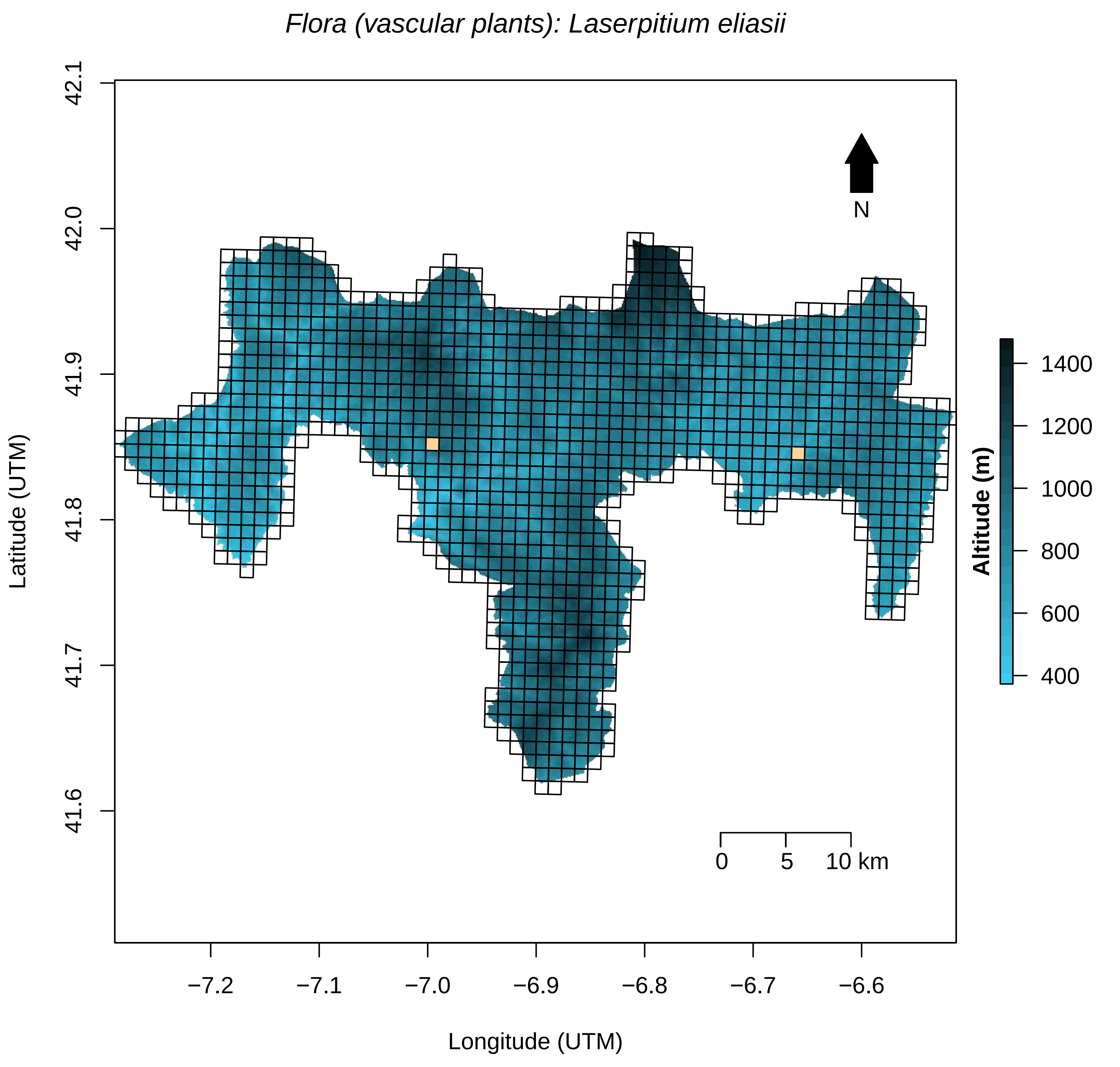

Flora (vascular plants): Laserpitium eliasii
Latitude (UTM)
42.1
42.0
41.9
41.8
41.7
41.6
Longitude (UTM)
−7.2
−7.1
−7.0
−6.9
−6.8
−6.7
−6.6
N
0
5
10 km
Altitude (m)
1400
1200
1000
800
600
400

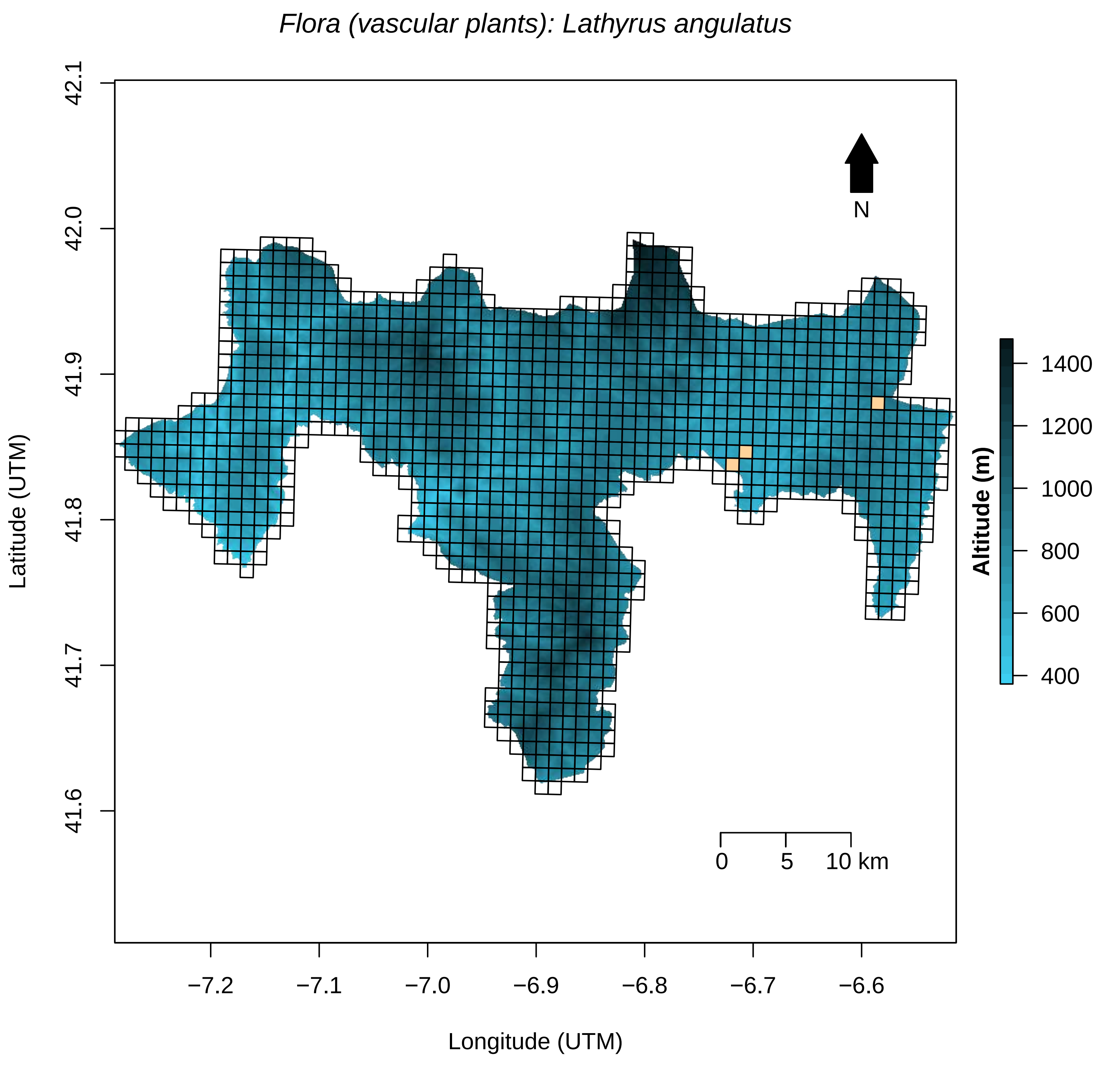

Flora (vascular plants): Lathyrus angulatus
Latitude (UTM)
Longitude (UTM)
42.1
42.0
41.9
41.8
41.7
41.6
−7.2
−7.1
−7.0
−6.9
−6.8
−6.7
−6.6
N
0
5
10 km
Altitude (m)
1400
1200
1000
800
600
400

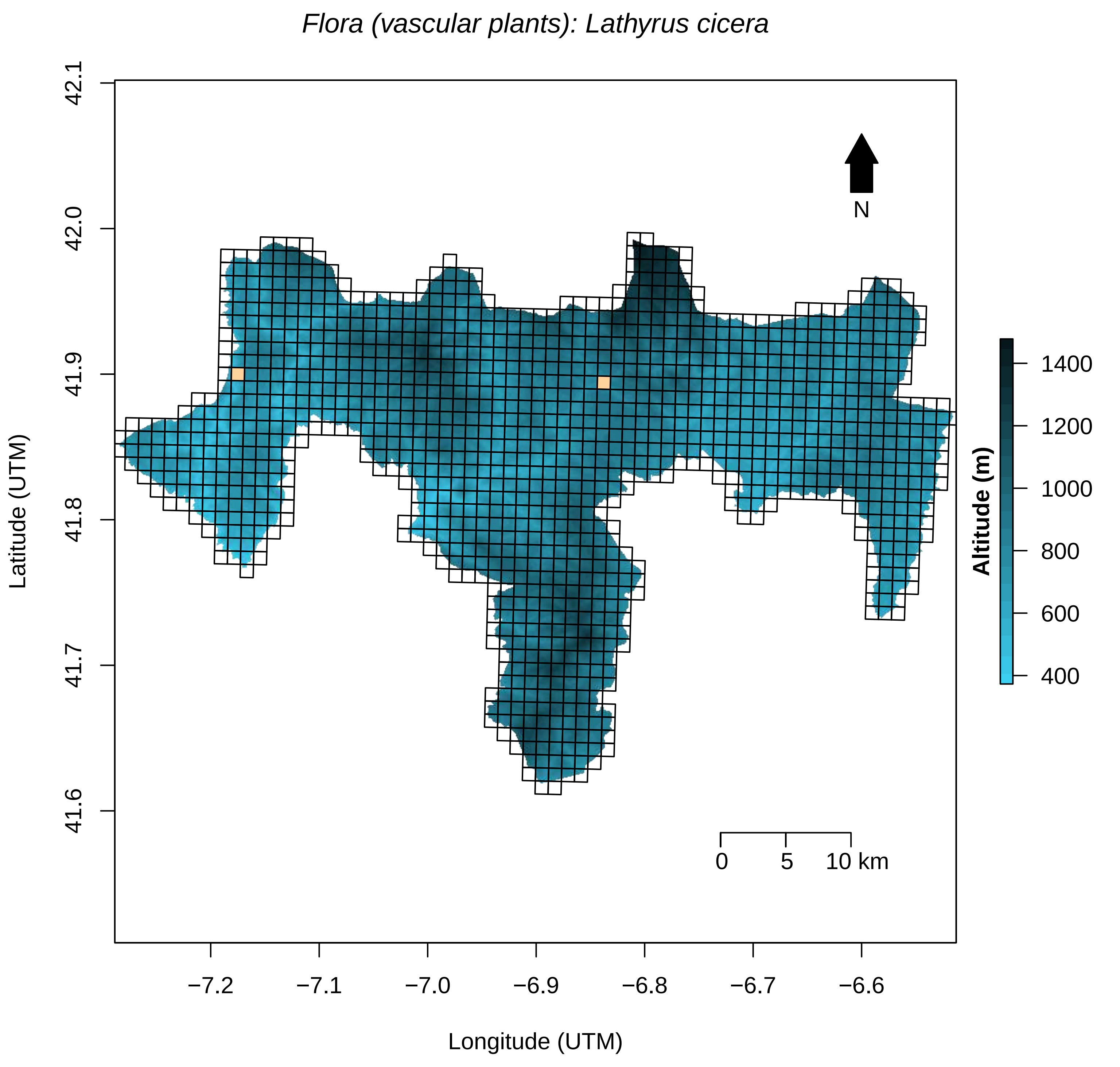
Flora (vascular plants): Lathyrus cicera
Latitude (UTM)
42.1
42.0
41.9
41.8
41.7
41.6
Longitude (UTM)
−7.2
−7.1
−7.0
−6.9
−6.8
−6.7
−6.6
N
0
5
10 km
Altitude (m)
1400
1200
1000
800
600
400

Flora (vascular plants): Lathyrus hirsutus

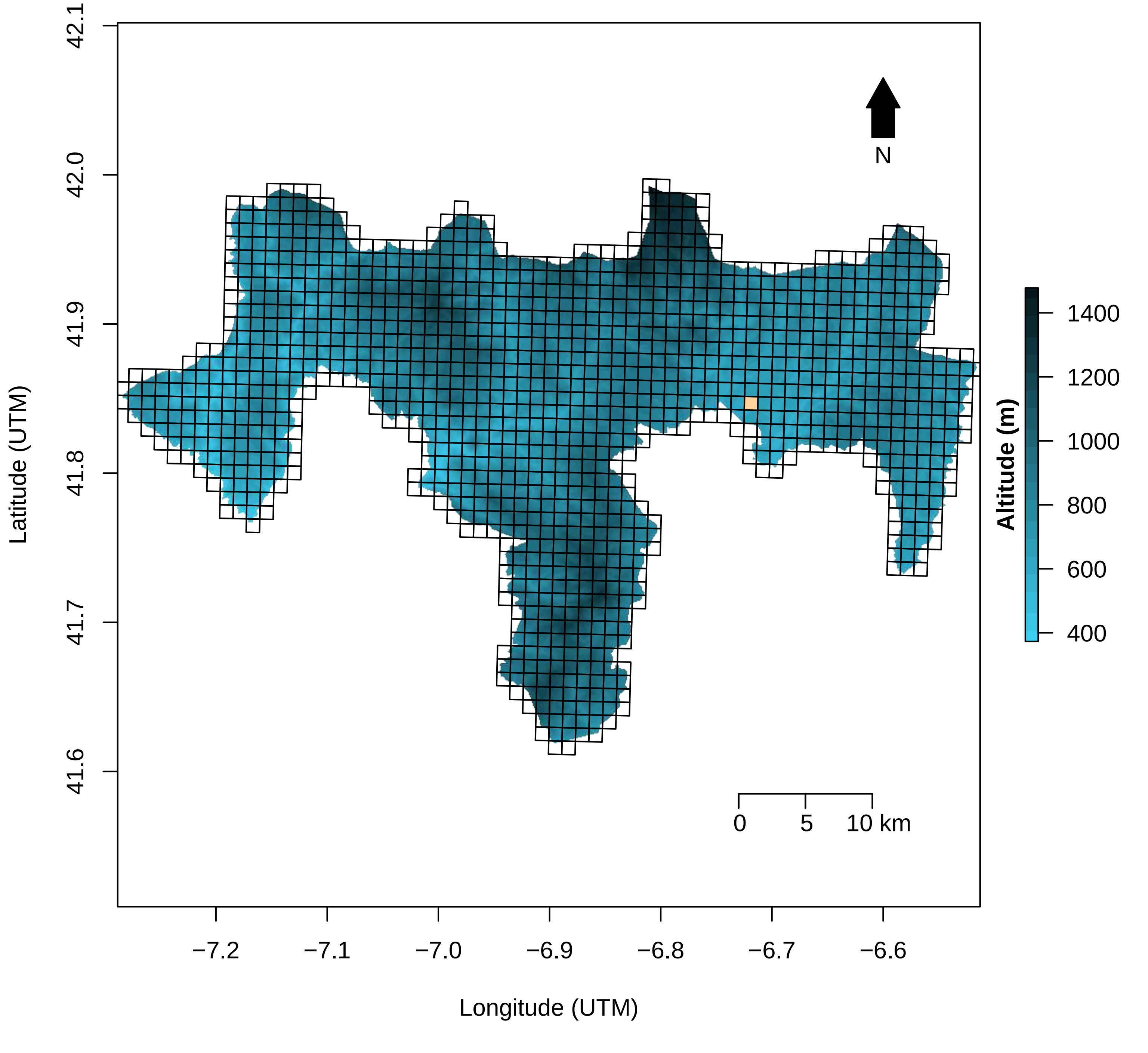

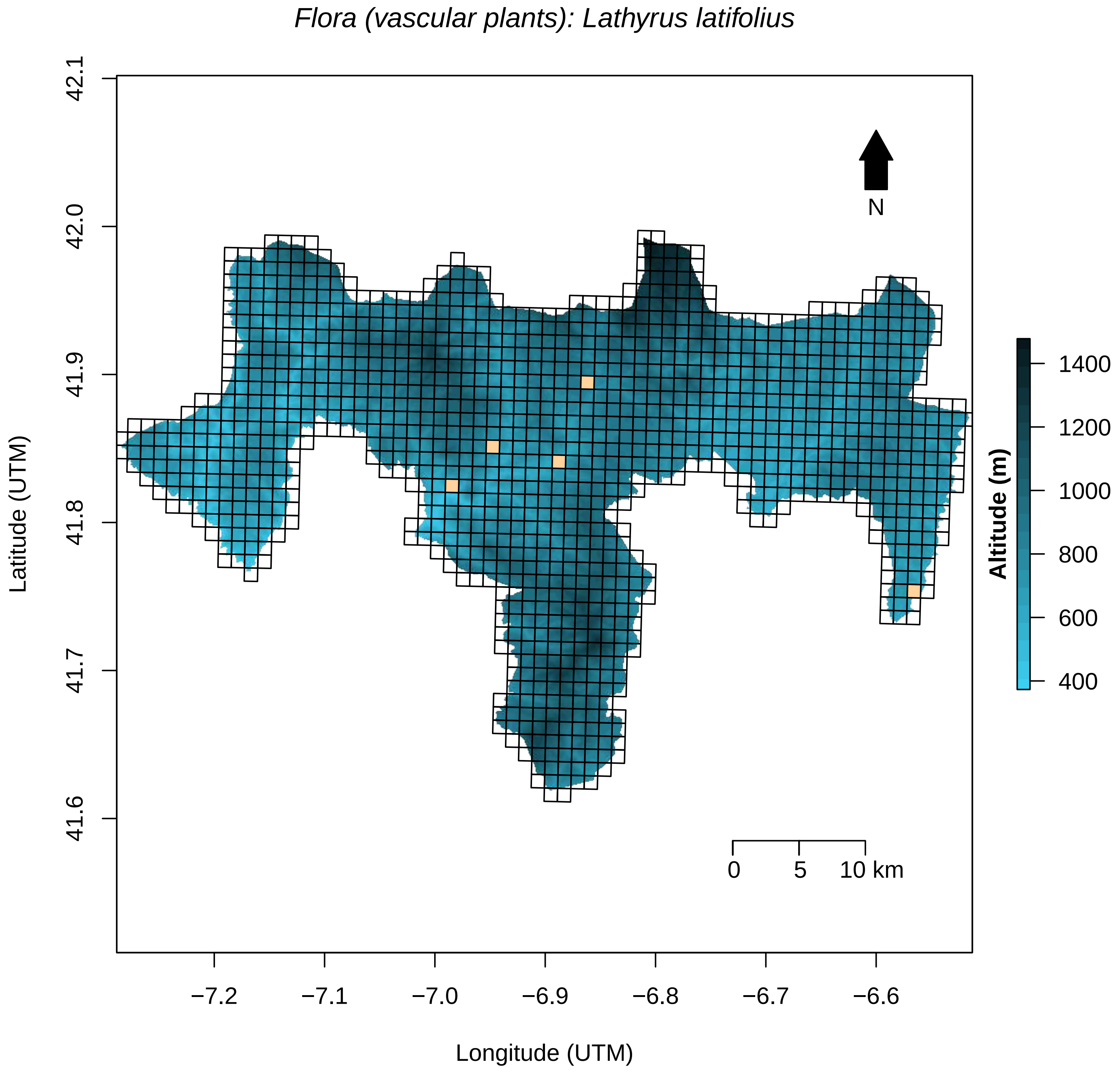

Flora (vascular plants): Lathyrus latifolius
N
0
5
10 km
Altitude (m)
400
600
800
1000
1200
1400
Latitude (UTM)
41.6
41.7
41.8
41.9
42.0
42.1
Longitude (UTM)
−7.2
−7.1
−7.0
−6.9
−6.8
−6.7
−6.6

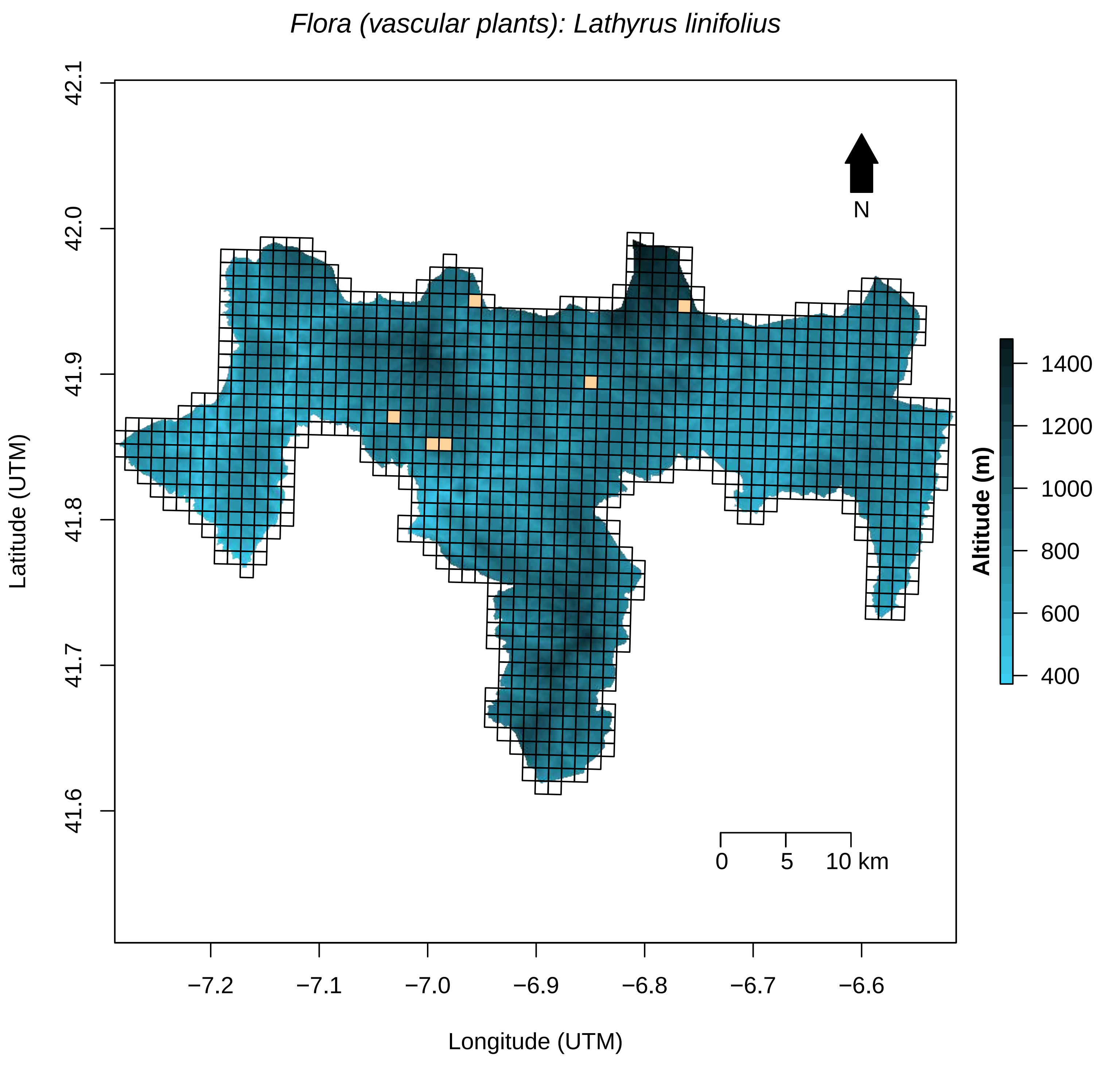

Flora (vascular plants): Lathyrus linifolius
42.1
42.0
41.9
41.8
41.7
41.6
Latitude (UTM)
−7.2
−7.1
−7.0
−6.9
−6.8
−6.7
−6.6
Longitude (UTM)
N
0
5
10 km
Altitude (m)
1400
1200
1000
800
600
400

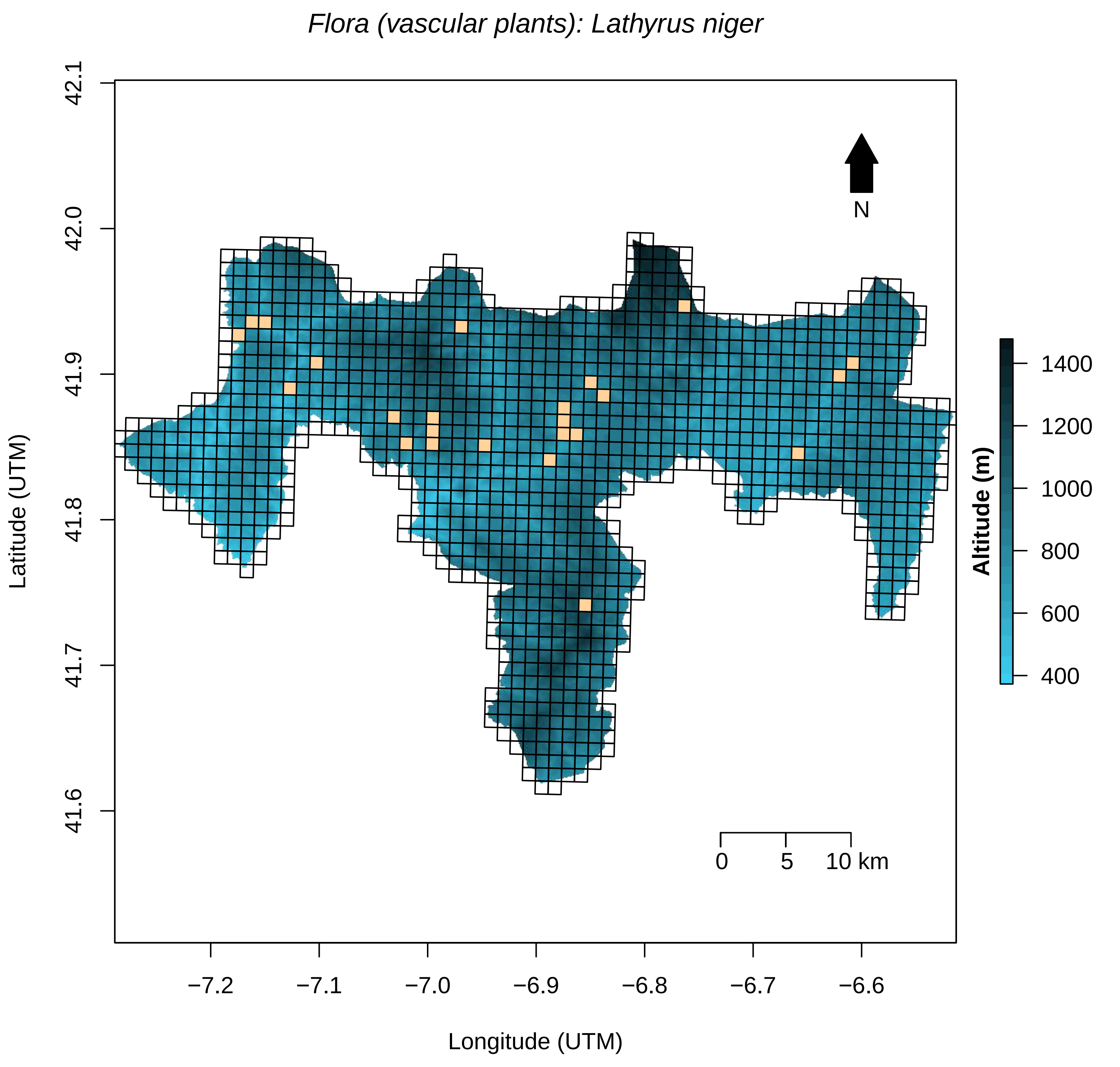
Flora (vascular plants): Lathyrus niger
Latitude (UTM)
Longitude (UTM)
42.1
42.0
41.9
41.8
41.7
41.6
−7.2
−7.1
−7.0
−6.9
−6.8
−6.7
−6.6
N
0
5
10 km
Altitude (m)
1400
1200
1000
800
600
400

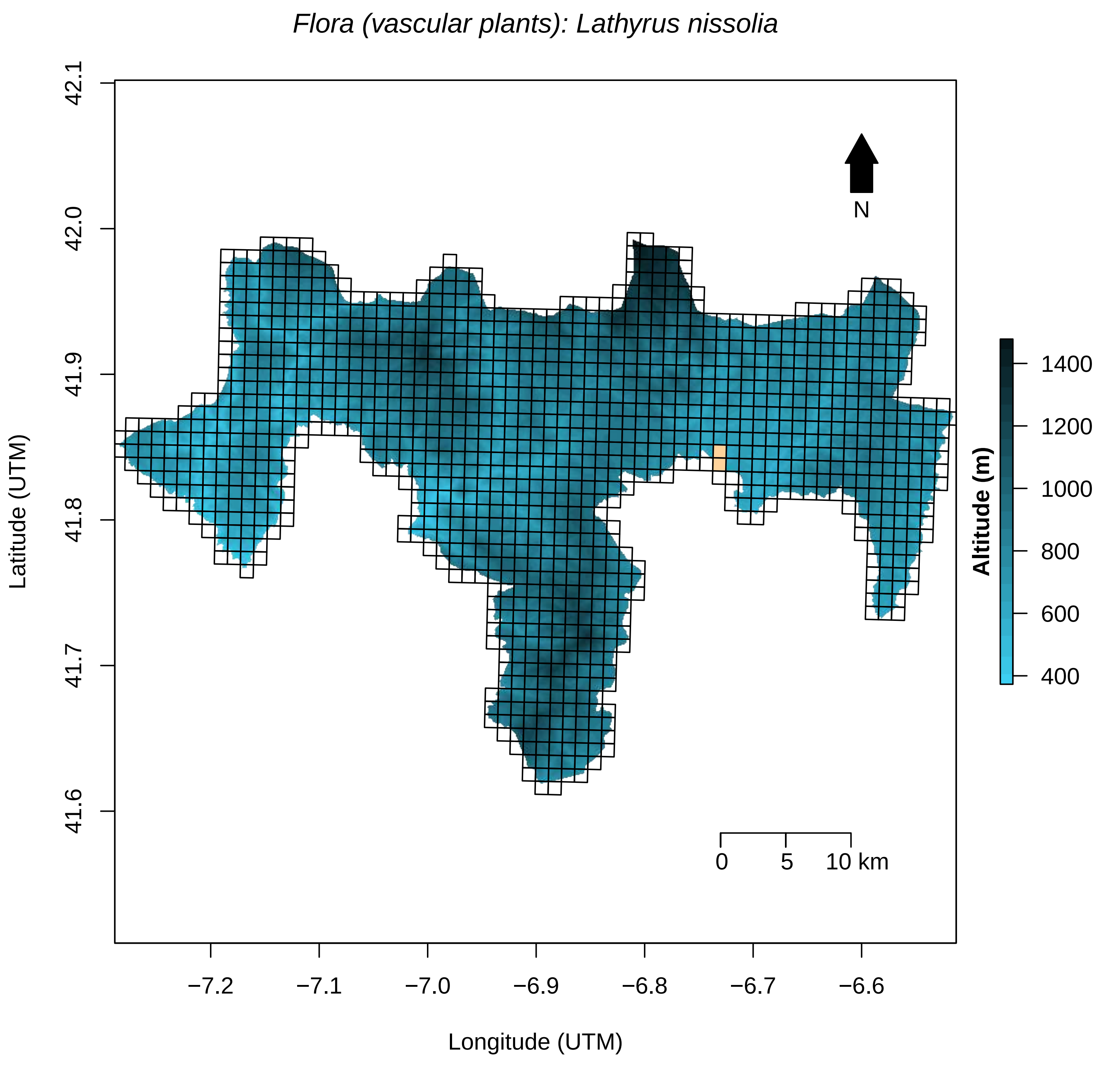

Flora (vascular plants): Lathyrus nissolia
N
0 5 10 km
Altitude (m)
1400
1200
1000
800
600
400
42.1
42.0
41.9
41.8
41.7
41.6
Latitude (UTM)
−7.2
−7.1
−7.0
−6.9
−6.8
−6.7
−6.6
Longitude (UTM)

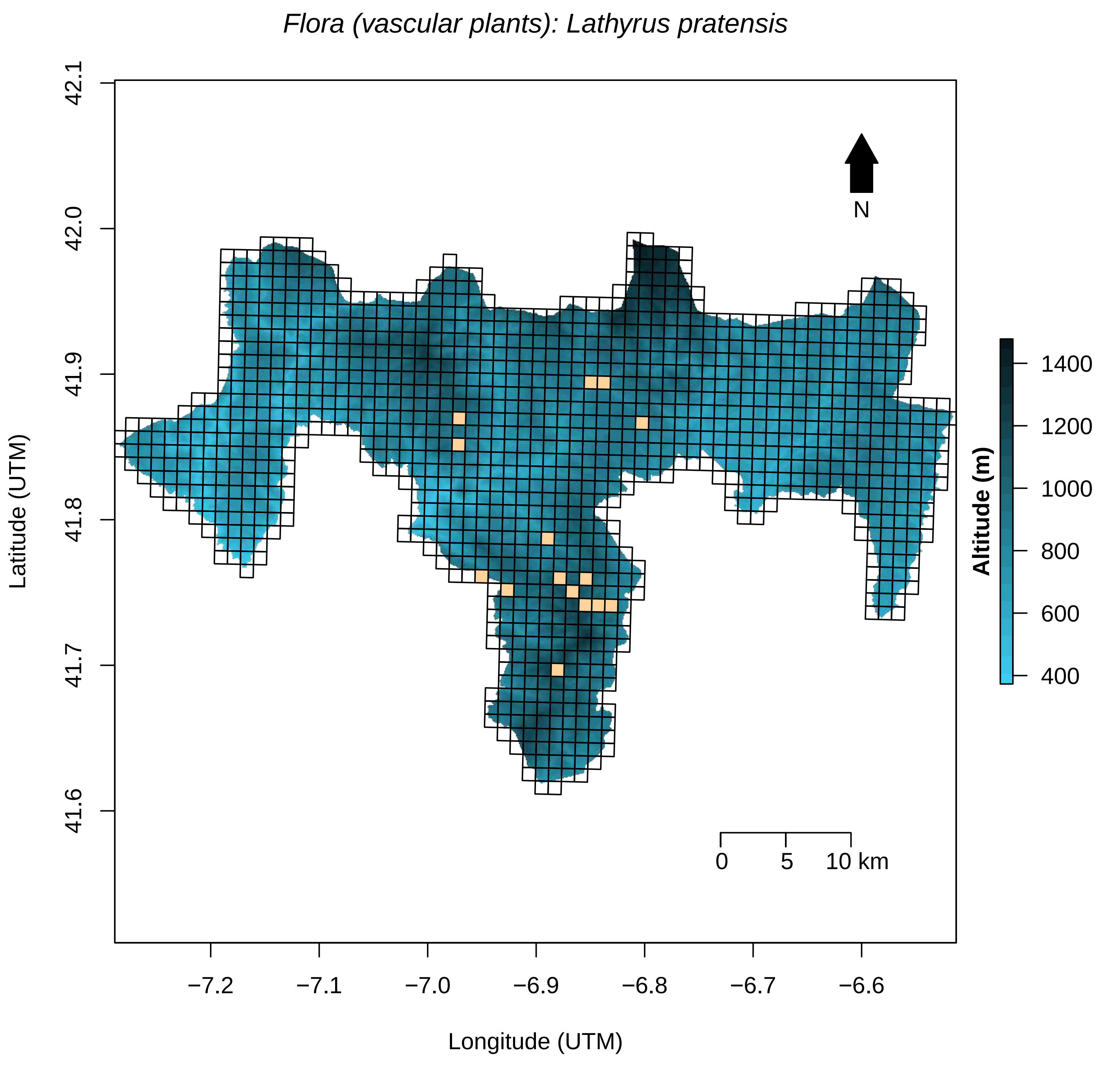

Flora (vascular plants): Lathyrus pratensis
42.1
42.0
41.9
41.8
41.7
41.6
Latitude (UTM)
−7.2
−7.1
−7.0
−6.9
−6.8
−6.7
−6.6
Longitude (UTM)
N
0
5
10 km
Altitude (m)
1400
1200
1000
800
600
400

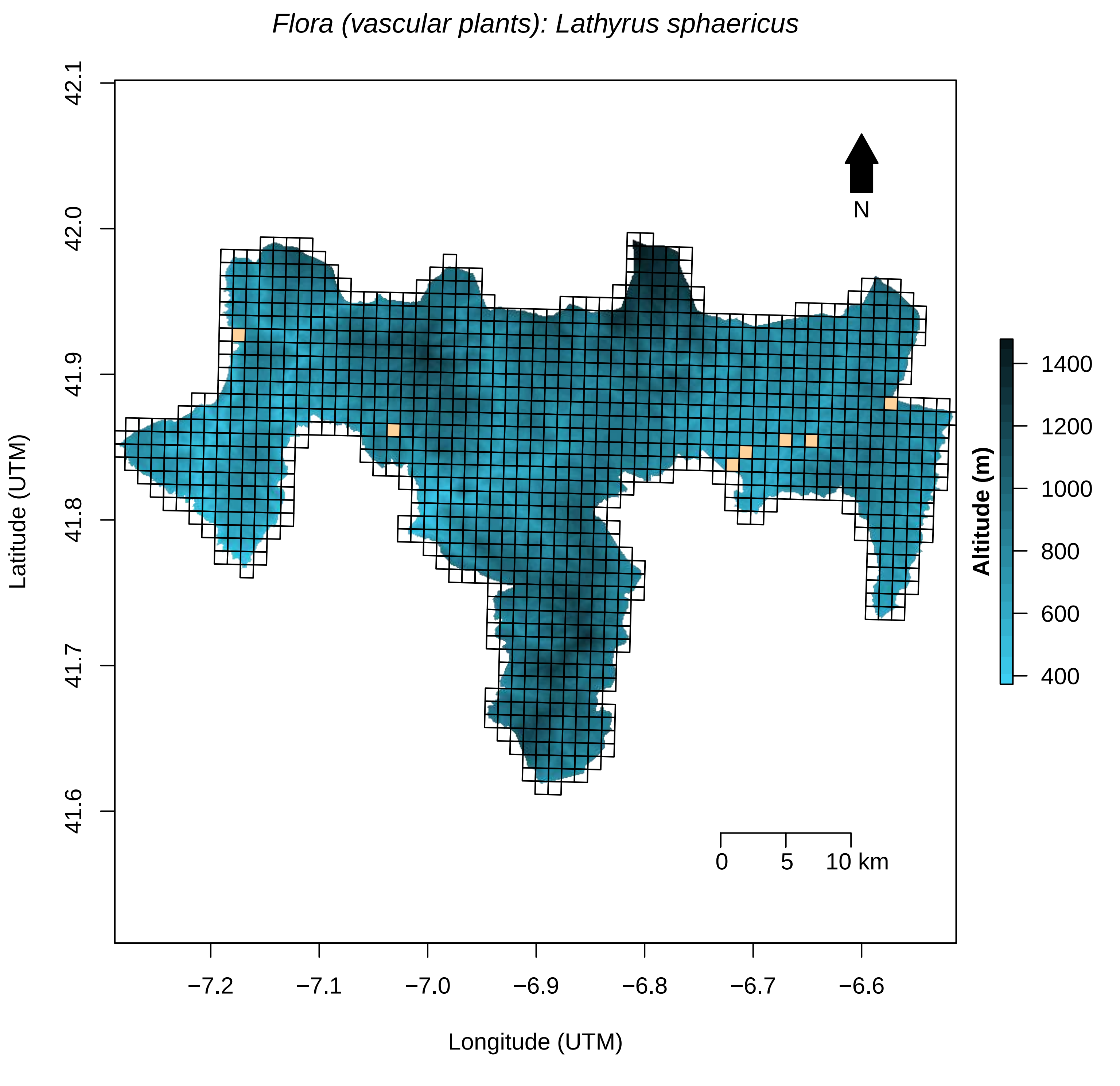
Flora (vascular plants): Lathyrus sphaericus
42.1
42.0
41.9
41.8
41.7
41.6
Latitude (UTM)
−7.2
−7.1
−7.0
−6.9
−6.8
−6.7
−6.6
Longitude (UTM)
N
0
5
10 km
Altitude (m)
1400
1200
1000
800
600
400

Flora (vascular plants): Lavandula pedunculata

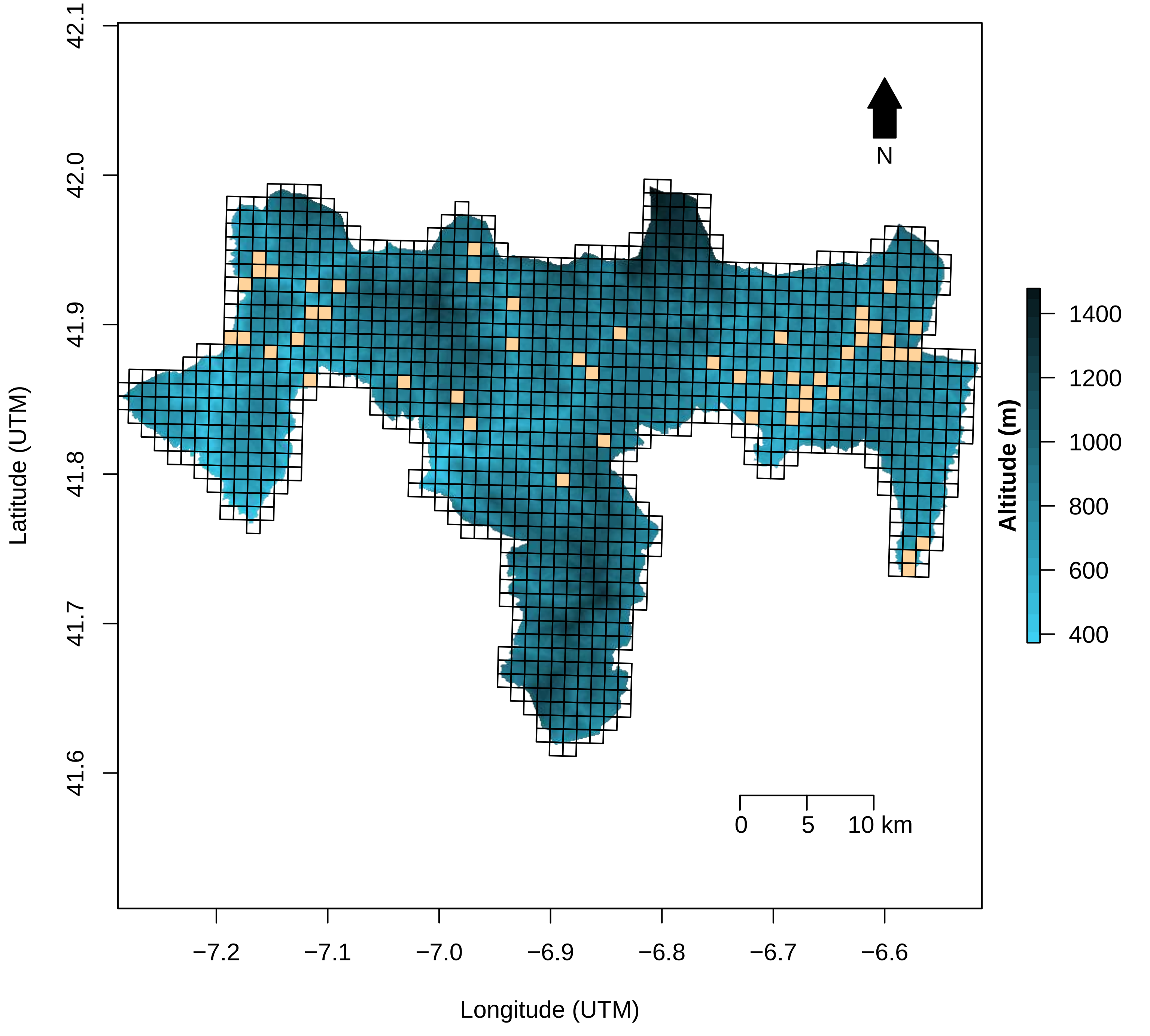

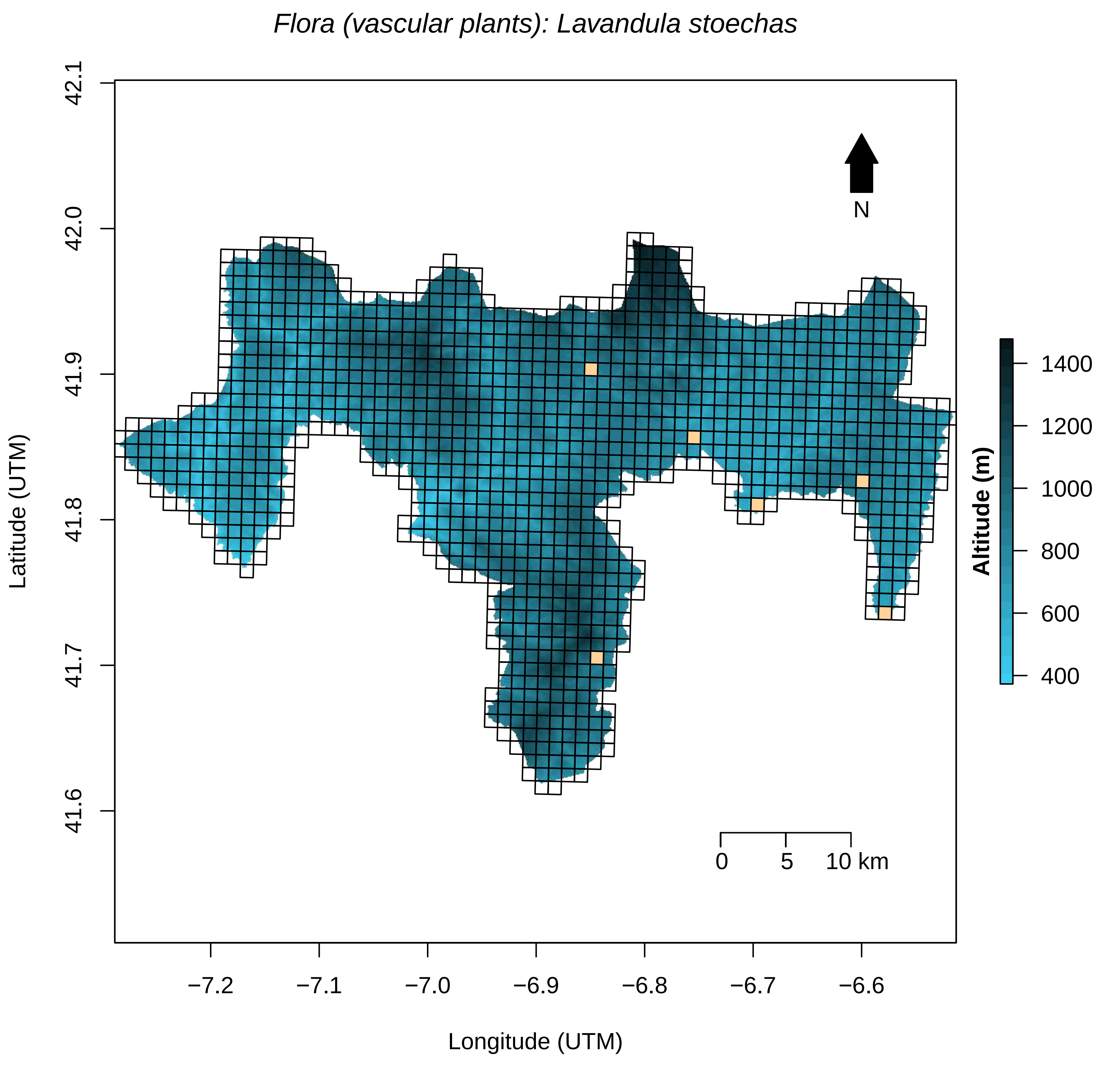
Flora (vascular plants): Lavandula stoechas
N
0
5
10 km
Latitude (UTM)
Longitude (UTM)
Altitude (m)
1400
1200
1000
800
600
400
42.1
42.0
41.9
41.8
41.7
41.6
−7.2
−7.1
−7.0
−6.9
−6.8
−6.7
−6.6

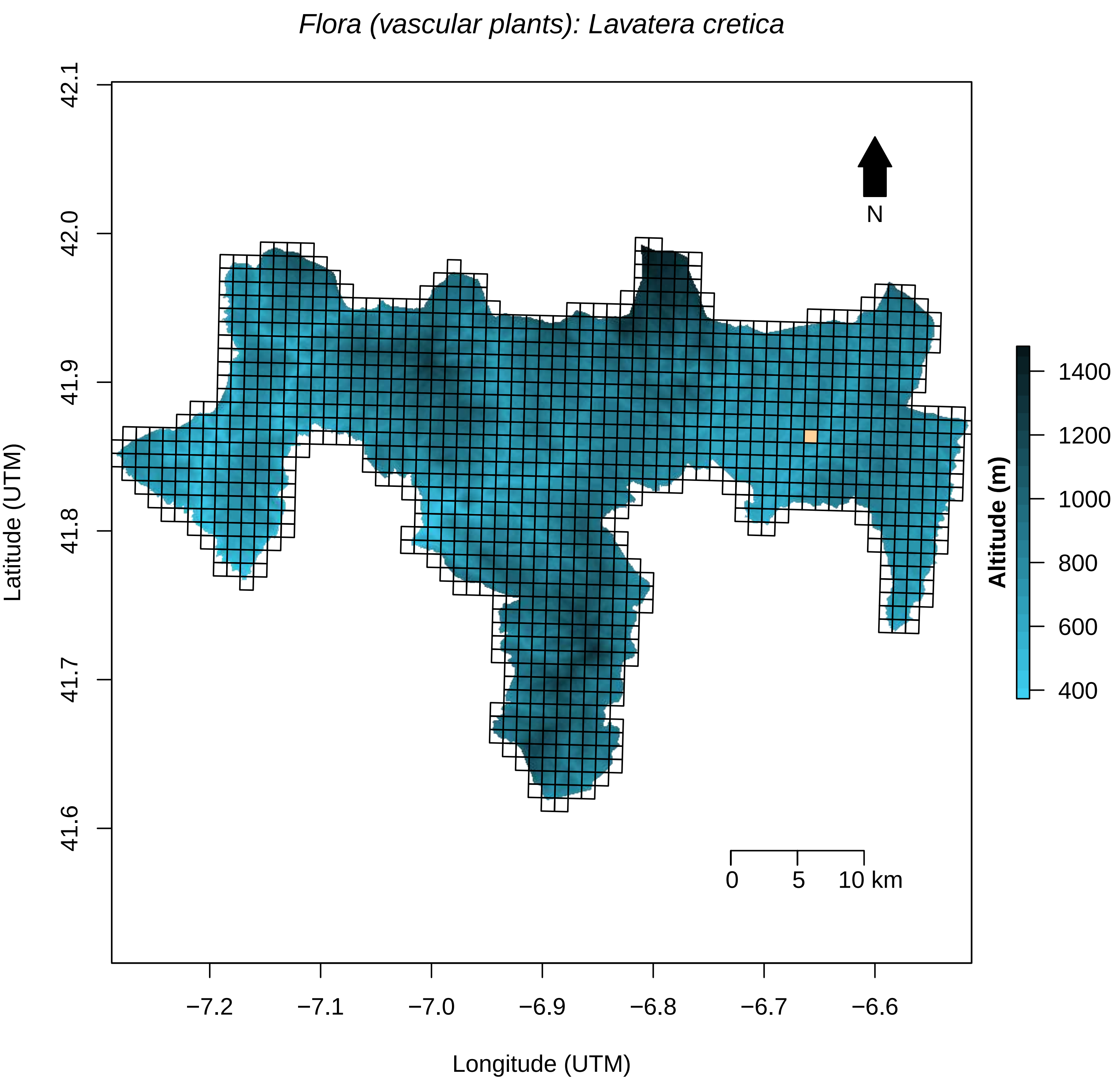

Flora (vascular plants): Lavatera cretica
42.1
42.0
41.9
41.8
41.7
41.6
Latitude (UTM)
−7.2
−7.1
−7.0
−6.9
−6.8
−6.7
−6.6
Longitude (UTM)
N
0
5
10 km
Altitude (m)
1400
1200
1000
800
600
400

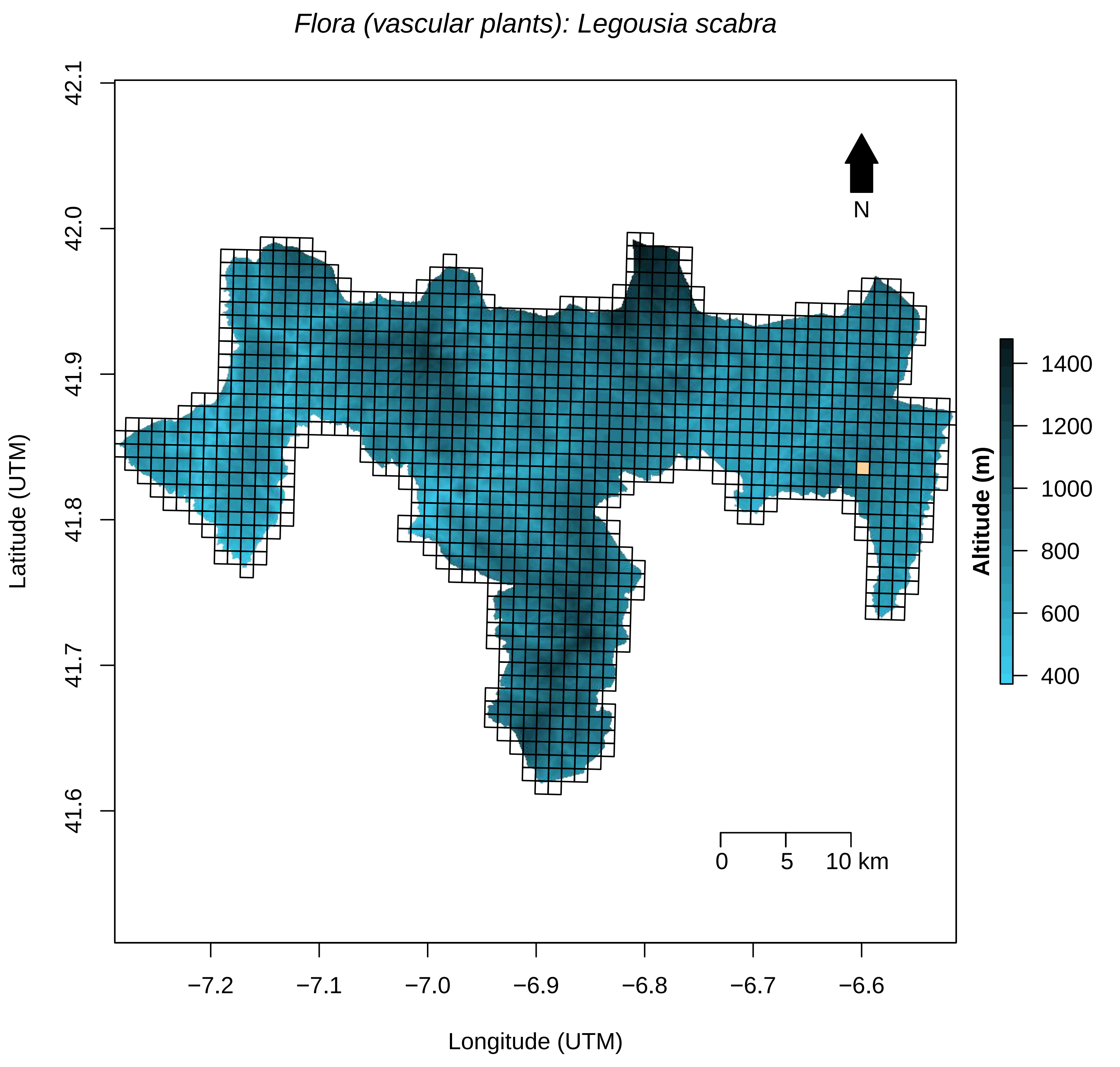
Flora (vascular plants): Legousia scabra
42.1
42.0
41.9
41.8
41.7
41.6
Latitude (UTM)
−7.2
−7.1
−7.0
−6.9
−6.8
−6.7
−6.6
Longitude (UTM)
N
0
5
10 km
Altitude (m)
1400
1200
1000
800
600
400

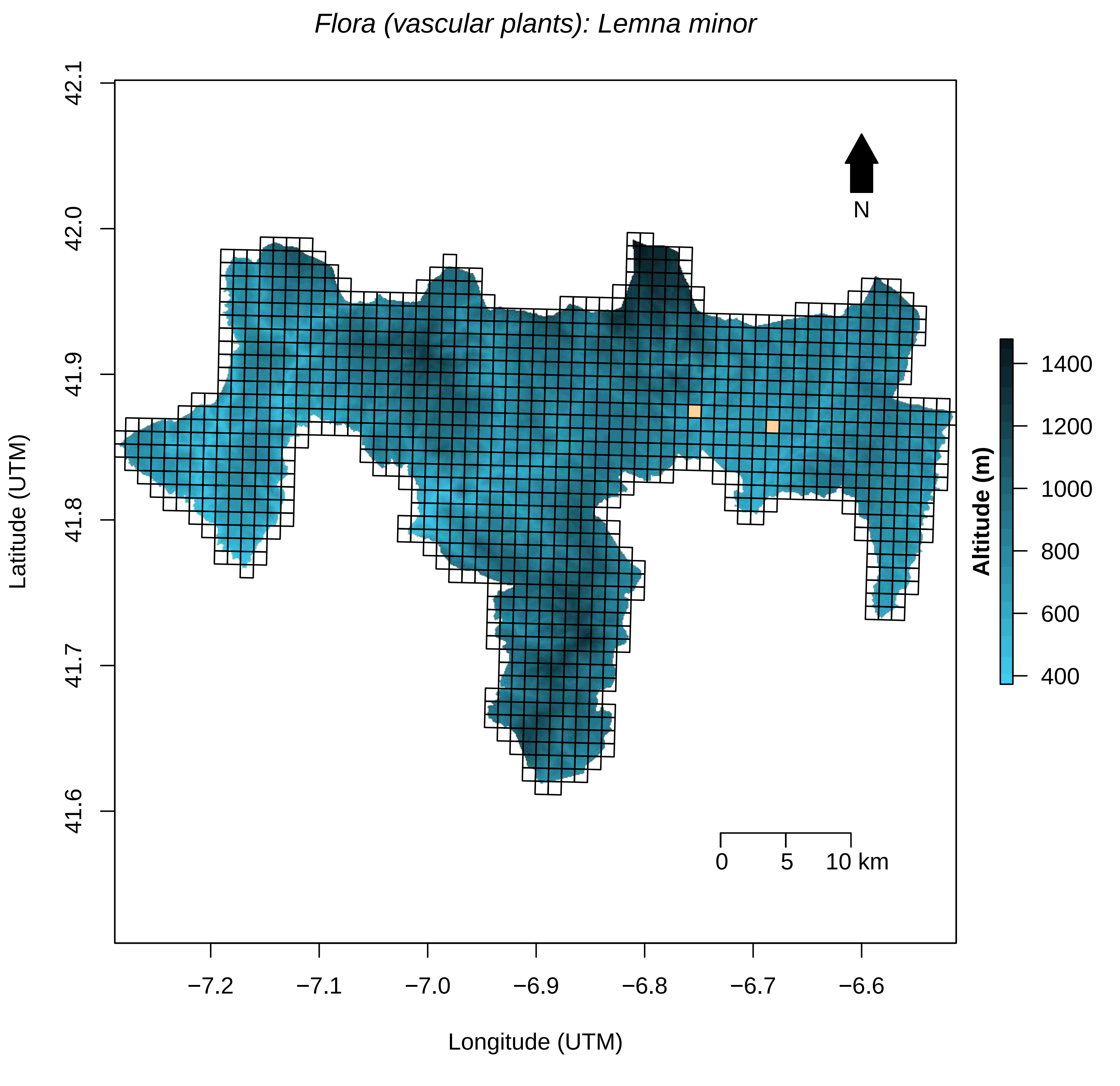

Flora (vascular plants): Lemna minor
42.1
42.0
41.9
41.8
41.7
41.6
Latitude (UTM)
−7.2
−7.1
−7.0
−6.9
−6.8
−6.7
−6.6
Longitude (UTM)
N
0
5
10 km
Altitude (m)
1400
1200
1000
800
600
400

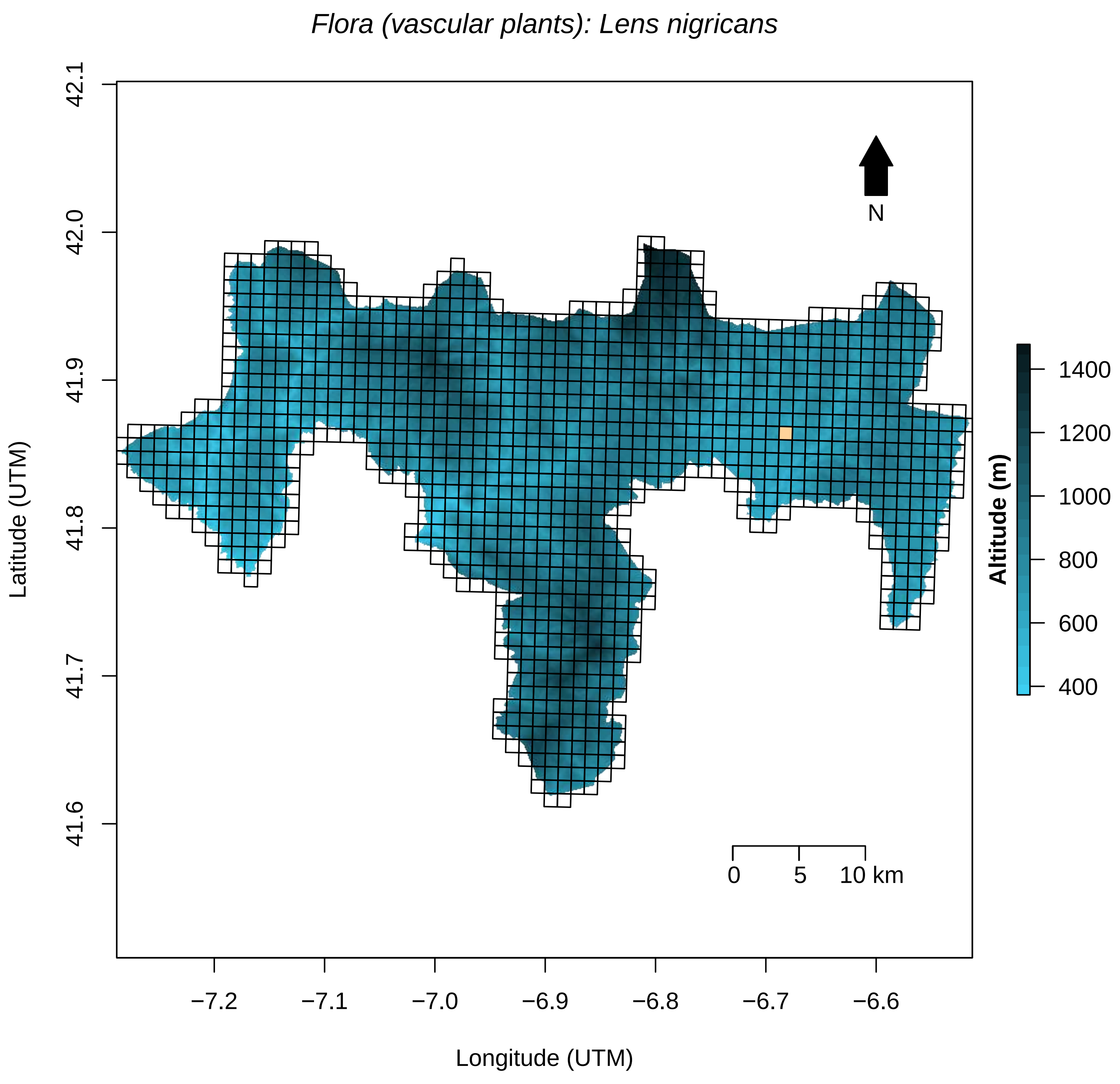
Flora (vascular plants): Lens nigricans
42.1
42.0
41.9
41.8
41.7
41.6
Latitude (UTM)
−7.2
−7.1
−7.0
−6.9
−6.8
−6.7
−6.6
Longitude (UTM)
N
0
5
10 km
Altitude (m)
1400
1200
1000
800
600
400

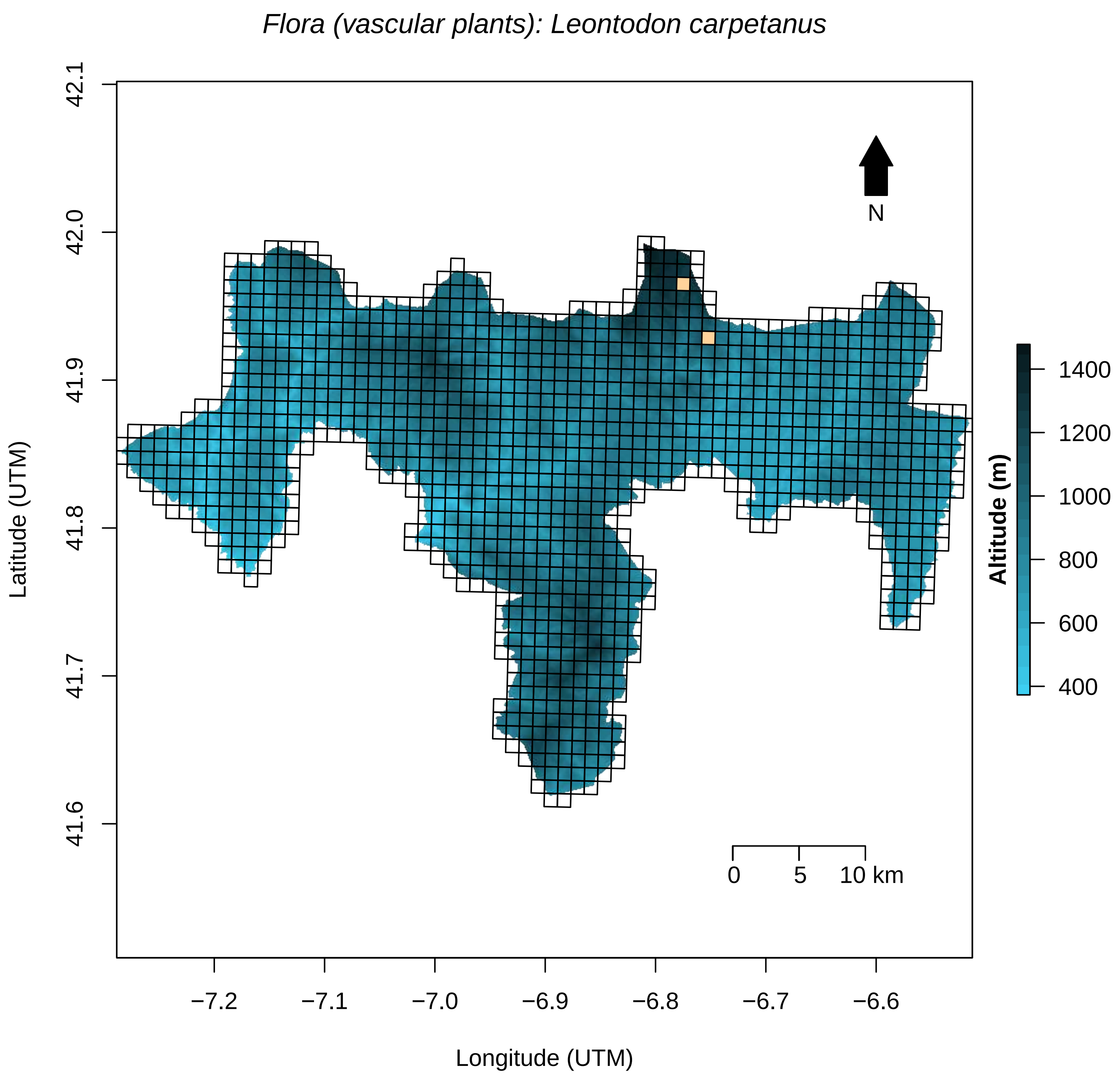
Flora (vascular plants): Leontodon carpetanus
42.1
42.0
41.9
41.8
41.7
41.6
Latitude (UTM)
N
0
5
10 km
−7.2
−7.1
−7.0
−6.9
−6.8
−6.7
−6.6
Longitude (UTM)
1400
1200
1000
800
600
400
Altitude (m)

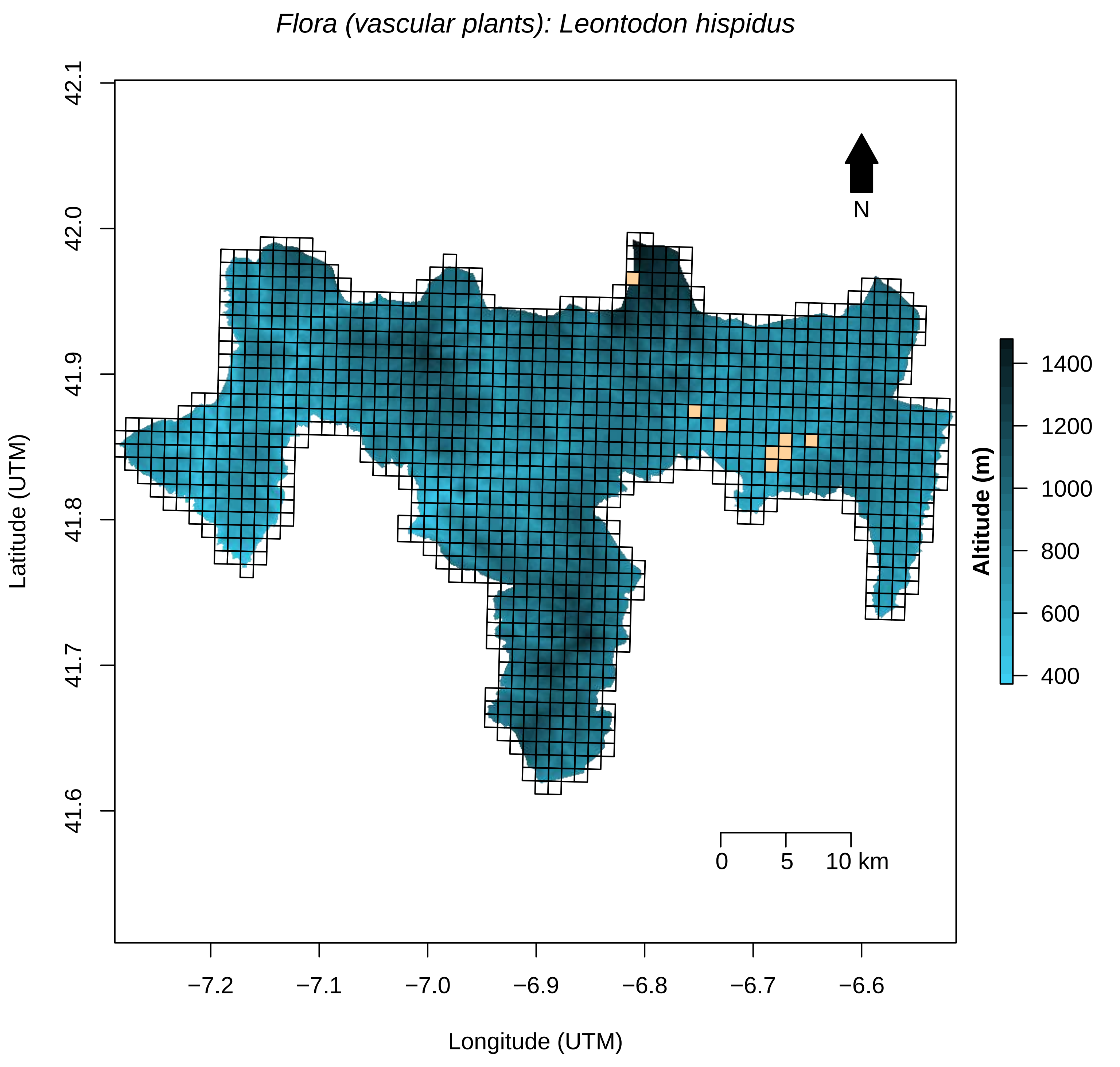

Flora (vascular plants): Leontodon hispidus
N
0
5
10 km
Altitude (m)
1400
1200
1000
800
600
400
Latitude (UTM)
42.1
42.0
41.9
41.8
41.7
41.6
Longitude (UTM)
−7.2
−7.1
−7.0
−6.9
−6.8
−6.7
−6.6

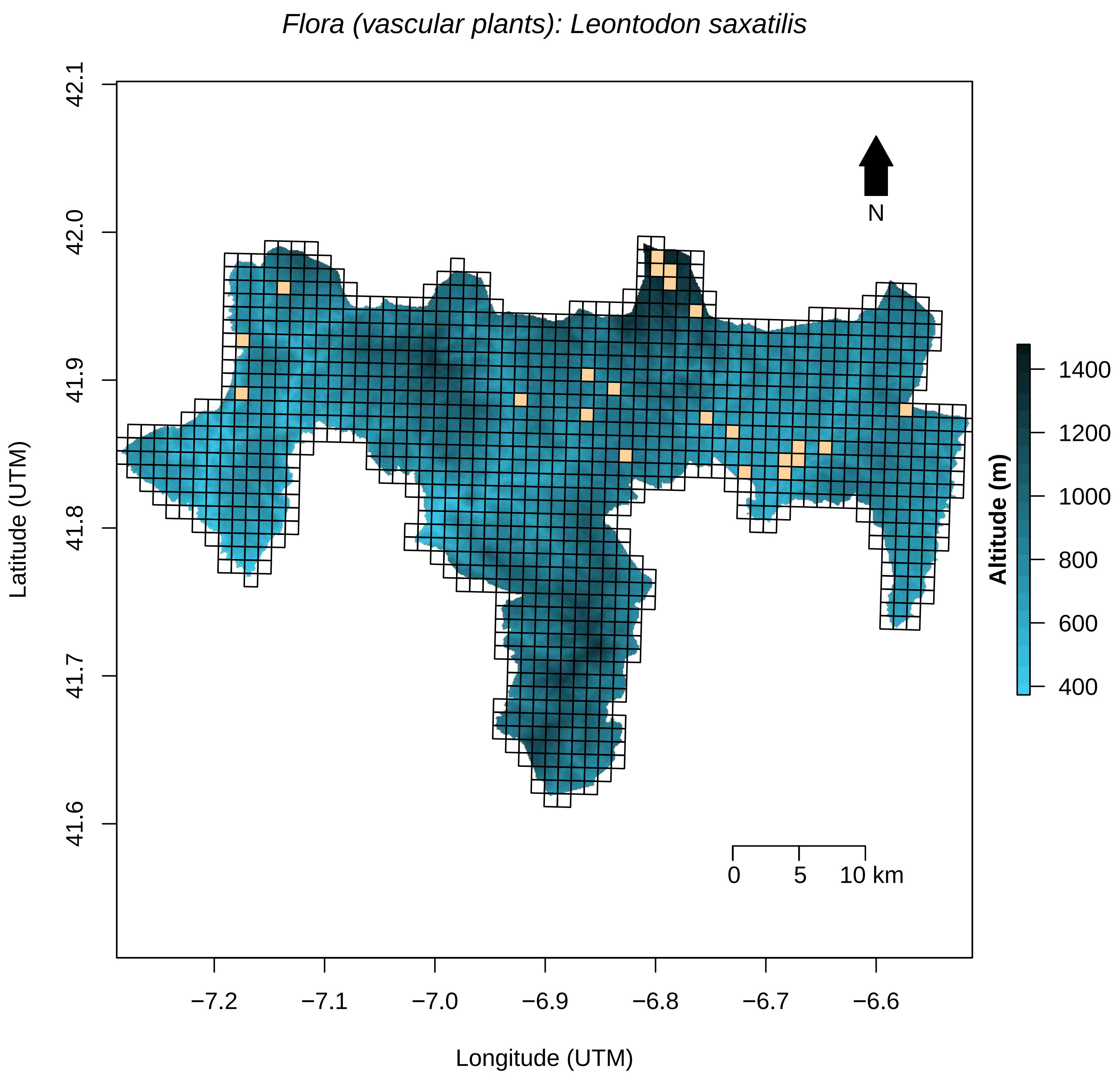

Flora (vascular plants): Leontodon saxatilis
42.1
42.0
41.9
41.8
41.7
41.6
Latitude (UTM)
−7.2
−7.1
−7.0
−6.9
−6.8
−6.7
−6.6
Longitude (UTM)
N
0
5
10 km
Altitude (m)
1400
1200
1000
800
600
400

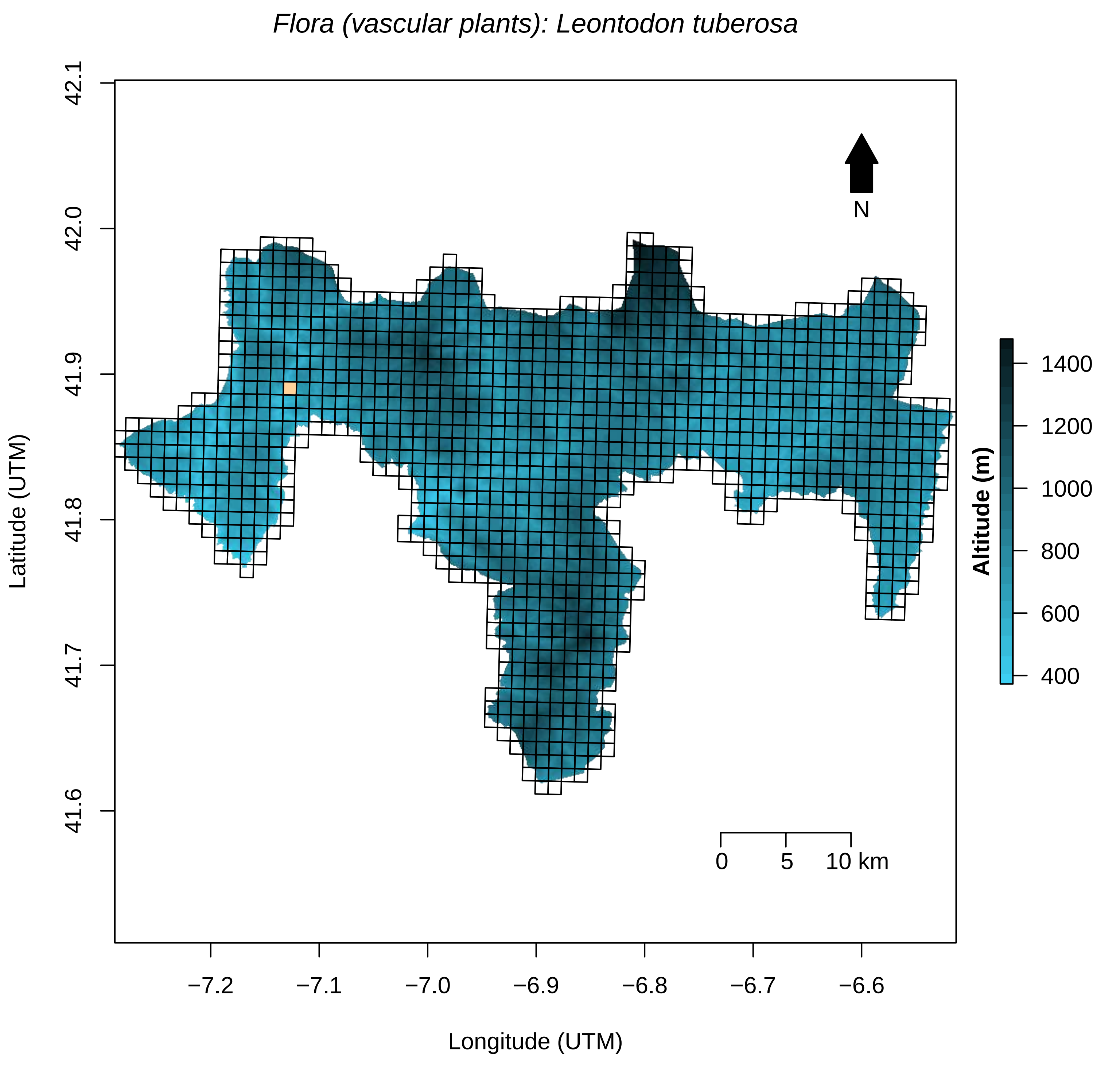

Flora (vascular plants): Leontodon tuberosa
N
0
5
10 km
Altitude (m)
1400
1200
1000
800
600
400
Latitude (UTM)
42.1
42.0
41.9
41.8
41.7
41.6
Longitude (UTM)
−7.2
−7.1
−7.0
−6.9
−6.8
−6.7
−6.6

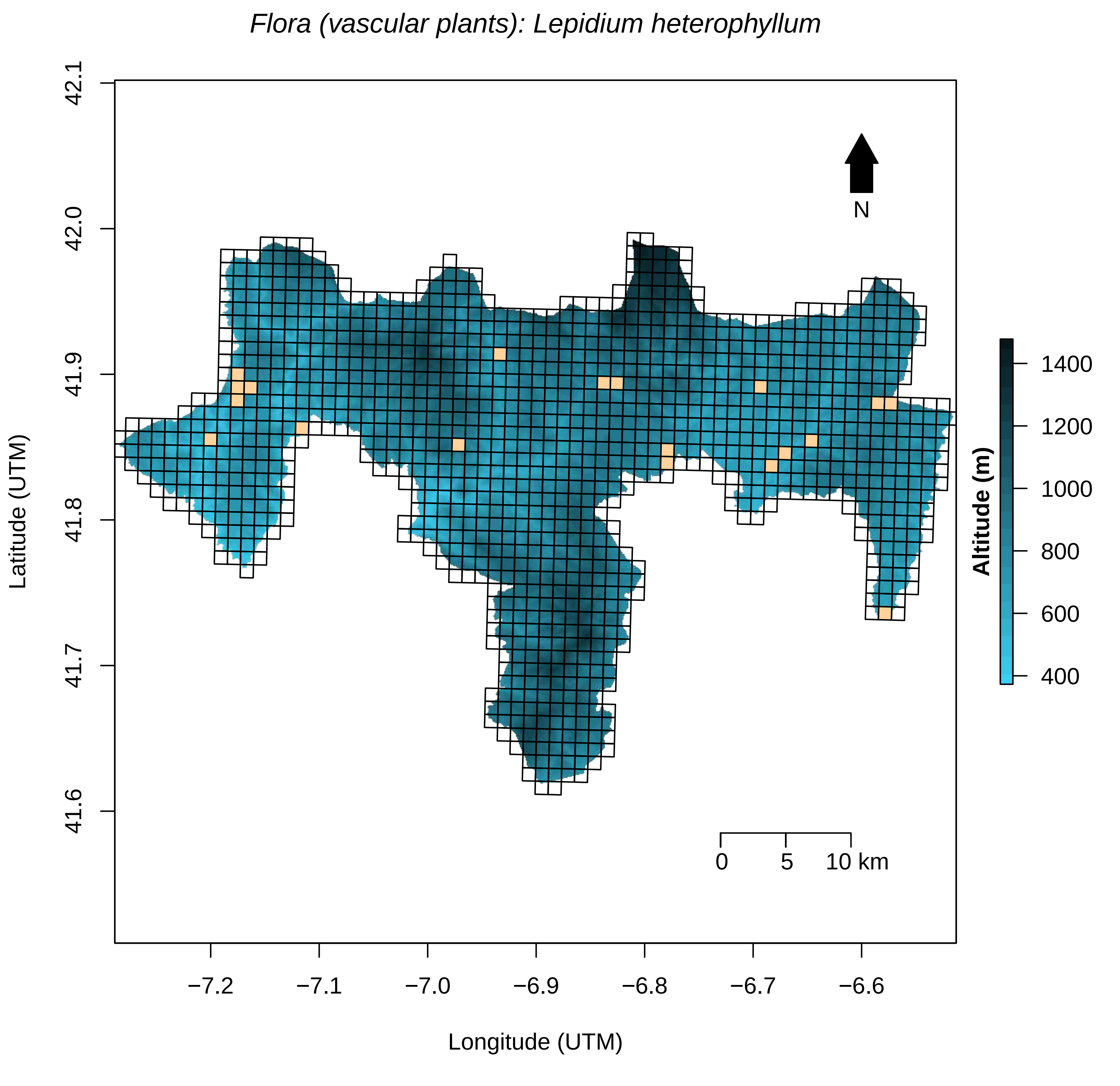

Flora (vascular plants): Lepidium heterophyllum
Latitude (UTM)
42.1
42.0
41.9
41.8
41.7
41.6
Longitude (UTM)
−7.2
−7.1
−7.0
−6.9
−6.8
−6.7
−6.6
N
0
5
10 km
Altitude (m)
1400
1200
1000
800
600
400

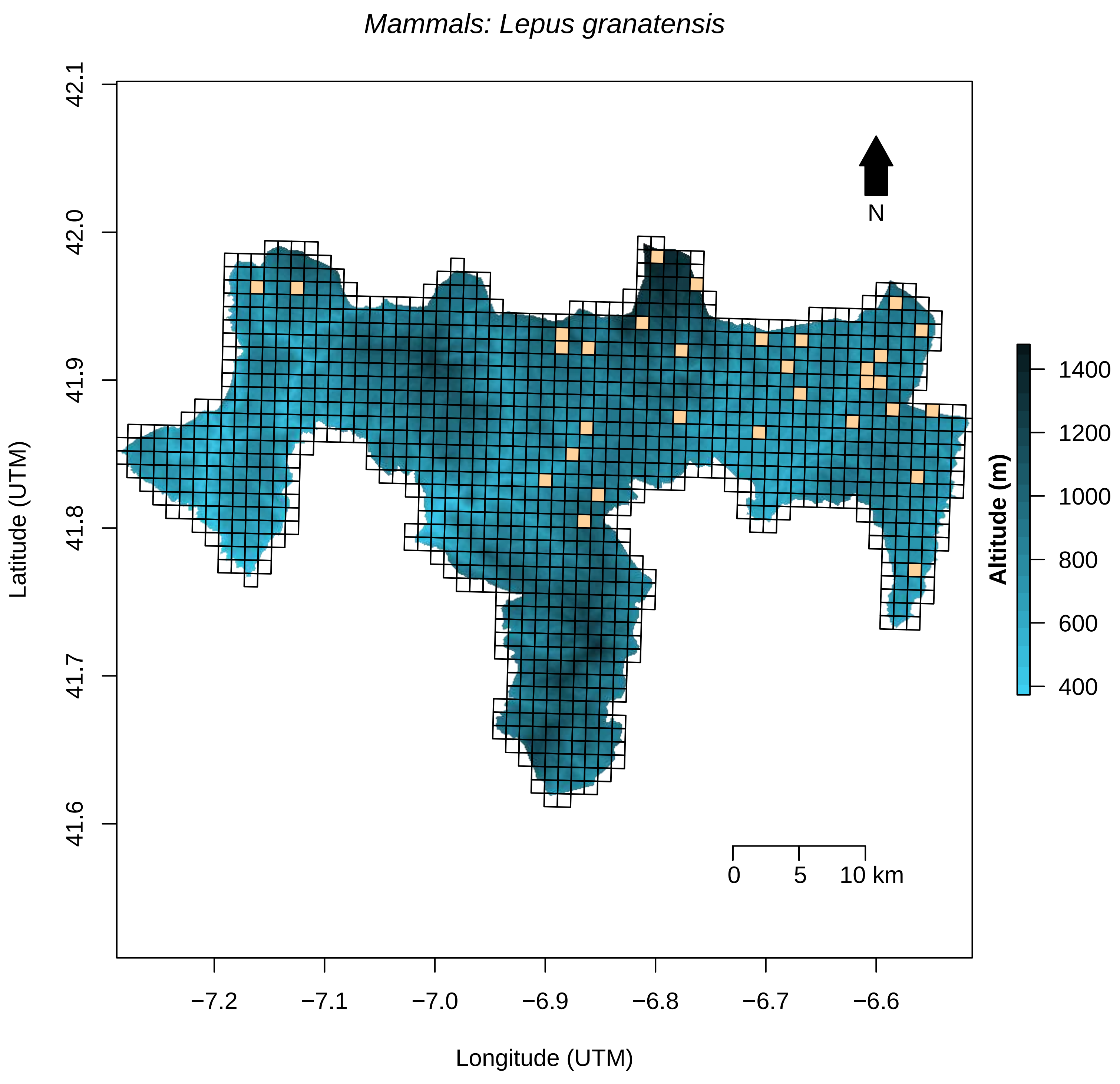

Mammals: Lepus granatensis
Latitude (UTM)
Longitude (UTM)
42.1
42.0
41.9
41.8
41.7
41.6
−7.2
−7.1
−7.0
−6.9
−6.8
−6.7
−6.6
N
0
5
10 km
Altitude (m)
1400
1200
1000
800
600
400

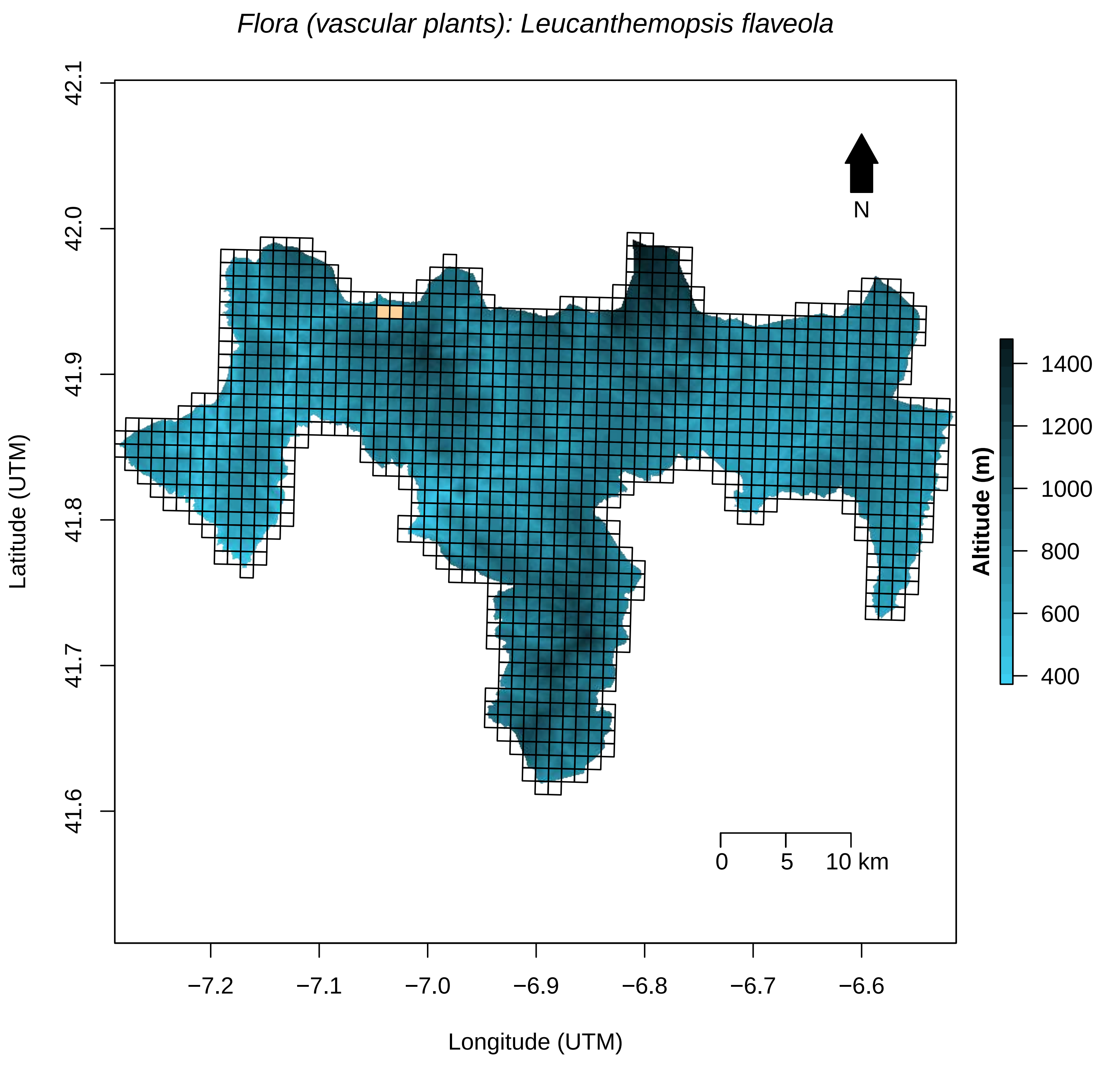
Flora (vascular plants): Leucanthemopsis flaveola
42.1
42.0
41.9
41.8
41.7
41.6
Latitude (UTM)
−7.2
−7.1
−7.0
−6.9
−6.8
−6.7
−6.6
Longitude (UTM)
N
0
5
10 km
Altitude (m)
1400
1200
1000
800
600
400

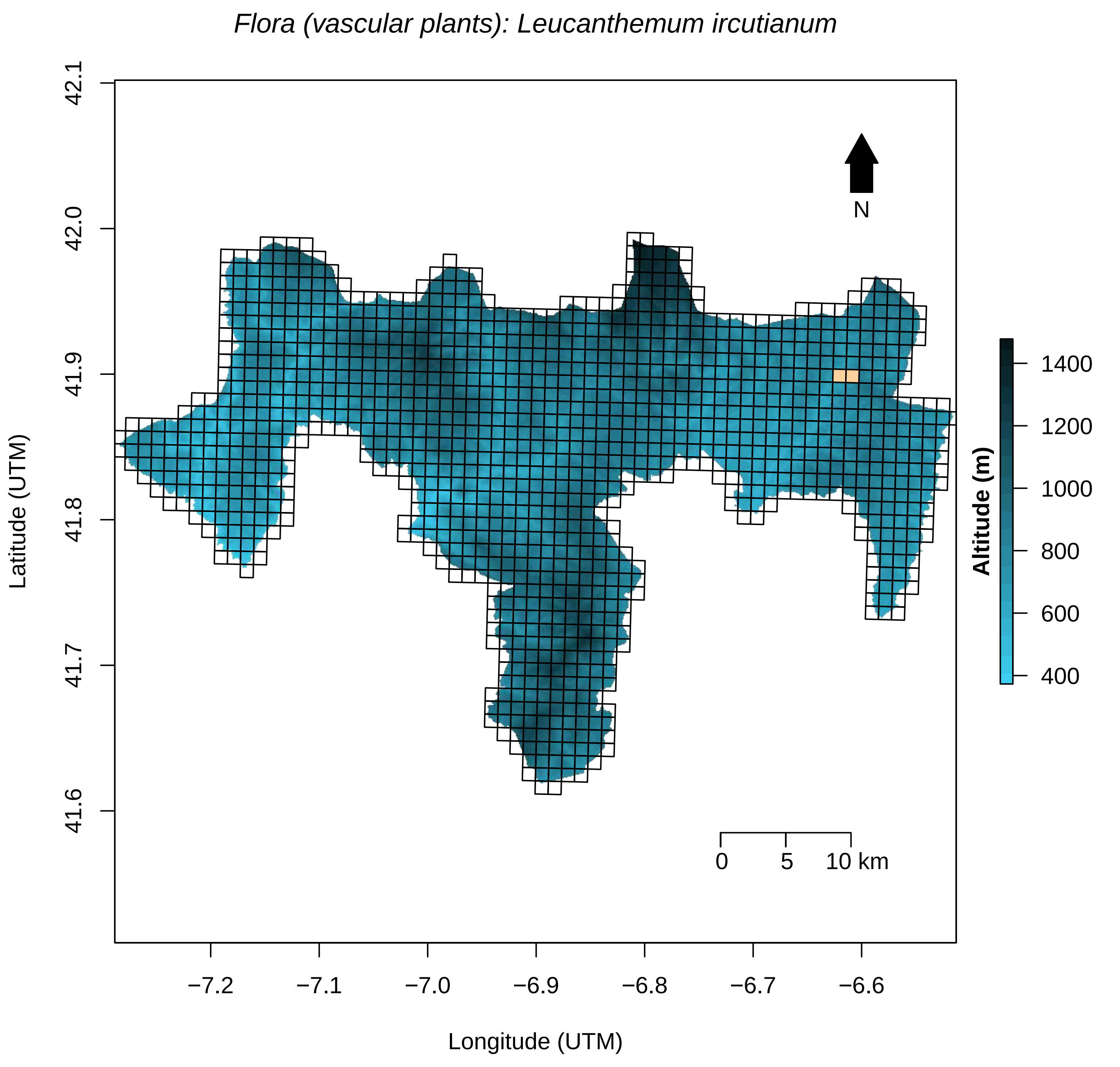
Flora (vascular plants): Leucanthemum ircutianum
42.1
42.0
41.9
41.8
41.7
41.6
Latitude (UTM)
−7.2
−7.1
−7.0
−6.9
−6.8
−6.7
−6.6
Longitude (UTM)
N
0
5
10 km
1400
1200
1000
800
600
400
Altitude (m)

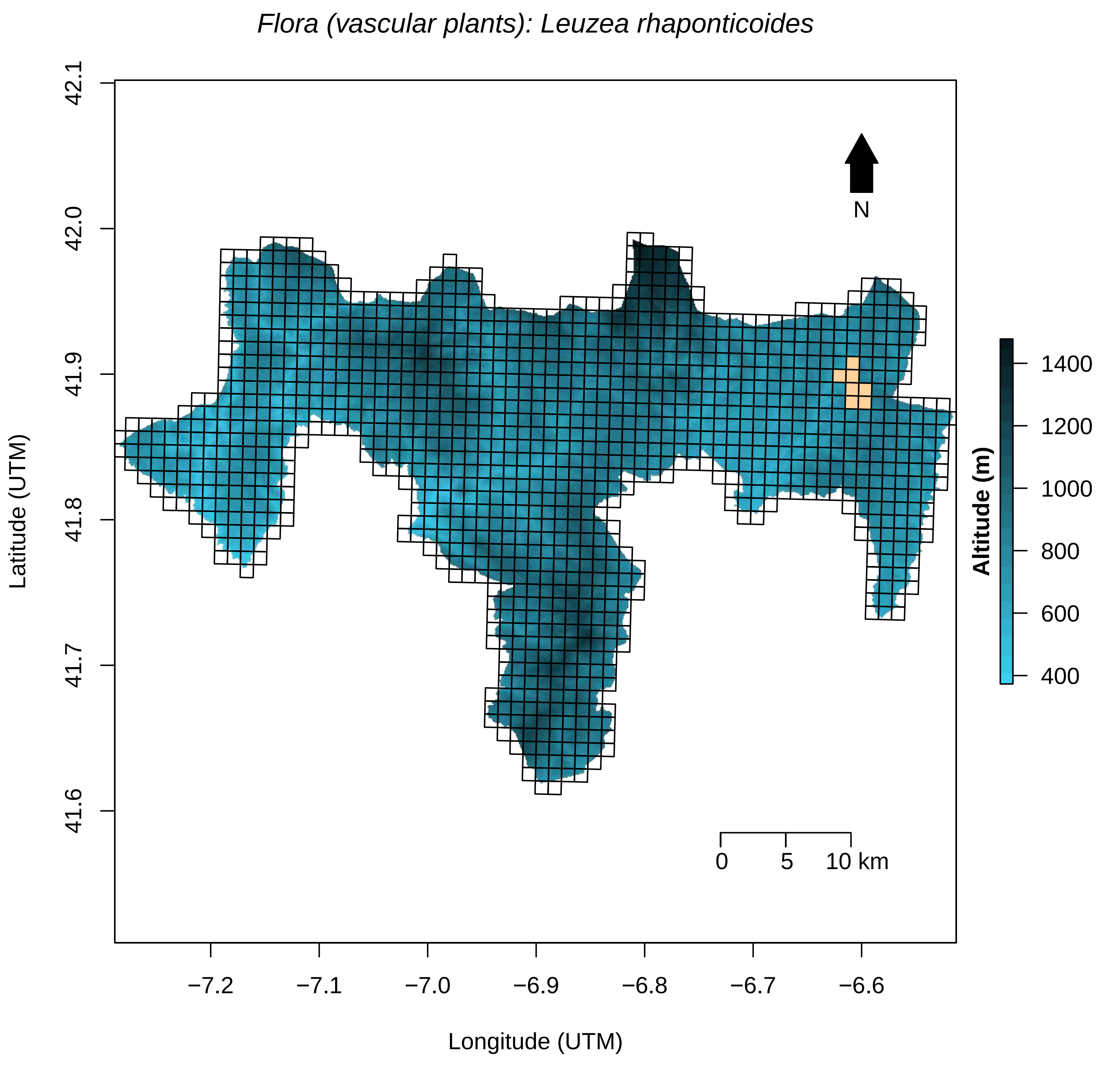

Flora (vascular plants): Leuzea rhaponticoides
N
0
5
10 km
Altitude (m)
1400
1200
1000
800
600
400
42.1
42.0
41.9
41.8
41.7
41.6
Latitude (UTM)
−7.2
−7.1
−7.0
−6.9
−6.8
−6.7
−6.6
Longitude (UTM)

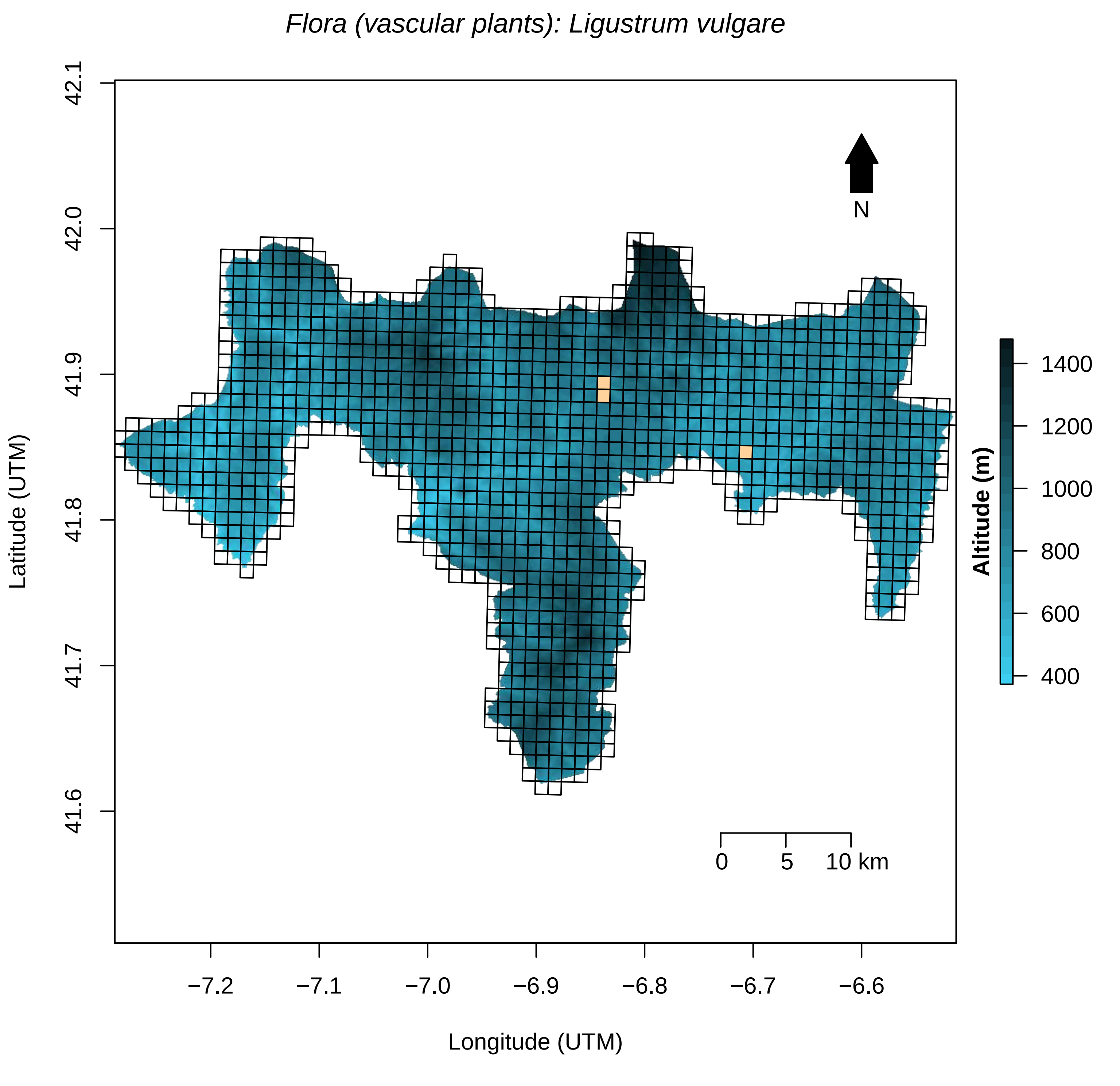

Flora (vascular plants): Ligustrum vulgare
42.1
42.0
41.9
41.8
41.7
41.6
Latitude (UTM)
N
0
5
10 km
−7.2
−7.1
−7.0
−6.9
−6.8
−6.7
−6.6
Longitude (UTM)
1400
1200
1000
800
600
400
Altitude (m)

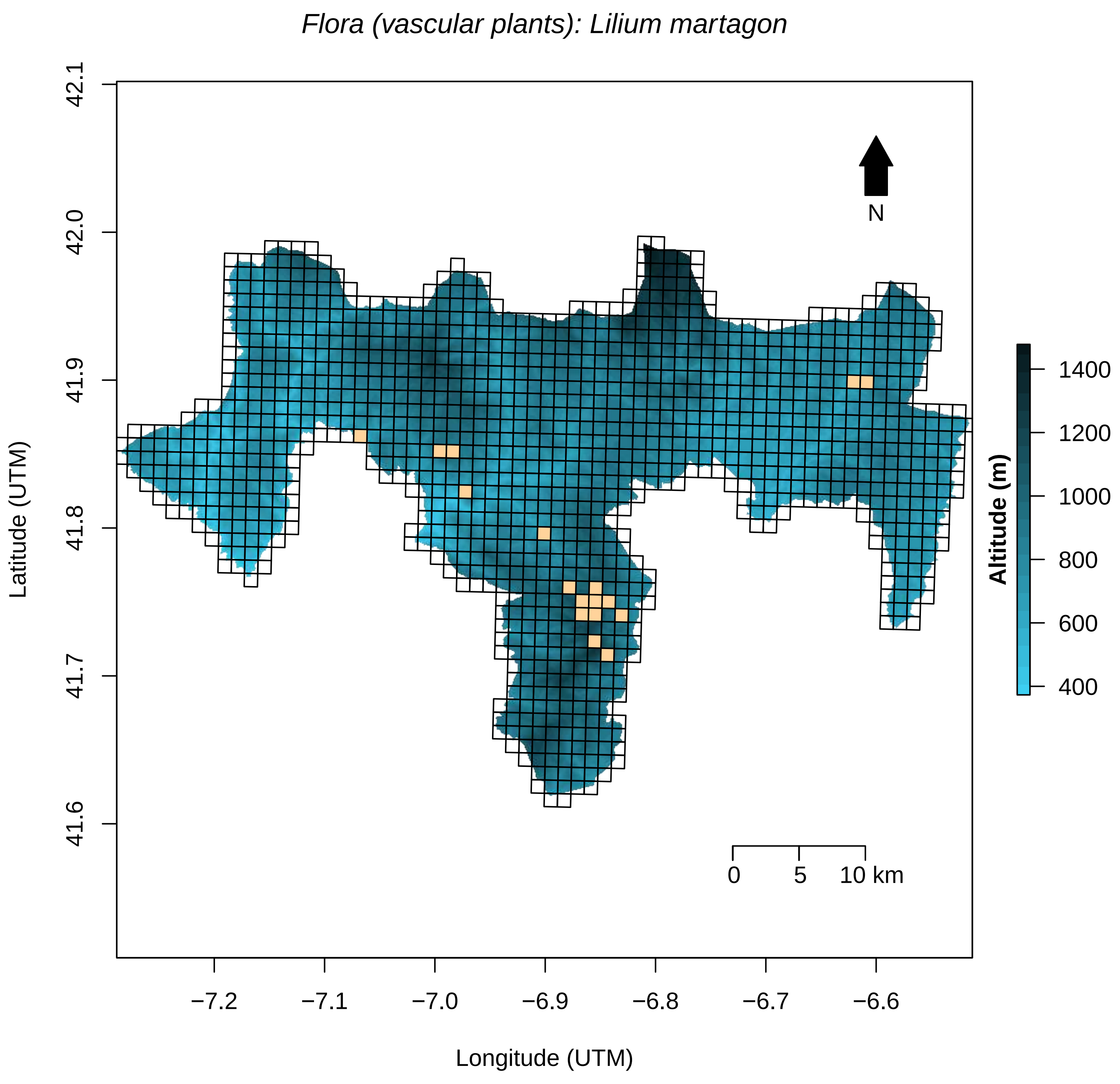

Flora (vascular plants): Lilium martagon
42.1
42.0
41.9
41.8
41.7
41.6
Latitude (UTM)
−7.2
−7.1
−7.0
−6.9
−6.8
−6.7
−6.6
Longitude (UTM)
N
0
5
10 km
1400
1200
1000
800
600
400
Altitude (m)

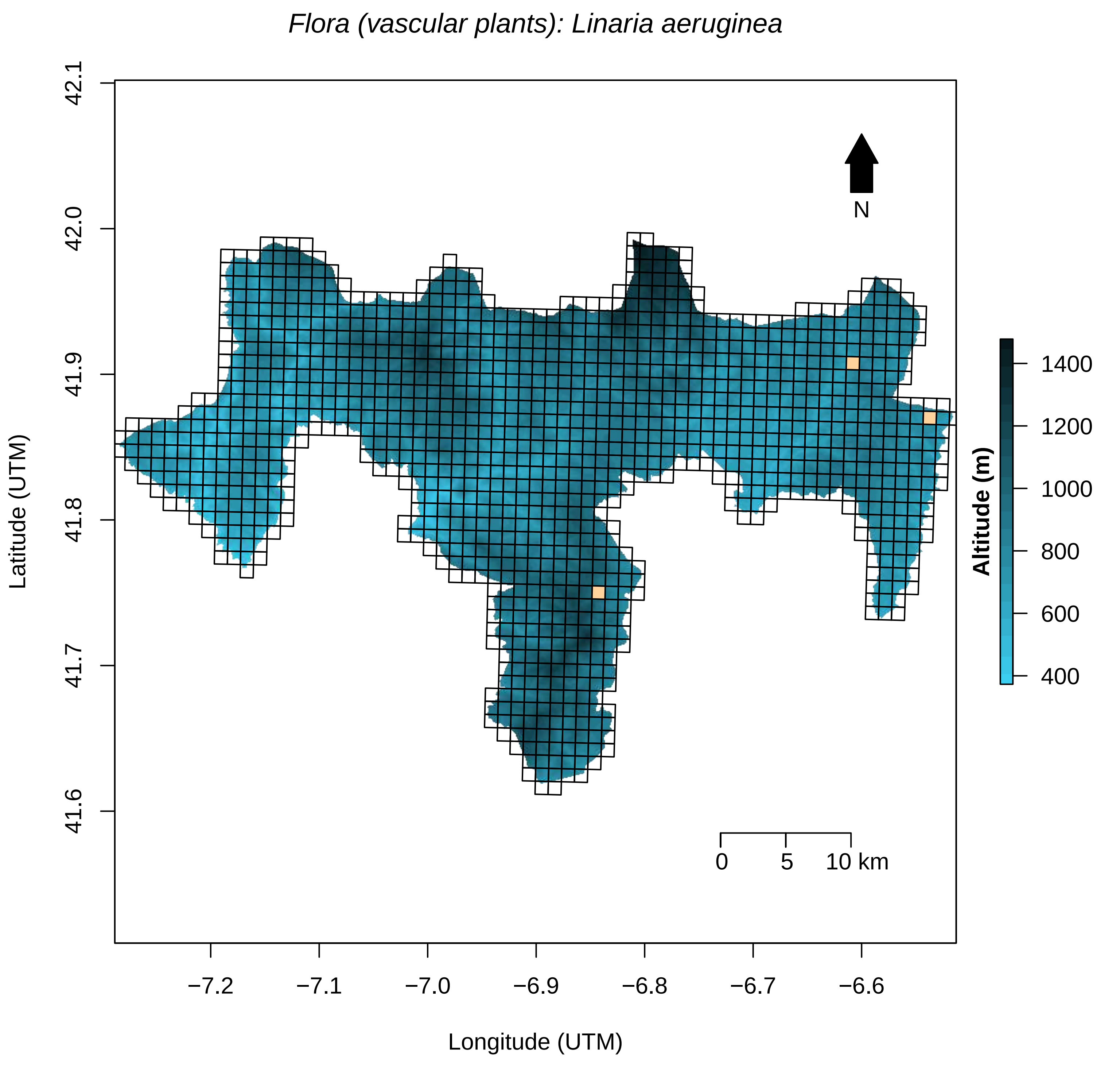

Flora (vascular plants): Linaria aeruginea
42.1
42.0
41.9
41.8
41.7
41.6
Latitude (UTM)
−7.2
−7.1
−7.0
−6.9
−6.8
−6.7
−6.6
Longitude (UTM)
N
0
5
10 km
1400
1200
1000
800
600
400
Altitude (m)

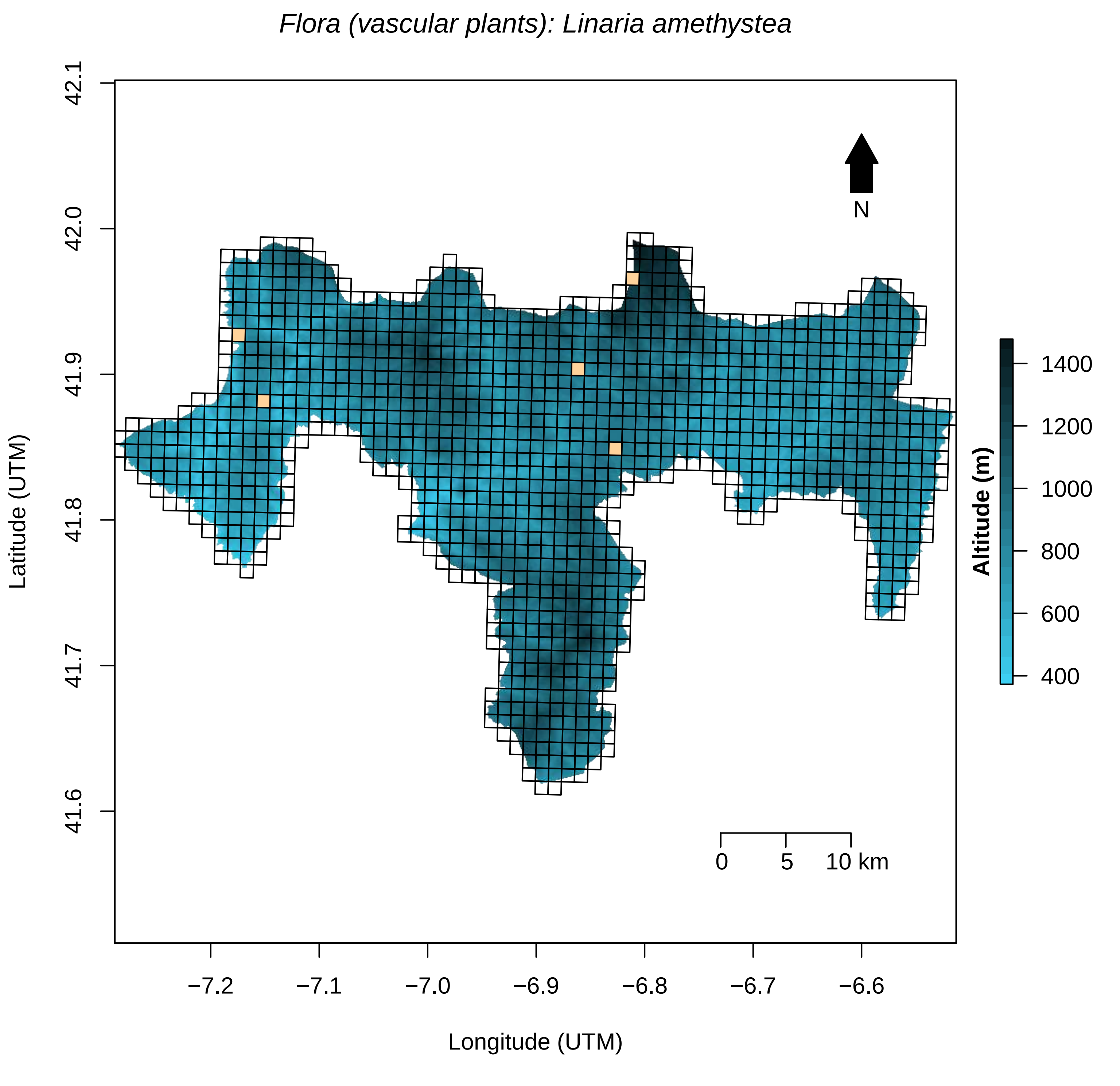

Flora (vascular plants): Linaria amethystea
42.1
42.0
41.9
41.8
41.7
41.6
Latitude (UTM)
−7.2
−7.1
−7.0
−6.9
−6.8
−6.7
−6.6
Longitude (UTM)
N
0
5
10 km
1400
1200
1000
800
600
400
Altitude (m)

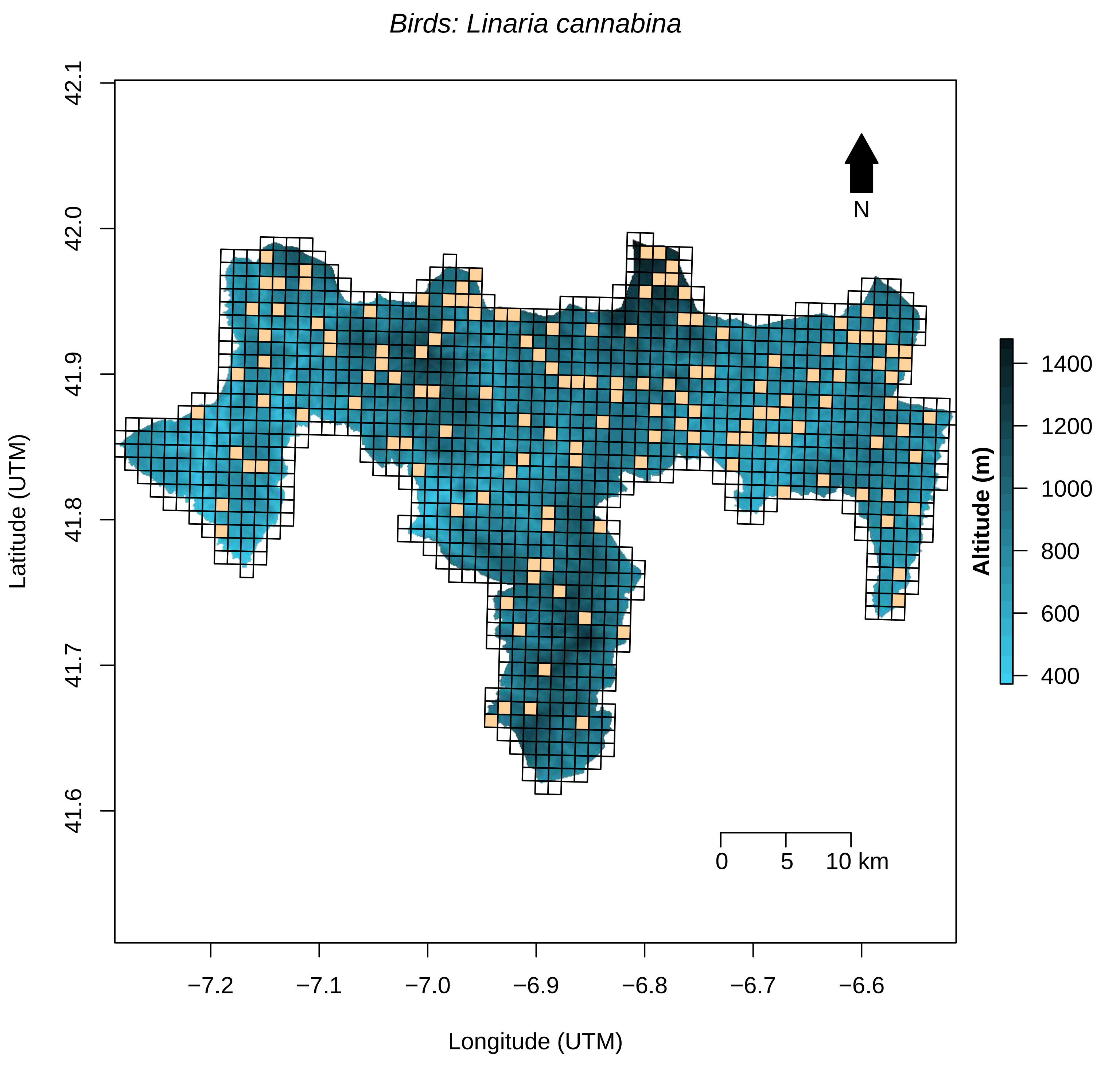

Birds: Linaria cannabina
Latitude (UTM)
42.1
42.0
41.9
41.8
41.7
41.6
Longitude (UTM)
−7.2
−7.1
−7.0
−6.9
−6.8
−6.7
−6.6
N
0
5
10 km
Altitude (m)
1400
1200
1000
800
600
400

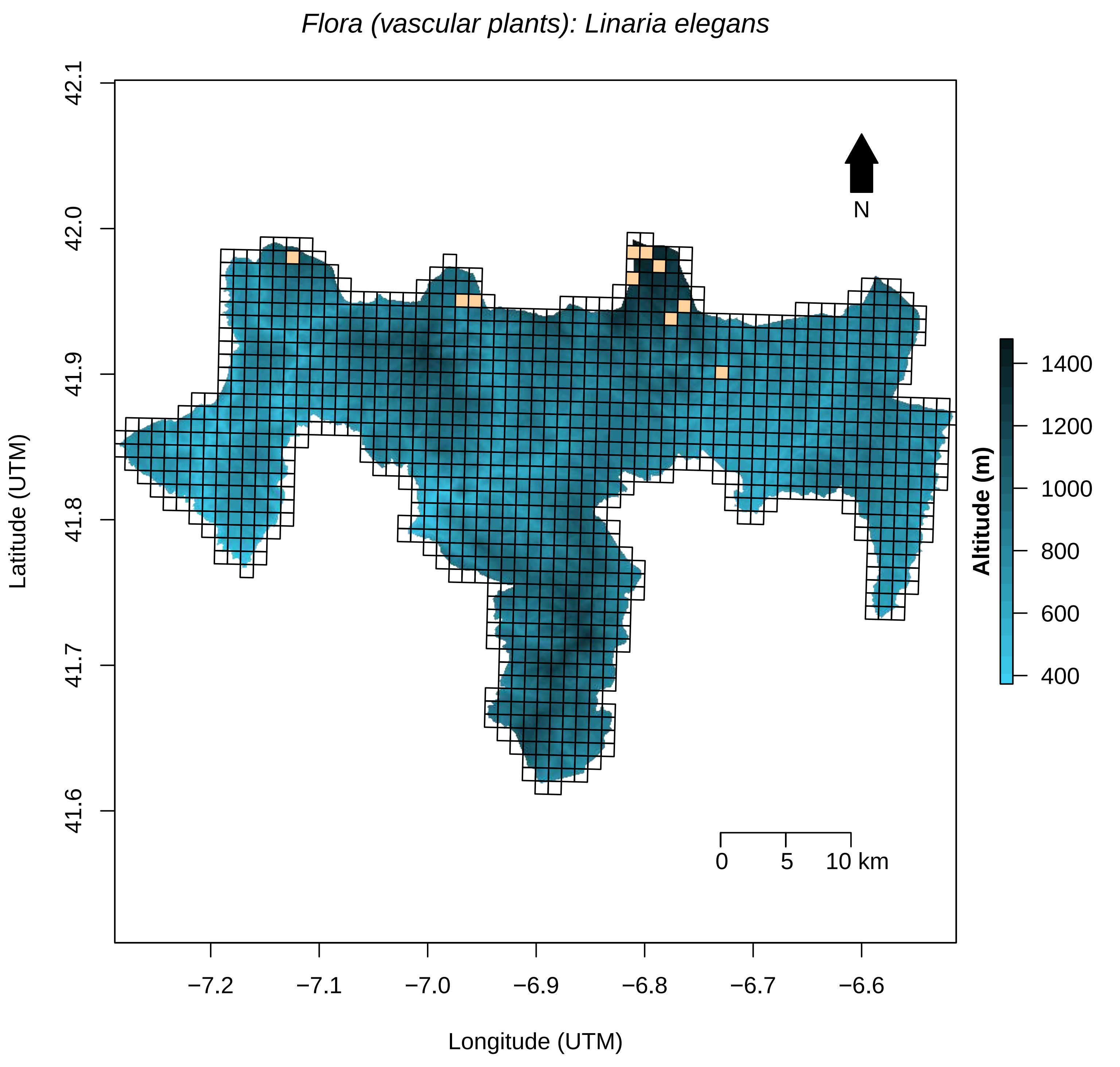

Flora (vascular plants): Linaria elegans
42.1
42.0
41.9
41.8
41.7
41.6
Latitude (UTM)
−7.2
−7.1
−7.0
−6.9
−6.8
−6.7
−6.6
Longitude (UTM)
N
0
5
10 km
Altitude (m)
1400
1200
1000
800
600
400

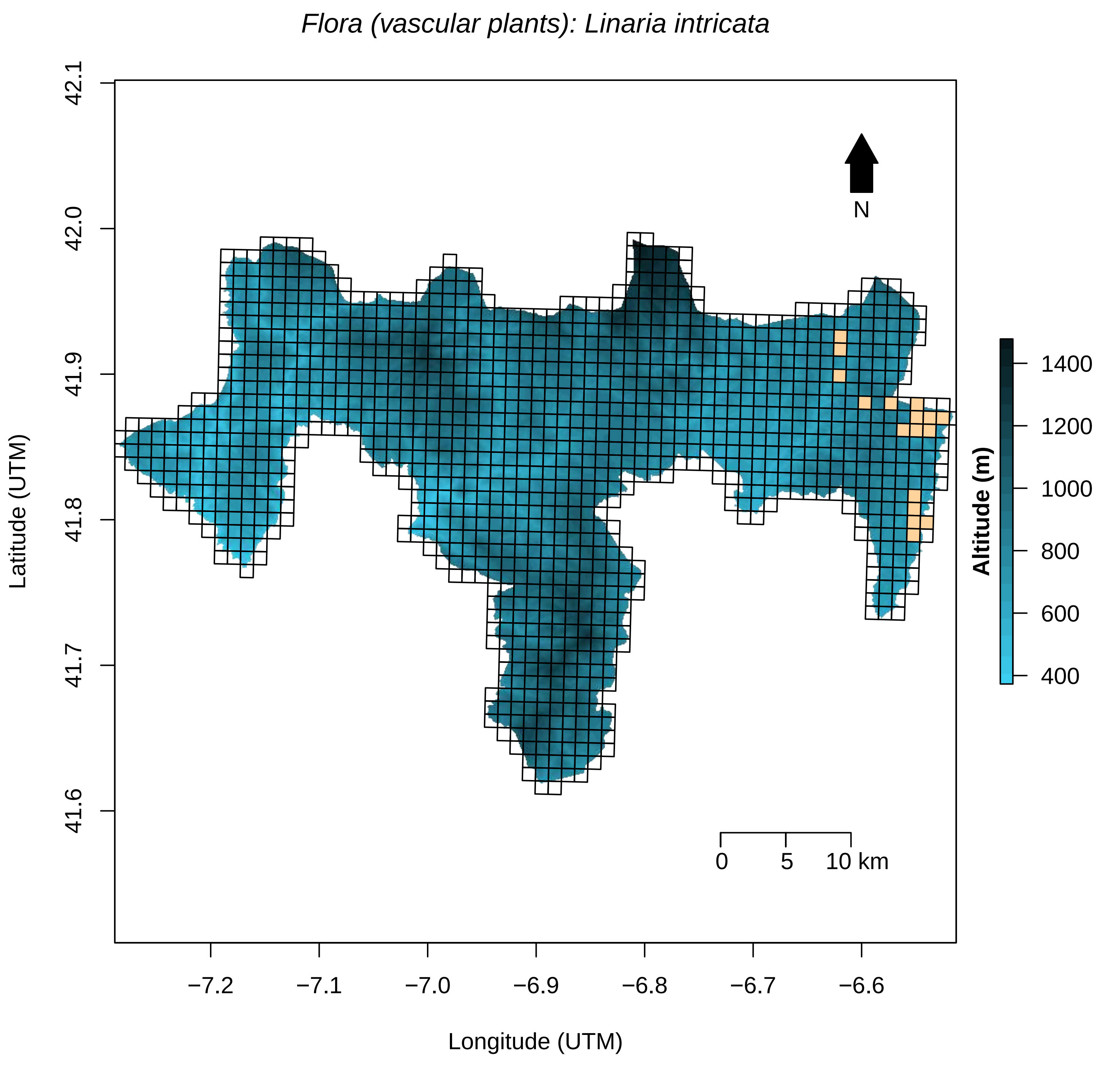

Flora (vascular plants): Linaria intricata
42.1
42.0
41.9
41.8
41.7
41.6
Latitude (UTM)
N
0
5
10 km
Altitude (m)
1400
1200
1000
800
600
400
−7.2
−7.1
−7.0
−6.9
−6.8
−6.7
−6.6
Longitude (UTM)

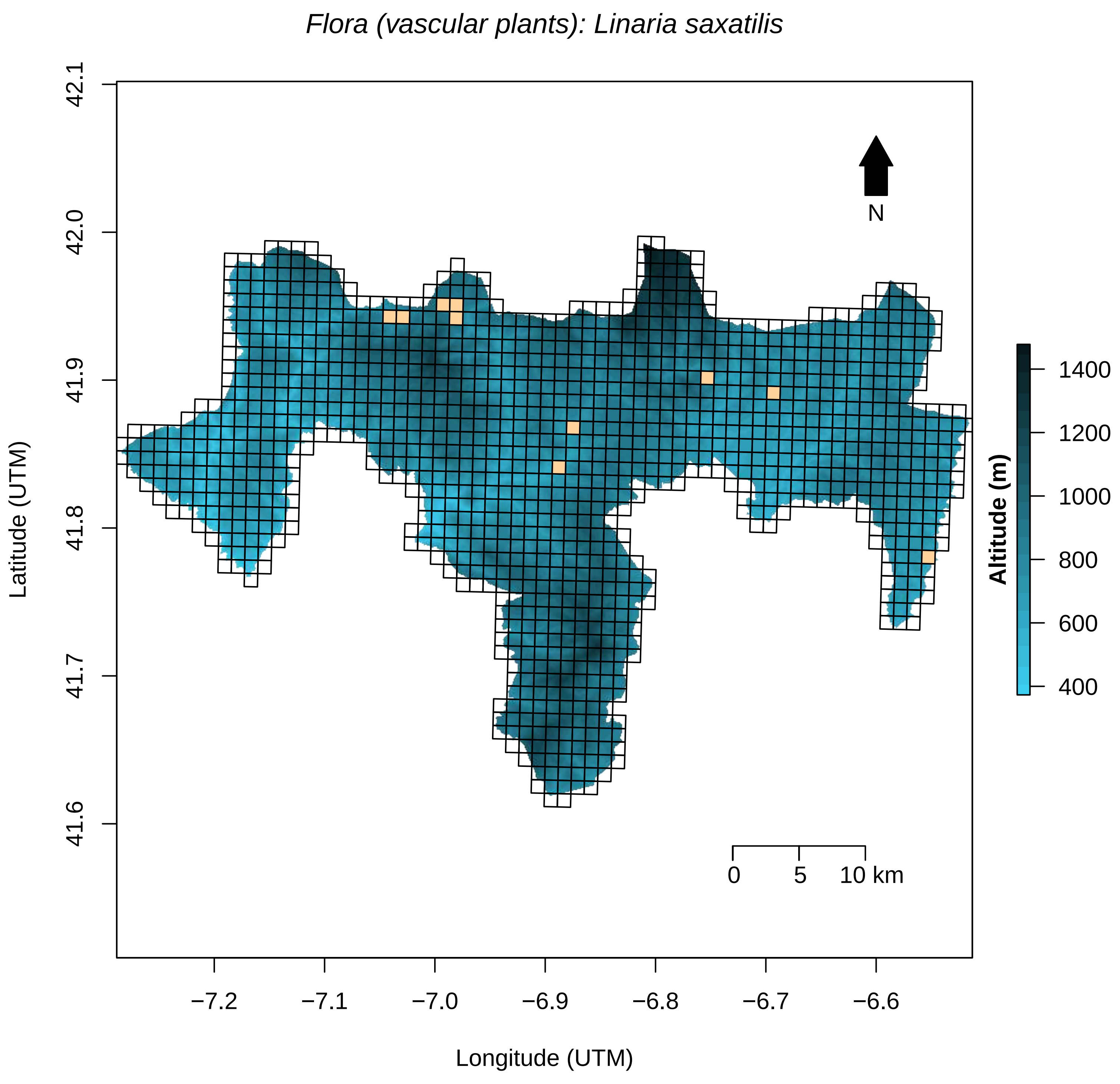

Flora (vascular plants): Linaria saxatilis
42.1
42.0
41.9
41.8
41.7
41.6
Latitude (UTM)
−7.2
−7.1
−7.0
−6.9
−6.8
−6.7
−6.6
Longitude (UTM)
N
0
5
10 km
Altitude (m)
1400
1200
1000
800
600
400

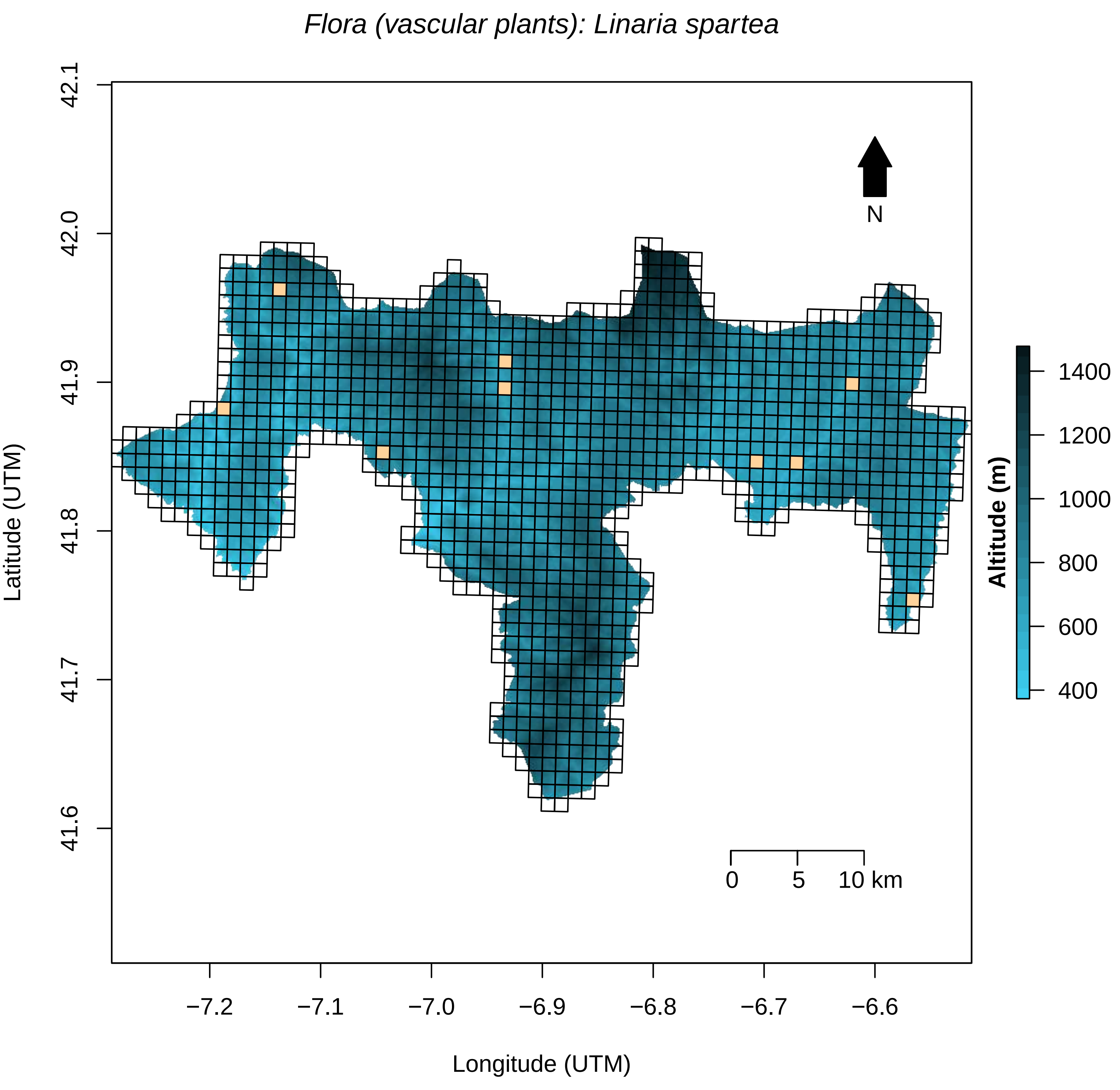

Flora (vascular plants): Linaria spartea
42.1
42.0
41.9
41.8
41.7
41.6
Latitude (UTM)
−7.2
−7.1
−7.0
−6.9
−6.8
−6.7
−6.6
Longitude (UTM)
N
0
5
10 km
Altitude (m)
1400
1200
1000
800
600
400

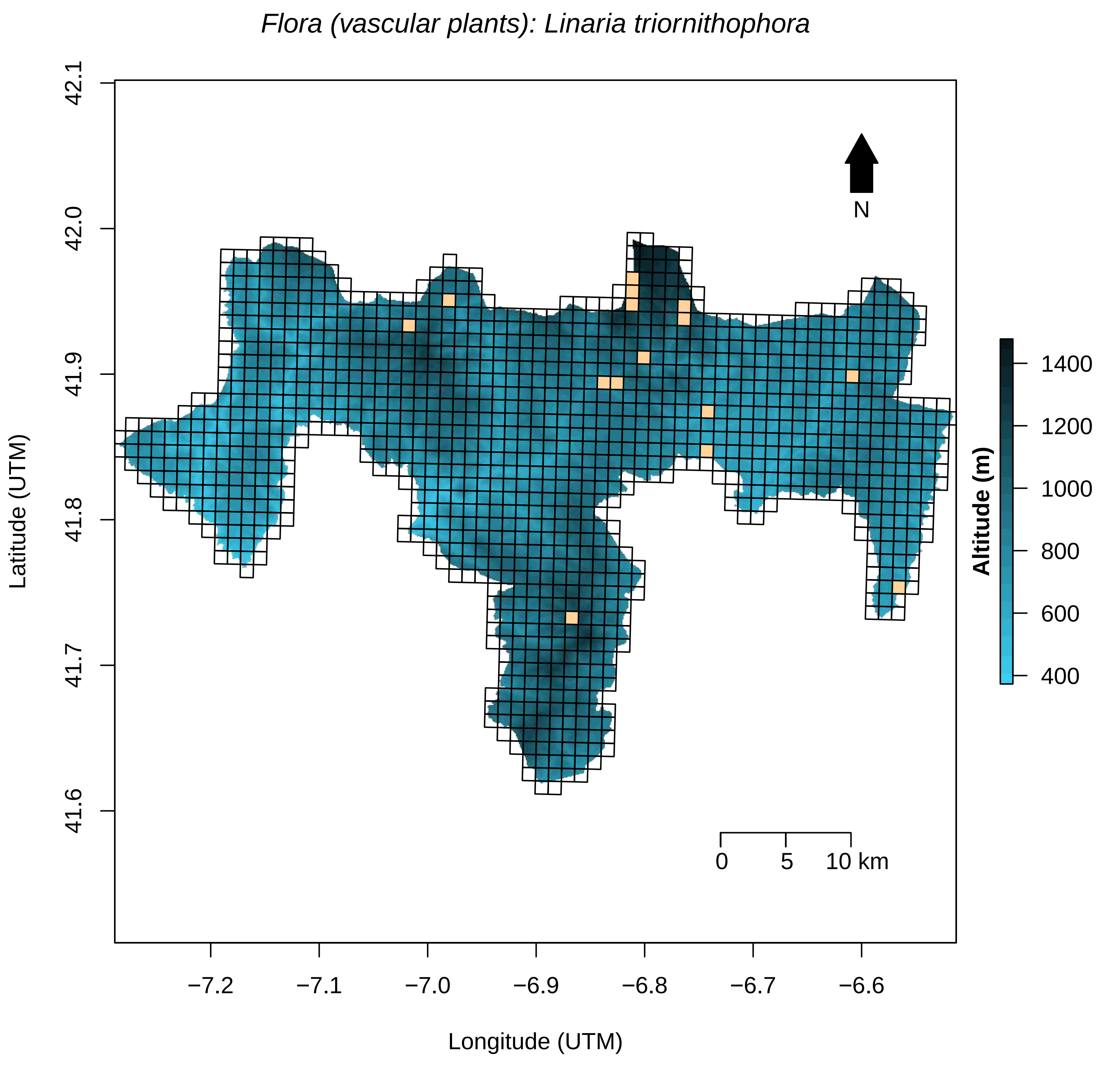

Flora (vascular plants): Linaria triornithophora
42.1
42.0
41.9
41.8
41.7
41.6
Latitude (UTM)
−7.2
−7.1
−7.0
−6.9
−6.8
−6.7
−6.6
Longitude (UTM)
N
0
5
10 km
Altitude (m)
1400
1200
1000
800
600
400

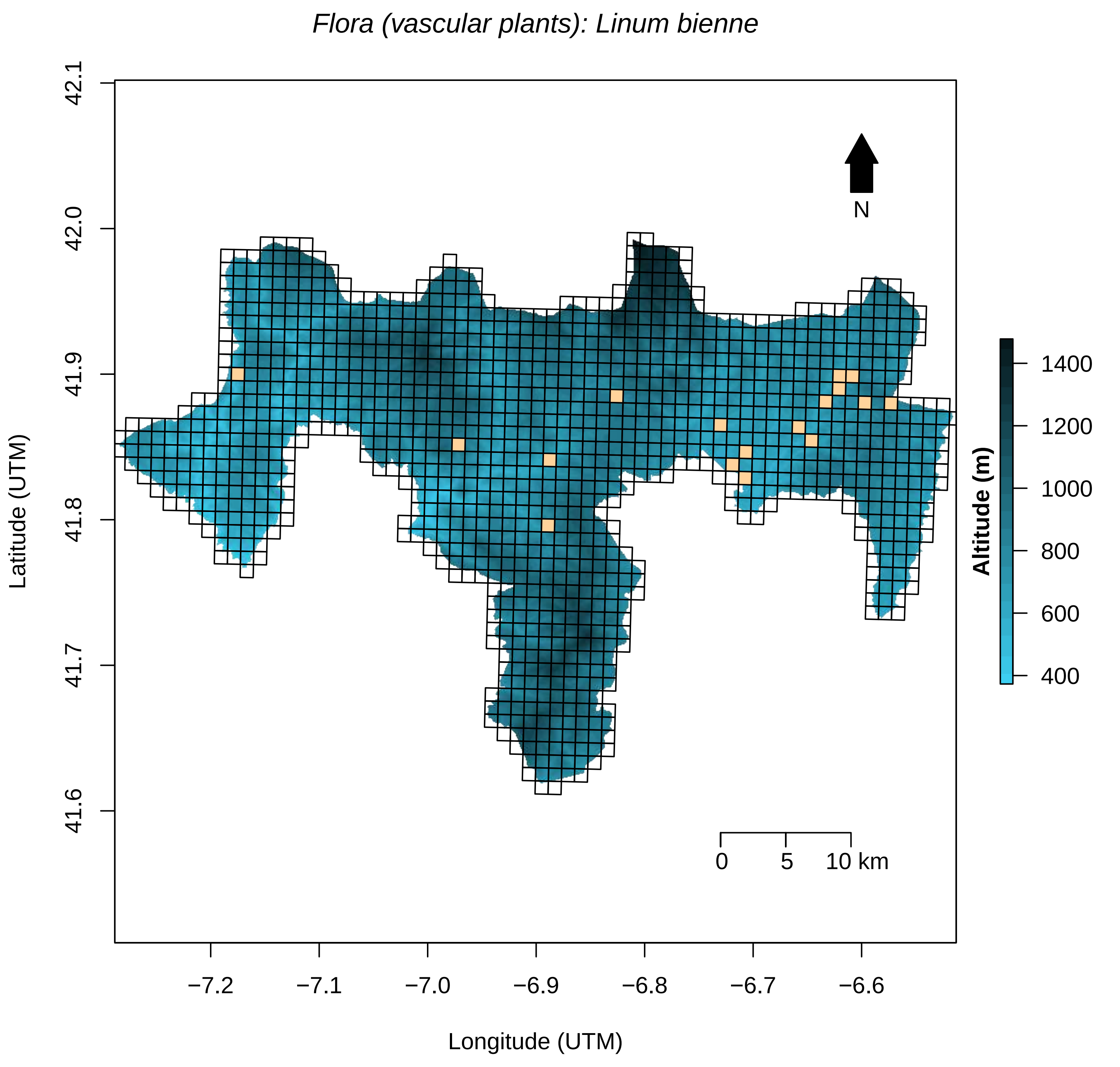

Flora (vascular plants): Linum bienne
Latitude (UTM)
42.1
42.0
41.9
41.8
41.7
41.6
Longitude (UTM)
−7.2
−7.1
−7.0
−6.9
−6.8
−6.7
−6.6
N
0
5
10 km
Altitude (m)
1400
1200
1000
800
600
400

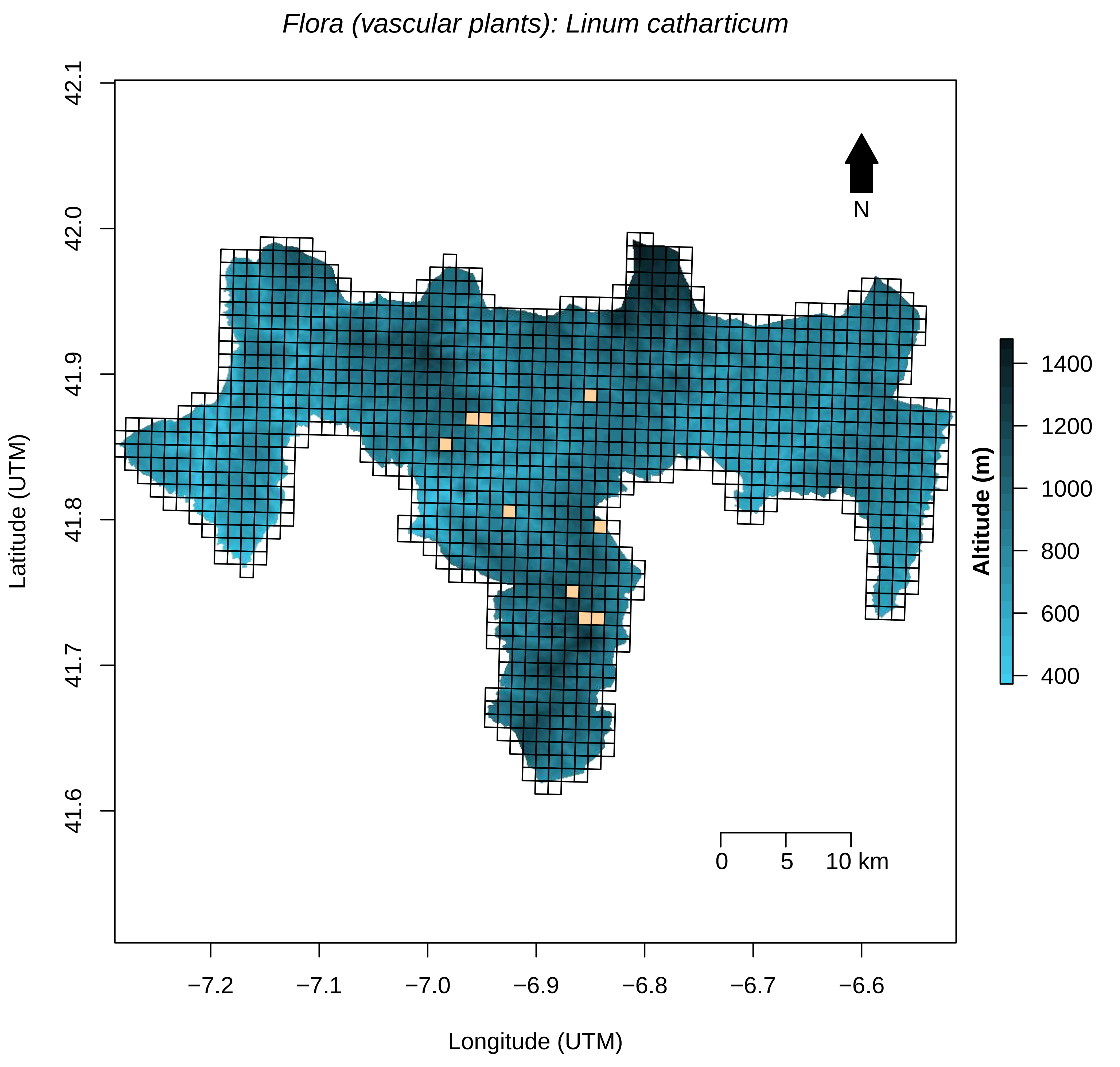

Flora (vascular plants): Linum catharticum
42.1
42.0
41.9
41.8
41.7
41.6
Latitude (UTM)
−7.2
−7.1
−7.0
−6.9
−6.8
−6.7
−6.6
Longitude (UTM)
N
0
5
10 km
1400
1200
1000
800
600
400
Altitude (m)

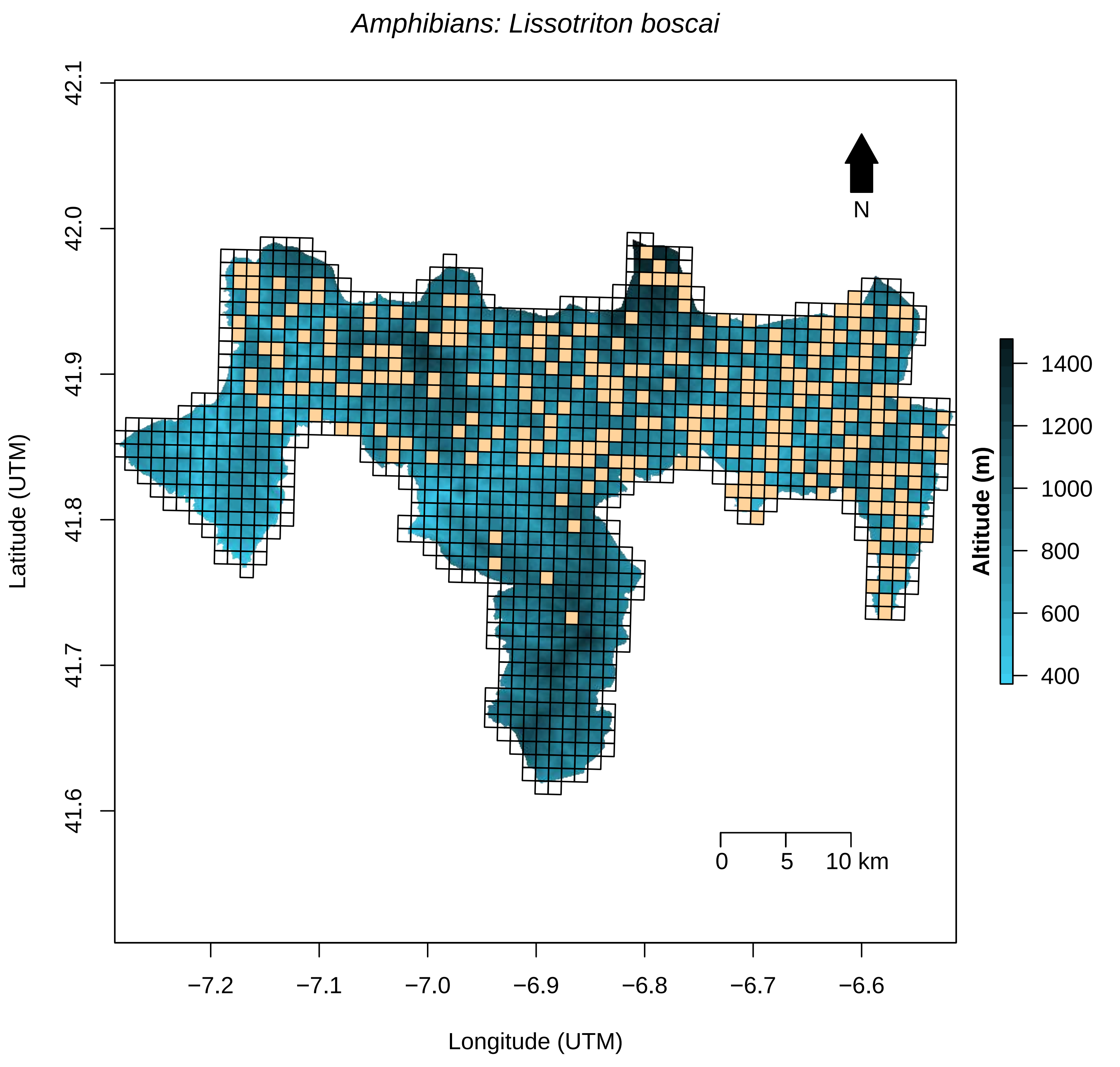
Amphibians: Lissotriton boscai
42.1
42.0
41.9
41.8
41.7
41.6
Latitude (UTM)
−7.2
−7.1
−7.0
−6.9
−6.8
−6.7
−6.6
Longitude (UTM)
N
0
5
10 km
Altitude (m)
1400
1200
1000
800
600
400

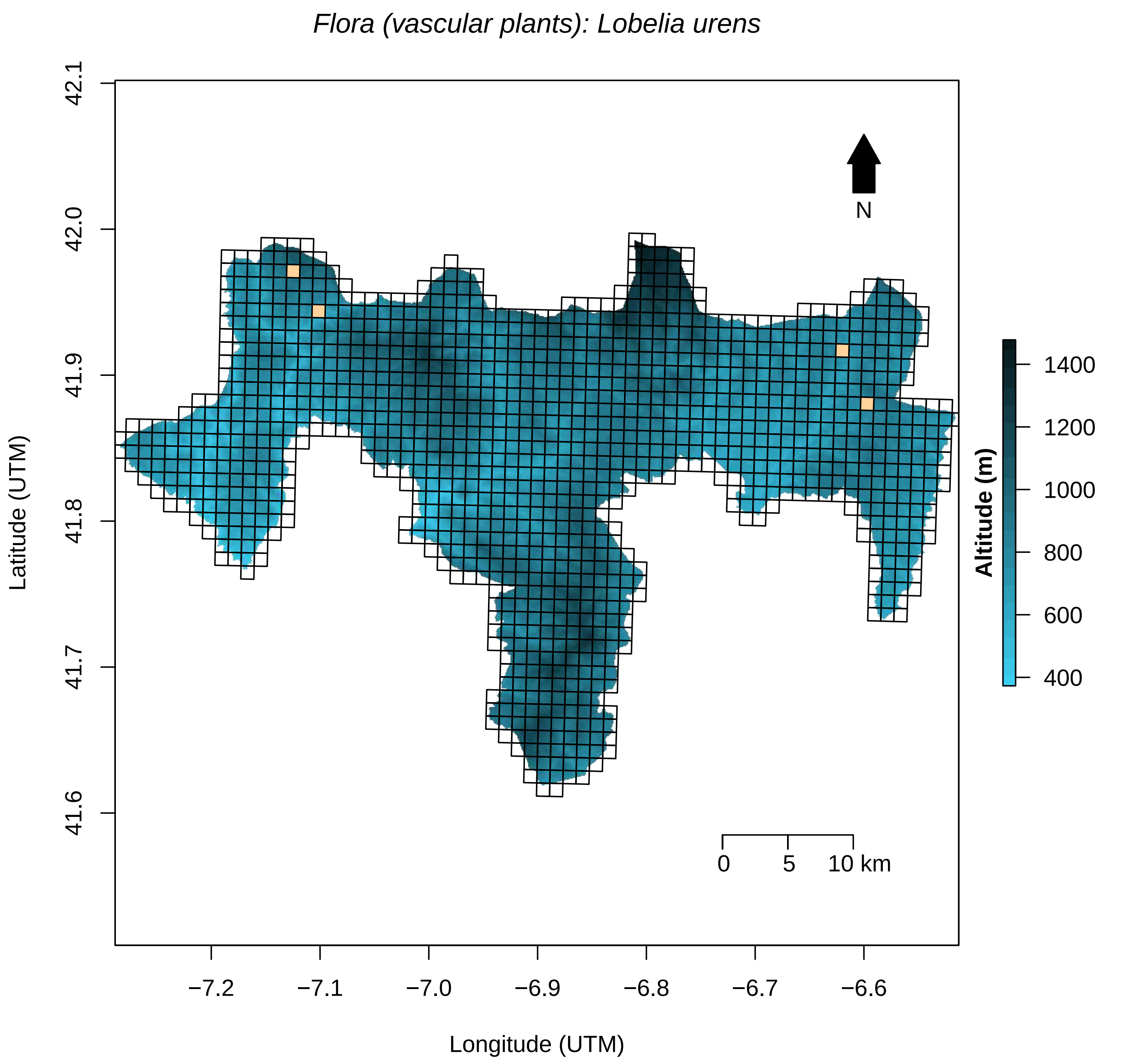

Flora (vascular plants): Lobelia urens
Latitude (UTM)
42.1
42.0
41.9
41.8
41.7
41.6
Longitude (UTM)
−7.2
−7.1
−7.0
−6.9
−6.8
−6.7
−6.6
N
0
5
10 km
Altitude (m)
1400
1200
1000
800
600
400

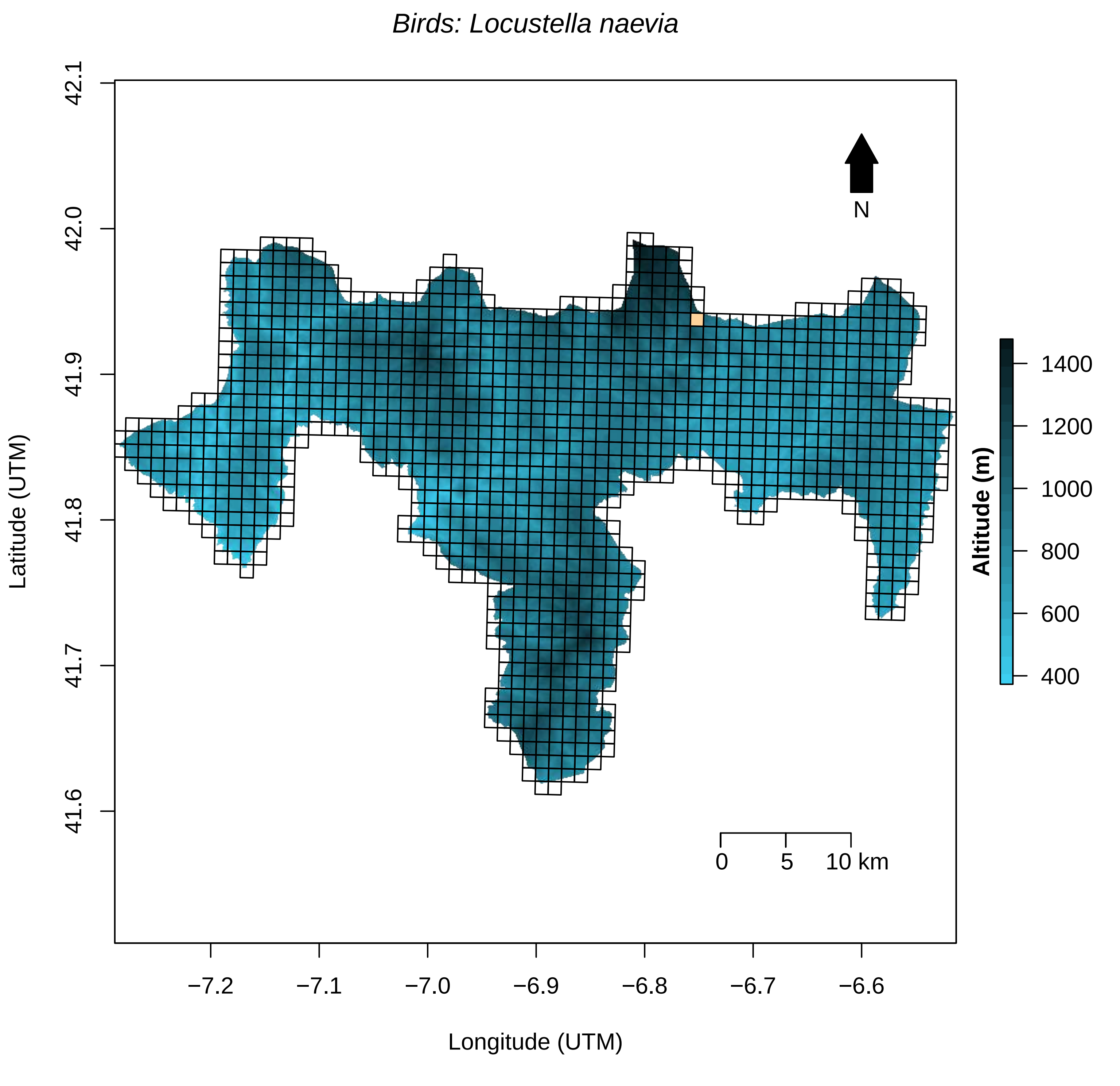

Birds: Locustella naevia
42.1
42.0
41.9
41.8
41.7
41.6
Latitude (UTM)
−7.2
−7.1
−7.0
−6.9
−6.8
−6.7
−6.6
Longitude (UTM)
N
0
5
10 km
Altitude (m)
1400
1200
1000
800
600
400

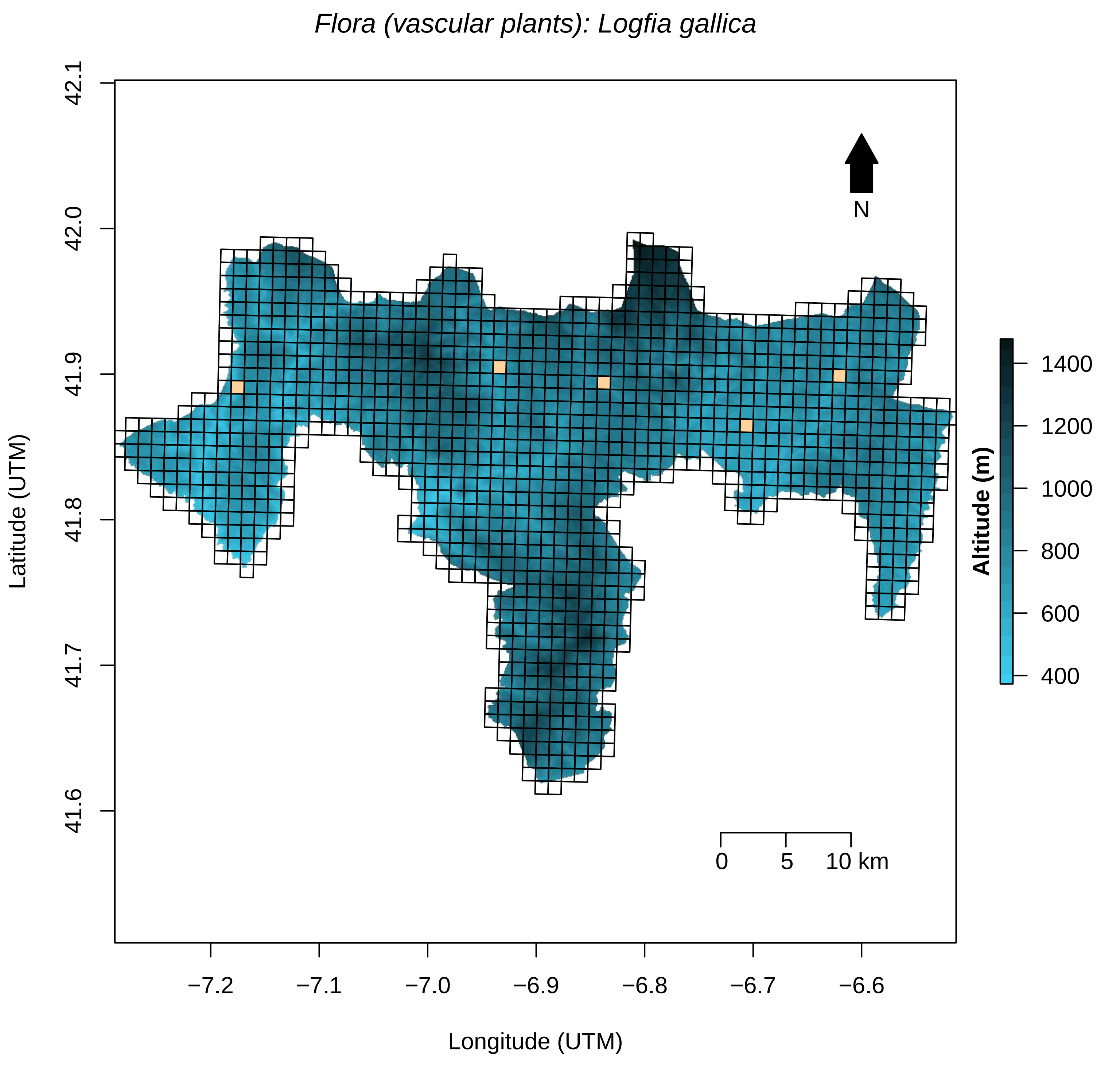
Flora (vascular plants): Logfia gallica
N
0 5 10 km
Altitude (m)
1400
1200
1000
800
600
400
Latitude (UTM)
42.1
42.0
41.9
41.8
41.7
41.6
Longitude (UTM)
−7.2
−7.1
−7.0
−6.9
−6.8
−6.7
−6.6

Flora (vascular plants): Logfia minima

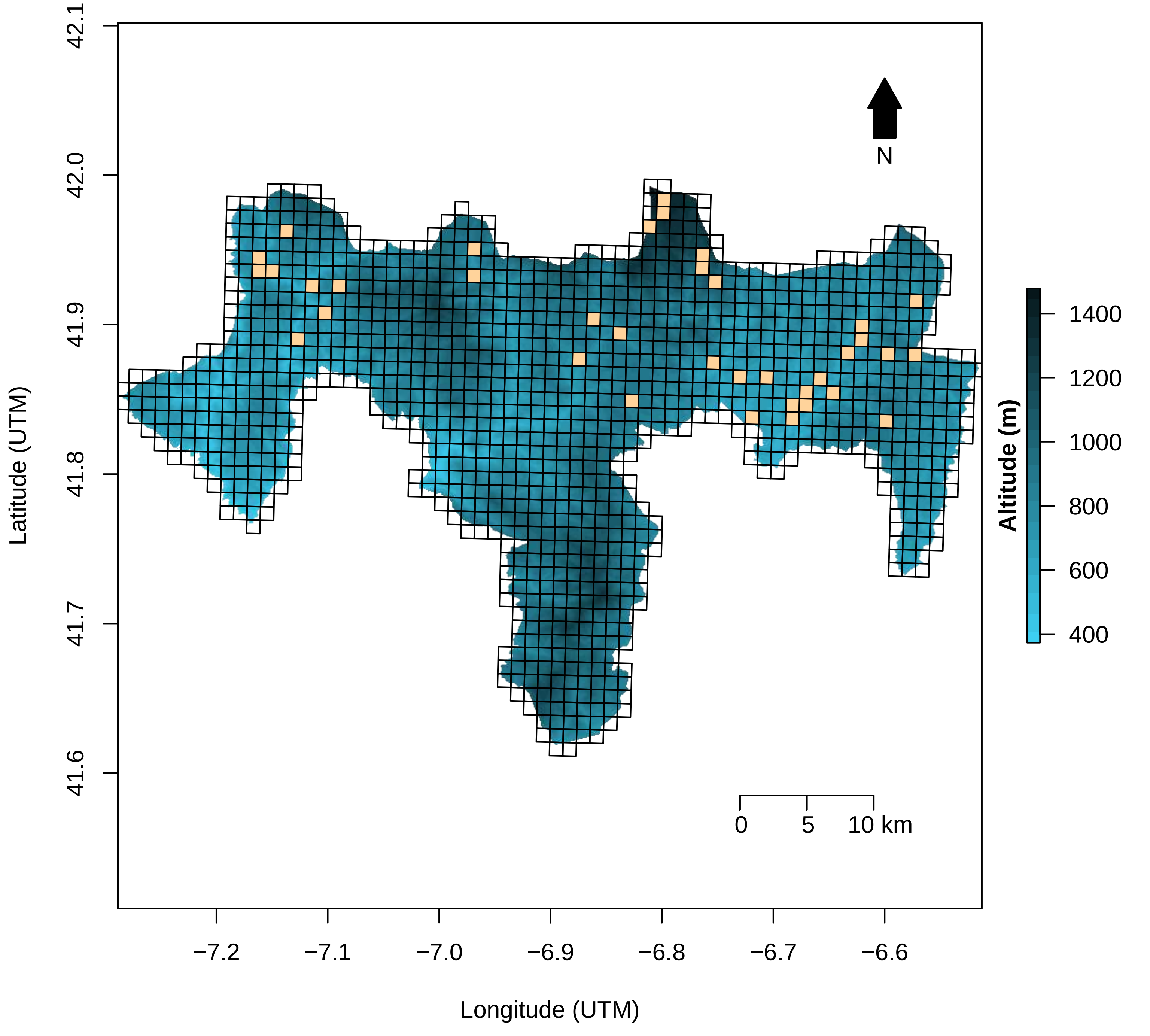

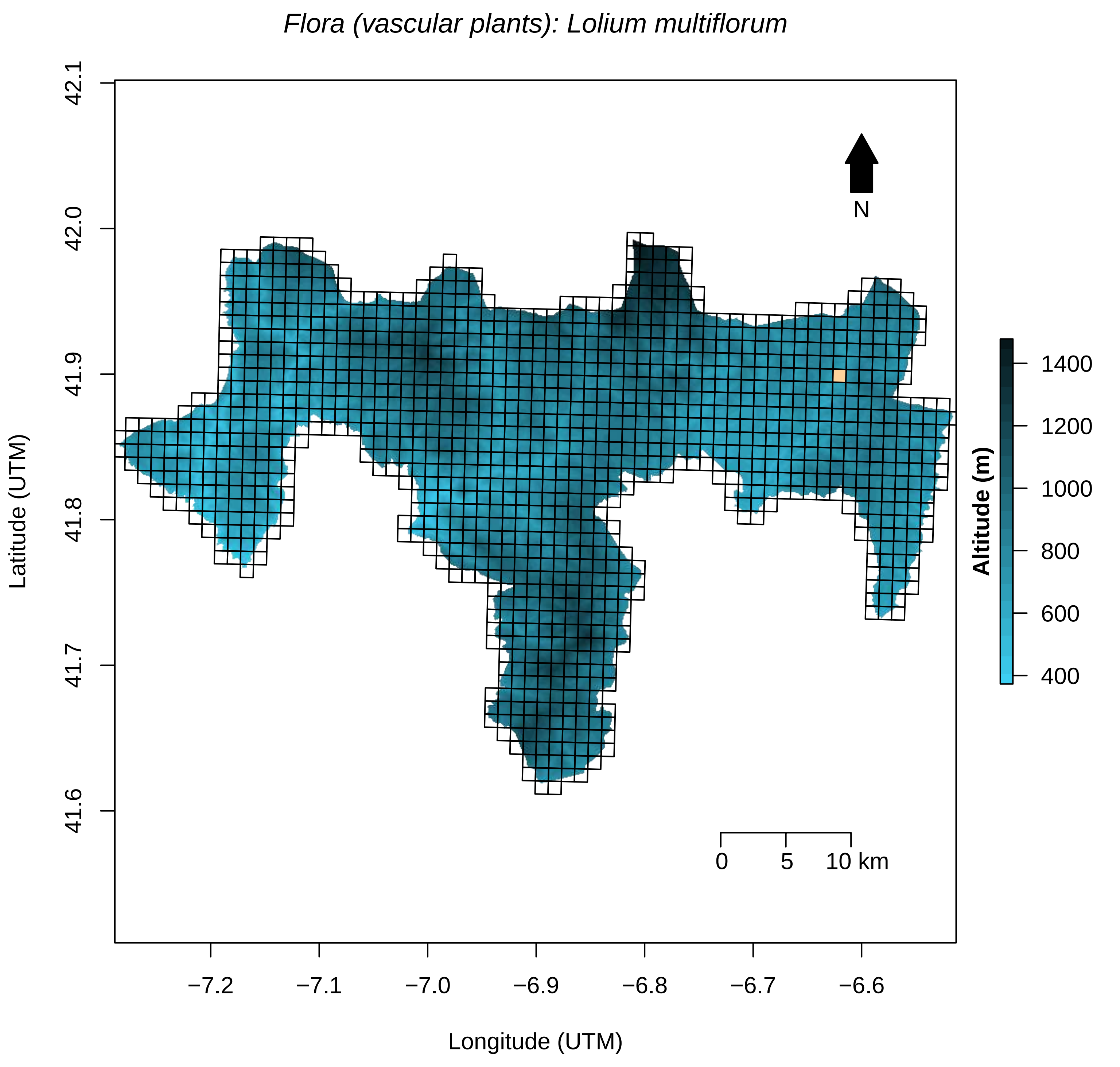
Flora (vascular plants): Lolium multiflorum
42.1
42.0
41.9
41.8
41.7
41.6
Latitude (UTM)
−7.2
−7.1
−7.0
−6.9
−6.8
−6.7
−6.6
Longitude (UTM)
N
0
5
10 km
Altitude (m)
1400
1200
1000
800
600
400

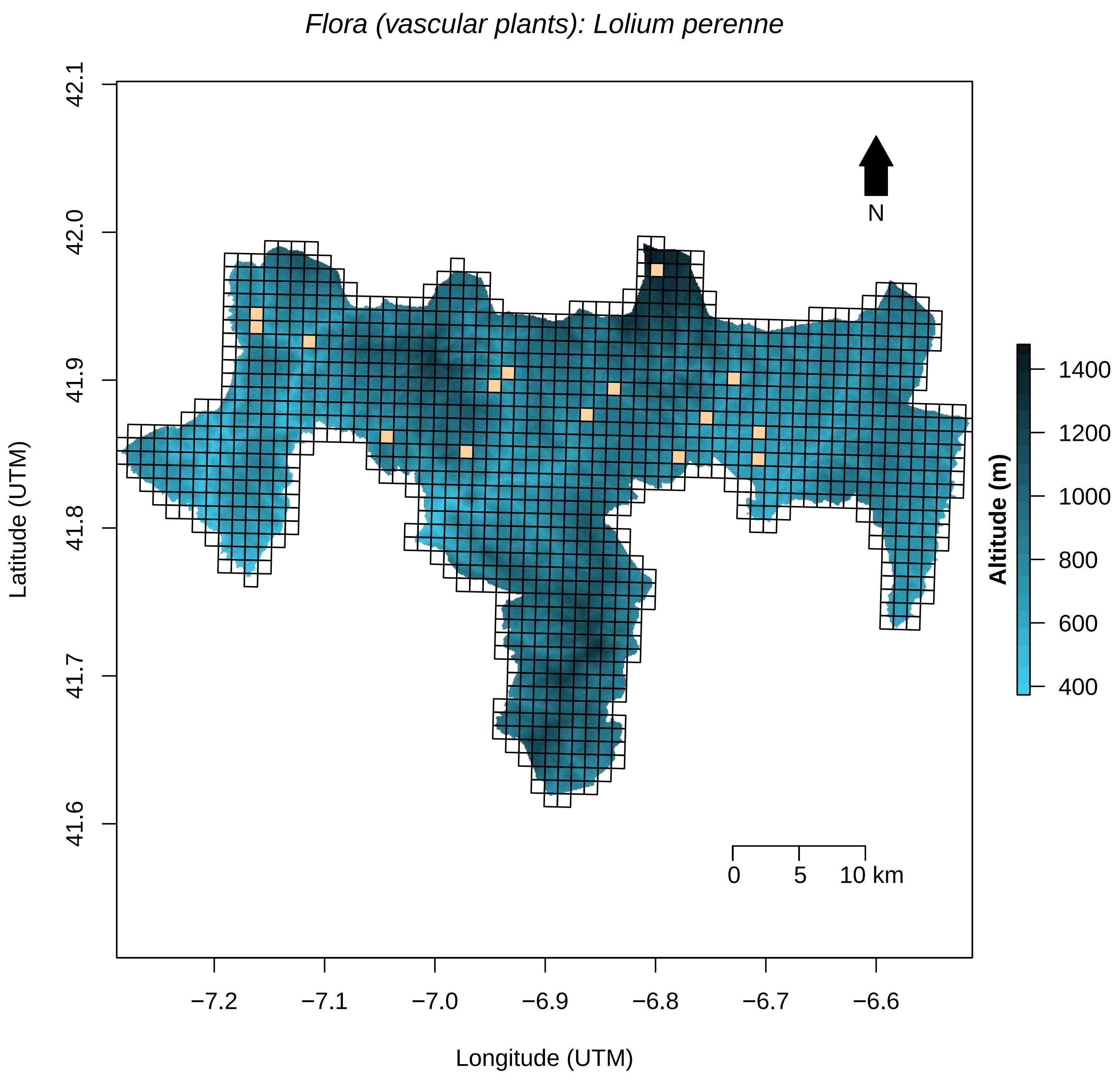

Flora (vascular plants): Lolium perenne
42.1
42.0
41.9
41.8
41.7
41.6
Latitude (UTM)
−7.2
−7.1
−7.0
−6.9
−6.8
−6.7
−6.6
Longitude (UTM)
N
0
5
10 km
Altitude (m)
1400
1200
1000
800
600
400

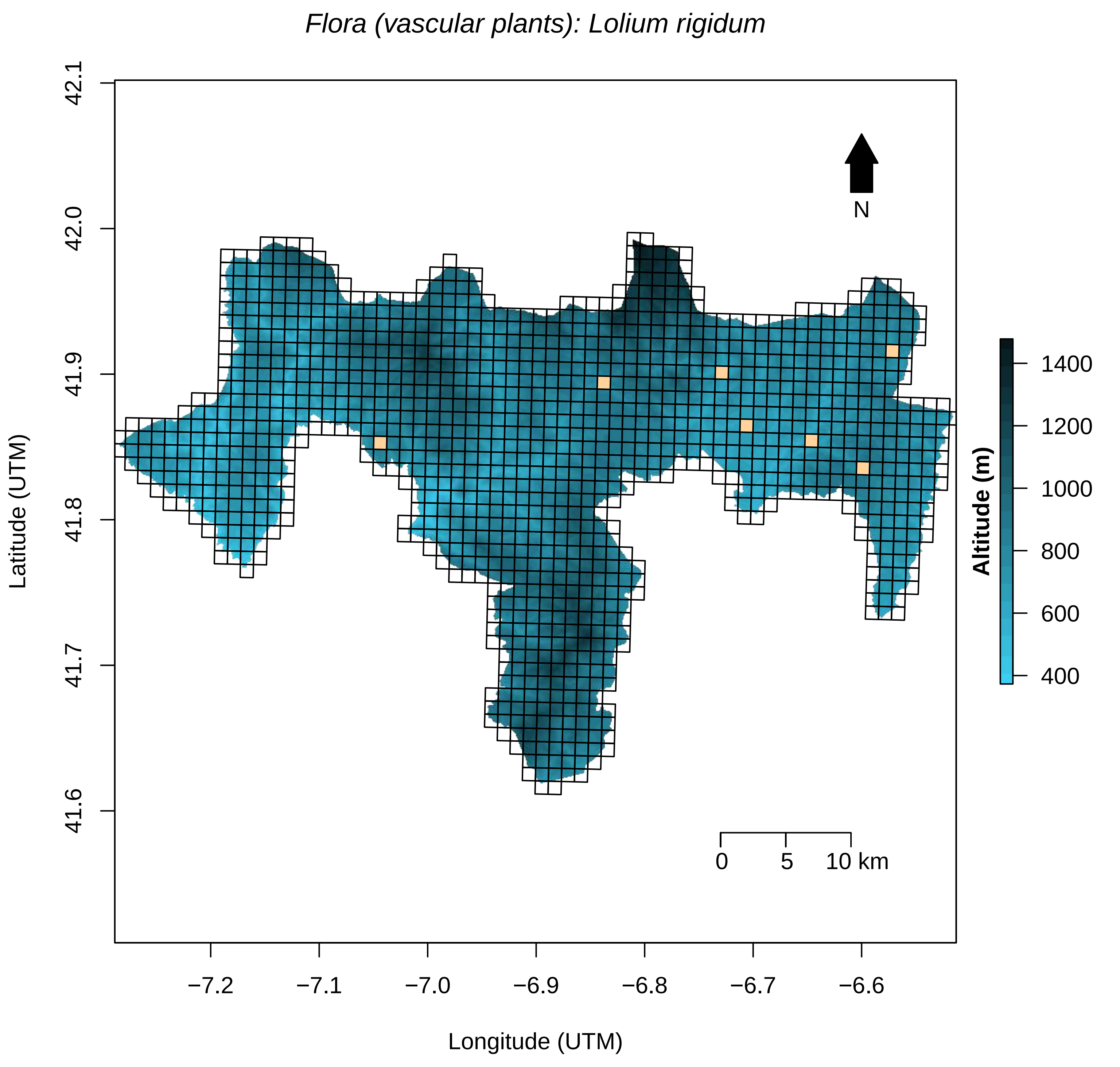

Flora (vascular plants): Lolium rigidum
42.1
42.0
41.9
41.8
41.7
41.6
Latitude (UTM)
−7.2
−7.1
−7.0
−6.9
−6.8
−6.7
−6.6
Longitude (UTM)
N
0
5
10 km
Altitude (m)
1400
1200
1000
800
600
400

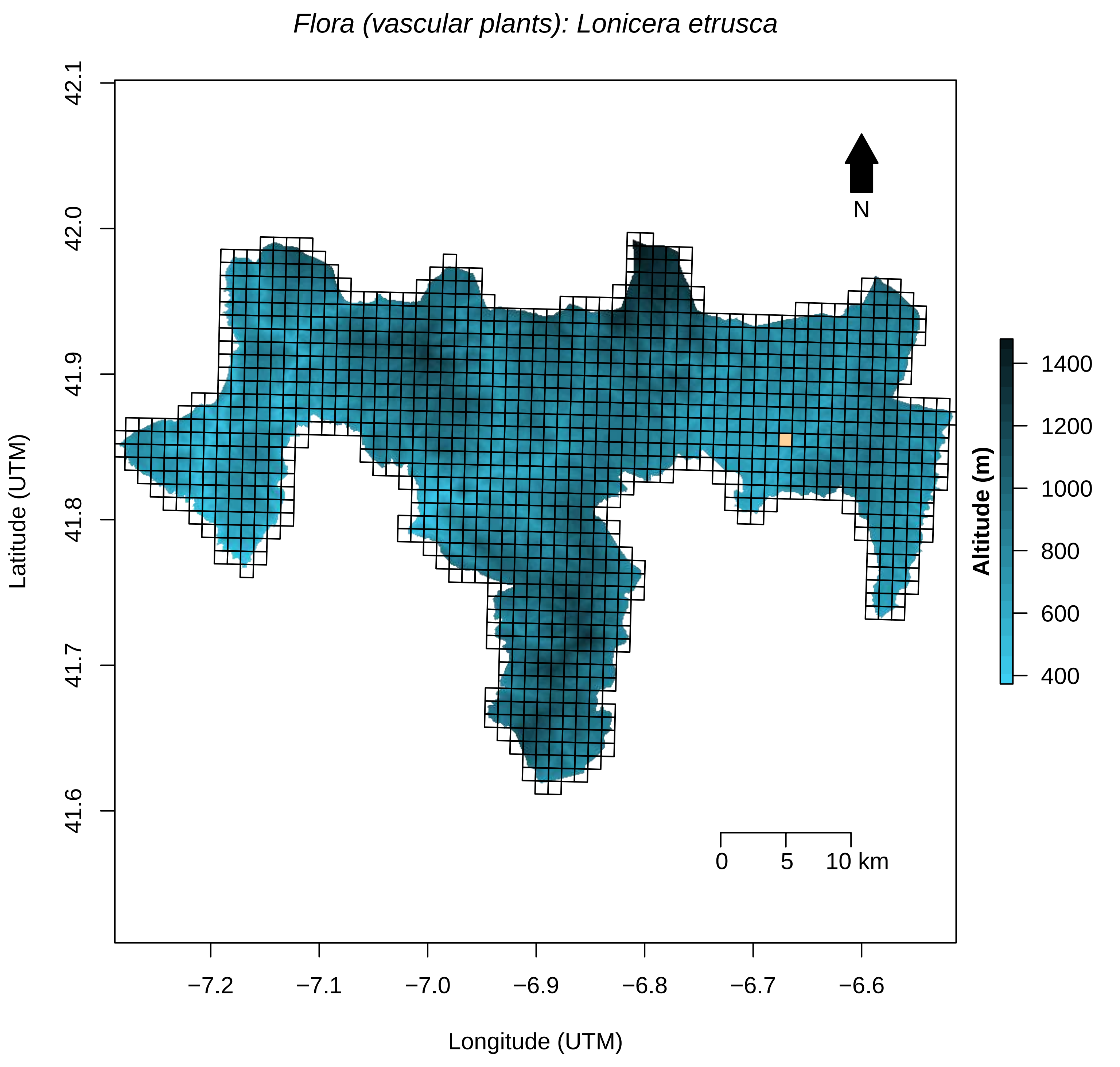
Flora (vascular plants): Lonicera etrusca
42.1
42.0
41.9
41.8
41.7
41.6
Latitude (UTM)
−7.2
−7.1
−7.0
−6.9
−6.8
−6.7
−6.6
Longitude (UTM)
N
0
5
10 km
Altitude (m)
1400
1200
1000
800
600
400

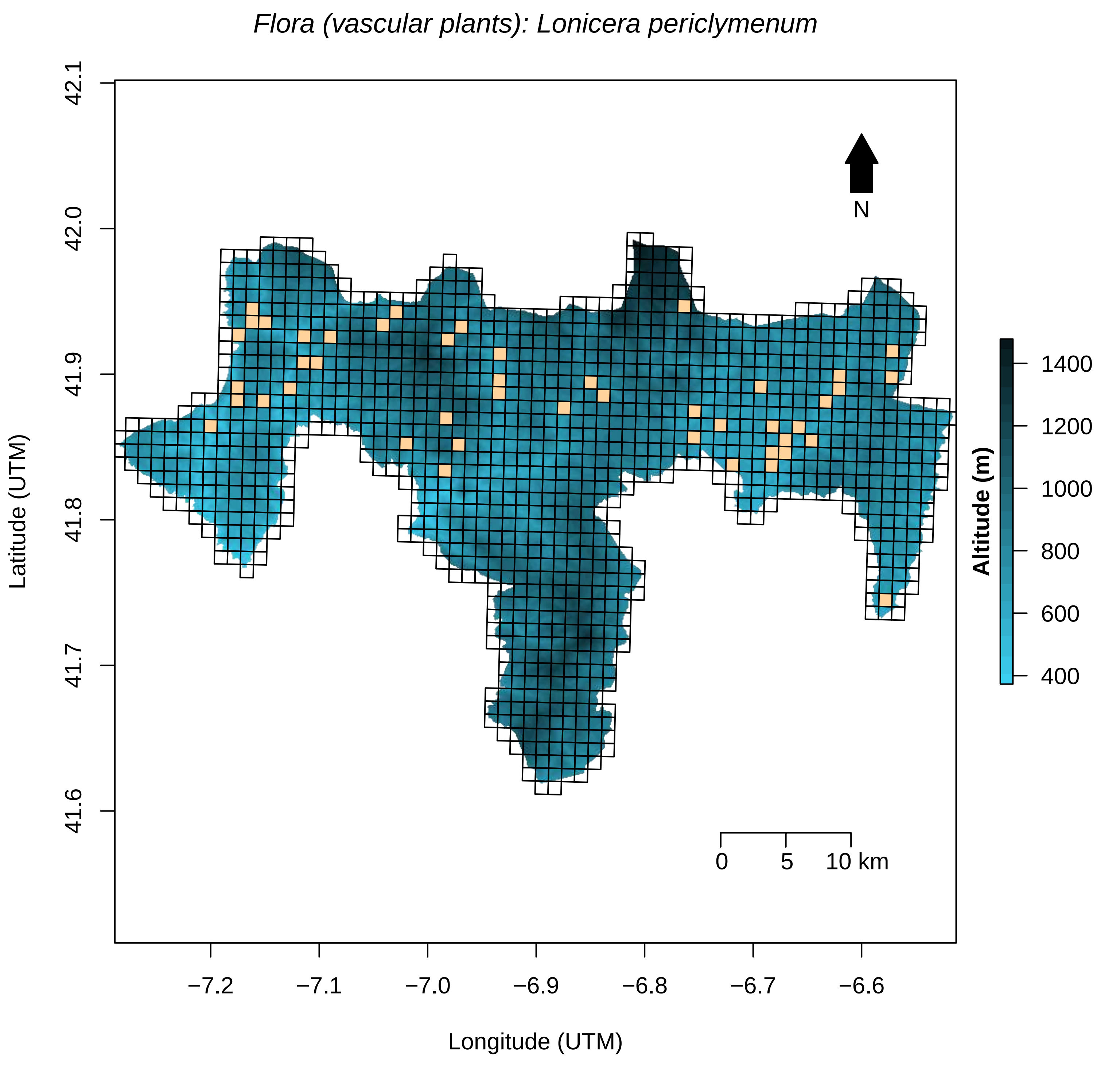

Flora (vascular plants): Lonicera periclymenum
42.1
42.0
41.9
41.8
41.7
41.6
Latitude (UTM)
−7.2
−7.1
−7.0
−6.9
−6.8
−6.7
−6.6
Longitude (UTM)
N
0
5
10 km
Altitude (m)
1400
1200
1000
800
600
400

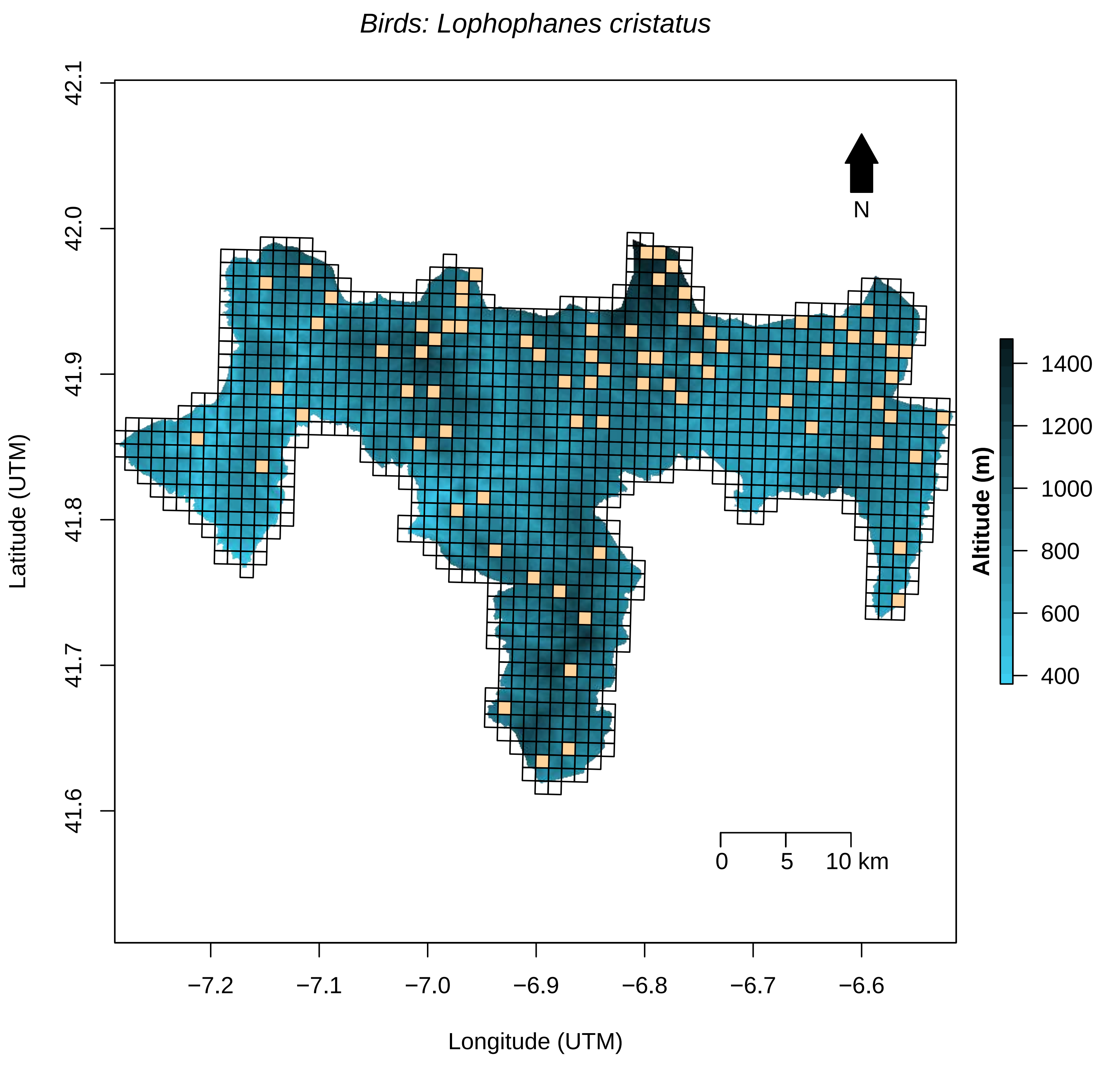

Birds: Lophophanes cristatus
Latitude (UTM)
Longitude (UTM)
42.1
42.0
41.9
41.8
41.7
41.6
−7.2
−7.1
−7.0
−6.9
−6.8
−6.7
−6.6
N
0
5
10 km
Altitude (m)
1400
1200
1000
800
600
400

Flora (vascular plants): Lotus angustissimus

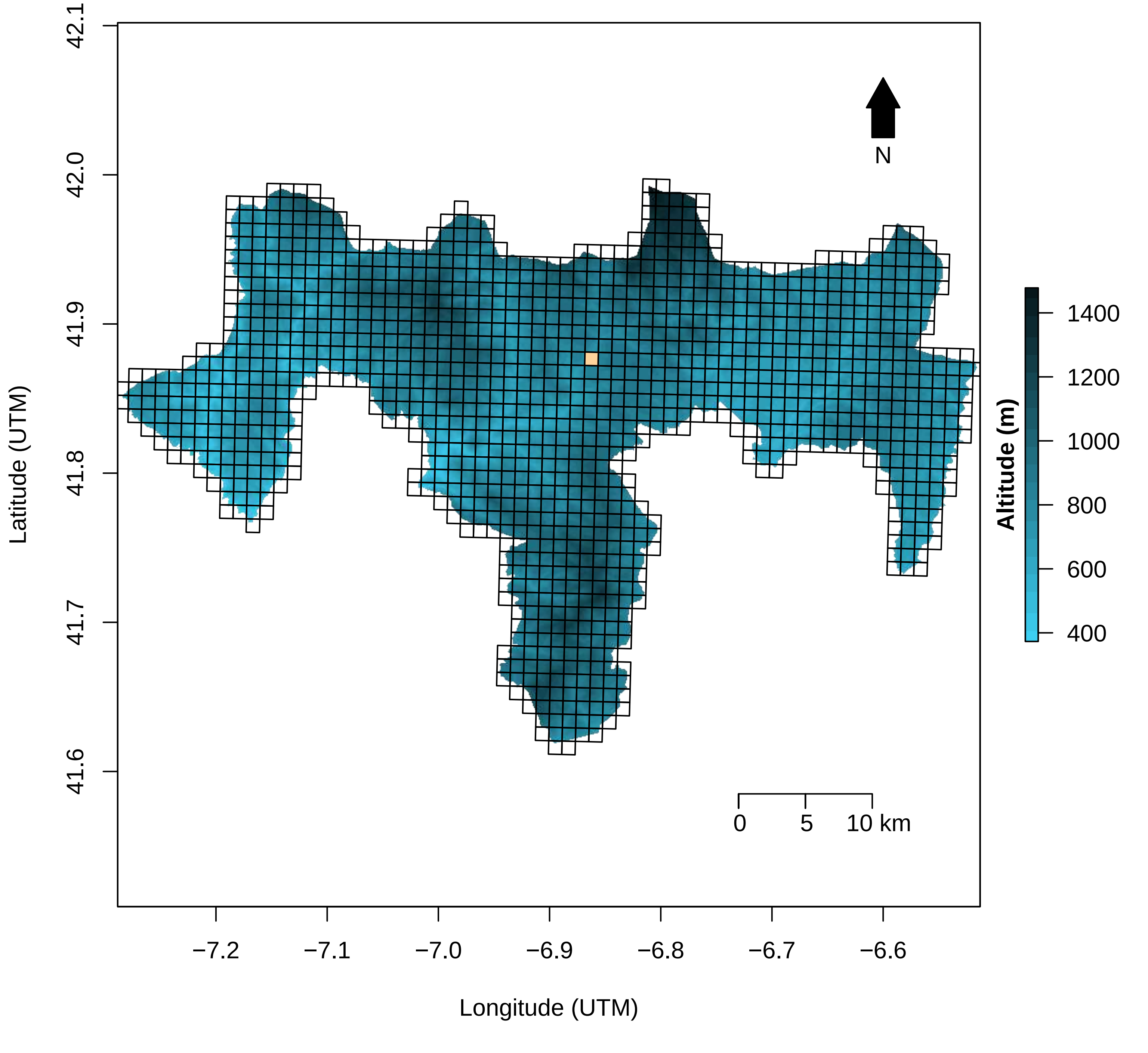

Flora (vascular plants): Lotus corniculatus

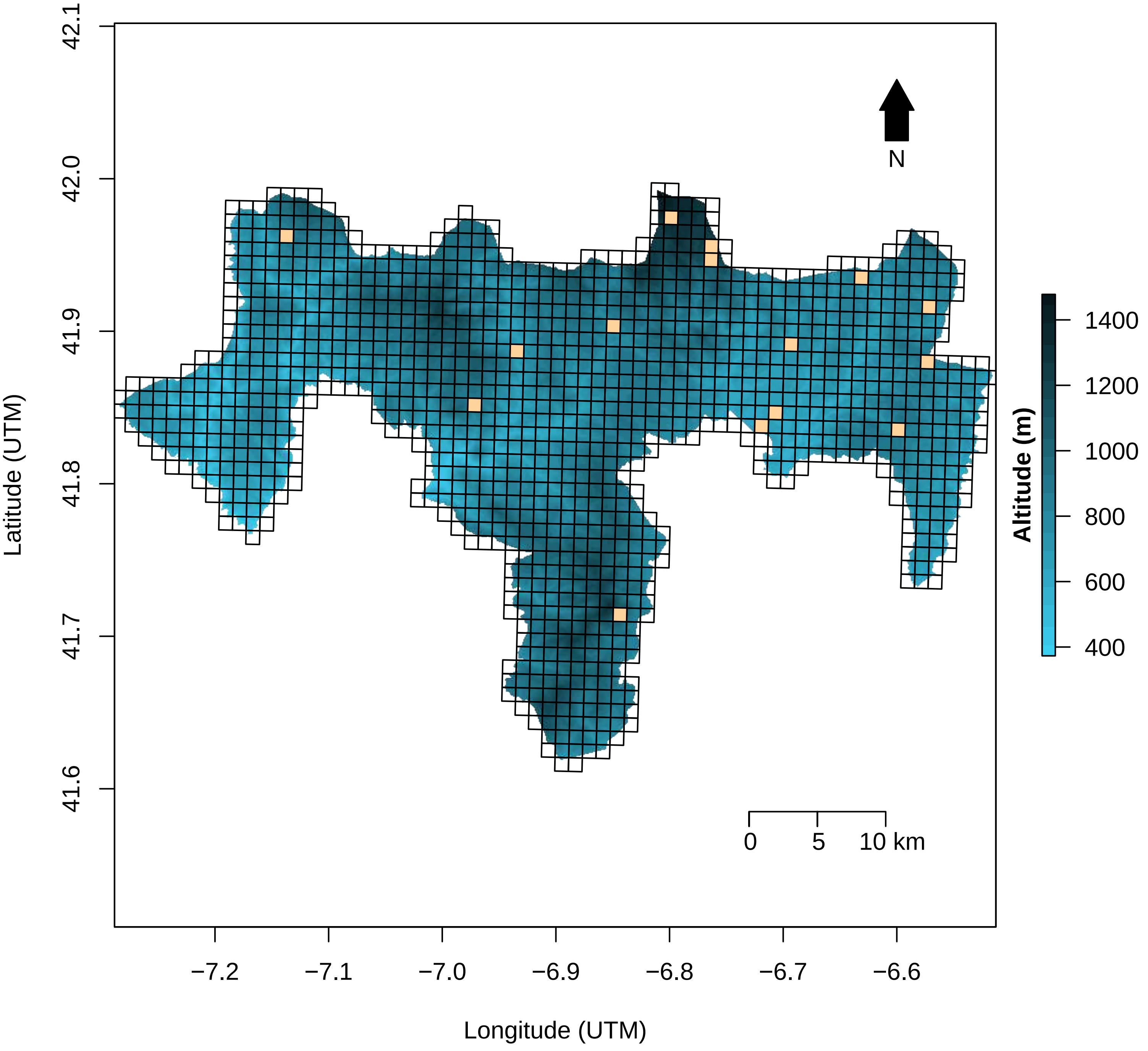

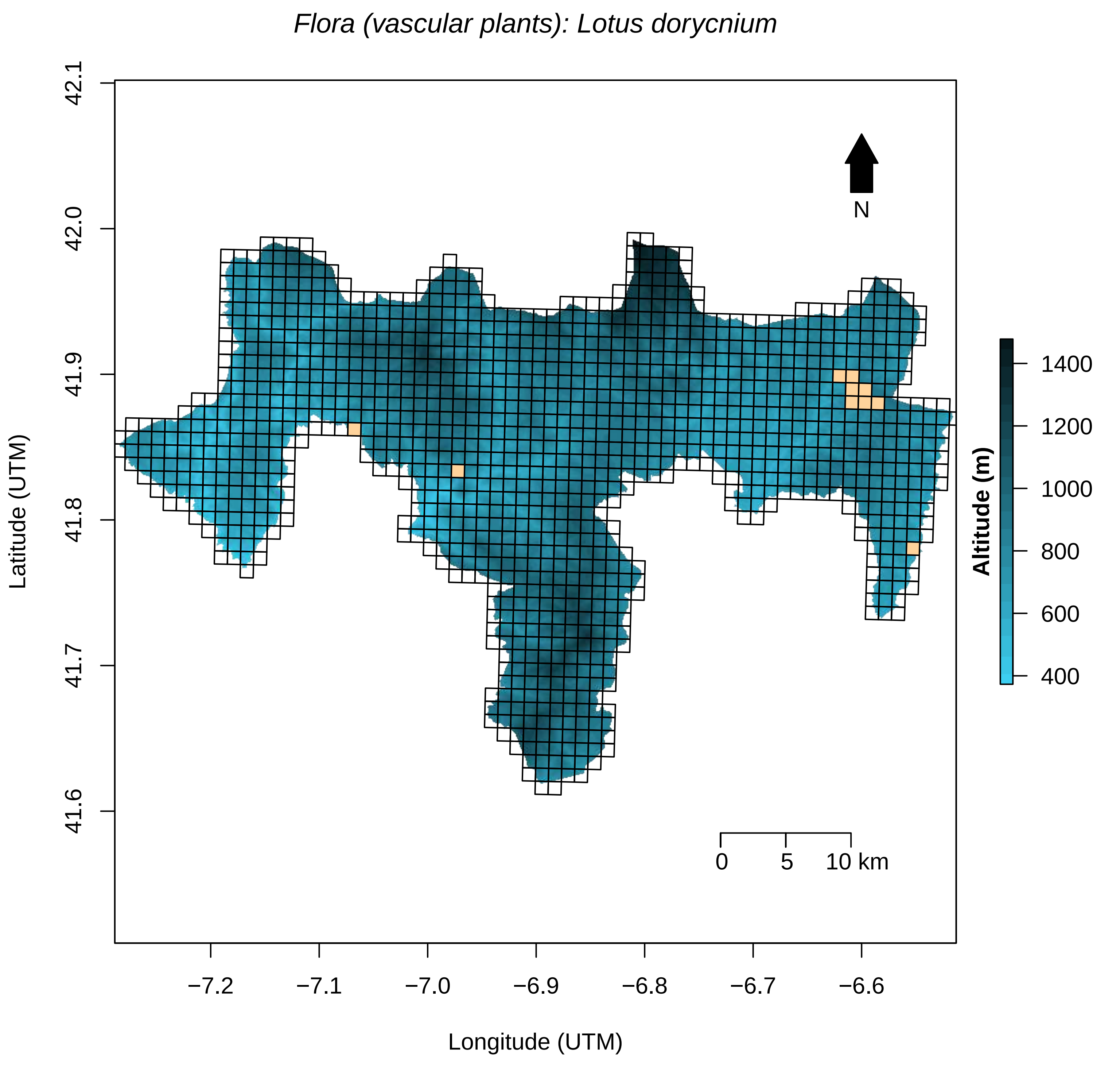

Flora (vascular plants): Lotus dorycnium
N
0
5
10 km
Altitude (m)
1400
1200
1000
800
600
400
Latitude (UTM)
42.1
42.0
41.9
41.8
41.7
41.6
Longitude (UTM)
−7.2
−7.1
−7.0
−6.9
−6.8
−6.7
−6.6

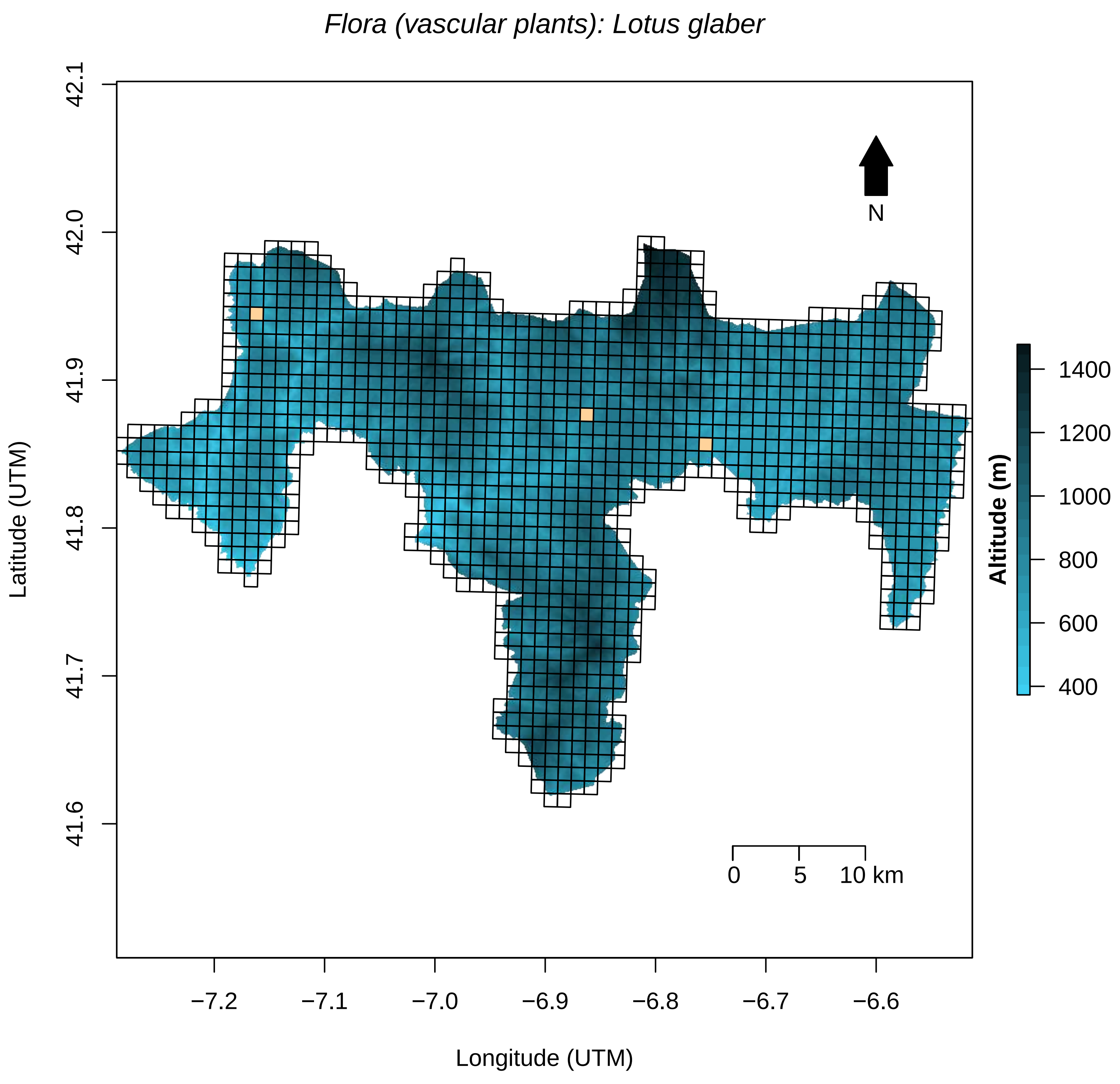

Flora (vascular plants): Lotus glaber
Latitude (UTM)
Longitude (UTM)
42.1
42.0
41.9
41.8
41.7
41.6
−7.2
−7.1
−7.0
−6.9
−6.8
−6.7
−6.6
N
0
5
10 km
Altitude (m)
1400
1200
1000
800
600
400

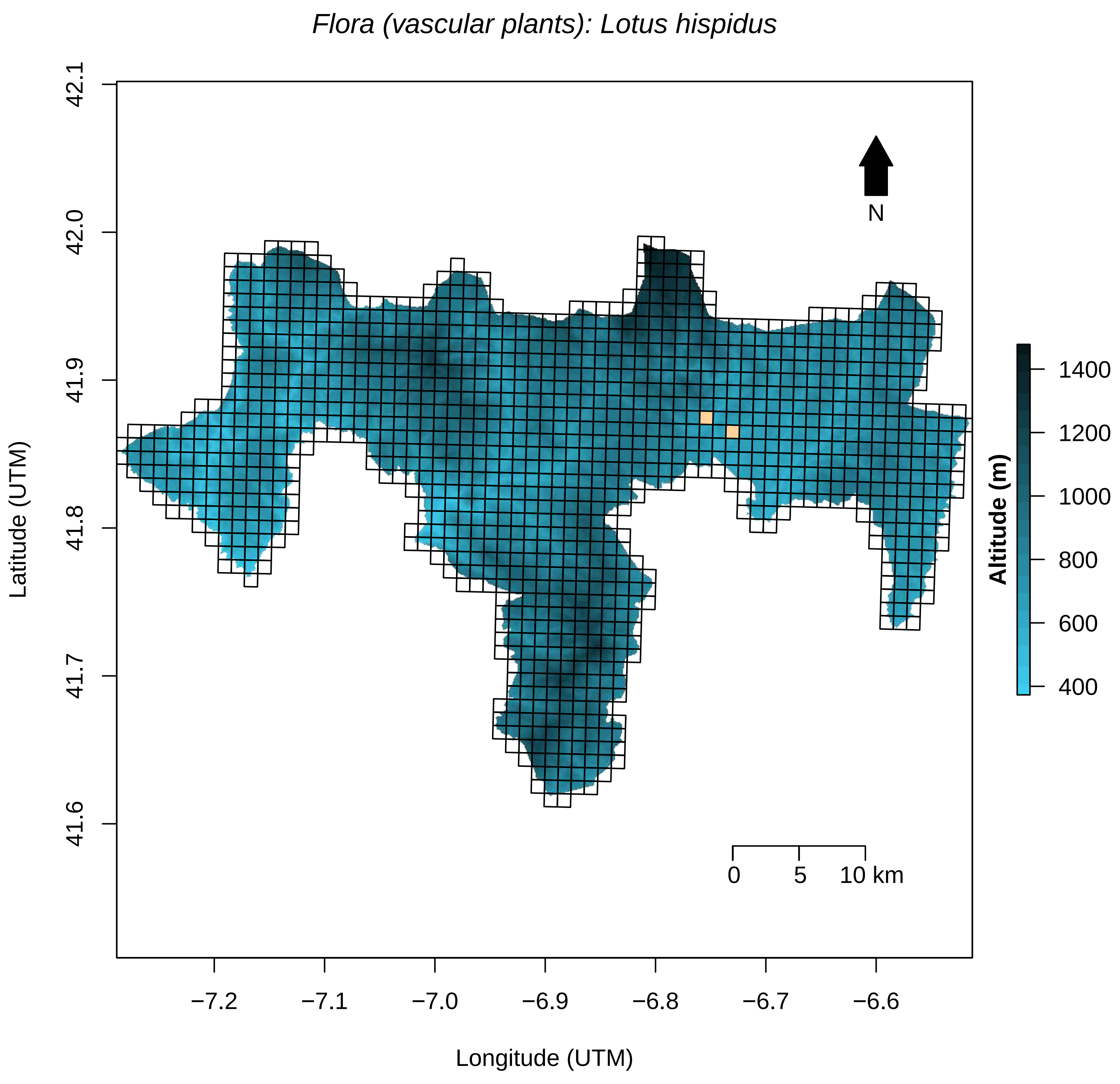

Flora (vascular plants): Lotus hispidus
42.1
42.0
41.9
41.8
41.7
41.6
Latitude (UTM)
−7.2
−7.1
−7.0
−6.9
−6.8
−6.7
−6.6
Longitude (UTM)
N
0
5
10 km
Altitude (m)
1400
1200
1000
800
600
400

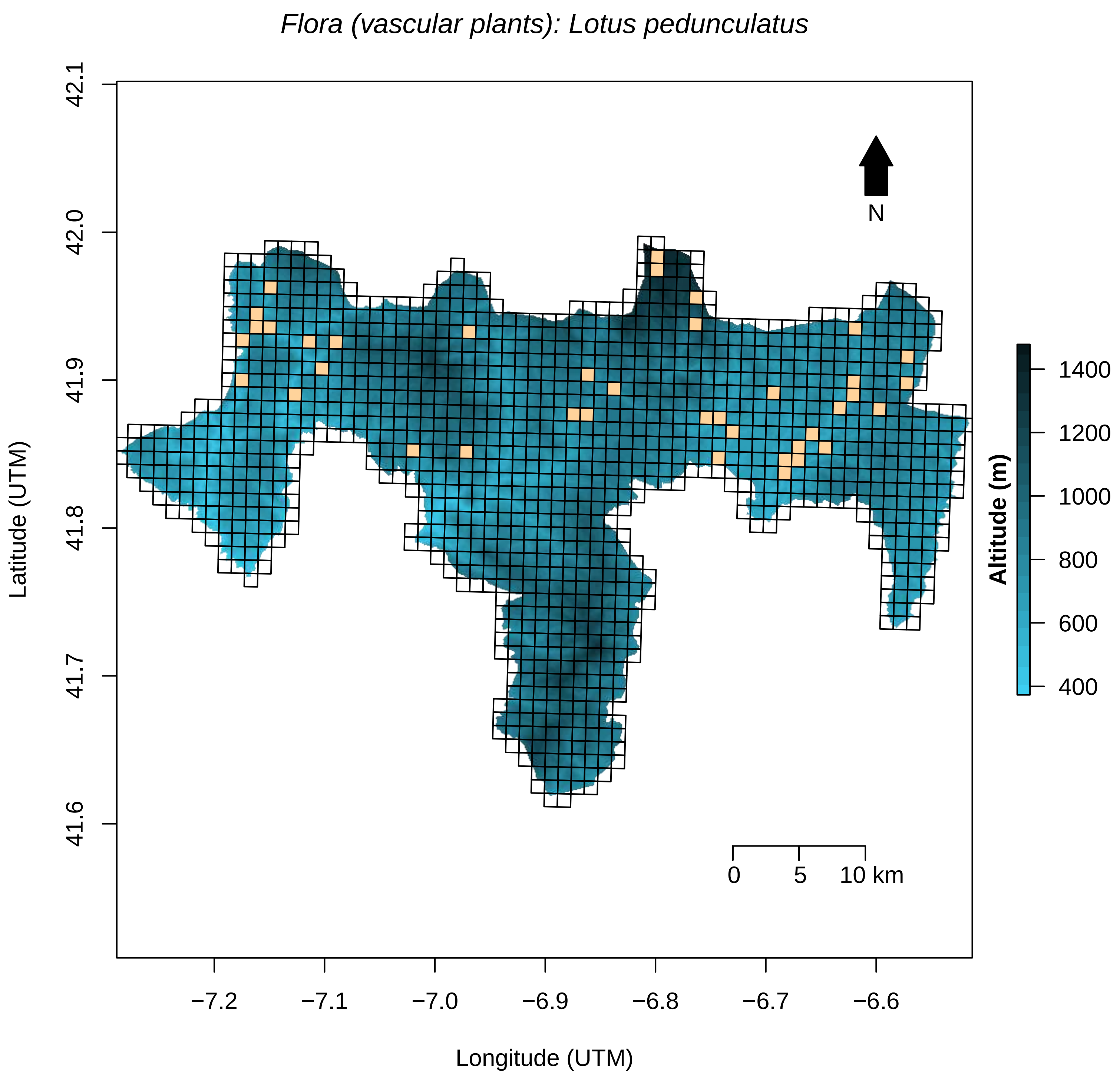

Flora (vascular plants): Lotus pedunculatus
42.1
42.0
41.9
41.8
41.7
41.6
Latitude (UTM)
−7.2
−7.1
−7.0
−6.9
−6.8
−6.7
−6.6
Longitude (UTM)
N
0
5
10 km
Altitude (m)
1400
1200
1000
800
600
400

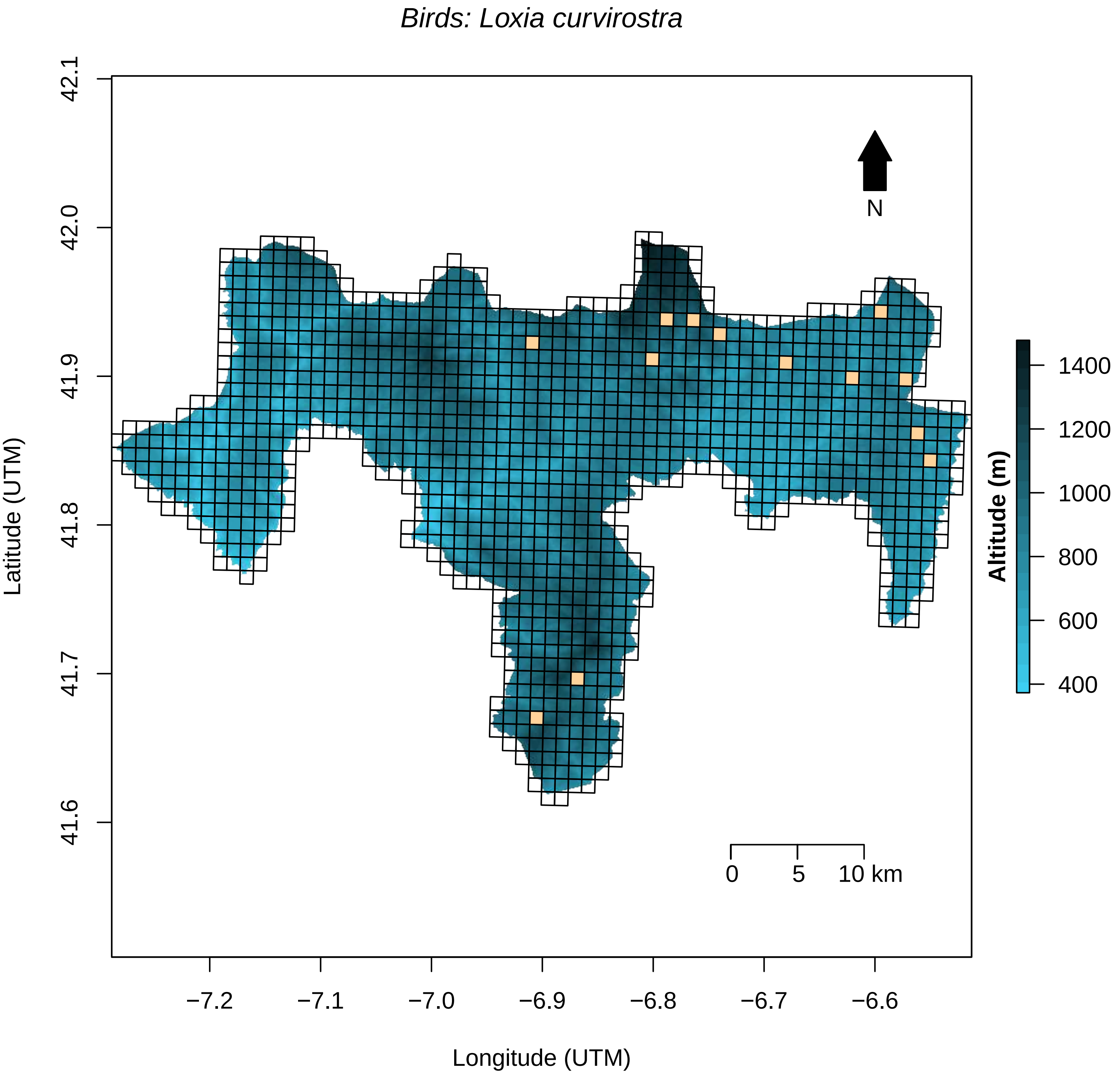

Birds: Loxia curvirostra
Latitude (UTM)
Longitude (UTM)
42.1
42.0
41.9
41.8
41.7
41.6
−7.2
−7.1
−7.0
−6.9
−6.8
−6.7
−6.6
N
0
5
10 km
Altitude (m)
1400
1200
1000
800
600
400

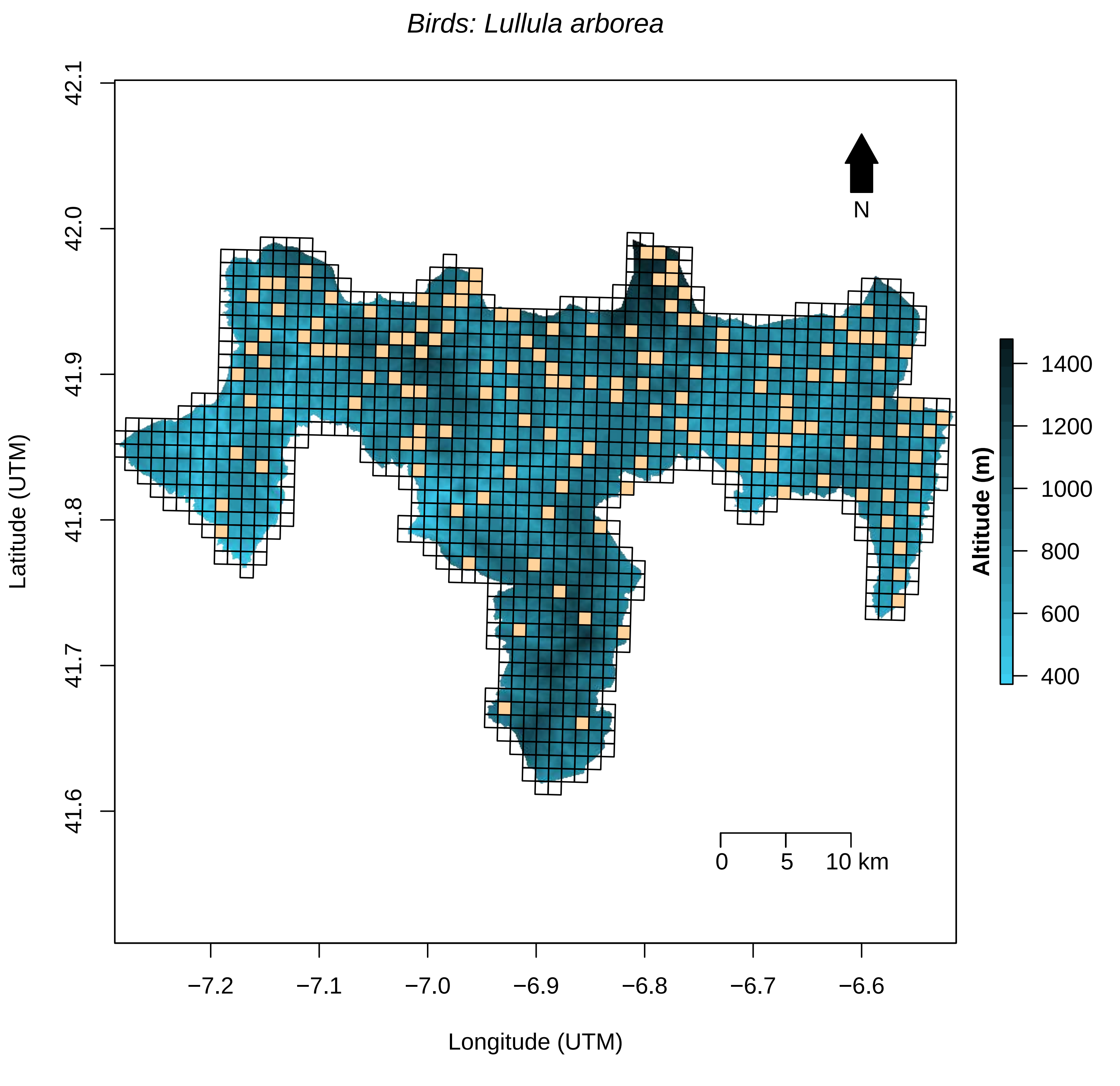

Birds: Lullula arborea
42.1
42.0
41.9
41.8
41.7
41.6
Latitude (UTM)
−7.2
−7.1
−7.0
−6.9
−6.8
−6.7
−6.6
Longitude (UTM)
N
0
5
10 km
1400
1200
1000
800
600
400
Altitude (m)

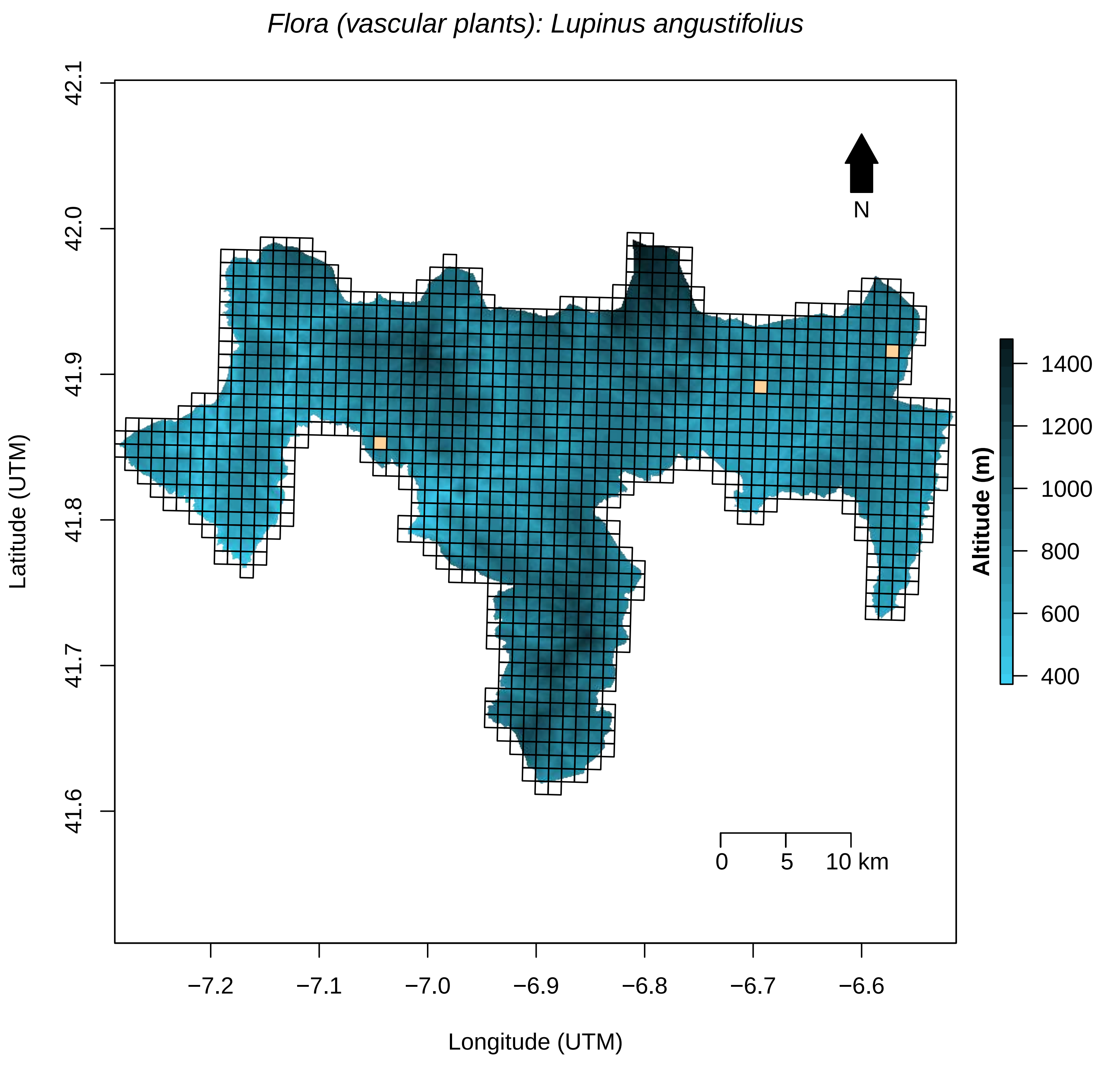

Flora (vascular plants): Lupinus angustifolius
N
0 5 10 km
Altitude (m)
1400
1200
1000
800
600
400
Latitude (UTM)
42.1
42.0
41.9
41.8
41.7
41.6
Longitude (UTM)
−7.2
−7.1
−7.0
−6.9
−6.8
−6.7
−6.6

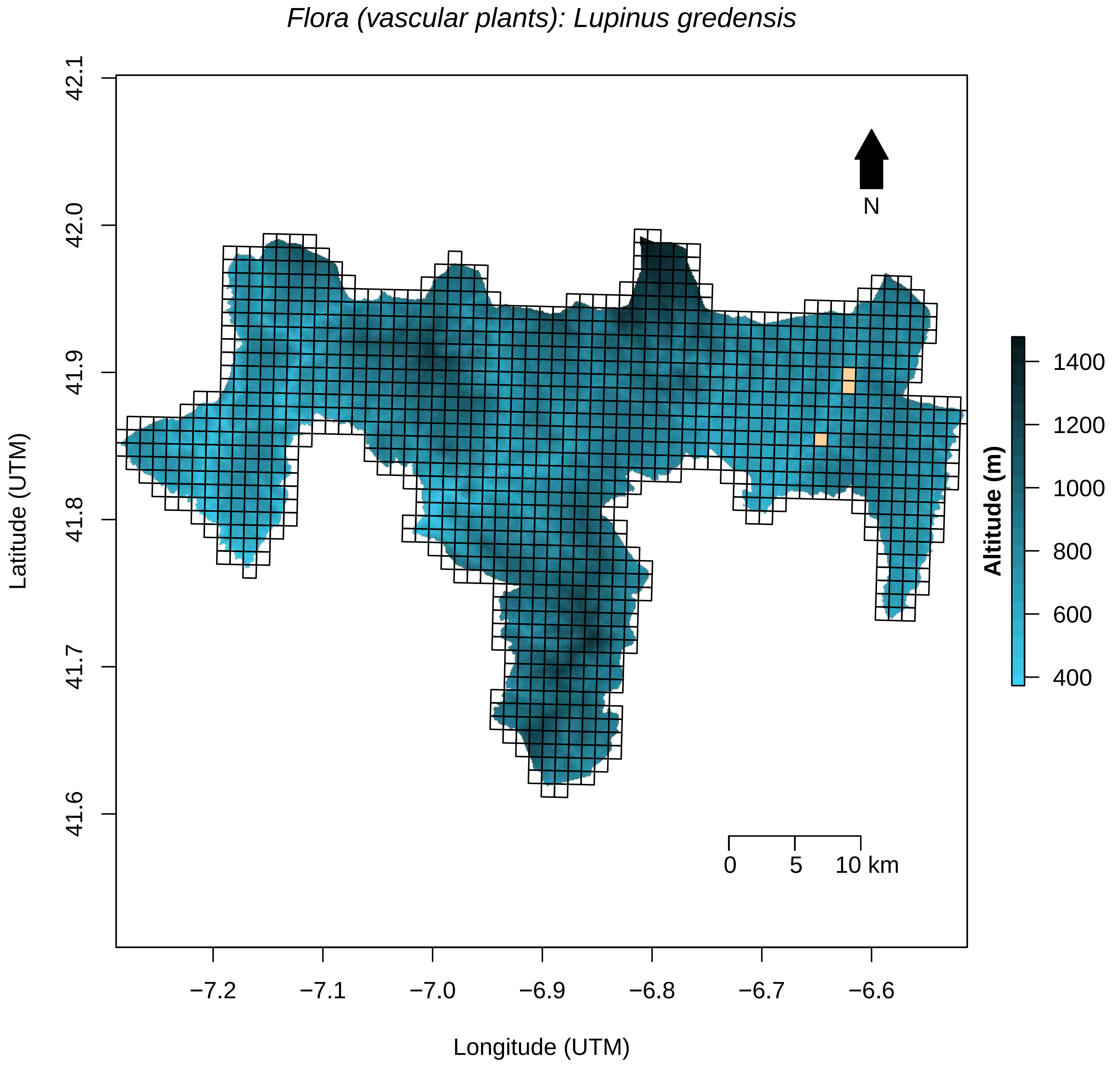

Flora (vascular plants): Lupinus gredensis
42.1
42.0
41.9
41.8
41.7
41.6
Latitude (UTM)
−7.2
−7.1
−7.0
−6.9
−6.8
−6.7
−6.6
Longitude (UTM)
N
0
5
10 km
Altitude (m)
1400
1200
1000
800
600
400

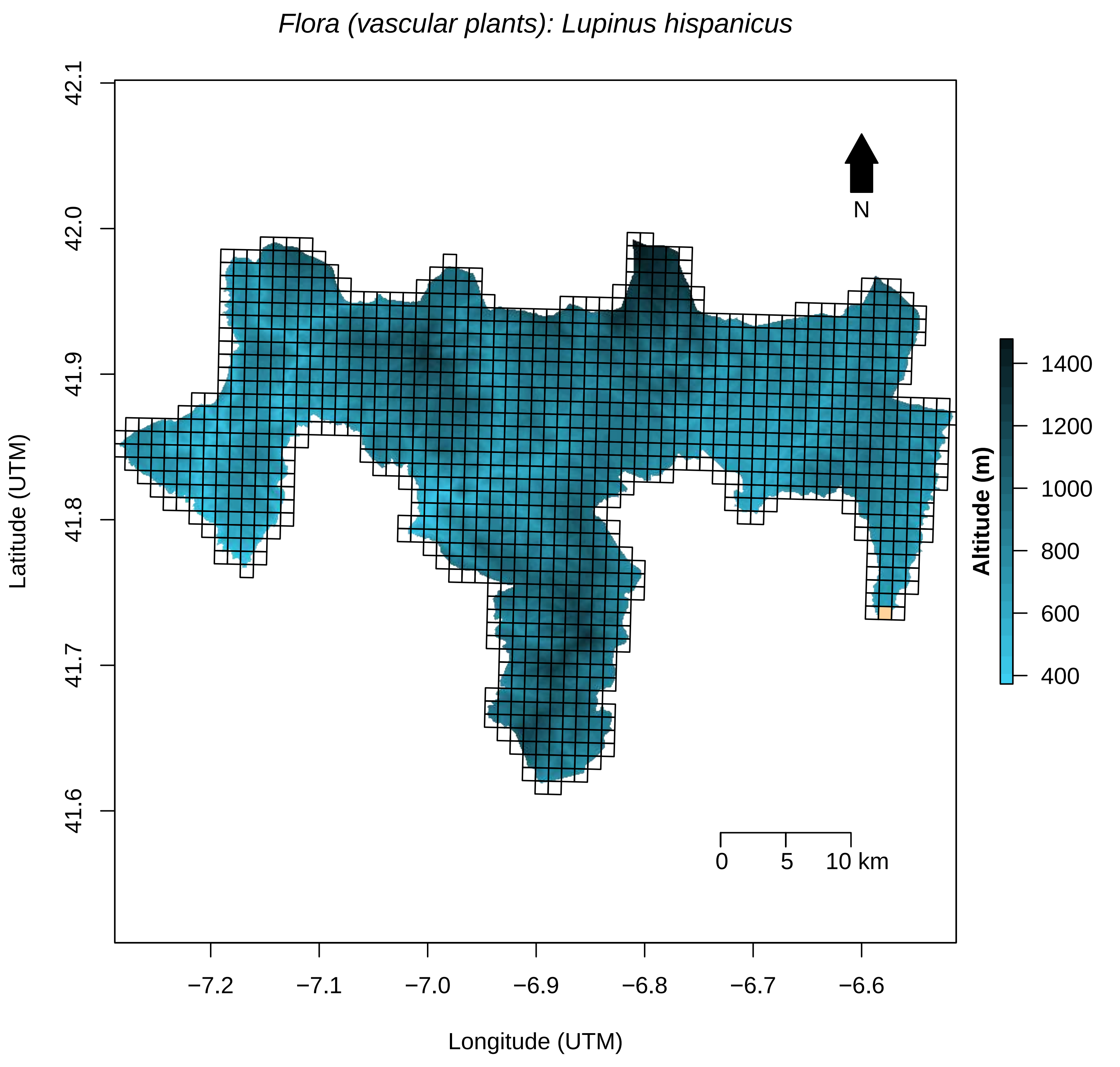

Flora (vascular plants): Lupinus hispanicus
42.1
42.0
41.9
41.8
41.7
41.6
Latitude (UTM)
−7.2
−7.1
−7.0
−6.9
−6.8
−6.7
−6.6
Longitude (UTM)
N
0
5
10 km
Altitude (m)
1400
1200
1000
800
600
400

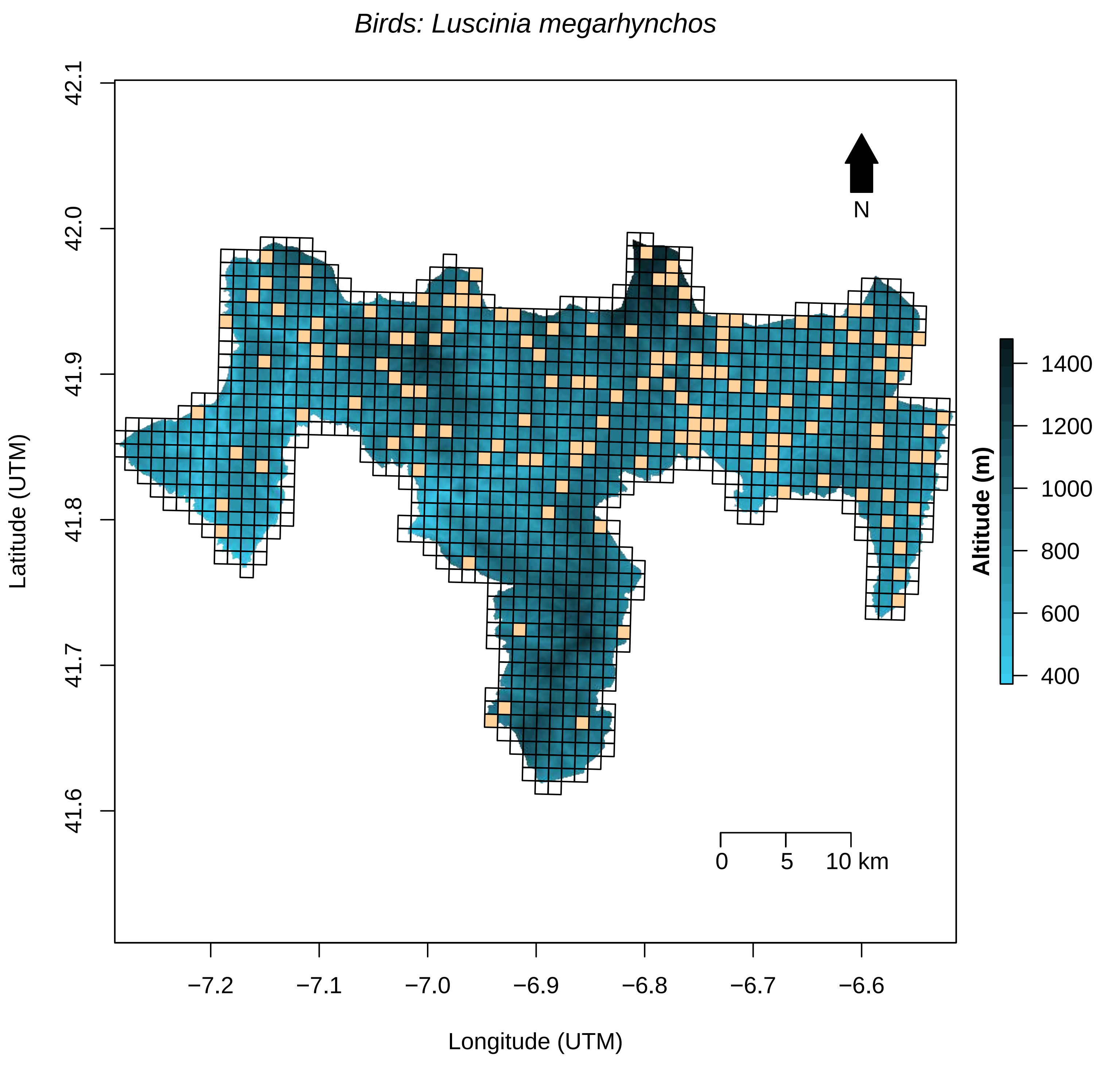

Birds: Luscinia megarhynchos
42.1
42.0
41.9
41.8
41.7
41.6
Latitude (UTM)
−7.2
−7.1
−7.0
−6.9
−6.8
−6.7
−6.6
Longitude (UTM)
N
0
5
10 km
1400
1200
1000
800
600
400
Altitude (m)

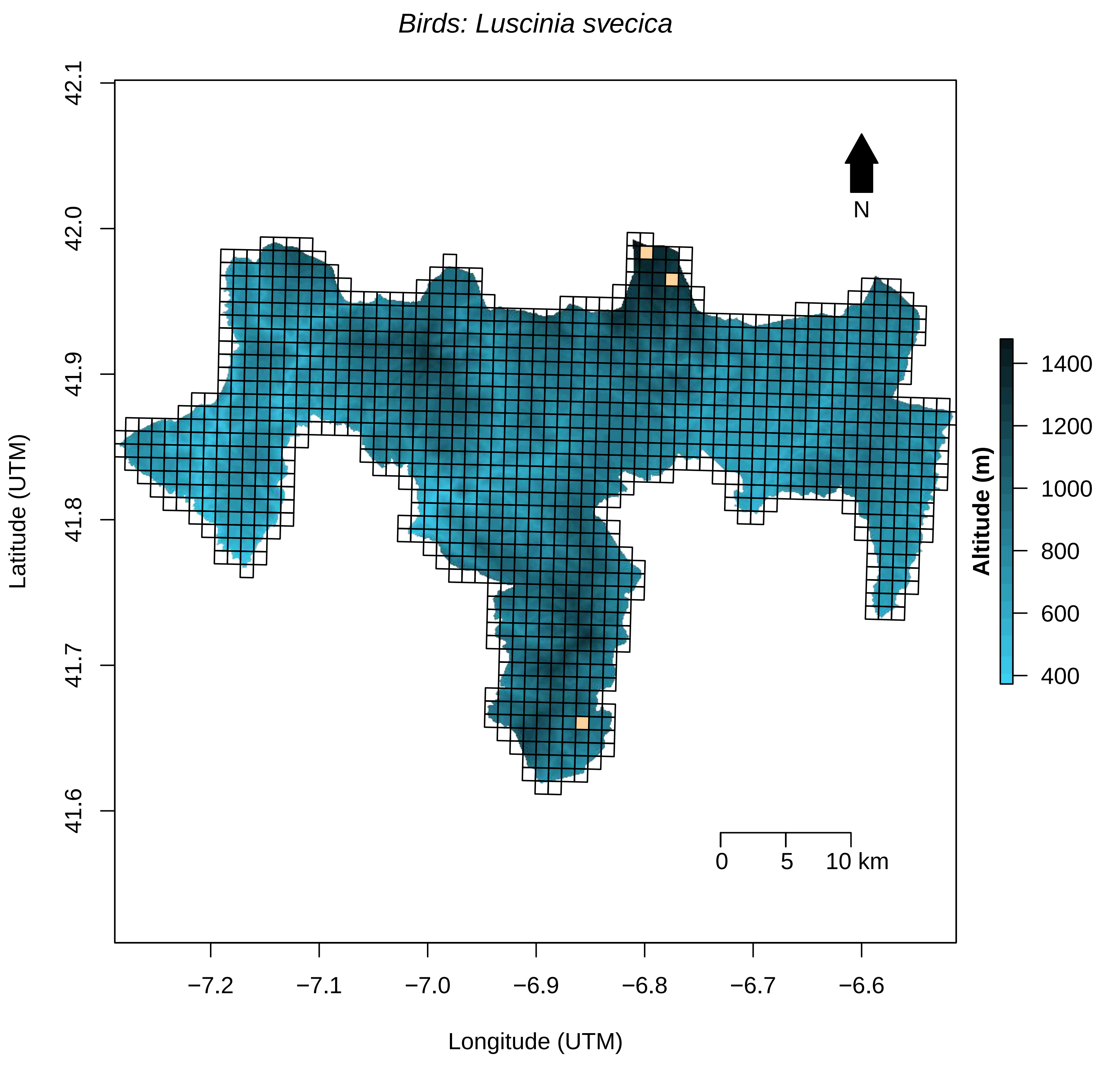
Birds: Luscinia svecica
42.1
42.0
41.9
41.8
41.7
41.6
Latitude (UTM)
N
0
5
10 km
−7.2
−7.1
−7.0
−6.9
−6.8
−6.7
−6.6
Longitude (UTM)
Altitude (m)
1400
1200
1000
800
600
400

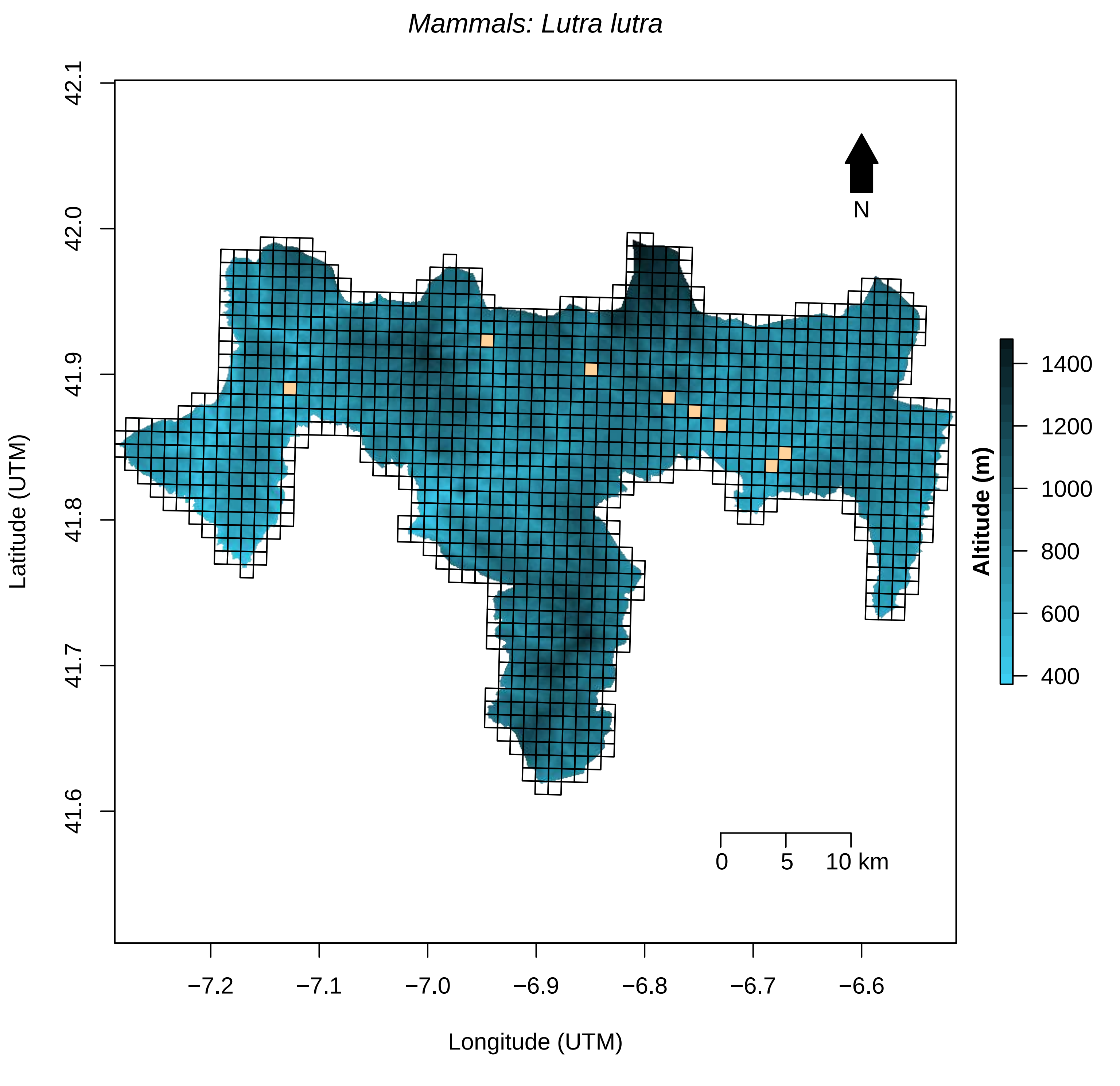
Mammals: Lutra lutra
42.1
42.0
41.9
41.8
41.7
41.6
Latitude (UTM)
−7.2
−7.1
−7.0
−6.9
−6.8
−6.7
−6.6
Longitude (UTM)
N
0
5
10 km
Altitude (m)
1400
1200
1000
800
600
400

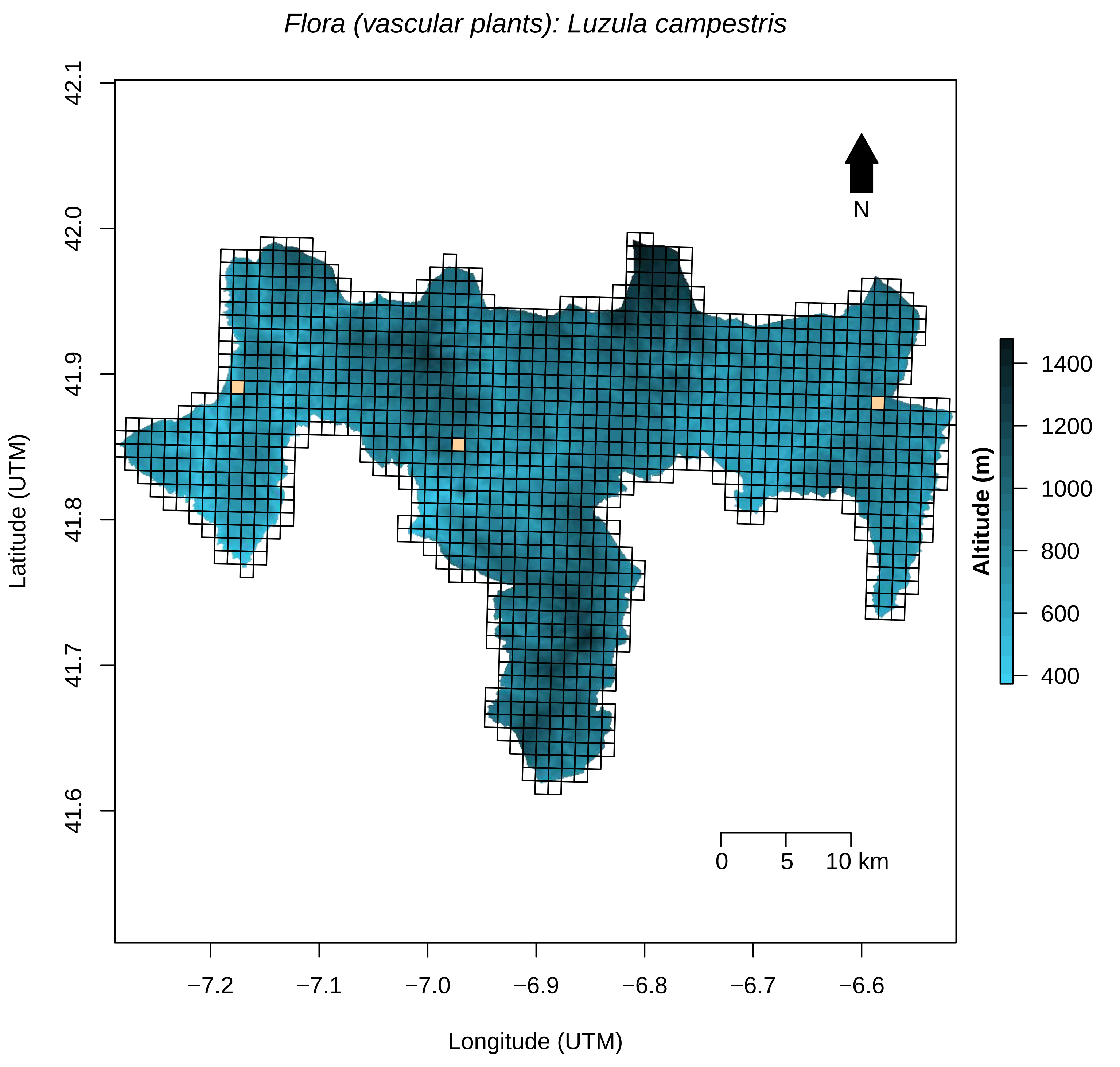

Flora (vascular plants): Luzula campestris
42.1
42.0
41.9
41.8
41.7
41.6
Latitude (UTM)
−7.2
−7.1
−7.0
−6.9
−6.8
−6.7
−6.6
Longitude (UTM)
N
0
5
10 km
1400
1200
1000
800
600
400
Altitude (m)

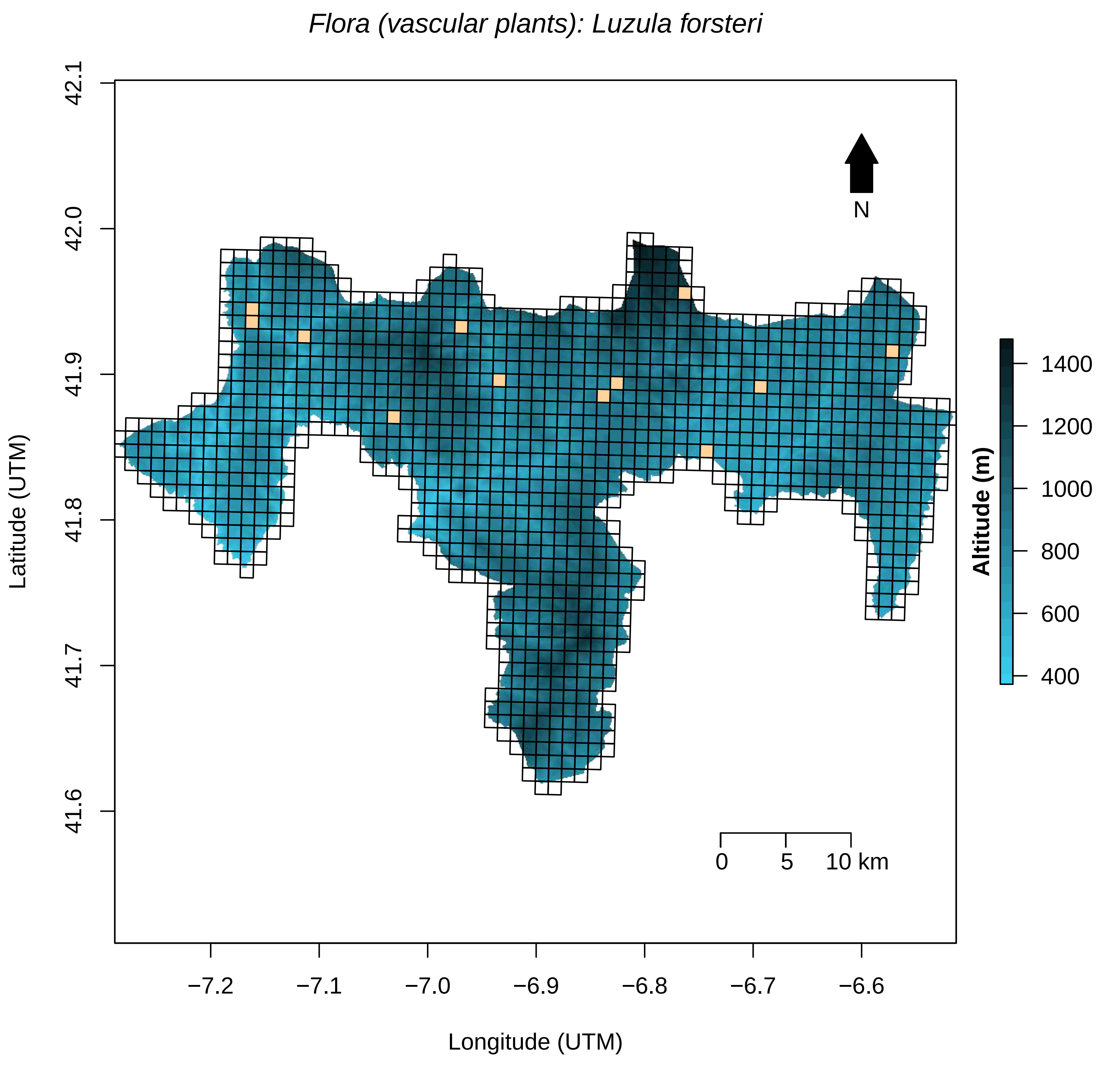

Flora (vascular plants): Luzula forsteri
Latitude (UTM)
42.1
42.0
41.9
41.8
41.7
41.6
Longitude (UTM)
−7.2
−7.1
−7.0
−6.9
−6.8
−6.7
−6.6
N
0
5
10 km
Altitude (m)
1400
1200
1000
800
600
400

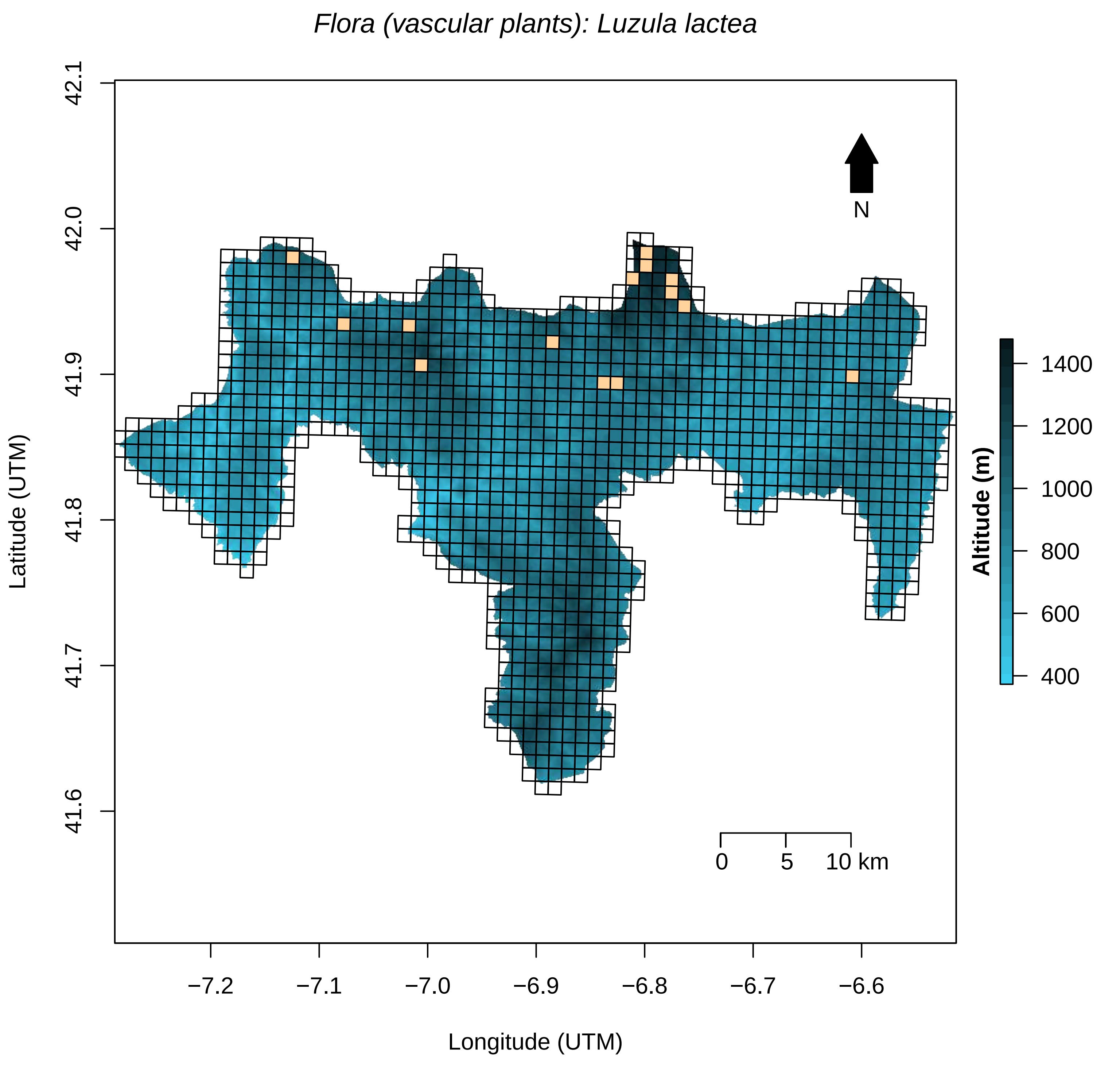
Flora (vascular plants): Luzula lactea
42.1
42.0
41.9
41.8
41.7
41.6
Latitude (UTM)
−7.2
−7.1
−7.0
−6.9
−6.8
−6.7
−6.6
Longitude (UTM)
N
0
5
10 km
Altitude (m)
1400
1200
1000
800
600
400

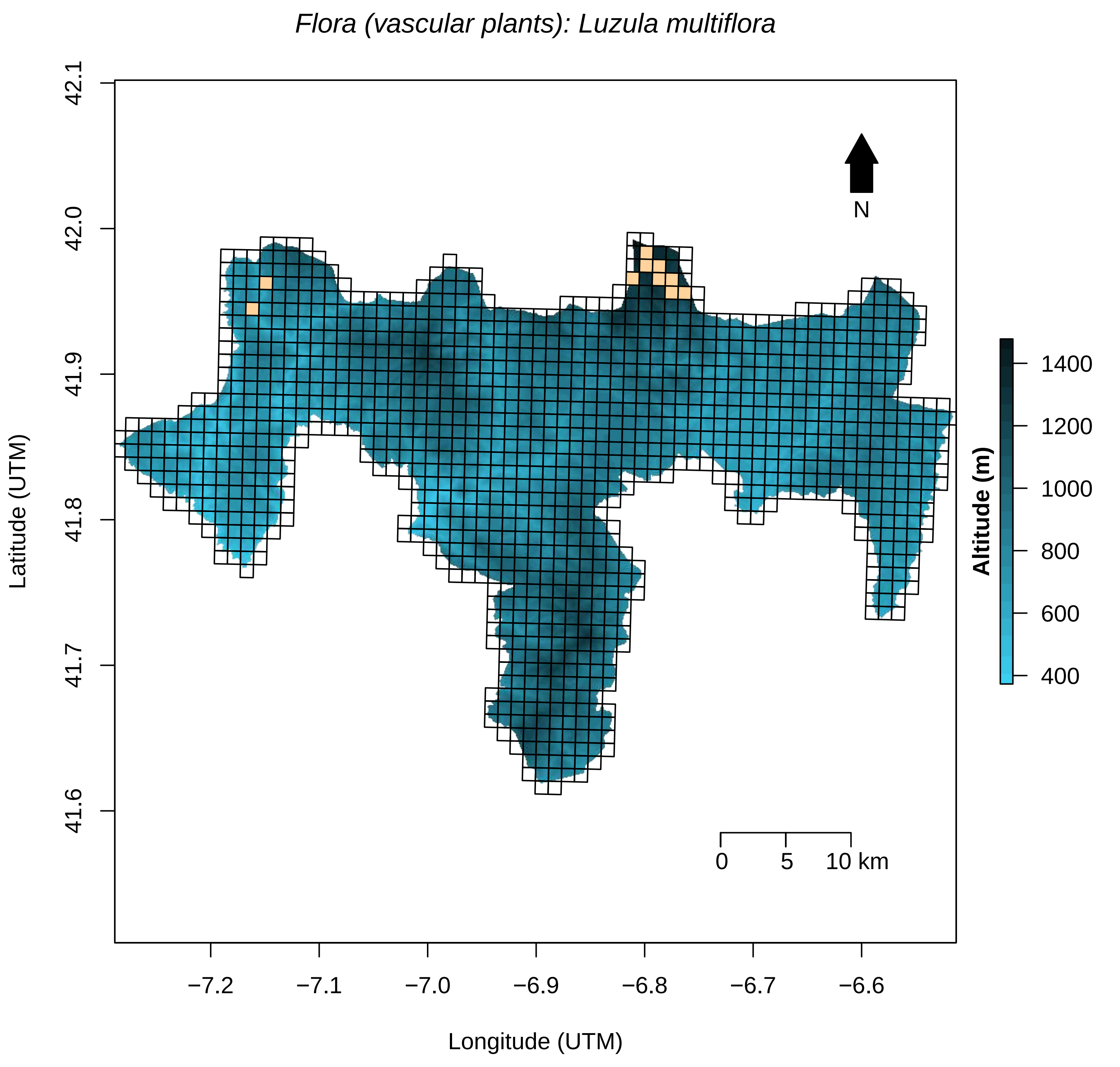

Flora (vascular plants): Luzula multiflora
Latitude (UTM)
42.1
42.0
41.9
41.8
41.7
41.6
Longitude (UTM)
−7.2
−7.1
−7.0
−6.9
−6.8
−6.7
−6.6
N
0
5
10 km
Altitude (m)
1400
1200
1000
800
600
400

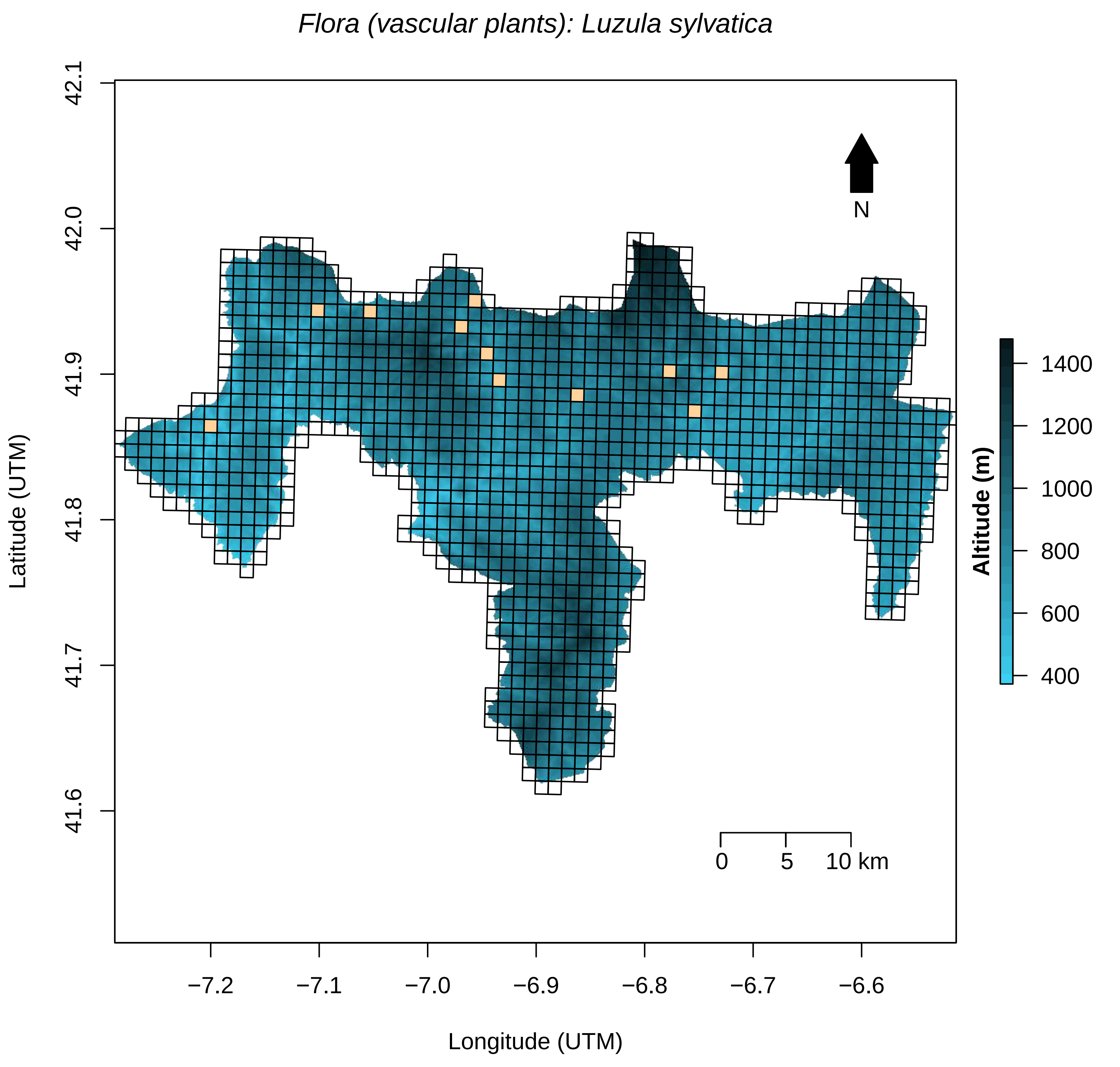
Flora (vascular plants): Luzula sylvatica
Latitude (UTM)
42.1
42.0
41.9
41.8
41.7
41.6
Longitude (UTM)
−7.2
−7.1
−7.0
−6.9
−6.8
−6.7
−6.6
N
0
5
10 km
Altitude (m)
1400
1200
1000
800
600
400

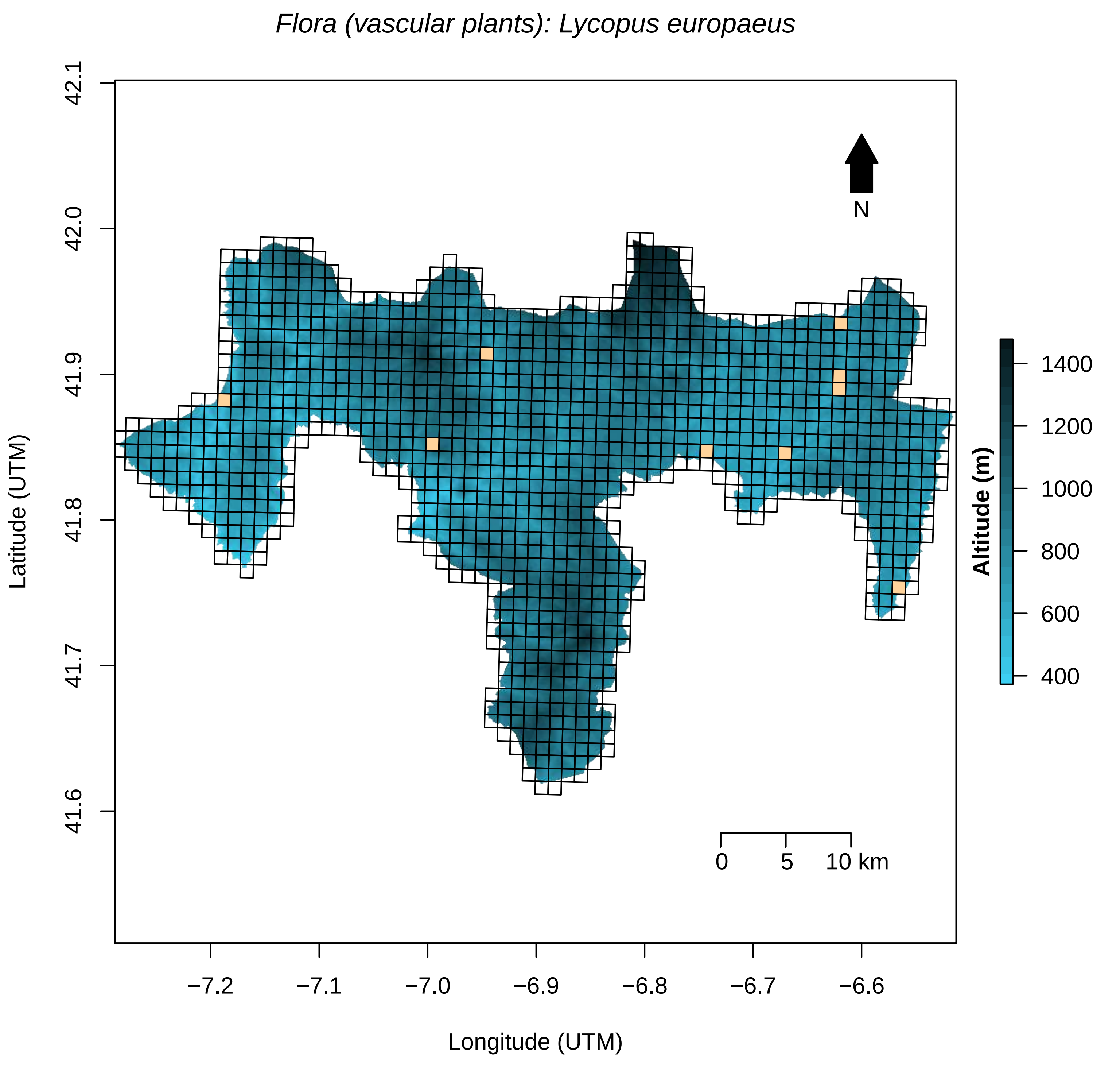
Flora (vascular plants): Lycopus europaeus
42.1
42.0
41.9
41.8
41.7
41.6
Latitude (UTM)
−7.2
−7.1
−7.0
−6.9
−6.8
−6.7
−6.6
Longitude (UTM)
N
0
5
10 km
1400
1200
1000
800
600
400
Altitude (m)

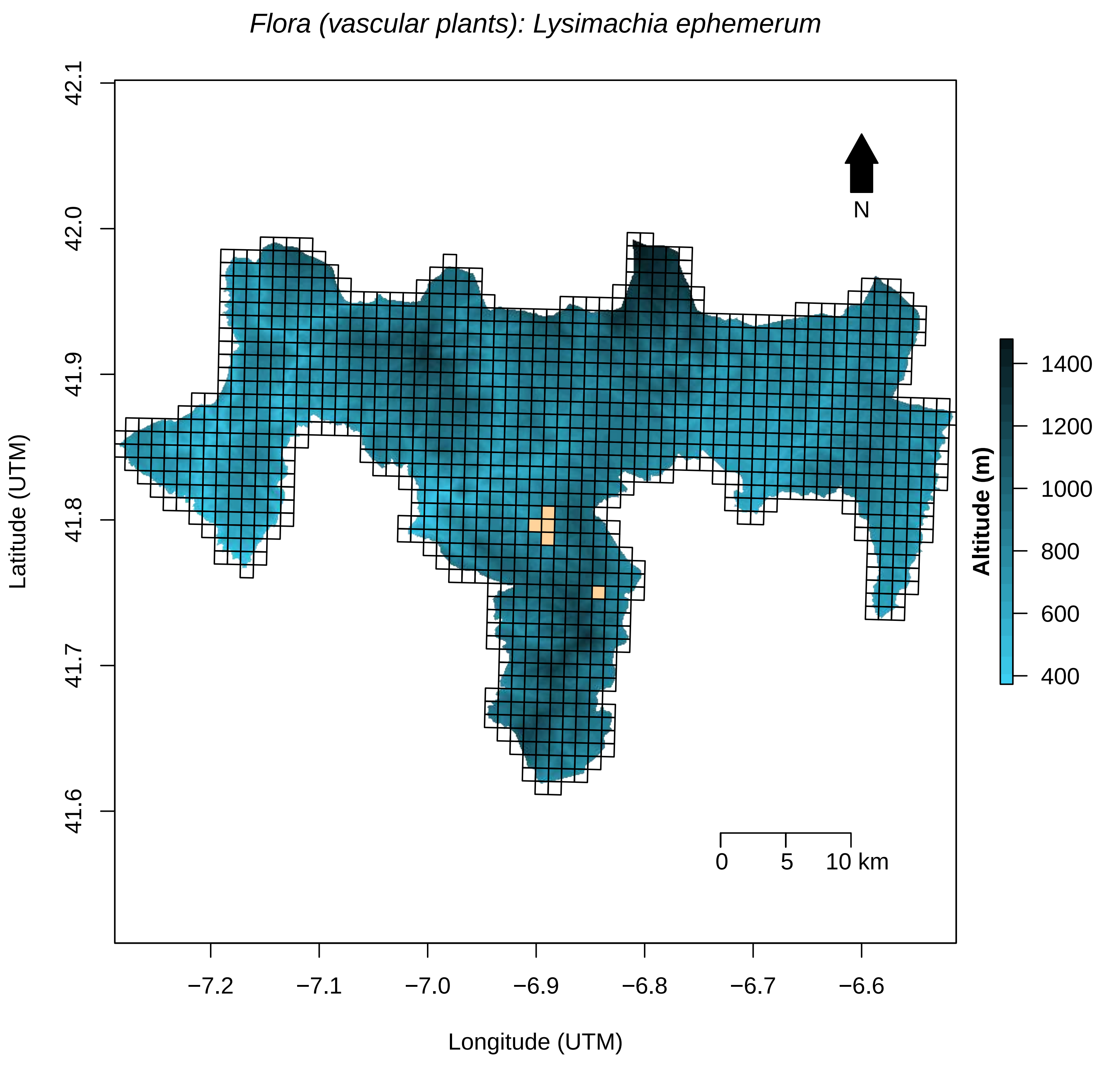

Flora (vascular plants): Lysimachia ephemerum
N
0
5
10 km
Altitude (m)
400
600
800
1000
1200
1400
Latitude (UTM)
41.6
41.7
41.8
41.9
42.0
42.1
Longitude (UTM)
−7.2
−7.1
−7.0
−6.9
−6.8
−6.7
−6.6

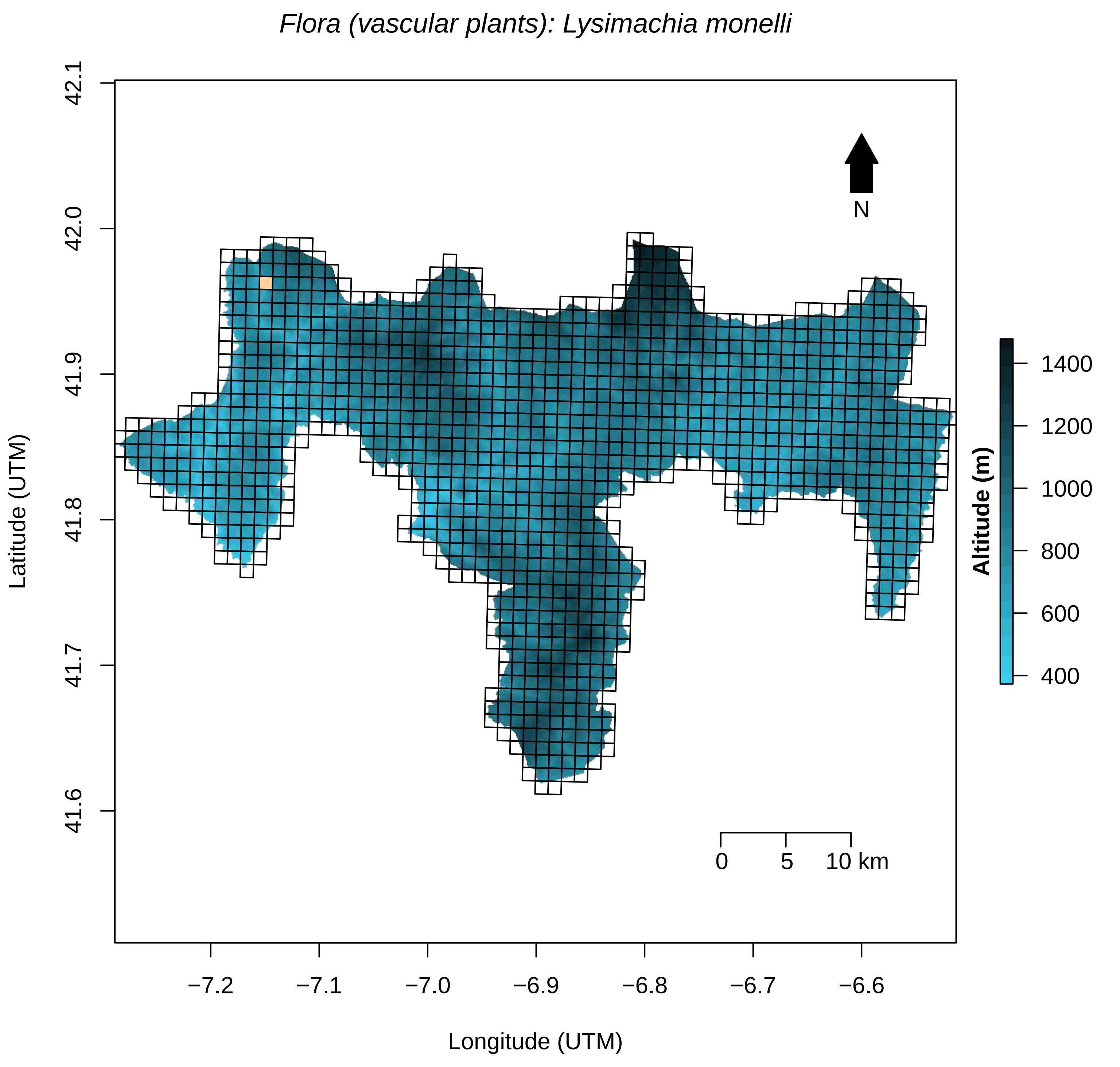

Flora (vascular plants): Lysimachia monelli
42.1
42.0
41.9
41.8
41.7
41.6
Latitude (UTM)
N
0 5 10 km
1400
1200
1000
800
600
400
Altitude (m)
−7.2
−7.1
−7.0
−6.9
−6.8
−6.7
−6.6
Longitude (UTM)

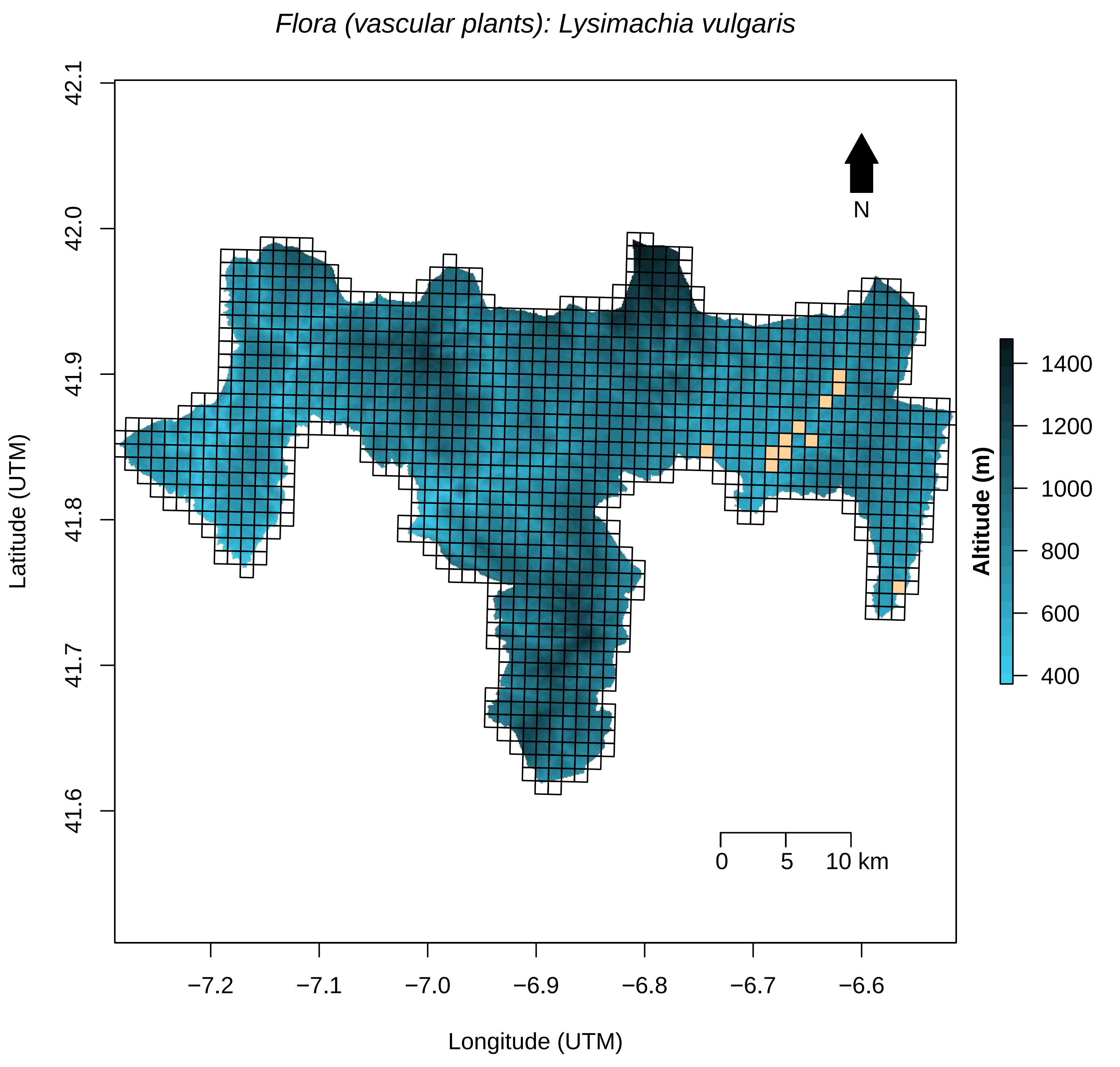

Flora (vascular plants): Lysimachia vulgaris
42.1
42.0
41.9
41.8
41.7
41.6
Latitude (UTM)
N
0
5
10 km
−7.2
−7.1
−7.0
−6.9
−6.8
−6.7
−6.6
Longitude (UTM)
1400
1200
1000
800
600
400
Altitude (m)

Flora (vascular plants): Lythrum portula

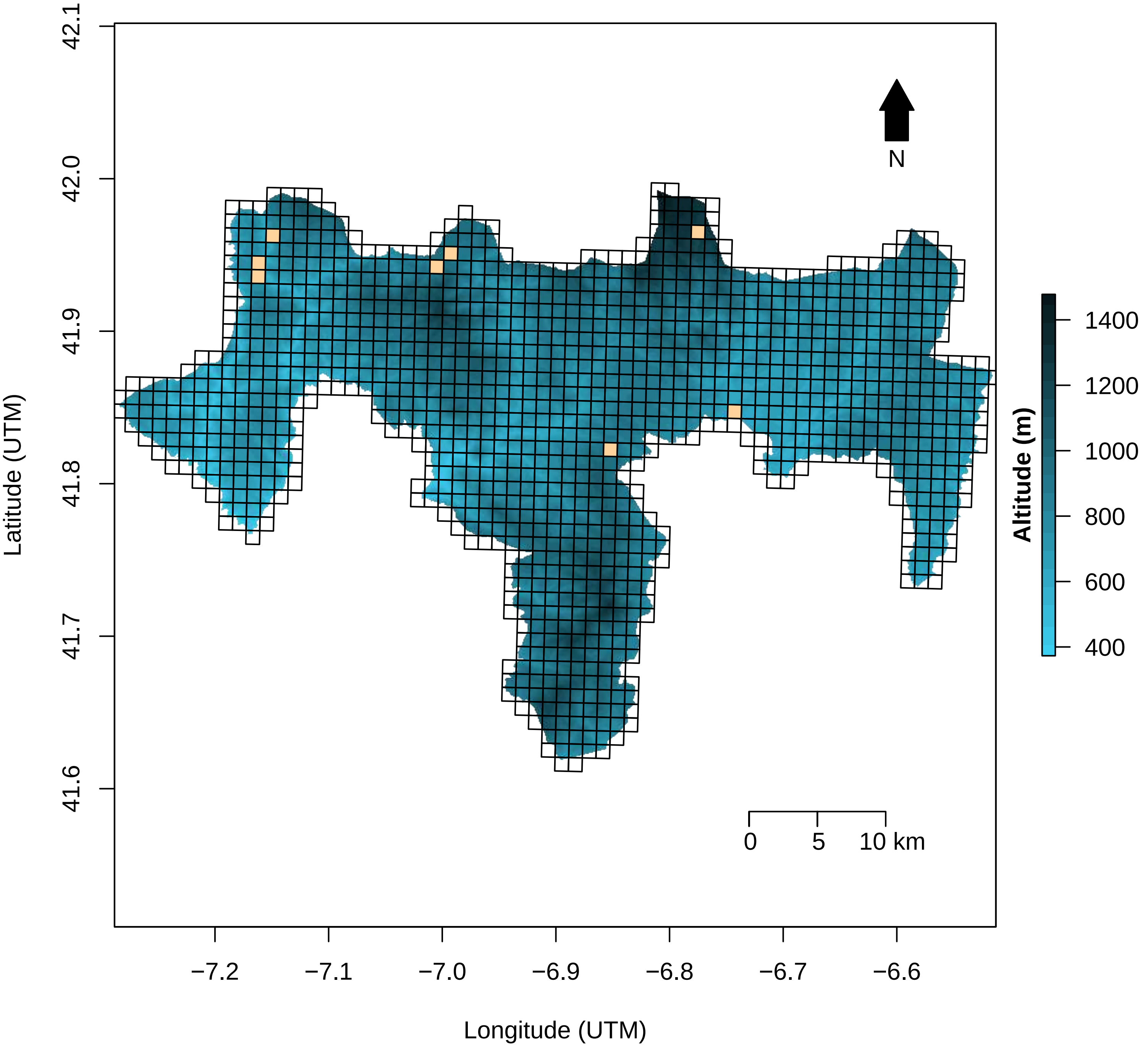

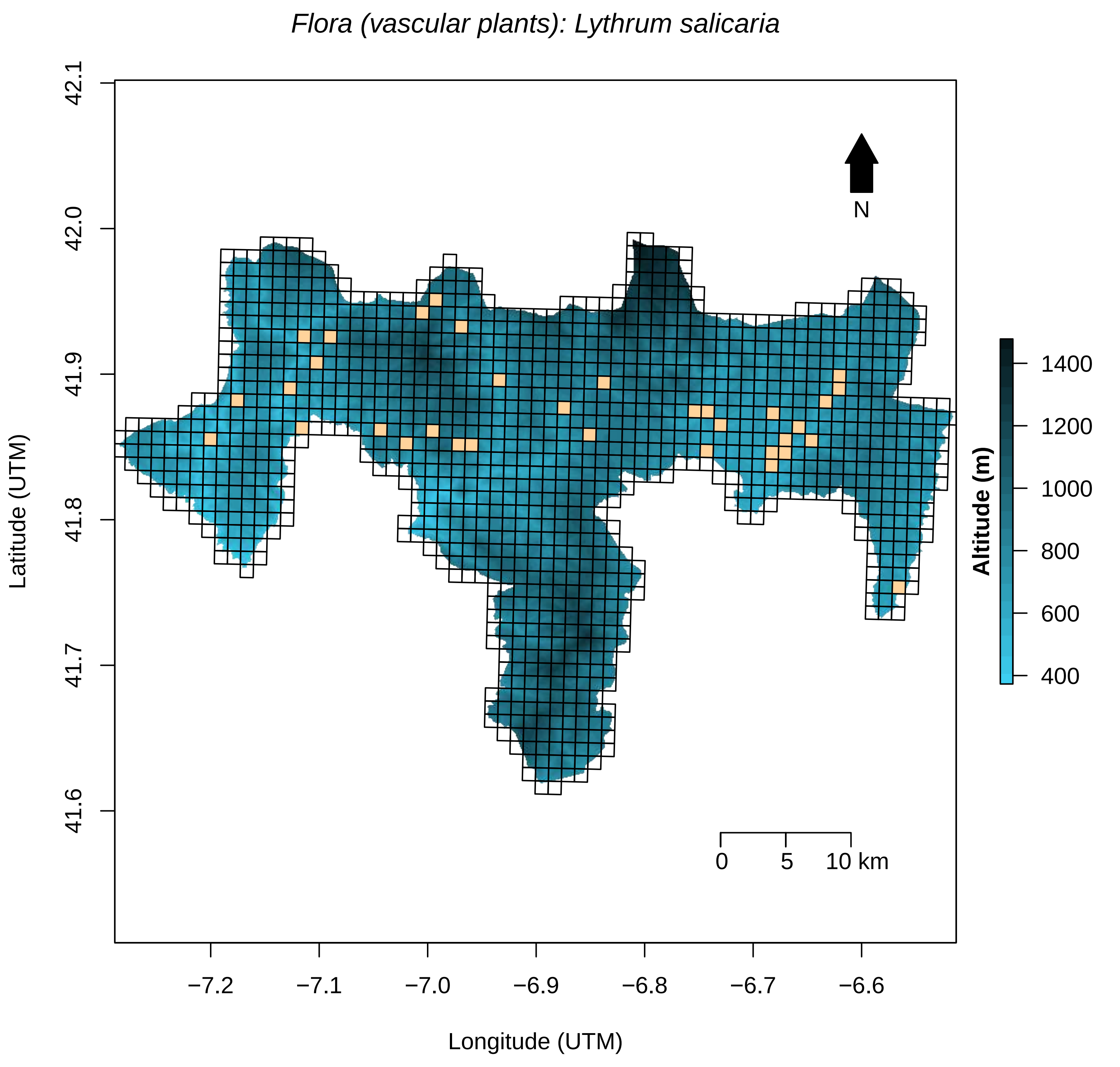

Flora (vascular plants): Lythrum salicaria
Latitude (UTM)
42.1
42.0
41.9
41.8
41.7
41.6
Longitude (UTM)
−7.2
−7.1
−7.0
−6.9
−6.8
−6.7
−6.6
N
0
5
10 km
Altitude (m)
1400
1200
1000
800
600
400

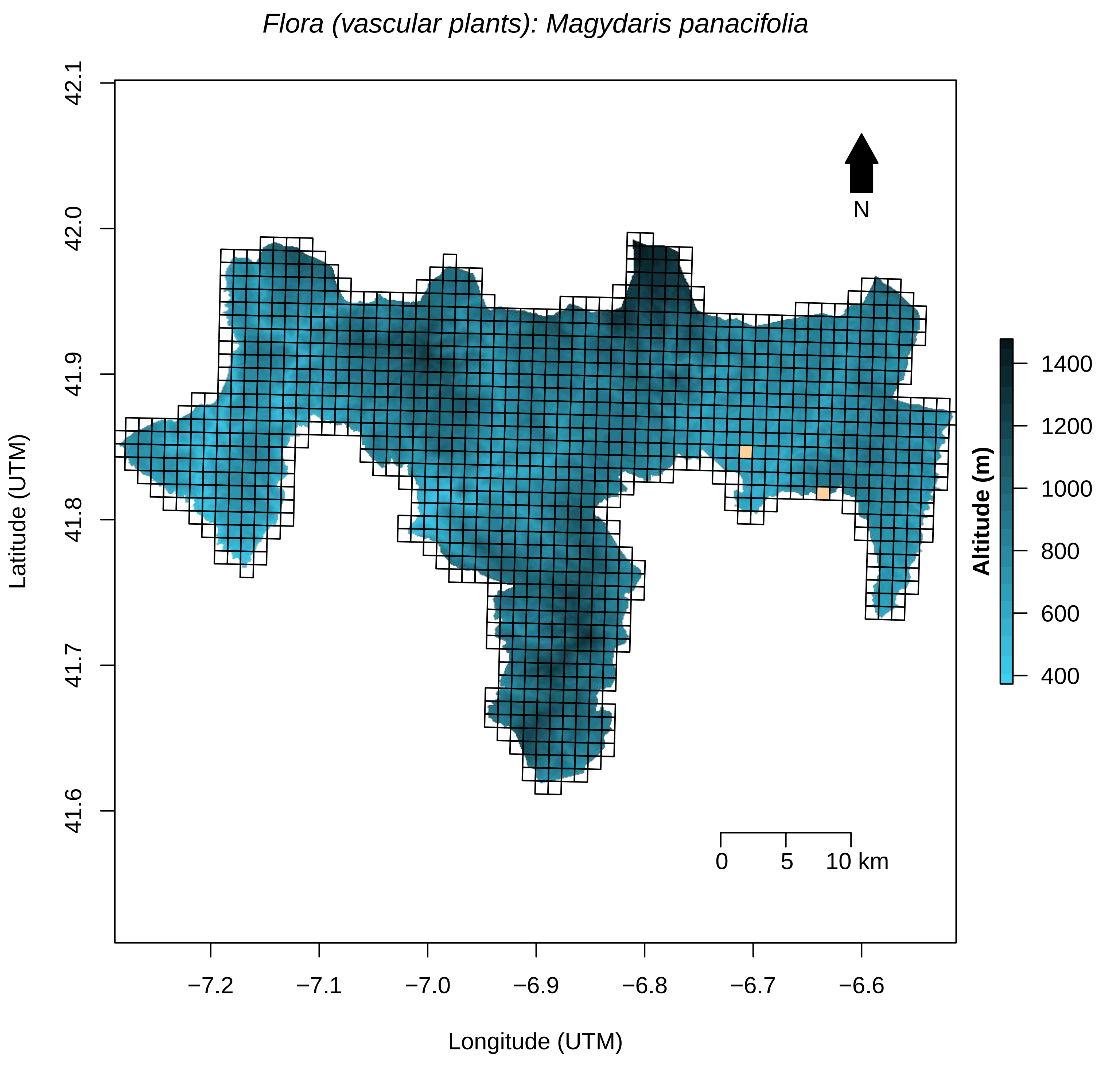
Flora (vascular plants): Magydaris panacifolia
42.1
42.0
41.9
41.8
41.7
41.6
Latitude (UTM)
−7.2
−7.1
−7.0
−6.9
−6.8
−6.7
−6.6
Longitude (UTM)
N
0
5
10 km
Altitude (m)
1400
1200
1000
800
600
400

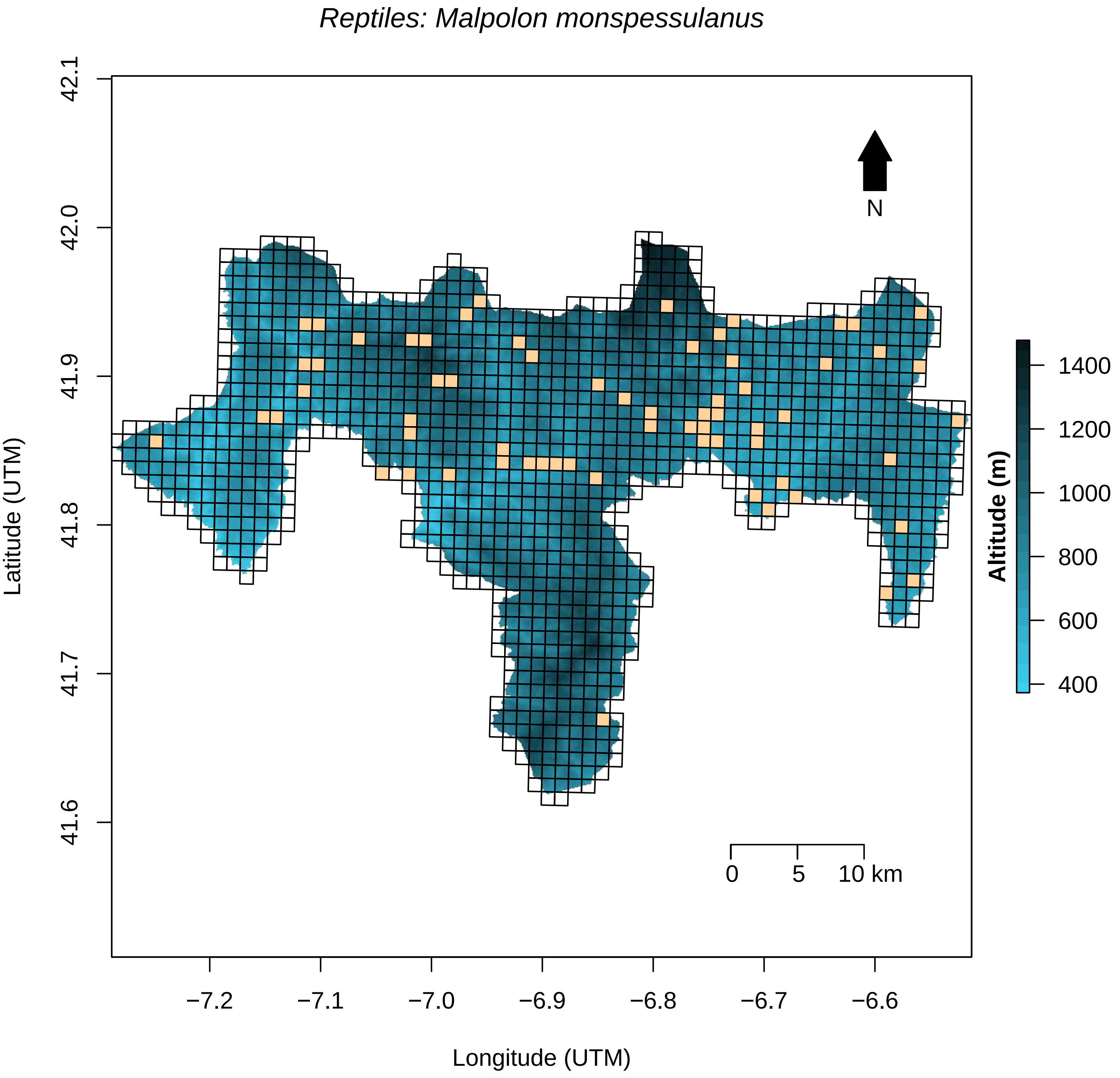

Reptiles: Malpolon monspessulanus
Latitude (UTM)
Longitude (UTM)
42.1
42.0
41.9
41.8
41.7
41.6
−7.2
−7.1
−7.0
−6.9
−6.8
−6.7
−6.6
N
0
5
10 km
Altitude (m)
1400
1200
1000
800
600
400

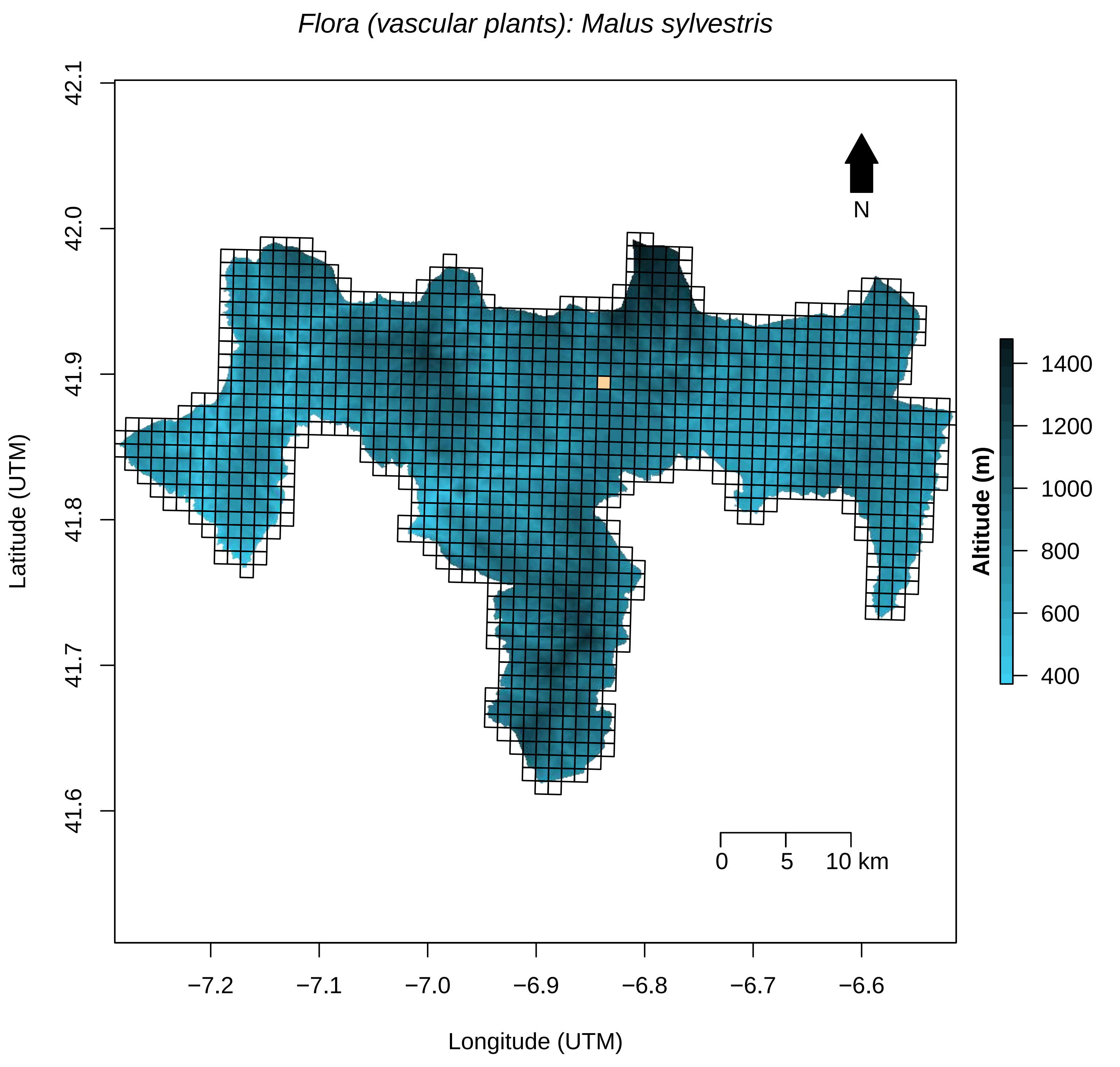

Flora (vascular plants): Malus sylvestris
42.1
42.0
41.9
41.8
41.7
41.6
Latitude (UTM)
−7.2
−7.1
−7.0
−6.9
−6.8
−6.7
−6.6
Longitude (UTM)
N
0
5
10 km
Altitude (m)
1400
1200
1000
800
600
400

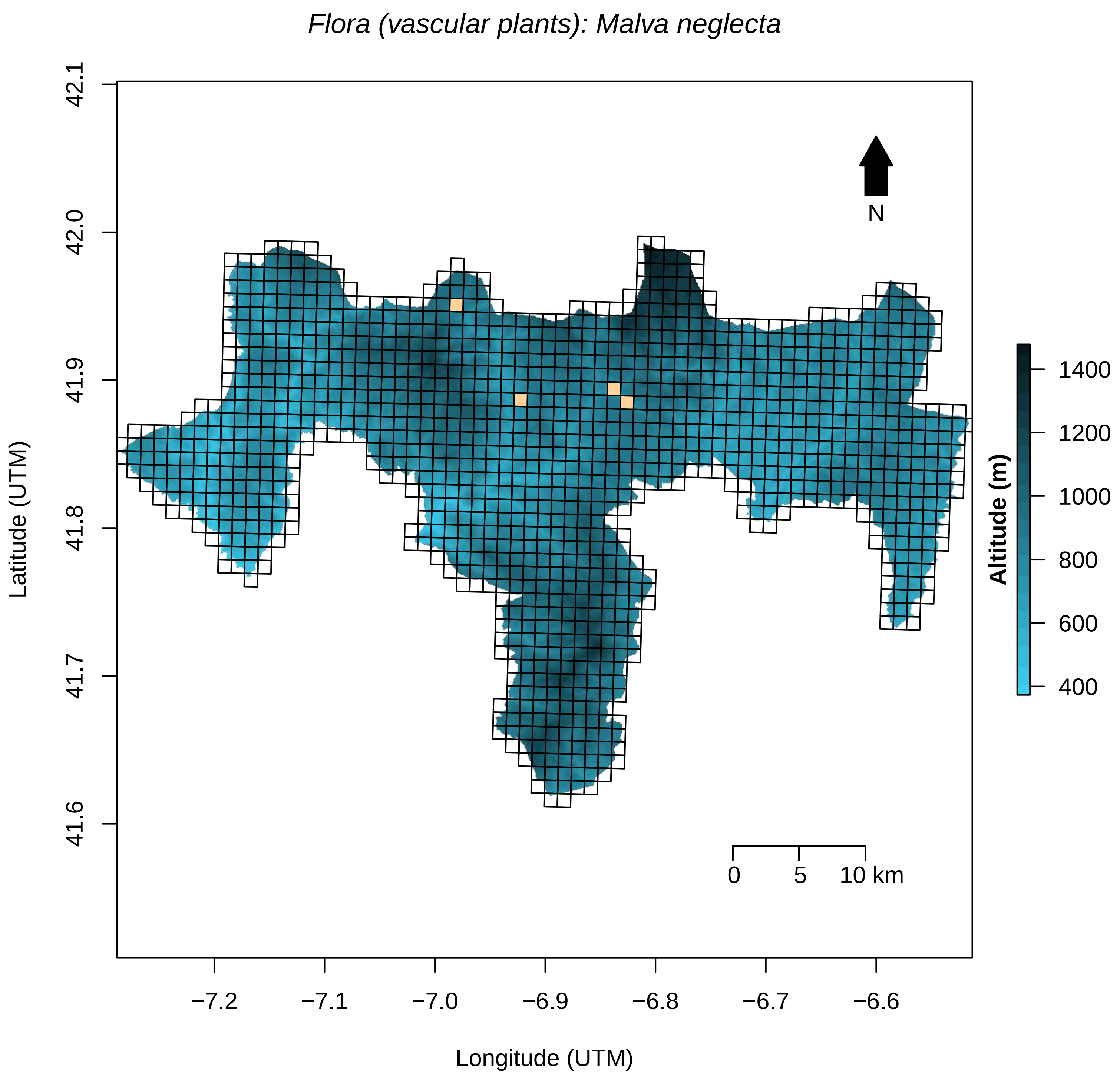

Flora (vascular plants): Malva neglecta
42.1
42.0
41.9
41.8
41.7
41.6
Latitude (UTM)
N
0
5
10 km
1400
1200
1000
800
600
400
Altitude (m)
−7.2
−7.1
−7.0
−6.9
−6.8
−6.7
−6.6
Longitude (UTM)

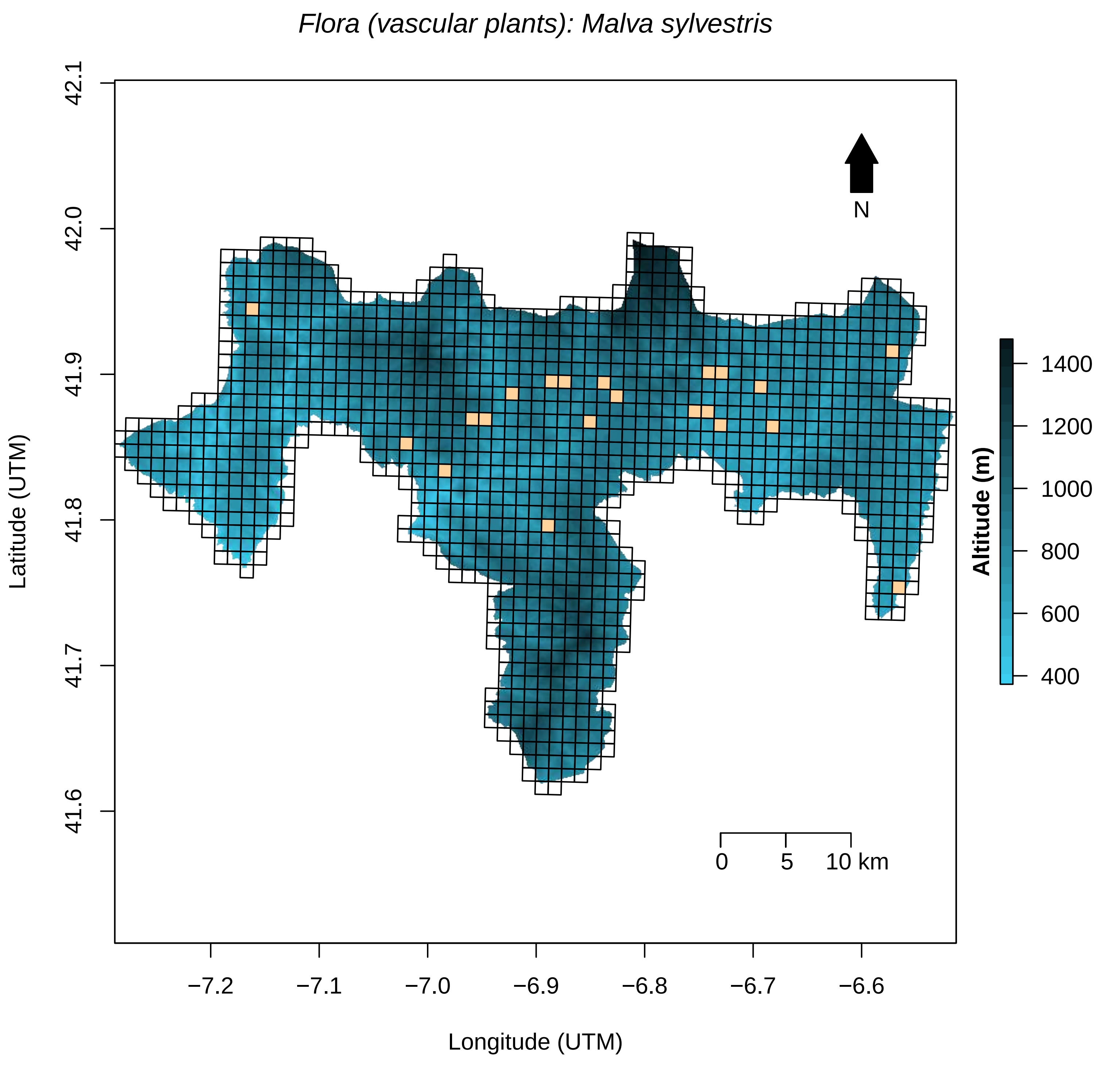

Flora (vascular plants): Malva sylvestris
Latitude (UTM)
42.1
42.0
41.9
41.8
41.7
41.6
Longitude (UTM)
−7.2
−7.1
−7.0
−6.9
−6.8
−6.7
−6.6
N
0
5
10 km
Altitude (m)
1400
1200
1000
800
600
400

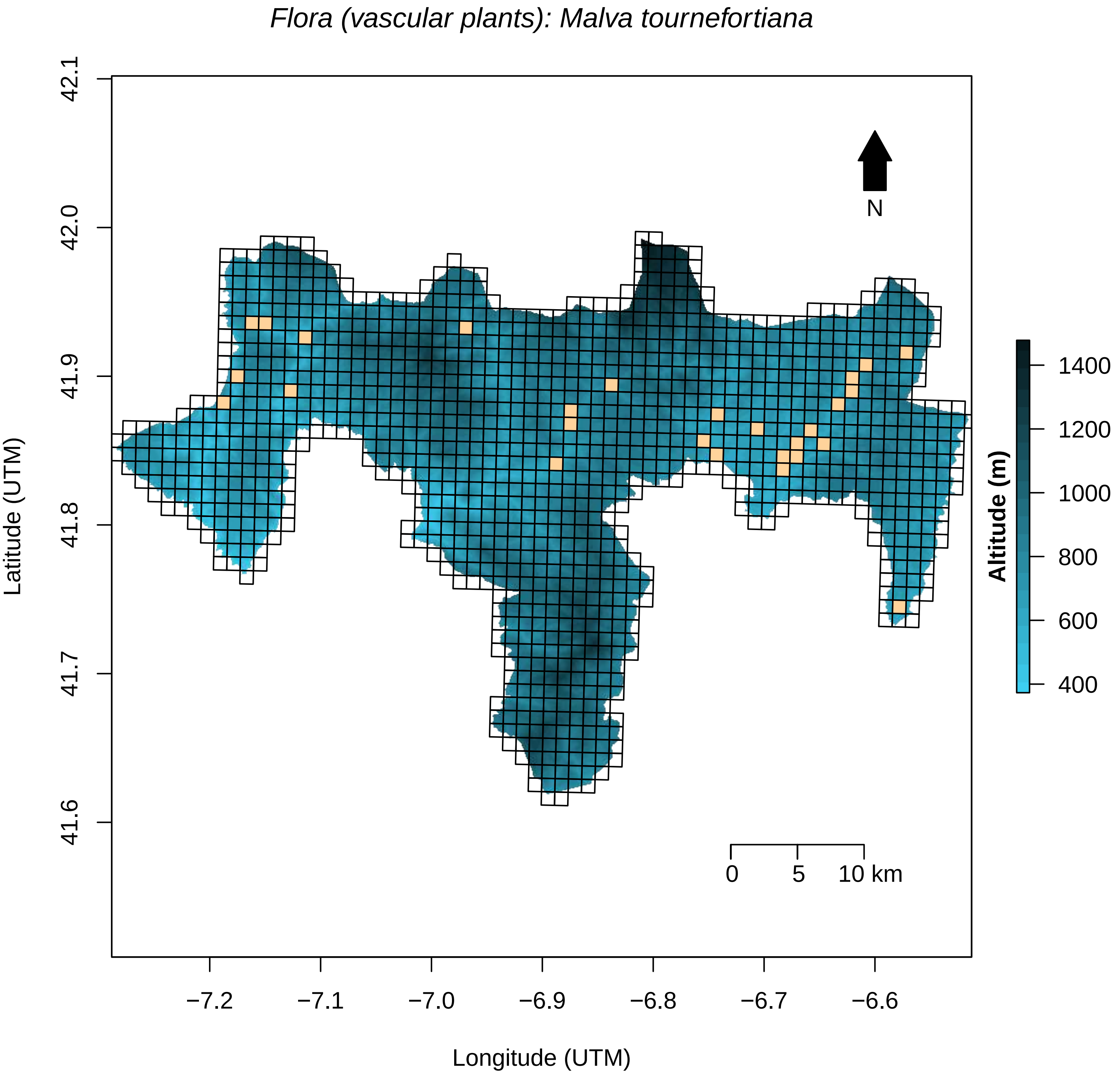
Flora (vascular plants): Malva tournefortiana
N
0 5 10 km
Altitude (m)
1400
1200
1000
800
600
400
42.1
42.0
41.9
41.8
41.7
41.6
Latitude (UTM)
−7.2
−7.1
−7.0
−6.9
−6.8
−6.7
−6.6
Longitude (UTM)

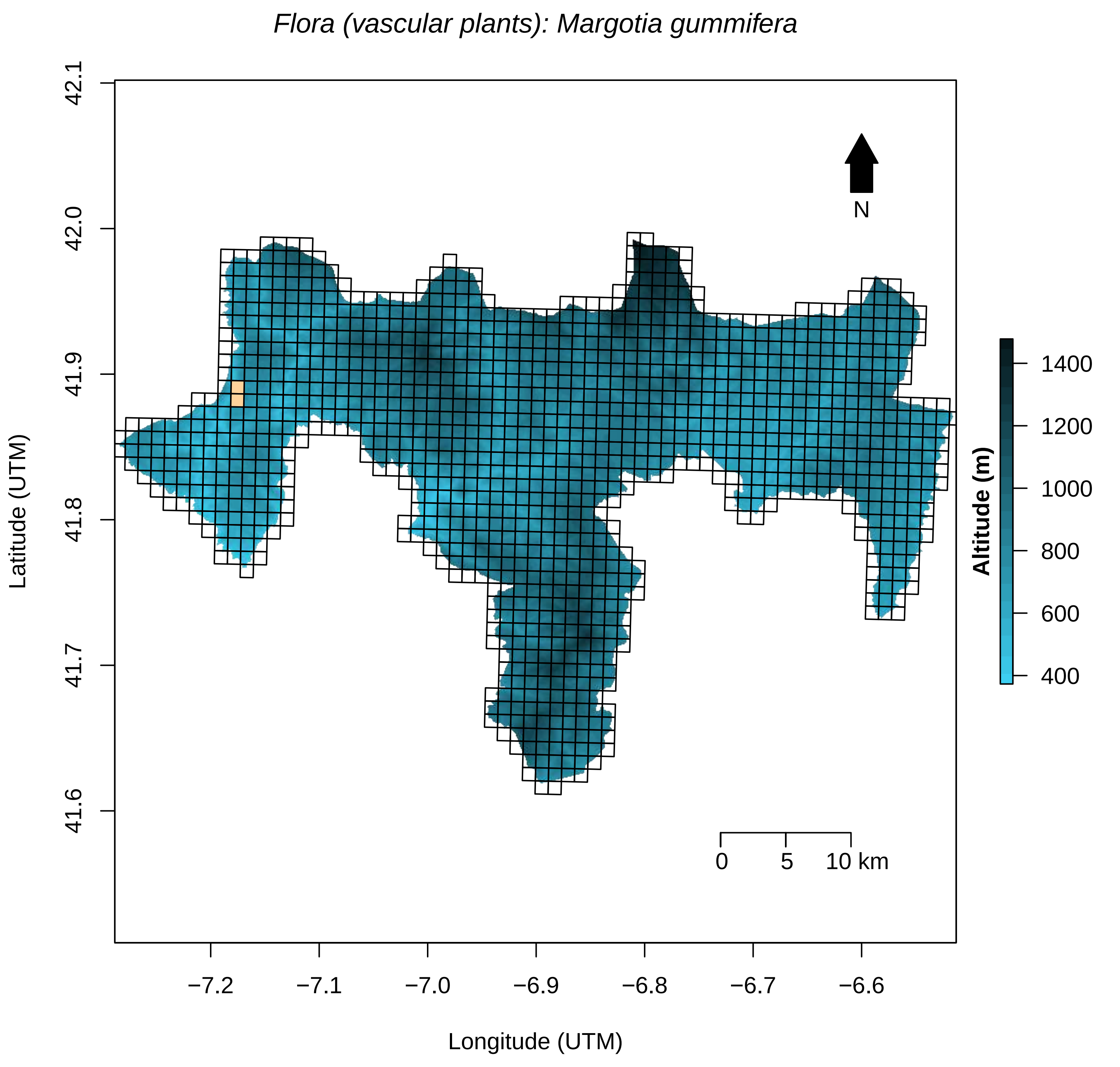
Flora (vascular plants): Margotia gummifera
42.1
42.0
41.9
41.8
41.7
41.6
Latitude (UTM)
−7.2
−7.1
−7.0
−6.9
−6.8
−6.7
−6.6
Longitude (UTM)
N
0
5
10 km
Altitude (m)
1400
1200
1000
800
600
400

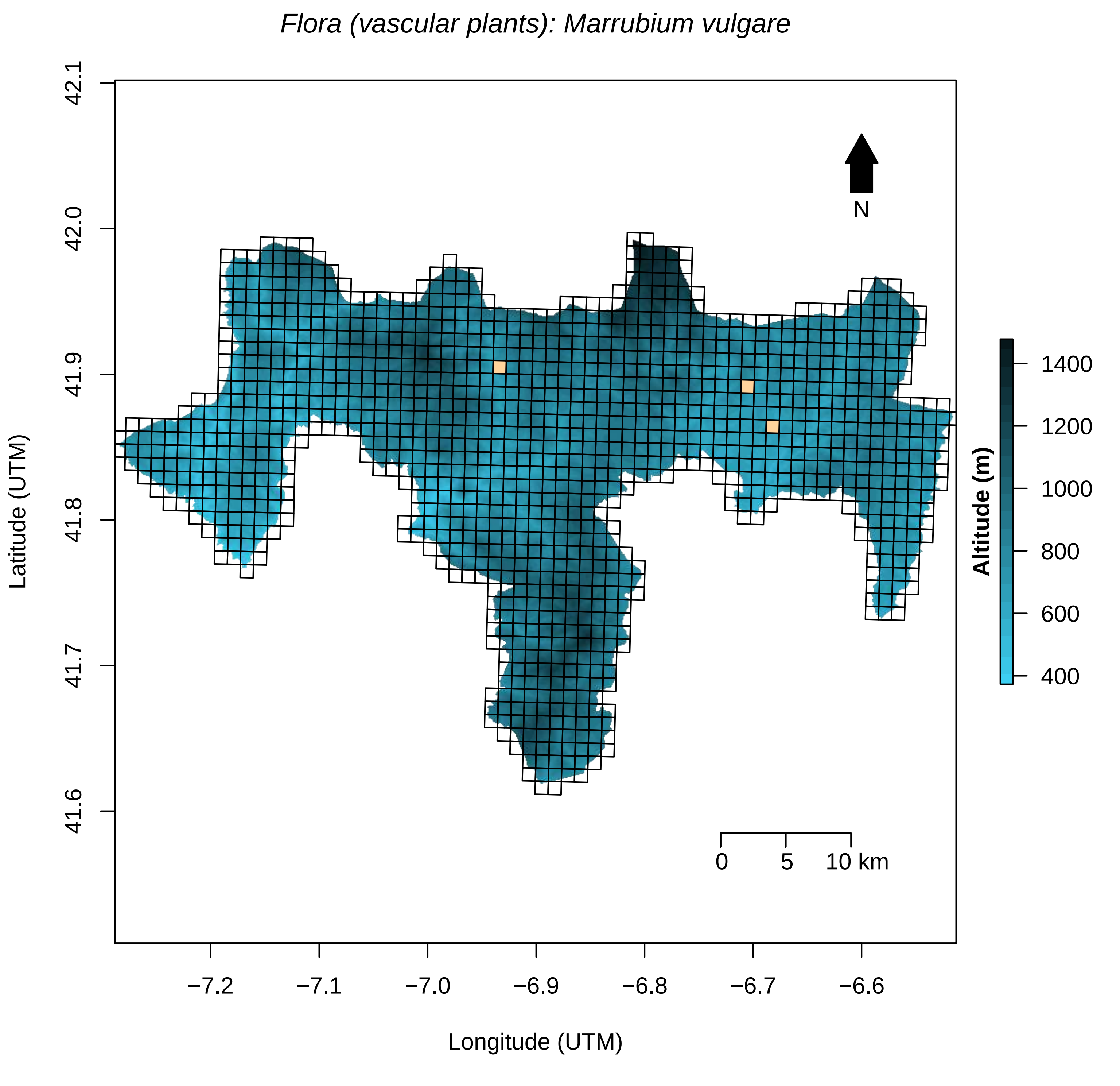

Flora (vascular plants): Marrubium vulgare
N
0
5
10 km
Altitude (m)
1400
1200
1000
800
600
400
42.1
42.0
41.9
41.8
41.7
41.6
Latitude (UTM)
−7.2
−7.1
−7.0
−6.9
−6.8
−6.7
−6.6
Longitude (UTM)

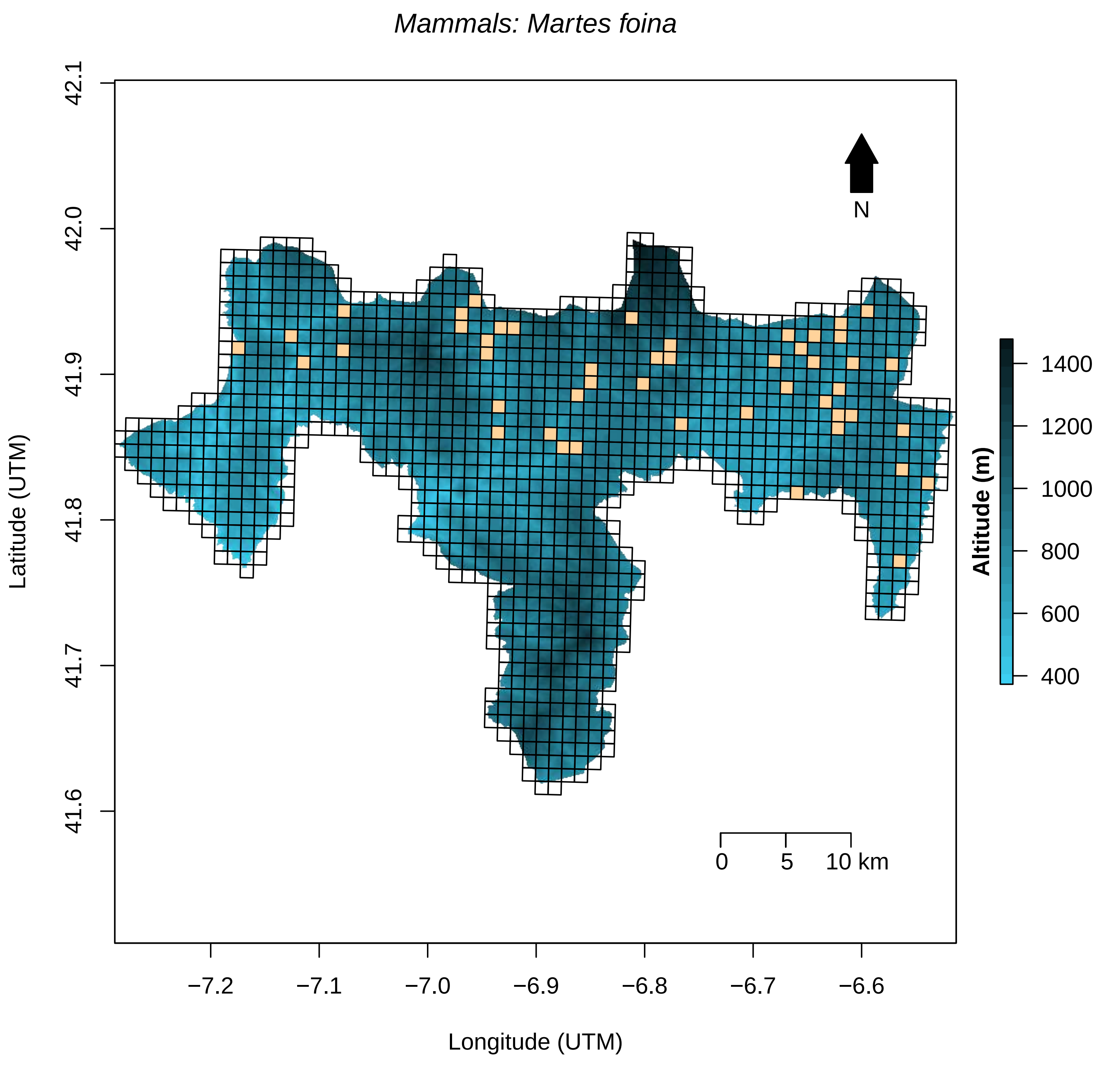

Mammals: Martes foina
42.1
42.0
41.9
41.8
41.7
41.6
Latitude (UTM)
−7.2
−7.1
−7.0
−6.9
−6.8
−6.7
−6.6
Longitude (UTM)
N
0
5
10 km
1400
1200
1000
800
600
400
Altitude (m)

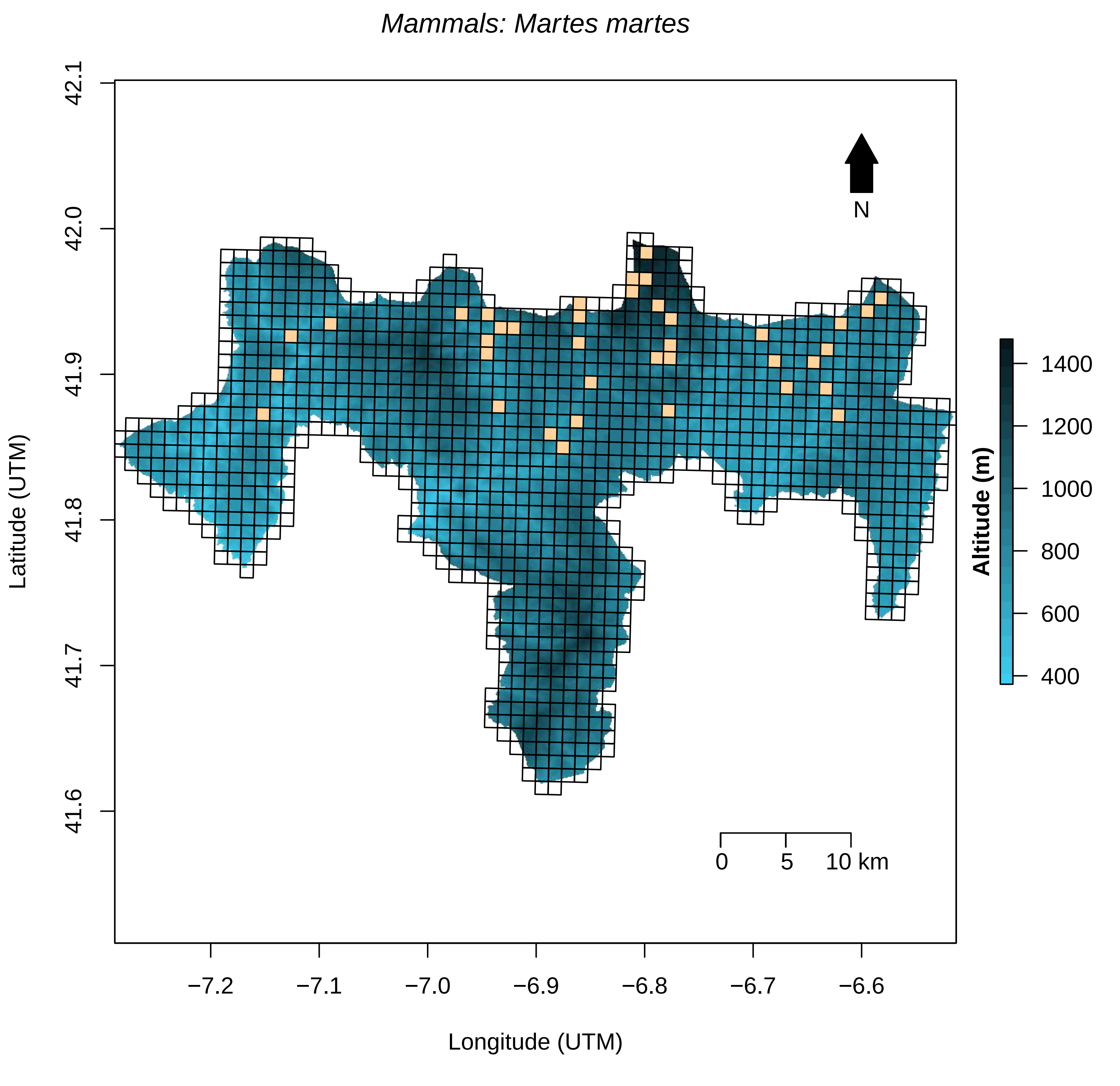

Mammals: Martes martes
42.1
42.0
41.9
41.8
41.7
41.6
Latitude (UTM)
−7.2
−7.1
−7.0
−6.9
−6.8
−6.7
−6.6
Longitude (UTM)
N
0
5
10 km
1400
1200
1000
800
600
400
Altitude (m)

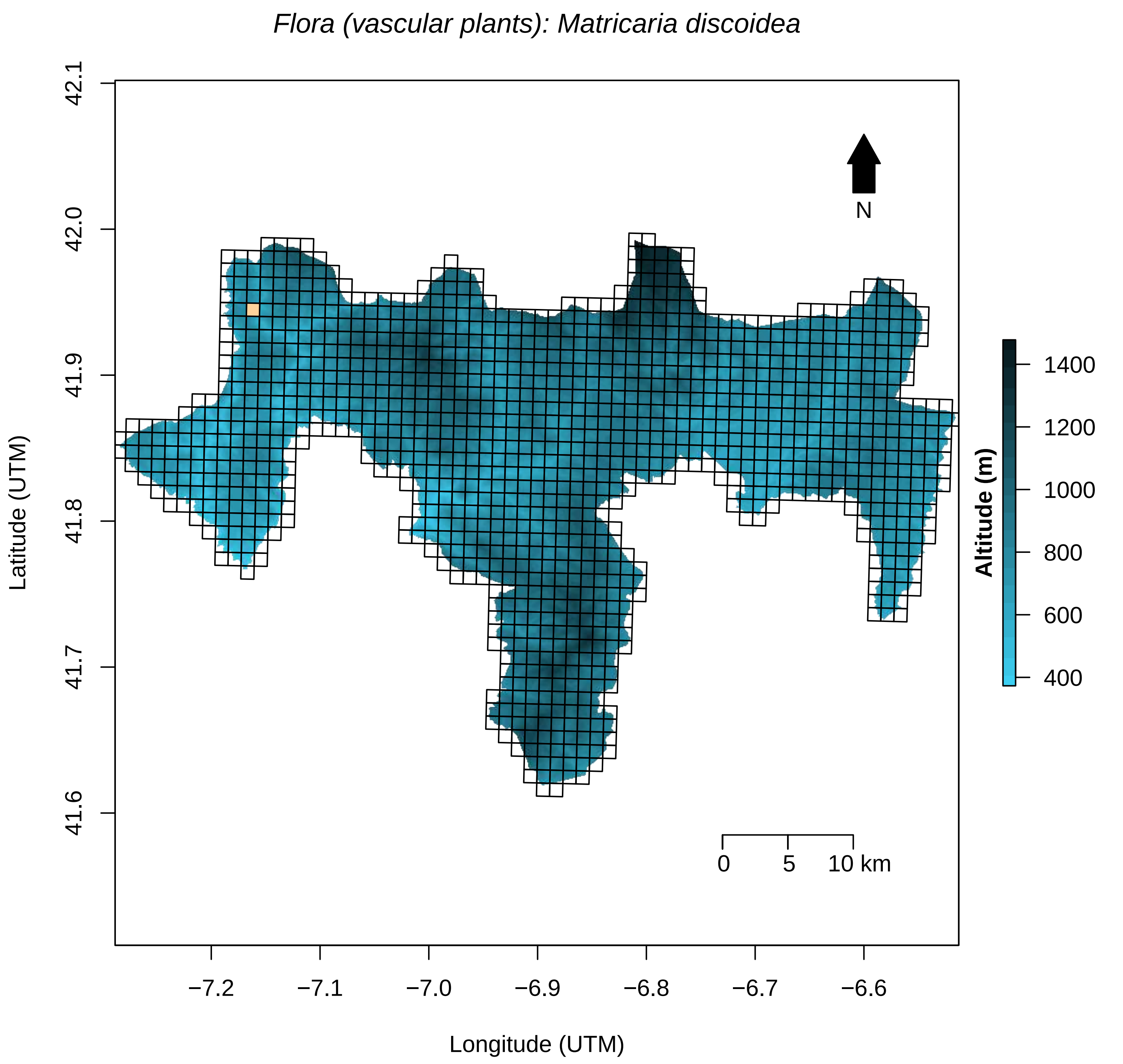

Flora (vascular plants): Matricaria discoidea
42.1
42.0
41.9
41.8
41.7
41.6
Latitude (UTM)
−7.2
−7.1
−7.0
−6.9
−6.8
−6.7
−6.6
Longitude (UTM)
N
0
5
10 km
1400
1200
1000
800
600
400
Altitude (m)

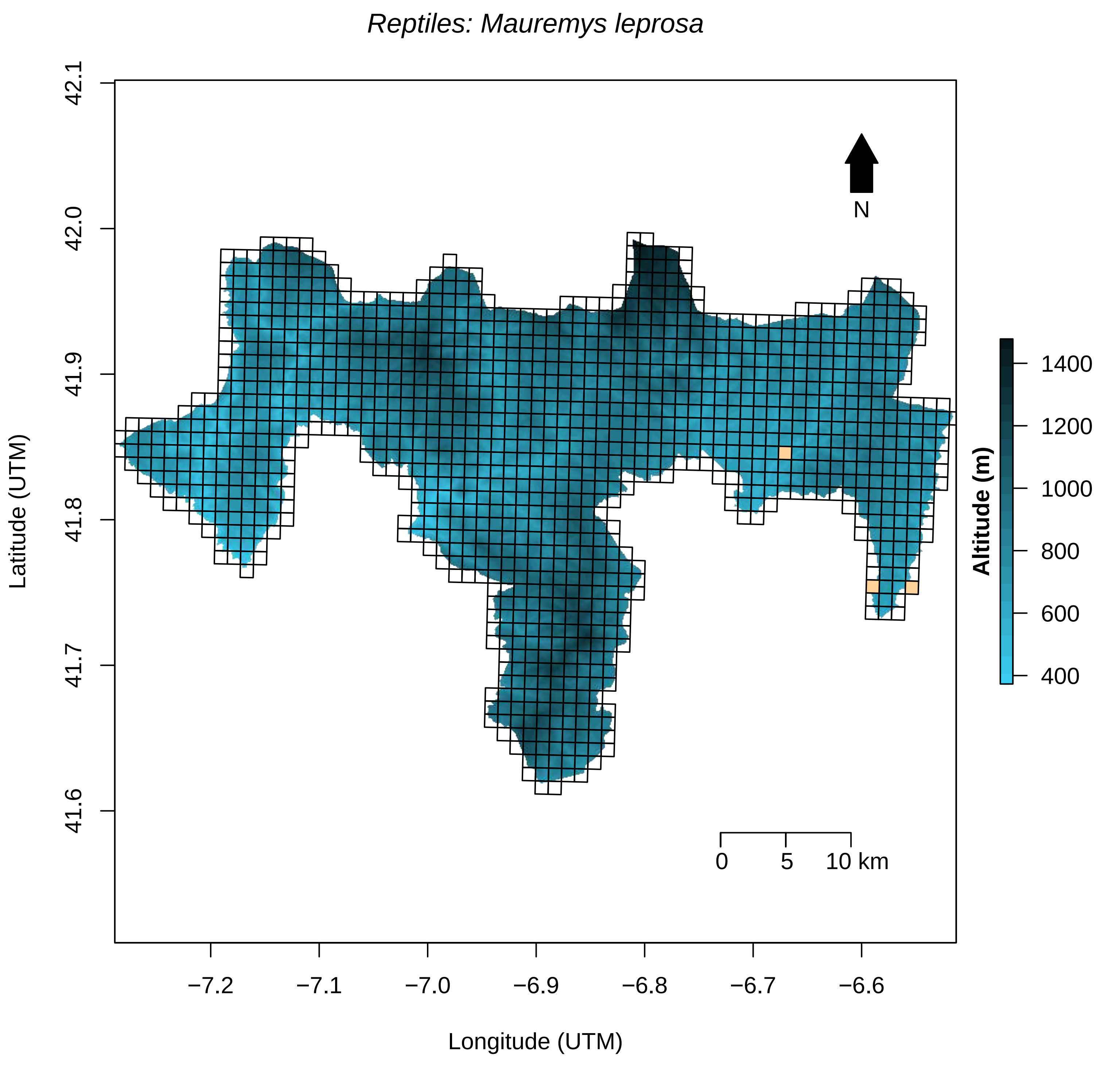
Reptiles: Mauremys leprosa
Latitude (UTM)
Longitude (UTM)
42.1
42.0
41.9
41.8
41.7
41.6
−7.2
−7.1
−7.0
−6.9
−6.8
−6.7
−6.6
N
0
5
10 km
Altitude (m)
1400
1200
1000
800
600
400

Flora (vascular plants): Medicago arabica

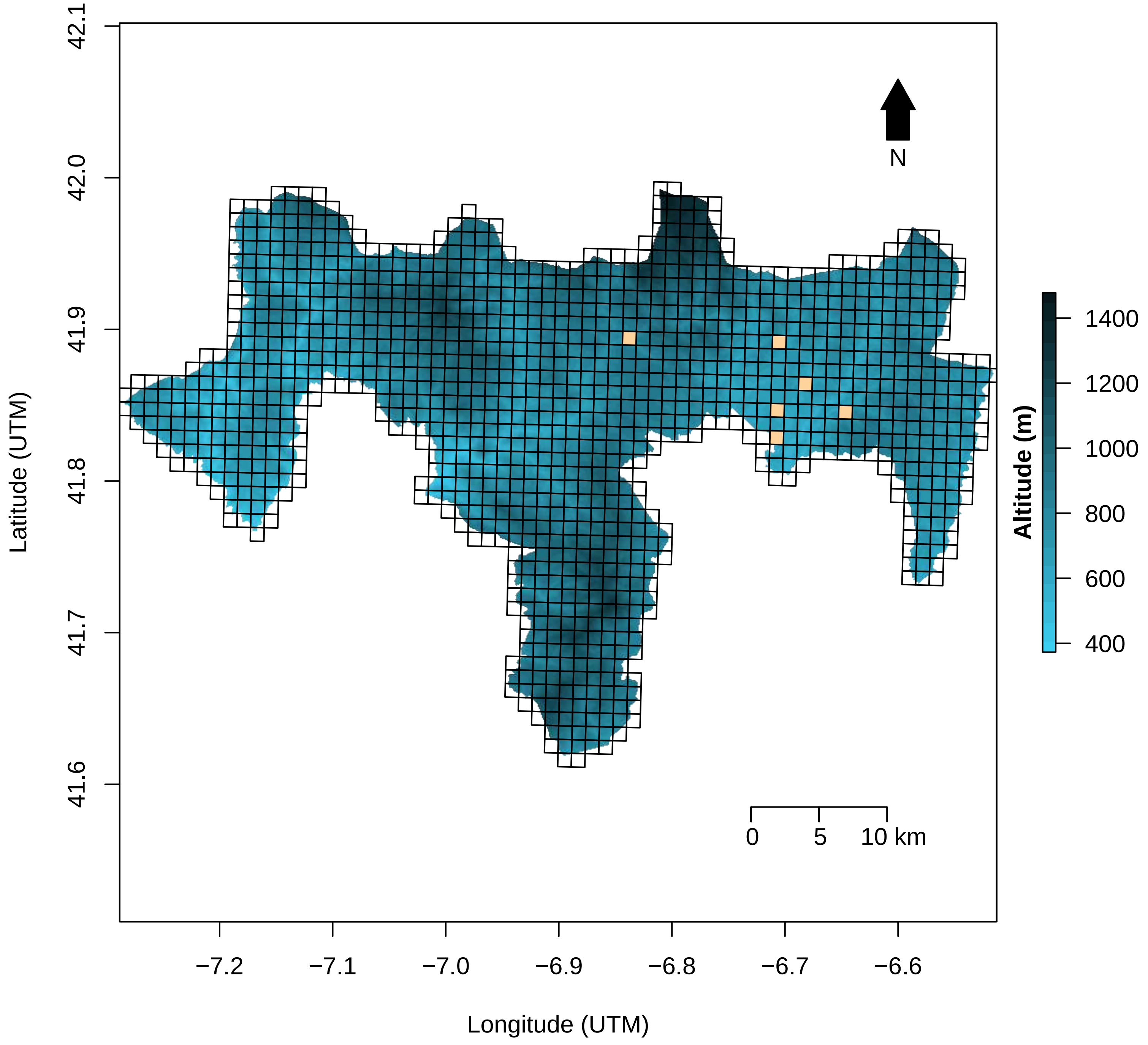

Flora (vascular plants): Medicago lupulina

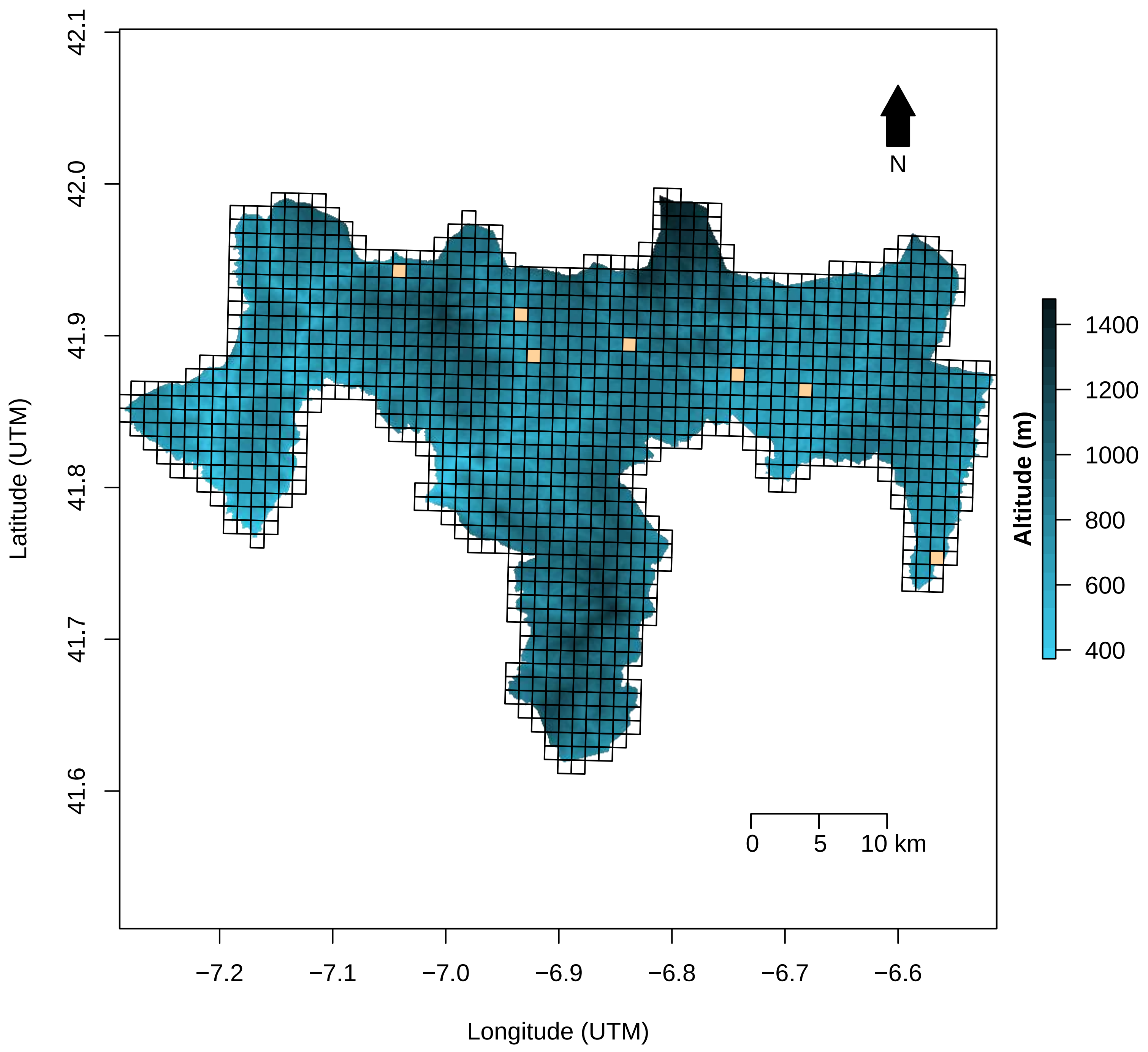

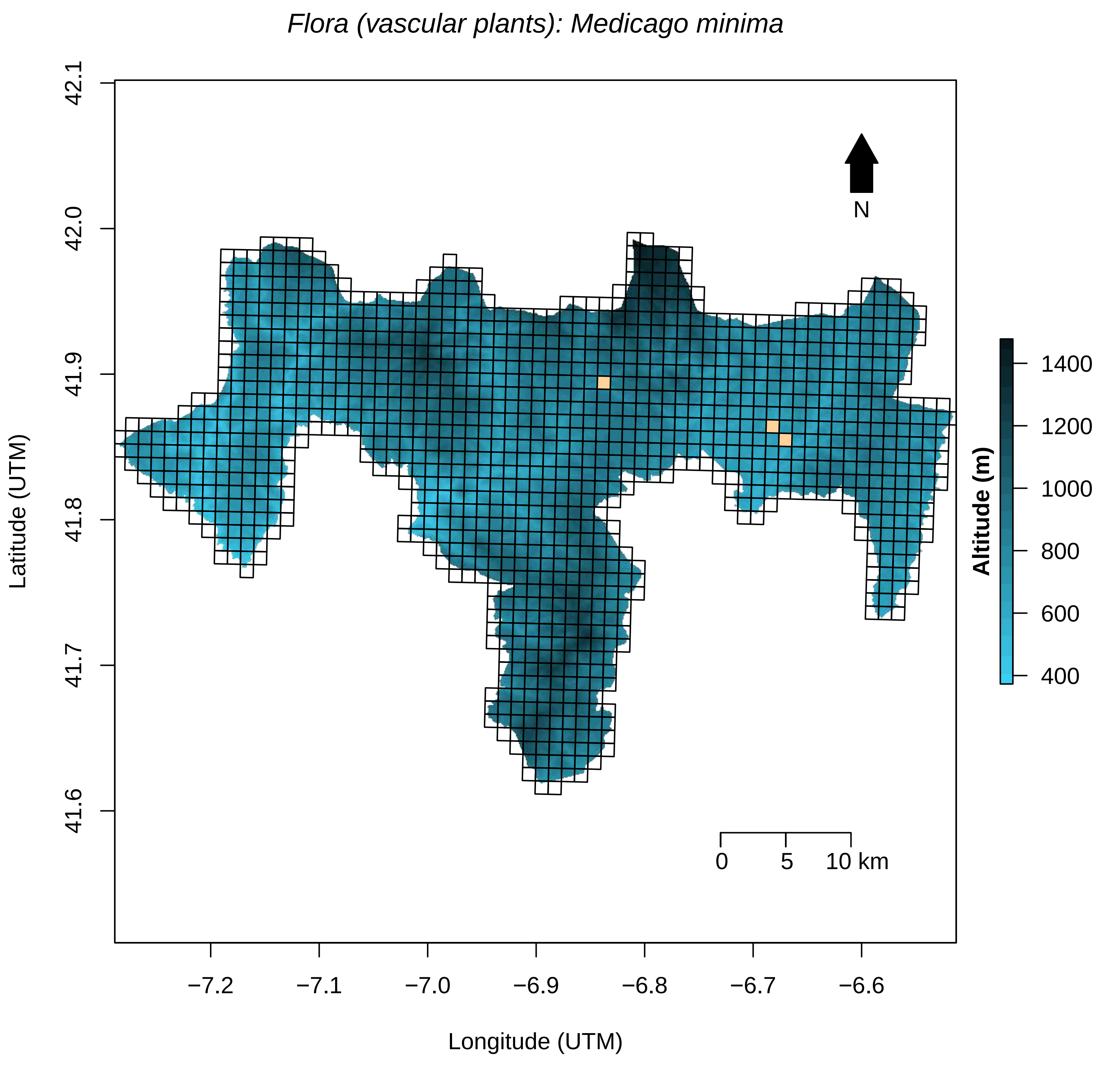

Flora (vascular plants): Medicago minima
N
0
5
10 km
Altitude (m)
1400
1200
1000
800
600
400
42.1
42.0
41.9
41.8
41.7
41.6
Latitude (UTM)
−7.2
−7.1
−7.0
−6.9
−6.8
−6.7
−6.6
Longitude (UTM)

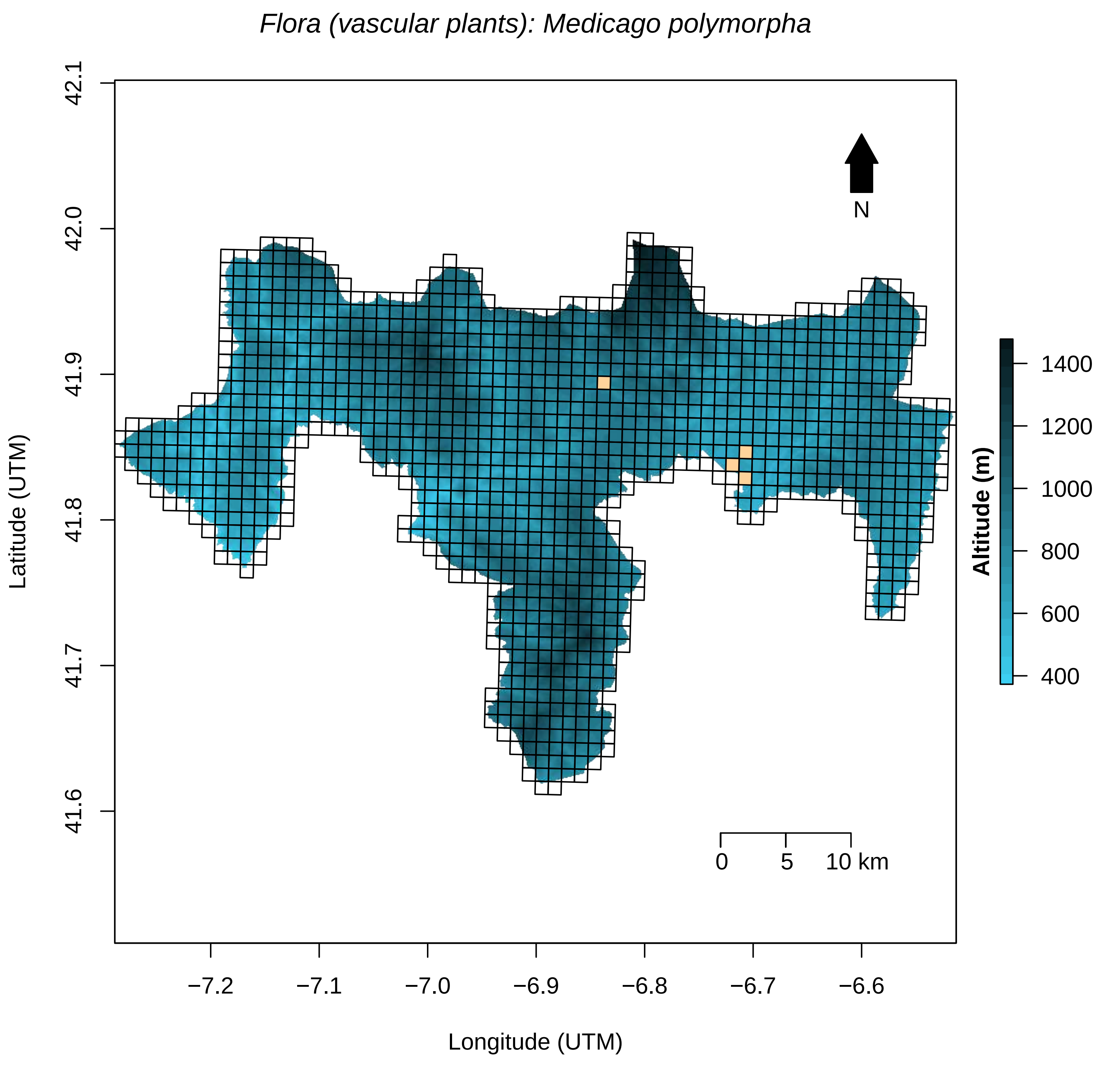

Flora (vascular plants): Medicago polymorpha
Latitude (UTM)
42.1
42.0
41.9
41.8
41.7
41.6
Longitude (UTM)
−7.2
−7.1
−7.0
−6.9
−6.8
−6.7
−6.6
N
0
5
10 km
Altitude (m)
1400
1200
1000
800
600
400

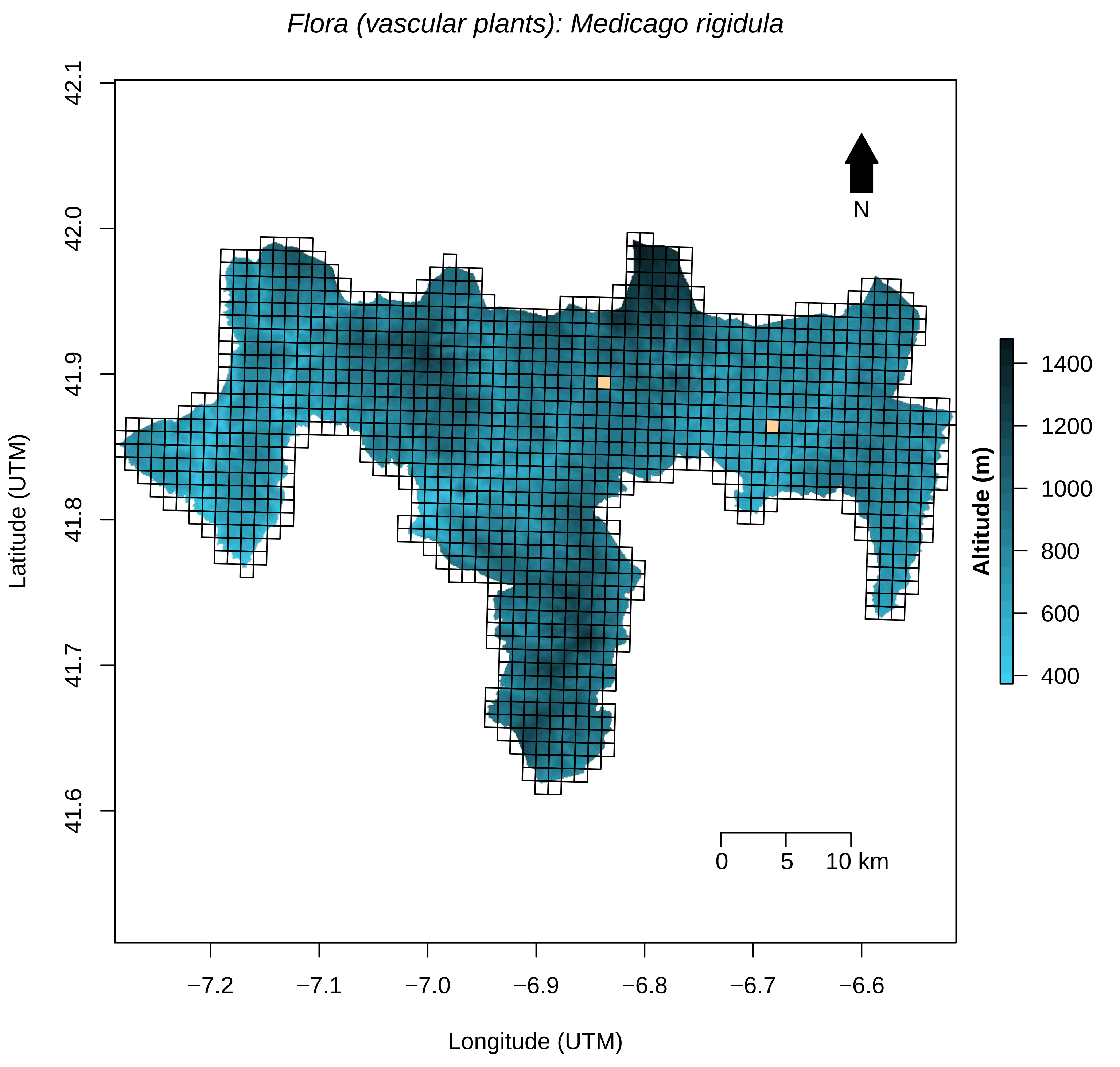

Flora (vascular plants): Medicago rigidula
42.1
42.0
41.9
41.8
41.7
41.6
Latitude (UTM)
−7.2
−7.1
−7.0
−6.9
−6.8
−6.7
−6.6
Longitude (UTM)
N
0
5
10 km
1400
1200
1000
800
600
400
Altitude (m)

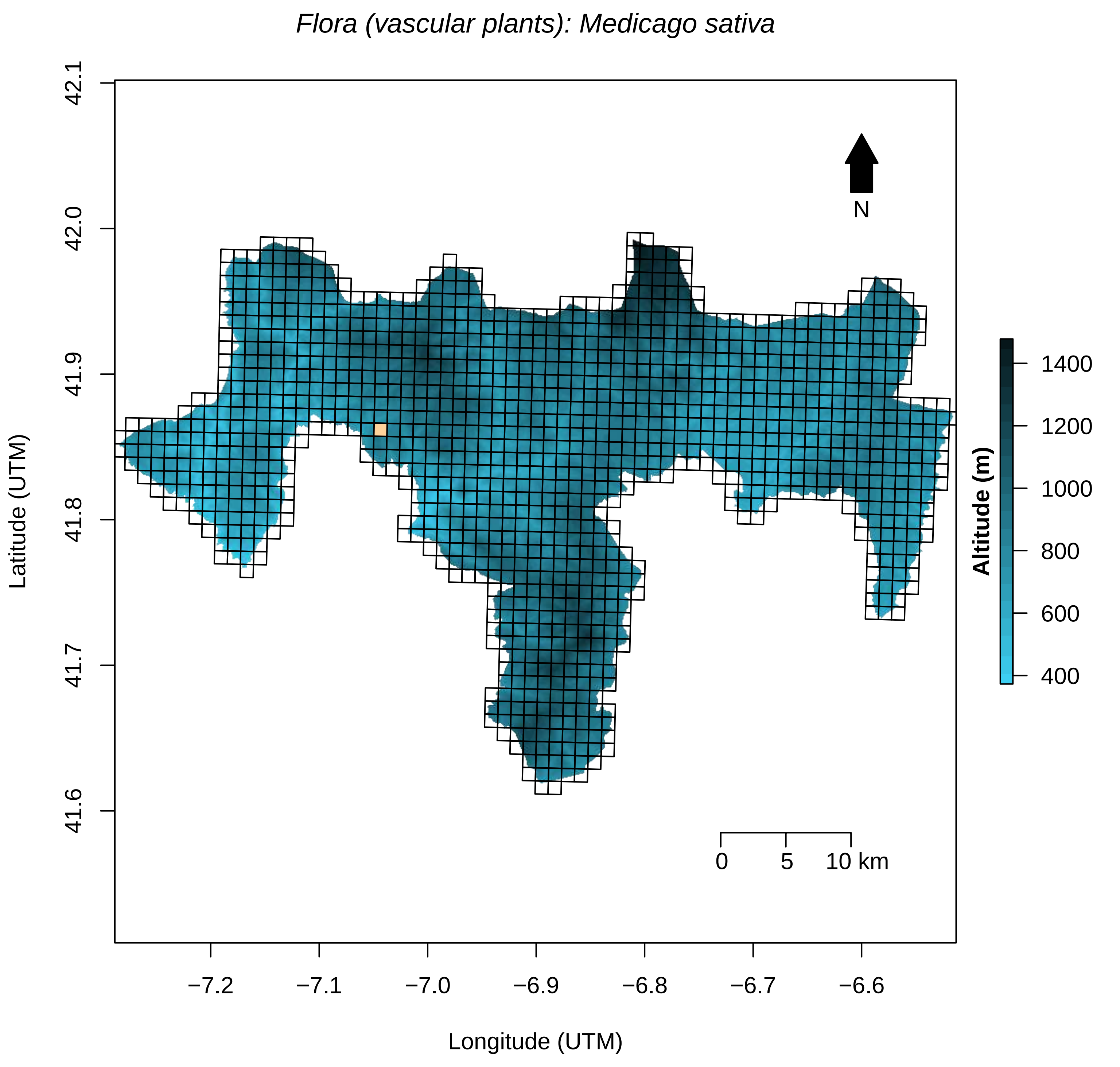

Flora (vascular plants): Medicago sativa
Latitude (UTM)
42.1
42.0
41.9
41.8
41.7
41.6
Longitude (UTM)
−7.2
−7.1
−7.0
−6.9
−6.8
−6.7
−6.6
N
0
5
10 km
Altitude (m)
1400
1200
1000
800
600
400

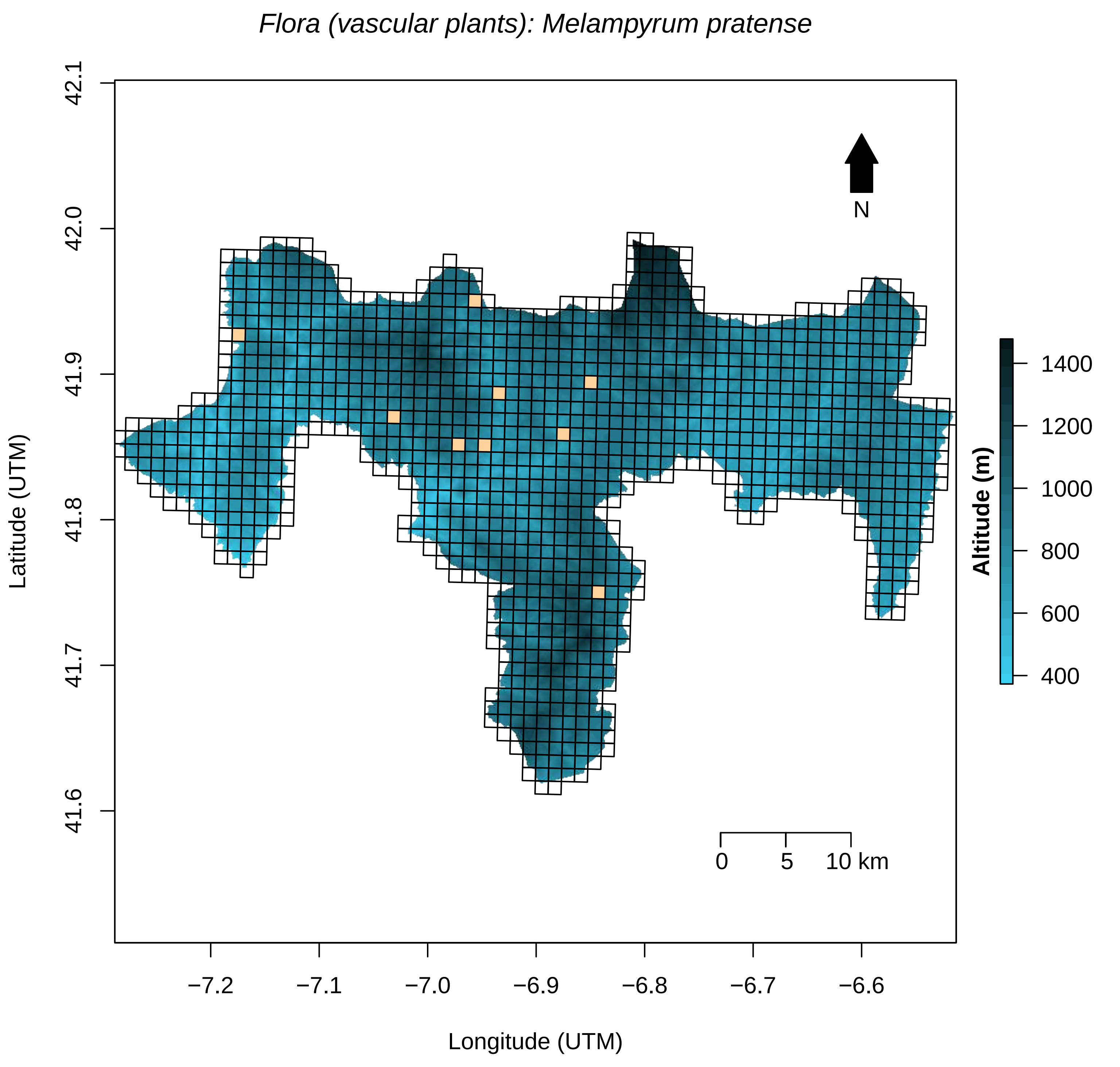

Flora (vascular plants): Melampyrum pratense
42.1
42.0
41.9
41.8
41.7
41.6
Latitude (UTM)
−7.2
−7.1
−7.0
−6.9
−6.8
−6.7
−6.6
Longitude (UTM)
N
0
5
10 km
Altitude (m)
1400
1200
1000
800
600
400

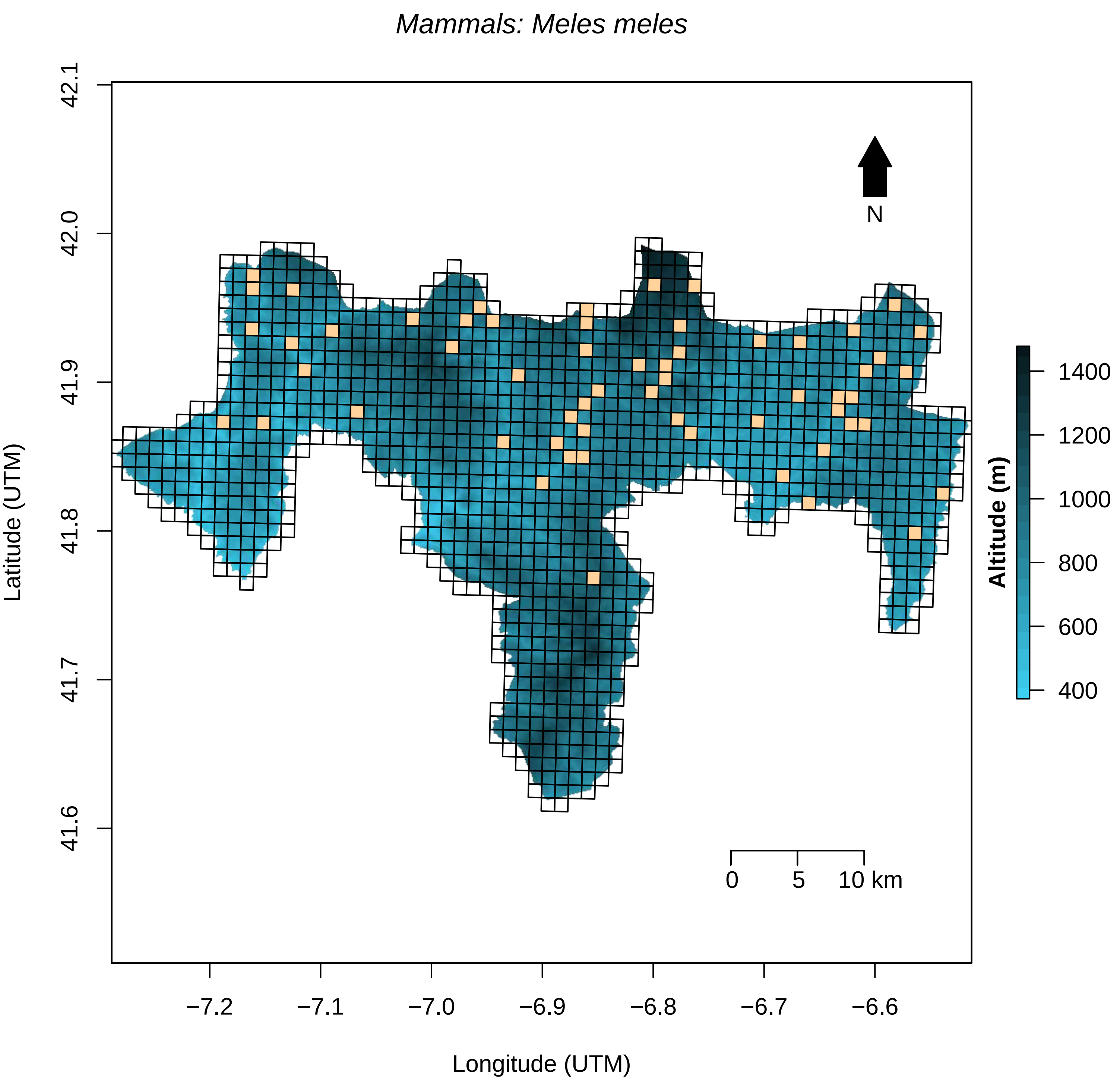

Mammals: Meles meles
42.1
42.0
41.9
41.8
41.7
41.6
Latitude (UTM)
−7.2
−7.1
−7.0
−6.9
−6.8
−6.7
−6.6
Longitude (UTM)
N
0
5
10 km
Altitude (m)
1400
1200
1000
800
600
400

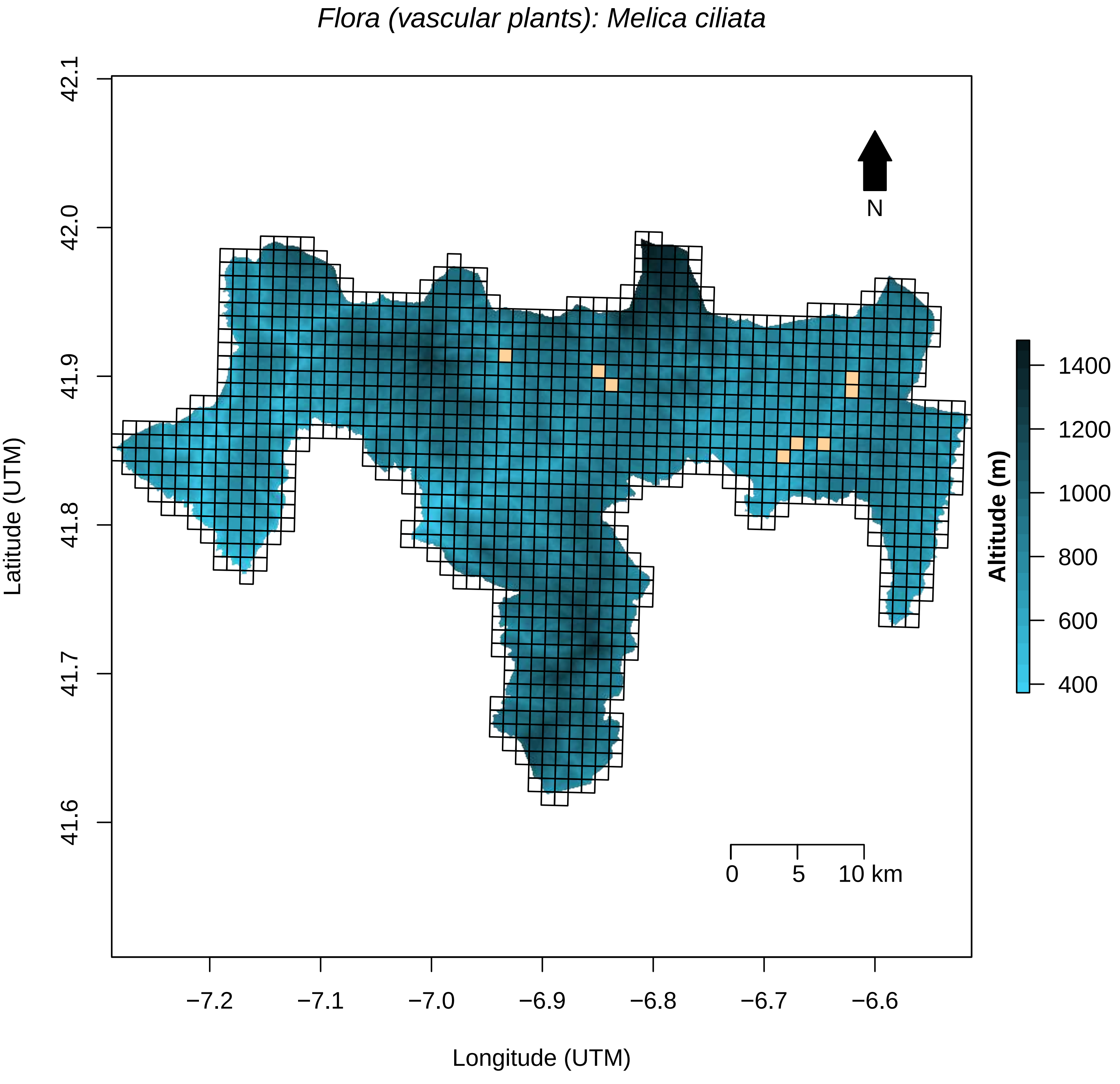

Flora (vascular plants): Melica ciliata
42.1
42.0
41.9
41.8
41.7
41.6
Latitude (UTM)
−7.2
−7.1
−7.0
−6.9
−6.8
−6.7
−6.6
Longitude (UTM)
N
0
5
10 km
1400
1200
1000
800
600
400
Altitude (m)

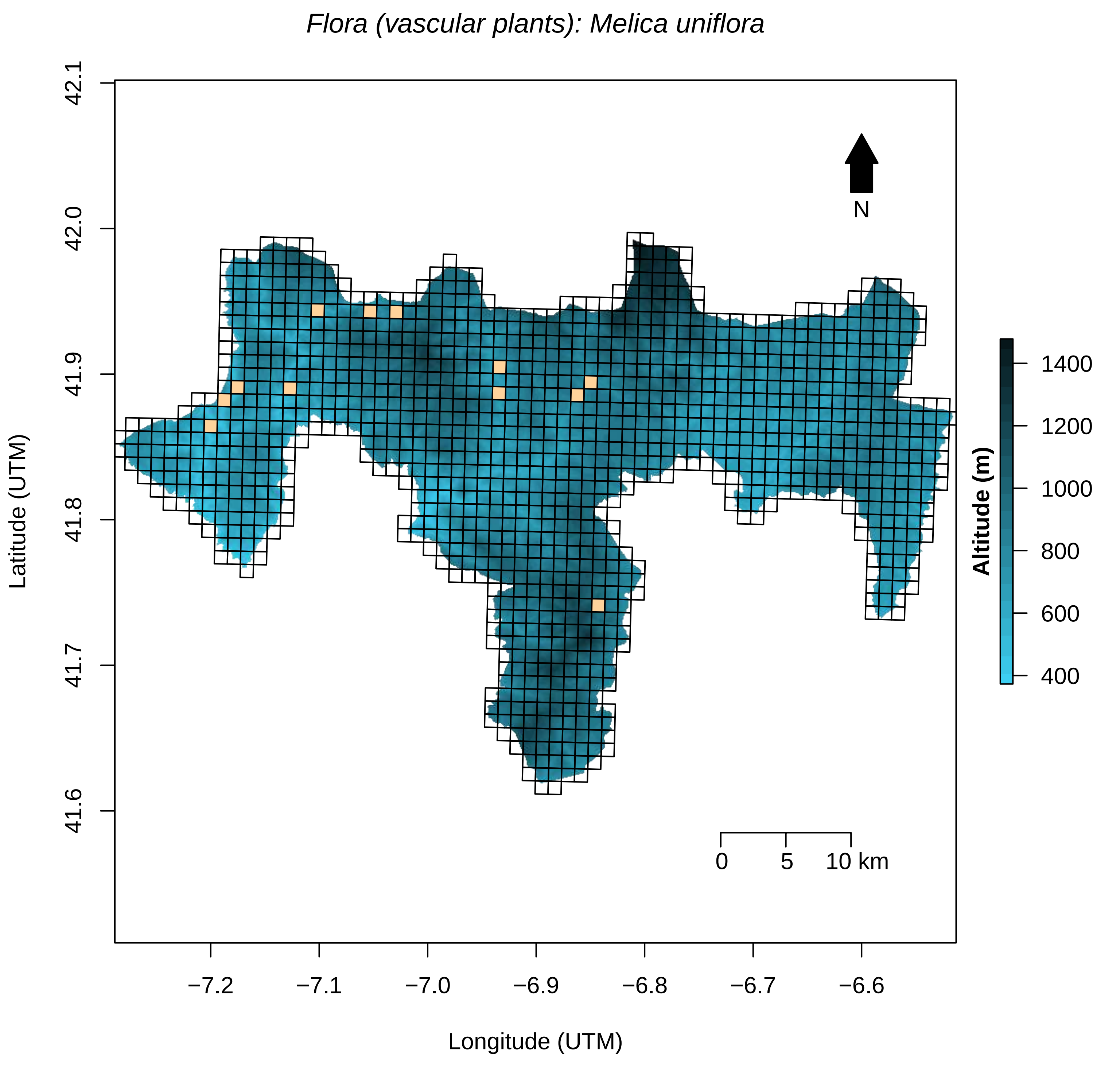

Flora (vascular plants): Melica uniflora
Latitude (UTM)
42.1
42.0
41.9
41.8
41.7
41.6
N
0
5
10 km
Altitude (m)
1400
1200
1000
800
600
400
−7.2
−7.1
−7.0
−6.9
−6.8
−6.7
−6.6
Longitude (UTM)

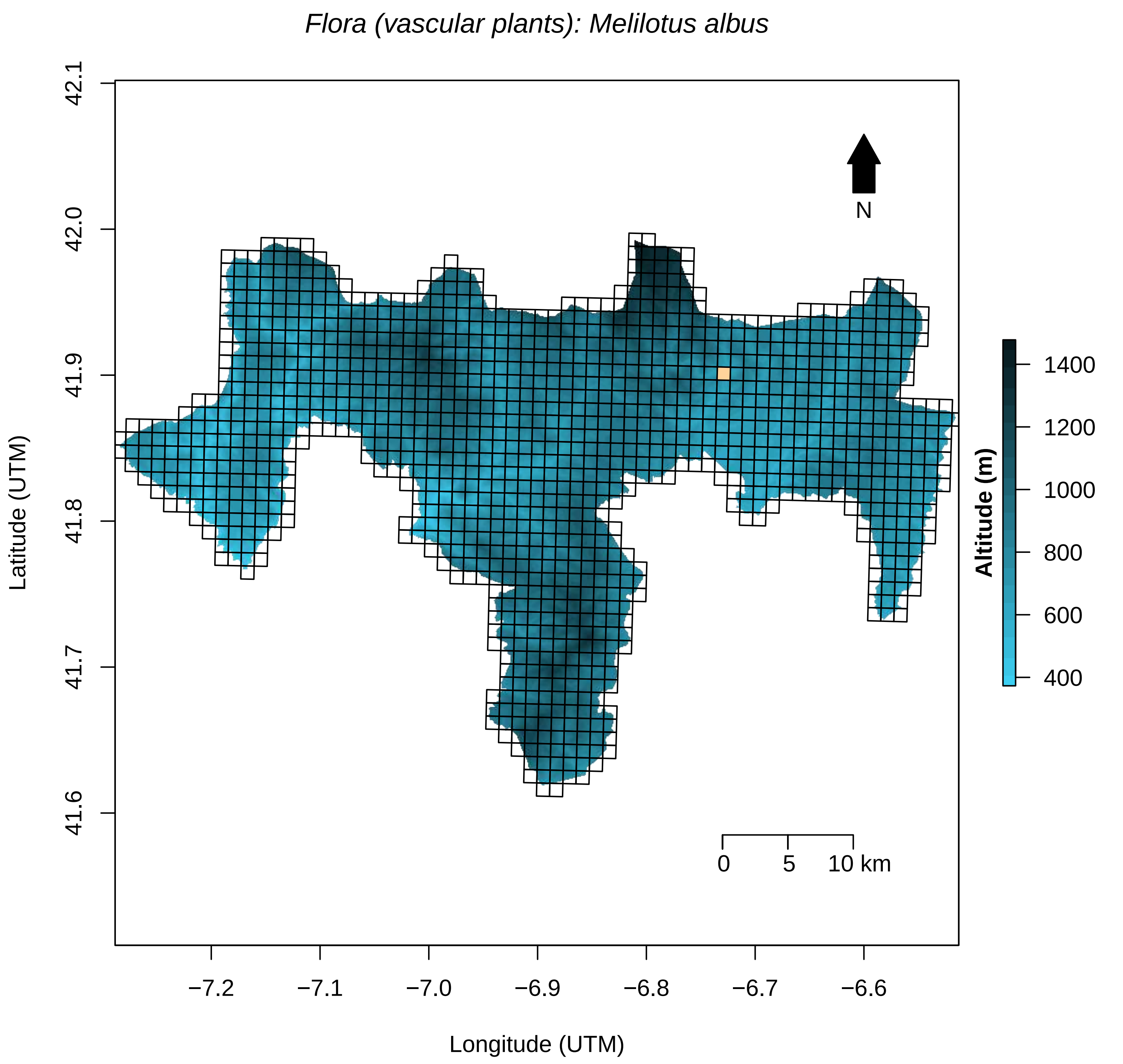

Flora (vascular plants): Melilotus albus
42.1
42.0
41.9
41.8
41.7
41.6
Latitude (UTM)
−7.2
−7.1
−7.0
−6.9
−6.8
−6.7
−6.6
Longitude (UTM)
N
0
5
10 km
Altitude (m)
1400
1200
1000
800
600
400

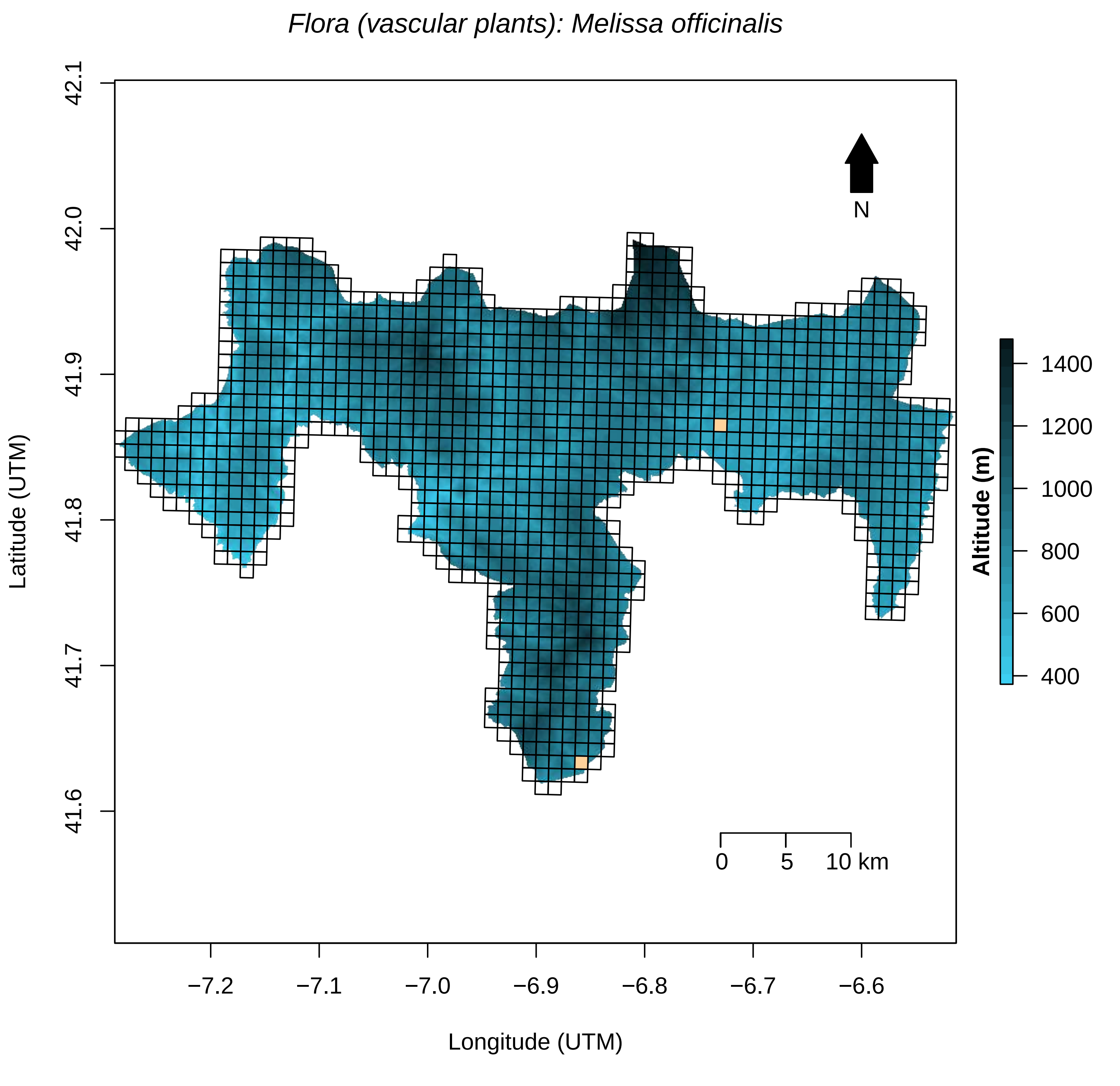

Flora (vascular plants): Melissa officinalis
42.1
42.0
41.9
41.8
41.7
41.6
Latitude (UTM)
−7.2
−7.1
−7.0
−6.9
−6.8
−6.7
−6.6
Longitude (UTM)
N
0
5
10 km
1400
1200
1000
800
600
400
Altitude (m)

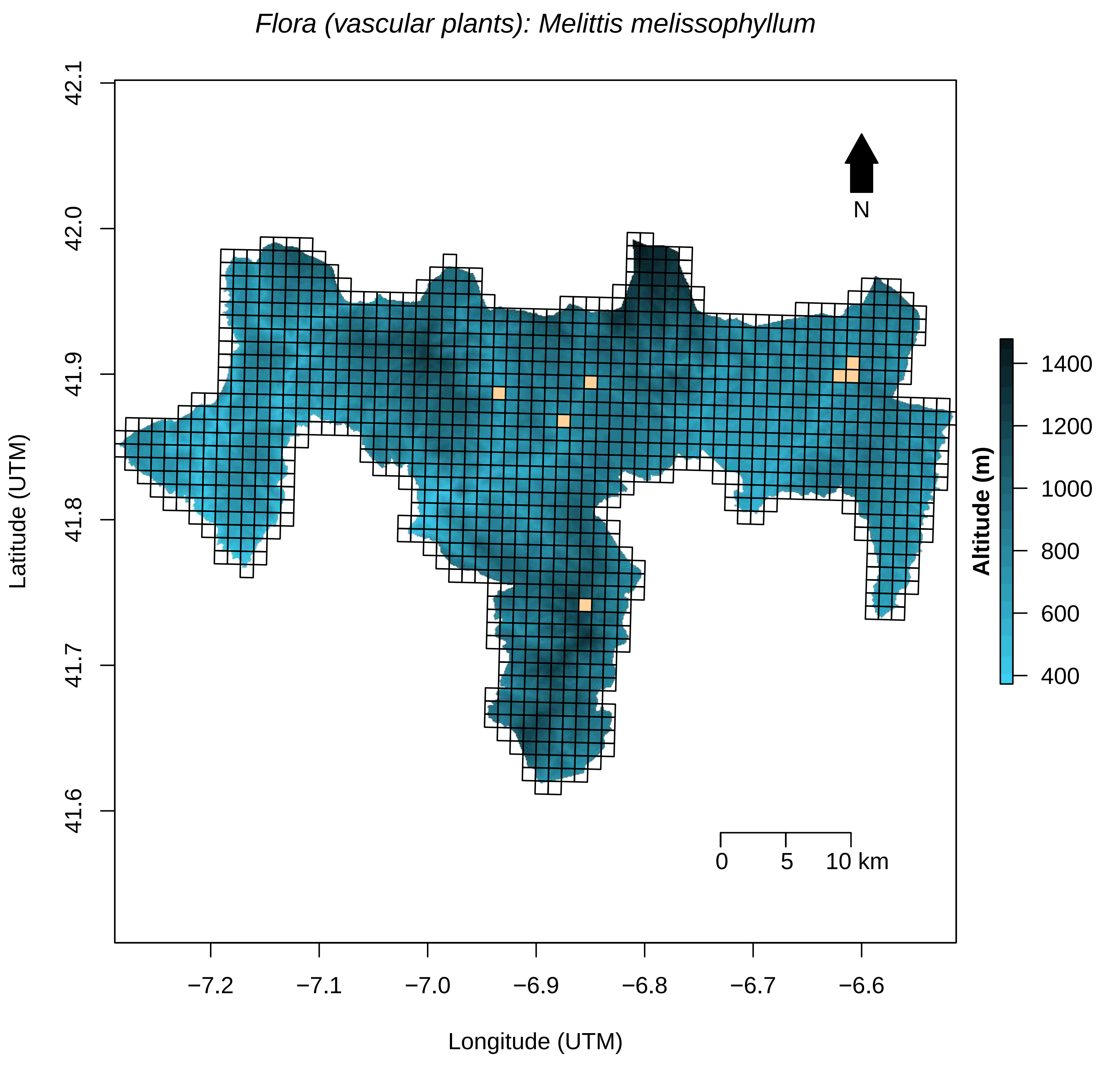

Flora (vascular plants): Melittis melissophyllum
N
0
5
10 km
Altitude (m)
1400
1200
1000
800
600
400
Latitude (UTM)
42.1
42.0
41.9
41.8
41.7
41.6
Longitude (UTM)
−7.2
−7.1
−7.0
−6.9
−6.8
−6.7
−6.6

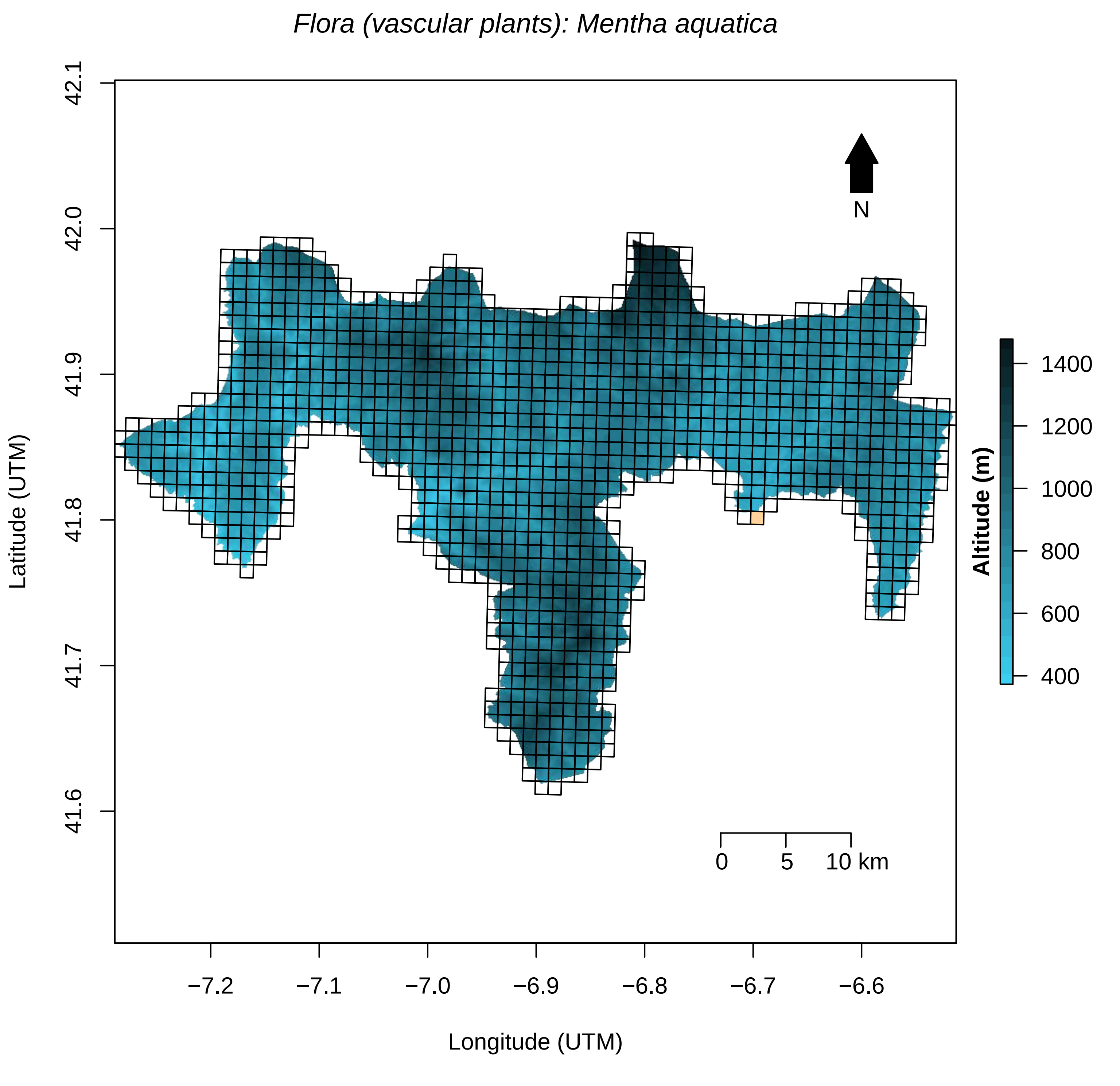

Flora (vascular plants): Mentha aquatica
Latitude (UTM)
Longitude (UTM)
42.1
42.0
41.9
41.8
41.7
41.6
−7.2
−7.1
−7.0
−6.9
−6.8
−6.7
−6.6
N
0
5
10 km
Altitude (m)
1400
1200
1000
800
600
400

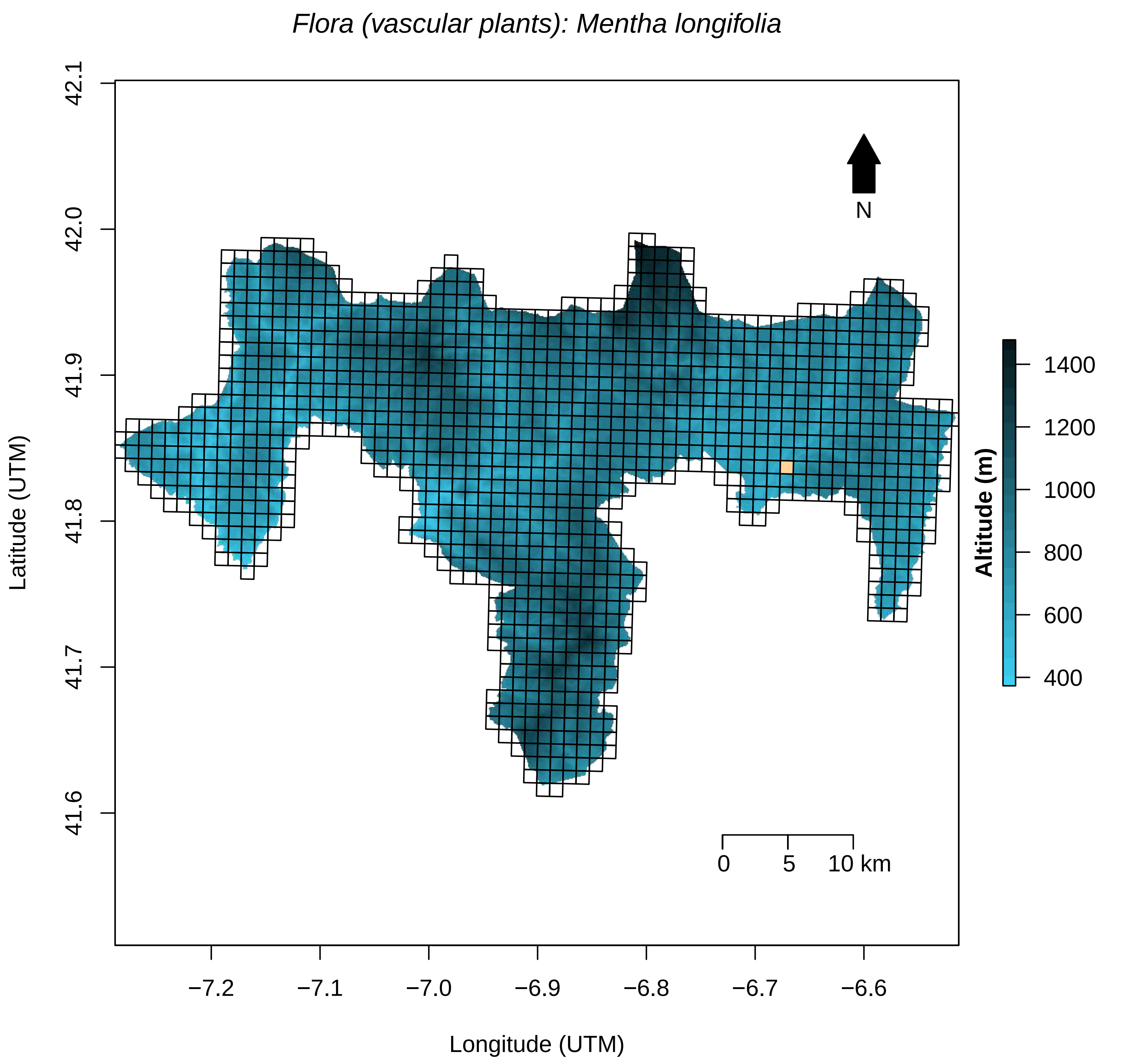

Flora (vascular plants): Mentha longifolia
42.1
42.0
41.9
41.8
41.7
41.6
Latitude (UTM)
−7.2
−7.1
−7.0
−6.9
−6.8
−6.7
−6.6
Longitude (UTM)
N
0
5
10 km
Altitude (m)
1400
1200
1000
800
600
400

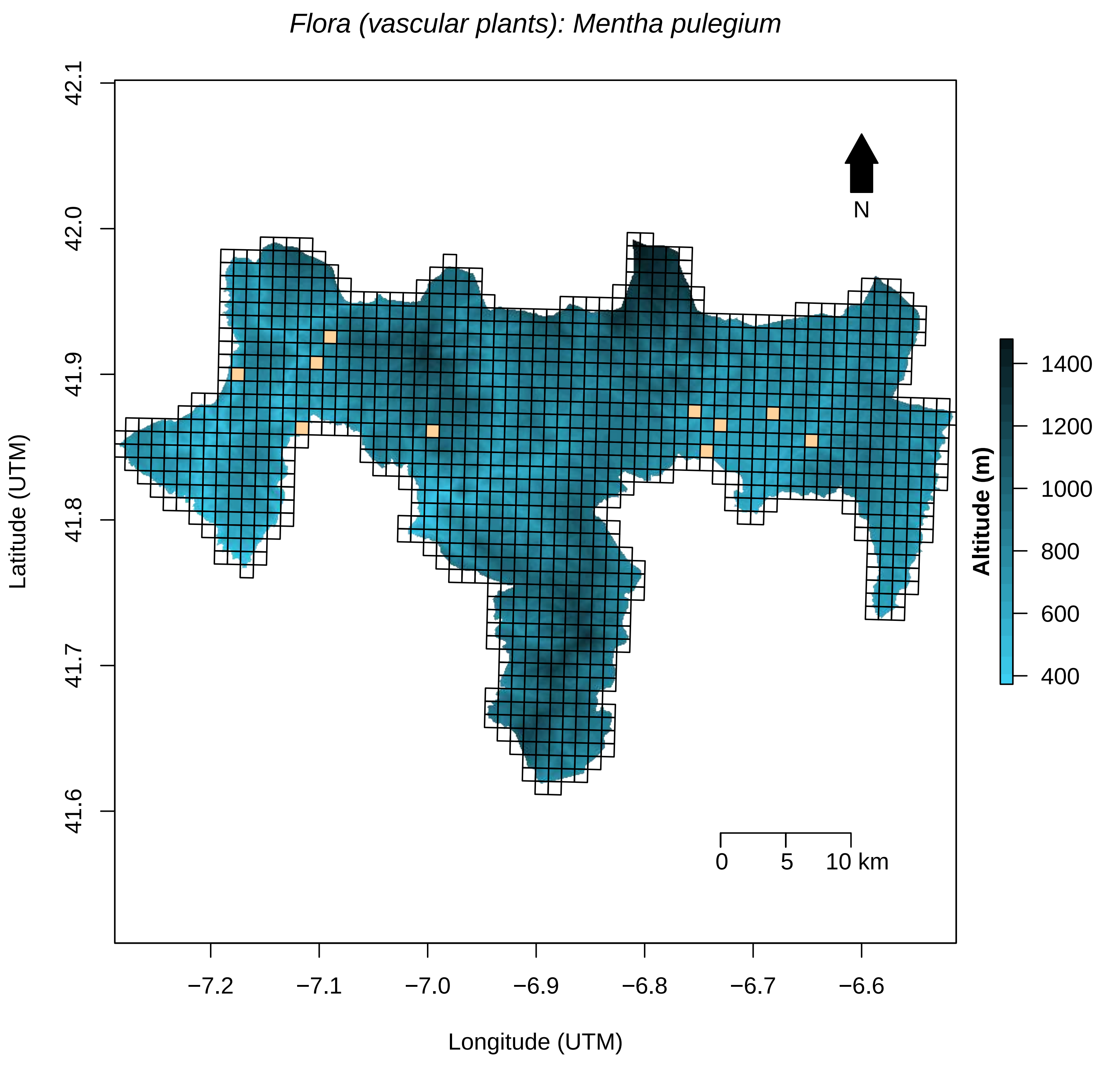

Flora (vascular plants): Mentha pulegium
42.1
42.0
41.9
41.8
41.7
41.6
Latitude (UTM)
−7.2
−7.1
−7.0
−6.9
−6.8
−6.7
−6.6
Longitude (UTM)
N
0
5
10 km
Altitude (m)
1400
1200
1000
800
600
400

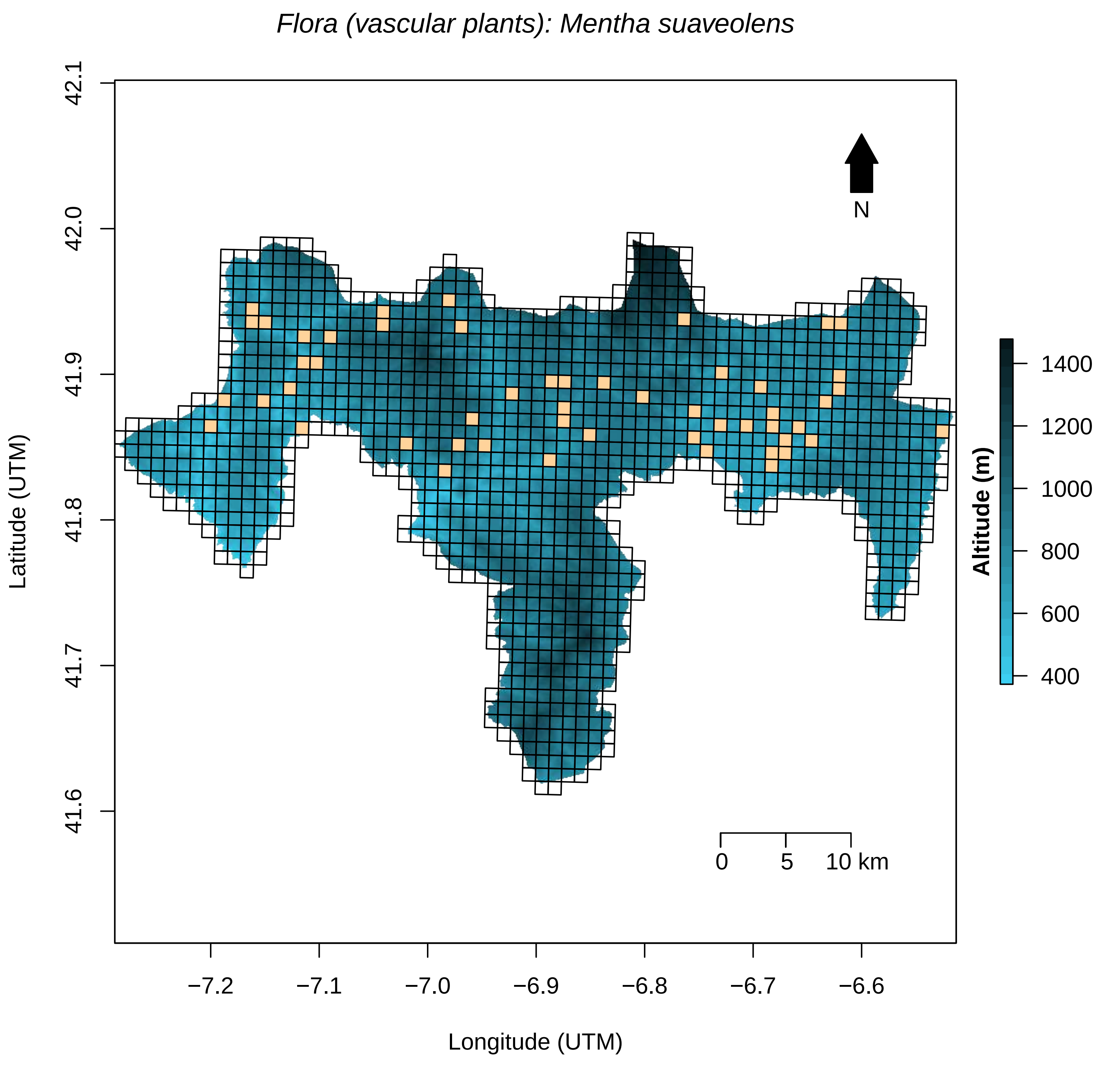

Flora (vascular plants): Mentha suaveolens
N
0 5 10 km
Altitude (m)
1400
1200
1000
800
600
400
Latitude (UTM)
42.1
42.0
41.9
41.8
41.7
41.6
Longitude (UTM)
−7.2
−7.1
−7.0
−6.9
−6.8
−6.7
−6.6

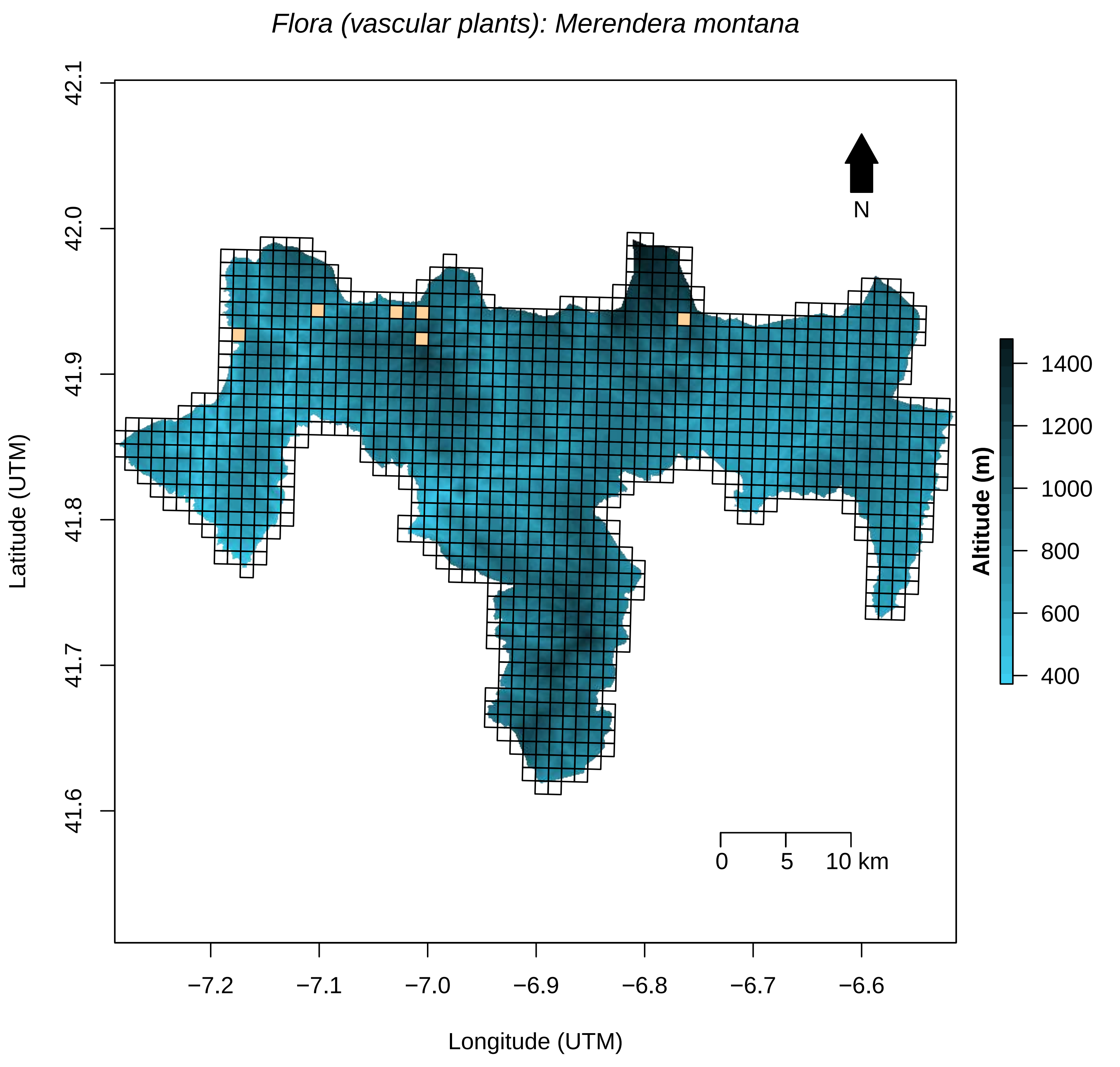

Flora (vascular plants): Merendera montana
42.1
42.0
41.9
41.8
41.7
41.6
Latitude (UTM)
−7.2
−7.1
−7.0
−6.9
−6.8
−6.7
−6.6
Longitude (UTM)
N
0
5
10 km
Altitude (m)
1400
1200
1000
800
600
400

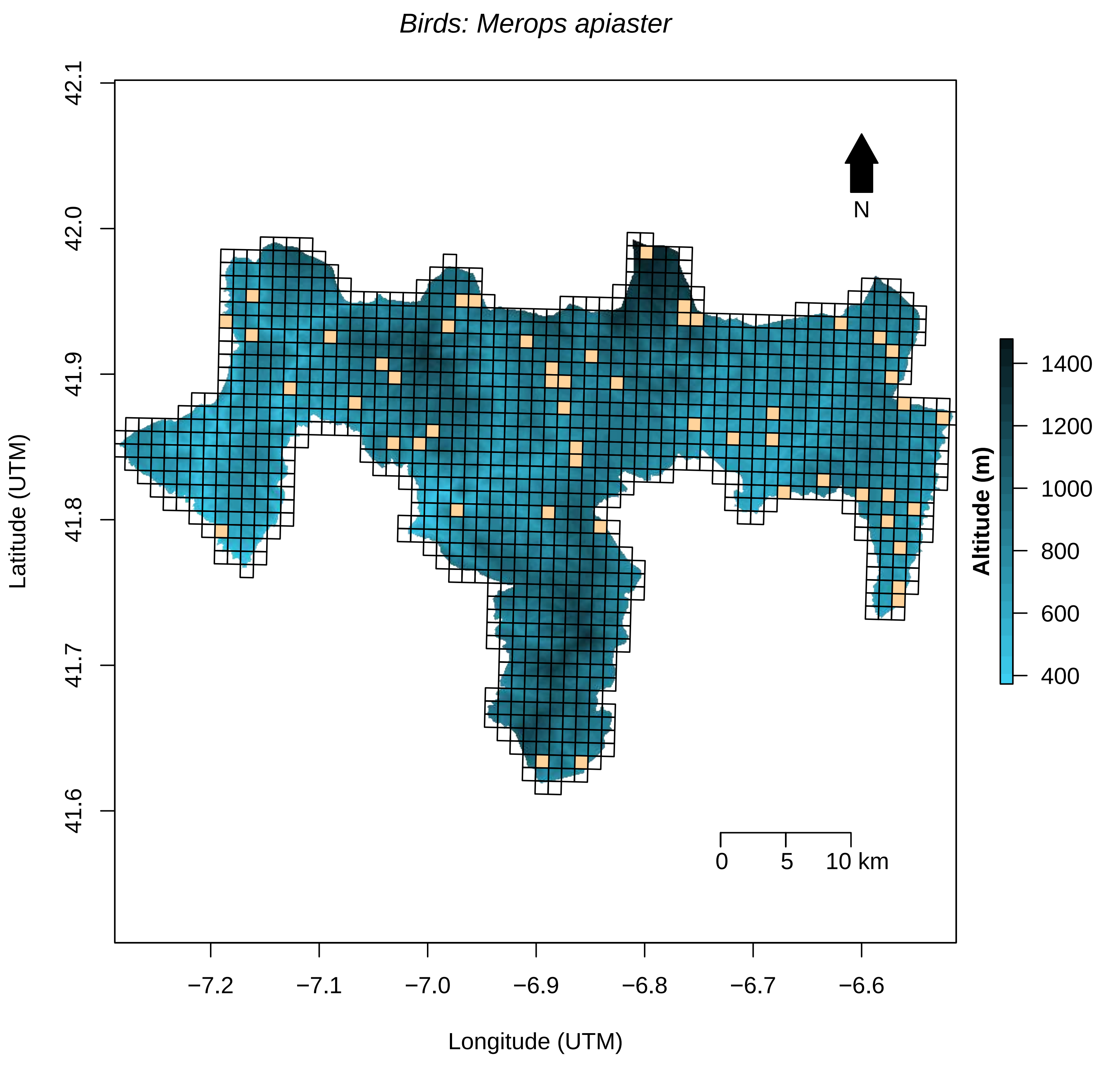

Birds: Merops apiaster
42.1
42.0
41.9
41.8
41.7
41.6
Latitude (UTM)
−7.2
−7.1
−7.0
−6.9
−6.8
−6.7
−6.6
Longitude (UTM)
N
0
5
10 km
1400
1200
1000
800
600
400
Altitude (m)

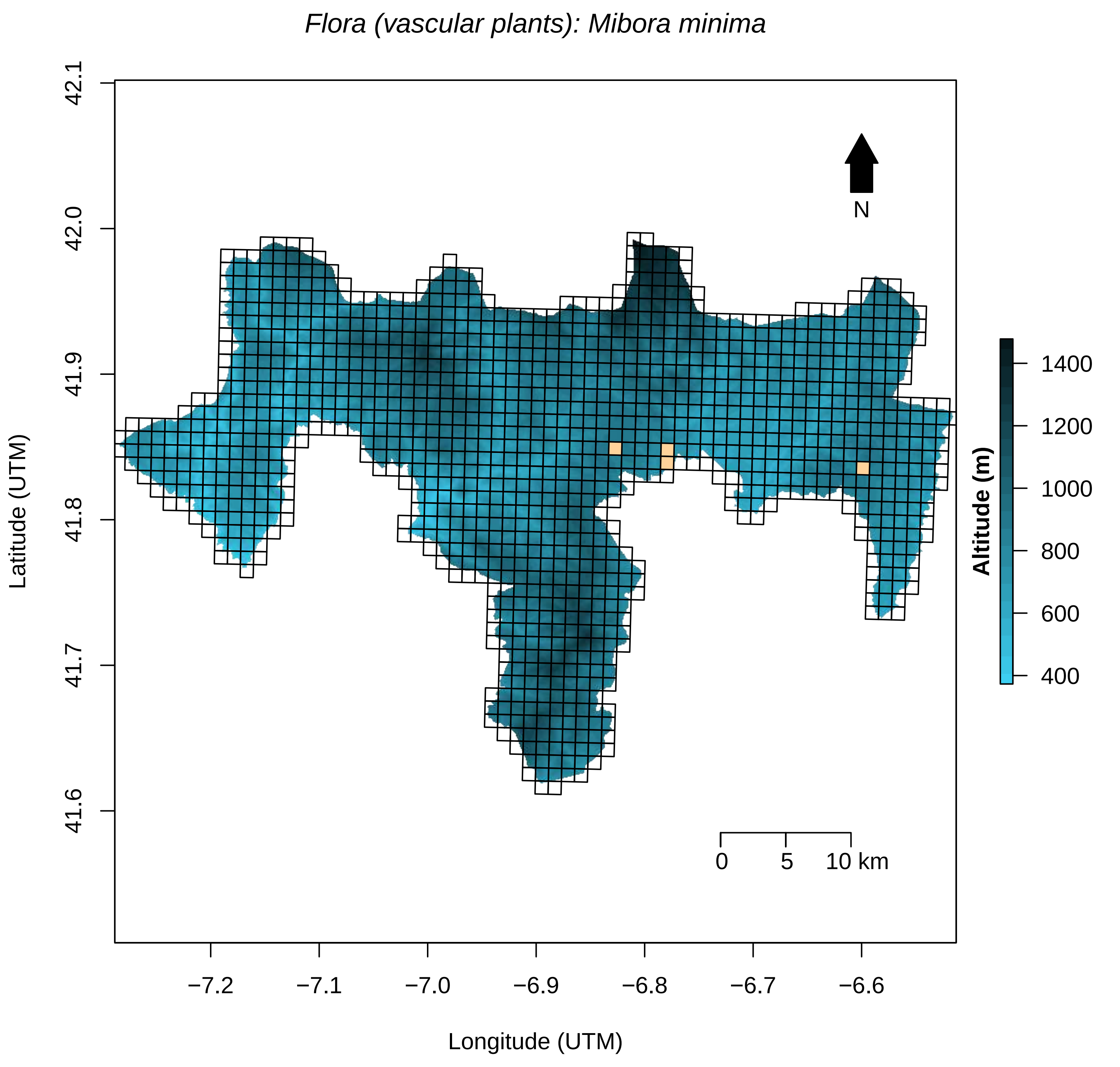

Flora (vascular plants): Mibora minima
42.1
42.0
41.9
41.8
41.7
41.6
Latitude (UTM)
−7.2
−7.1
−7.0
−6.9
−6.8
−6.7
−6.6
Longitude (UTM)
N
0
5
10 km
Altitude (m)
1400
1200
1000
800
600
400

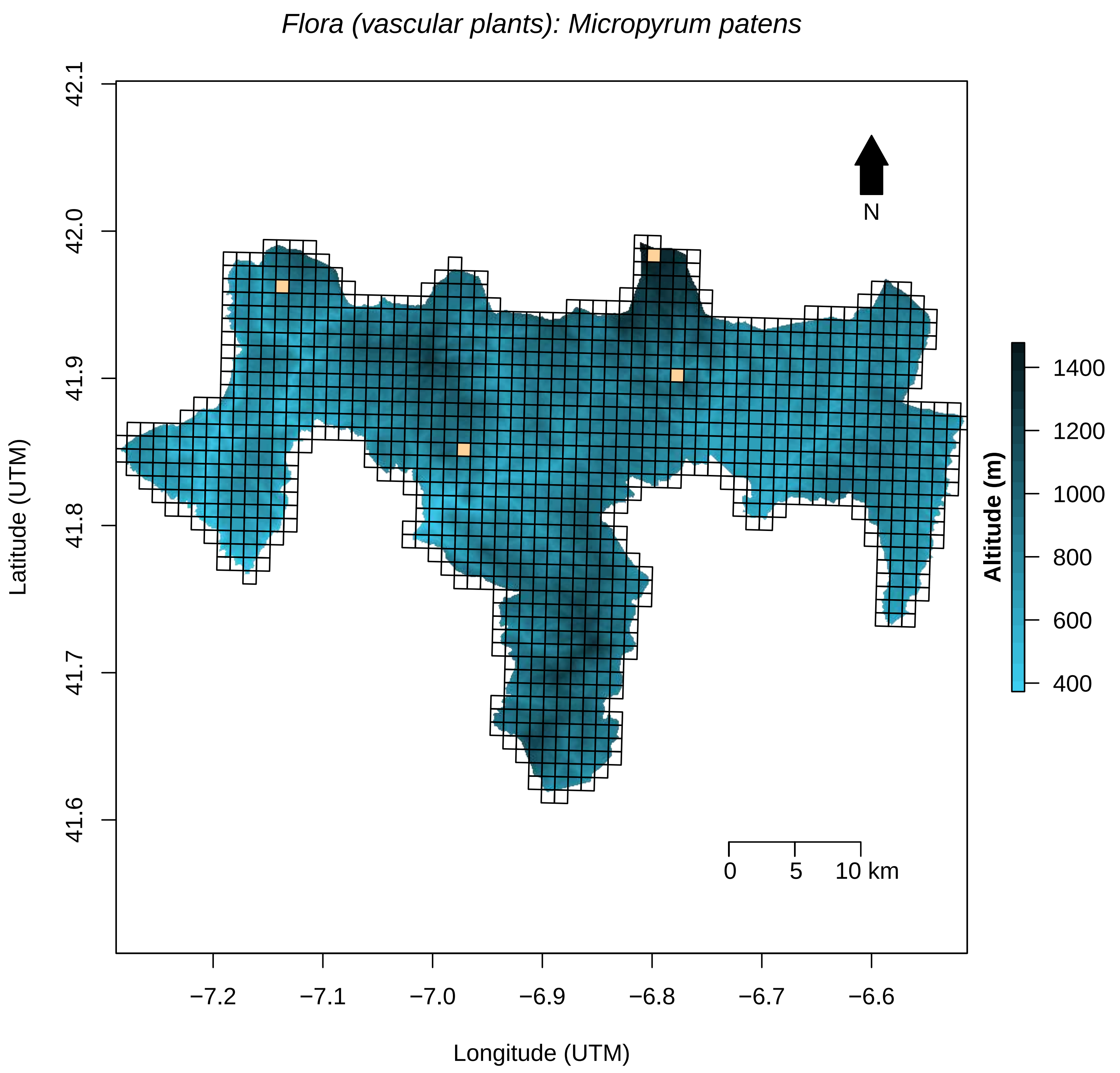
Flora (vascular plants): Micropyrum patens
Latitude (UTM)
42.1
42.0
41.9
41.8
41.7
41.6
Longitude (UTM)
−7.2
−7.1
−7.0
−6.9
−6.8
−6.7
−6.6
N
0
5
10 km
Altitude (m)
1400
1200
1000
800
600
400

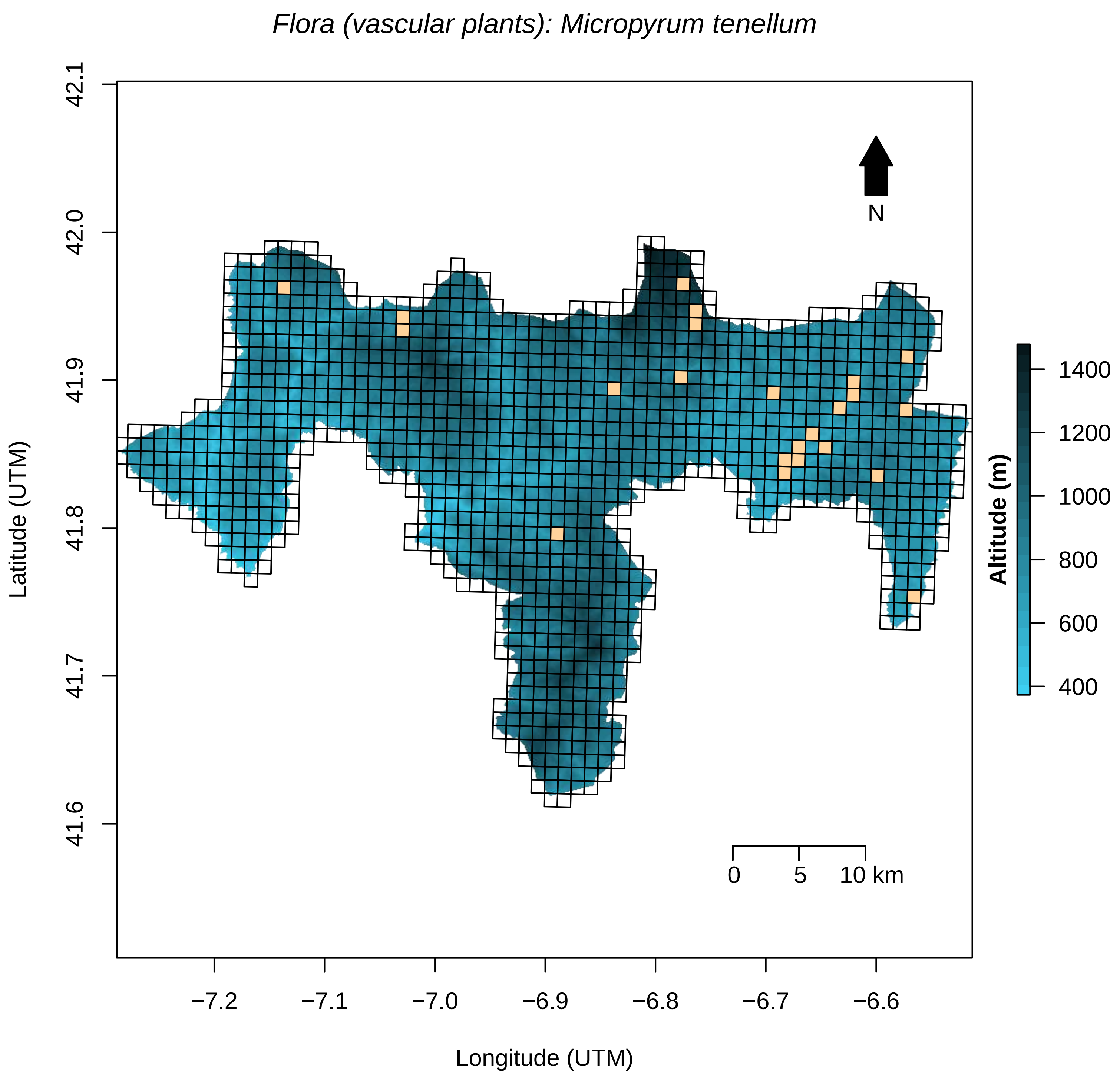
Flora (vascular plants): Micropyrum tenellum
N
0
5
10 km
42.1
42.0
41.9
41.8
41.7
41.6
Latitude (UTM)
−7.2
−7.1
−7.0
−6.9
−6.8
−6.7
−6.6
Longitude (UTM)
Altitude (m)
1400
1200
1000
800
600
400

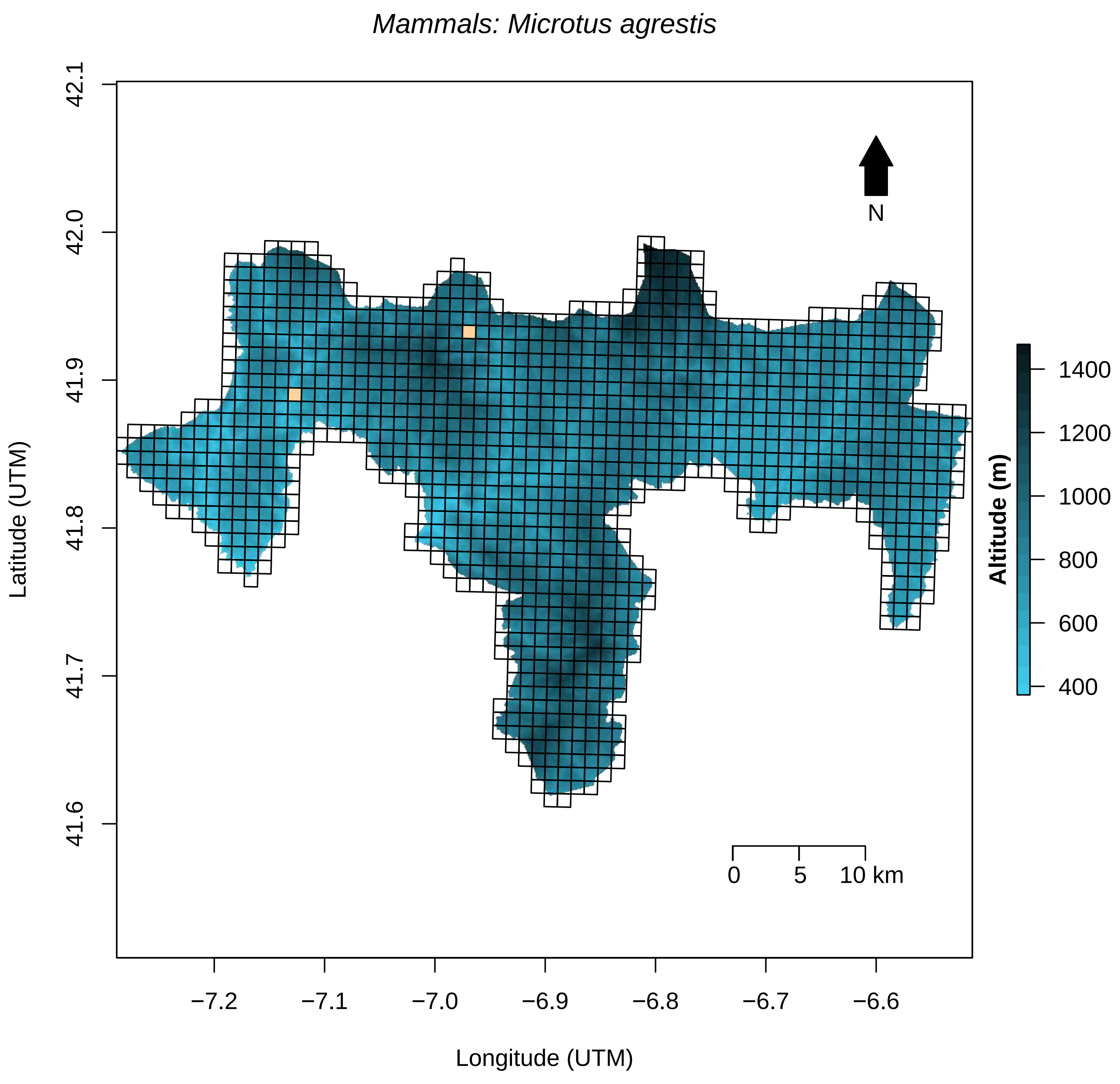

Mammals: Microtus agrestis
Latitude (UTM)
Longitude (UTM)
42.1
42.0
41.9
41.8
41.7
41.6
−7.2
−7.1
−7.0
−6.9
−6.8
−6.7
−6.6
N
0
5
10 km
Altitude (m)
1400
1200
1000
800
600
400

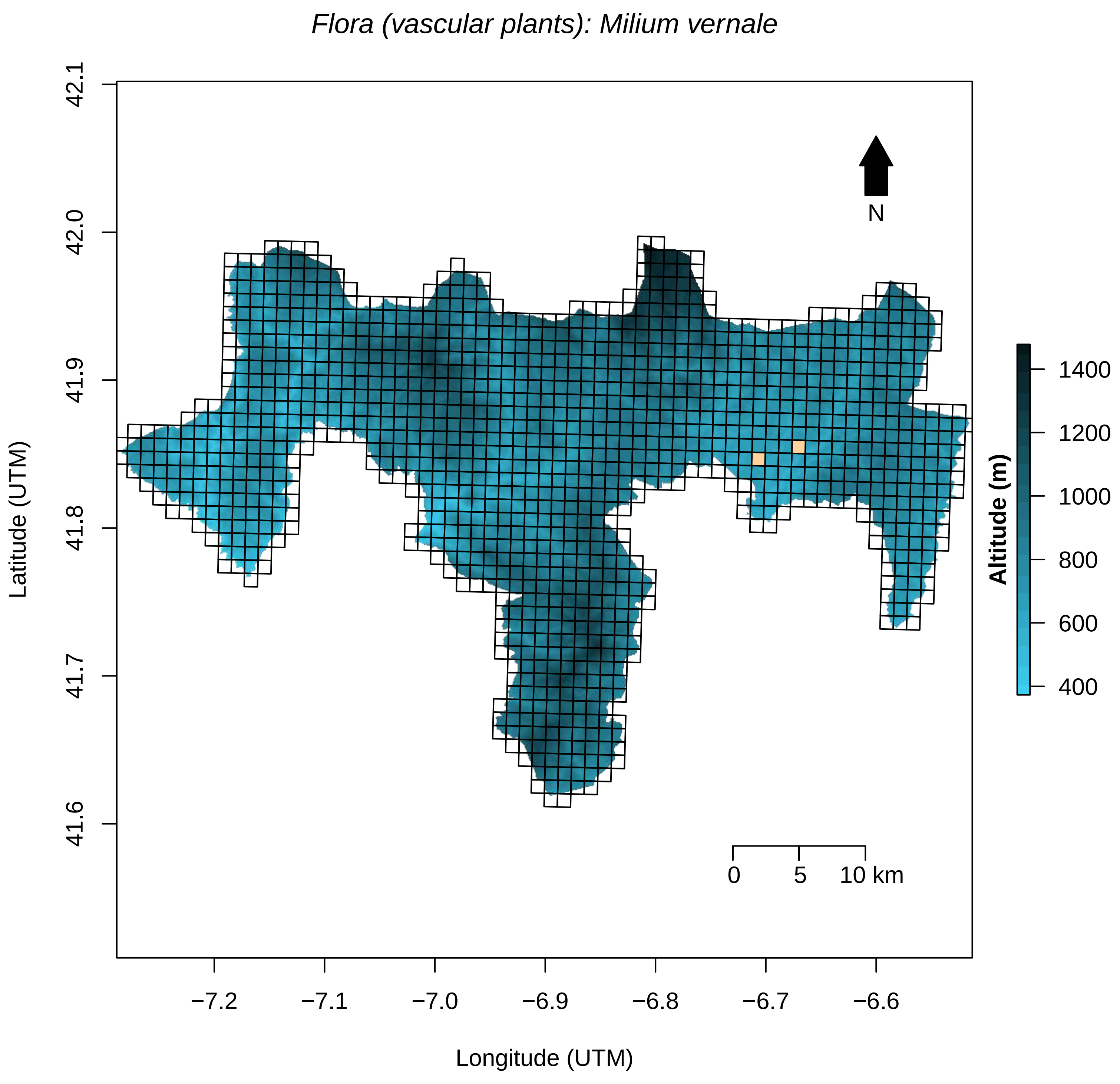
Flora (vascular plants): Milium vernale
42.1
42.0
41.9
41.8
41.7
41.6
Latitude (UTM)
−7.2
−7.1
−7.0
−6.9
−6.8
−6.7
−6.6
Longitude (UTM)
N
0
5
10 km
Altitude (m)
1400
1200
1000
800
600
400

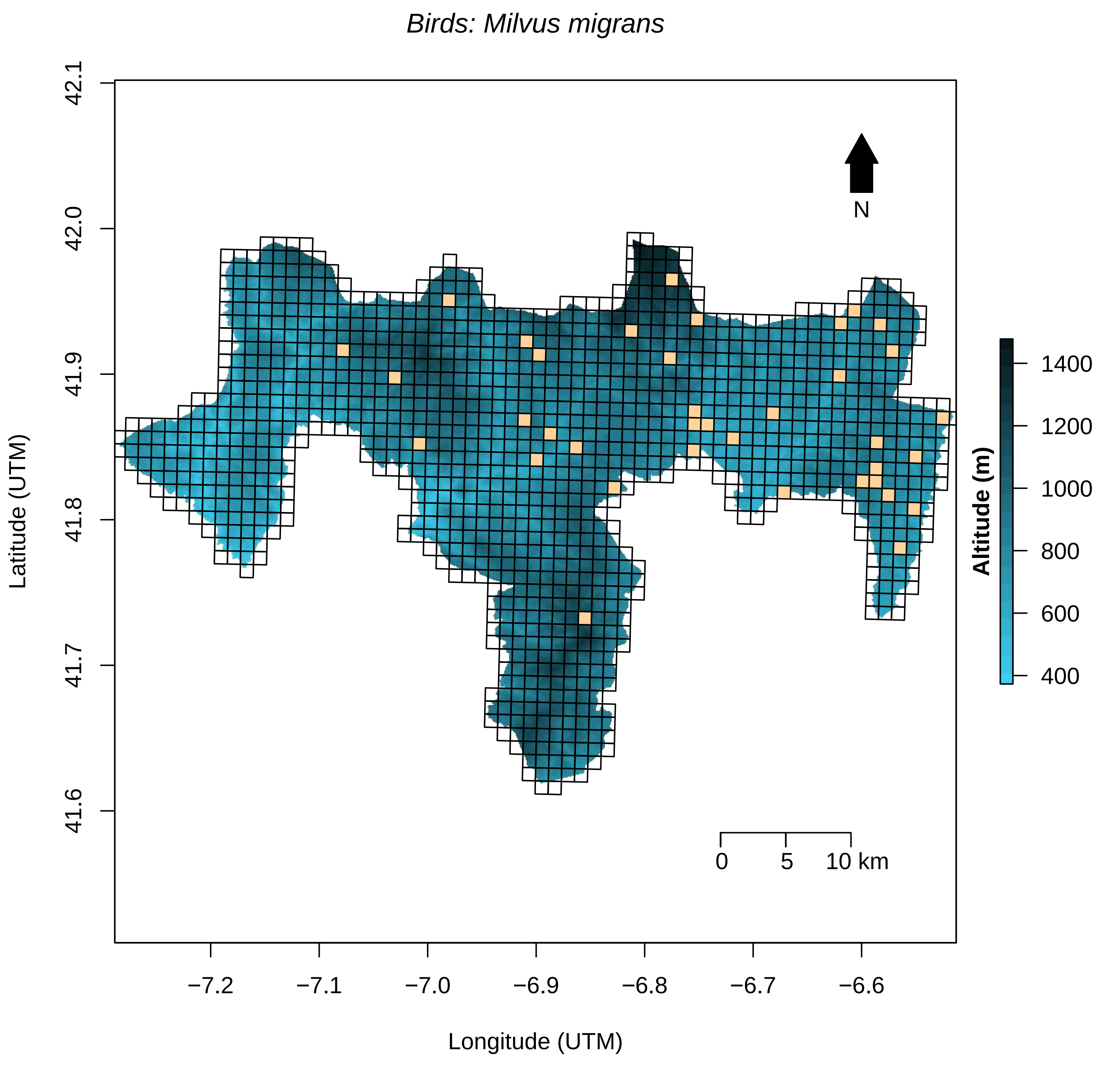

Birds: Milvus migrans
42.1
42.0
41.9
41.8
41.7
41.6
Latitude (UTM)
−7.2
−7.1
−7.0
−6.9
−6.8
−6.7
−6.6
Longitude (UTM)
N
0
5
10 km
Altitude (m)
1400
1200
1000
800
600
400

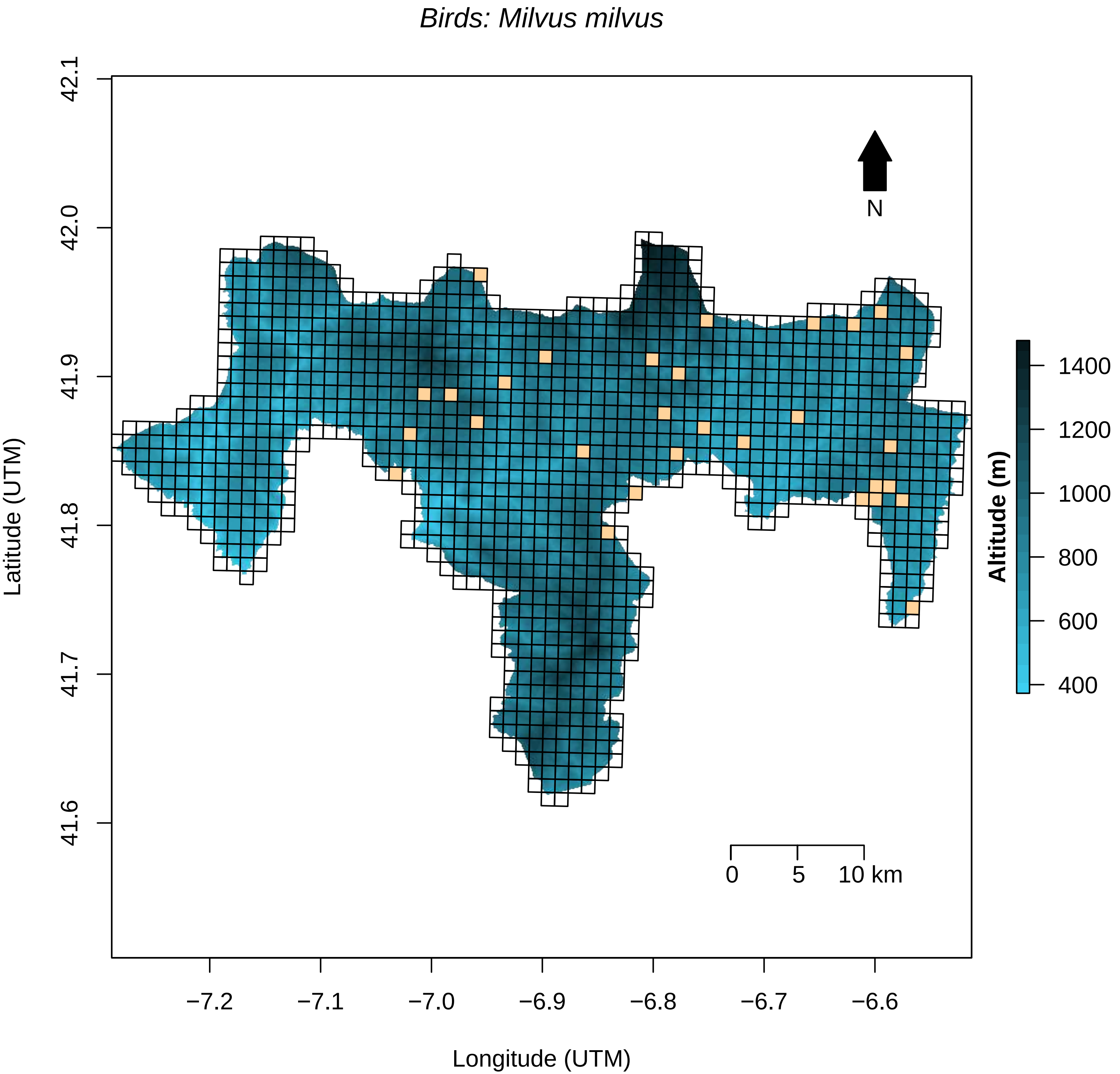

Birds: Milvus milvus
42.1
42.0
41.9
41.8
41.7
41.6
Latitude (UTM)
−7.2
−7.1
−7.0
−6.9
−6.8
−6.7
−6.6
Longitude (UTM)
N
0
5
10 km
1400
1200
1000
800
600
400
Altitude (m)

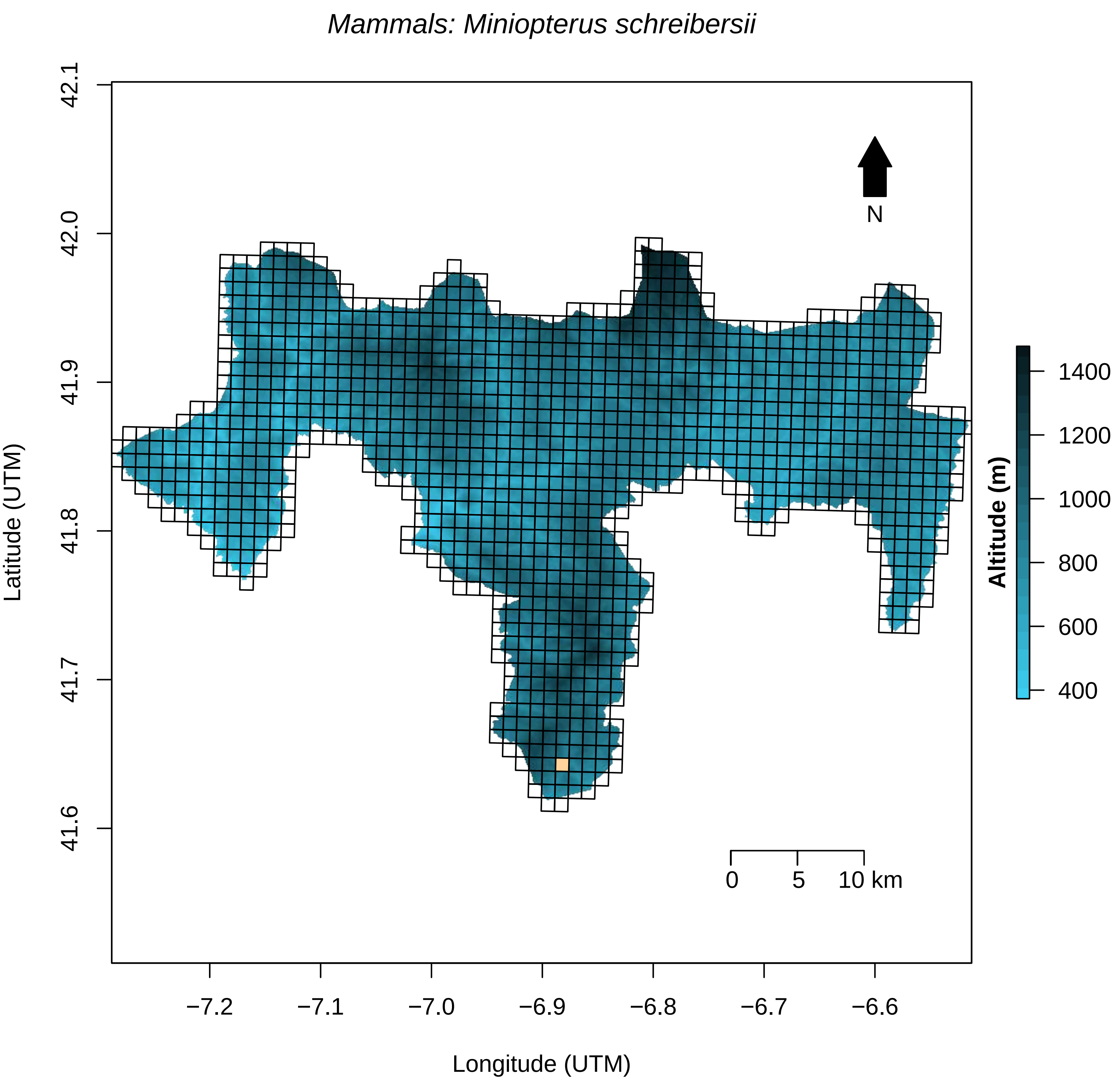

Mammals: Miniopterus schreibersii
N
0
5
10 km
Altitude (m)
400
600
800
1000
1200
1400
Latitude (UTM)
41.6
41.7
41.8
41.9
42.0
42.1
Longitude (UTM)
−7.2
−7.1
−7.0
−6.9
−6.8
−6.7
−6.6

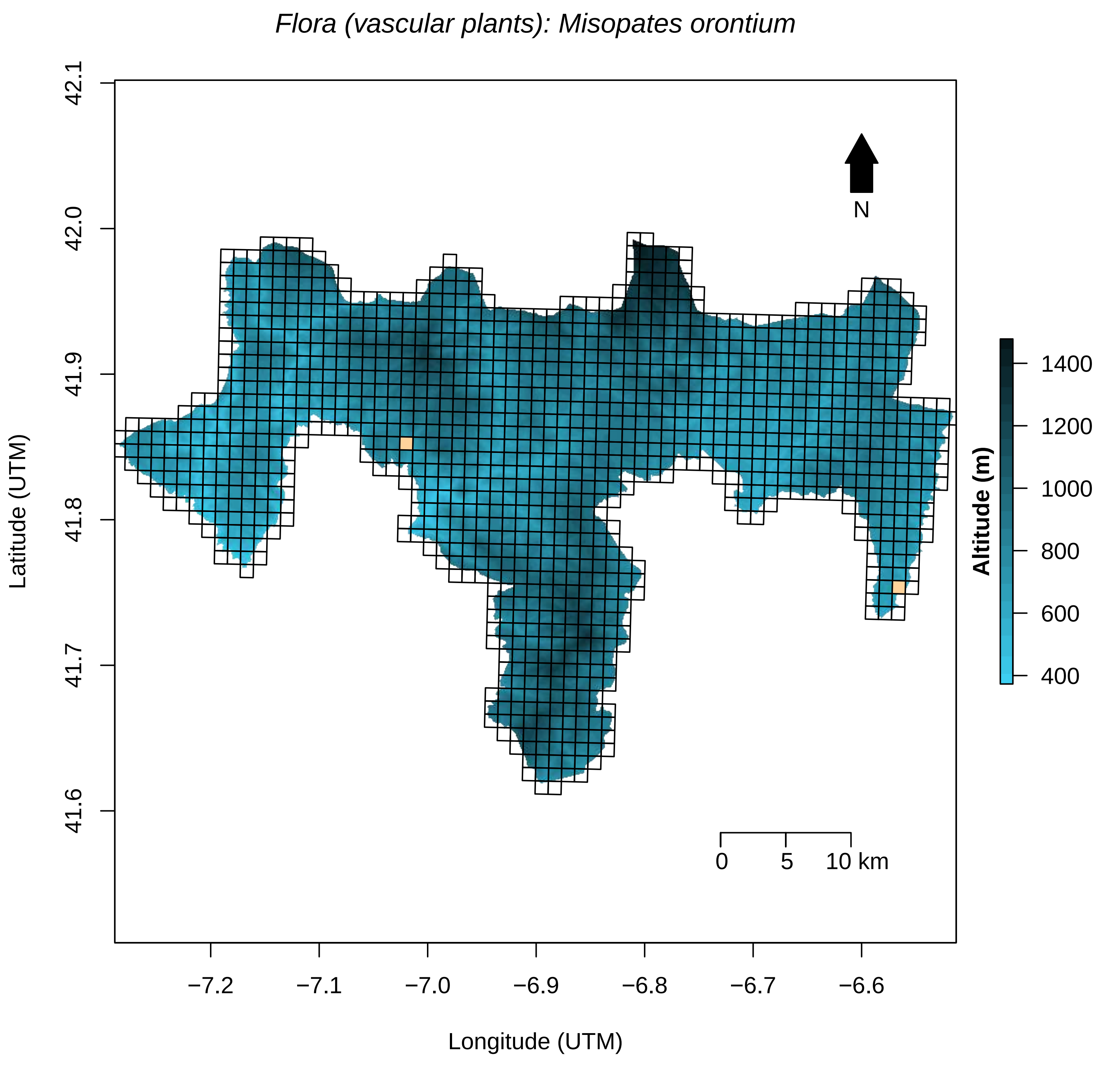
Flora (vascular plants): Misopates orontium
N
0 5 10 km
Altitude (m)
1400
1200
1000
800
600
400
42.1
42.0
41.9
41.8
41.7
41.6
Latitude (UTM)
−7.2
−7.1
−7.0
−6.9
−6.8
−6.7
−6.6
Longitude (UTM)

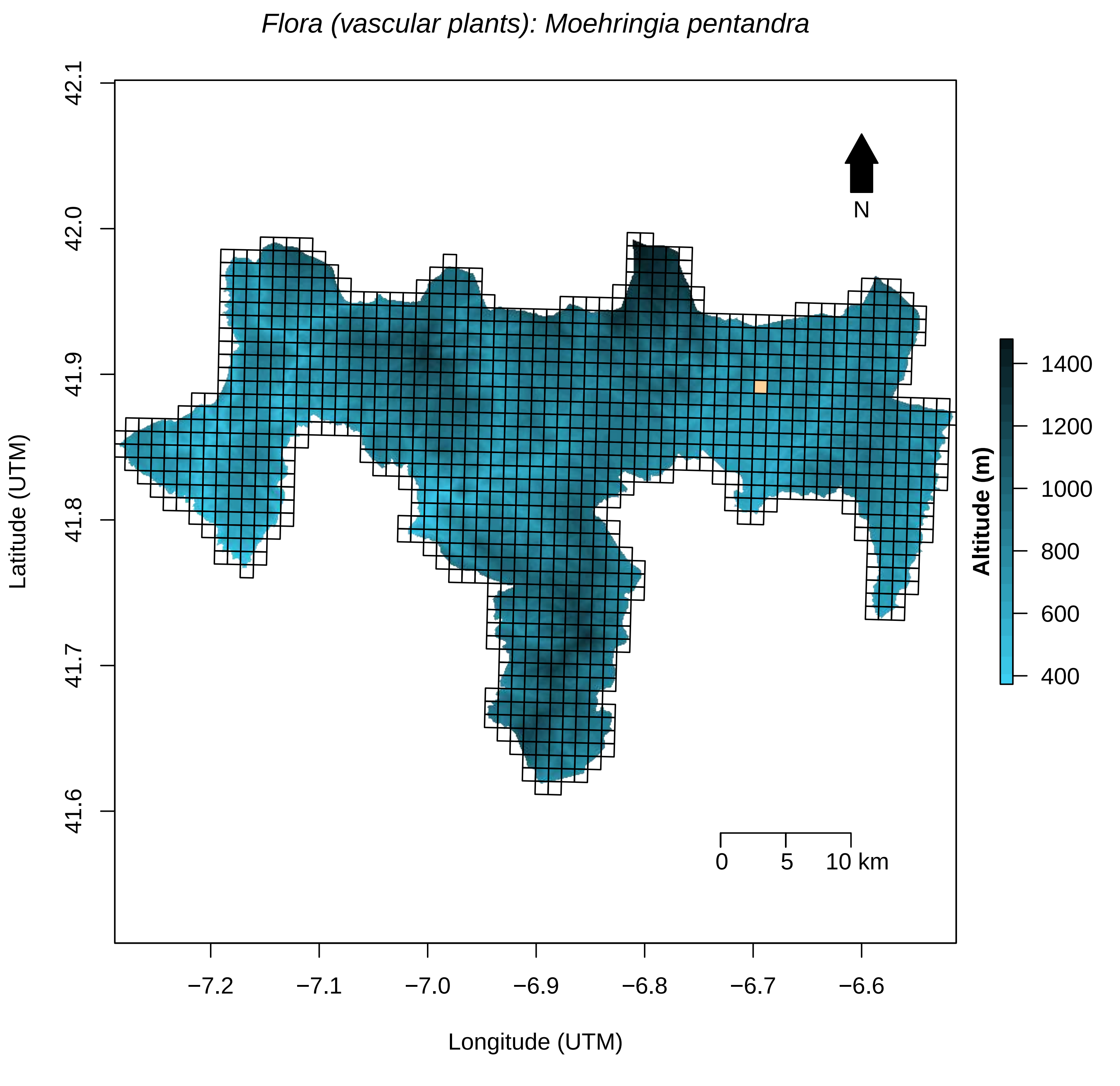

Flora (vascular plants): Moehringia pentandra
42.1
42.0
41.9
41.8
41.7
41.6
Latitude (UTM)
−7.2
−7.1
−7.0
−6.9
−6.8
−6.7
−6.6
Longitude (UTM)
N
0
5
10 km
1400
1200
1000
800
600
400
Altitude (m)

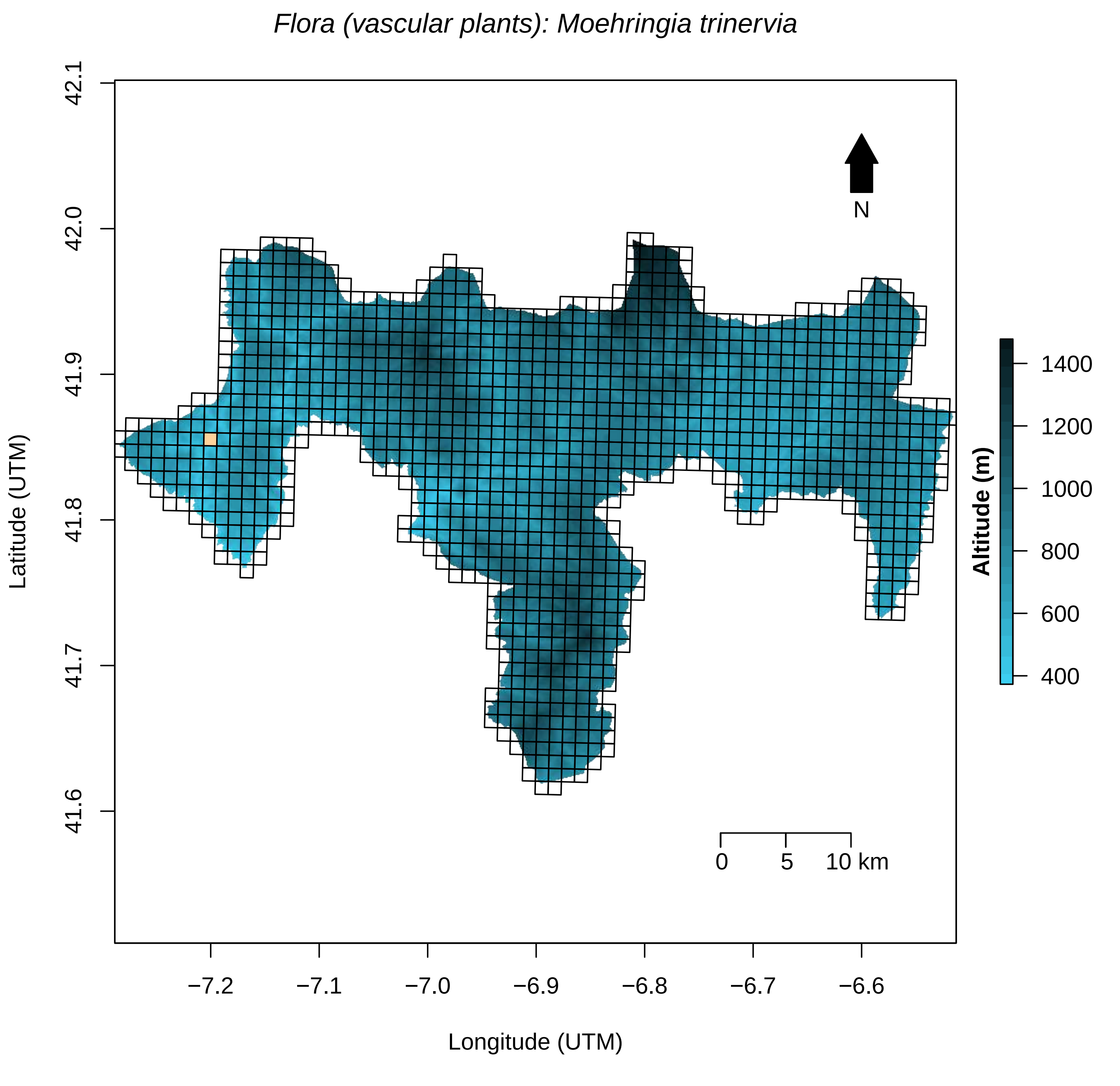

Flora (vascular plants): Moehringia trinervia
Latitude (UTM)
42.1
42.0
41.9
41.8
41.7
41.6
Longitude (UTM)
−7.2
−7.1
−7.0
−6.9
−6.8
−6.7
−6.6
N
0
5
10 km
Altitude (m)
1400
1200
1000
800
600
400

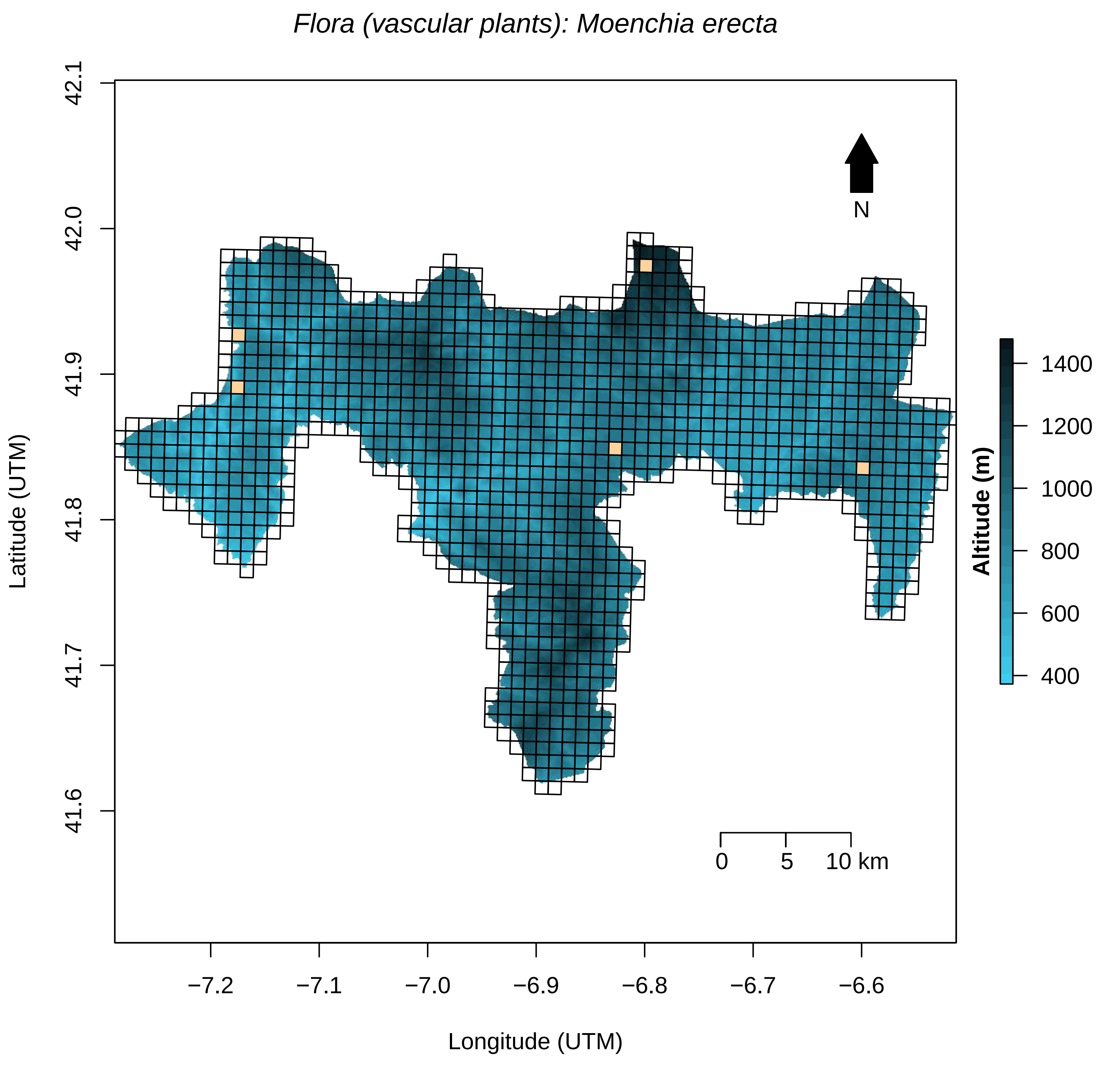

Flora (vascular plants): Moenchia erecta
42.1
42.0
41.9
41.8
41.7
41.6
Latitude (UTM)
−7.2
−7.1
−7.0
−6.9
−6.8
−6.7
−6.6
Longitude (UTM)
N
0
5
10 km
1400
1200
1000
800
600
400
Altitude (m)

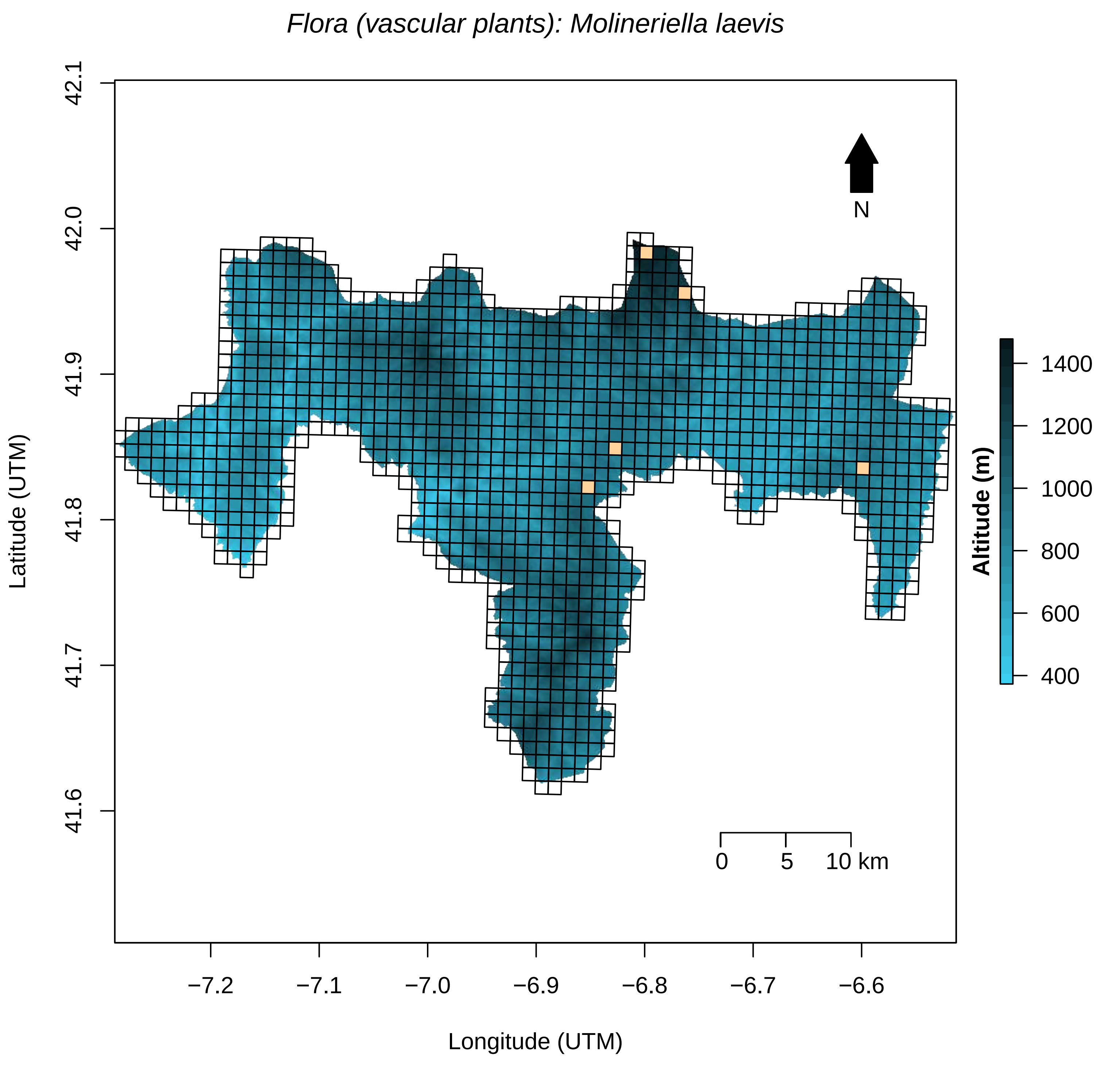

Flora (vascular plants): Molineriella laevis
N
0 5 10 km
Altitude (m)
1400
1200
1000
800
600
400
Latitude (UTM)
42.1
42.0
41.9
41.8
41.7
41.6
Longitude (UTM)
−7.2
−7.1
−7.0
−6.9
−6.8
−6.7
−6.6

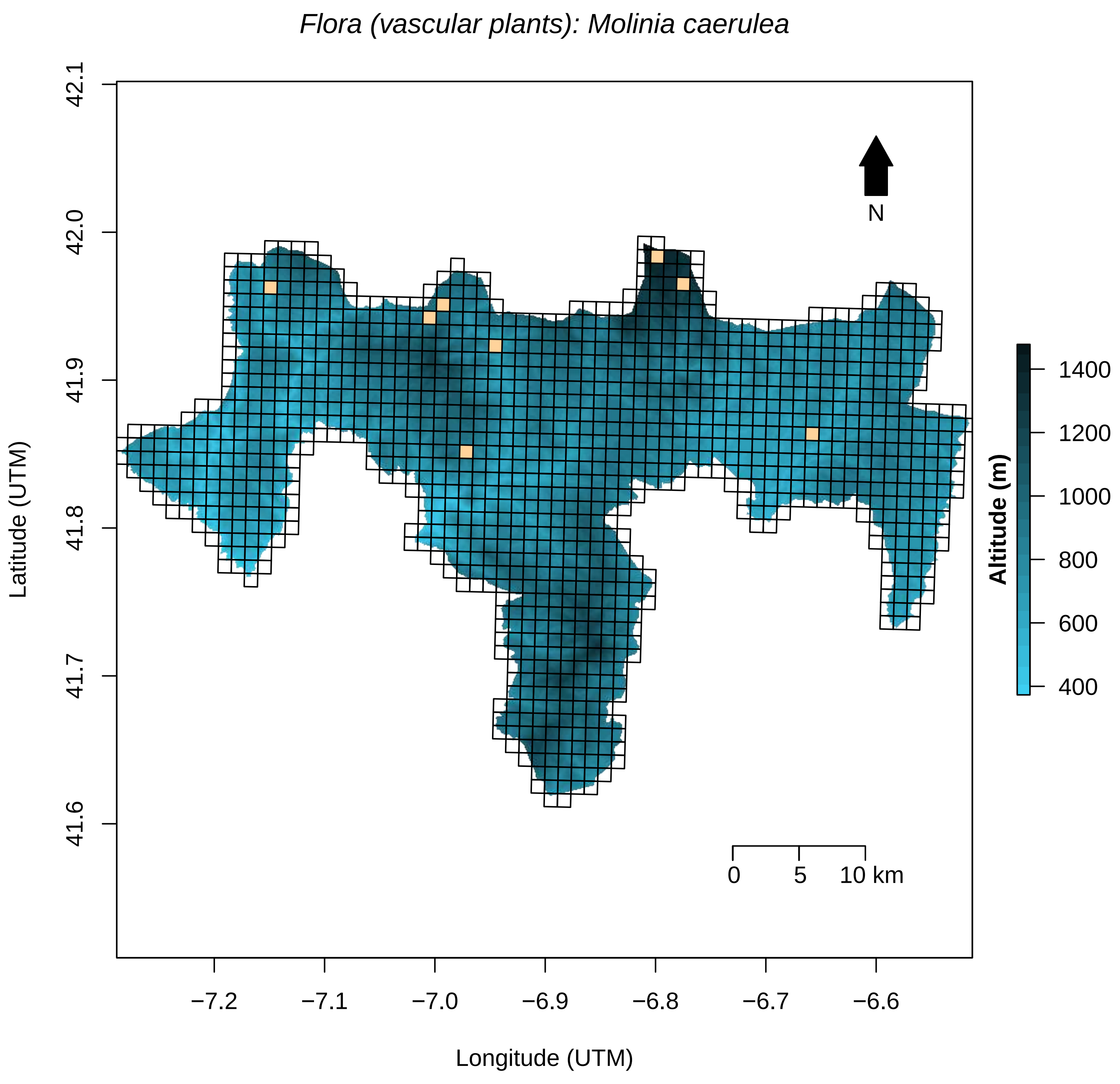

Flora (vascular plants): Molinia caerulea
Latitude (UTM)
Longitude (UTM)
42.1
42.0
41.9
41.8
41.7
41.6
−7.2
−7.1
−7.0
−6.9
−6.8
−6.7
−6.6
N
0
5
10 km
Altitude (m)
1400
1200
1000
800
600
400

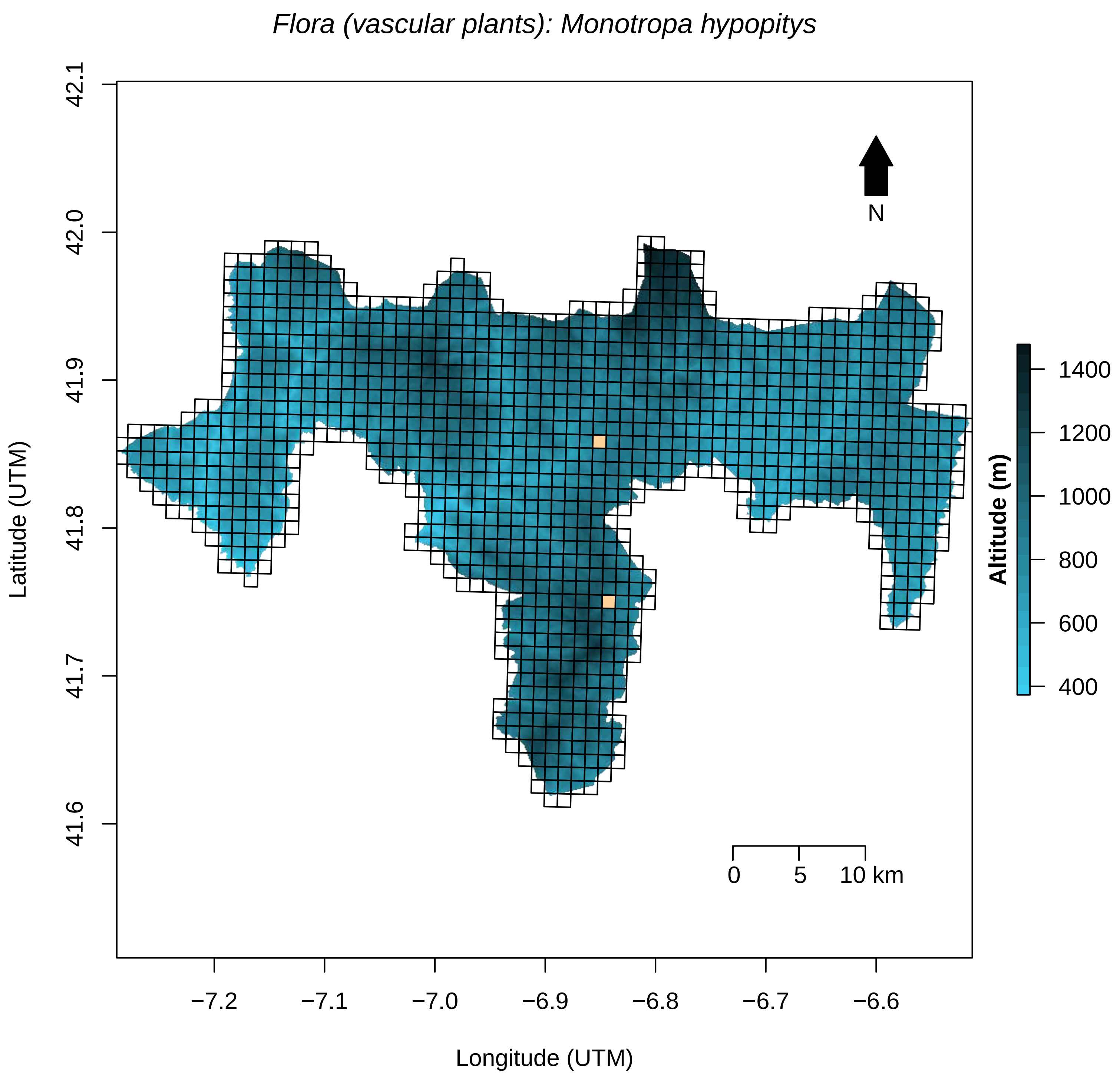

Flora (vascular plants): Monotropa hypopitys
42.1
42.0
41.9
41.8
41.7
41.6
Latitude (UTM)
−7.2
−7.1
−7.0
−6.9
−6.8
−6.7
−6.6
Longitude (UTM)
N
0
5
10 km
Altitude (m)
1400
1200
1000
800
600
400

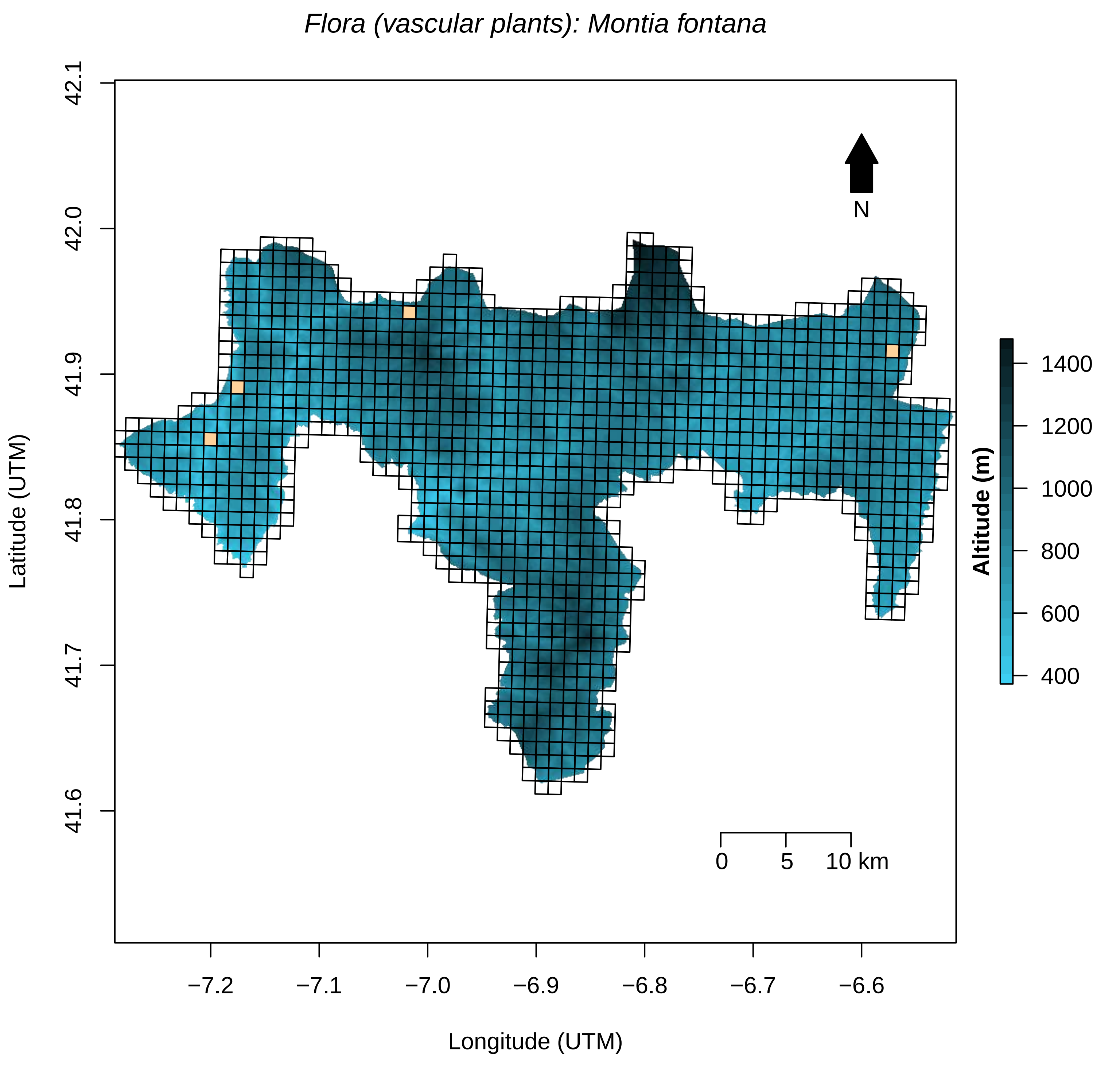
Flora (vascular plants): Montia fontana
42.1
42.0
41.9
41.8
41.7
41.6
Latitude (UTM)
−7.2
−7.1
−7.0
−6.9
−6.8
−6.7
−6.6
Longitude (UTM)
N
0
5
10 km
1400
1200
1000
800
600
400
Altitude (m)

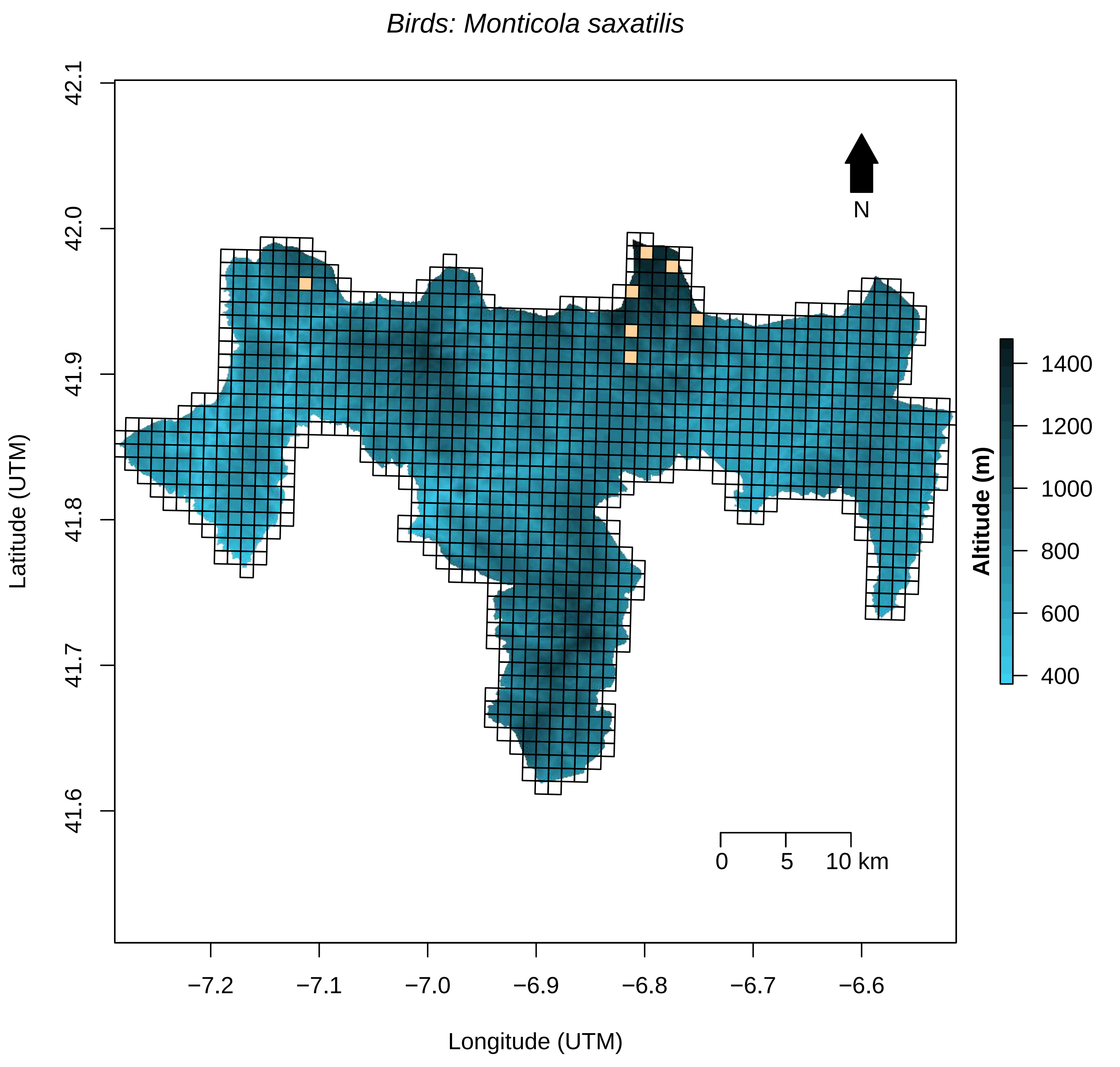
Birds: Monticola saxatilis
42.1
42.0
41.9
41.8
41.7
41.6
Latitude (UTM)
−7.2
−7.1
−7.0
−6.9
−6.8
−6.7
−6.6
Longitude (UTM)
N
0
5
10 km
Altitude (m)
1400
1200
1000
800
600
400

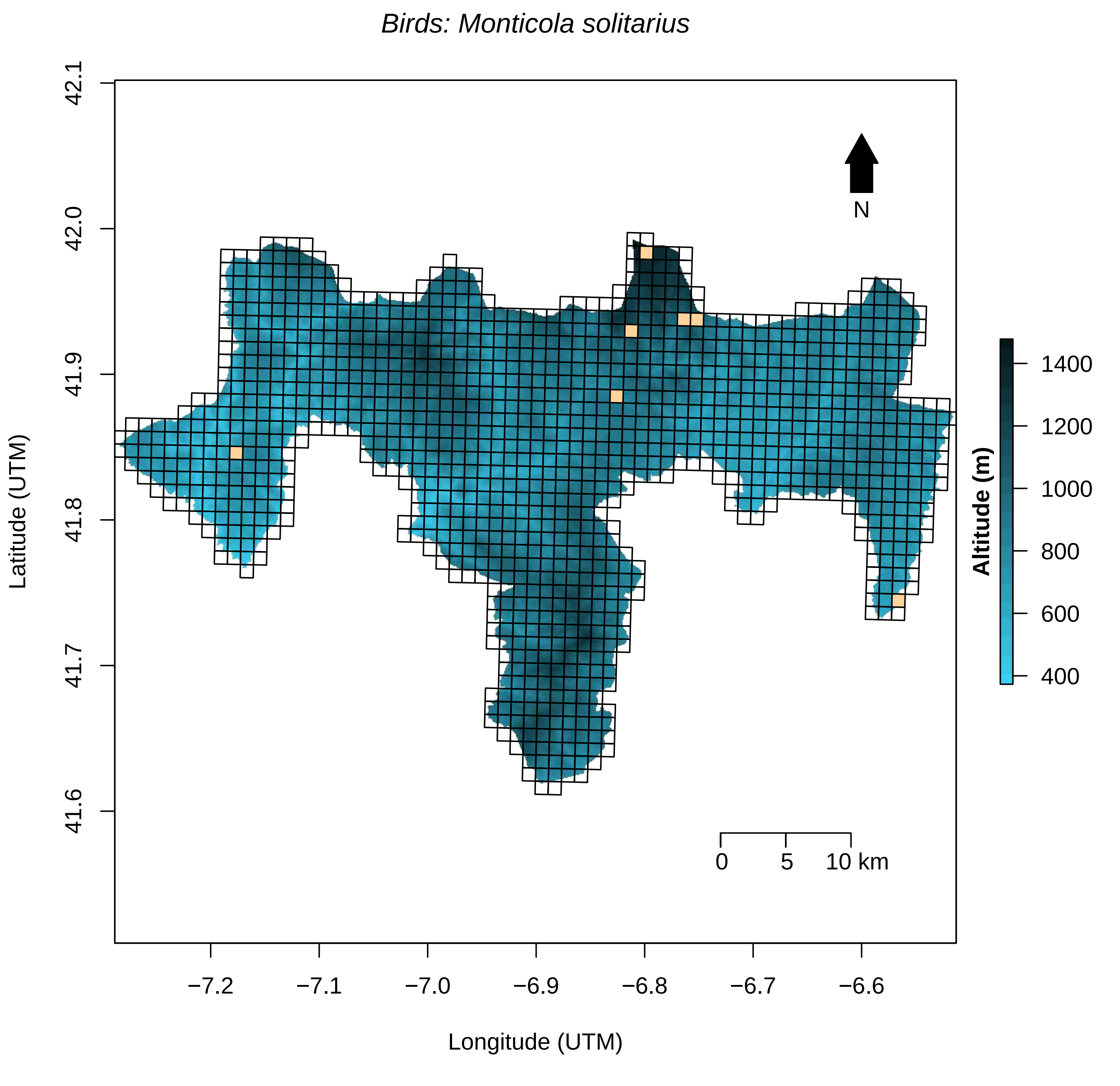

Birds: Monticola solitarius
42.1
42.0
41.9
41.8
41.7
41.6
Latitude (UTM)
−7.2
−7.1
−7.0
−6.9
−6.8
−6.7
−6.6
Longitude (UTM)
N
0
5
10 km
Altitude (m)
1400
1200
1000
800
600
400

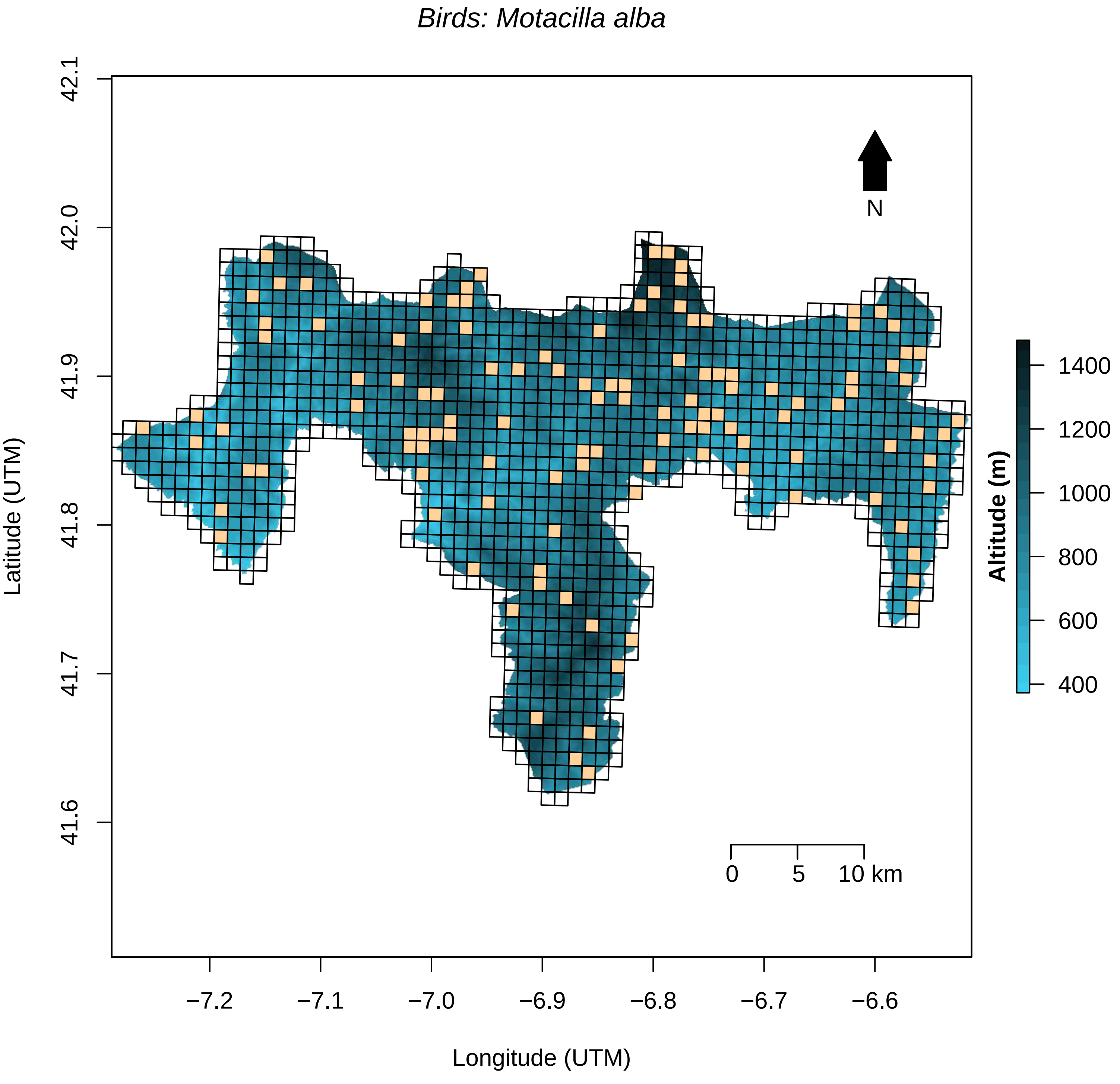
Birds: Motacilla alba
42.1
42.0
41.9
41.8
41.7
41.6
Latitude (UTM)
−7.2
−7.1
−7.0
−6.9
−6.8
−6.7
−6.6
Longitude (UTM)
N
0
5
10 km
Altitude (m)
1400
1200
1000
800
600
400

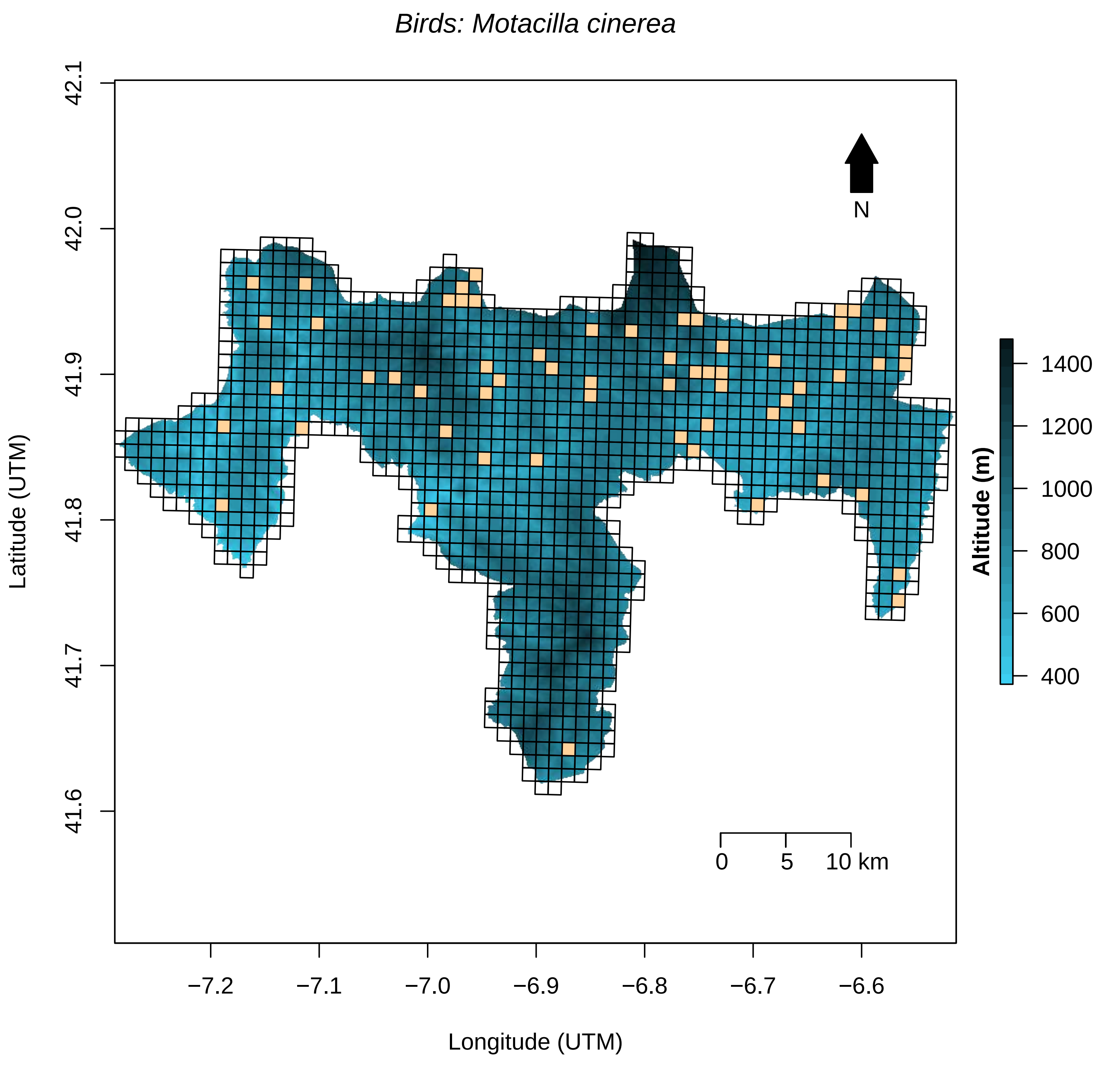

Birds: Motacilla cinerea
42.1
42.0
41.9
41.8
41.7
41.6
Latitude (UTM)
−7.2
−7.1
−7.0
−6.9
−6.8
−6.7
−6.6
Longitude (UTM)
N
0
5
10 km
1400
1200
1000
800
600
400
Altitude (m)

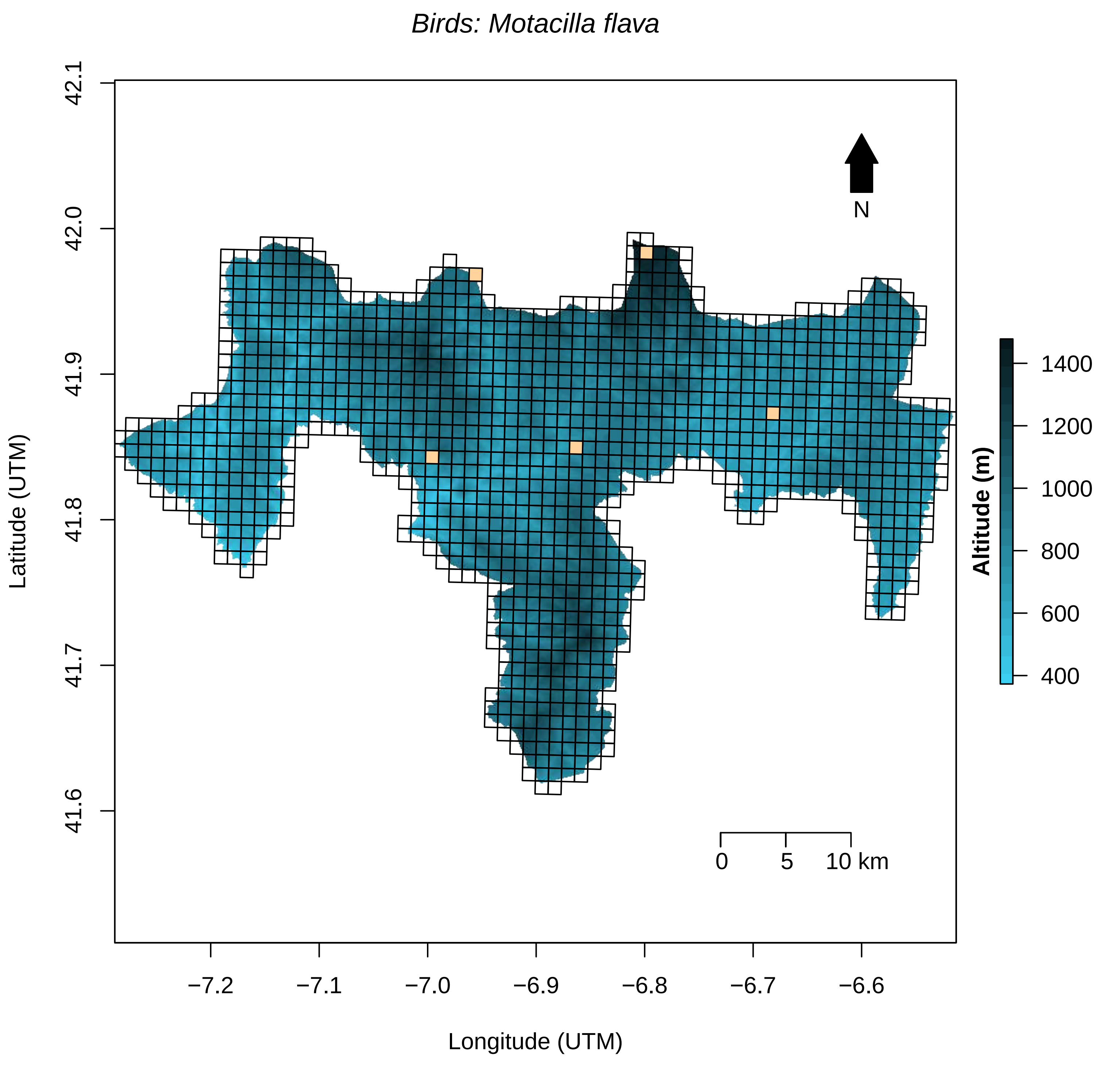

Birds: Motacilla flava
42.1
42.0
41.9
41.8
41.7
41.6
Latitude (UTM)
−7.2
−7.1
−7.0
−6.9
−6.8
−6.7
−6.6
Longitude (UTM)
N
0
5
10 km
1400
1200
1000
800
600
400
Altitude (m)

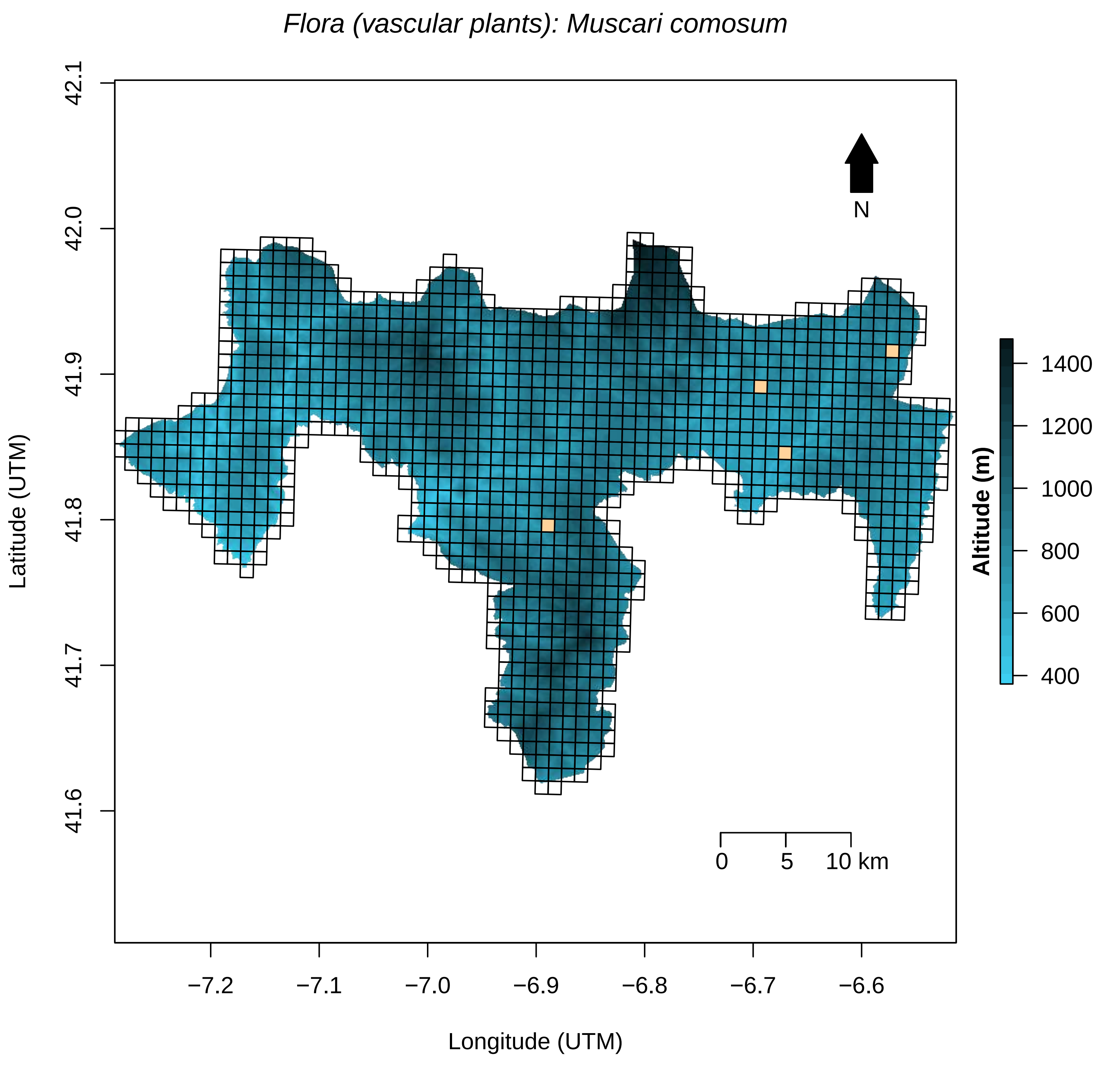

Flora (vascular plants): Muscari comosum
42.1
42.0
41.9
41.8
41.7
41.6
Latitude (UTM)
−7.2
−7.1
−7.0
−6.9
−6.8
−6.7
−6.6
Longitude (UTM)
N
0
5
10 km
Altitude (m)
1400
1200
1000
800
600
400

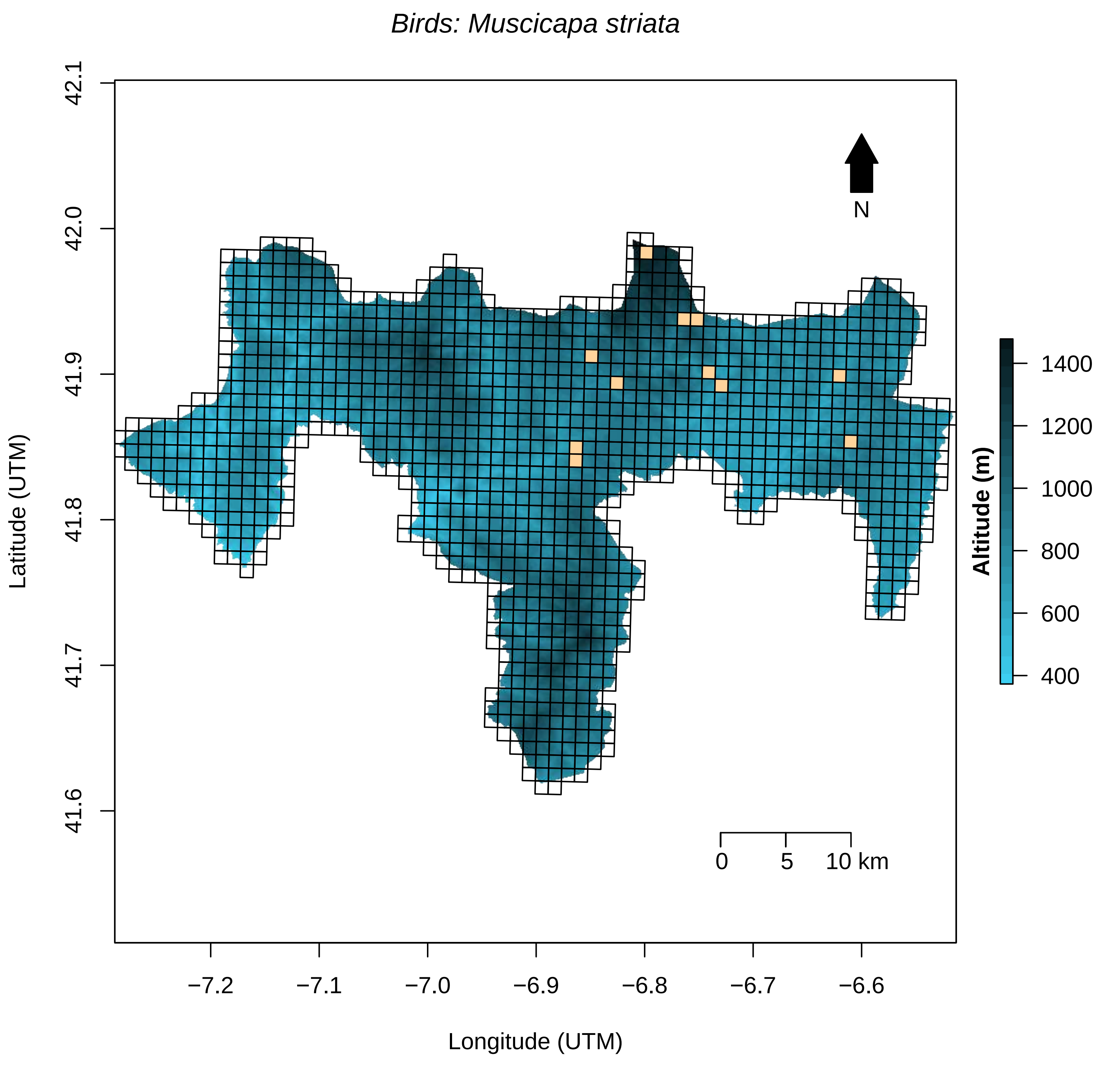
Birds: Muscicapa striata
N
0
5
10 km
Altitude (m)
1400
1200
1000
800
600
400
Latitude (UTM)
42.1
42.0
41.9
41.8
41.7
41.6
Longitude (UTM)
−7.2
−7.1
−7.0
−6.9
−6.8
−6.7
−6.6

Mammals: Mustela nivalis

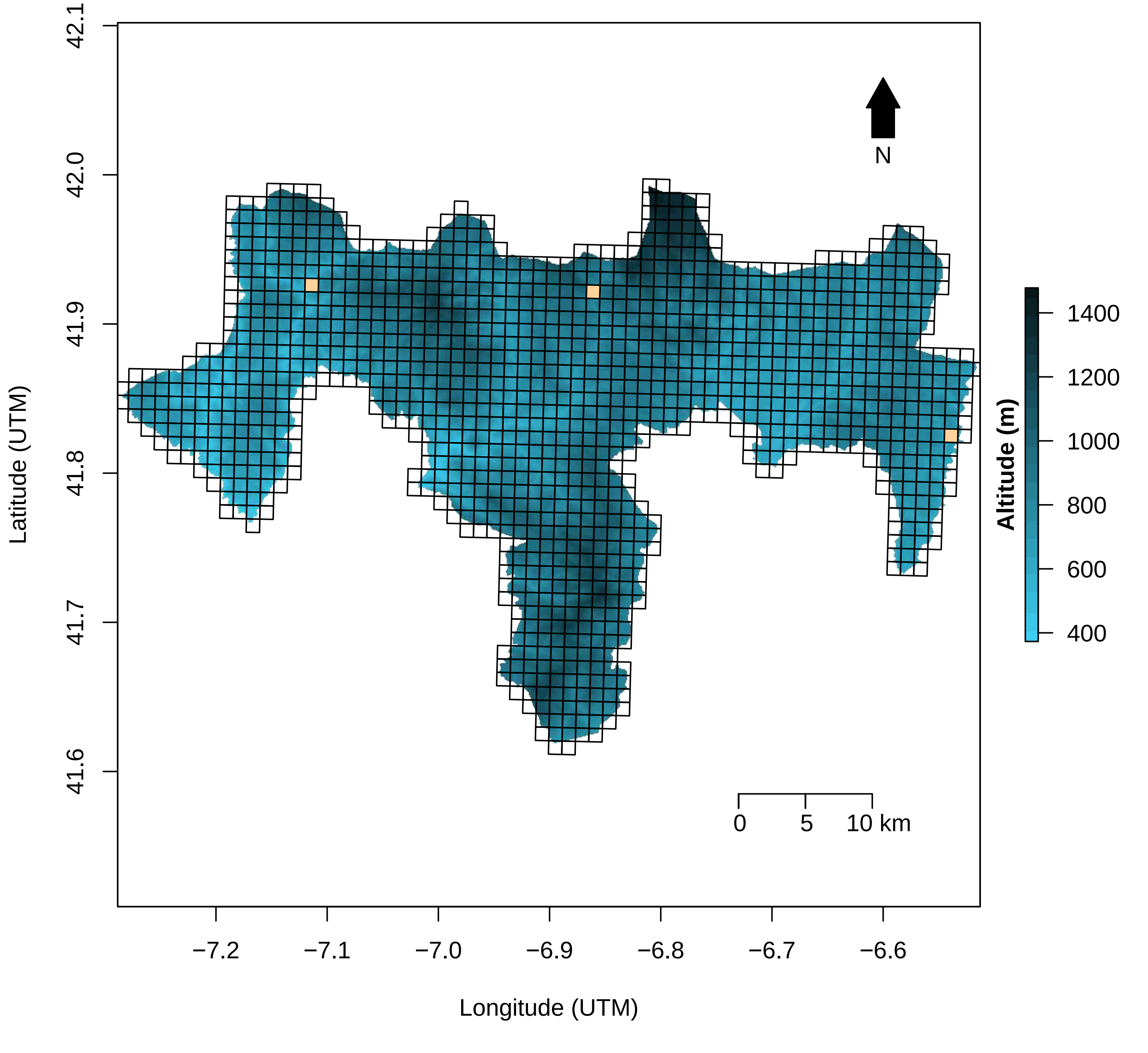

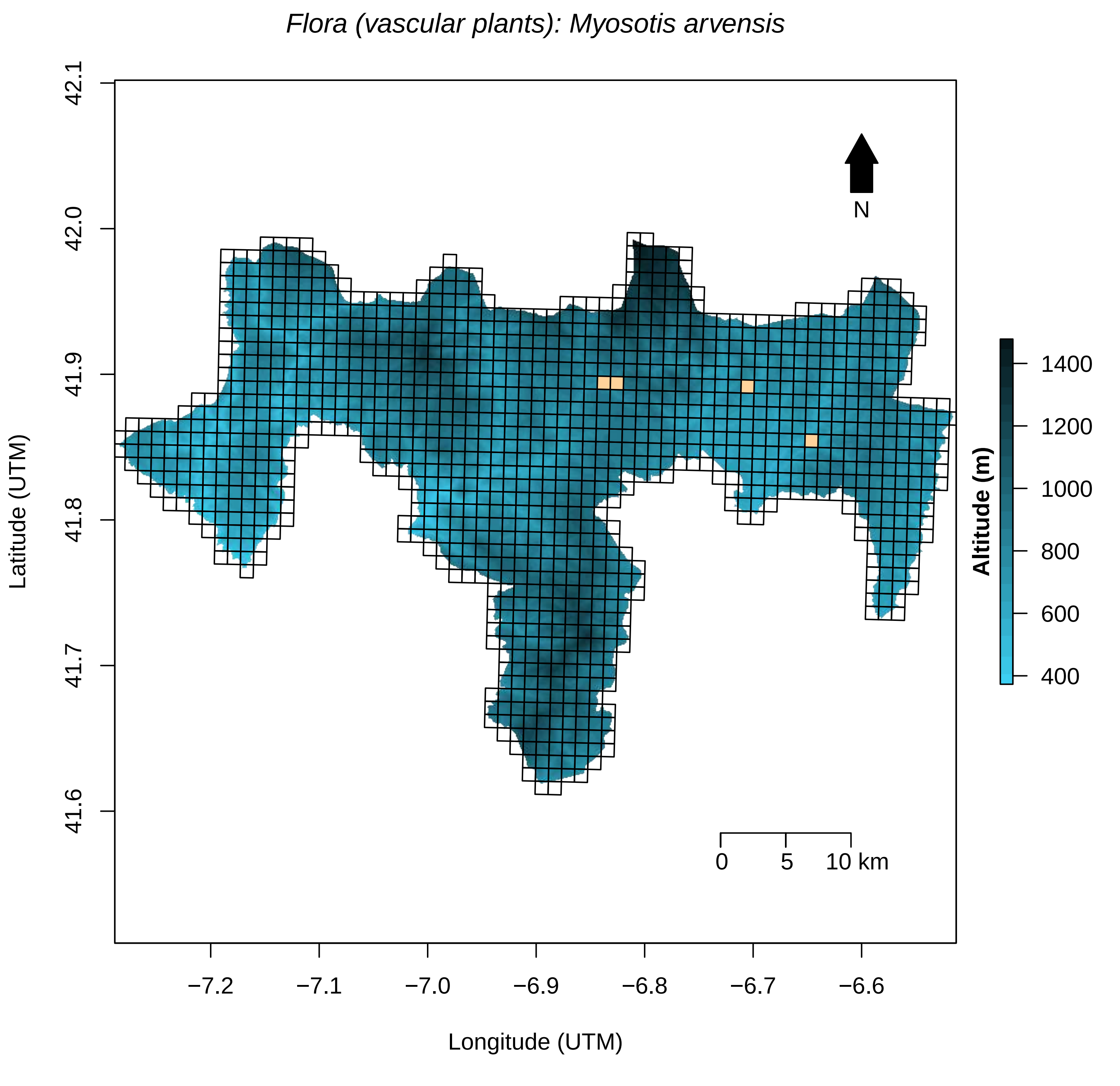

Flora (vascular plants): Myosotis arvensis
N
0
5
10 km
Altitude (m)
1400
1200
1000
800
600
400
Latitude (UTM)
42.1
42.0
41.9
41.8
41.7
41.6
Longitude (UTM)
−7.2
−7.1
−7.0
−6.9
−6.8
−6.7
−6.6

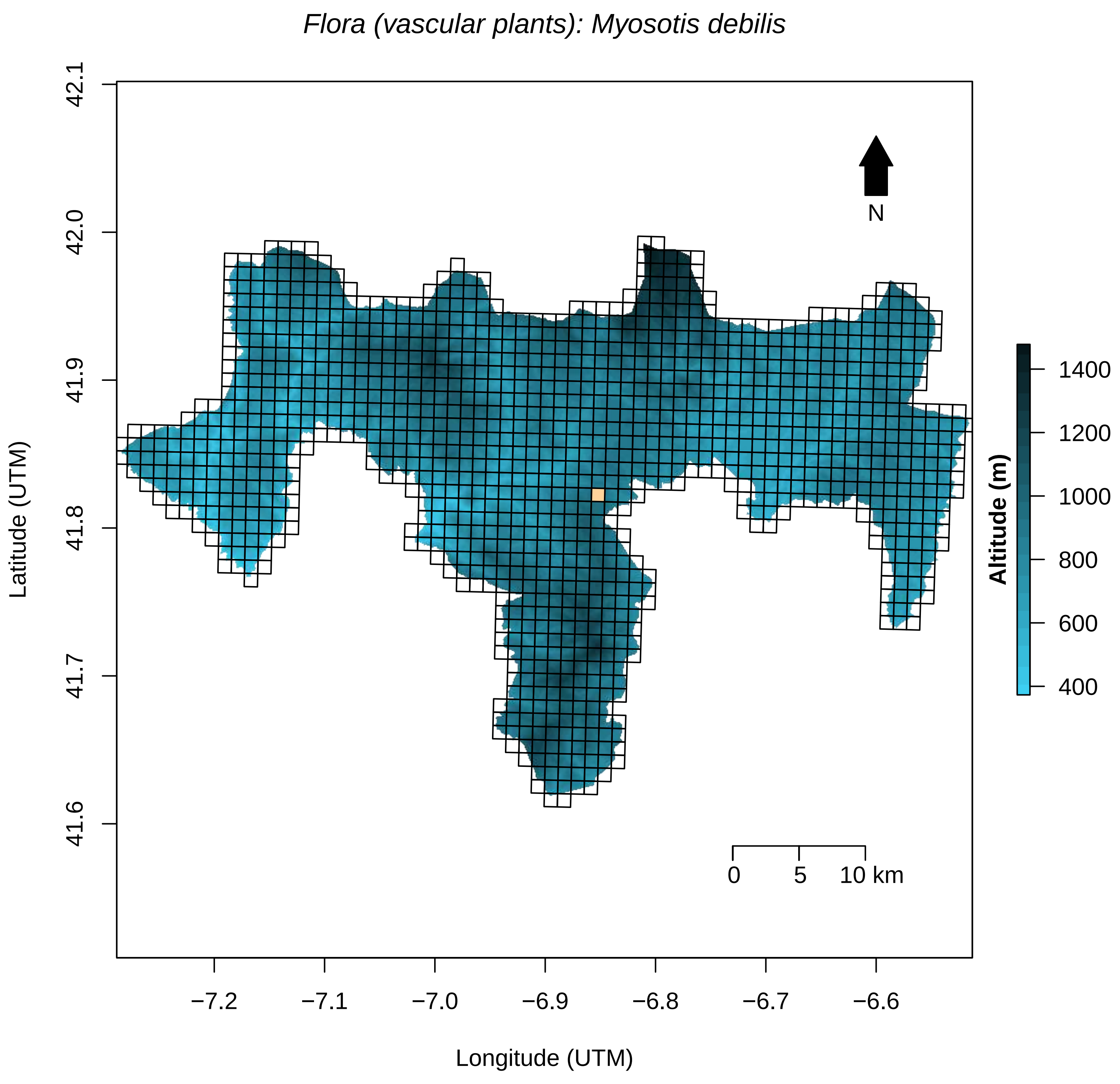

Flora (vascular plants): Myosotis debilis
42.1
42.0
41.9
41.8
41.7
41.6
Latitude (UTM)
−7.2
−7.1
−7.0
−6.9
−6.8
−6.7
−6.6
Longitude (UTM)
N
0
5
10 km
1400
1200
1000
800
600
400
Altitude (m)

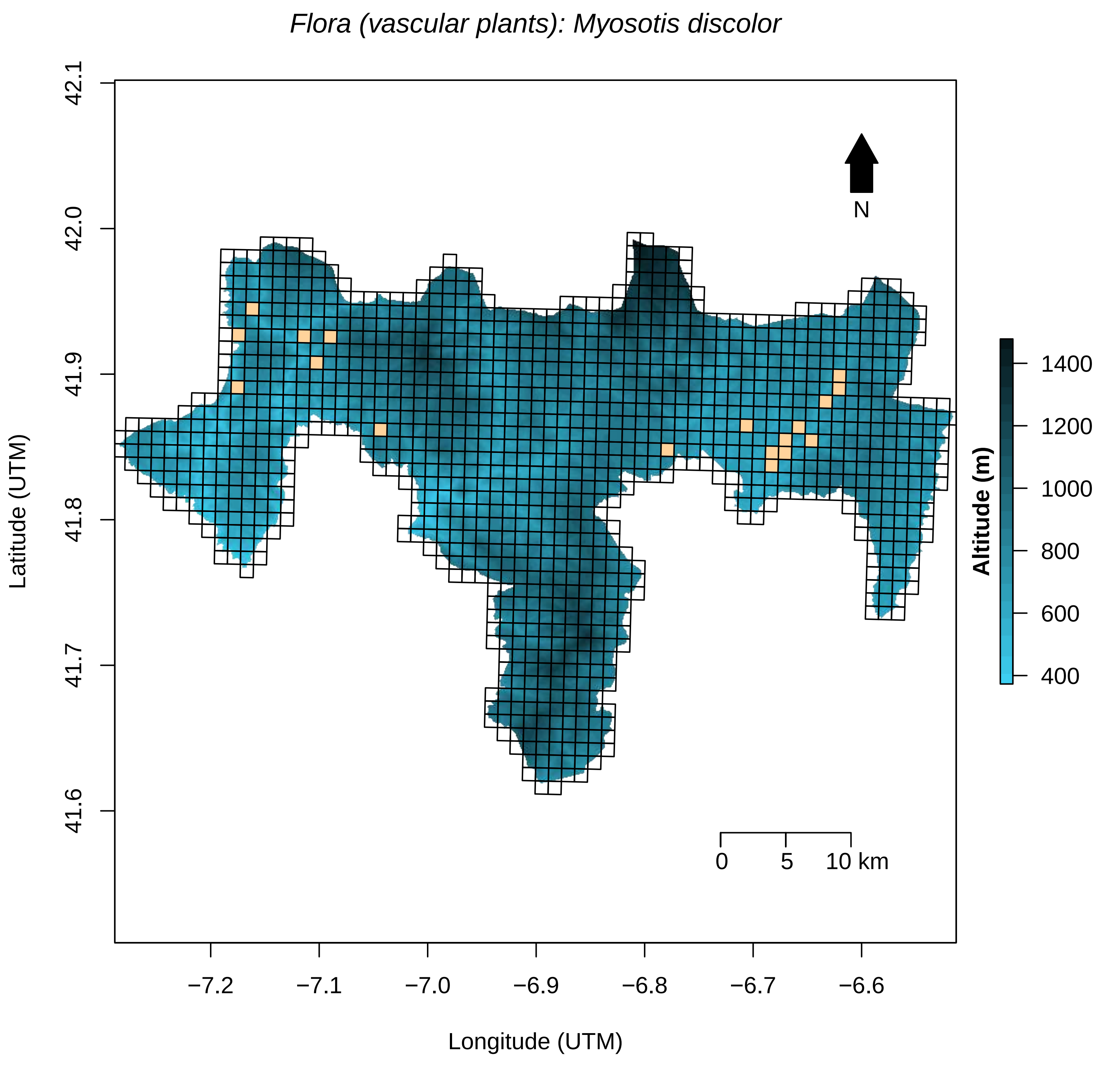

Flora (vascular plants): Myosotis discolor
Latitude (UTM)
42.1
42.0
41.9
41.8
41.7
41.6
Longitude (UTM)
−7.2
−7.1
−7.0
−6.9
−6.8
−6.7
−6.6
N
0
5
10 km
Altitude (m)
1400
1200
1000
800
600
400

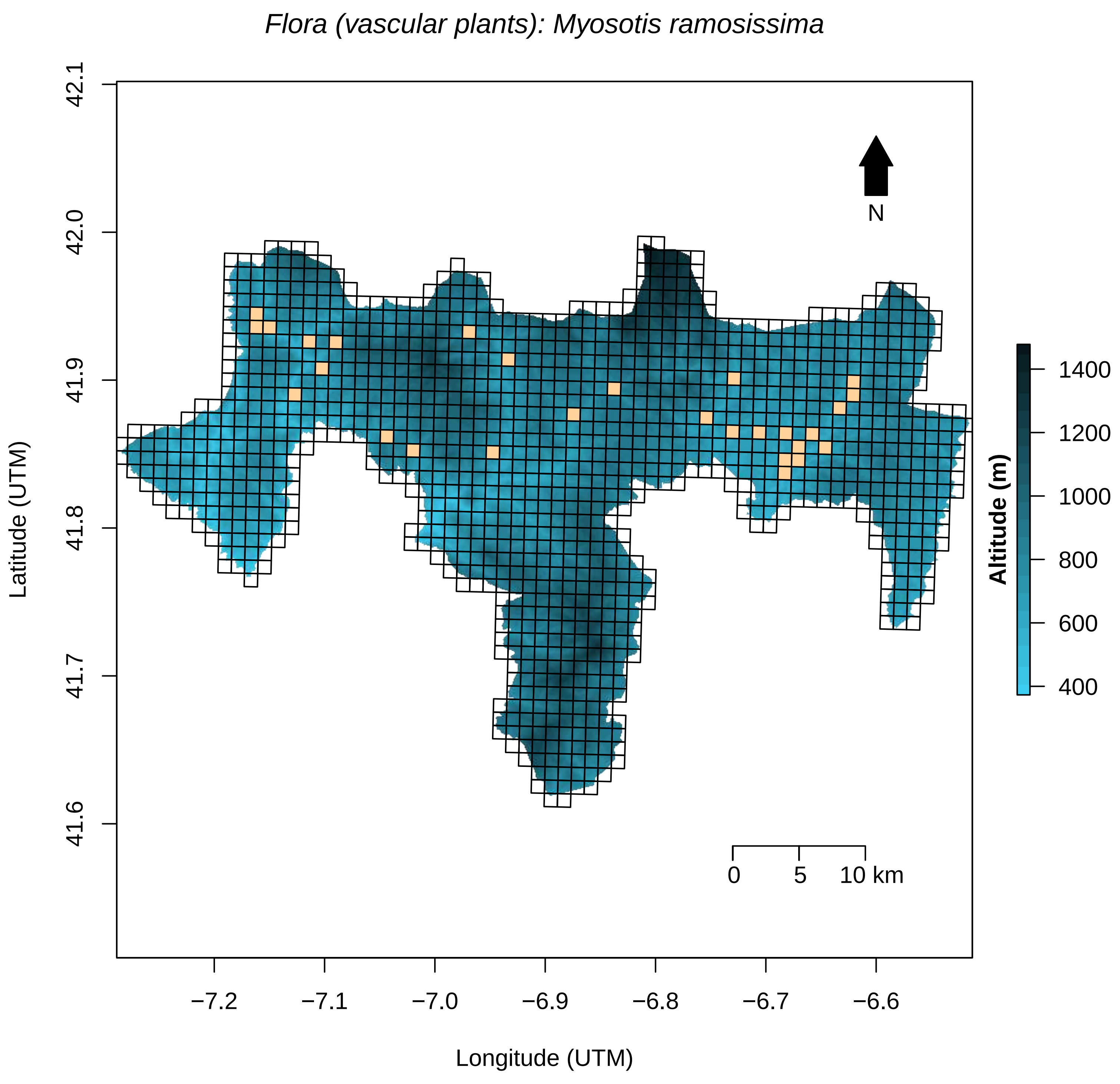

Flora (vascular plants): Myosotis ramosissima
Latitude (UTM)
Longitude (UTM)
42.1
42.0
41.9
41.8
41.7
41.6
−7.2
−7.1
−7.0
−6.9
−6.8
−6.7
−6.6
N
0
5
10 km
Altitude (m)
1400
1200
1000
800
600
400

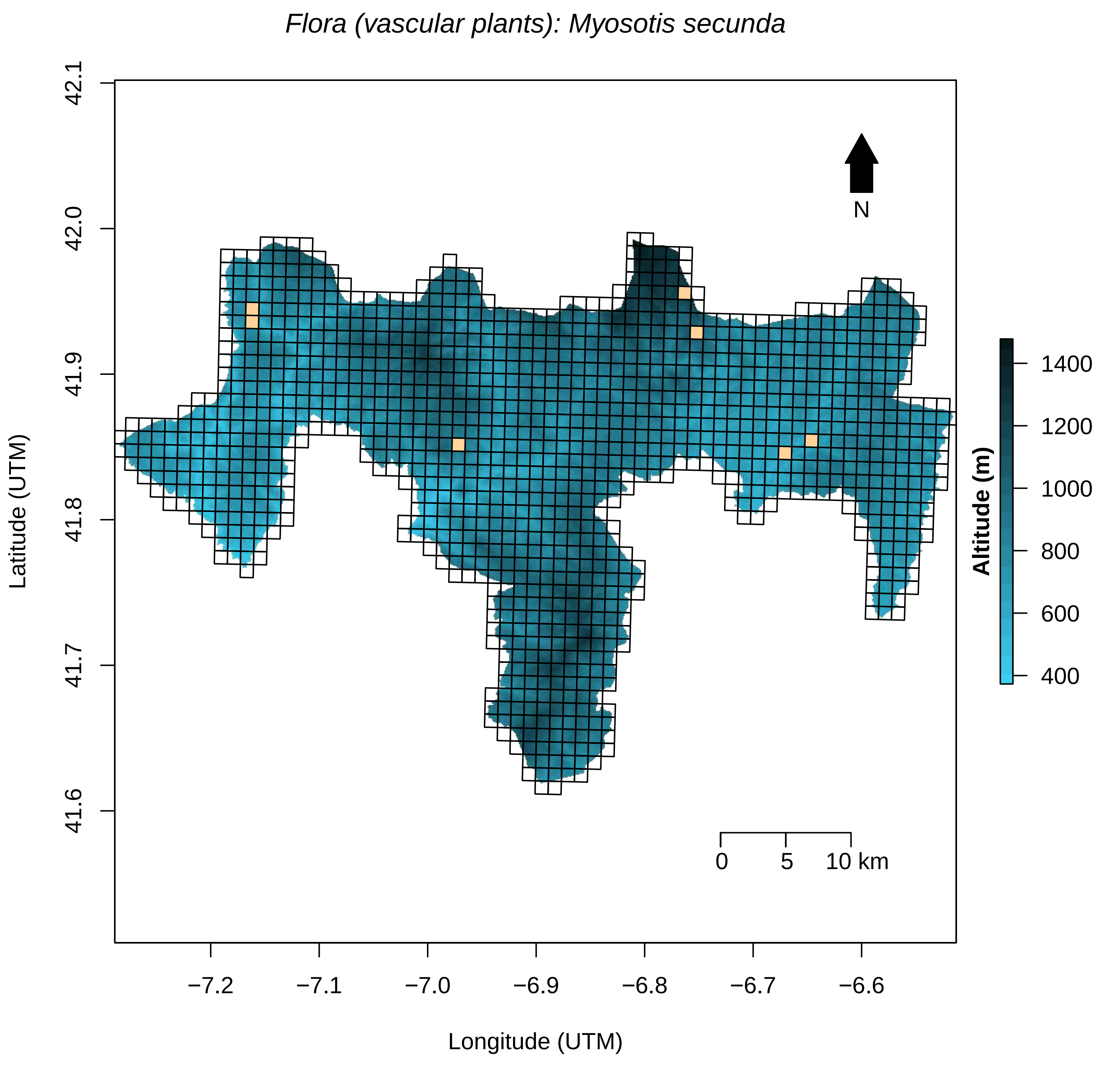

Flora (vascular plants): Myosotis secunda
42.1
42.0
41.9
41.8
41.7
41.6
Latitude (UTM)
−7.2
−7.1
−7.0
−6.9
−6.8
−6.7
−6.6
Longitude (UTM)
N
0
5
10 km
Altitude (m)
1400
1200
1000
800
600
400

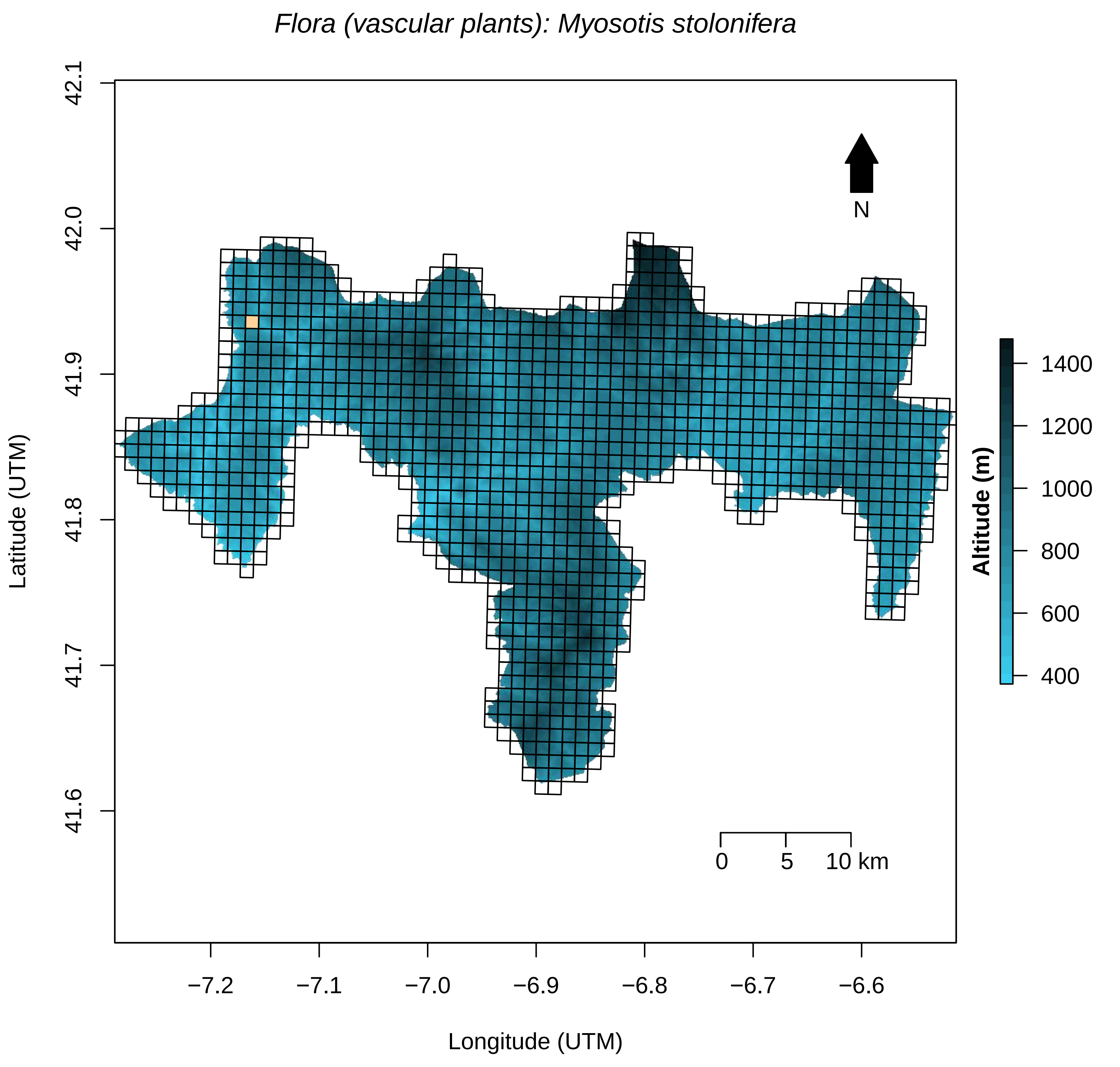

Flora (vascular plants): Myosotis stolonifera
42.1
42.0
41.9
41.8
41.7
41.6
Latitude (UTM)
−7.2
−7.1
−7.0
−6.9
−6.8
−6.7
−6.6
Longitude (UTM)
N
0
5
10 km
Altitude (m)
1400
1200
1000
800
600
400

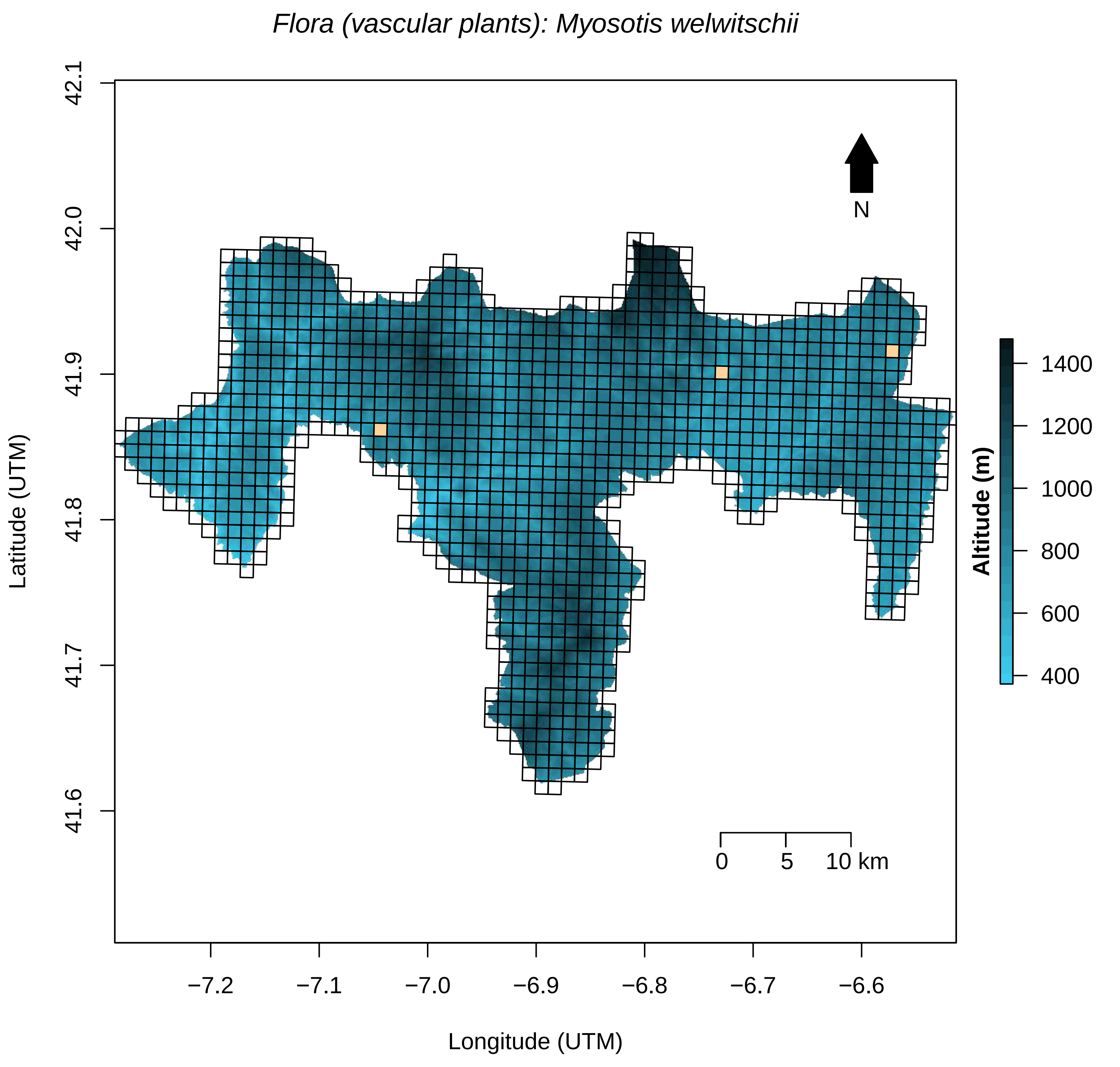

Flora (vascular plants): Myosotis welwitschii
42.1
42.0
41.9
41.8
41.7
41.6
Latitude (UTM)
−7.2
−7.1
−7.0
−6.9
−6.8
−6.7
−6.6
Longitude (UTM)
N
0
5
10 km
Altitude (m)
1400
1200
1000
800
600
400

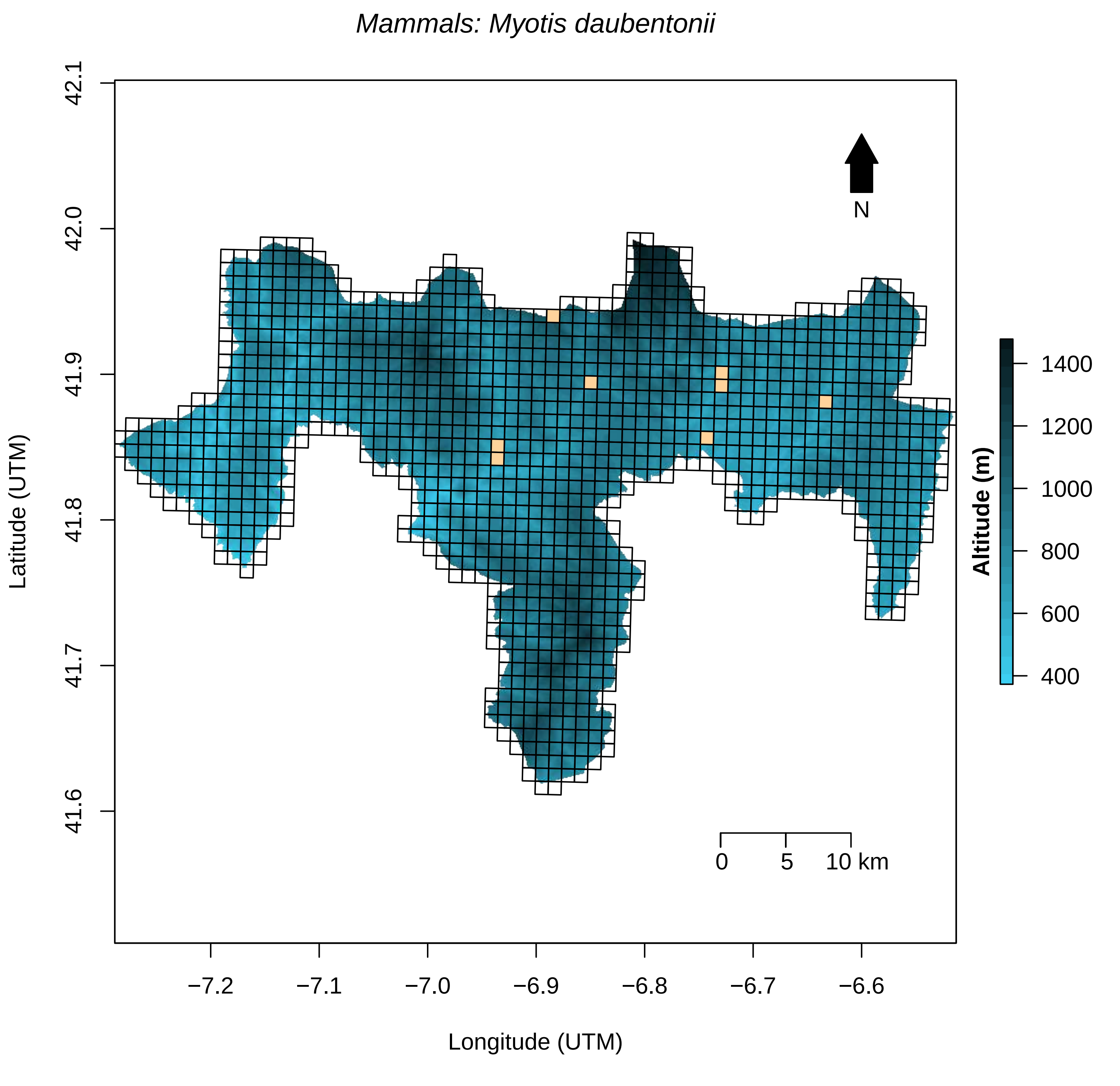
Mammals: Myotis daubentonii
42.1
42.0
41.9
41.8
41.7
41.6
Latitude (UTM)
−7.2
−7.1
−7.0
−6.9
−6.8
−6.7
−6.6
Longitude (UTM)
N
0
5
10 km
1400
1200
1000
800
600
400
Altitude (m)

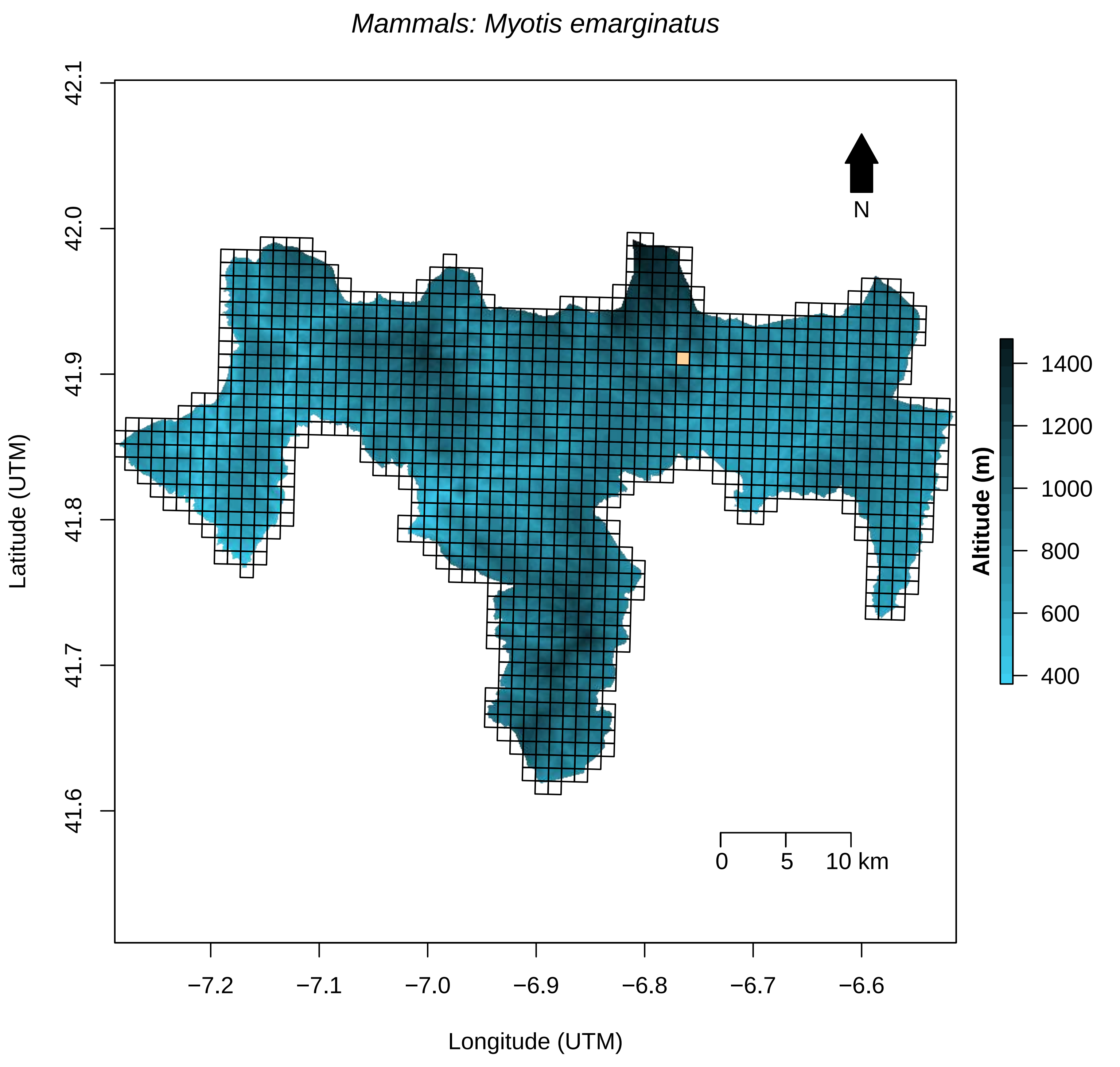

Mammals: Myotis emarginatus
N
0
5
10 km
Latitude (UTM)
Longitude (UTM)
42.1
42.0
41.9
41.8
41.7
41.6
−7.2
−7.1
−7.0
−6.9
−6.8
−6.7
−6.6
Altitude (m)
1400
1200
1000
800
600
400

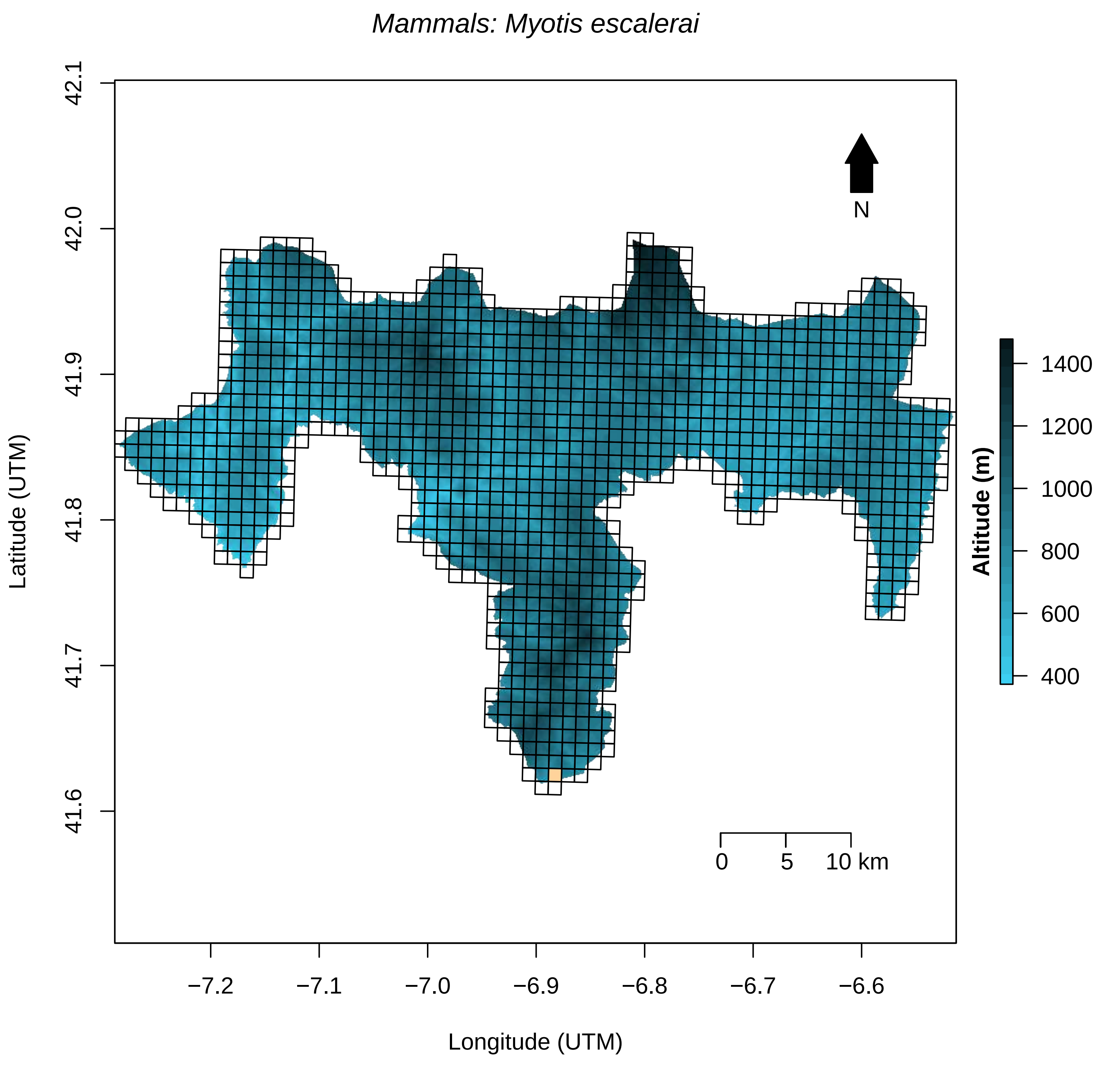

Mammals: Myotis escalerai
N
0
5
10 km
Altitude (m)
1400
1200
1000
800
600
400
Latitude (UTM)
42.1
42.0
41.9
41.8
41.7
41.6
Longitude (UTM)
−7.2
−7.1
−7.0
−6.9
−6.8
−6.7
−6.6

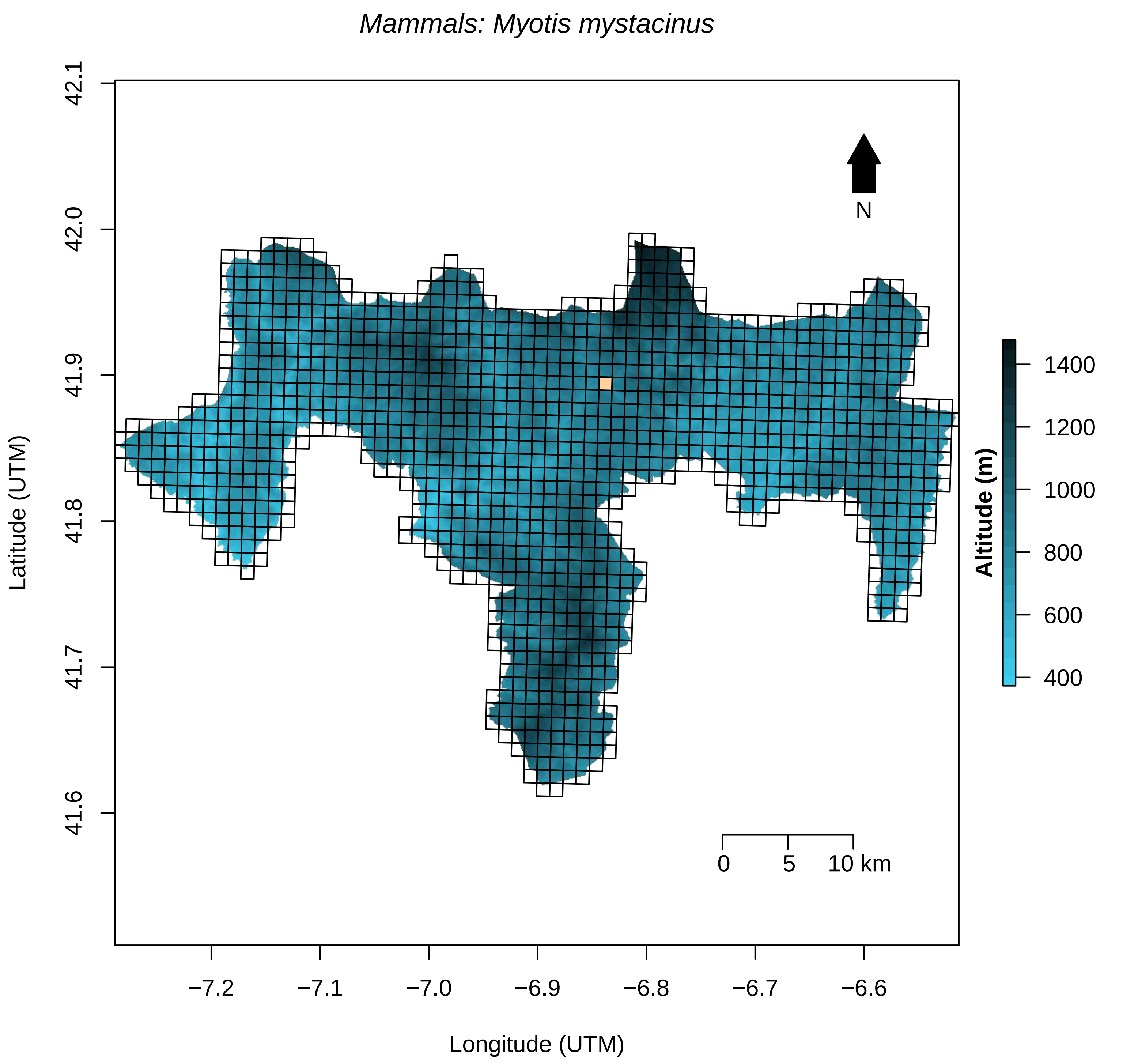

Mammals: Myotis mystacinus
42.1
42.0
41.9
41.8
41.7
41.6
Latitude (UTM)
−7.2
−7.1
−7.0
−6.9
−6.8
−6.7
−6.6
Longitude (UTM)
N
0
5
10 km
Altitude (m)
1400
1200
1000
800
600
400

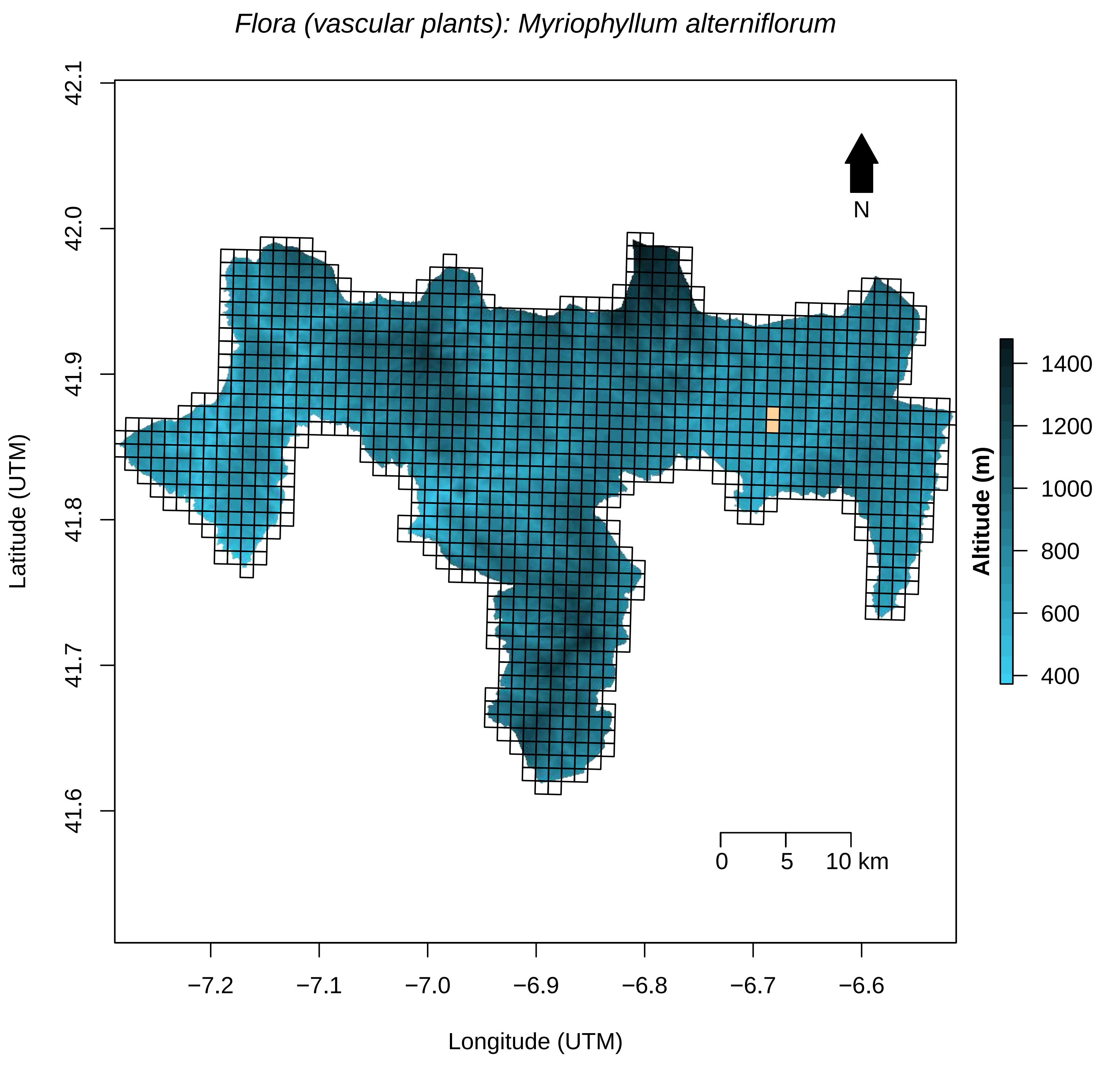
Flora (vascular plants): Myriophyllum alterniflorum
42.1
42.0
41.9
41.8
41.7
41.6
Latitude (UTM)
N
0 5 10 km
−7.2 −7.1 −7.0 −6.9 −6.8 −6.7 −6.6
Longitude (UTM)
1400
1200
1000
800
600
400
Altitude (m)

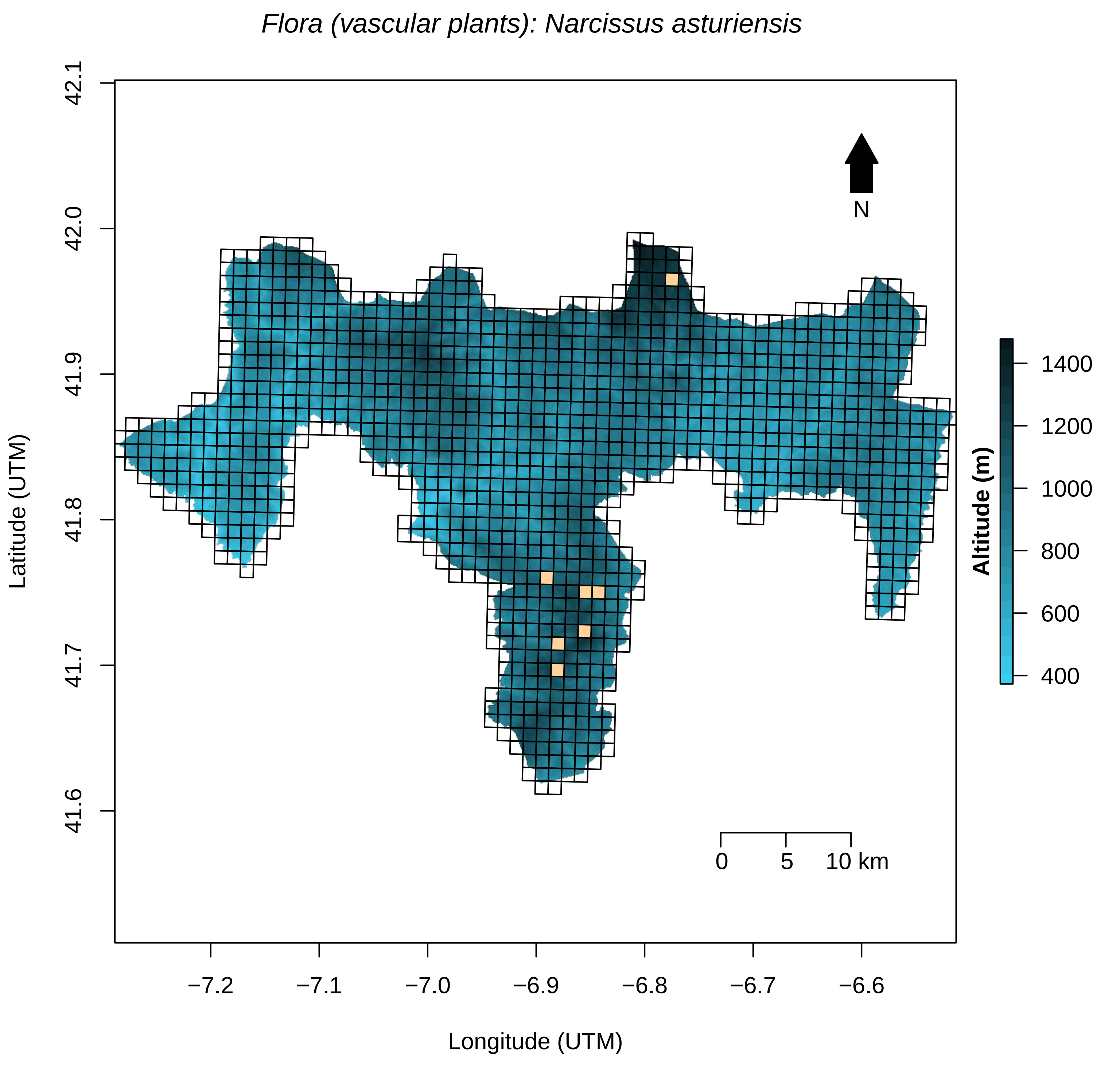

Flora (vascular plants): Narcissus asturiensis
N
0
5
10 km
Altitude (m)
1400
1200
1000
800
600
400
Latitude (UTM)
42.1
42.0
41.9
41.8
41.7
41.6
Longitude (UTM)
−7.2
−7.1
−7.0
−6.9
−6.8
−6.7
−6.6

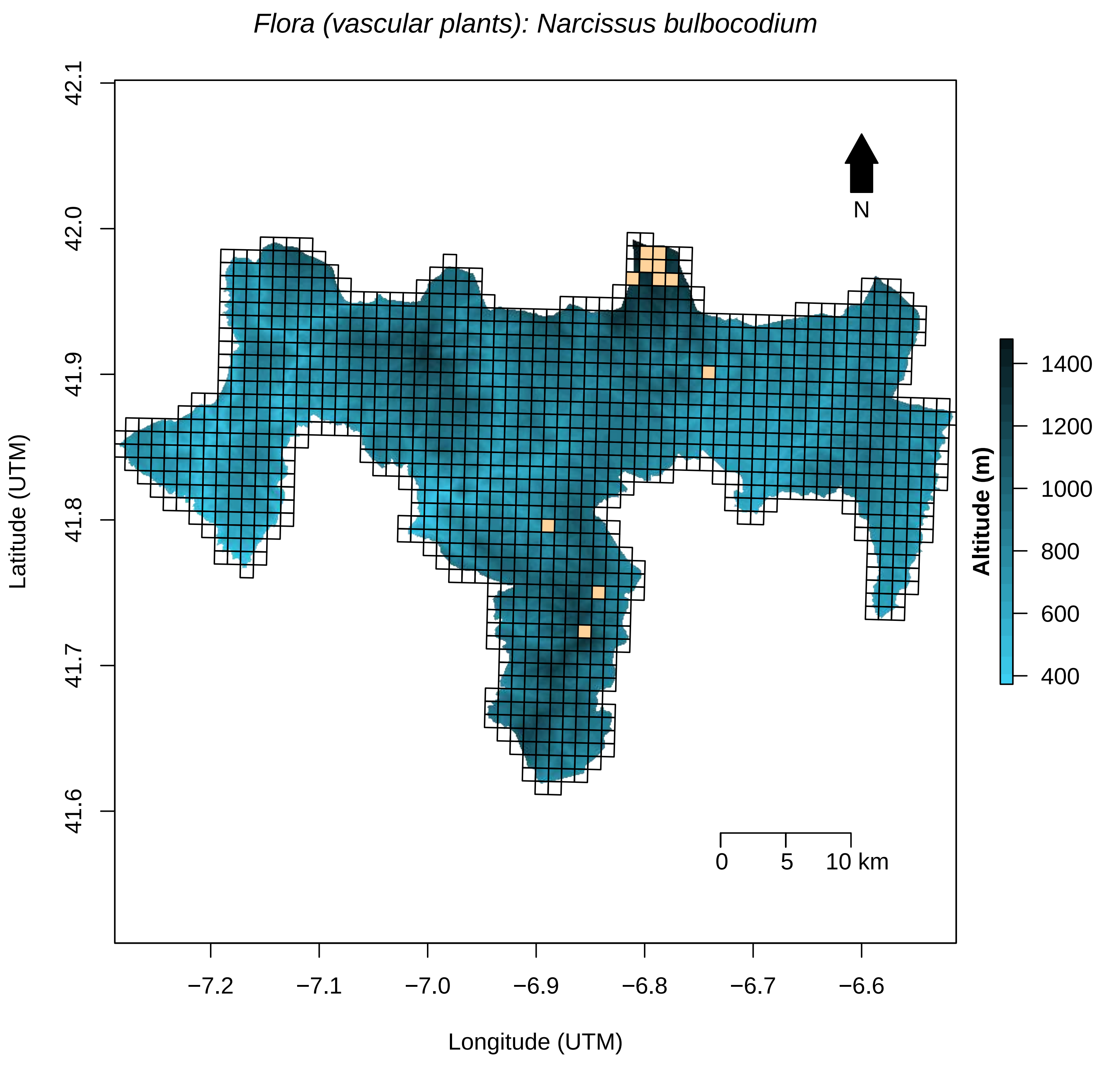

Flora (vascular plants): Narcissus bulbocodium
42.1
42.0
41.9
41.8
41.7
41.6
Latitude (UTM)
−7.2
−7.1
−7.0
−6.9
−6.8
−6.7
−6.6
Longitude (UTM)
N
0
5
10 km
Altitude (m)
1400
1200
1000
800
600
400

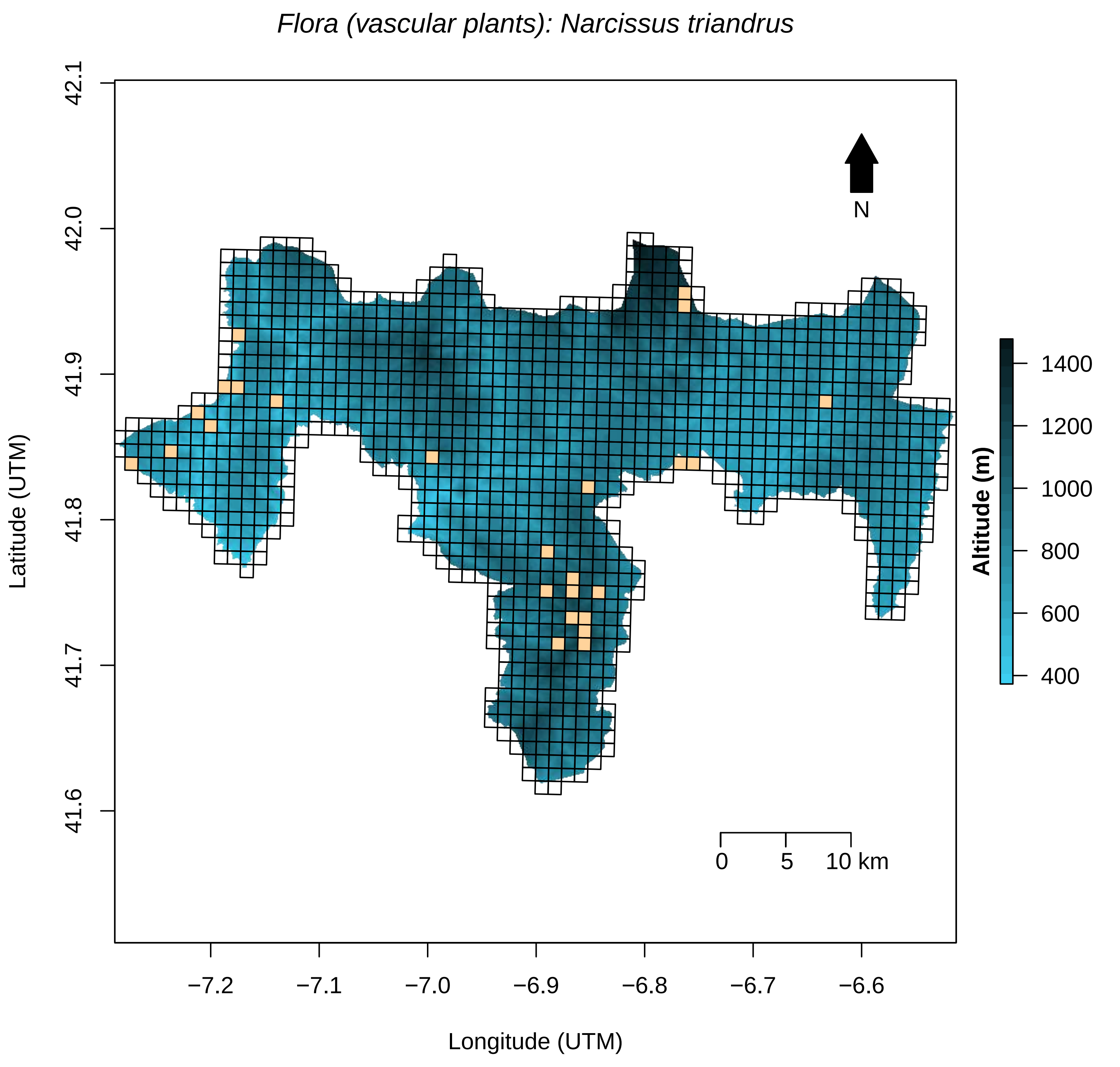

Flora (vascular plants): Narcissus triandrus
42.1
42.0
41.9
41.8
41.7
41.6
Latitude (UTM)
−7.2
−7.1
−7.0
−6.9
−6.8
−6.7
−6.6
Longitude (UTM)
N
0
5
10 km
Altitude (m)
1400
1200
1000
800
600
400

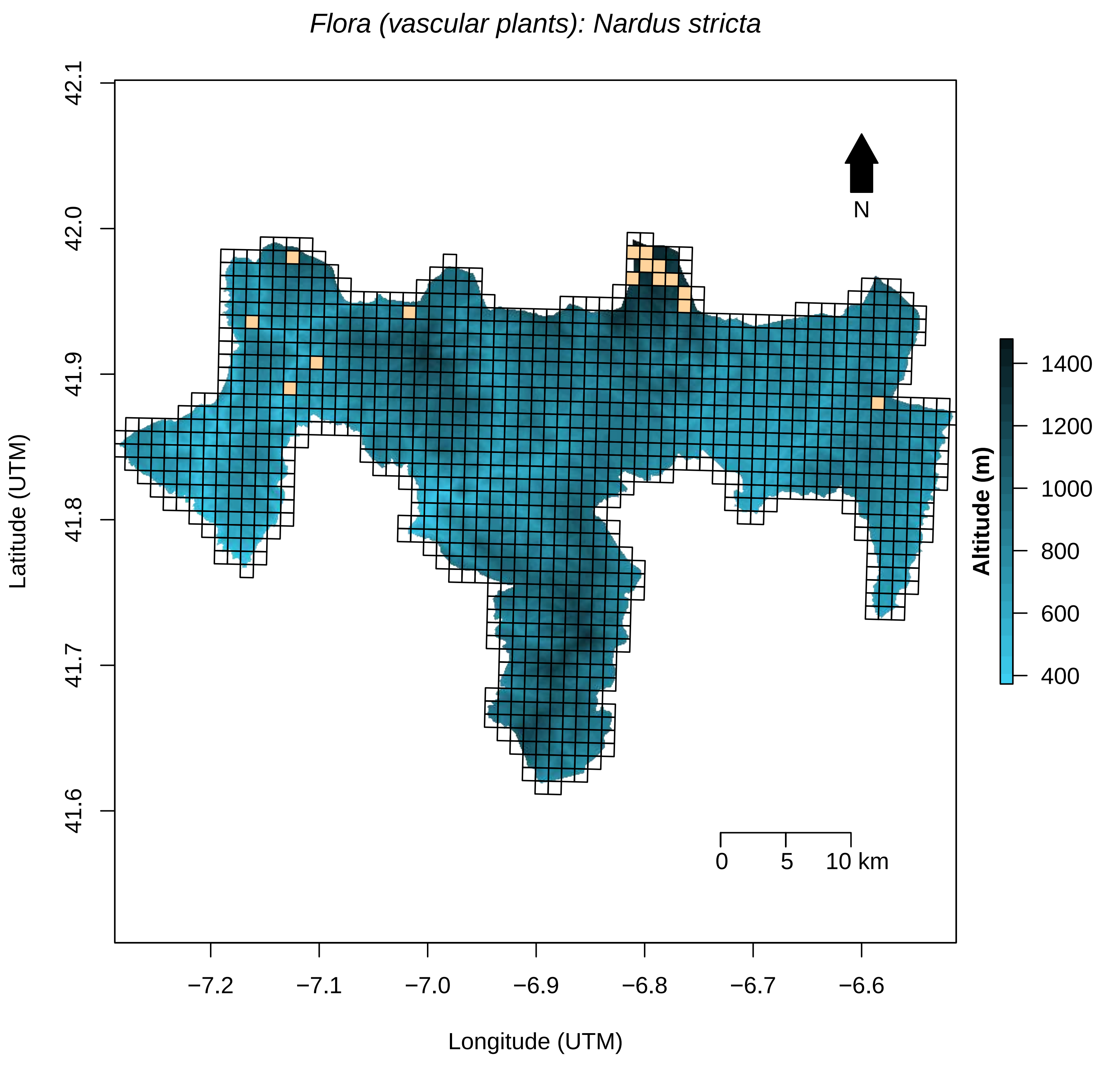

Flora (vascular plants): Nardus stricta
Latitude (UTM)
Longitude (UTM)
42.1
42.0
41.9
41.8
41.7
41.6
−7.2
−7.1
−7.0
−6.9
−6.8
−6.7
−6.6
N
0
5
10 km
Altitude (m)
1400
1200
1000
800
600
400

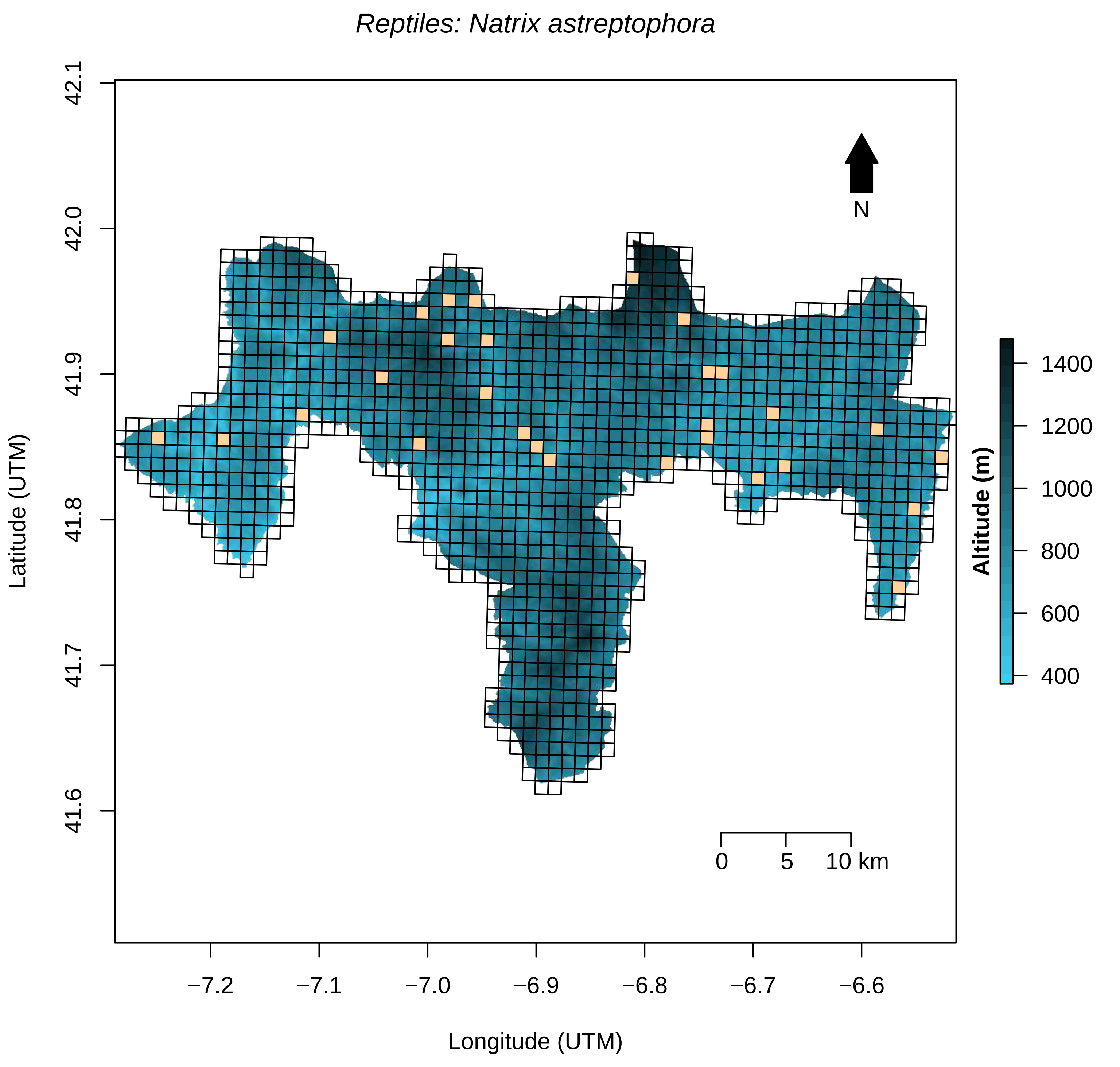
Reptiles: Natrix astreptophora
N
0 5 10 km
Altitude (m)
1400
1200
1000
800
600
400
42.1
42.0
41.9
41.8
41.7
41.6
Latitude (UTM)
−7.2
−7.1
−7.0
−6.9
−6.8
−6.7
−6.6
Longitude (UTM)

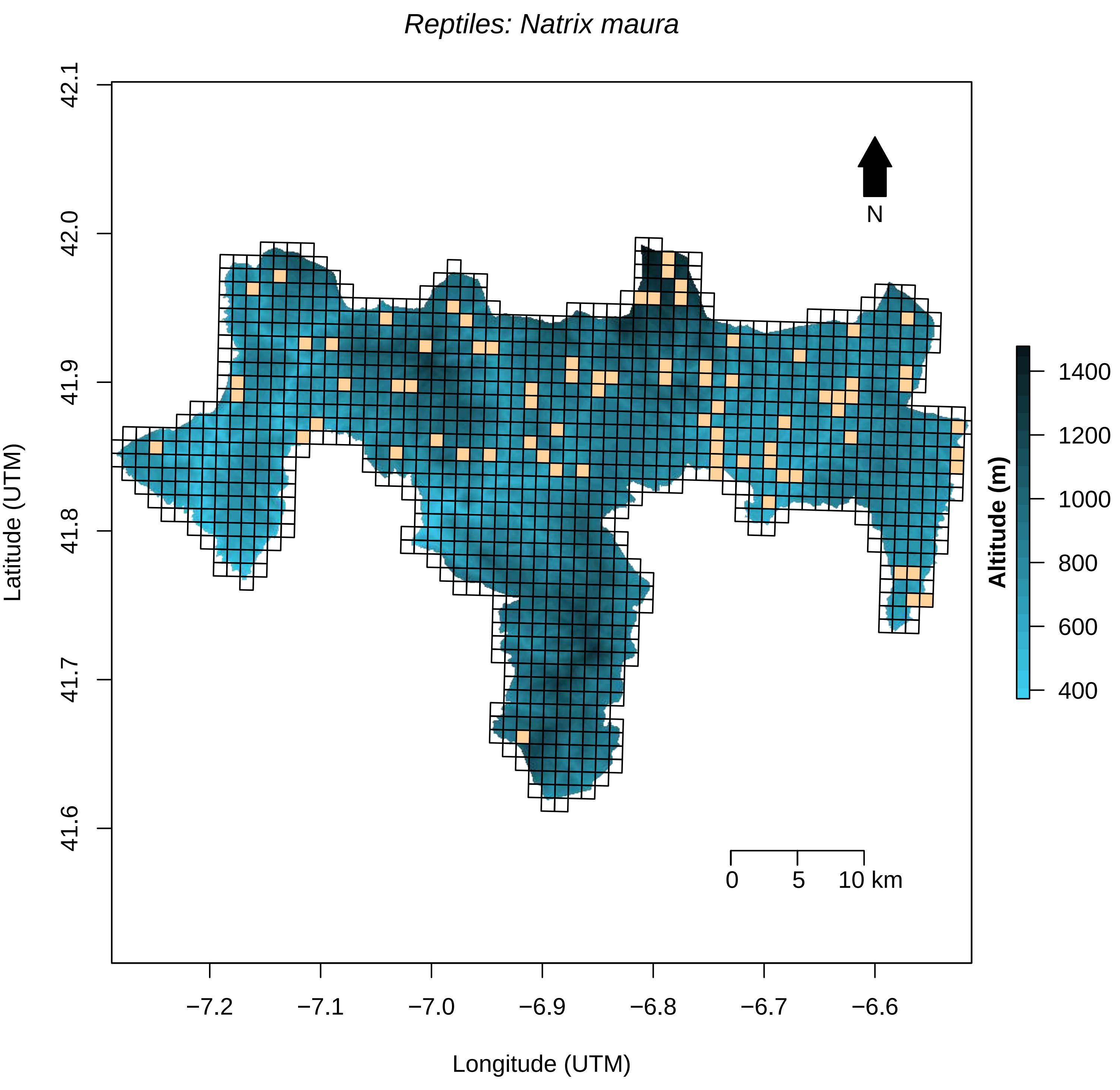
Reptiles: Natrix maura
Latitude (UTM)
Longitude (UTM)
42.1
42.0
41.9
41.8
41.7
41.6
−7.2
−7.1
−7.0
−6.9
−6.8
−6.7
−6.6
N
0
5
10 km
Altitude (m)
1400
1200
1000
800
600
400

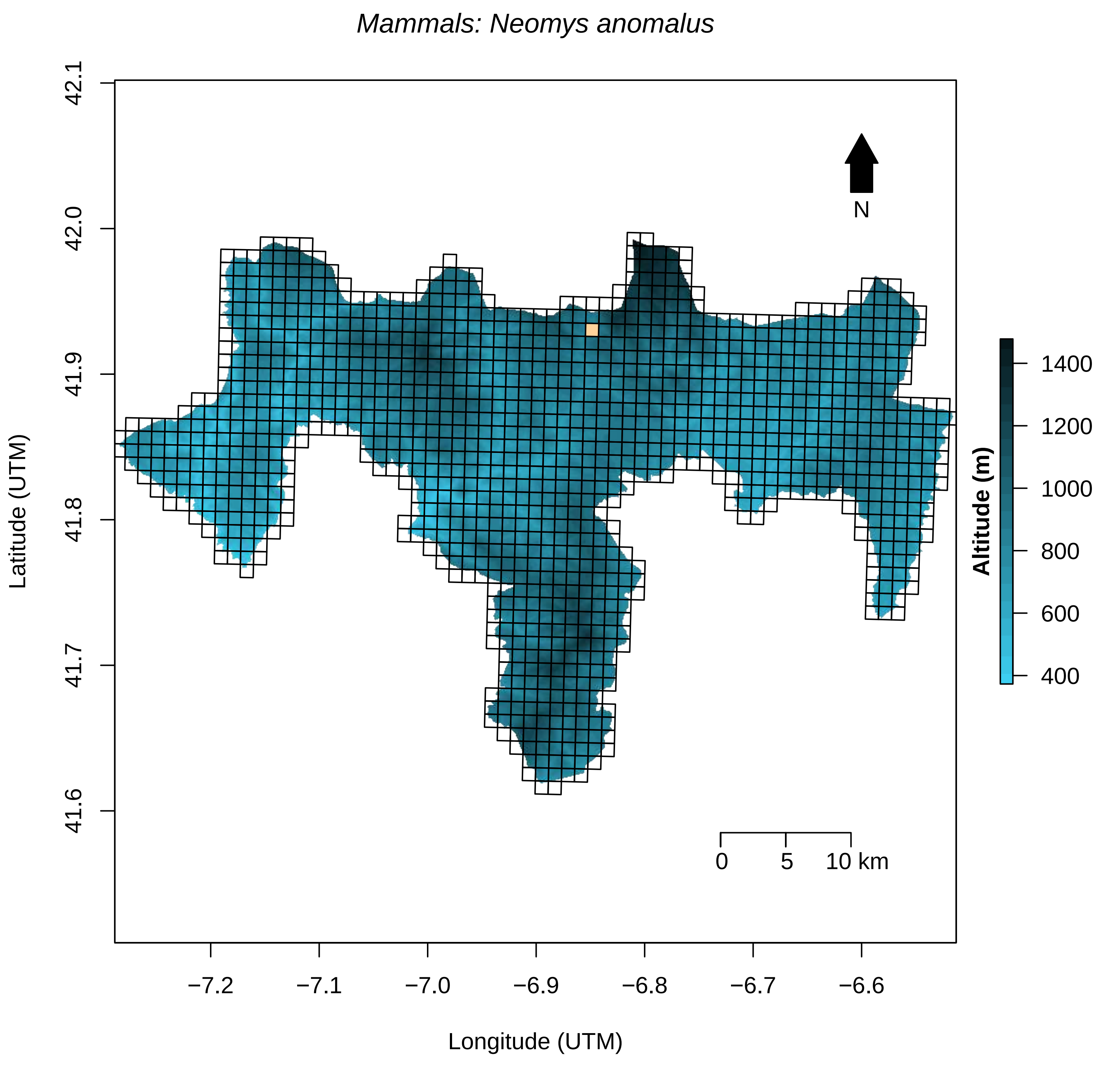

Mammals: Neomys anomalus
42.1
42.0
41.9
41.8
41.7
41.6
Latitude (UTM)
−7.2
−7.1
−7.0
−6.9
−6.8
−6.7
−6.6
Longitude (UTM)
N
0
5
10 km
Altitude (m)
1400
1200
1000
800
600
400

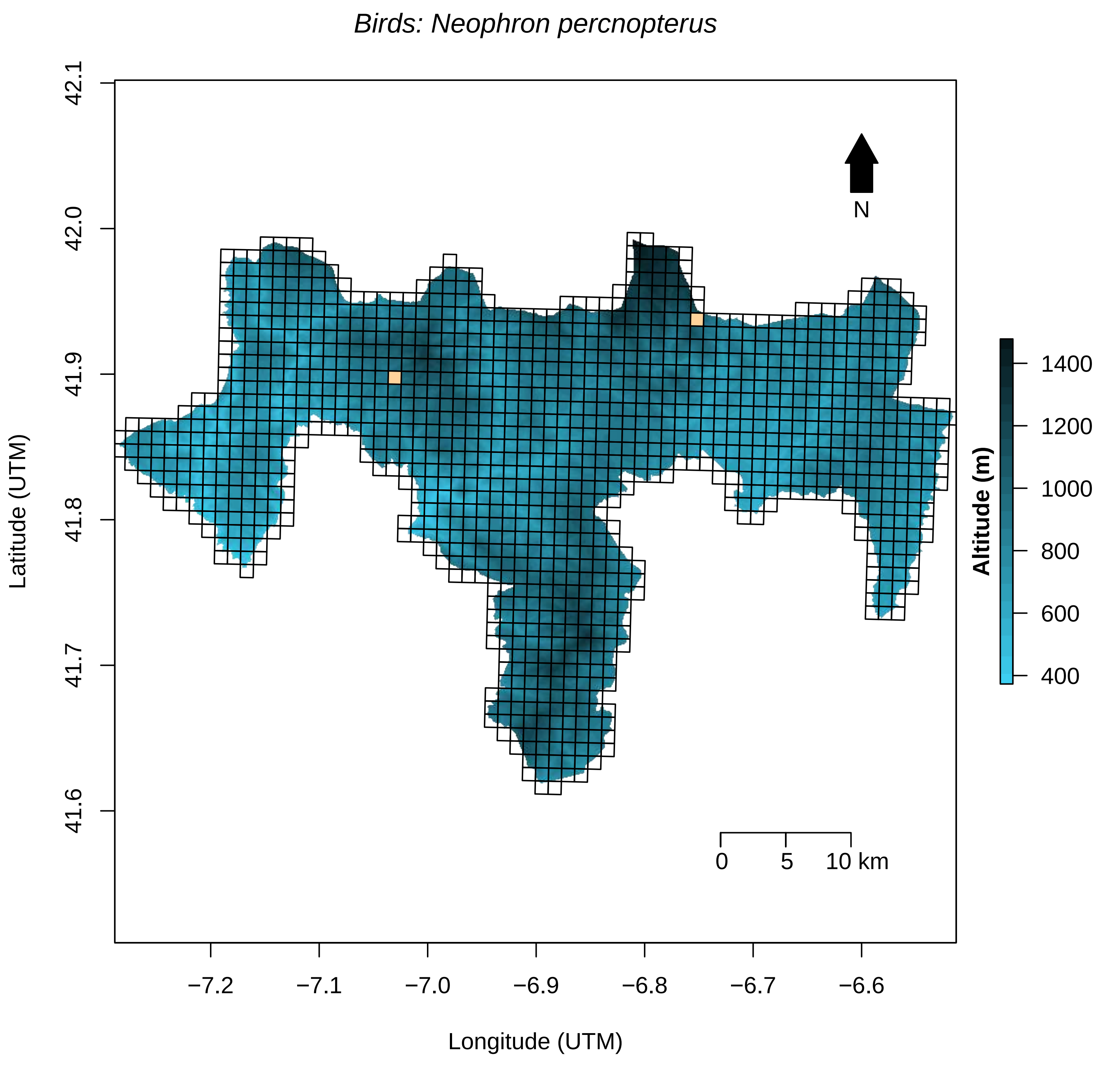

Birds: Neophron percnopterus
42.1
42.0
41.9
41.8
41.7
41.6
Latitude (UTM)
N
0
5
10 km
1400
1200
1000
800
600
400
Altitude (m)
−7.2
−7.1
−7.0
−6.9
−6.8
−6.7
−6.6
Longitude (UTM)

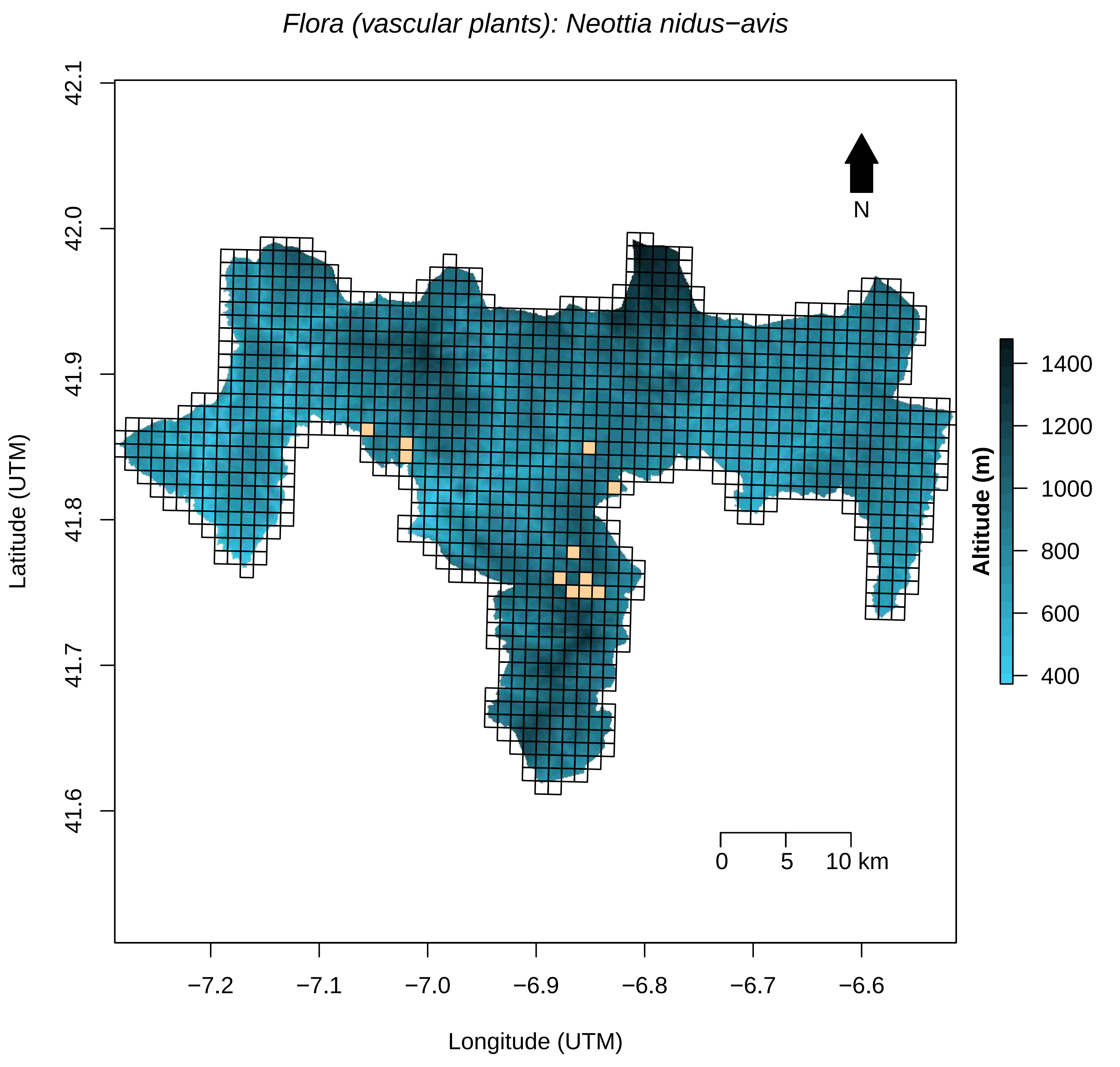

Flora (vascular plants): Neottia nidus−avis
42.1
42.0
41.9
41.8
41.7
41.6
Latitude (UTM)
−7.2
−7.1
−7.0
−6.9
−6.8
−6.7
−6.6
Longitude (UTM)
N
0
5
10 km
Altitude (m)
1400
1200
1000
800
600
400

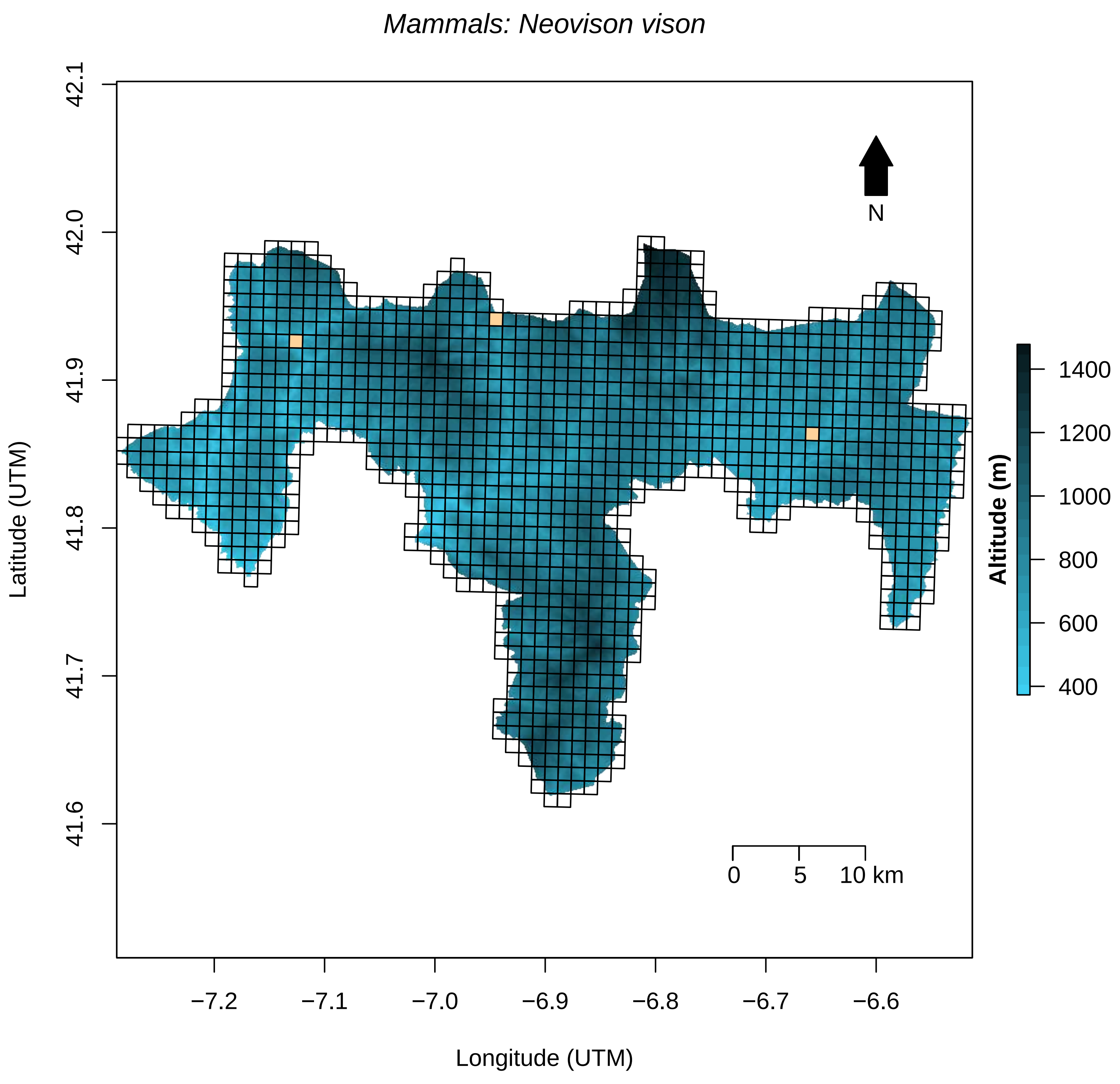

Mammals: Neovison vison
42.1
42.0
41.9
41.8
41.7
41.6
Latitude (UTM)
−7.2
−7.1
−7.0
−6.9
−6.8
−6.7
−6.6
Longitude (UTM)
N
0
5
10 km
Altitude (m)
1400
1200
1000
800
600
400

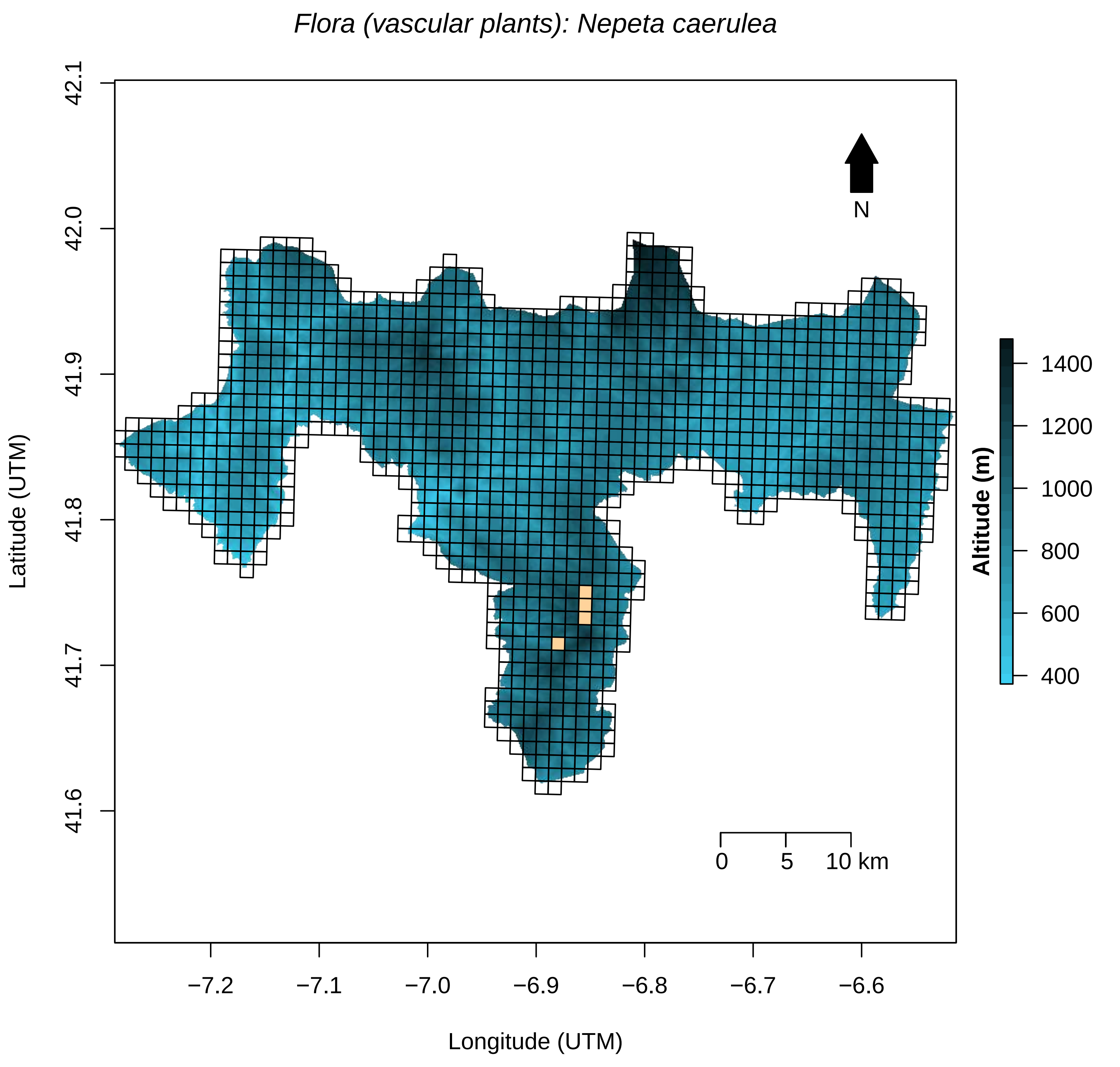
Flora (vascular plants): Nepeta caerulea
Latitude (UTM)
42.1
42.0
41.9
41.8
41.7
41.6
Longitude (UTM)
−7.2
−7.1
−7.0
−6.9
−6.8
−6.7
−6.6
N
0
5
10 km
Altitude (m)
1400
1200
1000
800
600
400

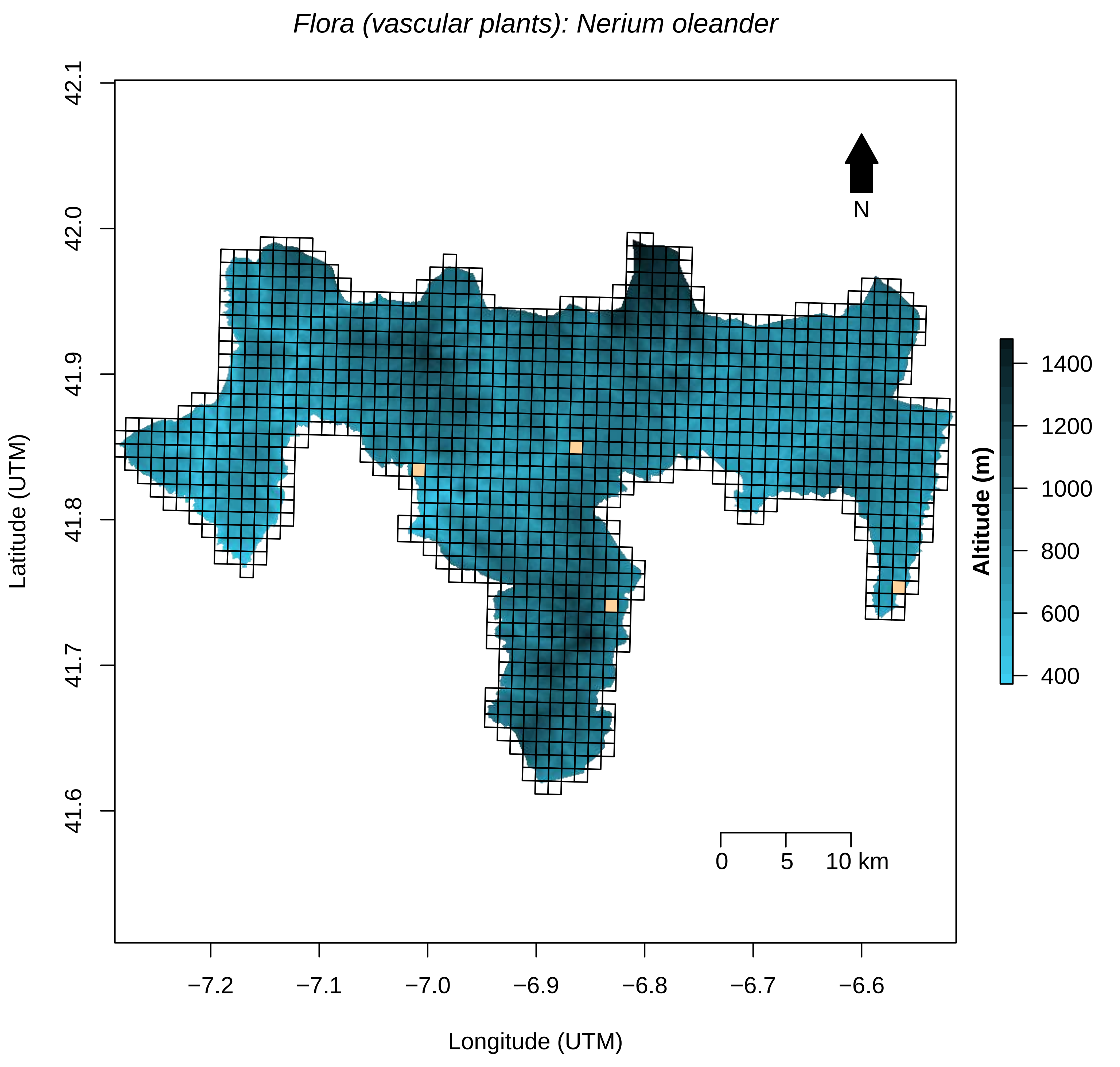

Flora (vascular plants): Nerium oleander
42.1
42.0
41.9
41.8
41.7
41.6
Latitude (UTM)
−7.2
−7.1
−7.0
−6.9
−6.8
−6.7
−6.6
Longitude (UTM)
N
0
5
10 km
Altitude (m)
1400
1200
1000
800
600
400

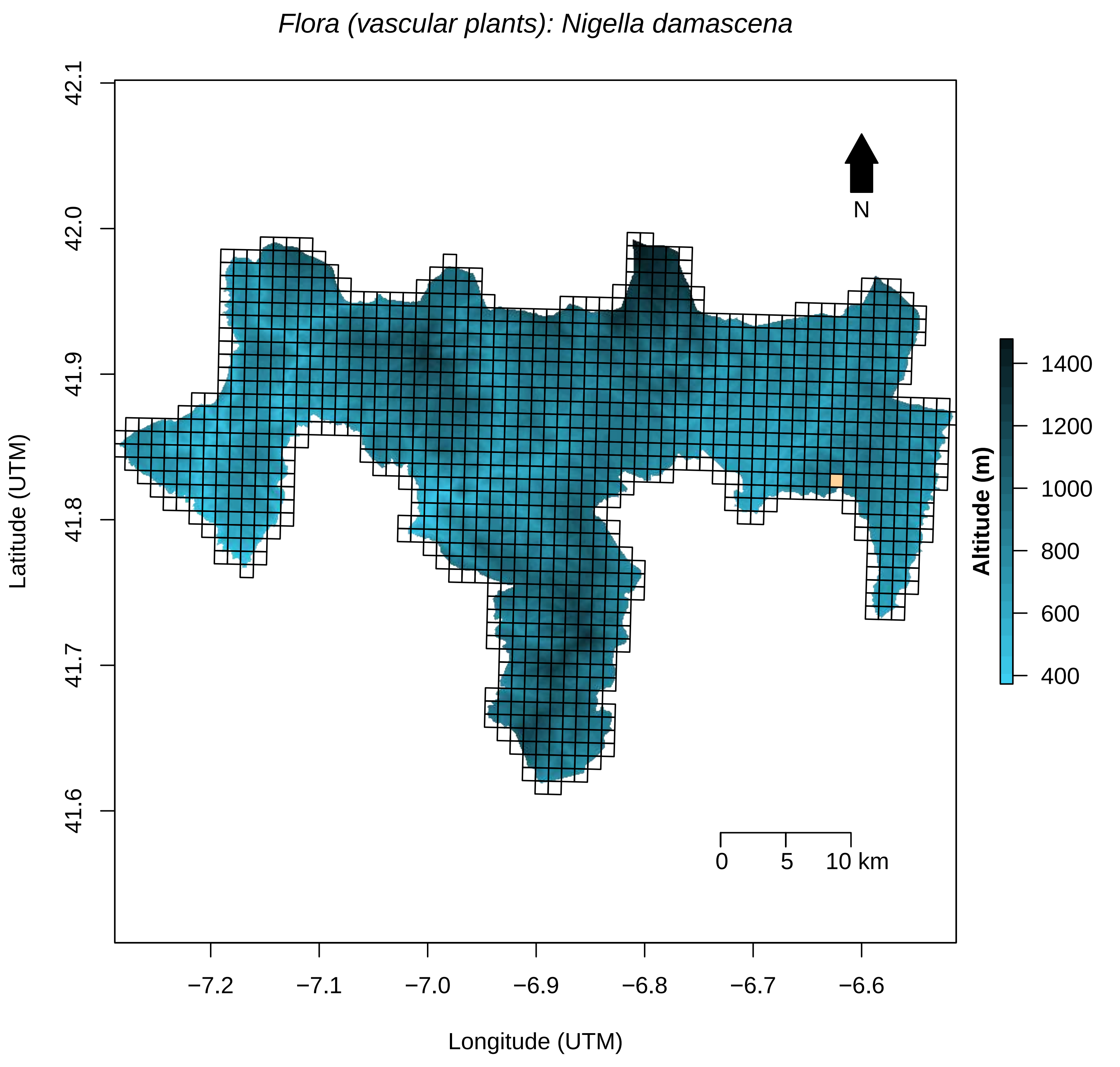

Flora (vascular plants): Nigella damascena
N
0 5 10 km
Latitude (UTM)
Longitude (UTM)
42.1
42.0
41.9
41.8
41.7
41.6
−7.2
−7.1
−7.0
−6.9
−6.8
−6.7
−6.6
Altitude (m)
1400
1200
1000
800
600
400

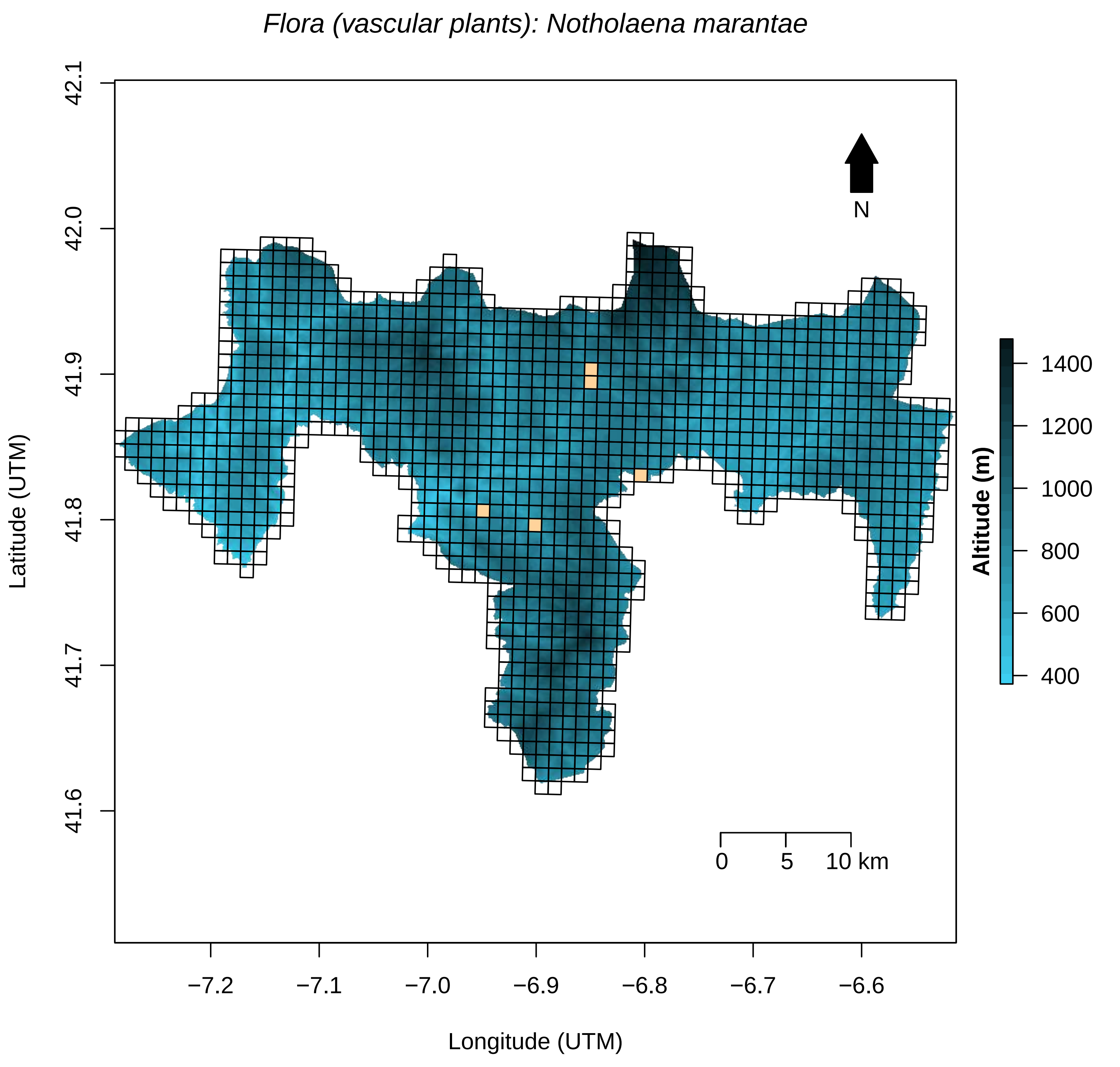

Flora (vascular plants): Notholaena marantae
N
0 5 10 km
Latitude (UTM)
Longitude (UTM)
Altitude (m)
42.1
42.0
41.9
41.8
41.7
41.6
−7.2
−7.1
−7.0
−6.9
−6.8
−6.7
−6.6
1400
1200
1000
800
600
400

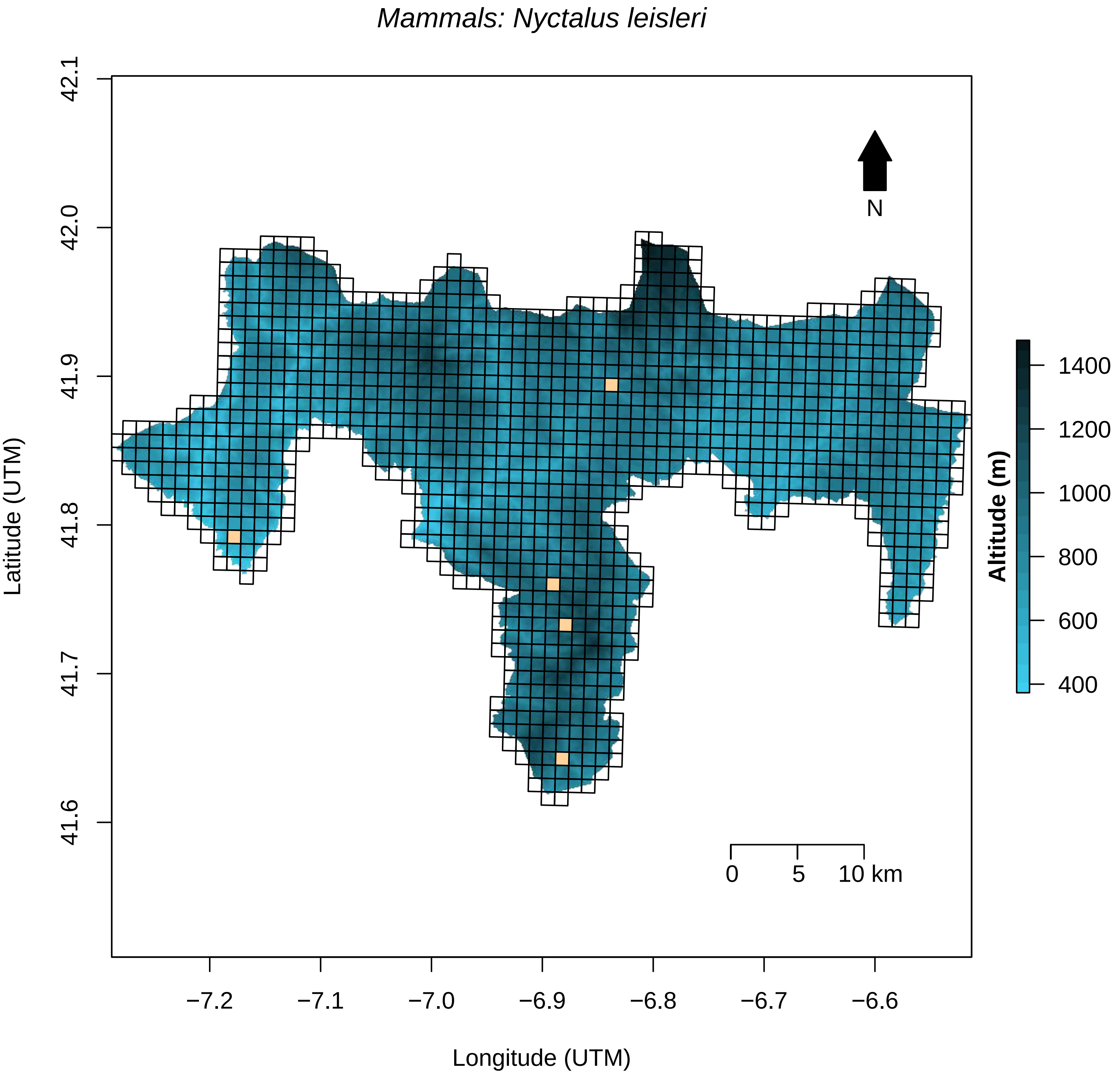

Mammals: Nyctalus leisleri
N
0
5
10 km
Altitude (m)
1400
1200
1000
800
600
400
42.1
42.0
41.9
41.8
41.7
41.6
Latitude (UTM)
−7.2
−7.1
−7.0
−6.9
−6.8
−6.7
−6.6
Longitude (UTM)

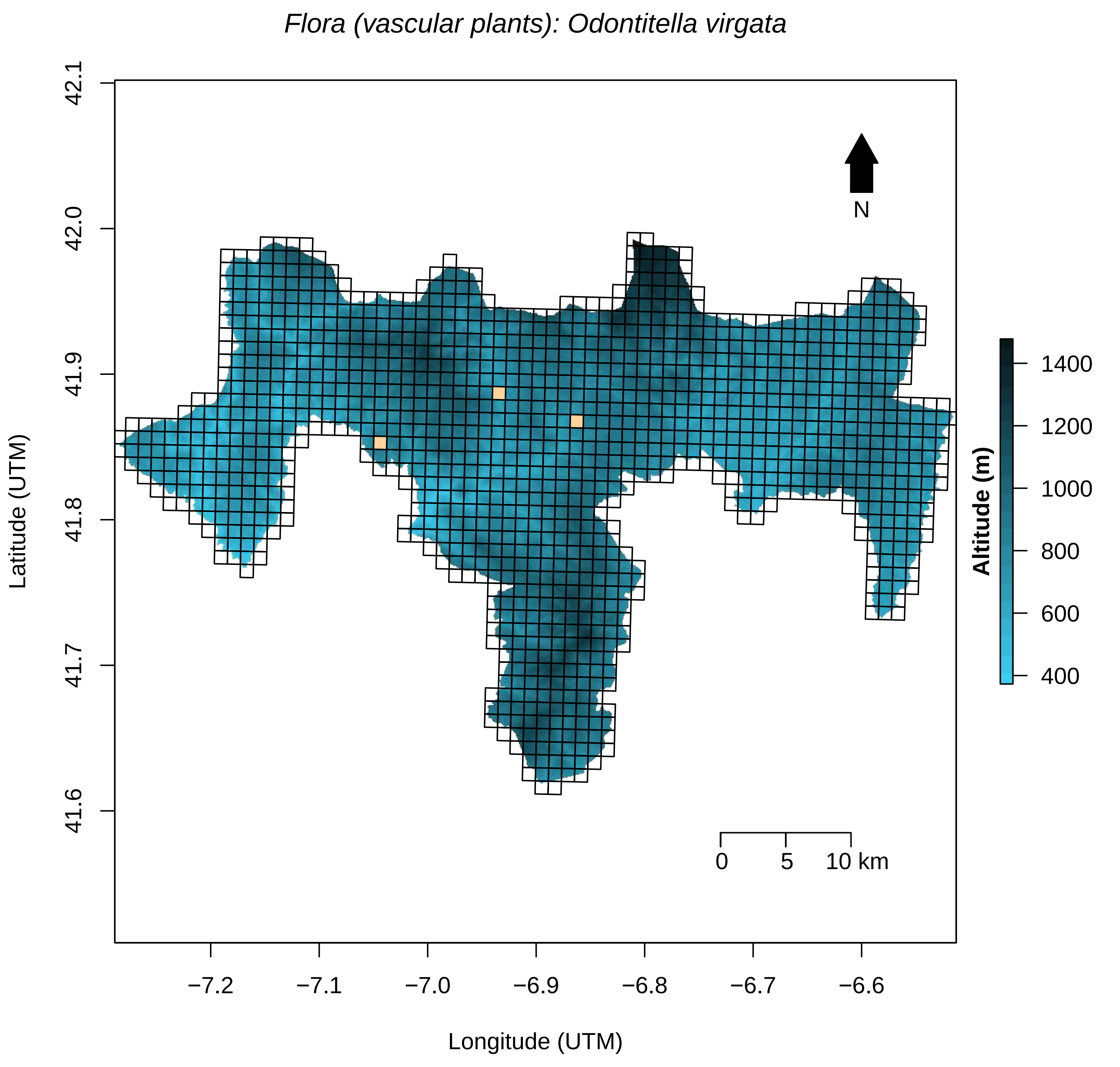

Flora (vascular plants): Odontitella virgata
N
0
5
10 km
Altitude (m)
1400
1200
1000
800
600
400
Latitude (UTM)
42.1
42.0
41.9
41.8
41.7
41.6
Longitude (UTM)
−7.2
−7.1
−7.0
−6.9
−6.8
−6.7
−6.6

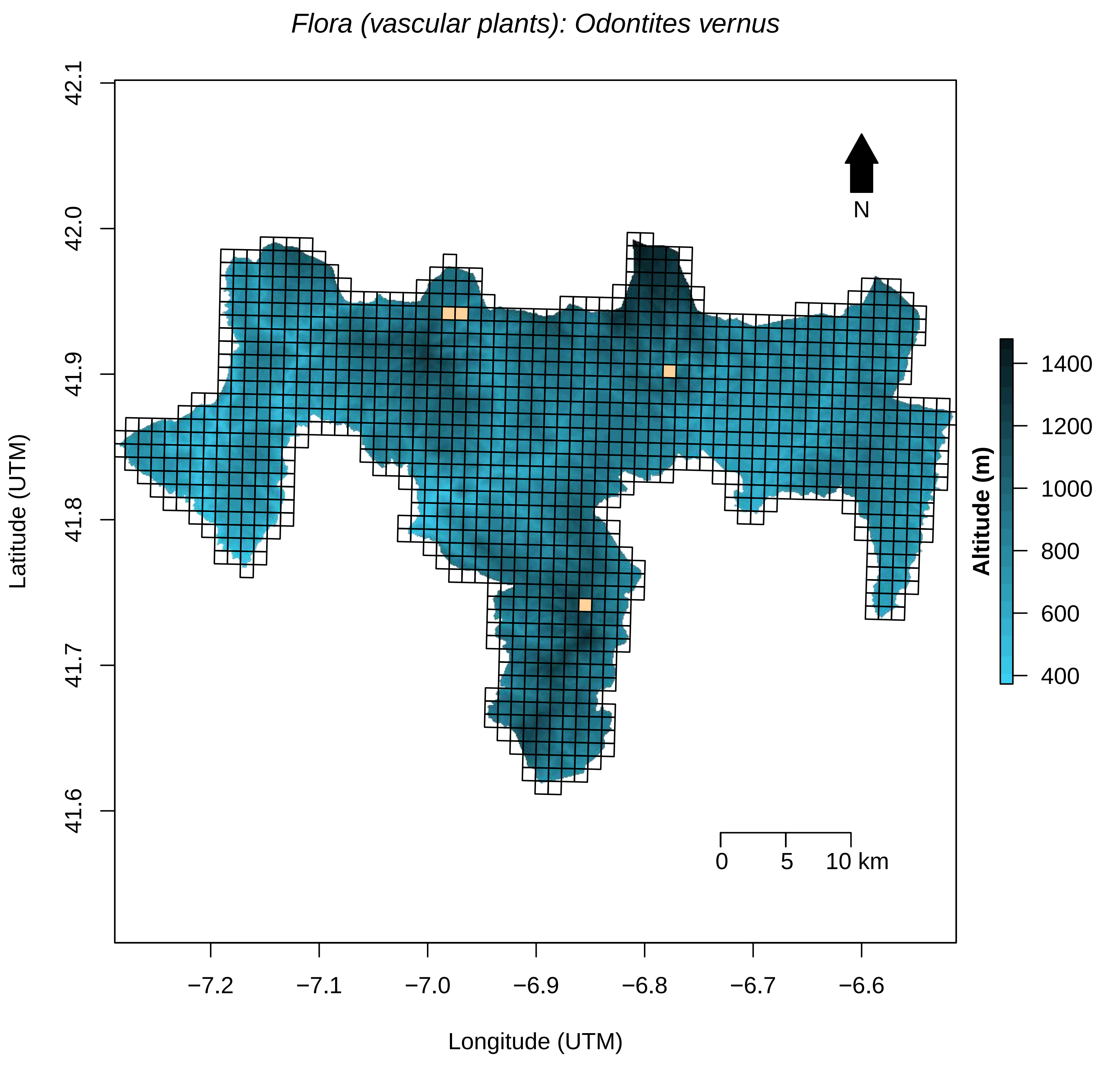
Flora (vascular plants): Odontites vernus
N
0
5
10 km
Altitude (m)
1400
1200
1000
800
600
400
42.1
42.0
41.9
41.8
41.7
41.6
Latitude (UTM)
−7.2
−7.1
−7.0
−6.9
−6.8
−6.7
−6.6
Longitude (UTM)

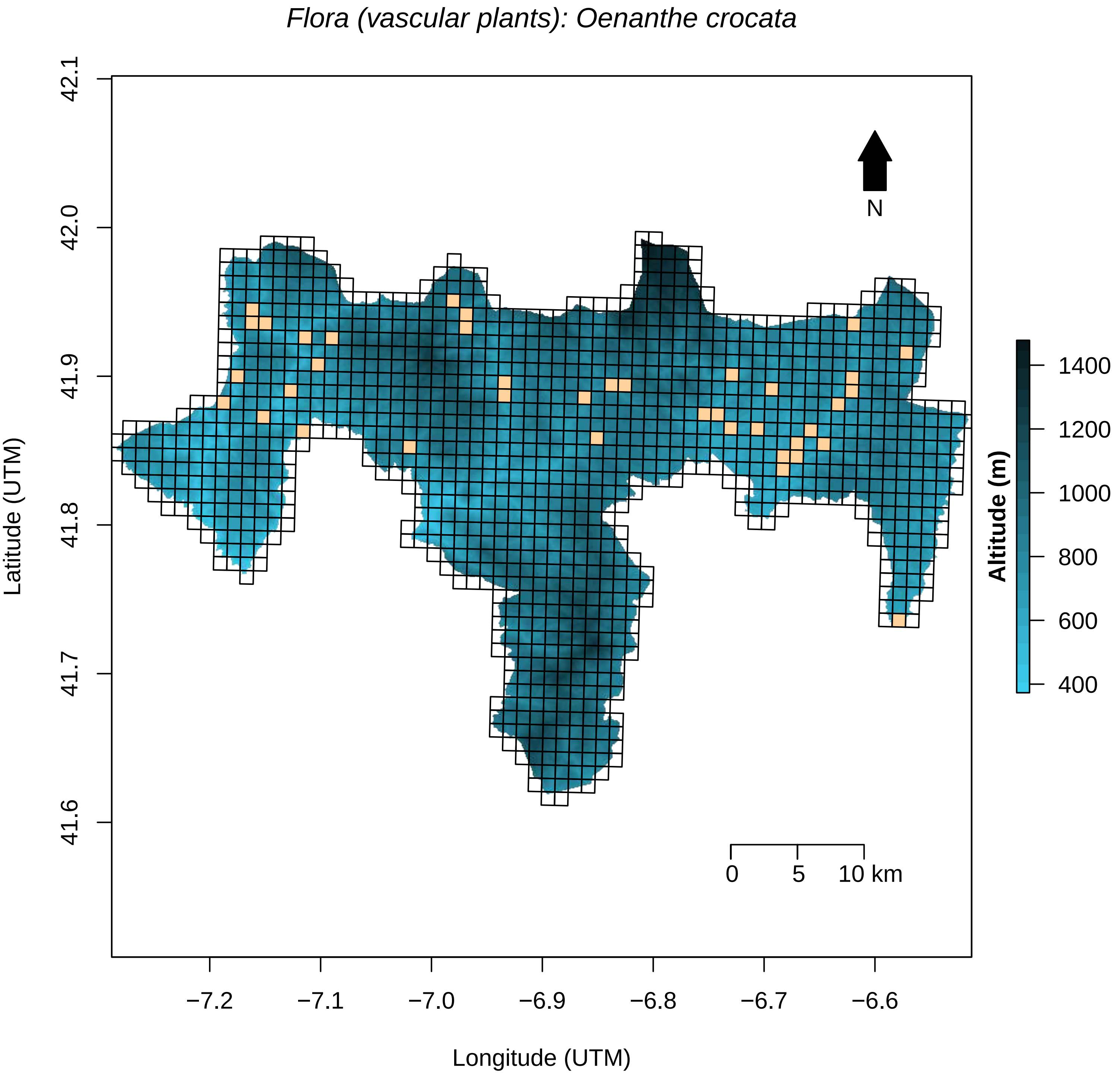

Flora (vascular plants): Oenanthe crocata
42.1
42.0
41.9
41.8
41.7
41.6
Latitude (UTM)
−7.2
−7.1
−7.0
−6.9
−6.8
−6.7
−6.6
Longitude (UTM)
N
0
5
10 km
Altitude (m)
1400
1200
1000
800
600
400

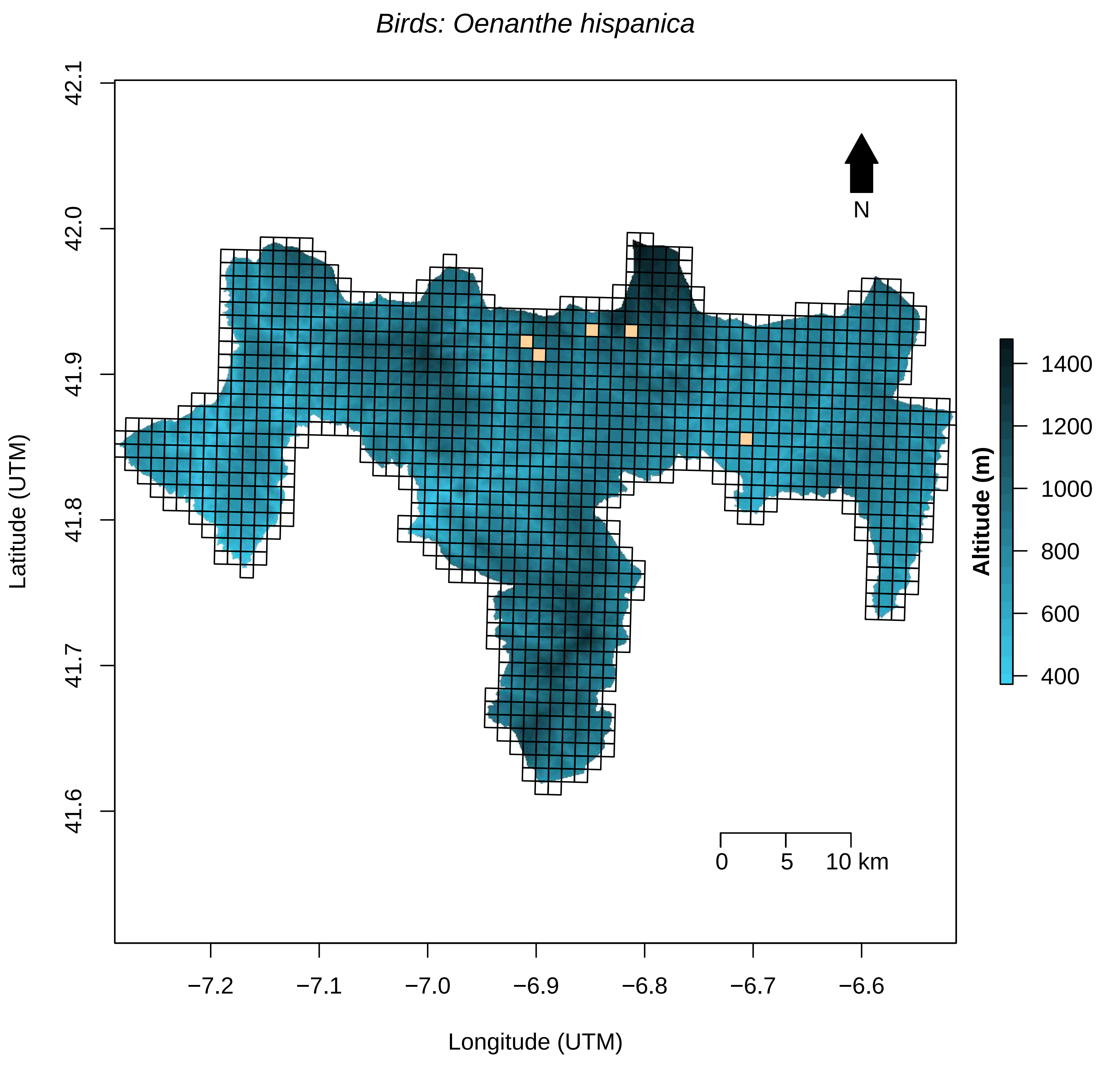
Birds: Oenanthe hispanica
42.1
42.0
41.9
41.8
41.7
41.6
Latitude (UTM)
−7.2
−7.1
−7.0
−6.9
−6.8
−6.7
−6.6
Longitude (UTM)
N
0
5
10 km
Altitude (m)
1400
1200
1000
800
600
400

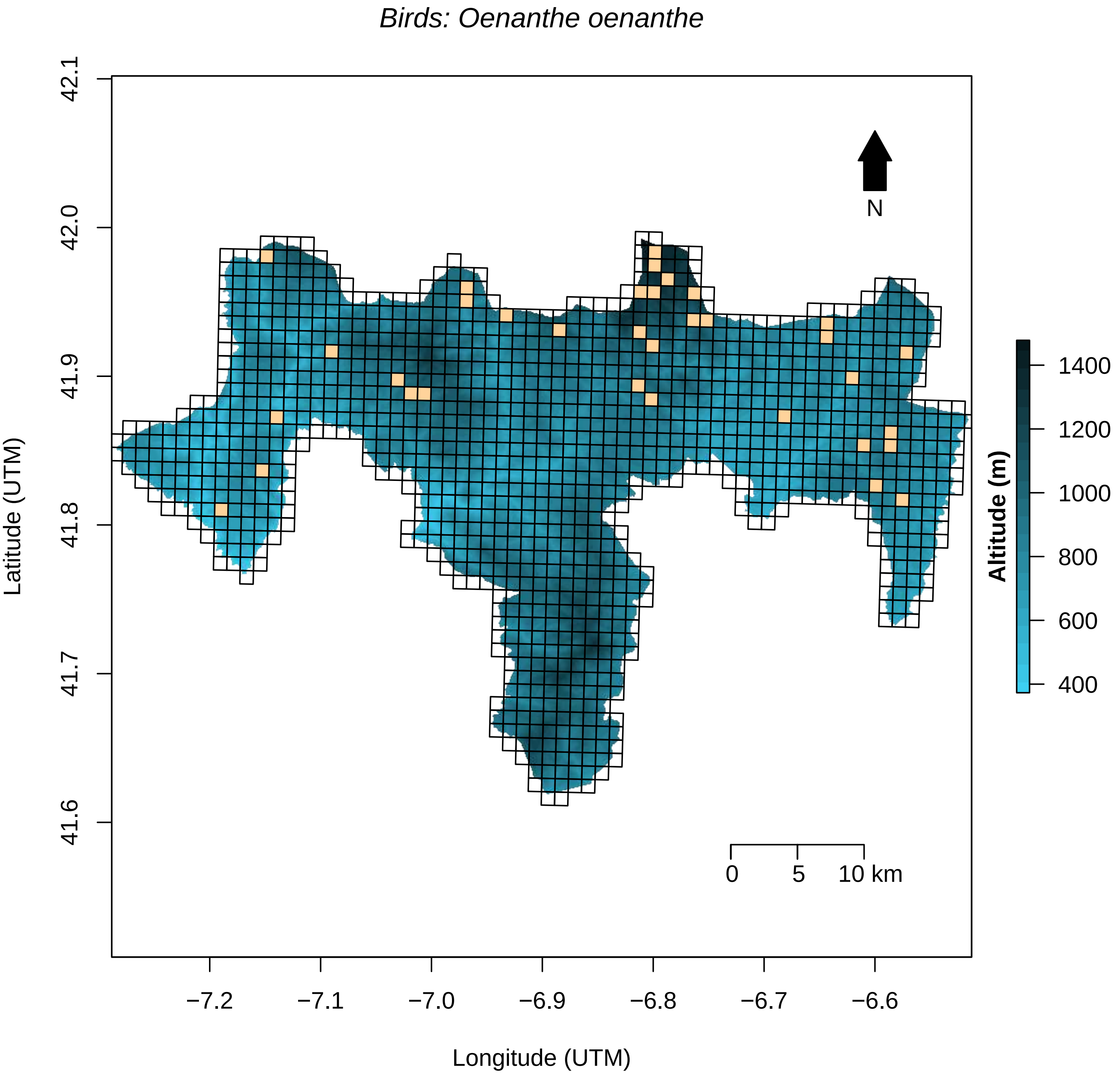

Birds: Oenanthe oenanthe
42.1
42.0
41.9
41.8
41.7
41.6
Latitude (UTM)
−7.2
−7.1
−7.0
−6.9
−6.8
−6.7
−6.6
Longitude (UTM)
N
0
5
10 km
Altitude (m)
1400
1200
1000
800
600
400

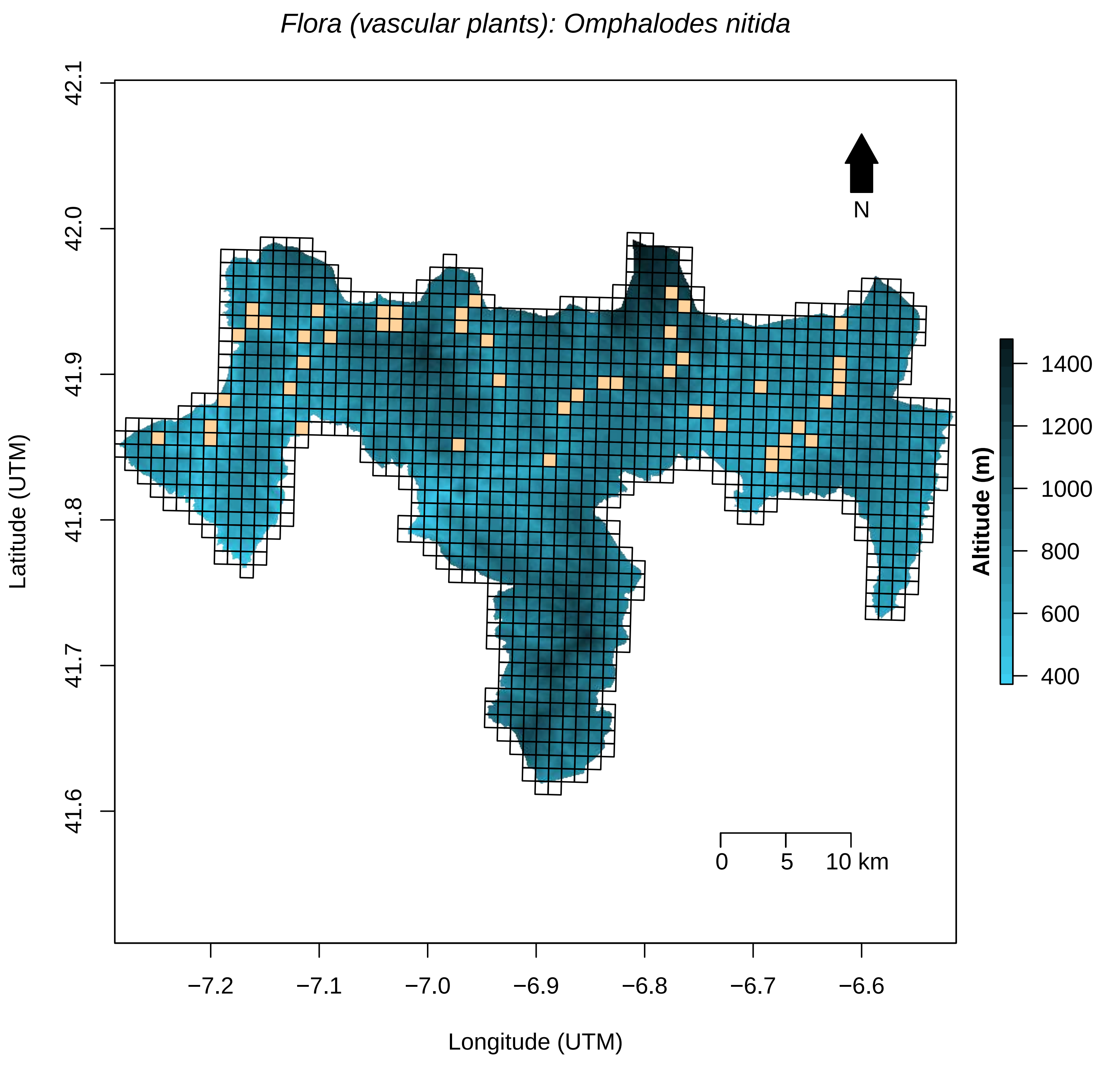

Flora (vascular plants): Omphalodes nitida
42.1
42.0
41.9
41.8
41.7
41.6
Latitude (UTM)
−7.2
−7.1
−7.0
−6.9
−6.8
−6.7
−6.6
Longitude (UTM)
N
0
5
10 km
Altitude (m)
1400
1200
1000
800
600
400

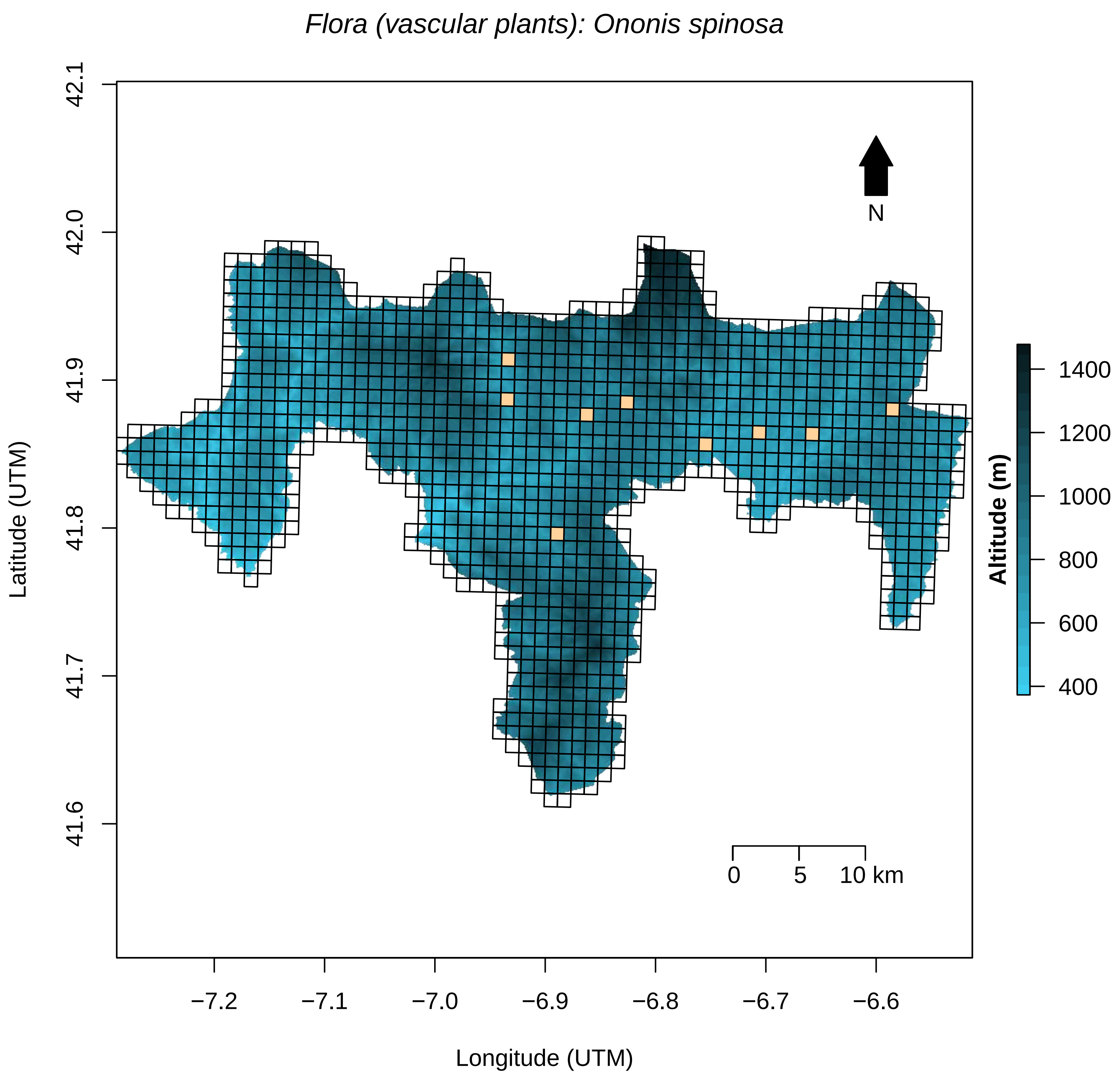

Flora (vascular plants): Ononis spinosa
42.1
42.0
41.9
41.8
41.7
41.6
Latitude (UTM)
−7.2
−7.1
−7.0
−6.9
−6.8
−6.7
−6.6
Longitude (UTM)
N
0
5
10 km
Altitude (m)
1400
1200
1000
800
600
400

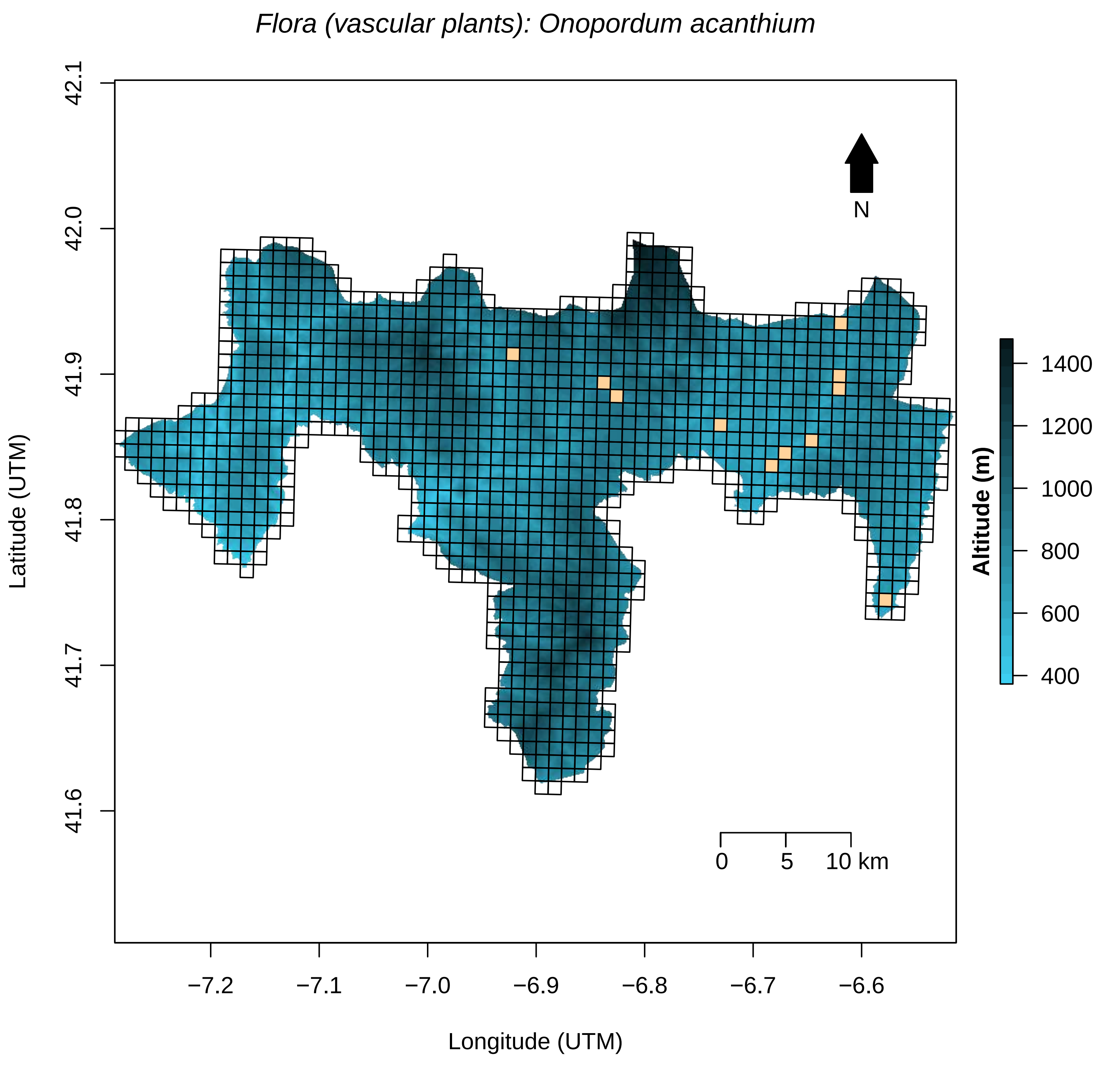
Flora (vascular plants): Onopordum acanthium
Latitude (UTM)
42.1
42.0
41.9
41.8
41.7
41.6
Longitude (UTM)
−7.2
−7.1
−7.0
−6.9
−6.8
−6.7
−6.6
N
0
5
10 km
Altitude (m)
1400
1200
1000
800
600
400

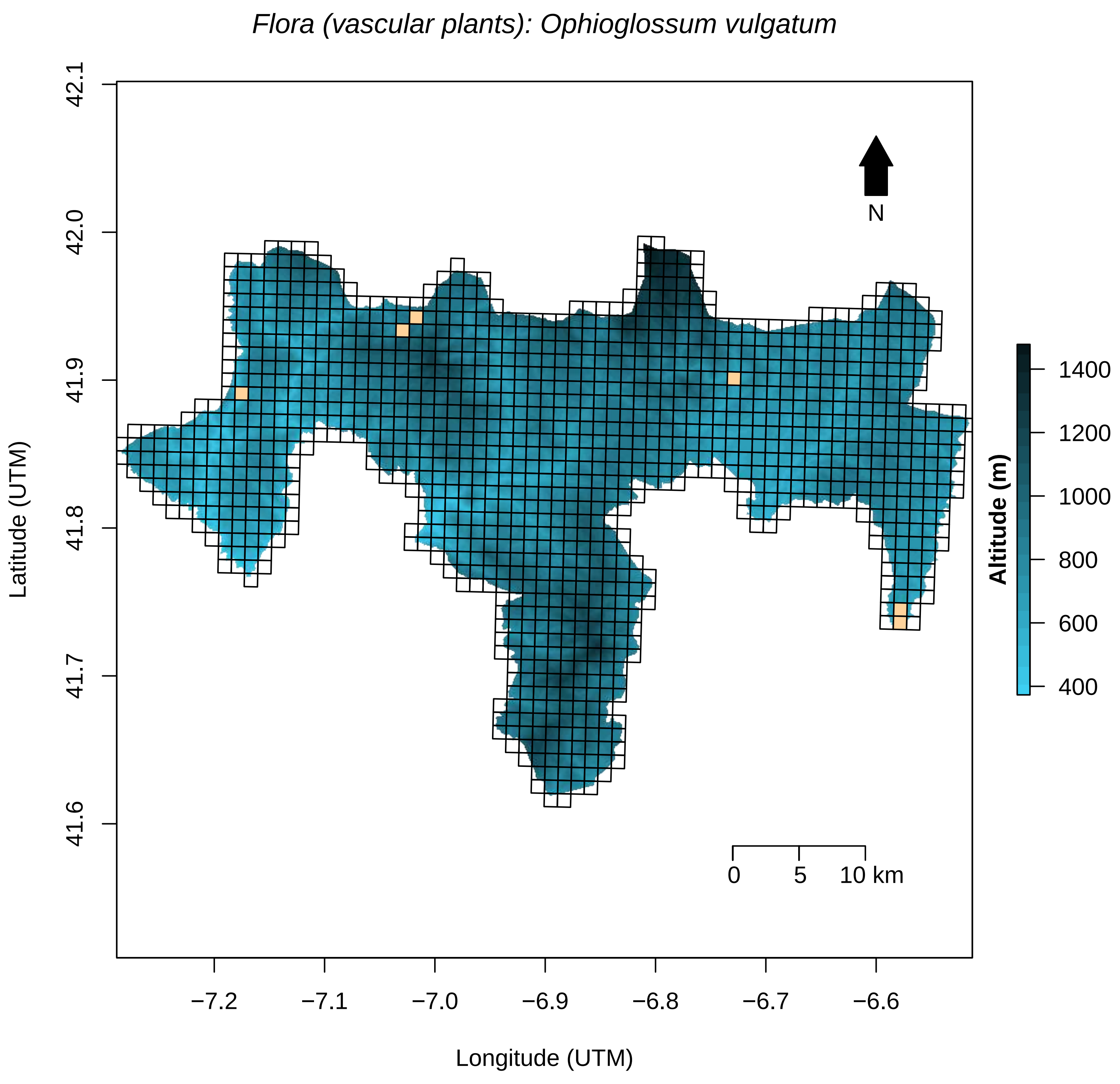

Flora (vascular plants): Ophioglossum vulgatum
N
0
5
10 km
Altitude (m)
1400
1200
1000
800
600
400
Latitude (UTM)
42.1
42.0
41.9
41.8
41.7
41.6
Longitude (UTM)
−7.2
−7.1
−7.0
−6.9
−6.8
−6.7
−6.6

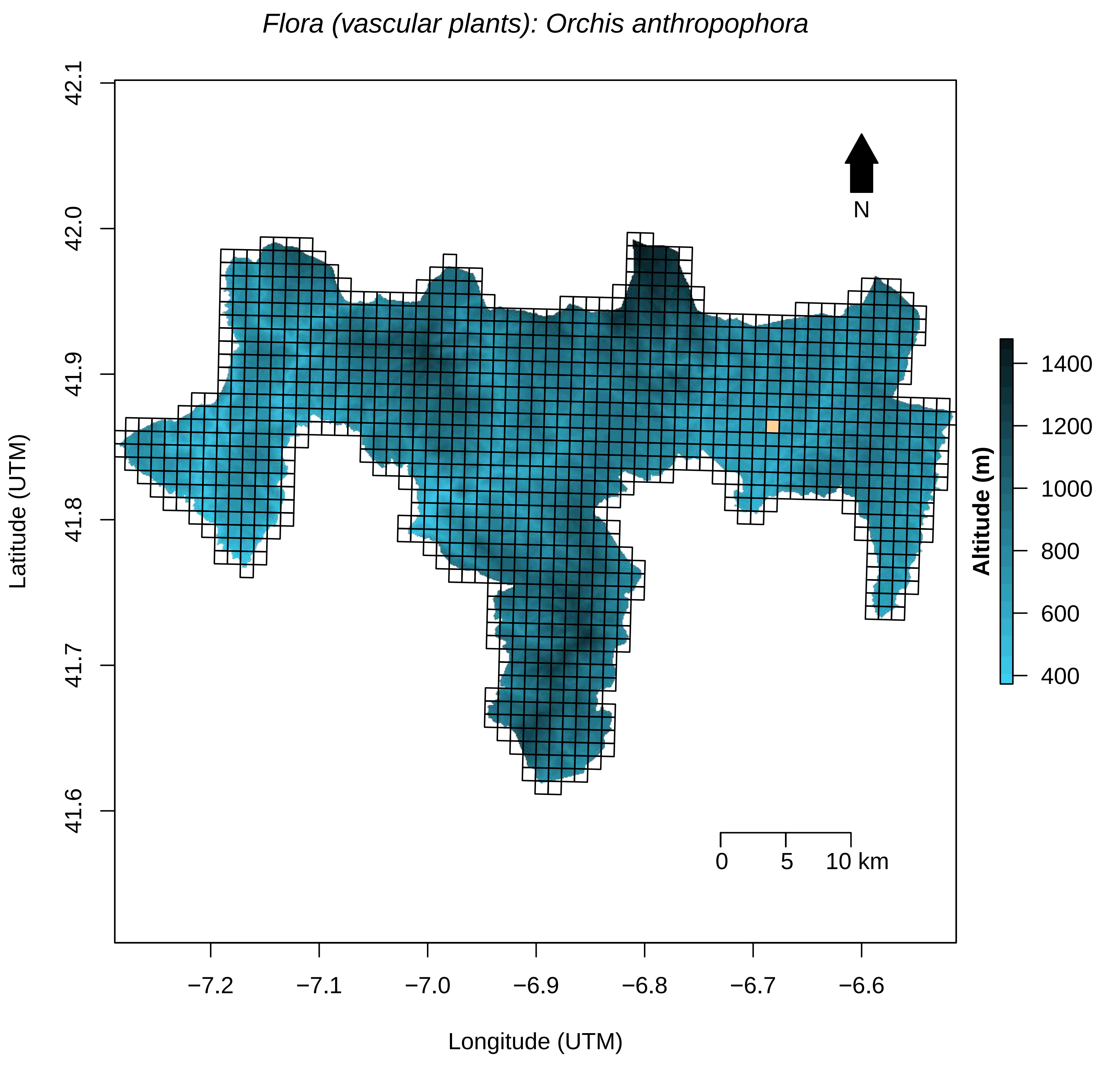

Flora (vascular plants): Orchis anthropophora
N
0
5
10 km
Altitude (m)
1400
1200
1000
800
600
400
Latitude (UTM)
42.1
42.0
41.9
41.8
41.7
41.6
Longitude (UTM)
−7.2
−7.1
−7.0
−6.9
−6.8
−6.7
−6.6

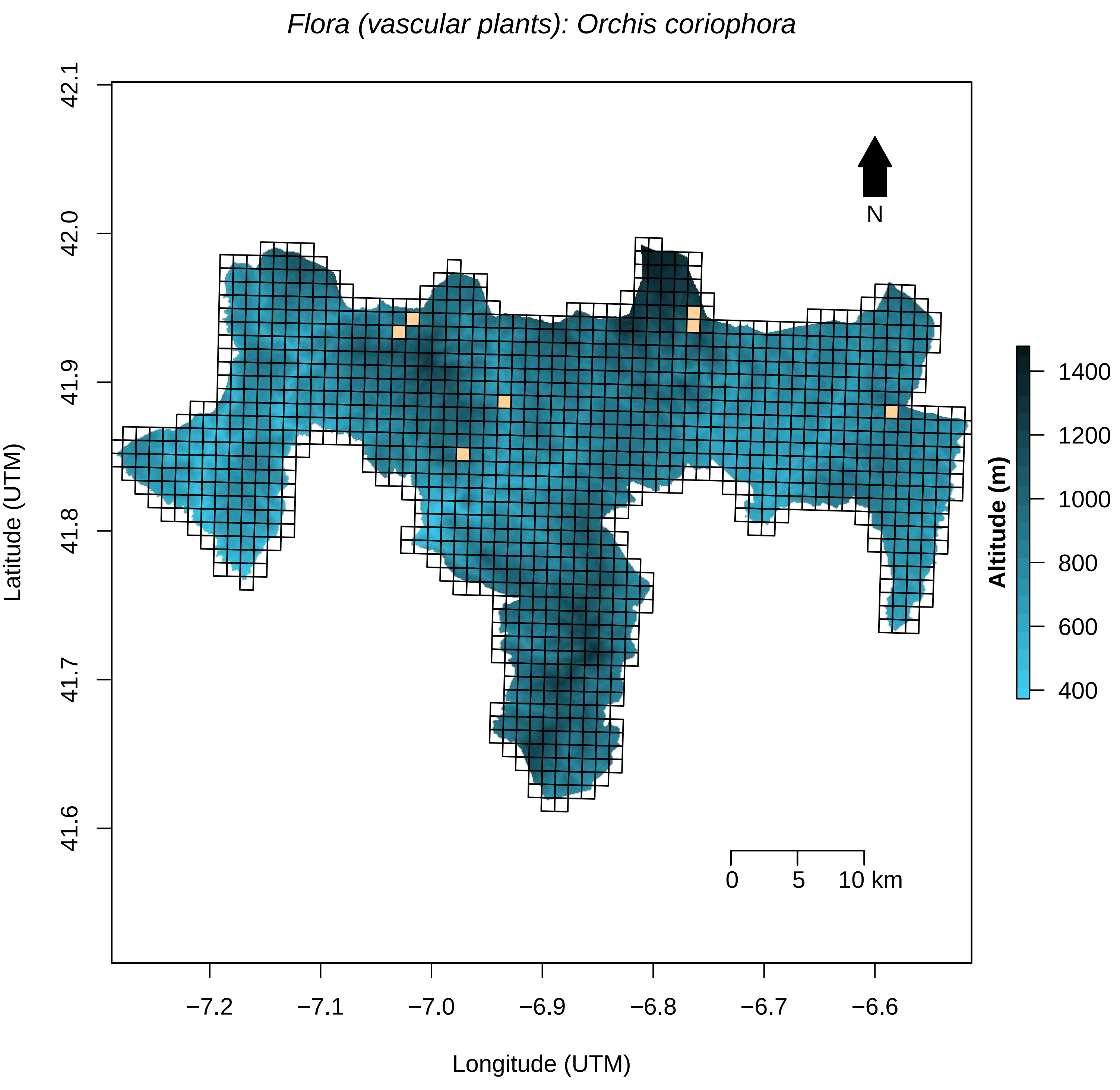

Flora (vascular plants): Orchis coriophora
42.1
42.0
41.9
41.8
41.7
41.6
Latitude (UTM)
−7.2
−7.1
−7.0
−6.9
−6.8
−6.7
−6.6
Longitude (UTM)
N
0
5
10 km
Altitude (m)
1400
1200
1000
800
600
400

Flora (vascular plants): Orchis langei

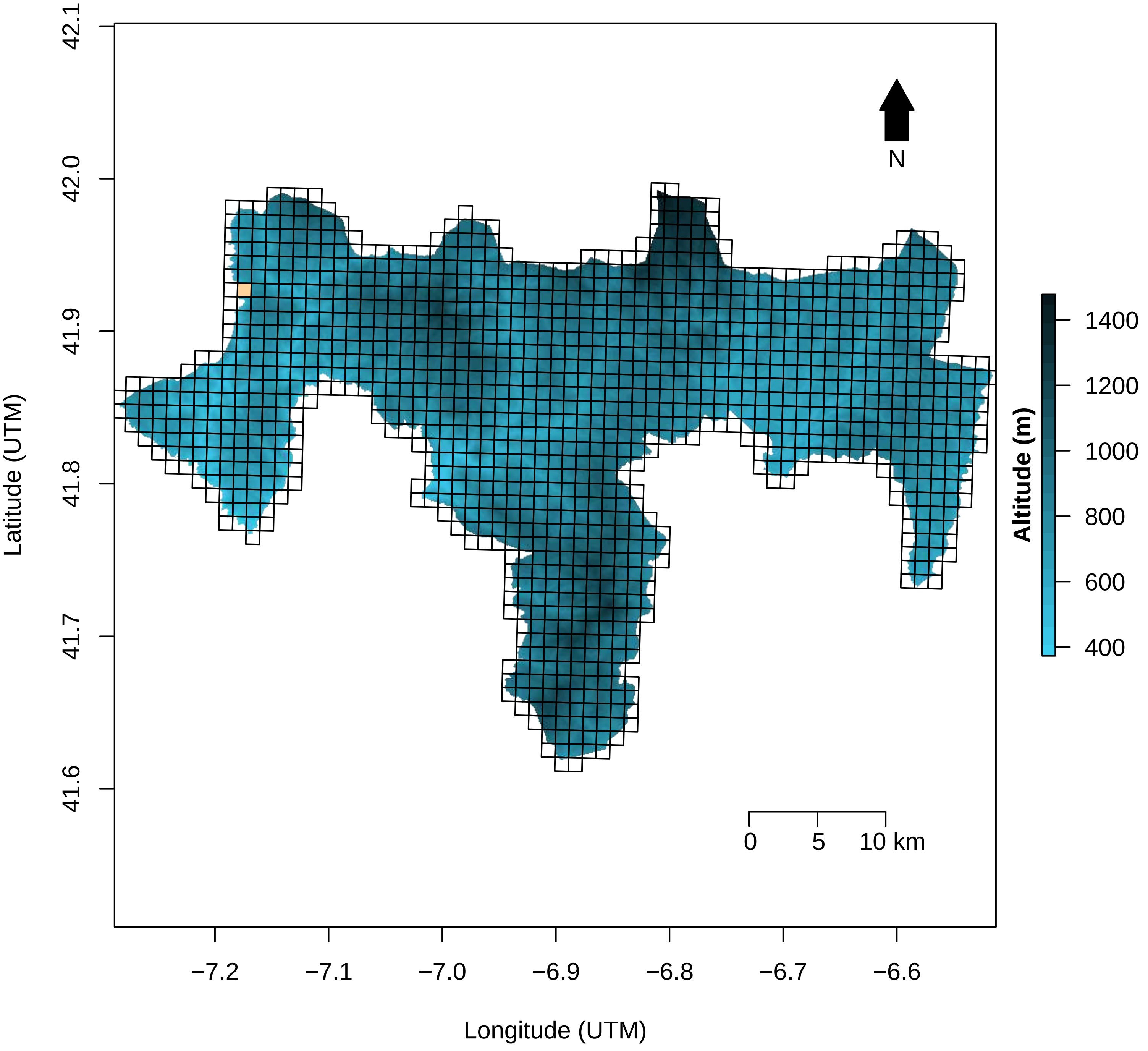

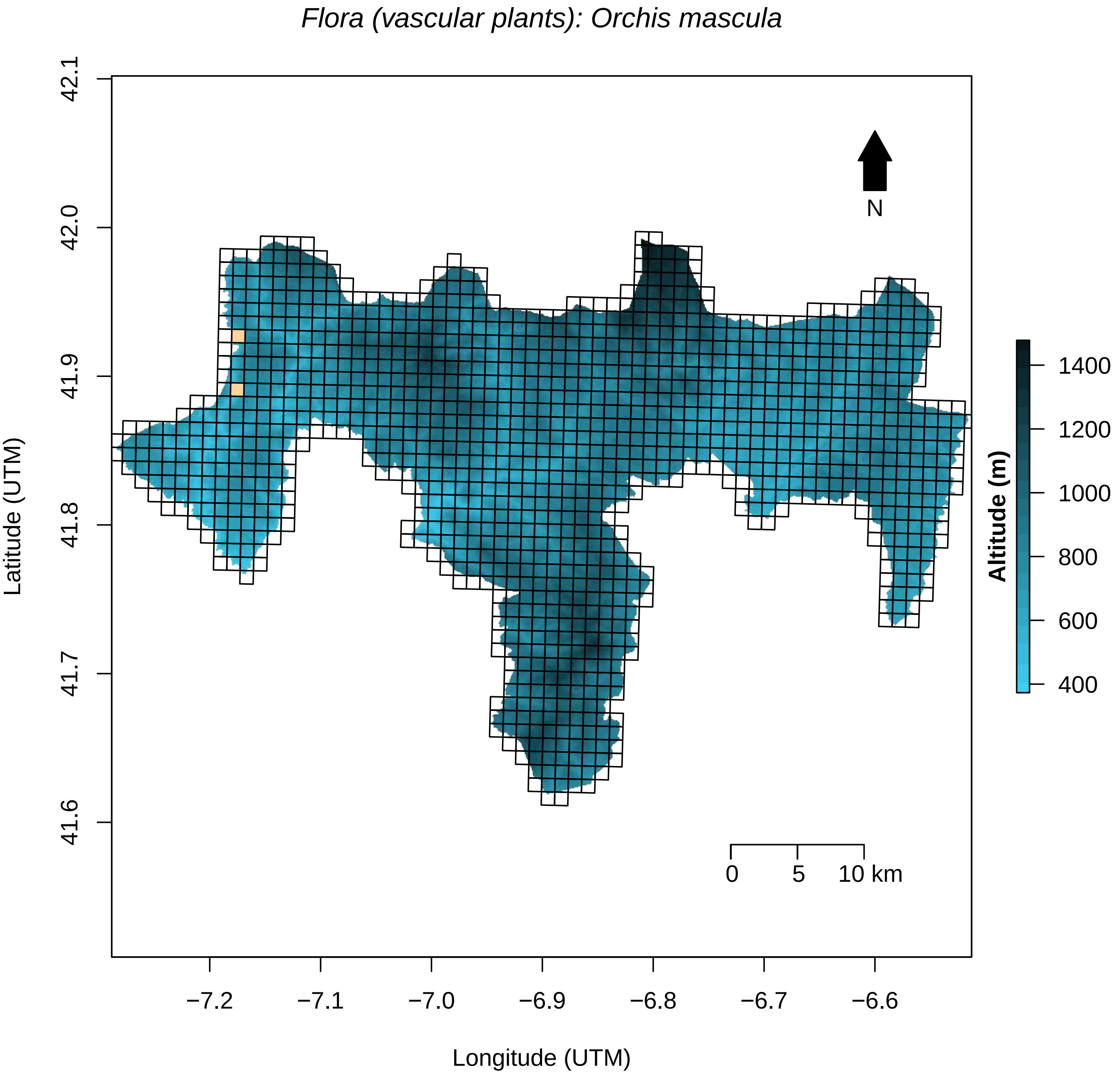

Flora (vascular plants): Orchis mascula
42.1
42.0
41.9
41.8
41.7
41.6
Latitude (UTM)
−7.2
−7.1
−7.0
−6.9
−6.8
−6.7
−6.6
Longitude (UTM)
N
0
5
10 km
1400
1200
1000
800
600
400
Altitude (m)

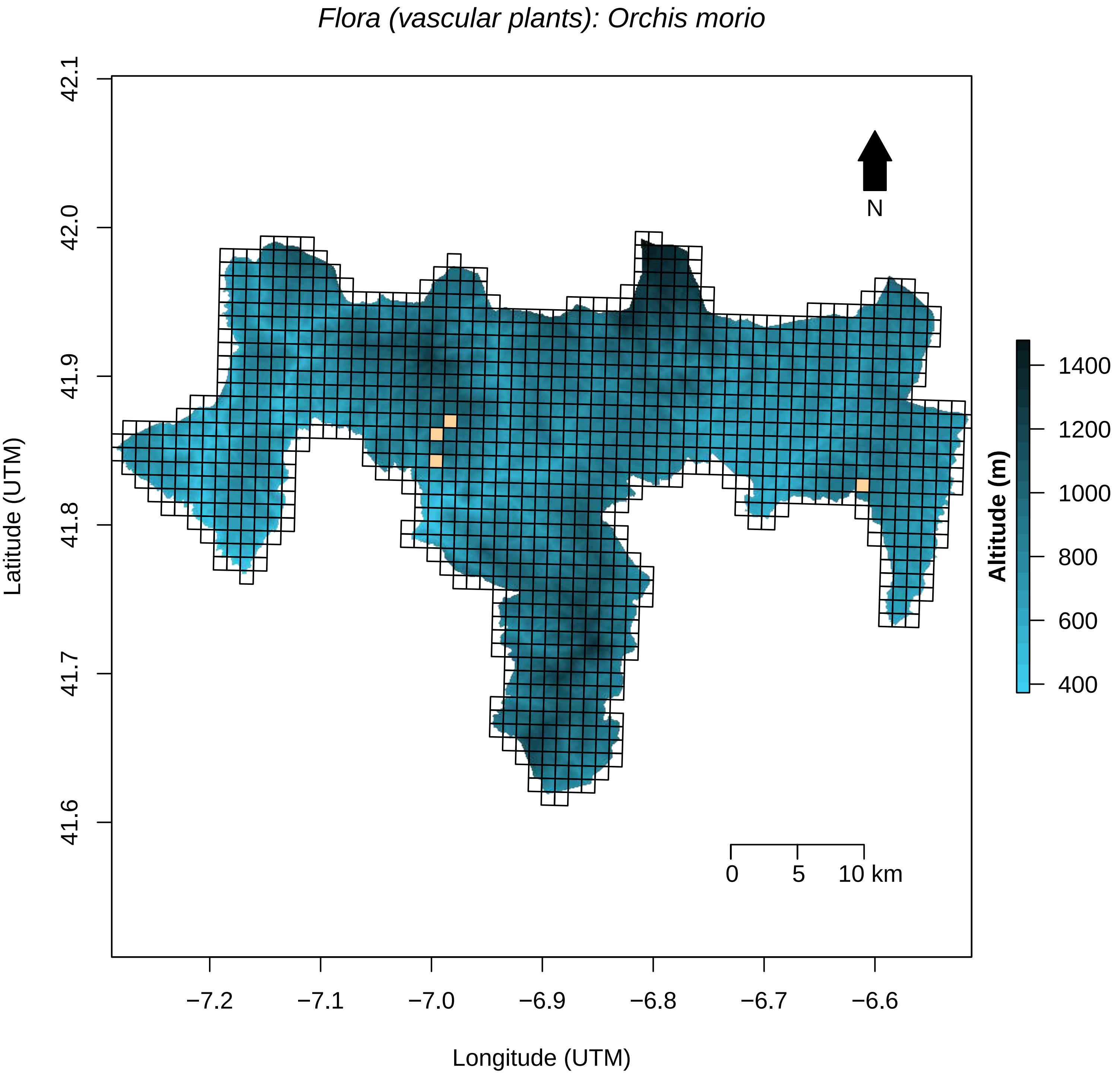

Flora (vascular plants): Orchis morio
Latitude (UTM)
42.1
42.0
41.9
41.8
41.7
41.6
Longitude (UTM)
−7.2
−7.1
−7.0
−6.9
−6.8
−6.7
−6.6
N
0
5
10 km
Altitude (m)
1400
1200
1000
800
600
400

Flora (vascular plants): Origanum vulgare

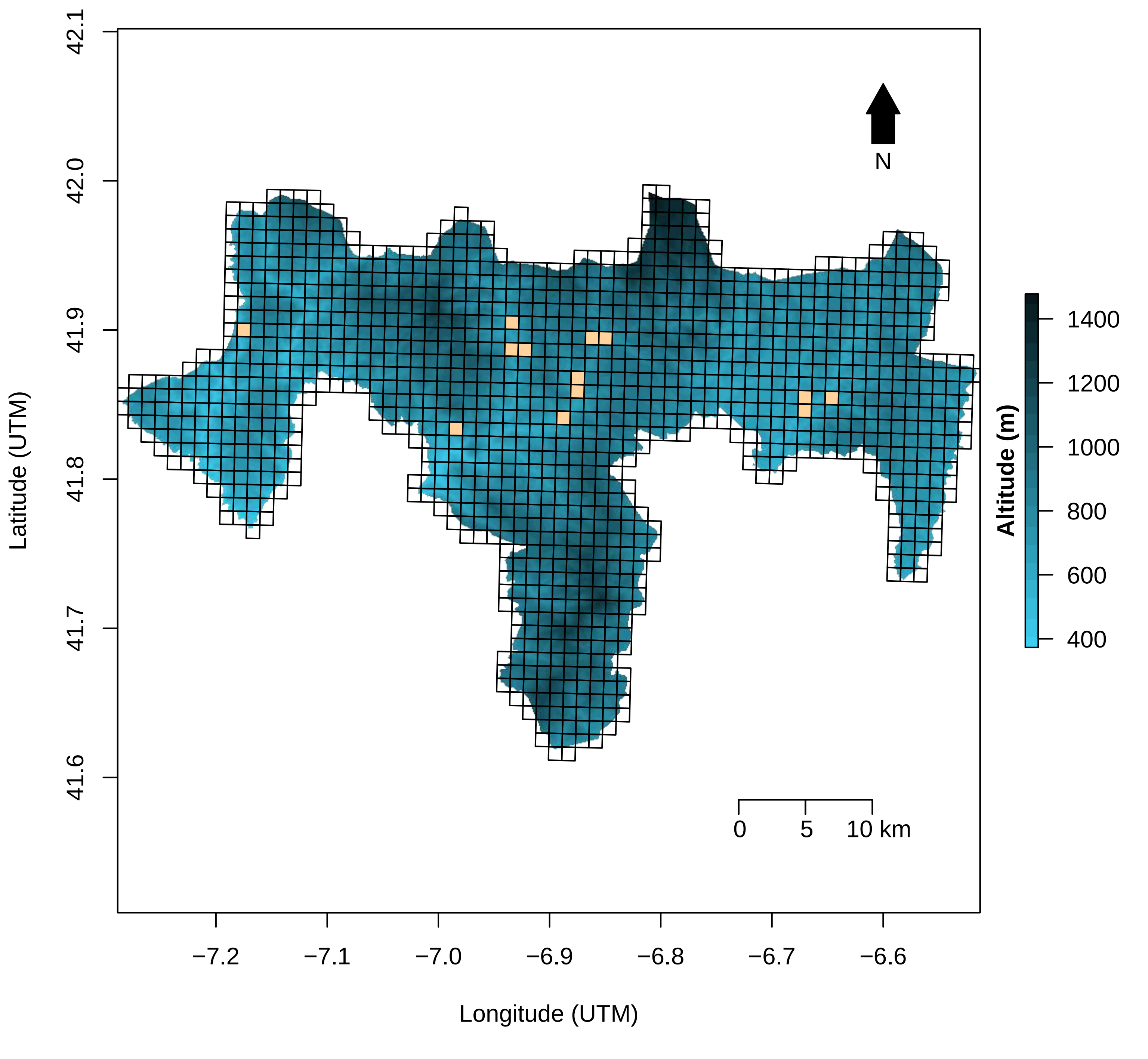

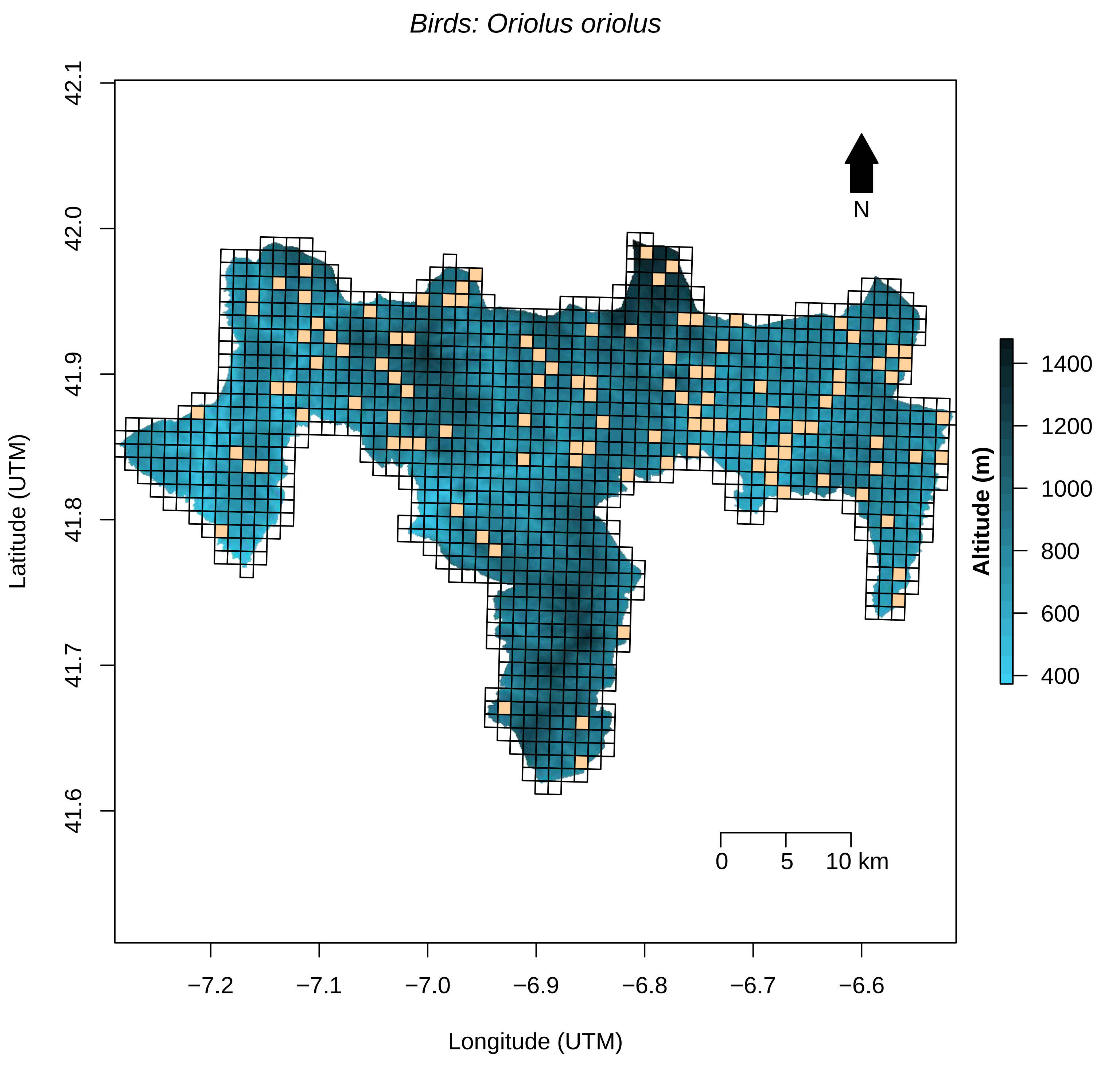

Birds: Oriolus oriolus
42.1
42.0
41.9
41.8
41.7
41.6
Latitude (UTM)
−7.2
−7.1
−7.0
−6.9
−6.8
−6.7
−6.6
Longitude (UTM)
N
0
5
10 km
1400
1200
1000
800
600
400
Altitude (m)

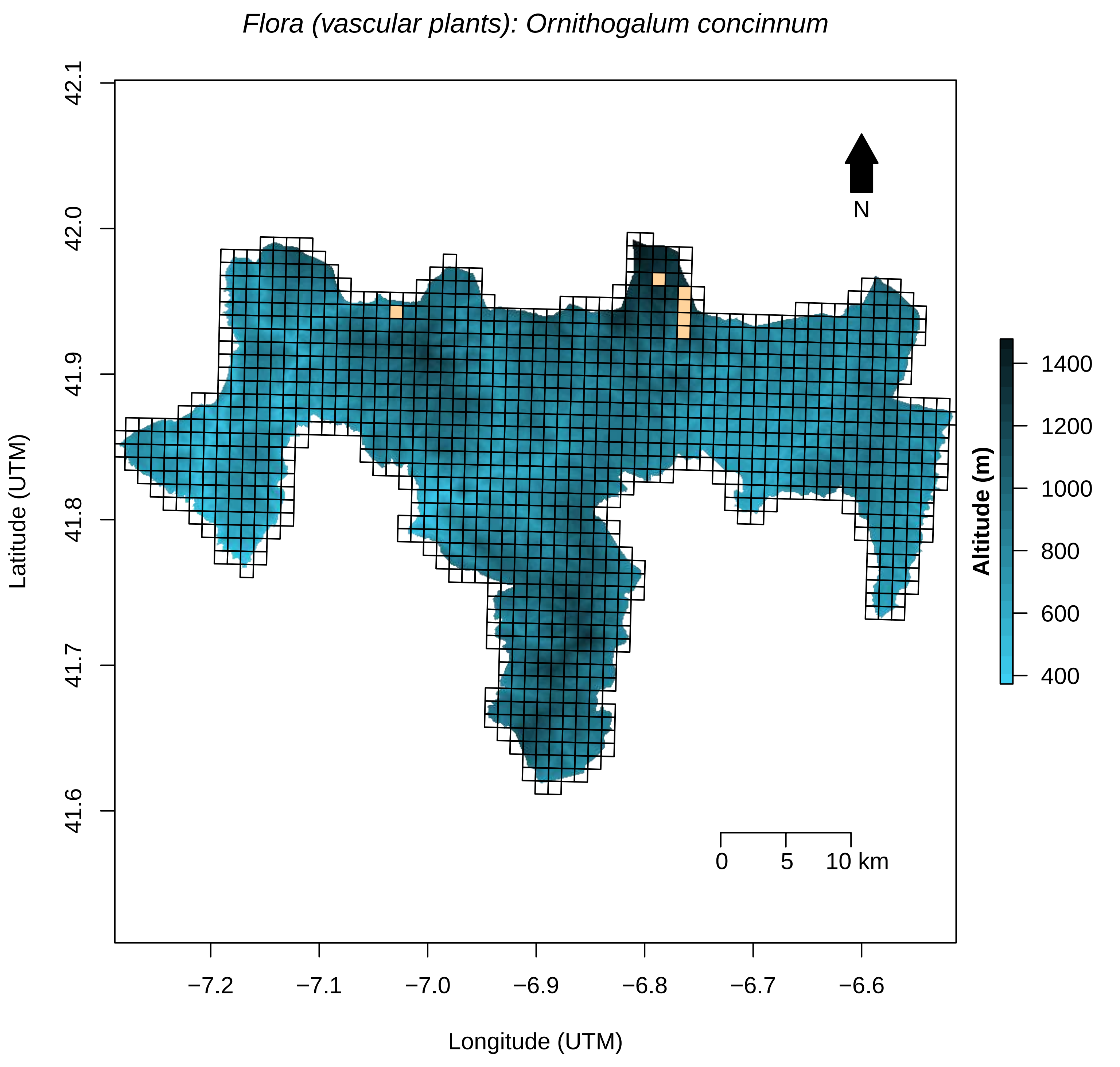
Flora (vascular plants): Ornithogalum concinnum
42.1
42.0
41.9
41.8
41.7
41.6
Latitude (UTM)
−7.2
−7.1
−7.0
−6.9
−6.8
−6.7
−6.6
Longitude (UTM)
N
0
5
10 km
1400
1200
1000
800
600
400
Altitude (m)

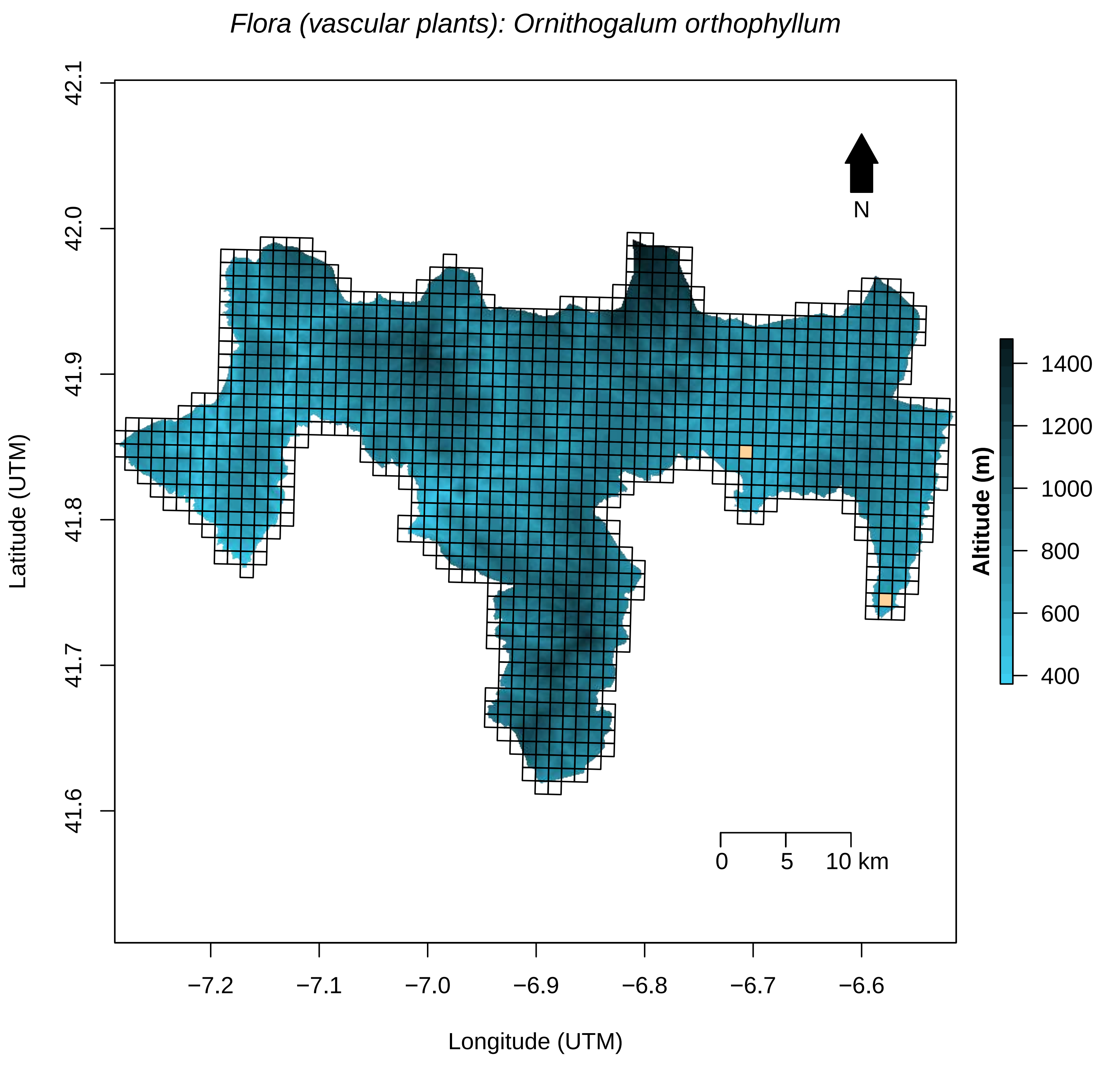
Flora (vascular plants): Ornithogalum orthophyllum
N
0 5 10 km
Altitude (m)
1400
1200
1000
800
600
400
Latitude (UTM)
42.1
42.0
41.9
41.8
41.7
41.6
Longitude (UTM)
−7.2
−7.1
−7.0
−6.9
−6.8
−6.7
−6.6

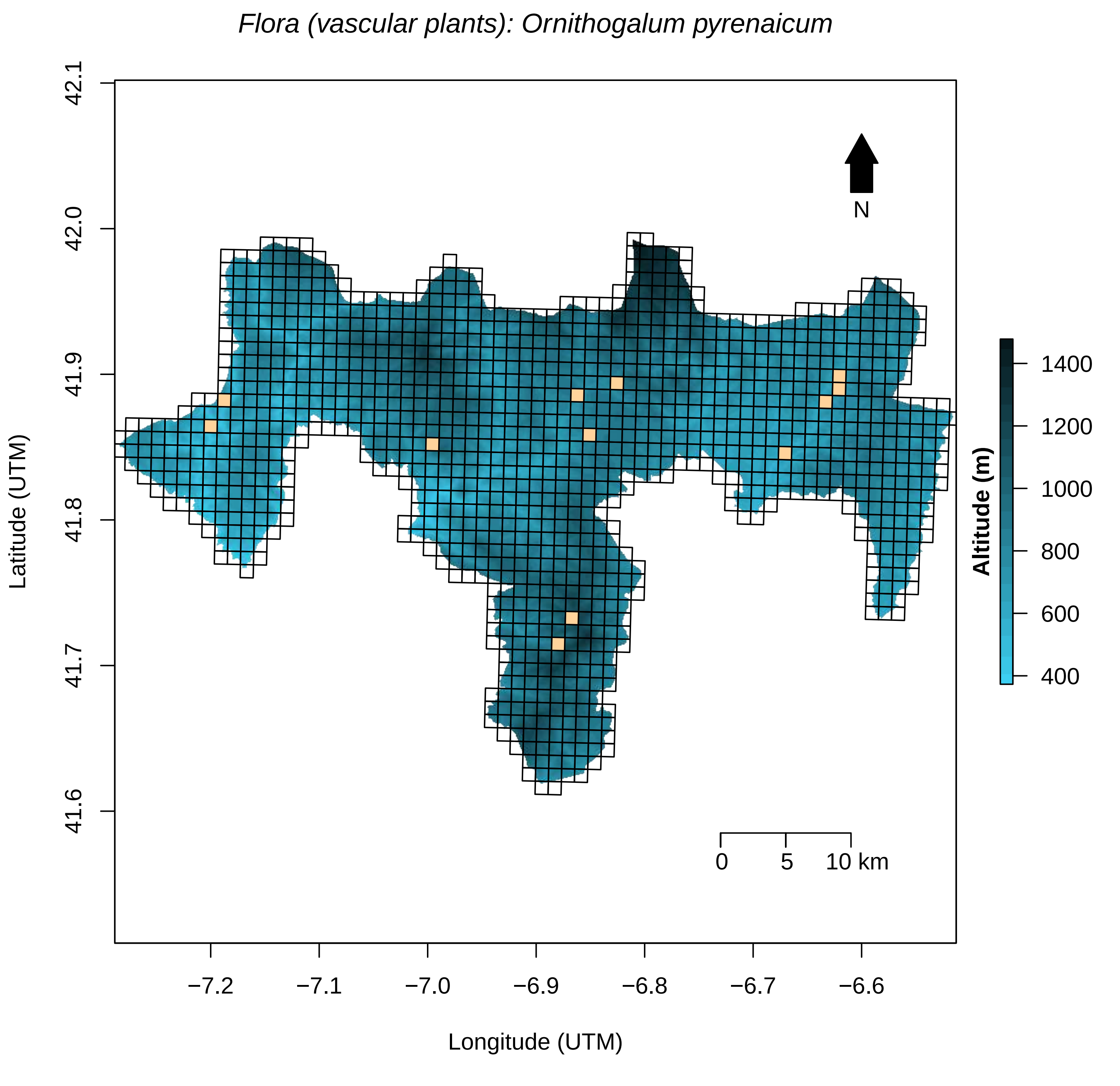
Flora (vascular plants): Ornithogalum pyrenaicum
42.1
42.0
41.9
41.8
41.7
41.6
Latitude (UTM)
−7.2
−7.1
−7.0
−6.9
−6.8
−6.7
−6.6
Longitude (UTM)
N
0
5
10 km
Altitude (m)
1400
1200
1000
800
600
400

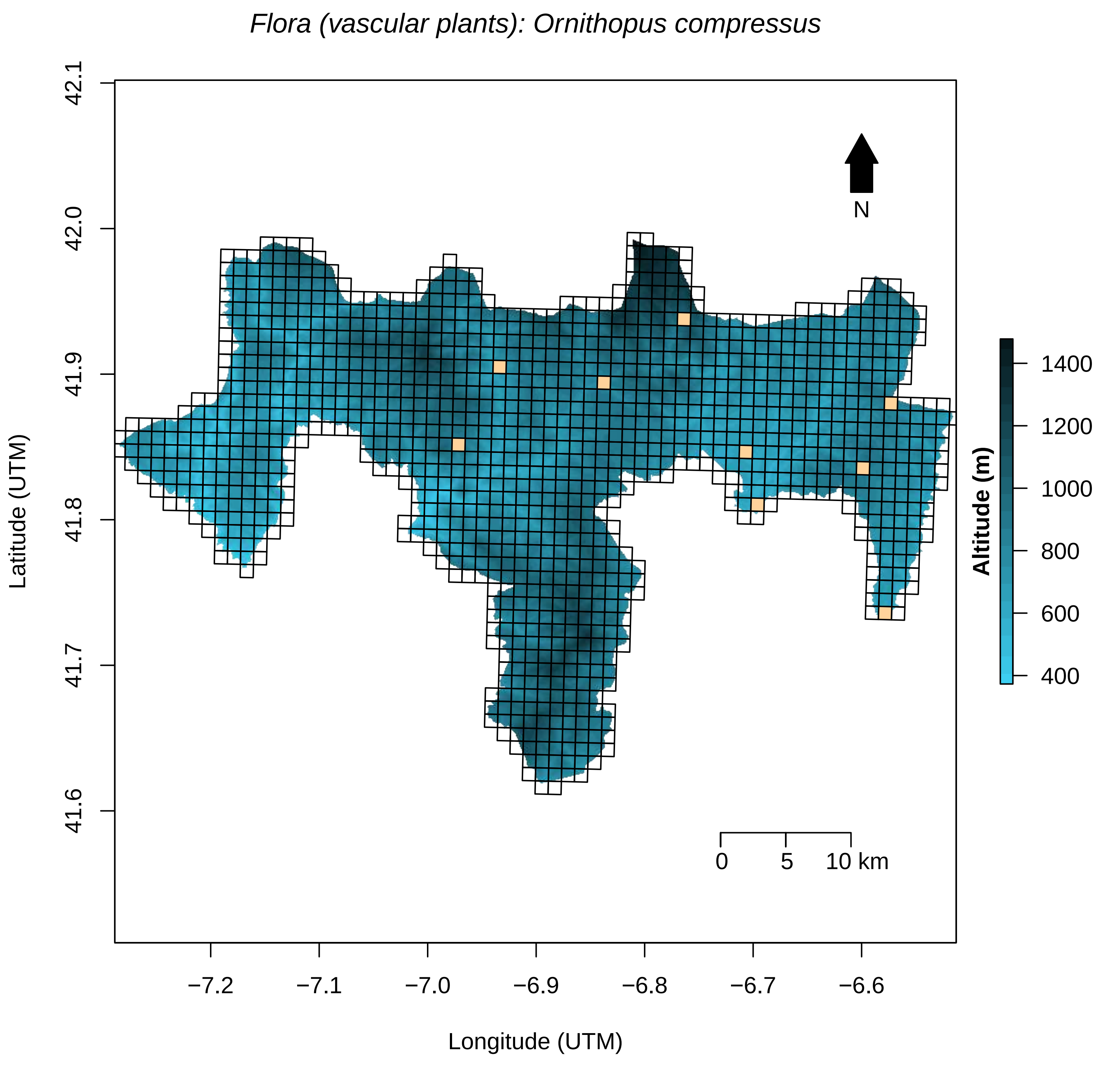
Flora (vascular plants): Ornithopus compressus
42.1
42.0
41.9
41.8
41.7
41.6
Latitude (UTM)
−7.2
−7.1
−7.0
−6.9
−6.8
−6.7
−6.6
Longitude (UTM)
N
0
5
10 km
Altitude (m)
1400
1200
1000
800
600
400

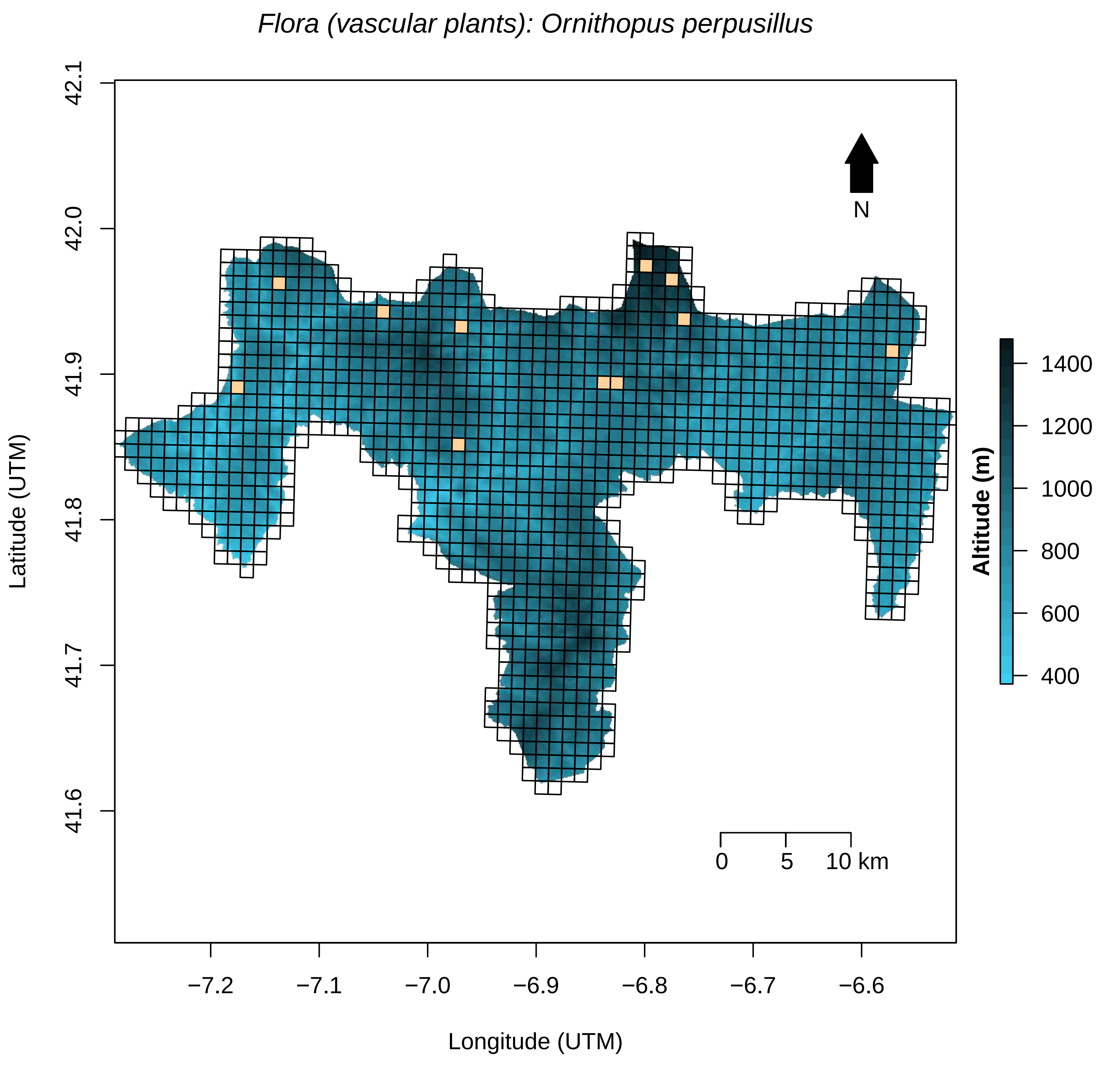

Flora (vascular plants): Ornithopus perpusillus
42.1
42.0
41.9
41.8
41.7
41.6
Latitude (UTM)
−7.2
−7.1
−7.0
−6.9
−6.8
−6.7
−6.6
Longitude (UTM)
N
0
5
10 km
Altitude (m)
1400
1200
1000
800
600
400

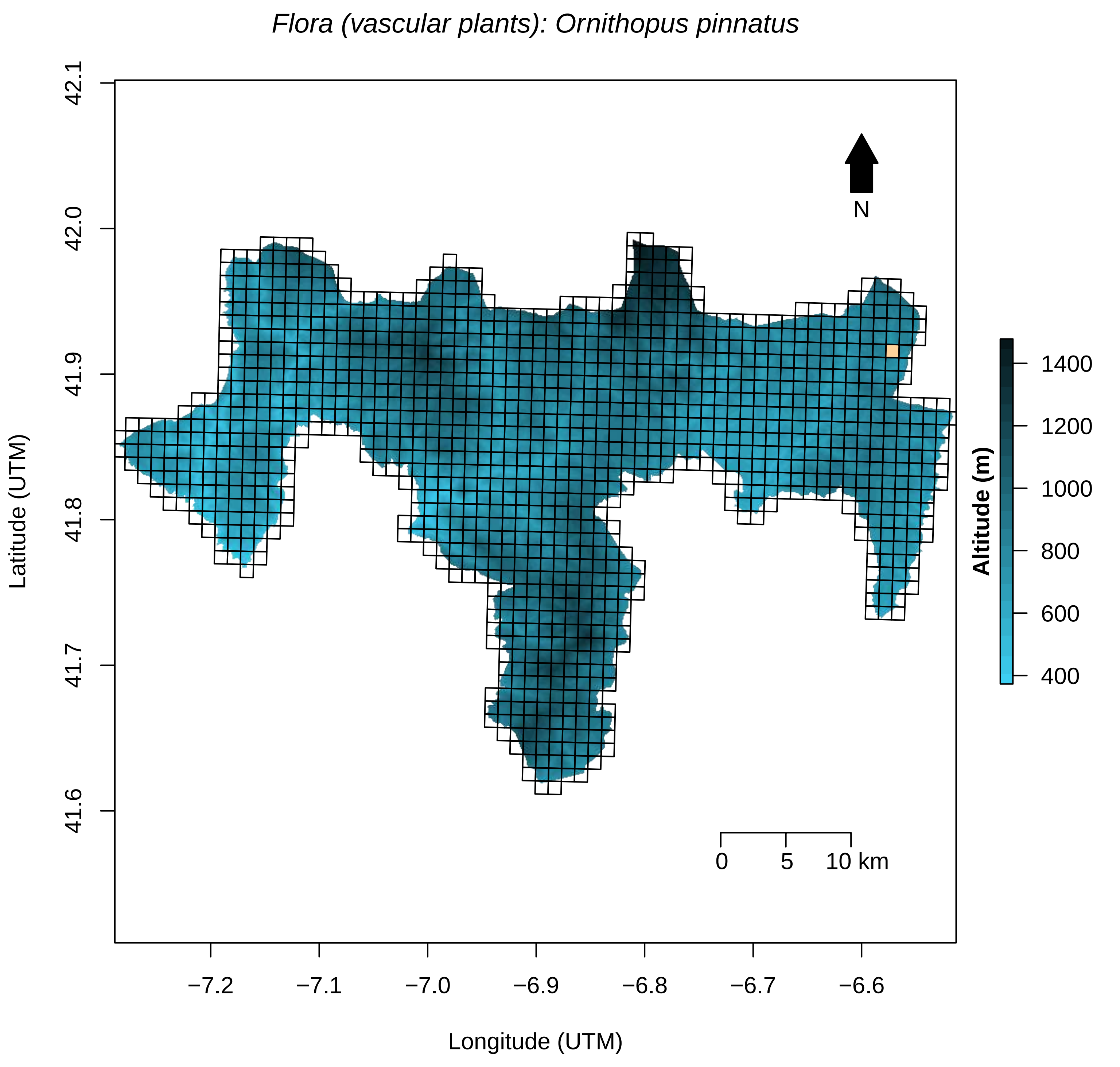

Flora (vascular plants): Ornithopus pinnatus
42.1
42.0
41.9
41.8
41.7
41.6
Latitude (UTM)
−7.2
−7.1
−7.0
−6.9
−6.8
−6.7
−6.6
Longitude (UTM)
N
0
5
10 km
Altitude (m)
1400
1200
1000
800
600
400

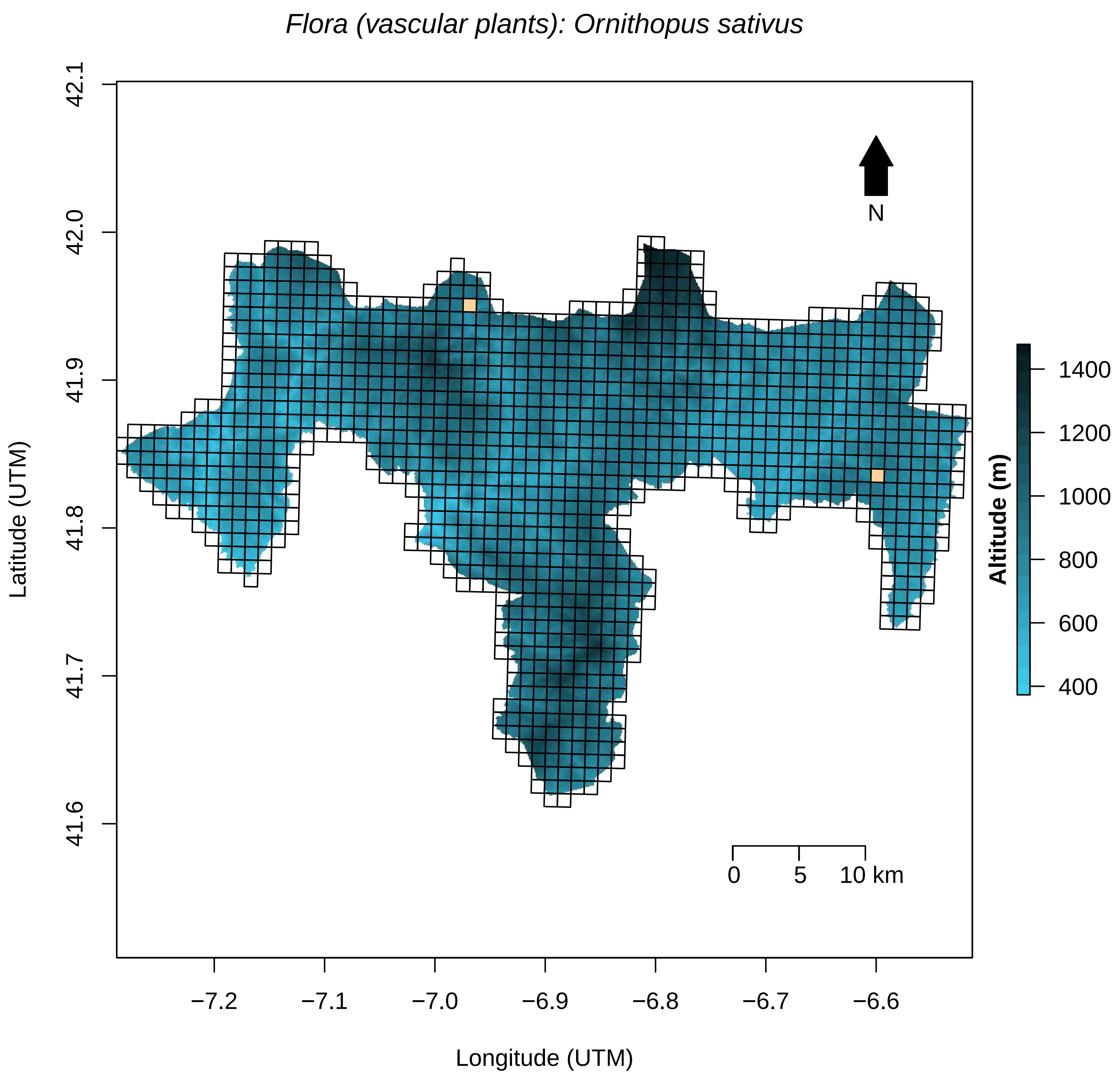

Flora (vascular plants): Ornithopus sativus
42.1
42.0
41.9
41.8
41.7
41.6
Latitude (UTM)
−7.2
−7.1
−7.0
−6.9
−6.8
−6.7
−6.6
Longitude (UTM)
N
0
5
10 km
1400
1200
1000
800
600
400
Altitude (m)

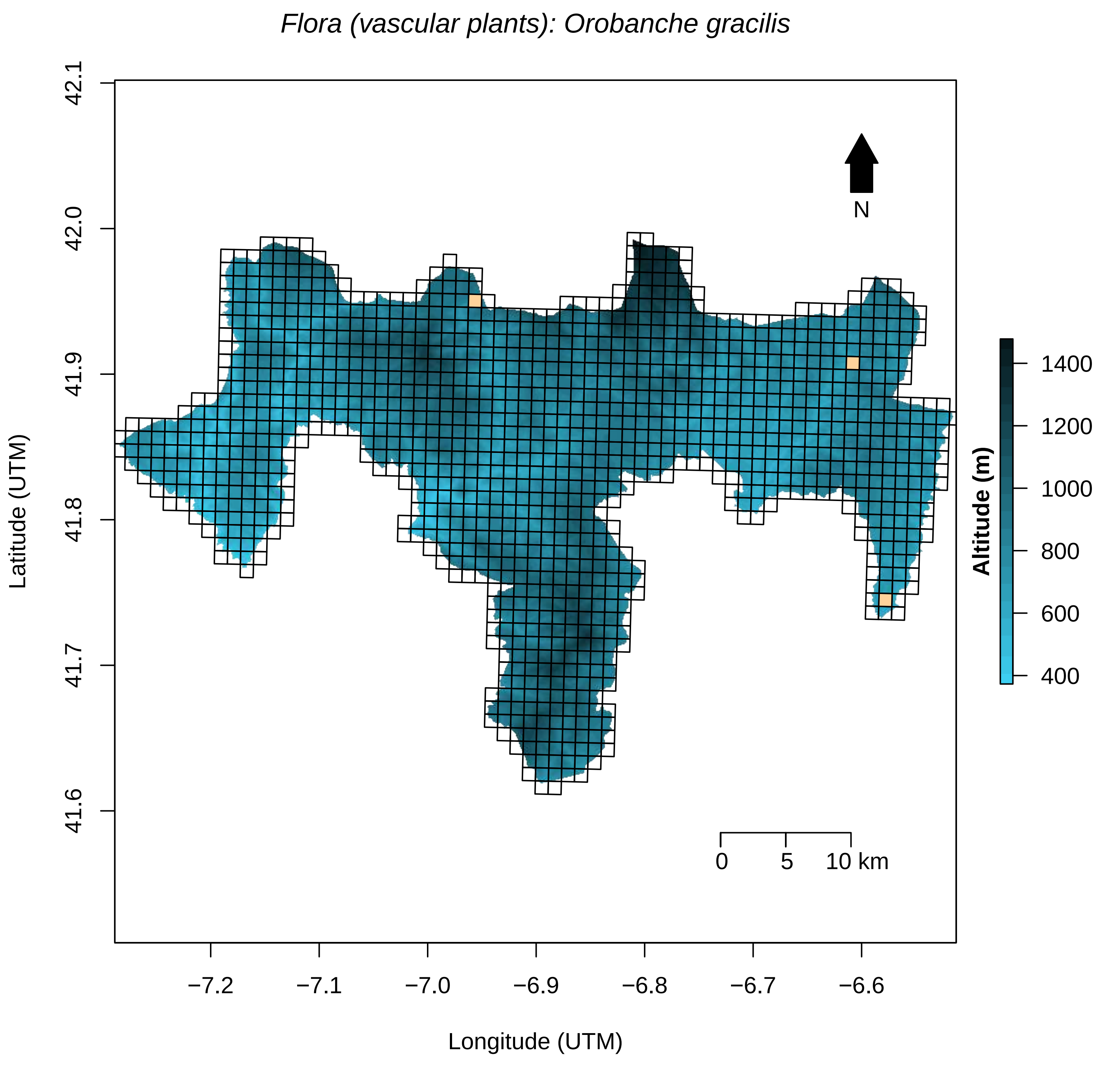

Flora (vascular plants): Orobanche gracilis
42.1
42.0
41.9
41.8
41.7
41.6
Latitude (UTM)
−7.2
−7.1
−7.0
−6.9
−6.8
−6.7
−6.6
Longitude (UTM)
N
0
5
10 km
Altitude (m)
1400
1200
1000
800
600
400

Flora (vascular plants): Orobanche hederae

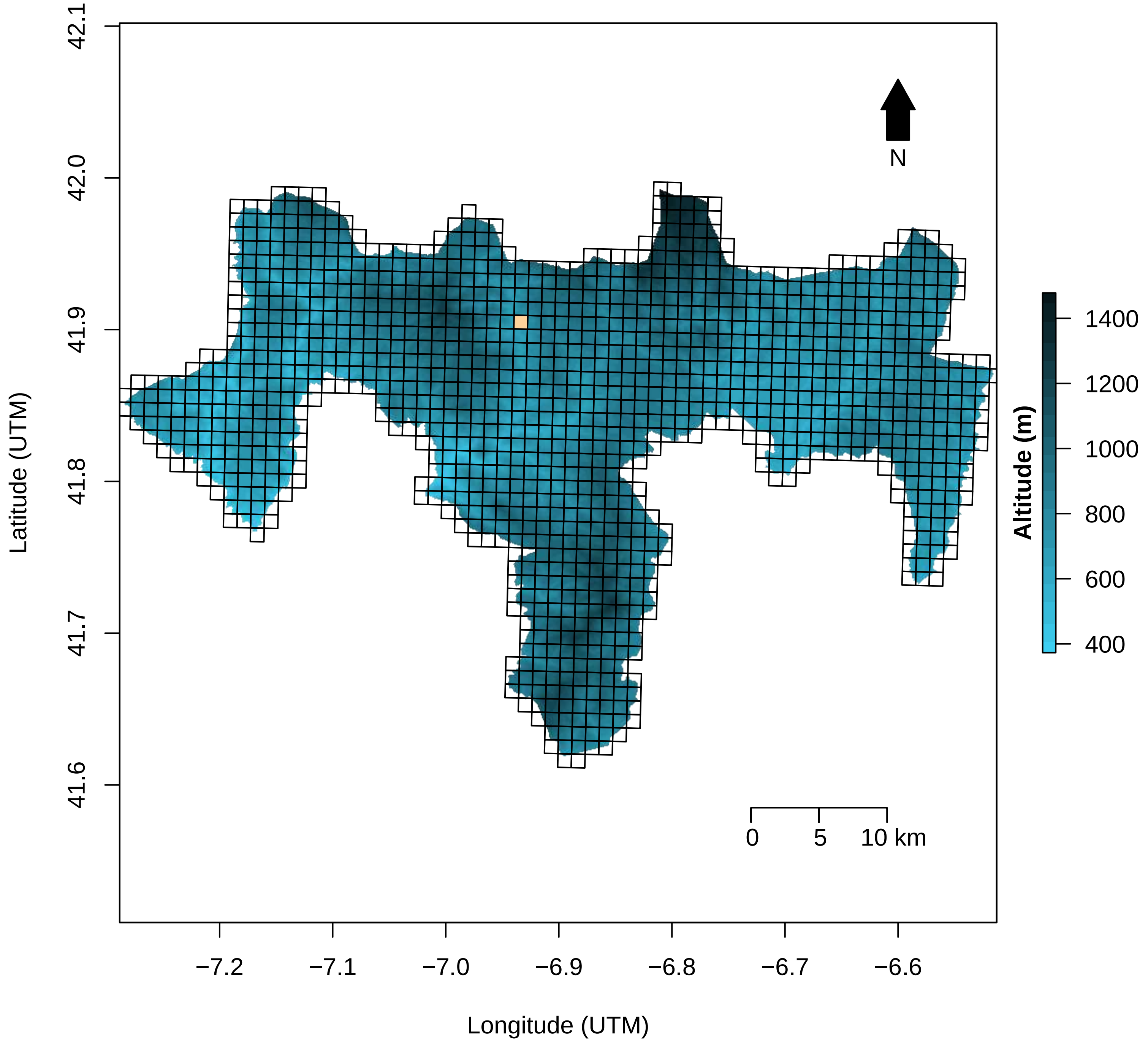

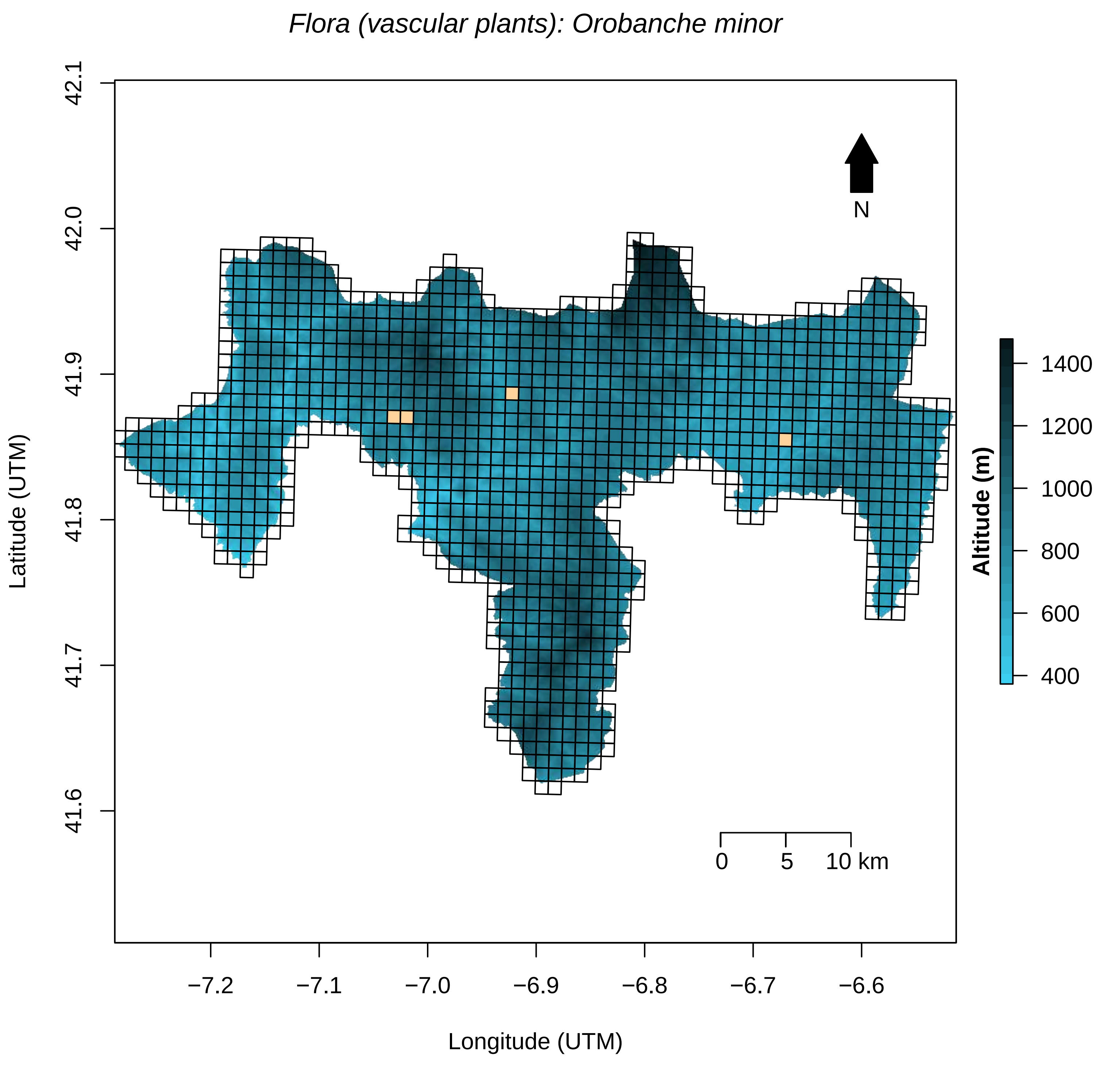
Flora (vascular plants): Orobanche minor
N
0
5
10 km
Altitude (m)
1400
1200
1000
800
600
400
Latitude (UTM)
42.1
42.0
41.9
41.8
41.7
41.6
Longitude (UTM)
−7.2
−7.1
−7.0
−6.9
−6.8
−6.7
−6.6

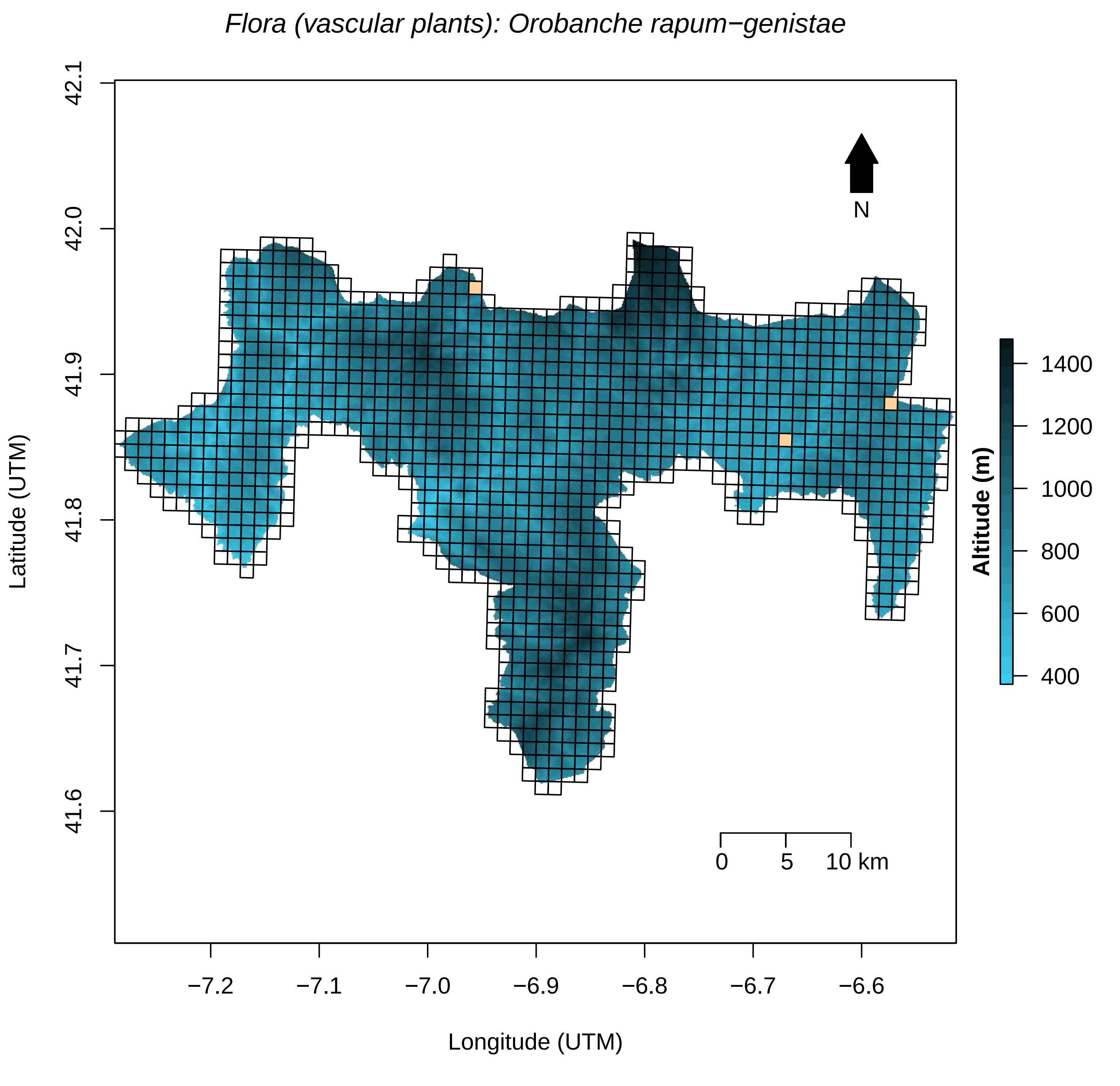

Flora (vascular plants): Orobanche rapum−genistae
42.1
42.0
41.9
41.8
41.7
41.6
Latitude (UTM)
N
0
5
10 km
−7.2
−7.1
−7.0
−6.9
−6.8
−6.7
−6.6
Longitude (UTM)
1400
1200
1000
800
600
400
Altitude (m)

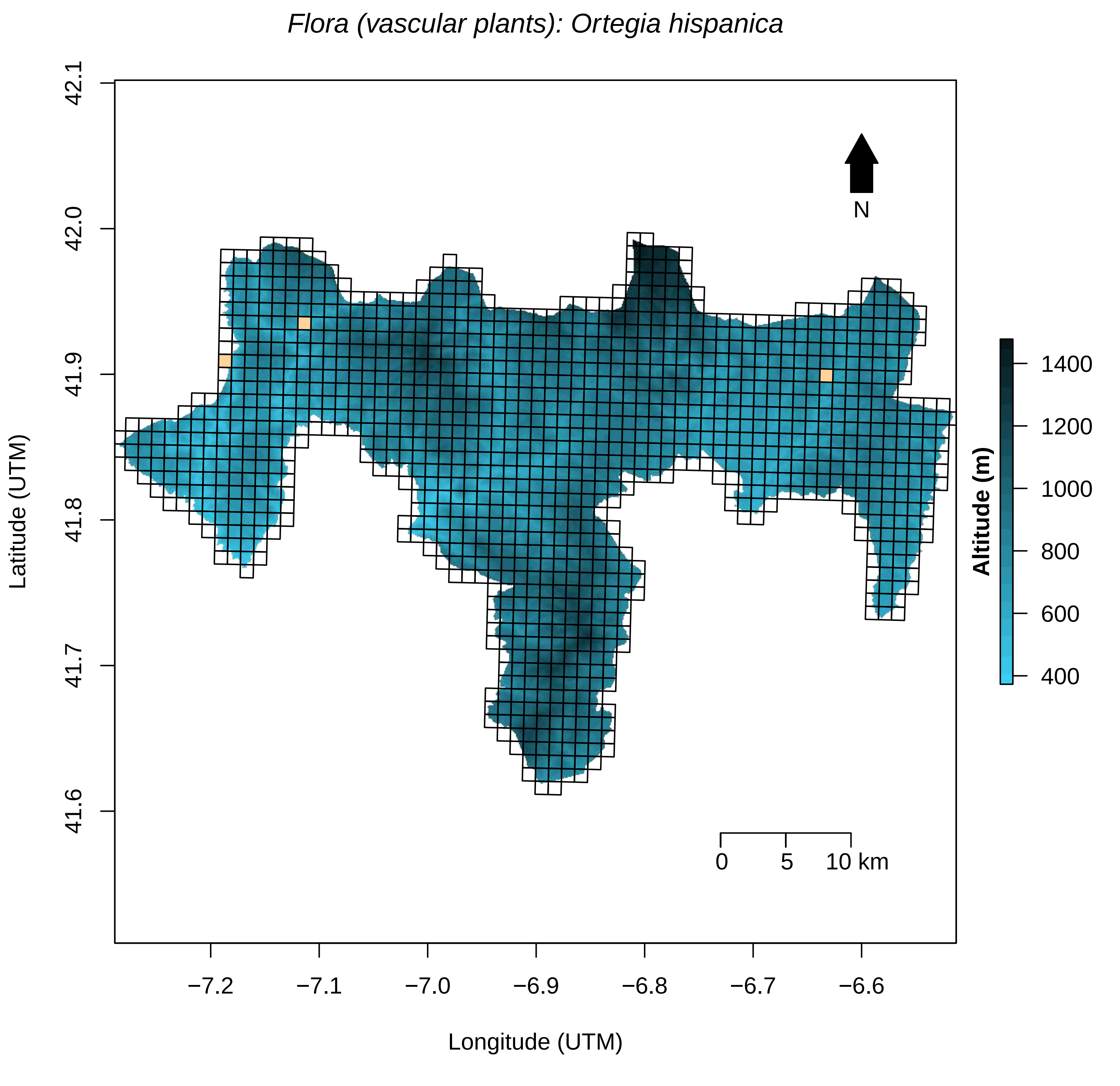

Flora (vascular plants): Ortegia hispanica
42.1
42.0
41.9
41.8
41.7
41.6
Latitude (UTM)
−7.2
−7.1
−7.0
−6.9
−6.8
−6.7
−6.6
Longitude (UTM)
N
0
5
10 km
Altitude (m)
1400
1200
1000
800
600
400

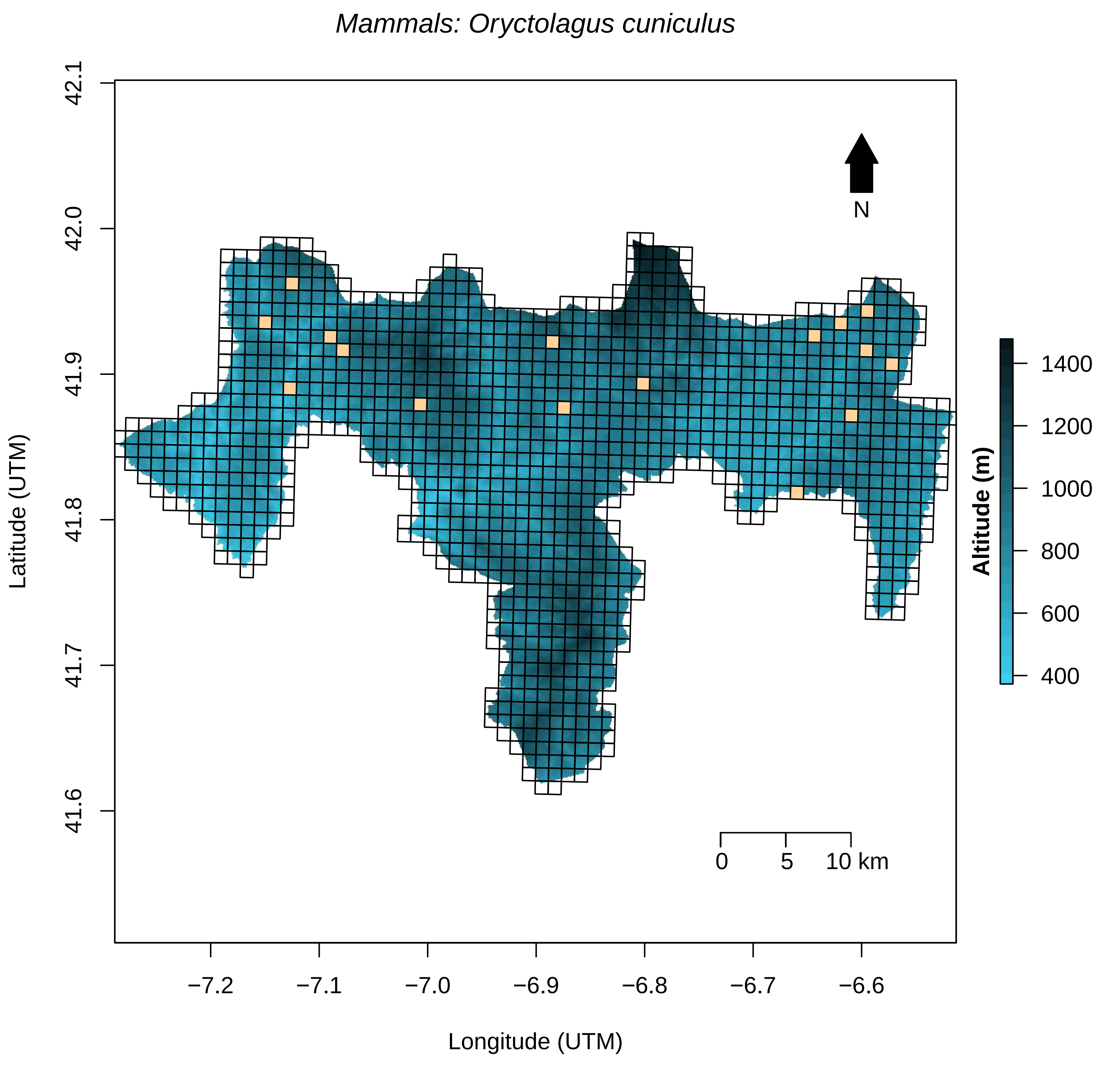

Mammals: Oryctolagus cuniculus
42.1
42.0
41.9
41.8
41.7
41.6
Latitude (UTM)
−7.2
−7.1
−7.0
−6.9
−6.8
−6.7
−6.6
Longitude (UTM)
N
0
5
10 km
Altitude (m)
1400
1200
1000
800
600
400

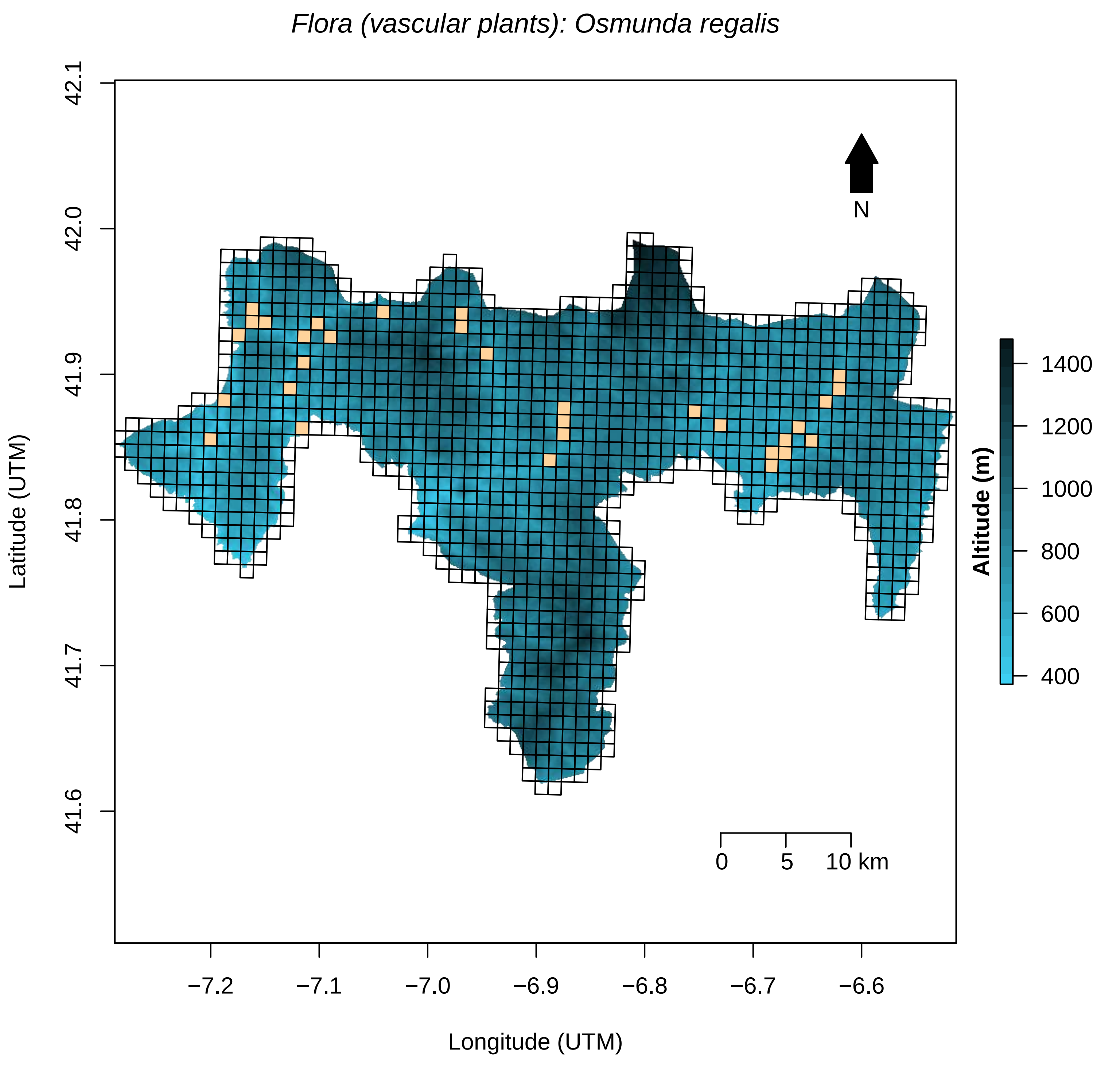
Flora (vascular plants): Osmunda regalis
Latitude (UTM)
Longitude (UTM)
42.1
42.0
41.9
41.8
41.7
41.6
−7.2
−7.1
−7.0
−6.9
−6.8
−6.7
−6.6
N
0
5
10 km
Altitude (m)
1400
1200
1000
800
600
400

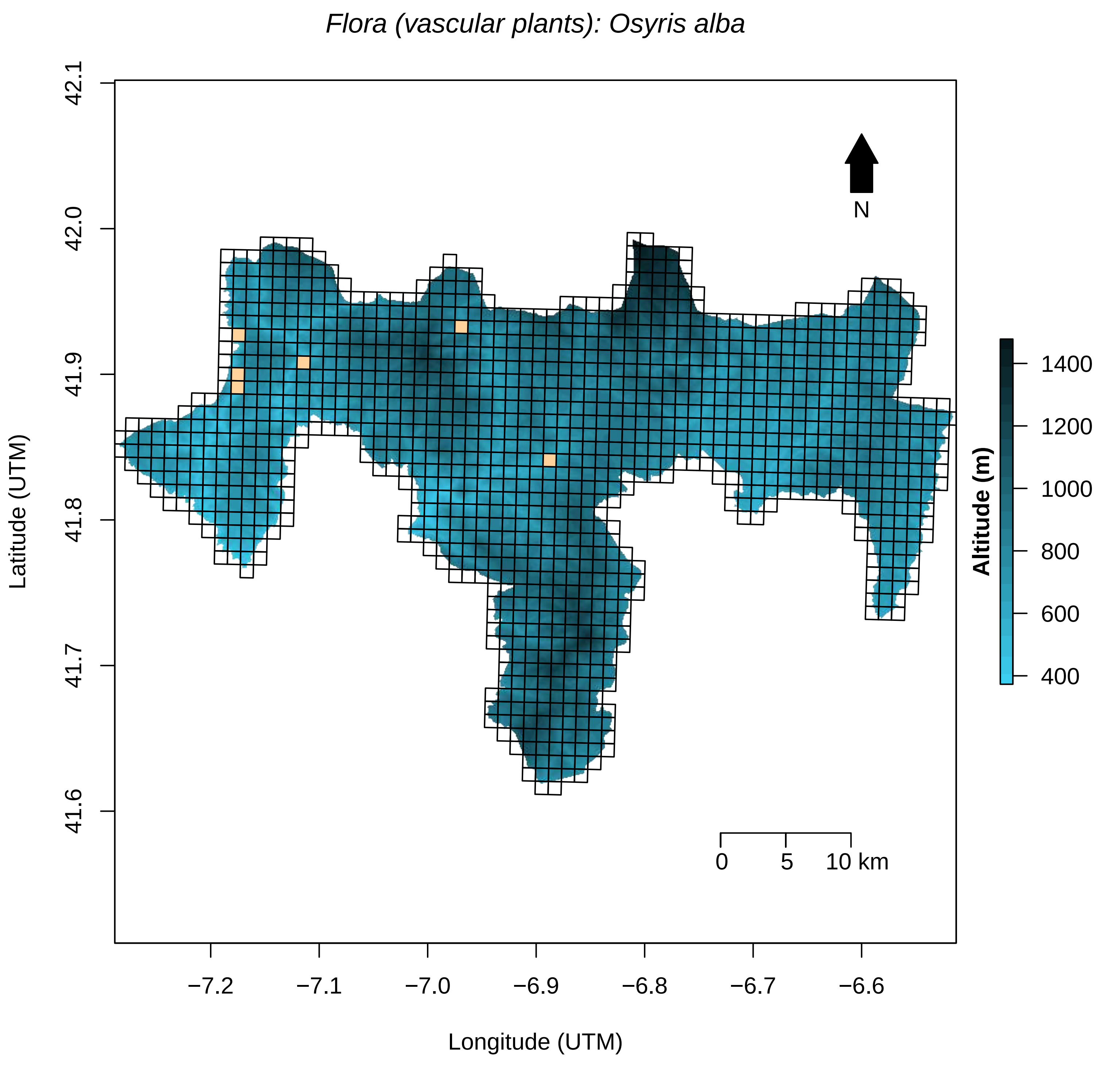

Flora (vascular plants): Osyris alba
42.1
42.0
41.9
41.8
41.7
41.6
Latitude (UTM)
−7.2
−7.1
−7.0
−6.9
−6.8
−6.7
−6.6
Longitude (UTM)
N
0
5
10 km
1400
1200
1000
800
600
400
Altitude (m)

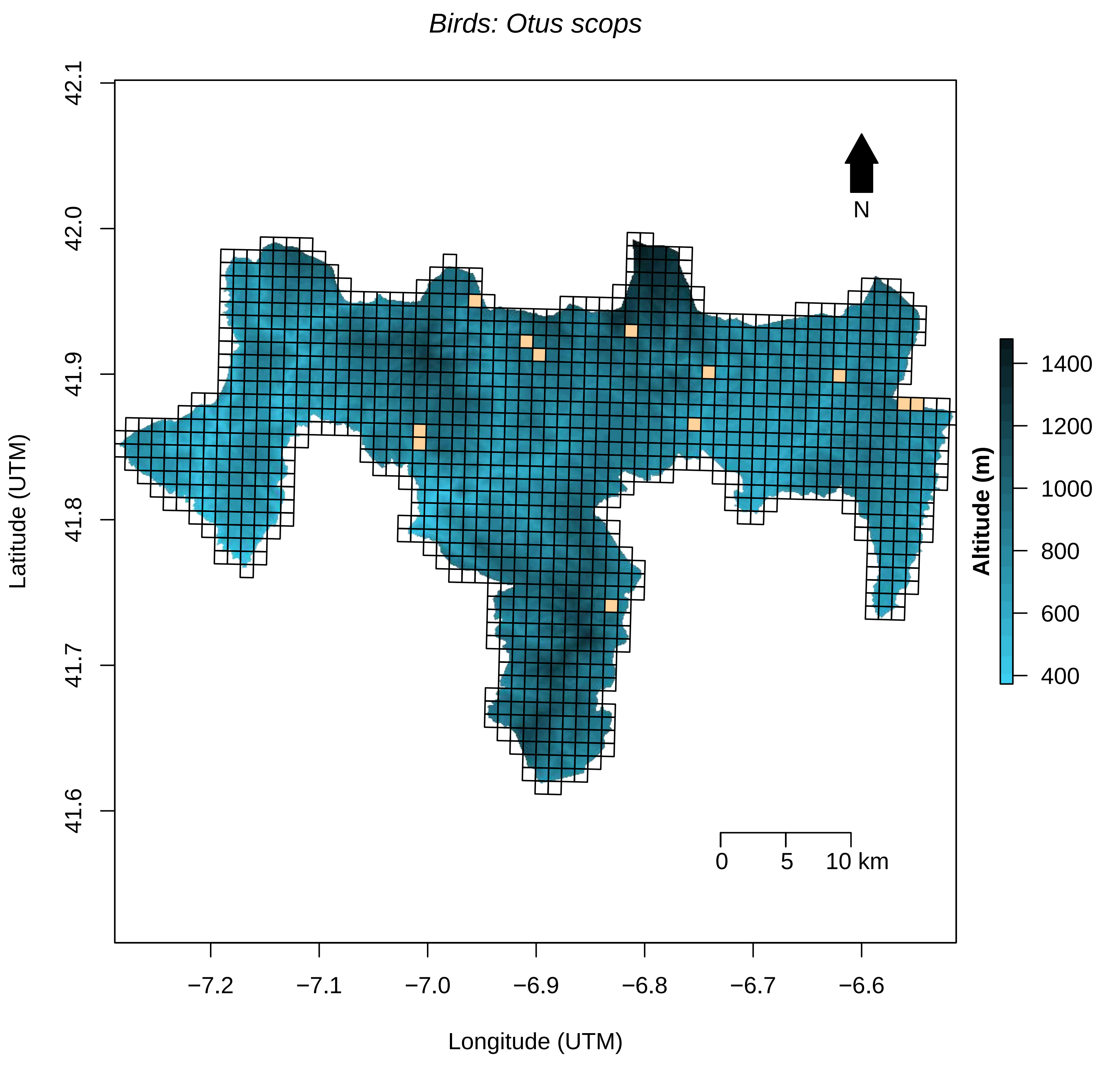

Birds: Otus scops
N
0
5
10 km
Altitude (m)
1400
1200
1000
800
600
400
Latitude (UTM)
42.1
42.0
41.9
41.8
41.7
41.6
Longitude (UTM)
−7.2
−7.1
−7.0
−6.9
−6.8
−6.7
−6.6

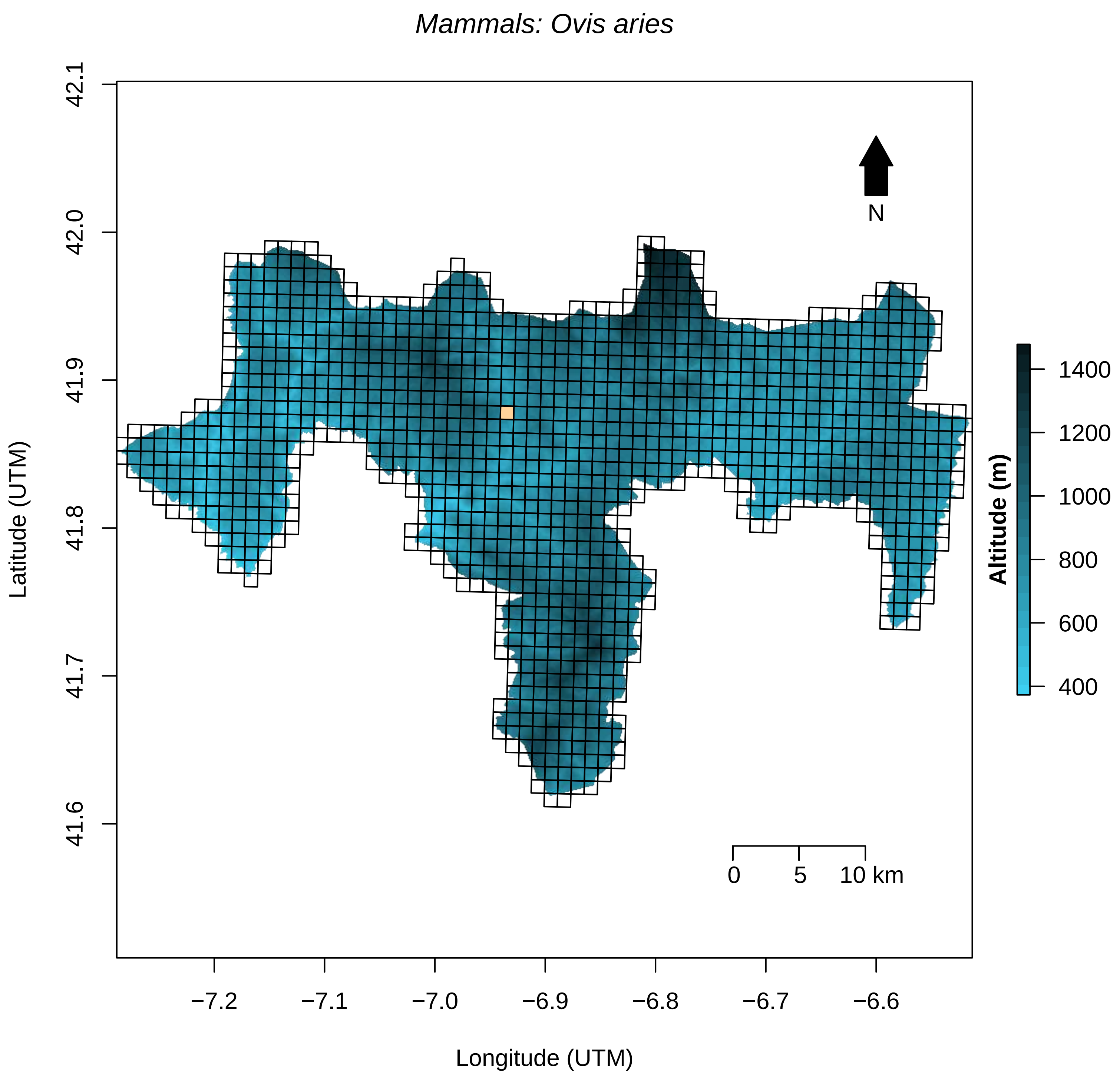

Mammals: Ovis aries
42.1
42.0
41.9
41.8
41.7
41.6
Latitude (UTM)
−7.2
−7.1
−7.0
−6.9
−6.8
−6.7
−6.6
Longitude (UTM)
N
0
5
10 km
Altitude (m)
1400
1200
1000
800
600
400

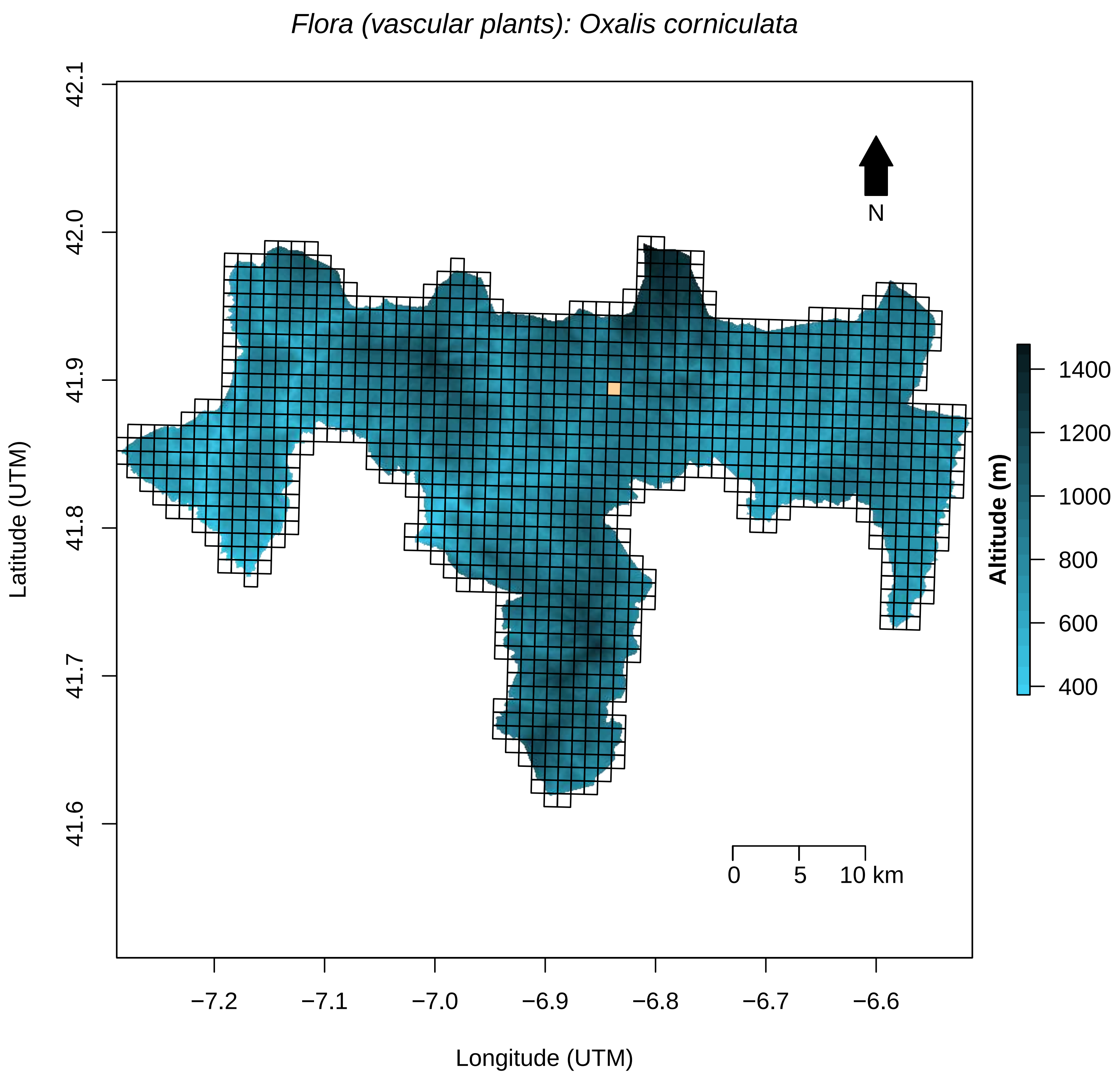

Flora (vascular plants): Oxalis corniculata
N
0
5
10 km
Altitude (m)
1400
1200
1000
800
600
400
42.1
42.0
41.9
41.8
41.7
41.6
Latitude (UTM)
−7.2
−7.1
−7.0
−6.9
−6.8
−6.7
−6.6
Longitude (UTM)

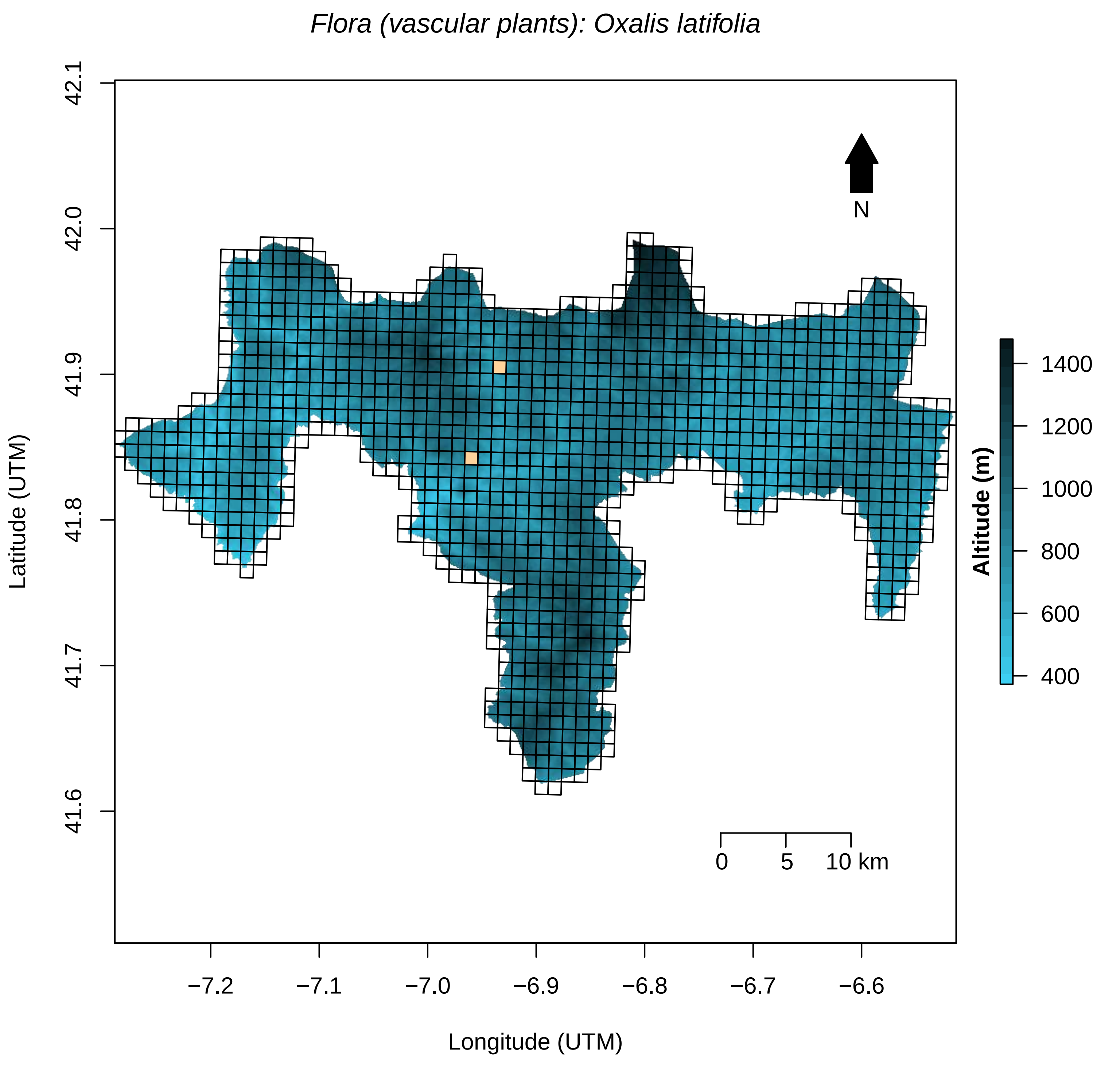

Flora (vascular plants): Oxalis latifolia
Latitude (UTM)
42.1
42.0
41.9
41.8
41.7
41.6
Longitude (UTM)
−7.2
−7.1
−7.0
−6.9
−6.8
−6.7
−6.6
N
0
5
10 km
Altitude (m)
1400
1200
1000
800
600
400

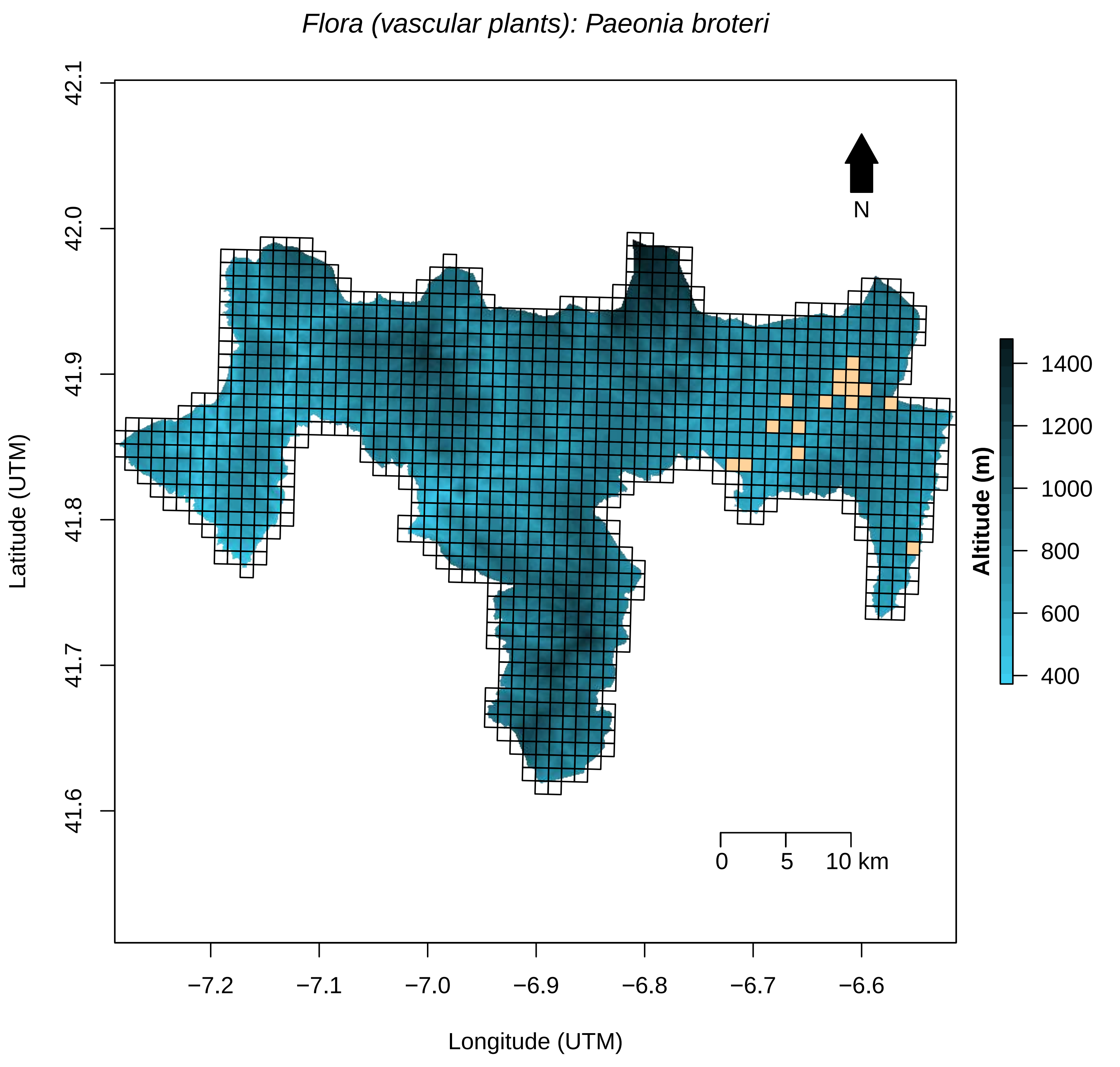

Flora (vascular plants): Paeonia broteri
42.1
42.0
41.9
41.8
41.7
41.6
Latitude (UTM)
−7.2
−7.1
−7.0
−6.9
−6.8
−6.7
−6.6
Longitude (UTM)
N
0
5
10 km
Altitude (m)
1400
1200
1000
800
600
400

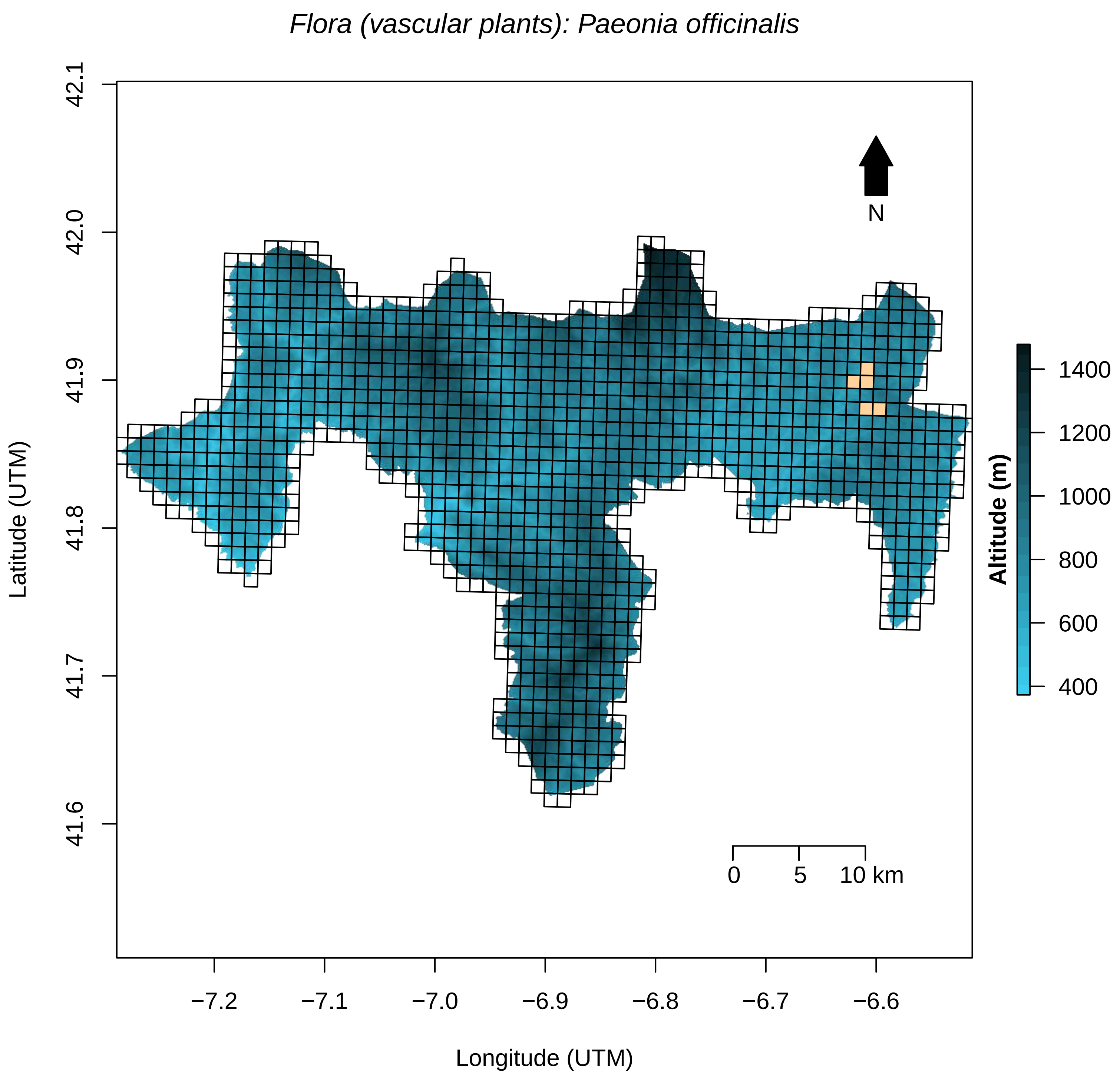
Flora (vascular plants): Paeonia officinalis
Latitude (UTM)
Longitude (UTM)
42.1
42.0
41.9
41.8
41.7
41.6
−7.2
−7.1
−7.0
−6.9
−6.8
−6.7
−6.6
N
0
5
10 km
Altitude (m)
1400
1200
1000
800
600
400

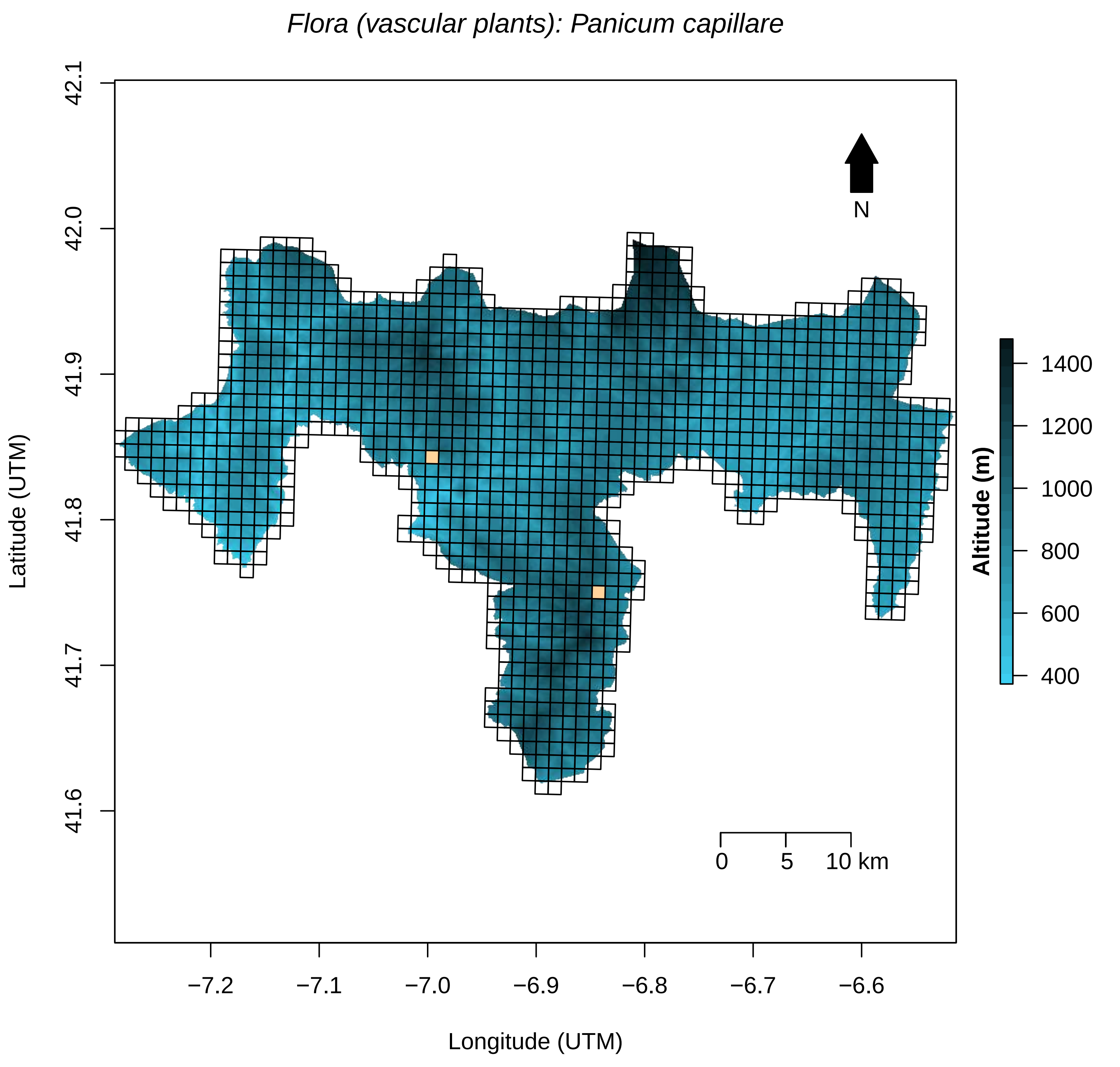

Flora (vascular plants): Panicum capillare
N
0
5
10 km
Altitude (m)
1400
1200
1000
800
600
400
Latitude (UTM)
42.1
42.0
41.9
41.8
41.7
41.6
Longitude (UTM)
−7.2
−7.1
−7.0
−6.9
−6.8
−6.7
−6.6

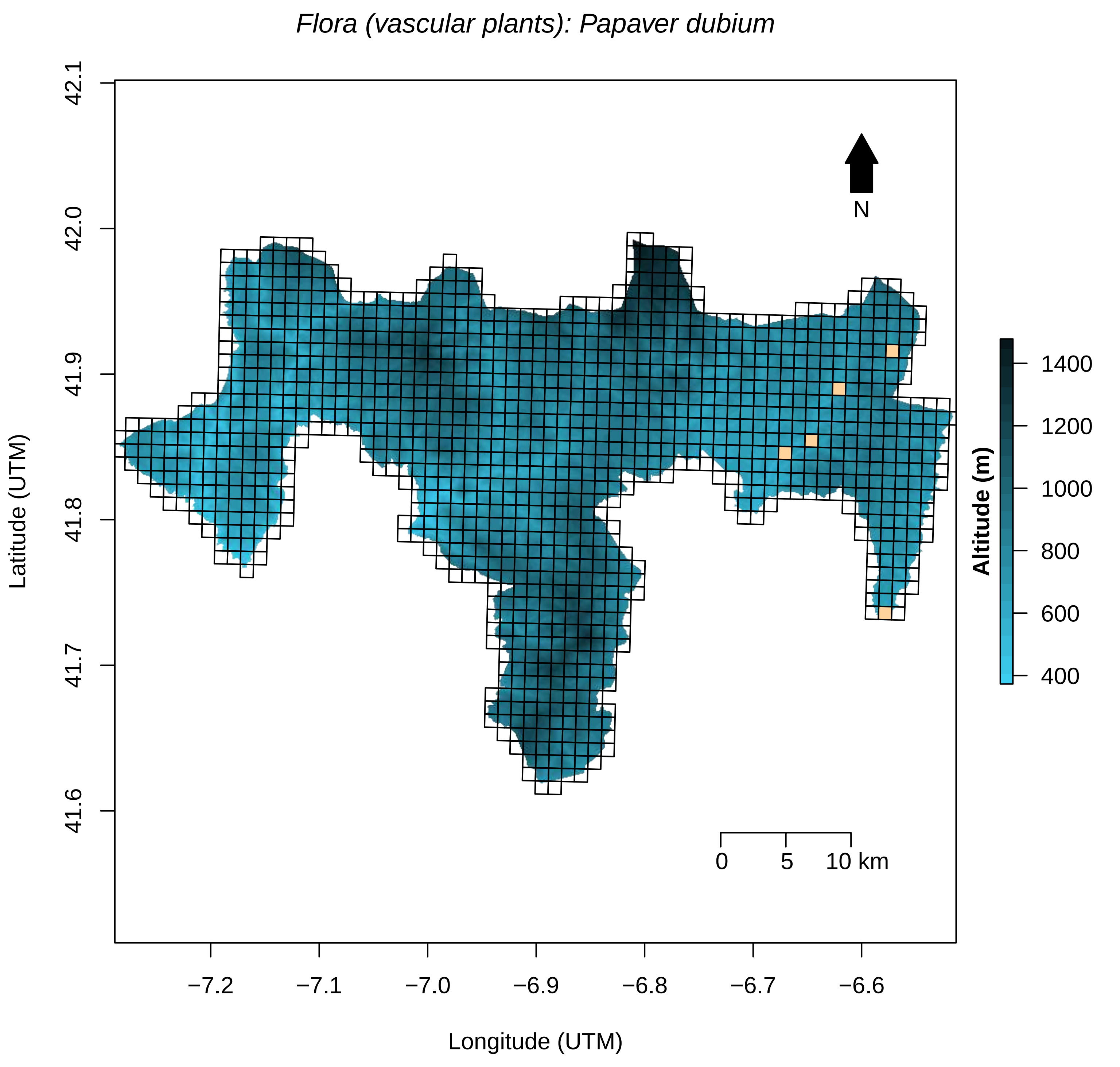

Flora (vascular plants): Papaver dubium
42.1
42.0
41.9
41.8
41.7
41.6
Latitude (UTM)
−7.2
−7.1
−7.0
−6.9
−6.8
−6.7
−6.6
Longitude (UTM)
N
0
5
10 km
Altitude (m)
1400
1200
1000
800
600
400

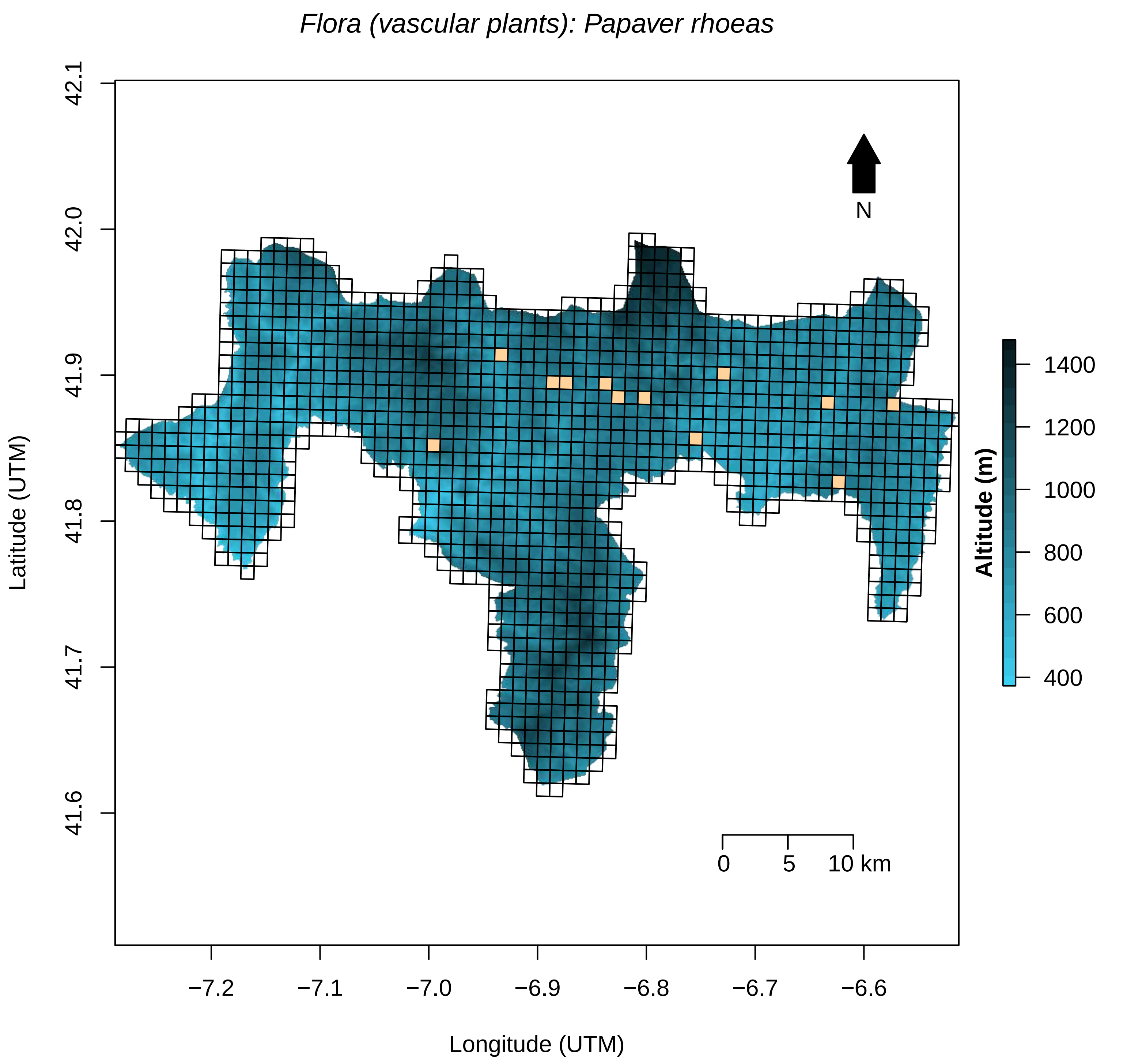

Flora (vascular plants): Papaver rhoeas
N
0
5
10 km
Altitude (m)
1400
1200
1000
800
600
400
42.1
42.0
41.9
41.8
41.7
41.6
Latitude (UTM)
−7.2
−7.1
−7.0
−6.9
−6.8
−6.7
−6.6
Longitude (UTM)

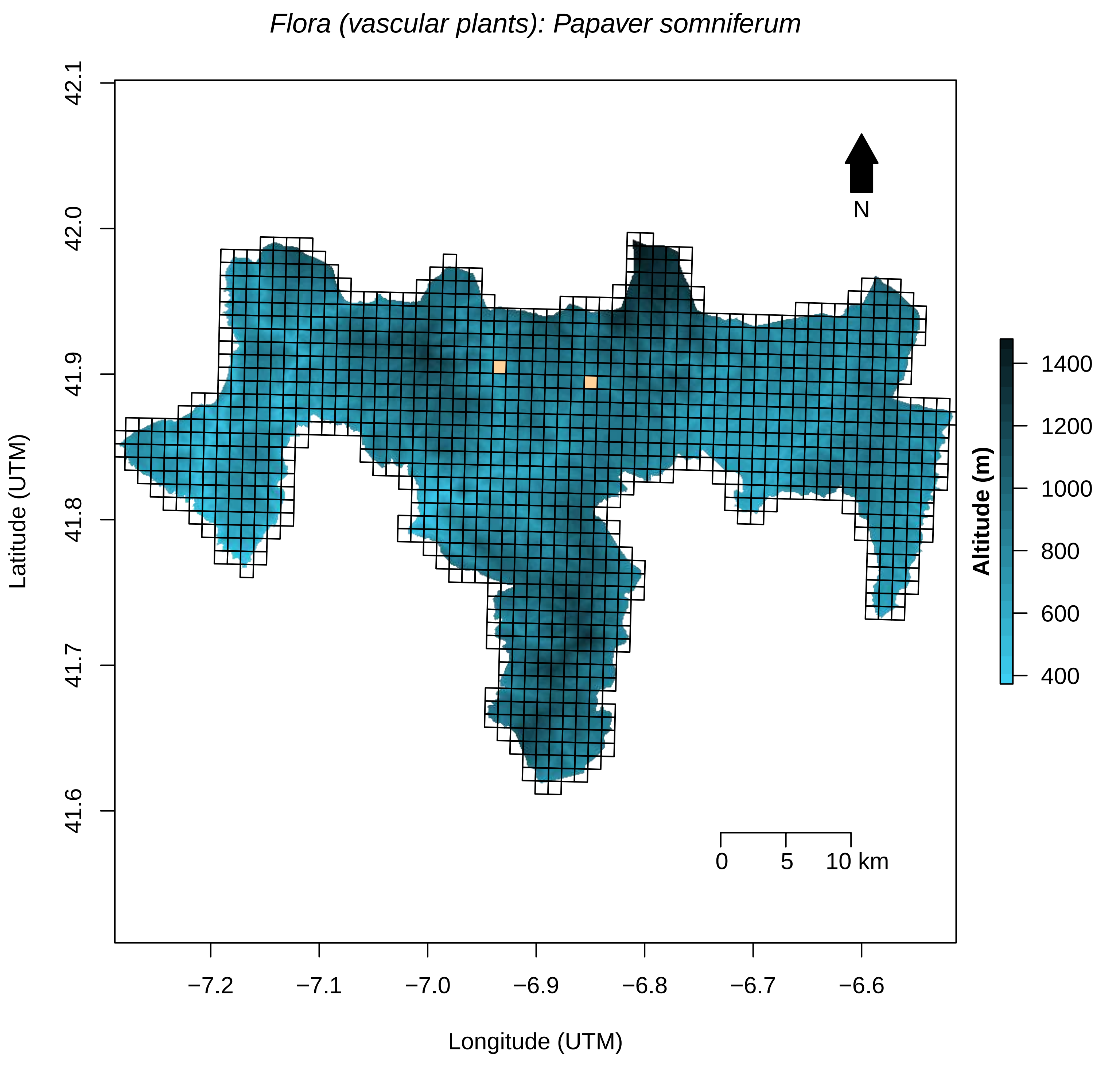

Flora (vascular plants): Papaver somniferum
42.1
42.0
41.9
41.8
41.7
41.6
Latitude (UTM)
−7.2
−7.1
−7.0
−6.9
−6.8
−6.7
−6.6
Longitude (UTM)
N
0
5
10 km
Altitude (m)
1400
1200
1000
800
600
400

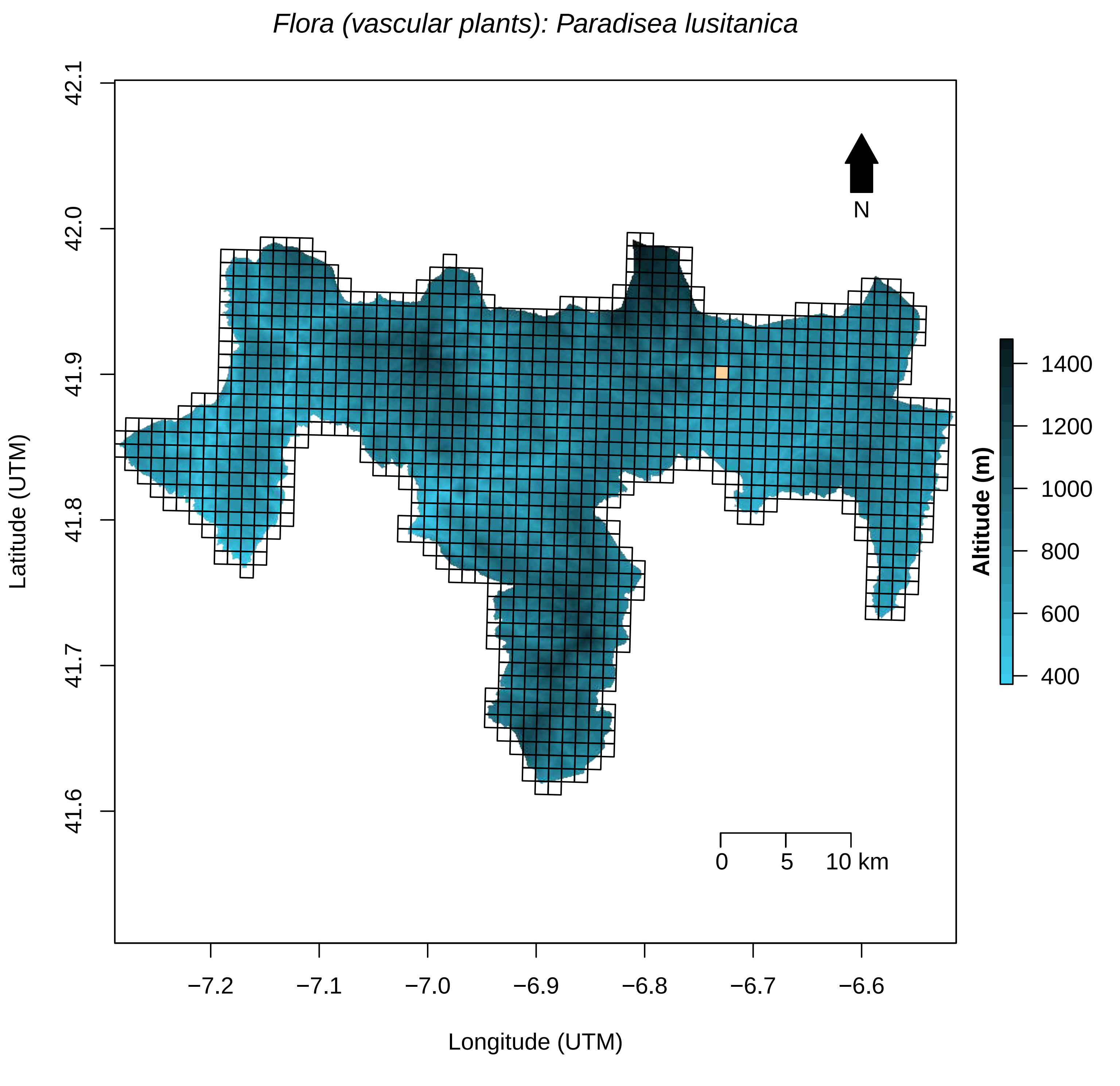

Flora (vascular plants): Paradisea lusitanica
N
0
5
10 km
Altitude (m)
1400
1200
1000
800
600
400
42.1
42.0
41.9
41.8
41.7
41.6
Latitude (UTM)
−7.2
−7.1
−7.0
−6.9
−6.8
−6.7
−6.6
Longitude (UTM)

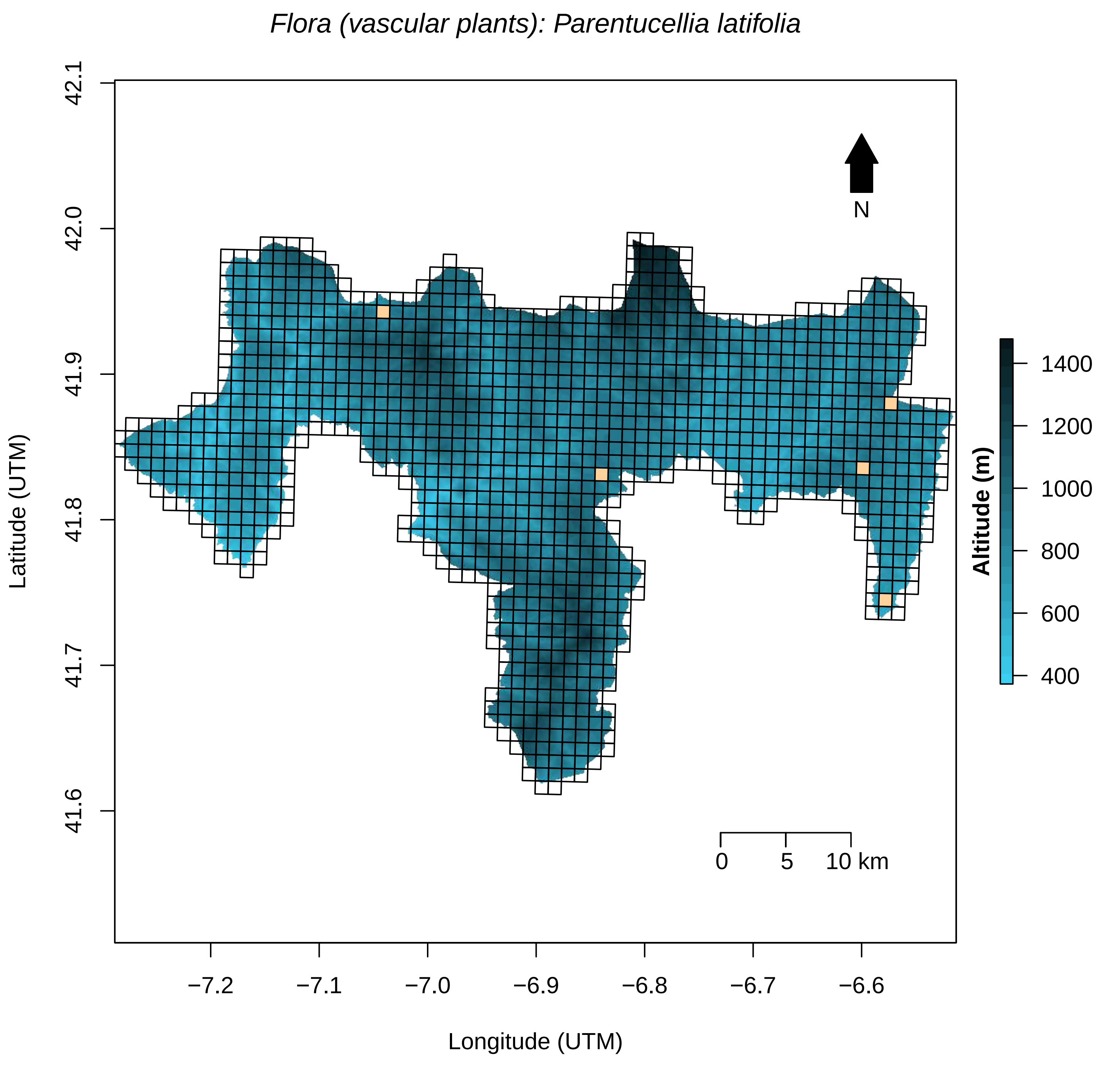

Flora (vascular plants): Parentucellia latifolia
42.1
42.0
41.9
41.8
41.7
41.6
Latitude (UTM)
−7.2
−7.1
−7.0
−6.9
−6.8
−6.7
−6.6
Longitude (UTM)
N
0
5
10 km
1400
1200
1000
800
600
400
Altitude (m)

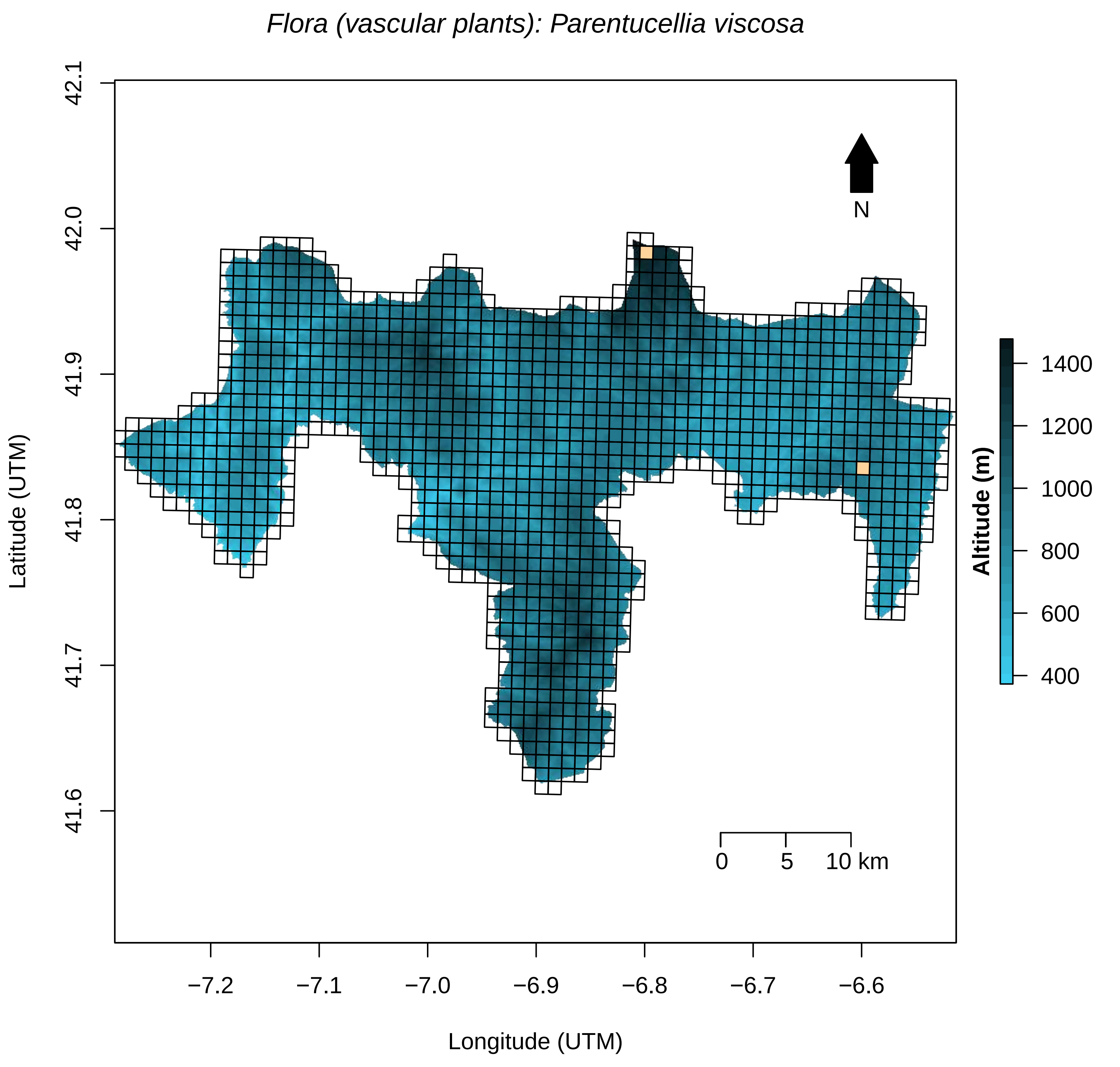

Flora (vascular plants): Parentucellia viscosa
42.1
42.0
41.9
41.8
41.7
41.6
Latitude (UTM)
−7.2
−7.1
−7.0
−6.9
−6.8
−6.7
−6.6
Longitude (UTM)
N
0
5
10 km
1400
1200
1000
800
600
400
Altitude (m)

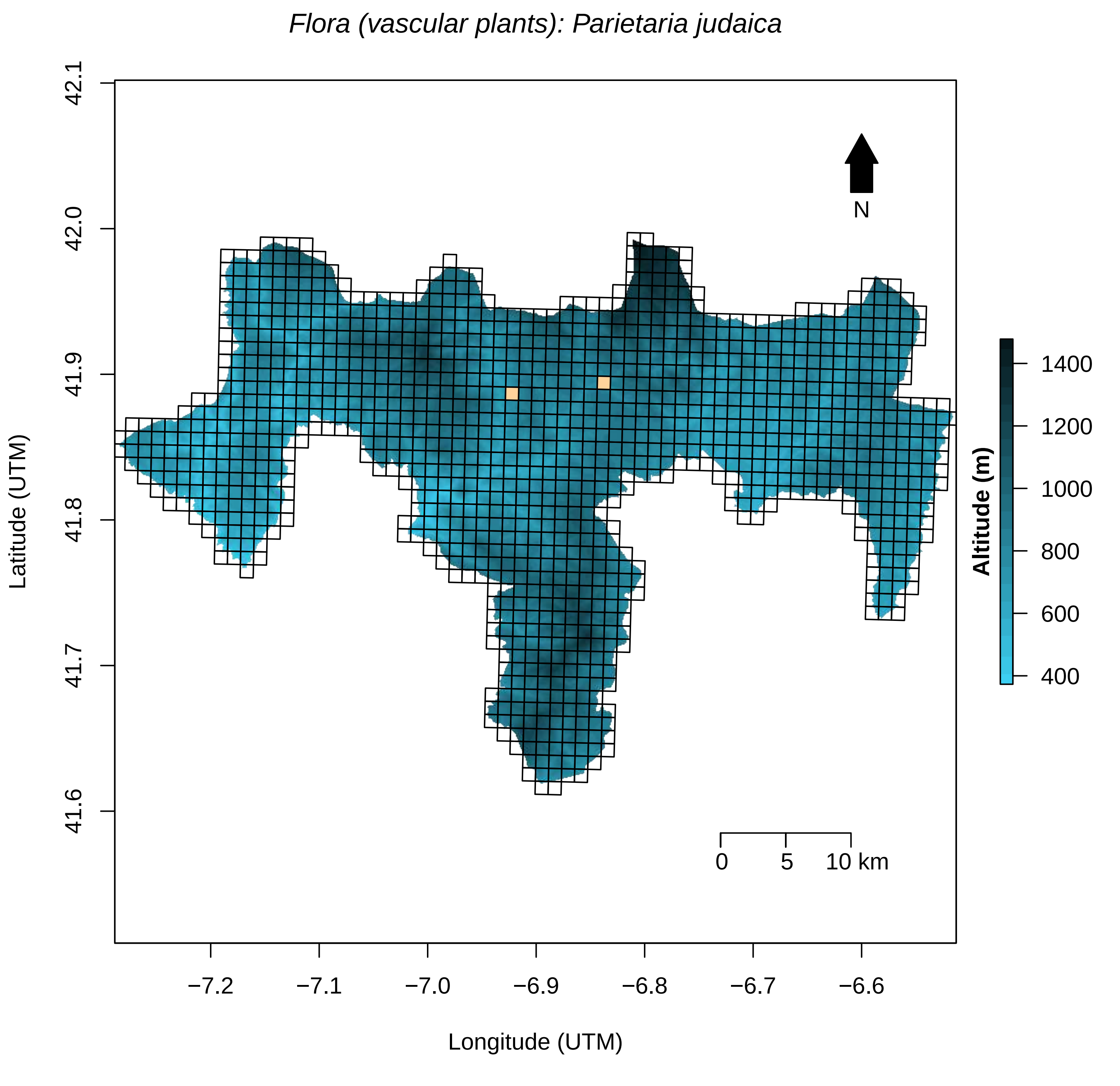

Flora (vascular plants): Parietaria judaica
42.1
42.0
41.9
41.8
41.7
41.6
Latitude (UTM)
−7.2
−7.1
−7.0
−6.9
−6.8
−6.7
−6.6
Longitude (UTM)
N
0
5
10 km
Altitude (m)
1400
1200
1000
800
600
400

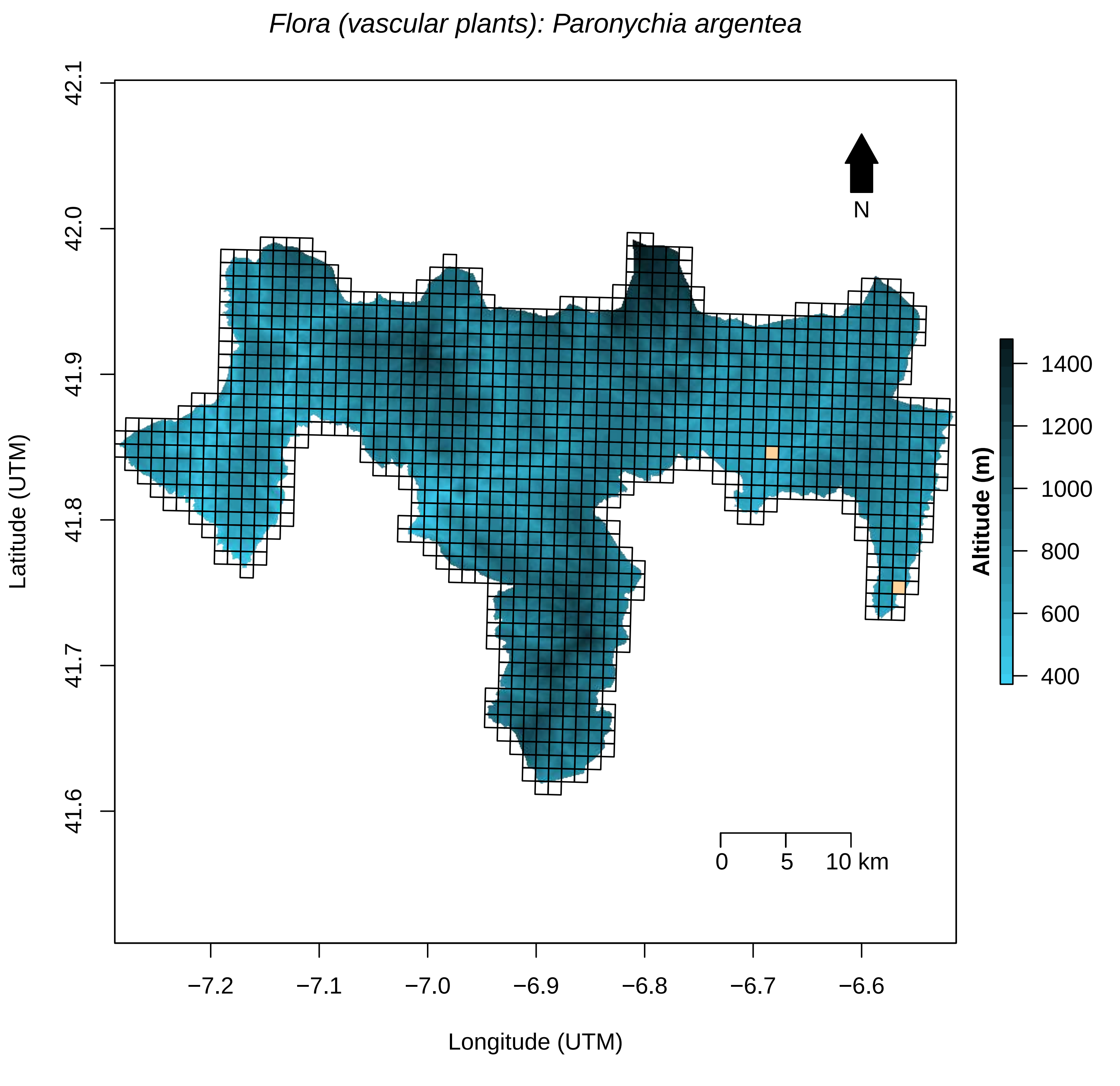
Flora (vascular plants): Paronychia argentea
42.1
42.0
41.9
41.8
41.7
41.6
Latitude (UTM)
−7.2
−7.1
−7.0
−6.9
−6.8
−6.7
−6.6
Longitude (UTM)
N
0
5
10 km
Altitude (m)
1400
1200
1000
800
600
400

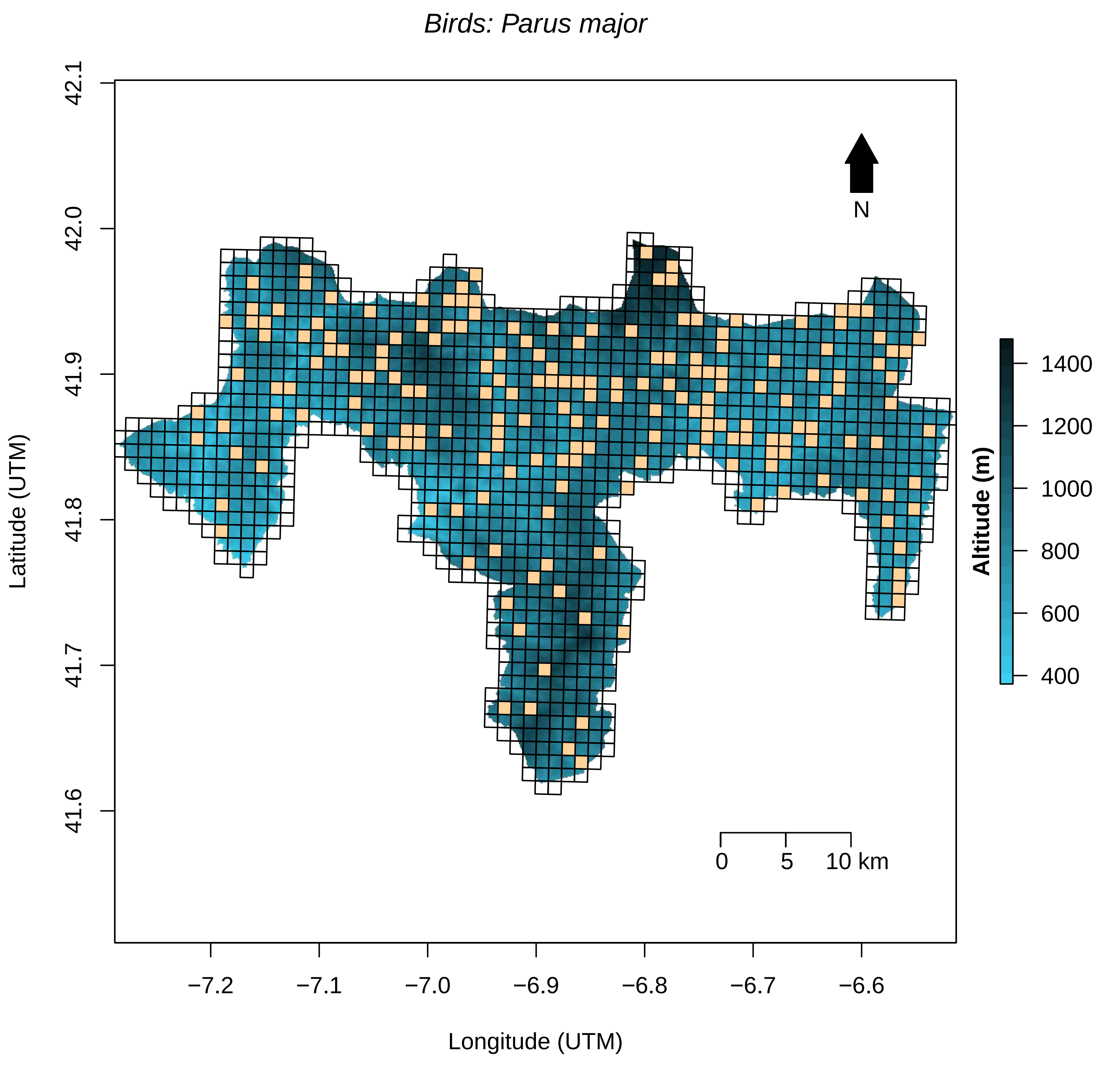
Birds: Parus major
N
0 5 10 km
Altitude (m)
1400
1200
1000
800
600
400
42.1
42.0
41.9
41.8
41.7
41.6
Latitude (UTM)
−7.2
−7.1
−7.0
−6.9
−6.8
−6.7
−6.6
Longitude (UTM)

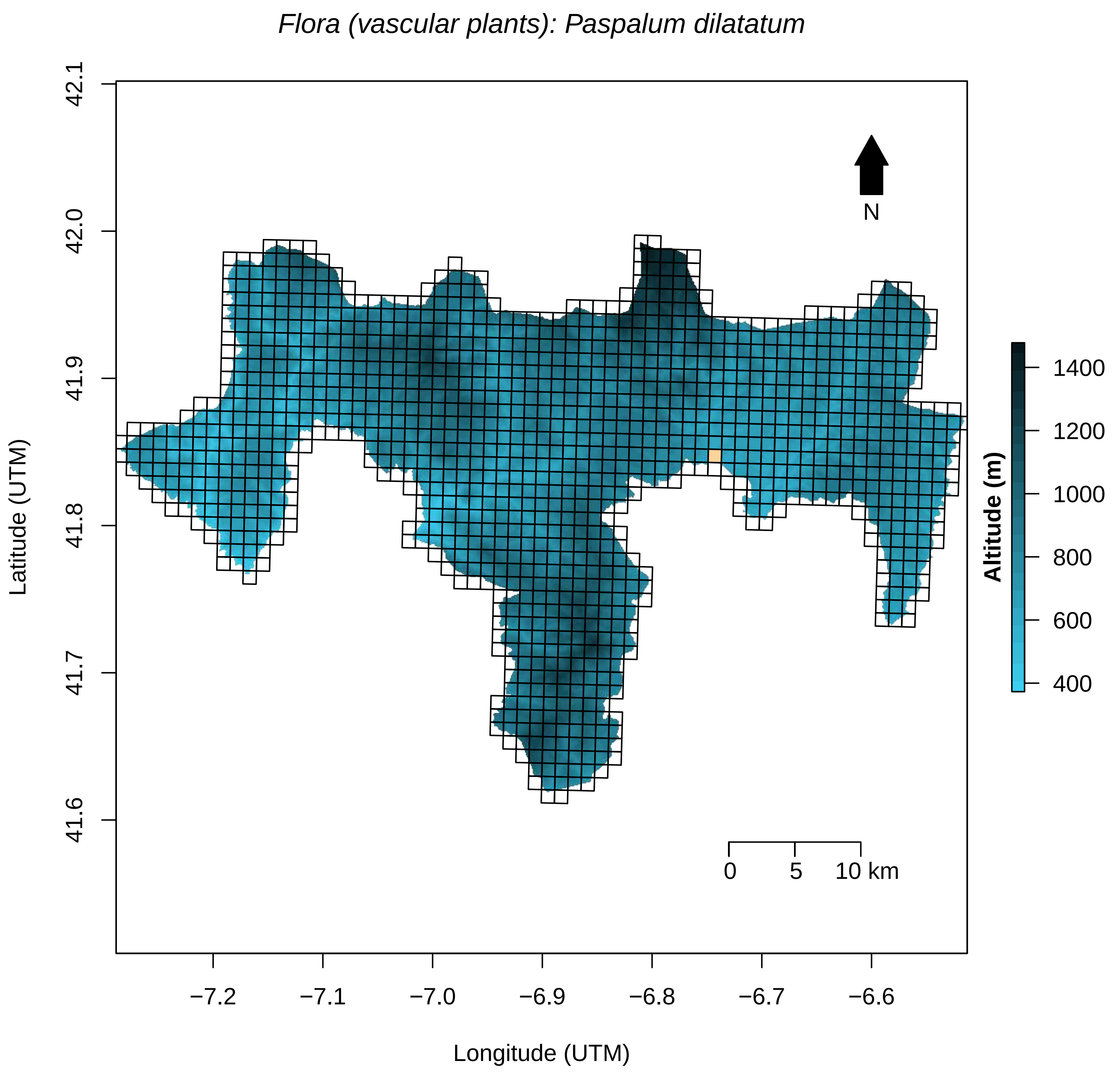

Flora (vascular plants): Paspalum dilatatum
Latitude (UTM)
42.1
42.0
41.9
41.8
41.7
41.6
Longitude (UTM)
−7.2
−7.1
−7.0
−6.9
−6.8
−6.7
−6.6
N
0
5
10 km
Altitude (m)
1400
1200
1000
800
600
400

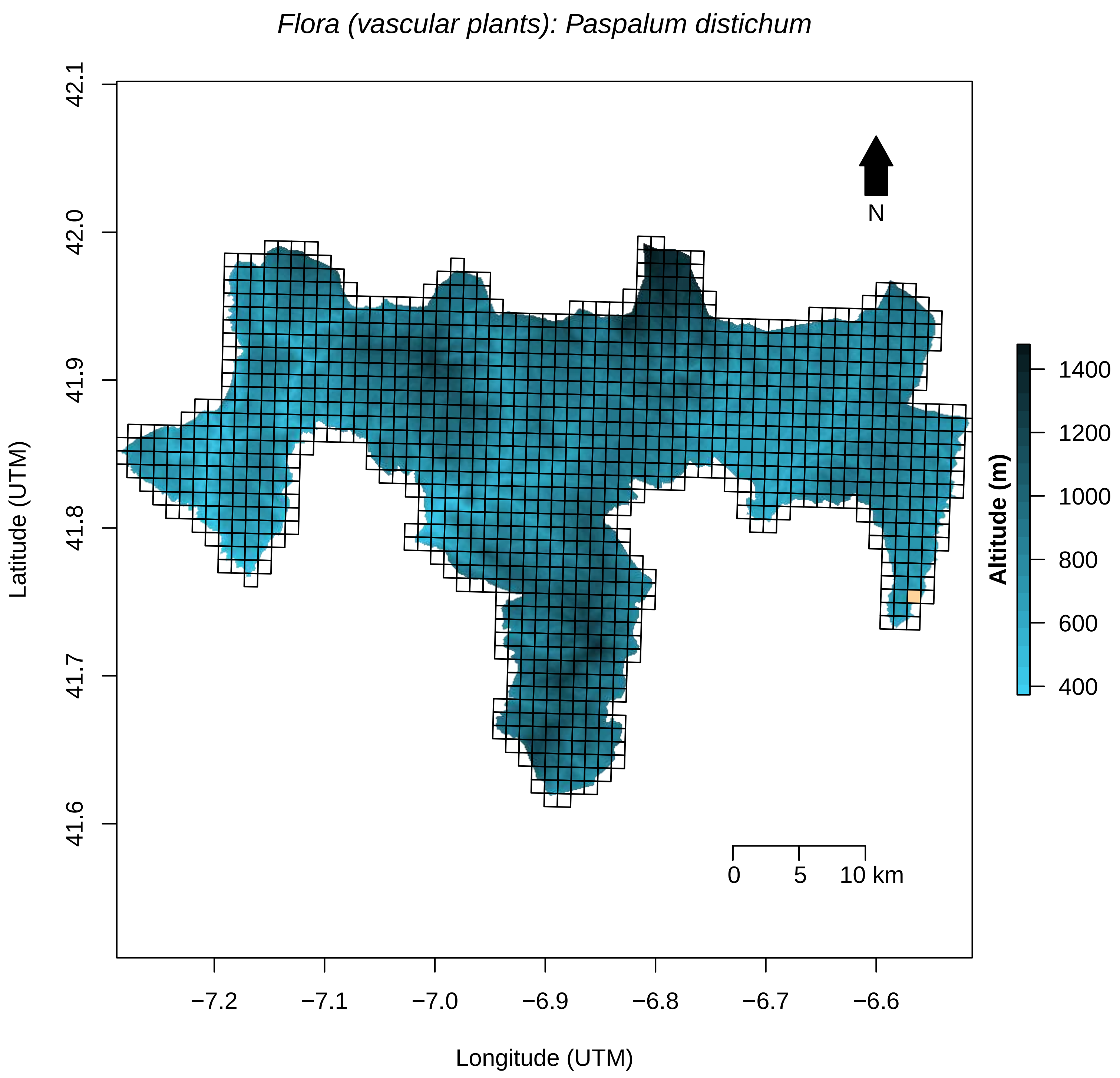

Flora (vascular plants): Paspalum distichum
Latitude (UTM)
42.1
42.0
41.9
41.8
41.7
41.6
Longitude (UTM)
−7.2
−7.1
−7.0
−6.9
−6.8
−6.7
−6.6
N
0
5
10 km
Altitude (m)
1400
1200
1000
800
600
400

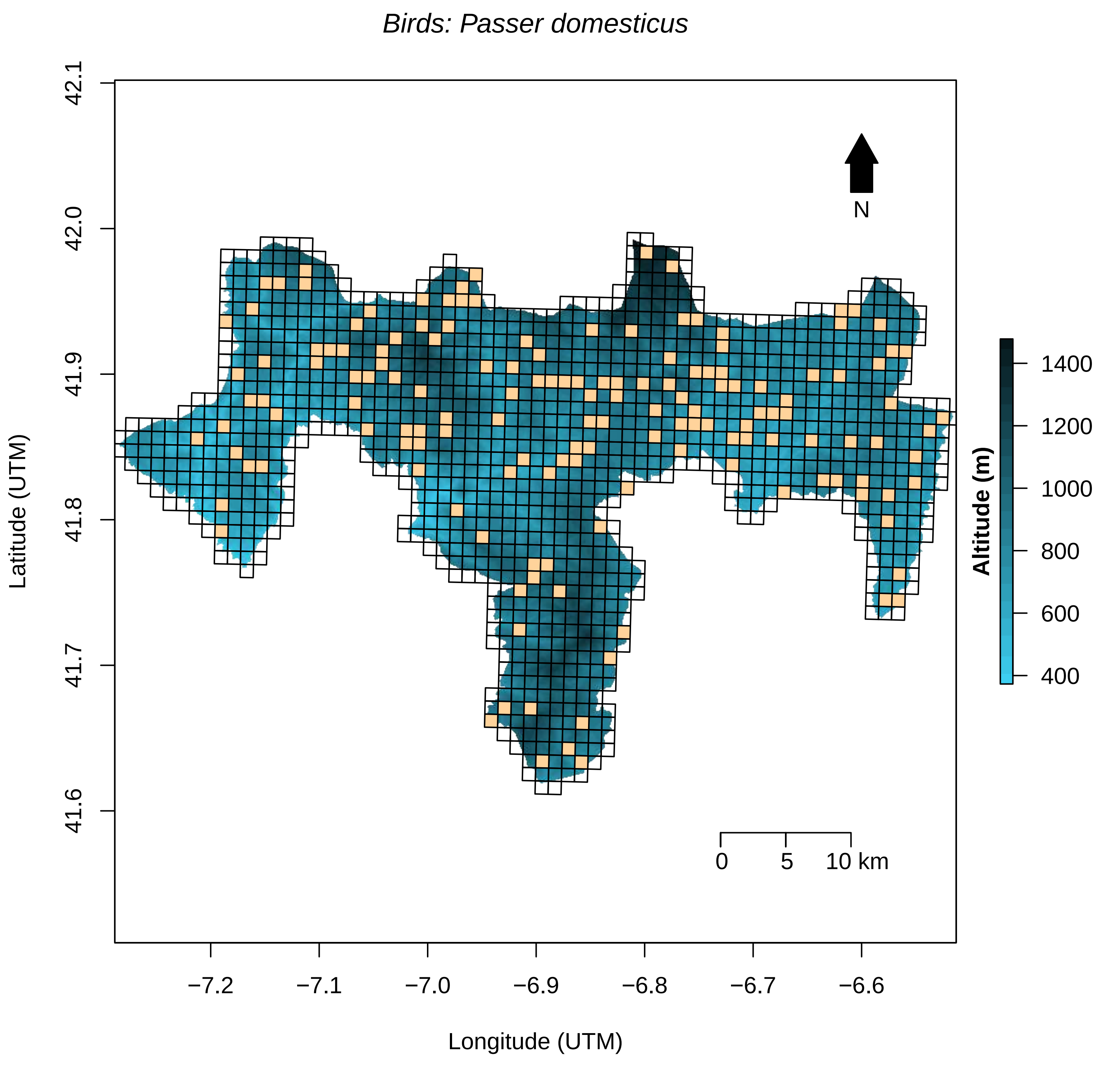
Birds: Passer domesticus
N
0
5
10 km
Altitude (m)
1400
1200
1000
800
600
400
Latitude (UTM)
42.1
42.0
41.9
41.8
41.7
41.6
Longitude (UTM)
−7.2
−7.1
−7.0
−6.9
−6.8
−6.7
−6.6

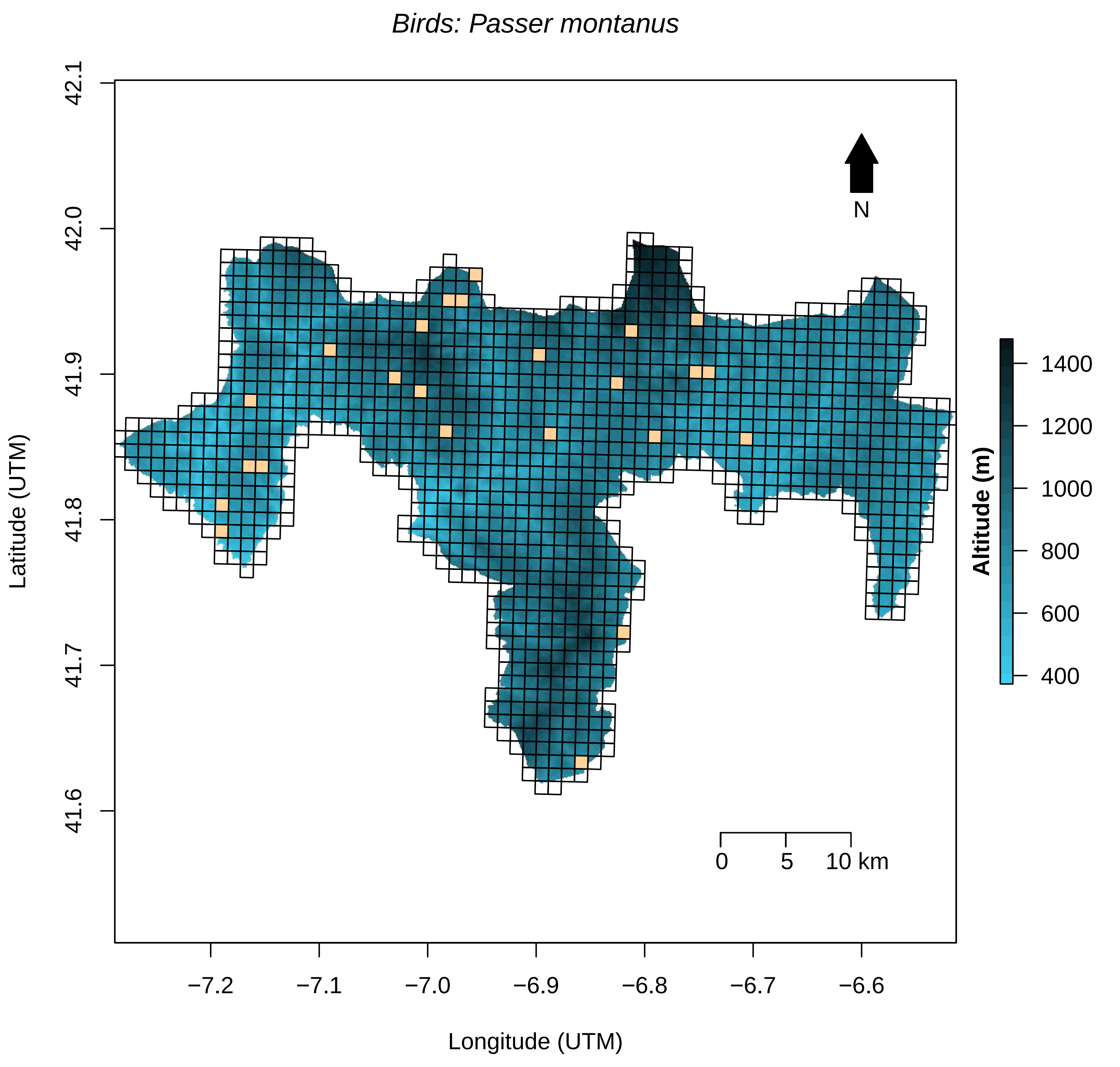
Birds: Passer montanus
42.1
42.0
41.9
41.8
41.7
41.6
Latitude (UTM)
−7.2
−7.1
−7.0
−6.9
−6.8
−6.7
−6.6
Longitude (UTM)
N
0
5
10 km
Altitude (m)
1400
1200
1000
800
600
400

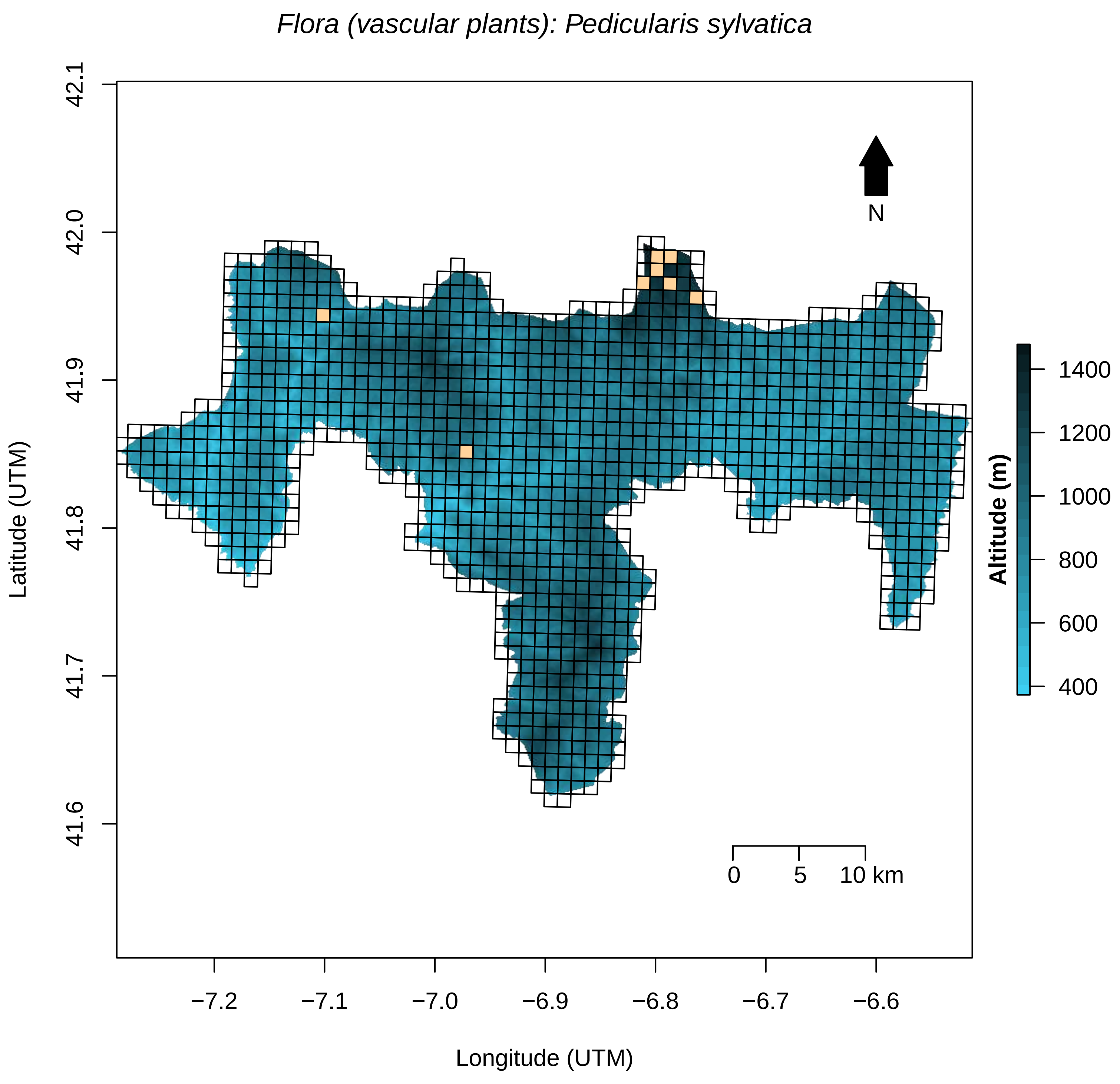

Flora (vascular plants): Pedicularis sylvatica
Latitude (UTM)
42.1
42.0
41.9
41.8
41.7
41.6
Longitude (UTM)
−7.2
−7.1
−7.0
−6.9
−6.8
−6.7
−6.6
N
0
5
10 km
Altitude (m)
1400
1200
1000
800
600
400

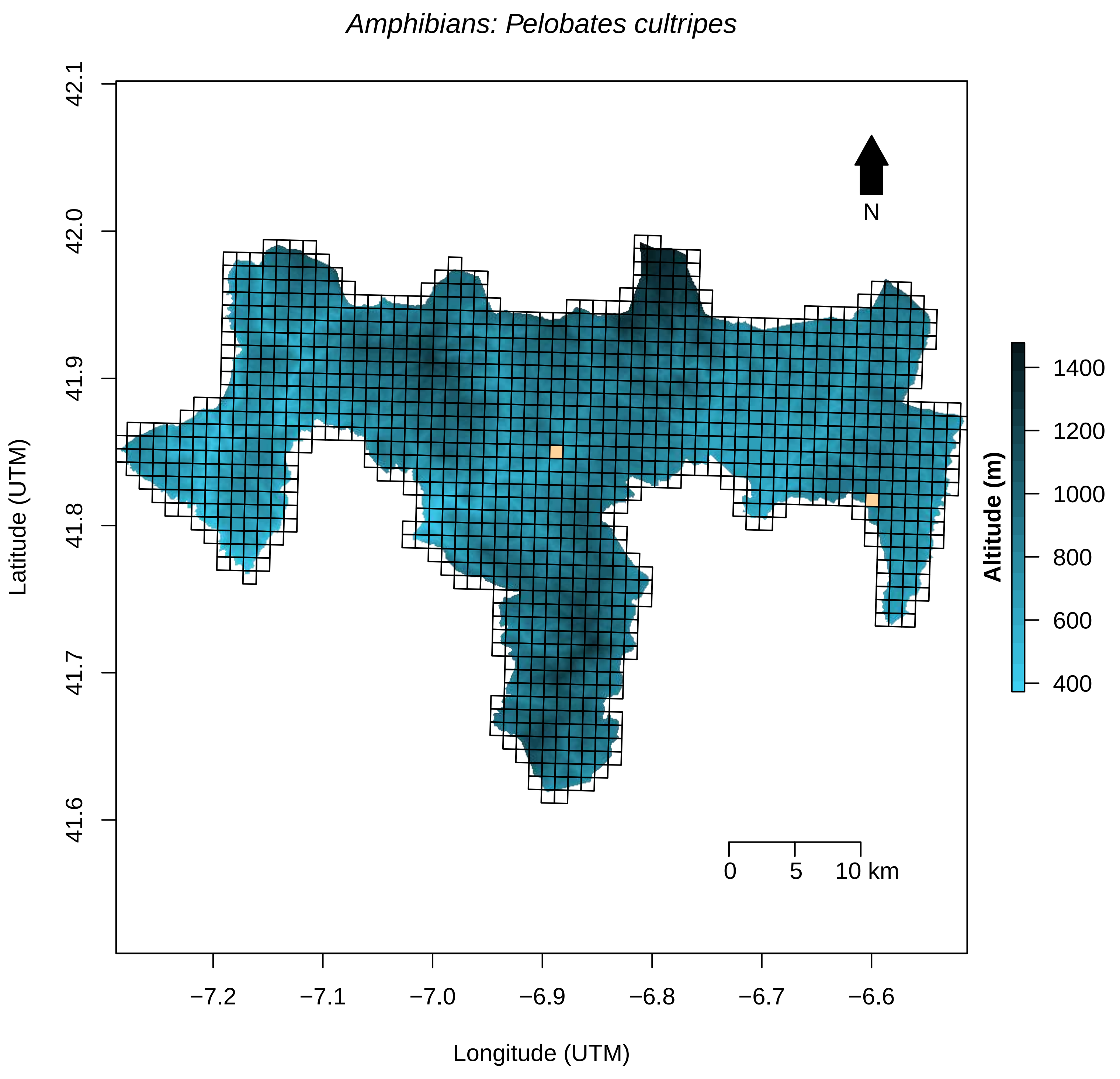

Amphibians: Pelobates cultripes
42.1
42.0
41.9
41.8
41.7
41.6
Latitude (UTM)
−7.2
−7.1
−7.0
−6.9
−6.8
−6.7
−6.6
Longitude (UTM)
N
0
5
10 km
Altitude (m)
1400
1200
1000
800
600
400

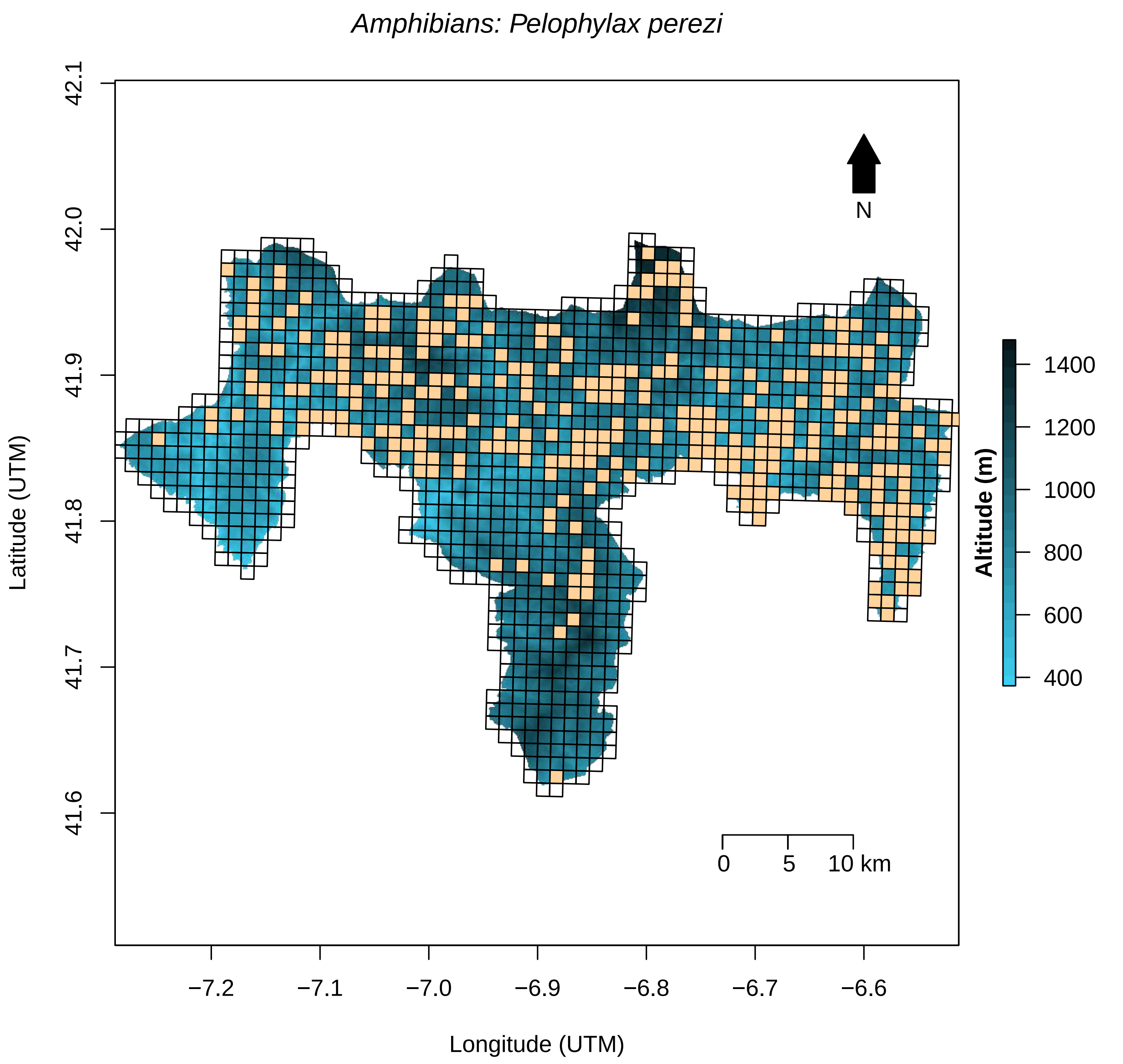

Amphibians: Pelophylax perezi
Latitude (UTM)
42.1
42.0
41.9
41.8
41.7
41.6
Longitude (UTM)
−7.2
−7.1
−7.0
−6.9
−6.8
−6.7
−6.6
N
0
5
10 km
Altitude (m)
1400
1200
1000
800
600
400

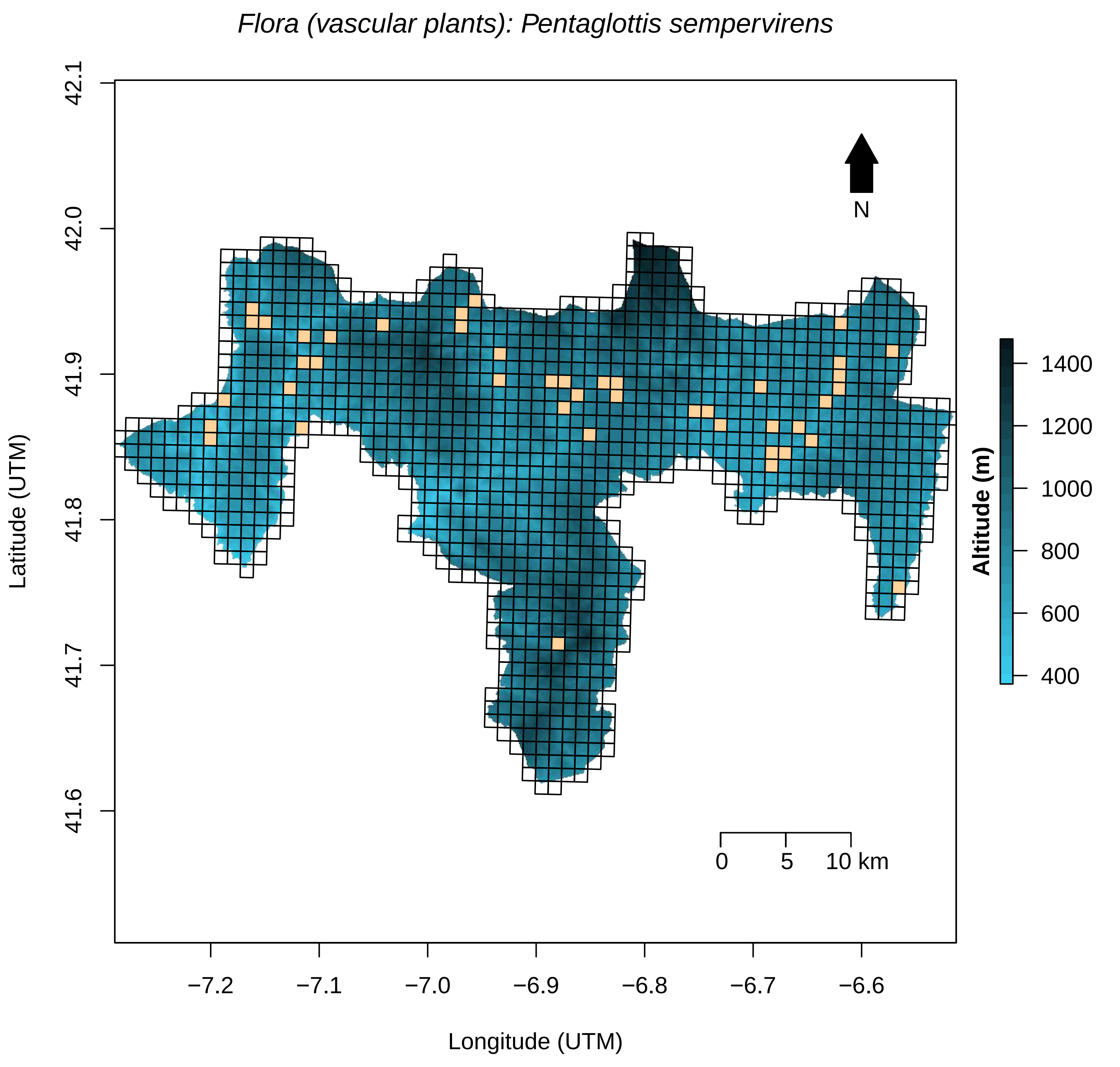

Flora (vascular plants): Pentaglottis sempervirens
42.1
42.0
41.9
41.8
41.7
41.6
Latitude (UTM)
−7.2
−7.1
−7.0
−6.9
−6.8
−6.7
−6.6
Longitude (UTM)
N
0
5
10 km
Altitude (m)
1400
1200
1000
800
600
400

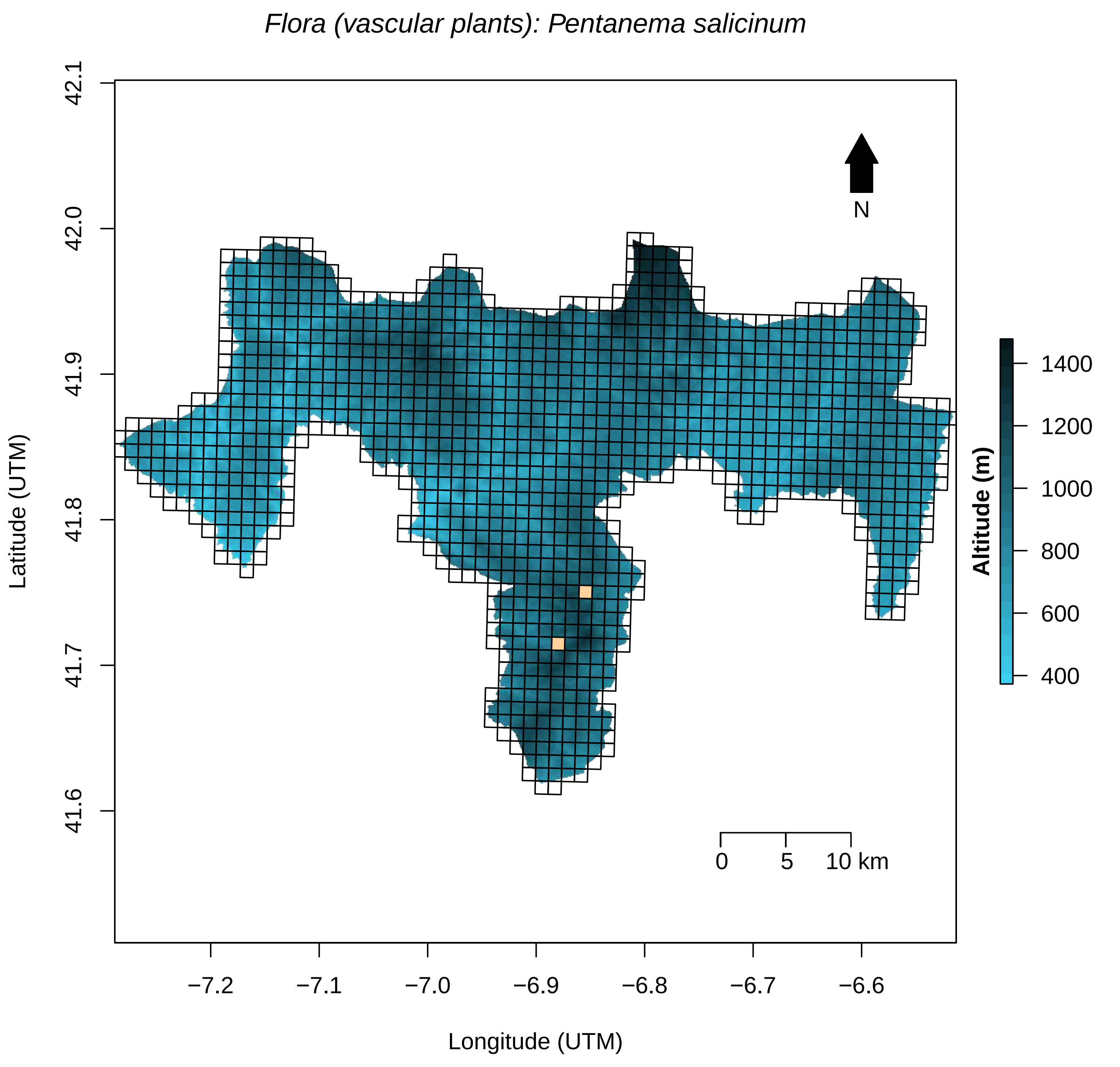

Flora (vascular plants): Pentanema salicinum
42.1
42.0
41.9
41.8
41.7
41.6
Latitude (UTM)
−7.2
−7.1
−7.0
−6.9
−6.8
−6.7
−6.6
Longitude (UTM)
N
0
5
10 km
Altitude (m)
1400
1200
1000
800
600
400

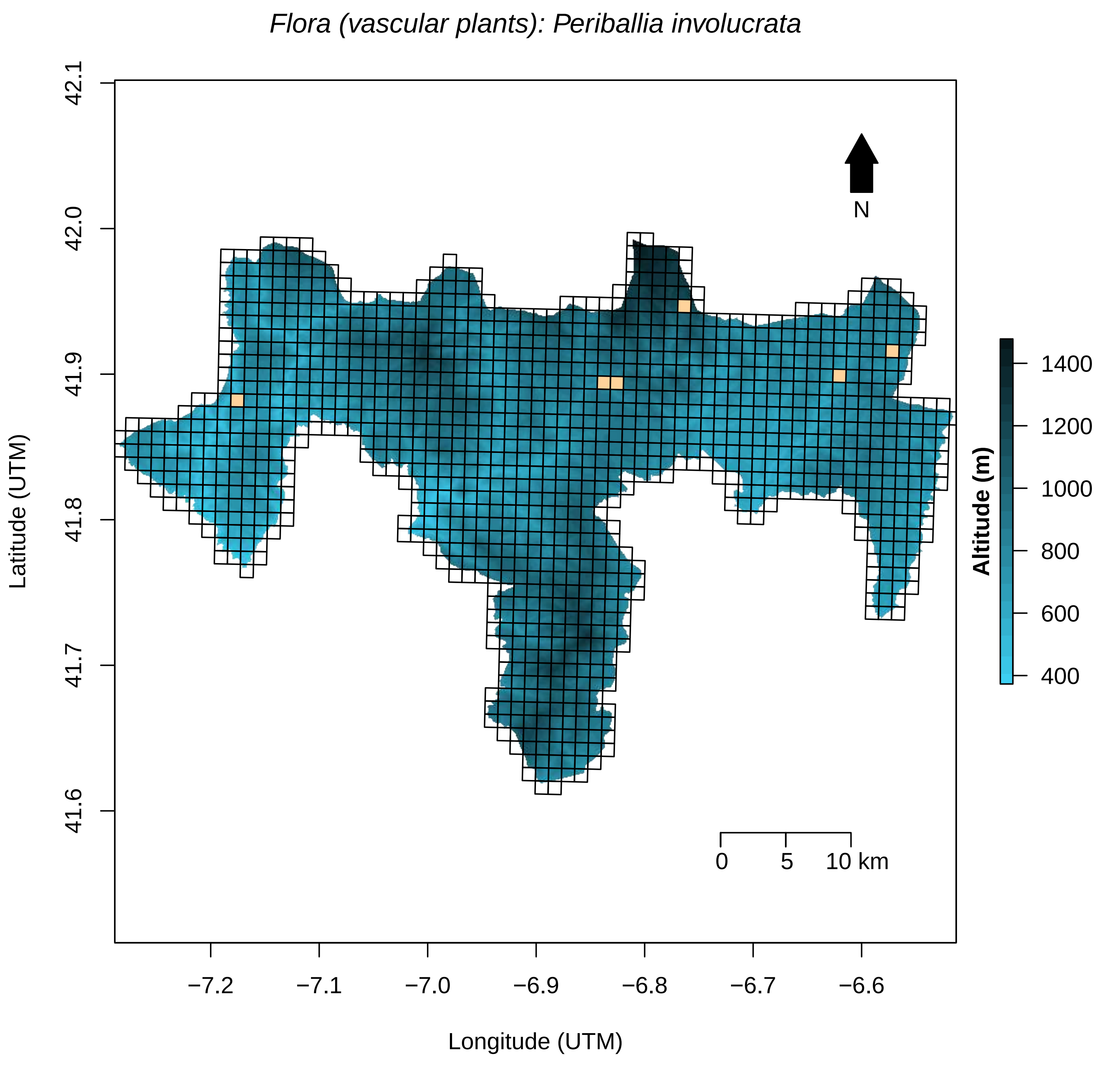
Flora (vascular plants): Periballia involucrata
42.1
42.0
41.9
41.8
41.7
41.6
N
0
5
10 km
−7.2
−7.1
−7.0
−6.9
−6.8
−6.7
−6.6
Longitude (UTM)
Latitude (UTM)
Altitude (m)
1400
1200
1000
800
600
400

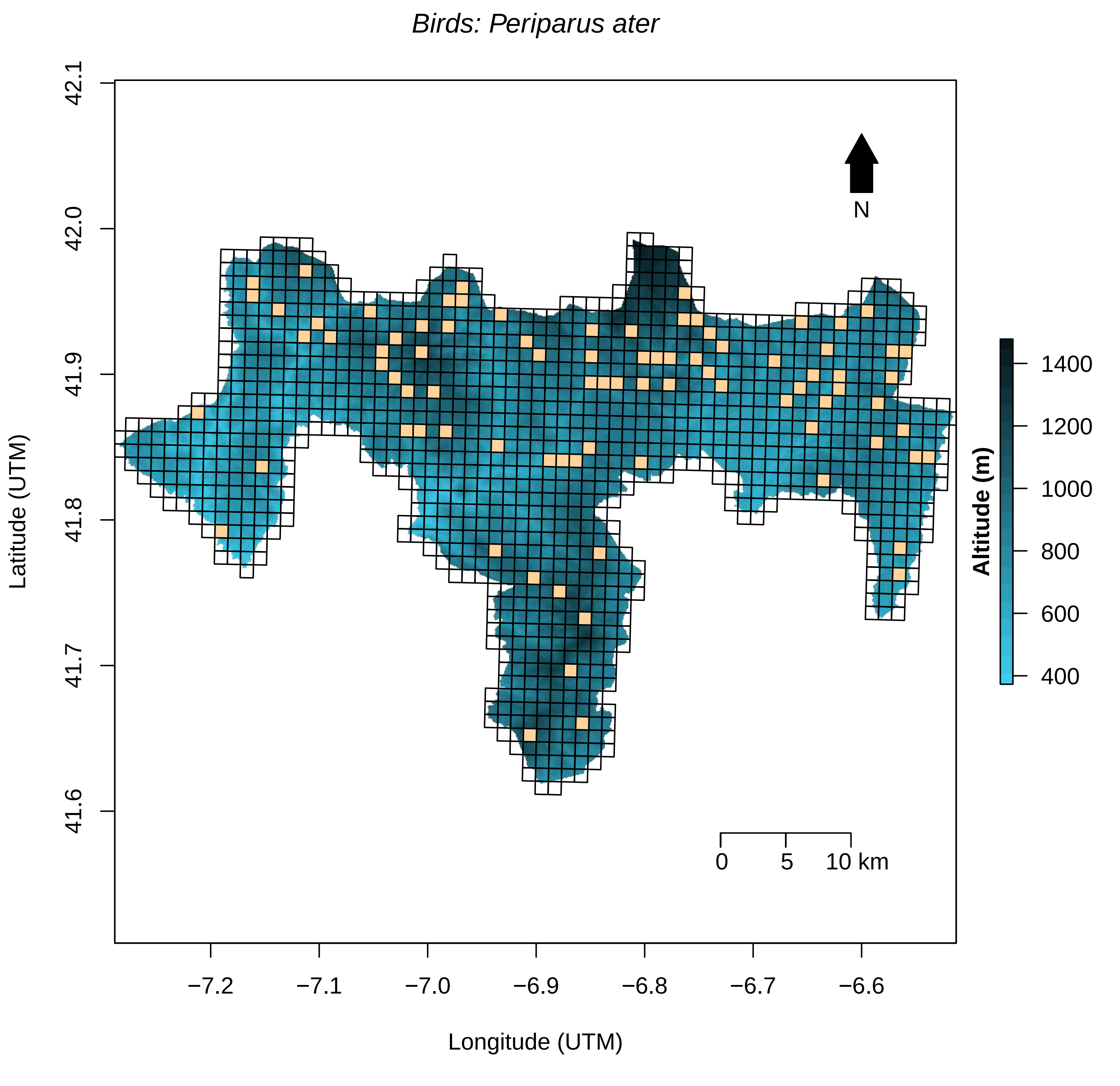

Birds: Periparus ater
Latitude (UTM)
Longitude (UTM)
42.1
42.0
41.9
41.8
41.7
41.6
−7.2
−7.1
−7.0
−6.9
−6.8
−6.7
−6.6
N
0
5
10 km
Altitude (m)
1400
1200
1000
800
600
400

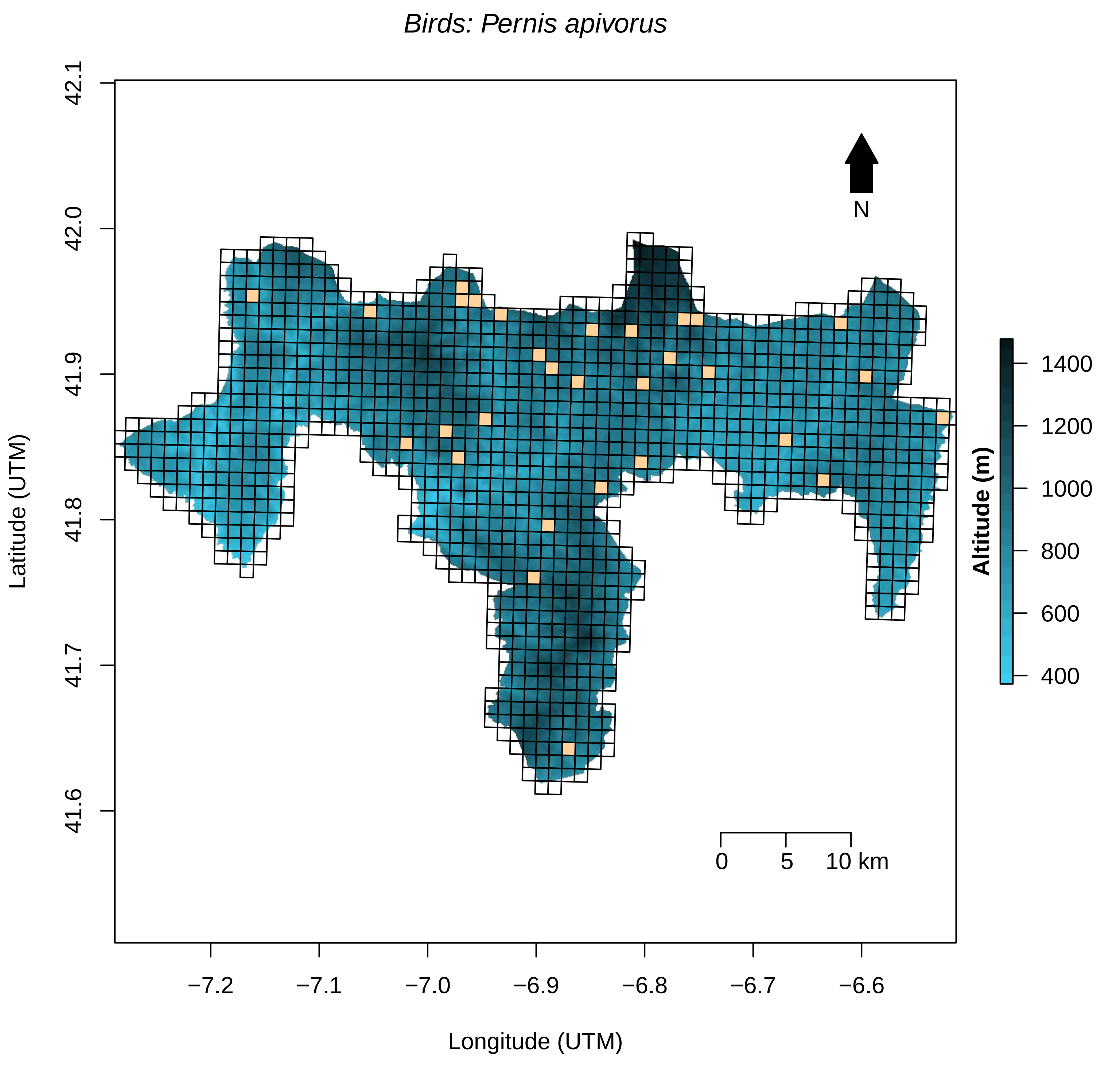

Birds: Pernis apivorus
N
0
5
10 km
Altitude (m)
1400
1200
1000
800
600
400
Latitude (UTM)
42.1
42.0
41.9
41.8
41.7
41.6
Longitude (UTM)
−7.2
−7.1
−7.0
−6.9
−6.8
−6.7
−6.6

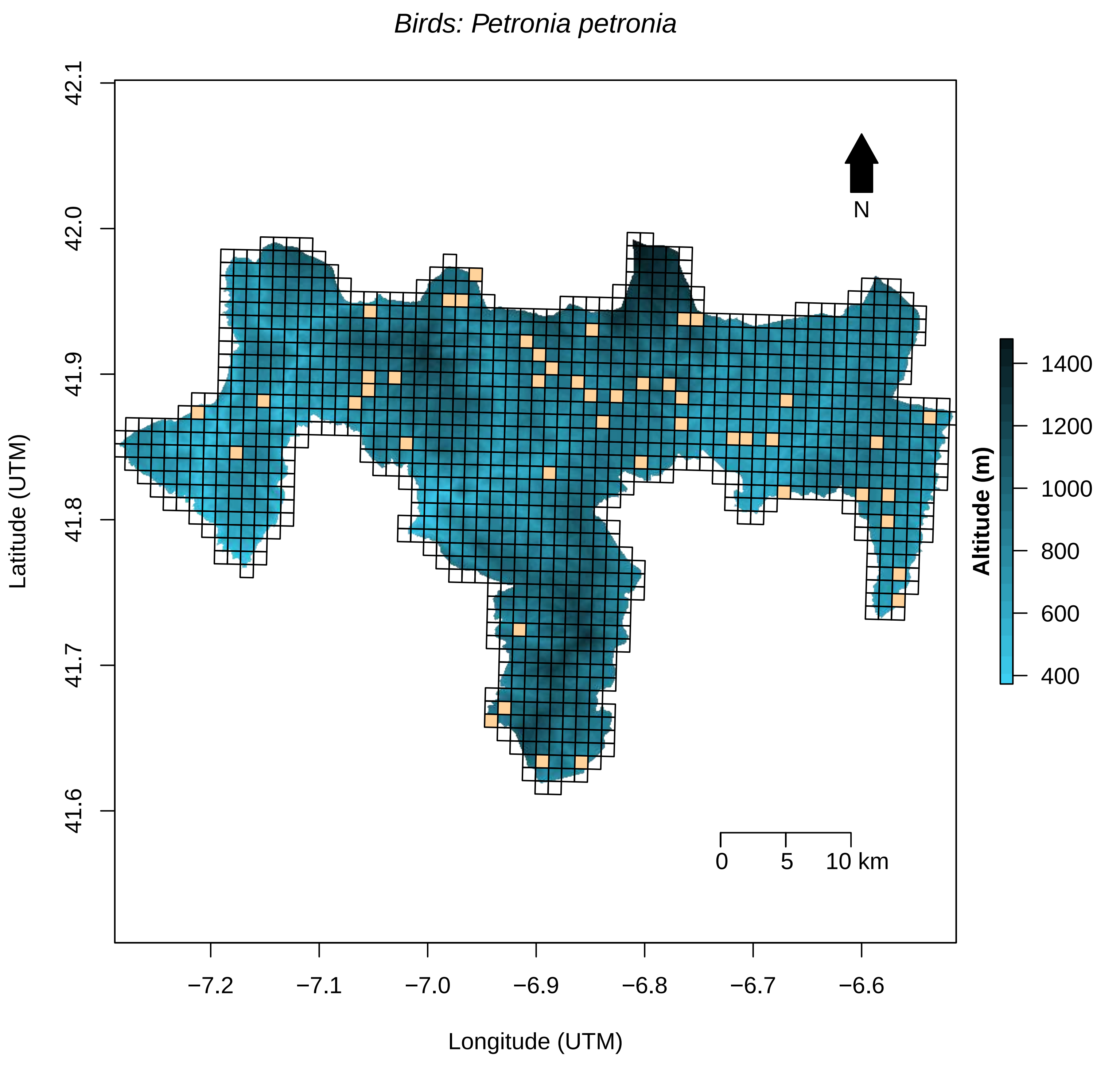
Birds: Petronia petronia
Latitude (UTM)
Longitude (UTM)
42.1
42.0
41.9
41.8
41.7
41.6
−7.2
−7.1
−7.0
−6.9
−6.8
−6.7
−6.6
N
0
5
10 km
Altitude (m)
1400
1200
1000
800
600
400

Flora (vascular plants): Petrorhagia nanteuilii

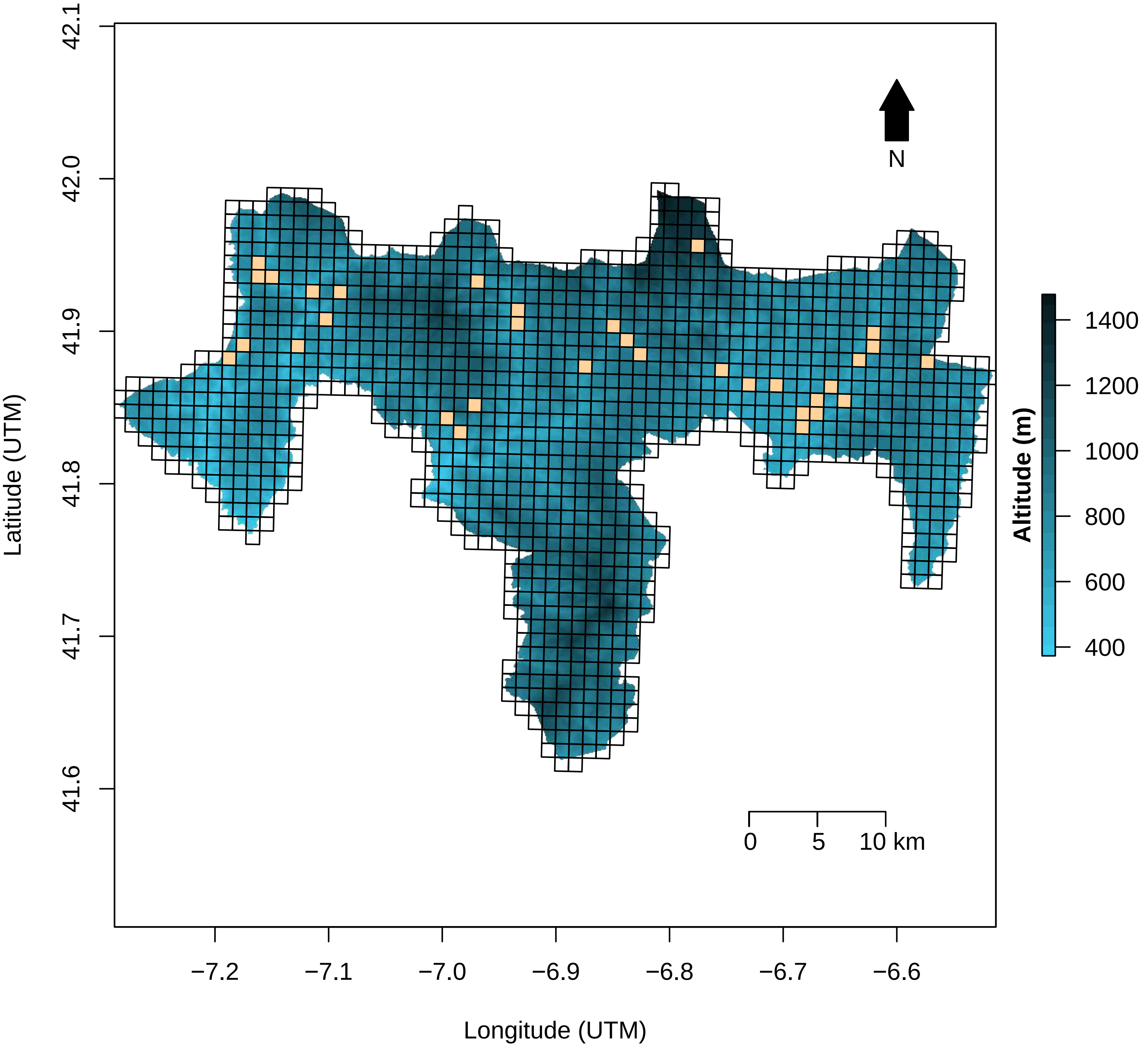

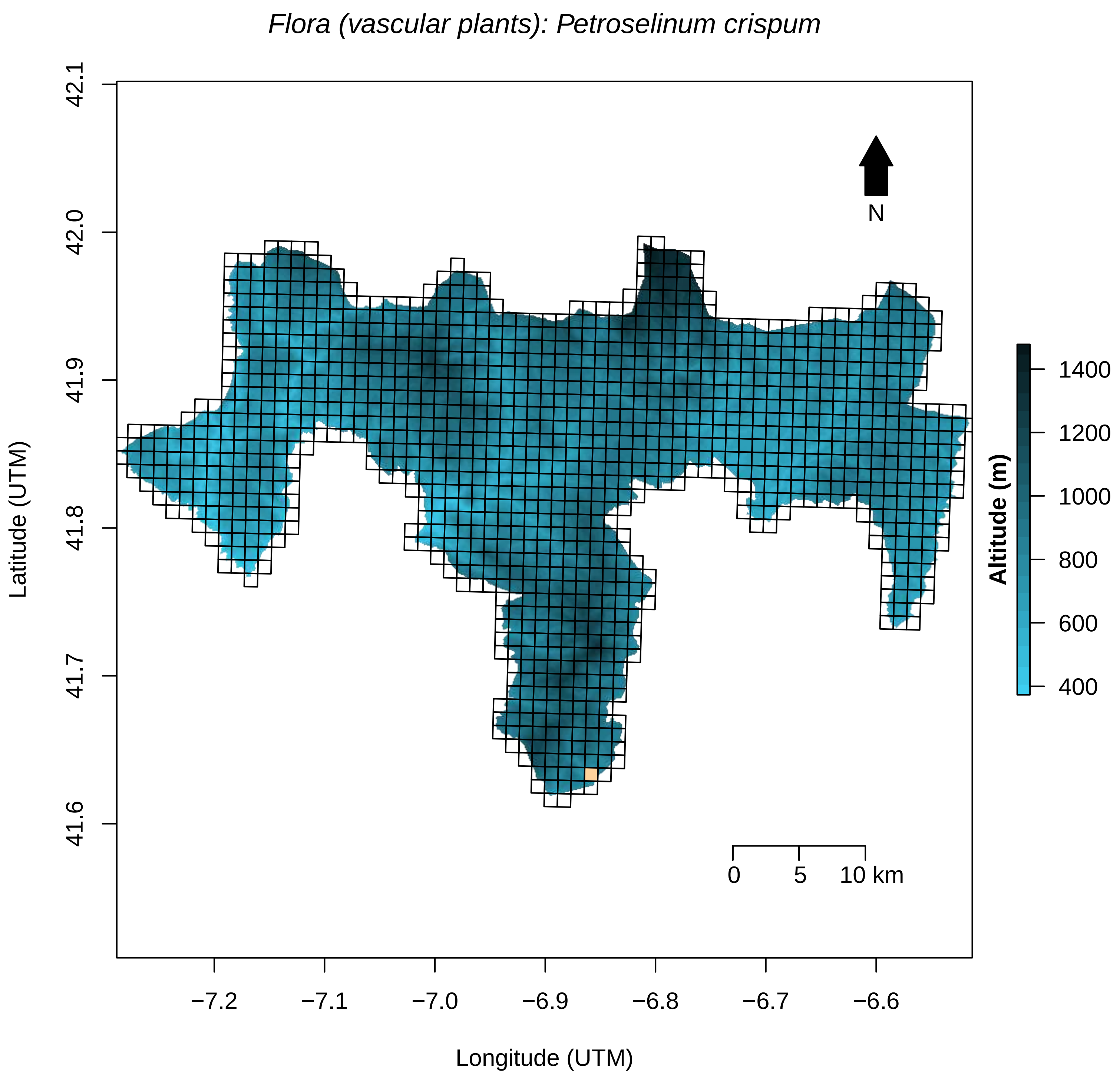

Flora (vascular plants): Petroselinum crispum
42.1
42.0
41.9
41.8
41.7
41.6
Latitude (UTM)
−7.2
−7.1
−7.0
−6.9
−6.8
−6.7
−6.6
Longitude (UTM)
N
0
5
10 km
Altitude (m)
1400
1200
1000
800
600
400

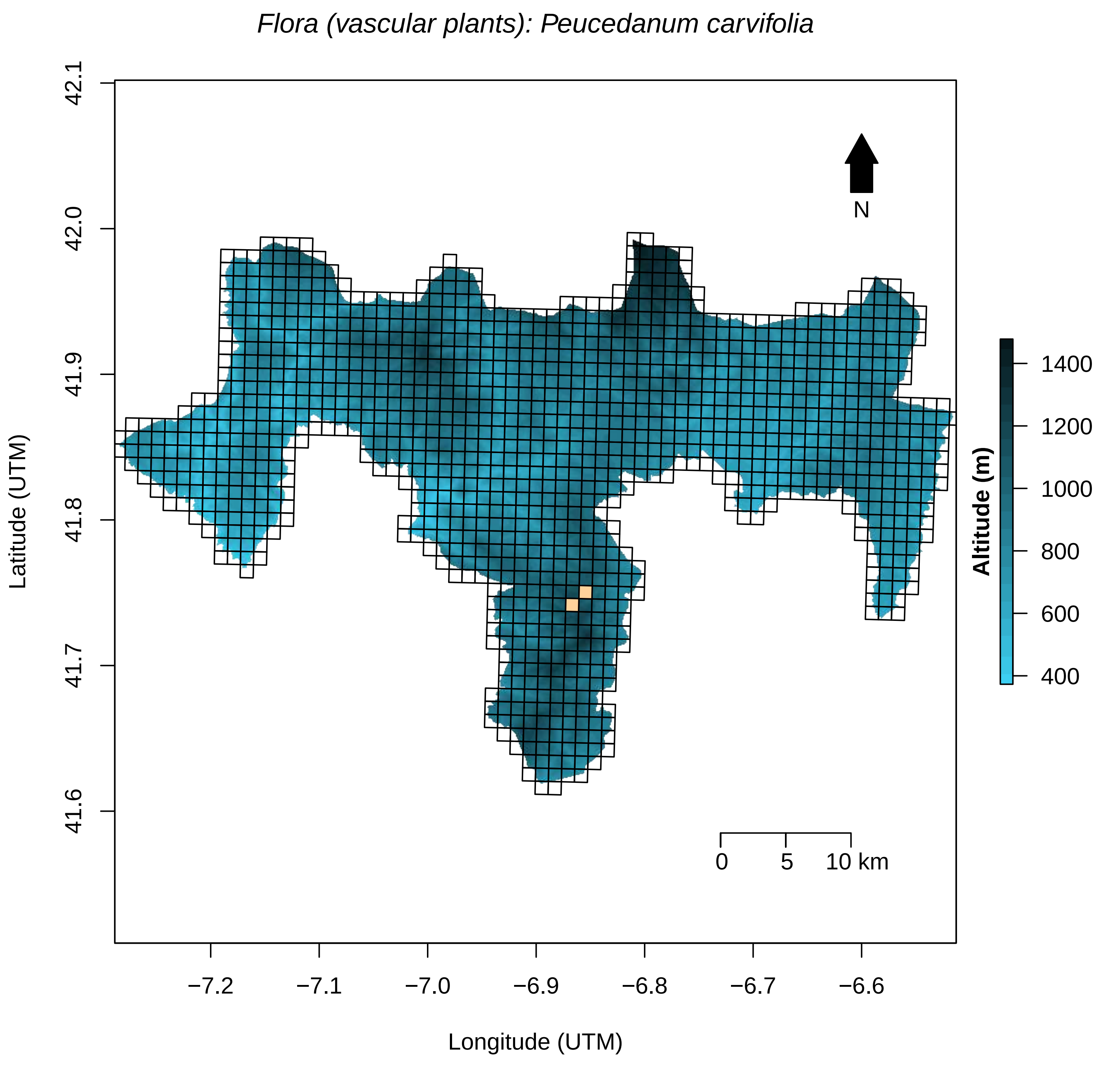

Flora (vascular plants): Peucedanum carvifolia
N
0
5
10 km
Altitude (m)
1400
1200
1000
800
600
400
42.1
42.0
41.9
41.8
41.7
41.6
Latitude (UTM)
−7.2
−7.1
−7.0
−6.9
−6.8
−6.7
−6.6
Longitude (UTM)

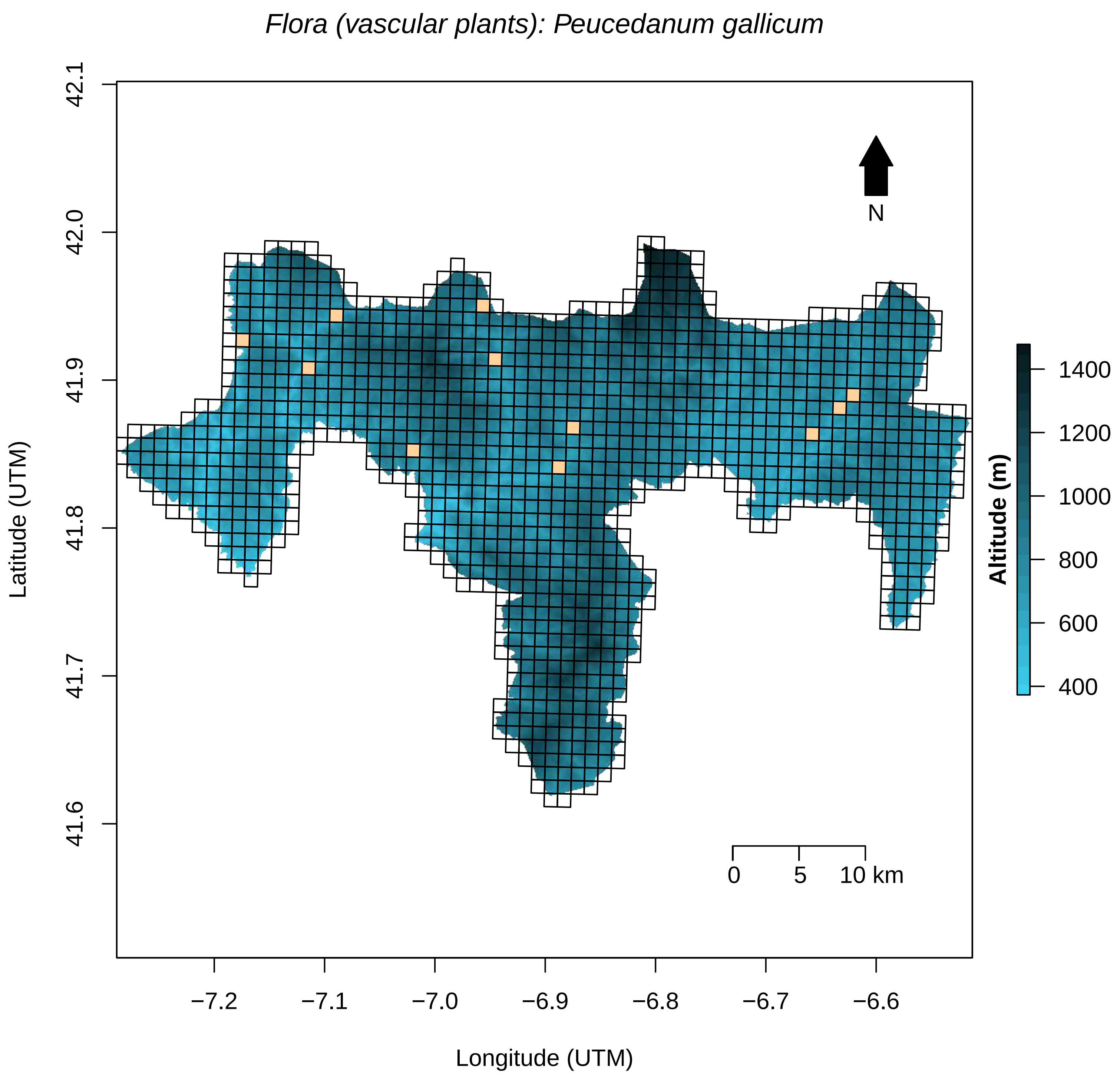
Flora (vascular plants): Peucedanum gallicum
42.1
42.0
41.9
41.8
41.7
41.6
Latitude (UTM)
−7.2
−7.1
−7.0
−6.9
−6.8
−6.7
−6.6
Longitude (UTM)
N
0
5
10 km
Altitude (m)
1400
1200
1000
800
600
400

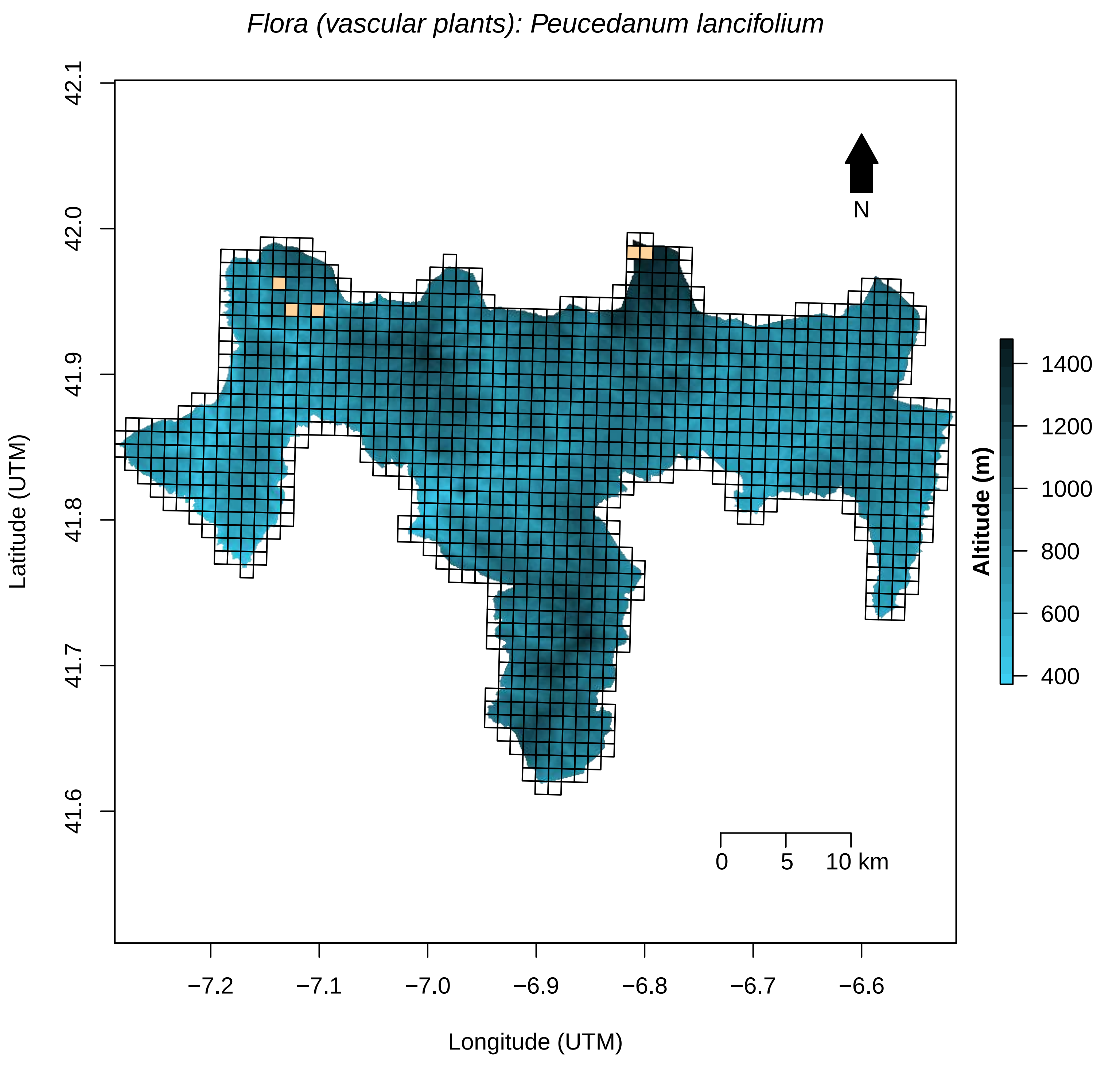

Flora (vascular plants): Peucedanum lancifolium
42.1
42.0
41.9
41.8
41.7
41.6
Latitude (UTM)
N
0
5
10 km
1400
1200
1000
800
600
400
Altitude (m)
−7.2
−7.1
−7.0
−6.9
−6.8
−6.7
−6.6
Longitude (UTM)

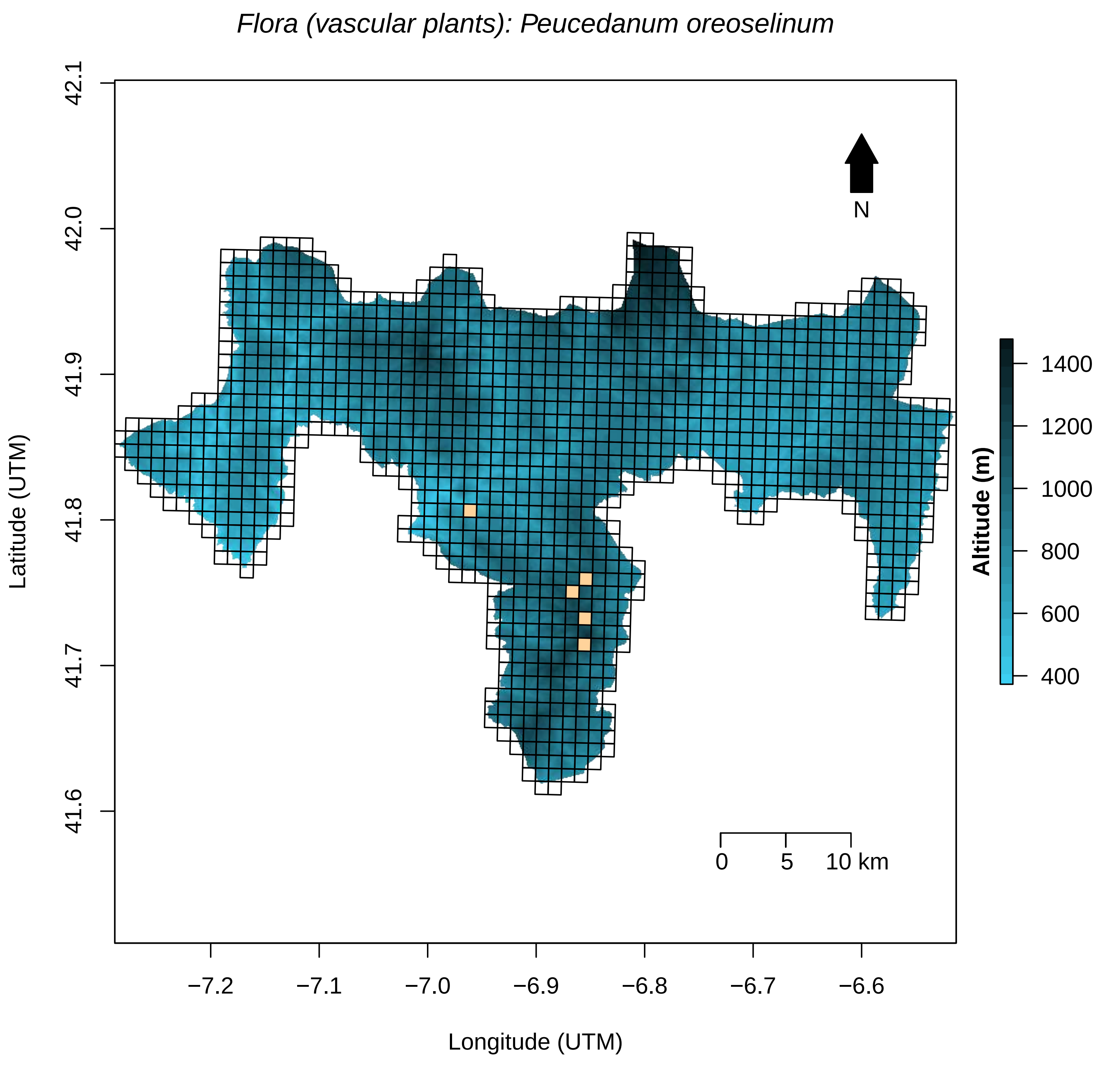

Flora (vascular plants): Peucedanum oreoselinum
42.1
42.0
41.9
41.8
41.7
41.6
Latitude (UTM)
−7.2
−7.1
−7.0
−6.9
−6.8
−6.7
−6.6
Longitude (UTM)
N
0
5
10 km
Altitude (m)
1400
1200
1000
800
600
400

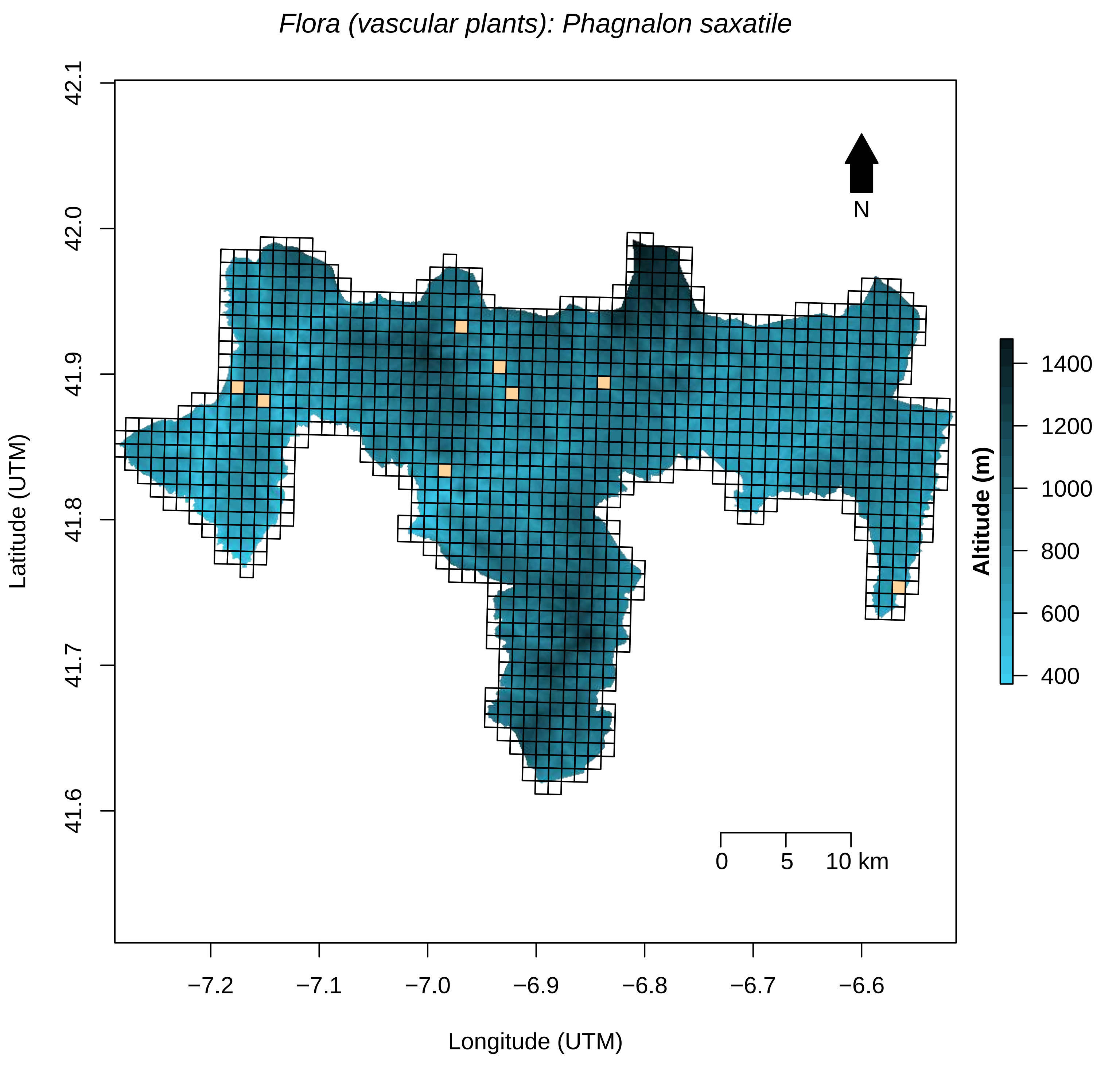

Flora (vascular plants): Phagnalon saxatile
42.1
42.0
41.9
41.8
41.7
41.6
Latitude (UTM)
−7.2
−7.1
−7.0
−6.9
−6.8
−6.7
−6.6
Longitude (UTM)
N
0
5
10 km
Altitude (m)
1400
1200
1000
800
600
400

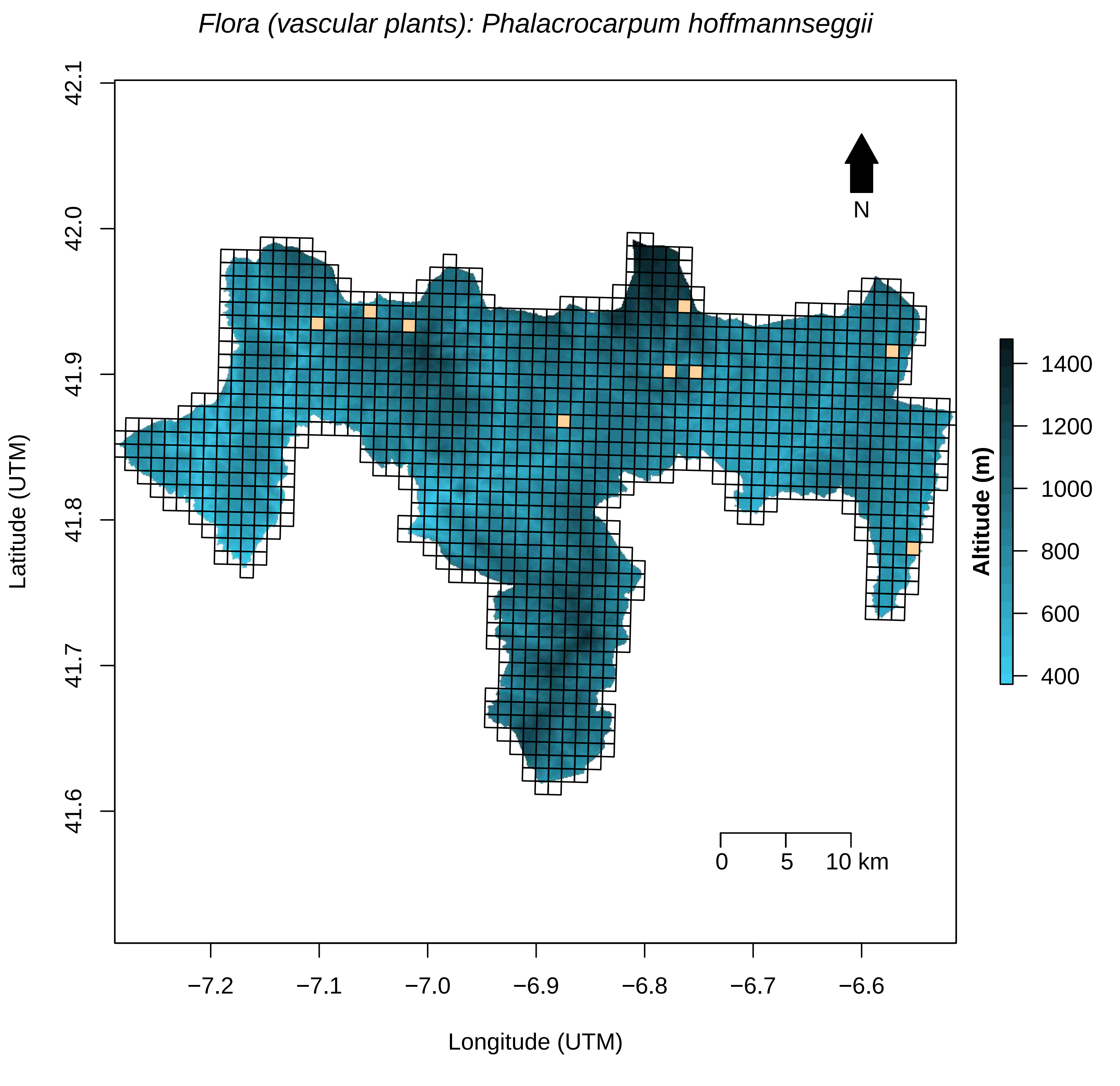

Flora (vascular plants): Phalacrocarpum hoffmannseggii
42.1
42.0
41.9
41.8
41.7
41.6
Latitude (UTM)
−7.2
−7.1
−7.0
−6.9
−6.8
−6.7
−6.6
Longitude (UTM)
N
0
5
10 km
Altitude (m)
1400
1200
1000
800
600
400

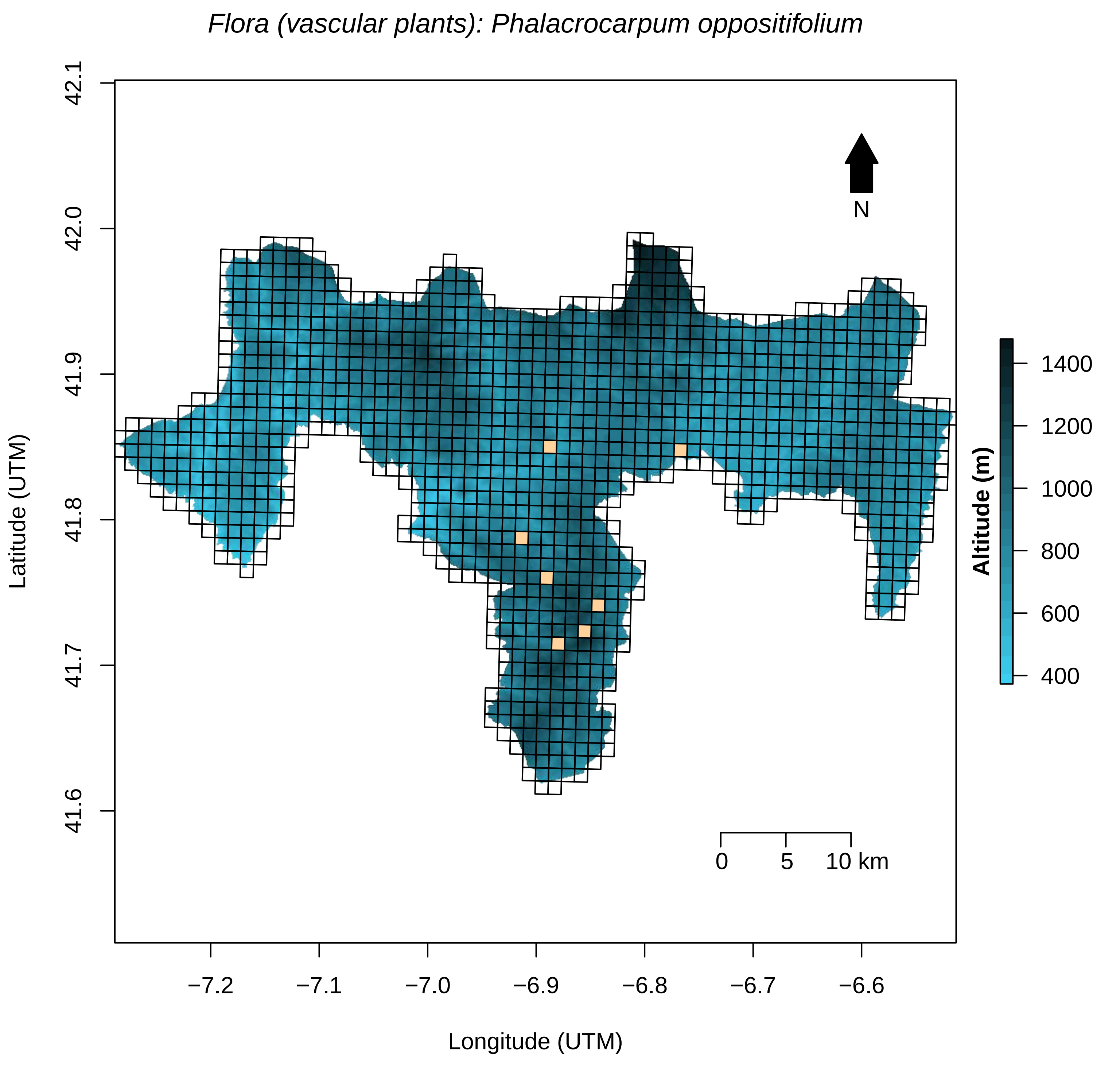
Flora (vascular plants): Phalacrocarpum oppositifolium
42.1
42.0
41.9
41.8
41.7
41.6
Latitude (UTM)
−7.2
−7.1
−7.0
−6.9
−6.8
−6.7
−6.6
Longitude (UTM)
N
0
5
10 km
1400
1200
1000
800
600
400
Altitude (m)

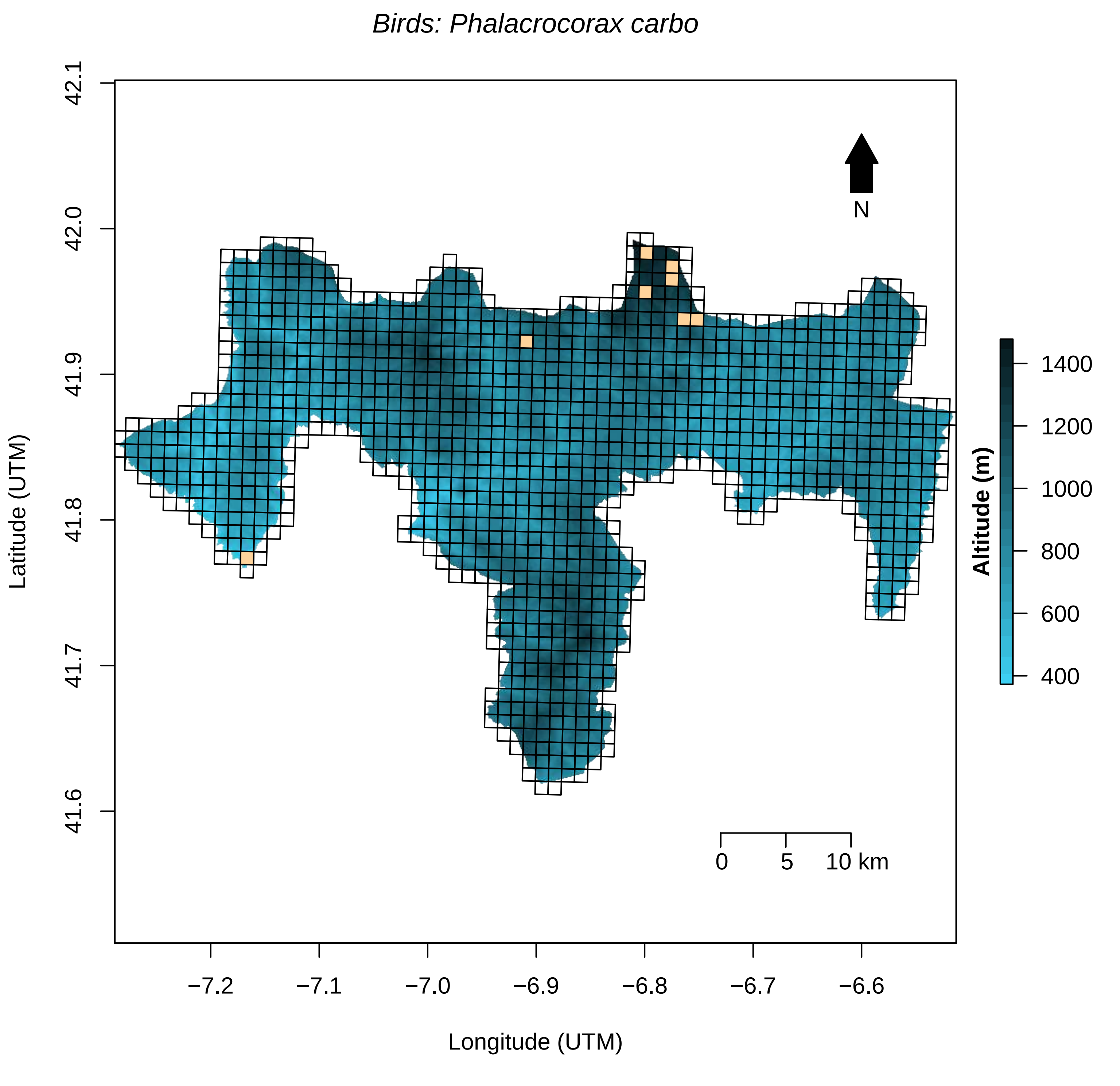

Birds: Phalacrocorax carbo
42.1
42.0
41.9
41.8
41.7
41.6
Latitude (UTM)
−7.2
−7.1
−7.0
−6.9
−6.8
−6.7
−6.6
Longitude (UTM)
N
0
5
10 km
1400
1200
1000
800
600
400
Altitude (m)

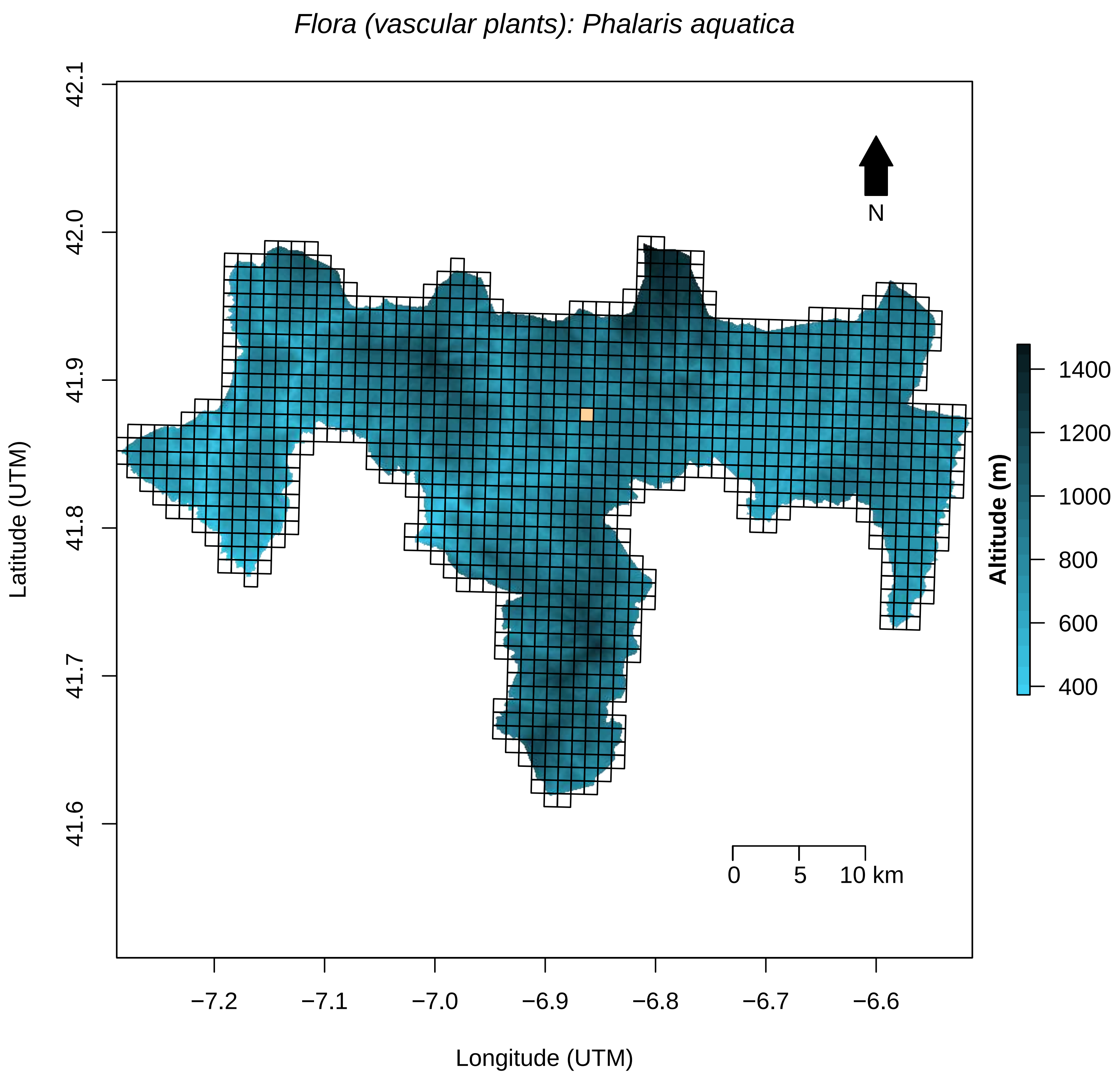

Flora (vascular plants): Phalaris aquatica
N
0
5
10 km
Altitude (m)
1400
1200
1000
800
600
400
Latitude (UTM)
42.1
42.0
41.9
41.8
41.7
41.6
Longitude (UTM)
−7.2
−7.1
−7.0
−6.9
−6.8
−6.7
−6.6

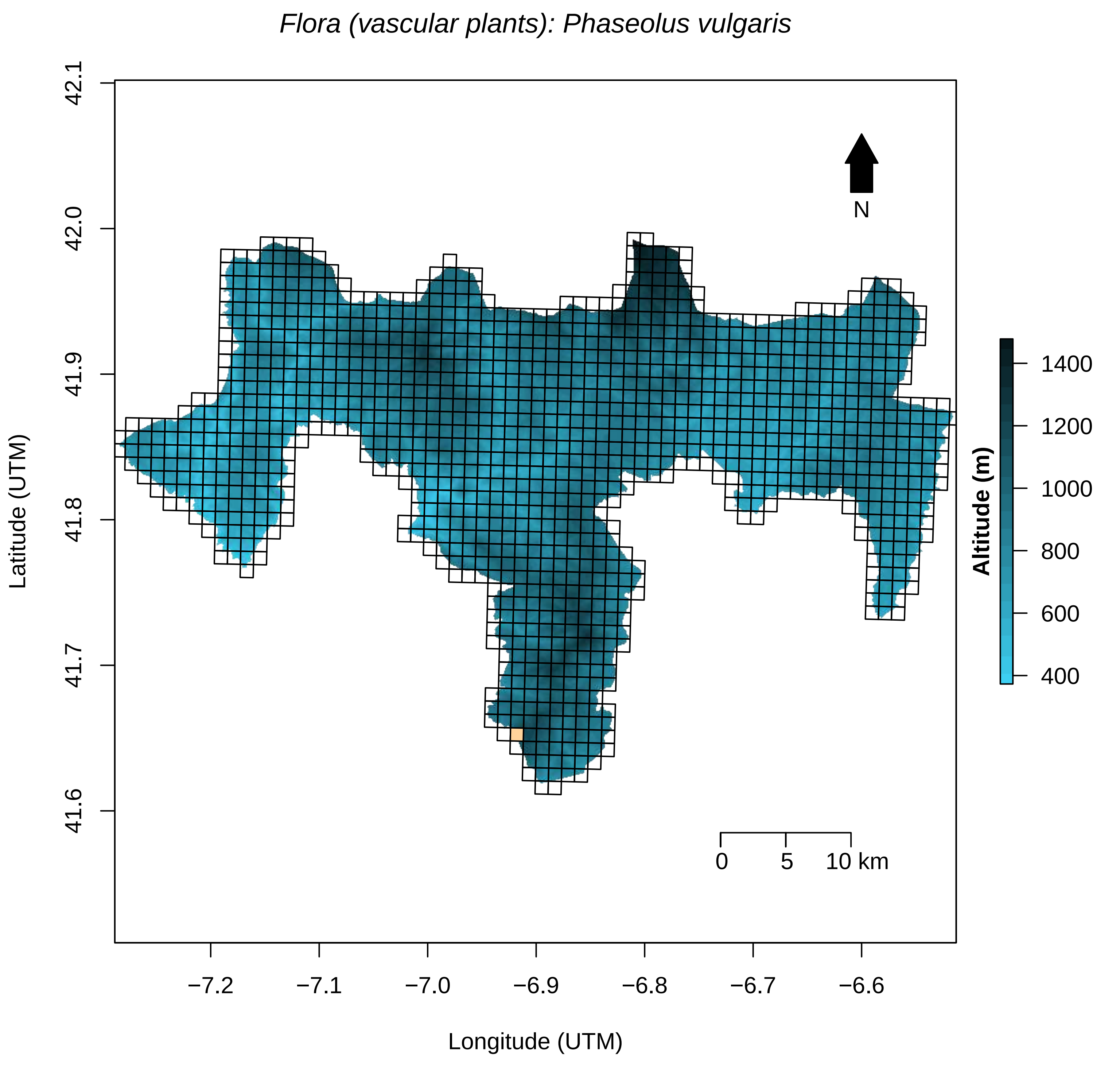

Flora (vascular plants): Phaseolus vulgaris
Latitude (UTM)
Longitude (UTM)
42.1
42.0
41.9
41.8
41.7
41.6
−7.2
−7.1
−7.0
−6.9
−6.8
−6.7
−6.6
N
0
5
10 km
Altitude (m)
1400
1200
1000
800
600
400

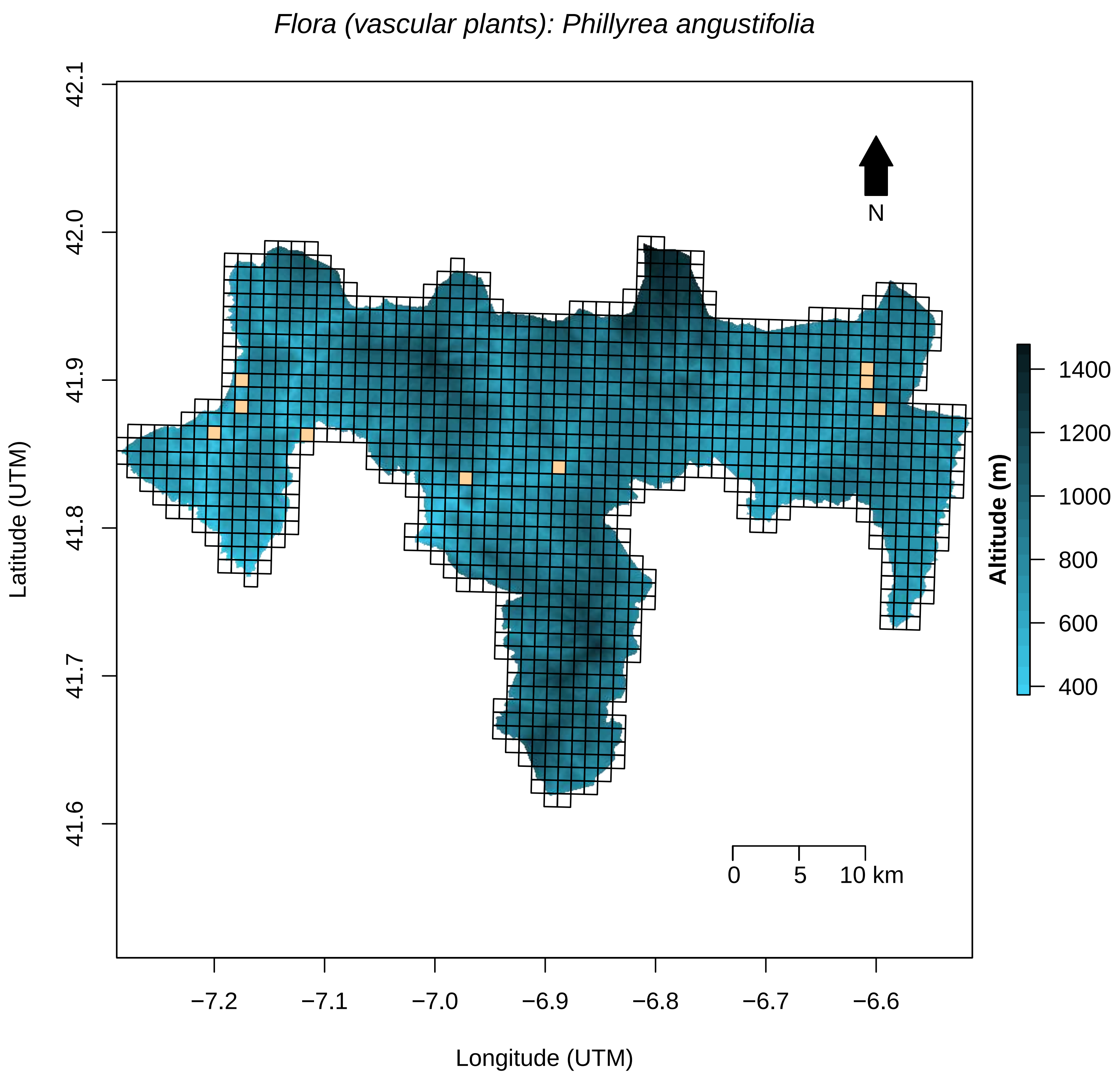

Flora (vascular plants): Phillyrea angustifolia
42.1
42.0
41.9
41.8
41.7
41.6
Latitude (UTM)
N
0 5 10 km
−7.2
−7.1
−7.0
−6.9
−6.8
−6.7
−6.6
Longitude (UTM)
Altitude (m)
1400
1200
1000
800
600
400

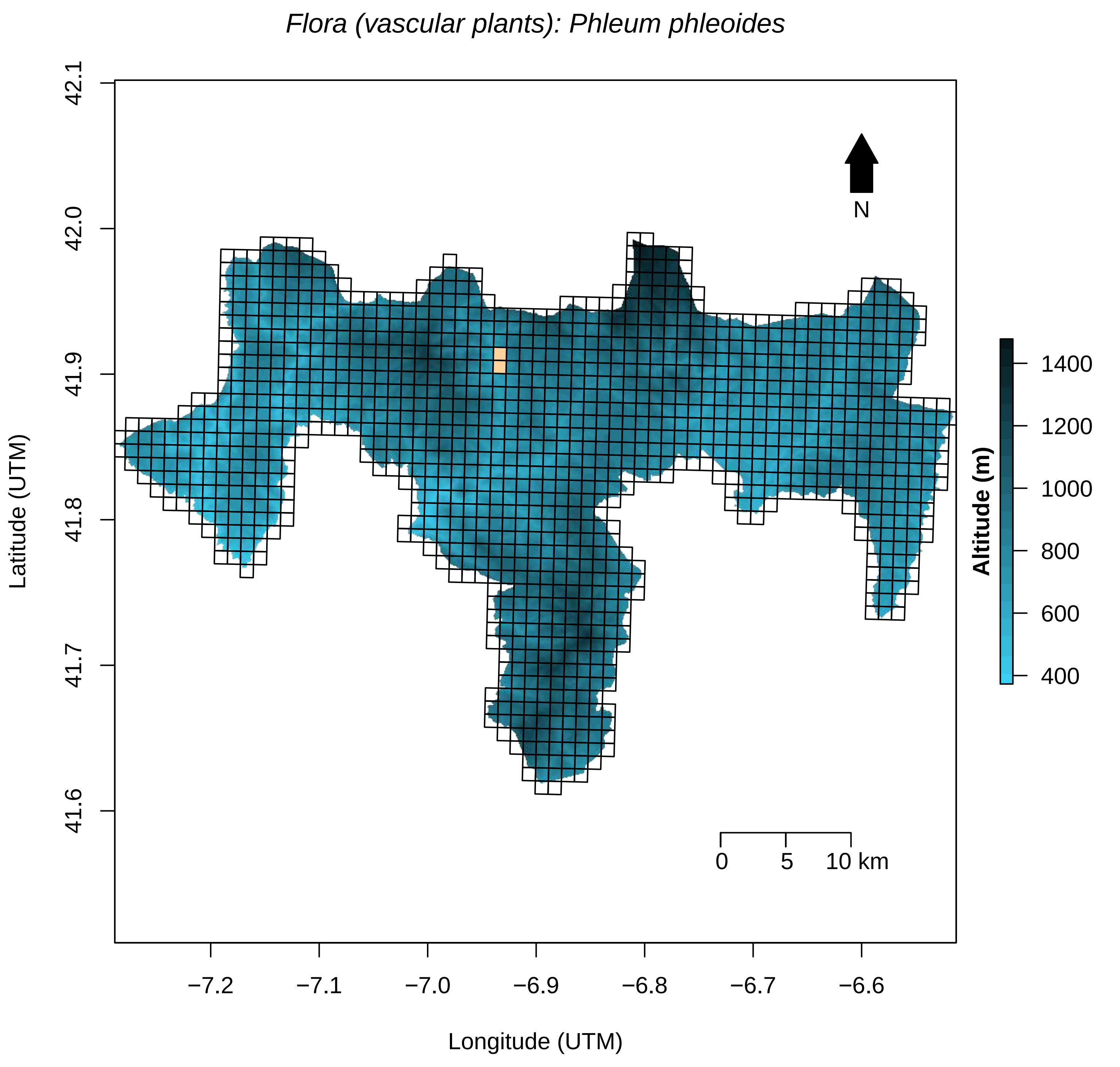

Flora (vascular plants): Phleum phleoides
N
0
5
10 km
42.1
42.0
41.9
41.8
41.7
41.6
Latitude (UTM)
−7.2
−7.1
−7.0
−6.9
−6.8
−6.7
−6.6
Longitude (UTM)
1400
1200
1000
800
600
400
Altitude (m)

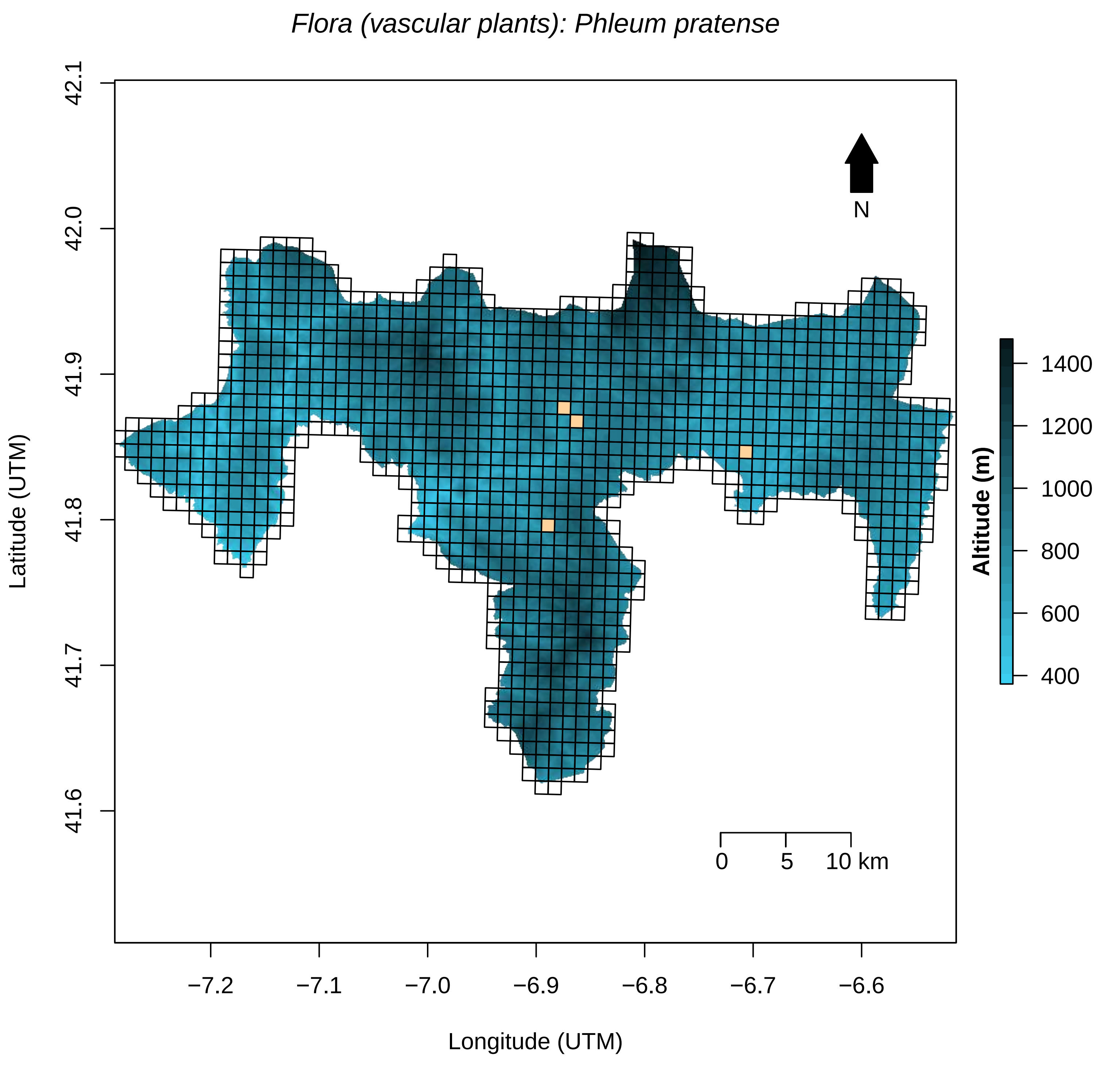
Flora (vascular plants): Phleum pratense
42.1
42.0
41.9
41.8
41.7
41.6
Latitude (UTM)
−7.2
−7.1
−7.0
−6.9
−6.8
−6.7
−6.6
Longitude (UTM)
N
0
5
10 km
Altitude (m)
1400
1200
1000
800
600
400

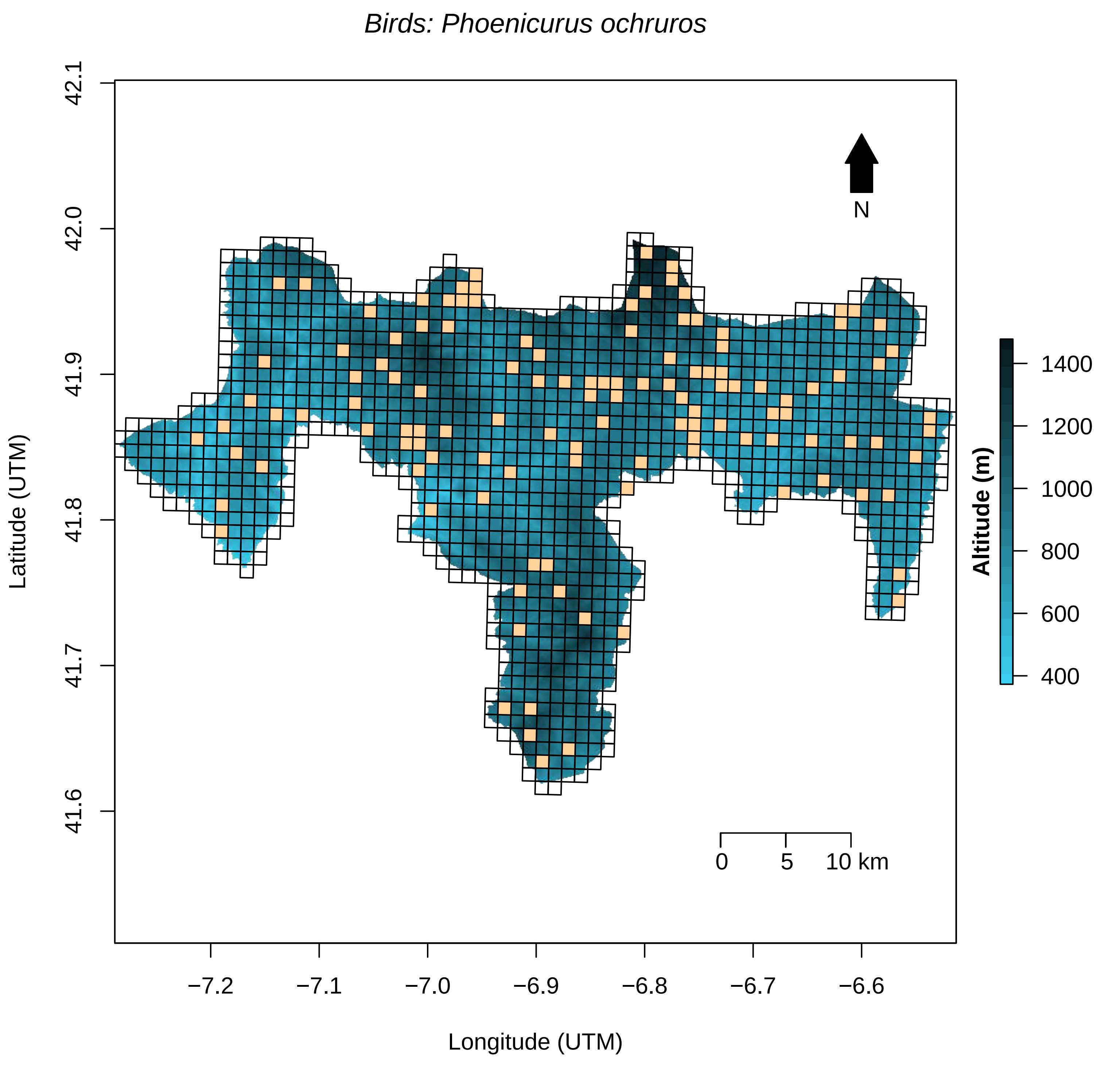
Birds: Phoenicurus ochruros
42.1
42.0
41.9
41.8
41.7
41.6
Latitude (UTM)
−7.2
−7.1
−7.0
−6.9
−6.8
−6.7
−6.6
Longitude (UTM)
N
0
5
10 km
Altitude (m)
1400
1200
1000
800
600
400

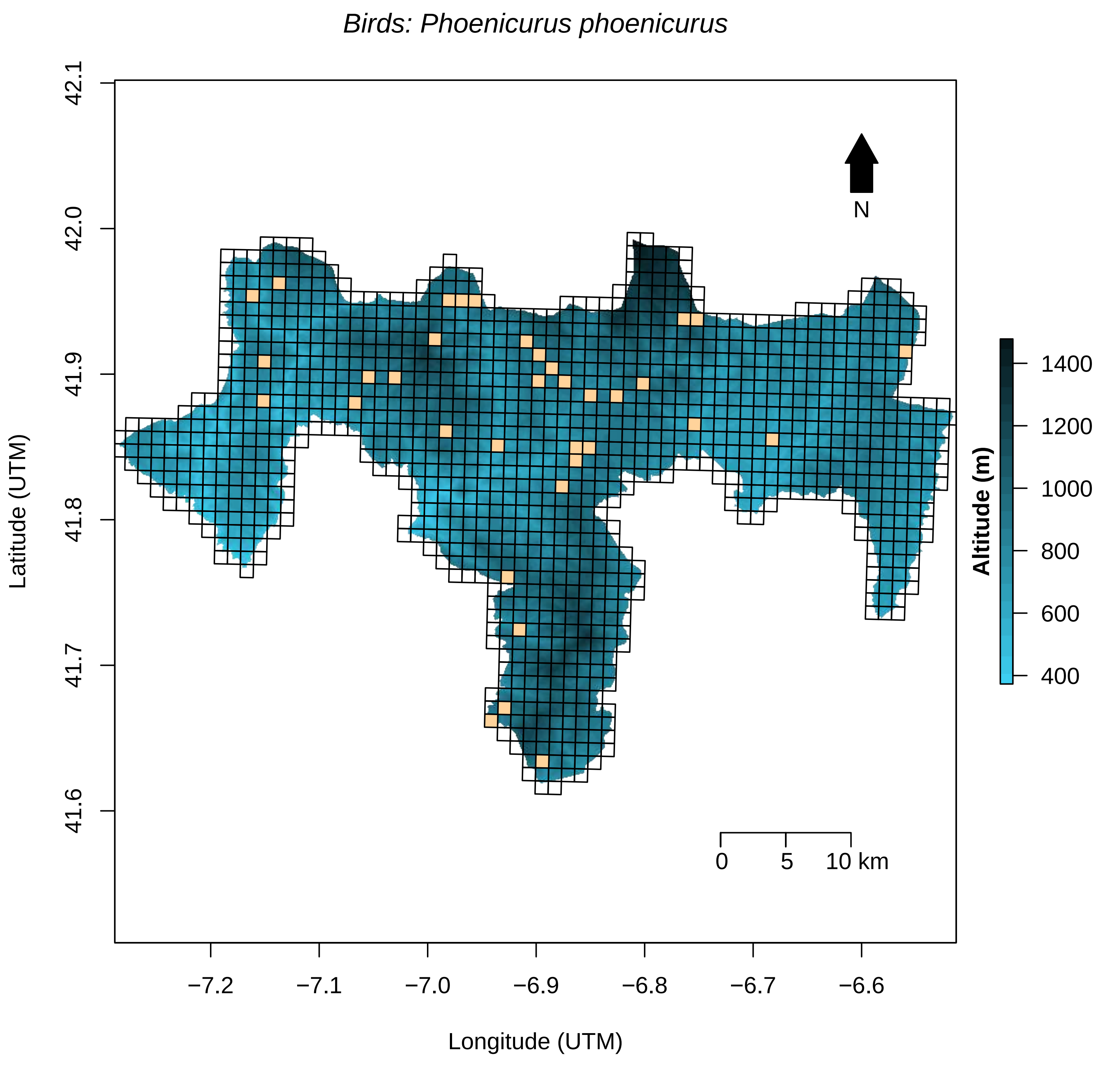

Birds: Phoenicurus phoenicurus
N
0 5 10 km
Altitude (m)
1400
1200
1000
800
600
400
42.1
42.0
41.9
41.8
41.7
41.6
Latitude (UTM)
−7.2
−7.1
−7.0
−6.9
−6.8
−6.7
−6.6
Longitude (UTM)

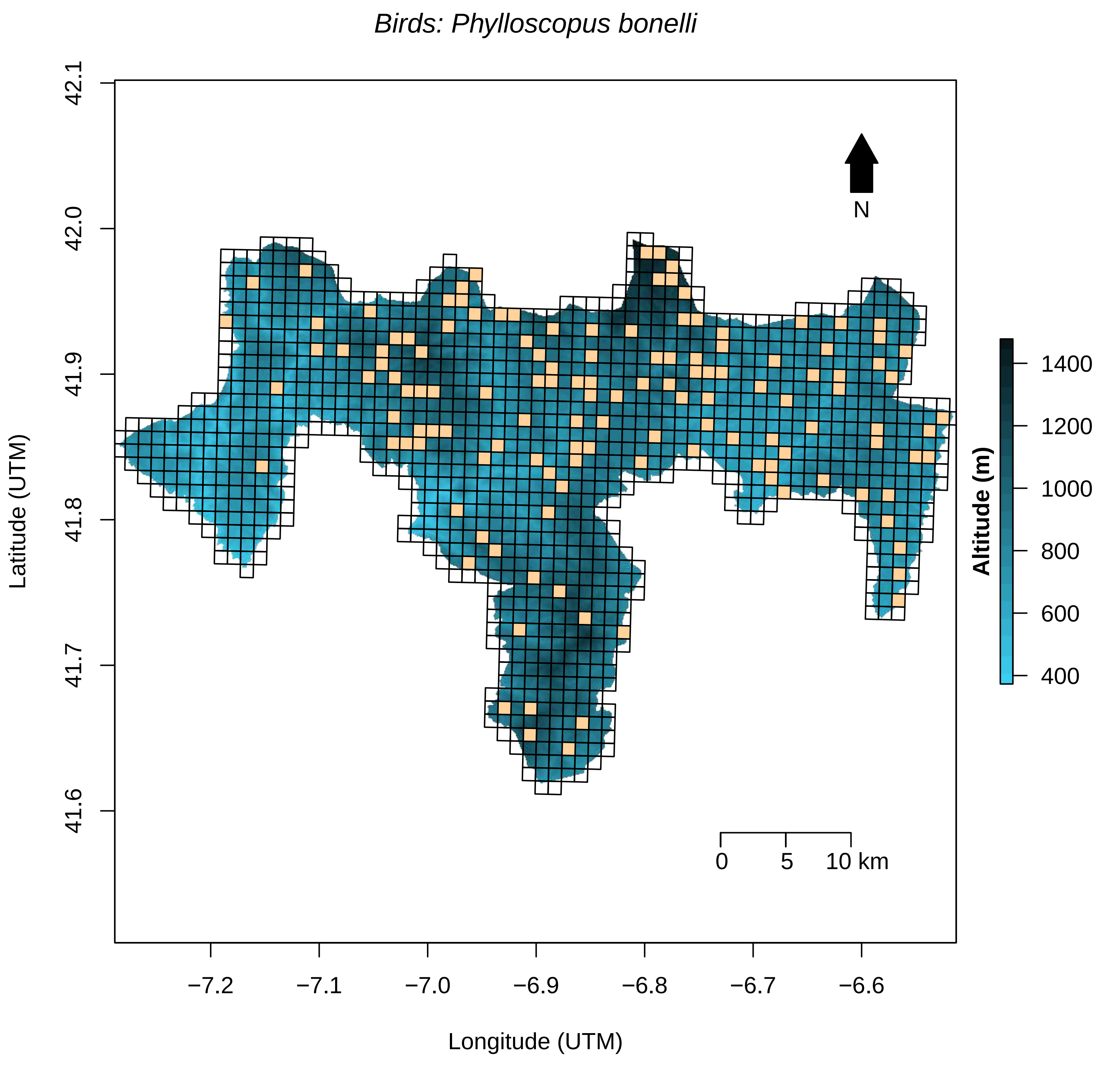

Birds: Phylloscopus bonelli
N
0
5
10 km
Altitude (m)
1400
1200
1000
800
600
400
Latitude (UTM)
42.1
42.0
41.9
41.8
41.7
41.6
Longitude (UTM)
−7.2
−7.1
−7.0
−6.9
−6.8
−6.7
−6.6

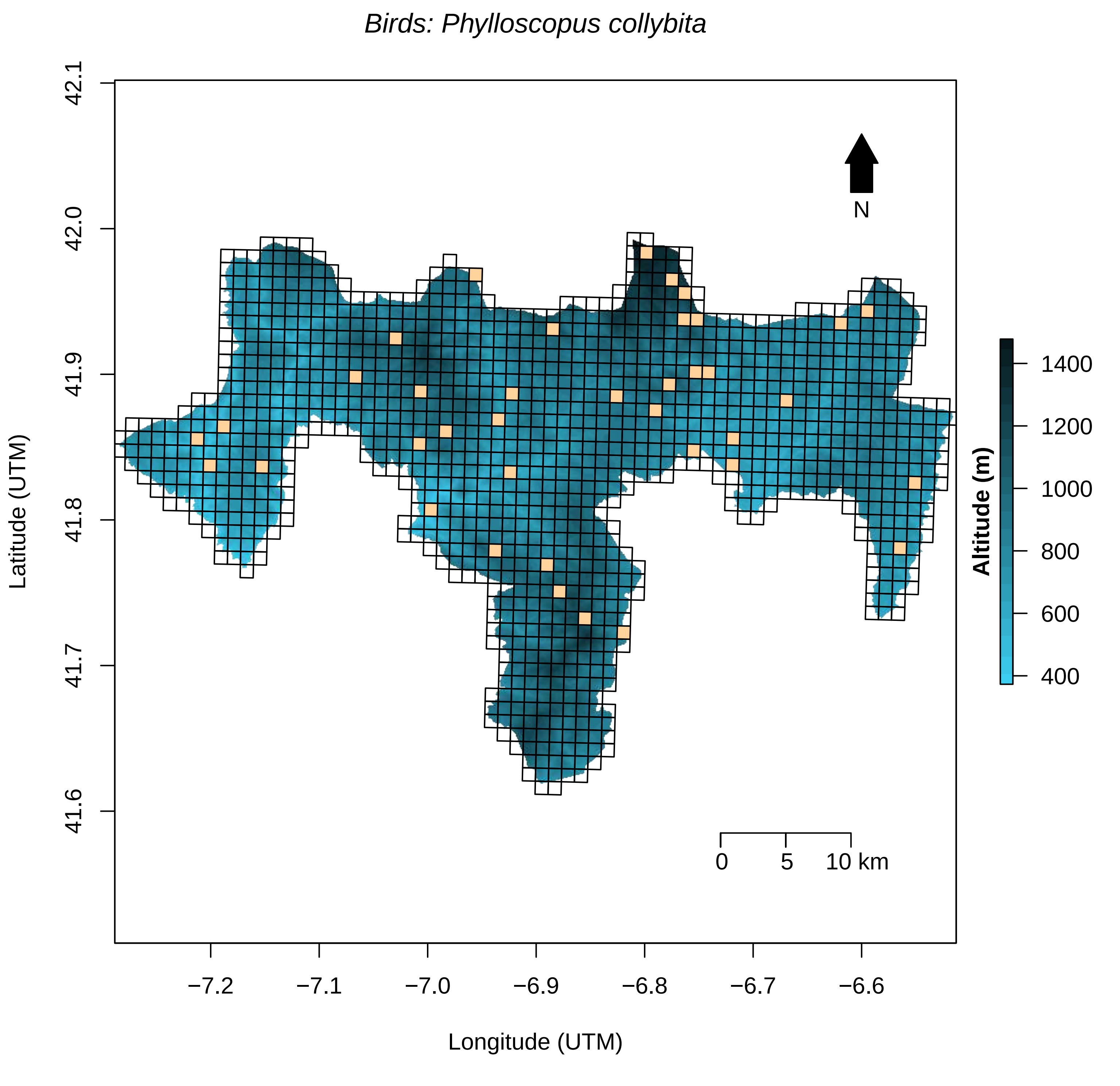

Birds: Phylloscopus collybita
42.1
42.0
41.9
41.8
41.7
41.6
Latitude (UTM)
−7.2
−7.1
−7.0
−6.9
−6.8
−6.7
−6.6
Longitude (UTM)
N
0
5
10 km
Altitude (m)
1400
1200
1000
800
600
400

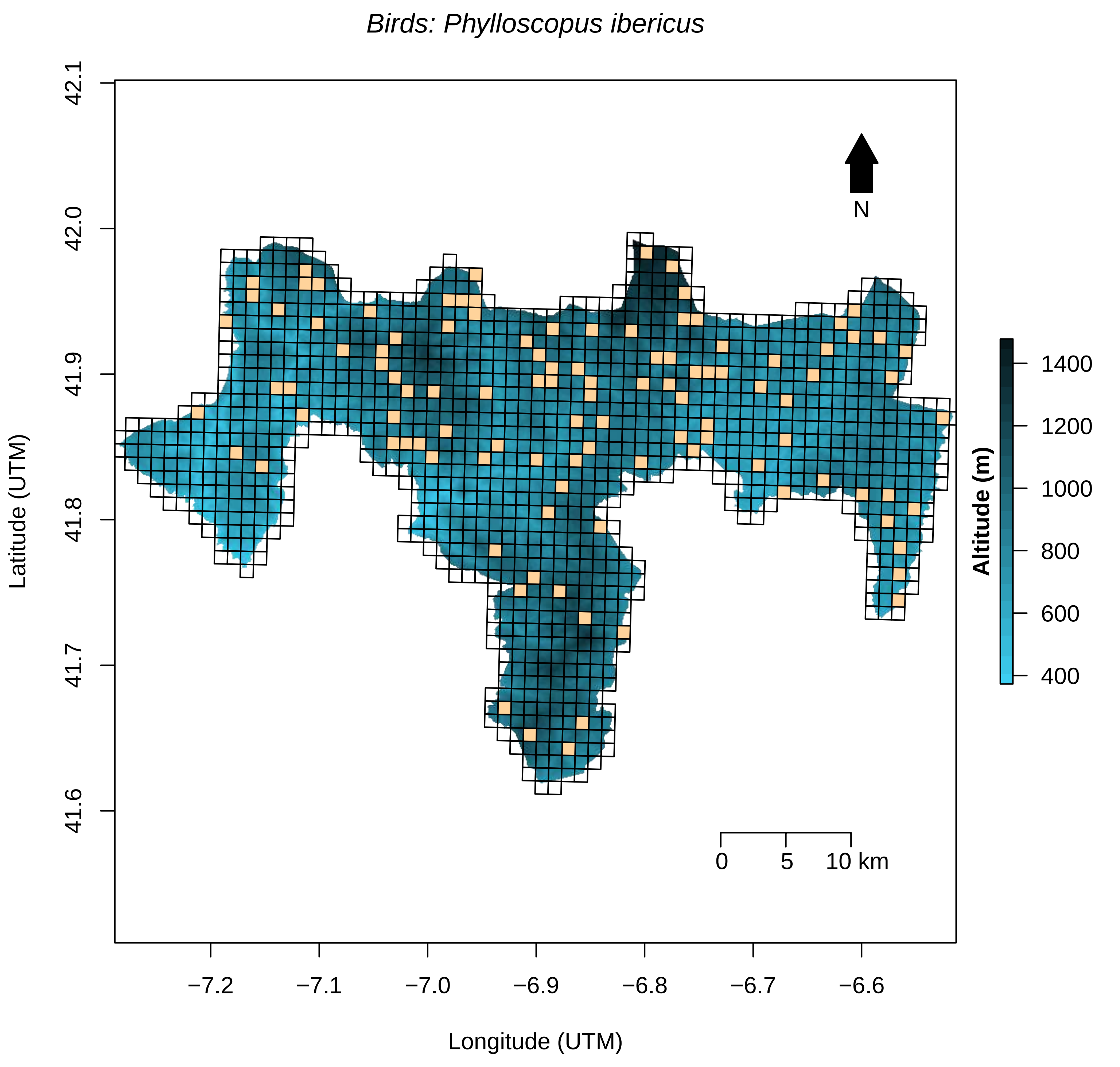

Birds: Phylloscopus ibericus
Latitude (UTM)
Longitude (UTM)
42.1
42.0
41.9
41.8
41.7
41.6
−7.2
−7.1
−7.0
−6.9
−6.8
−6.7
−6.6
N
0
5
10 km
Altitude (m)
1400
1200
1000
800
600
400

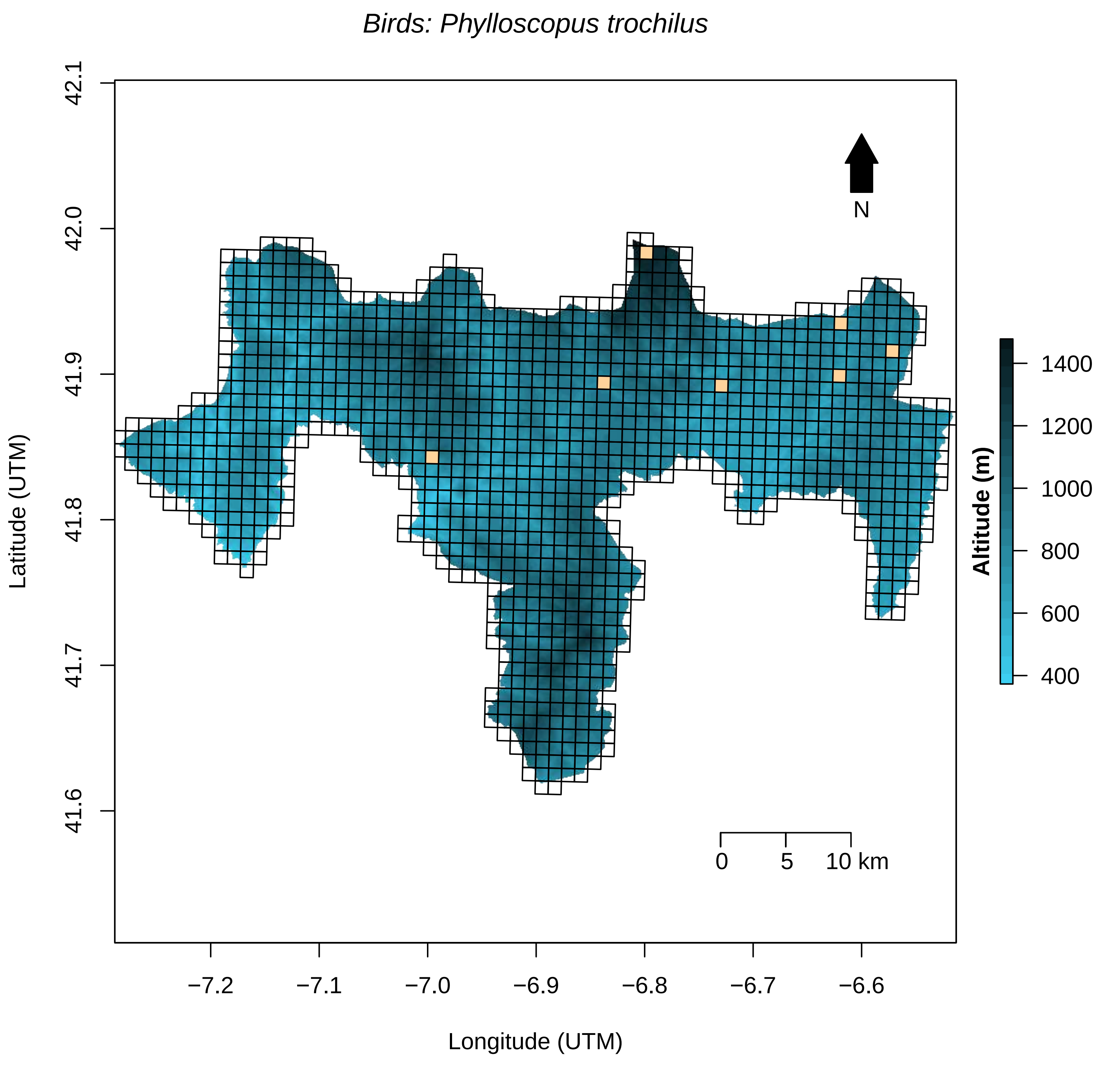

Birds: Phylloscopus trochilus
Latitude (UTM)
Longitude (UTM)
42.1
42.0
41.9
41.8
41.7
41.6
−7.2
−7.1
−7.0
−6.9
−6.8
−6.7
−6.6
N
0
5
10 km
Altitude (m)
1400
1200
1000
800
600
400

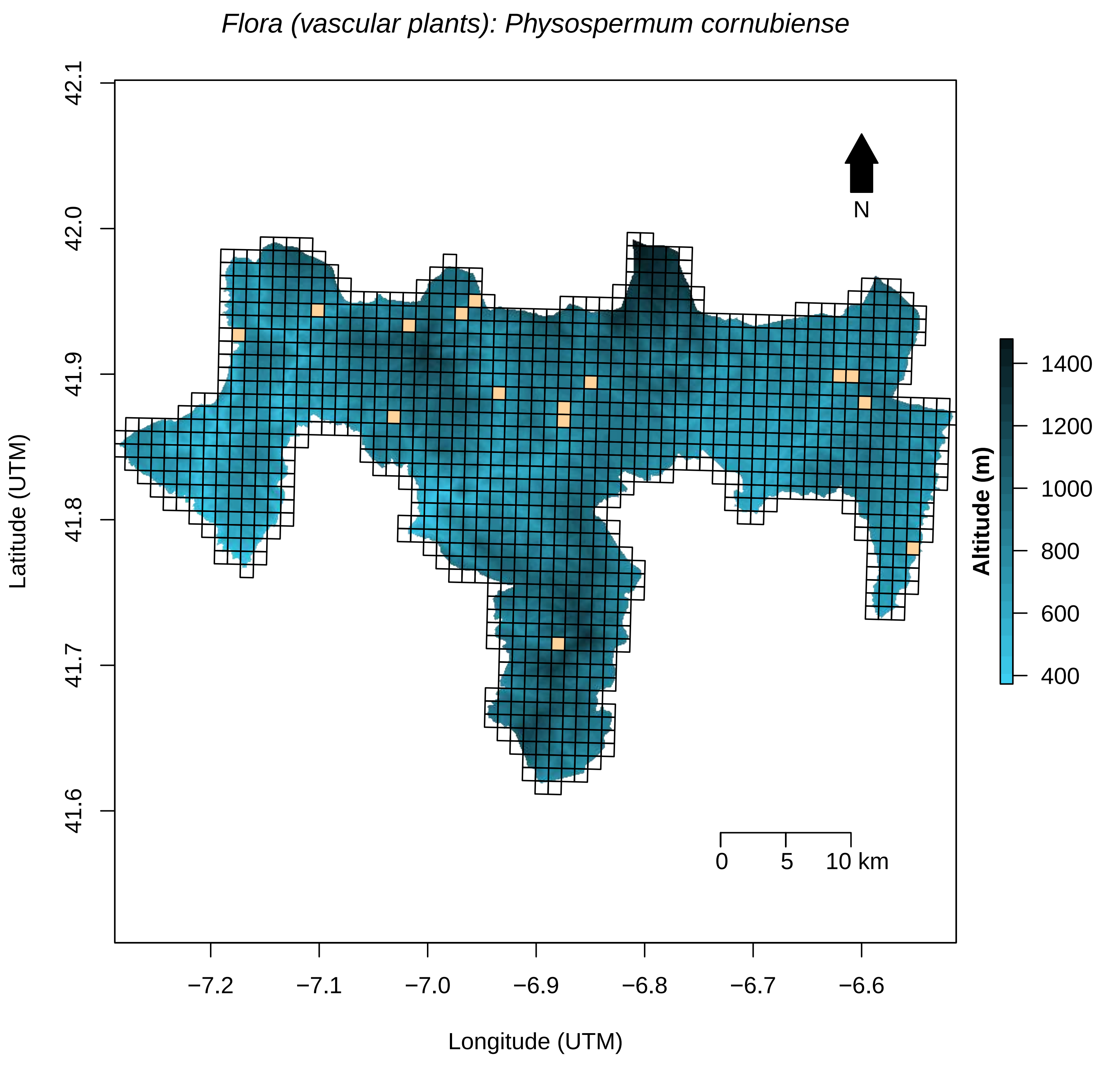
Flora (vascular plants): Physospermum cornubiense
42.1
42.0
41.9
41.8
41.7
41.6
Latitude (UTM)
−7.2
−7.1
−7.0
−6.9
−6.8
−6.7
−6.6
Longitude (UTM)
N
0
5
10 km
Altitude (m)
1400
1200
1000
800
600
400

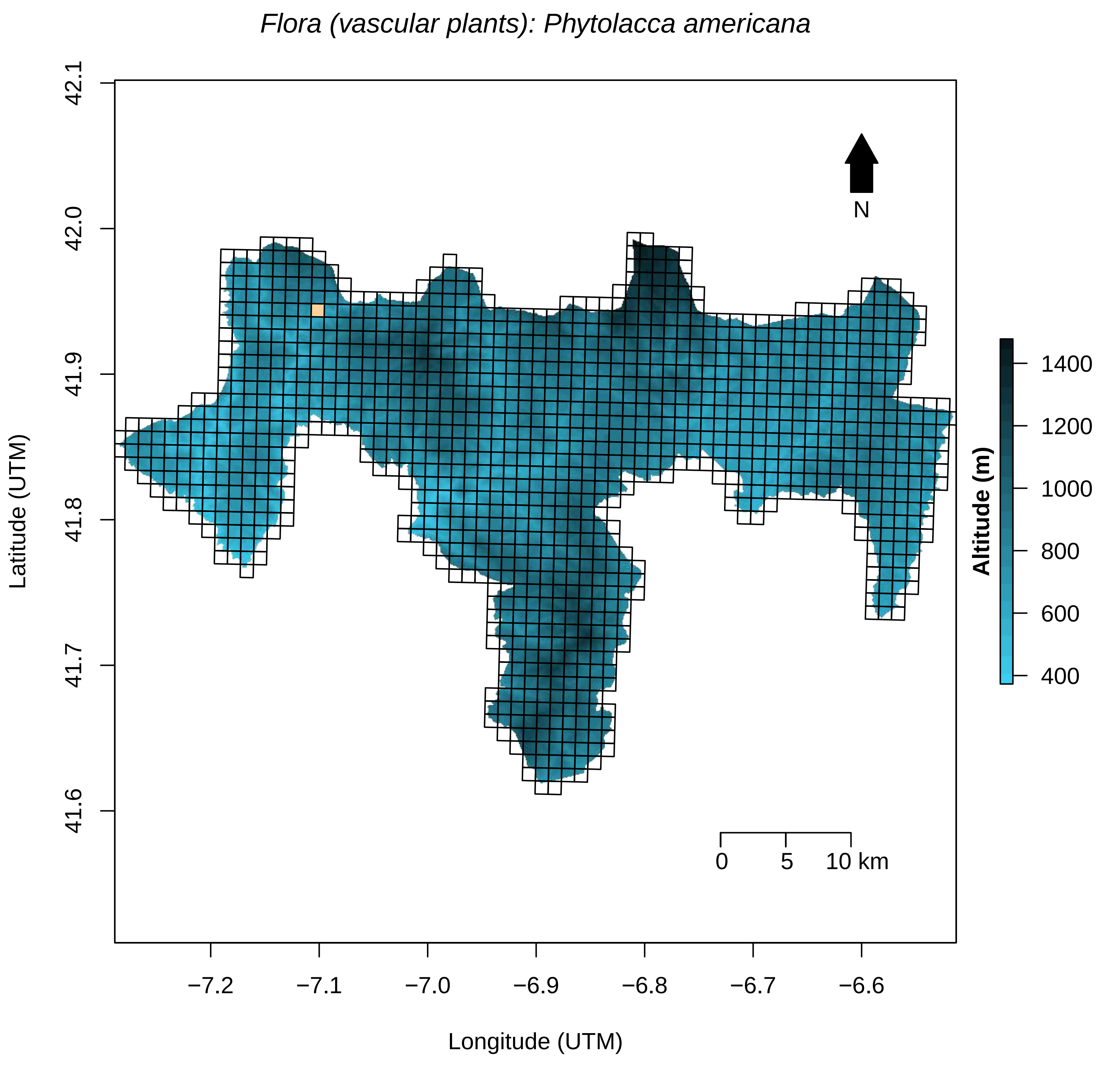
Flora (vascular plants): Phytolacca americana
42.1
42.0
41.9
41.8
41.7
41.6
Latitude (UTM)
−7.2
−7.1
−7.0
−6.9
−6.8
−6.7
−6.6
Longitude (UTM)
N
0
5
10 km
Altitude (m)
1400
1200
1000
800
600
400

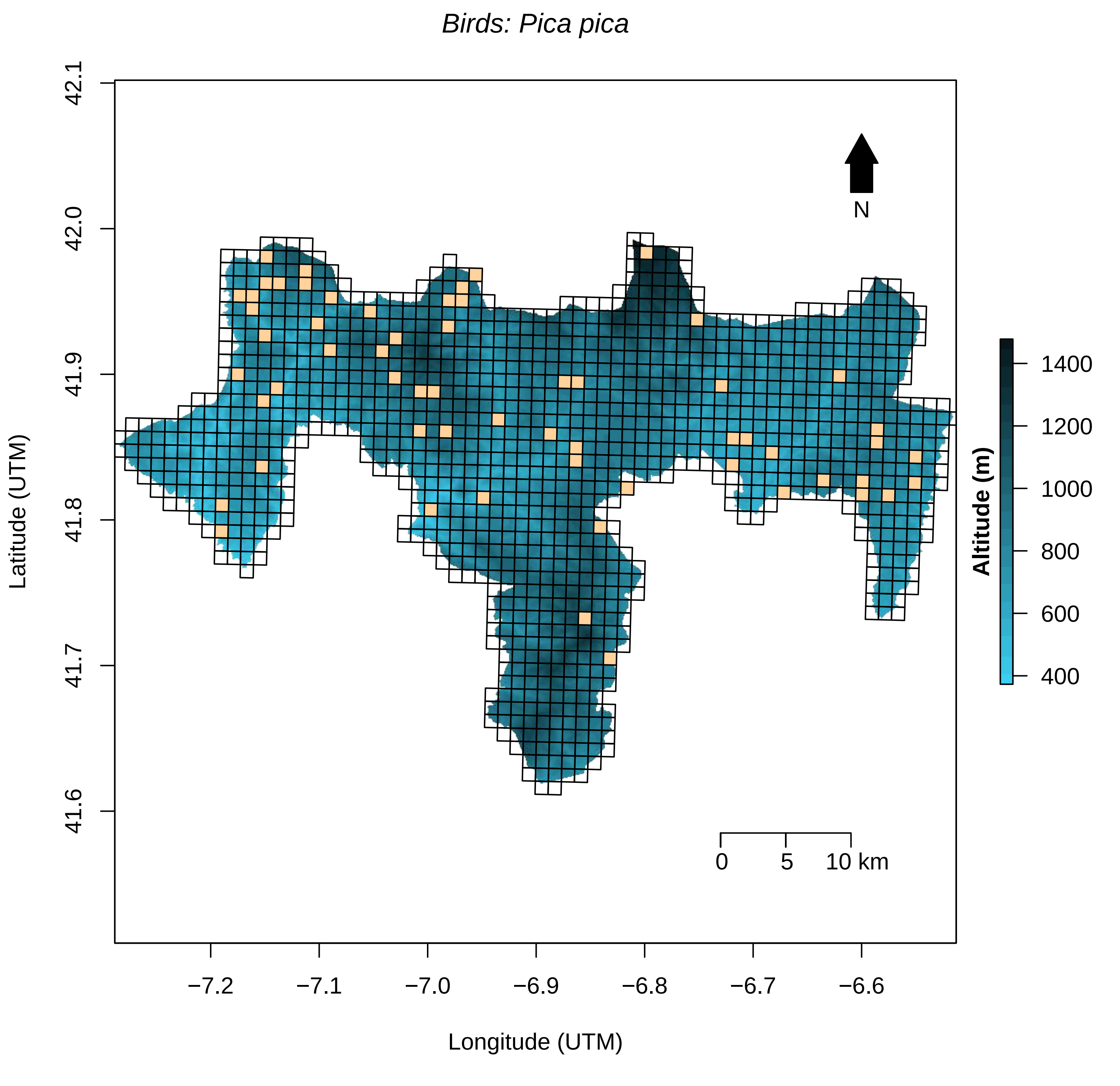

Birds: Pica pica
N
0 5 10 km
Latitude (UTM)
42.1
42.0
41.9
41.8
41.7
41.6
Longitude (UTM)
−7.2
−7.1
−7.0
−6.9
−6.8
−6.7
−6.6
Altitude (m)
1400
1200
1000
800
600
400

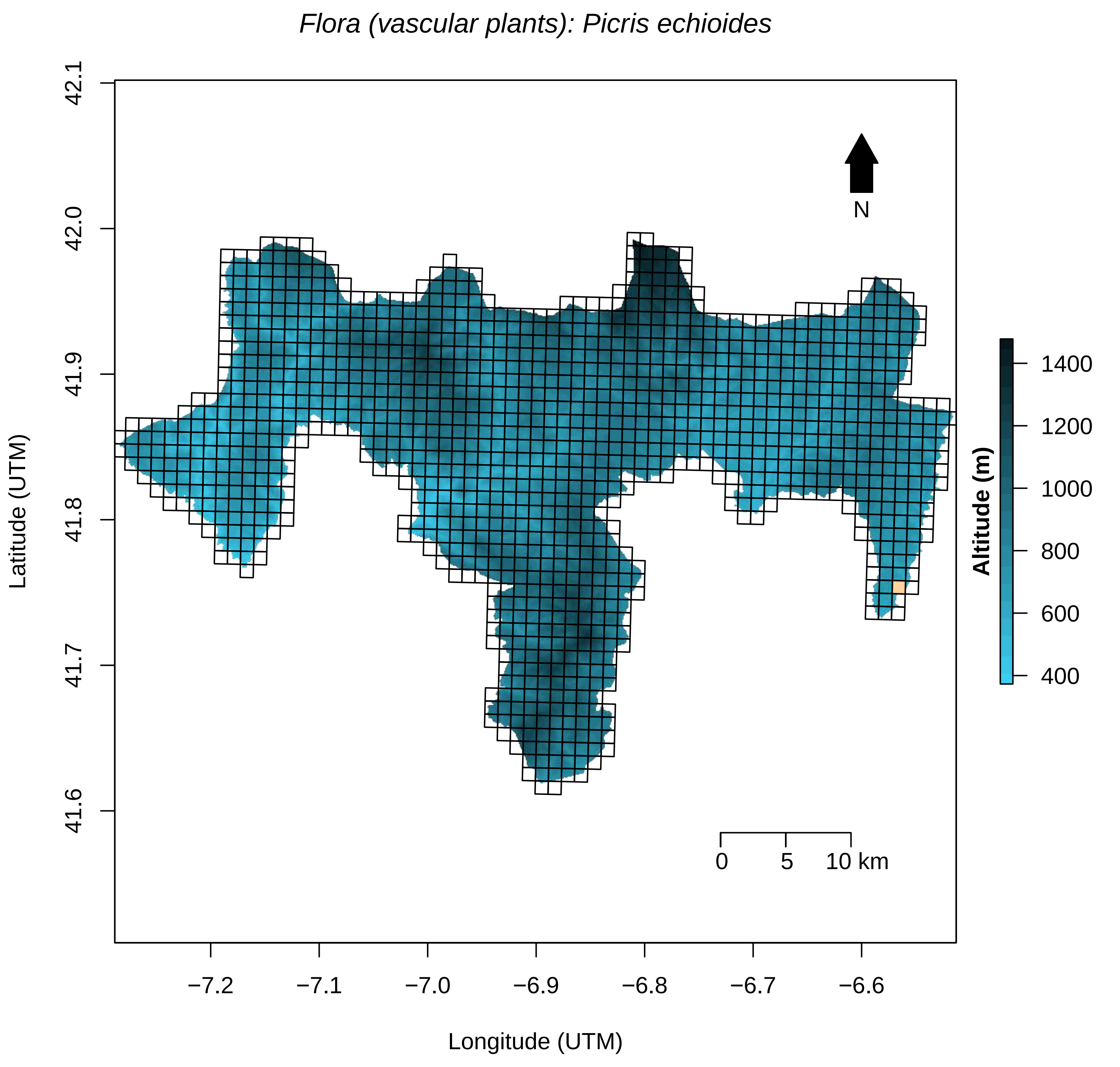
Flora (vascular plants): Picris echioides
Latitude (UTM)
42.1
42.0
41.9
41.8
41.7
41.6
N
0
5
10 km
−7.2
−7.1
−7.0
−6.9
−6.8
−6.7
−6.6
Longitude (UTM)
Altitude (m)
1400
1200
1000
800
600
400

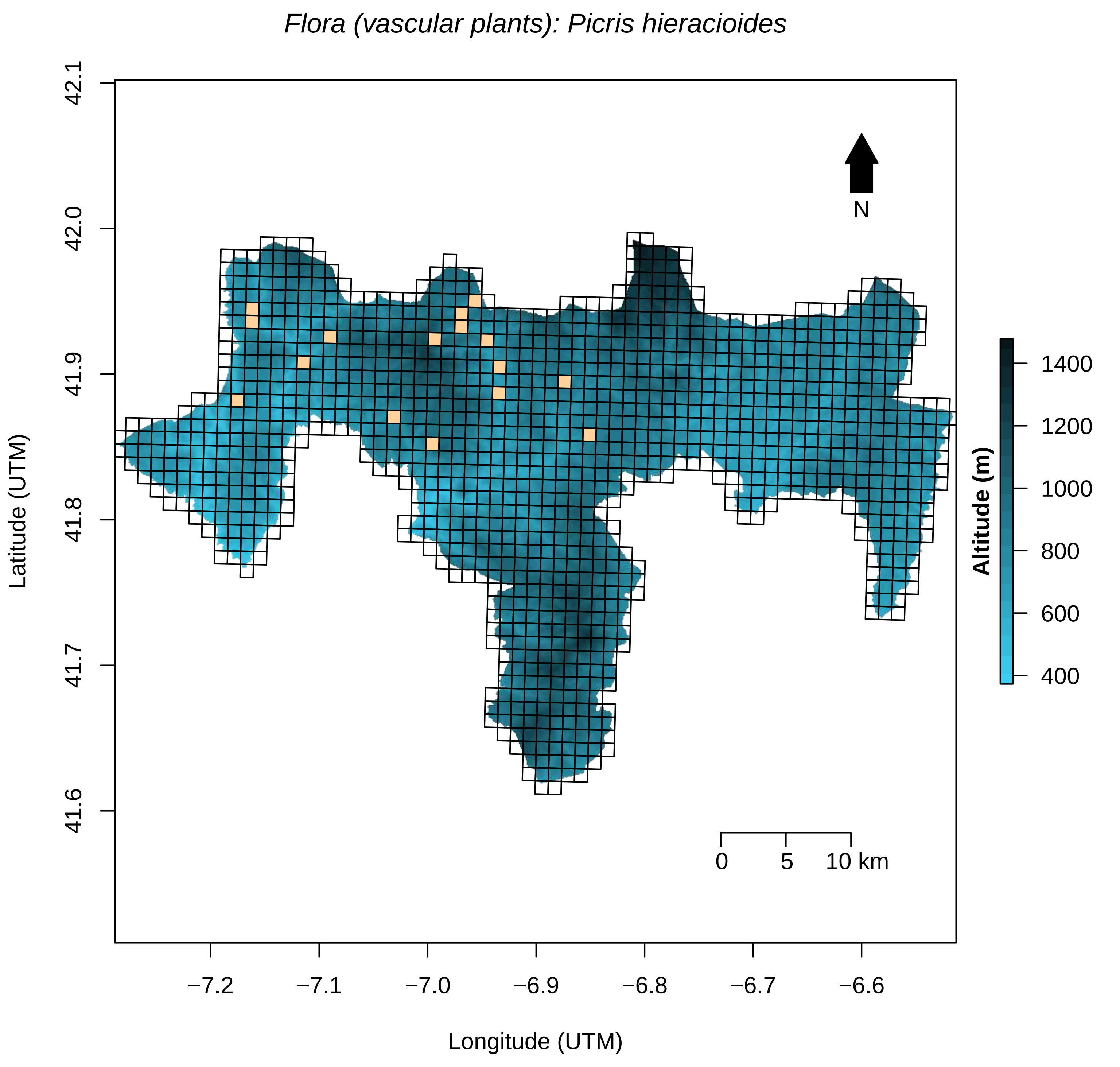
Flora (vascular plants): Picris hieracioides
N
0 5 10 km
Altitude (m)
1400
1200
1000
800
600
400
Latitude (UTM)
42.1
42.0
41.9
41.8
41.7
41.6
Longitude (UTM)
−7.2
−7.1
−7.0
−6.9
−6.8
−6.7
−6.6

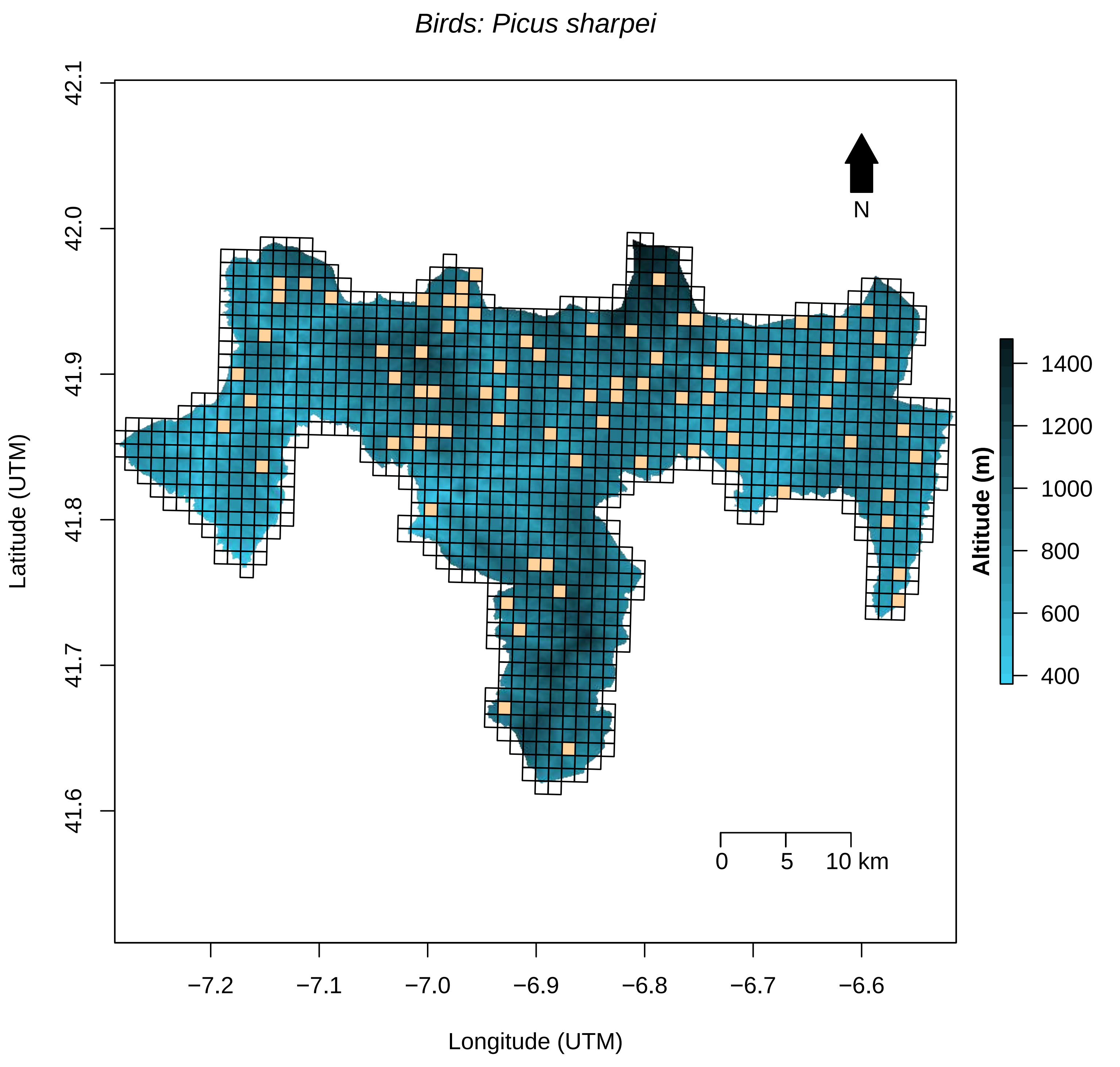

Birds: Picus sharpei
N
0 5 10 km
Altitude (m)
1400
1200
1000
800
600
400
Latitude (UTM)
42.1
42.0
41.9
41.8
41.7
41.6
Longitude (UTM)
−7.2
−7.1
−7.0
−6.9
−6.8
−6.7
−6.6

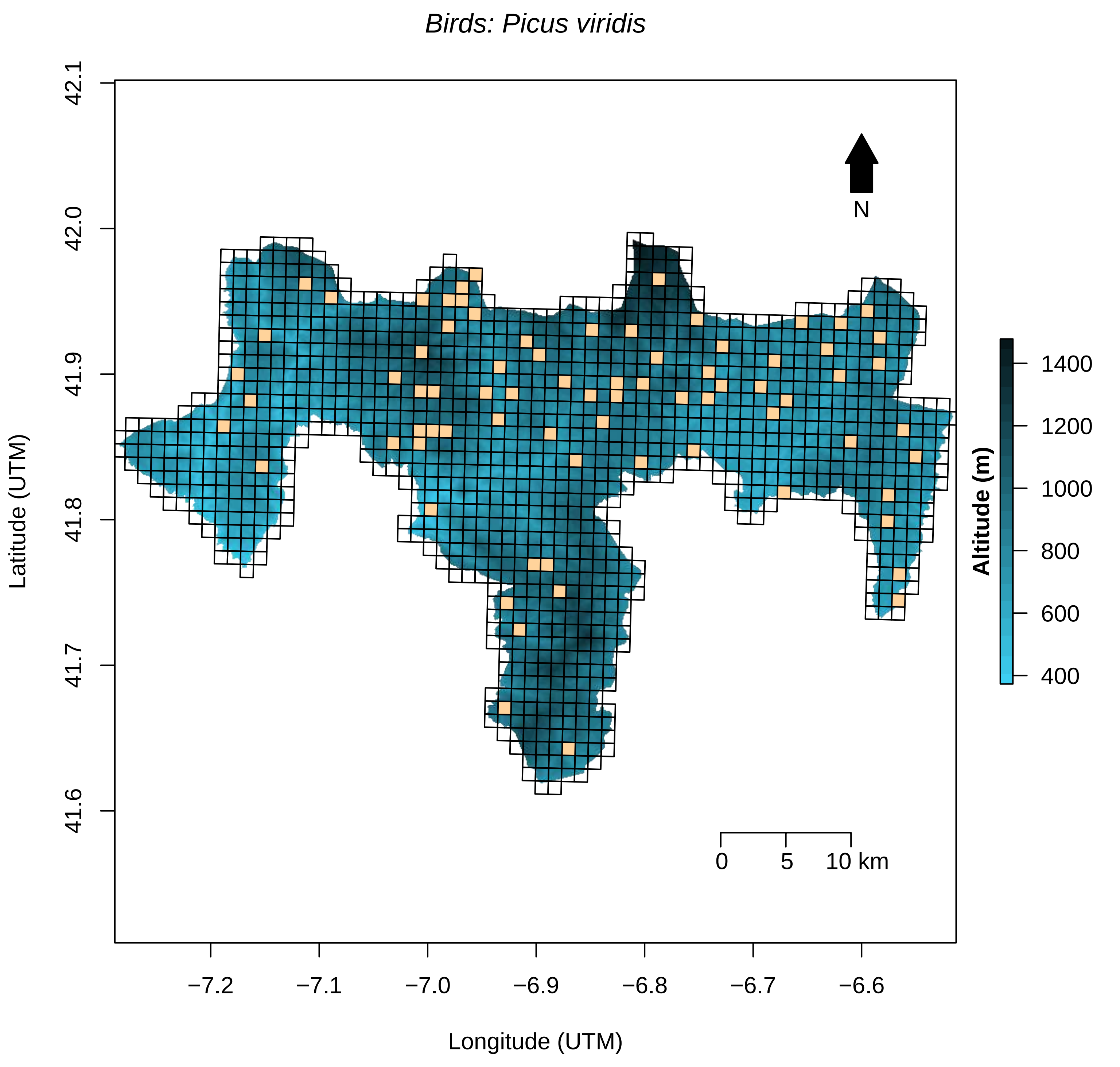

Birds: Picus viridis
42.1
42.0
41.9
41.8
41.7
41.6
Latitude (UTM)
−7.2
−7.1
−7.0
−6.9
−6.8
−6.7
−6.6
Longitude (UTM)
N
0
5
10 km
1400
1200
1000
800
600
400
Altitude (m)

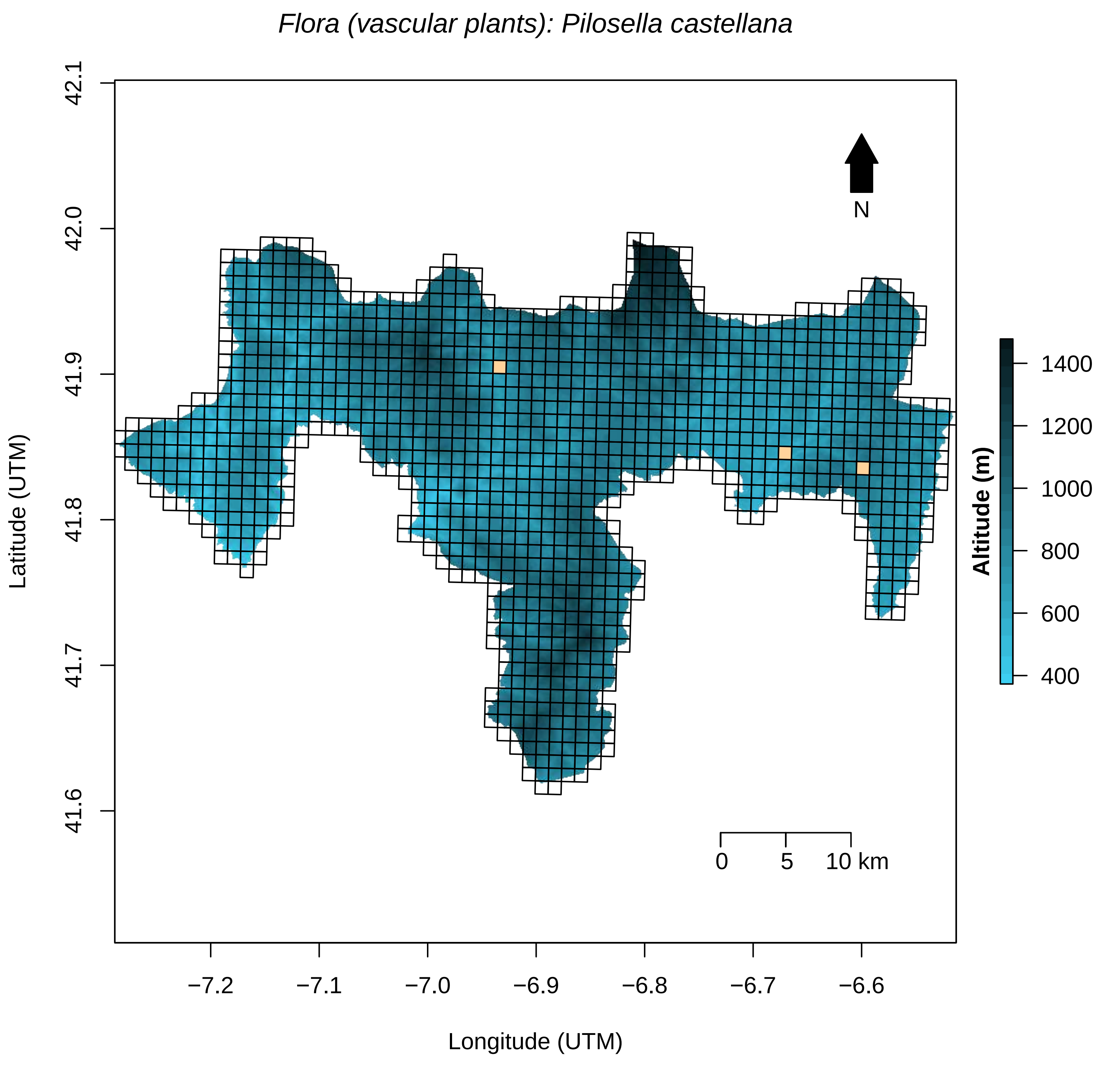
Flora (vascular plants): Pilosella castellana
N
0 5 10 km
Latitude (UTM)
Longitude (UTM)
Altitude (m)
42.1
42.0
41.9
41.8
41.7
41.6
−7.2
−7.1
−7.0
−6.9
−6.8
−6.7
−6.6
1400
1200
1000
800
600
400

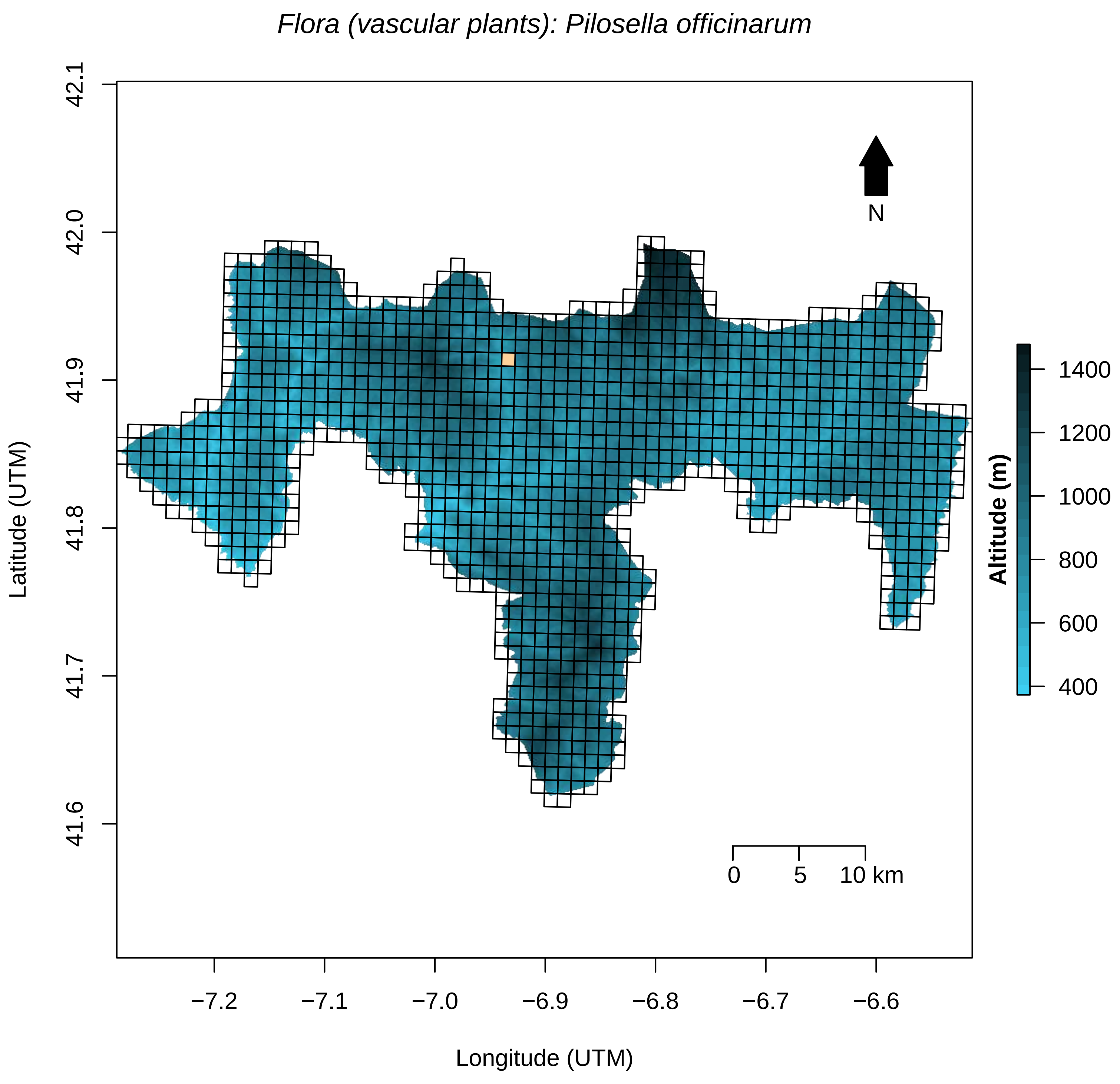

Flora (vascular plants): Pilosella officinarum
Latitude (UTM)
42.1
42.0
41.9
41.8
41.7
41.6
Longitude (UTM)
−7.2
−7.1
−7.0
−6.9
−6.8
−6.7
−6.6
N
0
5
10 km
Altitude (m)
1400
1200
1000
800
600
400

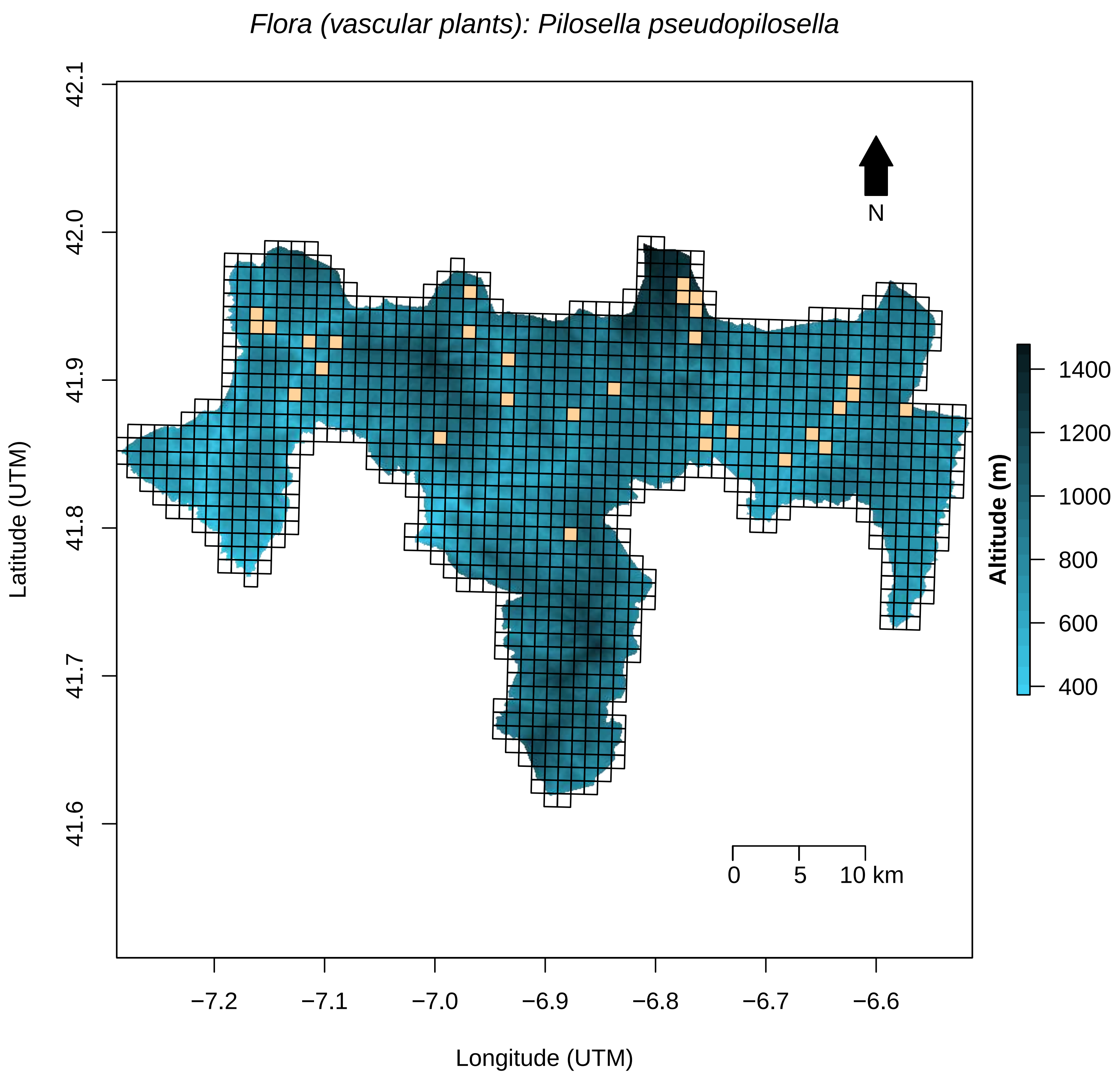
Flora (vascular plants): Pilosella pseudopilosella
Latitude (UTM)
Longitude (UTM)
42.1
42.0
41.9
41.8
41.7
41.6
−7.2
−7.1
−7.0
−6.9
−6.8
−6.7
−6.6
N
0
5
10 km
Altitude (m)
1400
1200
1000
800
600
400

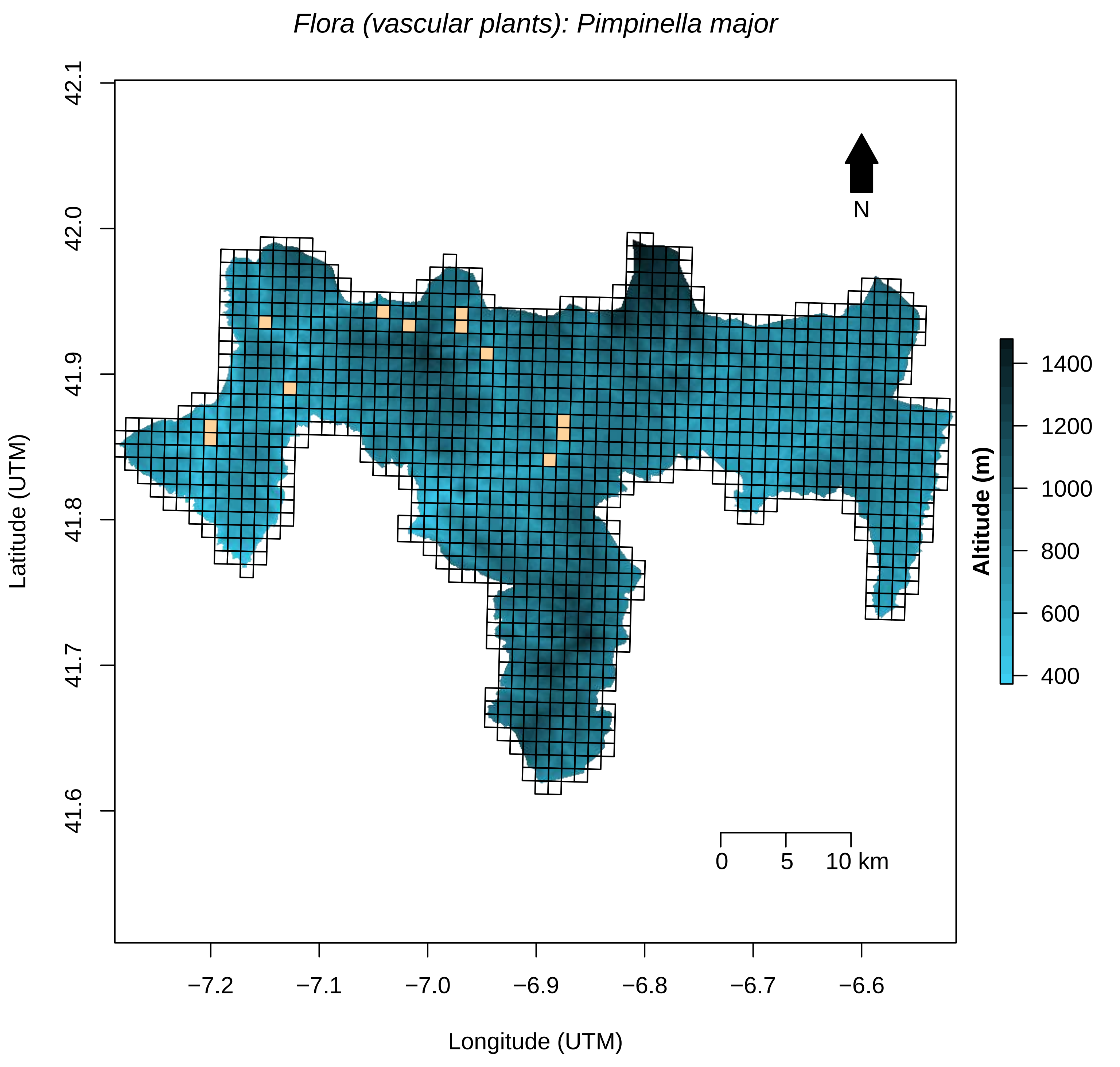

Flora (vascular plants): Pimpinella major
42.1
42.0
41.9
41.8
41.7
41.6
Latitude (UTM)
−7.2
−7.1
−7.0
−6.9
−6.8
−6.7
−6.6
Longitude (UTM)
N
0
5
10 km
Altitude (m)
1400
1200
1000
800
600
400

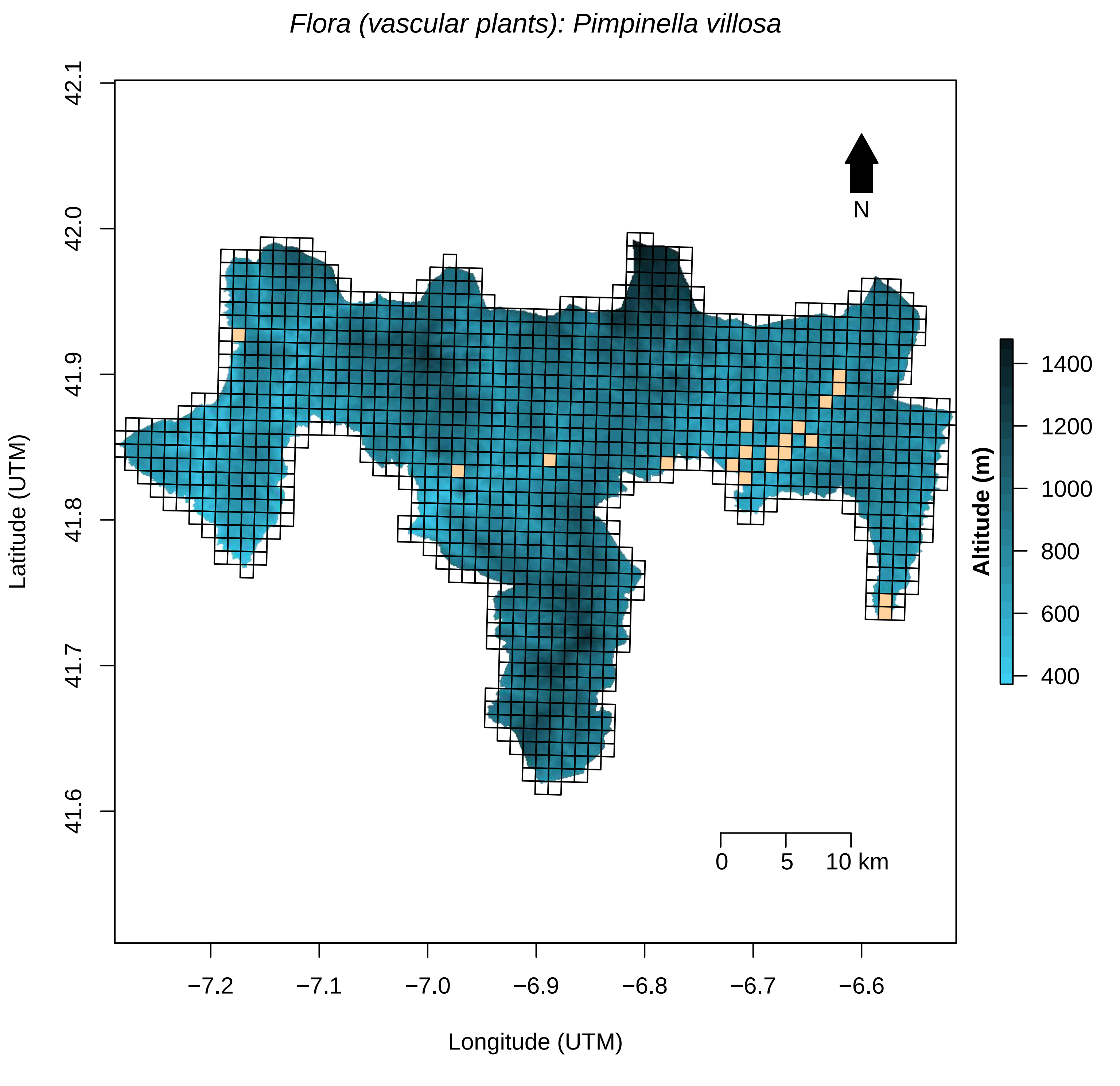
Flora (vascular plants): Pimpinella villosa
42.1
42.0
41.9
41.8
41.7
41.6
Latitude (UTM)
−7.2
−7.1
−7.0
−6.9
−6.8
−6.7
−6.6
Longitude (UTM)
N
0
5
10 km
Altitude (m)
1400
1200
1000
800
600
400

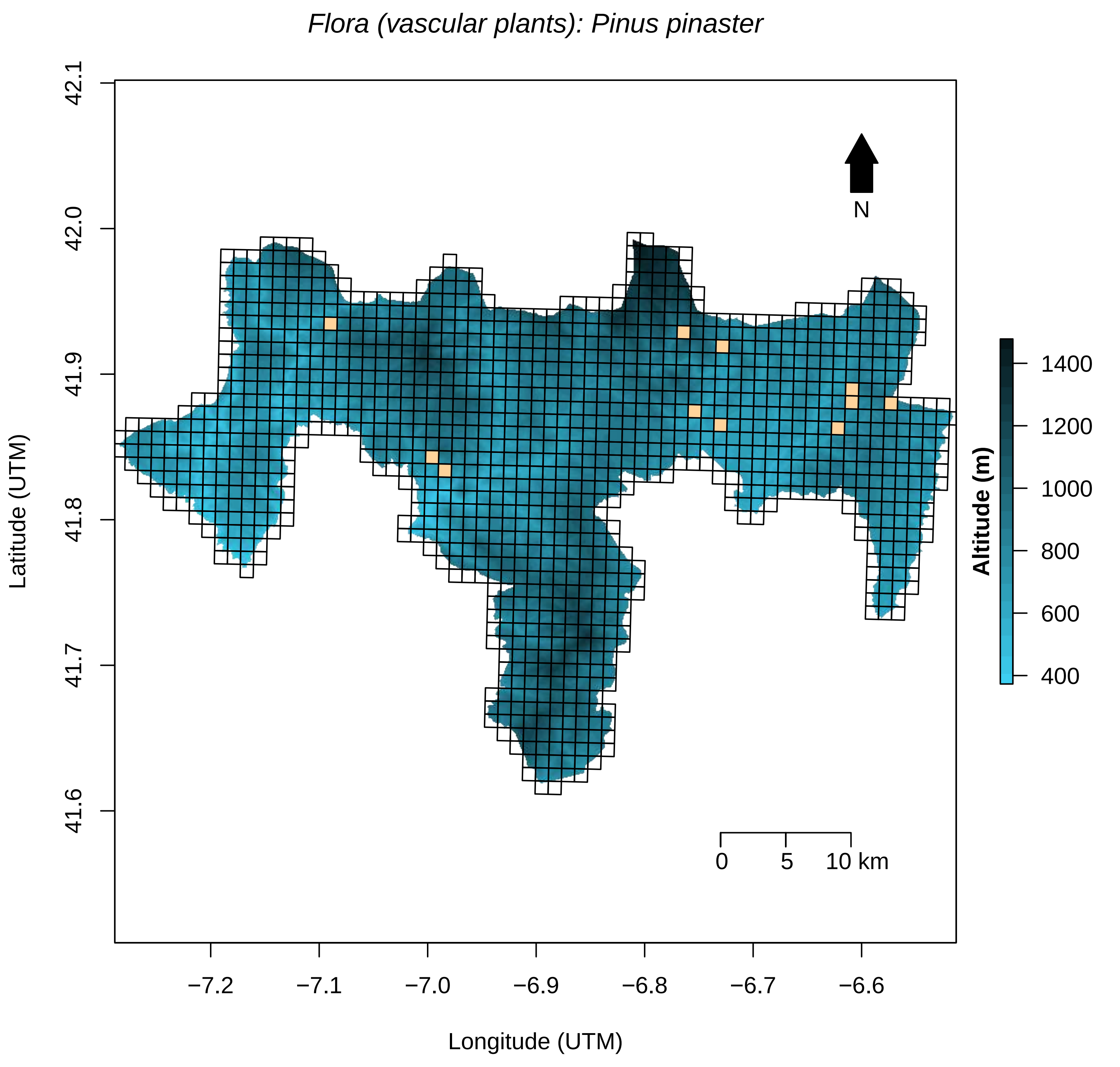

Flora (vascular plants): Pinus pinaster
Latitude (UTM)
42.1
42.0
41.9
41.8
41.7
41.6
N
0
5
10 km
−7.2
−7.1
−7.0
−6.9
−6.8
−6.7
−6.6
Longitude (UTM)
Altitude (m)
1400
1200
1000
800
600
400

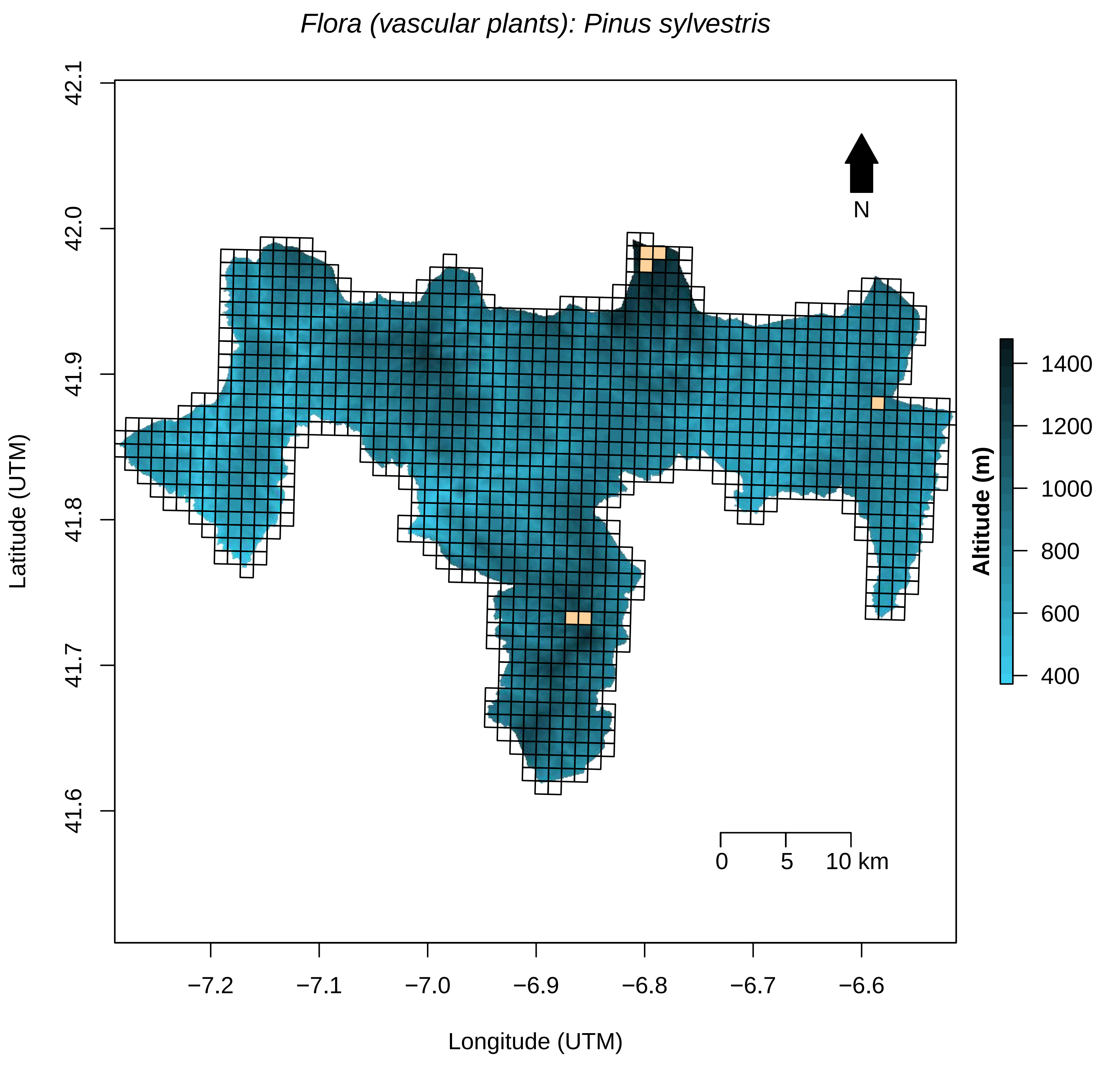
Flora (vascular plants): Pinus sylvestris
Latitude (UTM)
42.1
42.0
41.9
41.8
41.7
41.6
Longitude (UTM)
−7.2
−7.1
−7.0
−6.9
−6.8
−6.7
−6.6
N
0
5
10 km
Altitude (m)
1400
1200
1000
800
600
400

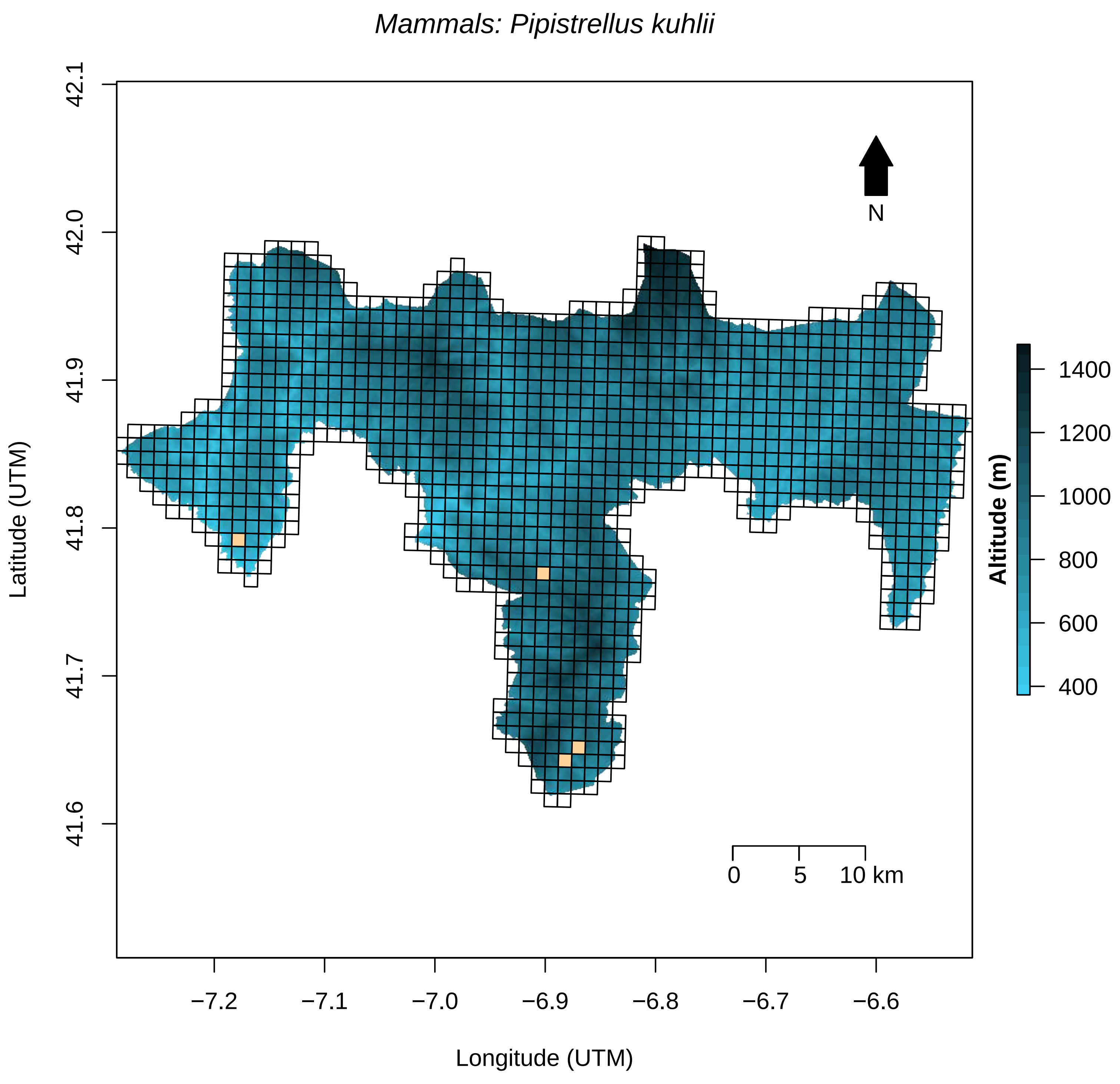

Mammals: Pipistrellus kuhlii
Latitude (UTM)
42.1
42.0
41.9
41.8
41.7
41.6
Longitude (UTM)
−7.2
−7.1
−7.0
−6.9
−6.8
−6.7
−6.6
N
0
5
10 km
Altitude (m)
1400
1200
1000
800
600
400

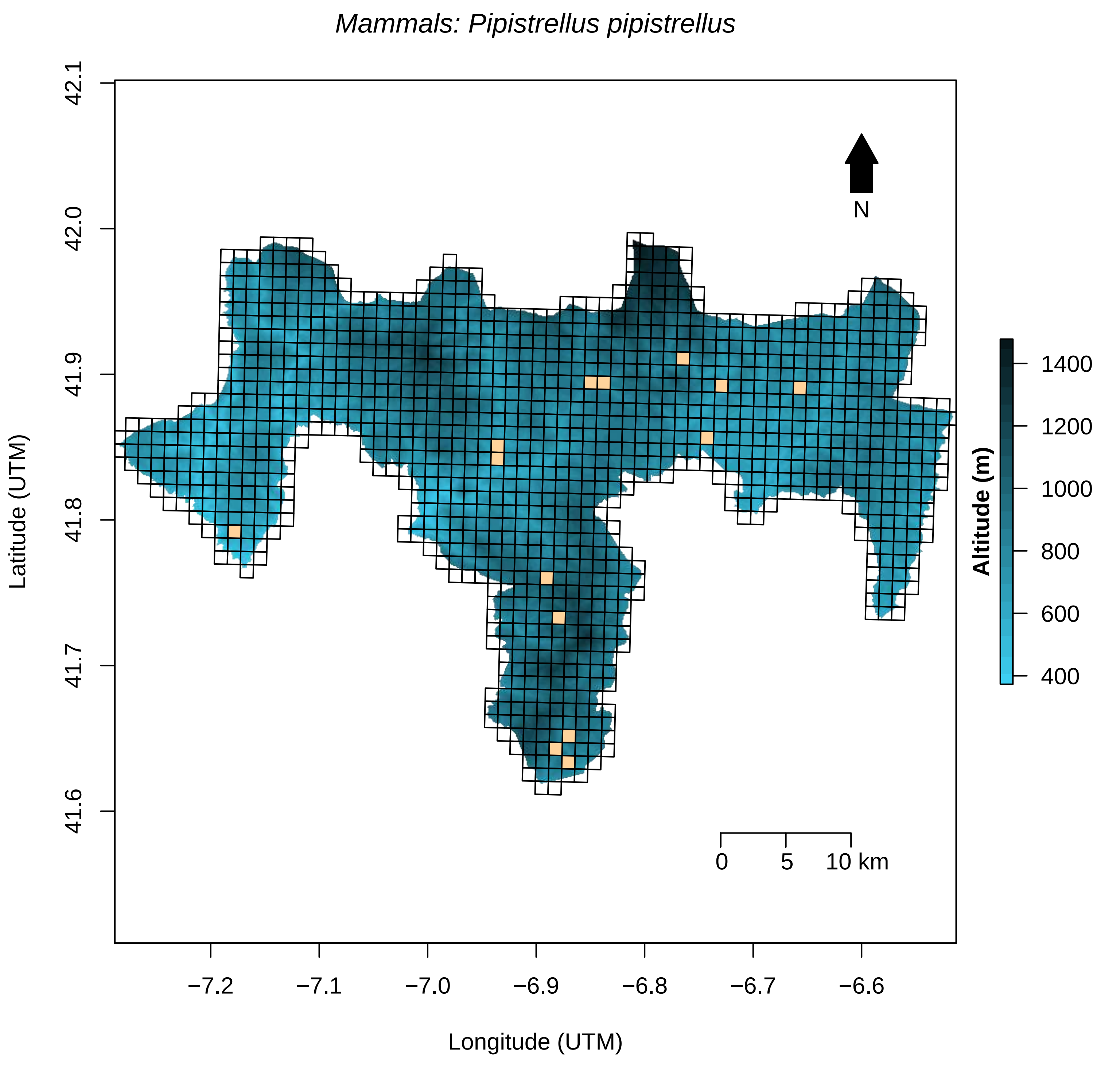

Mammals: Pipistrellus pipistrellus
42.1
42.0
41.9
41.8
41.7
41.6
Latitude (UTM)
−7.2
−7.1
−7.0
−6.9
−6.8
−6.7
−6.6
Longitude (UTM)
N
0
5
10 km
1400
1200
1000
800
600
400
Altitude (m)

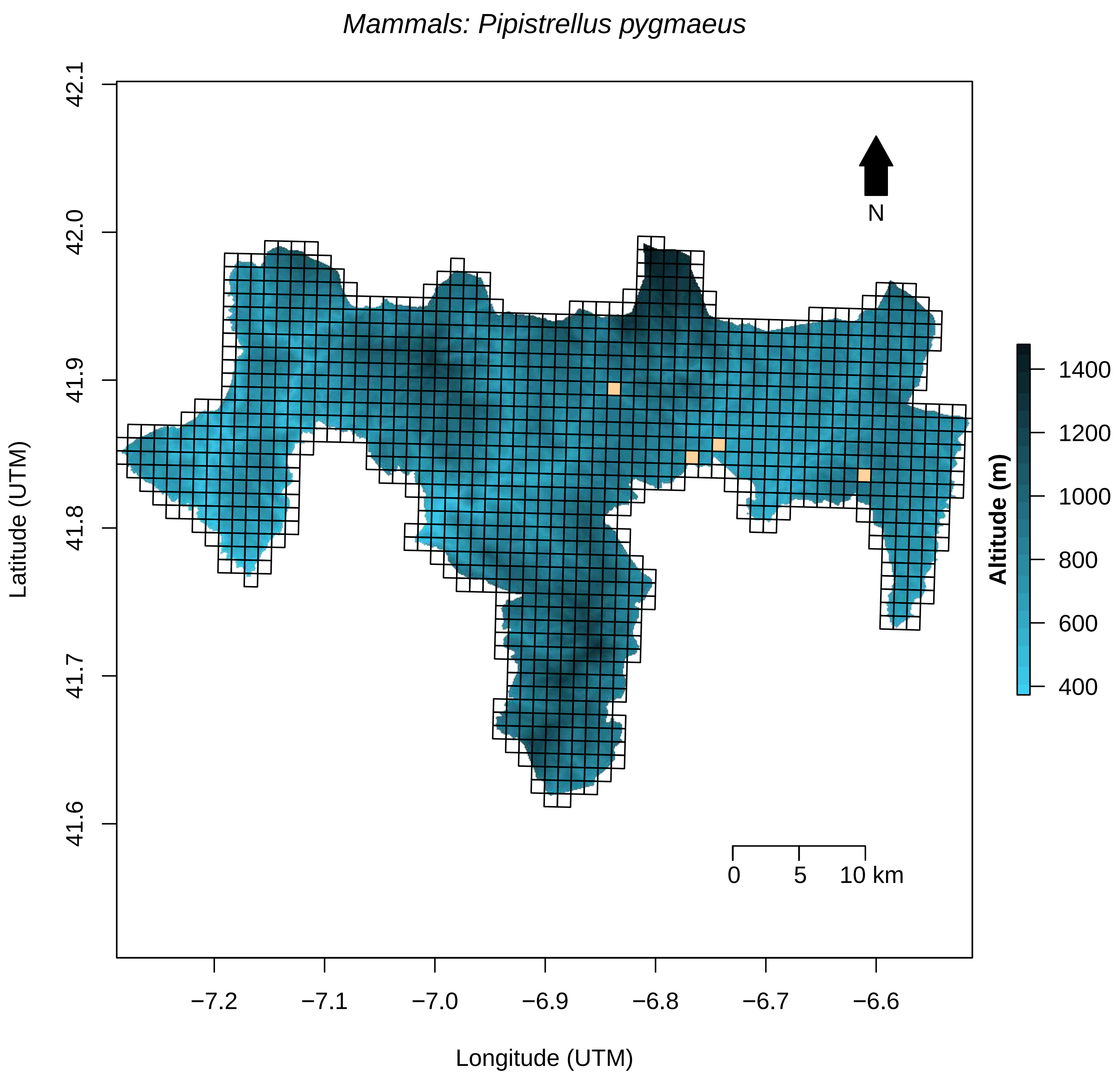
Mammals: Pipistrellus pygmaeus
Latitude (UTM)
42.1
42.0
41.9
41.8
41.7
41.6
−7.2
−7.1
−7.0
−6.9
−6.8
−6.7
−6.6
Longitude (UTM)
N
0
5
10 km
Altitude (m)
1400
1200
1000
800
600
400

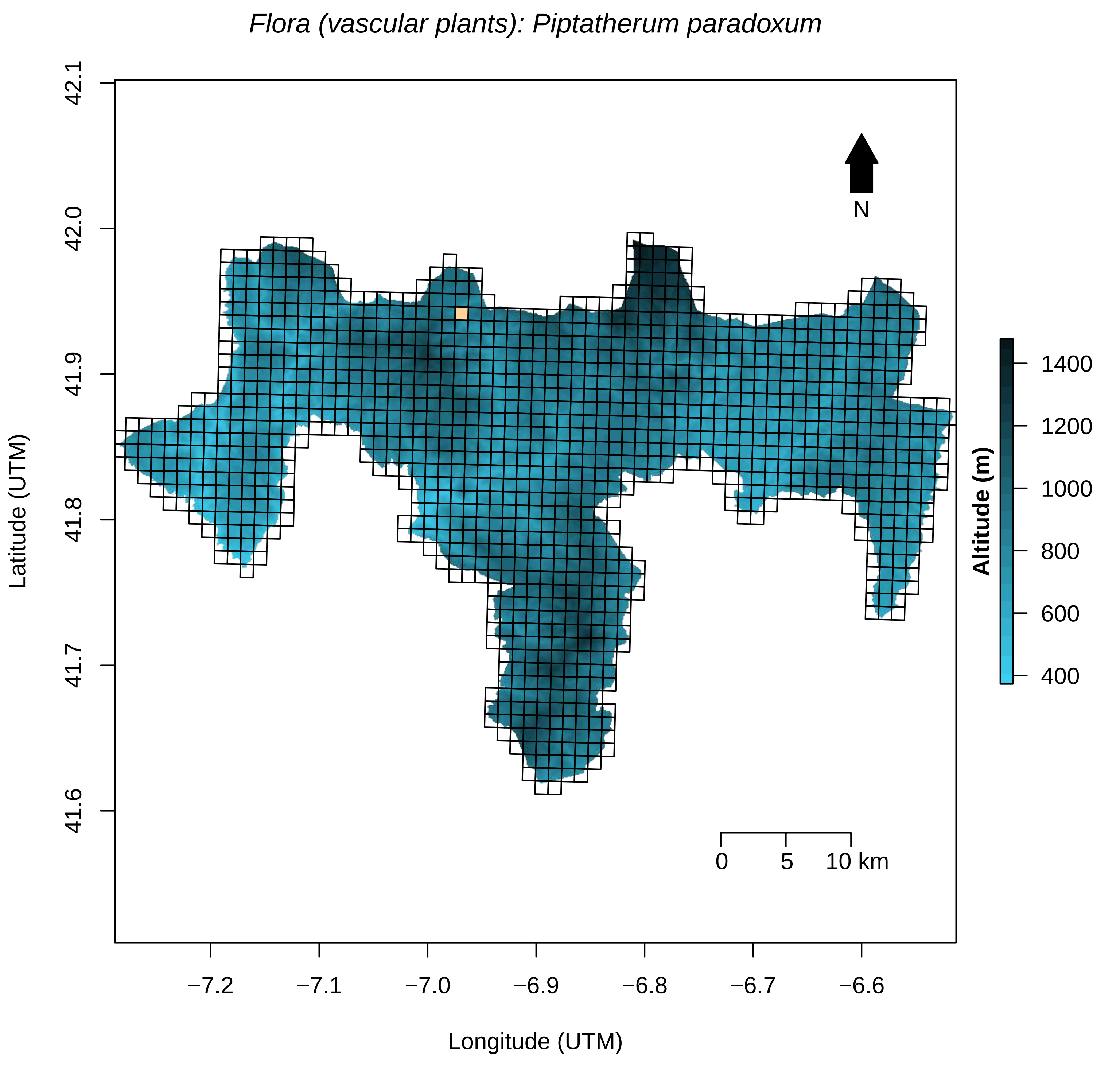
Flora (vascular plants): Piptatherum paradoxum
42.1
42.0
41.9
41.8
41.7
41.6
Latitude (UTM)
−7.2
−7.1
−7.0
−6.9
−6.8
−6.7
−6.6
Longitude (UTM)
N
0
5
10 km
Altitude (m)
1400
1200
1000
800
600
400

Flora (vascular plants): Pistacia terebinthus

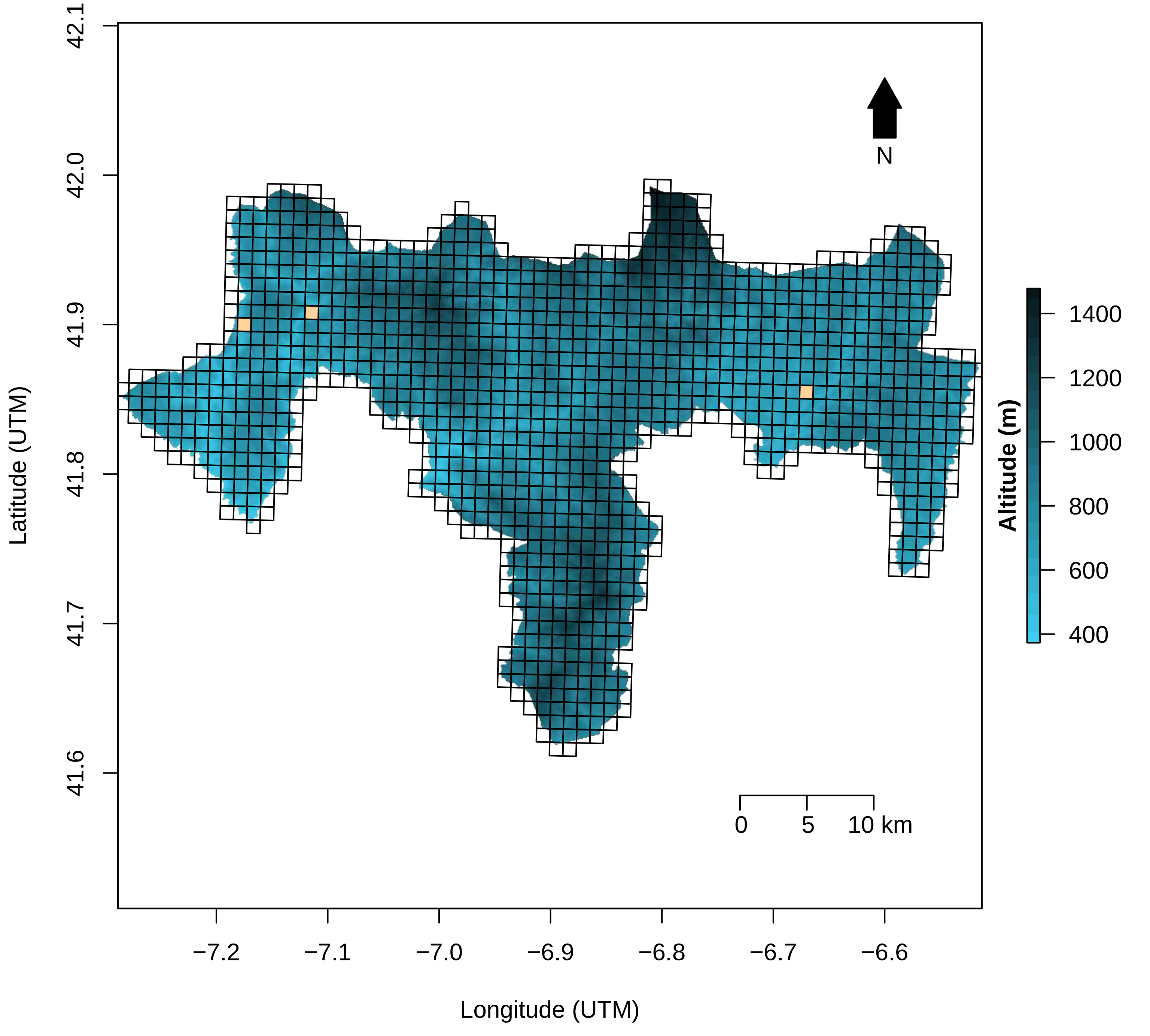

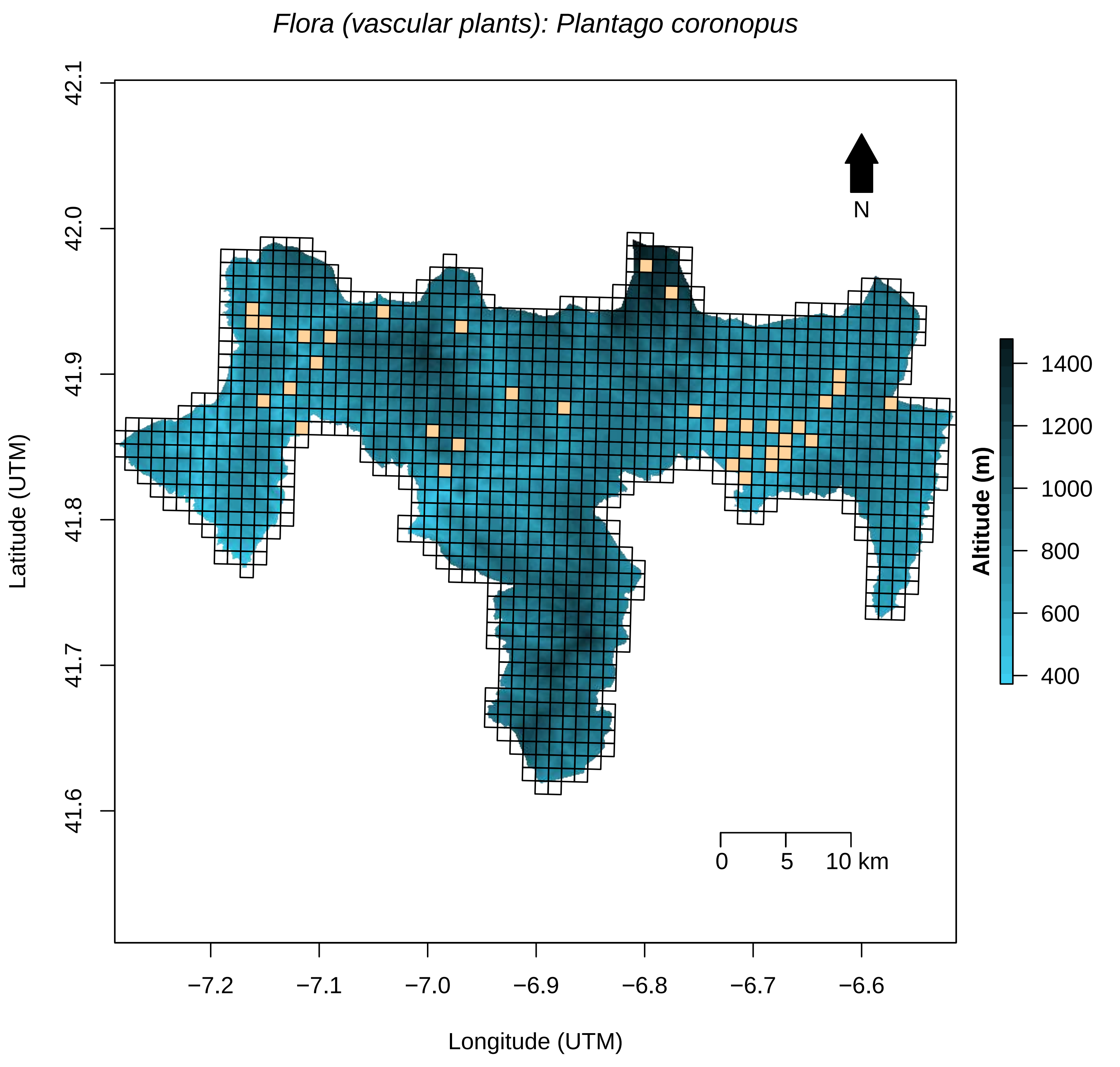

Flora (vascular plants): Plantago coronopus
N
0
5
10 km
Altitude (m)
1400
1200
1000
800
600
400
42.1
42.0
41.9
41.8
41.7
41.6
Latitude (UTM)
−7.2
−7.1
−7.0
−6.9
−6.8
−6.7
−6.6
Longitude (UTM)

Flora (vascular plants): Plantago holosteum

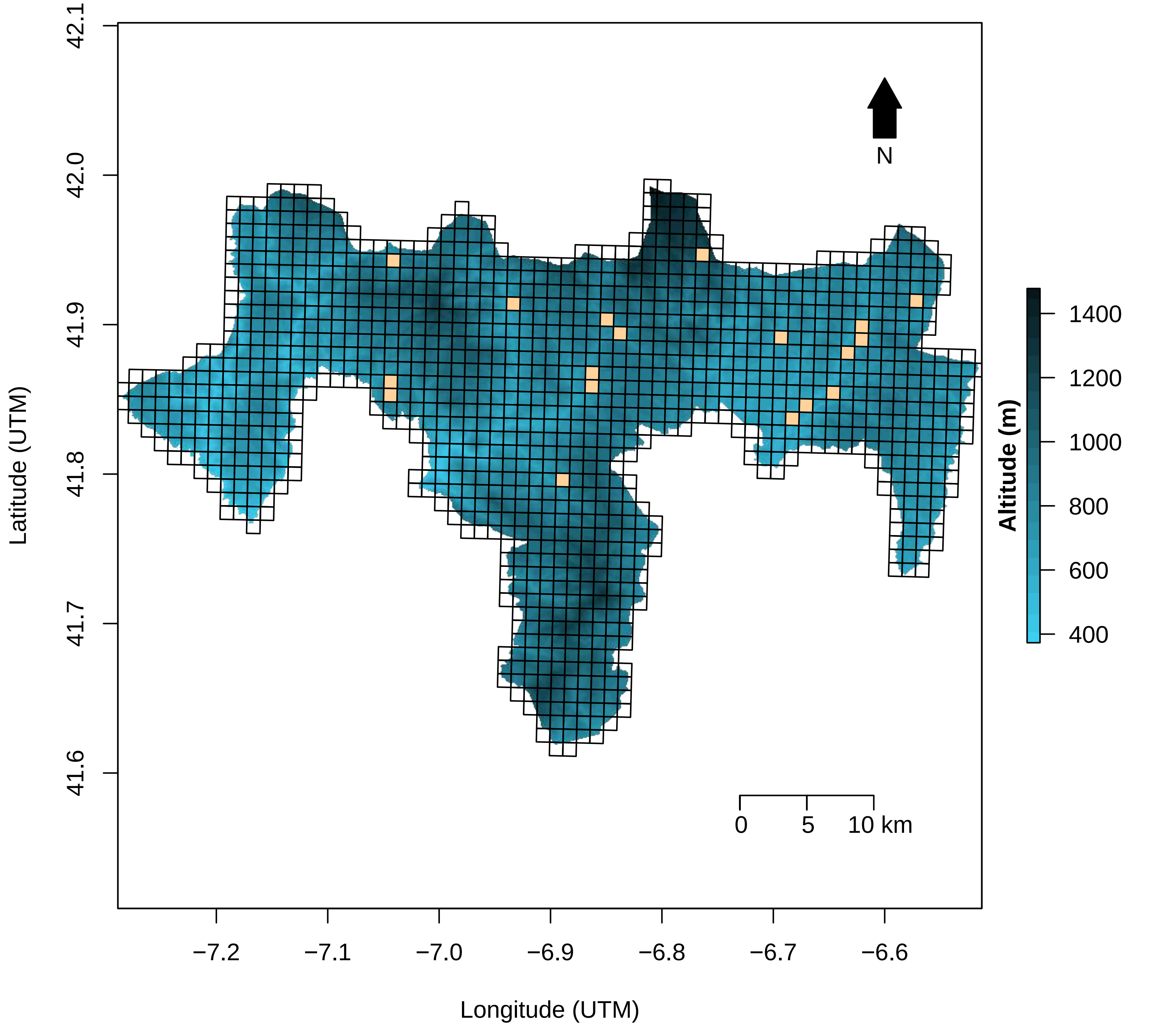

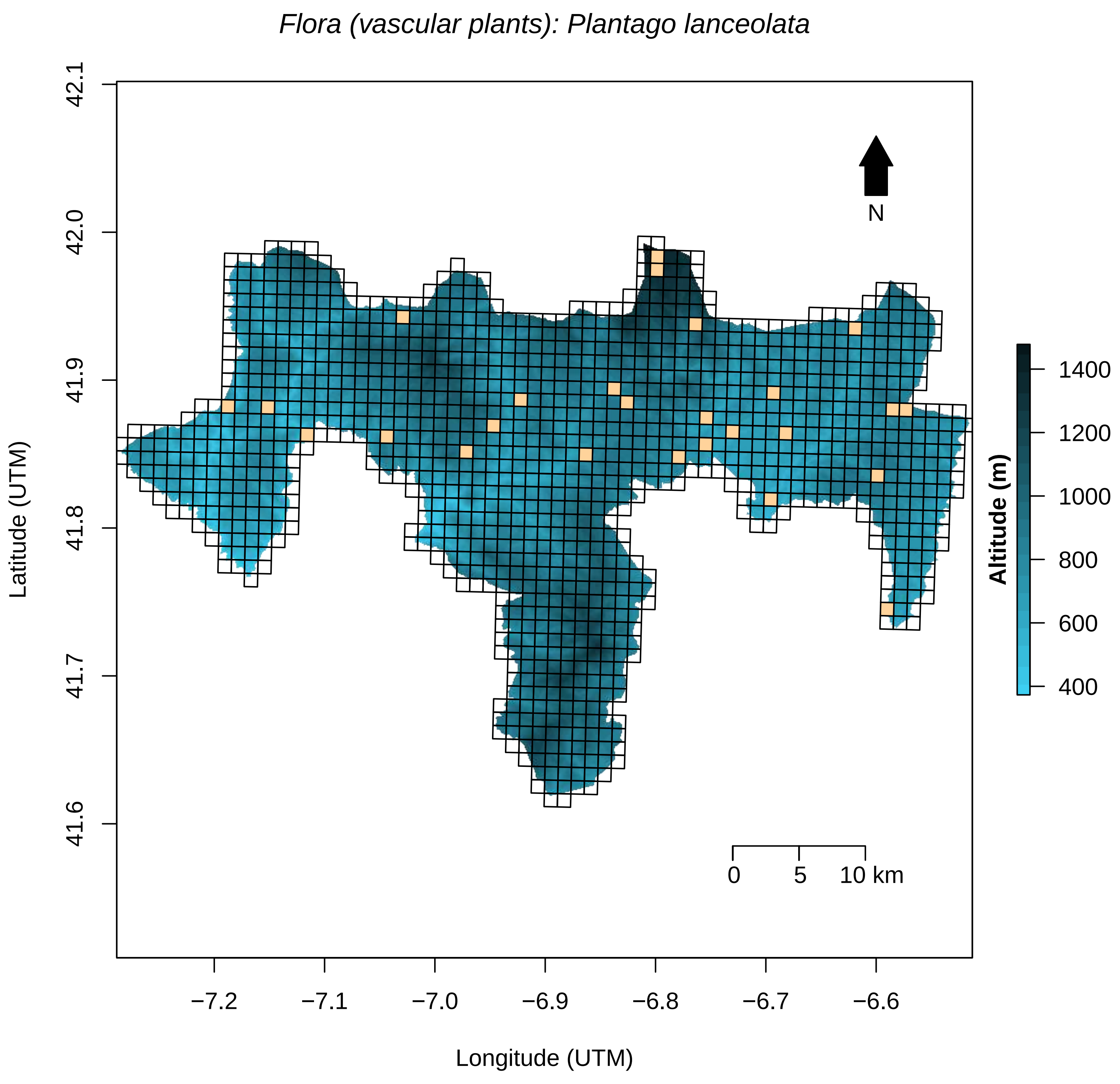

Flora (vascular plants): Plantago lanceolata
42.1
42.0
41.9
41.8
41.7
41.6
Latitude (UTM)
N
0
5
10 km
1400
1200
1000
800
600
400
Altitude (m)
−7.2
−7.1
−7.0
−6.9
−6.8
−6.7
−6.6
Longitude (UTM)

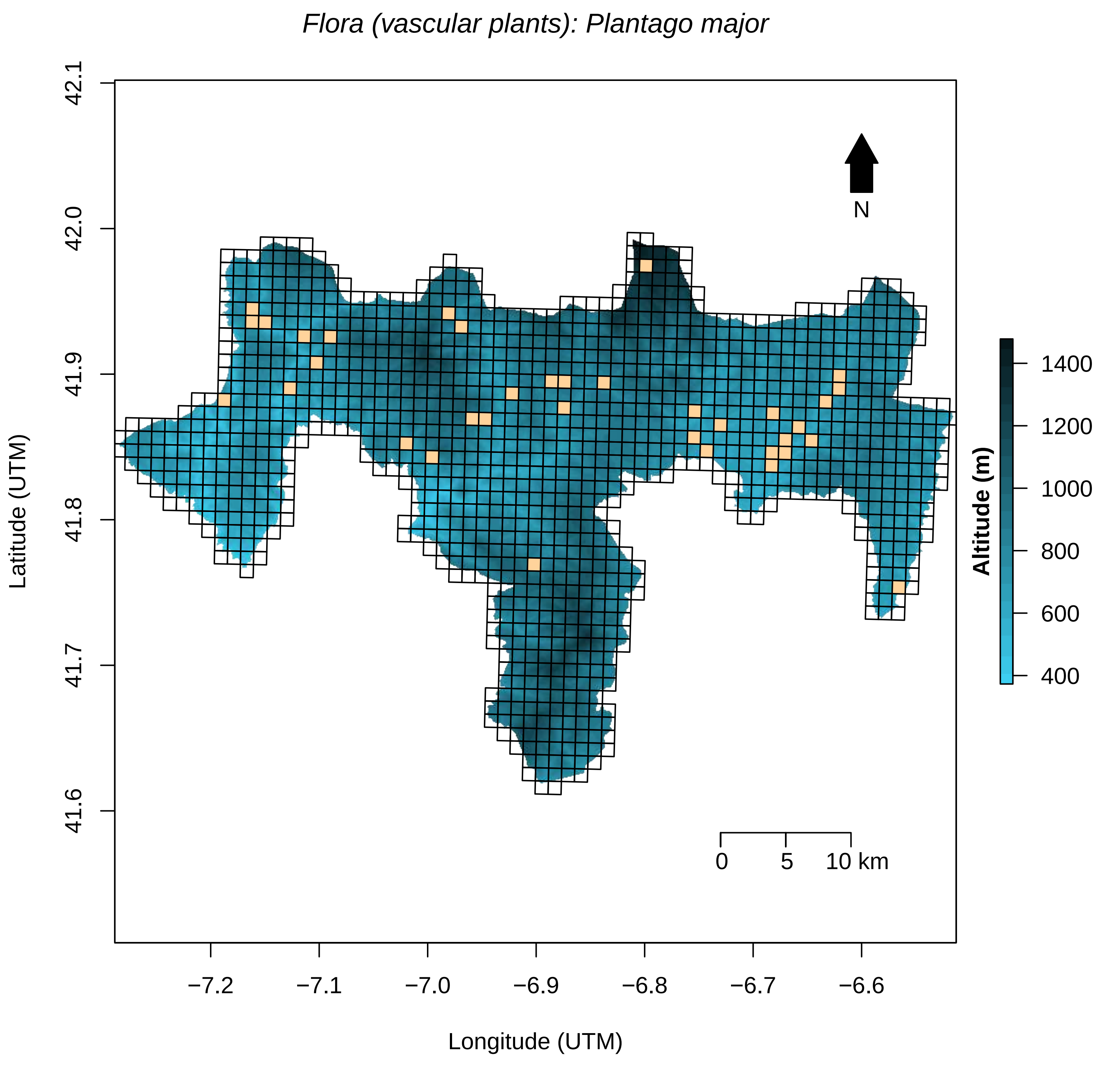

Flora (vascular plants): Plantago major
Latitude (UTM)
42.1
42.0
41.9
41.8
41.7
41.6
Longitude (UTM)
−7.2
−7.1
−7.0
−6.9
−6.8
−6.7
−6.6
N
0
5
10 km
Altitude (m)
1400
1200
1000
800
600
400

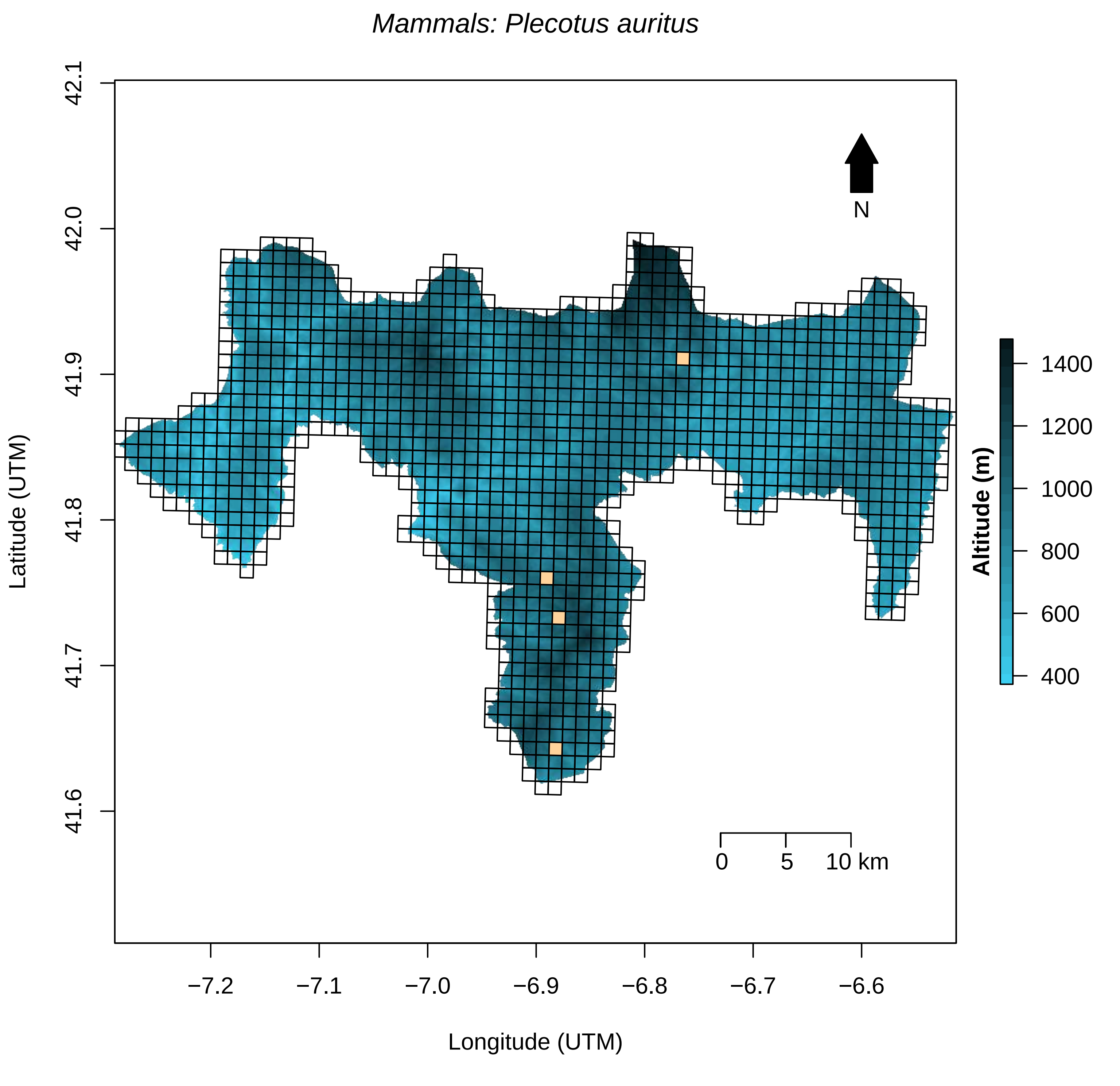

Mammals: Plecotus auritus
N
0 5 10 km
Latitude (UTM)
42.1
42.0
41.9
41.8
41.7
41.6
Longitude (UTM)
−7.2
−7.1
−7.0
−6.9
−6.8
−6.7
−6.6
Altitude (m)
1400
1200
1000
800
600
400

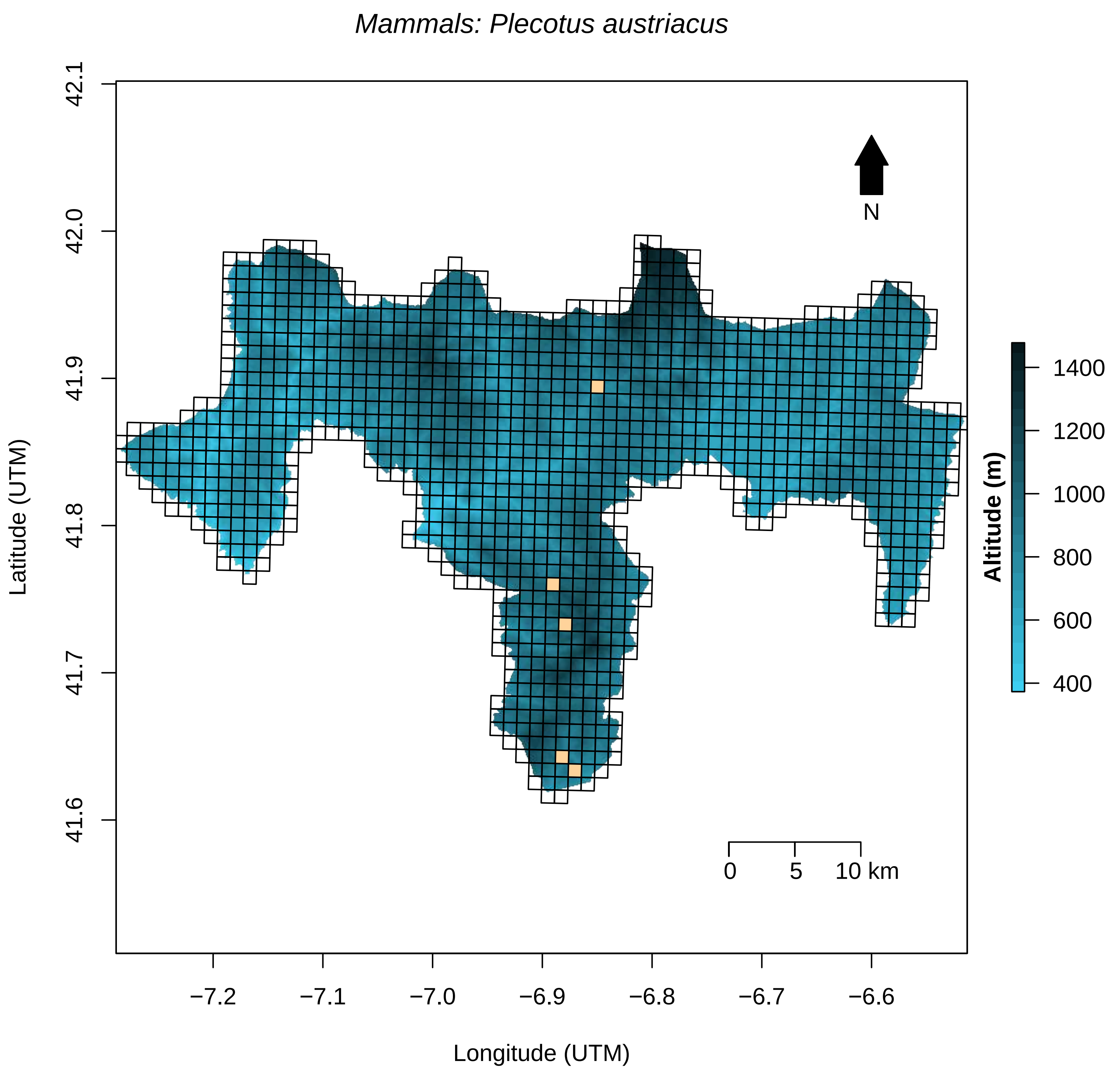

Mammals: Plecotus austriacus
42.1
42.0
41.9
41.8
41.7
41.6
Latitude (UTM)
−7.2
−7.1
−7.0
−6.9
−6.8
−6.7
−6.6
Longitude (UTM)
N
0
5
10 km
Altitude (m)
1400
1200
1000
800
600
400

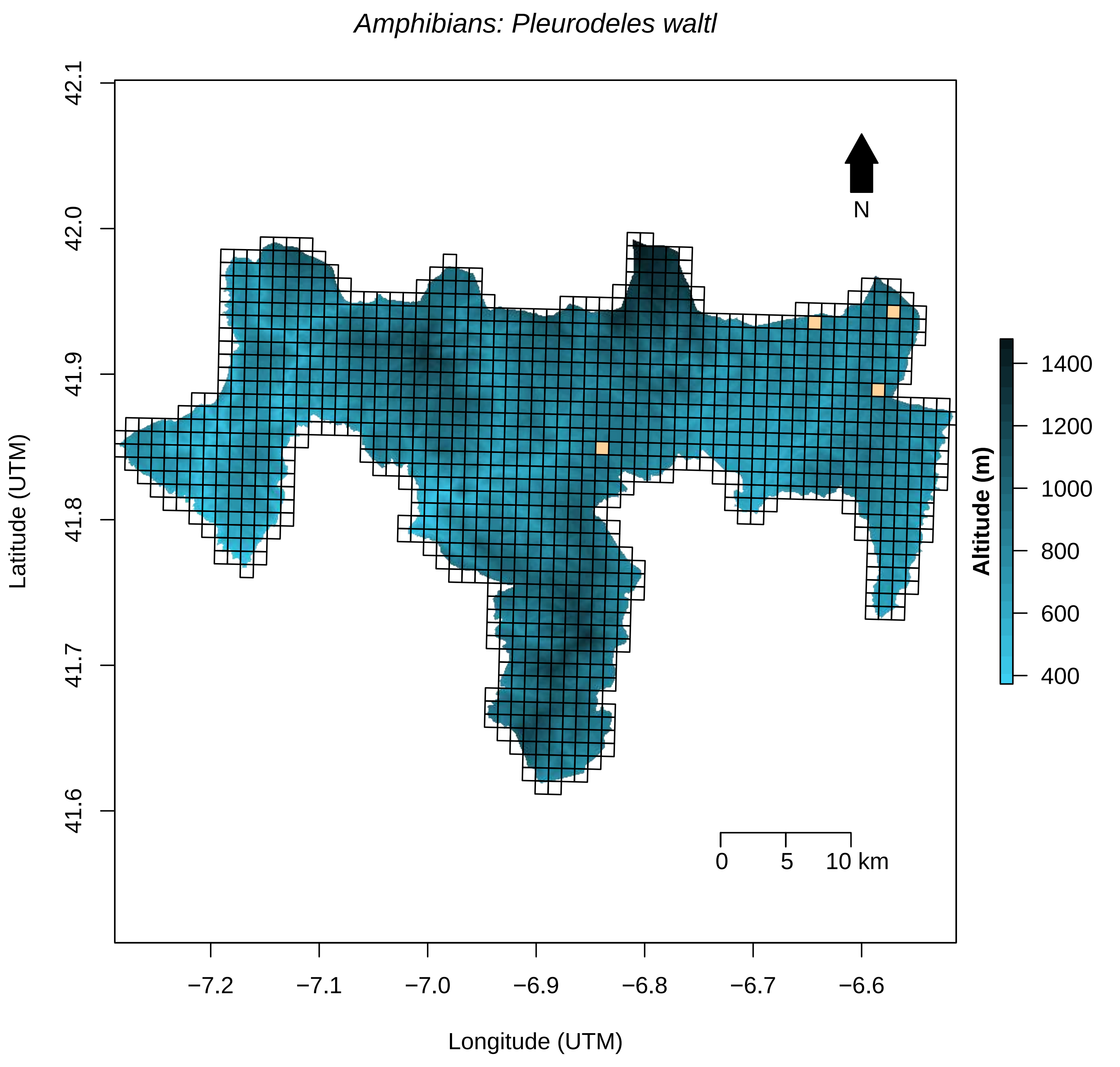

Amphibians: Pleurodeles waltl
N
0
5
10 km
Altitude (m)
1400
1200
1000
800
600
400
Latitude (UTM)
42.1
42.0
41.9
41.8
41.7
41.6
Longitude (UTM)
−7.2
−7.1
−7.0
−6.9
−6.8
−6.7
−6.6

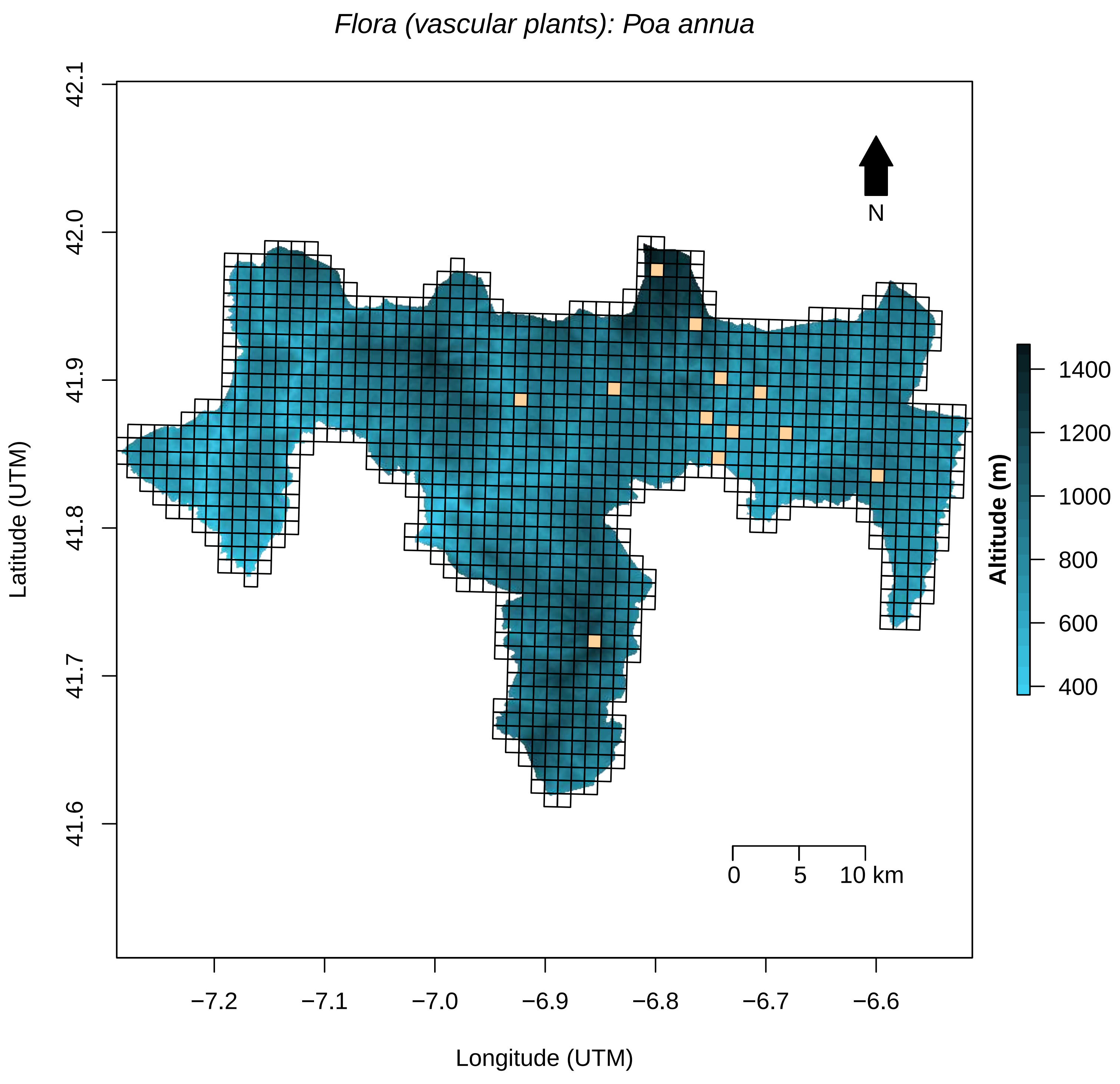

Flora (vascular plants): Poa annua
42.1
42.0
41.9
41.8
41.7
41.6
Latitude (UTM)
−7.2
−7.1
−7.0
−6.9
−6.8
−6.7
−6.6
Longitude (UTM)
N
0
5
10 km
Altitude (m)
1400
1200
1000
800
600
400

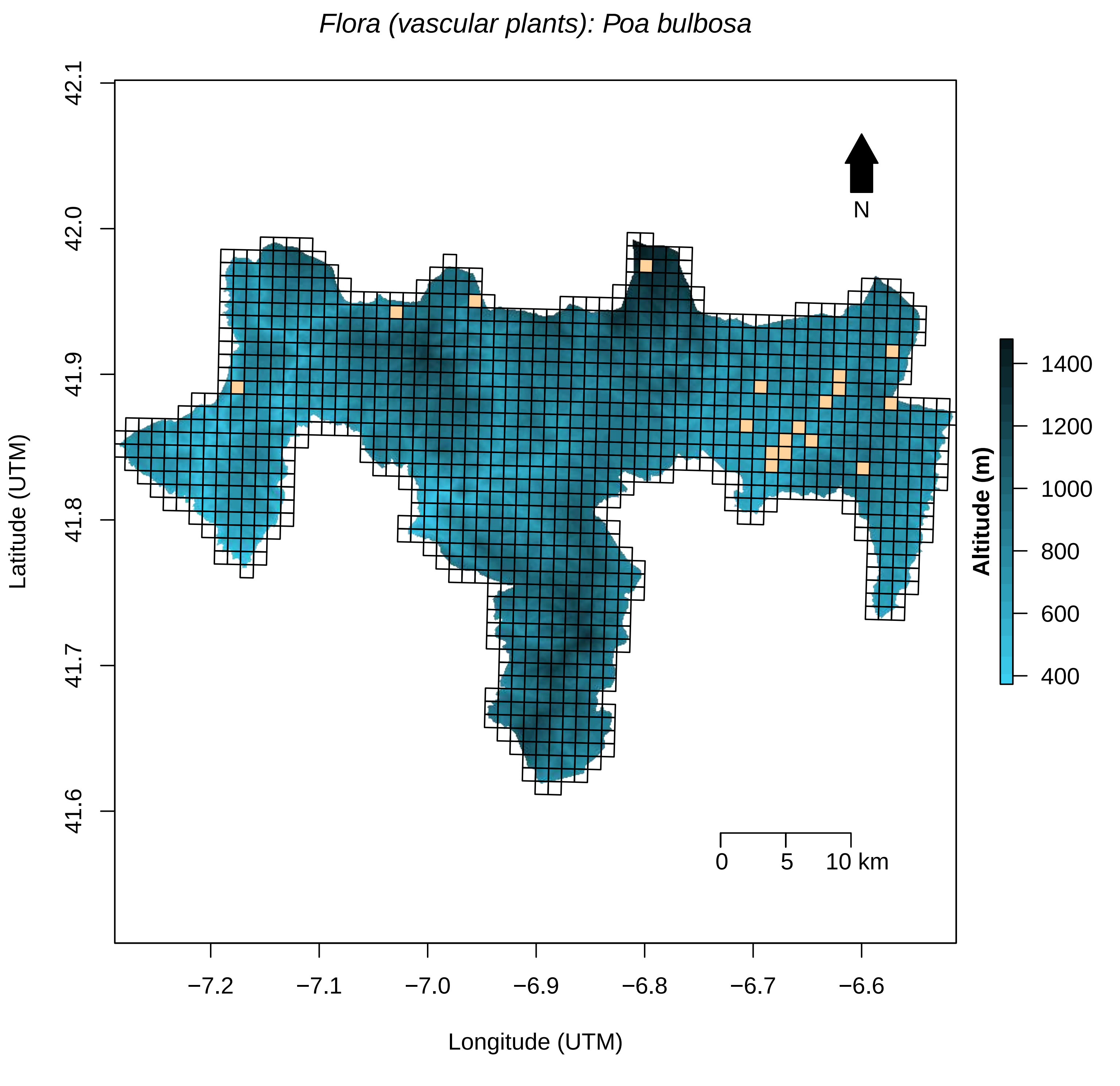

Flora (vascular plants): Poa bulbosa
42.1
42.0
41.9
41.8
41.7
41.6
Latitude (UTM)
−7.2
−7.1
−7.0
−6.9
−6.8
−6.7
−6.6
Longitude (UTM)
N
0
5
10 km
Altitude (m)
1400
1200
1000
800
600
400

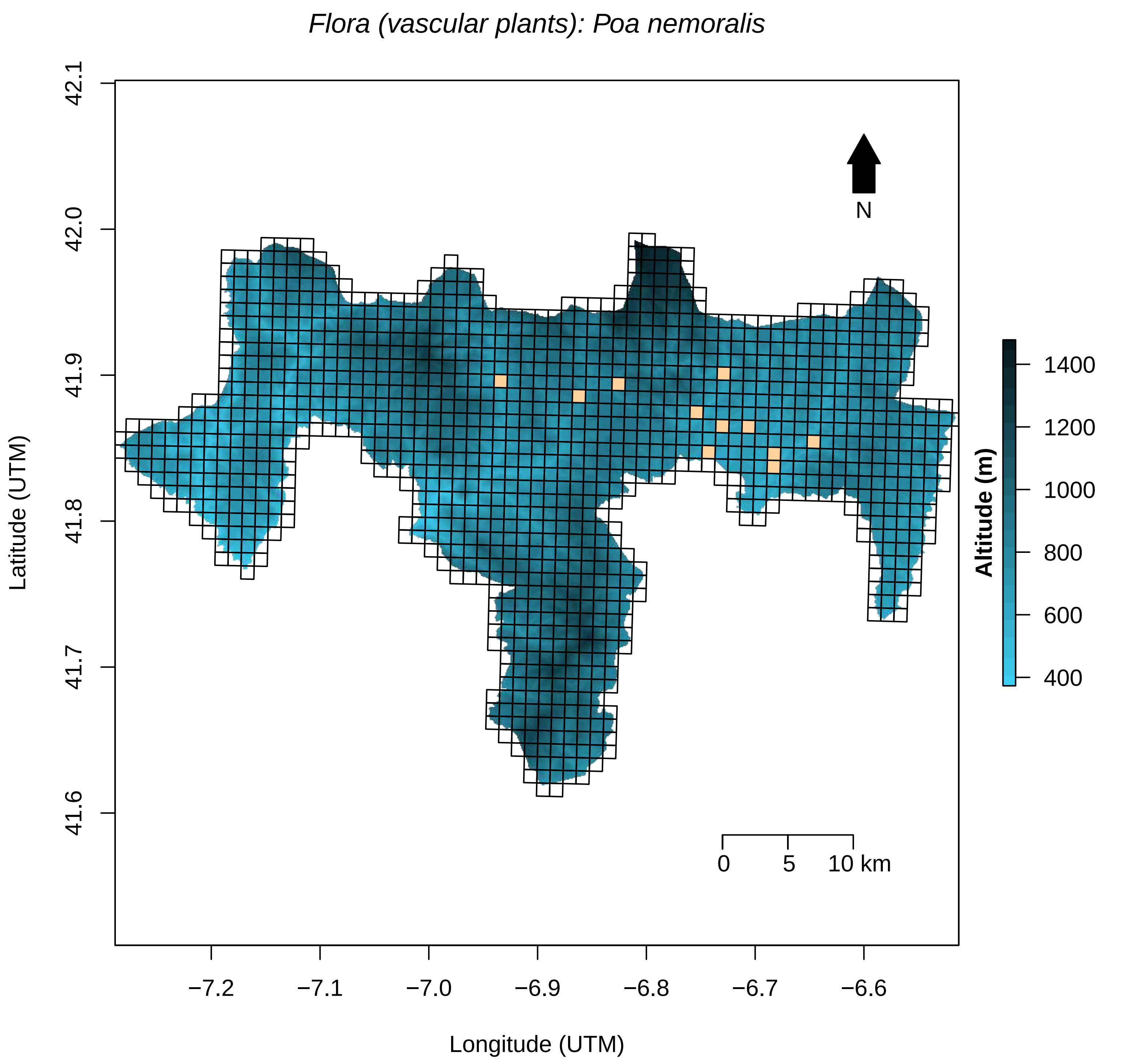

Flora (vascular plants): Poa nemoralis
42.1
42.0
41.9
41.8
41.7
41.6
Latitude (UTM)
−7.2
−7.1
−7.0
−6.9
−6.8
−6.7
−6.6
Longitude (UTM)
N
0
5
10 km
1400
1200
1000
800
600
400
Altitude (m)

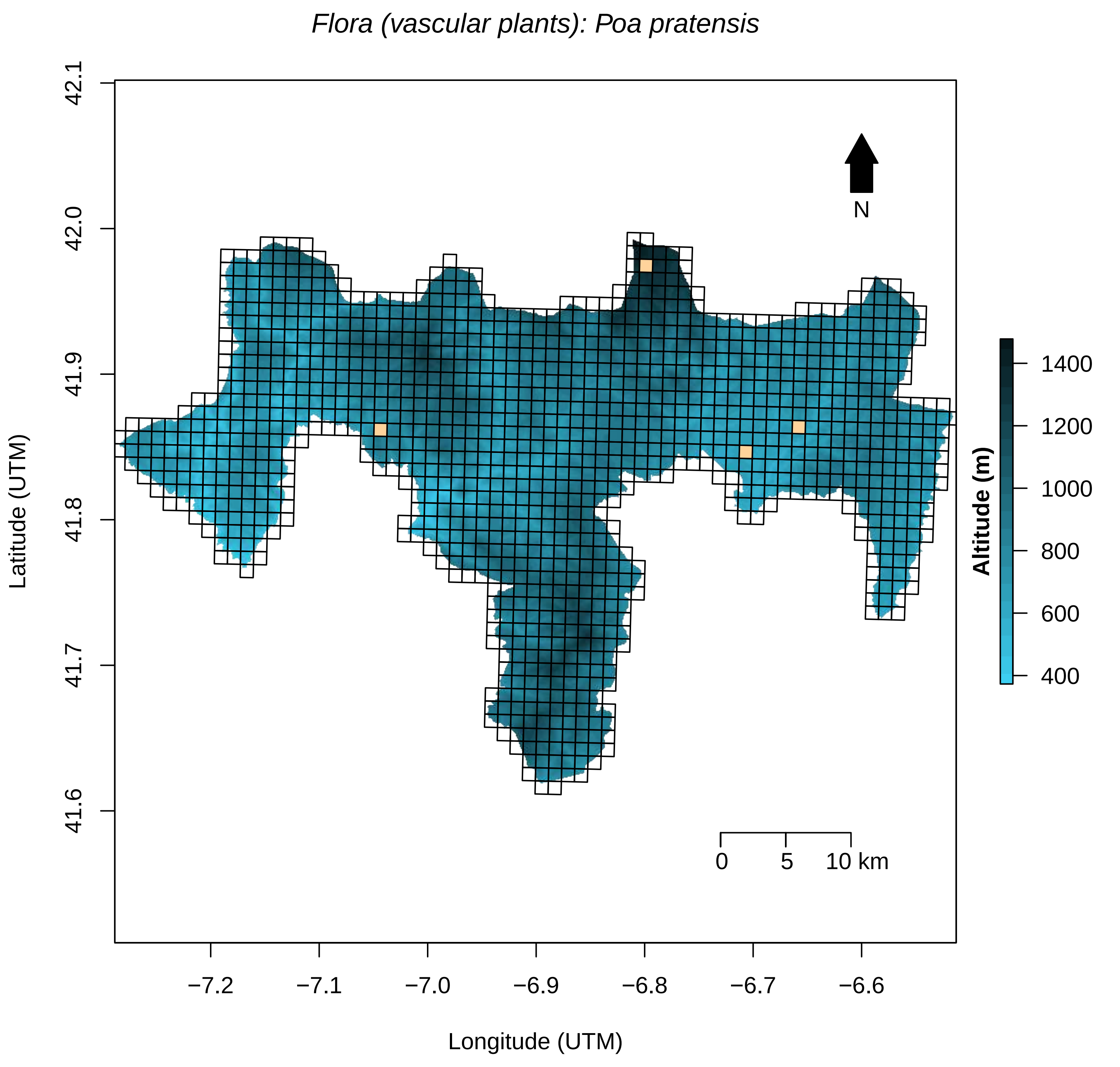

Flora (vascular plants): Poa pratensis
N
0
5
10 km
Altitude (m)
1400
1200
1000
800
600
400
Latitude (UTM)
42.1
42.0
41.9
41.8
41.7
41.6
Longitude (UTM)
−7.2
−7.1
−7.0
−6.9
−6.8
−6.7
−6.6

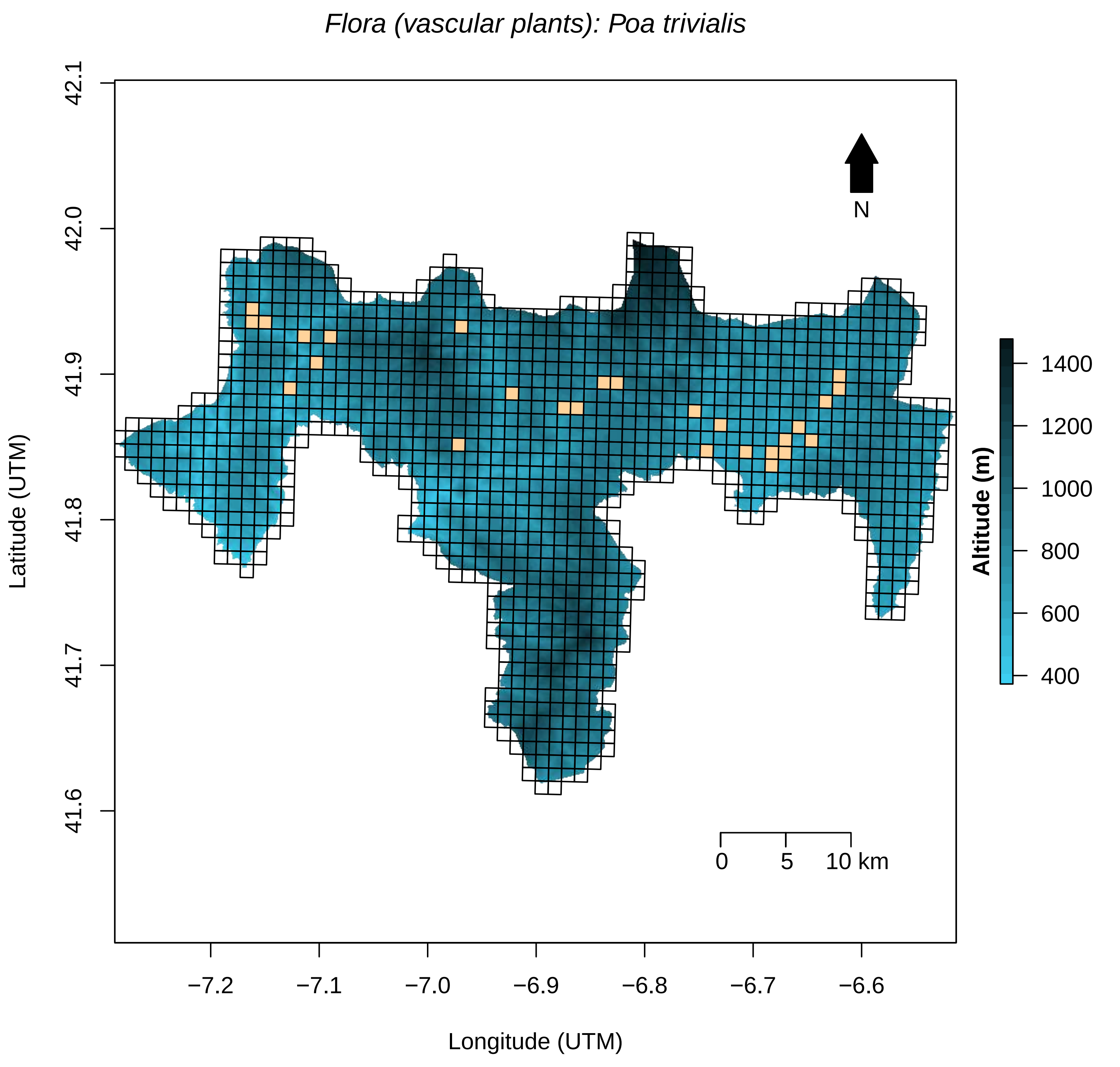

Flora (vascular plants): Poa trivialis
42.1
42.0
41.9
41.8
41.7
41.6
Latitude (UTM)
−7.2
−7.1
−7.0
−6.9
−6.8
−6.7
−6.6
Longitude (UTM)
N
0
5
10 km
1400
1200
1000
800
600
400
Altitude (m)

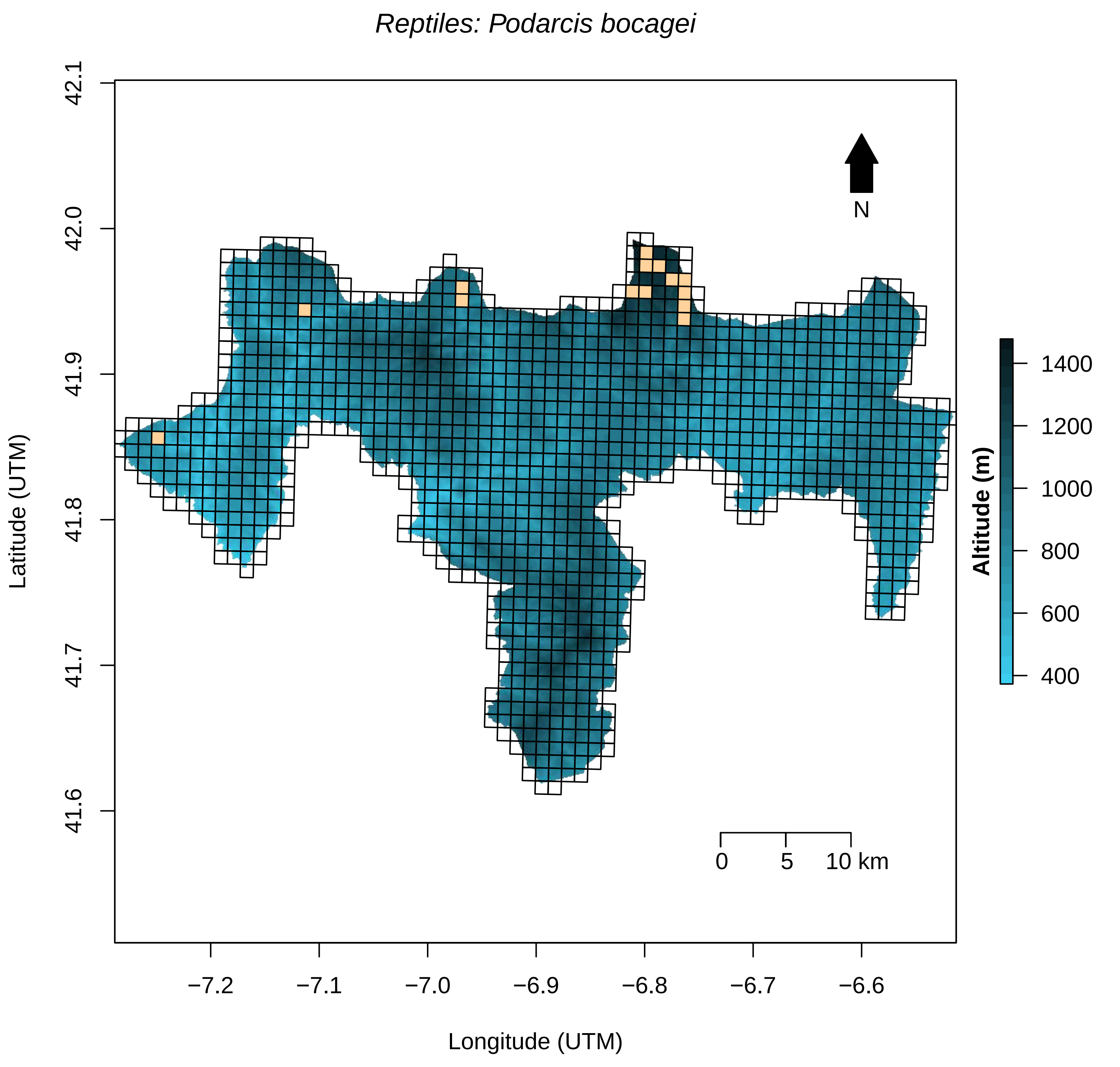

Reptiles: Podarcis bocagei
N
0
5
10 km
Latitude (UTM)
42.1
42.0
41.9
41.8
41.7
41.6
Longitude (UTM)
−7.2
−7.1
−7.0
−6.9
−6.8
−6.7
−6.6
Altitude (m)
1400
1200
1000
800
600
400

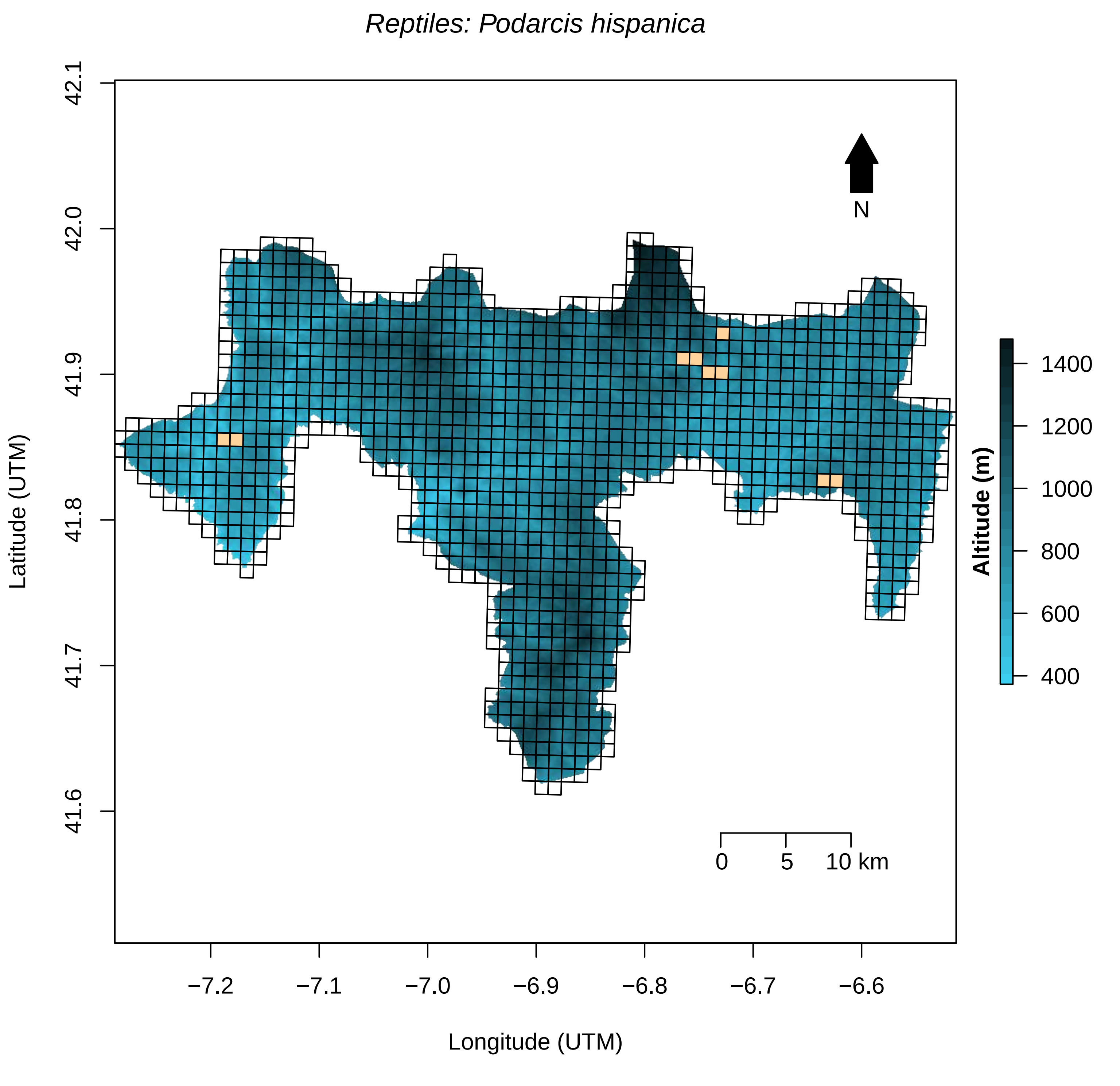

Reptiles: Podarcis hispanica
Latitude (UTM)
42.1
42.0
41.9
41.8
41.7
41.6
Longitude (UTM)
−7.2
−7.1
−7.0
−6.9
−6.8
−6.7
−6.6
N
0
5
10 km
Altitude (m)
1400
1200
1000
800
600
400

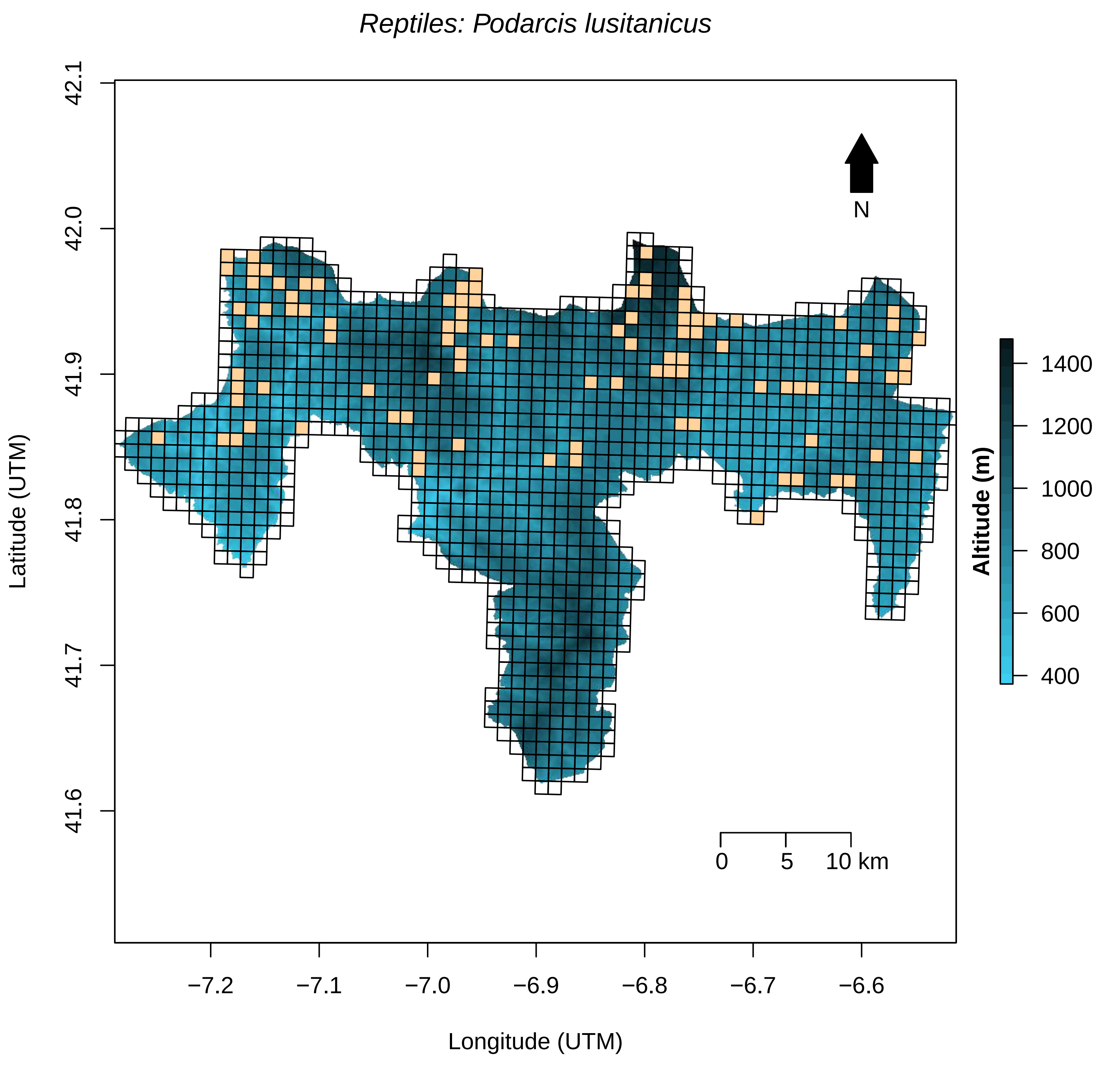
Reptiles: Podarcis lusitanicus
42.1
42.0
41.9
41.8
41.7
41.6
Latitude (UTM)
−7.2
−7.1
−7.0
−6.9
−6.8
−6.7
−6.6
Longitude (UTM)
N
0
5
10 km
Altitude (m)
1400
1200
1000
800
600
400

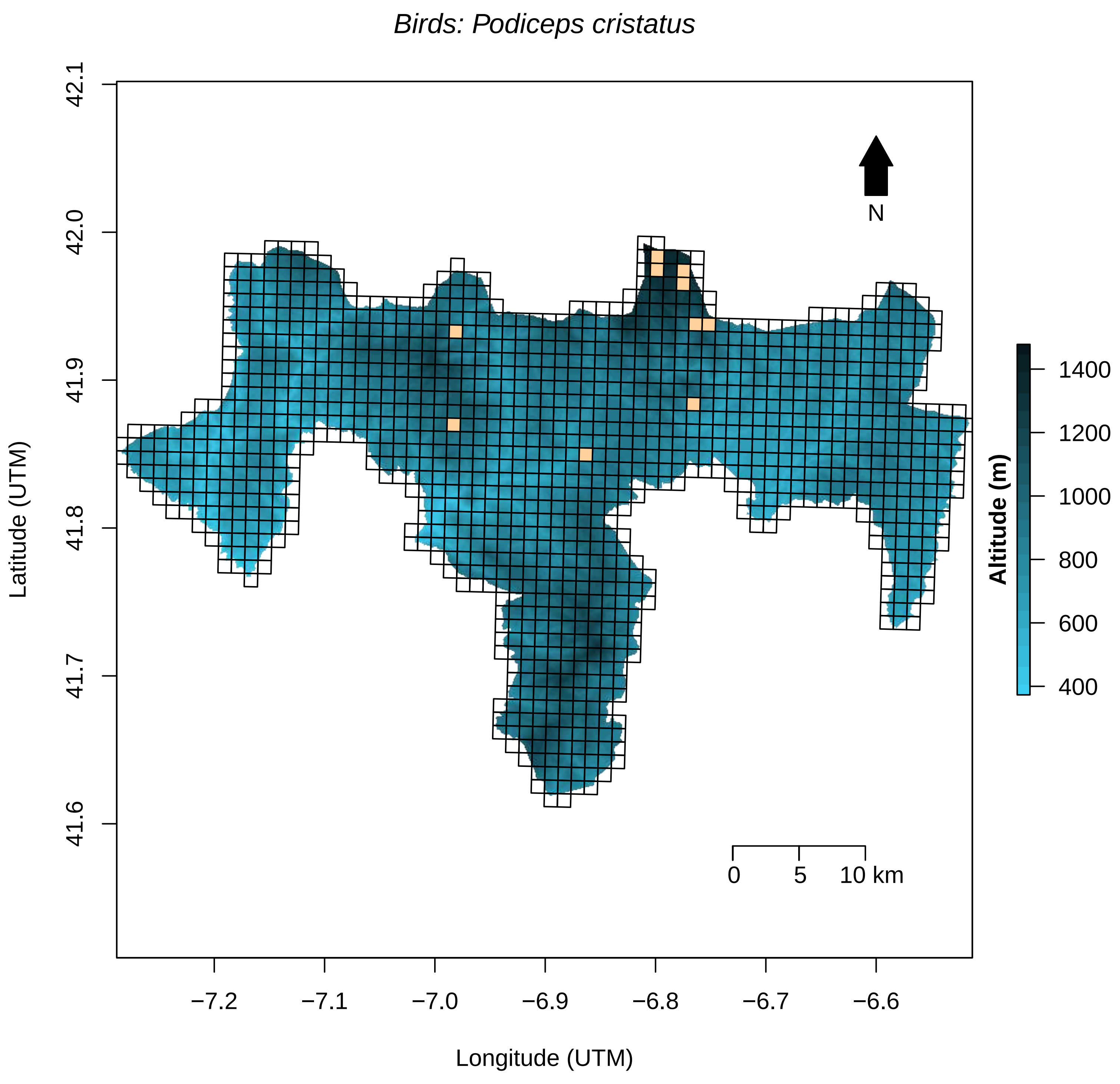

Birds: Podiceps cristatus
Latitude (UTM)
Longitude (UTM)
42.1
42.0
41.9
41.8
41.7
41.6
−7.2
−7.1
−7.0
−6.9
−6.8
−6.7
−6.6
N
0
5
10 km
Altitude (m)
1400
1200
1000
800
600
400

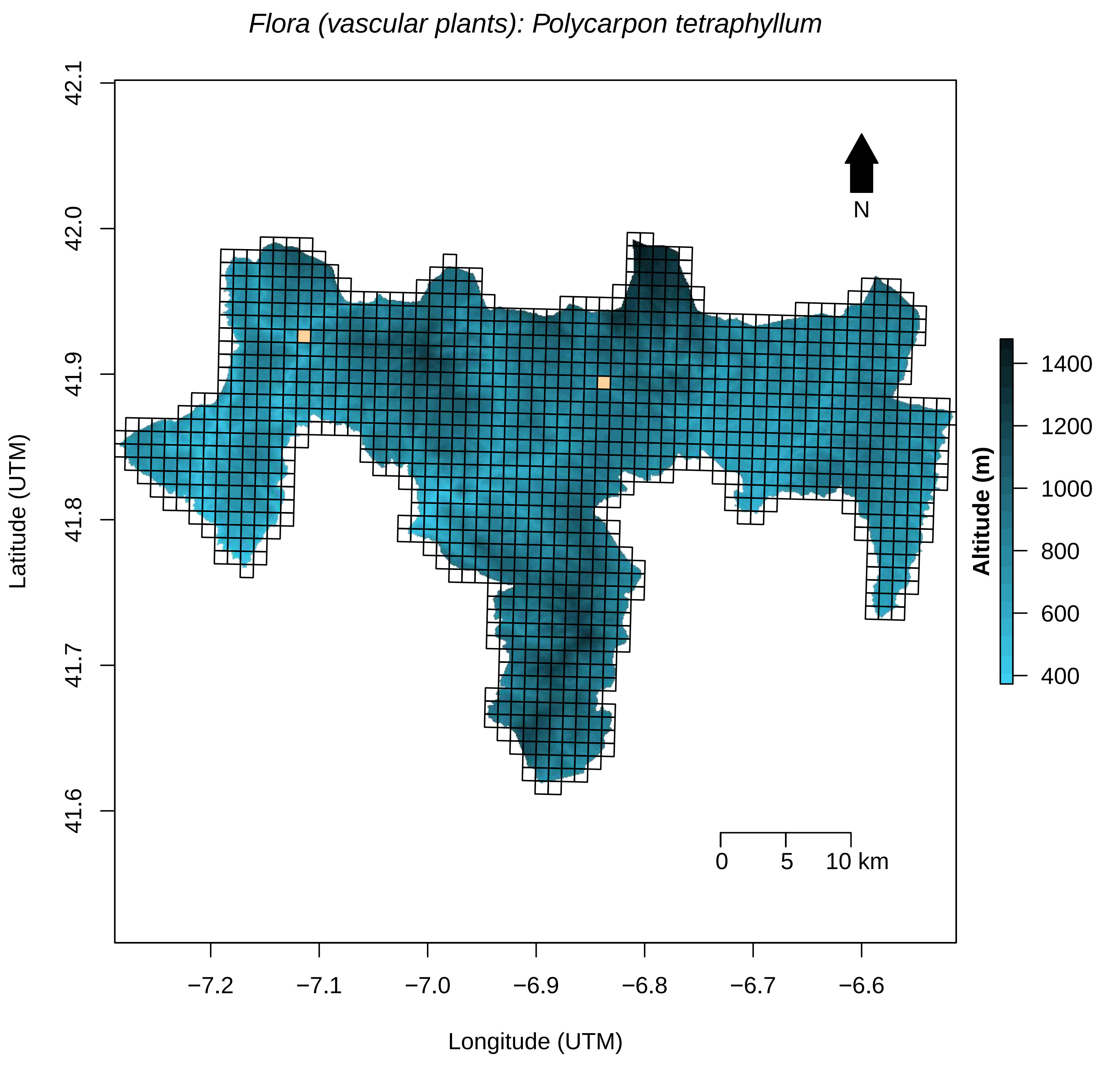

Flora (vascular plants): Polycarpon tetraphyllum
N
0 5 10 km
Altitude (m)
1400
1200
1000
800
600
400
42.1
42.0
41.9
41.8
41.7
41.6
−7.2
−7.1
−7.0
−6.9
−6.8
−6.7
−6.6
Latitude (UTM)
Longitude (UTM)

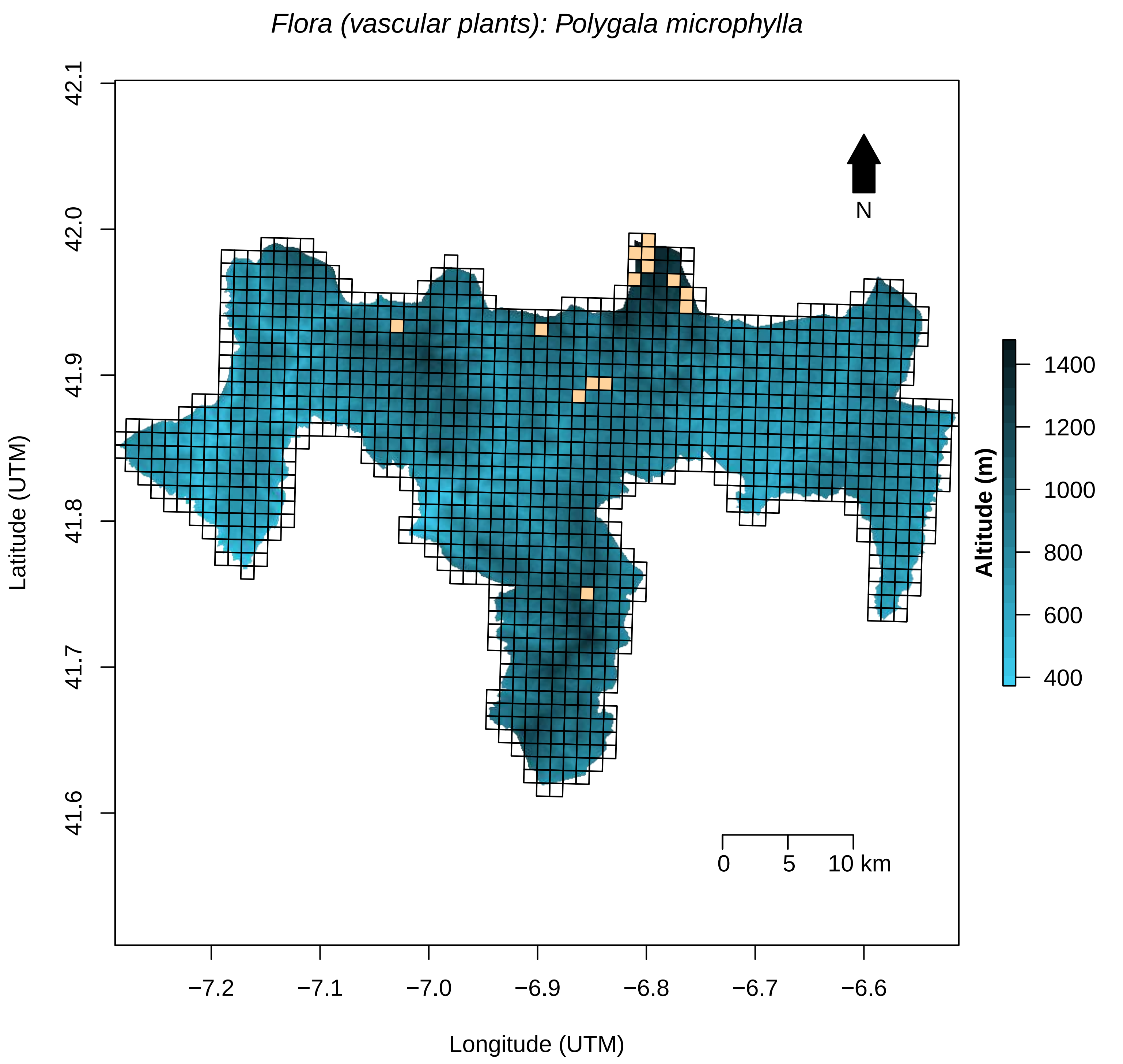

Flora (vascular plants): Polygala microphylla
42.1
42.0
41.9
41.8
41.7
41.6
Latitude (UTM)
−7.2
−7.1
−7.0
−6.9
−6.8
−6.7
−6.6
Longitude (UTM)
N
0
5
10 km
Altitude (m)
1400
1200
1000
800
600
400

Flora (vascular plants): Polygala serpyllifolia

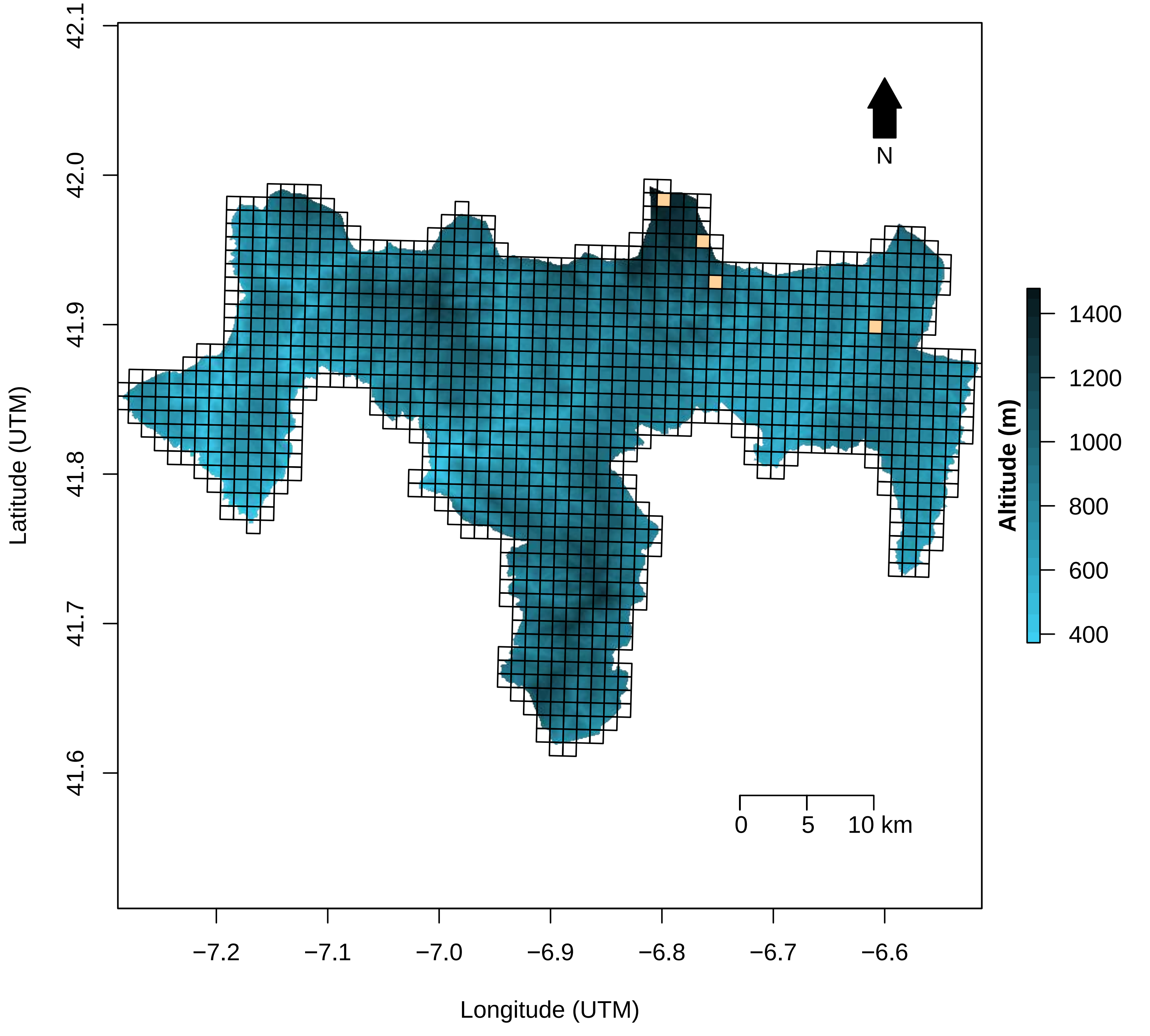

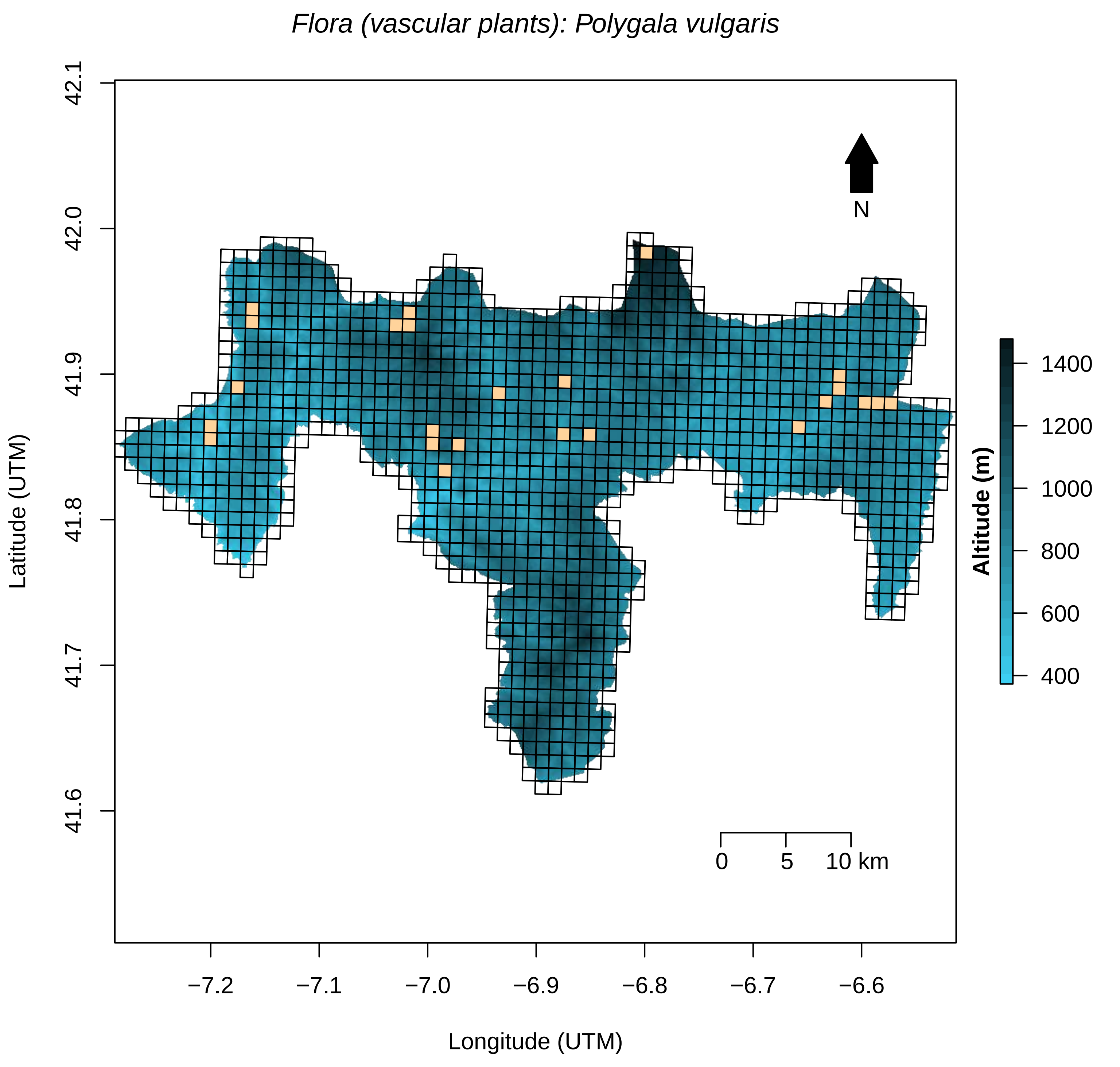

Flora (vascular plants): Polygala vulgaris
N
0
5
10 km
Altitude (m)
1400
1200
1000
800
600
400
Latitude (UTM)
42.1
42.0
41.9
41.8
41.7
41.6
−7.2
−7.1
−7.0
−6.9
−6.8
−6.7
−6.6
Longitude (UTM)

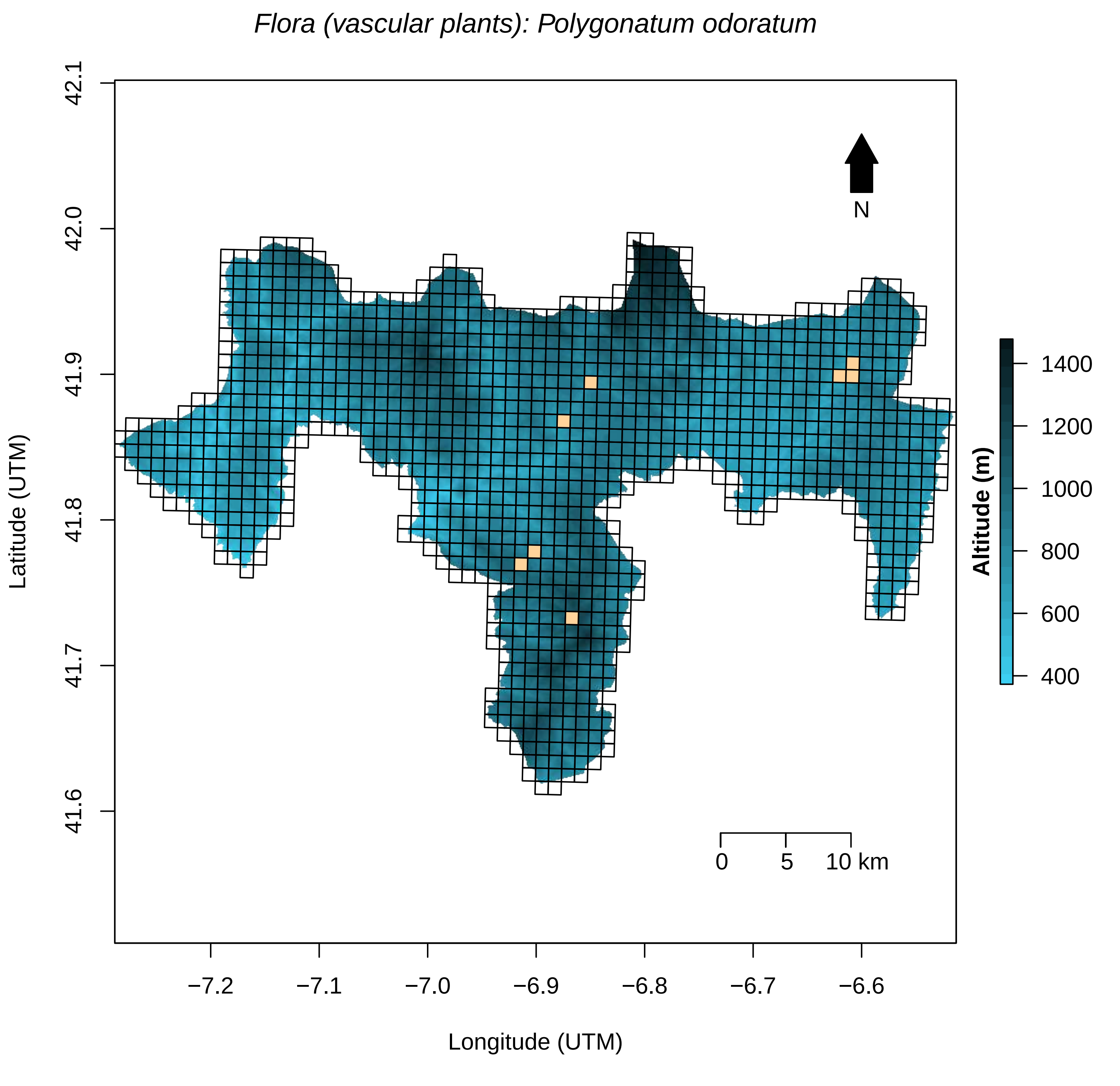

Flora (vascular plants): Polygonatum odoratum
N
0
5
10 km
Altitude (m)
1400
1200
1000
800
600
400
42.1
42.0
41.9
41.8
41.7
41.6
Latitude (UTM)
−7.2
−7.1
−7.0
−6.9
−6.8
−6.7
−6.6
Longitude (UTM)

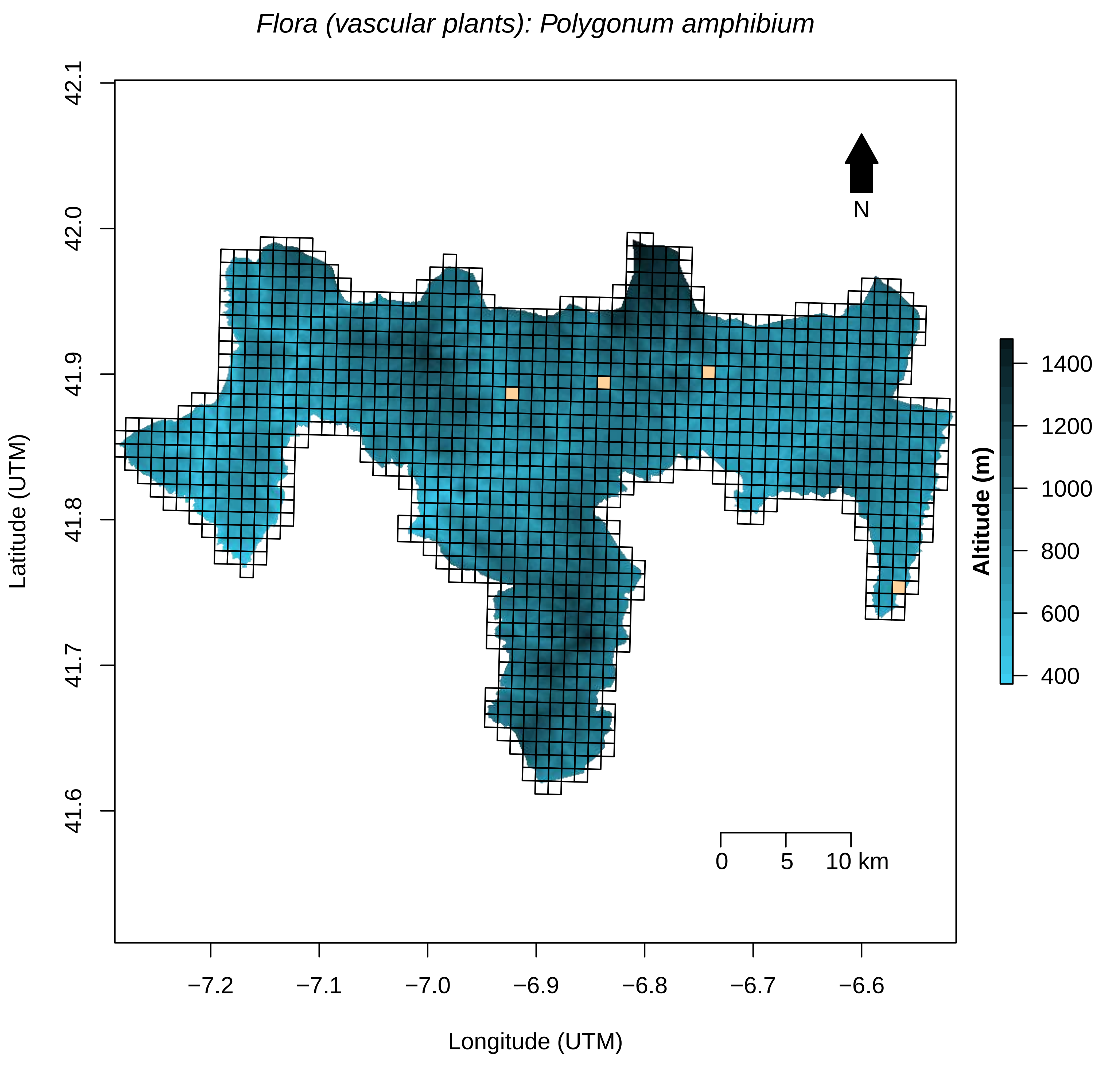

Flora (vascular plants): Polygonum amphibium
42.1
42.0
41.9
41.8
41.7
41.6
Latitude (UTM)
−7.2
−7.1
−7.0
−6.9
−6.8
−6.7
−6.6
Longitude (UTM)
N
0
5
10 km
1400
1200
1000
800
600
400
Altitude (m)

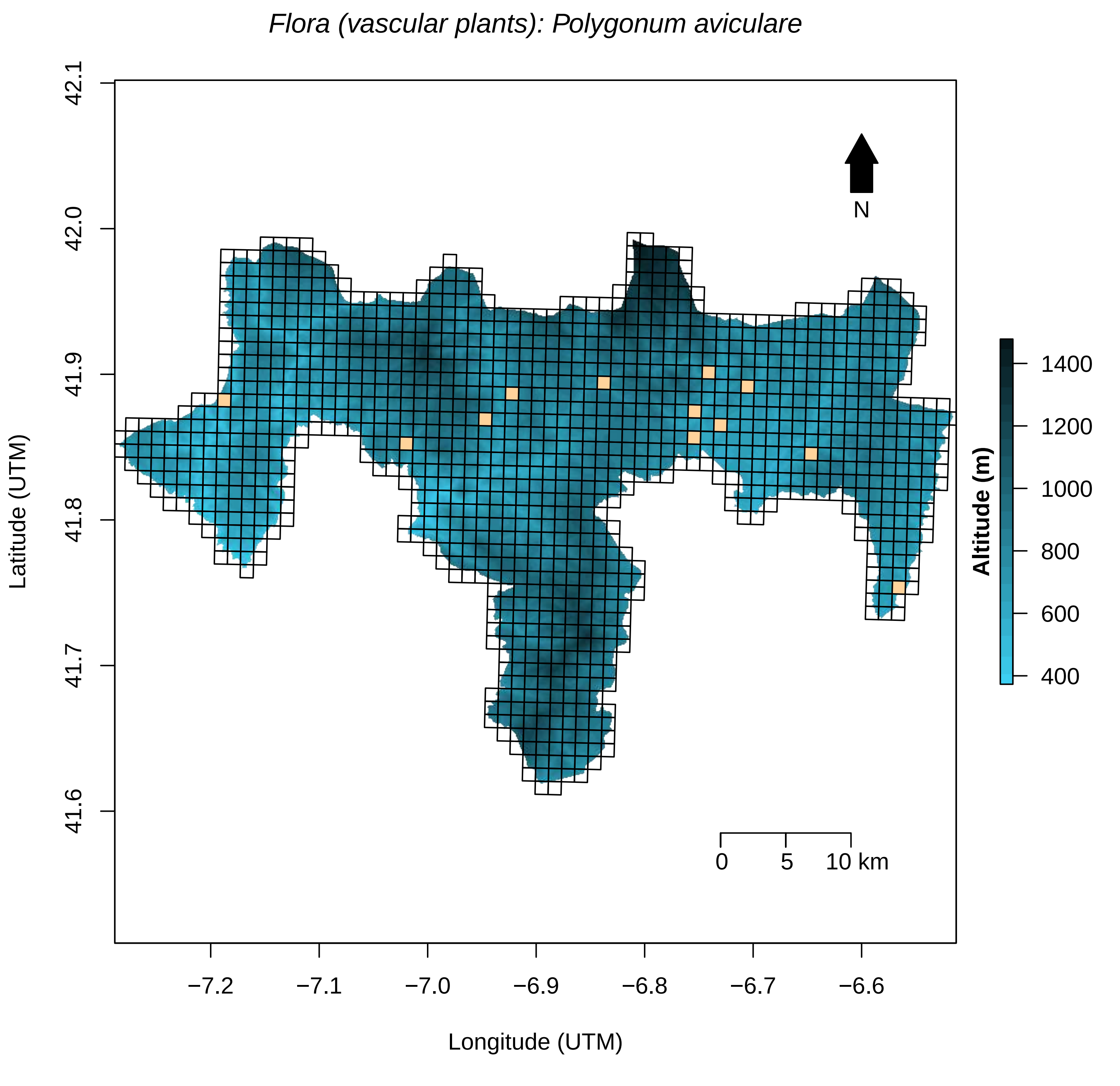

Flora (vascular plants): Polygonum aviculare
N
0 5 10 km
Altitude (m)
1400
1200
1000
800
600
400
Latitude (UTM)
42.1
42.0
41.9
41.8
41.7
41.6
Longitude (UTM)
−7.2
−7.1
−7.0
−6.9
−6.8
−6.7
−6.6

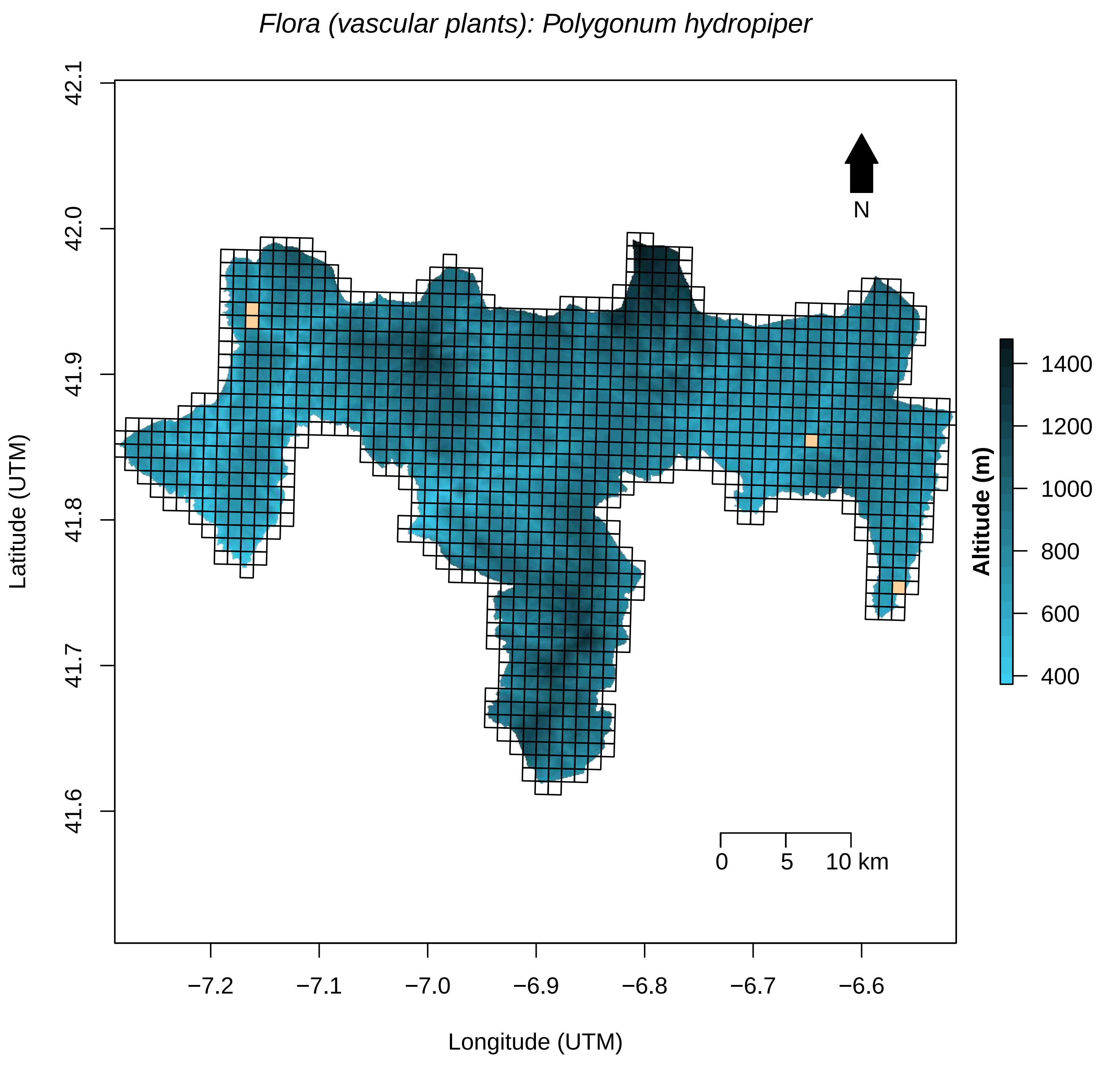

Flora (vascular plants): Polygonum hydropiper
42.1
42.0
41.9
41.8
41.7
41.6
Latitude (UTM)
−7.2
−7.1
−7.0
−6.9
−6.8
−6.7
−6.6
Longitude (UTM)
N
0
5
10 km
Altitude (m)
1400
1200
1000
800
600
400

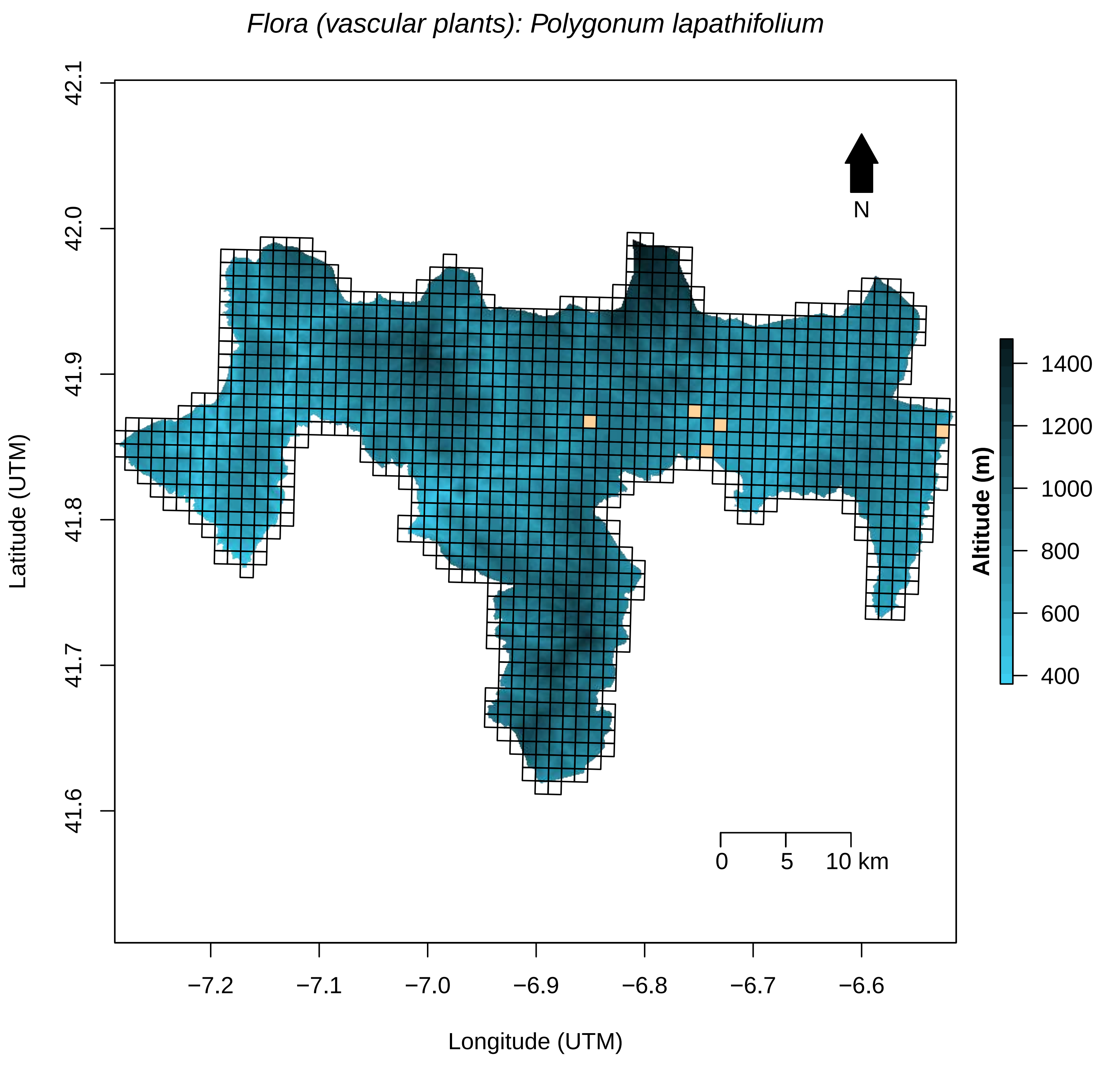

Flora (vascular plants): Polygonum lapathifolium
N
0
5
10 km
Altitude (m)
1400
1200
1000
800
600
400
Latitude (UTM)
42.1
42.0
41.9
41.8
41.7
41.6
Longitude (UTM)
−7.2
−7.1
−7.0
−6.9
−6.8
−6.7
−6.6

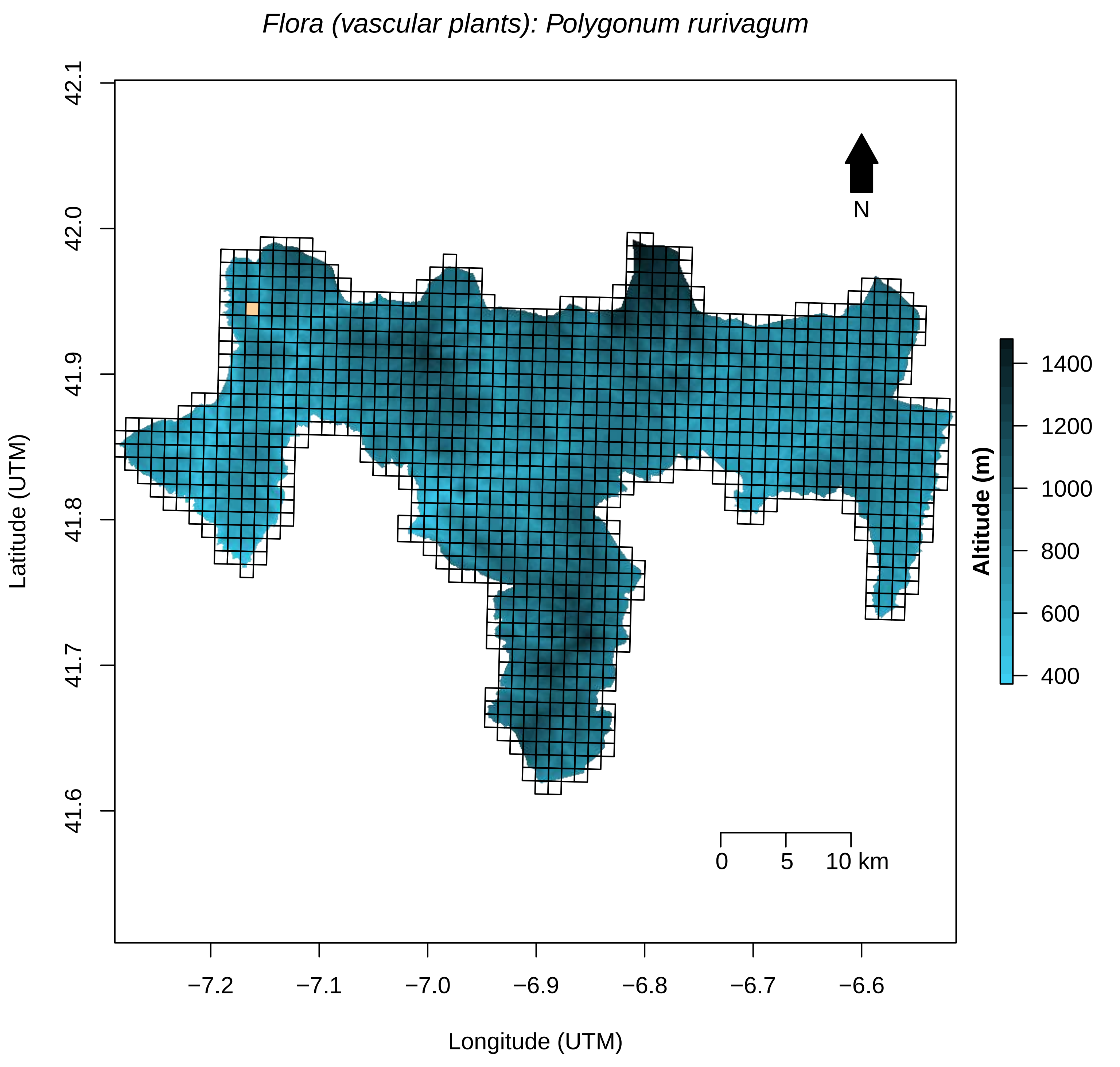

Flora (vascular plants): Polygonum rurivagum
N
0
5
10 km
Altitude (m)
1400
1200
1000
800
600
400
Latitude (UTM)
42.1
42.0
41.9
41.8
41.7
41.6
Longitude (UTM)
−7.2
−7.1
−7.0
−6.9
−6.8
−6.7
−6.6

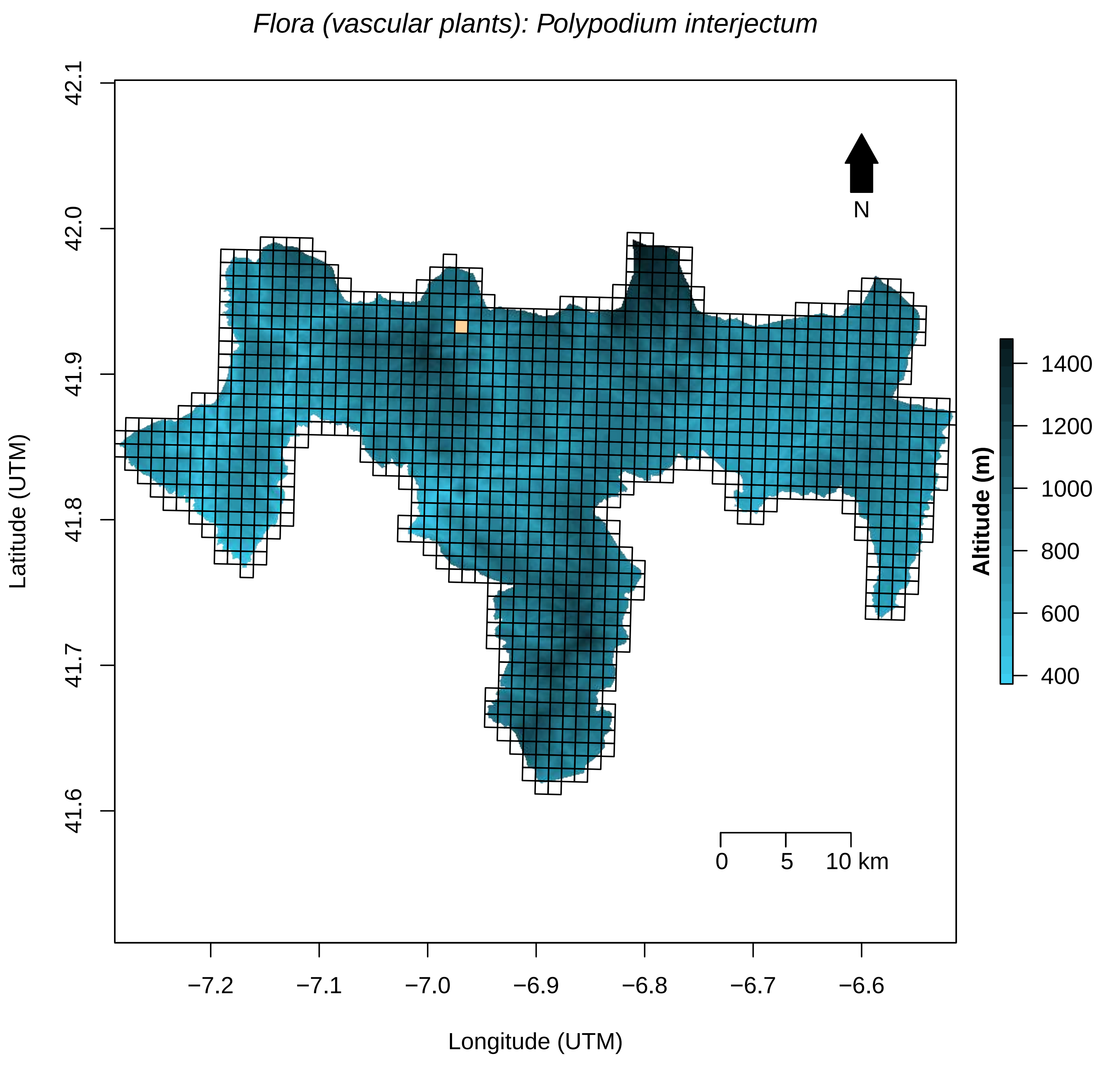
Flora (vascular plants): Polypodium interjectum
Latitude (UTM)
Longitude (UTM)
42.1
42.0
41.9
41.8
41.7
41.6
−7.2
−7.1
−7.0
−6.9
−6.8
−6.7
−6.6
N
0
5
10 km
Altitude (m)
1400
1200
1000
800
600
400

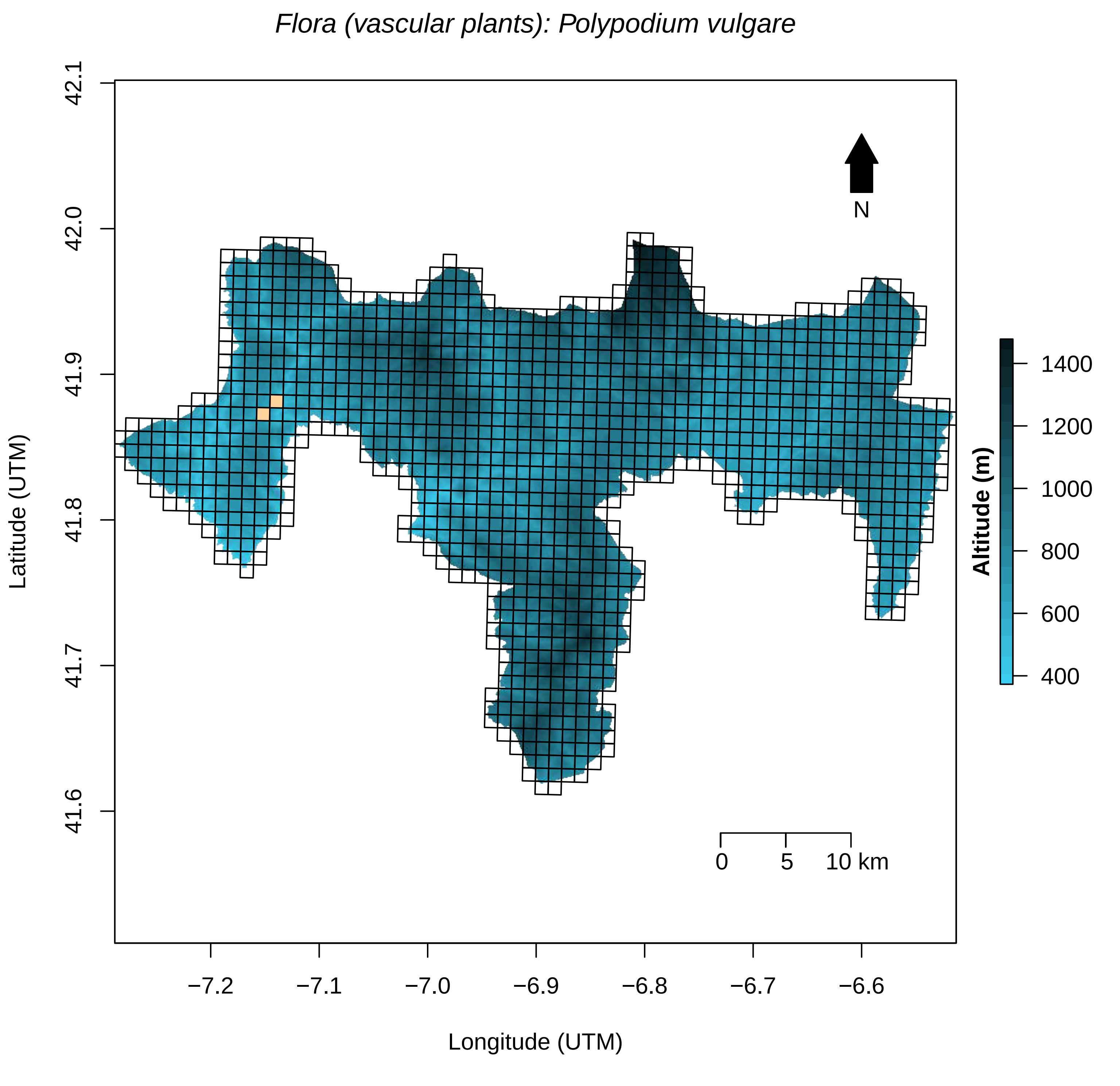

Flora (vascular plants): Polypodium vulgare
N
0
5
10 km
Altitude (m)
1400
1200
1000
800
600
400
Latitude (UTM)
42.1
42.0
41.9
41.8
41.7
41.6
Longitude (UTM)
−7.2
−7.1
−7.0
−6.9
−6.8
−6.7
−6.6

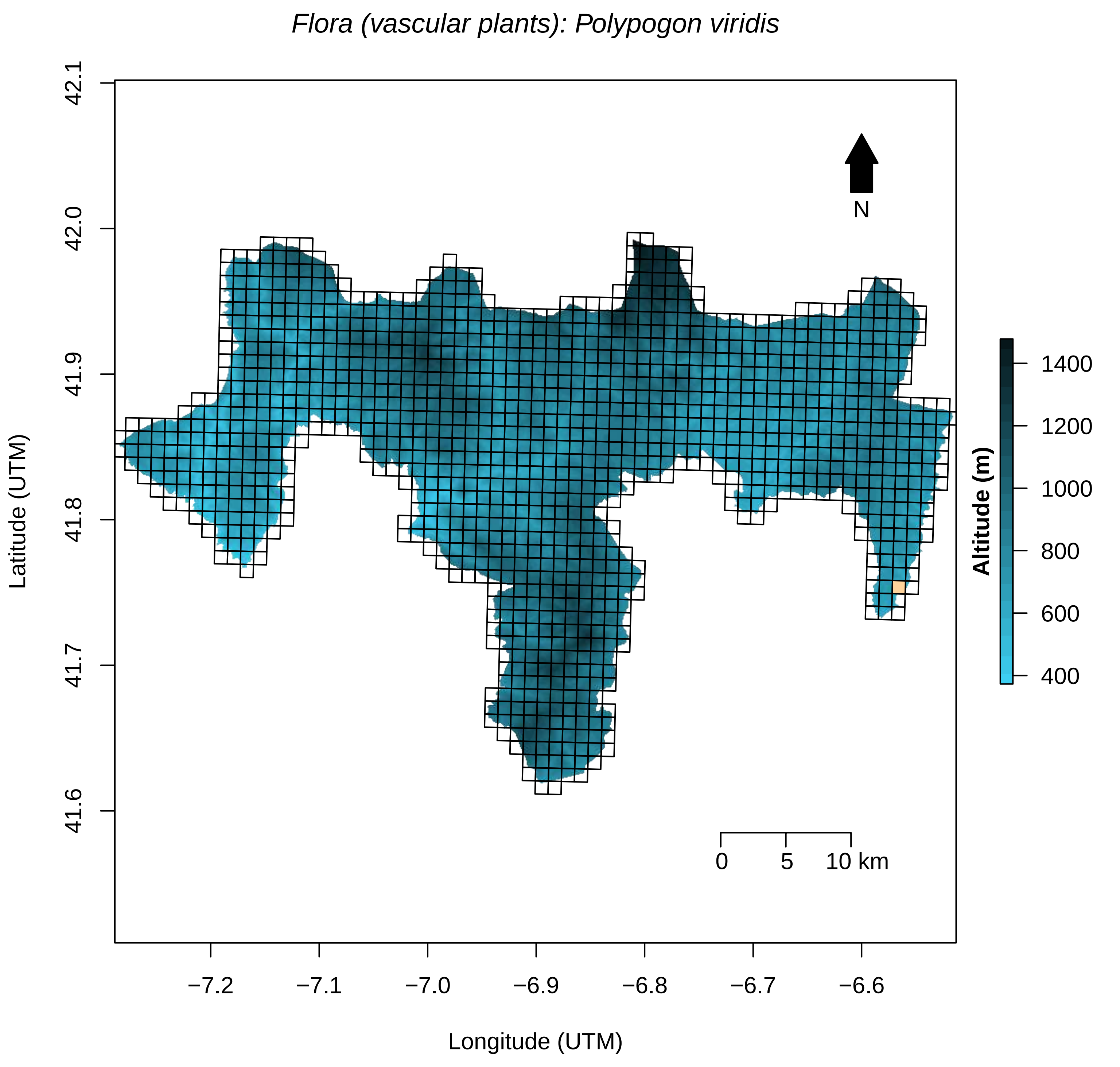
Flora (vascular plants): Polypogon viridis
42.1
42.0
41.9
41.8
41.7
41.6
Latitude (UTM)
N
0
5
10 km
Altitude (m)
1400
1200
1000
800
600
400
−7.2
−7.1
−7.0
−6.9
−6.8
−6.7
−6.6
Longitude (UTM)

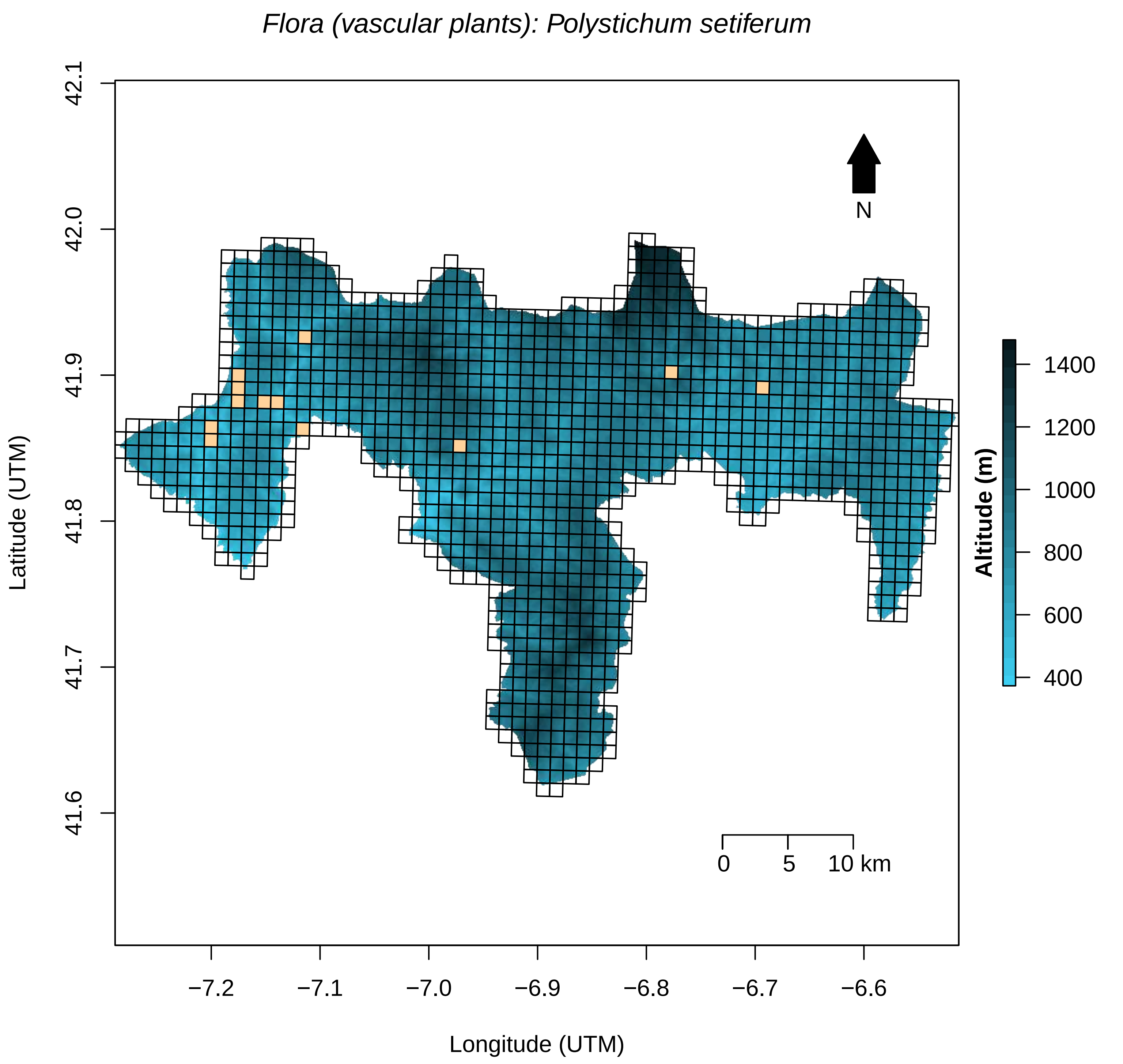

Flora (vascular plants): Polystichum setiferum
42.1
42.0
41.9
41.8
41.7
41.6
Latitude (UTM)
−7.2
−7.1
−7.0
−6.9
−6.8
−6.7
−6.6
Longitude (UTM)
N
0
5
10 km
1400
1200
1000
800
600
400
Altitude (m)

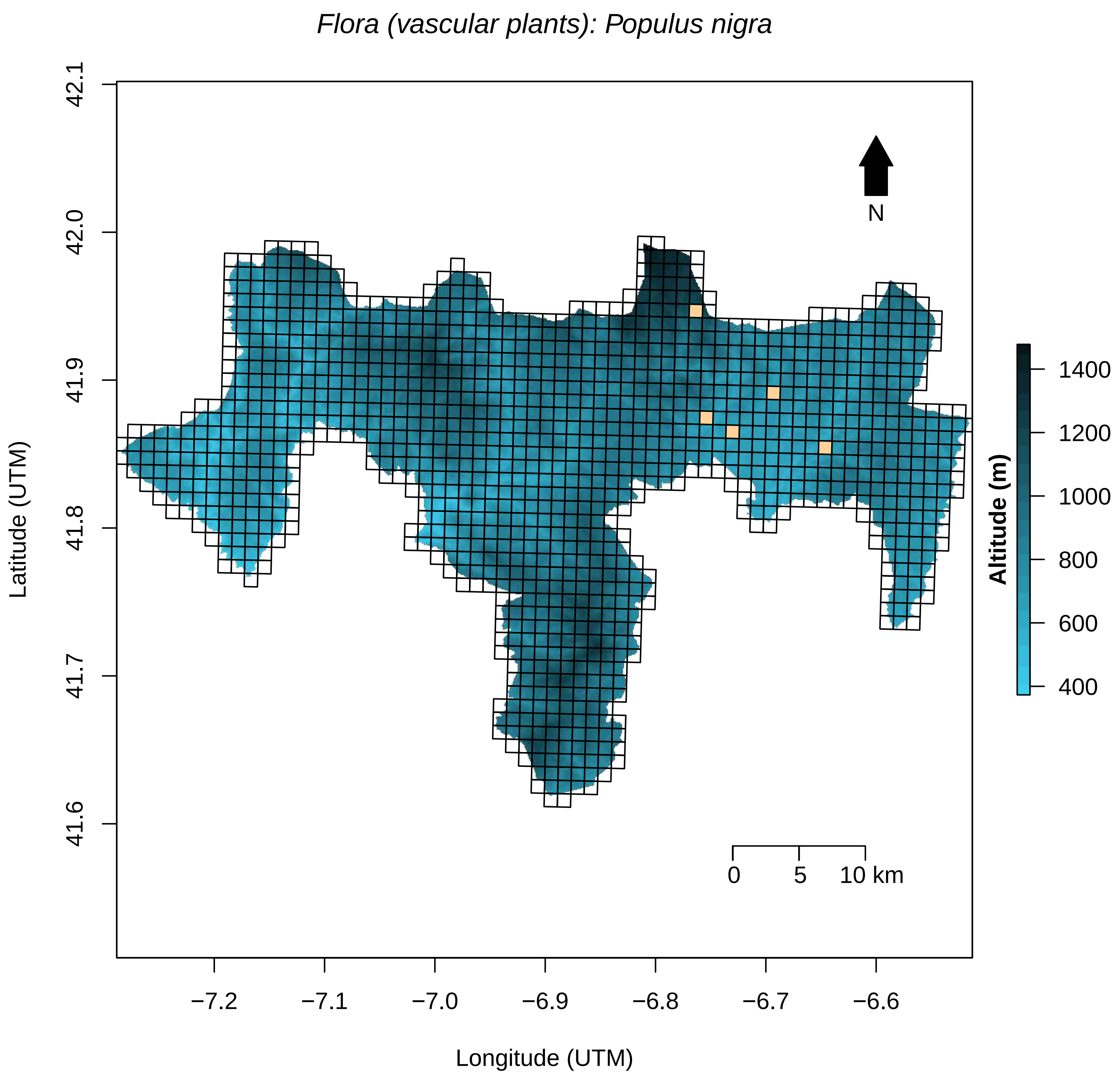

Flora (vascular plants): Populus nigra
N
0
5
10 km
42.1
42.0
41.9
41.8
41.7
41.6
Latitude (UTM)
−7.2
−7.1
−7.0
−6.9
−6.8
−6.7
−6.6
Longitude (UTM)
1400
1200
1000
800
600
400
Altitude (m)

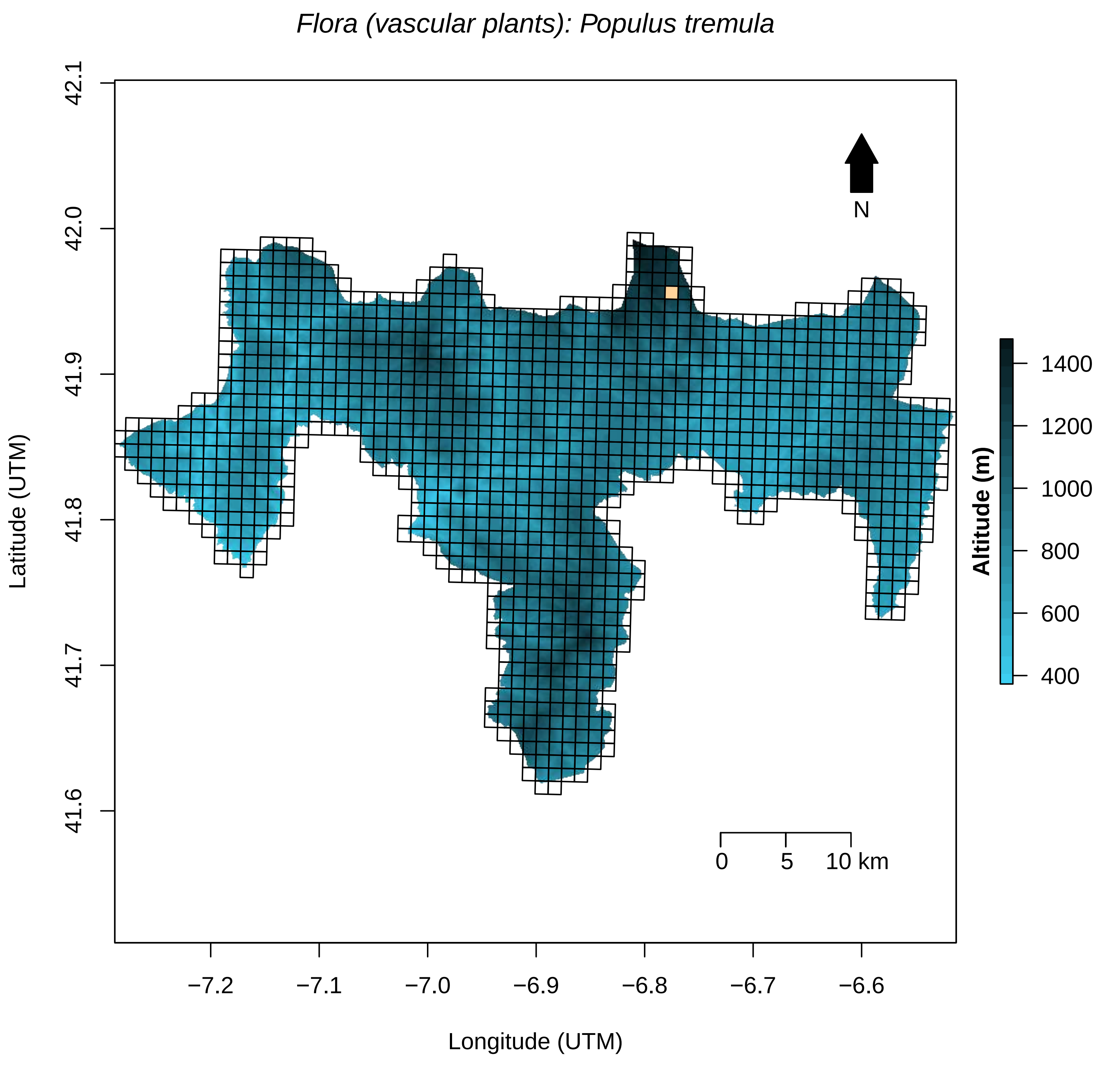

Flora (vascular plants): Populus tremula
Latitude (UTM)
Longitude (UTM)
42.1
42.0
41.9
41.8
41.7
41.6
−7.2
−7.1
−7.0
−6.9
−6.8
−6.7
−6.6
N
0
5
10 km
Altitude (m)
1400
1200
1000
800
600
400

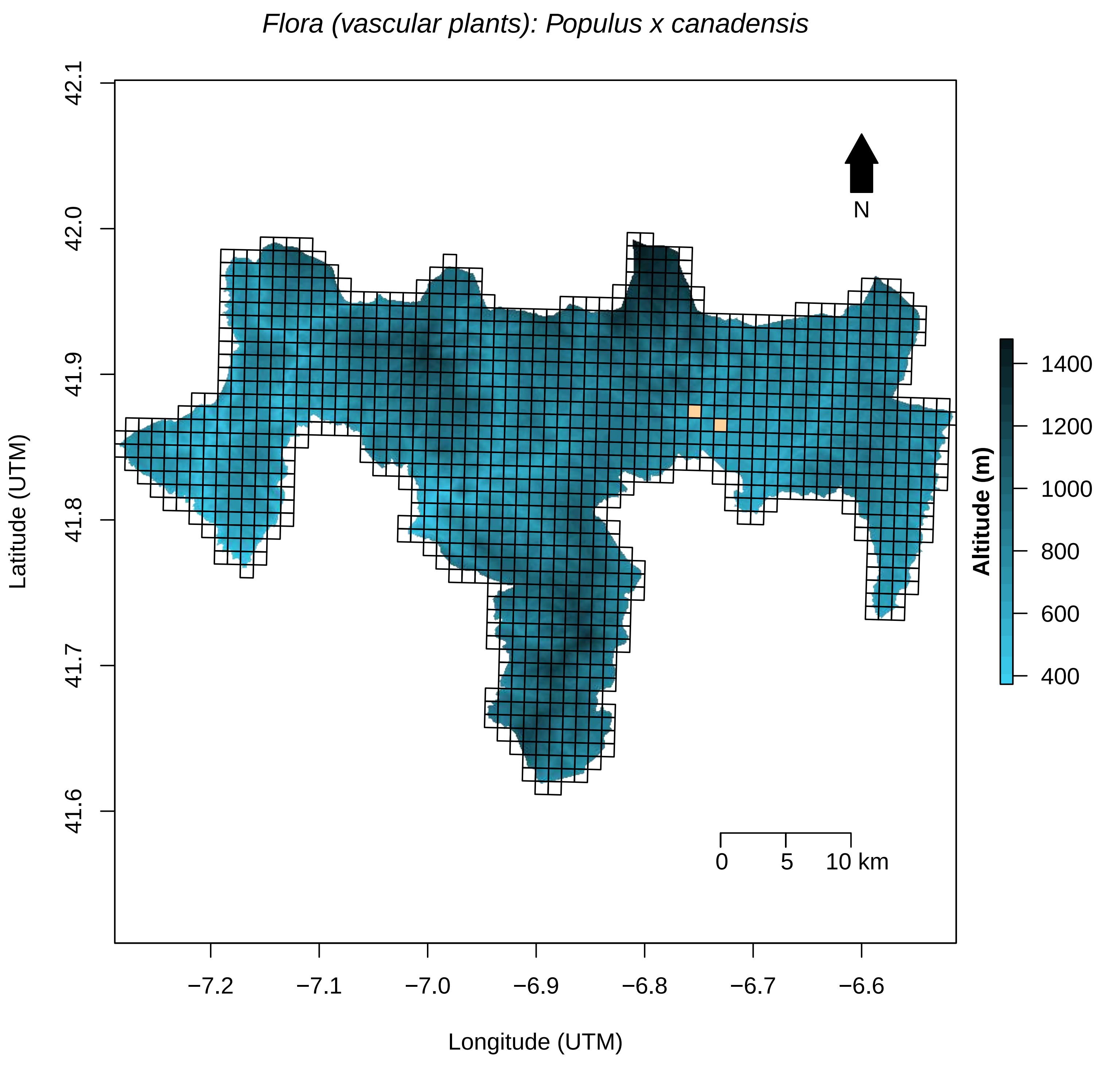
Flora (vascular plants): Populus x canadensis
42.1
42.0
41.9
41.8
41.7
41.6
Latitude (UTM)
−7.2
−7.1
−7.0
−6.9
−6.8
−6.7
−6.6
Longitude (UTM)
N
0
5
10 km
1400
1200
1000
800
600
400
Altitude (m)

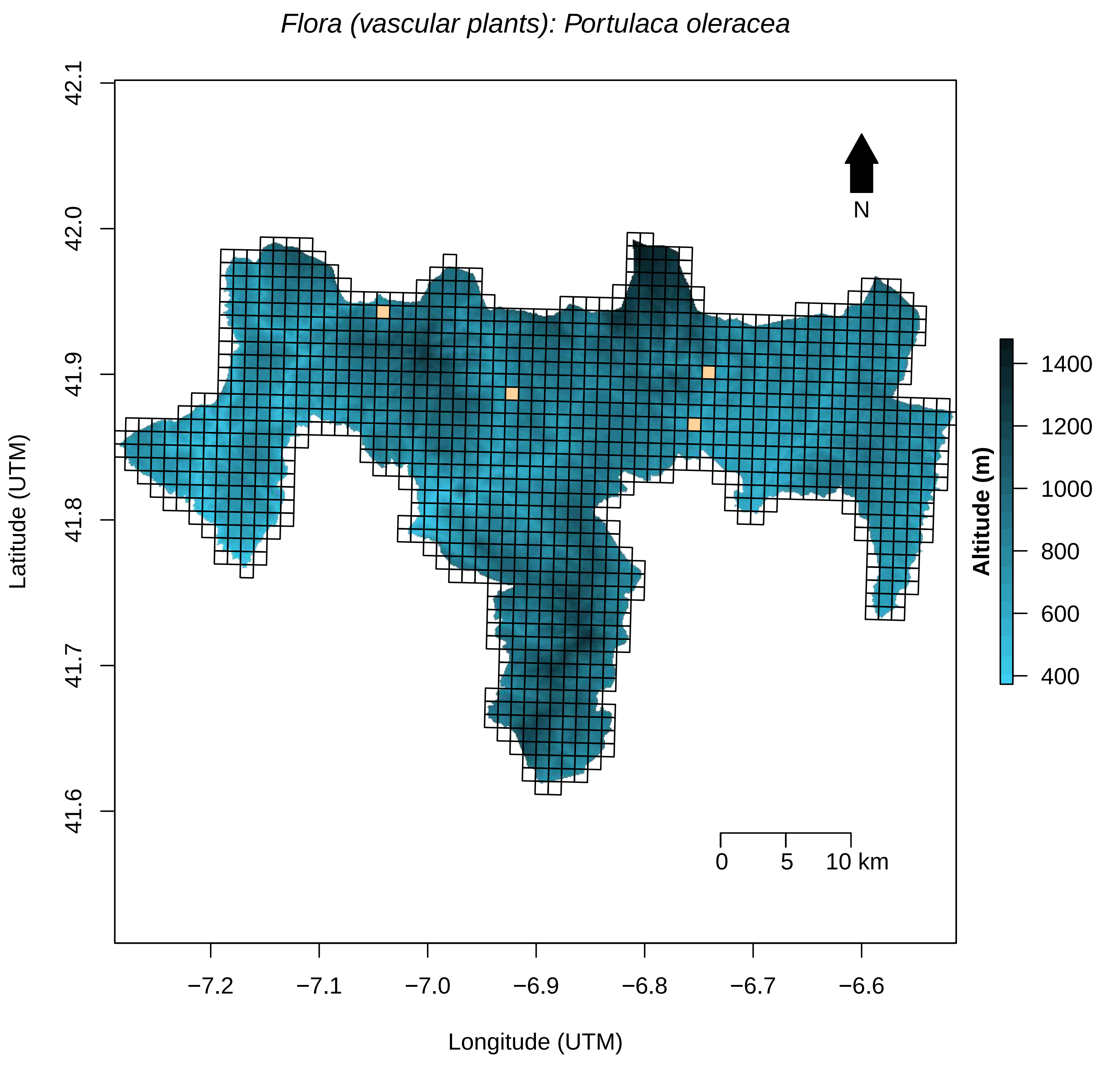

Flora (vascular plants): Portulaca oleracea
N
0 5 10 km
Latitude (UTM)
Longitude (UTM)
Altitude (m)
42.1
42.0
41.9
41.8
41.7
41.6
−7.2
−7.1
−7.0
−6.9
−6.8
−6.7
−6.6
1400
1200
1000
800
600
400

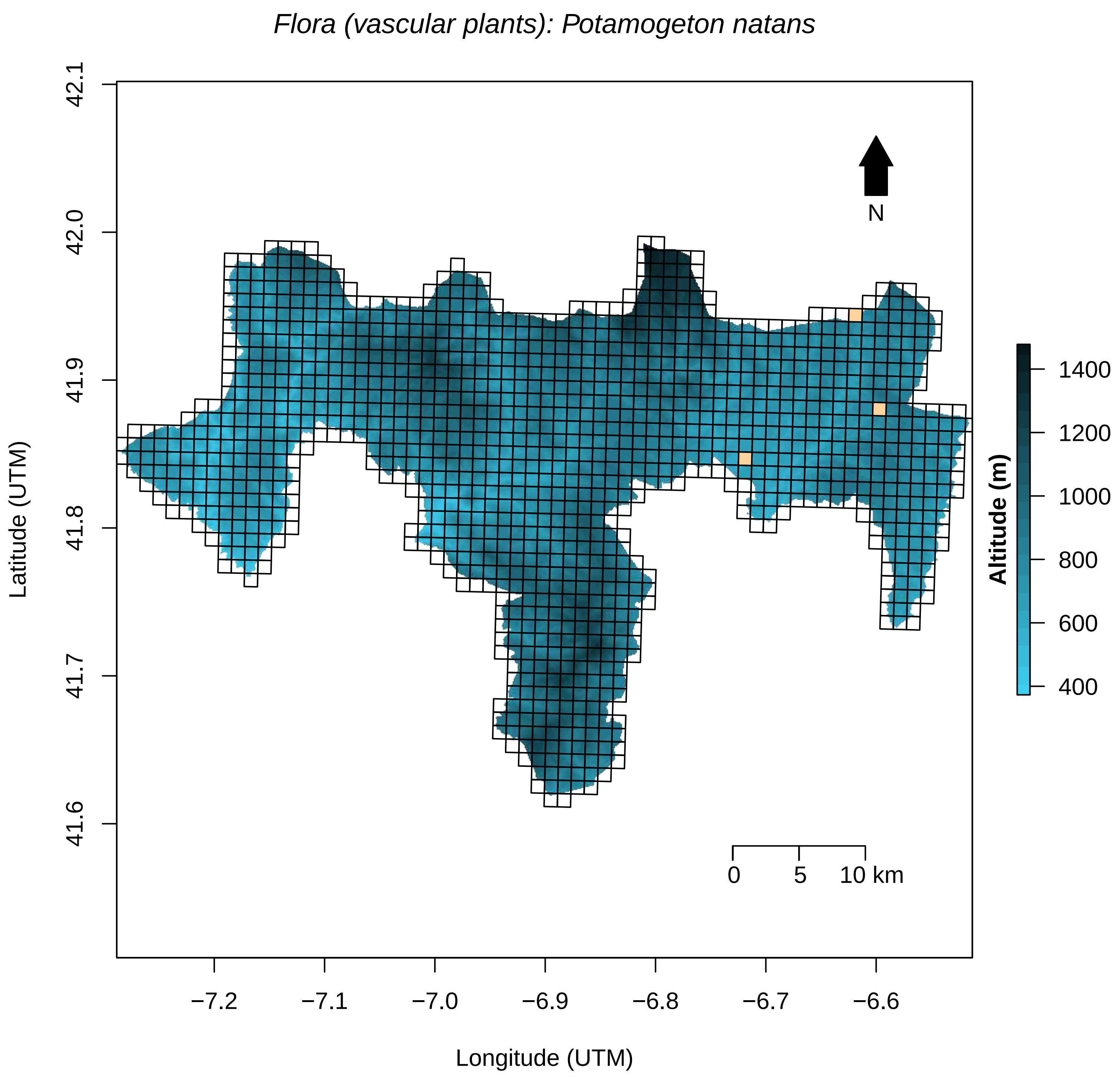
Flora (vascular plants): Potamogeton natans
42.1
42.0
41.9
41.8
41.7
41.6
Latitude (UTM)
−7.2
−7.1
−7.0
−6.9
−6.8
−6.7
−6.6
Longitude (UTM)
N
0
5
10 km
Altitude (m)
1400
1200
1000
800
600
400

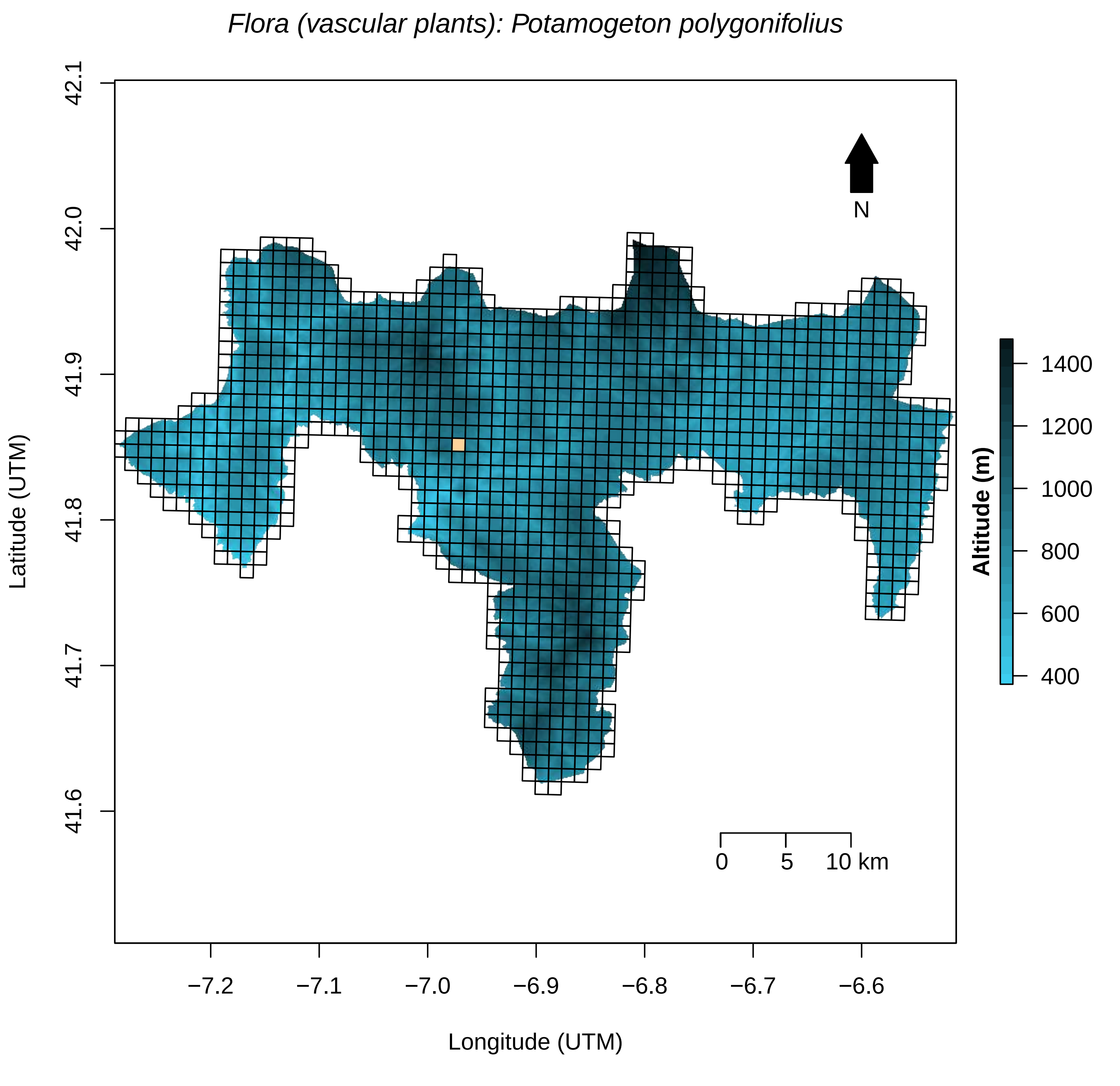

Flora (vascular plants): Potamogeton polygonifolius
42.1
42.0
41.9
41.8
41.7
41.6
Latitude (UTM)
−7.2
−7.1
−7.0
−6.9
−6.8
−6.7
−6.6
Longitude (UTM)
N
0
5
10 km
1400
1200
1000
800
600
400
Altitude (m)

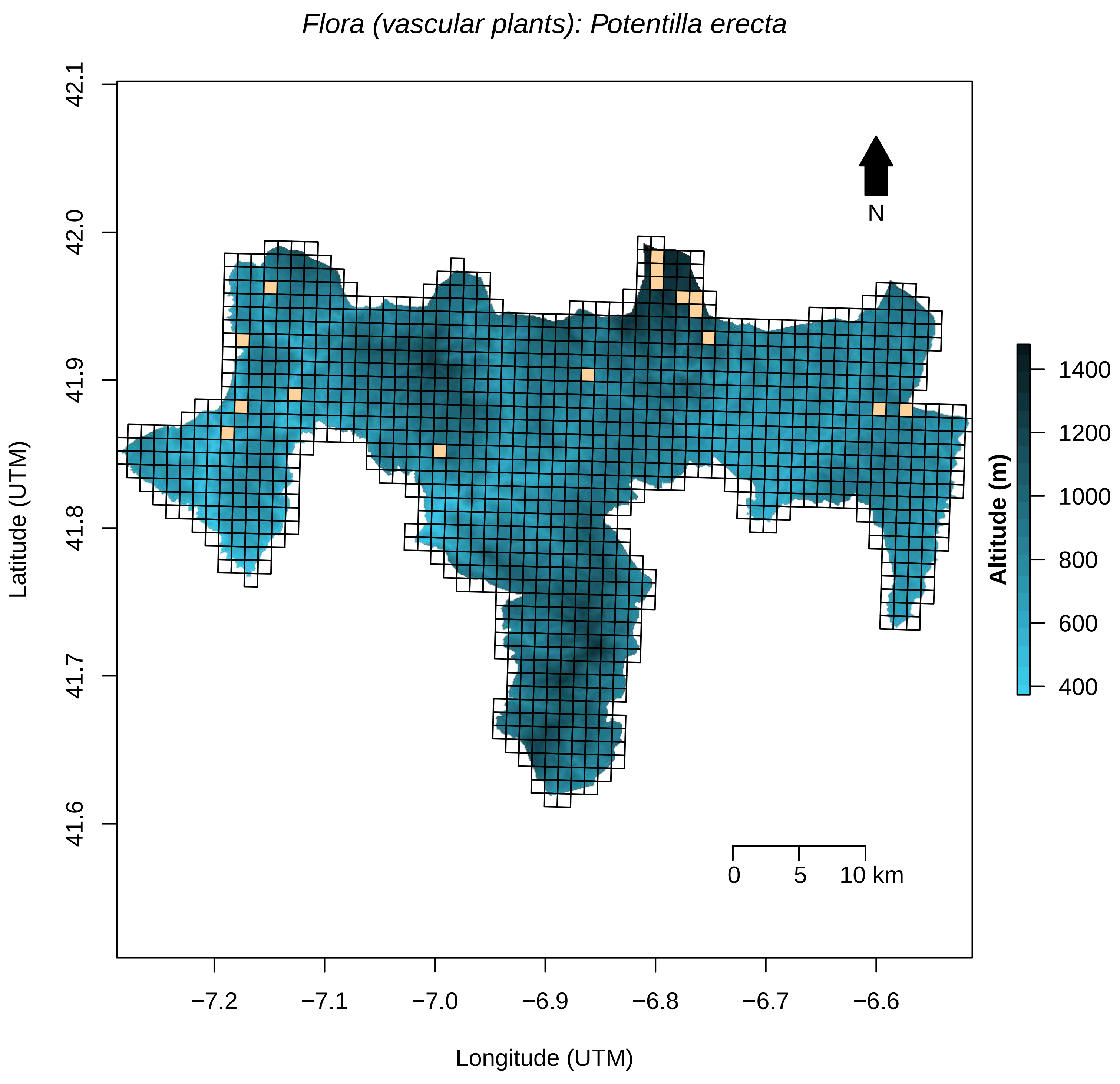

Flora (vascular plants): Potentilla erecta
42.1
42.0
41.9
41.8
41.7
41.6
Latitude (UTM)
−7.2
−7.1
−7.0
−6.9
−6.8
−6.7
−6.6
Longitude (UTM)
N
0
5
10 km
1400
1200
1000
800
600
400
Altitude (m)

Flora (vascular plants): Potentilla neumanniana

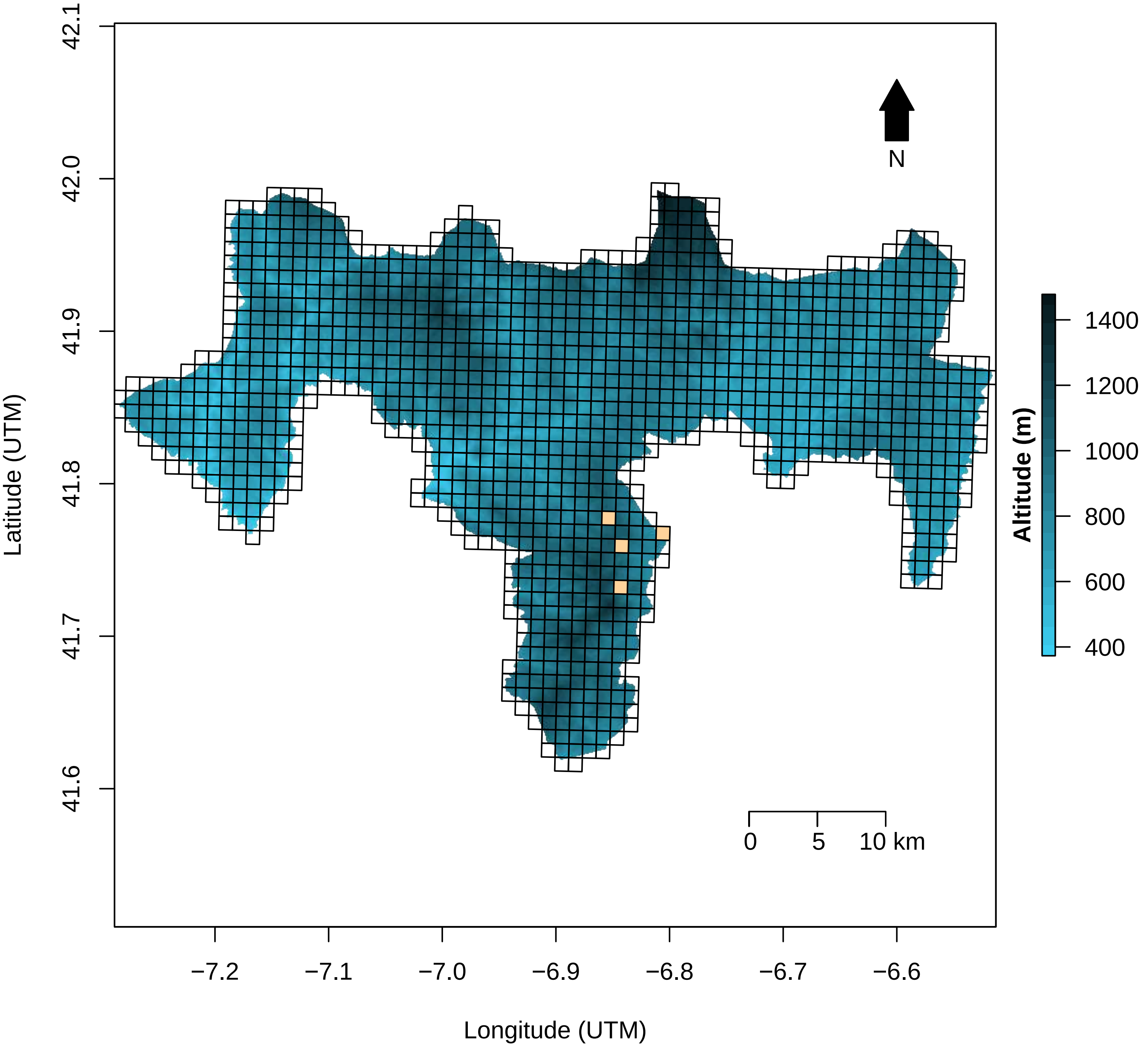

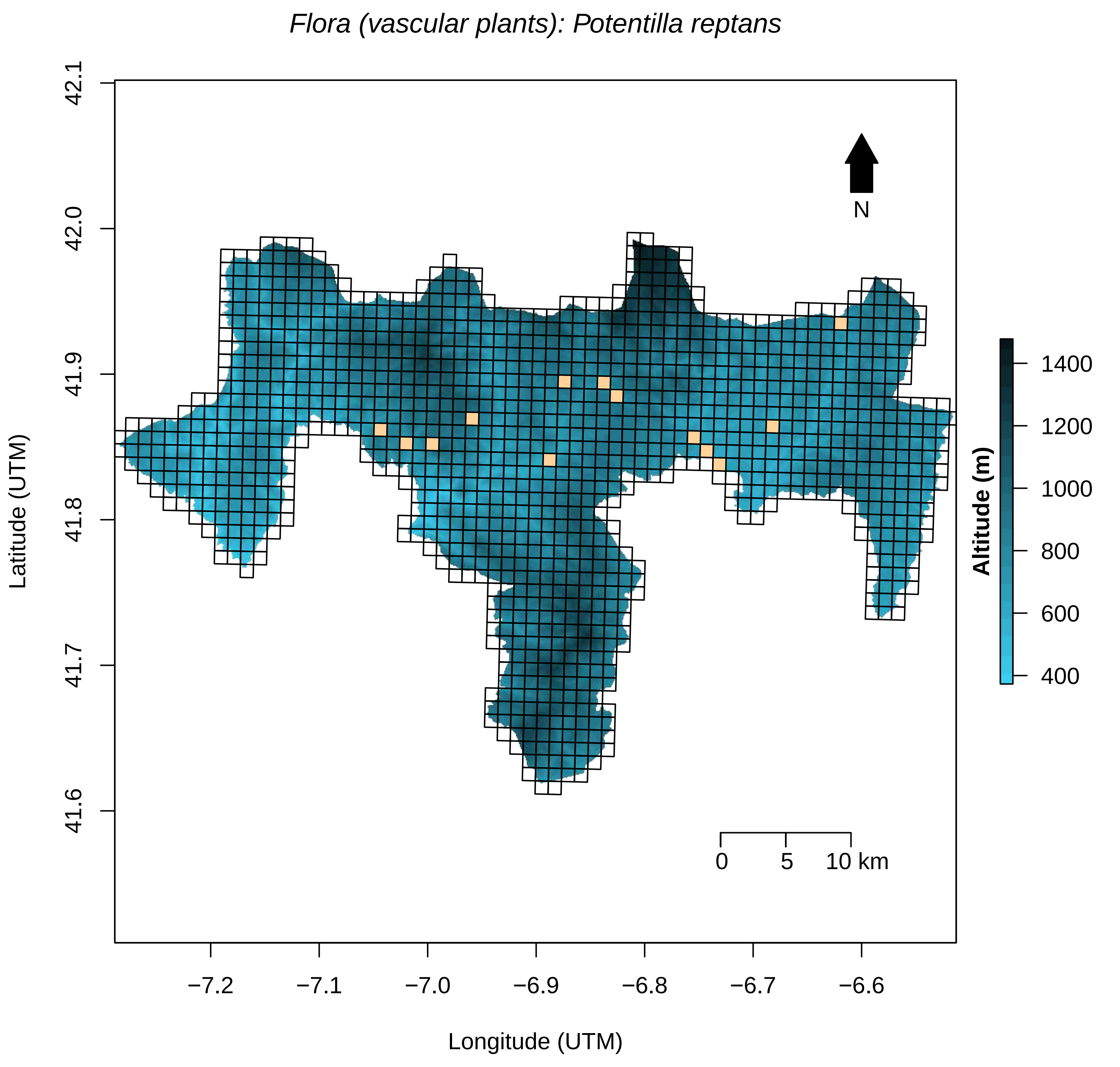

Flora (vascular plants): Potentilla reptans
42.1
42.0
41.9
41.8
41.7
41.6
Latitude (UTM)
−7.2
−7.1
−7.0
−6.9
−6.8
−6.7
−6.6
Longitude (UTM)
N
0
5
10 km
1400
1200
1000
800
600
400
Altitude (m)

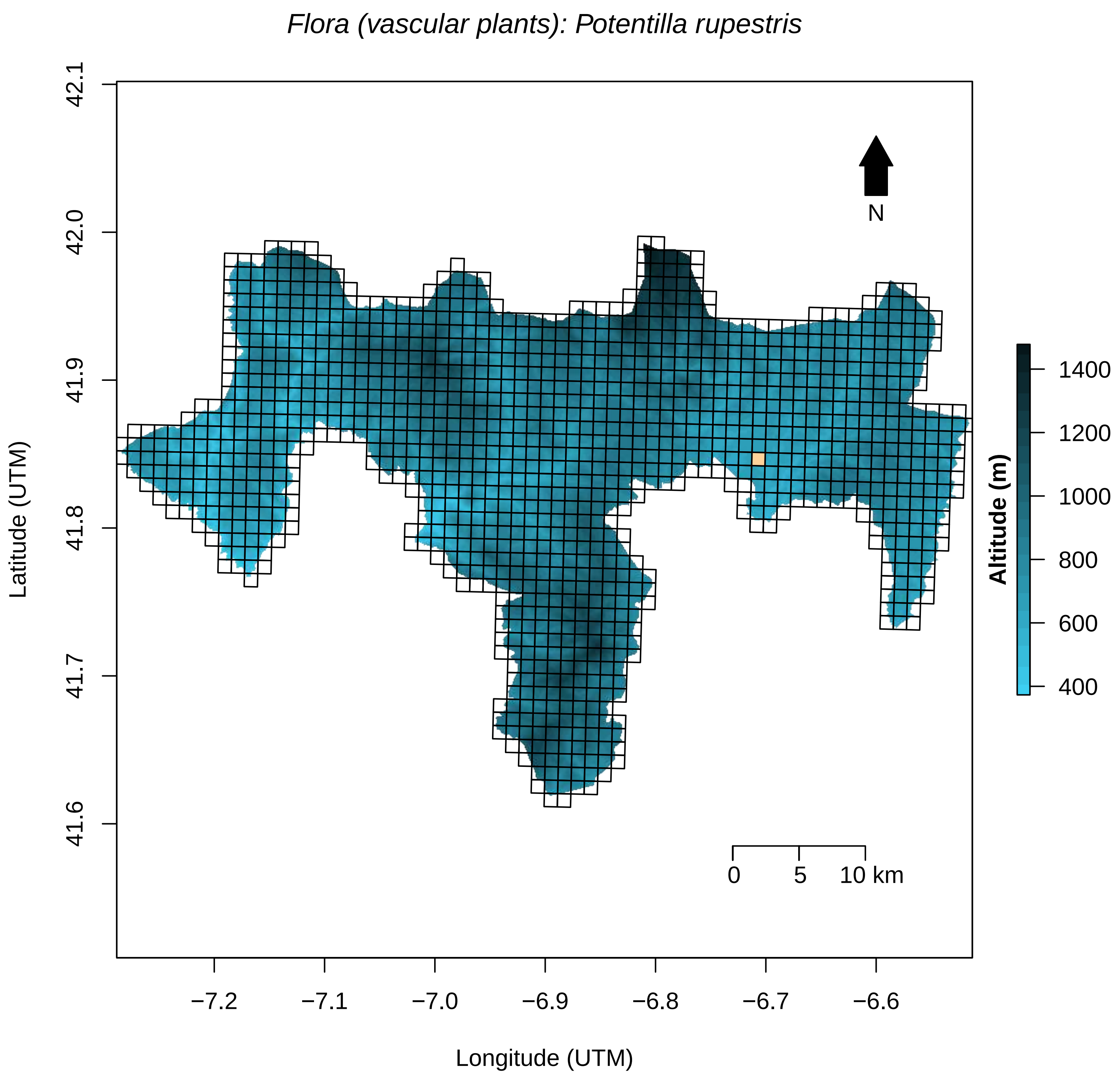

Flora (vascular plants): Potentilla rupestris
N
0
5
10 km
42.1
42.0
41.9
41.8
41.7
41.6
Latitude (UTM)
−7.2
−7.1
−7.0
−6.9
−6.8
−6.7
−6.6
Longitude (UTM)
Altitude (m)
1400
1200
1000
800
600
400

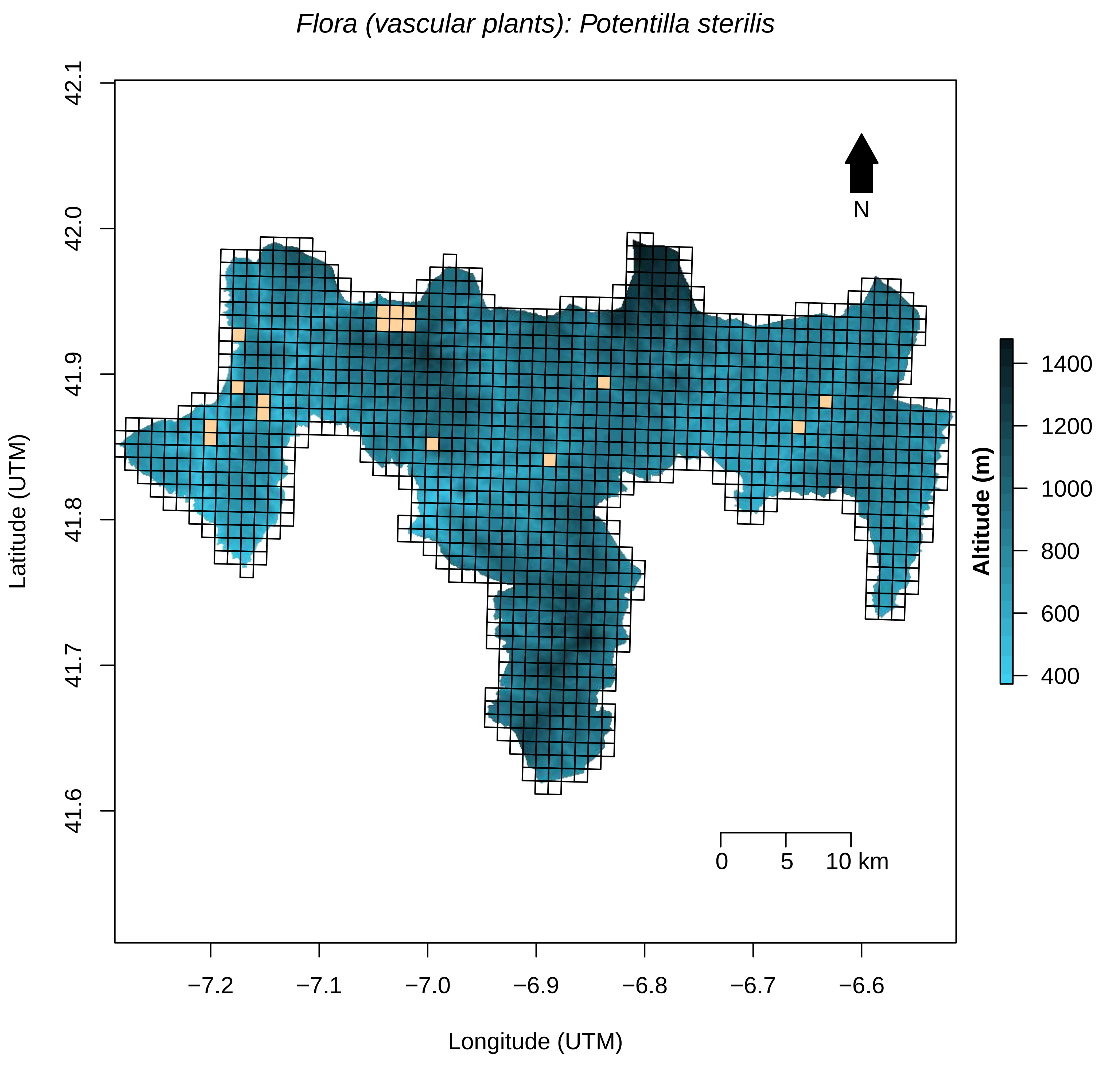

Flora (vascular plants): Potentilla sterilis
N
0 5 10 km
Altitude (m)
1400
1200
1000
800
600
400
Latitude (UTM)
42.1
42.0
41.9
41.8
41.7
41.6
Longitude (UTM)
−7.2
−7.1
−7.0
−6.9
−6.8
−6.7
−6.6

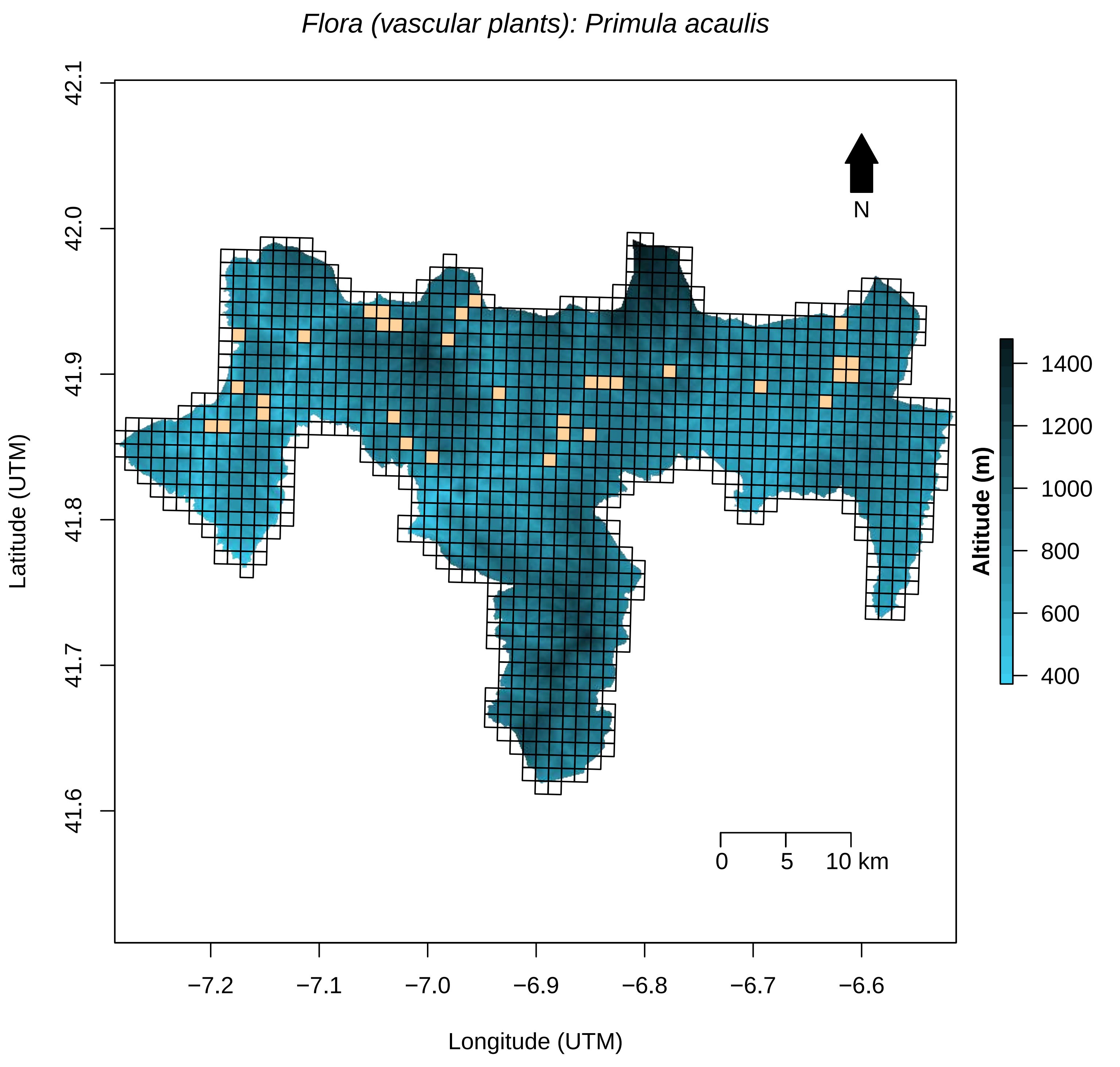

Flora (vascular plants): Primula acaulis
42.1
42.0
41.9
41.8
41.7
41.6
Latitude (UTM)
−7.2
−7.1
−7.0
−6.9
−6.8
−6.7
−6.6
Longitude (UTM)
N
0
5
10 km
Altitude (m)
1400
1200
1000
800
600
400

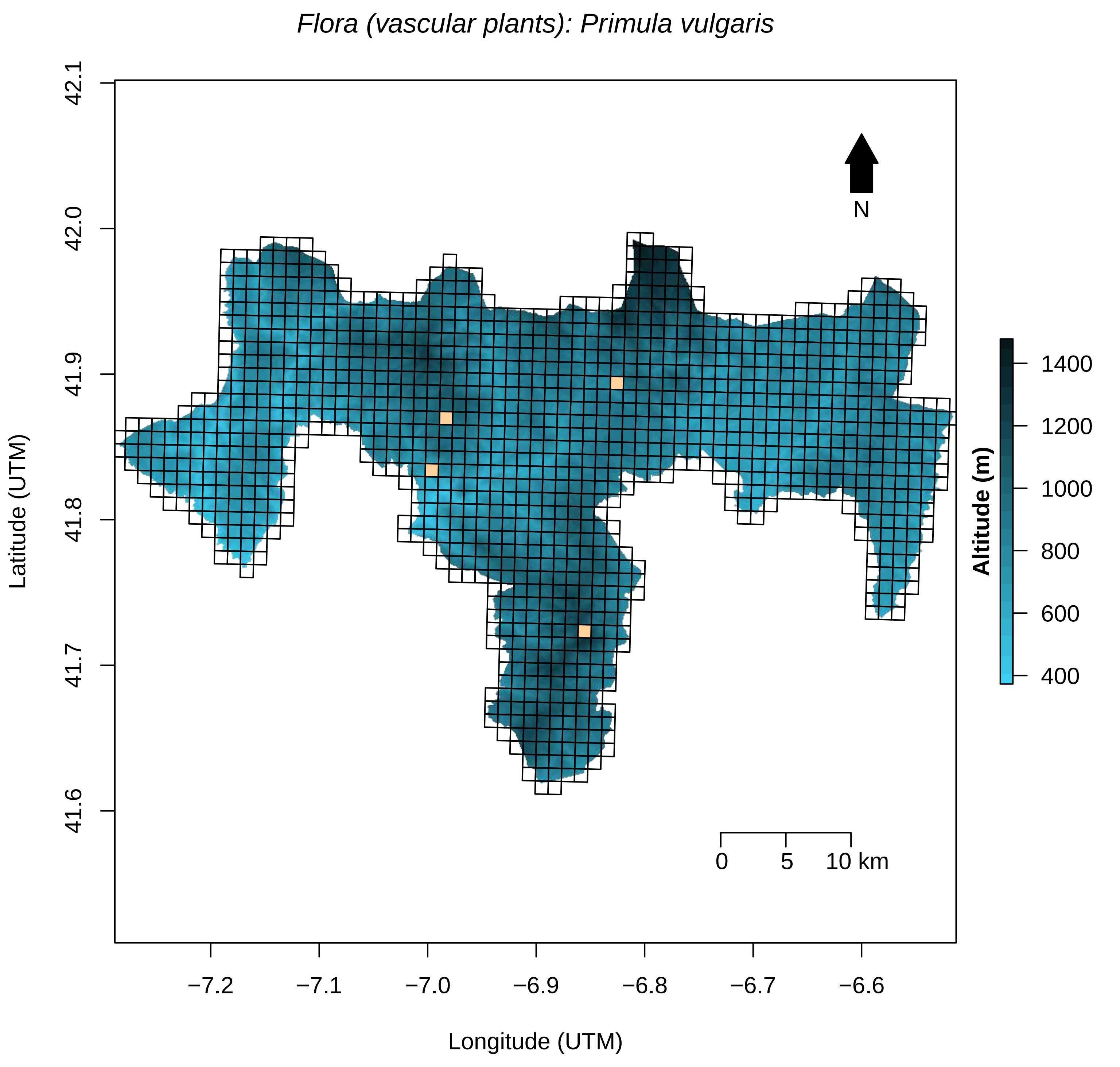

Flora (vascular plants): Primula vulgaris
N
0
5
10 km
Altitude (m)
1400
1200
1000
800
600
400
42.1
42.0
41.9
41.8
41.7
41.6
Latitude (UTM)
−7.2
−7.1
−7.0
−6.9
−6.8
−6.7
−6.6
Longitude (UTM)

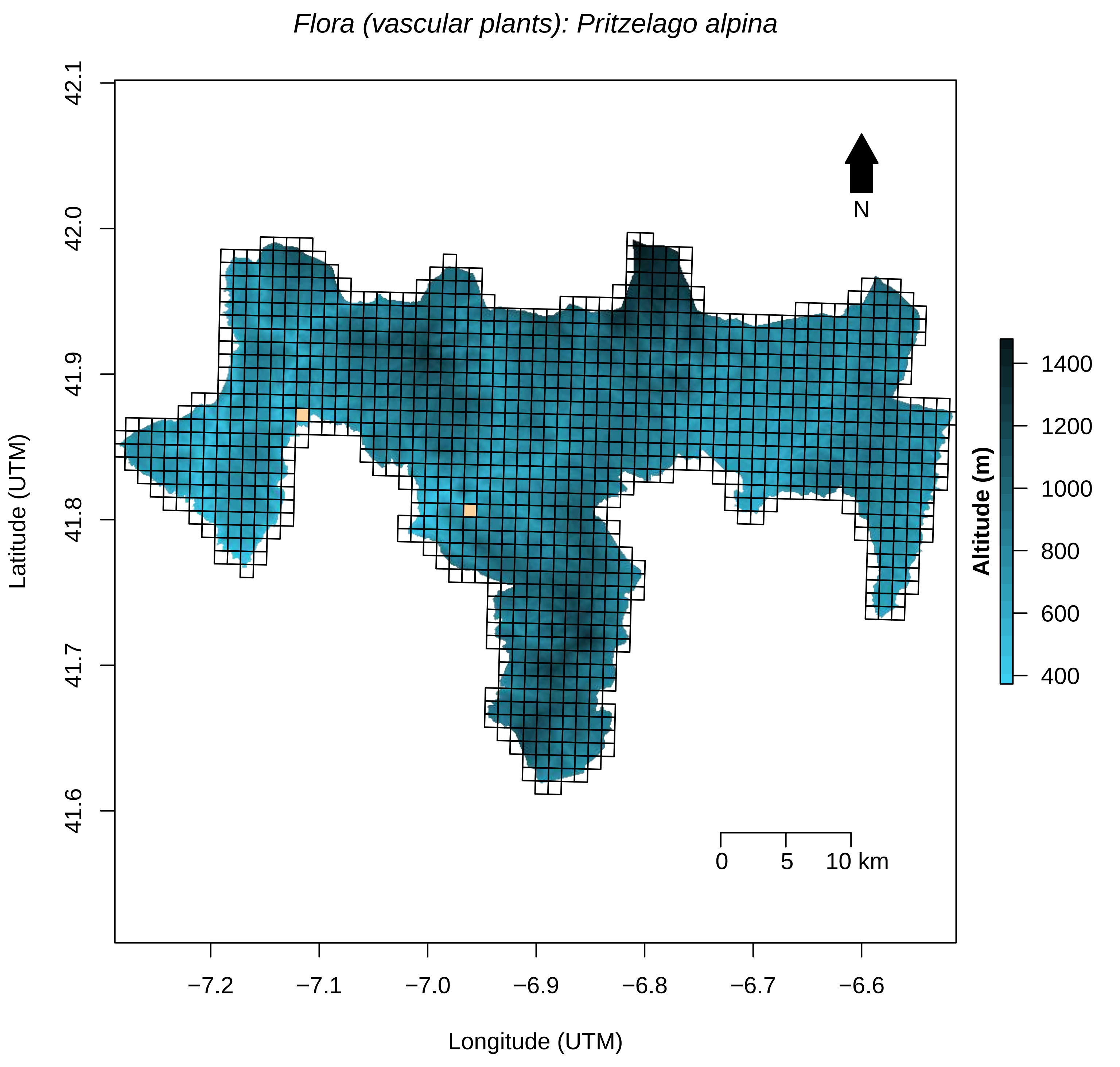
Flora (vascular plants): Pritzelago alpina
N
0
5
10 km
Latitude (UTM)
42.1
42.0
41.9
41.8
41.7
41.6
Longitude (UTM)
−7.2
−7.1
−7.0
−6.9
−6.8
−6.7
−6.6
Altitude (m)
1400
1200
1000
800
600
400

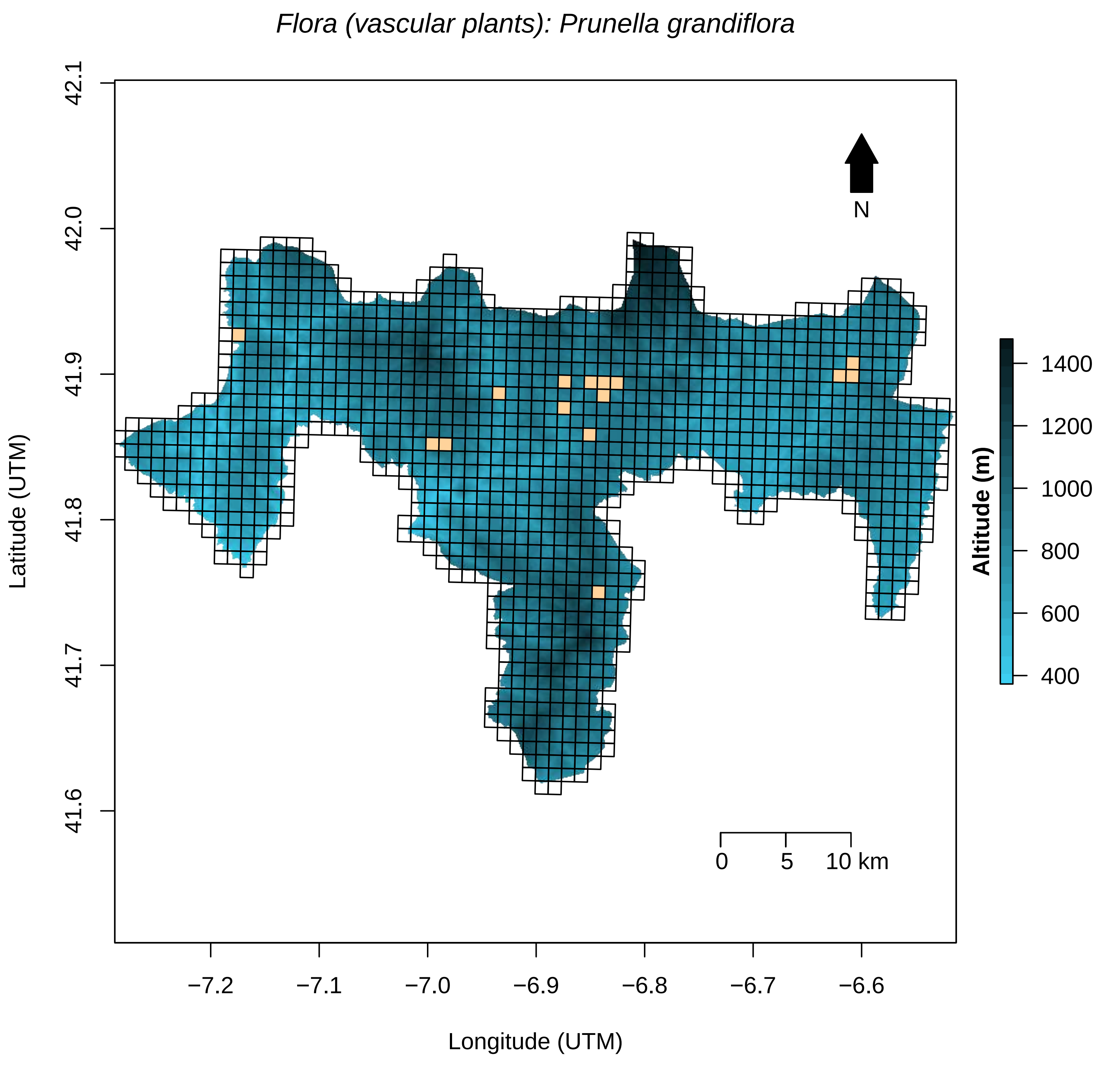
Flora (vascular plants): Prunella grandiflora
Latitude (UTM)
Longitude (UTM)
42.1
42.0
41.9
41.8
41.7
41.6
−7.2
−7.1
−7.0
−6.9
−6.8
−6.7
−6.6
N
0
5
10 km
Altitude (m)
1400
1200
1000
800
600
400

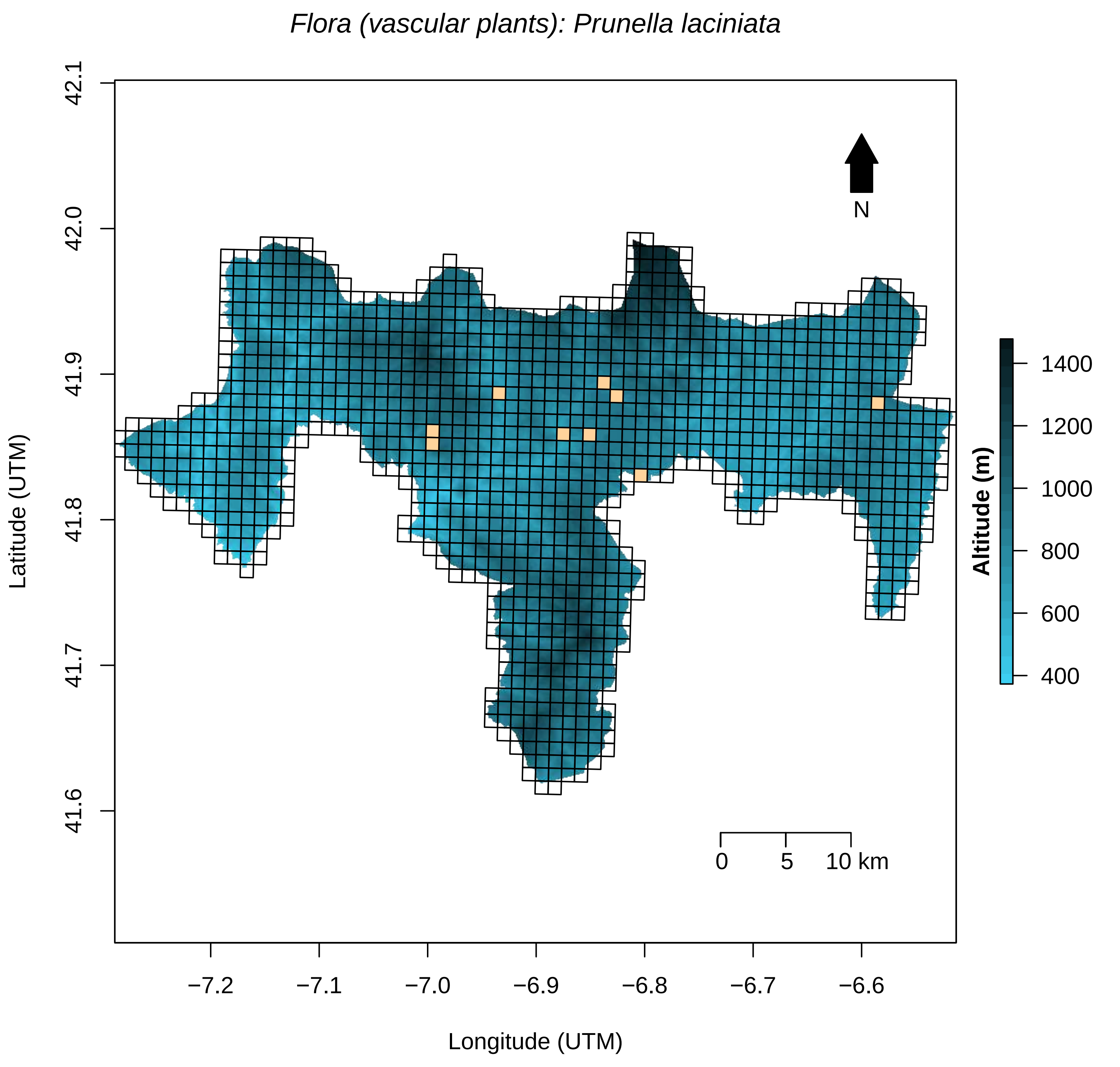

Flora (vascular plants): Prunella laciniata
42.1
42.0
41.9
41.8
41.7
41.6
Latitude (UTM)
N
0
5
10 km
−7.2
−7.1
−7.0
−6.9
−6.8
−6.7
−6.6
Longitude (UTM)
1400
1200
1000
800
600
400
Altitude (m)

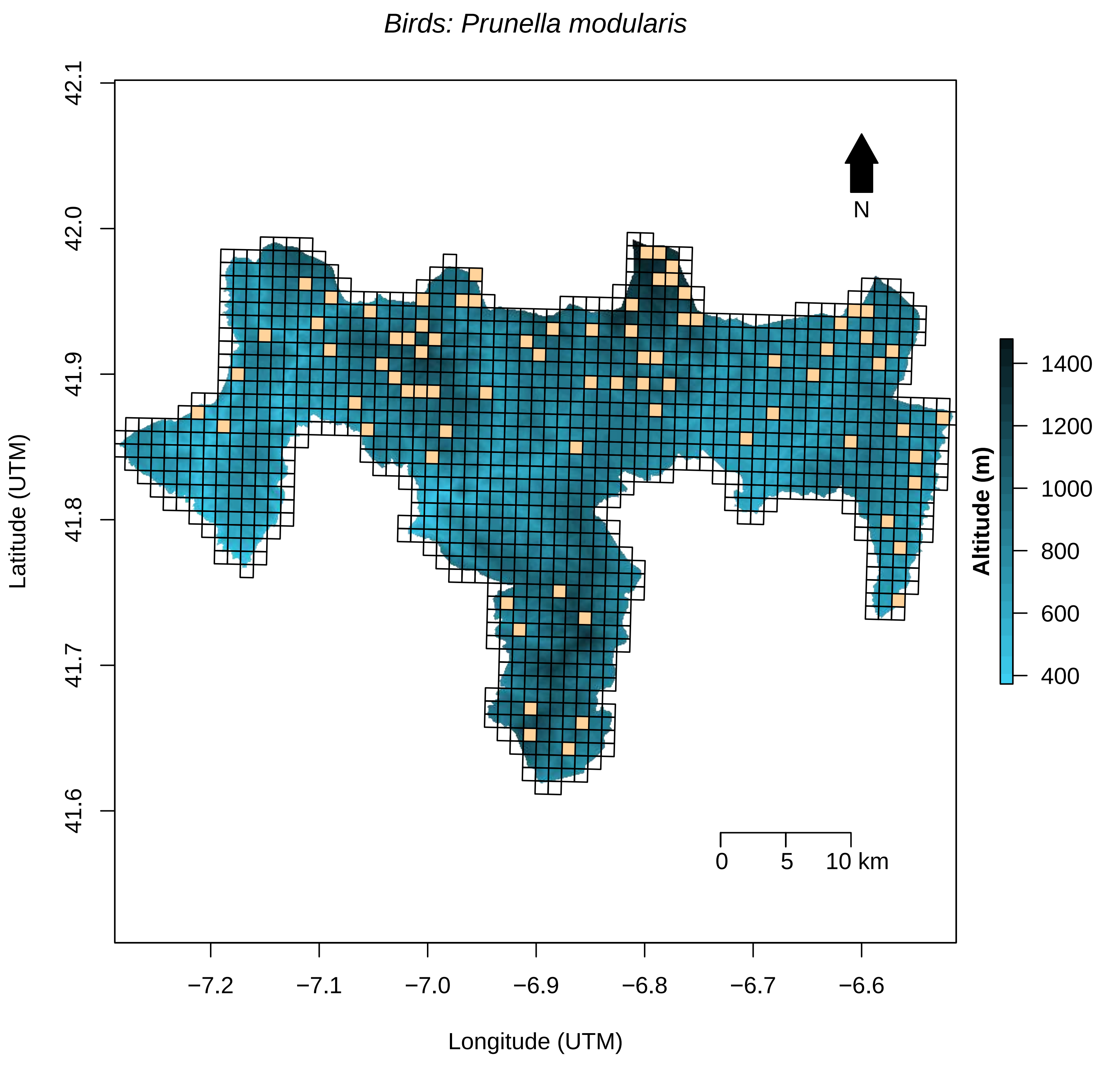

Birds: Prunella modularis
N
0 5 10 km
Altitude (m)
1400
1200
1000
800
600
400
Latitude (UTM)
42.1
42.0
41.9
41.8
41.7
41.6
Longitude (UTM)
−7.2
−7.1
−7.0
−6.9
−6.8
−6.7
−6.6

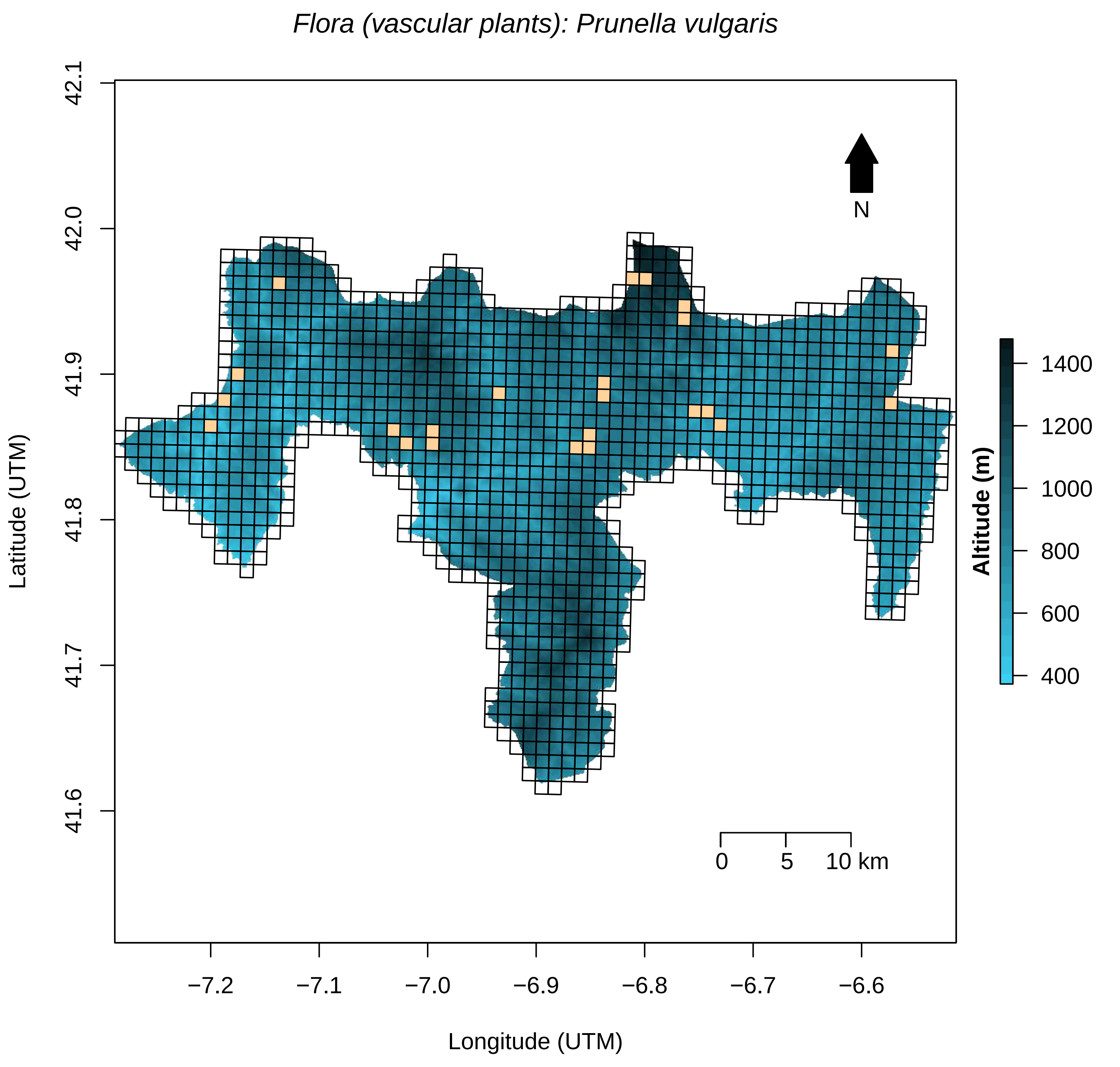

Flora (vascular plants): Prunella vulgaris
42.1
42.0
41.9
41.8
41.7
41.6
Latitude (UTM)
−7.2
−7.1
−7.0
−6.9
−6.8
−6.7
−6.6
Longitude (UTM)
N
0
5
10 km
Altitude (m)
1400
1200
1000
800
600
400

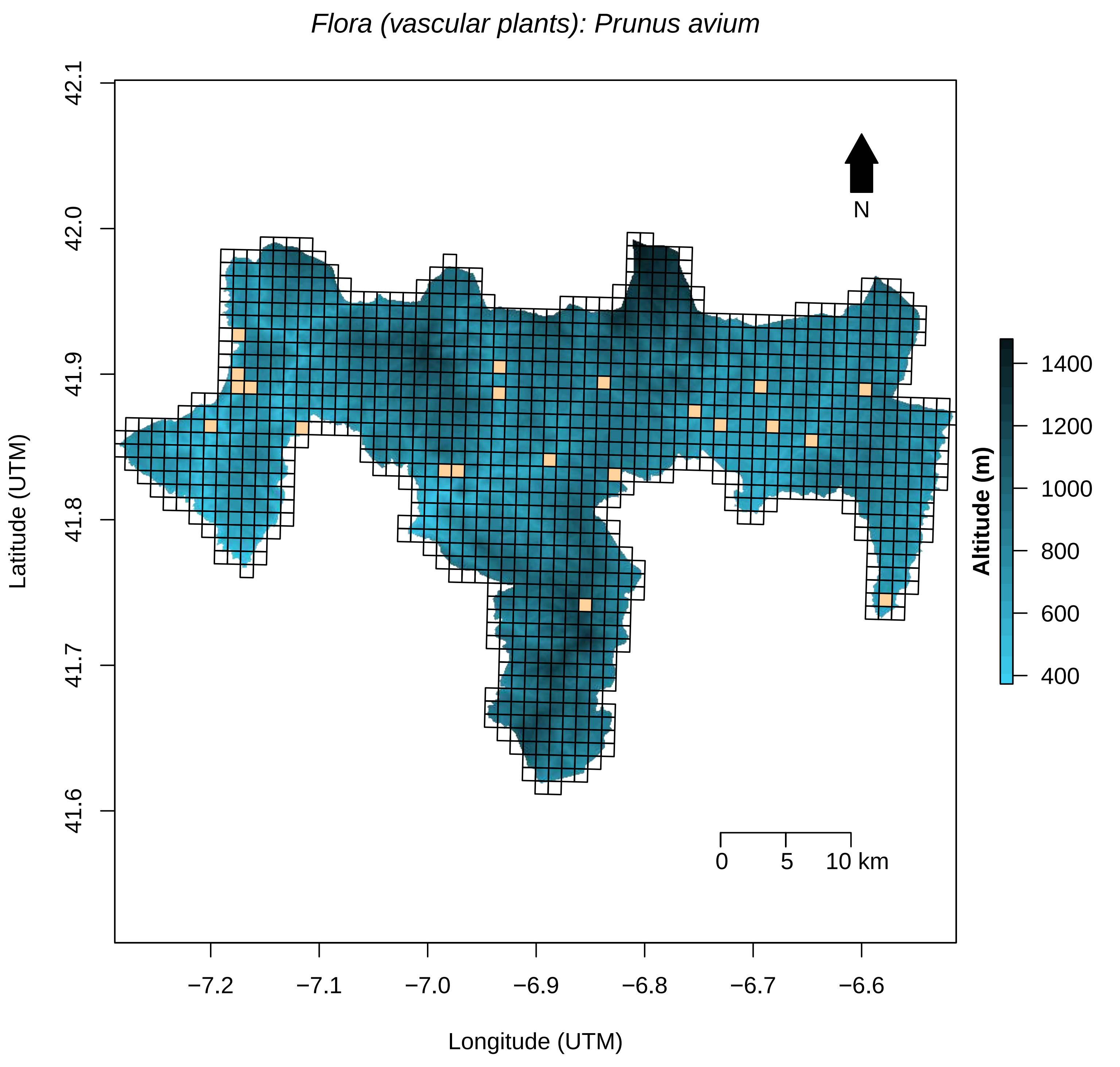

Flora (vascular plants): Prunus avium
42.1
42.0
41.9
41.8
41.7
41.6
Latitude (UTM)
N
0 5 10 km
−7.2 −7.1 −7.0 −6.9 −6.8 −6.7 −6.6
Longitude (UTM)
1400
1200
1000
800
600
400
Altitude (m)

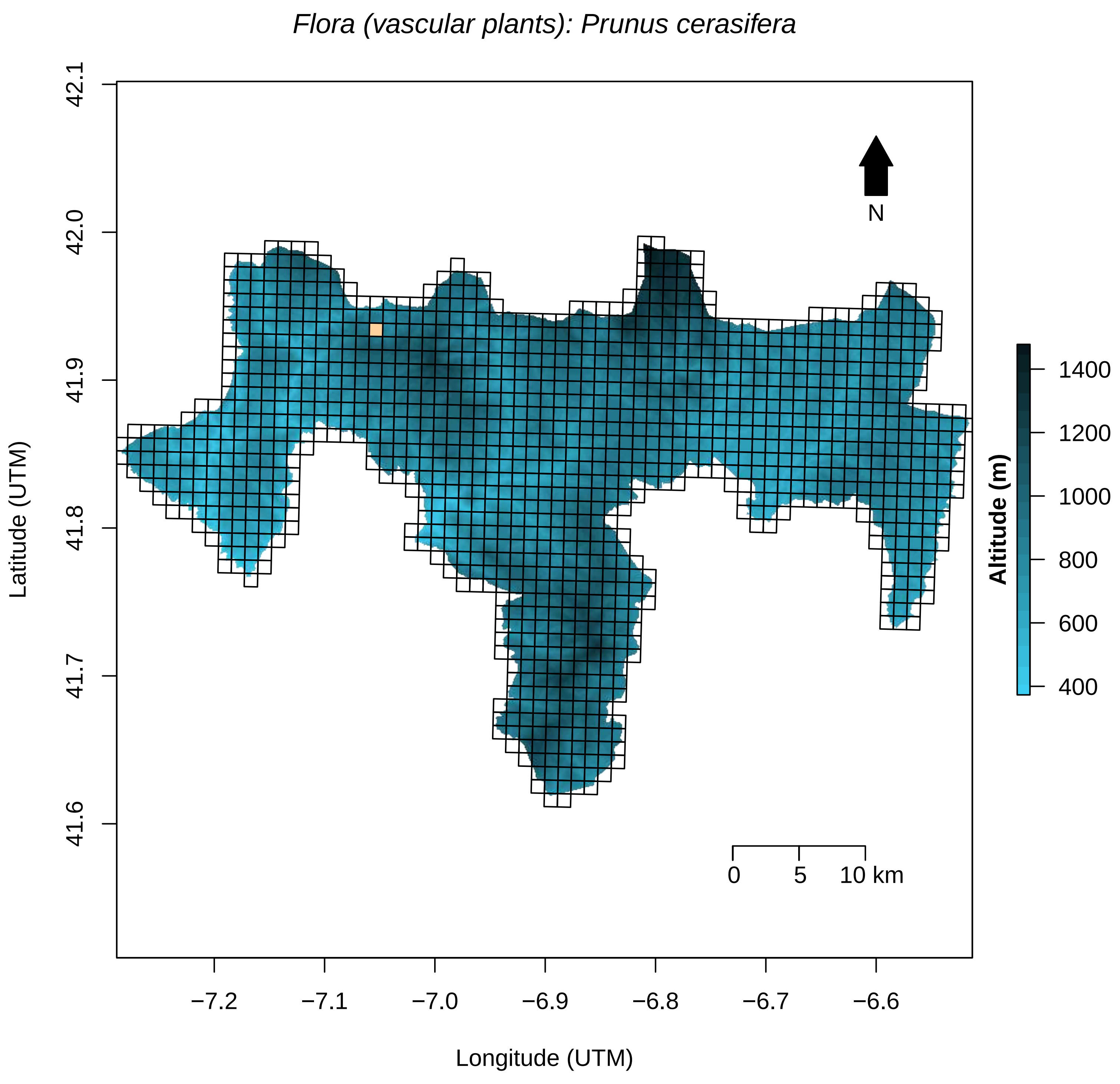

Flora (vascular plants): Prunus cerasifera
42.1
42.0
41.9
41.8
41.7
41.6
Latitude (UTM)
−7.2
−7.1
−7.0
−6.9
−6.8
−6.7
−6.6
Longitude (UTM)
N
0
5
10 km
Altitude (m)
1400
1200
1000
800
600
400

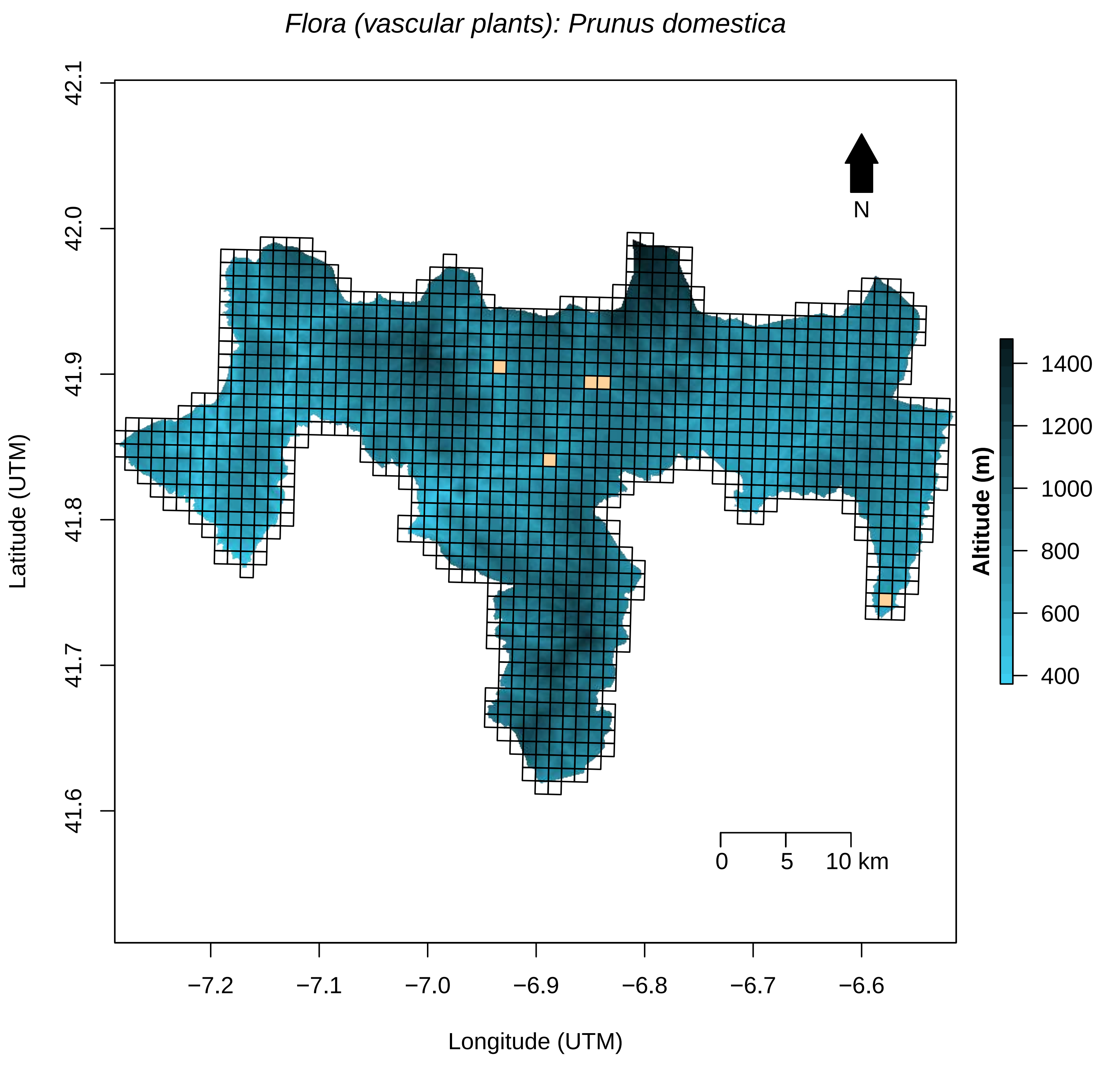
Flora (vascular plants): Prunus domestica
42.1
42.0
41.9
41.8
41.7
41.6
Latitude (UTM)
N
0
5
10 km
−7.2
−7.1
−7.0
−6.9
−6.8
−6.7
−6.6
Longitude (UTM)
Altitude (m)
1400
1200
1000
800
600
400

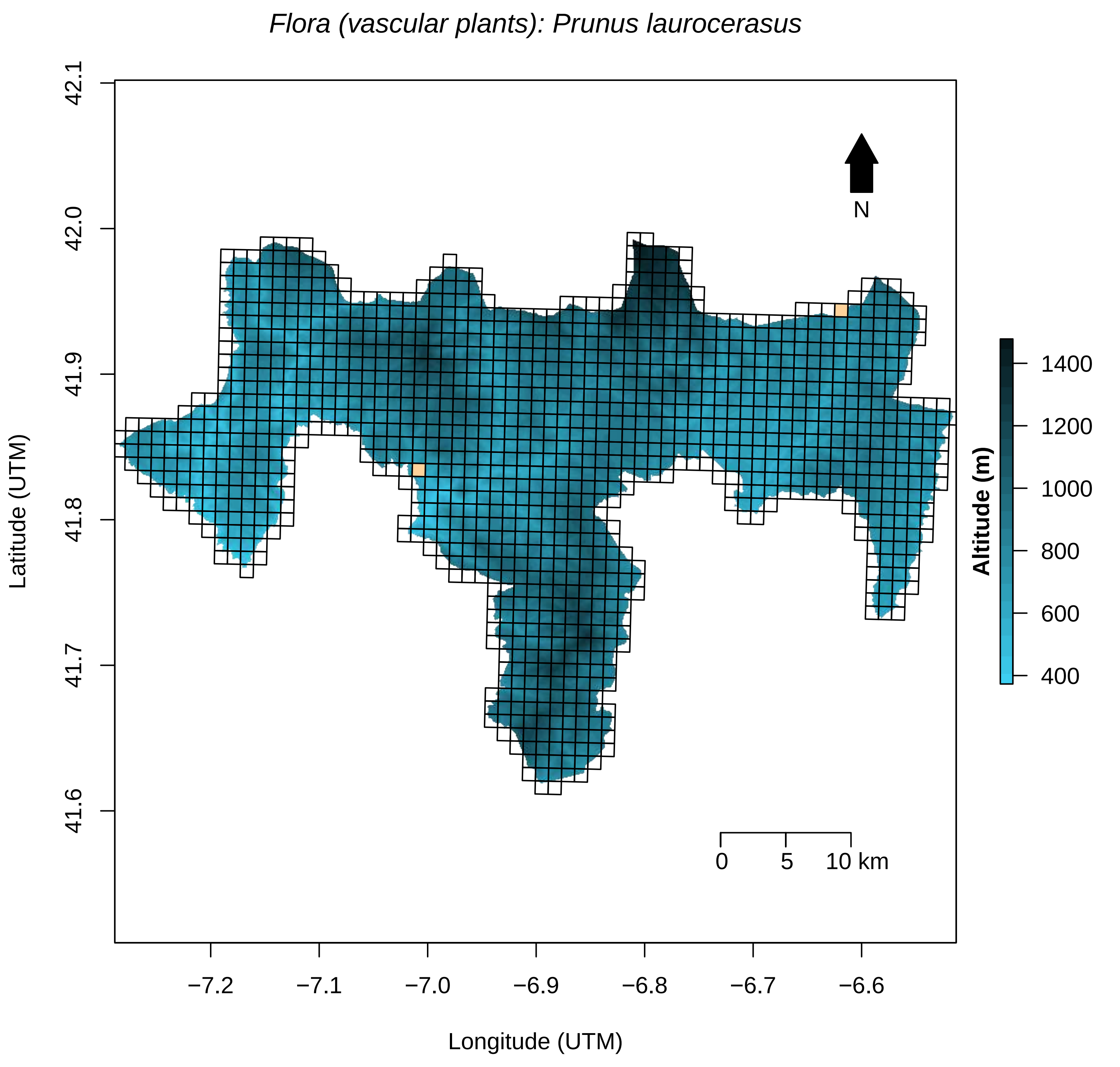

Flora (vascular plants): Prunus laurocerasus
42.1
42.0
41.9
41.8
41.7
41.6
Latitude (UTM)
−7.2
−7.1
−7.0
−6.9
−6.8
−6.7
−6.6
Longitude (UTM)
N
0
5
10 km
1400
1200
1000
800
600
400
Altitude (m)

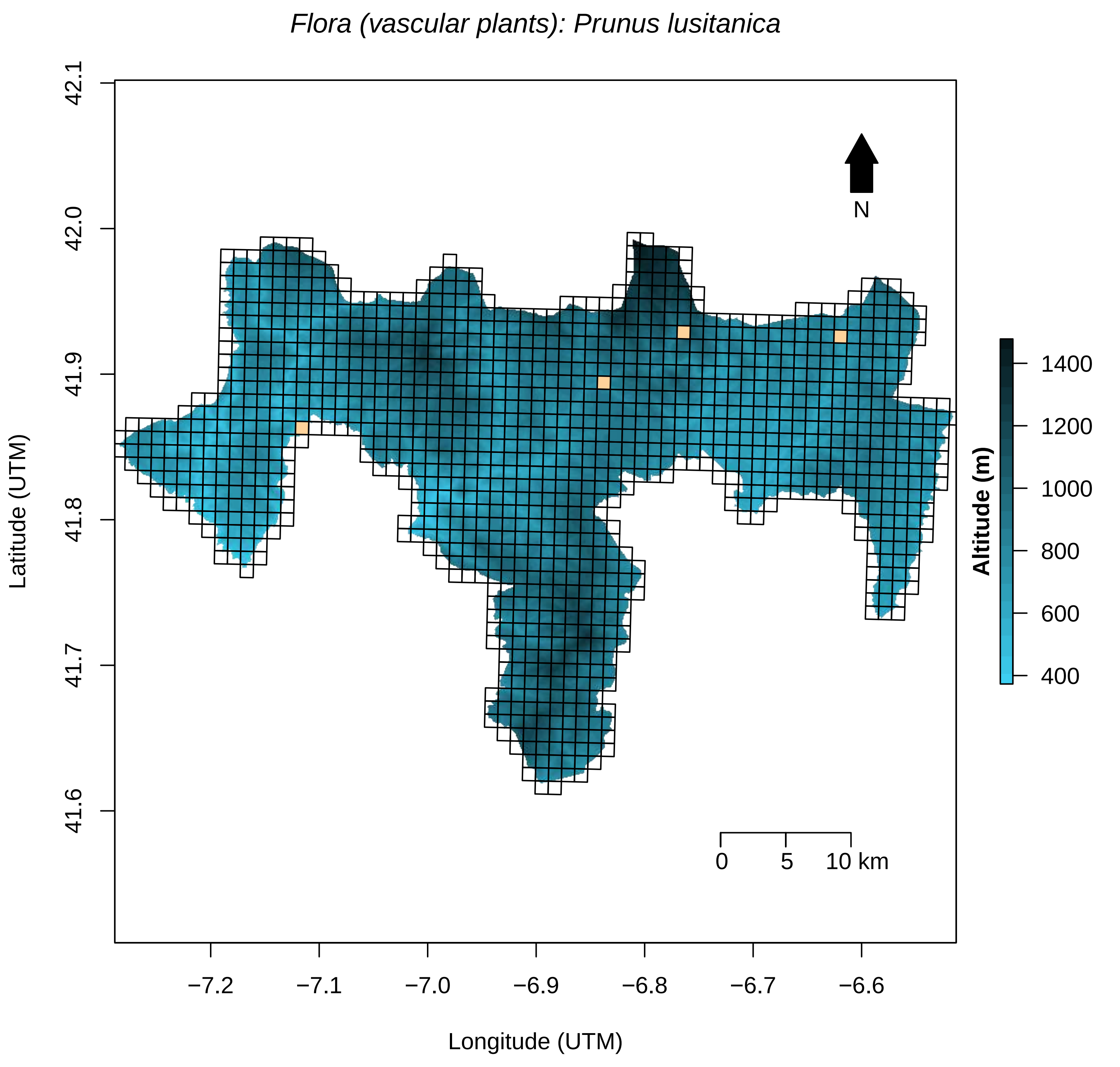

Flora (vascular plants): Prunus lusitanica
42.1
42.0
41.9
41.8
41.7
41.6
Latitude (UTM)
−7.2
−7.1
−7.0
−6.9
−6.8
−6.7
−6.6
Longitude (UTM)
N
0
5
10 km
Altitude (m)
1400
1200
1000
800
600
400

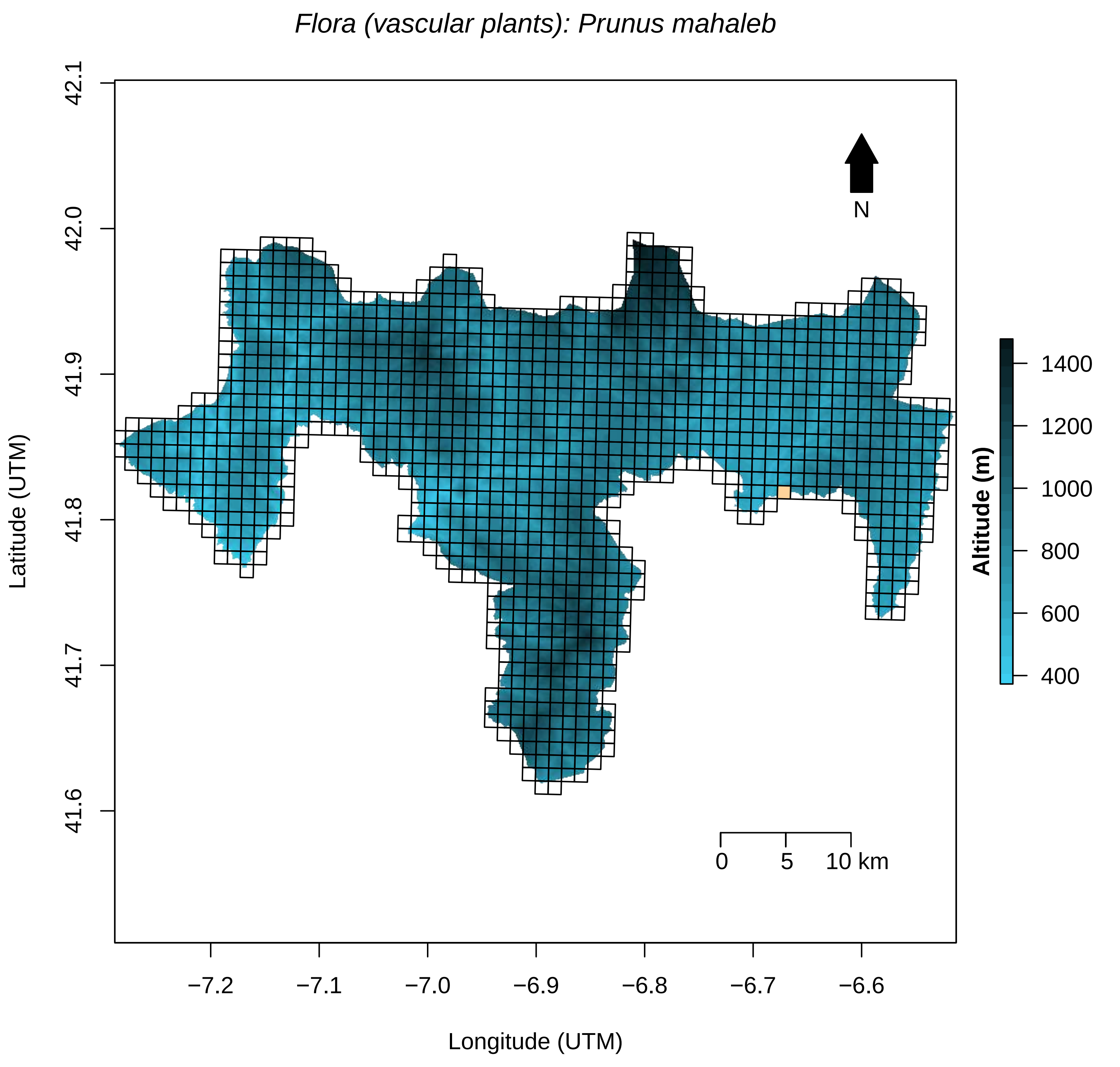

Flora (vascular plants): Prunus mahaleb
42.1
42.0
41.9
41.8
41.7
41.6
Latitude (UTM)
−7.2
−7.1
−7.0
−6.9
−6.8
−6.7
−6.6
Longitude (UTM)
N
0
5
10 km
1400
1200
1000
800
600
400
Altitude (m)

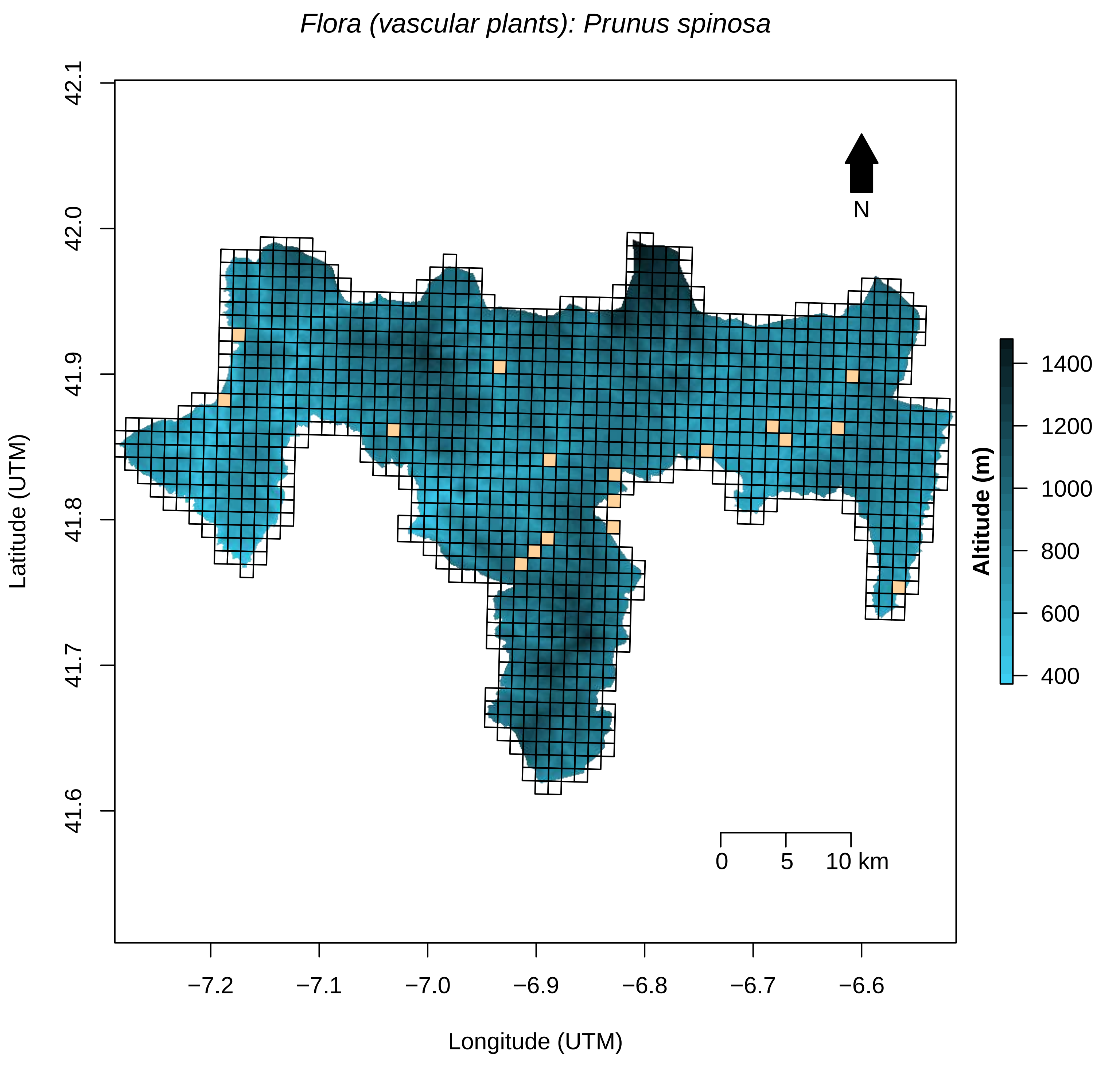
Flora (vascular plants): Prunus spinosa
N
0
5
10 km
Altitude (m)
1400
1200
1000
800
600
400
Latitude (UTM)
42.1
42.0
41.9
41.8
41.7
41.6
Longitude (UTM)
−7.2
−7.1
−7.0
−6.9
−6.8
−6.7
−6.6

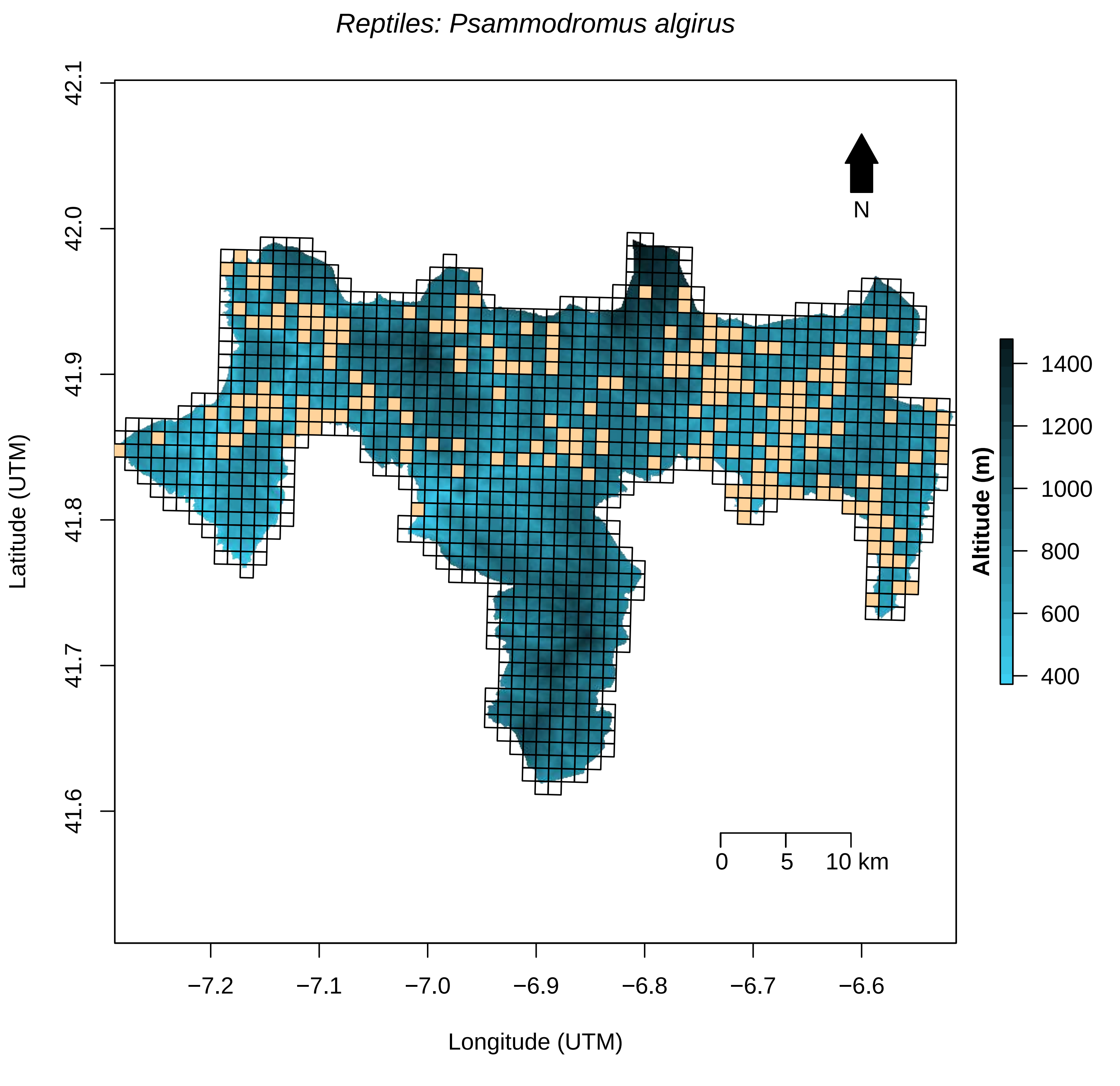
Reptiles: Psammodromus algirus
42.1
42.0
41.9
41.8
41.7
41.6
Latitude (UTM)
−7.2
−7.1
−7.0
−6.9
−6.8
−6.7
−6.6
Longitude (UTM)
N
0
5
10 km
1400
1200
1000
800
600
400
Altitude (m)

Reptiles: Psammodromus occidentalis

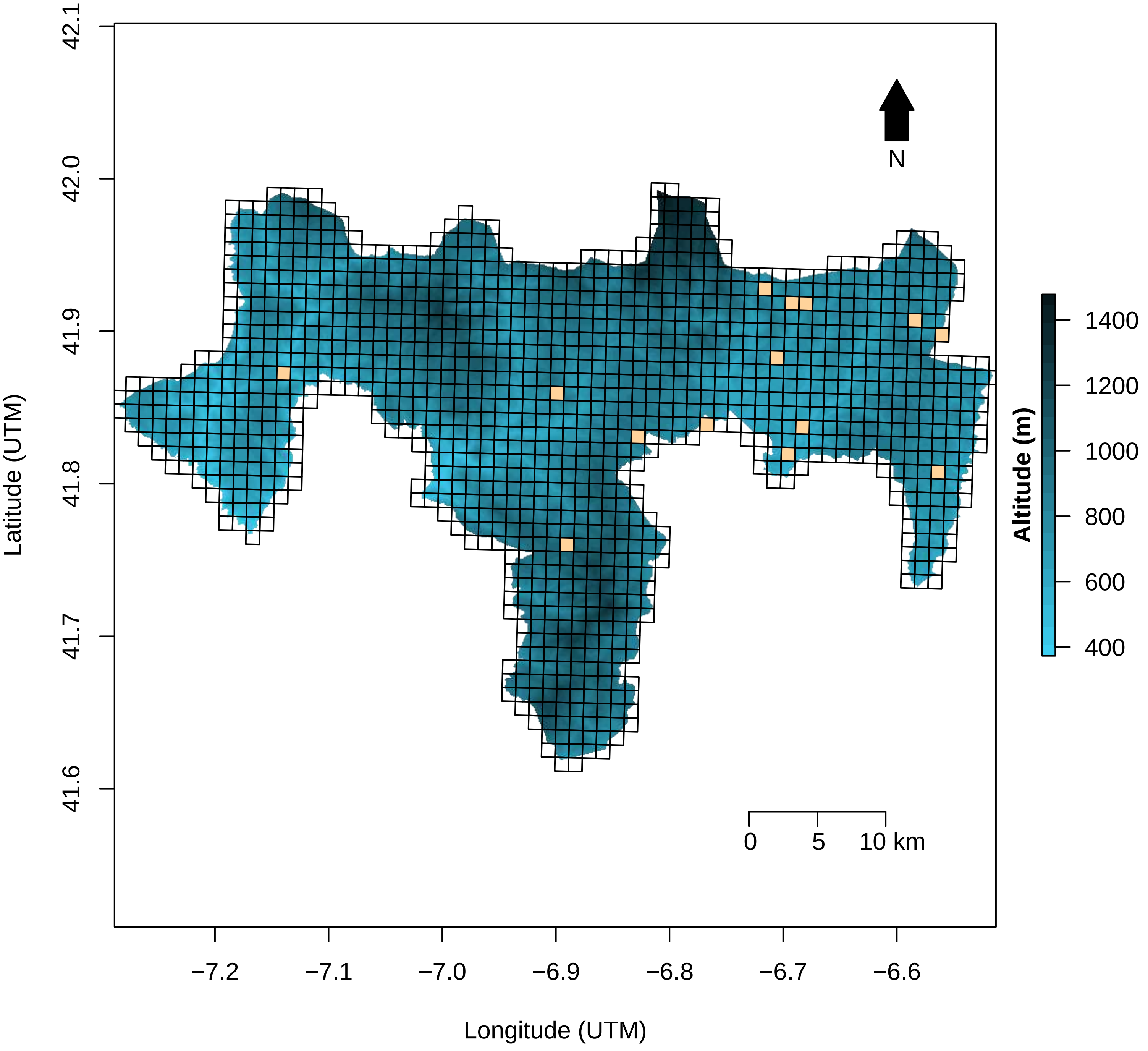

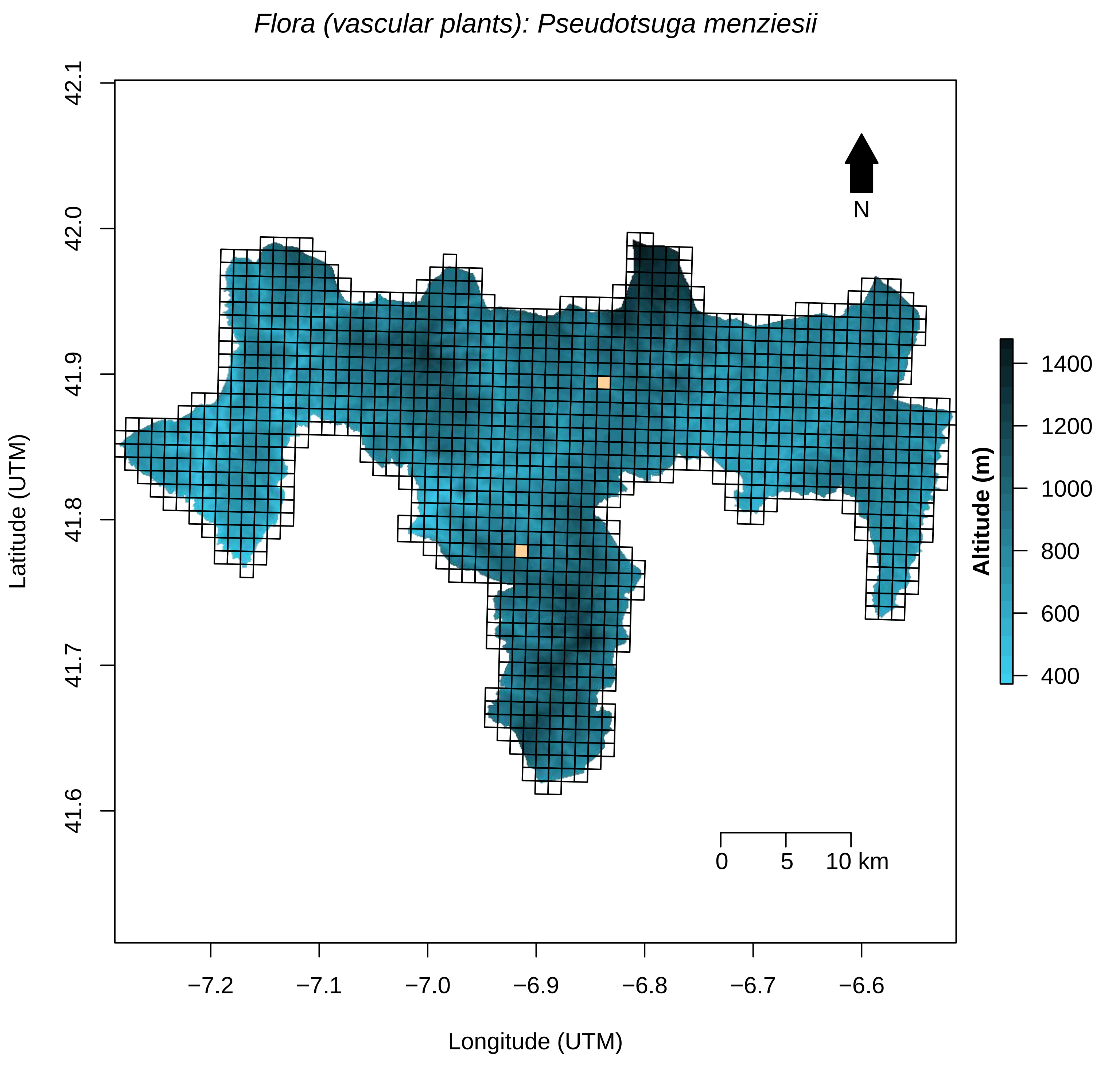
Flora (vascular plants): Pseudotsuga menziesii
Latitude (UTM)
42.1
42.0
41.9
41.8
41.7
41.6
Longitude (UTM)
−7.2
−7.1
−7.0
−6.9
−6.8
−6.7
−6.6
N
0
5
10 km
Altitude (m)
1400
1200
1000
800
600
400

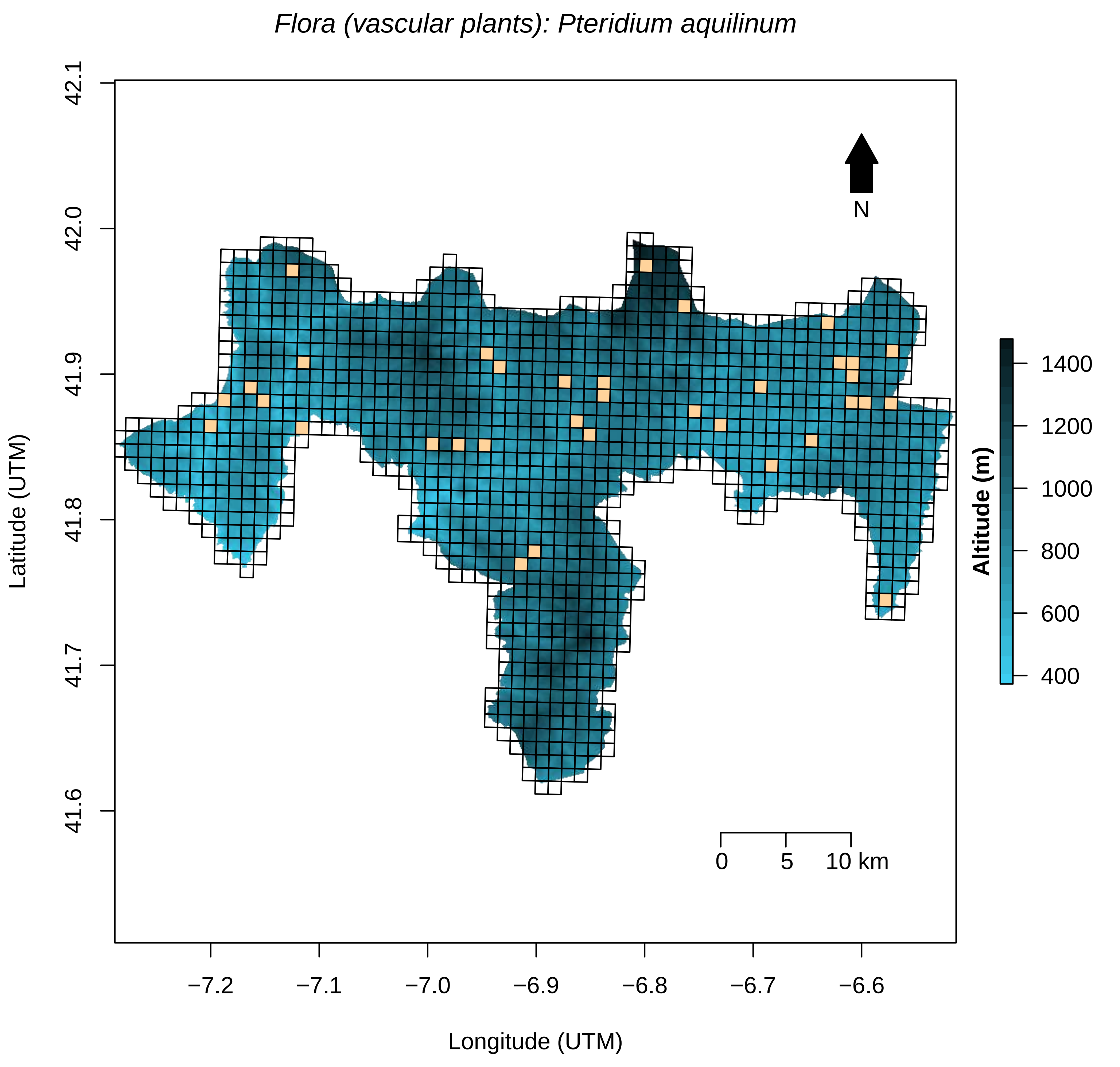

Flora (vascular plants): Pteridium aquilinum
N
0
5
10 km
Altitude (m)
1400
1200
1000
800
600
400
Latitude (UTM)
42.1
42.0
41.9
41.8
41.7
41.6
Longitude (UTM)
−7.2
−7.1
−7.0
−6.9
−6.8
−6.7
−6.6

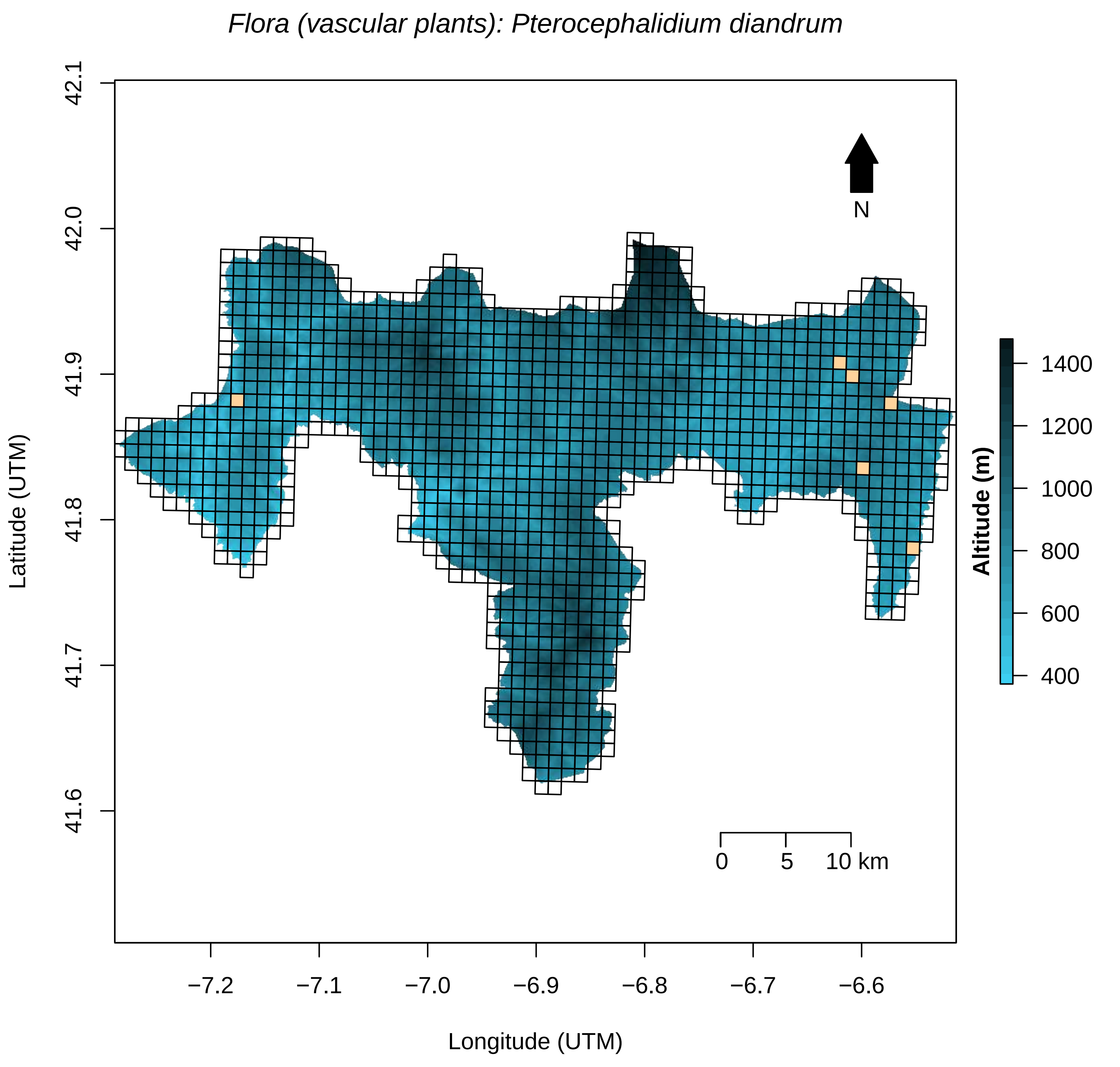

Flora (vascular plants): Pterocephalidium diandrum
N
0 5 10 km
Altitude (m)
1400
1200
1000
800
600
400
Latitude (UTM)
42.1
42.0
41.9
41.8
41.7
41.6
Longitude (UTM)
−7.2
−7.1
−7.0
−6.9
−6.8
−6.7
−6.6

Flora (vascular plants): Pterospartum tridentatum

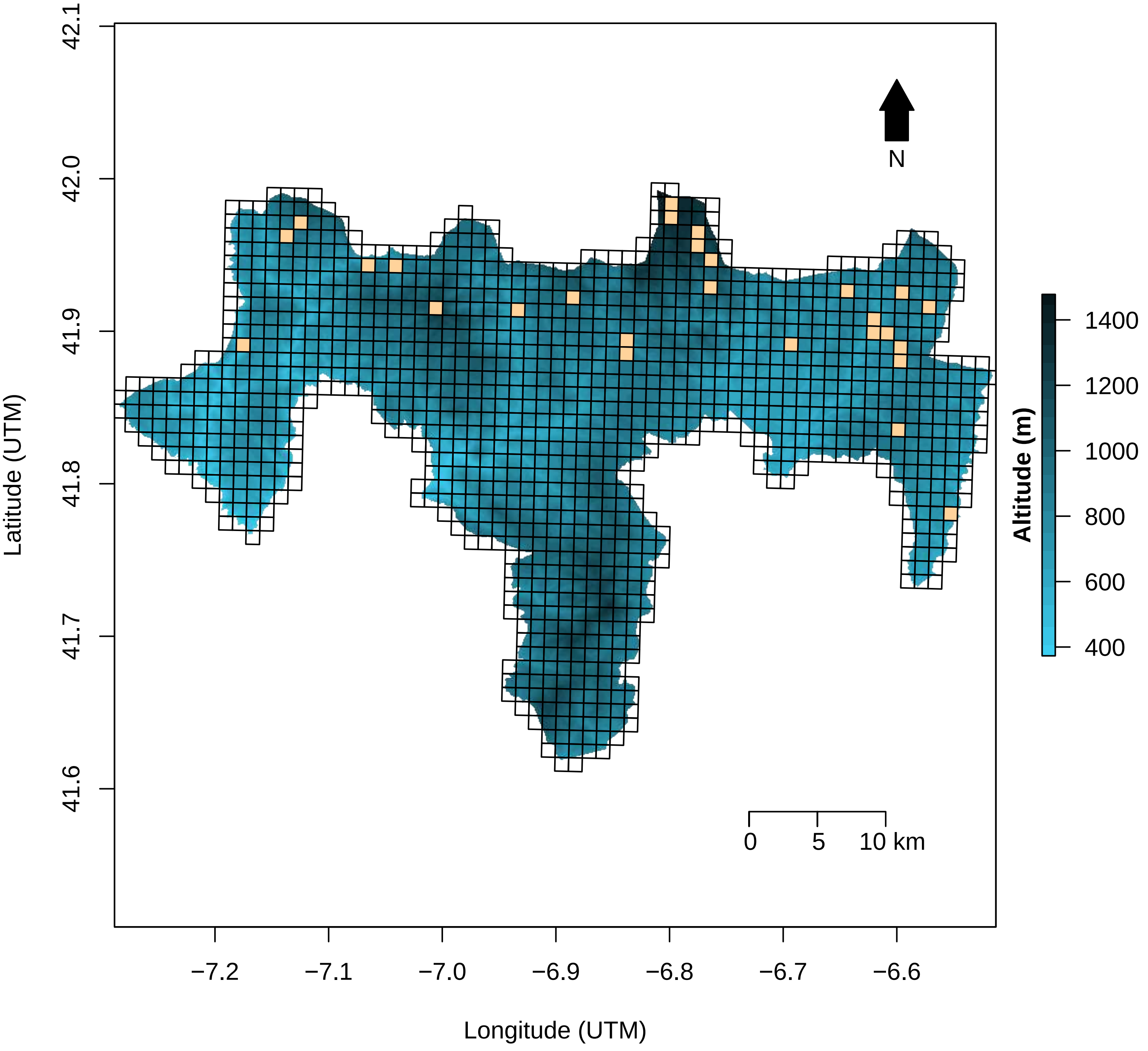

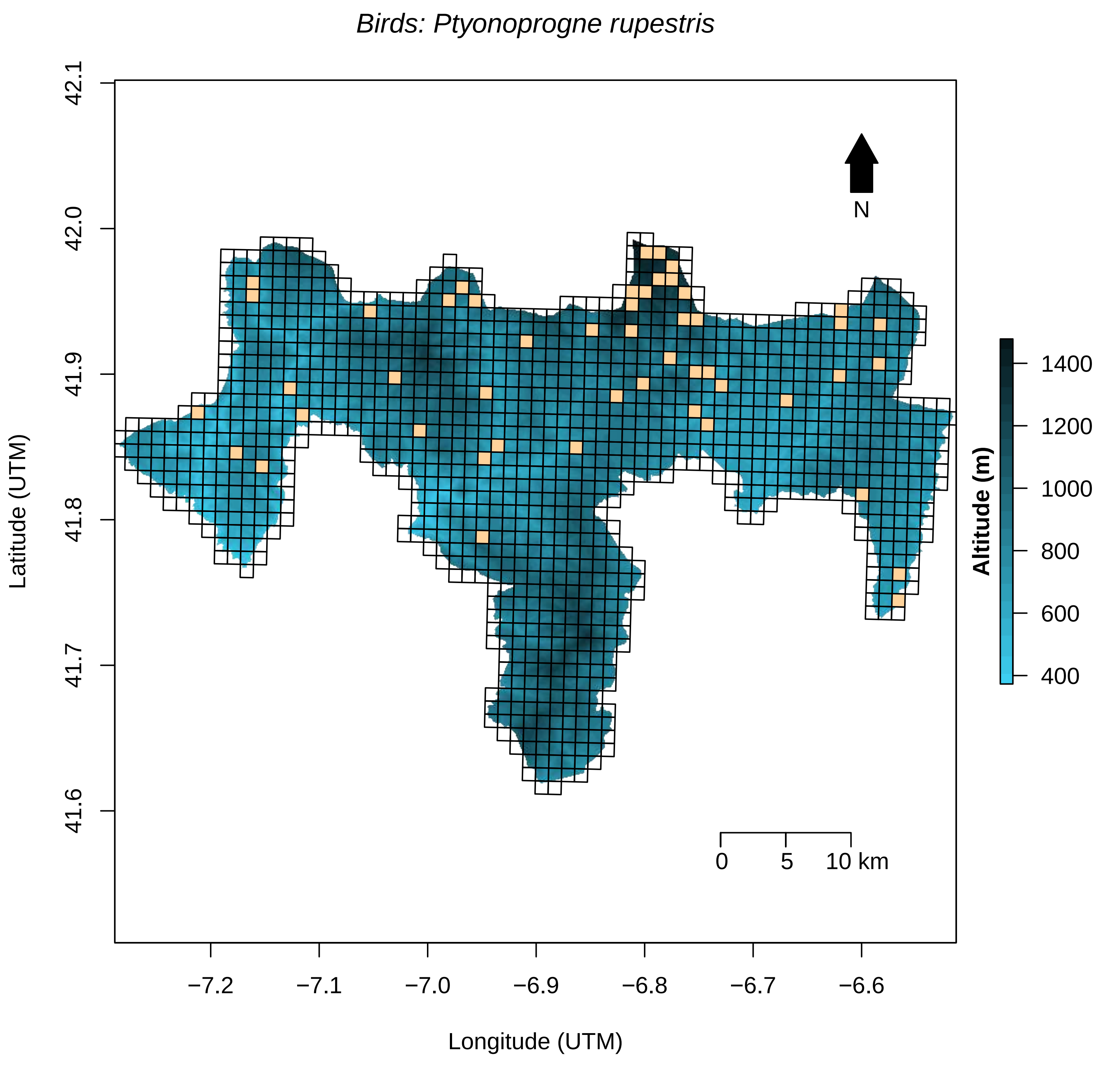

Birds: Ptyonoprogne rupestris
N
0
5
10 km
Altitude (m)
1400
1200
1000
800
600
400
Latitude (UTM)
42.1
42.0
41.9
41.8
41.7
41.6
Longitude (UTM)
−7.2
−7.1
−7.0
−6.9
−6.8
−6.7
−6.6

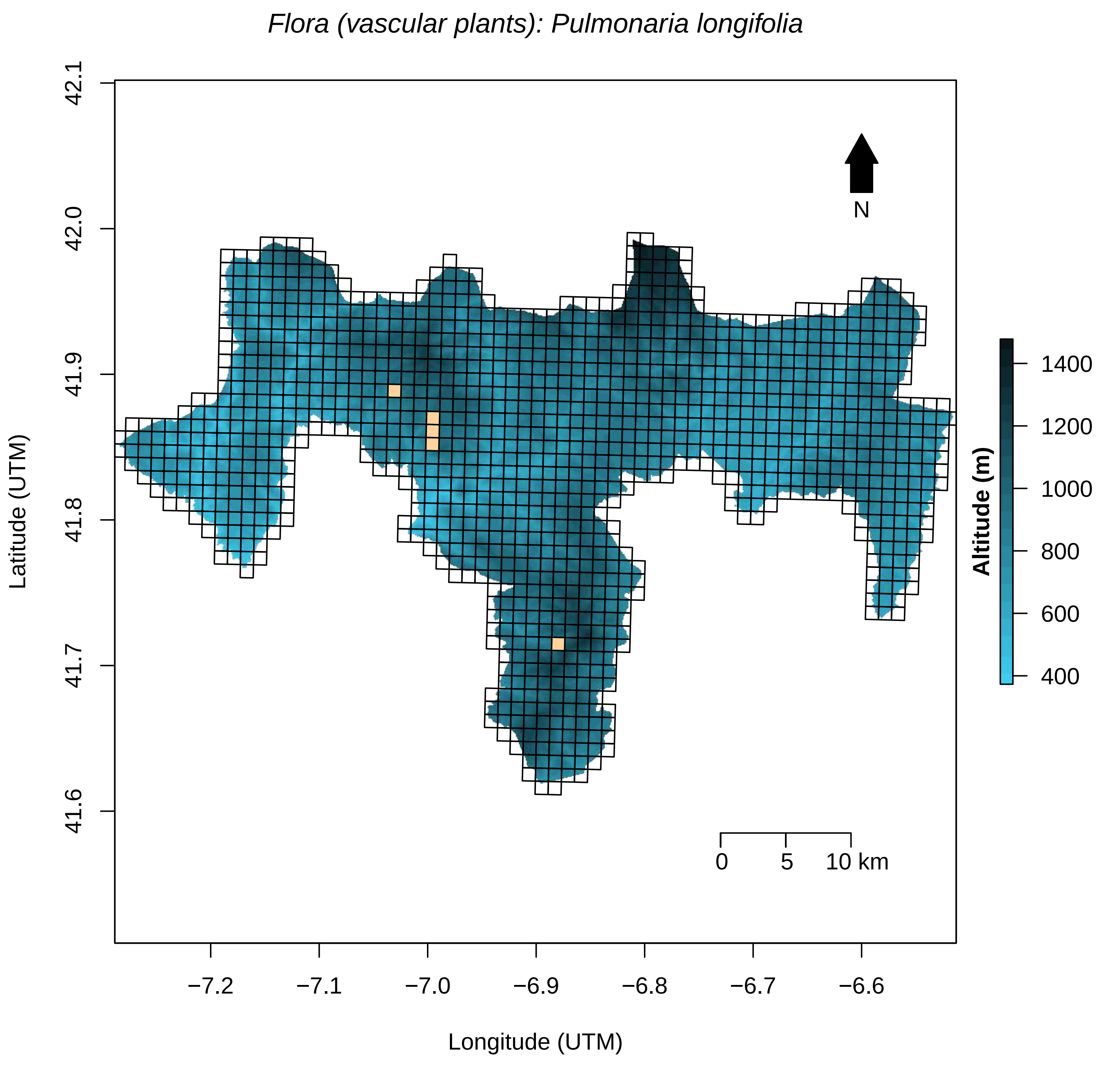

Flora (vascular plants): Pulmonaria longifolia
Latitude (UTM)
42.1
42.0
41.9
41.8
41.7
41.6
Longitude (UTM)
−7.2
−7.1
−7.0
−6.9
−6.8
−6.7
−6.6
N
0
5
10 km
Altitude (m)
1400
1200
1000
800
600
400

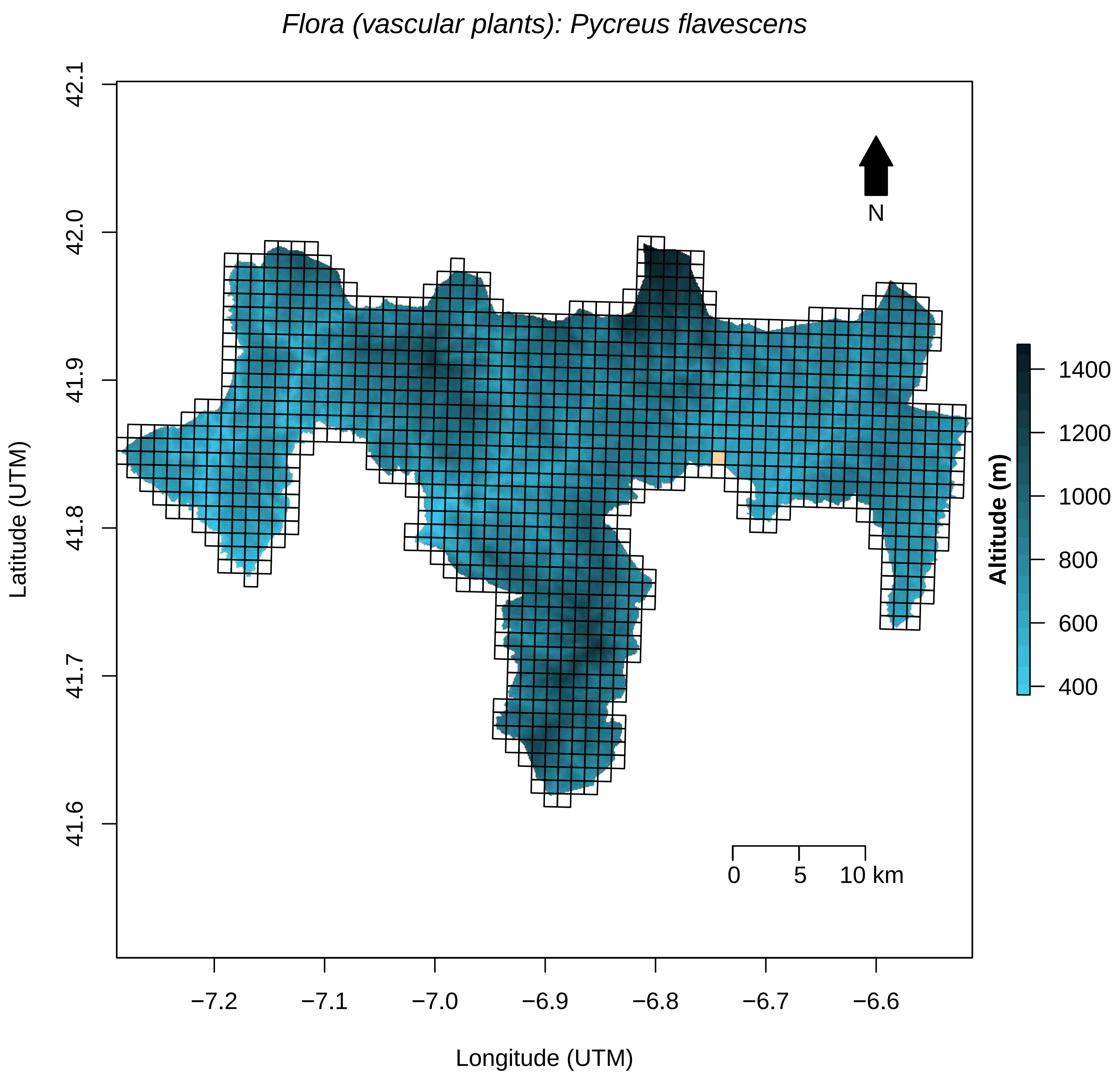

Flora (vascular plants): Pycreus flavescens
42.1
42.0
41.9
41.8
41.7
41.6
Latitude (UTM)
−7.2
−7.1
−7.0
−6.9
−6.8
−6.7
−6.6
Longitude (UTM)
N
0
5
10 km
Altitude (m)
1400
1200
1000
800
600
400

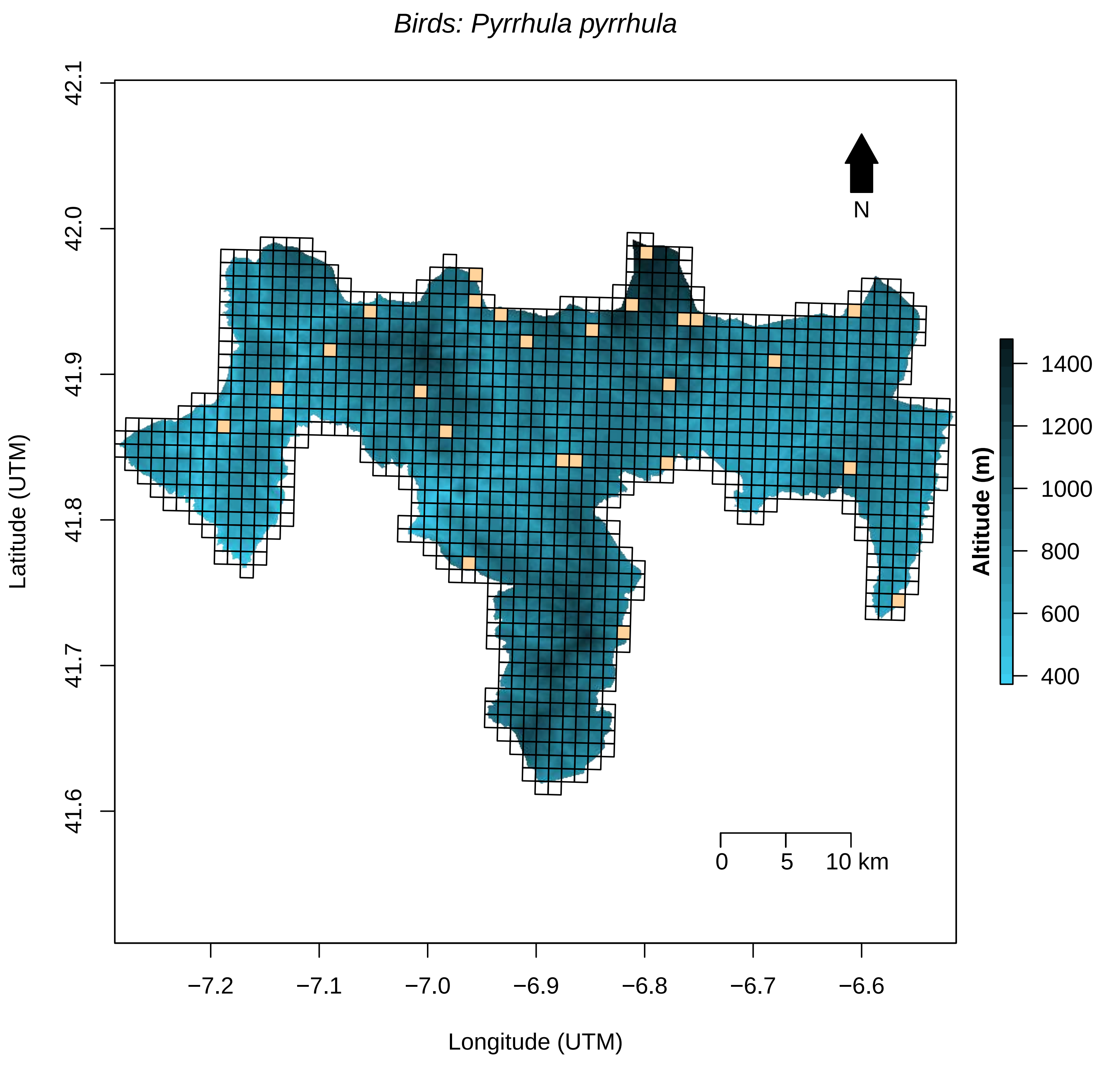
Birds: Pyrrhula pyrrhula
Latitude (UTM)
Longitude (UTM)
42.1
42.0
41.9
41.8
41.7
41.6
−7.2
−7.1
−7.0
−6.9
−6.8
−6.7
−6.6
N
0
5
10 km
Altitude (m)
1400
1200
1000
800
600
400

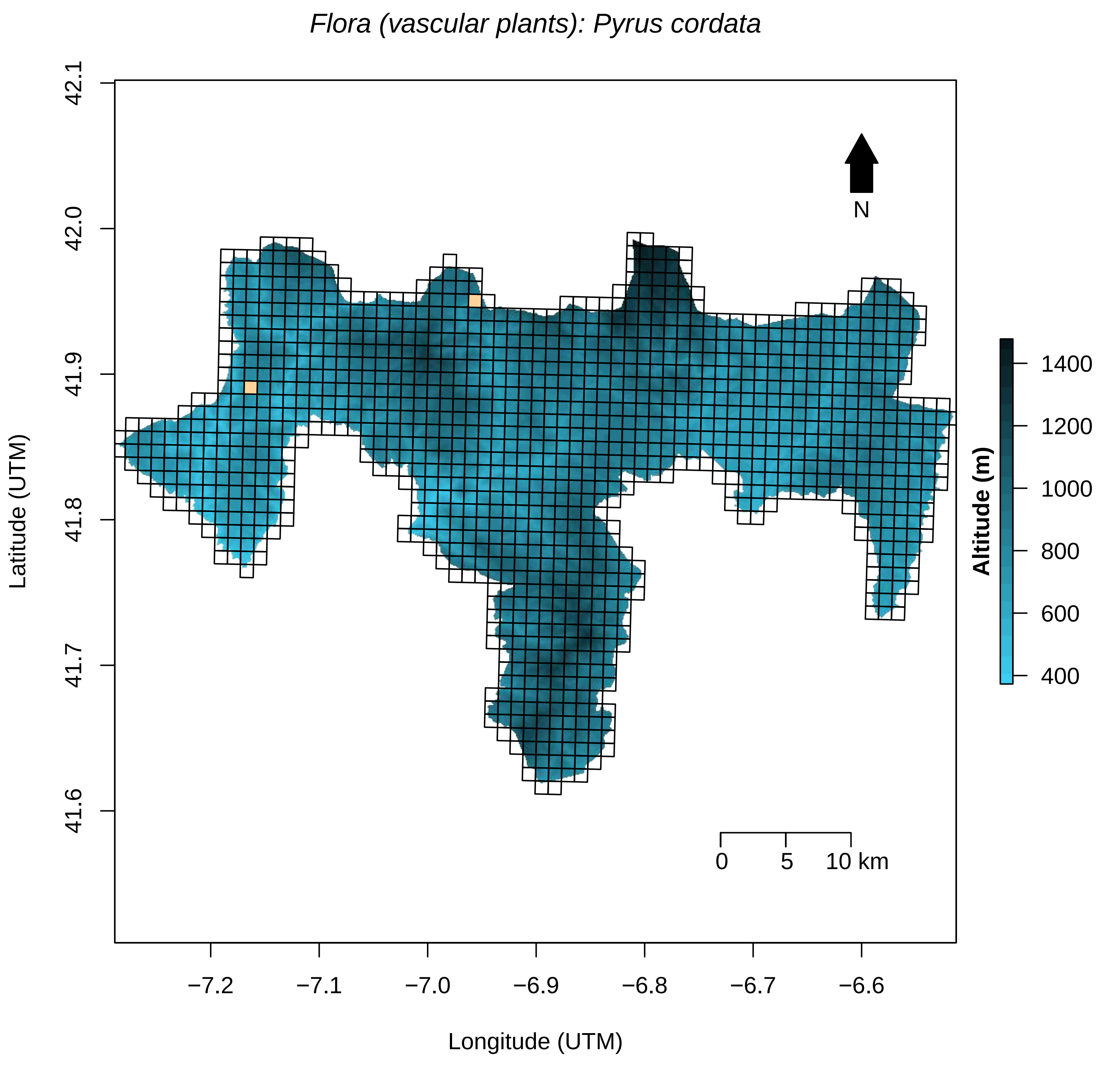
Flora (vascular plants): Pyrus cordata
N
0
5
10 km
Altitude (m)
1400
1200
1000
800
600
400
42.1
42.0
41.9
41.8
41.7
41.6
−7.2
−7.1
−7.0
−6.9
−6.8
−6.7
−6.6
Latitude (UTM)
Longitude (UTM)

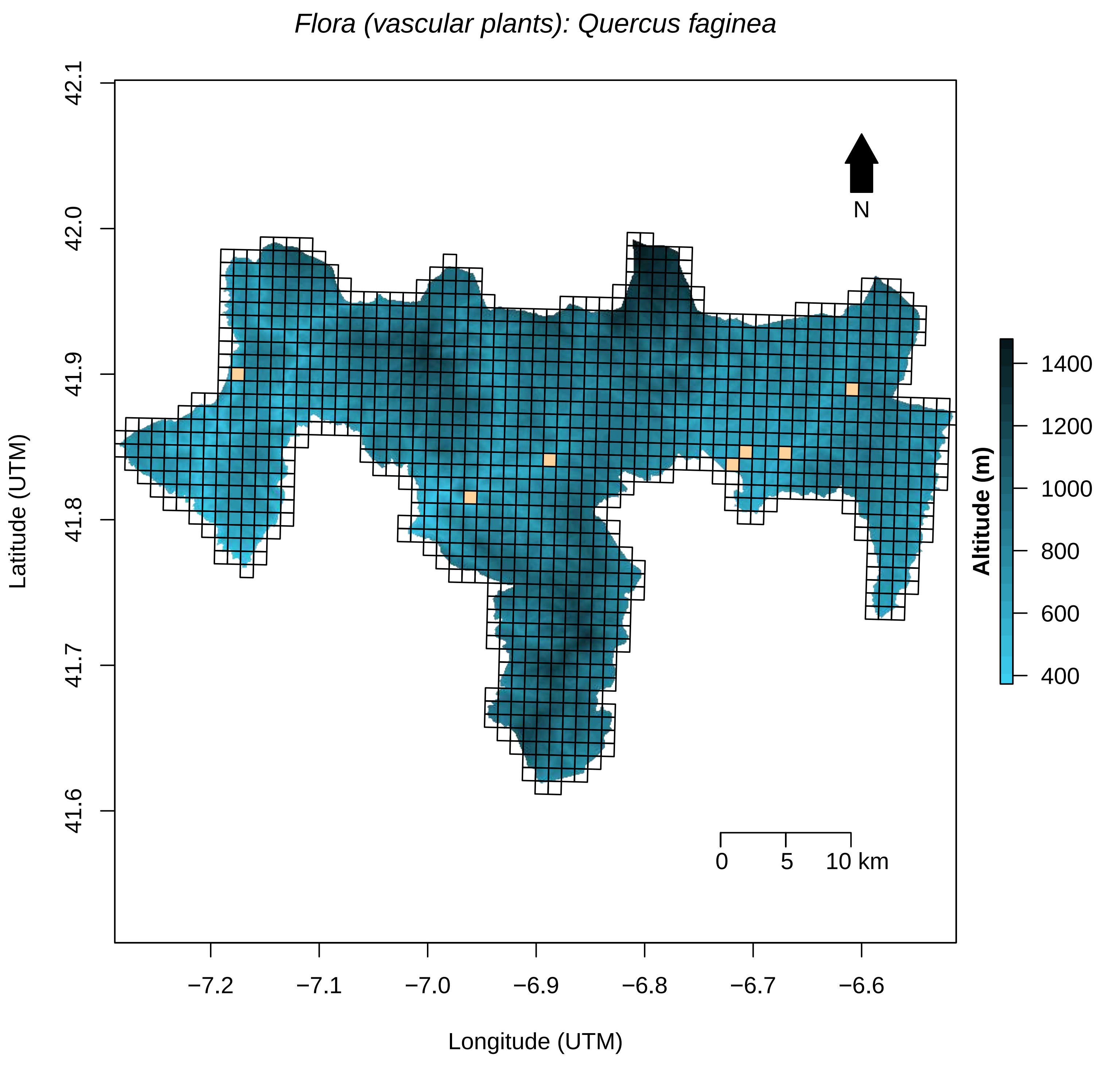

Flora (vascular plants): Quercus faginea
N
0
5
10 km
Altitude (m)
1400
1200
1000
800
600
400
42.1
42.0
41.9
41.8
41.7
41.6
Latitude (UTM)
−7.2
−7.1
−7.0
−6.9
−6.8
−6.7
−6.6
Longitude (UTM)

Flora (vascular plants): Quercus ilex

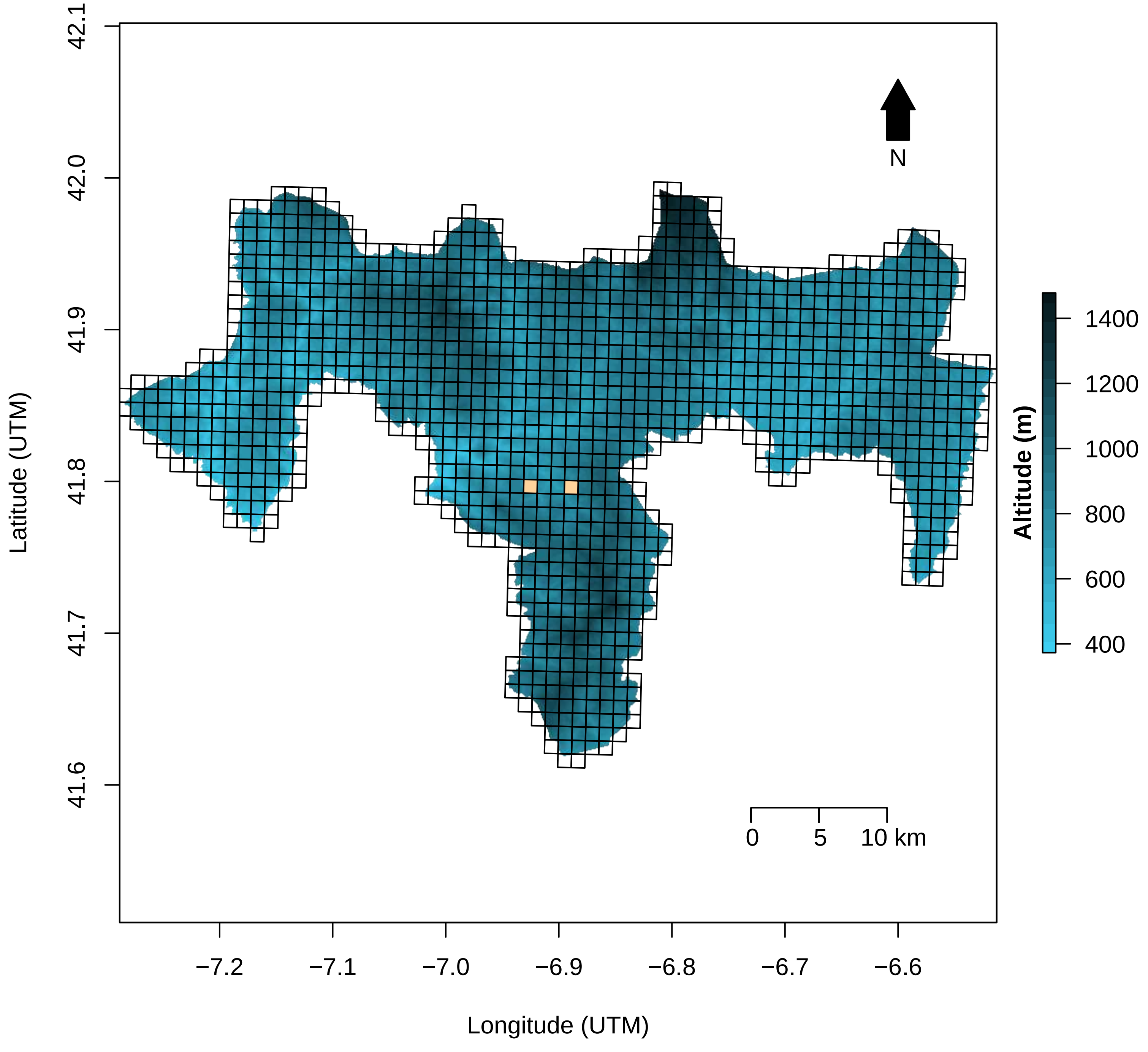

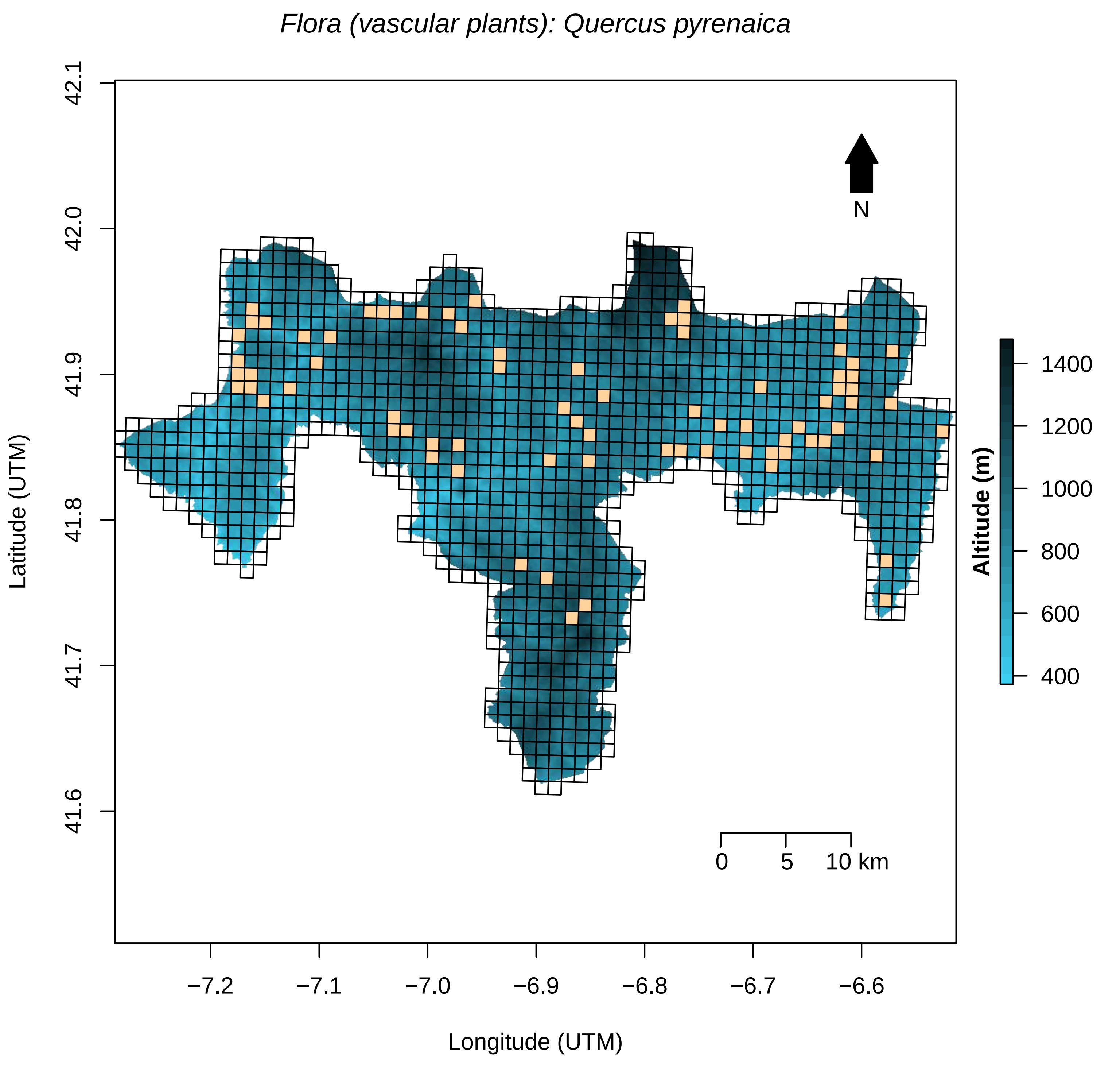

Flora (vascular plants): Quercus pyrenaica
Latitude (UTM)
Longitude (UTM)
42.1
42.0
41.9
41.8
41.7
41.6
−7.2
−7.1
−7.0
−6.9
−6.8
−6.7
−6.6
N
0
5
10 km
Altitude (m)
1400
1200
1000
800
600
400

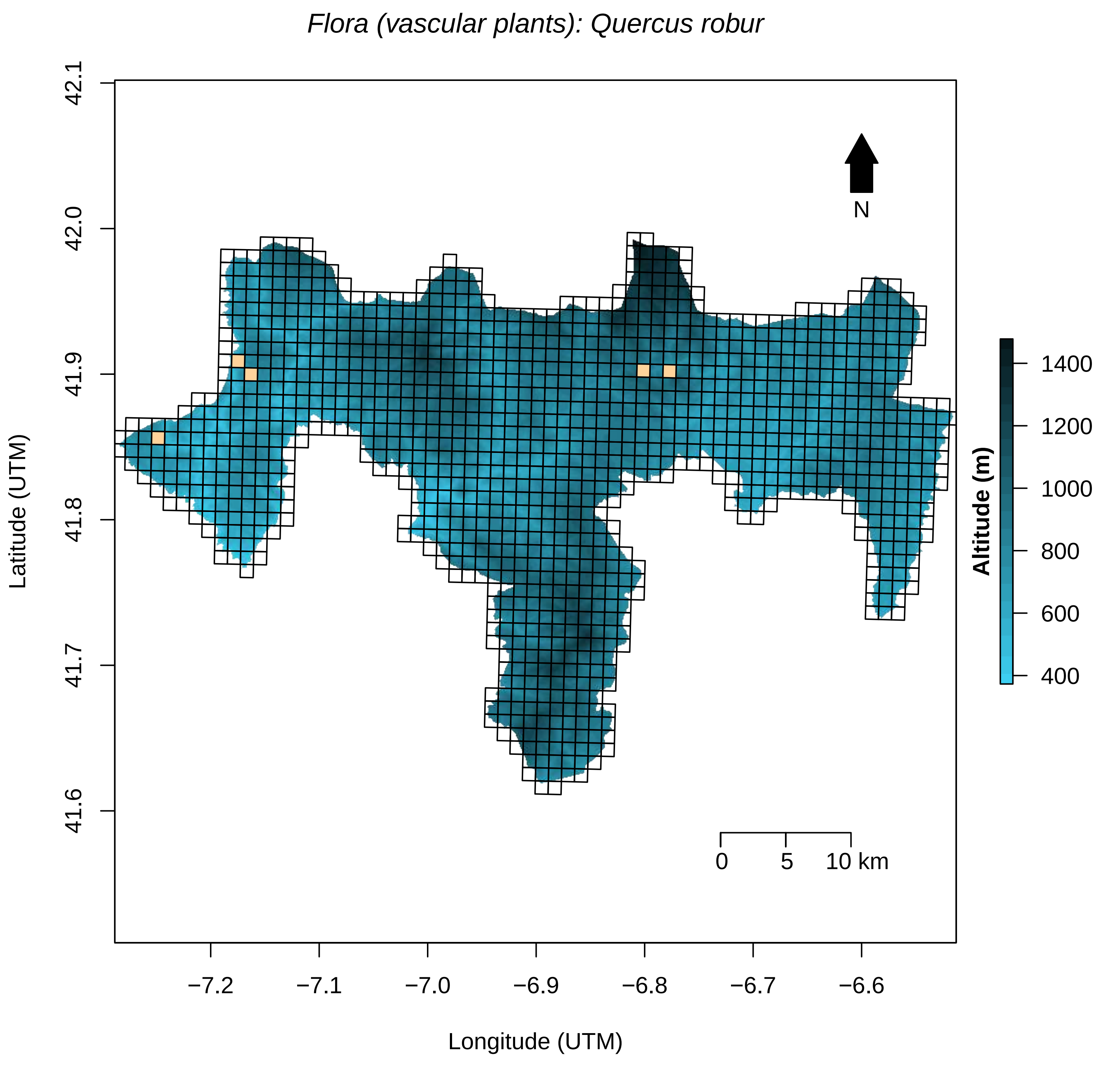
Flora (vascular plants): Quercus robur
42.1
42.0
41.9
41.8
41.7
41.6
Latitude (UTM)
−7.2
−7.1
−7.0
−6.9
−6.8
−6.7
−6.6
Longitude (UTM)
N
0
5
10 km
Altitude (m)
1400
1200
1000
800
600
400

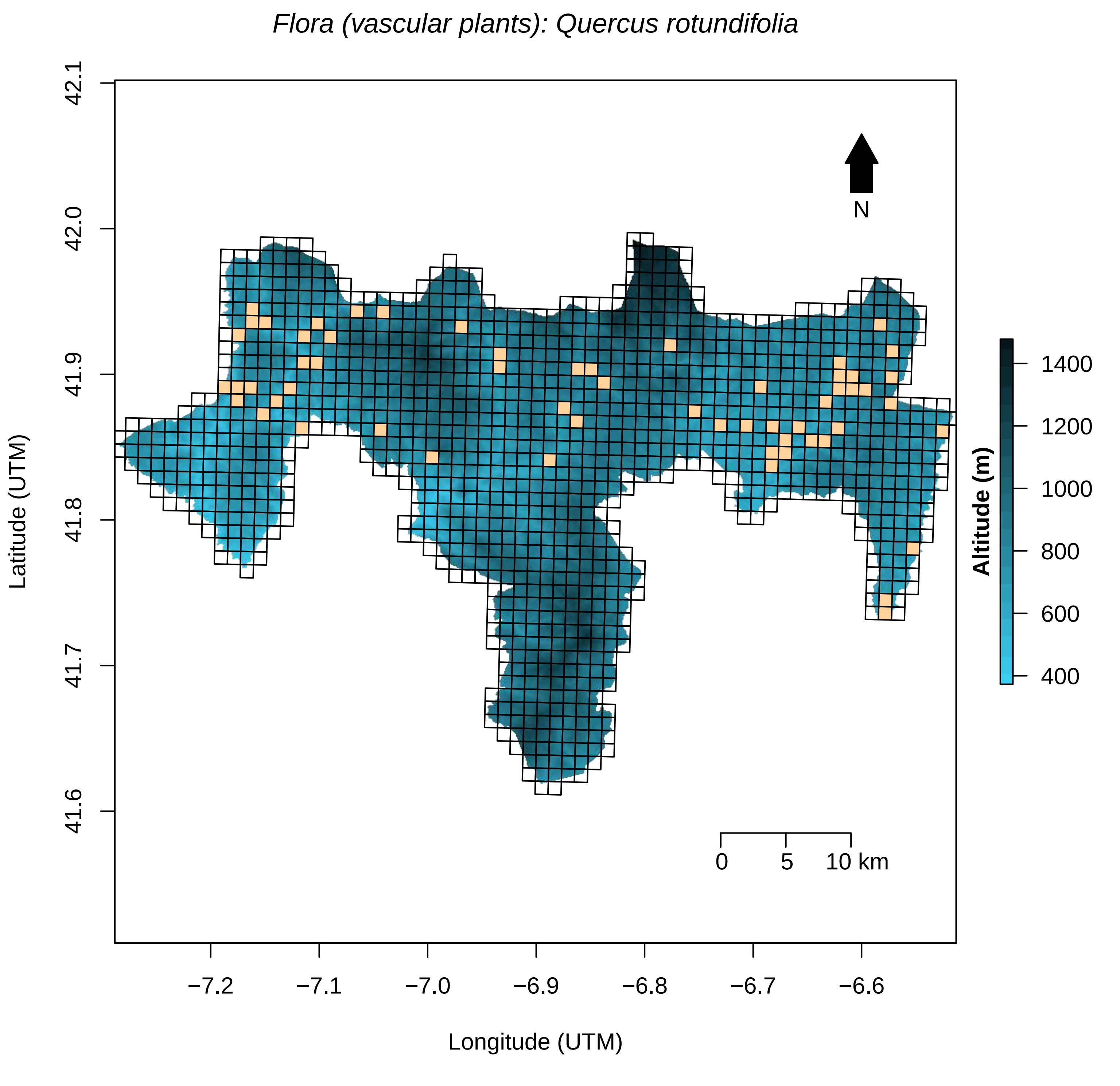

Flora (vascular plants): Quercus rotundifolia
42.1
42.0
41.9
41.8
41.7
41.6
Latitude (UTM)
−7.2
−7.1
−7.0
−6.9
−6.8
−6.7
−6.6
Longitude (UTM)
N
0
5
10 km
Altitude (m)
1400
1200
1000
800
600
400

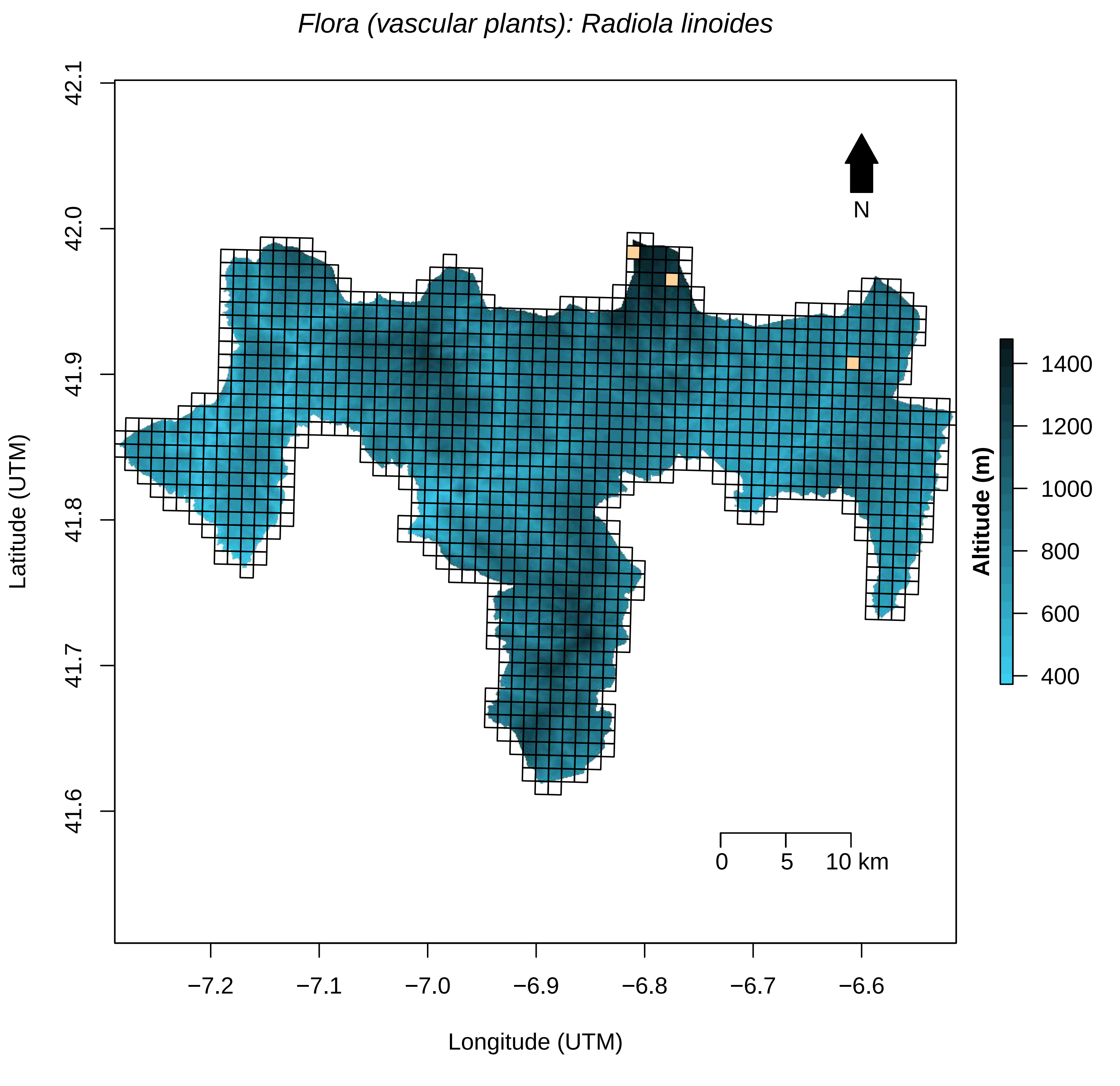

Flora (vascular plants): Radiola linoides
42.1
42.0
41.9
41.8
41.7
41.6
Latitude (UTM)
−7.2
−7.1
−7.0
−6.9
−6.8
−6.7
−6.6
Longitude (UTM)
N
0
5
10 km
Altitude (m)
1400
1200
1000
800
600
400

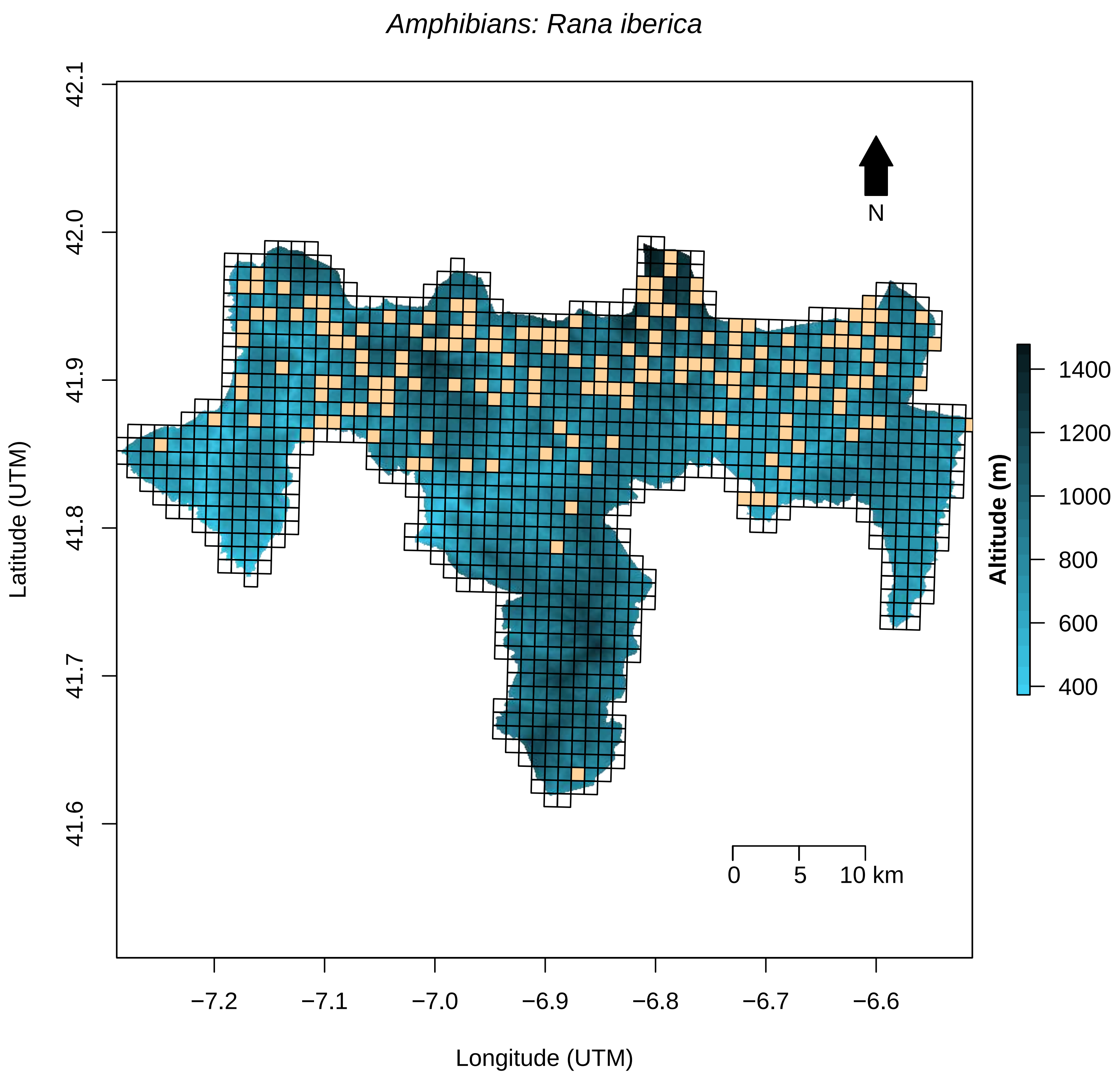

Amphibians: Rana iberica
42.1
42.0
41.9
41.8
41.7
41.6
Latitude (UTM)
−7.2
−7.1
−7.0
−6.9
−6.8
−6.7
−6.6
Longitude (UTM)
N
0
5
10 km
1400
1200
1000
800
600
400
Altitude (m)

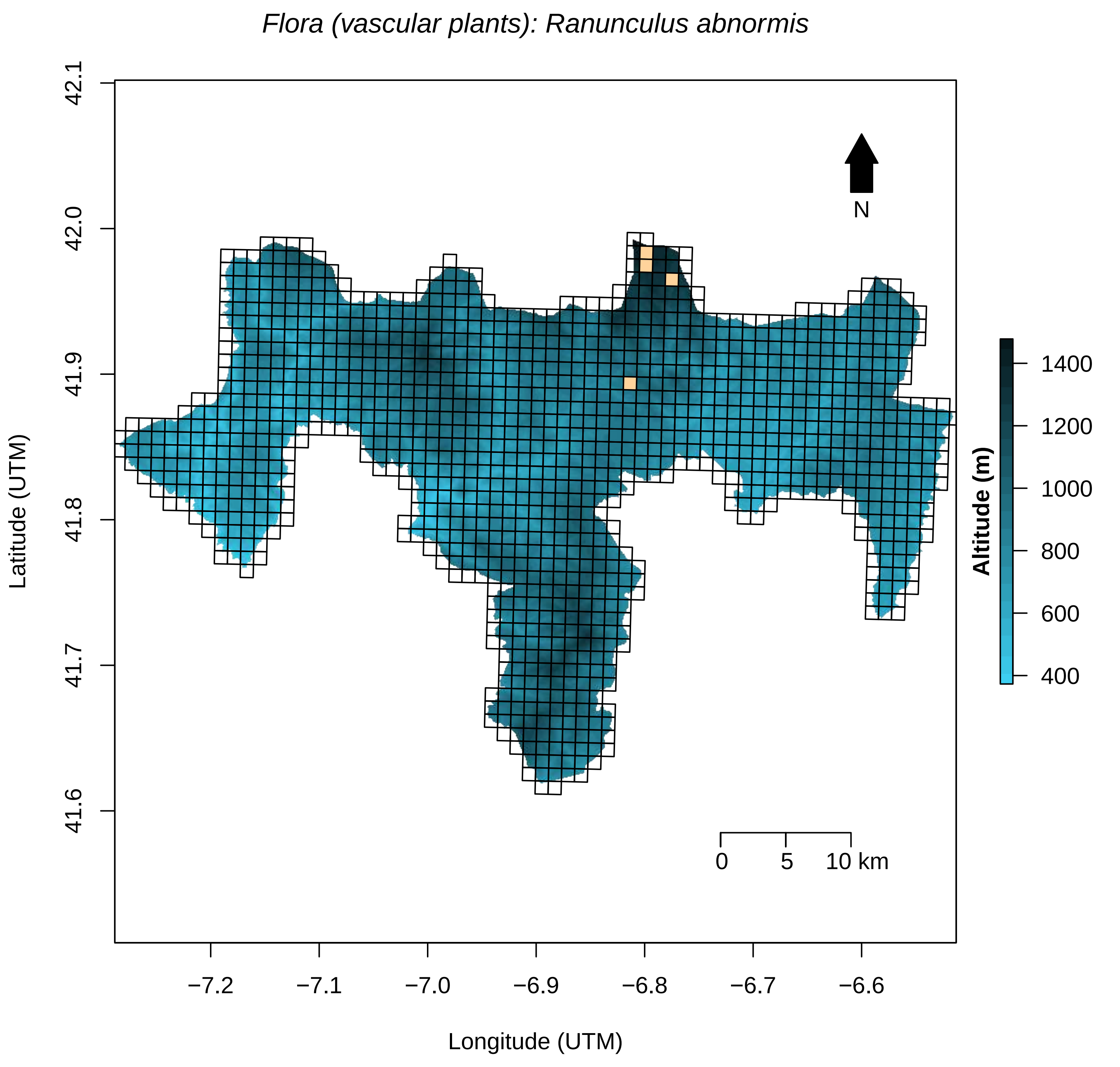
Flora (vascular plants): Ranunculus abnormis
N
0
5
10 km
Latitude (UTM)
42.1
42.0
41.9
41.8
41.7
41.6
Longitude (UTM)
−7.2
−7.1
−7.0
−6.9
−6.8
−6.7
−6.6
Altitude (m)
1400
1200
1000
800
600
400

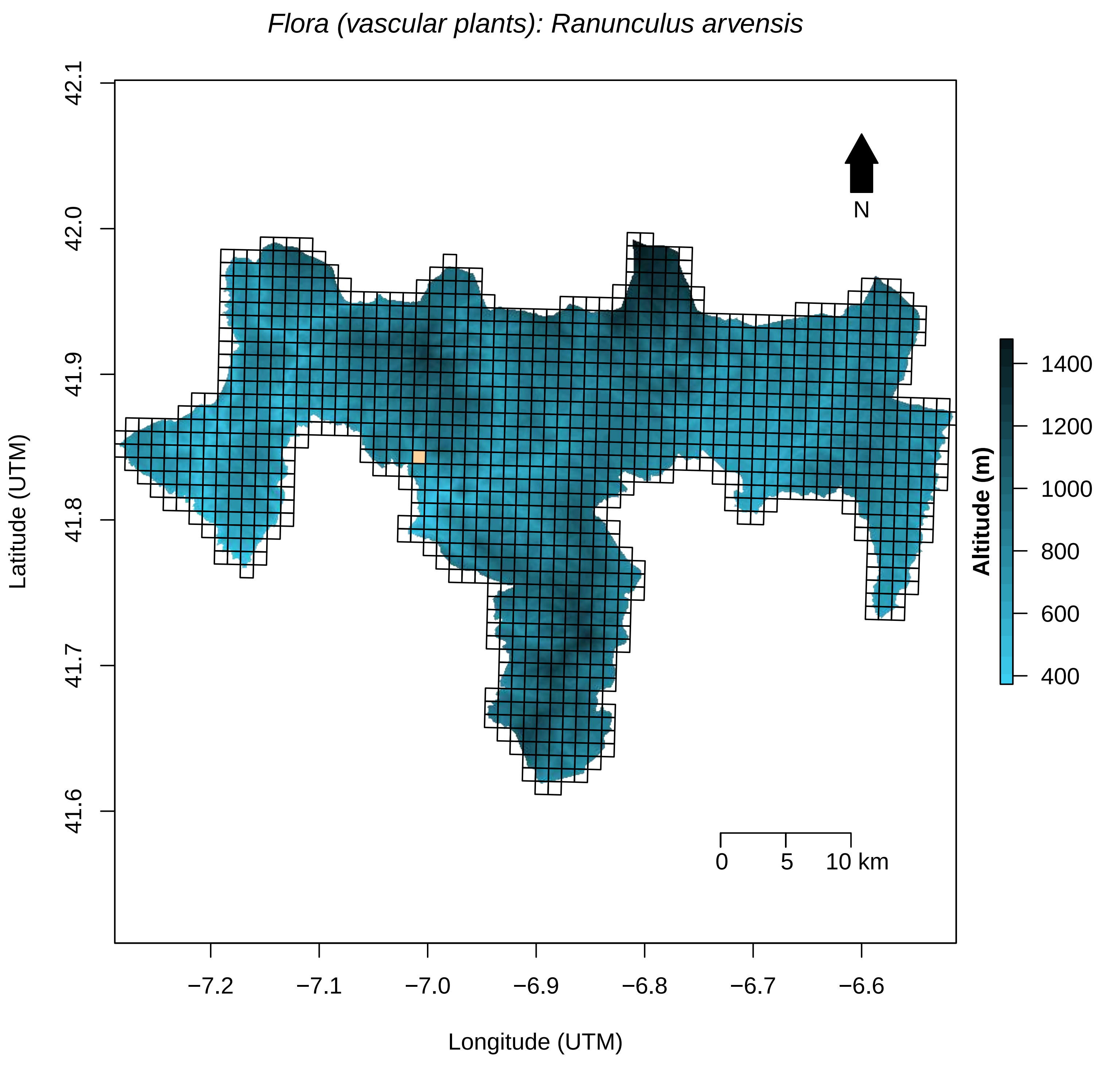

Flora (vascular plants): Ranunculus arvensis
42.1
42.0
41.9
41.8
41.7
41.6
Latitude (UTM)
N
0
5
10 km
−7.2
−7.1
−7.0
−6.9
−6.8
−6.7
−6.6
Longitude (UTM)
1400
1200
1000
800
600
400
Altitude (m)

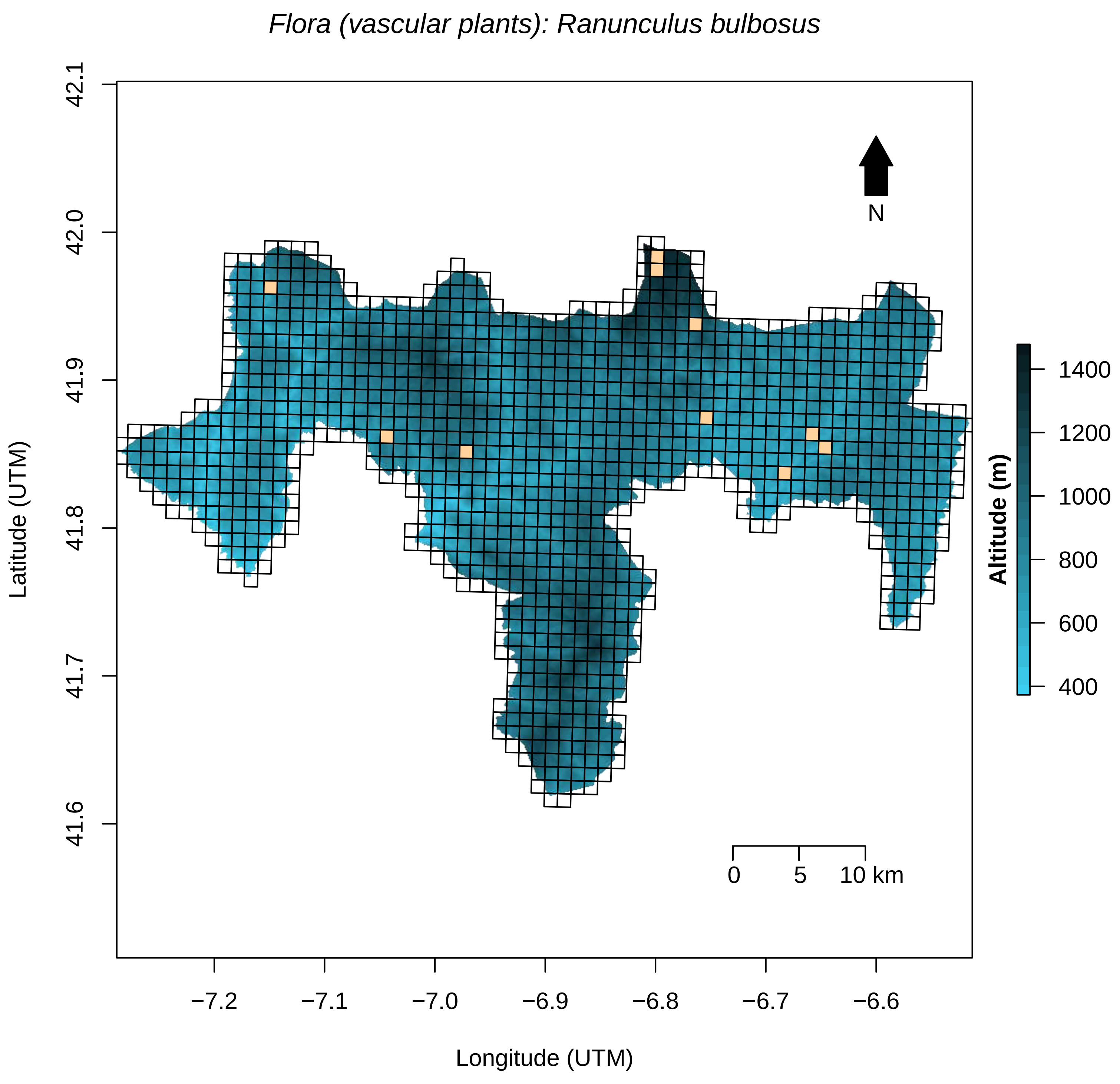

Flora (vascular plants): Ranunculus bulbosus
Latitude (UTM)
42.1
42.0
41.9
41.8
41.7
41.6
Longitude (UTM)
−7.2
−7.1
−7.0
−6.9
−6.8
−6.7
−6.6
N
0
5
10 km
Altitude (m)
1400
1200
1000
800
600
400

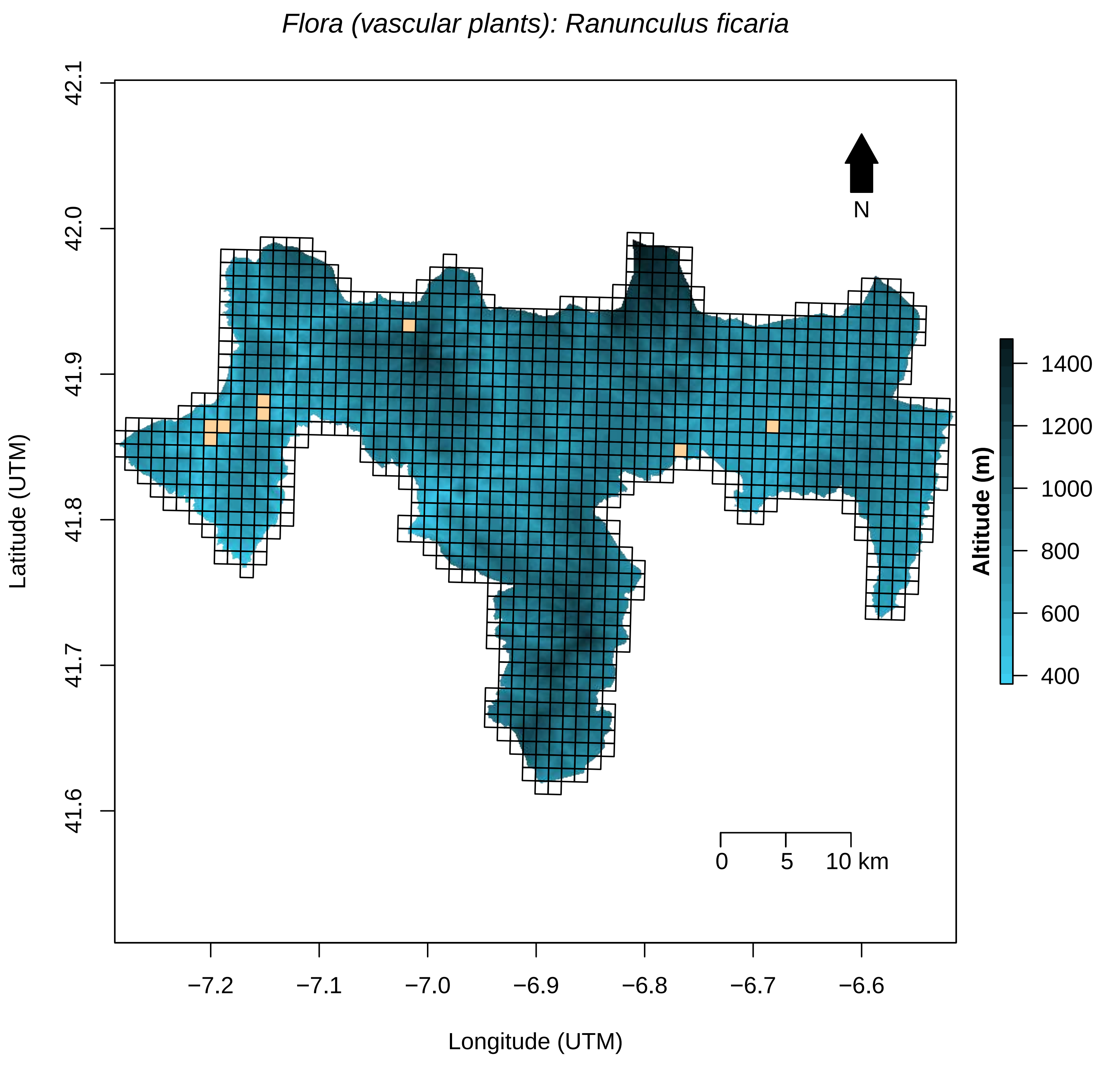

Flora (vascular plants): Ranunculus ficaria
42.1
42.0
41.9
41.8
41.7
41.6
Latitude (UTM)
N
0
5
10 km
1400
1200
1000
800
600
400
Altitude (m)
−7.2
−7.1
−7.0
−6.9
−6.8
−6.7
−6.6
Longitude (UTM)

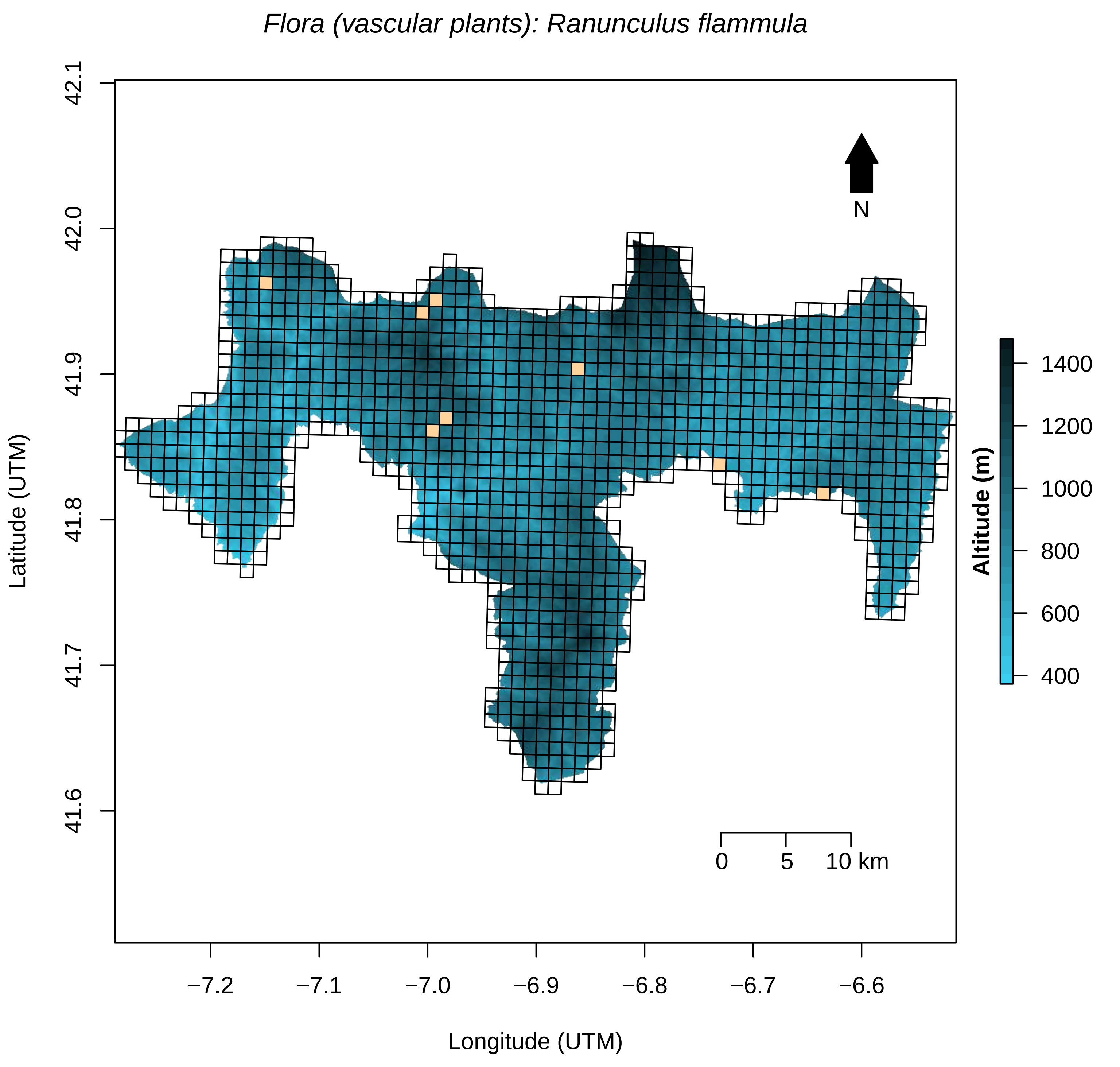

Flora (vascular plants): Ranunculus flammula
42.1
42.0
41.9
41.8
41.7
41.6
Latitude (UTM)
−7.2
−7.1
−7.0
−6.9
−6.8
−6.7
−6.6
Longitude (UTM)
N
0
5
10 km
Altitude (m)
1400
1200
1000
800
600
400

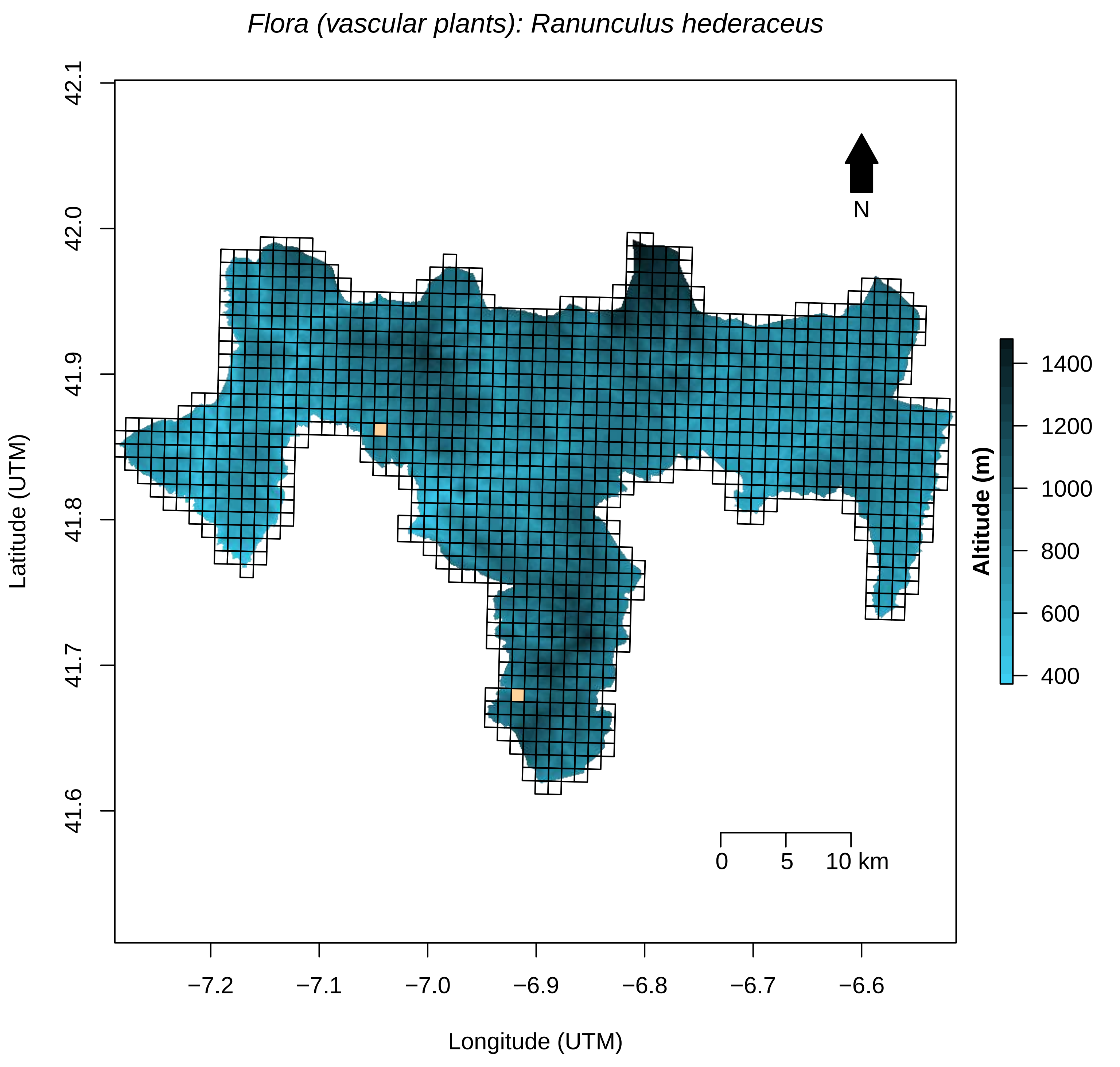

Flora (vascular plants): Ranunculus hederaceus
42.1
42.0
41.9
41.8
41.7
41.6
Latitude (UTM)
−7.2
−7.1
−7.0
−6.9
−6.8
−6.7
−6.6
Longitude (UTM)
N
0
5
10 km
1400
1200
1000
800
600
400
Altitude (m)

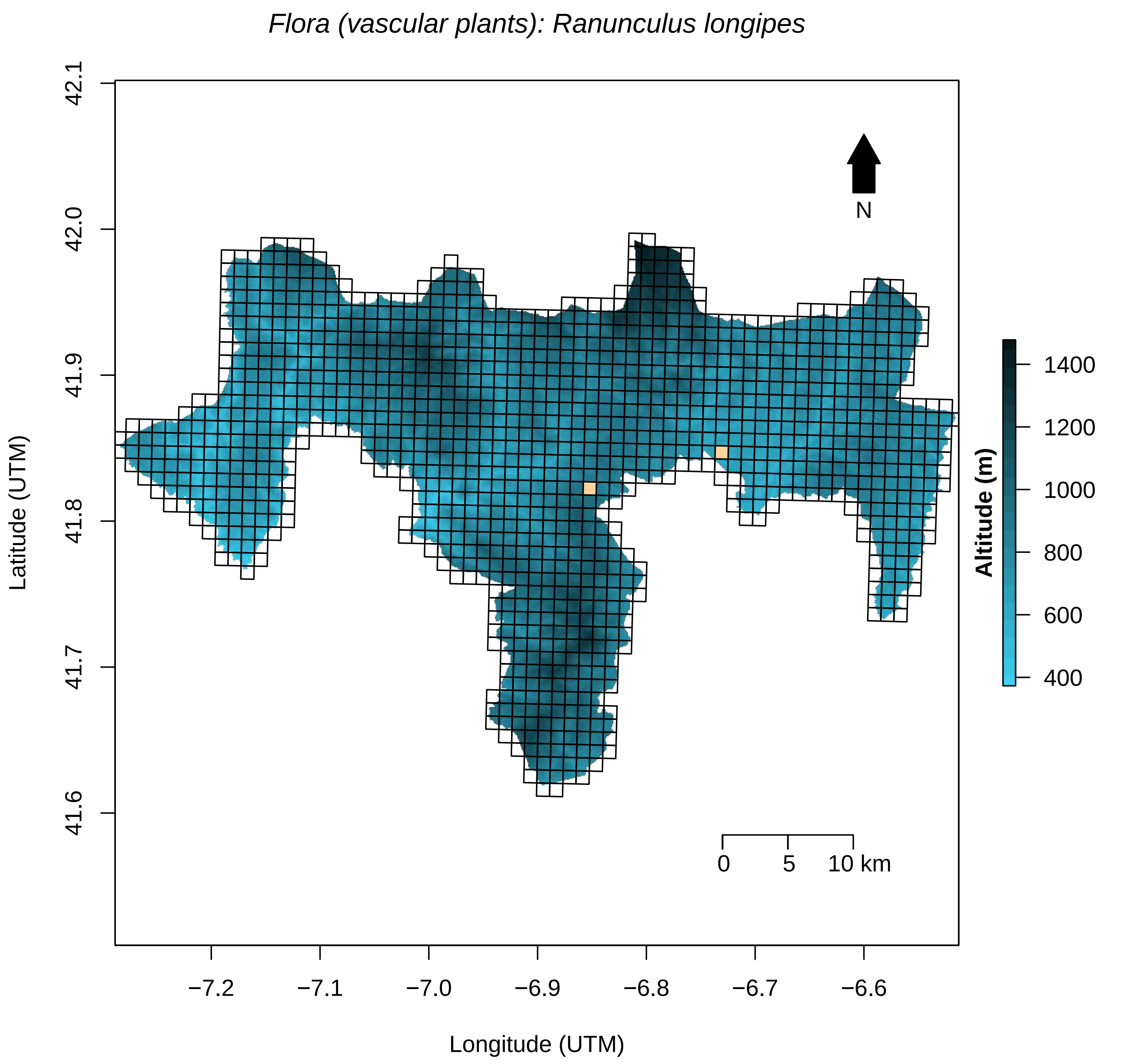

Flora (vascular plants): Ranunculus longipes
N
0
5
10 km
Altitude (m)
1400
1200
1000
800
600
400
Latitude (UTM)
42.1
42.0
41.9
41.8
41.7
41.6
Longitude (UTM)
−7.2
−7.1
−7.0
−6.9
−6.8
−6.7
−6.6

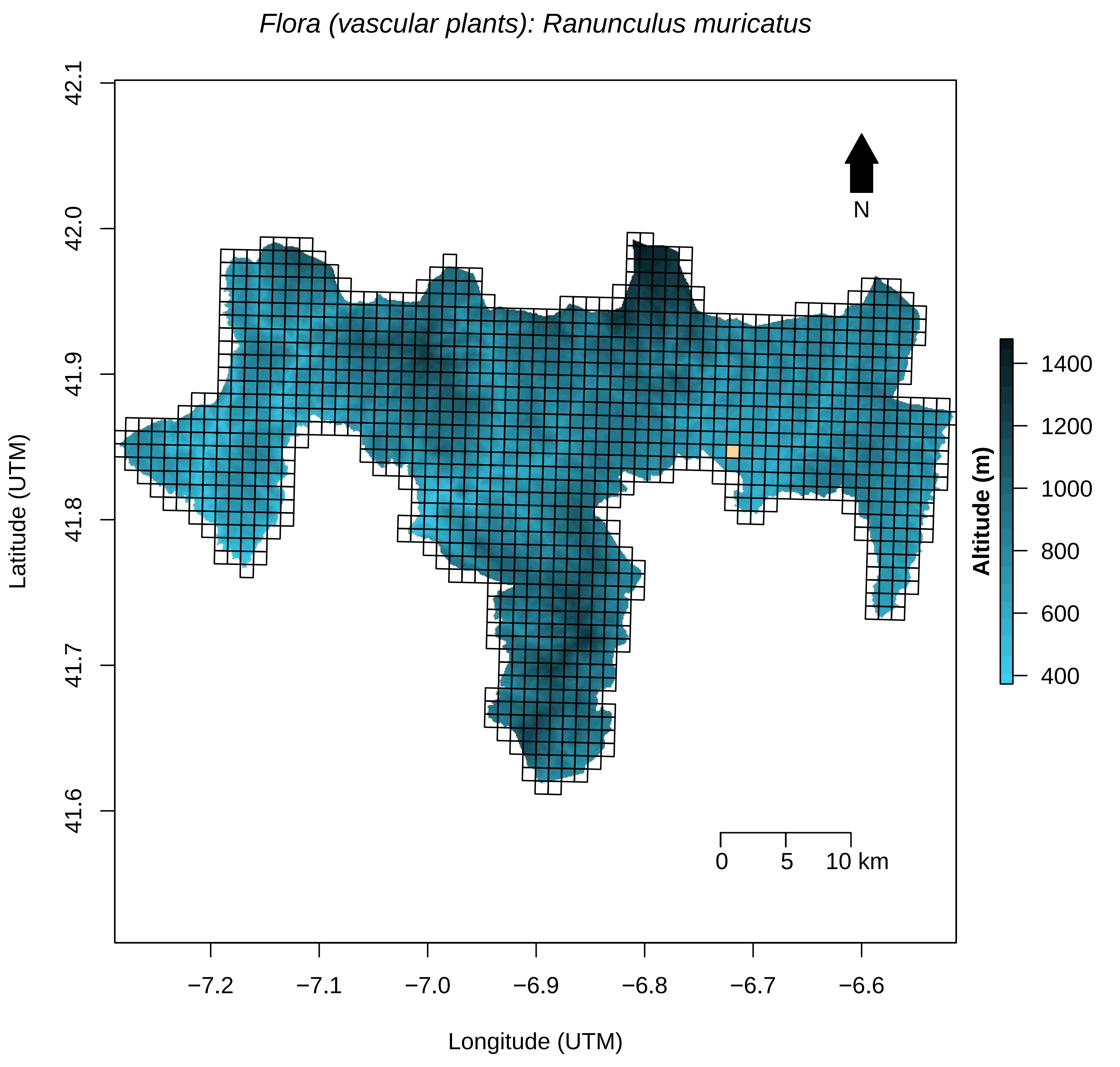

Flora (vascular plants): Ranunculus muricatus
42.1
42.0
41.9
41.8
41.7
41.6
Latitude (UTM)
−7.2
−7.1
−7.0
−6.9
−6.8
−6.7
−6.6
Longitude (UTM)
N
0
5
10 km
1400
1200
1000
800
600
400
Altitude (m)

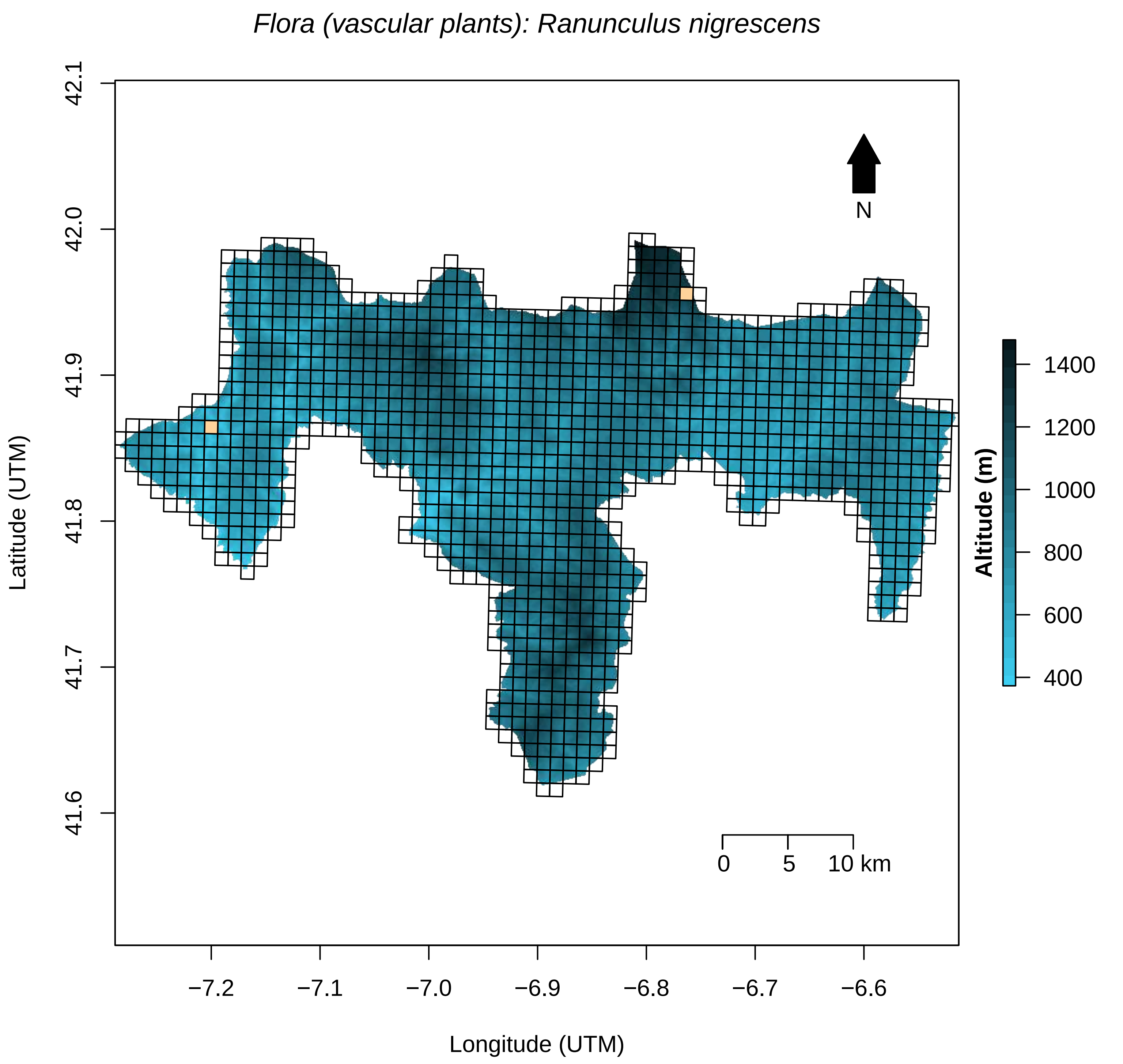

Flora (vascular plants): Ranunculus nigrescens
42.1
42.0
41.9
41.8
41.7
41.6
Latitude (UTM)
N
0
5
10 km
−7.2
−7.1
−7.0
−6.9
−6.8
−6.7
−6.6
Longitude (UTM)
Altitude (m)
1400
1200
1000
800
600
400

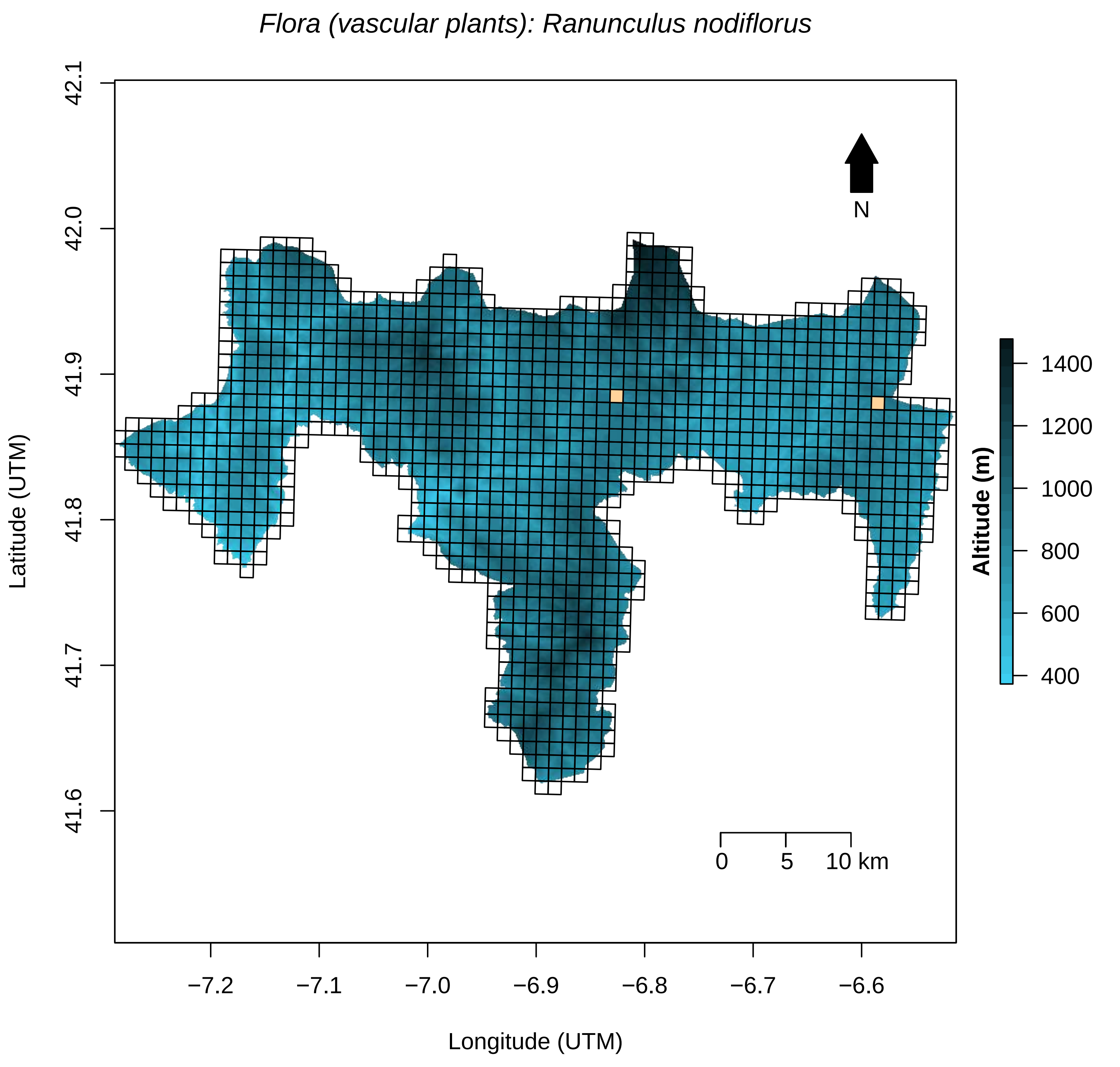

Flora (vascular plants): Ranunculus nodiflorus
42.1
42.0
41.9
41.8
41.7
41.6
Latitude (UTM)
−7.2
−7.1
−7.0
−6.9
−6.8
−6.7
−6.6
Longitude (UTM)
N
0
5
10 km
Altitude (m)
1400
1200
1000
800
600
400

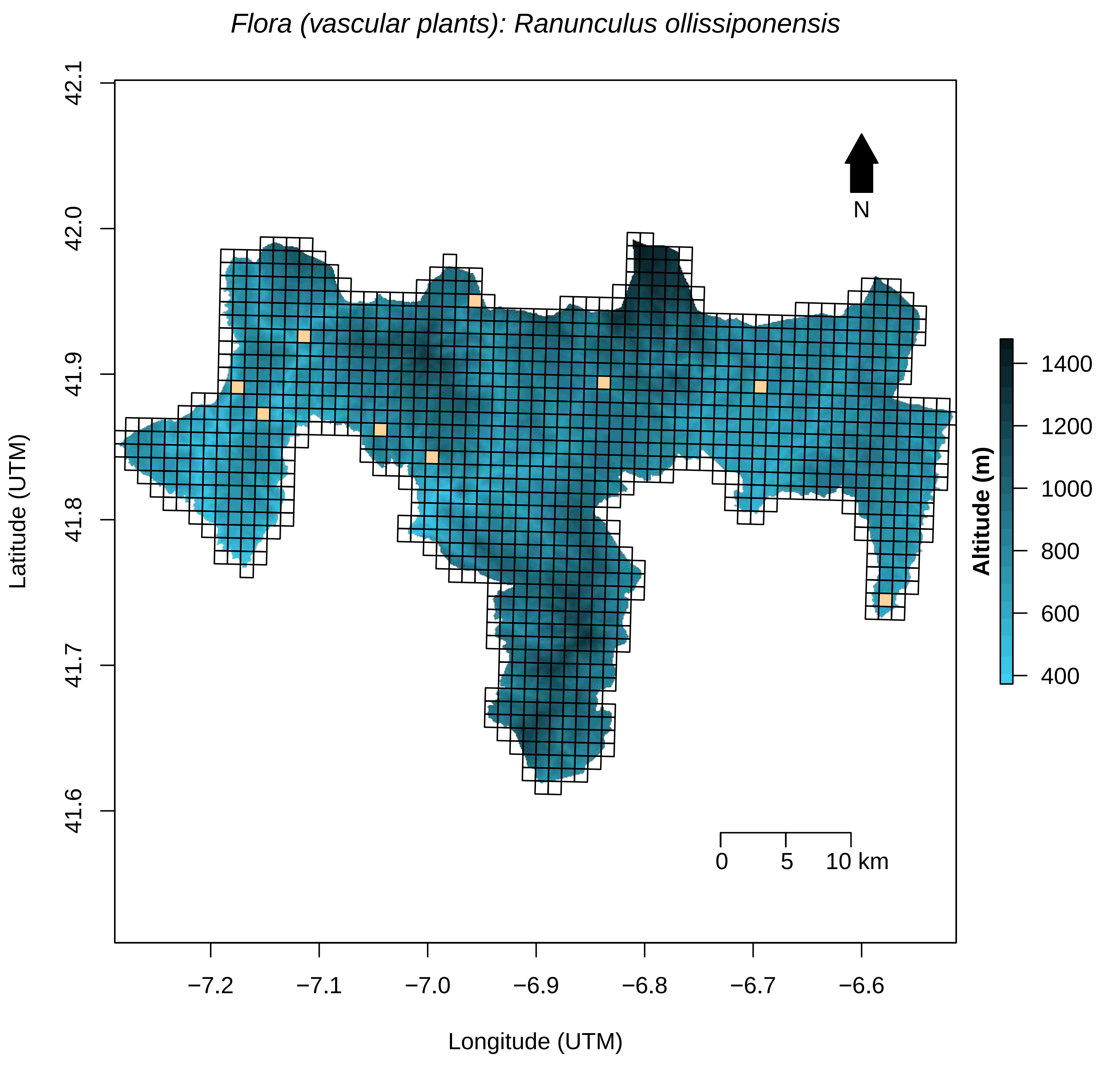

Flora (vascular plants): Ranunculus ollissiponensis
N
0 5 10 km
Altitude (m)
1400
1200
1000
800
600
400
Latitude (UTM)
42.1
42.0
41.9
41.8
41.7
41.6
Longitude (UTM)
−7.2
−7.1
−7.0
−6.9
−6.8
−6.7
−6.6

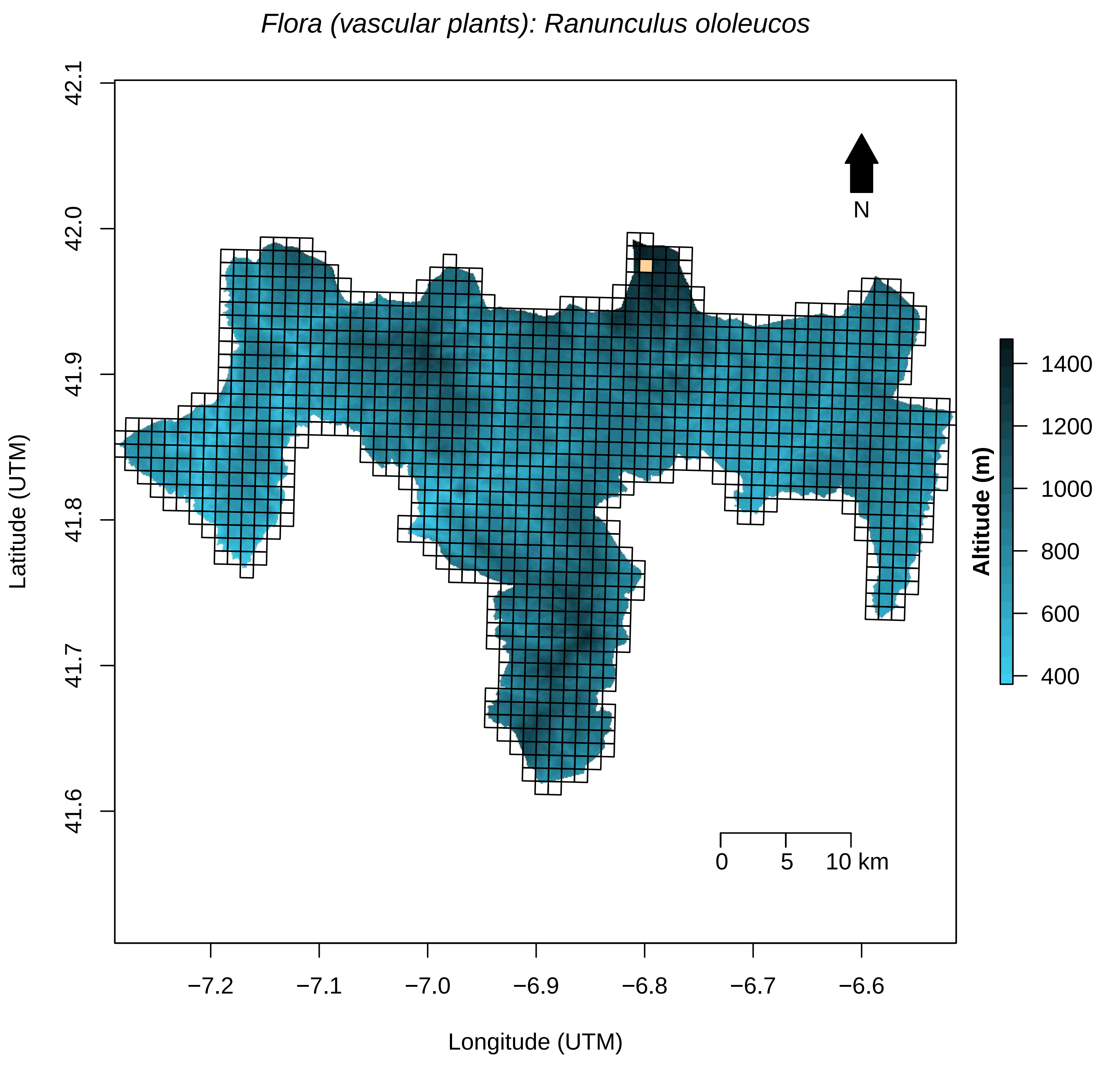

Flora (vascular plants): Ranunculus ololeucos
42.1
42.0
41.9
41.8
41.7
41.6
Latitude (UTM)
−7.2
−7.1
−7.0
−6.9
−6.8
−6.7
−6.6
Longitude (UTM)
N
0
5
10 km
Altitude (m)
1400
1200
1000
800
600
400

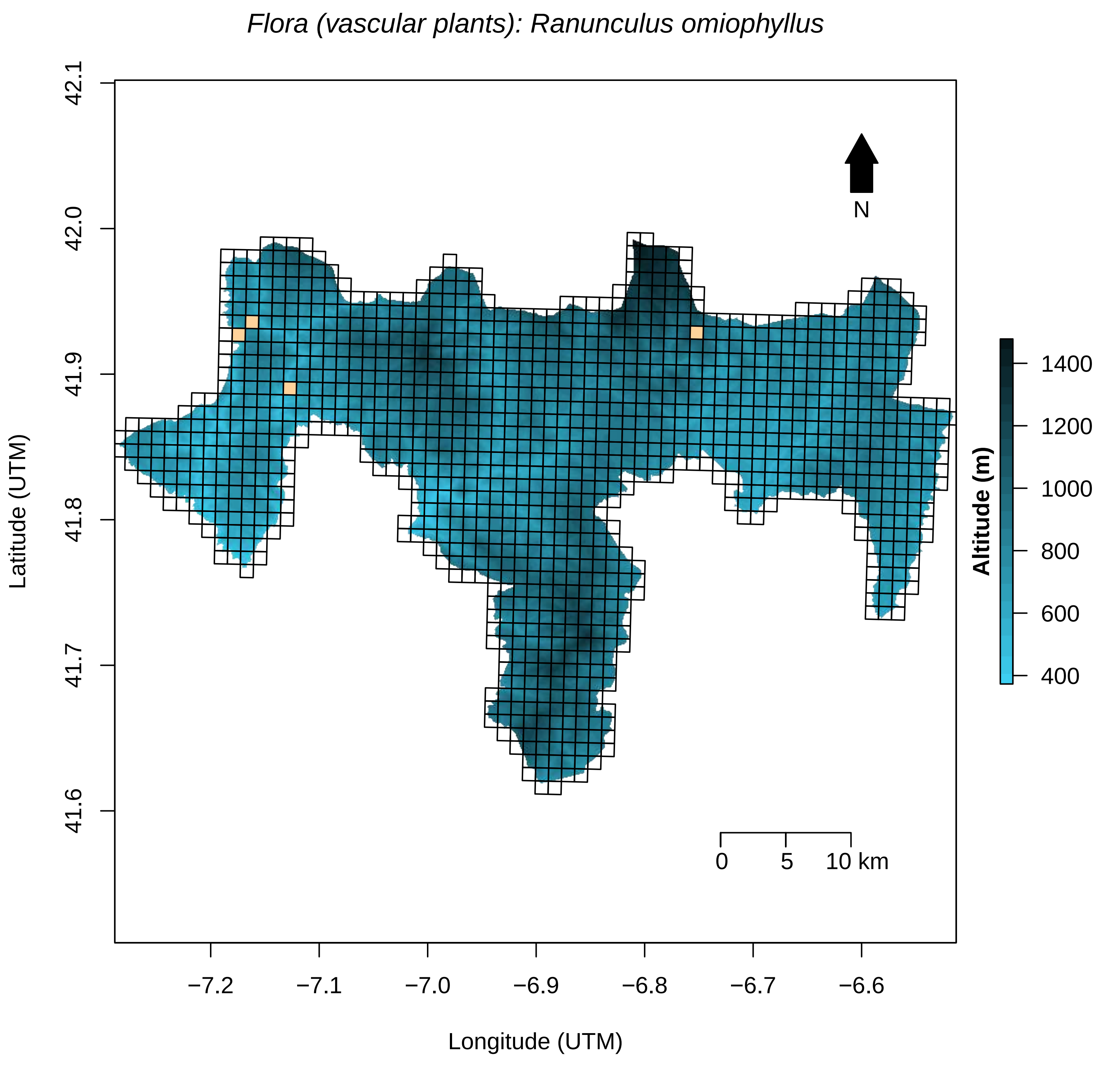
Flora (vascular plants): Ranunculus omiophyllus
42.1
42.0
41.9
41.8
41.7
41.6
Latitude (UTM)
−7.2
−7.1
−7.0
−6.9
−6.8
−6.7
−6.6
Longitude (UTM)
N
0
5
10 km
Altitude (m)
1400
1200
1000
800
600
400

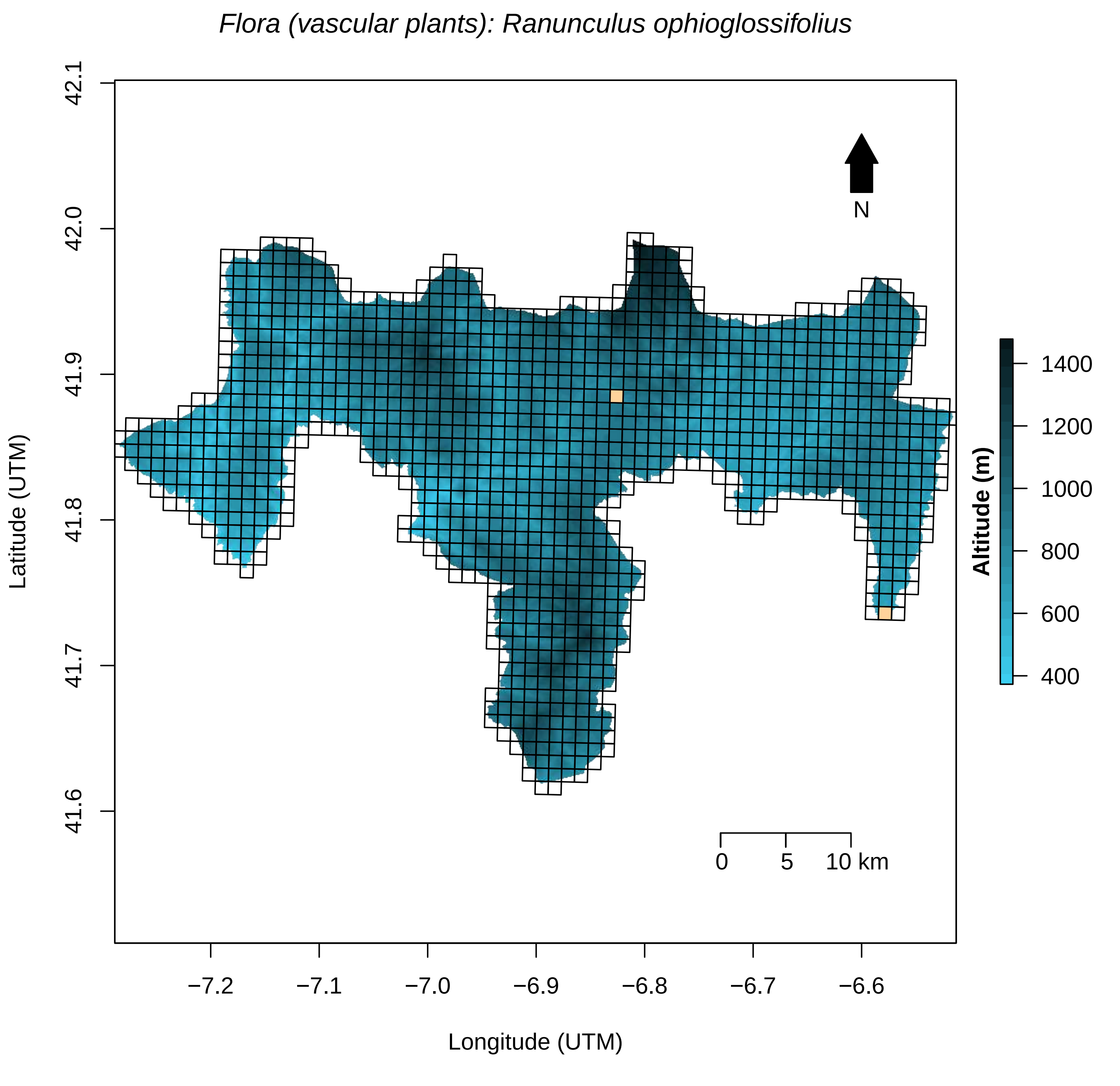
Flora (vascular plants): Ranunculus ophioglossifolius
42.1
42.0
41.9
41.8
41.7
41.6
Latitude (UTM)
−7.2
−7.1
−7.0
−6.9
−6.8
−6.7
−6.6
Longitude (UTM)
N
0
5
10 km
1400
1200
1000
800
600
400
Altitude (m)

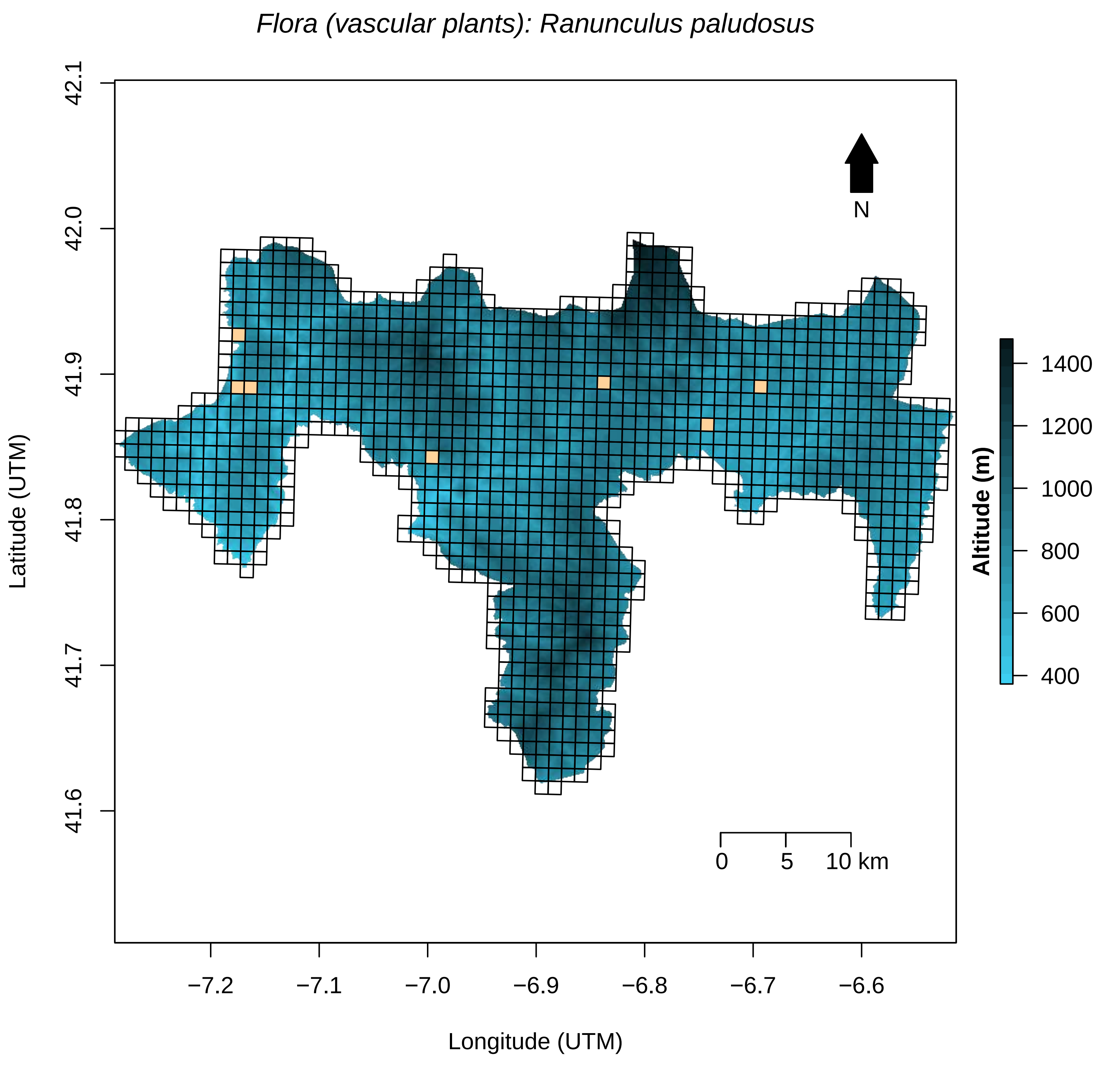

Flora (vascular plants): Ranunculus paludosus
N
0 5 10 km
Altitude (m)
1400
1200
1000
800
600
400
42.1
42.0
41.9
41.8
41.7
41.6
Latitude (UTM)
−7.2
−7.1
−7.0
−6.9
−6.8
−6.7
−6.6
Longitude (UTM)

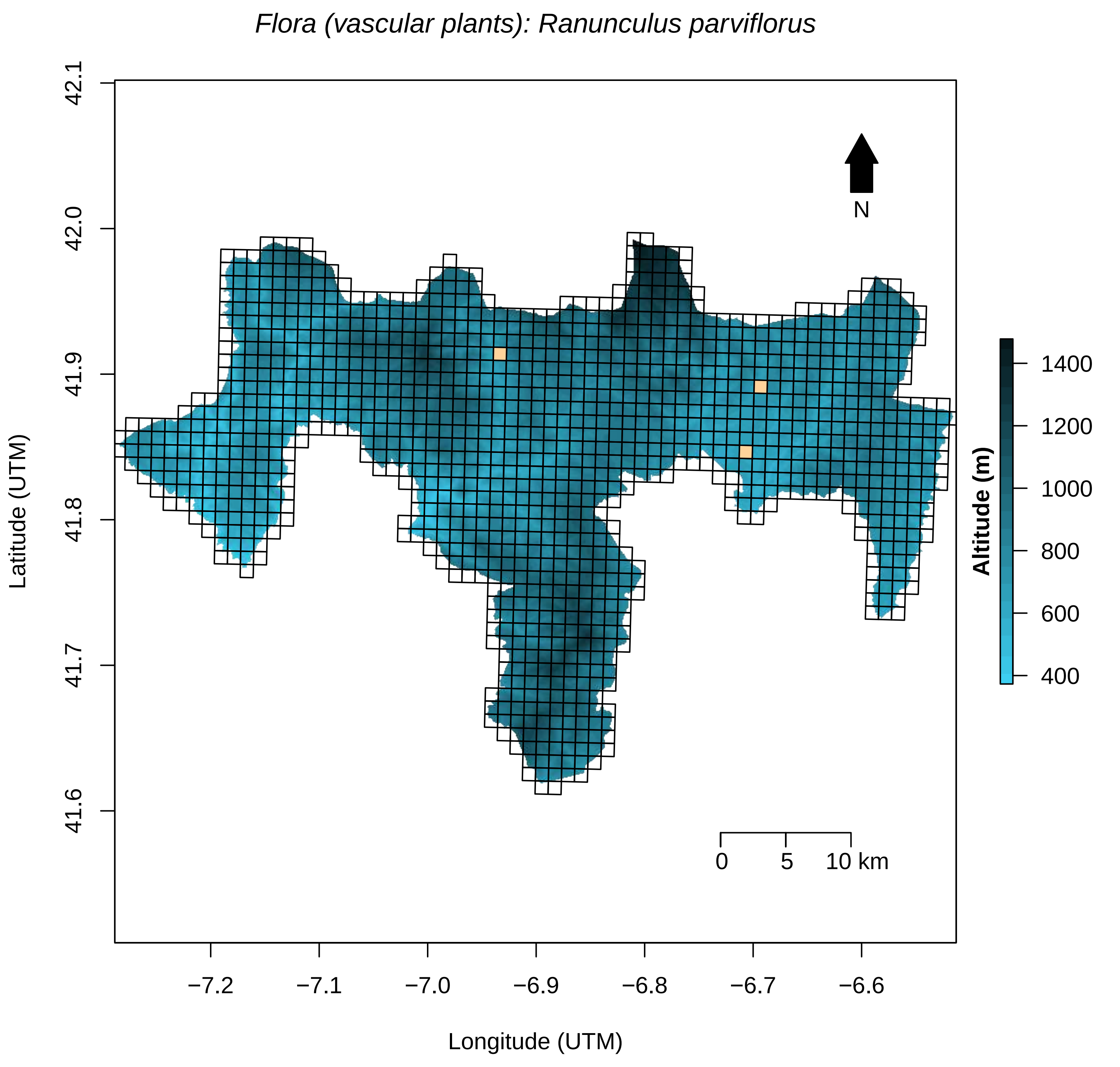
Flora (vascular plants): Ranunculus parviflorus
N
0
5
10 km
Altitude (m)
1400
1200
1000
800
600
400
42.1
42.0
41.9
41.8
41.7
41.6
−7.2
−7.1
−7.0
−6.9
−6.8
−6.7
−6.6
Latitude (UTM)
Longitude (UTM)

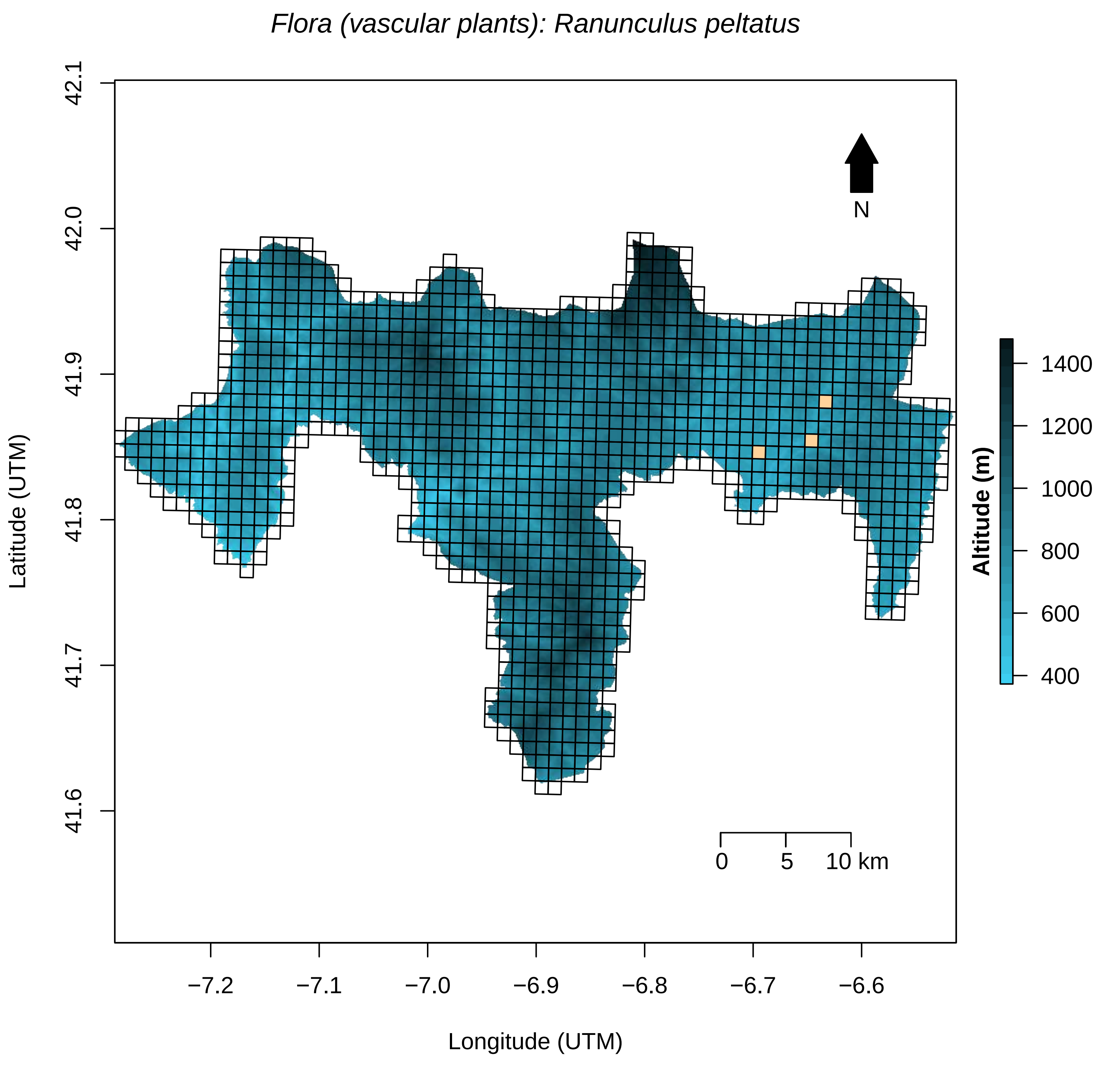

Flora (vascular plants): Ranunculus peltatus
42.1
42.0
41.9
41.8
41.7
41.6
Latitude (UTM)
−7.2
−7.1
−7.0
−6.9
−6.8
−6.7
−6.6
Longitude (UTM)
N
0
5
10 km
Altitude (m)
1400
1200
1000
800
600
400

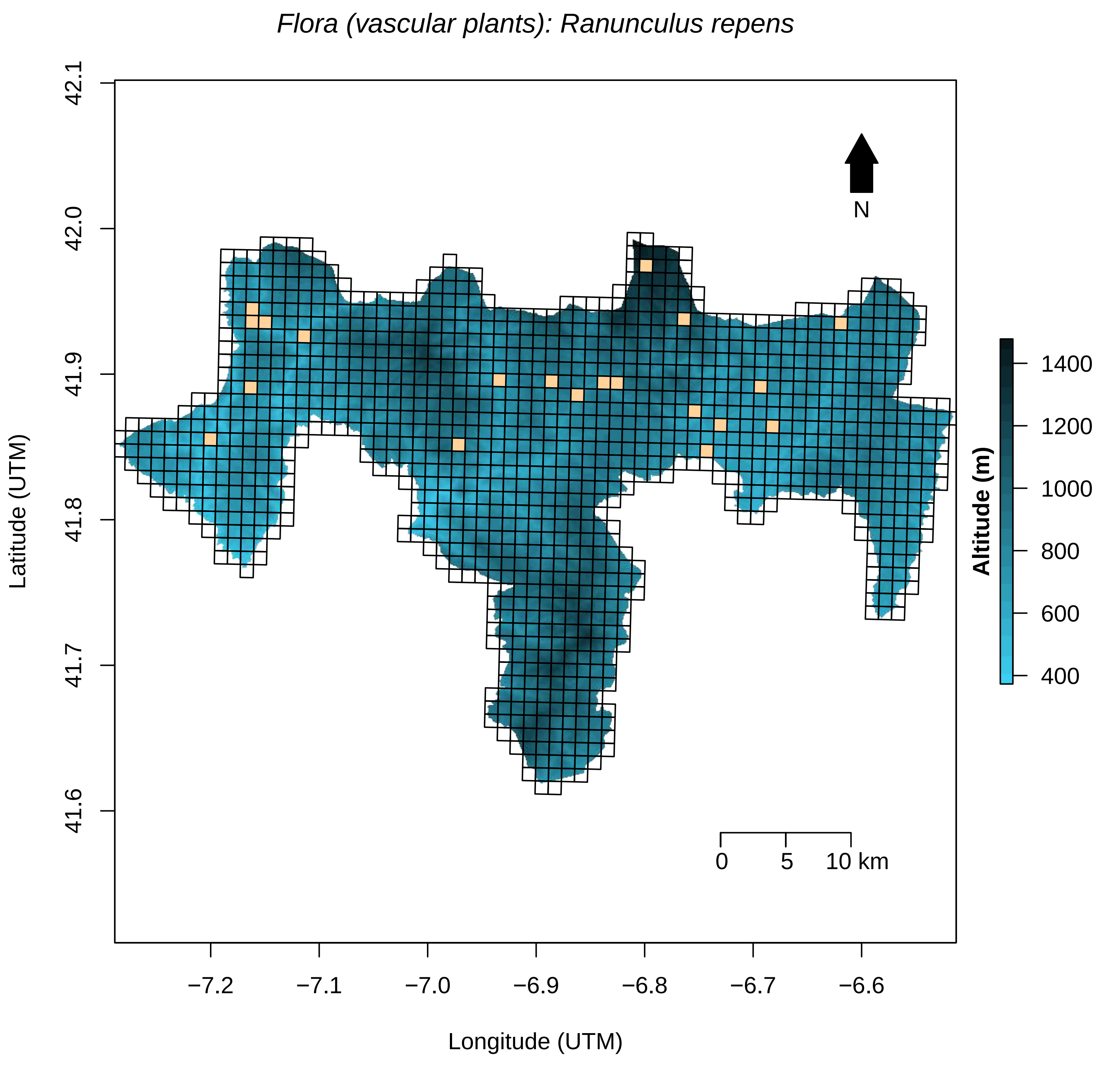

Flora (vascular plants): Ranunculus repens
42.1
42.0
41.9
41.8
41.7
41.6
Latitude (UTM)
−7.2
−7.1
−7.0
−6.9
−6.8
−6.7
−6.6
Longitude (UTM)
N
0
5
10 km
Altitude (m)
1400
1200
1000
800
600
400

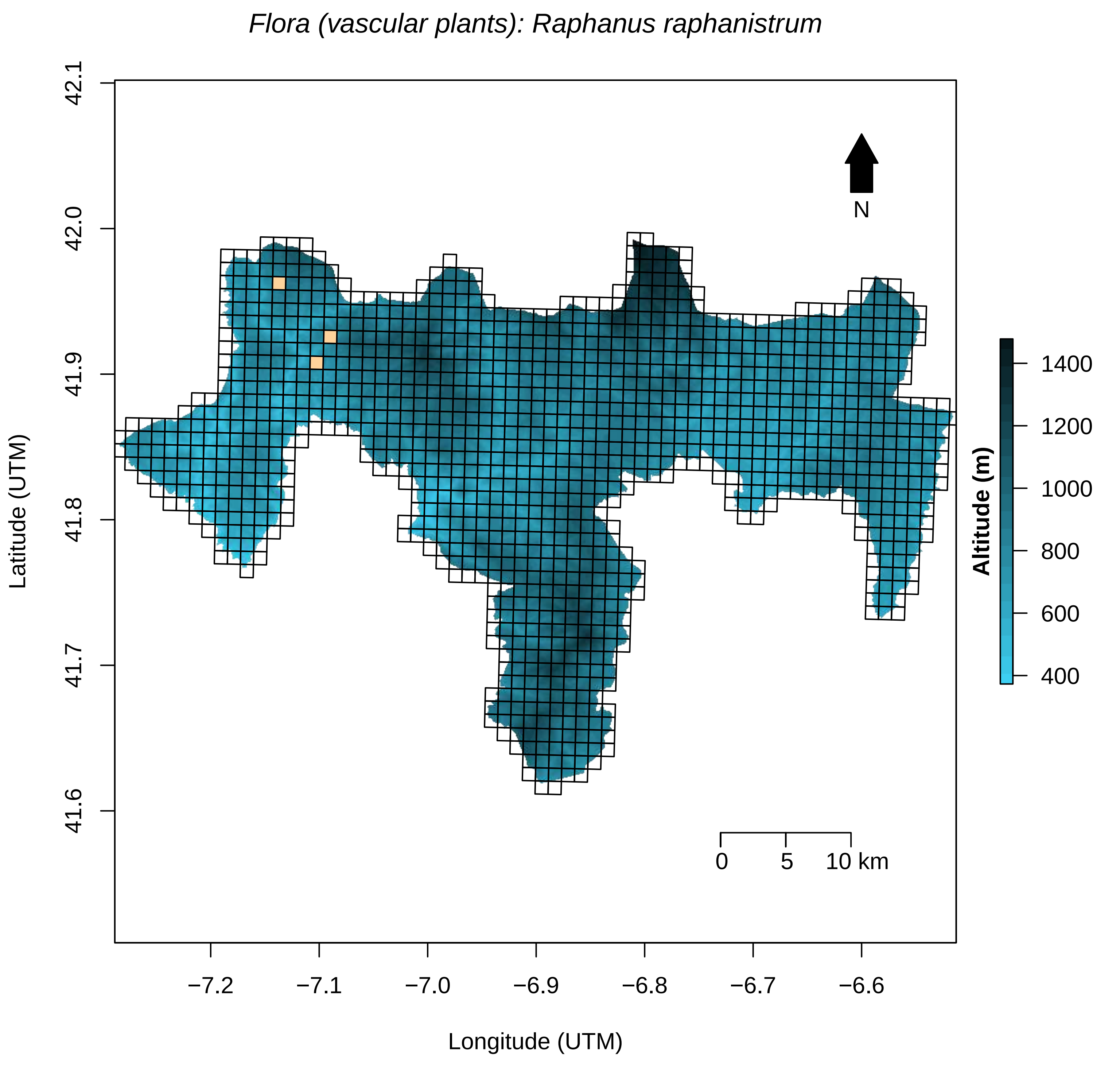

Flora (vascular plants): Raphanus raphanistrum
N
0
5
10 km
Latitude (UTM)
42.1
42.0
41.9
41.8
41.7
41.6
Longitude (UTM)
−7.2
−7.1
−7.0
−6.9
−6.8
−6.7
−6.6
Altitude (m)
1400
1200
1000
800
600
400

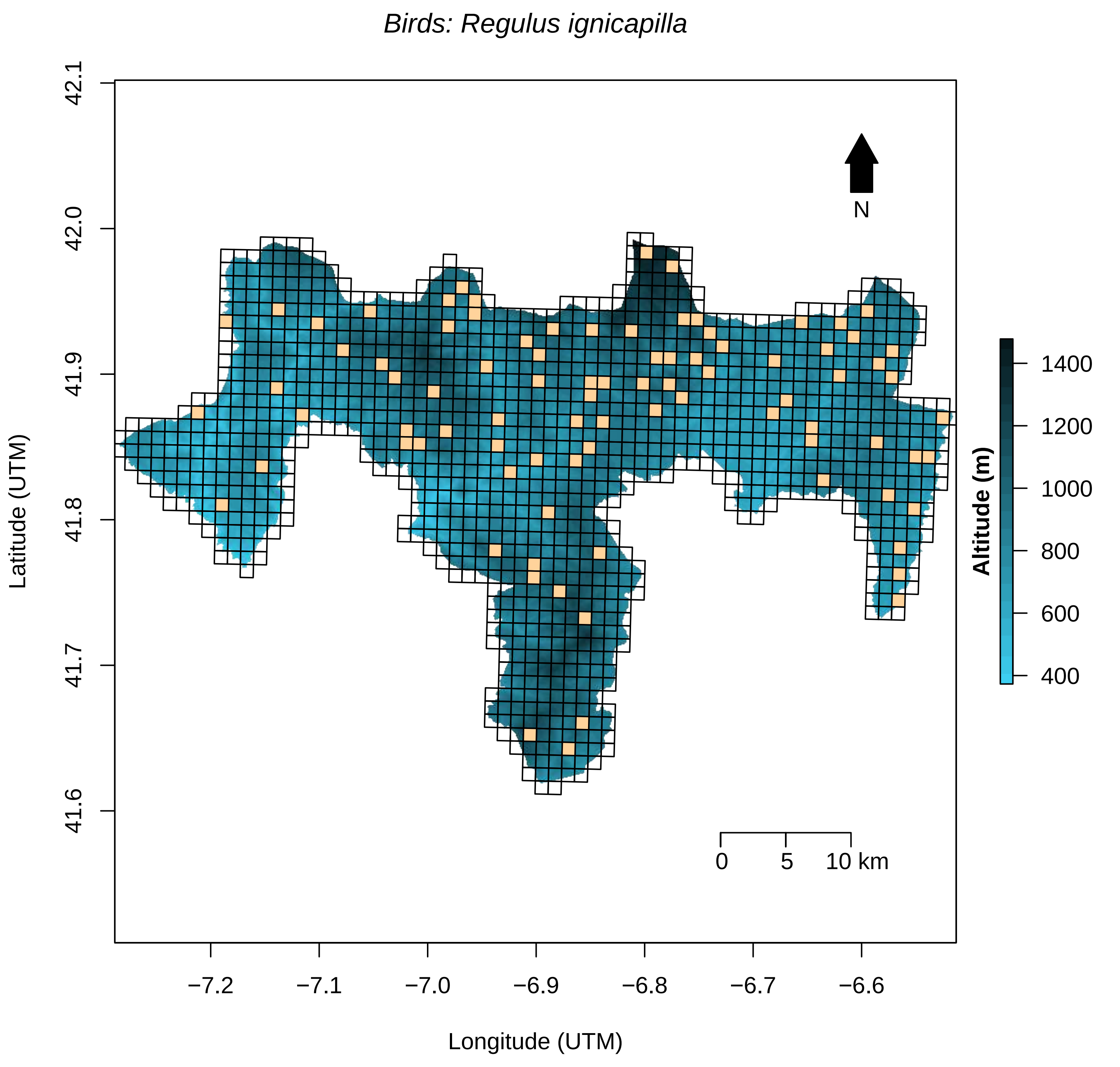

Birds: Regulus ignicapilla
N
0 5 10 km
Latitude (UTM)
42.1
42.0
41.9
41.8
41.7
41.6
Longitude (UTM)
−7.2
−7.1
−7.0
−6.9
−6.8
−6.7
−6.6
Altitude (m)
1400
1200
1000
800
600
400

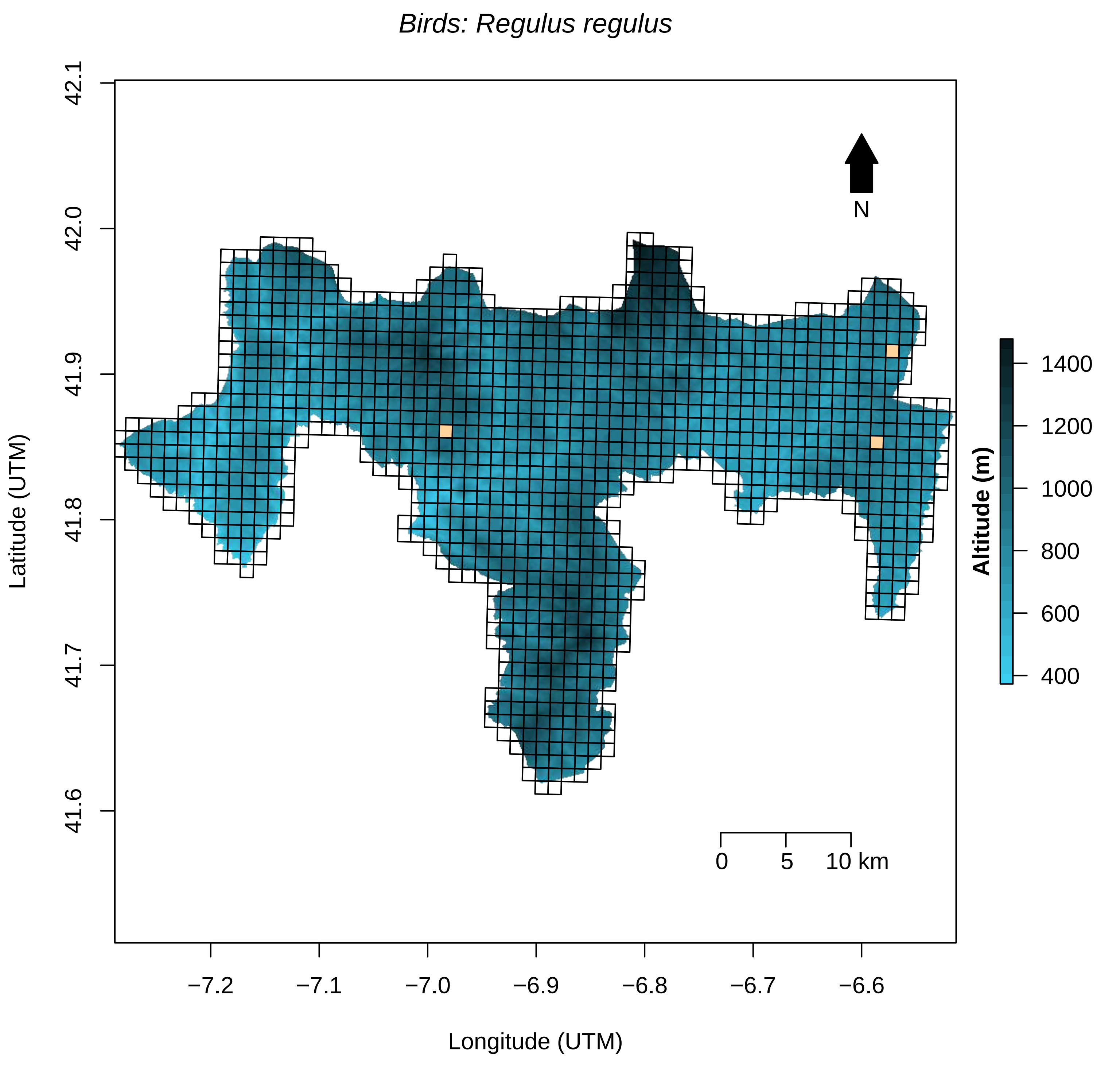
Birds: Regulus regulus
42.1
42.0
41.9
41.8
41.7
41.6
Latitude (UTM)
−7.2
−7.1
−7.0
−6.9
−6.8
−6.7
−6.6
Longitude (UTM)
N
0
5
10 km
1400
1200
1000
800
600
400
Altitude (m)

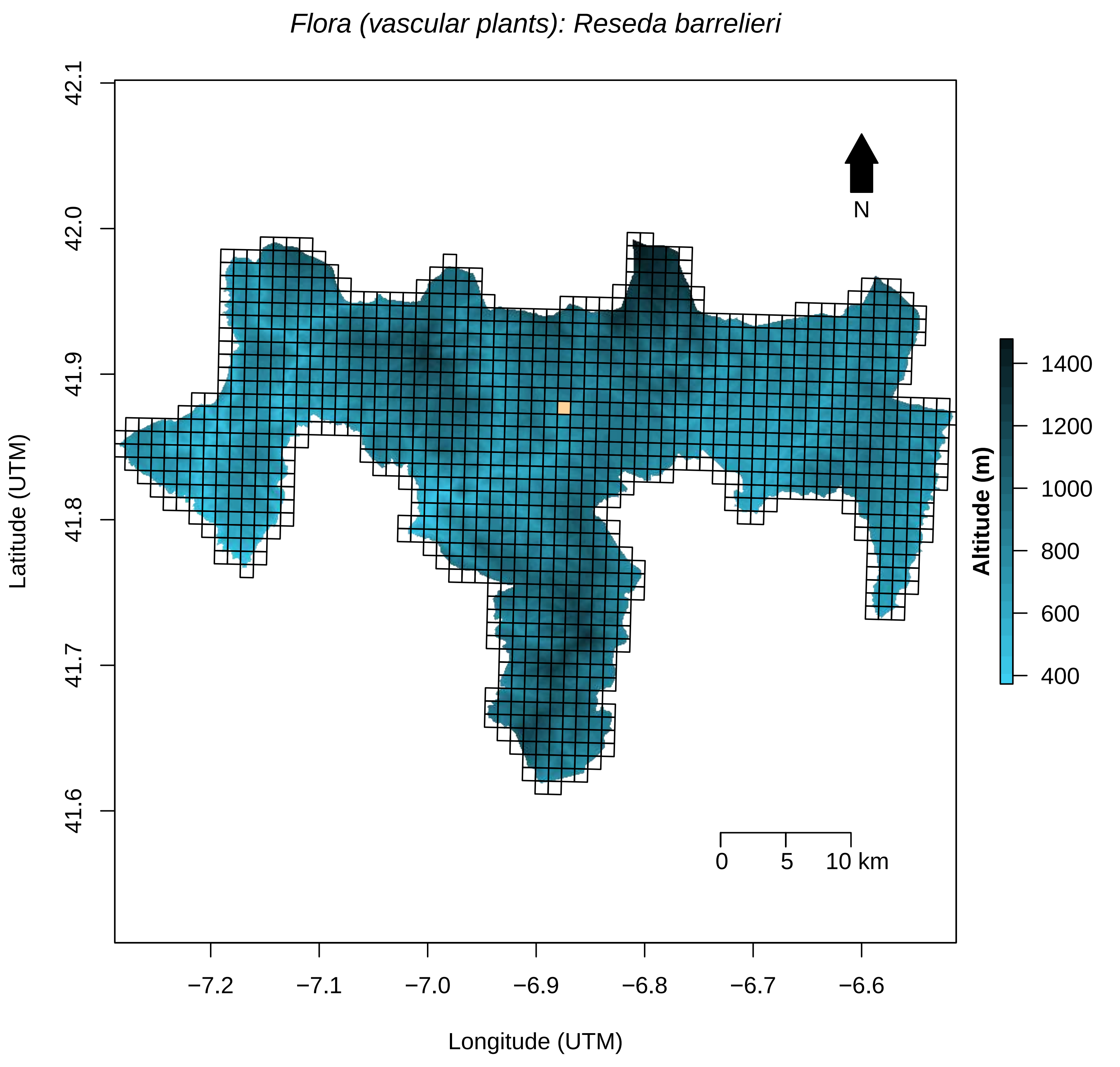

Flora (vascular plants): Reseda barrelieri
N
0
5
10 km
Altitude (m)
1400
1200
1000
800
600
400
Latitude (UTM)
42.1
42.0
41.9
41.8
41.7
41.6
Longitude (UTM)
−7.2
−7.1
−7.0
−6.9
−6.8
−6.7
−6.6

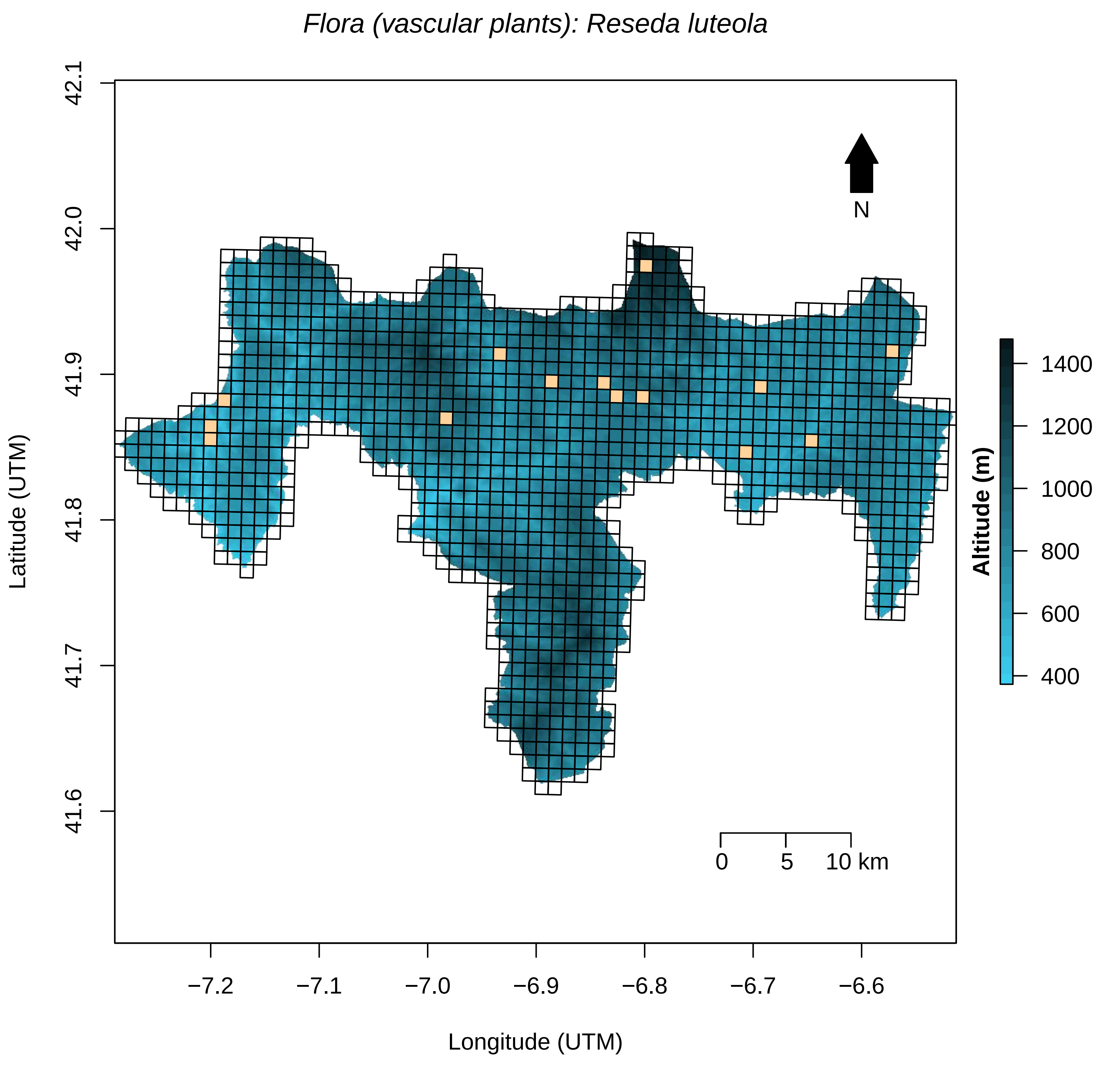

Flora (vascular plants): Reseda luteola
N
0
5
10 km
Latitude (UTM)
Longitude (UTM)
Altitude (m)
42.1
42.0
41.9
41.8
41.7
41.6
−7.2
−7.1
−7.0
−6.9
−6.8
−6.7
−6.6
1400
1200
1000
800
600
400

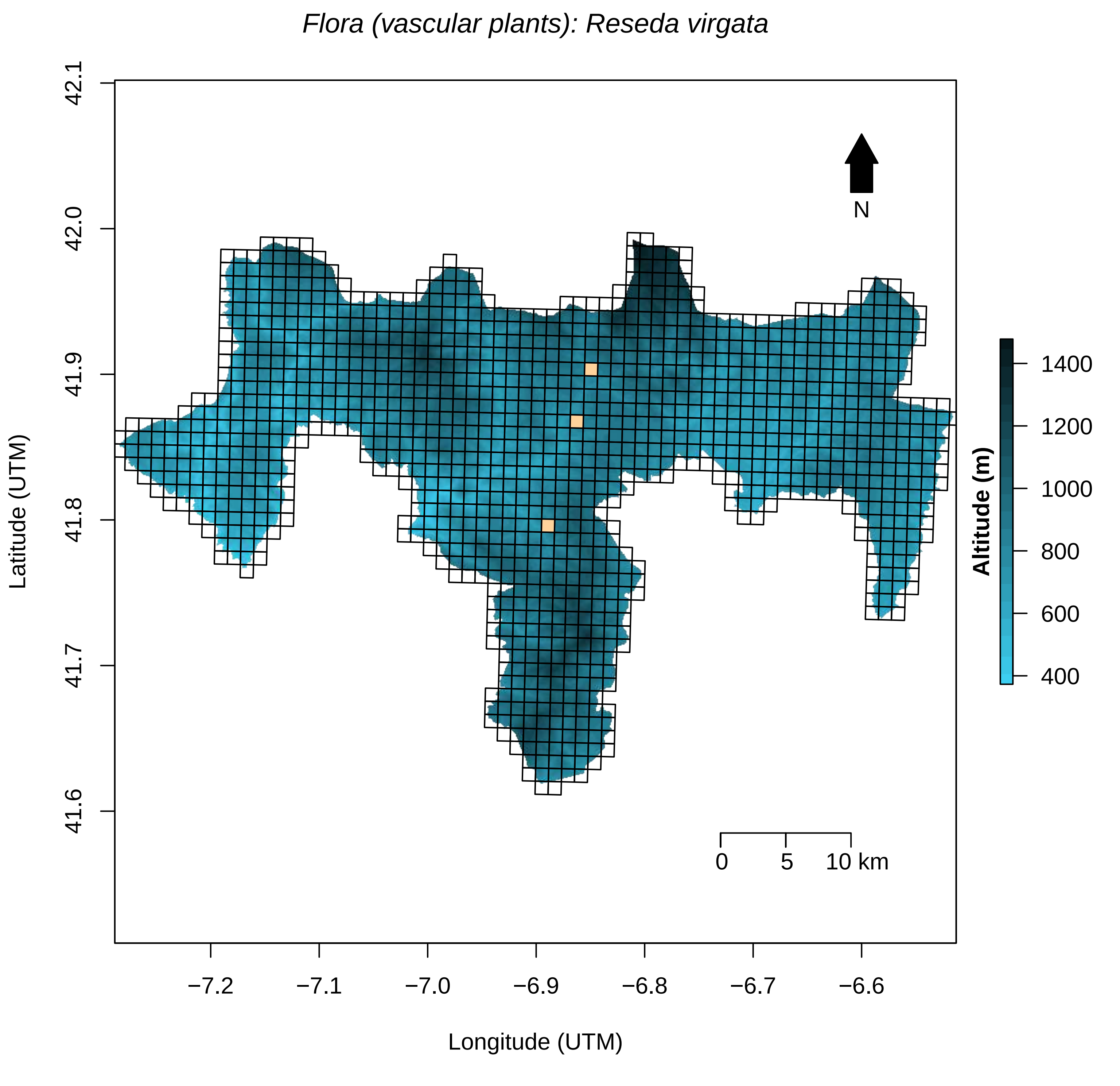

Flora (vascular plants): Reseda virgata
42.1
42.0
41.9
41.8
41.7
41.6
Latitude (UTM)
−7.2
−7.1
−7.0
−6.9
−6.8
−6.7
−6.6
Longitude (UTM)
N
0
5
10 km
1400
1200
1000
800
600
400
Altitude (m)

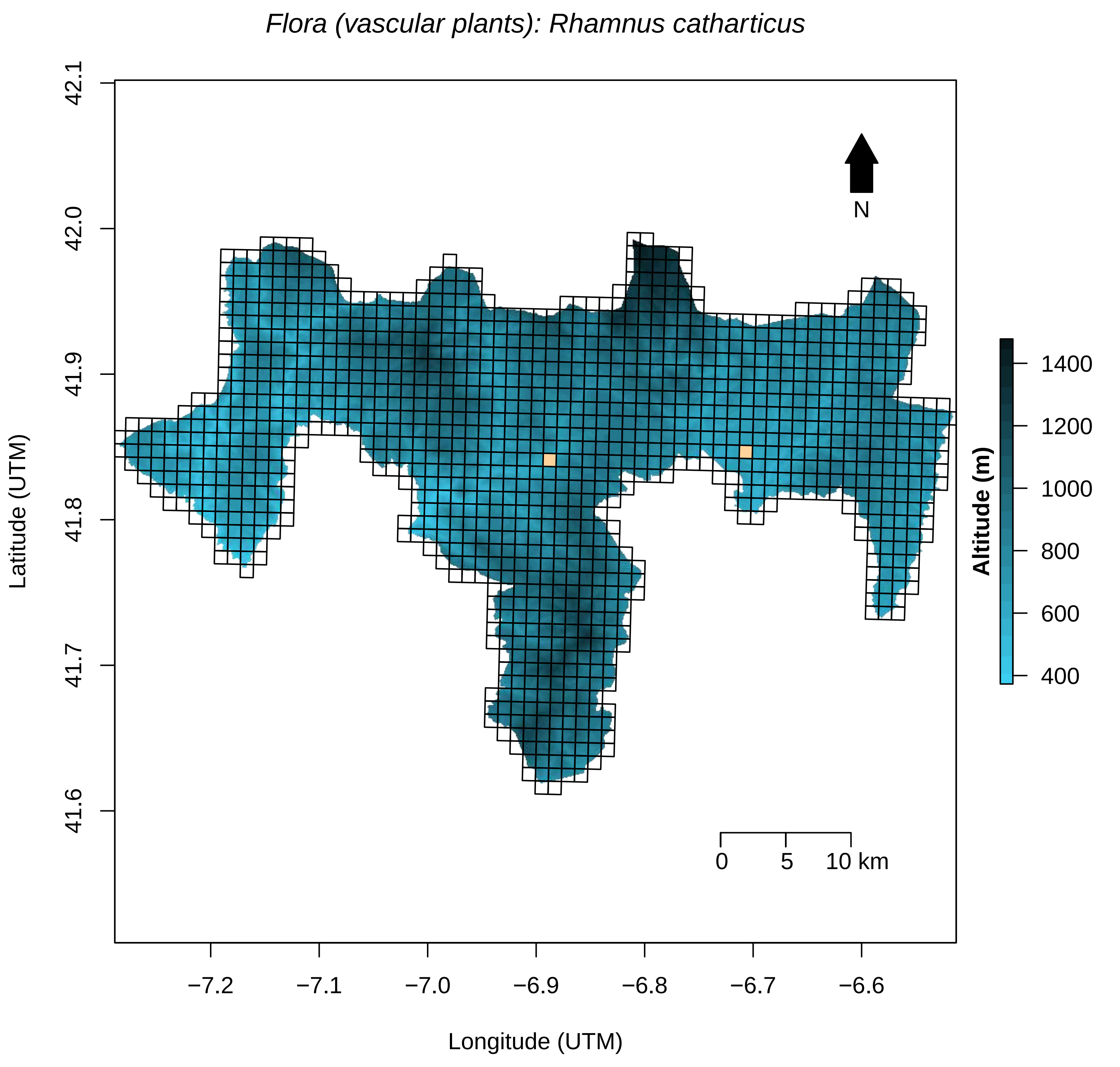

Flora (vascular plants): Rhamnus catharticus
N
0
5
10 km
Altitude (m)
1400
1200
1000
800
600
400
42.1
42.0
41.9
41.8
41.7
41.6
Latitude (UTM)
−7.2
−7.1
−7.0
−6.9
−6.8
−6.7
−6.6
Longitude (UTM)

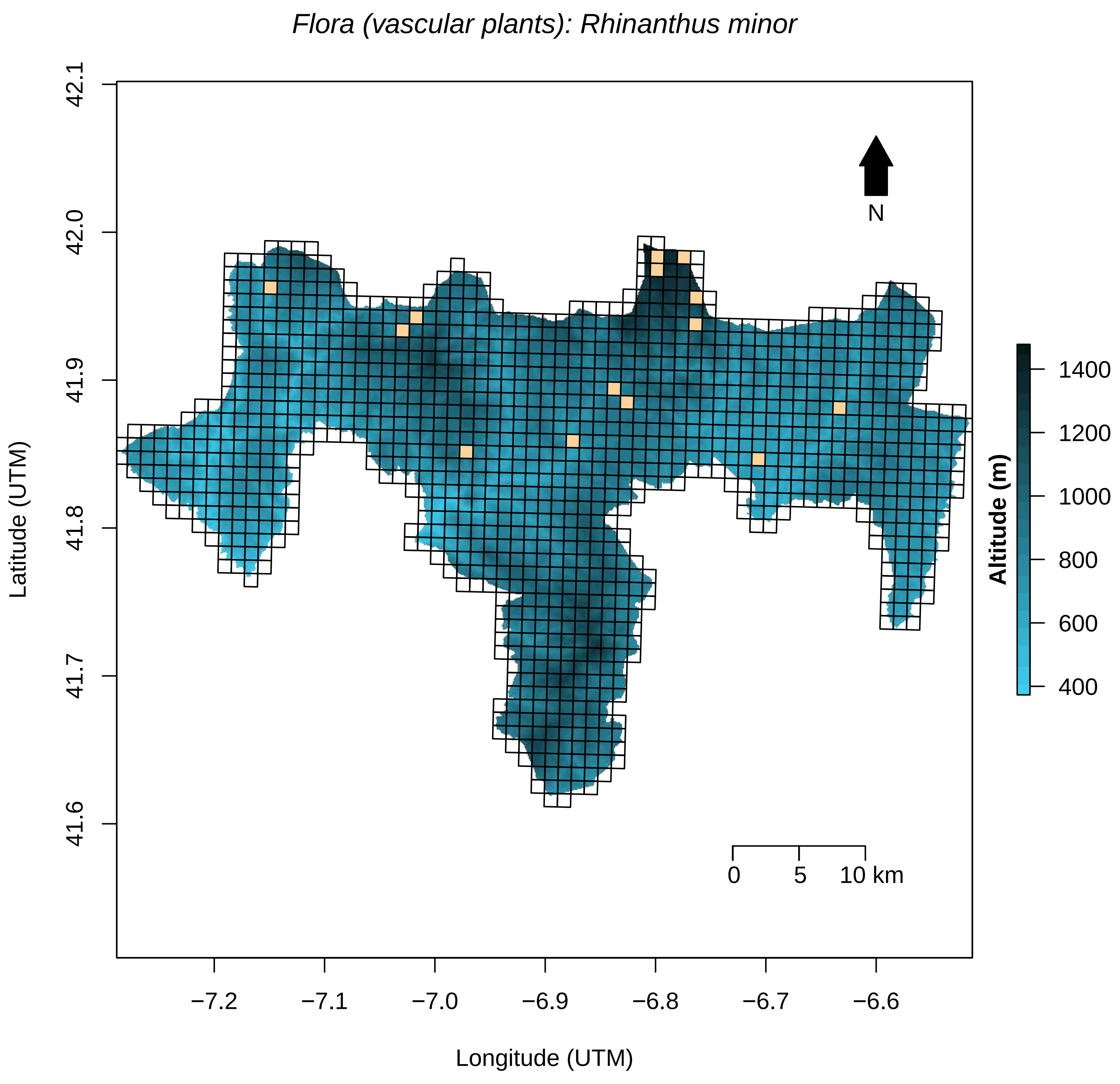
Flora (vascular plants): Rhinanthus minor
Latitude (UTM)
Longitude (UTM)
42.1
42.0
41.9
41.8
41.7
41.6
−7.2
−7.1
−7.0
−6.9
−6.8
−6.7
−6.6
N
0
5
10 km
Altitude (m)
1400
1200
1000
800
600
400

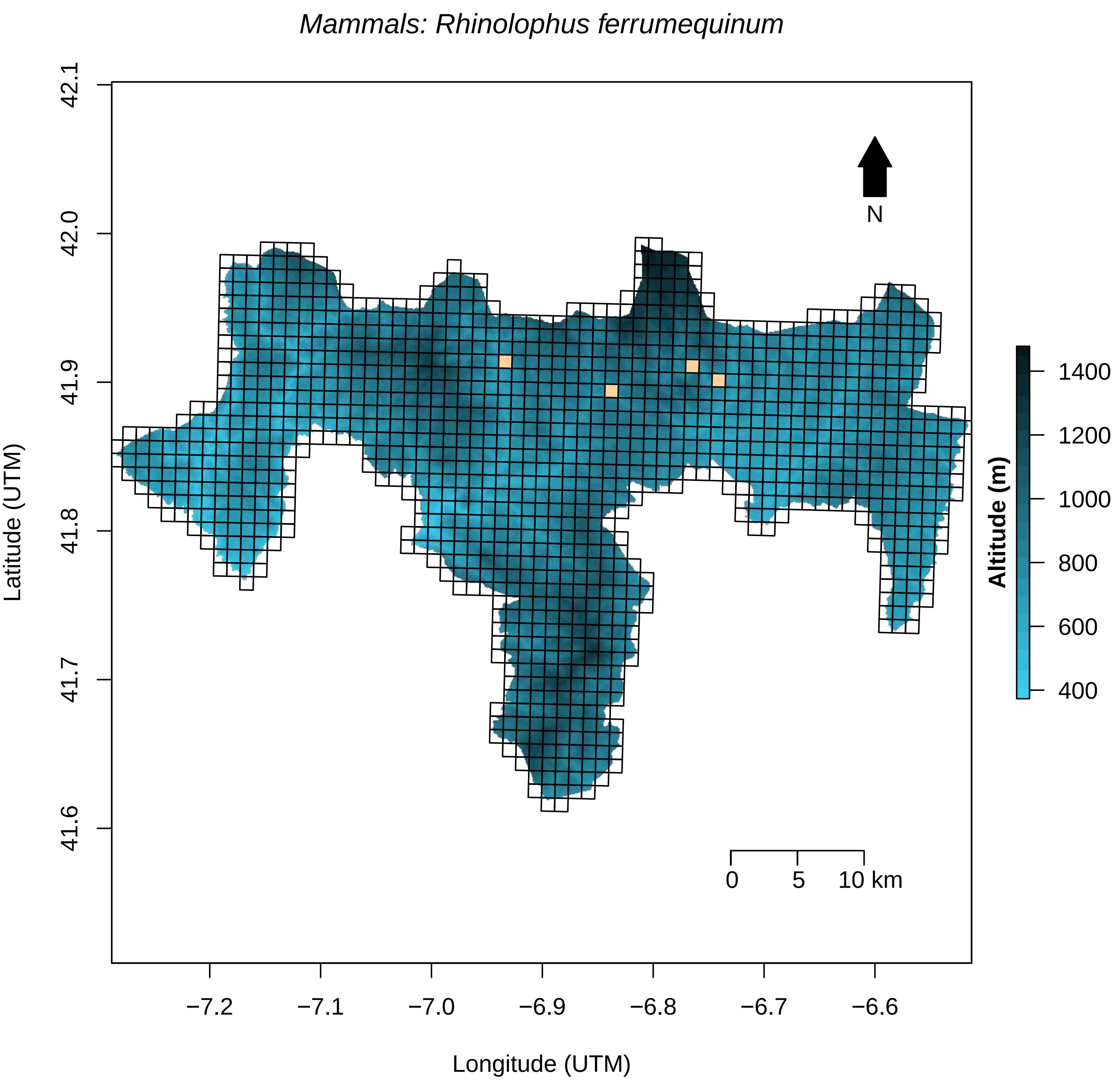

Mammals: Rhinolophus ferrumequinum
42.1
42.0
41.9
41.8
41.7
41.6
Latitude (UTM)
−7.2
−7.1
−7.0
−6.9
−6.8
−6.7
−6.6
Longitude (UTM)
N
0
5
10 km
Altitude (m)
1400
1200
1000
800
600
400

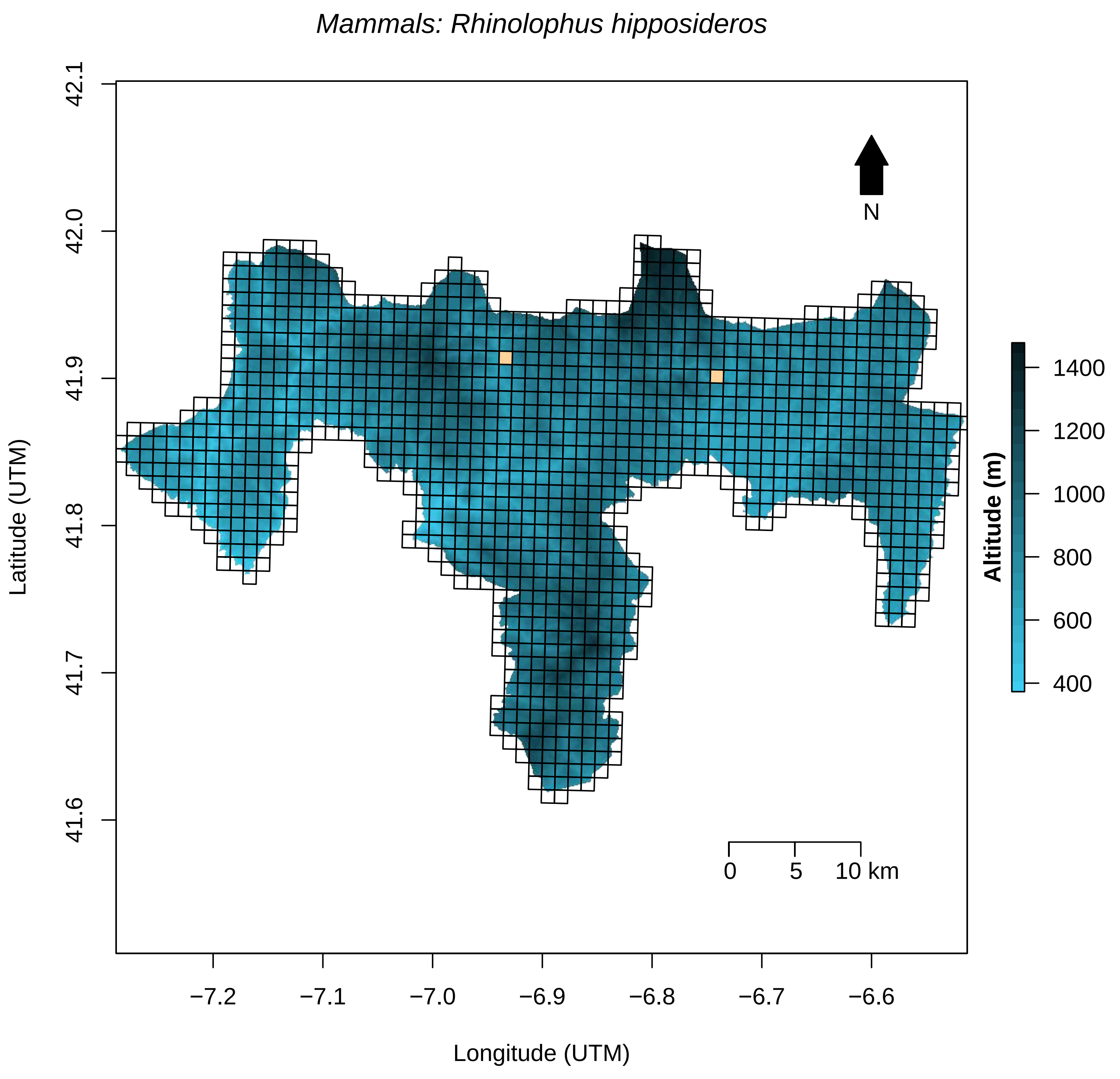

Mammals: Rhinolophus hipposideros
42.1
42.0
41.9
41.8
41.7
41.6
Latitude (UTM)
−7.2
−7.1
−7.0
−6.9
−6.8
−6.7
−6.6
Longitude (UTM)
N
0
5
10 km
Altitude (m)
1400
1200
1000
800
600
400

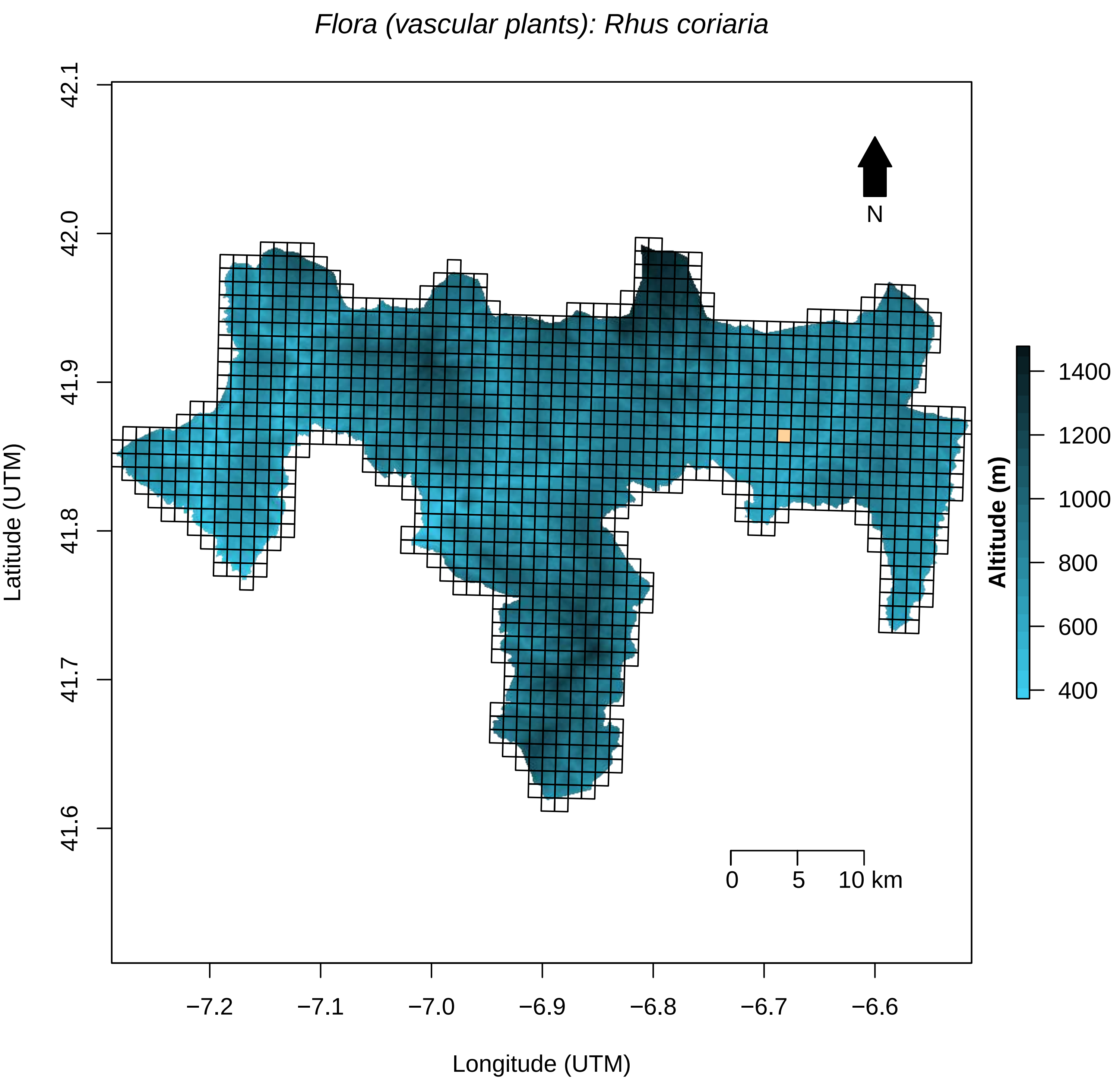

Flora (vascular plants): Rhus coriaria
N
0
5
10 km
Altitude (m)
1400
1200
1000
800
600
400
42.1
42.0
41.9
41.8
41.7
41.6
Latitude (UTM)
−7.2
−7.1
−7.0
−6.9
−6.8
−6.7
−6.6
Longitude (UTM)

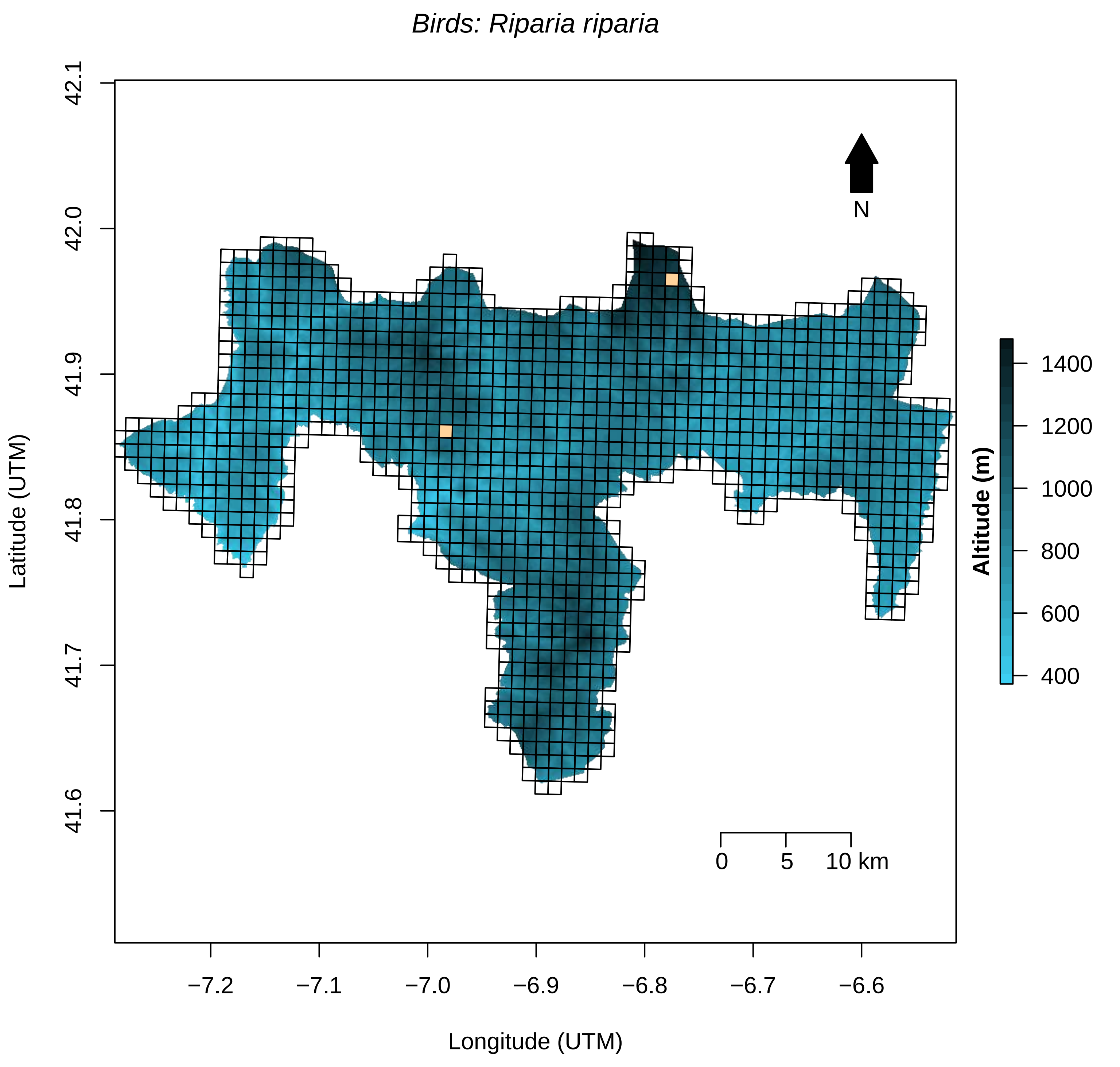

Birds: Riparia riparia
N
0 5 10 km
Altitude (m)
1400
1200
1000
800
600
400
42.1
42.0
41.9
41.8
41.7
41.6
Latitude (UTM)
−7.2
−7.1
−7.0
−6.9
−6.8
−6.7
−6.6
Longitude (UTM)

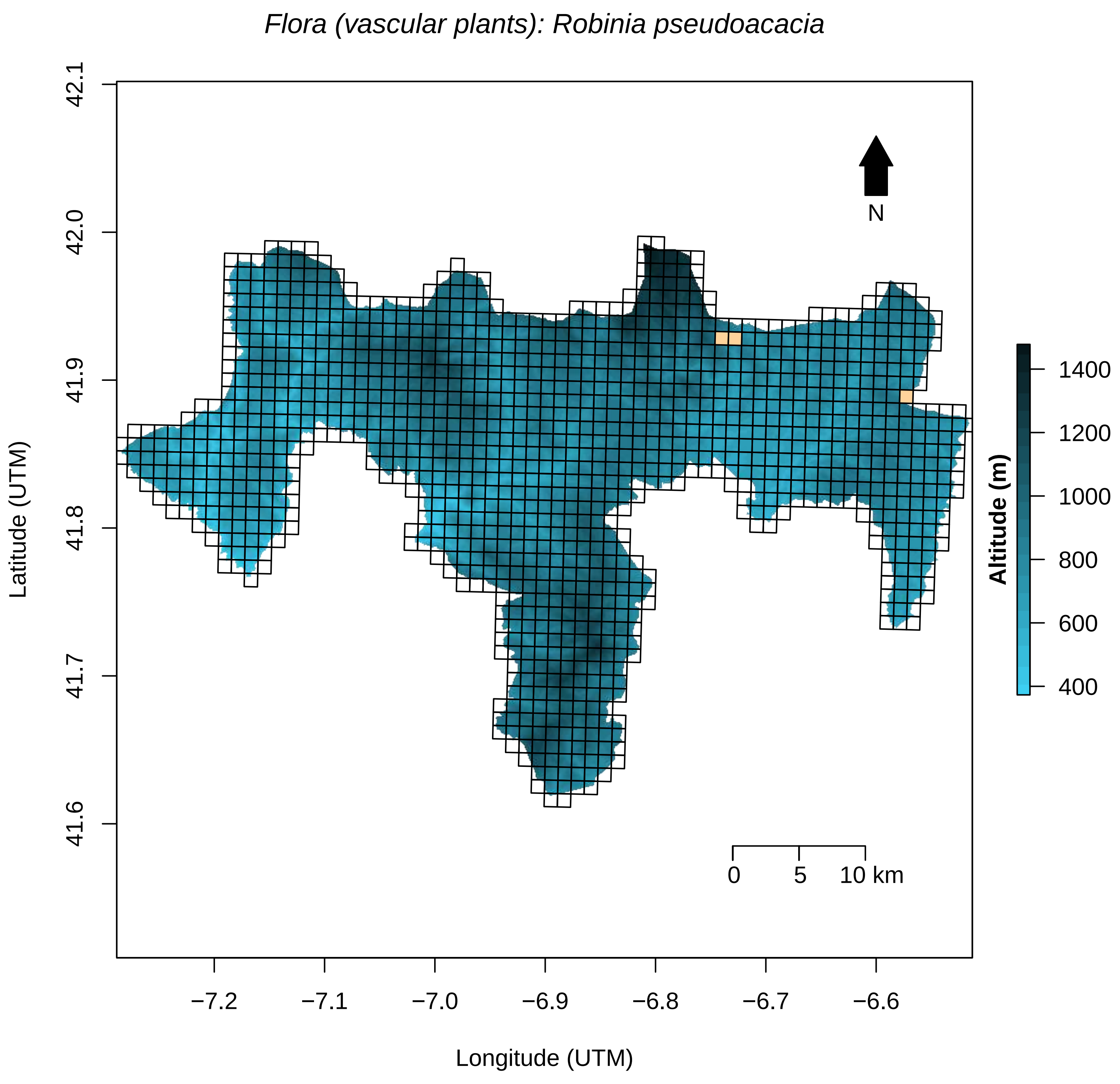

Flora (vascular plants): Robinia pseudoacacia
42.1
42.0
41.9
41.8
41.7
41.6
Latitude (UTM)
−7.2
−7.1
−7.0
−6.9
−6.8
−6.7
−6.6
Longitude (UTM)
N
0
5
10 km
Altitude (m)
1400
1200
1000
800
600
400

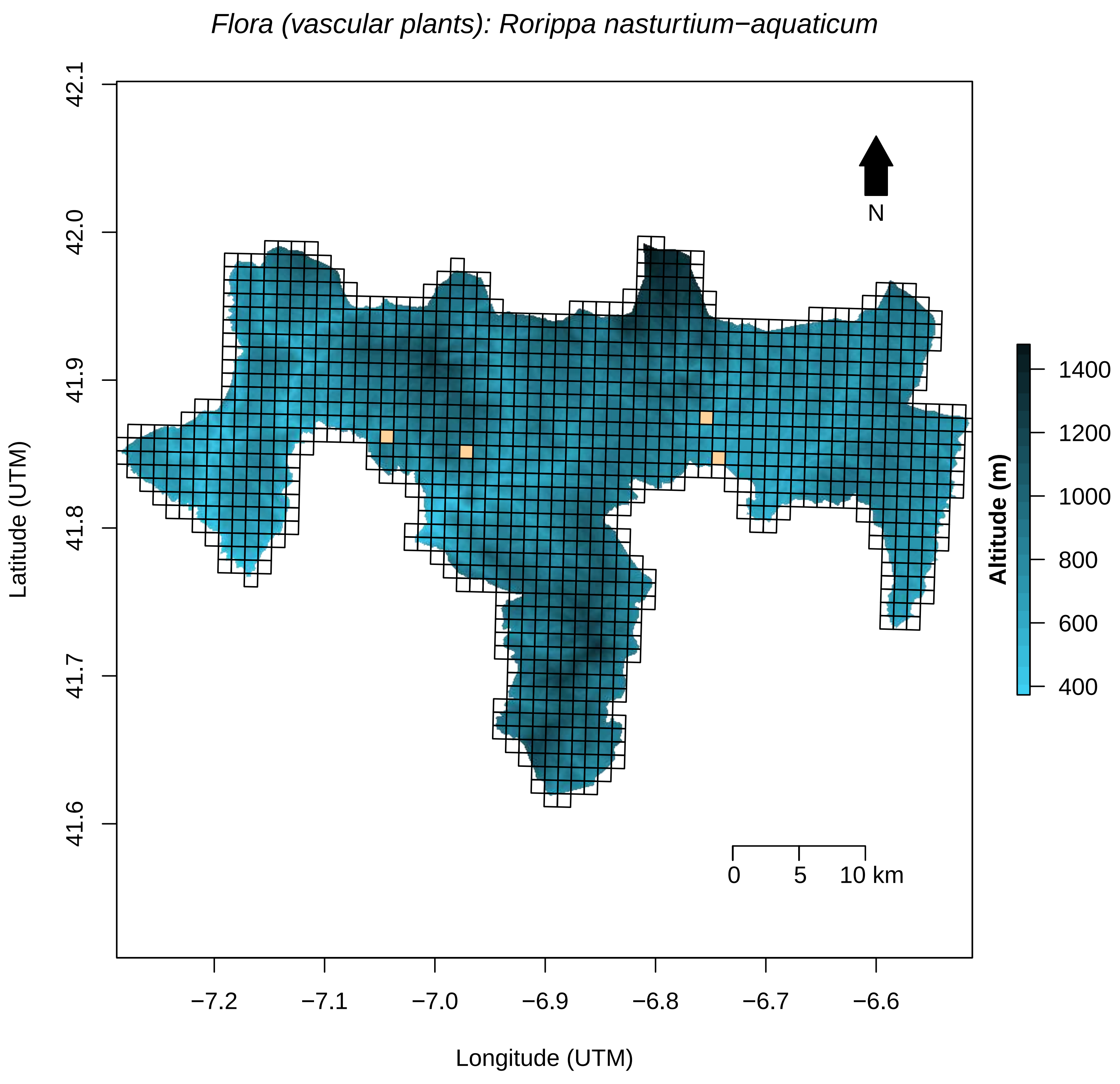

Flora (vascular plants): Rorippa nasturtium−aquaticum
42.1
42.0
41.9
41.8
41.7
41.6
Latitude (UTM)
−7.2
−7.1
−7.0
−6.9
−6.8
−6.7
−6.6
Longitude (UTM)
N
0
5
10 km
Altitude (m)
1400
1200
1000
800
600
400

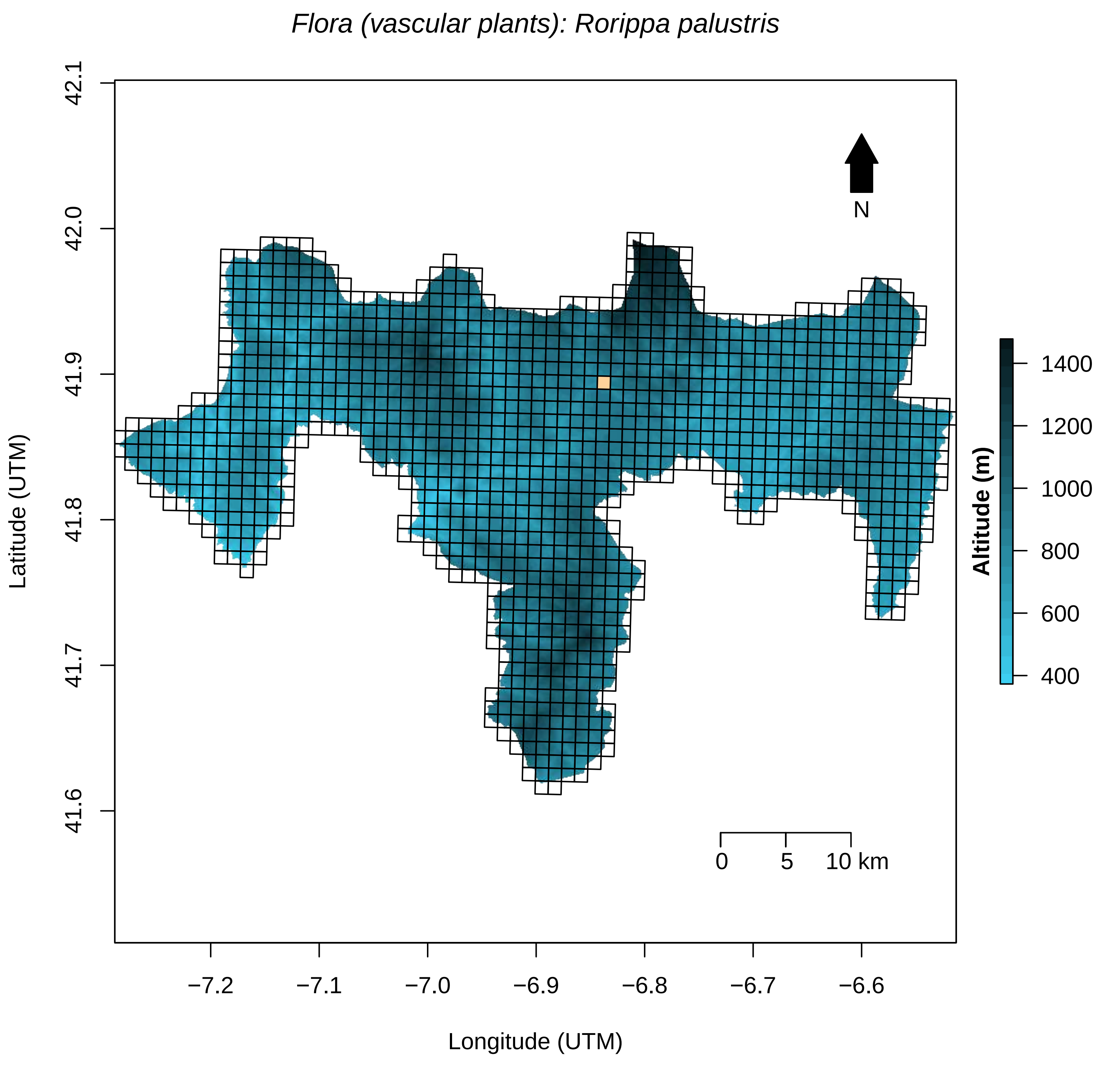

Flora (vascular plants): Rorippa palustris
N
0
5
10 km
Altitude (m)
1400
1200
1000
800
600
400
42.1
42.0
41.9
41.8
41.7
41.6
Latitude (UTM)
−7.2
−7.1
−7.0
−6.9
−6.8
−6.7
−6.6
Longitude (UTM)

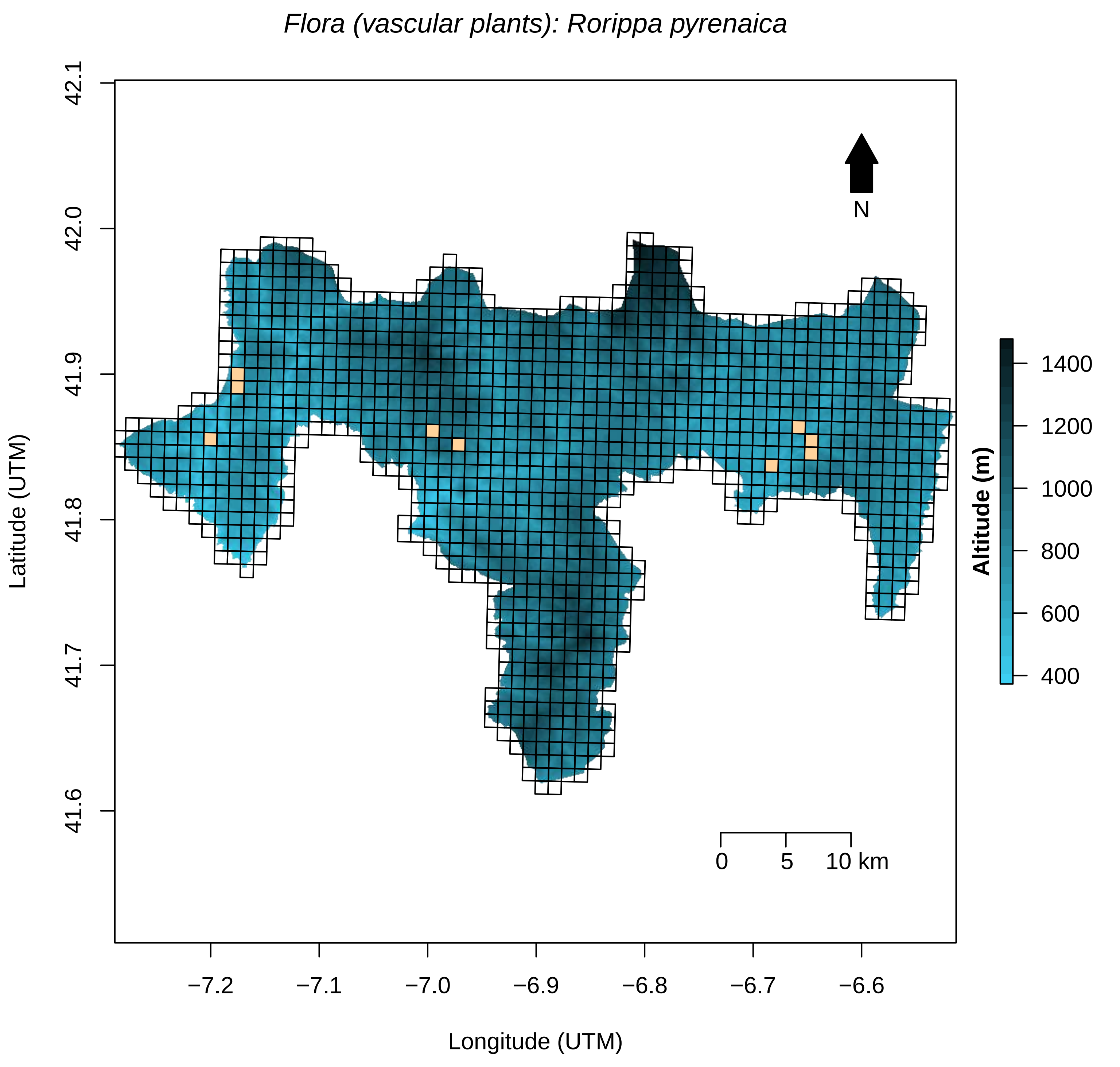

Flora (vascular plants): Rorippa pyrenaica
N
0 5 10 km
Altitude (m)
1400
1200
1000
800
600
400
Latitude (UTM)
42.1
42.0
41.9
41.8
41.7
41.6
Longitude (UTM)
−7.2
−7.1
−7.0
−6.9
−6.8
−6.7
−6.6

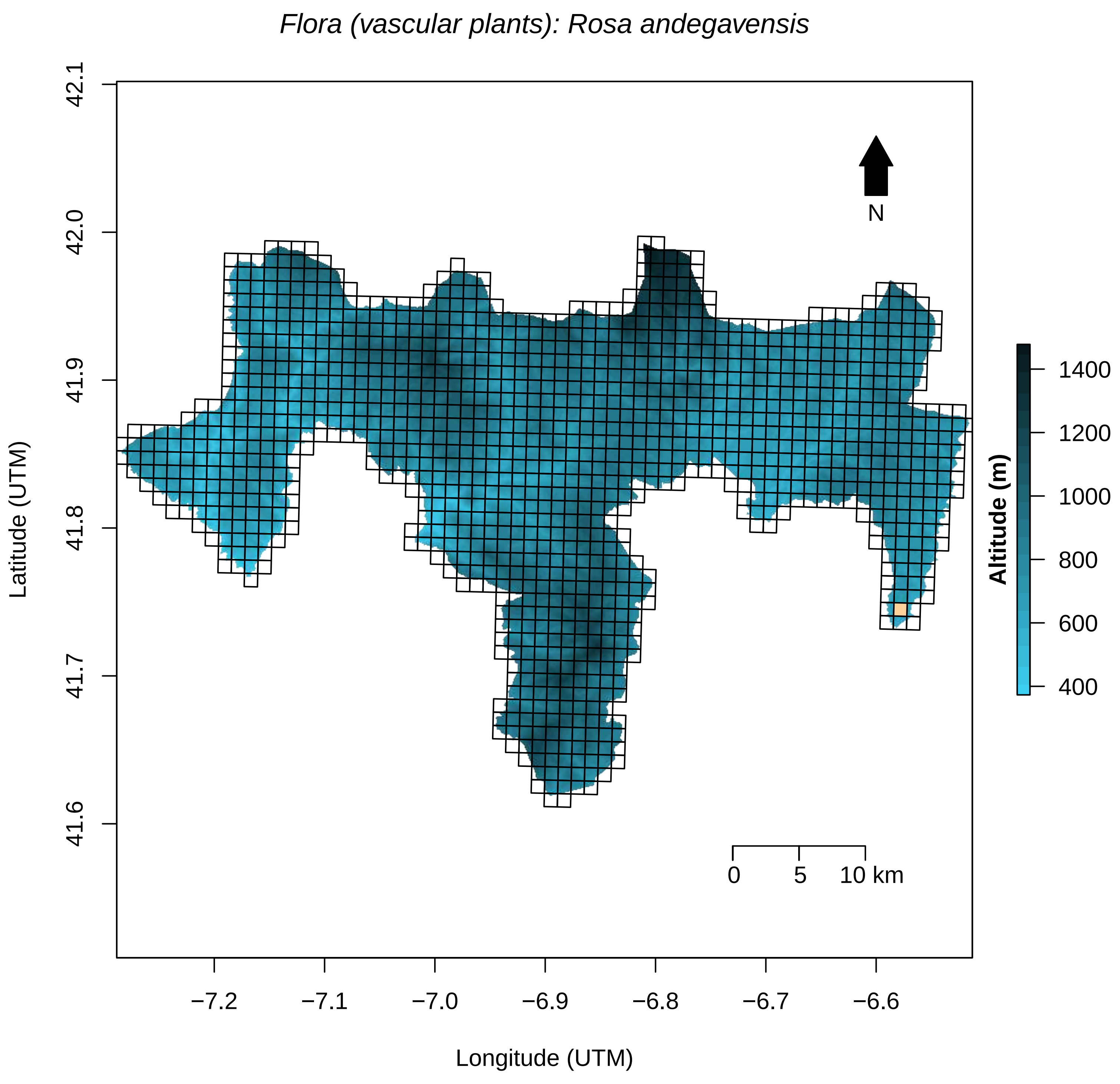
Flora (vascular plants): Rosa andegavensis
N
0 5 10 km
Latitude (UTM)
42.1
42.0
41.9
41.8
41.7
41.6
Longitude (UTM)
−7.2
−7.1
−7.0
−6.9
−6.8
−6.7
−6.6
Altitude (m)
1400
1200
1000
800
600
400

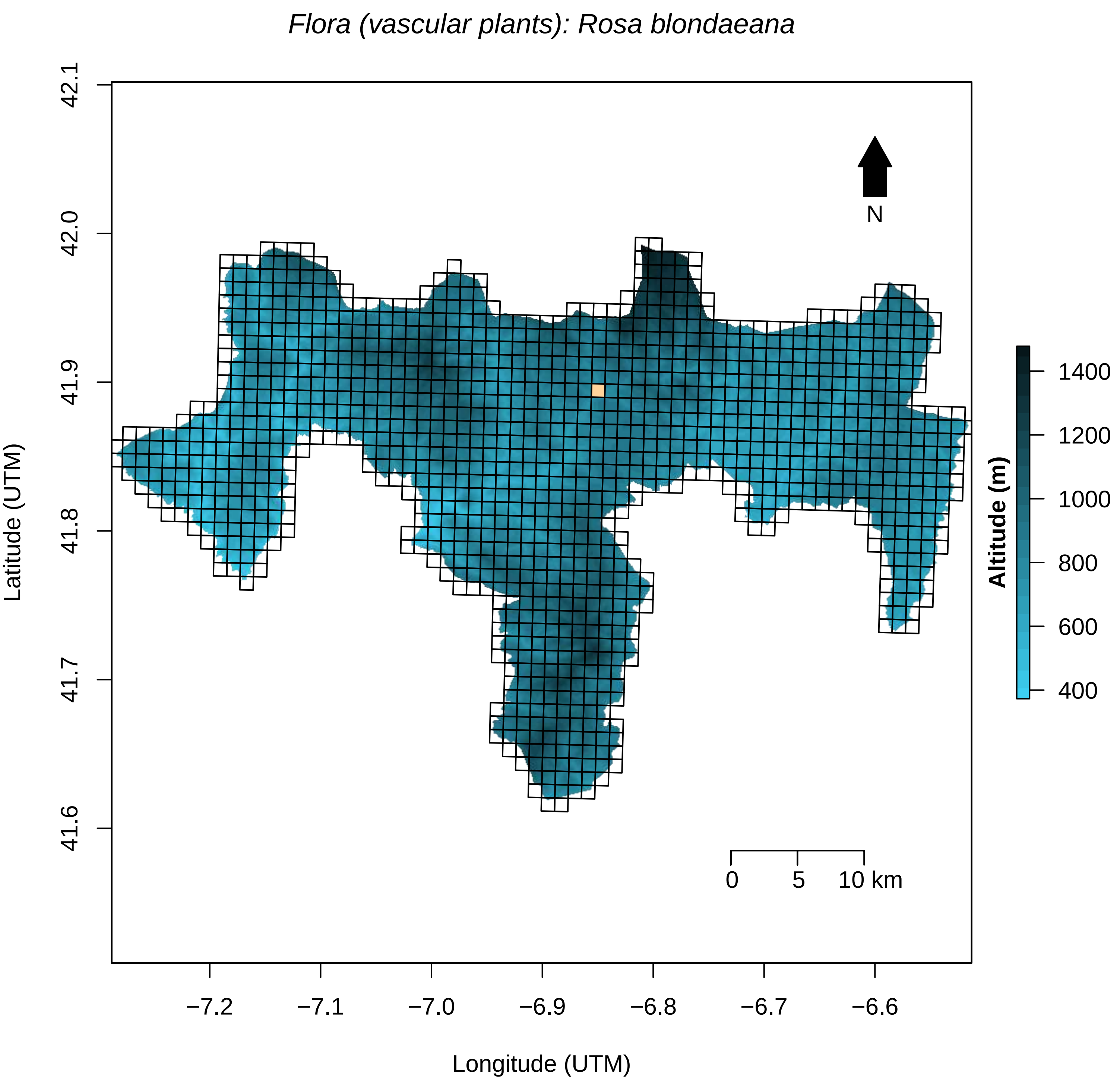

Flora (vascular plants): Rosa blondaeana
42.1
42.0
41.9
41.8
41.7
41.6
Latitude (UTM)
−7.2
−7.1
−7.0
−6.9
−6.8
−6.7
−6.6
Longitude (UTM)
N
0
5
10 km
Altitude (m)
1400
1200
1000
800
600
400

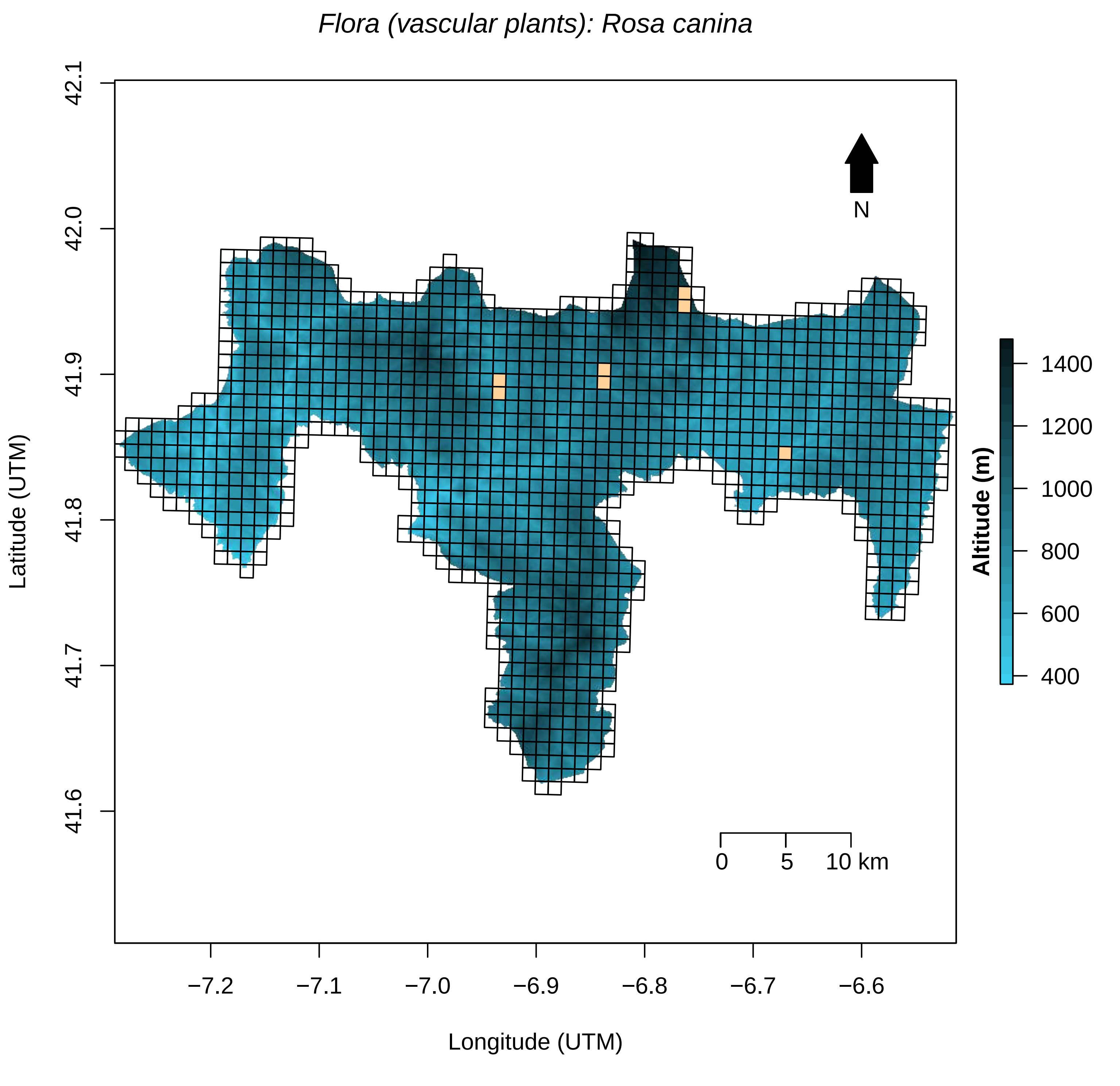
Flora (vascular plants): Rosa canina
42.1
42.0
41.9
41.8
41.7
41.6
Latitude (UTM)
−7.2
−7.1
−7.0
−6.9
−6.8
−6.7
−6.6
Longitude (UTM)
N
0
5
10 km
Altitude (m)
1400
1200
1000
800
600
400

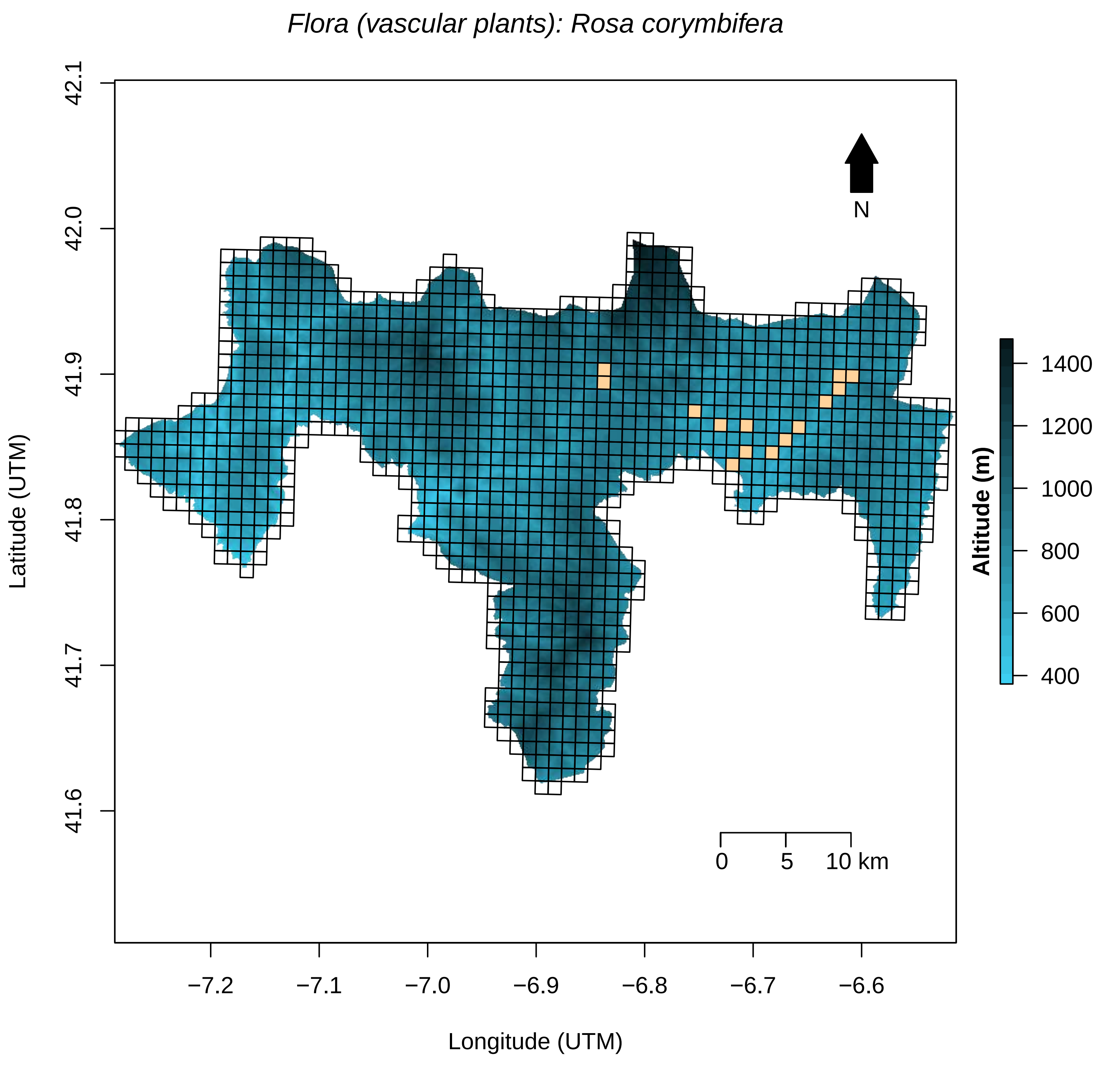

Flora (vascular plants): Rosa corymbifera
42.1
42.0
41.9
41.8
41.7
41.6
Latitude (UTM)
N
0
5
10 km
1400
1200
1000
800
600
400
Altitude (m)
−7.2
−7.1
−7.0
−6.9
−6.8
−6.7
−6.6
Longitude (UTM)

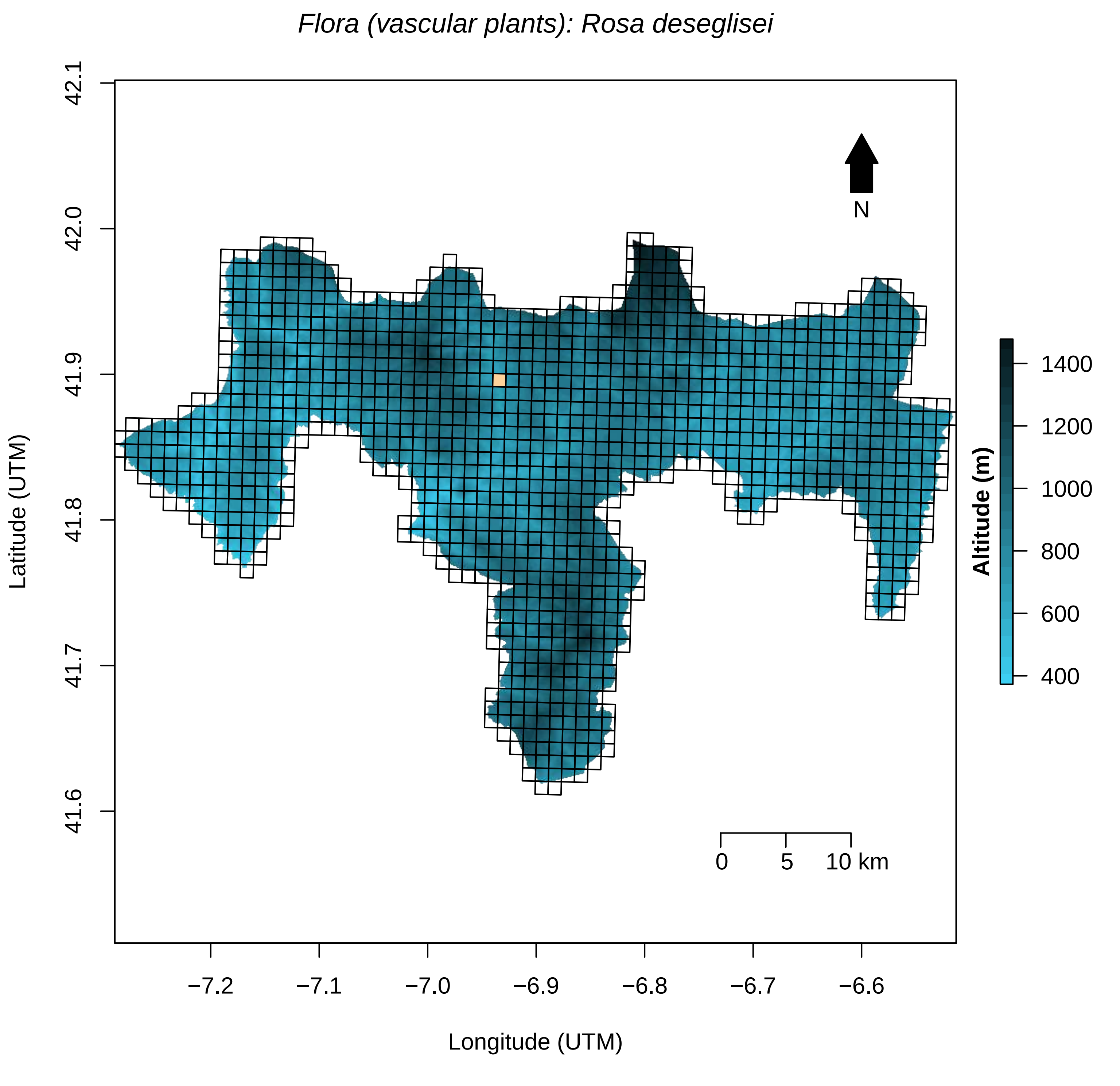

Flora (vascular plants): Rosa deseglisei
42.1
42.0
41.9
41.8
41.7
41.6
Latitude (UTM)
−7.2
−7.1
−7.0
−6.9
−6.8
−6.7
−6.6
Longitude (UTM)
N
0
5
10 km
Altitude (m)
1400
1200
1000
800
600
400

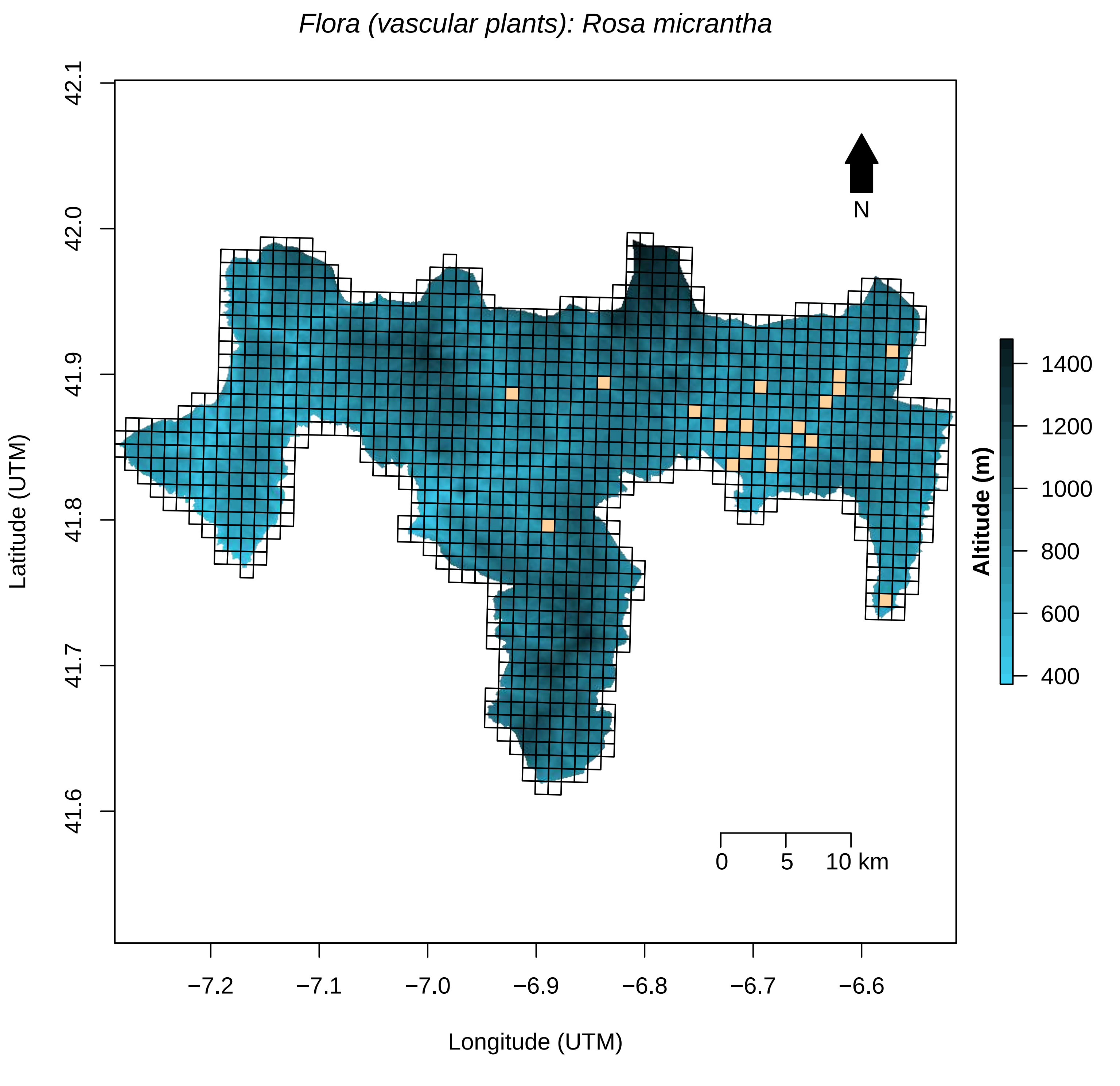

Flora (vascular plants): Rosa micrantha
N
0
5
10 km
Altitude (m)
1400
1200
1000
800
600
400
42.1
42.0
41.9
41.8
41.7
41.6
Latitude (UTM)
−7.2
−7.1
−7.0
−6.9
−6.8
−6.7
−6.6
Longitude (UTM)

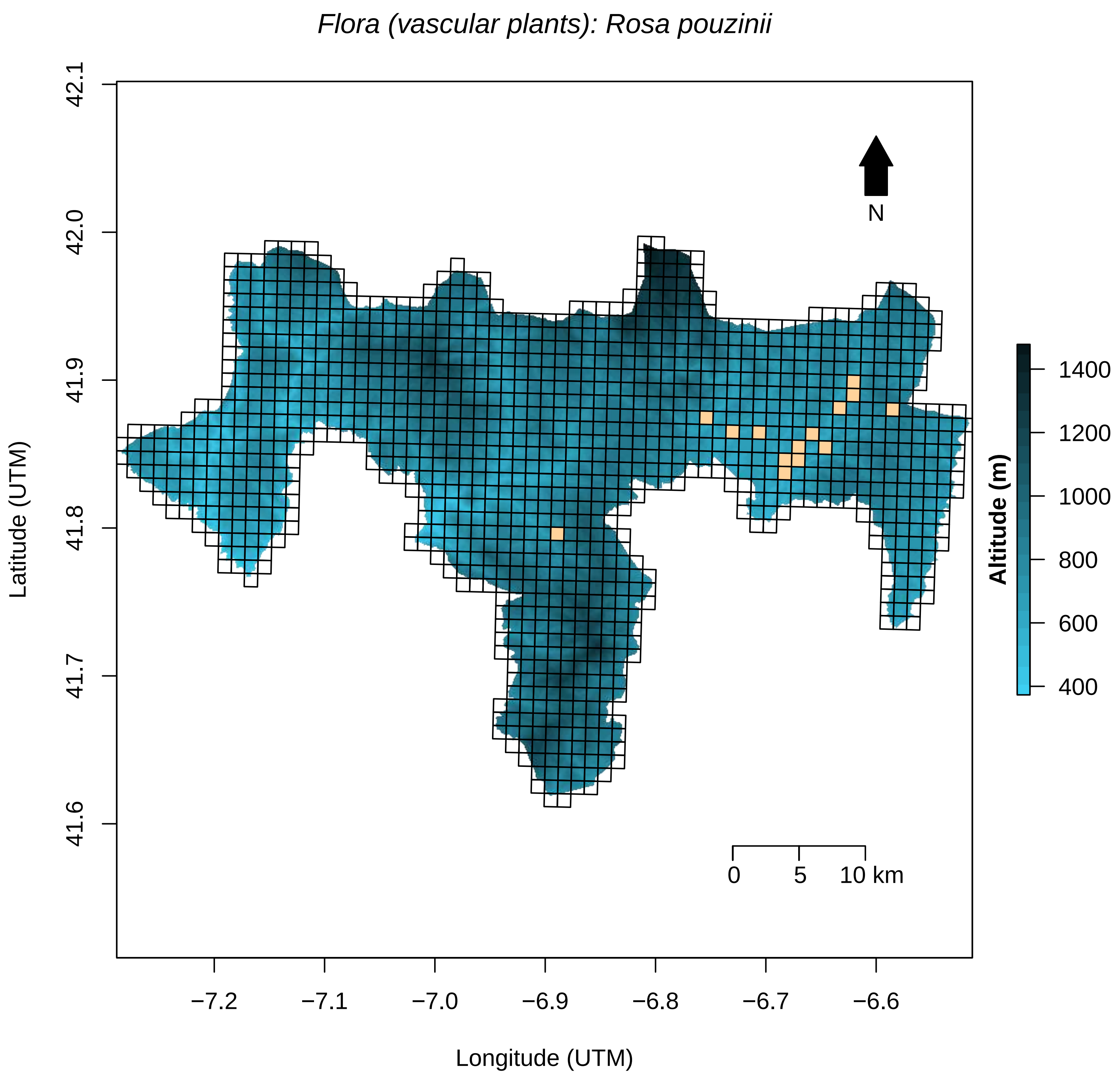

Flora (vascular plants): Rosa pouzinii
N
0
5
10 km
Altitude (m)
400
600
800
1000
1200
1400
Latitude (UTM)
41.6
41.7
41.8
41.9
42.0
42.1
Longitude (UTM)
−7.2
−7.1
−7.0
−6.9
−6.8
−6.7
−6.6

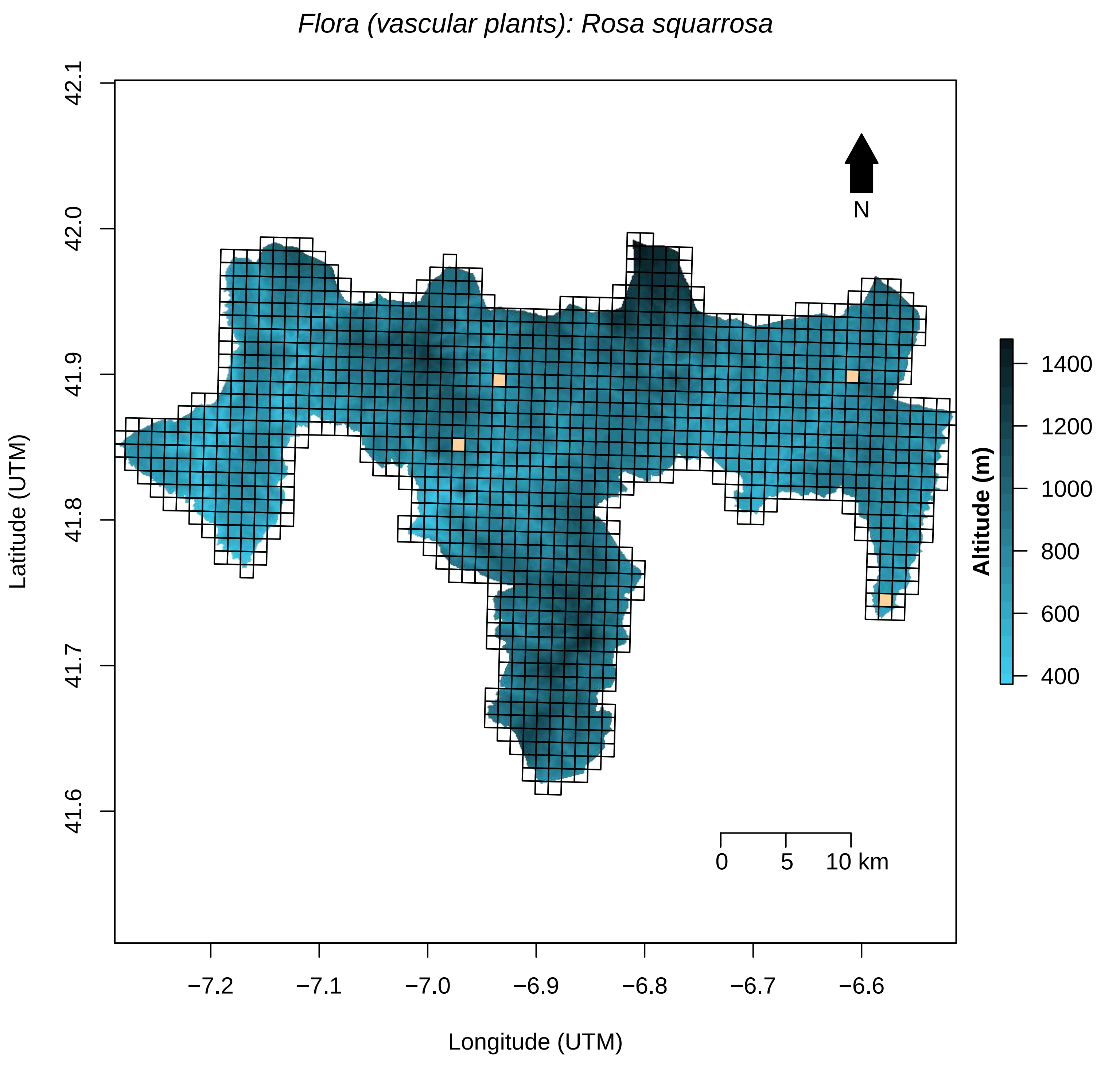

Flora (vascular plants): Rosa squarrosa
Latitude (UTM)
42.1
42.0
41.9
41.8
41.7
41.6
N
0
5
10 km
−7.2
−7.1
−7.0
−6.9
−6.8
−6.7
−6.6
Longitude (UTM)
Altitude (m)
1400
1200
1000
800
600
400

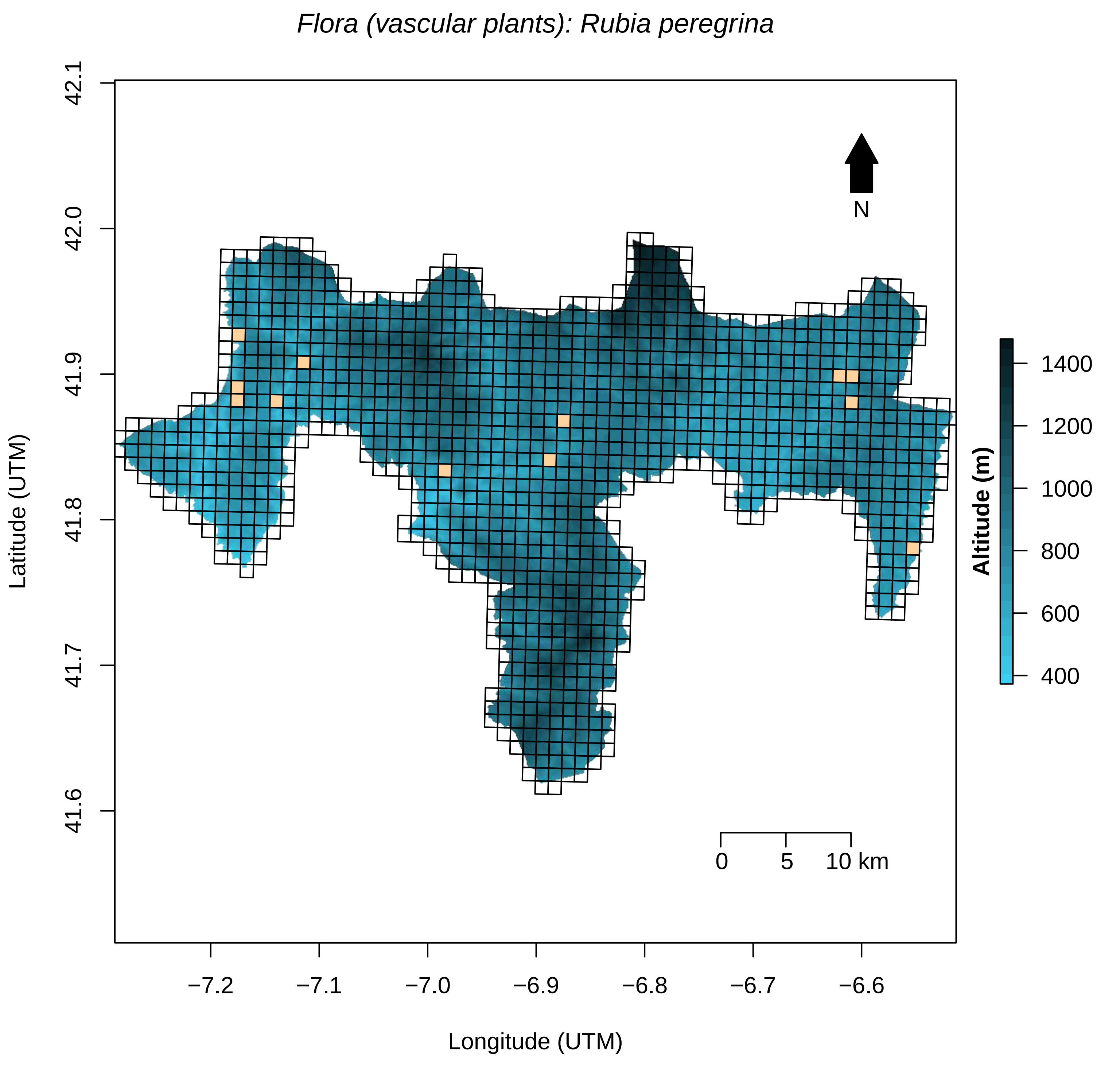
Flora (vascular plants): Rubia peregrina
Latitude (UTM)
Longitude (UTM)
42.1
42.0
41.9
41.8
41.7
41.6
−7.2
−7.1
−7.0
−6.9
−6.8
−6.7
−6.6
N
0
5
10 km
Altitude (m)
1400
1200
1000
800
600
400

Flora (vascular plants): Rubus brigantinus

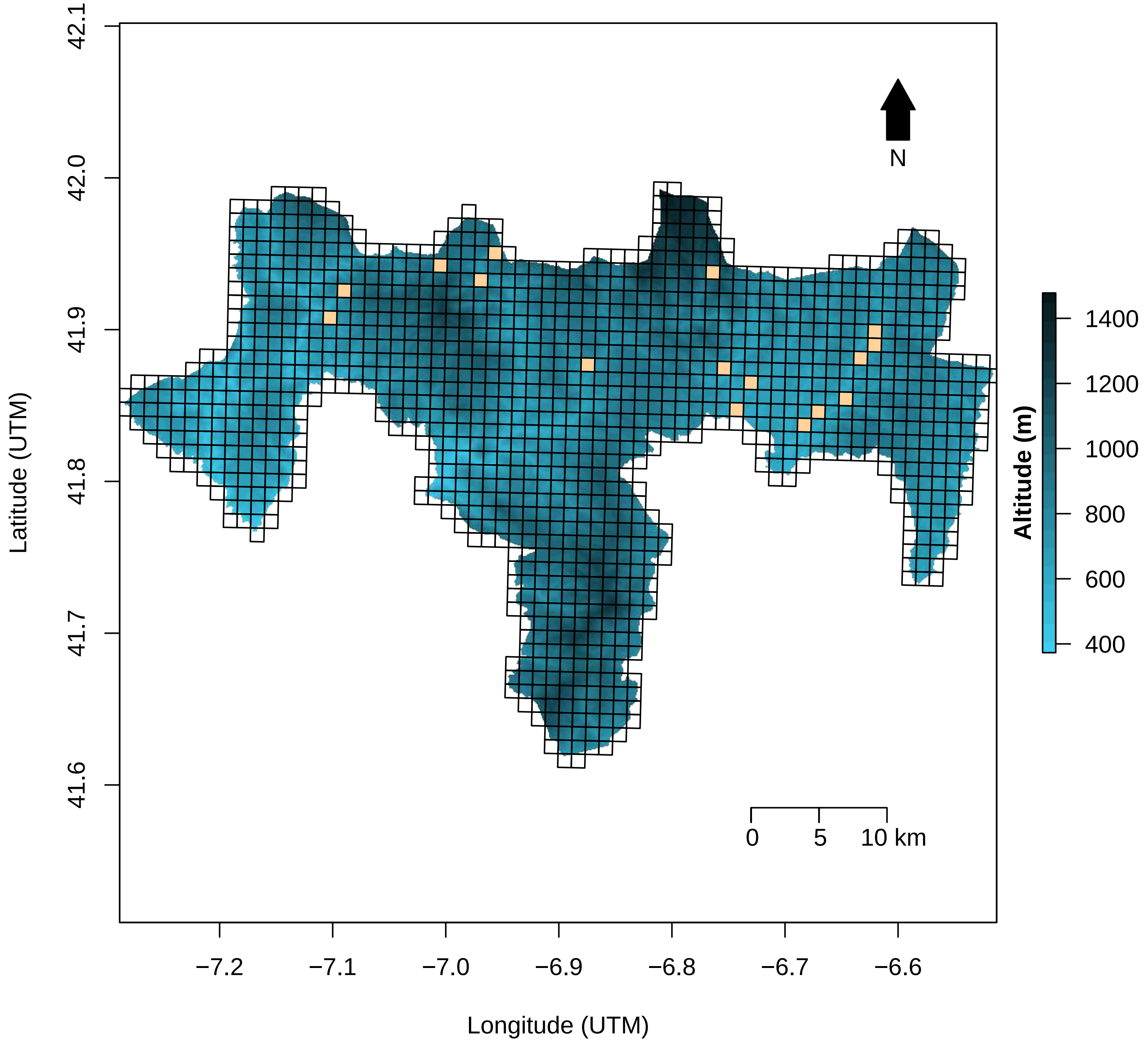

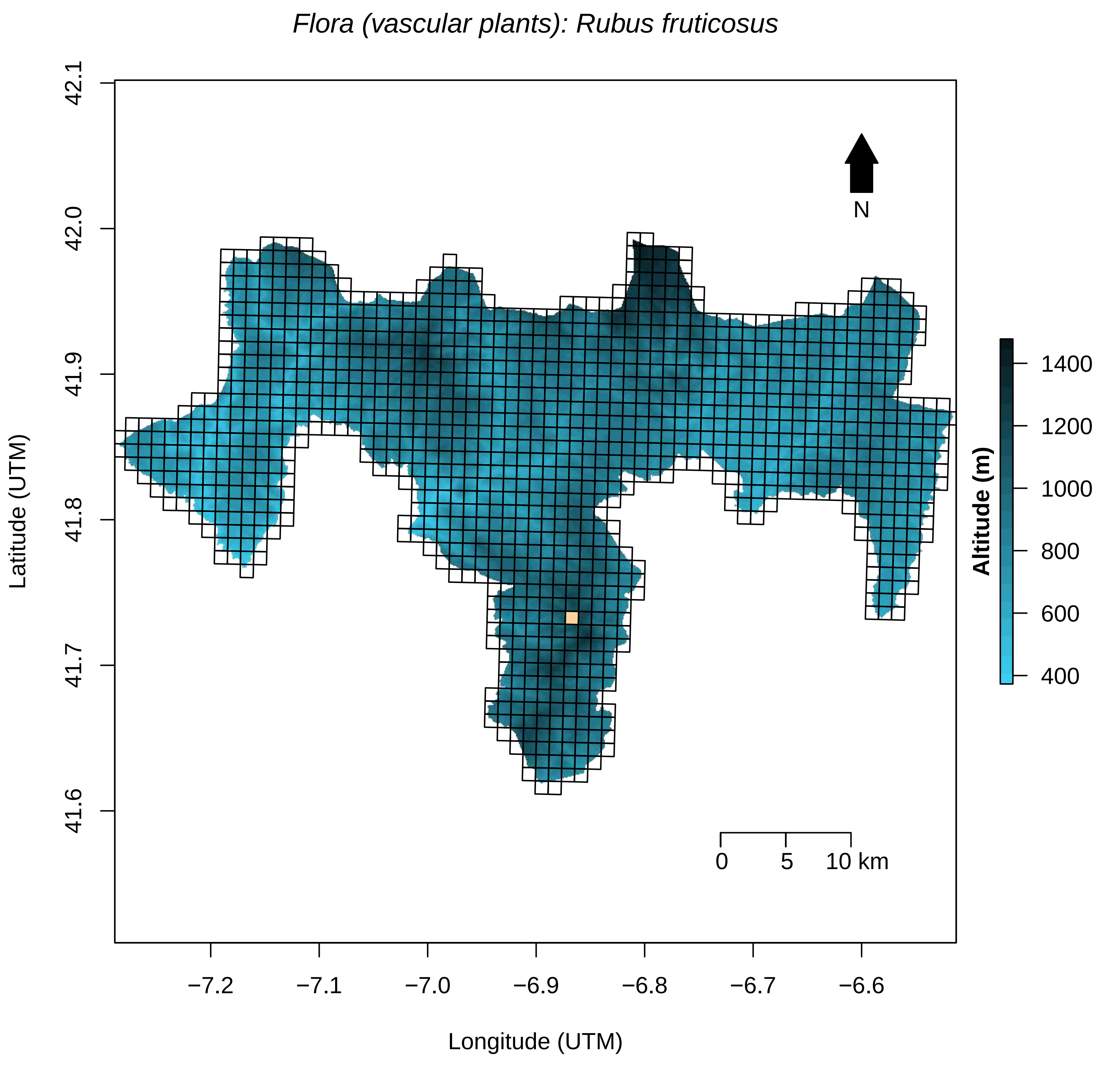

Flora (vascular plants): Rubus fruticosus
N
0
5
10 km
Latitude (UTM)
Longitude (UTM)
Altitude (m)
42.1
42.0
41.9
41.8
41.7
41.6
−7.2
−7.1
−7.0
−6.9
−6.8
−6.7
−6.6
1400
1200
1000
800
600
400

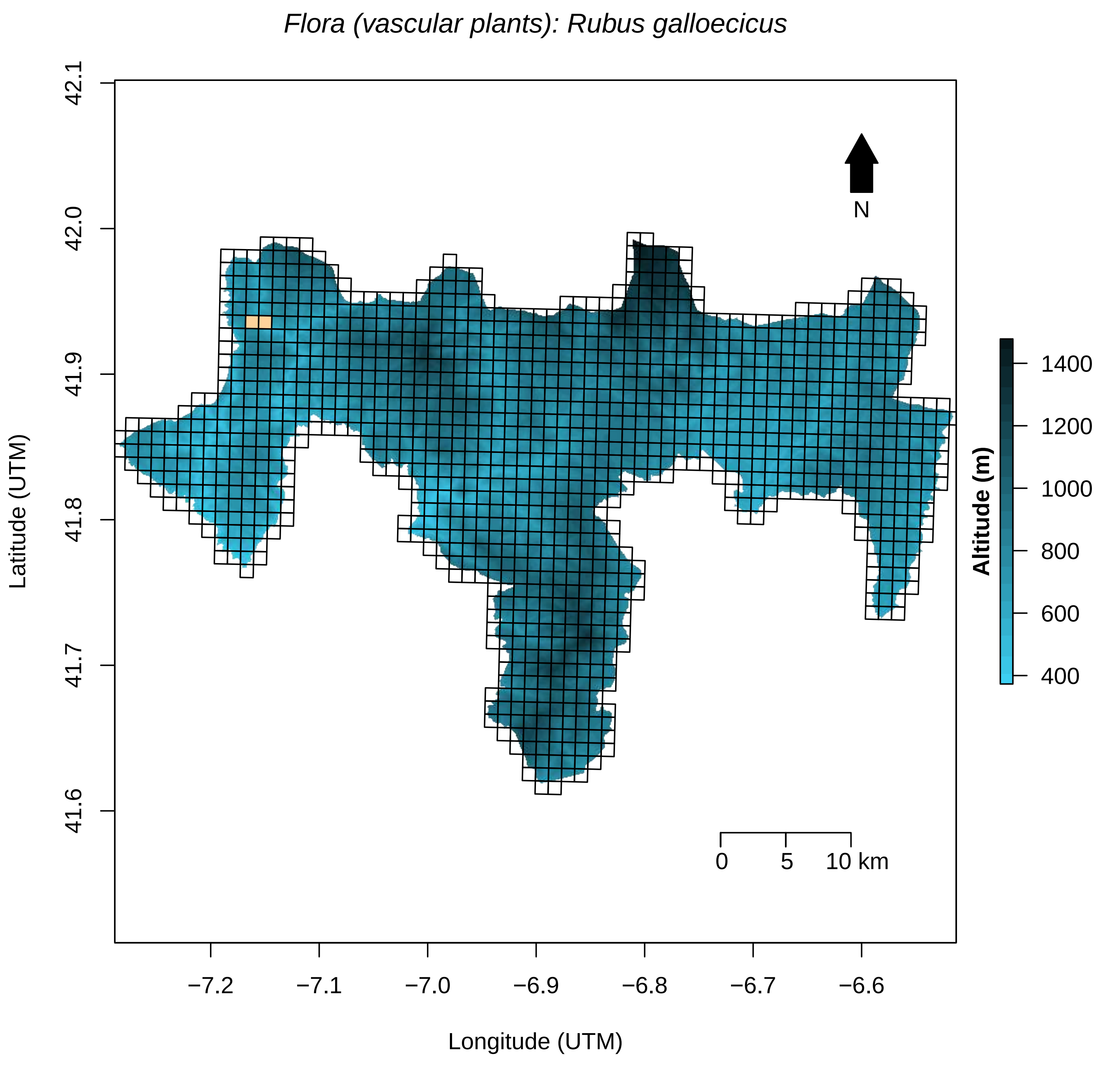

Flora (vascular plants): Rubus galloecicus
42.1
42.0
41.9
41.8
41.7
41.6
Latitude (UTM)
N
0 5 10 km
−7.2
−7.1
−7.0
−6.9
−6.8
−6.7
−6.6
Longitude (UTM)
Altitude (m)
1400
1200
1000
800
600
400

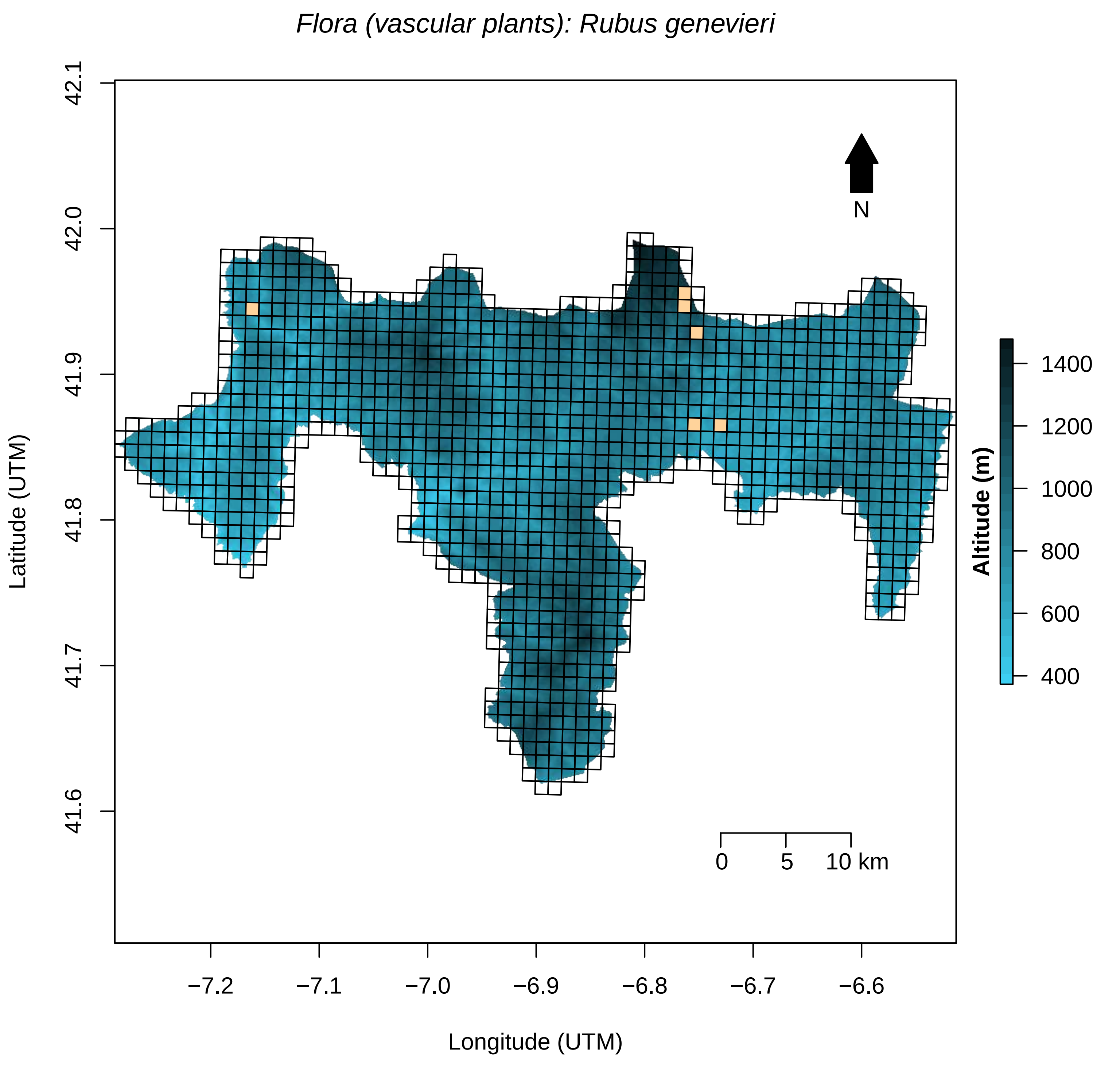

Flora (vascular plants): Rubus genevieri
Latitude (UTM)
42.1
42.0
41.9
41.8
41.7
41.6
Longitude (UTM)
−7.2
−7.1
−7.0
−6.9
−6.8
−6.7
−6.6
N
0
5
10 km
Altitude (m)
1400
1200
1000
800
600
400

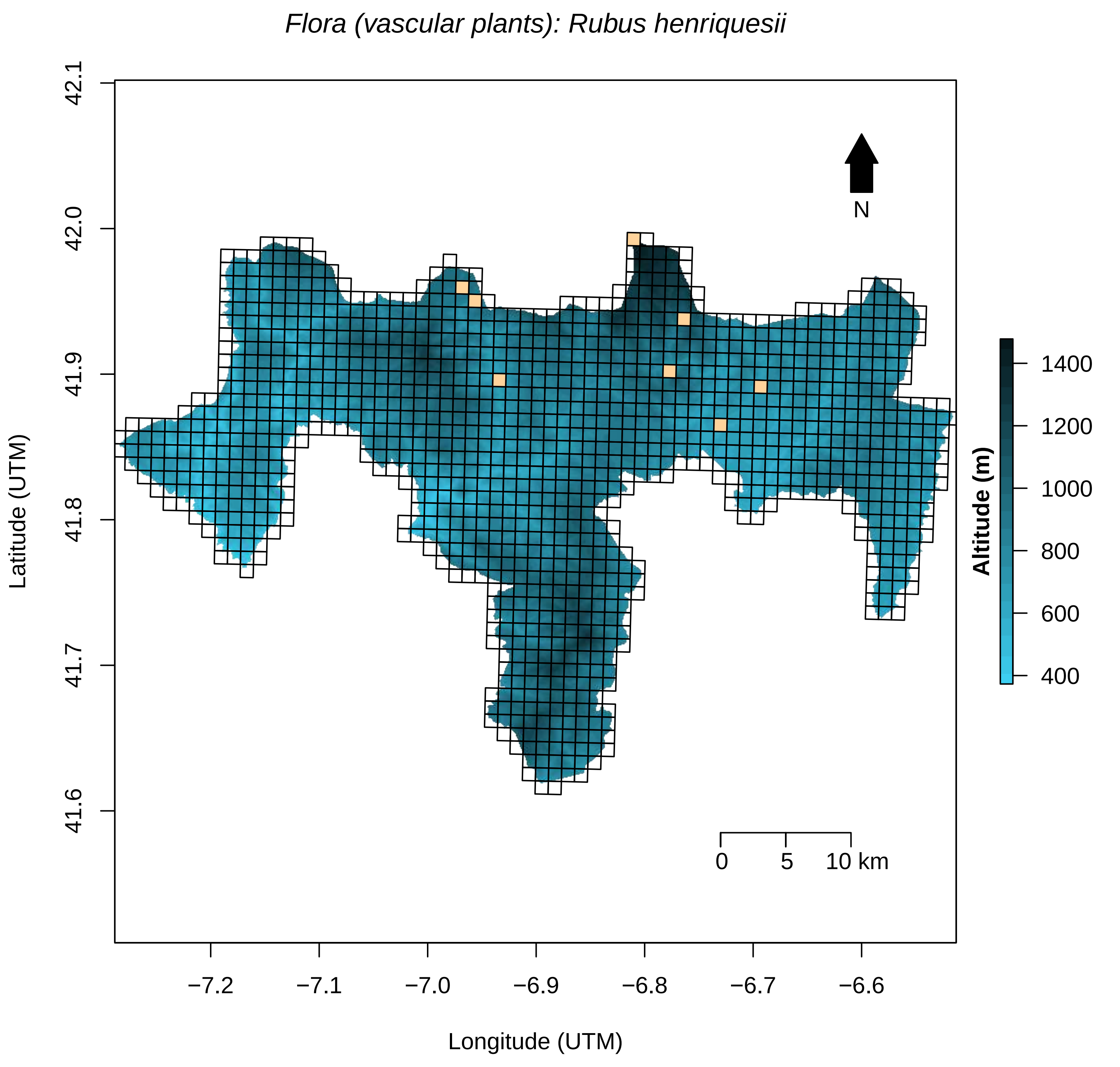

Flora (vascular plants): Rubus henriquesii
42.1
42.0
41.9
41.8
41.7
41.6
Latitude (UTM)
−7.2
−7.1
−7.0
−6.9
−6.8
−6.7
−6.6
Longitude (UTM)
N
0
5
10 km
Altitude (m)
1400
1200
1000
800
600
400

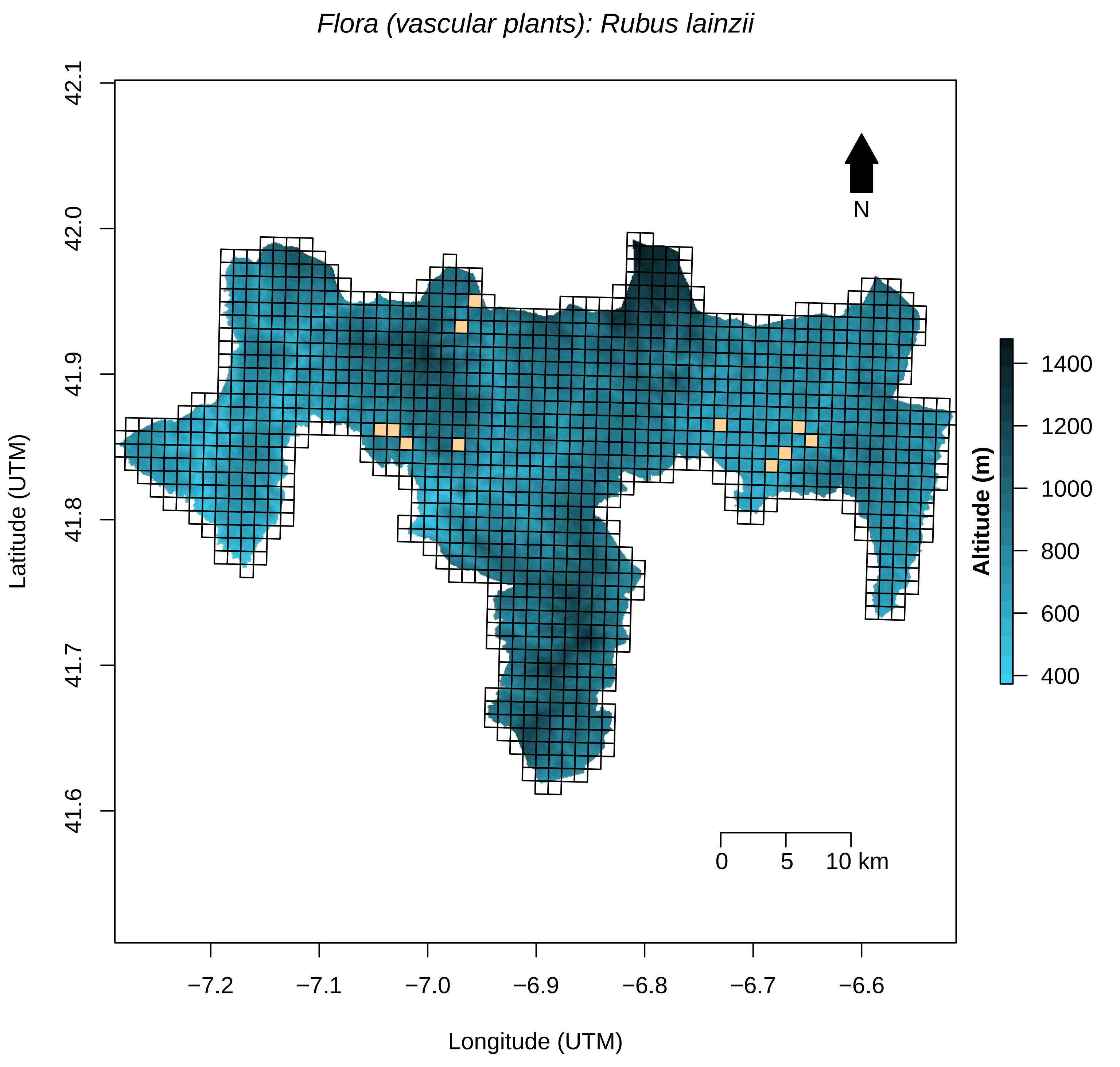
Flora (vascular plants): Rubus lainzii
42.1
42.0
41.9
41.8
41.7
41.6
Latitude (UTM)
−7.2
−7.1
−7.0
−6.9
−6.8
−6.7
−6.6
Longitude (UTM)
N
0
5
10 km
Altitude (m)
1400
1200
1000
800
600
400

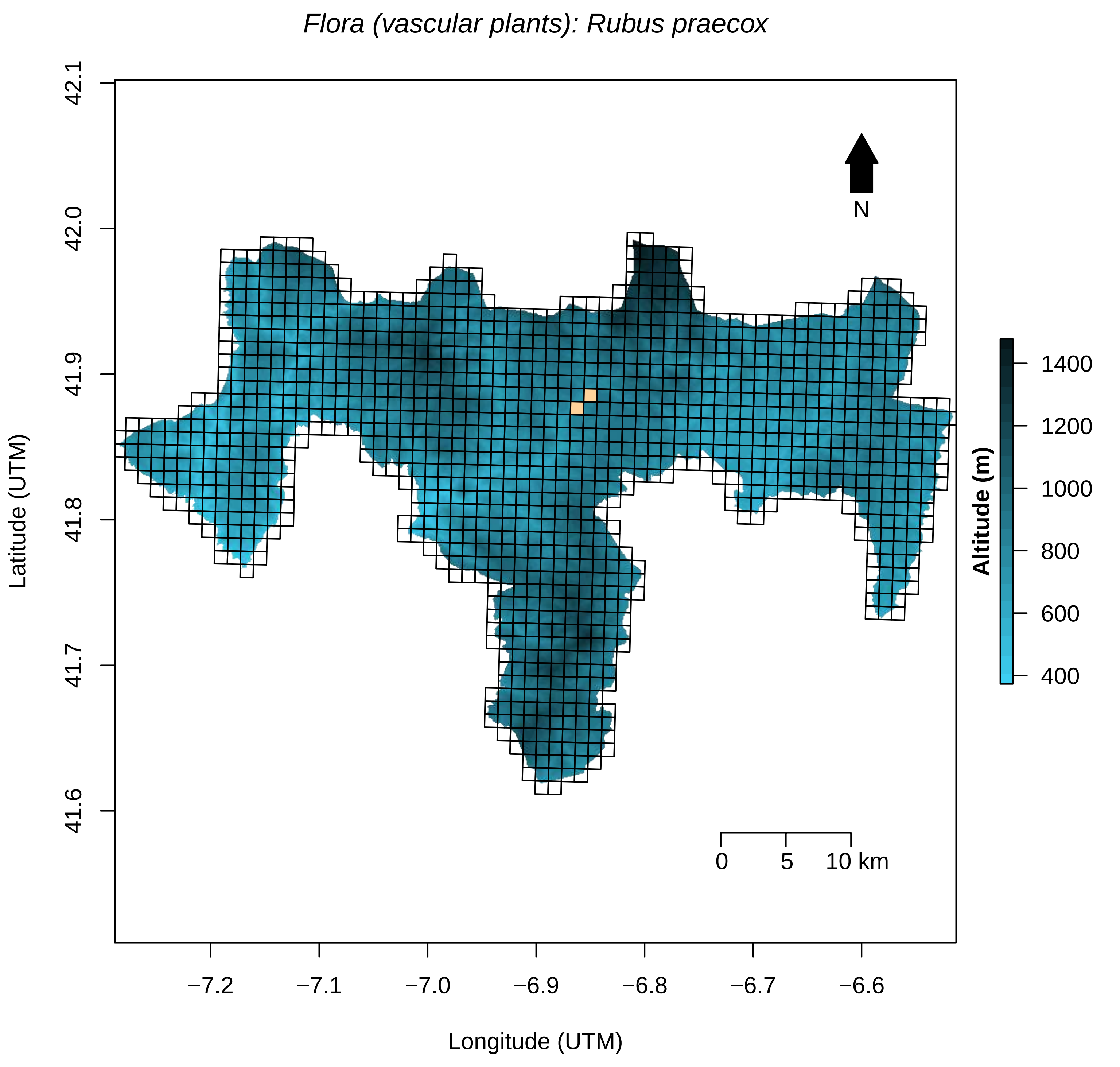
Flora (vascular plants): Rubus praecox
N
0 5 10 km
Altitude (m)
1400
1200
1000
800
600
400
42.1
42.0
41.9
41.8
41.7
41.6
Latitude (UTM)
−7.2
−7.1
−7.0
−6.9
−6.8
−6.7
−6.6
Longitude (UTM)

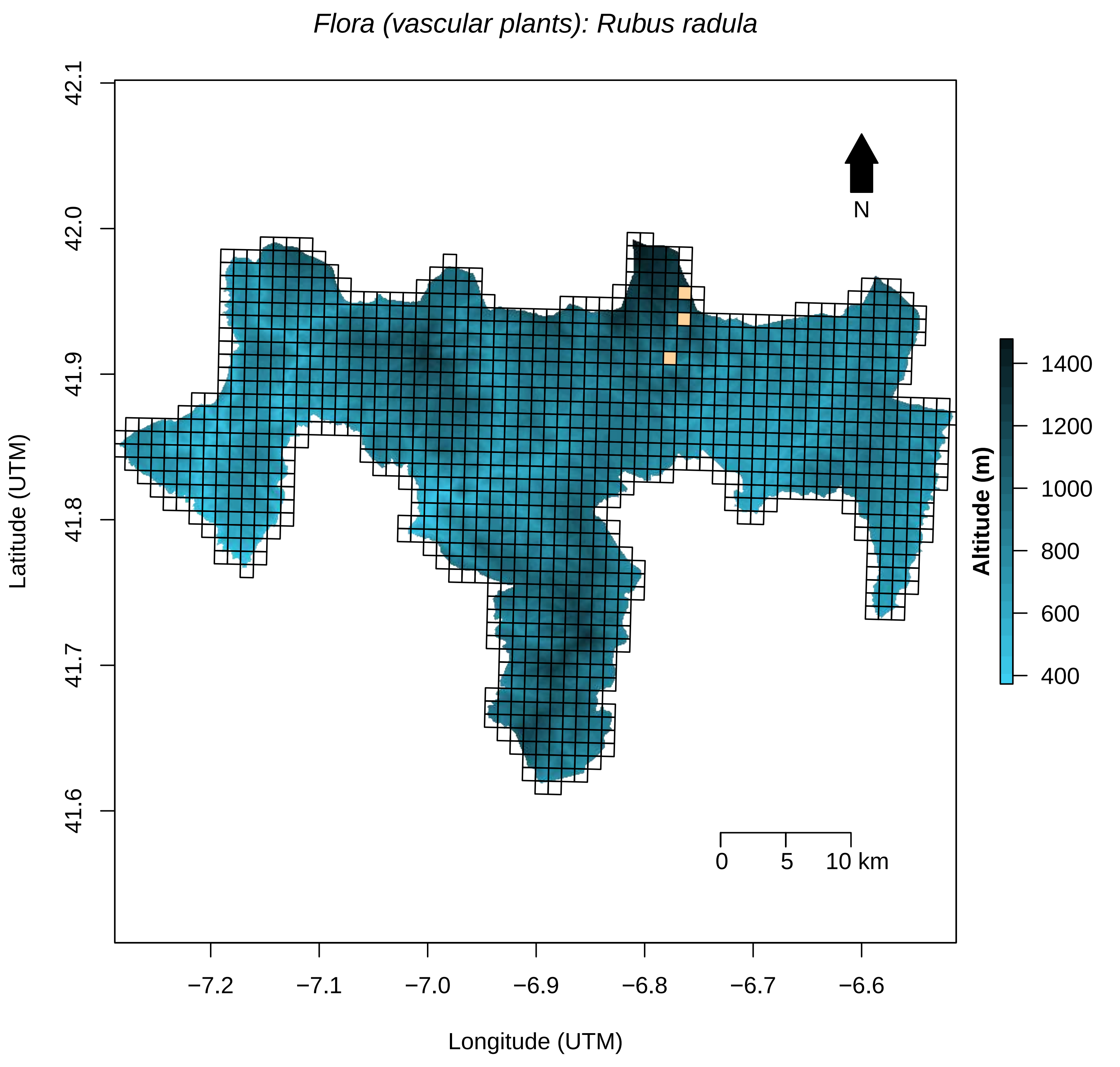
Flora (vascular plants): Rubus radula
Latitude (UTM)
Longitude (UTM)
42.1
42.0
41.9
41.8
41.7
41.6
−7.2
−7.1
−7.0
−6.9
−6.8
−6.7
−6.6
N
0
5
10 km
Altitude (m)
1400
1200
1000
800
600
400

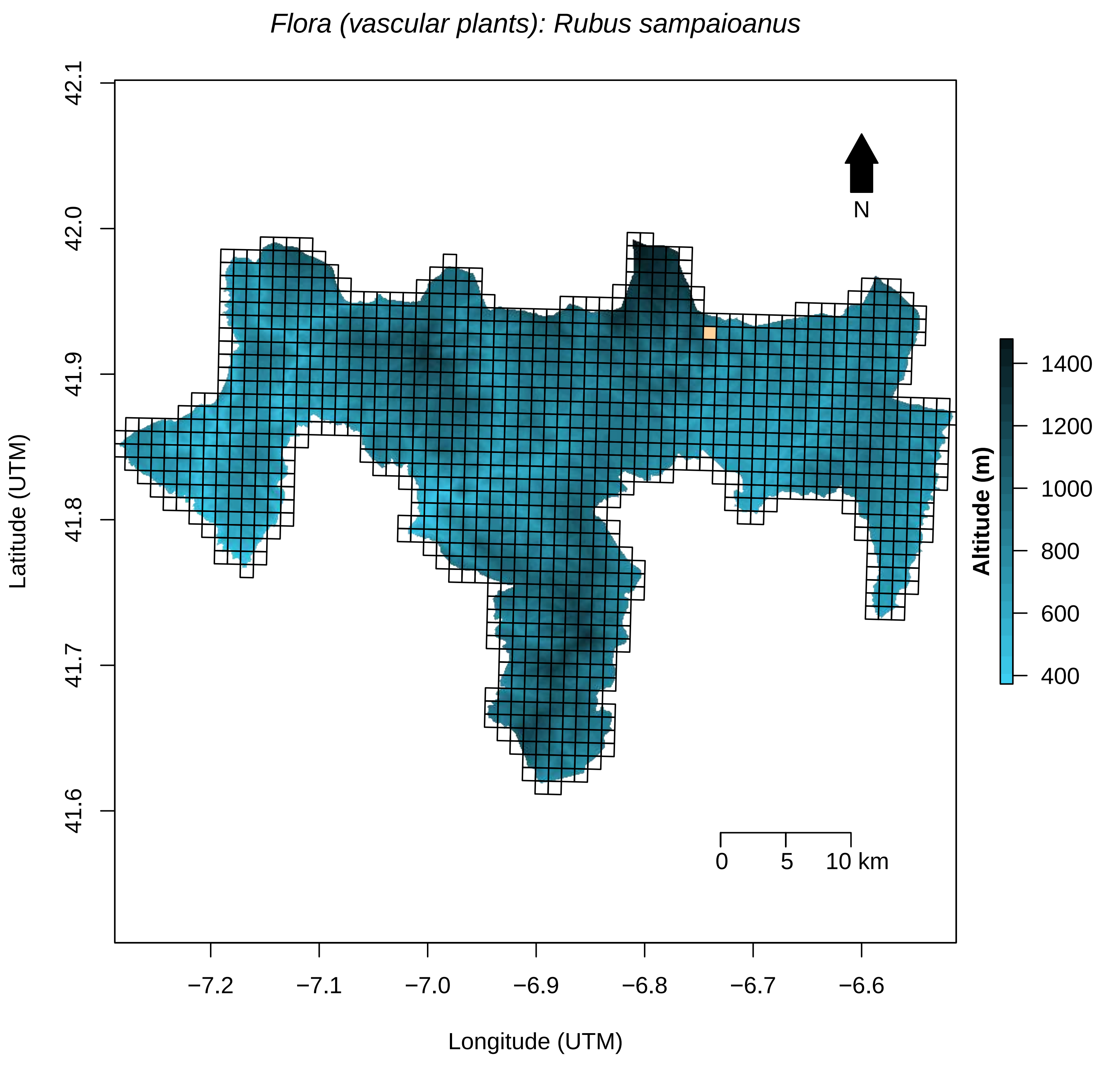

Flora (vascular plants): Rubus sampaioanus
42.1
42.0
41.9
41.8
41.7
41.6
Latitude (UTM)
−7.2
−7.1
−7.0
−6.9
−6.8
−6.7
−6.6
Longitude (UTM)
N
0
5
10 km
1400
1200
1000
800
600
400
Altitude (m)

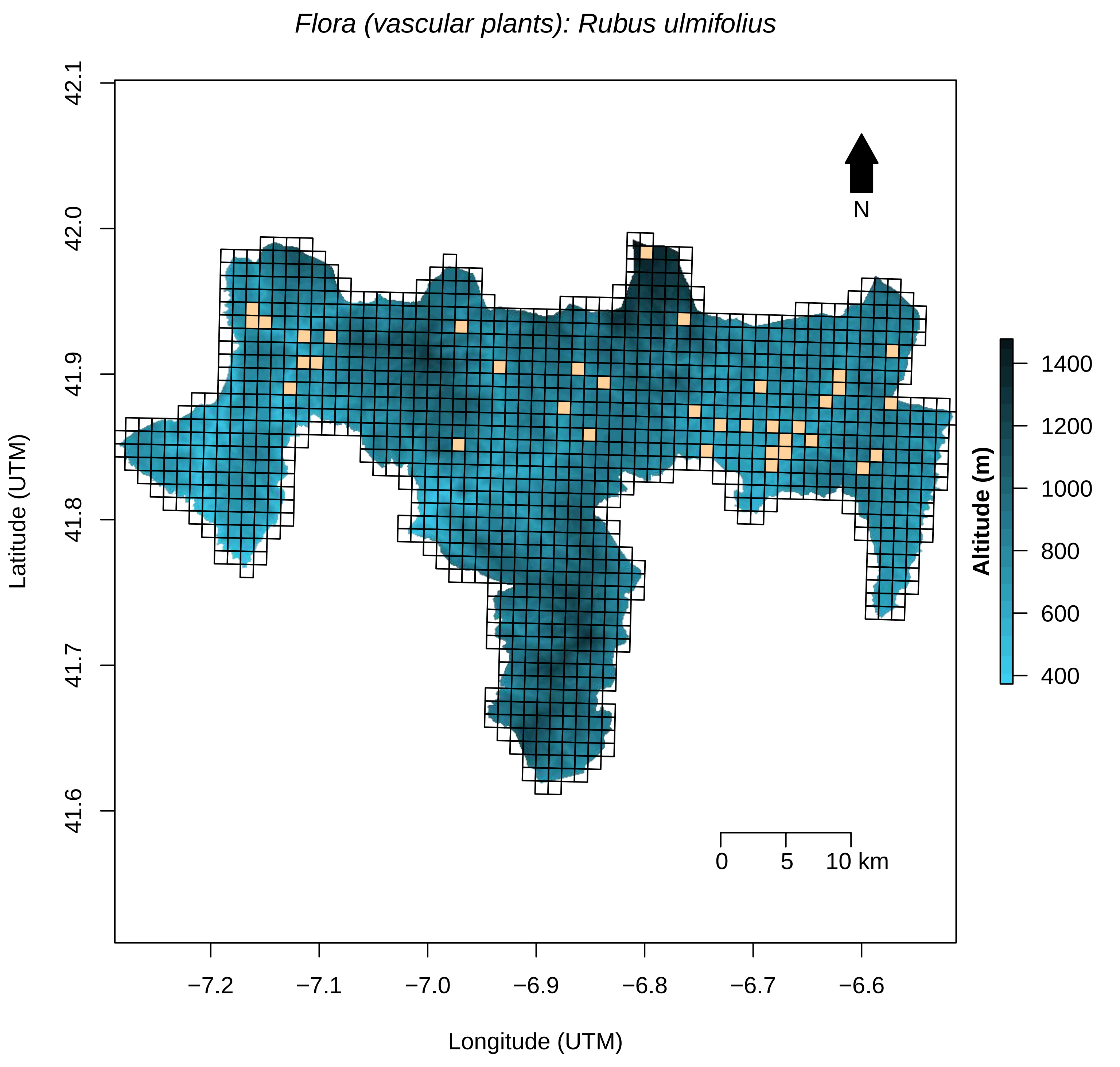

Flora (vascular plants): Rubus ulmifolius
N
0
5
10 km
Altitude (m)
1400
1200
1000
800
600
400
42.1
42.0
41.9
41.8
41.7
41.6
Latitude (UTM)
−7.2
−7.1
−7.0
−6.9
−6.8
−6.7
−6.6
Longitude (UTM)

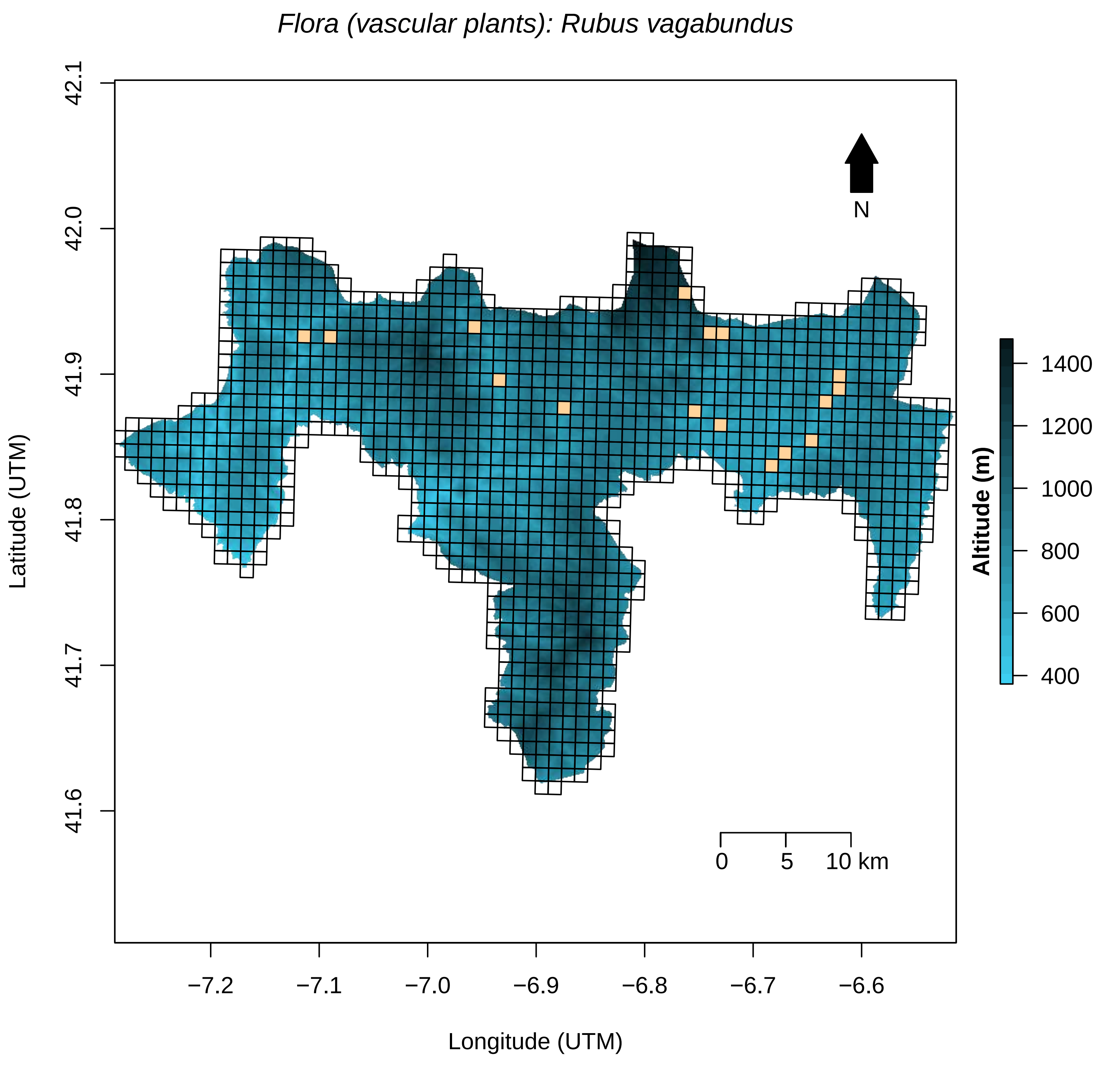

Flora (vascular plants): Rubus vagabundus
42.1
42.0
41.9
41.8
41.7
41.6
Latitude (UTM)
−7.2
−7.1
−7.0
−6.9
−6.8
−6.7
−6.6
Longitude (UTM)
N
0
5
10 km
Altitude (m)
1400
1200
1000
800
600
400

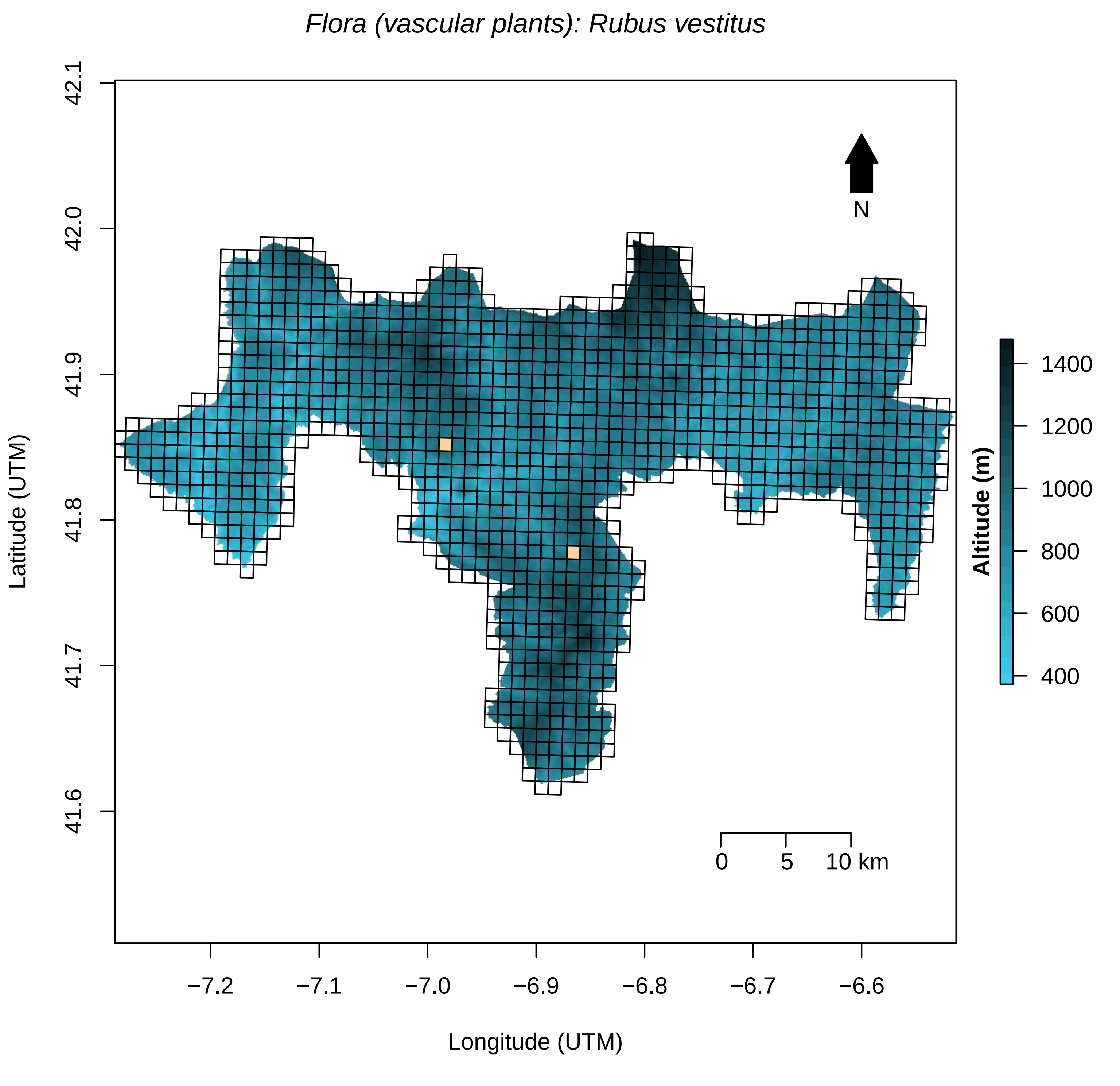
Flora (vascular plants): Rubus vestitus
Latitude (UTM)
42.1
42.0
41.9
41.8
41.7
41.6
N
0
5
10 km
Altitude (m)
1400
1200
1000
800
600
400
−7.2
−7.1
−7.0
−6.9
−6.8
−6.7
−6.6
Longitude (UTM)

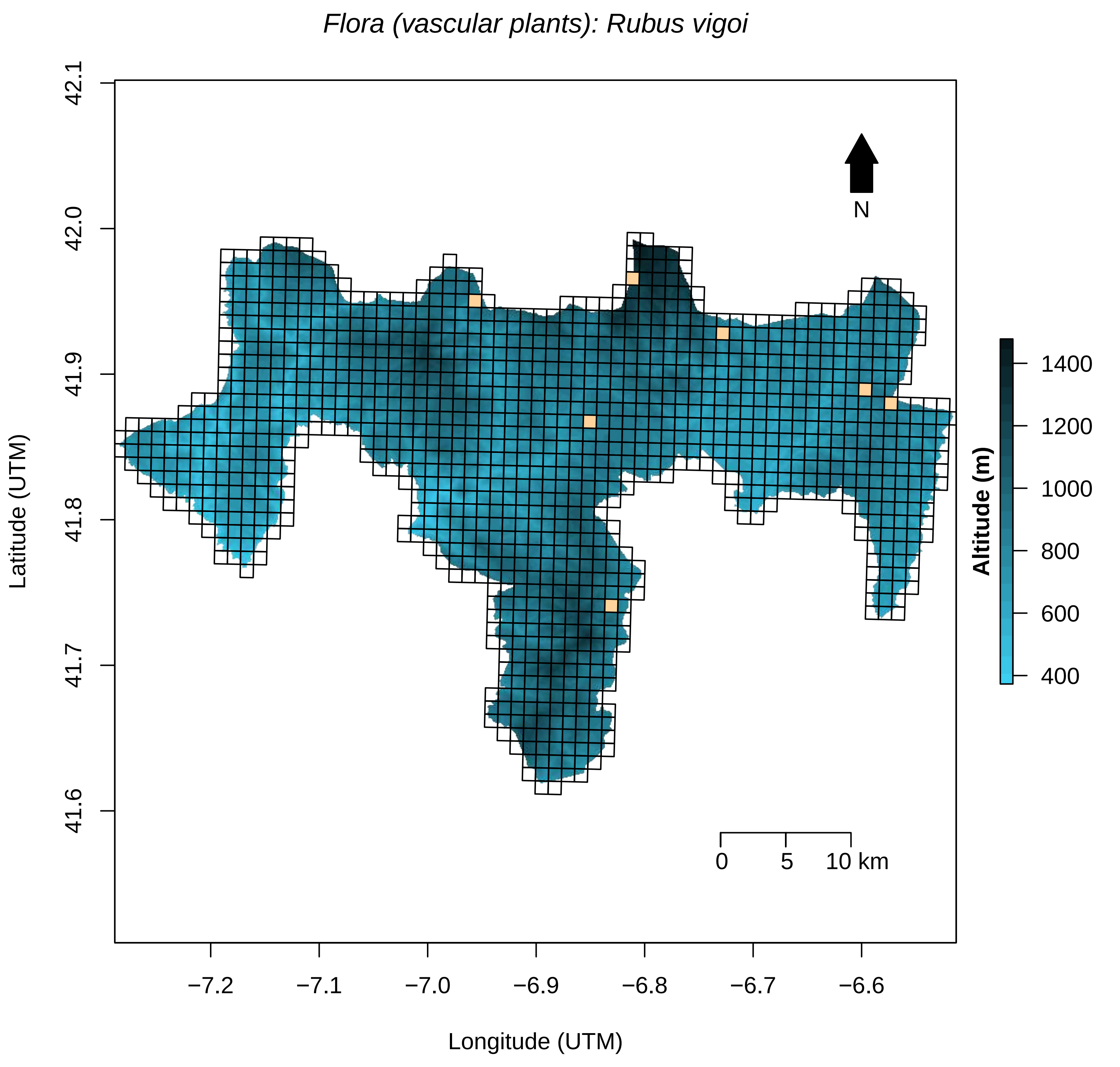
Flora (vascular plants): Rubus vigoi
42.1
42.0
41.9
41.8
41.7
41.6
Latitude (UTM)
−7.2
−7.1
−7.0
−6.9
−6.8
−6.7
−6.6
Longitude (UTM)
N
0
5
10 km
Altitude (m)
1400
1200
1000
800
600
400

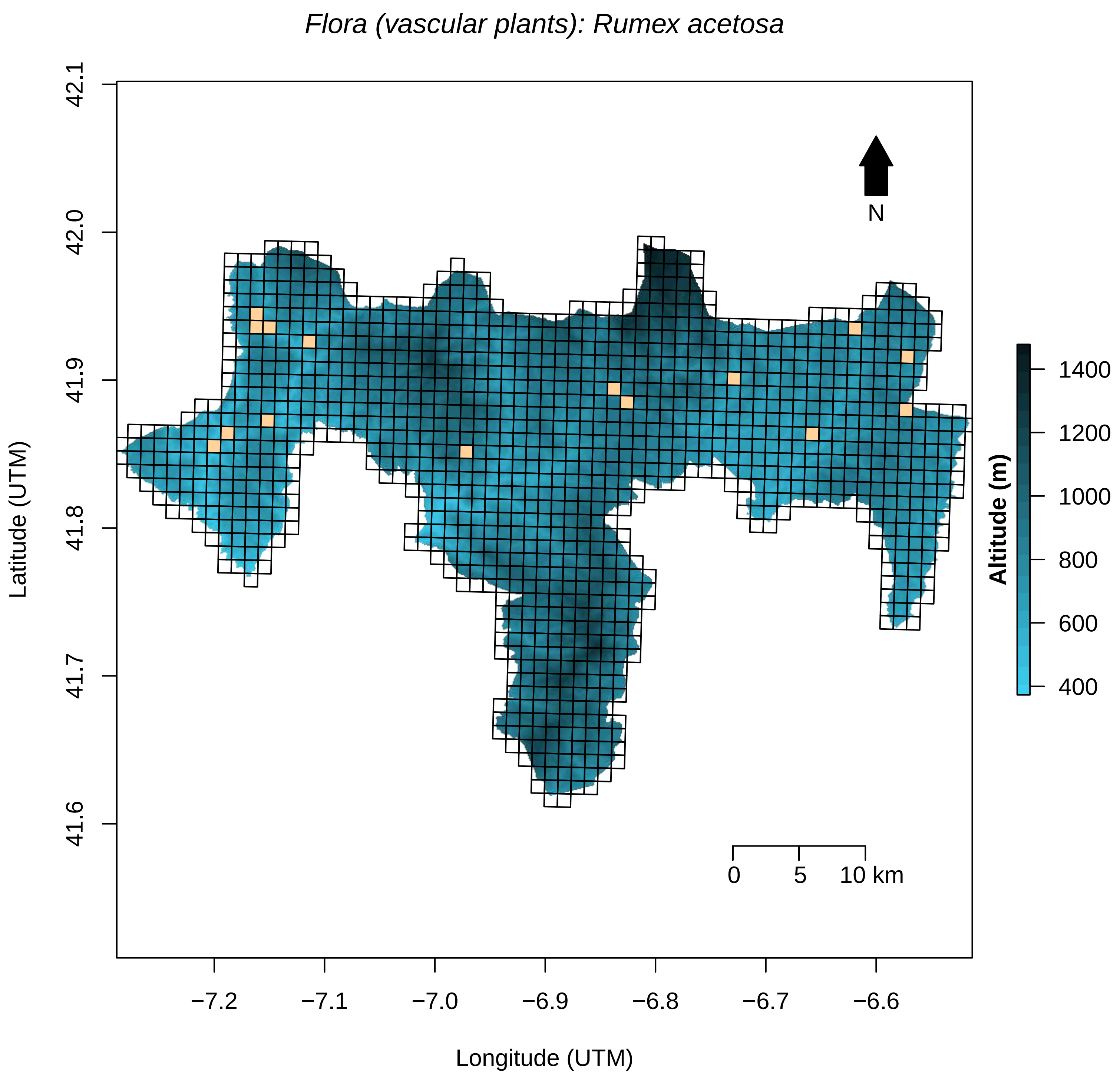

Flora (vascular plants): Rumex acetosa
42.1
42.0
41.9
41.8
41.7
41.6
Latitude (UTM)
−7.2
−7.1
−7.0
−6.9
−6.8
−6.7
−6.6
Longitude (UTM)
N
0
5
10 km
Altitude (m)
1400
1200
1000
800
600
400

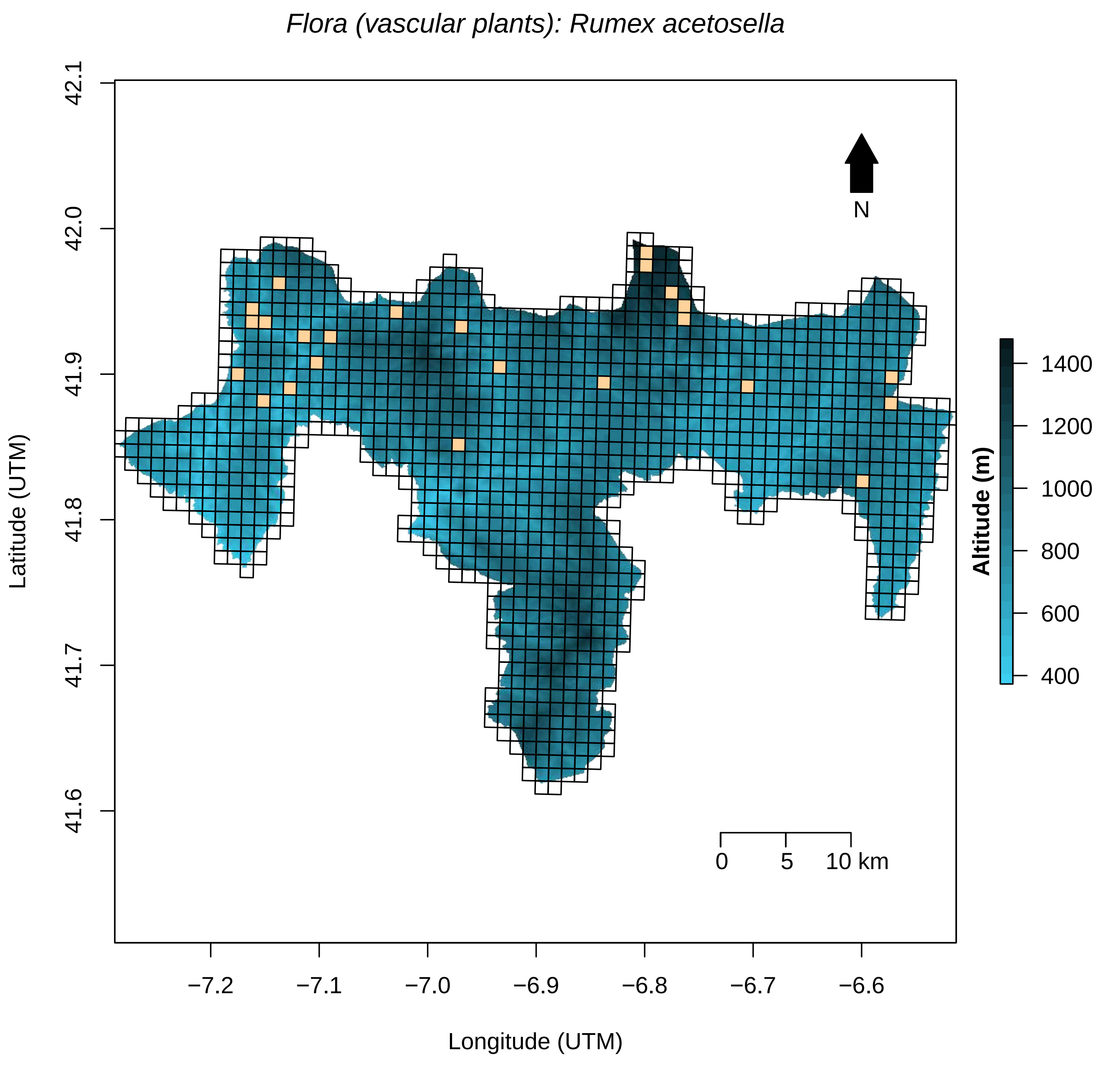

Flora (vascular plants): Rumex acetosella
Latitude (UTM)
Longitude (UTM)
42.1
42.0
41.9
41.8
41.7
41.6
−7.2
−7.1
−7.0
−6.9
−6.8
−6.7
−6.6
N
0
5
10 km
Altitude (m)
1400
1200
1000
800
600
400

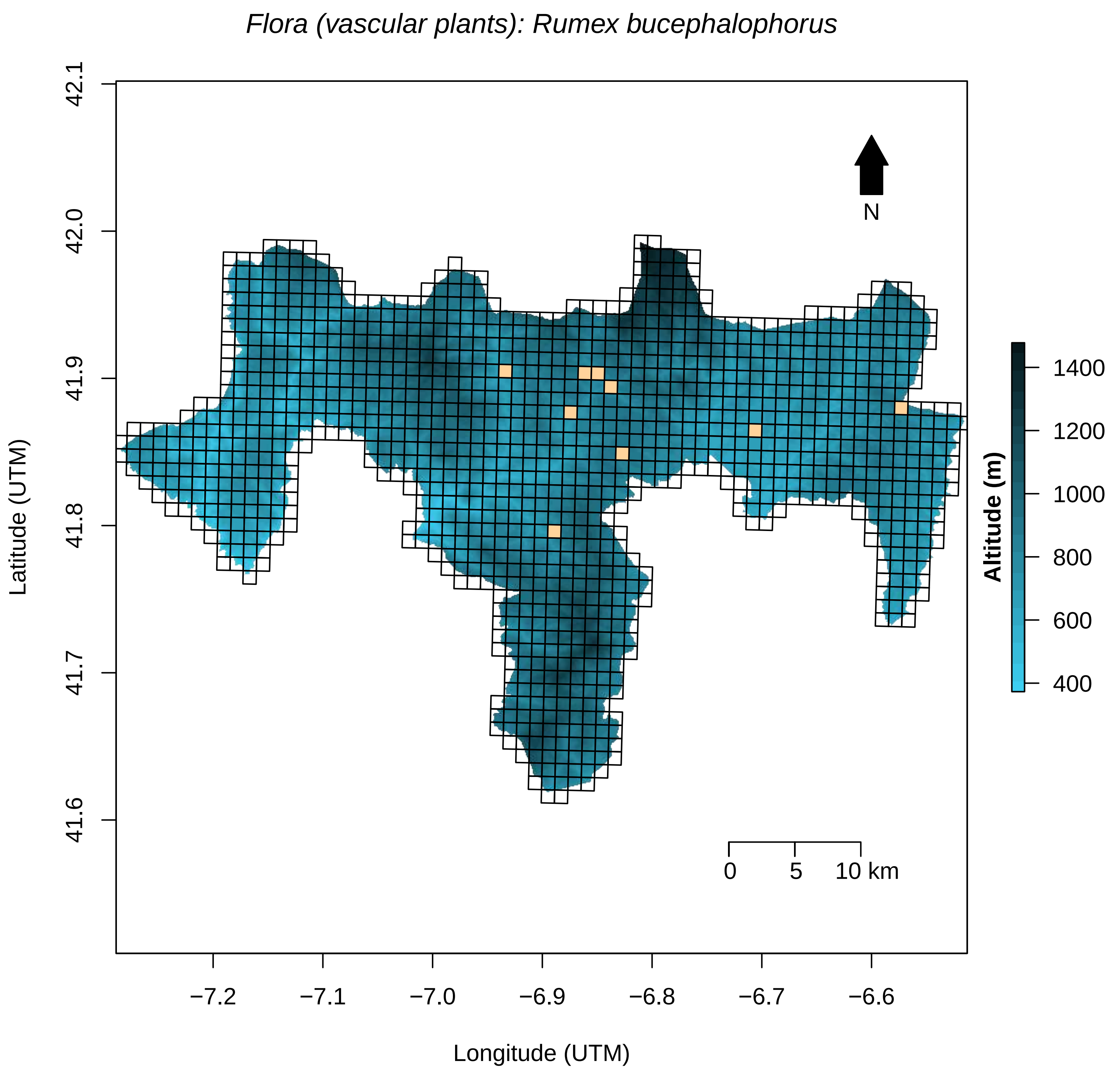
Flora (vascular plants): Rumex bucephalophorus
42.1
42.0
41.9
41.8
41.7
41.6
Latitude (UTM)
N
0 5 10 km
−7.2 −7.1 −7.0 −6.9 −6.8 −6.7 −6.6
Longitude (UTM)
Altitude (m)
1400
1200
1000
800
600
400

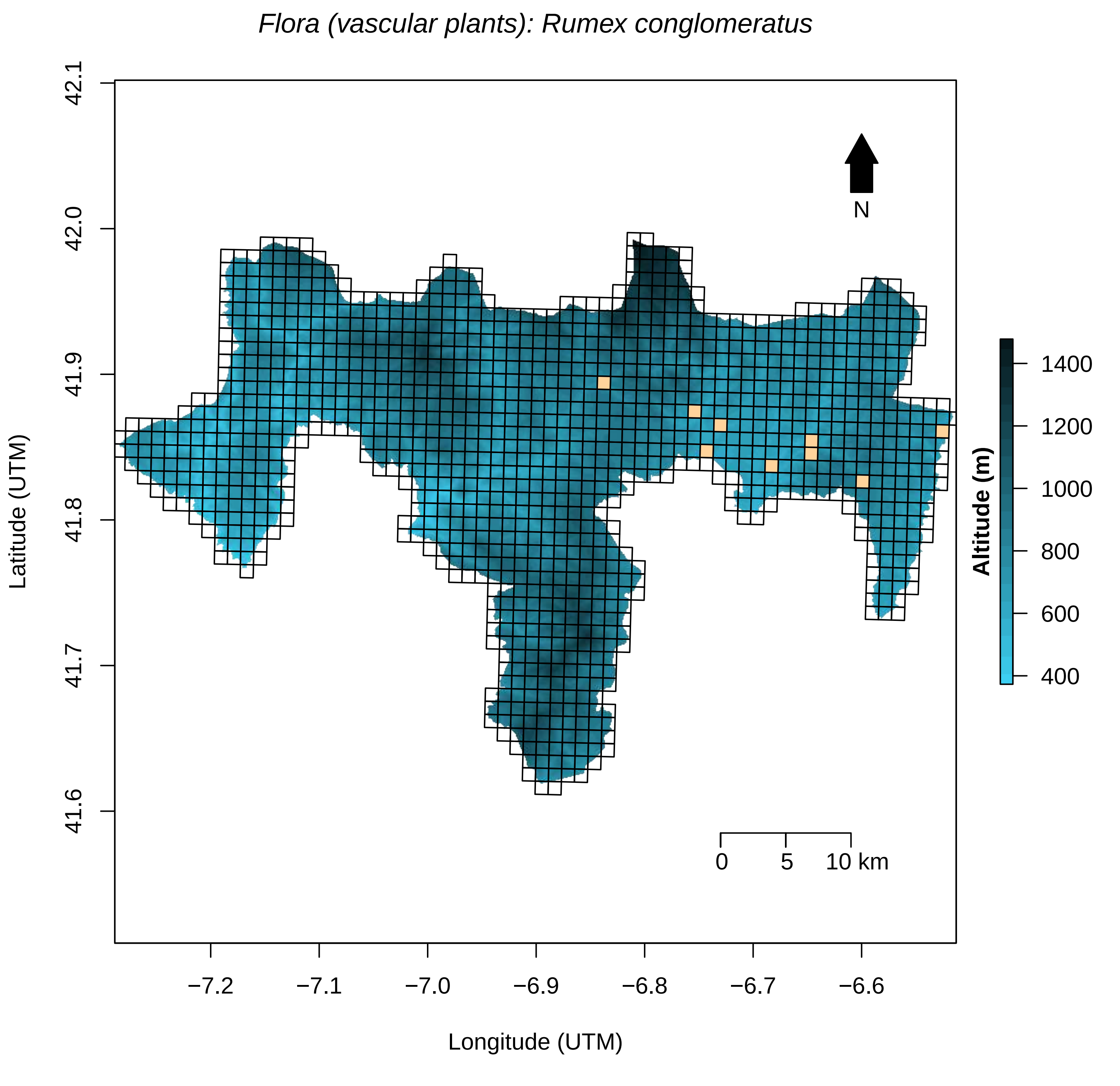

Flora (vascular plants): Rumex conglomeratus
N
0
5
10 km
Altitude (m)
1400
1200
1000
800
600
400
Latitude (UTM)
42.1
42.0
41.9
41.8
41.7
41.6
Longitude (UTM)
−7.2
−7.1
−7.0
−6.9
−6.8
−6.7
−6.6

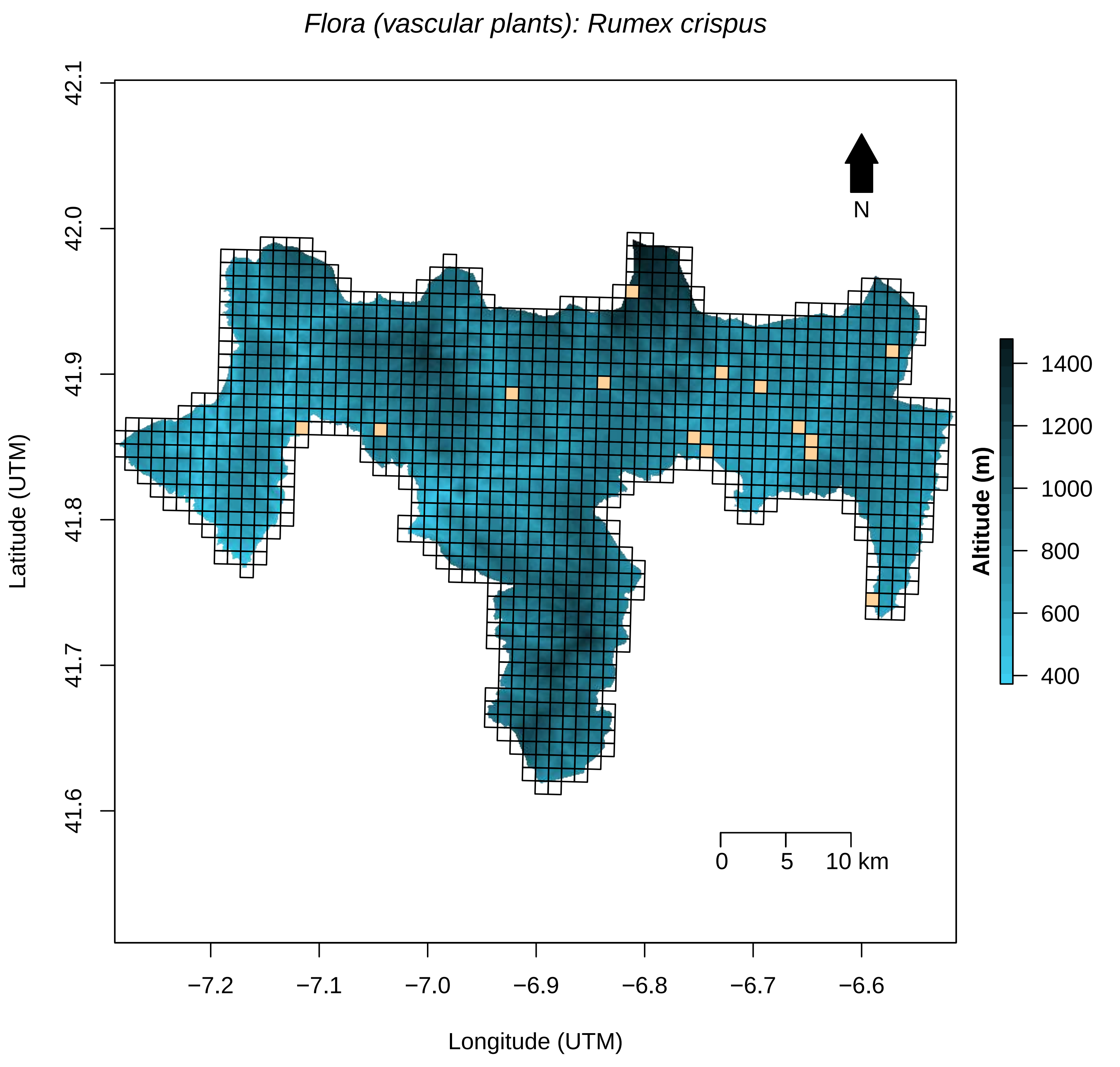

Flora (vascular plants): Rumex crispus
42.1
42.0
41.9
41.8
41.7
41.6
Latitude (UTM)
−7.2
−7.1
−7.0
−6.9
−6.8
−6.7
−6.6
Longitude (UTM)
N
0
5
10 km
1400
1200
1000
800
600
400
Altitude (m)

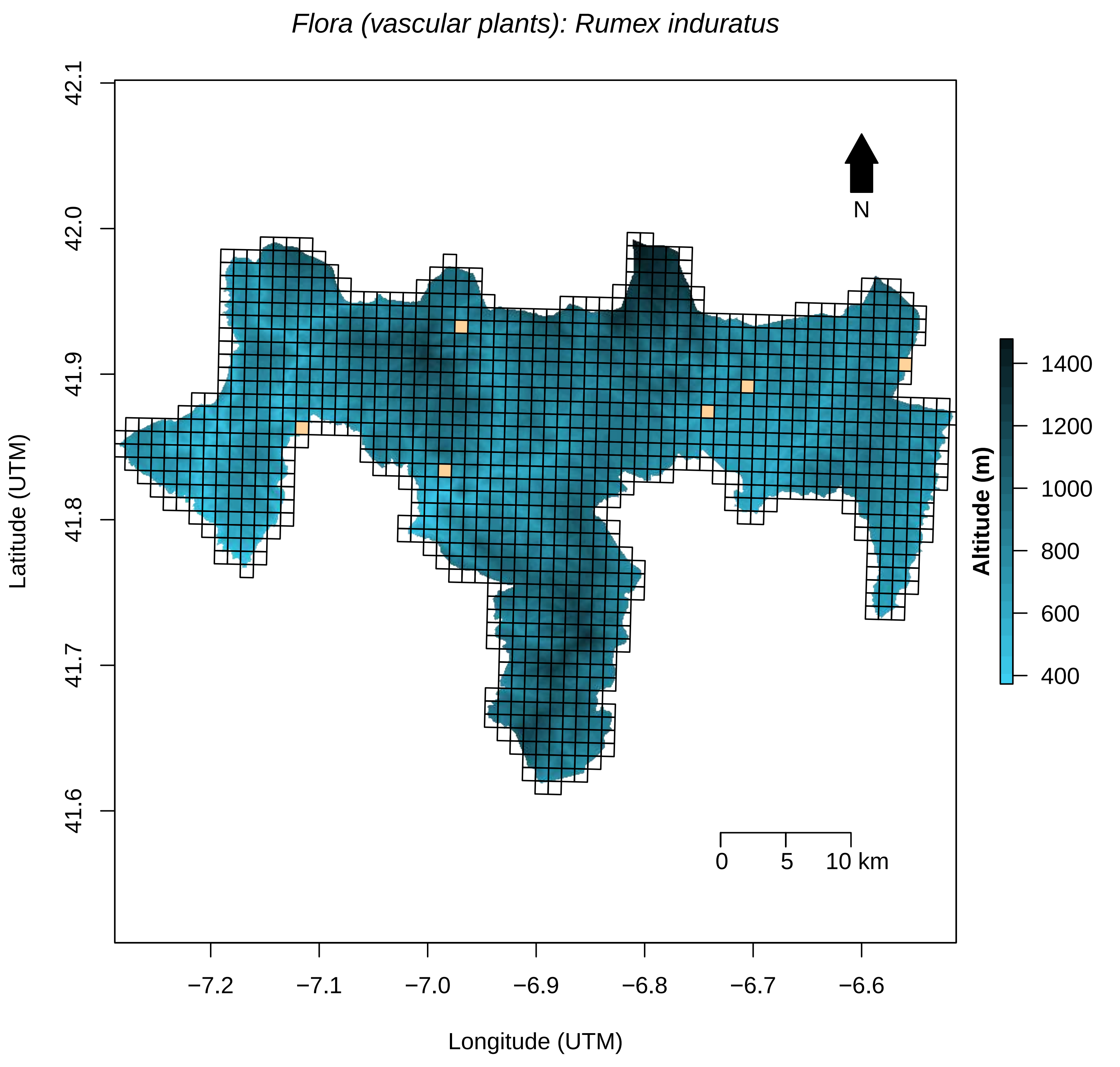

Flora (vascular plants): Rumex induratus
Latitude (UTM)
42.1
42.0
41.9
41.8
41.7
41.6
Longitude (UTM)
−7.2
−7.1
−7.0
−6.9
−6.8
−6.7
−6.6
N
0
5
10 km
Altitude (m)
1400
1200
1000
800
600
400

Flora (vascular plants): Rumex longifolius

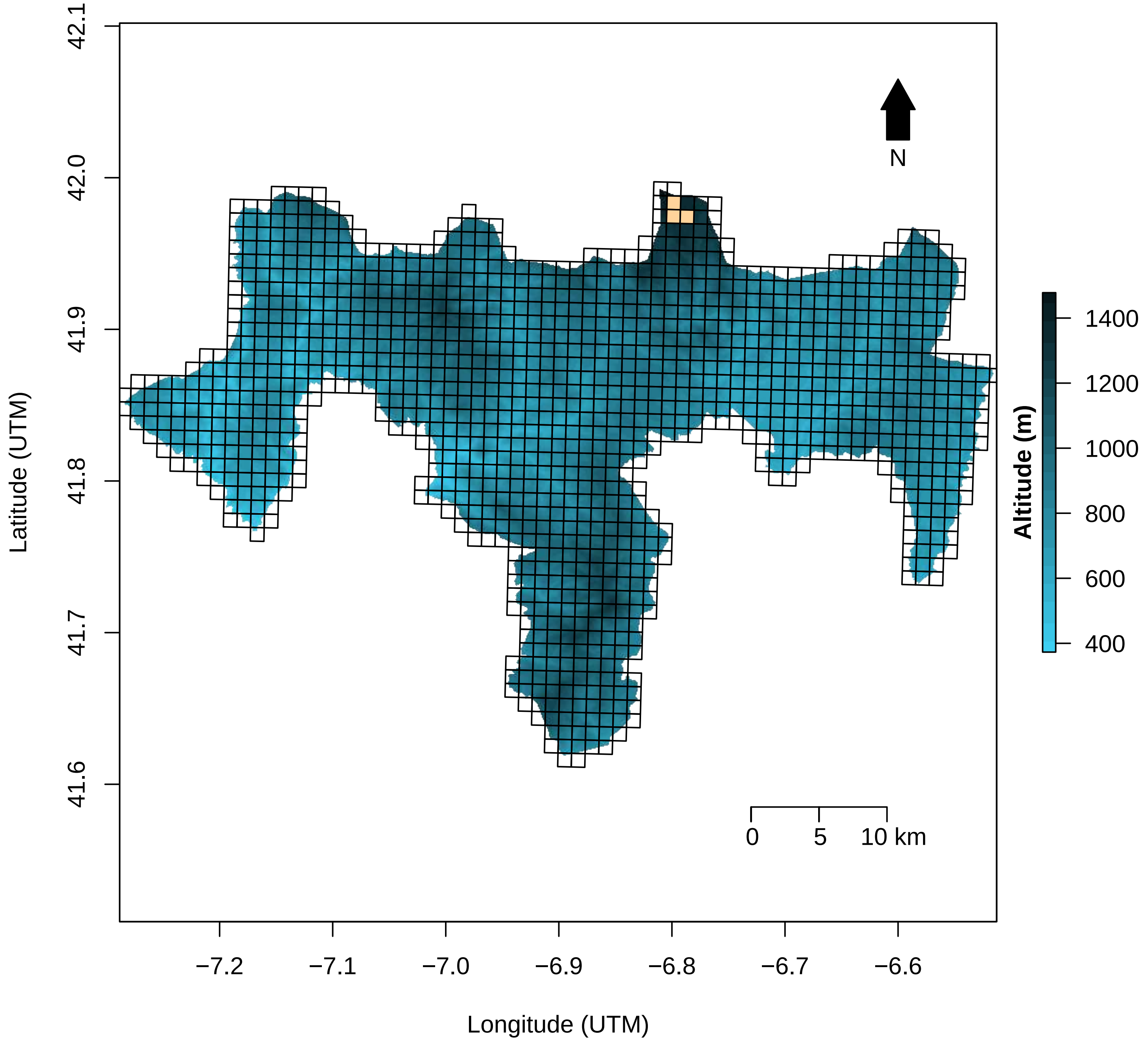

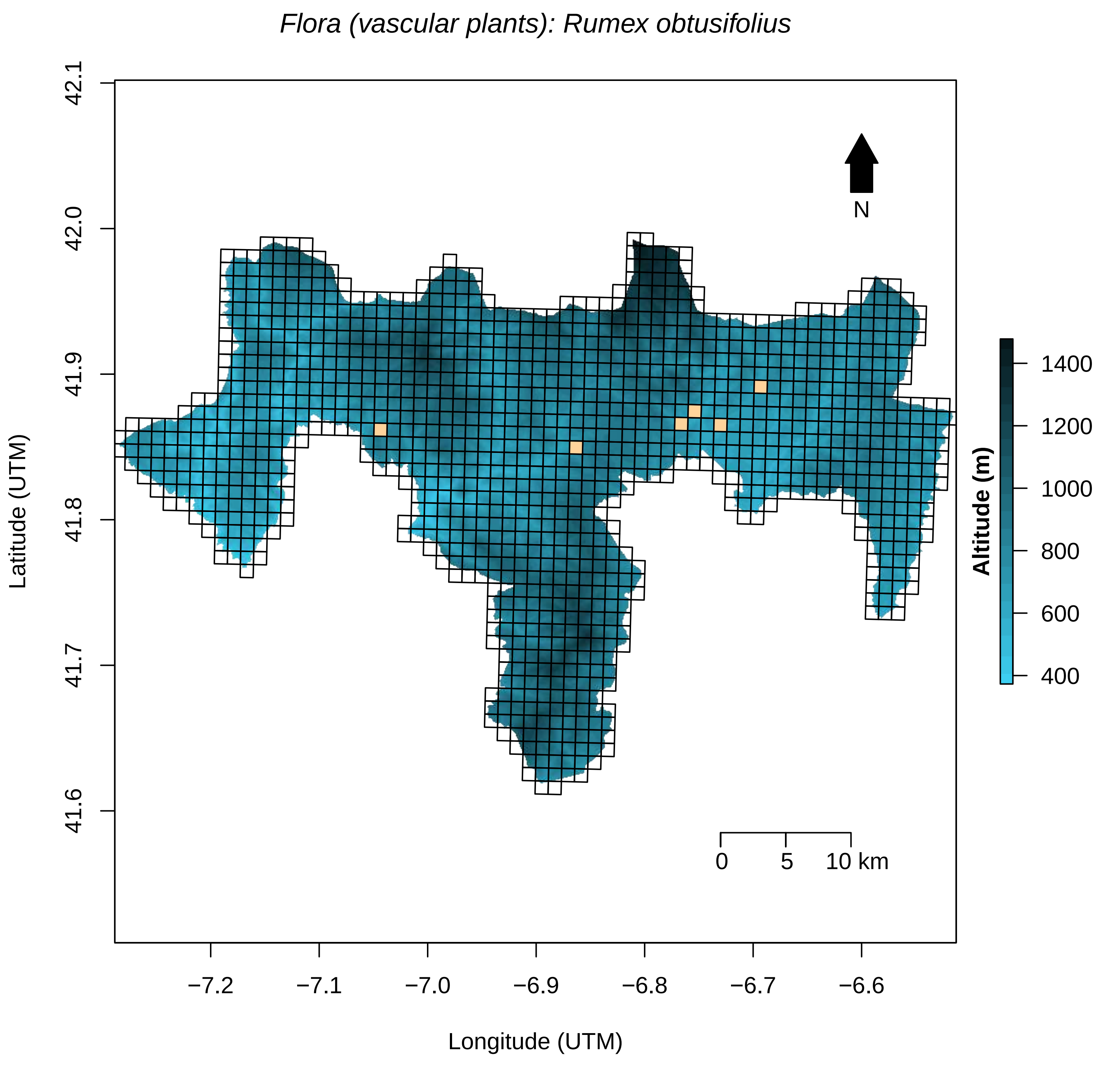

Flora (vascular plants): Rumex obtusifolius
Latitude (UTM)
42.1
42.0
41.9
41.8
41.7
41.6
N
0
5
10 km
−7.2
−7.1
−7.0
−6.9
−6.8
−6.7
−6.6
Longitude (UTM)
Altitude (m)
1400
1200
1000
800
600
400

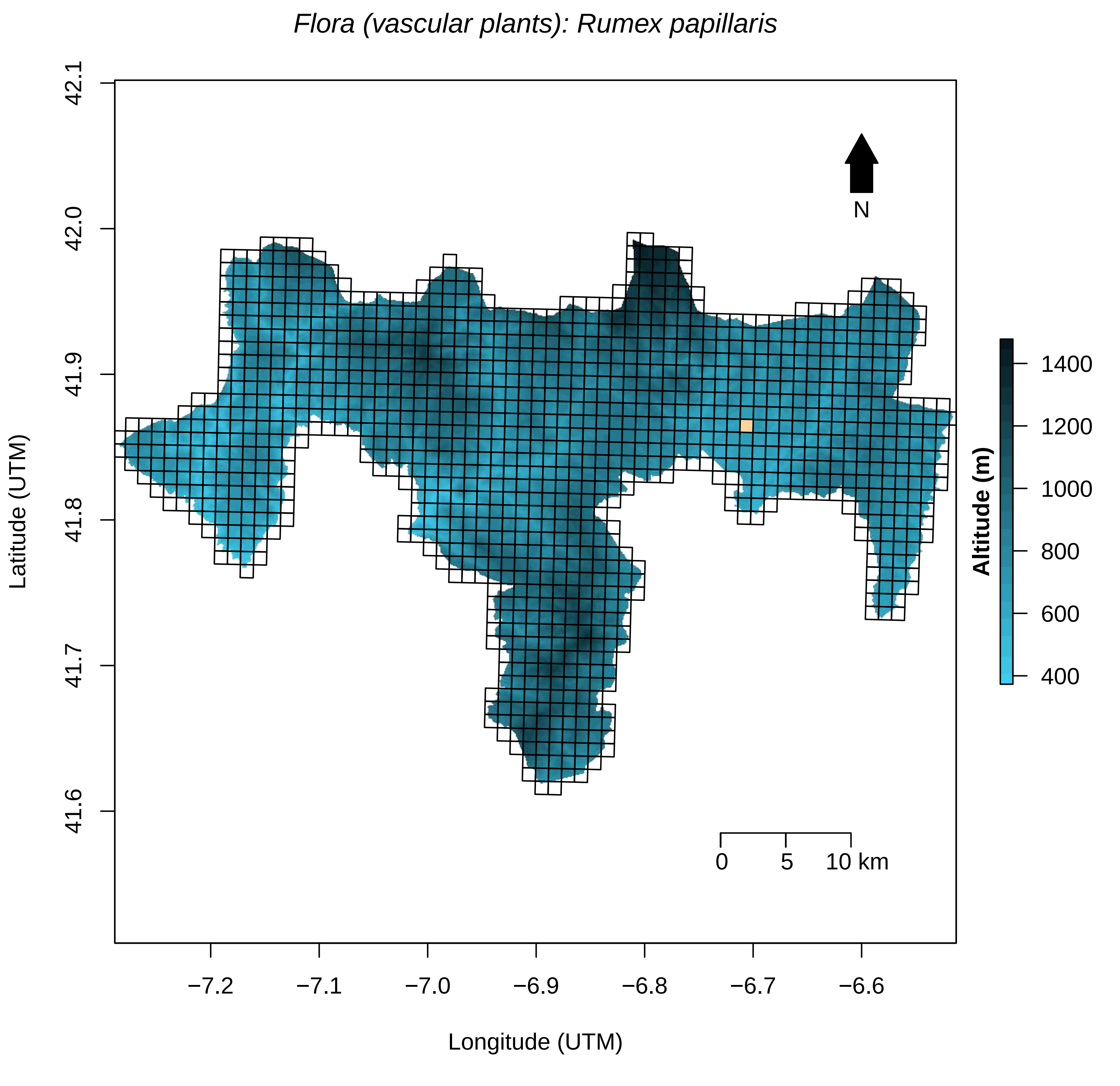

Flora (vascular plants): Rumex papillaris
42.1
42.0
41.9
41.8
41.7
41.6
Latitude (UTM)
−7.2
−7.1
−7.0
−6.9
−6.8
−6.7
−6.6
Longitude (UTM)
N
0
5
10 km
Altitude (m)
1400
1200
1000
800
600
400

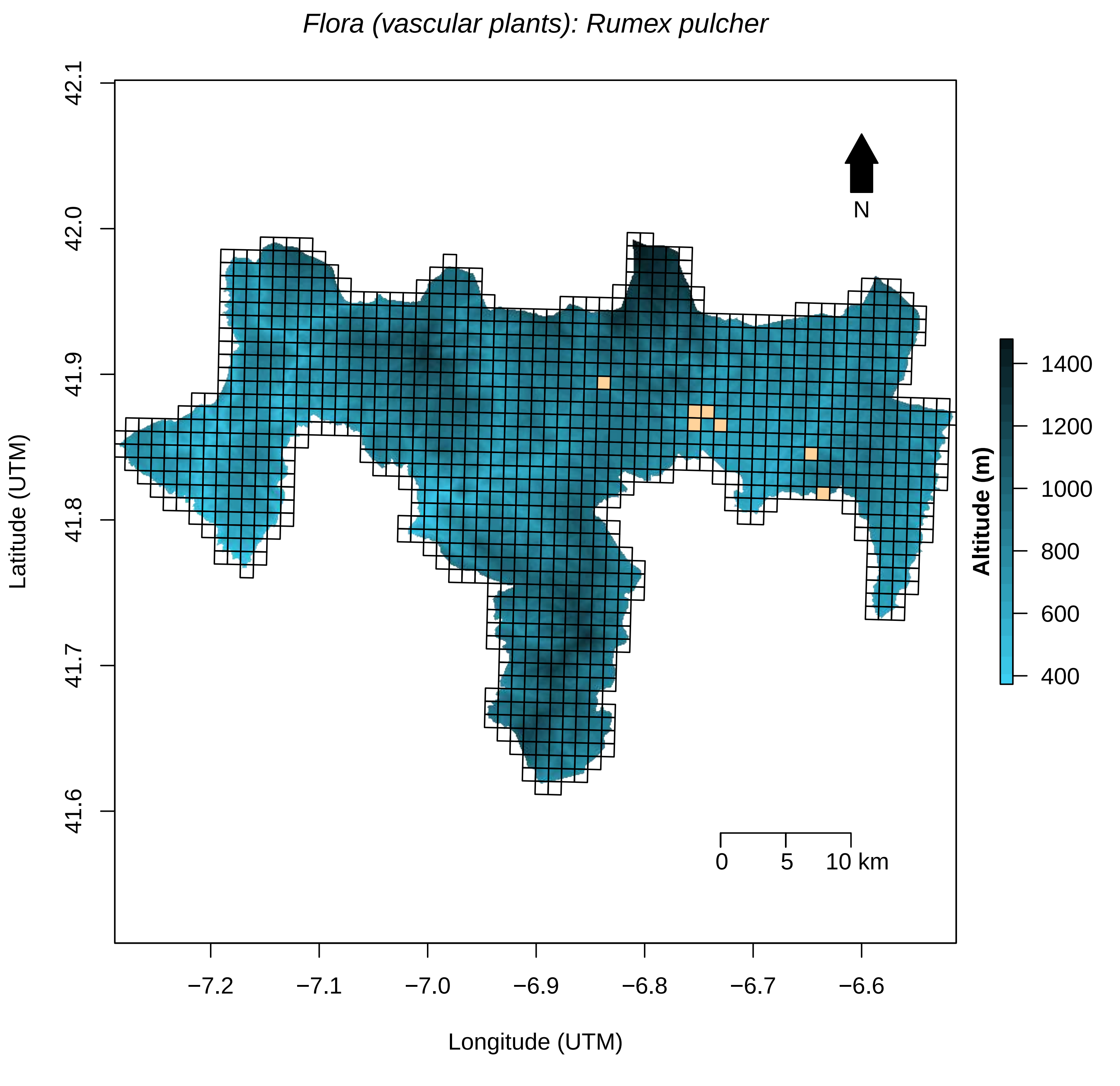
Flora (vascular plants): Rumex pulcher
42.1
42.0
41.9
41.8
41.7
41.6
Latitude (UTM)
−7.2
−7.1
−7.0
−6.9
−6.8
−6.7
−6.6
Longitude (UTM)
N
0
5
10 km
1400
1200
1000
800
600
400
Altitude (m)

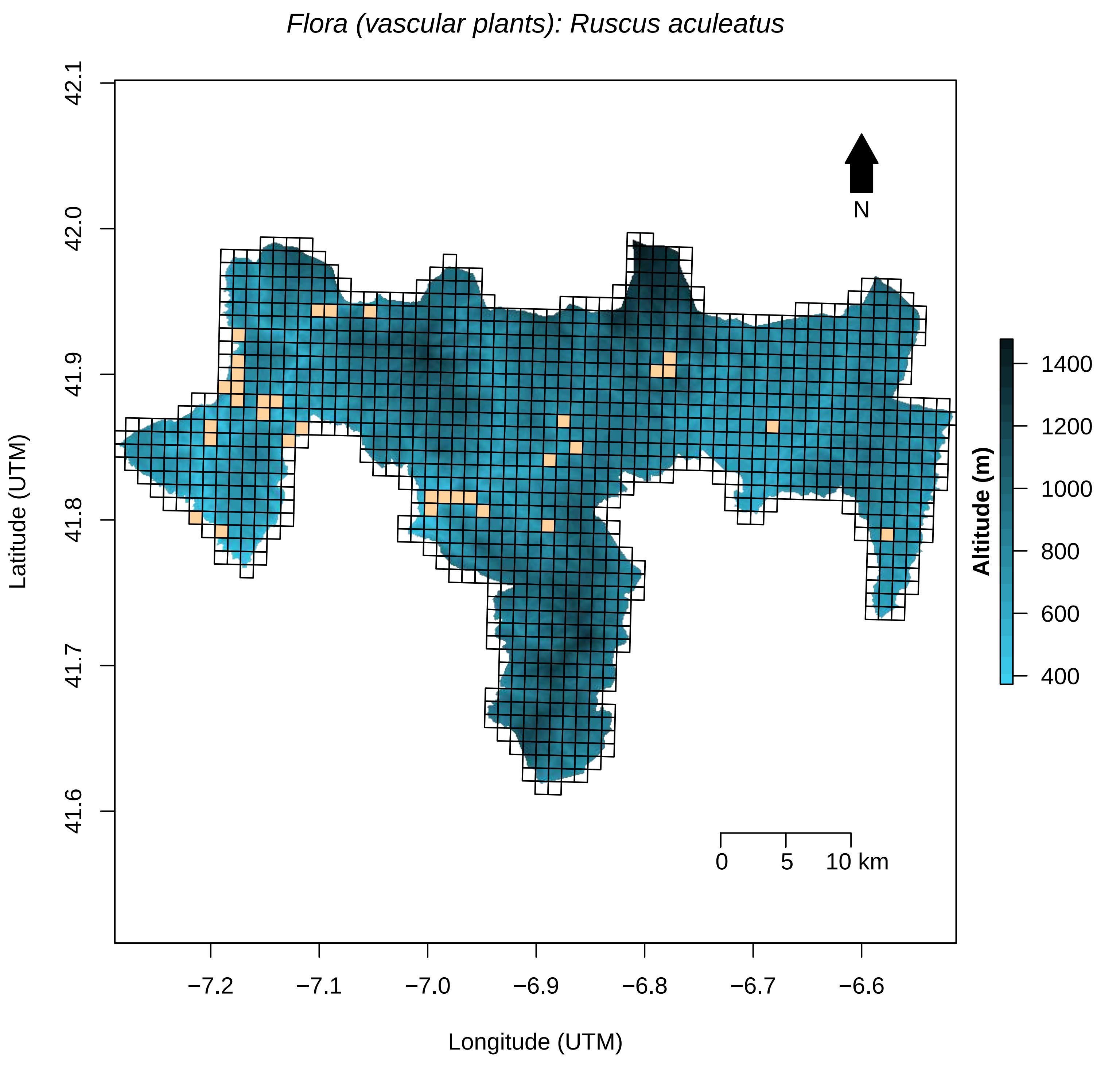
Flora (vascular plants): Ruscus aculeatus
N
0
5
10 km
Latitude (UTM)
Longitude (UTM)
Altitude (m)
42.1
42.0
41.9
41.8
41.7
41.6
−7.2
−7.1
−7.0
−6.9
−6.8
−6.7
−6.6
1400
1200
1000
800
600
400

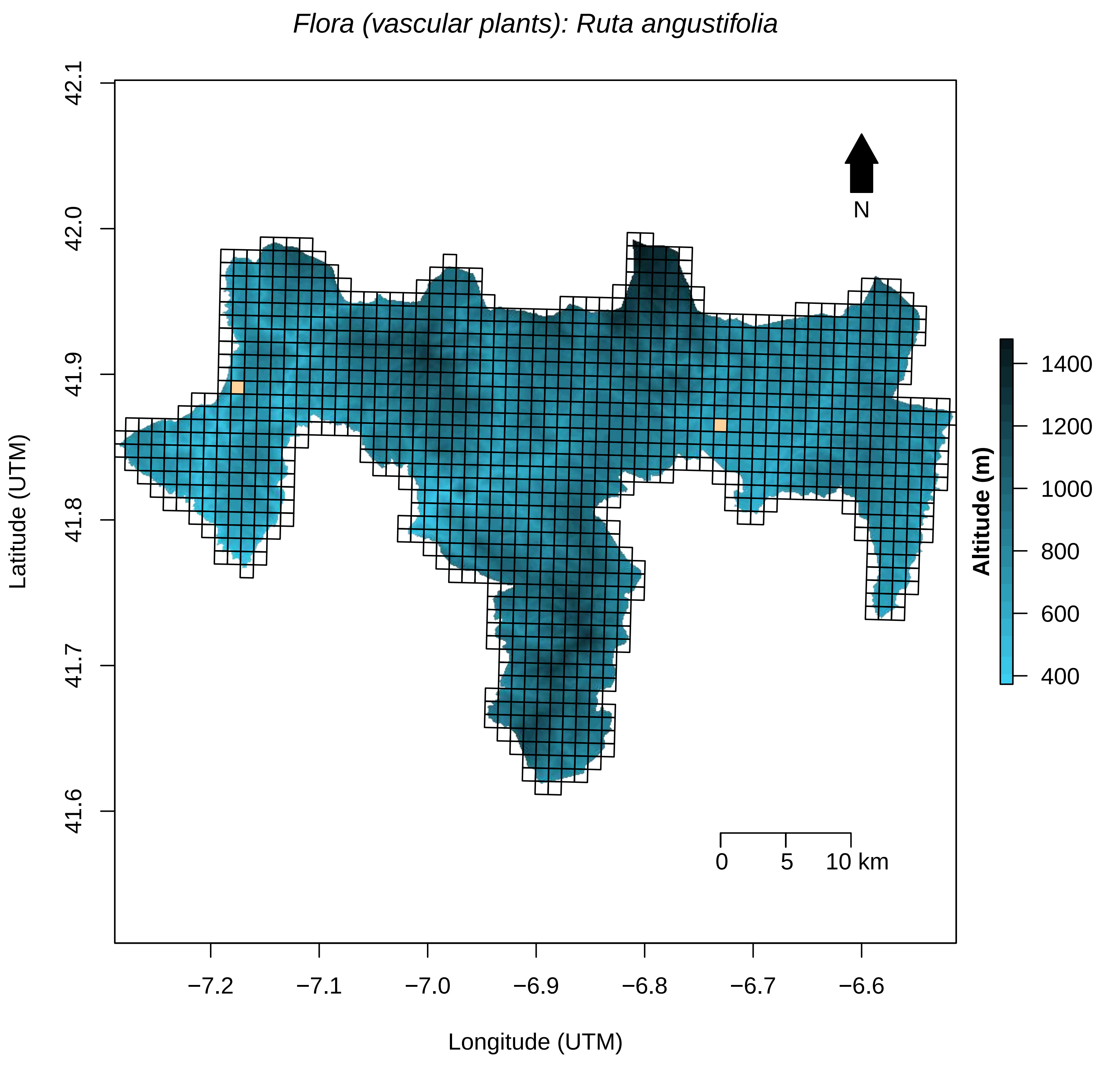
Flora (vascular plants): Ruta angustifolia
42.1
42.0
41.9
41.8
41.7
41.6
Latitude (UTM)
−7.2
−7.1
−7.0
−6.9
−6.8
−6.7
−6.6
Longitude (UTM)
N
0
5
10 km
1400
1200
1000
800
600
400
Altitude (m)

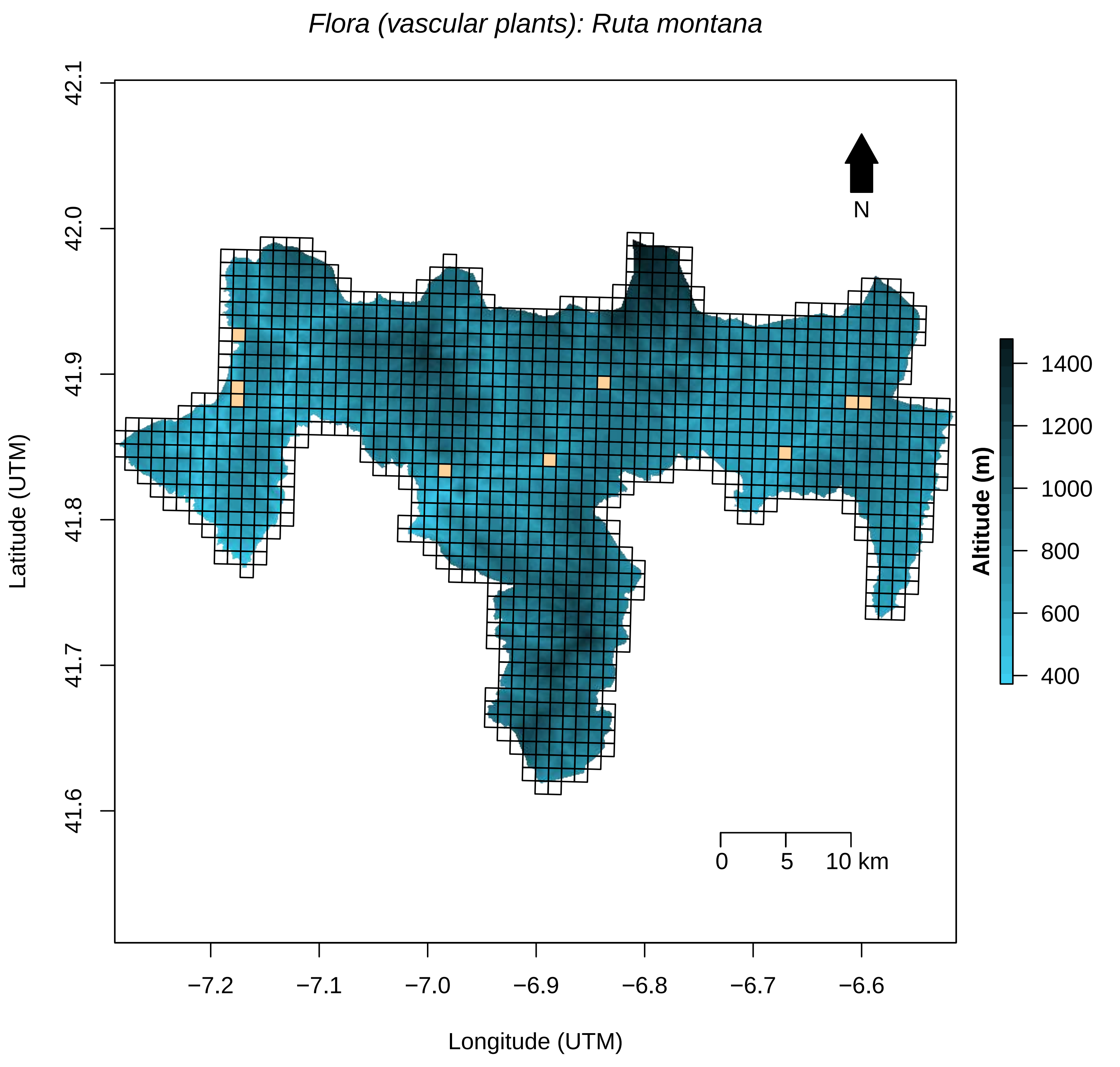
Flora (vascular plants): Ruta montana
N
0
5
10 km
Altitude (m)
1400
1200
1000
800
600
400
42.1
42.0
41.9
41.8
41.7
41.6
Latitude (UTM)
−7.2
−7.1
−7.0
−6.9
−6.8
−6.7
−6.6
Longitude (UTM)

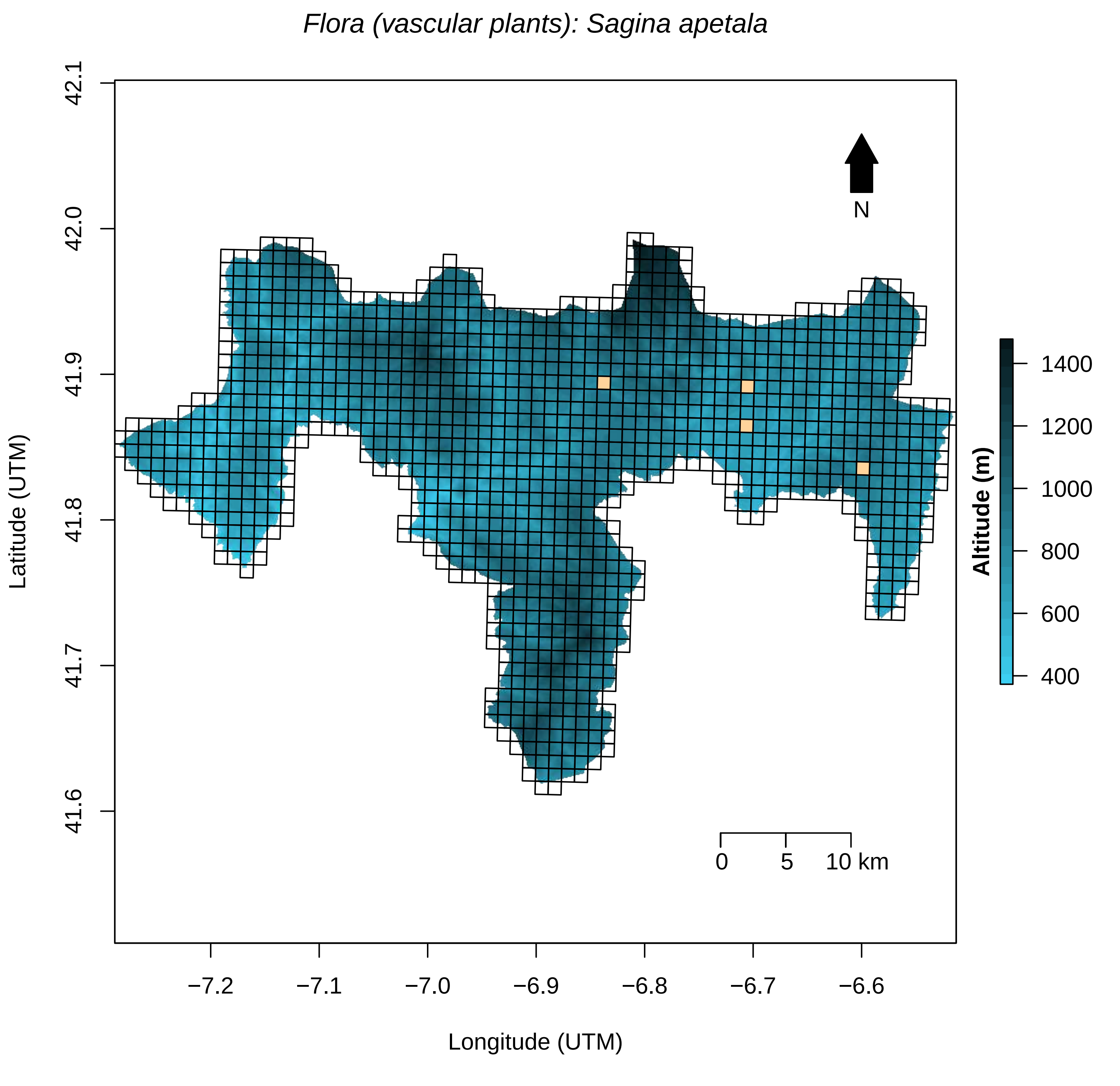
Flora (vascular plants): Sagina apetala
42.1
42.0
41.9
41.8
41.7
41.6
Latitude (UTM)
−7.2
−7.1
−7.0
−6.9
−6.8
−6.7
−6.6
Longitude (UTM)
N
0
5
10 km
Altitude (m)
1400
1200
1000
800
600
400

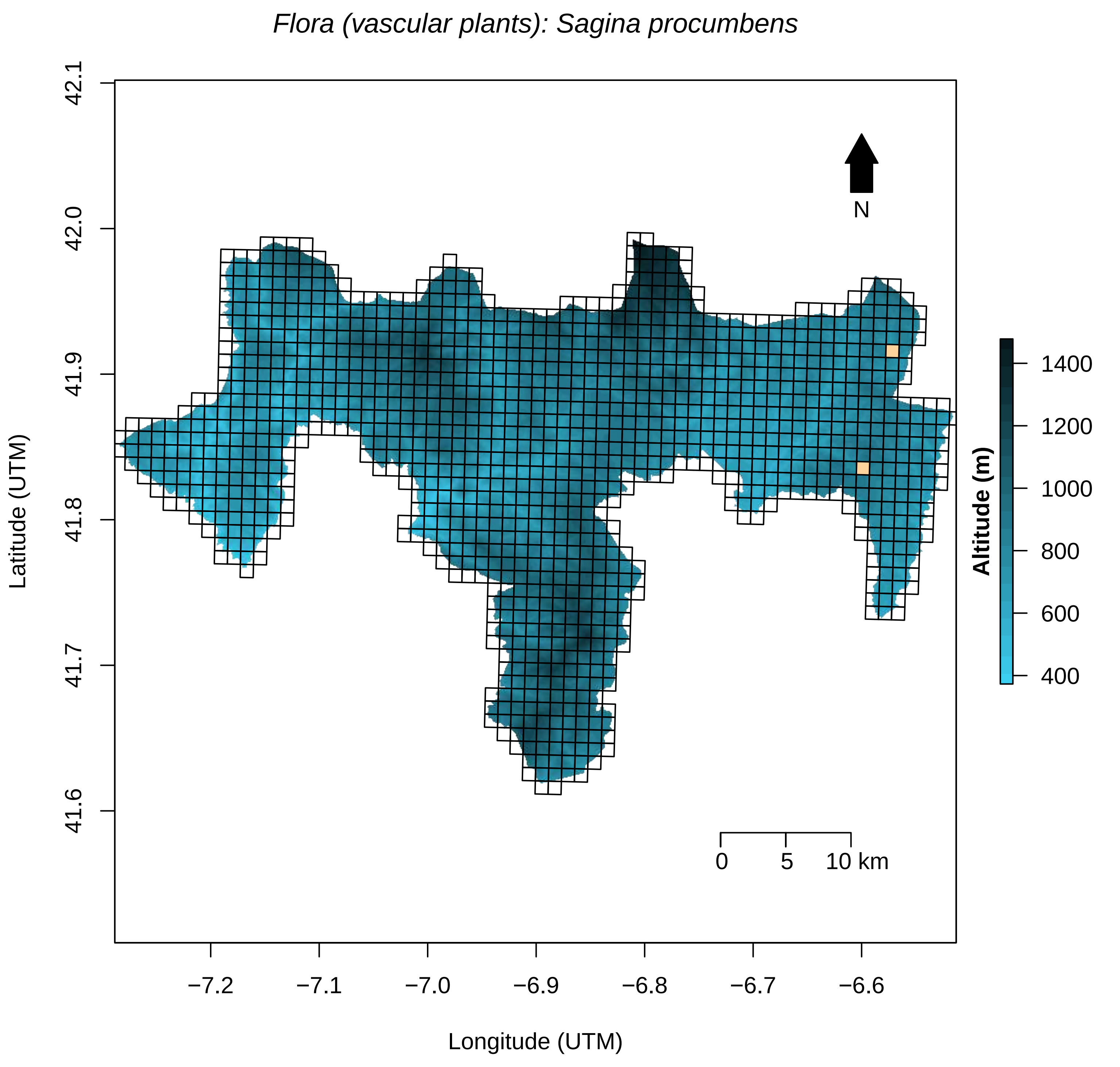

Flora (vascular plants): Sagina procumbens
N
0
5
10 km
Altitude (m)
1400
1200
1000
800
600
400
Latitude (UTM)
42.1
42.0
41.9
41.8
41.7
41.6
Longitude (UTM)
−7.2
−7.1
−7.0
−6.9
−6.8
−6.7
−6.6

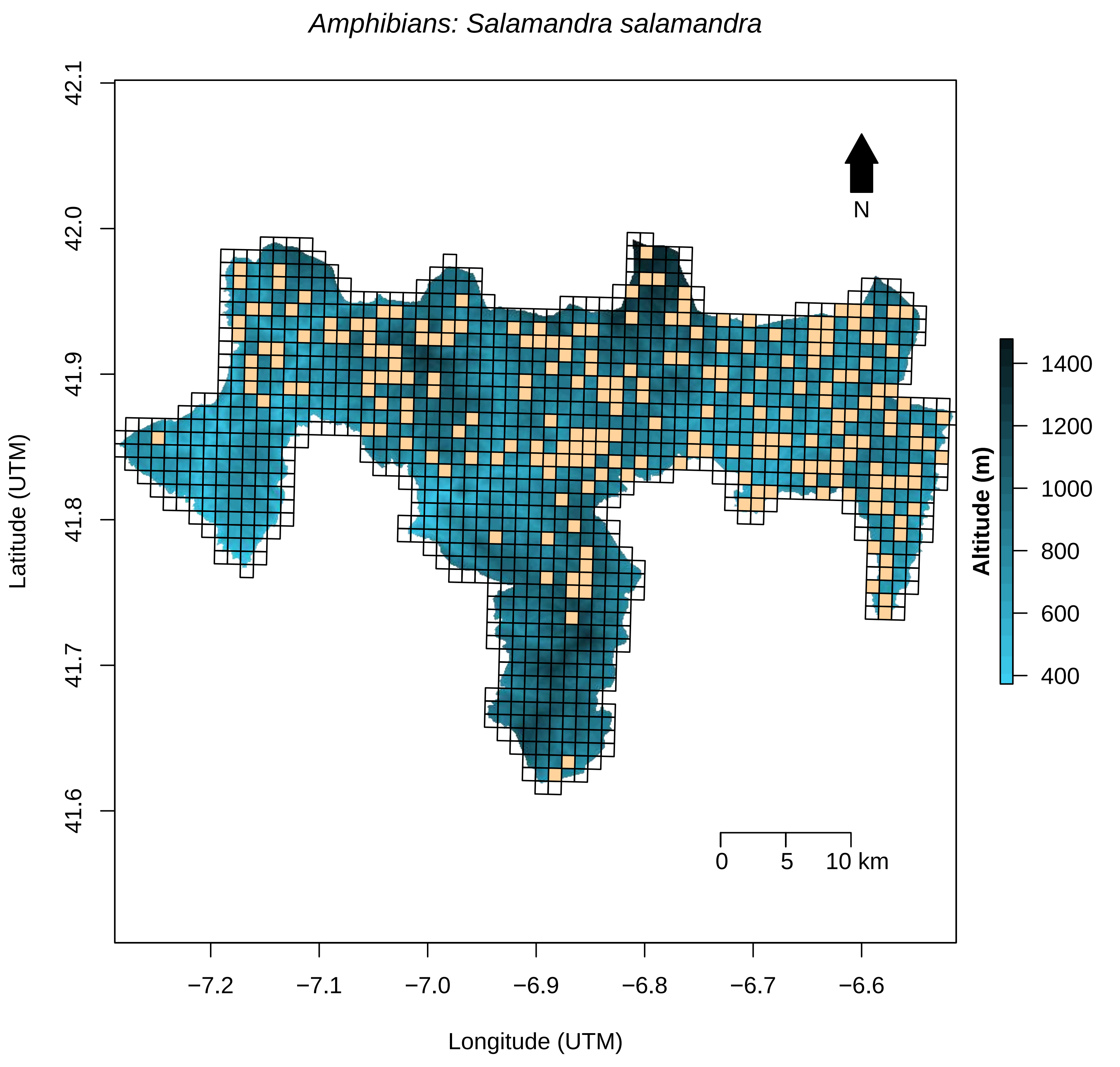

Amphibians: Salamandra salamandra
42.1
42.0
41.9
41.8
41.7
41.6
Latitude (UTM)
−7.2
−7.1
−7.0
−6.9
−6.8
−6.7
−6.6
Longitude (UTM)
N
0
5
10 km
Altitude (m)
1400
1200
1000
800
600
400

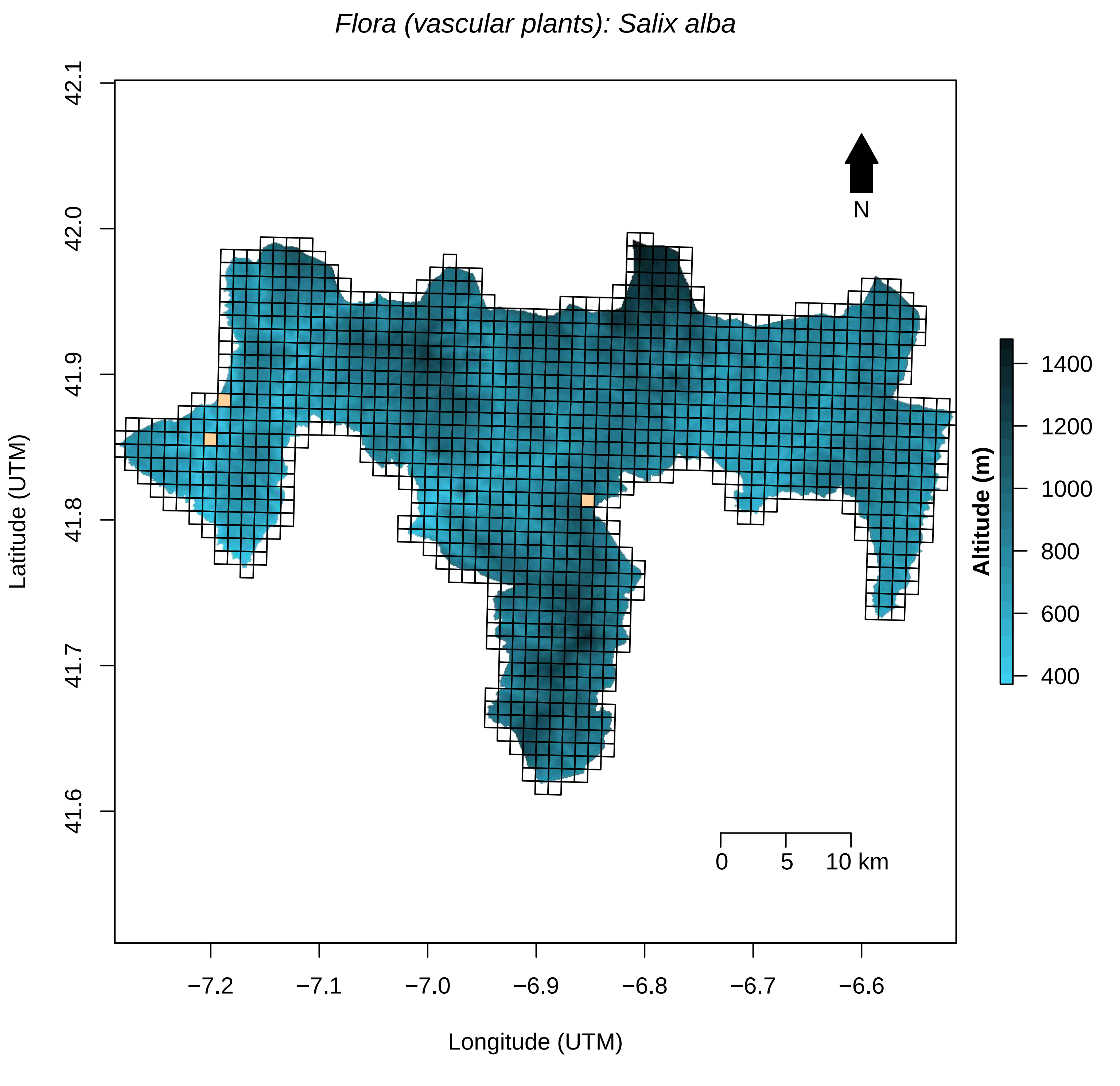
Flora (vascular plants): Salix alba
Latitude (UTM)
42.1
42.0
41.9
41.8
41.7
41.6
Longitude (UTM)
−7.2
−7.1
−7.0
−6.9
−6.8
−6.7
−6.6
N
0
5
10 km
Altitude (m)
1400
1200
1000
800
600
400

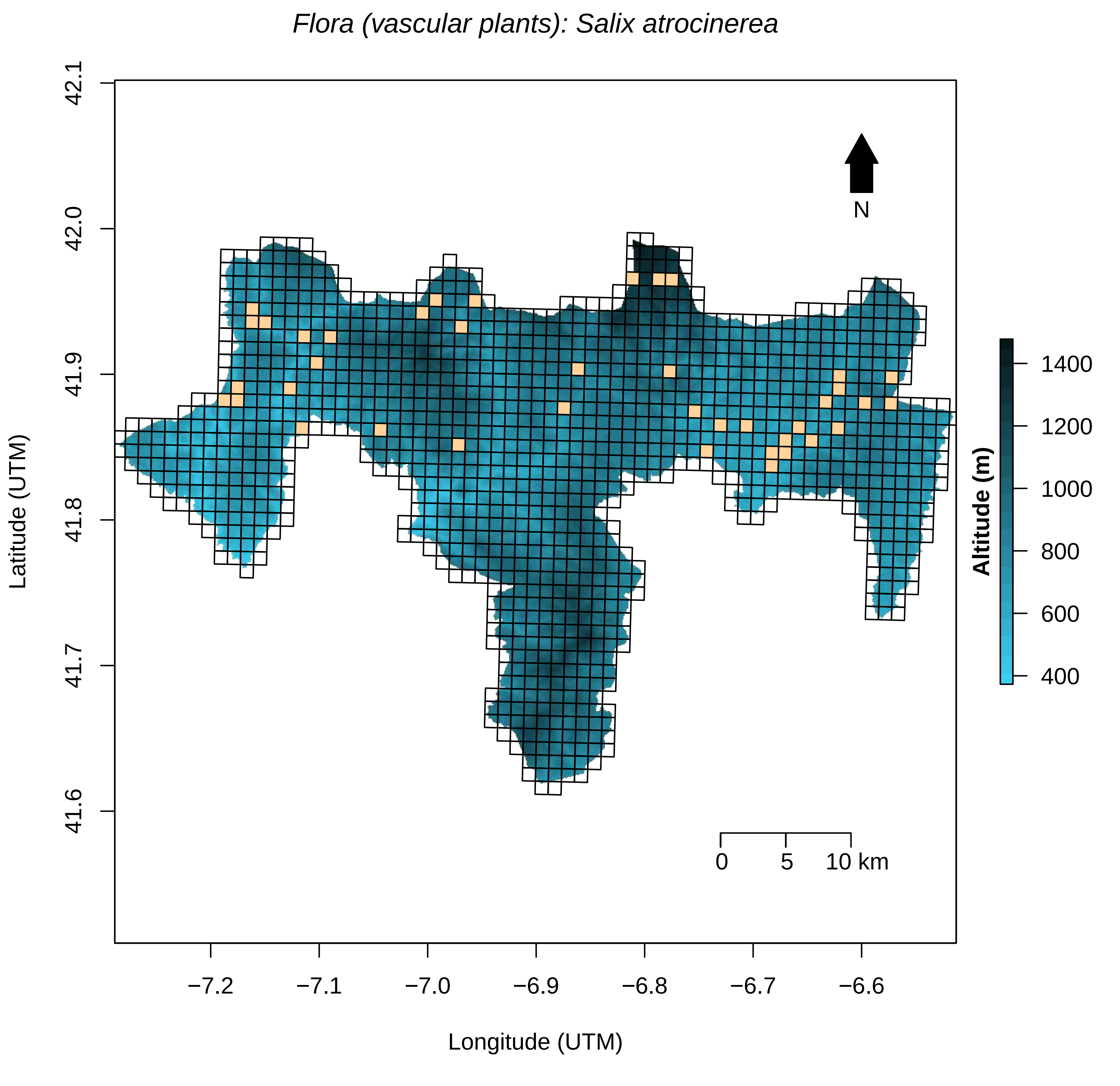
Flora (vascular plants): Salix atrocinerea
Latitude (UTM)
Longitude (UTM)
42.1
42.0
41.9
41.8
41.7
41.6
−7.2
−7.1
−7.0
−6.9
−6.8
−6.7
−6.6
N
0
5
10 km
Altitude (m)
1400
1200
1000
800
600
400

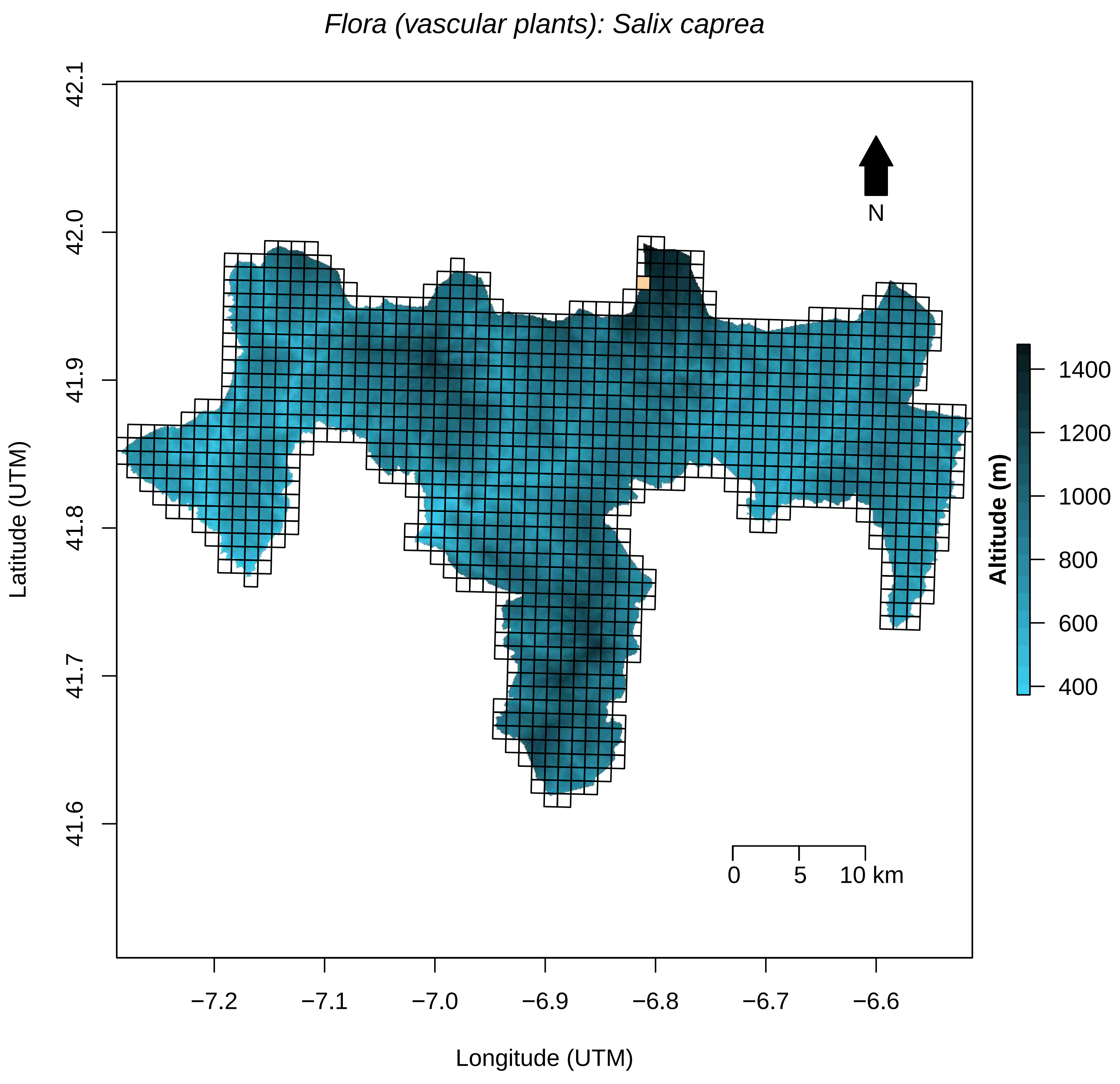

Flora (vascular plants): Salix caprea
42.1
42.0
41.9
41.8
41.7
41.6
Latitude (UTM)
−7.2
−7.1
−7.0
−6.9
−6.8
−6.7
−6.6
Longitude (UTM)
N
0
5
10 km
Altitude (m)
1400
1200
1000
800
600
400

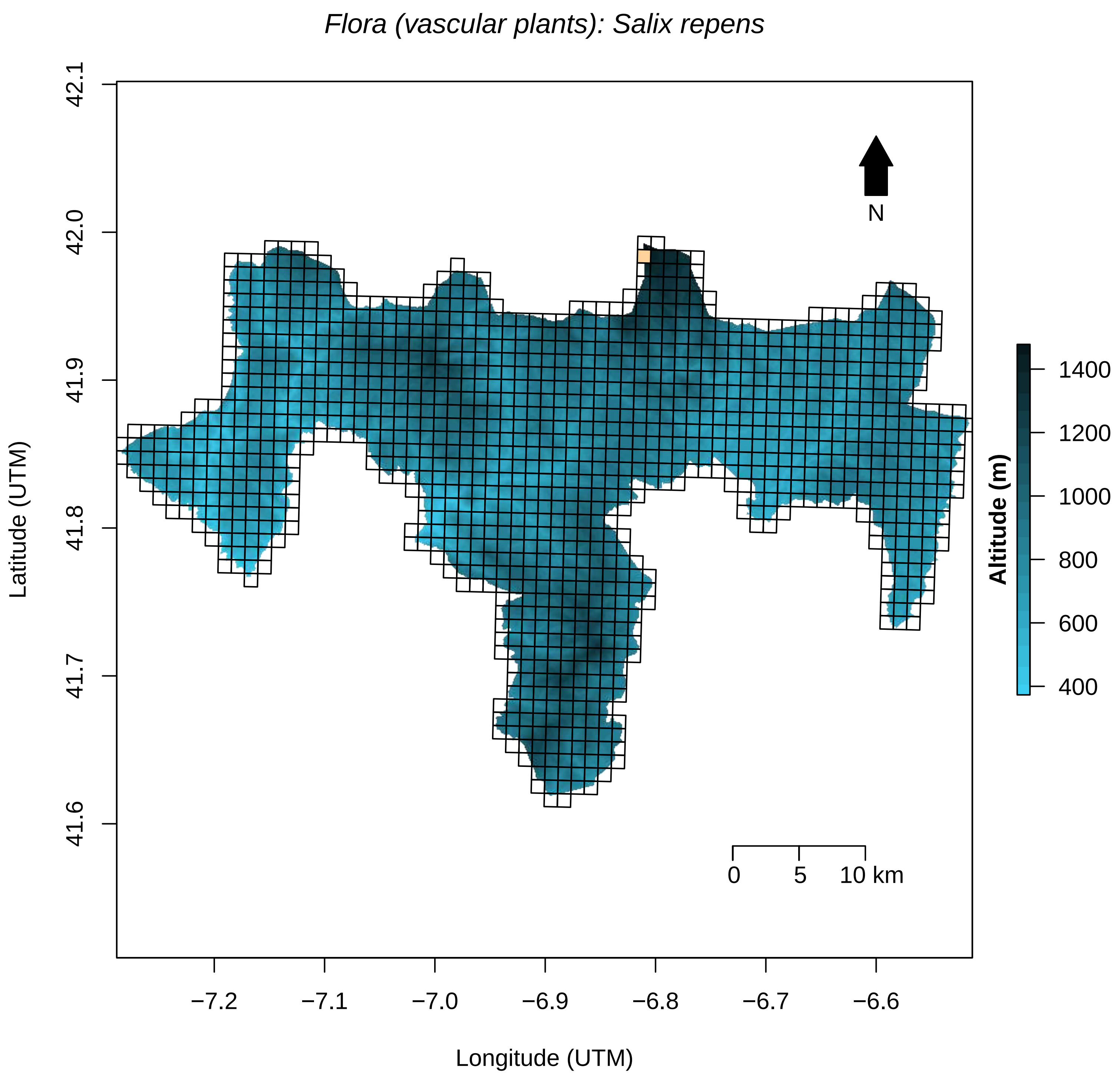

Flora (vascular plants): Salix repens
42.1
42.0
41.9
41.8
41.7
41.6
Latitude (UTM)
−7.2
−7.1
−7.0
−6.9
−6.8
−6.7
−6.6
Longitude (UTM)
N
0
5
10 km
Altitude (m)
1400
1200
1000
800
600
400

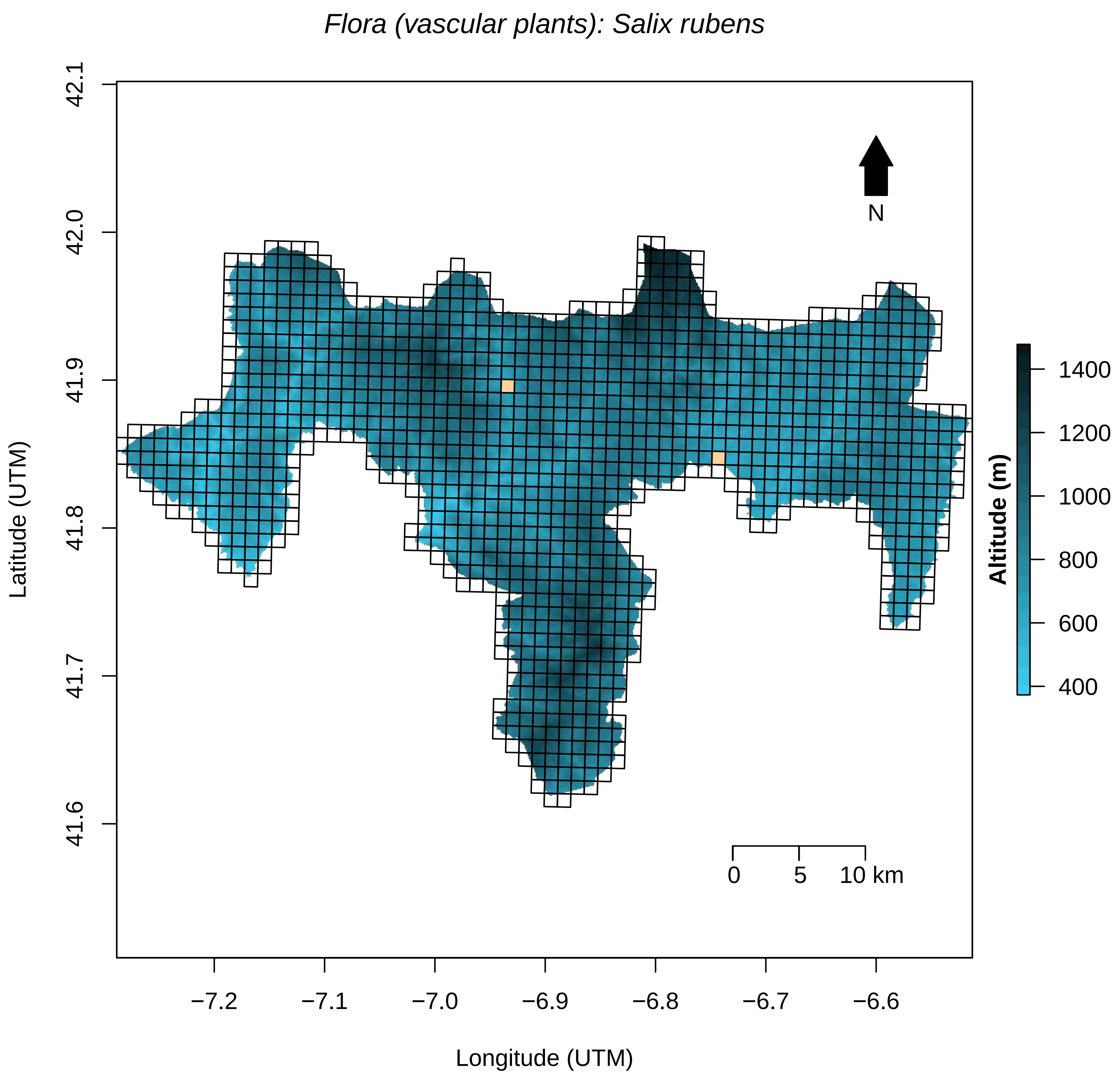
Flora (vascular plants): Salix rubens
Latitude (UTM)
Longitude (UTM)
42.1
42.0
41.9
41.8
41.7
41.6
−7.2
−7.1
−7.0
−6.9
−6.8
−6.7
−6.6
N
0
5
10 km
Altitude (m)
1400
1200
1000
800
600
400

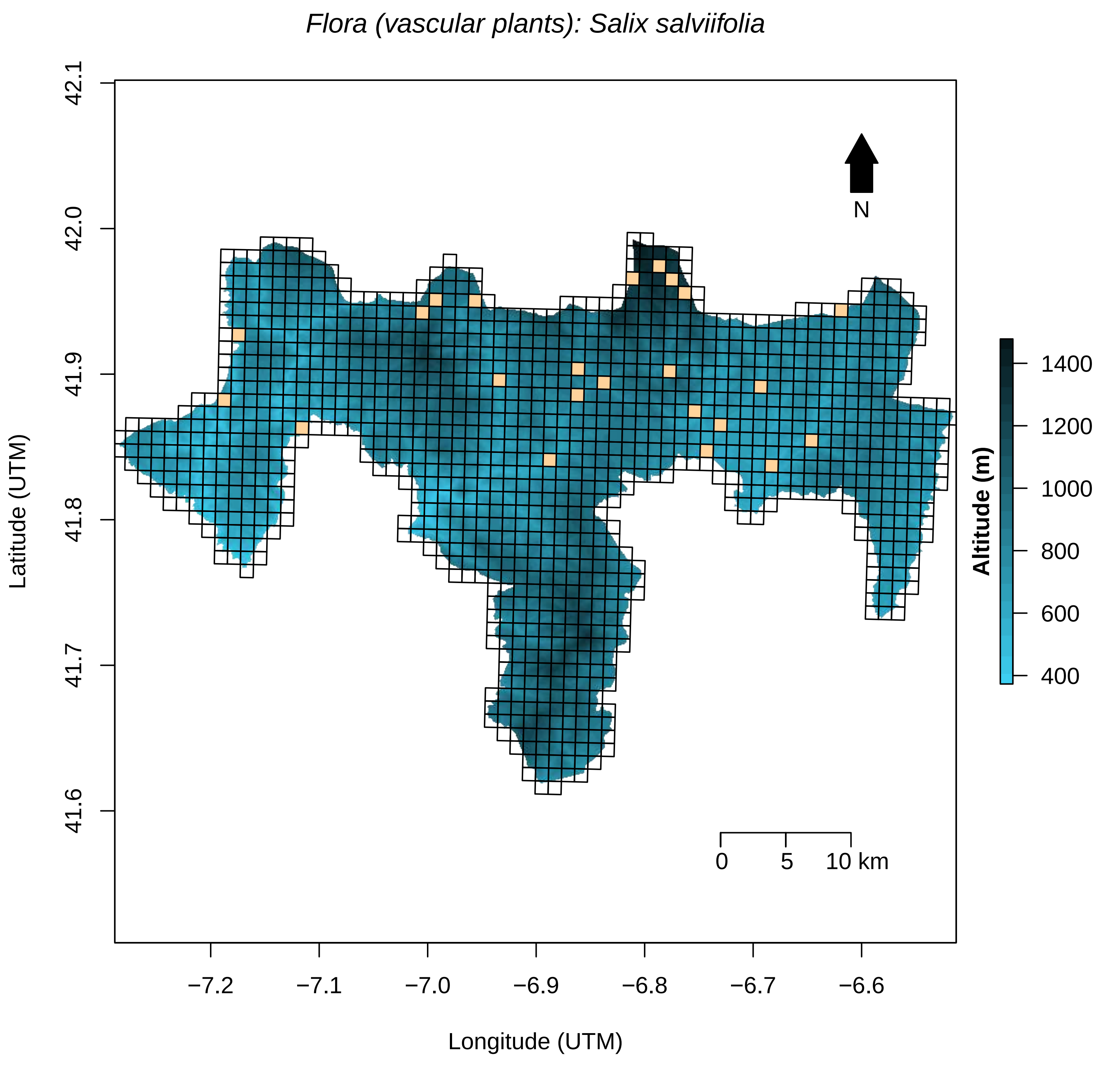
Flora (vascular plants): Salix salviifolia
42.1
42.0
41.9
41.8
41.7
41.6
Latitude (UTM)
−7.2
−7.1
−7.0
−6.9
−6.8
−6.7
−6.6
Longitude (UTM)
N
0
5
10 km
Altitude (m)
1400
1200
1000
800
600
400

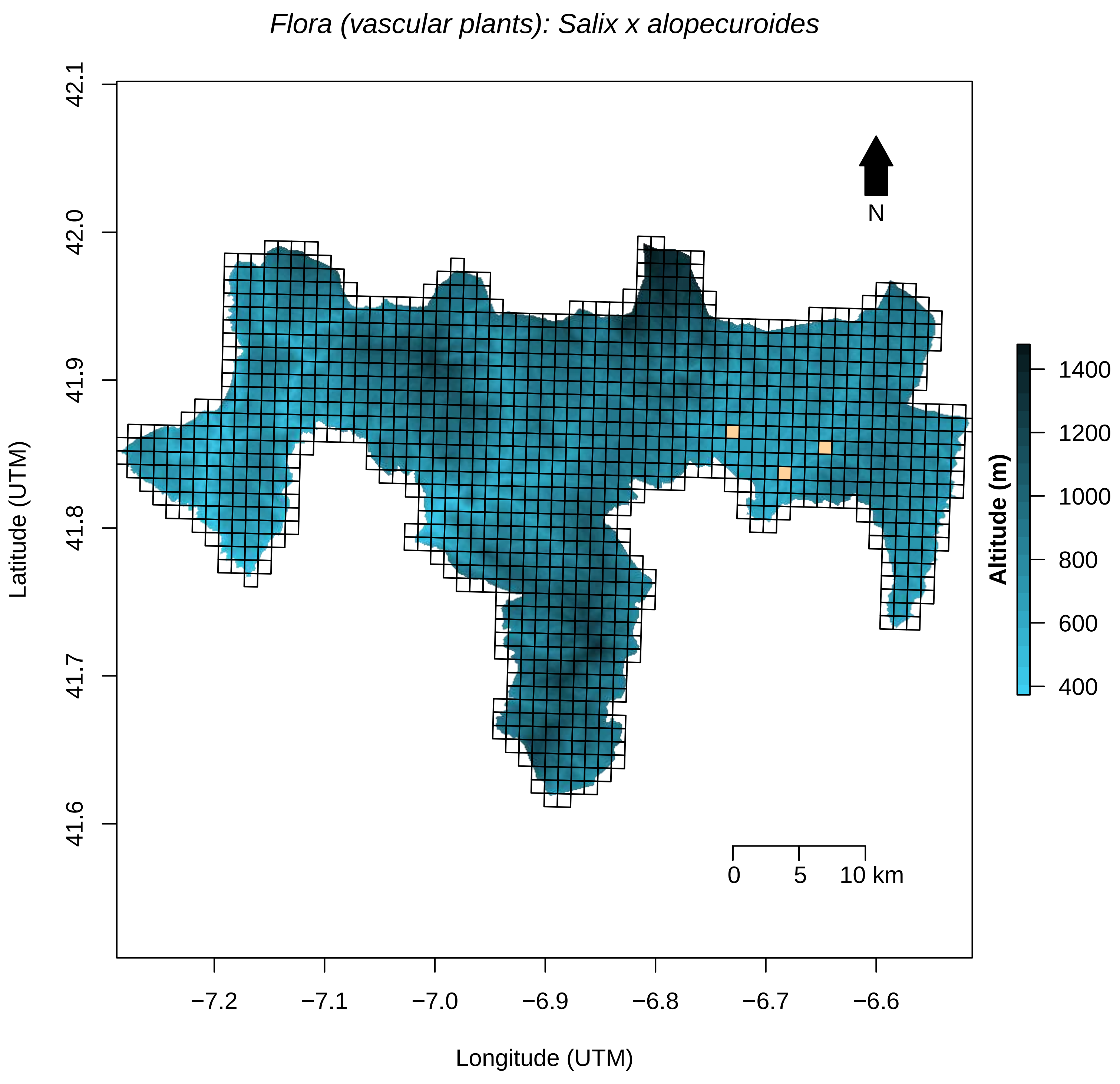

Flora (vascular plants): Salix x alopecuroides
Latitude (UTM)
42.1
42.0
41.9
41.8
41.7
41.6
Longitude (UTM)
−7.2
−7.1
−7.0
−6.9
−6.8
−6.7
−6.6
N
0
5
10 km
Altitude (m)
1400
1200
1000
800
600
400

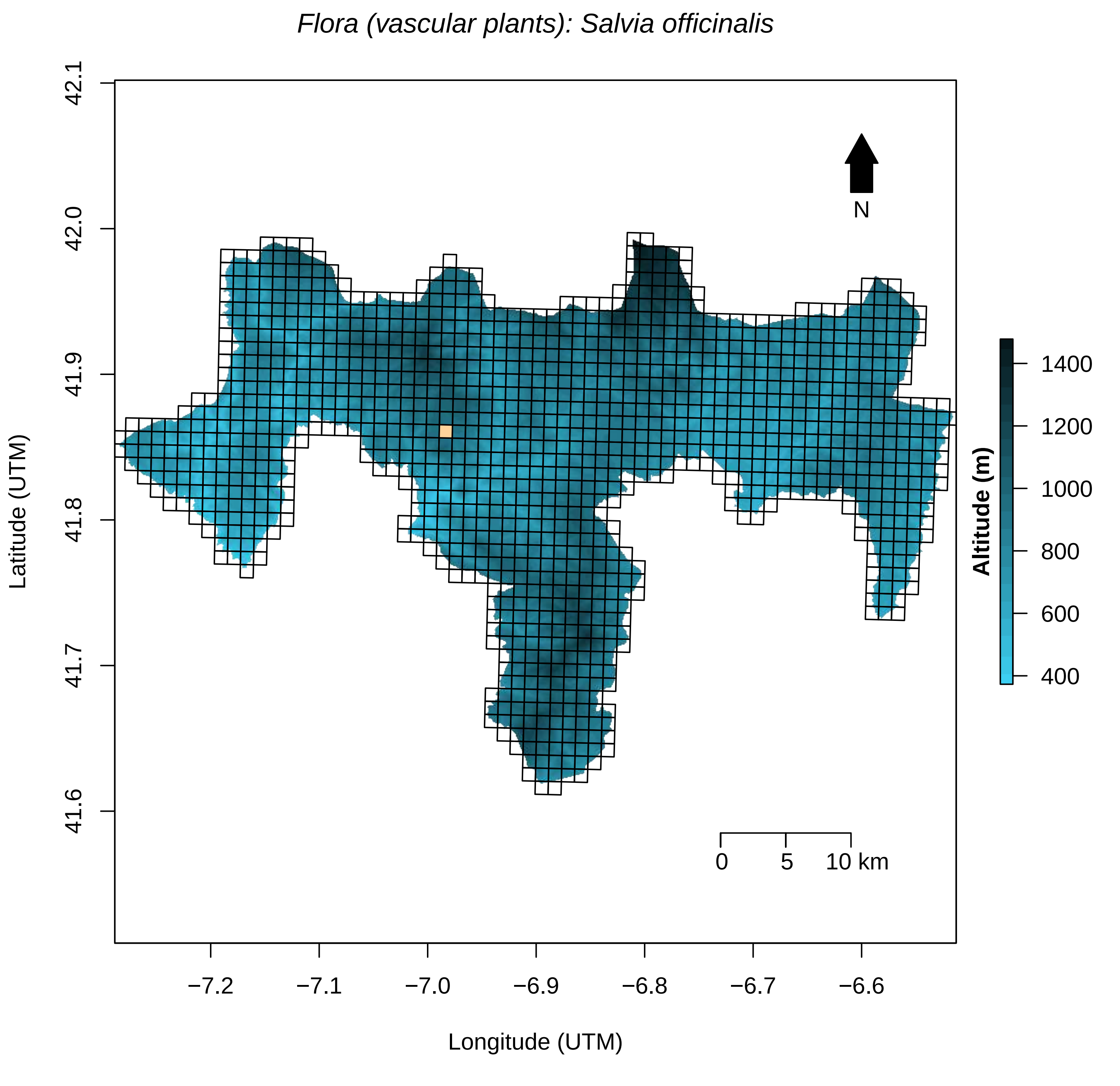

Flora (vascular plants): Salvia officinalis
N
0
5
10 km
Altitude (m)
1400
1200
1000
800
600
400
Latitude (UTM)
42.1
42.0
41.9
41.8
41.7
41.6
Longitude (UTM)
−7.2
−7.1
−7.0
−6.9
−6.8
−6.7
−6.6

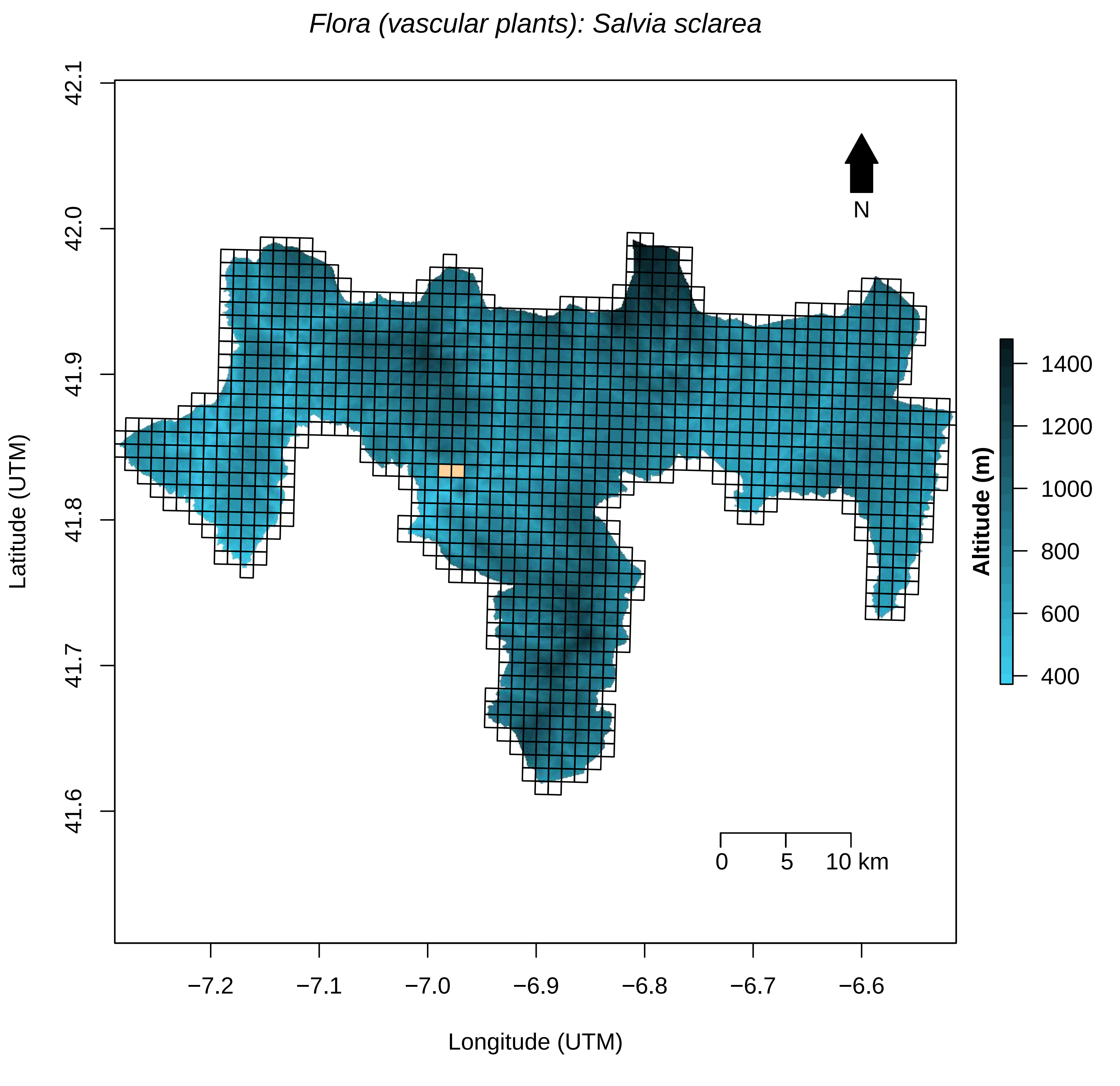
Flora (vascular plants): Salvia sclarea
42.1
42.0
41.9
41.8
41.7
41.6
Latitude (UTM)
−7.2
−7.1
−7.0
−6.9
−6.8
−6.7
−6.6
Longitude (UTM)
N
0
5
10 km
Altitude (m)
1400
1200
1000
800
600
400

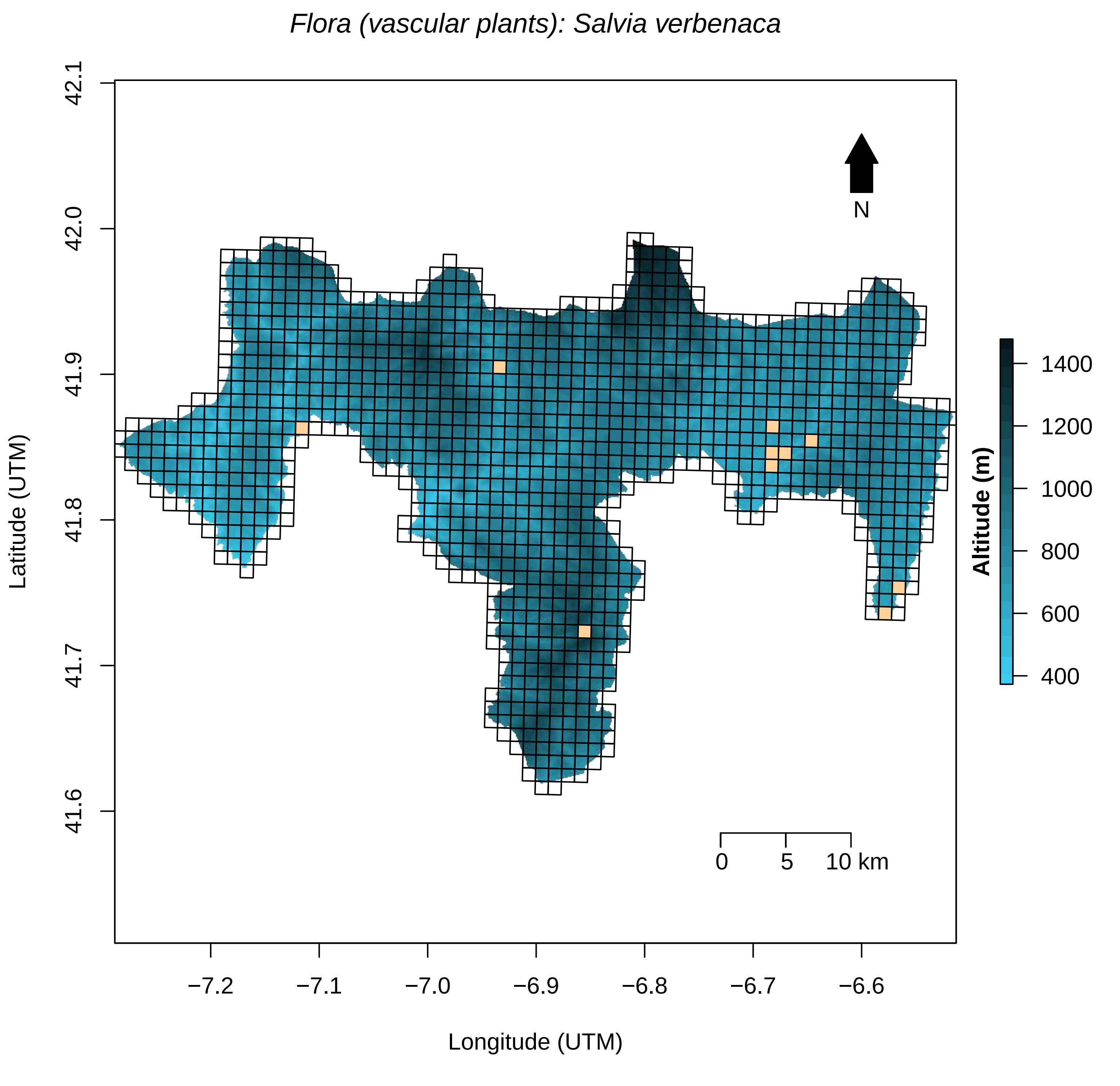
Flora (vascular plants): Salvia verbenaca
Latitude (UTM)
Longitude (UTM)
42.1
42.0
41.9
41.8
41.7
41.6
−7.2
−7.1
−7.0
−6.9
−6.8
−6.7
−6.6
N
0
5
10 km
Altitude (m)
1400
1200
1000
800
600
400

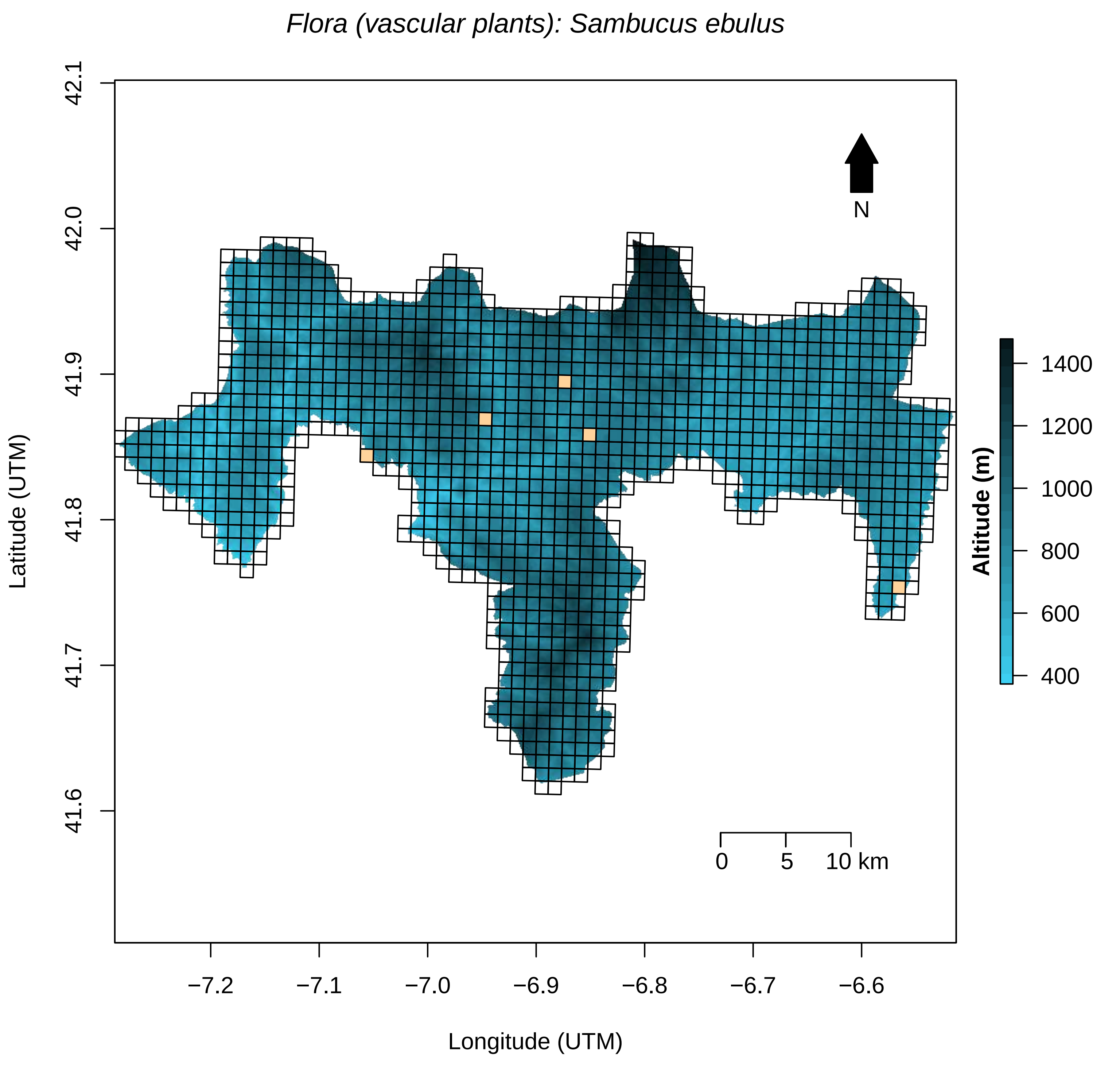

Flora (vascular plants): Sambucus ebulus
42.1
42.0
41.9
41.8
41.7
41.6
Latitude (UTM)
−7.2
−7.1
−7.0
−6.9
−6.8
−6.7
−6.6
Longitude (UTM)
N
0
5
10 km
Altitude (m)
1400
1200
1000
800
600
400

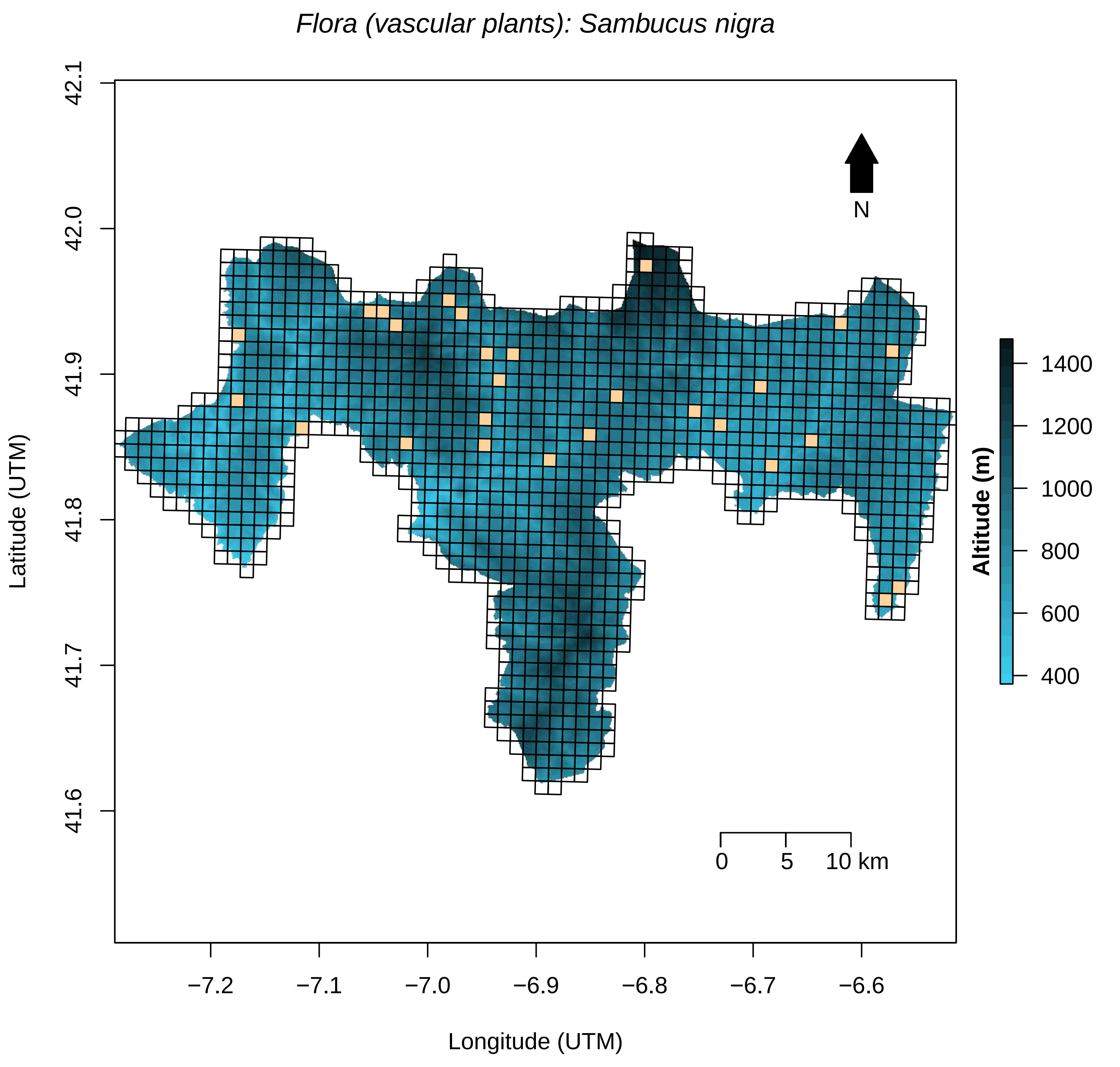

Flora (vascular plants): Sambucus nigra
42.1
42.0
41.9
41.8
41.7
41.6
Latitude (UTM)
−7.2
−7.1
−7.0
−6.9
−6.8
−6.7
−6.6
Longitude (UTM)
N
0
5
10 km
1400
1200
1000
800
600
400
Altitude (m)

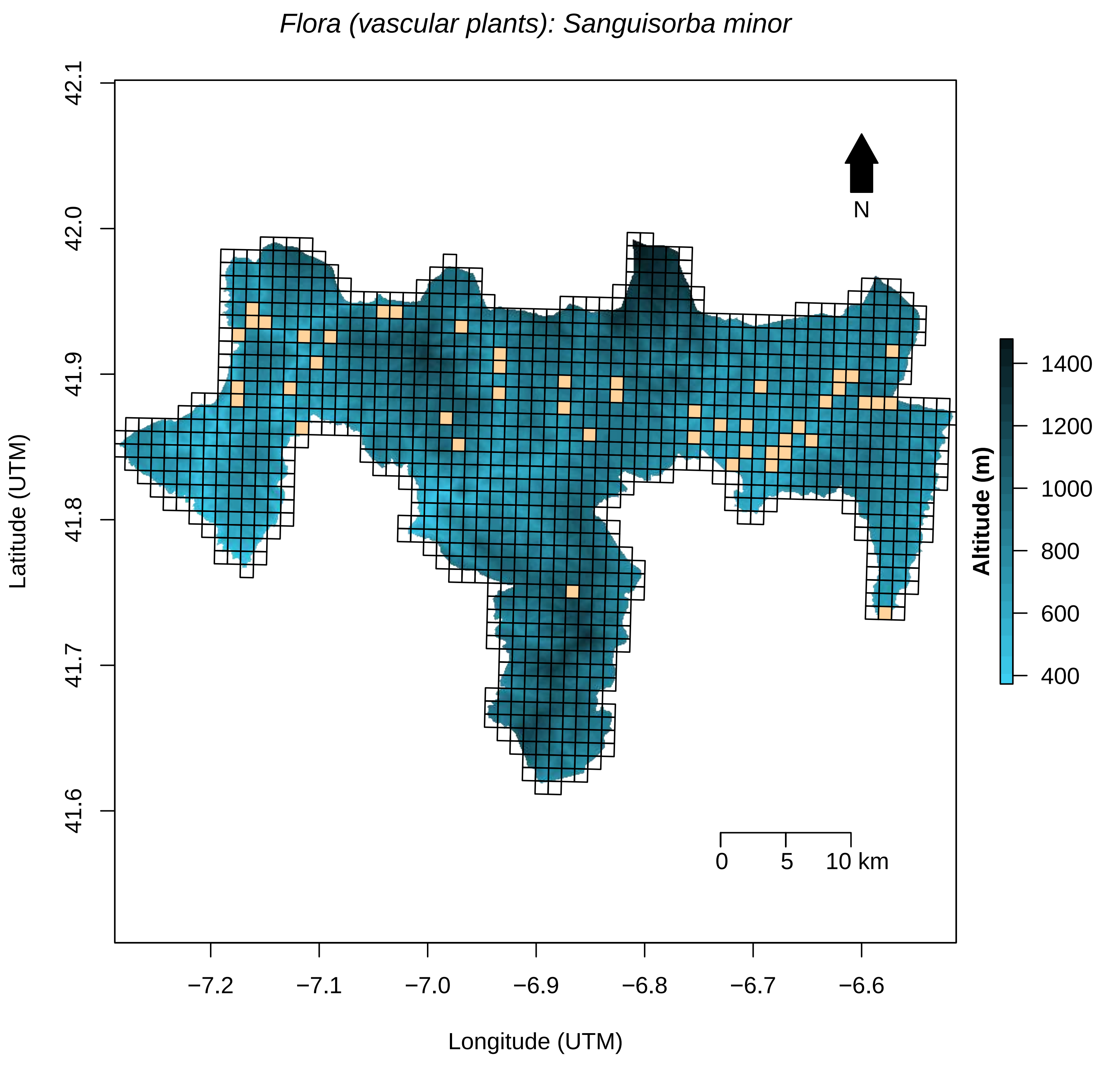

Flora (vascular plants): Sanguisorba minor
42.1
42.0
41.9
41.8
41.7
41.6
Latitude (UTM)
−7.2
−7.1
−7.0
−6.9
−6.8
−6.7
−6.6
Longitude (UTM)
N
0
5
10 km
1400
1200
1000
800
600
400
Altitude (m)

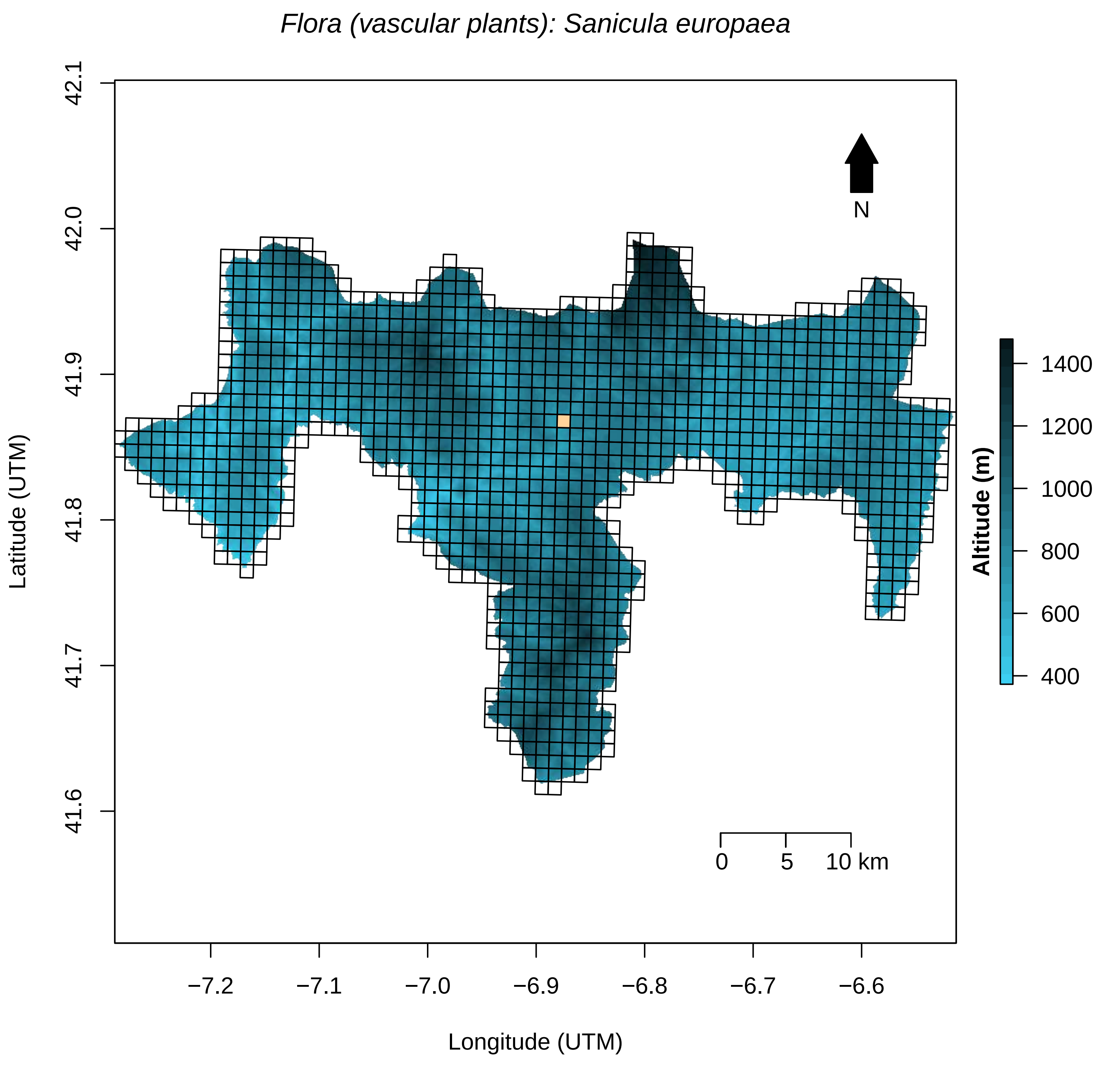
Flora (vascular plants): Sanicula europaea
N
0
5
10 km
Altitude (m)
1400
1200
1000
800
600
400
42.1
42.0
41.9
41.8
41.7
41.6
Latitude (UTM)
−7.2
−7.1
−7.0
−6.9
−6.8
−6.7
−6.6
Longitude (UTM)

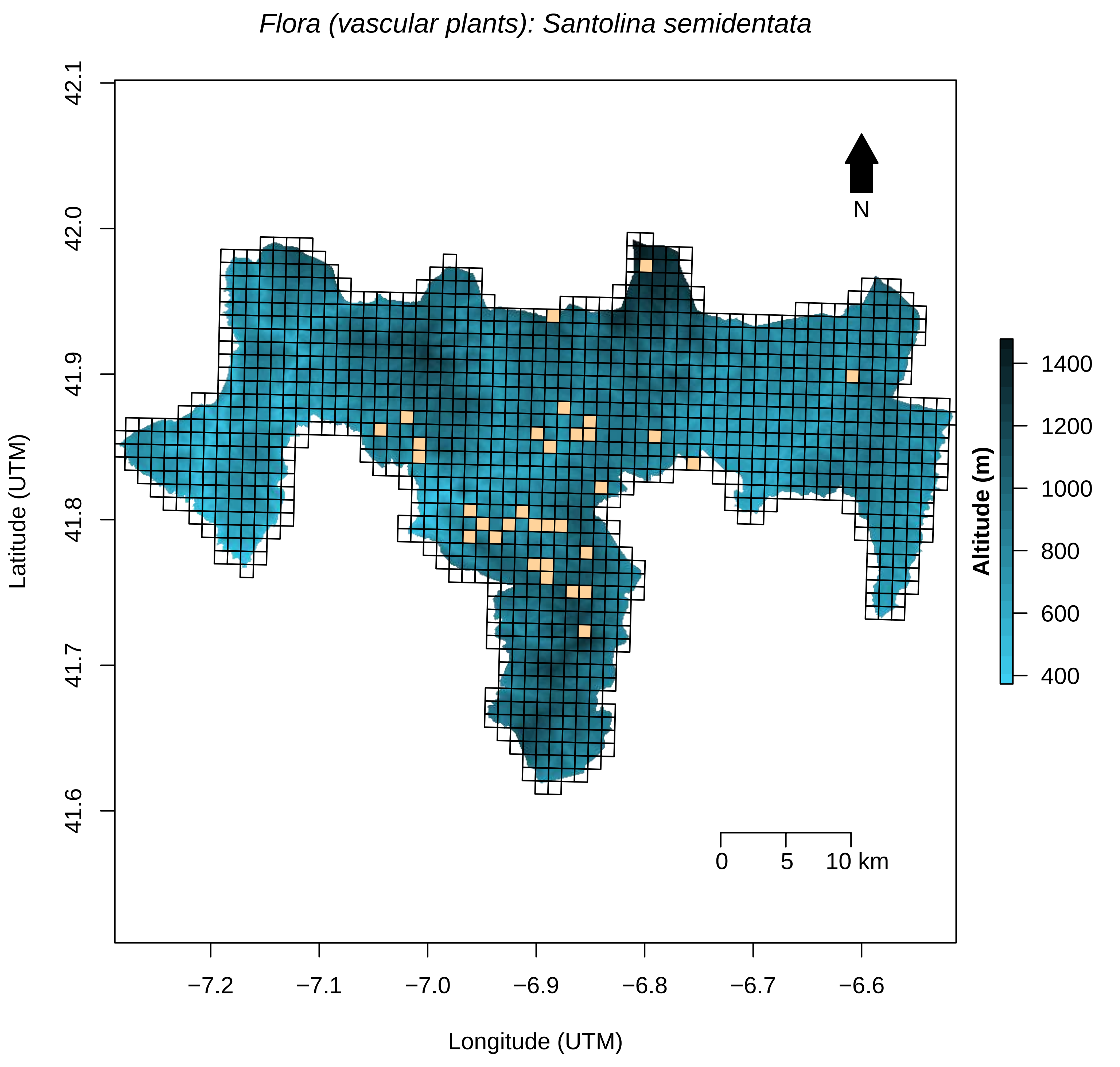

Flora (vascular plants): Santolina semidentata
N
0
5
10 km
Altitude (m)
1400
1200
1000
800
600
400
Latitude (UTM)
42.1
42.0
41.9
41.8
41.7
41.6
Longitude (UTM)
−7.2
−7.1
−7.0
−6.9
−6.8
−6.7
−6.6

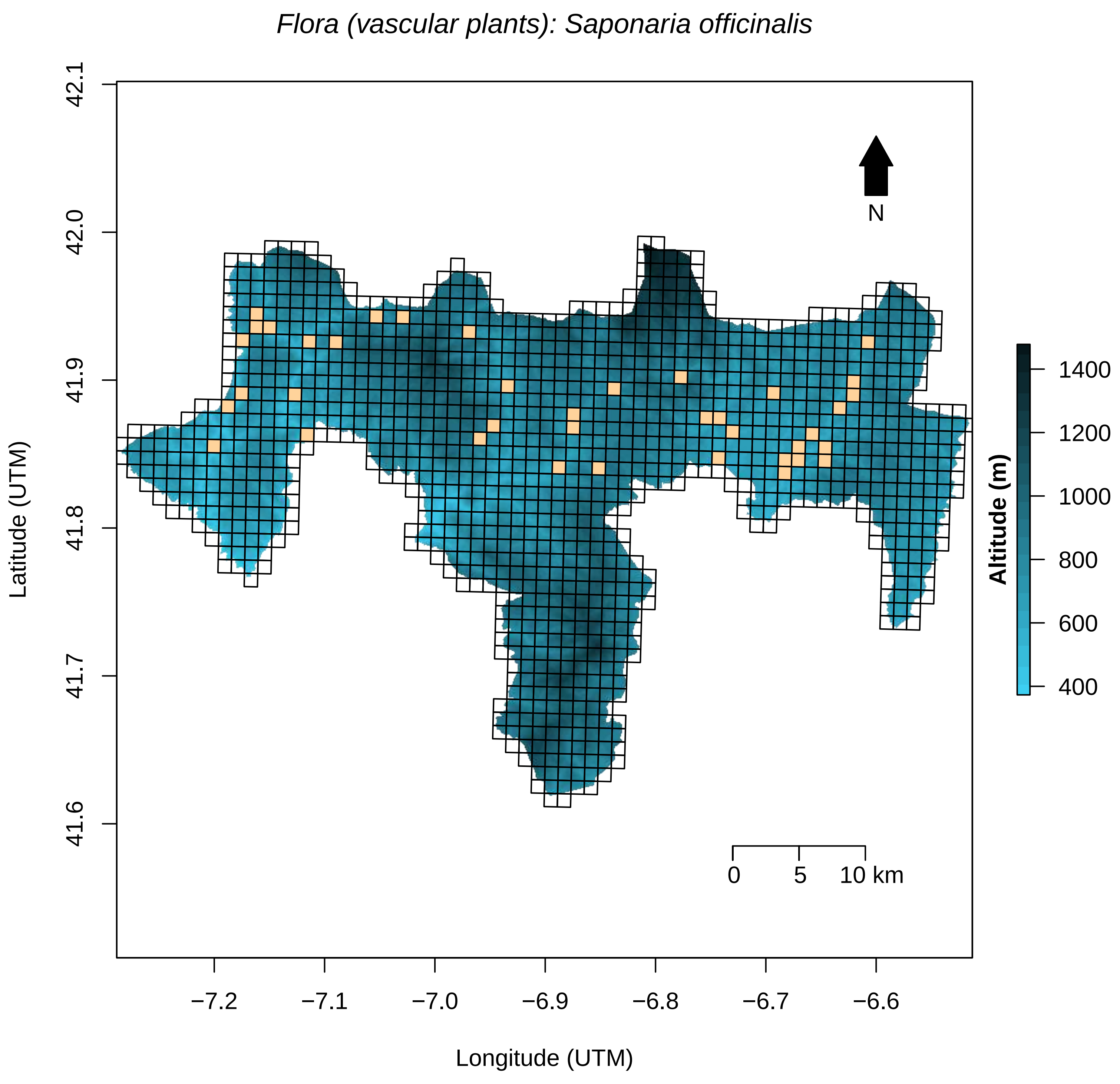

Flora (vascular plants): Saponaria officinalis
Latitude (UTM)
42.1
42.0
41.9
41.8
41.7
41.6
Longitude (UTM)
−7.2
−7.1
−7.0
−6.9
−6.8
−6.7
−6.6
N
0
5
10 km
Altitude (m)
1400
1200
1000
800
600
400

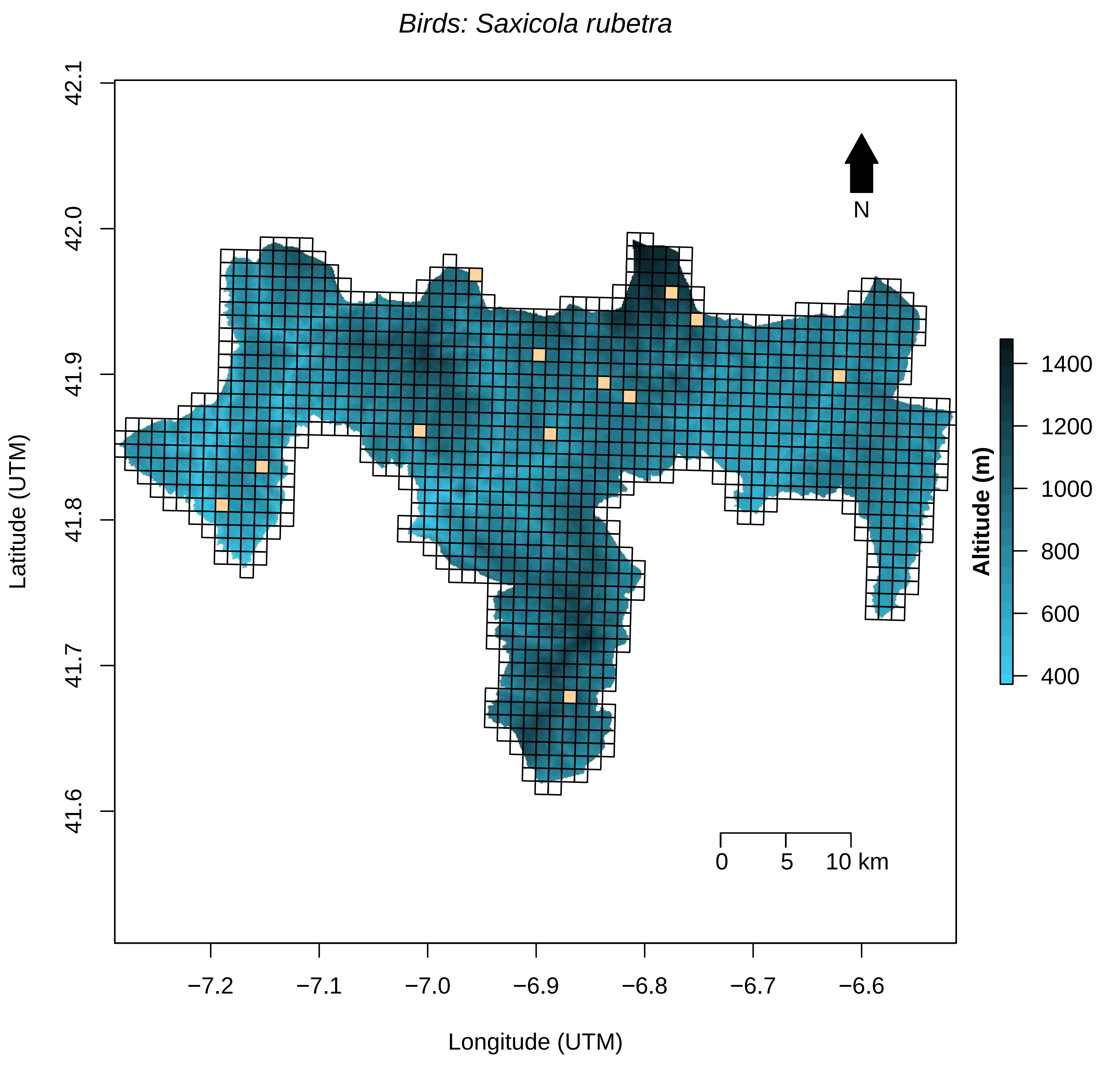

Birds: Saxicola rubetra
42.1
42.0
41.9
41.8
41.7
41.6
Latitude (UTM)
N
0
5
10 km
−7.2
−7.1
−7.0
−6.9
−6.8
−6.7
−6.6
Longitude (UTM)
1400
1200
1000
800
600
400
Altitude (m)

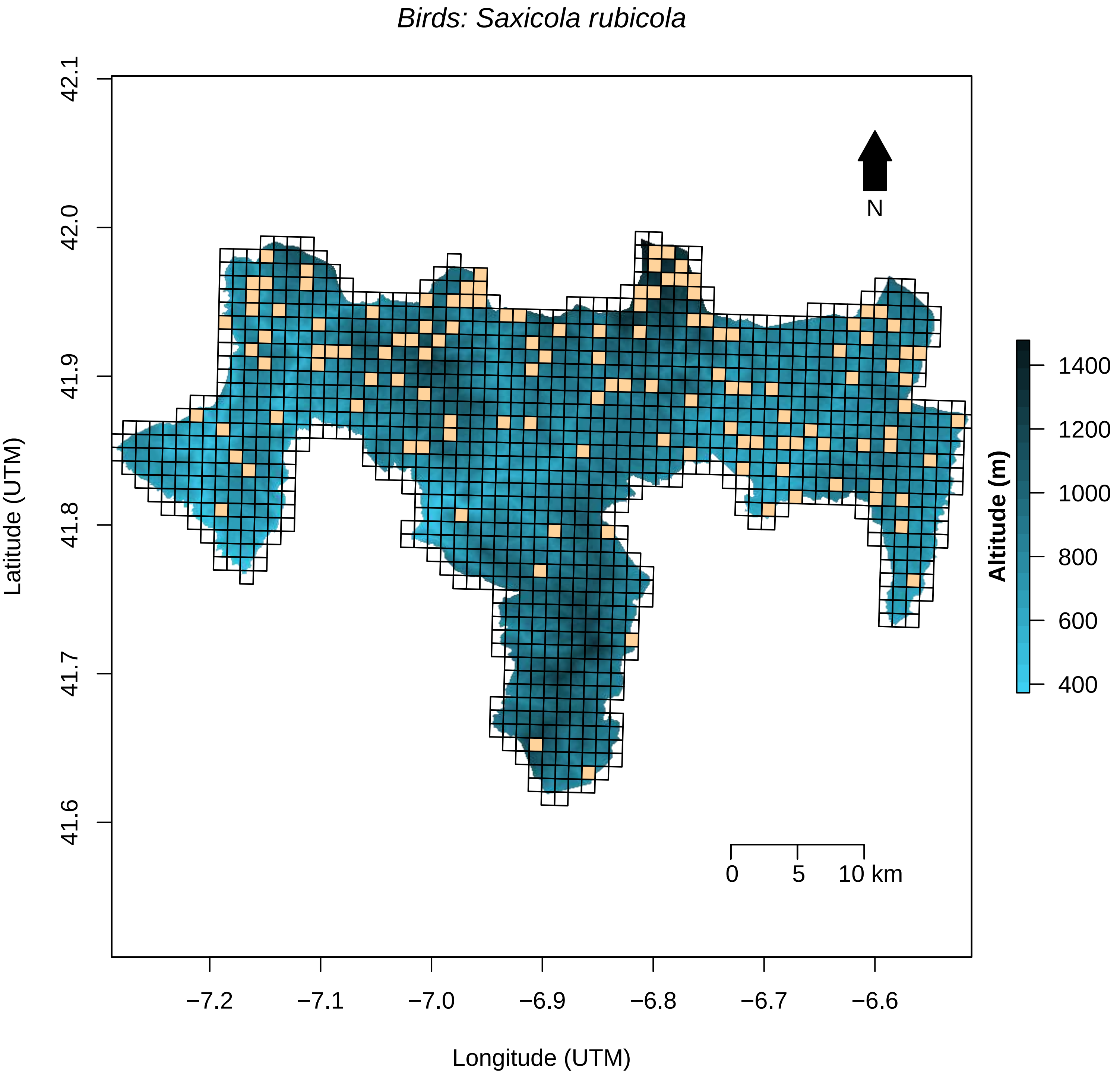

Birds: Saxicola rubicola
N
0
5
10 km
Latitude (UTM)
42.1
42.0
41.9
41.8
41.7
41.6
−7.2
−7.1
−7.0
−6.9
−6.8
−6.7
−6.6
Longitude (UTM)
Altitude (m)
1400
1200
1000
800
600
400

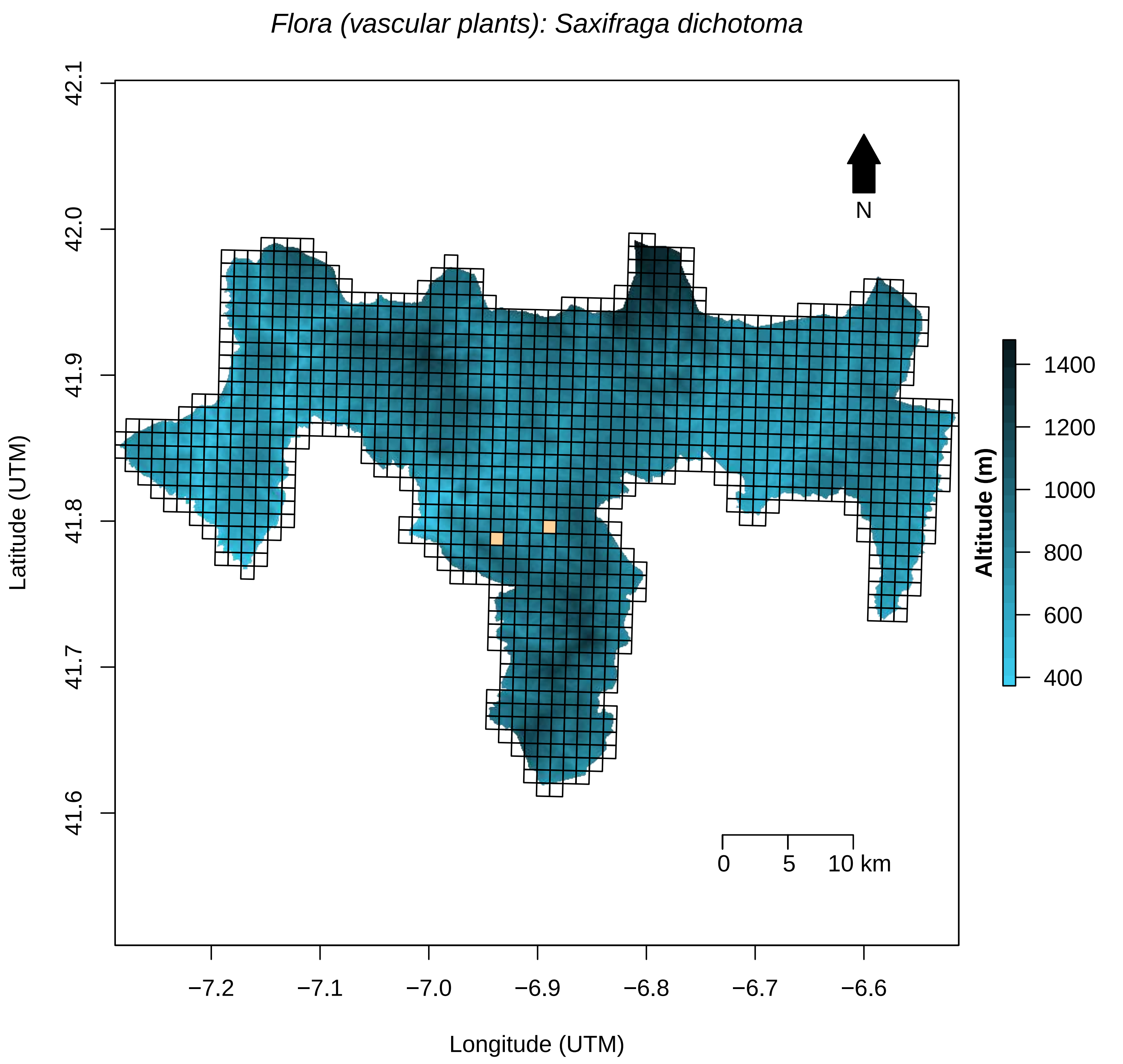

Flora (vascular plants): Saxifraga dichotoma
N
0
5
10 km
Altitude (m)
1400
1200
1000
800
600
400
Latitude (UTM)
42.1
42.0
41.9
41.8
41.7
41.6
Longitude (UTM)
−7.2
−7.1
−7.0
−6.9
−6.8
−6.7
−6.6

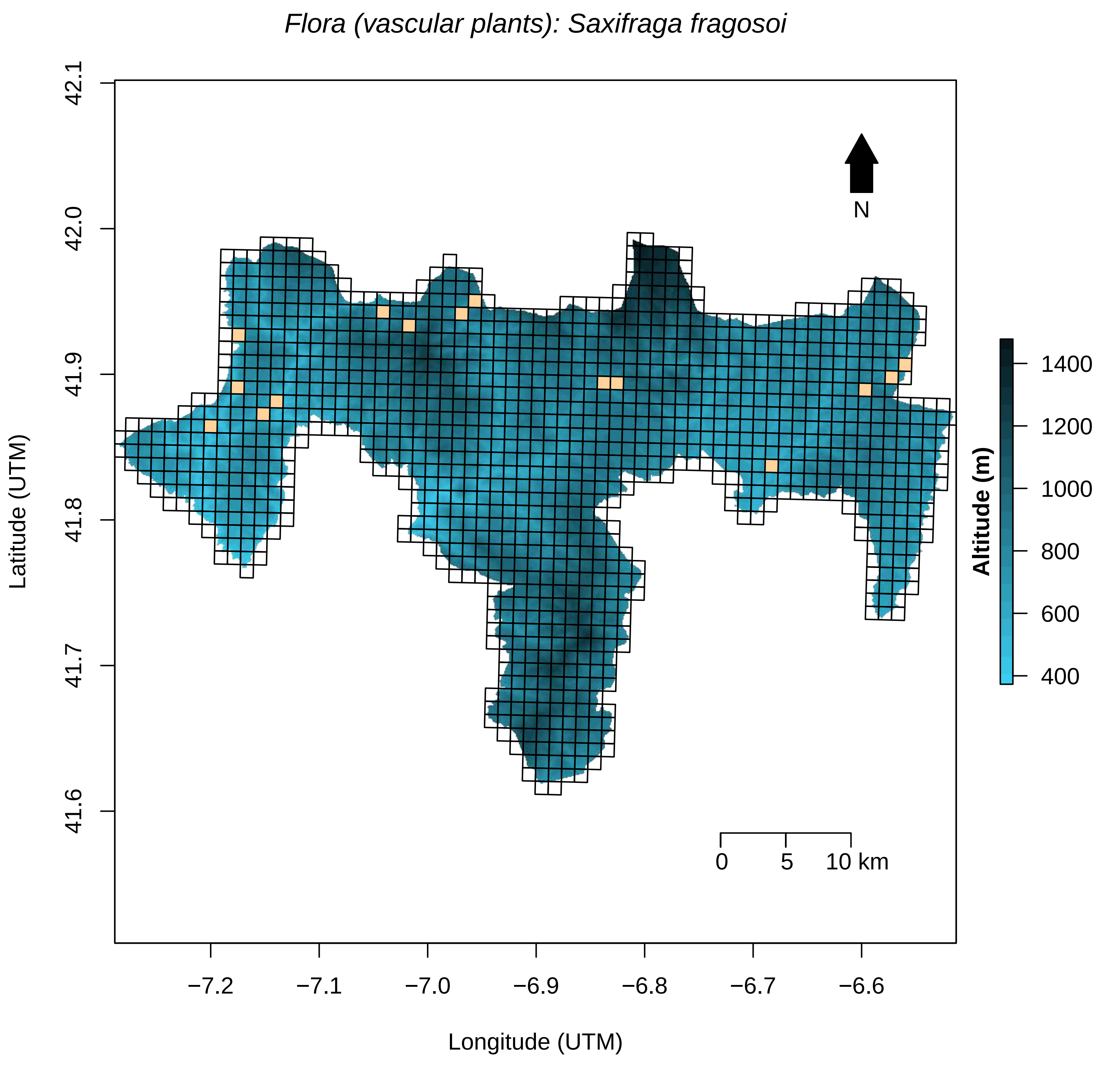
Flora (vascular plants): Saxifraga fragosoi
Latitude (UTM)
42.1
42.0
41.9
41.8
41.7
41.6
Longitude (UTM)
−7.2
−7.1
−7.0
−6.9
−6.8
−6.7
−6.6
N
0
5
10 km
Altitude (m)
1400
1200
1000
800
600
400

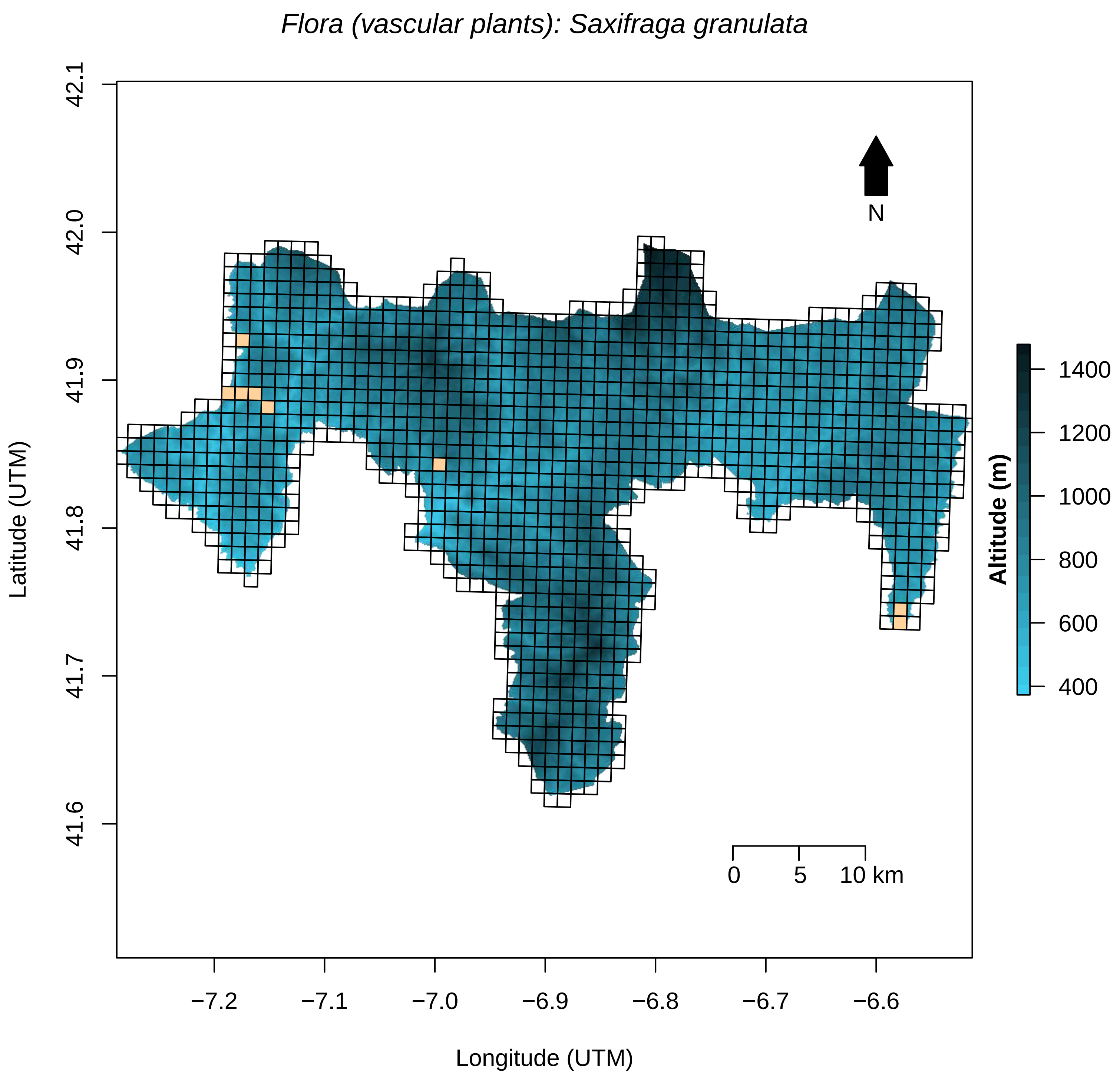

Flora (vascular plants): Saxifraga granulata
Latitude (UTM)
42.1
42.0
41.9
41.8
41.7
41.6
Longitude (UTM)
−7.2
−7.1
−7.0
−6.9
−6.8
−6.7
−6.6
N
0
5
10 km
Altitude (m)
1400
1200
1000
800
600
400

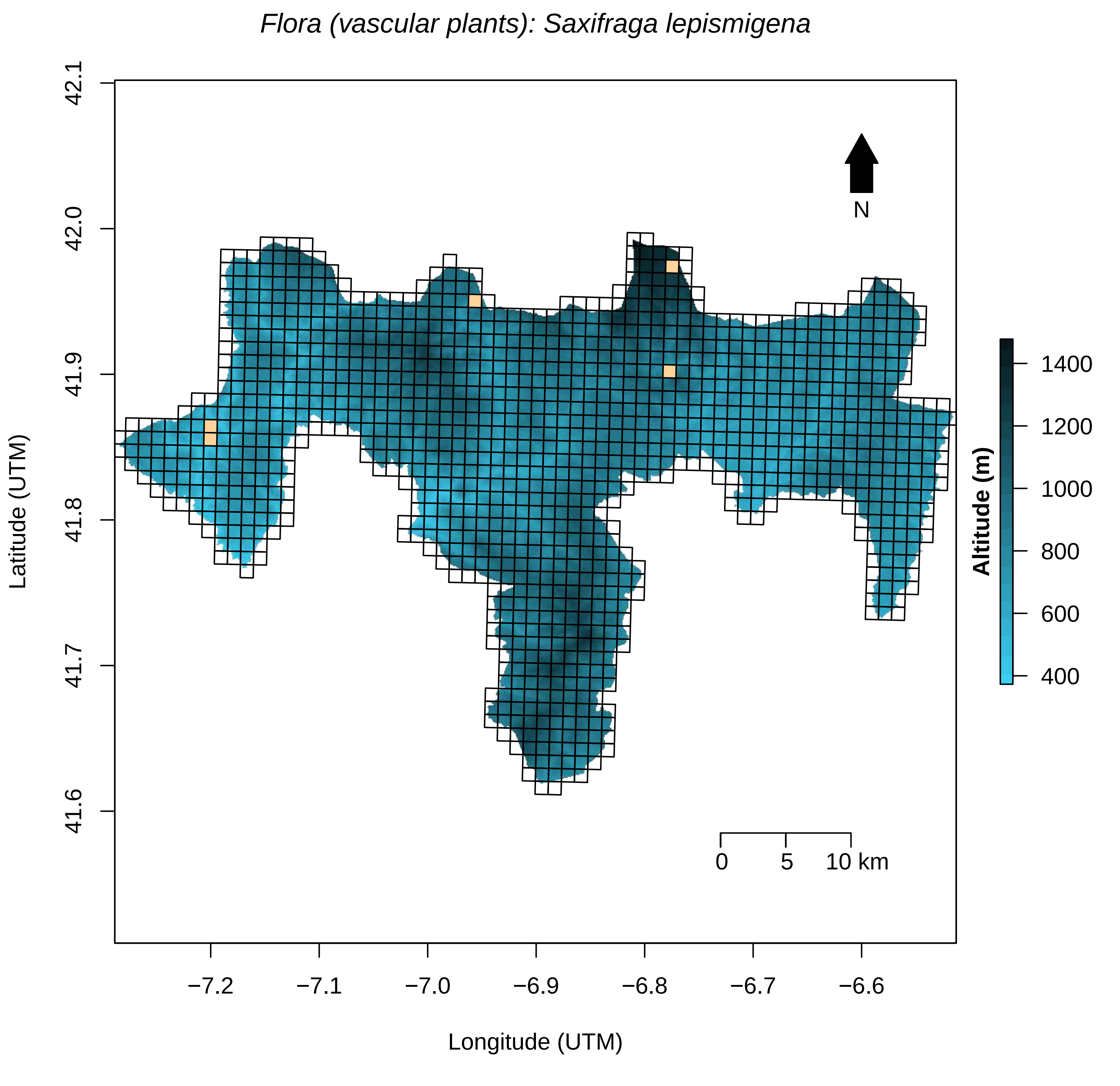

Flora (vascular plants): Saxifraga lepismigena
42.1
42.0
41.9
41.8
41.7
41.6
Latitude (UTM)
−7.2
−7.1
−7.0
−6.9
−6.8
−6.7
−6.6
Longitude (UTM)
N
0
5
10 km
Altitude (m)
1400
1200
1000
800
600
400

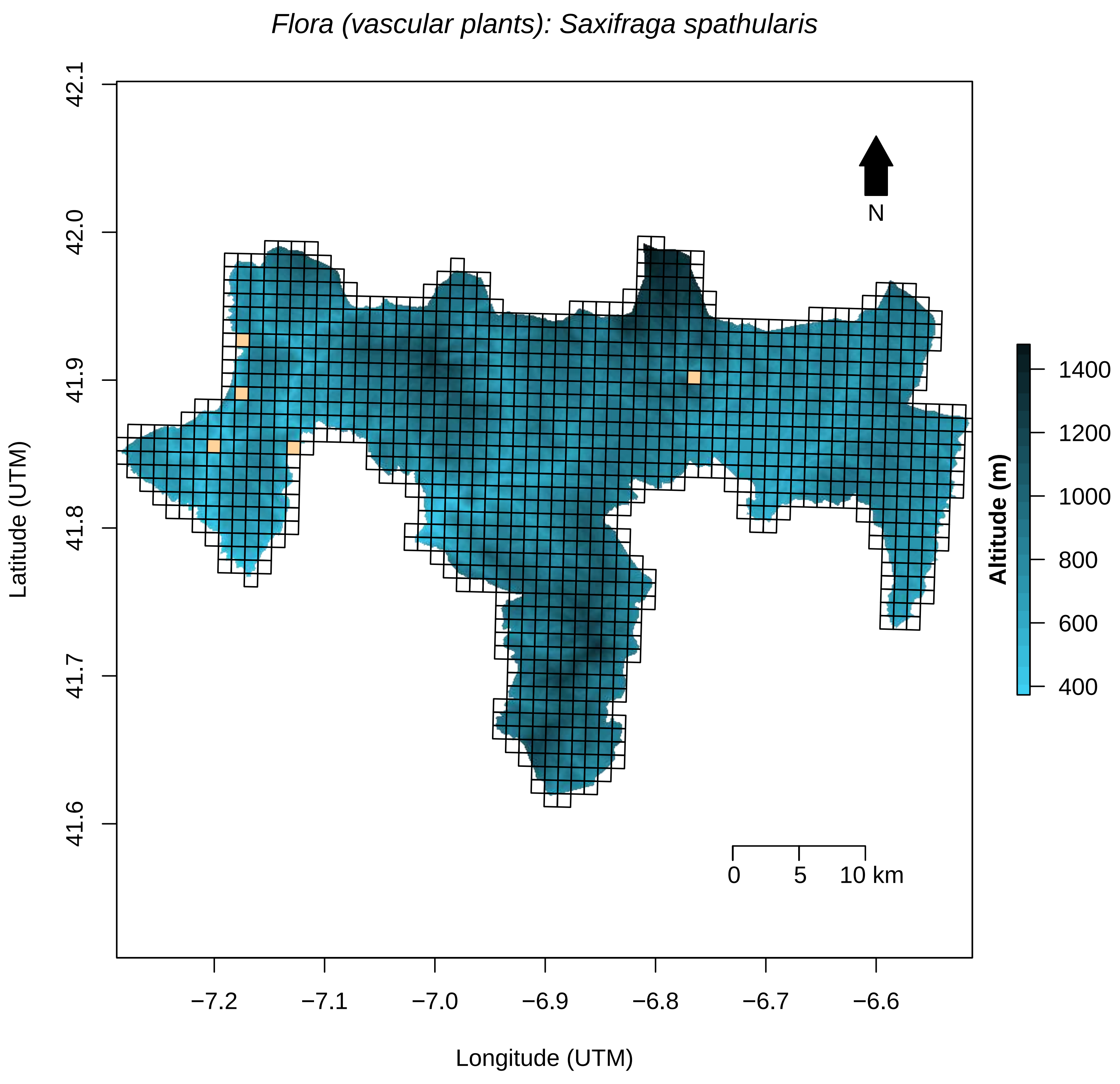

Flora (vascular plants): Saxifraga spathularis
Latitude (UTM)
42.1
42.0
41.9
41.8
41.7
41.6
Longitude (UTM)
−7.2
−7.1
−7.0
−6.9
−6.8
−6.7
−6.6
N
0
5
10 km
Altitude (m)
1400
1200
1000
800
600
400

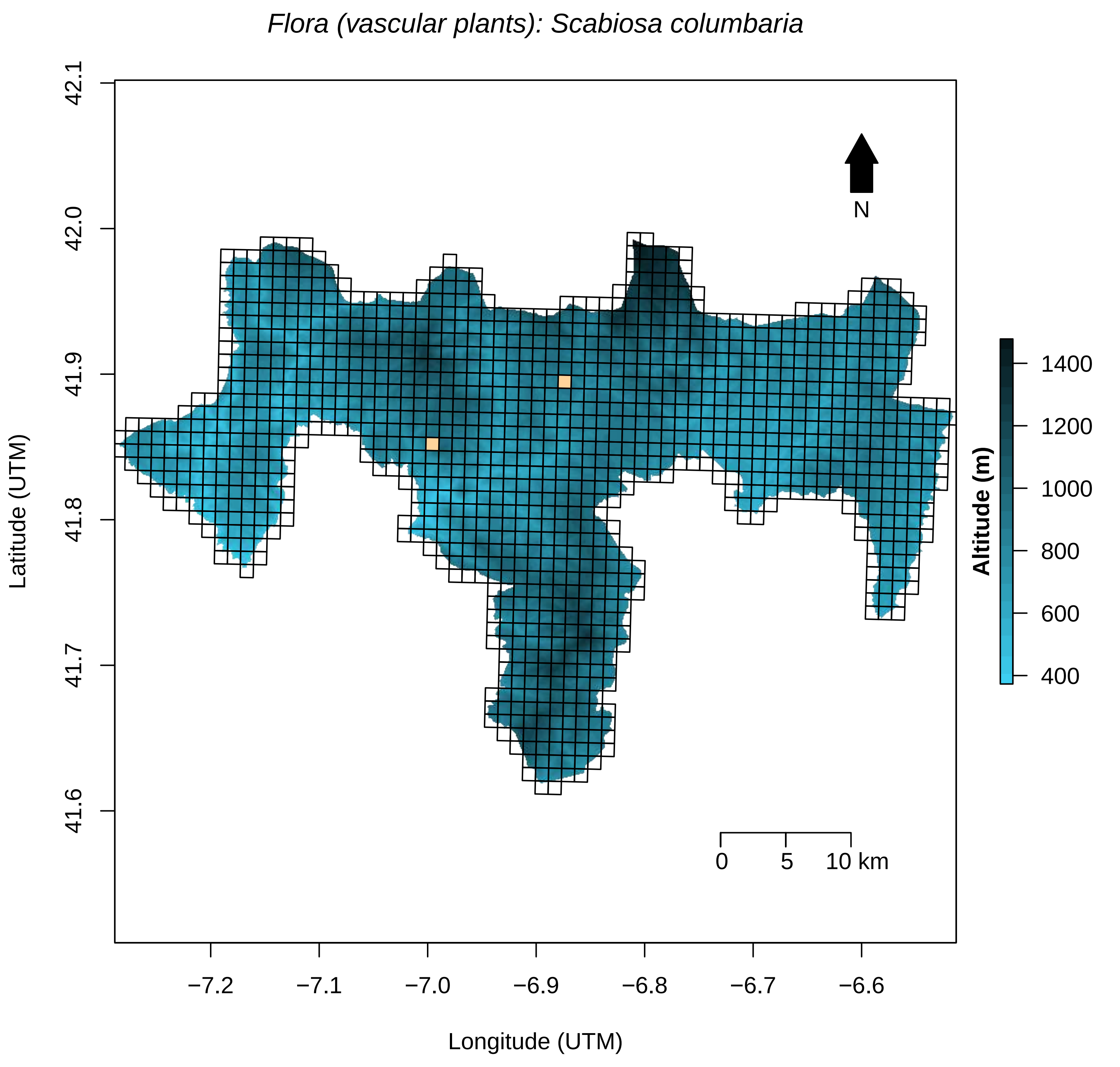
Flora (vascular plants): Scabiosa columbaria
Latitude (UTM)
42.1
42.0
41.9
41.8
41.7
41.6
Longitude (UTM)
−7.2
−7.1
−7.0
−6.9
−6.8
−6.7
−6.6
N
0
5
10 km
Altitude (m)
1400
1200
1000
800
600
400

Flora (vascular plants): Scilla autumnalis

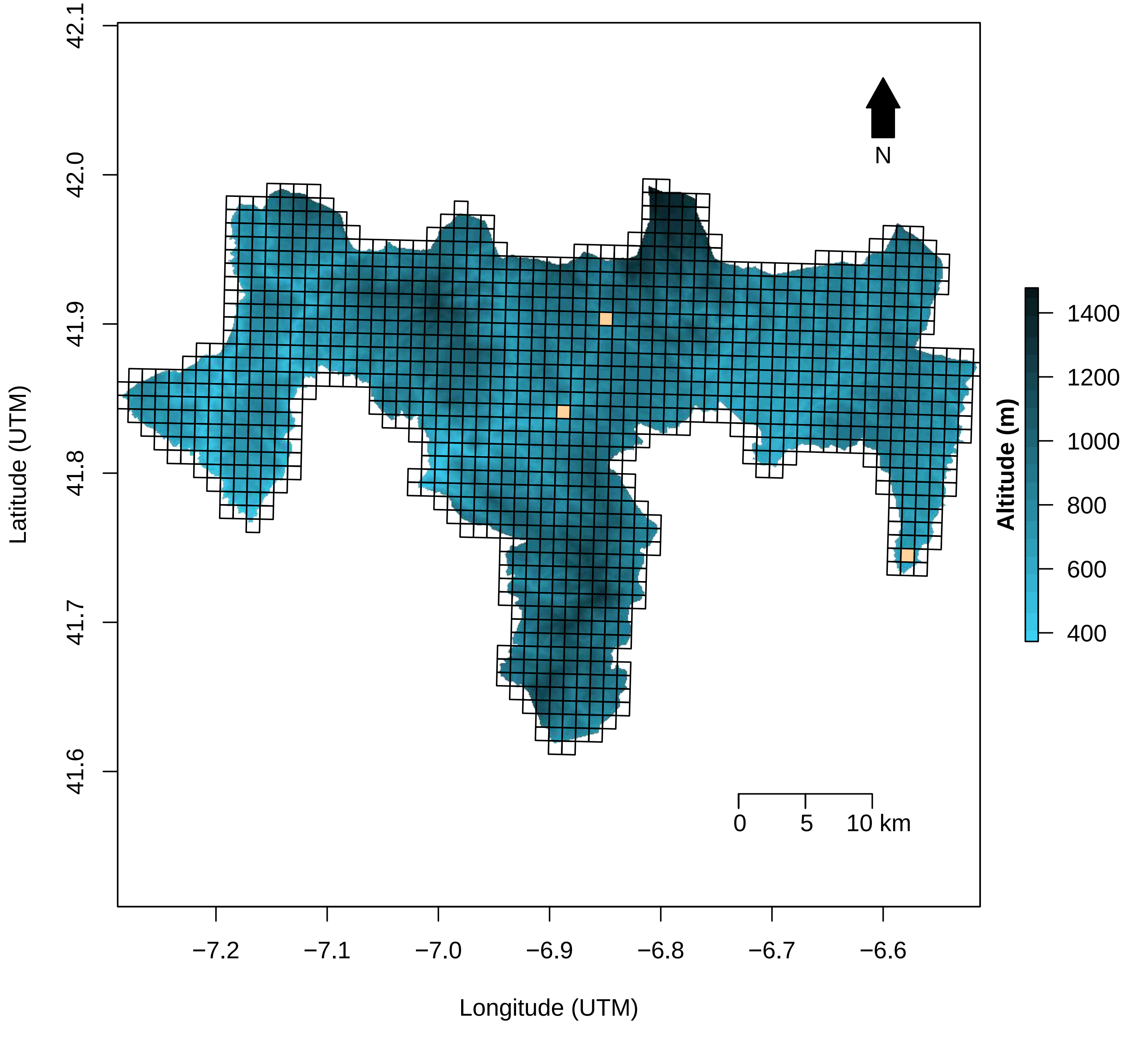

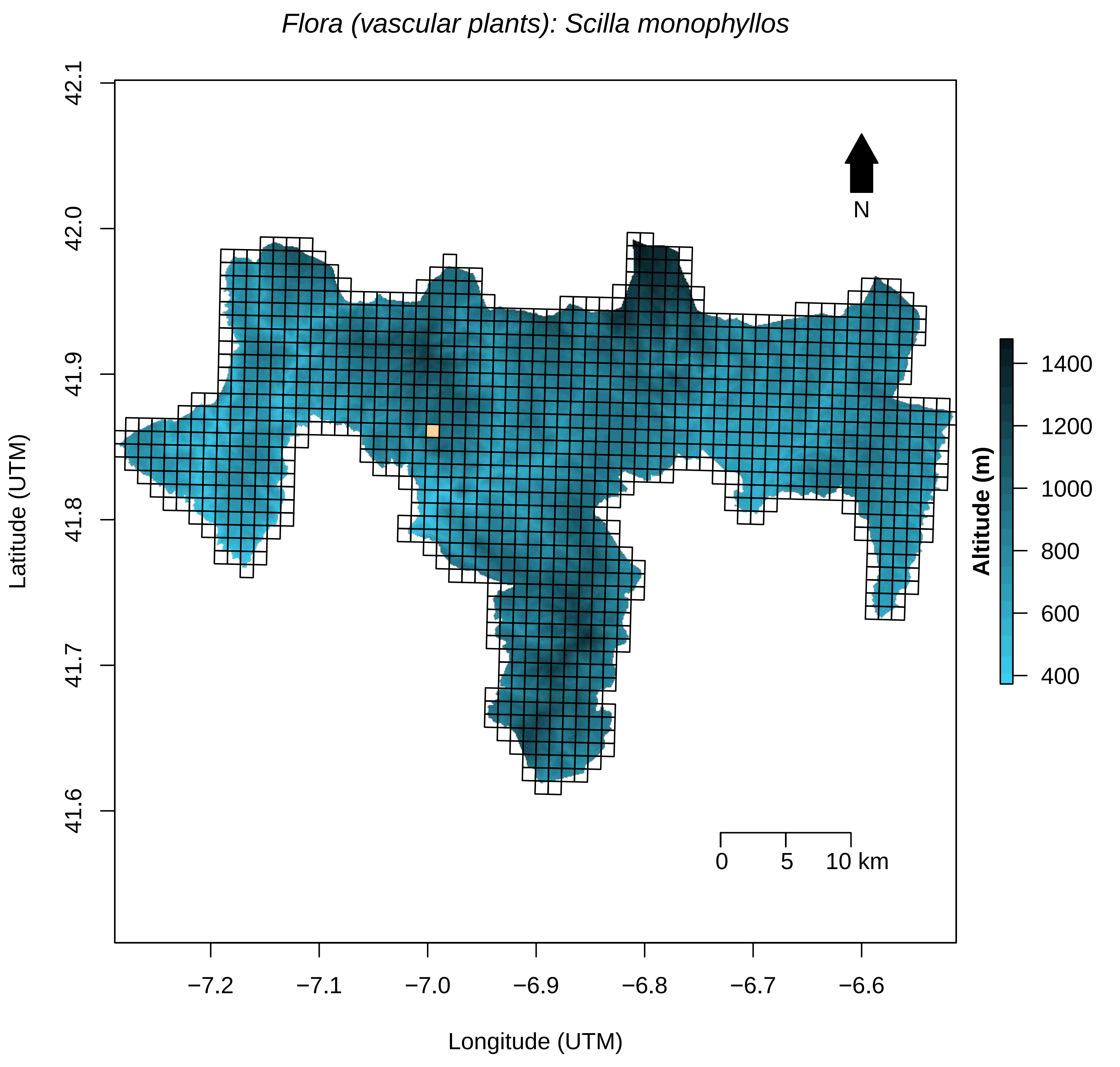

Flora (vascular plants): Scilla monophyllos
42.1
42.0
41.9
41.8
41.7
41.6
Latitude (UTM)
−7.2
−7.1
−7.0
−6.9
−6.8
−6.7
−6.6
Longitude (UTM)
N
0
5
10 km
Altitude (m)
1400
1200
1000
800
600
400

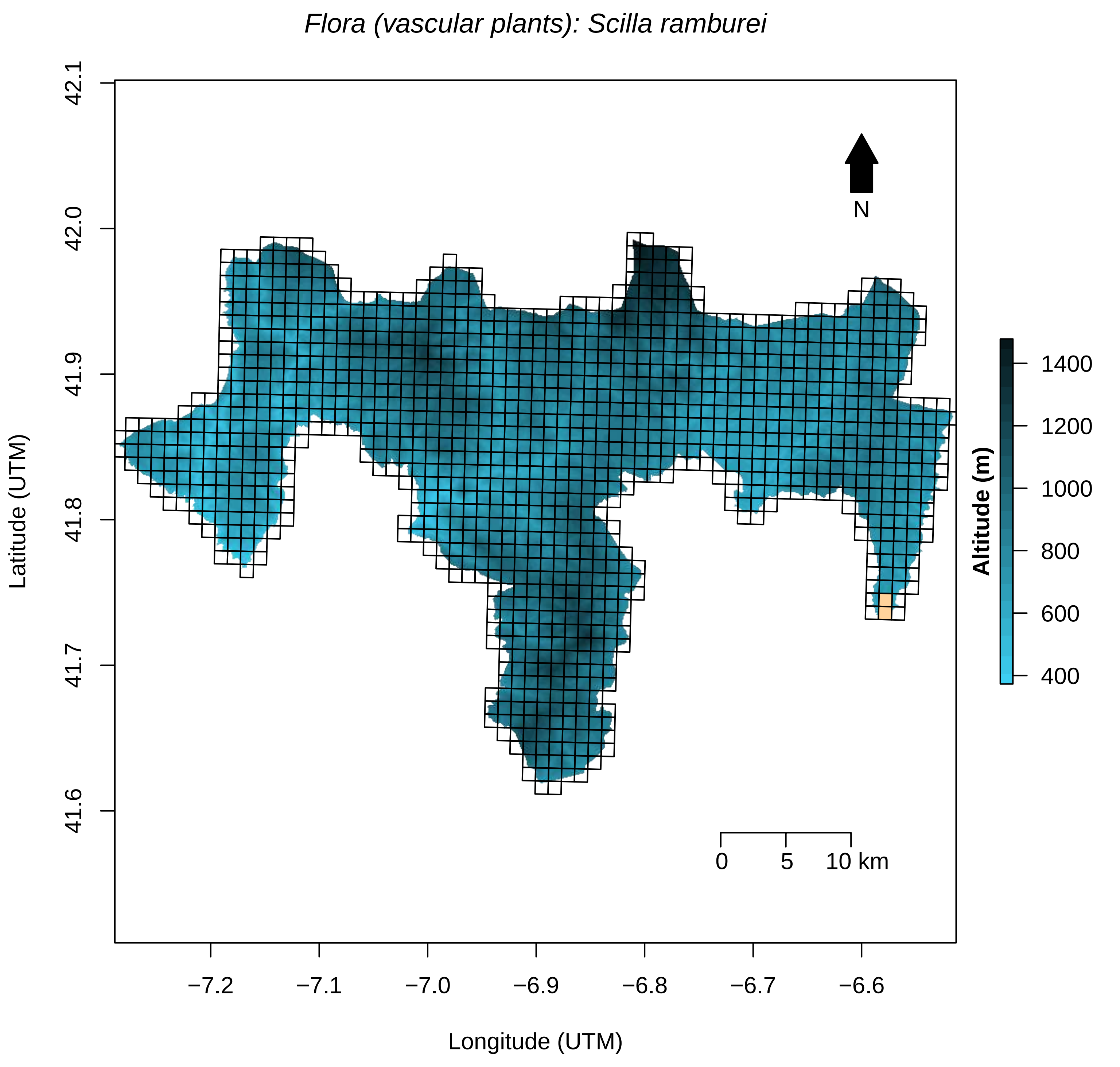
Flora (vascular plants): Scilla ramburei
42.1
42.0
41.9
41.8
41.7
41.6
Latitude (UTM)
−7.2
−7.1
−7.0
−6.9
−6.8
−6.7
−6.6
Longitude (UTM)
N
0
5
10 km
Altitude (m)
1400
1200
1000
800
600
400

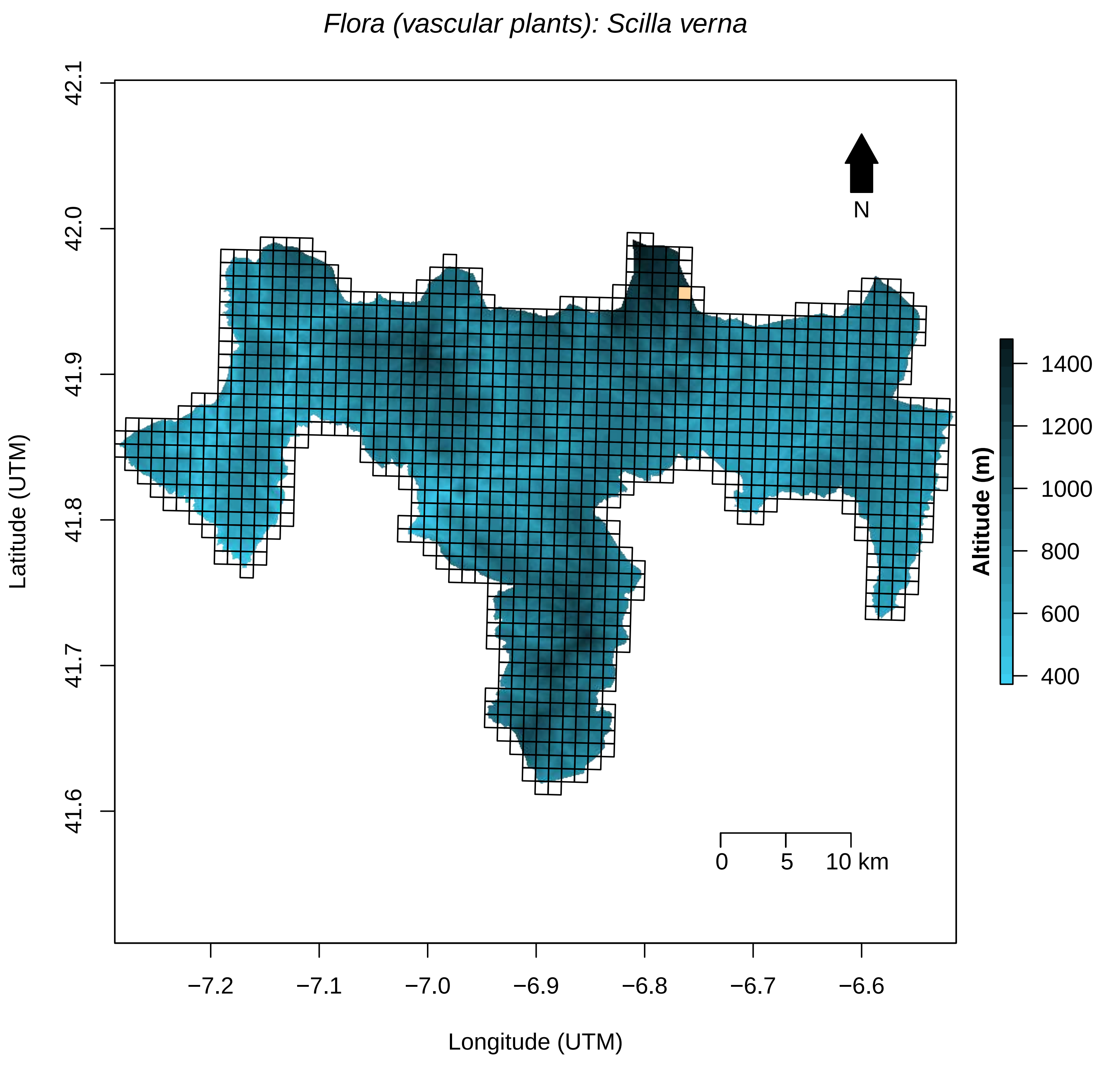
Flora (vascular plants): Scilla verna
N
0
5
10 km
Altitude (m)
1400
1200
1000
800
600
400
Latitude (UTM)
42.1
42.0
41.9
41.8
41.7
41.6
Longitude (UTM)
−7.2
−7.1
−7.0
−6.9
−6.8
−6.7
−6.6

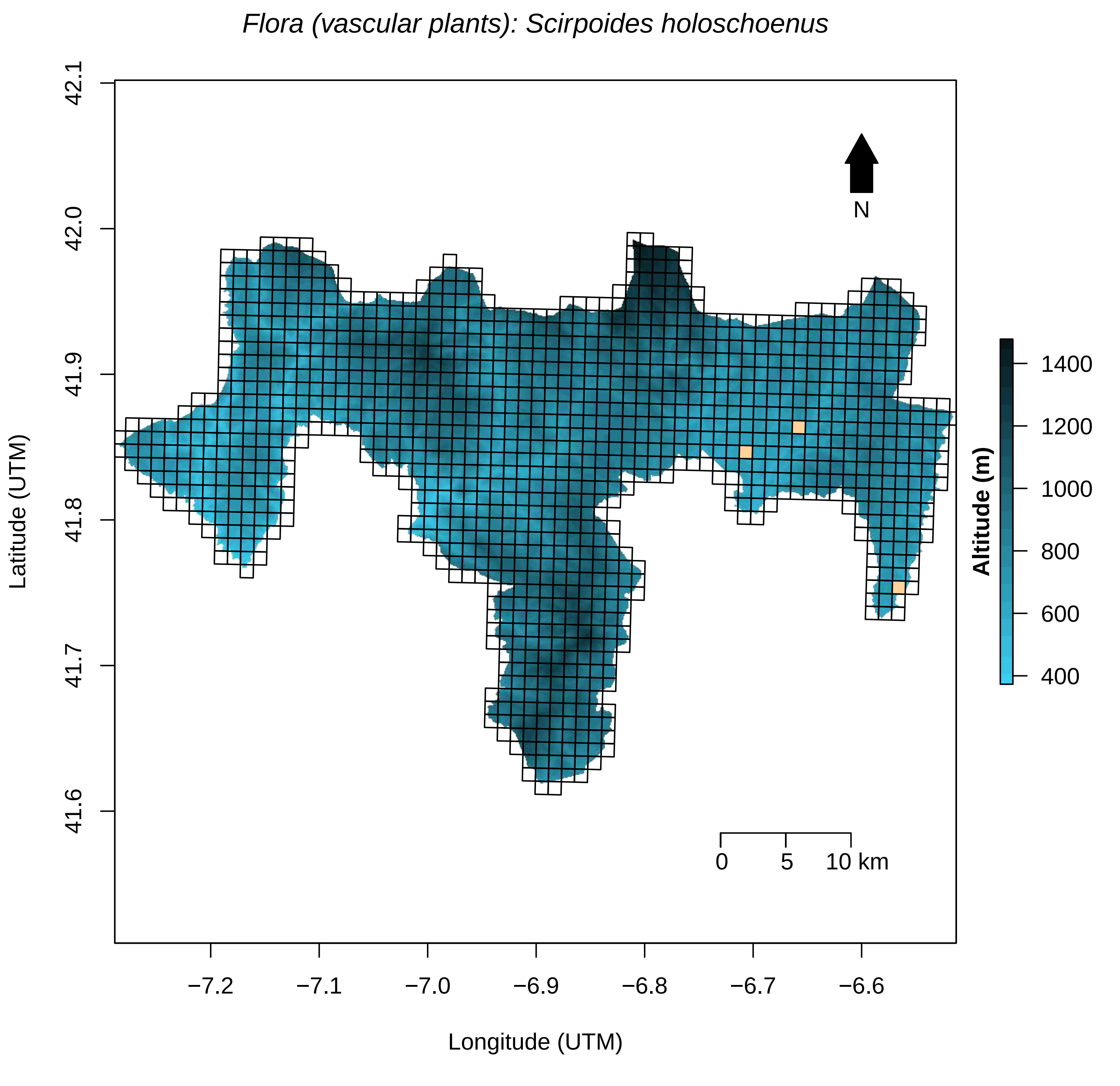
Flora (vascular plants): Scirpoides holoschoenus
N
0
5
10 km
Altitude (m)
1400
1200
1000
800
600
400
Latitude (UTM)
42.1
42.0
41.9
41.8
41.7
41.6
Longitude (UTM)
−7.2
−7.1
−7.0
−6.9
−6.8
−6.7
−6.6

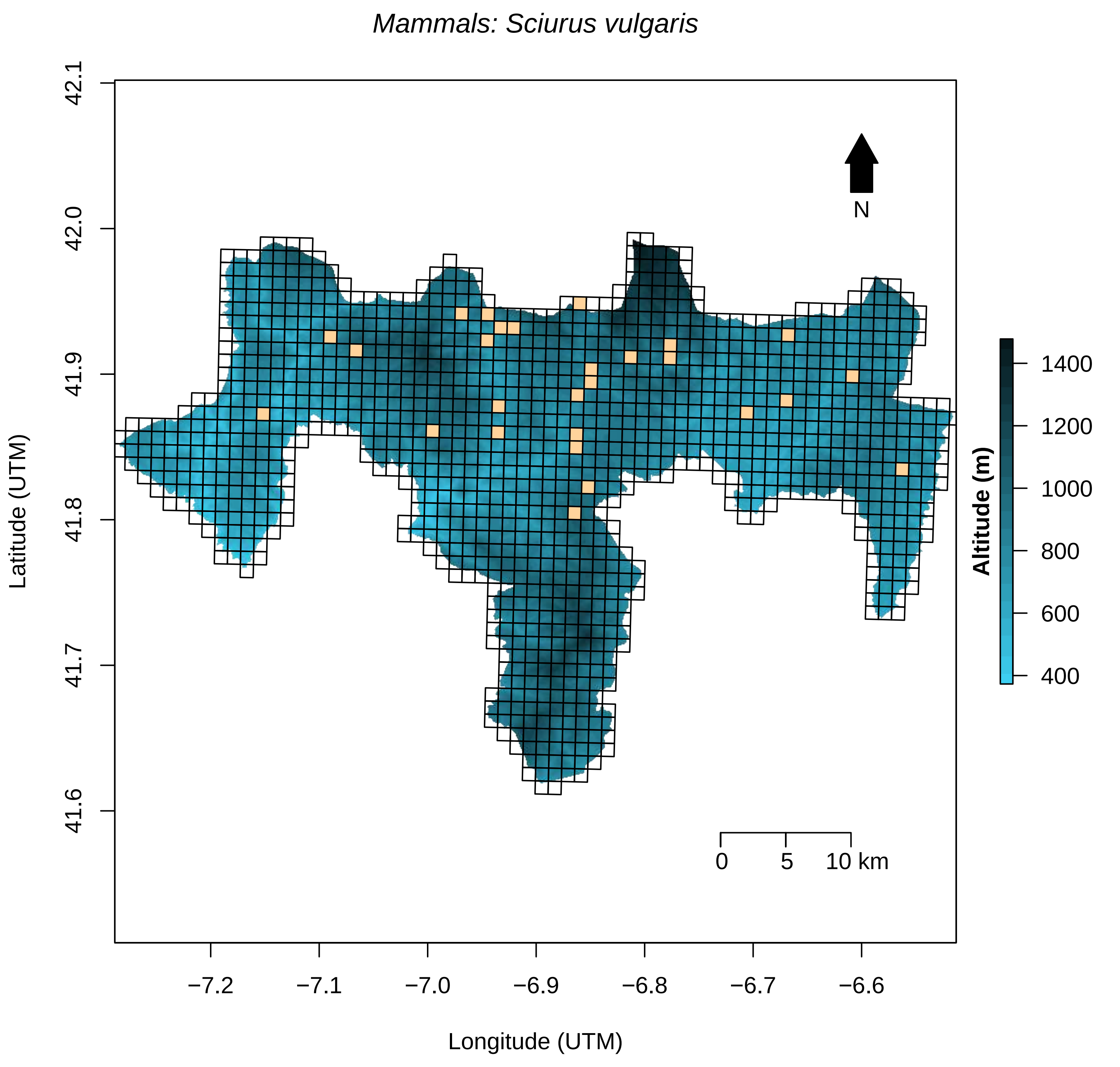
Mammals: Sciurus vulgaris
42.1
42.0
41.9
41.8
41.7
41.6
Latitude (UTM)
−7.2
−7.1
−7.0
−6.9
−6.8
−6.7
−6.6
Longitude (UTM)
N
0
5
10 km
Altitude (m)
1400
1200
1000
800
600
400

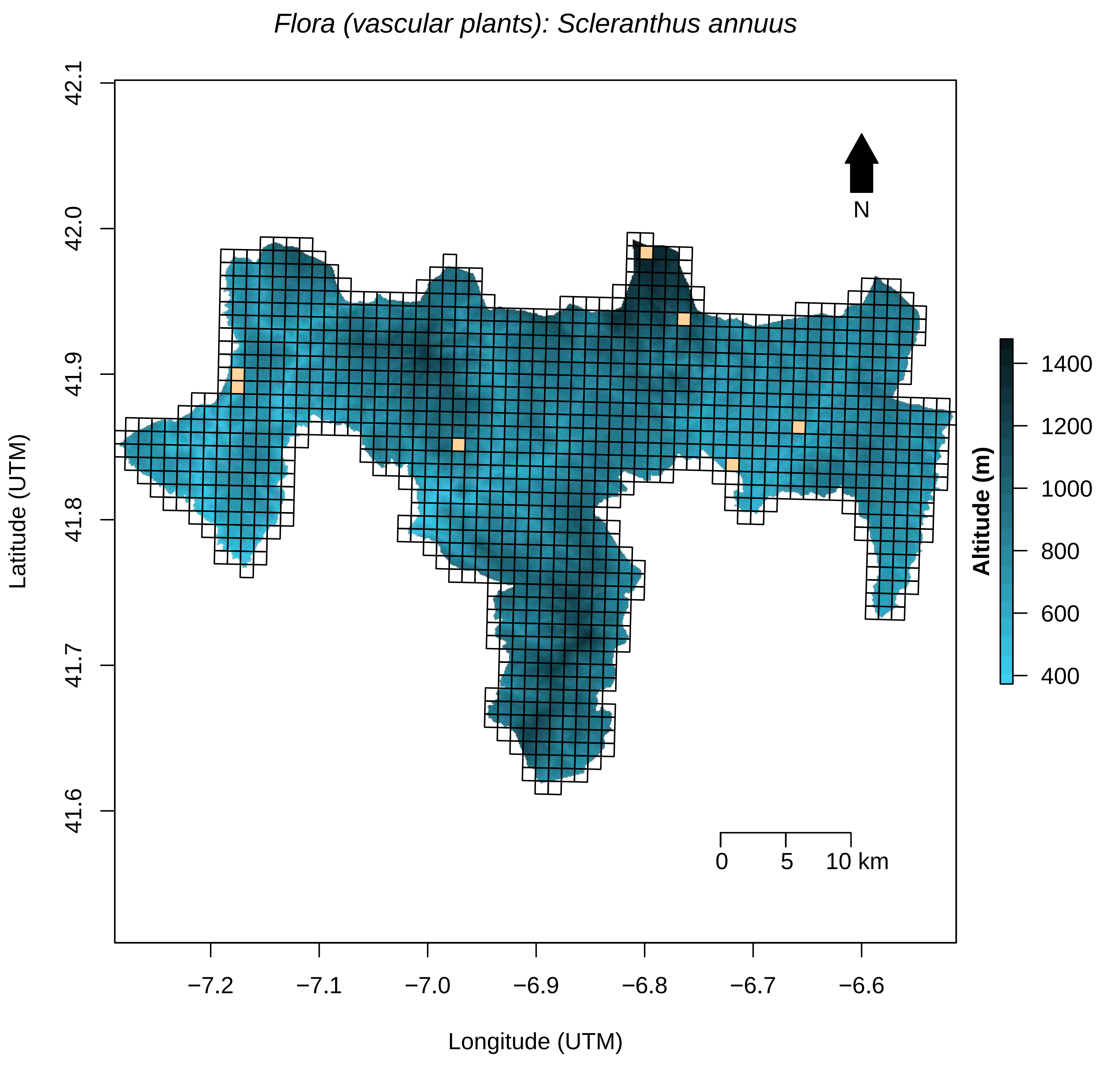

Flora (vascular plants): Scleranthus annuus
42.1
42.0
41.9
41.8
41.7
41.6
Latitude (UTM)
N
0
5
10 km
Altitude (m)
1400
1200
1000
800
600
400
−7.2
−7.1
−7.0
−6.9
−6.8
−6.7
−6.6
Longitude (UTM)

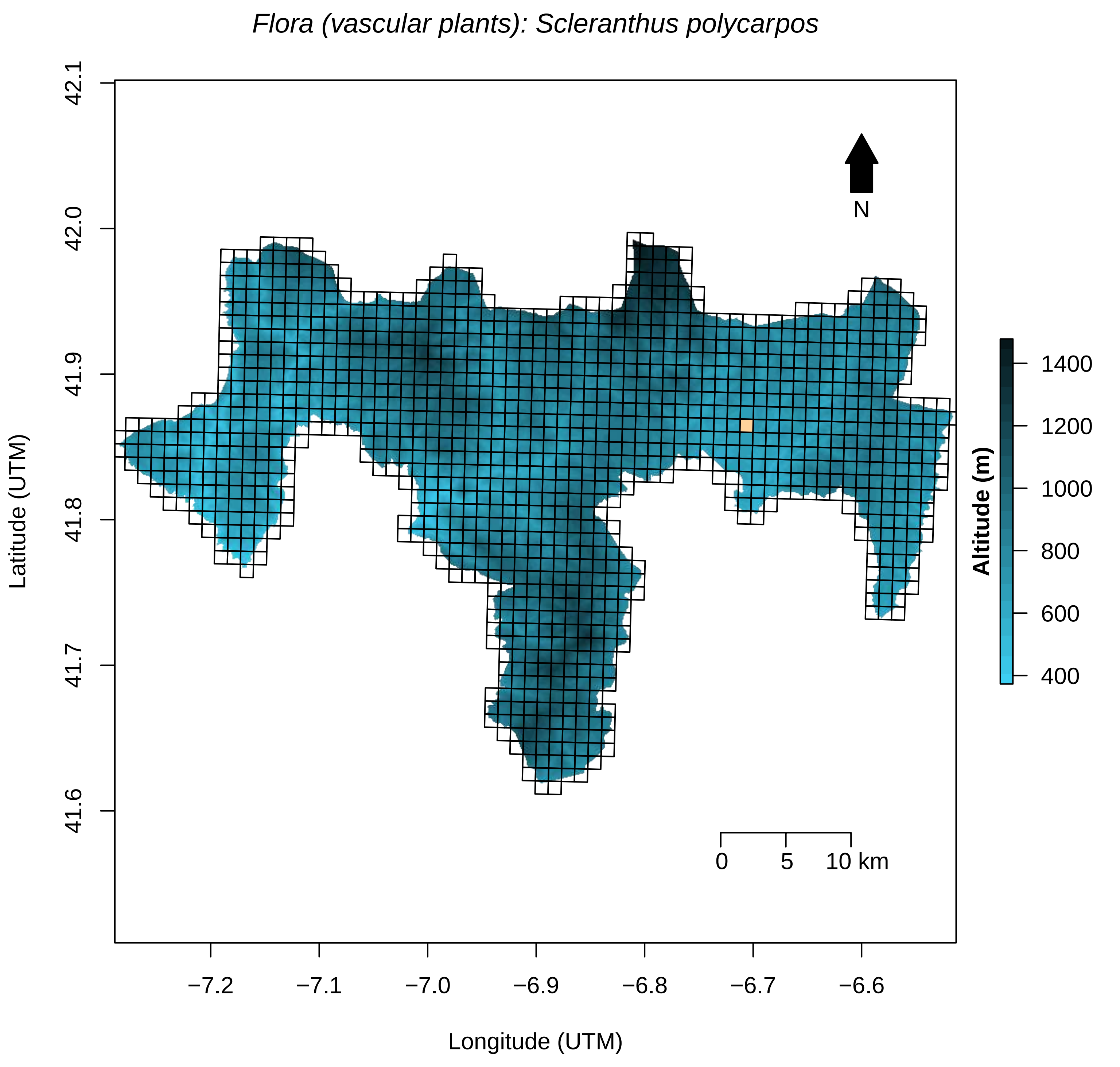

Flora (vascular plants): Scleranthus polycarpos
N
0 5 10 km
Altitude (m)
1400
1200
1000
800
600
400
42.1
42.0
41.9
41.8
41.7
41.6
Latitude (UTM)
−7.2
−7.1
−7.0
−6.9
−6.8
−6.7
−6.6
Longitude (UTM)

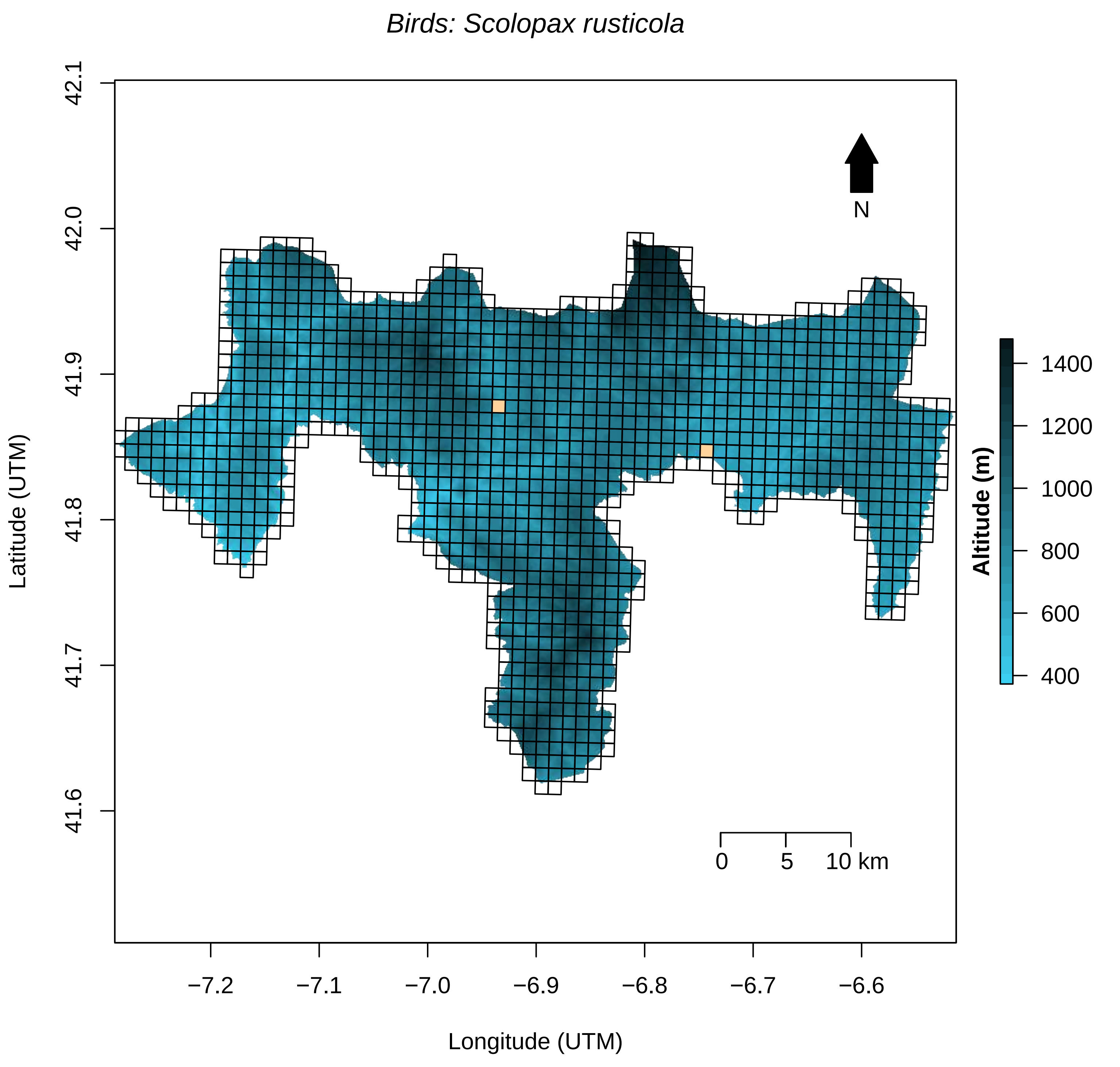
Birds: Scolopax rusticola
Latitude (UTM)
Longitude (UTM)
42.1
42.0
41.9
41.8
41.7
41.6
−7.2
−7.1
−7.0
−6.9
−6.8
−6.7
−6.6
N
0
5
10 km
Altitude (m)
1400
1200
1000
800
600
400

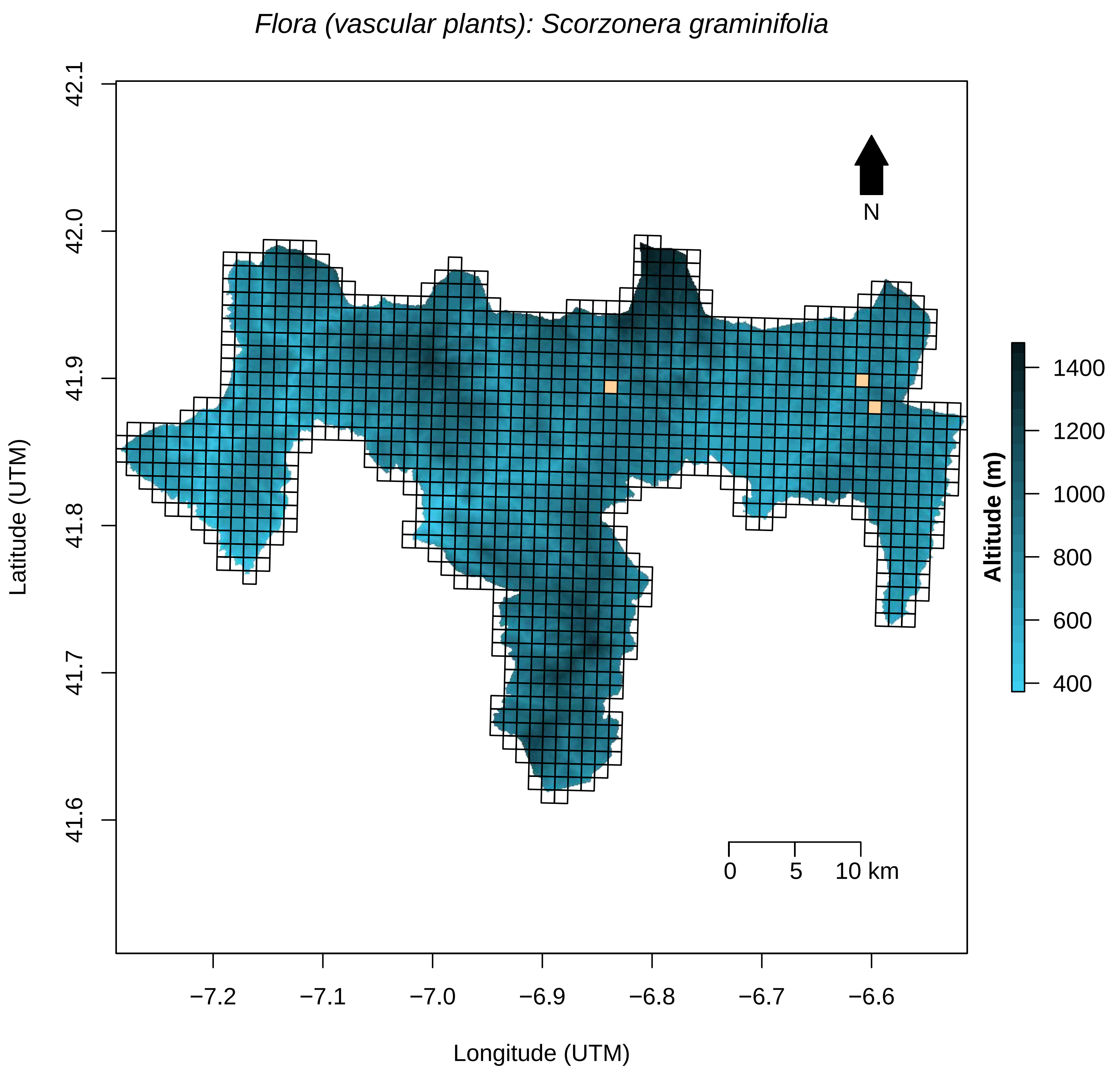
Flora (vascular plants): Scorzonera graminifolia
Latitude (UTM)
Longitude (UTM)
42.1
42.0
41.9
41.8
41.7
41.6
−7.2
−7.1
−7.0
−6.9
−6.8
−6.7
−6.6
N
0
5
10 km
Altitude (m)
1400
1200
1000
800
600
400

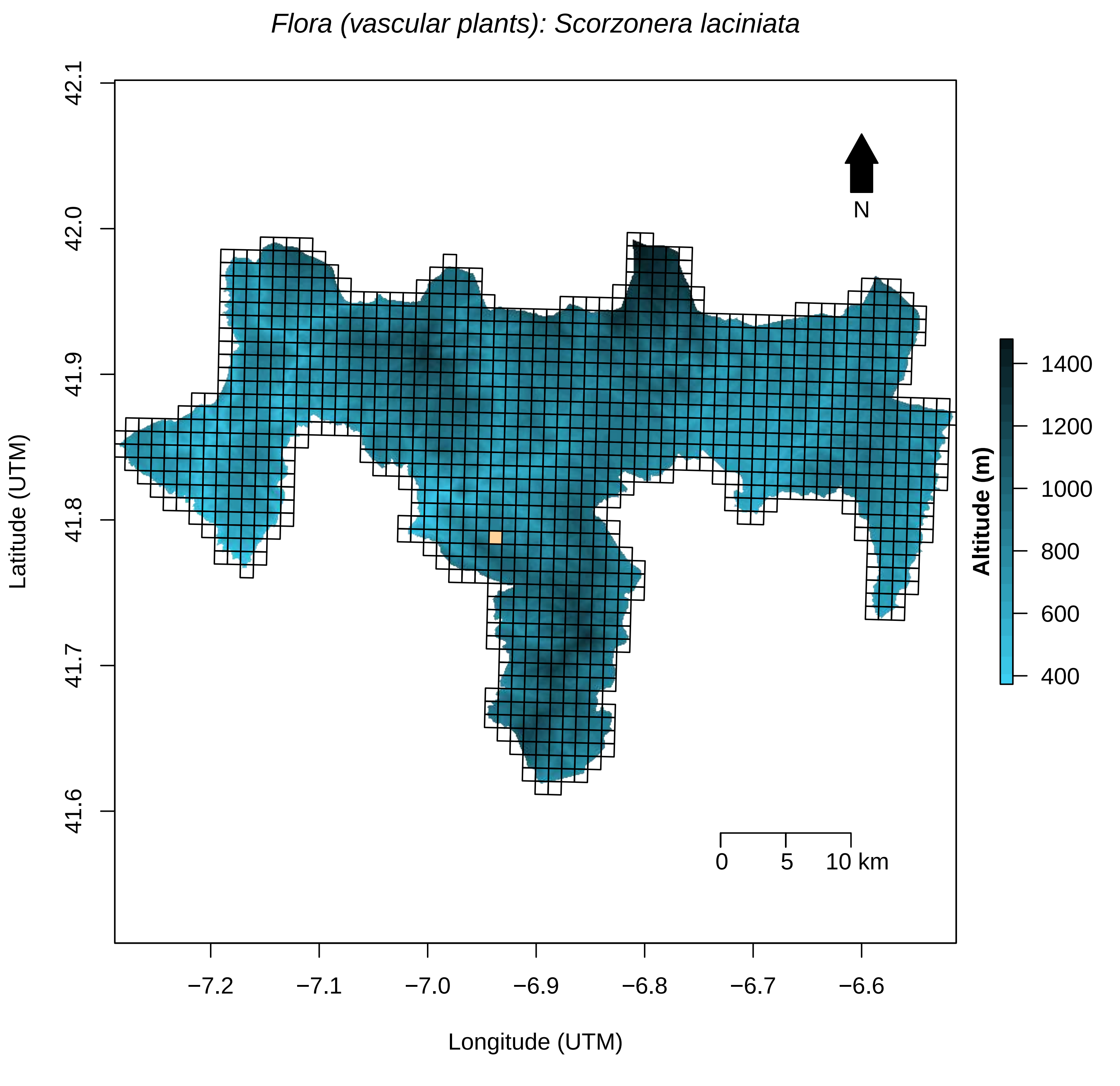
Flora (vascular plants): Scorzonera laciniata
42.1
42.0
41.9
41.8
41.7
41.6
Latitude (UTM)
−7.2
−7.1
−7.0
−6.9
−6.8
−6.7
−6.6
Longitude (UTM)
N
0
5
10 km
Altitude (m)
1400
1200
1000
800
600
400

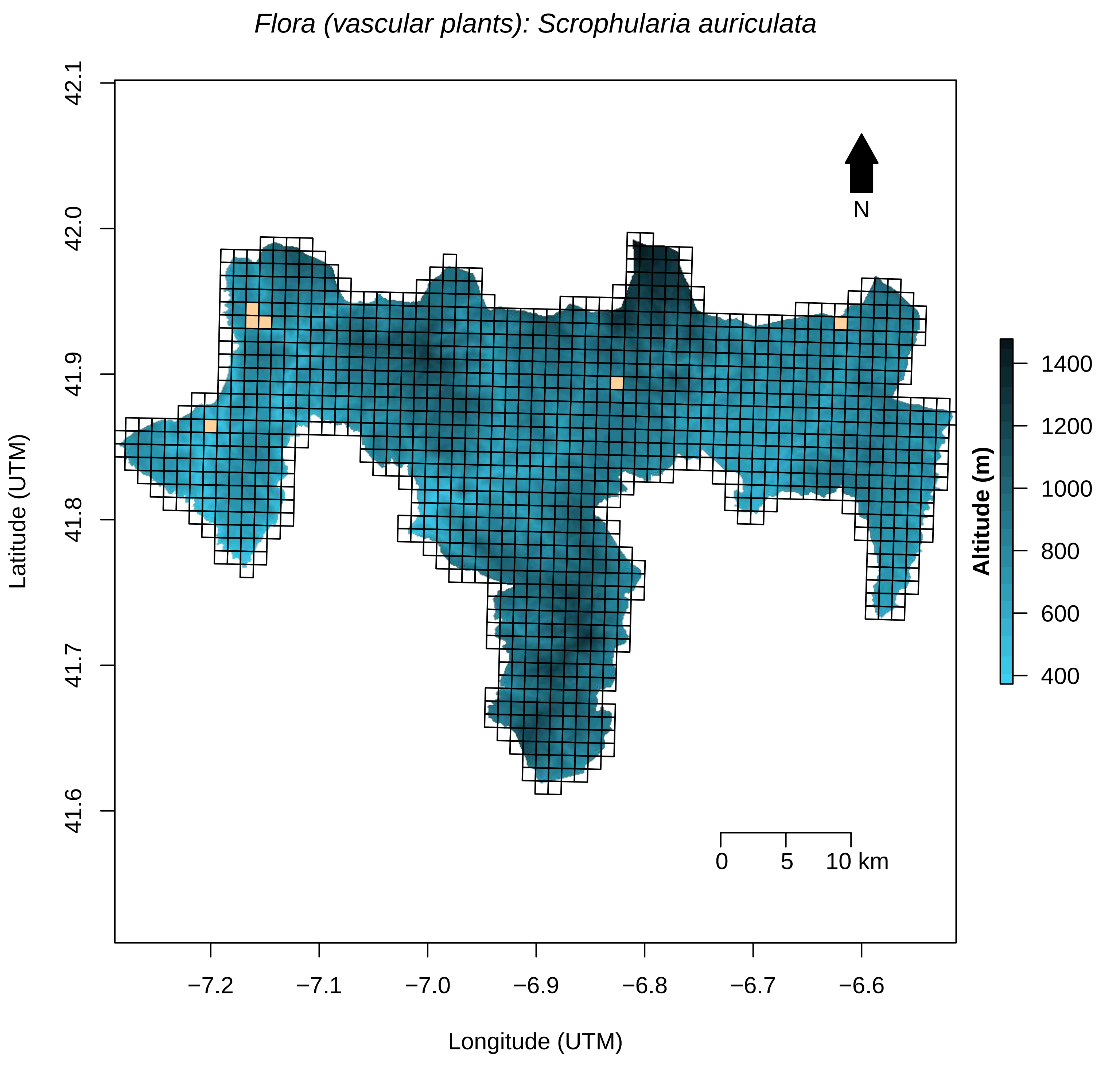
Flora (vascular plants): Scrophularia auriculata
42.1
42.0
41.9
41.8
41.7
41.6
Latitude (UTM)
N
0 5 10 km
1400
1200
1000
800
600
400
Altitude (m)
−7.2
−7.1
−7.0
−6.9
−6.8
−6.7
−6.6
Longitude (UTM)

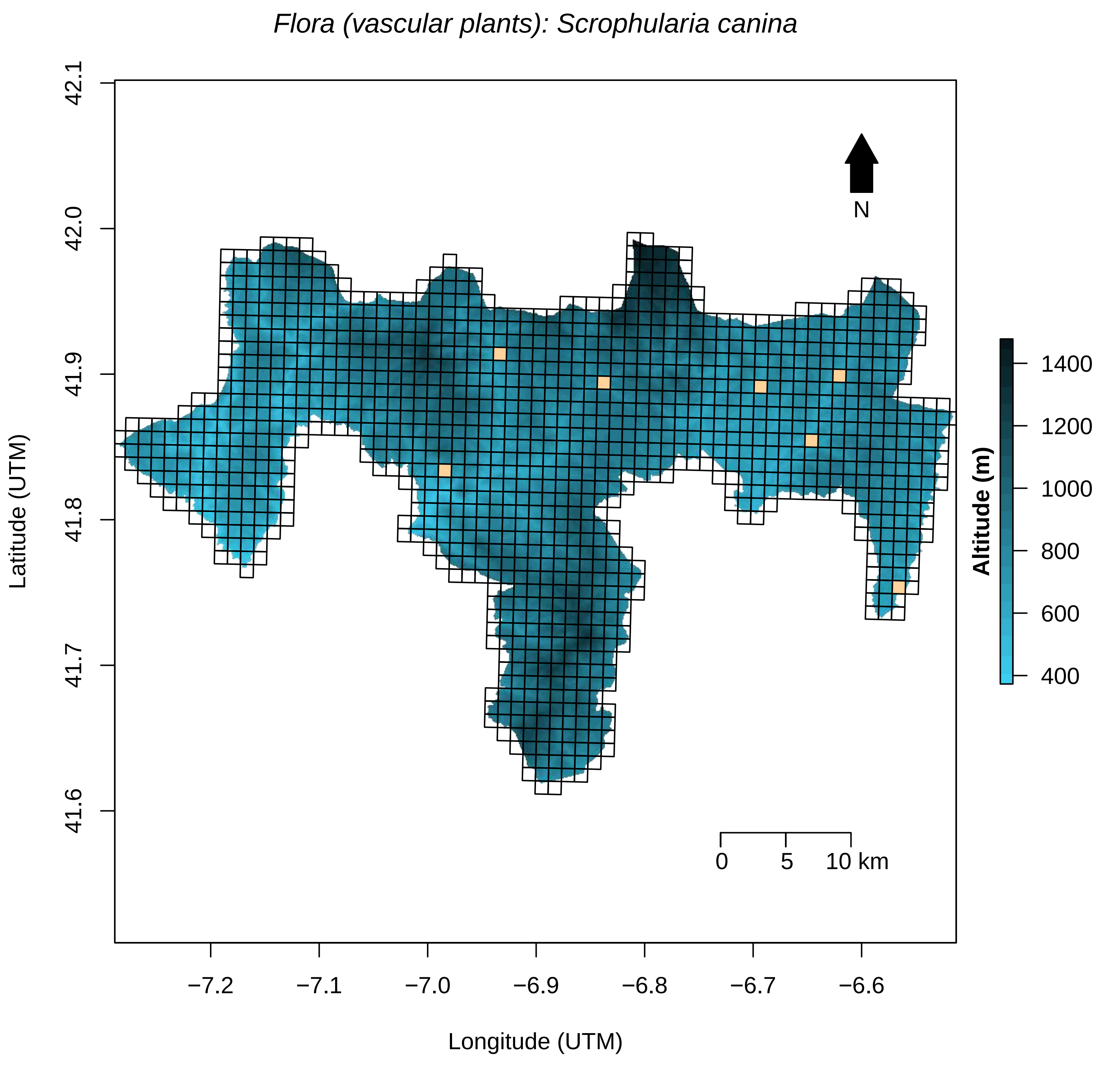
Flora (vascular plants): Scrophularia canina
N
0
5
10 km
Altitude (m)
1400
1200
1000
800
600
400
42.1
42.0
41.9
41.8
41.7
41.6
Latitude (UTM)
−7.2
−7.1
−7.0
−6.9
−6.8
−6.7
−6.6
Longitude (UTM)

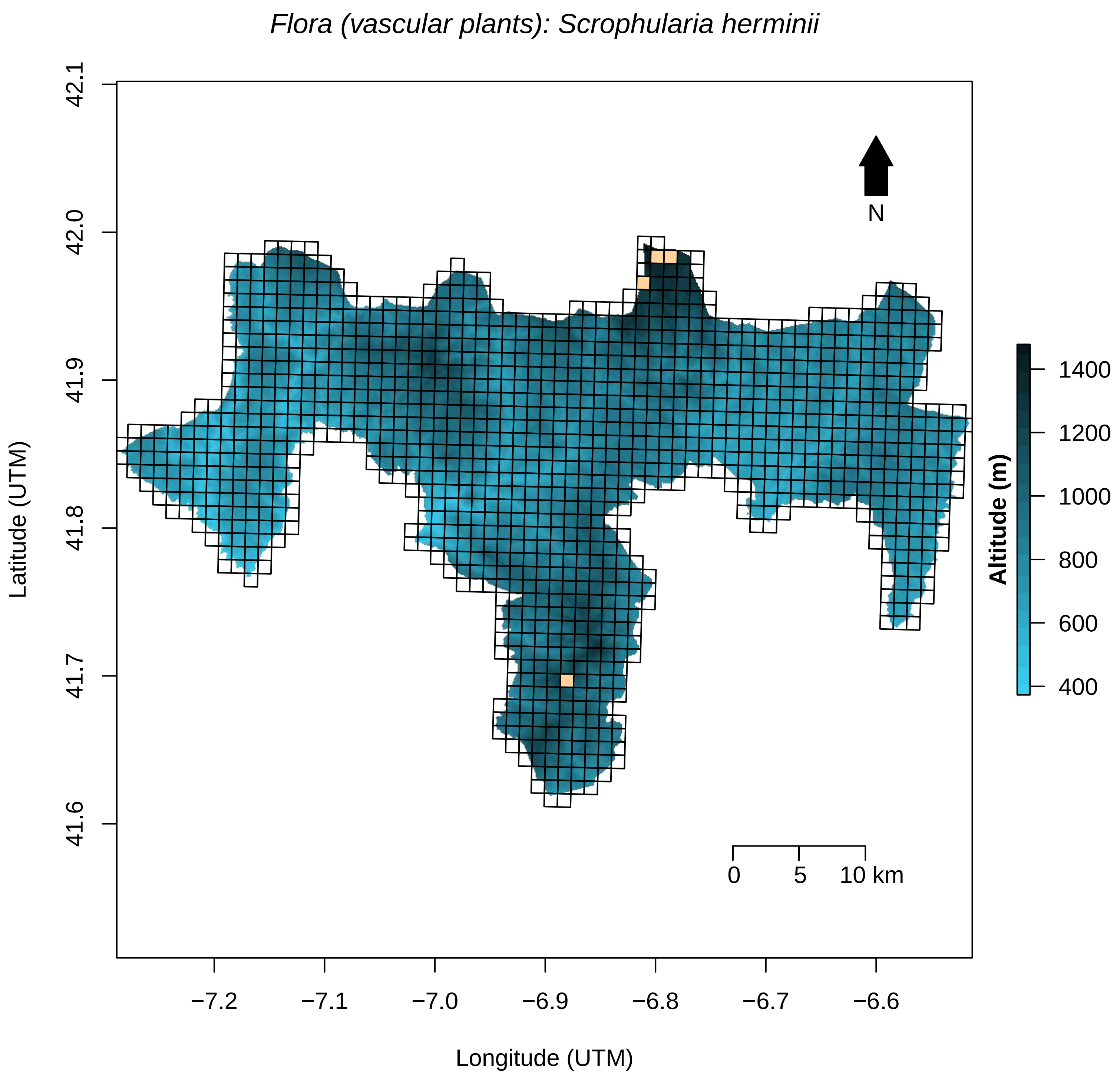

Flora (vascular plants): Scrophularia herminii
42.1
42.0
41.9
41.8
41.7
41.6
Latitude (UTM)
−7.2
−7.1
−7.0
−6.9
−6.8
−6.7
−6.6
Longitude (UTM)
N
0
5
10 km
1400
1200
1000
800
600
400
Altitude (m)

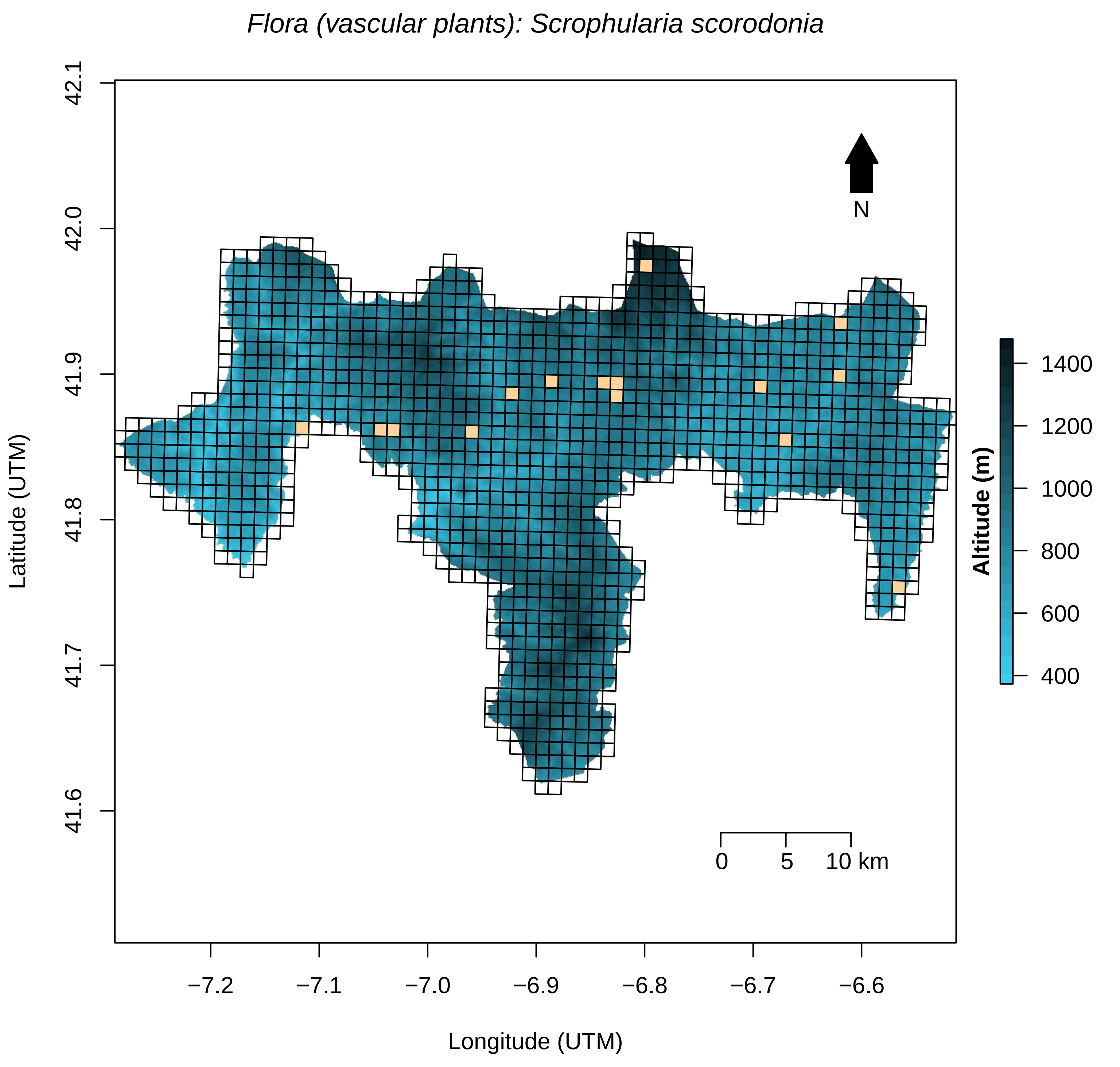
Flora (vascular plants): Scrophularia scorodonia
42.1
42.0
41.9
41.8
41.7
41.6
Latitude (UTM)
−7.2
−7.1
−7.0
−6.9
−6.8
−6.7
−6.6
Longitude (UTM)
N
0
5
10 km
Altitude (m)
1400
1200
1000
800
600
400

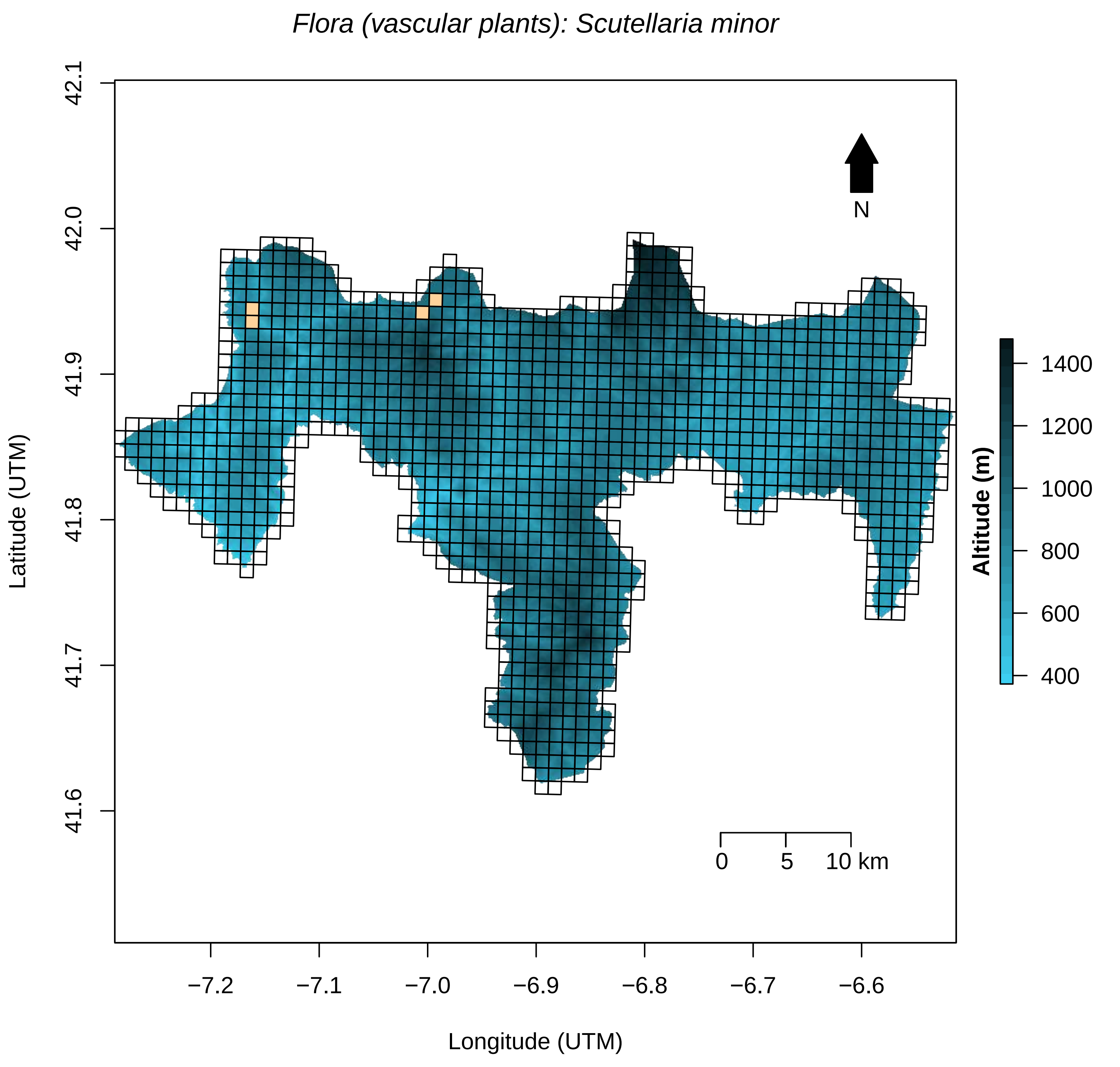
Flora (vascular plants): Scutellaria minor
42.1
42.0
41.9
41.8
41.7
41.6
Latitude (UTM)
−7.2
−7.1
−7.0
−6.9
−6.8
−6.7
−6.6
Longitude (UTM)
N
0
5
10 km
Altitude (m)
1400
1200
1000
800
600
400

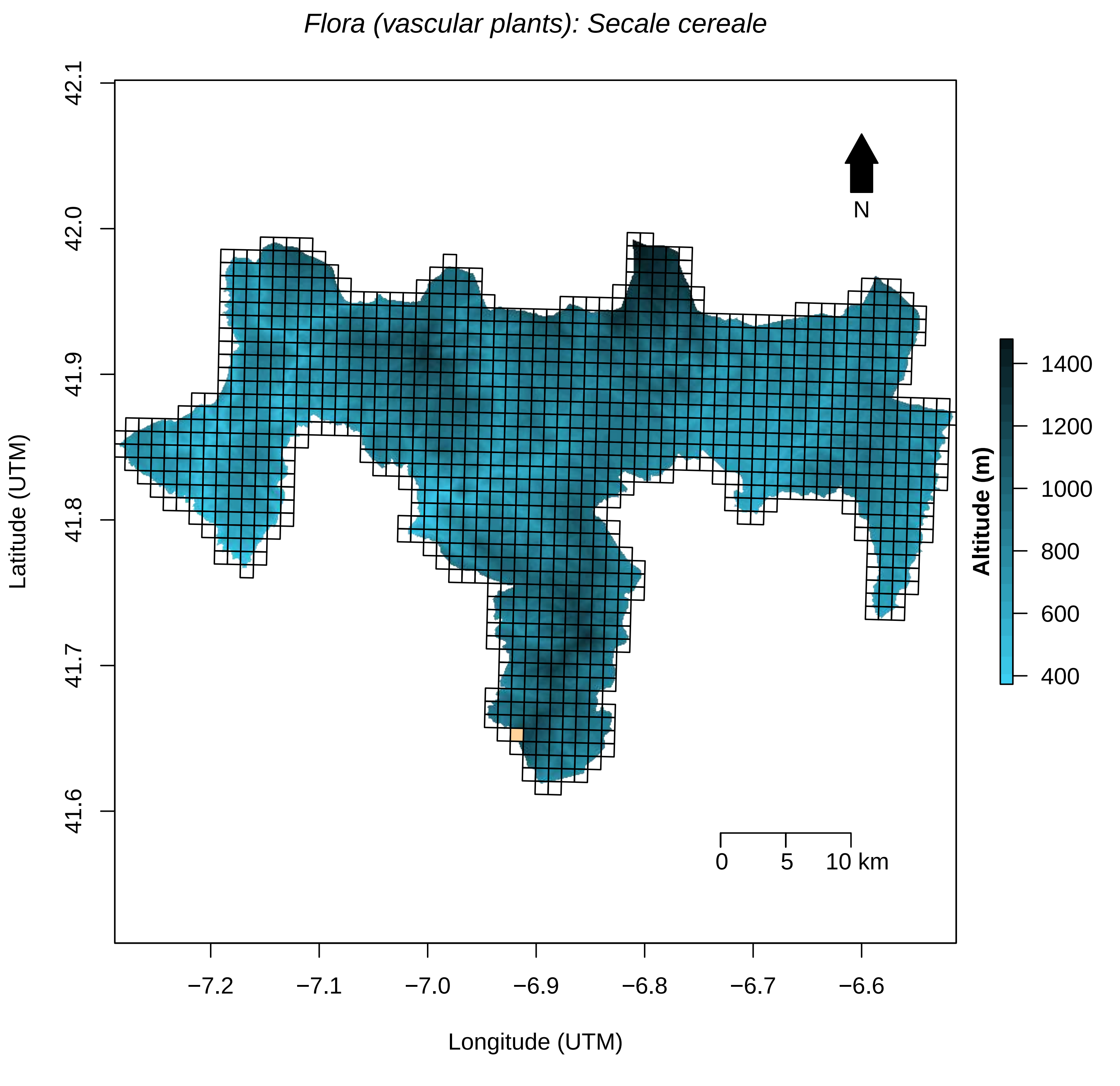

Flora (vascular plants): Secale cereale
Latitude (UTM)
42.1
42.0
41.9
41.8
41.7
41.6
Longitude (UTM)
−7.2
−7.1
−7.0
−6.9
−6.8
−6.7
−6.6
N
0
5
10 km
Altitude (m)
1400
1200
1000
800
600
400

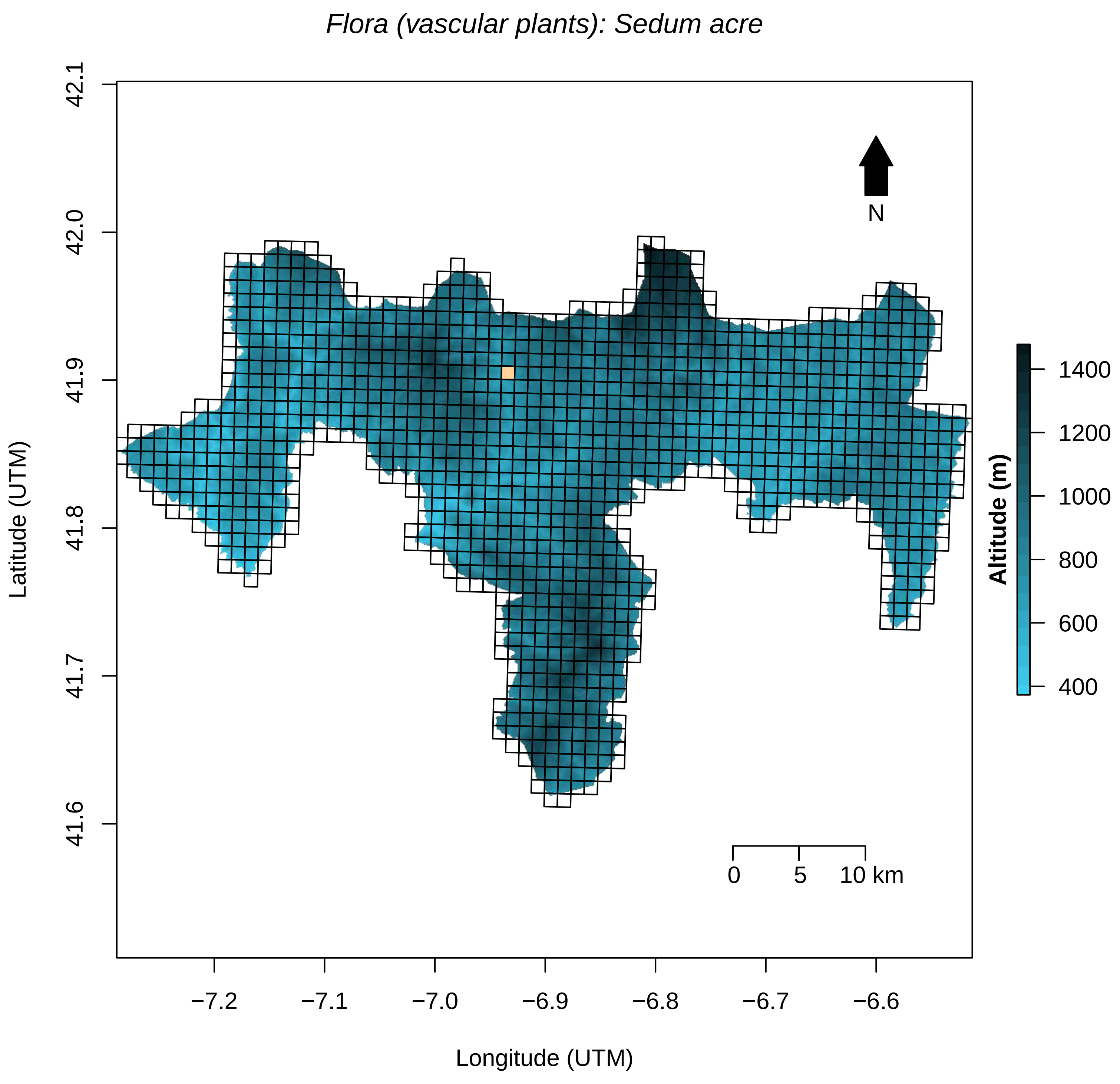
Flora (vascular plants): Sedum acre
N
0
5
10 km
Altitude (m)
1400
1200
1000
800
600
400
42.1
42.0
41.9
41.8
41.7
41.6
Latitude (UTM)
−7.2
−7.1
−7.0
−6.9
−6.8
−6.7
−6.6
Longitude (UTM)

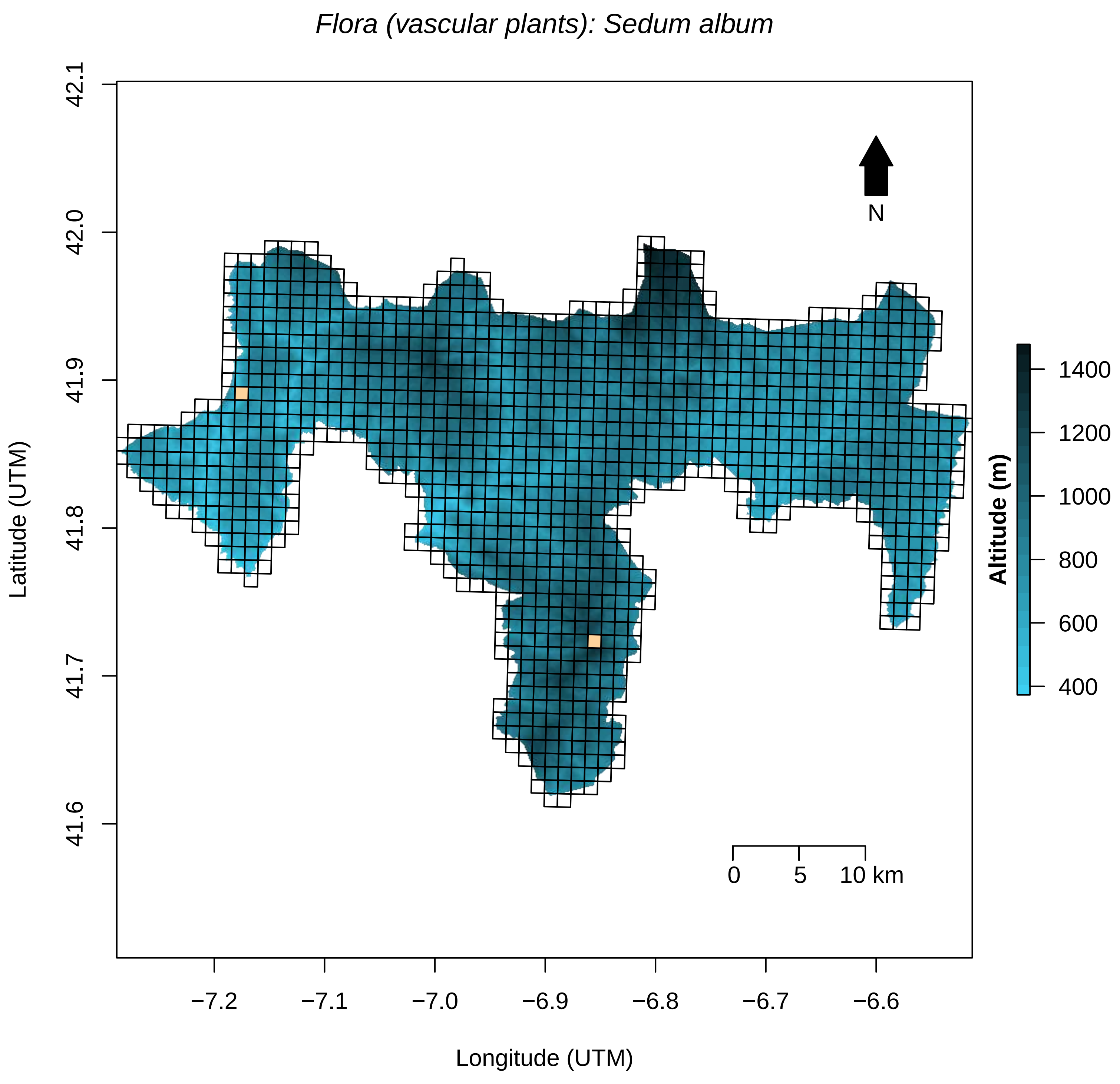
Flora (vascular plants): Sedum album
42.1
42.0
41.9
41.8
41.7
41.6
Latitude (UTM)
−7.2
−7.1
−7.0
−6.9
−6.8
−6.7
−6.6
Longitude (UTM)
N
0
5
10 km
Altitude (m)
1400
1200
1000
800
600
400

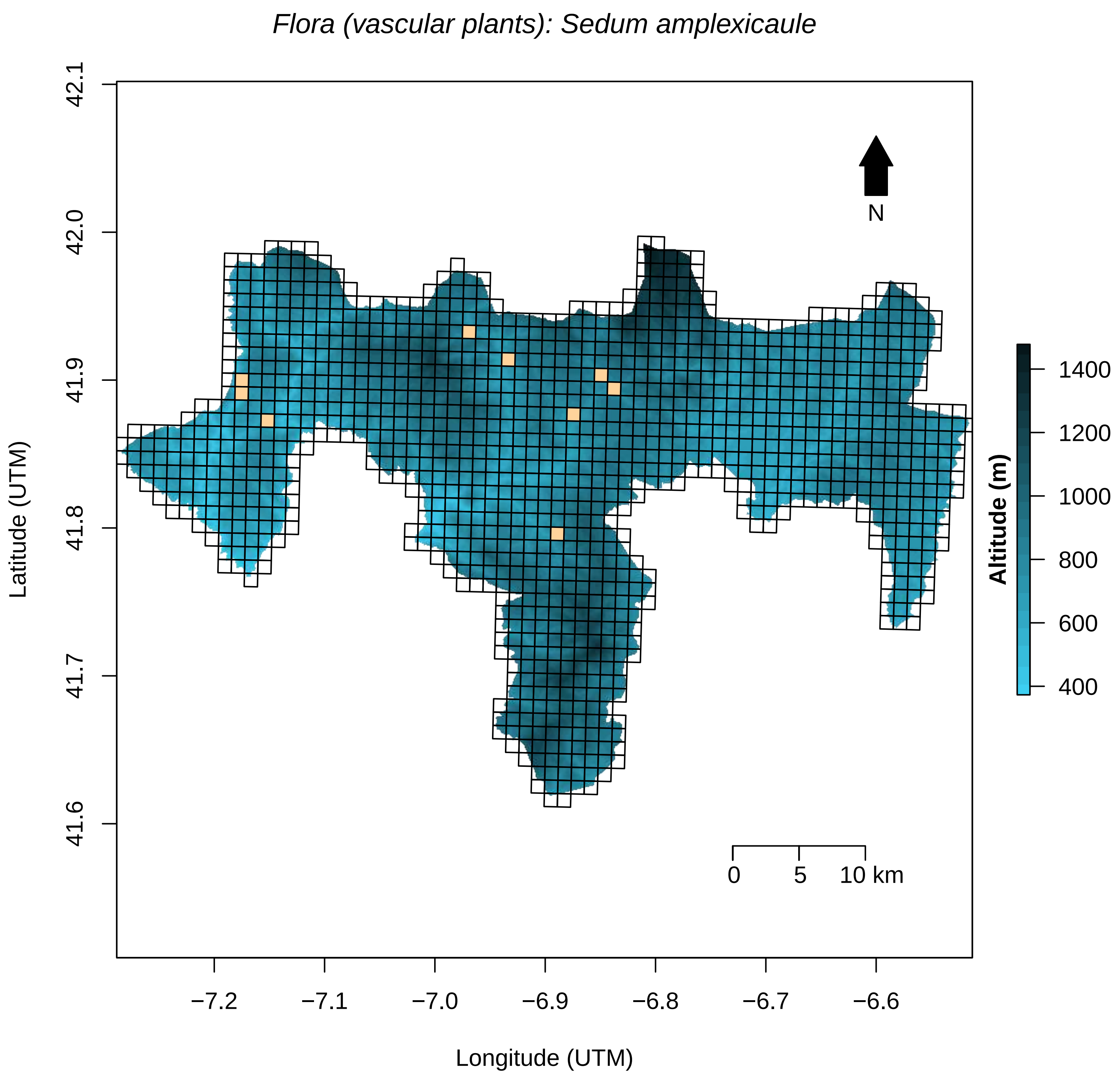

Flora (vascular plants): Sedum amplexicaule
42.1
42.0
41.9
41.8
41.7
41.6
Latitude (UTM)
−7.2
−7.1
−7.0
−6.9
−6.8
−6.7
−6.6
Longitude (UTM)
N
0
5
10 km
Altitude (m)
1400
1200
1000
800
600
400

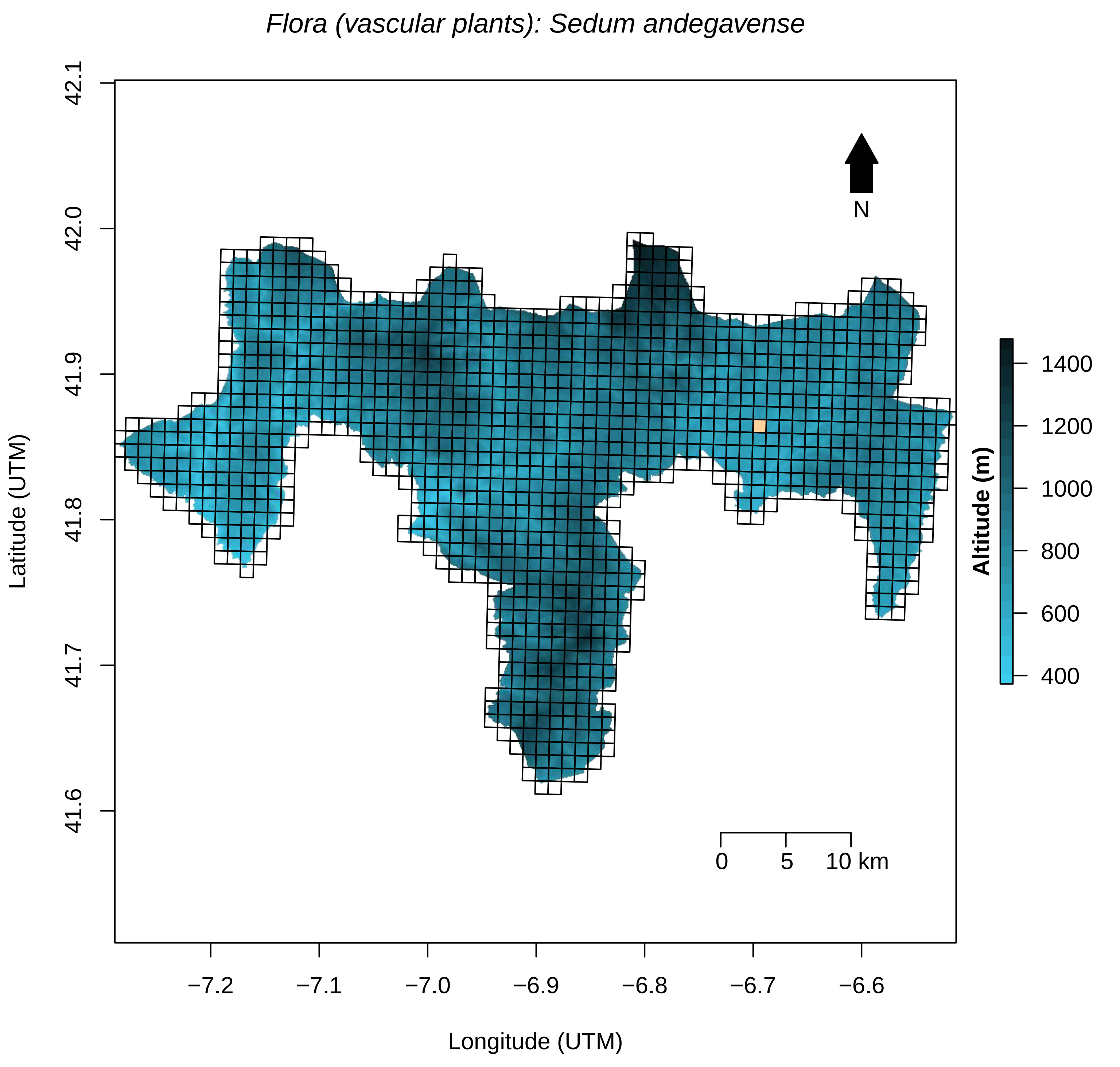

Flora (vascular plants): Sedum andegavense
Latitude (UTM)
42.1
42.0
41.9
41.8
41.7
41.6
Longitude (UTM)
−7.2
−7.1
−7.0
−6.9
−6.8
−6.7
−6.6
N
0
5
10 km
Altitude (m)
1400
1200
1000
800
600
400

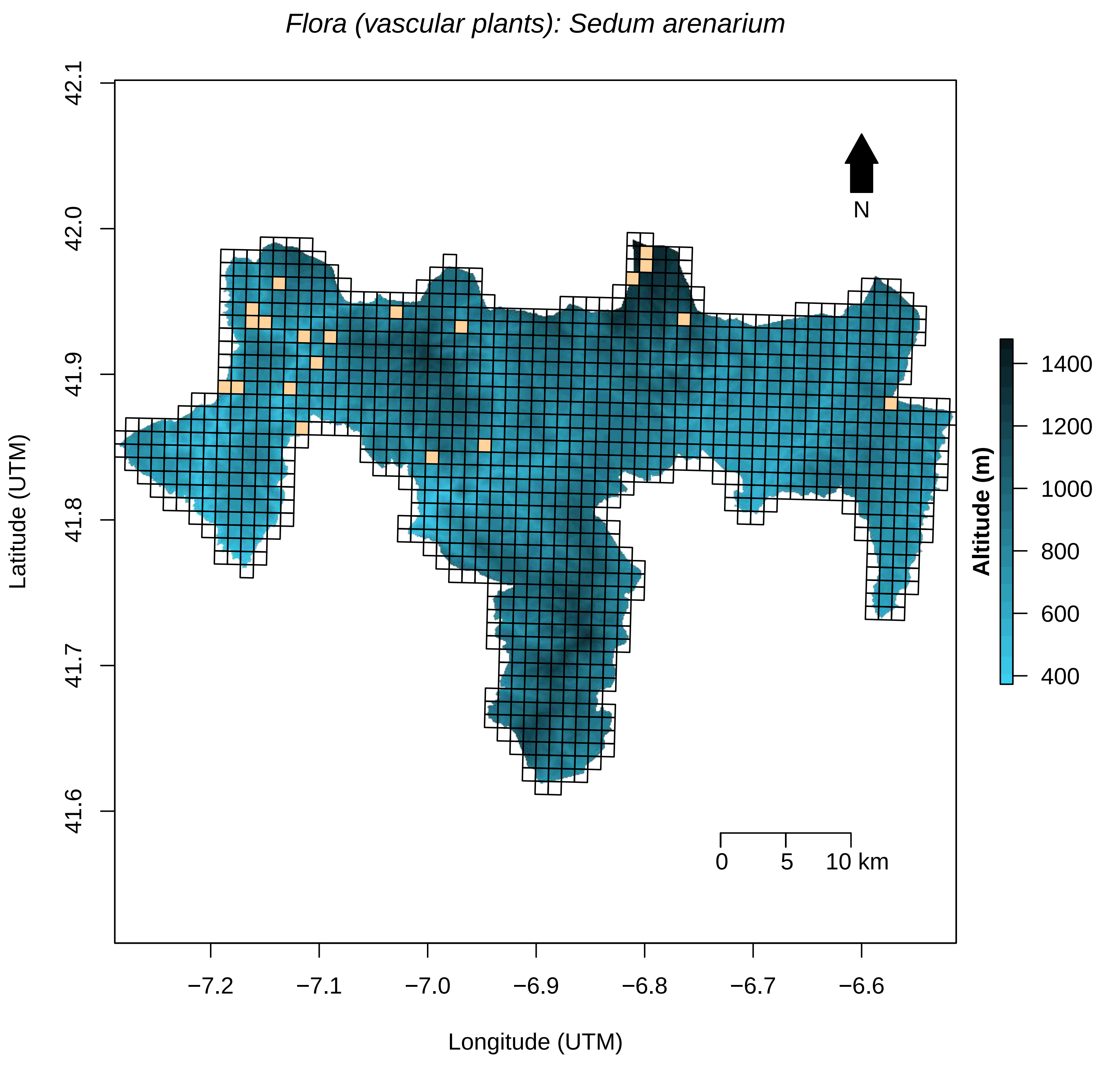
Flora (vascular plants): Sedum arenarium
42.1
42.0
41.9
41.8
41.7
41.6
Latitude (UTM)
−7.2
−7.1
−7.0
−6.9
−6.8
−6.7
−6.6
Longitude (UTM)
N
0
5
10 km
Altitude (m)
1400
1200
1000
800
600
400

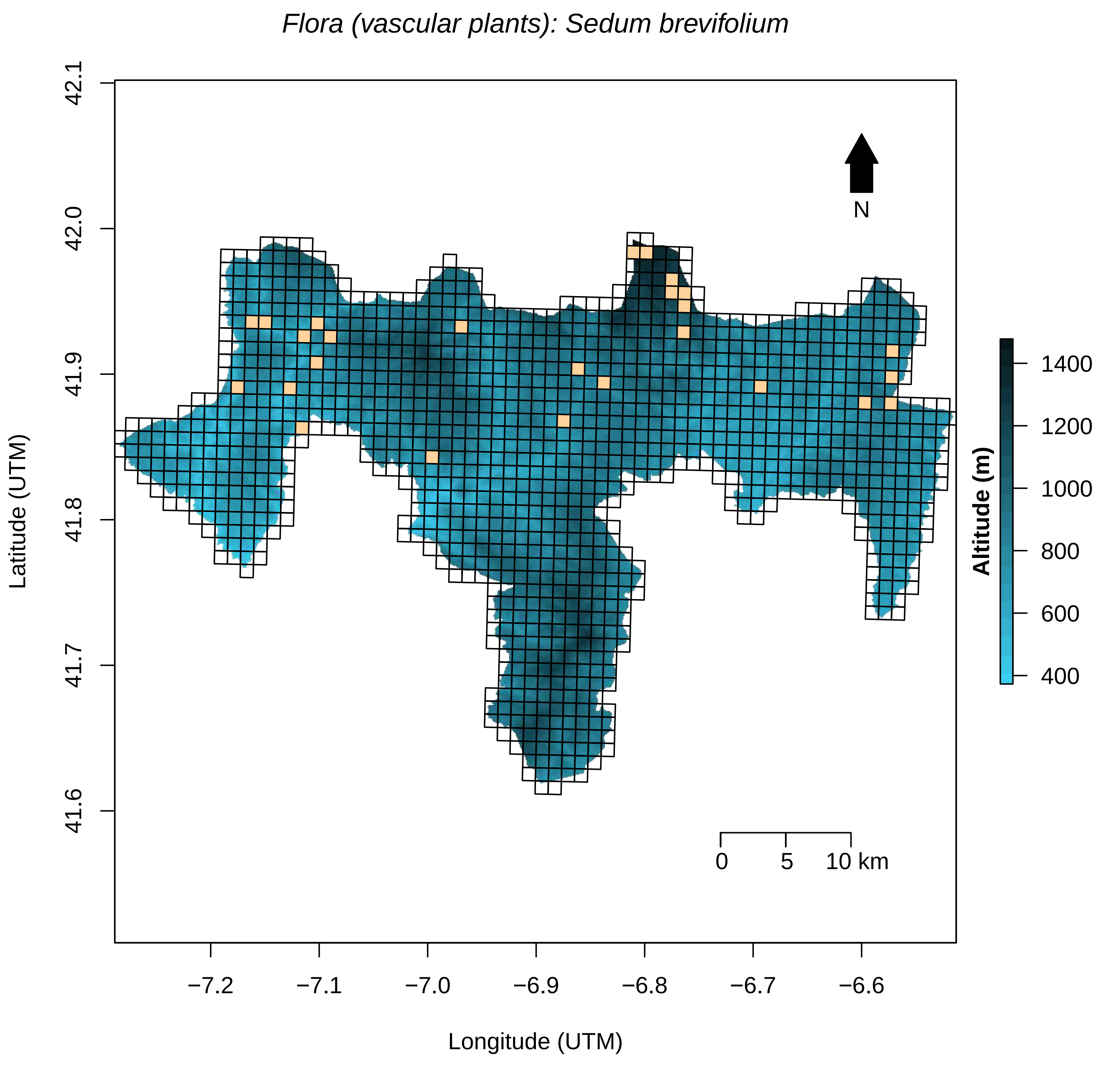
Flora (vascular plants): Sedum brevifolium
42.1
42.0
41.9
41.8
41.7
41.6
Latitude (UTM)
−7.2
−7.1
−7.0
−6.9
−6.8
−6.7
−6.6
Longitude (UTM)
N
0
5
10 km
Altitude (m)
1400
1200
1000
800
600
400

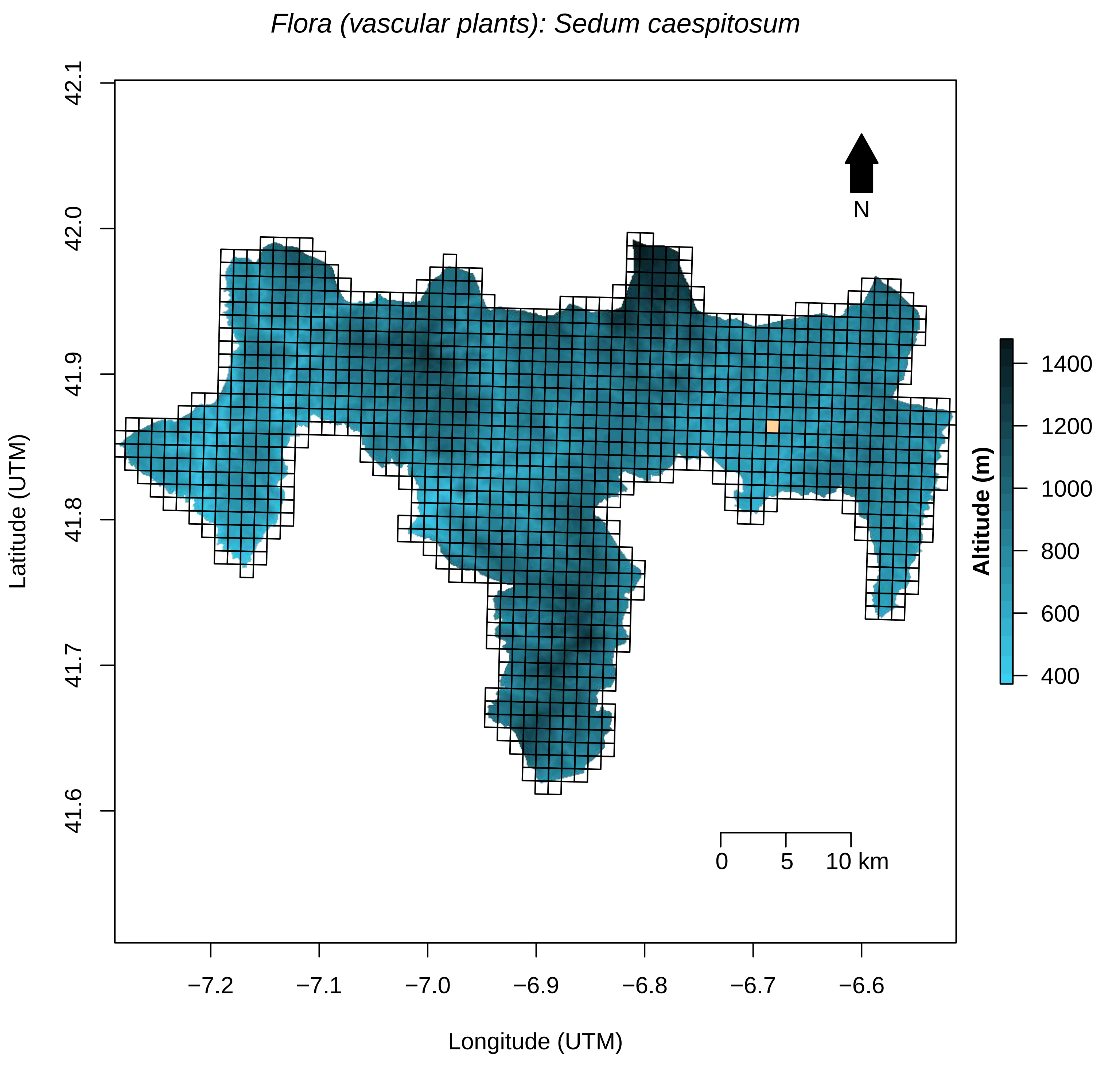

Flora (vascular plants): Sedum caespitosum
N
0
5
10 km
Latitude (UTM)
Longitude (UTM)
Altitude (m)
42.1
42.0
41.9
41.8
41.7
41.6
−7.2
−7.1
−7.0
−6.9
−6.8
−6.7
−6.6
1400
1200
1000
800
600
400

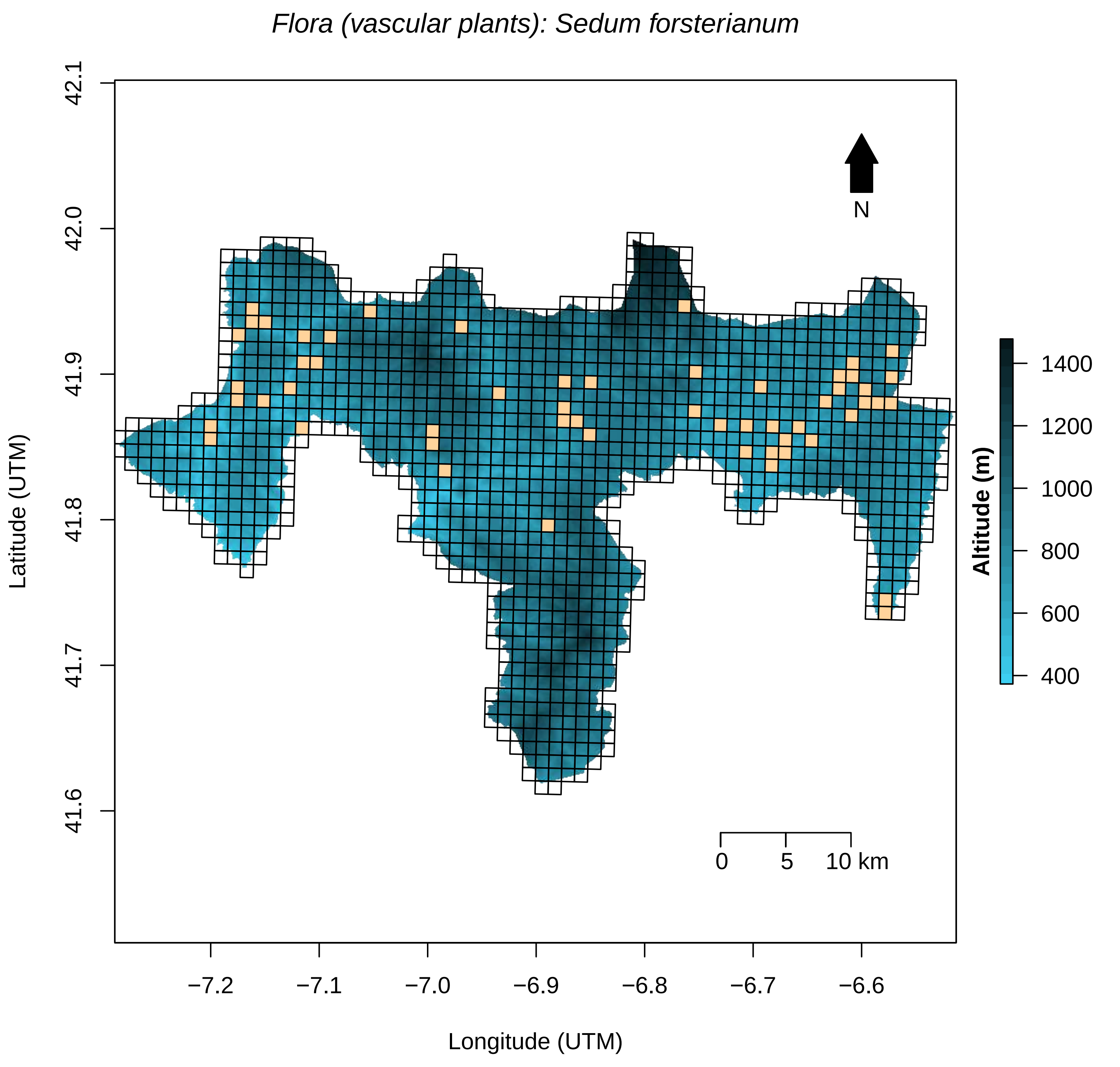
Flora (vascular plants): Sedum forsterianum
Latitude (UTM)
42.1
42.0
41.9
41.8
41.7
41.6
Longitude (UTM)
−7.2
−7.1
−7.0
−6.9
−6.8
−6.7
−6.6
N
0
5
10 km
Altitude (m)
1400
1200
1000
800
600
400

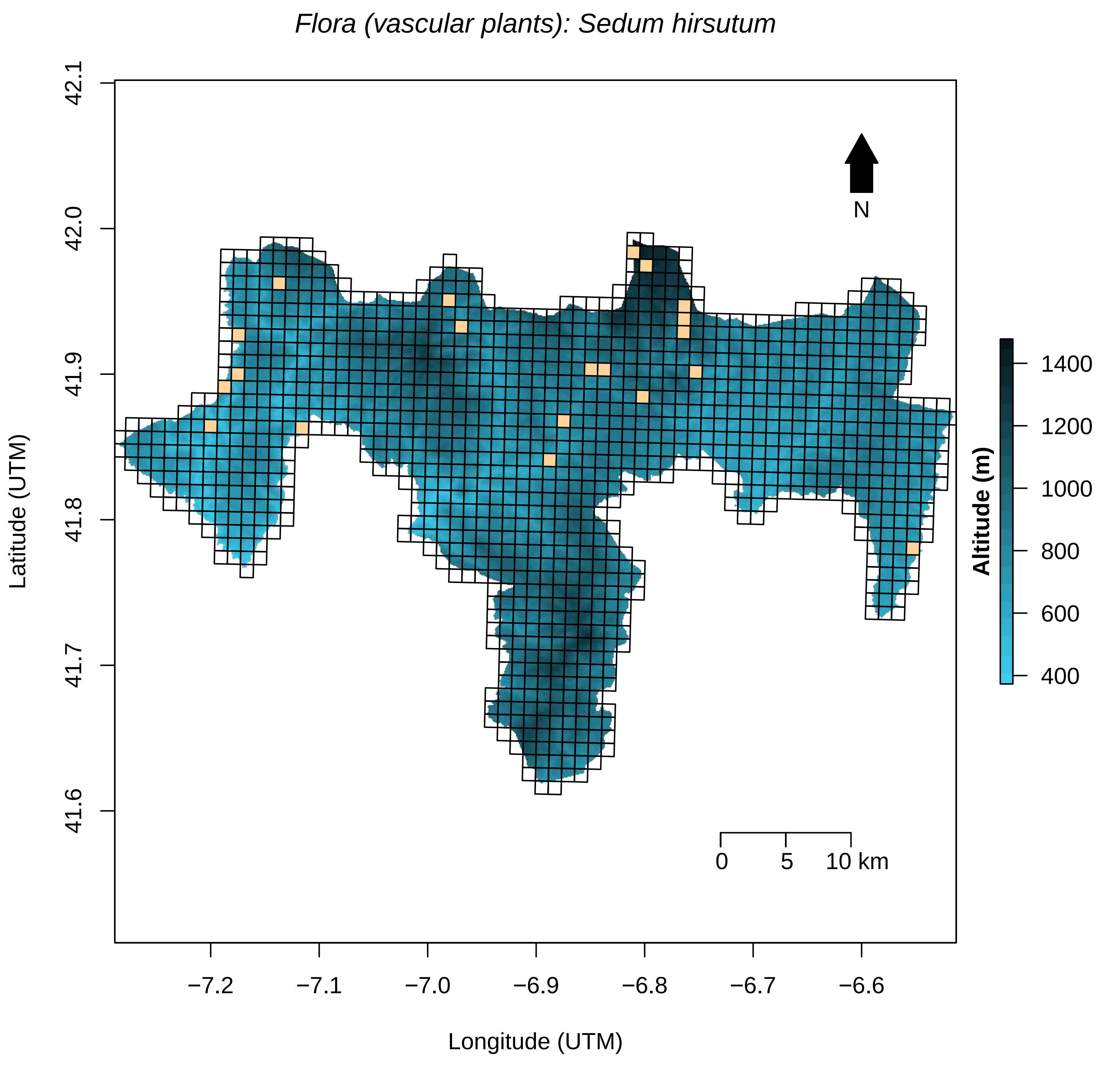

Flora (vascular plants): Sedum hirsutum
Latitude (UTM)
Longitude (UTM)
42.1
42.0
41.9
41.8
41.7
41.6
−7.2
−7.1
−7.0
−6.9
−6.8
−6.7
−6.6
N
0
5
10 km
Altitude (m)
1400
1200
1000
800
600
400

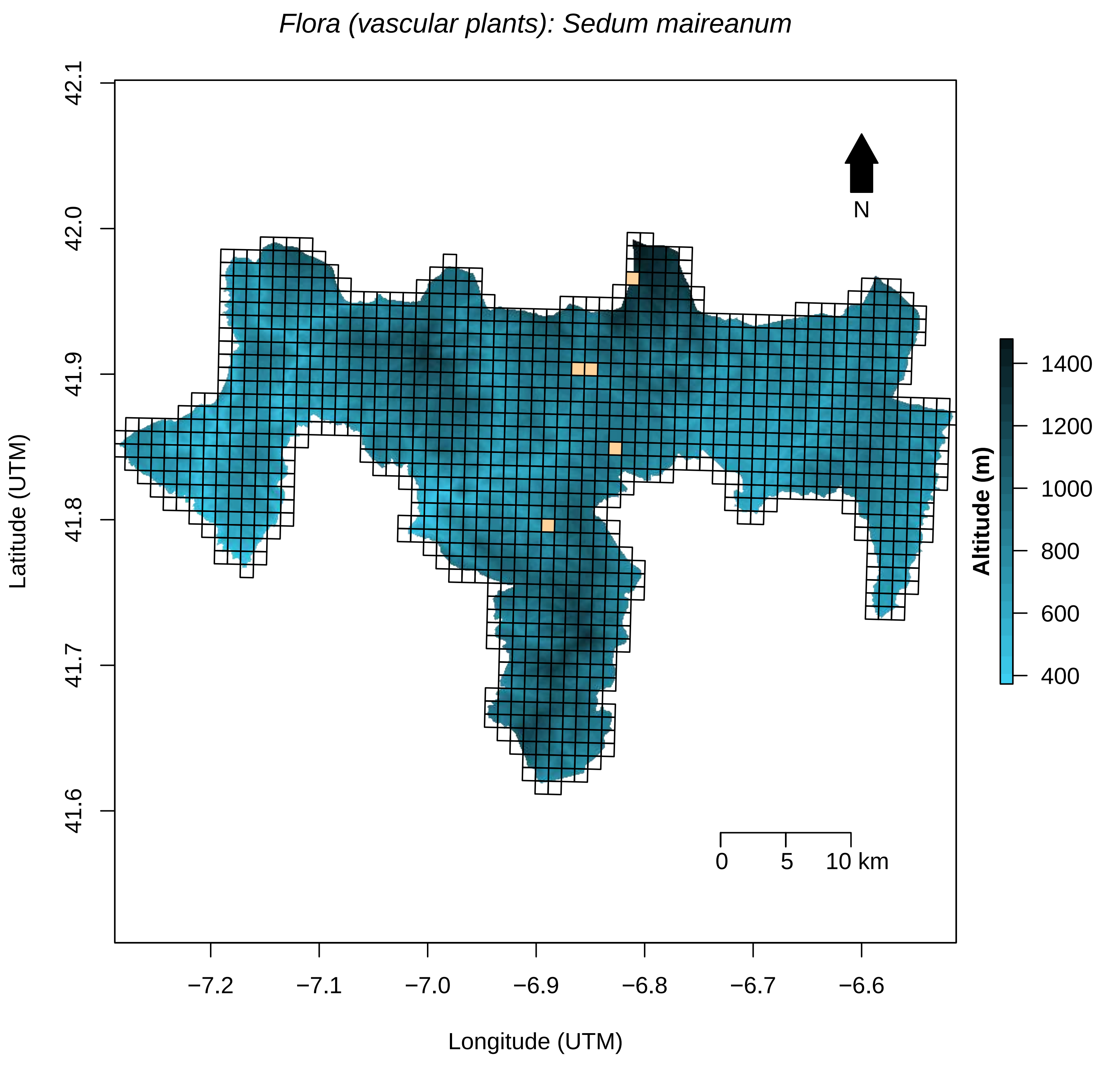
Flora (vascular plants): Sedum maireanum
N
0
5
10 km
Altitude (m)
1400
1200
1000
800
600
400
Latitude (UTM)
42.1
42.0
41.9
41.8
41.7
41.6
Longitude (UTM)
−7.2
−7.1
−7.0
−6.9
−6.8
−6.7
−6.6

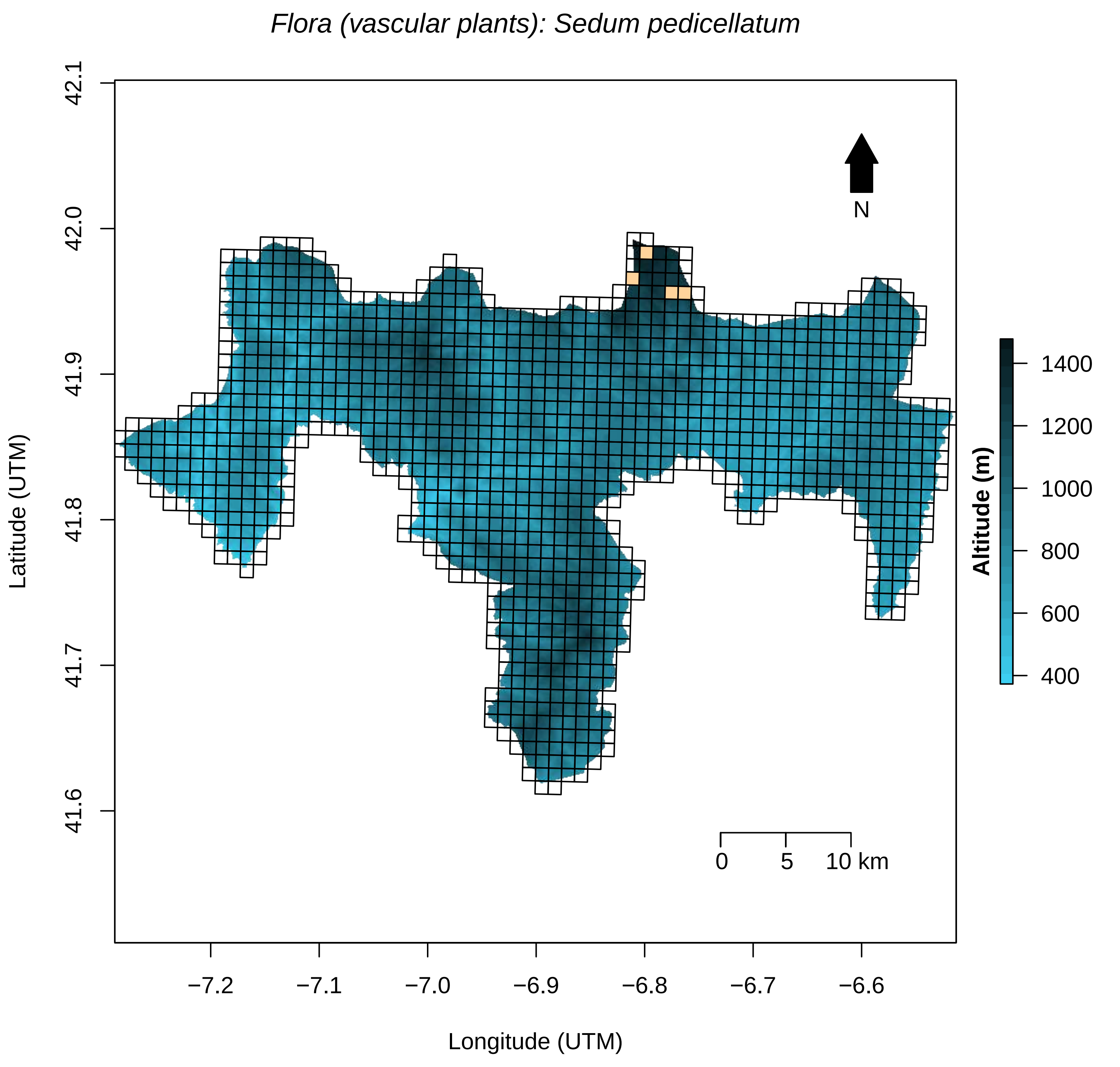

Flora (vascular plants): Sedum pedicellatum
N
0
5
10 km
Altitude (m)
1400
1200
1000
800
600
400
Latitude (UTM)
42.1
42.0
41.9
41.8
41.7
41.6
Longitude (UTM)
−7.2
−7.1
−7.0
−6.9
−6.8
−6.7
−6.6

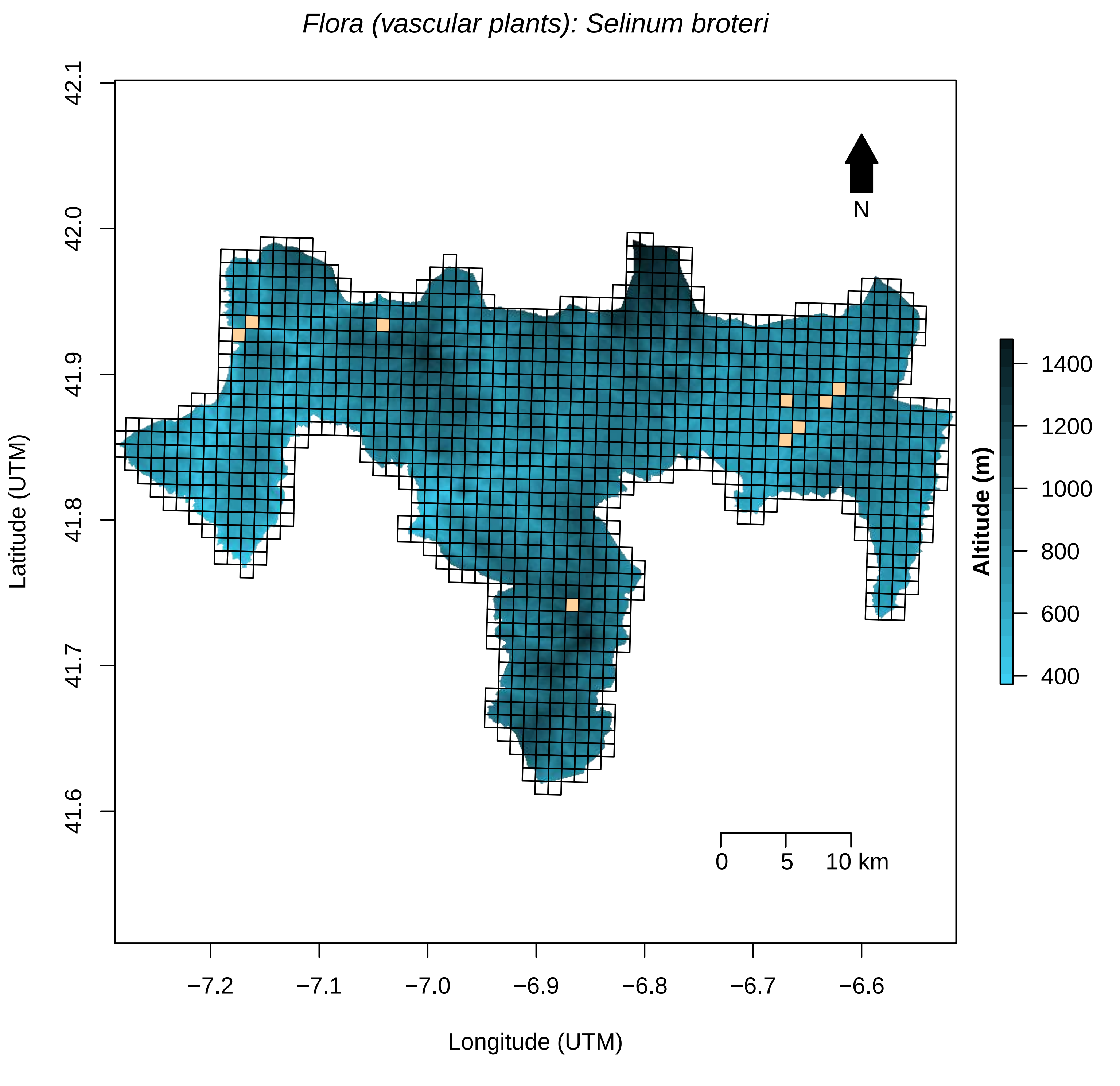

Flora (vascular plants): Selinum broteri
42.1
42.0
41.9
41.8
41.7
41.6
Latitude (UTM)
−7.2
−7.1
−7.0
−6.9
−6.8
−6.7
−6.6
Longitude (UTM)
N
0
5
10 km
Altitude (m)
1400
1200
1000
800
600
400

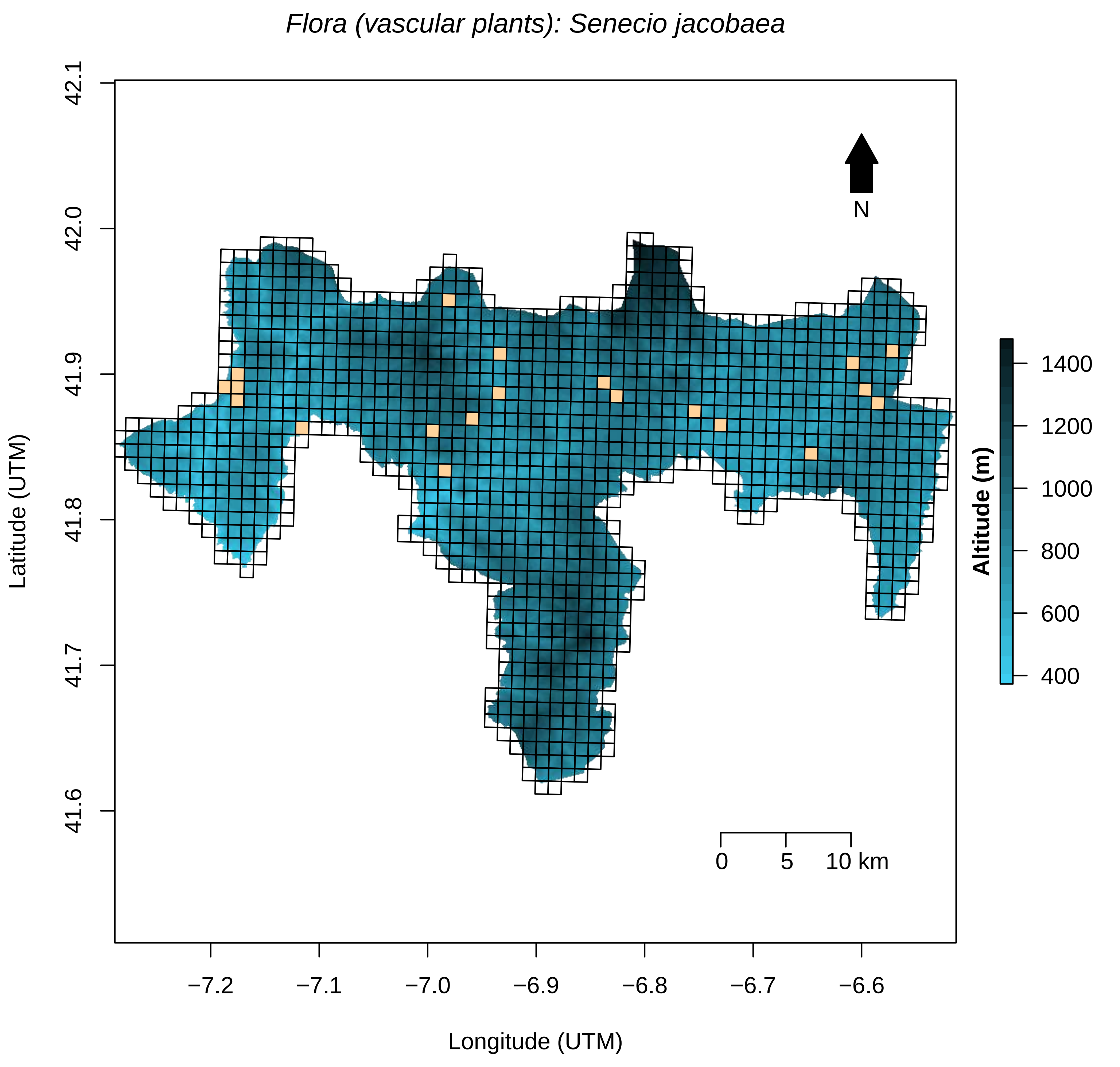
Flora (vascular plants): Senecio jacobaea
N
0
5
10 km
Altitude (m)
1400
1200
1000
800
600
400
Latitude (UTM)
42.1
42.0
41.9
41.8
41.7
41.6
Longitude (UTM)
−7.2
−7.1
−7.0
−6.9
−6.8
−6.7
−6.6

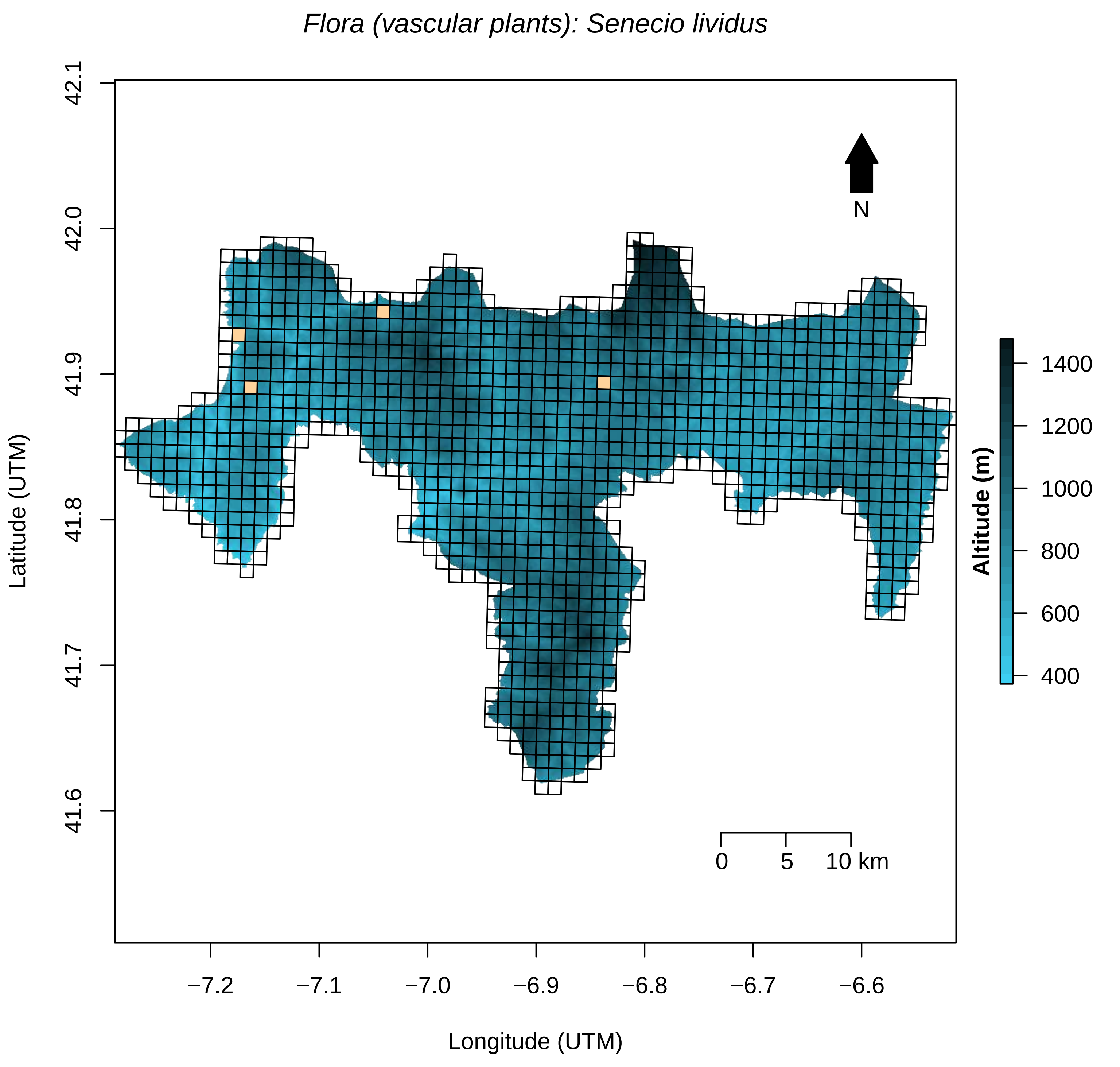
Flora (vascular plants): Senecio lividus
Latitude (UTM)
Longitude (UTM)
42.1
42.0
41.9
41.8
41.7
41.6
−7.2
−7.1
−7.0
−6.9
−6.8
−6.7
−6.6
N
0
5
10 km
Altitude (m)
1400
1200
1000
800
600
400

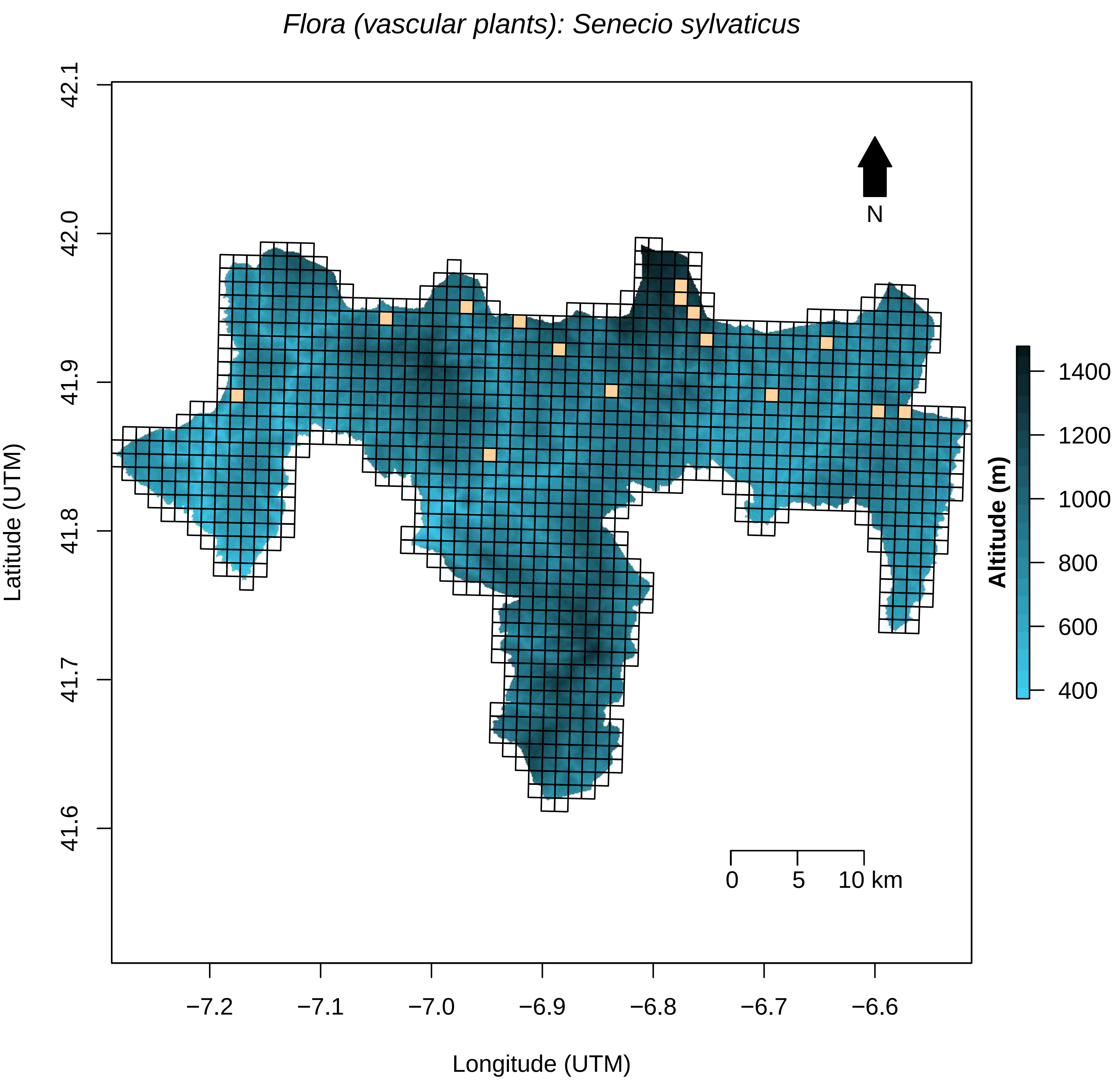

Flora (vascular plants): Senecio sylvaticus
42.1
42.0
41.9
41.8
41.7
41.6
Latitude (UTM)
−7.2
−7.1
−7.0
−6.9
−6.8
−6.7
−6.6
Longitude (UTM)
N
0
5
10 km
Altitude (m)
1400
1200
1000
800
600
400

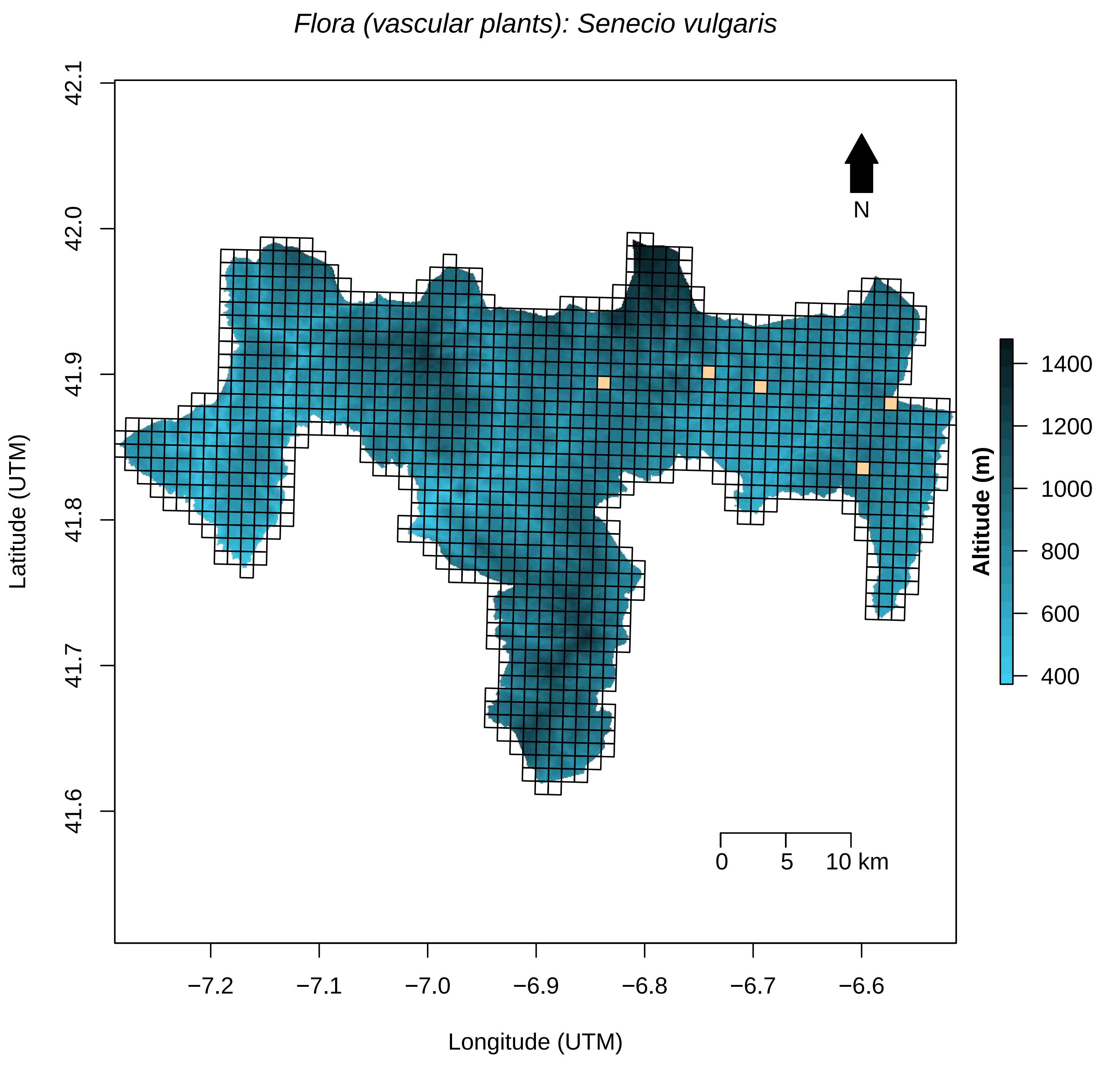

Flora (vascular plants): Senecio vulgaris
42.1
42.0
41.9
41.8
41.7
41.6
Latitude (UTM)
−7.2
−7.1
−7.0
−6.9
−6.8
−6.7
−6.6
Longitude (UTM)
N
0
5
10 km
Altitude (m)
1400
1200
1000
800
600
400

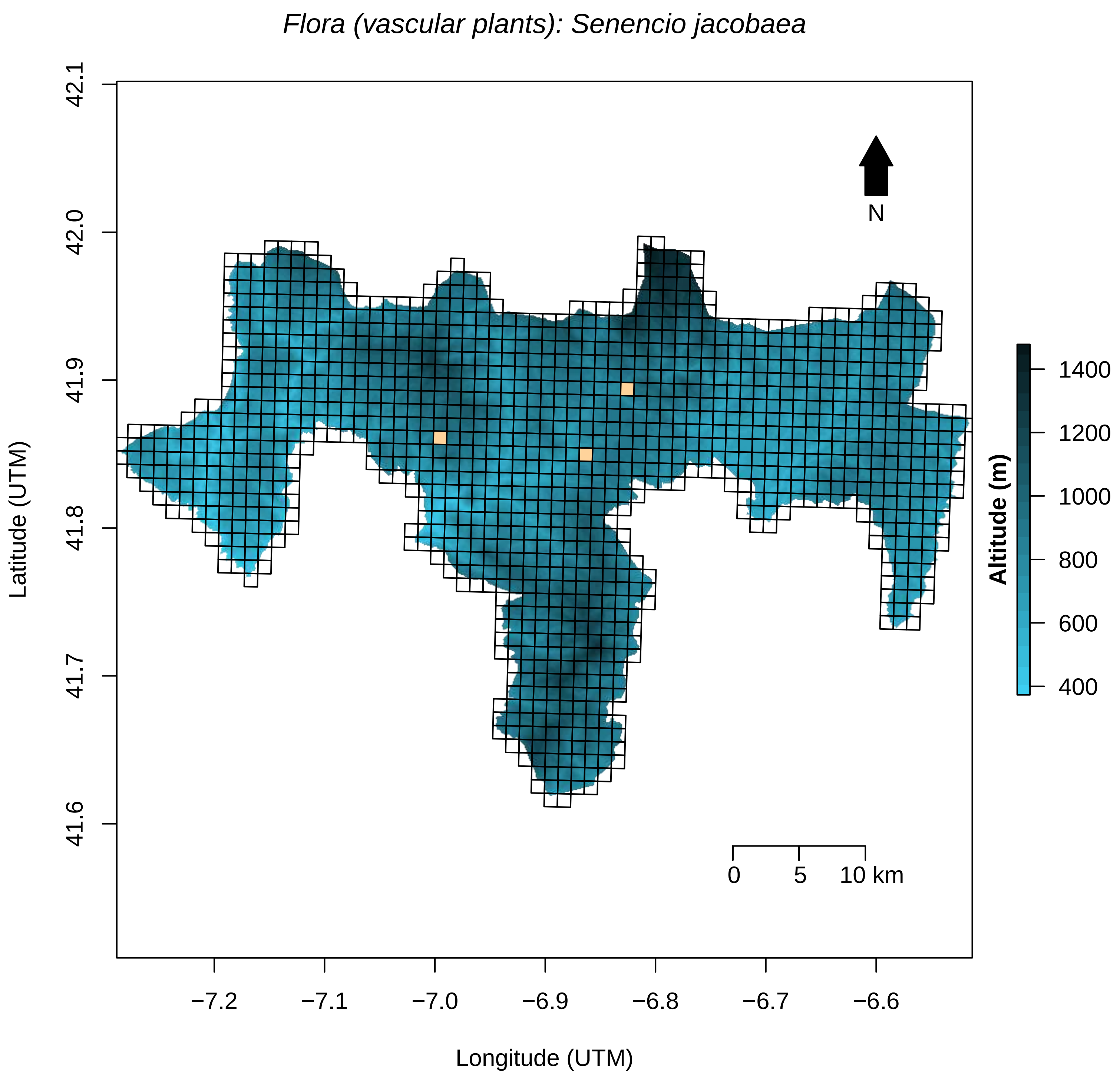

Flora (vascular plants): Senencio jacobaea
42.1
42.0
41.9
41.8
41.7
41.6
Latitude (UTM)
−7.2
−7.1
−7.0
−6.9
−6.8
−6.7
−6.6
Longitude (UTM)
N
0
5
10 km
Altitude (m)
1400
1200
1000
800
600
400

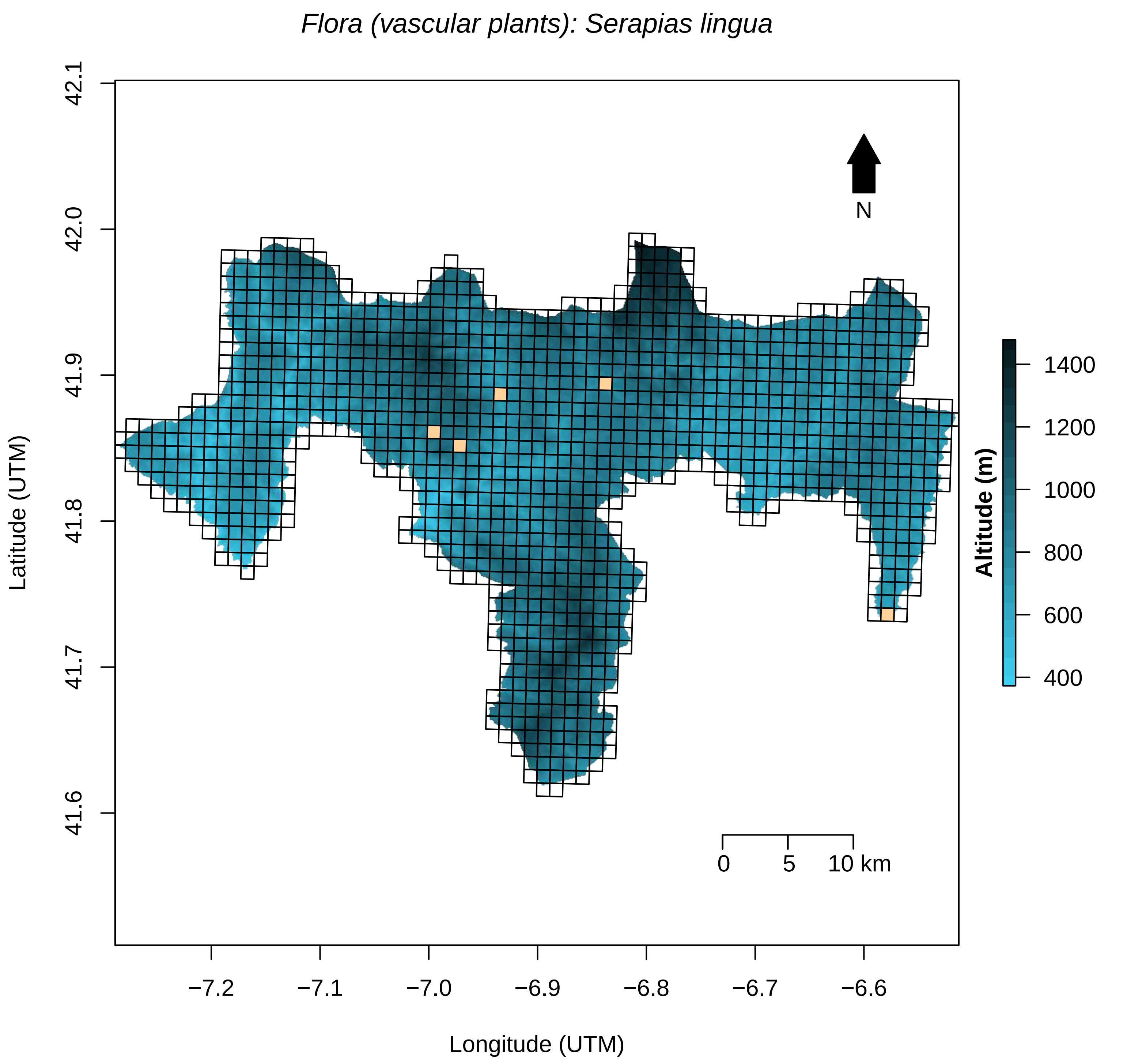
Flora (vascular plants): Serapias lingua
42.1
42.0
41.9
41.8
41.7
41.6
Latitude (UTM)
−7.2
−7.1
−7.0
−6.9
−6.8
−6.7
−6.6
Longitude (UTM)
N
0
5
10 km
Altitude (m)
1400
1200
1000
800
600
400

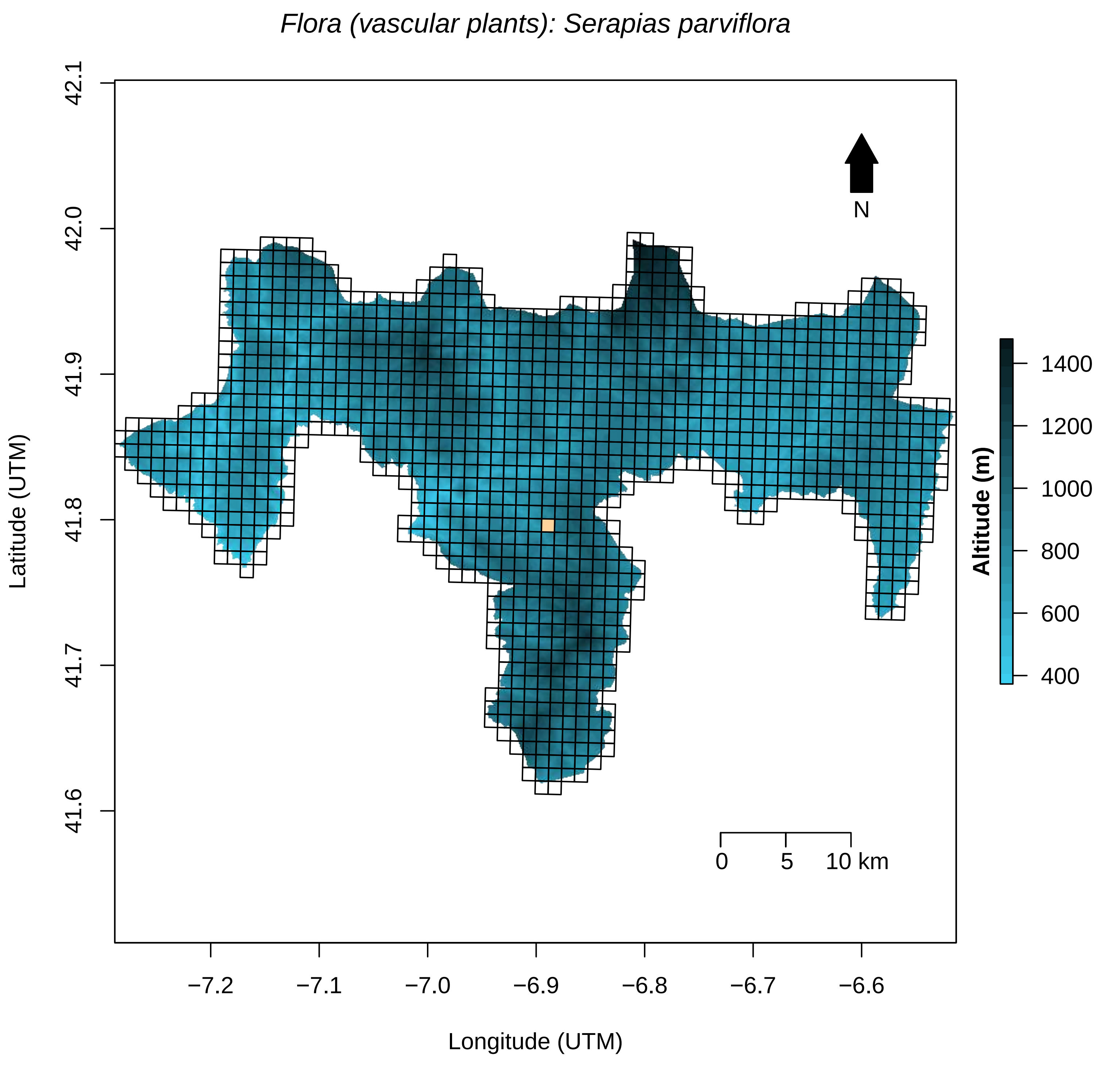
Flora (vascular plants): Serapias parviflora
42.1
42.0
41.9
41.8
41.7
41.6
Latitude (UTM)
−7.2
−7.1
−7.0
−6.9
−6.8
−6.7
−6.6
Longitude (UTM)
N
0
5
10 km
Altitude (m)
1400
1200
1000
800
600
400

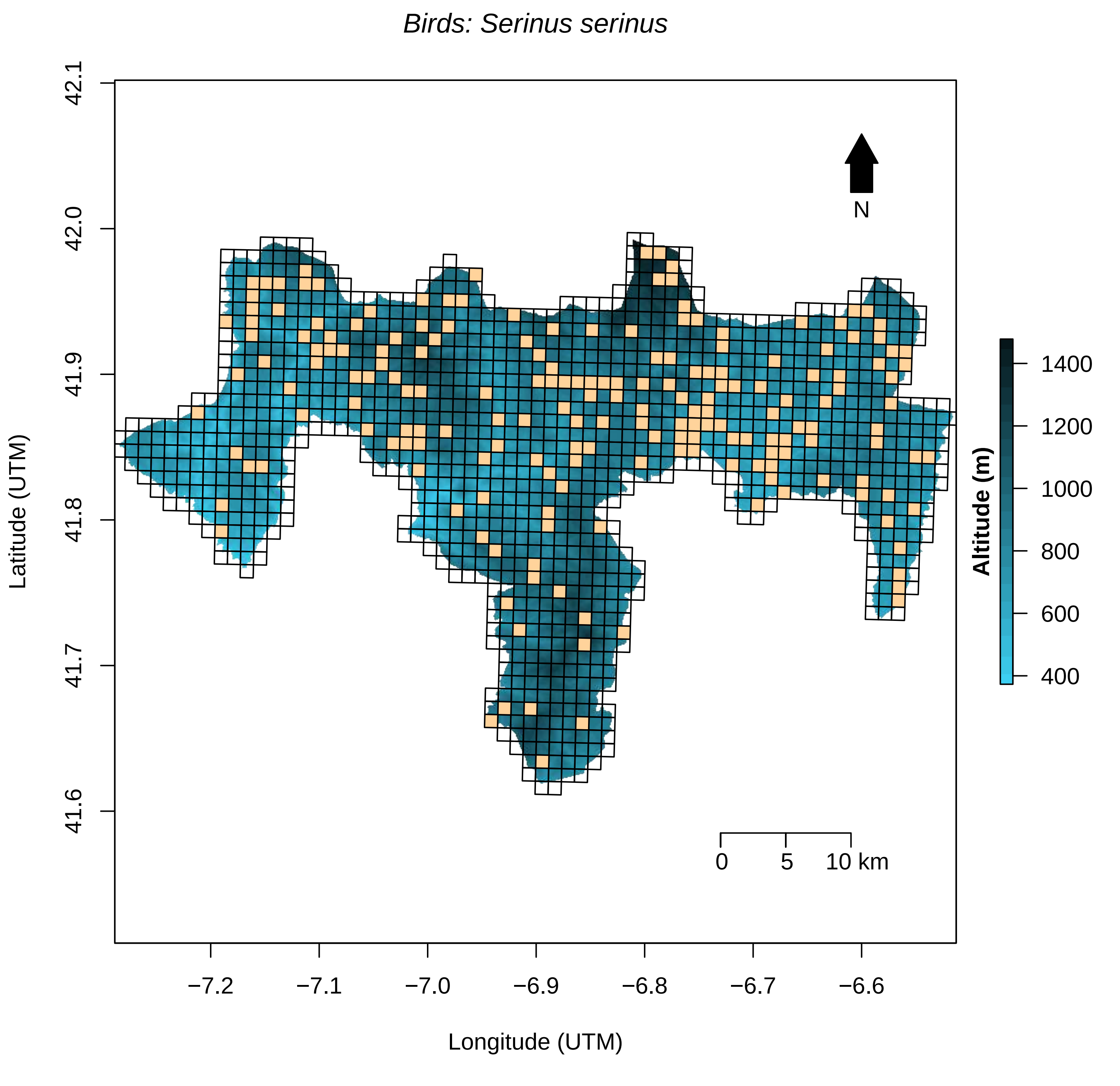

Birds: Serinus serinus
N
Latitude (UTM)
42.1
42.0
41.9
41.8
41.7
41.6
Longitude (UTM)
−7.2
−7.1
−7.0
−6.9
−6.8
−6.7
−6.6
0
5
10 km
Altitude (m)
1400
1200
1000
800
600
400

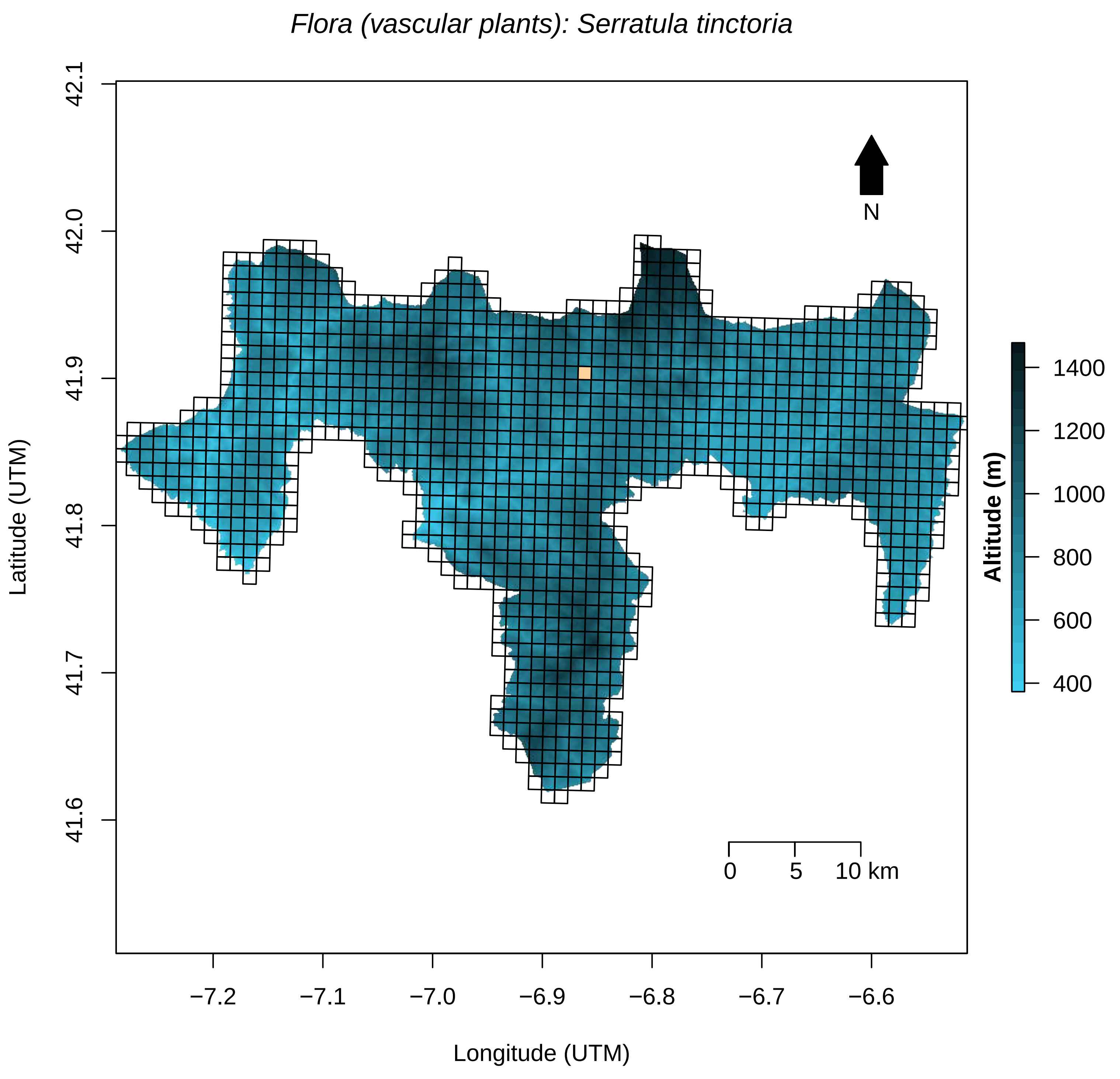
Flora (vascular plants): Serratula tinctoria
N
0
5
10 km
Altitude (m)
1400
1200
1000
800
600
400
Latitude (UTM)
42.1
42.0
41.9
41.8
41.7
41.6
Longitude (UTM)
−7.2
−7.1
−7.0
−6.9
−6.8
−6.7
−6.6

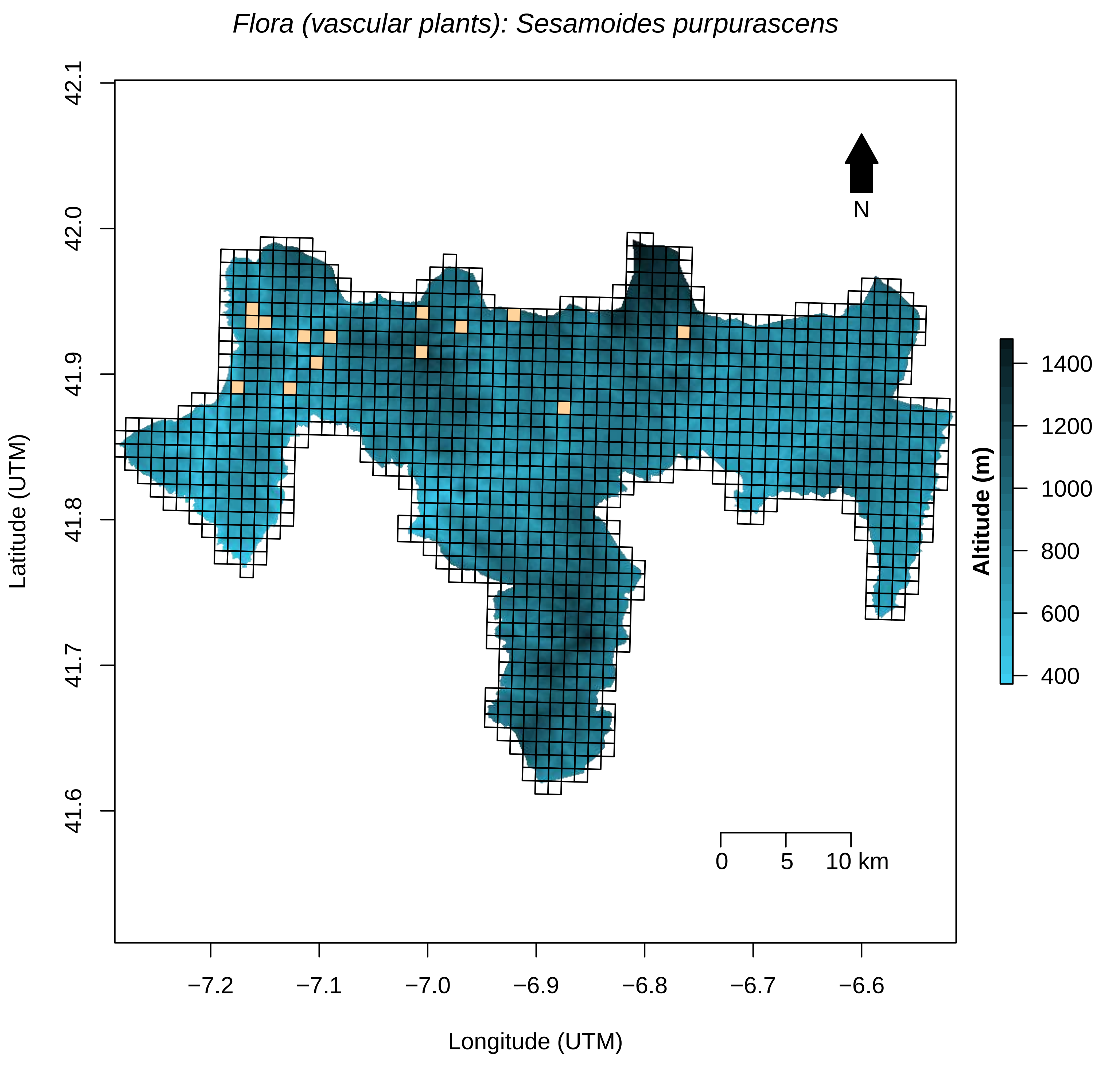
Flora (vascular plants): Sesamoides purpurascens
Latitude (UTM)
Longitude (UTM)
42.1
42.0
41.9
41.8
41.7
41.6
−7.2
−7.1
−7.0
−6.9
−6.8
−6.7
−6.6
N
0
5
10 km
Altitude (m)
1400
1200
1000
800
600
400

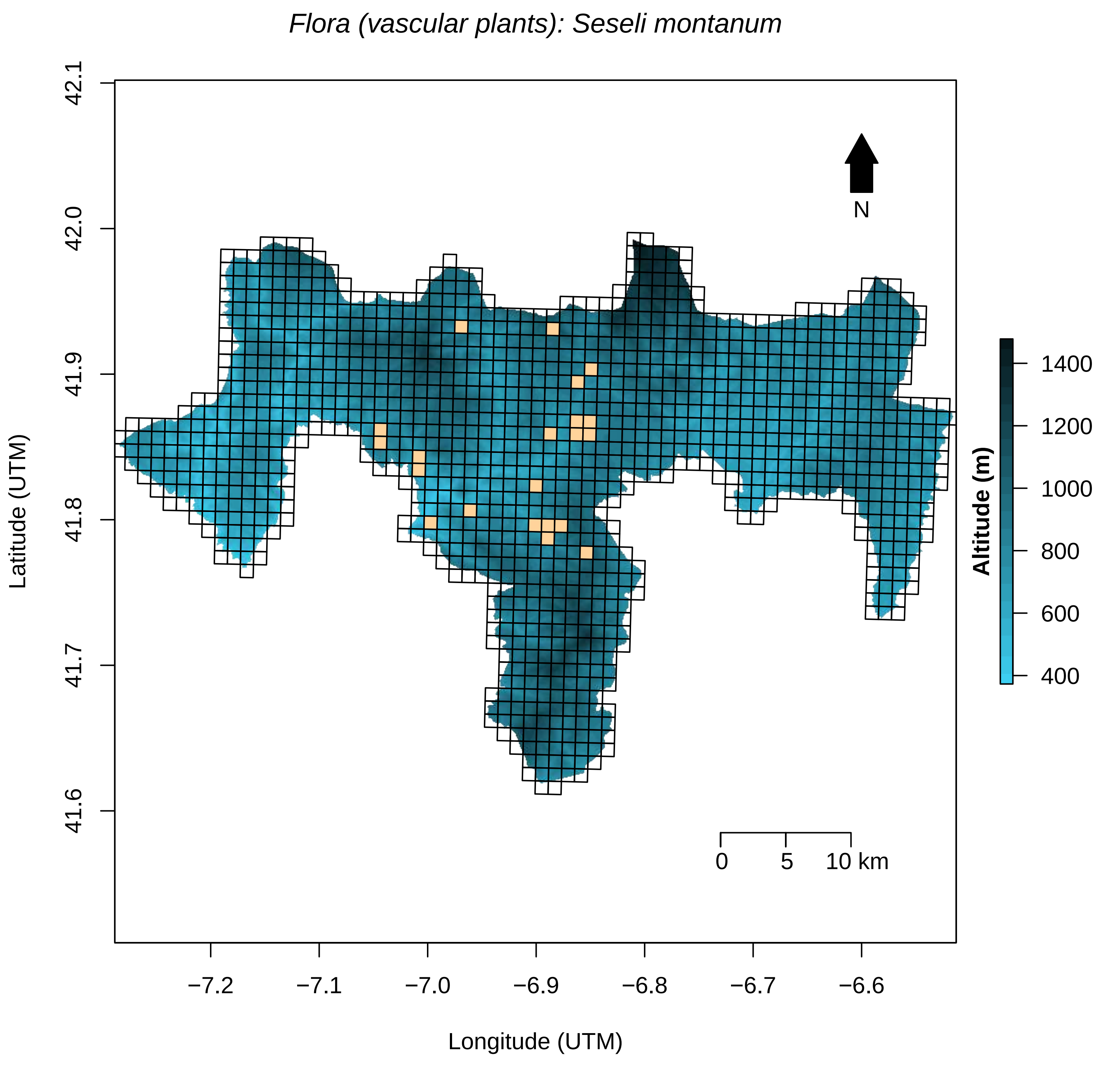

Flora (vascular plants): Seseli montanum
42.1
42.0
41.9
41.8
41.7
41.6
Latitude (UTM)
−7.2
−7.1
−7.0
−6.9
−6.8
−6.7
−6.6
Longitude (UTM)
N
0
5
10 km
Altitude (m)
1400
1200
1000
800
600
400

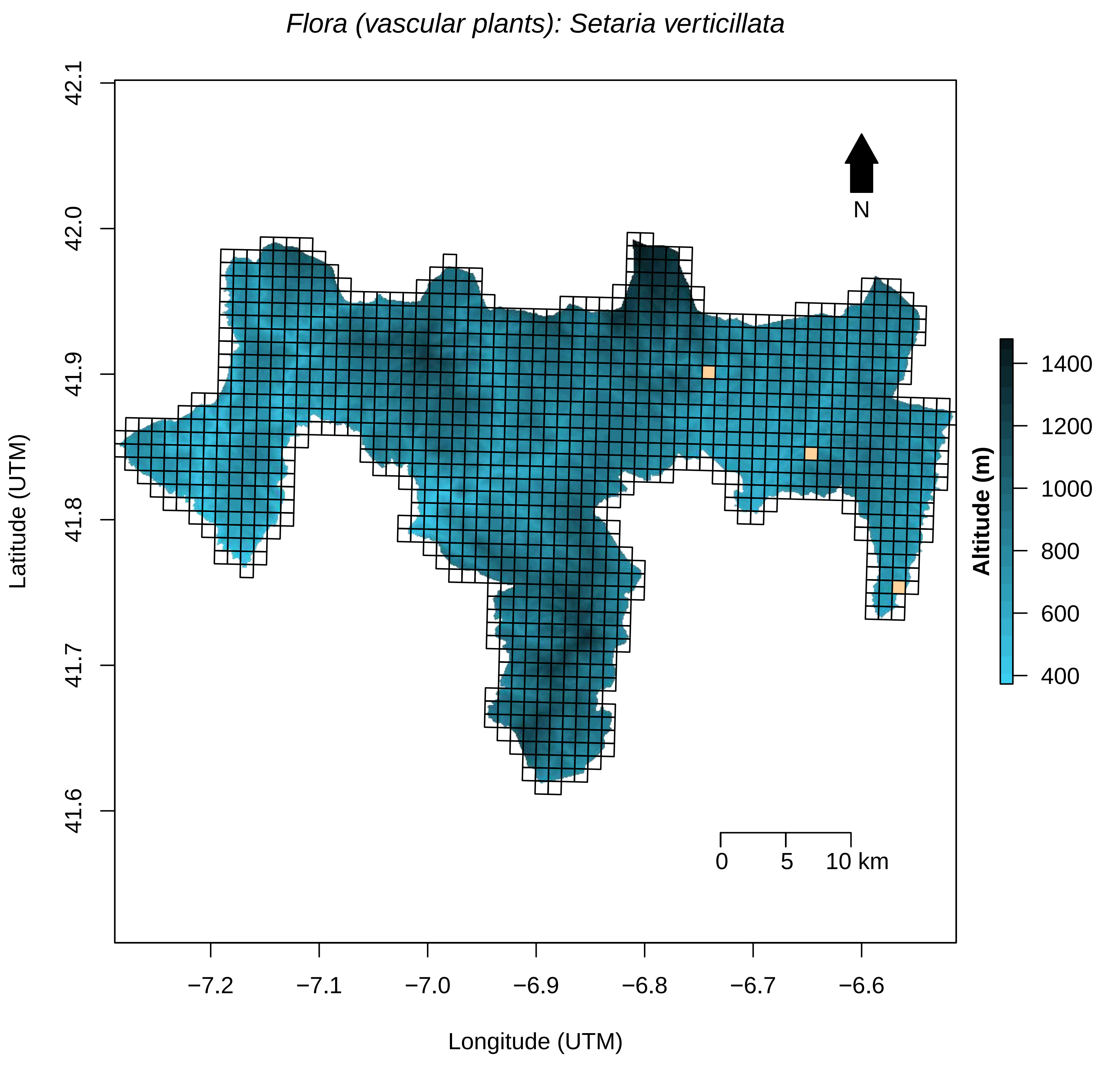
Flora (vascular plants): Setaria verticillata
N
0 5 10 km
Altitude (m)
1400
1200
1000
800
600
400
Latitude (UTM)
42.1
42.0
41.9
41.8
41.7
41.6
Longitude (UTM)
−7.2
−7.1
−7.0
−6.9
−6.8
−6.7
−6.6

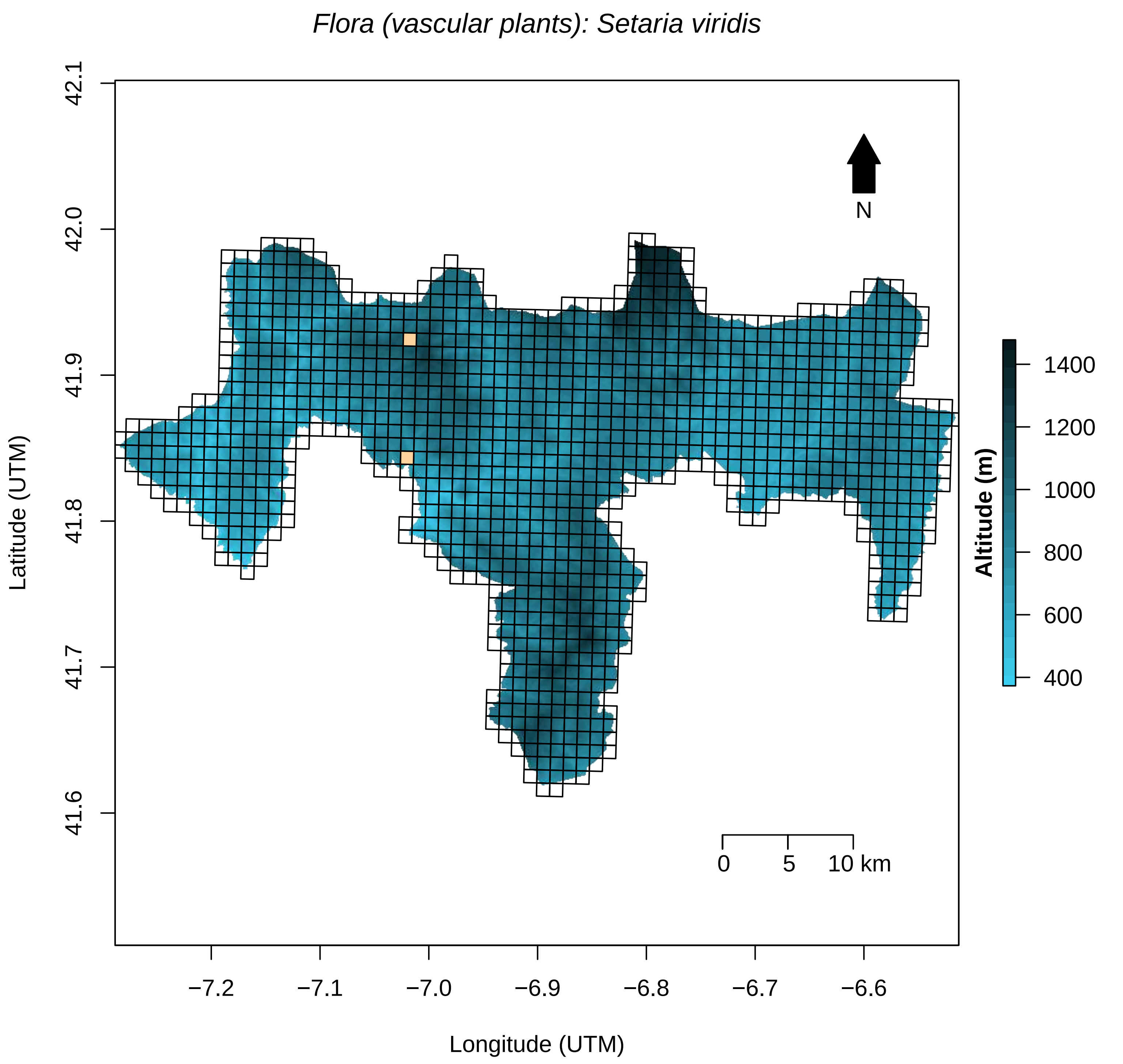
Flora (vascular plants): Setaria viridis
N
0
5
10 km
Altitude (m)
1400
1200
1000
800
600
400
42.1
42.0
41.9
41.8
41.7
41.6
Latitude (UTM)
−7.2
−7.1
−7.0
−6.9
−6.8
−6.7
−6.6
Longitude (UTM)

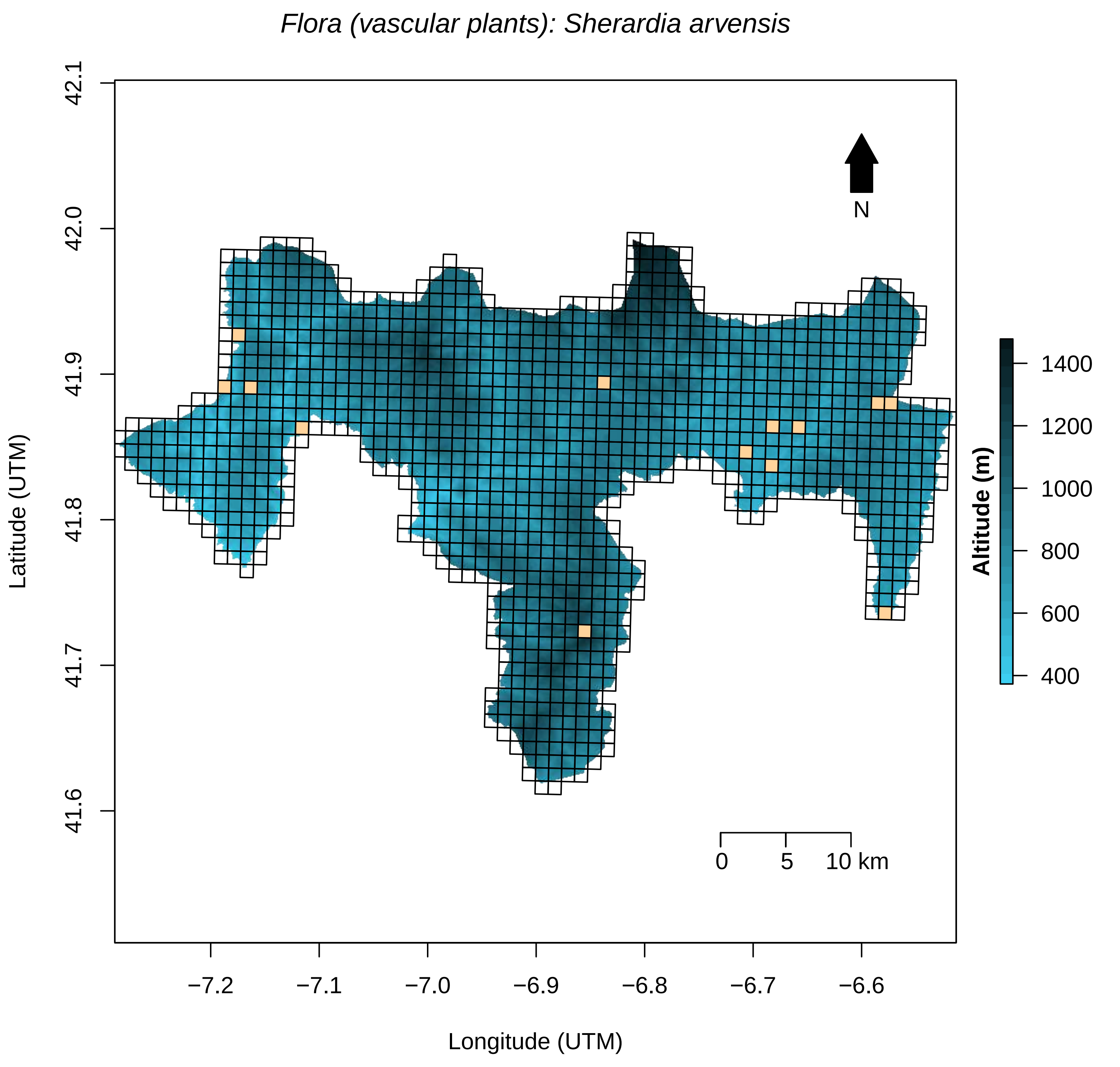
Flora (vascular plants): Sherardia arvensis
42.1
42.0
41.9
41.8
41.7
41.6
Latitude (UTM)
−7.2
−7.1
−7.0
−6.9
−6.8
−6.7
−6.6
Longitude (UTM)
N
0
5
10 km
1400
1200
1000
800
600
400
Altitude (m)

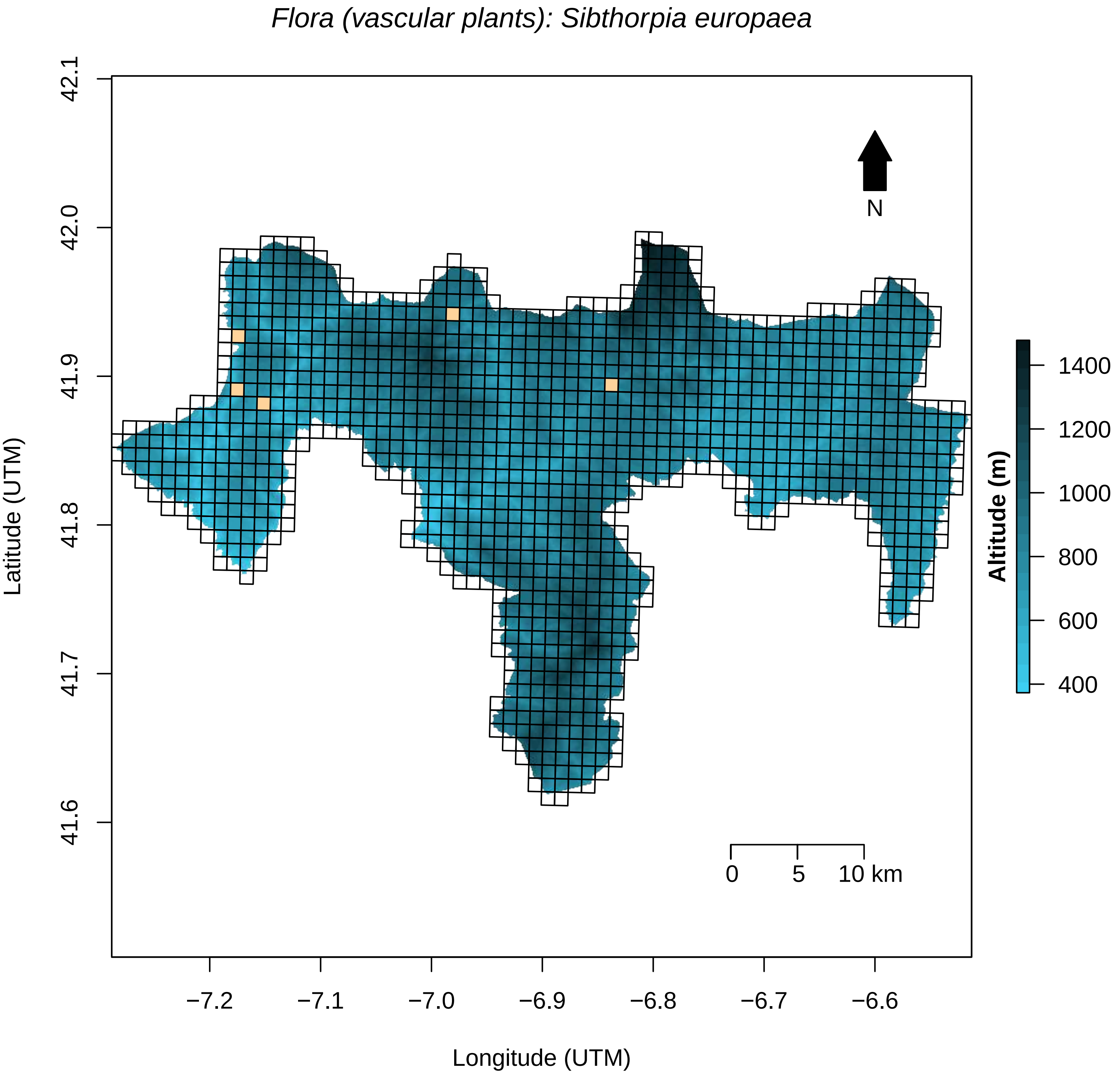
Flora (vascular plants): Sibthorpia europaea
42.1
42.0
41.9
41.8
41.7
41.6
Latitude (UTM)
−7.2
−7.1
−7.0
−6.9
−6.8
−6.7
−6.6
Longitude (UTM)
N
0
5
10 km
Altitude (m)
1400
1200
1000
800
600
400

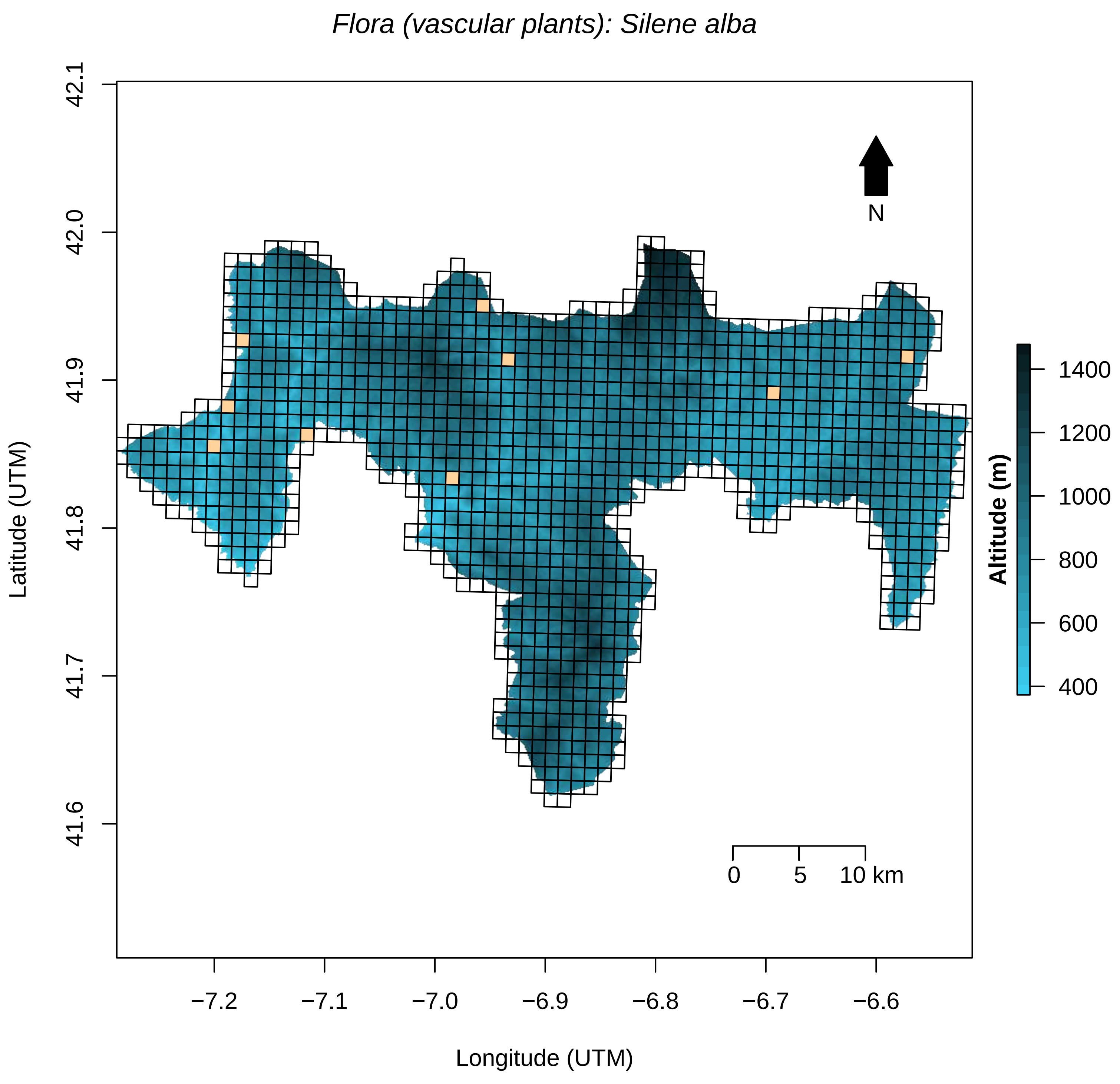

Flora (vascular plants): Silene alba
42.1
42.0
41.9
41.8
41.7
41.6
Latitude (UTM)
−7.2
−7.1
−7.0
−6.9
−6.8
−6.7
−6.6
Longitude (UTM)
N
0
5
10 km
Altitude (m)
1400
1200
1000
800
600
400

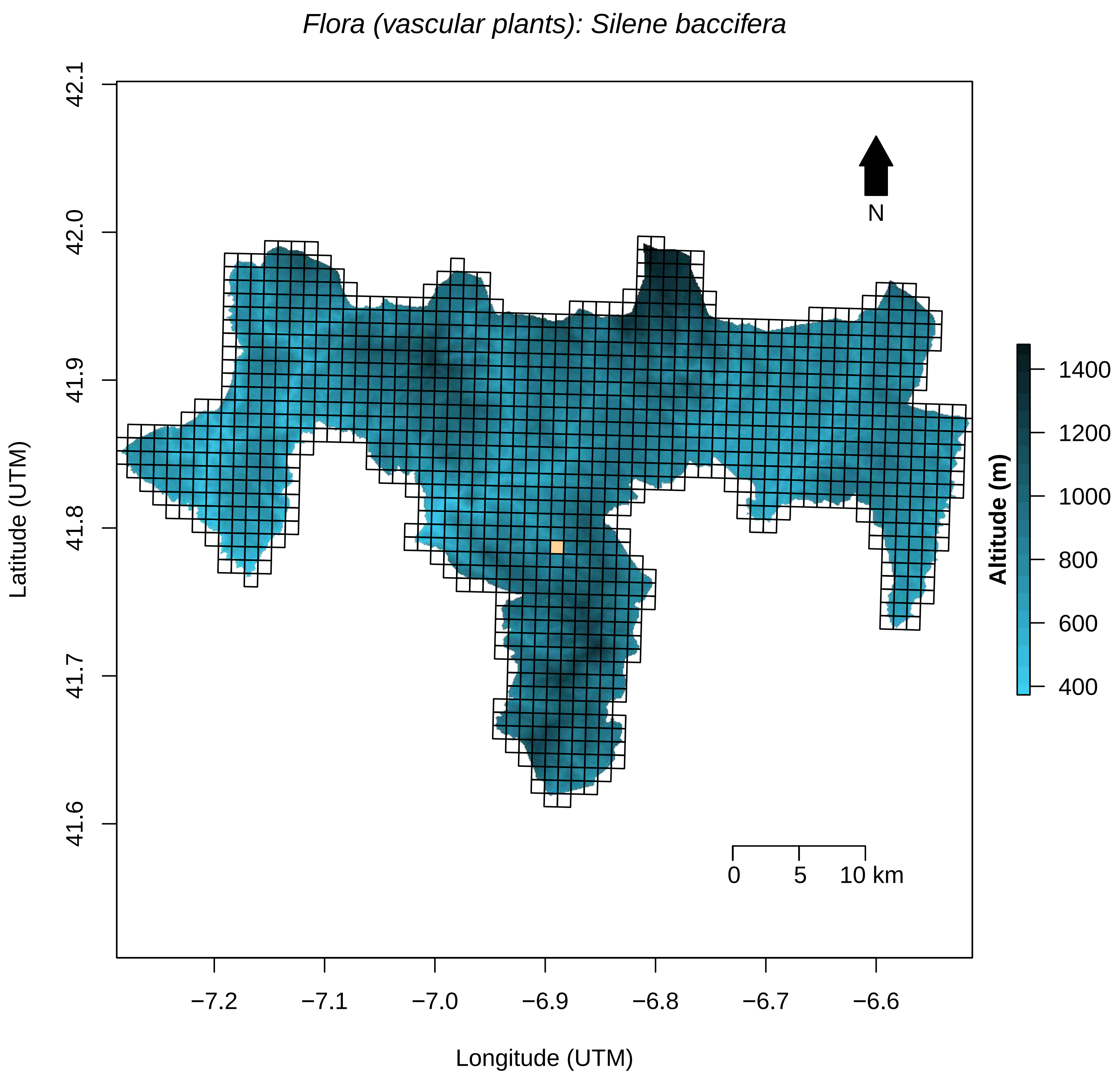

Flora (vascular plants): Silene baccifera
42.1
42.0
41.9
41.8
41.7
41.6
Latitude (UTM)
−7.2
−7.1
−7.0
−6.9
−6.8
−6.7
−6.6
Longitude (UTM)
N
0
5
10 km
Altitude (m)
1400
1200
1000
800
600
400

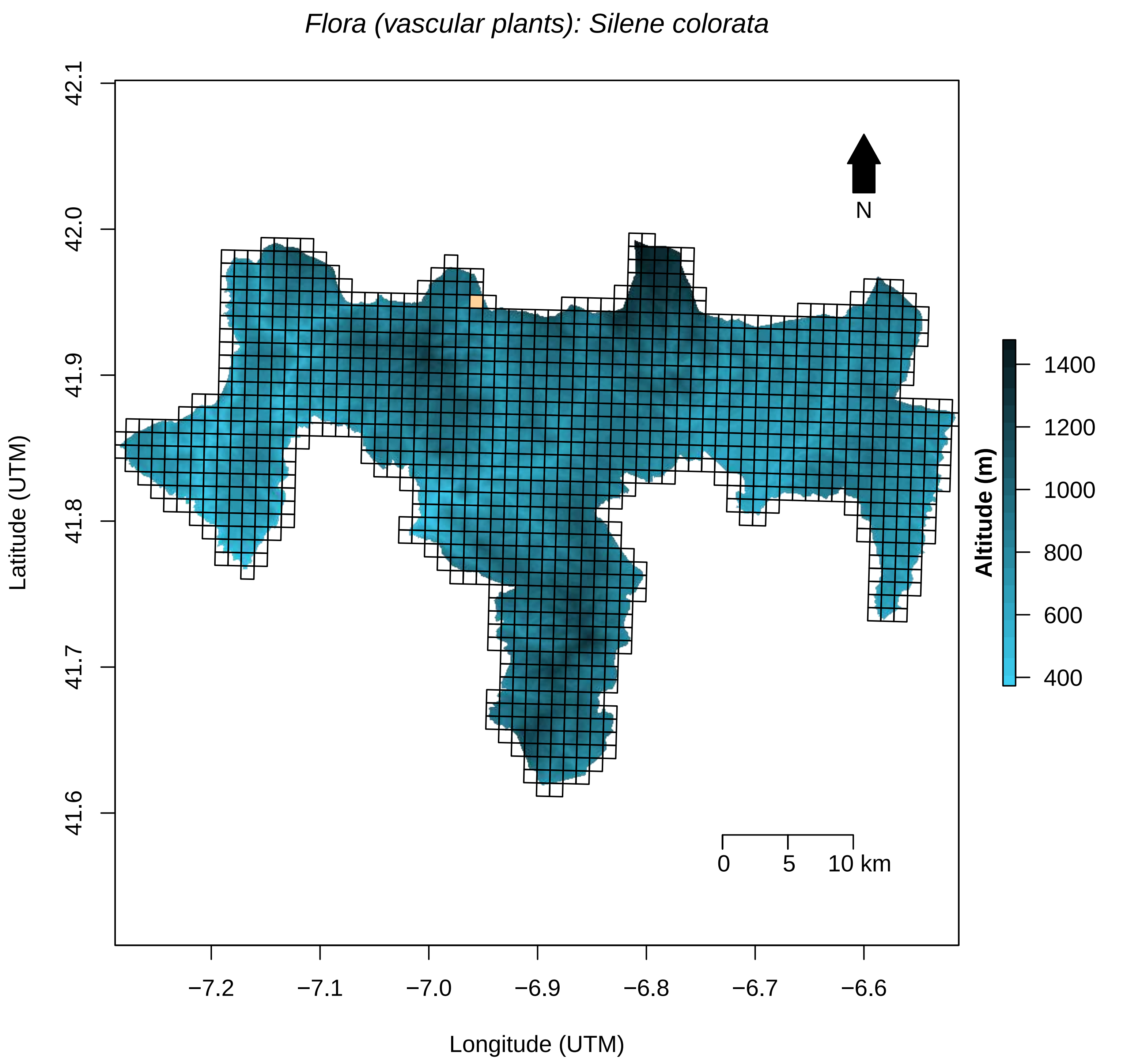
Flora (vascular plants): Silene colorata
Latitude (UTM)
42.1
42.0
41.9
41.8
41.7
41.6
Longitude (UTM)
−7.2
−7.1
−7.0
−6.9
−6.8
−6.7
−6.6
N
0
5
10 km
Altitude (m)
1400
1200
1000
800
600
400

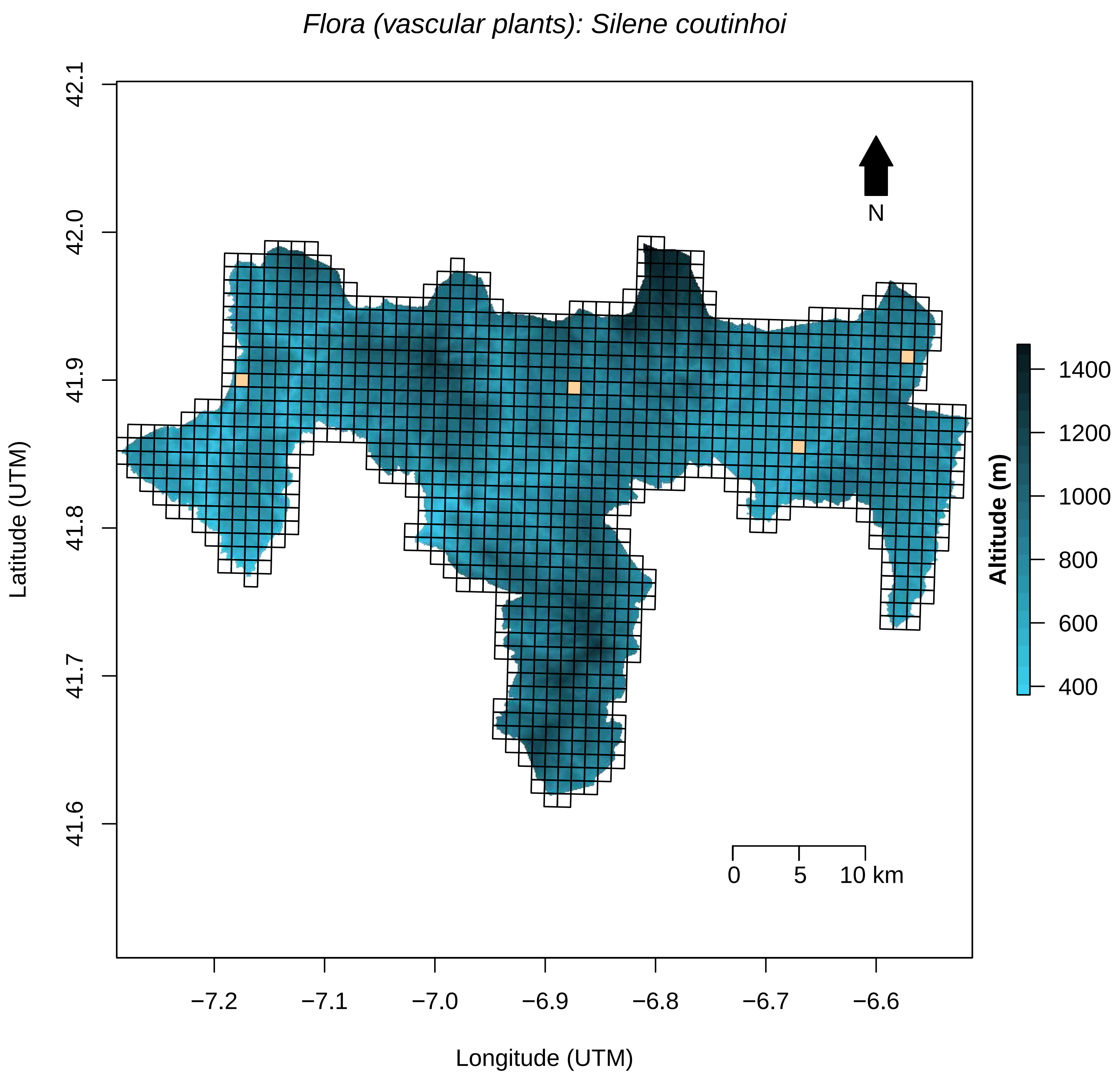
Flora (vascular plants): Silene coutinhoi
Latitude (UTM)
Longitude (UTM)
42.1
42.0
41.9
41.8
41.7
41.6
−7.2
−7.1
−7.0
−6.9
−6.8
−6.7
−6.6
N
0
5
10 km
Altitude (m)
1400
1200
1000
800
600
400

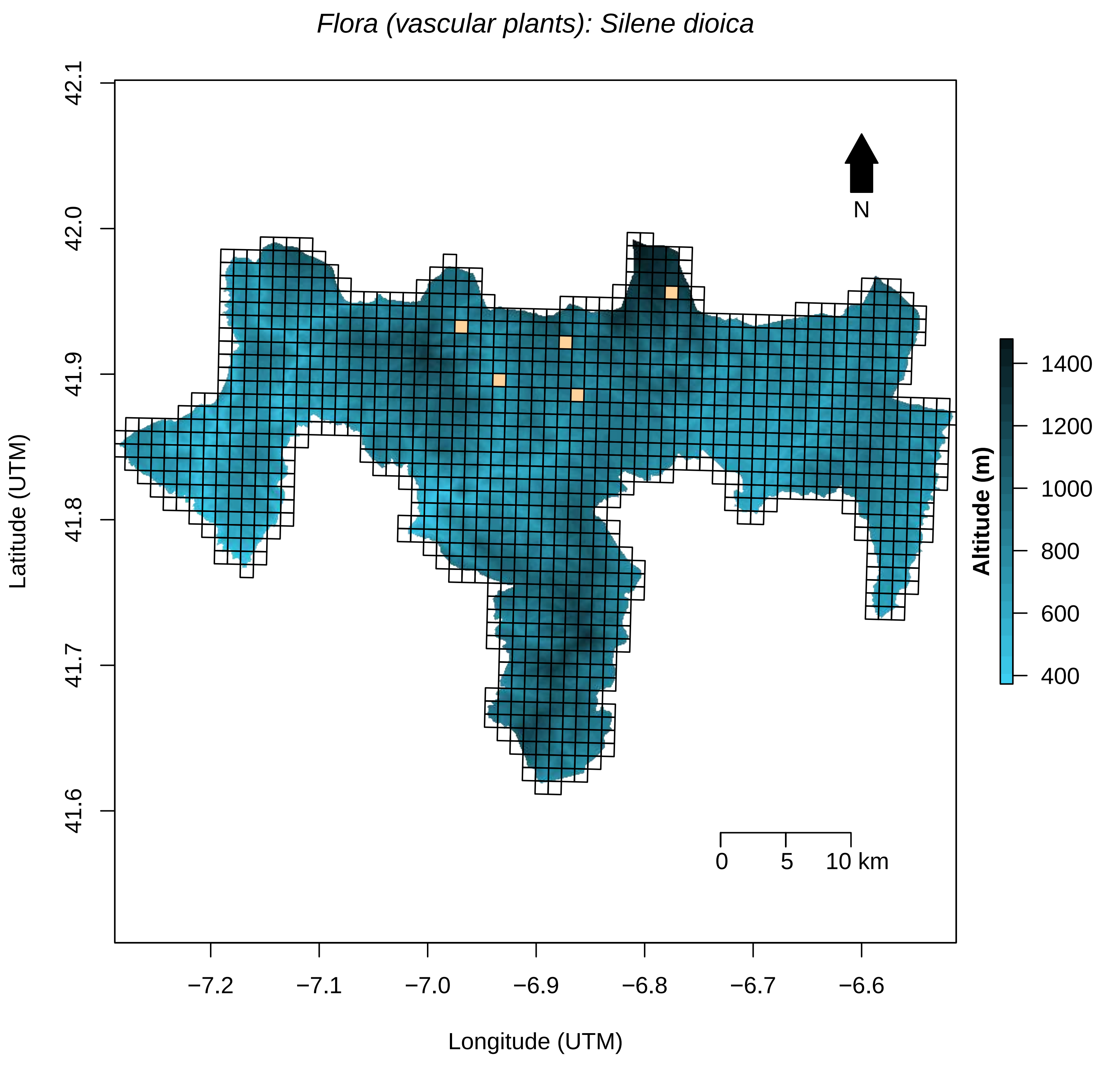
Flora (vascular plants): Silene dioica
Latitude (UTM)
42.1
42.0
41.9
41.8
41.7
41.6
Longitude (UTM)
−7.2
−7.1
−7.0
−6.9
−6.8
−6.7
−6.6
N
0
5
10 km
Altitude (m)
1400
1200
1000
800
600
400

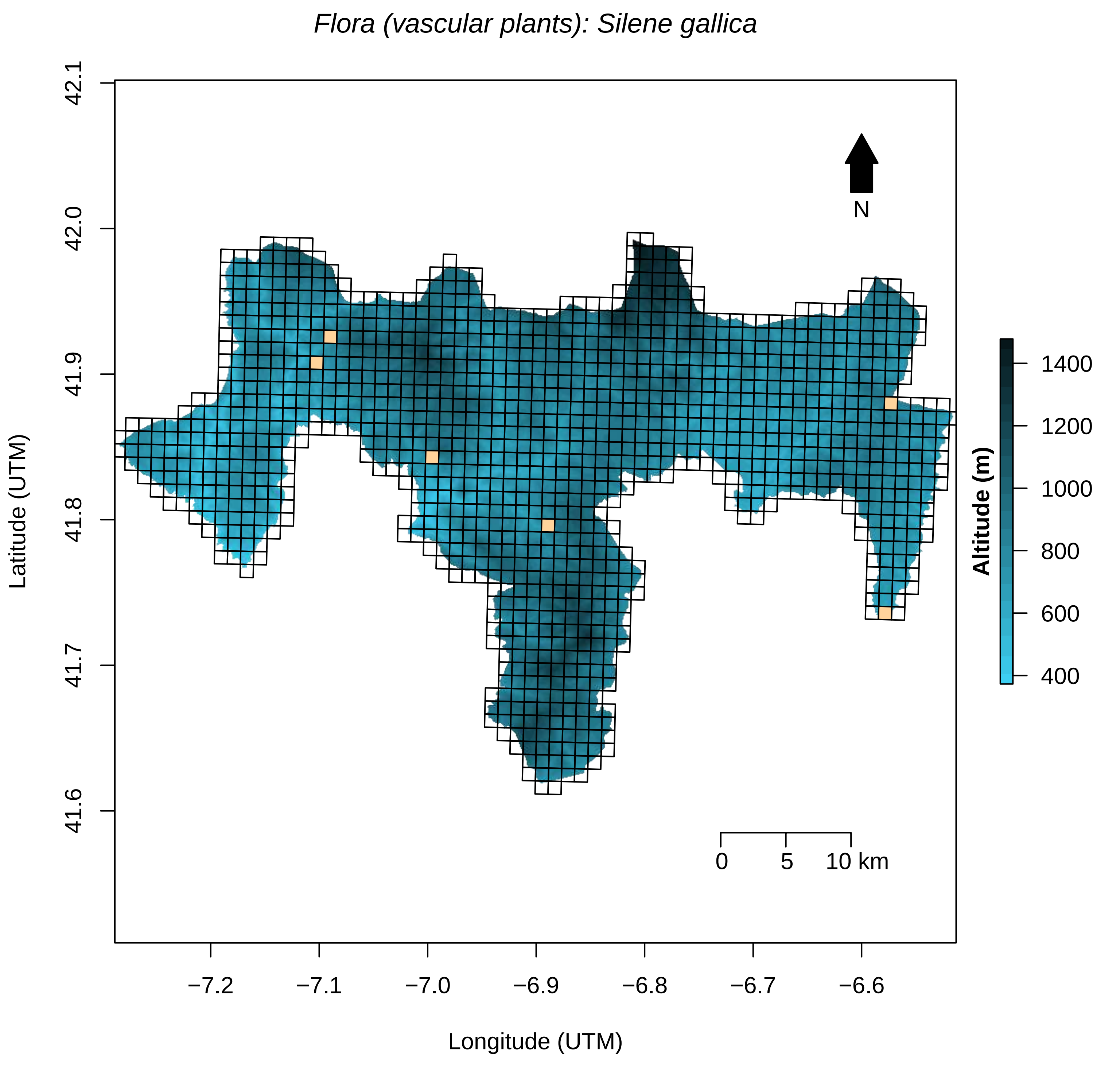

Flora (vascular plants): Silene gallica
42.1
42.0
41.9
41.8
41.7
41.6
Latitude (UTM)
N
0
5
10 km
−7.2
−7.1
−7.0
−6.9
−6.8
−6.7
−6.6
Longitude (UTM)
1400
1200
1000
800
600
400
Altitude (m)

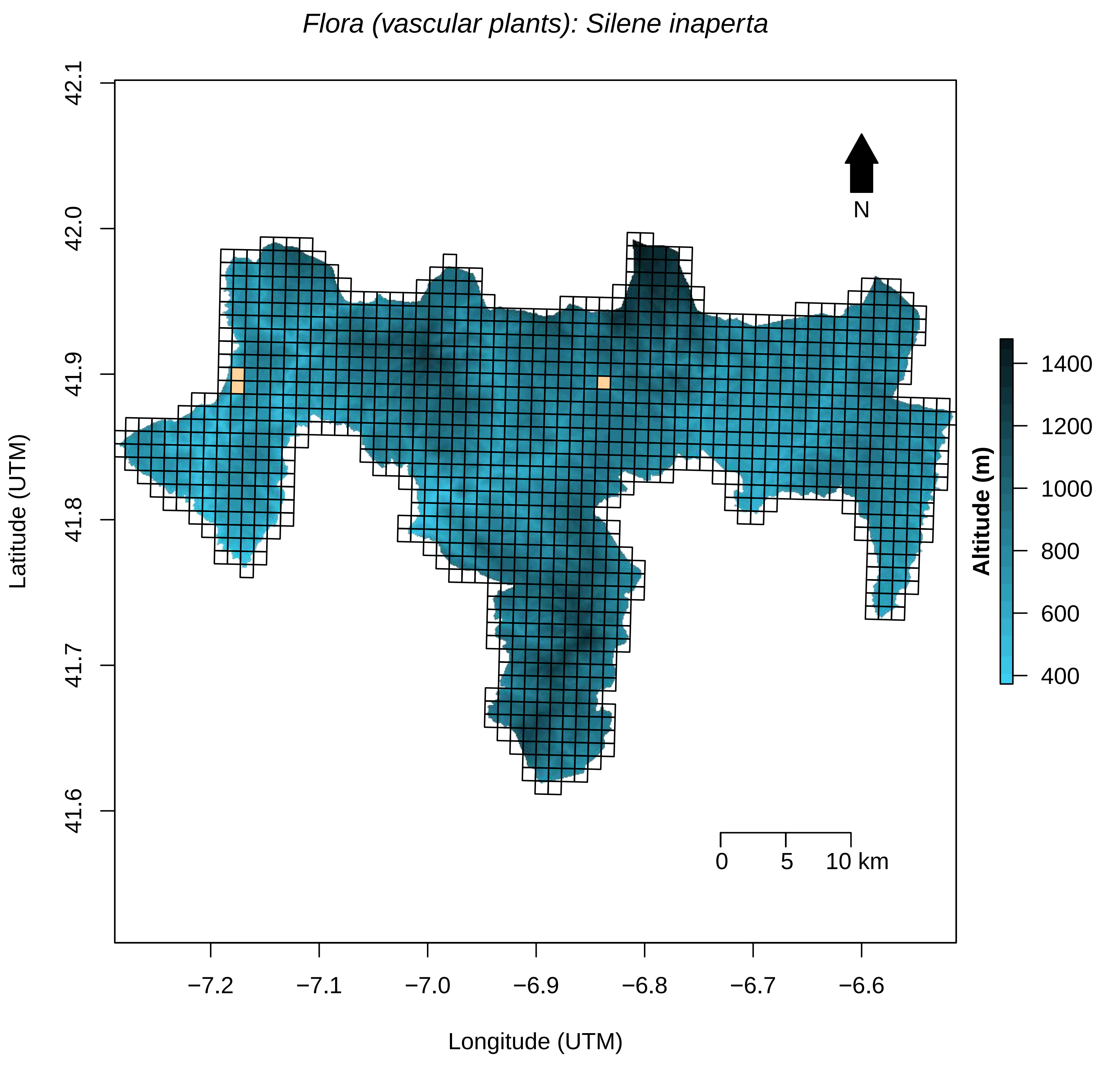

Flora (vascular plants): Silene inaperta
42.1
42.0
41.9
41.8
41.7
41.6
Latitude (UTM)
−7.2
−7.1
−7.0
−6.9
−6.8
−6.7
−6.6
Longitude (UTM)
N
0
5
10 km
Altitude (m)
1400
1200
1000
800
600
400

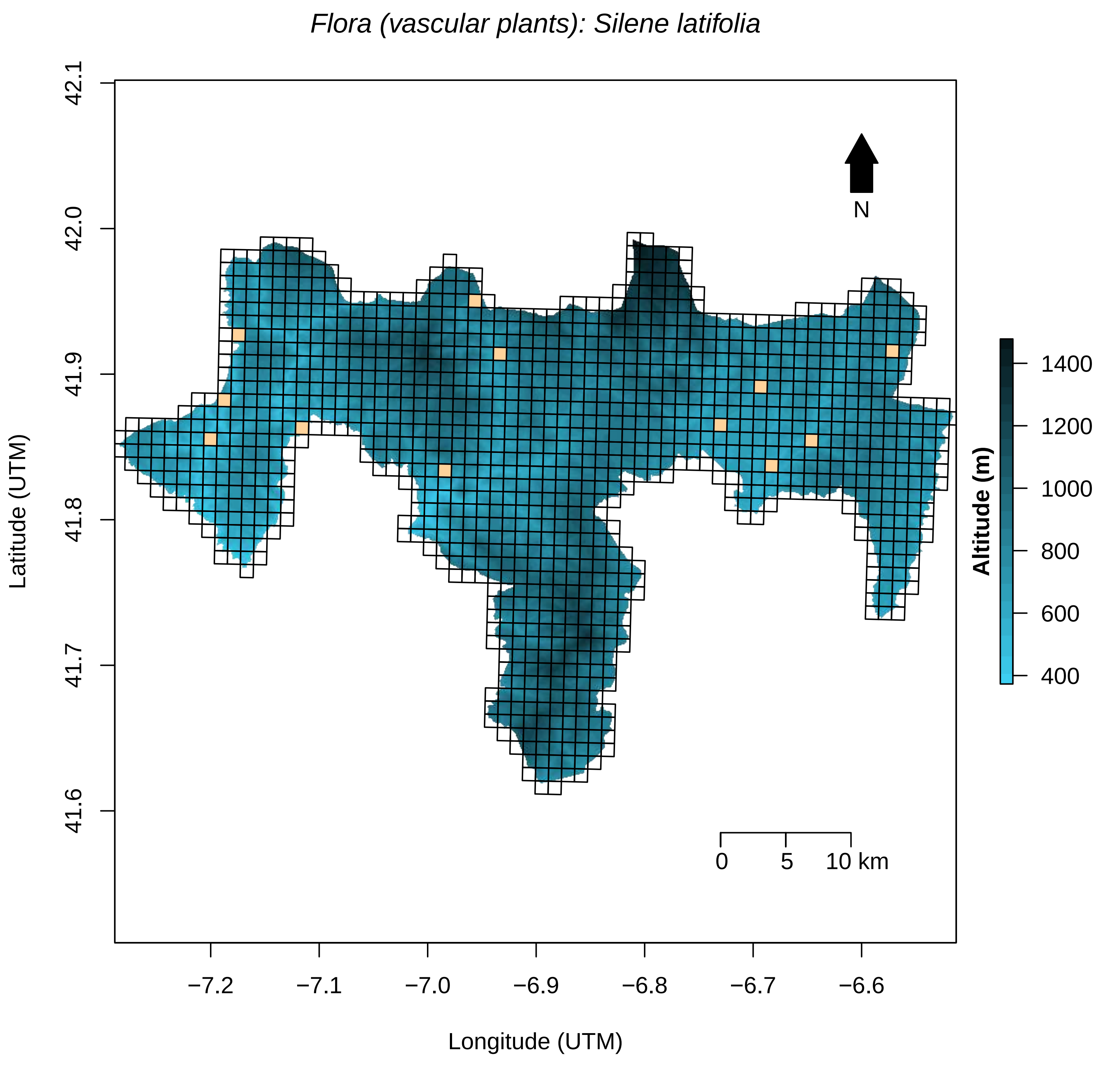
Flora (vascular plants): Silene latifolia
42.1
42.0
41.9
41.8
41.7
41.6
Latitude (UTM)
N
0
5
10 km
−7.2
−7.1
−7.0
−6.9
−6.8
−6.7
−6.6
Longitude (UTM)
1400
1200
1000
800
600
400
Altitude (m)

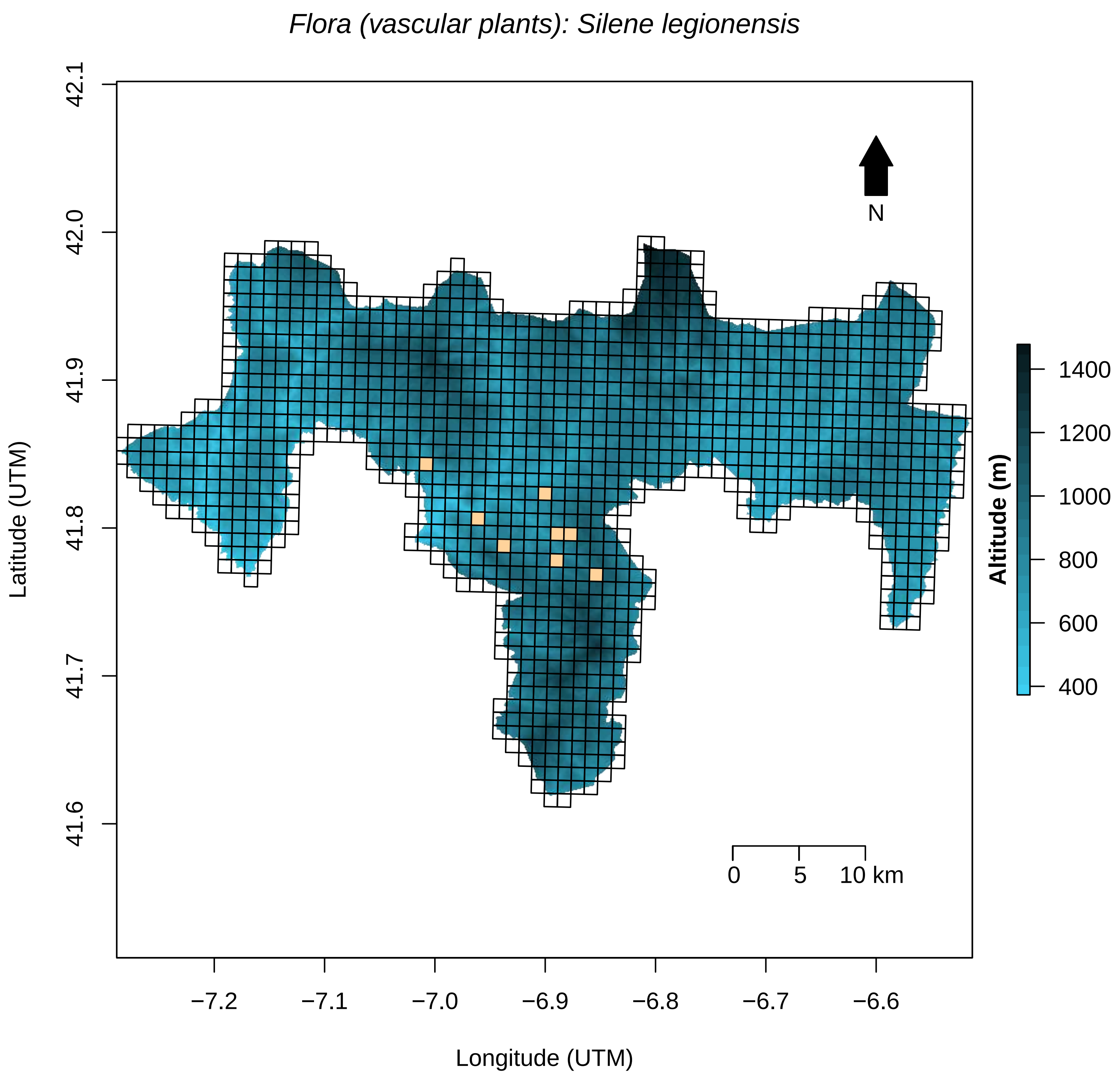

Flora (vascular plants): Silene legionensis
42.1
42.0
41.9
41.8
41.7
41.6
Latitude (UTM)
−7.2
−7.1
−7.0
−6.9
−6.8
−6.7
−6.6
Longitude (UTM)
N
0
5
10 km
Altitude (m)
1400
1200
1000
800
600
400

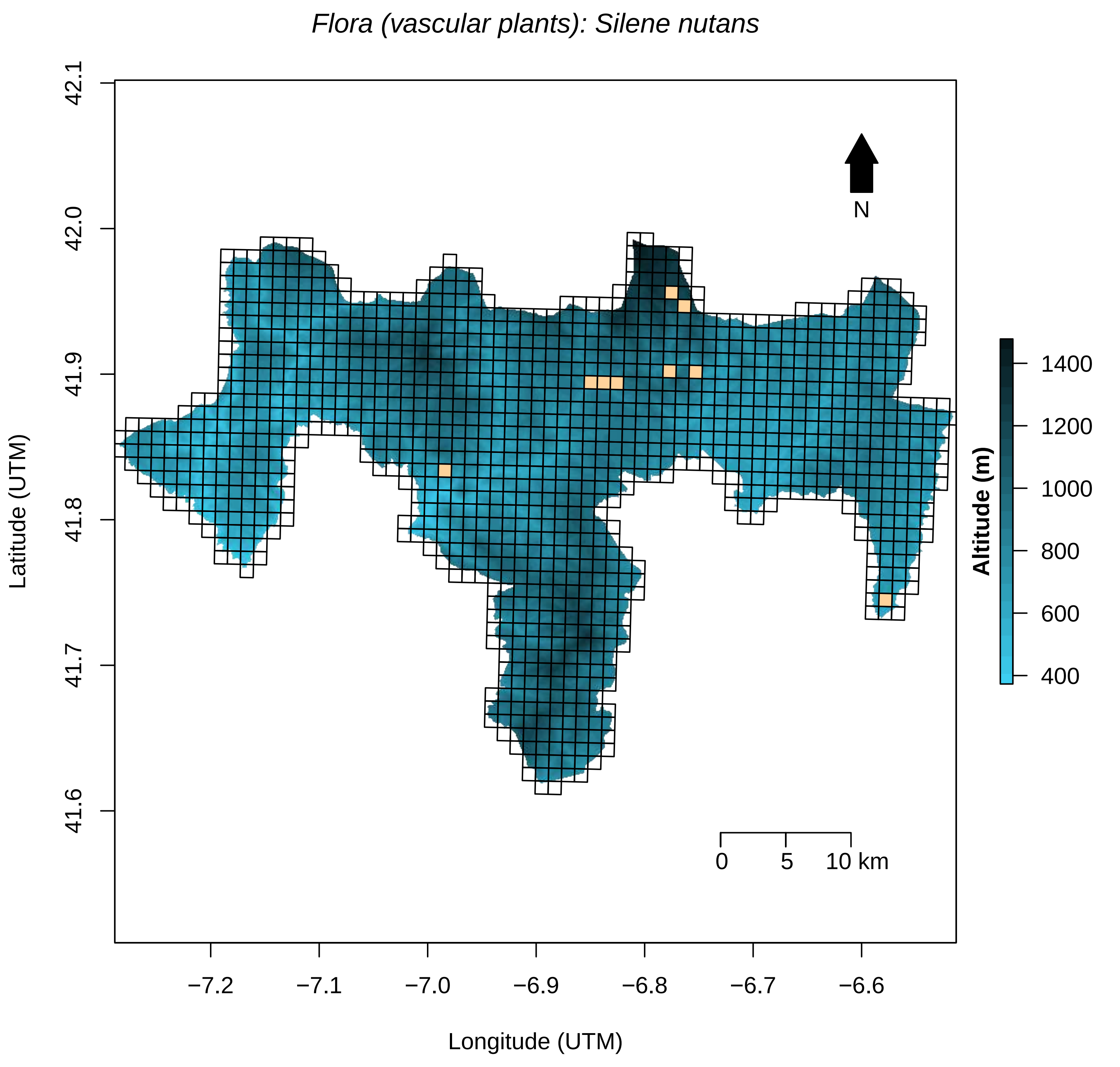
Flora (vascular plants): Silene nutans
N
0
5
10 km
Altitude (m)
1400
1200
1000
800
600
400
42.1
42.0
41.9
41.8
41.7
41.6
Latitude (UTM)
−7.2
−7.1
−7.0
−6.9
−6.8
−6.7
−6.6
Longitude (UTM)

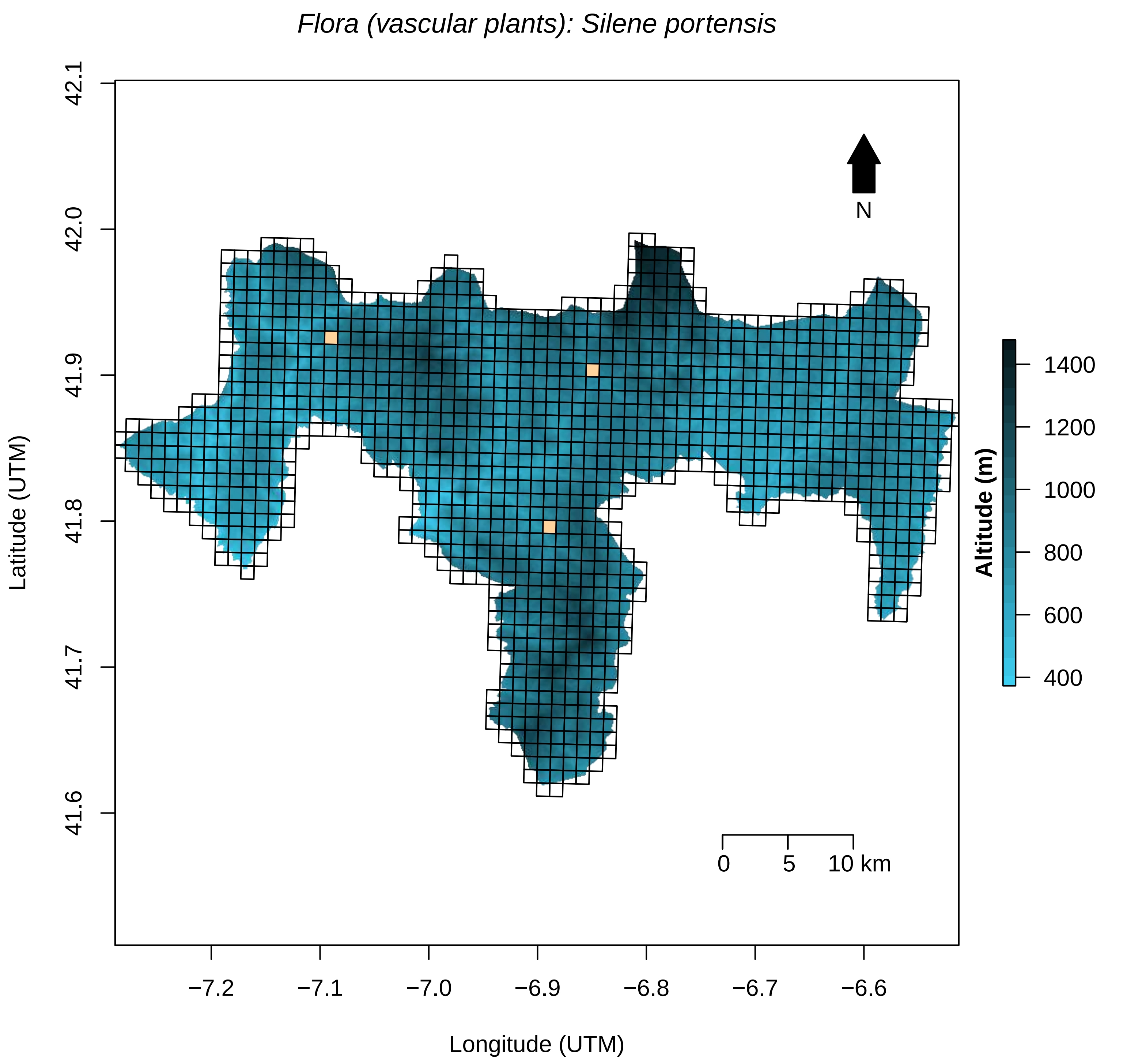
Flora (vascular plants): Silene portensis
N
0
5
10 km
Altitude (m)
1400
1200
1000
800
600
400
Latitude (UTM)
42.1
42.0
41.9
41.8
41.7
41.6
Longitude (UTM)
−7.2
−7.1
−7.0
−6.9
−6.8
−6.7
−6.6

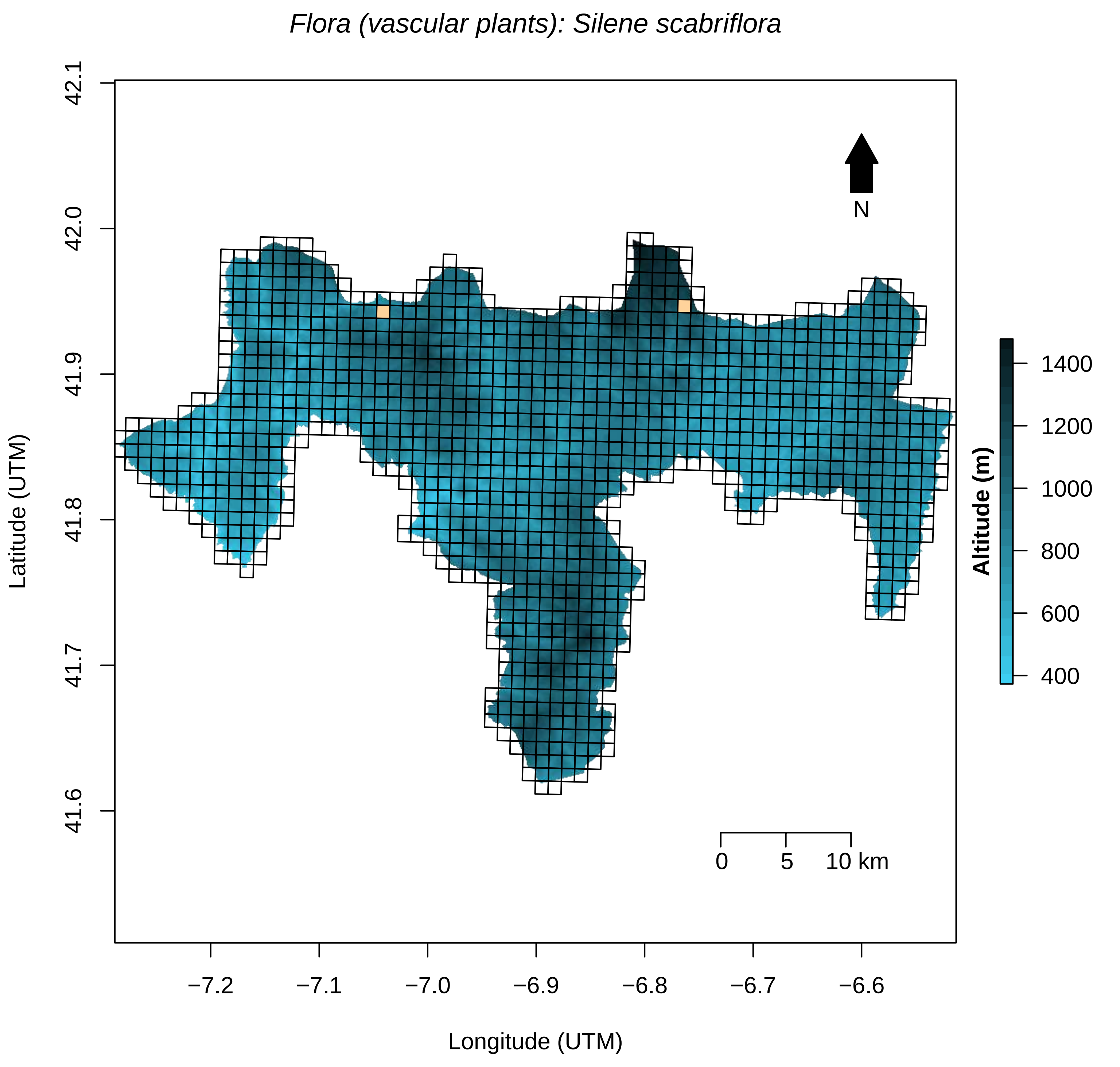
Flora (vascular plants): Silene scabriflora
42.1
42.0
41.9
41.8
41.7
41.6
Latitude (UTM)
−7.2
−7.1
−7.0
−6.9
−6.8
−6.7
−6.6
Longitude (UTM)
N
0
5
10 km
Altitude (m)
1400
1200
1000
800
600
400

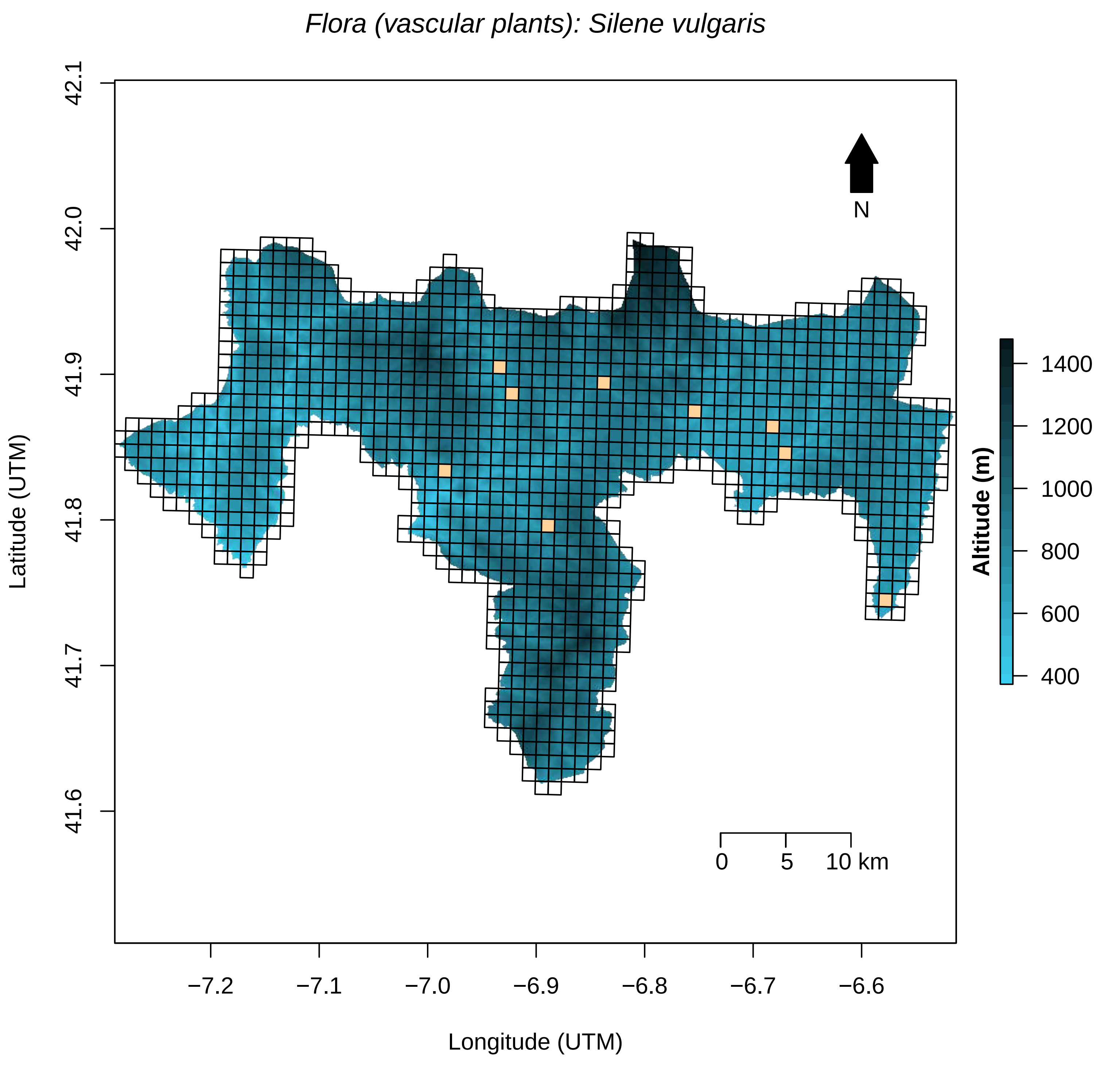

Flora (vascular plants): Silene vulgaris
Latitude (UTM)
42.1
42.0
41.9
41.8
41.7
41.6
Longitude (UTM)
−7.2
−7.1
−7.0
−6.9
−6.8
−6.7
−6.6
N
0
5
10 km
Altitude (m)
1400
1200
1000
800
600
400

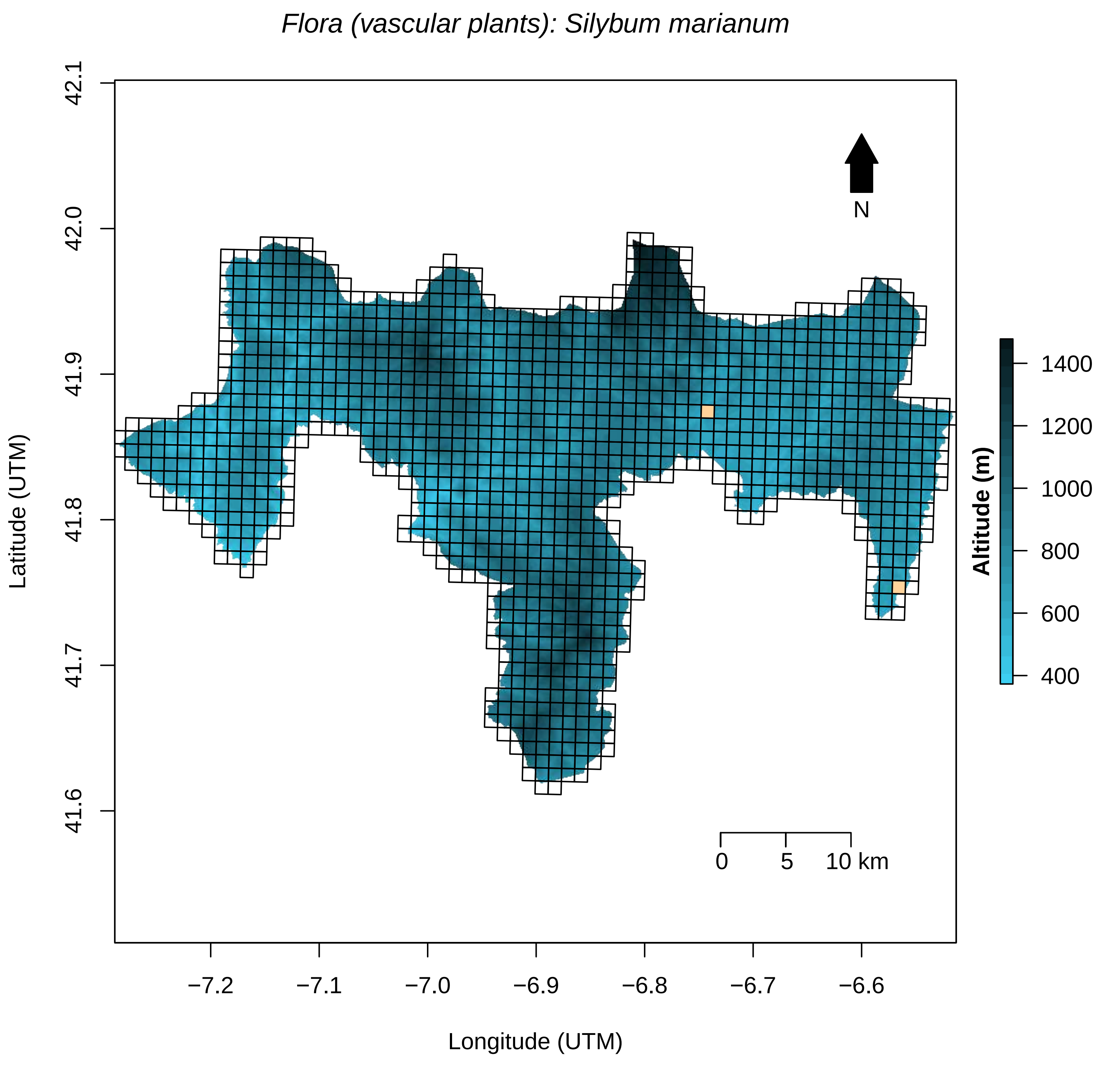

Flora (vascular plants): Silybum marianum
42.1
42.0
41.9
41.8
41.7
41.6
Latitude (UTM)
−7.2
−7.1
−7.0
−6.9
−6.8
−6.7
−6.6
Longitude (UTM)
N
0
5
10 km
Altitude (m)
1400
1200
1000
800
600
400

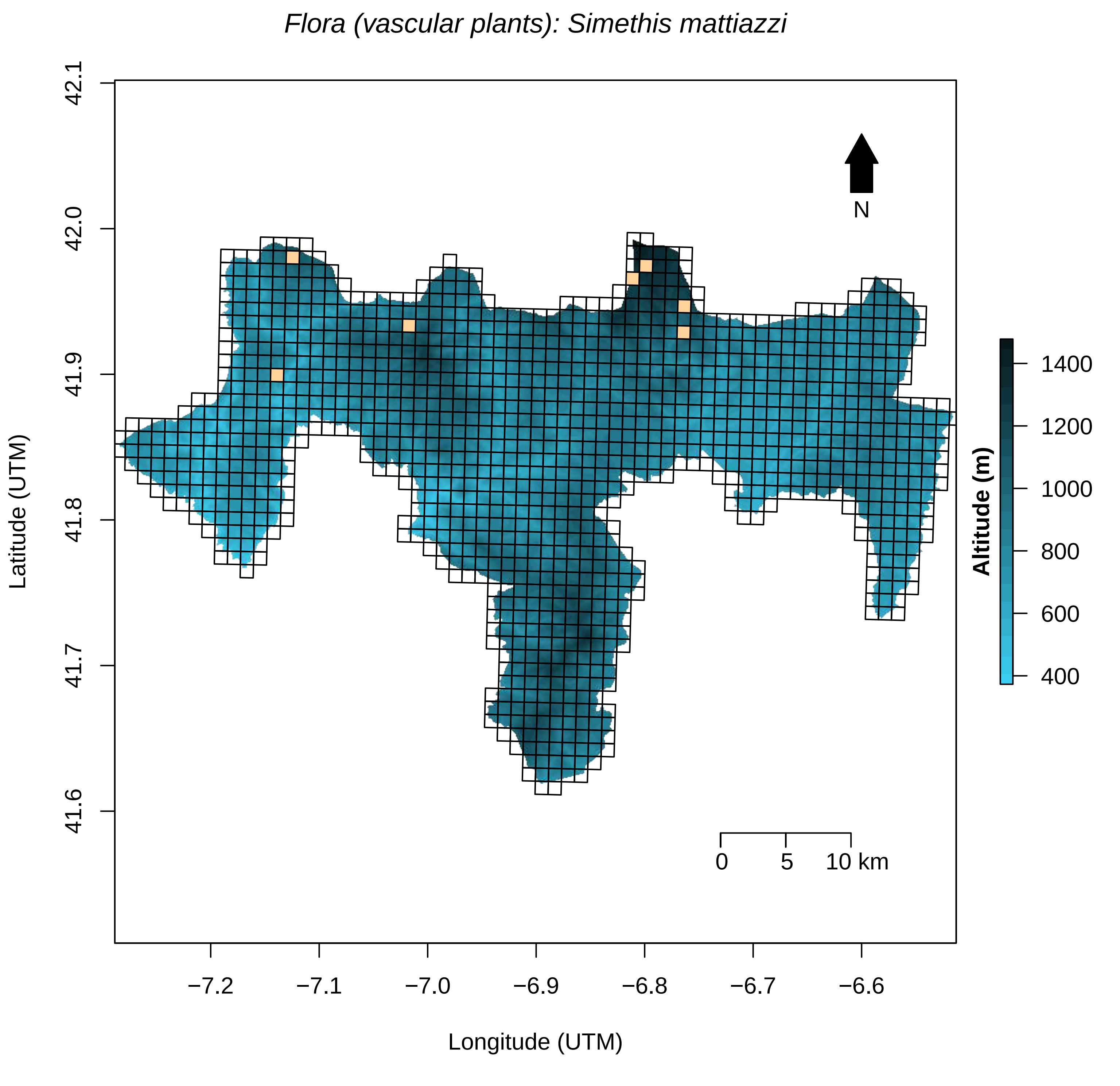

Flora (vascular plants): Simethis mattiazzi
42.1
42.0
41.9
41.8
41.7
41.6
Latitude (UTM)
−7.2
−7.1
−7.0
−6.9
−6.8
−6.7
−6.6
Longitude (UTM)
N
0
5
10 km
Altitude (m)
1400
1200
1000
800
600
400

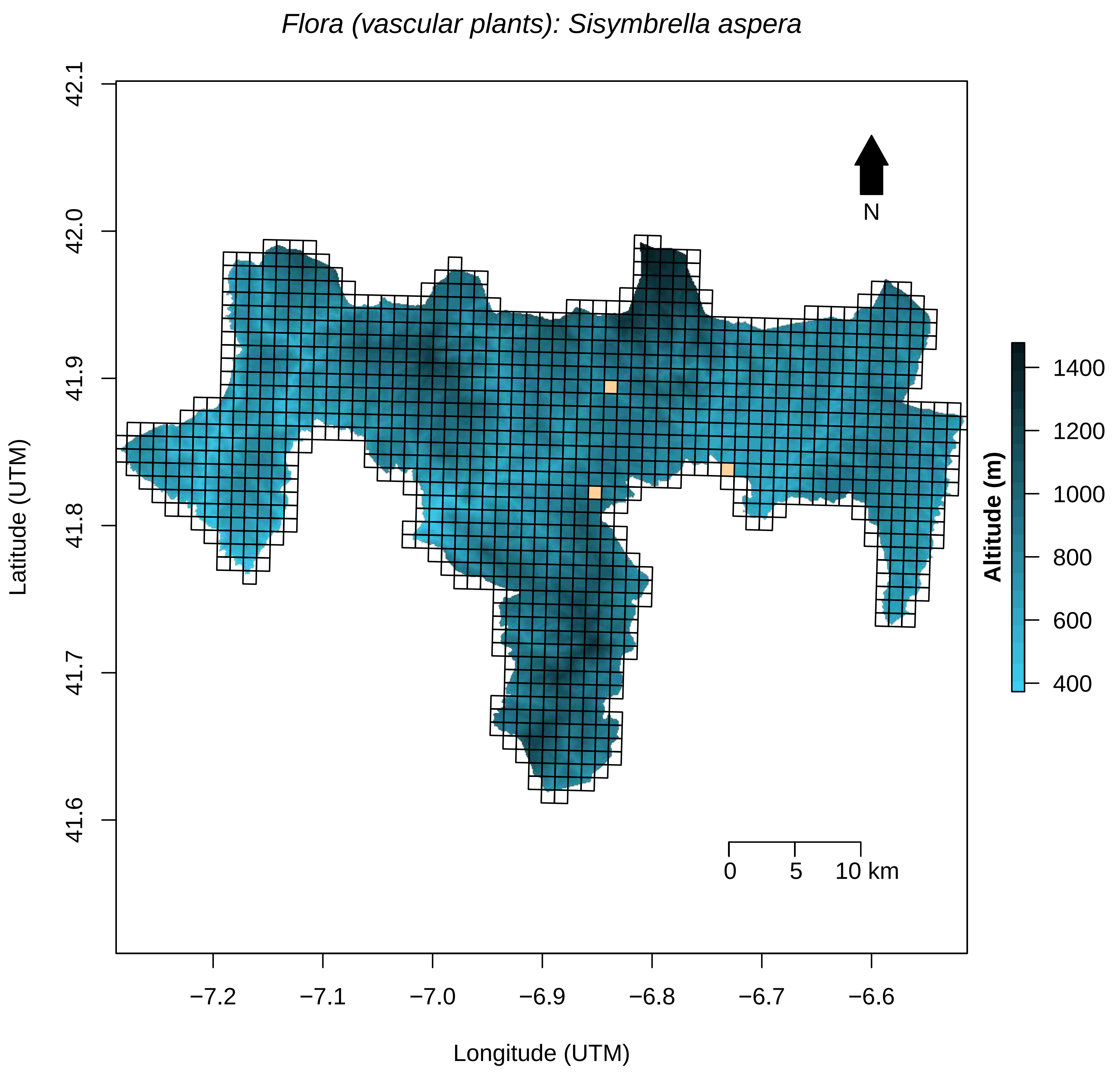

Flora (vascular plants): Sisymbrella aspera
42.1
42.0
41.9
41.8
41.7
41.6
Latitude (UTM)
−7.2
−7.1
−7.0
−6.9
−6.8
−6.7
−6.6
Longitude (UTM)
N
0
5
10 km
Altitude (m)
1400
1200
1000
800
600
400

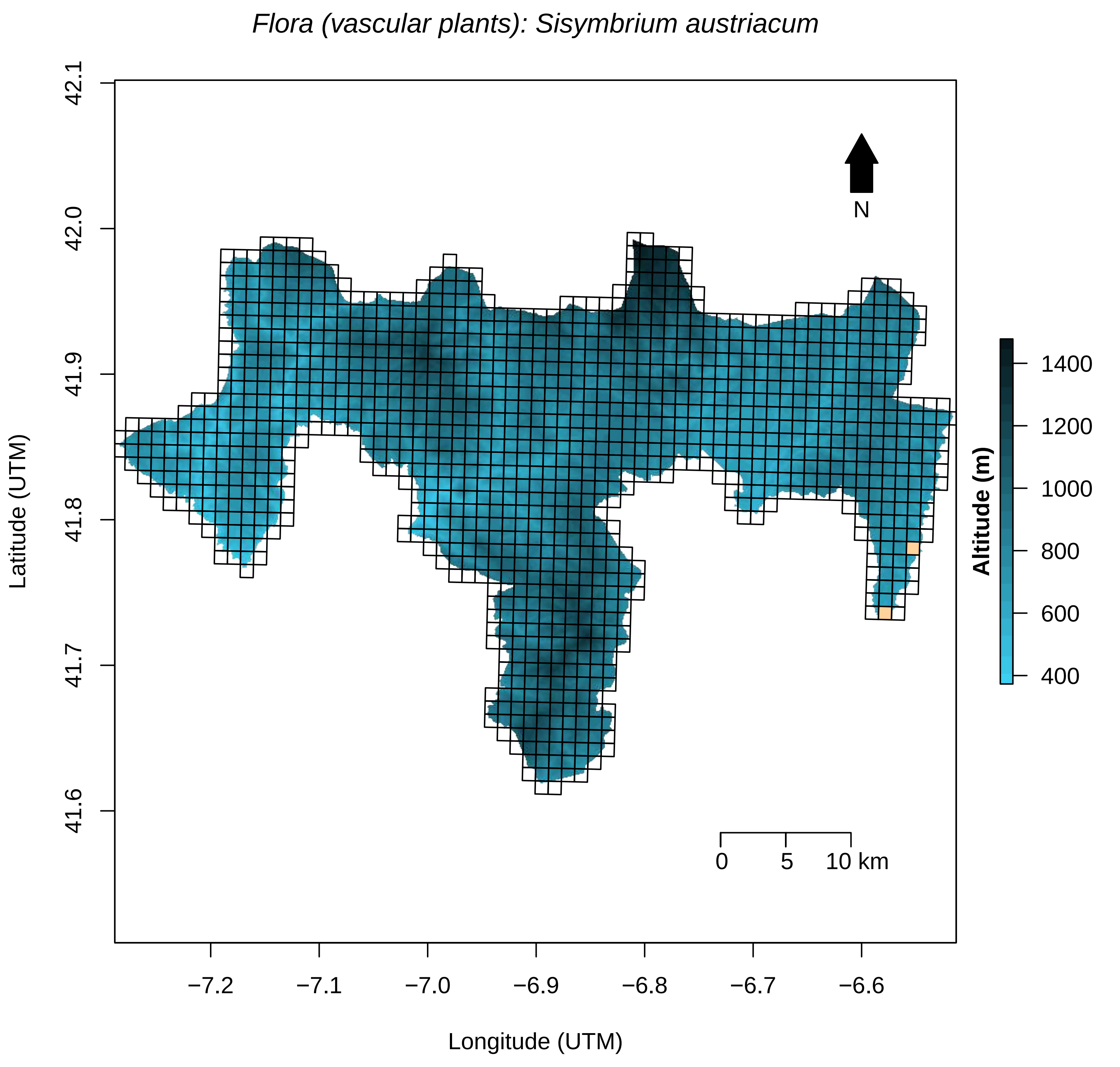
Flora (vascular plants): Sisymbrium austriacum
N
0
5
10 km
Altitude (m)
400
600
800
1000
1200
1400
Latitude (UTM)
41.6
41.7
41.8
41.9
42.0
42.1
Longitude (UTM)
−7.2
−7.1
−7.0
−6.9
−6.8
−6.7
−6.6

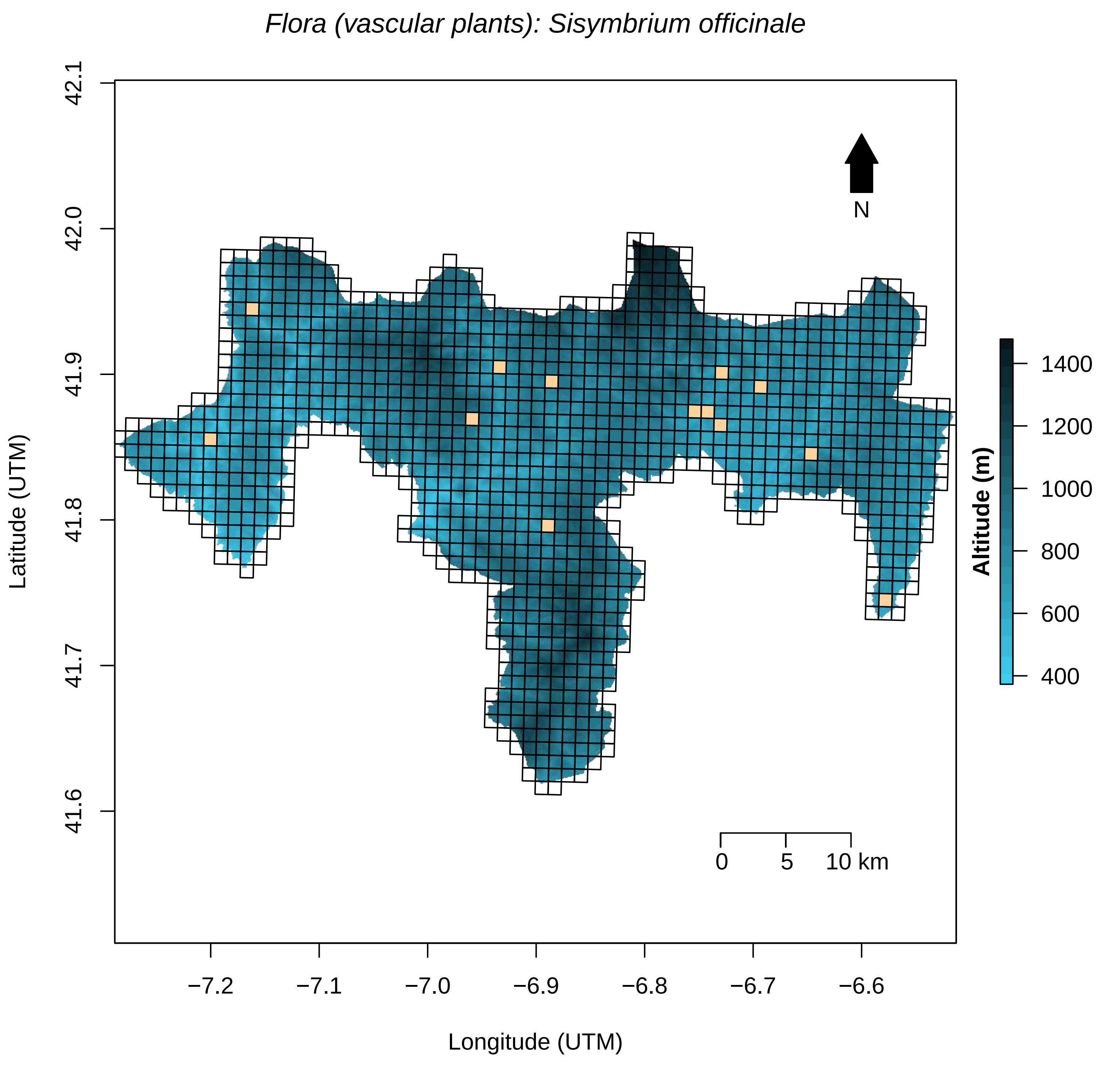
Flora (vascular plants): Sisymbrium officinale
N
0 5 10 km
Altitude (m)
1400
1200
1000
800
600
400
42.1
42.0
41.9
41.8
41.7
41.6
Latitude (UTM)
−7.2
−7.1
−7.0
−6.9
−6.8
−6.7
−6.6
Longitude (UTM)

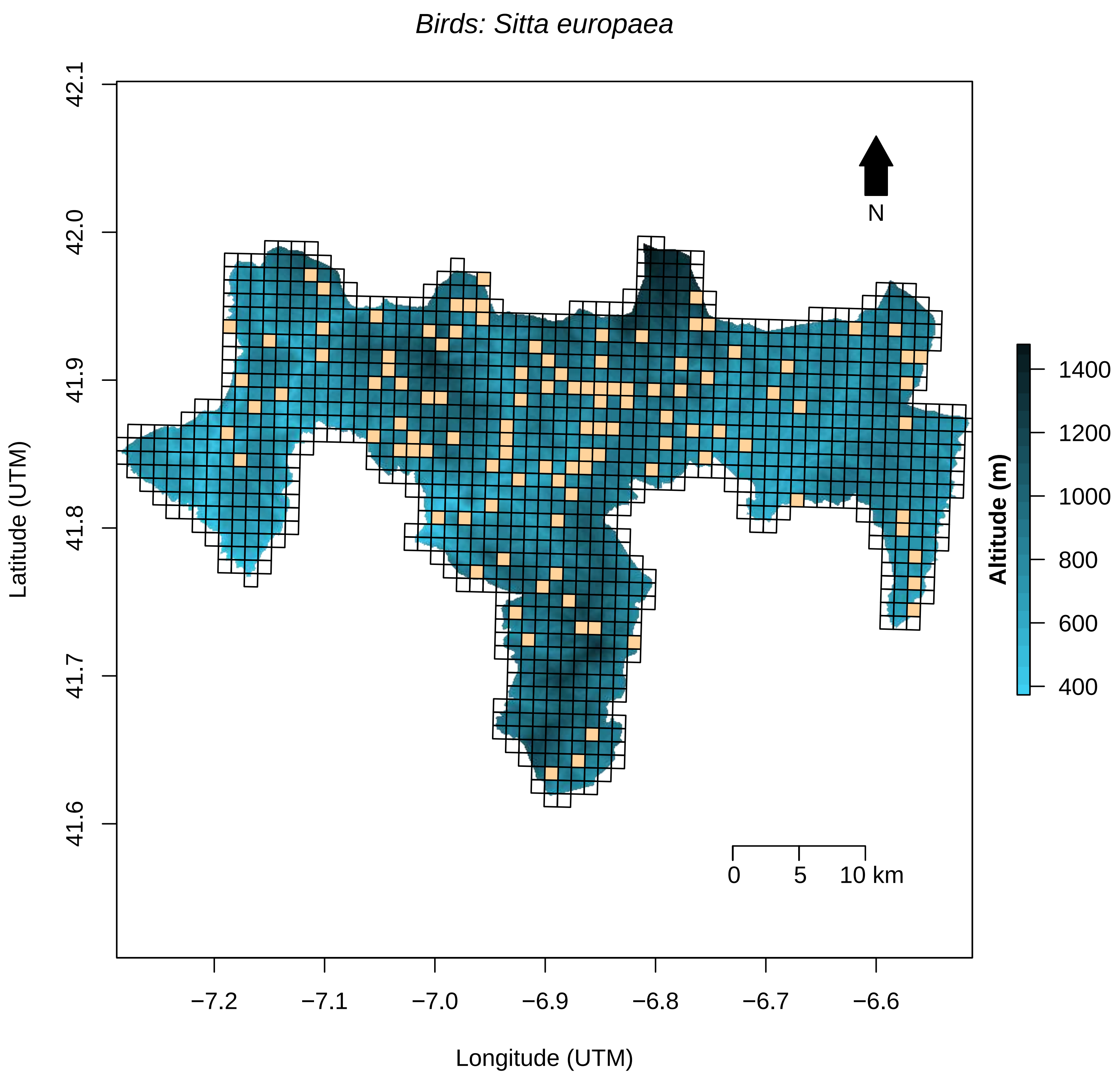

Birds: Sitta europaea
42.1
42.0
41.9
41.8
41.7
41.6
Latitude (UTM)
−7.2
−7.1
−7.0
−6.9
−6.8
−6.7
−6.6
Longitude (UTM)
N
0
5
10 km
Altitude (m)
1400
1200
1000
800
600
400

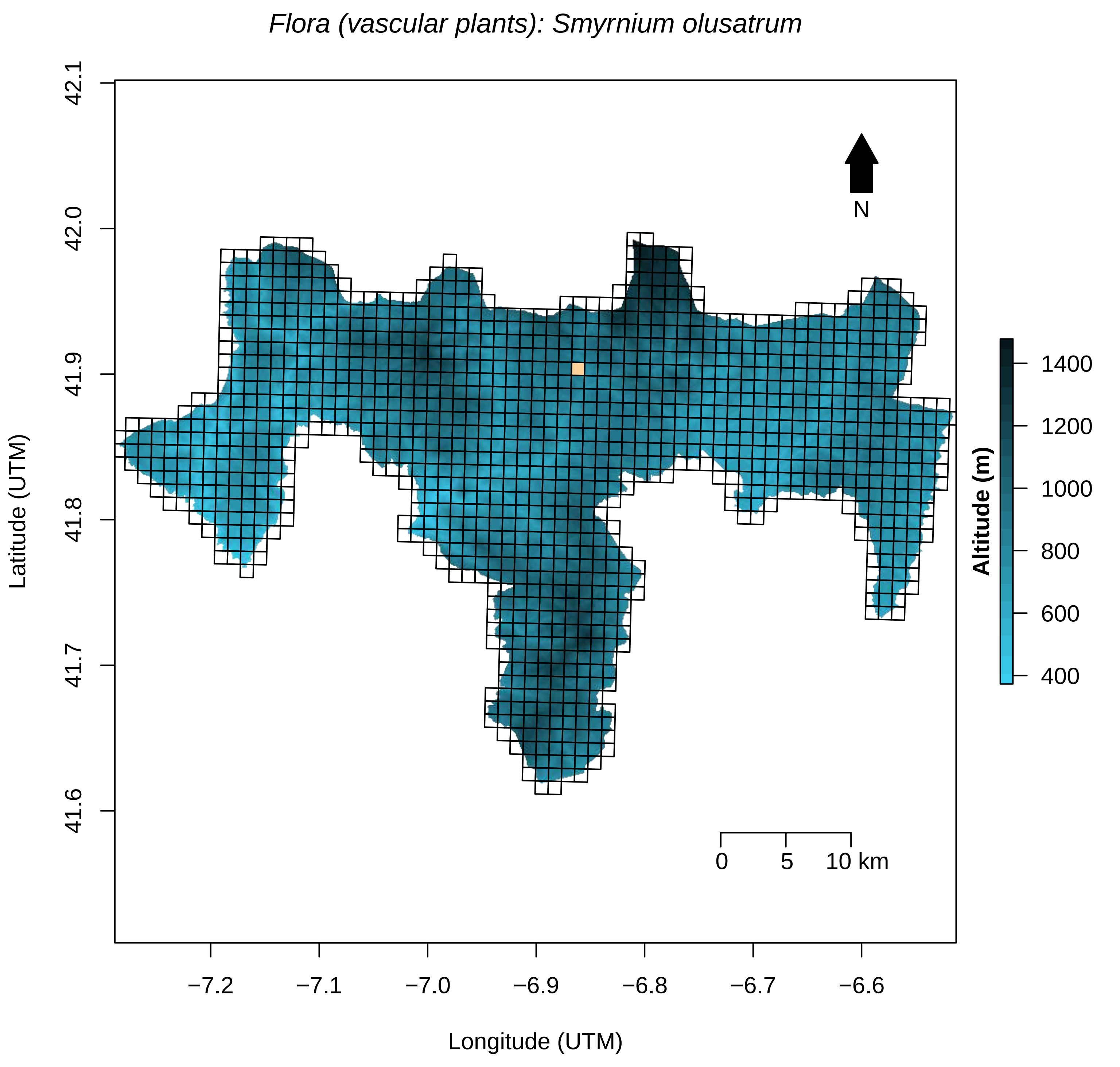

Flora (vascular plants): Smyrnium olusatrum
42.1
42.0
41.9
41.8
41.7
41.6
Latitude (UTM)
−7.2
−7.1
−7.0
−6.9
−6.8
−6.7
−6.6
Longitude (UTM)
N
0
5
10 km
Altitude (m)
1400
1200
1000
800
600
400

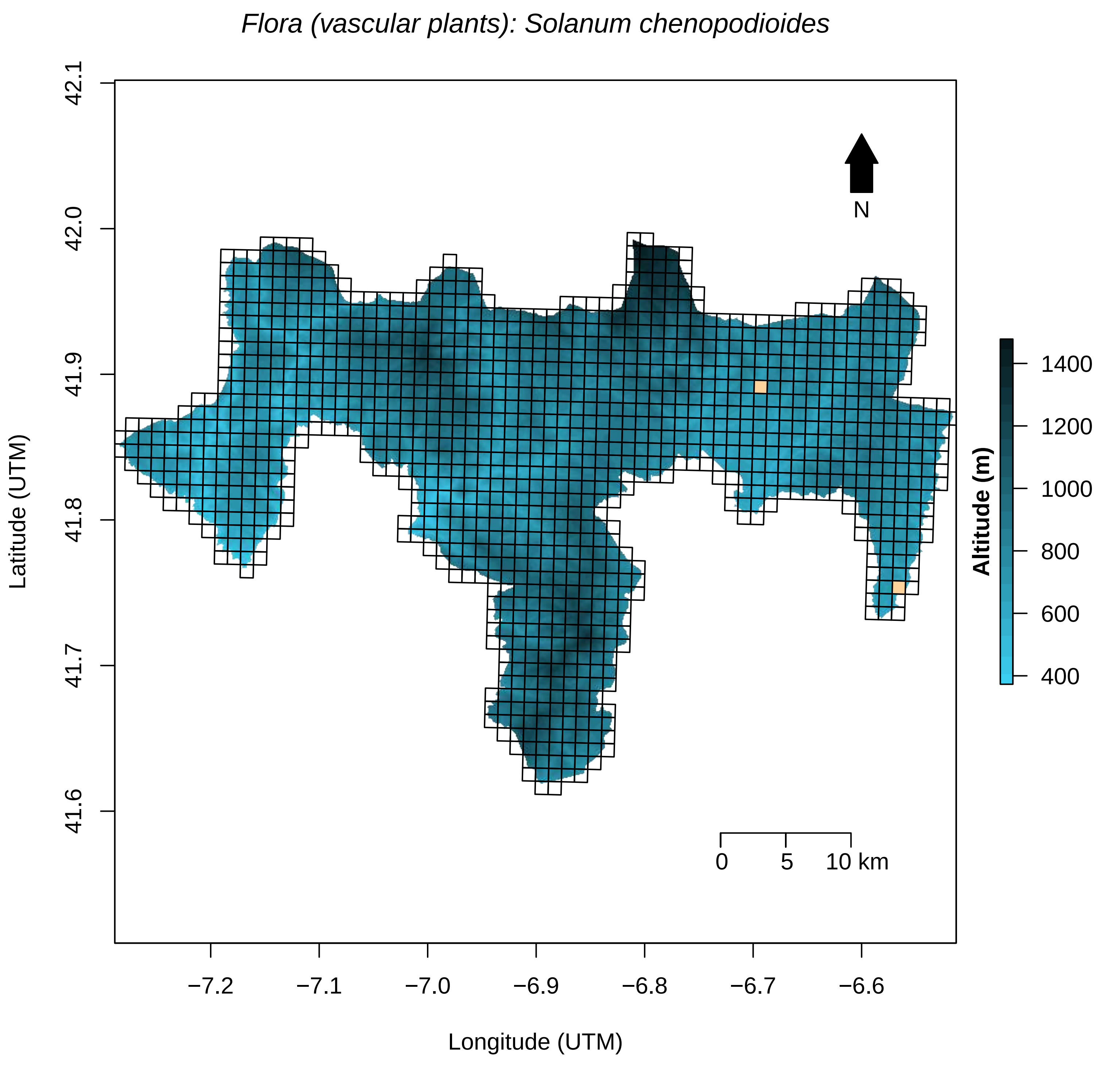

Flora (vascular plants): Solanum chenopodioides
42.1
42.0
41.9
41.8
41.7
41.6
Latitude (UTM)
−7.2
−7.1
−7.0
−6.9
−6.8
−6.7
−6.6
Longitude (UTM)
N
0
5
10 km
1400
1200
1000
800
600
400
Altitude (m)

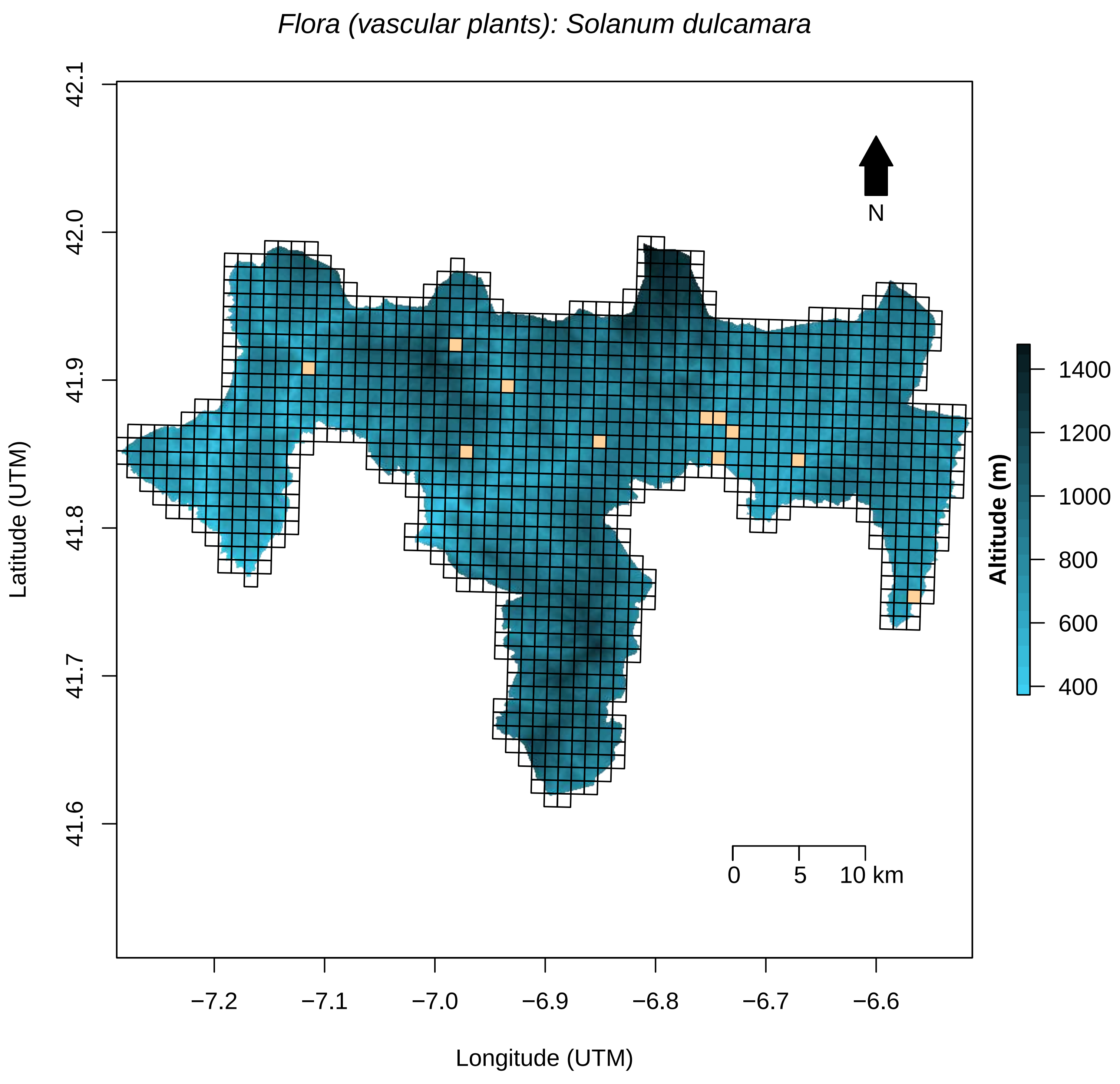

Flora (vascular plants): Solanum dulcamara
Latitude (UTM)
42.1
42.0
41.9
41.8
41.7
41.6
Longitude (UTM)
−7.2
−7.1
−7.0
−6.9
−6.8
−6.7
−6.6
N
0
5
10 km
Altitude (m)
1400
1200
1000
800
600
400

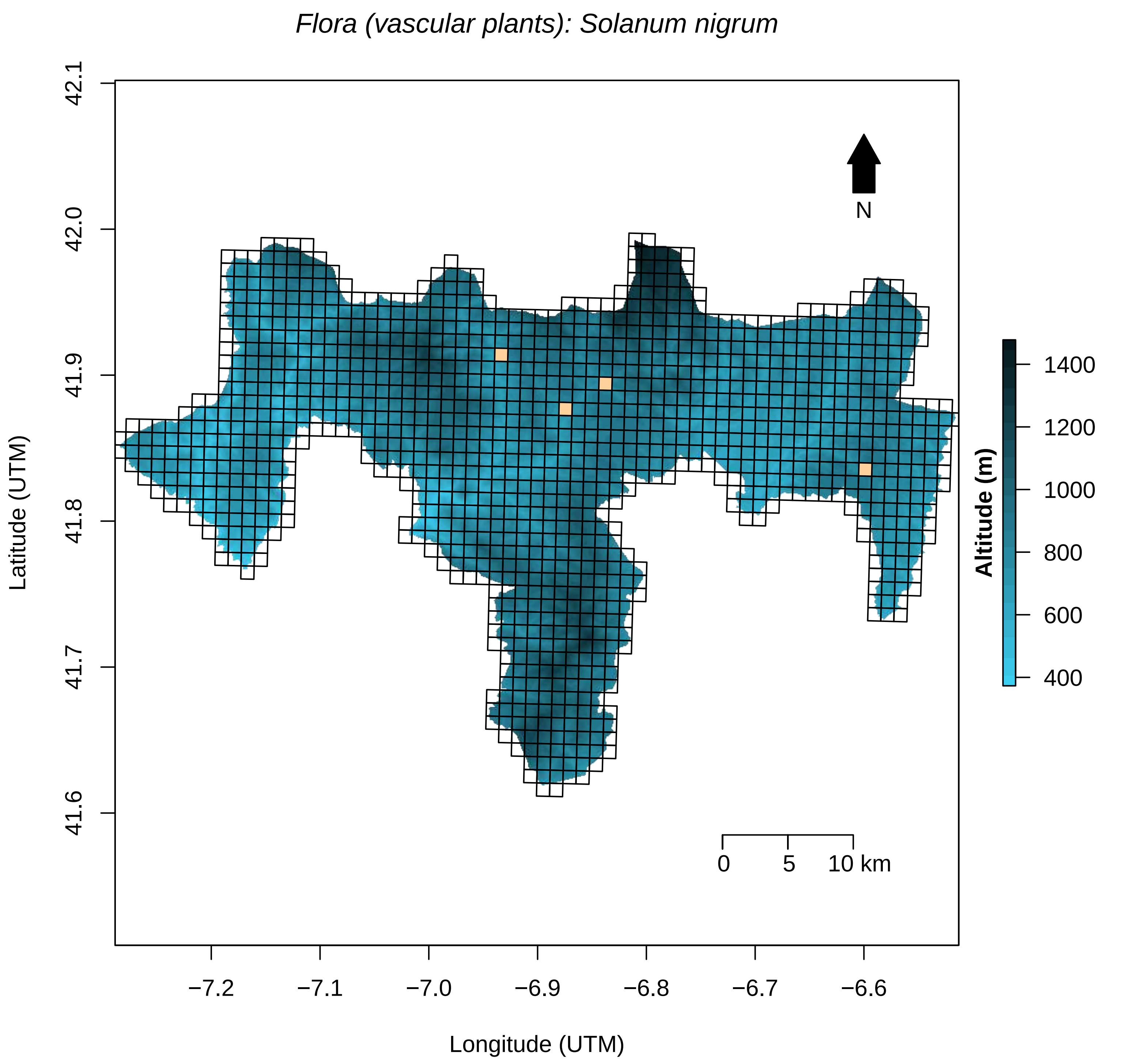
Flora (vascular plants): Solanum nigrum
42.1
42.0
41.9
41.8
41.7
41.6
Latitude (UTM)
−7.2
−7.1
−7.0
−6.9
−6.8
−6.7
−6.6
Longitude (UTM)
N
0
5
10 km
1400
1200
1000
800
600
400
Altitude (m)

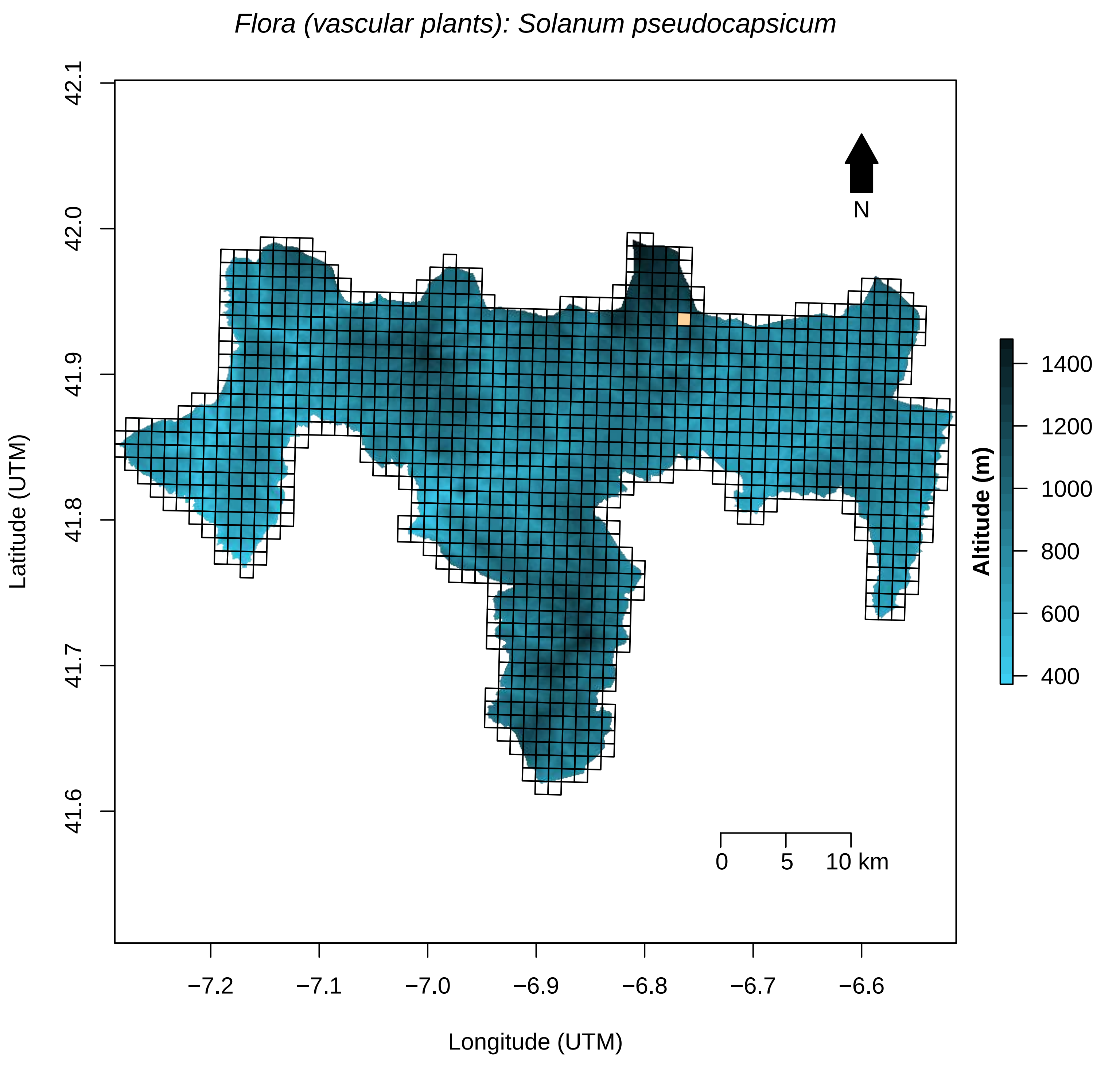

Flora (vascular plants): Solanum pseudocapsicum
42.1
42.0
41.9
41.8
41.7
41.6
Latitude (UTM)
−7.2
−7.1
−7.0
−6.9
−6.8
−6.7
−6.6
Longitude (UTM)
N
0
5
10 km
Altitude (m)
1400
1200
1000
800
600
400

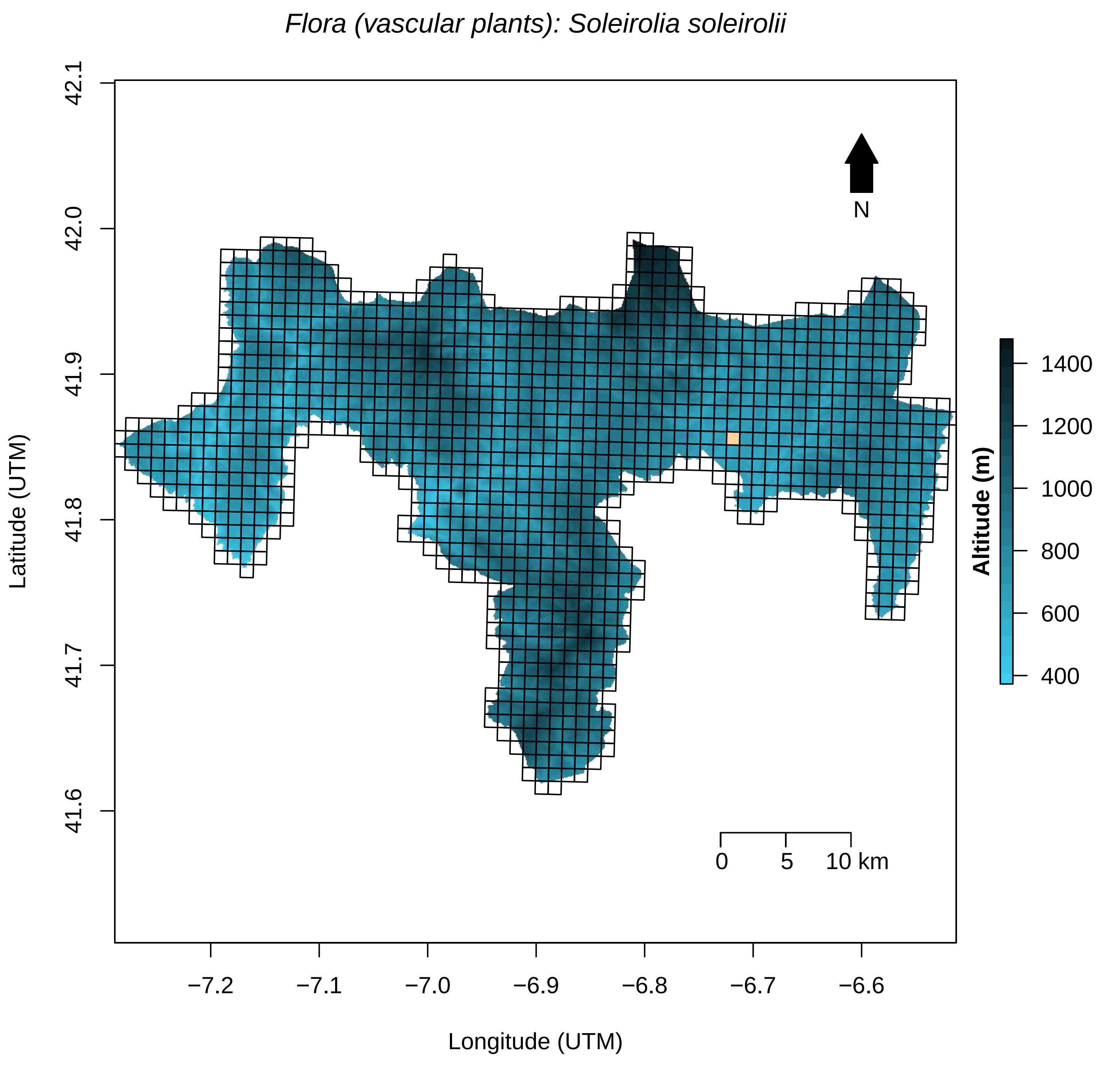

Flora (vascular plants): Soleirolia soleirolii
42.1
42.0
41.9
41.8
41.7
41.6
Latitude (UTM)
−7.2
−7.1
−7.0
−6.9
−6.8
−6.7
−6.6
Longitude (UTM)
N
0
5
10 km
Altitude (m)
1400
1200
1000
800
600
400

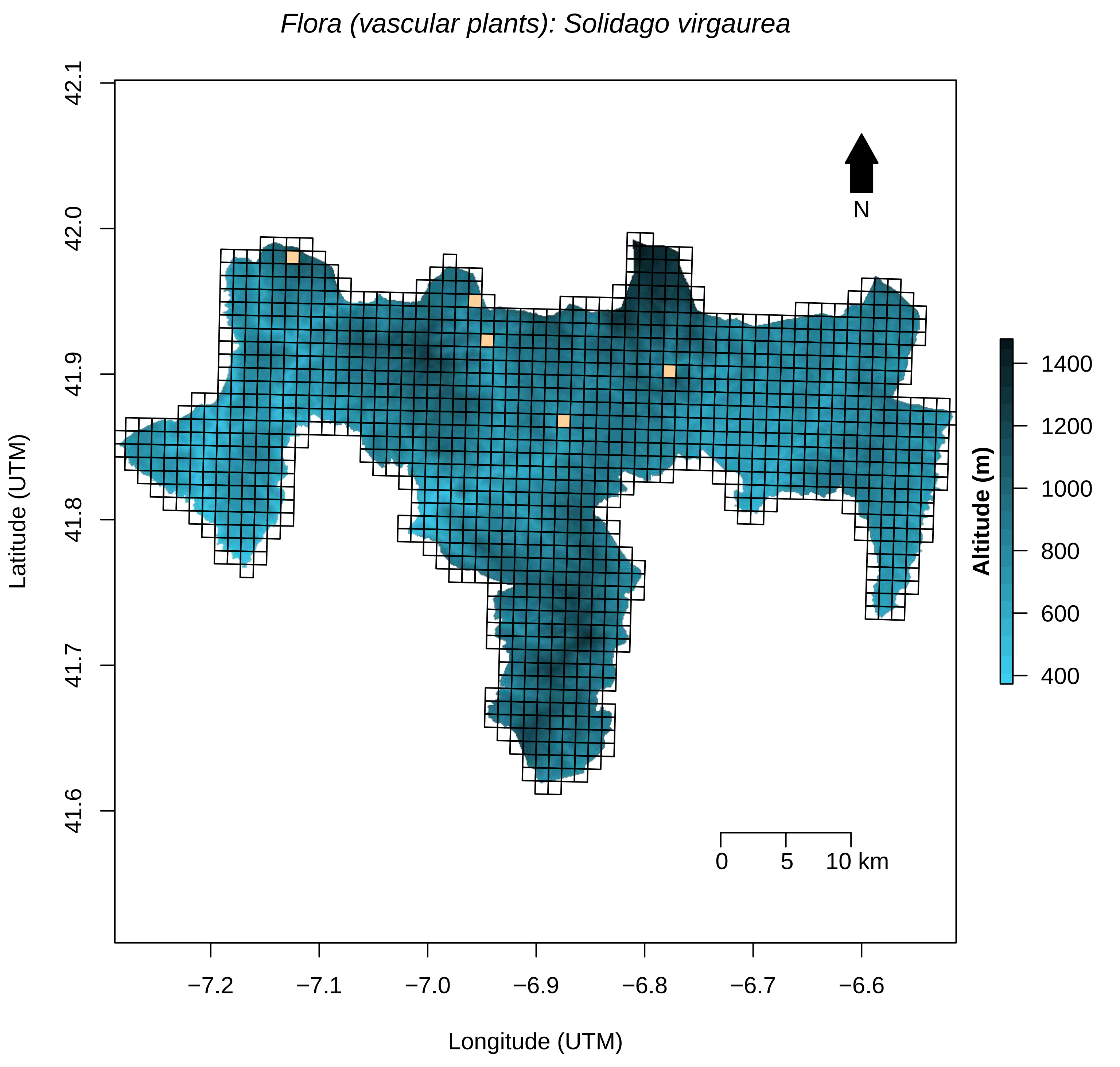
Flora (vascular plants): Solidago virgaurea
Latitude (UTM)
42.1
42.0
41.9
41.8
41.7
41.6
Longitude (UTM)
−7.2
−7.1
−7.0
−6.9
−6.8
−6.7
−6.6
N
0
5
10 km
Altitude (m)
1400
1200
1000
800
600
400

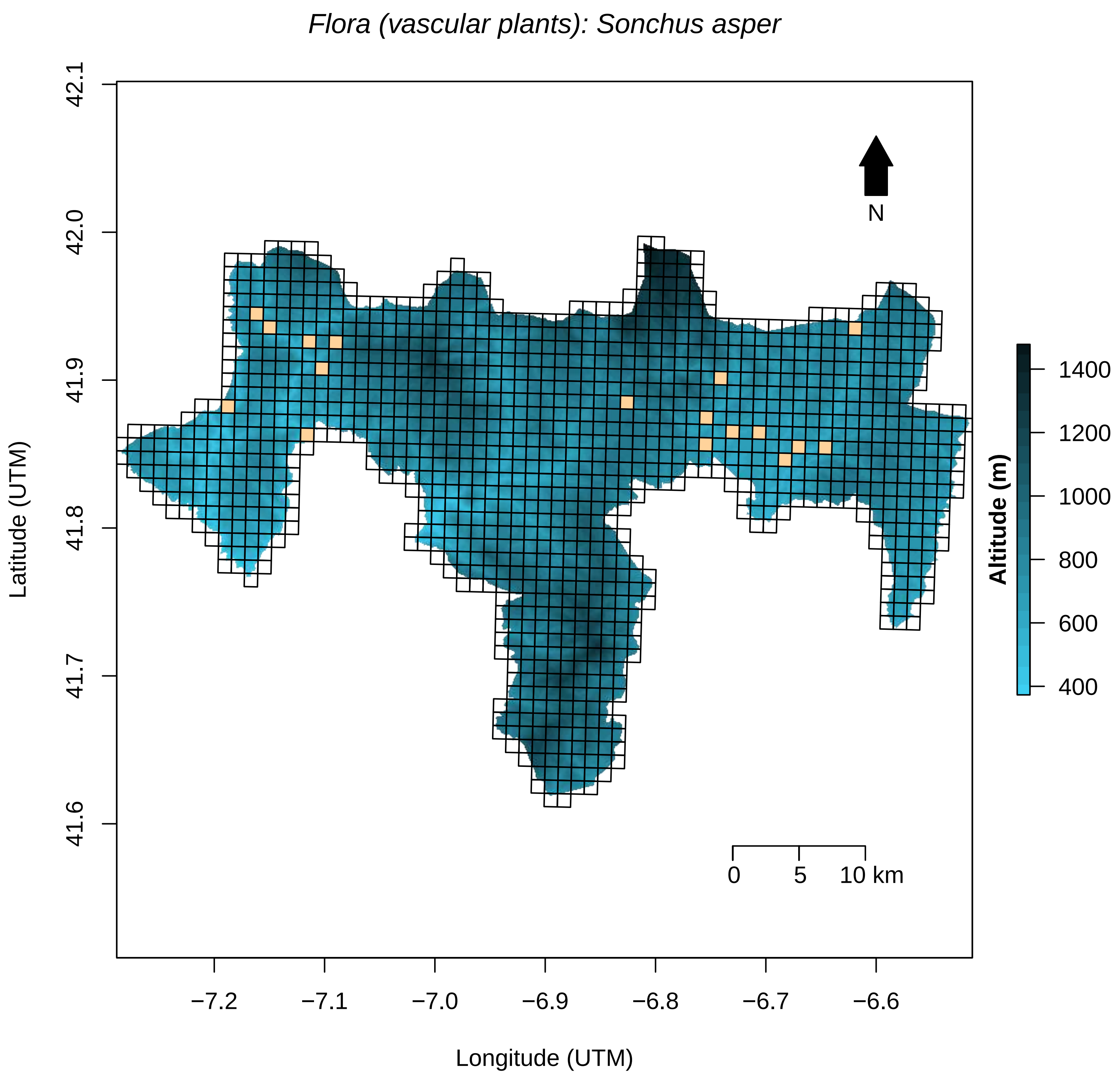

Flora (vascular plants): Sonchus asper
N
0 5 10 km
Latitude (UTM)
Longitude (UTM)
Altitude (m)
42.1
42.0
41.9
41.8
41.7
41.6
−7.2
−7.1
−7.0
−6.9
−6.8
−6.7
−6.6
1400
1200
1000
800
600
400

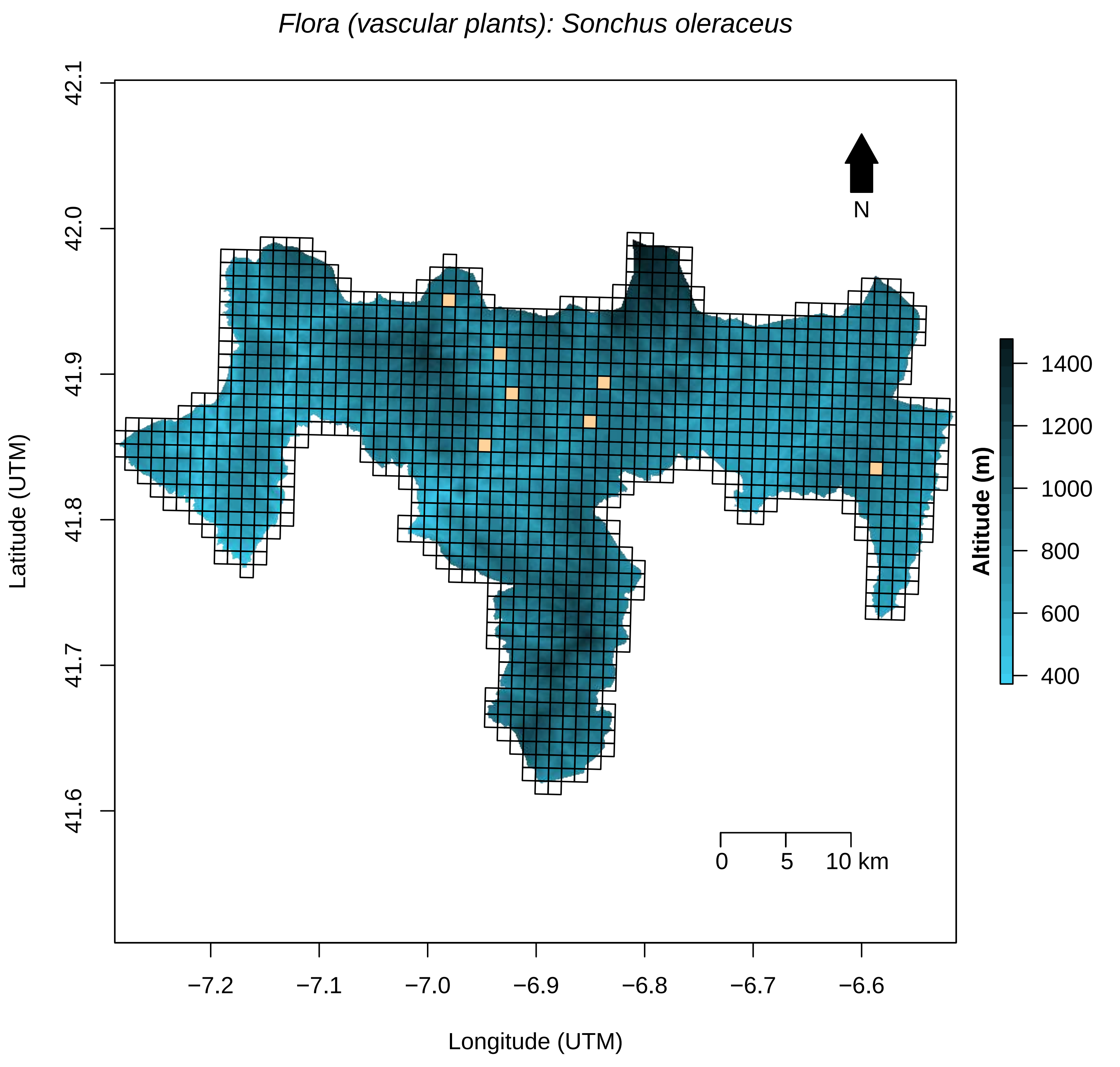
Flora (vascular plants): Sonchus oleraceus
42.1
42.0
41.9
41.8
41.7
41.6
Latitude (UTM)
−7.2
−7.1
−7.0
−6.9
−6.8
−6.7
−6.6
Longitude (UTM)
N
0
5
10 km
1400
1200
1000
800
600
400
Altitude (m)

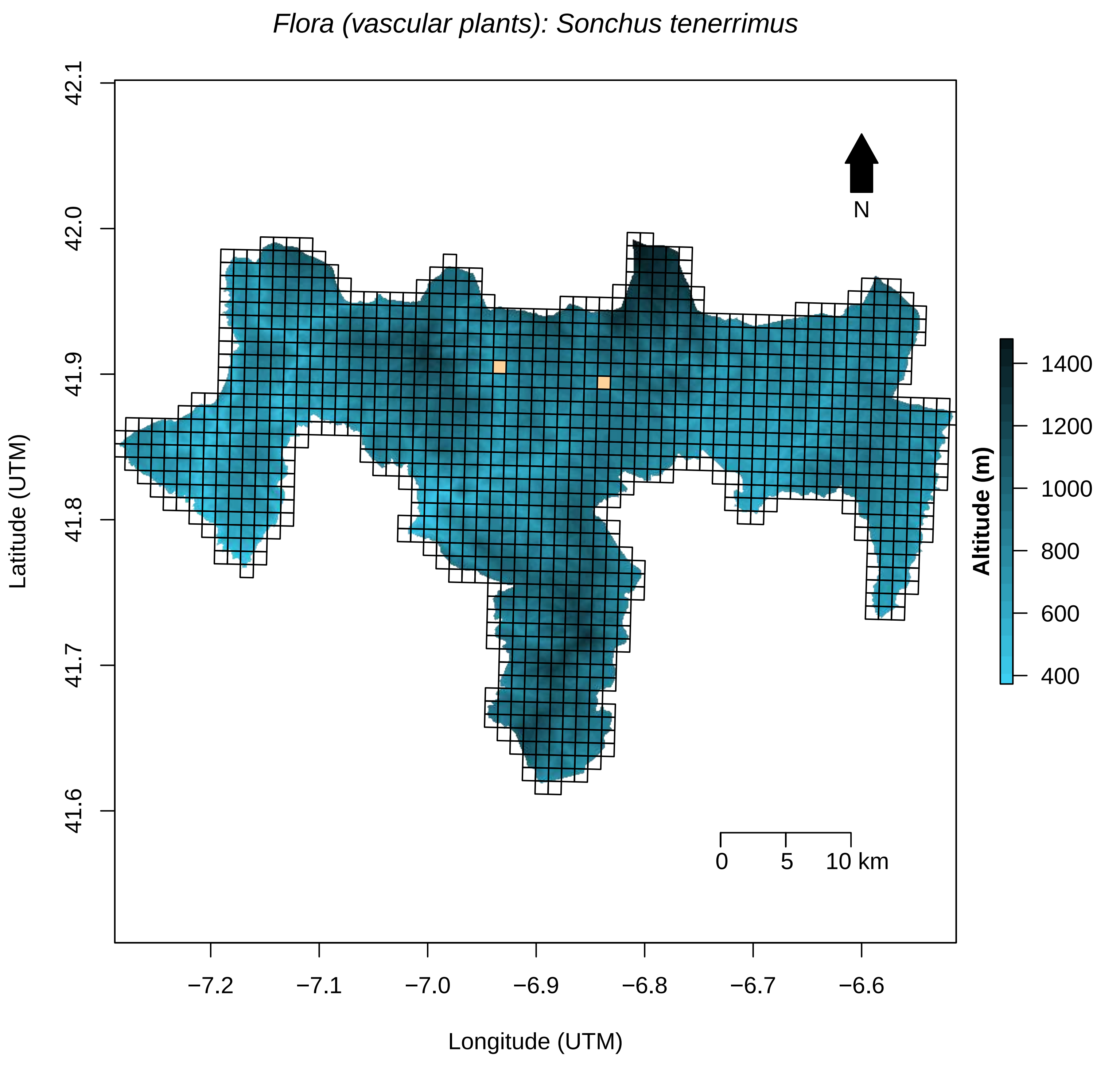

Flora (vascular plants): Sonchus tenerrimus
N
0 5 10 km
Altitude (m)
1400
1200
1000
800
600
400
Latitude (UTM)
42.1
42.0
41.9
41.8
41.7
41.6
Longitude (UTM)
−7.2
−7.1
−7.0
−6.9
−6.8
−6.7
−6.6

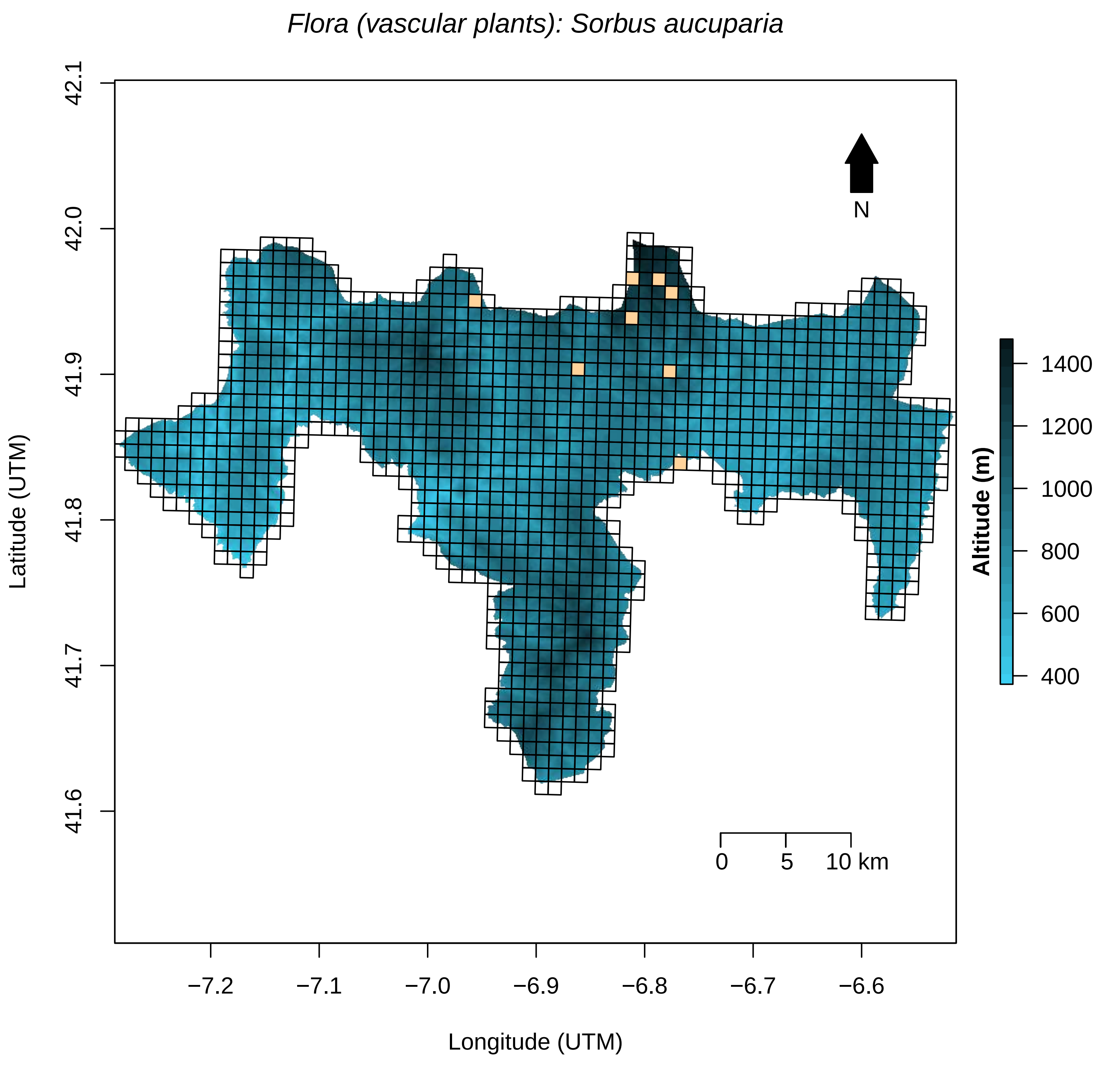

Flora (vascular plants): Sorbus aucuparia
42.1
42.0
41.9
41.8
41.7
41.6
Latitude (UTM)
−7.2
−7.1
−7.0
−6.9
−6.8
−6.7
−6.6
Longitude (UTM)
N
0
5
10 km
Altitude (m)
1400
1200
1000
800
600
400

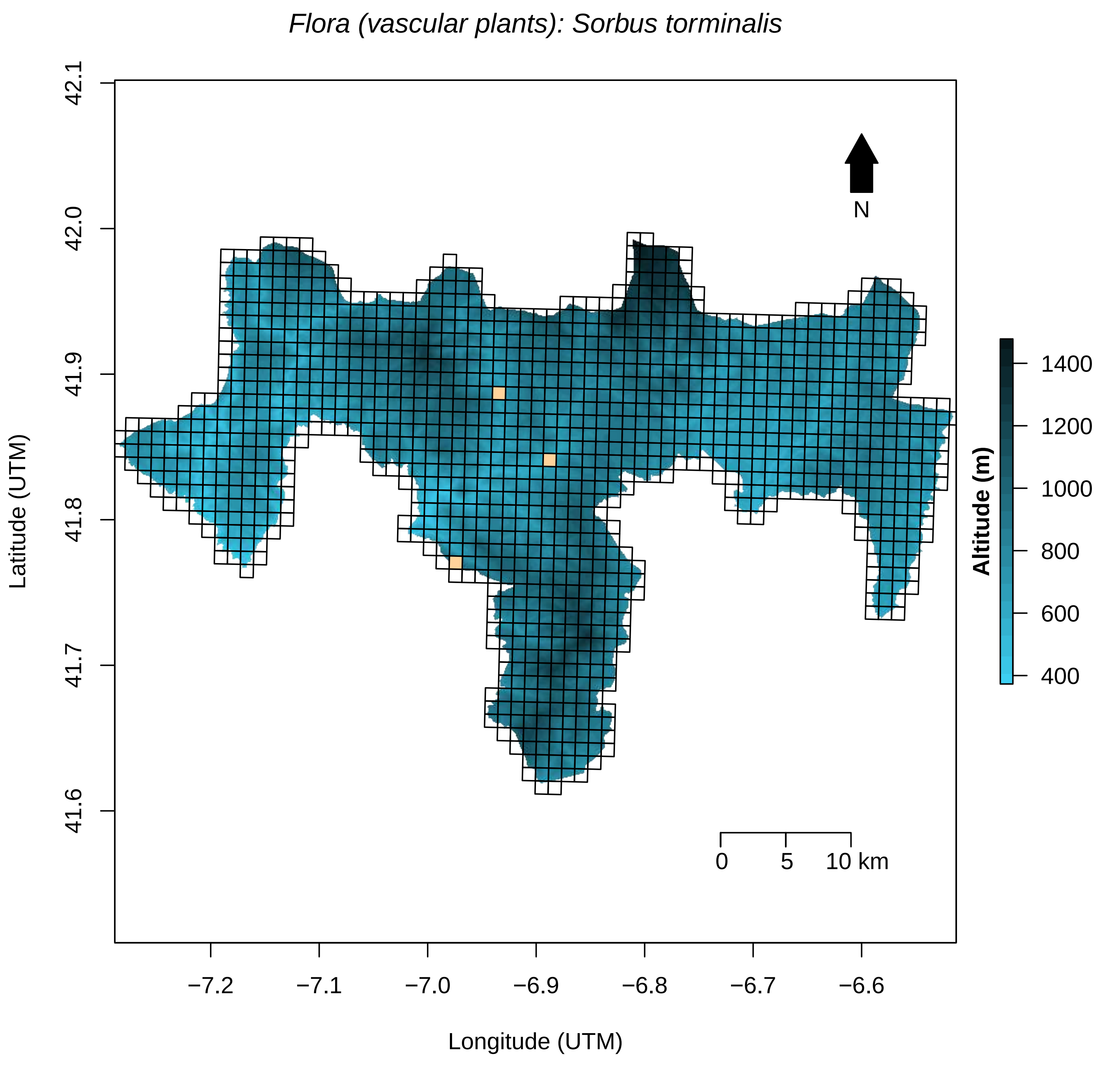
Flora (vascular plants): Sorbus torminalis
Latitude (UTM)
Longitude (UTM)
42.1
42.0
41.9
41.8
41.7
41.6
−7.2
−7.1
−7.0
−6.9
−6.8
−6.7
−6.6
N
0
5
10 km
Altitude (m)
1400
1200
1000
800
600
400

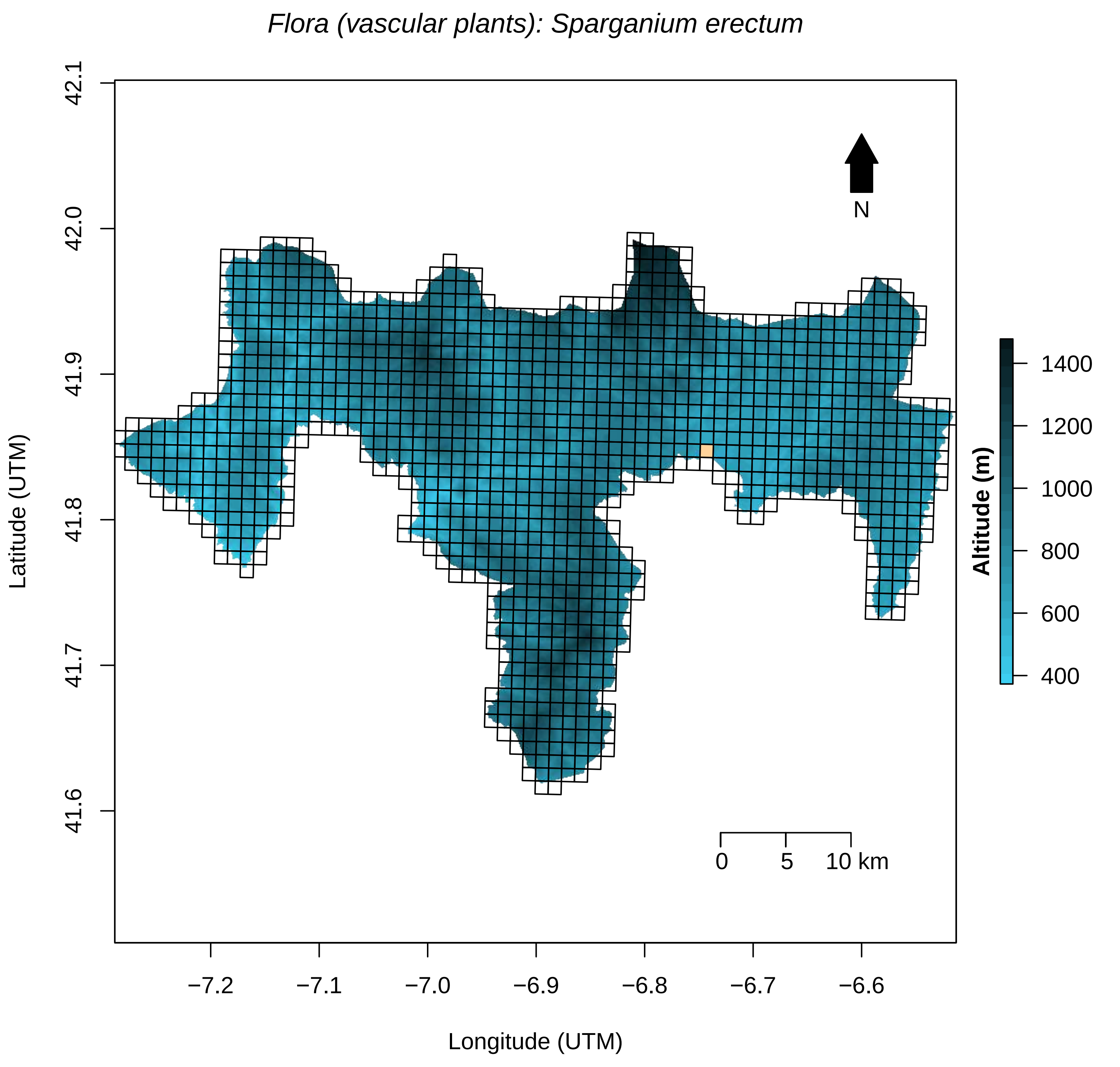

Flora (vascular plants): Sparganium erectum
42.1
42.0
41.9
41.8
41.7
41.6
Latitude (UTM)
N
0
5
10 km
1400
1200
1000
800
600
400
Altitude (m)
−7.2
−7.1
−7.0
−6.9
−6.8
−6.7
−6.6
Longitude (UTM)

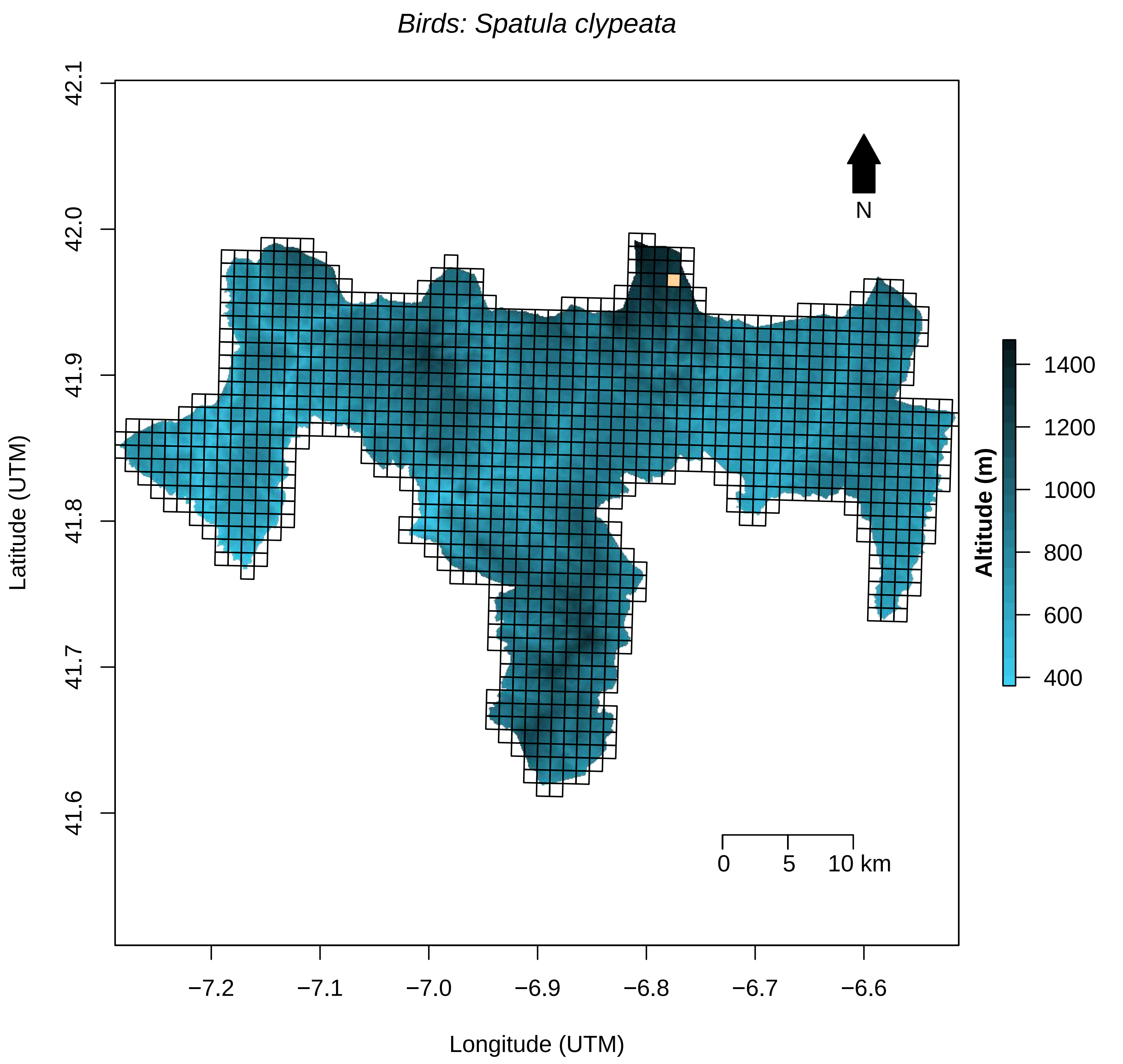

Birds: Spatula clypeata
42.1
42.0
41.9
41.8
41.7
41.6
Latitude (UTM)
−7.2
−7.1
−7.0
−6.9
−6.8
−6.7
−6.6
Longitude (UTM)
N
0
5
10 km
Altitude (m)
1400
1200
1000
800
600
400

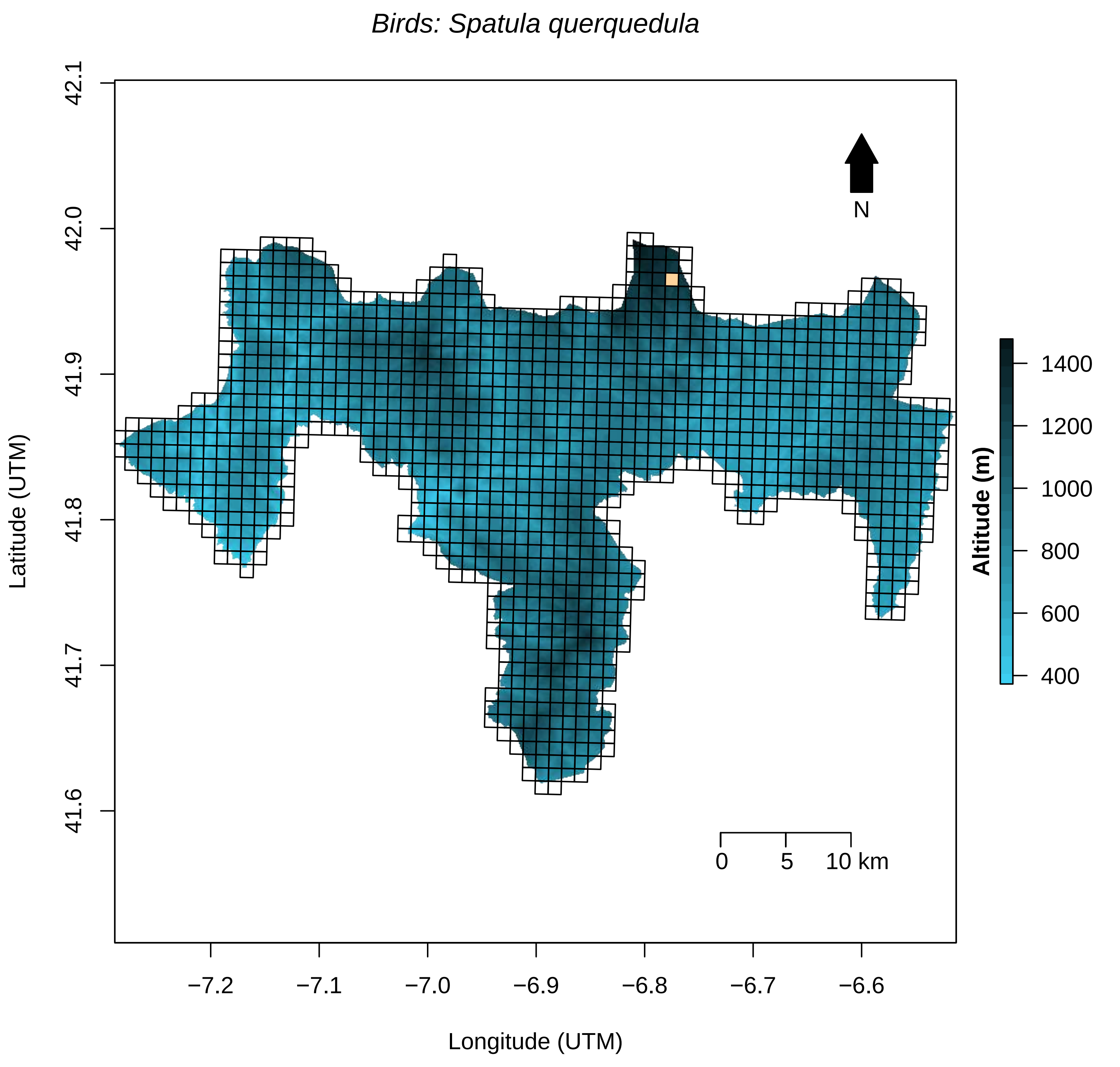

Birds: Spatula querquedula
N
0
5
10 km
Altitude (m)
1400
1200
1000
800
600
400
42.1
42.0
41.9
41.8
41.7
41.6
Latitude (UTM)
−7.2
−7.1
−7.0
−6.9
−6.8
−6.7
−6.6
Longitude (UTM)

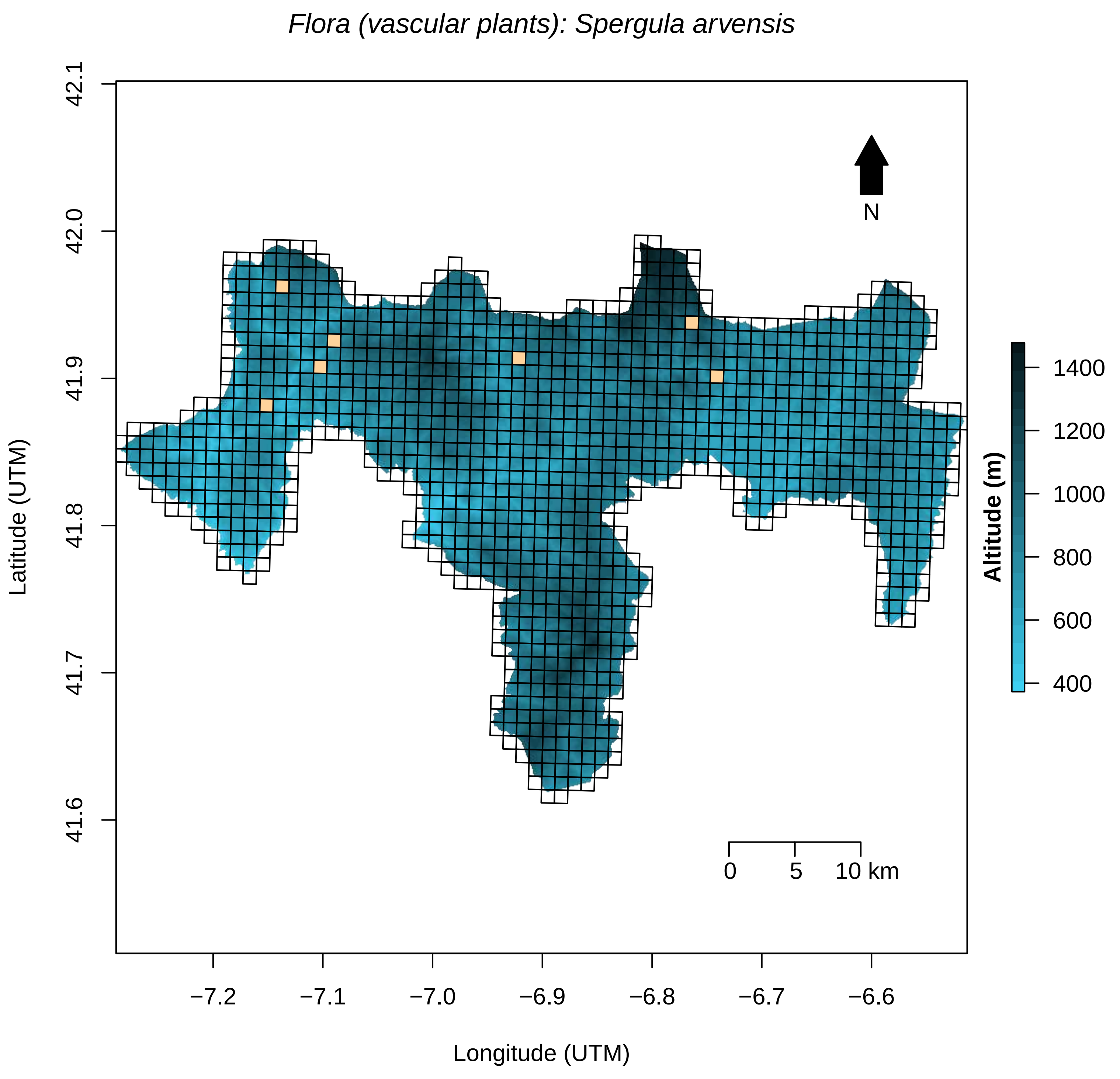
Flora (vascular plants): Spergula arvensis
42.1
42.0
41.9
41.8
41.7
41.6
Latitude (UTM)
−7.2
−7.1
−7.0
−6.9
−6.8
−6.7
−6.6
Longitude (UTM)
N
0
5
10 km
Altitude (m)
1400
1200
1000
800
600
400

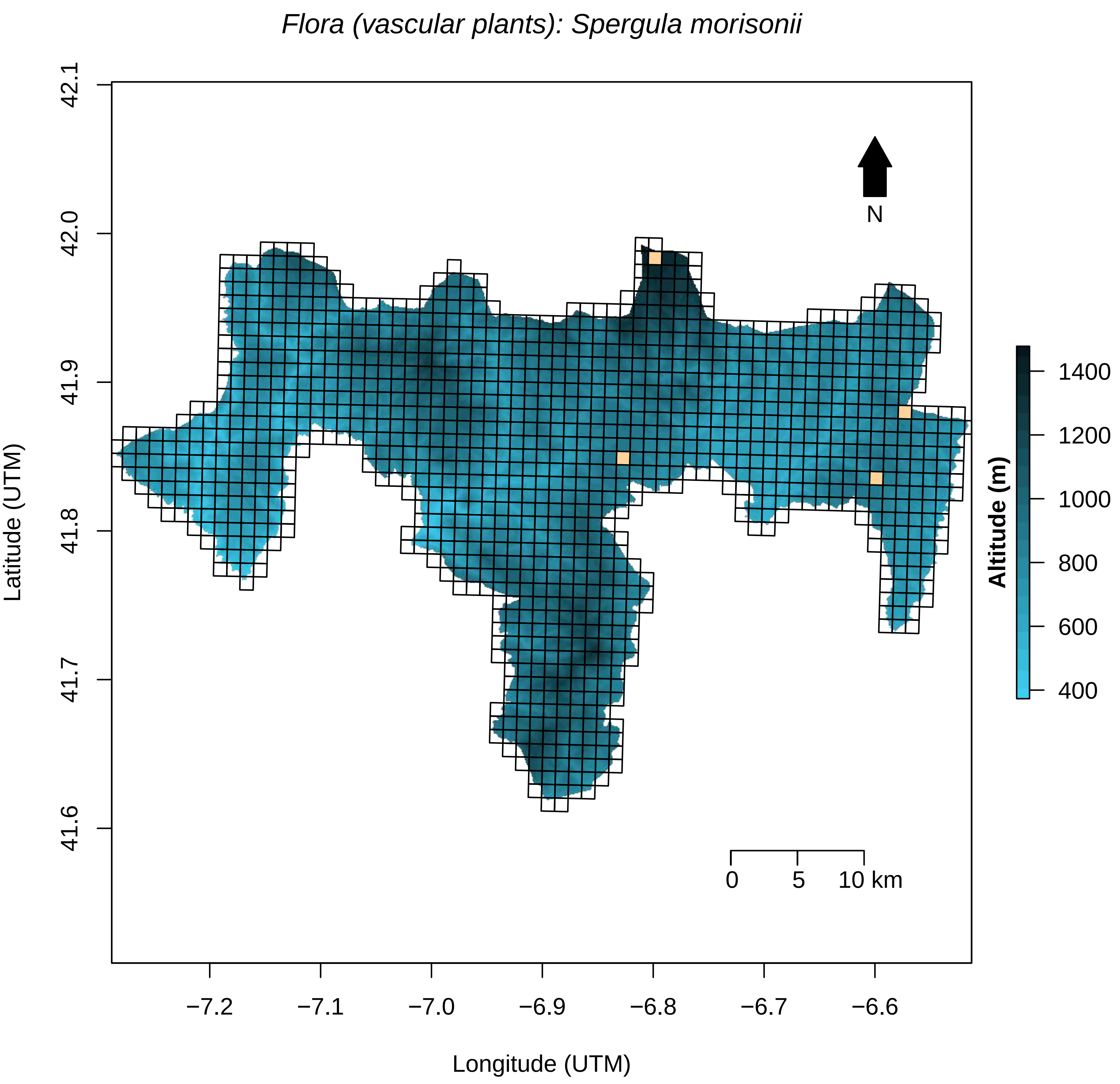

Flora (vascular plants): Spergula morisonii
Latitude (UTM)
Longitude (UTM)
42.1
42.0
41.9
41.8
41.7
41.6
−7.2
−7.1
−7.0
−6.9
−6.8
−6.7
−6.6
N
0
5
10 km
Altitude (m)
1400
1200
1000
800
600
400

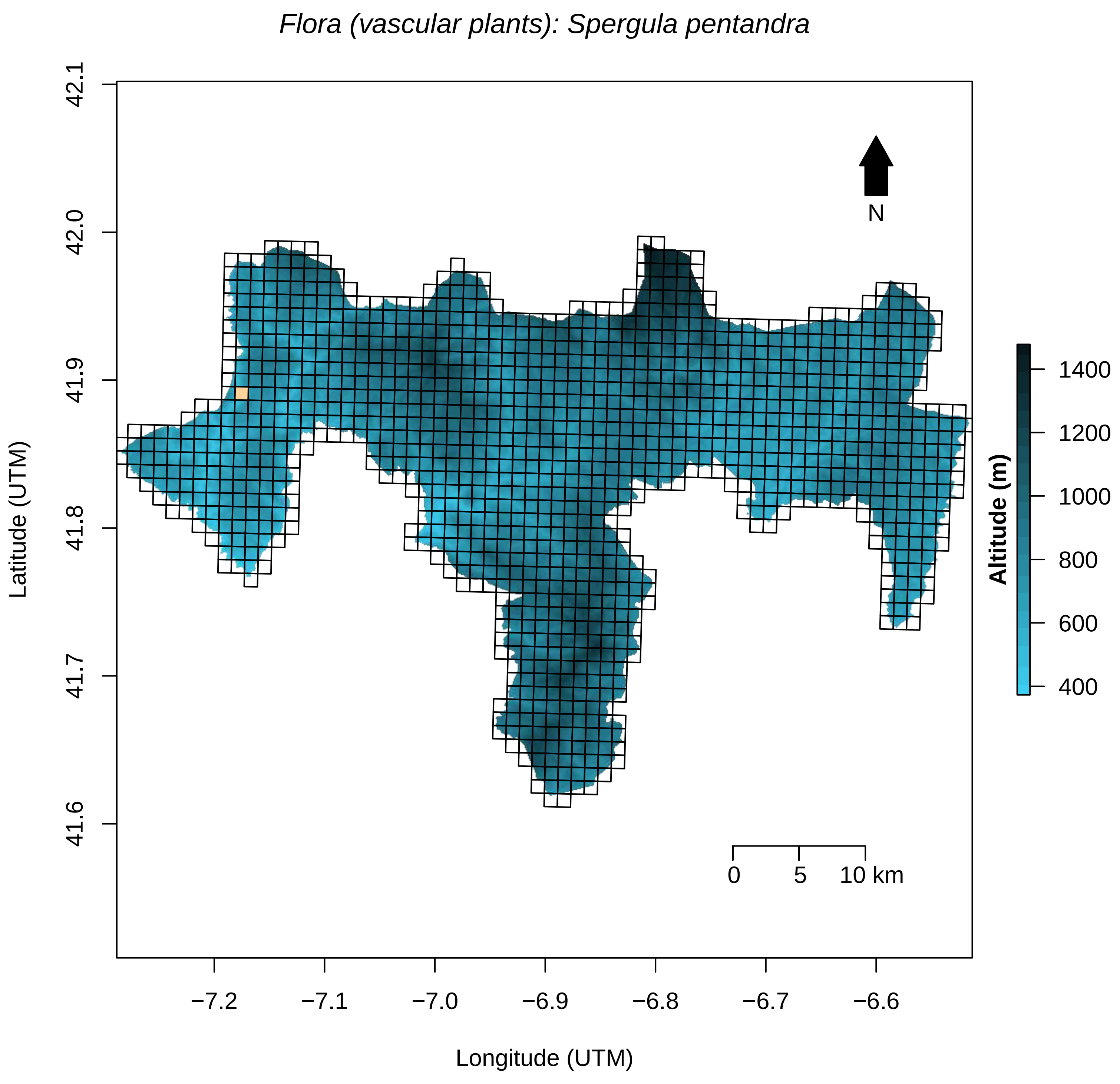

Flora (vascular plants): Spergula pentandra
42.1
42.0
41.9
41.8
41.7
41.6
Latitude (UTM)
−7.2
−7.1
−7.0
−6.9
−6.8
−6.7
−6.6
Longitude (UTM)
N
0
5
10 km
Altitude (m)
1400
1200
1000
800
600
400

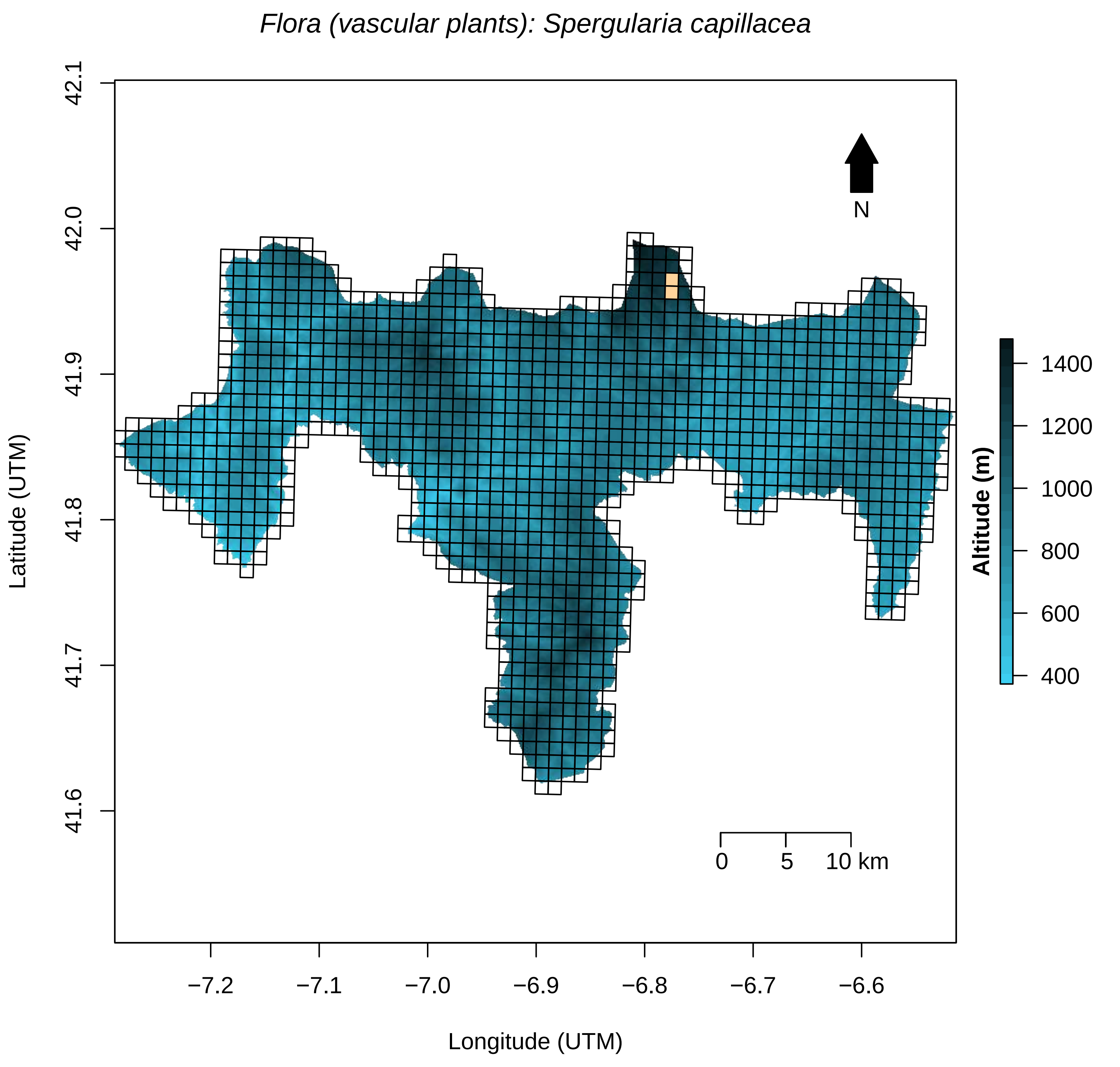
Flora (vascular plants): Spergularia capillacea
N
0
5
10 km
Altitude (m)
1400
1200
1000
800
600
400
Latitude (UTM)
42.1
42.0
41.9
41.8
41.7
41.6
Longitude (UTM)
−7.2
−7.1
−7.0
−6.9
−6.8
−6.7
−6.6

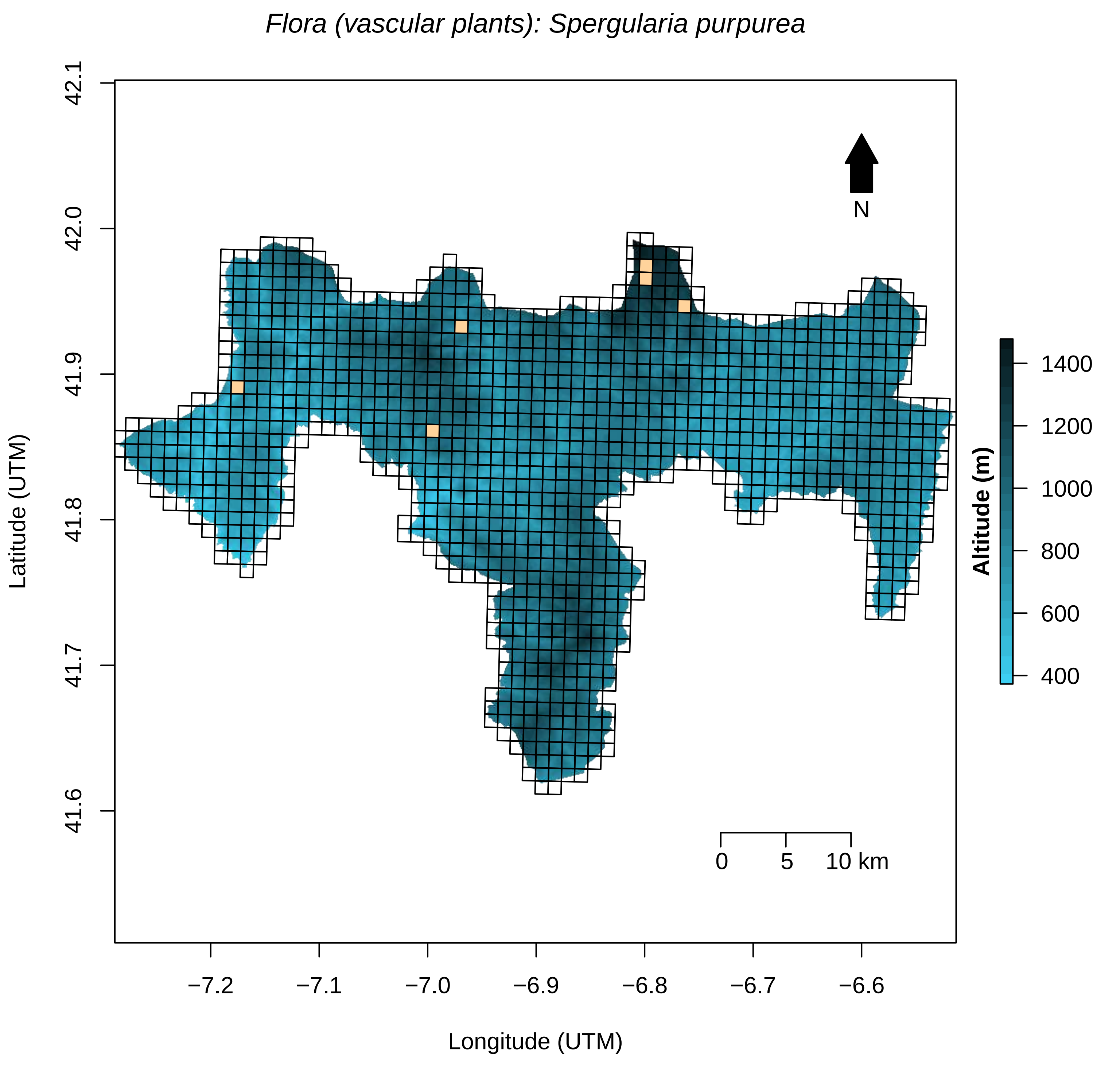

Flora (vascular plants): Spergularia purpurea
42.1
42.0
41.9
41.8
41.7
41.6
Latitude (UTM)
−7.2
−7.1
−7.0
−6.9
−6.8
−6.7
−6.6
Longitude (UTM)
N
0
5
10 km
1400
1200
1000
800
600
400
Altitude (m)

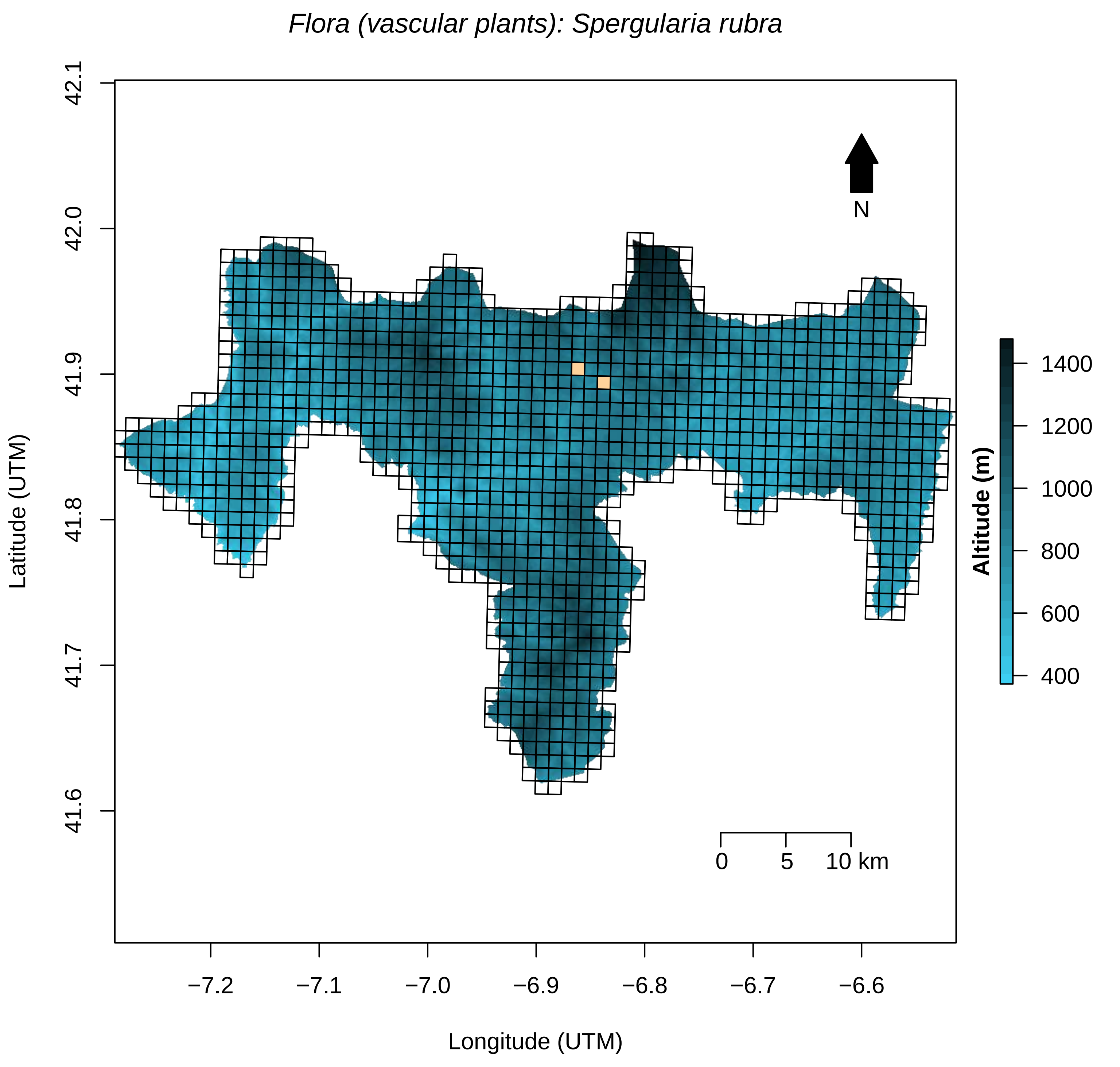

Flora (vascular plants): Spergularia rubra
N
0
5
10 km
Altitude (m)
1400
1200
1000
800
600
400
42.1
42.0
41.9
41.8
41.7
41.6
Latitude (UTM)
−7.2
−7.1
−7.0
−6.9
−6.8
−6.7
−6.6
Longitude (UTM)

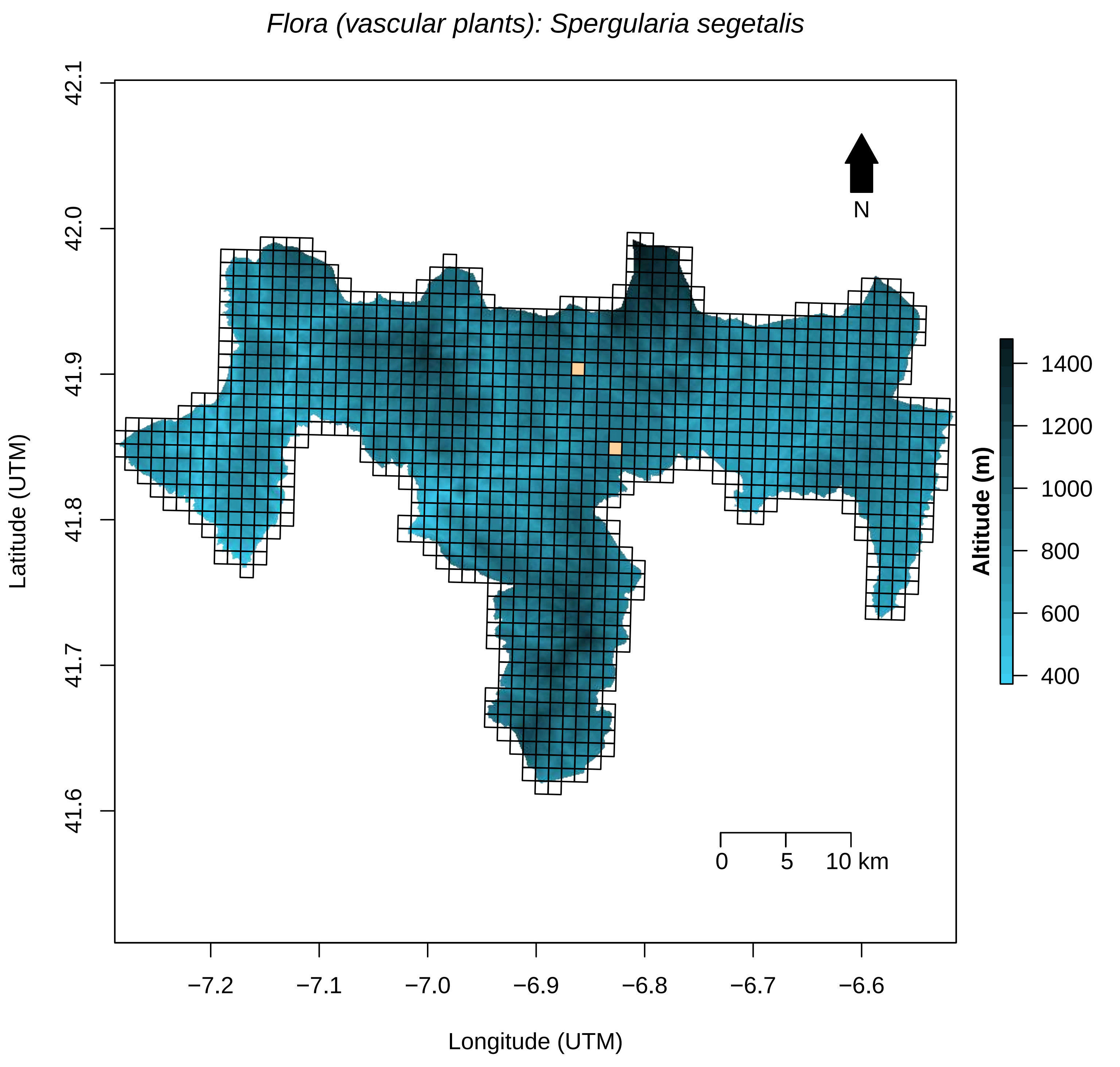

Flora (vascular plants): Spergularia segetalis
42.1
42.0
41.9
41.8
41.7
41.6
Latitude (UTM)
−7.2
−7.1
−7.0
−6.9
−6.8
−6.7
−6.6
Longitude (UTM)
N
0
5
10 km
Altitude (m)
1400
1200
1000
800
600
400

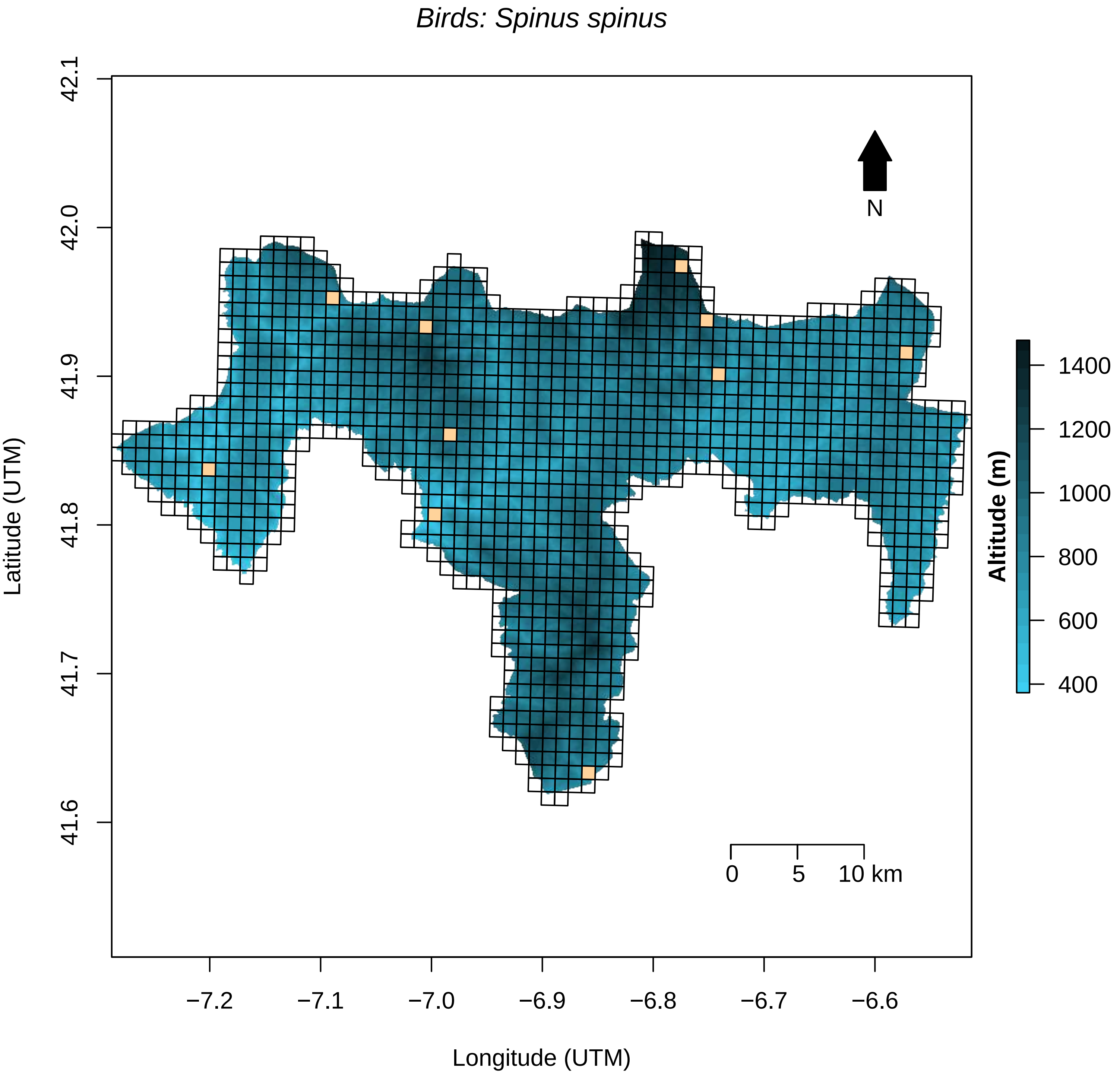
Birds: Spinus spinus
N
0
5
10 km
Altitude (m)
1400
1200
1000
800
600
400
Latitude (UTM)
42.1
42.0
41.9
41.8
41.7
41.6
Longitude (UTM)
−7.2
−7.1
−7.0
−6.9
−6.8
−6.7
−6.6

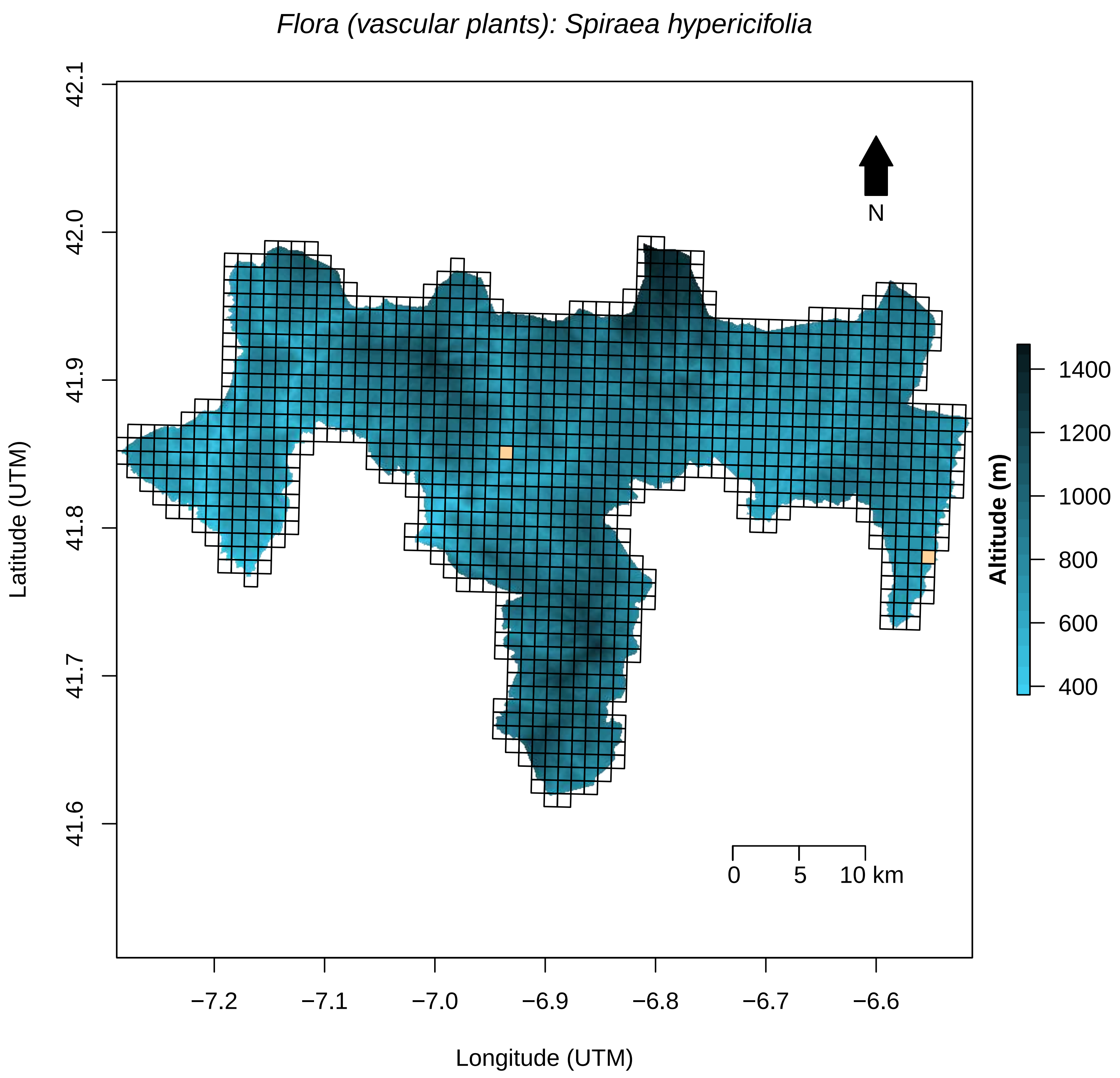

Flora (vascular plants): Spiraea hypericifolia
N
0 5 10 km
Altitude (m)
1400
1200
1000
800
600
400
Latitude (UTM)
42.1
42.0
41.9
41.8
41.7
41.6
Longitude (UTM)
−7.2
−7.1
−7.0
−6.9
−6.8
−6.7
−6.6

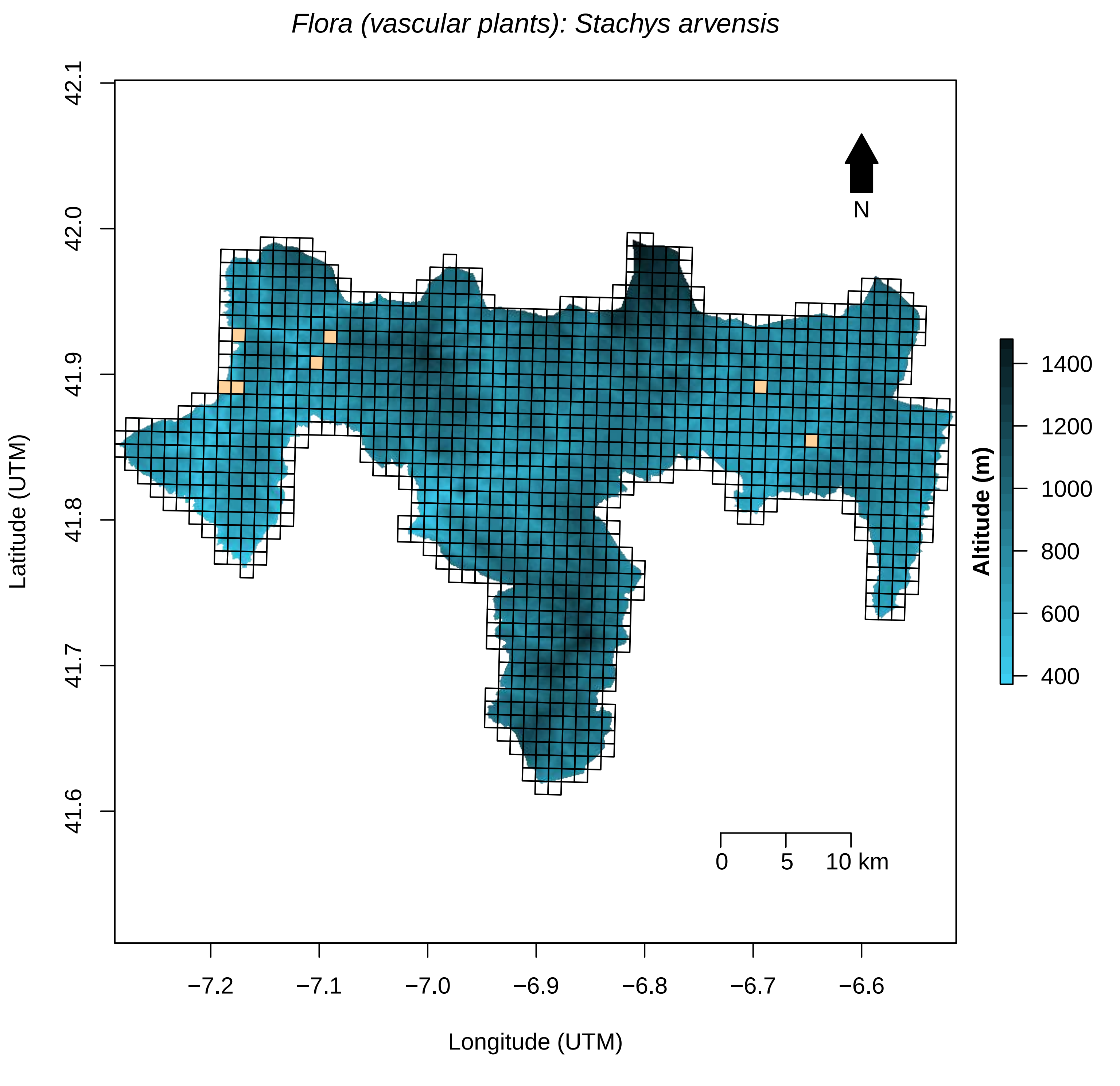
Flora (vascular plants): Stachys arvensis
42.1
42.0
41.9
41.8
41.7
41.6
Latitude (UTM)
−7.2
−7.1
−7.0
−6.9
−6.8
−6.7
−6.6
Longitude (UTM)
N
0
5
10 km
Altitude (m)
1400
1200
1000
800
600
400

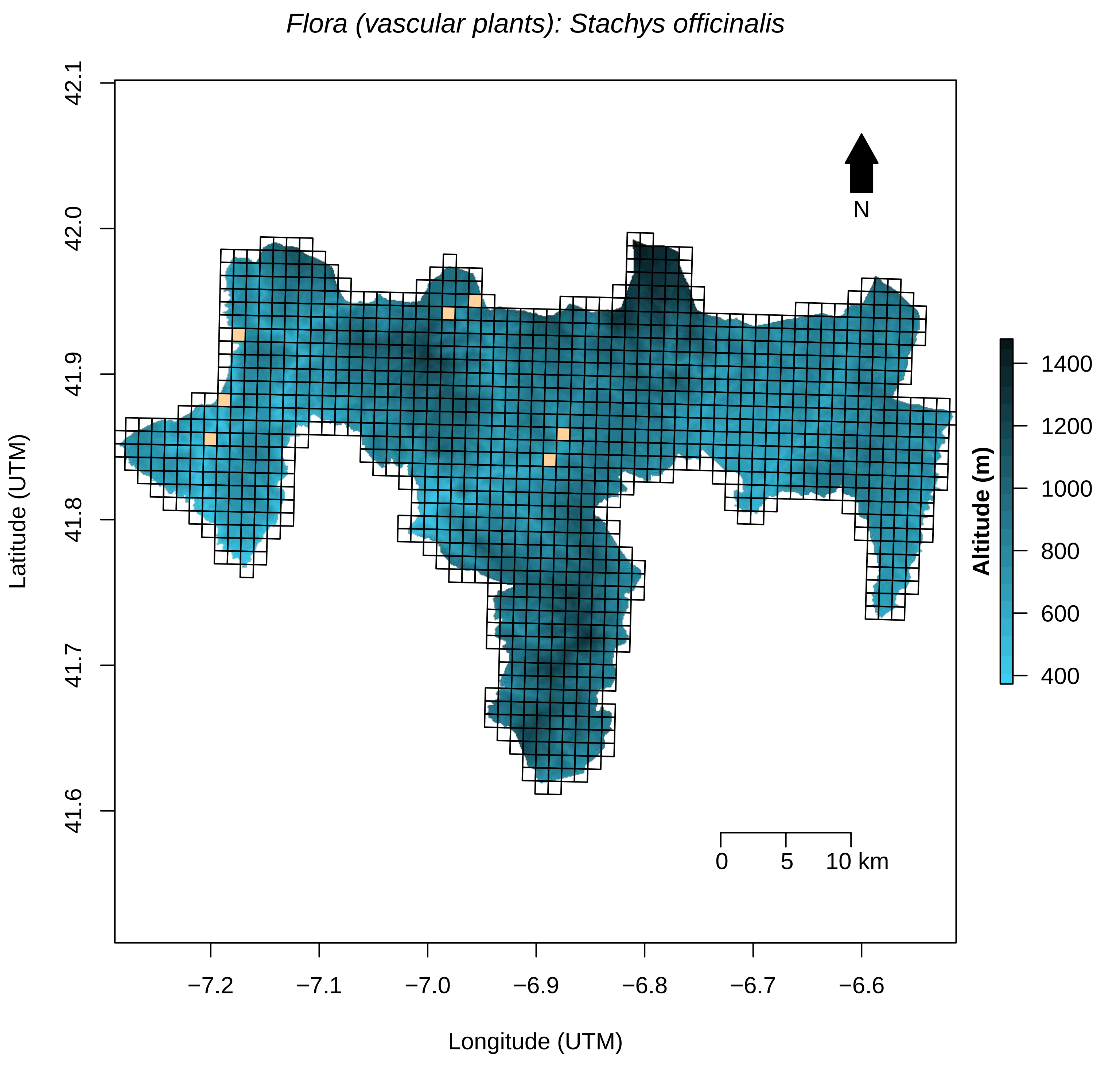

Flora (vascular plants): Stachys officinalis
42.1
42.0
41.9
41.8
41.7
41.6
Latitude (UTM)
−7.2
−7.1
−7.0
−6.9
−6.8
−6.7
−6.6
Longitude (UTM)
N
0
5
10 km
Altitude (m)
1400
1200
1000
800
600
400

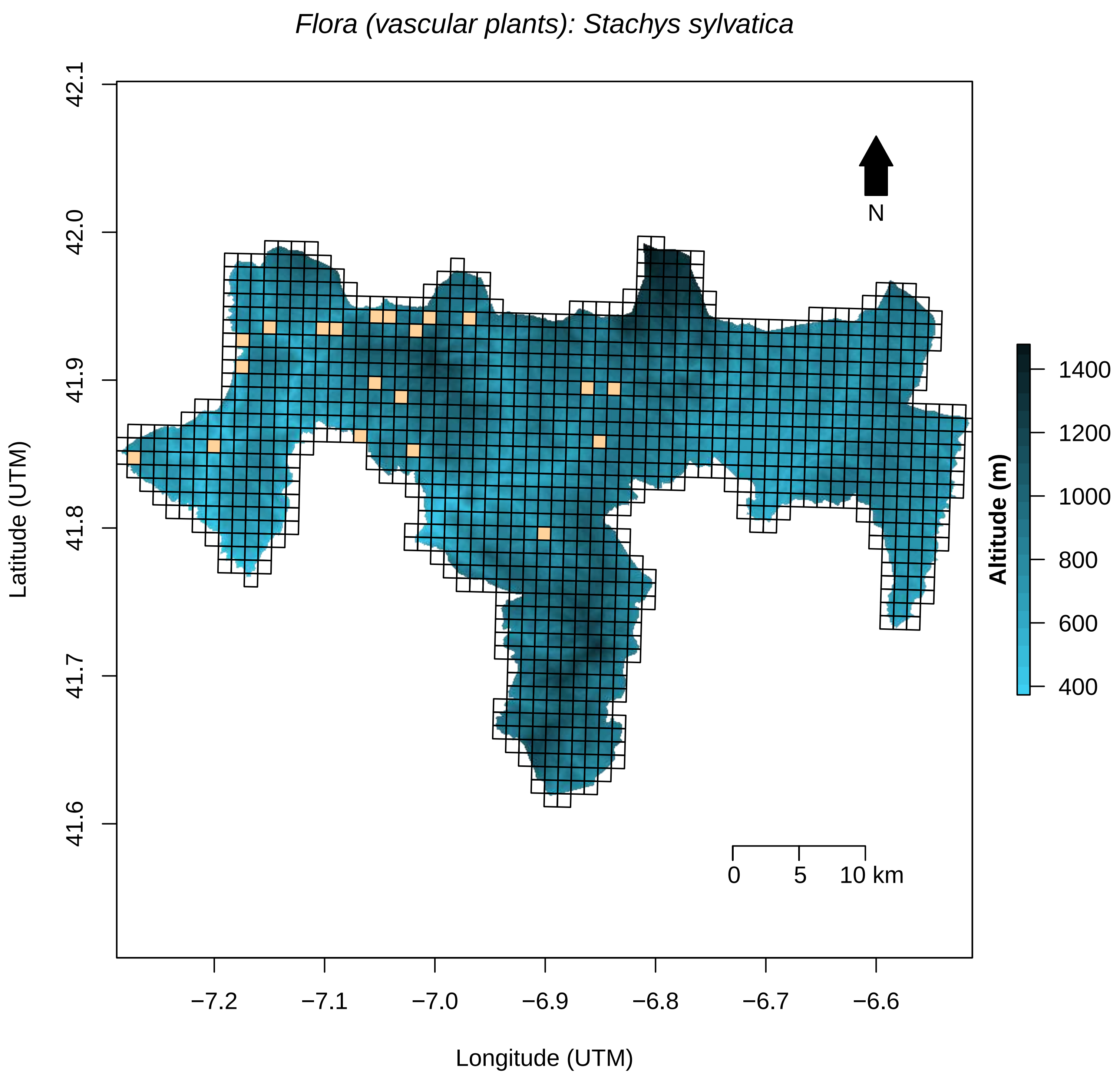

Flora (vascular plants): Stachys sylvatica
42.1
42.0
41.9
41.8
41.7
41.6
Latitude (UTM)
−7.2
−7.1
−7.0
−6.9
−6.8
−6.7
−6.6
Longitude (UTM)
N
0
5
10 km
Altitude (m)
1400
1200
1000
800
600
400

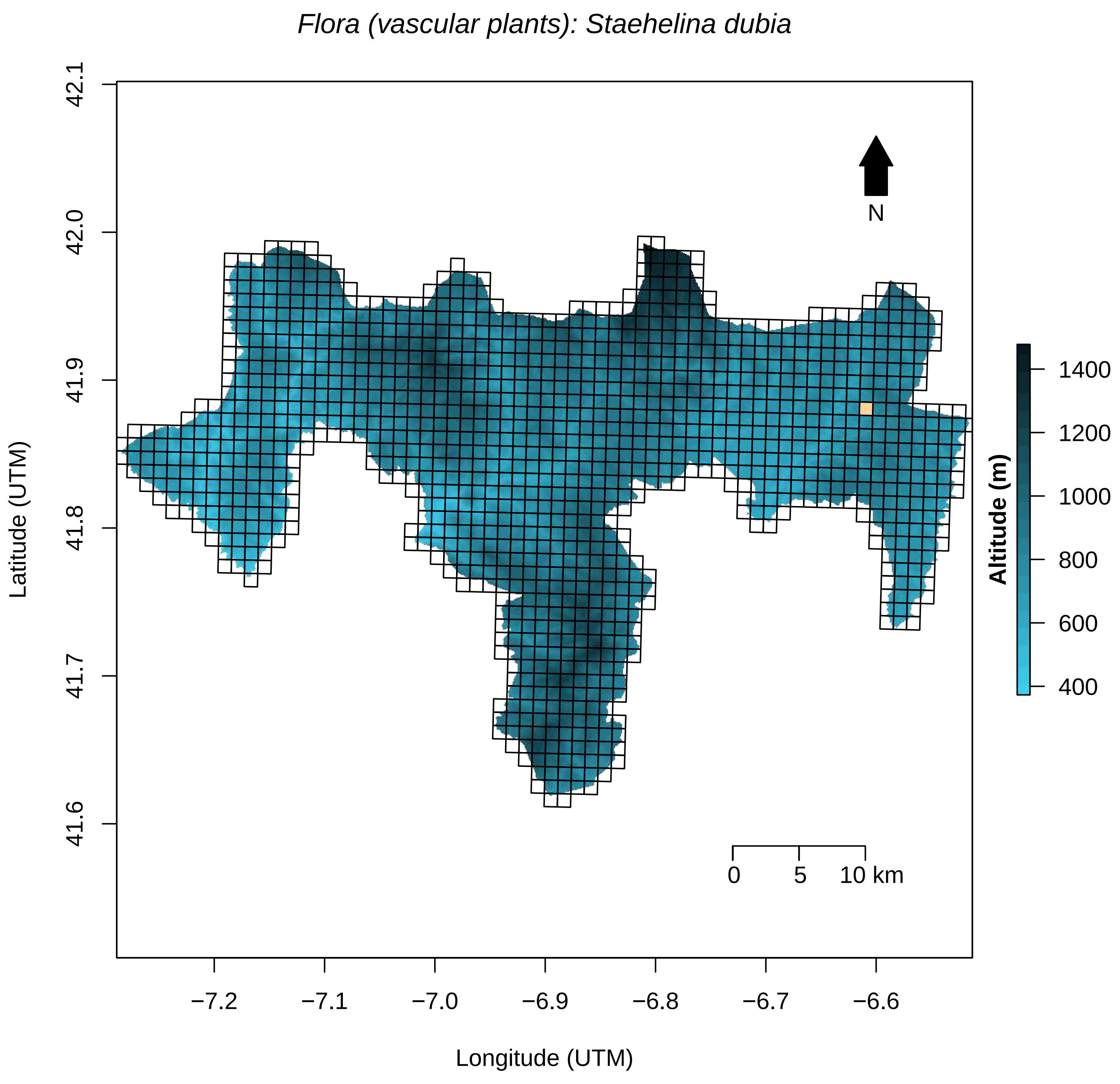
Flora (vascular plants): Staehelina dubia
N
0 5 10 km
Altitude (m)
1400
1200
1000
800
600
400
Latitude (UTM)
42.1
42.0
41.9
41.8
41.7
41.6
Longitude (UTM)
−7.2
−7.1
−7.0
−6.9
−6.8
−6.7
−6.6

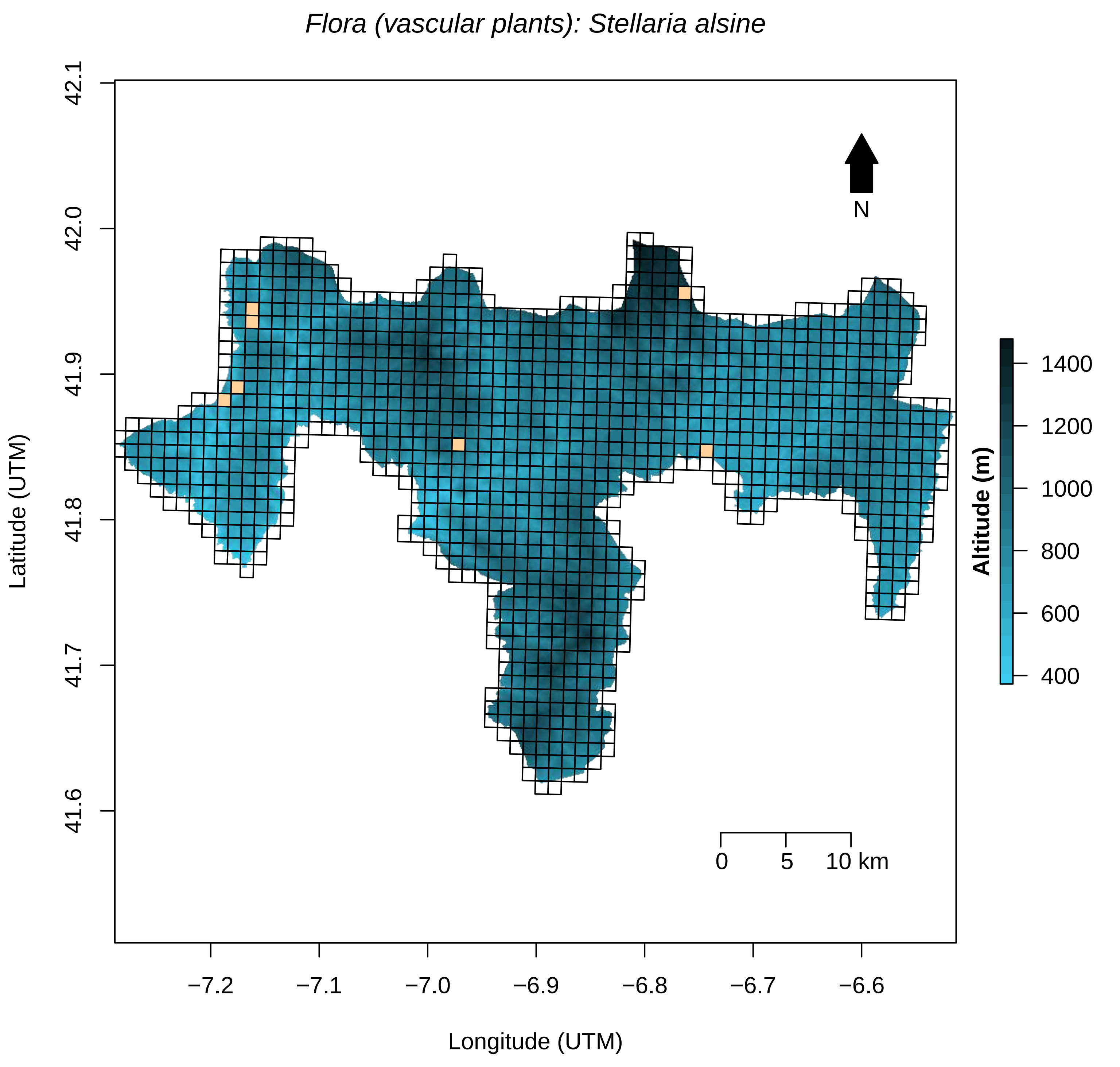

Flora (vascular plants): Stellaria alsine
42.1
42.0
41.9
41.8
41.7
41.6
Latitude (UTM)
−7.2
−7.1
−7.0
−6.9
−6.8
−6.7
−6.6
Longitude (UTM)
N
0
5
10 km
Altitude (m)
1400
1200
1000
800
600
400

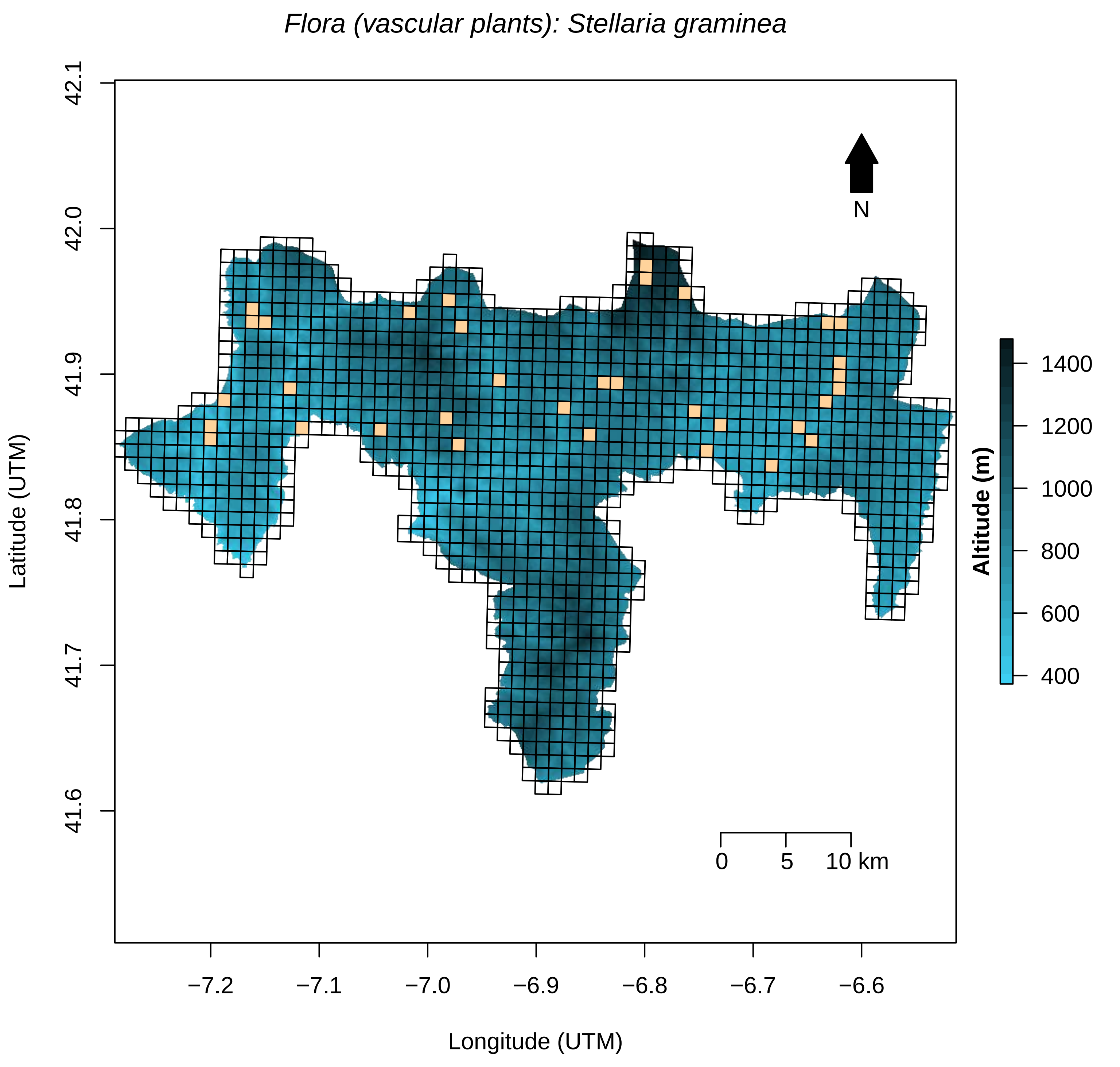

Flora (vascular plants): Stellaria graminea
42.1
42.0
41.9
41.8
41.7
41.6
Latitude (UTM)
N
0
5
10 km
−7.2
−7.1
−7.0
−6.9
−6.8
−6.7
−6.6
Longitude (UTM)
Altitude (m)
1400
1200
1000
800
600
400

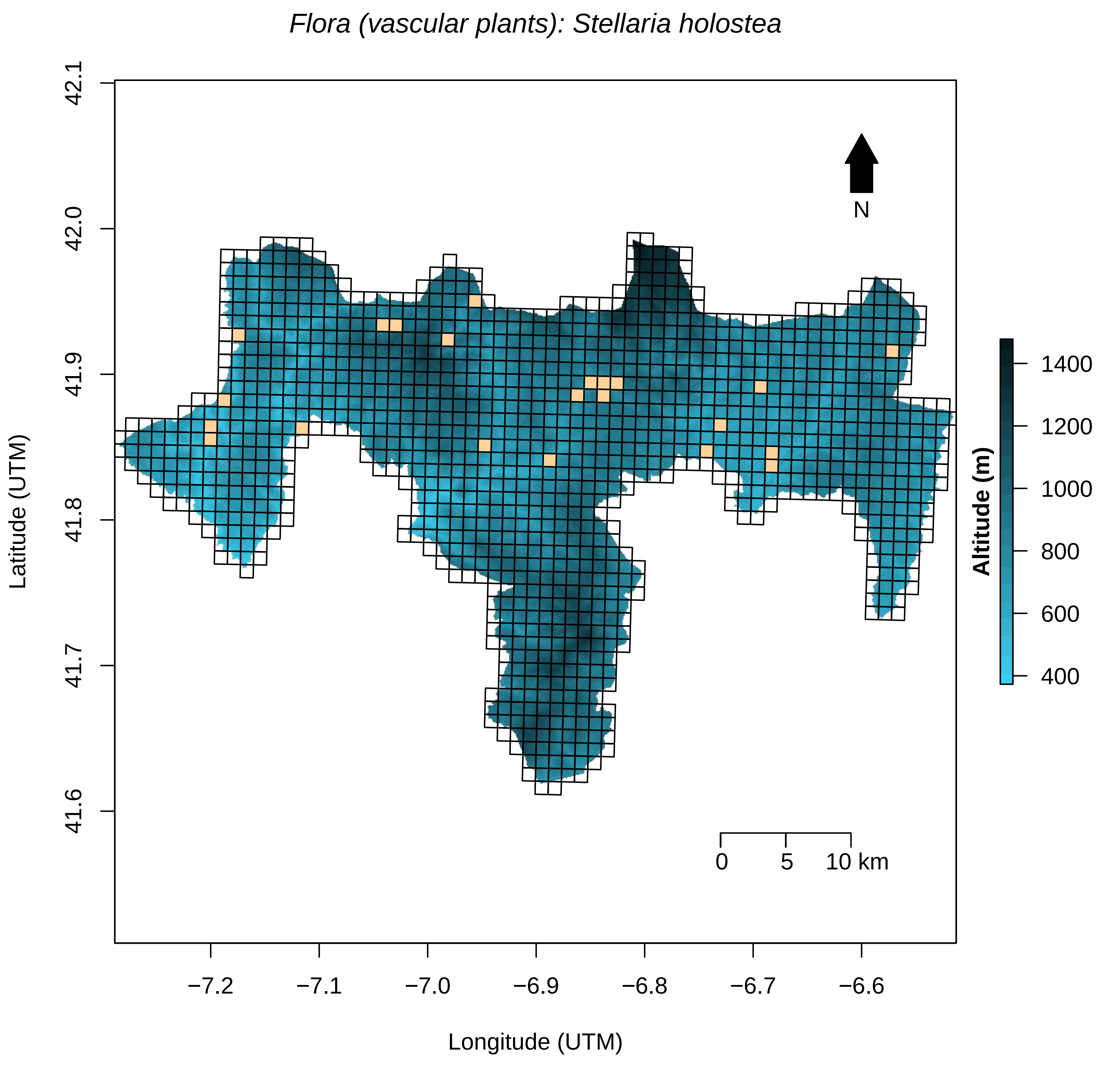

Flora (vascular plants): Stellaria holostea
42.1
42.0
41.9
41.8
41.7
41.6
Latitude (UTM)
−7.2
−7.1
−7.0
−6.9
−6.8
−6.7
−6.6
Longitude (UTM)
N
0
5
10 km
Altitude (m)
1400
1200
1000
800
600
400

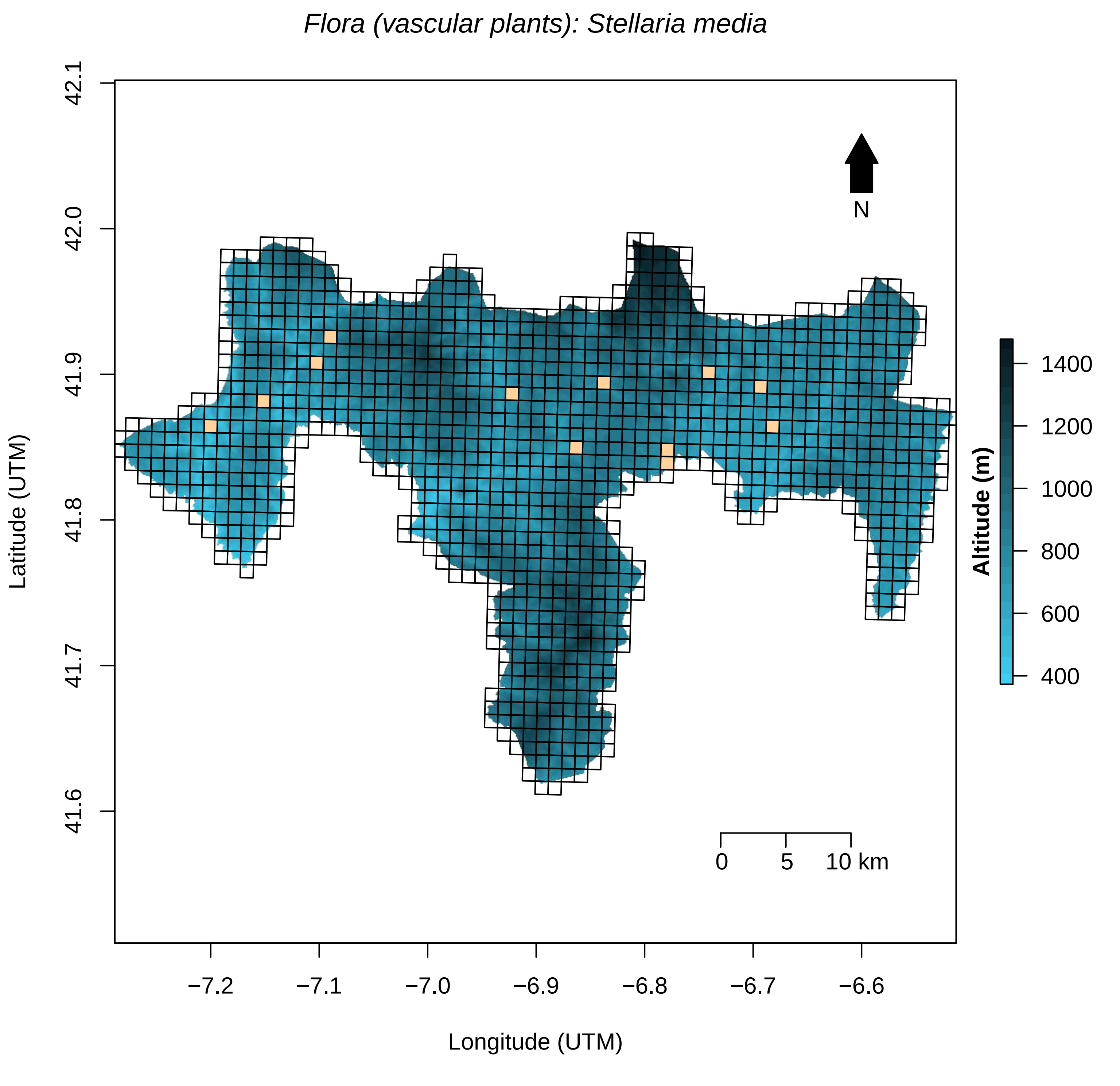
Flora (vascular plants): Stellaria media
N
0 5 10 km
Altitude (m)
1400
1200
1000
800
600
400
42.1
42.0
41.9
41.8
41.7
41.6
Latitude (UTM)
−7.2 −7.1 −7.0 −6.9 −6.8 −6.7 −6.6
Longitude (UTM)

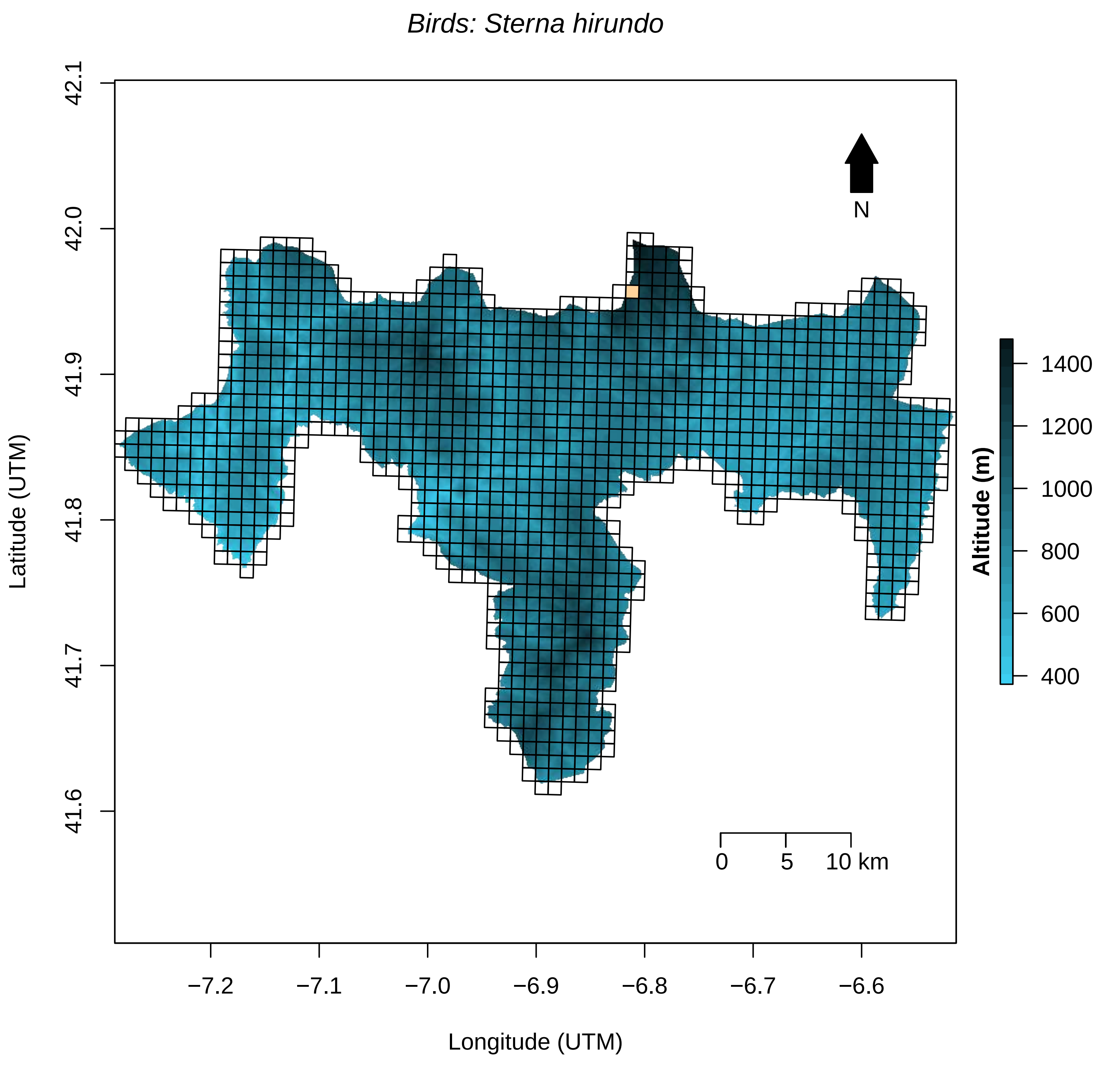

Birds: Sterna hirundo
N
0
5
10 km
Altitude (m)
1400
1200
1000
800
600
400
Latitude (UTM)
42.1
42.0
41.9
41.8
41.7
41.6
Longitude (UTM)
−7.2
−7.1
−7.0
−6.9
−6.8
−6.7
−6.6

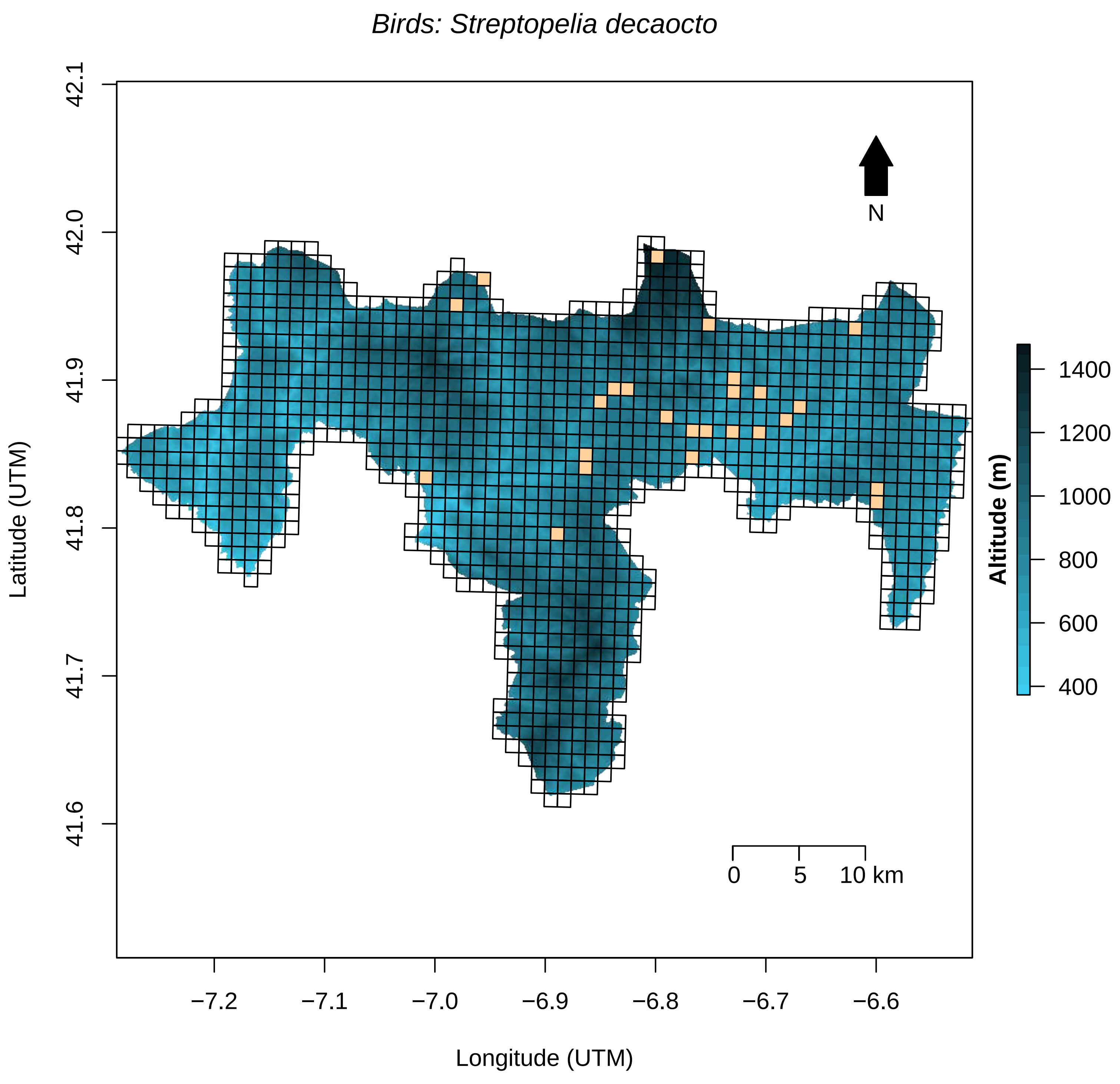

Birds: Streptopelia decaocto
42.1
42.0
41.9
41.8
41.7
41.6
Latitude (UTM)
N
0
5
10 km
−7.2
−7.1
−7.0
−6.9
−6.8
−6.7
−6.6
Longitude (UTM)
Altitude (m)
1400
1200
1000
800
600
400

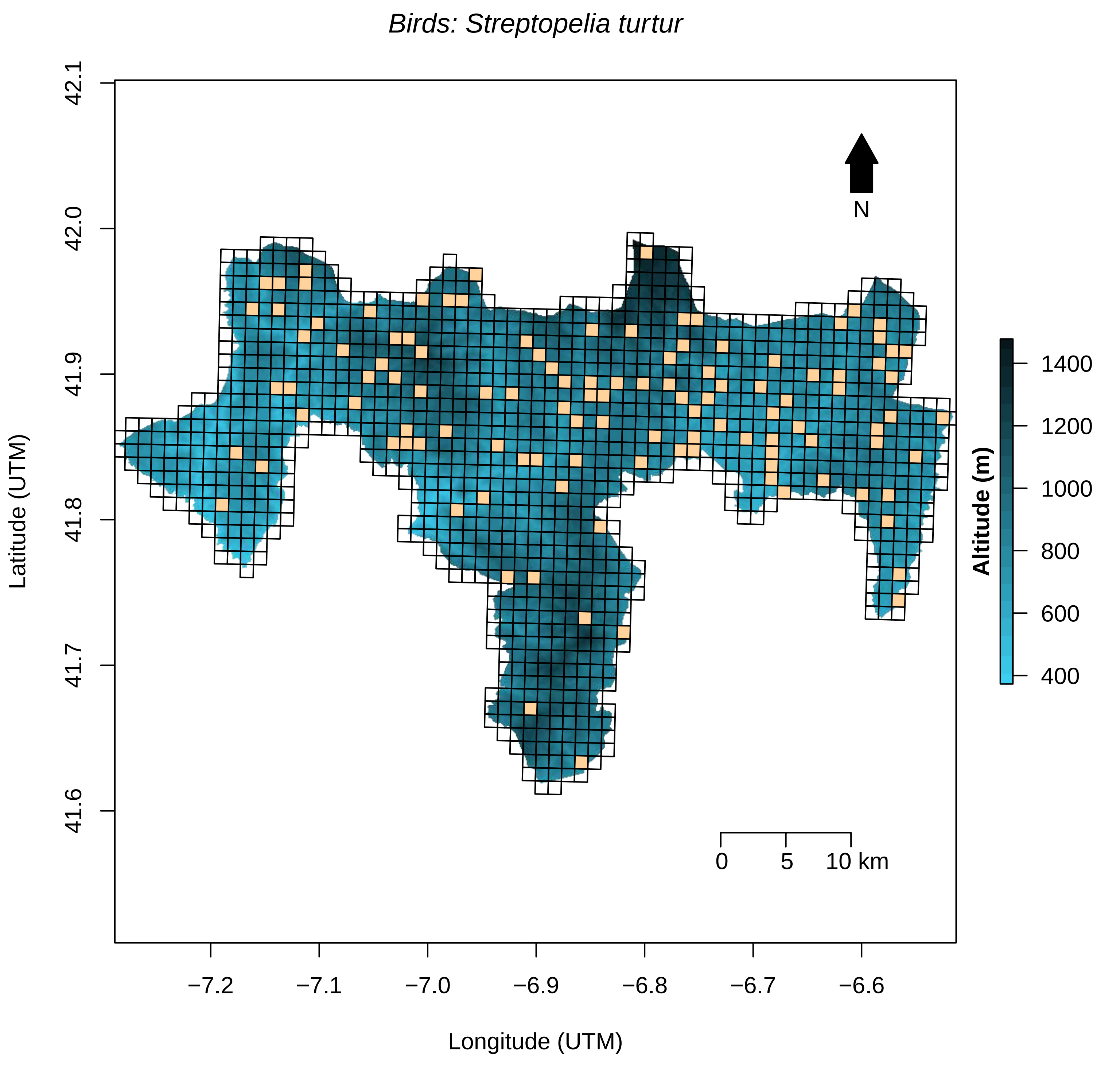

Birds: Streptopelia turtur
N
0 5 10 km
Altitude (m)
400
600
800
1000
1200
1400
Latitude (UTM)
42.1
42.0
41.9
41.8
41.7
41.6
Longitude (UTM)
−7.2
−7.1
−7.0
−6.9
−6.8
−6.7
−6.6

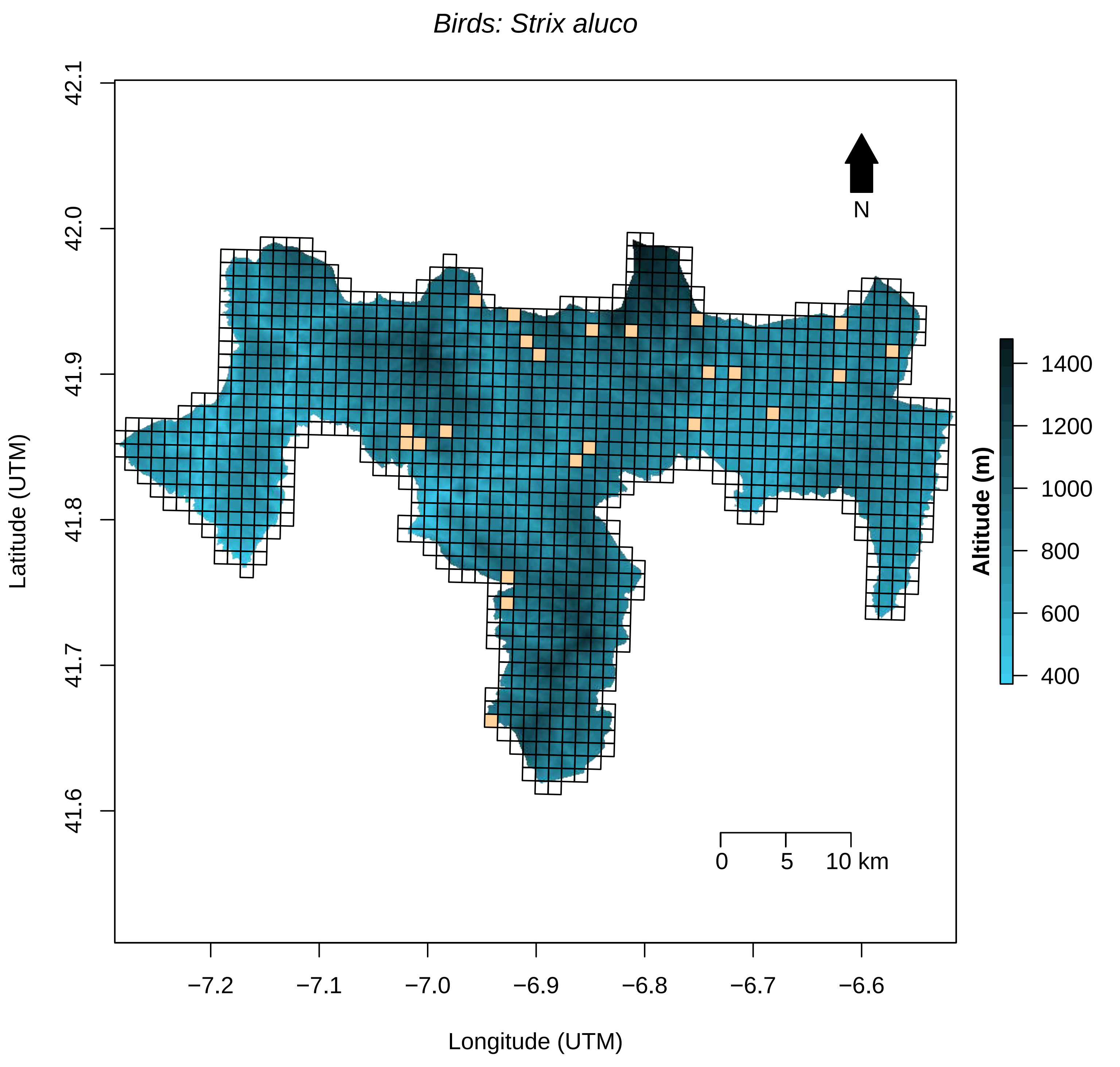
Birds: Strix aluco
N
0 5 10 km
Altitude (m)
1400
1200
1000
800
600
400
Latitude (UTM)
42.1
42.0
41.9
41.8
41.7
41.6
Longitude (UTM)
−7.2
−7.1
−7.0
−6.9
−6.8
−6.7
−6.6

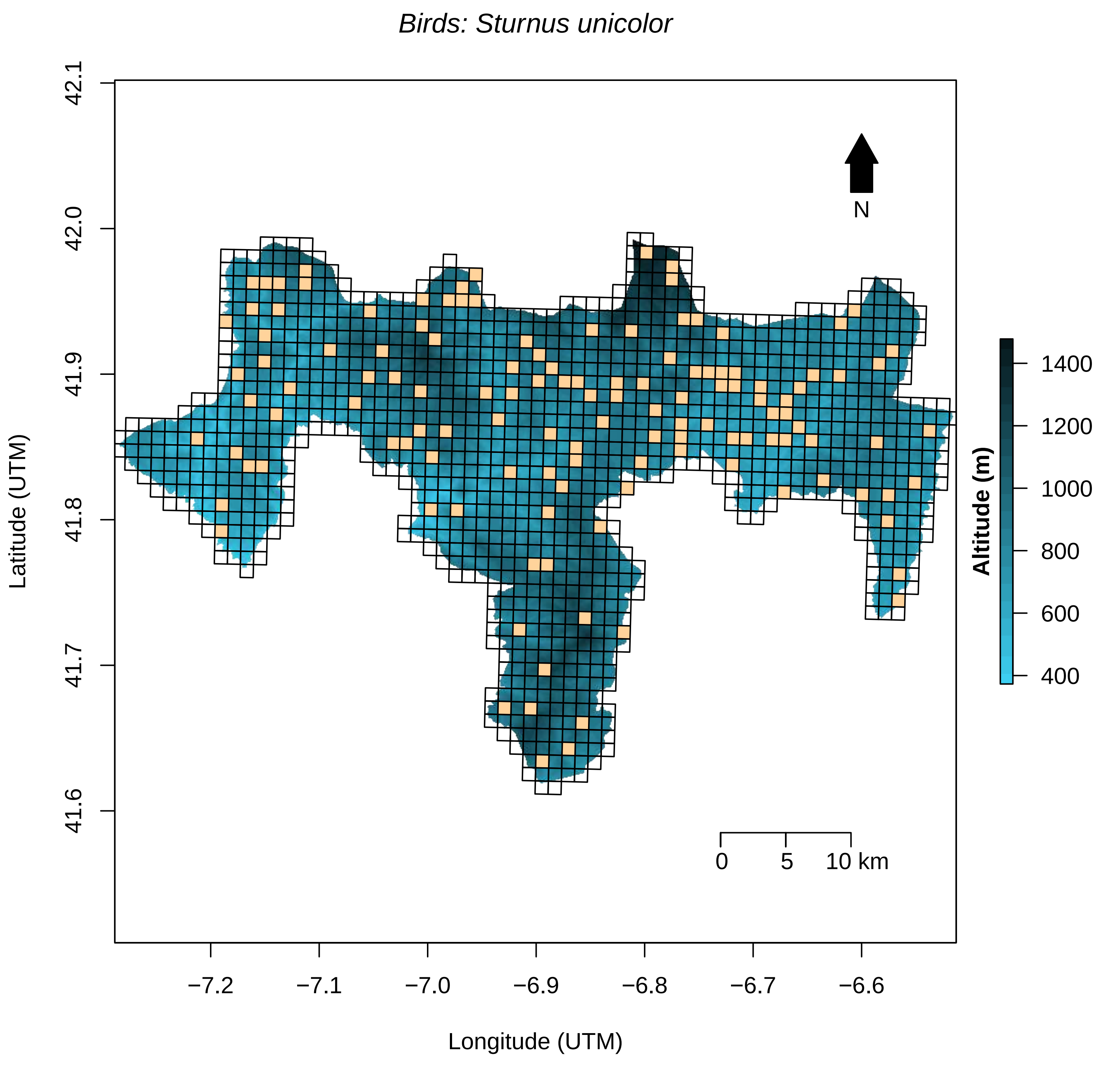
Birds: Sturnus unicolor
Latitude (UTM)
42.1
42.0
41.9
41.8
41.7
41.6
Longitude (UTM)
−7.2
−7.1
−7.0
−6.9
−6.8
−6.7
−6.6
N
0
5
10 km
Altitude (m)
1400
1200
1000
800
600
400

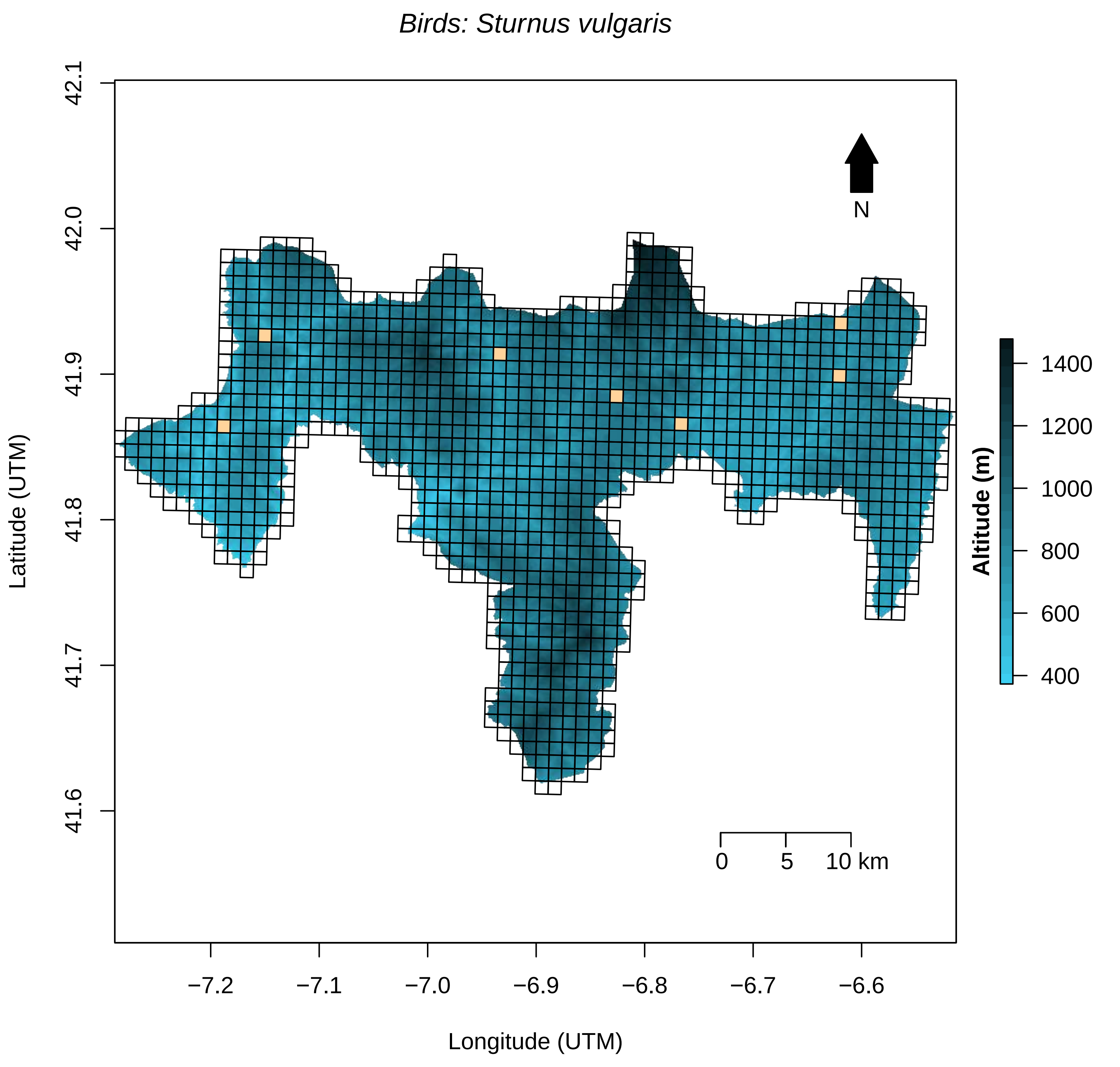

Birds: Sturnus vulgaris
Latitude (UTM)
41.6
41.7
41.8
41.9
42.0
42.1
Longitude (UTM)
−7.2
−7.1
−7.0
−6.9
−6.8
−6.7
−6.6
N
0
5
10 km
Altitude (m)
400
600
800
1000
1200
1400

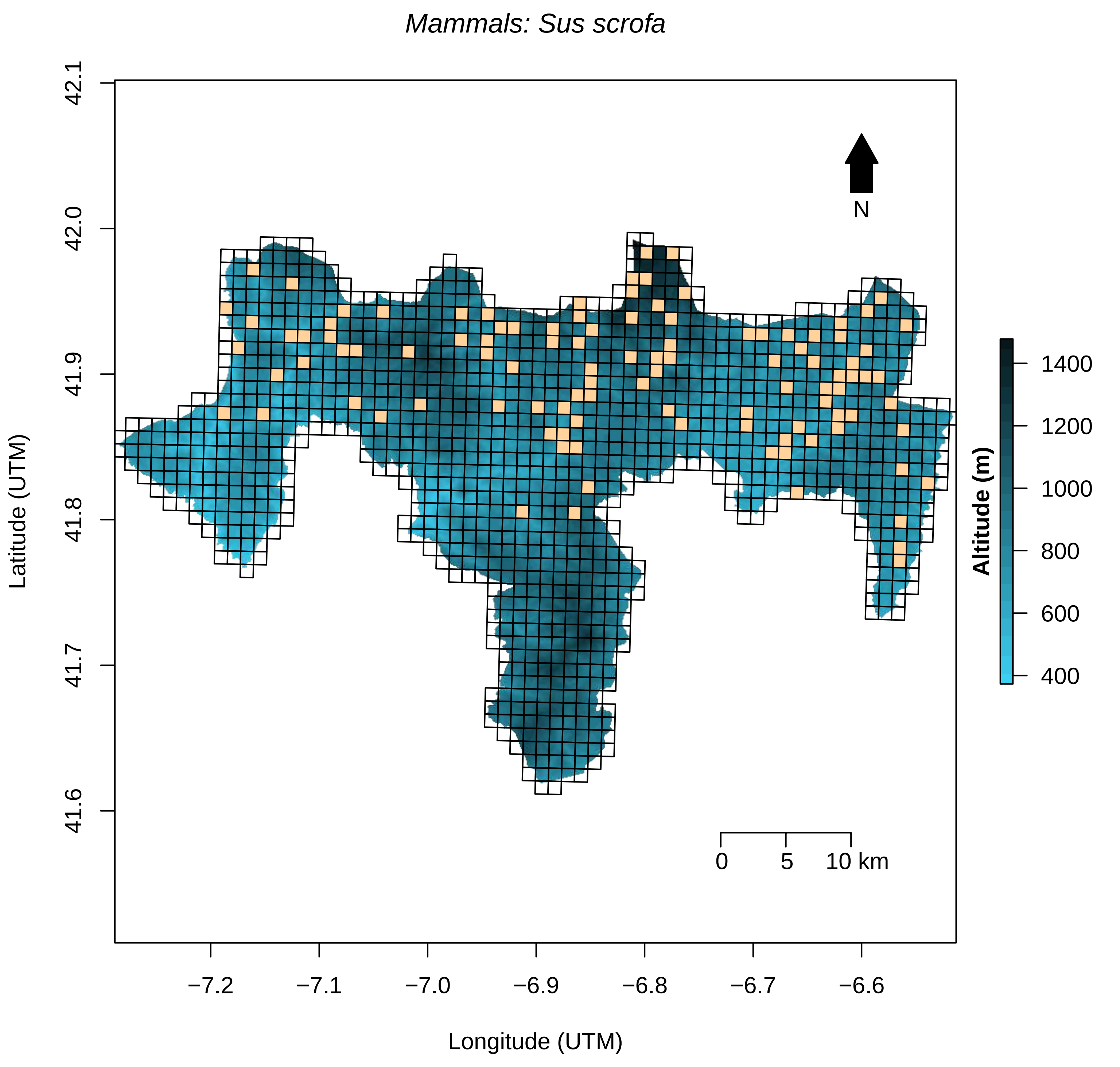

Mammals: Sus scrofa
Latitude (UTM)
Longitude (UTM)
42.1
42.0
41.9
41.8
41.7
41.6
−7.2
−7.1
−7.0
−6.9
−6.8
−6.7
−6.6
N
0
5
10 km
Altitude (m)
1400
1200
1000
800
600
400

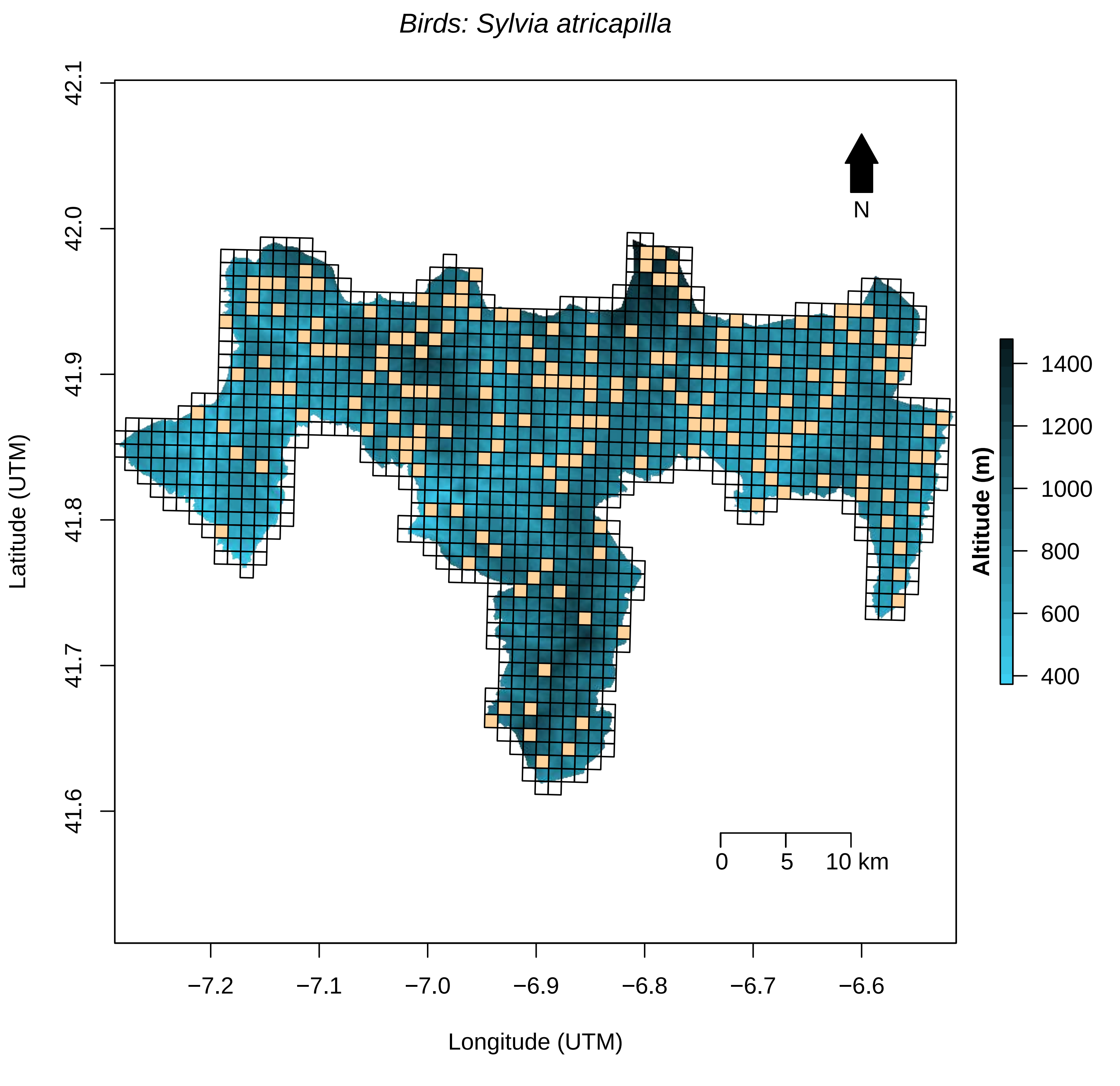
Birds: Sylvia atricapilla
42.1
42.0
41.9
41.8
41.7
41.6
Latitude (UTM)
−7.2
−7.1
−7.0
−6.9
−6.8
−6.7
−6.6
Longitude (UTM)
N
0
5
10 km
Altitude (m)
1400
1200
1000
800
600
400

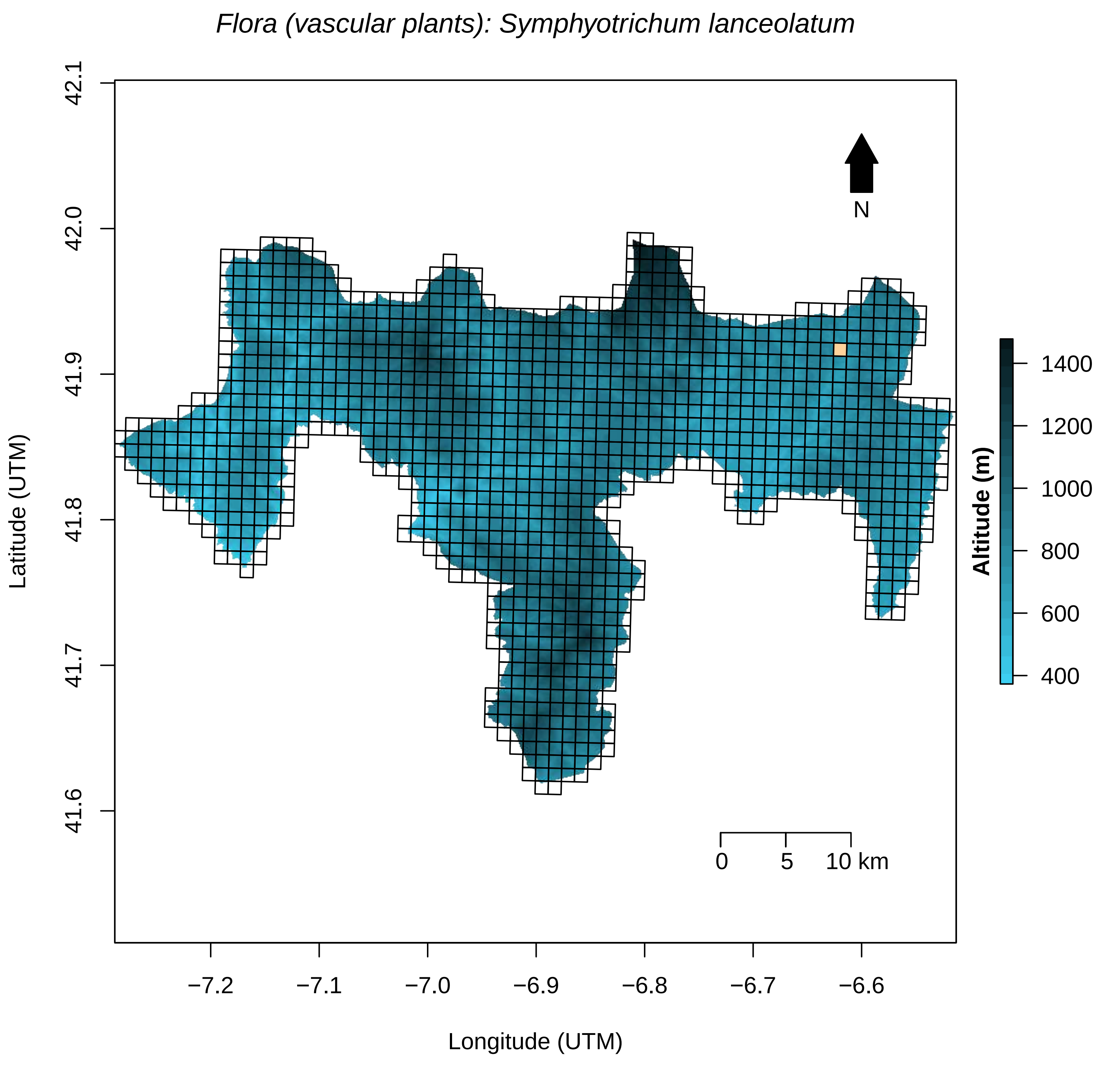

Flora (vascular plants): Symphyotrichum lanceolatum
42.1
42.0
41.9
41.8
41.7
41.6
Latitude (UTM)
−7.2
−7.1
−7.0
−6.9
−6.8
−6.7
−6.6
Longitude (UTM)
N
0
5
10 km
Altitude (m)
1400
1200
1000
800
600
400

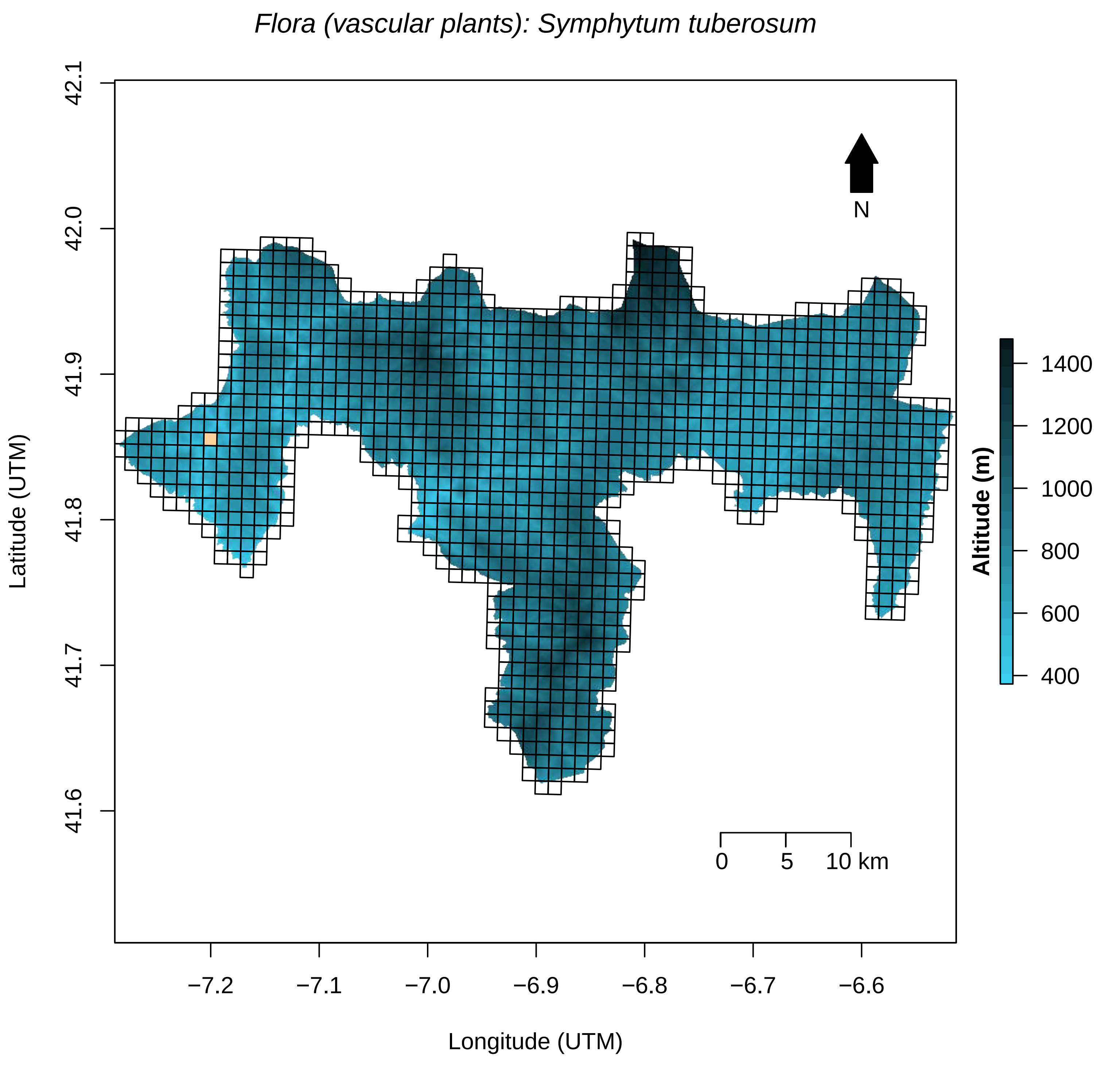

Flora (vascular plants): Symphytum tuberosum
42.1
42.0
41.9
41.8
41.7
41.6
Latitude (UTM)
−7.2
−7.1
−7.0
−6.9
−6.8
−6.7
−6.6
Longitude (UTM)
N
0
5
10 km
1400
1200
1000
800
600
400
Altitude (m)

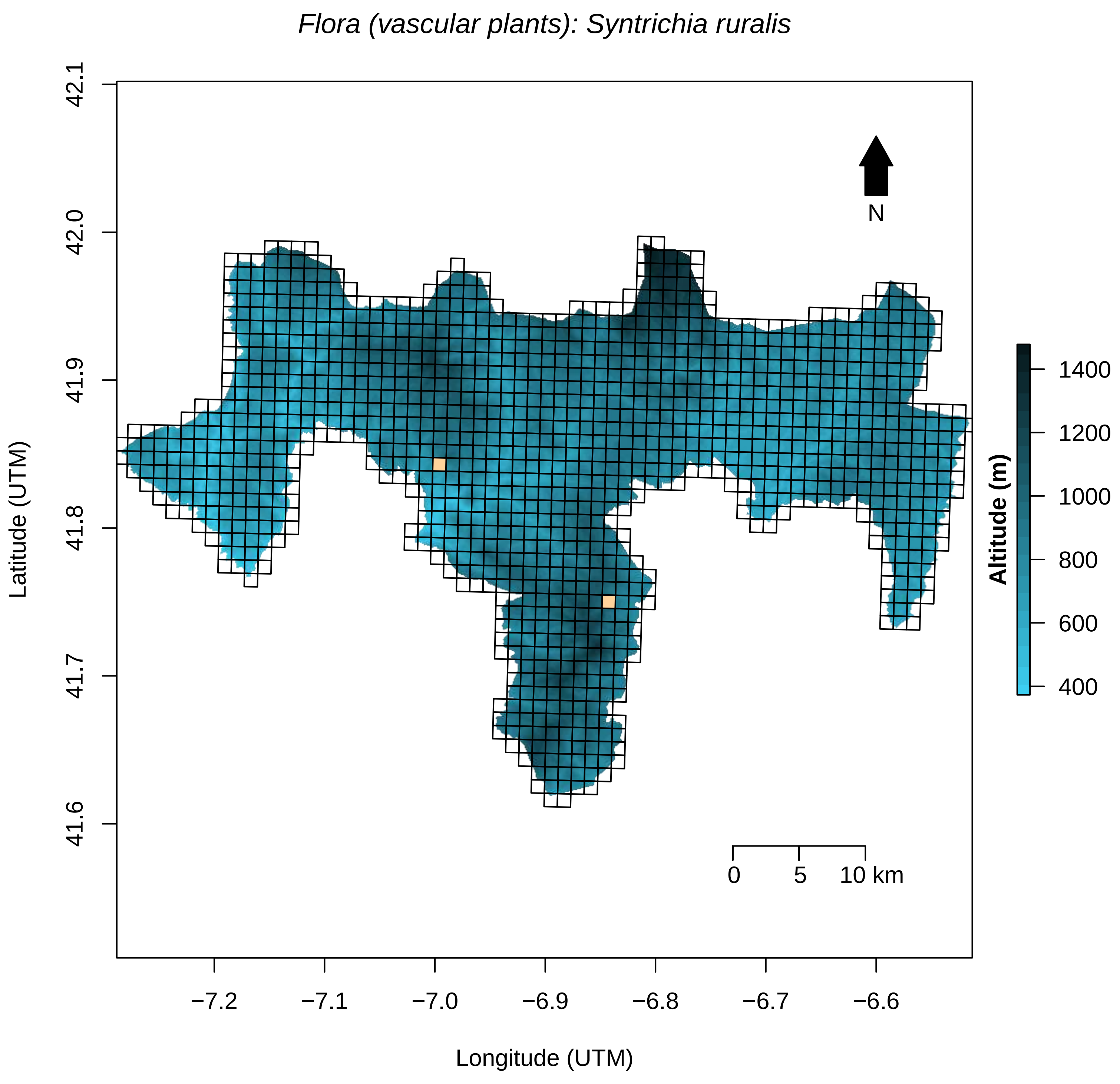
Flora (vascular plants): Syntrichia ruralis
N
0 5 10 km
Altitude (m)
1400
1200
1000
800
600
400
Latitude (UTM)
42.1
42.0
41.9
41.8
41.7
41.6
−7.2 −7.1 −7.0 −6.9 −6.8 −6.7 −6.6
Longitude (UTM)

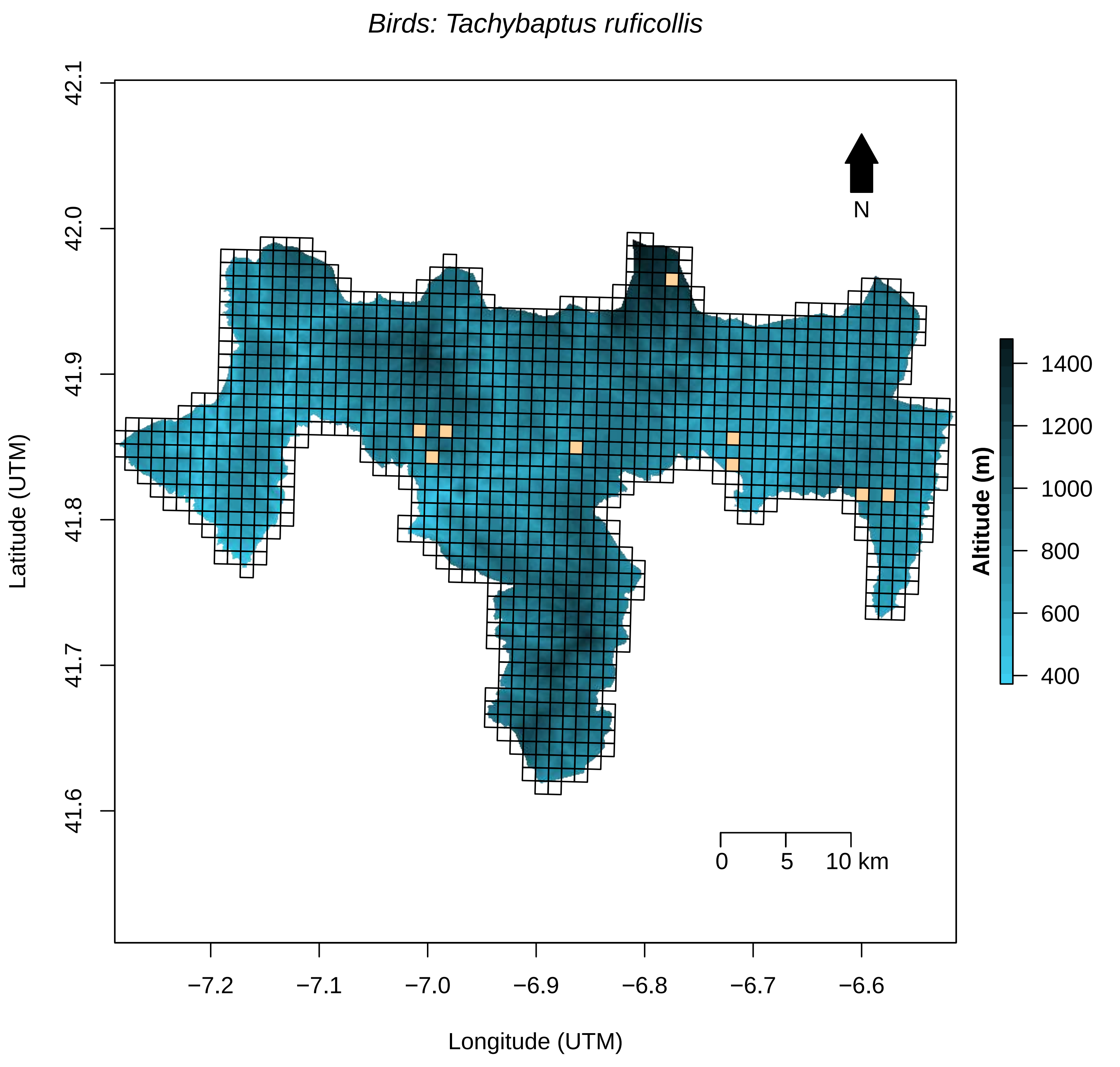

Birds: Tachybaptus ruficollis
N
0
5
10 km
Altitude (m)
1400
1200
1000
800
600
400
Latitude (UTM)
42.1
42.0
41.9
41.8
41.7
41.6
Longitude (UTM)
−7.2
−7.1
−7.0
−6.9
−6.8
−6.7
−6.6

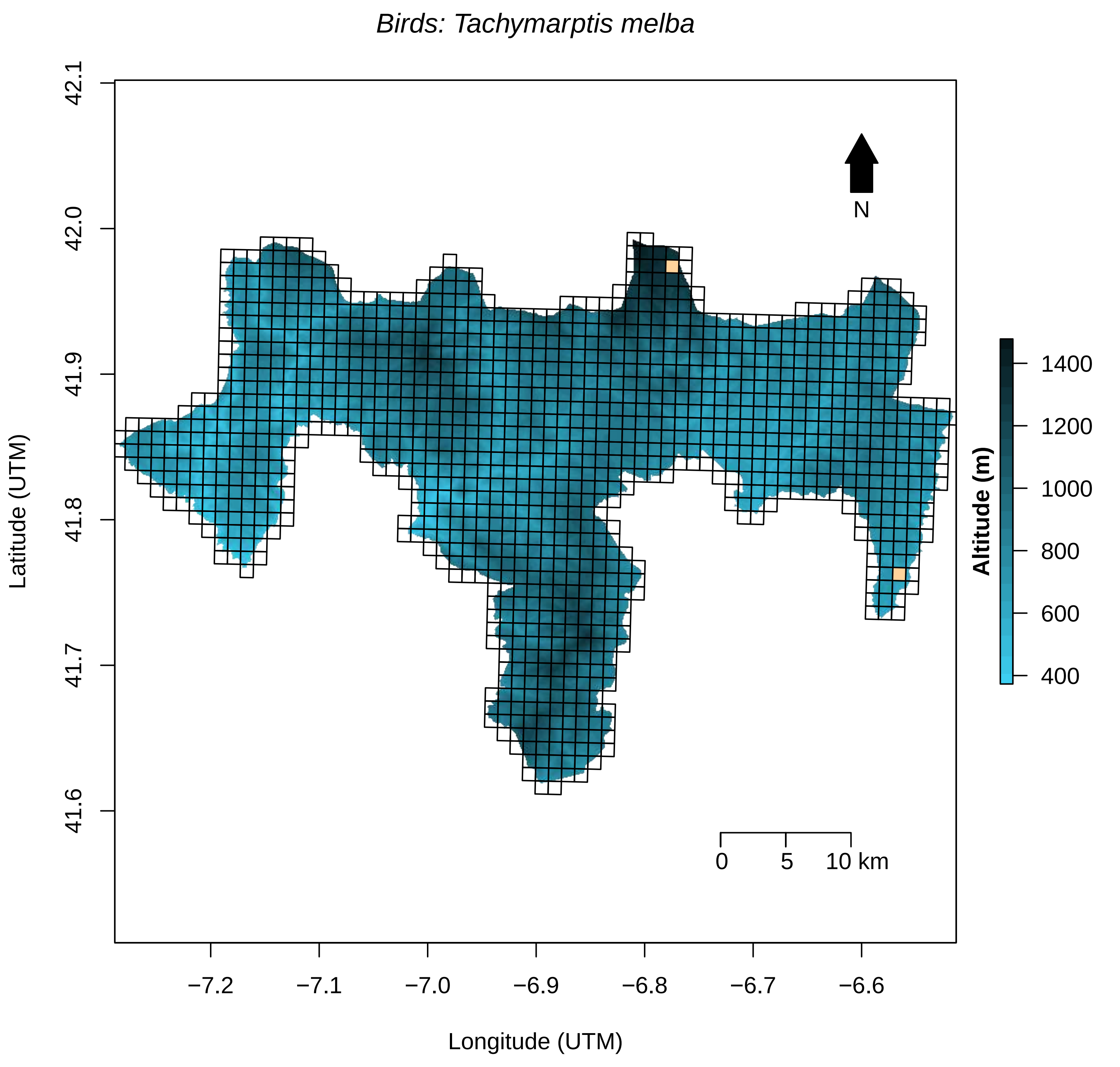
Birds: Tachymarptis melba
N
0
5
10 km
42.1
42.0
41.9
41.8
41.7
41.6
Latitude (UTM)
−7.2
−7.1
−7.0
−6.9
−6.8
−6.7
−6.6
Longitude (UTM)
1400
1200
1000
800
600
400
Altitude (m)

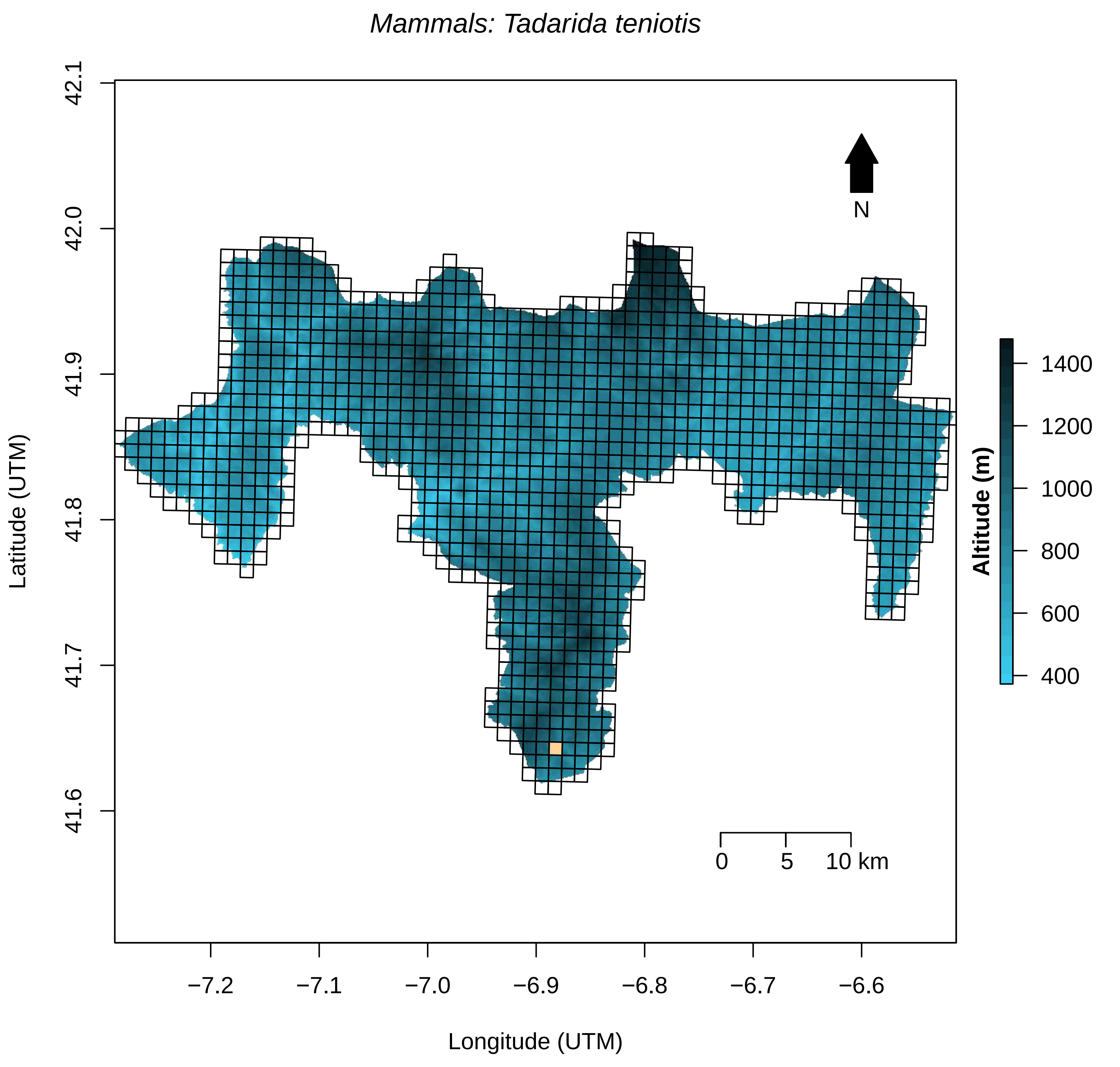
Mammals: Tadarida teniotis
42.1
42.0
41.9
41.8
41.7
41.6
Latitude (UTM)
−7.2
−7.1
−7.0
−6.9
−6.8
−6.7
−6.6
Longitude (UTM)
N
0
5
10 km
Altitude (m)
1400
1200
1000
800
600
400

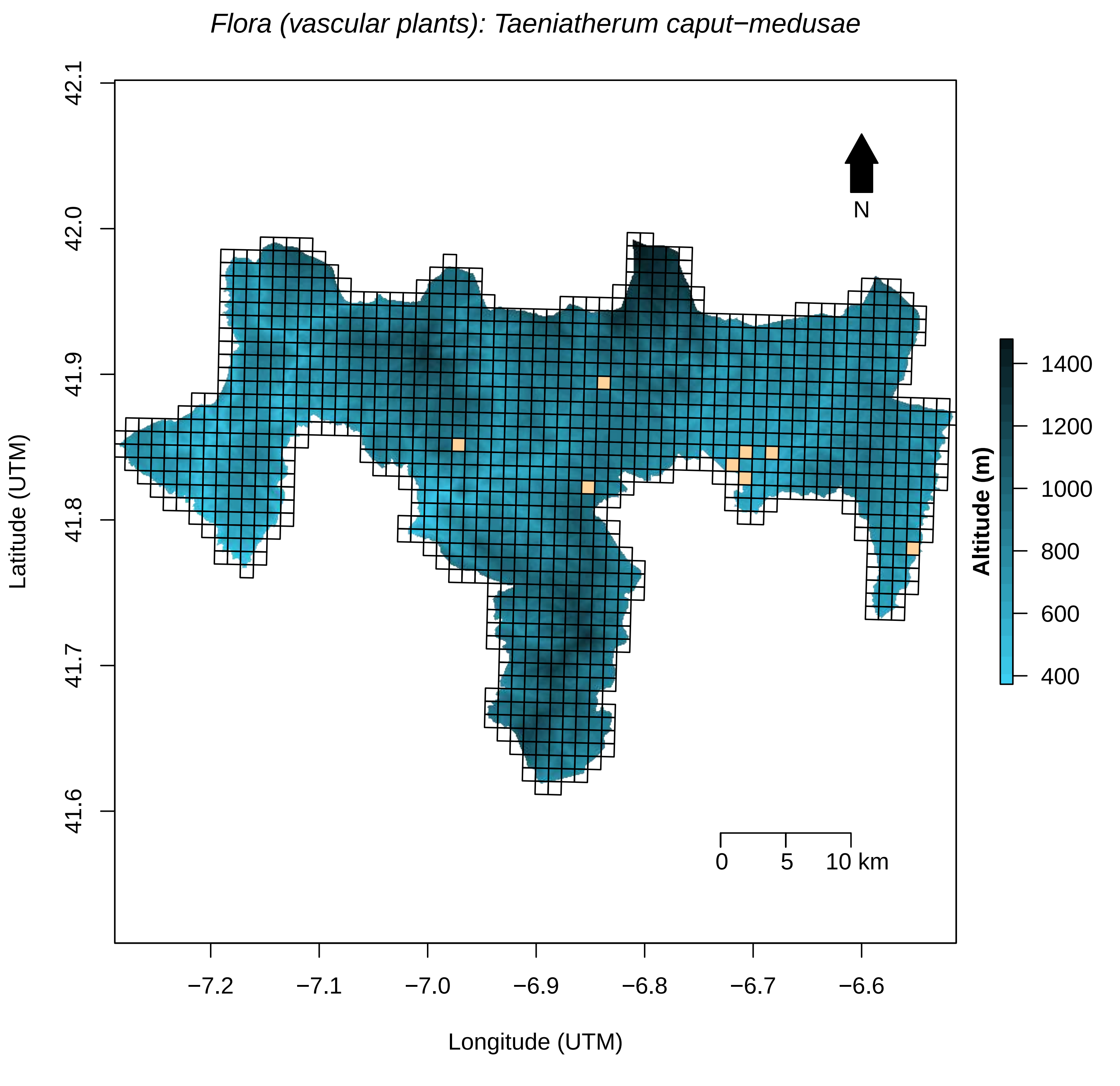
Flora (vascular plants): Taeniatherum caput−medusae
42.1
42.0
41.9
41.8
41.7
41.6
Latitude (UTM)
−7.2
−7.1
−7.0
−6.9
−6.8
−6.7
−6.6
Longitude (UTM)
N
0
5
10 km
Altitude (m)
1400
1200
1000
800
600
400

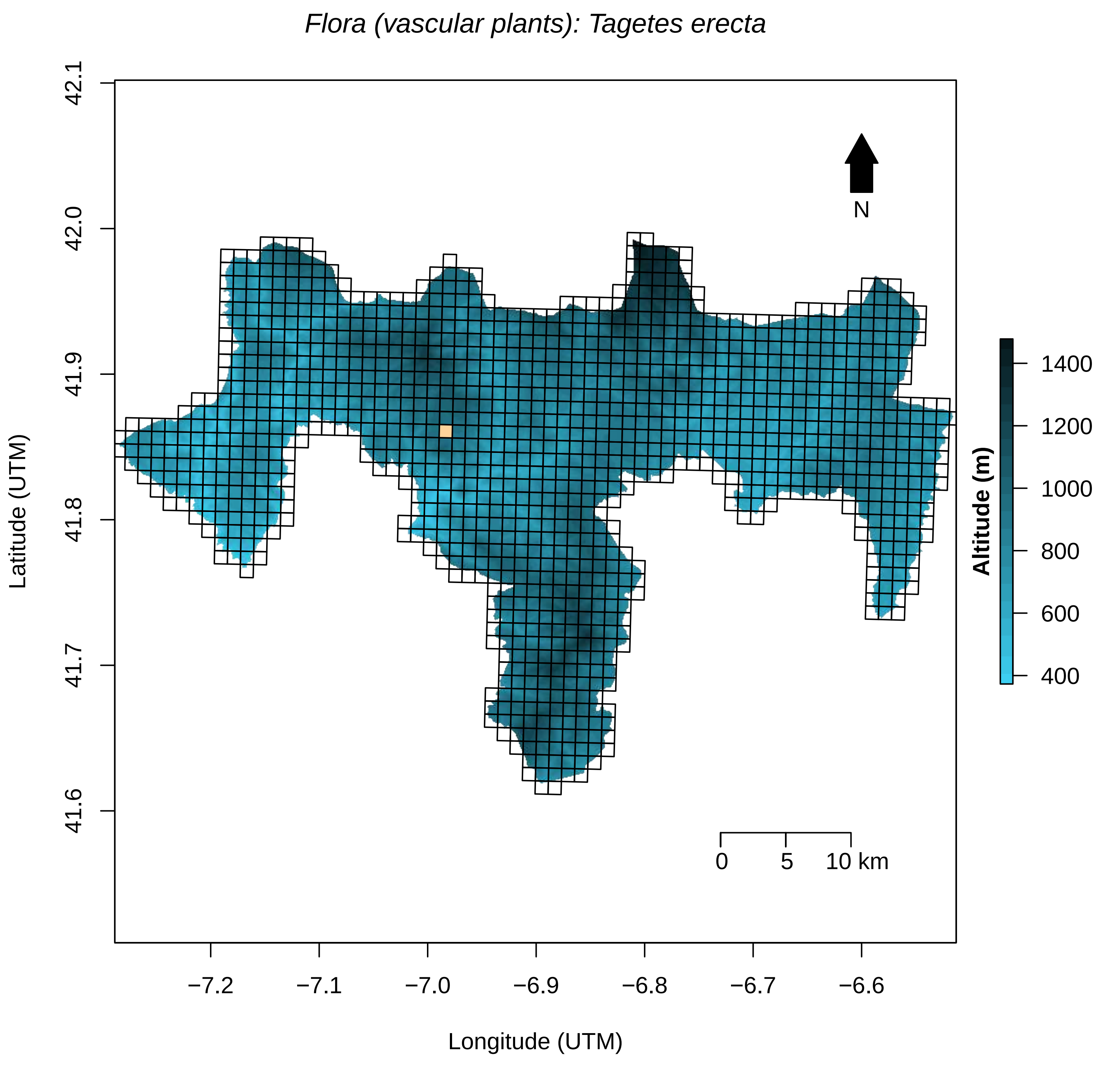

Flora (vascular plants): Tagetes erecta
Latitude (UTM)
42.1
42.0
41.9
41.8
41.7
41.6
N
0
5
10 km
Altitude (m)
1400
1200
1000
800
600
400
−7.2
−7.1
−7.0
−6.9
−6.8
−6.7
−6.6
Longitude (UTM)

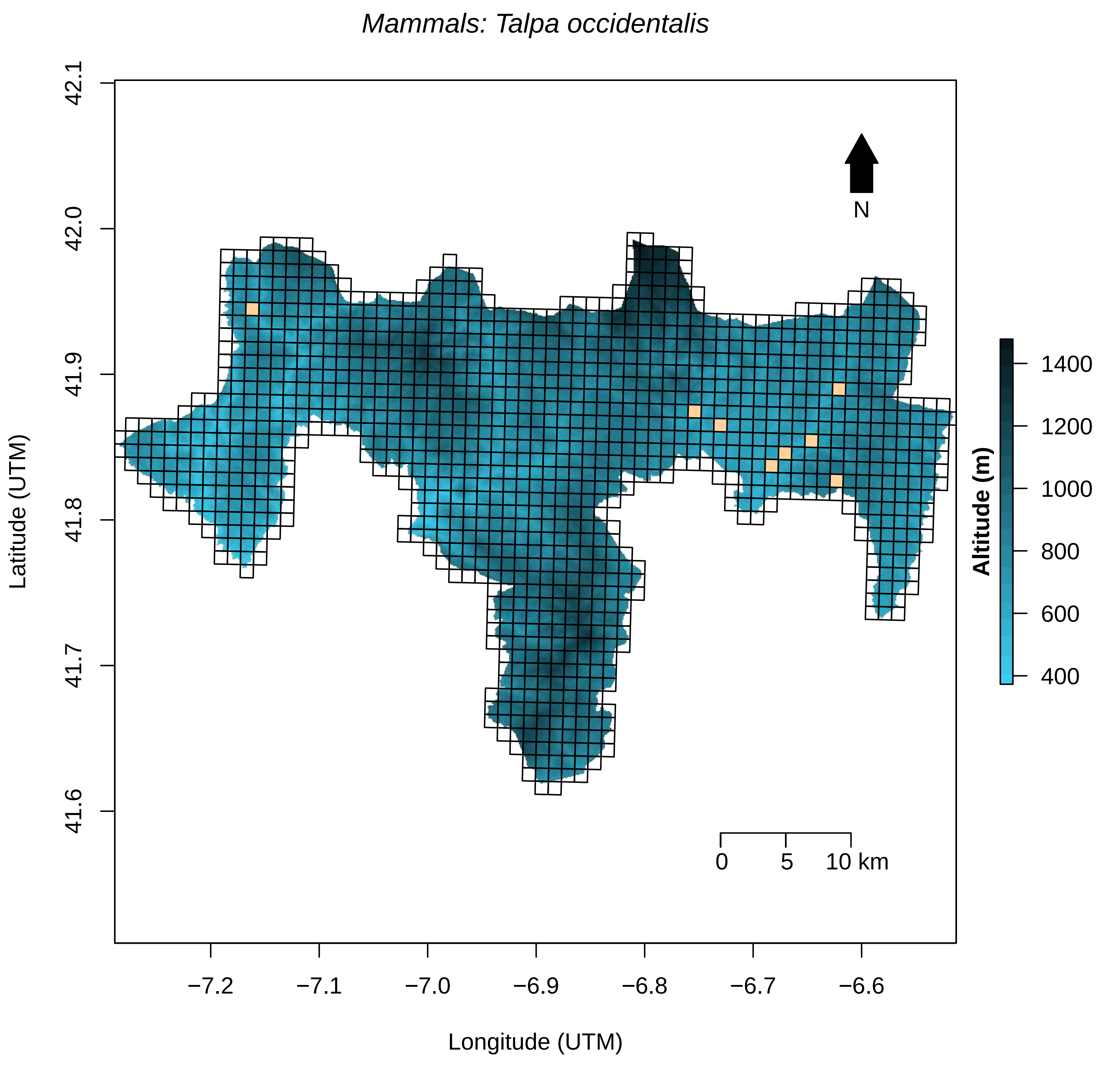

Mammals: Talpa occidentalis
Latitude (UTM)
Longitude (UTM)
42.1
42.0
41.9
41.8
41.7
41.6
−7.2
−7.1
−7.0
−6.9
−6.8
−6.7
−6.6
N
0
5
10 km
Altitude (m)
1400
1200
1000
800
600
400

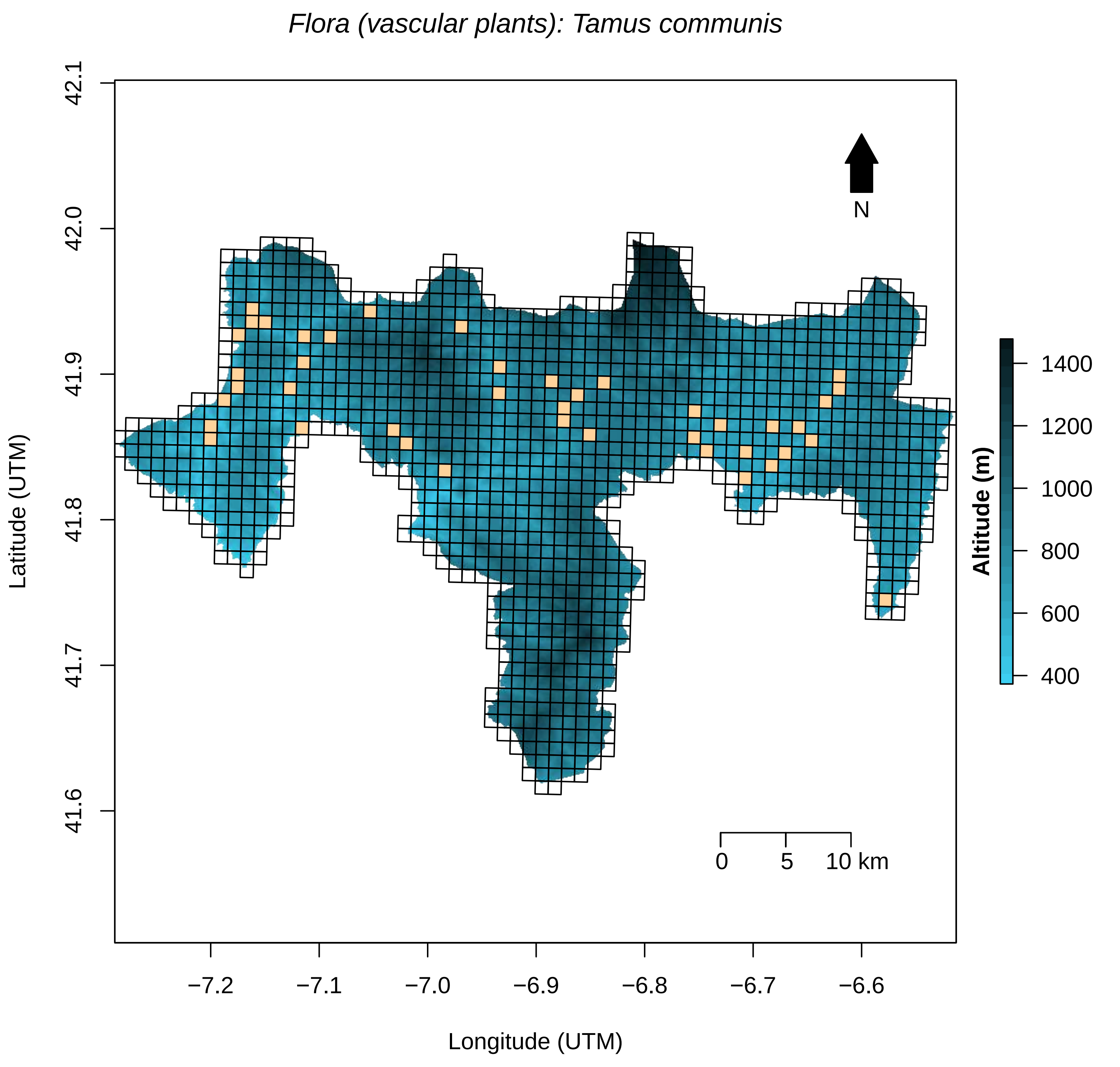

Flora (vascular plants): Tamus communis
42.1
42.0
41.9
41.8
41.7
41.6
Latitude (UTM)
−7.2
−7.1
−7.0
−6.9
−6.8
−6.7
−6.6
Longitude (UTM)
N
0
5
10 km
Altitude (m)
1400
1200
1000
800
600
400

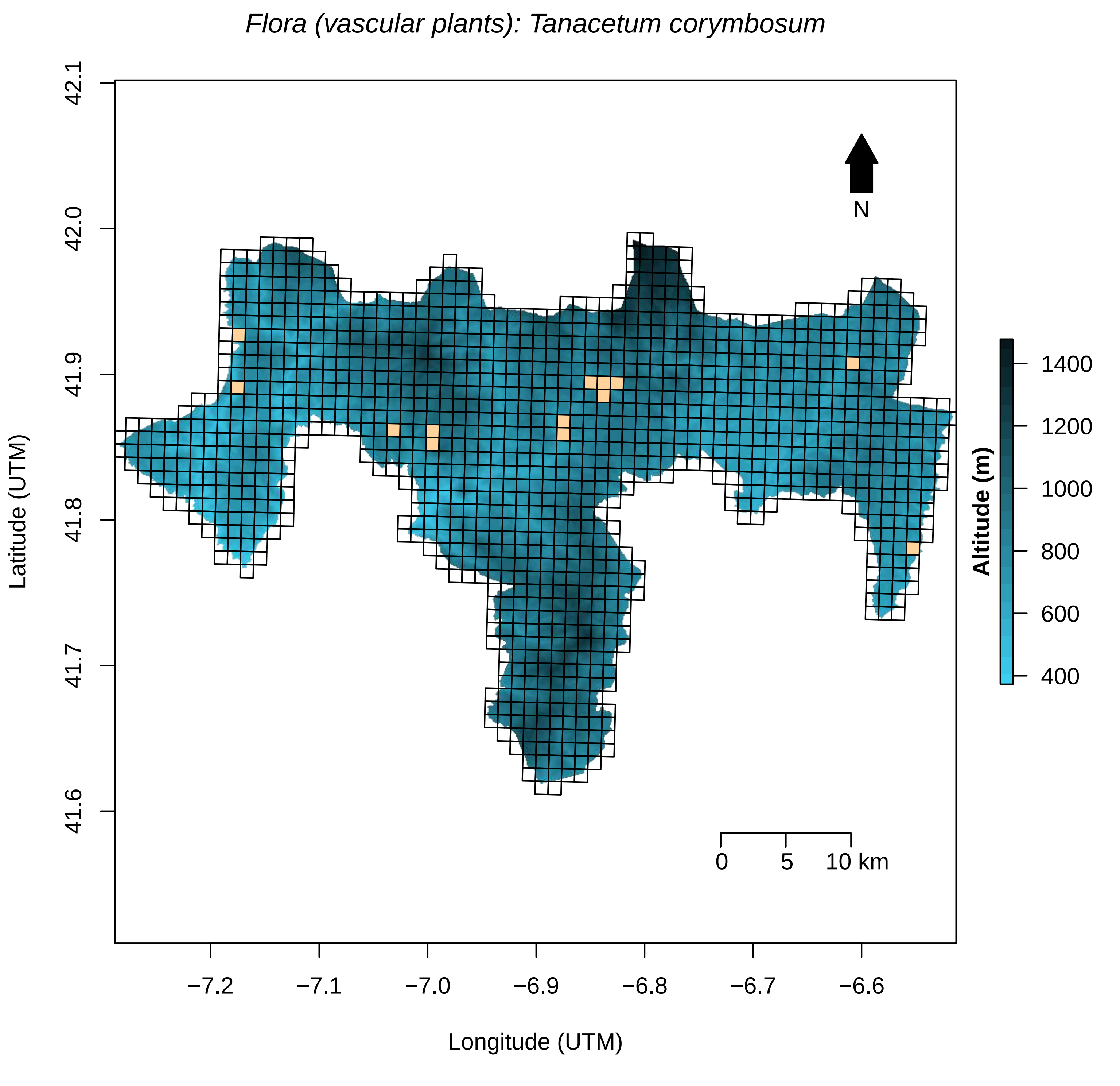

Flora (vascular plants): Tanacetum corymbosum
Latitude (UTM)
42.1
42.0
41.9
41.8
41.7
41.6
Longitude (UTM)
−7.2
−7.1
−7.0
−6.9
−6.8
−6.7
−6.6
N
0
5
10 km
Altitude (m)
1400
1200
1000
800
600
400

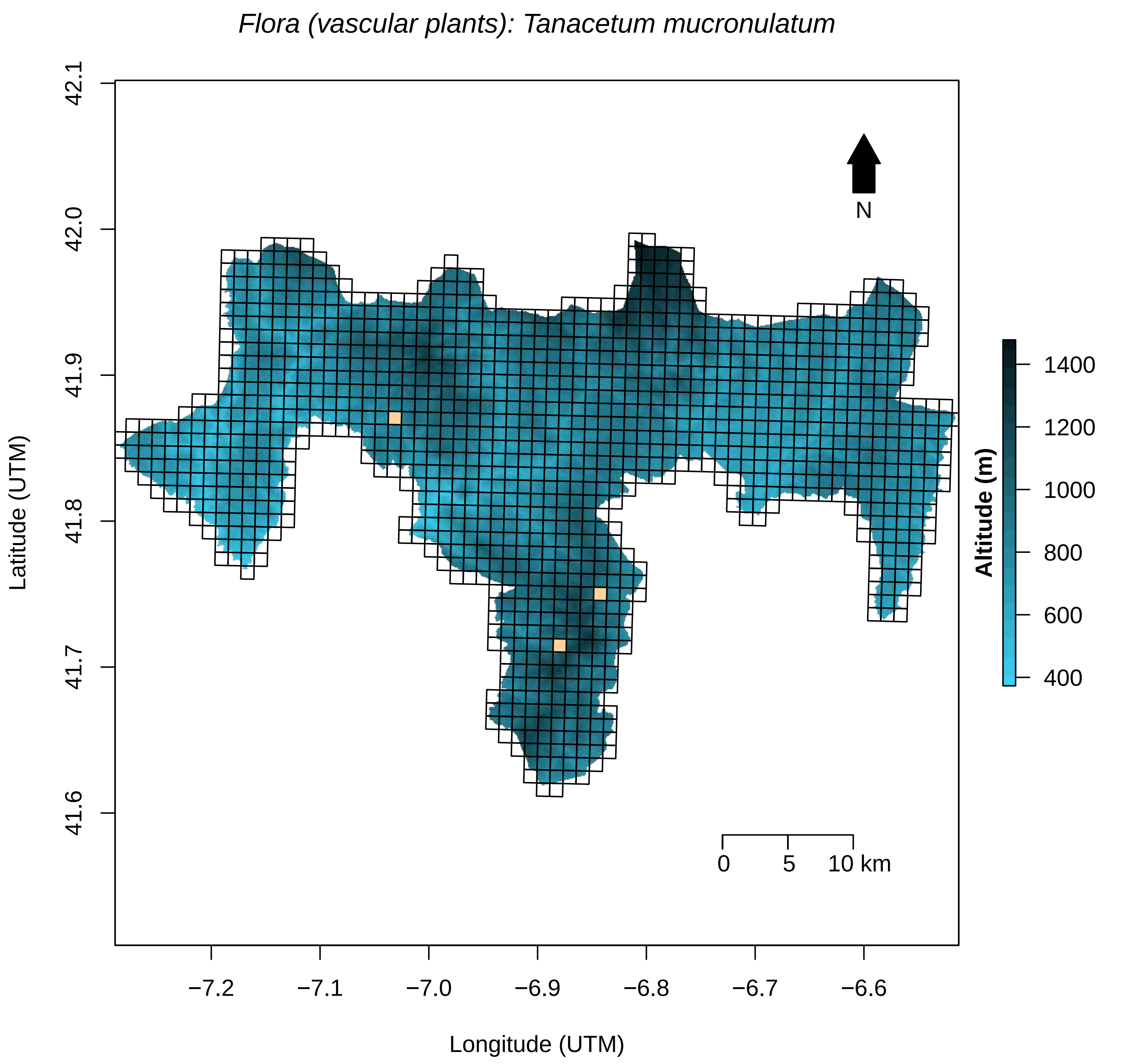
Flora (vascular plants): Tanacetum mucronulatum
42.1
42.0
41.9
41.8
41.7
41.6
Latitude (UTM)
−7.2
−7.1
−7.0
−6.9
−6.8
−6.7
−6.6
Longitude (UTM)
N
0
5
10 km
Altitude (m)
1400
1200
1000
800
600
400

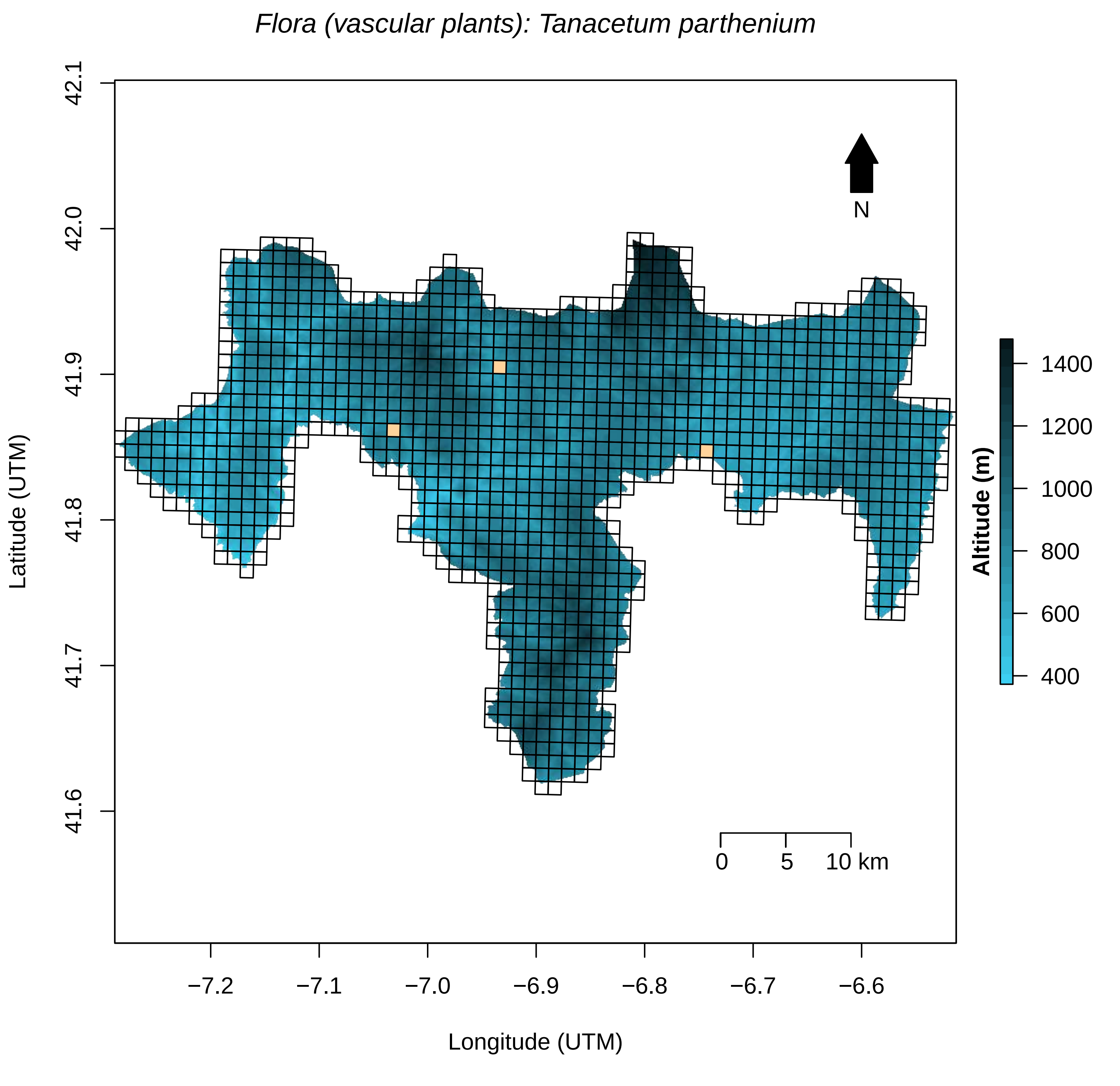

Flora (vascular plants): Tanacetum parthenium
42.1
42.0
41.9
41.8
41.7
41.6
Latitude (UTM)
−7.2
−7.1
−7.0
−6.9
−6.8
−6.7
−6.6
Longitude (UTM)
N
0
5
10 km
Altitude (m)
1400
1200
1000
800
600
400

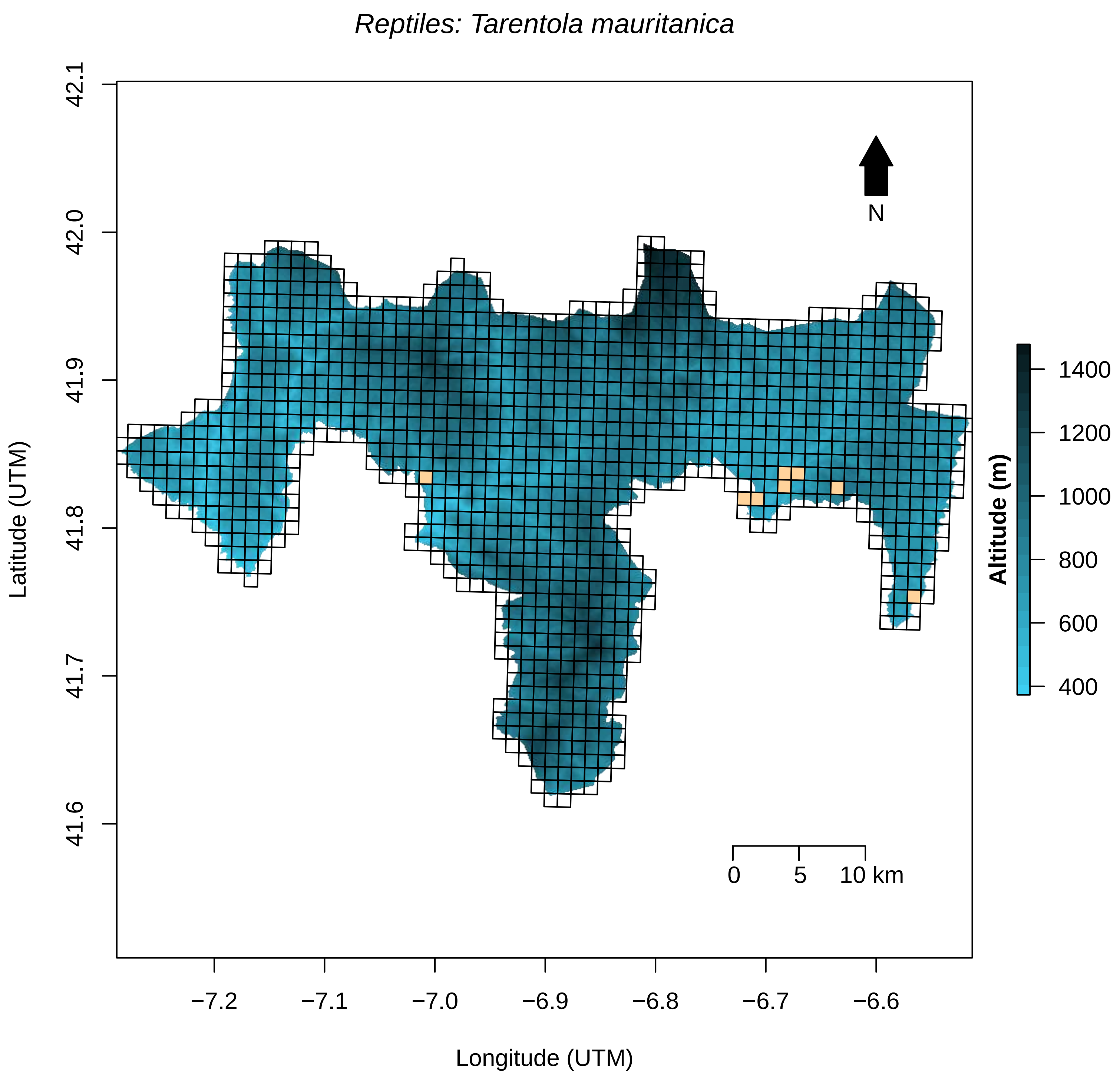

Reptiles: Tarentola mauritanica
42.1
42.0
41.9
41.8
41.7
41.6
Latitude (UTM)
−7.2
−7.1
−7.0
−6.9
−6.8
−6.7
−6.6
Longitude (UTM)
N
0
5
10 km
Altitude (m)
1400
1200
1000
800
600
400

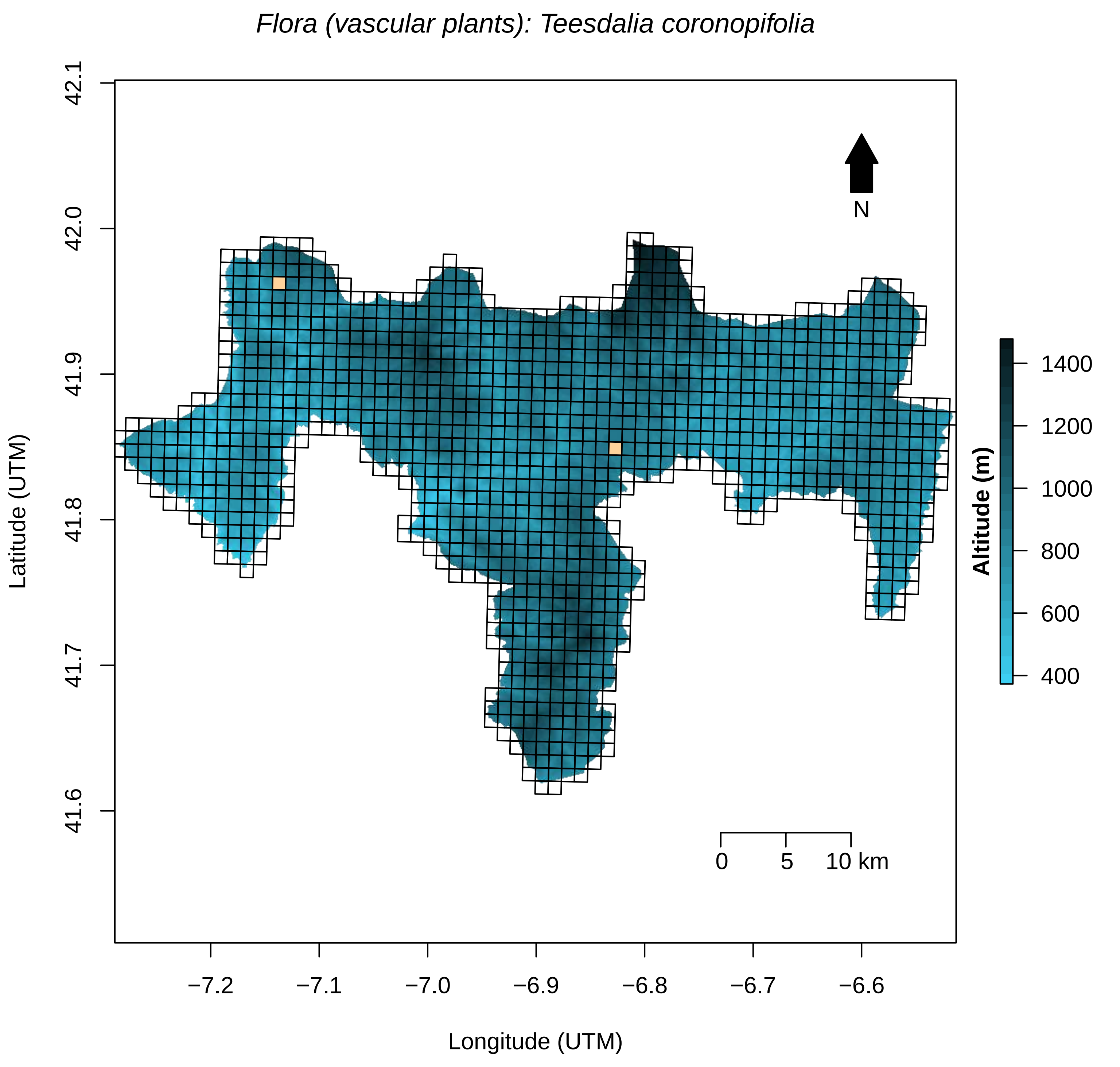
Flora (vascular plants): Teesdalia coronopifolia
42.1
42.0
41.9
41.8
41.7
41.6
Latitude (UTM)
−7.2
−7.1
−7.0
−6.9
−6.8
−6.7
−6.6
Longitude (UTM)
N
0
5
10 km
1400
1200
1000
800
600
400
Altitude (m)

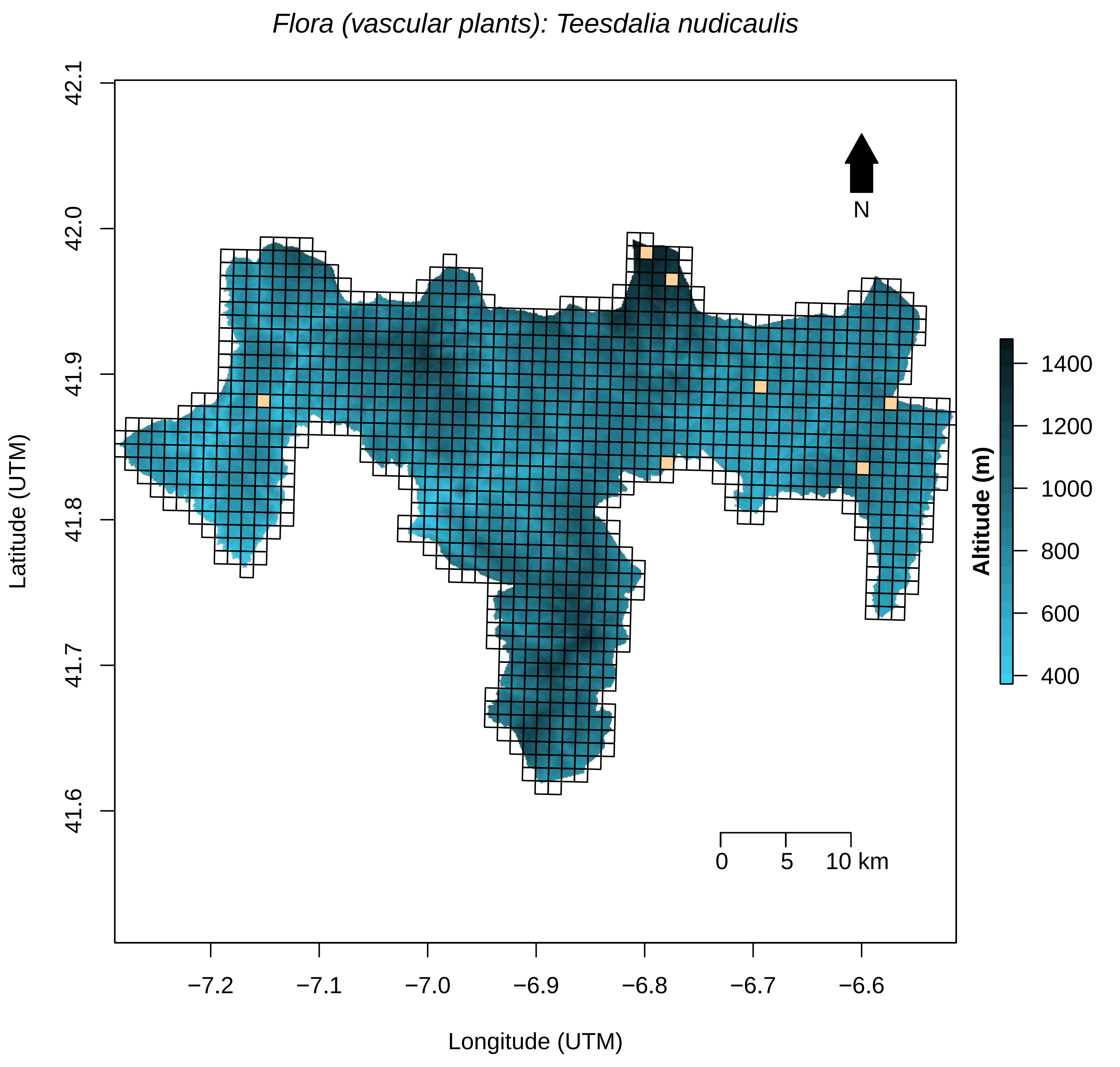

Flora (vascular plants): Teesdalia nudicaulis
42.1
42.0
41.9
41.8
41.7
41.6
Latitude (UTM)
−7.2
−7.1
−7.0
−6.9
−6.8
−6.7
−6.6
Longitude (UTM)
N
0
5
10 km
1400
1200
1000
800
600
400
Altitude (m)

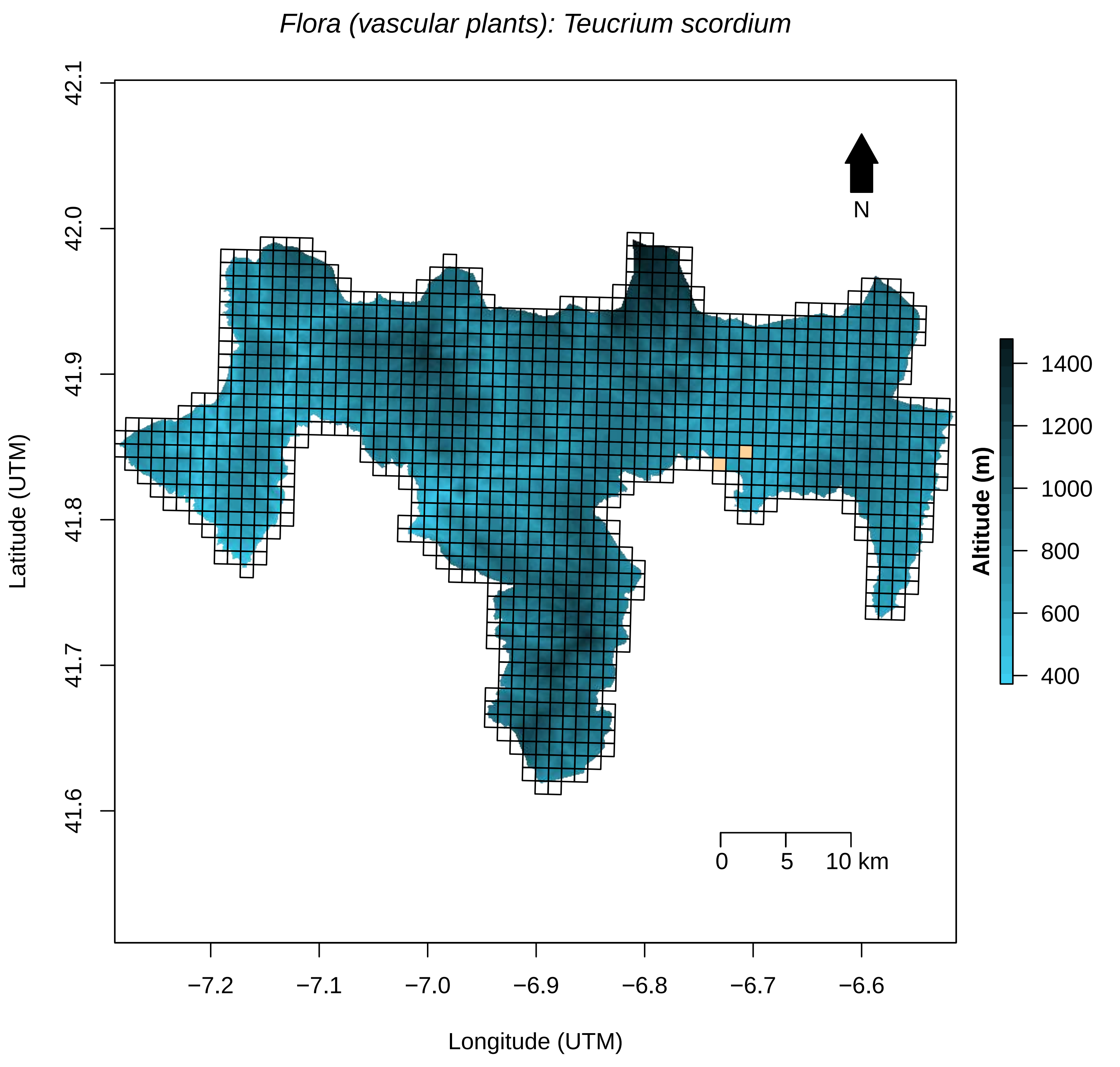

Flora (vascular plants): Teucrium scordium
42.1
42.0
41.9
41.8
41.7
41.6
Latitude (UTM)
−7.2
−7.1
−7.0
−6.9
−6.8
−6.7
−6.6
Longitude (UTM)
N
0
5
10 km
Altitude (m)
1400
1200
1000
800
600
400

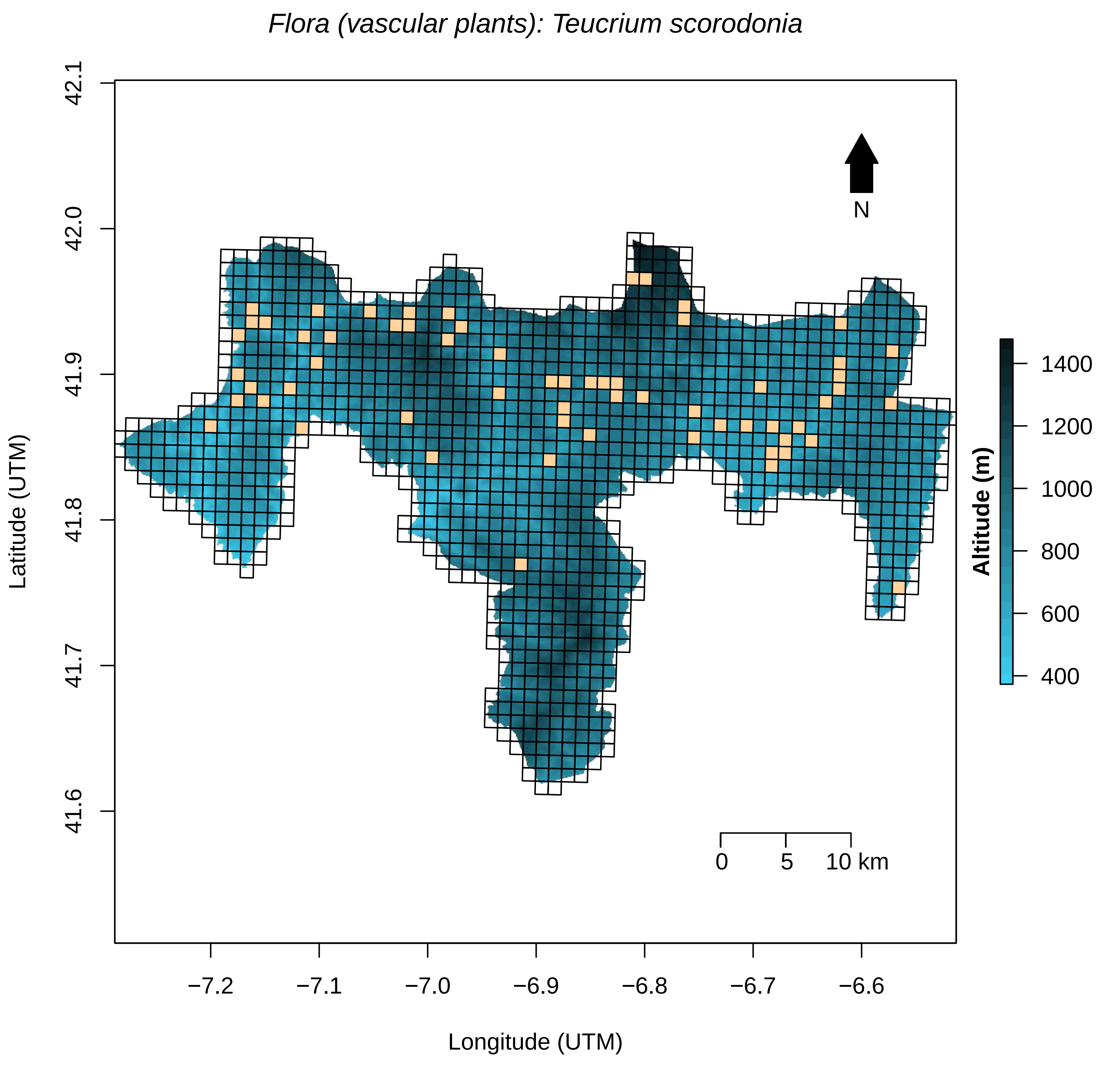

Flora (vascular plants): Teucrium scorodonia
42.1
42.0
41.9
41.8
41.7
41.6
Latitude (UTM)
−7.2
−7.1
−7.0
−6.9
−6.8
−6.7
−6.6
Longitude (UTM)
N
0
5
10 km
Altitude (m)
1400
1200
1000
800
600
400

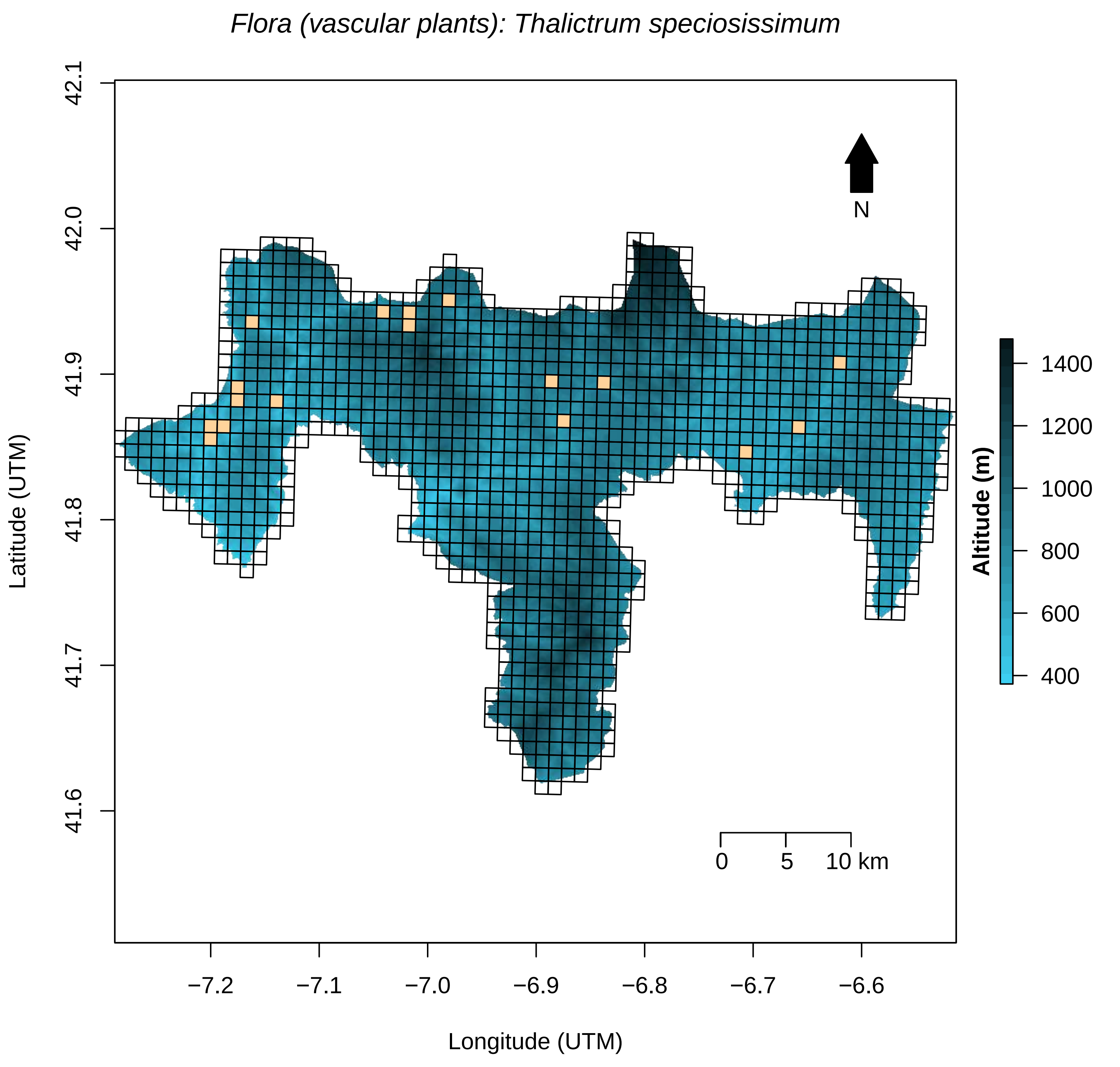

Flora (vascular plants): Thalictrum speciosissimum
Latitude (UTM)
42.1
42.0
41.9
41.8
41.7
41.6
−7.2
−7.1
−7.0
−6.9
−6.8
−6.7
−6.6
Longitude (UTM)
N
0
5
10 km
Altitude (m)
1400
1200
1000
800
600
400

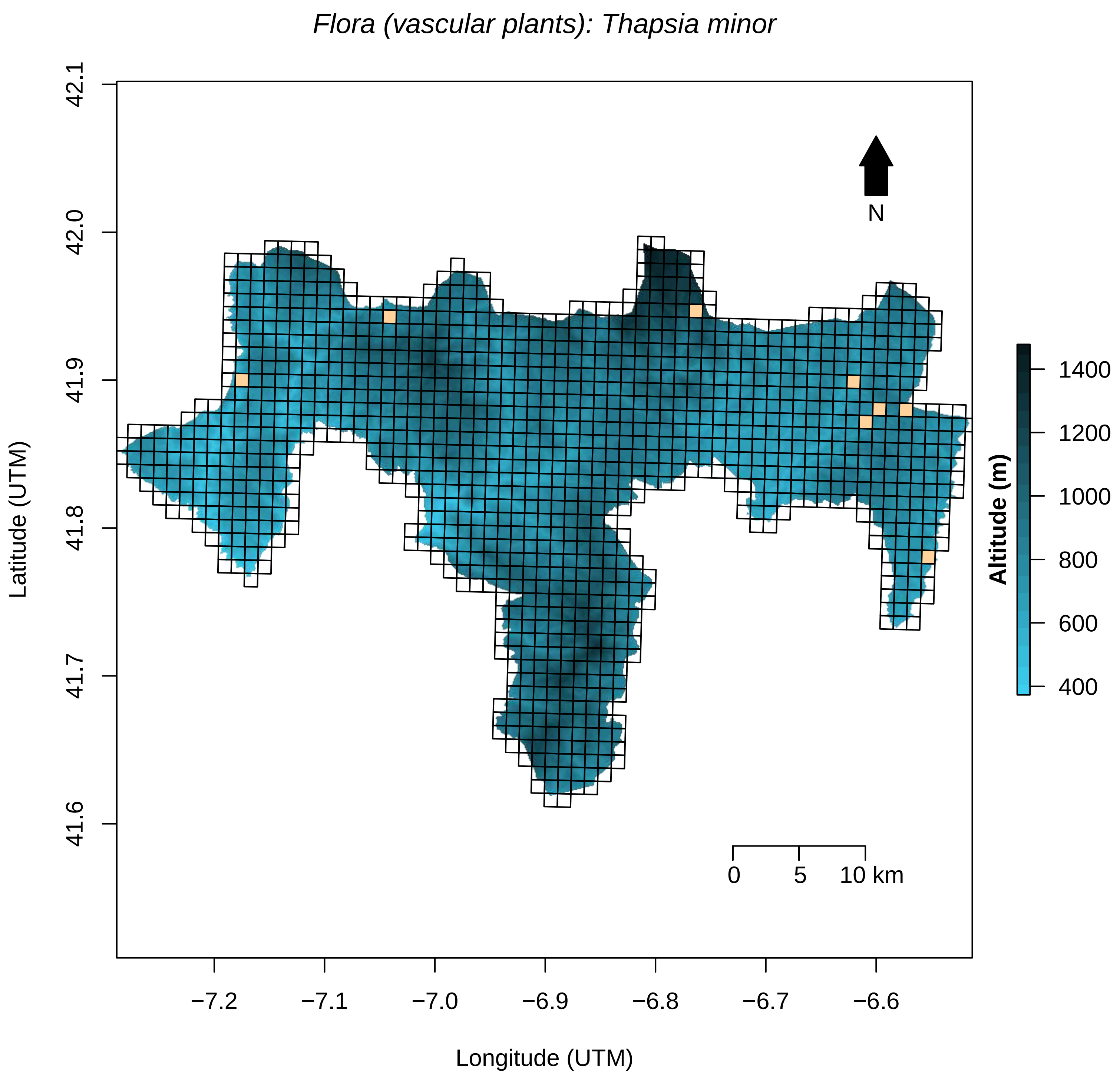

Flora (vascular plants): Thapsia minor
42.1
42.0
41.9
41.8
41.7
41.6
Latitude (UTM)
−7.2
−7.1
−7.0
−6.9
−6.8
−6.7
−6.6
Longitude (UTM)
N
0
5
10 km
Altitude (m)
1400
1200
1000
800
600
400

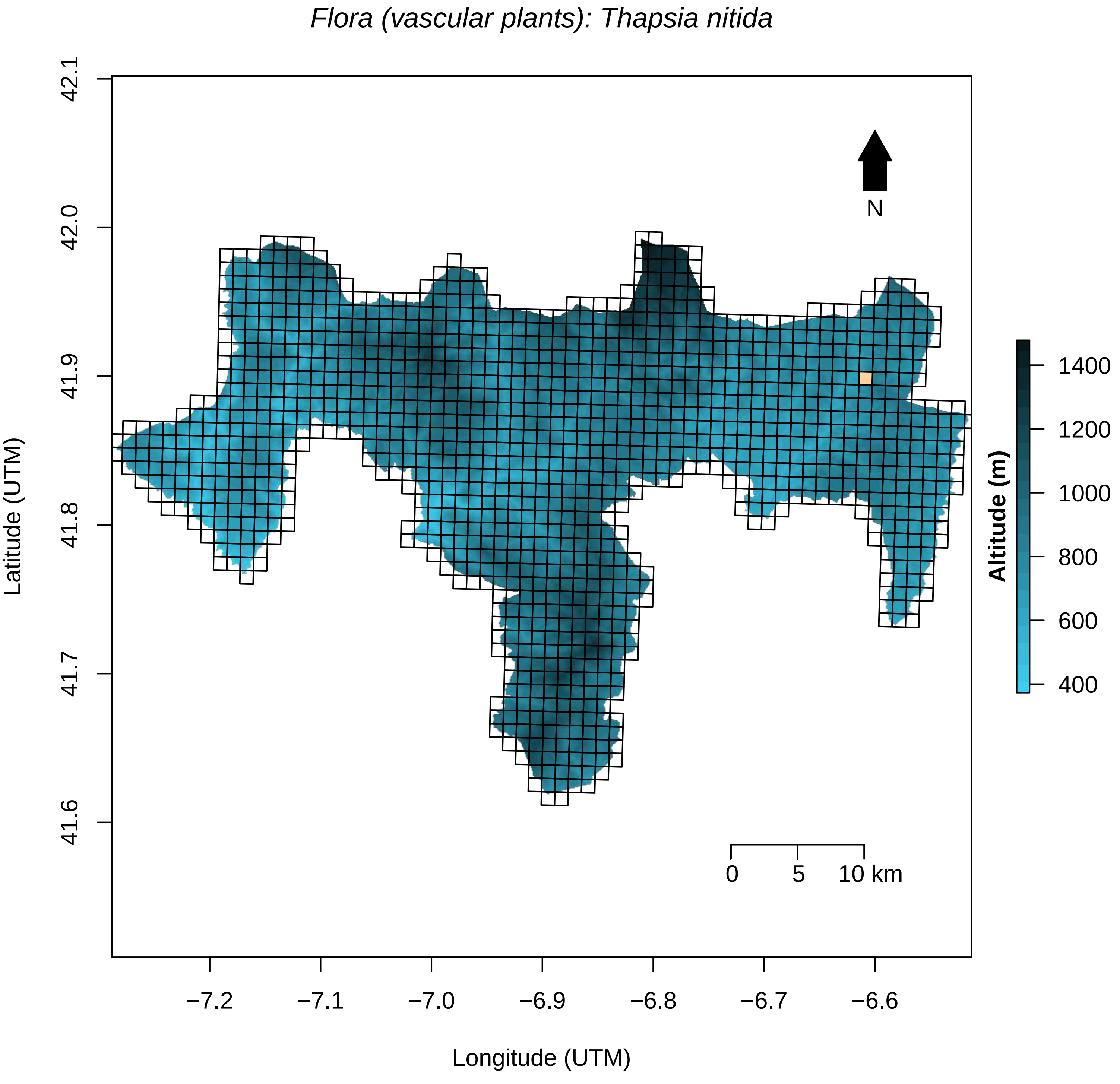

Flora (vascular plants): Thapsia nitida
42.1
42.0
41.9
41.8
41.7
41.6
Latitude (UTM)
N
0
5
10 km
−7.2
−7.1
−7.0
−6.9
−6.8
−6.7
−6.6
Longitude (UTM)
1400
1200
1000
800
600
400
Altitude (m)

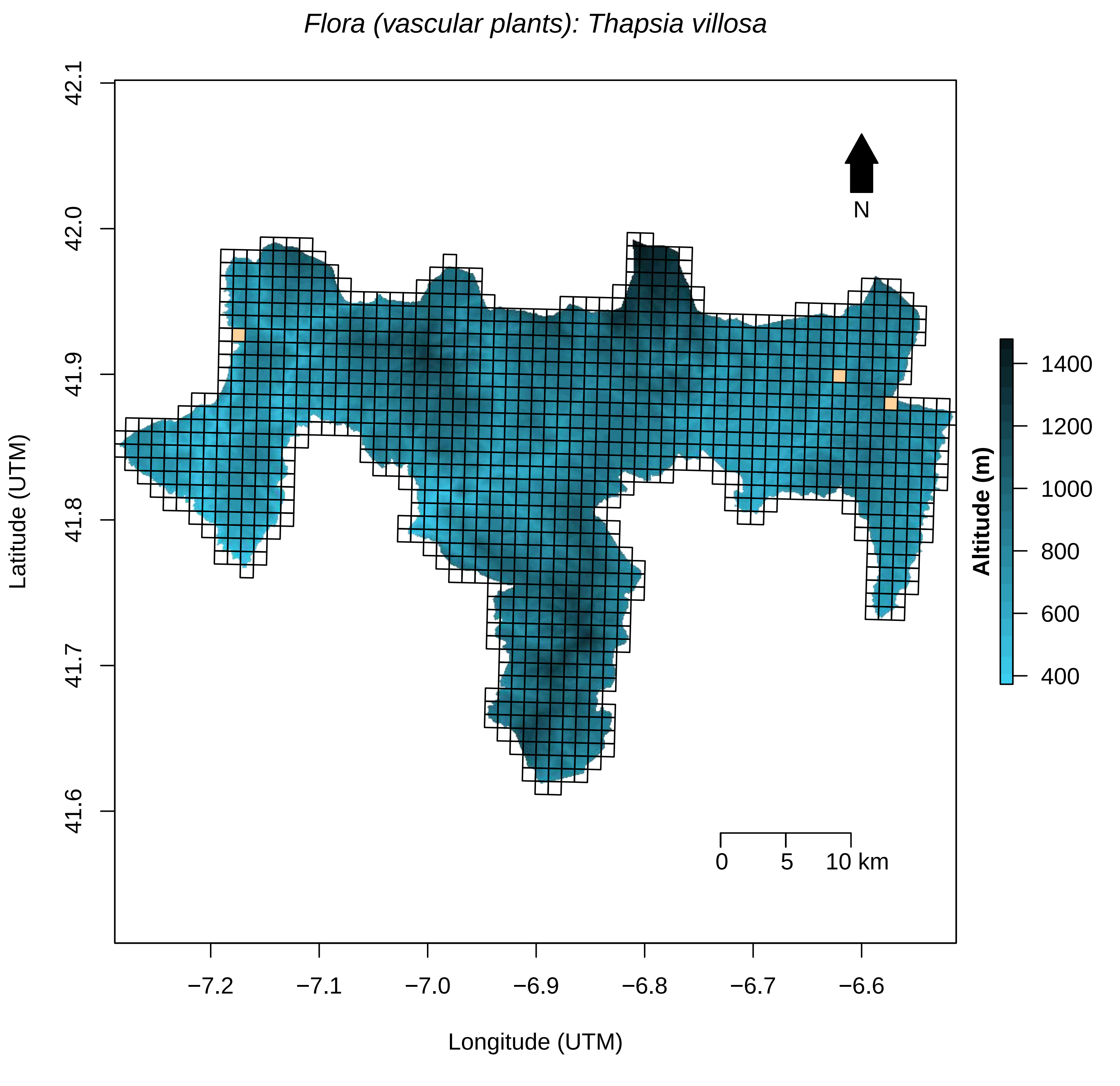
Flora (vascular plants): Thapsia villosa
42.1
42.0
41.9
41.8
41.7
41.6
Latitude (UTM)
−7.2
−7.1
−7.0
−6.9
−6.8
−6.7
−6.6
Longitude (UTM)
N
0
5
10 km
Altitude (m)
1400
1200
1000
800
600
400

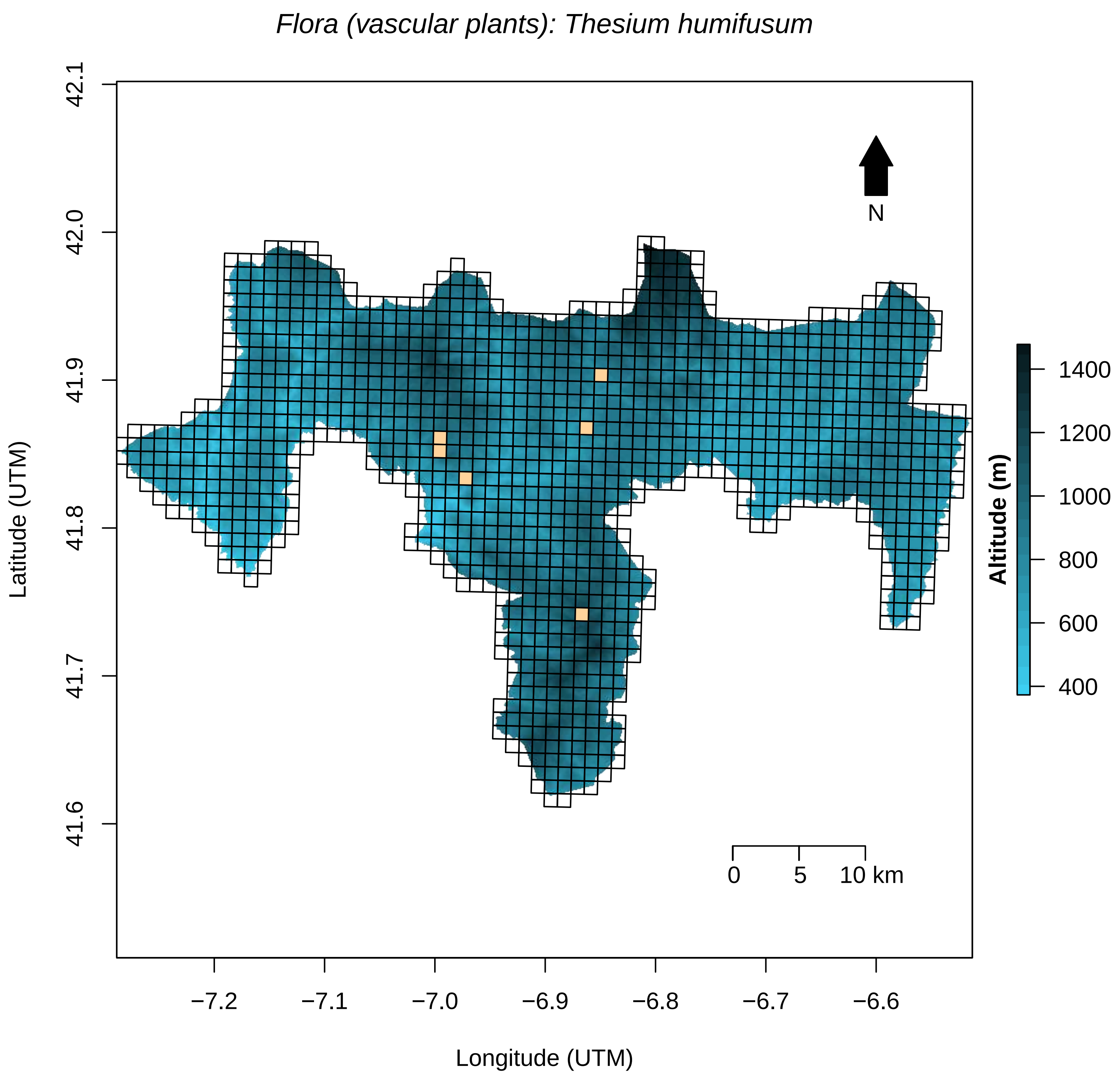

Flora (vascular plants): Thesium humifusum
42.1
42.0
41.9
41.8
41.7
41.6
Latitude (UTM)
−7.2
−7.1
−7.0
−6.9
−6.8
−6.7
−6.6
Longitude (UTM)
N
0
5
10 km
Altitude (m)
1400
1200
1000
800
600
400

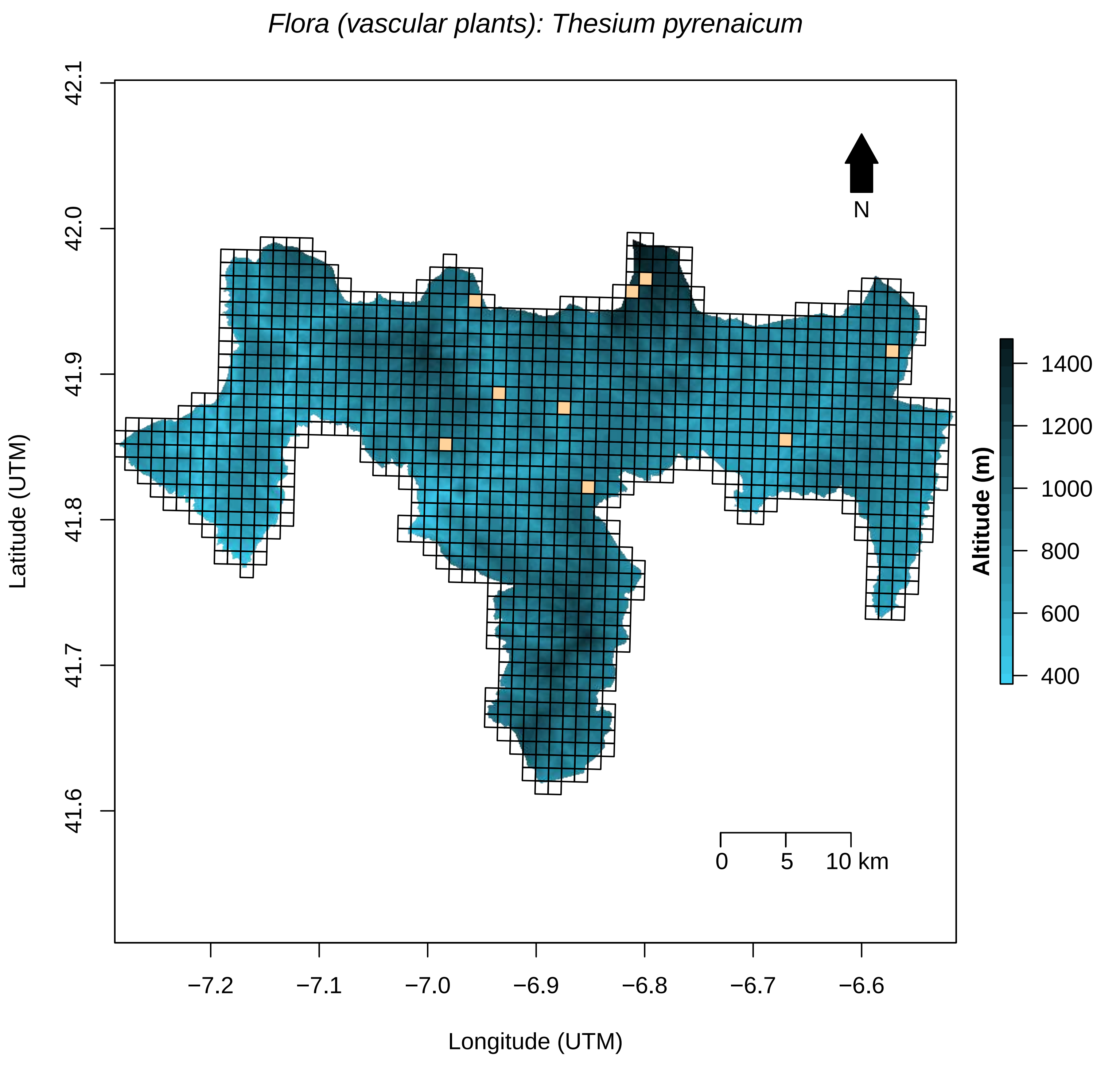

Flora (vascular plants): Thesium pyrenaicum
Latitude (UTM)
42.1
42.0
41.9
41.8
41.7
41.6
Longitude (UTM)
−7.2
−7.1
−7.0
−6.9
−6.8
−6.7
−6.6
N
0
5
10 km
Altitude (m)
1400
1200
1000
800
600
400

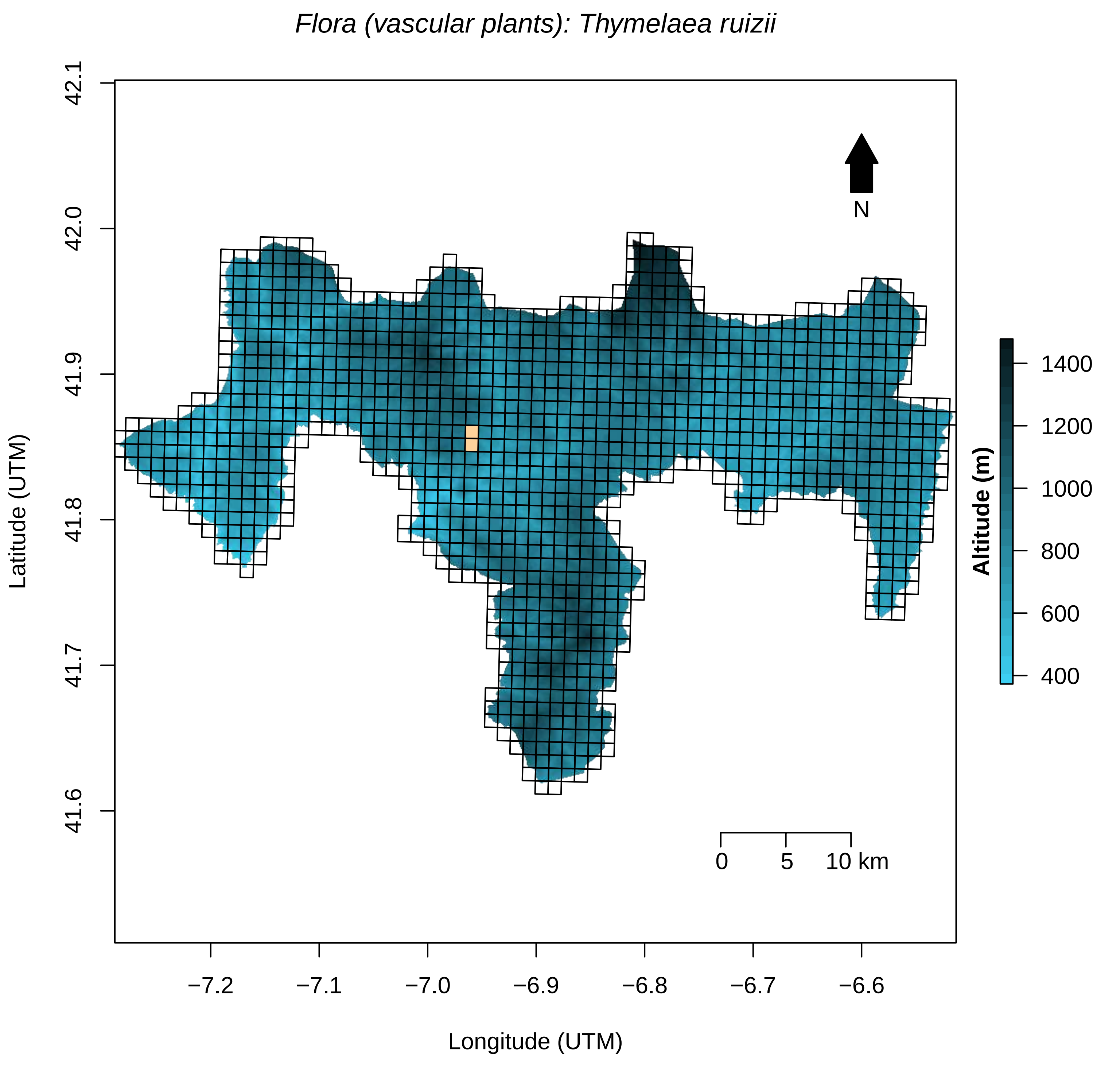

Flora (vascular plants): Thymelaea ruizii
42.1
42.0
41.9
41.8
41.7
41.6
Latitude (UTM)
−7.2
−7.1
−7.0
−6.9
−6.8
−6.7
−6.6
Longitude (UTM)
N
0
5
10 km
Altitude (m)
1400
1200
1000
800
600
400

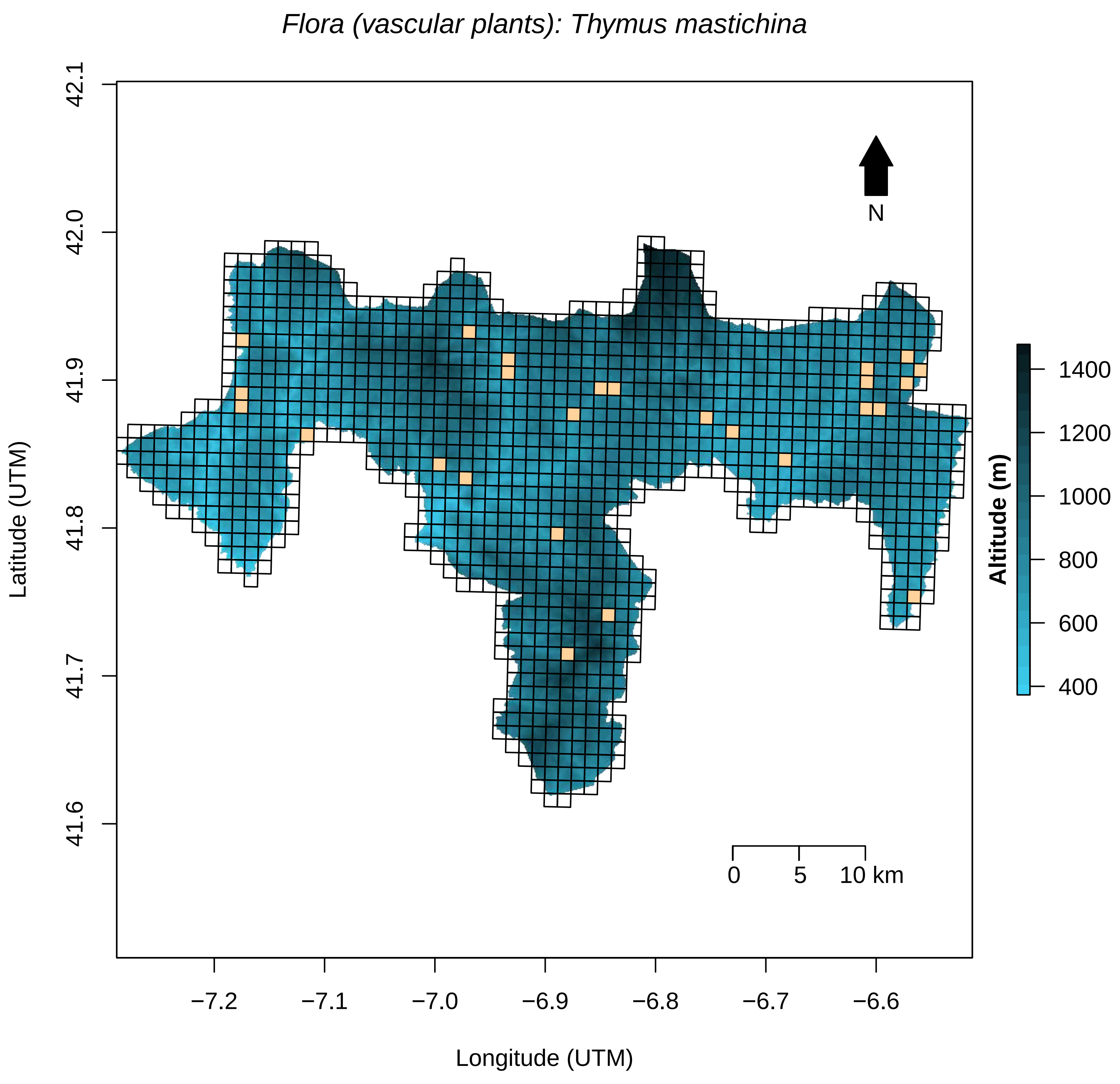

Flora (vascular plants): Thymus mastichina
42.1
42.0
41.9
41.8
41.7
41.6
Latitude (UTM)
N
0
5
10 km
−7.2
−7.1
−7.0
−6.9
−6.8
−6.7
−6.6
Longitude (UTM)
1400
1200
1000
800
600
400
Altitude (m)

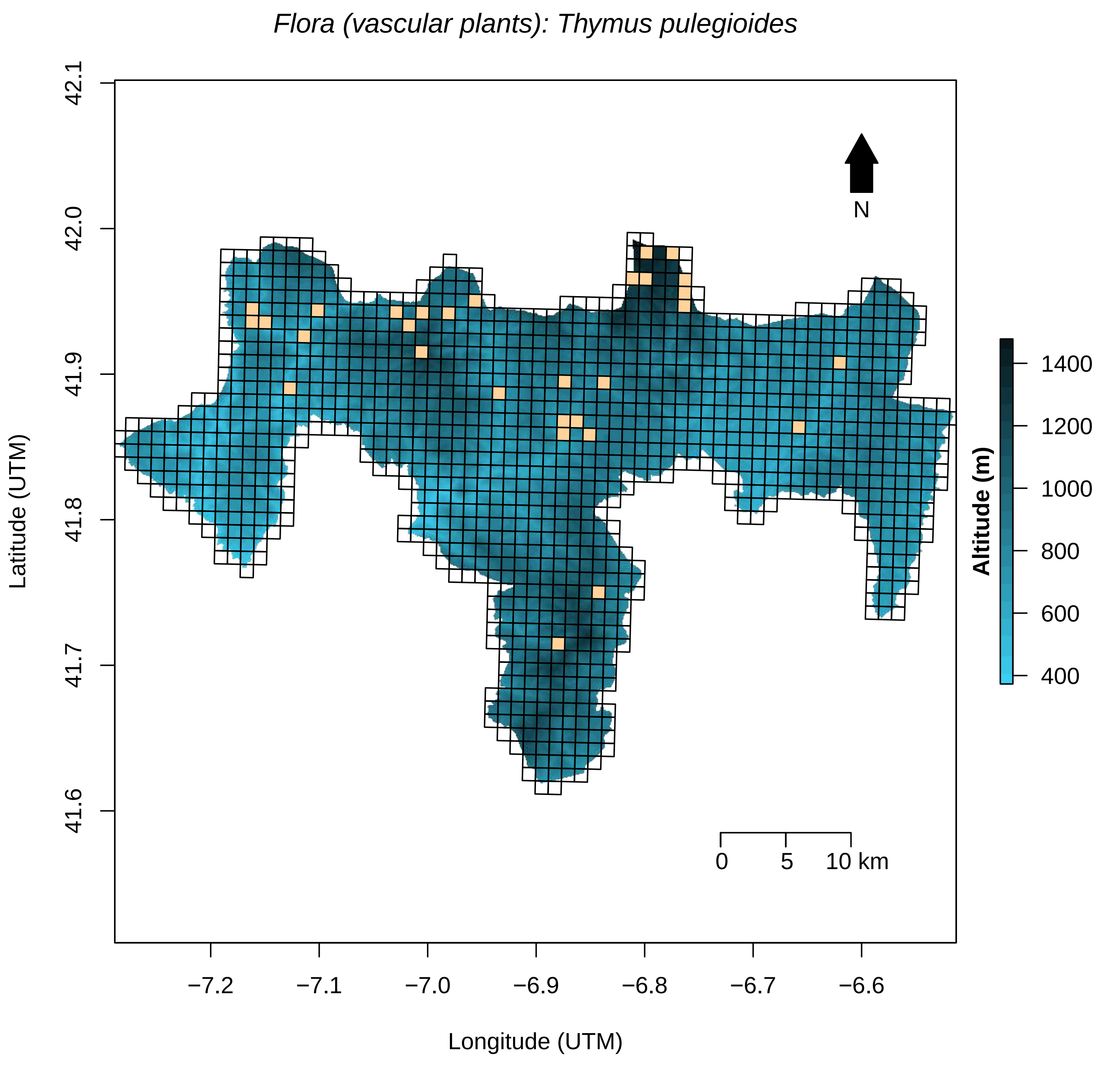
Flora (vascular plants): Thymus pulegioides
N
0
5
10 km
Altitude (m)
1400
1200
1000
800
600
400
Latitude (UTM)
42.1
42.0
41.9
41.8
41.7
41.6
Longitude (UTM)
−7.2
−7.1
−7.0
−6.9
−6.8
−6.7
−6.6

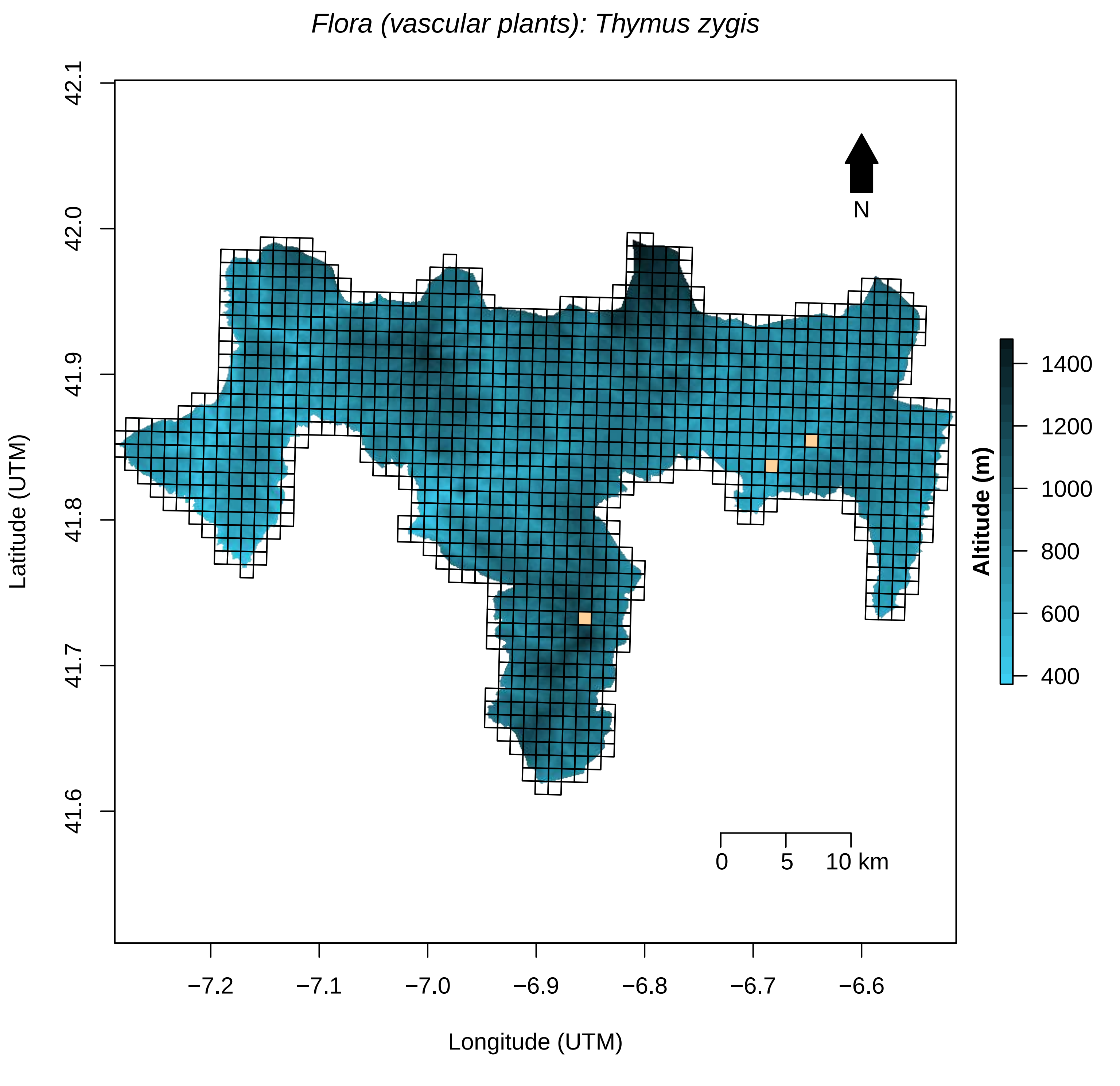
Flora (vascular plants): Thymus zygis
42.1
42.0
41.9
41.8
41.7
41.6
Latitude (UTM)
N
0
5
10 km
−7.2
−7.1
−7.0
−6.9
−6.8
−6.7
−6.6
Longitude (UTM)
Altitude (m)
1400
1200
1000
800
600
400

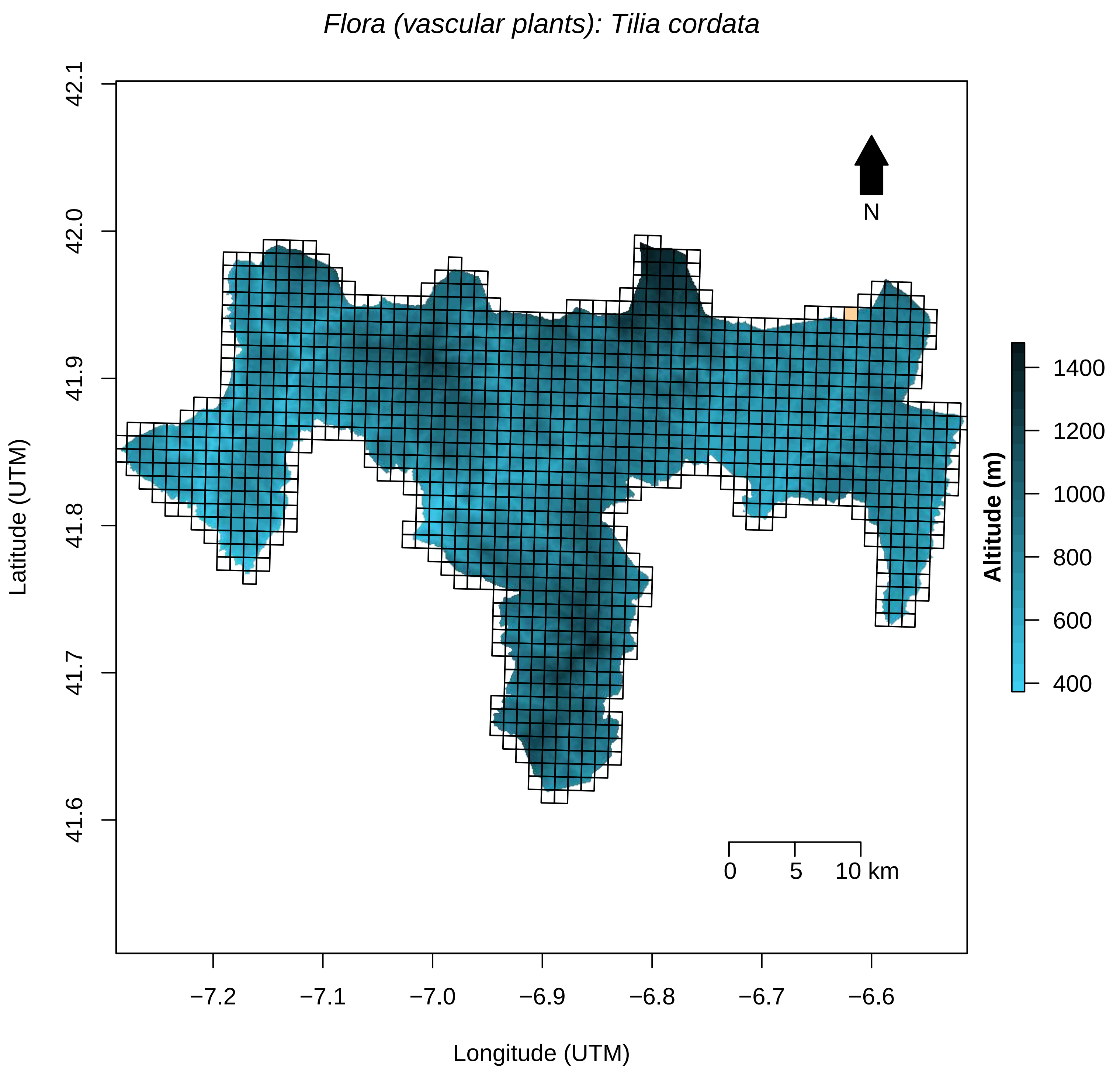
Flora (vascular plants): Tilia cordata
N
0
5
10 km
Altitude (m)
1400
1200
1000
800
600
400
42.1
42.0
41.9
41.8
41.7
41.6
Latitude (UTM)
−7.2
−7.1
−7.0
−6.9
−6.8
−6.7
−6.6
Longitude (UTM)

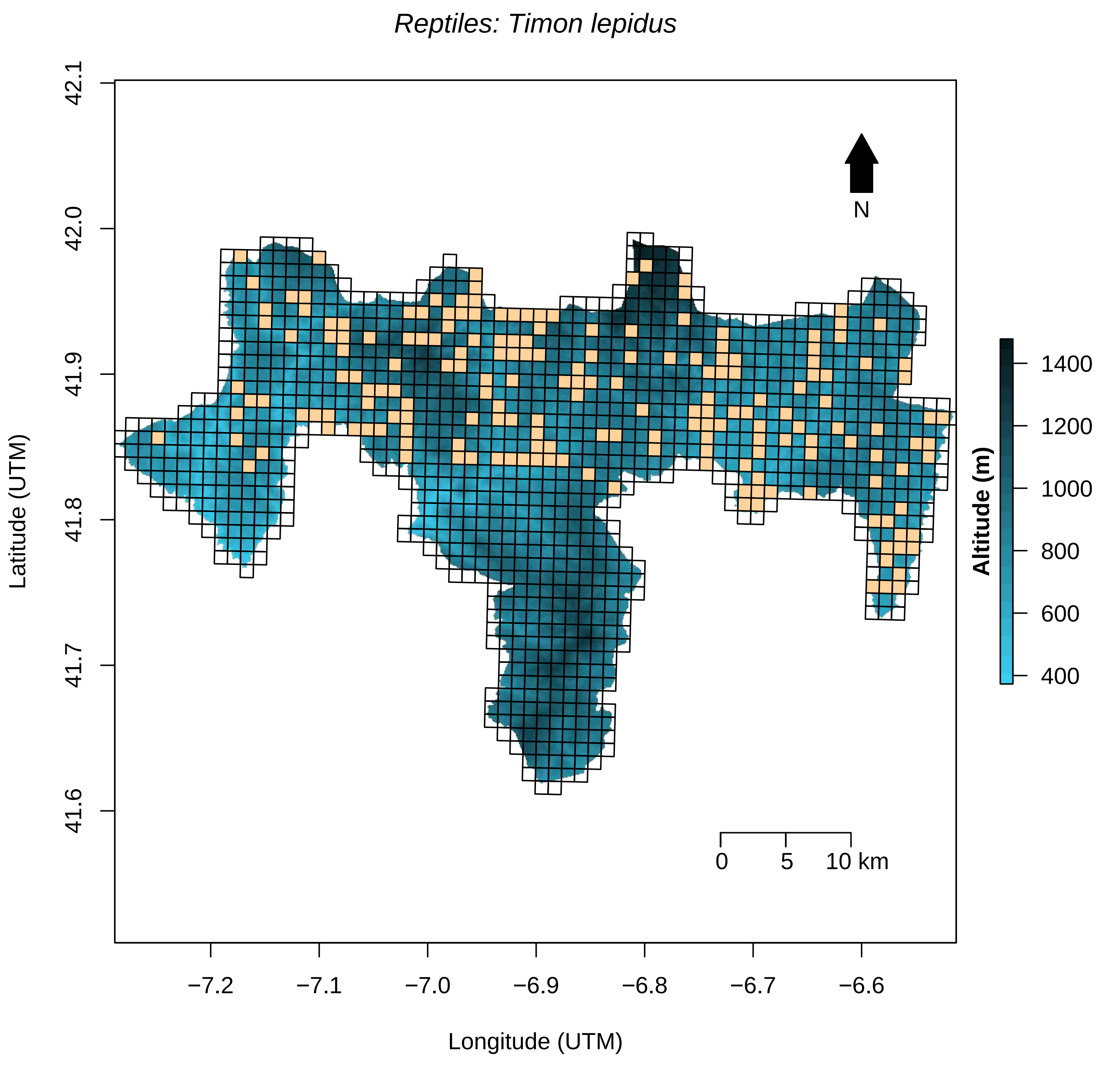

Reptiles: Timon lepidus
N
0 5 10 km
Altitude (m)
1400
1200
1000
800
600
400
Latitude (UTM)
42.1
42.0
41.9
41.8
41.7
41.6
Longitude (UTM)
−7.2
−7.1
−7.0
−6.9
−6.8
−6.7
−6.6

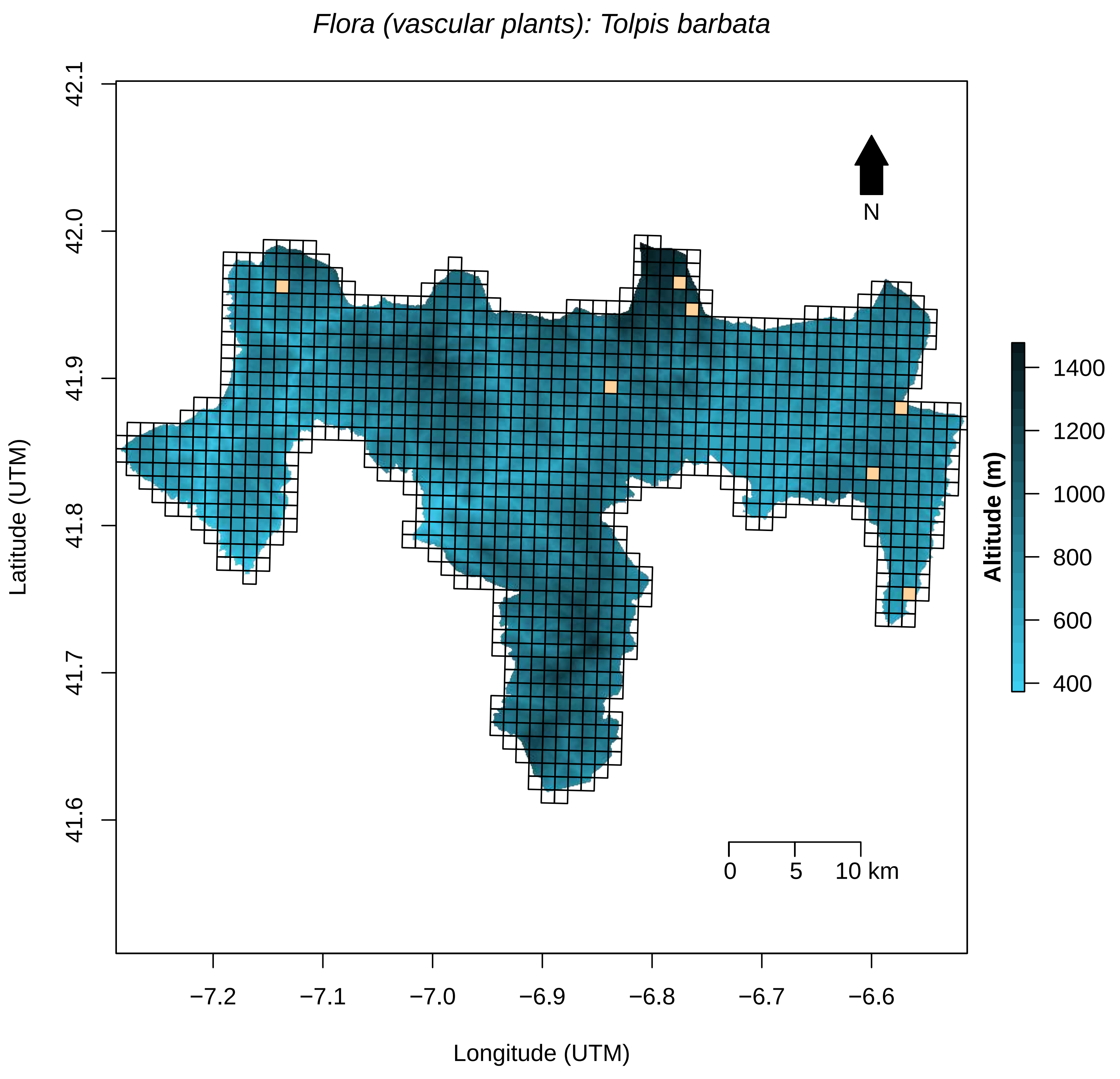

Flora (vascular plants): Tolpis barbata
42.1
42.0
41.9
41.8
41.7
41.6
Latitude (UTM)
−7.2
−7.1
−7.0
−6.9
−6.8
−6.7
−6.6
Longitude (UTM)
N
0
5
10 km
Altitude (m)
1400
1200
1000
800
600
400

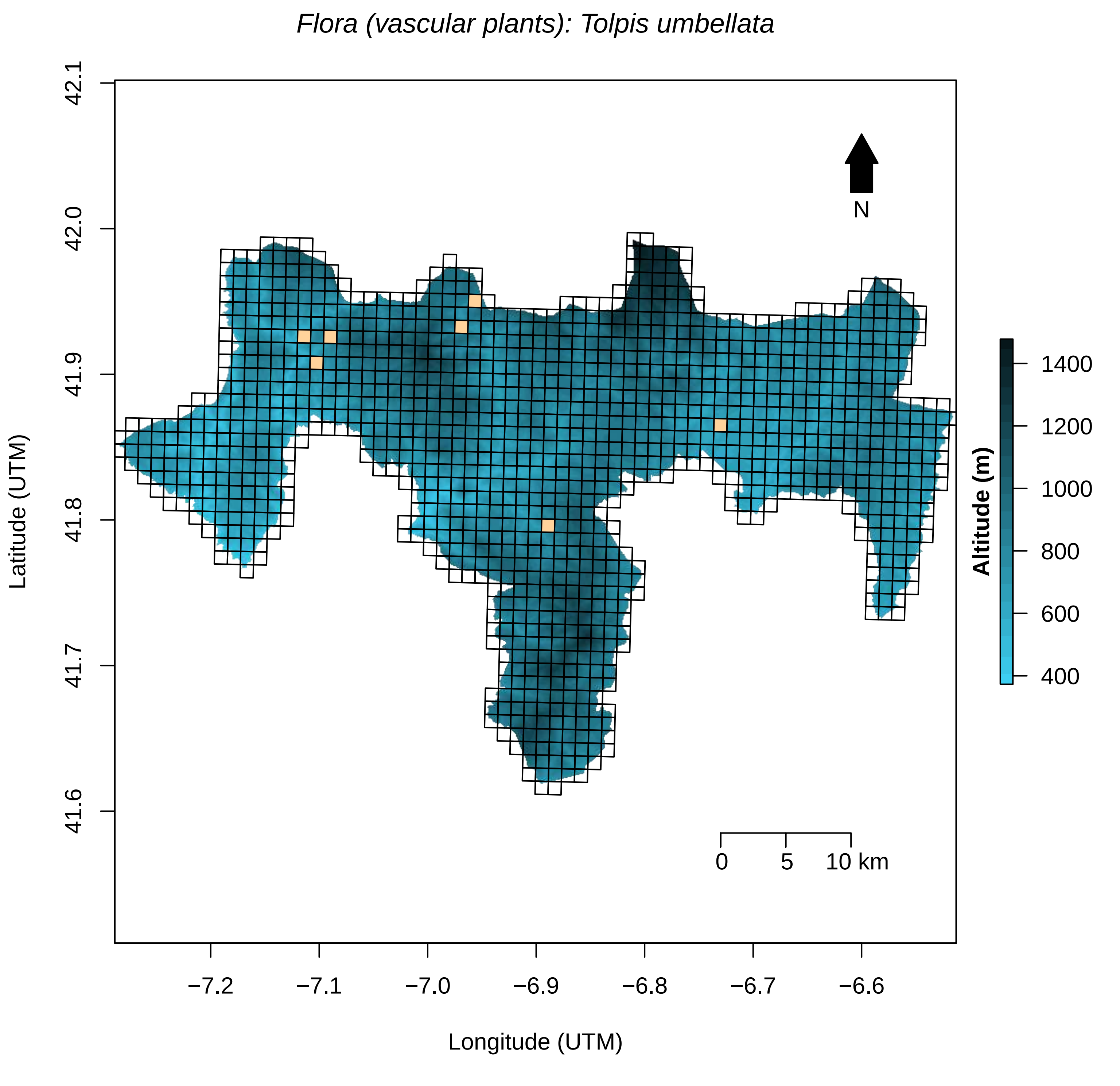
Flora (vascular plants): Tolpis umbellata
42.1
42.0
41.9
41.8
41.7
41.6
Latitude (UTM)
−7.2
−7.1
−7.0
−6.9
−6.8
−6.7
−6.6
Longitude (UTM)
N
0
5
10 km
Altitude (m)
1400
1200
1000
800
600
400

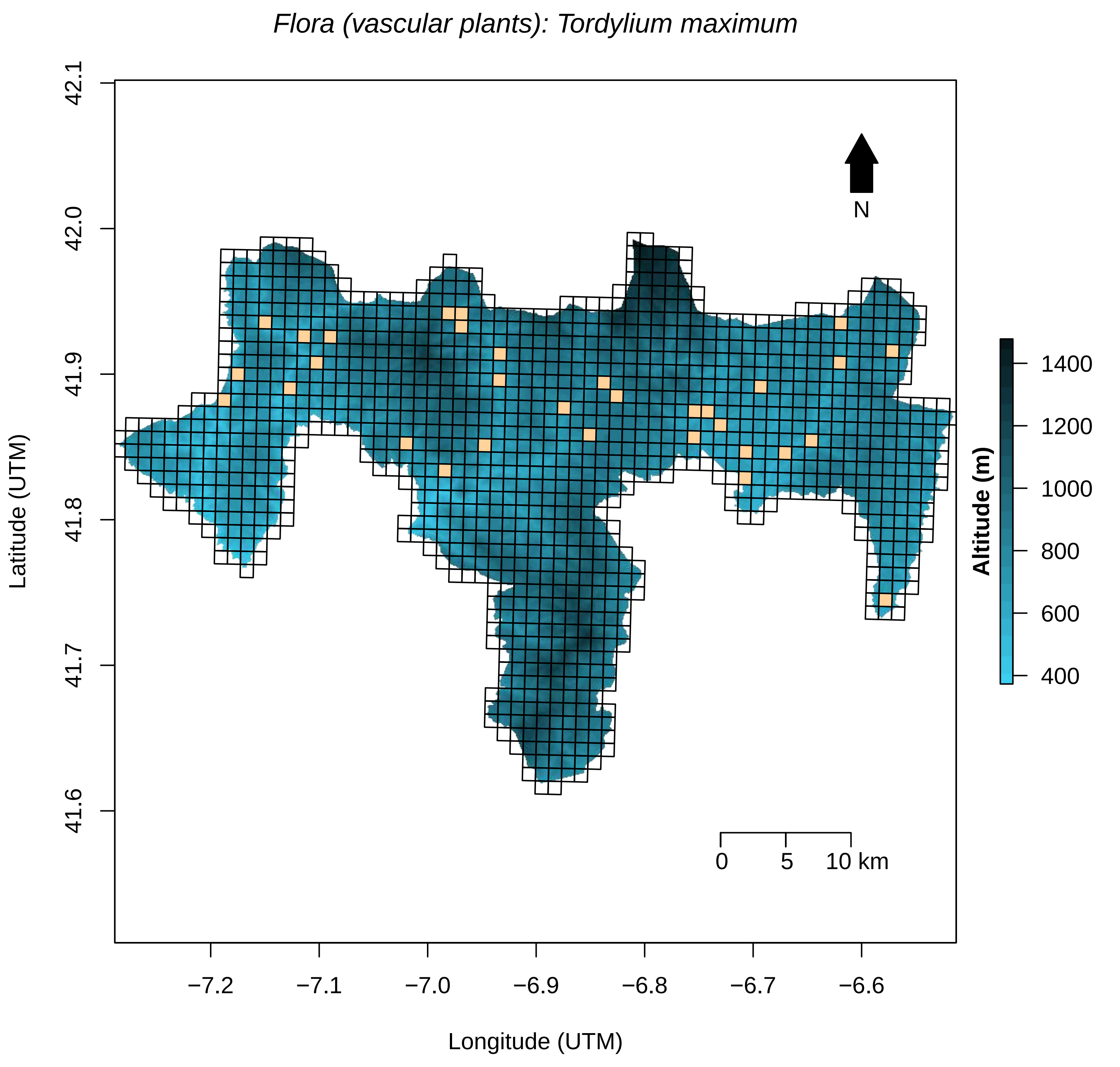

Flora (vascular plants): Tordylium maximum
42.1
42.0
41.9
41.8
41.7
41.6
Latitude (UTM)
−7.2
−7.1
−7.0
−6.9
−6.8
−6.7
−6.6
Longitude (UTM)
N
0
5
10 km
Altitude (m)
1400
1200
1000
800
600
400

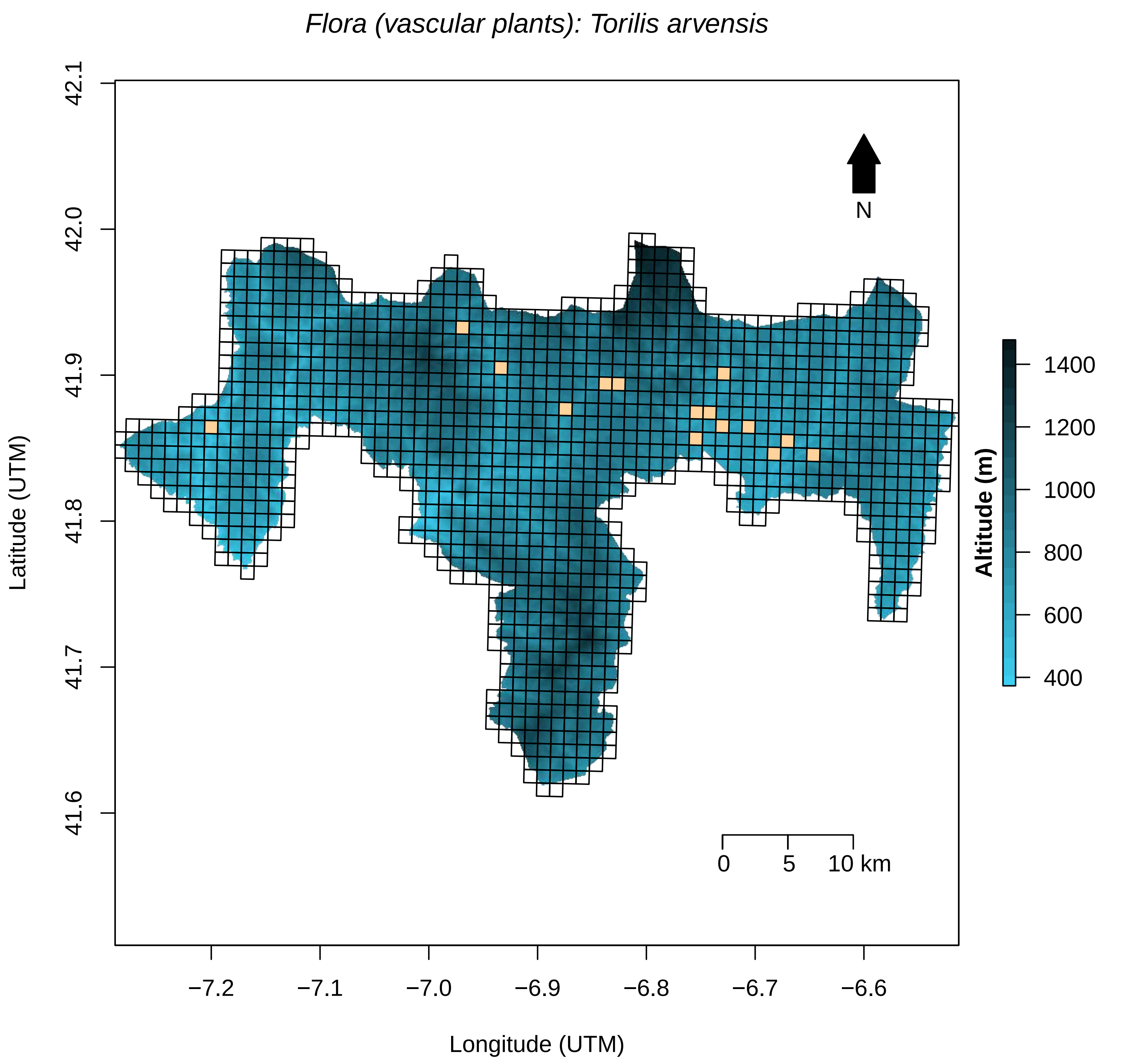
Flora (vascular plants): Torilis arvensis
Latitude (UTM)
42.1
42.0
41.9
41.8
41.7
41.6
Longitude (UTM)
−7.2
−7.1
−7.0
−6.9
−6.8
−6.7
−6.6
N
0
5
10 km
Altitude (m)
1400
1200
1000
800
600
400

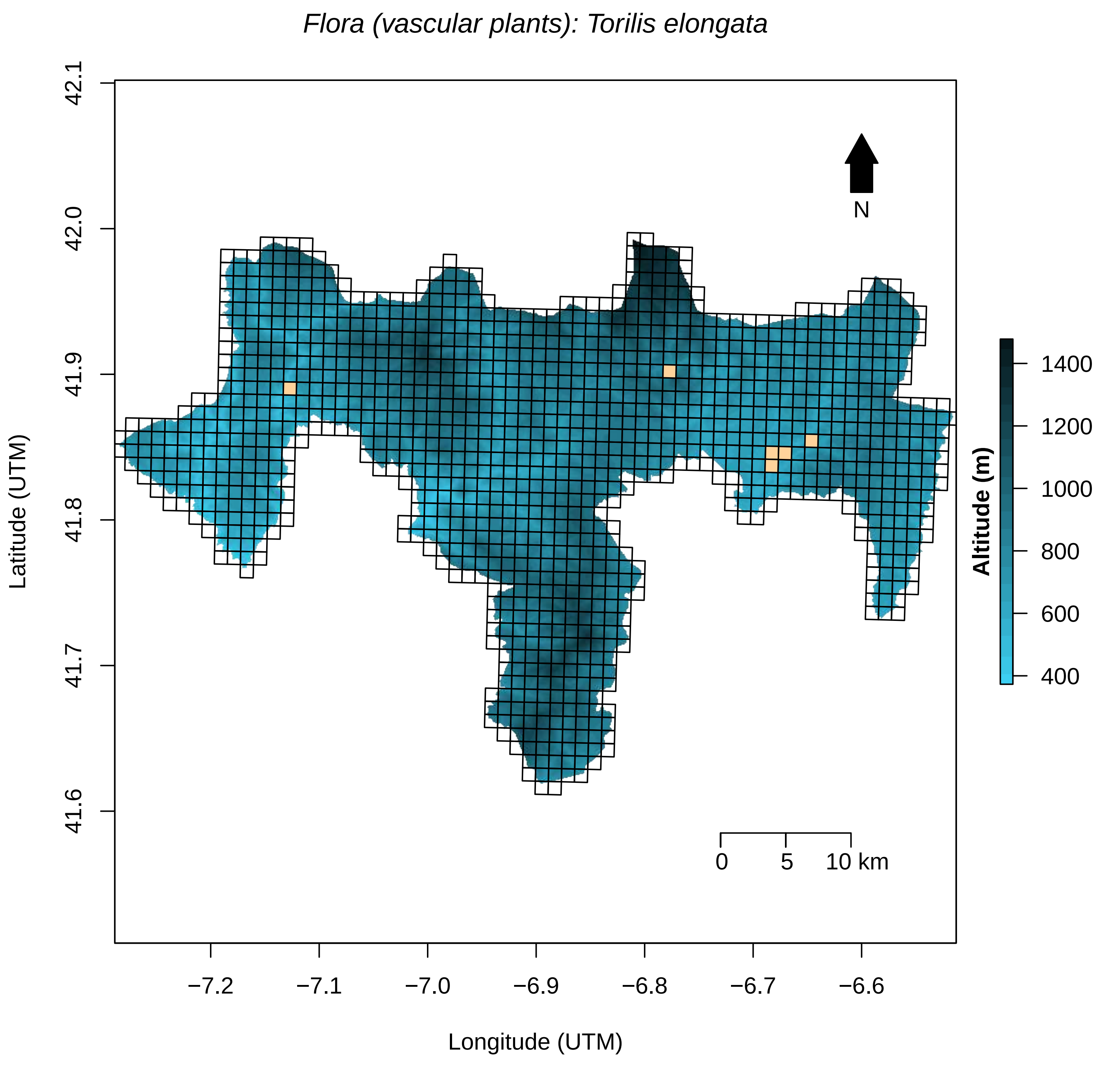

Flora (vascular plants): Torilis elongata
42.1
42.0
41.9
41.8
41.7
41.6
Latitude (UTM)
−7.2
−7.1
−7.0
−6.9
−6.8
−6.7
−6.6
Longitude (UTM)
N
0
5
10 km
Altitude (m)
1400
1200
1000
800
600
400

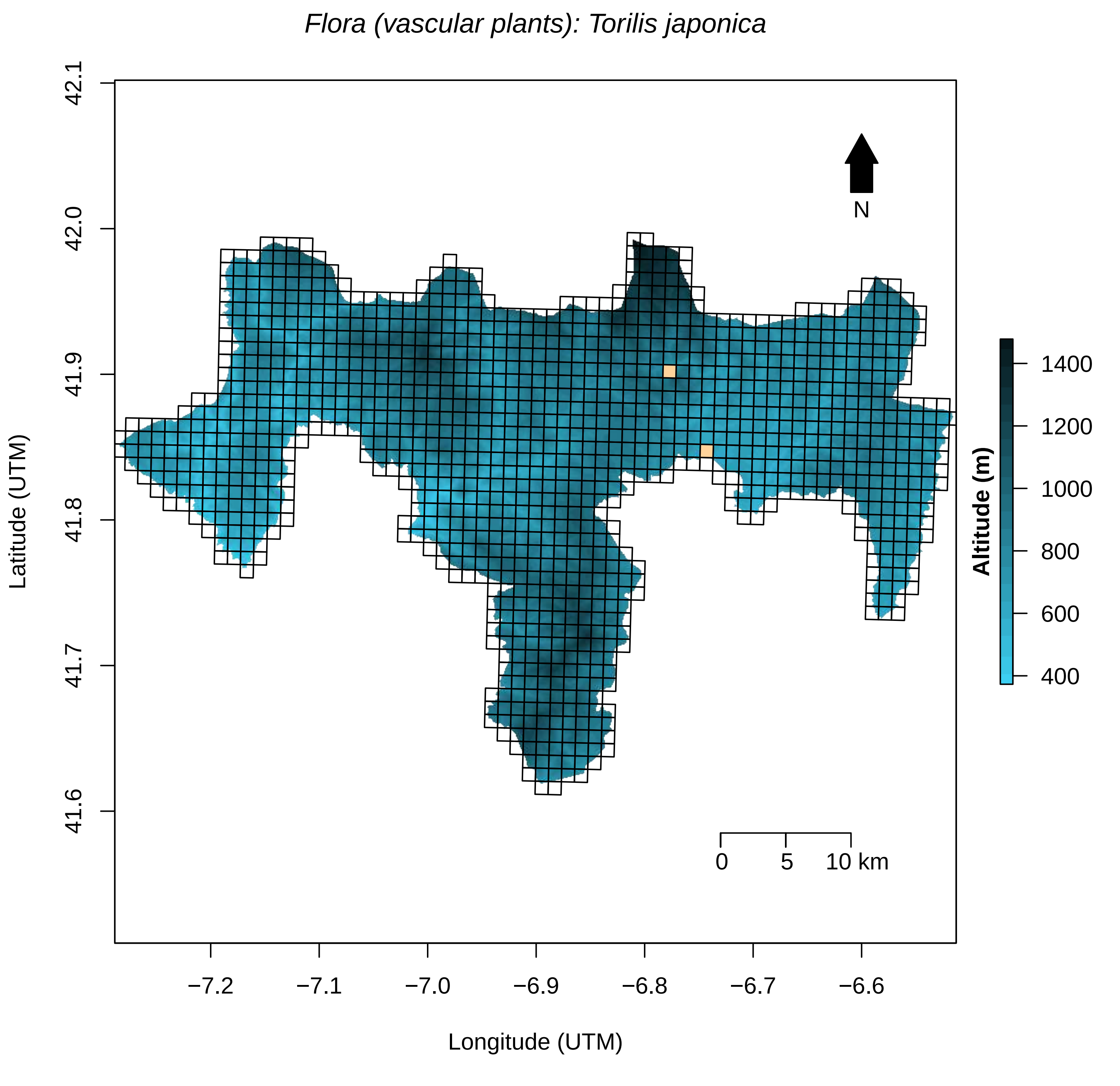

Flora (vascular plants): Torilis japonica
N
0
5
10 km
1400
1200
1000
800
600
400
Altitude (m)
42.1
42.0
41.9
41.8
41.7
41.6
Latitude (UTM)
−7.2
−7.1
−7.0
−6.9
−6.8
−6.7
−6.6
Longitude (UTM)

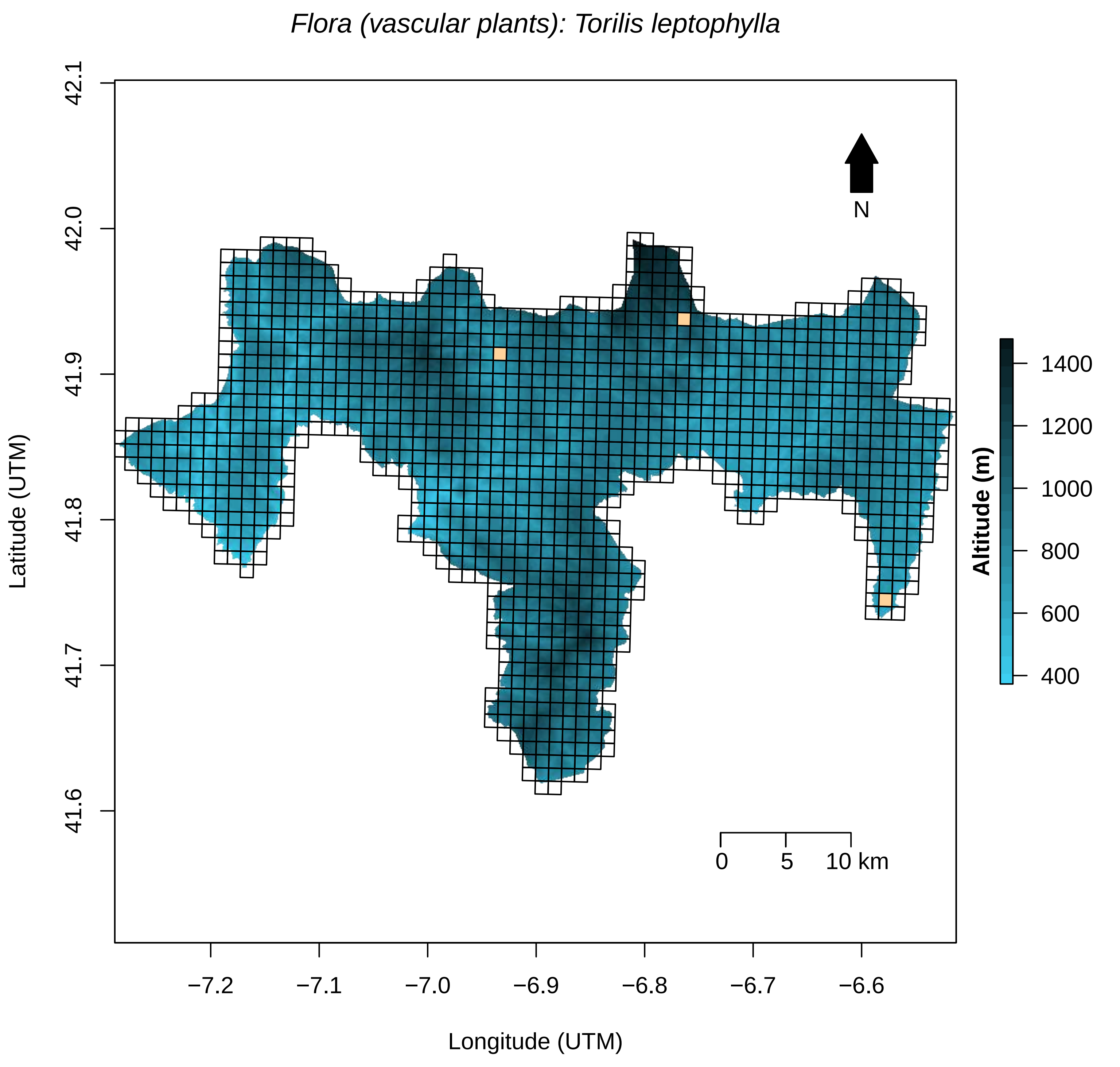

Flora (vascular plants): Torilis leptophylla
N
0 5 10 km
Latitude (UTM)
42.1
42.0
41.9
41.8
41.7
41.6
Longitude (UTM)
−7.2
−7.1
−7.0
−6.9
−6.8
−6.7
−6.6
Altitude (m)
1400
1200
1000
800
600
400

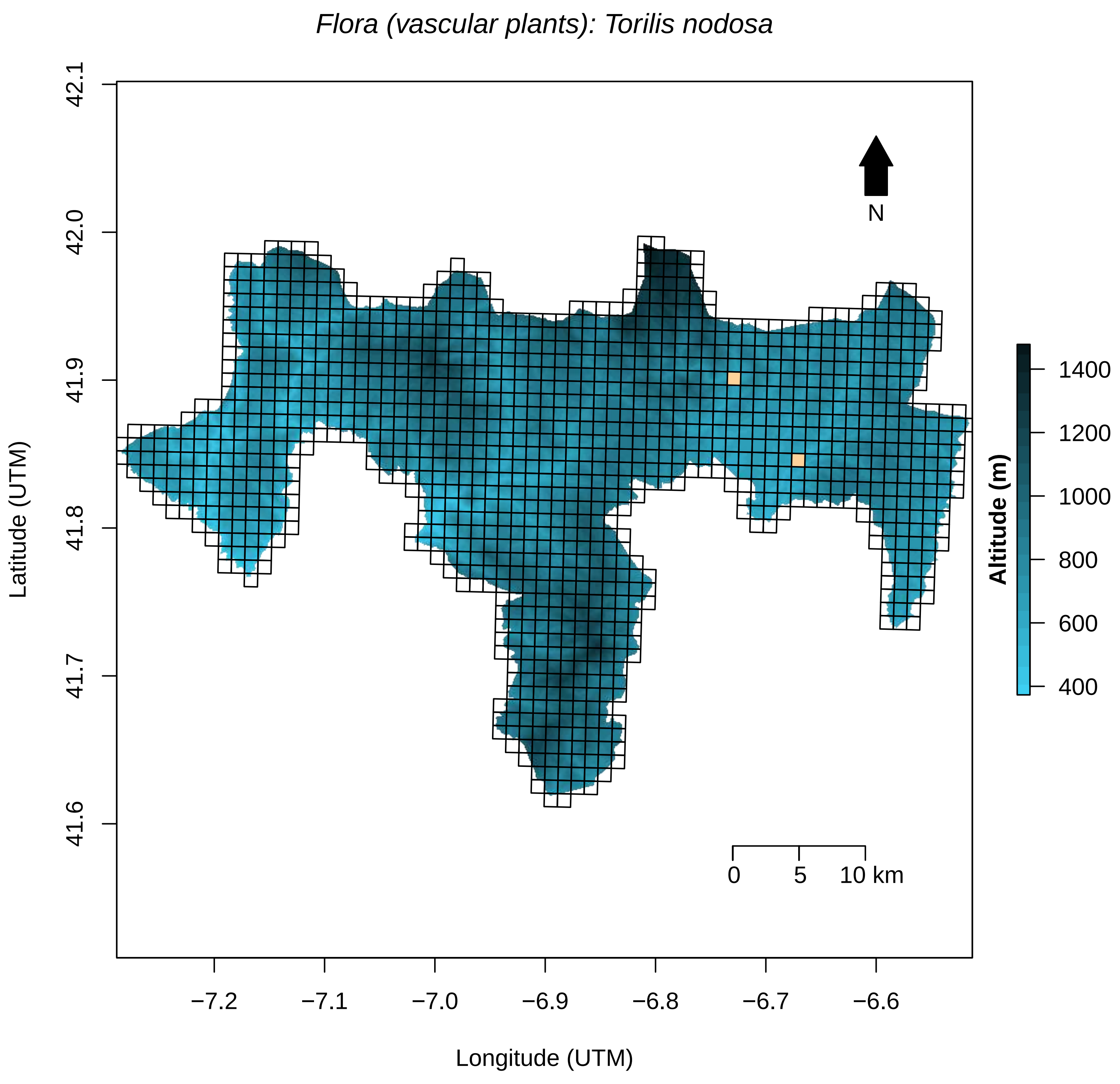

Flora (vascular plants): Torilis nodosa
42.1
42.0
41.9
41.8
41.7
41.6
Latitude (UTM)
−7.2
−7.1
−7.0
−6.9
−6.8
−6.7
−6.6
Longitude (UTM)
N
0
5
10 km
Altitude (m)
1400
1200
1000
800
600
400

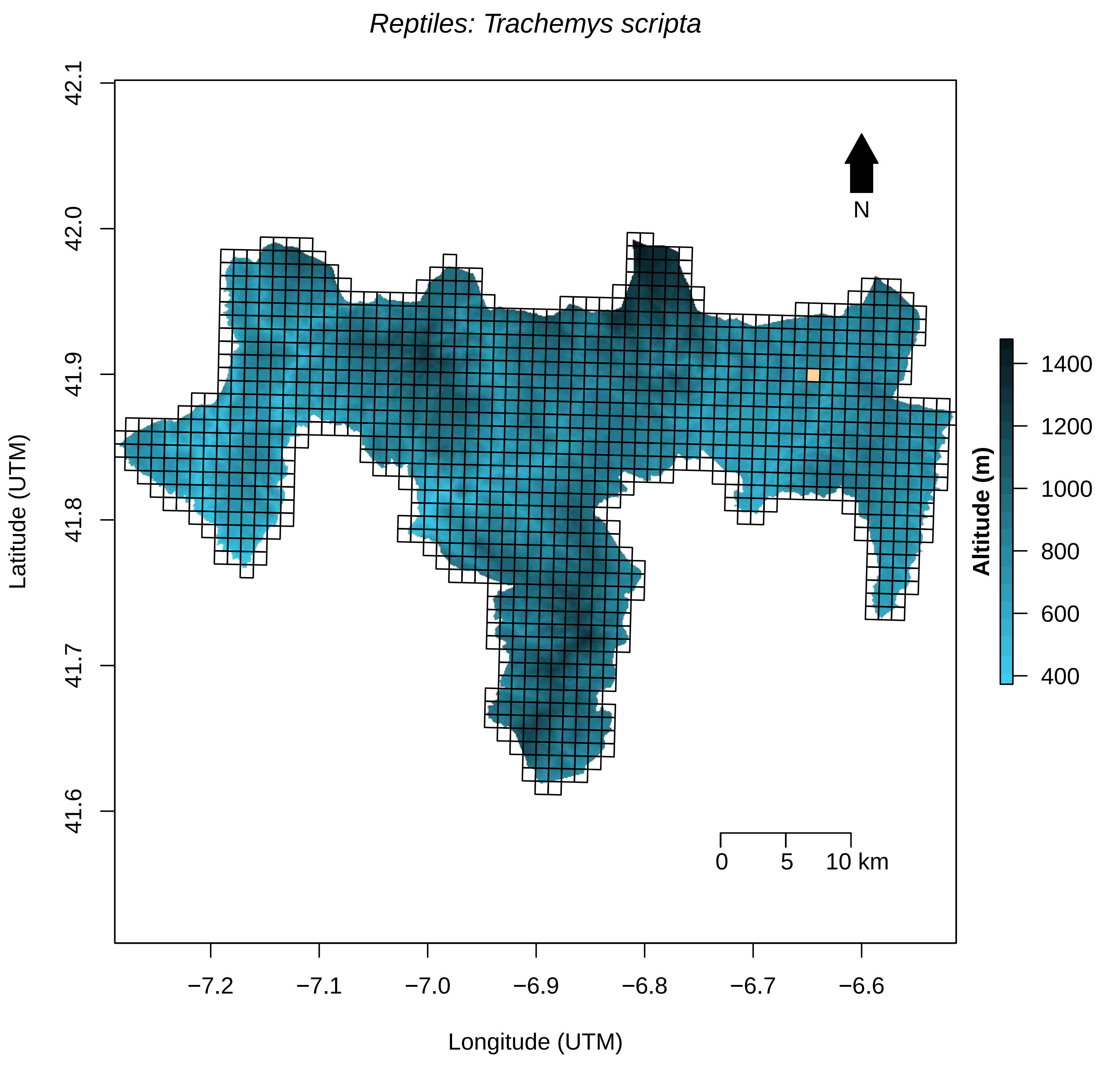
Reptiles: Trachemys scripta
42.1
42.0
41.9
41.8
41.7
41.6
Latitude (UTM)
−7.2
−7.1
−7.0
−6.9
−6.8
−6.7
−6.6
Longitude (UTM)
N
0
5
10 km
Altitude (m)
1400
1200
1000
800
600
400

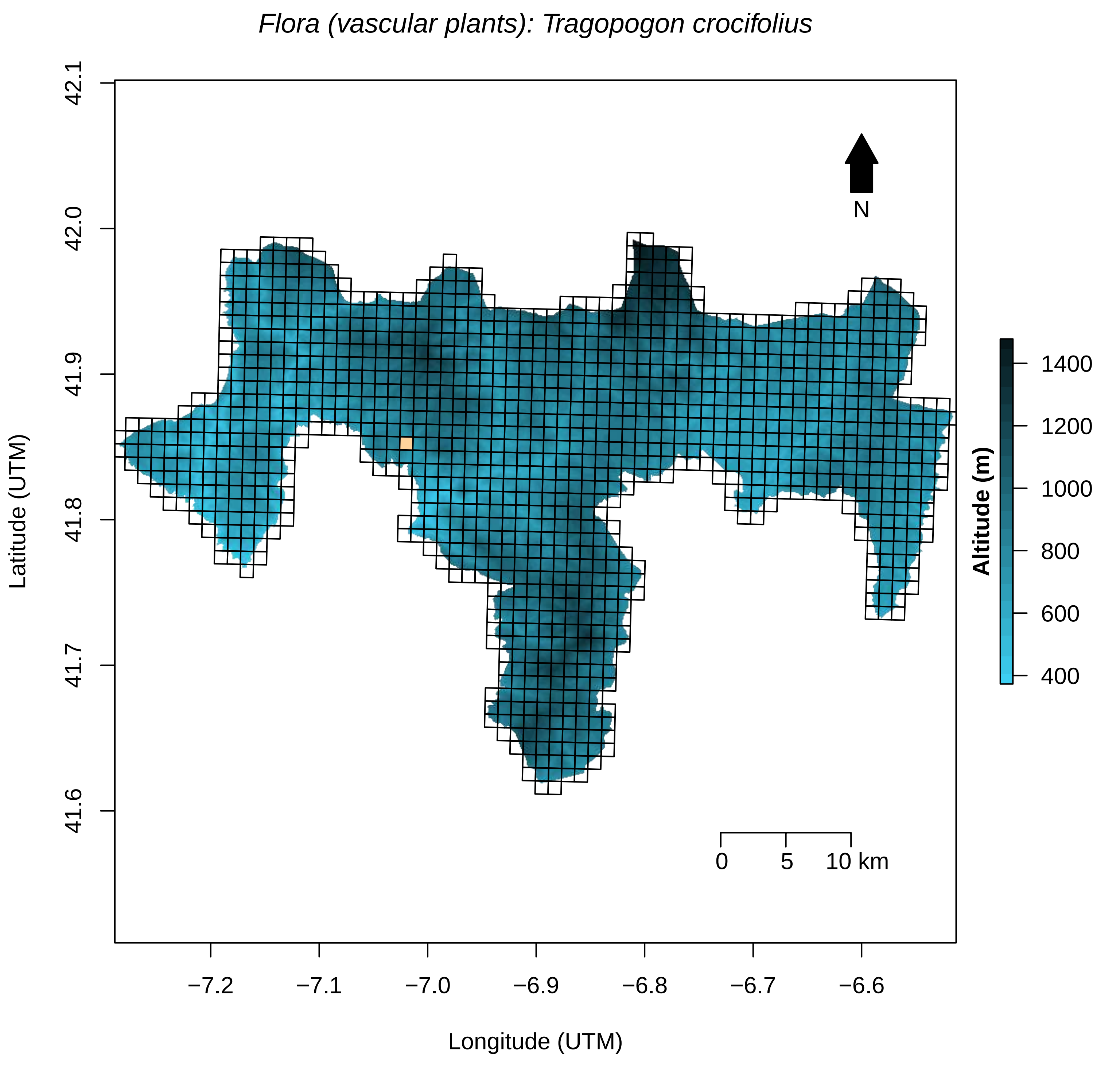

Flora (vascular plants): Tragopogon crocifolius
42.1
42.0
41.9
41.8
41.7
41.6
Latitude (UTM)
−7.2
−7.1
−7.0
−6.9
−6.8
−6.7
−6.6
Longitude (UTM)
N
0
5
10 km
1400
1200
1000
800
600
400
Altitude (m)

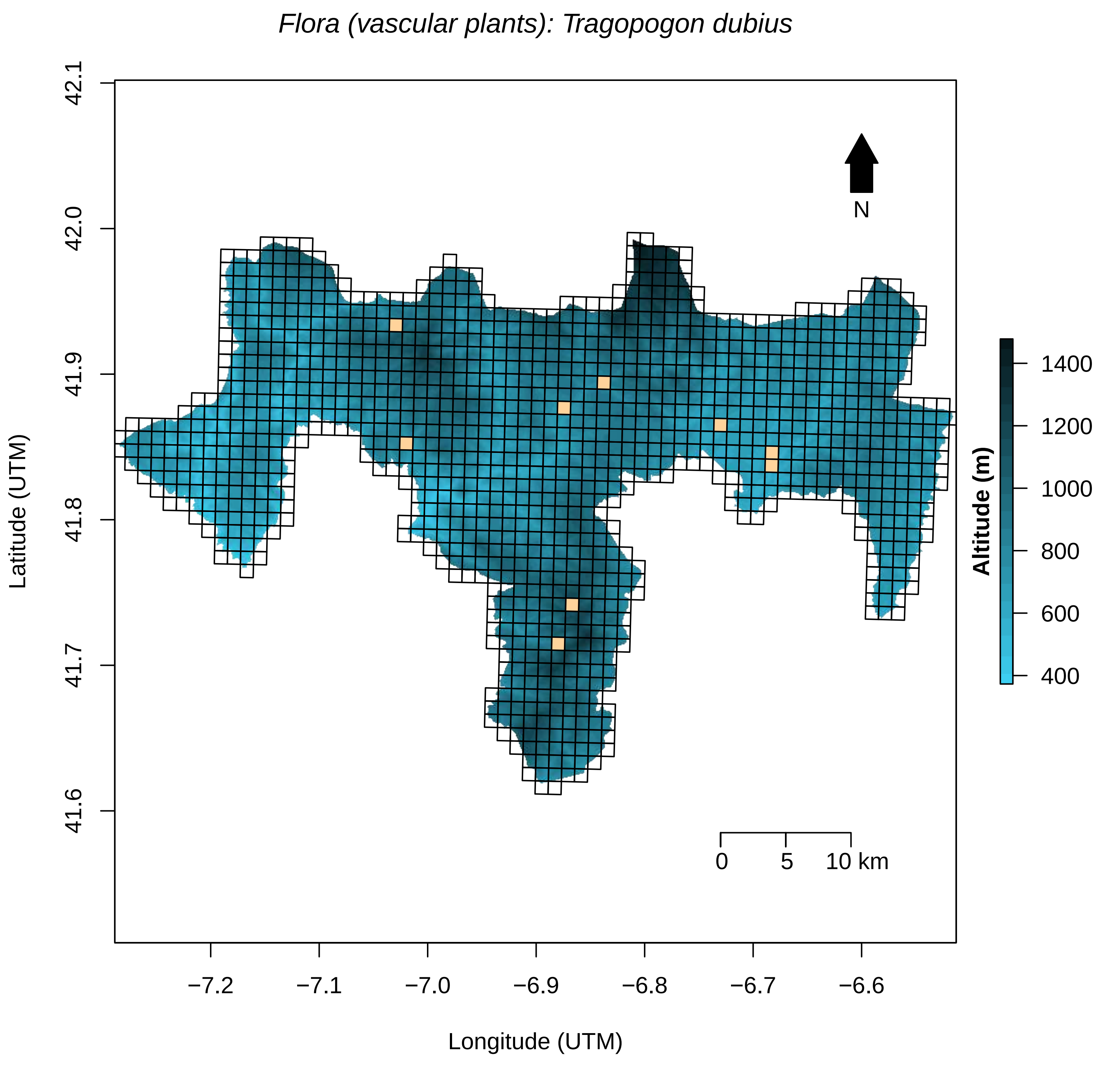

Flora (vascular plants): Tragopogon dubius
42.1
42.0
41.9
41.8
41.7
41.6
Latitude (UTM)
−7.2
−7.1
−7.0
−6.9
−6.8
−6.7
−6.6
Longitude (UTM)
N
0
5
10 km
Altitude (m)
1400
1200
1000
800
600
400

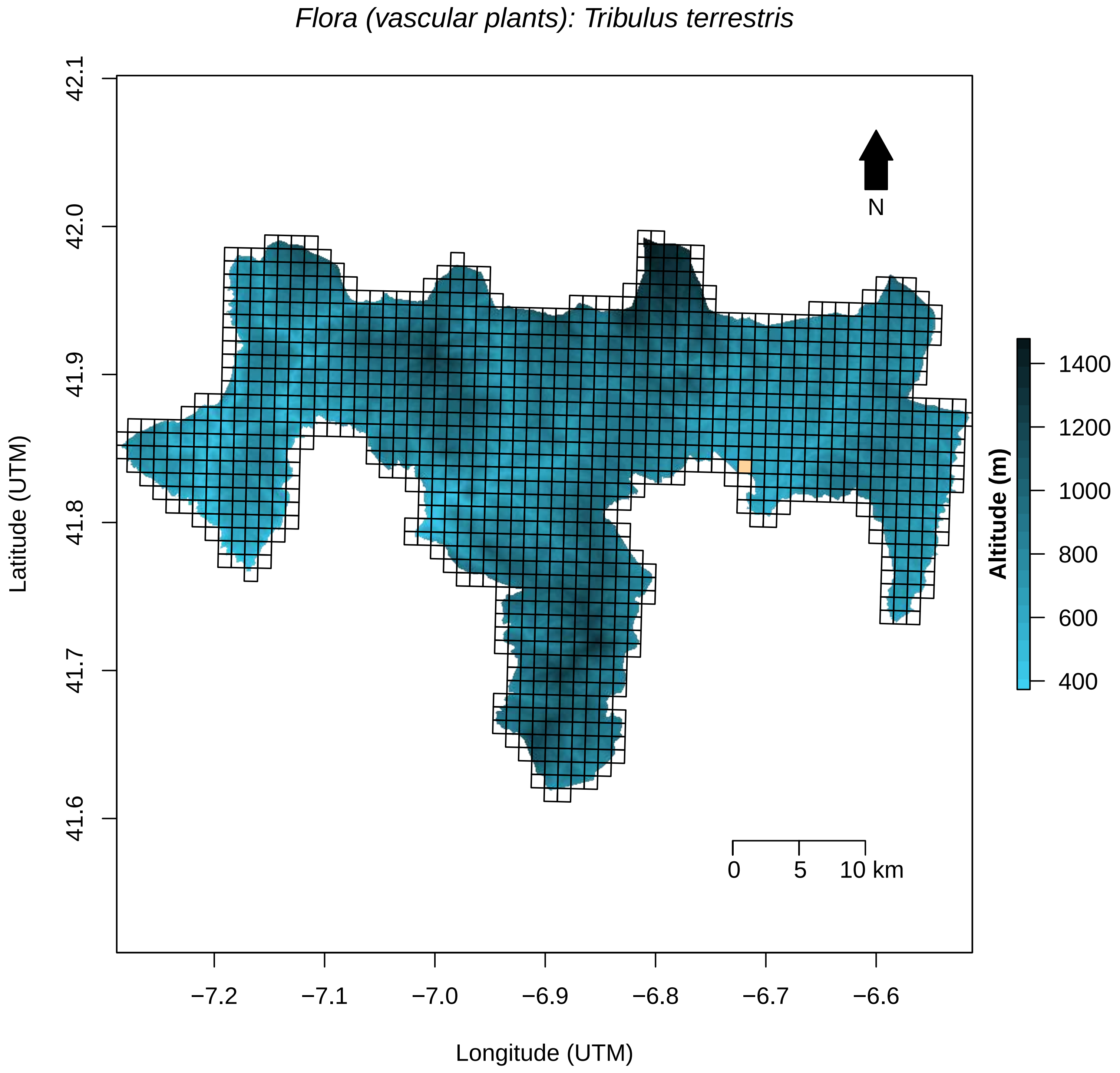

Flora (vascular plants): Tribulus terrestris
42.1
42.0
41.9
41.8
41.7
41.6
Latitude (UTM)
−7.2
−7.1
−7.0
−6.9
−6.8
−6.7
−6.6
Longitude (UTM)
N
0
5
10 km
Altitude (m)
1400
1200
1000
800
600
400

Flora (vascular plants): Trichophorum cespitosum

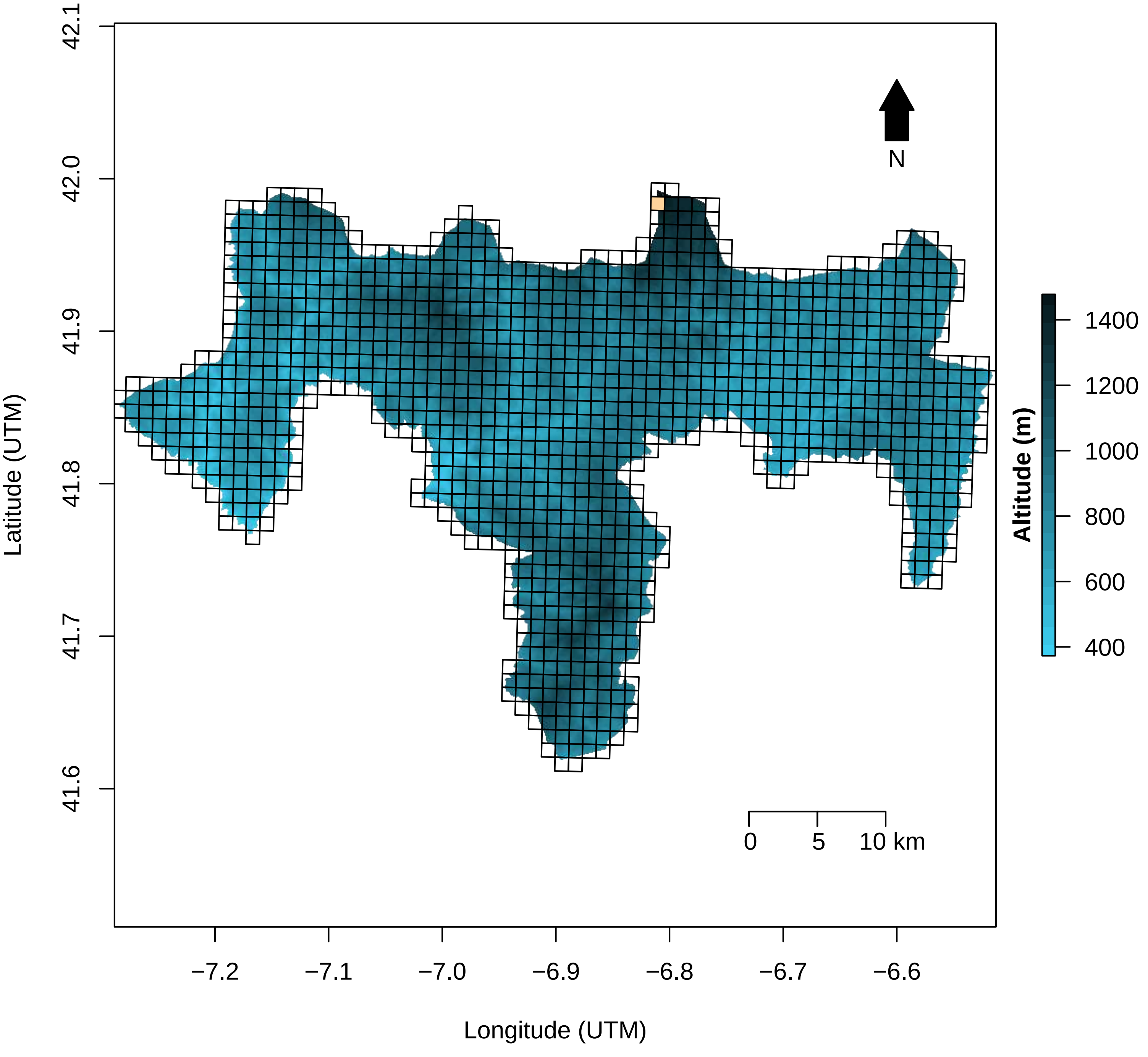

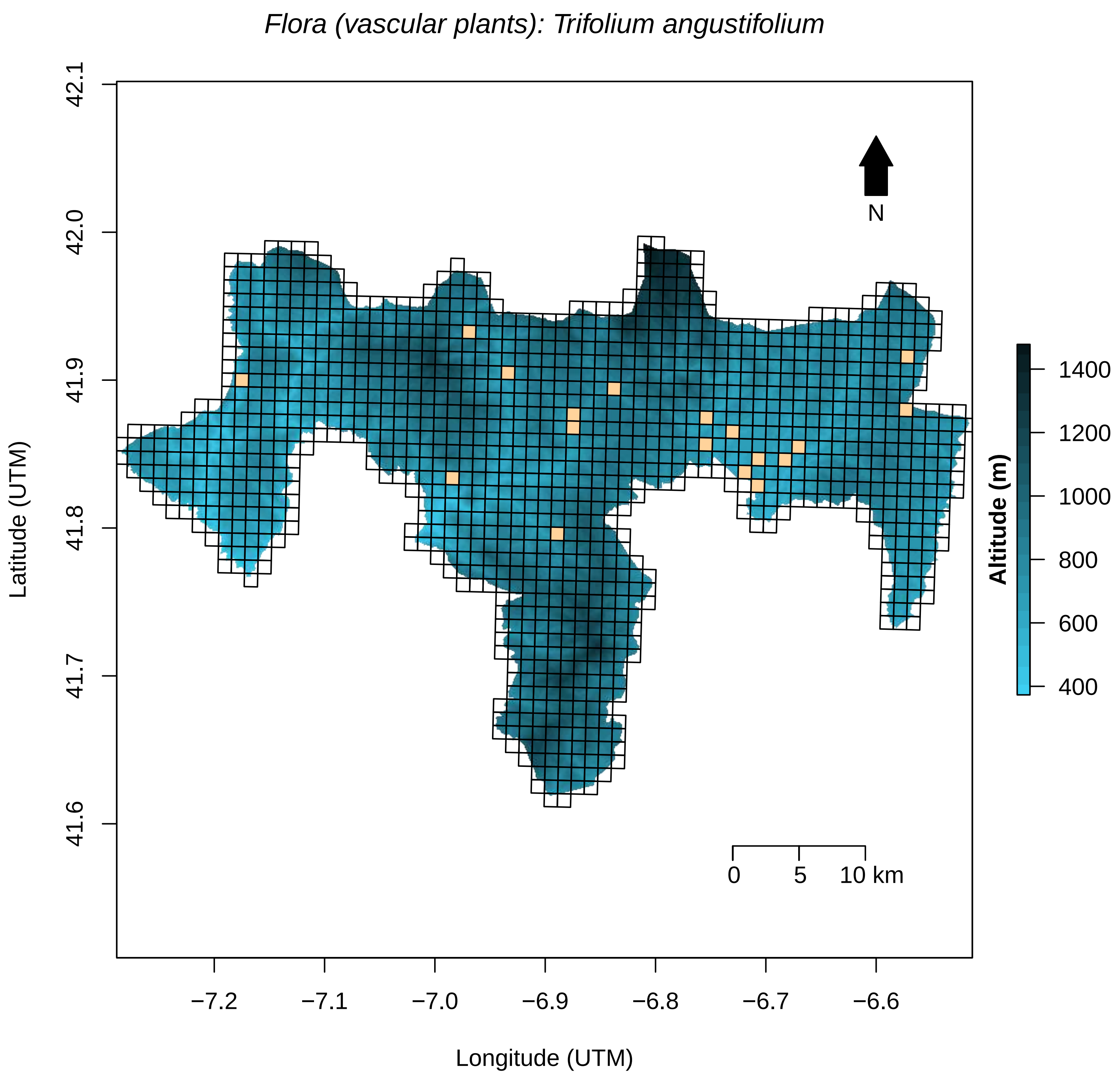

Flora (vascular plants): Trifolium angustifolium
42.1
42.0
41.9
41.8
41.7
41.6
Latitude (UTM)
−7.2
−7.1
−7.0
−6.9
−6.8
−6.7
−6.6
Longitude (UTM)
N
0
5
10 km
1400
1200
1000
800
600
400
Altitude (m)

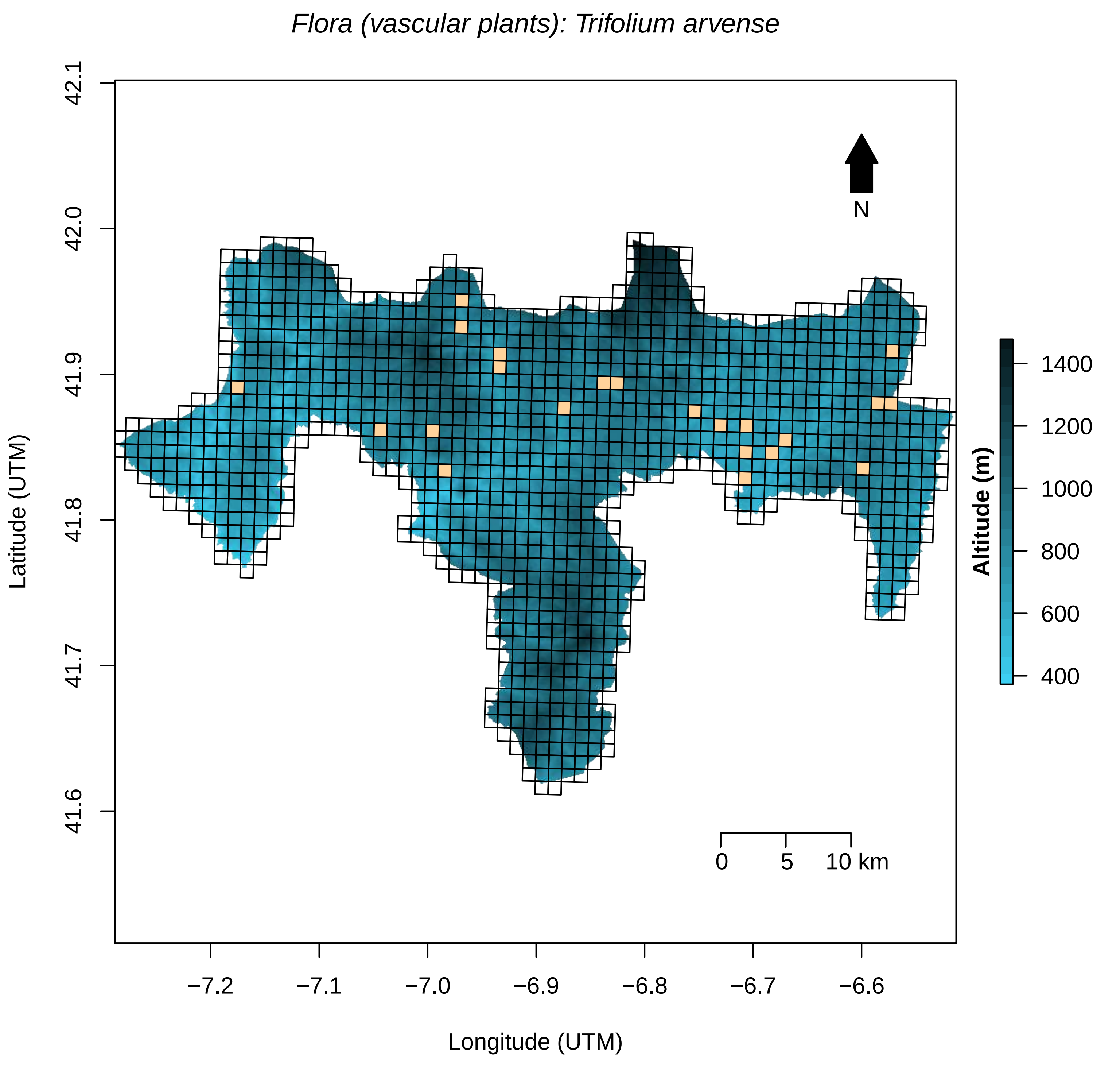

Flora (vascular plants): Trifolium arvense
42.1
42.0
41.9
41.8
41.7
41.6
Latitude (UTM)
−7.2
−7.1
−7.0
−6.9
−6.8
−6.7
−6.6
Longitude (UTM)
N
0
5
10 km
1400
1200
1000
800
600
400
Altitude (m)

Flora (vascular plants): Trifolium bocconei

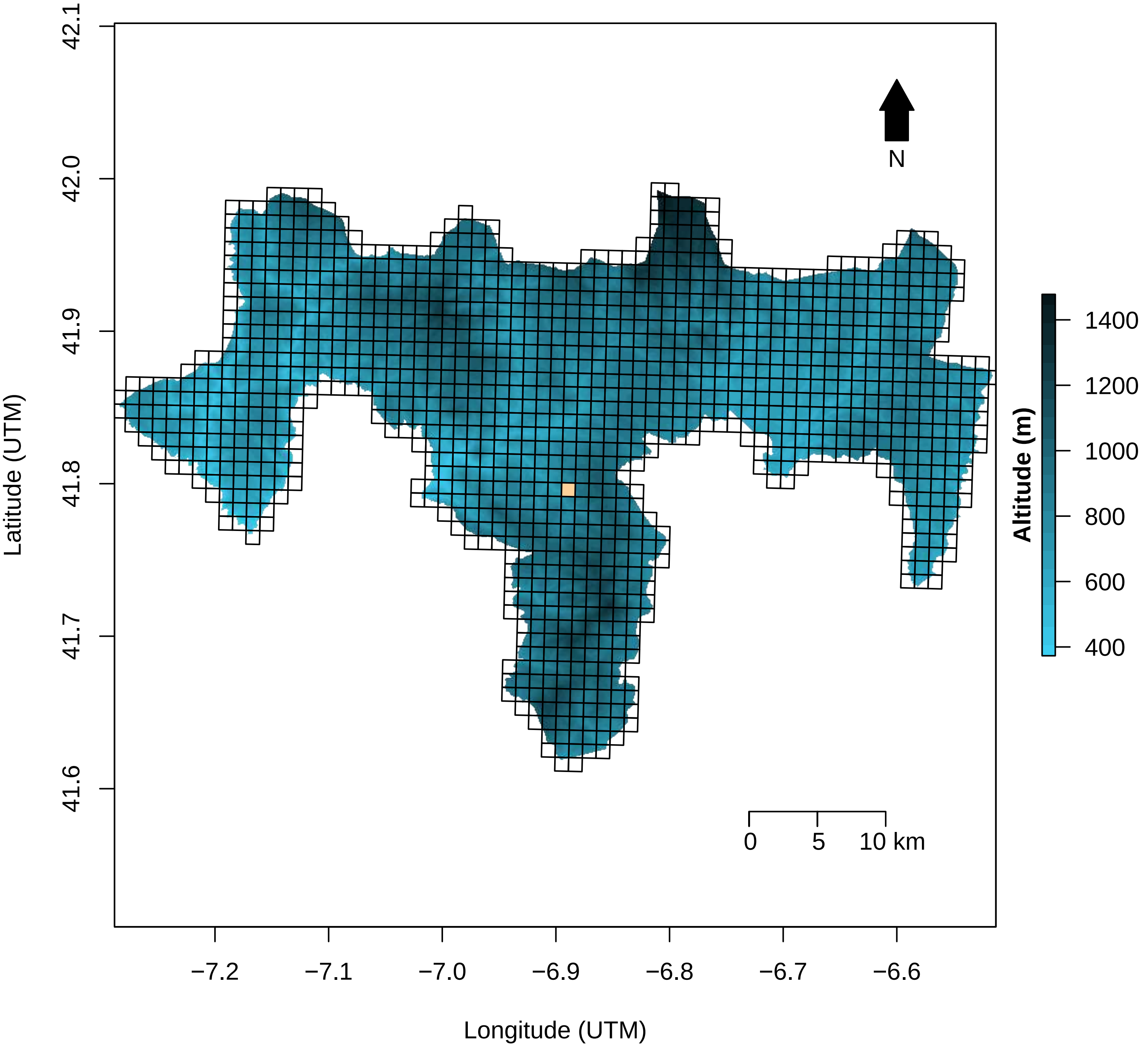

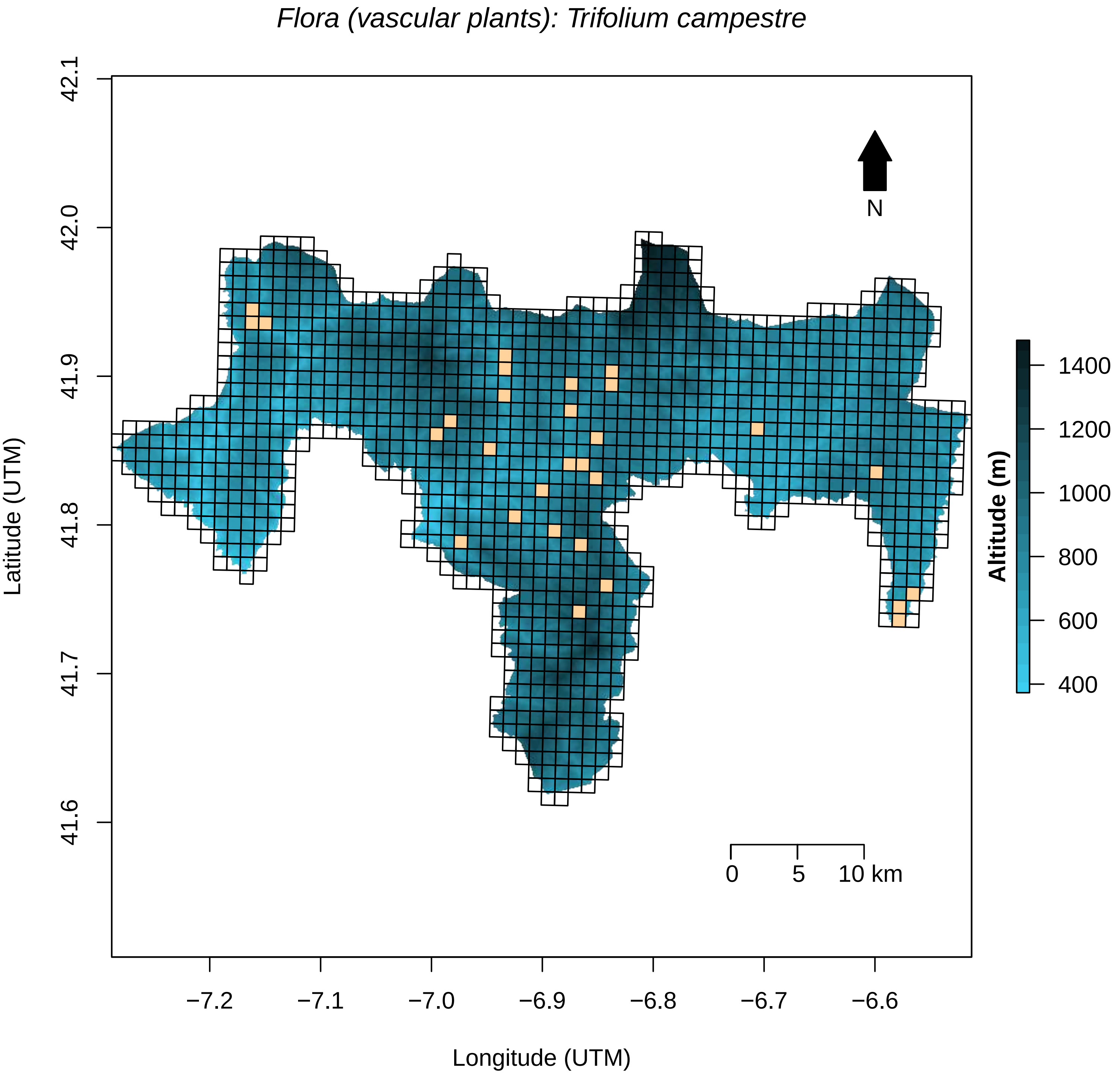
Flora (vascular plants): Trifolium campestre
N
0
5
10 km
Altitude (m)
1400
1200
1000
800
600
400
Latitude (UTM)
42.1
42.0
41.9
41.8
41.7
41.6
Longitude (UTM)
−7.2
−7.1
−7.0
−6.9
−6.8
−6.7
−6.6

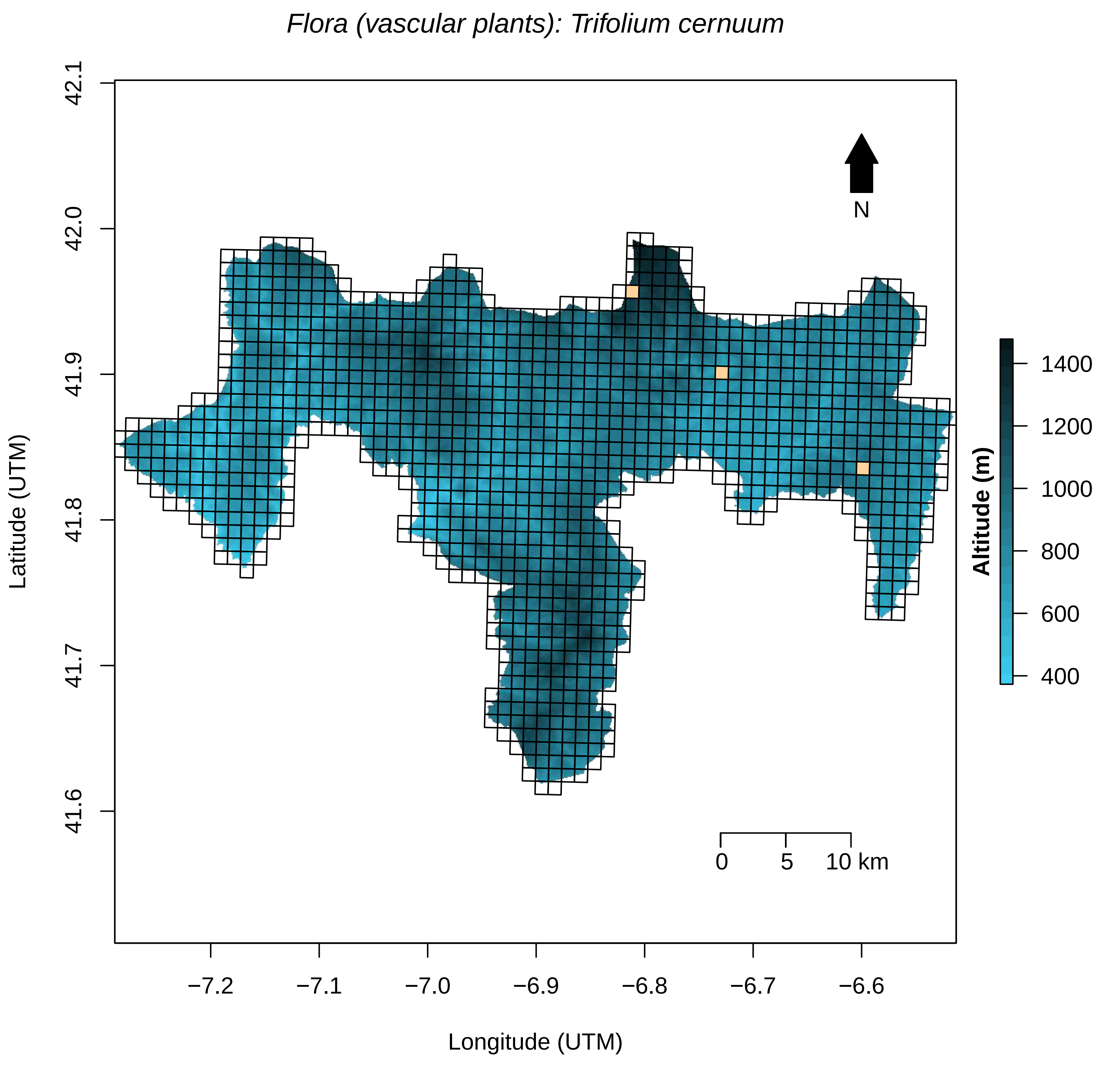
Flora (vascular plants): Trifolium cernuum
42.1
42.0
41.9
41.8
41.7
41.6
Latitude (UTM)
−7.2
−7.1
−7.0
−6.9
−6.8
−6.7
−6.6
Longitude (UTM)
N
0
5
10 km
1400
1200
1000
800
600
400
Altitude (m)

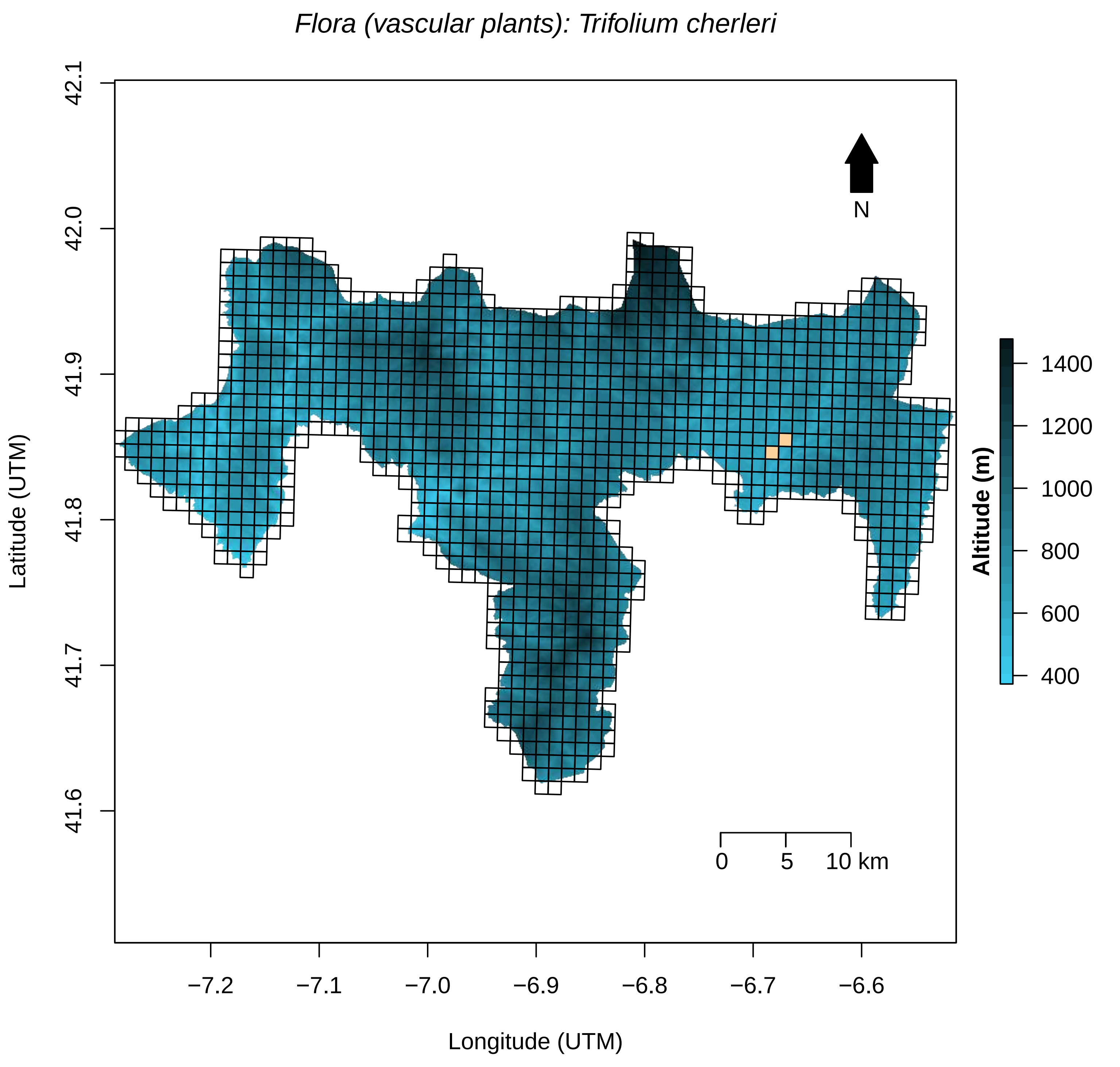

Flora (vascular plants): Trifolium cherleri
N
0
5
10 km
Altitude (m)
1400
1200
1000
800
600
400
42.1
42.0
41.9
41.8
41.7
41.6
Latitude (UTM)
−7.2
−7.1
−7.0
−6.9
−6.8
−6.7
−6.6
Longitude (UTM)

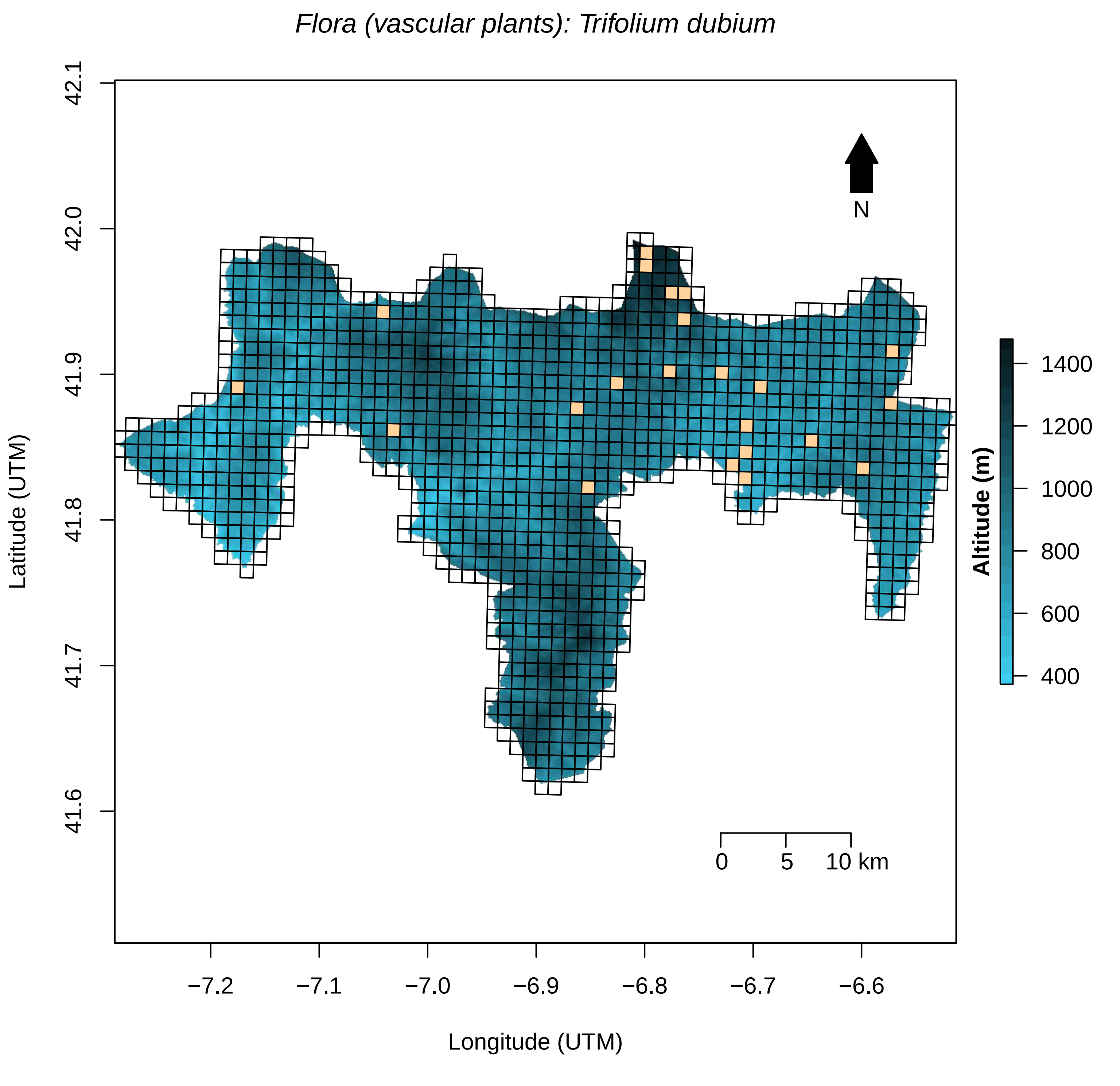
Flora (vascular plants): Trifolium dubium
42.1
42.0
41.9
41.8
41.7
41.6
Latitude (UTM)
−7.2
−7.1
−7.0
−6.9
−6.8
−6.7
−6.6
Longitude (UTM)
N
0
5
10 km
Altitude (m)
1400
1200
1000
800
600
400

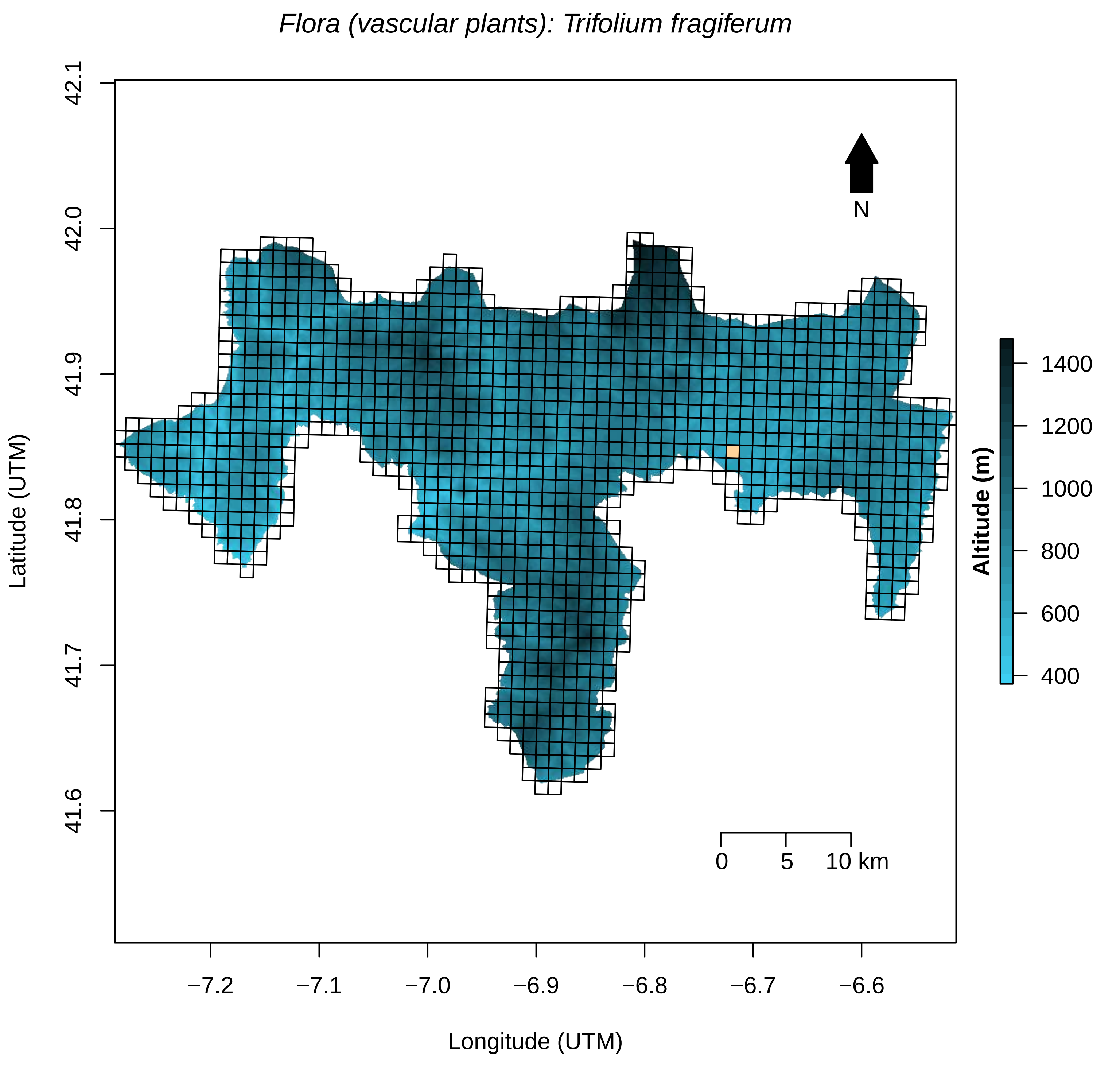

Flora (vascular plants): Trifolium fragiferum
42.1
42.0
41.9
41.8
41.7
41.6
Latitude (UTM)
−7.2
−7.1
−7.0
−6.9
−6.8
−6.7
−6.6
Longitude (UTM)
N
0 5 10 km
Altitude (m)
1400
1200
1000
800
600
400

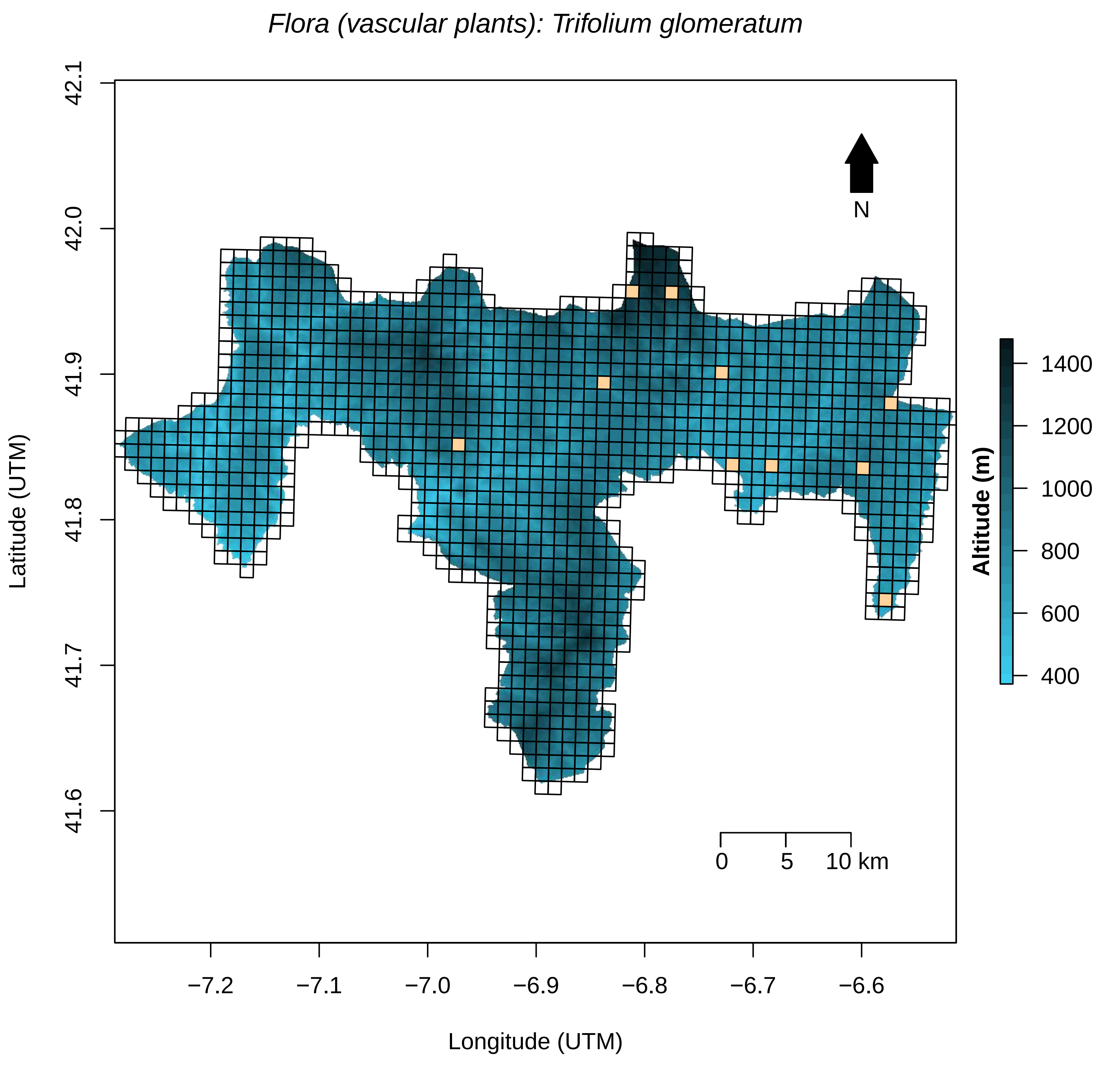

Flora (vascular plants): Trifolium glomeratum
N
0 5 10 km
Altitude (m)
1400
1200
1000
800
600
400
41.6
41.7
41.8
41.9
42.0
42.1
Latitude (UTM)
−7.2
−7.1
−7.0
−6.9
−6.8
−6.7
−6.6
Longitude (UTM)

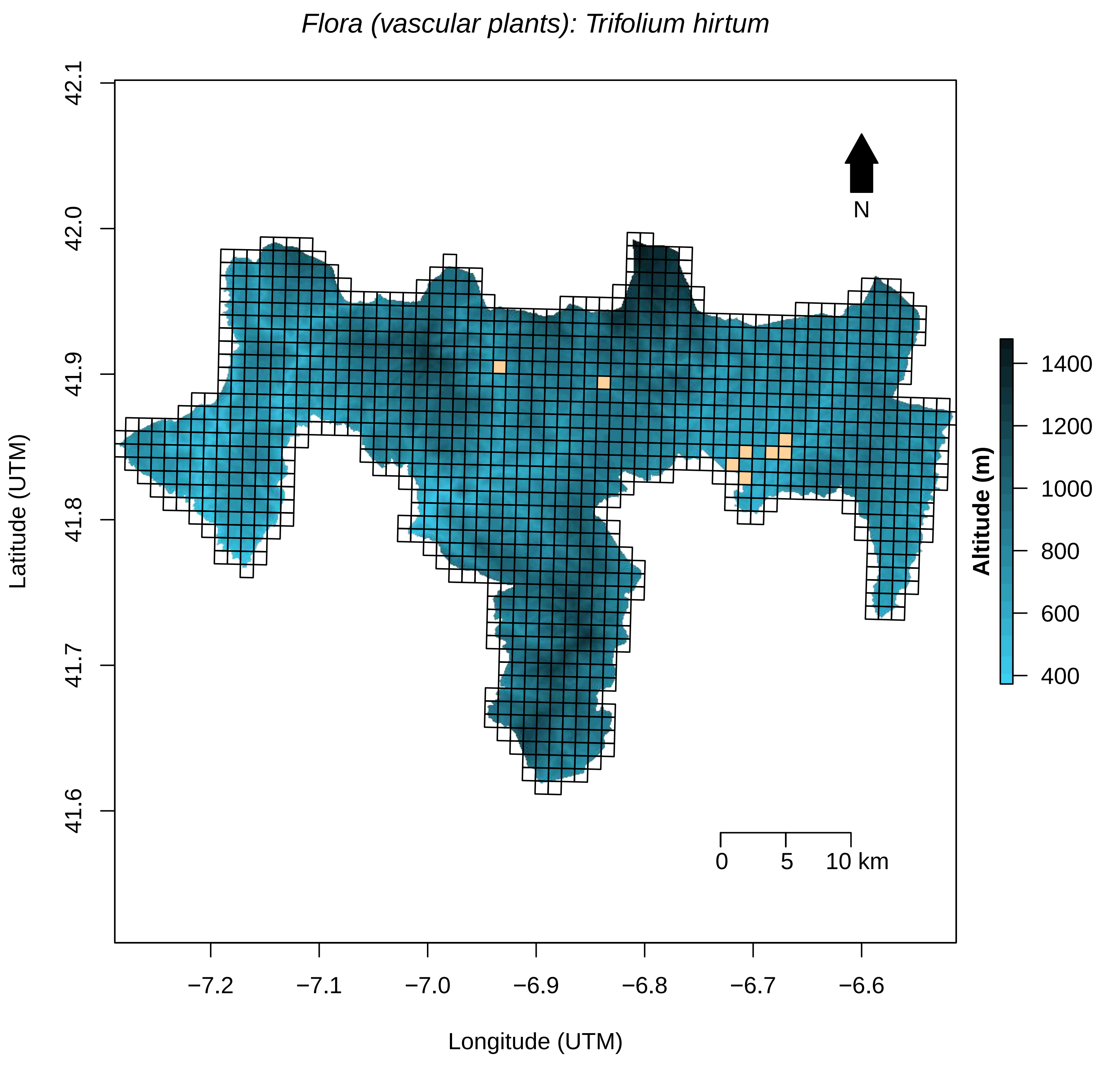
Flora (vascular plants): Trifolium hirtum
Latitude (UTM)
Longitude (UTM)
42.1
42.0
41.9
41.8
41.7
41.6
−7.2
−7.1
−7.0
−6.9
−6.8
−6.7
−6.6
N
0
5
10 km
Altitude (m)
1400
1200
1000
800
600
400

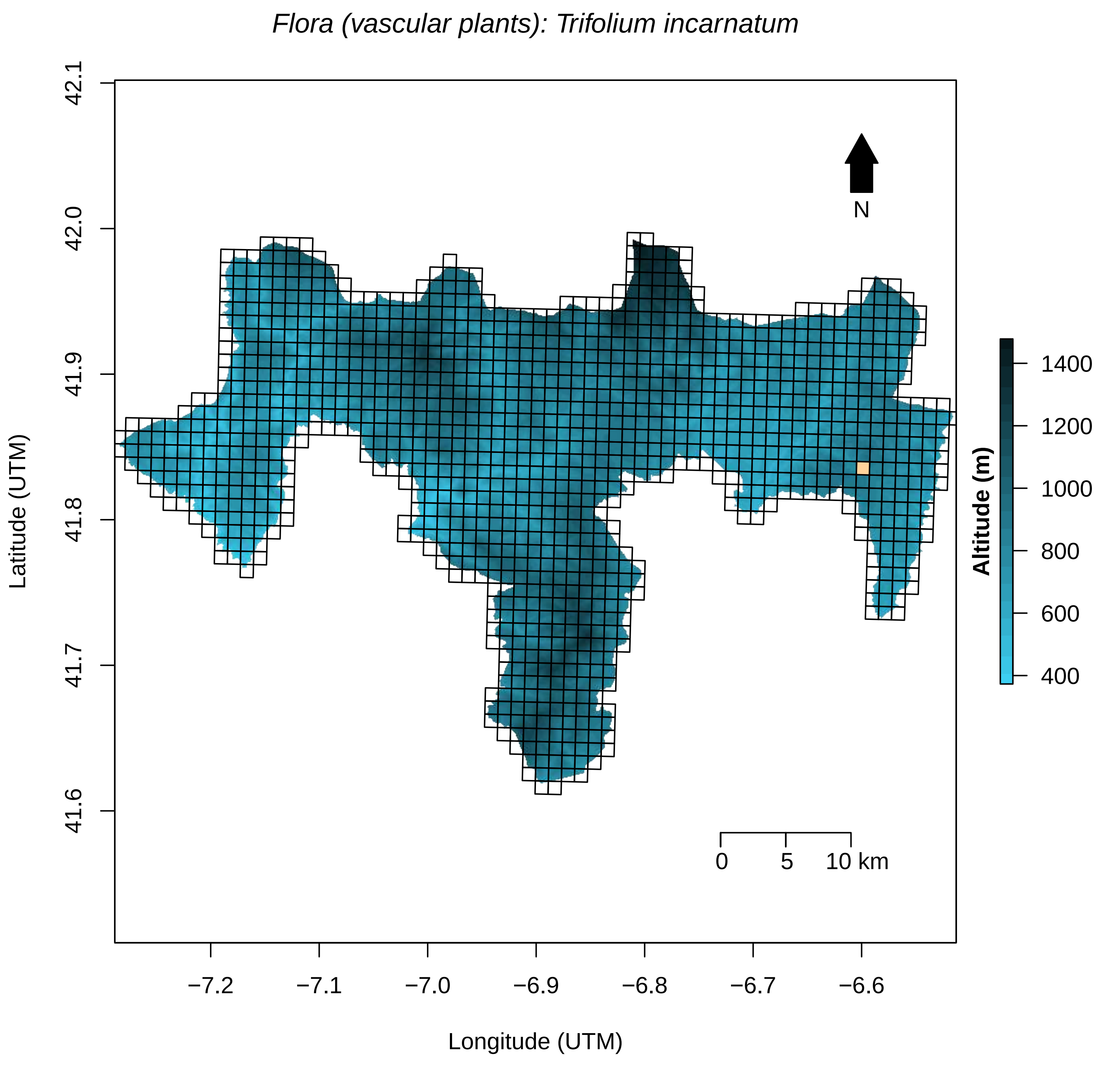
Flora (vascular plants): Trifolium incarnatum
N
0 5 10 km
Altitude (m)
1400
1200
1000
800
600
400
Latitude (UTM)
42.1
42.0
41.9
41.8
41.7
41.6
Longitude (UTM)
−7.2
−7.1
−7.0
−6.9
−6.8
−6.7
−6.6

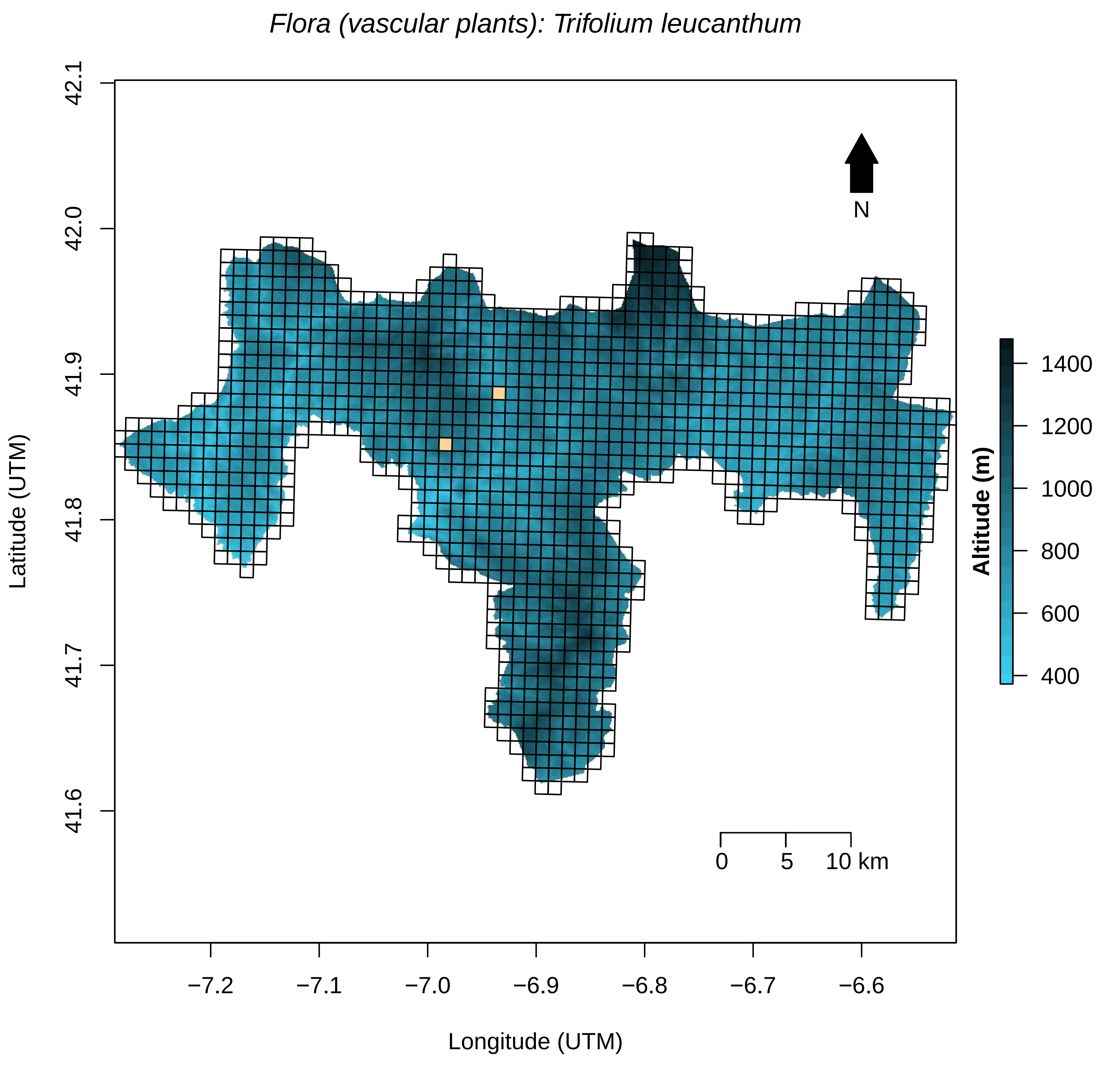
Flora (vascular plants): Trifolium leucanthum
Latitude (UTM)
42.1
42.0
41.9
41.8
41.7
41.6
Longitude (UTM)
−7.2
−7.1
−7.0
−6.9
−6.8
−6.7
−6.6
N
0
5
10 km
Altitude (m)
1400
1200
1000
800
600
400

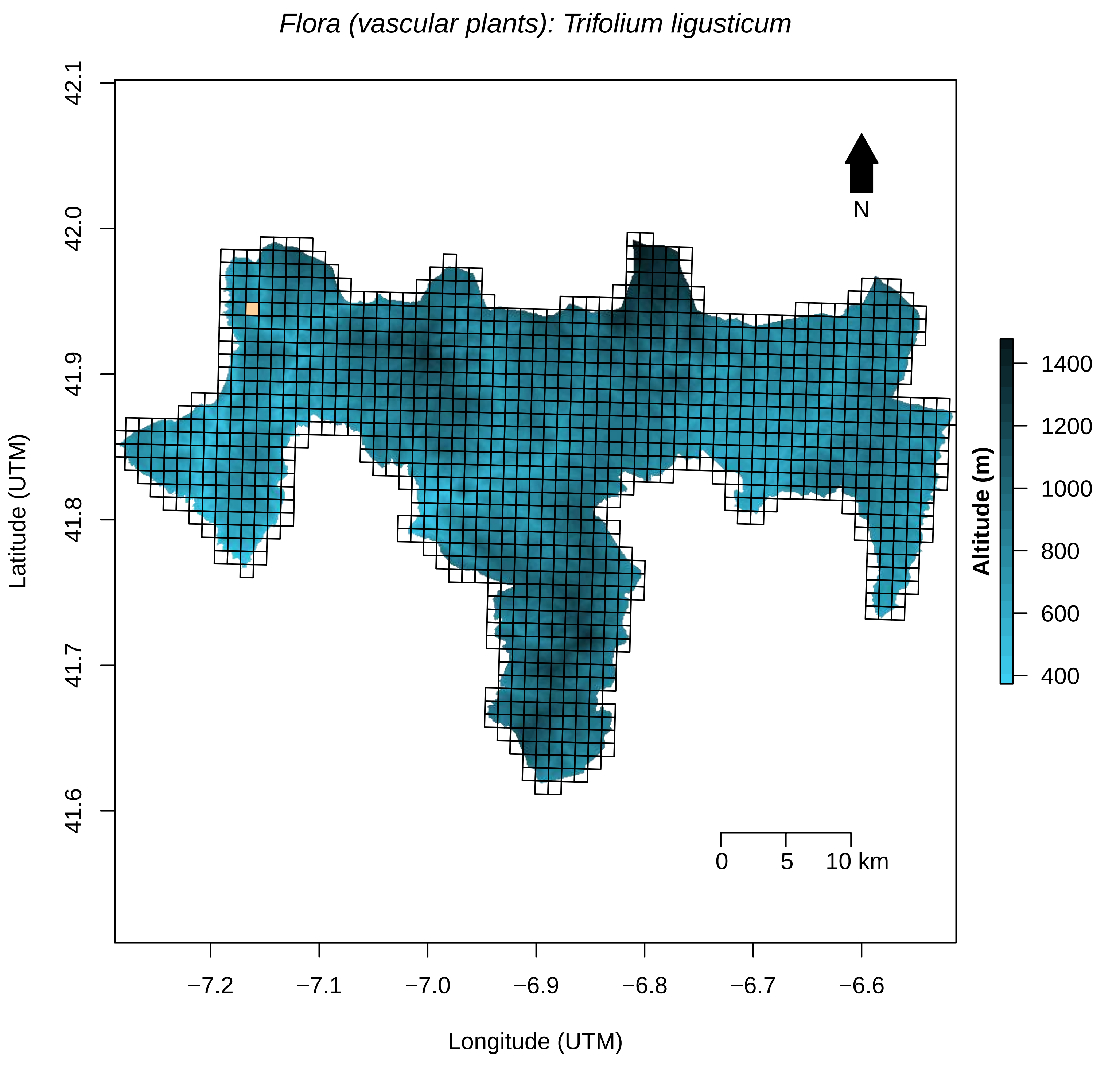

Flora (vascular plants): Trifolium ligusticum
42.1
42.0
41.9
41.8
41.7
41.6
Latitude (UTM)
−7.2
−7.1
−7.0
−6.9
−6.8
−6.7
−6.6
Longitude (UTM)
N
0
5
10 km
Altitude (m)
1400
1200
1000
800
600
400

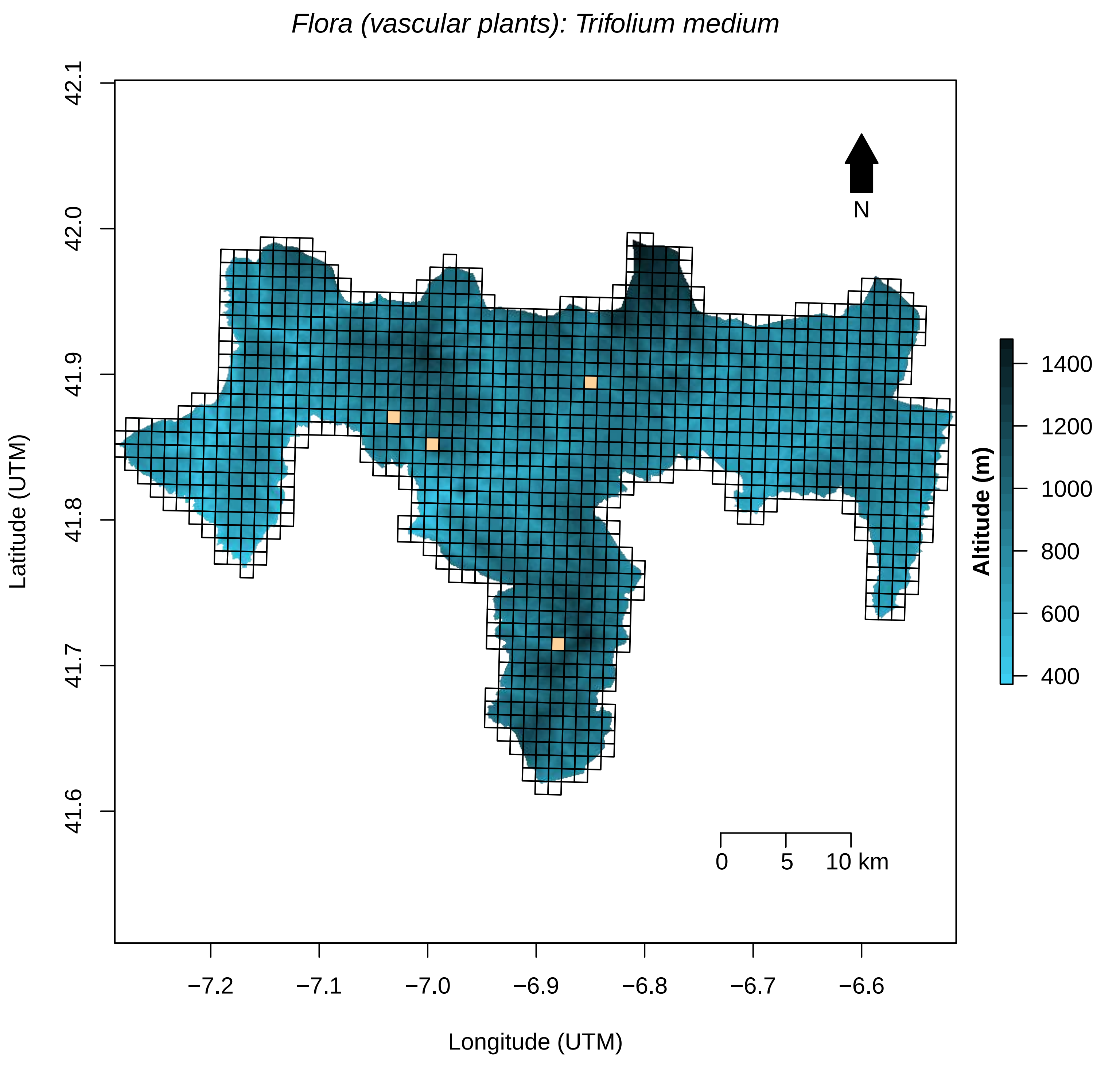
Flora (vascular plants): Trifolium medium
42.1
42.0
41.9
41.8
41.7
41.6
Latitude (UTM)
−7.2
−7.1
−7.0
−6.9
−6.8
−6.7
−6.6
Longitude (UTM)
N
0
5
10 km
Altitude (m)
1400
1200
1000
800
600
400

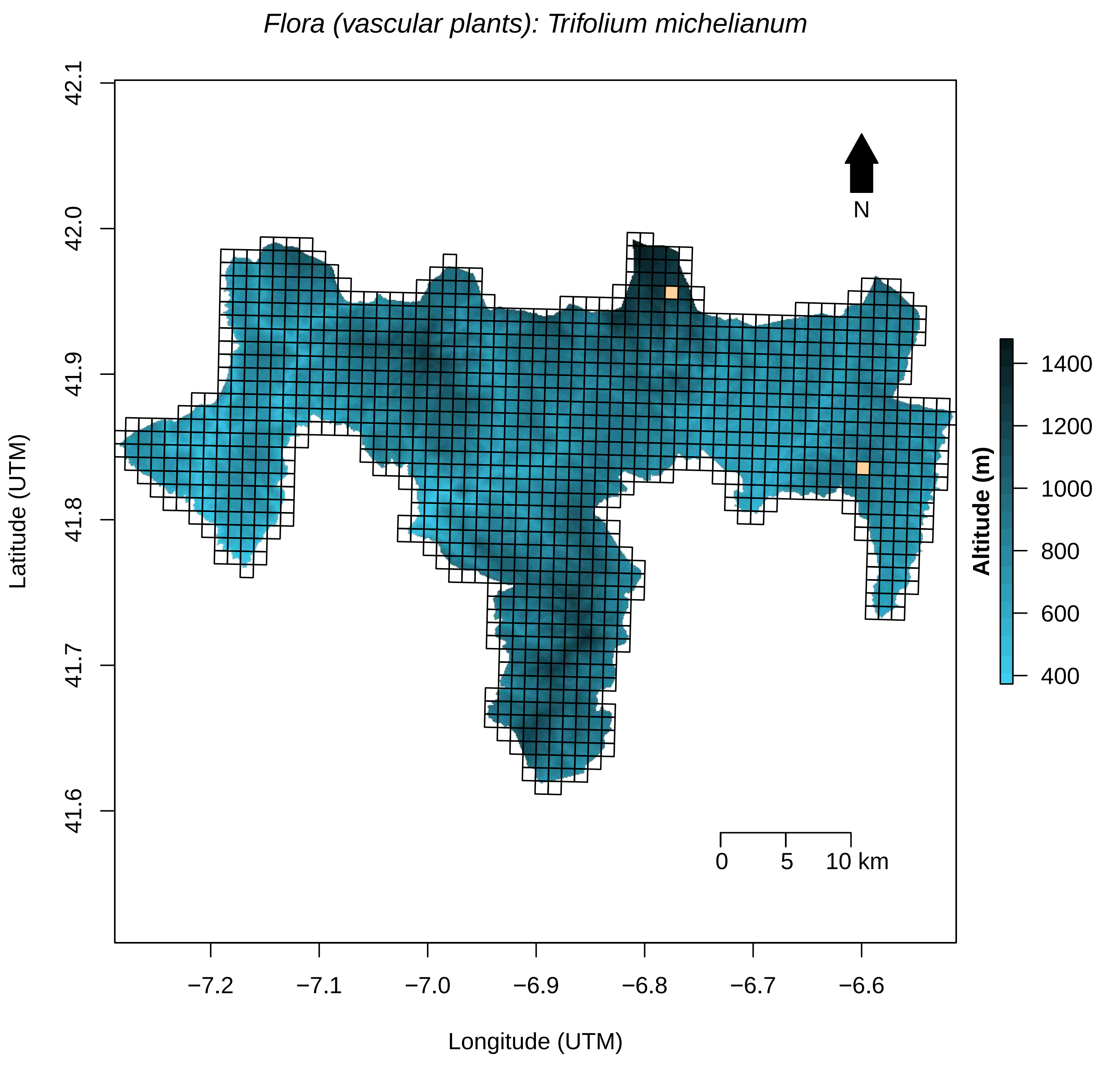

Flora (vascular plants): Trifolium michelianum
42.1
42.0
41.9
41.8
41.7
41.6
Latitude (UTM)
−7.2
−7.1
−7.0
−6.9
−6.8
−6.7
−6.6
Longitude (UTM)
N
0
5
10 km
1400
1200
1000
800
600
400
Altitude (m)

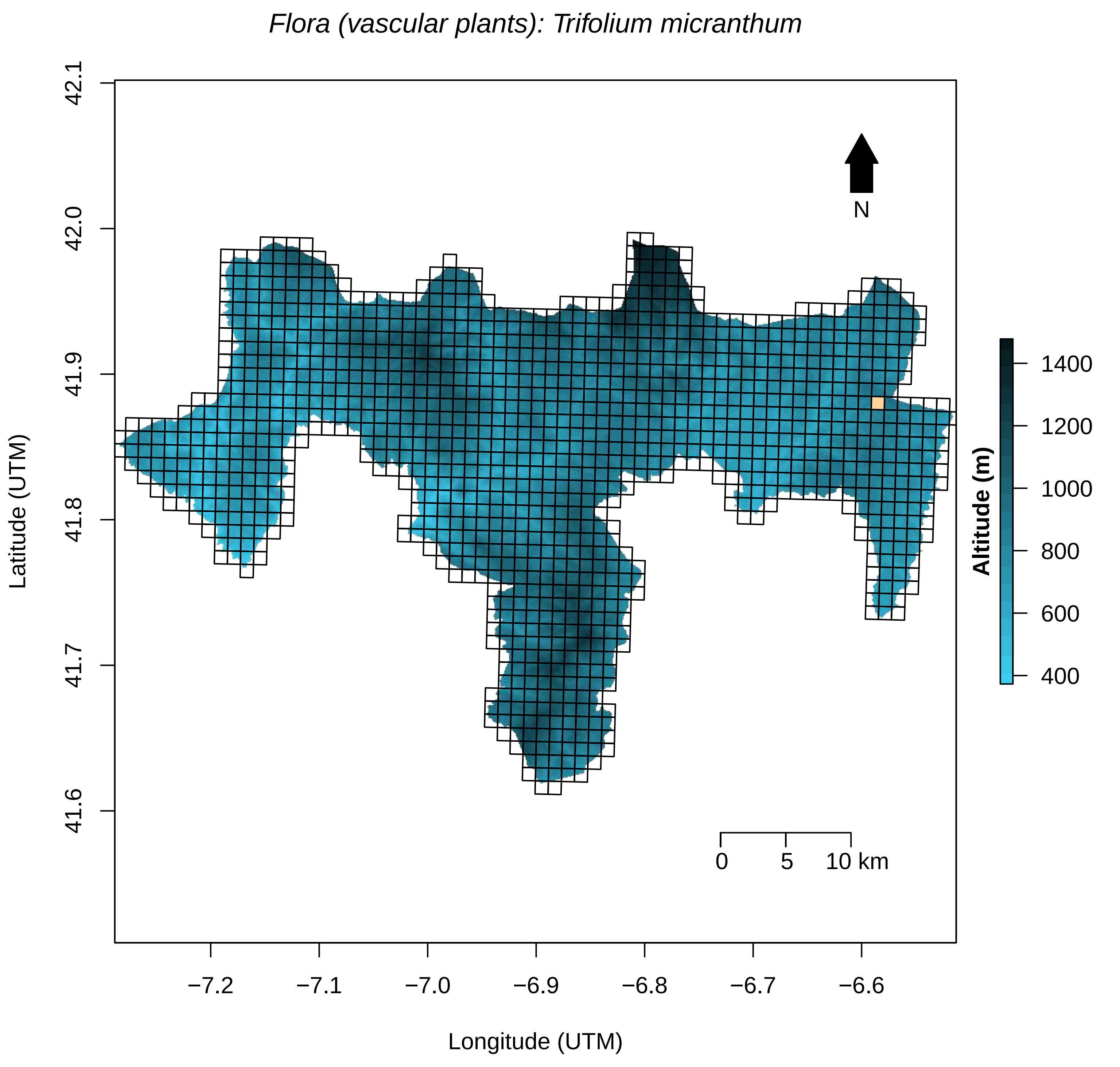

Flora (vascular plants): Trifolium micranthum
N
0 5 10 km
Altitude (m)
1400
1200
1000
800
600
400
Latitude (UTM)
42.1
42.0
41.9
41.8
41.7
41.6
Longitude (UTM)
−7.2
−7.1
−7.0
−6.9
−6.8
−6.7
−6.6

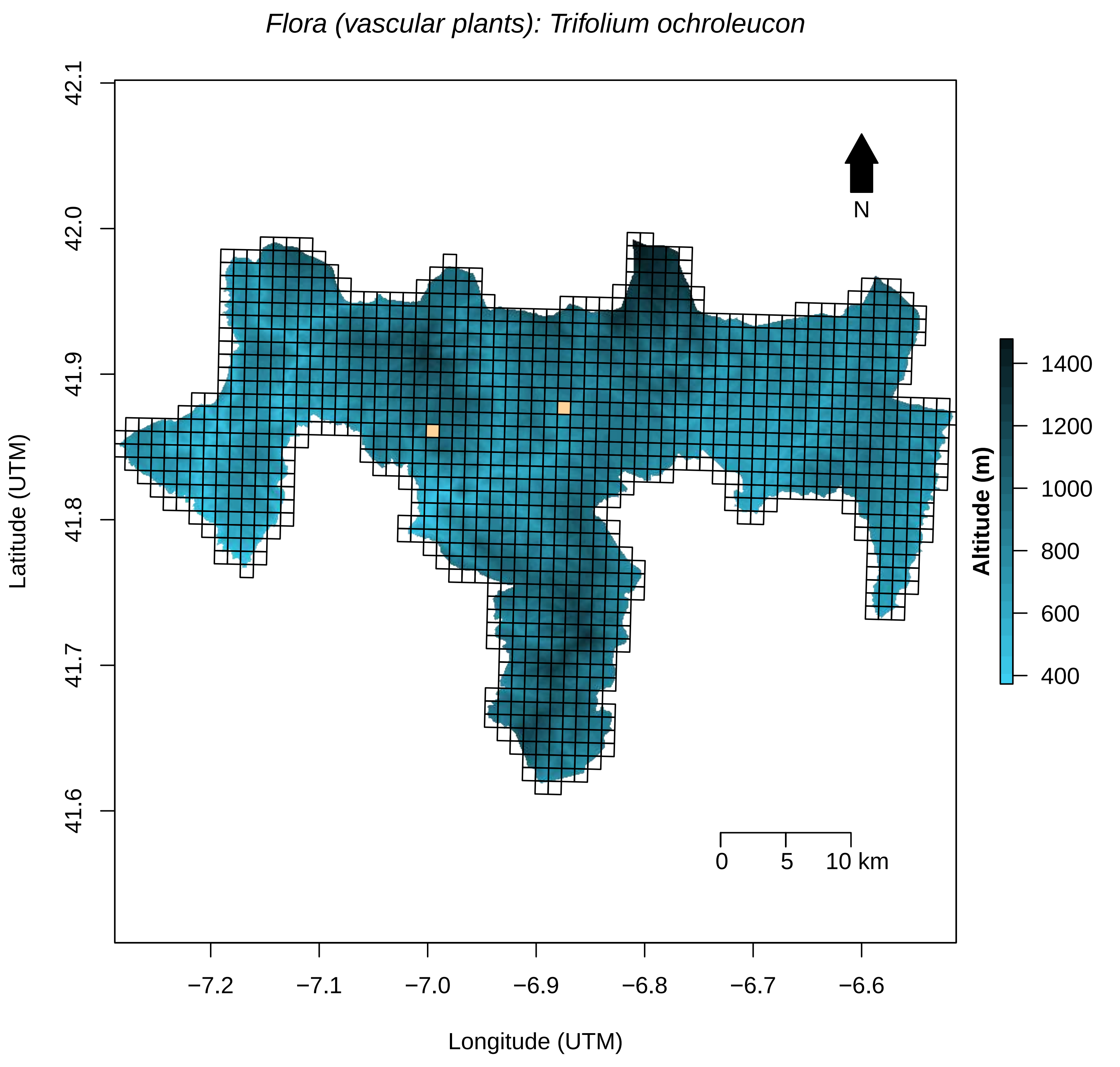
Flora (vascular plants): Trifolium ochroleucon
N
0
5
10 km
Altitude (m)
1400
1200
1000
800
600
400
Latitude (UTM)
42.1
42.0
41.9
41.8
41.7
41.6
Longitude (UTM)
−7.2
−7.1
−7.0
−6.9
−6.8
−6.7
−6.6

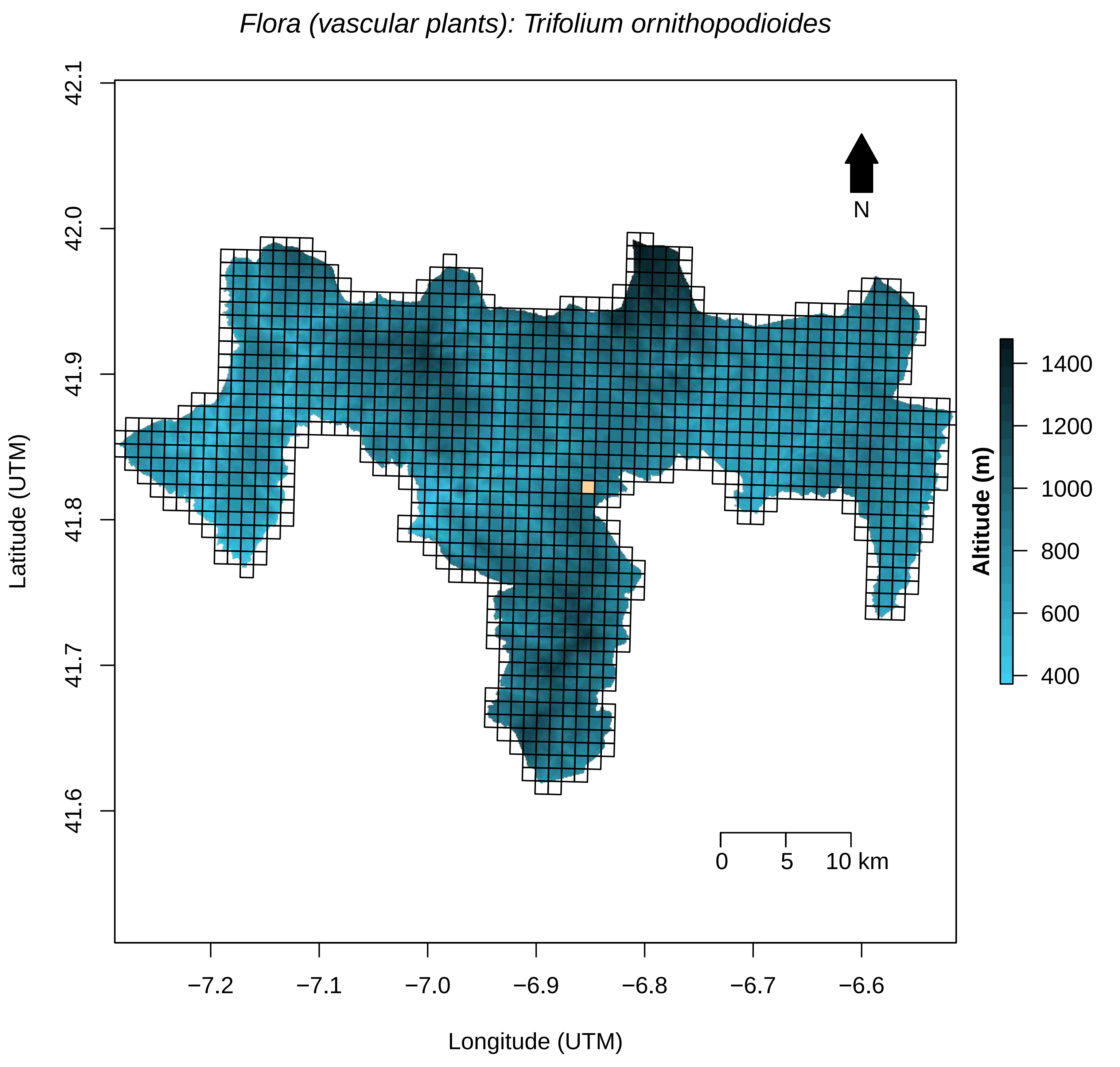

Flora (vascular plants): Trifolium ornithopodioides
42.1
42.0
41.9
41.8
41.7
41.6
Latitude (UTM)
N
0
5
10 km
−7.2
−7.1
−7.0
−6.9
−6.8
−6.7
−6.6
Longitude (UTM)
Altitude (m)
1400
1200
1000
800
600
400

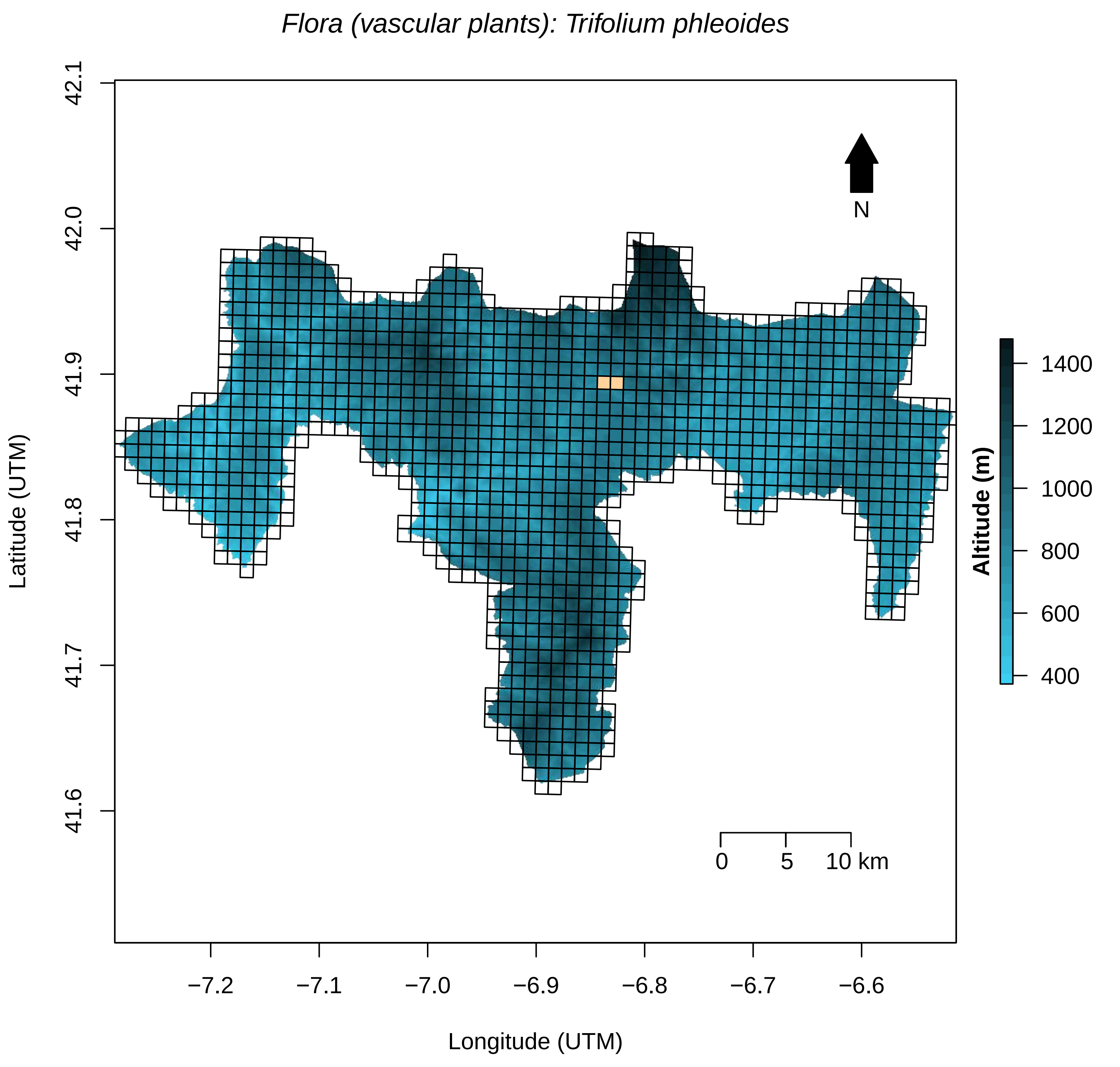

Flora (vascular plants): Trifolium phleoides
42.1
42.0
41.9
41.8
41.7
41.6
Latitude (UTM)
−7.2
−7.1
−7.0
−6.9
−6.8
−6.7
−6.6
Longitude (UTM)
N
0
5
10 km
Altitude (m)
1400
1200
1000
800
600
400

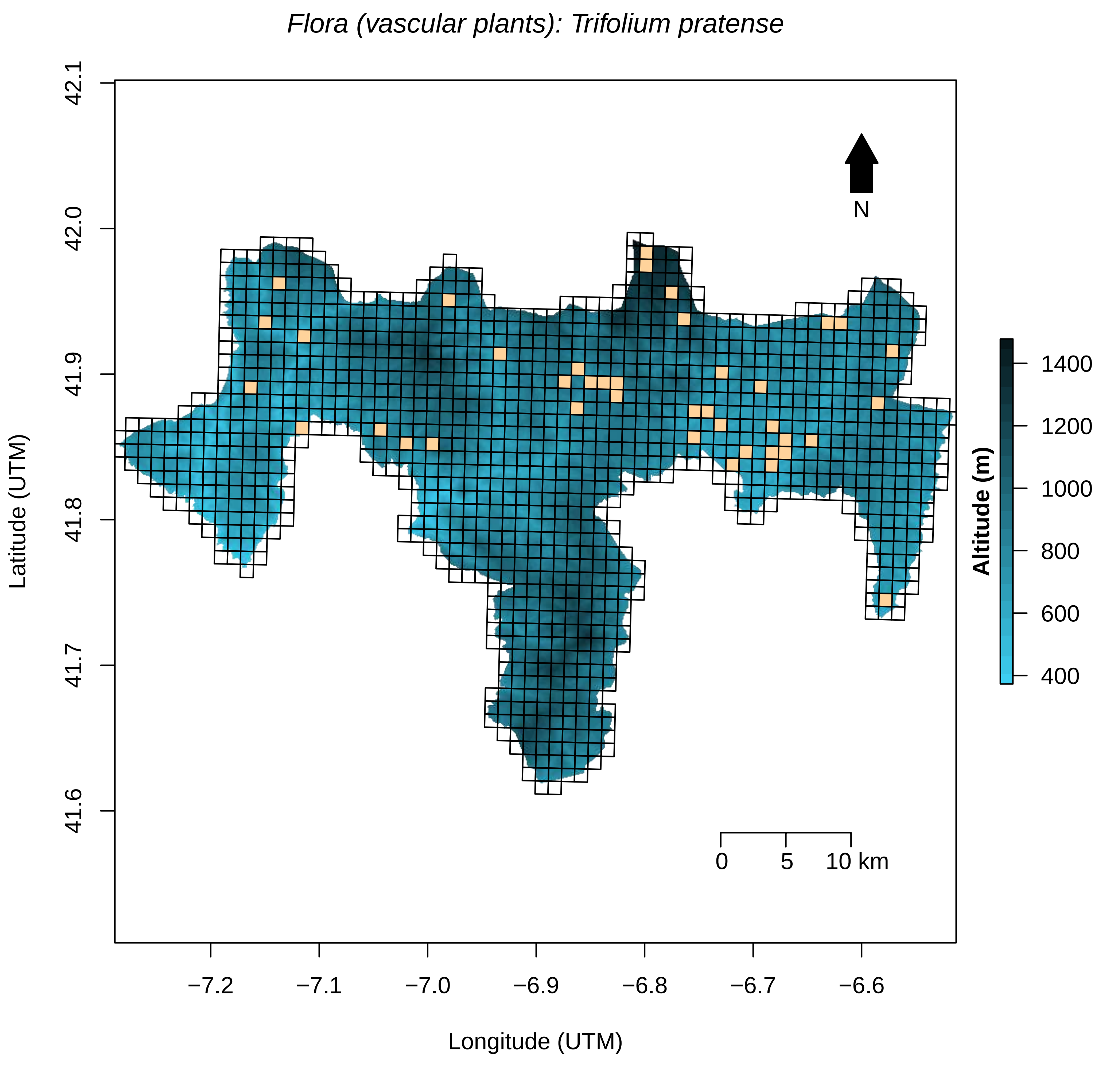
Flora (vascular plants): Trifolium pratense
42.1
42.0
41.9
41.8
41.7
41.6
Latitude (UTM)
−7.2
−7.1
−7.0
−6.9
−6.8
−6.7
−6.6
Longitude (UTM)
N
0
5
10 km
Altitude (m)
1400
1200
1000
800
600
400

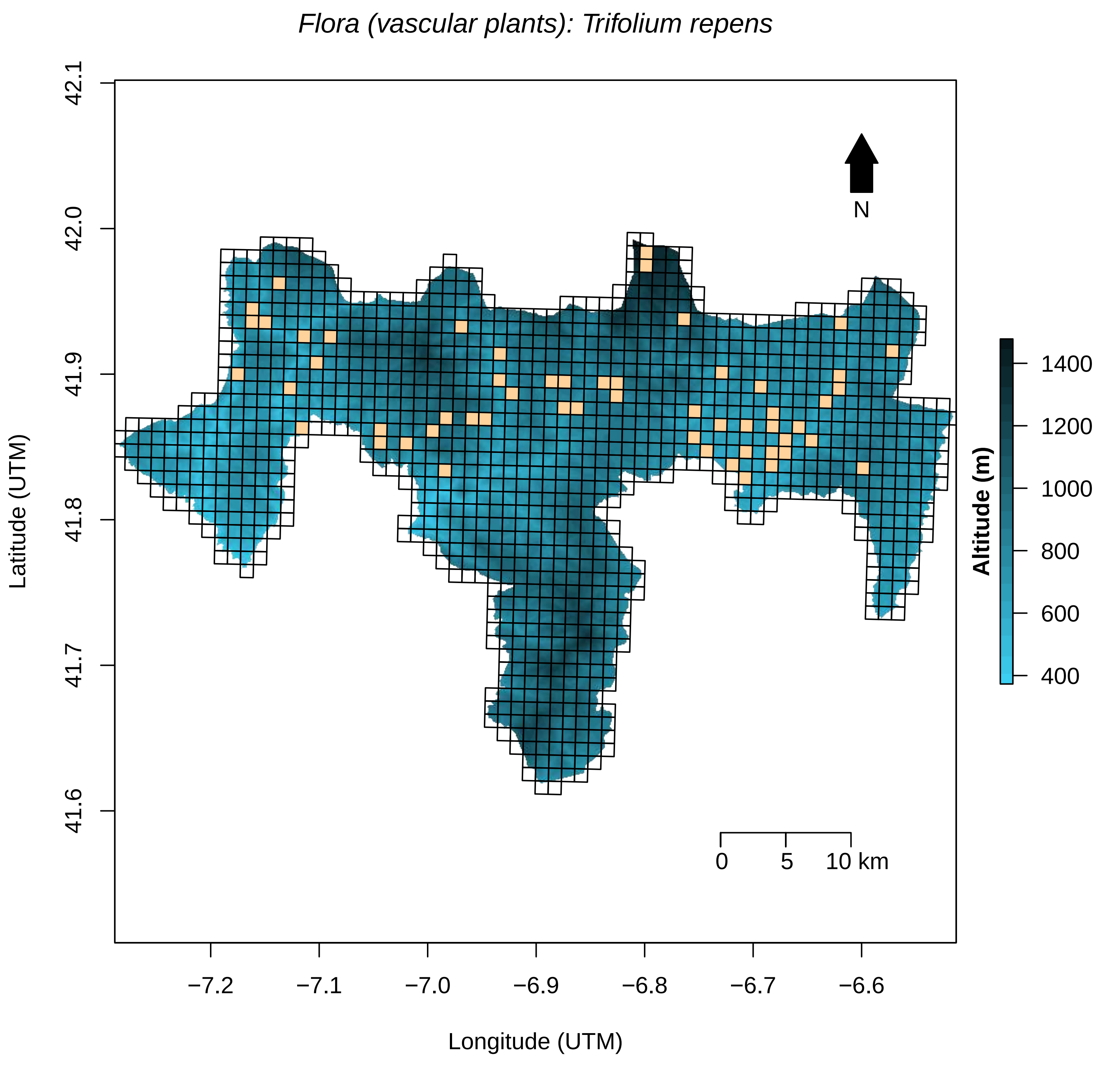

Flora (vascular plants): Trifolium repens
N
0
5
10 km
Altitude (m)
1400
1200
1000
800
600
400
Latitude (UTM)
42.1
42.0
41.9
41.8
41.7
41.6
Longitude (UTM)
−7.2
−7.1
−7.0
−6.9
−6.8
−6.7
−6.6

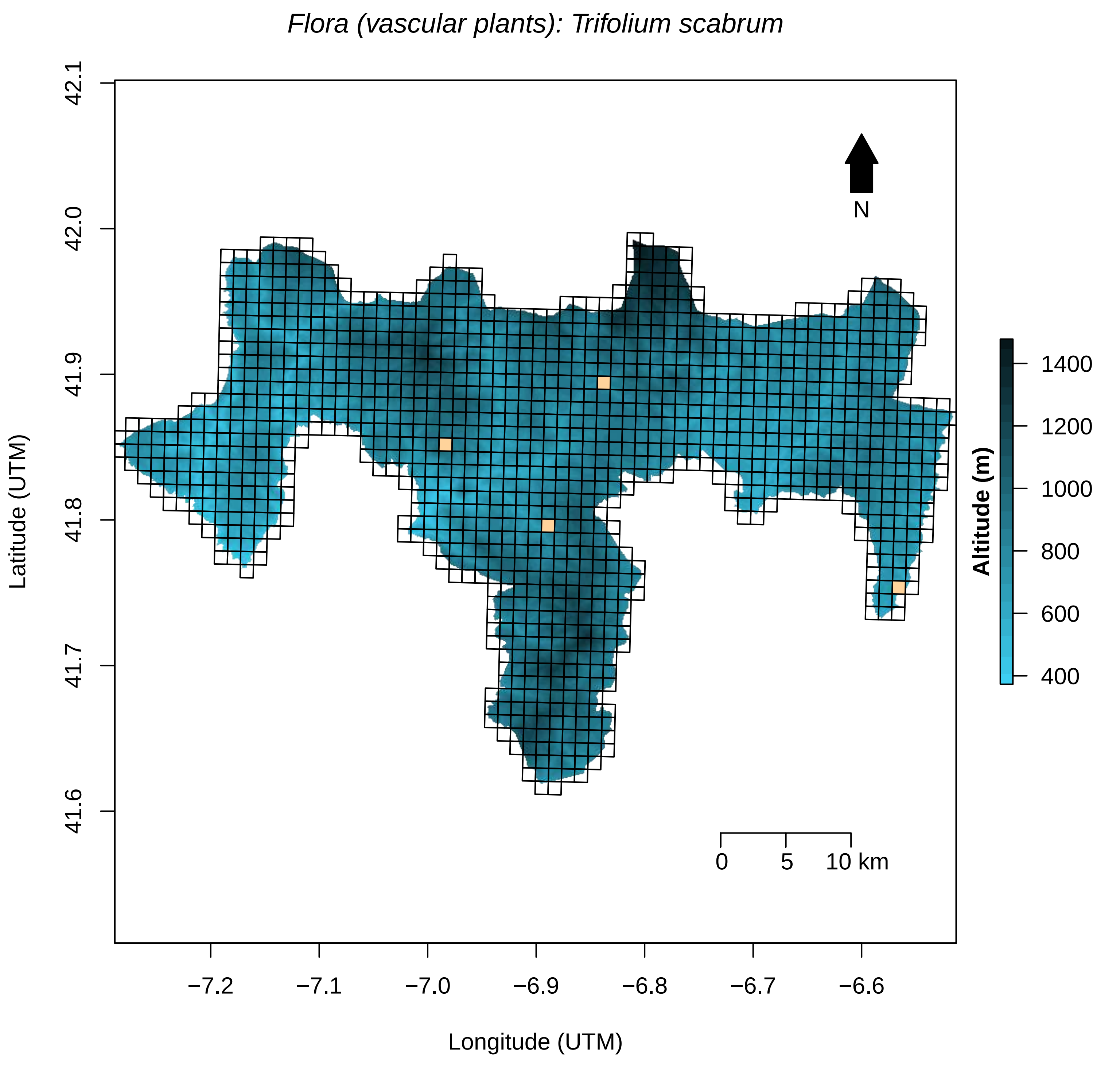
Flora (vascular plants): Trifolium scabrum
42.1
42.0
41.9
41.8
41.7
41.6
Latitude (UTM)
−7.2
−7.1
−7.0
−6.9
−6.8
−6.7
−6.6
Longitude (UTM)
N
0
5
10 km
Altitude (m)
1400
1200
1000
800
600
400

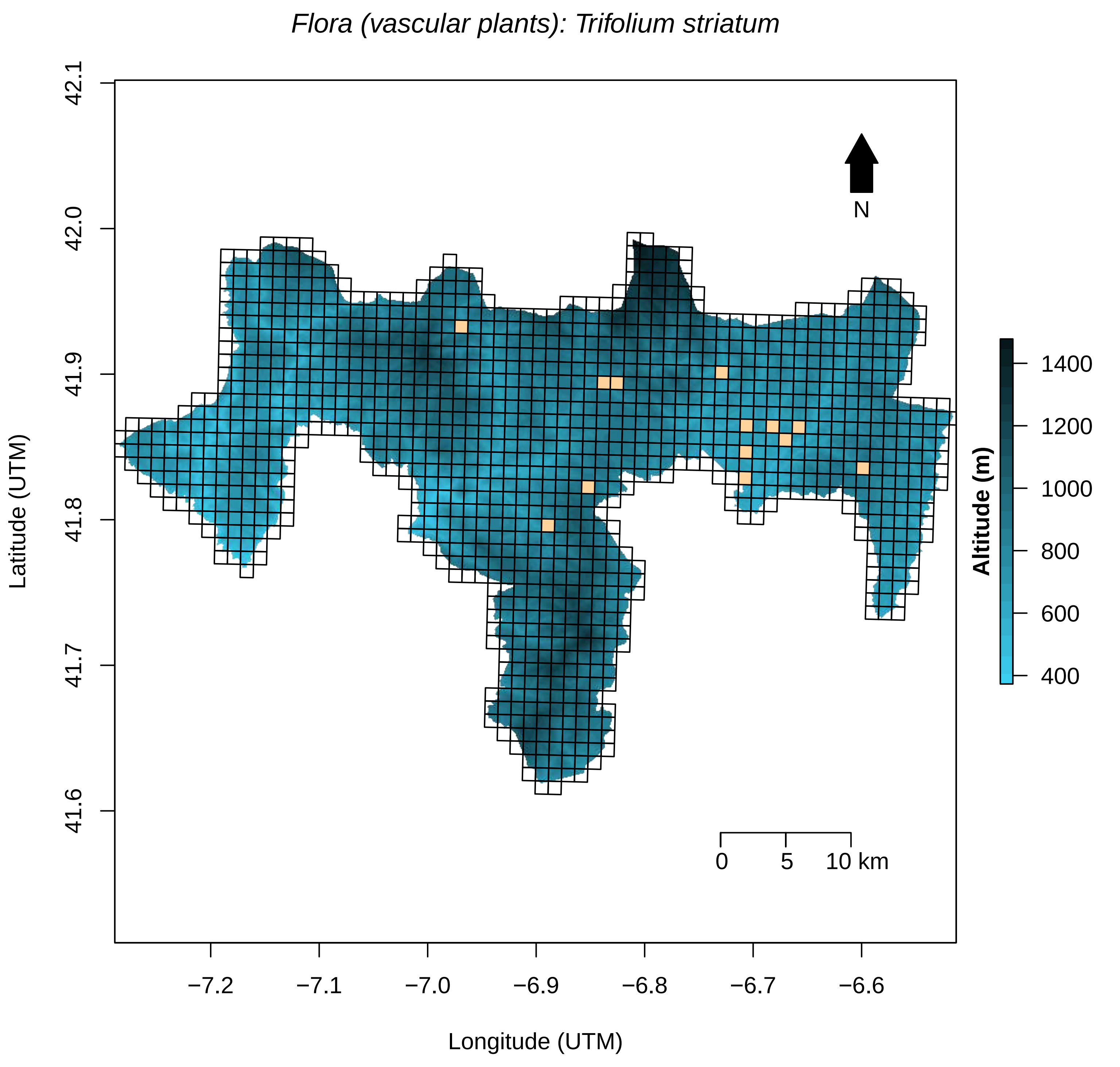

Flora (vascular plants): Trifolium striatum
Latitude (UTM)
Longitude (UTM)
42.1
42.0
41.9
41.8
41.7
41.6
−7.2
−7.1
−7.0
−6.9
−6.8
−6.7
−6.6
N
0
5
10 km
Altitude (m)
1400
1200
1000
800
600
400

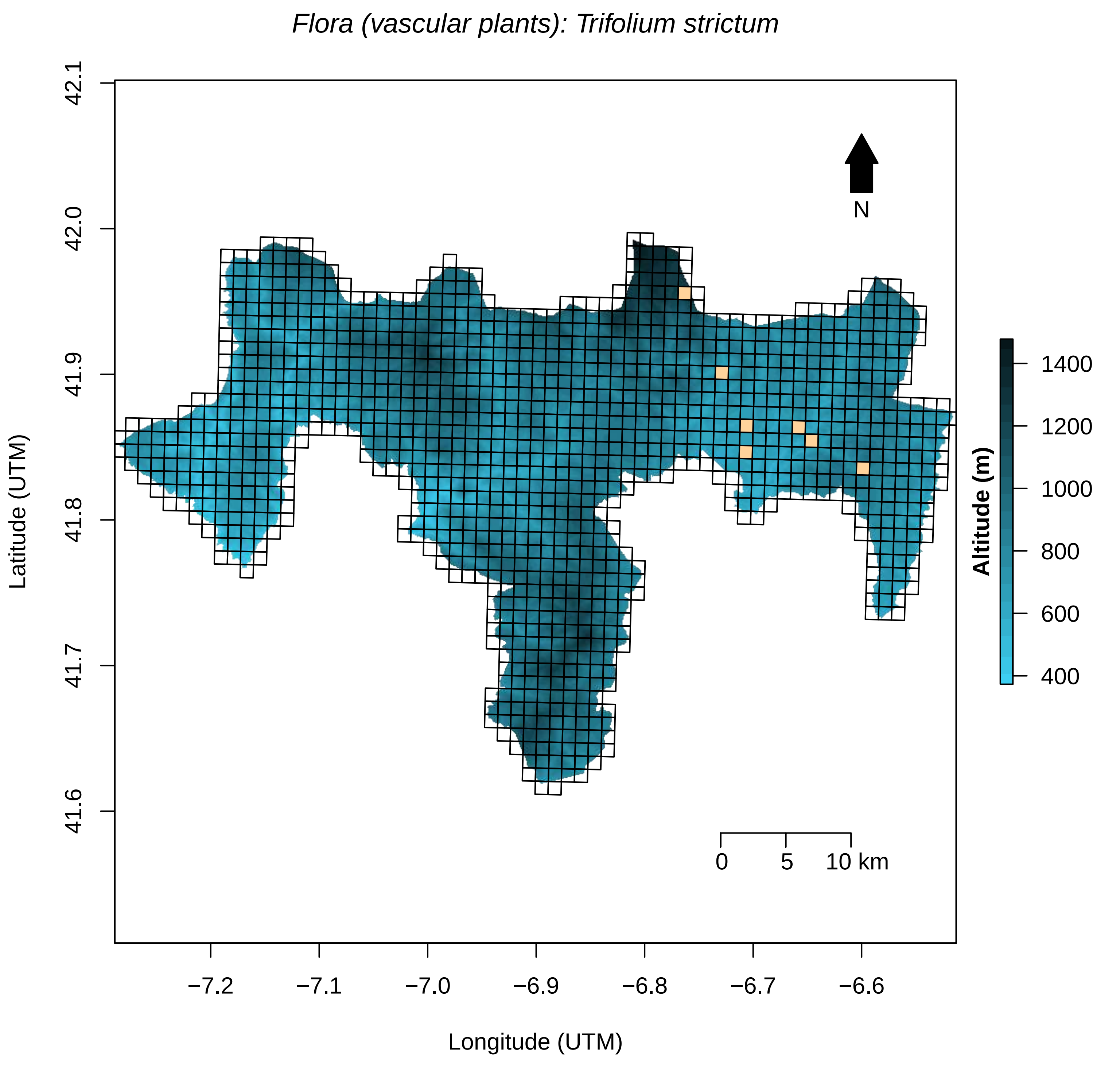
Flora (vascular plants): Trifolium strictum
N
0
5
10 km
Altitude (m)
1400
1200
1000
800
600
400
Latitude (UTM)
42.1
42.0
41.9
41.8
41.7
41.6
Longitude (UTM)
−7.2
−7.1
−7.0
−6.9
−6.8
−6.7
−6.6

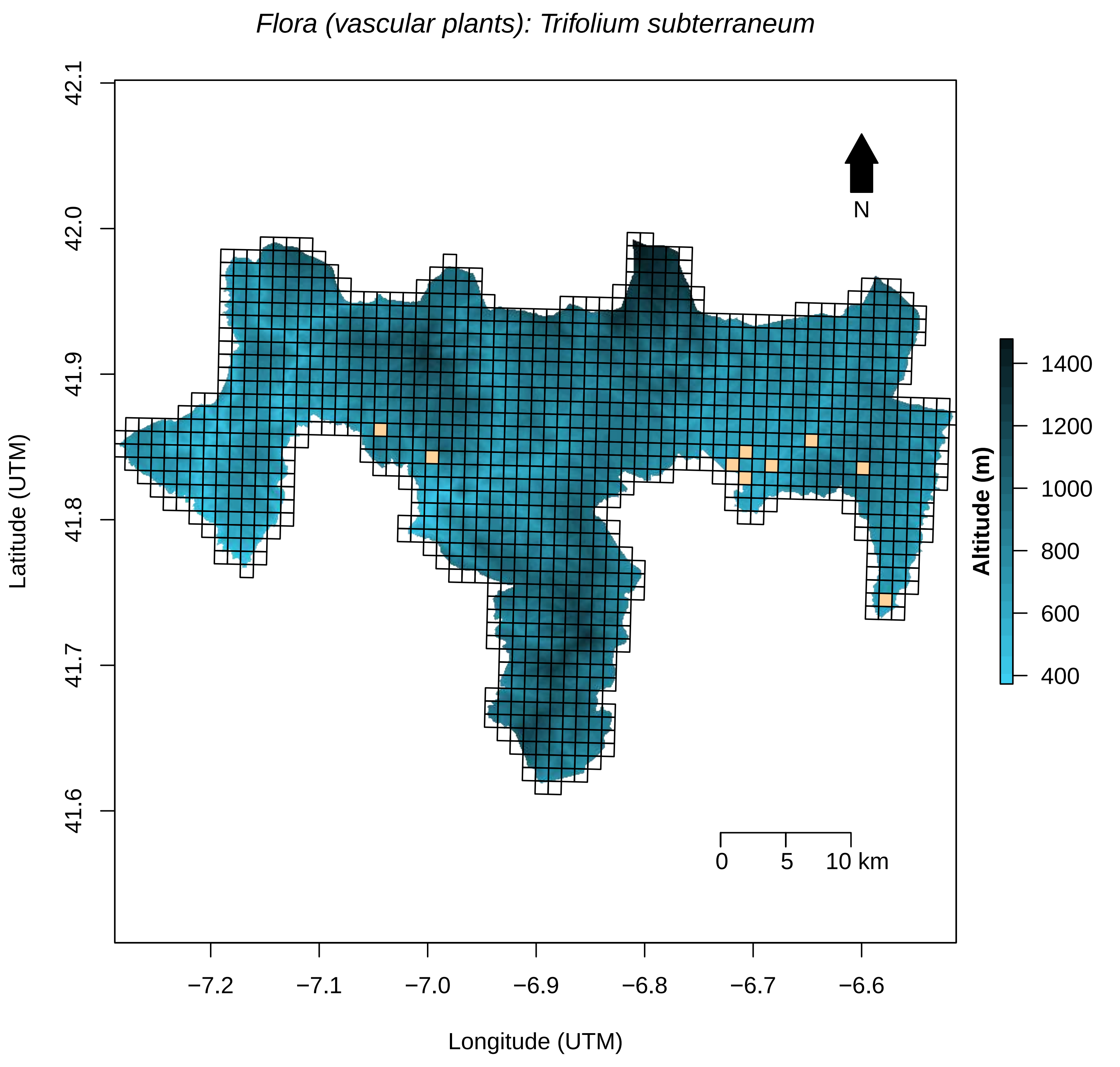

Flora (vascular plants): Trifolium subterraneum
N
0
5
10 km
Latitude (UTM)
Longitude (UTM)
Altitude (m)
1400
1200
1000
800
600
400
42.1
42.0
41.9
41.8
41.7
41.6
−7.2
−7.1
−7.0
−6.9
−6.8
−6.7
−6.6

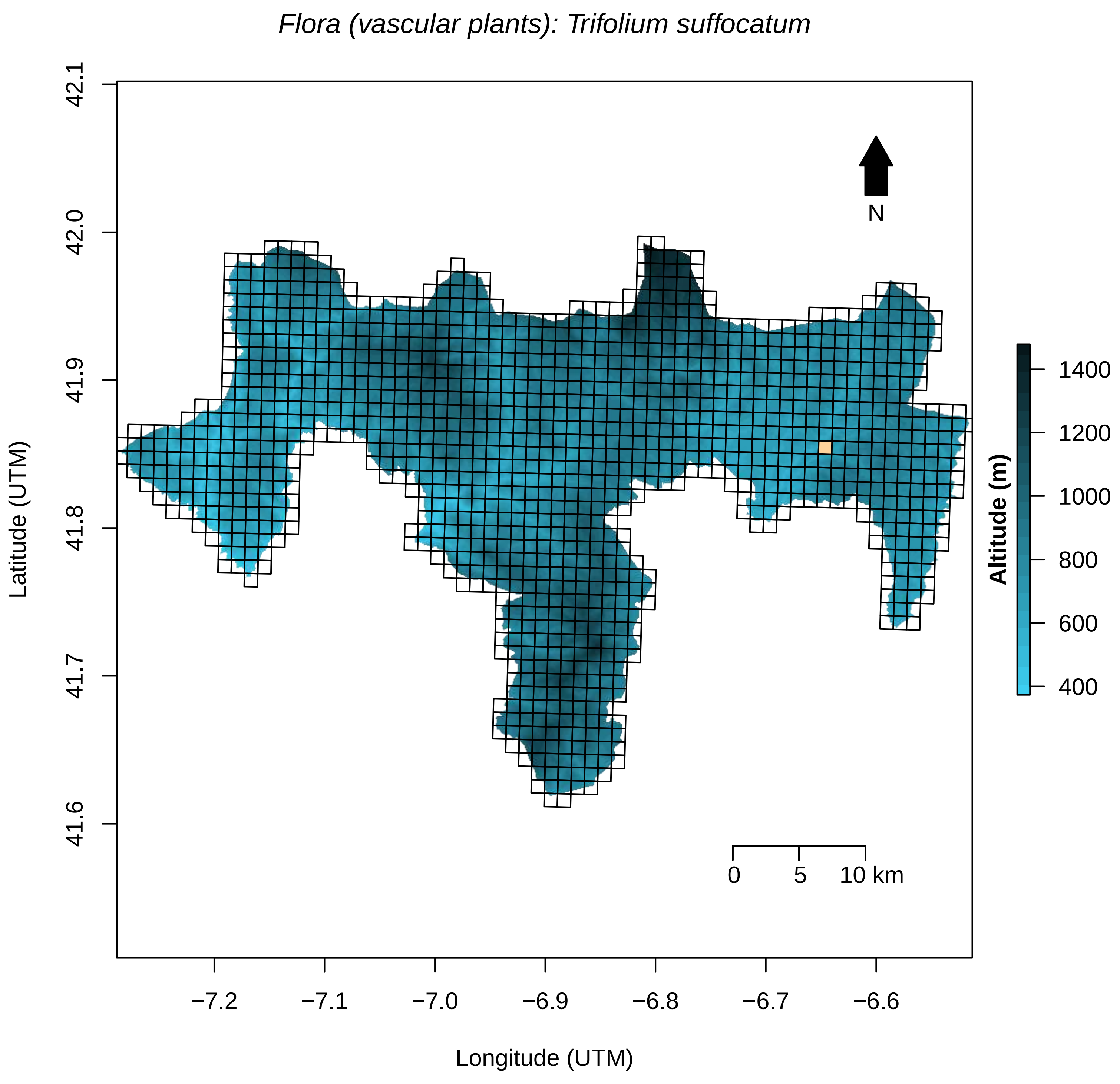

Flora (vascular plants): Trifolium suffocatum
Latitude (UTM)
42.1
42.0
41.9
41.8
41.7
41.6
Longitude (UTM)
−7.2
−7.1
−7.0
−6.9
−6.8
−6.7
−6.6
N
0
5
10 km
Altitude (m)
1400
1200
1000
800
600
400

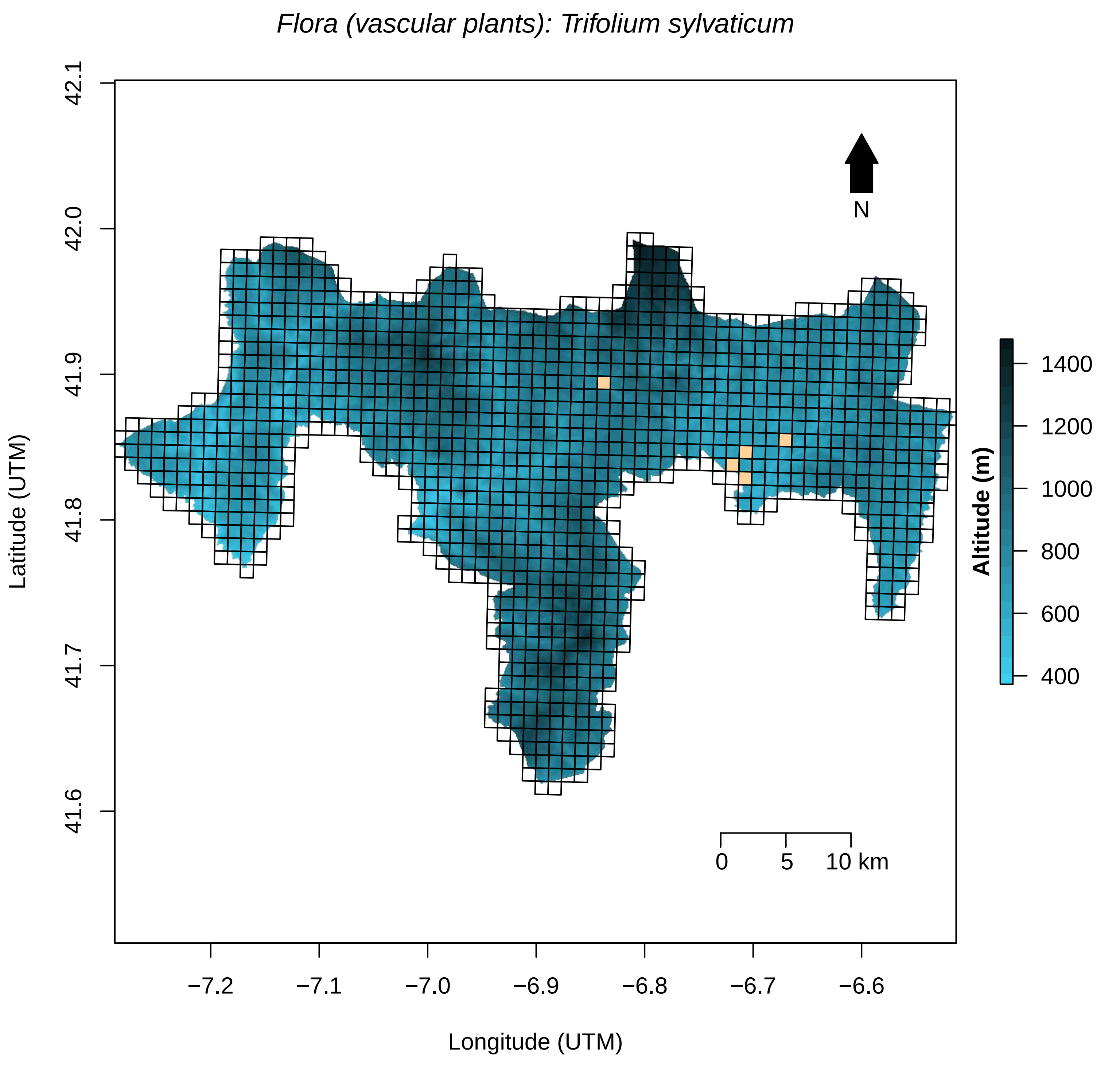
Flora (vascular plants): Trifolium sylvaticum
Latitude (UTM)
Longitude (UTM)
42.1
42.0
41.9
41.8
41.7
41.6
−7.2
−7.1
−7.0
−6.9
−6.8
−6.7
−6.6
N
0
5
10 km
Altitude (m)
1400
1200
1000
800
600
400

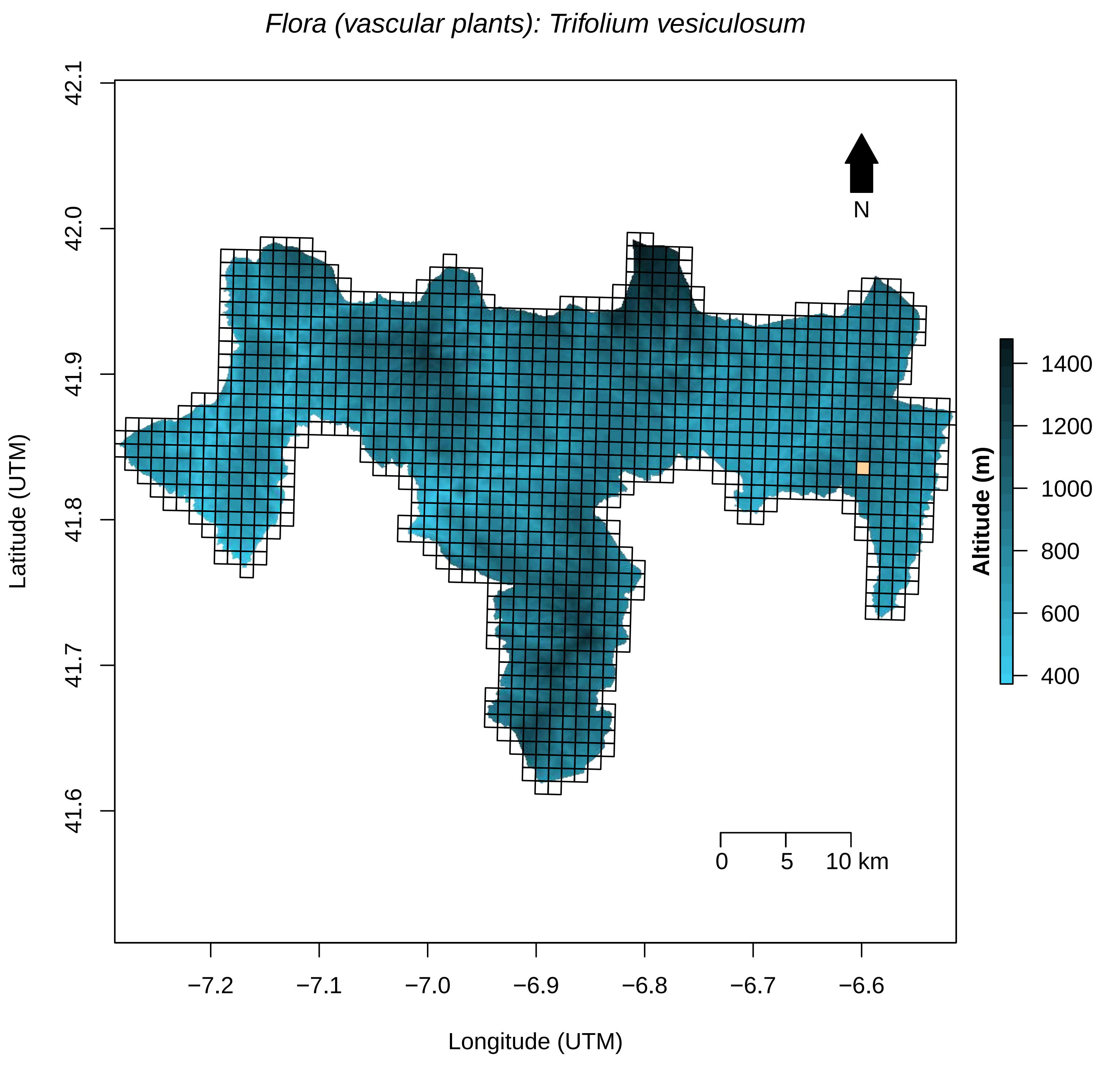

Flora (vascular plants): Trifolium vesiculosum
N
0 5 10 km
Altitude (m)
1400
1200
1000
800
600
400
42.1
42.0
41.9
41.8
41.7
41.6
Latitude (UTM)
−7.2
−7.1
−7.0
−6.9
−6.8
−6.7
−6.6
Longitude (UTM)

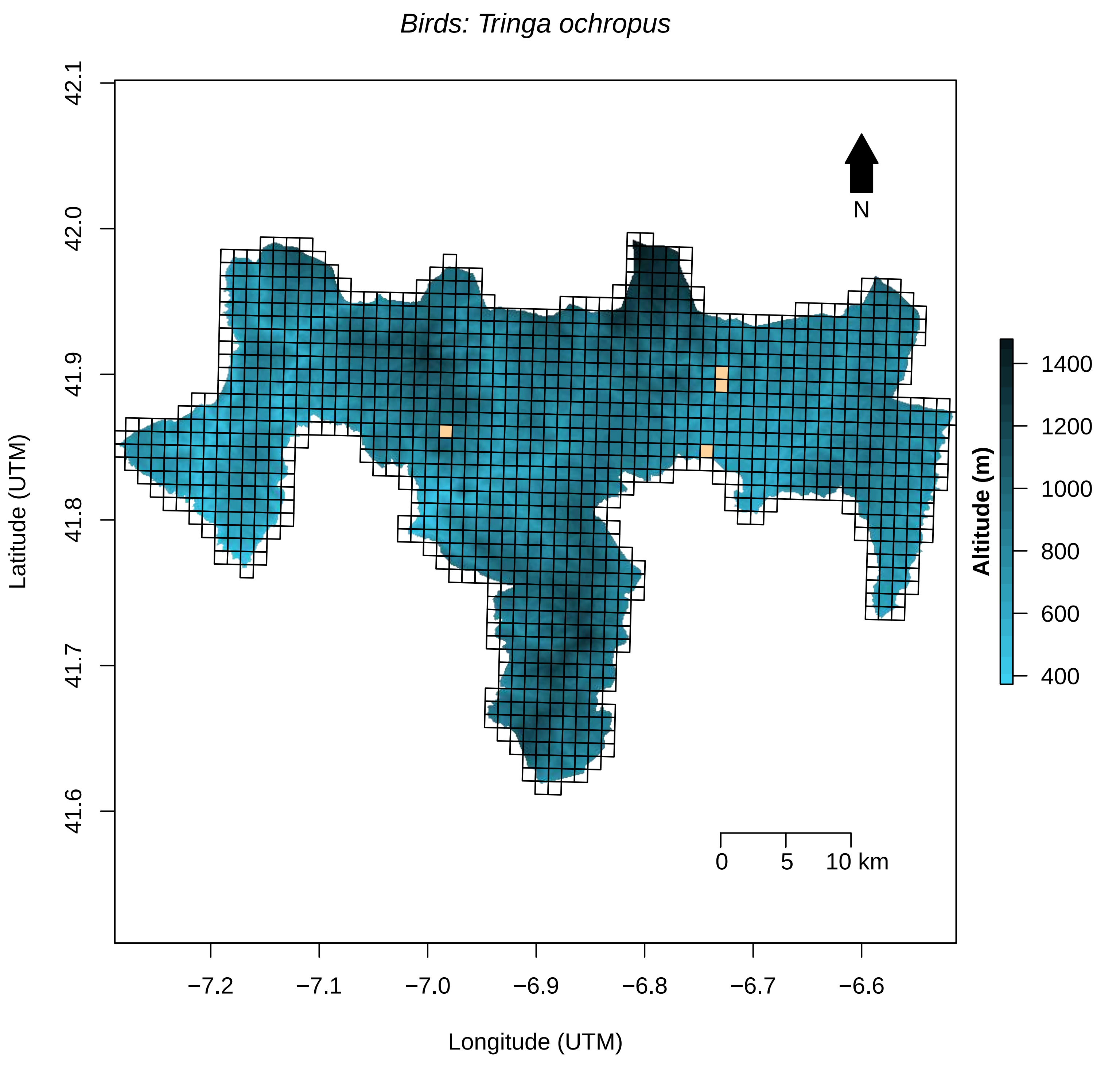

Birds: Tringa ochropus
N
0
5
10 km
Altitude (m)
1400
1200
1000
800
600
400
42.1
42.0
41.9
41.8
41.7
41.6
Latitude (UTM)
−7.2
−7.1
−7.0
−6.9
−6.8
−6.7
−6.6
Longitude (UTM)

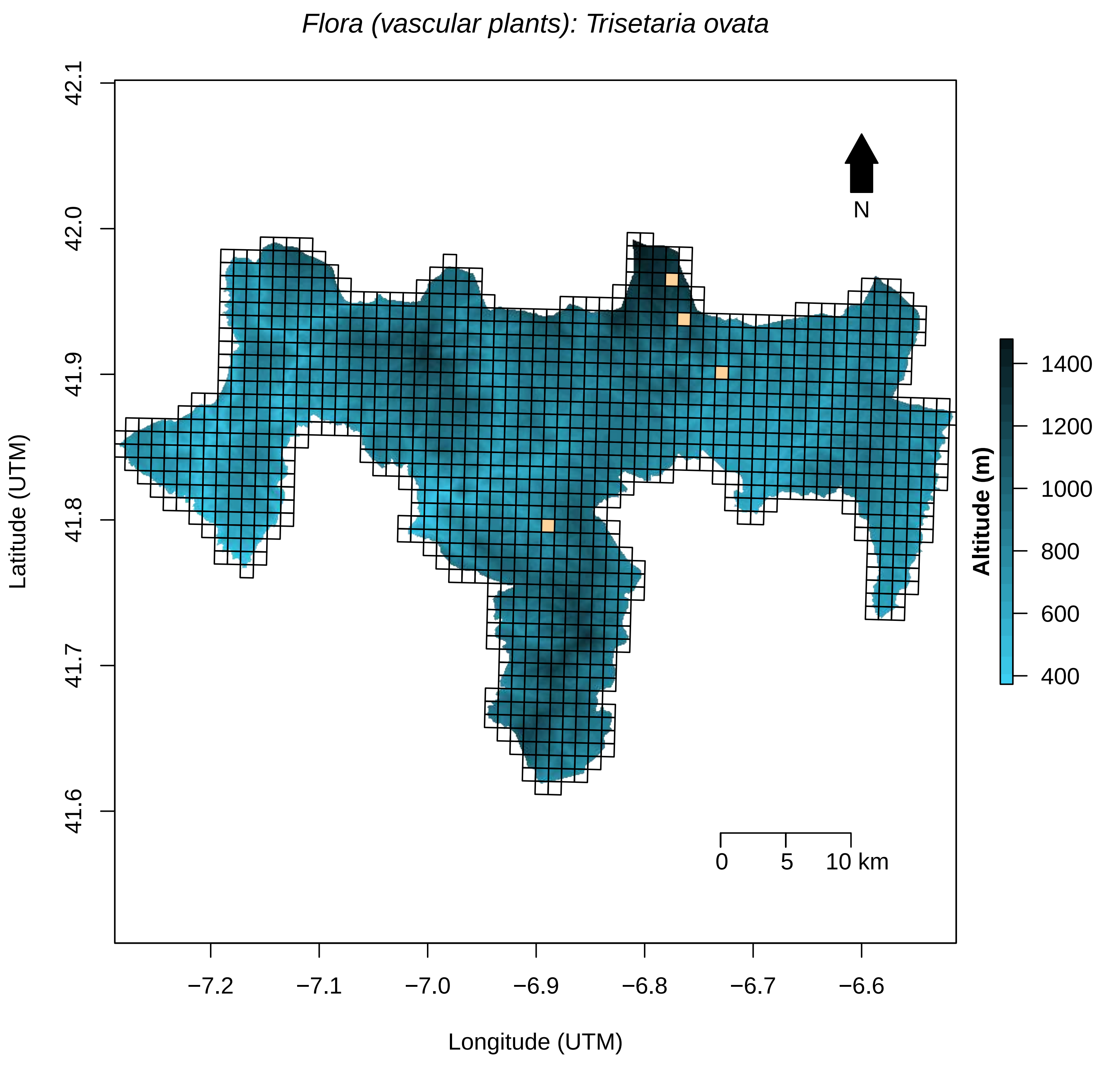

Flora (vascular plants): Trisetaria ovata
N
0
5
10 km
42.1
42.0
41.9
41.8
41.7
41.6
Latitude (UTM)
−7.2
−7.1
−7.0
−6.9
−6.8
−6.7
−6.6
Longitude (UTM)
Altitude (m)
1400
1200
1000
800
600
400

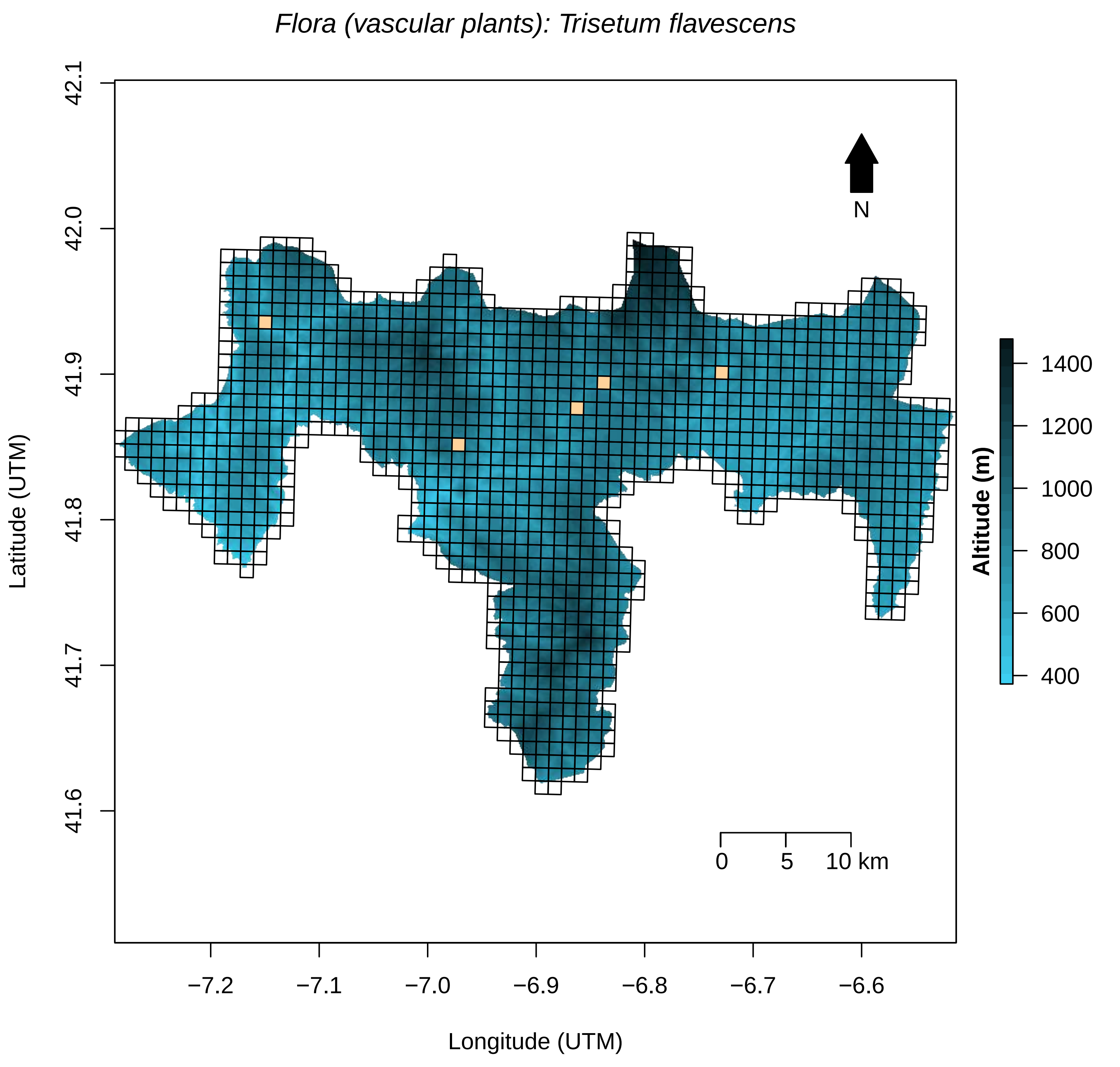

Flora (vascular plants): Trisetum flavescens
42.1
42.0
41.9
41.8
41.7
41.6
Latitude (UTM)
−7.2
−7.1
−7.0
−6.9
−6.8
−6.7
−6.6
Longitude (UTM)
N
0
5
10 km
Altitude (m)
1400
1200
1000
800
600
400

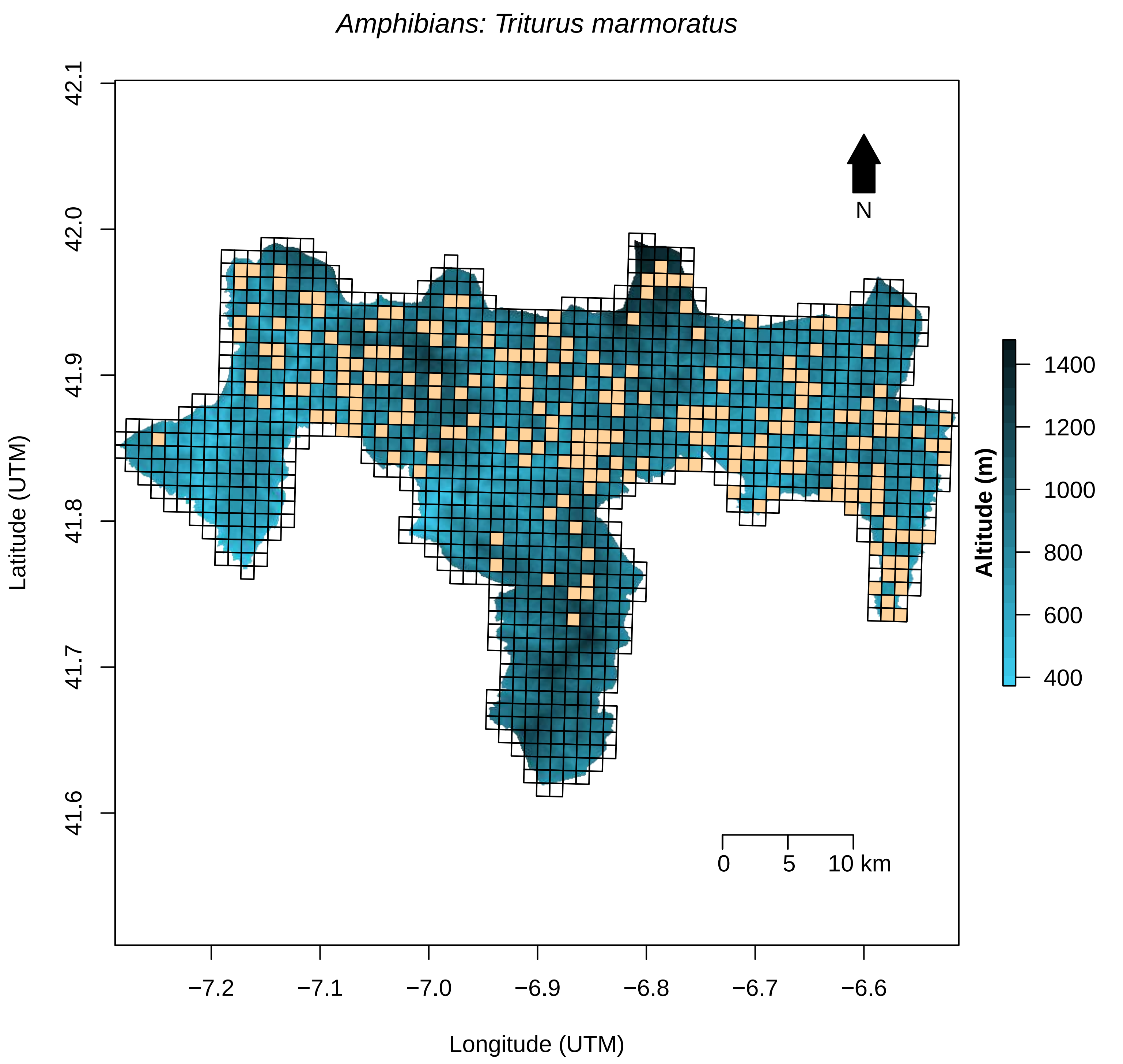

Amphibians: Triturus marmoratus
42.1
42.0
41.9
41.8
41.7
41.6
Latitude (UTM)
−7.2
−7.1
−7.0
−6.9
−6.8
−6.7
−6.6
Longitude (UTM)
N
0
5
10 km
1400
1200
1000
800
600
400
Altitude (m)

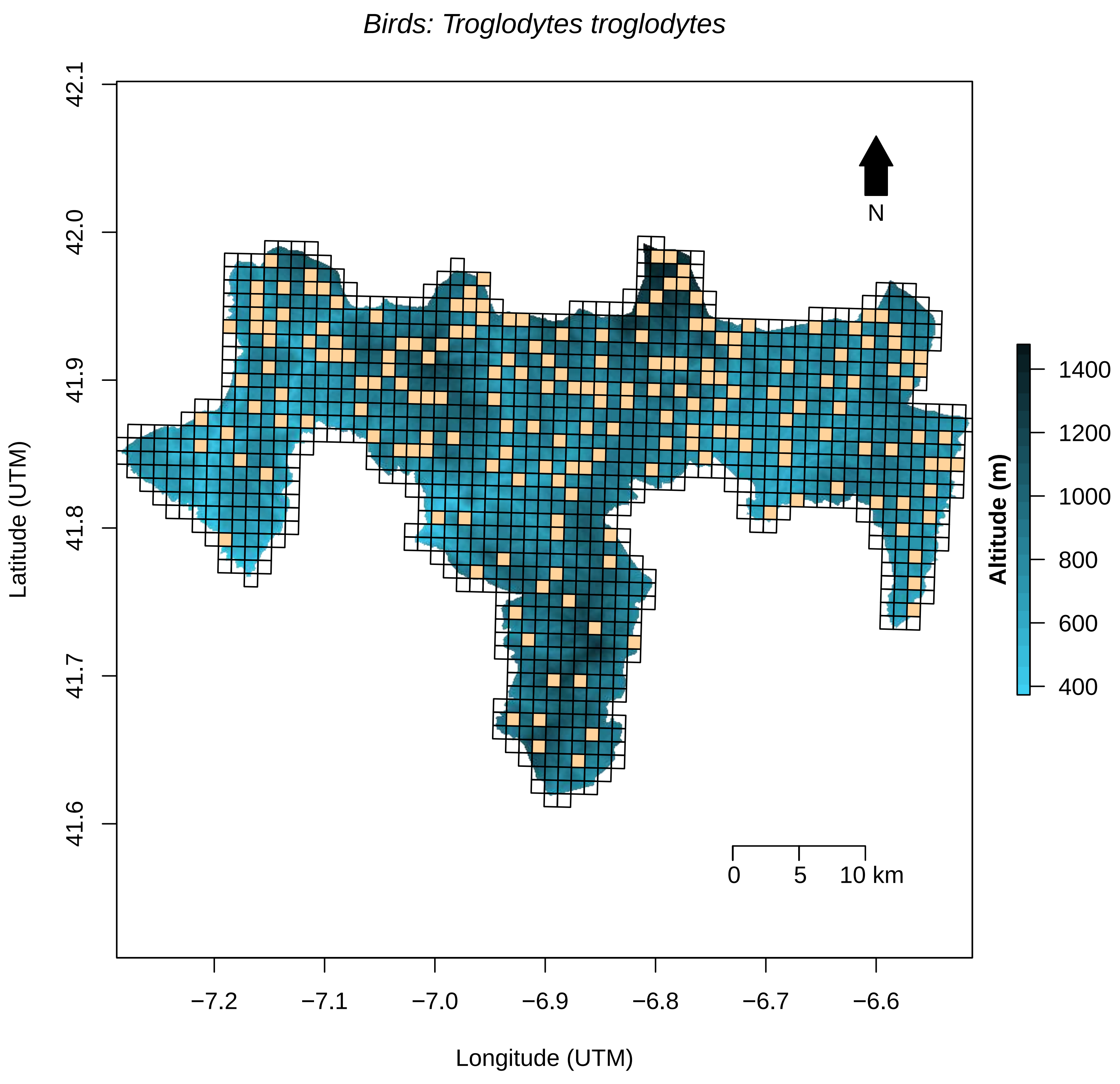

Birds: Troglodytes troglodytes
42.1
42.0
41.9
41.8
41.7
41.6
Latitude (UTM)
−7.2
−7.1
−7.0
−6.9
−6.8
−6.7
−6.6
Longitude (UTM)
N
0
5
10 km
1400
1200
1000
800
600
400
Altitude (m)

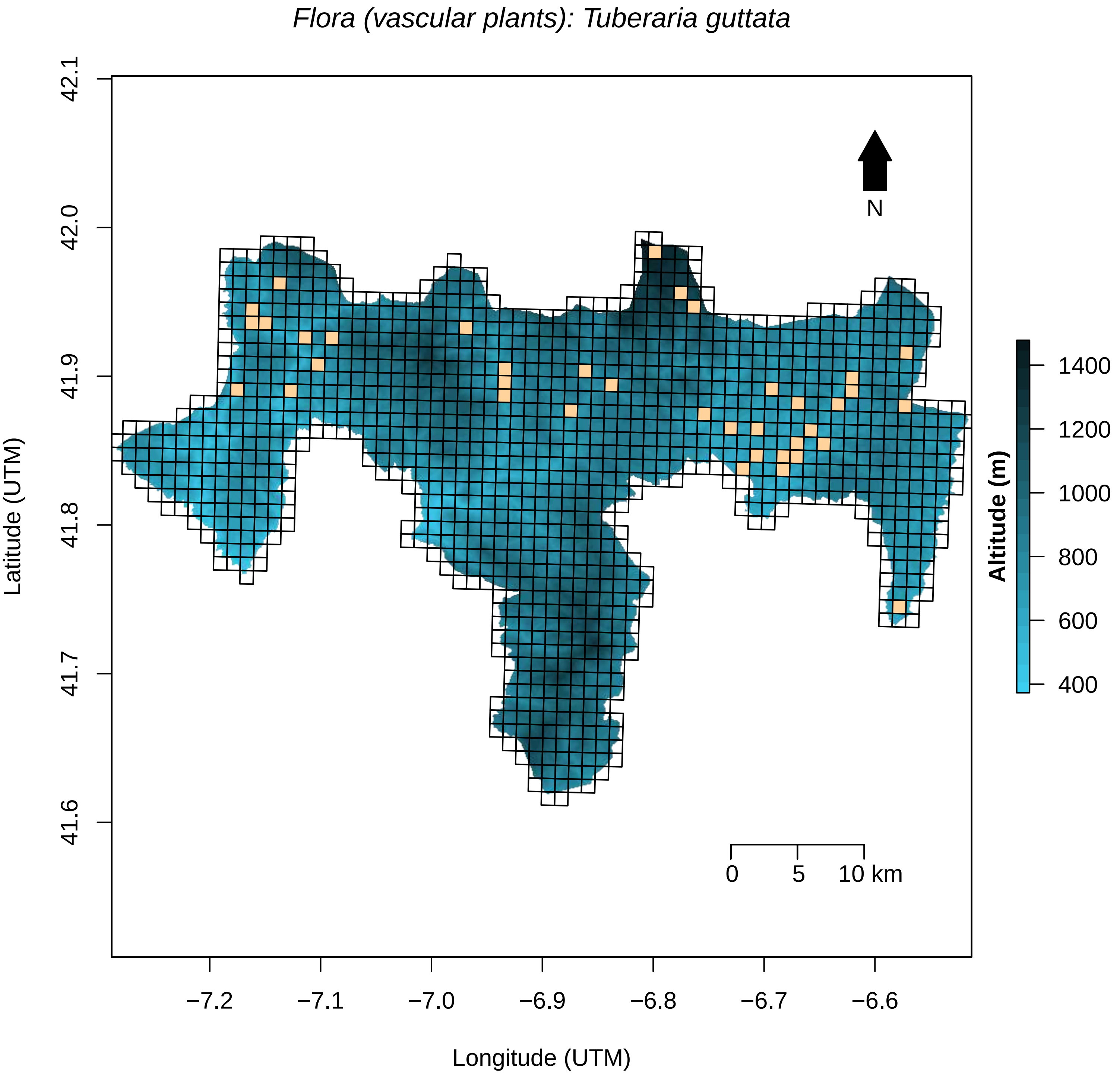

Flora (vascular plants): Tuberaria guttata
Latitude (UTM)
42.1
42.0
41.9
41.8
41.7
41.6
Longitude (UTM)
−7.2
−7.1
−7.0
−6.9
−6.8
−6.7
−6.6
N
0
5
10 km
Altitude (m)
1400
1200
1000
800
600
400

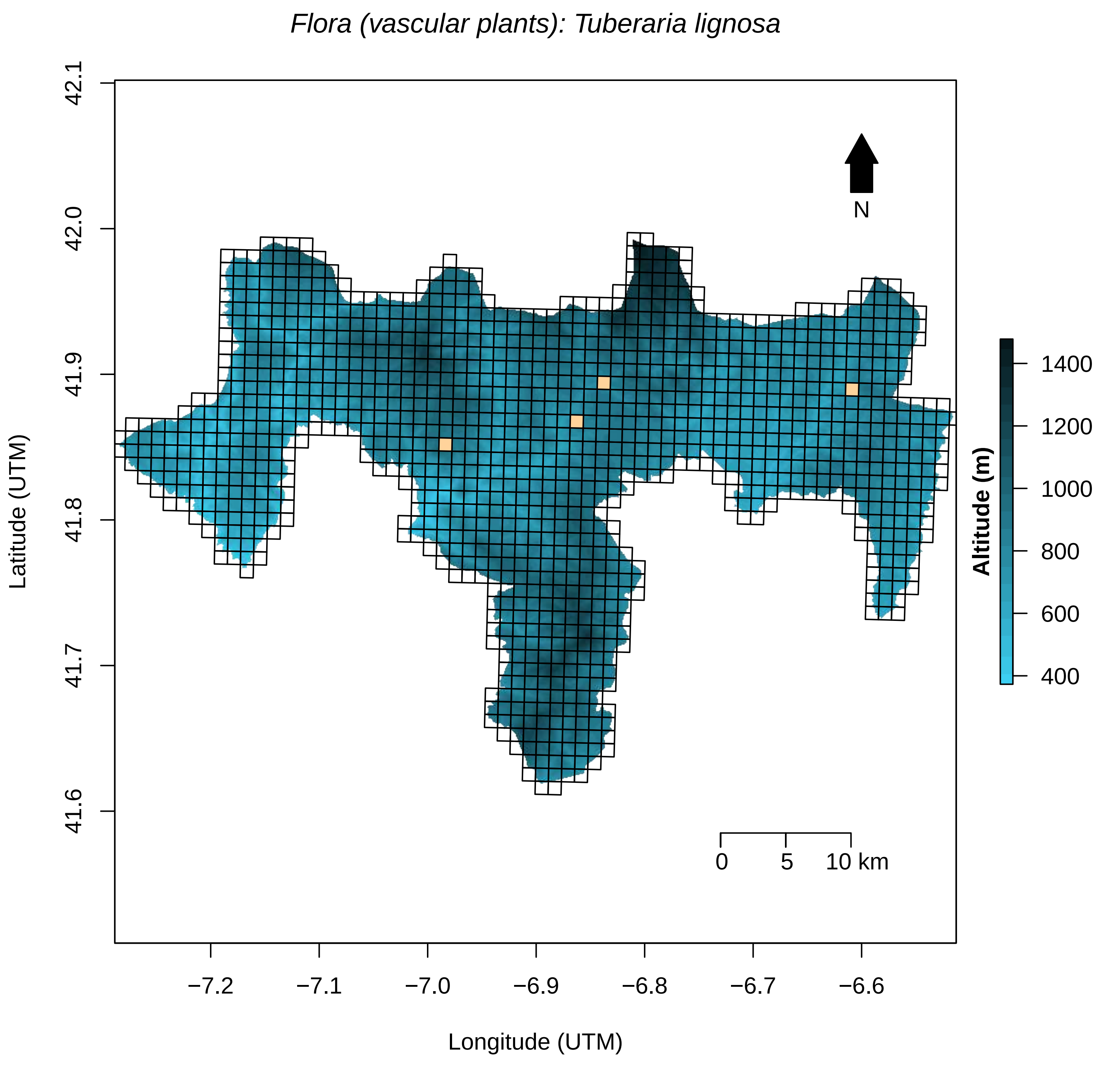

Flora (vascular plants): Tuberaria lignosa
42.1
42.0
41.9
41.8
41.7
41.6
Latitude (UTM)
−7.2
−7.1
−7.0
−6.9
−6.8
−6.7
−6.6
Longitude (UTM)
N
0
5
10 km
1400
1200
1000
800
600
400
Altitude (m)

Flora (vascular plants): Tuberaria plantaginea

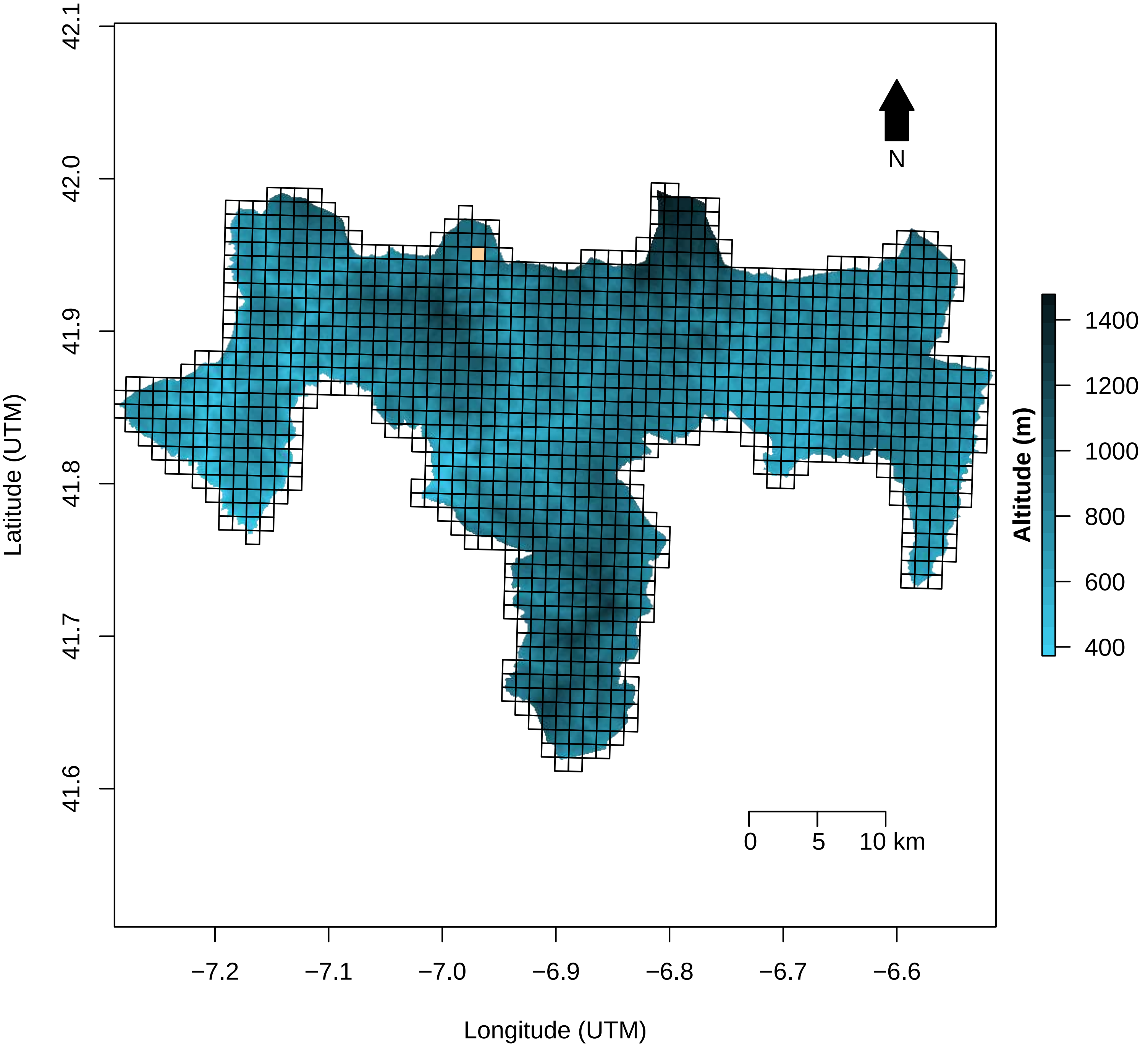

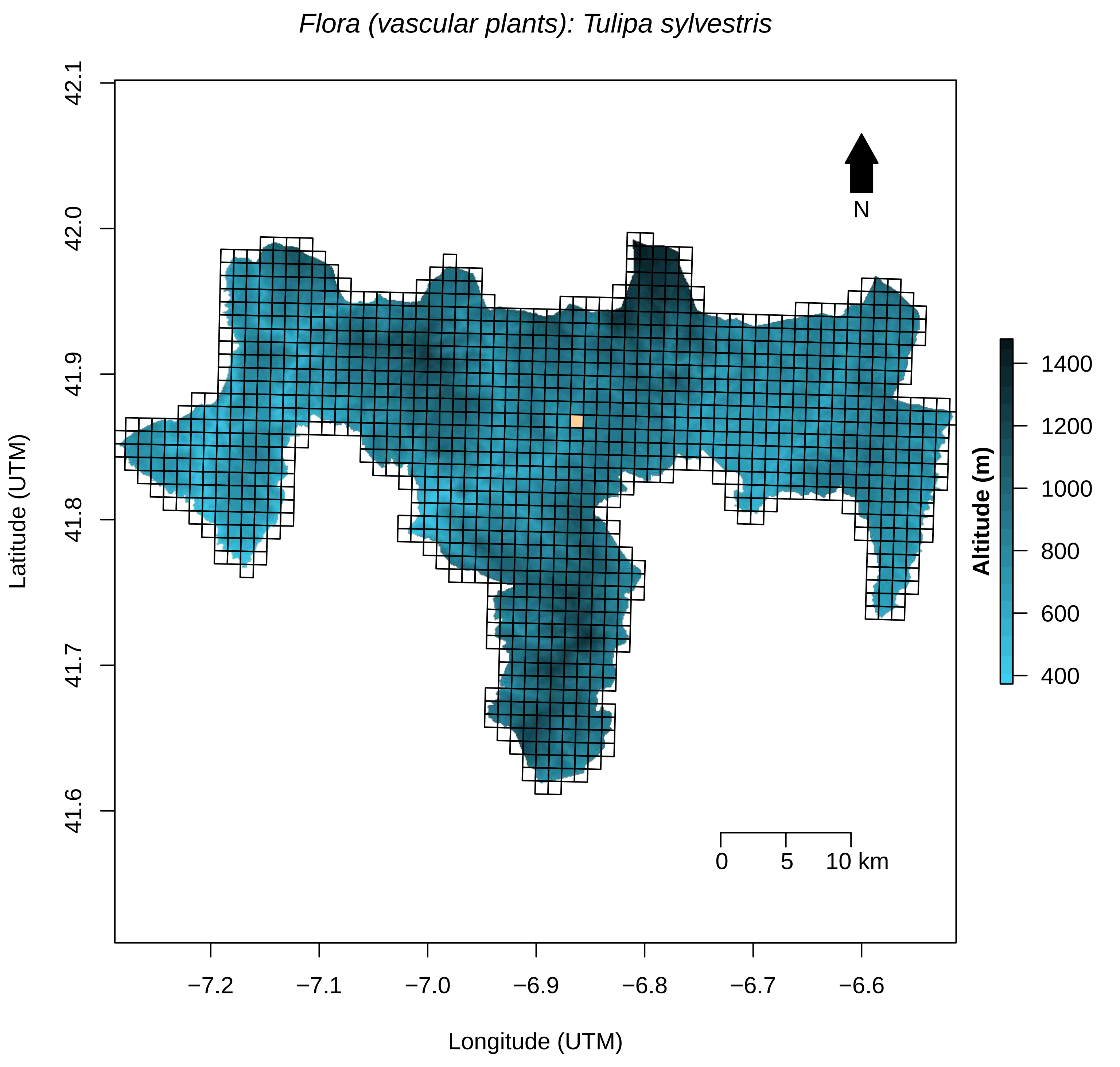

Flora (vascular plants): Tulipa sylvestris
42.1
42.0
41.9
41.8
41.7
41.6
Latitude (UTM)
−7.2
−7.1
−7.0
−6.9
−6.8
−6.7
−6.6
Longitude (UTM)
N
0
5
10 km
Altitude (m)
1400
1200
1000
800
600
400

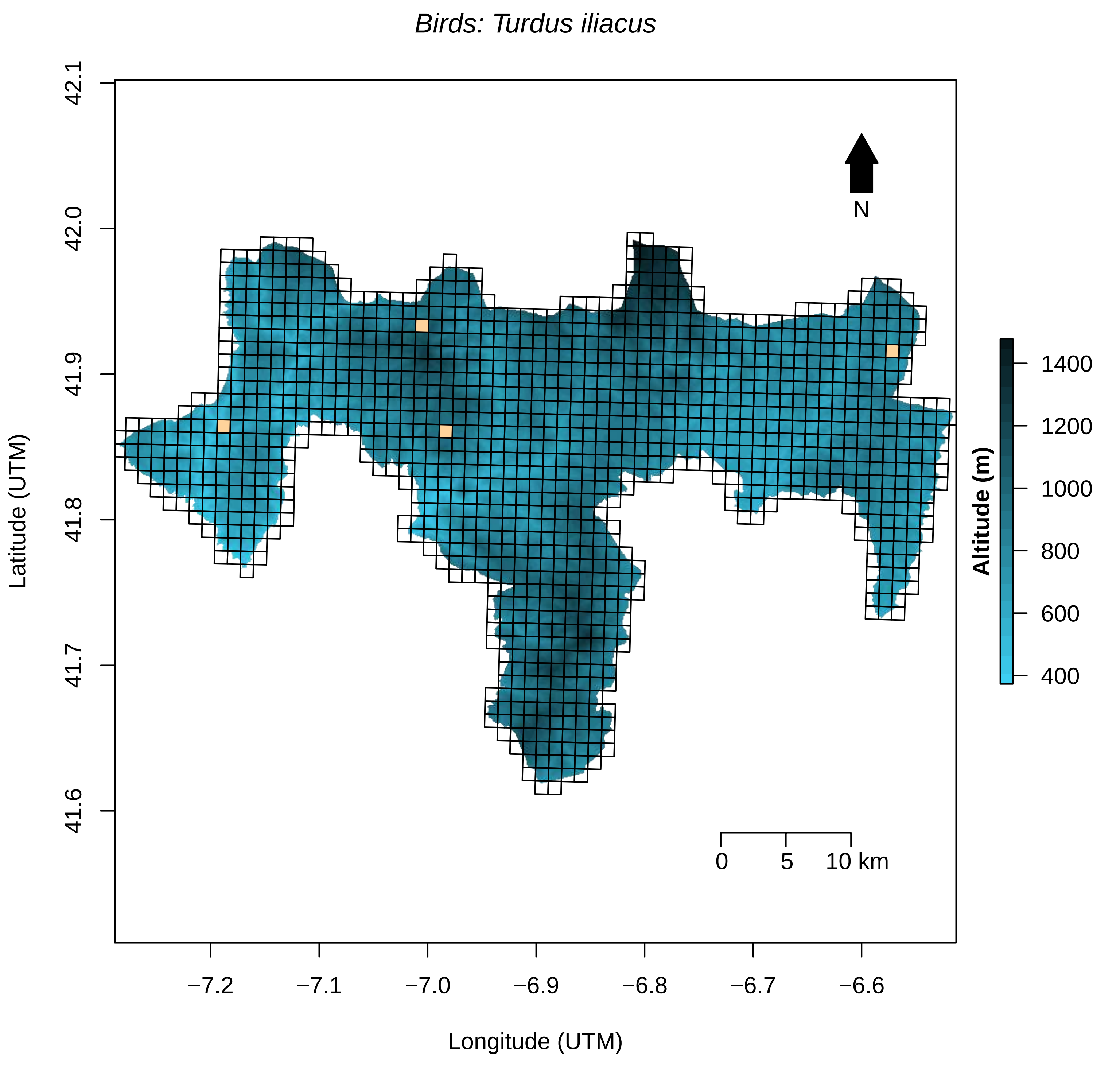
Birds: Turdus iliacus
42.1
42.0
41.9
41.8
41.7
41.6
Latitude (UTM)
−7.2
−7.1
−7.0
−6.9
−6.8
−6.7
−6.6
Longitude (UTM)
N
0
5
10 km
Altitude (m)
1400
1200
1000
800
600
400

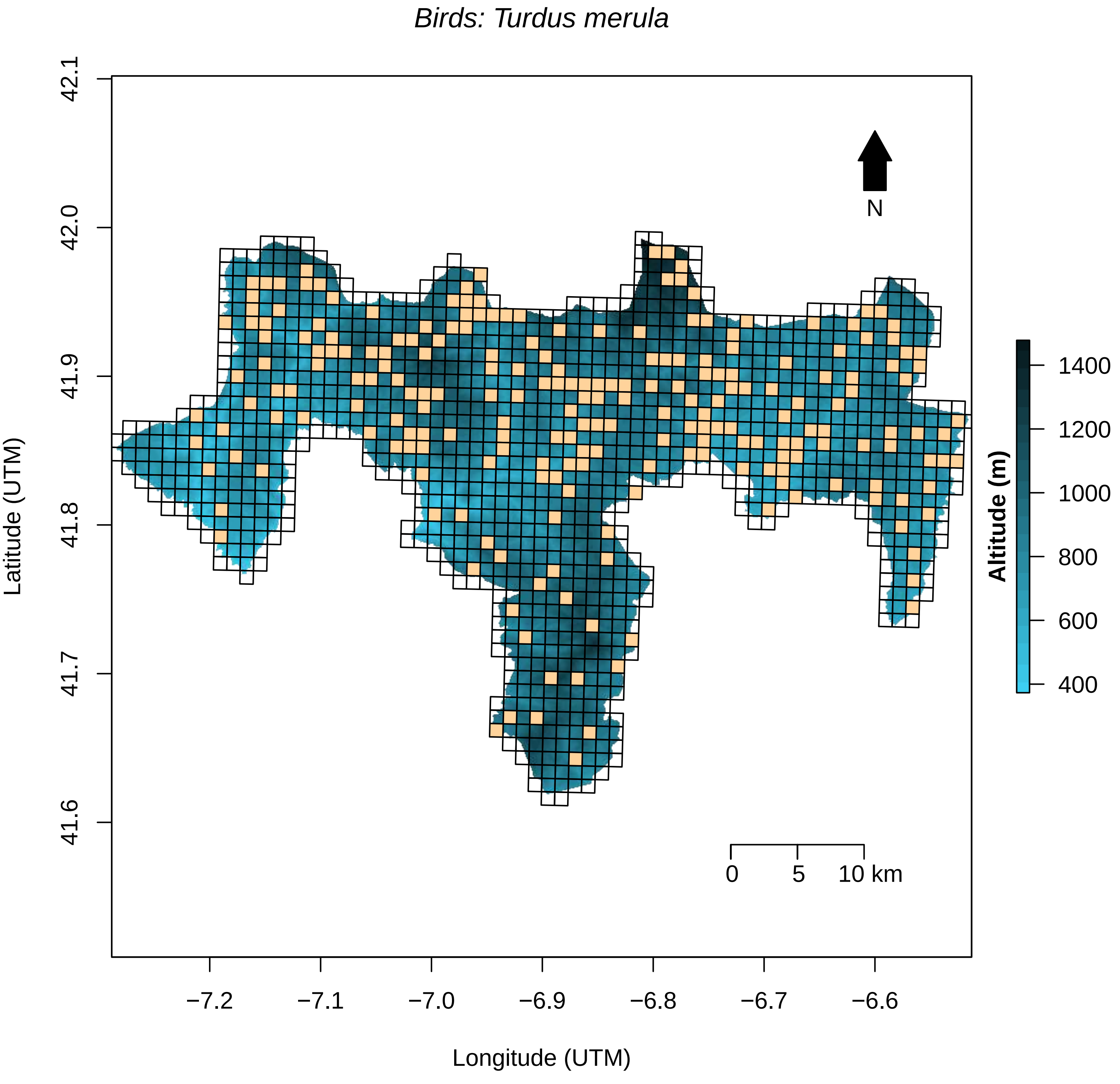
Birds: Turdus merula
N
0
5
10 km
Altitude (m)
1400
1200
1000
800
600
400
Latitude (UTM)
42.1
42.0
41.9
41.8
41.7
41.6
Longitude (UTM)
−7.2
−7.1
−7.0
−6.9
−6.8
−6.7
−6.6

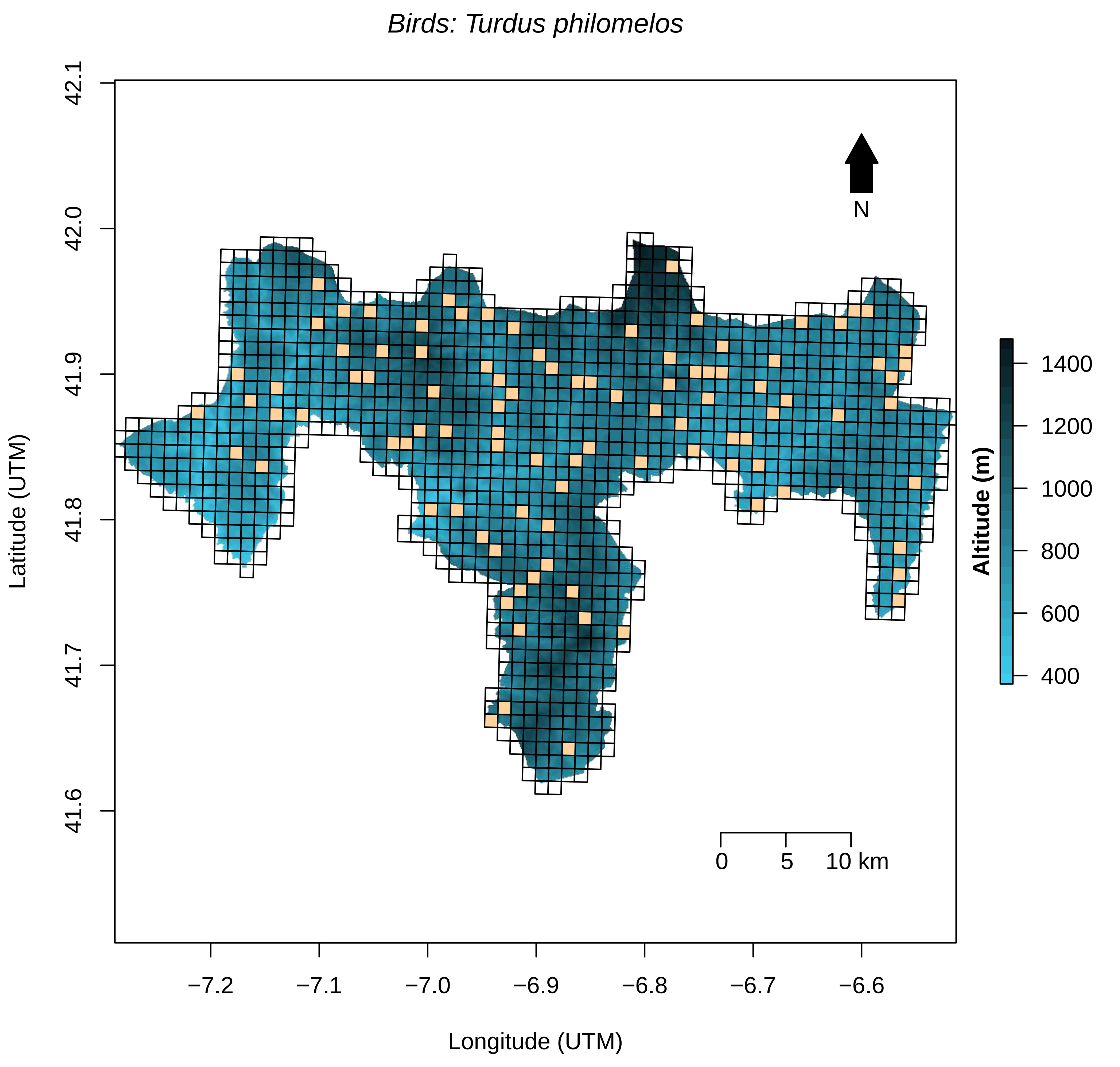

Birds: Turdus philomelos
Latitude (UTM)
42.1
42.0
41.9
41.8
41.7
41.6
Longitude (UTM)
−7.2
−7.1
−7.0
−6.9
−6.8
−6.7
−6.6
N
0
5
10 km
Altitude (m)
1400
1200
1000
800
600
400

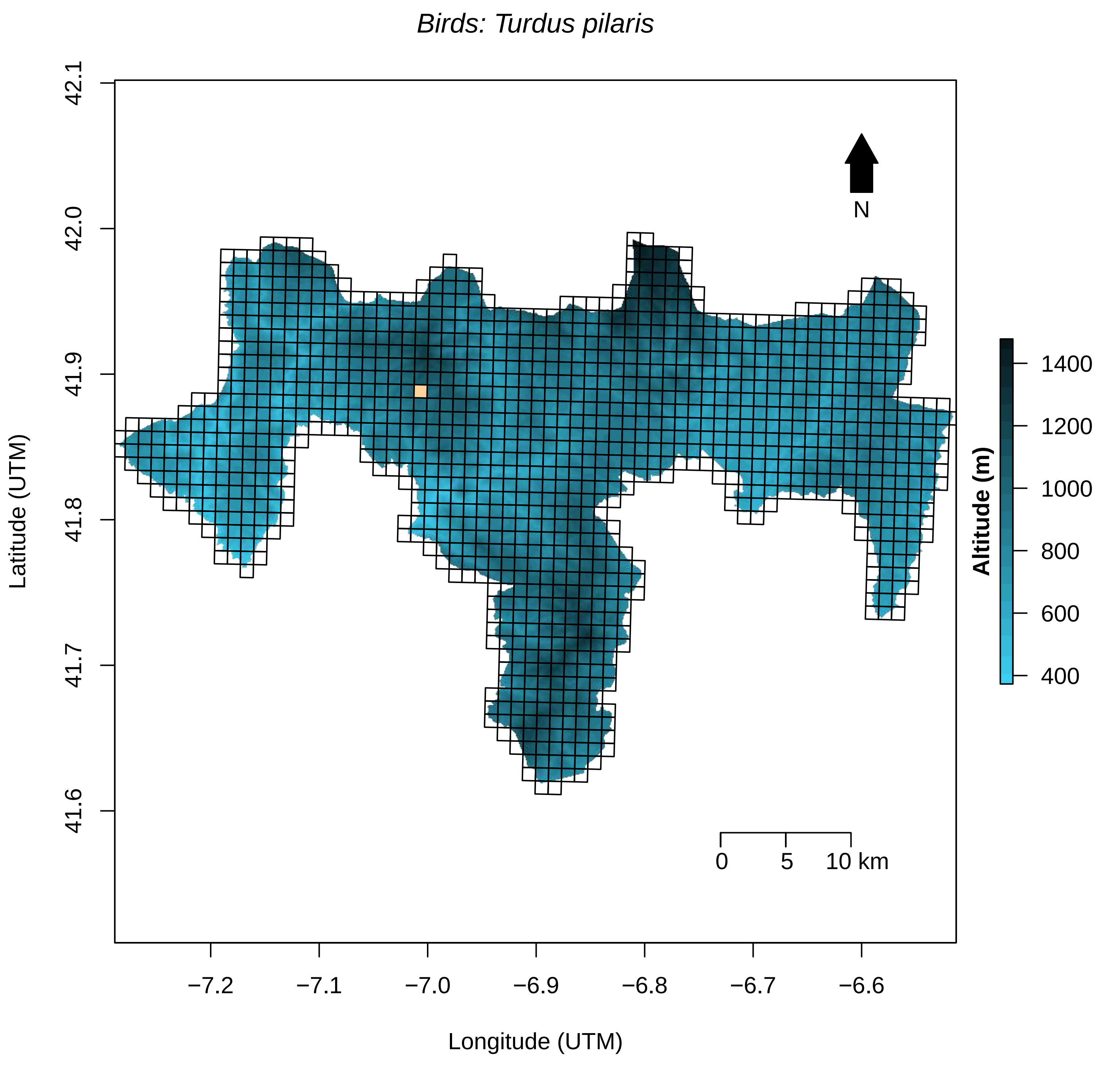
Birds: Turdus pilaris
42.1
42.0
41.9
41.8
41.7
41.6
Latitude (UTM)
−7.2
−7.1
−7.0
−6.9
−6.8
−6.7
−6.6
Longitude (UTM)
N
0
5
10 km
Altitude (m)
1400
1200
1000
800
600
400

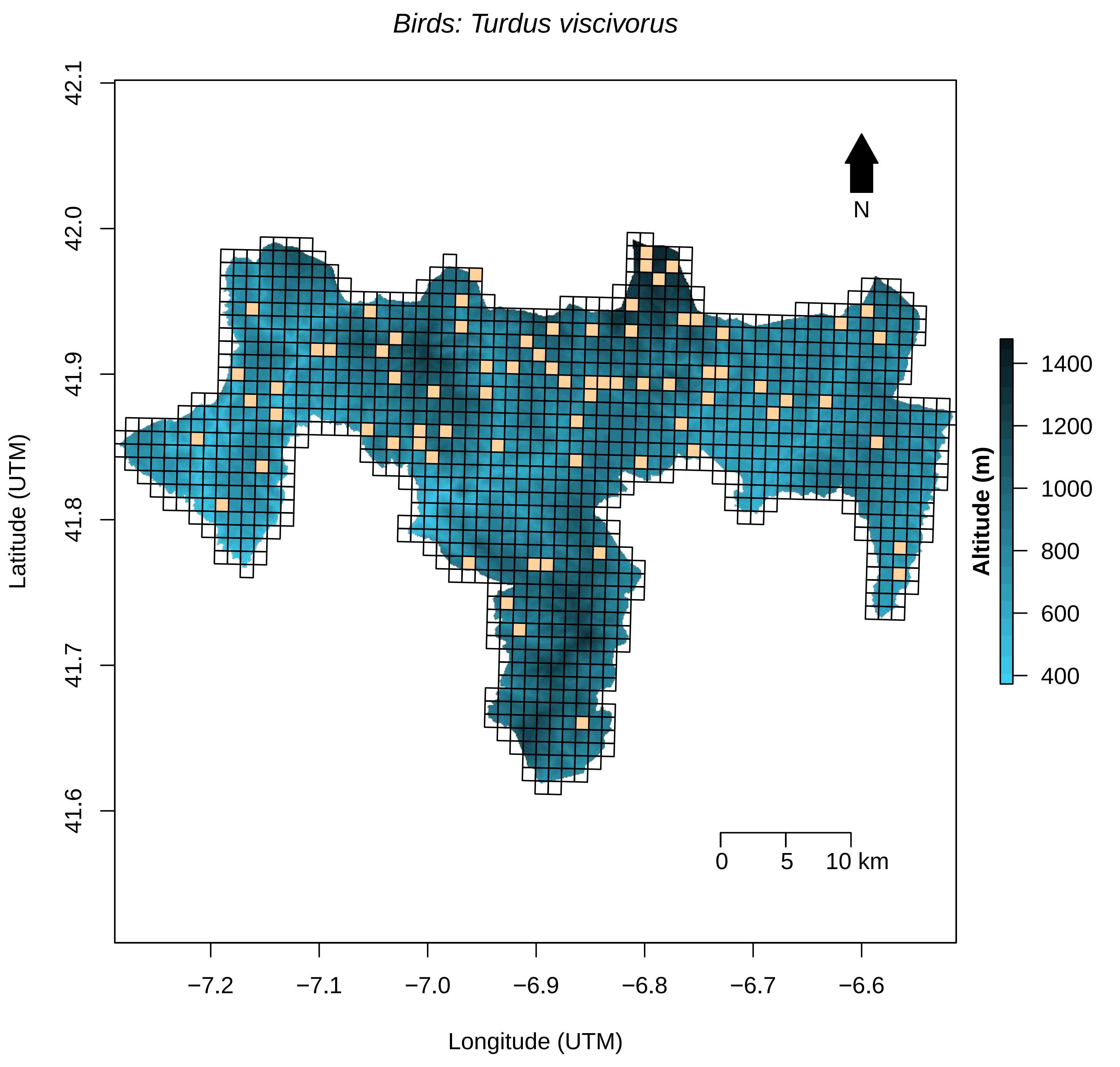

Birds: Turdus viscivorus
N
0 5 10 km
Altitude (m)
400
600
800
1000
1200
1400
Latitude (UTM)
41.6
41.7
41.8
41.9
42.0
42.1
Longitude (UTM)
−7.2
−7.1
−7.0
−6.9
−6.8
−6.7
−6.6

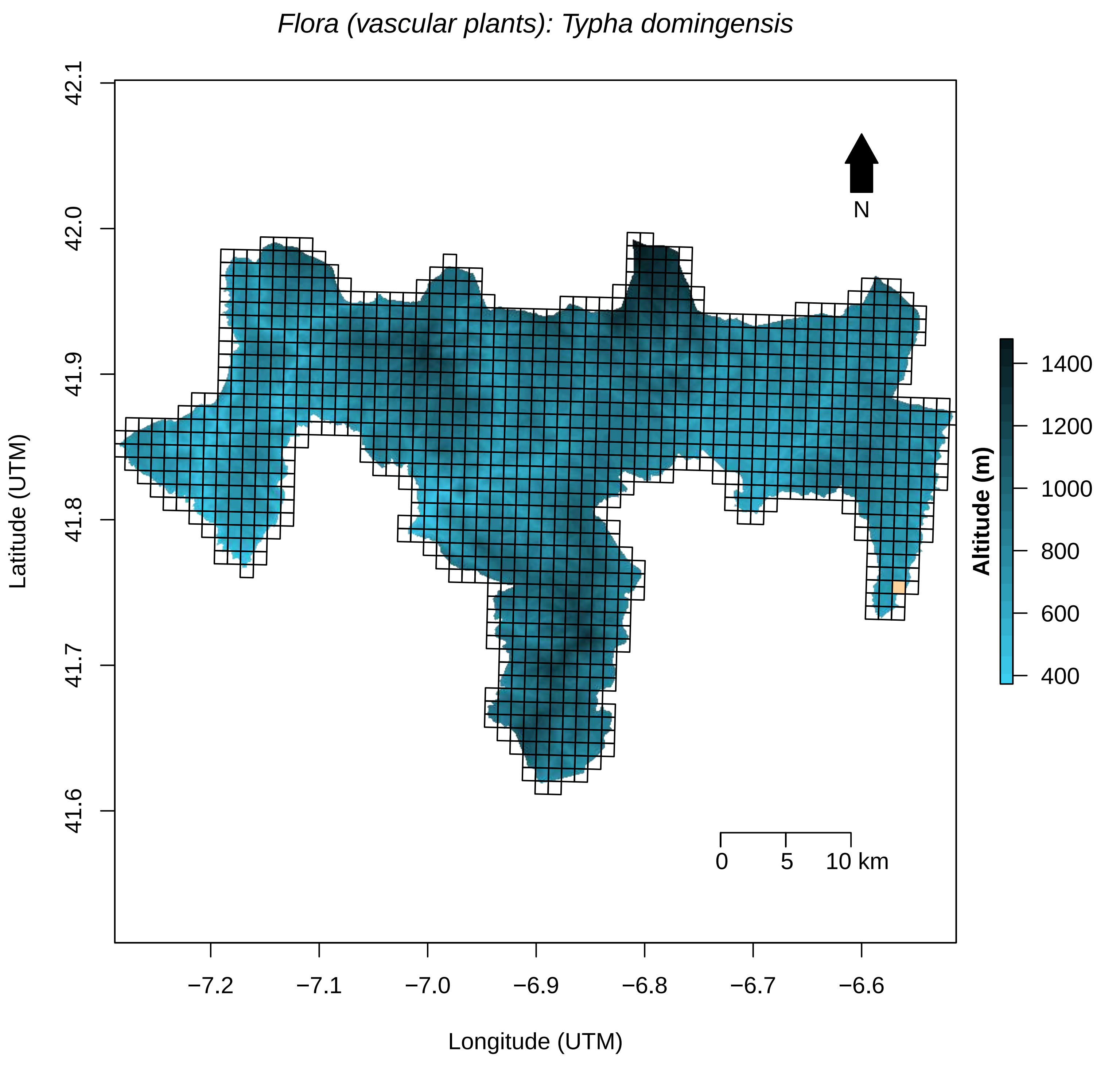
Flora (vascular plants): Typha domingensis
Latitude (UTM)
Longitude (UTM)
42.1
42.0
41.9
41.8
41.7
41.6
−7.2
−7.1
−7.0
−6.9
−6.8
−6.7
−6.6
N
0
5
10 km
Altitude (m)
1400
1200
1000
800
600
400

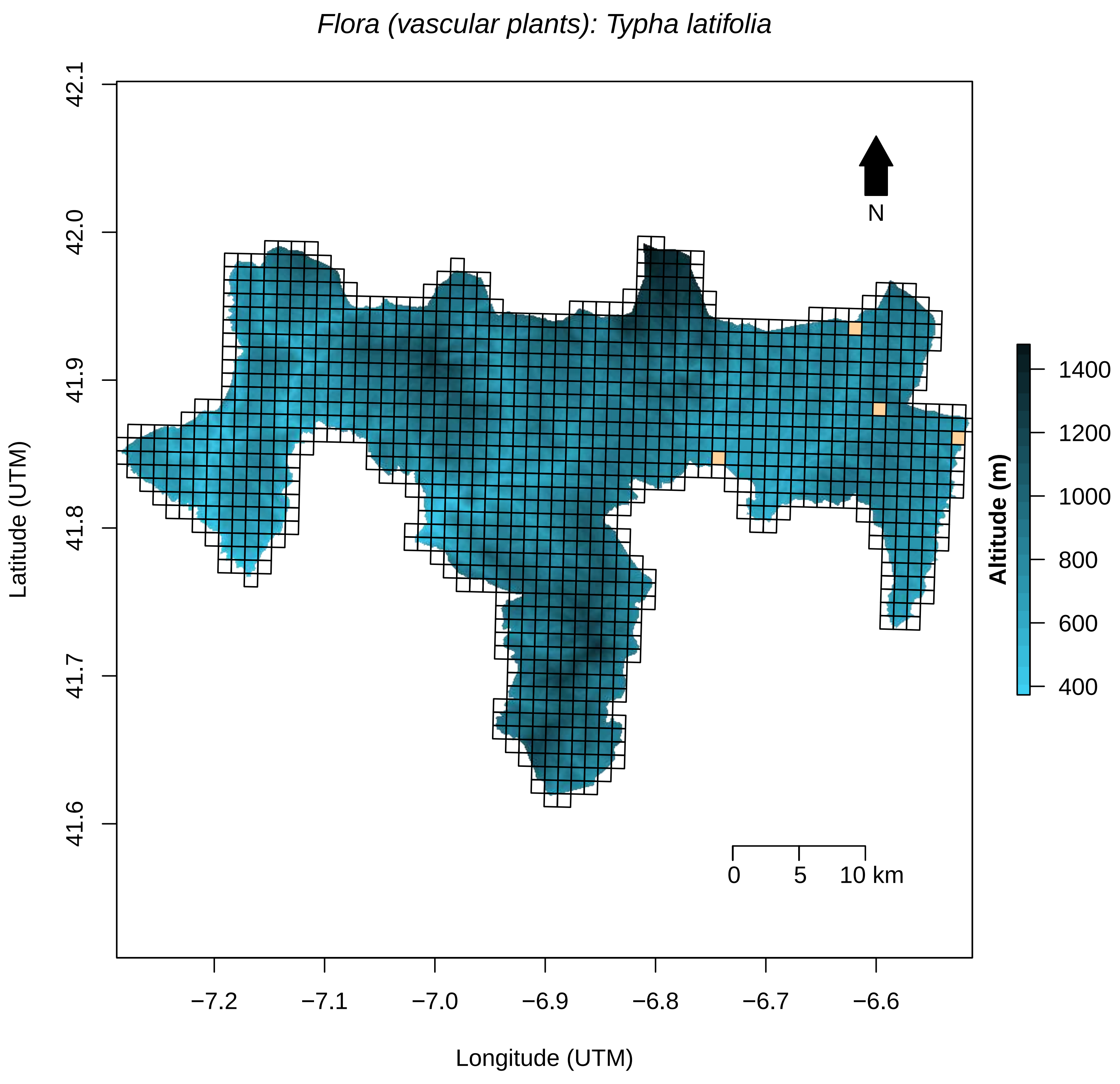

Flora (vascular plants): Typha latifolia
42.1
42.0
41.9
41.8
41.7
41.6
Latitude (UTM)
−7.2
−7.1
−7.0
−6.9
−6.8
−6.7
−6.6
Longitude (UTM)
N
0
5
10 km
Altitude (m)
1400
1200
1000
800
600
400

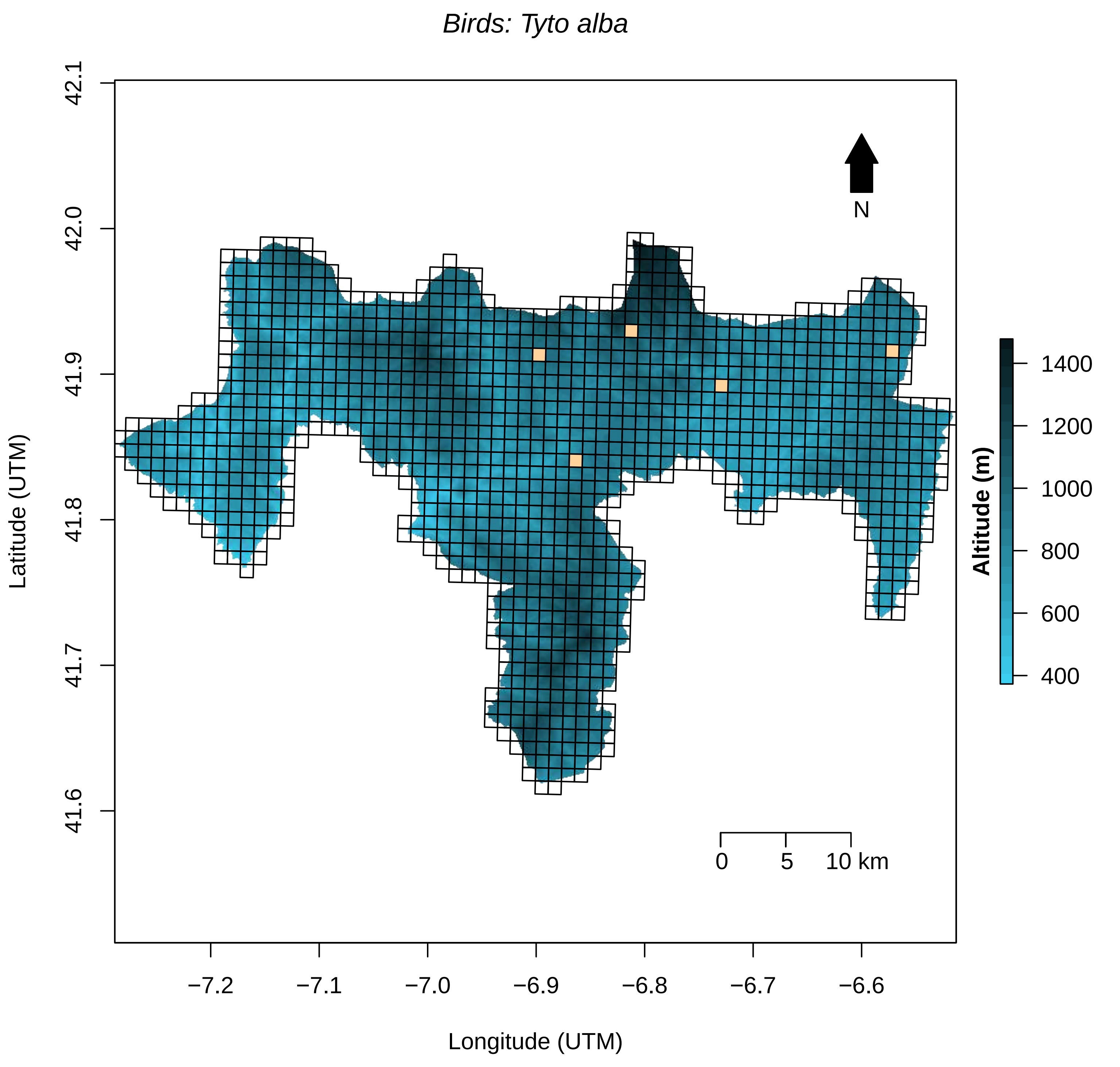

Birds: Tyto alba
Latitude (UTM)
42.1
42.0
41.9
41.8
41.7
41.6
Longitude (UTM)
−7.2
−7.1
−7.0
−6.9
−6.8
−6.7
−6.6
N
0
5
10 km
Altitude (m)
1400
1200
1000
800
600
400

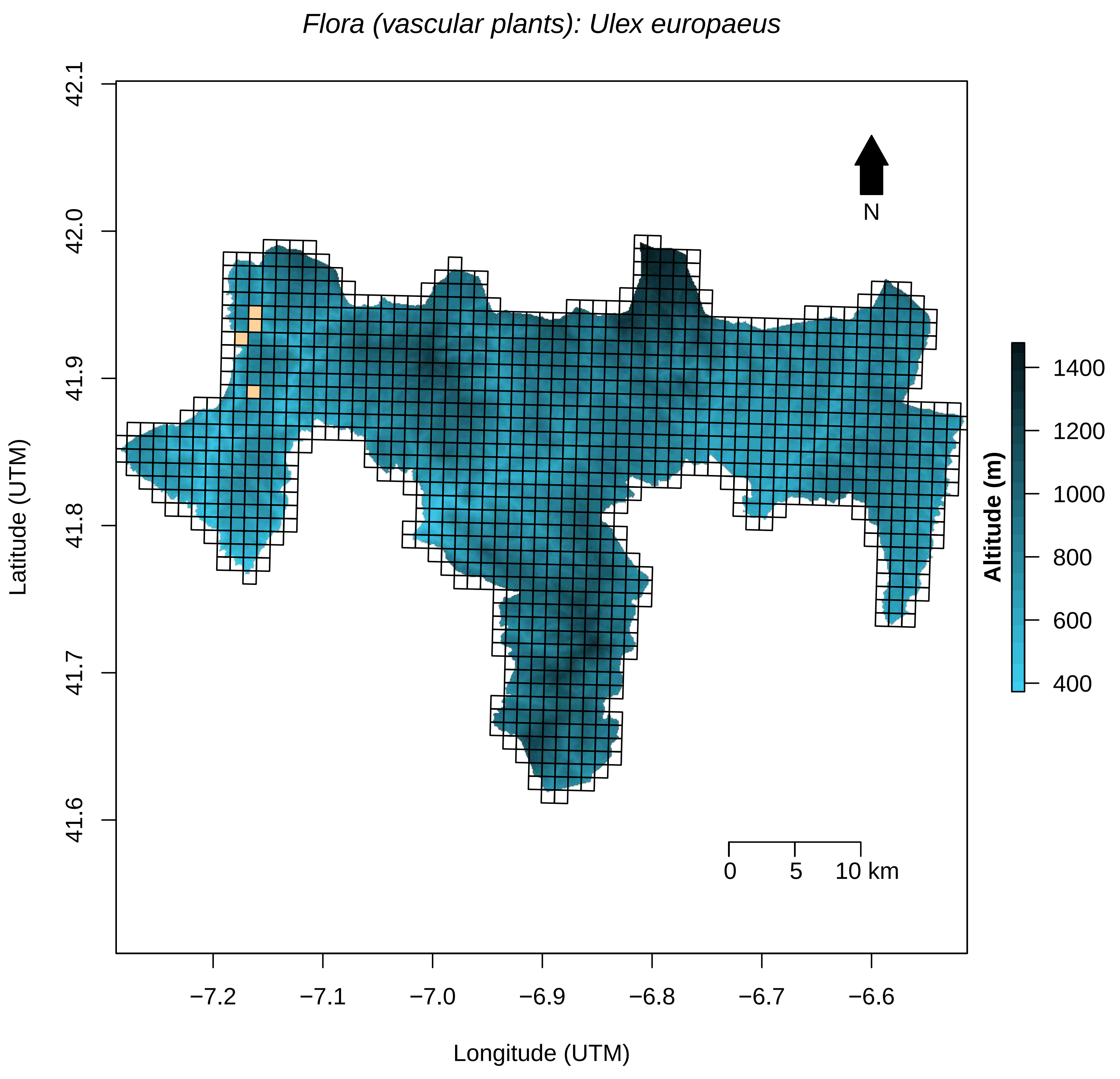

Flora (vascular plants): Ulex europaeus
42.1
42.0
41.9
41.8
41.7
41.6
Latitude (UTM)
−7.2
−7.1
−7.0
−6.9
−6.8
−6.7
−6.6
Longitude (UTM)
N
0
5
10 km
Altitude (m)
1400
1200
1000
800
600
400

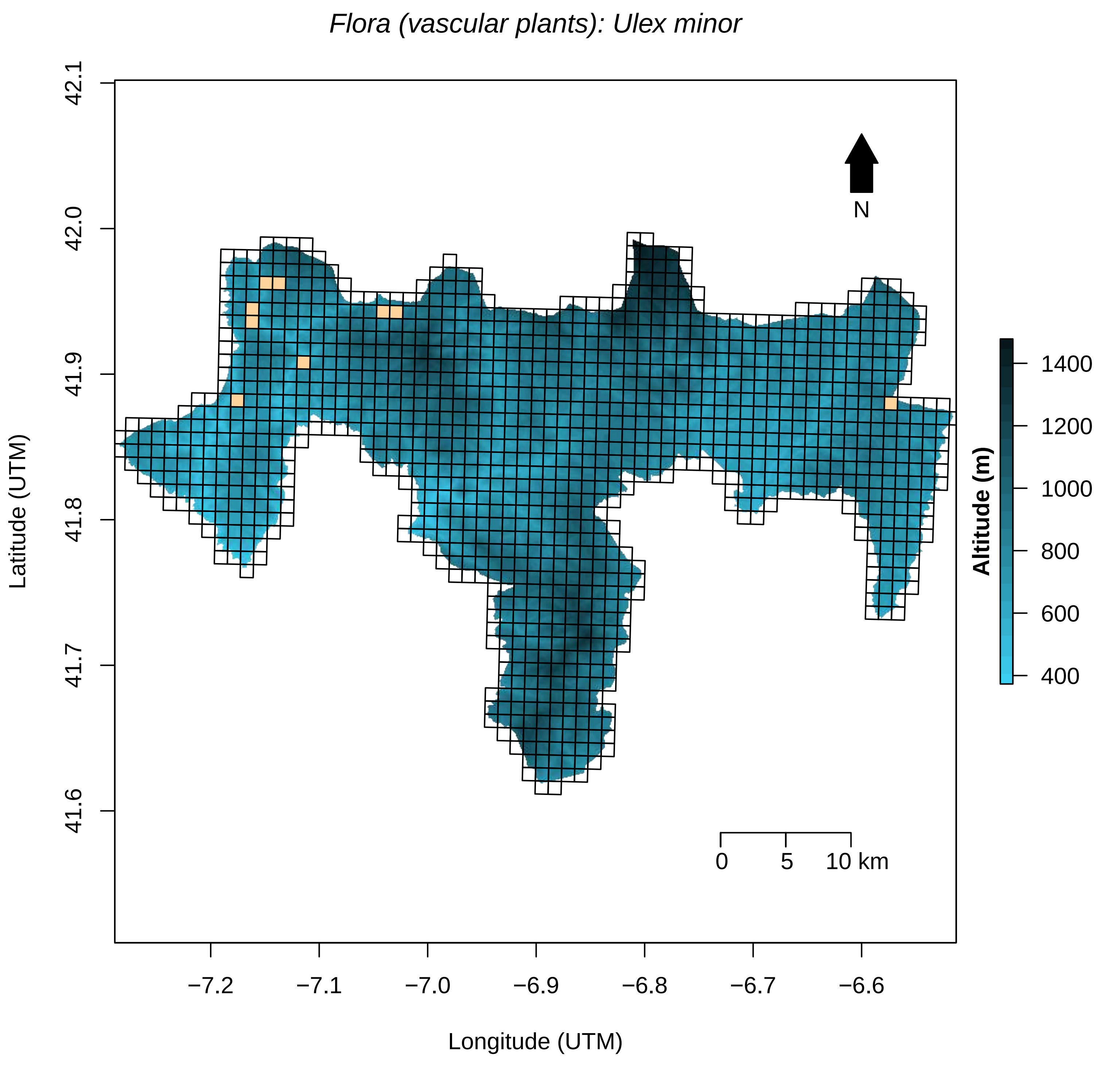
Flora (vascular plants): Ulex minor
Latitude (UTM)
Longitude (UTM)
42.1
42.0
41.9
41.8
41.7
41.6
−7.2
−7.1
−7.0
−6.9
−6.8
−6.7
−6.6
N
0
5
10 km
Altitude (m)
1400
1200
1000
800
600
400

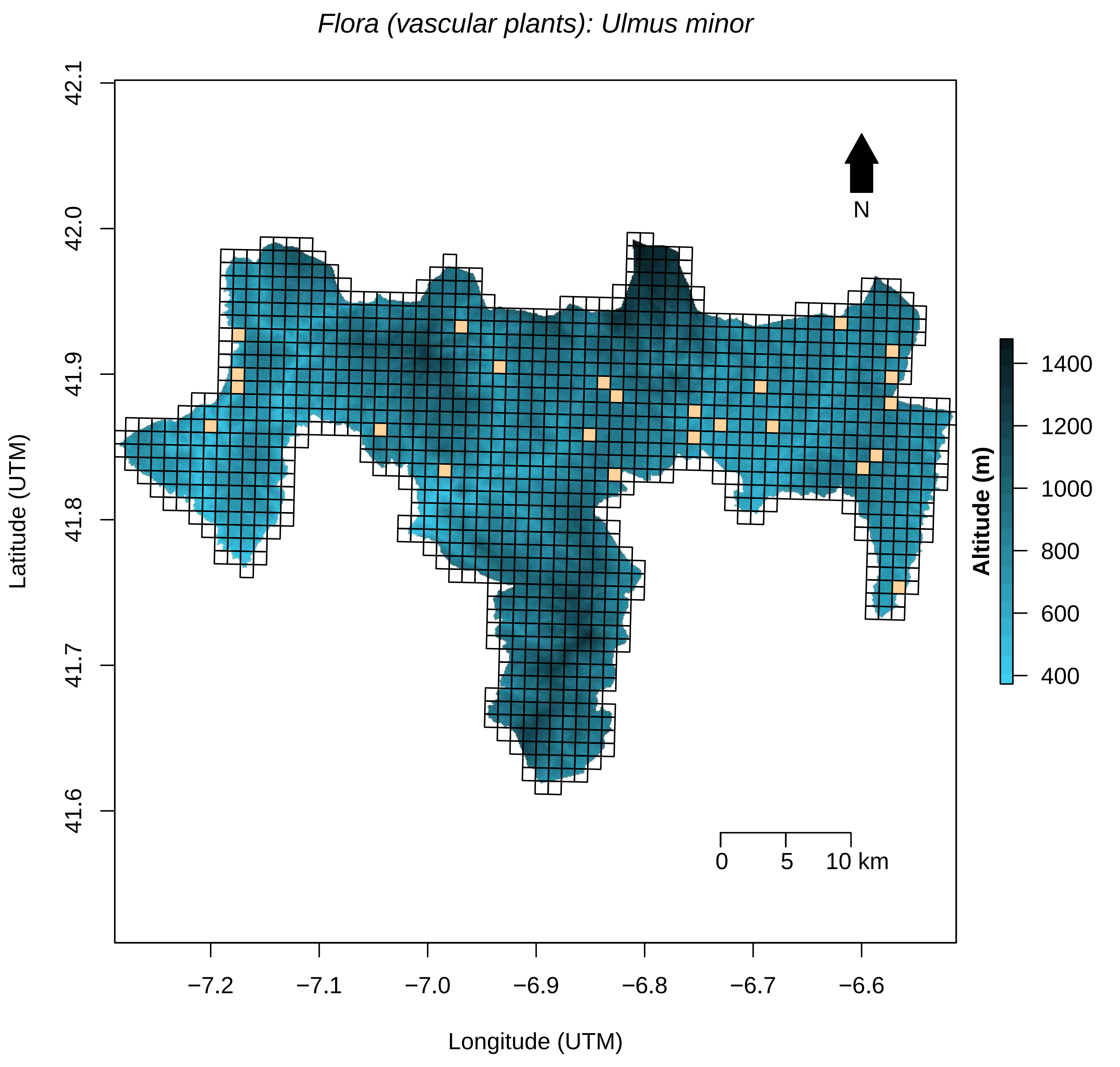
Flora (vascular plants): Ulmus minor
N
0 5 10 km
Altitude (m)
400
600
800
1000
1200
1400
Latitude (UTM)
42.1
42.0
41.9
41.8
41.7
41.6
Longitude (UTM)
−7.2
−7.1
−7.0
−6.9
−6.8
−6.7
−6.6

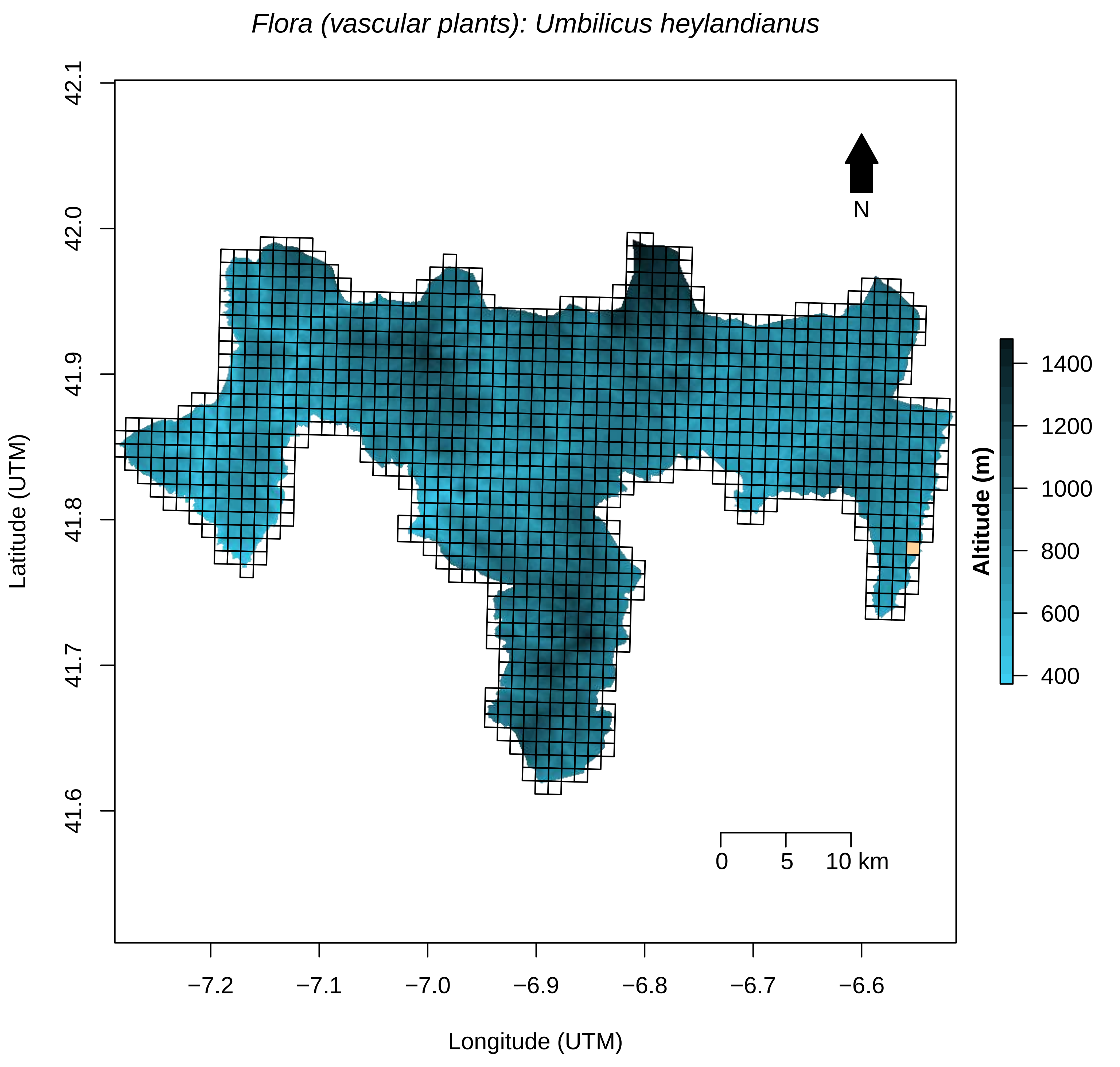
Flora (vascular plants): Umbilicus heylandianus
42.1
42.0
41.9
41.8
41.7
41.6
Latitude (UTM)
−7.2
−7.1
−7.0
−6.9
−6.8
−6.7
−6.6
Longitude (UTM)
N
0
5
10 km
1400
1200
1000
800
600
400
Altitude (m)

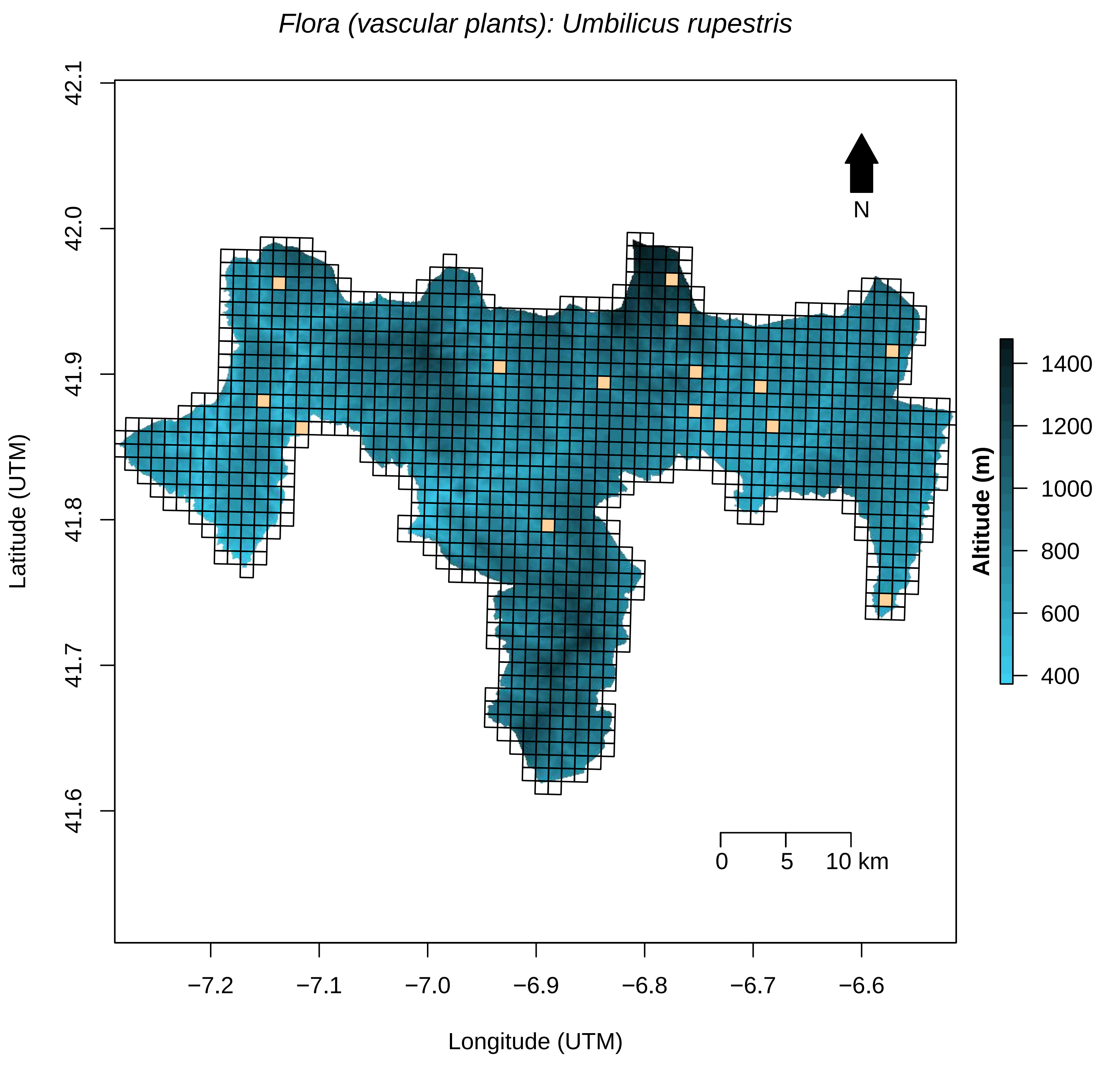

Flora (vascular plants): Umbilicus rupestris
42.1
42.0
41.9
41.8
41.7
41.6
Latitude (UTM)
−7.2
−7.1
−7.0
−6.9
−6.8
−6.7
−6.6
Longitude (UTM)
N
0
5
10 km
Altitude (m)
1400
1200
1000
800
600
400

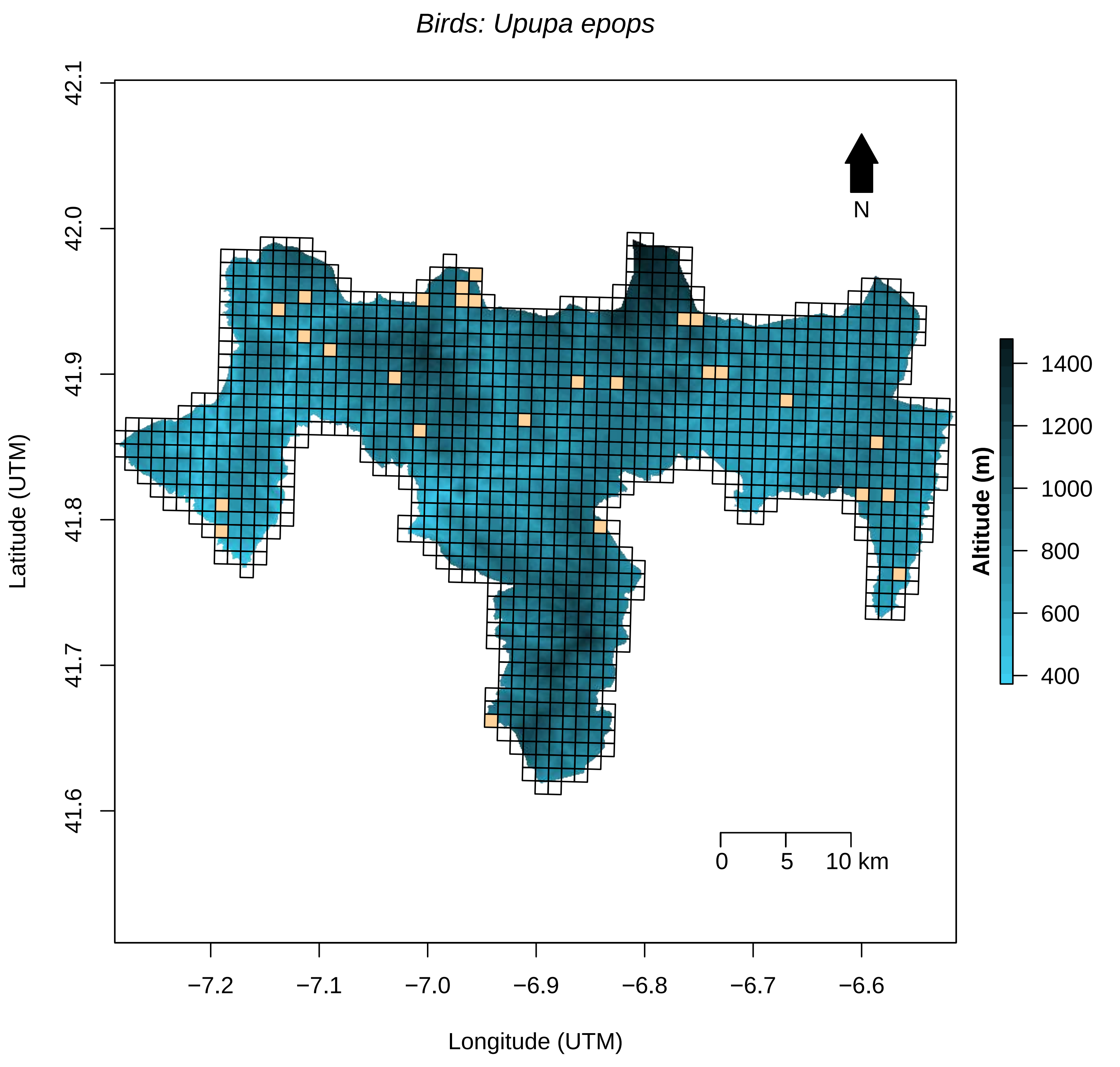

Birds: Upupa epops
42.1
42.0
41.9
41.8
41.7
41.6
Latitude (UTM)
−7.2
−7.1
−7.0
−6.9
−6.8
−6.7
−6.6
Longitude (UTM)
N
0
5
10 km
Altitude (m)
1400
1200
1000
800
600
400

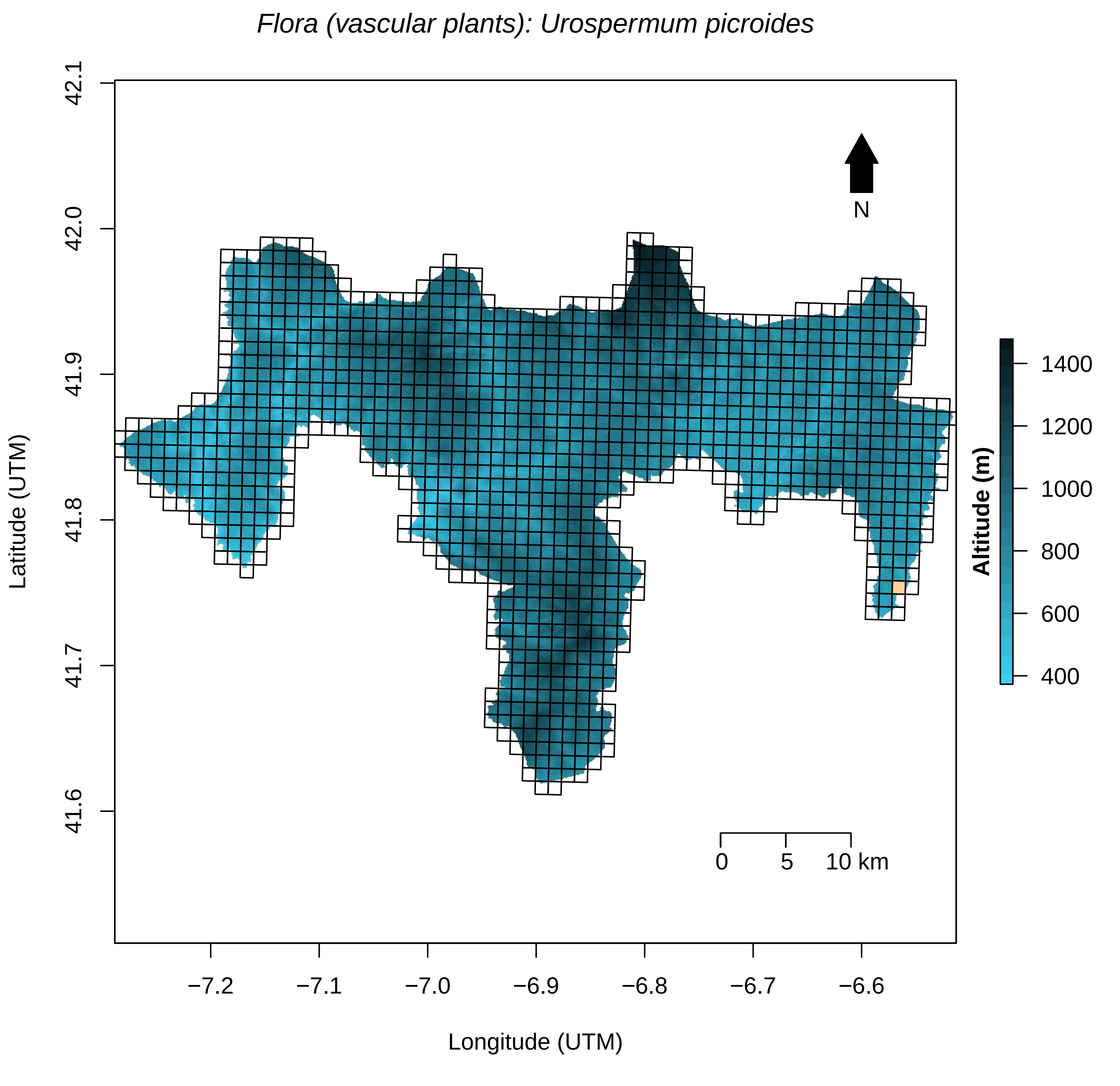
Flora (vascular plants): Urospermum picroides
42.1
42.0
41.9
41.8
41.7
41.6
Latitude (UTM)
−7.2
−7.1
−7.0
−6.9
−6.8
−6.7
−6.6
Longitude (UTM)
N
0
5
10 km
1400
1200
1000
800
600
400
Altitude (m)

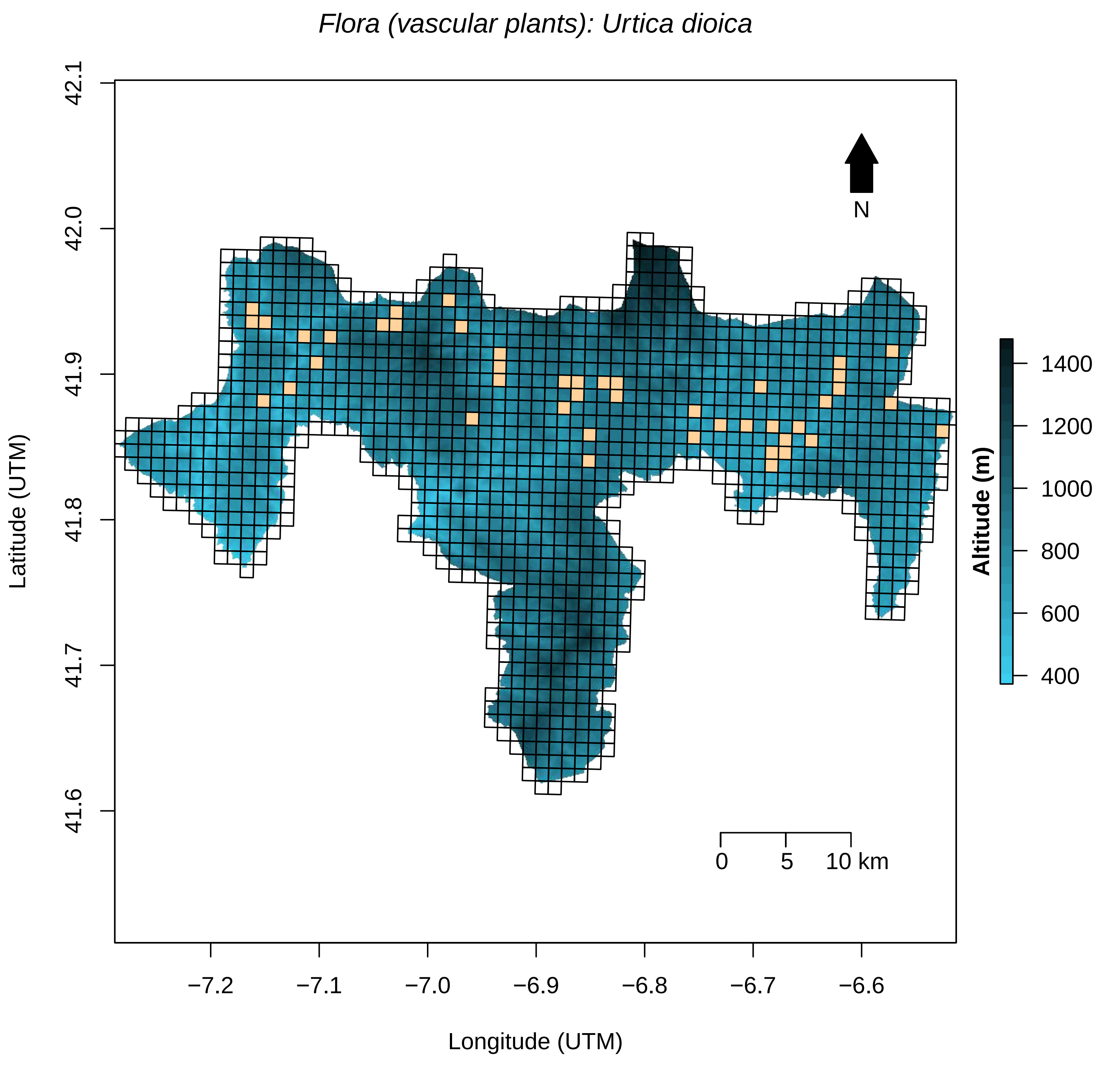
Flora (vascular plants): Urtica dioica
42.1
42.0
41.9
41.8
41.7
41.6
Latitude (UTM)
−7.2
−7.1
−7.0
−6.9
−6.8
−6.7
−6.6
Longitude (UTM)
N
0
5
10 km
Altitude (m)
1400
1200
1000
800
600
400

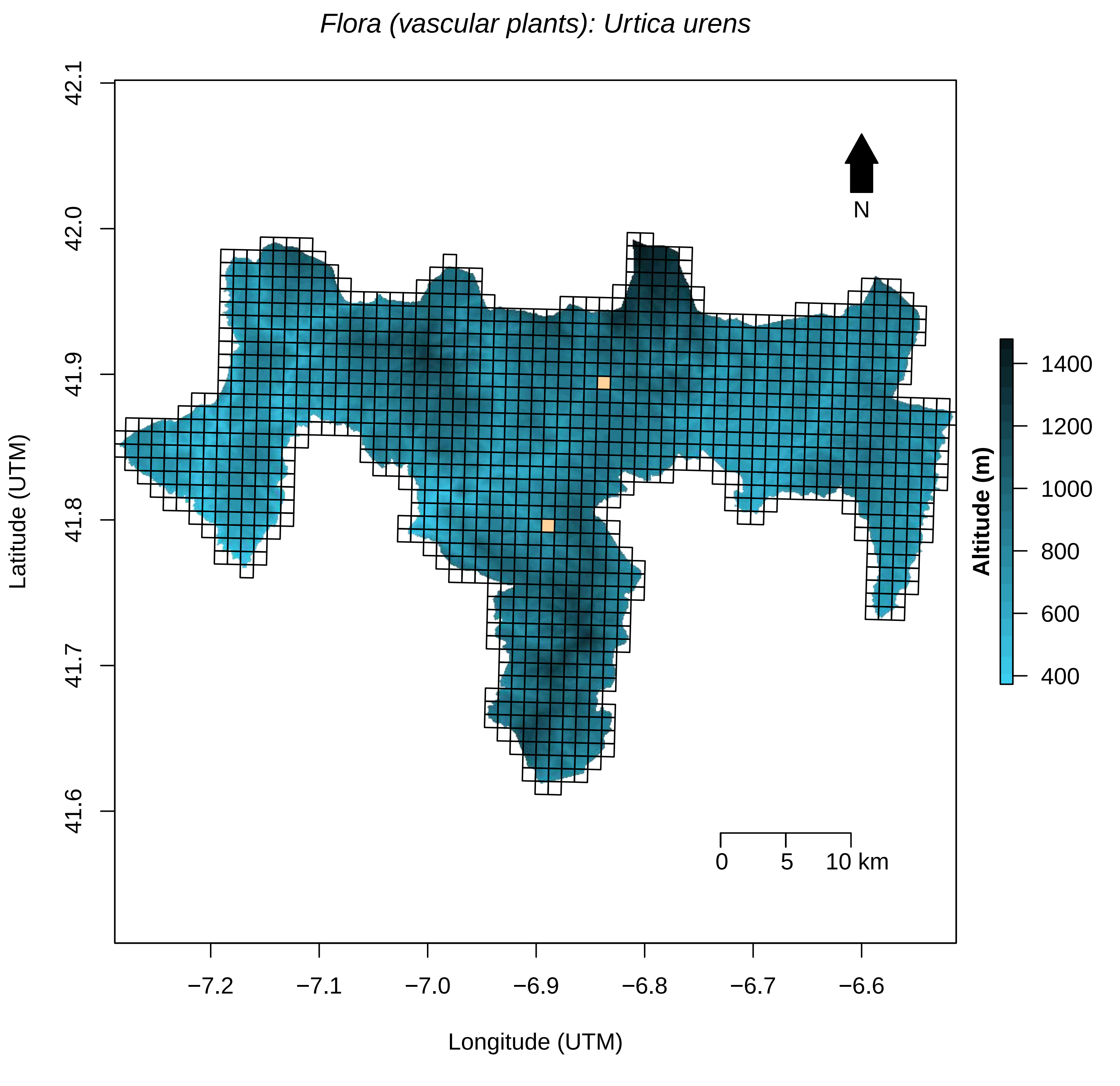

Flora (vascular plants): Urtica urens
42.1
42.0
41.9
41.8
41.7
41.6
Latitude (UTM)
−7.2
−7.1
−7.0
−6.9
−6.8
−6.7
−6.6
Longitude (UTM)
N
0
5
10 km
Altitude (m)
1400
1200
1000
800
600
400

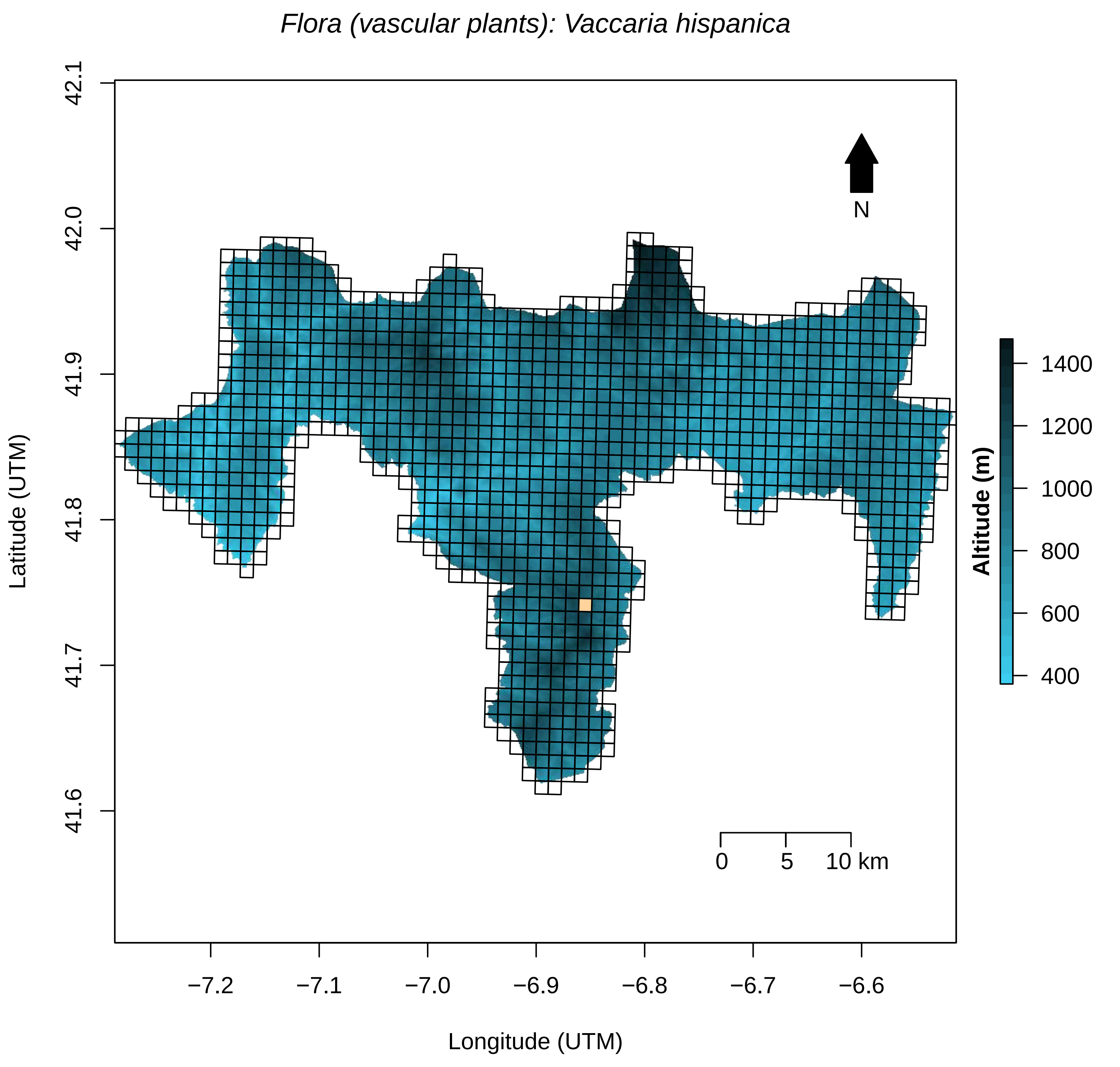

Flora (vascular plants): Vaccaria hispanica
42.1
42.0
41.9
41.8
41.7
41.6
Latitude (UTM)
−7.2
−7.1
−7.0
−6.9
−6.8
−6.7
−6.6
Longitude (UTM)
N
0
5
10 km
Altitude (m)
1400
1200
1000
800
600
400

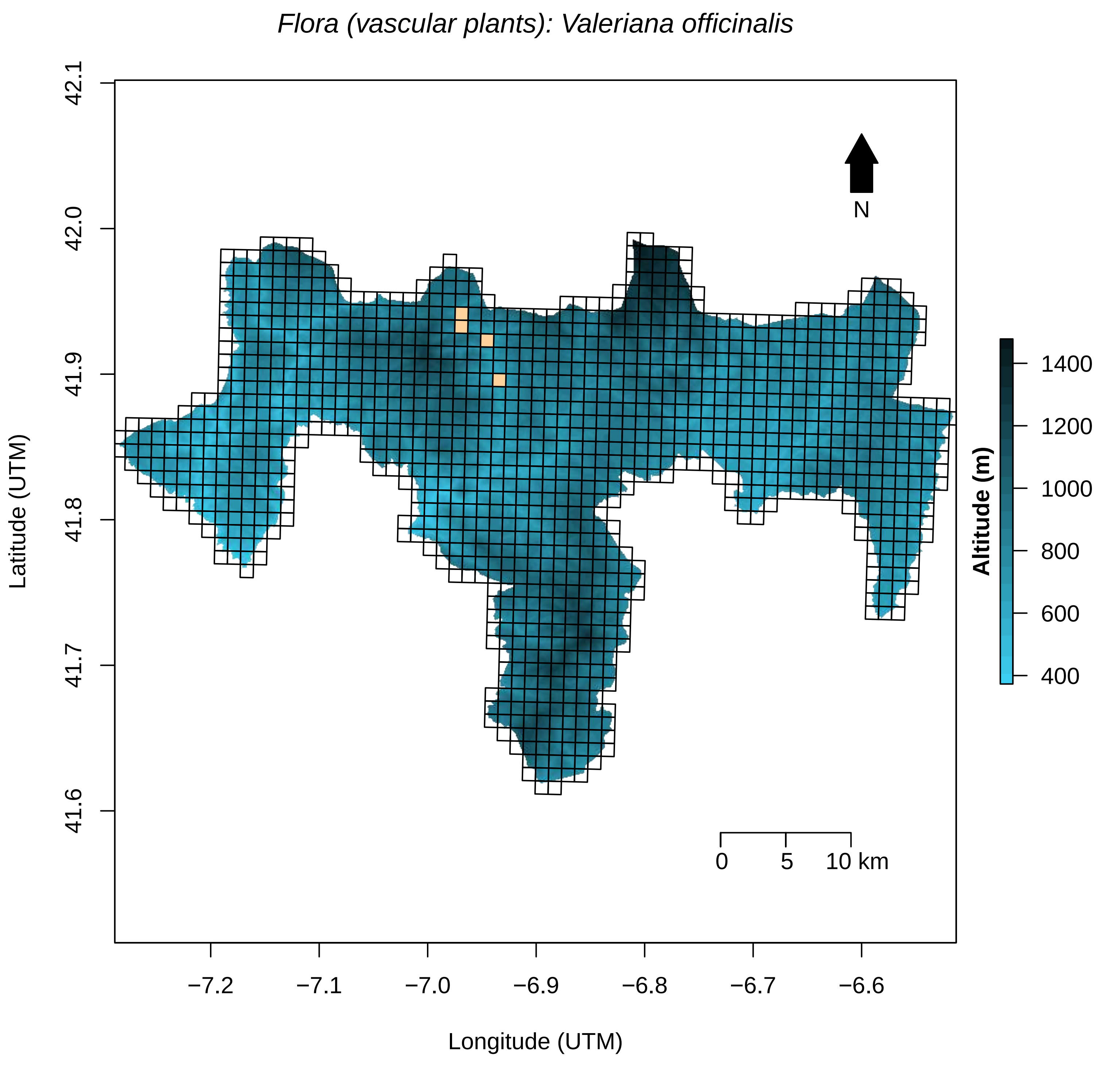

Flora (vascular plants): Valeriana officinalis
42.1
42.0
41.9
41.8
41.7
41.6
Latitude (UTM)
N
0
5
10 km
−7.2
−7.1
−7.0
−6.9
−6.8
−6.7
−6.6
Longitude (UTM)
1400
1200
1000
800
600
400
Altitude (m)

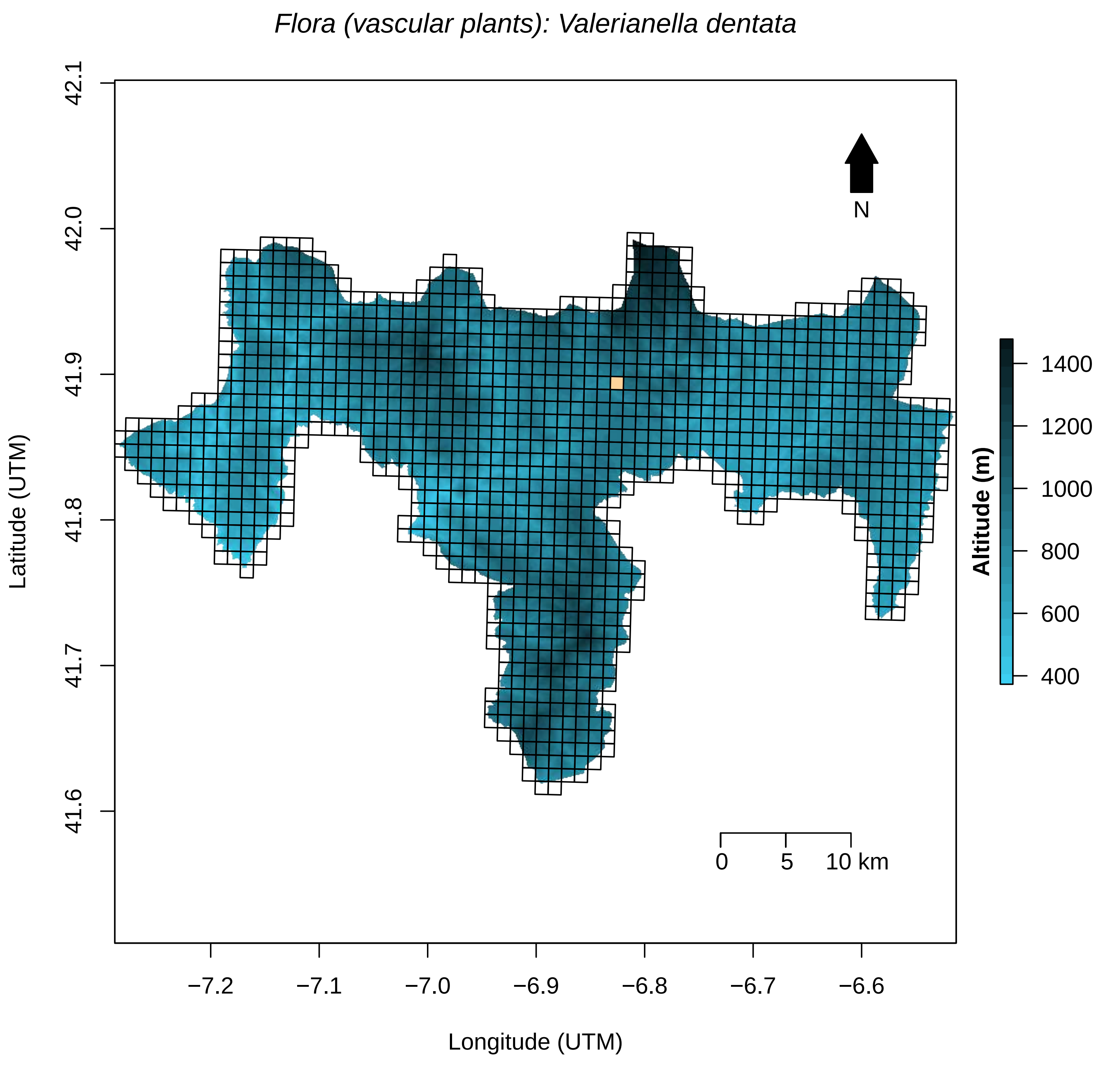

Flora (vascular plants): Valerianella dentata
Latitude (UTM)
42.1
42.0
41.9
41.8
41.7
41.6
Longitude (UTM)
−7.2
−7.1
−7.0
−6.9
−6.8
−6.7
−6.6
N
0
5
10 km
Altitude (m)
1400
1200
1000
800
600
400

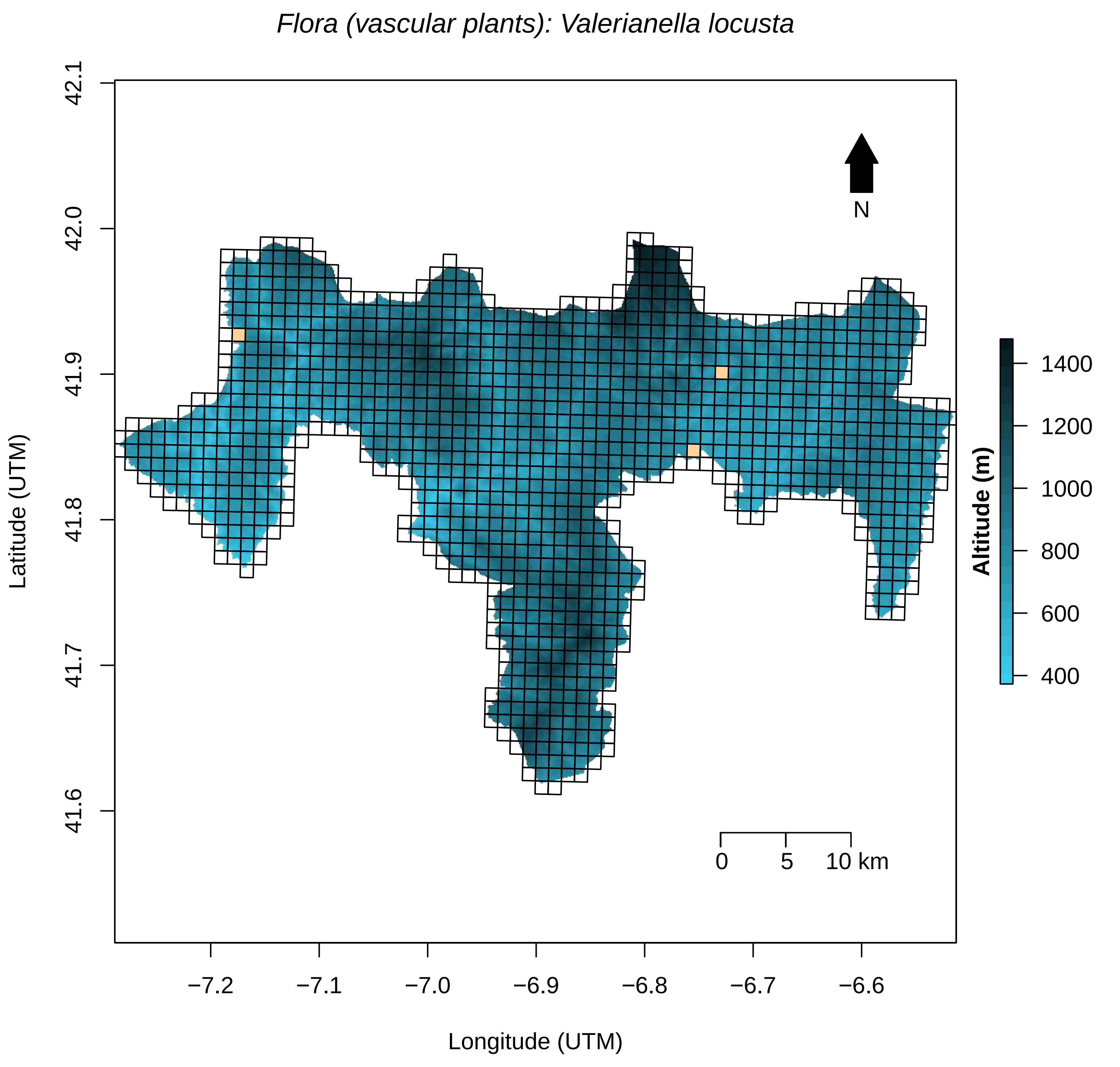

Flora (vascular plants): Valerianella locusta
Latitude (UTM)
Longitude (UTM)
42.1
42.0
41.9
41.8
41.7
41.6
−7.2
−7.1
−7.0
−6.9
−6.8
−6.7
−6.6
N
0
5
10 km
Altitude (m)
1400
1200
1000
800
600
400

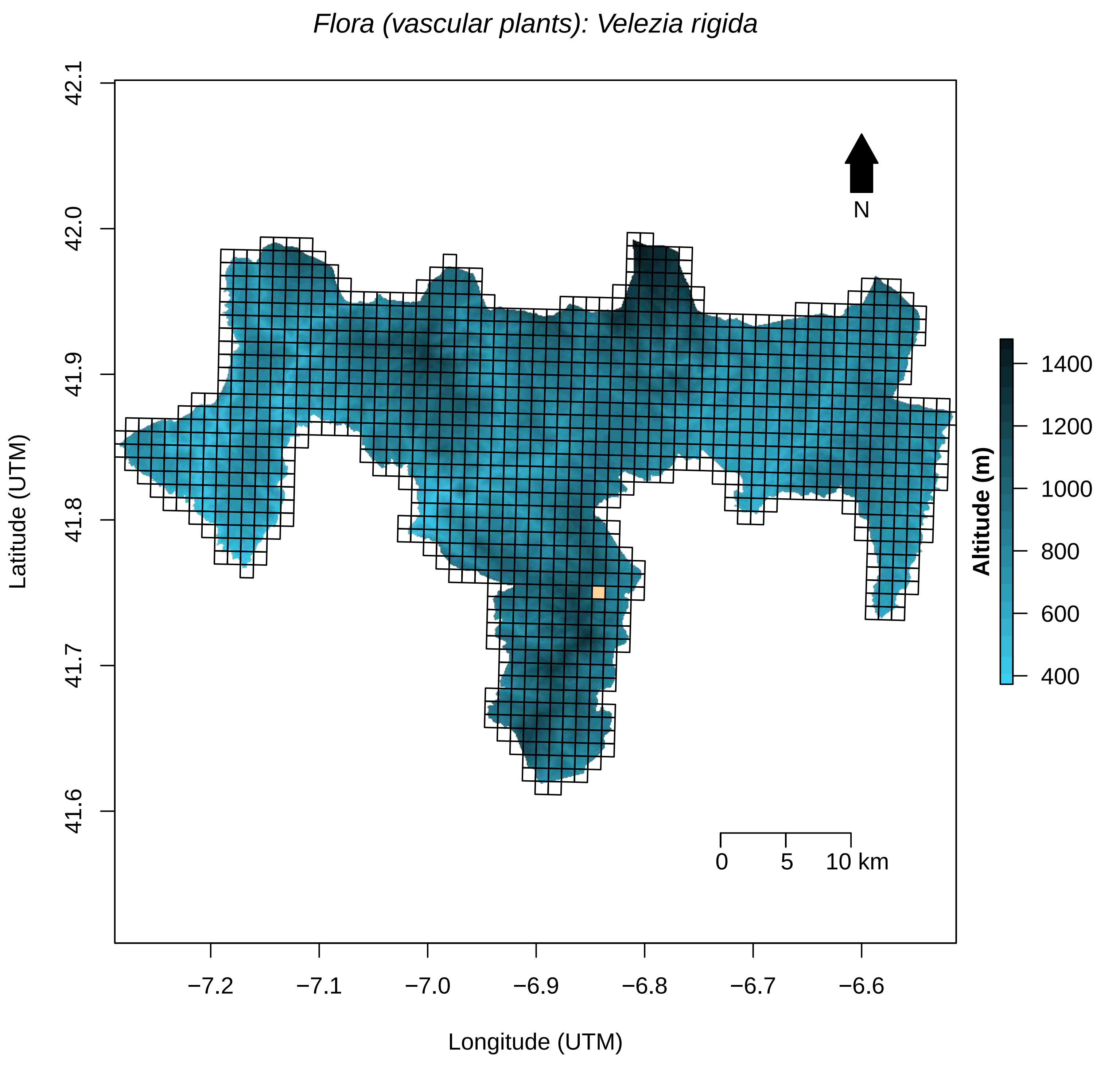
Flora (vascular plants): Velezia rigida
Latitude (UTM)
Longitude (UTM)
42.1
42.0
41.9
41.8
41.7
41.6
−7.2
−7.1
−7.0
−6.9
−6.8
−6.7
−6.6
N
0
5
10 km
Altitude (m)
1400
1200
1000
800
600
400

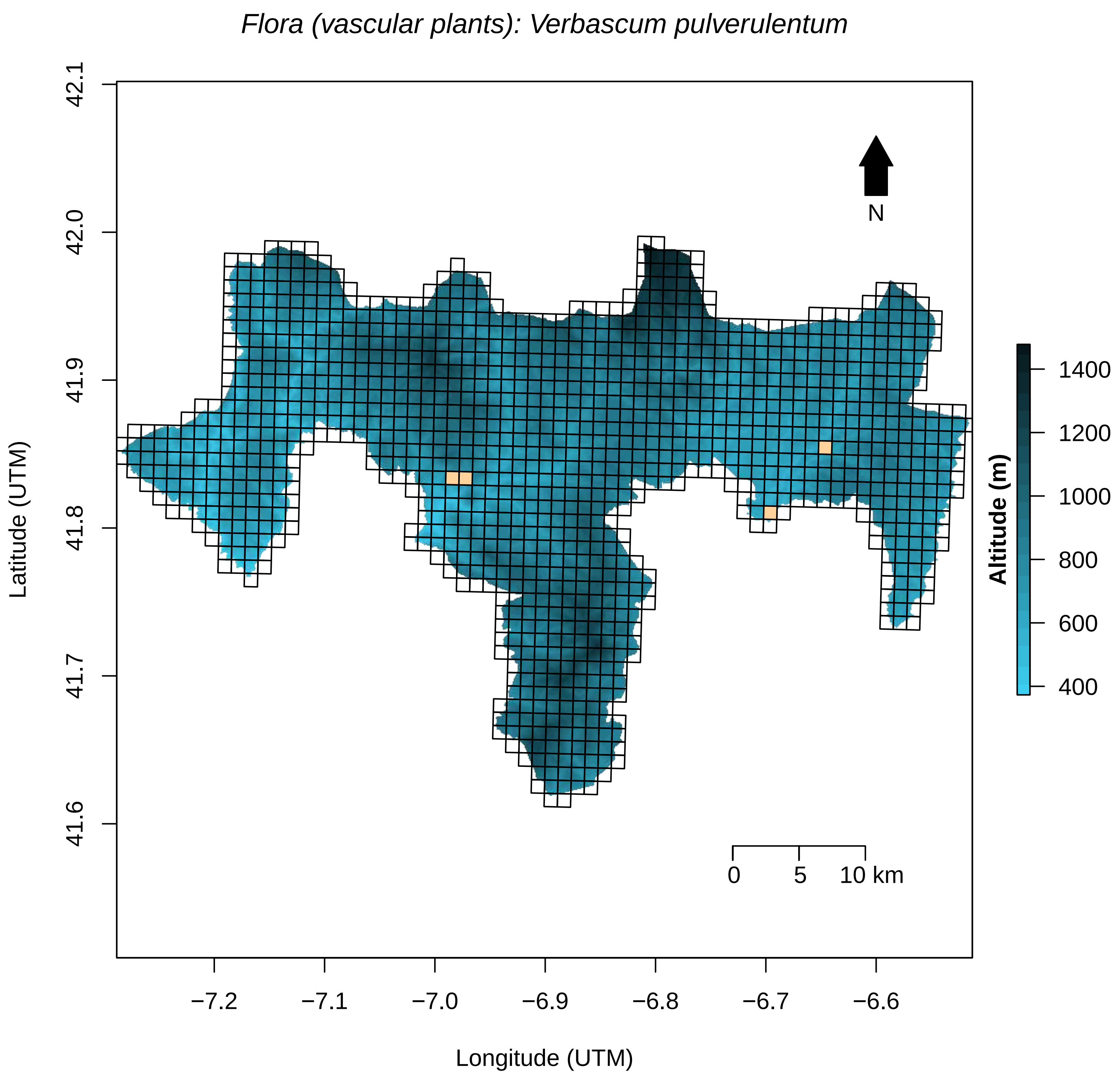
Flora (vascular plants): Verbascum pulverulentum
42.1
42.0
41.9
41.8
41.7
41.6
Latitude (UTM)
−7.2
−7.1
−7.0
−6.9
−6.8
−6.7
−6.6
Longitude (UTM)
N
0
5
10 km
Altitude (m)
1400
1200
1000
800
600
400

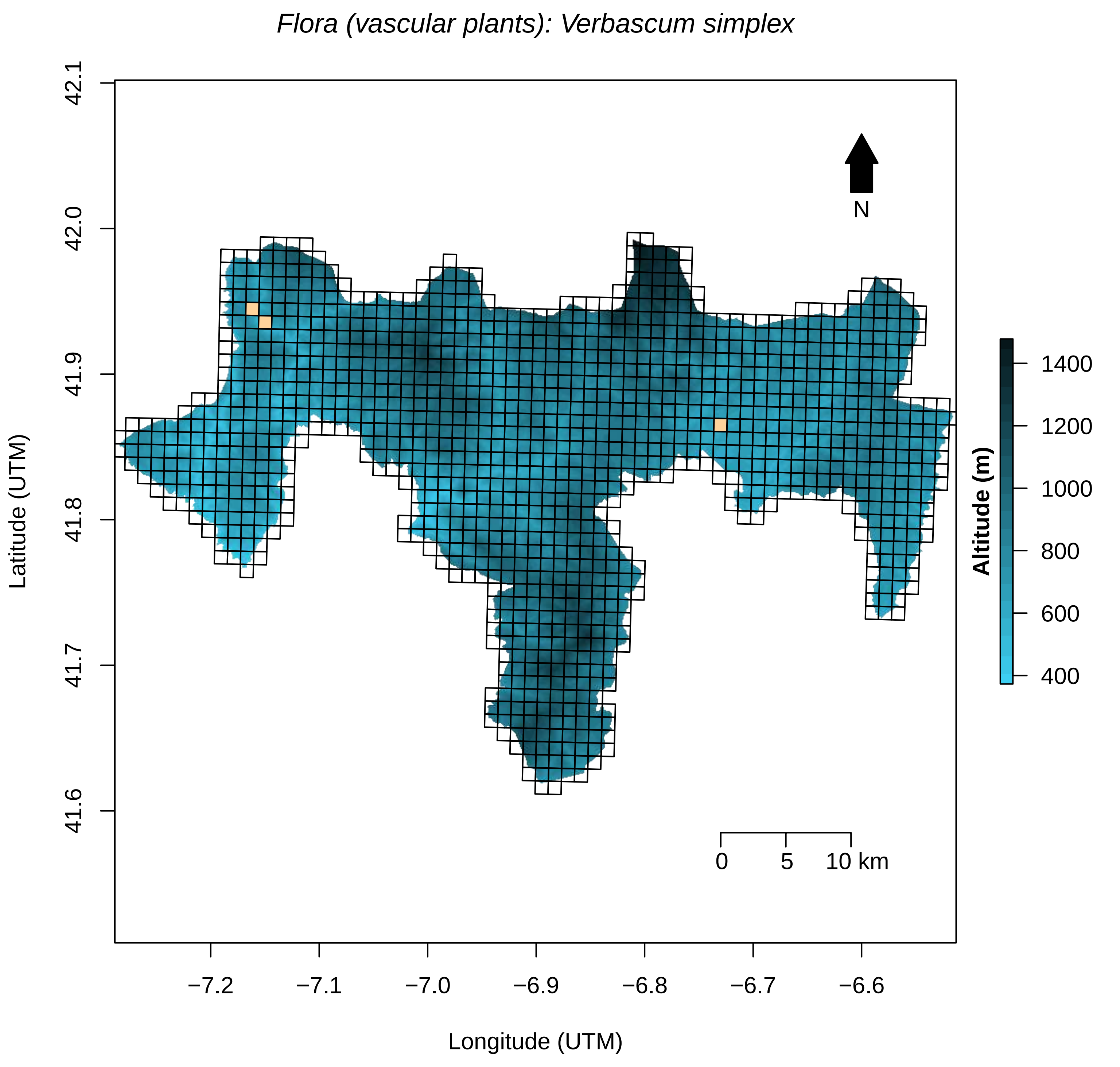
Flora (vascular plants): Verbascum simplex
42.1
42.0
41.9
41.8
41.7
41.6
Latitude (UTM)
−7.2
−7.1
−7.0
−6.9
−6.8
−6.7
−6.6
Longitude (UTM)
N
0
5
10 km
Altitude (m)
1400
1200
1000
800
600
400

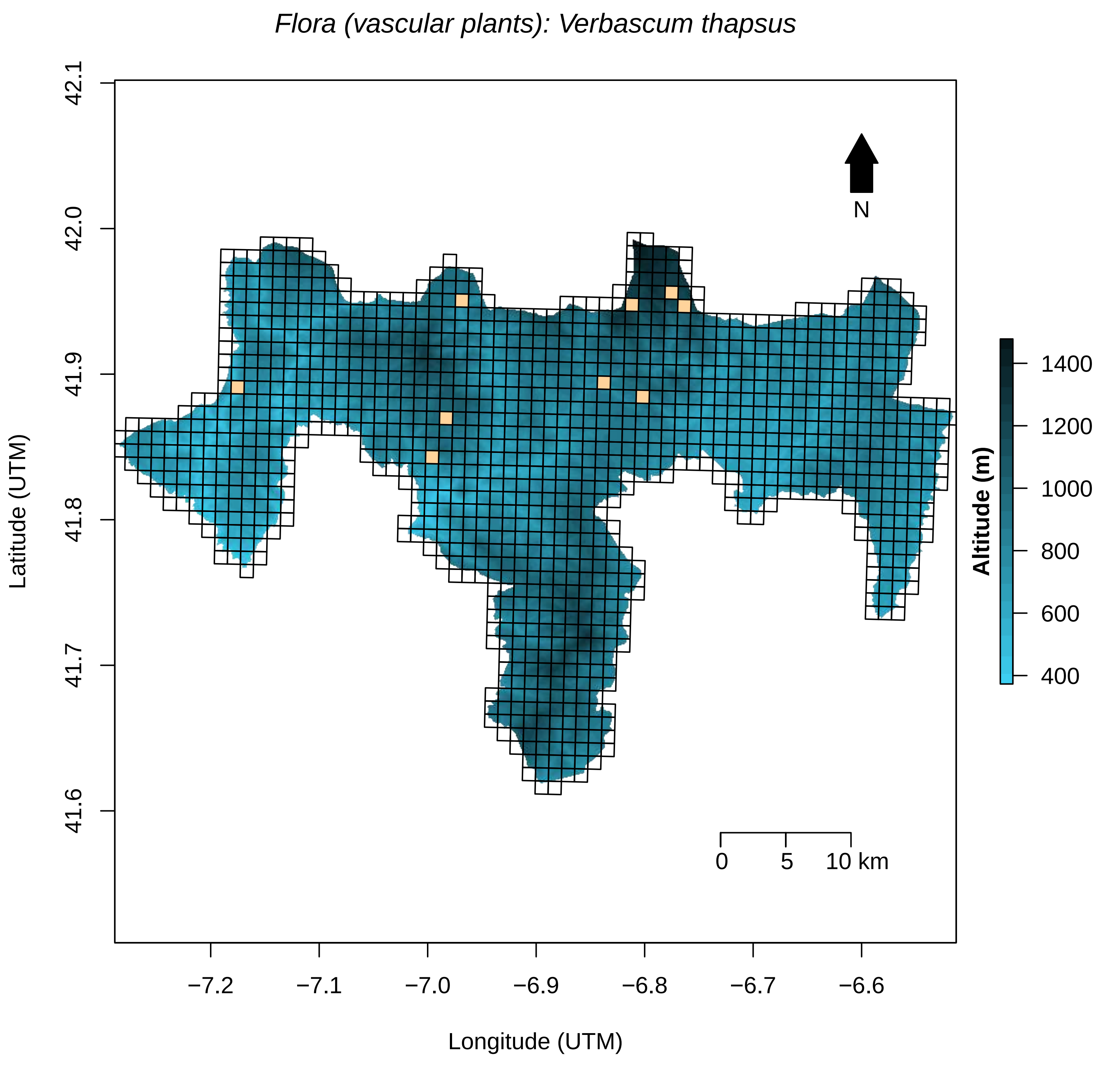
Flora (vascular plants): Verbascum thapsus
Latitude (UTM)
Longitude (UTM)
42.1
42.0
41.9
41.8
41.7
41.6
−7.2
−7.1
−7.0
−6.9
−6.8
−6.7
−6.6
N
0
5
10 km
Altitude (m)
1400
1200
1000
800
600
400

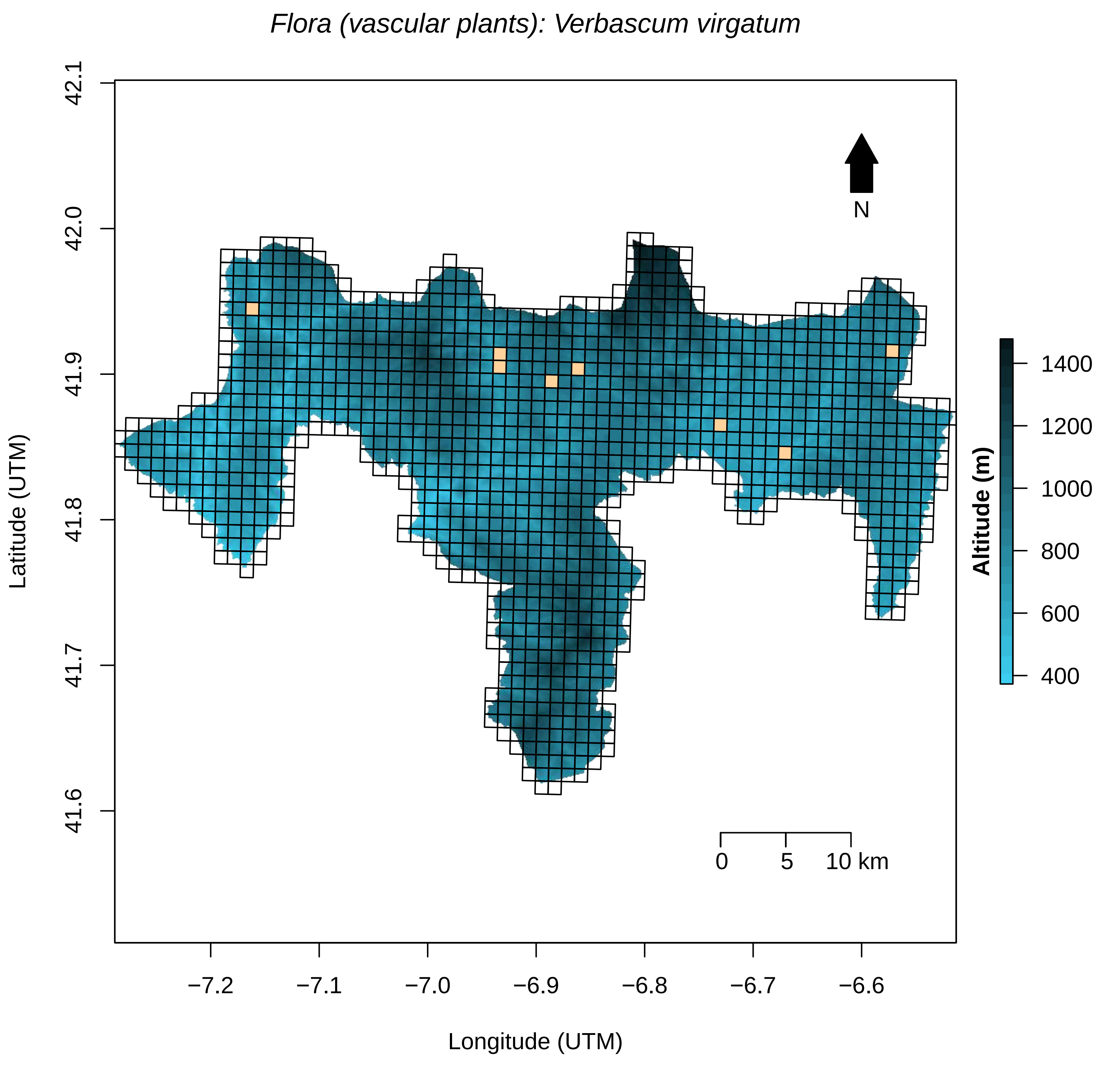
Flora (vascular plants): Verbascum virgatum
Latitude (UTM)
42.1
42.0
41.9
41.8
41.7
41.6
Longitude (UTM)
−7.2
−7.1
−7.0
−6.9
−6.8
−6.7
−6.6
N
0
5
10 km
Altitude (m)
1400
1200
1000
800
600
400

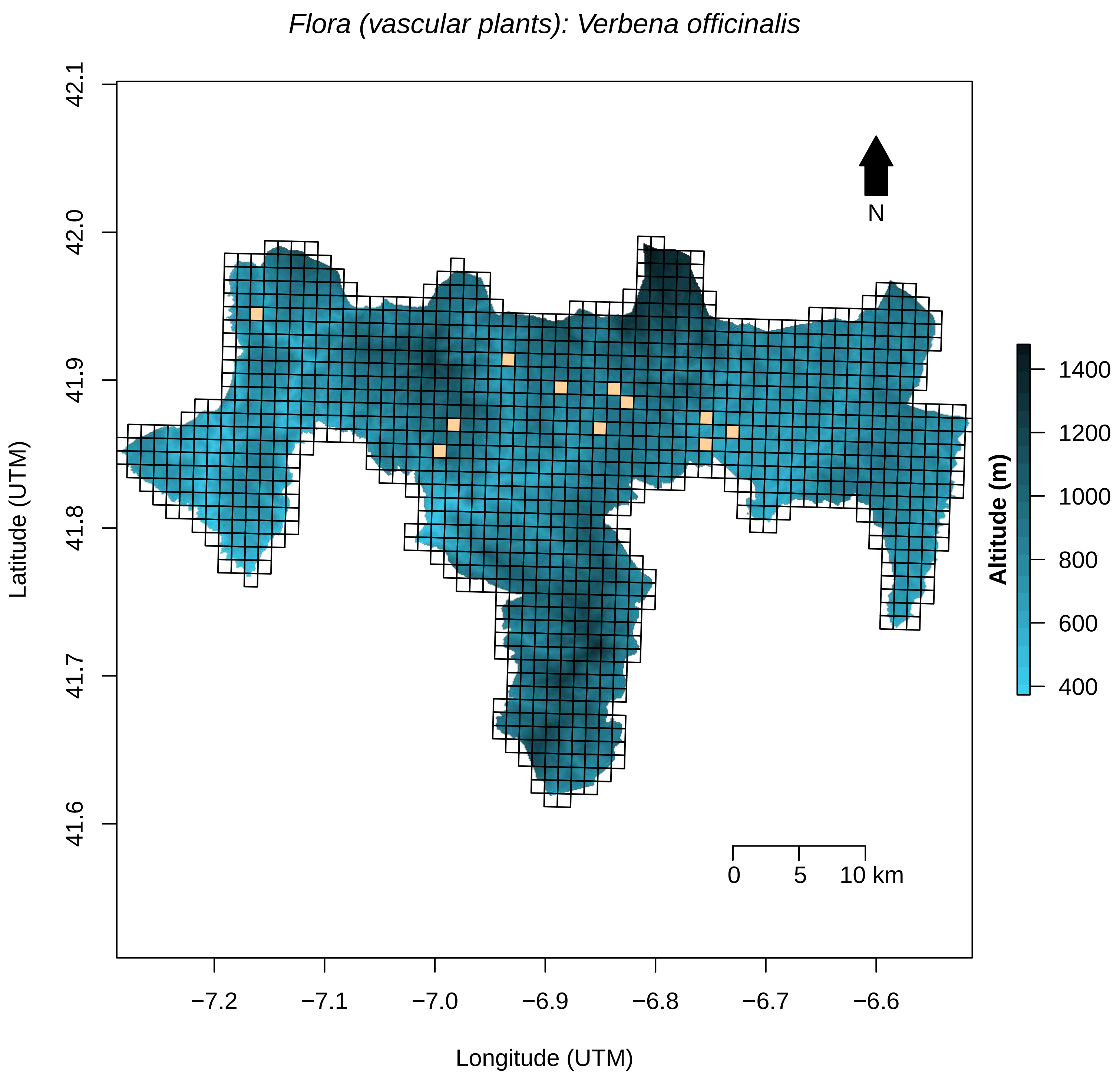

Flora (vascular plants): Verbena officinalis
Latitude (UTM)
42.1
42.0
41.9
41.8
41.7
41.6
Longitude (UTM)
−7.2
−7.1
−7.0
−6.9
−6.8
−6.7
−6.6
N
0
5
10 km
Altitude (m)
1400
1200
1000
800
600
400

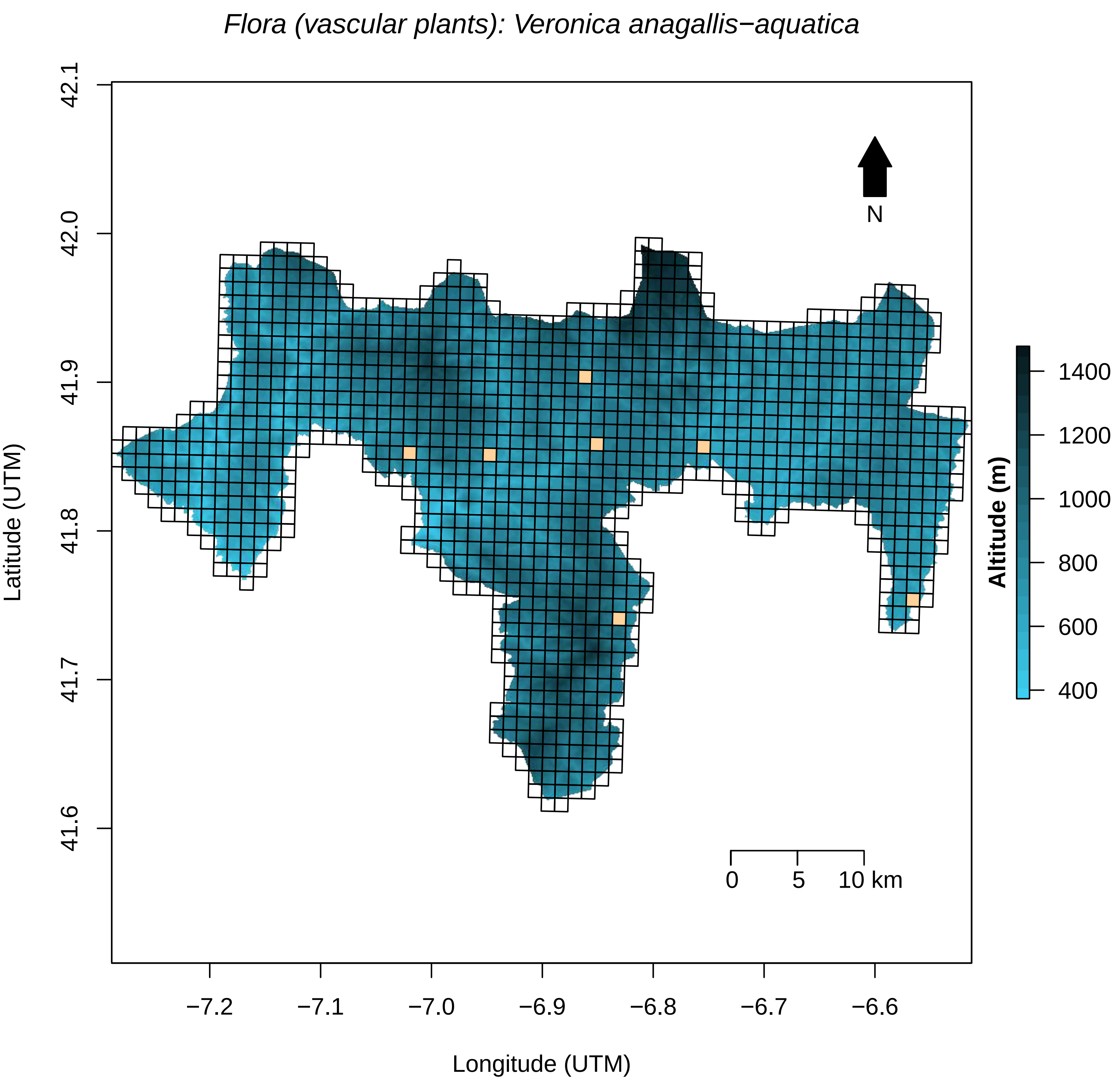

Flora (vascular plants): Veronica anagallis−aquatica
42.1
42.0
41.9
41.8
41.7
41.6
Latitude (UTM)
−7.2
−7.1
−7.0
−6.9
−6.8
−6.7
−6.6
Longitude (UTM)
N
0
5
10 km
Altitude (m)
1400
1200
1000
800
600
400

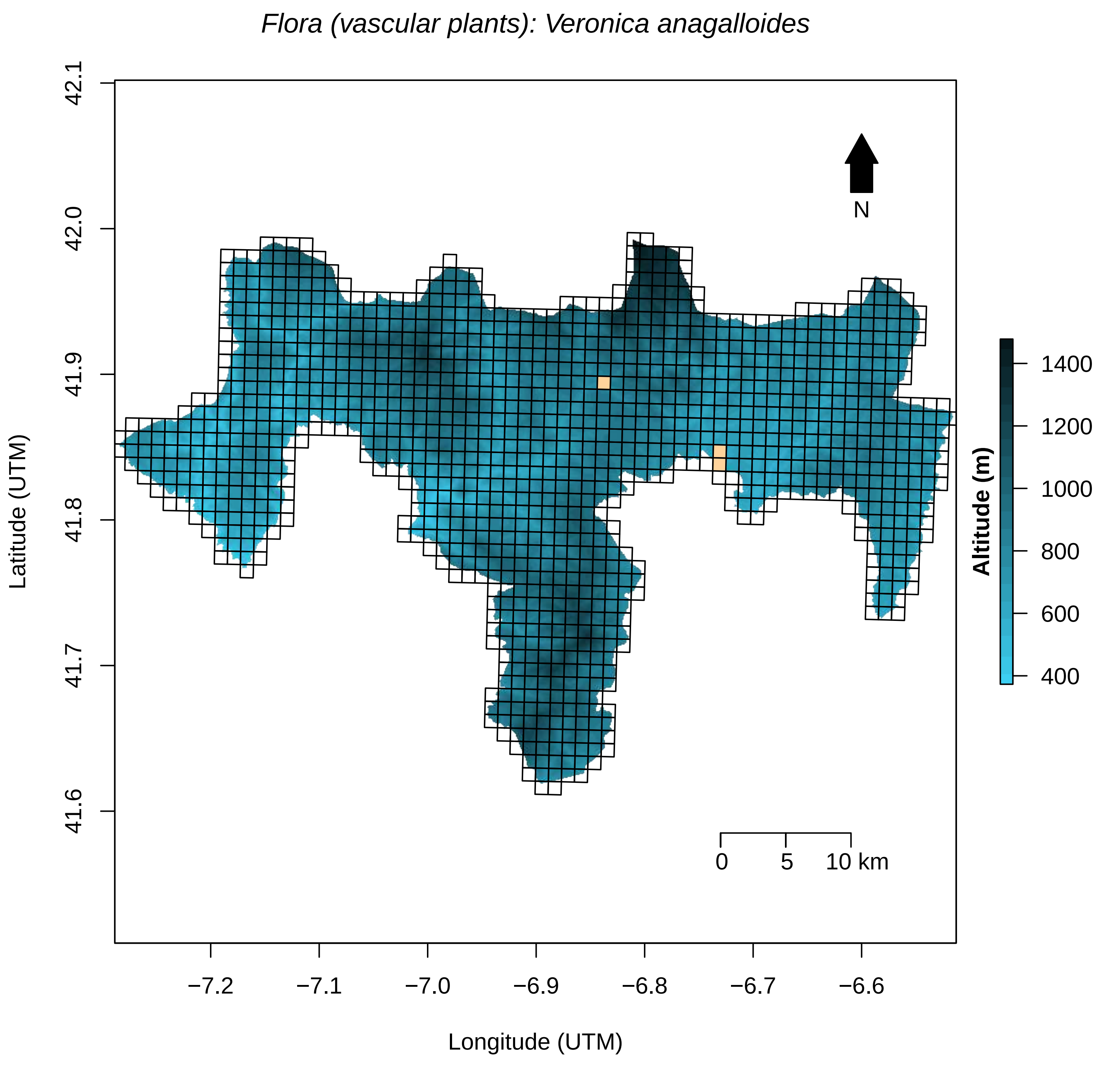

Flora (vascular plants): Veronica anagalloides
Latitude (UTM)
42.1
42.0
41.9
41.8
41.7
41.6
N
0
5
10 km
Altitude (m)
1400
1200
1000
800
600
400
−7.2
−7.1
−7.0
−6.9
−6.8
−6.7
−6.6
Longitude (UTM)

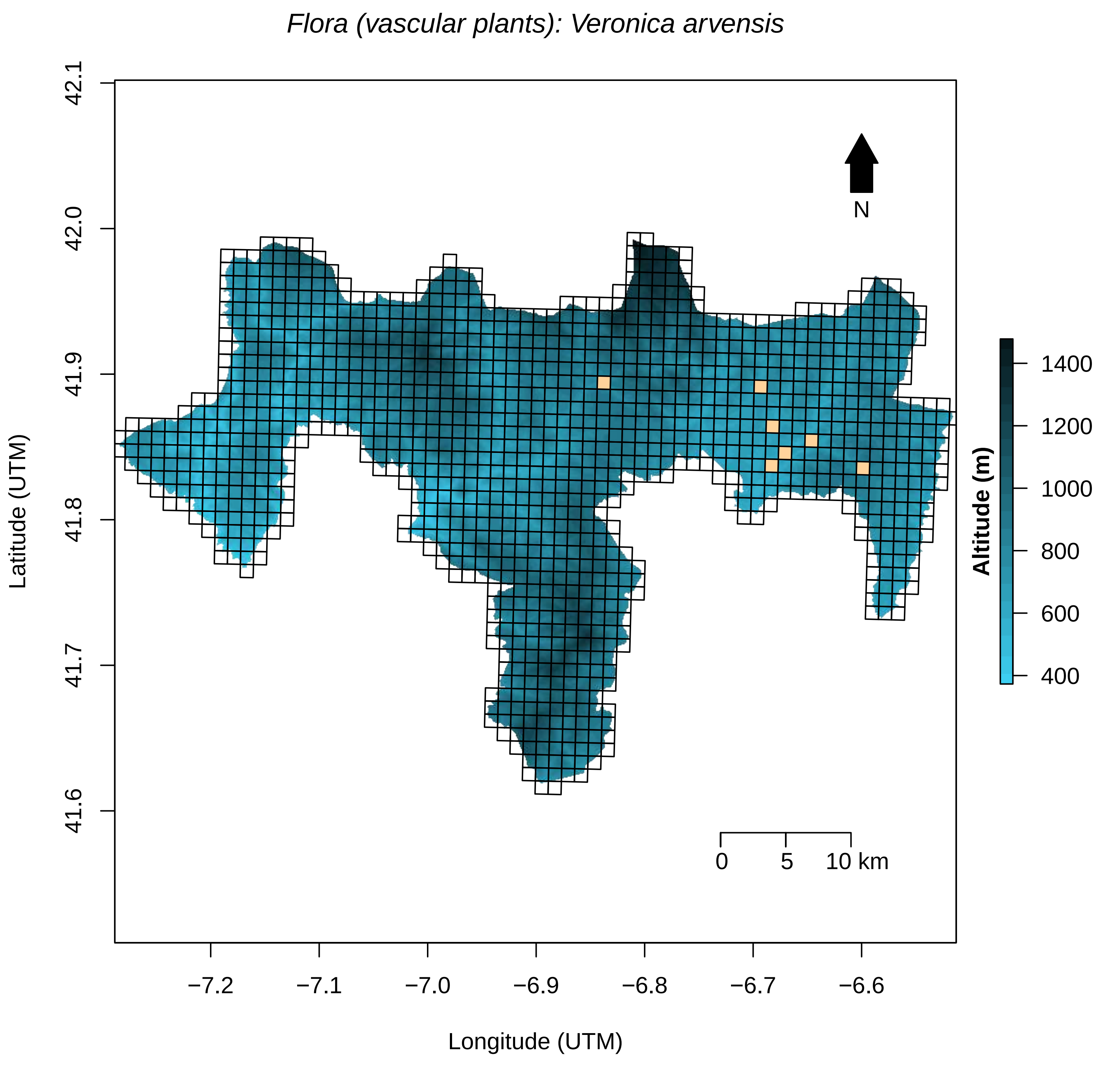
Flora (vascular plants): Veronica arvensis
Latitude (UTM)
Longitude (UTM)
42.1
42.0
41.9
41.8
41.7
41.6
−7.2
−7.1
−7.0
−6.9
−6.8
−6.7
−6.6
N
0
5
10 km
Altitude (m)
1400
1200
1000
800
600
400

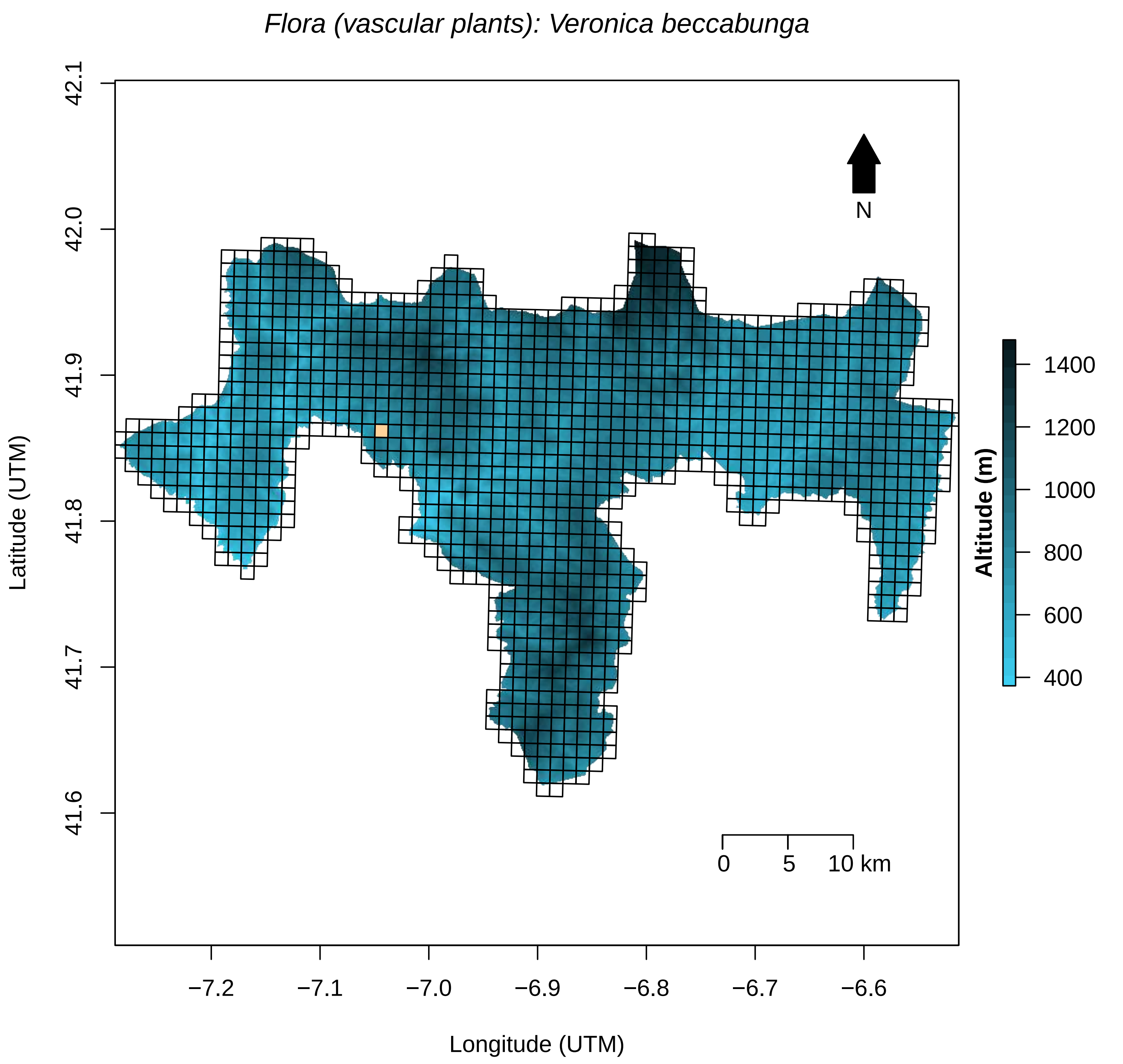

Flora (vascular plants): Veronica beccabunga
Latitude (UTM)
42.1
42.0
41.9
41.8
41.7
41.6
Longitude (UTM)
−7.2
−7.1
−7.0
−6.9
−6.8
−6.7
−6.6
N
0
5
10 km
Altitude (m)
1400
1200
1000
800
600
400

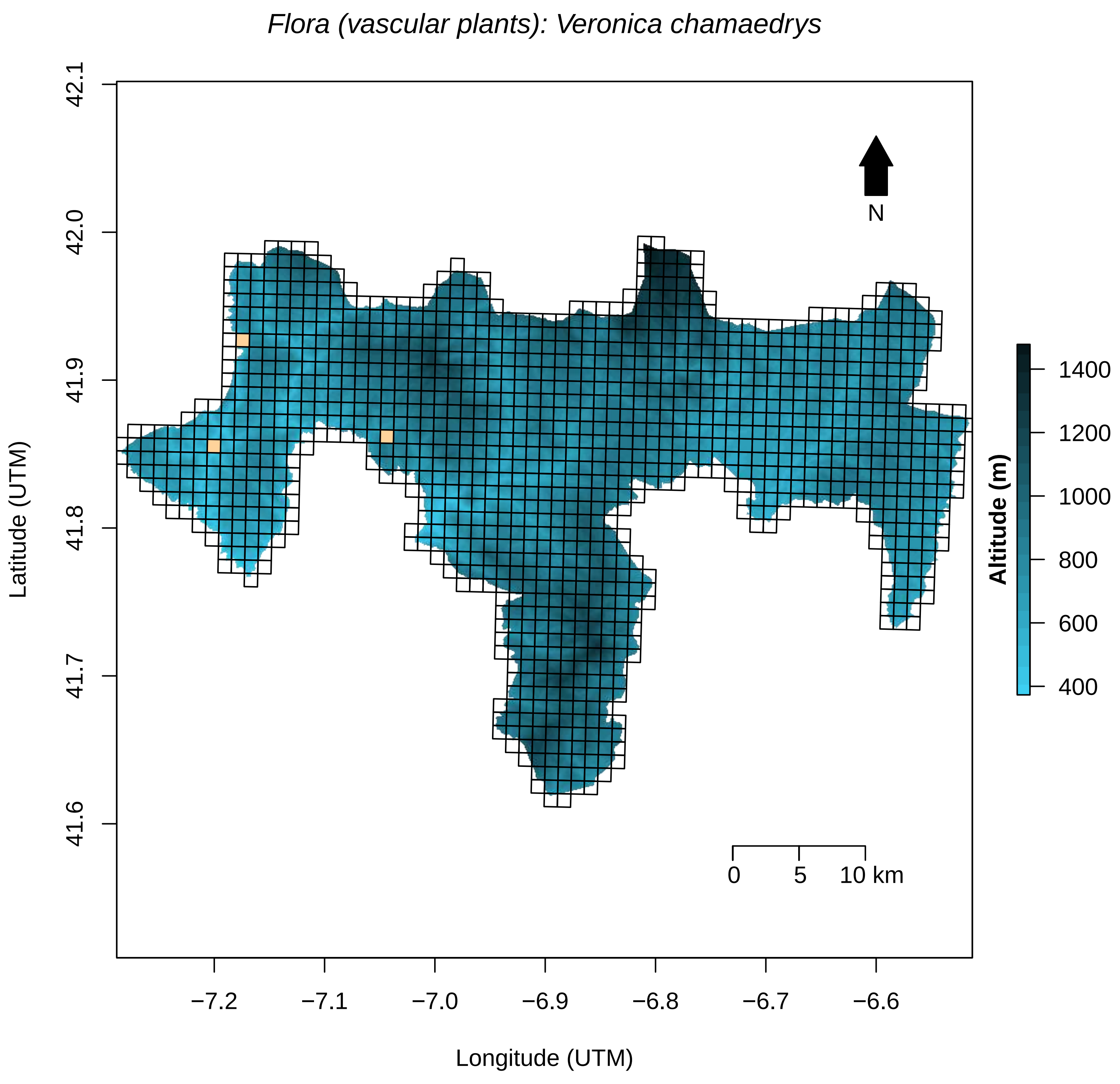

Flora (vascular plants): Veronica chamaedrys
Latitude (UTM)
Longitude (UTM)
42.1
42.0
41.9
41.8
41.7
41.6
−7.2
−7.1
−7.0
−6.9
−6.8
−6.7
−6.6
N
0
5
10 km
Altitude (m)
1400
1200
1000
800
600
400

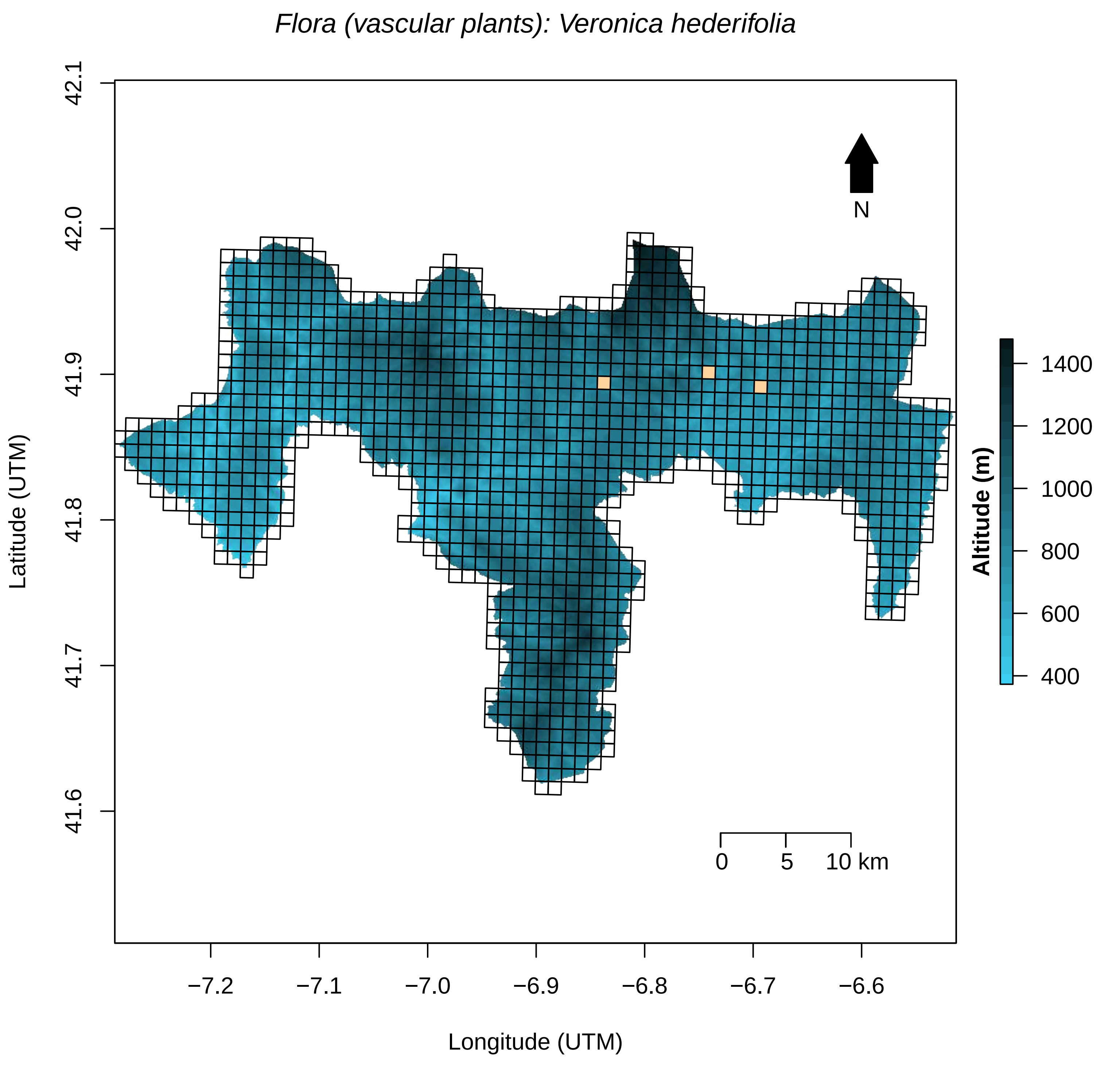

Flora (vascular plants): Veronica hederifolia
N
0
5
10 km
Altitude (m)
1400
1200
1000
800
600
400
42.1
42.0
41.9
41.8
41.7
41.6
Latitude (UTM)
−7.2
−7.1
−7.0
−6.9
−6.8
−6.7
−6.6
Longitude (UTM)

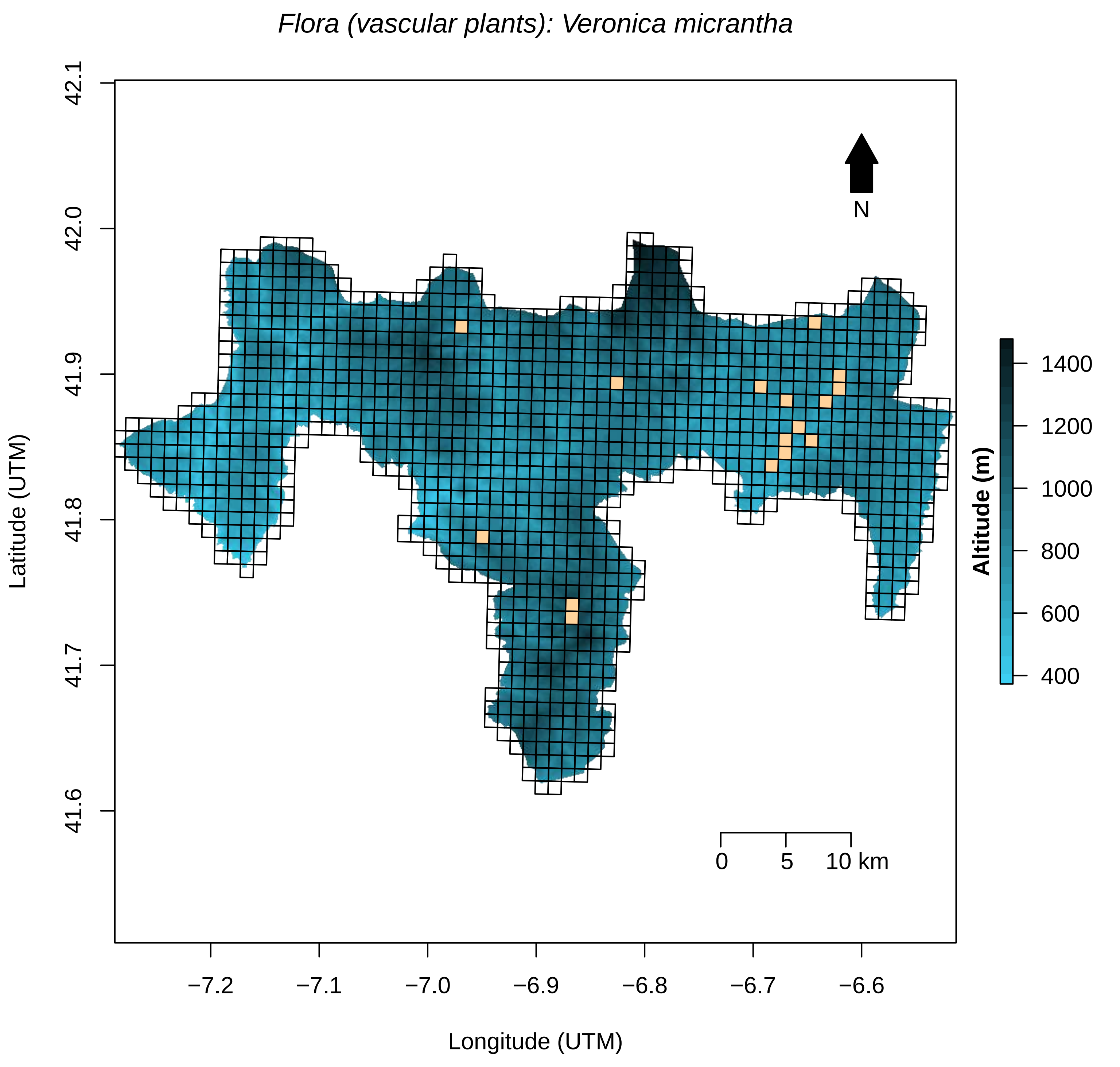

Flora (vascular plants): Veronica micrantha
42.1
42.0
41.9
41.8
41.7
41.6
Latitude (UTM)
−7.2
−7.1
−7.0
−6.9
−6.8
−6.7
−6.6
Longitude (UTM)
N
0
5
10 km
Altitude (m)
1400
1200
1000
800
600
400

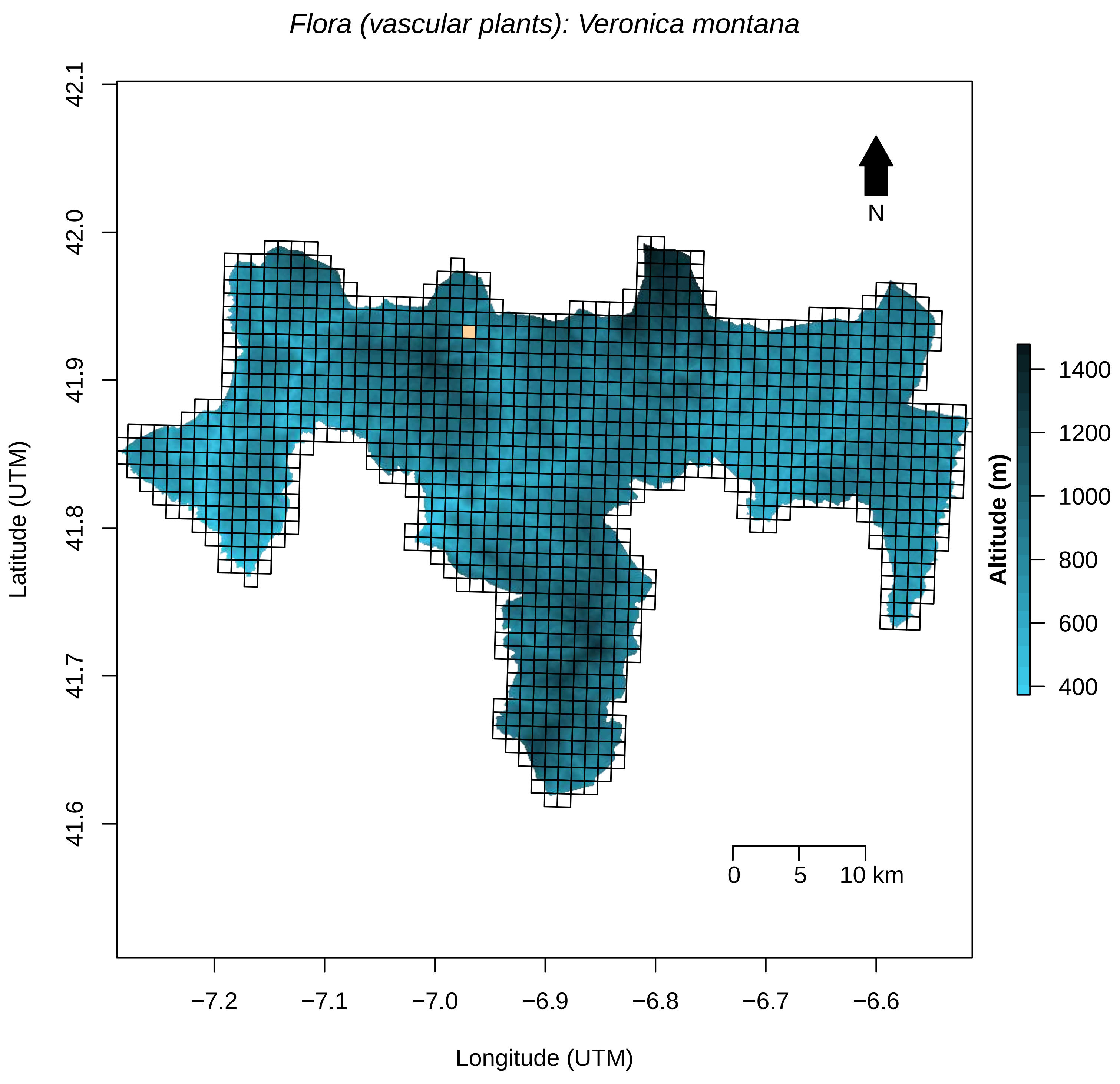

Flora (vascular plants): Veronica montana
42.1
42.0
41.9
41.8
41.7
41.6
Latitude (UTM)
−7.2
−7.1
−7.0
−6.9
−6.8
−6.7
−6.6
Longitude (UTM)
N
0
5
10 km
Altitude (m)
1400
1200
1000
800
600
400

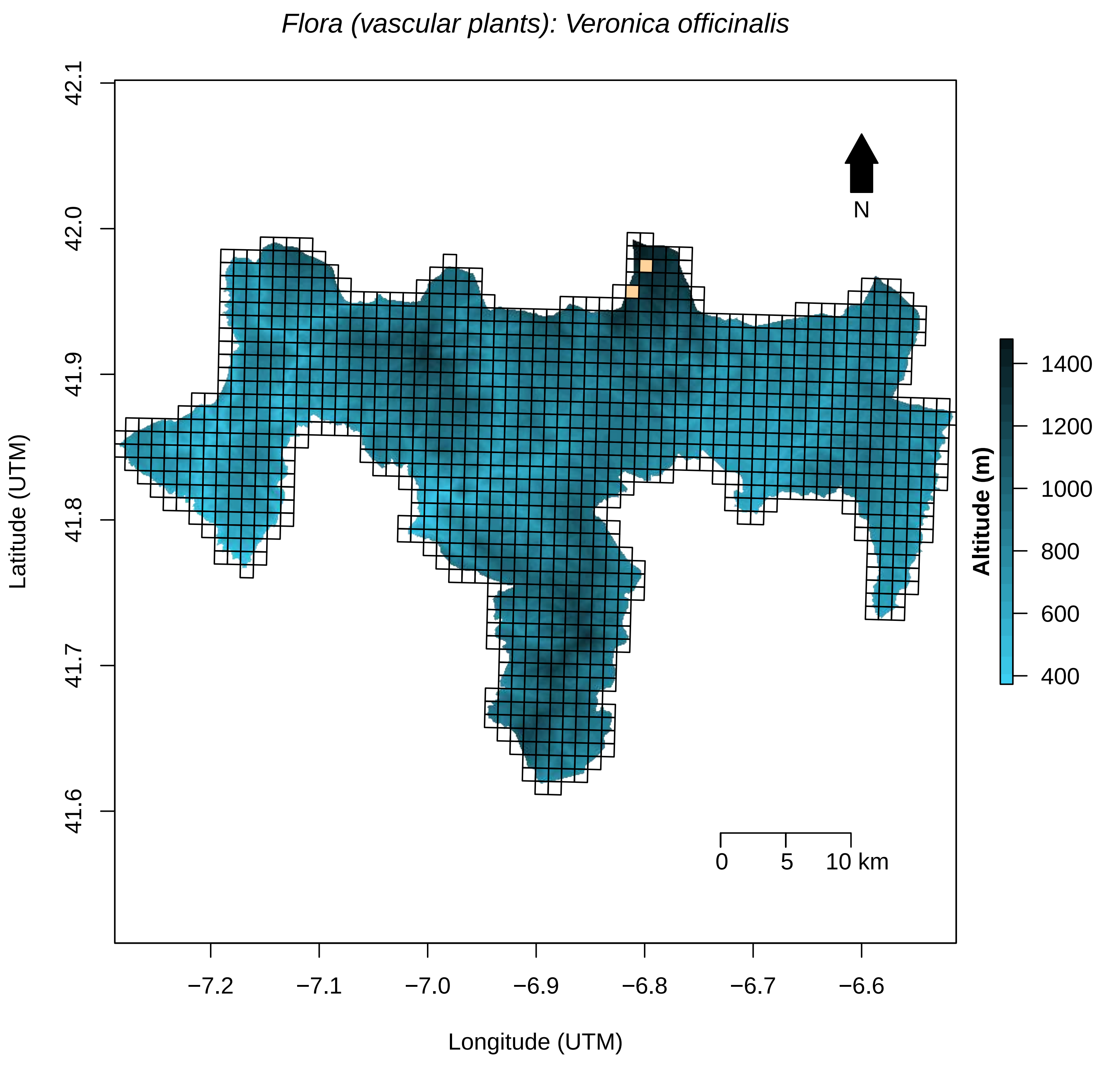

Flora (vascular plants): Veronica officinalis
Latitude (UTM)
Longitude (UTM)
Altitude (m)
N
0
5
10 km
42.1
42.0
41.9
41.8
41.7
41.6
−7.2
−7.1
−7.0
−6.9
−6.8
−6.7
−6.6
1400
1200
1000
800
600
400

Flora (vascular plants): Veronica peregrina

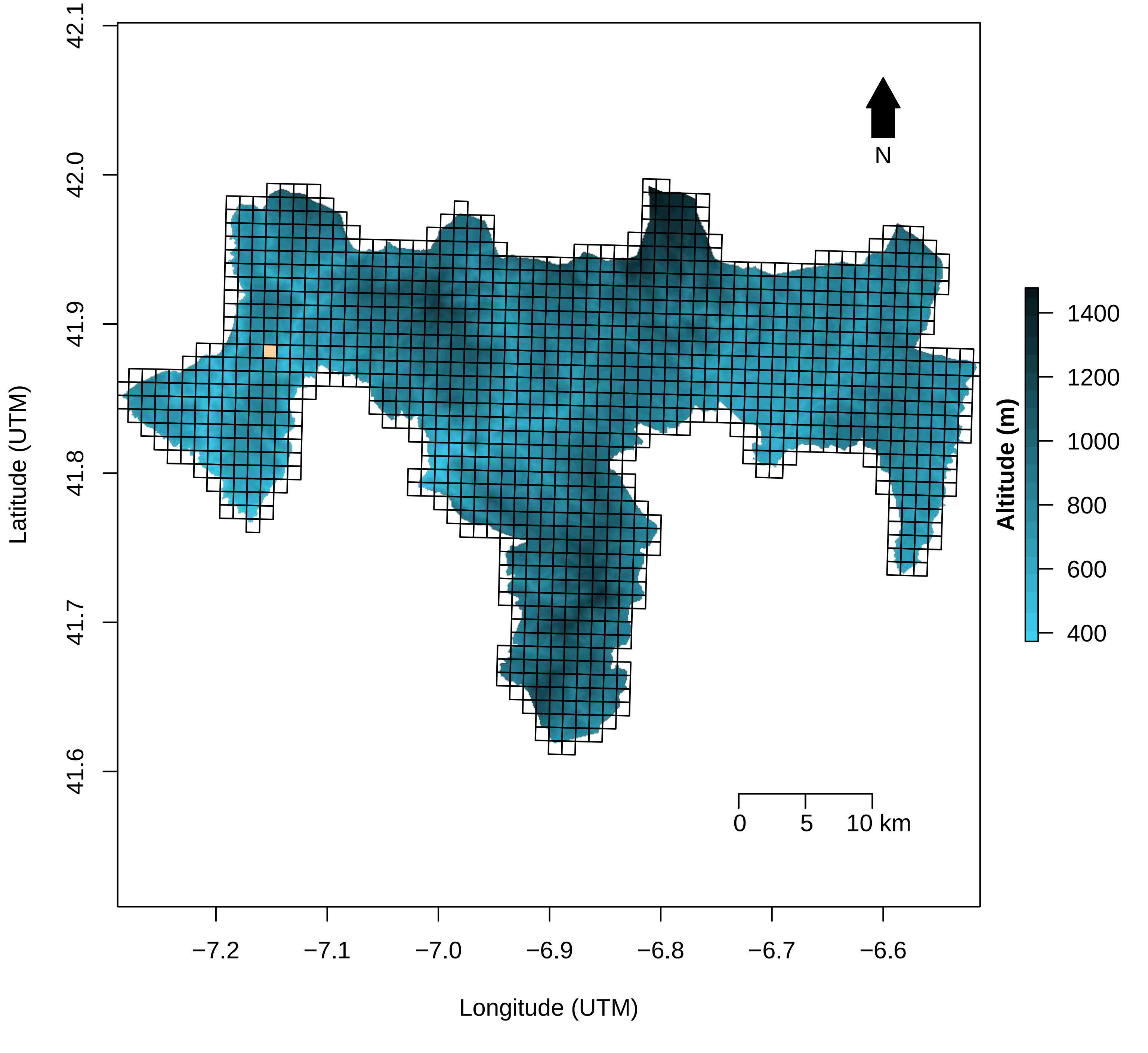

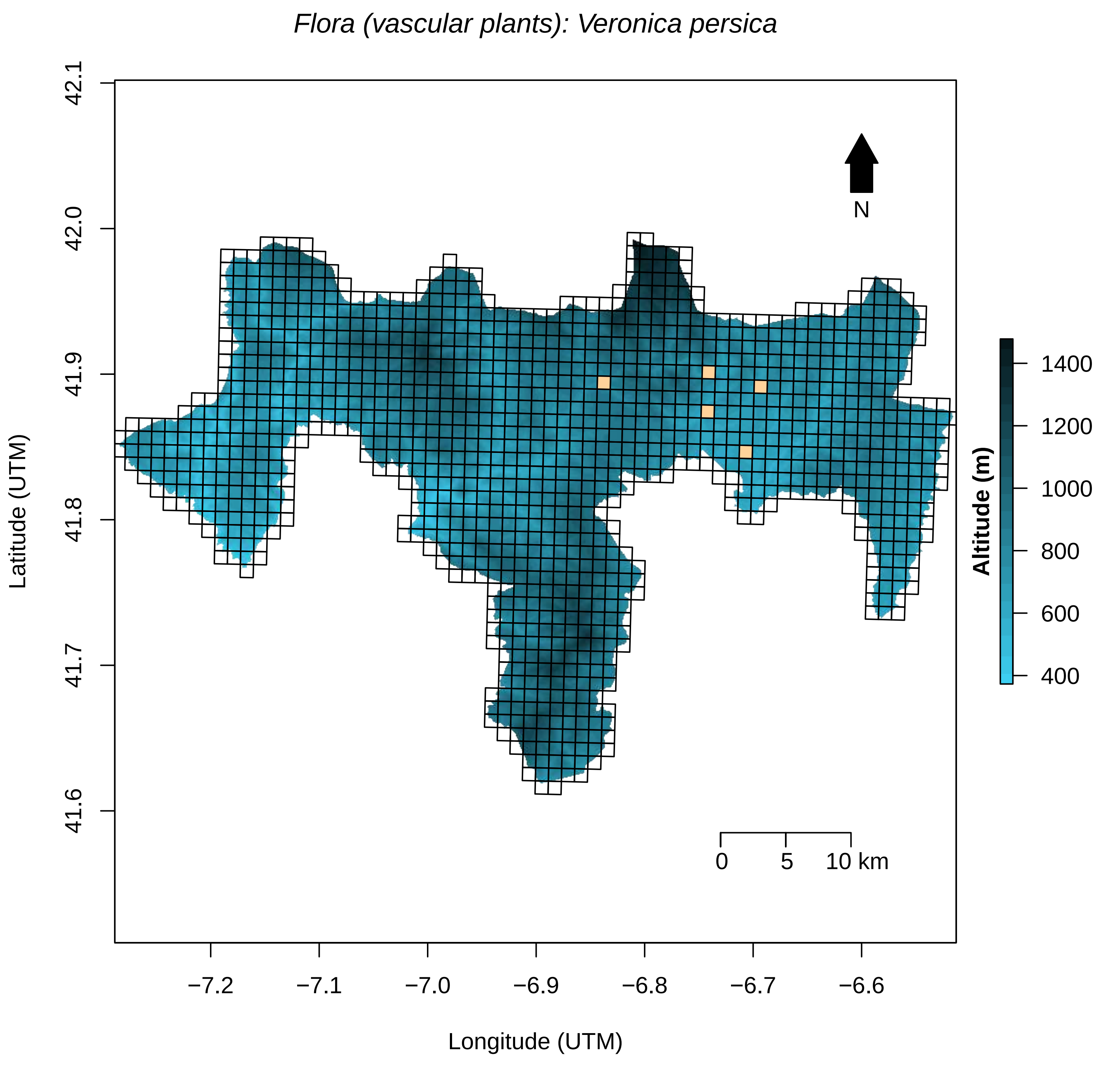

Flora (vascular plants): Veronica persica
N
0 5 10 km
Altitude (m)
1400
1200
1000
800
600
400
Latitude (UTM)
42.1
42.0
41.9
41.8
41.7
41.6
Longitude (UTM)
−7.2
−7.1
−7.0
−6.9
−6.8
−6.7
−6.6

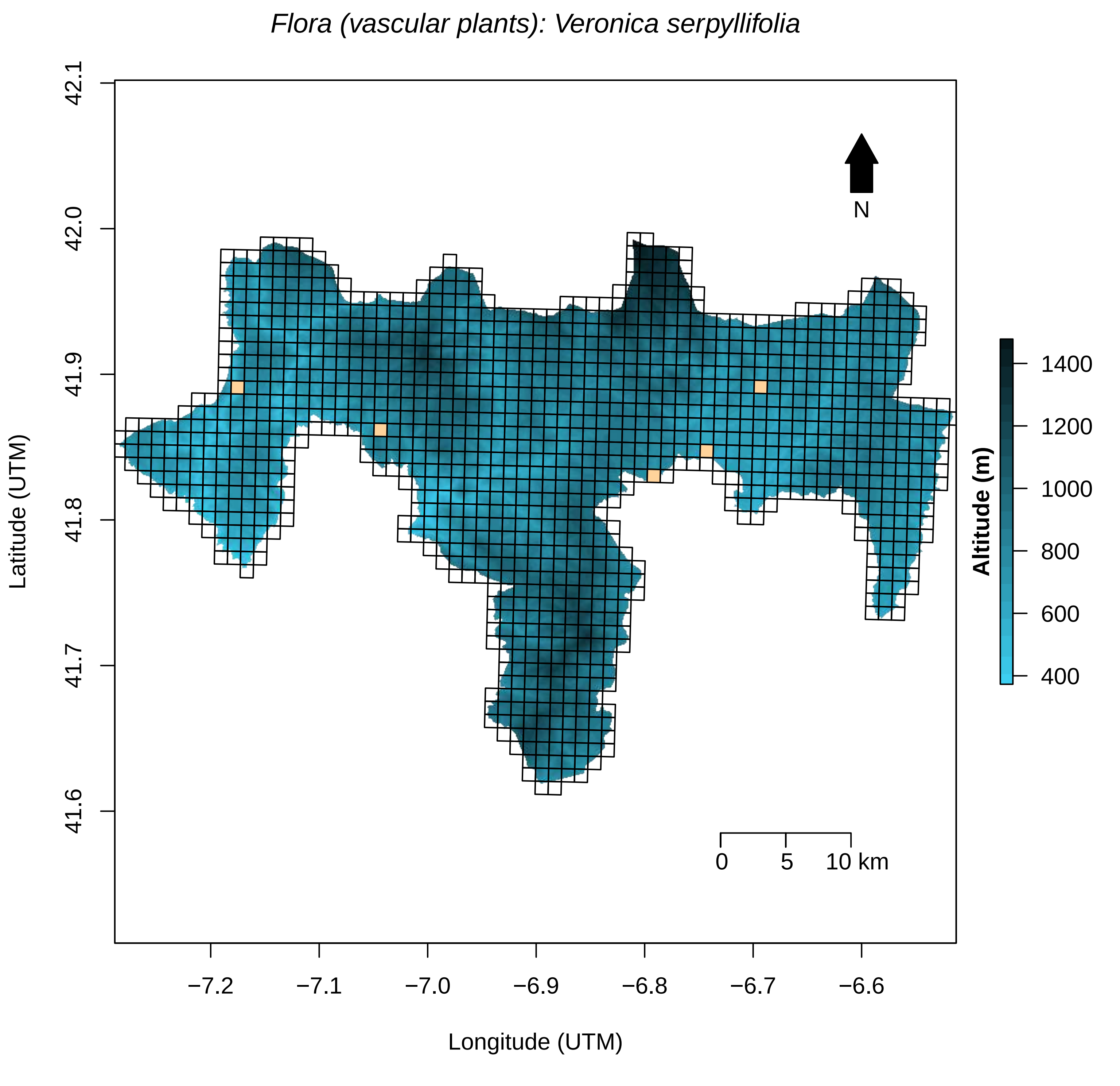

Flora (vascular plants): Veronica serpyllifolia
42.1
42.0
41.9
41.8
41.7
41.6
Latitude (UTM)
N
0
5
10 km
1400
1200
1000
800
600
400
Altitude (m)
−7.2
−7.1
−7.0
−6.9
−6.8
−6.7
−6.6
Longitude (UTM)

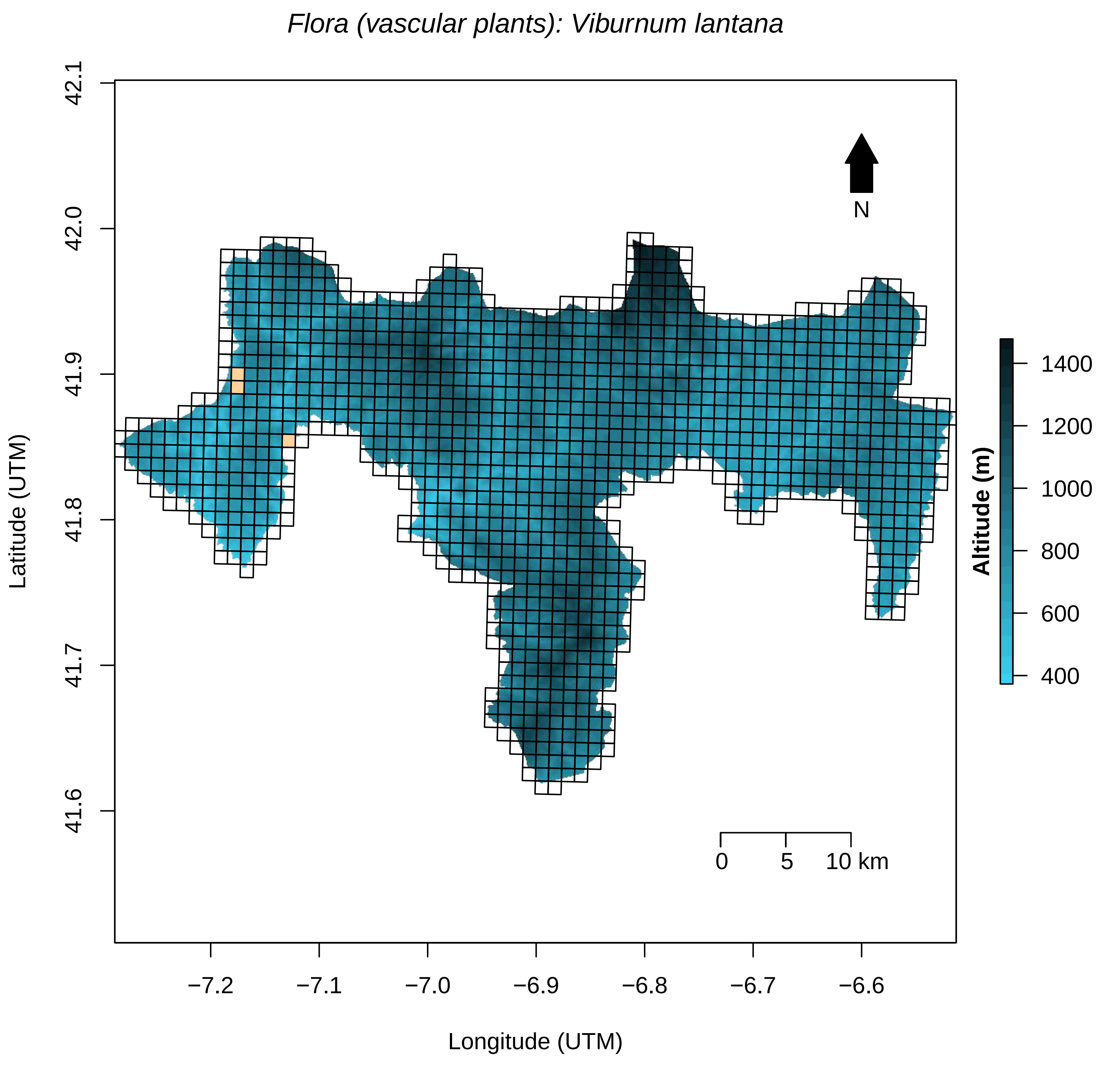

Flora (vascular plants): Viburnum lantana
42.1
42.0
41.9
41.8
41.7
41.6
Latitude (UTM)
−7.2
−7.1
−7.0
−6.9
−6.8
−6.7
−6.6
Longitude (UTM)
N
0
5
10 km
1400
1200
1000
800
600
400
Altitude (m)

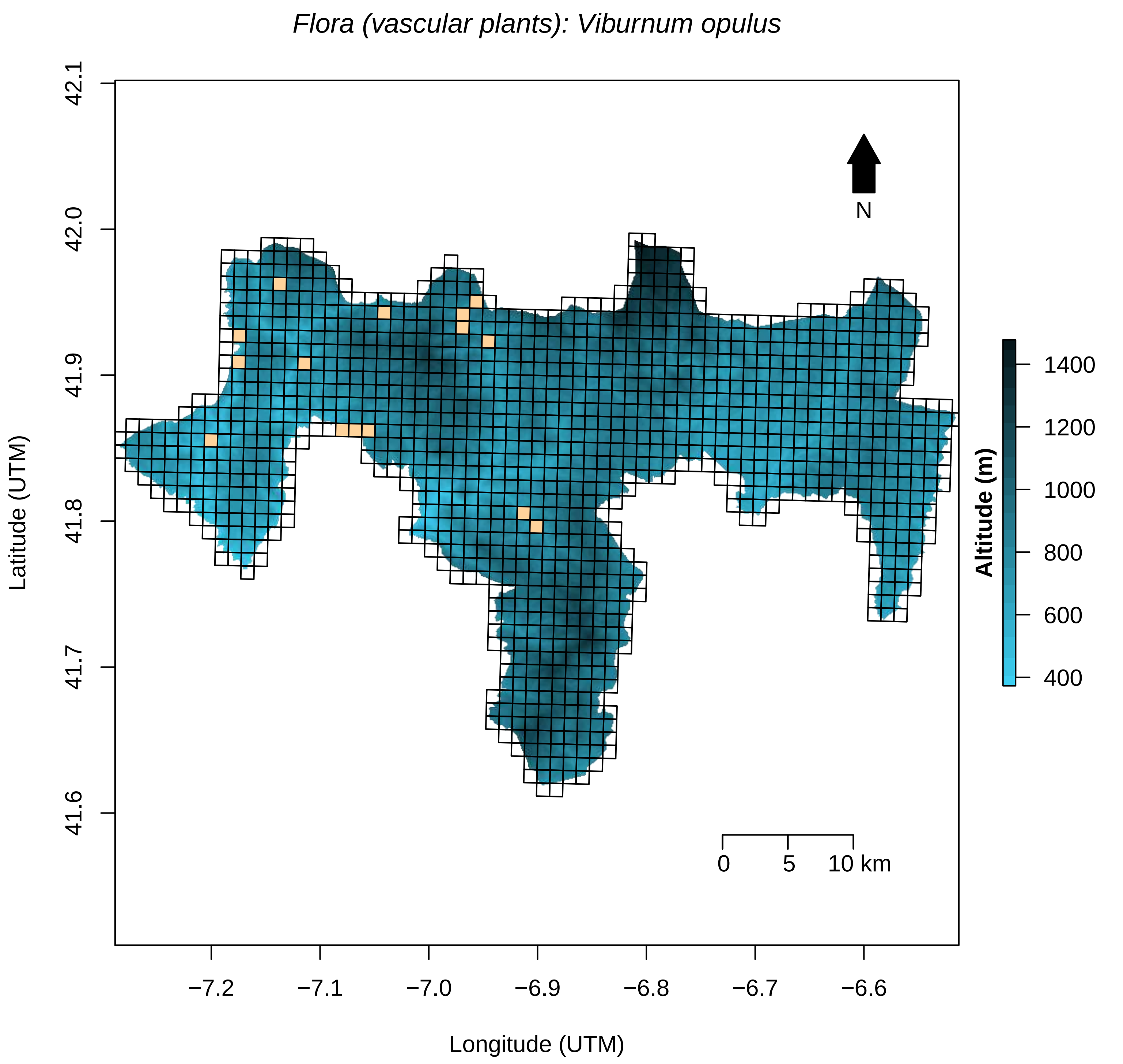
Flora (vascular plants): Viburnum opulus
42.1
42.0
41.9
41.8
41.7
41.6
Latitude (UTM)
−7.2
−7.1
−7.0
−6.9
−6.8
−6.7
−6.6
Longitude (UTM)
N
0
5
10 km
Altitude (m)
1400
1200
1000
800
600
400

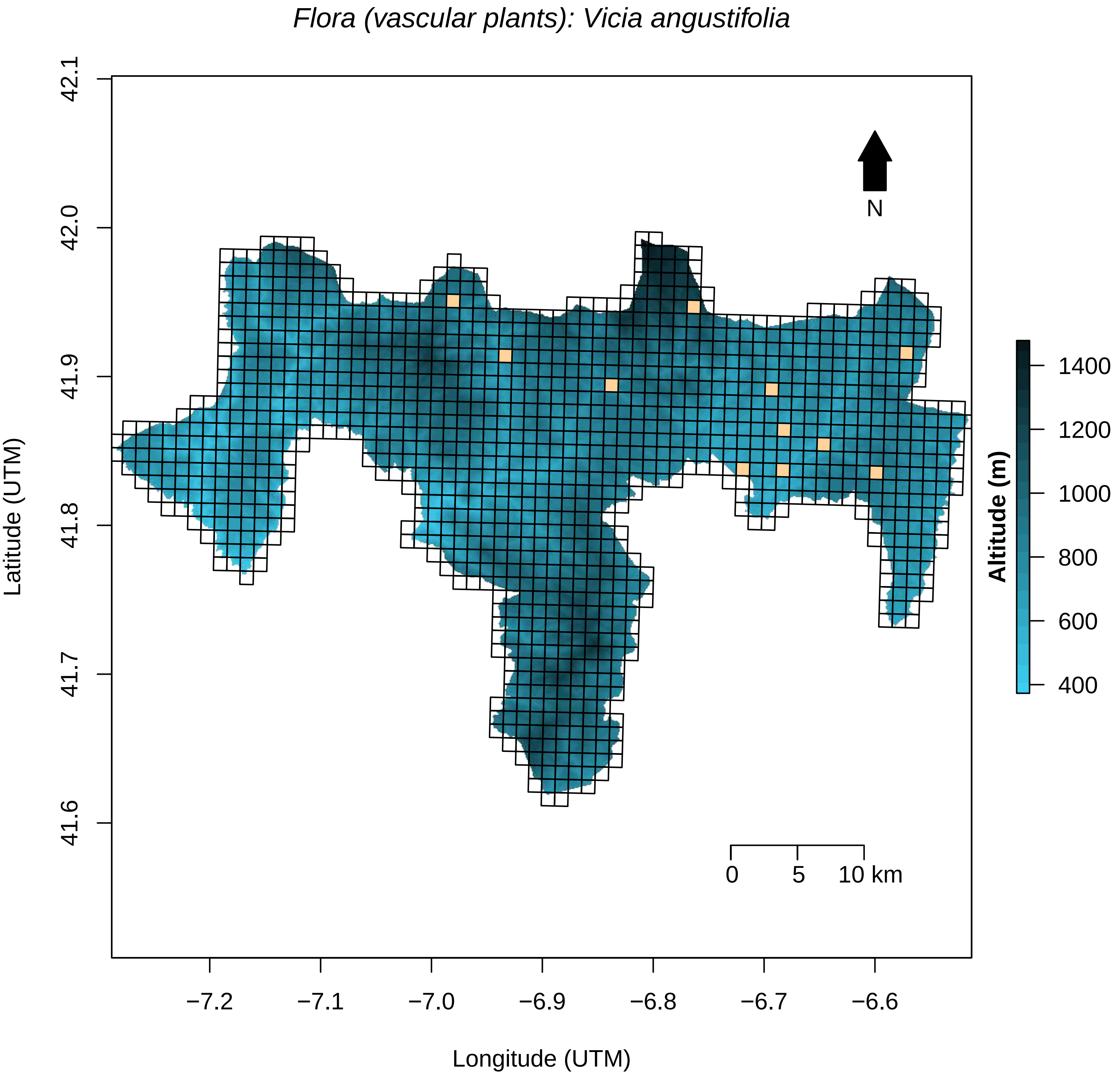

Flora (vascular plants): Vicia angustifolia
42.1
42.0
41.9
41.8
41.7
41.6
Latitude (UTM)
−7.2
−7.1
−7.0
−6.9
−6.8
−6.7
−6.6
Longitude (UTM)
N
0
5
10 km
1400
1200
1000
800
600
400
Altitude (m)

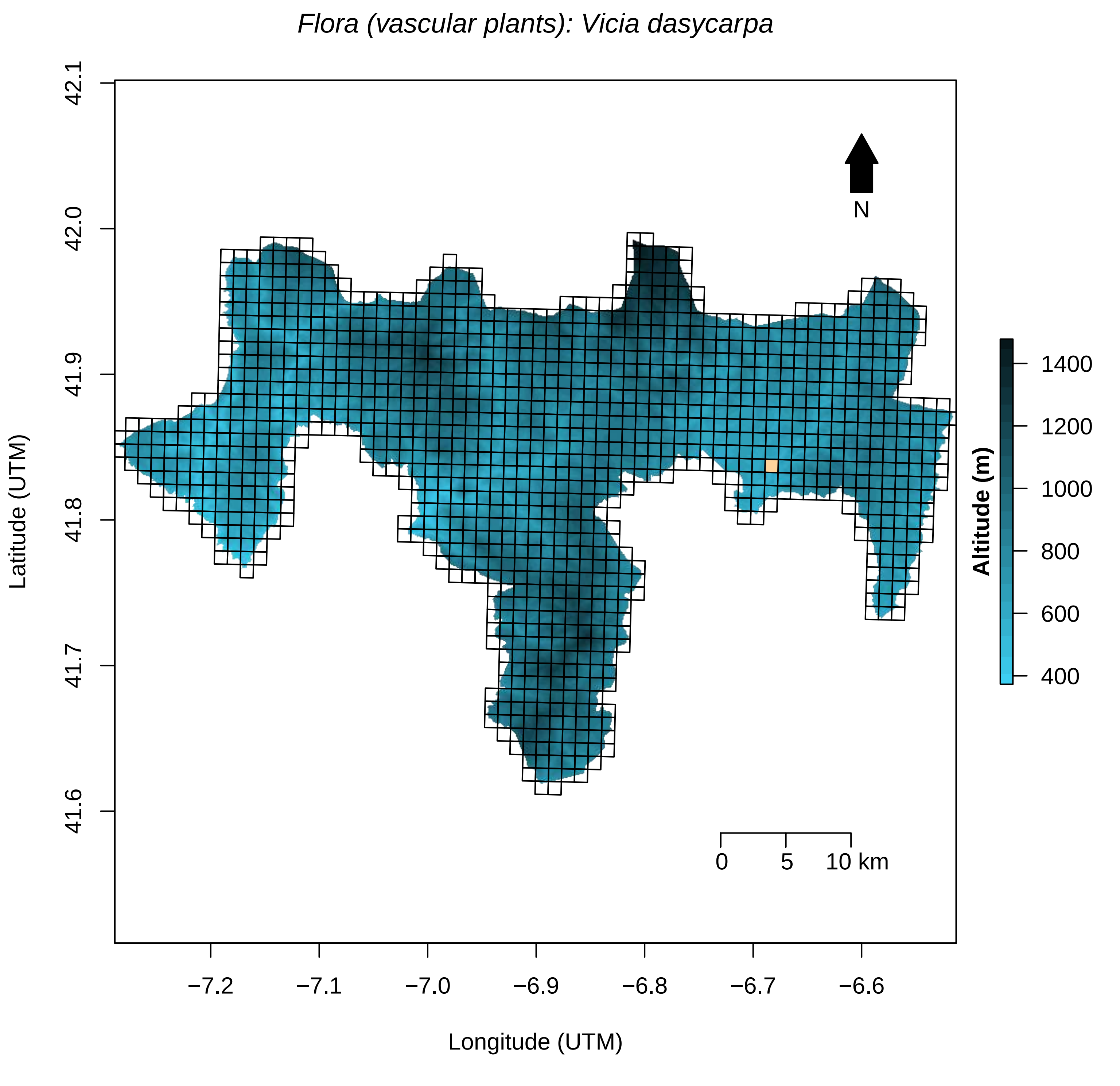

Flora (vascular plants): Vicia dasycarpa
N
0
5
10 km
Altitude (m)
1400
1200
1000
800
600
400
Latitude (UTM)
42.1
42.0
41.9
41.8
41.7
41.6
Longitude (UTM)
−7.2
−7.1
−7.0
−6.9
−6.8
−6.7
−6.6

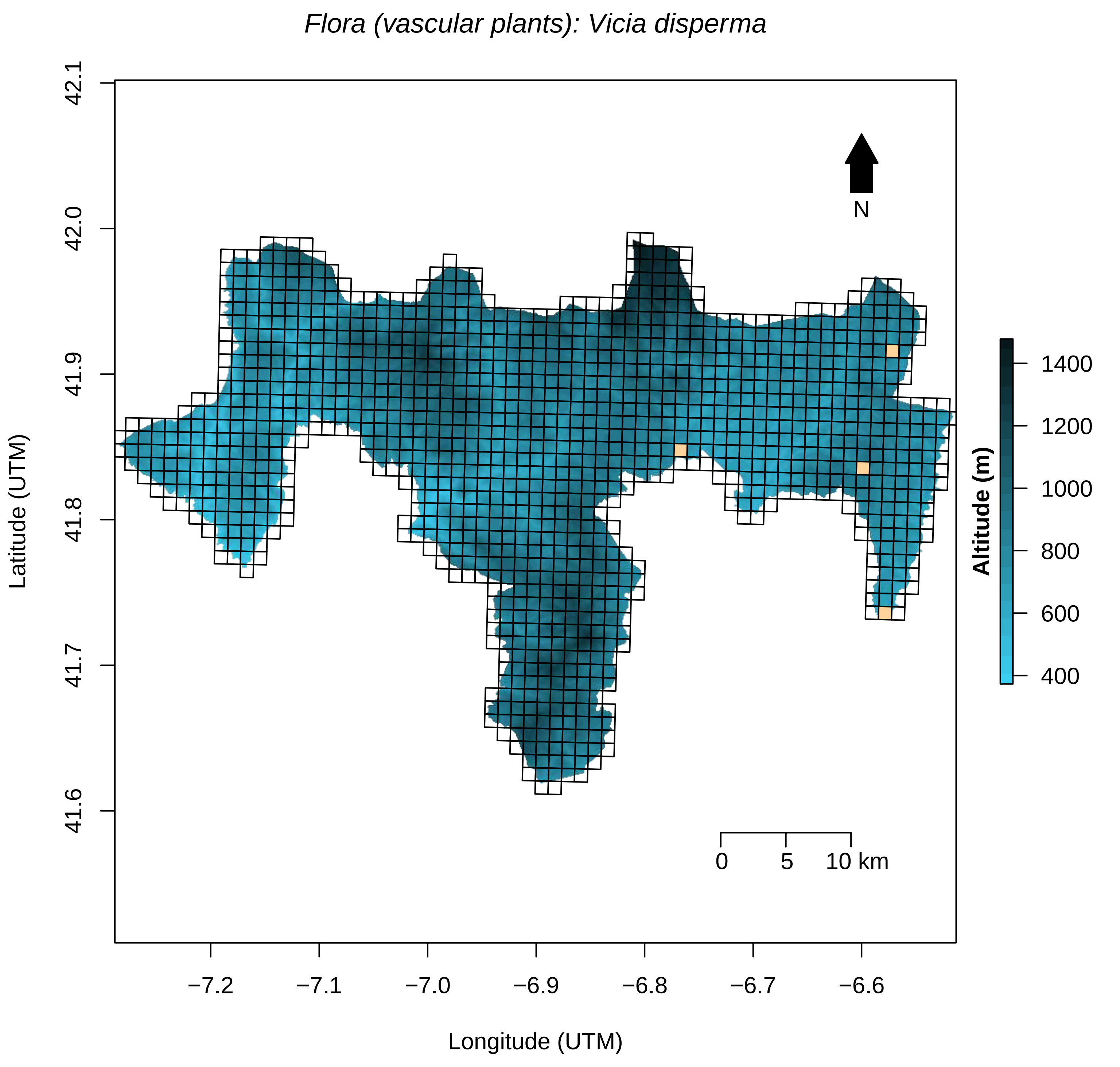
Flora (vascular plants): Vicia disperma
42.1
42.0
41.9
41.8
41.7
41.6
Latitude (UTM)
−7.2
−7.1
−7.0
−6.9
−6.8
−6.7
−6.6
Longitude (UTM)
N
0
5
10 km
Altitude (m)
1400
1200
1000
800
600
400

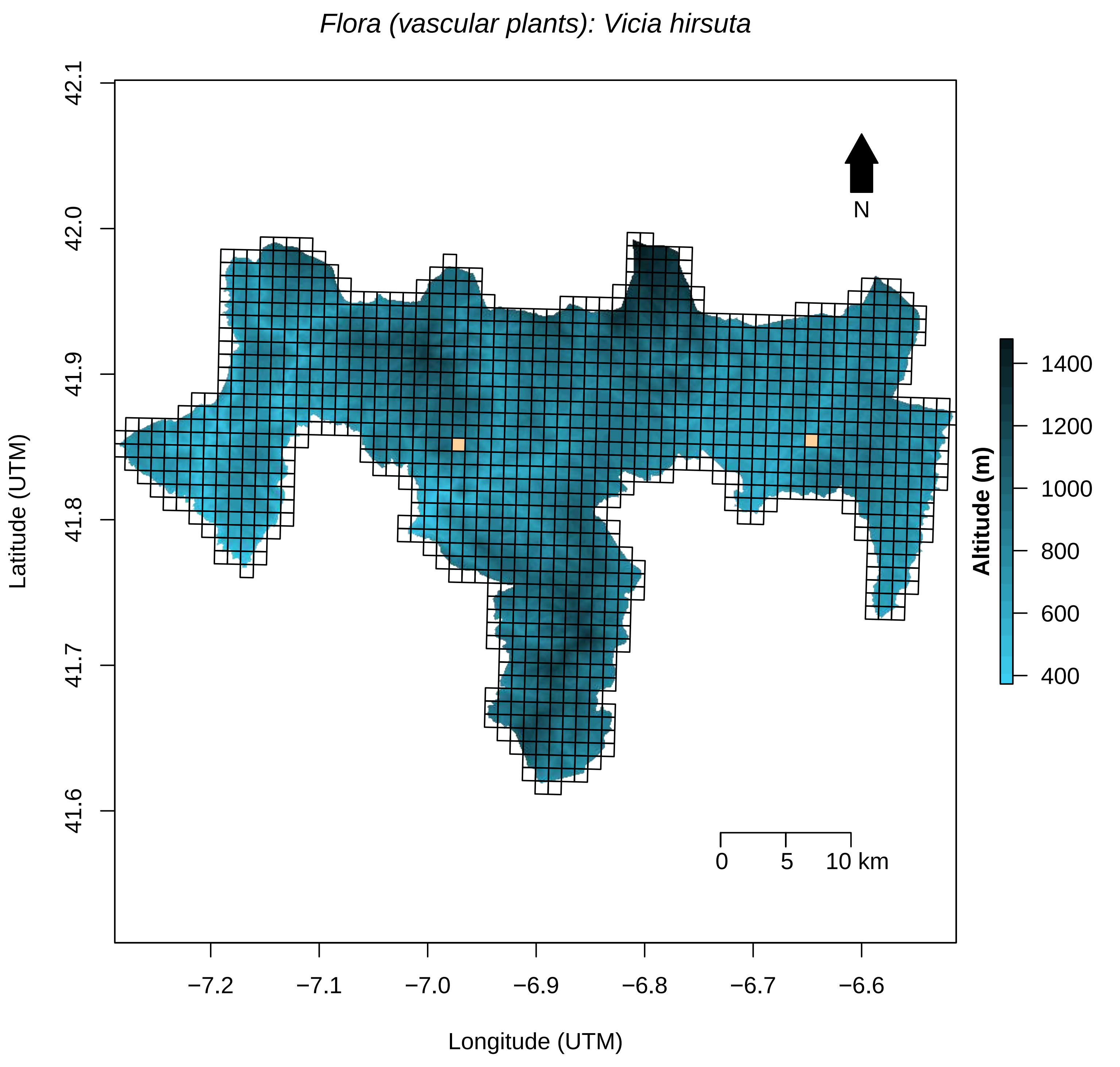
Flora (vascular plants): Vicia hirsuta
42.1
42.0
41.9
41.8
41.7
41.6
Latitude (UTM)
−7.2
−7.1
−7.0
−6.9
−6.8
−6.7
−6.6
Longitude (UTM)
N
0
5
10 km
Altitude (m)
1400
1200
1000
800
600
400

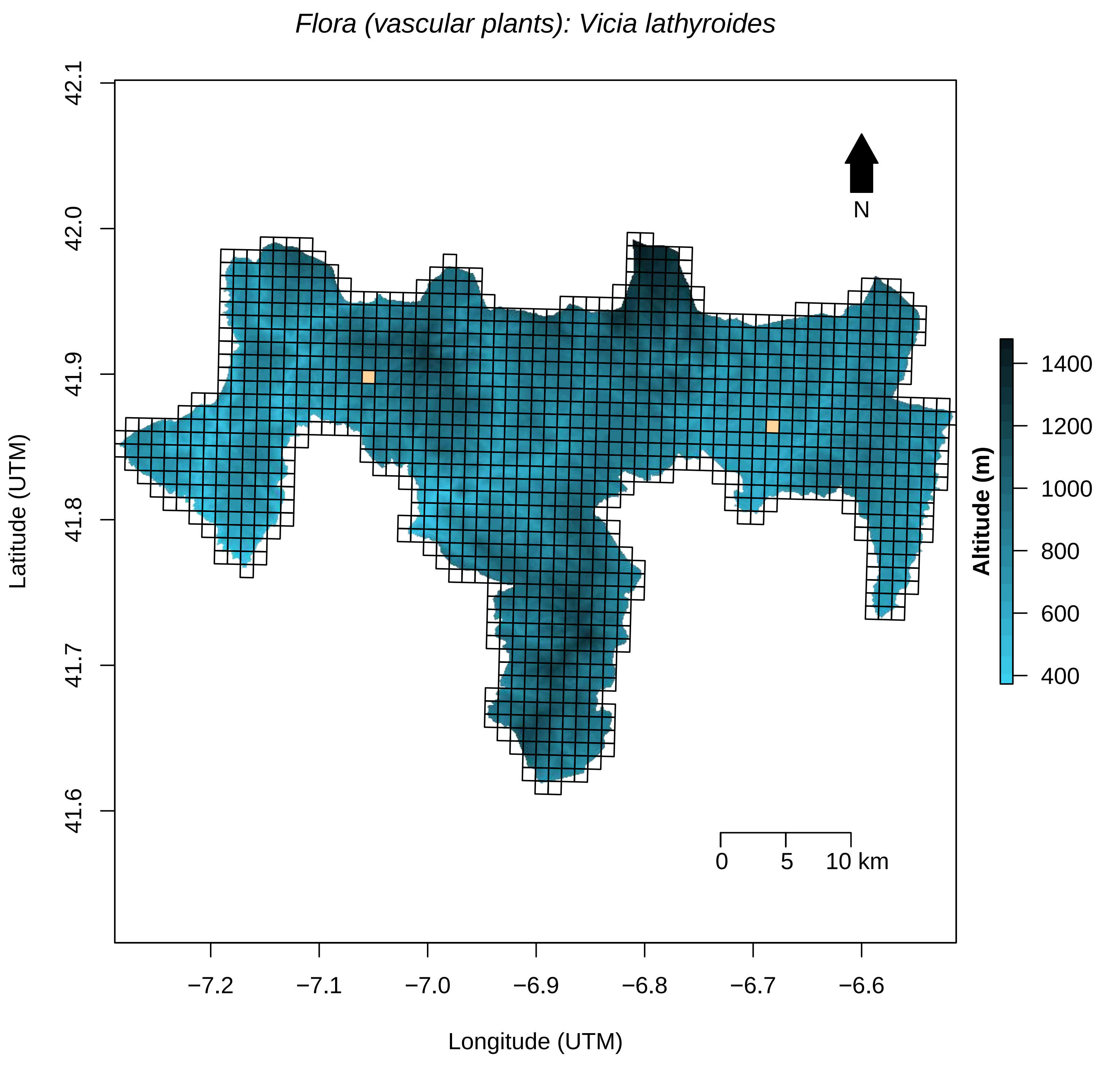

Flora (vascular plants): Vicia lathyroides
42.1
42.0
41.9
41.8
41.7
41.6
Latitude (UTM)
N
0
5
10 km
−7.2
−7.1
−7.0
−6.9
−6.8
−6.7
−6.6
Longitude (UTM)
Altitude (m)
1400
1200
1000
800
600
400

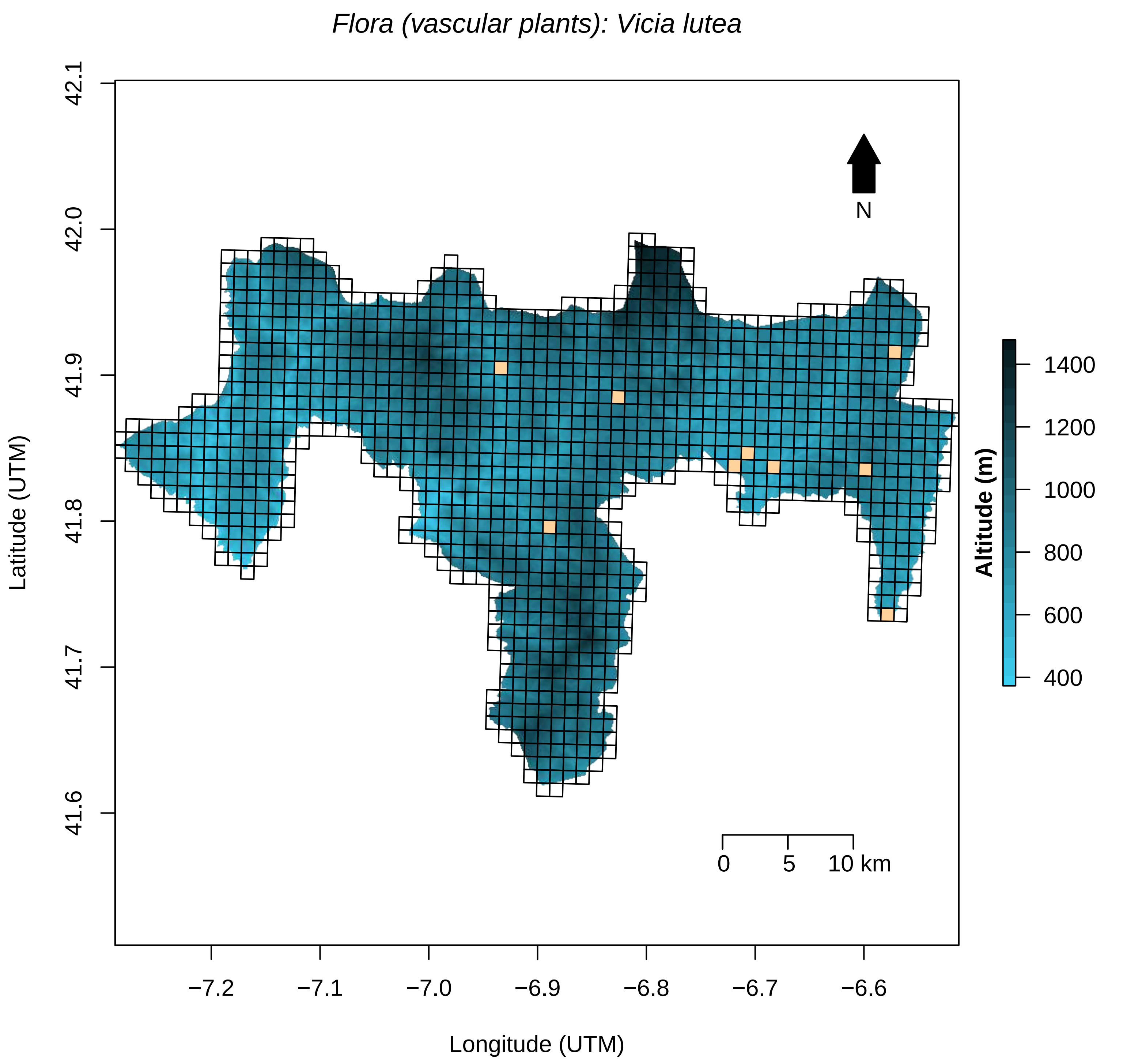
Flora (vascular plants): Vicia lutea
N
0
5
10 km
Altitude (m)
1400
1200
1000
800
600
400
42.1
42.0
41.9
41.8
41.7
41.6
Latitude (UTM)
−7.2
−7.1
−7.0
−6.9
−6.8
−6.7
−6.6
Longitude (UTM)

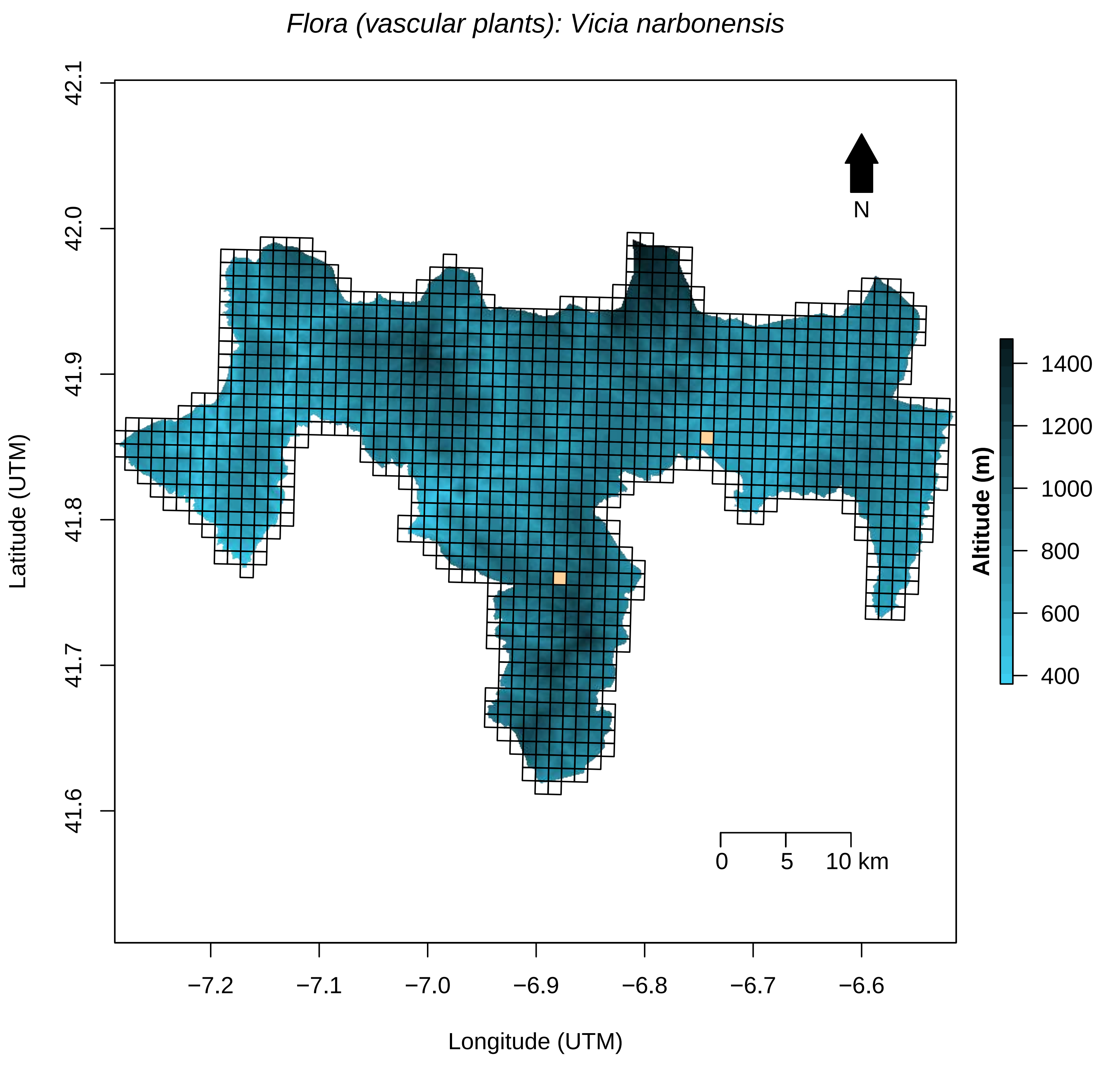

Flora (vascular plants): Vicia narbonensis
42.1
42.0
41.9
41.8
41.7
41.6
Latitude (UTM)
−7.2
−7.1
−7.0
−6.9
−6.8
−6.7
−6.6
Longitude (UTM)
N
0
5
10 km
1400
1200
1000
800
600
400
Altitude (m)

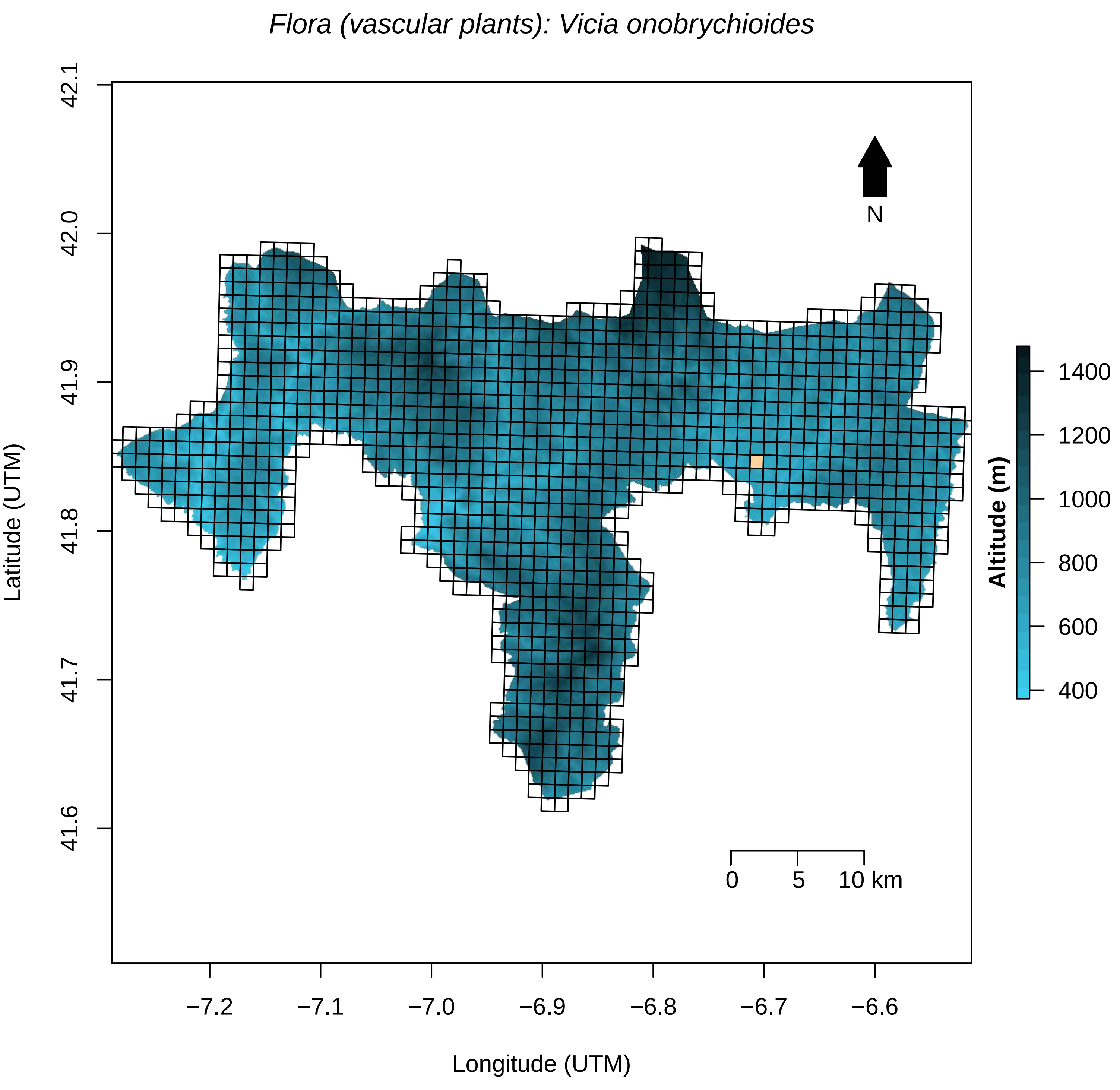

Flora (vascular plants): Vicia onobrychioides
42.1
42.0
41.9
41.8
41.7
41.6
Latitude (UTM)
−7.2
−7.1
−7.0
−6.9
−6.8
−6.7
−6.6
Longitude (UTM)
N
0
5
10 km
Altitude (m)
1400
1200
1000
800
600
400

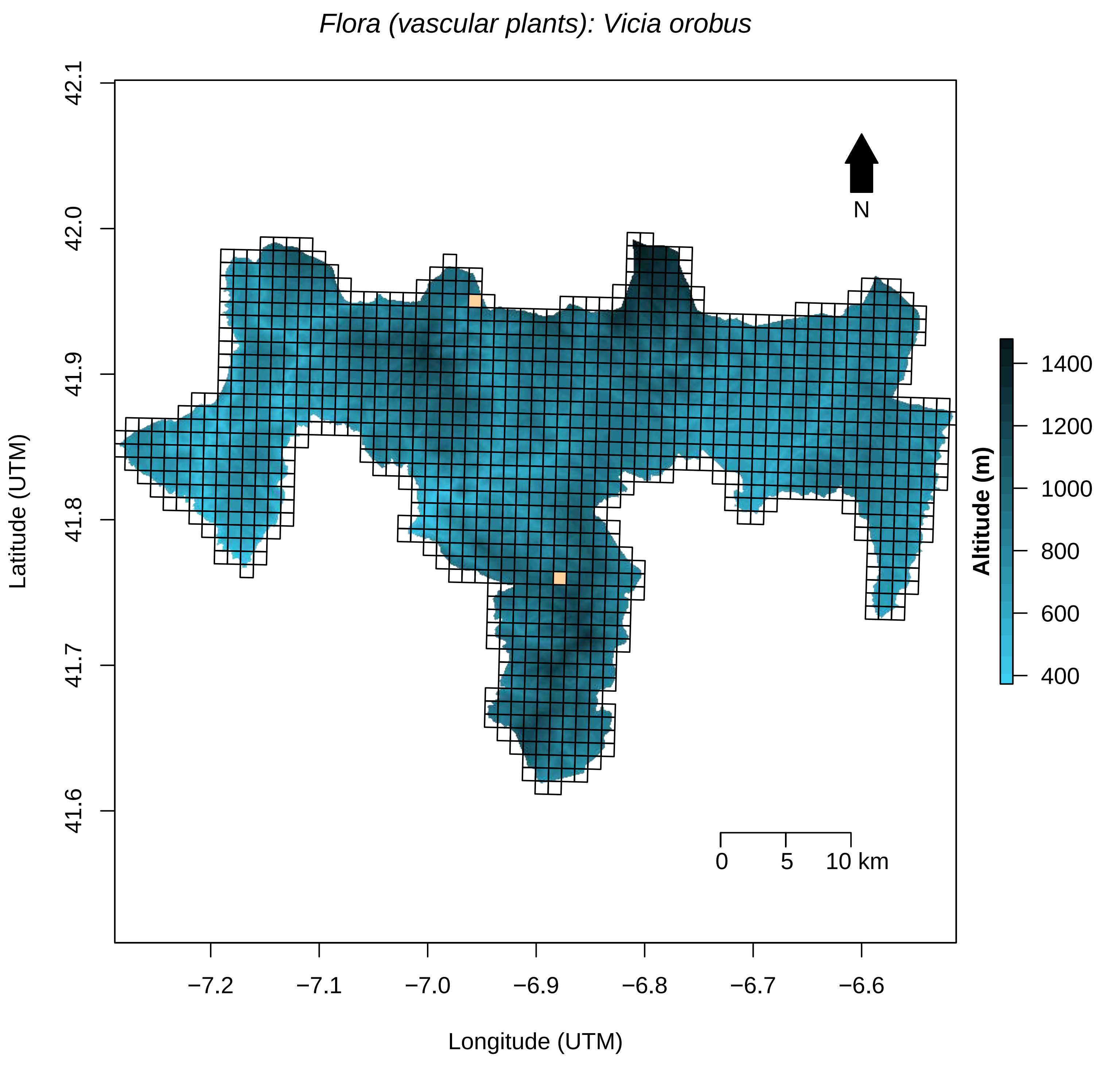
Flora (vascular plants): Vicia orobus
N
0
5
10 km
Altitude (m)
1400
1200
1000
800
600
400
42.1
42.0
41.9
41.8
41.7
41.6
Latitude (UTM)
−7.2
−7.1
−7.0
−6.9
−6.8
−6.7
−6.6
Longitude (UTM)

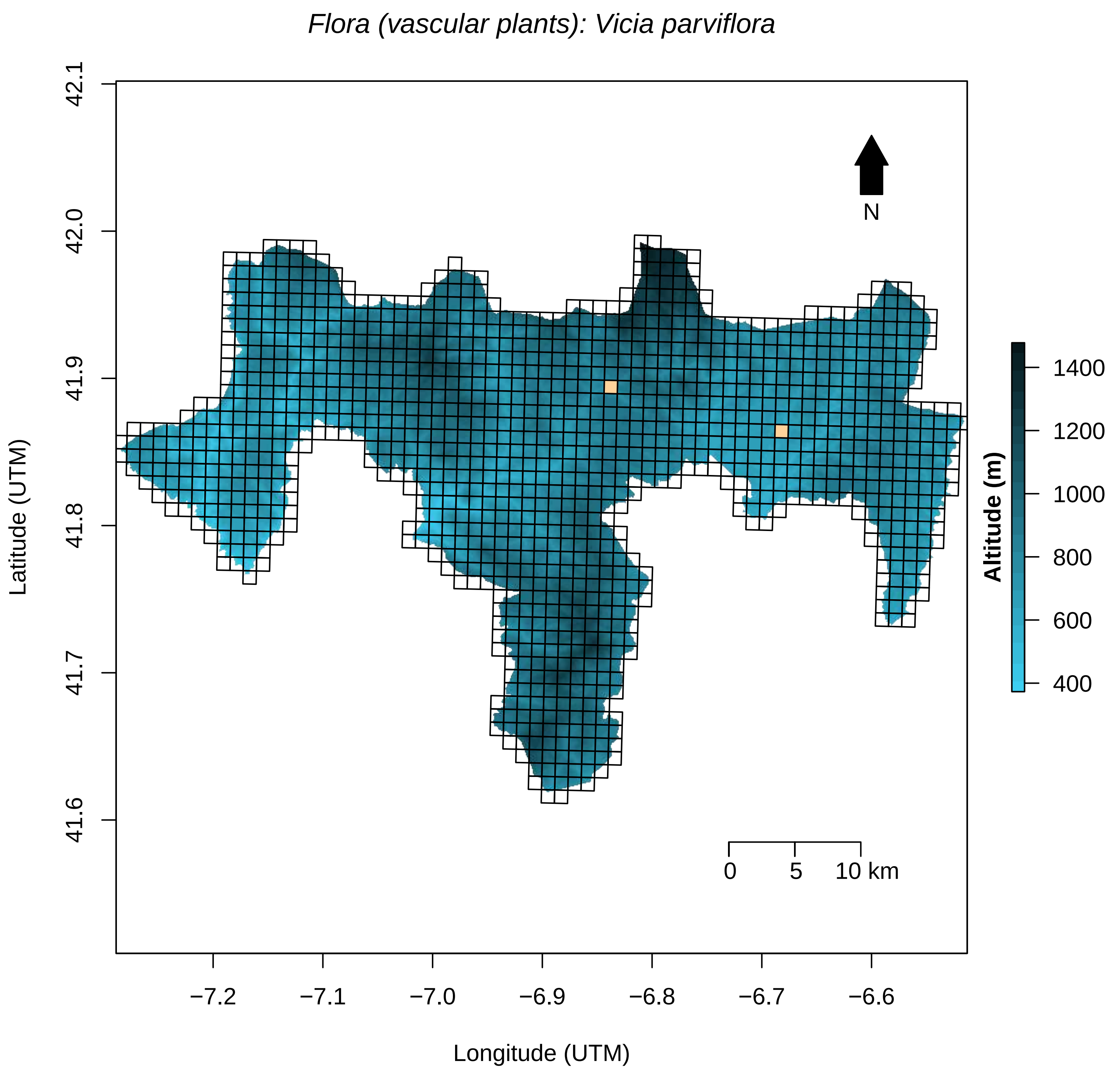

Flora (vascular plants): Vicia parviflora
N
0
5
10 km
Latitude (UTM)
42.1
42.0
41.9
41.8
41.7
41.6
Longitude (UTM)
−7.2
−7.1
−7.0
−6.9
−6.8
−6.7
−6.6
Altitude (m)
1400
1200
1000
800
600
400

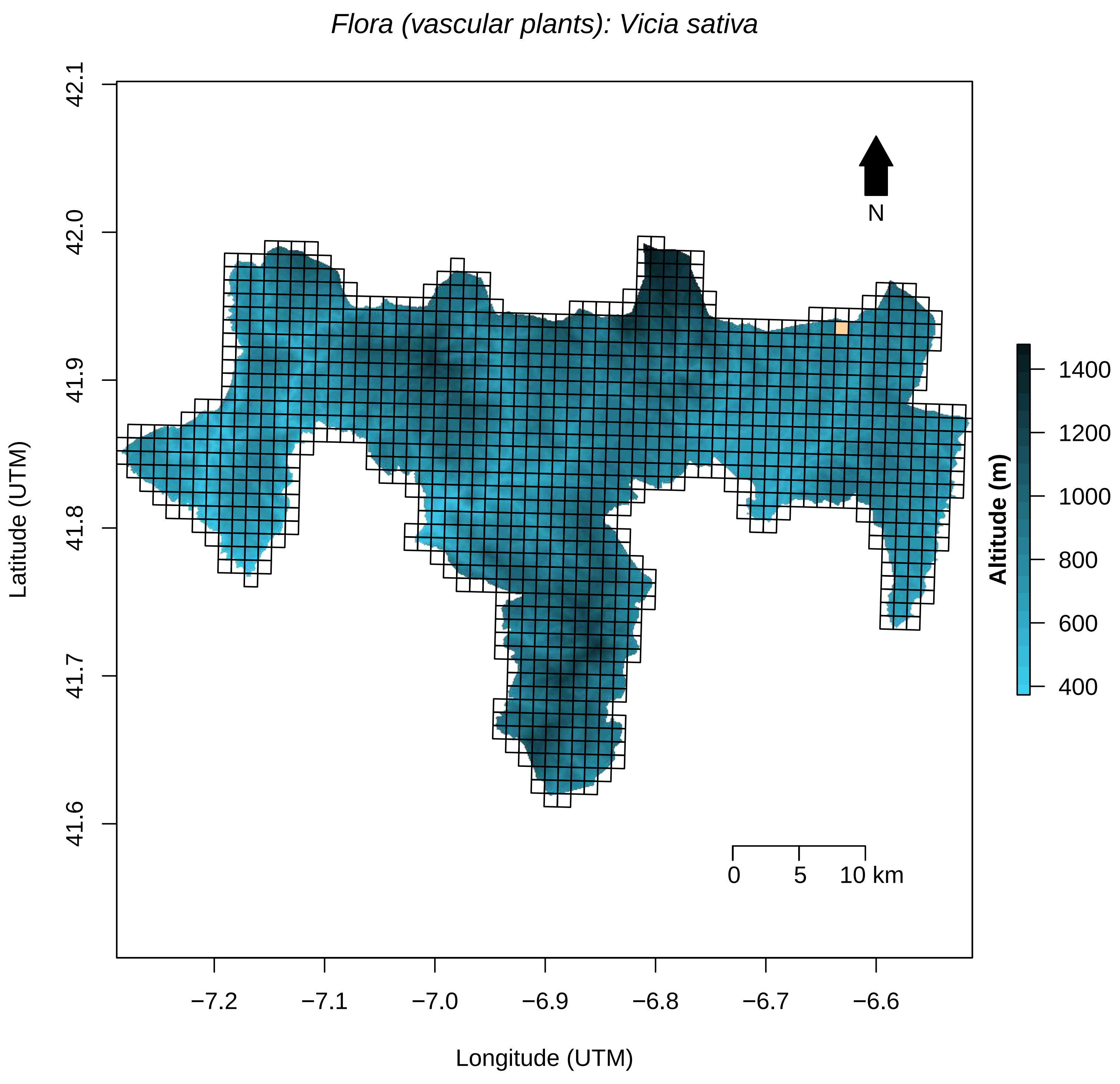

Flora (vascular plants): Vicia sativa
N
0 5 10 km
Altitude (m)
1400
1200
1000
800
600
400
42.1
42.0
41.9
41.8
41.7
41.6
Latitude (UTM)
−7.2
−7.1
−7.0
−6.9
−6.8
−6.7
−6.6
Longitude (UTM)

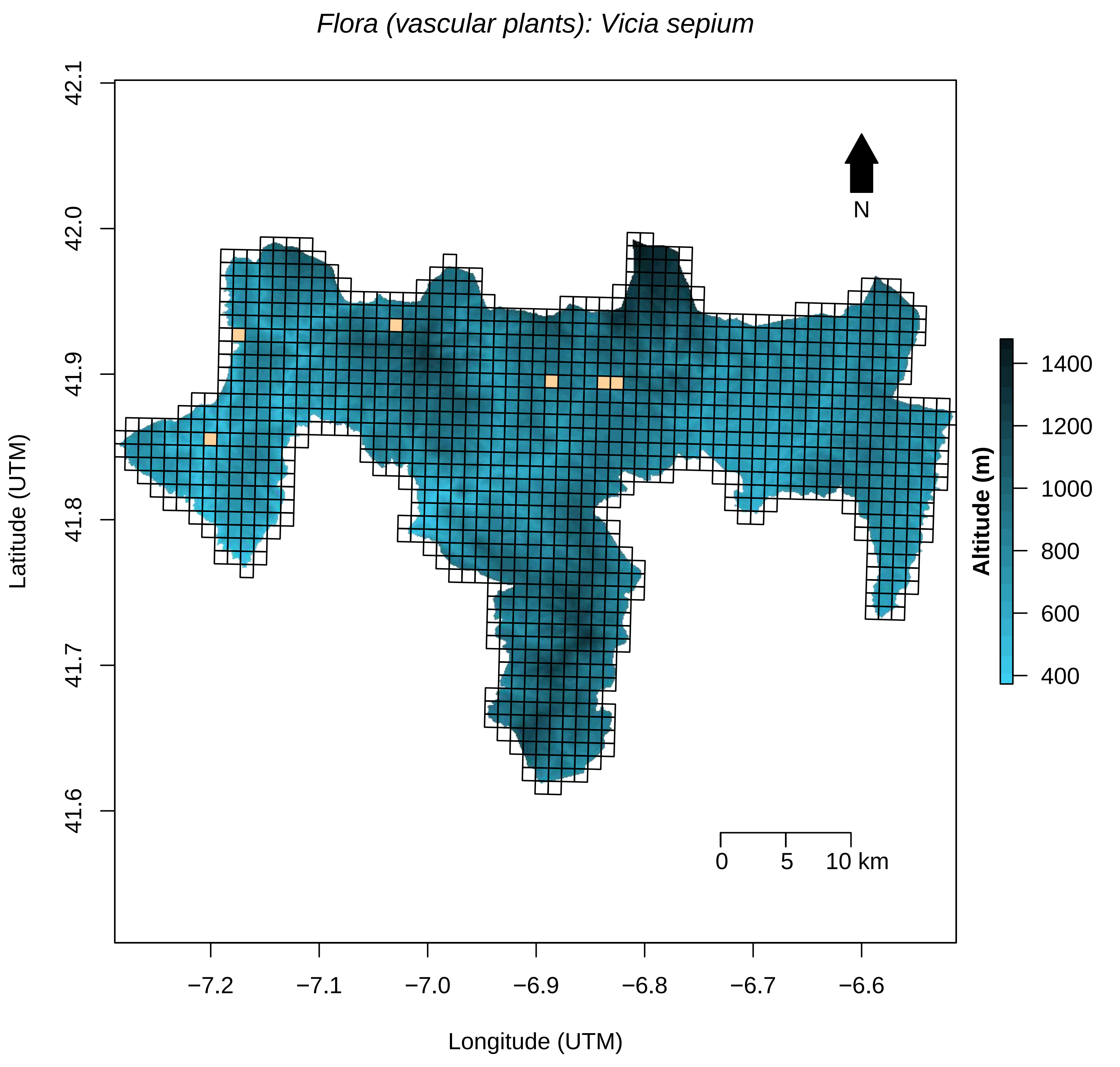
Flora (vascular plants): Vicia sepium
42.1
42.0
41.9
41.8
41.7
41.6
Latitude (UTM)
−7.2
−7.1
−7.0
−6.9
−6.8
−6.7
−6.6
Longitude (UTM)
N
0
5
10 km
Altitude (m)
1400
1200
1000
800
600
400

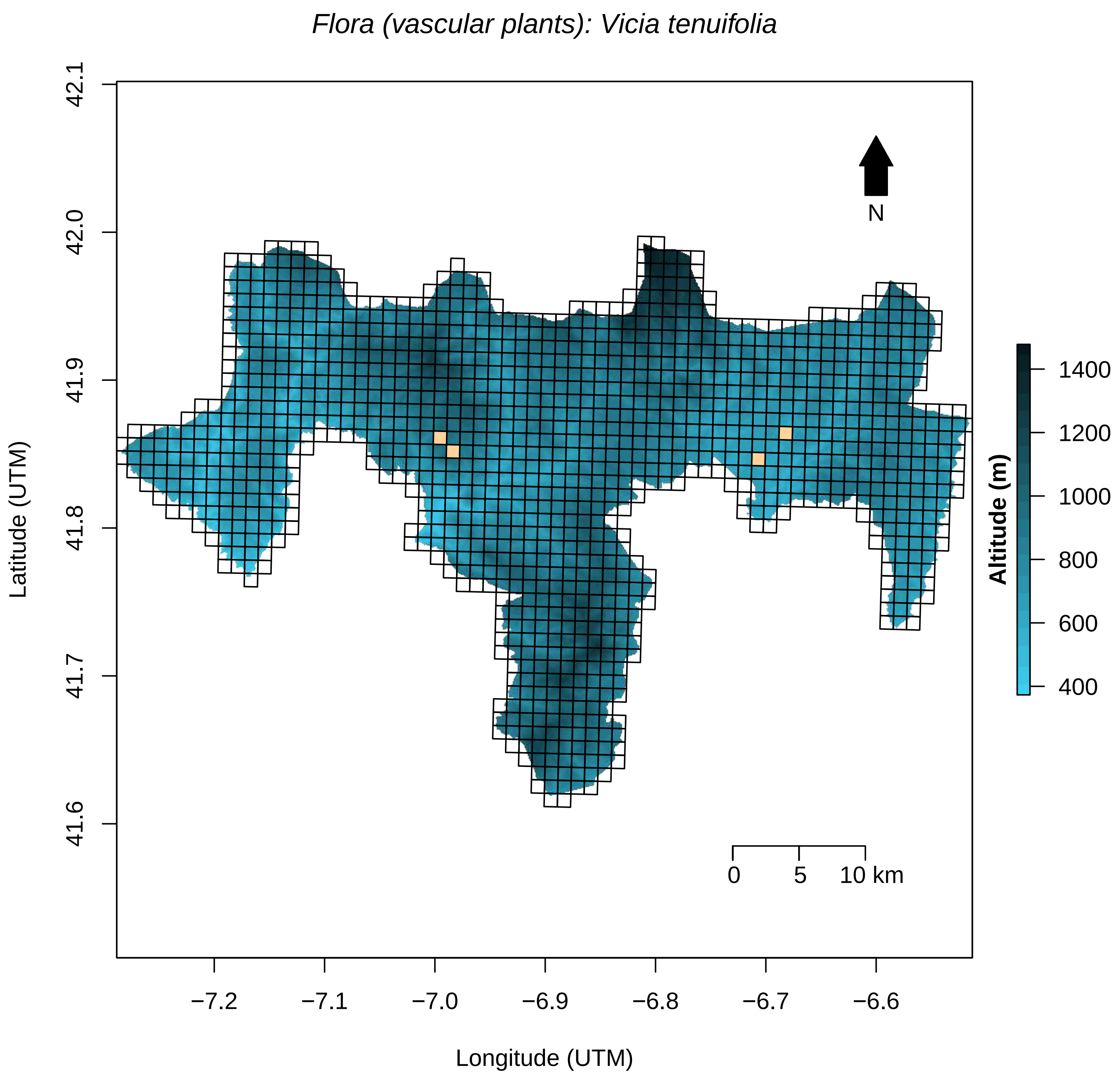

Flora (vascular plants): Vicia tenuifolia
Latitude (UTM)
Longitude (UTM)
42.1
42.0
41.9
41.8
41.7
41.6
−7.2
−7.1
−7.0
−6.9
−6.8
−6.7
−6.6
N
0
5
10 km
Altitude (m)
1400
1200
1000
800
600
400

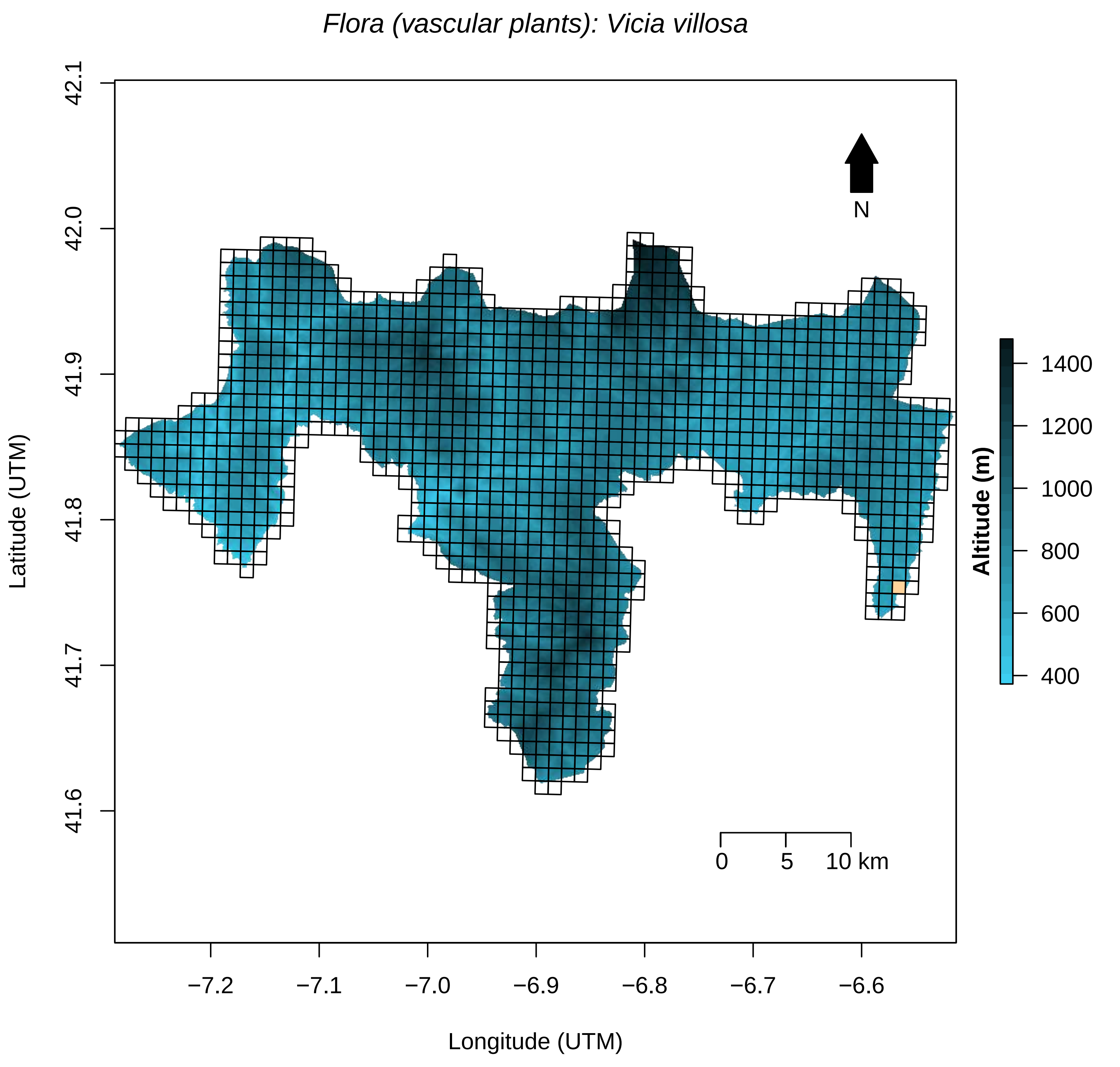

Flora (vascular plants): Vicia villosa
N
0
5
10 km
Altitude (m)
1400
1200
1000
800
600
400
Latitude (UTM)
42.1
42.0
41.9
41.8
41.7
41.6
Longitude (UTM)
−7.2
−7.1
−7.0
−6.9
−6.8
−6.7
−6.6

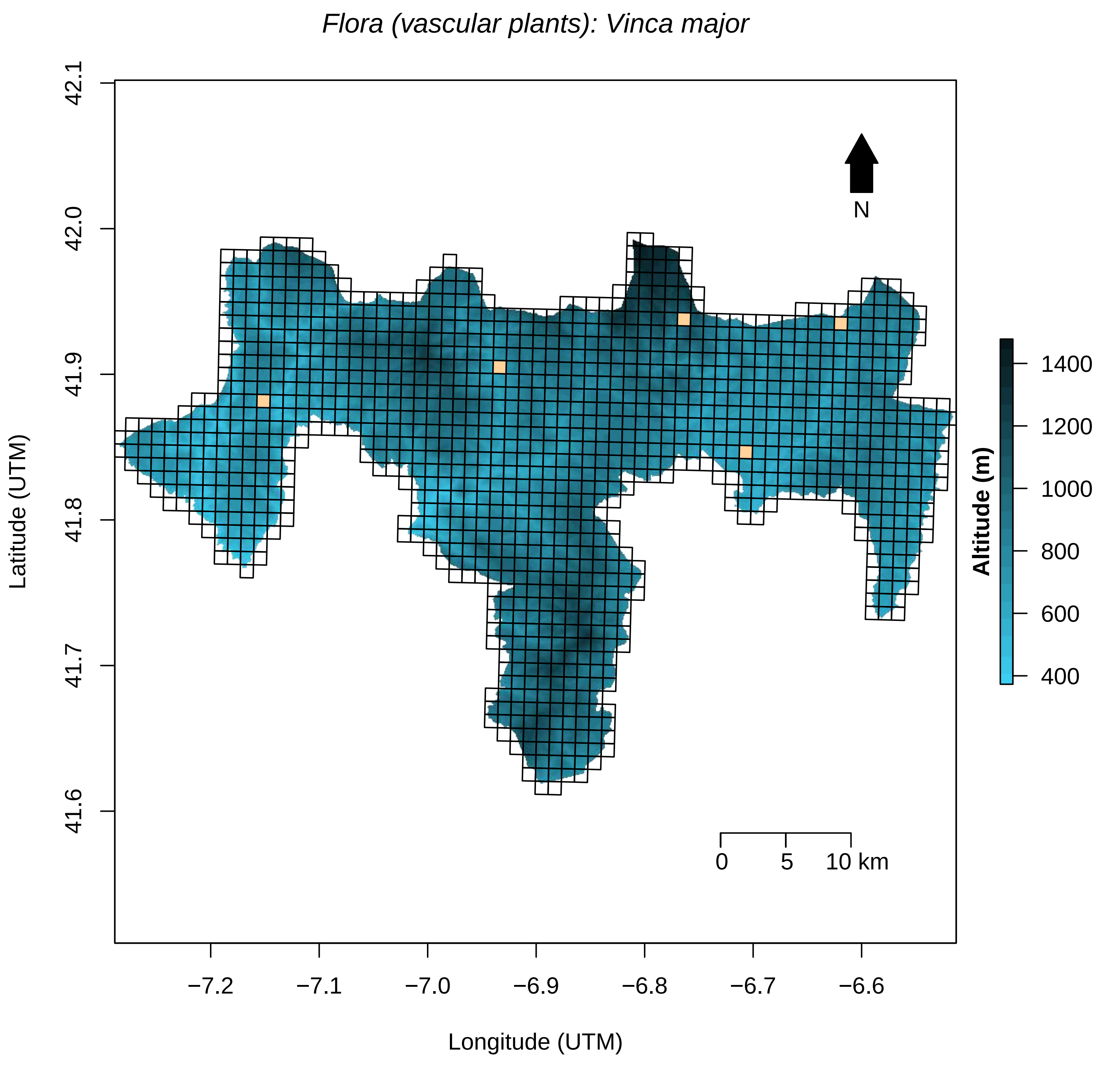

Flora (vascular plants): Vinca major
42.1
42.0
41.9
41.8
41.7
41.6
Latitude (UTM)
−7.2
−7.1
−7.0
−6.9
−6.8
−6.7
−6.6
Longitude (UTM)
N
0
5
10 km
Altitude (m)
1400
1200
1000
800
600
400

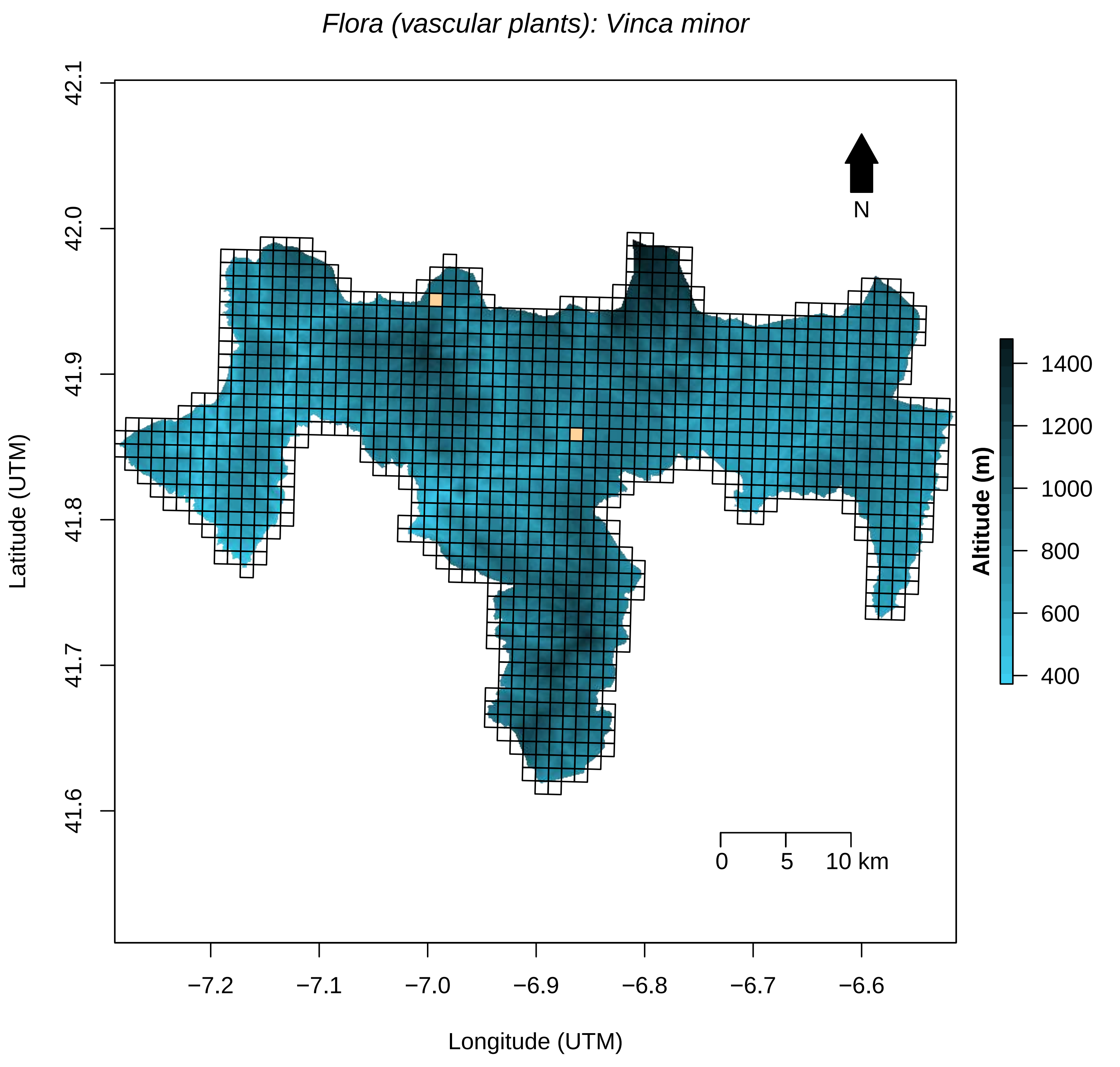

Flora (vascular plants): Vinca minor
42.1
42.0
41.9
41.8
41.7
41.6
Latitude (UTM)
−7.2
−7.1
−7.0
−6.9
−6.8
−6.7
−6.6
Longitude (UTM)
N
0
5
10 km
1400
1200
1000
800
600
400
Altitude (m)

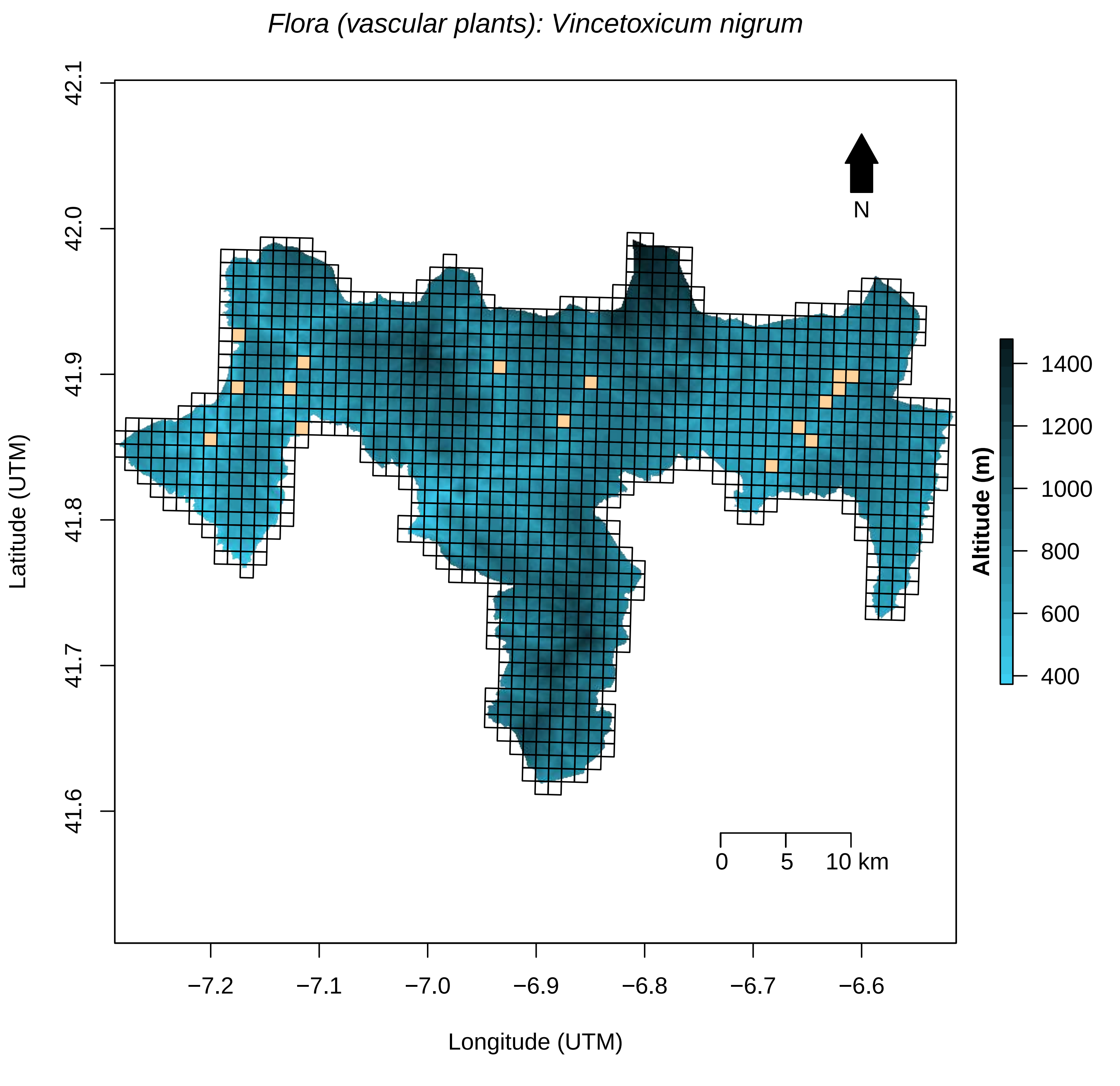

Flora (vascular plants): Vincetoxicum nigrum
42.1
42.0
41.9
41.8
41.7
41.6
Latitude (UTM)
−7.2
−7.1
−7.0
−6.9
−6.8
−6.7
−6.6
Longitude (UTM)
N
0
5
10 km
Altitude (m)
1400
1200
1000
800
600
400

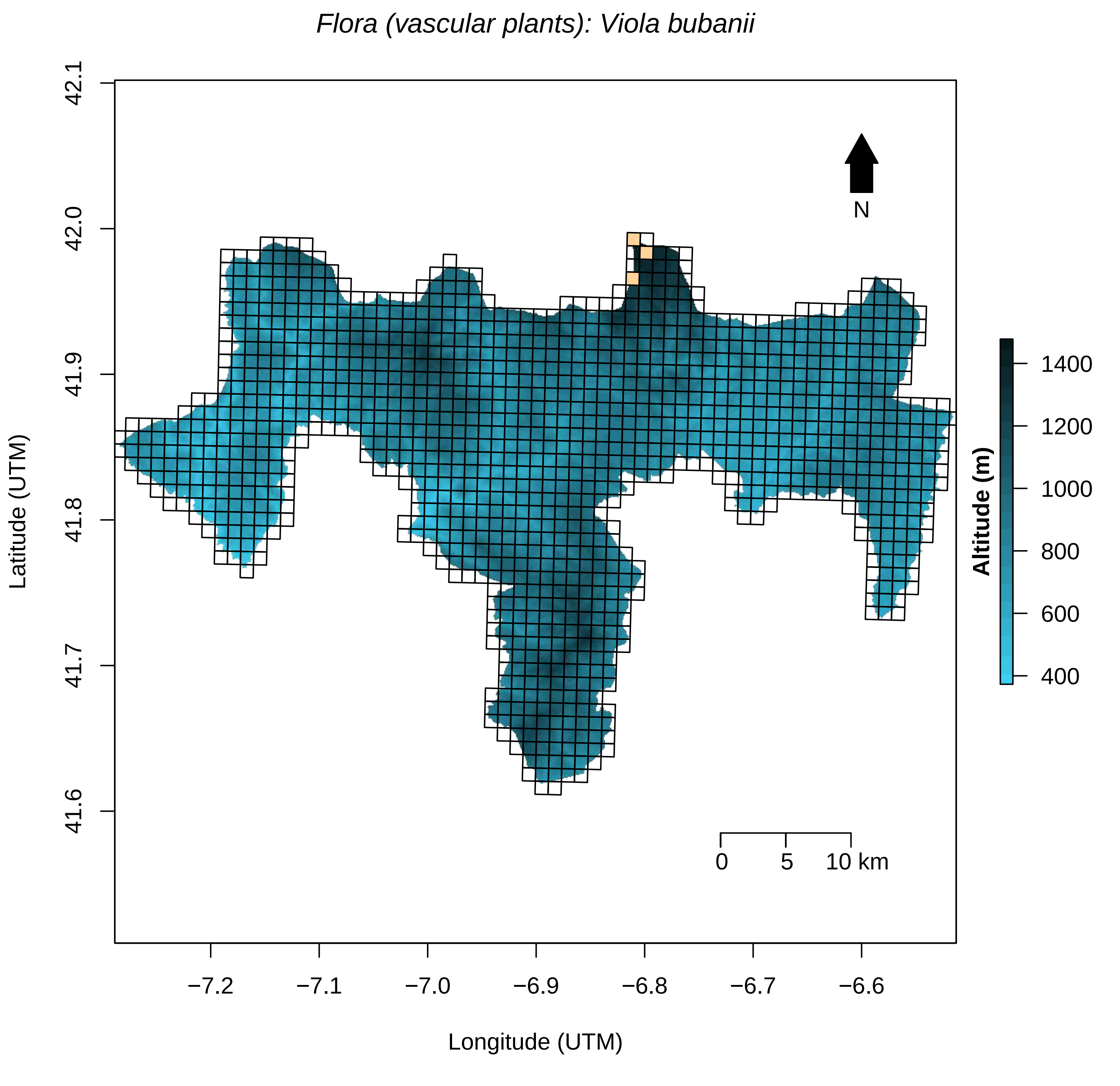
Flora (vascular plants): Viola bubanii
42.1
42.0
41.9
41.8
41.7
41.6
Latitude (UTM)
−7.2
−7.1
−7.0
−6.9
−6.8
−6.7
−6.6
Longitude (UTM)
N
0
5
10 km
Altitude (m)
1400
1200
1000
800
600
400

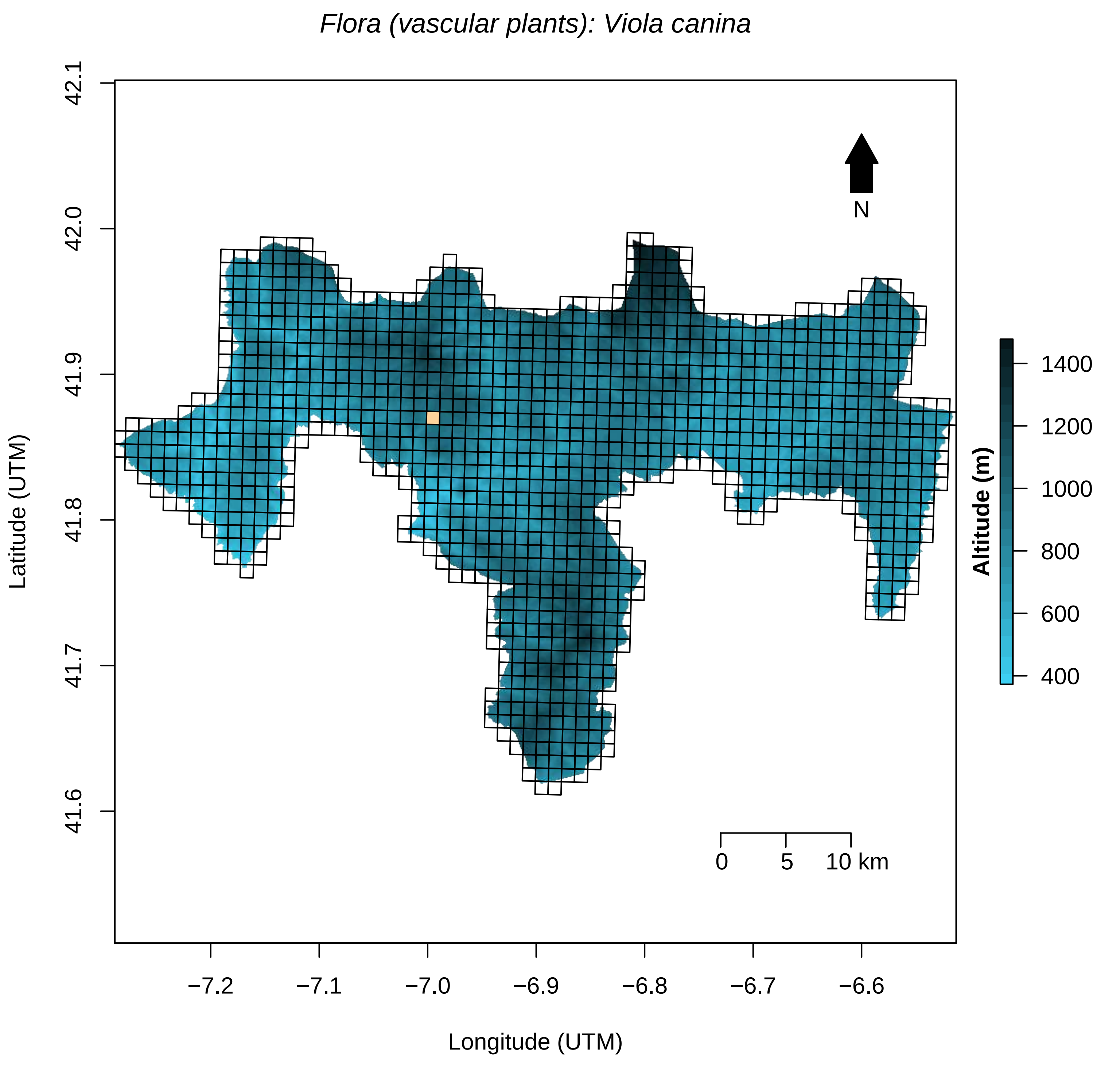
Flora (vascular plants): Viola canina
42.1
42.0
41.9
41.8
41.7
41.6
Latitude (UTM)
−7.2
−7.1
−7.0
−6.9
−6.8
−6.7
−6.6
Longitude (UTM)
N
0
5
10 km
Altitude (m)
1400
1200
1000
800
600
400

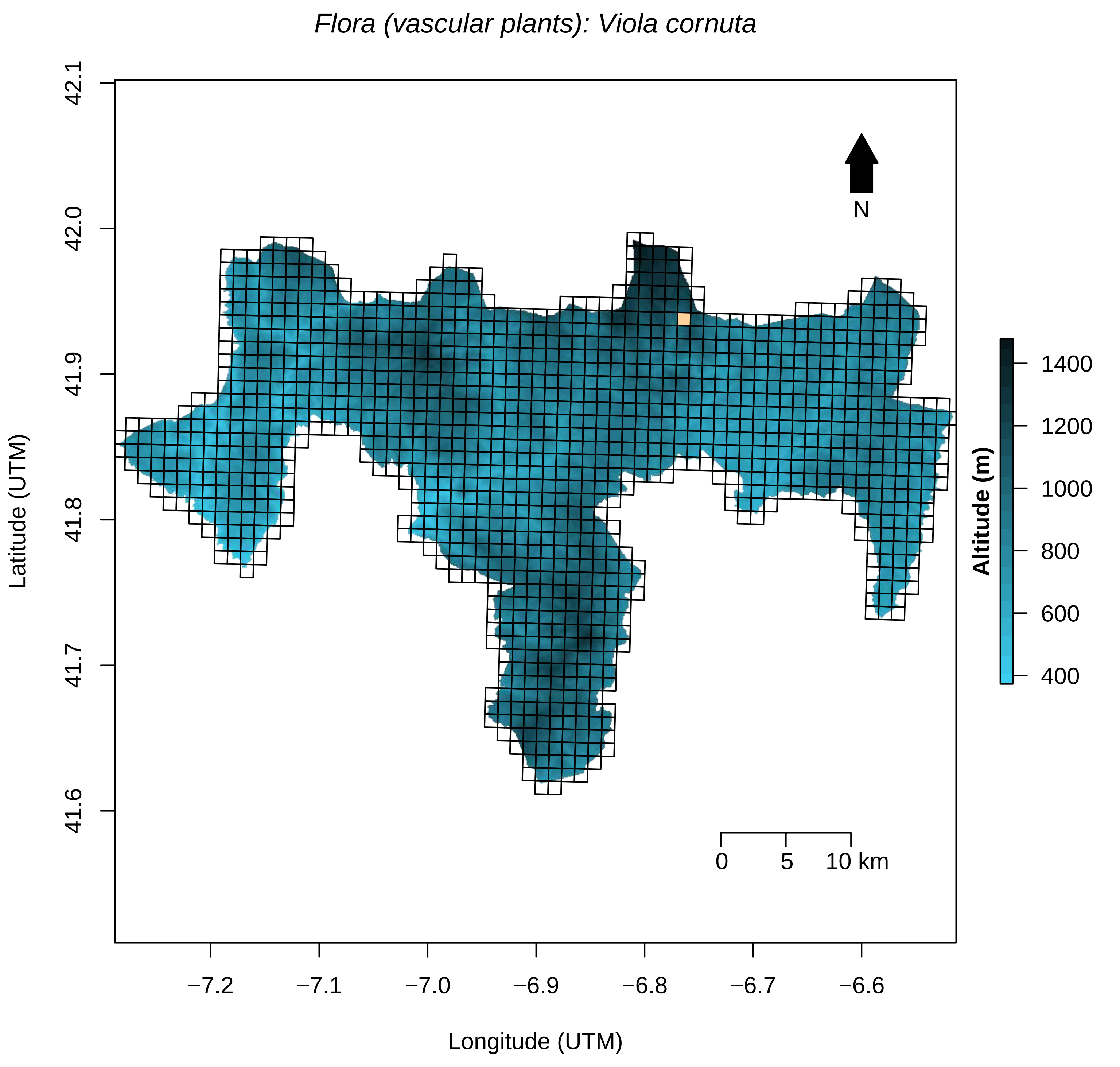

Flora (vascular plants): Viola cornuta
N
0
5
10 km
Latitude (UTM)
Longitude (UTM)
Altitude (m)
42.1
42.0
41.9
41.8
41.7
41.6
−7.2
−7.1
−7.0
−6.9
−6.8
−6.7
−6.6
1400
1200
1000
800
600
400

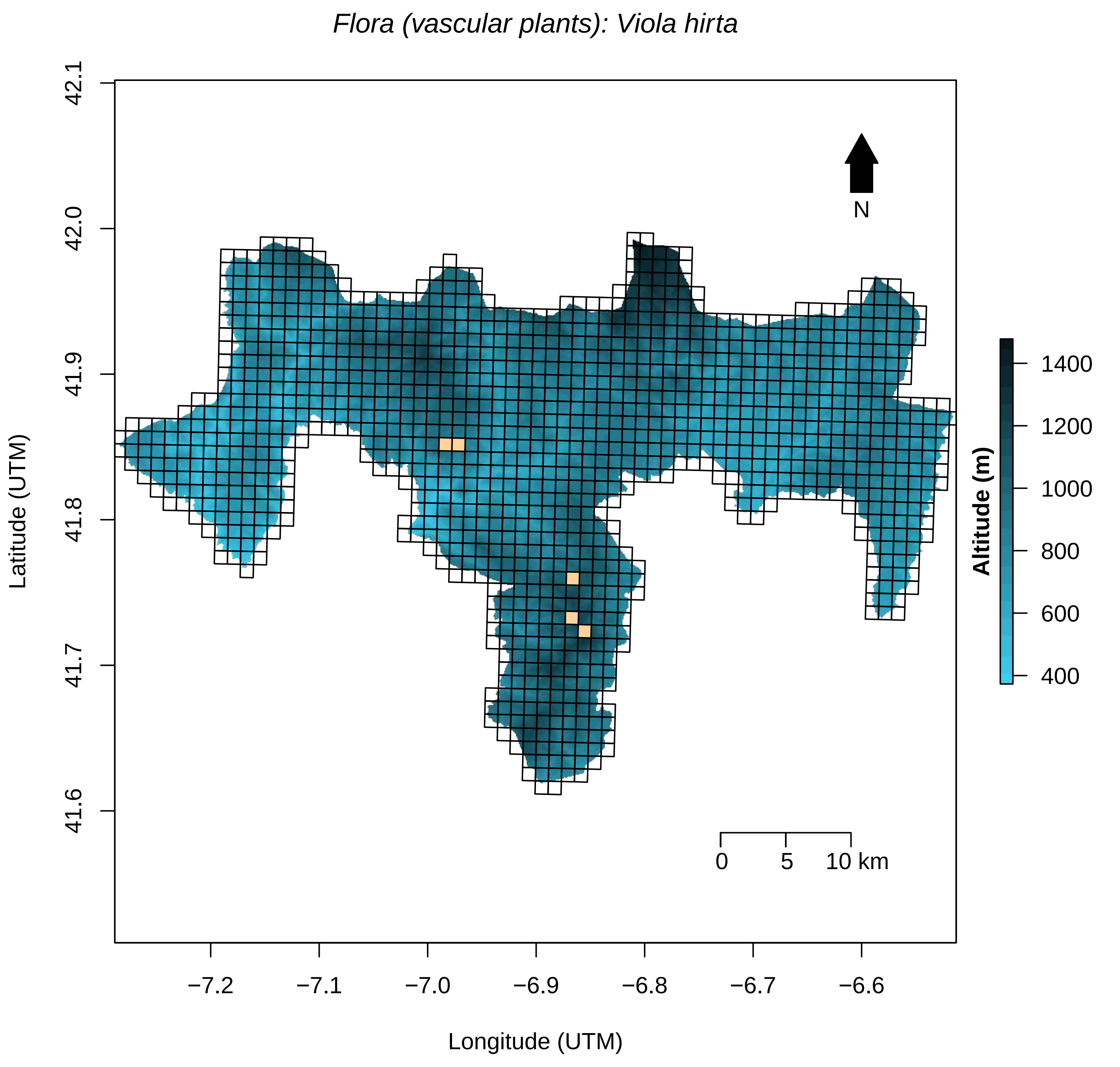
Flora (vascular plants): Viola hirta
42.1
42.0
41.9
41.8
41.7
41.6
Latitude (UTM)
−7.2
−7.1
−7.0
−6.9
−6.8
−6.7
−6.6
Longitude (UTM)
N
0
5
10 km
Altitude (m)
1400
1200
1000
800
600
400

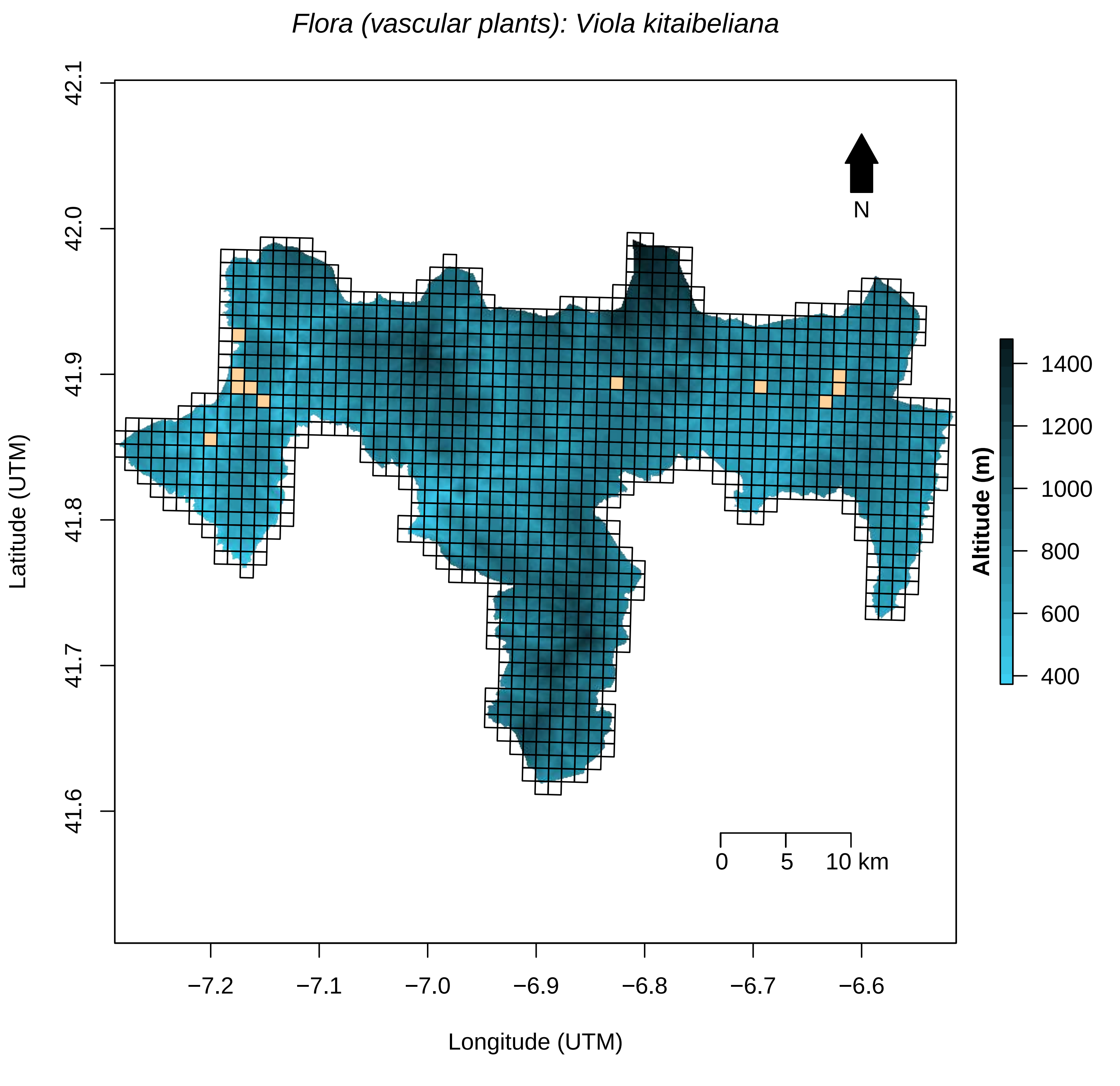
Flora (vascular plants): Viola kitaibeliana
42.1
42.0
41.9
41.8
41.7
41.6
Latitude (UTM)
−7.2
−7.1
−7.0
−6.9
−6.8
−6.7
−6.6
Longitude (UTM)
N
0
5
10 km
Altitude (m)
1400
1200
1000
800
600
400

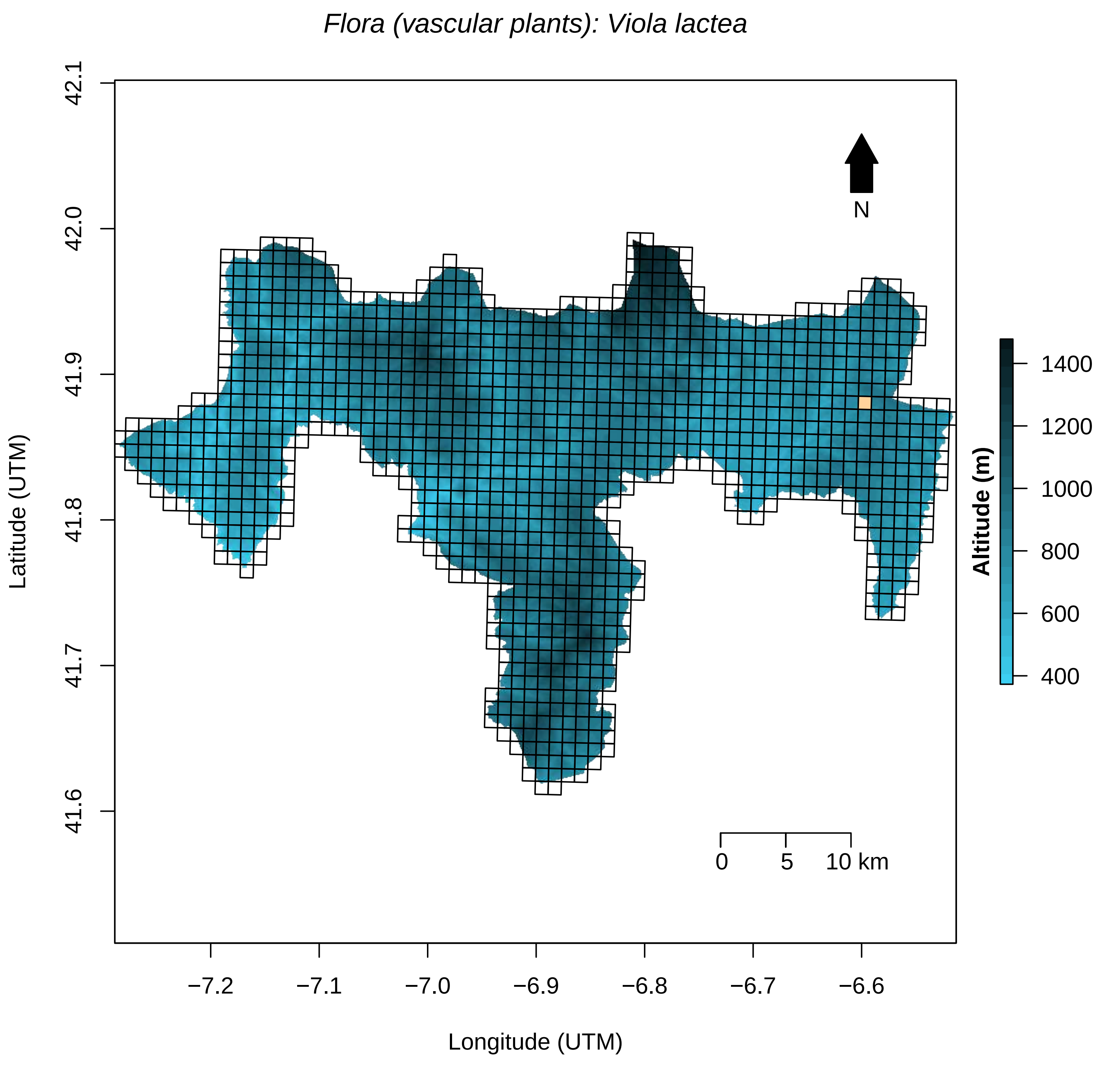

Flora (vascular plants): Viola lactea
N
0
5
10 km
Altitude (m)
1400
1200
1000
800
600
400
Latitude (UTM)
42.1
42.0
41.9
41.8
41.7
41.6
Longitude (UTM)
−7.2
−7.1
−7.0
−6.9
−6.8
−6.7
−6.6

Flora (vascular plants): Viola palustris

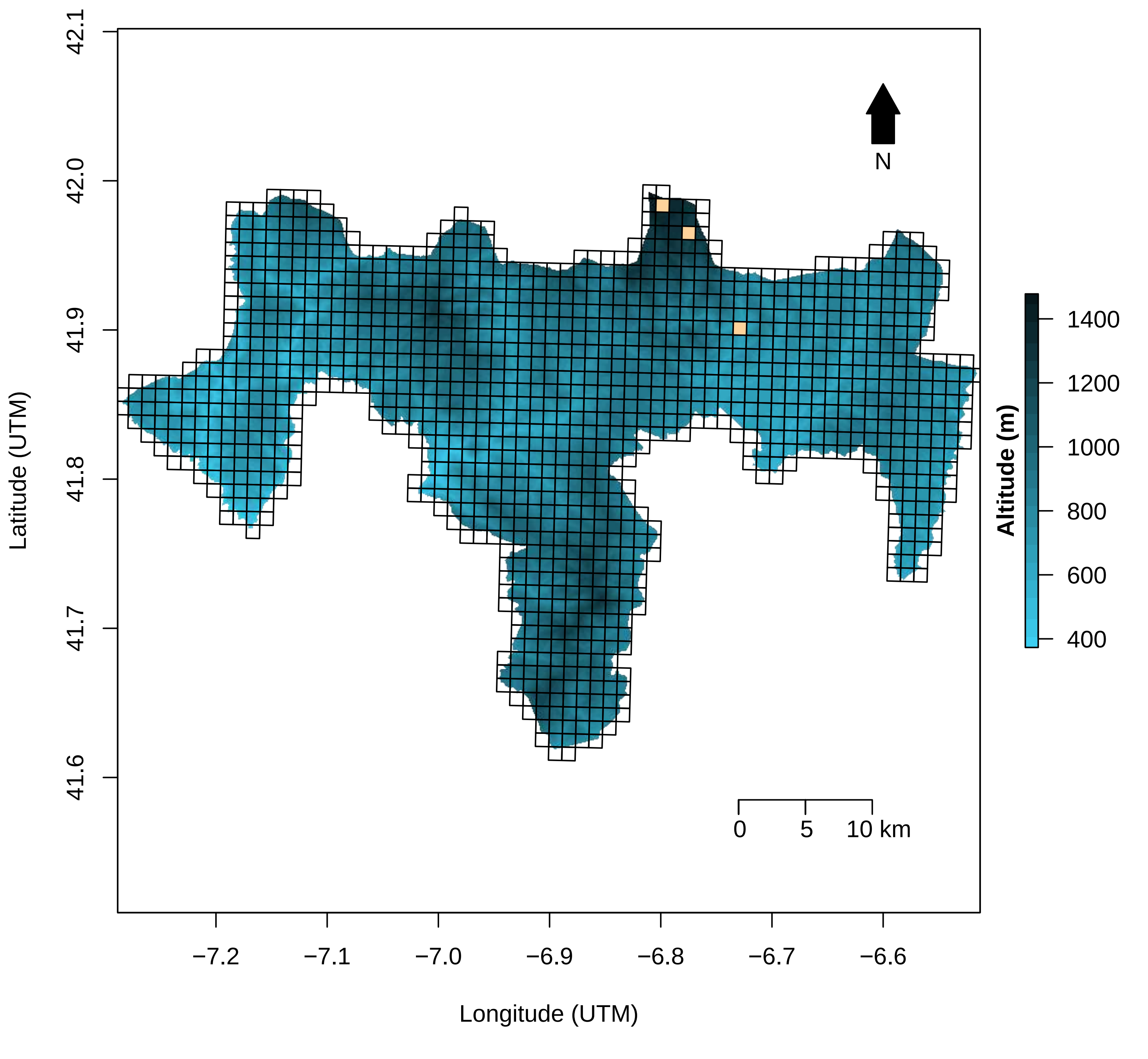

Flora (vascular plants): Viola parvula

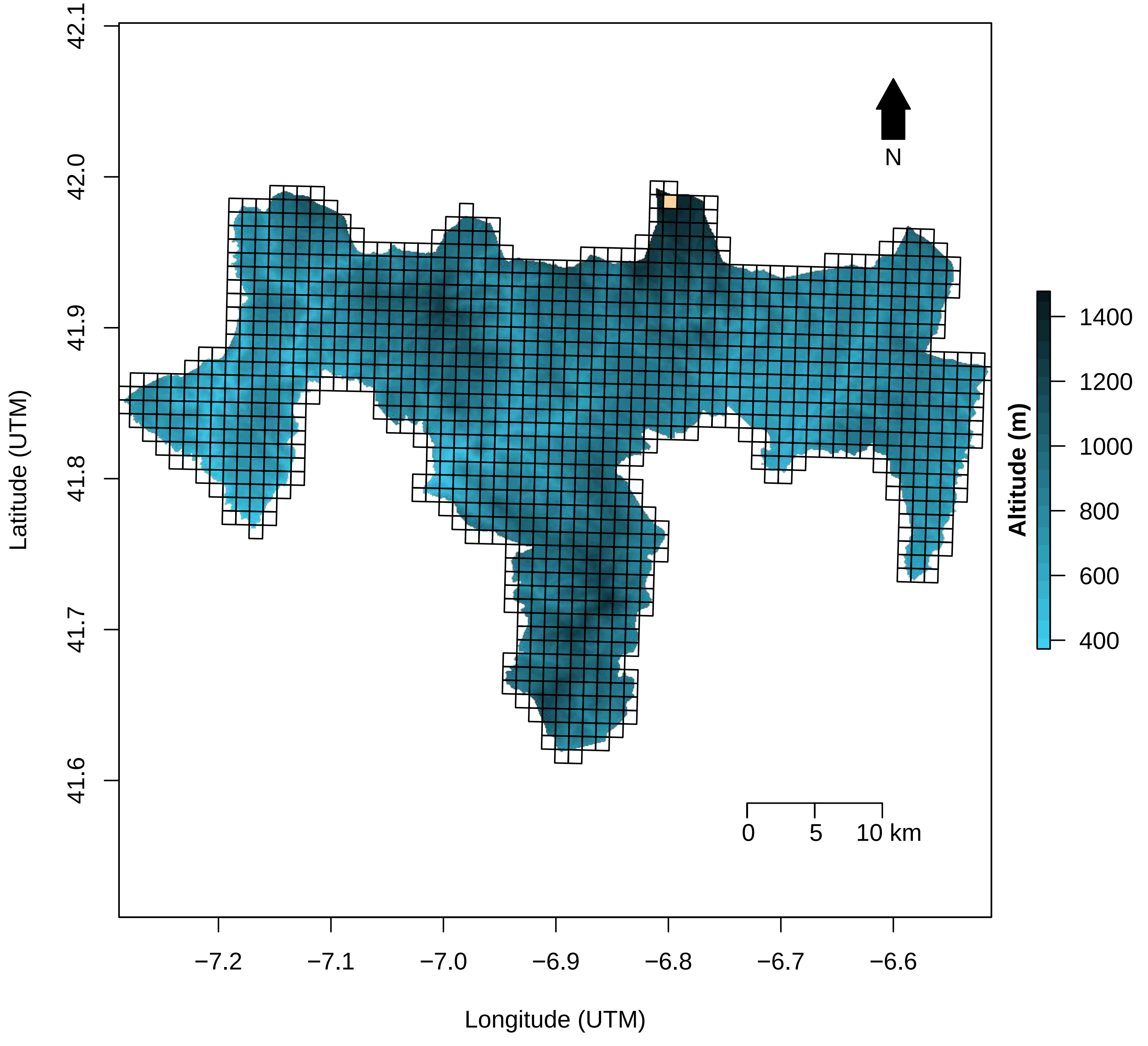

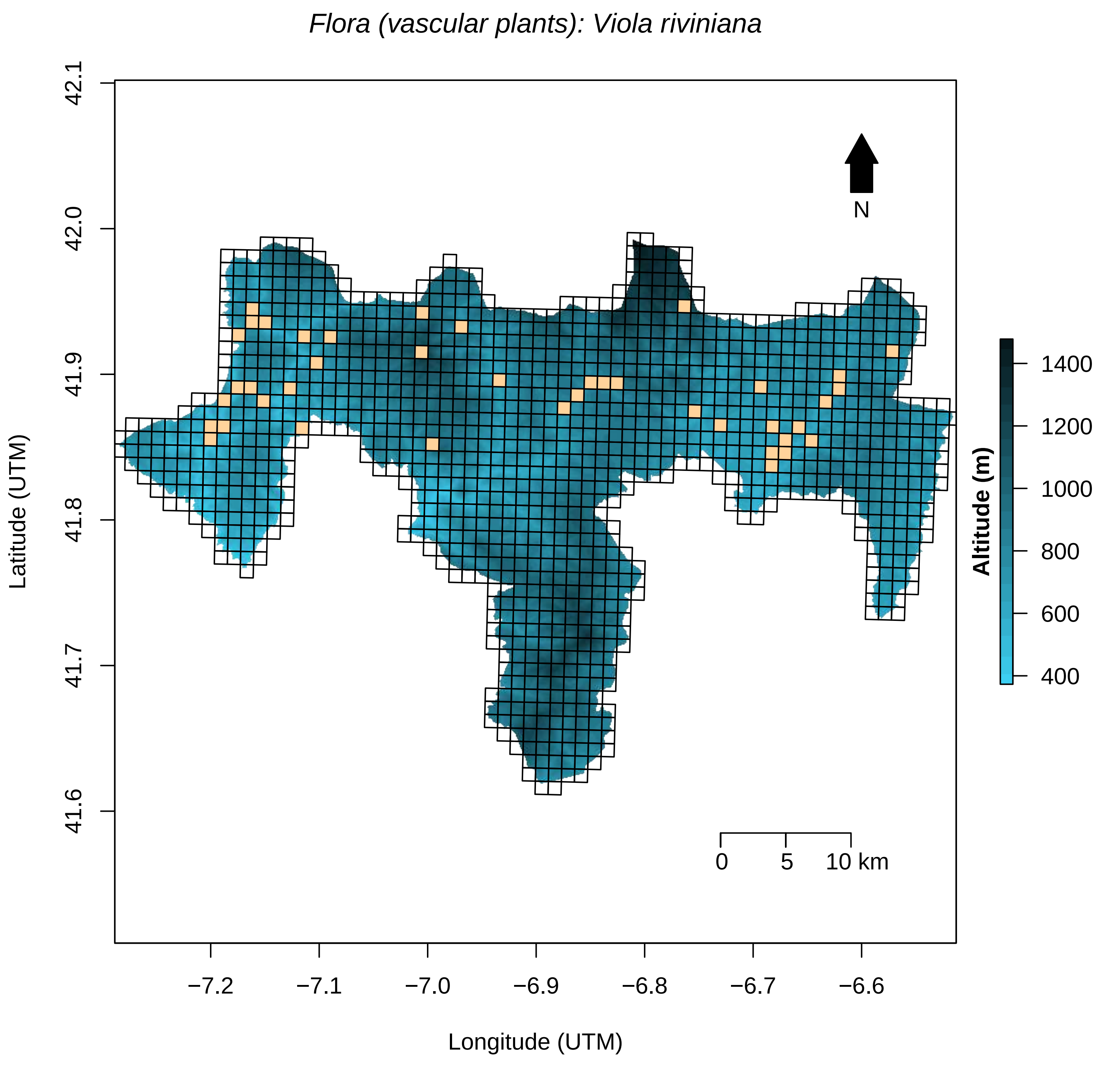

Flora (vascular plants): Viola riviniana
42.1
42.0
41.9
41.8
41.7
41.6
Latitude (UTM)
−7.2
−7.1
−7.0
−6.9
−6.8
−6.7
−6.6
Longitude (UTM)
N
0
5
10 km
Altitude (m)
1400
1200
1000
800
600
400

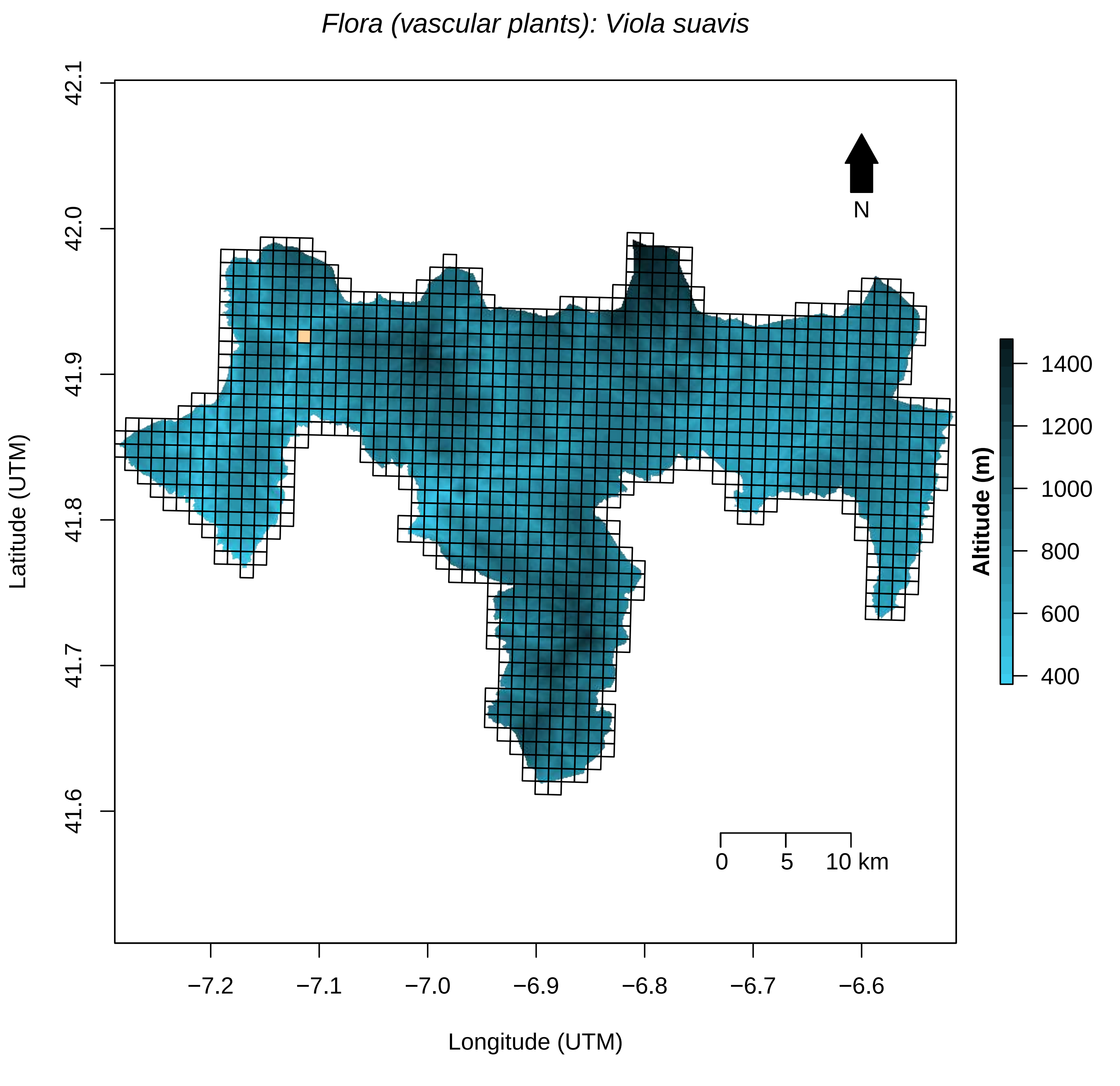

Flora (vascular plants): Viola suavis
Latitude (UTM)
Longitude (UTM)
42.1
42.0
41.9
41.8
41.7
41.6
−7.2
−7.1
−7.0
−6.9
−6.8
−6.7
−6.6
N
0
5
10 km
Altitude (m)
1400
1200
1000
800
600
400

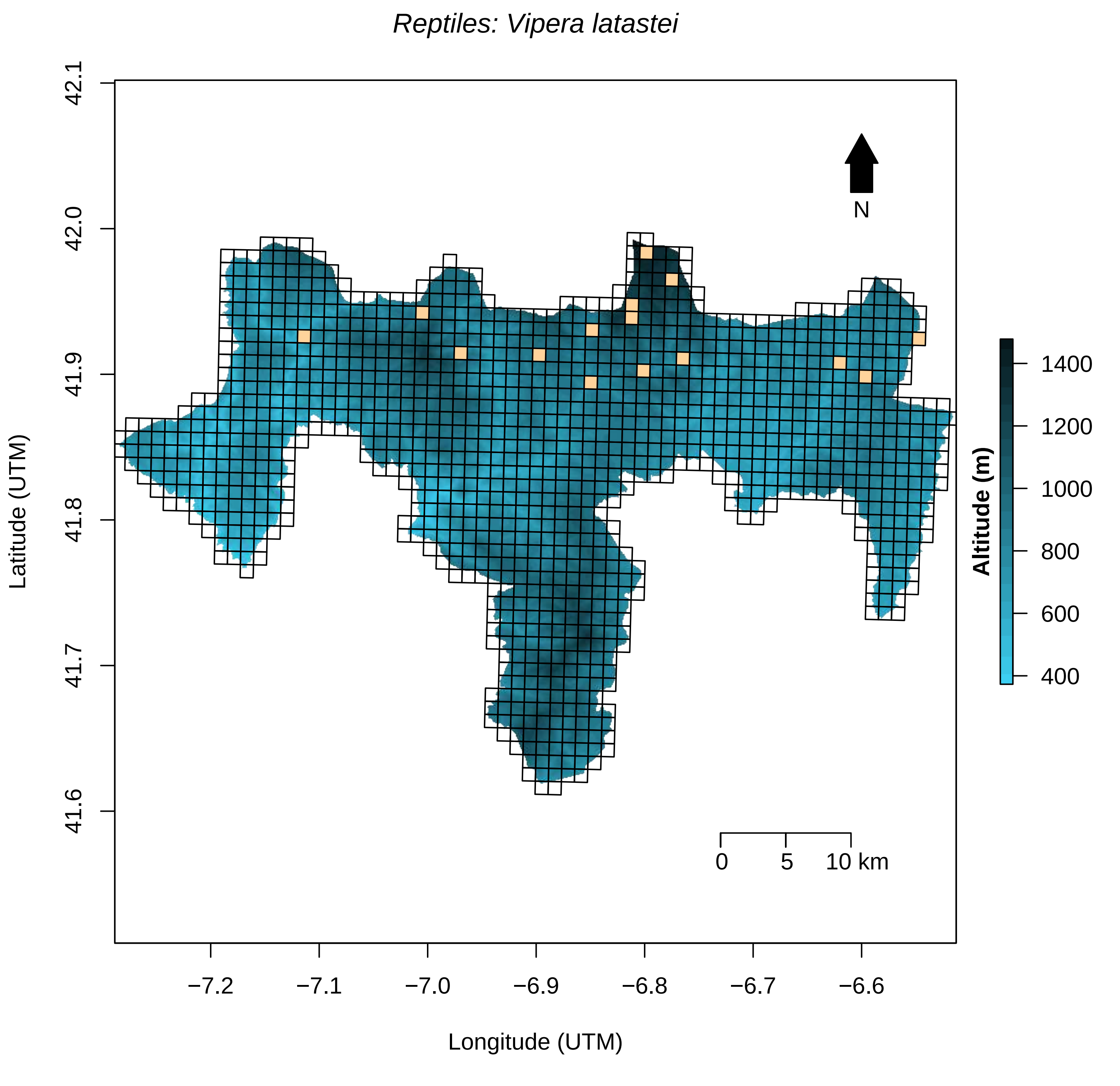

Reptiles: Vipera latastei
42.1
42.0
41.9
41.8
41.7
41.6
Latitude (UTM)
−7.2
−7.1
−7.0
−6.9
−6.8
−6.7
−6.6
Longitude (UTM)
N
0
5
10 km
Altitude (m)
1400
1200
1000
800
600
400

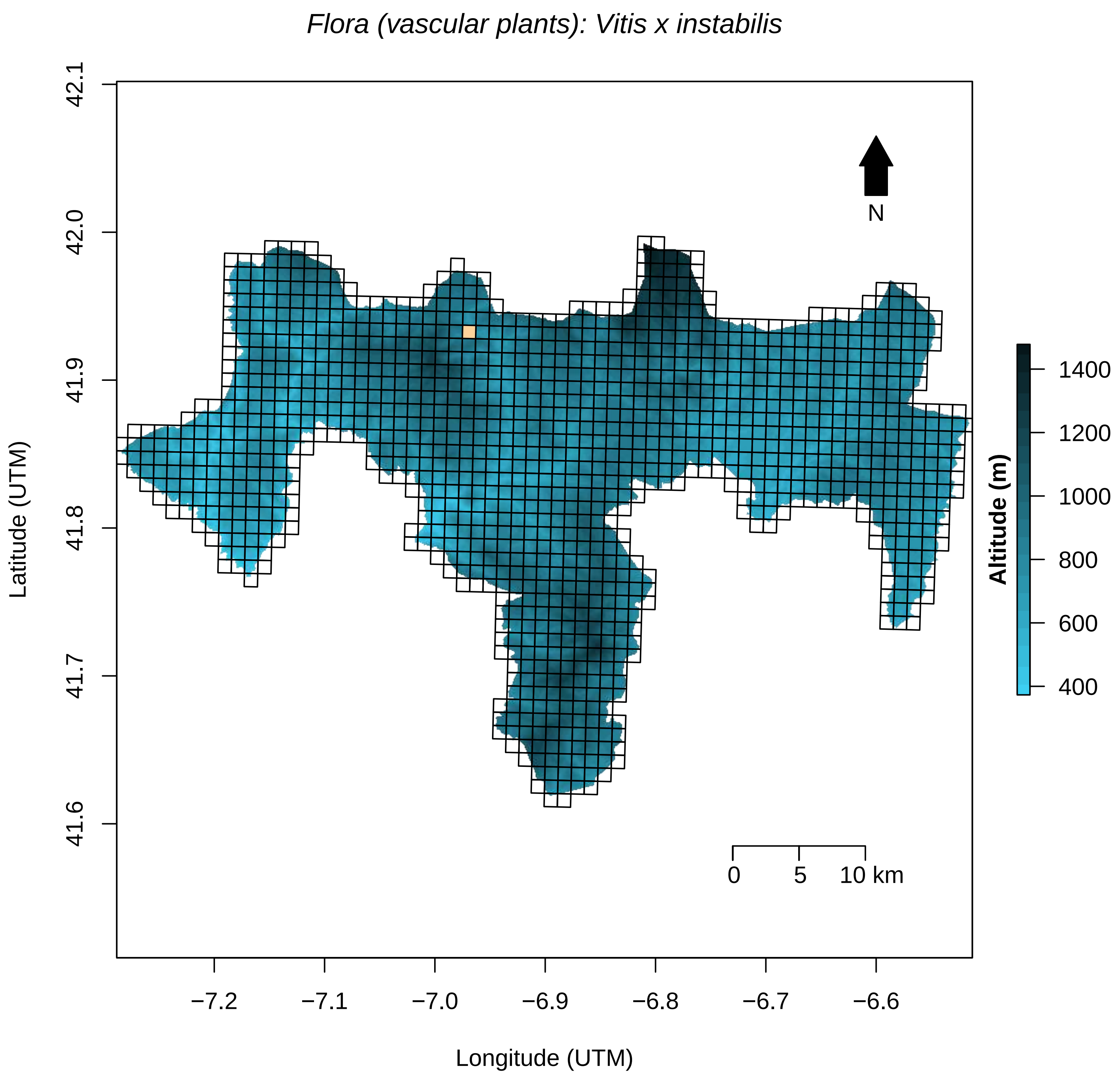
Flora (vascular plants): Vitis x instabilis
42.1
42.0
41.9
41.8
41.7
41.6
Latitude (UTM)
N
0 5 10 km
−7.2 −7.1 −7.0 −6.9 −6.8 −6.7 −6.6
Longitude (UTM)
Altitude (m)
1400
1200
1000
800
600
400

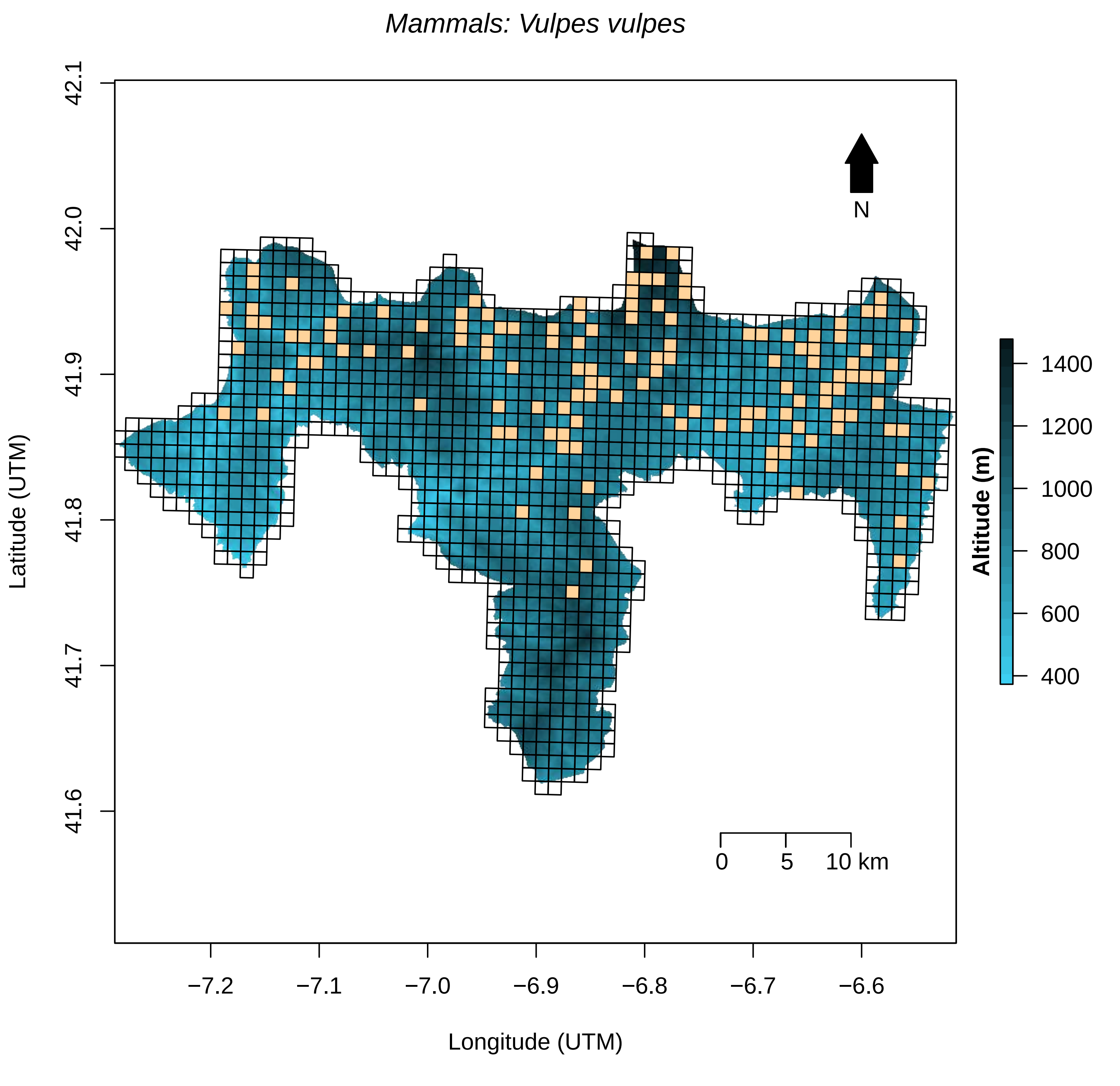

Mammals: Vulpes vulpes
N
0
5
10 km
Altitude (m)
1400
1200
1000
800
600
400
Latitude (UTM)
42.1
42.0
41.9
41.8
41.7
41.6
Longitude (UTM)
−7.2
−7.1
−7.0
−6.9
−6.8
−6.7
−6.6

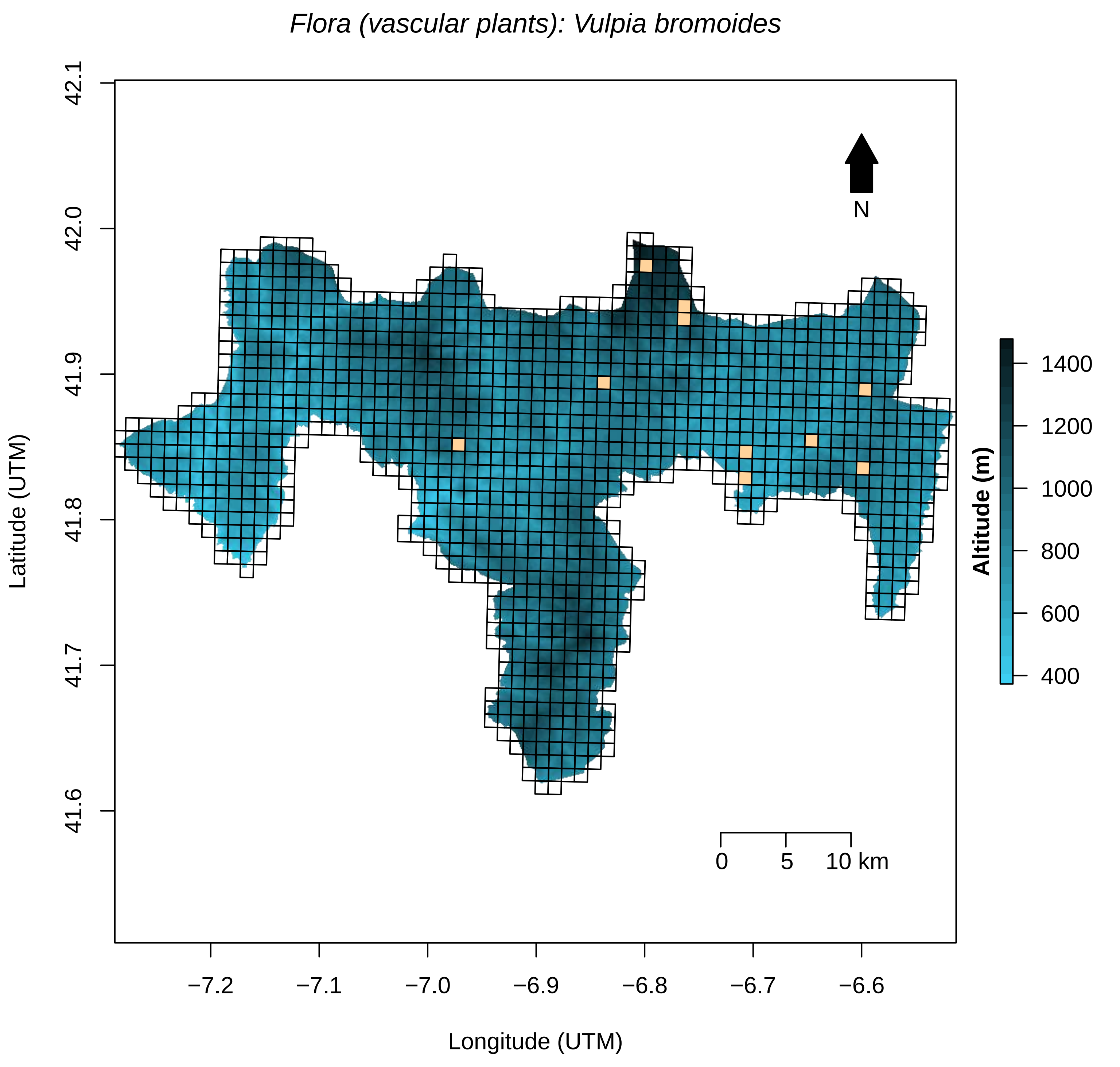
Flora (vascular plants): Vulpia bromoides
42.1
42.0
41.9
41.8
41.7
41.6
Latitude (UTM)
−7.2
−7.1
−7.0
−6.9
−6.8
−6.7
−6.6
Longitude (UTM)
N
0
5
10 km
Altitude (m)
1400
1200
1000
800
600
400

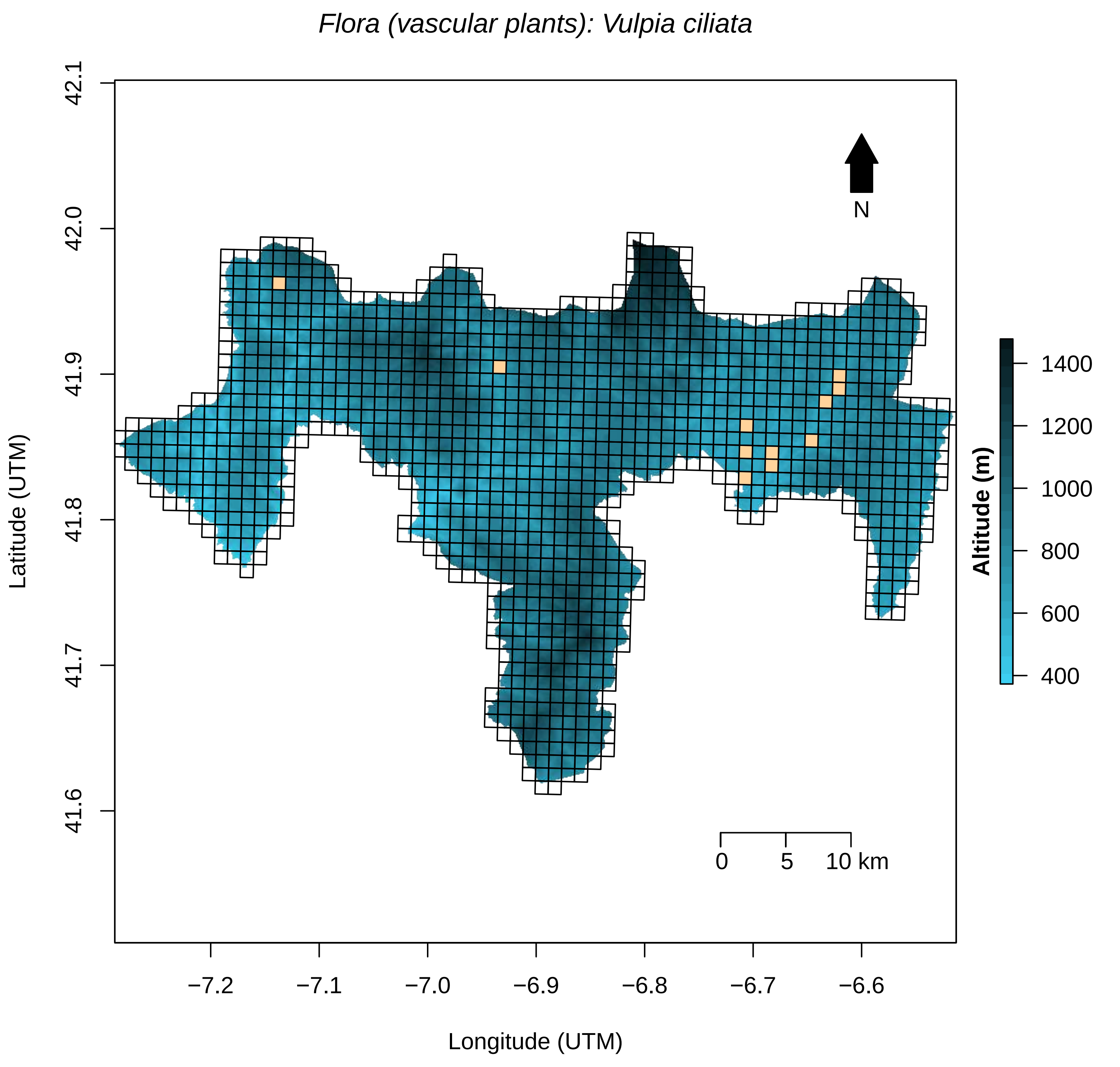

Flora (vascular plants): Vulpia ciliata
Latitude (UTM)
42.1
42.0
41.9
41.8
41.7
41.6
N
0
5
10 km
−7.2
−7.1
−7.0
−6.9
−6.8
−6.7
−6.6
Longitude (UTM)
Altitude (m)
1400
1200
1000
800
600
400

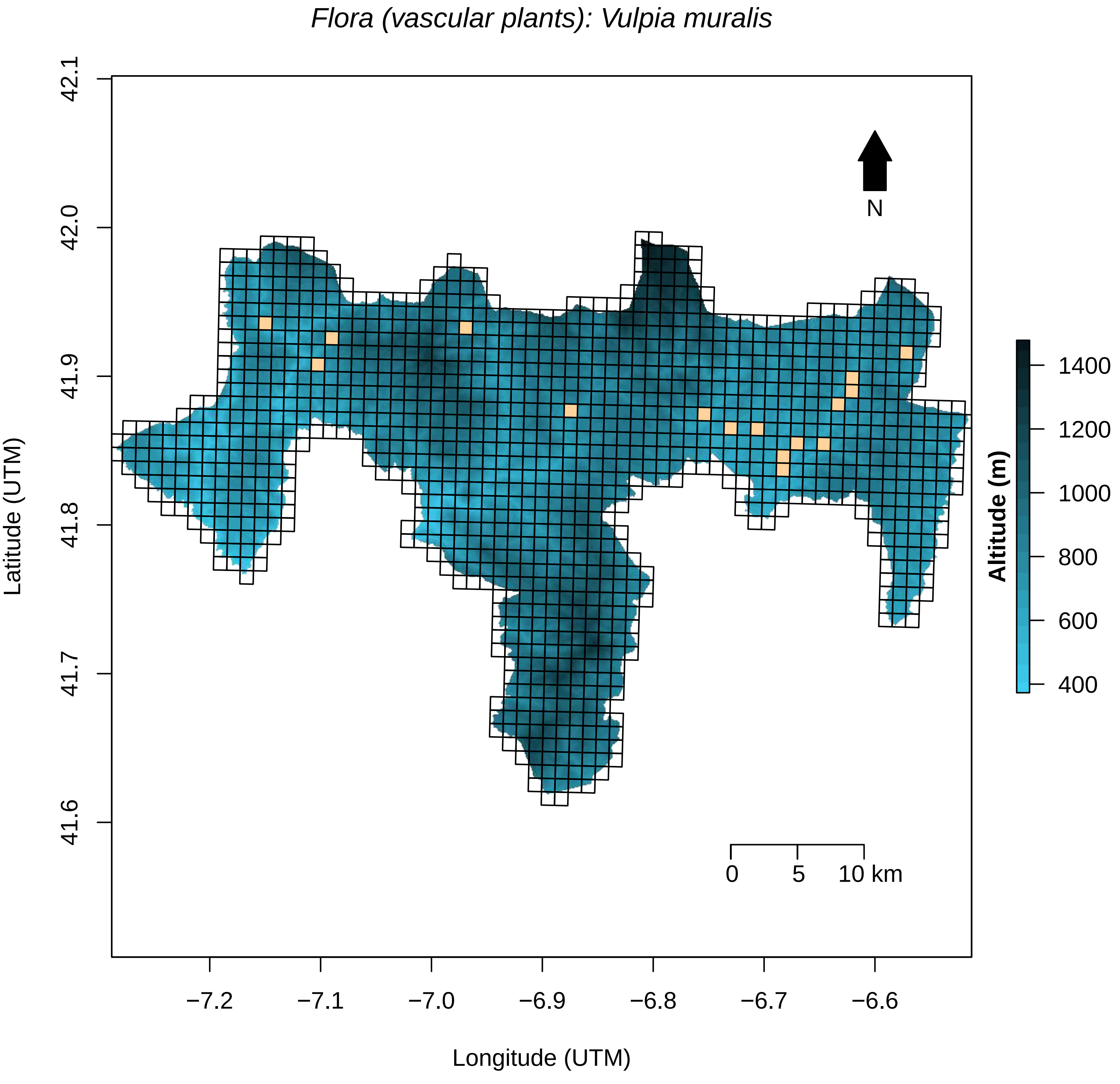

Flora (vascular plants): Vulpia muralis
Latitude (UTM)
Longitude (UTM)
42.1
42.0
41.9
41.8
41.7
41.6
−7.2
−7.1
−7.0
−6.9
−6.8
−6.7
−6.6
N
0
5
10 km
Altitude (m)
1400
1200
1000
800
600
400

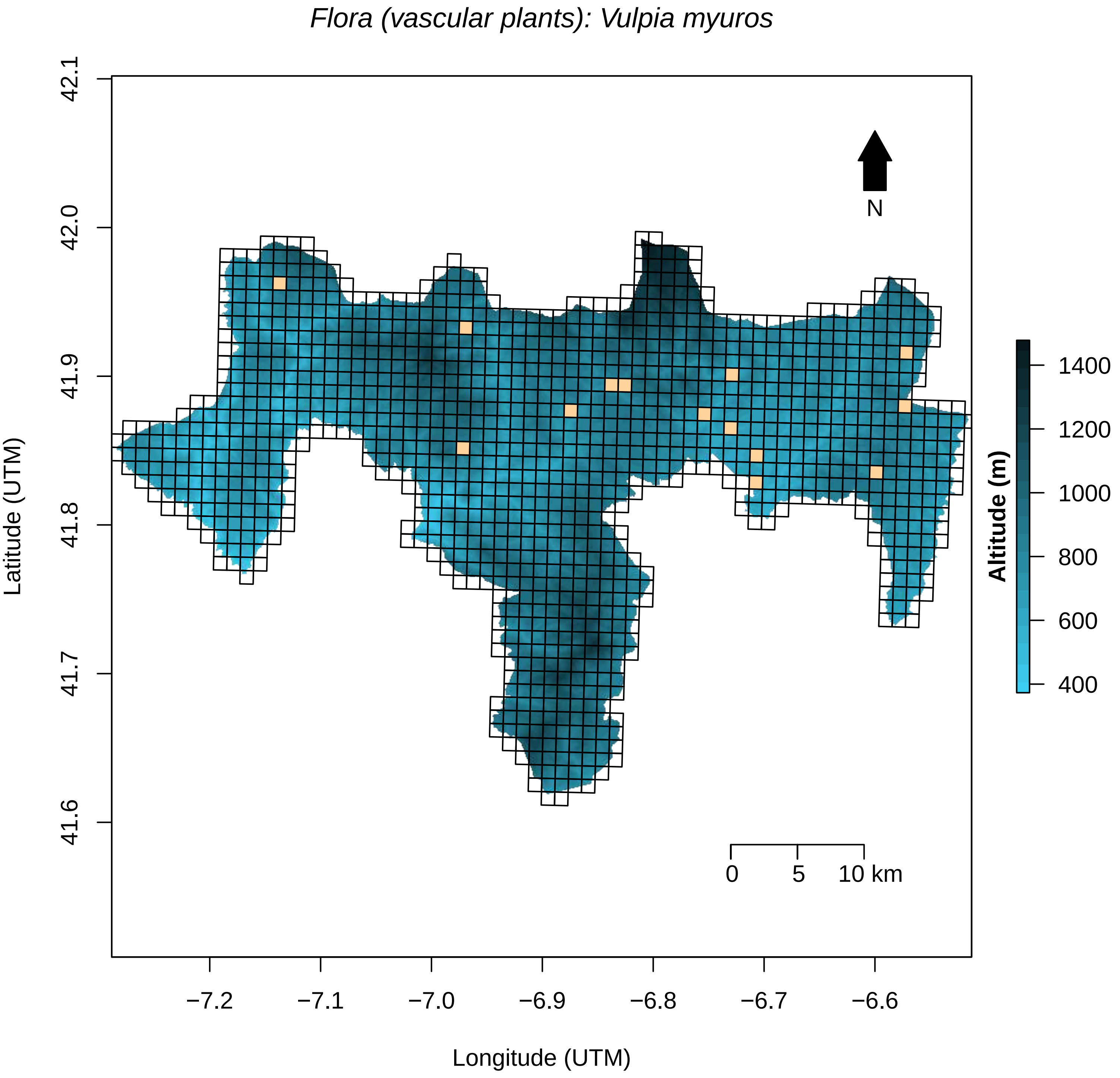
Flora (vascular plants): Vulpia myuros
N
0
5
10 km
Altitude (m)
1400
1200
1000
800
600
400
42.1
42.0
41.9
41.8
41.7
41.6
Latitude (UTM)
−7.2
−7.1
−7.0
−6.9
−6.8
−6.7
−6.6
Longitude (UTM)

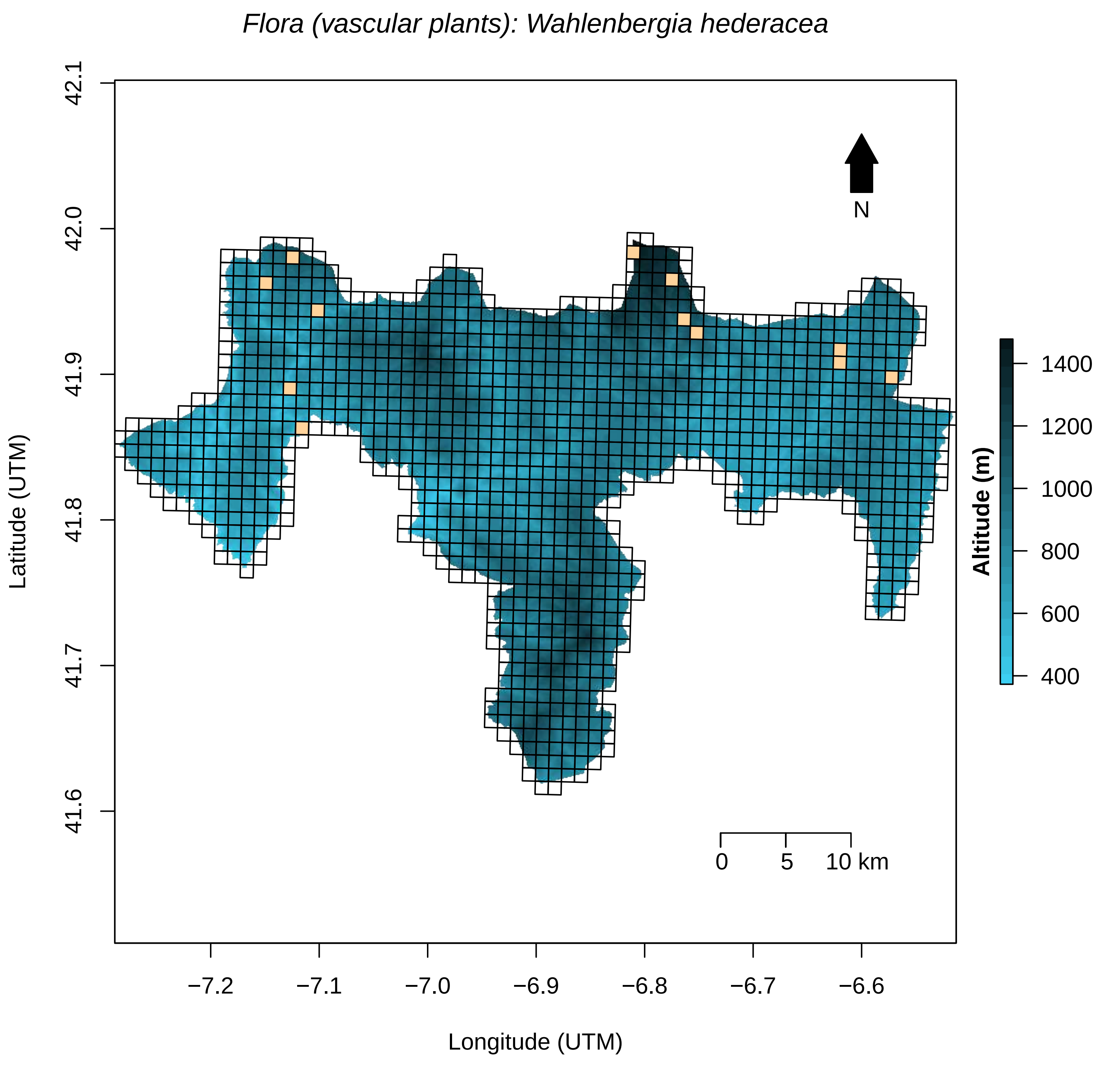
Flora (vascular plants): Wahlenbergia hederacea
Latitude (UTM)
42.1
42.0
41.9
41.8
41.7
41.6
Longitude (UTM)
−7.2
−7.1
−7.0
−6.9
−6.8
−6.7
−6.6
N
0
5
10 km
Altitude (m)
1400
1200
1000
800
600
400

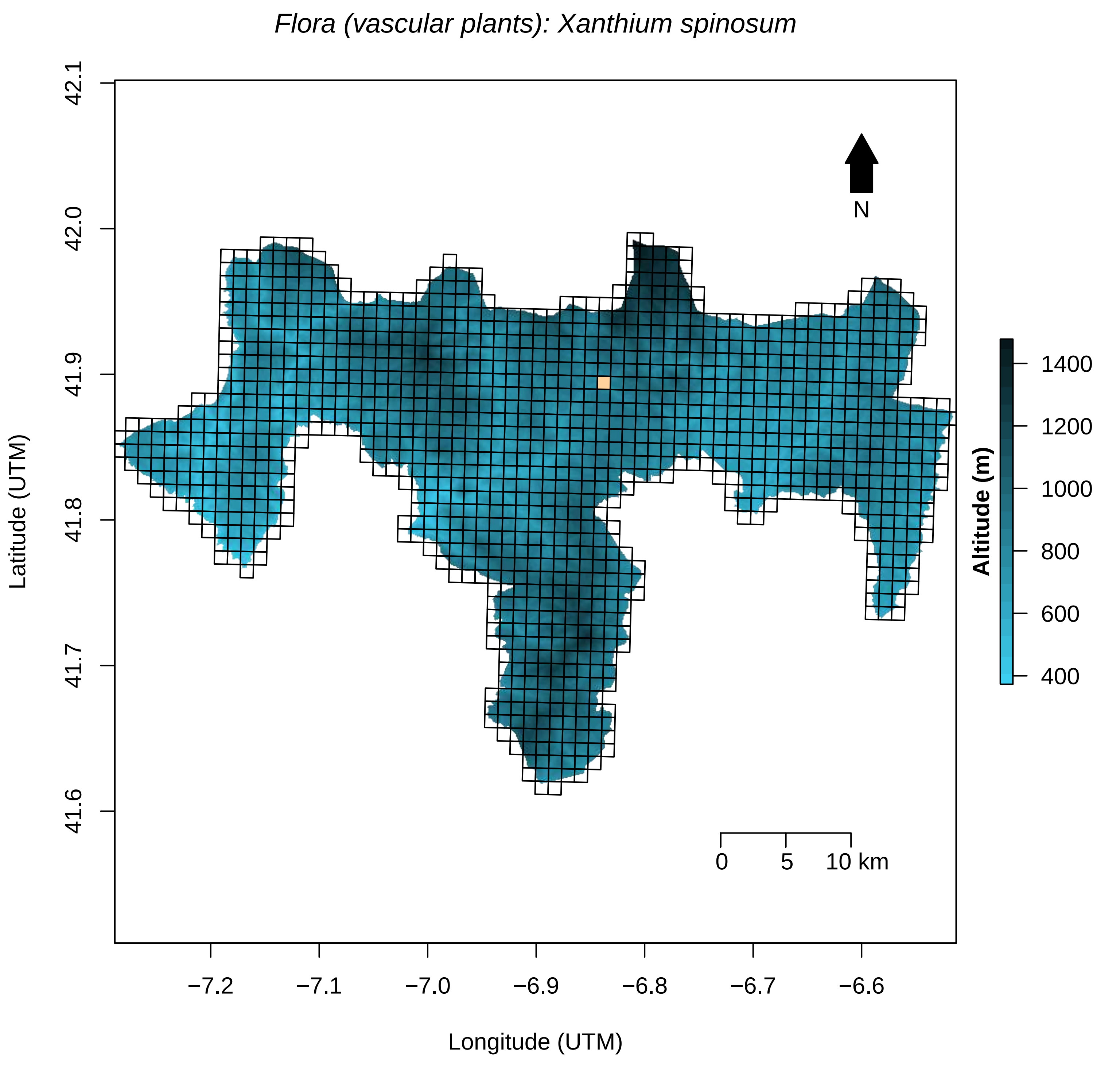
Flora (vascular plants): Xanthium spinosum
N
0 5 10 km
Latitude (UTM)
42.1
42.0
41.9
41.8
41.7
41.6
Longitude (UTM)
−7.2
−7.1
−7.0
−6.9
−6.8
−6.7
−6.6
Altitude (m)
1400
1200
1000
800
600
400

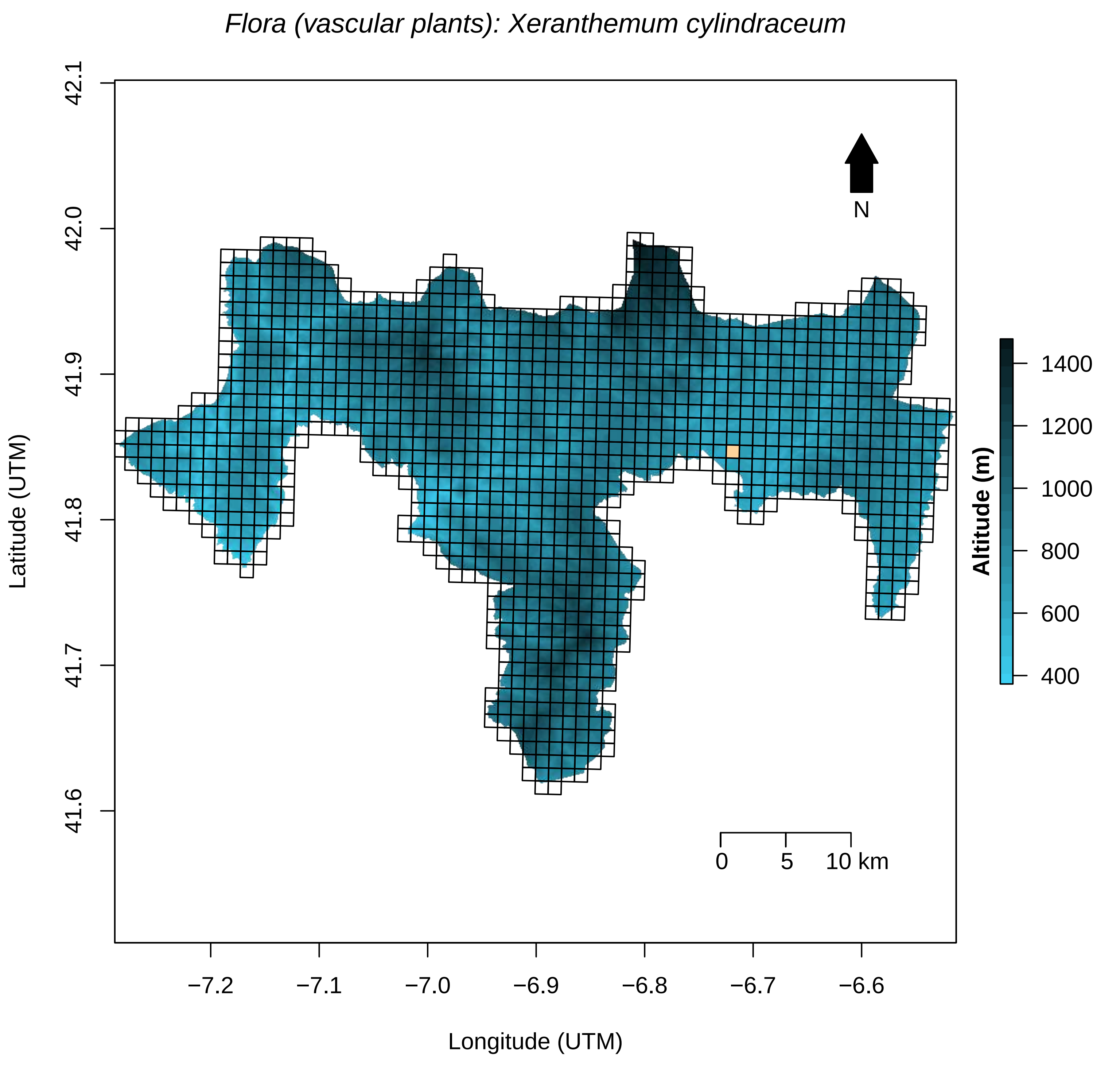

Flora (vascular plants): Xeranthemum cylindraceum
N
0
5
10 km
Altitude (m)
1400
1200
1000
800
600
400
Latitude (UTM)
42.1
42.0
41.9
41.8
41.7
41.6
Longitude (UTM)
−7.2
−7.1
−7.0
−6.9
−6.8
−6.7
−6.6

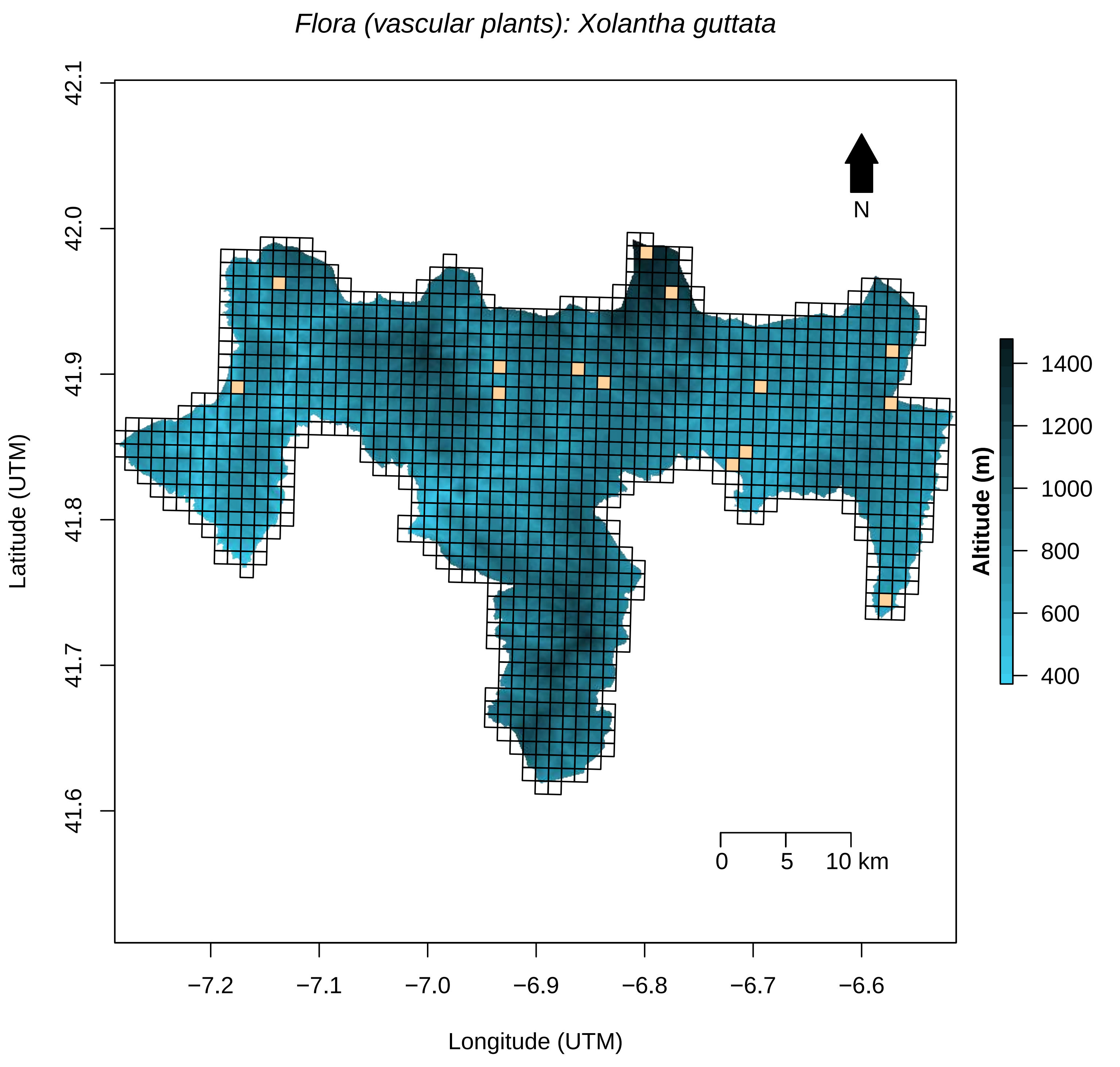
Flora (vascular plants): Xolantha guttata
42.1
42.0
41.9
41.8
41.7
41.6
Latitude (UTM)
−7.2
−7.1
−7.0
−6.9
−6.8
−6.7
−6.6
Longitude (UTM)
N
0
5
10 km
1400
1200
1000
800
600
400
Altitude (m)

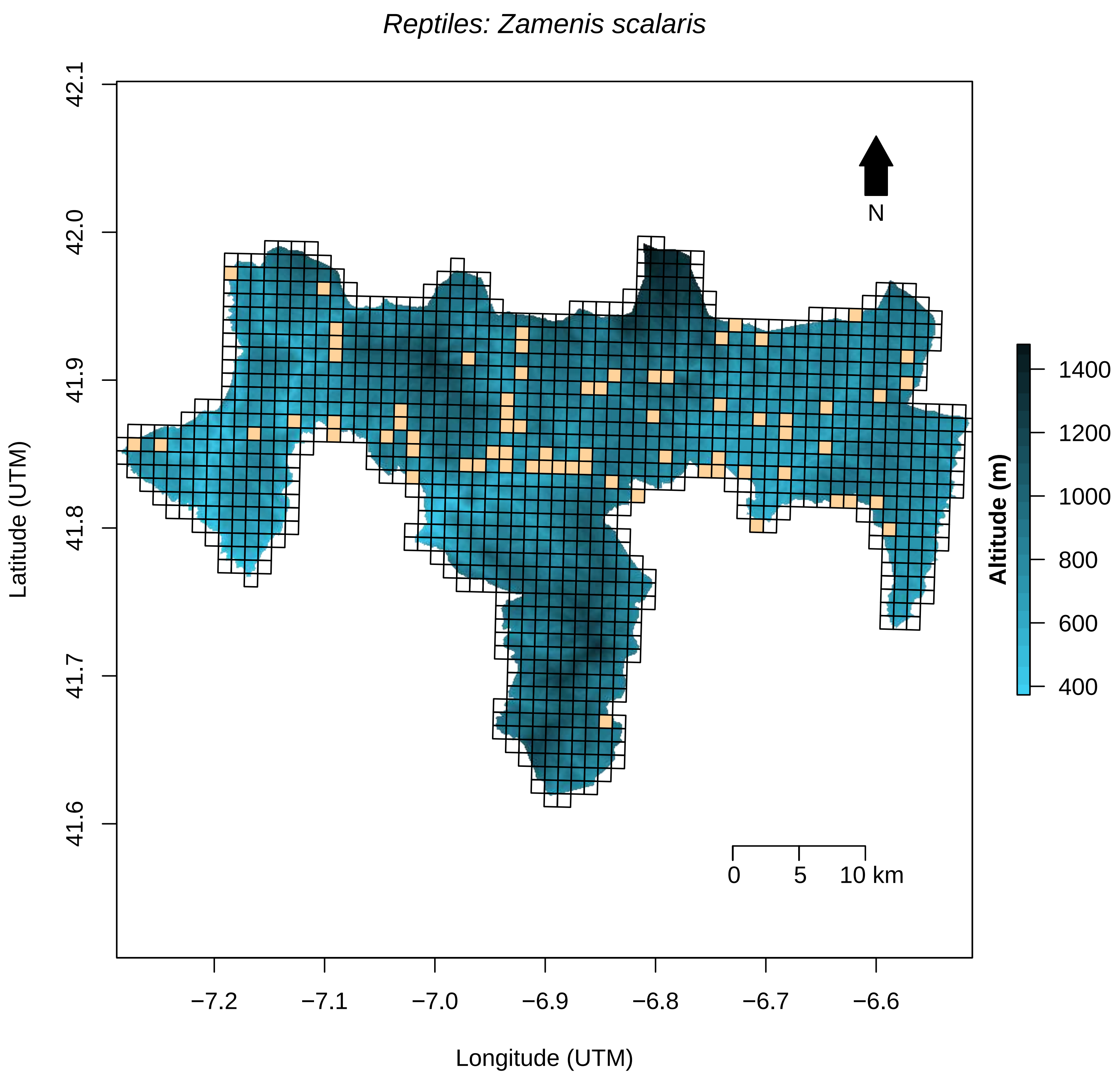
Reptiles: Zamenis scalaris
Latitude (UTM)
Longitude (UTM)
42.1
42.0
41.9
41.8
41.7
41.6
−7.2
−7.1
−7.0
−6.9
−6.8
−6.7
−6.6
N
0
5
10 km
Altitude (m)
1400
1200
1000
800
600
400

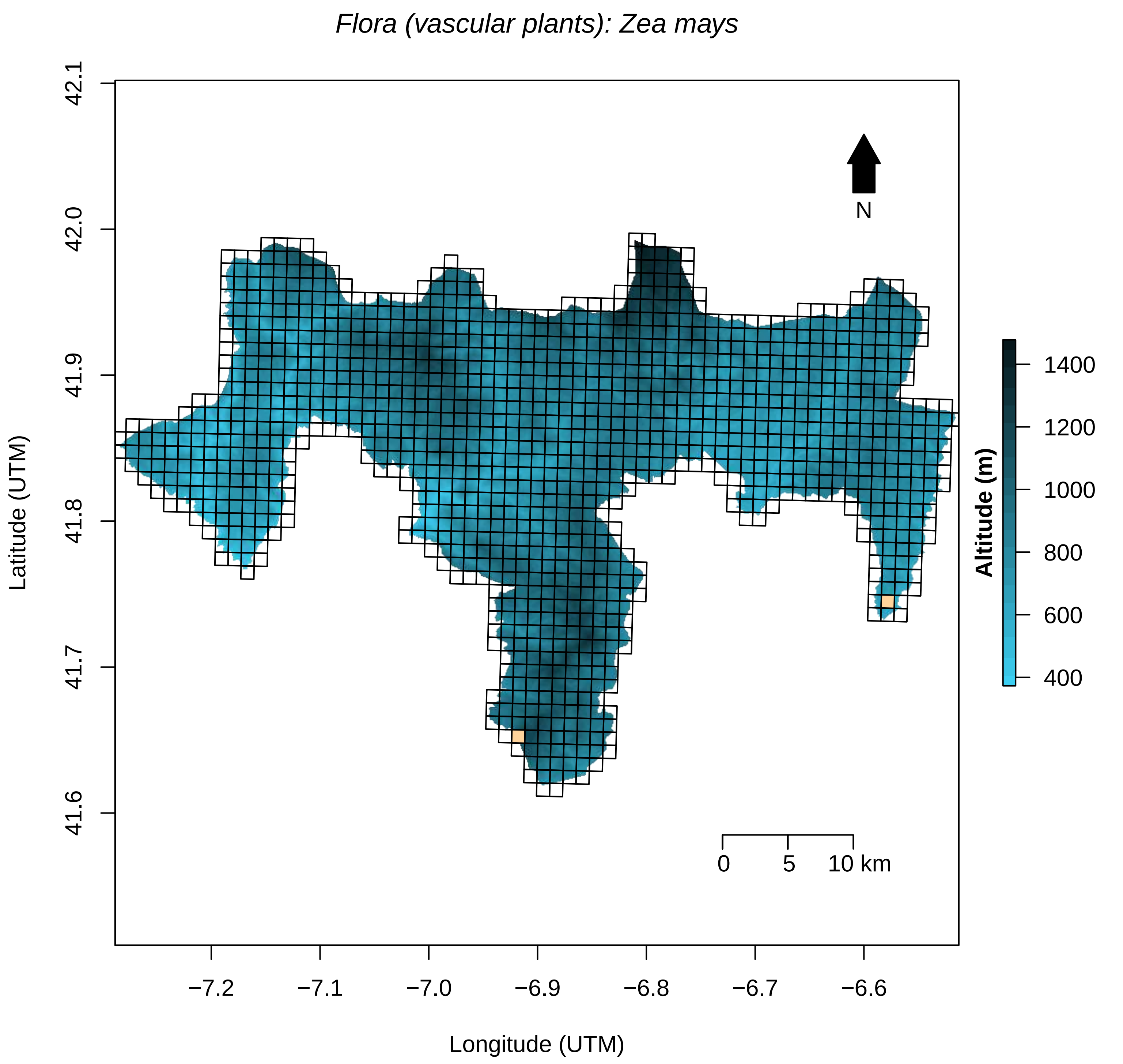

Flora (vascular plants): Zea mays
42.1
42.0
41.9
41.8
41.7
41.6
Latitude (UTM)
−7.2
−7.1
−7.0
−6.9
−6.8
−6.7
−6.6
Longitude (UTM)
N
0
5
10 km
Altitude (m)
1400
1200
1000
800
600
400

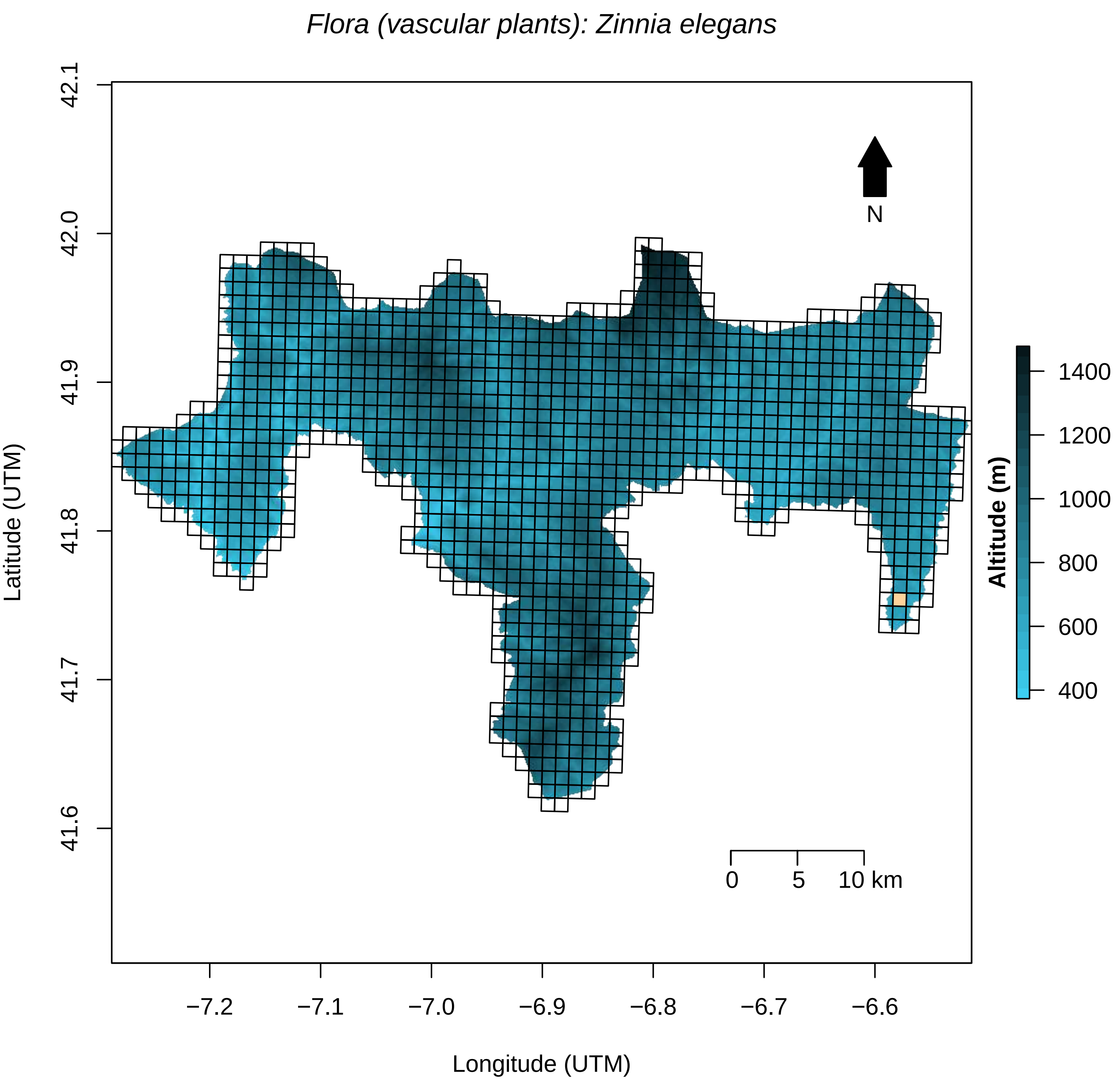
Flora (vascular plants): Zinnia elegans
N
0 5 10 km
Altitude (m)
1400
1200
1000
800
600
400
Latitude (UTM)
42.1
42.0
41.9
41.8
41.7
41.6
Longitude (UTM)
−7.2
−7.1
−7.0
−6.9
−6.8
−6.7
−6.6
